# Global, regional, and national mortality among young people aged 10–24 years, 1950–2019: a systematic analysis for the Global Burden of Disease Study 2019

**DOI:** 10.1016/S0140-6736(21)01546-4

**Published:** 2021-10-30

**Authors:** GBD 2019 Adolescent Mortality Collaborators, Joseph L Ward, Peter S Azzopardi, Kate Louise Francis, John S Santelli, Vegard Skirbekk, Susan M Sawyer, Nicholas J Kassebaum, Ali H Mokdad, Simon I Hay, Foad Abd-Allah, Amir Abdoli, Mohammad Abdollahi, Aidin Abedi, Hassan Abolhassani, Lucas Guimarães Abreu, Michael R M Abrigo, Eman Abu-Gharbieh, Abdelrahman I Abushouk, Oladimeji M Adebayo, Victor Adekanmbi, Davoud Adham, Shailesh M Advani, Khashayar Afshari, Anurag Agrawal, Tauseef Ahmad, Keivan Ahmadi, Anwar E Ahmed, Budi Aji, Blessing Akombi-Inyang, Fares Alahdab, Ziyad Al-Aly, Khurshid Alam, Fahad Mashhour Alanezi, Turki M Alanzi, Jacqueline Elizabeth Alcalde-Rabanal, Biresaw Wassihun Alemu, Samar Al-Hajj, Robert Kaba Alhassan, Saqib Ali, Gianfranco Alicandro, Mehran Alijanzadeh, Syed Mohamed Aljunid, Amir Almasi-Hashiani, Nihad A Almasri, Hesham M Al-Mekhlafi, Jordi Alonso, Rajaa M Al-Raddadi, Khalid A Altirkawi, Nelson Alvis-Guzman, Azmeraw T Amare, Saeed Amini, Arya Aminorroaya, Arianna Maever L Amit, Dickson A Amugsi, Robert Ancuceanu, Deanna Anderlini, Catalina Liliana Andrei, Sofia Androudi, Fereshteh Ansari, Iman Ansari, Carl Abelardo T Antonio, Davood Anvari, Razique Anwer, Seth Christopher Yaw Appiah, Jalal Arabloo, Morteza Arab-Zozani, Johan Ärnlöv, Malke Asaad, Mehran Asadi-Aliabadi, Ali A Asadi-Pooya, Maha Moh'd Wahbi Atout, Marcel Ausloos, Elvis Korku Avenyo, Leticia Avila-Burgos, Beatriz Paulina Ayala Quintanilla, Getinet Ayano, Yared Asmare Aynalem, Samad Azari, Zelalem Nigussie Azene, Mohammad Hossein Bakhshaei, Shankar M Bakkannavar, Maciej Banach, Palash Chandra Banik, Miguel A Barboza, Suzanne Lyn Barker-Collo, Till Winfried Bärnighausen, Sanjay Basu, Bernhard T Baune, Mohsen Bayati, Neeraj Bedi, Ettore Beghi, Tariku Tesfaye Bekuma, Arielle Wilder Bell, Michelle L Bell, Corina Benjet, Isabela M Bensenor, Abadi Kidanemariam Berhe, Kidanemaryam Berhe, Adam E Berman, Akshaya Srikanth Bhagavathula, Nikha Bhardwaj, Pankaj Bhardwaj, Krittika Bhattacharyya, Suraj Bhattarai, Zulfiqar A Bhutta, Ali Bijani, Boris Bikbov, Antonio Biondi, Tesega Tesega Mengistu Birhanu, Raaj Kishore Biswas, Somayeh Bohlouli, Srinivasa Rao Bolla, Archith Boloor, Rohan Borschmann, Soufiane Boufous, Nicola Luigi Bragazzi, Dejana Braithwaite, Nicholas J K Breitborde, Hermann Brenner, Gabrielle B Britton, Richard A Burns, Sharath Burugina Nagaraja, Zahid A Butt, Florentino Luciano Caetano dos Santos, Luis Alberto Cámera, Ismael R Campos-Nonato, Julio Cesar Campuzano Rincon, Rosario Cárdenas, Giulia Carreras, Juan J Carrero, Felix Carvalho, Joao Mauricio Castaldelli-Maia, Carlos A Castañeda-Orjuela, Giulio Castelpietra, Ferrán Catalá-López, Ester Cerin, Joht Singh Chandan, Hsing-Yi Chang, Jung-Chen Chang, Jaykaran Charan, Vijay Kumar Chattu, Sarika Chaturvedi, Jee-Young Jasmine Choi, Mohiuddin Ahsanul Kabir Chowdhury, Devasahayam J Christopher, Dinh-Toi Chu, Michael T Chung, Sheng-Chia Chung, Flavia M Cicuttini, Traian Vasile Constantin, Vera Marisa Costa, Saad M A Dahlawi, Haijiang Dai, Xiaochen Dai, Giovanni Damiani, Lalit Dandona, Rakhi Dandona, Parnaz Daneshpajouhnejad, Aso Mohammad Darwesh, Claudio Alberto Dávila-Cervantes, Kairat Davletov, Fernando Pio De la Hoz, Diego De Leo, Nikolaos Dervenis, Rupak Desai, Assefa Desalew, Keshab Deuba, Samath Dhamminda Dharmaratne, Govinda Prasad Dhungana, Mostafa Dianatinasab, Diana Dias da Silva, Daniel Diaz, Alireza Didarloo, Shirin Djalalinia, Fariba Dorostkar, Chirag P Doshi, Leila Doshmangir, Kerrie E Doyle, Andre Rodrigues Duraes, Mohammad Ebrahimi Kalan, Sanam Ebtehaj, David Edvardsson, Maha El Tantawi, Islam Y Elgendy, Shaimaa I El-Jaafary, Aisha Elsharkawy, Babak Eshrati, Sharareh Eskandarieh, Saman Esmaeilnejad, Firooz Esmaeilzadeh, Sadaf Esteghamati, Andre Faro, Farshad Farzadfar, Nazir Fattahi, Valery L Feigin, Tomas Y Ferede, Seyed-Mohammad Fereshtehnejad, Eduarda Fernandes, Pietro Ferrara, Irina Filip, Florian Fischer, James L Fisher, Nataliya A Foigt, Morenike Oluwatoyin Folayan, Artem Alekseevich Fomenkov, Masoud Foroutan, Takeshi Fukumoto, Mohamed M Gad, Abhay Motiramji Gaidhane, Silvano Gallus, Teshome Gebre, Ketema Bizuwork Gebremedhin, Gebreamlak Gebremedhn Gebremeskel, Leake Gebremeskel, Assefa Ayalew Gebreslassie, Hailay Abrha Gesesew, Keyghobad Ghadiri, Mansour Ghafourifard, Farhad Ghamari, Ahmad Ghashghaee, Syed Amir Gilani, Elena V Gnedovskaya, Myron Anthony Godinho, Mahaveer Golechha, Srinivas Goli, Philimon N Gona, Sameer Vali Gopalani, Giuseppe Gorini, Michal Grivna, Mohammed Ibrahim Mohialdeen Gubari, Harish Chander Gugnani, Rafael Alves Guimarães, Yuming Guo, Rajeev Gupta, Juanita A Haagsma, Nima Hafezi-Nejad, Teklehaimanot Gereziher Haile, Arvin Haj-Mirzaian, Arya Haj-Mirzaian, Brian J Hall, Randah R Hamadeh, Kanaan Hamagharib Abdullah, Samer Hamidi, Demelash Woldeyohannes Handiso, Asif Hanif, Graeme J Hankey, Hamidreza Haririan, Josep Maria Haro, Ahmed I Hasaballah, Abdiwahab Hashi, Amr Hassan, Soheil Hassanipour, Hadi Hassankhani, Khezar Hayat, Reza Heidari-Soureshjani, Claudiu Herteliu, Fatemeh Heydarpour, Hung Chak Ho, Michael K Hole, Ramesh Holla, Praveen Hoogar, Mostafa Hosseini, Mehdi Hosseinzadeh, Mihaela Hostiuc, Sorin Hostiuc, Mowafa Househ, Mohamed Hsairi, Tanvir M Huda, Ayesha Humayun, Rabia Hussain, Bing-Fang Hwang, Ivo Iavicoli, Segun Emmanuel Ibitoye, Olayinka Stephen Ilesanmi, Irena M Ilic, Milena D Ilic, Leeberk Raja Inbaraj, Nirun Intarut, Usman Iqbal, Seyed Sina Naghibi Irvani, M Mofizul Islam, Sheikh Mohammed Shariful Islam, Hiroyasu Iso, Rebecca Q Ivers, Mohammad Ali Jahani, Mihajlo Jakovljevic, Amir Jalali, Manthan Dilipkumar Janodia, Tahereh Javaheri, Panniyammakal Jeemon, Ensiyeh Jenabi, Ravi Prakash Jha, Vivekanand Jha, John S Ji, Jost B Jonas, Kelly M Jones, Farahnaz Joukar, Jacek Jerzy Jozwiak, Petur B Juliusson, Mikk Jürisson, Ali Kabir, Zubair Kabir, Leila R Kalankesh, Rohollah Kalhor, Naser Kamyari, Tanuj Kanchan, André Karch, Salah Eddin Karimi, Supreet Kaur, Gbenga A Kayode, Peter Njenga Keiyoro, Nauman Khalid, Mohammad Khammarnia, Maseer Khan, Md Nuruzzaman Khan, Khaled Khatab, Mona M Khater, Mahalaqua Nazli Khatib, Maryam Khayamzadeh, Habibolah Khazaie, Abdullah T Khoja, Christian Kieling, Young-Eun Kim, Yun Jin Kim, Ruth W Kimokoti, Adnan Kisa, Sezer Kisa, Mika Kivimäki, Ali Koolivand, Soewarta Kosen, Ai Koyanagi, Kewal Krishan, Nuworza Kugbey, G Anil Kumar, Manasi Kumar, Nithin Kumar, Om P Kurmi, Dian Kusuma, Carlo La Vecchia, Ben Lacey, Dharmesh Kumar Lal, Ratilal Lalloo, Qing Lan, Iván Landires, Van Charles Lansingh, Anders O Larsson, Savita Lasrado, Zohra S Lassi, Paolo Lauriola, Paul H Lee, Shaun Wen Huey Lee, James Leigh, Matilde Leonardi, Janni Leung, Miriam Levi, Sonia Lewycka, Bingyu Li, Ming-Chieh Li, Shanshan Li, Lee-Ling Lim, Stephen S Lim, Xuefeng Liu, Stefan Lorkowski, Paulo A Lotufo, Raimundas Lunevicius, Ralph Maddison, Phetole Walter Mahasha, Mokhtar Mahdavi Mahdavi, Morteza Mahmoudi, Azeem Majeed, Afshin Maleki, Reza Malekzadeh, Deborah Carvalho Malta, Abdullah A Mamun, Borhan Mansouri, Mohammad Ali Mansournia, Gabriel Martinez, Jose Martinez-Raga, Francisco Rogerlândio Martins-Melo, Amanda J Mason-Jones, Seyedeh Zahra Masoumi, Manu Raj Mathur, Pallab K Maulik, John J McGrath, Man Mohan Mehndiratta, Fereshteh Mehri, Peter T N Memiah, Walter Mendoza, Ritesh G Menezes, Endalkachew Worku Mengesha, Atte Meretoja, Tuomo J Meretoja, Tomislav Mestrovic, Bartosz Miazgowski, Tomasz Miazgowski, Irmina Maria Michalek, Ted R Miller, GK Mini, Andreea Mirica, Erkin M Mirrakhimov, Hamed Mirzaei, Maryam Mirzaei, Babak Moazen, Dara K Mohammad, Shadieh Mohammadi, Abdollah Mohammadian-Hafshejani, Noushin Mohammadifard, Reza Mohammadpourhodki, Shafiu Mohammed, Lorenzo Monasta, Ghobad Moradi, Maziar Moradi-Lakeh, Rahmatollah Moradzadeh, Paula Moraga, Shane Douglas Morrison, Abbas Mosapour, Amin Mousavi Khaneghah, Ulrich Otto Mueller, Moses K Muriithi, Christopher J L Murray, Saravanan Muthupandian, Mehdi Naderi, Ahamarshan Jayaraman Nagarajan, Mohsen Naghavi, Mukhammad David Naimzada, Vinay Nangia, Vinod C Nayak, Javad Nazari, Rawlance Ndejjo, Ionut Negoi, Ruxandra Irina Negoi, Henok Biresaw Netsere, Georges Nguefack-Tsague, Diep Ngoc Nguyen, Huong Lan Thi Nguyen, Jing Nie, Dina Nur Anggraini Ningrum, Chukwudi A Nnaji, Shuhei Nomura, Jean Jacques Noubiap, Christoph Nowak, Virginia Nuñez-Samudio, Felix Akpojene Ogbo, Onome Bright Oghenetega, In-Hwan Oh, Morteza Oladnabi, Andrew T Olagunju, Bolajoko Olubukunola Olusanya, Jacob Olusegun Olusanya, Ahmed Omar Bali, Muktar Omer Omer, Obinna E Onwujekwe, Alberto Ortiz, Adrian Otoiu, Nikita Otstavnov, Stanislav S Otstavnov, Simon Øverland, Mayowa O Owolabi, Mahesh P A, Jagadish Rao Padubidri, Keyvan Pakshir, Raffaele Palladino, Adrian Pana, Songhomitra Panda-Jonas, Anamika Pandey, Carlo Irwin Able Panelo, Eun-Kee Park, Scott B Patten, Amy E Peden, Veincent Christian Filipino Pepito, Emmanuel K Peprah, Jeevan Pereira, Konrad Pesudovs, Hai Quang Pham, Michael R Phillips, Michael A Piradov, Meghdad Pirsaheb, Maarten J Postma, Faheem Hyder Pottoo, Hadi Pourjafar, Akram Pourshams, Sergio I Prada, Elisabetta Pupillo, Zahiruddin Quazi Syed, Mohammad Hasan Rabiee, Navid Rabiee, Amir Radfar, Ata Rafiee, Alberto Raggi, Fakher Rahim, Vafa Rahimi-Movaghar, Mohammad Hifz Ur Rahman, Muhammad Aziz Rahman, Kiana Ramezanzadeh, Chhabi Lal Ranabhat, Sowmya J Rao, Vahid Rashedi, Prateek Rastogi, Priya Rathi, David Laith Rawaf, Salman Rawaf, Lal Rawal, Reza Rawassizadeh, Andre M N Renzaho, Negar Rezaei, Nima Rezaei, Mohammad sadegh Rezai, Seyed Mohammad Riahi, Jennifer Rickard, Leonardo Roever, Luca Ronfani, Gregory A Roth, Enrico Rubagotti, Susan Fred Rumisha, Godfrey M Rwegerera, Siamak Sabour, Perminder S Sachdev, Basema Saddik, Ehsan Sadeghi, Sahar Saeedi Moghaddam, Rajesh Sagar, Amirhossein Sahebkar, Mohammad Ali Sahraian, S Mohammad Sajadi, Marwa Rashad Salem, Hamideh Salimzadeh, Abdallah M Samy, Juan Sanabria, Milena M Santric-Milicevic, Sivan Yegnanarayana Iyer Saraswathy, Nizal Sarrafzadegan, Arash Sarveazad, Thirunavukkarasu Sathish, Davide Sattin, Deepak Saxena, Sonia Saxena, Silvia Schiavolin, David C Schwebel, Falk Schwendicke, Subramanian Senthilkumaran, Sadaf G Sepanlou, Feng Sha, Omid Shafaat, Saeed Shahabi, Amira A Shaheen, Masood Ali Shaikh, Saeed Shakiba, MohammadBagher Shamsi, Mohammed Shannawaz, Kiomars Sharafi, Aziz Sheikh, Sara Sheikhbahaei, B Suresh Kumar Shetty, Peilin Shi, Mika Shigematsu, Jae Il Shin, Rahman Shiri, Kerem Shuval, Soraya Siabani, Inga Dora Sigfusdottir, Rannveig Sigurvinsdottir, Diego Augusto Santos Silva, João Pedro Silva, Biagio Simonetti, Jasvinder A Singh, Virendra Singh, Abiy H Sinke, Valentin Yurievich Skryabin, Helen Slater, Emma U R Smith, Mohammad Reza Sobhiyeh, Eugene Sobngwi, Amin Soheili, Oluwaseyi Dolapo Somefun, Muluken Bekele Sorrie, Ireneous N Soyiri, Chandrashekhar T Sreeramareddy, Dan J Stein, Mark A Stokes, Agus Sudaryanto, Iyad Sultan, Rafael Tabarés-Seisdedos, Takahiro Tabuchi, Santosh Kumar Tadakamadla, Amir Taherkhani, Animut Tagele Tamiru, Md Ismail Tareque, Kavumpurathu Raman Thankappan, Rekha Thapar, Nihal Thomas, Mariya Vladimirovna Titova, Marcello Tonelli, Marcos Roberto Tovani-Palone, Bach Xuan Tran, Ravensara S Travillian, Alexander C Tsai, Aristidis Tsatsakis, Lorainne Tudor Car, Riaz Uddin, Brigid Unim, Bhaskaran Unnikrishnan, Era Upadhyay, Marco Vacante, Sahel Valadan Tahbaz, Pascual R Valdez, Santosh Varughese, Tommi Juhani Vasankari, Narayanaswamy Venketasubramanian, Paul J Villeneuve, Francesco S Violante, Vasily Vlassov, Theo Vos, Giang Thu Vu, Yasir Waheed, Richard G Wamai, Yafeng Wang, Yanzhong Wang, Yuan-Pang Wang, Ronny Westerman, Nuwan Darshana Wickramasinghe, Ai-Min Wu, Chenkai Wu, Seyed Hossein Yahyazadeh Jabbari, Kazumasa Yamagishi, Yuichiro Yano, Sanni Yaya, Vahid Yazdi-Feyzabadi, Yordanos Gizachew Yeshitila, Paul Yip, Naohiro Yonemoto, Seok-Jun Yoon, Mustafa Z Younis, Taraneh Yousefinezhadi, Chuanhua Yu, Yong Yu, Deniz Yuce, Syed Saoud Zaidi, Sojib Bin Zaman, Mohammad Zamani, Maryam Zamanian, Hadi Zarafshan, Ahmad Zarei, Mikhail Sergeevich Zastrozhin, Yunquan Zhang, Zhi-Jiang Zhang, Xiu-Ju George Zhao, Cong Zhu, George C Patton, Russell M Viner

## Abstract

**Background:**

Documentation of patterns and long-term trends in mortality in young people, which reflect huge changes in demographic and social determinants of adolescent health, enables identification of global investment priorities for this age group. We aimed to analyse data on the number of deaths, years of life lost, and mortality rates by sex and age group in people aged 10–24 years in 204 countries and territories from 1950 to 2019 by use of estimates from the Global Burden of Diseases, Injuries, and Risk Factors Study (GBD) 2019.

**Methods:**

We report trends in estimated total numbers of deaths and mortality rate per 100 000 population in young people aged 10–24 years by age group (10–14 years, 15–19 years, and 20–24 years) and sex in 204 countries and territories between 1950 and 2019 for all causes, and between 1980 and 2019 by cause of death. We analyse variation in outcomes by region, age group, and sex, and compare annual rate of change in mortality in young people aged 10–24 years with that in children aged 0–9 years from 1990 to 2019. We then analyse the association between mortality in people aged 10–24 years and socioeconomic development using the GBD Socio-demographic Index (SDI), a composite measure based on average national educational attainment in people older than 15 years, total fertility rate in people younger than 25 years, and income per capita. We assess the association between SDI and all-cause mortality in 2019, and analyse the ratio of observed to expected mortality by SDI using the most recent available data release (2017).

**Findings:**

In 2019 there were 1·49 million deaths (95% uncertainty interval 1·39–1·59) worldwide in people aged 10–24 years, of which 61% occurred in males. 32·7% of all adolescent deaths were due to transport injuries, unintentional injuries, or interpersonal violence and conflict; 32·1% were due to communicable, nutritional, or maternal causes; 27·0% were due to non-communicable diseases; and 8·2% were due to self-harm. Since 1950, deaths in this age group decreased by 30·0% in females and 15·3% in males, and sex-based differences in mortality rate have widened in most regions of the world. Geographical variation has also increased, particularly in people aged 10–14 years. Since 1980, communicable and maternal causes of death have decreased sharply as a proportion of total deaths in most GBD super-regions, but remain some of the most common causes in sub-Saharan Africa and south Asia, where more than half of all adolescent deaths occur. Annual percentage decrease in all-cause mortality rate since 1990 in adolescents aged 15–19 years was 1·3% in males and 1·6% in females, almost half that of males aged 1–4 years (2·4%), and around a third less than in females aged 1–4 years (2·5%). The proportion of global deaths in people aged 0–24 years that occurred in people aged 10–24 years more than doubled between 1950 and 2019, from 9·5% to 21·6%.

**Interpretation:**

Variation in adolescent mortality between countries and by sex is widening, driven by poor progress in reducing deaths in males and older adolescents. Improving global adolescent mortality will require action to address the specific vulnerabilities of this age group, which are being overlooked. Furthermore, indirect effects of the COVID-19 pandemic are likely to jeopardise efforts to improve health outcomes including mortality in young people aged 10–24 years. There is an urgent need to respond to the changing global burden of adolescent mortality, address inequities where they occur, and improve the availability and quality of primary mortality data in this age group.

**Funding:**

Bill & Melinda Gates Foundation.

## Introduction

The most recent systematic global analysis to focus on mortality in young people aged 10–24 years is now more than a decade old.^1^ Since then, there have been huge changes to patterns of health risk, population growth, and improvements in the availability and quality of mortality estimates. There has also been wider recognition of the importance of adolescent health to global development,^2^, ^3^ and in harnessing the demographic dividend resulting from forecasted population change.^4^ Adolescents are now included within the UN Every Woman Every Child agenda,^5^ the Countdown to 2030 collaboration to track progress towards the Sustainable Development Goals (SDGs),^6^ and the work of the Global Financing Facility.^7^ Despite this progress, there is also growing concern that global adolescent health priorities are still neglected.^8^, ^9^ Global progress to improve health outcomes for young people has been slow, and major challenges remain in addressing social determinants of adolescent health and mortality, such as unmet contraception need, child marriage, and access to quality secondary education.^8^ Furthermore, the COVID-19 pandemic has ongoing consequences on the health of young people that are not fully understood. Although case fatality and morbidity due to COVID-19 is lower in young people than in older adults,^10^ this age group is particularly susceptible to indirect effects of the pandemic, with ongoing disruption to education and employment likely to further hinder progress in health.^11^

**Research in context**

**Evidence before this study**

We searched PubMed and Embase for studies to answer the research question: “What are current global patterns and previous trends in mortality in adolescents and young people”. We used the following terms in titles or abstracts: (“adolescent” OR “young people” AND (“trends” OR “patterns”) AND (“death” OR “mortality”) AND (“world” OR “global” OR “international”). We found one study that focused on global mortality patterns in adolescents (10–24 years), published in 2009. Using data from 2004, this study reported the considerable burden of global adolescent mortality, and analysed geographic variation in outcomes, but did not explore trends over time. Two further studies reported trends in global mortality or years of life lost (YLLs) in adolescents and young people from 1990. As part of wider analyses of patterns of disease and risk factors in people aged 10–24 years, these studies found considerable variation in improvements in mortality and YLLs by country, age group, and sex. We identified other studies that examined adolescent deaths, but these either included smaller age groups (10–14 years, 10–19 years, or 15–24 years), focused on one region or group of countries, or were part of a wider study of mortality in other age groups and were not focused on adolescents. Although these studies provide useful insights into adolescent mortality, they do not provide a comprehensive global analysis of trends and burden of deaths in this age group.

**Added value of this study**

This is a comprehensive update of global mortality in young people aged 10–24 years, and the first analysis we are aware of to include estimates for 204 countries and territories, including all members and associate members of WHO. We found huge changes to adolescent mortality over the study period and increasing variation in outcomes. Mortality in people aged 10–24 years is increasing in many regions, particularly in males, and sex differences have widened in most regions of the world. Adolescent mortality rates were strongly associated with level of development. Demographic change and differences in mortality improvements are shifting the global burden of adolescent mortality towards sub-Saharan Africa, however, where communicable and maternal causes of death continue to predominate. We were able to identify countries with good adolescent mortality outcomes relative to their level of socioeconomic development, which can inform global health strategies for this age group.

**Implications of all the available evidence**

Despite increased recognition of the importance of adolescent health to global development and future economic prosperity, these findings highlight an ongoing failure to adequately respond to health risks during the adolescent years, and of funding levels that remain insufficient. This is compounded by rapid demographic change in low-resource settings where mortality hazards for adolescents remain high. Indirect effects of the COVID-19 pandemic threaten to restrict progress even further. Approaches must be developed to address growing inequities in mortality in this age group, and to focus on regions of the world and causes of death where improvements have stagnated. To monitor progress reliably, improvements in the quality and availability of adolescent health data are also urgently required.

Mortality is a fundamental indicator of health^12^ and mortality estimates provide a proxy for broader health outcomes with little or no high quality data. This is particularly pertinent for young people, where data scarcity presents a barrier to understanding health needs in many countries,^13^, ^14^ and the process of determining which metrics best capture adolescent health priorities is ongoing.^15^ Earlier work^1^ illustrated that global adolescent mortality is greater than previously recognised and that improvements lagged behind those seen in younger children,^16^ despite a high proportion of adolescent deaths being from preventable causes.^17^

Mortality is included in the indicator set for monitoring future progress in adolescent health. All-cause mortality in people aged 10–19 years is an indicator within the Global Strategy for Women's, Children's and Adolescents’ Health 2016–2030,^5^, ^18^ and SDG 3 includes indicators for causes of death that are highly relevant to young people including maternal mortality (death due to complications during or after pregnancy; SDG indicator 3.1.1), suicide (3.4.2), and deaths due to road traffic injury (3.6.1).^19^

Agencies that provide adolescent mortality estimates for a broad range of countries include the UN Population Division, Department of Economic and Social Affairs,^20^ WHO,^21^ US Census Bureau,^22^ and the UN Inter-agency Group for Child Mortality Estimation.^23^ However, the ongoing Global Burden of Diseases, Injuries, and Risk Factors Study (GBD) provides the only source of frequently updated, age-specific mortality estimates (including uncertainty intervals [UIs]) covering almost all countries and territories in the world.^12^ Furthermore, the availability of estimates in GBD 2019 of all-cause mortality from 1950, and cause-specific estimates from 1980,^12^ provides a unique opportunity to analyse long-run trends over time, and so identify both successes and areas requiring global and national investment.

We use GBD 2019 estimates^12^ to analyse current and long-term trends in mortality in young people aged 10–24 years globally and in 204 countries and territories. We use this age range to capture the social, biological, and neurocognitive transitions that occur during this stage of the life course,^24^ and use the terms adolescent and young person synonymously. This manuscript was produced as part of the GBD Collaborator Network and in accordance with the GBD Protocol.

## Methods

### Overview

We used data provided by the Institute for Health Metrics and Evaluation GBD 2019.^12^ Estimates were available for number of deaths, years of life lost (YLLs) and mortality rate per 100 000 population with UIs by sex and age group in 204 countries and territories. Mortality estimates for all causes were available from 1950 to 2019, and by cause of death from 1980 to 2019. GBD 2019 estimations are based on primary data from 86 249 sources, including civil registrations, vital statistics, censuses, disease notifications systems, and household surveys. The GBD 2019 capstone papers and appendices describe these methods in detail,^12^ including procedures to standardise primary sources, redistribute non-specific or implausible causes of death, adjust for large spikes in mortality due to conflicts or natural disaster, and model estimates for locations that lack primary data. GBD 2019 complies with the Guidelines for Accurate and Transparent Health Estimates Reporting statement.^25^

GBD 2019 provides estimates for 281 underlying causes of death across a 4-level hierarchy, shown in appendix 1 (table S2). Level 1 consists of three mutually exclusive and collectively exhaustive categories: communicable, maternal, neonatal, and nutritional diseases; non-communicable diseases; and injuries. Level 2 distinguishes these groups into 21 causes of death, (eg, neoplasms), with level 3 and then level 4 specifying underlying cause in greater detail. We report causes at level 2 of this hierarchy, but modified this to examine leading causes of death in young people aged 10–24 years in more detail. Firstly, we separated the level 2 group self-harm and interpersonal violence into two groups (self-harm [ie, suicide] and interpersonal violence and conflict). Secondly, due to the importance of maternal deaths in this age group, we separated the level 2 group maternal and neonatal disorders into its two level 3 causes (maternal disorders and neonatal disorders).

GBD 2019 mortality estimates were available for 204 countries and territories (hereafter referred to as countries), which includes all members and associate members of WHO. Estimates were also available for 21 GBD regions and 7 GBD super-regions (defined by both geography and income status). Here we primarily report outcomes by GBD super-region, with country-level and region-level estimates available in appendix 2. The seven GBD super-regions are central Europe, eastern Europe, and central Asia; Latin America and the Caribbean; southeast Asia, east Asia, and Oceania; north Africa and the Middle East; south Asia; sub-Saharan Africa; and high income. Note that separate from the high-income GBD super-region, there are countries defined as high income by the World Bank in all other GBD super-regions except sub-Saharan Africa and south Asia. However, when we discuss high-income countries in this analysis we are referring to the GBD high-income super-region. The list of countries included in the analysis, their GBD region, GBD super-region and World Bank income classification for 2021 is found in appendix 1 (table S1).

### Analyses

We describe current and long-term trends in all-cause mortality rate and number of deaths from 1950 to 2019, by cause of death from 1980 to 2019; and by sex in people aged 10–14 years, 15–19 years, and 20–24 years. We analyse relative risk for mortality between age groups by dividing the mortality rate in people aged 20–24 years and people aged 15–19 years by the mortality rate in adolescents aged 10–14 years. We analyse variation in outcomes by sex by calculating the ratio of male to female all-cause mortality rate. We analyse between-country variation by calculating the ratio for all-cause mortality rate in people aged 10–24 years in the 90th to 10th centile countries globally to account for extreme values in some location-years.

We then analyse the relationship between mortality rate per 100 000 population in 2019 and country development status using the GBD Socio-demographic Index (SDI).^26^ The SDI is a summary indicator of social and economic conditions that are strongly correlated with health outcomes. An index value between 0 and 1 is defined for three components: average national educational attainment in those older than 15 years, total fertility rate in those younger than 25 years, and lag-distributed income per capita, using the observed minima and maxima over the estimation period for each component to set the scales. The composite SDI is the geometric mean of these indices for each location-year.^26^ We analyse the strength of the association between SDI and all-cause mortality rate per 100 000 population by use of Spearman's correlation coefficients. We then identified countries with mortality in young people that is lower or higher relative to their level of development. We did this by calculating the expected value of YLLs by age group and sex based solely on SDI for that location-year using a generalised additive model with a Loess smoother on SDI.^26^ We then calculated the ratio of observed YLLs to the expected value according to country SDI. We calculate YLLs as the sum of each death multiplied by the standard life expectancy at each age using GBD's standard life table. Note these data were only available from 1990 to 2017.

**Table:** Deaths in people aged 10–24 years in 2019 by sex and GBD super-region

	**Total deaths**			**Rate per 100** **000 people**	
	Estimate	95% uncertainty interval	Percent of global total	Estimate	95% uncertainty interval
**Females**					
Global	581 311	531 162–634 825	100%	63·99	58·47–69·88
Central Europe, eastern Europe, and central Asia	13 032	12 042–14 207	2·2%	36·70	33·92–40·01
High income	19 715	19 532–19 913	3·4%	21·28	21·09–21·50
Latin America and Caribbean	33 376	29 924–37 078	5·7%	46·19	41·41–51·31
North Africa and Middle East	38 862	34 148–45 205	6·7%	49·81	43·77–57·95
South Asia	224 137	196 768–253 694	38·6%	88·05	77·30–99·67
Southeast Asia, east Asia, and Oceania	68 295	61 737–75 149	11·7%	34·82	31·47–38·31
Sub-Saharan Africa	183 893	159 437–213 930	31·6%	102·57	88·93–119·33
**Males**					
Global	909 678	846 675–974 477	100%	95·41	88·80–102·20
Central Europe, eastern Europe, and central Asia	31 263	28 845–33 776	3·4%	83·56	77·10–90·28
High income	48 600	48 045–49 176	5·3%	49·85	49·28–50·44
Latin America and Caribbean	96 676	87 477–107 076	10·6%	130·62	118·19–144·67
North Africa and Middle East	73 335	64 813–83 549	8·1%	88·07	77·84–100·34
South Asia	253 999	224 664–286 215	27·9%	93·64	82·82–105·51
Southeast Asia, east Asia, and Oceania	157 271	141 742–173 199	17·3%	73·31	66·07–80·74
Sub-Saharan Africa	248 533	220 401–279 643	27·3%	141·60	125·57–159·32

Finally, we assess variation in progress to reduce mortality across the early life course, comparing trends and current mortality burden in young people aged 10–24 years with infants younger than 1 year and children aged 1–4 years and 5–9 years. We first report annual rates of change between 1990 and 2019 for all-cause mortality rate for each country by 5-year age groups. We used the β coefficient from linear regression models of mortality rate per 100 000 population against time for each country to estimate annual rate of change (expressed as a percentage). We used these models rather than estimates at the start and end of each period to account for large fluctuations in country mortality due to war or natural disasters. We then identified countries with large differences in mortality performance by age group by comparing country-level all-cause mortality percentile in adolescents with that seen in children younger than 5 years, as this age group has been the focus of global programming and experienced good mortality declines in recent years. Finally, we describe how the proportion of deaths in people younger than 25 years that occur in those aged 10–24 years changed between 1950 and 2019. All analyses were done in Stata 16 (StataCorp, College Station TX, USA).

### Role of the funding source

The funder of this study had no role in study design, data collection, data analysis, data interpretation, or writing of the report.

## Results

The table shows the number of global deaths and mortality rate per 100 000 population in 2019 by sex and GBD super-region for young people aged 10–24 years. Data by age group (10–14 years, 15–19 years, and 20–24 years) can be found in appendix 1 (tables S3–S5), and country-level estimates are available in appendix 2 (tables S1–S233).

Within a total global population of 1·86 billion people aged 10–24 years, there were around 1·49 million deaths in 2019 (95% UI 1·39–1·59). Just under half of these deaths occurred in people aged 20–24 years (692 000, 95% UI 645 000–738 000), a third occurred in people aged 15–19 years (499 000, 465 000–536 000), and a fifth occurred in people aged 10–14 years (299 000, 276 000–325 000]. 51% of all people aged 10–24 years in 2019 were male. 61·0% of all deaths in people aged 10–24 years were in males (910 000, 847 000–974 000), with this proportion increasing with age (56·9% of deaths in people aged 10–14 years; 60·5% of deaths in people aged 15–19 years, and 63·2% of deaths in people aged 20–24 years). Most adolescent deaths in both sexes occurred in south Asia. The fewest adolescent deaths in both sexes were reported in Central Europe, eastern Europe, and central Asia. Mortality rates in 2019 increased with age across adolescence in all regions of the world, particularly in males, but with considerable variation. Within GBD super-regions, the relative risk of mortality in young adulthood (20–24 years) compared with early adolescence (10–14 years) ranged from 6·6 in the high-income GBD super-region in males to 1·9 in north Africa and the Middle East in females.

In 2019, the mortality rate per 100 000 in young people aged 10–14 years ranged from 7·32 (95% UI 6·90–7·78) in Denmark to 187·25 (165·74–217·44) in Central African Republic in males, and from 6·01 (5·79–6·25) in Denmark to 115·73 (107·50–129·23) in Central African Republic in females. The mortality rate in young people age 15–19 years ranged from 22·55 (21·50–23·72) in Denmark to 370·28 (305·42–448·95) in Lesotho in males, and 12·15 (12·02–12·29) in Japan to 248·02 (169·99–348·35) in Lesotho in females. The mortality rate in people aged 20–24 years ranged from 36·24 (35·48–37·05) in Singapore to 559·77 (434·53–670·69) in Lesotho in males and 14·44 (14·05–14·85) in Singapore to 445·22 (303·38–636·53) in Lesotho in females (appendix 1, figures S6–S11; appendix 2, tables S1–S233).

Globally, 32·7% of deaths in young people aged 10–24 years in 2019 were due to transport injuries, unintentional injuries, or interpersonal violence or conflict; 32·1% were due to communicable, nutritional, or maternal causes; 27·0% were due to non-communicable diseases; and 8·2% were due to self-harm. However, there was large variation in the leading causes of adolescent death by GBD super-region, sex, and age. In males aged 10–14 years, the most common cause of death was unintentional injury in all GBD super-regions except high income, where neoplasms were the leading cause of death, and south Asia and sub-Saharan Africa, where enteric infections were the leading cause of death. In males aged 15–24 years, the most common cause of death was transport injury in all GBD super-regions except Latin America and the Caribbean, where the leading cause of death was interpersonal violence and conflict, and central Europe, eastern Europe and central Asia, where the leading cause of death was self-harm (appendix 1, tables S50–S112).

The leading causes of death in females aged 10–14 years were neoplasms (Latin America and the Caribbean; central Europe, eastern Europe, and central Asia; and high income), unintentional injury (southeast Asia, east Asia, and Oceania), enteric infections (south Asia) and transport injuries (north Africa and the Middle East). In females aged 15–19 years, the leading causes of death were transport injuries (high income; north Africa and the Middle East; and southeast Asia, east Asia, and Oceania), self-harm (south Asia and central Europe, eastern Europe, and central Asia), interpersonal violence and conflict (Latin America and the Caribbean). The most frequent causes of death in females aged 20–24 years were transport injuries (high income), neoplasms (southeast Asia, east Asia, and Oceania and central Europe, eastern Europe, and central Asia), interpersonal violence and conflict (Latin America and the Caribbean), cardiovascular diseases (north Africa and the Middle East), and self-harm (south Asia). HIV/AIDS and sexually transmitted infections were the leading causes of death in sub-Saharan Africa in females in all adolescent age groups. Although not the leading cause in any GBD super-region, maternal death was still in the three most common causes of death in females aged 20–24 years in sub-Saharan Africa, north Africa and the Middle East, and south Asia, and was the fourth most common cause of death in Latin America and the Caribbean.

**Figure 1:**
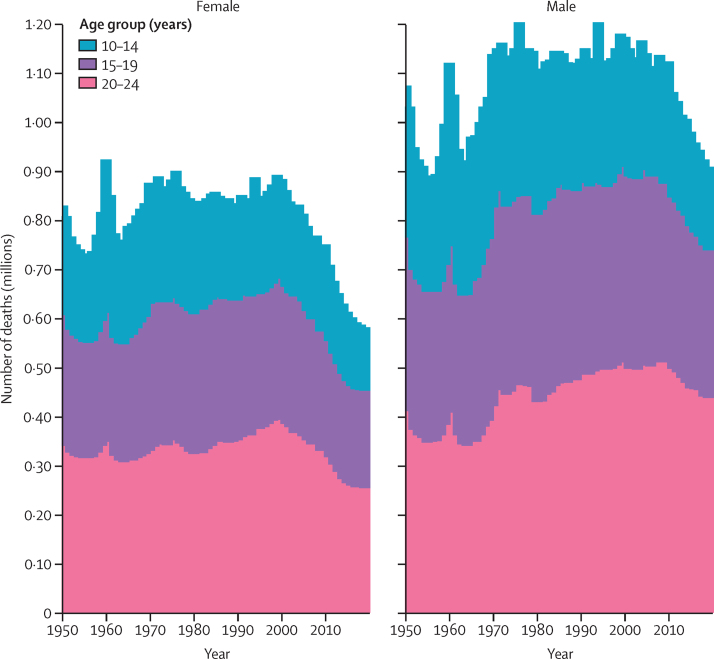
Global number of deaths in people aged 10–24 years from 1950 to 2019 by sex and age group

Annual global deaths in adolescents aged 10–24 years reduced by 21·7% between 1950 and 2019, during which time the world population in this age group increased by 157% (figure 1; appendix 1, figure S2). The largest decrease in deaths was in females (30·0%, *vs* 15·3% in males) despite similar population growth (154% in females and 161% in males). There were also large differences in trends in deaths by age group; in children aged 10–14 years, deaths decreased by 37·5% in males and 37·9% in females; in people aged 15–19 years, deaths decreased by 17·8% in males and 27·9% in females; and in people aged 20–24 years, deaths decreased by 26·9% in females and increased by 0·8% in males.

In young people aged 10–14 years, deaths decreased between 1950 and 2019 in all GBD super-regions except sub-Saharan Africa, where deaths increased by 140·6% in males and 144·3% in females. The largest decrease in deaths since 1950 in young people aged 10–14 years were in high-income countries (86·0% reduction in males and 87·3% reduction in females). In males aged 15–24 years, deaths increased in all GBD super-regions except high income; central Europe, eastern Europe, and central Asia; and southeast Asia, east Asia, and Oceania. The greatest increase in deaths in males aged 15–24 years was in sub-Saharan Africa, where deaths increased by 180·5% in males aged 15–19 years and 218·2% in males aged 20–24 years. The greatest decrease in deaths in males aged 15–24 years was in the high-income GBD super-region, where deaths decreased by 72·6% in males aged 15–19 years and 66·0% in males aged 20–24 years. In females aged 15–24 years, deaths decreased in all GBD super-regions except north Africa and the Middle East and sub-Saharan Africa. The greatest increase in deaths in females aged 15–24 years was in sub-Saharan Africa in both females aged 15–19 years (175·7% increase) and 20–24 years (164·3% increase). The greatest decrease in deaths in females aged 15–24 years was in high-income countries in both females aged 15–19 years (81·0% decrease) and 20–24 years (79·9% decrease).

**Figure 2:**
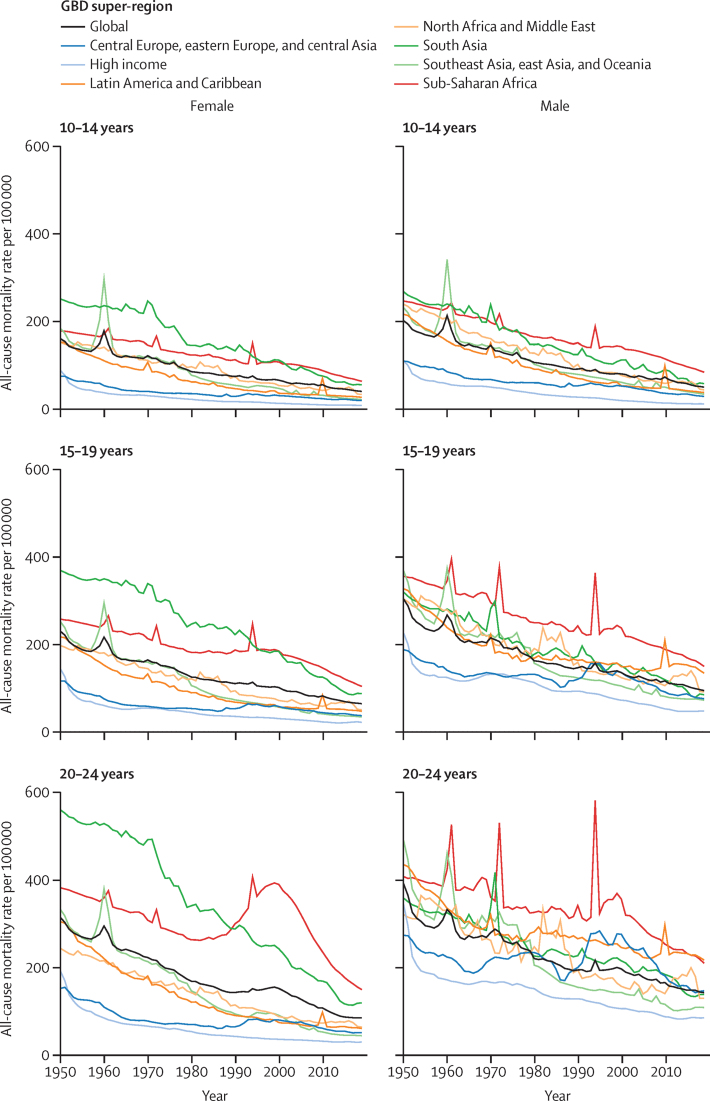
Mortality rate per 100 000 population in people aged 10–24 years from 1950 to 2019 by sex and GBD super-region GBD=Global Burden of Diseases, Injuries, and Risk Factors Study.

Figure 2 shows mortality rate per 100 000 population in people aged 10–14 years, 15–19 years, and 20–24 years by sex and GBD super-region between 1950 and 2019. In people aged 10–14 years, global mortality rates reduced by 74·6% in males and 74·3% in females, with the greatest relative change in mortality in high-income countries (reduction of 88·2% in males and 89·0% in females). The GBD super-region with the smallest relative change in mortality rate was sub-Saharan Africa, with reductions of 65·4% in males and 63·9% in females. All other regions had a 72–88% reduction in mortality rate over this period in males and females aged 10–14 years.

Global mortality rate per 100 000 population in people aged 15–19 years decreased by 68·7% in males and 71·7% in females between 1950 and 2019. The region with the greatest relative reduction in mortality in this age group was southeast Asia, east Asia, and Oceania, where the mortality rate declined by 80·1% in males and 86·1% in females. The region with the lowest relative reduction in mortality rate was sub-Saharan Africa, with relative reductions of 57·8% in males and 59·4% in females.

Global mortality rate per 100 000 population in people aged 20–24 years reduced by 63·4% in males and 72·7% in females between 1950 and 2019. Relative declines in mortality were greatest in southeast Asia, east Asia, and Oceania, with reductions of 77·7% in males and 86·4% in females. In males aged 20–24 years, the lowest relative decline was in central Europe, eastern Europe, and central Asia, where mortality rates reduced by 46·1% since 1950. Among females aged 20–24 years, the lowest relative reduction in mortality was seen in sub-Saharan Africa, where mortality rates decreased by 60·7%.

In 2019, the ratio of male to female mortality rate per 100 000 population was 1·2 for people aged 10–14 years, 1·5 for people aged 15–19 years, and 1·7 for people aged 20–24 years (appendix 1, figure S20). Mortality rate per 100 000 people aged 10–24 years was higher in males in all regions of the world except south Asia, where outcomes were similar. Sex differences in mortality were greatest in Latin America and the Caribbean in people aged 20–24 years, where the mortality rate per 100 000 in males was more than three times that in females. The ratio of male to female mortality rate increased between 1950 and 2019 for older adolescents aged 15–24 years in all GBD super-regions except high income and central Europe, eastern Europe, and central Asia, where it has reduced since the mid-1990s.

**Figure 3:**
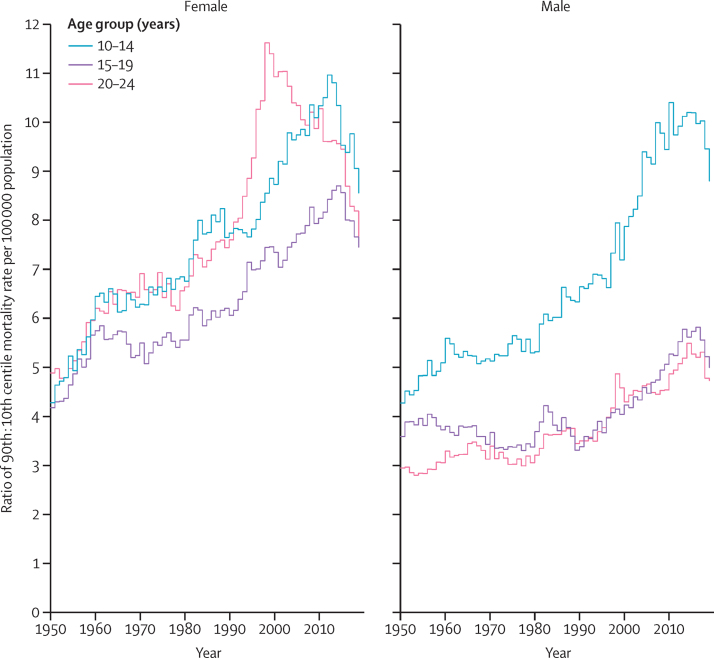
Ratio of mortality rate per 100 000 population in the 90th centile country to 10th centile country in people aged 10–24 years between 1950 and 2019 by sex and age group

Inequality in mortality rate per 100 000 population between countries was reported using the ratio of mortality rate for the highest decile country (90th centile) to lowest decile country (10th centile) in each year. In 2019, variations in mortality by country were greatest in adolescents aged 10–14 years, with the mortality rate in the 90th centile country around 9 times higher than that in the 10th centile country for both males and females. Inequality in outcomes by country has also increased over time for all age groups since 1950, although this seems to have decreased in females aged 20–24 years since around 2000 (figure 3).

The percentage of total deaths by cause from 1980 to 2019 in each GBD super-region is shown in figure 4 for young people aged 10–14 years, 15–19 years, and 20–24 years. Bump charts that rank cause of death between 1980 and 2019 for each age group, sex, and GBD super-region are shown in appendix 1 (figures S50–S112).

**Figure 4:**
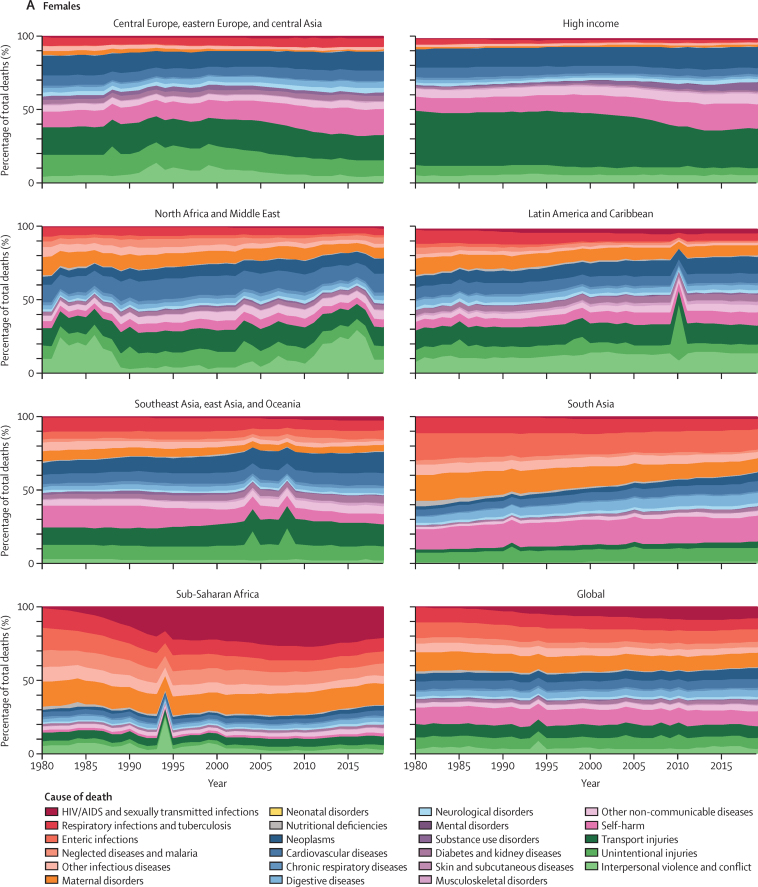

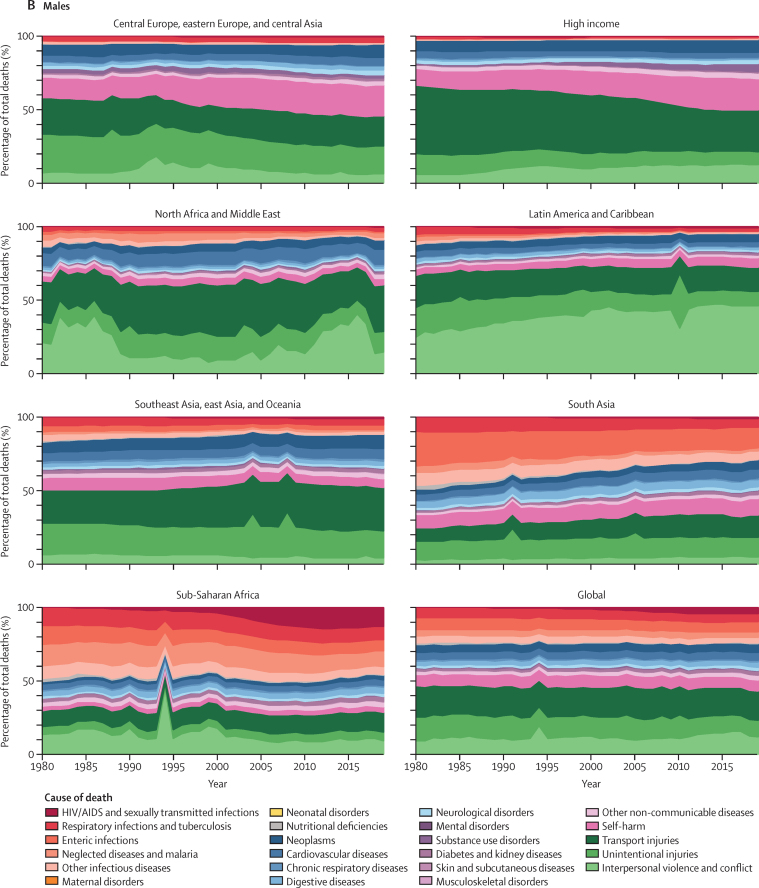
Percentage of total deaths by cause in people aged 15–19 years from 1980 to 2019 by sex and GBD super-region (A) Females (B) Males. GBD=Global Burden of Diseases, Injuries, and Risk Factors Study.

We report large reductions in the contribution of communicable and maternal causes to total adolescent deaths between 1980 and 2019 in Latin America and the Caribbean; south Asia; southeast Asia, east Asia, and the Caribbean; and north Africa and the Middle East. Furthermore, although maternal deaths still contribute a substantial proportion of global deaths in females aged 20–24 years, this has fallen substantially since 1980. Maternal deaths halved as a proportion of total deaths between 1980 and 2019 in southeast Asia, east Asia, and Oceania; north Africa and the Middle East; and central Europe, eastern Europe, and central Asia, with large reductions also seen in south Asia and Latin America and the Caribbean. However, in sub-Saharan Africa, the five most common causes of death in 2019 in females aged 10–24 years were communicable or maternal causes, with communicable causes contributing to three of the top five causes in males. HIV/AIDS and sexually transmitted diseases have been the leading causes of death in this GBD super-region in females aged 15–24 years since the early 1990s, and in females aged 10–14 years since the early 2000s.

In central Europe, eastern Europe, and central Asia, self-harm rose from the third highest cause of death in males aged 15–24 years in 1980 to be the leading cause of death (now contributing to more than 20% of all deaths), and from fourth highest to the leading cause of death in females aged 15–19 years and second highest cause of death in females aged 20–24 years. In males aged 10–14 years, self-harm is now the fourth most common cause of death in this super-region (almost 9% of all deaths). By contrast, in southeast Asia, east Asia, and Oceania, self-harm in females aged 15–24 years fell from the leading cause of death in 1980 (around 15% of all deaths), to sixth highest in females aged 15–19 years and seventh highest in females aged 20–24 years in 2019 (around 7% of all deaths in both age groups). In the high-income GBD super-region, deaths due to substance misuse disorders in young adults aged 20–24 years rose from around 1% of total deaths in 1980 to 14–16% in 2019 in both sexes, with substantial increases also seen in people aged 15–19 years.

**Figure 5:**
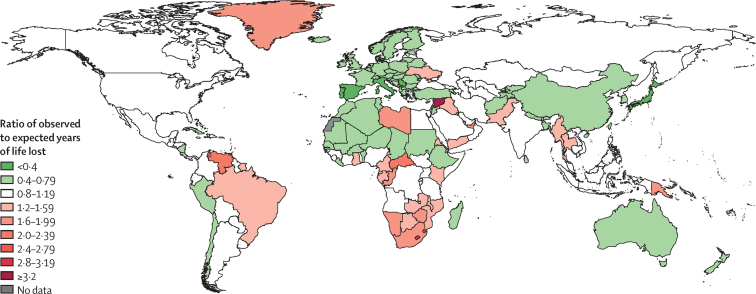
Ratio of observed to expected years of life lost by Sociodemographic Index from all causes in people aged 15–19 years in 2017 from 195 countries

Increasing SDI in 2019 was strongly associated with lower all-cause mortality rate per 100 000 people in all adolescent age groups (appendix 1, table S6). Figure 5 shows the ratio of observed to expected YLLs by SDI from all causes for people aged 15–19 years in 2017. Equivalent figures for people aged 10–14 years and 20–24 years are shown in appendix 1 (figures S26–28), with estimates from 1990 to 2017 for all countries shown in appendix 1 (figures S29–S49). Observed YLLs from all causes in people aged 15–19 years were at least 20% higher than expected by level of SDI in 41 countries, including both Brazil and Pakistan, which collectively contribute to around 10% of deaths in this age group. In people aged 15–19 years, the ratio of observed to expected YLLs in 2017 was highest in Syria (4·02), but from 1990 to 2010 (before the start of the civil war), observed YLLs in Syria were consistently around 30% lower than expected by level of SDI. 84 countries had observed YLLs in people aged 15–19 years at least 20% lower than expected by level of SDI. The countries with the lowest ratio of observed to expected YLLs in this age group were the Maldives (0·30), Spain (0·33), and Singapore (0·35). Other notable countries included China (0·49) and Ethiopia (0·67), which together contribute more than 7% of global deaths in people aged 15–19 years.

**Figure 6:**
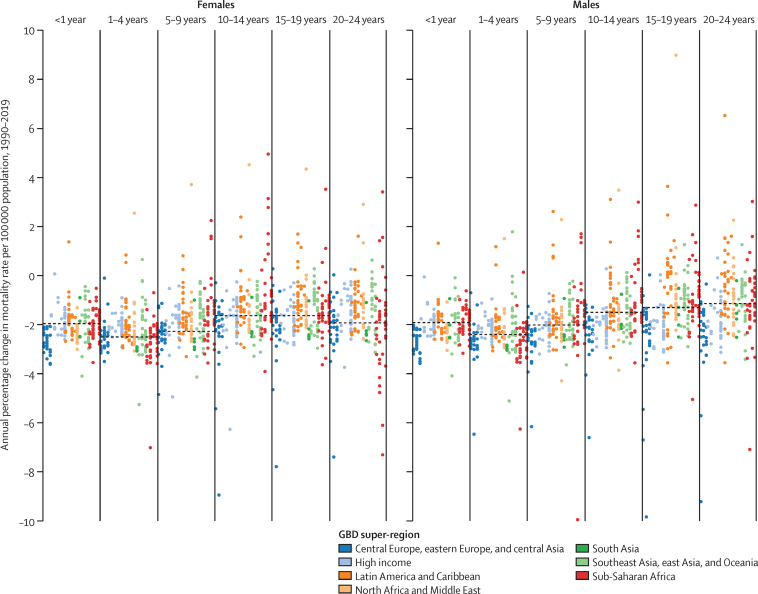
Annual percentage change in mortality rate per 100 000 population in people aged 0–24 years 1990–2019 by sex, age group, and GBD super-region The dashed line represents global annualised percentage change in mortality rate. Each coloured dot represents the percentage change in a single year for each country, coloured by GBD super-region. Data for Rwanda (both sexes, 15–19 years) and Bosnia and Herzegovina (females aged 1–4 years) are excluded as the range was restricted to 10% annual change. GBD=Global Burden of Diseases, Injuries, and Risk Factors Study.

Annual percentage decline in mortality rate per 100 000 population in infants younger than 1 year and children and young people aged 1–4 years, 5–9 years, 10–14 years, 15–19 years, and 20–24 years by sex, country, and globally between 1990 and 2019 is shown in figure 6. Rates of decline were highest in children aged 1–4 years and lowest in young people aged 15–24 years. In children aged 1–4 years, global mortality rates decreased by around 2·4% per year since 1990 in both males and females, compared with people aged 15–19 years, in whom mortality rates decreased by 1·3% in males and 1·6% in females. The range in mortality rate change between countries was also greater in adolescents than in younger children (appendix 1, figure S24).

**Figure 7:**
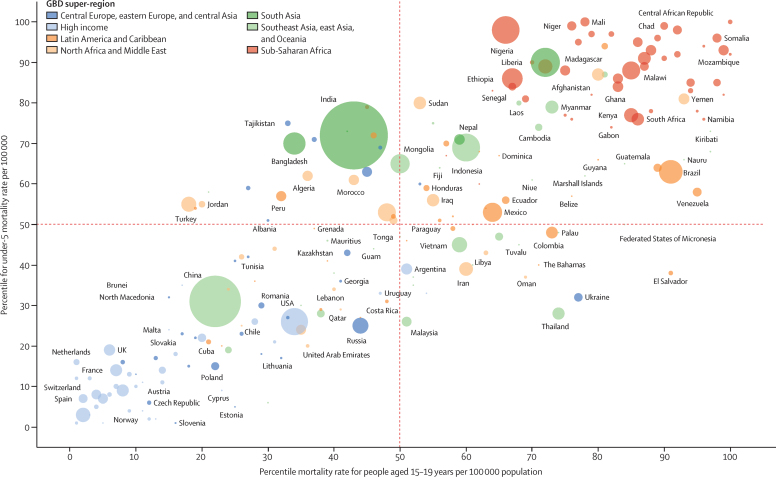
Percentiles of mortality rate per 100 000 population in males aged younger than 5 years and 15–19 years in 2019 by country and GBD super-region Size of each mark is proportional to the population of males aged 15–19 years. Due to space constraints, not all countries are labelled. GBD=Global Burden of Diseases, Injuries, and Risk Factors Study.

Mortality rate performance varied within countries for different age groups, particularly in males. Figure 7 shows mortality rate percentile for males younger than 5 years compared with males aged 15–19 years for each country in 2019, with the equivalent figure for females shown in appendix 1 (figure S25). Although in most countries the mortality rate percentiles for these age groups are similar, there are notable exceptions. For example, in Brazil and Venezuela, mortality rates for males younger than 5 years are around the 60th centile but in the 90th centile for males aged 15–19 years. Similarly, mortality rates in Ukraine and Thailand are around the 30th centile for children younger than 5 years, but above the 75th centile for young people aged 15–19 years. By contrast, the mortality rate in India for people younger than 5 years is around the 70th centile, and between the 30th and 40th centiles for those aged 15–19 years.

**Figure 8:**
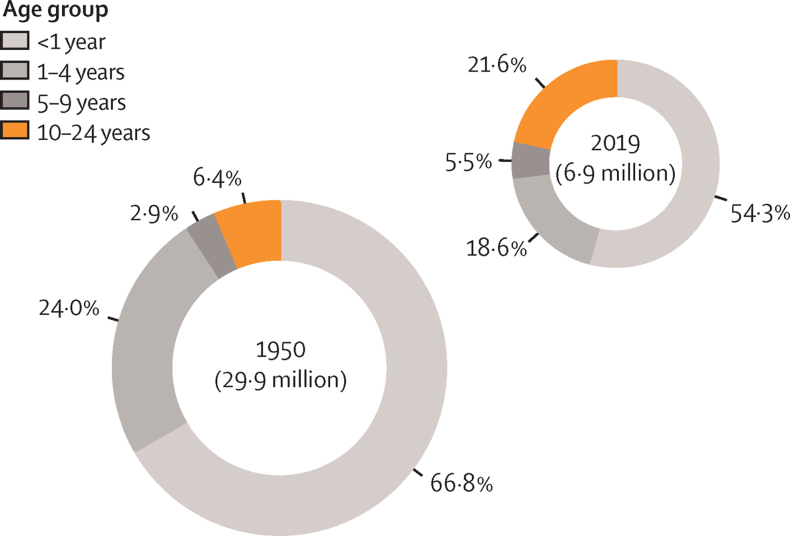
Proportion of deaths in people younger than 25 years occurring in adolescents aged 10–24 years in 1950 and 2019 The size of each plot is proportional to the total number of deaths in people younger than 25 years in that year.

Figure 8 shows the proportion of global deaths in people aged 0–24 years that occur in people aged 10–24 years. This proportion increased from 9·5% in 1950 to 21·6% by 2019 and increased in all GBD super-regions between 1950 and 2019, with the greatest change seen in Latin America and the Caribbean in males (from 7·5% to 39·2%). In the high-income GBD super-region, deaths in adolescence now account for more than half of all deaths before 25 years of age. In nine countries (Estonia, Thailand, Saudi Arabia, Finland, Puerto Rico, Slovenia, Monaco, Cook Islands, and Andorra), more than 70% of deaths in males younger than 25 years now occur in people aged 10–24 years.

## Discussion

Despite marked reductions in total numbers of deaths and mortality rates for adolescents over the past 70 years, improvements have lagged behind those seen in younger children, variation in outcomes between countries has increased, and inequities by sex have worsened. Global numbers of deaths in adolescence remain high, at around 4000 each day, most of which are from preventable causes.

We found widening variation in all-cause mortality between countries, particularly during early adolescence, and huge differences in the leading causes of death between different regions of the world, as others have reported.^17^, ^27^ Variation in all-cause mortality between countries seems likely to increase further, as population growth in this age group is highest in countries with the worst mortality outcomes (appendix 1, figures S21–S23). Around 20% of people aged 10–24 years live in sub-Saharan Africa, and this is set to rise to a third by 2050,^4^ which presents huge challenges to the improvement of adolescent mortality. Of the ten countries with the highest mortality rates in this age group, eight are in sub-Saharan Africa, and decreases have been far slower than in other GBD super-regions. These changes are shifting the global burden of adolescent mortality towards sub-Saharan Africa, where 29·0% of deaths in adolescents now occur, compared with 8·1% in 1950. Sub-Saharan Africa has already replaced south Asia as the main contributor to global deaths in males aged 10–19 years, and is set to do the same in males aged 20–24 years and females aged 10–19 years if current trends continue.

We found marked sex differences in adolescent mortality, with notably higher mortality and slower rates of decline in young men than in young women. Indeed, more adolescent males died in 2019 than adolescent females in 1950, and inequalities in mortality by sex also seem to be widening in many regions of the world. These differences reflect the increasing burden of deaths due to injuries and violence in this age group, particularly in Latin America and the Caribbean, and the rise in deaths due to substance misuse in high-income settings, which predominantly affect young men. Addressing inequities in access to health services, the social conditions in which young women live, and the impact of gender-based violence are fundamental to improving adolescent health globally and have understandably been the focus of previous programming.^19^ However, inequitable gender norms are also damaging to adolescent males, and advancing the health of all adolescents requires action to reduce inequities in outcomes wherever they occur.

Consistent with previous studies,^14^, ^16^, ^17^, ^27^ we found declines in adolescent mortality to have been slower than in younger children, particularly in males (annualised decrease in mortality in males aged 1–4 years almost twice that in males aged 15–19 years, and annualised decrease in mortality in females aged 1–4 years about 50% higher than in females aged 15–19 years).

A previous analysis^16^ of long-term mortality trends using WHO estimates found that differences in mortality reduction by age resulted in people aged 15–24 years replacing those aged 1–4 years as the group with the highest mortality burden in many countries. 15–19 years was highlighted as the age group with the slowest mortality decline among people aged 0–19 years using GBD data,^27^ and Masquelier and colleagues^14^ found that the decrease in mortality in children aged 5–9 years exceeded that in young people aged 10–14 years between 1990 and 2016 using estimates from the UN.^14^ The continued neglect of this age group is highlighted by several (mostly middle-income) countries such as Brazil and Mexico having good outcomes for children aged 0–4 years but persistently high adolescent mortality (figure 7), which suggests that more adolescent-specific interventions are needed in these settings. Almost a quarter of deaths in people aged 0–24 years now occur in people aged 10–24 years, and this proportion has more than doubled since 1950. In high-income countries, these account for almost half of deaths before 25 years of age, and in countries with particularly high mortality due to violence, adolescent deaths now account for up to three-quarters of early life course mortality.

This poor progress in reducing mortality might reflect the omission of adolescents from most global health investments. Adolescents were largely absent from the Millennium Development Goal (MDG) agenda, and although they will have benefited from public health interventions aimed at other groups, they have not had the accelerated decreases in mortality seen in infants and younger children that is attributed to MDG programming.^27^ The SDGs include indicators that are highly relevant to young people, but do not provide a comprehensive mechanism to address the unique health needs of this age group,^27^ and a list of metrics to best capture global adolescent health priorities has not yet been defined.^15^ The 2016 Global Strategy for Women's, Children and Adolescents’ Health and other initiatives have increased recognition of the crucial role of adolescents to the sustainable development agenda and global targets for Universal Health Coverage.^5^ However, investment remains inadequate, and specific challenges to improvement of adolescent health outcomes and achieving core Universal Health Coverage indicators in this age group continue to be overlooked.^8^, ^9^

These factors are compounded by limited evidence for effective adolescent health interventions needed to inform investment.^28^ A key focus to improve outcomes in this age group should be to address these knowledge gaps and better establish which interventions work, guided by identifying and measuring key indicators that capture adolescent health priorities.^15^ Investment in existing evidenced-based actions to prevent causes of death that predominate during adolescence is also likely to improve outcomes, but is currently inadequate. This should include improving water safety; preventing unintentional injuries;^29^ and targeting key behavioural, legal, and structural risk factors for road traffic deaths.^30^, ^31^ However, global increases in injury prevention spending have been lower than those on other public health interventions^32^ and progress towards reducing road traffic deaths in line with SDG targets is insufficient.^30^ Self-harm has emerged as a leading cause of adolescent mortality, and now accounts for around 20% of all deaths in people aged 15–24 years in the high-income and central Europe, eastern Europe, and central Asia GBD super-regions. Self-harm is also the leading cause of death in south Asia for females aged 15–24 years and second highest cause for males aged 20–24 years. Although understanding these trends is complex and solutions to improve outcomes need to be country-specific, investment in evidence-based interventions to improve mental health in this age group^33^, ^34^ and measures to restrict access to firearms and chemicals used in suicide are likely to be beneficial.^35^, ^36^ However, global improvements to the quality and accessibility of mental health services have been slow, disproportionately affecting adolescents and young people.^34^ Furthermore, strategies to improve outcomes for communicable and maternal causes of death, which still contribute to around a third of global deaths in people aged 10–24 years, also need to be specifically tailored for this age group.^37^ Although evidence-based interventions around adolescent sexual and reproductive health are available, these are mainly focused on high-income countries, and knowledge gaps remain.^38^

We found several large spikes in mortality over the study period, particularly in older male adolescents, as a result of violent conflict and natural disasters. Deaths due to interpersonal violence are also impeding progress in reduction of all-cause mortality in males in many countries, particularly in Latin America and the Caribbean where there has been little to no improvement in all-cause mortality in males aged 15–24 years over the past 20 years. Global strategies to improve adolescent health outcomes must include efforts to mitigate the effect of interpersonal violence and conflicts on young people. Existing humanitarian response guidance highlights specific vulnerabilities of adolescents during natural disasters and conflict.^39^ However, the evidence base to manage these in low-income and middle-income countries remains weak,^40^ and further work is required to understand adolescent health needs in these situations.

Reducing mortality in young people also requires an understanding of the broader social determinants of adolescent health, and how structural changes such as rapid urbanisation and technological and economic development might affect young people differently from other age groups. The importance of primary education to population health is well described, but national progress in secondary education is also associated with large improvements in all-cause mortality and other important health outcomes for young people.^41^ The increasing numbers of adolescents growing up in urban settings could extend opportunities for education, in addition to potential economic benefits for young people and their families. However, rapid, unplanned urbanisation can also increase health risks that are pertinent to adolescents, including those related to injury, separation from family support through migration, exposure to violence, substance misuse, and unsafe employment.^2^, ^42^ The effect of other macro-level health determinants seems to differ across the early life course, with national wealth a weaker predictor of mortality in adolescents than it is in young children.^43^ Indeed, for some common causes of death in adolescents (eg, road injuries), rapid economic growth can result in a transient increase in mortality, as the introduction of safety legislation and appropriate infrastructure might lag behind rising demand for transportation.^44^, ^45^ By contrast, inequality in income distribution within countries seems to be pervasively harmful throughout the early life course, and thus might be of greater relative importance to outcomes during adolescence.^43^

Our data are limited by factors inherent in the production of GBD mortality estimates, and by the availability of authoritative mortality data for adolescents. Global coverage of civil registration systems is of varying quality, and progress to improve these systems has been minimal.^46^ Primary data sources for adolescents are particularly scarce, as attempts to develop alternative methods to measure mortality have focused on other groups.^13^ Data availability and accuracy are further impeded by ongoing conflicts and associated migration in many countries. Where data are available, there are often long delays in reporting outcomes. Analysis of the global adolescent mortality burden is therefore reliant on modelled data, and the estimates we report here need to be viewed within that context. Limitations within the GBD estimation process and the paucity of mortality data for this age group are reflected in wide UIs for many time periods, locations, and causes of death.

Using alternative data sources with different modelling techniques might have provided different results. Estimates for global number of deaths in people aged 10–24 years in 2019 produced by the UN Interagency Group for Child Mortality Estimation were 8–19% higher than GBD 2019 (appendix 1, figures S113–S115), and 10–20% lower than those in the World Population Prospects report.^20^ Although variation in available mortality estimates for high-income countries have been highlighted,^47^ the main discrepancies are within sub-Saharan Africa, where the future global burden of mortality in adolescents will be concentrated. This further highlights the need to expand primary data collection for adolescent health outcomes in this region.

We used SDI to identify countries with lower or higher adolescent mortality than would be expected from country income per capita, years in education, and fertility younger than 25 years; indicators for socioeconomic development that are particularly relevant for adolescents. However, other disaggregated metrics of development could provide additional insights, and further detailed analyses are required to explore the contribution of trends in key social determinants on adolescent mortality, which are likely to vary in different regions of the world. We report estimates at the country level, and future analyses of adolescent mortality should include sub-national trends. Where these are available within GBD they have wide variation in outcomes. Using level 2 of the GBD cause of death hierarchy allows a description of high-level trends in mortality in young people, but further analyses using more granular cause of death data are needed to increase understanding of the patterns we describe. Analysis of associations between health-care quality and adolescent mortality by use of the Healthcare Access and Quality Index^48^ provided by GBD could provide further insights, and looking beyond mortality and describing key trends in morbidity in young people should also be the focus of future study.

This analysis does not include the impact of the COVID-19 pandemic on young people. Although susceptibility to SARS-CoV-2 in young people is similar to that in older groups,^49^ case fatality has been much lower than in older adults,^10^ and the direct effect on mortality in this age group is likely to be small. More concerning are the indirect effects of COVID-19 on the future health of young people, particularly through disruption to health services caused by the pandemic, the impact on opportunities for education and employment and related consequences to health and nutrition, and the exacerbation of existing inequalities.^50^ The ongoing closure of schools and universities in many countries will have lasting effects on the health of young people. Further, this age group are more likely to work in sectors vulnerable to restrictions introduced during the pandemic than are older adults, and at a greater risk of losing employment and reporting reduced earnings. There is some evidence to suggest that young people have been more affected by worsening mental health than older adults, and concern that suicide risk in early adolescence might have increased.^51^, ^52^ Broader effects of the pandemic to key adolescent health determinants are likely to include those related to economic instability, conflict, and failing to prioritise the climate crisis.^53^ The extent to which these determinants will affect wellbeing in young people, and prospects to improve health outcomes including mortality, are not understood and warrant further study.

Our findings reveal a persistant failure by policy makers to adequately address global health risks during adolescence or respond to changes in the global burden of early life course mortality. Despite increased understanding of the importance of adolescents to global development, funding remains insufficient and the challenge to improve outcomes is increasing. Investment in this age group builds on health improvements achieved in younger children, will affect future adult health trajectories and those of the next generation,^3^ and will be an important determinant of future economic development.^2^, ^17^, ^54^ Renewed emphasis is urgently needed to reduce inequities in outcomes in this age group, improve the availability and quality of primary data, and establish mechanisms to use these data to better inform global health policy, focusing on regions of the world where mortality is increasing.

## Data sharing

To download the data used in these analyses, please visit the Global Health Data Exchange at http://ghdx.healthdata.org/gbd-2019

## Declaration of interests

R Ancuceanu reports consulting fees from AbbVie and AstraZeneca; payment or honoraria for lectures, presentations, speaker's bureaus, manuscript writing or educational events from Sandoz, AbbVie, and B Braun; and support for attending meetings and/or travel from AbbVie and AstraZeneca, all outside the submitted work. J Ärnlöv reports payment or honoraria for lectures, presentations, speaker's bureaus, manuscript writing or educational events from AstraZeneca and Novartis; and payment for expert testimony from AstraZeneca and Boehringer Ingelheim, all outside the submitted work. M Ausloos reports grants or contracts from Romanian National Authority for Scientific Research and Innovation, CNDS-UEFISCDI, project number PN-III-P4-ID-PCCF-2016-0084, outside the submitted work. T Bärnighausen reports research grants from the European Union (Horizon 2020 and EIT Health), German Research Foundation (DFG), US National Institutes of Health, German Ministry of Education and Research, Alexander von Humboldt Foundation, Else-Kröner-Fresenius-Foundation, Wellcome Trust, Bill & Melinda Gates Foundation, KfW, UNAIDS, and WHO; consulting fees for KfW on the OSCAR initiative in Vietnam; and participation on a Data Safety Monitoring Board or Advisory Board through the NIH-funded study “Healthy Options” (PIs: Smith Fawzi, Kaaya), Chair, Data Safety and Monitoring Board (DSMB), German National Committee on the “Future of Public Health Research and Education”, Chair of the scientific advisory board to the EDCTP Evaluation, Member of the UNAIDS Evaluation Expert Advisory Committee, National Institutes of Health Study Section Member on Population and Public Health Approaches to HIV/AIDS (PPAH), US National Academies of Sciences, Engineering, and Medicine's Committee for the “Evaluation of Human Resources for Health in the Republic of Rwanda under the President's Emergency Plan for AIDS Relief (PEPFAR)”, University of Pennsylvania (UPenn) Population Aging Research Center (PARC) External Advisory Board Member; and Leadership or fiduciary role in other board, society, committee or advocacy group, paid or unpaid as Co-chair of the Global Health Hub Germany (which was initiated by the German Ministry of Health), all outside the submitted work. M Bell reports research funding and payments to institution from Wellcome Trust, NIH, Yale Climate Change and Health Center, Robert Wood Johnson Foundation, Yale Women Faculty Forum, EPA, and High Tide Foundation; honorarium for proposal review from NIH and Johns Hopkins University; honorarium from mentoring program from University of Montana; and travel expenses to give seminars from University of Illinois at Champaign, Johns Hopkins University, Ohio State University, Royal Society London, Atmospheric Chemistry Gordon Research Conference, New York School of Medicine, and Global Research Laboratory (Seoul), Seoul National University, all outside the submitted work. Z Bhutta reports grants or contracts from the Bill & Melinda Gates foundation, outside the submitted work. I Filip reports financial support from Avicenna Medical and Clinical Research Institute, outside the submitted work. B Hall reports stocks that are unrelated to the paper, outside the submitted work. G Hankey reports consulting fees from Bayer for stroke prevention advisory boards; payment or honoraria for lectures, presentations, speakers bureaus, manuscript writing or educational events from the American Heart Association, Medscape, and Bristol Myers Squibb; and participation on a Data Safety Monitoring Board or Advisory Board with AC Immune, all outside the submitted work. C Herteliu reports grants or contracts from Romanian National Authority for Scientific Research and Innovation, CNDS-UEFISCDI, project number PN-III-P4-ID-PCCF-2016-0084, research grant (Oct 2018–Sept 2022) “Understanding and modelling time-space patterns of psychology-related inequalities and polarization”, and project number PN-III-P2-2.1-SOL-2020-2-0351, research grant (June 2021–Oct 2021) “Approaches within public health management in the context of COVID-19 pandemic”, and from the Ministry of Labour and Social Justice Romania, project number 30/PSCD/2018, research grant (Sept 2018–June 2019) “Agenda for skills Romania 2020–2025”, all outside the submitted work. S Islam reports grants or contracts from NHMRC and National Heart Foundation of Australia Fellowships, outside the submitted work. R Ivers reports support for the present manuscript from the National Health and Medical Research Council of NSW through salary funding via a senior research fellowship. V Jha reports grants or contracts from GSK, Baxter Healthcare, and AstraZeneca, outside the submitted work. J Jozwiak reports payment or honoraria for lectures, presentations, speaker's bureaus, manuscript writing or educational events from Teva, Amgen, Synexus, Boehringer Ingelheim, ALAB Laboratories, and Zentiva, all outside the submitted work. C Kieling reports grants or contracts from MQ: Transforming Mental Health, UK Academy of Medical Sciences, UK Royal Academy of Engineering, and US National Institute of Mental Health; and royalties or licences from Manole, outside the submitted work. K Krishan reports non-financial support from UGC Centre of Advanced Study, CAS II, Department of Anthropology, Panjab University, Chandigarh, India, outside the submitted work. S Lorkowski reports grants or contracts from Akcea Therapeutics Germany; consulting fees from Danone, Swedish Orphan Biovitrum (SOBI), and Upfield; payment or honoraria for lectures, presentations, speaker's bureaus, manuscript writing or educational events from Akcea Therapeutics Germany, AMARIN Germany, Amedes Holding, AMGEN, Berlin-Chemie, Boehringer Ingelheim Pharma, Daiichi Sankyo Deutschland, Danone, Hubert Burda Media Holding, Lilly Deutschland, Novo Nordisk Pharma, Roche Pharma, Sanofi-Aventis, SYNLAB Holding Deutschland & SYNLAB Akademie; participation on a Data Safety Monitoring Board or Advisory Board with Akcea Therapeutics Germany, AMGEN, Daiichi Sankyo Deutschland, and Sanofi-Aventis, all outside the submitted work. R Maddison reports grants or contracts from NHMRC Ideas Grant and National Heart Foundation of Australia Vanguard Grant, outside the submitted work. M Mahmoudi reports placement as a co-founder and director of the Academic Parity Movement, a non-profit organisation dedicated to addressing academic discrimination, violence and incivility; and receives royalties/honoraria for his published books, plenary lectures, and licensed patents, outside the submitted work. S Nomura reports grant support for the present manuscript from the Ministry of Education, Culture, Sports, Science and Technology (MEXT). C Nowak reports employment with Diamyd Medical AB (Stockholm, Sweden) which develops a treatment for type 1 diabetes, outside the submitted work. A Ortiz reports grants or contracts from Sanofi, Mundipharma, and AstraZeneca; and payment or honoraria for lectures, presentations, speakers bureaus, manuscript writing or educational events from Astellas, Astrazeneca, Amicus, Amgen, Fresenius Medical Care, Bayer, Sanofi-Genzyme, Menarini, Kyowa Kirin, Alexion, Otsuka and Vifor Fresenius Medical Care Renal Pharma. C Panelo reports grants or contracts from USAID Philippines as Chief of Party of USAID's ProtectHealth, a financing policy support project assisting the Philippine Government in implementing universal health care with focus on tuberculosis, family planning and HIV, outside the submitted work. M Postma reports leadership or fiduciary role in other board, society, committee or advocacy group, unpaid as member of UK's JCVI. A Radfar reports financial support from Avicenna Medical and Clinical Research Institute. P Sachdev reports an investigator grant from the National Health and Medical Research Council of Australia, paid to the University; participation on a Data Safety Monitoring Board or Advisory Board with the Advisory Committee for Biogen Australia; and leadership or fiduciary role in other board, society, committee or advocacy group, paid or unpaid as honorary director of International Neuropsychiatric Association and executive member of VASCOG; outside the submitted work. J Singh reports consulting fees from Crealta/Horizon, Medisys, Fidia, Two labs Inc, Adept Field Solutions, Clinical Care options, Clearview healthcare partners, Putnam associates, Focus forward, Navigant consulting, Spherix, MedIQ, UBM LLC, Trio Health, Medscape, WebMD, and Practice Point communications; and the National Institutes of Health and the American College of Rheumatology; payment or honoraria for lectures, presentations, speakers bureaus, manuscript writing or educational events from Simply Speaking; support for attending meetings and/or travel from OMERACT, an international organisation that develops measures for clinical trials and receives arm's length funding from 12 pharmaceutical companies, when traveling bi-annually to OMERACT meetings; participation on a Data Safety Monitoring Board or Advisory Board as a member of the FDA Arthritis Advisory Committee; leadership or fiduciary role in other board, society, committee or advocacy group, paid or unpaid, with OMERACT as a member of the steering committee, with the Veterans Affairs Rheumatology Field Advisory Committee as a member, and with the UAB Cochrane Musculoskeletal Group Satellite Center on Network Meta-analysis as a director and editor; and stock or stock options in TPT Global Tech, Vaxart pharmaceuticals, Charlotte's Web Holdings Inc and previously owned stock options in Amarin, Viking, and Moderna pharmaceuticals, all outside the submitted work. H Slater reports grants or contracts awarded for co-developing a digital platform with young people with musculoskeletal pain to support their care from the Washington Department of Health, 2018–2021. D Stein reports personal fees from Lundbeck, Takeda, Johnson & Johnson and Servier, all outside the submitted work. M Stokes reports payment or honoraria for lectures, presentations, speakers bureaus, manuscript writing or educational events from the Autism Teaching Institute; leadership or fiduciary role in other board, society, committee or advocacy group, paid or unpaid as Vice President of Kidsafe Victoria and Board Member of Australasian Society for Autism Research, all outside the submitted work. S Tadakamadla reports grants or contracts from National Health and Medical Research Council, Australia, Early Career Fellowship. A Tsai reports stipend for work as Editor-in-Chief of Social Science and Medicine: Mental Health from Elsevier, Inc. R Uddin is supported by an Alfred Deakin Postdoctoral Research Fellowship, Deakin University, Australia and reports support for attending meetings and/or travel accommodation reimbursement from Deakin University Institute for Physical Activity and Nutrition, all outside of the submitted work. All other authors declare no competing interests.
