# Department of Error

**DOI:** 10.1016/S0140-6736(22)00320-8

**Published:** 2022-02-26

*GBD 2019 Adolescent Mortality Collaborators. Global, regional, and national mortality among young people aged 10–24 years, 1950–2019: a systematic analysis for the Global Burden of Disease Study 2019.* Lancet *2021; **398:** 1593–618*—In figure 8 of this Article, the total deaths and proportion in each age group in 1950 were incorrect. These corrections have been made to the online version as of Feb 24, 2022.

