# Supplementary material for: Global, regional, and national mortality among young people aged 10–24 years, 1950–2019: a systematic analysis for the Global Burden of Disease Study 2019
Source: Lancet. 2021 Oct 30;398(10311):1593–618. doi: 10.1016/S0140-6736(21)01546-4 (PMC8576274; doi:10.1016/S0140-6736(21)01546-4)
Supplement: Supplementary appendix [file mmc1.pdf]

THE LANCET

Supplementary appendix

This appendix formed part of the original submission and has been peer reviewed. We post it as supplied by the authors.

Supplement to: GBD 2019 Adolescent Mortality Collaborators. Global, regional, and national mortality among young people aged 10–24 years, 1950–2019: a systematic analysis for the Global Burden of Disease Study 2019. *Lancet* 2021; **398:** 1593–618.

Global, regional, and national mortality among young people aged 10 to 24 years, 1950-2019: a systematic analysis for the Global Burden of Disease Study 2019

Supplementary Material 1

List of figures

Figure S1: World map of 21 GBD regions within 7 GBD super-regions 6
Figure S2: Population trends in 10-14, 15-19 and 20-24 year olds 1950 - 2019 by sex................................19
Figure S3: Main contributors to global number of deaths in 10-24 year olds in 2019 (both sexes).............20
Figure S4: Numbers of deaths in 10-14, 15-19 and 20-24 year olds by sex and GBD super-region between 1950 and 2019...24
Figure S5: Proportion of deaths in 10-14, 15-19 and 20-24 occurring in each GBD super-region by sex and between 1950 and 2019 25
Figure S6: Mortality rate per 100,000 in 204 countries in 2019 in 10-14 olds by sex 26
Figure S7: Mortality rate per 100,000 in 204 countries in 2019 in 15-19 olds by sex 27
Figure S8: Mortality rate per 100,000 in 204 countries in 2019 in 20-24 olds by sex 28
Figure S9: Global rank for mortality rate per 100,000 in 204 countries in 2019 in 10-14 olds by sex 29
Figure S10: Global rank for mortality rate per 100,000 in 204 countries in 2019 in 15-19 olds by sex 30
Figure S11: Global rank for mortality rate per 100,000 in 204 countries in 2019 in 20-24 olds by sex 31
Figure S12: Proportion of deaths in 0-24 occurring in 10-24 year olds by GBD super-region 1950 and 2019 33
Figure S13: Proportion of deaths in 0-24 occurring in 10-24 year olds in Central and Eastern Europe, Central Asia GBD super-region in 2019 34
Figure S14: Proportion of deaths in 0-24 occurring in 10-24 year olds in High Income GBD super-region in 2019 35
Figure S15: Proportion of deaths in 0-24 occurring in 10-24 year olds in Latin America and Caribbean GBD super-region in 2019...36
Figure S16: Proportion of deaths in 0-24 occurring in 10-24 year olds in North Africa and Middle East GBD super-region in 2019...37
Figure S17: Proportion of deaths in 0-24 occurring in 10-24 year olds in South Asia GBD super-region in 2019 37
Figure S18: Proportion of deaths in 0-24 occurring in 10-24 year olds in Southeast Asia, East Asia, and Oceania GBD super-region in 2019 38
Figure S19: Proportion of deaths in 0-24 occurring in 10-24 year olds in Sub-Saharan Africa GBD super-region in 2019 39
Figure S20: Ratio of male to female all-cause mortality rate per 100 000 by GBD super-region 1950 – 2019 40
Figure S21: All-cause mortality in 2019 and annual percentage change in population 1990 – 2019 in 10-14 year olds by sex 41
Figure S22: All-cause mortality in 2019 and annual percentage change in population 1990 – 2019 in 15-19 year olds by sex 42
Figure S23: All-cause mortality in 2019 and annual percentage change in population 1990 – 2019 in 20-24 year olds by sex 43
Figure S24: Distribution of annual percentage change in all-cause mortality rate per 100 000 in 204 countries 1990 – 2019 in 1-4 and 15-19 year olds by sex 44
Figure S25: All-cause mortality rate per 100,000 percentile for 0-5 against 15-19 year old females in 204 countries by GBD super-region in 2019...45

Figure S26: Ratio of observed : expected all-cause mortality rate per 100,000 in 2017 amongst 10-14 year olds (both sexes)......46
Figure S27: Ratio of observed : expected all-cause mortality rate per 100,000 in 2017 amongst 15-19 year olds (both sexes)......47
Figure S28: Ratio of observed : expected all-cause mortality rate per 100,000 in 2017 amongst 20-24 year olds (both sexes)......48
Figure S29: Ratio of observed : expected all-cause mortality rate per 100,000, 1990 - 2017 amongst 10-14 year olds (both sexes) in Central and Eastern Europe, Central Asia GBD super-region 49
Figure S30: Ratio of observed : expected all-cause mortality rate per 100,000, 1990 - 2017 amongst 15-19 year olds (both sexes) in Central and Eastern Europe, Central Asia GBD super-region 50
Figure S31: Ratio of observed : expected all-cause mortality rate per 100,000, 1990 - 2017 amongst 20-24 year olds (both sexes) in Central and Eastern Europe, Central Asia GBD super-region 51
Figure S32: Ratio of observed : expected all-cause mortality rate per 100,000, 1990 - 2017 amongst 10-14 year olds (both sexes) in High Income GBD super-region 52
Figure S33: Ratio of observed : expected all-cause mortality rate per 100,000, 1990 - 2017 amongst 15-19 year olds (both sexes) in High Income GBD super-region 53
Figure S34: Ratio of observed : expected all-cause mortality rate per 100,000, 1990 - 2017 amongst 20-24 year olds (both sexes) in High Income GBD super-region 54
Figure S35: Ratio of observed : expected all-cause mortality rate per 100,000, 1990 - 2017 amongst 10-14 year olds (both sexes) in Latin America and Caribbean GBD super-region......55
Figure S36: Ratio of observed : expected all-cause mortality rate per 100,000, 1990 - 2017 amongst 15-19 year olds (both sexes) in Latin America and Caribbean GBD super-region......56
Figure S37: Ratio of observed : expected all-cause mortality rate per 100,000, 1990 - 2017 amongst 20-24 year olds (both sexes) in Latin America and Caribbean GBD super-region......57
Figure S38: Ratio of observed : expected all-cause mortality rate per 100,000, 1990 - 2017 amongst 10-14 year olds (both sexes) in North Africa and Middle East GBD super-region......58
Figure S39: Ratio of observed : expected all-cause mortality rate per 100,000, 1990 - 2017 amongst 15-19 year olds (both sexes) in North Africa and Middle East GBD super-region......59
Figure S40: Ratio of observed : expected all-cause mortality rate per 100,000, 1990 - 2017 amongst 20-24 year olds (both sexes) in North Africa and Middle East GBD super-region......60
Figure S41: Ratio of observed : expected all-cause mortality rate per 100,000, 1990 - 2017 amongst 10-14 year olds (both sexes) in South Asia GBD super-region 61
Figure S42: Ratio of observed : expected all-cause mortality rate per 100,000, 1990 - 2017 amongst 15-19 year olds (both sexes) in South Asia GBD super-region 61
Figure S43: Ratio of observed : expected all-cause mortality rate per 100,000, 1990 - 2017 amongst 20-24 year olds (both sexes) in South Asia GBD super-region 61
Figure S44: Ratio of observed : expected all-cause mortality rate per 100,000, 1990 - 2017 amongst 10-14 year olds (both sexes) in Southeast Asia, East Asia, and Oceania GBD super-region......63
Figure S45: Ratio of observed : expected all-cause mortality rate per 100,000, 1990 - 2017 amongst 15-19 year olds (both sexes) in Southeast Asia, East Asia, and Oceania GBD super-region......64
Figure S46: Ratio of observed : expected all-cause mortality rate per 100,000, 1990 - 2017 amongst 20-24 year olds (both sexes) in Southeast Asia, East Asia, and Oceania GBD super-region......65
Figure S47: Ratio of observed : expected all-cause mortality rate per 100,000, 1990 - 2017 amongst 10-14 year olds (both sexes) in Sub-Saharan Africa GBD super-region 66
Figure S48: Ratio of observed : expected all-cause mortality rate per 100,000, 1990 - 2017 amongst 15-19 year olds (both sexes) in Sub-Saharan Africa GBD super-region 68
Figure S49: Ratio of observed : expected all-cause mortality rate per 100,000, 1990 - 2017 amongst 20-24 year olds (both sexes) in Sub-Saharan Africa GBD super-region 70
Figure S50: Global deaths by cause group in 10-14, 15-19, 20-24and 10-24 in 2019 by sex 72
Figure S51: Percentage of total deaths by cause group in 10-14 males 1980 – 2019......73
Figure S52: Percentage of total deaths by cause group in 10-14 females 1980 – 2019......74

Figure S53: Percentage of total deaths by cause group in 15-19 males 1980 – 2019 75
Figure S54: Percentage of total deaths by cause group in 15-19 females 1980 – 2019 76
Figure S55: Percentage of total deaths by cause group in 20-24 males 1980 – 2019 77
Figure S56: Percentage of total deaths by cause group in 20-24 females 1980 – 2019 78
Figure S57: Mortality rate per 100,000 population by cause of death in 10-24 year olds 1980 – 2019: Central Europe, Eastern Europe and Central Asia GBD super-region 79
Figure S58: Number of deaths by cause of death in 10-24 year olds 1980 – 2019: Central Europe, Eastern Europe and Central Asia GBD super-region 80
Figure S59: Rank of number of deaths by cause group 1980 – 2019: Central Europe, Eastern Europe and Central Asia GBD super-region. 10-14 year old males. 81
Figure S60: Rank of number of deaths by cause group 1980 – 2019: Central Europe, Eastern Europe and Central Asia GBD super-region. 10-14 year old females. 82
Figure S61: Rank of number of deaths by cause group 1980 – 2019: Central Europe, Eastern Europe and Central Asia GBD super-region. 15-19 year old males. 83
Figure S62: Rank of number of deaths by cause group 1980 – 2019: Central Europe, Eastern Europe and Central Asia GBD super-region. 15-19 year old females. 84
Figure S63: Rank of number of deaths by cause group 1980 – 2019: Central Europe, Eastern Europe and Central Asia GBD super-region. 20-24 year old males. 85
Figure S64: Rank of number of deaths by cause group 1980 – 2019: Central Europe, Eastern Europe and Central Asia GBD super-region. 20-24 year old females. 86
Figure S65: Mortality rate per 100,000 population by cause of death in 10-24 year olds 1980 – 2019: High Income GBD super-region 87
Figure S66: Number of deaths by cause in 10-24 year olds 1980 – 2019: High Income GBD super-region 88
Figure S67: Rank of number of deaths by cause group 1980 – 2019: High Income GBD super-region. 10-14 year old males. 89
Figure S68: Rank of number of deaths by cause group 1980 – 2019: High Income GBD super-region. 10-14 year old females. 90
Figure S69: Rank of number of deaths by cause group 1980 – 2019: High Income GBD super-region. 15-19 year old males. 91
Figure S70: Rank of number of deaths by cause group 1980 – 2019: High Income GBD super-region. 15-19 year old females. 92
Figure S71: Rank of number of deaths by cause group 1980 – 2019: High Income GBD super-region. 20-24 year old males. 93
Figure S72: Rank of number of deaths by cause group 1980 – 2019: High Income GBD super-region. 20-24 year old females. 94
Figure S73: Mortality rate per 100,000 population by cause of death in 10-24 year olds 1980 – 2019: Latin America and the Caribbean GBD super-region 95
Figure S74: Number of deaths by cause in 10-24 year olds 1980 – 2019: Latin America and the Caribbean GBD super-region 96
Figure S75: Rank of number of deaths by cause group 1980 – 2019: Latin America and the Caribbean GBD super-region. 10-14 year old males. 97
Figure S76: Rank of number of deaths by cause group 1980 – 2019: Latin America and the Caribbean GBD super-region. 10-14 year old females. 98
Figure S77: Rank of number of deaths by cause group 1980 – 2019: Latin America and the Caribbean GBD super-region. 15-19 year old males. 99
Figure S78: Rank of number of deaths by cause group 1980 – 2019: Latin America and the Caribbean GBD super-region. 15-19 year old females. 100
Figure S79: Rank of number of deaths by cause group 1980 – 2019: Latin America and the Caribbean GBD super-region. 20-24 year old males. 101
Figure S80: Rank of number of deaths by cause group 1980 – 2019: Latin America and the Caribbean GBD super-region. 20-24 year old females. 102

Figure S81: Mortality rate per 100,000 population by cause of death in 10-24 year olds 1980 – 2019: North Africa and the Middle East GBD super-region 103
Figure S82: Number of deaths by cause in 10-24 year olds 1980 – 2019: North Africa and the Middle East GBD super-region 104
Figure S83: Rank of number of deaths by cause group 1980 – 2019: North Africa and the Middle East GBD super-region. 10-14 year old males........ 105
Figure S84: Rank of number of deaths by cause group 1980 – 2019: North Africa and the Middle East GBD super-region. 10-14 year old females. 106
Figure S85: Rank of number of deaths by cause group 1980 – 2019: North Africa and the Middle East GBD super-region. 15-19 year old males........ 107
Figure S86: Rank of number of deaths by cause group 1980 – 2019: North Africa and the Middle East GBD super-region. 15-19 year old females. 108
Figure S87: Rank of number of deaths by cause group 1980 – 2019: North Africa and the Middle East GBD super-region. 20-24 year old males........ 109
Figure S88: Rank of number of deaths by cause group 1980 – 2019: North Africa and the Middle East GBD super-region. 20-24 year old females. 110
Figure S89: Mortality rate per 100,000 population by cause of death in 10-24 year olds 1980 – 2019: South Asia GBD super-region 111
Figure S90: Number of deaths by cause in 10-24 year olds 1980 – 2019: South Asia GBD super-region 112
Figure S91: Rank of number of deaths by cause group 1980 – 2019: South Asia GBD super-region. 10-14 year old males. 113
Figure S92: Rank of number of deaths by cause group 1980 – 2019: South Asia GBD super-region. 10-14 year old females. 114
Figure S93: Rank of number of deaths by cause group 1980 – 2019: South Asia GBD super-region. 15-19 year old males. 115
Figure S94: Rank of number of deaths by cause group 1980 – 2019: South Asia GBD super-region. 15-19 year old females. 116
Figure S95: Rank of number of deaths by cause group 1980 – 2019: South Asia GBD super-region. 20-24 year old males. 117
Figure S96: Rank of number of deaths by cause group 1980 – 2019: South Asia GBD super-region. 20-24 year old females. 118
Figure S97: Mortality rate per 100,000 population by cause of death in 10-24 year olds 1980 – 2019: Southeast Asia, East Asia and Oceania GBD super-region 119
Figure S98: Number of deaths by cause in 10-24 year olds 1980 – 2019: Southeast Asia, East Asia and Oceania GBD super-region........ 120
Figure S99: Rank of number of deaths by cause group 1980 – 2019: Southeast Asia, East Asia and Oceania GBD super-region. 10-14 year old males........ 121
Figure S100: Rank of number of deaths by cause group 1980 – 2019: Southeast Asia, East Asia and Oceania GBD super-region. 10-14 year old females........ 122
Figure S101: Rank of number of deaths by cause group 1980 – 2019: Southeast Asia, East Asia and Oceania GBD super-region. 15-19 year old males........ 123
Figure S102: Rank of number of deaths by cause group 1980 – 2019: Southeast Asia, East Asia and Oceania GBD super-region. 15-19 year old females........ 124
Figure S103: Rank of number of deaths by cause group 1980 – 2019: Southeast Asia, East Asia and Oceania GBD super-region. 20-24 year old males........ 125
Figure S104: Rank of number of deaths by cause group 1980 – 2019: Southeast Asia, East Asia and Oceania GBD super-region. 20-24 year old females........ 126
Figure S105: Mortality rate per 100,000 population by cause of death in 10-24 year olds 1980 – 2019: Sub Saharan Africa super-region 127
Figure S106: Number of deaths by cause in 10-24 year olds 1980 – 2019: Sub Saharan Africa super-region 128

Figure S107: Rank of number of deaths by cause group 1980 – 2019: Sub Saharan Africa GBD super-region. 10-14 year old males. 129
Figure S108: Rank of number of deaths by cause group 1980 – 2019: Sub Saharan Africa GBD super-region. 10-14 year old females. 130
Figure S109: Rank of number of deaths by cause group 1980 – 2019: Sub Saharan Africa GBD super-region. 15-19 year old males. 131
Figure S110: Rank of number of deaths by cause group 1980 – 2019: Sub Saharan Africa GBD super-region. 15-19 year old females. 132
Figure S111: Rank of number of deaths by cause group 1980 – 2019: Sub Saharan Africa GBD super-region. 20-24 year old males. 133
Figure S112: Rank of number of deaths by cause group 1980 – 2019: Sub Saharan Africa GBD super-region. 20-24 year old females. 134
Figure 113: Differences in estimates for global number of deaths between GBD 2019 and UN IGME* 10 to 14 both sexes 135
Figure 114: Differences in estimates for global number of deaths between GBD 2019 and UN IGME* 15 to 19 both sexes 135
Figure 115: Differences in estimates for global number of deaths between GBD 2019 and UN IGME* 20 to 24 both sexes 136

List of tables

Table S1: List of GBD 2019 countries and territories by GBD super-region, GBD Region and World Bank Income Group....7
Table S2: Global Burden of Disease 2019 Cause Hierarchy....13
Table S3: Number of deaths and all-cause mortality rate per 100,00 in 10-14 by sex in 2019 21
Table S4: Number of deaths and all-cause mortality rate per 100,00 in 15-19 by sex in 2019 22
Table S5: Number of deaths and all-cause mortality rate per 100,00 in 20-24 by sex in 2019 23
Table S6: Spearman's correlation coefficient (ρ) for association between country Sociodemographic Index (SDI) and all-cause mortality rate per 100,000 in 204 countries and territories by age group and sex in 1950, 1985 and 2019....32

Figure S1: World map of 21 GBD regions within 7 GBD super-regions

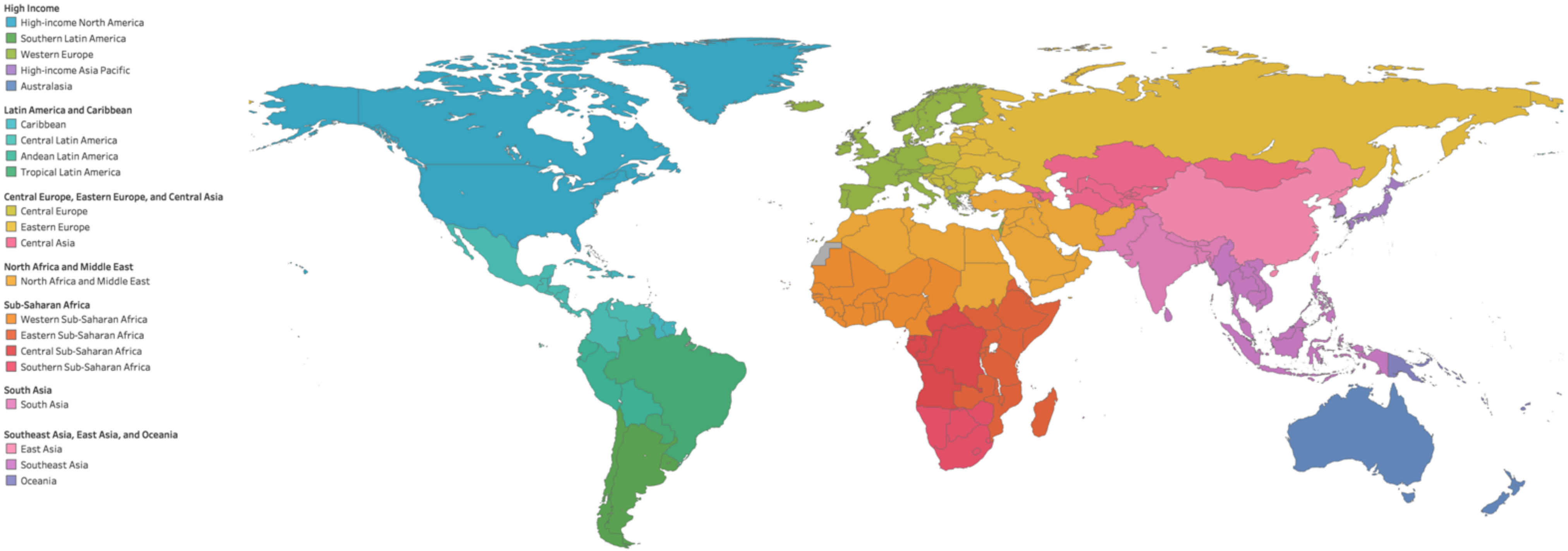

Table S1: List of GBD 2019 countries and territories by GBD super-region, GBD Region and World Bank Income Group

Location Name	Super Region	Region	Income Classification
**Global**			
**Central Europe, Eastern Europe, and Central Asia**			
Central Asia	Central Europe, Eastern Europe, and Central Asia		
Armenia	Central Europe, Eastern Europe, and Central Asia	Central Asia	Upper middle income
Azerbaijan	Central Europe, Eastern Europe, and Central Asia	Central Asia	Upper middle income
Georgia	Central Europe, Eastern Europe, and Central Asia	Central Asia	Upper middle income
Kazakhstan	Central Europe, Eastern Europe, and Central Asia	Central Asia	Upper middle income
Kyrgyzstan	Central Europe, Eastern Europe, and Central Asia	Central Asia	Lower middle income
Mongolia	Central Europe, Eastern Europe, and Central Asia	Central Asia	Lower middle income
Tajikistan	Central Europe, Eastern Europe, and Central Asia	Central Asia	Low income
Turkmenistan	Central Europe, Eastern Europe, and Central Asia	Central Asia	Upper middle income
Uzbekistan	Central Europe, Eastern Europe, and Central Asia	Central Asia	Lower middle income
Central Europe	Central Europe, Eastern Europe, and Central Asia		
Albania	Central Europe, Eastern Europe, and Central Asia	Central Europe	Upper middle income
Bosnia and Herzegovina	Central Europe, Eastern Europe, and Central Asia	Central Europe	Upper middle income
Bulgaria	Central Europe, Eastern Europe, and Central Asia	Central Europe	Upper middle income
Croatia	Central Europe, Eastern Europe, and Central Asia	Central Europe	High income
Czechia	Central Europe, Eastern Europe, and Central Asia	Central Europe	High income
Hungary	Central Europe, Eastern Europe, and Central Asia	Central Europe	High income
Montenegro	Central Europe, Eastern Europe, and Central Asia	Central Europe	Upper middle income
North Macedonia	Central Europe, Eastern Europe, and Central Asia	Central Europe	Upper middle income
Poland	Central Europe, Eastern Europe, and Central Asia	Central Europe	High income
Romania	Central Europe, Eastern Europe, and Central Asia	Central Europe	High income
Serbia	Central Europe, Eastern Europe, and Central Asia	Central Europe	Upper middle income
Slovakia	Central Europe, Eastern Europe, and Central Asia	Central Europe	High income
Slovenia	Central Europe, Eastern Europe, and Central Asia	Central Europe	High income
Eastern Europe	Central Europe, Eastern Europe, and Central Asia		
Belarus	Central Europe, Eastern Europe, and Central Asia	Eastern Europe	Upper middle income
Estonia	Central Europe, Eastern Europe, and Central Asia	Eastern Europe	High income
Latvia	Central Europe, Eastern Europe, and Central Asia	Eastern Europe	High income
Lithuania	Central Europe, Eastern Europe, and Central Asia	Eastern Europe	High income
Republic of Moldova	Central Europe, Eastern Europe, and Central Asia	Eastern Europe	Lower middle income
Russian Federation	Central Europe, Eastern Europe, and Central Asia	Eastern Europe	Upper middle income
Ukraine	Central Europe, Eastern Europe, and Central Asia	Eastern Europe	Lower middle income
**High-income**			
Australasia	High-income		

Location Name	Super Region	Region	Income Classification
Australia	High-income	Australasia	High income
New Zealand	High-income	Australasia	High income
High-income Asia Pacific	High-income		
Brunei Darussalam	High-income	High-income Asia Pacific	High income
Japan	High-income	High-income Asia Pacific	High income
Republic of Korea	High-income	High-income Asia Pacific	High income
Singapore	High-income	High-income Asia Pacific	High income
High-income North America	High-income		
Canada	High-income	High-income North America	High income
Greenland	High-income	High-income North America	High income
United States of America	High-income	High-income North America	High income
Southern Latin America	High-income		
Argentina	High-income	Southern Latin America	Upper middle income
Chile	High-income	Southern Latin America	High income
Uruguay	High-income	Southern Latin America	High income
Western Europe	High-income		
Andorra	High-income	Western Europe	High income
Austria	High-income	Western Europe	High income
Belgium	High-income	Western Europe	High income
Cyprus	High-income	Western Europe	High income
Denmark	High-income	Western Europe	High income
Finland	High-income	Western Europe	High income
France	High-income	Western Europe	High income
Germany	High-income	Western Europe	High income
Greece	High-income	Western Europe	High income
Iceland	High-income	Western Europe	High income
Ireland	High-income	Western Europe	High income
Israel	High-income	Western Europe	High income
Italy	High-income	Western Europe	High income
Luxembourg	High-income	Western Europe	High income
Malta	High-income	Western Europe	High income
Monaco	High-income	Western Europe	High income
Netherlands	High-income	Western Europe	High income
Norway	High-income	Western Europe	High income
Portugal	High-income	Western Europe	High income
San Marino	High-income	Western Europe	High income
Spain	High-income	Western Europe	High income
Sweden	High-income	Western Europe	High income
Switzerland	High-income	Western Europe	High income
United Kingdom	High-income	Western Europe	High income

Location Name	Super Region	Region	Income Classification
**Latin America and Caribbean**	Latin America and Caribbean		
Andean Latin America	Latin America and Caribbean		
Bolivia (Plurinational State of)	Latin America and Caribbean	Andean Latin America	Lower middle income
Ecuador	Latin America and Caribbean	Andean Latin America	Upper middle income
Peru	Latin America and Caribbean	Andean Latin America	Upper middle income
Caribbean	Latin America and Caribbean		
Antigua and Barbuda	Latin America and Caribbean	Caribbean	High income
Bahamas	Latin America and Caribbean	Caribbean	High income
Barbados	Latin America and Caribbean	Caribbean	High income
Belize	Latin America and Caribbean	Caribbean	Upper middle income
Bermuda	Latin America and Caribbean	Caribbean	High income
Cuba	Latin America and Caribbean	Caribbean	Upper middle income
Dominica	Latin America and Caribbean	Caribbean	Upper middle income
Dominican Republic	Latin America and Caribbean	Caribbean	Upper middle income
Grenada	Latin America and Caribbean	Caribbean	Upper middle income
Guyana	Latin America and Caribbean	Caribbean	Upper middle income
Haiti	Latin America and Caribbean	Caribbean	Low income
Jamaica	Latin America and Caribbean	Caribbean	Upper middle income
Puerto Rico	Latin America and Caribbean	Caribbean	High income
Saint Kitts and Nevis	Latin America and Caribbean	Caribbean	High income
Saint Lucia	Latin America and Caribbean	Caribbean	Upper middle income
Saint Vincent and the Grenadines	Latin America and Caribbean	Caribbean	Upper middle income
Suriname	Latin America and Caribbean	Caribbean	Upper middle income
Trinidad and Tobago	Latin America and Caribbean	Caribbean	High income
United States Virgin Islands	Latin America and Caribbean	Caribbean	High income
Central Latin America	Latin America and Caribbean		
Colombia	Latin America and Caribbean	Central Latin America	Upper middle income
Costa Rica	Latin America and Caribbean	Central Latin America	Upper middle income
El Salvador	Latin America and Caribbean	Central Latin America	Lower middle income
Guatemala	Latin America and Caribbean	Central Latin America	Upper middle income
Honduras	Latin America and Caribbean	Central Latin America	Lower middle income
Mexico	Latin America and Caribbean	Central Latin America	Upper middle income
Nicaragua	Latin America and Caribbean	Central Latin America	Lower middle income
Panama	Latin America and Caribbean	Central Latin America	High income
Venezuela (Bolivarian Republic of)	Latin America and Caribbean	Central Latin America	Upper middle income
Tropical Latin America	*Latin America and Caribbean*		
Brazil	Latin America and Caribbean	Tropical Latin America	Upper middle income
Paraguay	Latin America and Caribbean	Tropical Latin America	Upper middle income
**North Africa and Middle East**			
Afghanistan	North Africa and Middle East	North Africa and Middle East	Low income

Location Name	Super Region	Region	Income Classification
Algeria	North Africa and Middle East	North Africa and Middle East	Lower middle income
Bahrain	North Africa and Middle East	North Africa and Middle East	High income
Egypt	North Africa and Middle East	North Africa and Middle East	Lower middle income
Iran (Islamic Republic of)	North Africa and Middle East	North Africa and Middle East	Upper middle income
Iraq	North Africa and Middle East	North Africa and Middle East	Upper middle income
Jordan	North Africa and Middle East	North Africa and Middle East	Upper middle income
Kuwait	North Africa and Middle East	North Africa and Middle East	High income
Lebanon	North Africa and Middle East	North Africa and Middle East	Upper middle income
Libya	North Africa and Middle East	North Africa and Middle East	Upper middle income
Morocco	North Africa and Middle East	North Africa and Middle East	Lower middle income
Oman	North Africa and Middle East	North Africa and Middle East	High income
Palestine	North Africa and Middle East	North Africa and Middle East	Lower middle income
Qatar	North Africa and Middle East	North Africa and Middle East	High income
Saudi Arabia	North Africa and Middle East	North Africa and Middle East	High income
Sudan	North Africa and Middle East	North Africa and Middle East	Low income
Syrian Arab Republic	North Africa and Middle East	North Africa and Middle East	Low income
Tunisia	North Africa and Middle East	North Africa and Middle East	Lower middle income
Turkey	North Africa and Middle East	North Africa and Middle East	Upper middle income
United Arab Emirates	North Africa and Middle East	North Africa and Middle East	High income
Yemen	North Africa and Middle East	North Africa and Middle East	Low income
**South Asia**			
Bangladesh	South Asia	South Asia	Lower middle income
Bhutan	South Asia	South Asia	Lower middle income
India	South Asia	South Asia	Lower middle income
Nepal	South Asia	South Asia	Lower middle income
Pakistan	South Asia	South Asia	Lower middle income
**Southeast Asia, East Asia, and Oceania**			
East Asia	Southeast Asia, East Asia, and Oceania		
China	Southeast Asia, East Asia, and Oceania	East Asia	Upper middle income
Democratic People's Republic of Korea	Southeast Asia, East Asia, and Oceania	East Asia	Low income
Taiwan (Province of China)	Southeast Asia, East Asia, and Oceania	East Asia	High income
Oceania	Southeast Asia, East Asia, and Oceania		
American Samoa	Southeast Asia, East Asia, and Oceania	Oceania	Upper middle income
Cook Islands	Southeast Asia, East Asia, and Oceania	Oceania	
Fiji	Southeast Asia, East Asia, and Oceania	Oceania	Upper middle income
Guam	Southeast Asia, East Asia, and Oceania	Oceania	High income
Kiribati	Southeast Asia, East Asia, and Oceania	Oceania	Lower middle income
Marshall Islands	Southeast Asia, East Asia, and Oceania	Oceania	Upper middle income
Micronesia (Federated States of)	Southeast Asia, East Asia, and Oceania	Oceania	Lower middle income
Nauru	Southeast Asia, East Asia, and Oceania	Oceania	High income

Location Name	Super Region	Region	Income Classification
Niue	Southeast Asia, East Asia, and Oceania	Oceania	
Northern Mariana Islands	Southeast Asia, East Asia, and Oceania	Oceania	High income
Palau	Southeast Asia, East Asia, and Oceania	Oceania	High income
Papua New Guinea	Southeast Asia, East Asia, and Oceania	Oceania	Lower middle income
Samoa	Southeast Asia, East Asia, and Oceania	Oceania	Upper middle income
Solomon Islands	Southeast Asia, East Asia, and Oceania	Oceania	Lower middle income
Tokelau	Southeast Asia, East Asia, and Oceania	Oceania	
Tonga	Southeast Asia, East Asia, and Oceania	Oceania	Upper middle income
Tuvalu	Southeast Asia, East Asia, and Oceania	Oceania	Upper middle income
Vanuatu	Southeast Asia, East Asia, and Oceania	Oceania	Lower middle income
Southeast Asia	Southeast Asia, East Asia, and Oceania		
Cambodia	Southeast Asia, East Asia, and Oceania	Southeast Asia	Lower middle income
Indonesia	Southeast Asia, East Asia, and Oceania	Southeast Asia	Upper middle income
Lao People's Democratic Republic	Southeast Asia, East Asia, and Oceania	Southeast Asia	Lower middle income
Malaysia	Southeast Asia, East Asia, and Oceania	Southeast Asia	Upper middle income
Maldives	Southeast Asia, East Asia, and Oceania	Southeast Asia	Upper middle income
Mauritius	Southeast Asia, East Asia, and Oceania	Southeast Asia	High income
Myanmar	Southeast Asia, East Asia, and Oceania	Southeast Asia	Lower middle income
Philippines	Southeast Asia, East Asia, and Oceania	Southeast Asia	Lower middle income
Seychelles	Southeast Asia, East Asia, and Oceania	Southeast Asia	High income
Sri Lanka	Southeast Asia, East Asia, and Oceania	Southeast Asia	Lower middle income
Thailand	Southeast Asia, East Asia, and Oceania	Southeast Asia	Upper middle income
Timor-Leste	Southeast Asia, East Asia, and Oceania	Southeast Asia	Lower middle income
Viet Nam	Southeast Asia, East Asia, and Oceania	Southeast Asia	Lower middle income
**Sub-Saharan Africa**			
Central Sub-Saharan Africa	Sub-Saharan Africa		
Angola	Sub-Saharan Africa	Central Sub-Saharan Africa	Lower middle income
Central African Republic	Sub-Saharan Africa	Central Sub-Saharan Africa	Low income
Congo	Sub-Saharan Africa	Central Sub-Saharan Africa	Lower middle income
Democratic Republic of the Congo	Sub-Saharan Africa	Central Sub-Saharan Africa	Low income
Equatorial Guinea	Sub-Saharan Africa	Central Sub-Saharan Africa	Upper middle income
Gabon	Sub-Saharan Africa	Central Sub-Saharan Africa	Upper middle income
Eastern Sub-Saharan Africa	Sub-Saharan Africa		
Burundi	Sub-Saharan Africa	Eastern Sub-Saharan Africa	Low income
Comoros	Sub-Saharan Africa	Eastern Sub-Saharan Africa	Lower middle income
Djibouti	Sub-Saharan Africa	Eastern Sub-Saharan Africa	Lower middle income
Eritrea	Sub-Saharan Africa	Eastern Sub-Saharan Africa	Low income
Ethiopia	Sub-Saharan Africa	Eastern Sub-Saharan Africa	Low income
Kenya	Sub-Saharan Africa	Eastern Sub-Saharan Africa	Lower middle income
Madagascar	Sub-Saharan Africa	Eastern Sub-Saharan Africa	Low income

Location Name	Super Region	Region	Income Classification
Malawi	Sub-Saharan Africa	Eastern Sub-Saharan Africa	Low income
Mozambique	Sub-Saharan Africa	Eastern Sub-Saharan Africa	Low income
Rwanda	Sub-Saharan Africa	Eastern Sub-Saharan Africa	Low income
Somalia	Sub-Saharan Africa	Eastern Sub-Saharan Africa	Low income
South Sudan	Sub-Saharan Africa	Eastern Sub-Saharan Africa	Low income
Uganda	Sub-Saharan Africa	Eastern Sub-Saharan Africa	Low income
United Republic of Tanzania	Sub-Saharan Africa	Eastern Sub-Saharan Africa	Lower middle income
Zambia	Sub-Saharan Africa	Eastern Sub-Saharan Africa	Lower middle income
Southern Sub-Saharan Africa	Sub-Saharan Africa		
Botswana	Sub-Saharan Africa	Southern Sub-Saharan Africa	Upper middle income
Eswatini	Sub-Saharan Africa	Southern Sub-Saharan Africa	Lower middle income
Lesotho	Sub-Saharan Africa	Southern Sub-Saharan Africa	Lower middle income
Namibia	Sub-Saharan Africa	Southern Sub-Saharan Africa	Upper middle income
South Africa	Sub-Saharan Africa	Southern Sub-Saharan Africa	Upper middle income
Zimbabwe	Sub-Saharan Africa	Southern Sub-Saharan Africa	Lower middle income
Western Sub-Saharan Africa	Sub-Saharan Africa		
Benin	Sub-Saharan Africa	Western Sub-Saharan Africa	Lower middle income
Burkina Faso	Sub-Saharan Africa	Western Sub-Saharan Africa	Low income
Cabo Verde	Sub-Saharan Africa	Western Sub-Saharan Africa	Lower middle income
Cameroon	Sub-Saharan Africa	Western Sub-Saharan Africa	Lower middle income
Chad	Sub-Saharan Africa	Western Sub-Saharan Africa	Low income
Cote d'Ivoire	Sub-Saharan Africa	Western Sub-Saharan Africa	Lower middle income
Gambia	Sub-Saharan Africa	Western Sub-Saharan Africa	Low income
Ghana	Sub-Saharan Africa	Western Sub-Saharan Africa	Lower middle income
Guinea	Sub-Saharan Africa	Western Sub-Saharan Africa	Low income
Guinea-Bissau	Sub-Saharan Africa	Western Sub-Saharan Africa	Low income
Liberia	Sub-Saharan Africa	Western Sub-Saharan Africa	Low income
Mali	Sub-Saharan Africa	Western Sub-Saharan Africa	Low income
Mauritania	Sub-Saharan Africa	Western Sub-Saharan Africa	Lower middle income
Niger	Sub-Saharan Africa	Western Sub-Saharan Africa	Low income
Nigeria	Sub-Saharan Africa	Western Sub-Saharan Africa	Lower middle income
Sao Tome and Principe	Sub-Saharan Africa	Western Sub-Saharan Africa	Lower middle income
Senegal	Sub-Saharan Africa	Western Sub-Saharan Africa	Lower middle income
Sierra Leone	Sub-Saharan Africa	Western Sub-Saharan Africa	Low income
Togo	Sub-Saharan Africa	Western Sub-Saharan Africa	Low income

Table S2: Global Burden of Disease 2019 Cause Hierarchy

Cause ID	Cause Name	Hierarchy Level	Cause Outline
294	All causes	0	Total
295	Communicable, maternal, neonatal, and nutritional diseases	1	A
955	HIV/AIDS and sexually transmitted infections	2	A.1
298	HIV/AIDS	3	A.1.1
948	HIV/AIDS - Drug-susceptible Tuberculosis	4	A.1.1.1
949	HIV/AIDS - Multidrug-resistant Tuberculosis without extensive drug resistance	4	A.1.1.2
950	HIV/AIDS - Extensively drug-resistant Tuberculosis	4	A.1.1.3
300	HIV/AIDS resulting in other diseases	4	A.1.1.4
393	Sexually transmitted infections excluding HIV	3	A.1.2
394	Syphilis	4	A.1.2.1
395	Chlamydial infection	4	A.1.2.2
396	Gonococcal infection	4	A.1.2.3
399	Other sexually transmitted infections	4	A.1.2.6
956	Respiratory infections and tuberculosis	2	A.2
297	Tuberculosis	3	A.2.1
934	Drug-susceptible tuberculosis	4	A.2.1.2
946	Multidrug-resistant tuberculosis without extensive drug resistance	4	A.2.1.3
947	Extensively drug-resistant tuberculosis	4	A.2.1.4
322	Lower respiratory infections	3	A.2.2
328	Upper respiratory infections	3	A.2.3
329	Otitis media	3	A.2.4
957	Enteric infections	2	A.3
302	Diarrheal diseases	3	A.3.1
958	Typhoid and paratyphoid	3	A.3.2
319	Typhoid fever	4	A.3.2.1
320	Paratyphoid fever	4	A.3.2.2
959	Invasive Non-typhoidal Salmonella (iNTS)	3	A.3.3
321	Other intestinal infectious diseases	3	A.3.4
344	Neglected tropical diseases and malaria	2	A.4
345	Malaria	3	A.4.1
346	Chagas disease	3	A.4.2
347	Leishmaniasis	3	A.4.3
348	Visceral leishmaniasis	4	A.4.3.1
350	African trypanosomiasis	3	A.4.4
351	Schistosomiasis	3	A.4.5
352	Cysticercosis	3	A.4.6
353	Cystic echinococcosis	3	A.4.7
357	Dengue	3	A.4.11
358	Yellow fever	3	A.4.12
359	Rabies	3	A.4.13
360	Intestinal nematode infections	3	A.4.14
361	Ascariasis	4	A.4.14.1
843	Ebola	3	A.4.17
935	Zika virus	3	A.4.18

Cause ID	Cause Name	Hierarchy Level	Cause Outline
365	Other neglected tropical diseases	3	A.4.20
961	Other infectious diseases	2	A.5
332	Meningitis	3	A.5.1
337	Encephalitis	3	A.5.2
338	Diphtheria	3	A.5.3
339	Whooping cough	3	A.5.4
340	Tetanus	3	A.5.5
341	Measles	3	A.5.6
342	Varicella and herpes zoster	3	A.5.7
400	Acute hepatitis	3	A.5.8
401	Acute hepatitis A	4	A.5.8.1
402	Acute hepatitis B	4	A.5.8.2
403	Acute hepatitis C	4	A.5.8.3
404	Acute hepatitis E	4	A.5.8.4
408	Other unspecified infectious diseases	3	A.5.9
962	Maternal and neonatal disorders	2	A.6
366	Maternal disorders	3	A.6.1
367	Maternal hemorrhage	4	A.6.1.1
368	Maternal sepsis and other maternal infections	4	A.6.1.2
369	Maternal hypertensive disorders	4	A.6.1.3
370	Maternal obstructed labor and uterine rupture	4	A.6.1.4
995	Maternal abortion and miscarriage	4	A.6.1.5
374	Ectopic pregnancy	4	A.6.1.6
375	Indirect maternal deaths	4	A.6.1.7
376	Late maternal deaths	4	A.6.1.8
741	Maternal deaths aggravated by HIV/AIDS	4	A.6.1.9
379	Other maternal disorders	4	A.6.1.10
380	Neonatal disorders	3	A.6.2
381	Neonatal preterm birth	4	A.6.2.1
382	Neonatal encephalopathy due to birth asphyxia and trauma	4	A.6.2.2
383	Neonatal sepsis and other neonatal infections	4	A.6.2.3
384	Hemolytic disease and other neonatal jaundice	4	A.6.2.4
385	Other neonatal disorders	4	A.6.2.5
386	Nutritional deficiencies	2	A.7
387	Protein-energy malnutrition	3	A.7.1
391	Other nutritional deficiencies	3	A.7.5
409	Non-communicable diseases	1	B
410	Neoplasms	2	B.1
444	Lip and oral cavity cancer	3	B.1.1
447	Nasopharynx cancer	3	B.1.2
450	Other pharynx cancer	3	B.1.3
411	Esophageal cancer	3	B.1.4
414	Stomach cancer	3	B.1.5
441	Colon and rectum cancer	3	B.1.6
417	Liver cancer	3	B.1.7
418	Liver cancer due to hepatitis B	4	B.1.7.1
419	Liver cancer due to hepatitis C	4	B.1.7.2
420	Liver cancer due to alcohol use	4	B.1.7.3

Cause ID	Cause Name	Hierarchy Level	Cause Outline
996	Liver cancer due to NASH	4	B.1.7.4
1021	Liver cancer due to other causes	4	B.1.7.5
453	Gallbladder and biliary tract cancer	3	B.1.8
456	Pancreatic cancer	3	B.1.9
423	Larynx cancer	3	B.1.10
426	Tracheal, bronchus, and lung cancer	3	B.1.11
459	Malignant skin melanoma	3	B.1.12
462	Non-melanoma skin cancer	3	B.1.13
849	Non-melanoma skin cancer (squamous-cell carcinoma)	4	B.1.13.1
429	Breast cancer	3	B.1.14
432	Cervical cancer	3	B.1.15
435	Uterine cancer	3	B.1.16
465	Ovarian cancer	3	B.1.17
438	Prostate cancer	3	B.1.18
468	Testicular cancer	3	B.1.19
471	Kidney cancer	3	B.1.20
474	Bladder cancer	3	B.1.21
477	Brain and central nervous system cancer	3	B.1.22
480	Thyroid cancer	3	B.1.23
483	Mesothelioma	3	B.1.24
484	Hodgkin lymphoma	3	B.1.25
485	Non-Hodgkin lymphoma	3	B.1.26
486	Multiple myeloma	3	B.1.27
487	Leukemia	3	B.1.28
845	Acute lymphoid leukemia	4	B.1.28.1
846	Chronic lymphoid leukemia	4	B.1.28.2
847	Acute myeloid leukemia	4	B.1.28.3
848	Chronic myeloid leukemia	4	B.1.28.4
943	Other leukemia	4	B.1.28.5
1022	Other malignant neoplasms	3	B.1.29
490	Other neoplasms	3	B.1.30
964	Myelodysplastic, myeloproliferative, and other hematopoietic neoplasms	4	B.1.30.1
491	Cardiovascular diseases	2	B.2
492	Rheumatic heart disease	3	B.2.1
493	Ischemic heart disease	3	B.2.2
494	Stroke	3	B.2.3
495	Ischemic stroke	4	B.2.3.1
496	Intracerebral hemorrhage	4	B.2.3.2
497	Subarachnoid hemorrhage	4	B.2.3.3
498	Hypertensive heart disease	3	B.2.4
504	Non-rheumatic valvular heart disease	3	B.2.5
968	Non-rheumatic calcific aortic valve disease	4	B.2.5.1
969	Non-rheumatic degenerative mitral valve disease	4	B.2.5.2
970	Other non-rheumatic valve diseases	4	B.2.5.3
499	Cardiomyopathy and myocarditis	3	B.2.6
942	Myocarditis	4	B.2.6.1
938	Alcoholic cardiomyopathy	4	B.2.6.2
944	Other cardiomyopathy	4	B.2.6.3

Cause ID	Cause Name	Hierarchy Level	Cause Outline
500	Atrial fibrillation and flutter	3	B.2.8
501	Aortic aneurysm	3	B.2.9
502	Peripheral artery disease	3	B.2.10
503	Endocarditis	3	B.2.11
1023	Other cardiovascular and circulatory diseases	3	B.2.12
508	Chronic respiratory diseases	2	B.3
509	Chronic obstructive pulmonary disease	3	B.3.1
510	Pneumoconiosis	3	B.3.2
511	Silicosis	4	B.3.2.1
512	Asbestosis	4	B.3.2.2
513	Coal workers pneumoconiosis	4	B.3.2.3
514	Other pneumoconiosis	4	B.3.2.4
515	Asthma	3	B.3.3
516	Interstitial lung disease and pulmonary sarcoidosis	3	B.3.4
520	Other chronic respiratory diseases	3	B.3.5
526	Digestive diseases	2	B.4
521	Cirrhosis and other chronic liver diseases	3	B.4.1
522	Cirrhosis and other chronic liver diseases due to hepatitis B	4	B.4.1.1
523	Cirrhosis and other chronic liver diseases due to hepatitis C	4	B.4.1.2
524	Cirrhosis and other chronic liver diseases due to alcohol use	4	B.4.1.3
971	Cirrhosis and other chronic liver diseases due to NAFLD	4	B.4.1.4
525	Cirrhosis and other chronic liver diseases due to other causes	4	B.4.1.5
992	Upper digestive system diseases	3	B.4.2
527	Peptic ulcer disease	4	B.4.2.1
528	Gastritis and duodenitis	4	B.4.2.2
529	Appendicitis	3	B.4.3
530	Paralytic ileus and intestinal obstruction	3	B.4.4
531	Inguinal, femoral, and abdominal hernia	3	B.4.5
532	Inflammatory bowel disease	3	B.4.6
533	Vascular intestinal disorders	3	B.4.7
534	Gallbladder and biliary diseases	3	B.4.8
535	Pancreatitis	3	B.4.9
541	Other digestive diseases	3	B.4.10
542	Neurological disorders	2	B.5
543	Alzheimer's disease and other dementias	3	B.5.1
544	Parkinson's disease	3	B.5.2
545	Idiopathic epilepsy	3	B.5.3
546	Multiple sclerosis	3	B.5.4
554	Motor neuron disease	3	B.5.5
557	Other neurological disorders	3	B.5.7
558	Mental disorders	2	B.6
572	Eating disorders	3	B.6.5
573	Anorexia nervosa	4	B.6.5.1
574	Bulimia nervosa	4	B.6.5.2
973	Substance use disorders	2	B.7
560	Alcohol use disorders	3	B.7.1
561	Drug use disorders	3	B.7.2

Cause ID	Cause Name	Hierarchy Level	Cause Outline
562	Opioid use disorders	4	B.7.2.1
563	Cocaine use disorders	4	B.7.2.2
564	Amphetamine use disorders	4	B.7.2.3
566	Other drug use disorders	4	B.7.2.5
974	Diabetes and kidney diseases	2	B.8
587	Diabetes mellitus	3	B.8.1
975	Diabetes mellitus type 1	4	B.8.1.1
976	Diabetes mellitus type 2	4	B.8.1.2
589	Chronic kidney disease	3	B.8.2
997	Chronic kidney disease due to diabetes mellitus type 1	4	B.8.2.1
998	Chronic kidney disease due to diabetes mellitus type 2	4	B.8.2.2
591	Chronic kidney disease due to hypertension	4	B.8.2.3
592	Chronic kidney disease due to glomerulonephritis	4	B.8.2.4
593	Chronic kidney disease due to other and unspecified causes	4	B.8.2.5
588	Acute glomerulonephritis	3	B.8.3
653	Skin and subcutaneous diseases	2	B.9
980	Bacterial skin diseases	3	B.9.3
656	Cellulitis	4	B.9.3.1
657	Pyoderma	4	B.9.3.2
665	Decubitus ulcer	3	B.9.11
668	Other skin and subcutaneous diseases	3	B.9.12
626	Musculoskeletal disorders	2	B.11
627	Rheumatoid arthritis	3	B.11.1
639	Other musculoskeletal disorders	3	B.11.6
640	Other non-communicable diseases	2	B.12
641	Congenital birth defects	3	B.12.1
642	Neural tube defects	4	B.12.1.1
643	Congenital heart anomalies	4	B.12.1.2
644	Orofacial clefts	4	B.12.1.3
645	Down syndrome	4	B.12.1.4
648	Other chromosomal abnormalities	4	B.12.1.7
649	Congenital musculoskeletal and limb anomalies	4	B.12.1.8
650	Urogenital congenital anomalies	4	B.12.1.9
651	Digestive congenital anomalies	4	B.12.1.10
652	Other congenital birth defects	4	B.12.1.11
594	Urinary diseases and male infertility	3	B.12.2
595	Urinary tract infections and interstitial nephritis	4	B.12.2.1
596	Urolithiasis	4	B.12.2.2
602	Other urinary diseases	4	B.12.2.5
603	Gynecological diseases	3	B.12.3
604	Uterine fibroids	4	B.12.3.1
607	Endometriosis	4	B.12.3.4
608	Genital prolapse	4	B.12.3.5
612	Other gynecological diseases	4	B.12.3.7
613	Hemoglobinopathies and hemolytic anemias	3	B.12.4
614	Thalassemias	4	B.12.4.1
615	Sickle cell disorders	4	B.12.4.3
616	G6PD deficiency	4	B.12.4.5

Cause ID	Cause Name	Hierarchy Level	Cause Outline
618	Other hemoglobinopathies and hemolytic anemias	4	B.12.4.7
619	Endocrine, metabolic, blood, and immune disorders	3	B.12.5
686	Sudden infant death syndrome	3	B.12.7
687	Injuries	1	C
688	Transport injuries	2	C.1
689	Road injuries	3	C.1.1
690	Pedestrian road injuries	4	C.1.1.1
691	Cyclist road injuries	4	C.1.1.2
692	Motorcyclist road injuries	4	C.1.1.3
693	Motor vehicle road injuries	4	C.1.1.4
694	Other road injuries	4	C.1.1.5
695	Other transport injuries	3	C.1.2
696	Unintentional injuries	2	C.2
697	Falls	3	C.2.1
698	Drowning	3	C.2.2
699	Fire, heat, and hot substances	3	C.2.3
700	Poisonings	3	C.2.4
701	Poisoning by carbon monoxide	4	C.2.4.1
703	Poisoning by other means	4	C.2.4.2
704	Exposure to mechanical forces	3	C.2.5
705	Unintentional firearm injuries	4	C.2.5.1
707	Other exposure to mechanical forces	4	C.2.5.2
708	Adverse effects of medical treatment	3	C.2.6
709	Animal contact	3	C.2.7
710	Venomous animal contact	4	C.2.7.1
711	Non-venomous animal contact	4	C.2.7.2
712	Foreign body	3	C.2.8
713	Pulmonary aspiration and foreign body in airway	4	C.2.8.1
715	Foreign body in other body part	4	C.2.8.3
842	Environmental heat and cold exposure	3	C.2.9
729	Exposure to forces of nature	3	C.2.10
716	Other unintentional injuries	3	C.2.11
717	Self-harm and interpersonal violence	2	C.3
718	Self-harm	3	C.3.1
721	Self-harm by firearm	4	C.3.1.1
723	Self-harm by other specified means	4	C.3.1.2
724	Interpersonal violence	3	C.3.2
725	Physical violence by firearm	4	C.3.2.1
726	Physical violence by sharp object	4	C.3.2.2
727	Physical violence by other means	4	C.3.2.4
945	Conflict and terrorism	3	C.3.3
854	Executions and police conflict	3	C.3.4

Figure S2: Population trends in 10-14, 15-19 and 20-24 year olds 1950 - 2019 by sex

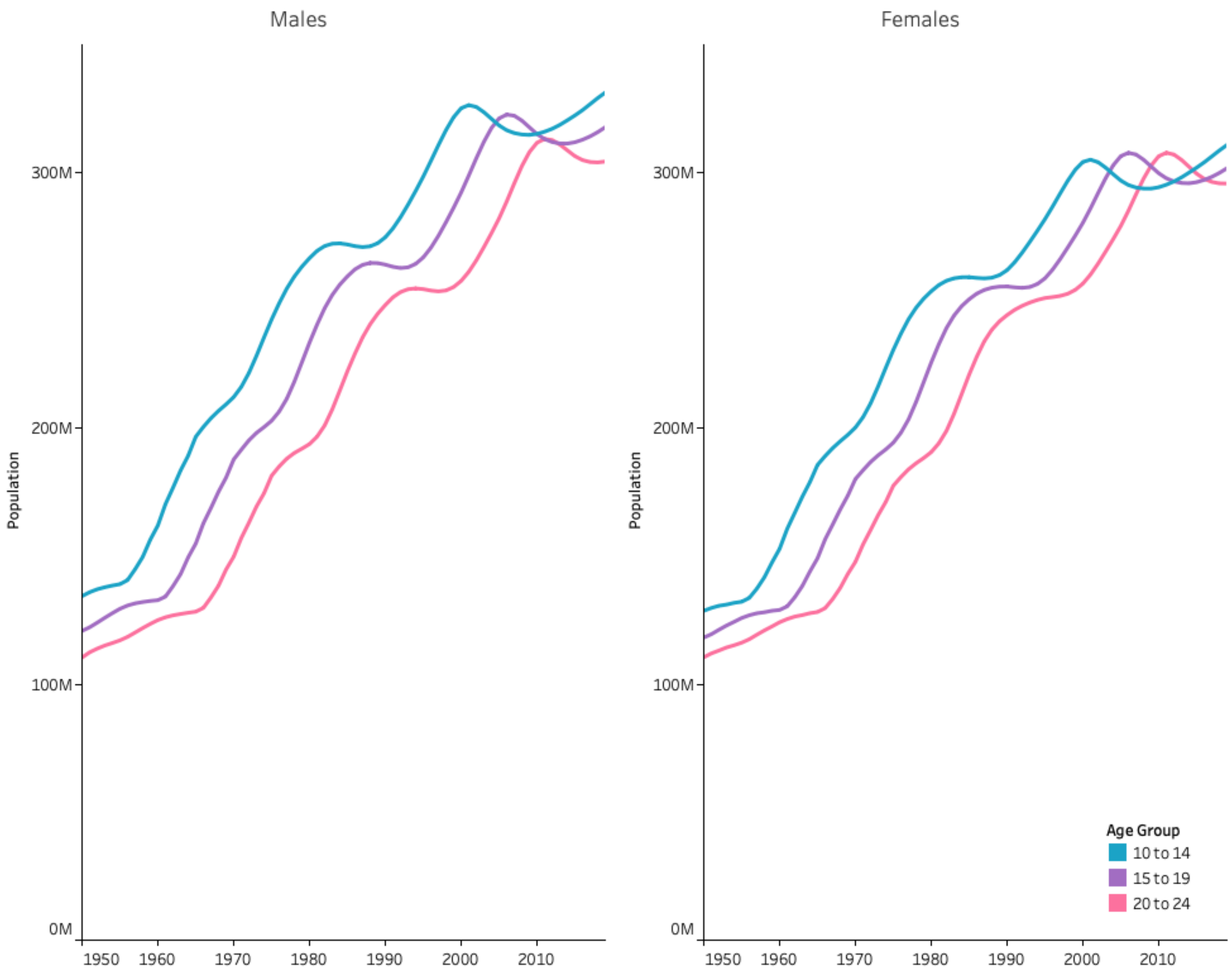

Figure S3: Main contributors to global number of deaths in 10-24 year olds in 2019 (both sexes)

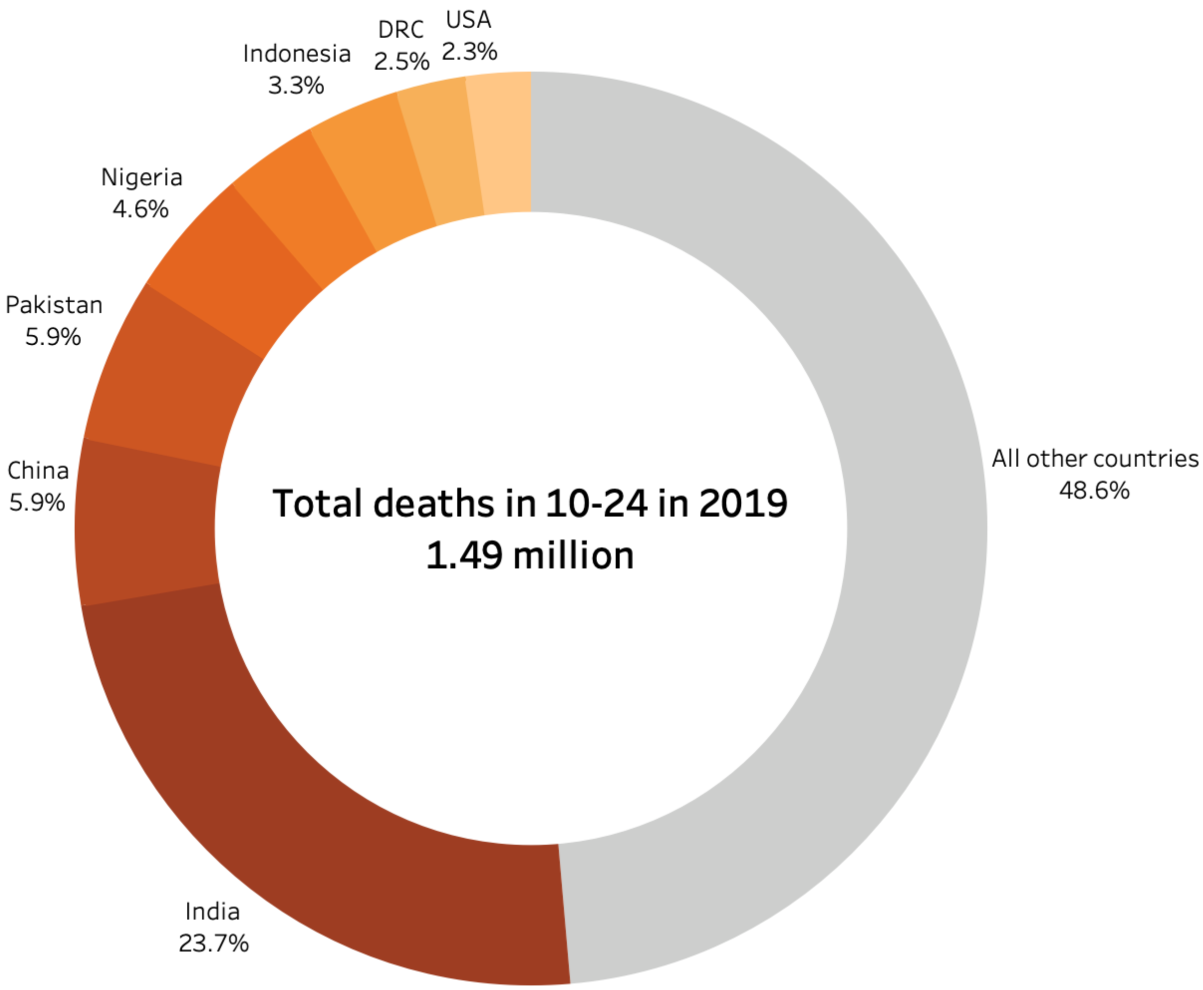

Table S3: Number of deaths and all-cause mortality rate per 100,00 in 10-14 by sex in 2019

	Number of deaths			Rate per 100,000	
10 to 14	Estimate	Uncertainty Interval	Percent of global total	Estimate	Uncertainty Interval
**Males**					
Global	170,079	[156,649 - 184,434]		51.33	[47.28 - 55.66]
Central Europe, Eastern Europe, and Central Asia	3,993	[3,705 - 4,324]	2.3	30.15	[27.97 - 32.65]
High-income	4,164	[4,064 - 4,266]	2.4	13.12	[12.81 - 13.44]
Latin America and Caribbean	9,684	[8,543 - 10,995]	5.7	39.12	[34.51 - 44.41]
North Africa and Middle East	13,167	[11,720 - 14,929]	7.7	45.05	[40.10 - 51.08]
South Asia	55,467	[50,132 - 61,412]	32.6	59.34	[53.64 - 65.70]
Southeast Asia, East Asia, and Oceania	24,644	[22,572 - 26,861]	14.5	35.21	[32.25 - 38.38]
Sub-Saharan Africa	58,958	[52,234 - 66,984]	34.7	85.56	[75.80 - 97.20]
**Females**					
Global	129,193	[119,022 - 140,301]		41.56	[38.29 - 45.13]
Central Europe, Eastern Europe, and Central Asia	2,580	[2,412 - 2,762]	2.0	20.66	[19.31 - 22.11]
High-income	2,962	[2,930 - 2,998]	2.3	9.80	[9.69 - 9.91]
Latin America and Caribbean	6,690	[6,045 - 7,379]	5.2	28.15	[25.44 - 31.05]
North Africa and Middle East	9,472	[8,580 - 10,642]	7.3	34.60	[31.34 - 38.88]
South Asia	48,902	[44,407 - 53,786]	37.9	56.72	[51.50 - 62.38]
Southeast Asia, East Asia, and Oceania	14,297	[13,177 - 15,402]	11.1	22.97	[21.17 - 24.74]
Sub-Saharan Africa	44,290	[38,964 - 50,696]	34.3	64.65	[56.87 - 74.00]

Table S4: Number of deaths and all-cause mortality rate per 100,00 in 15-19 by sex in 2019

	Number of deaths			Rate per 100,000	
15 to 19	Estimate	Uncertainty Interval	Percent of global total	Estimate	Uncertainty Interval
Males					
Global	302,328	[279,932 - 325,494]		95.14	[88.09 - 102.43]
Central Europe, Eastern Europe, and Central Asia	9,150	[8,377 - 9,977]	3.0	76.75	[70.27 - 83.69]
High-income	15,636	[15,389 - 15,889]	5.2	48.44	[47.68 - 49.23]
Latin America and Caribbean	33,289	[29,635 - 37,364]	11.0	135.09	[120.26 - 151.62]
North Africa and Middle East	25,297	[22,255 - 28,643]	8.4	92.48	[81.36 - 104.72]
South Asia	78,767	[68,836 - 89,881]	26.1	85.69	[74.89 - 97.78]
Southeast Asia, East Asia, and Oceania	52,172	[46,911 - 57,685]	17.3	73.26	[65.87 - 81.00]
Sub-Saharan Africa	88,017	[77,466 - 98,969]	29.1	150.58	[132.53 - 169.32]
Females					
Global	196,977	[181,494 - 215,401]		65.28	[60.15 - 71.38]
Central Europe, Eastern Europe, and Central Asia	4,287	[4,004 - 4,600]	2.2	37.99	[35.48 - 40.76]
High-income	6,921	[6,859 - 6,989]	3.5	22.64	[22.44 - 22.87]
Latin America and Caribbean	11,489	[10,359 - 12,668]	5.8	47.96	[43.24 - 52.88]
North Africa and Middle East	13,137	[11,580 - 15,199]	6.7	51.32	[45.23 - 59.37]
South Asia	75,919	[68,799 - 83,687]	38.5	88.44	[80.14 - 97.48]
Southeast Asia, East Asia, and Oceania	22,732	[20,807 - 24,618]	11.5	35.02	[32.05 - 37.92]
Sub-Saharan Africa	62,491	[53,649 - 73,311]	31.7	104.86	[90.02 - 123.02]

Table S5: Number of deaths and all-cause mortality rate per 100,00 in 20-24 by sex in 2019

	Number of deaths			Rate per 100,000	
20 to 24	Estimate	Uncertainty Interval	Percent of global total	Estimate	Uncertainty Interval
Males					
Global	437,271	[408,762 - 465,081]		143.67	[134.30 - 152.80]
Central Europe, Eastern Europe, and Central Asia	18,120	[16,704 - 19,526]	4.1	147.96	[136.40 - 159.45]
High-income	28,800	[28,573 - 29,031]	6.6	86.02	[85.34 - 86.71]
Latin America and Caribbean	53,703	[49,195 - 58,710]	12.3	218.21	[199.89 - 238.55]
North Africa and Middle East	34,871	[30,822 - 39,910]	8.0	130.65	[115.48 - 149.53]
South Asia	119,765	[104,790 - 136,024]	27.4	139.47	[122.03 - 158.41]
Southeast Asia, East Asia, and Oceania	80,455	[72,303 - 88,906]	18.4	109.74	[98.62 - 121.27]
Sub-Saharan Africa	101,557	[90,061 - 114,073]	23.2	210.88	[187.01 - 236.87]
Females					
Global	255,141	[229,825 - 281,183]		86.26	[77.70 - 95.07]
Central Europe, Eastern Europe, and Central Asia	6,165	[5,611 - 6,842]	2.4	52.55	[47.82 - 58.32]
High-income	9,833	[9,738 - 9,934]	3.9	30.89	[30.59 - 31.21]
Latin America and Caribbean	15,197	[13,471 - 17,063]	6.0	61.93	[54.90 - 69.54]
North Africa and Middle East	16,253	[13,892 - 19,441]	6.4	64.91	[55.49 - 77.65]
South Asia	99,315	[83,110 - 116,886]	38.9	120.42	[100.77 - 141.72]
Southeast Asia, East Asia, and Oceania	31,266	[27,586 - 35,144]	12.3	45.32	[39.99 - 50.94]
Sub-Saharan Africa	77,112	[65,979 - 90,524]	30.2	150.67	[128.92 - 176.88]

Figure S4: Numbers of deaths in 10-14, 15-19 and 20-24 year olds by sex and GBD super-region between 1950 and 2019

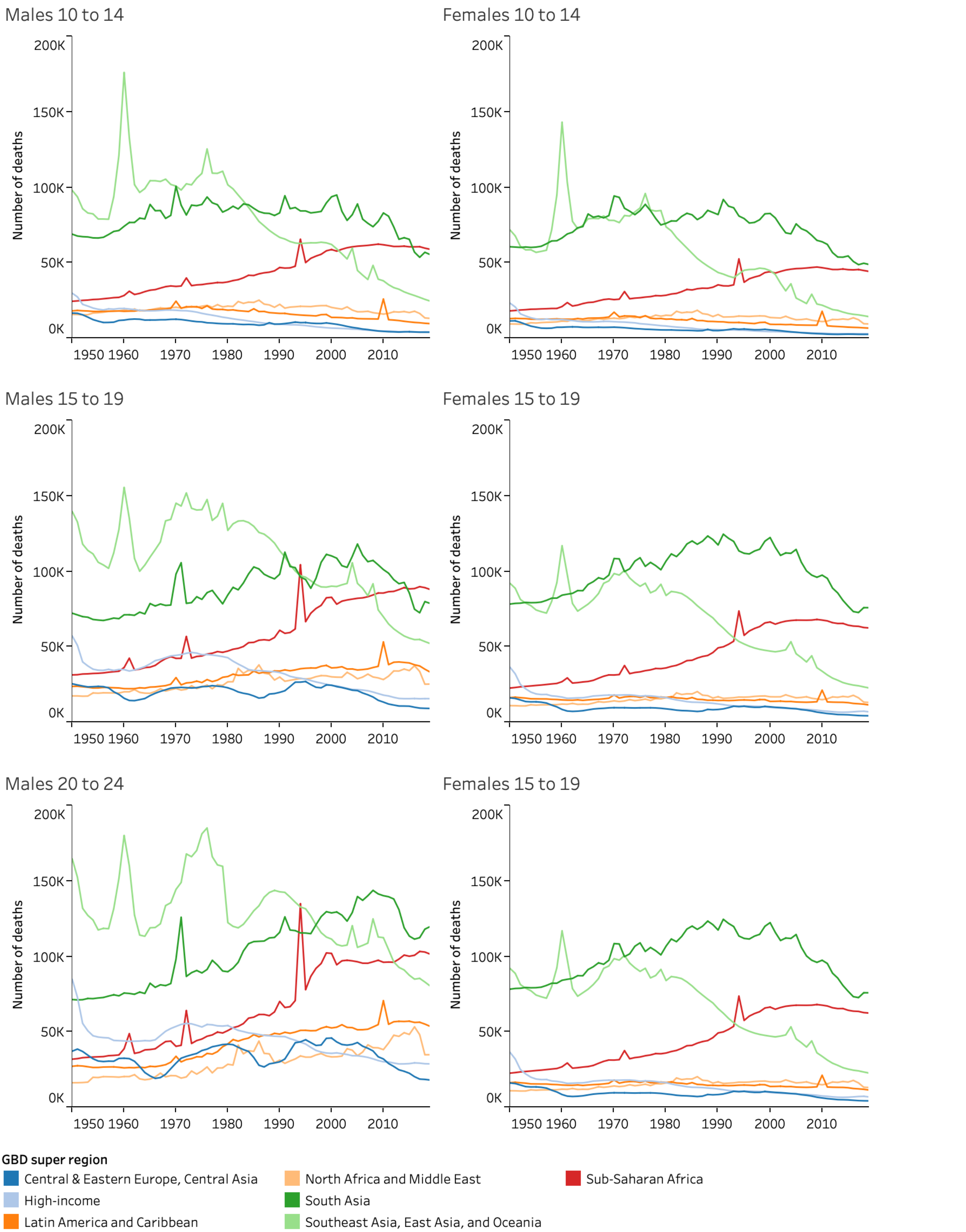

Figure S5: Proportion of deaths in 10-14, 15-19 and 20-24 occurring in each GBD super-region by sex and between 1950 and 2019

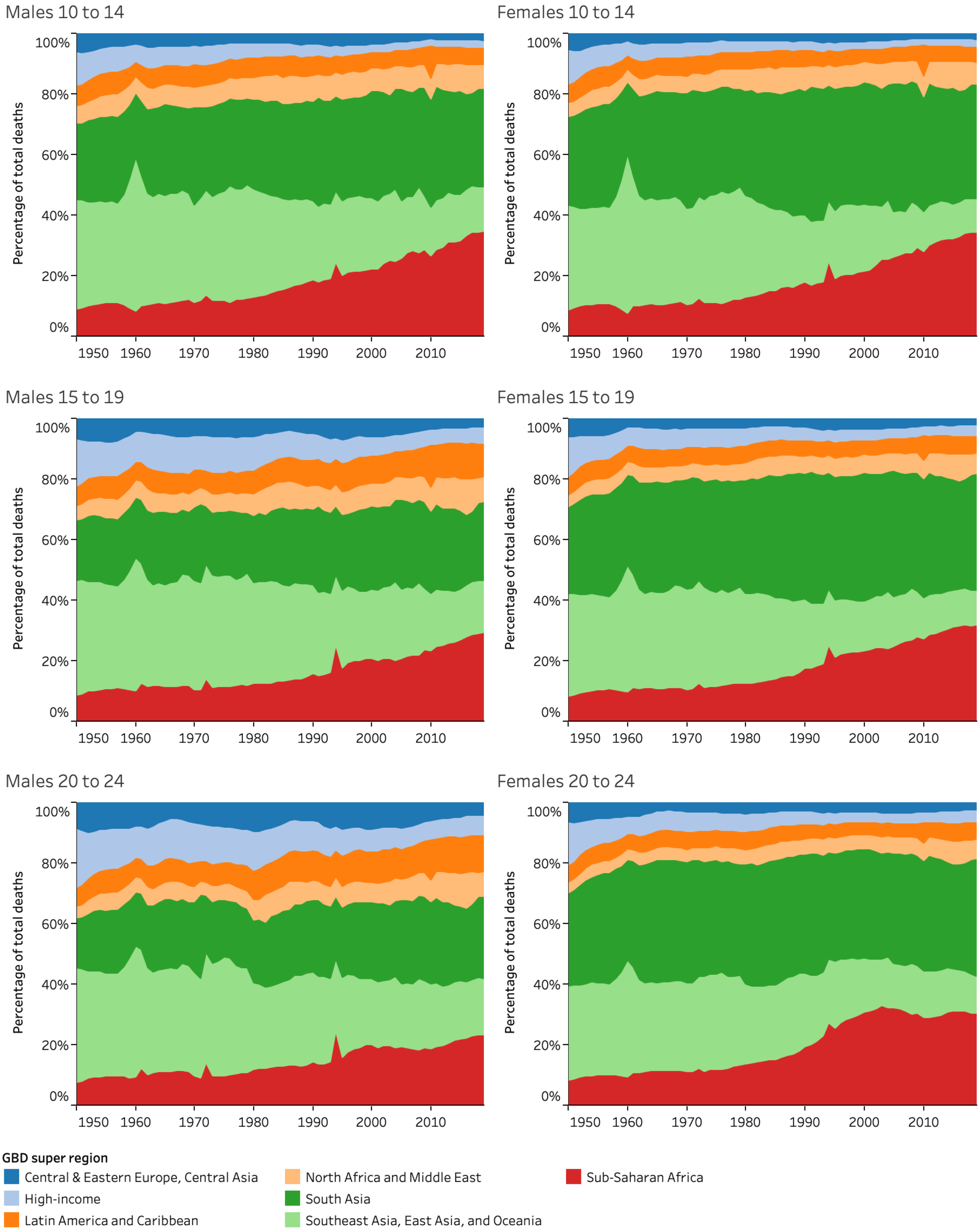

Figure S6: Mortality rate per 100,000 in 204 countries in 2019 in 10-14 olds by sex

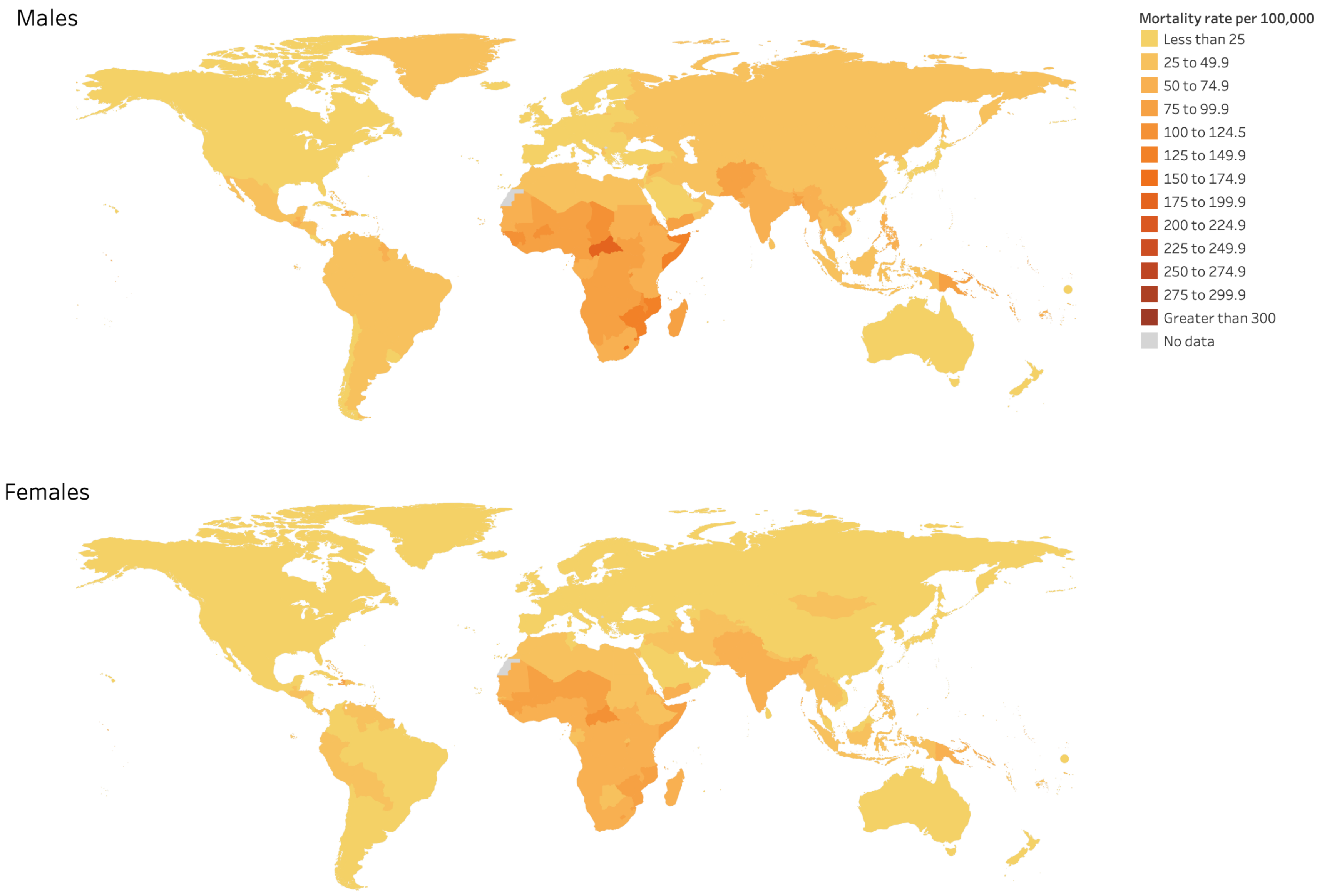

Figure S7: Mortality rate per 100,000 in 204 countries in 2019 in 15-19 olds by sex

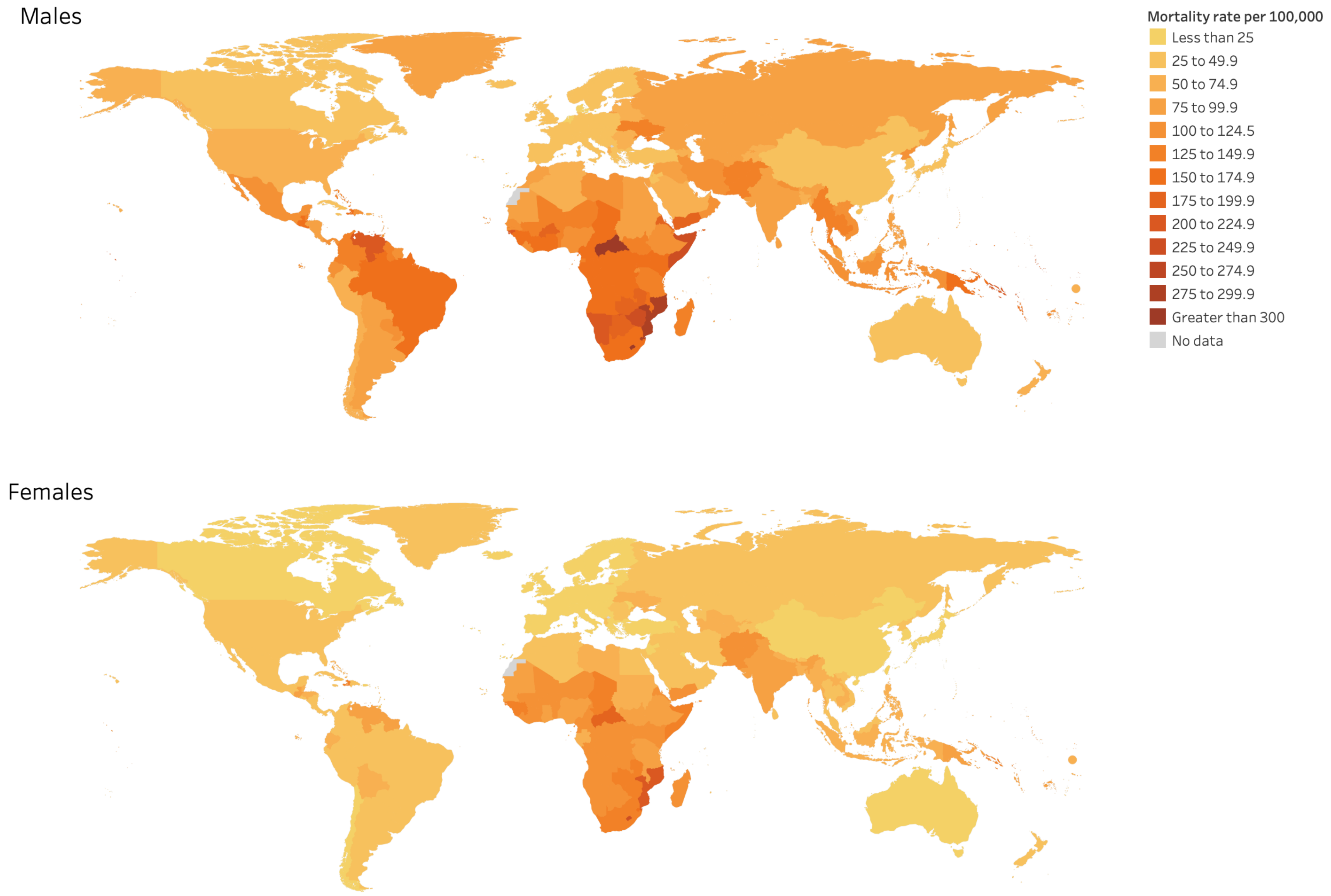

Figure S8: Mortality rate per 100,000 in 204 countries in 2019 in 20-24 olds by sex

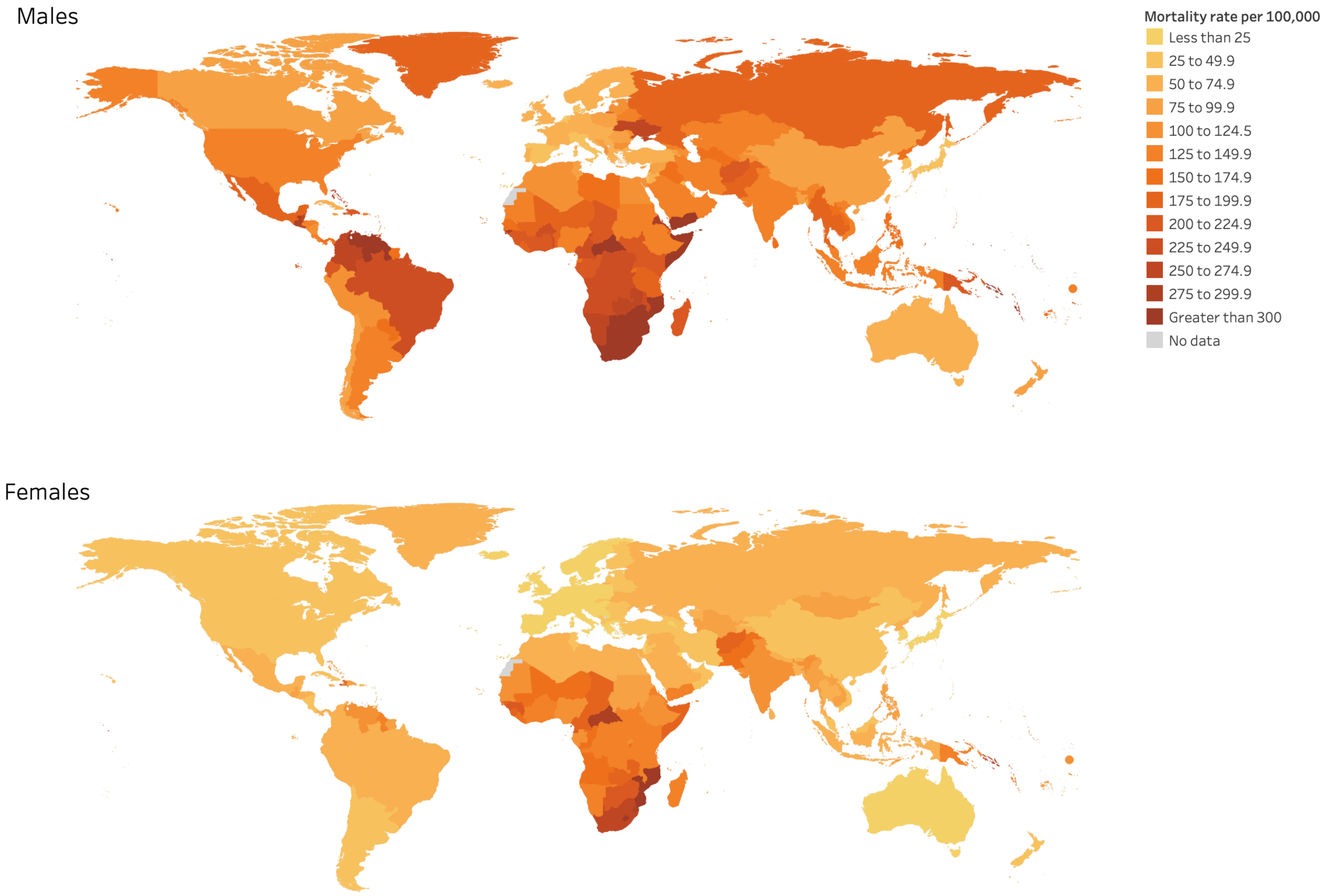

Figure S9: Global rank for mortality rate per 100,000 in 204 countries in 2019 in 10-14 olds by sex

Males

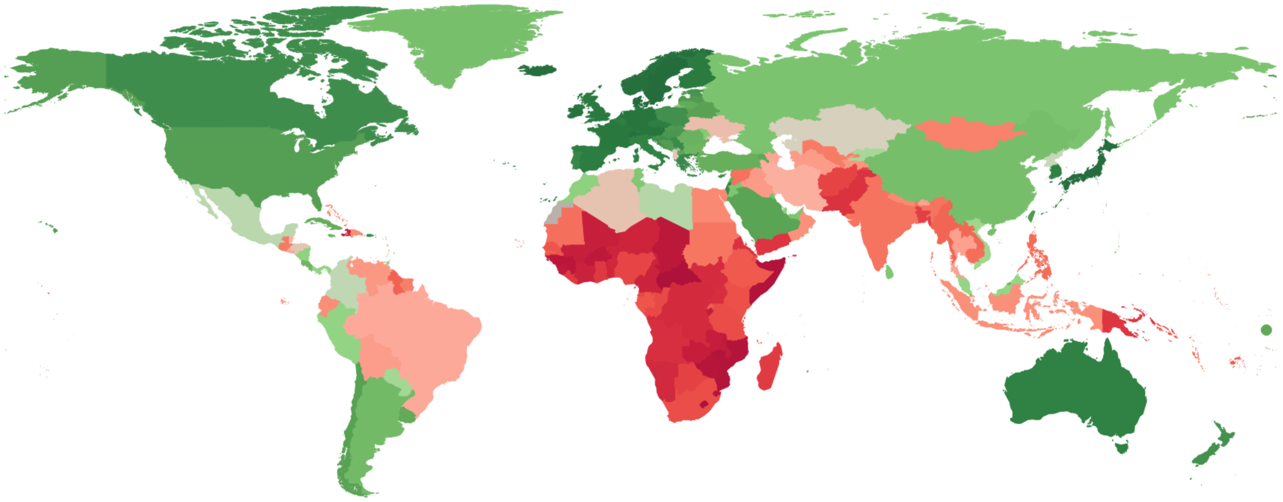

Females

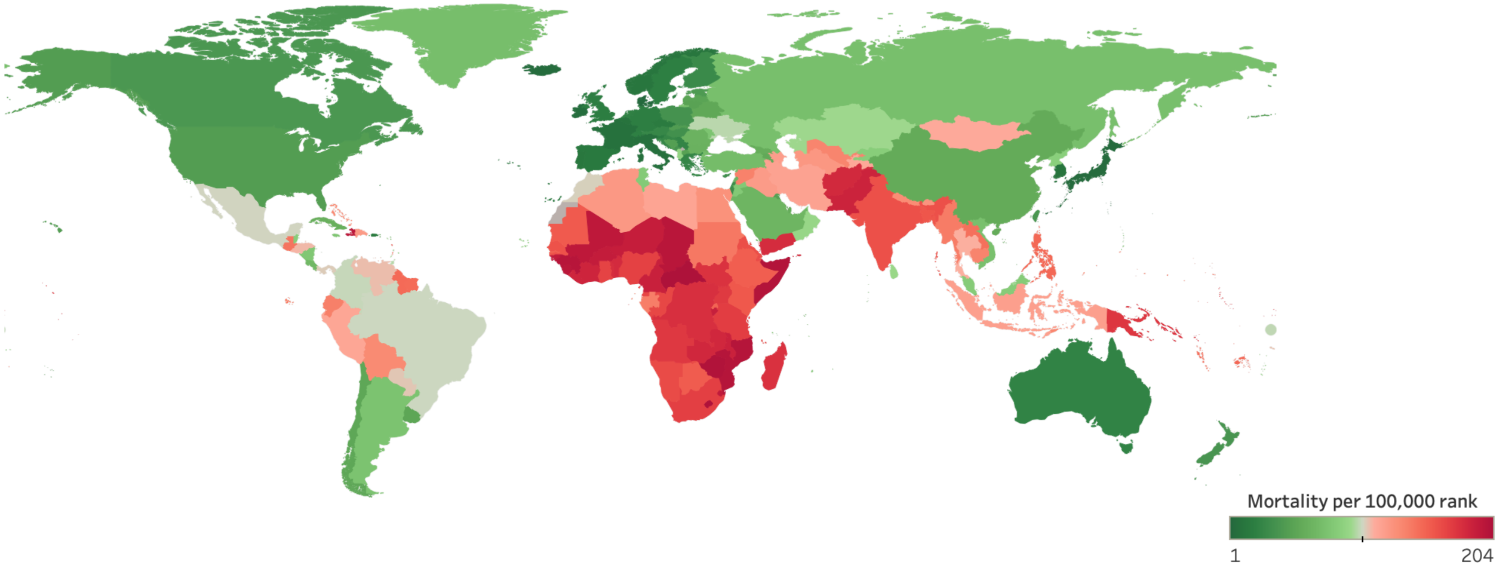

Figure S10: Global rank for mortality rate per 100,000 in 204 countries in 2019 in 15-19 olds by sex

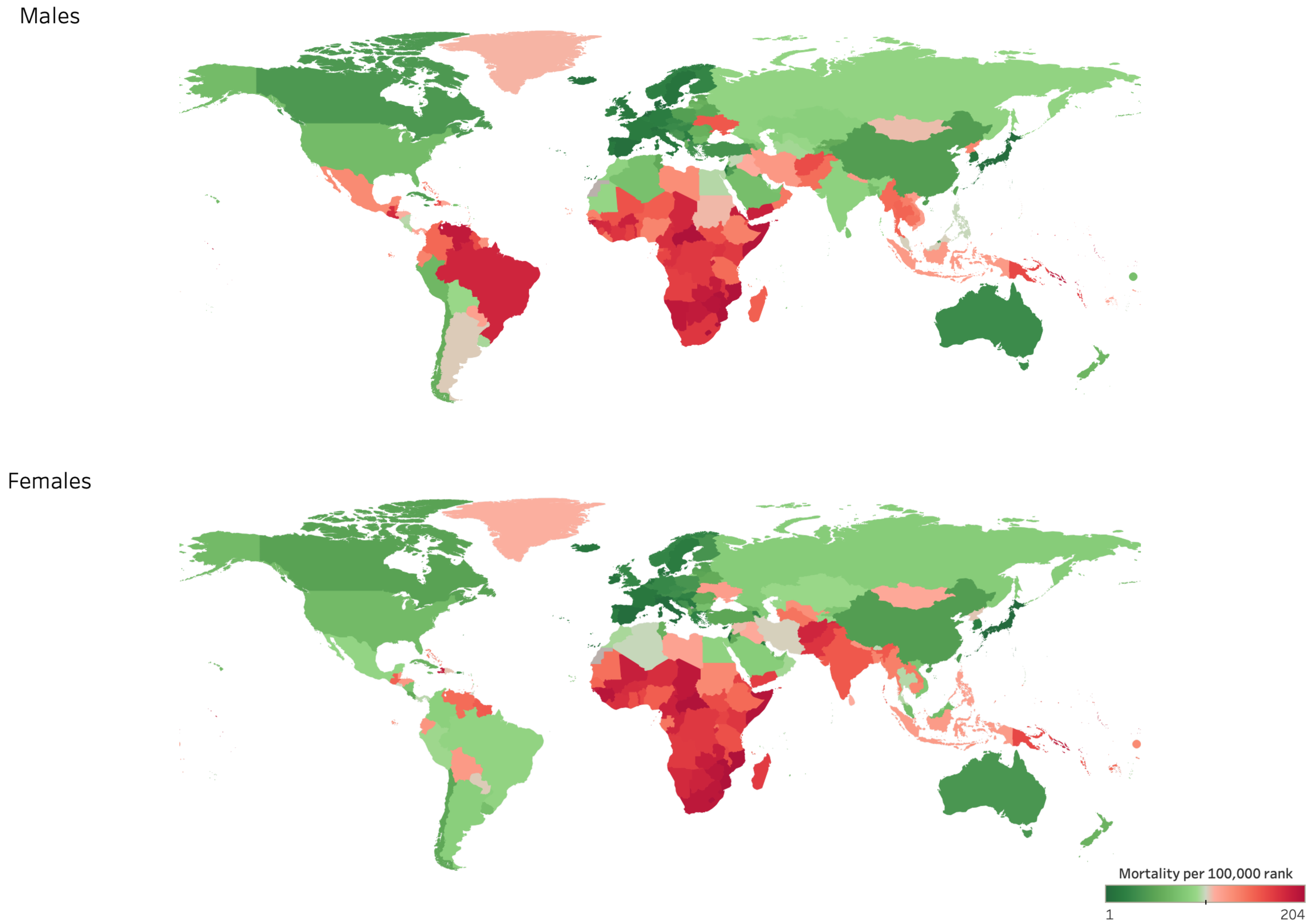

Figure S11: Global rank for mortality rate per 100,000 in 204 countries in 2019 in 20-24 olds by sex

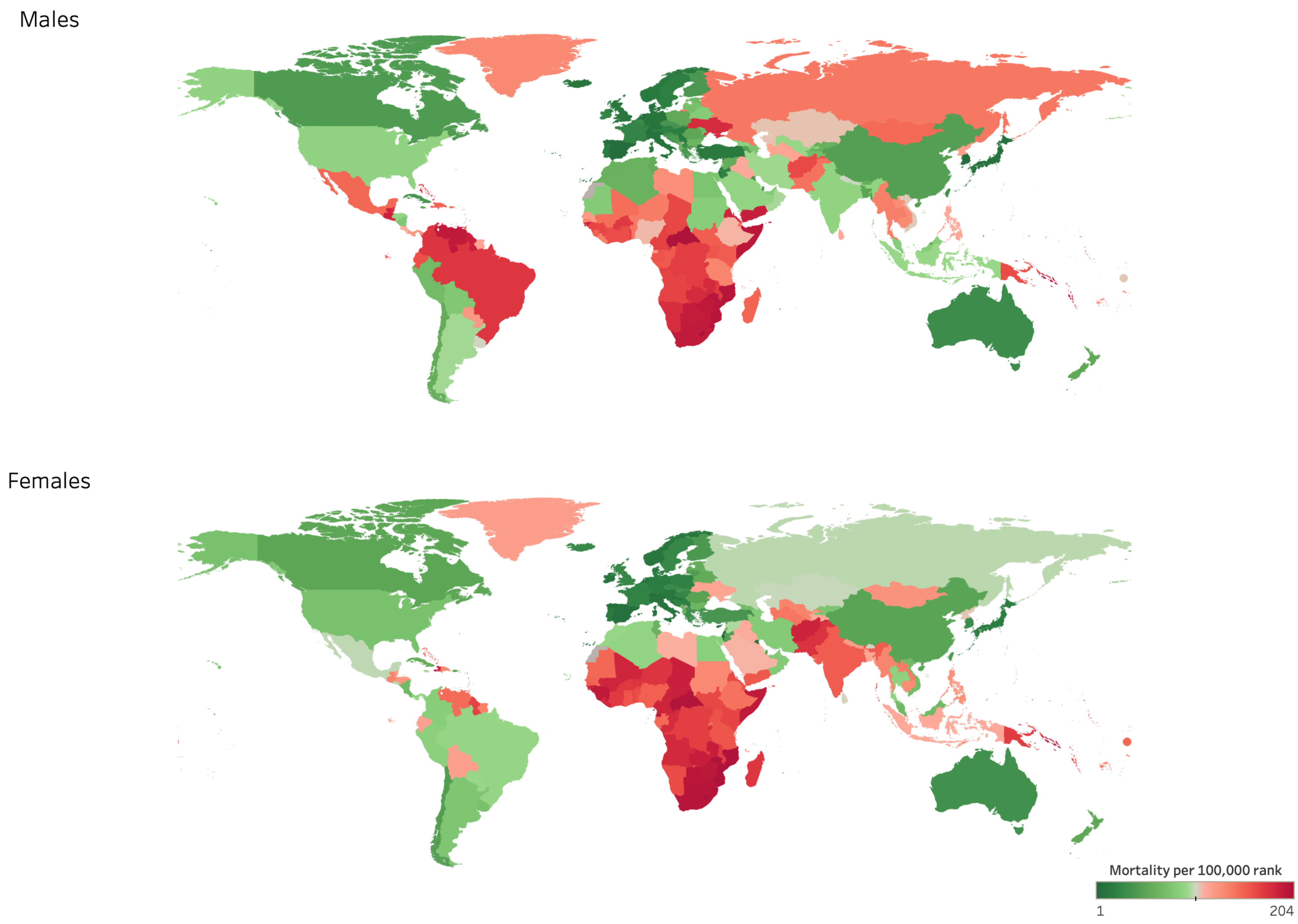

Table S6: Spearman's correlation coefficient (ρ) for association between country Sociodemographic Index (SDI) and all-cause mortality rate per 100,000 in 204 countries and territories by age group and sex in 1950, 1985 and 2019.

Age	Sex	Year	ρ
10 to 14	male	1950	-0.74
		1985	-0.89
		2019	-0.89
	female	1950	-0.73
		1985	-0.92
		2019	-0.89
	both	1950	-0.74
		1985	-0.91
		2019	-0.90
15 to 19	male	1950	-0.69
		1985	-0.75
		2019	-0.80
	female	1950	-0.67
		1985	-0.89
		2019	-0.84
	both	1950	-0.70
		1985	-0.83
		2019	-0.83
20 to 24	male	1950	-0.53
		1985	-0.67
		2019	-0.70
	female	1950	-0.63
		1985	-0.89
		2019	-0.83
	both	1950	-0.60
		1985	-0.79
		2019	-0.77
10 to 24	male	1950	-0.65
		1985	-0.75
		2019	-0.77
	female	1950	-0.66
		1985	-0.90
		2019	-0.85
	both	1950	-0.67
		1985	-0.83
		2019	-0.82

Figure S12: Proportion of deaths in 0-24 occurring in 10-24 year olds by GBD super-region 1950 and 2019

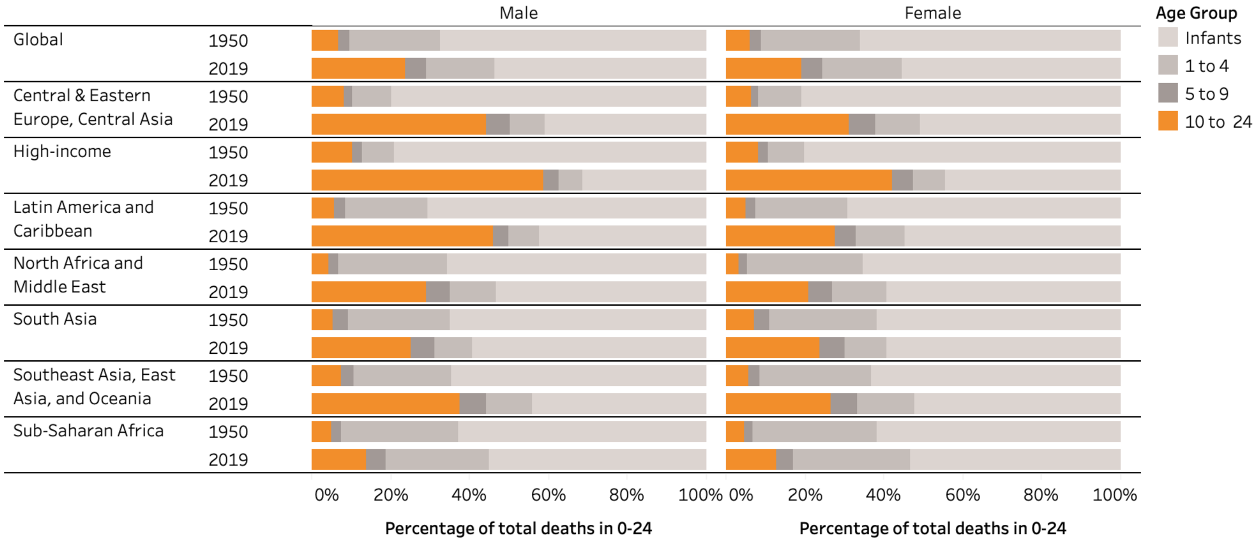

Figure S13: Proportion of deaths in 0-24 occurring in 10-24 year olds in Central and Eastern Europe, Central Asia GBD super-region in 2019

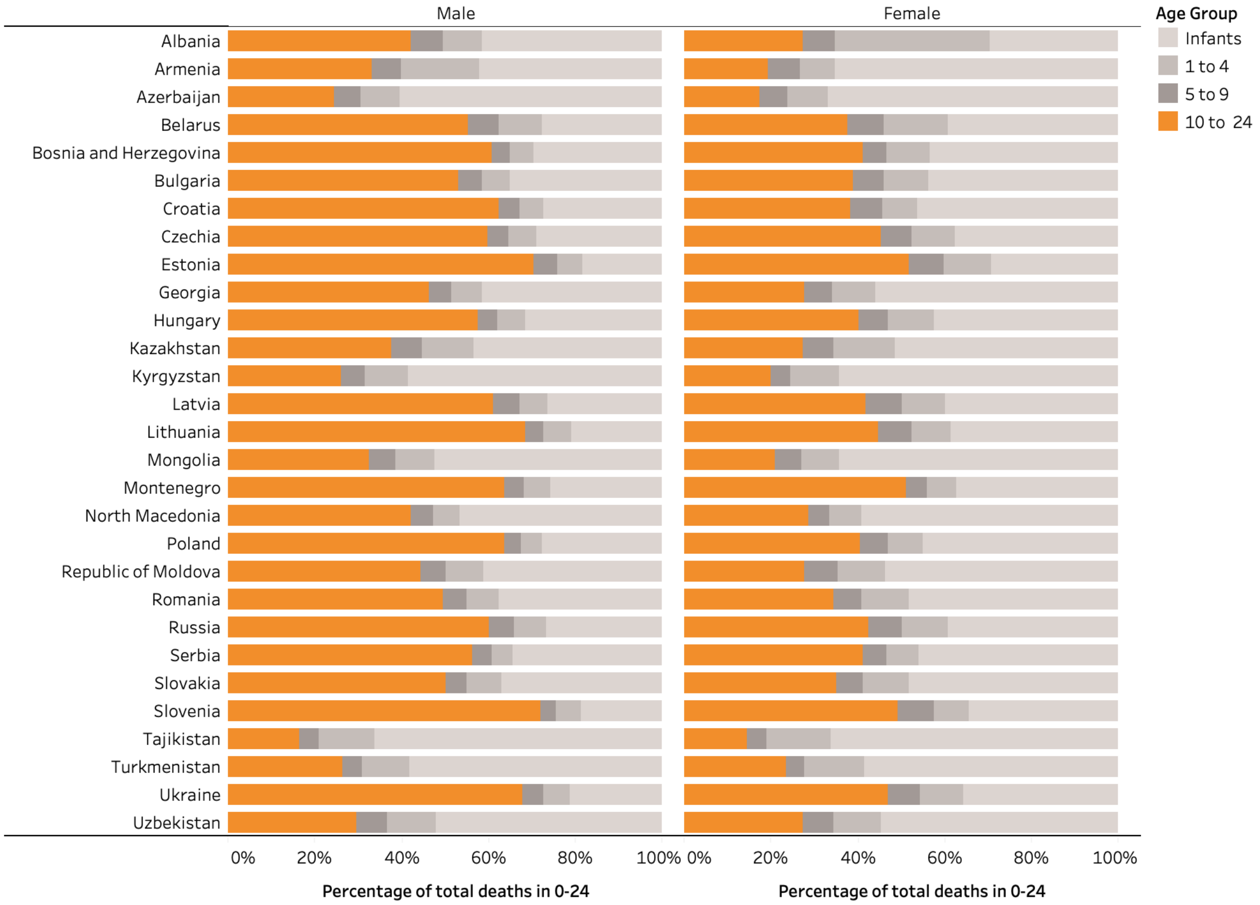

Figure S14: Proportion of deaths in 0-24 occurring in 10-24 year olds in High Income GBD super-region in 2019

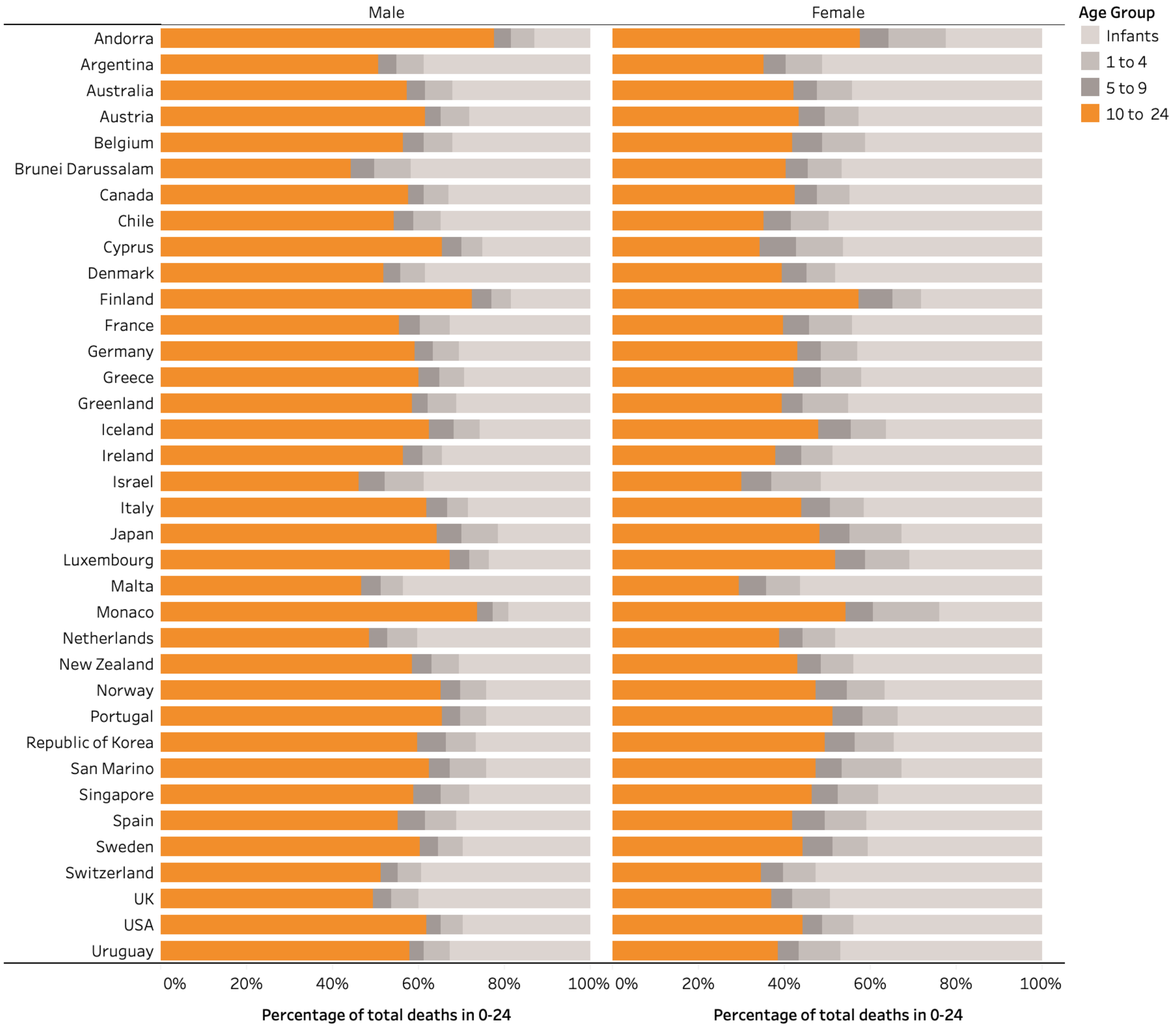

Figure S15: Proportion of deaths in 0-24 occurring in 10-24 year olds in Latin America and Caribbean GBD super-region in 2019

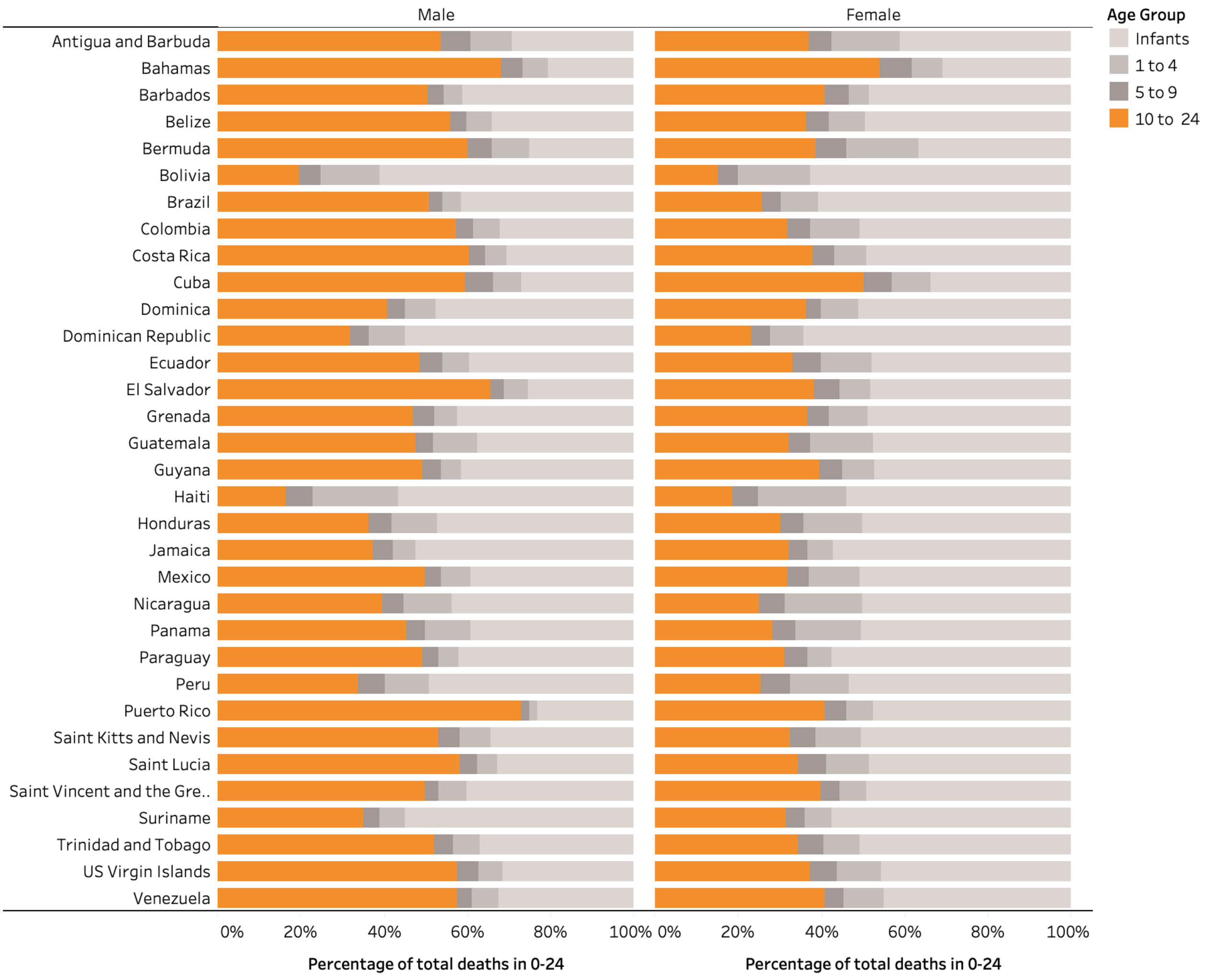

Figure S16: Proportion of deaths in 0-24 occurring in 10-24 year olds in North Africa and Middle East GBD super-region in 2019

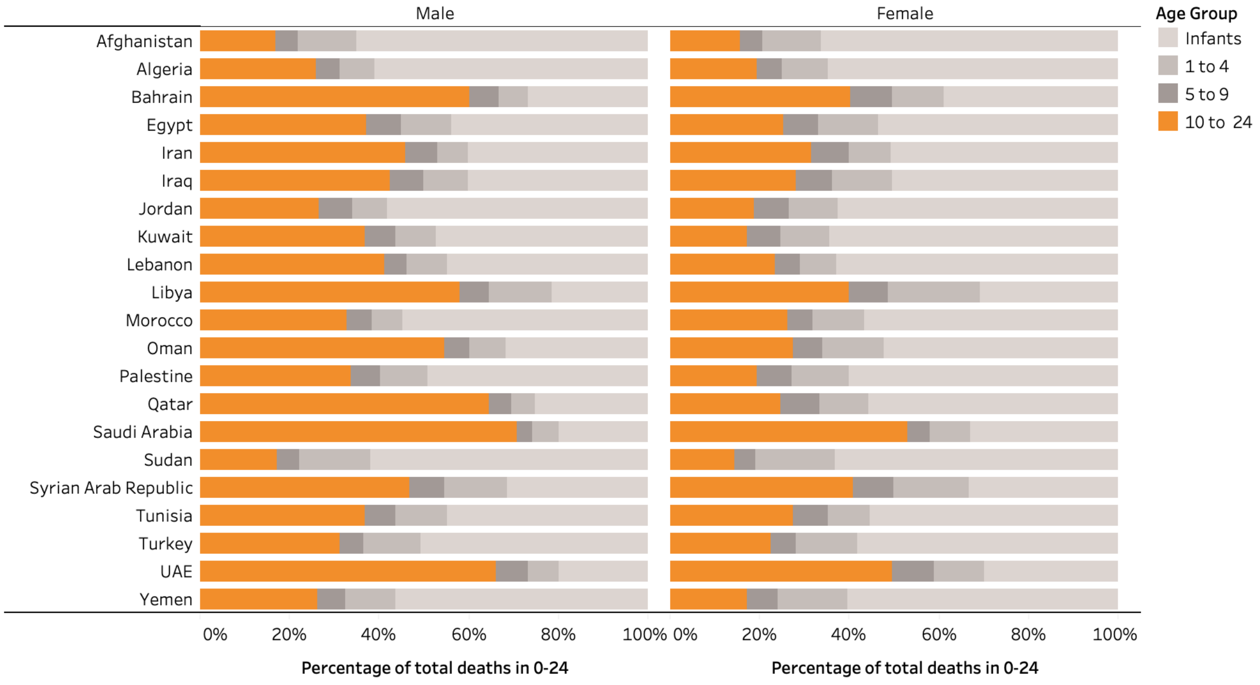

Figure S17: Proportion of deaths in 0-24 occurring in 10-24 year olds in South Asia GBD super-region in 2019

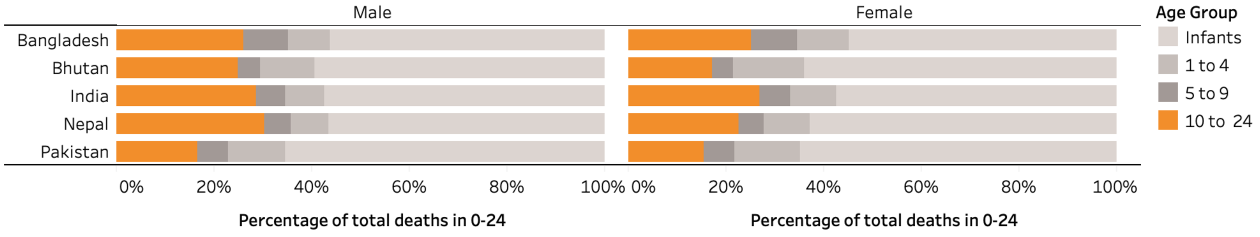

Figure S18: Proportion of deaths in 0-24 occurring in 10-24 year olds in Southeast Asia, East Asia, and Oceania GBD super-region in 2019

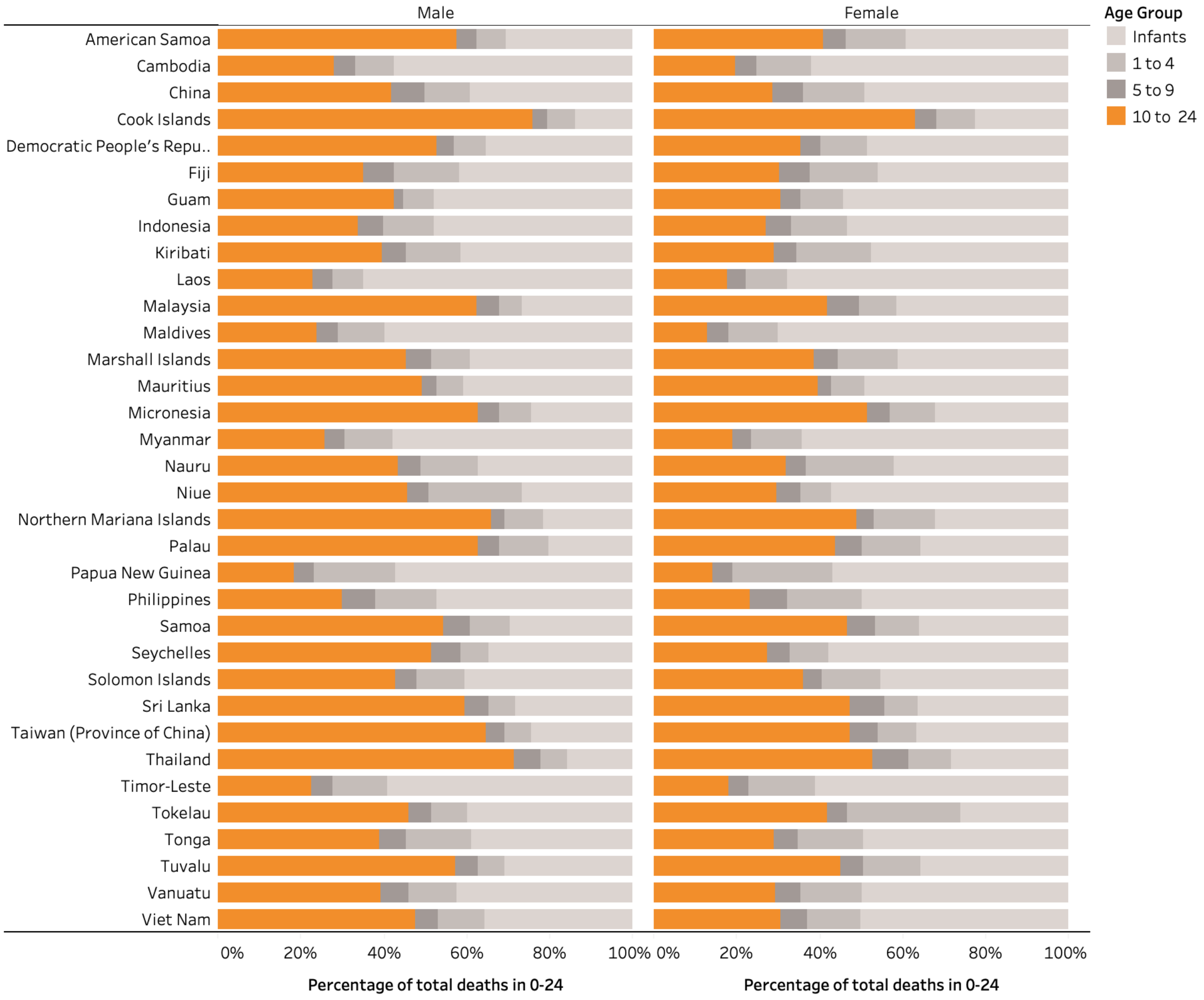

Figure S19: Proportion of deaths in 0-24 occurring in 10-24 year olds in Sub-Saharan Africa GBD super-region in 2019

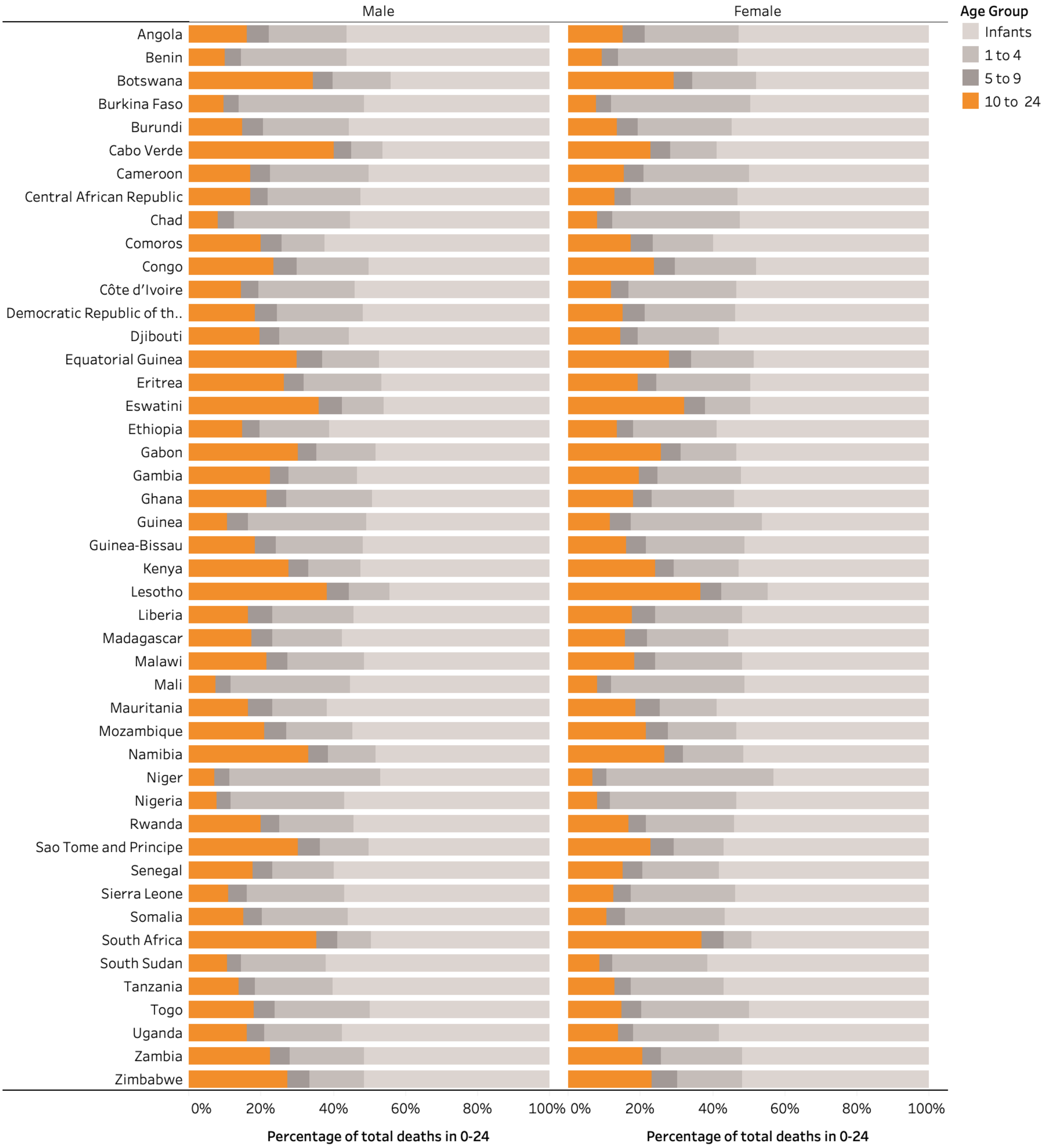

Figure S20: Ratio of male to female all-cause mortality rate per 100 000 by GBD super-region 1950 – 2019

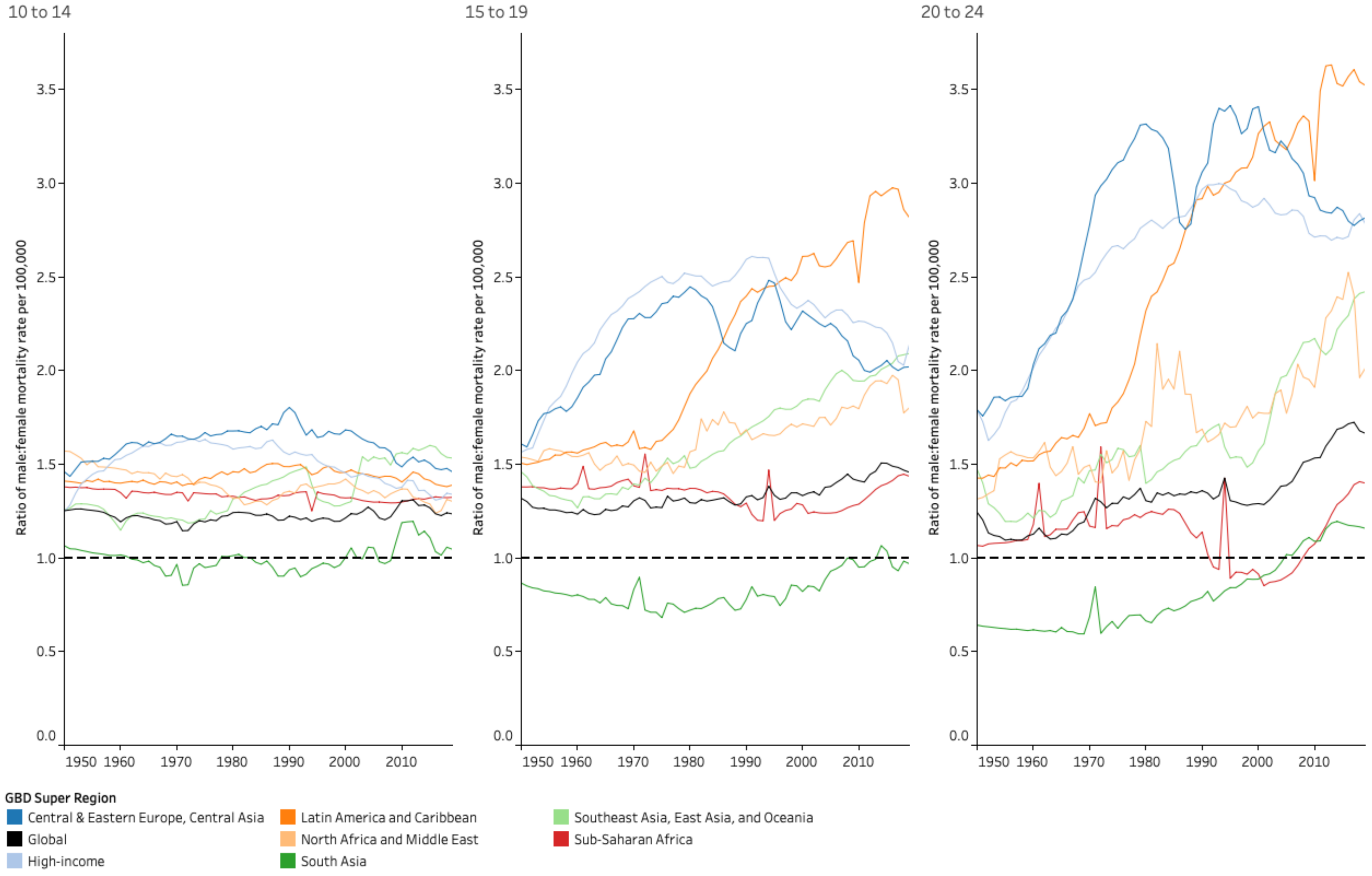

Figure S21: All-cause mortality in 2019 and annual percentage change in population 1990 – 2019 in 10-14 year olds by sex

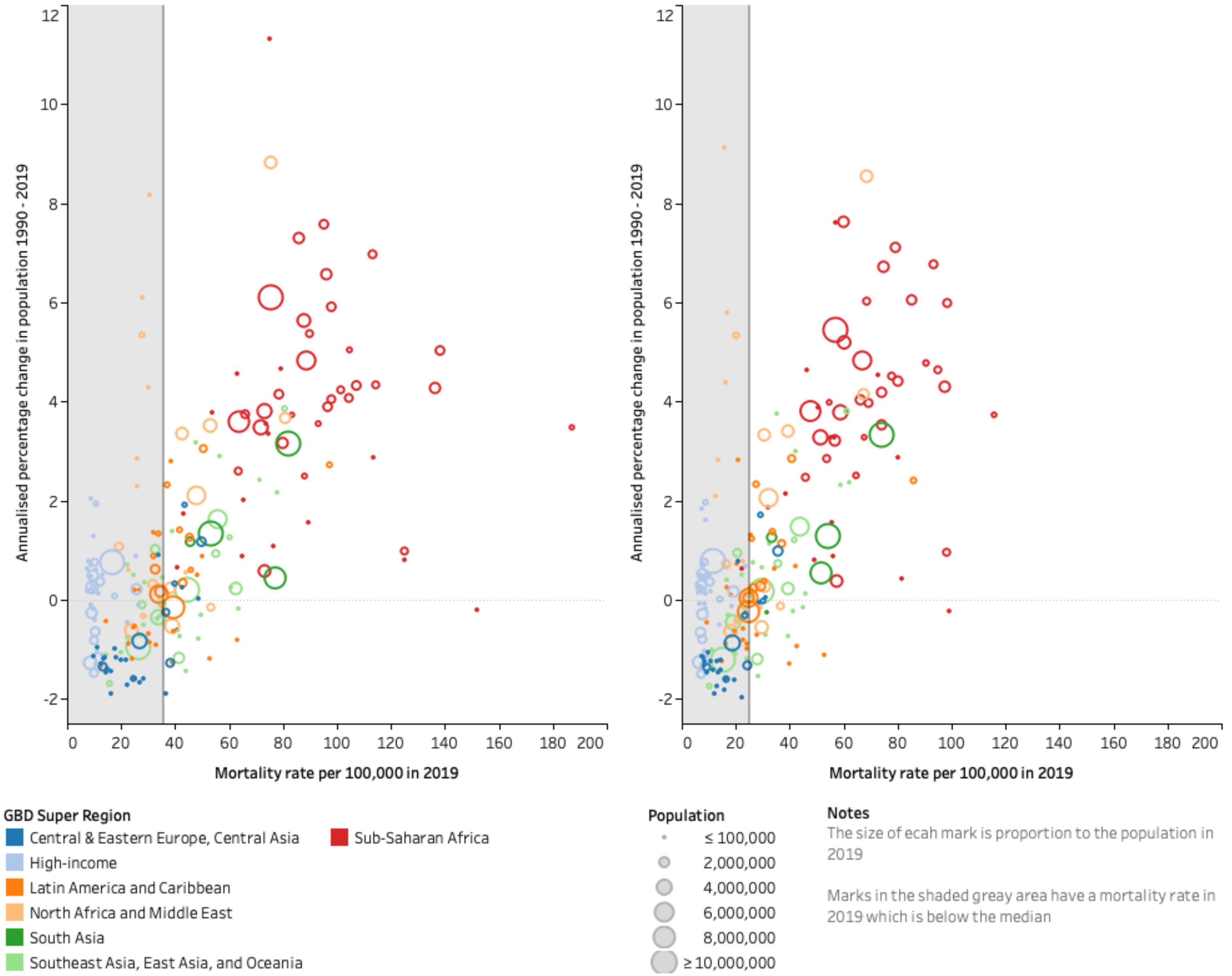

Figure S22: All-cause mortality in 2019 and annual percentage change in population 1990 – 2019 in 15-19 year olds by sex

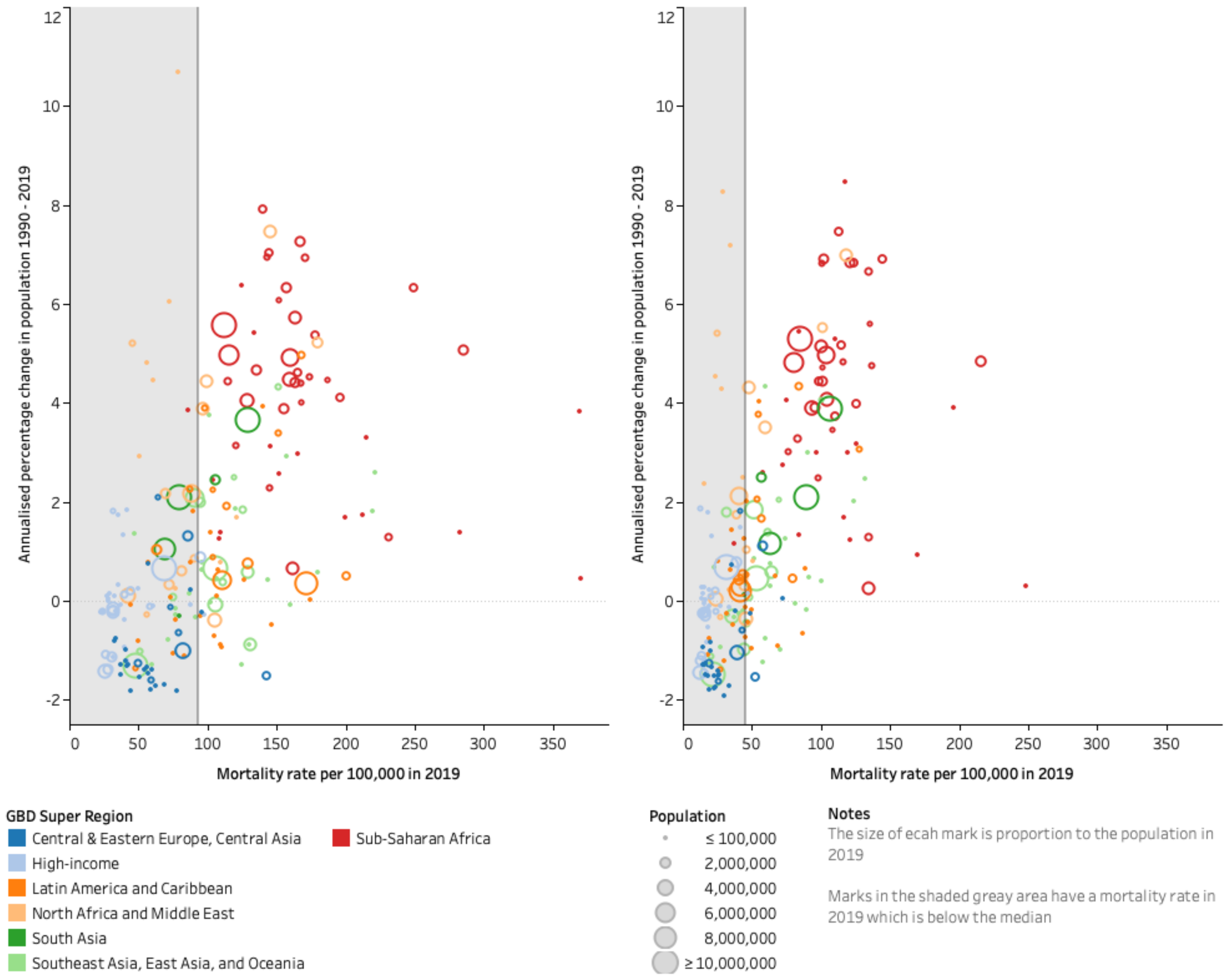

Figure S23: All-cause mortality in 2019 and annual percentage change in population 1990 – 2019 in 20-24 year olds by sex

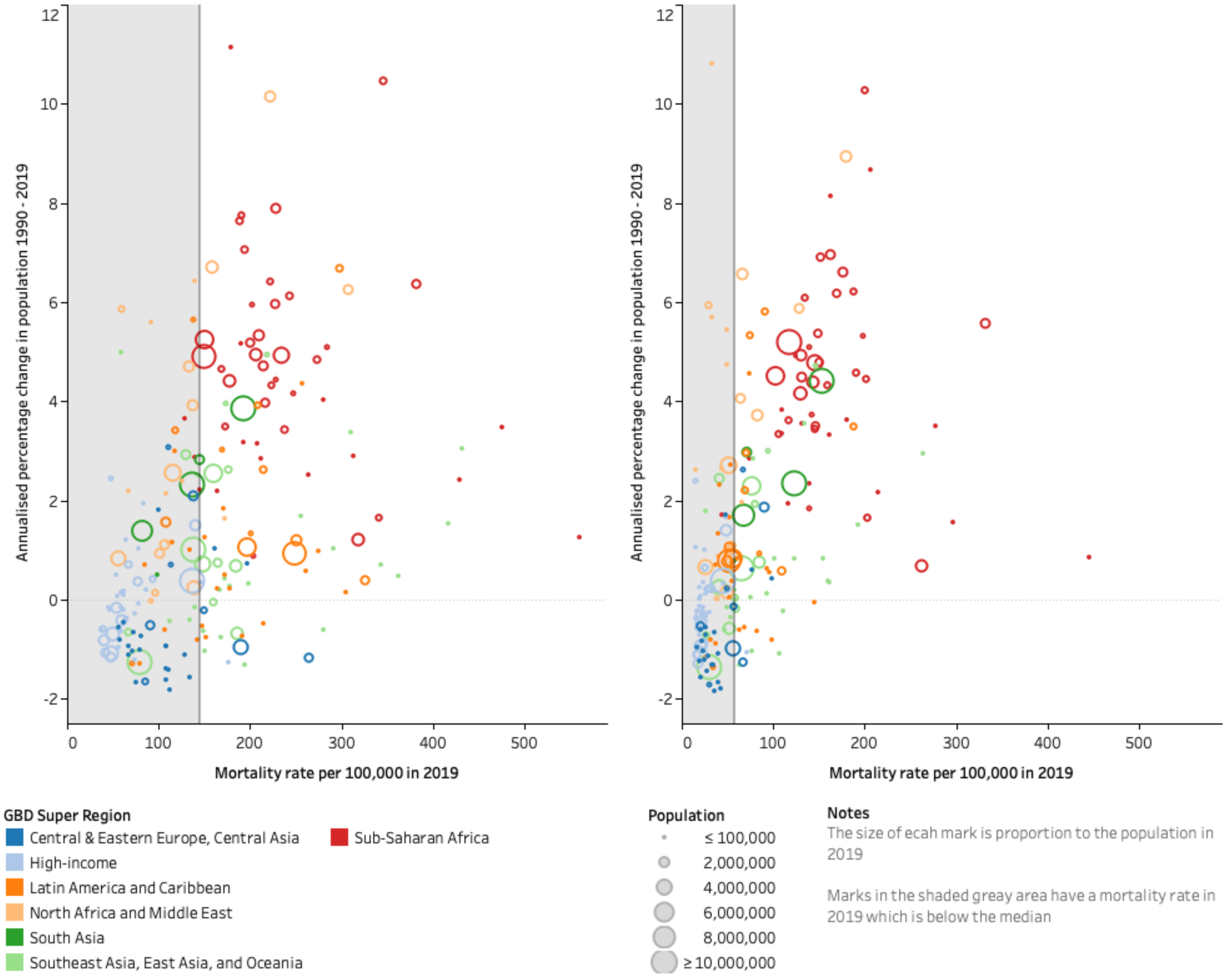

Figure S24: Distribution of annual percentage change in all-cause mortality rate per 100 000 in 204 countries 1990 – 2019 in 1-4 and 15-19 year olds by sex

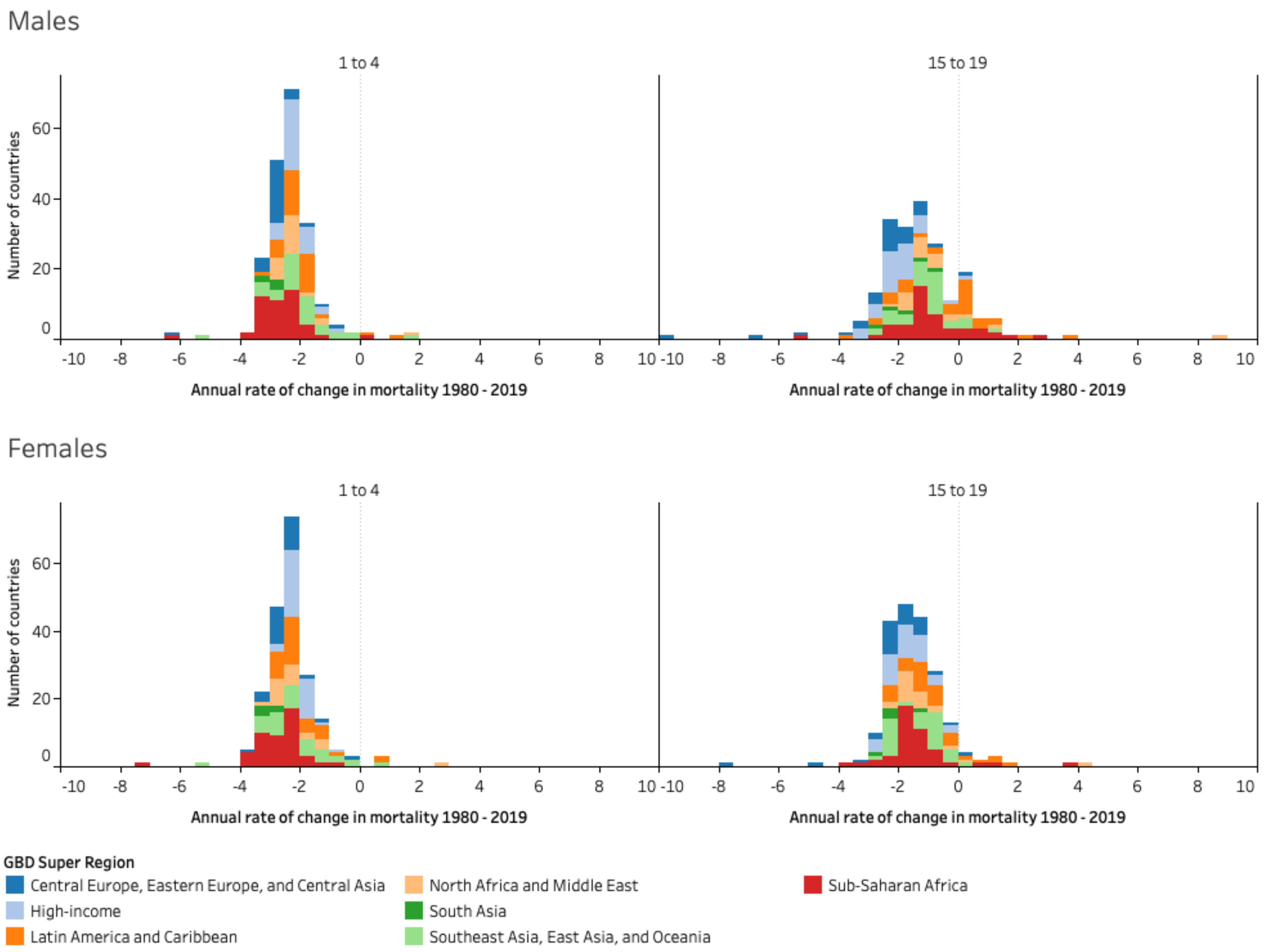

Rwanda (15-19) and Bonsia & Herzegovina (1-4 females) are exlcuded as range in rate of change restricted to 10% annual decrease in mortality rate

Figure S25: All-cause mortality rate per 100,000 percentile for 0-5 against 15-19 year old females in 204 countries by GBD super-region in 2019

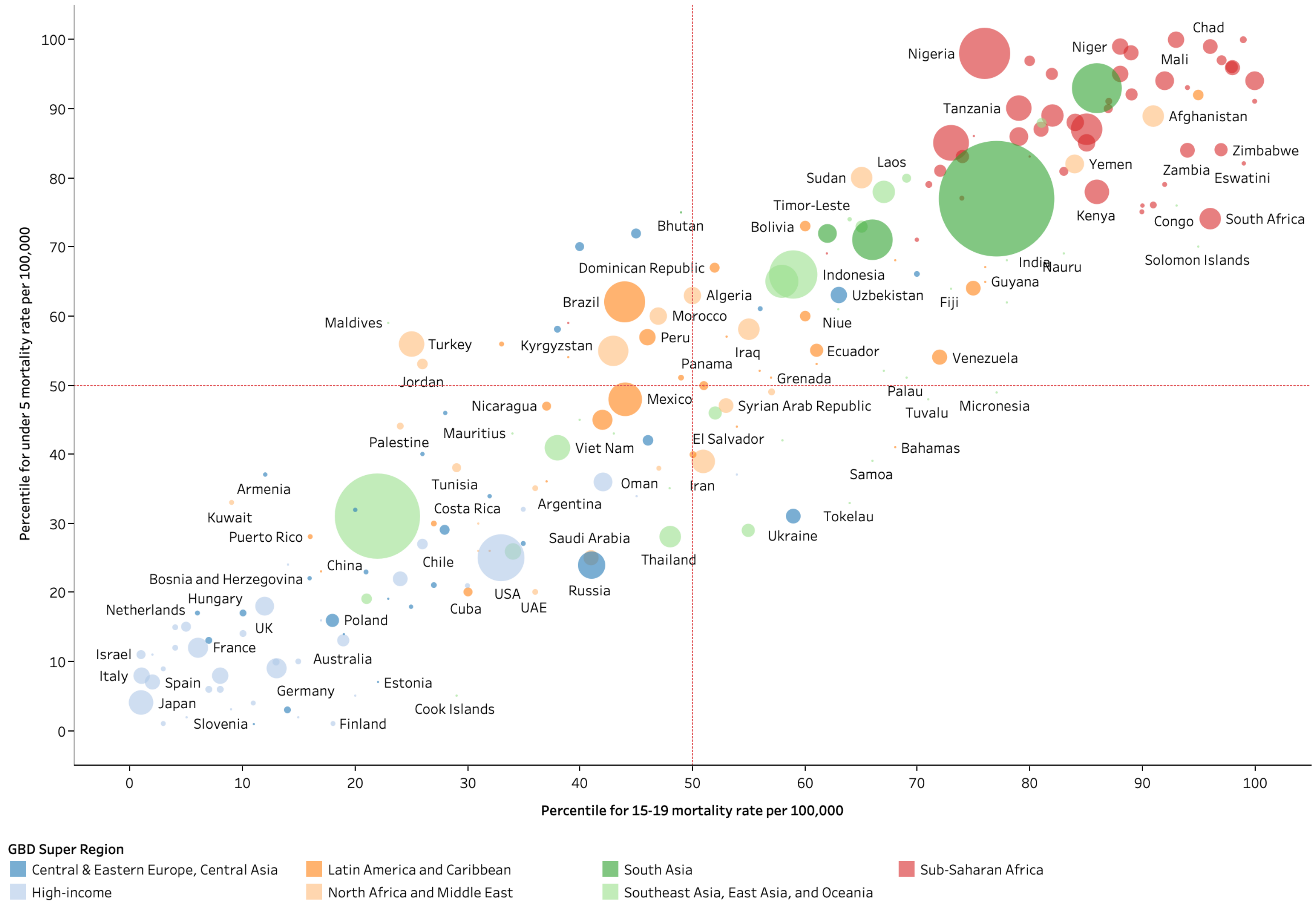

Notes: The size of each mark is proportion to population aged 15 to 19. Due to space constraints, not all countries are labelled.

Figure S26: Ratio of observed : expected all-cause mortality rate per 100,000 in 2017 amongst 10-14 year olds (both sexes)

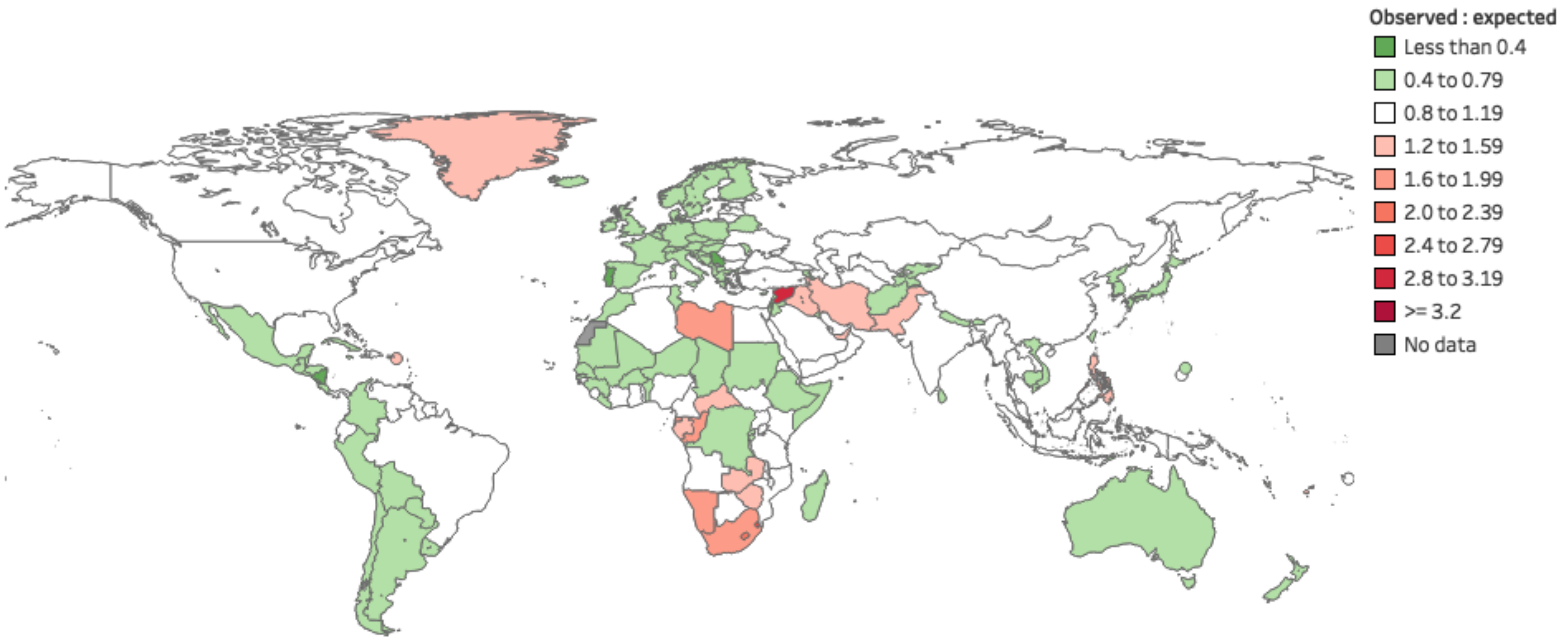

Figure S27: Ratio of observed : expected all-cause mortality rate per 100,000 in 2017 amongst 15-19 year olds (both sexes)

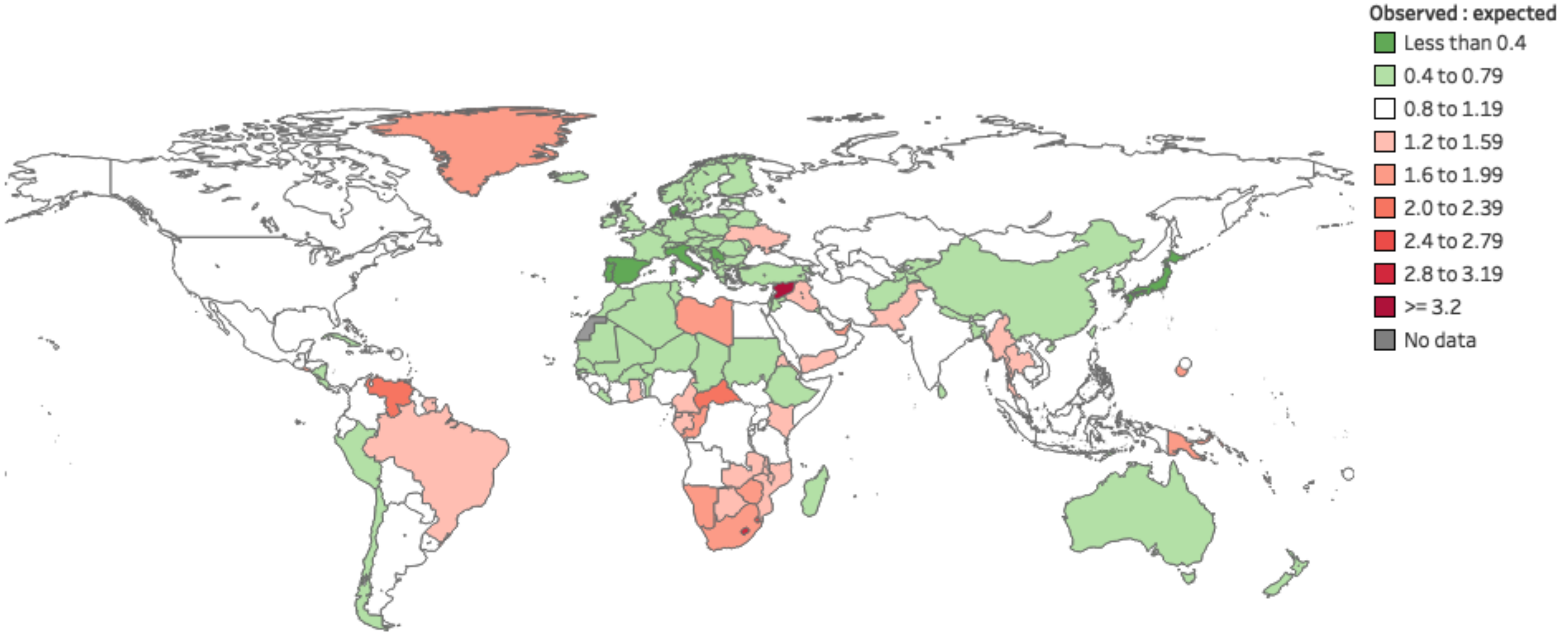

Figure S28: Ratio of observed : expected all-cause mortality rate per 100,000 in 2017 amongst 20-24 year olds (both sexes)

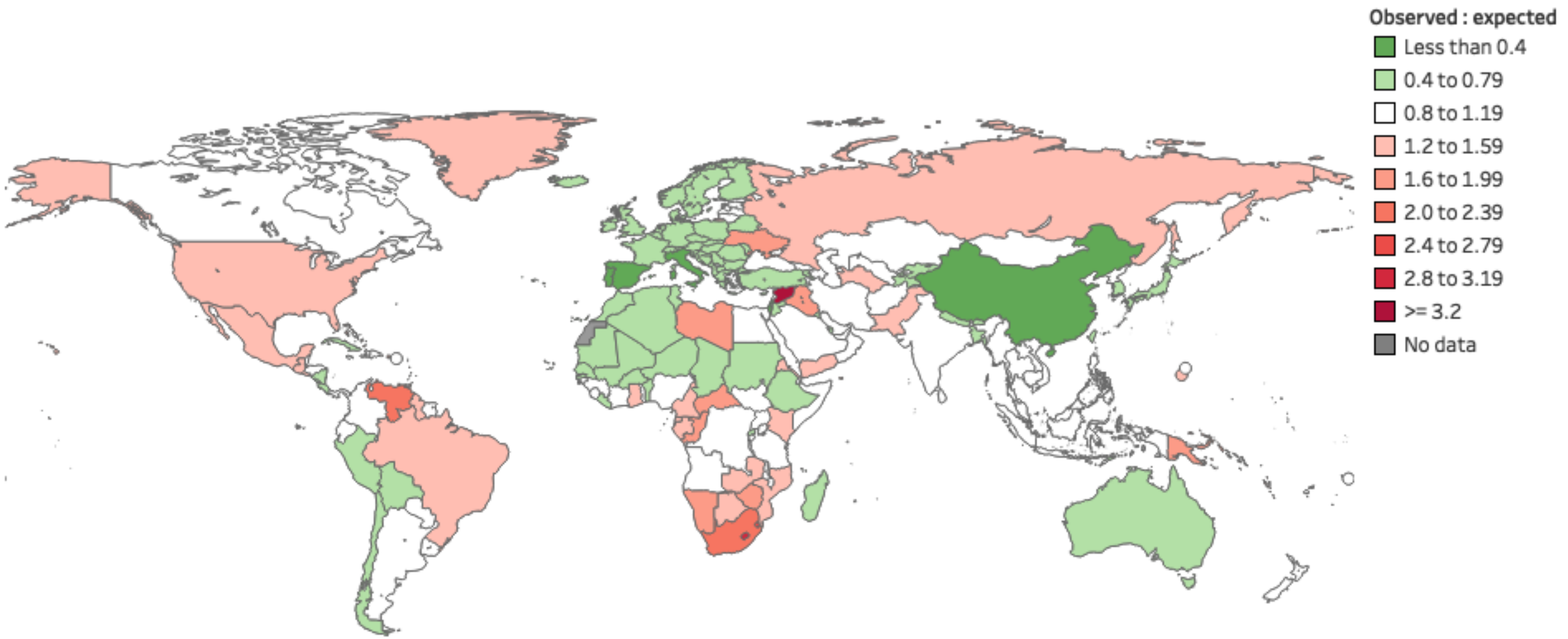

Figure S29: Ratio of observed : expected all-cause mortality rate per 100,000, 1990 - 2017 amongst 10-14 year olds (both sexes) in Central and Eastern Europe, Central Asia GBD super-region

GBD region	Country	1990	1995	2000	2005	2010	2017
Central Asia	Armenia	0.64	0.55	0.45	0.44	0.70	0.58
	Azerbaijan	1.05	1.26	1.11	1.14	1.11	1.14
	Georgia	1.02	0.86	0.51	0.44	0.69	0.81
	Kazakhstan	1.14	1.29	1.28	1.41	1.15	1.11
	Kyrgyzstan	1.09	0.98	0.89	0.86	0.61	0.75
	Mongolia	0.93	1.46	1.12	1.17	1.20	1.20
	Tajikistan	0.75	0.93	0.68	0.63	0.66	0.75
	Turkmenistan	1.06	1.21	1.18	1.20	1.10	1.13
	Uzbekistan	0.74	0.87	0.98	0.97	0.91	0.96
Central Europe	Albania	0.75	0.74	0.82	0.88	0.81	0.74
	Bosnia and Herzegovina	0.30	2.16	0.37	0.42	0.37	0.47
	Bulgaria	0.99	1.09	0.91	1.10	1.10	0.80
	Croatia	0.72	1.14	0.72	0.60	0.56	0.50
	Czech Republic	0.73	0.89	0.87	0.85	0.76	0.55
	Hungary	0.73	0.78	0.81	0.67	0.66	0.61
	Macedonia	0.66	0.60	0.59	0.61	0.46	0.57
	Montenegro	0.74	0.72	0.72	0.68	0.67	0.77
	Poland	0.71	0.71	0.72	0.77	0.75	0.62
	Romania	1.12	1.17	1.70	1.12	1.00	0.97
	Serbia	0.71	0.65	0.57	0.53	0.37	0.38
	Slovakia	0.73	0.83	0.94	0.68	0.95	0.67
	Slovenia	0.81	0.83	0.65	0.74	0.44	0.73
Eastern Europe	Belarus	0.89	0.80	0.83	0.76	0.88	0.64
	Estonia	1.48	1.59	1.14	0.87	1.00	0.83
	Latvia	1.59	1.51	1.12	1.17	1.17	0.87
	Lithuania	1.17	1.18	1.05	1.02	1.04	1.06
	Moldova	0.92	0.97	0.83	0.79	0.80	0.68
	Russian Federation	1.29	1.71	1.56	1.57	1.41	1.15
	Ukraine	1.13	1.17	1.02	0.80	0.71	1.15

Observed : expected
- Less than 0.4
- 0.4 to 0.79
- 0.8 to 1.19
- 1.2 to 1.59
- 1.6 to 1.99
- 2.0 to 2.39
- 2.4 to 2.79
- 2.8 to 3.19
- >= 3.2
- No data

Figure S30: Ratio of observed : expected all-cause mortality rate per 100,000, 1990 - 2017 amongst 15-19 year olds (both sexes) in Central and Eastern Europe, Central Asia GBD super-region

GBD region	Country	1990	1995	2000	2005	2010	2017
Central Asia	Armenia	0.62	1.05	0.51	0.47	0.69	0.56
	Azerbaijan	0.99	1.02	0.82	0.74	0.79	0.89
	Georgia	0.85	0.81	0.53	0.52	0.71	0.82
	Kazakhstan	1.15	1.45	1.38	1.47	1.16	0.96
	Kyrgyzstan	1.03	0.99	0.87	0.90	0.71	0.62
	Mongolia	1.08	1.38	0.98	1.01	0.94	0.94
	Tajikistan	0.68	0.94	0.69	0.66	0.71	0.77
	Turkmenistan	1.03	1.28	1.14	1.27	1.02	1.10
	Uzbekistan	0.68	0.77	0.89	0.89	0.79	0.84
Central Europe	Albania	0.64	0.64	0.71	0.64	0.58	0.59
	Bosnia and Herzegovina	0.34	3.30	0.44	0.45	0.41	0.52
	Bulgaria	0.92	0.98	0.90	0.73	0.86	0.78
	Croatia	0.86	1.30	0.84	0.80	0.61	0.52
	Czech Republic	0.86	1.01	0.88	0.77	0.70	0.54
	Hungary	0.95	0.76	0.66	0.69	0.59	0.51
	Macedonia	0.63	0.54	0.58	0.52	0.52	0.62
	Montenegro	0.57	0.55	0.74	0.63	0.45	0.48
	Poland	0.88	0.89	0.82	0.79	0.81	0.67
	Romania	0.90	0.87	0.85	0.90	0.84	0.71
	Serbia	0.77	0.78	0.68	0.59	0.45	0.38
	Slovakia	0.83	0.81	0.73	0.72	0.65	0.59
	Slovenia	0.94	1.14	0.98	0.79	0.53	0.61
Eastern Europe	Belarus	1.01	1.18	1.07	1.00	0.98	0.65
	Estonia	1.72	1.75	1.26	1.20	0.83	0.78
	Latvia	1.70	1.67	1.29	1.27	0.92	0.83
	Lithuania	1.39	1.48	1.32	1.36	1.18	0.80
	Moldova	1.00	1.03	0.83	0.85	0.86	0.67
	Russian Federation	1.51	2.35	2.18	1.92	1.59	1.13
	Ukraine	1.16	1.45	1.18	1.00	0.78	1.50

Observed : expected
- Less than 0.4
- 0.4 to 0.79
- 0.8 to 1.19
- 1.2 to 1.59
- 1.6 to 1.99
- 2.0 to 2.39
- 2.4 to 2.79
- 2.8 to 3.19
- >= 3.2
- No data

Figure S31: Ratio of observed : expected all-cause mortality rate per 100,000, 1990 - 2017 amongst 20-24 year olds (both sexes) in Central and Eastern Europe, Central Asia GBD super-region

GBD region	Country	1990	1995	2000	2005	2010	2017
Central Asia	Armenia	0.73	0.84	0.42	0.41	0.52	0.53
	Azerbaijan	1.03	1.01	0.78	0.74	0.74	0.81
	Georgia	1.00	0.96	0.59	0.65	0.88	0.90
	Kazakhstan	1.26	1.83	1.76	2.12	1.51	1.09
	Kyrgyzstan	1.15	1.17	1.11	1.03	0.75	0.58
	Mongolia	1.05	1.60	1.37	1.20	1.06	1.00
	Tajikistan	0.73	1.00	0.74	0.72	0.76	0.75
	Turkmenistan	1.07	1.27	1.28	1.41	1.14	1.22
	Uzbekistan	0.71	0.81	0.98	0.97	0.80	0.84
Central Europe	Albania	0.67	0.69	0.78	0.66	0.60	0.66
	Bosnia and Herzegovina	0.39	3.77	0.45	0.43	0.44	0.52
	Bulgaria	0.84	0.95	0.86	0.81	0.78	0.75
	Croatia	1.00	1.36	0.80	0.77	0.68	0.50
	Czech Republic	0.85	0.87	0.87	0.80	0.75	0.64
	Hungary	0.96	0.77	0.69	0.66	0.56	0.48
	Macedonia	0.52	0.56	0.55	0.46	0.45	0.48
	Montenegro	0.81	0.74	0.72	0.70	0.55	0.65
	Poland	0.92	0.86	0.84	0.82	0.83	0.74
	Romania	0.89	0.89	0.74	0.69	0.85	0.69
	Serbia	0.79	0.76	0.68	0.62	0.48	0.41
	Slovakia	0.80	0.75	0.78	0.74	0.72	0.66
	Slovenia	1.04	1.05	0.95	0.92	0.67	0.60
Eastern Europe	Belarus	1.14	1.40	1.39	1.40	1.22	0.80
	Estonia	1.61	2.05	1.67	1.60	1.07	0.97
	Latvia	1.47	1.97	1.70	1.39	0.99	0.88
	Lithuania	1.34	1.75	1.62	1.71	1.42	0.92
	Moldova	1.18	1.14	0.91	0.91	0.86	0.66
	Russian Federation	1.55	2.74	3.00	2.77	2.08	1.52
	Ukraine	1.26	1.77	1.60	1.36	1.07	1.67

Observed : expected
- Less than 0.4
- 0.4 to 0.79
- 0.8 to 1.19
- 1.2 to 1.59
- 1.6 to 1.99
- 2.0 to 2.39
- 2.4 to 2.79
- 2.8 to 3.19
- >= 3.2
- No data

Figure S32: Ratio of observed : expected all-cause mortality rate per 100,000, 1990 - 2017 amongst 10-14 year olds (both sexes) in High Income GBD super-region

GBD region	Country	1990	1995	2000	2005	2010	2017
Australasia	Australia	0.83	0.87	0.76	0.59	0.59	0.63
	New Zealand	0.98	1.00	1.03	0.78	0.81	0.60
High-income Asia Pacific	Brunei	1.96	1.66	1.59	1.67	1.76	1.72
	Japan	0.68	0.80	0.61	0.55	0.52	0.48
	Singapore	0.80	0.85	0.71	0.63	0.63	0.53
	South Korea	1.55	1.37	0.94	0.78	0.76	0.54
High-income North America	Canada	1.04	0.99	0.85	0.83	0.70	0.82
	Greenland	2.60	2.42	2.20	2.02	1.94	1.55
	United States	1.11	1.15	0.98	0.94	0.82	0.88
Southern Latin America	Argentina	0.69	0.73	0.71	0.76	0.77	0.73
	Chile	0.67	0.63	0.58	0.62	0.63	0.63
	Uruguay	0.64	0.62	0.57	0.60	0.60	0.64
Western Europe	Andorra	0.82	0.72	0.65	0.66	0.63	0.57
	Austria	0.68	0.77	0.75	0.64	0.59	0.49
	Belgium	0.97	1.07	0.88	0.72	0.69	0.49
	Cyprus	0.49	0.58	0.75	0.78	0.57	0.50
	Denmark	1.08	0.99	1.01	0.76	0.54	0.46
	Finland	0.94	0.92	0.81	0.74	0.61	0.70
	France	0.79	0.75	0.77	0.60	0.56	0.48
	Germany	0.83	0.76	0.68	0.63	0.56	0.52
	Greece	0.62	0.64	0.58	0.68	0.63	0.45
	Iceland	1.18	2.11	0.75	0.85	0.84	0.78
	Ireland	0.73	0.82	0.76	0.73	0.59	0.57
	Israel	0.59	0.62	0.69	0.70	0.57	0.52
	Italy	0.80	0.90	0.73	0.62	0.56	0.52
	Luxembourg	0.90	1.10	1.10	0.81	0.83	0.69
	Malta	0.40	0.52	0.58	0.53	0.67	0.71
	Netherlands	1.07	1.00	0.93	0.83	0.71	0.61
	Norway	0.97	0.91	0.72	0.81	0.76	0.71
	Portugal	0.89	0.88	0.75	0.62	0.48	0.39
	Spain	0.76	0.70	0.71	0.60	0.52	0.41
	Sweden	0.70	0.61	0.69	0.68	0.57	0.52
	Switzerland	1.05	1.03	0.90	0.71	0.57	0.55
	United Kingdom	0.68	0.65	0.60	0.58	0.48	0.49

Observed : expected
- Less than 0.4
- 0.4 to 0.79
- 0.8 to 1.19
- 1.2 to 1.59
- 1.6 to 1.99
- 2.0 to 2.39
- 2.4 to 2.79
- 2.8 to 3.19
- >= 3.2
- No data

Figure S33: Ratio of observed : expected all-cause mortality rate per 100,000, 1990 - 2017 amongst 15-19 year olds (both sexes) in High Income GBD super-region

GBD region	Country	1990	1995	2000	2005	2010	2017
Australasia	Australia	1.13	0.98	0.97	0.73	0.71	0.66
	New Zealand	1.60	1.45	1.20	1.23	1.04	0.79
High-income Asia Pacific	Brunei	1.32	1.13	1.11	1.15	1.21	1.27
	Japan	0.76	0.72	0.61	0.54	0.46	0.40
	Singapore	0.62	0.63	0.44	0.45	0.41	0.35
	South Korea	1.28	1.28	0.83	0.58	0.58	0.47
High-income North America	Canada	1.16	1.05	0.96	0.91	0.78	0.82
	Greenland	3.94	3.50	3.06	2.57	2.33	1.69
	United States	1.45	1.46	1.22	1.21	0.96	1.02
Southern Latin America	Argentina	0.80	0.92	0.96	0.90	1.00	1.03
	Chile	0.78	0.77	0.69	0.72	0.74	0.69
	Uruguay	0.88	1.00	0.87	0.83	0.96	0.97
Western Europe	Andorra	0.84	0.72	0.61	0.59	0.54	0.48
	Austria	1.13	1.20	1.06	0.97	0.74	0.59
	Belgium	1.08	1.03	1.00	0.73	0.61	0.50
	Cyprus	0.80	1.07	1.10	1.03	0.79	0.66
	Denmark	0.81	0.87	0.90	0.74	0.55	0.38
	Finland	1.27	0.96	0.96	0.85	0.76	0.59
	France	0.89	0.84	0.85	0.69	0.59	0.42
	Germany	1.01	0.96	0.83	0.62	0.53	0.51
	Greece	0.73	0.78	0.79	0.70	0.66	0.47
	Iceland	1.15	1.08	0.69	0.57	0.57	0.58
	Ireland	0.79	0.82	1.06	0.96	0.74	0.47
	Israel	0.67	0.69	0.72	0.61	0.44	0.44
	Italy	0.90	0.88	0.75	0.64	0.52	0.39
	Luxembourg	1.75	1.17	1.01	0.74	0.65	0.59
	Malta	0.57	0.58	0.64	0.54	0.43	0.39
	Netherlands	0.74	0.74	0.75	0.54	0.46	0.41
	Norway	1.04	0.95	1.08	0.80	0.74	0.57
	Portugal	1.17	1.02	0.88	0.64	0.47	0.38
	Spain	0.93	0.69	0.74	0.65	0.42	0.33
	Sweden	0.79	0.59	0.63	0.56	0.53	0.42
	Switzerland	1.25	1.05	0.80	0.68	0.57	0.44
	United Kingdom	0.78	0.72	0.68	0.60	0.51	0.45

Observed : expected
- Less than 0.4
- 0.4 to 0.79
- 0.8 to 1.19
- 1.2 to 1.59
- 1.6 to 1.99
- 2.0 to 2.39
- 2.4 to 2.79
- 2.8 to 3.19
- >= 3.2
- No data

Figure S34: Ratio of observed : expected all-cause mortality rate per 100,000, 1990 - 2017 amongst 20-24 year olds (both sexes) in High Income GBD super-region

GBD region	Country	1990	1995	2000	2005	2010	2017
Australasia	Australia	1.09	1.02	1.00	0.79	0.69	0.66
	New Zealand	1.48	1.30	1.03	0.95	0.92	0.80
High-income Asia Pacific	Brunei	1.38	1.19	1.17	1.25	1.32	1.38
	Japan	0.67	0.67	0.63	0.61	0.60	0.50
	Singapore	0.58	0.58	0.55	0.50	0.49	0.41
	South Korea	1.09	1.04	0.77	0.61	0.61	0.46
High-income North America	Canada	1.00	0.93	0.88	0.88	0.80	0.80
	Greenland	3.71	2.96	2.50	2.28	2.11	1.33
	United States	1.27	1.27	1.17	1.30	1.18	1.39
Southern Latin America	Argentina	0.75	0.88	0.92	0.83	0.91	0.95
	Chile	0.83	0.80	0.74	0.70	0.76	0.67
	Uruguay	0.69	0.78	0.85	0.73	0.91	0.98
Western Europe	Andorra	0.87	0.76	0.70	0.68	0.65	0.61
	Austria	0.97	1.01	0.90	0.80	0.76	0.51
	Belgium	1.11	1.10	1.11	0.78	0.74	0.57
	Cyprus	0.84	0.92	1.08	1.04	0.84	0.72
	Denmark	0.84	0.81	0.77	0.66	0.59	0.56
	Finland	1.25	0.94	1.03	0.94	1.00	0.76
	France	1.09	0.98	0.88	0.76	0.66	0.54
	Germany	0.90	0.88	0.77	0.59	0.52	0.50
	Greece	0.79	0.79	0.86	0.87	0.74	0.55
	Iceland	0.87	0.83	0.81	0.74	0.56	0.61
	Ireland	0.81	0.96	1.05	0.94	0.78	0.59
	Israel	0.62	0.73	0.68	0.69	0.49	0.38
	Italy	0.84	0.83	0.77	0.65	0.51	0.39
	Luxembourg	1.37	1.24	0.92	0.91	0.69	0.65
	Malta	0.54	0.54	0.52	0.63	0.58	0.47
	Netherlands	0.66	0.66	0.68	0.53	0.47	0.44
	Norway	0.84	0.77	1.03	0.98	0.82	0.56
	Portugal	1.07	1.10	0.91	0.65	0.55	0.39
	Spain	1.02	0.79	0.69	0.57	0.39	0.32
	Sweden	0.69	0.60	0.67	0.69	0.58	0.58
	Switzerland	1.30	1.20	0.87	0.67	0.48	0.42
	United Kingdom	0.68	0.68	0.68	0.60	0.54	0.51

Observed : expected
Less than 0.4
0.4 to 0.79
0.8 to 1.19
1.2 to 1.59
1.6 to 1.99
2.0 to 2.39
2.4 to 2.79
2.8 to 3.19
>= 3.2
No data

Figure S35: Ratio of observed : expected all-cause mortality rate per 100,000, 1990 - 2017 amongst 10-14 year olds (both sexes) in Latin America and Caribbean GBD super-region

GBD region	Country	1990	1995	2000	2005	2010	2017
Andean Latin America	Bolivia	0.97	0.92	0.88	0.89	0.82	0.78
	Ecuador	1.04	0.94	1.07	1.18	1.10	1.04
	Peru	1.19	0.99	0.93	0.82	0.90	0.78
Caribbean	Antigua and Barbuda	0.73	0.85	0.74	0.78	0.83	0.87
	Barbados	1.07	0.81	1.23	1.46	1.48	1.52
	Belize	0.56	0.61	0.82	0.68	0.71	0.67
	Bermuda	1.40	1.09	0.86	0.83	0.79	0.56
	Cuba	0.80	0.73	0.62	0.55	0.53	0.58
	Dominica	0.82	0.99	1.05	1.36	1.63	1.90
	Dominican Republic	0.64	0.62	0.59	0.69	0.74	0.81
	Grenada	0.56	0.61	0.64	0.70	0.84	0.89
	Guyana	0.75	0.65	0.80	0.96	0.99	0.89
	Haiti	0.99	0.90	0.89	0.95	22.81	0.80
	Jamaica	0.60	0.60	0.59	0.56	0.66	0.72
	Puerto Rico	0.66	0.91	0.65	0.56	0.55	2.82
	Saint Lucia	0.57	0.67	0.72	0.90	0.79	0.78
	St. Vincent & the Grenadines	0.48	0.80	0.82	1.02	1.20	1.27
	Suriname	0.96	0.92	1.02	1.08	1.00	1.17
	The Bahamas	1.03	0.98	1.16	1.26	1.25	1.24
	Trinidad and Tobago	1.04	0.91	0.90	0.94	0.99	0.94
	Virgin Islands, U.S.	0.96	1.18	0.93	0.95	1.02	1.23
Central Latin America	Colombia	0.82	0.87	0.80	0.65	0.71	0.67
	Costa Rica	0.46	0.50	0.46	0.48	0.54	0.51
	El Salvador	0.85	0.74	0.69	0.74	0.74	0.77
	Guatemala	1.06	0.82	0.78	0.79	0.72	0.74
	Honduras	0.82	0.73	0.71	0.69	0.64	0.57
	Mexico	0.80	0.70	0.70	0.70	0.73	0.76
	Nicaragua	0.55	0.51	0.49	0.46	0.39	0.35
	Panama	0.74	0.74	0.68	0.74	0.87	0.83
	Venezuela	0.77	0.88	0.77	0.70	0.86	0.91
Tropical Latin America	Brazil	0.76	0.79	0.74	0.79	0.86	0.86
	Paraguay	0.49	0.60	0.65	0.76	0.65	0.65

Observed : expected
- Less than 0.4
- 0.4 to 0.79
- 0.8 to 1.19
- 1.2 to 1.59
- 1.6 to 1.99
- 2.0 to 2.39
- 2.4 to 2.79
- 2.8 to 3.19
- >= 3.2
- No data

Figure S36: Ratio of observed : expected all-cause mortality rate per 100,000, 1990 - 2017 amongst 15-19 year olds (both sexes) in Latin America and Caribbean GBD super-region

GBD region	Country	1990	1995	2000	2005	2010	2017
Andean Latin America	Bolivia	1.08	1.01	0.97	0.92	0.86	0.83
	Ecuador	1.07	1.19	1.15	1.34	1.41	1.11
	Peru	1.18	1.02	0.86	0.78	0.81	0.70
Caribbean	Antigua and Barbuda	0.72	0.81	0.83	0.89	1.03	1.03
	Barbados	0.99	1.26	1.27	1.15	0.98	0.94
	Belize	0.56	0.99	1.23	1.12	1.23	1.16
	Bermuda	1.11	0.94	0.75	0.68	0.65	0.62
	Cuba	1.01	1.00	0.65	0.58	0.48	0.54
	Dominica	0.95	1.11	1.16	1.21	1.20	1.37
	Dominican Republic	0.66	0.67	0.65	0.74	0.79	0.93
	Grenada	0.68	0.70	0.69	0.72	0.86	0.68
	Guyana	0.91	0.90	1.09	1.29	1.22	1.13
	Haiti	1.20	1.10	0.99	1.04	17.06	0.99
	Jamaica	0.37	0.51	0.59	0.52	0.73	0.87
	Puerto Rico	1.11	1.52	1.28	1.07	1.05	1.84
	Saint Lucia	0.77	0.80	0.88	0.88	0.79	0.85
	St. Vincent & the Grenadines	0.58	0.69	0.87	0.79	0.86	1.04
	Suriname	1.18	1.18	0.99	0.99	1.28	1.26
	The Bahamas	0.95	1.19	1.29	1.17	1.34	1.35
	Trinidad and Tobago	1.03	1.02	0.94	1.39	1.43	1.17
	Virgin Islands, U.S.	1.31	1.39	1.15	1.10	1.10	1.19
Central Latin America	Colombia	1.53	1.77	1.85	1.23	1.20	1.04
	Costa Rica	0.56	0.68	0.65	0.63	0.73	0.70
	El Salvador	1.40	1.40	1.37	1.54	1.52	1.61
	Guatemala	1.15	1.13	1.22	1.34	1.27	1.18
	Honduras	0.87	0.86	0.87	0.88	0.87	0.81
	Mexico	0.95	0.89	0.81	0.81	1.14	1.13
	Nicaragua	0.60	0.70	0.67	0.66	0.57	0.54
	Panama	0.85	0.91	0.86	0.90	1.24	1.09
	Venezuela	1.17	1.50	1.75	1.67	2.05	2.22
Tropical Latin America	Brazil	1.07	1.14	1.19	1.27	1.34	1.51
	Paraguay	0.54	0.80	0.88	0.93	1.05	0.97

Observed : expected

- Less than 0.4
- 0.4 to 0.79
- 0.8 to 1.19
- 1.2 to 1.59
- 1.6 to 1.99
- 2.0 to 2.39
- 2.4 to 2.79
- 2.8 to 3.19
- >= 3.2
- No data

Figure S37: Ratio of observed : expected all-cause mortality rate per 100,000, 1990 - 2017 amongst 20-24 year olds (both sexes) in Latin America and Caribbean GBD super-region

GBD region	Country	1990	1995	2000	2005	2010	2017
Andean Latin America	Bolivia	1.12	1.04	0.96	0.86	0.79	0.75
	Ecuador	1.04	1.09	1.16	1.41	1.48	1.19
	Peru	1.13	1.00	0.86	0.76	0.78	0.65
Caribbean	Antigua and Barbuda	0.78	0.82	0.77	0.82	0.94	0.96
	Barbados	1.01	1.09	0.99	1.05	1.00	0.93
	Belize	0.71	1.14	1.49	1.43	1.34	1.20
	Bermuda	1.13	1.00	1.34	0.74	0.72	0.66
	Cuba	0.95	0.88	0.66	0.55	0.47	0.51
	Dominica	1.04	1.17	1.14	1.25	1.47	1.50
	Dominican Republic	0.77	0.81	0.82	0.90	0.94	1.08
	Grenada	0.86	0.78	0.71	0.64	0.60	0.55
	Guyana	1.03	1.23	1.45	1.48	1.35	1.35
	Haiti	1.39	1.44	1.16	1.09	12.11	1.00
	Jamaica	0.40	0.59	0.79	0.60	0.88	1.06
	Puerto Rico	1.31	1.75	1.53	1.73	1.68	2.21
	Saint Lucia	0.94	0.92	1.07	0.98	0.95	1.02
	St. Vincent & the Grenadines	0.65	0.81	1.08	1.04	1.05	1.14
	Suriname	1.21	1.29	1.21	1.32	1.39	1.04
	The Bahamas	1.31	1.62	1.66	1.51	1.79	1.82
	Trinidad and Tobago	1.17	1.15	1.24	1.59	1.85	1.43
	Virgin Islands, U.S.	1.20	1.26	1.13	1.13	1.10	1.19
Central Latin America	Colombia	1.70	1.76	1.93	1.40	1.31	1.18
	Costa Rica	0.54	0.63	0.65	0.62	0.74	0.76
	El Salvador	1.54	1.37	1.37	1.55	1.35	1.44
	Guatemala	1.25	1.34	1.33	1.44	1.35	1.29
	Honduras	1.21	1.08	1.09	1.08	1.07	0.99
	Mexico	0.95	0.91	0.78	0.78	1.14	1.24
	Nicaragua	0.62	0.68	0.66	0.67	0.57	0.56
	Panama	0.84	1.01	0.90	0.94	1.23	1.04
	Venezuela	1.18	1.46	1.82	1.74	2.14	2.23
Tropical Latin America	Brazil	1.09	1.20	1.21	1.21	1.31	1.39
	Paraguay	0.54	0.76	0.76	0.97	0.94	0.90

Observed : expected
Less than 0.4
0.4 to 0.79
0.8 to 1.19
1.2 to 1.59
1.6 to 1.99
2.0 to 2.39
2.4 to 2.79
2.8 to 3.19
>= 3.2
No data

Figure S38: Ratio of observed : expected all-cause mortality rate per 100,000, 1990 - 2017 amongst 10-14 year olds (both sexes) in North Africa and Middle East GBD super-region

GBD region	Country	1990	1995	2000	2005	2010	2017
North Africa and Middle East	Afghanistan	0.71	0.71	0.67	0.58	0.63	0.54
	Algeria	0.90	1.14	1.21	1.04	1.07	1.00
	Bahrain	0.90	0.77	1.04	0.60	0.57	0.56
	Egypt	1.11	1.09	0.95	0.97	0.92	0.90
	Iran	1.99	1.12	1.23	1.46	1.59	1.20
	Iraq	1.11	1.10	1.12	1.16	1.08	1.51
	Jordan	1.22	1.18	1.14	1.03	0.65	0.67
	Kuwait	1.85	0.60	0.76	0.78	0.84	0.75
	Lebanon	0.89	0.68	0.63	0.59	0.62	0.70
	Libya	1.22	1.37	1.51	1.31	2.04	2.00
	Morocco	0.88	0.89	0.82	0.73	0.72	0.75
	Oman	0.89	0.89	0.85	0.75	1.09	1.12
	Palestine	0.53	0.45	0.87	0.86	0.55	0.39
	Qatar	1.09	1.16	1.17	1.15	1.01	0.89
	Saudi Arabia	0.60	0.67	0.75	0.84	0.82	0.89
	Sudan	0.68	0.63	0.63	0.62	0.66	0.72
	Syria	0.78	0.76	0.68	0.62	0.62	3.14
	Tunisia	0.69	0.69	0.67	0.69	0.70	0.62
	Turkey	0.72	0.74	0.62	0.60	0.66	0.85
	United Arab Emirates	1.26	1.38	1.65	1.36	1.60	1.47
	Yemen	0.58	0.57	0.55	0.55	0.60	0.88

Observed : expected

- Less than 0.4
- 0.4 to 0.79
- 0.8 to 1.19
- 1.2 to 1.59
- 1.6 to 1.99
- 2.0 to 2.39
- 2.4 to 2.79
- 2.8 to 3.19
- >= 3.2
- No data

Figure S39: Ratio of observed : expected all-cause mortality rate per 100,000, 1990 - 2017 amongst 15-19 year olds (both sexes) in North Africa and Middle East GBD super-region

GBD region	Country	1990	1995	2000	2005	2010	2017
North Africa and Middle East	Afghanistan	0.86	0.90	0.89	0.80	0.90	0.78
	Algeria	0.83	1.05	1.04	0.85	0.78	0.74
	Bahrain	0.76	0.77	0.94	0.64	0.46	0.44
	Egypt	0.94	0.91	0.84	0.92	0.90	0.96
	Iran	1.76	1.11	1.15	1.28	1.30	1.17
	Iraq	1.02	1.10	1.12	1.11	0.98	1.53
	Jordan	0.84	0.81	0.74	0.66	0.57	0.52
	Kuwait	1.92	0.71	0.82	0.77	0.67	0.60
	Lebanon	1.46	0.84	0.78	0.75	0.81	0.88
	Libya	1.04	1.09	1.22	1.10	1.54	1.86
	Morocco	0.77	0.81	0.76	0.73	0.74	0.77
	Oman	0.98	0.97	0.97	0.84	1.12	0.90
	Palestine	0.67	0.55	1.23	1.17	0.64	0.46
	Qatar	1.22	1.13	1.22	1.35	1.00	0.83
	Saudi Arabia	0.64	0.67	0.74	0.86	0.97	1.08
	Sudan	0.74	0.66	0.72	0.69	0.72	0.77
	Syria	0.73	0.76	0.74	0.68	0.67	4.02
	Tunisia	0.57	0.59	0.64	0.63	0.59	0.55
	Turkey	0.66	0.68	0.51	0.51	0.52	0.76
	United Arab Emirates	1.28	1.27	1.28	2.66	2.30	1.61
	Yemen	0.65	0.65	0.64	0.64	0.71	1.22

Observed : expected
- Less than 0.4
- 0.4 to 0.79
- 0.8 to 1.19
- 1.2 to 1.59
- 1.6 to 1.99
- 2.0 to 2.39
- 2.4 to 2.79
- 2.8 to 3.19
- >= 3.2
- No data

Figure S40: Ratio of observed : expected all-cause mortality rate per 100,000, 1990 - 2017 amongst 20-24 year olds (both sexes) in North Africa and Middle East GBD super-region

GBD region	Country	1990	1995	2000	2005	2010	2017
North Africa and Middle East	Afghanistan	0.96	0.94	0.96	0.85	1.01	0.82
	Algeria	0.74	0.98	0.97	0.78	0.72	0.68
	Bahrain	0.53	0.66	0.95	0.55	0.43	0.39
	Egypt	0.70	0.67	0.69	0.77	0.76	0.81
	Iran	1.35	0.89	0.92	1.03	1.13	0.96
	Iraq	0.99	1.06	1.05	1.05	0.95	1.65
	Jordan	0.78	0.75	0.69	0.62	0.51	0.49
	Kuwait	1.93	0.60	0.70	0.67	0.56	0.52
	Lebanon	1.42	0.74	0.67	0.63	0.71	0.88
	Libya	0.94	0.99	1.11	0.97	1.54	1.95
	Morocco	0.77	0.76	0.77	0.70	0.71	0.74
	Oman	0.96	0.94	0.95	0.92	1.07	0.97
	Palestine	0.67	0.51	1.29	1.21	0.61	0.42
	Qatar	0.91	0.92	0.94	0.89	0.81	0.69
	Saudi Arabia	0.60	0.61	0.67	0.77	0.84	1.12
	Sudan	0.78	0.70	0.75	0.73	0.71	0.73
	Syria	0.48	0.47	0.45	0.44	0.46	4.35
	Tunisia	0.43	0.55	0.57	0.58	0.55	0.52
	Turkey	0.62	0.70	0.50	0.42	0.43	0.68
	United Arab Emirates	0.80	0.88	0.98	1.04	1.15	1.00
	Yemen	0.69	0.69	0.68	0.67	0.70	1.25

Observed : expected
- Less than 0.4
- 0.4 to 0.79
- 0.8 to 1.19
- 1.2 to 1.59
- 1.6 to 1.99
- 2.0 to 2.39
- 2.4 to 2.79
- 2.8 to 3.19
- >= 3.2
- No data

Figure S41: Ratio of observed : expected all-cause mortality rate per 100,000, 1990 - 2017 amongst 10-14 year olds (both sexes) in South Asia GBD super-region

GBD region	Country	1990	1995	2000	2005	2010	2017
South Asia	Bangladesh	0.99	0.93	0.88	0.82	1.06	0.86
	Bhutan	0.82	0.79	1.06	0.72	0.71	0.70
	India	1.08	1.04	1.19	1.15	1.07	0.87
	Nepal	0.62	0.56	0.50	0.51	0.50	0.49
	Pakistan	0.91	1.20	1.18	1.70	1.32	1.23

Observed : expected
- Less than 0.4
- 0.4 to 0.79
- 0.8 to 1.19
- 1.2 to 1.59
- 1.6 to 1.99
- 2.0 to 2.39
- 2.4 to 2.79
- 2.8 to 3.19
- >= 3.2
- No data

Figure S42: Ratio of observed : expected all-cause mortality rate per 100,000, 1990 - 2017 amongst 15-19 year olds (both sexes) in South Asia GBD super-region

GBD region	Country	1990	1995	2000	2005	2010	2017
South Asia	Bangladesh	0.86	0.78	0.71	0.70	0.71	0.64
	Bhutan	0.83	0.81	0.99	0.73	0.72	0.74
	India	1.20	1.14	1.25	1.25	1.18	0.83
	Nepal	0.68	0.62	0.56	0.59	0.60	0.65
	Pakistan	1.06	1.21	1.24	1.66	1.40	1.31

Observed : expected
- Less than 0.4
- 0.4 to 0.79
- 0.8 to 1.19
- 1.2 to 1.59
- 1.6 to 1.99
- 2.0 to 2.39
- 2.4 to 2.79
- 2.8 to 3.19
- >= 3.2
- No data

Figure S43: Ratio of observed : expected all-cause mortality rate per 100,000, 1990 - 2017 amongst 20-24 year olds (both sexes) in South Asia GBD super-region

GBD region	Country	1990	1995	2000	2005	2010	2017
**South Asia**	Bangladesh	0.69	0.62	0.54	0.52	0.50	0.48
	Bhutan	0.87	0.83	0.91	0.70	0.67	0.67
	India	1.05	1.06	1.17	1.10	1.13	0.90
	Nepal	0.72	0.65	0.58	0.59	0.58	0.59
	Pakistan	0.90	1.16	1.22	1.51	1.32	1.22

Observed : expected

- Less than 0.4
- 0.4 to 0.79
- 0.8 to 1.19
- 1.2 to 1.59
- 1.6 to 1.99
- 2.0 to 2.39
- 2.4 to 2.79
- 2.8 to 3.19
- >= 3.2
- No data

Figure S44: Ratio of observed : expected all-cause mortality rate per 100,000, 1990 - 2017 amongst 10-14 year olds (both sexes) in Southeast Asia, East Asia, and Oceania GBD super-region

GBD region	Country	1990	1995	2000	2005	2010	2017
East Asia	China	0.94	1.14	1.14	0.73	0.94	1.07
	North Korea	0.60	1.28	1.26	0.68	0.67	0.61
	Taiwan	1.06	1.14	0.91	0.84	0.79	0.80
Oceania	American Samoa	0.77	0.78	0.80	0.91	0.84	0.83
	Fiji	0.93	1.12	1.40	1.22	1.22	1.35
	FSOM	0.88	0.90	0.90	0.84	0.84	0.83
	Guam	1.23	1.03	1.11	1.16	1.09	1.17
	Kiribati	0.92	0.90	0.91	0.90	0.86	0.82
	Marshall Islands	0.68	0.89	0.96	0.98	0.97	0.93
	Northern Mariana Islands	0.82	0.80	0.77	0.81	0.74	0.77
	Papua New Guinea	1.00	0.99	0.99	1.03	1.05	1.04
	Samoa	1.00	0.81	0.70	0.74	0.57	0.57
	Solomon Islands	0.62	0.63	0.66	0.63	0.63	0.62
	Tonga	0.66	0.68	0.72	0.76	0.80	0.78
	Vanuatu	0.68	0.70	0.73	0.77	0.78	0.79
Southeast Asia	Cambodia	0.73	0.75	0.74	0.69	0.72	0.65
	Indonesia	0.98	1.00	0.97	0.93	0.94	0.96
	Laos	1.12	1.11	1.05	0.97	0.90	0.87
	Malaysia	0.87	0.88	0.95	0.91	0.95	1.12
	Maldives	0.75	0.58	0.72	0.72	0.51	0.44
	Mauritius	0.64	0.61	0.65	0.56	0.64	0.81
	Myanmar	1.03	1.01	0.99	1.01	1.00	0.99
	Philippines	1.38	1.26	1.23	1.19	1.17	1.28
	Seychelles	0.70	0.82	0.95	0.70	0.66	0.86
	Sri Lanka	1.00	0.96	0.82	0.70	1.04	0.57
	Thailand	1.10	1.22	1.04	1.07	1.01	1.06
	Timor-Leste	0.76	0.79	0.59	0.59	0.63	0.65
	Vietnam	0.59	0.61	0.58	0.59	0.61	0.58

Observed : expected
- Less than 0.4
- 0.4 to 0.79
- 0.8 to 1.19
- 1.2 to 1.59
- 1.6 to 1.99
- 2.0 to 2.39
- 2.4 to 2.79
- 2.8 to 3.19
- >= 3.2
- No data

FSOM: Federated States of Miconesia

Figure S45: Ratio of observed : expected all-cause mortality rate per 100,000, 1990 - 2017 amongst 15-19 year olds (both sexes) in Southeast Asia, East Asia, and Oceania GBD super-region

GBD region	Country	1990	1995	2000	2005	2010	2017
East Asia	China	0.66	0.69	0.67	0.60	0.54	0.49
	North Korea	0.78	1.11	1.13	0.87	0.87	0.97
	Taiwan	1.35	1.33	1.08	0.92	0.71	0.70
Oceania	American Samoa	1.11	1.11	1.15	1.23	1.10	1.09
	Fiji	1.12	1.17	1.33	1.30	1.15	1.18
	FSOM	1.36	1.39	1.36	1.29	1.28	1.26
	Guam	1.28	1.28	1.35	1.44	1.50	1.62
	Kiribati	1.26	1.35	1.32	1.34	1.30	1.29
	Marshall Islands	1.22	1.56	1.67	1.69	1.66	1.55
	Northern Mariana Islands	1.06	1.05	0.95	1.04	1.02	1.05
	Papua New Guinea	1.57	1.56	1.58	1.63	1.65	1.66
	Samoa	1.09	1.06	1.11	1.10	0.95	0.93
	Solomon Islands	1.00	1.02	1.07	1.03	1.04	1.03
	Tonga	1.01	1.04	1.12	1.20	1.21	1.16
	Vanuatu	1.14	1.17	1.20	1.25	1.26	1.27
Southeast Asia	Cambodia	0.87	0.92	0.89	0.83	0.86	0.87
	Indonesia	1.07	1.08	1.05	1.01	1.02	1.04
	Laos	1.27	1.25	1.20	1.11	1.05	0.97
	Malaysia	0.98	1.17	1.07	0.98	1.06	1.12
	Maldives	0.75	0.48	0.51	0.47	0.35	0.30
	Mauritius	0.74	0.70	0.67	0.62	0.68	0.86
	Myanmar	1.21	1.21	1.18	1.19	1.16	1.23
	Philippines	1.08	0.94	0.96	0.98	1.00	1.03
	Seychelles	0.69	0.79	0.93	0.85	0.80	0.95
	Sri Lanka	1.33	1.35	1.20	0.92	1.26	0.74
	Thailand	1.41	1.95	1.49	1.35	1.25	1.25
	Timor-Leste	0.84	0.93	0.67	0.66	0.73	0.79
	Vietnam	0.71	0.76	0.82	0.95	1.00	0.95

Observed : expected
- Less than 0.4
- 0.4 to 0.79
- 0.8 to 1.19
- 1.2 to 1.59
- 1.6 to 1.99
- 2.0 to 2.39
- 2.4 to 2.79
- 2.8 to 3.19
- >= 3.2
- No data

FSOM: Federated States of Miconesia

Figure S46: Ratio of observed : expected all-cause mortality rate per 100,000, 1990 - 2017 amongst 20-24 year olds (both sexes) in Southeast Asia, East Asia, and Oceania GBD super-region

GBD region	Country	1990	1995	2000	2005	2010	2017
East Asia	China	0.70	0.85	0.73	0.52	0.52	0.39
	North Korea	0.69	0.94	0.98	0.80	0.80	0.86
	Taiwan	1.02	1.07	0.87	0.84	0.65	0.65
Oceania	American Samoa	1.02	1.06	1.12	1.22	1.14	1.16
	Fiji	1.07	1.10	1.28	1.14	1.05	1.11
	FSOM	1.35	1.38	1.38	1.35	1.36	1.40
	Guam	1.32	1.28	1.25	1.19	1.24	1.26
	Kiribati	1.30	1.40	1.42	1.45	1.44	1.42
	Marshall Islands	1.80	1.78	1.87	1.97	1.98	1.89
	Northern Mariana Islands	1.11	1.07	0.86	1.07	1.05	1.08
	Papua New Guinea	1.79	1.77	1.81	1.91	1.90	1.85
	Samoa	1.03	1.03	0.99	0.96	0.84	0.83
	Solomon Islands	1.06	1.07	1.13	1.08	1.08	1.09
	Tonga	0.89	0.90	0.98	1.08	1.11	1.10
	Vanuatu	1.27	1.29	1.29	1.32	1.33	1.34
Southeast Asia	Cambodia	0.93	1.03	1.04	0.90	0.86	0.81
	Indonesia	1.08	1.08	1.04	0.97	0.96	0.94
	Laos	1.34	1.30	1.25	1.14	1.06	0.95
	Malaysia	0.92	1.00	0.94	0.85	0.87	0.98
	Maldives	0.73	0.52	0.47	0.35	0.34	0.30
	Mauritius	0.64	0.72	0.62	0.70	0.78	0.89
	Myanmar	1.28	1.30	1.28	1.28	1.17	1.19
	Philippines	1.14	0.98	1.02	1.05	1.04	1.07
	Seychelles	0.88	0.87	0.83	0.87	0.82	1.10
	Sri Lanka	1.41	1.43	1.30	1.00	1.60	0.84
	Thailand	1.19	2.03	1.80	1.24	1.03	1.14
	Timor-Leste	0.90	1.02	0.70	0.66	0.68	0.71
	Vietnam	0.70	0.72	0.73	0.83	0.89	0.88

Observed : expected
- Less than 0.4
- 0.4 to 0.79
- 0.8 to 1.19
- 1.2 to 1.59
- 1.6 to 1.99
- 2.0 to 2.39
- 2.4 to 2.79
- 2.8 to 3.19
- >= 3.2
- No data

FSOM: Federated States of Miconesia

Figure S47: Ratio of observed : expected all-cause mortality rate per 100,000, 1990 - 2017 amongst 10-14 year olds (both sexes) in Sub-Saharan Africa GBD super-region

GBD region	Country	1990	1995	2000	2005	2010	2017
Central Sub-Saharan Africa	Angola	1.12	1.05	1.10	0.90	0.85	0.91
	CAR	0.98	1.05	1.22	1.30	1.41	1.46
	Congo	1.12	1.39	1.84	1.57	1.51	1.64
	DRC	0.93	0.94	0.90	0.77	0.75	0.80
	Equatorial Guinea	0.95	1.03	1.05	1.17	1.38	1.94
	Gabon	1.05	1.12	1.19	1.29	1.55	1.59
Eastern Sub-Saharan Africa	Burundi	1.04	1.86	1.81	1.52	0.81	0.70
	Comoros	0.71	0.71	0.68	0.66	0.63	0.61
	Djibouti	0.81	0.77	0.73	0.77	0.83	0.86
	Eritrea	3.93	0.96	1.16	1.02	1.00	0.86
	Ethiopia	0.87	0.69	0.83	0.66	0.63	0.55
	Kenya	0.68	0.74	0.85	1.01	1.09	1.01
	Madagascar	1.23	1.12	0.86	0.62	0.60	0.59
	Malawi	0.81	0.76		1.05	1.26	1.11
	Mozambique	0.72	0.65	0.66	0.73	0.94	1.18
	Rwanda	1.17	1.42	1.11	0.89	0.79	0.69
	Somalia	0.83	0.73	0.68	0.64	0.73	0.68
	South Sudan	0.81	0.71	0.71	0.61	0.66	0.81
	Tanzania	0.77	0.78	0.85	0.84	0.87	0.96
	Uganda	0.60	0.75	0.98	0.99	0.94	1.05
	Zambia	1.00	1.02	1.22	1.35	1.42	1.47
Southern Sub-Saharan Africa	Botswana	0.80	0.83	0.90	1.15	1.40	1.05
	Lesotho	0.76	0.82	1.08	1.62	1.86	2.24
	Namibia	0.96	0.97	1.26	1.45	1.62	1.88
	South Africa	1.12	1.21	1.79	2.02	2.54	1.67
	Swaziland	0.93	0.98	1.26	1.90	2.60	2.72
	Zimbabwe	0.63	0.76	1.33	1.83	1.57	1.51

Observed : expected
- Less than 0.4
- 0.4 to 0.79
- 0.8 to 1.19
- 1.2 to 1.59
- 1.6 to 1.99
- 2.0 to 2.39
- 2.4 to 2.79
- 2.8 to 3.19
- >= 3.2
- No data

**Western Sub-Saharan Africa**	Benin	0.74	0.72	0.71	0.71	0.75	0.71
	Burkina Faso	0.66	0.68	0.74	0.73	0.68	0.62
	Cameroon	0.90	0.99	1.00	1.00	1.09	1.16
	Cape Verde	0.43	0.49	0.51	0.54	0.57	0.55
	Chad	0.60	0.59	0.66	0.64	0.64	0.62
	Cote d'Ivoire	0.77	0.88	1.05	1.10	1.20	0.89
	Ghana	0.96	0.96	1.00	1.05	1.11	1.19
	Guinea	0.71	0.71	0.75	0.71	0.73	0.73
	Guinea-Bissau	0.88	0.86	0.86	0.86	0.84	0.99
	Liberia	1.35	1.03	0.68	0.61	0.61	0.62
	Mali	0.73	0.69	0.70	0.62	0.61	0.60
	Mauritania	0.71	0.73	0.72	0.68	0.65	0.64
	Niger	0.68	0.68	0.61	0.53	0.49	0.45
	Nigeria	1.03	1.02	1.08	1.12	1.13	1.06
	Sao Tome and Principe	0.75	0.76	0.77	0.75	0.73	0.69
	Senegal	0.67	0.70	0.68	0.60	0.56	0.54
	Sierra Leone	0.83	1.11	0.94	0.87	0.85	0.84
	The Gambia	0.60	0.60	0.60	0.62	0.63	0.68
	Togo	0.74	0.75	0.78	0.88	0.95	0.93

DRC: Democratic Republic of the Congo
CAR Central African Republic

Figure S48: Ratio of observed : expected all-cause mortality rate per 100,000, 1990 - 2017 amongst 15-19 year olds (both sexes) in Sub-Saharan Africa GBD super-region

GBD region	Country	1990	1995	2000	2005	2010	2017
Central Sub-Saharan Africa	Angola	1.27	1.18	1.29	1.04	0.99	1.07
	CAR	1.29	1.36	1.48	1.60	1.75	2.02
	Congo	1.66	1.80	2.08	2.02	1.93	1.88
	DRC	1.07	1.08	1.04	0.91	0.89	0.97
	Equatorial Guinea	1.16	1.23	1.21	1.28	1.37	1.55
	Gabon	1.36	1.47	1.57	1.49	1.45	1.41
Eastern Sub-Saharan Africa	Burundi	1.32	2.70	2.55	2.00	1.00	0.92
	Comoros	0.83	0.83	0.79	0.77	0.73	0.72
	Djibouti	0.92	0.89	0.87	0.90	0.88	1.04
	Eritrea	6.38	1.43	1.78	1.47	1.42	1.33
	Ethiopia	1.09	0.84	1.04	0.69	0.75	0.67
	Kenya	0.82	0.97	1.05	1.17	1.38	1.33
	Madagascar	1.13	1.05	0.89	0.75	0.71	0.72
	Malawi	0.92	0.98		1.16	1.55	1.48
	Mozambique	0.78	0.74	0.78	0.85	1.16	1.46
	Rwanda	1.51	1.90	1.39	0.92	0.91	0.83
	Somalia	1.02	0.89	0.81	0.78	0.89	0.86
	South Sudan	0.89	0.81	0.86	0.71	0.80	1.03
	Tanzania	0.87	0.87	0.84	0.92	1.00	0.97
	Uganda	0.67	0.72	1.02	1.25	1.26	1.05
	Zambia	1.25	1.32	1.28	1.52	1.74	1.56
Southern Sub-Saharan Africa	Botswana	1.13	1.27	1.40	1.37	1.32	1.25
	Lesotho	1.02	1.27	1.64	2.16	2.56	2.83
	Namibia	1.28	1.44	1.93	1.88	1.73	1.76
	South Africa	1.18	1.37	2.44	2.34	2.34	1.84
	Swaziland	1.25	1.66	2.31	2.57	3.08	2.44
	Zimbabwe	1.05	1.26	1.69	2.43	2.35	1.69

Observed : expected
- Less than 0.4
- 0.4 to 0.79
- 0.8 to 1.19
- 1.2 to 1.59
- 1.6 to 1.99
- 2.0 to 2.39
- 2.4 to 2.79
- 2.8 to 3.19
- >= 3.2
- No data

**Western Sub-Saharan Africa**	Benin	0.73	0.77	0.79	0.77	0.80	0.79
	Burkina Faso	0.69	0.67	0.71	0.79	0.77	0.71
	Cameroon	1.01	1.15	1.20	1.16	1.19	1.31
	Cape Verde	0.44	0.51	0.54	0.58	0.62	0.62
	Chad	0.61	0.60	0.70	0.67	0.69	0.69
	Cote d'Ivoire	0.92	1.02	1.11	1.28	1.25	1.01
	Ghana	1.07	1.08	1.12	1.25	1.32	1.29
	Guinea	0.66	0.69	0.81	0.77	0.82	0.83
	Guinea-Bissau	1.01	1.01	1.01	1.03	1.03	1.14
	Liberia	1.70	1.22	0.69	0.64	0.67	0.72
	Mali	0.72	0.68	0.70	0.58	0.57	0.58
	Mauritania	0.83	0.81	0.76	0.72	0.69	0.69
	Niger	0.59	0.59	0.55	0.50	0.48	0.47
	Nigeria	0.91	0.93	0.96	0.91	0.94	0.89
	Sao Tome and Principe	0.69	0.73	0.81	0.84	0.85	0.87
	Senegal	0.73	0.76	0.73	0.67	0.65	0.65
	Sierra Leone	0.78	1.25	1.00	0.93	0.91	0.87
	The Gambia	0.69	0.71	0.74	0.79	0.77	0.85
	Togo	0.81	0.88	0.91	1.00	1.06	1.17

DRC: Democratic Republic of the Congo
CAR Central African Republic

Figure S49: Ratio of observed : expected all-cause mortality rate per 100,000, 1990 - 2017 amongst 20-24 year olds (both sexes) in Sub-Saharan Africa GBD super-region

GBD region	Country	1990	1995	2000	2005	2010	2017
Central Sub-Saharan Africa	Angola	1.36	1.26	1.43	1.19	1.13	1.10
	CAR	1.60	1.74	1.88	1.77	1.71	1.99
	Congo	2.10	2.12	2.15	1.77	1.78	1.75
	DRC	1.23	1.23	1.17	0.99	0.93	0.95
	Equatorial Guinea	1.24	1.36	1.42	1.51	1.61	1.47
	Gabon	1.48	1.69	2.15	1.98	1.57	1.31
Eastern Sub-Saharan Africa	Burundi	1.47	3.25	3.14	2.29	0.87	0.86
	Comoros	0.88	0.89	0.83	0.80	0.75	0.70
	Djibouti	1.00	0.95	1.07	1.13	0.94	0.88
	Eritrea	7.33	1.74	2.25	1.75	1.51	1.32
	Ethiopia	1.22	1.05	1.25	0.68	0.60	0.59
	Kenya	1.05	1.60	1.71	1.50	1.38	1.28
	Madagascar	1.05	0.99	0.88	0.83	0.77	0.75
	Malawi	1.27	1.72		1.39	1.22	1.09
	Mozambique	0.85	0.92	1.13	1.26	1.42	1.48
	Rwanda	1.69	2.21	1.71	1.06	0.84	0.76
	Somalia	1.12	0.97	0.89	0.85	0.96	0.93
	South Sudan	0.96	0.90	1.00	0.81	0.86	1.11
	Tanzania	1.23	1.35	1.23	1.06	0.92	0.85
	Uganda	1.20	1.02	1.07	1.11	1.16	0.94
	Zambia	1.85	2.29	2.03	1.86	1.74	1.39
Southern Sub-Saharan Africa	Botswana	1.64	2.36	3.59	3.07	2.09	1.49
	Lesotho	1.19	1.93	3.10	3.75	3.64	3.20
	Namibia	1.51	2.00	3.13	3.13	2.27	1.72
	South Africa	1.89	2.25	4.52	5.01	3.88	2.40
	Swaziland	1.25	2.42	4.76	5.04	4.99	2.70
	Zimbabwe	2.03	3.04	3.18	3.36	2.45	1.62

Observed : expected
- Less than 0.4
- 0.4 to 0.79
- 0.8 to 1.19
- 1.2 to 1.59
- 1.6 to 1.99
- 2.0 to 2.39
- 2.4 to 2.79
- 2.8 to 3.19
- >= 3.2
- No data

**Western Sub-Saharan Africa**	Benin	0.75	0.83	0.89	0.84	0.82	0.79
	Burkina Faso	0.84	0.81	0.75	0.75	0.75	0.71
	Cameroon	1.10	1.33	1.56	1.52	1.35	1.23
	Cape Verde	0.50	0.57	0.61	0.62	0.59	0.62
	Chad	0.67	0.71	0.81	0.74	0.71	0.70
	Cote d'Ivoire	1.25	1.43	1.32	1.33	1.22	1.09
	Ghana	1.21	1.27	1.28	1.34	1.33	1.22
	Guinea	0.69	0.74	0.90	0.88	0.91	0.86
	Guinea-Bissau	1.09	1.10	1.16	1.23	1.20	1.13
	Liberia	1.98	1.41	0.83	0.74	0.68	0.70
	Mali	0.77	0.74	0.79	0.63	0.59	0.59
	Mauritania	0.88	0.85	0.80	0.74	0.68	0.64
	Niger	0.60	0.61	0.59	0.53	0.50	0.48
	Nigeria	0.94	1.10	1.20	1.11	1.03	0.96
	Sao Tome and Principe	0.71	0.75	0.82	0.87	0.88	0.85
	Senegal	0.77	0.83	0.81	0.73	0.68	0.63
	Sierra Leone	0.78	1.32	1.07	1.05	1.03	0.90
	The Gambia	0.73	0.77	0.86	0.94	0.89	0.85
	Togo	0.88	1.05	1.21	1.24	1.05	1.01

DRC: Democratic Republic of the Congo
CAR Central African Republic

Figure S50: Global deaths by cause group in 10-14, 15-19, 20-24and 10-24 in 2019 by sex

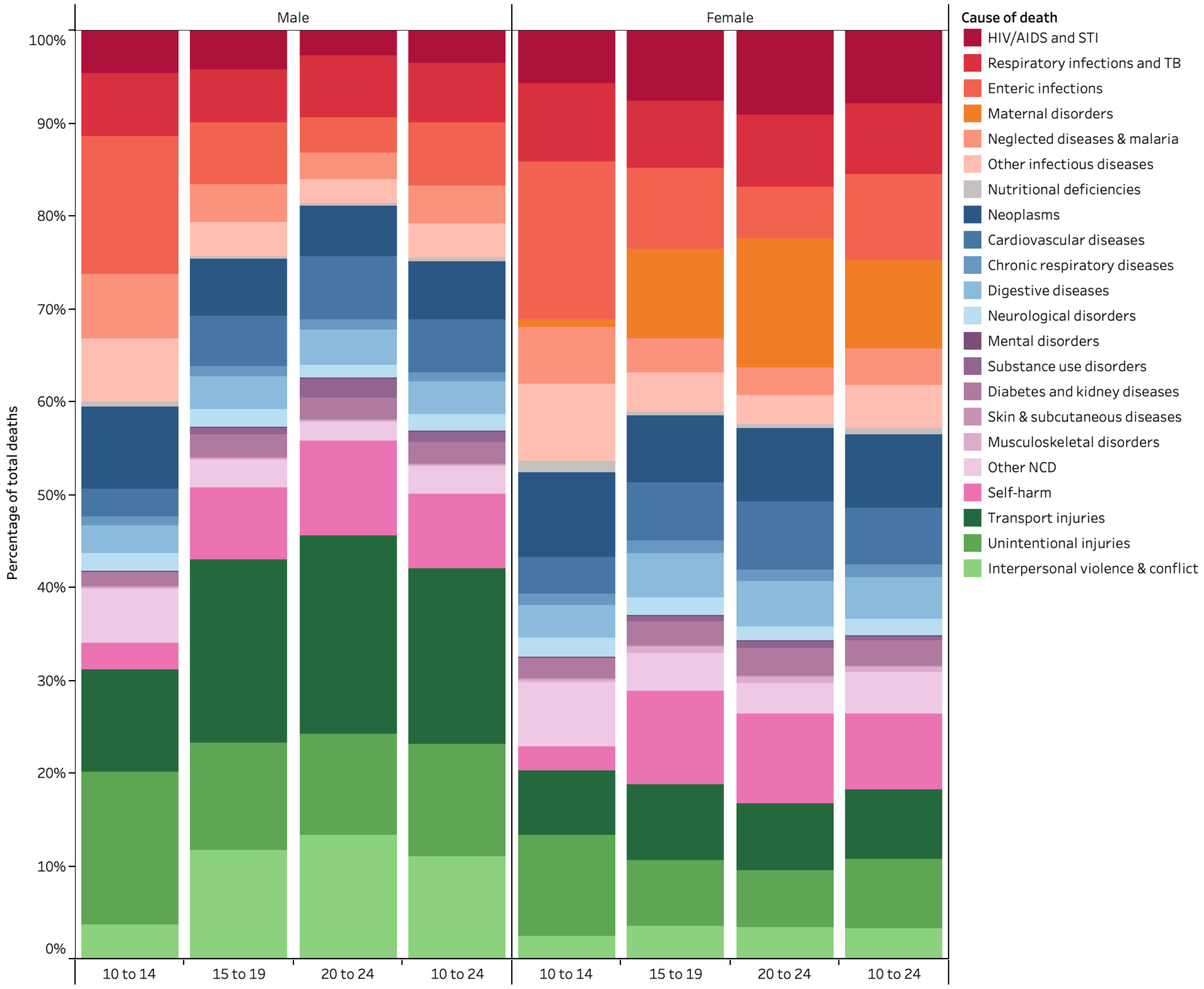

Figure S51: Percentage of total deaths by cause group in 10-14 males 1980 – 2019

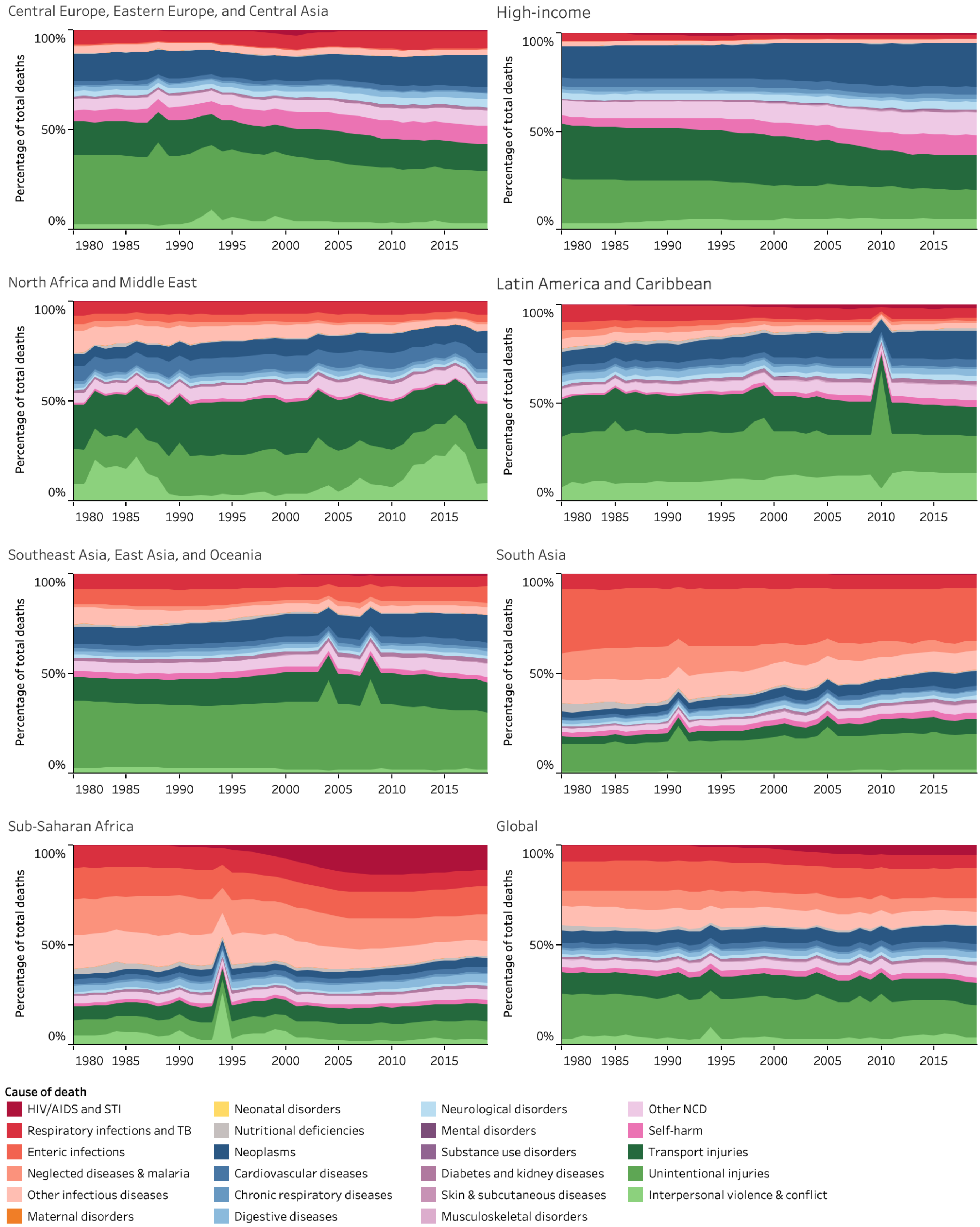

Figure S52: Percentage of total deaths by cause group in 10-14 females 1980 – 2019

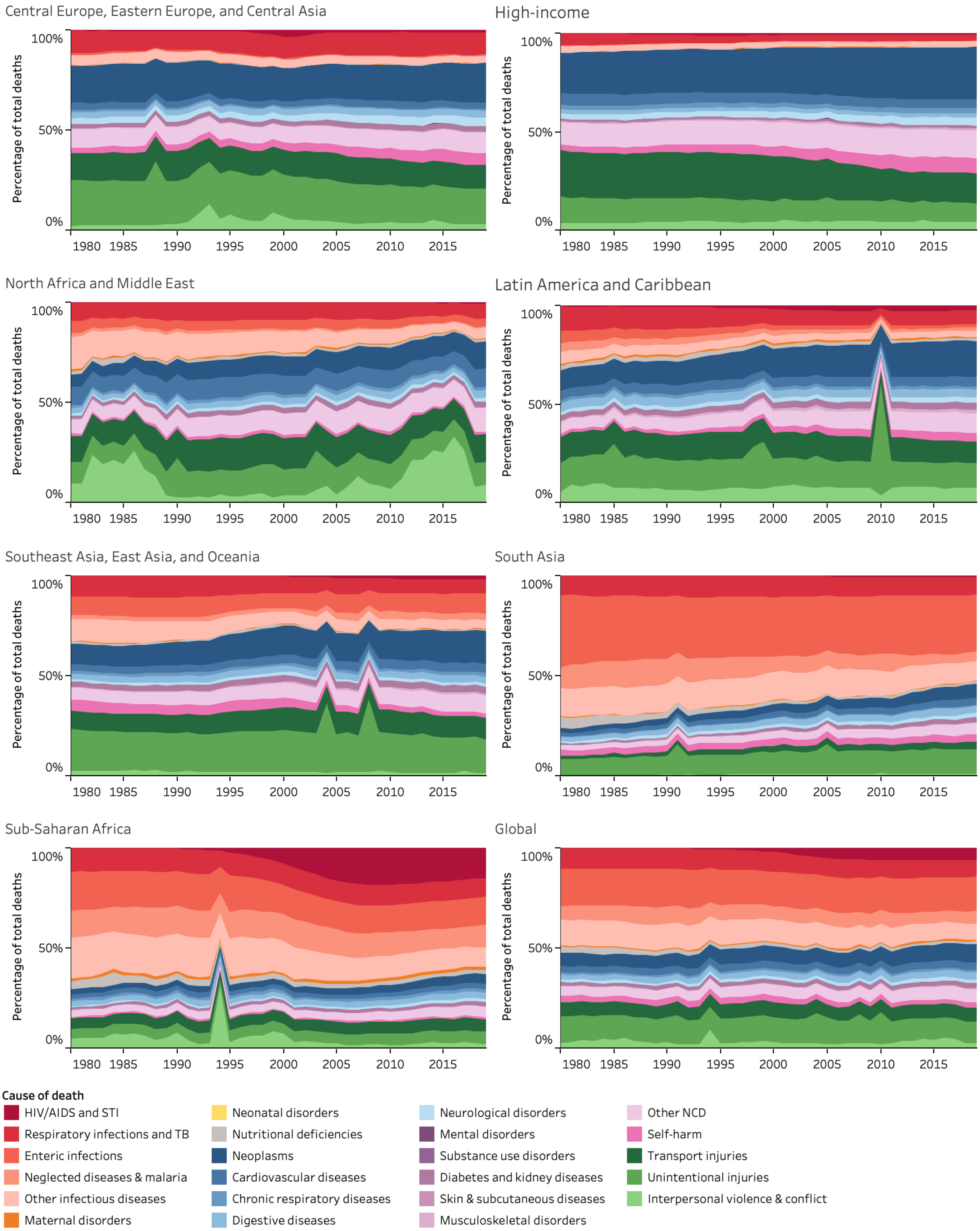

Figure S53: Percentage of total deaths by cause group in 15-19 males 1980 – 2019

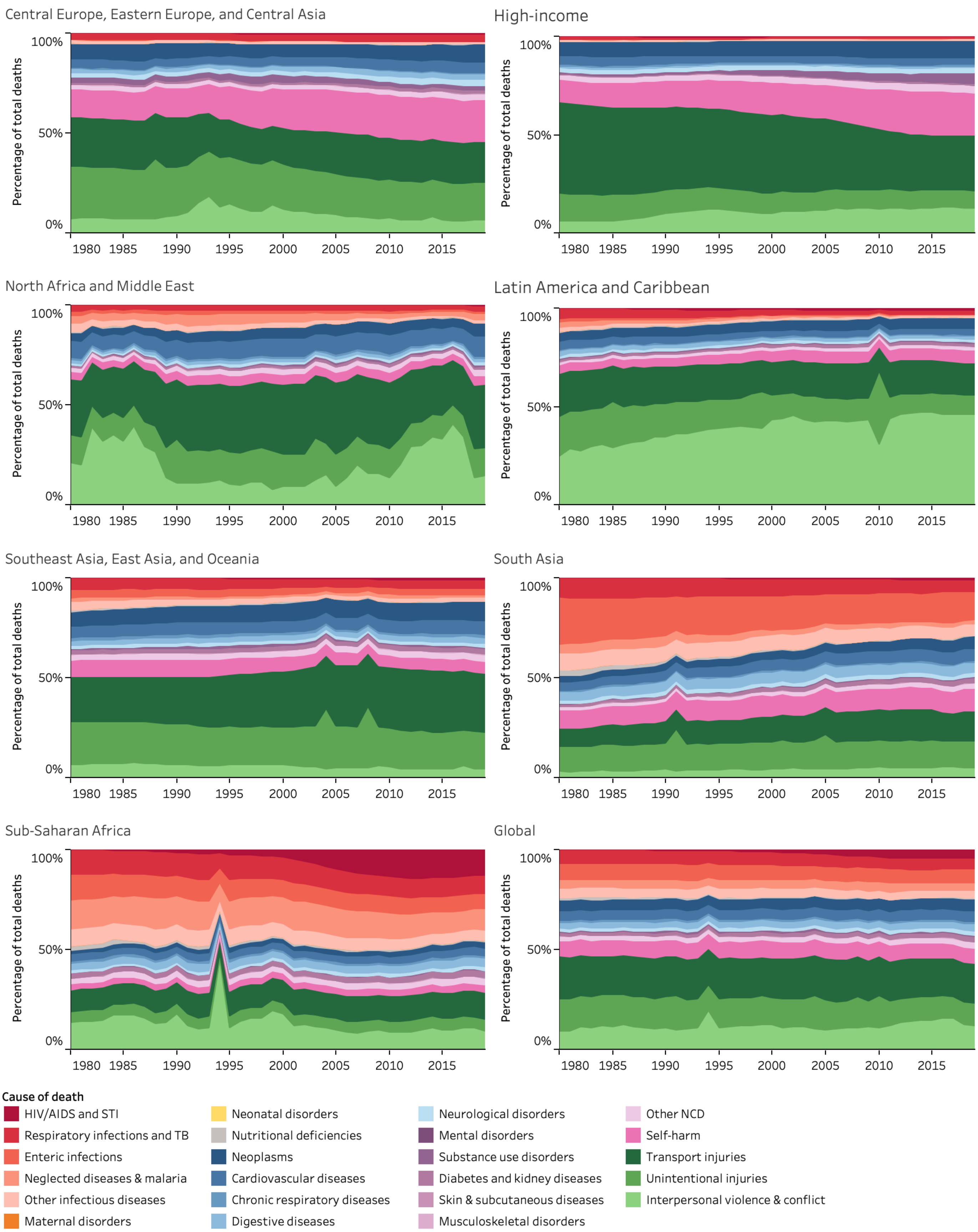

Figure S54: Percentage of total deaths by cause group in 15-19 females 1980 – 2019

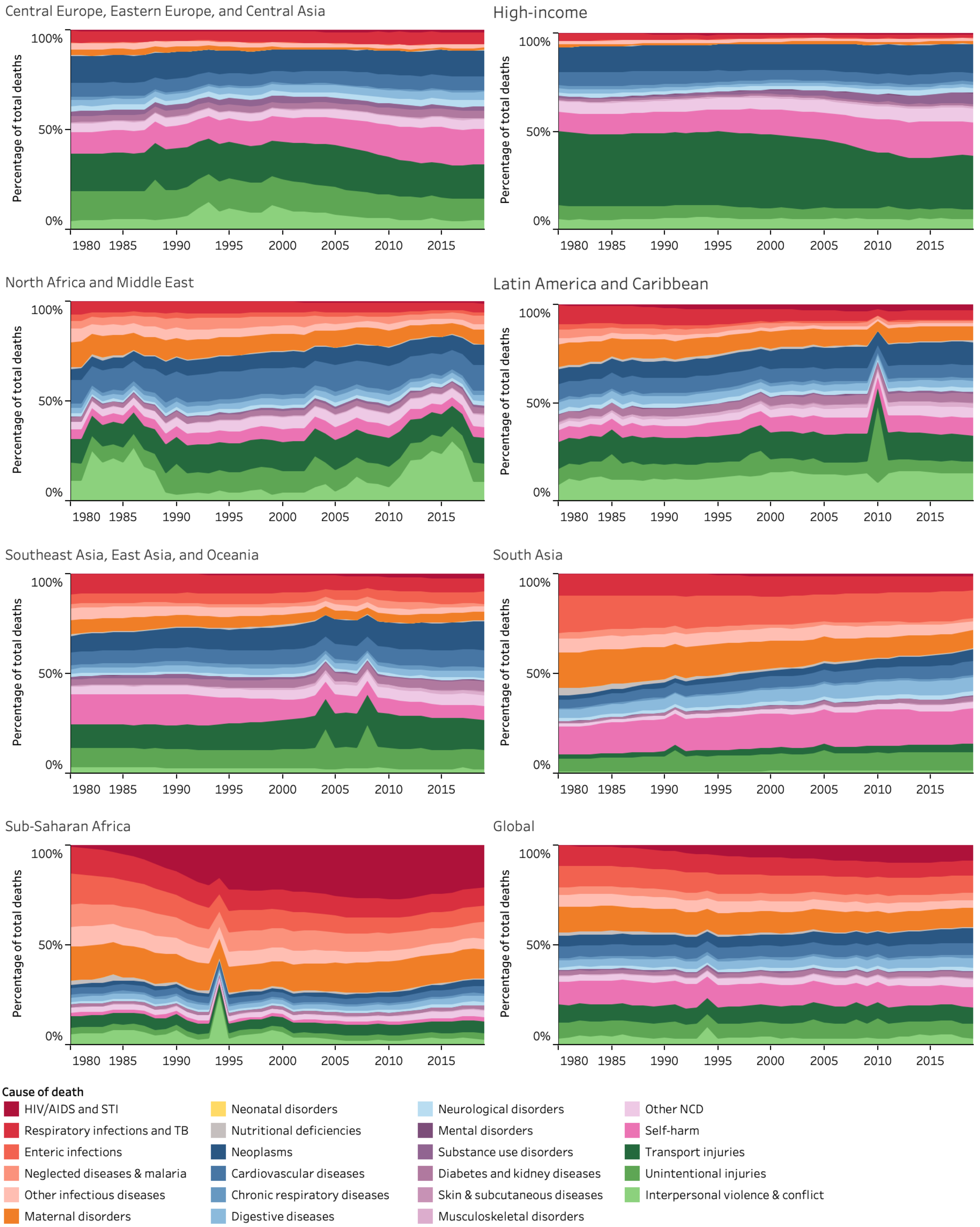

Figure S55: Percentage of total deaths by cause group in 20-24 males 1980 – 2019

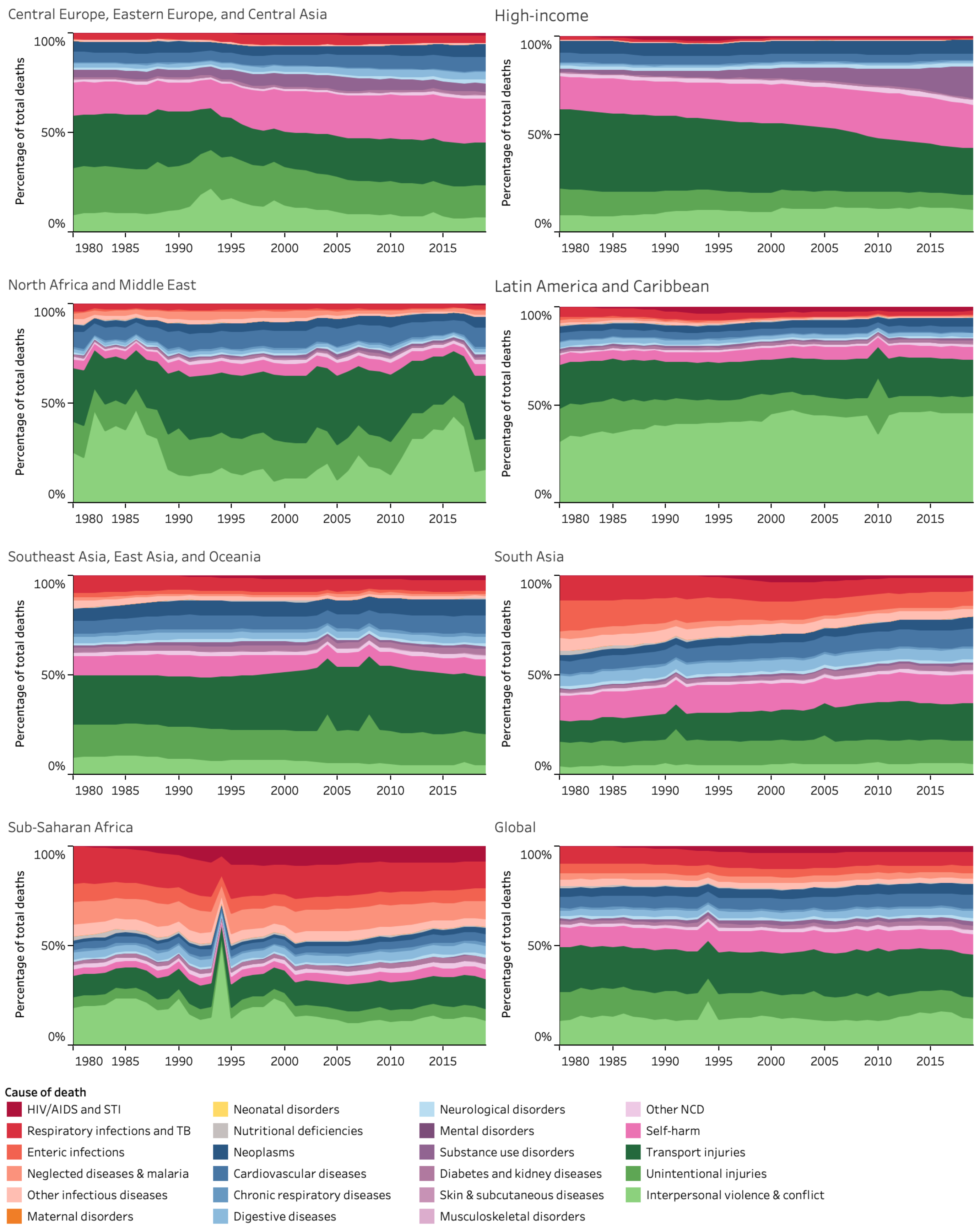

Figure S56: Percentage of total deaths by cause group in 20-24 females 1980 – 2019

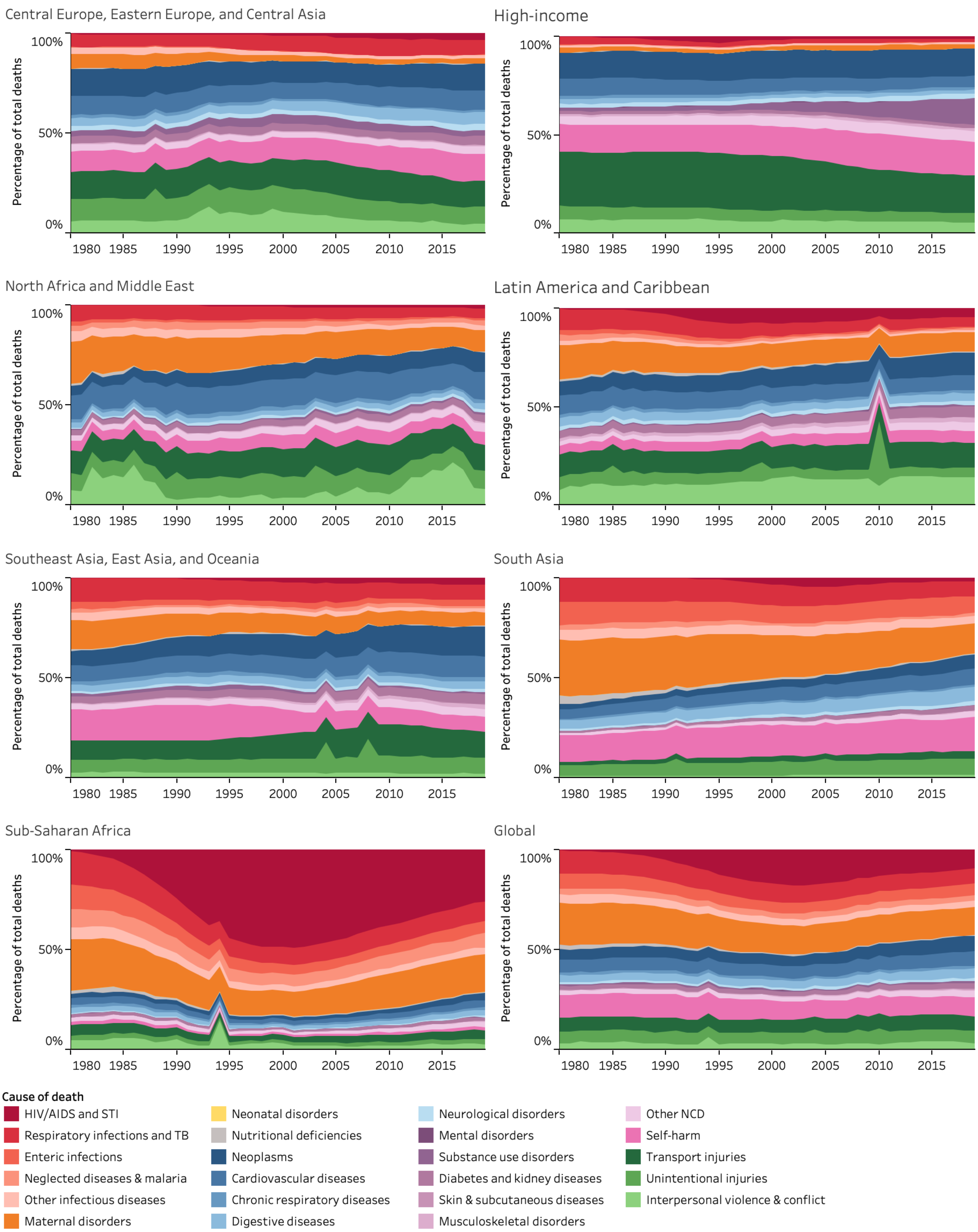

Figure S57: Mortality rate per 100,000 population by cause of death in 10-24 year olds 1980 – 2019: Central Europe, Eastern Europe and Central Asia GBD super-region

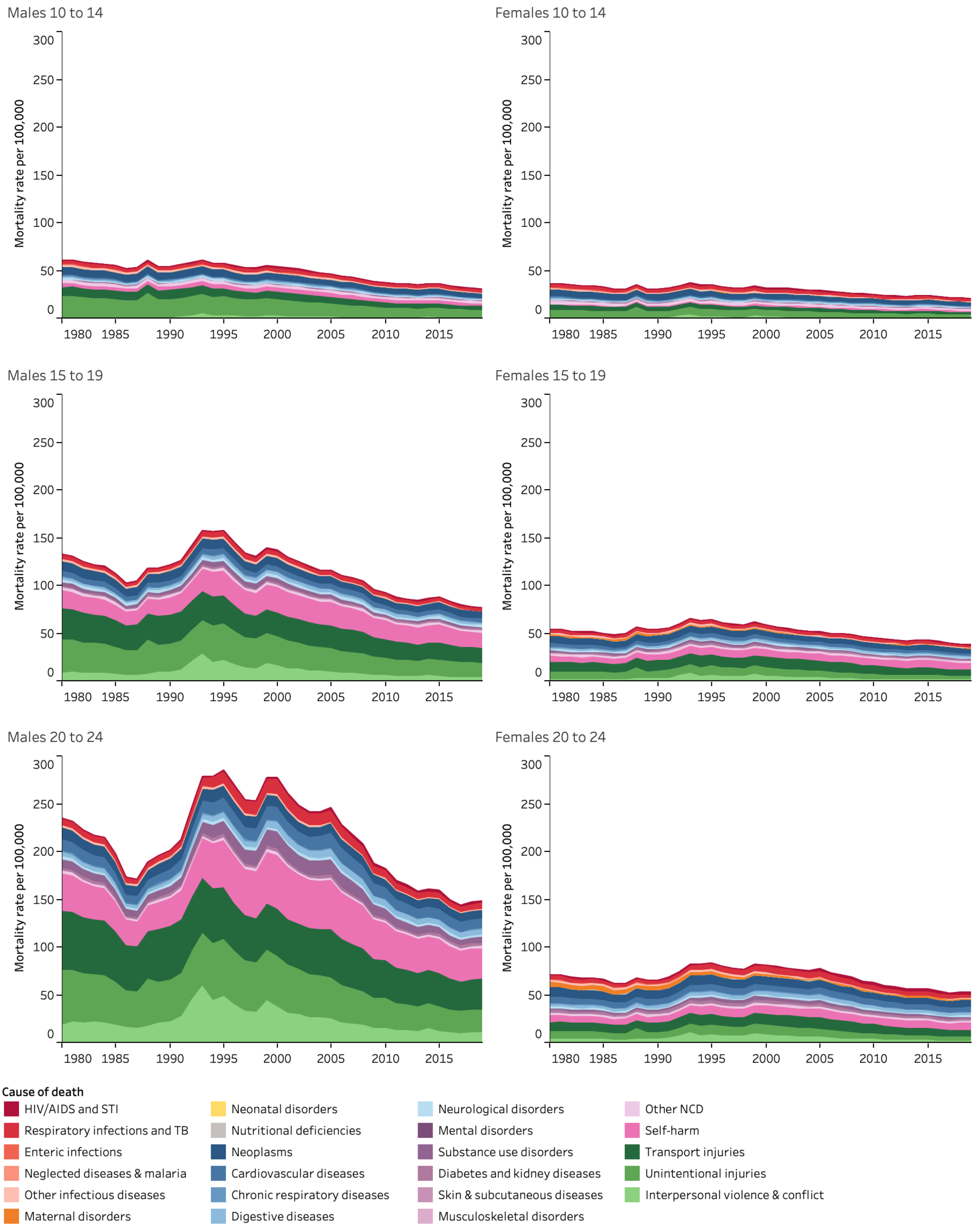

Figure S58: Number of deaths by cause of death in 10-24 year olds 1980 – 2019: Central Europe, Eastern Europe and Central Asia GBD super-region

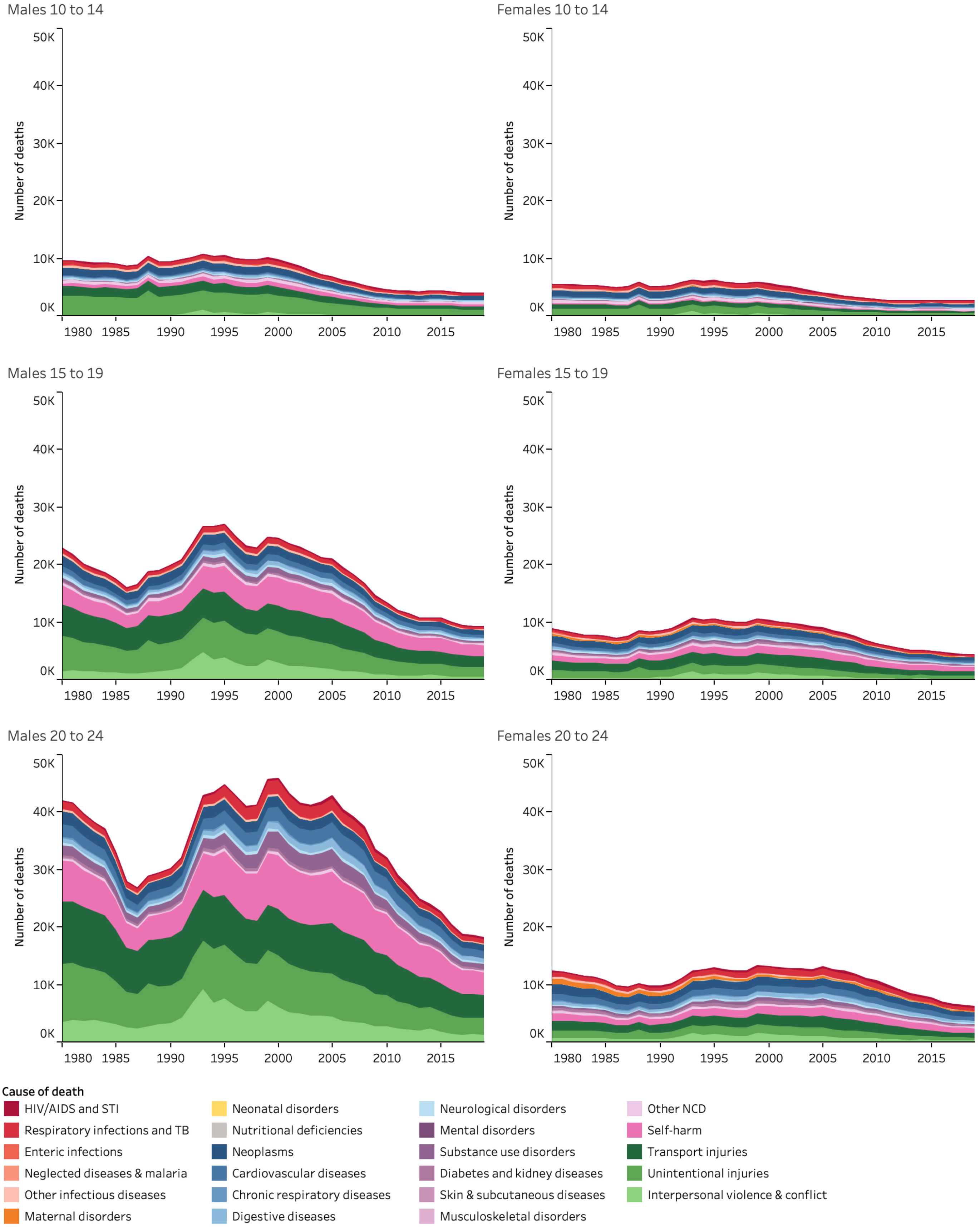

Figure S59: Rank of number of deaths by cause group 1980 – 2019: Central Europe, Eastern Europe and Central Asia GBD super-region. 10-14 year old males.

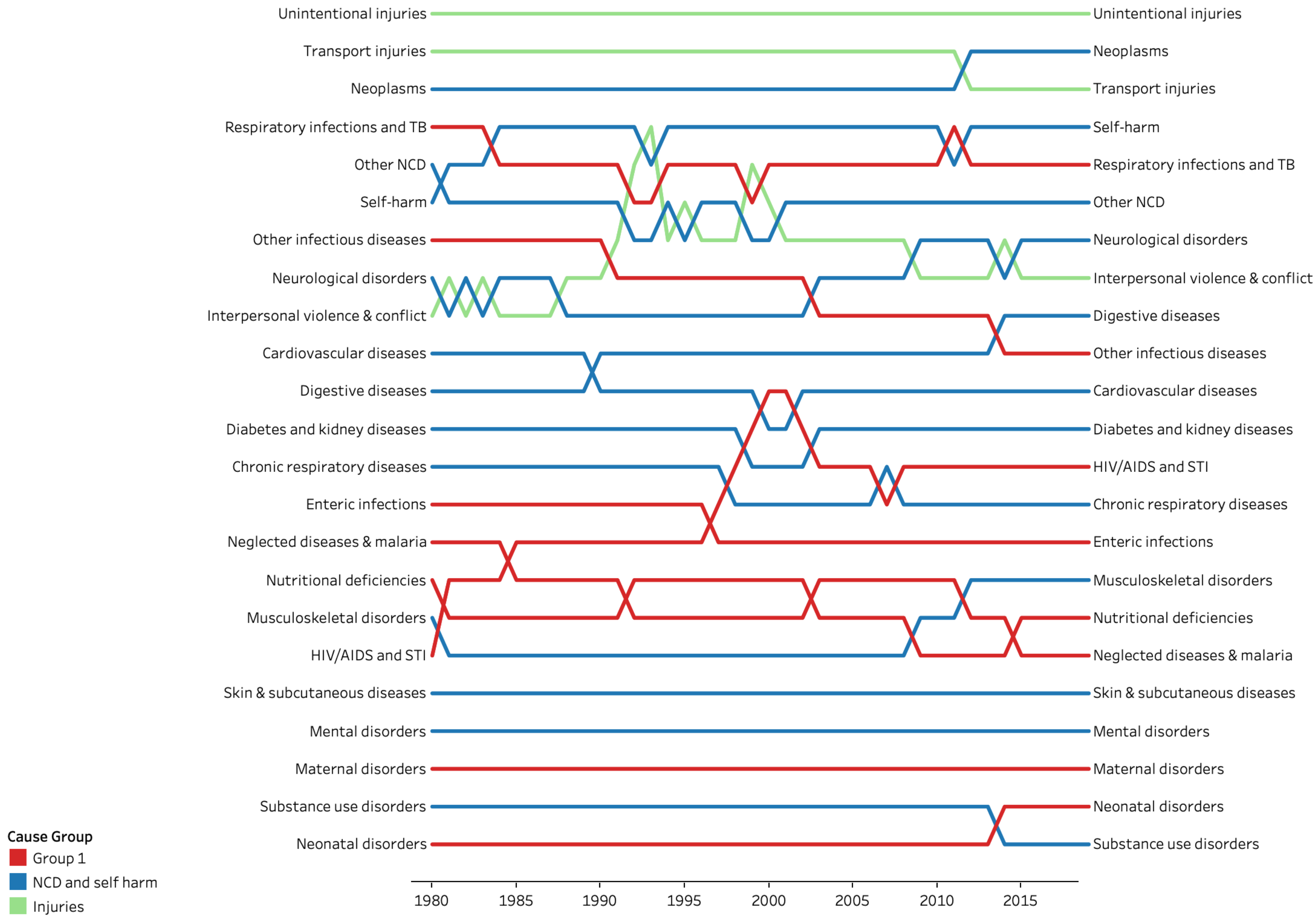

Figure S60: Rank of number of deaths by cause group 1980 – 2019: Central Europe, Eastern Europe and Central Asia GBD super-region. 10-14 year old females.

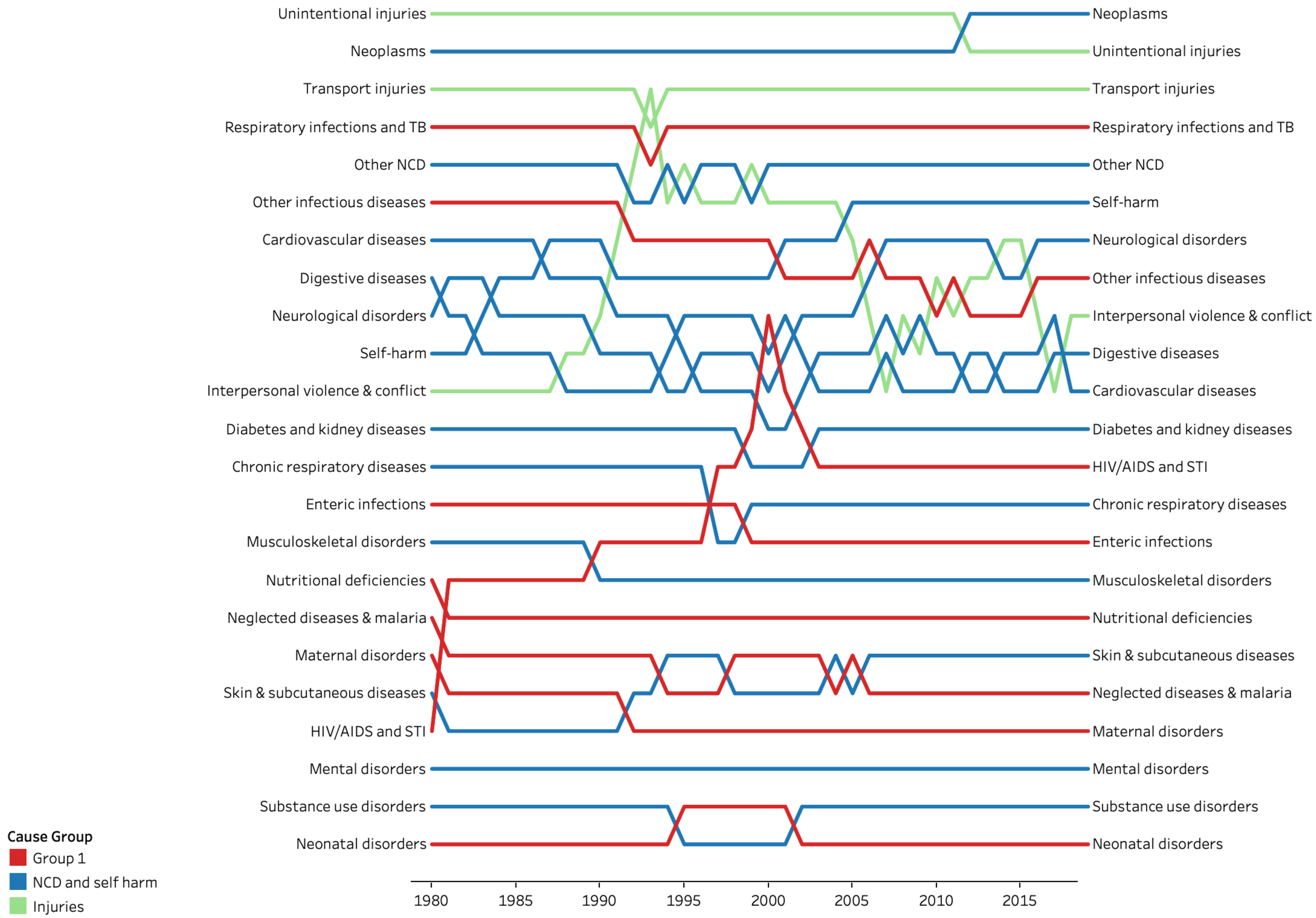

Figure S61: Rank of number of deaths by cause group 1980 – 2019: Central Europe, Eastern Europe and Central Asia GBD super-region. 15-19 year old males.

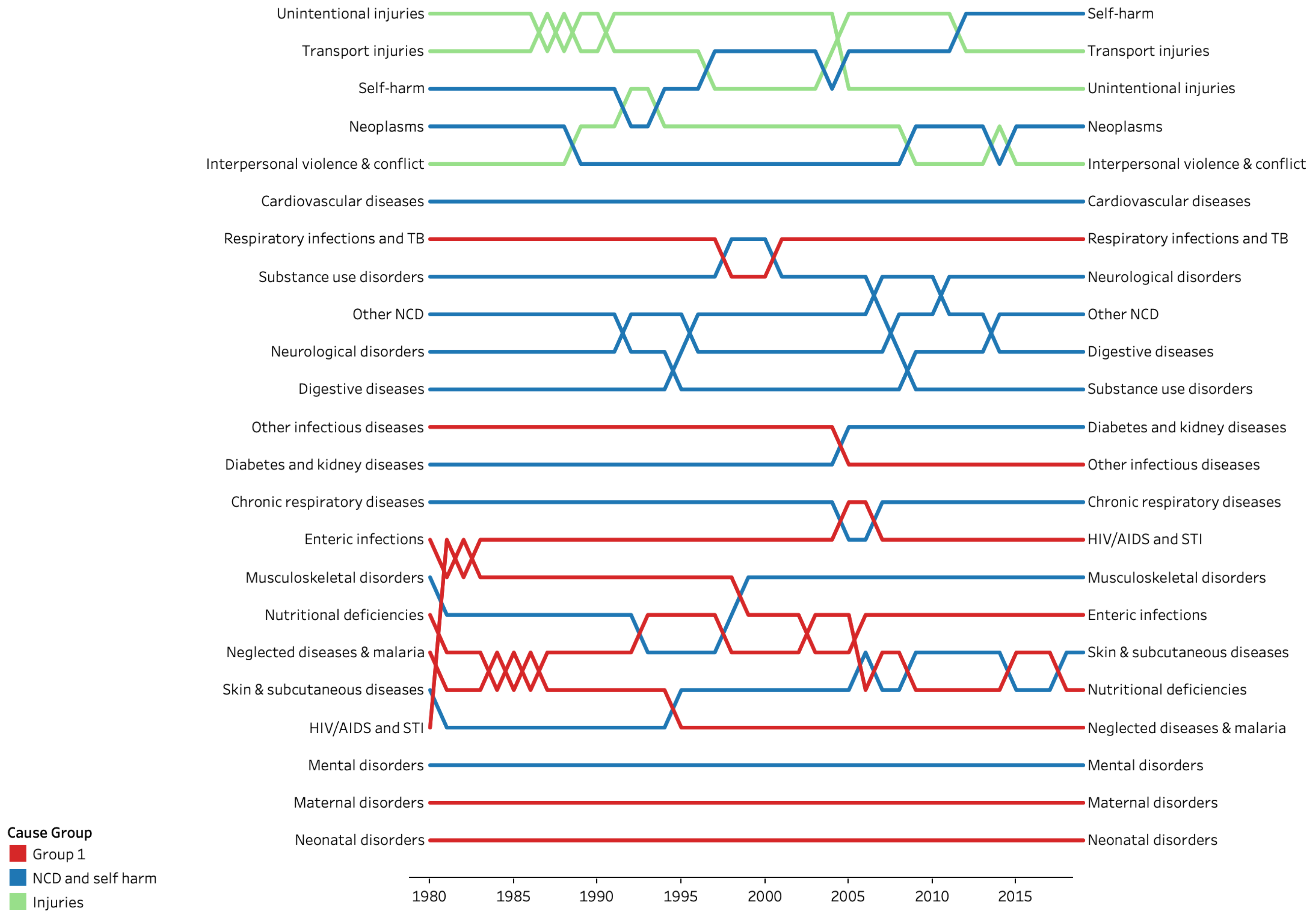

Figure S62: Rank of number of deaths by cause group 1980 – 2019: Central Europe, Eastern Europe and Central Asia GBD super-region. 15-19 year old females.

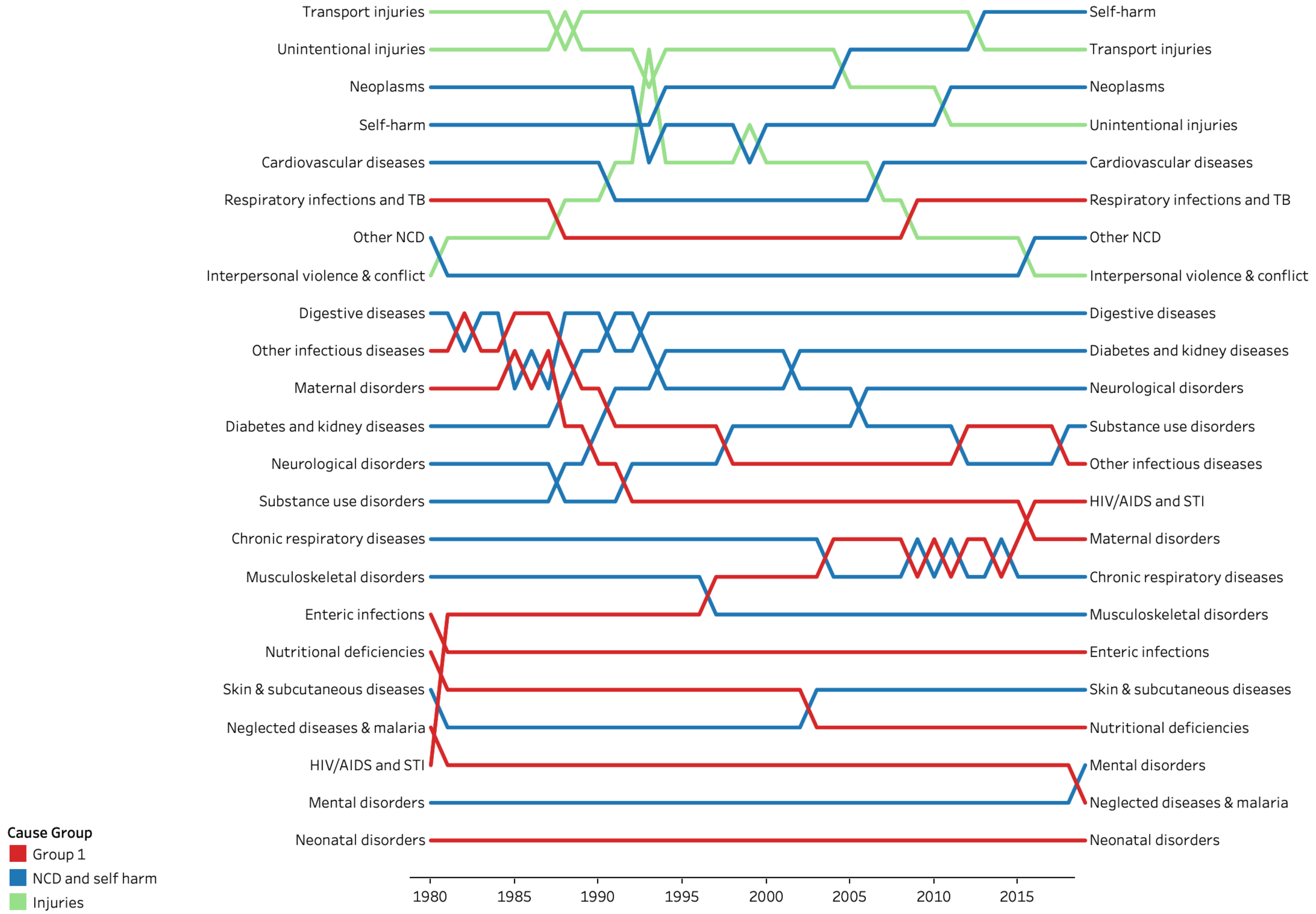

Figure S63: Rank of number of deaths by cause group 1980 – 2019: Central Europe, Eastern Europe and Central Asia GBD super-region. 20-24 year old males.

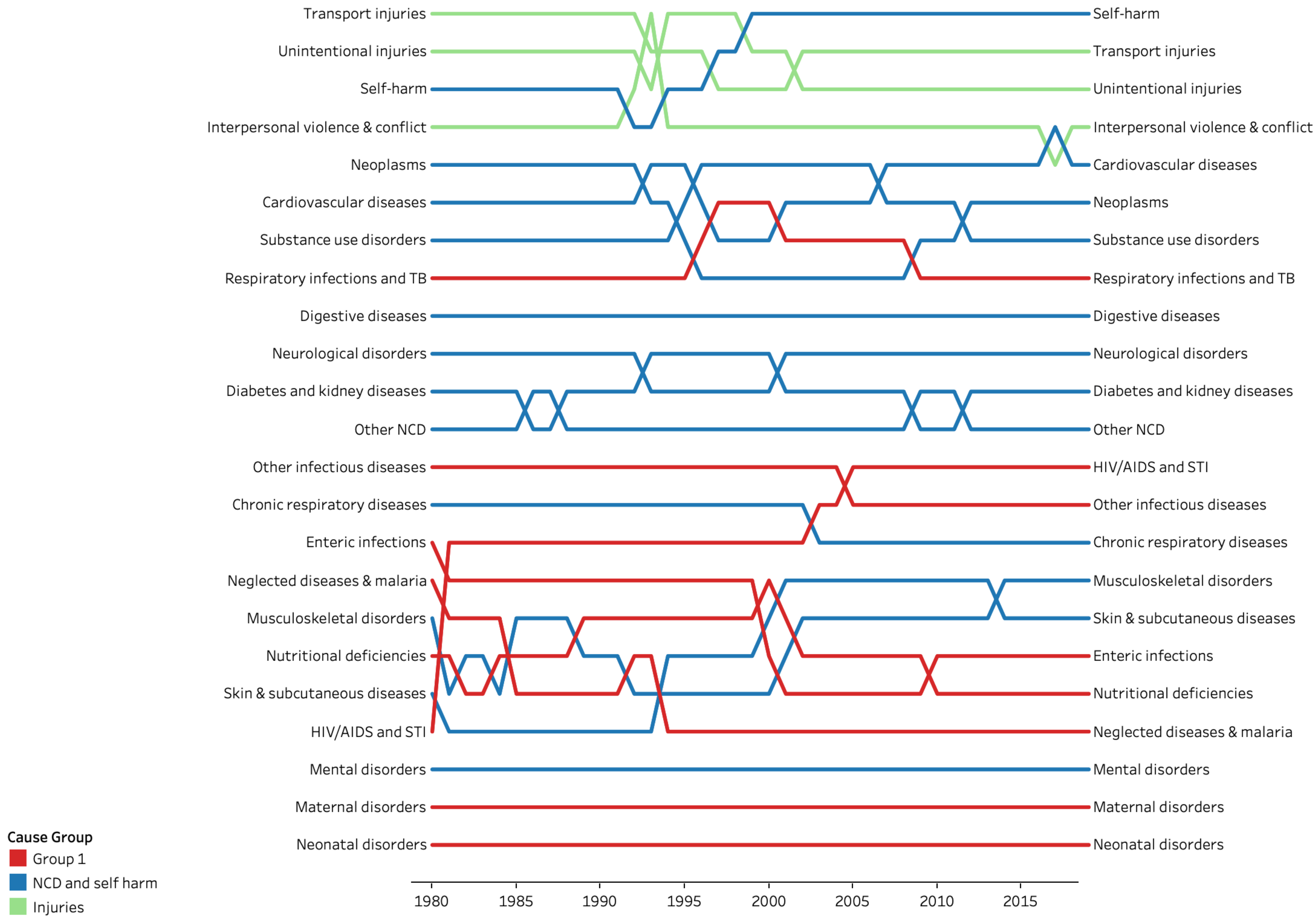

Figure S64: Rank of number of deaths by cause group 1980 – 2019: Central Europe, Eastern Europe and Central Asia GBD super-region. 20-24 year old females.

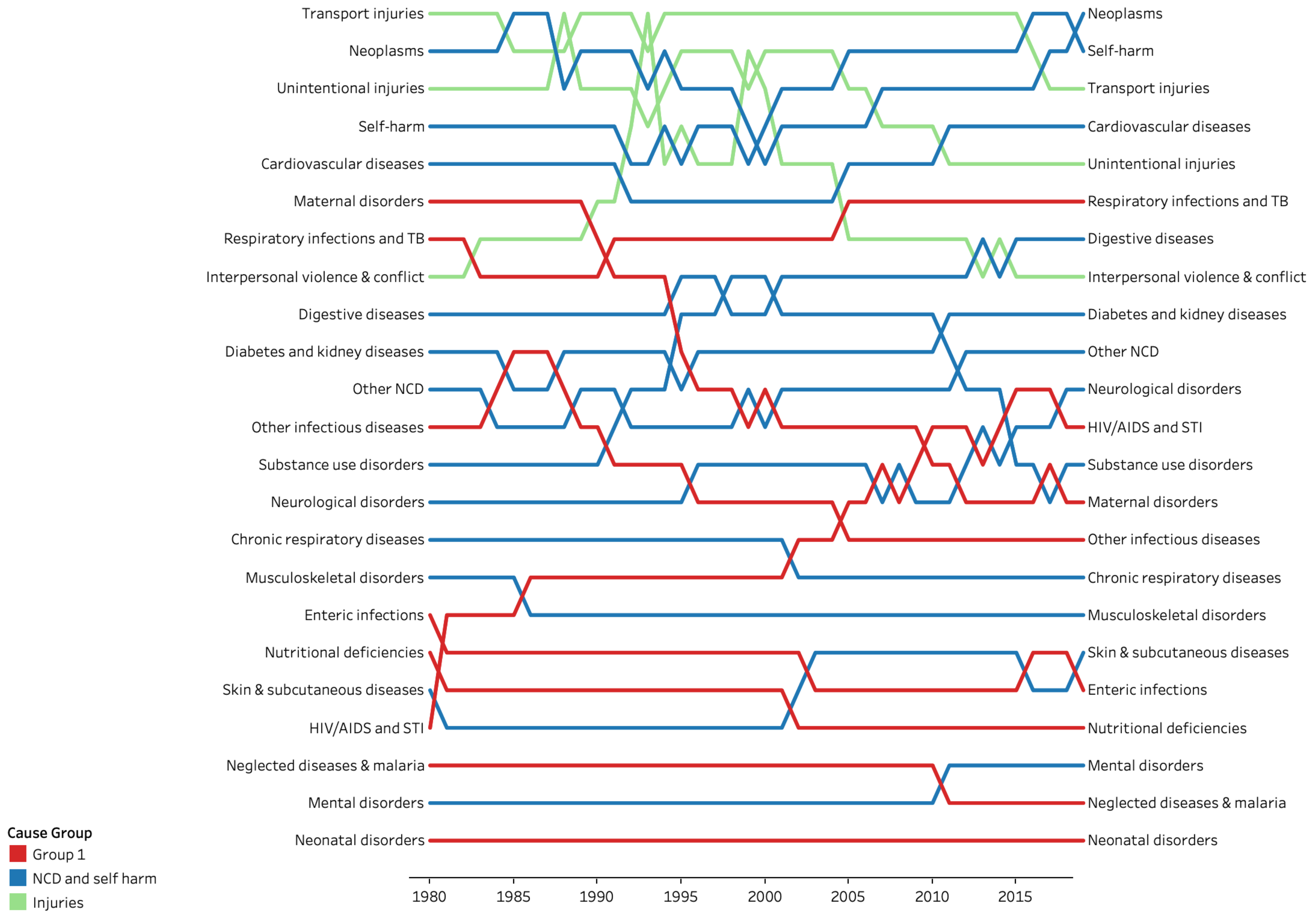

Figure S65: Mortality rate per 100,000 population by cause of death in 10-24 year olds 1980 – 2019: High Income GBD super-region

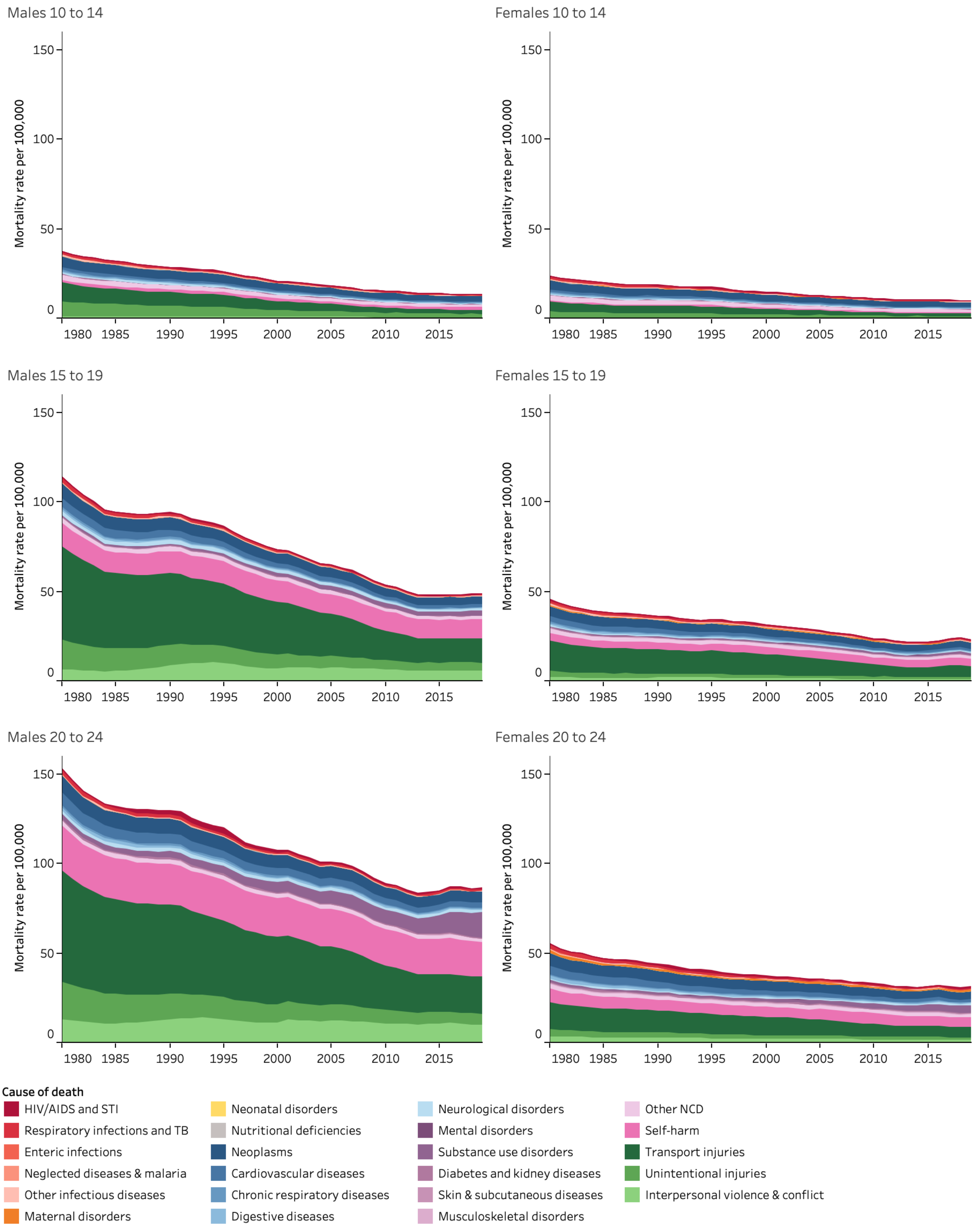

Figure S66: Number of deaths by cause in 10-24 year olds 1980 – 2019: High Income GBD super-region

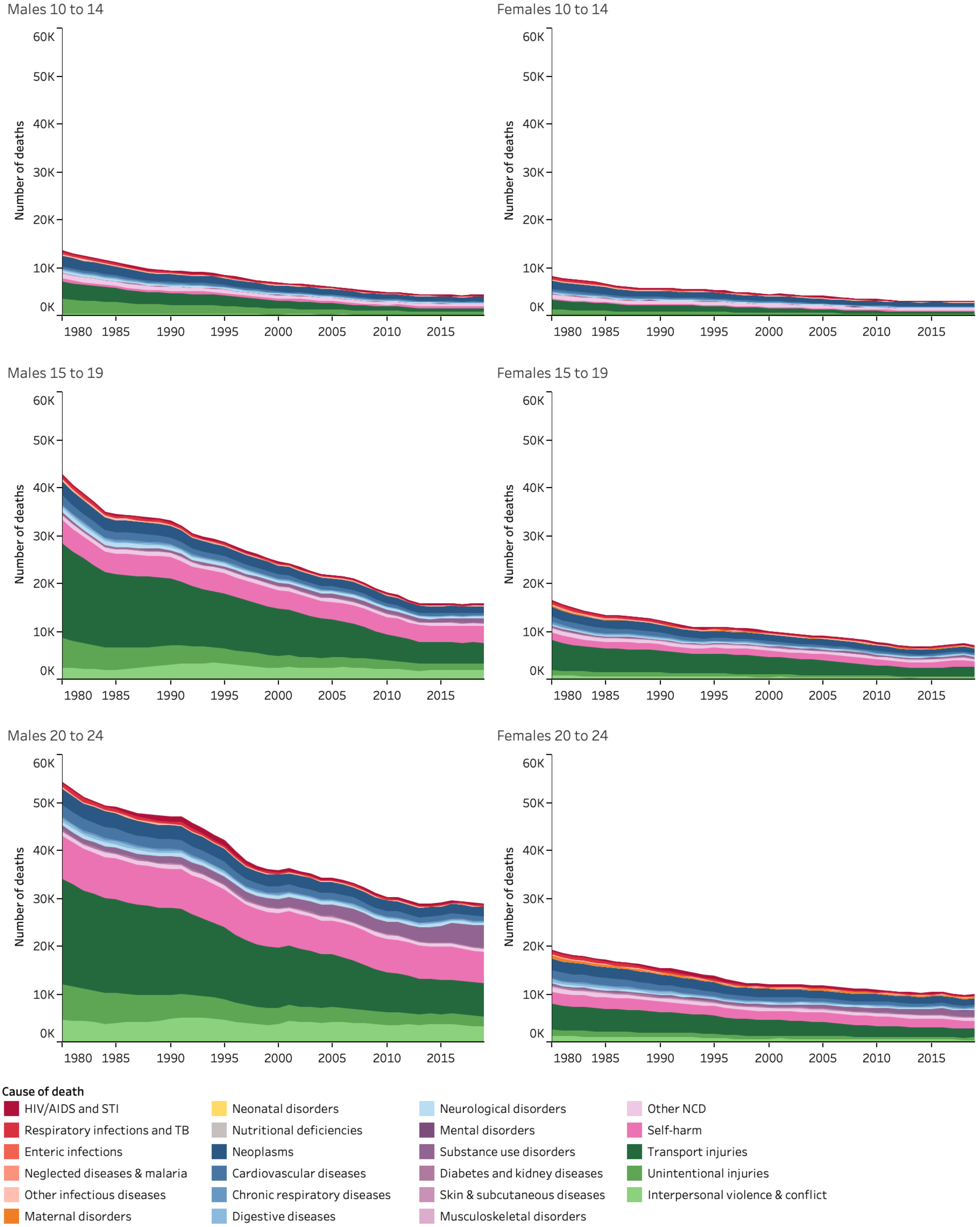

Figure S67: Rank of number of deaths by cause group 1980 – 2019: High Income GBD super-region. 10-14 year old males.

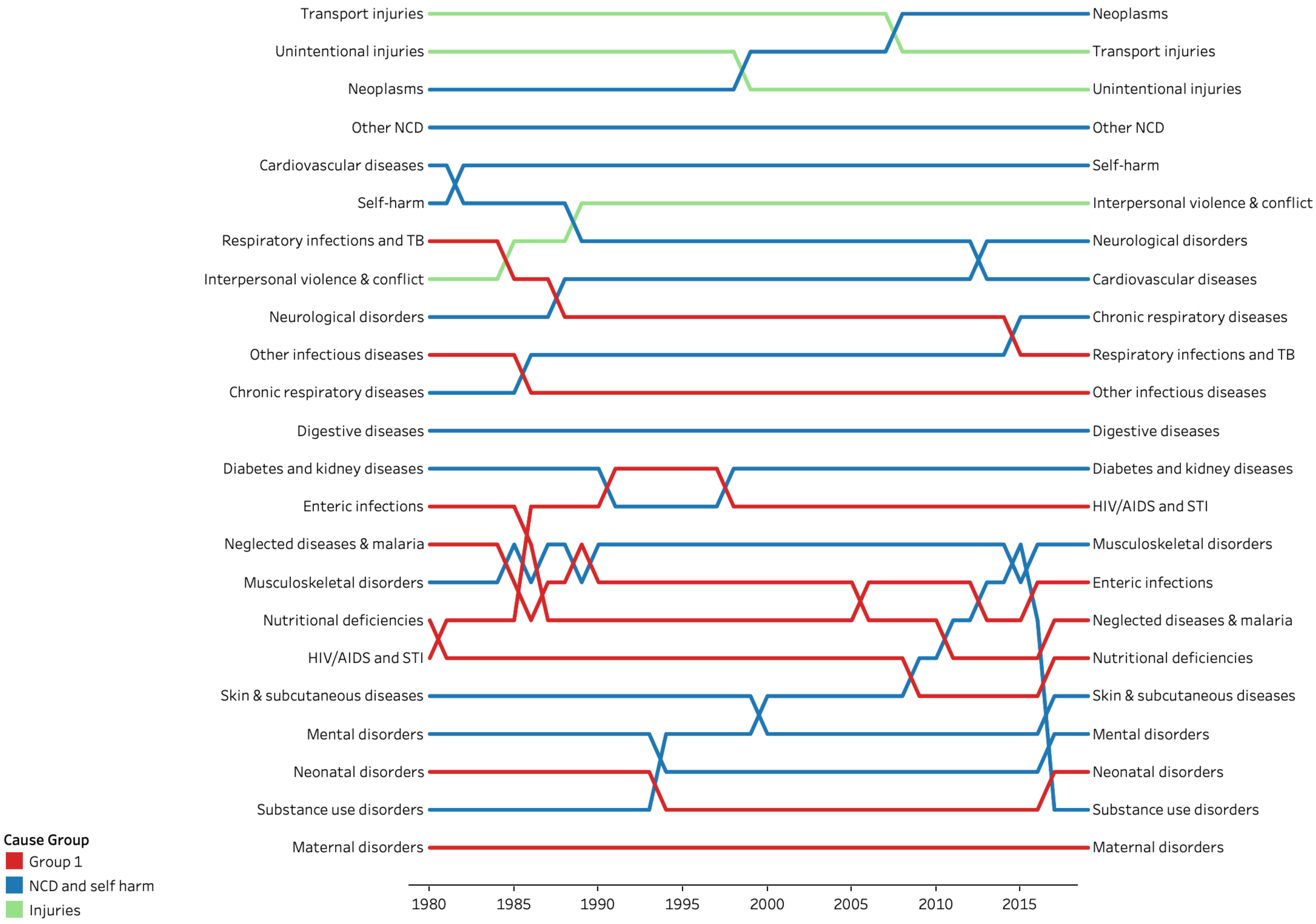

Figure S68: Rank of number of deaths by cause group 1980 – 2019: High Income GBD super-region. 10-14 year old females.

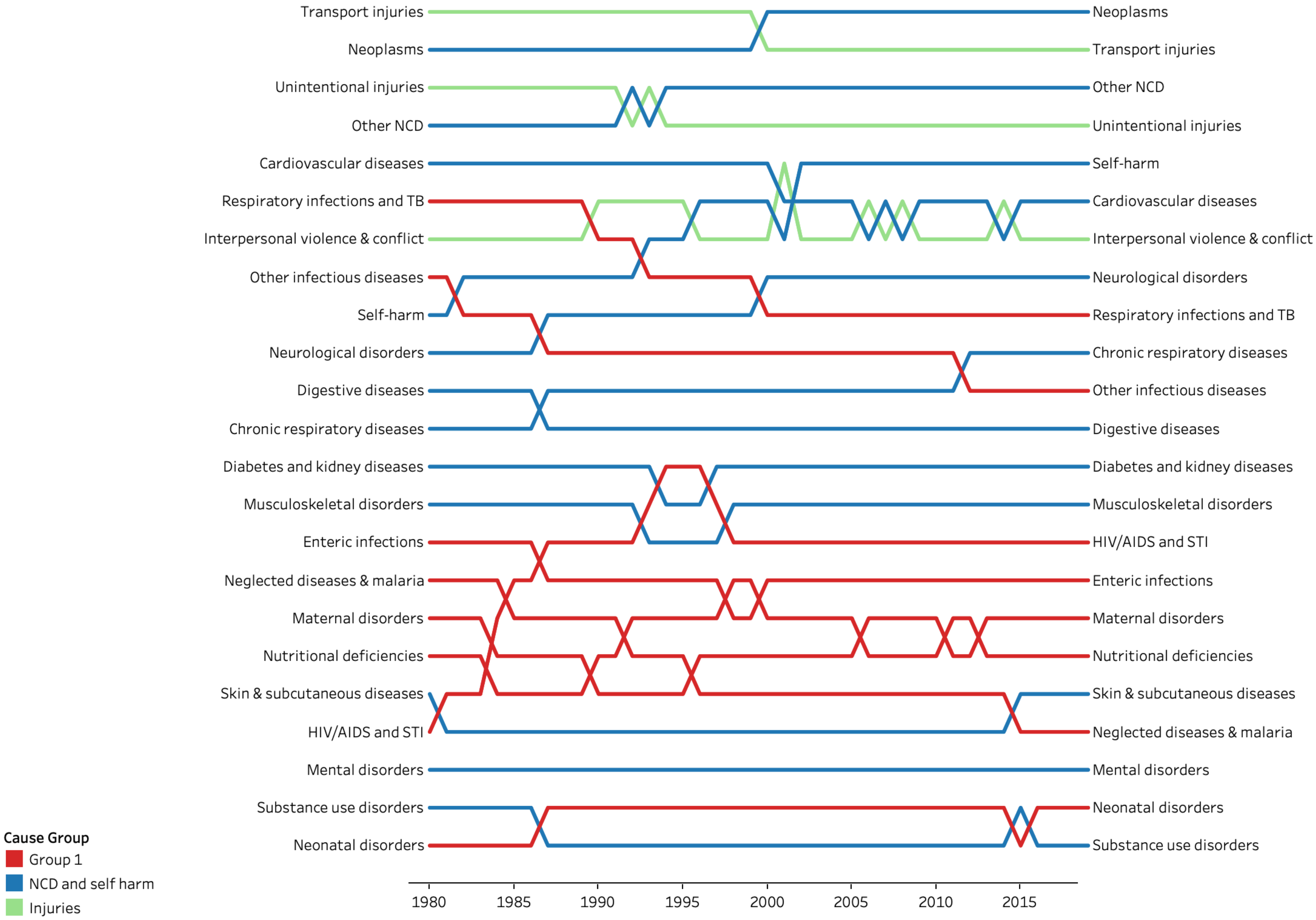

Figure S69: Rank of number of deaths by cause group 1980 – 2019: High Income GBD super-region. 15-19 year old males.

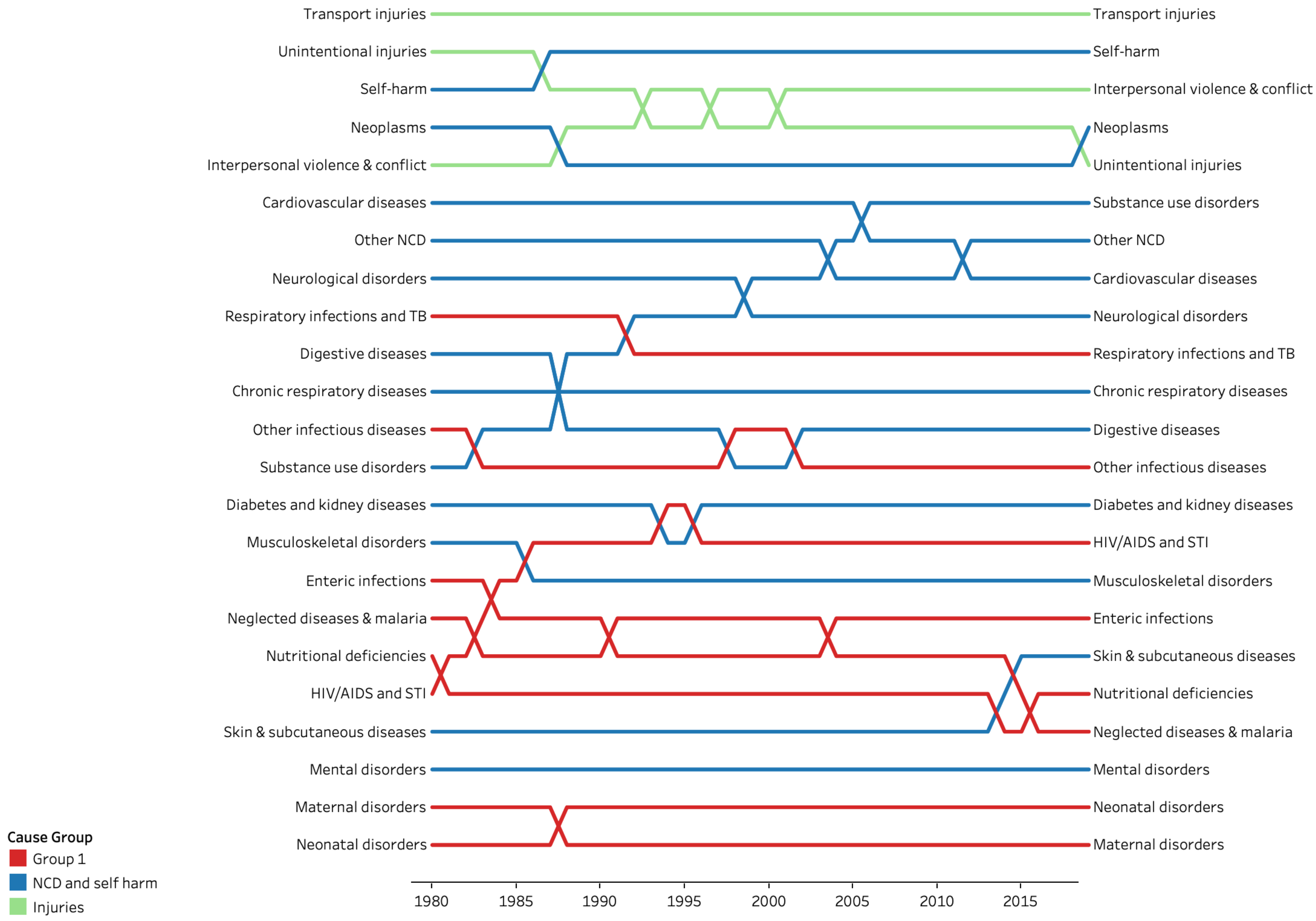

Figure S70: Rank of number of deaths by cause group 1980 – 2019: High Income GBD super-region. 15-19 year old females.

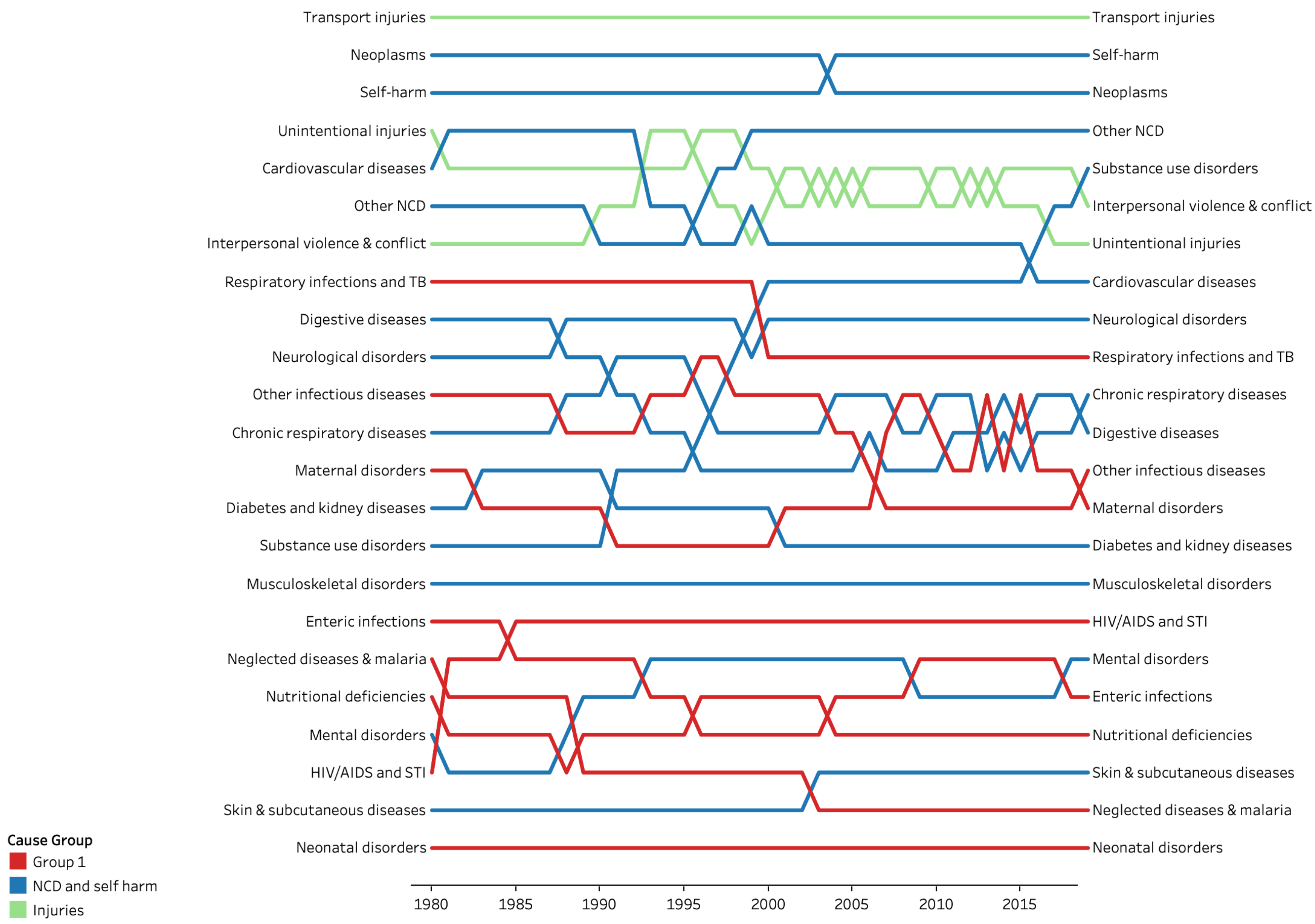

Figure S71: Rank of number of deaths by cause group 1980 – 2019: High Income GBD super-region. 20-24 year old males.

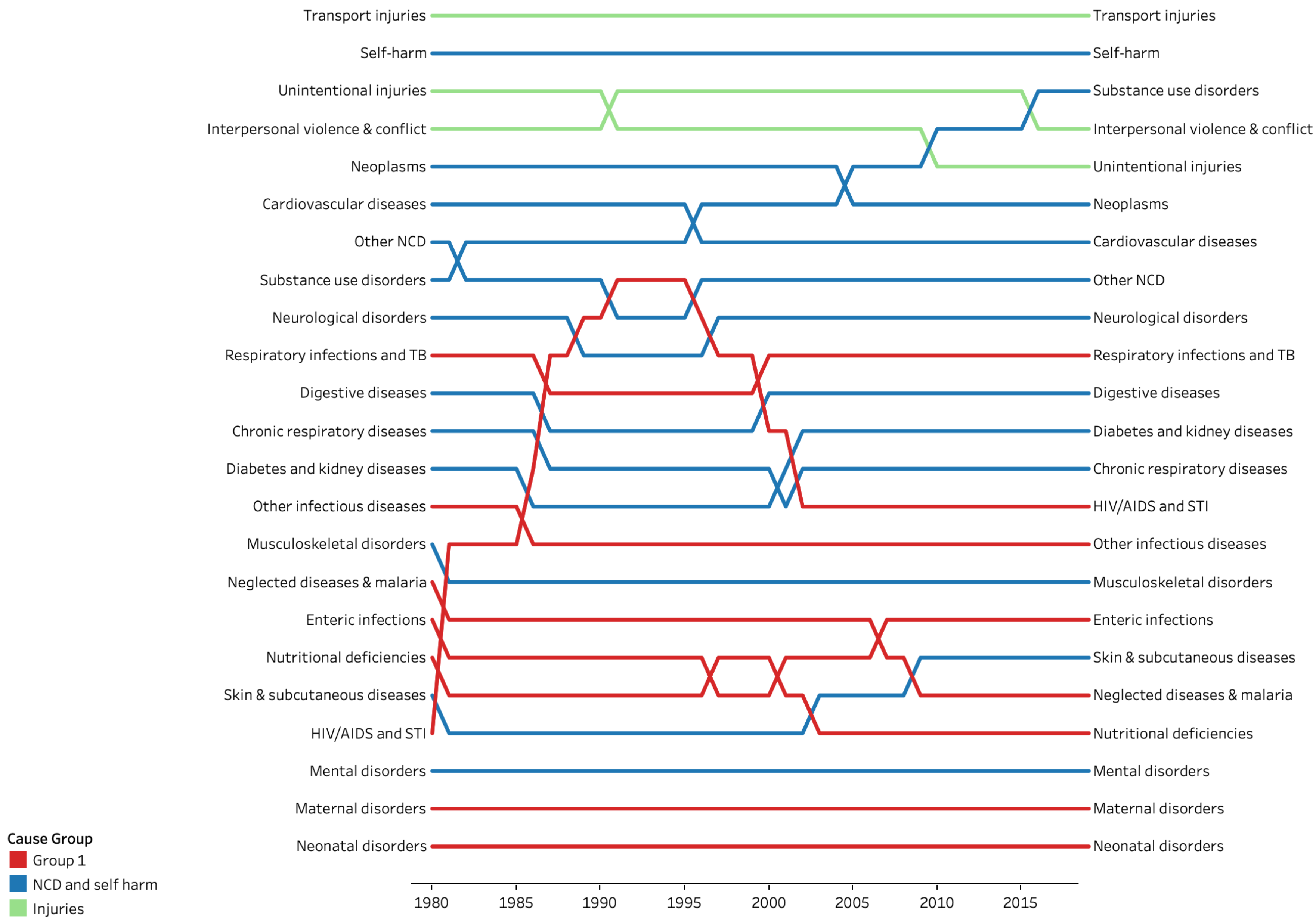

Figure S72: Rank of number of deaths by cause group 1980 – 2019: High Income GBD super-region. 20-24 year old females.

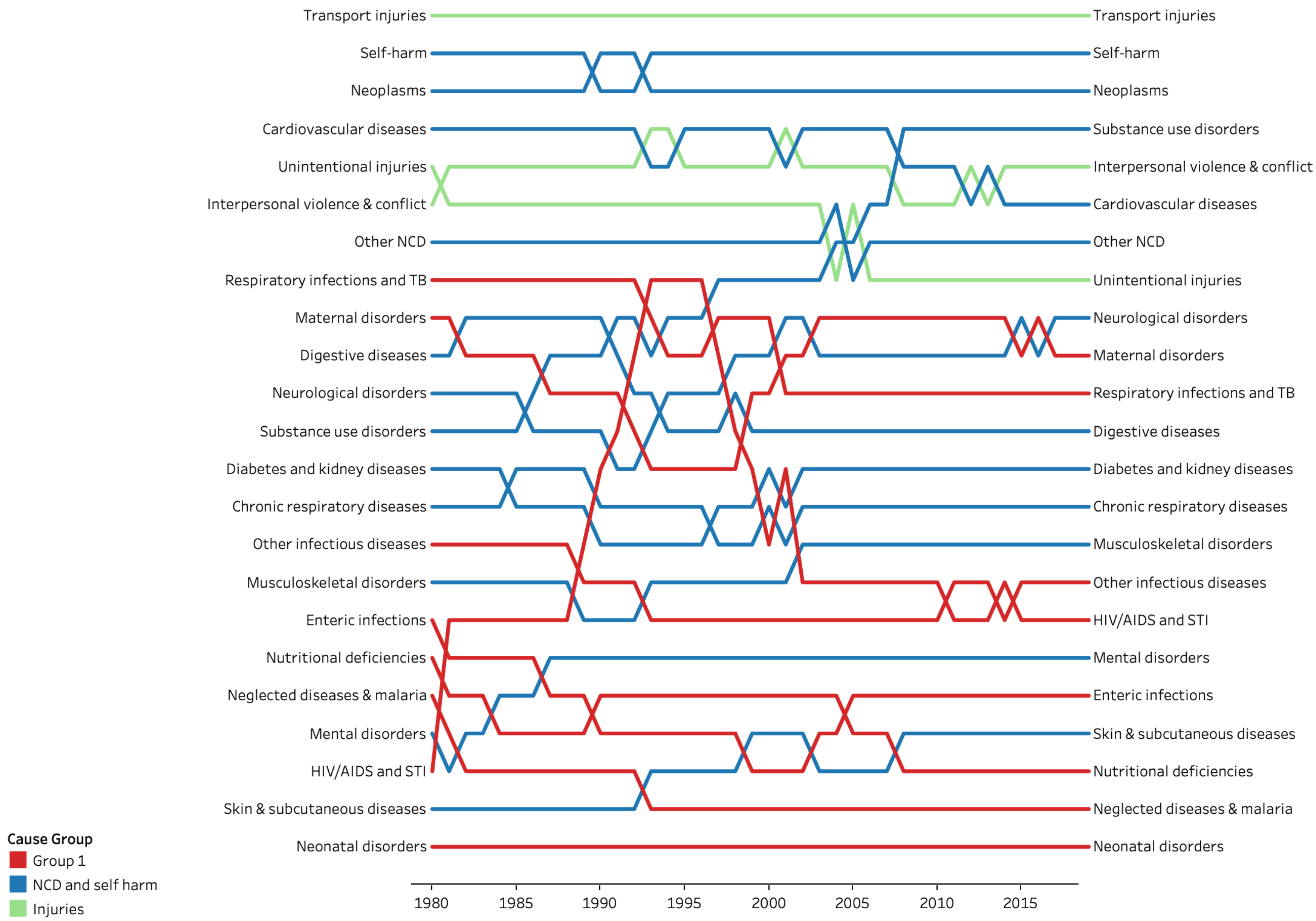

Figure S73: Mortality rate per 100,000 population by cause of death in 10-24 year olds 1980 – 2019: Latin America and the Caribbean GBD super-region

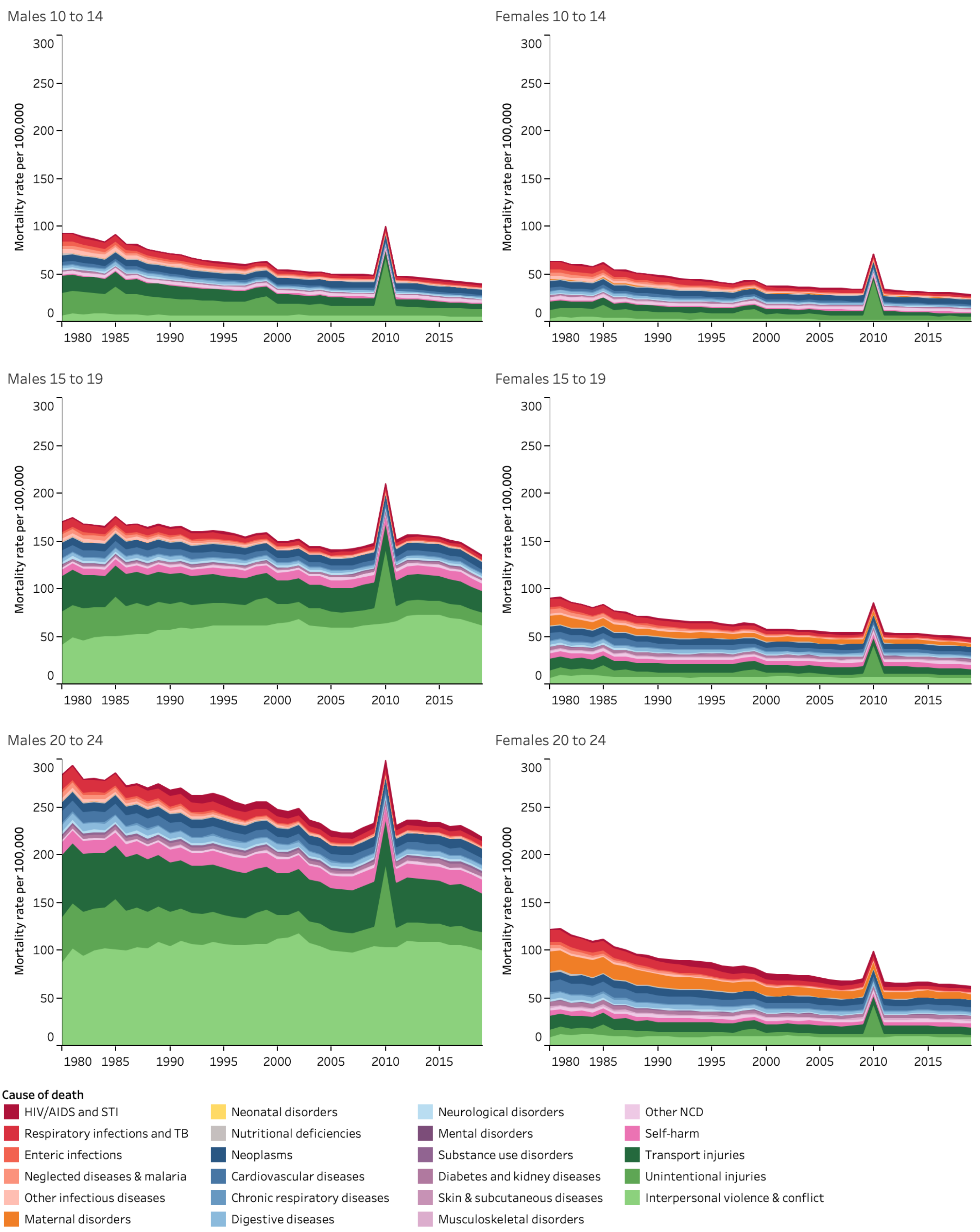

Figure S74: Number of deaths by cause in 10-24 year olds 1980 – 2019: Latin America and the Caribbean GBD super-region

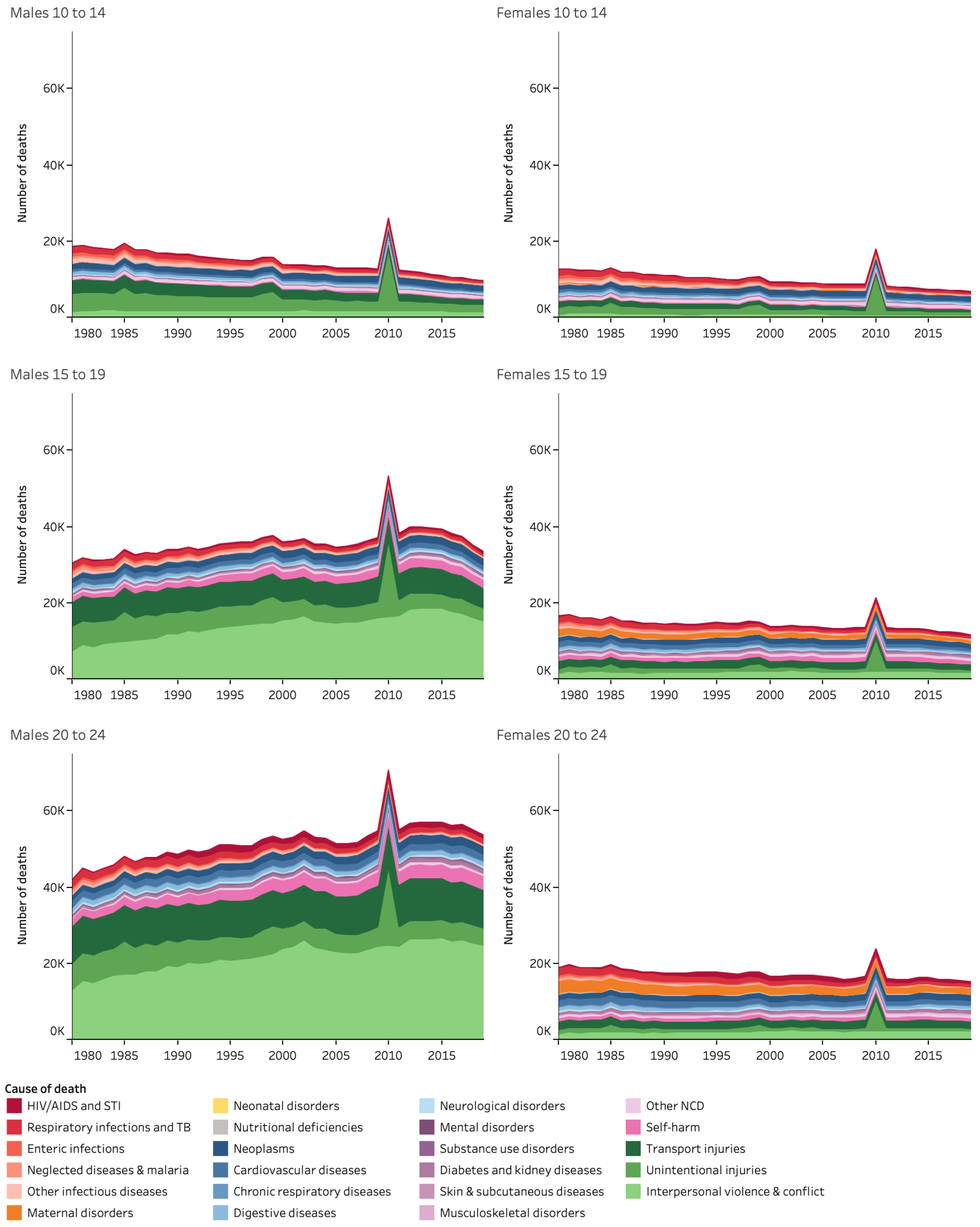

Figure S75: Rank of number of deaths by cause group 1980 – 2019: Latin America and the Caribbean GBD super-region. 10-14 year old males.

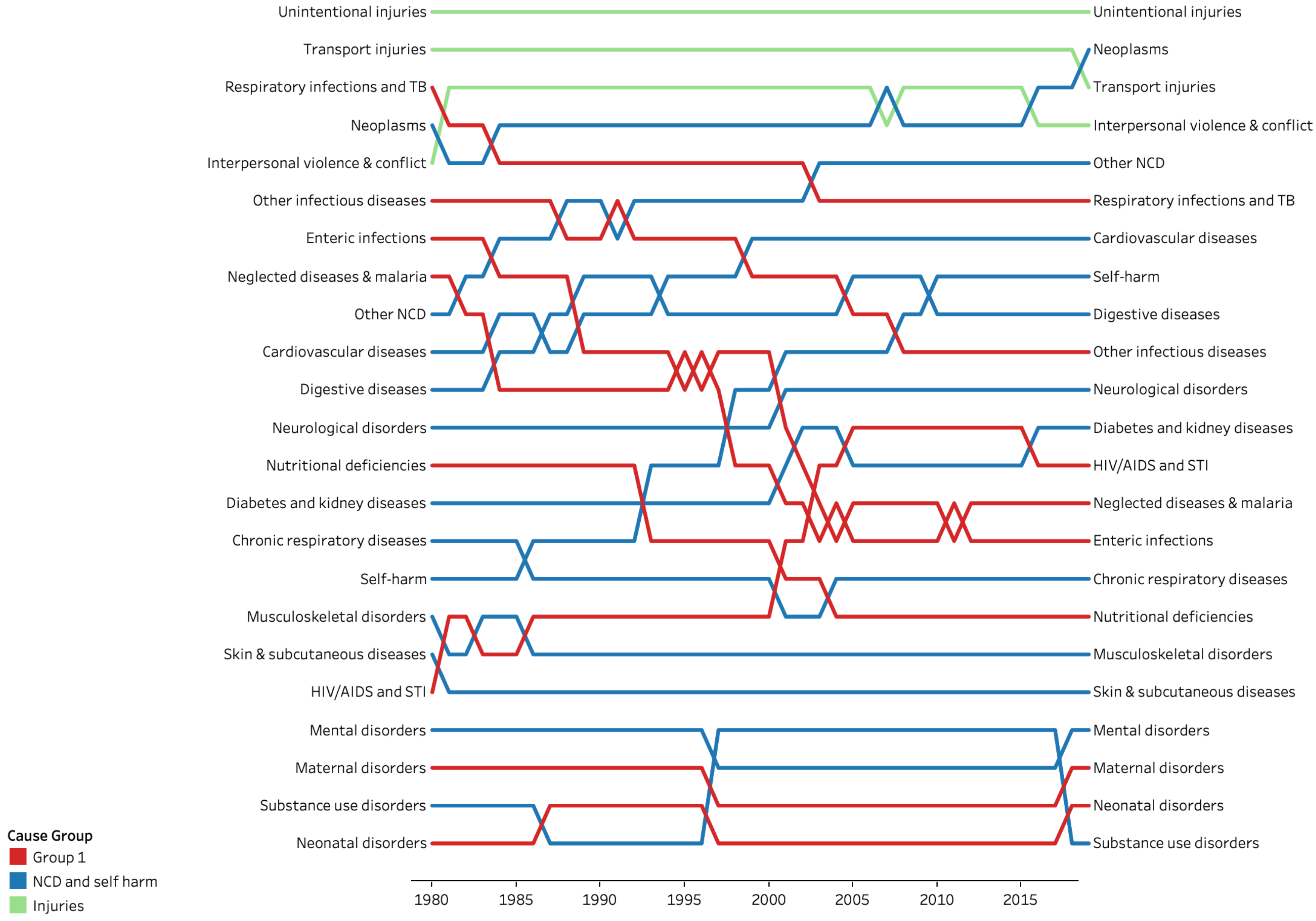

Figure S76: Rank of number of deaths by cause group 1980 – 2019: Latin America and the Caribbean GBD super-region. 10-14 year old females.

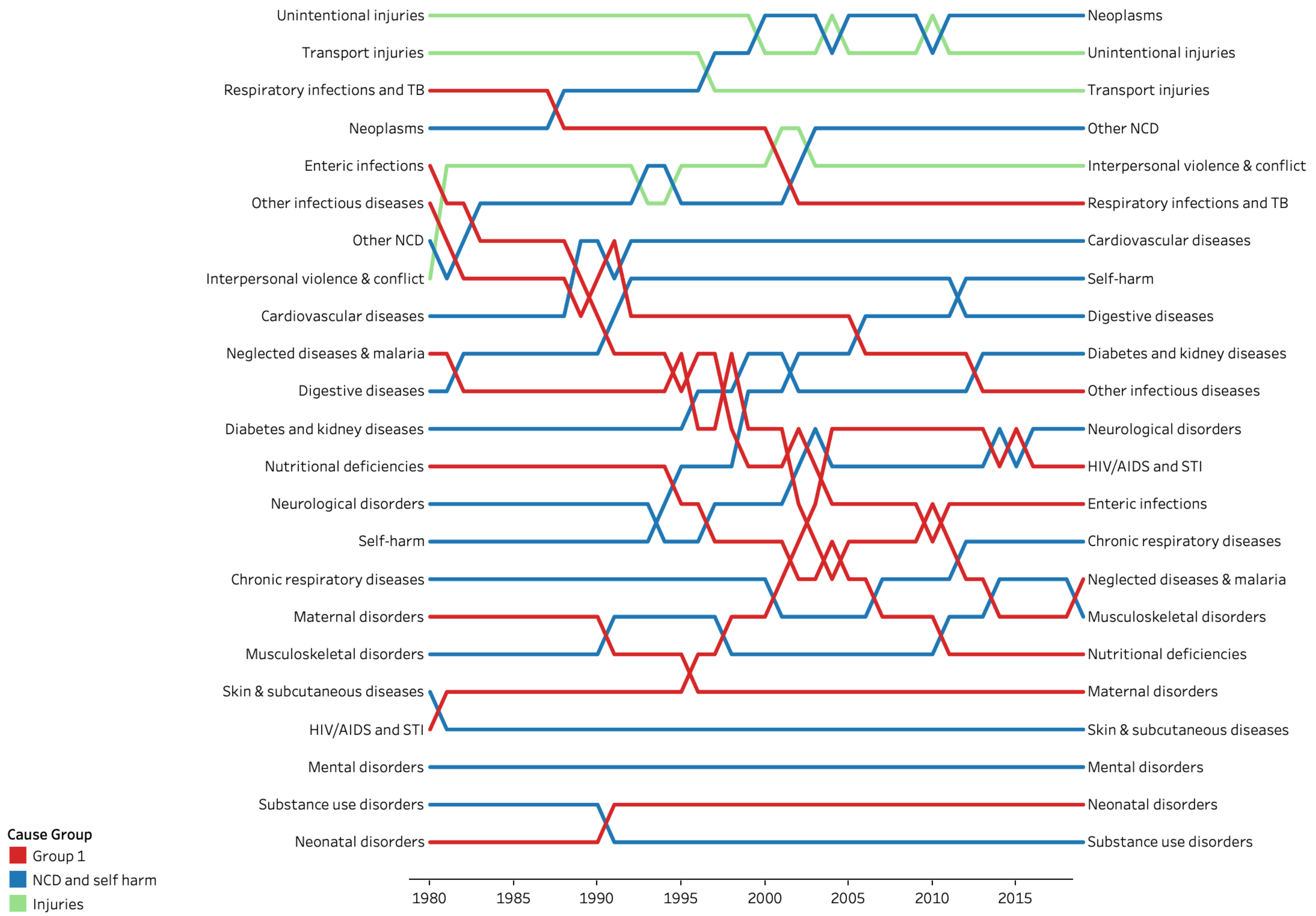

Figure S77: Rank of number of deaths by cause group 1980 – 2019: Latin America and the Caribbean GBD super-region. 15-19 year old males.

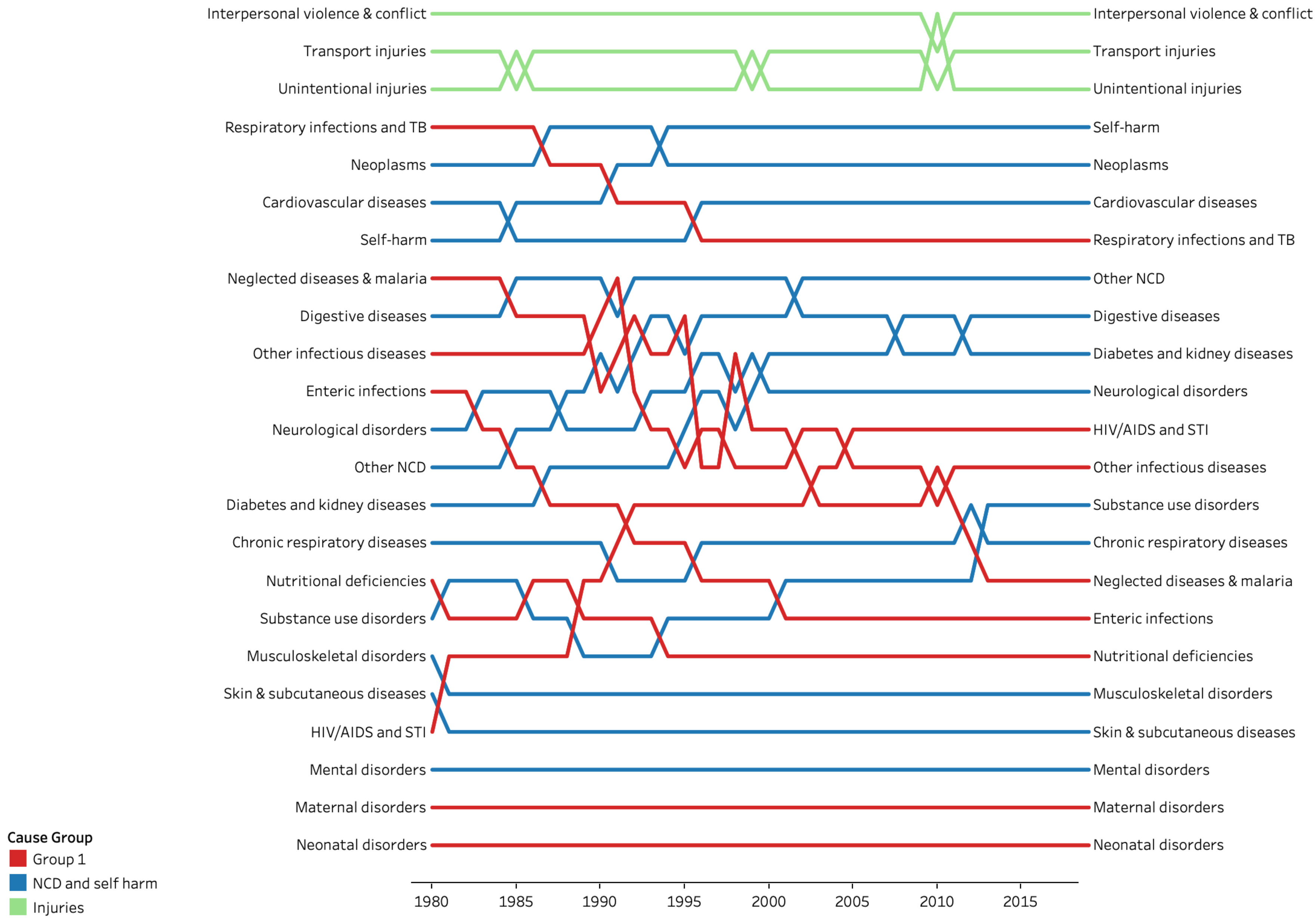

Figure S78: Rank of number of deaths by cause group 1980 – 2019: Latin America and the Caribbean GBD super-region. 15-19 year old females.

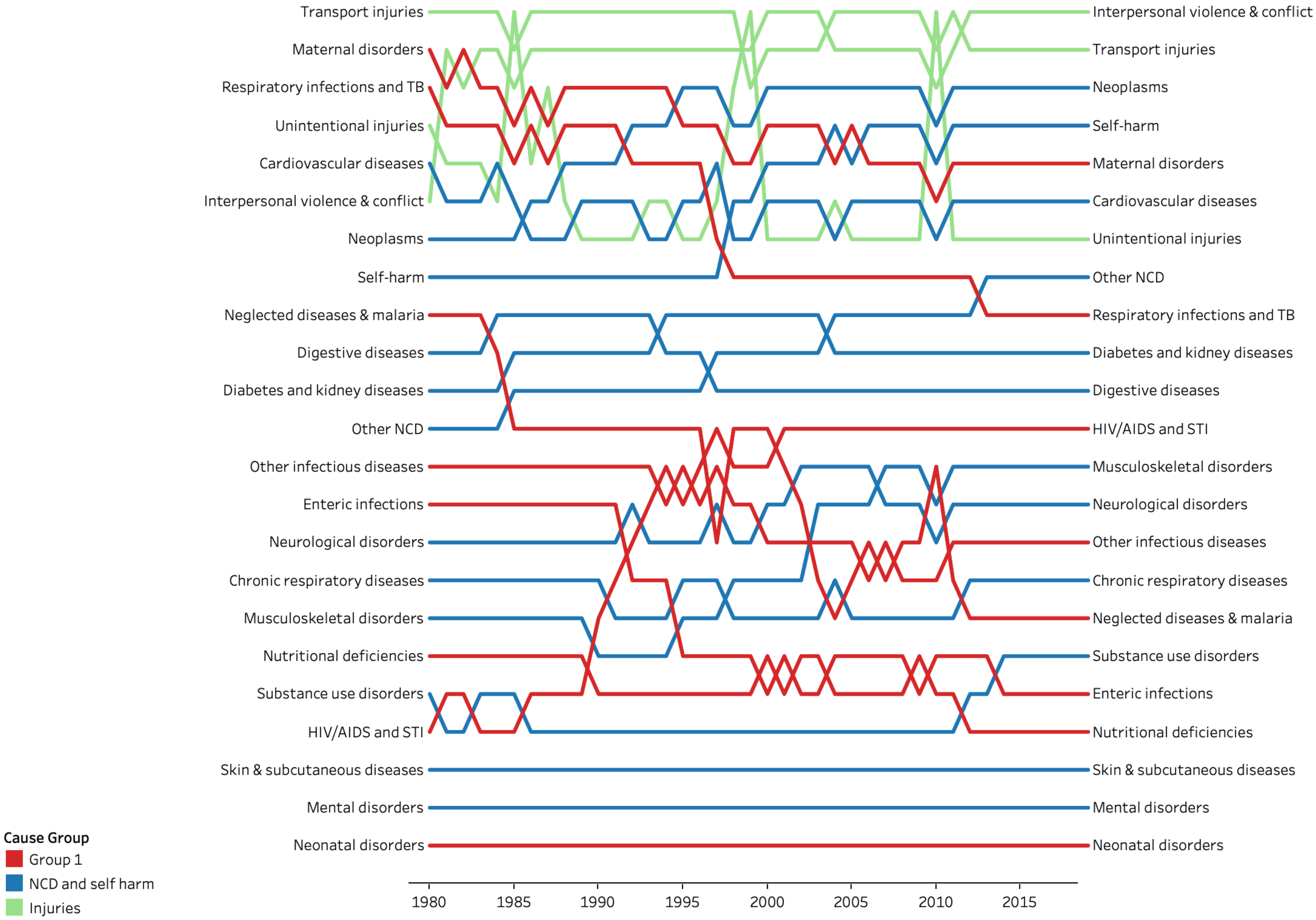

Figure S79: Rank of number of deaths by cause group 1980 – 2019: Latin America and the Caribbean GBD super-region. 20-24 year old males.

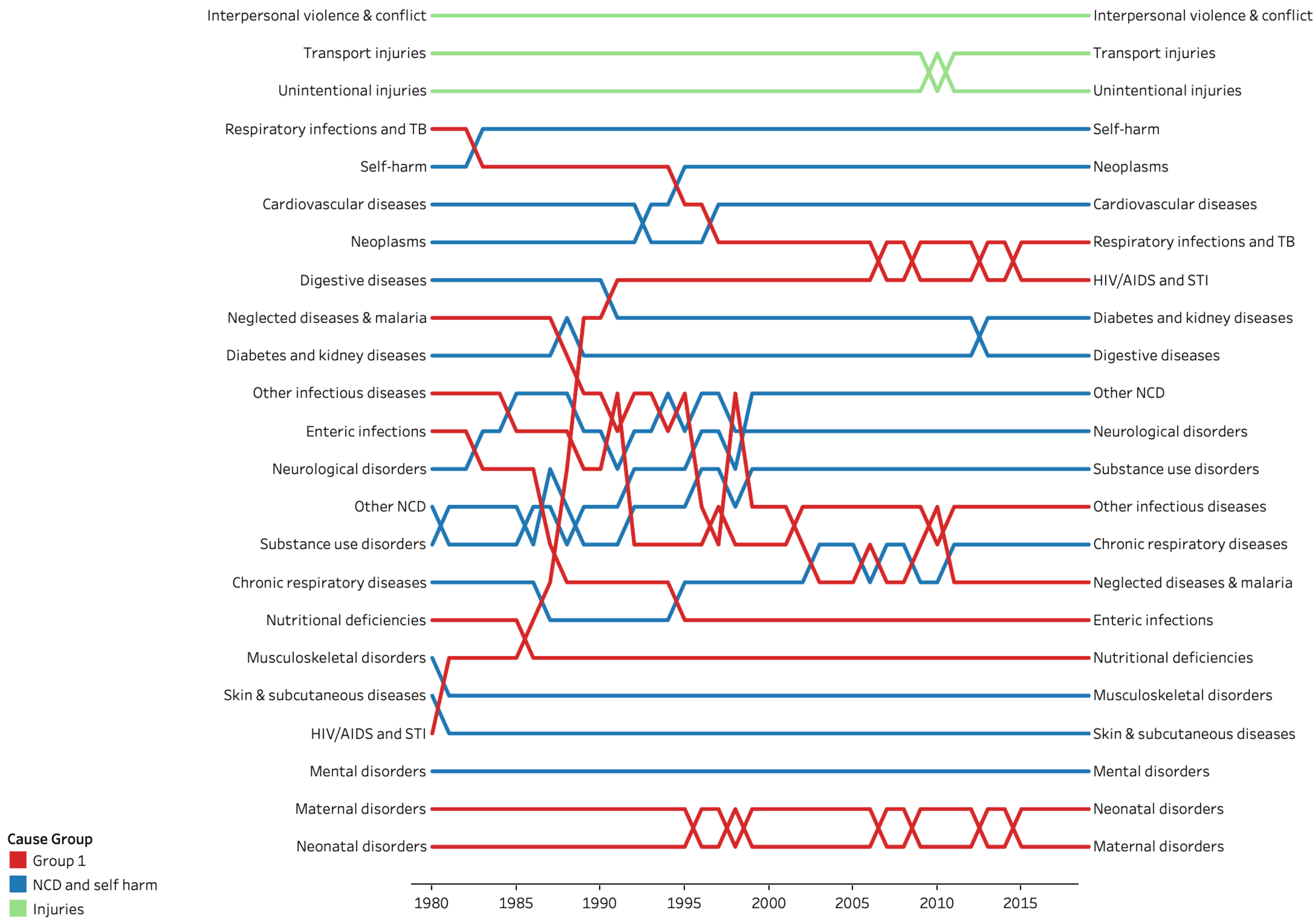

Figure S80: Rank of number of deaths by cause group 1980 – 2019: Latin America and the Caribbean GBD super-region. 20-24 year old females.

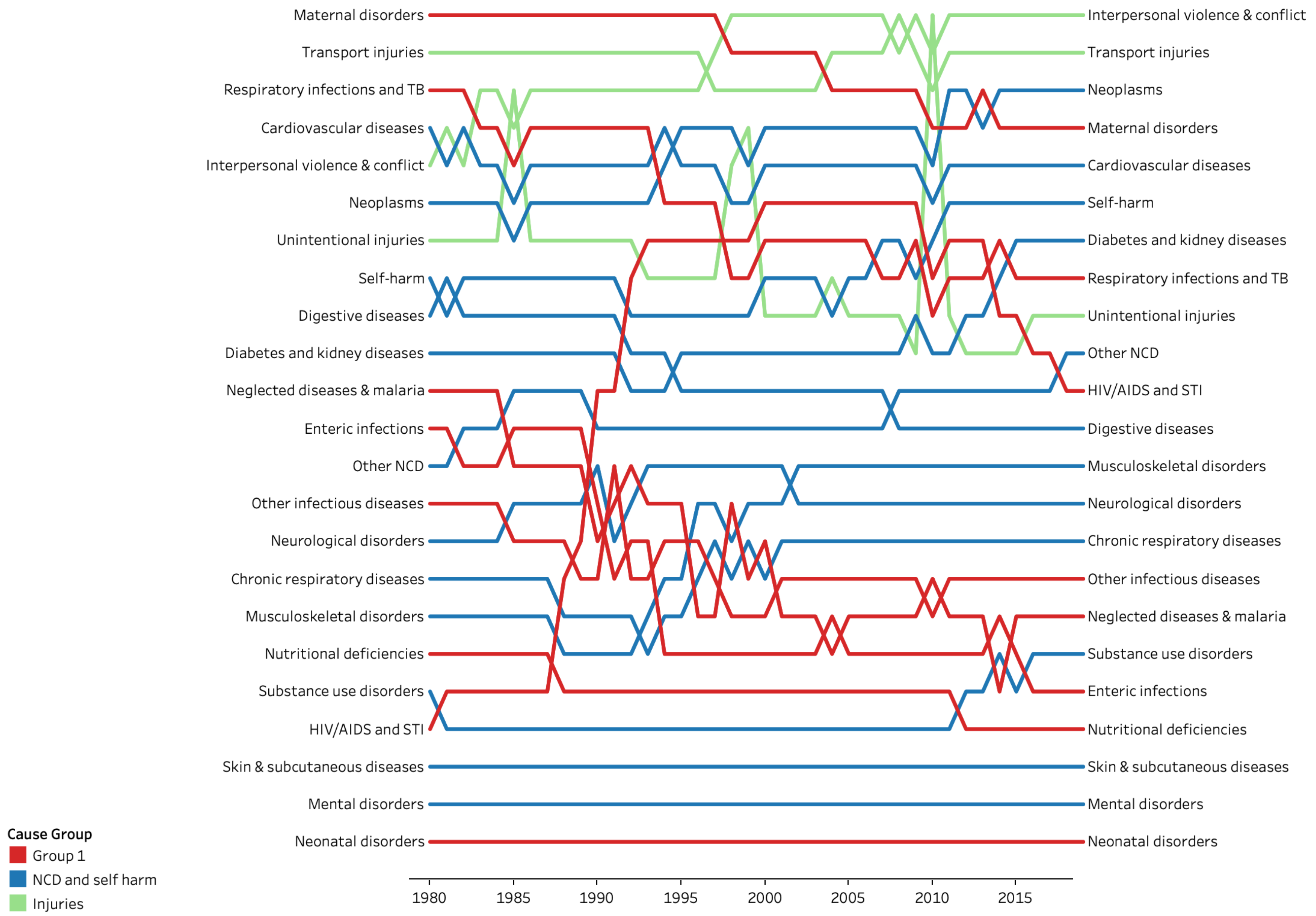

Figure S81: Mortality rate per 100,000 population by cause of death in 10-24 year olds 1980 – 2019: North Africa and the Middle East GBD super-region

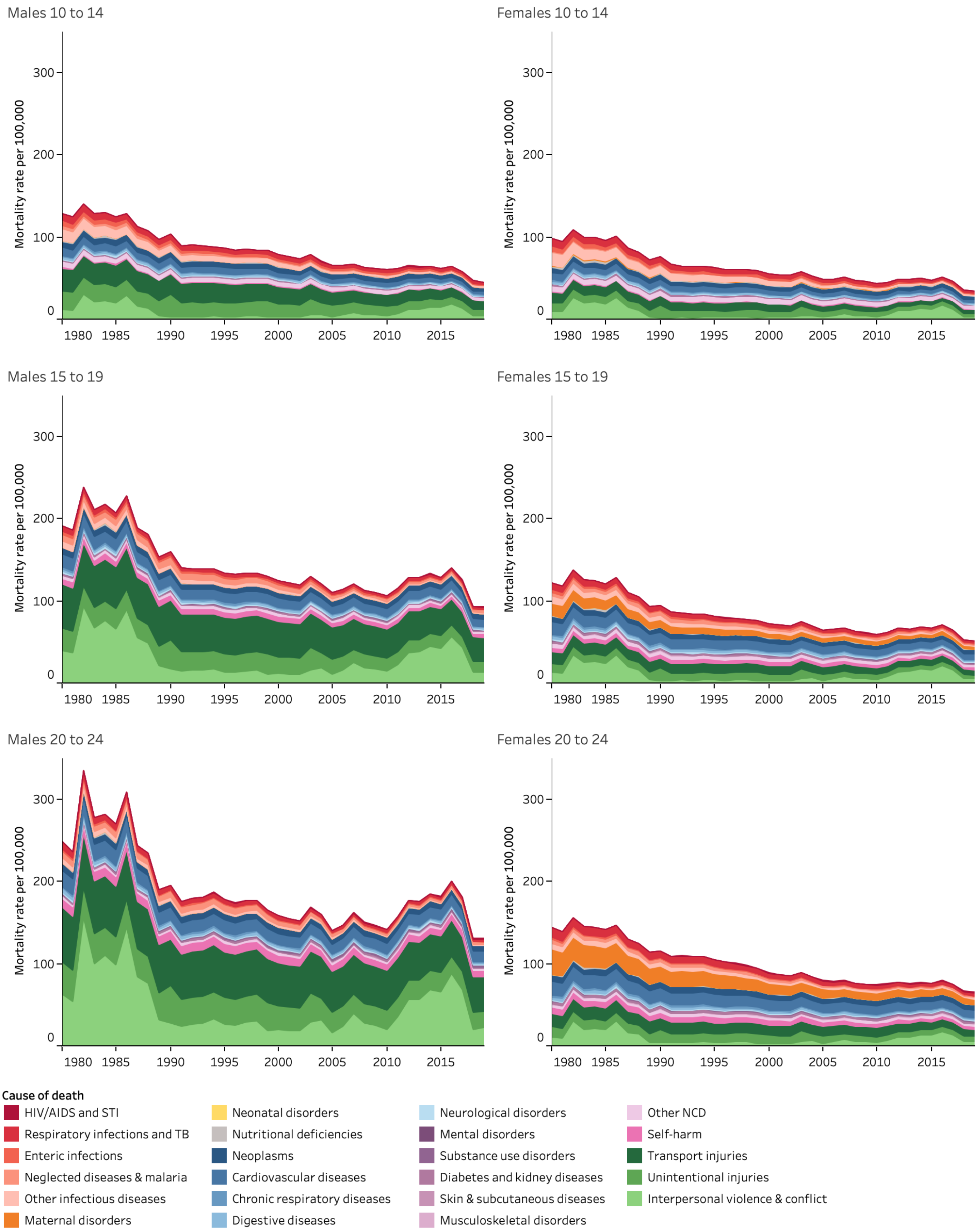

Figure S82: Number of deaths by cause in 10-24 year olds 1980 – 2019: North Africa and the Middle East GBD super-region

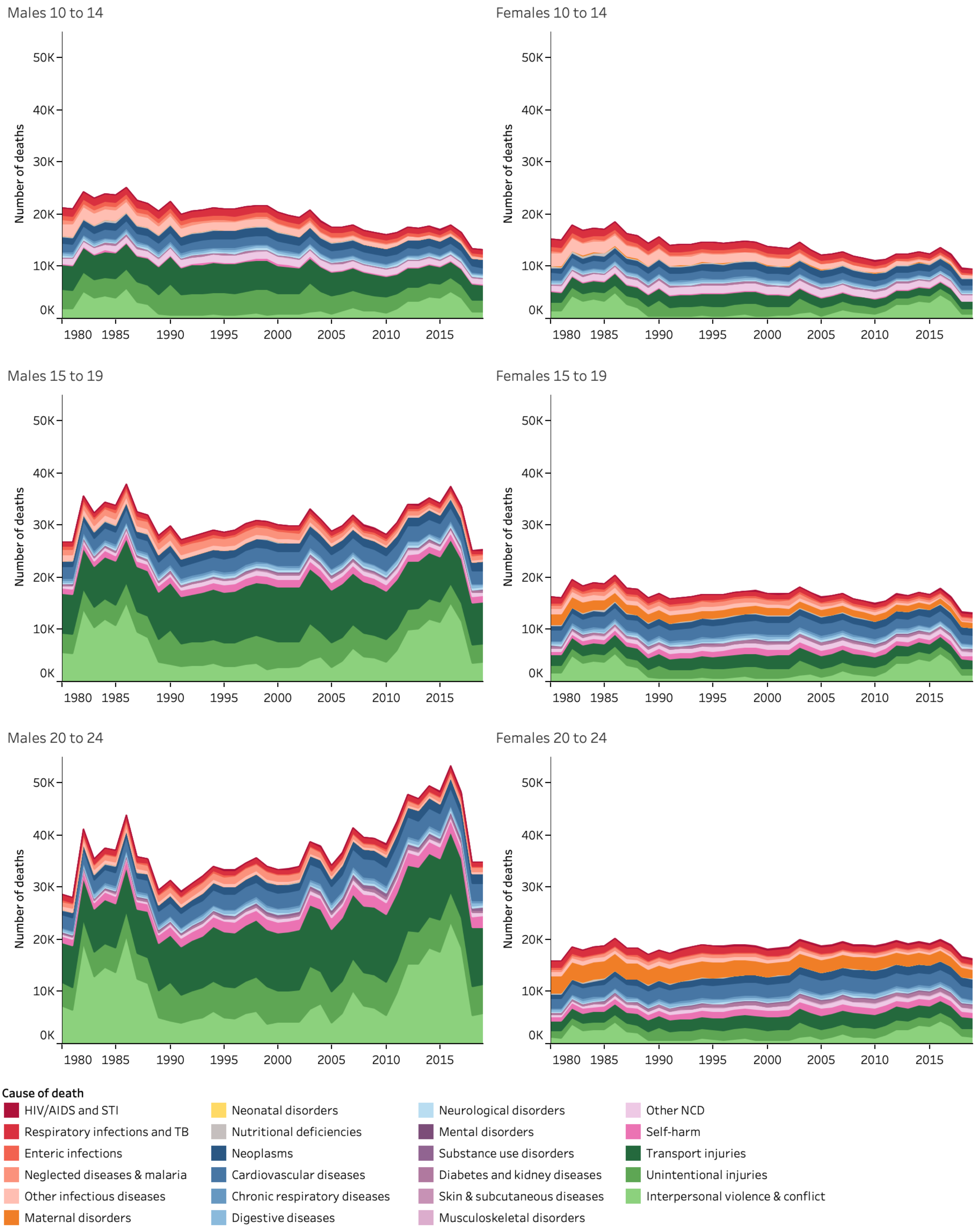

Figure S83: Rank of number of deaths by cause group 1980 – 2019: North Africa and the Middle East GBD super-region. 10-14 year old males.

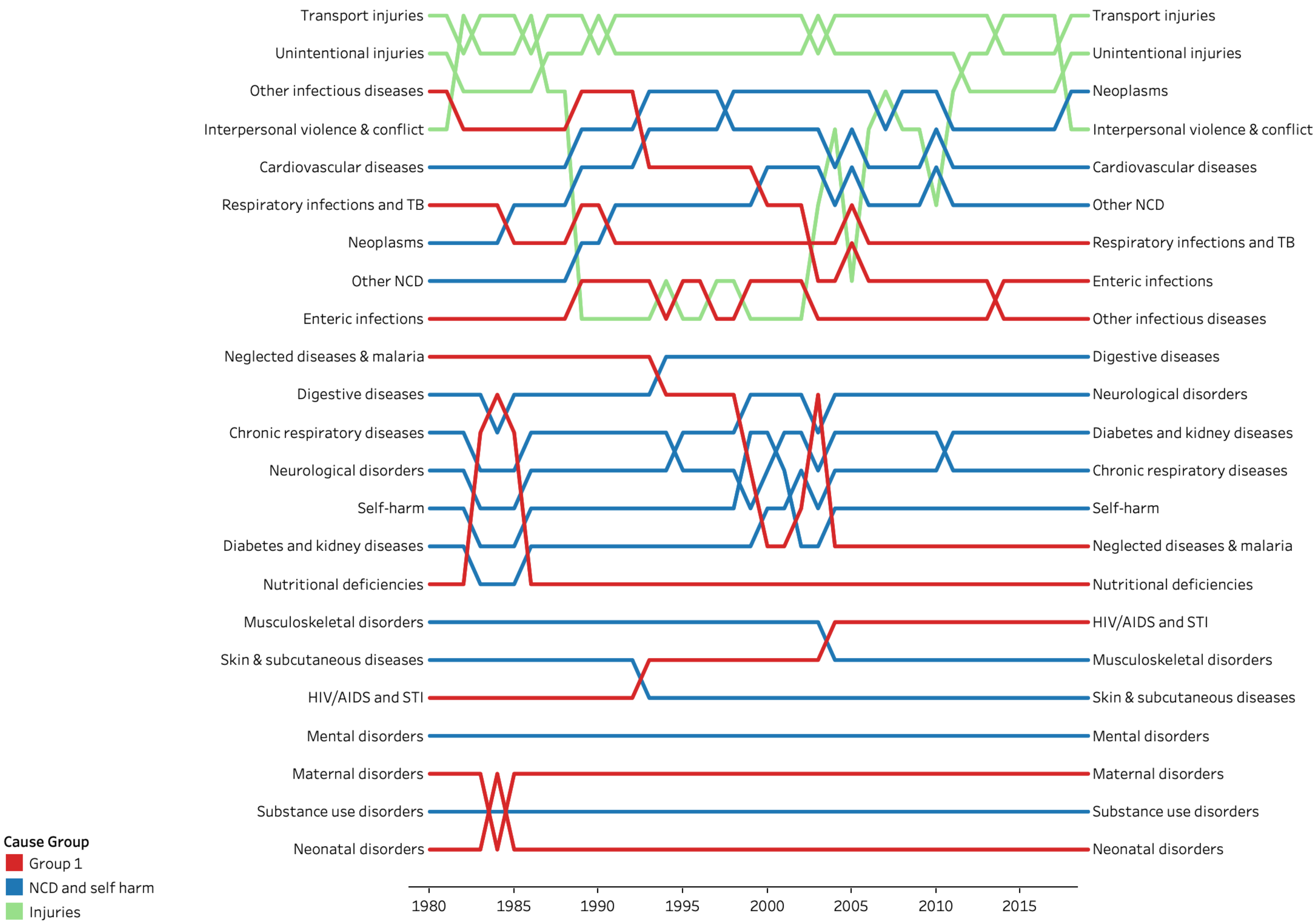

Figure S84: Rank of number of deaths by cause group 1980 – 2019: North Africa and the Middle East GBD super-region. 10-14 year old females.

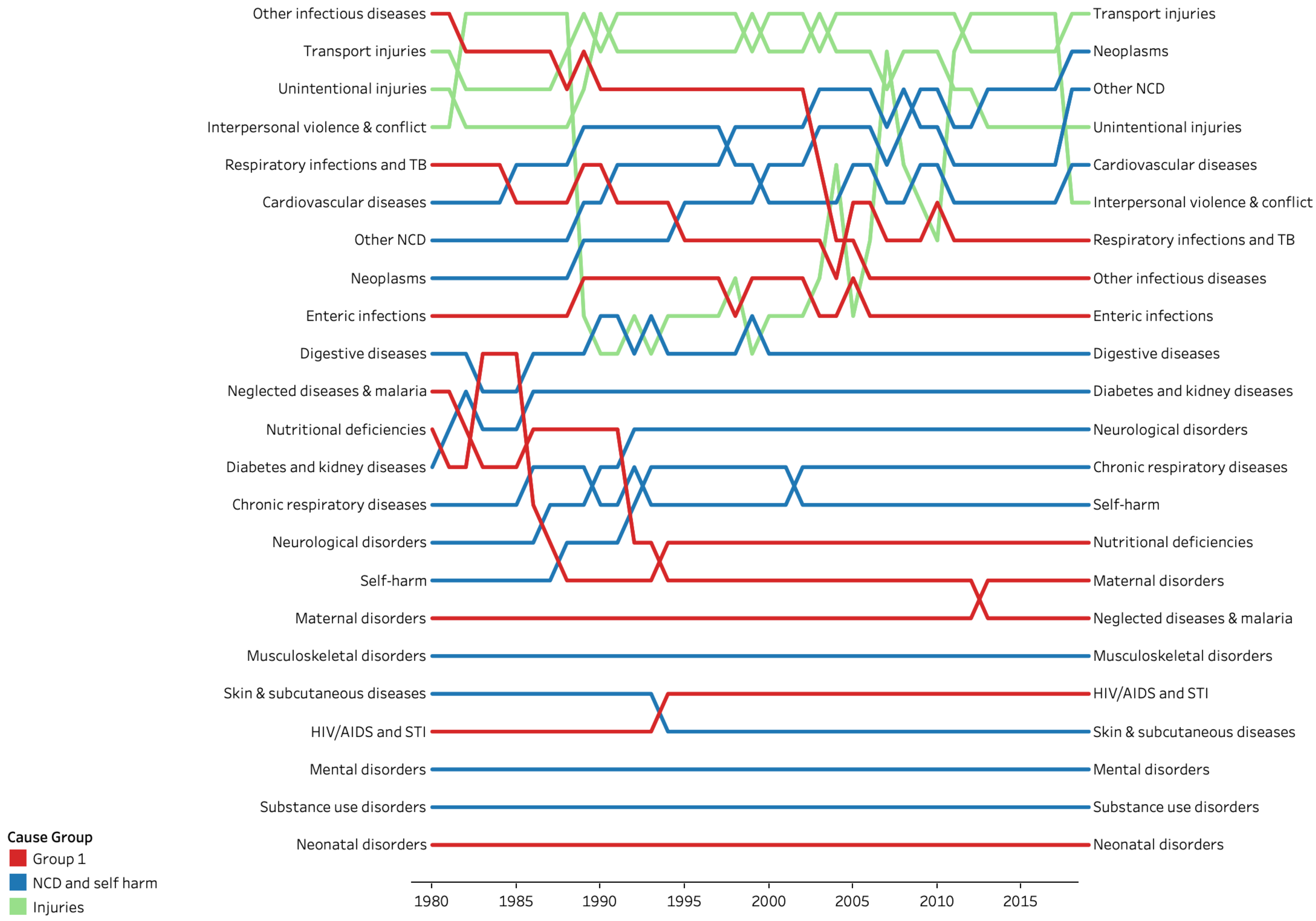

Figure S85: Rank of number of deaths by cause group 1980 – 2019: North Africa and the Middle East GBD super-region. 15-19 year old males.

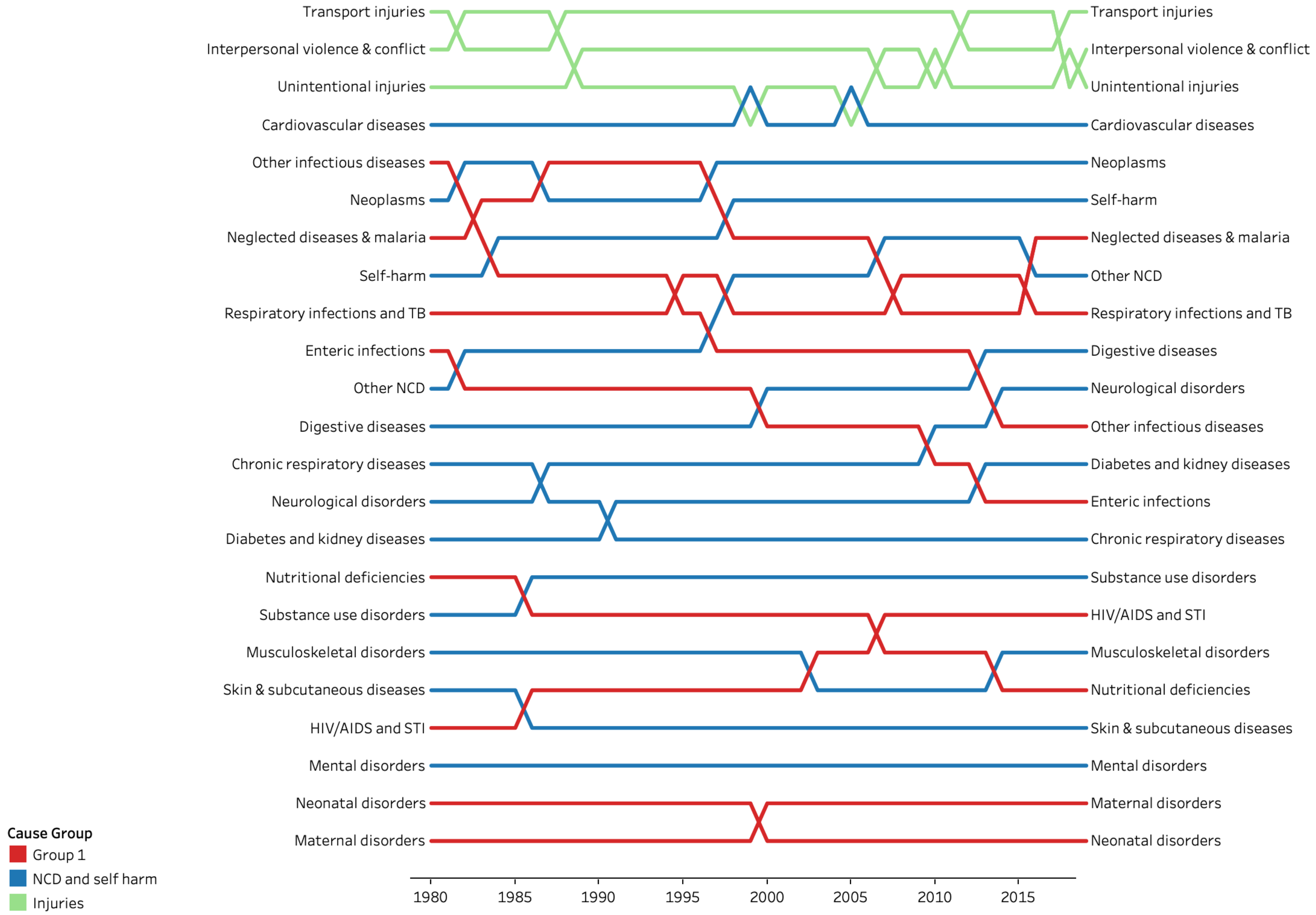

Figure S86: Rank of number of deaths by cause group 1980 – 2019: North Africa and the Middle East GBD super-region. 15-19 year old females.

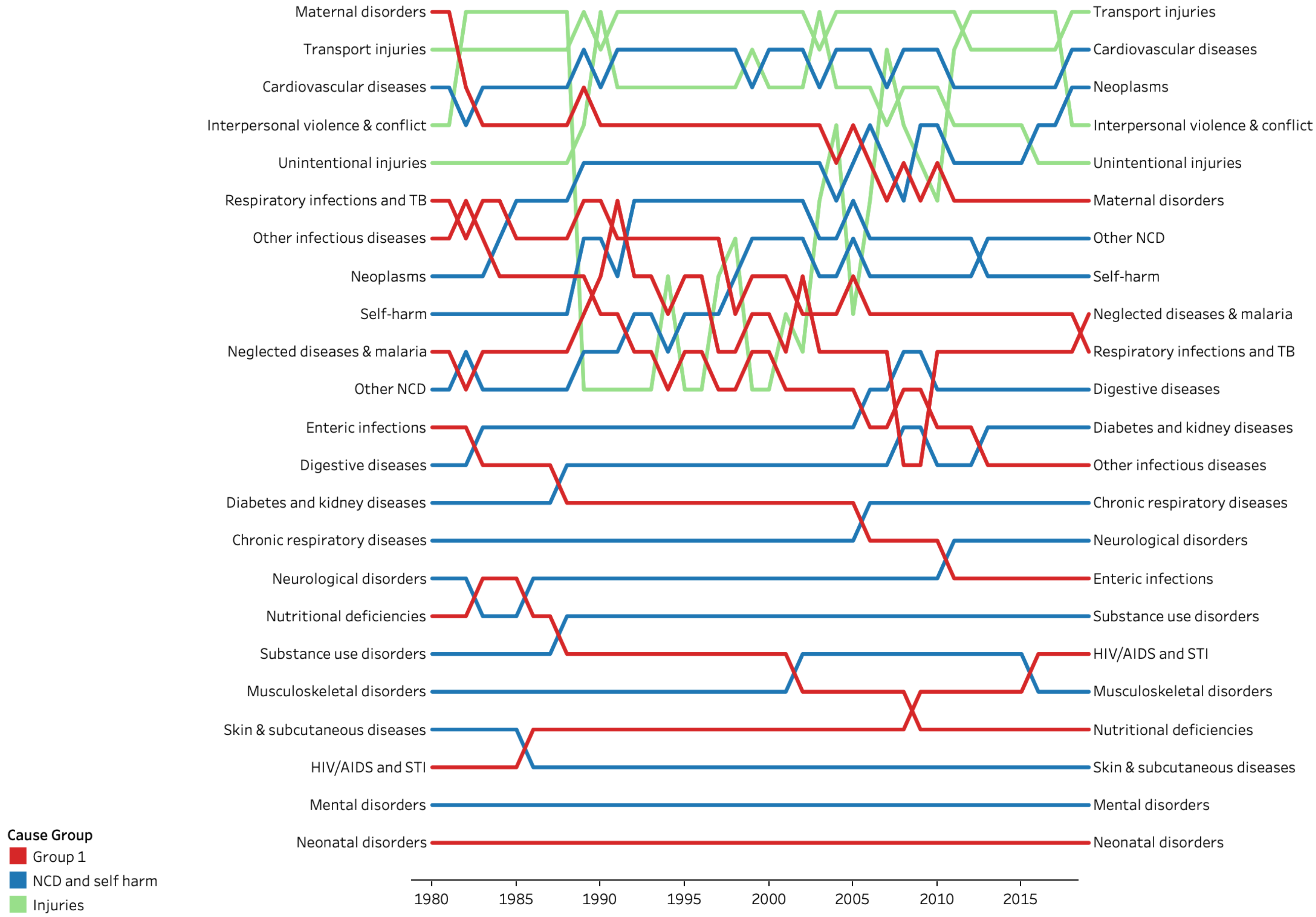

Figure S87: Rank of number of deaths by cause group 1980 – 2019: North Africa and the Middle East GBD super-region. 20-24 year old males.

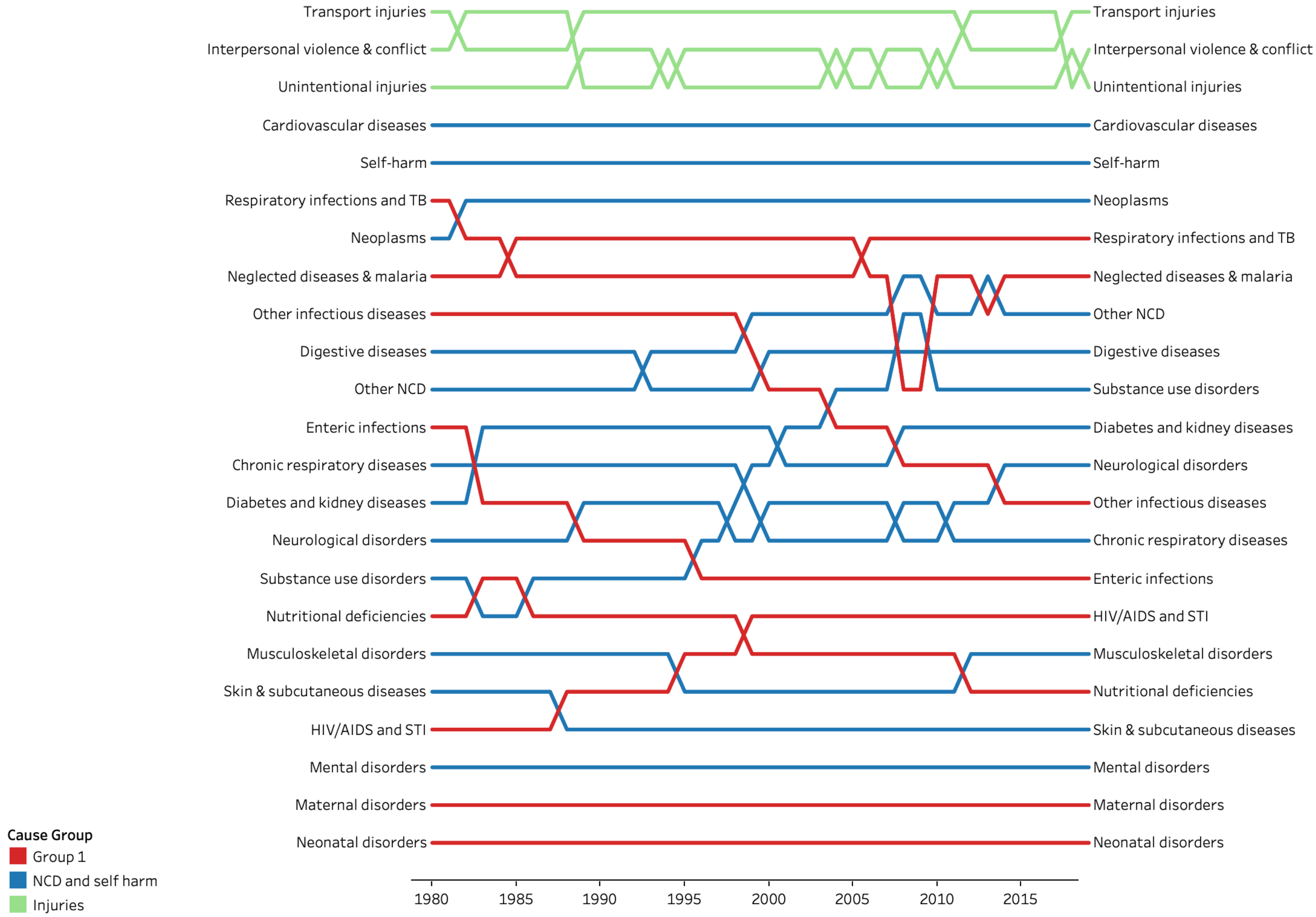

Figure S88: Rank of number of deaths by cause group 1980 – 2019: North Africa and the Middle East GBD super-region. 20-24 year old females.

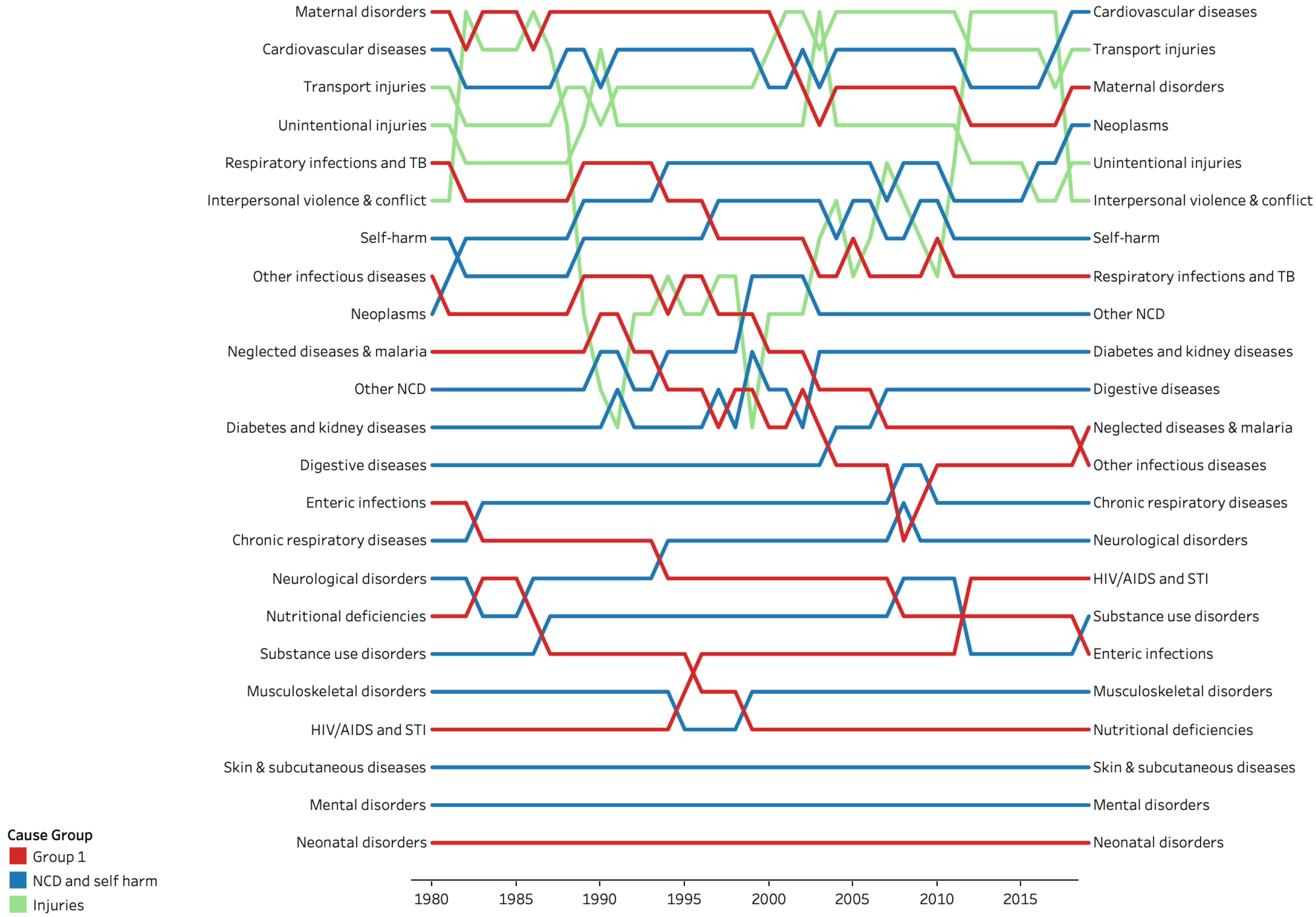

Figure S89: Mortality rate per 100,000 population by cause of death in 10-24 year olds 1980 – 2019: South Asia GBD super-region

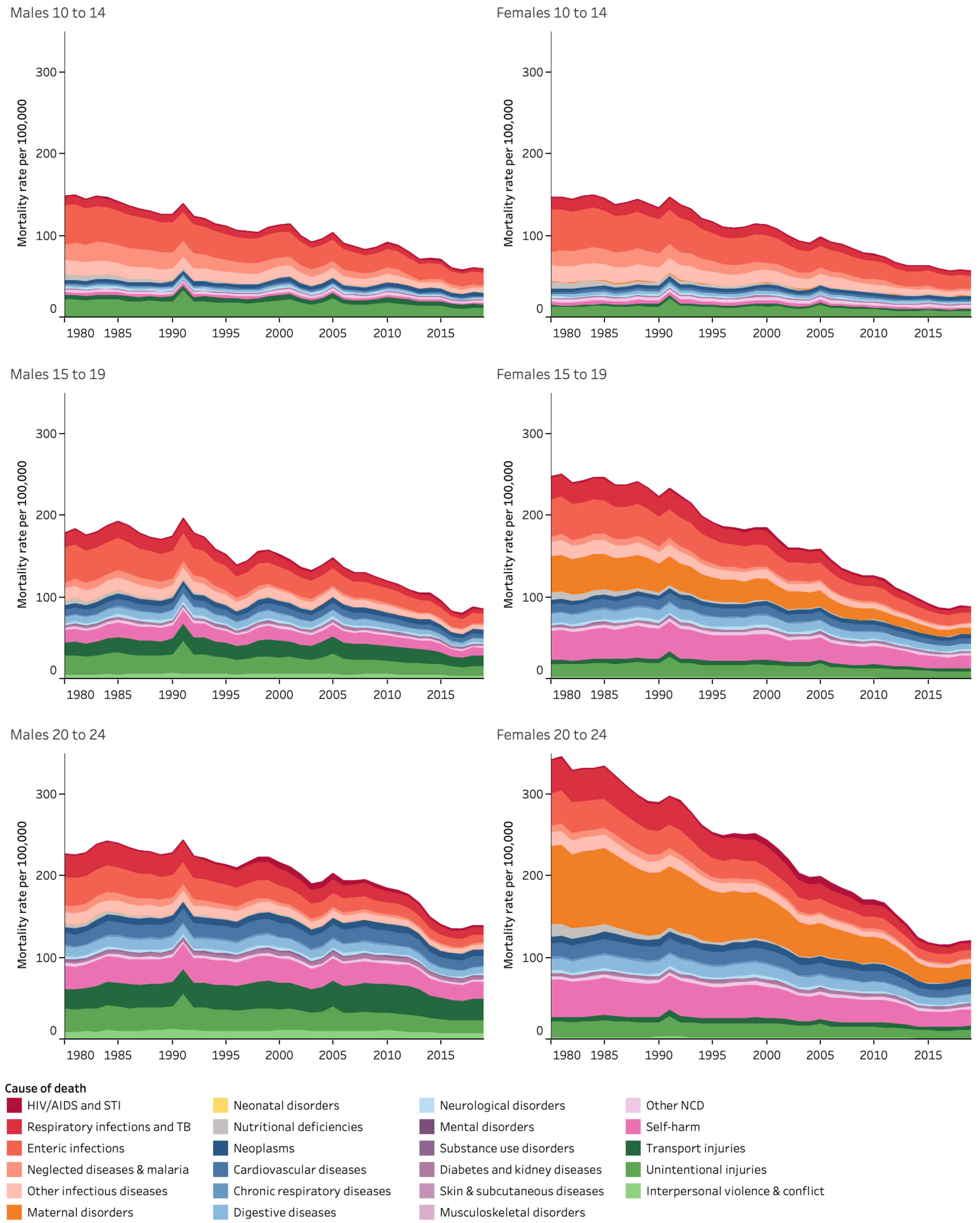

Figure S90: Number of deaths by cause in 10-24 year olds 1980 – 2019: South Asia GBD super-region

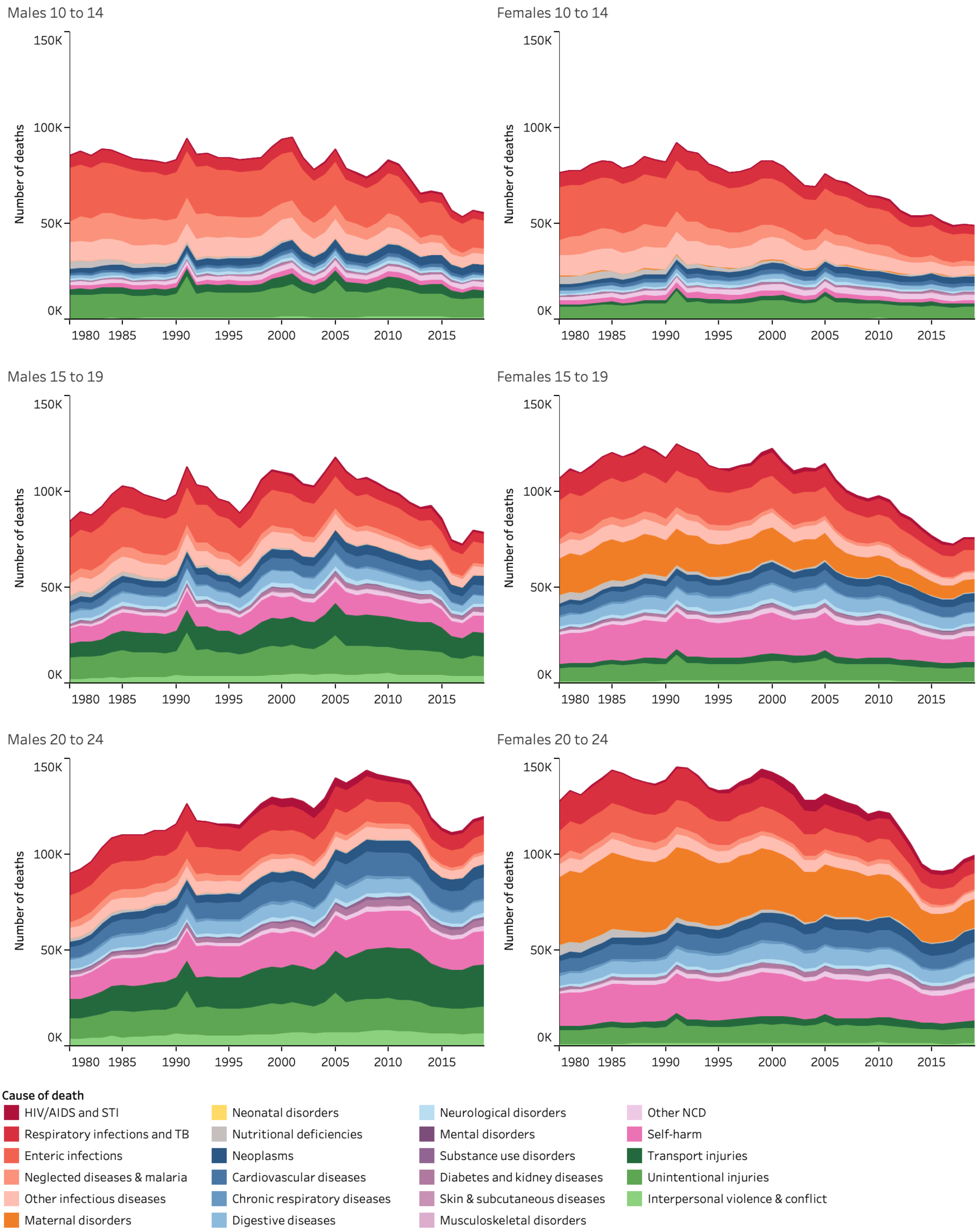

Figure S91: Rank of number of deaths by cause group 1980 – 2019: South Asia GBD super-region. 10-14 year old males.

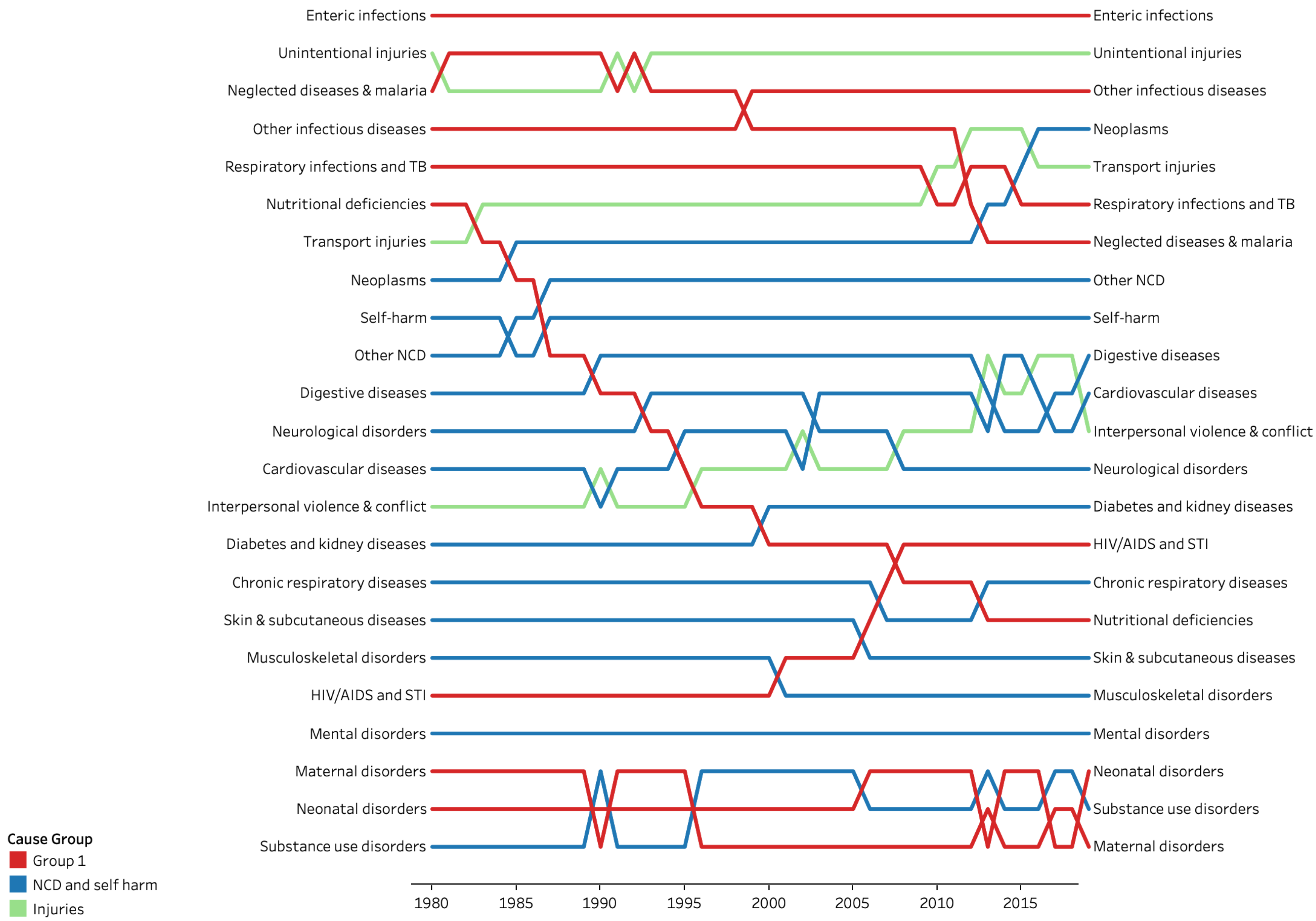

Figure S92: Rank of number of deaths by cause group 1980 – 2019: South Asia GBD super-region. 10-14 year old females.

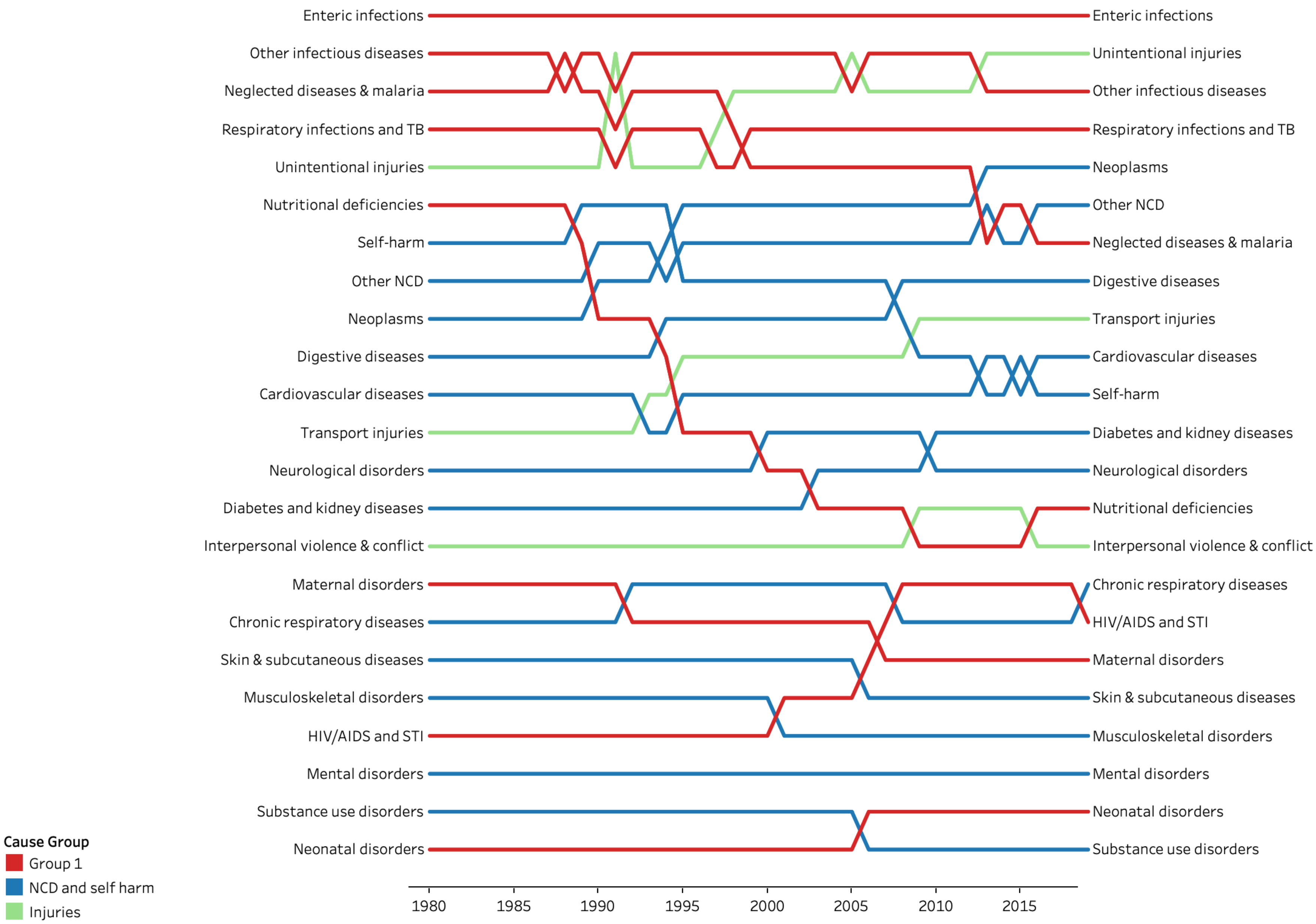

Figure S93: Rank of number of deaths by cause group 1980 – 2019: South Asia GBD super-region. 15-19 year old males.

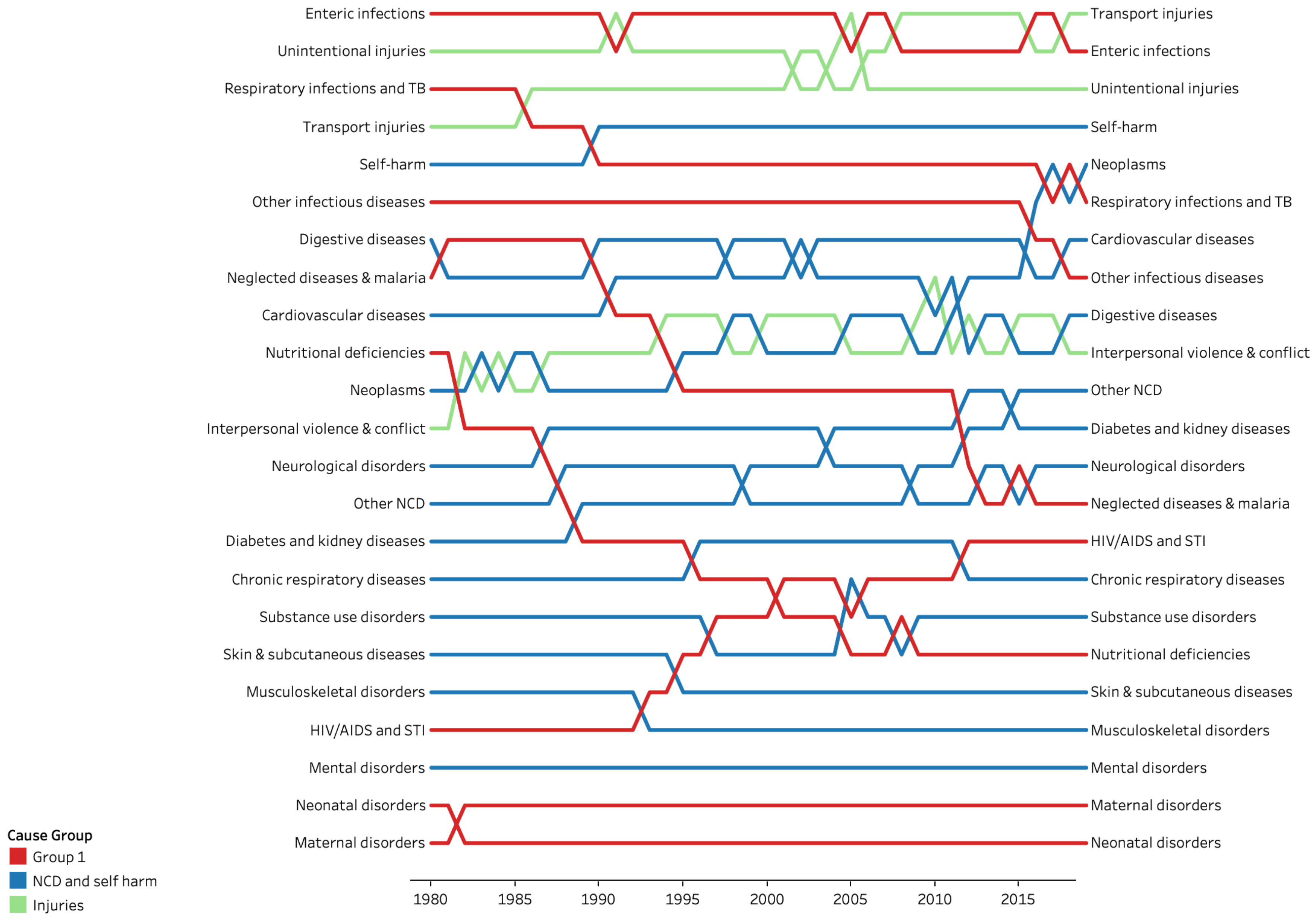

Figure S94: Rank of number of deaths by cause group 1980 – 2019: South Asia GBD super-region. 15-19 year old females.

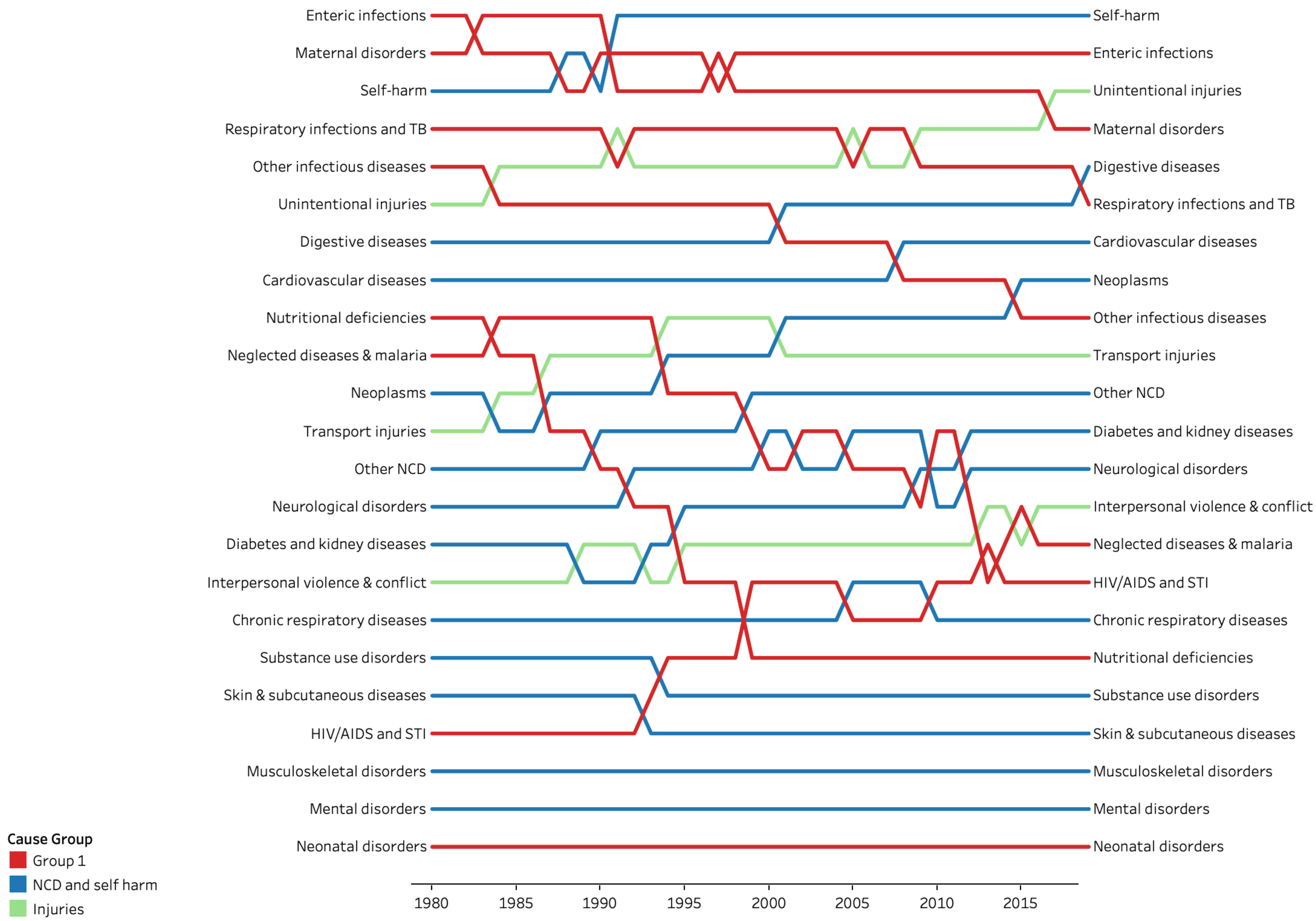

Figure S95: Rank of number of deaths by cause group 1980 – 2019: South Asia GBD super-region. 20-24 year old males.

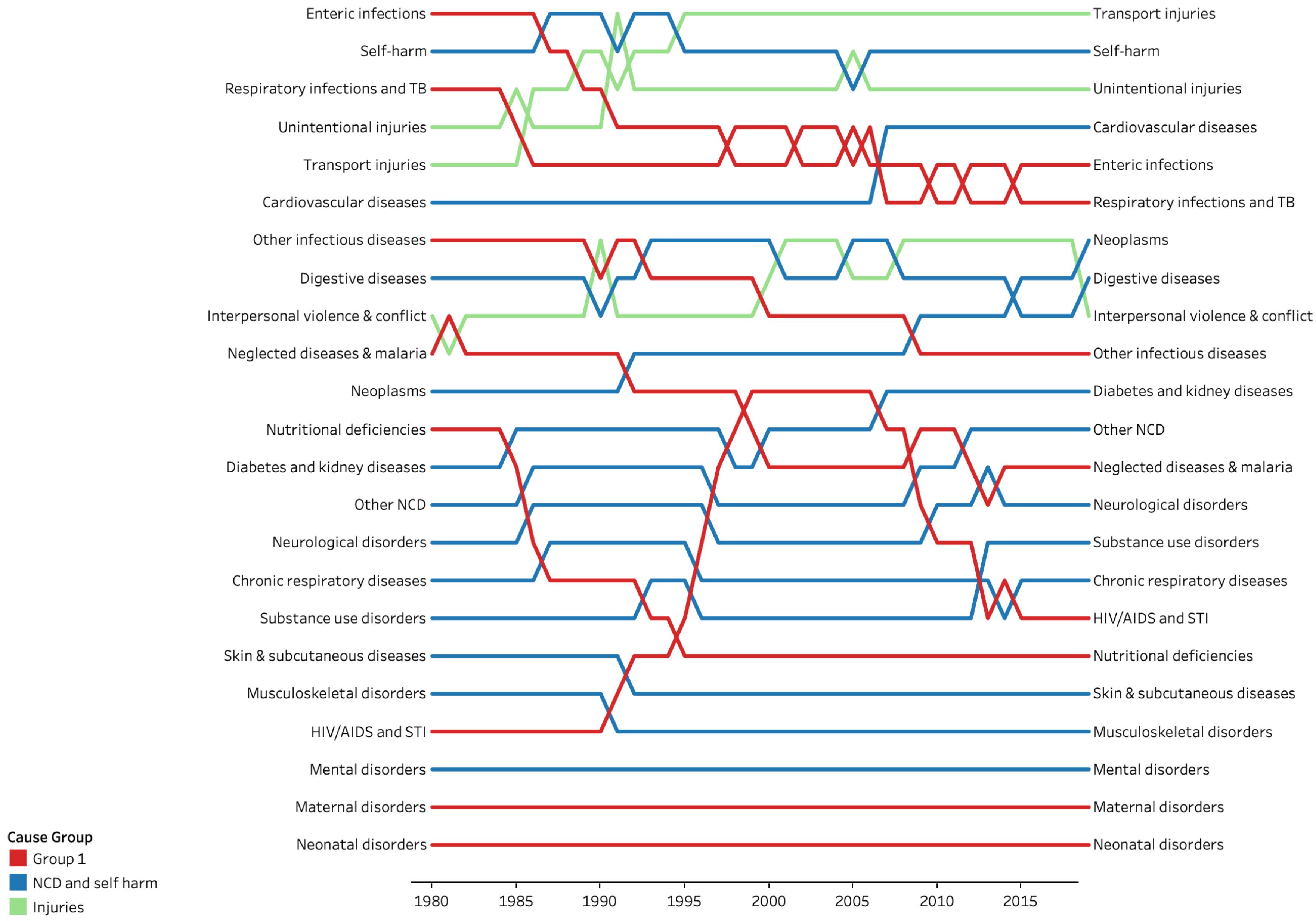

Figure S96: Rank of number of deaths by cause group 1980 – 2019: South Asia GBD super-region. 20-24 year old females.

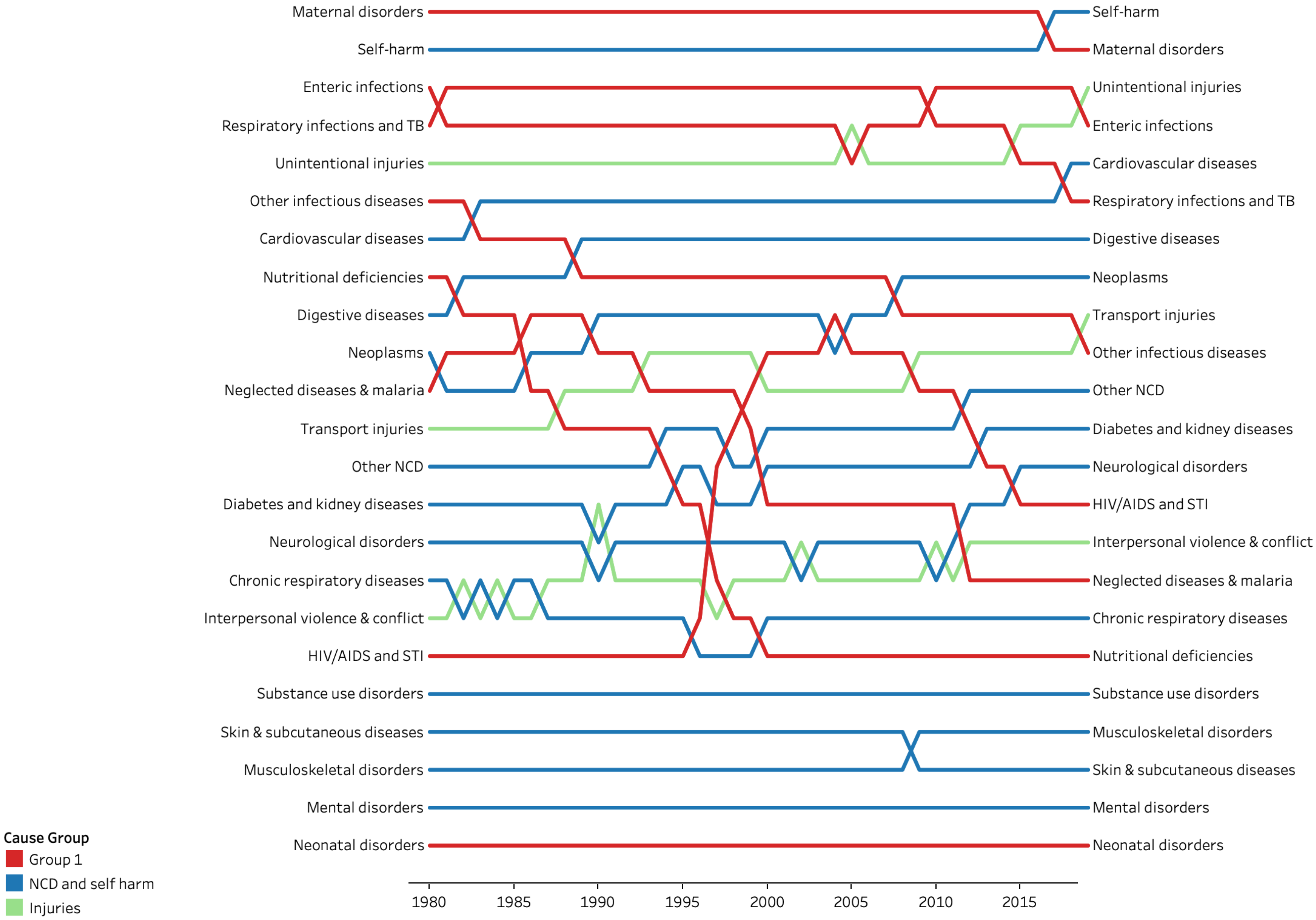

Figure S97: Mortality rate per 100,000 population by cause of death in 10-24 year olds 1980 – 2019: Southeast Asia, East Asia and Oceania GBD super-region

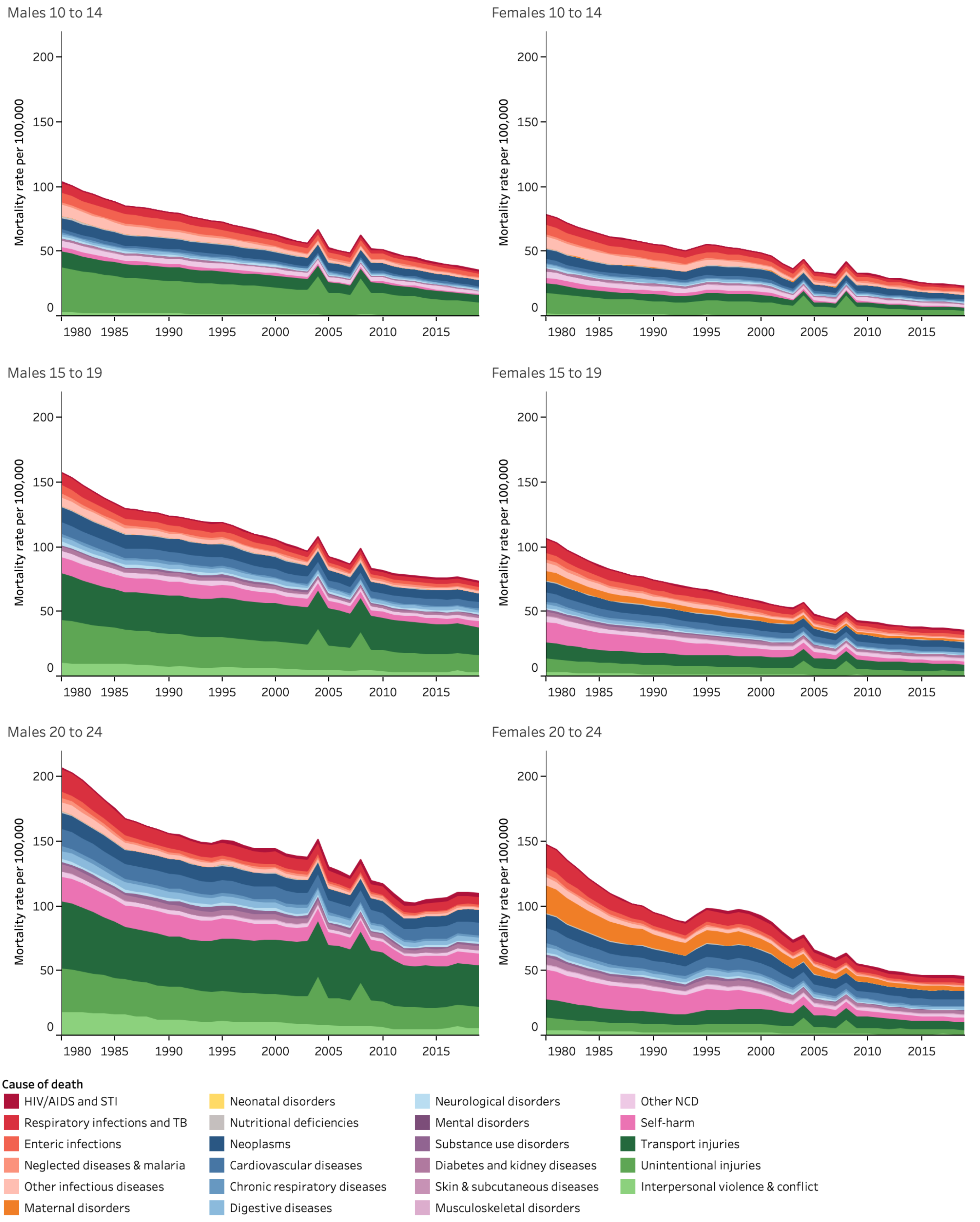

Figure S98: Number of deaths by cause in 10-24 year olds 1980 – 2019: Southeast Asia, East Asia and Oceania GBD super-region

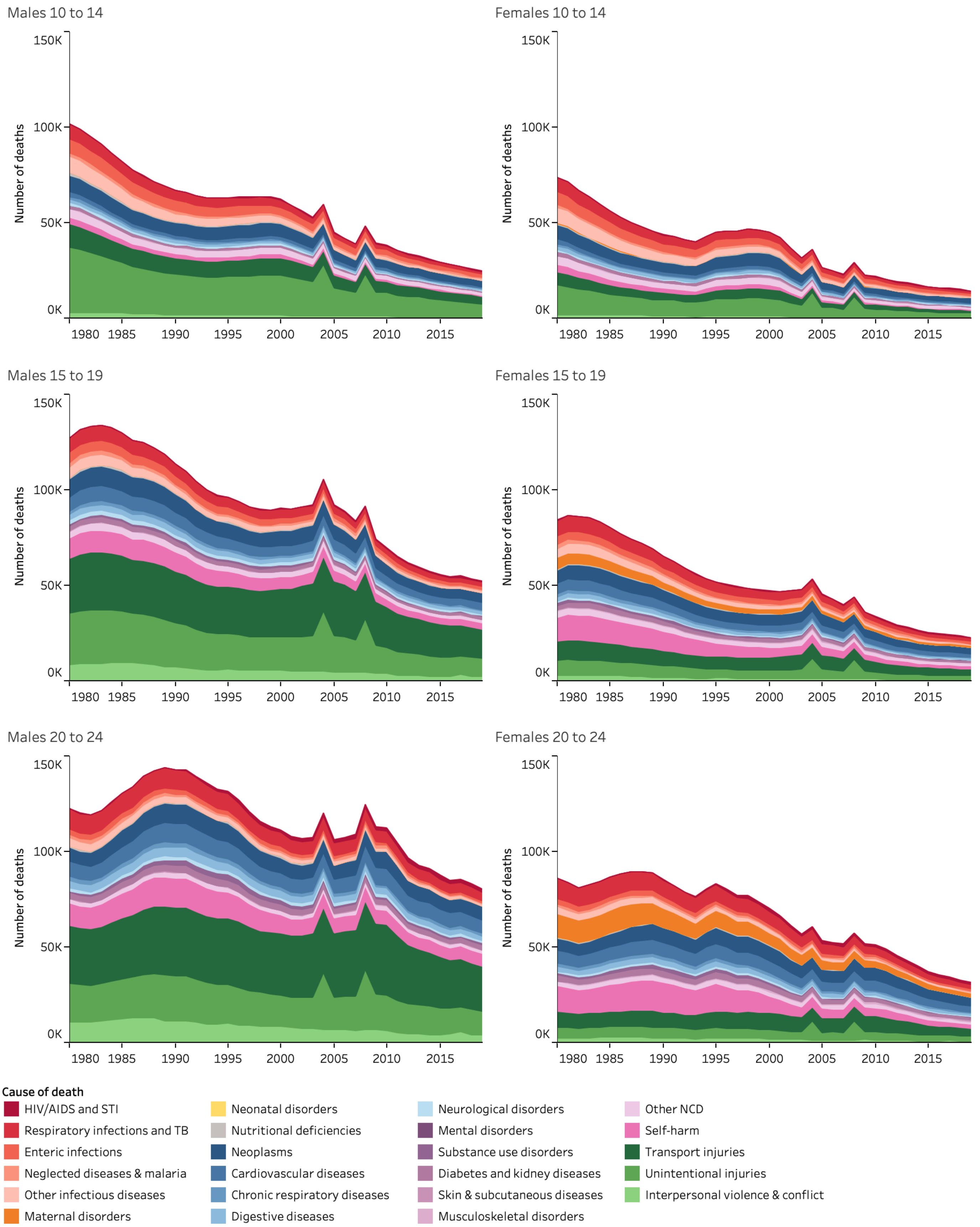

Figure S99: Rank of number of deaths by cause group 1980 – 2019: Southeast Asia, East Asia and Oceania GBD super-region. 10-14 year old males.

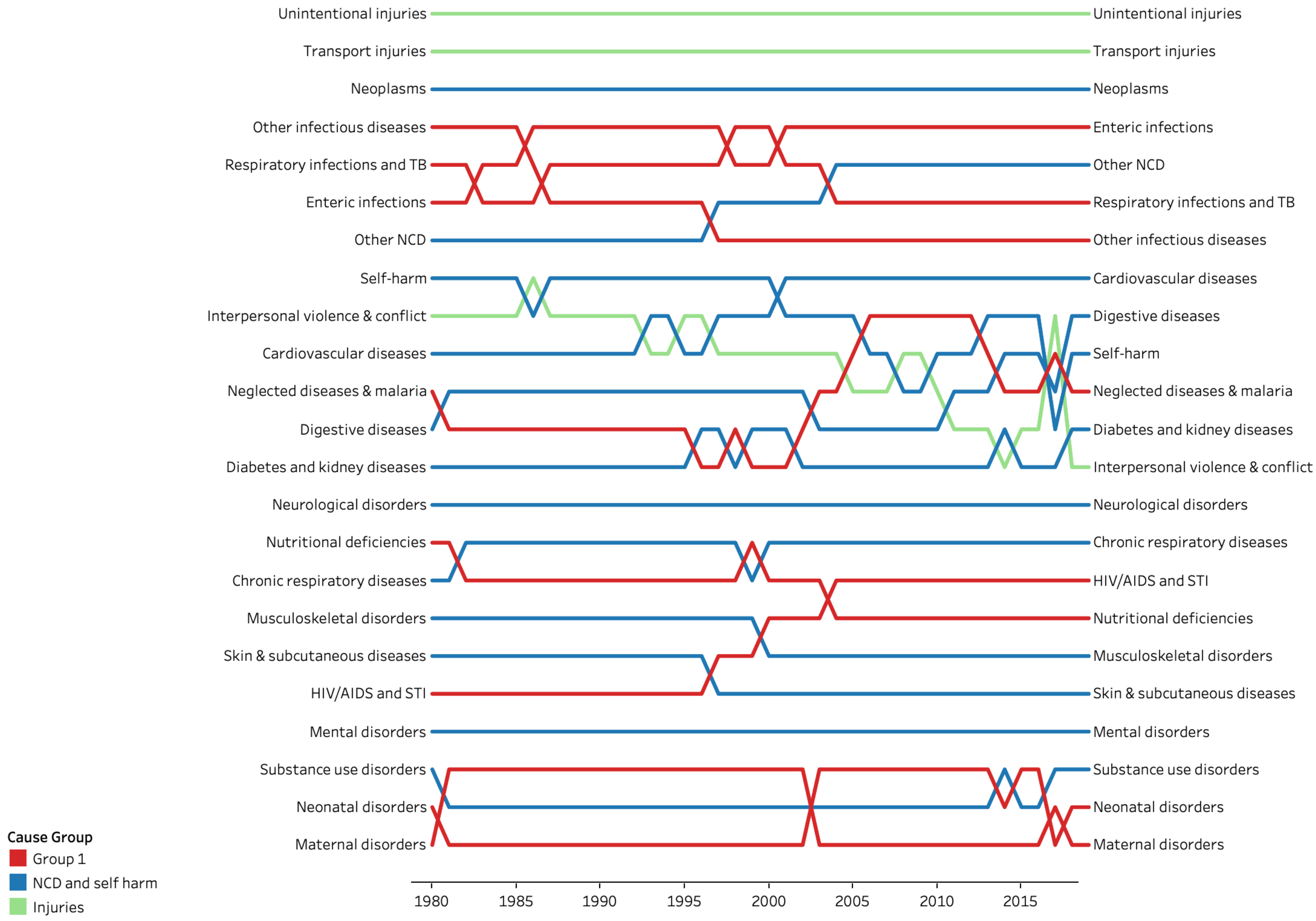

Figure S100: Rank of number of deaths by cause group 1980 – 2019: Southeast Asia, East Asia and Oceania GBD super-region. 10-14 year old females.

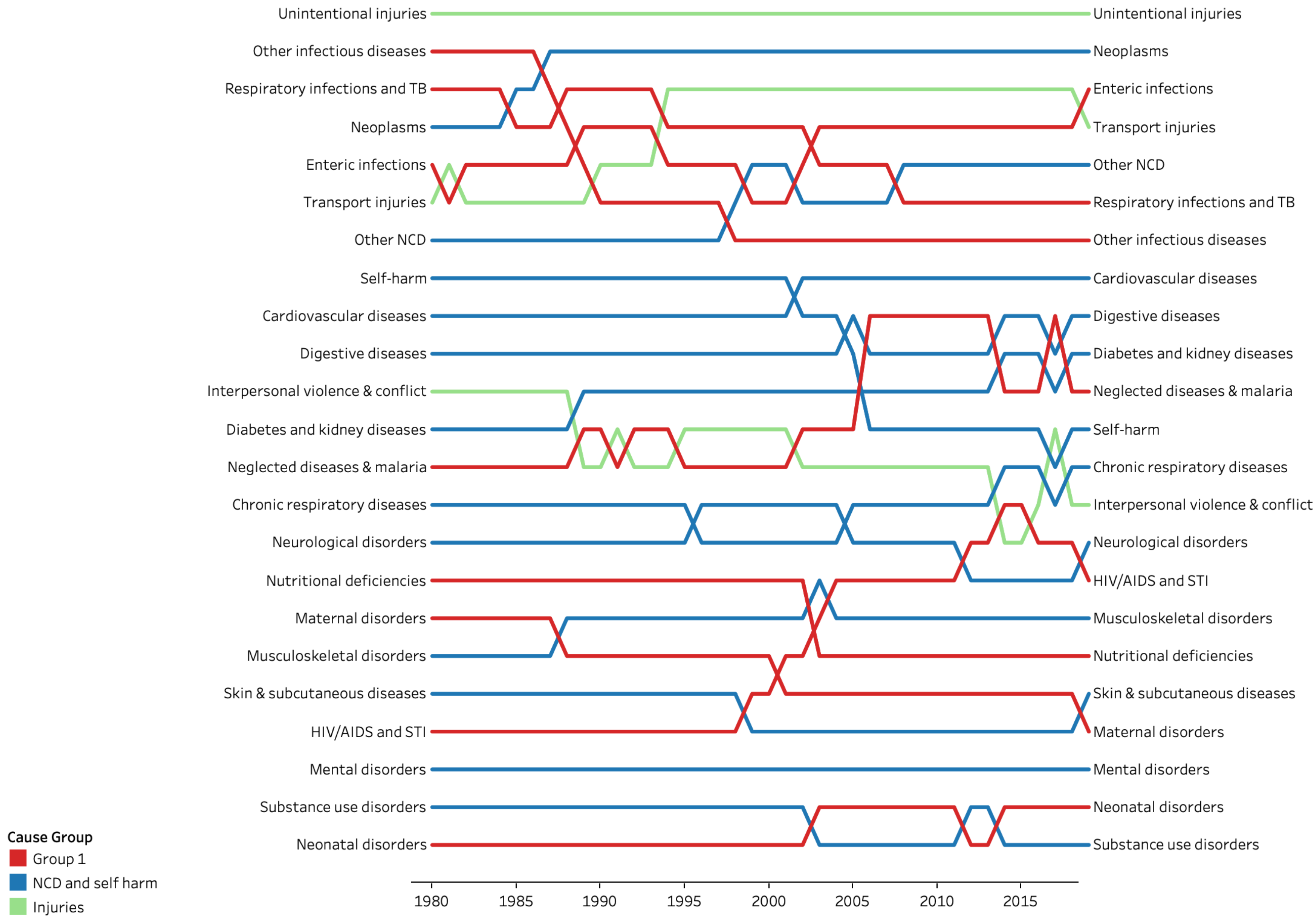

Figure S101: Rank of number of deaths by cause group 1980 – 2019: Southeast Asia, East Asia and Oceania GBD super-region. 15-19 year old males.

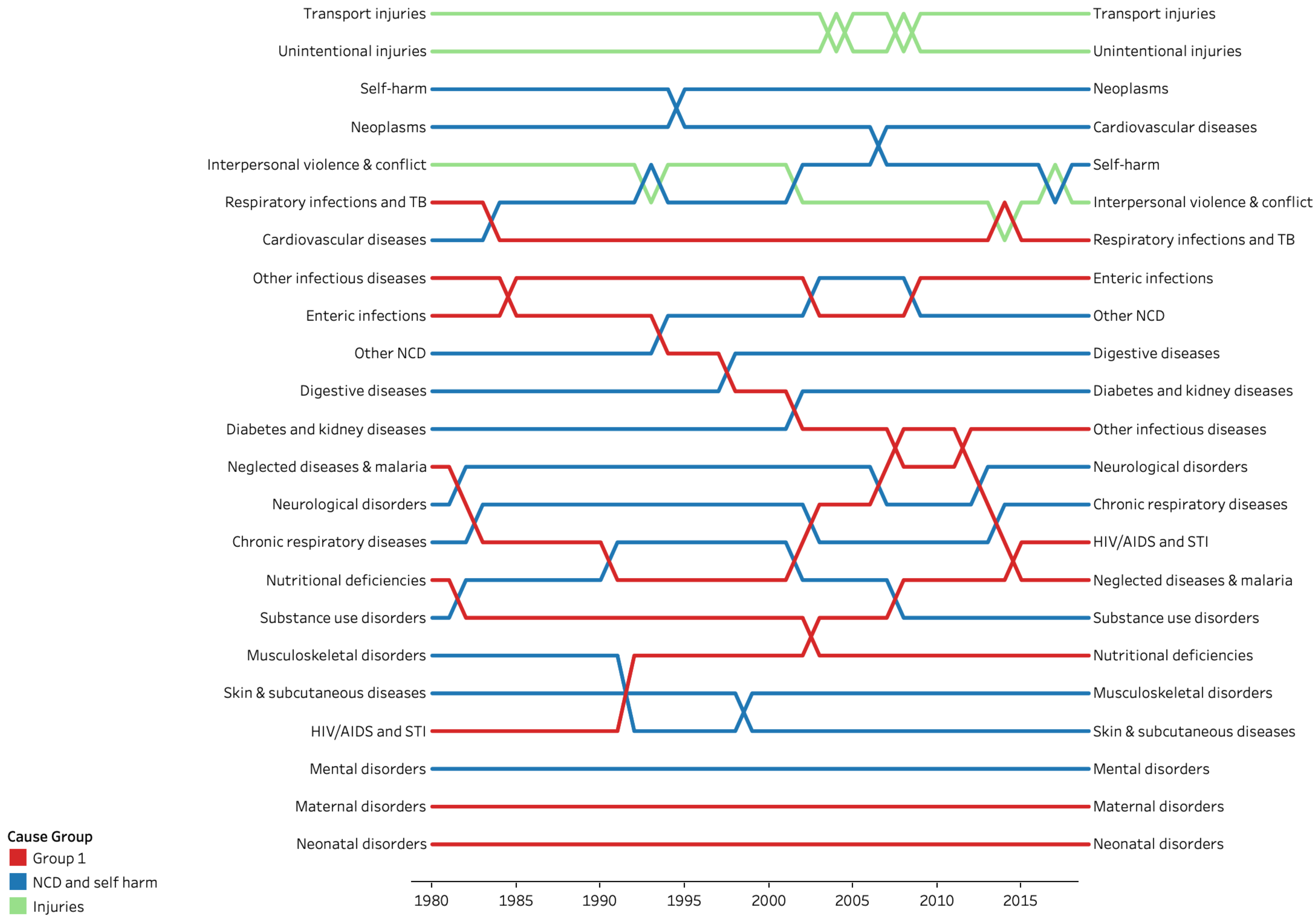

Figure S102: Rank of number of deaths by cause group 1980 – 2019: Southeast Asia, East Asia and Oceania GBD super-region. 15-19 year old females.

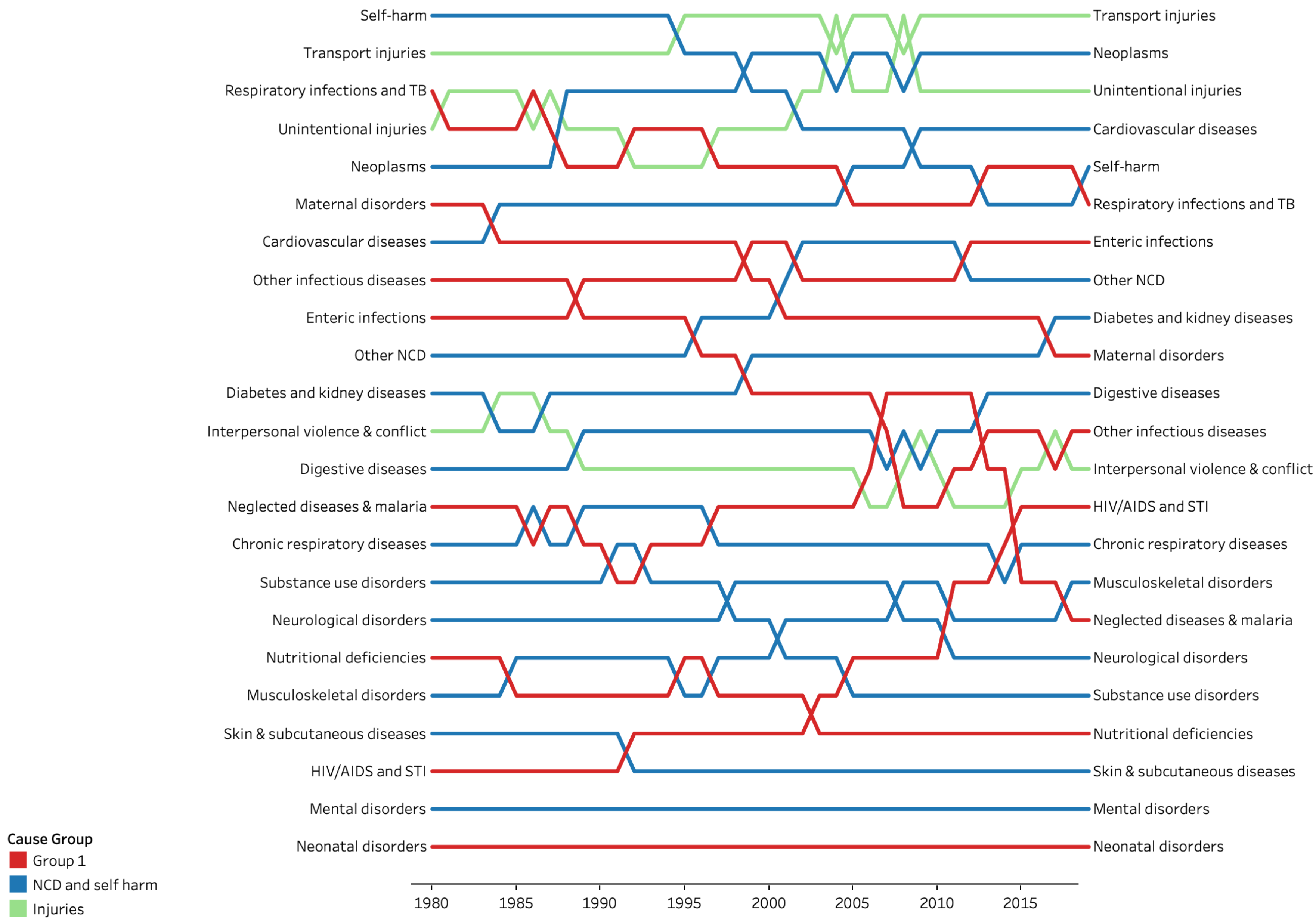

Figure S103: Rank of number of deaths by cause group 1980 – 2019: Southeast Asia, East Asia and Oceania GBD super-region. 20-24 year old males.

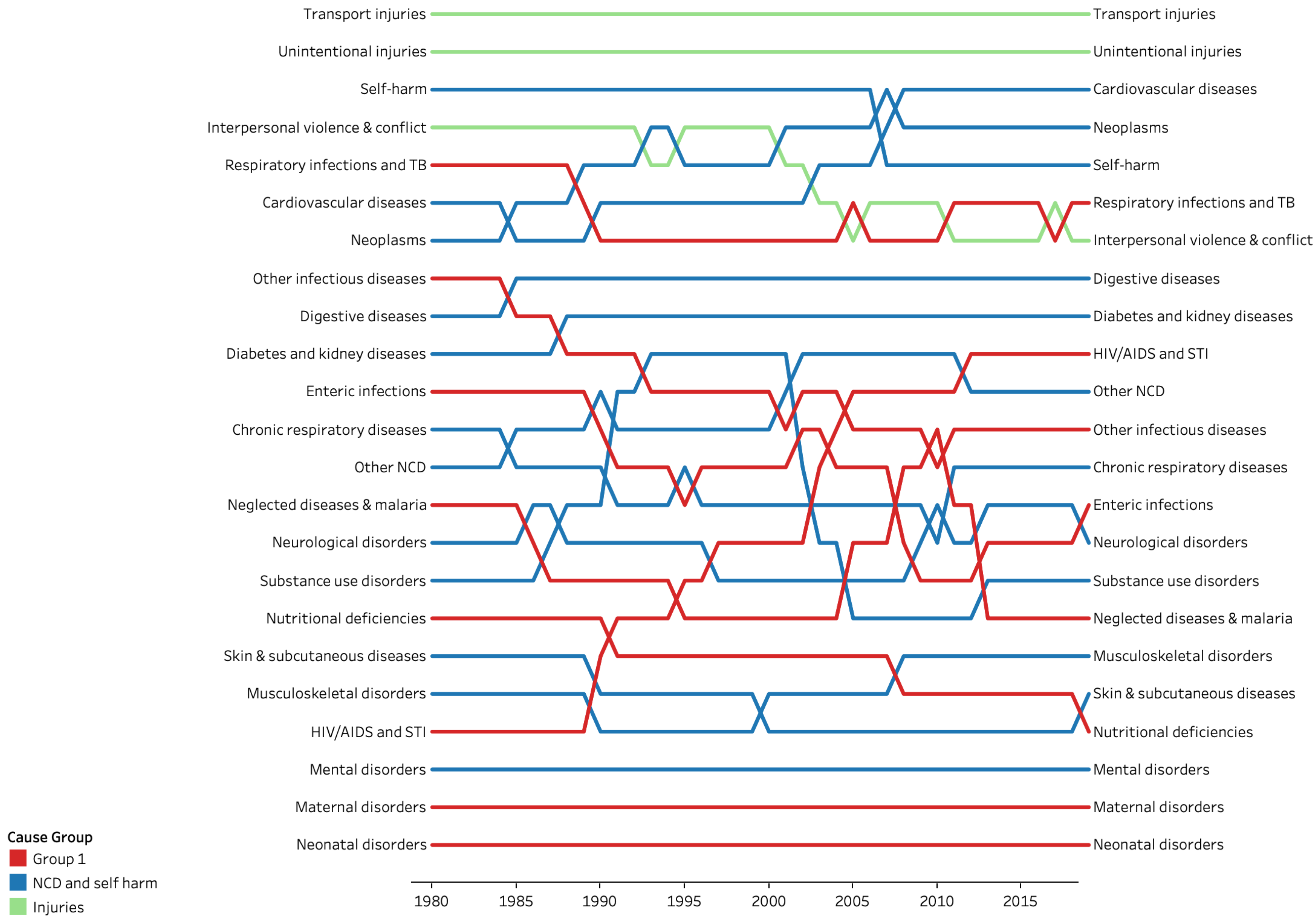

Figure S104: Rank of number of deaths by cause group 1980 – 2019: Southeast Asia, East Asia and Oceania GBD super-region. 20-24 year old females.

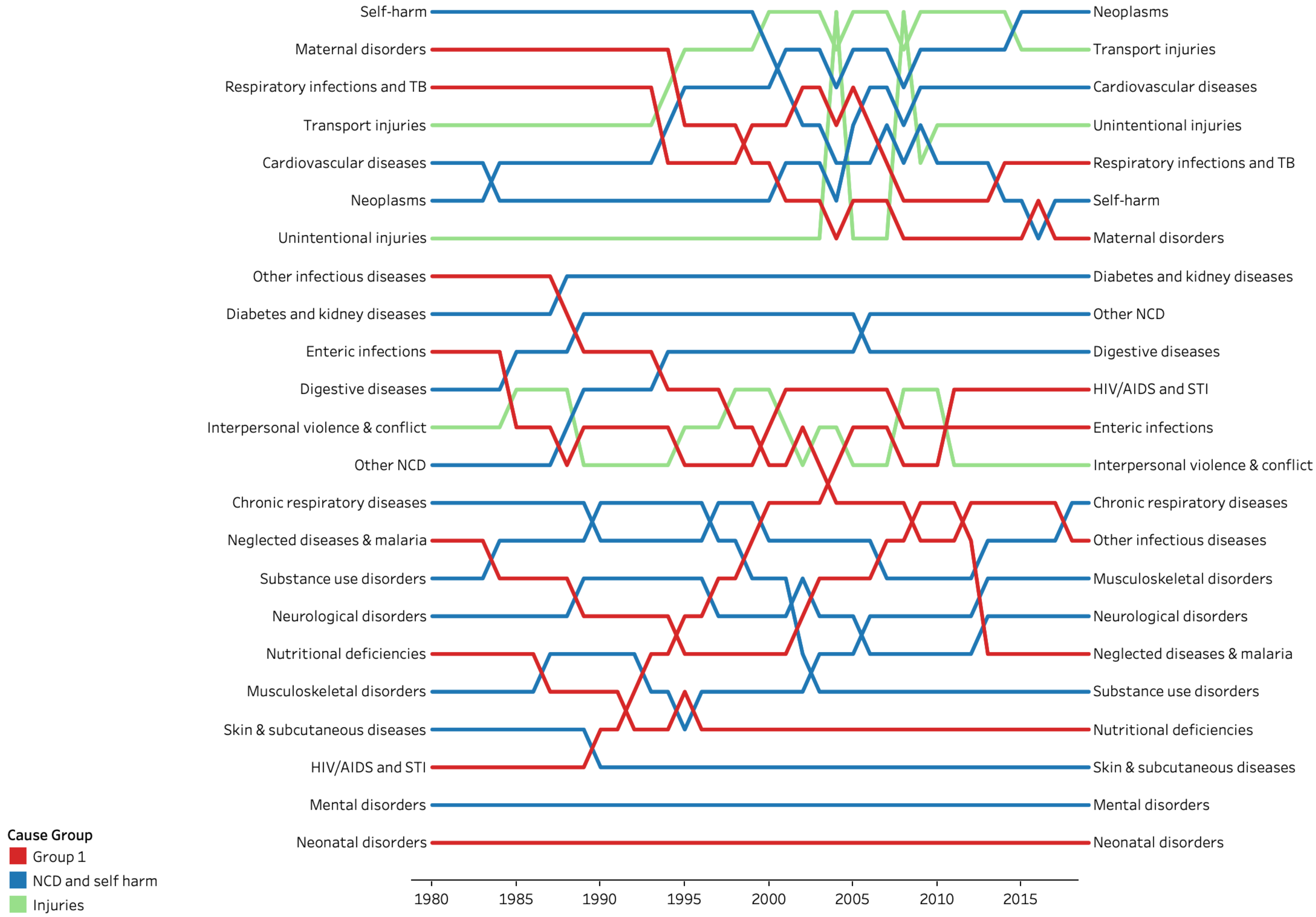

Figure S105: Mortality rate per 100,000 population by cause of death in 10-24 year olds 1980 – 2019: Sub Saharan Africa super-region

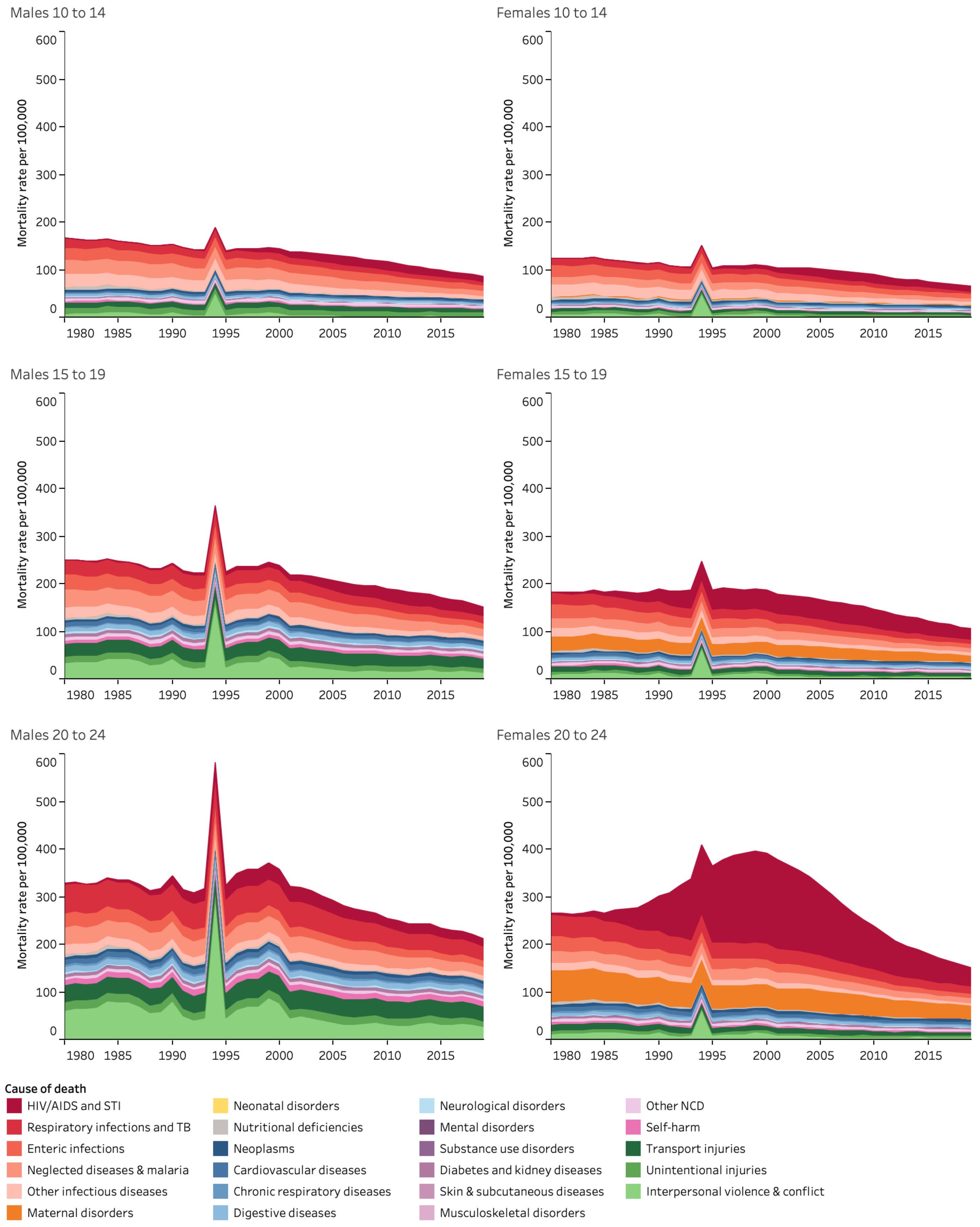

Figure S106: Number of deaths by cause in 10-24 year olds 1980 – 2019: Sub Saharan Africa super-region

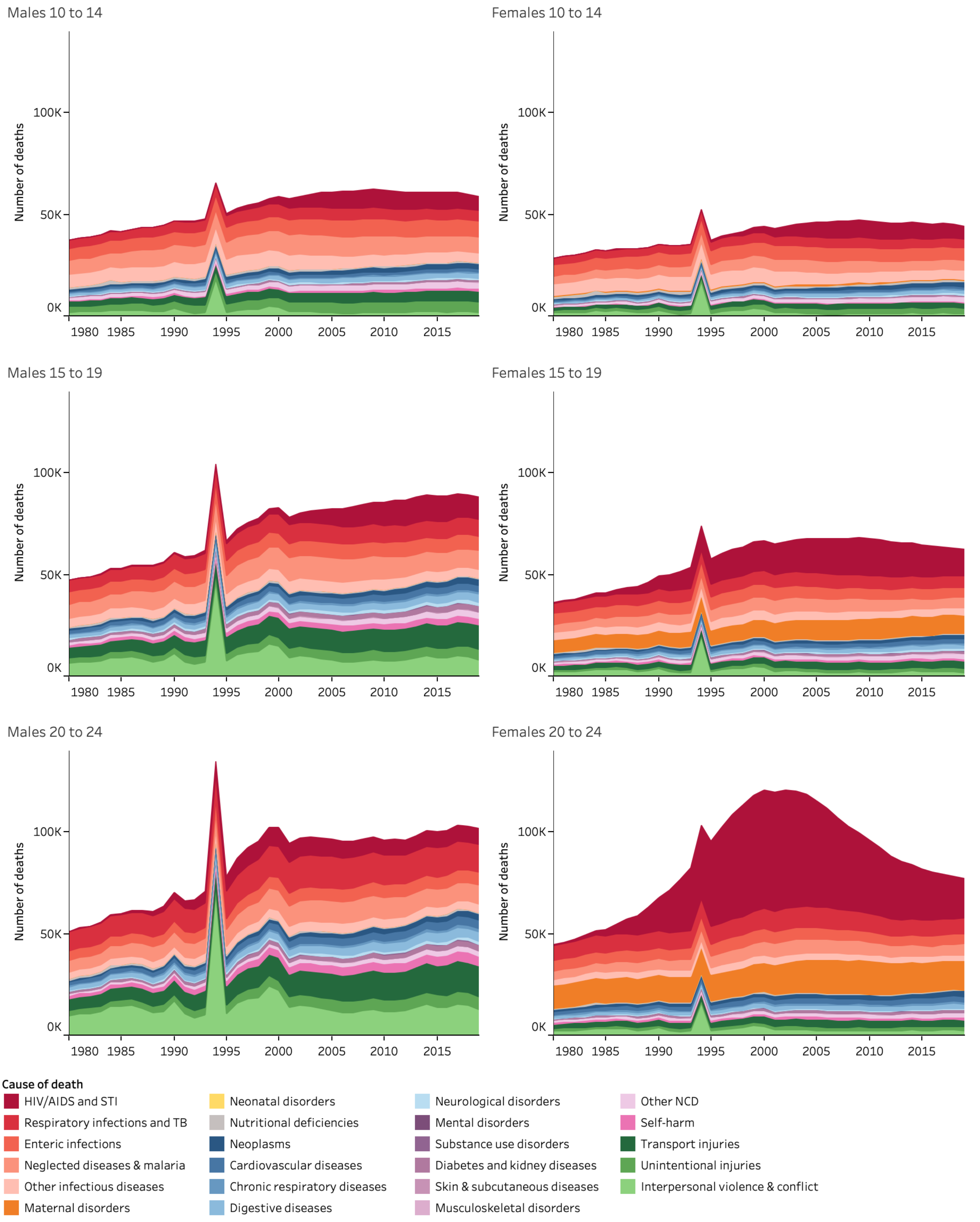

Figure S107: Rank of number of deaths by cause group 1980 – 2019: Sub Saharan Africa GBD super-region. 10-14 year old males.

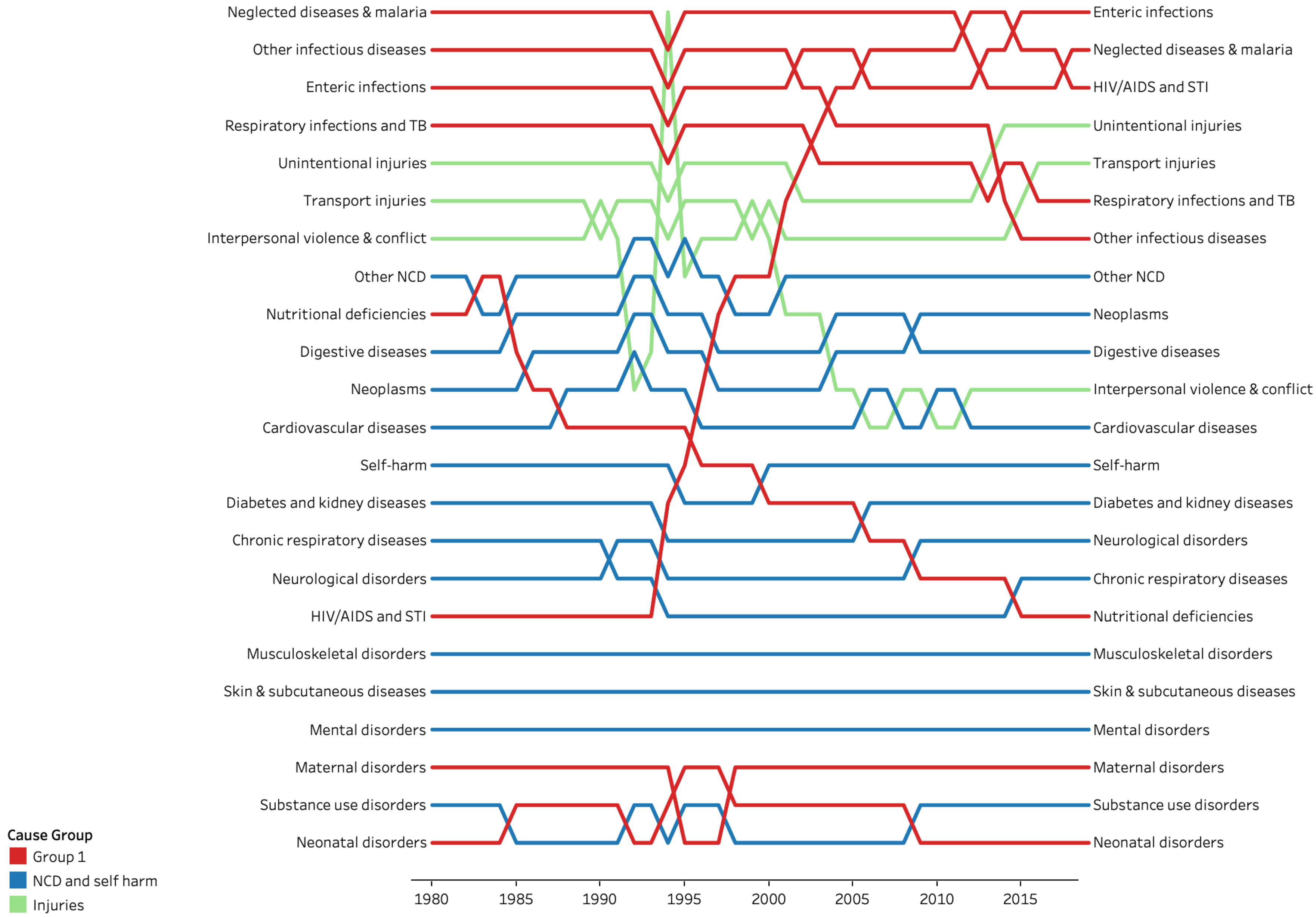

Figure S108: Rank of number of deaths by cause group 1980 – 2019: Sub Saharan Africa GBD super-region. 10-14 year old females.

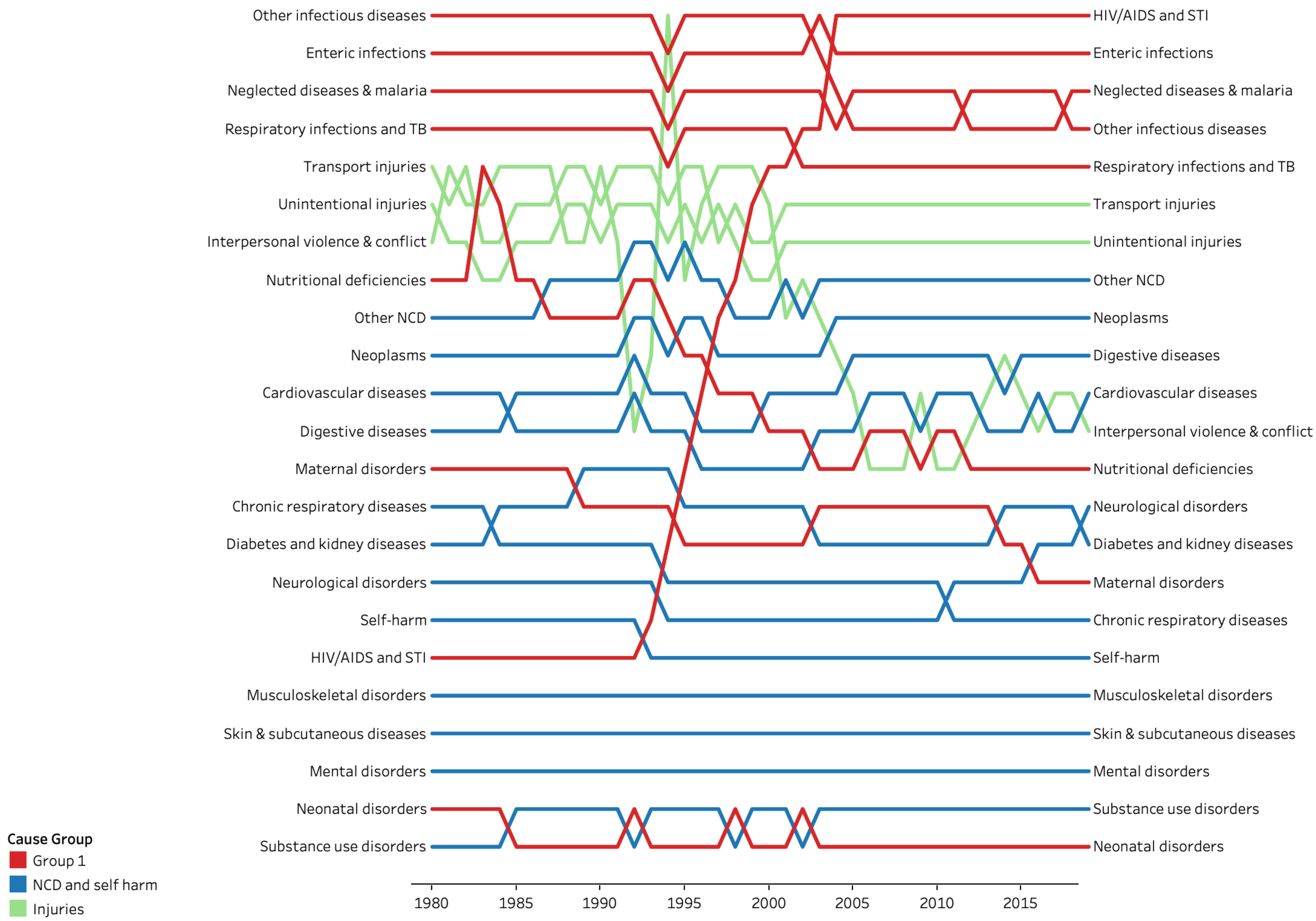

Figure S109: Rank of number of deaths by cause group 1980 – 2019: Sub Saharan Africa GBD super-region. 15-19 year old males.

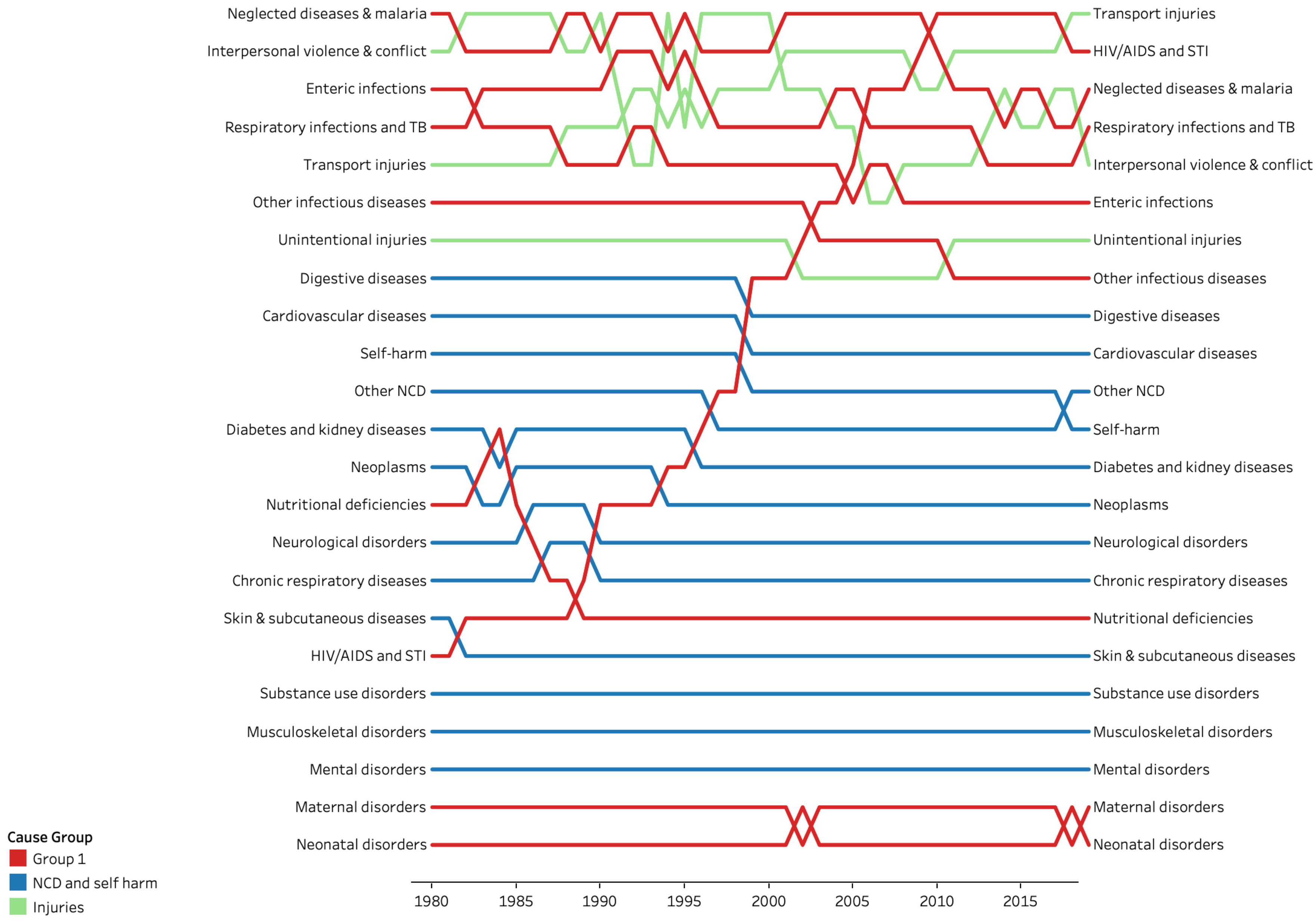

Figure S110: Rank of number of deaths by cause group 1980 – 2019: Sub Saharan Africa GBD super-region. 15-19 year old females.

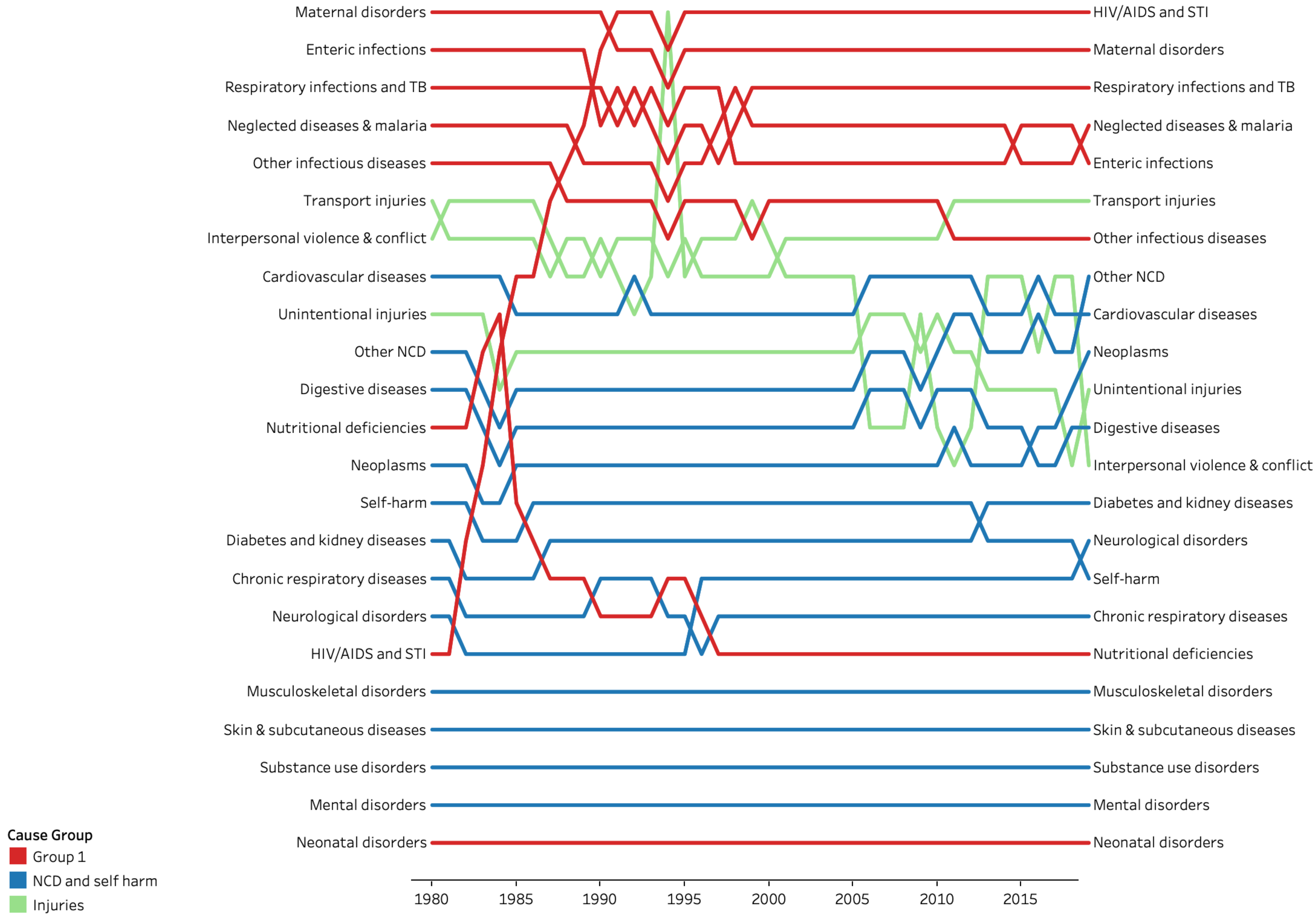

Figure S111: Rank of number of deaths by cause group 1980 – 2019: Sub Saharan Africa GBD super-region. 20-24 year old males.

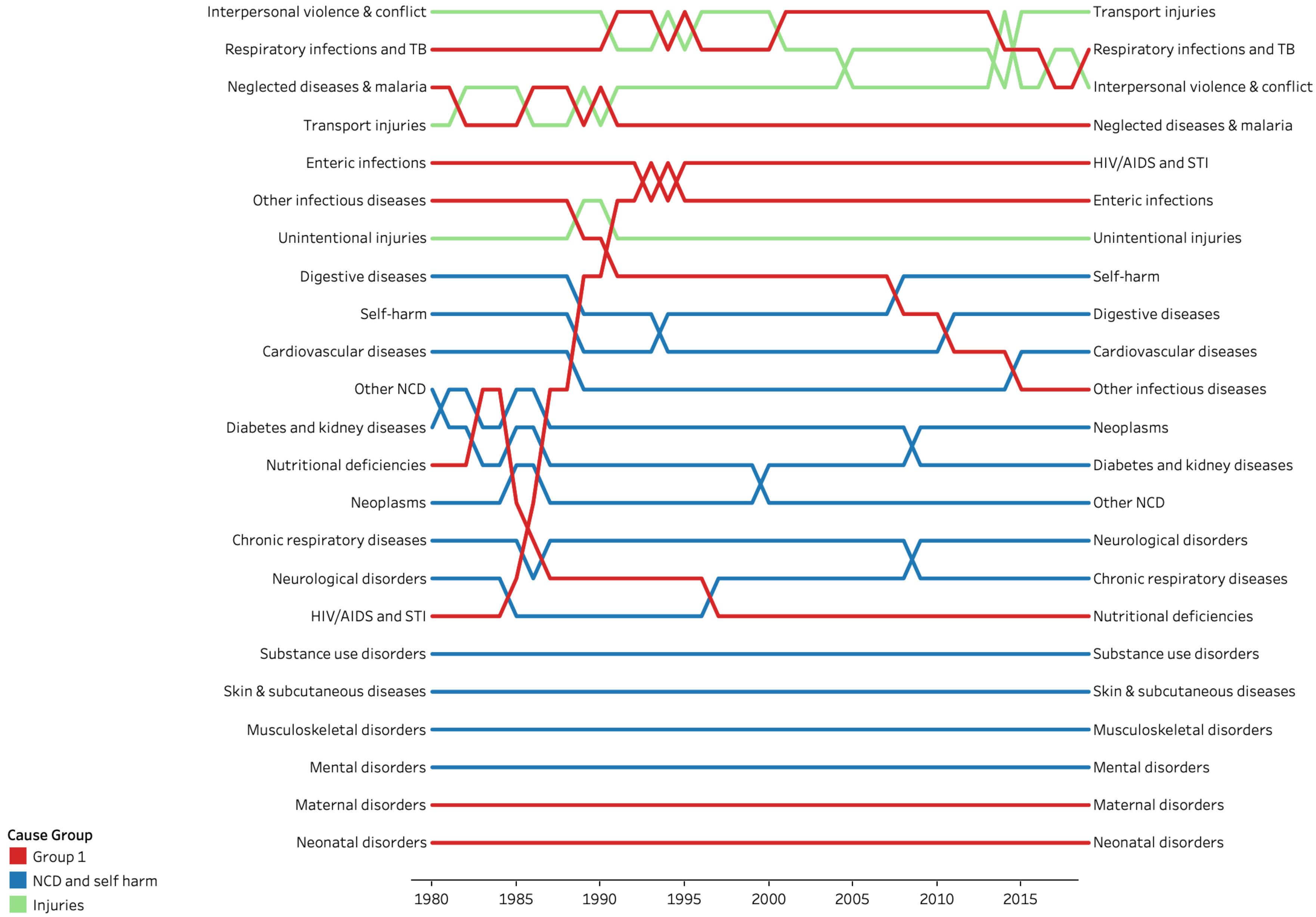

Figure S112: Rank of number of deaths by cause group 1980 – 2019: Sub Saharan Africa GBD super-region. 20-24 year old females.

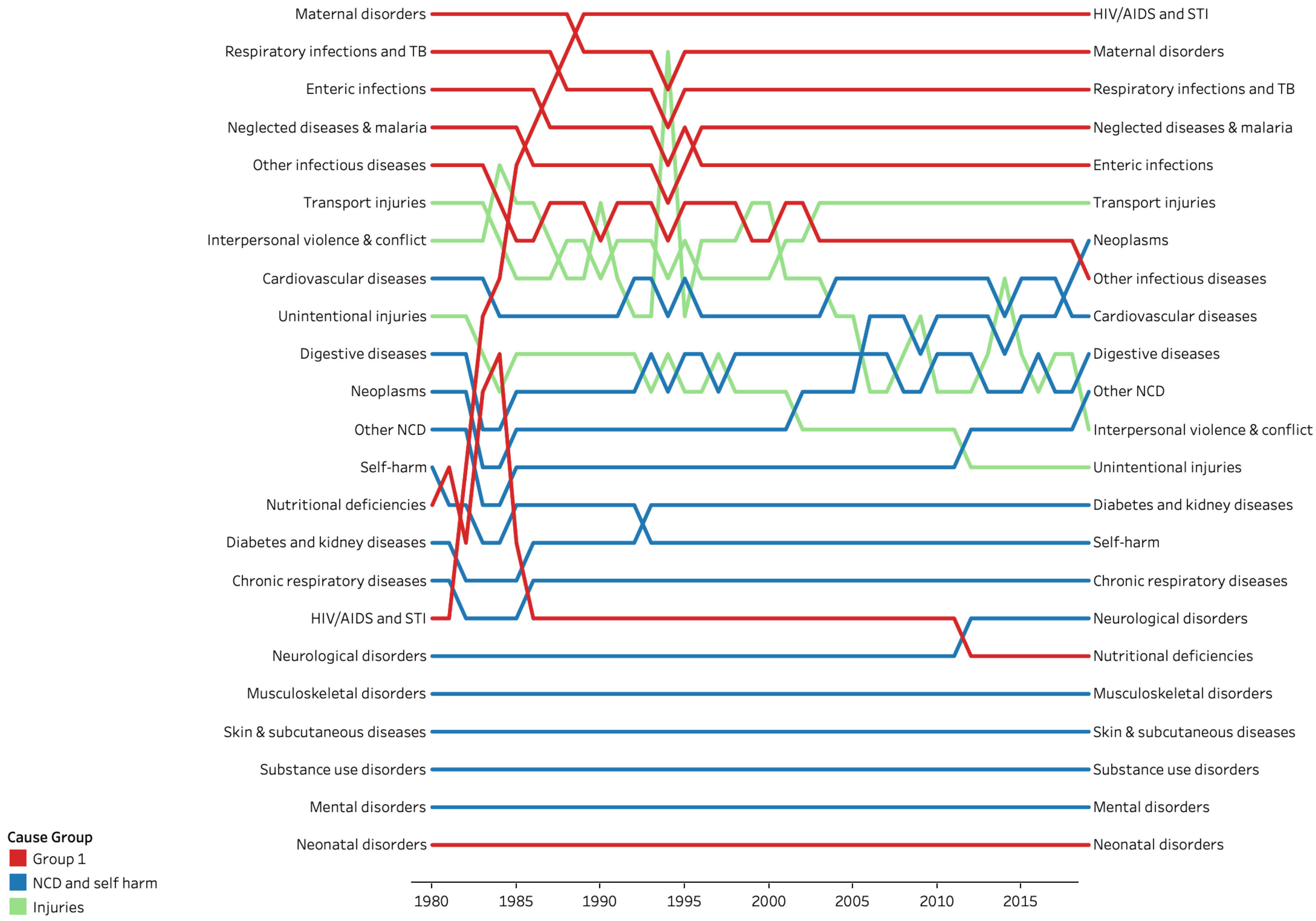

Figure 113: Differences in estimates for global number of deaths between GBD 2019 and UN IGME* 10 to 14 both sexes

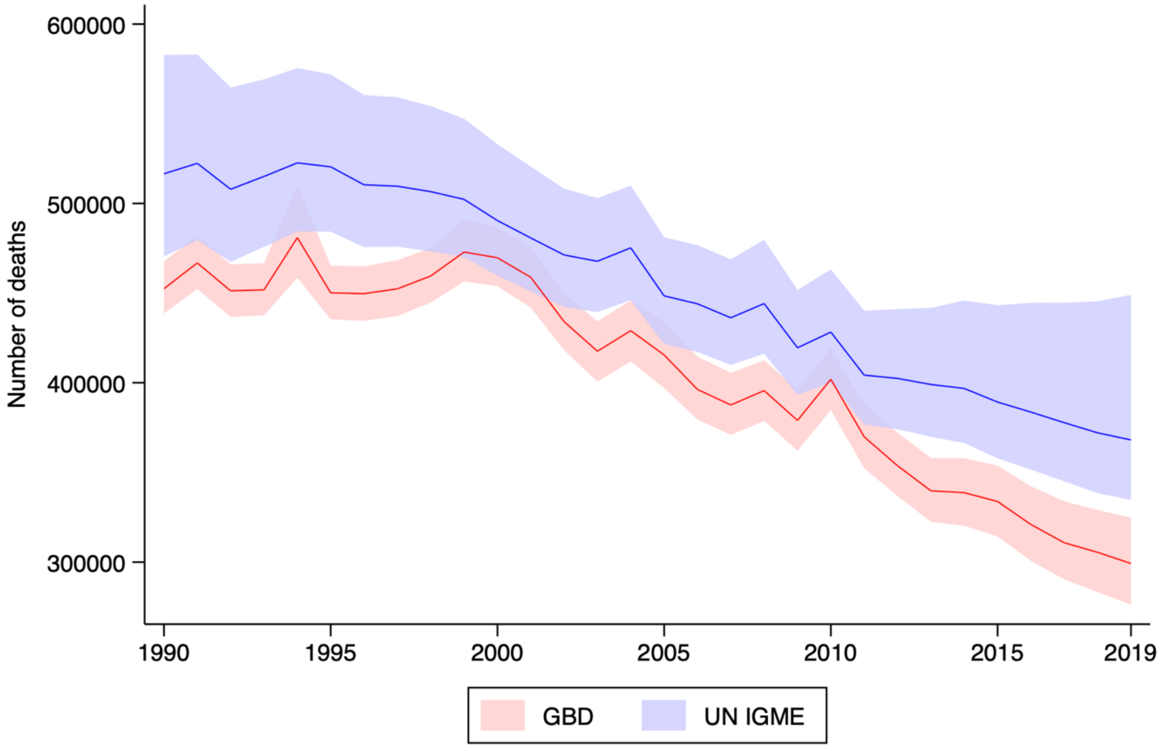

UN Inter-agency group for child mortality estimation

Figure 114: Differences in estimates for global number of deaths between GBD 2019 and UN IGME* 15 to 19 both sexes

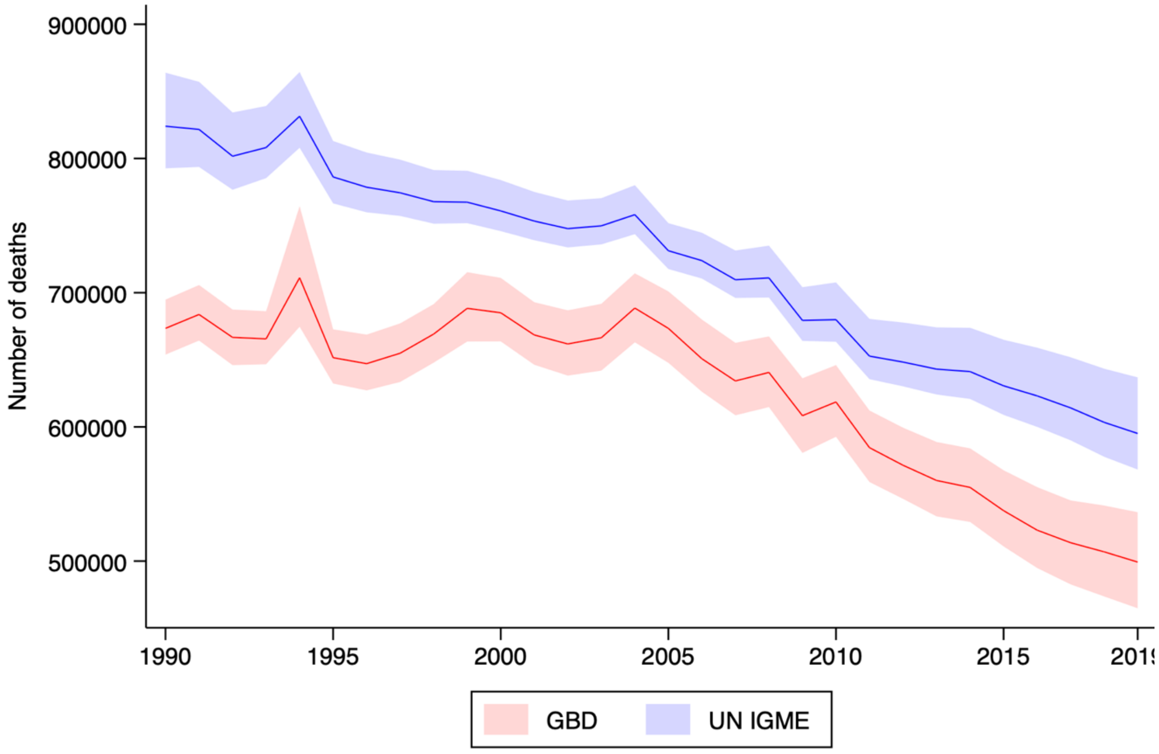

UN Inter-agency group for child mortality estimation

Figure 115: Differences in estimates for global number of deaths between GBD 2019 and UN IGME* 20 to 24 both sexes

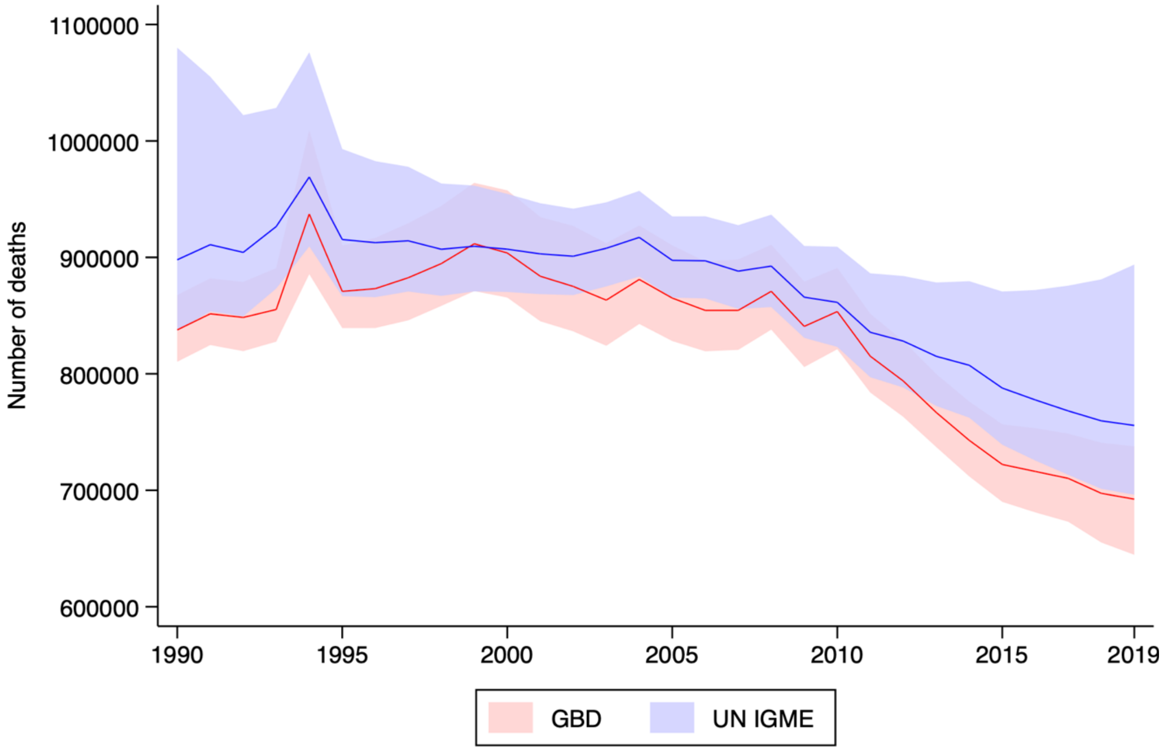

**UN Inter-agency group for child mortality estimation*
