# Supplementary material for: Global, regional, and national mortality among young people aged 10–24 years, 1950–2019: a systematic analysis for the Global Burden of Disease Study 2019
Source: Lancet. 2021 Oct 30;398(10311):1593–618. doi: 10.1016/S0140-6736(21)01546-4 (PMC8576274; doi:10.1016/S0140-6736(21)01546-4)
Supplement: Supplementary appendix [file mmc2.pdf]

THE LANCET

Supplementary appendix

This appendix formed part of the original submission and has been peer reviewed. We post it as supplied by the authors.

Supplement to: GBD 2019 Adolescent Mortality Collaborators. Global, regional, and national mortality among young people aged 10–24 years, 1950–2019: a systematic analysis for the Global Burden of Disease Study 2019. *Lancet* 2021; **398:** 1593–618.

Global, regional, and national mortality among young people aged 10 to 24 years, 1950-2019: a systematic analysis for the Global Burden of Disease Study 2019

Supplementary material 2

Figures showing mortality rate per 100,000 and numbers of death in 10-14, 15-19 and 20-24 by sex for all causes (1950 – 2019) and by cause of death (1980 – 2019) Globally and in 7 GBD super regions, 21 GBD regions and 204 countries and territories (pages 16-708)

Tables showing all-cause mortality rate per 100,000 and number of deaths in 10-14, 15-19 and 20-24 by sex 1950 – 2019 Globally and in 7 GBD super regions, 21 GBD regions and 204 countries and territories (pages 711 – 941)

Figures are labelled

a) All-cause mortality rate per 100,000 population in 10-24 by sex 1950-2019
b) Numbers of death from all causes in 10-24 by sex 1950-2019
c) Mortality rate per 100,000 population by cause in 10-24 by sex 1980-2019
d) Number of deaths by cause in 10-24 by sex 1980-2019

Figure S1: Global....16
Figure S2: Southeast Asia, East Asia, and Oceania....19
Figure S3: East Asia....22
Figure S4: China....25
Figure S5: Democratic People's Republic of Korea....28
Figure S6: Taiwan (Province of China)....31
Figure S7: Southeast Asia....34
Figure S8: Cambodia....37
Figure S9: Indonesia....40
Figure S10: Lao People's Democratic Republic....43
Figure S11: Malaysia....46
Figure S12: Maldives....49
Figure S13: Myanmar....52
Figure S14: Philippines....55
Figure S15: Sri Lanka....58
Figure S16: Thailand....61
Figure S17: Timor-Leste....64
Figure S18: Viet Nam....67
Figure S19: Oceania....70
Figure S20: Fiji....73
Figure S21: Kiribati....76
Figure S22: Marshall Islands....79
Figure S23: Micronesia (Federated States of)....82
Figure S24: Papua New Guinea....85
Figure S25: Samoa....88
Figure S26: Solomon Islands....91
Figure S27: Tonga....94

Figure S28: Vanuatu 97
Figure S29: Central Europe, Eastern Europe, and Central Asia 100
Figure S30: Central Asia 105
Figure S31: Armenia 108
Figure S32: Azerbaijan 111
Figure S33: Georgia 114
Figure S34: Kazakhstan 117
Figure S35: Kyrgyzstan 120
Figure S36: Mongolia 123
Figure S37: Tajikistan 126
Figure S38: Turkmenistan 129
Figure S39: Uzbekistan 132
Figure S40: Central Europe 135
Figure S41: Albania 138
Figure S42: Bosnia and Herzegovina 141
Figure S43: Bulgaria 144
Figure S44: Croatia 147
Figure S45: Czechia 150
Figure S46: Hungary 153
Figure S47: North Macedonia 156
Figure S48: Montenegro 159
Figure S49: Poland 162
Figure S50: Romania 165
Figure S51: Serbia 168
Figure S52: Slovakia 171
Figure S53: Slovenia 174
Figure S54: Eastern Europe 177
Figure S55: Belarus 180
Figure S56: Estonia 183
Figure S57: Latvia 186
Figure S58: Lithuania 189
Figure S59: Republic of Moldova 192
Figure S60: Russian Federation 195
Figure S61: Ukraine 198
Figure S62: High-income 201
Figure S63: High-income Asia Pacific 204
Figure S64: Brunei Darussalam 207
Figure S65: Japan 210
Figure S66: Republic of Korea 213
Figure S67: Singapore 216
Figure S68: Australasia 219
Figure S69: Australia 222
Figure S70: New Zealand 225
Figure S71: Western Europe 228
Figure S72: Andorra 231
Figure S73: Austria 234
Figure S74: Belgium 237
Figure S75: Cyprus 240
Figure S76: Denmark 243

Figure S77: Finland....246
Figure S78: France....249
Figure S79: Germany....252
Figure S80: Greece....255
Figure S81: Iceland....258
Figure S82: Ireland....261
Figure S83: Israel....264
Figure S84: Italy....267
Figure S85: Luxembourg....270
Figure S86: Malta....273
Figure S87: Netherlands....276
Figure S88: Norway....279
Figure S89: Portugal....282
Figure S90: Spain....285
Figure S91: Sweden....288
Figure S92: Switzerland....291
Figure S93: United Kingdom....294
Figure S94: Southern Latin America....297
Figure S95: Argentina....300
Figure S96: Chile....303
Figure S97: Uruguay....306
Figure S98: High-income North America....309
Figure S99: Canada....312
Figure S100: United States of America....315
Figure S101: Latin America and Caribbean....318
Figure S102: Caribbean....321
Figure S103: Antigua and Barbuda....324
Figure S104: Bahamas....327
Figure S105: Barbados....330
Figure S106: Belize....333
Figure S107: Cuba....336
Figure S108: Dominica....339
Figure S109: Dominican Republic....342
Figure S110: Grenada....345
Figure S111: Guyana....348
Figure S112: Haiti....351
Figure S113: Jamaica....354
Figure S114: Saint Lucia....357
Figure S115: Saint Vincent and the Grenadines....360
Figure S116: Suriname....363
Figure S117: Trinidad and Tobago....366
Figure S118: Andean Latin America....369
Figure S119: Bolivia (Plurinational State of)....372
Figure S120: Ecuador....375
Figure S121: Peru....378
Figure S122: Central Latin America....381
Figure S123: Colombia....384
Figure S124: Costa Rica....387
Figure S125: El Salvador....390

Figure S126: Guatemala.......393
Figure S127: Honduras.......396
Figure S128: Mexico.......399
Figure S129: Nicaragua.......402
Figure S130: Panama.......405
Figure S131: Venezuela (Bolivarian Republic of).......408
Figure S132: Tropical Latin America.......411
Figure S133: Brazil.......414
Figure S134: Paraguay.......417
Figure S135: North Africa and Middle East.......420
Figure S136: Algeria.......423
Figure S137: Bahrain.......426
Figure S138: Egypt.......429
Figure S139: Iran (Islamic Republic of).......432
Figure S140: Iraq.......435
Figure S141: Jordan.......438
Figure S142: Kuwait.......441
Figure S143: Lebanon.......444
Figure S144: Libya.......447
Figure S145: Morocco.......450
Figure S146: Palestine.......453
Figure S147: Oman.......456
Figure S148: Qatar.......459
Figure S149: Saudi Arabia.......462
Figure S150: Syrian Arab Republic.......465
Figure S151: Tunisia.......468
Figure S152: Turkey.......471
Figure S153: United Arab Emirates.......474
Figure S154: Yemen.......477
Figure S155: South Asia.......480
Figure S156: Afghanistan.......483
Figure S157: Bangladesh.......486
Figure S158: Bhutan.......489
Figure S159: India.......492
Figure S160: Nepal.......495
Figure S161: Pakistan.......498
Figure S162: Sub-Saharan Africa.......501
Figure S163: Central Sub-Saharan Africa.......504
Figure S164: Angola.......507
Figure S165: Central African Republic.......510
Figure S166: Congo.......513
Figure S167: Democratic Republic of the Congo.......516
Figure S168: Equatorial Guinea.......519
Figure S169: Gabon.......522
Figure S170: Eastern Sub-Saharan Africa.......525
Figure S171: Burundi.......528
Figure S172: Comoros.......531
Figure S173: Djibouti.......534
Figure S174: Eritrea.......537

Figure S175: Ethiopia 540
Figure S176: Kenya 543
Figure S177: Madagascar 546
Figure S178: Malawi 549
Figure S179: Mauritius 552
Figure S180: Mozambique 555
Figure S181: Rwanda 558
Figure S182: Seychelles 561
Figure S183: Somalia 564
Figure S184: United Republic of Tanzania 567
Figure S185: Uganda 570
Figure S186: Zambia 573
Figure S187: Southern Sub-Saharan Africa 576
Figure S188: Botswana 579
Figure S189: Lesotho 582
Figure S190: Namibia 585
Figure S191: South Africa 588
Figure S192: Eswatini 591
Figure S193: Zimbabwe 594
Figure S194: Western Sub-Saharan Africa 597
Figure S195: Benin 600
Figure S196: Burkina Faso 603
Figure S197: Cameroon 606
Figure S198: Cabo Verde 609
Figure S199: Chad 612
Figure S200: Côte d'Ivoire 615
Figure S201: Gambia 618
Figure S202: Ghana 621
Figure S203: Guinea 624
Figure S204: Guinea-Bissau 627
Figure S205: Liberia 630
Figure S206: Mali 633
Figure S207: Mauritania 636
Figure S208: Niger 639
Figure S209: Nigeria 642
Figure S210: Sao Tome and Principe 645
Figure S211: Senegal 648
Figure S212: Sierra Leone 651
Figure S213: Togo 654
Figure S214: American Samoa 657
Figure S215: Bermuda 660
Figure S216: Cook Islands 663
Figure S217: Greenland 666
Figure S218: Guam 669
Figure S219: Monaco 672
Figure S220: Nauru 675
Figure S221: Niue 678
Figure S222: Northern Mariana Islands 681
Figure S223: Palau 684

Figure S224: Puerto Rico 687
Figure S225: Saint Kitts and Nevis 690
Figure S226: San Marino 693
Figure S227: Tokelau 696
Figure S228: Tuvalu 699
Figure S229: United States Virgin Islands 702
Figure S230: South Sudan 705
Figure S231: Sudan 708

Tables of all-cause mortality rate and number of deaths by location, sex and age 1950-2019

Table S1: All-cause mortality rate per 100,000 population and number of deaths in 10-24 by sex 1950 - 2019: Global 711
Table S2: All-cause mortality rate per 100,000 population and number of deaths in 10-24 by sex 1950 - 2019: Southeast Asia, East Asia, and Oceania 712
Table S3: All-cause mortality rate per 100,000 population and number of deaths in 10-24 by sex 1950 - 2019: East Asia 713
Table S4: All-cause mortality rate per 100,000 population and number of deaths in 10-24 by sex 1950 - 2019: China 714
Table S5: All-cause mortality rate per 100,000 population and number of deaths in 10-24 by sex 1950 - 2019: Democratic People's Republic of Korea 715
Table S6: All-cause mortality rate per 100,000 population and number of deaths in 10-24 by sex 1950 - 2019: Taiwan (Province of China) 716
Table S7: All-cause mortality rate per 100,000 population and number of deaths in 10-24 by sex 1950 - 2019: Southeast Asia 717
Table S8: All-cause mortality rate per 100,000 population and number of deaths in 10-24 by sex 1950 - 2019: Cambodia 718
Table S9: All-cause mortality rate per 100,000 population and number of deaths in 10-24 by sex 1950 - 2019: Indonesia 719
Table S10: All-cause mortality rate per 100,000 population and number of deaths in 10-24 by sex 1950 - 2019: Lao People's Democratic Republic 720
Table S11: All-cause mortality rate per 100,000 population and number of deaths in 10-24 by sex 1950 - 2019: Malaysia 721
Table S12: All-cause mortality rate per 100,000 population and number of deaths in 10-24 by sex 1950 - 2019: Maldives 722
Table S13: All-cause mortality rate per 100,000 population and number of deaths in 10-24 by sex 1950 - 2019: Myanmar 723
Table S14: All-cause mortality rate per 100,000 population and number of deaths in 10-24 by sex 1950 - 2019: Philippines 724
Table S15: All-cause mortality rate per 100,000 population and number of deaths in 10-24 by sex 1950 - 2019: Sri Lanka 725
Table S16: All-cause mortality rate per 100,000 population and number of deaths in 10-24 by sex 1950 - 2019: Thailand 726
Table S17: All-cause mortality rate per 100,000 population and number of deaths in 10-24 by sex 1950 - 2019: Timor-Leste 727
Table S18: All-cause mortality rate per 100,000 population and number of deaths in 10-24 by sex 1950 - 2019: Viet Nam 728
Table S19: All-cause mortality rate per 100,000 population and number of deaths in 10-24 by sex 1950 - 2019: Oceania 729

Table S20: All-cause mortality rate per 100,000 population and number of deaths in 10-24 by sex 1950 - 2019: Fiji 730
Table S21: All-cause mortality rate per 100,000 population and number of deaths in 10-24 by sex 1950 - 2019: Kiribati 731
Table S22: All-cause mortality rate per 100,000 population and number of deaths in 10-24 by sex 1950 - 2019: Marshall Islands 732
Table S23: All-cause mortality rate per 100,000 population and number of deaths in 10-24 by sex 1950 - 2019: Micronesia (Federated States of) 733
Table S24: All-cause mortality rate per 100,000 population and number of deaths in 10-24 by sex 1950 - 2019: Papua New Guinea 734
Table S25: All-cause mortality rate per 100,000 population and number of deaths in 10-24 by sex 1950 - 2019: Samoa 735
Table S26: All-cause mortality rate per 100,000 population and number of deaths in 10-24 by sex 1950 - 2019: Solomon Islands 736
Table S27: All-cause mortality rate per 100,000 population and number of deaths in 10-24 by sex 1950 - 2019: Tonga 737
Table S28: All-cause mortality rate per 100,000 population and number of deaths in 10-24 by sex 1950 - 2019: Vanuatu 738
Table S29: All-cause mortality rate per 100,000 population and number of deaths in 10-24 by sex 1950 - 2019: Central Europe, Eastern Europe, and Central Asia 739
Table S30: All-cause mortality rate per 100,000 population and number of deaths in 10-24 by sex 1950 - 2019: Central Asia 740
Table S31: All-cause mortality rate per 100,000 population and number of deaths in 10-24 by sex 1950 - 2019: Armenia 741
Table S32: All-cause mortality rate per 100,000 population and number of deaths in 10-24 by sex 1950 - 2019: Azerbaijan 742
Table S33: All-cause mortality rate per 100,000 population and number of deaths in 10-24 by sex 1950 - 2019: Georgia 743
Table S34: All-cause mortality rate per 100,000 population and number of deaths in 10-24 by sex 1950 - 2019: Kazakhstan 744
Table S35: All-cause mortality rate per 100,000 population and number of deaths in 10-24 by sex 1950 - 2019: Kyrgyzstan 745
Table S36: All-cause mortality rate per 100,000 population and number of deaths in 10-24 by sex 1950 - 2019: Mongolia 746
Table S37: All-cause mortality rate per 100,000 population and number of deaths in 10-24 by sex 1950 - 2019: Tajikistan 747
Table S38: All-cause mortality rate per 100,000 population and number of deaths in 10-24 by sex 1950 - 2019: Turkmenistan 748
Table S39: All-cause mortality rate per 100,000 population and number of deaths in 10-24 by sex 1950 - 2019: Uzbekistan 749
Table S40: All-cause mortality rate per 100,000 population and number of deaths in 10-24 by sex 1950 - 2019: Central Europe 750
Table S41: All-cause mortality rate per 100,000 population and number of deaths in 10-24 by sex 1950 - 2019: Albania 751
Table S42: All-cause mortality rate per 100,000 population and number of deaths in 10-24 by sex 1950 - 2019: Bosnia and Herzegovina 752
Table S43: All-cause mortality rate per 100,000 population and number of deaths in 10-24 by sex 1950 - 2019: Bulgaria 753

Table S44: All-cause mortality rate per 100,000 population and number of deaths in 10-24 by sex 1950 - 2019: Croatia 754
Table S45: All-cause mortality rate per 100,000 population and number of deaths in 10-24 by sex 1950 - 2019: Czechia 755
Table S46: All-cause mortality rate per 100,000 population and number of deaths in 10-24 by sex 1950 - 2019: Hungary 756
Table S47: All-cause mortality rate per 100,000 population and number of deaths in 10-24 by sex 1950 - 2019: North Macedonia 757
Table S48: All-cause mortality rate per 100,000 population and number of deaths in 10-24 by sex 1950 - 2019: Montenegro 758
Table S49: All-cause mortality rate per 100,000 population and number of deaths in 10-24 by sex 1950 - 2019: Poland 759
Table S50: All-cause mortality rate per 100,000 population and number of deaths in 10-24 by sex 1950 - 2019: Romania 760
Table S51: All-cause mortality rate per 100,000 population and number of deaths in 10-24 by sex 1950 - 2019: Serbia 761
Table S52: All-cause mortality rate per 100,000 population and number of deaths in 10-24 by sex 1950 - 2019: Slovakia 762
Table S53: All-cause mortality rate per 100,000 population and number of deaths in 10-24 by sex 1950 - 2019: Slovenia 763
Table S54: All-cause mortality rate per 100,000 population and number of deaths in 10-24 by sex 1950 - 2019: Eastern Europe 764
Table S55: All-cause mortality rate per 100,000 population and number of deaths in 10-24 by sex 1950 - 2019: Belarus 765
Table S56: All-cause mortality rate per 100,000 population and number of deaths in 10-24 by sex 1950 - 2019: Estonia 766
Table S57: All-cause mortality rate per 100,000 population and number of deaths in 10-24 by sex 1950 - 2019: Latvia 767
Table S58: All-cause mortality rate per 100,000 population and number of deaths in 10-24 by sex 1950 - 2019: Lithuania 768
Table S59: All-cause mortality rate per 100,000 population and number of deaths in 10-24 by sex 1950 - 2019: Republic of Moldova 769
Table S60: All-cause mortality rate per 100,000 population and number of deaths in 10-24 by sex 1950 - 2019: Russian Federation 770
Table S61: All-cause mortality rate per 100,000 population and number of deaths in 10-24 by sex 1950 - 2019: Ukraine 771
Table S62: All-cause mortality rate per 100,000 population and number of deaths in 10-24 by sex 1950 - 2019: High-income 772
Table S63: All-cause mortality rate per 100,000 population and number of deaths in 10-24 by sex 1950 - 2019: High-income Asia Pacific 773
Table S64: All-cause mortality rate per 100,000 population and number of deaths in 10-24 by sex 1950 - 2019: Brunei Darussalam 774
Table S65: All-cause mortality rate per 100,000 population and number of deaths in 10-24 by sex 1950 - 2019: Japan 775
Table S66: All-cause mortality rate per 100,000 population and number of deaths in 10-24 by sex 1950 - 2019: Republic of Korea 776
Table S67: All-cause mortality rate per 100,000 population and number of deaths in 10-24 by sex 1950 - 2019: Singapore 777

Table S68: All-cause mortality rate per 100,000 population and number of deaths in 10-24 by sex 1950 - 2019: Australasia 778
Table S69: All-cause mortality rate per 100,000 population and number of deaths in 10-24 by sex 1950 - 2019: Australia 779
Table S70: All-cause mortality rate per 100,000 population and number of deaths in 10-24 by sex 1950 - 2019: New Zealand 780
Table S71: All-cause mortality rate per 100,000 population and number of deaths in 10-24 by sex 1950 - 2019: Western Europe 781
Table S72: All-cause mortality rate per 100,000 population and number of deaths in 10-24 by sex 1950 - 2019: Andorra 782
Table S73: All-cause mortality rate per 100,000 population and number of deaths in 10-24 by sex 1950 - 2019: Austria 783
Table S74: All-cause mortality rate per 100,000 population and number of deaths in 10-24 by sex 1950 - 2019: Belgium 784
Table S75: All-cause mortality rate per 100,000 population and number of deaths in 10-24 by sex 1950 - 2019: Cyprus 785
Table S76: All-cause mortality rate per 100,000 population and number of deaths in 10-24 by sex 1950 - 2019: Denmark 786
Table S77: All-cause mortality rate per 100,000 population and number of deaths in 10-24 by sex 1950 - 2019: Finland 787
Table S78: All-cause mortality rate per 100,000 population and number of deaths in 10-24 by sex 1950 - 2019: France 788
Table S79: All-cause mortality rate per 100,000 population and number of deaths in 10-24 by sex 1950 - 2019: Germany 789
Table S80: All-cause mortality rate per 100,000 population and number of deaths in 10-24 by sex 1950 - 2019: Greece 790
Table S81: All-cause mortality rate per 100,000 population and number of deaths in 10-24 by sex 1950 - 2019: Iceland 791
Table S82: All-cause mortality rate per 100,000 population and number of deaths in 10-24 by sex 1950 - 2019: Ireland 792
Table S83: All-cause mortality rate per 100,000 population and number of deaths in 10-24 by sex 1950 - 2019: Israel 793
Table S84: All-cause mortality rate per 100,000 population and number of deaths in 10-24 by sex 1950 - 2019: Italy 794
Table S85: All-cause mortality rate per 100,000 population and number of deaths in 10-24 by sex 1950 - 2019: Luxembourg 795
Table S86: All-cause mortality rate per 100,000 population and number of deaths in 10-24 by sex 1950 - 2019: Malta 796
Table S87: All-cause mortality rate per 100,000 population and number of deaths in 10-24 by sex 1950 - 2019: Netherlands 797
Table S88: All-cause mortality rate per 100,000 population and number of deaths in 10-24 by sex 1950 - 2019: Norway 798
Table S89: All-cause mortality rate per 100,000 population and number of deaths in 10-24 by sex 1950 - 2019: Portugal 799
Table S90: All-cause mortality rate per 100,000 population and number of deaths in 10-24 by sex 1950 - 2019: Spain 800
Table S91: All-cause mortality rate per 100,000 population and number of deaths in 10-24 by sex 1950 - 2019: Sweden 801

Table S92: All-cause mortality rate per 100,000 population and number of deaths in 10-24 by sex 1950 - 2019: Switzerland 802
Table S93: All-cause mortality rate per 100,000 population and number of deaths in 10-24 by sex 1950 - 2019: United Kingdom 803
Table S94: All-cause mortality rate per 100,000 population and number of deaths in 10-24 by sex 1950 - 2019: Southern Latin America 804
Table S95: All-cause mortality rate per 100,000 population and number of deaths in 10-24 by sex 1950 - 2019: Argentina 805
Table S96: All-cause mortality rate per 100,000 population and number of deaths in 10-24 by sex 1950 - 2019: Chile 806
Table S97: All-cause mortality rate per 100,000 population and number of deaths in 10-24 by sex 1950 - 2019: Uruguay 807
Table S98: All-cause mortality rate per 100,000 population and number of deaths in 10-24 by sex 1950 - 2019: High-income North America 808
Table S99: All-cause mortality rate per 100,000 population and number of deaths in 10-24 by sex 1950 - 2019: Canada 809
Table S100: All-cause mortality rate per 100,000 population and number of deaths in 10-24 by sex 1950 - 2019: United States of America 810
Table S101: All-cause mortality rate per 100,000 population and number of deaths in 10-24 by sex 1950 - 2019: Latin America and Caribbean 811
Table S102: All-cause mortality rate per 100,000 population and number of deaths in 10-24 by sex 1950 - 2019: Caribbean 812
Table S103: All-cause mortality rate per 100,000 population and number of deaths in 10-24 by sex 1950 - 2019: Antigua and Barbuda 813
Table S104: All-cause mortality rate per 100,000 population and number of deaths in 10-24 by sex 1950 - 2019: Bahamas 814
Table S105: All-cause mortality rate per 100,000 population and number of deaths in 10-24 by sex 1950 - 2019: Barbados 815
Table S106: All-cause mortality rate per 100,000 population and number of deaths in 10-24 by sex 1950 - 2019: Belize 816
Table S107: All-cause mortality rate per 100,000 population and number of deaths in 10-24 by sex 1950 - 2019: Cuba 817
Table S108: All-cause mortality rate per 100,000 population and number of deaths in 10-24 by sex 1950 - 2019: Dominica 818
Table S109: All-cause mortality rate per 100,000 population and number of deaths in 10-24 by sex 1950 - 2019: Dominican Republic 819
Table S110: All-cause mortality rate per 100,000 population and number of deaths in 10-24 by sex 1950 - 2019: Grenada 820
Table S111: All-cause mortality rate per 100,000 population and number of deaths in 10-24 by sex 1950 - 2019: Guyana 821
Table S112: All-cause mortality rate per 100,000 population and number of deaths in 10-24 by sex 1950 - 2019: Haiti 822
Table S113: All-cause mortality rate per 100,000 population and number of deaths in 10-24 by sex 1950 - 2019: Jamaica 823
Table S114: All-cause mortality rate per 100,000 population and number of deaths in 10-24 by sex 1950 - 2019: Saint Lucia 824
Table S115: All-cause mortality rate per 100,000 population and number of deaths in 10-24 by sex 1950 - 2019: Saint Vincent and the Grenadines 825

Table S116: All-cause mortality rate per 100,000 population and number of deaths in 10-24 by sex 1950 - 2019: Suriname 826
Table S117: All-cause mortality rate per 100,000 population and number of deaths in 10-24 by sex 1950 - 2019: Trinidad and Tobago 827
Table S118: All-cause mortality rate per 100,000 population and number of deaths in 10-24 by sex 1950 - 2019: Andean Latin America 828
Table S119: All-cause mortality rate per 100,000 population and number of deaths in 10-24 by sex 1950 - 2019: Bolivia (Plurinational State of) 829
Table S120: All-cause mortality rate per 100,000 population and number of deaths in 10-24 by sex 1950 - 2019: Ecuador 830
Table S121: All-cause mortality rate per 100,000 population and number of deaths in 10-24 by sex 1950 - 2019: Peru 831
Table S122: All-cause mortality rate per 100,000 population and number of deaths in 10-24 by sex 1950 - 2019: Central Latin America 832
Table S123: All-cause mortality rate per 100,000 population and number of deaths in 10-24 by sex 1950 - 2019: Colombia 833
Table S124: All-cause mortality rate per 100,000 population and number of deaths in 10-24 by sex 1950 - 2019: Costa Rica 834
Table S125: All-cause mortality rate per 100,000 population and number of deaths in 10-24 by sex 1950 - 2019: El Salvador 835
Table S126: All-cause mortality rate per 100,000 population and number of deaths in 10-24 by sex 1950 - 2019: Guatemala 836
Table S127: All-cause mortality rate per 100,000 population and number of deaths in 10-24 by sex 1950 - 2019: Honduras 837
Table S128: All-cause mortality rate per 100,000 population and number of deaths in 10-24 by sex 1950 - 2019: Mexico 838
Table S129: All-cause mortality rate per 100,000 population and number of deaths in 10-24 by sex 1950 - 2019: Nicaragua 839
Table S130: All-cause mortality rate per 100,000 population and number of deaths in 10-24 by sex 1950 - 2019: Panama 840
Table S131: All-cause mortality rate per 100,000 population and number of deaths in 10-24 by sex 1950 - 2019: Venezuela (Bolivarian Republic of) 841
Table S132: All-cause mortality rate per 100,000 population and number of deaths in 10-24 by sex 1950 - 2019: Tropical Latin America 842
Table S133: All-cause mortality rate per 100,000 population and number of deaths in 10-24 by sex 1950 - 2019: Brazil 843
Table S134: All-cause mortality rate per 100,000 population and number of deaths in 10-24 by sex 1950 - 2019: Paraguay 844
Table S135: All-cause mortality rate per 100,000 population and number of deaths in 10-24 by sex 1950 - 2019: North Africa and Middle East 845
Table S137: All-cause mortality rate per 100,000 population and number of deaths in 10-24 by sex 1950 - 2019: Algeria 846
Table S138: All-cause mortality rate per 100,000 population and number of deaths in 10-24 by sex 1950 - 2019: Bahrain 847
Table S139: All-cause mortality rate per 100,000 population and number of deaths in 10-24 by sex 1950 - 2019: Egypt 848
Table S140: All-cause mortality rate per 100,000 population and number of deaths in 10-24 by sex 1950 - 2019: Iran (Islamic Republic of) 849

Table S141: All-cause mortality rate per 100,000 population and number of deaths in 10-24 by sex 1950 - 2019: Iraq 850
Table S142: All-cause mortality rate per 100,000 population and number of deaths in 10-24 by sex 1950 - 2019: Jordan 851
Table S143: All-cause mortality rate per 100,000 population and number of deaths in 10-24 by sex 1950 - 2019: Kuwait 852
Table S144: All-cause mortality rate per 100,000 population and number of deaths in 10-24 by sex 1950 - 2019: Lebanon 853
Table S145: All-cause mortality rate per 100,000 population and number of deaths in 10-24 by sex 1950 - 2019: Libya 854
Table S146: All-cause mortality rate per 100,000 population and number of deaths in 10-24 by sex 1950 - 2019: Morocco 855
Table S147: All-cause mortality rate per 100,000 population and number of deaths in 10-24 by sex 1950 - 2019: Palestine 856
Table S148: All-cause mortality rate per 100,000 population and number of deaths in 10-24 by sex 1950 - 2019: Oman 857
Table S149: All-cause mortality rate per 100,000 population and number of deaths in 10-24 by sex 1950 - 2019: Qatar 858
Table S150: All-cause mortality rate per 100,000 population and number of deaths in 10-24 by sex 1950 - 2019: Saudi Arabia 859
Table S151: All-cause mortality rate per 100,000 population and number of deaths in 10-24 by sex 1950 - 2019: Syrian Arab Republic 860
Table S152: All-cause mortality rate per 100,000 population and number of deaths in 10-24 by sex 1950 - 2019: Tunisia 861
Table S153: All-cause mortality rate per 100,000 population and number of deaths in 10-24 by sex 1950 - 2019: Turkey 862
Table S154: All-cause mortality rate per 100,000 population and number of deaths in 10-24 by sex 1950 - 2019: United Arab Emirates 863
Table S155: All-cause mortality rate per 100,000 population and number of deaths in 10-24 by sex 1950 - 2019: Yemen 864
Table S157: All-cause mortality rate per 100,000 population and number of deaths in 10-24 by sex 1950 - 2019: South Asia 865
Table S158: All-cause mortality rate per 100,000 population and number of deaths in 10-24 by sex 1950 - 2019: Afghanistan 866
Table S159: All-cause mortality rate per 100,000 population and number of deaths in 10-24 by sex 1950 - 2019: Bangladesh 867
Table S160: All-cause mortality rate per 100,000 population and number of deaths in 10-24 by sex 1950 - 2019: Bhutan 868
Table S161: All-cause mortality rate per 100,000 population and number of deaths in 10-24 by sex 1950 - 2019: India 869
Table S162: All-cause mortality rate per 100,000 population and number of deaths in 10-24 by sex 1950 - 2019: Nepal 870
Table S163: All-cause mortality rate per 100,000 population and number of deaths in 10-24 by sex 1950 - 2019: Pakistan 871
Table S164: All-cause mortality rate per 100,000 population and number of deaths in 10-24 by sex 1950 - 2019: Sub-Saharan Africa 872
Table S165: All-cause mortality rate per 100,000 population and number of deaths in 10-24 by sex 1950 - 2019: Central Sub-Saharan Africa 873

Table S166: All-cause mortality rate per 100,000 population and number of deaths in 10-24 by sex 1950 - 2019: Angola 874
Table S167: All-cause mortality rate per 100,000 population and number of deaths in 10-24 by sex 1950 - 2019: Central African Republic 875
Table S168: All-cause mortality rate per 100,000 population and number of deaths in 10-24 by sex 1950 - 2019: Congo 876
Table S169: All-cause mortality rate per 100,000 population and number of deaths in 10-24 by sex 1950 - 2019: Democratic Republic of the Congo 877
Table S170: All-cause mortality rate per 100,000 population and number of deaths in 10-24 by sex 1950 - 2019: Equatorial Guinea 878
Table S171: All-cause mortality rate per 100,000 population and number of deaths in 10-24 by sex 1950 - 2019: Gabon 879
Table S172: All-cause mortality rate per 100,000 population and number of deaths in 10-24 by sex 1950 - 2019: Eastern Sub-Saharan Africa 880
Table S173: All-cause mortality rate per 100,000 population and number of deaths in 10-24 by sex 1950 - 2019: Burundi 881
Table S174: All-cause mortality rate per 100,000 population and number of deaths in 10-24 by sex 1950 - 2019: Comoros 882
Table S175: All-cause mortality rate per 100,000 population and number of deaths in 10-24 by sex 1950 - 2019: Djibouti 883
Table S176: All-cause mortality rate per 100,000 population and number of deaths in 10-24 by sex 1950 - 2019: Eritrea 884
Table S177: All-cause mortality rate per 100,000 population and number of deaths in 10-24 by sex 1950 - 2019: Ethiopia 885
Table S178: All-cause mortality rate per 100,000 population and number of deaths in 10-24 by sex 1950 - 2019: Kenya 886
Table S179: All-cause mortality rate per 100,000 population and number of deaths in 10-24 by sex 1950 - 2019: Madagascar 887
Table S180: All-cause mortality rate per 100,000 population and number of deaths in 10-24 by sex 1950 - 2019: Malawi 888
Table S181: All-cause mortality rate per 100,000 population and number of deaths in 10-24 by sex 1950 - 2019: Mauritius 889
Table S182: All-cause mortality rate per 100,000 population and number of deaths in 10-24 by sex 1950 - 2019: Mozambique 890
Table S183: All-cause mortality rate per 100,000 population and number of deaths in 10-24 by sex 1950 - 2019: Rwanda 891
Table S184: All-cause mortality rate per 100,000 population and number of deaths in 10-24 by sex 1950 - 2019: Seychelles 892
Table S185: All-cause mortality rate per 100,000 population and number of deaths in 10-24 by sex 1950 - 2019: Somalia 893
Table S186: All-cause mortality rate per 100,000 population and number of deaths in 10-24 by sex 1950 - 2019: United Republic of Tanzania 894
Table S187: All-cause mortality rate per 100,000 population and number of deaths in 10-24 by sex 1950 - 2019: Uganda 895
Table S188: All-cause mortality rate per 100,000 population and number of deaths in 10-24 by sex 1950 - 2019: Zambia 896
Table S189: All-cause mortality rate per 100,000 population and number of deaths in 10-24 by sex 1950 - 2019: Southern Sub-Saharan Africa 897

Table S190: All-cause mortality rate per 100,000 population and number of deaths in 10-24 by sex 1950 - 2019: Botswana 898
Table S191: All-cause mortality rate per 100,000 population and number of deaths in 10-24 by sex 1950 - 2019: Lesotho 899
Table S192: All-cause mortality rate per 100,000 population and number of deaths in 10-24 by sex 1950 - 2019: Namibia 900
Table S193: All-cause mortality rate per 100,000 population and number of deaths in 10-24 by sex 1950 - 2019: South Africa 901
Table S194: All-cause mortality rate per 100,000 population and number of deaths in 10-24 by sex 1950 - 2019: Eswatini 902
Table S195: All-cause mortality rate per 100,000 population and number of deaths in 10-24 by sex 1950 - 2019: Zimbabwe 903
Table S196: All-cause mortality rate per 100,000 population and number of deaths in 10-24 by sex 1950 - 2019: Western Sub-Saharan Africa 904
Table S197: All-cause mortality rate per 100,000 population and number of deaths in 10-24 by sex 1950 - 2019: Benin 905
Table S198: All-cause mortality rate per 100,000 population and number of deaths in 10-24 by sex 1950 - 2019: Burkina Faso 906
Table S199: All-cause mortality rate per 100,000 population and number of deaths in 10-24 by sex 1950 - 2019: Cameroon 907
Table S200: All-cause mortality rate per 100,000 population and number of deaths in 10-24 by sex 1950 - 2019: Cabo Verde 908
Table S201: All-cause mortality rate per 100,000 population and number of deaths in 10-24 by sex 1950 - 2019: Chad 909
Table S202: All-cause mortality rate per 100,000 population and number of deaths in 10-24 by sex 1950 - 2019: Côte d'Ivoire 910
Table S203: All-cause mortality rate per 100,000 population and number of deaths in 10-24 by sex 1950 - 2019: Gambia 911
Table S204: All-cause mortality rate per 100,000 population and number of deaths in 10-24 by sex 1950 - 2019: Ghana 912
Table S205: All-cause mortality rate per 100,000 population and number of deaths in 10-24 by sex 1950 - 2019: Guinea 913
Table S206: All-cause mortality rate per 100,000 population and number of deaths in 10-24 by sex 1950 - 2019: Guinea-Bissau 914
Table S207: All-cause mortality rate per 100,000 population and number of deaths in 10-24 by sex 1950 - 2019: Liberia 915
Table S208: All-cause mortality rate per 100,000 population and number of deaths in 10-24 by sex 1950 - 2019: Mali 916
Table S209: All-cause mortality rate per 100,000 population and number of deaths in 10-24 by sex 1950 - 2019: Mauritania 917
Table S210: All-cause mortality rate per 100,000 population and number of deaths in 10-24 by sex 1950 - 2019: Niger 918
Table S211: All-cause mortality rate per 100,000 population and number of deaths in 10-24 by sex 1950 - 2019: Nigeria 919
Table S212: All-cause mortality rate per 100,000 population and number of deaths in 10-24 by sex 1950 - 2019: Sao Tome and Principe 920
Table S213: All-cause mortality rate per 100,000 population and number of deaths in 10-24 by sex 1950 - 2019: Senegal 921

Table S214: All-cause mortality rate per 100,000 population and number of deaths in 10-24 by sex 1950 - 2019: Sierra Leone 922
Table S215: All-cause mortality rate per 100,000 population and number of deaths in 10-24 by sex 1950 - 2019: Togo 923
Table S216: All-cause mortality rate per 100,000 population and number of deaths in 10-24 by sex 1950 - 2019: American Samoa 924
Table S217: All-cause mortality rate per 100,000 population and number of deaths in 10-24 by sex 1950 - 2019: Bermuda 925
Table S218: All-cause mortality rate per 100,000 population and number of deaths in 10-24 by sex 1950 - 2019: Cook Islands 926
Table S219: All-cause mortality rate per 100,000 population and number of deaths in 10-24 by sex 1950 - 2019: Greenland 927
Table S220: All-cause mortality rate per 100,000 population and number of deaths in 10-24 by sex 1950 - 2019: Guam 928
Table S221: All-cause mortality rate per 100,000 population and number of deaths in 10-24 by sex 1950 - 2019: Monaco 929
Table S222: All-cause mortality rate per 100,000 population and number of deaths in 10-24 by sex 1950 - 2019: Nauru 930
Table S223: All-cause mortality rate per 100,000 population and number of deaths in 10-24 by sex 1950 - 2019: Niue 931
Table S224: All-cause mortality rate per 100,000 population and number of deaths in 10-24 by sex 1950 - 2019: Northern Mariana Islands 932
Table S225: All-cause mortality rate per 100,000 population and number of deaths in 10-24 by sex 1950 - 2019: Palau 933
Table S226: All-cause mortality rate per 100,000 population and number of deaths in 10-24 by sex 1950 - 2019: Puerto Rico 934
Table S227: All-cause mortality rate per 100,000 population and number of deaths in 10-24 by sex 1950 - 2019: Saint Kitts and Nevis 935
Table S228: All-cause mortality rate per 100,000 population and number of deaths in 10-24 by sex 1950 - 2019: San Marino 936
Table S229: All-cause mortality rate per 100,000 population and number of deaths in 10-24 by sex 1950 - 2019: Tokelau 937
Table S230: All-cause mortality rate per 100,000 population and number of deaths in 10-24 by sex 1950 - 2019: Tuvalu 938
Table S231: All-cause mortality rate per 100,000 population and number of deaths in 10-24 by sex 1950 - 2019: United States Virgin Islands 939
Table S232: All-cause mortality rate per 100,000 population and number of deaths in 10-24 by sex 1950 - 2019: South Sudan 940
Table S233: All-cause mortality rate per 100,000 population and number of deaths in 10-24 by sex 1950 - 2019: Sudan 941

Figure S1: Global

Figure S1a All-cause mortality rate per 100,000 in 10-24 1950 – 2019: Global

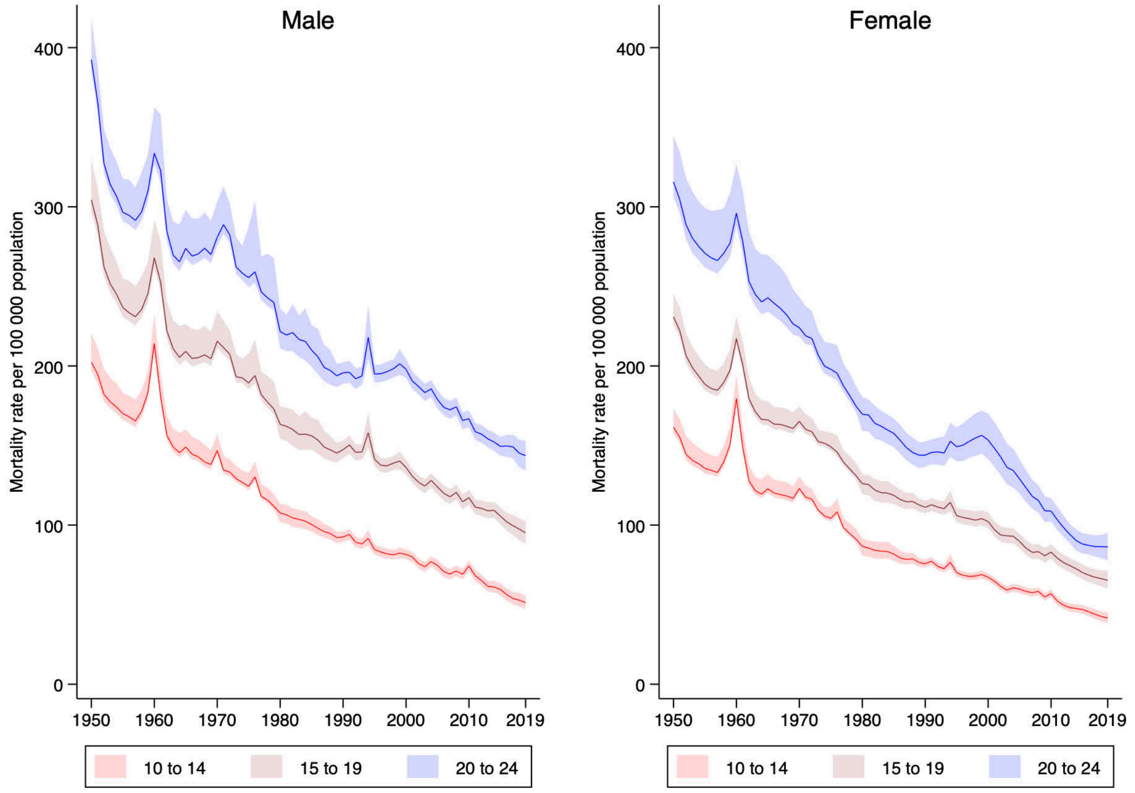

Figure S1b Number of deaths from all causes in 10-24 1950 – 2019: Global

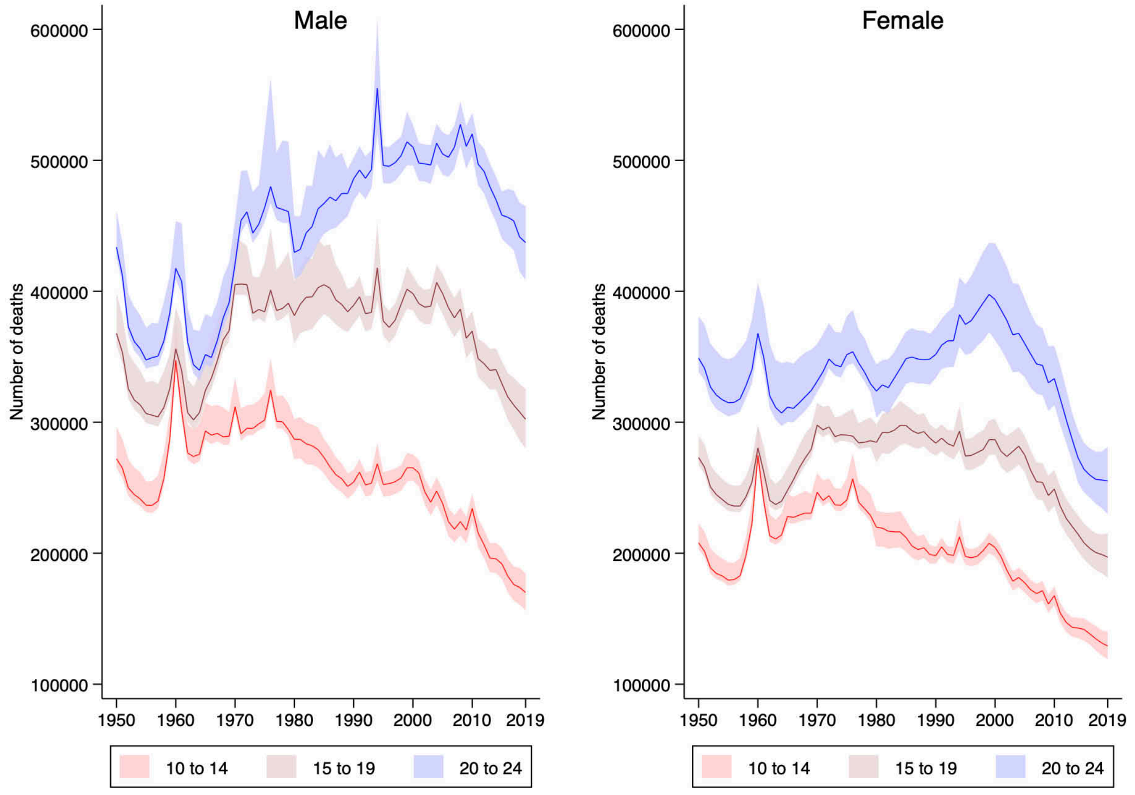

Figure S1c Mortality rate per 100,000 in 10-24 1980 – 2019 by cause: Global

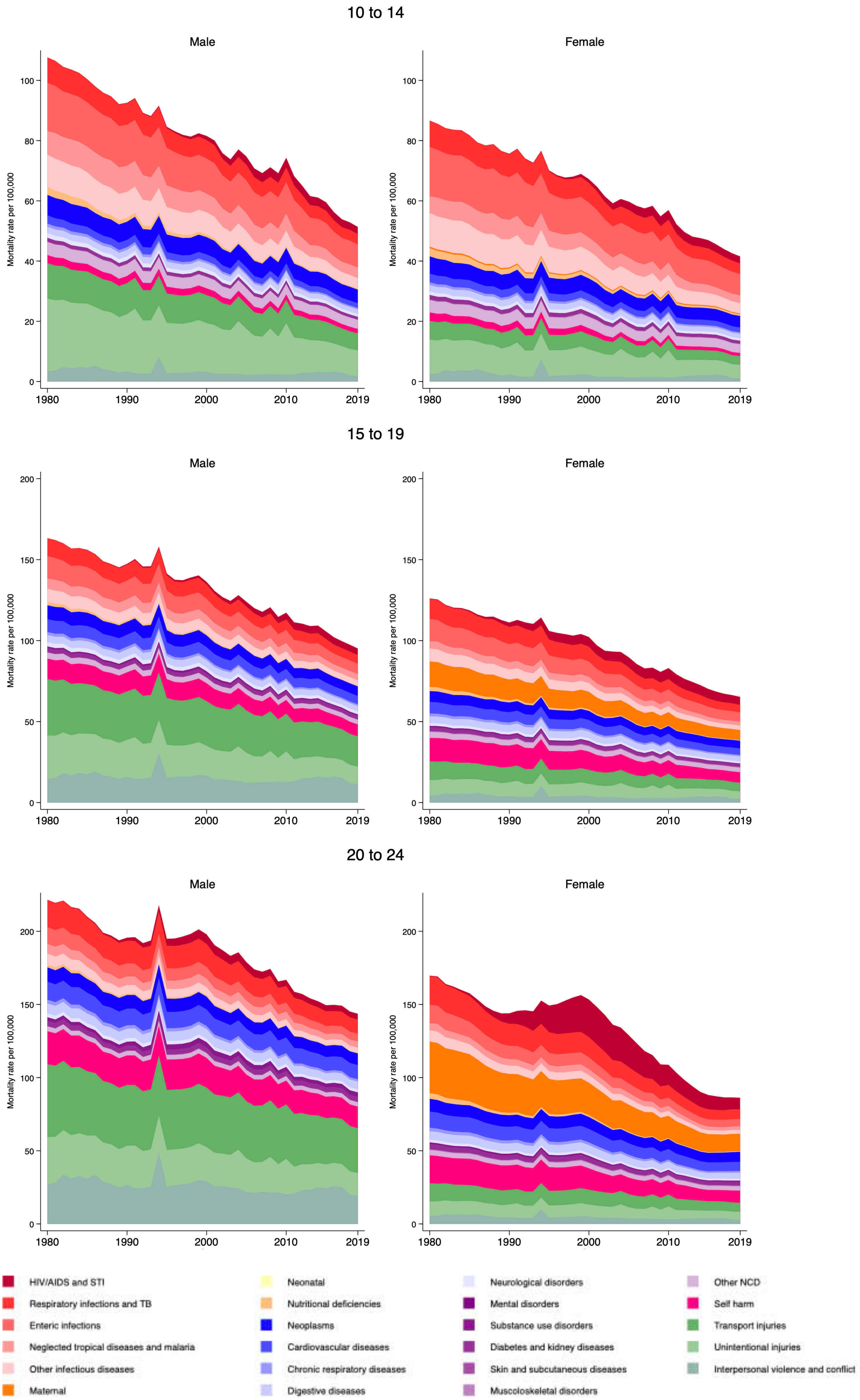

Figure S1d Number of deaths in 10-24 1980 – 2019 by cause: Global

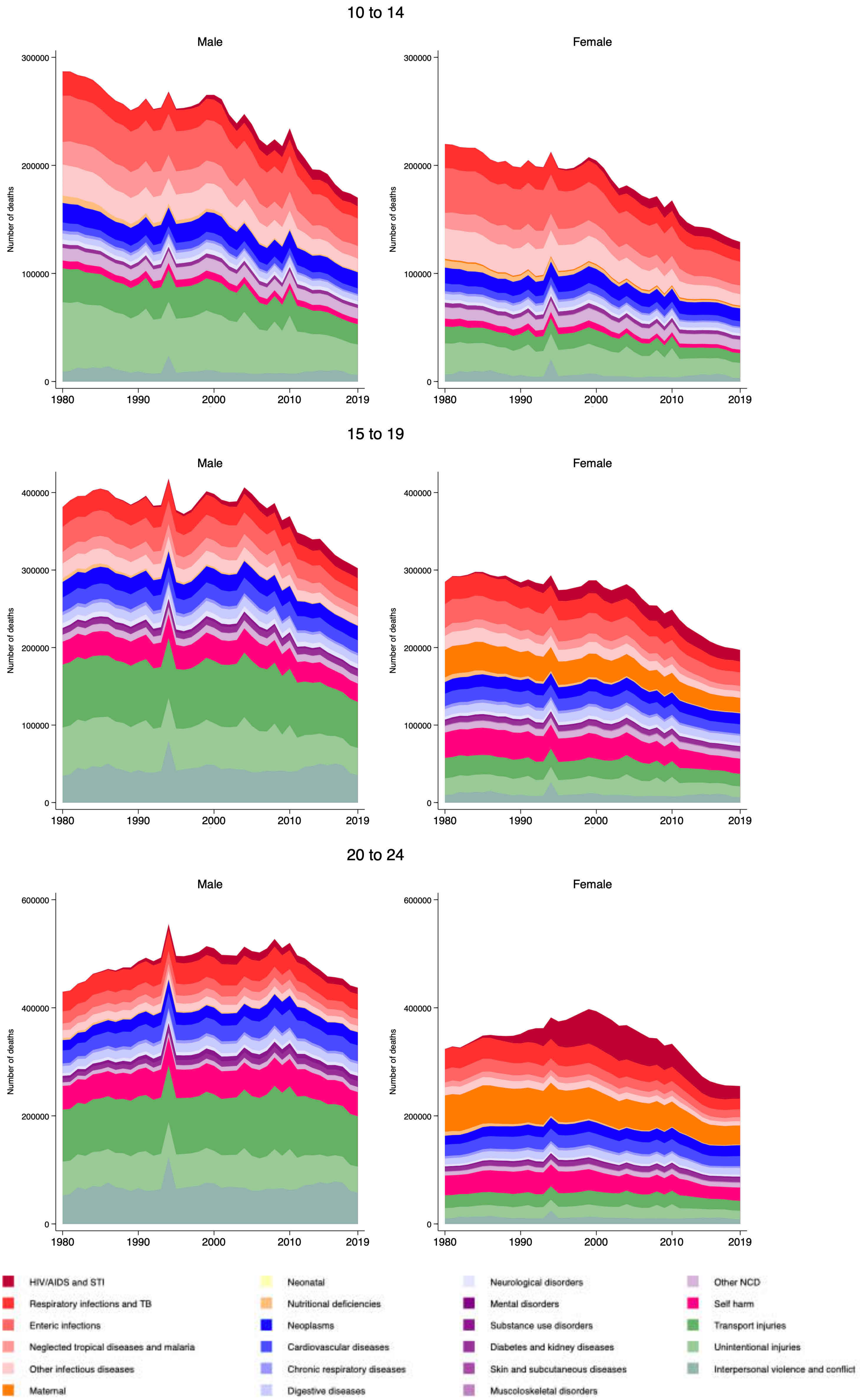

Figure S2: Southeast Asia, East Asia, and Oceania

Figure S2a All-cause mortality rate per 100,000 in 10-24 1950 – 2019: Southeast Asia, East Asia, and Oceania

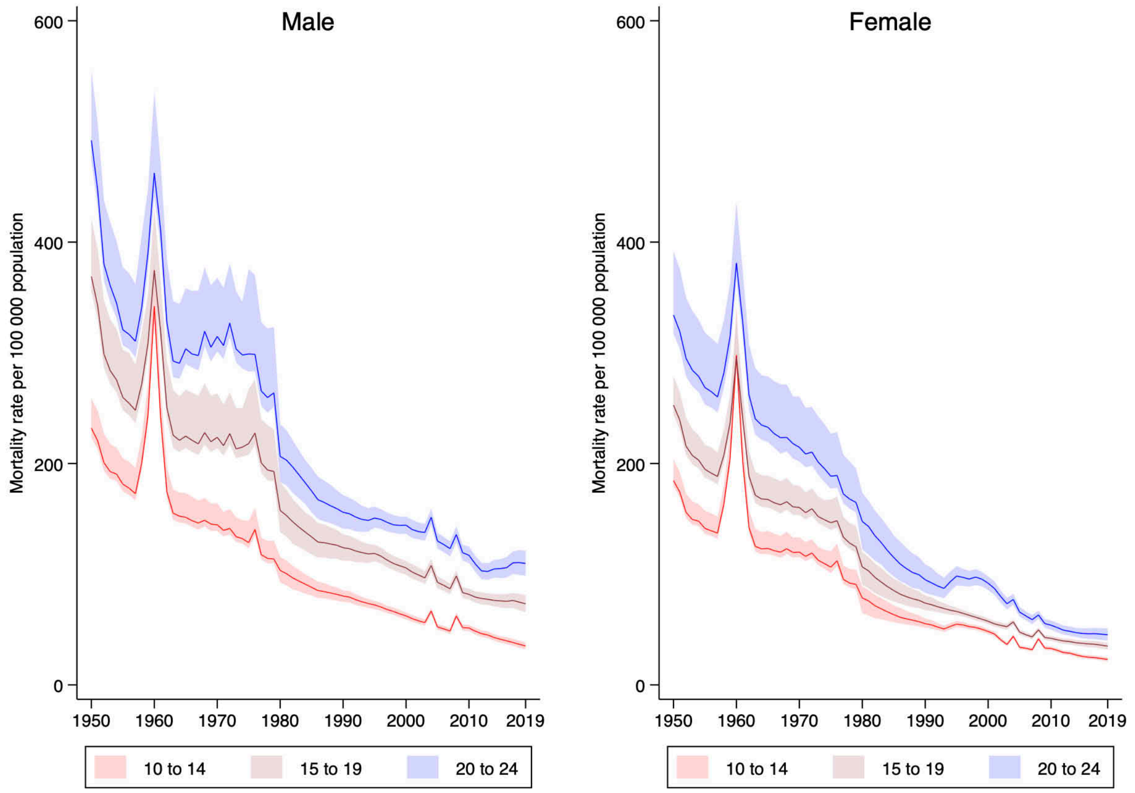

Figure S2b Number of deaths from all causes in 10-24 1950 – 2019: Southeast Asia, East Asia, and Oceania

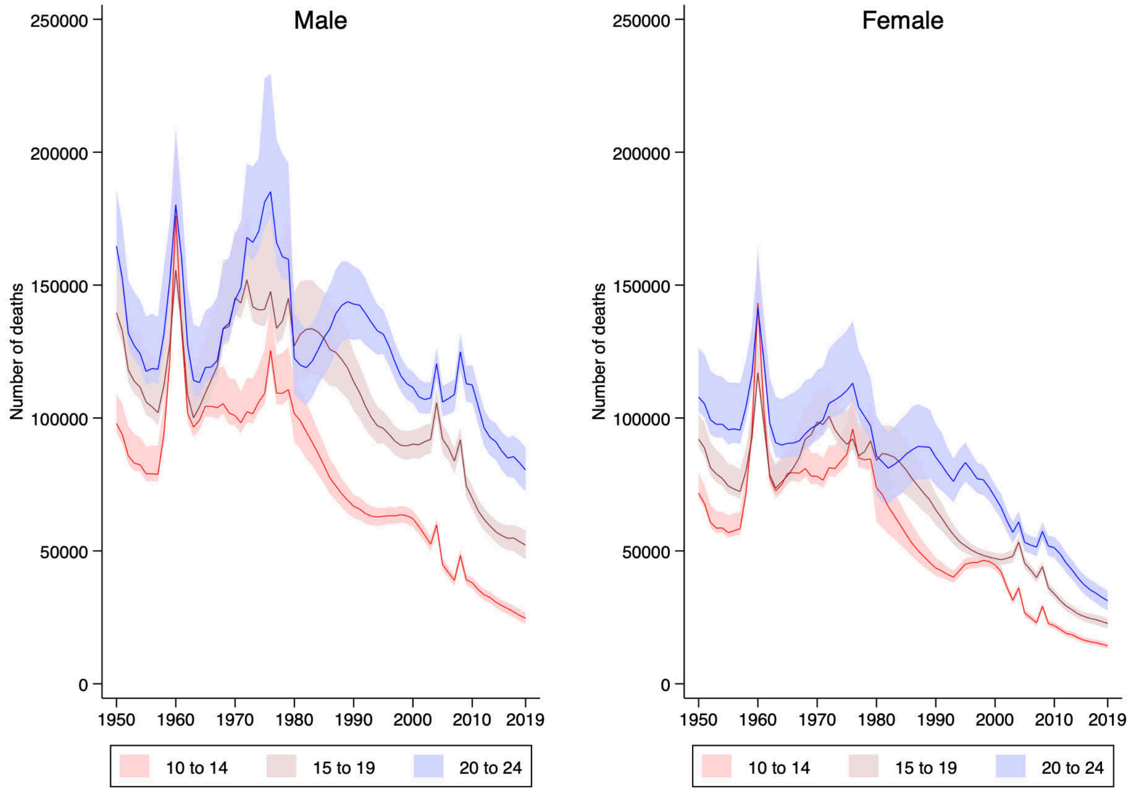

Figure S2c Mortality rate per 100,000 in 10-24 1980 – 2019 by cause: Southeast Asia, East Asia, and Oceania

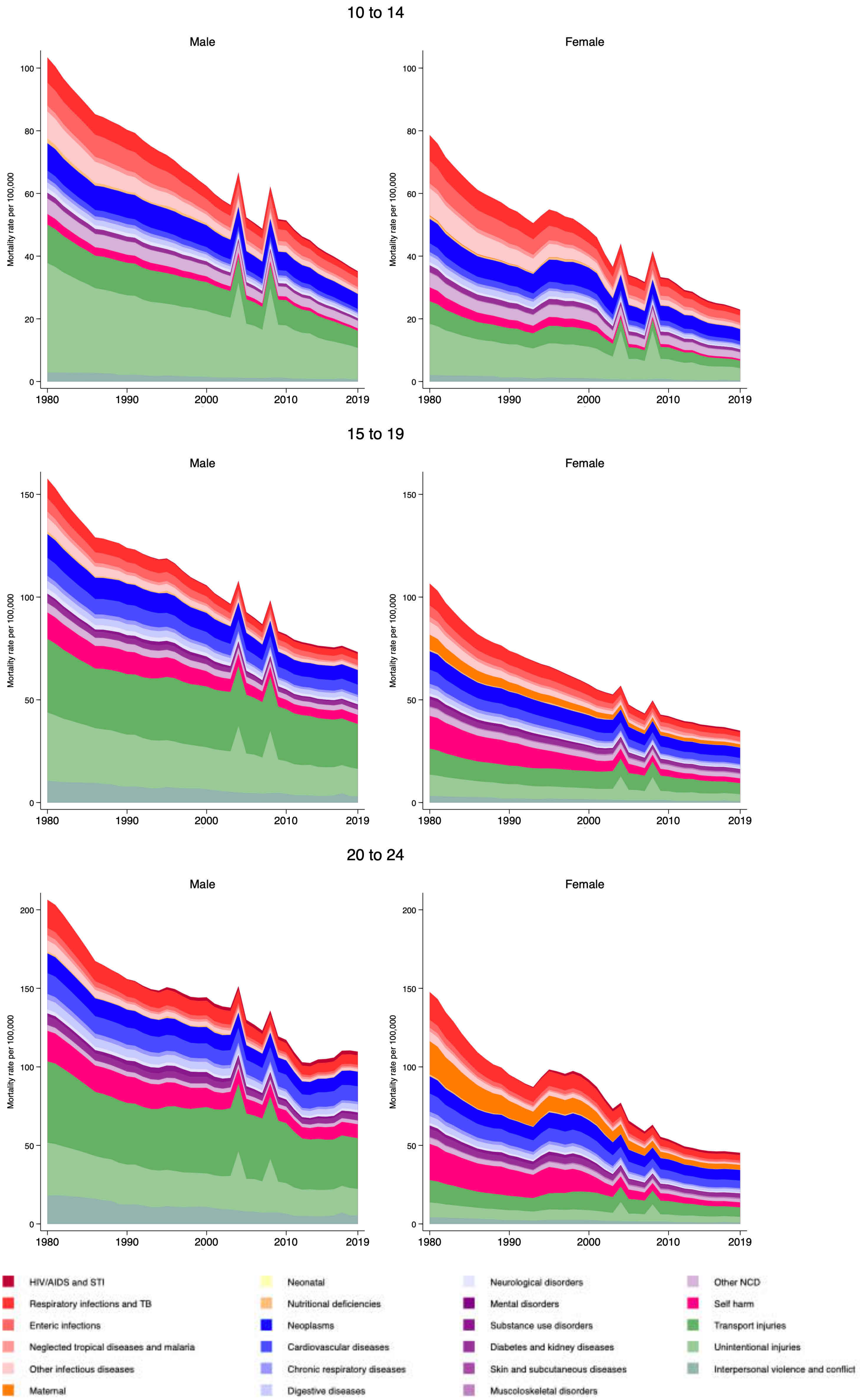

Figure S2d Number of deaths in 10-24 1980 – 2019 by cause: Southeast Asia, East Asia, and Oceania

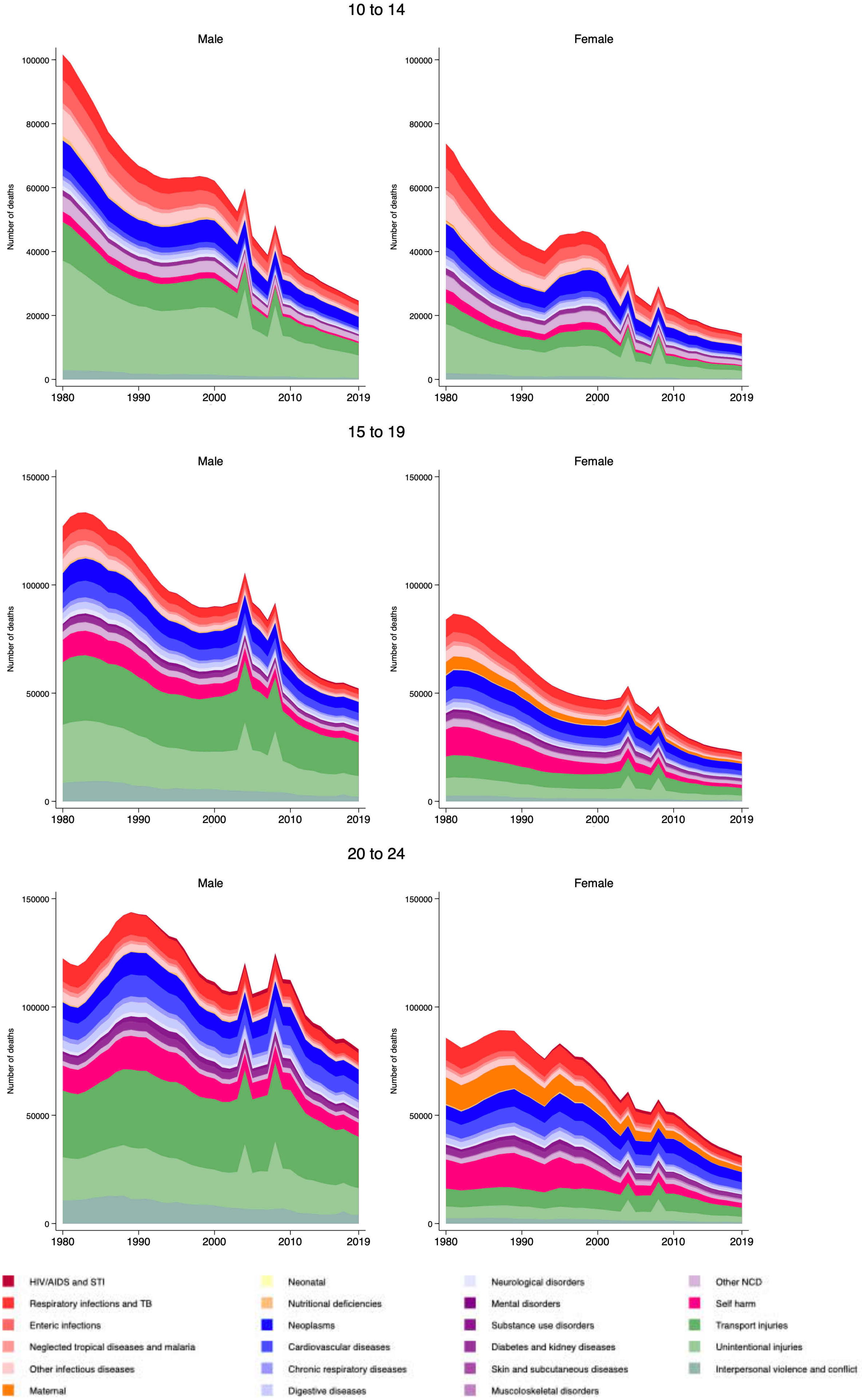

Figure S3: East Asia

Figure S3a All-cause mortality rate per 100,000 in 10-24 1950 – 2019: East Asia

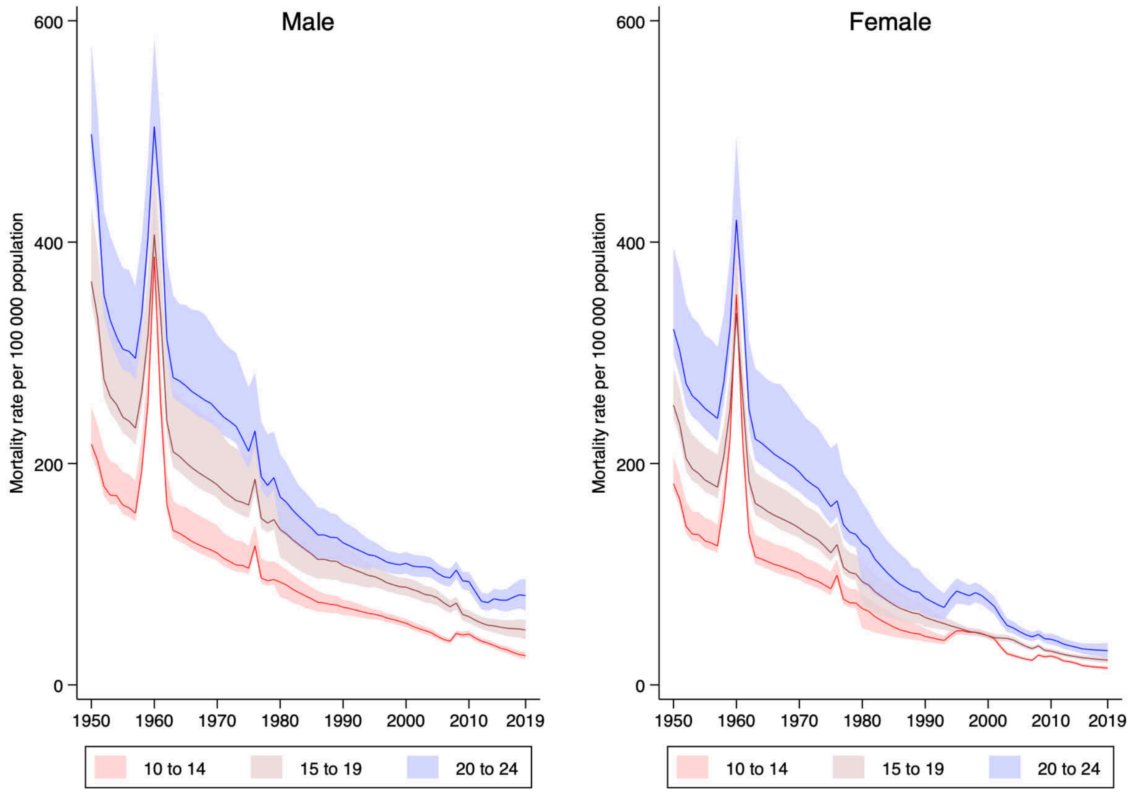

Figure S3b Number of deaths from all causes in 10-24 1950 – 2019: East Asia

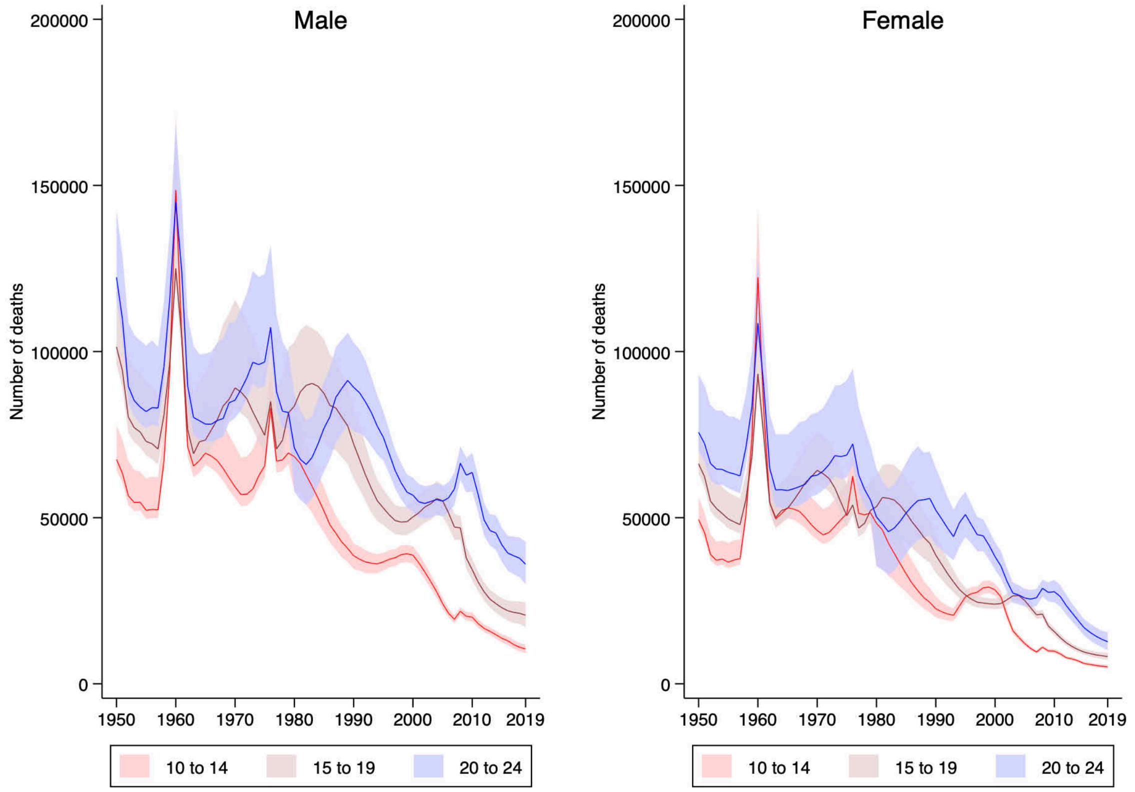

Figure S3c Mortality rate per 100,000 in 10-24 1980 – 2019 by cause: East Asia

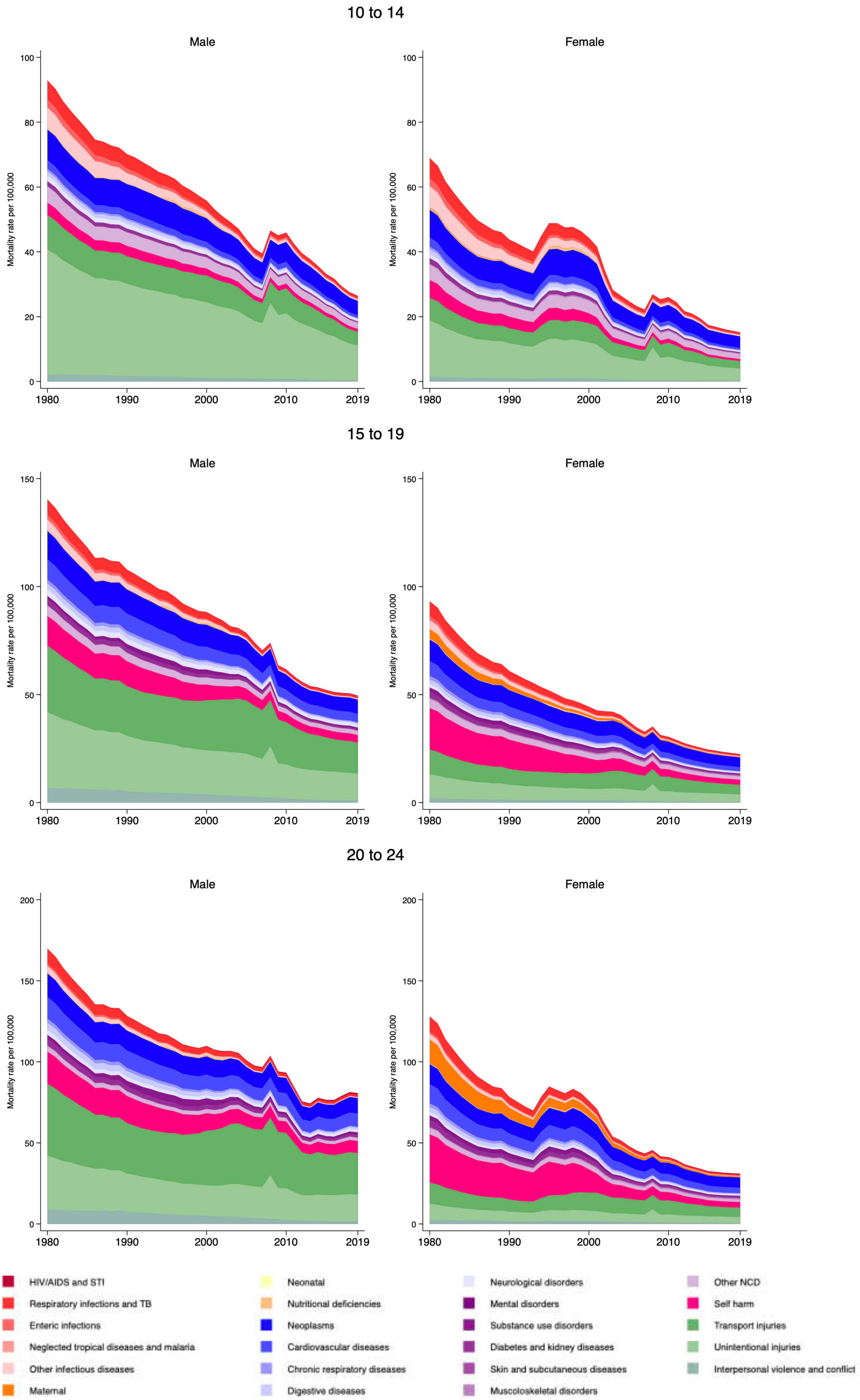

Figure S3d Number of deaths in 10-24 1980 – 2019 by cause: East Asia

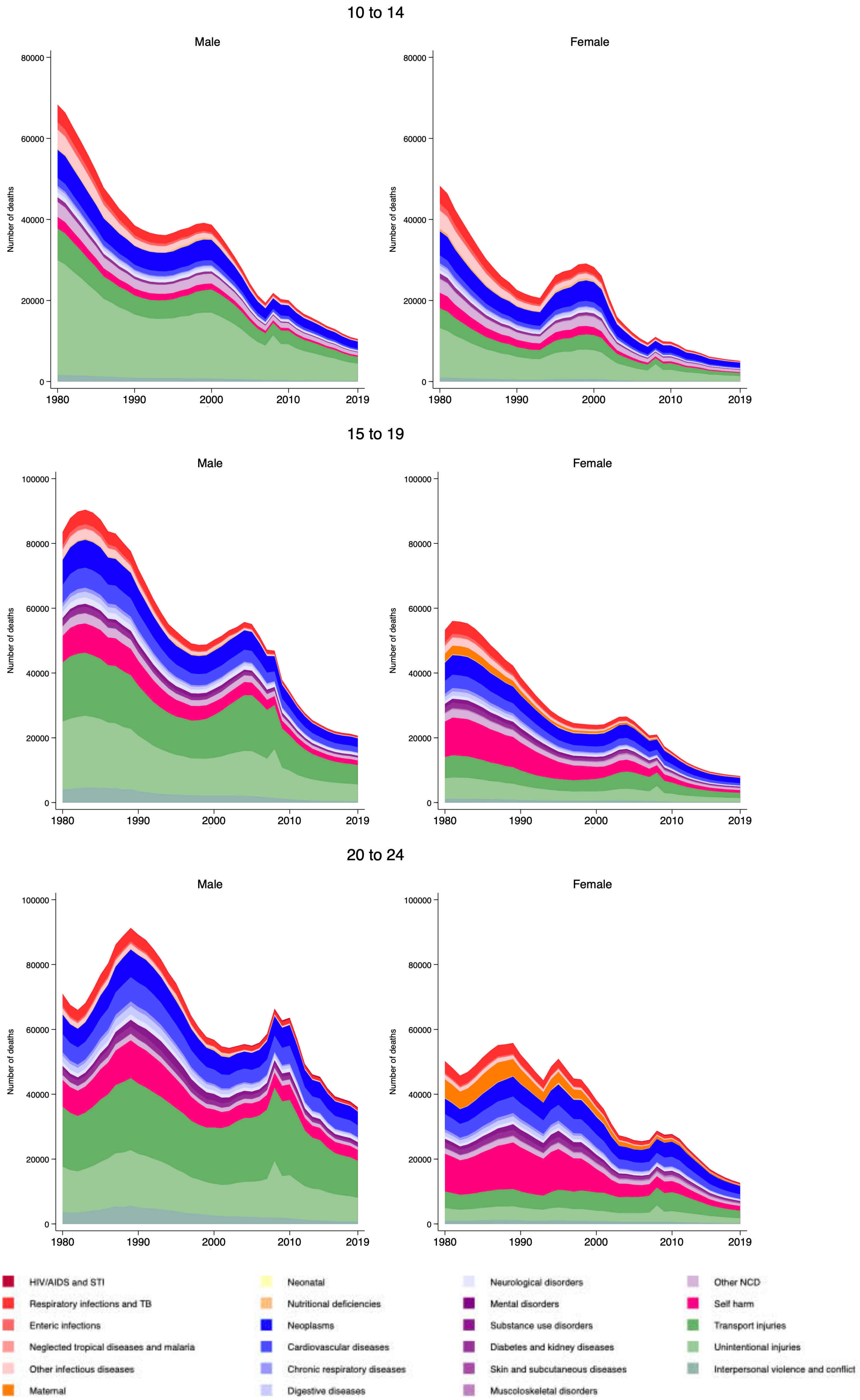

Figure S4: China

Figure S4a All-cause mortality rate per 100,000 in 10-24 1950 – 2019: China

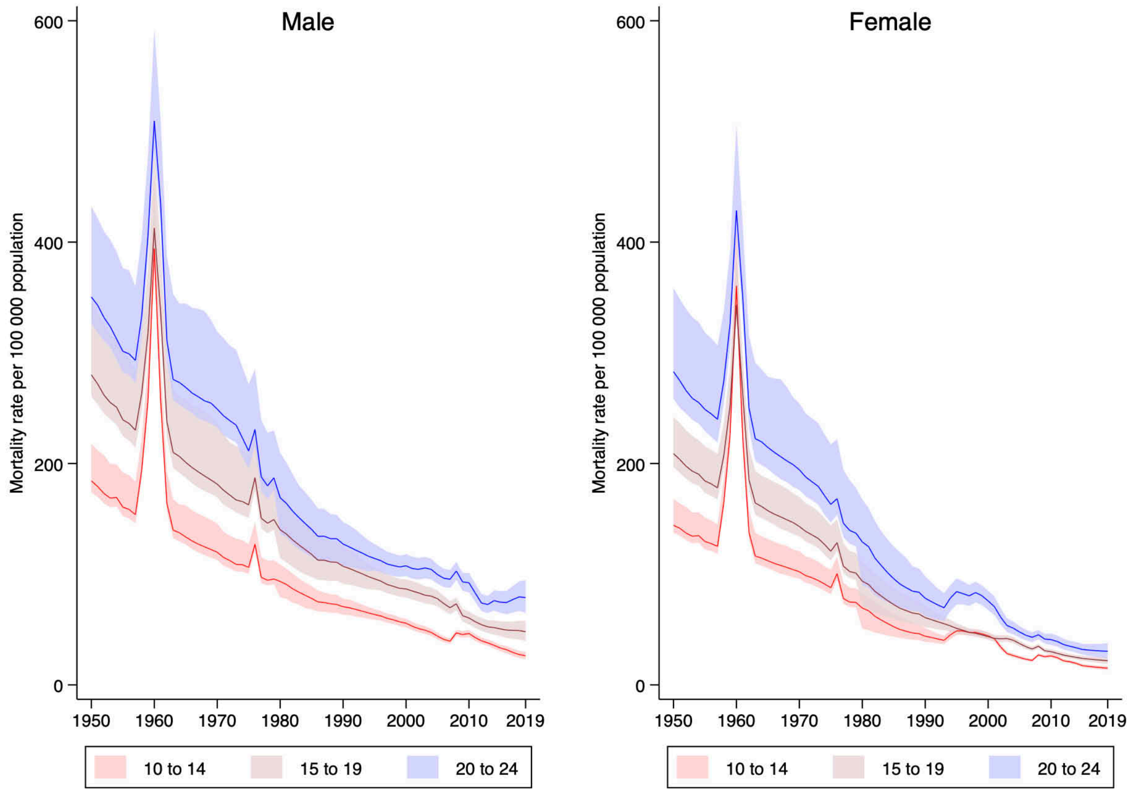

Figure S4b Number of deaths from all causes in 10-24 1950 – 2019: China

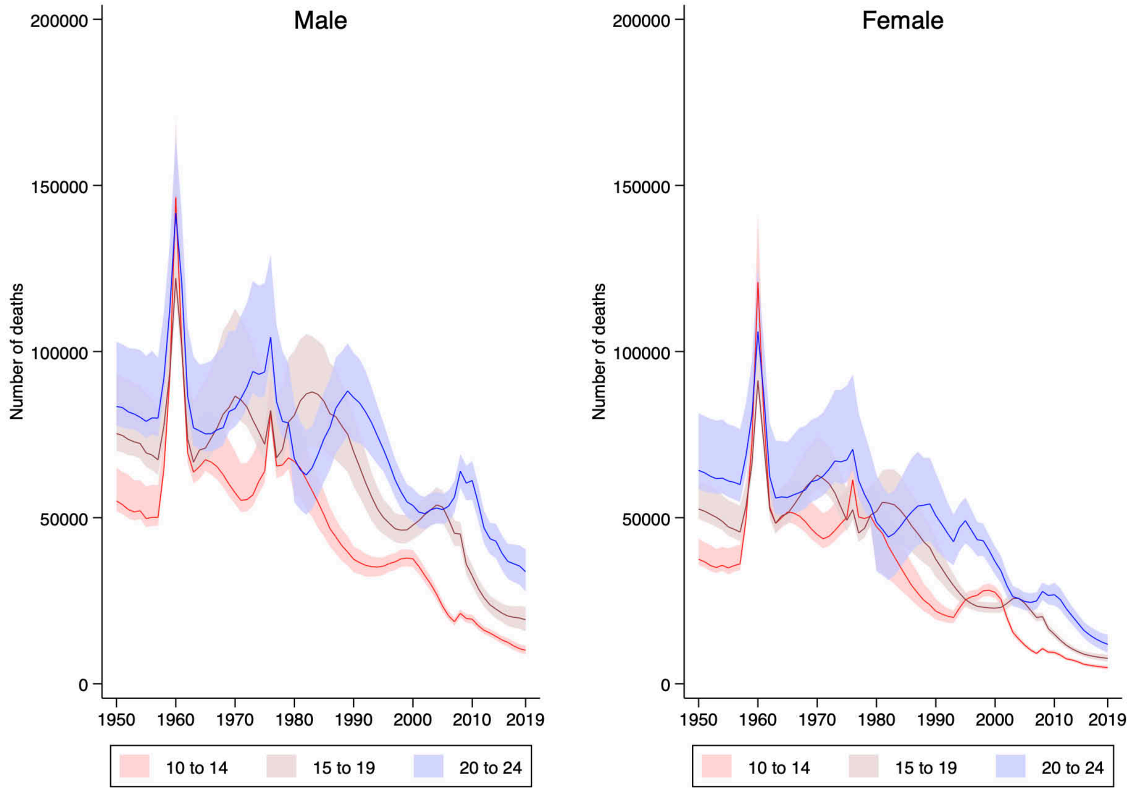

Figure S4c Mortality rate per 100,000 in 10-24 1980 – 2019 by cause: China

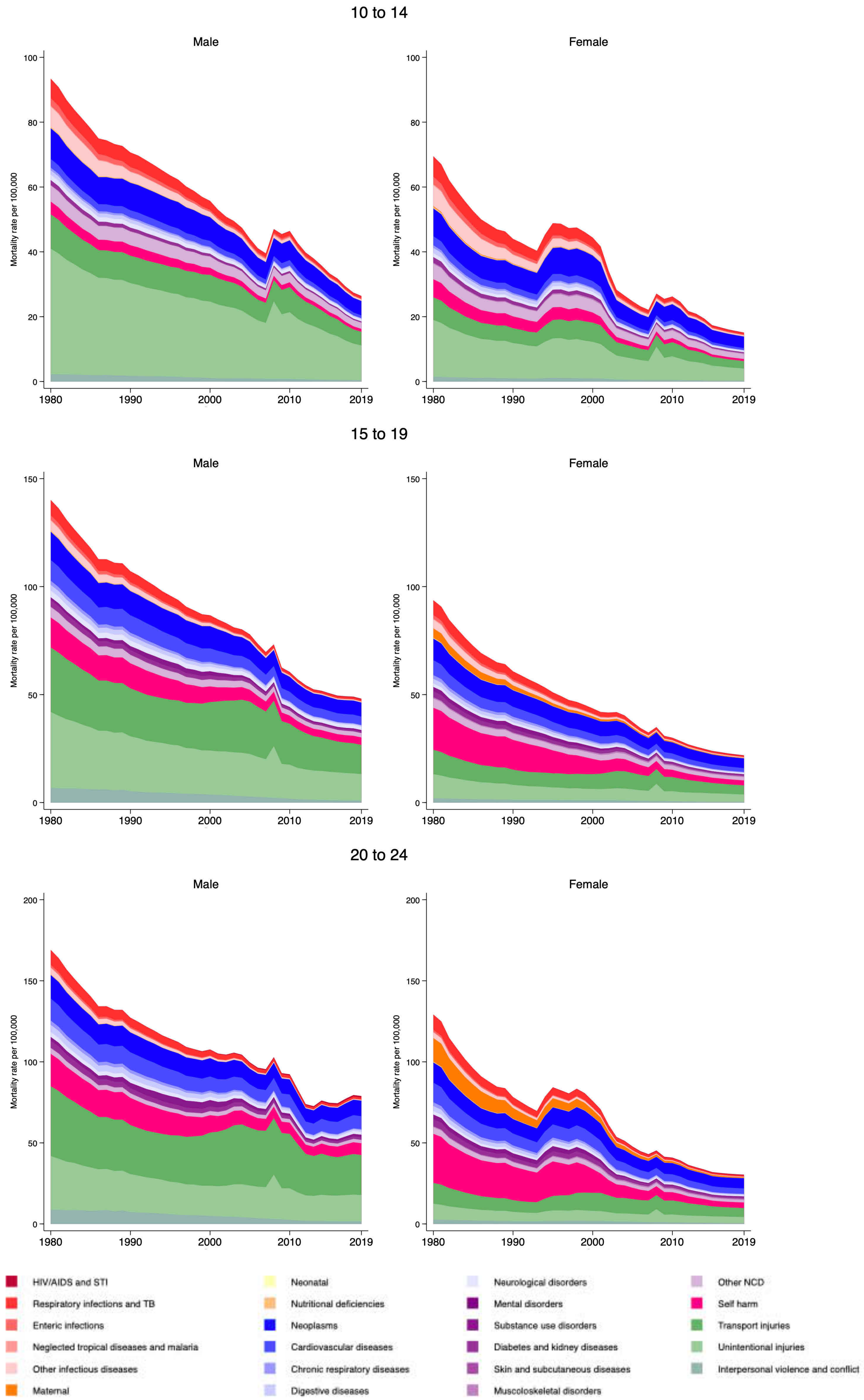

Figure S4d Number of deaths in 10-24 1980 – 2019 by cause: China

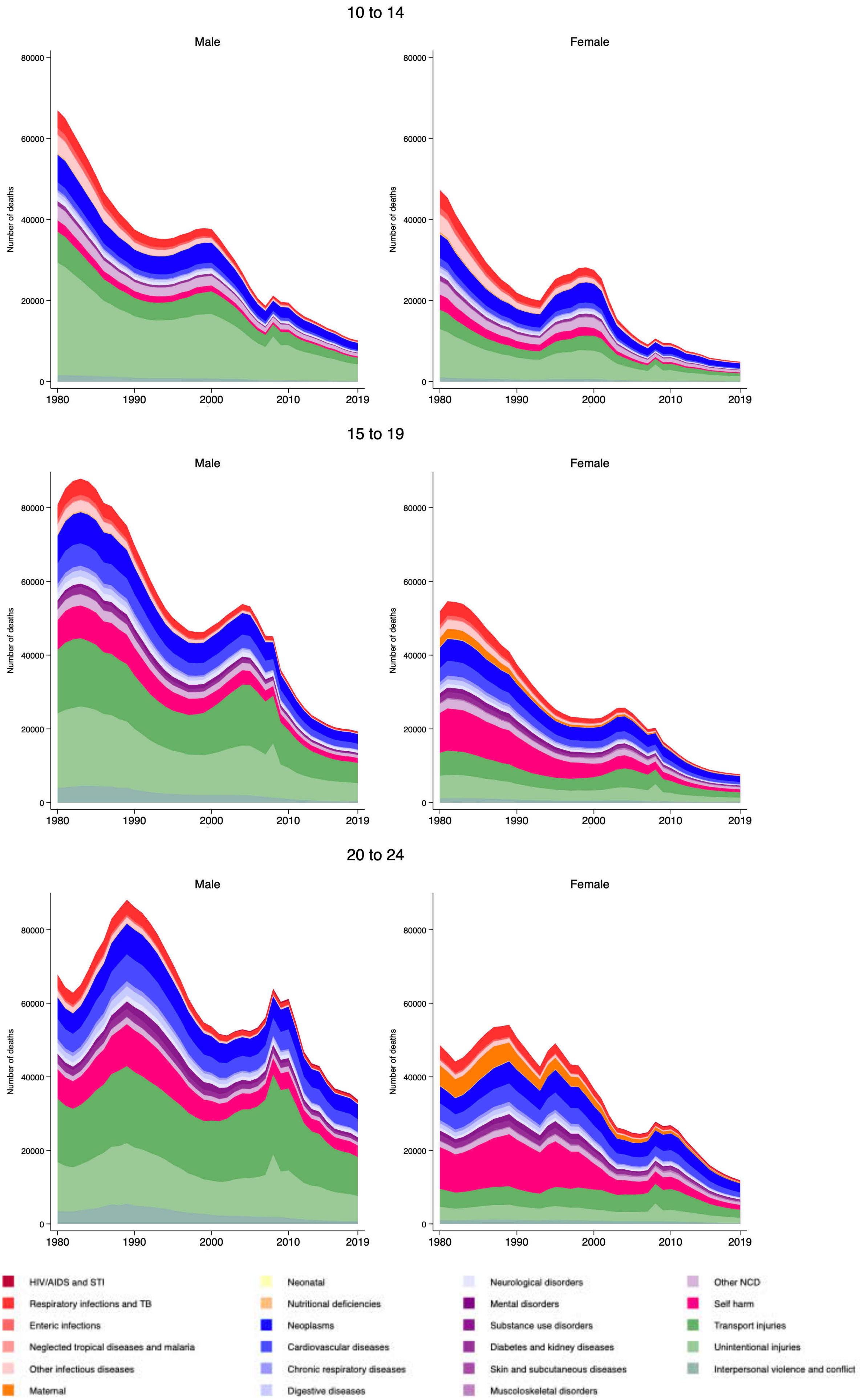

Figure S5: Democratic People's Republic of Korea

Figure S5a All-cause mortality rate per 100,000 in 10-24 1950 – 2019: Democratic People's Republic of Korea

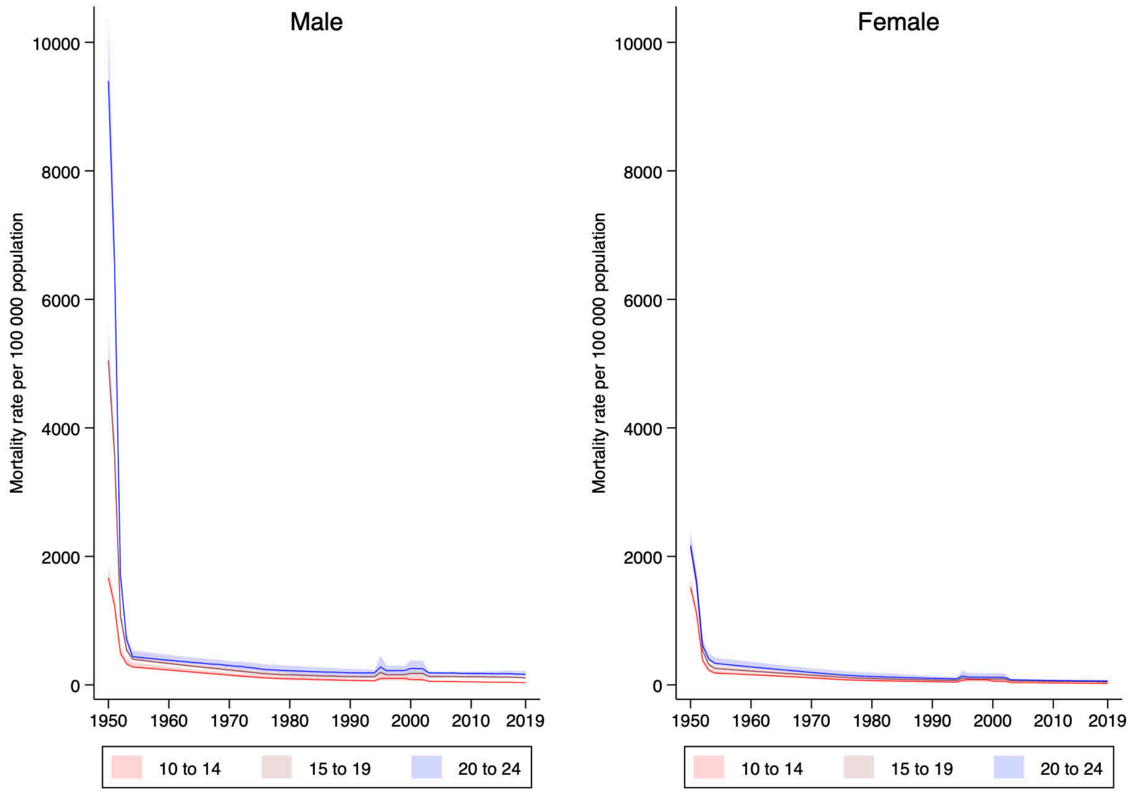

Figure S5b Number of deaths from all causes in 10-24 1950 – 2019: Democratic People's Republic of Korea

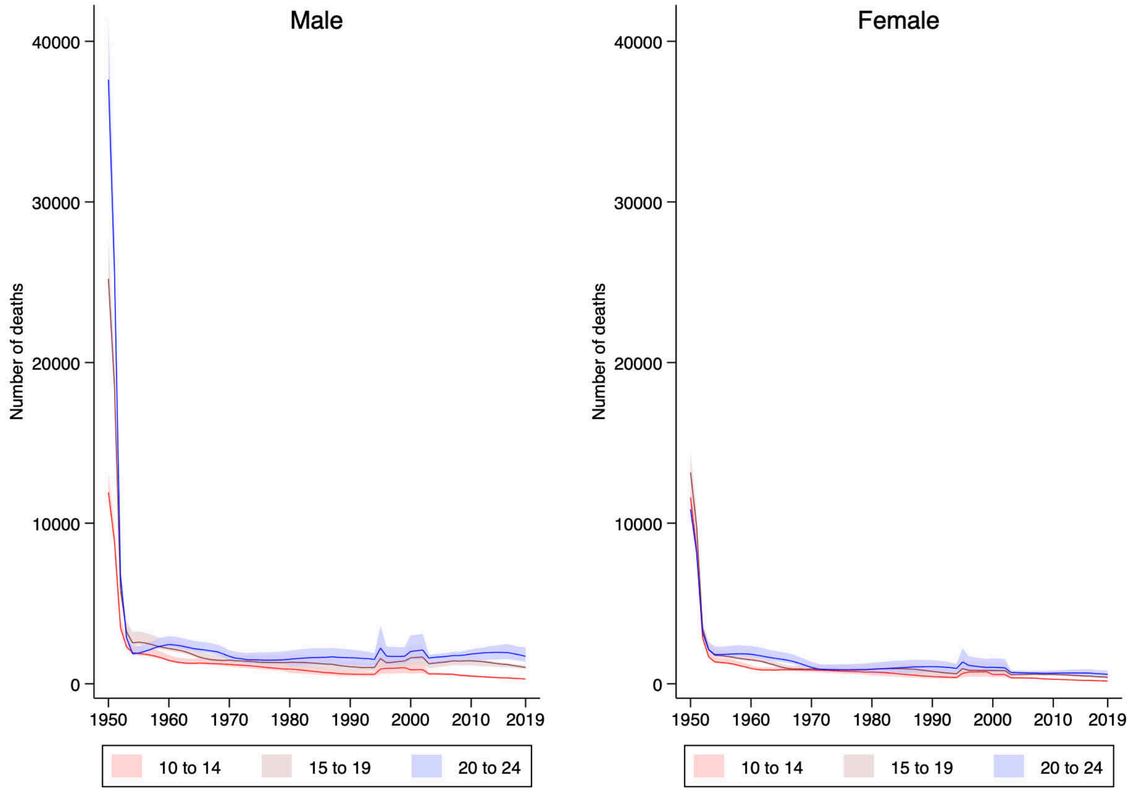

Figure S5c Mortality rate per 100,000 in 10-24 1980 – 2019 by cause: Democratic People's Republic of Korea

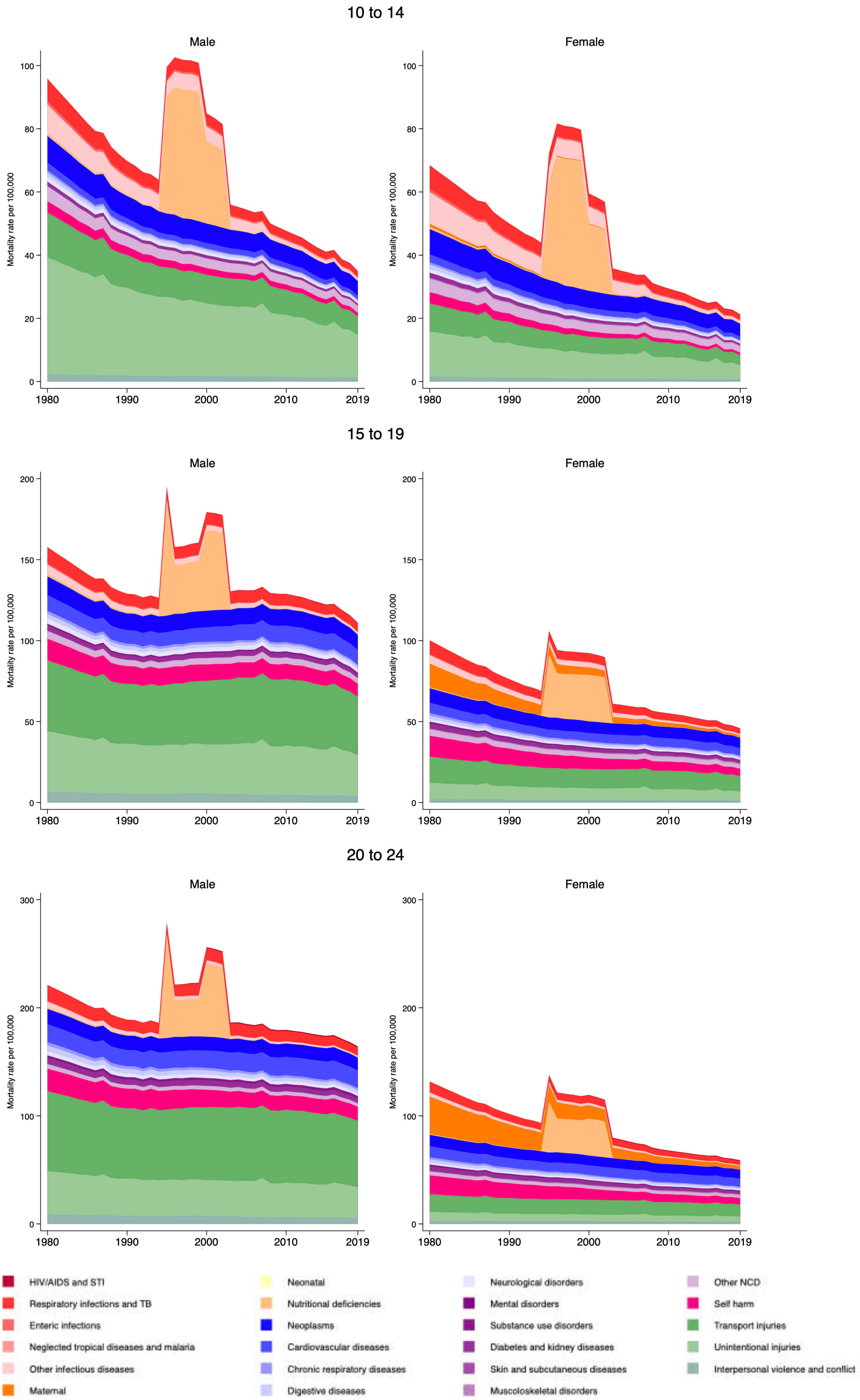

Figure S5d Number of deaths in 10-24 1980 – 2019 by cause: Democratic People's Republic of Korea

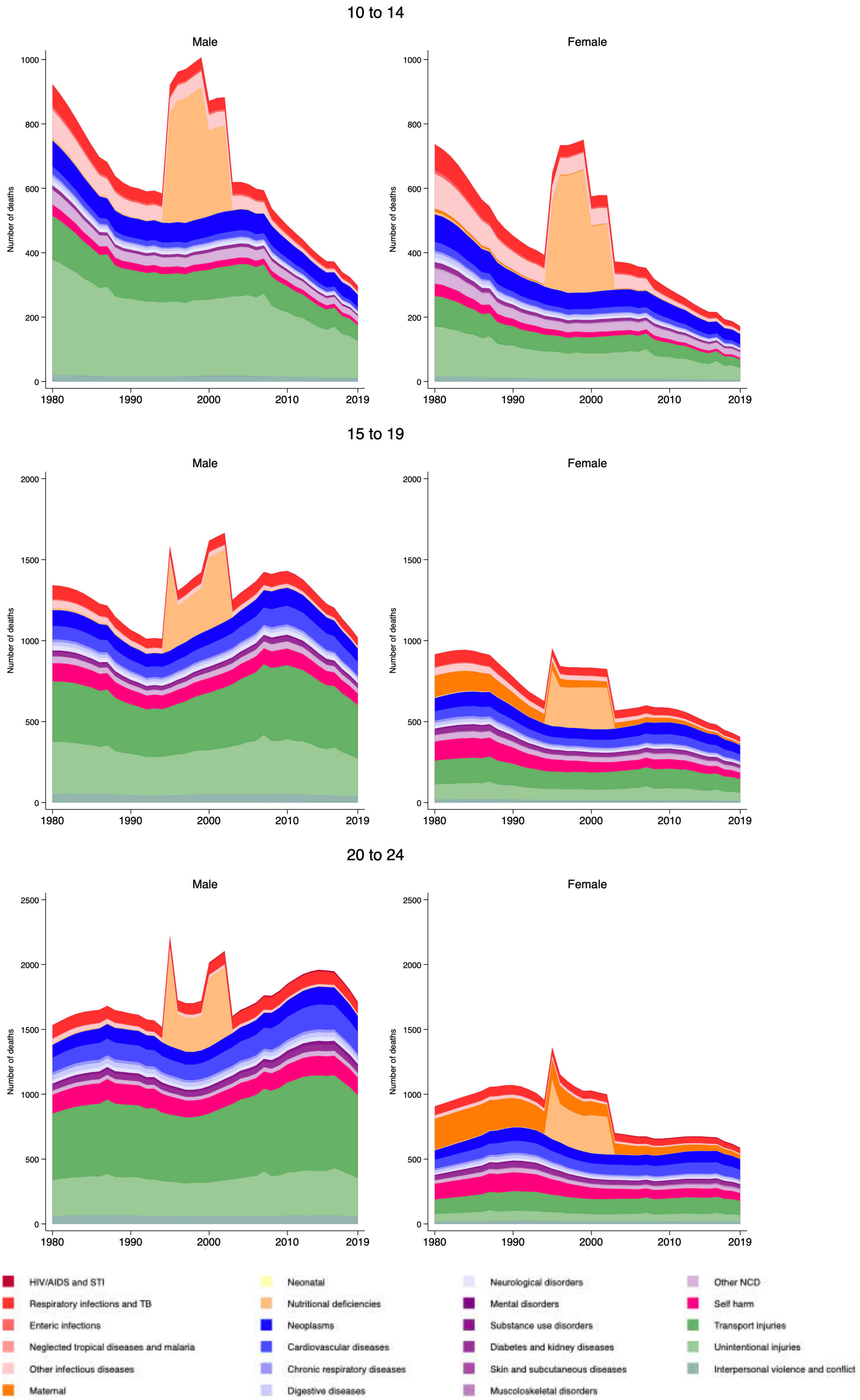

Figure S6: Taiwan (Province of China)

Figure S6a All-cause mortality rate per 100,000 in 10-24 1950 – 2019: Taiwan (Province of China)

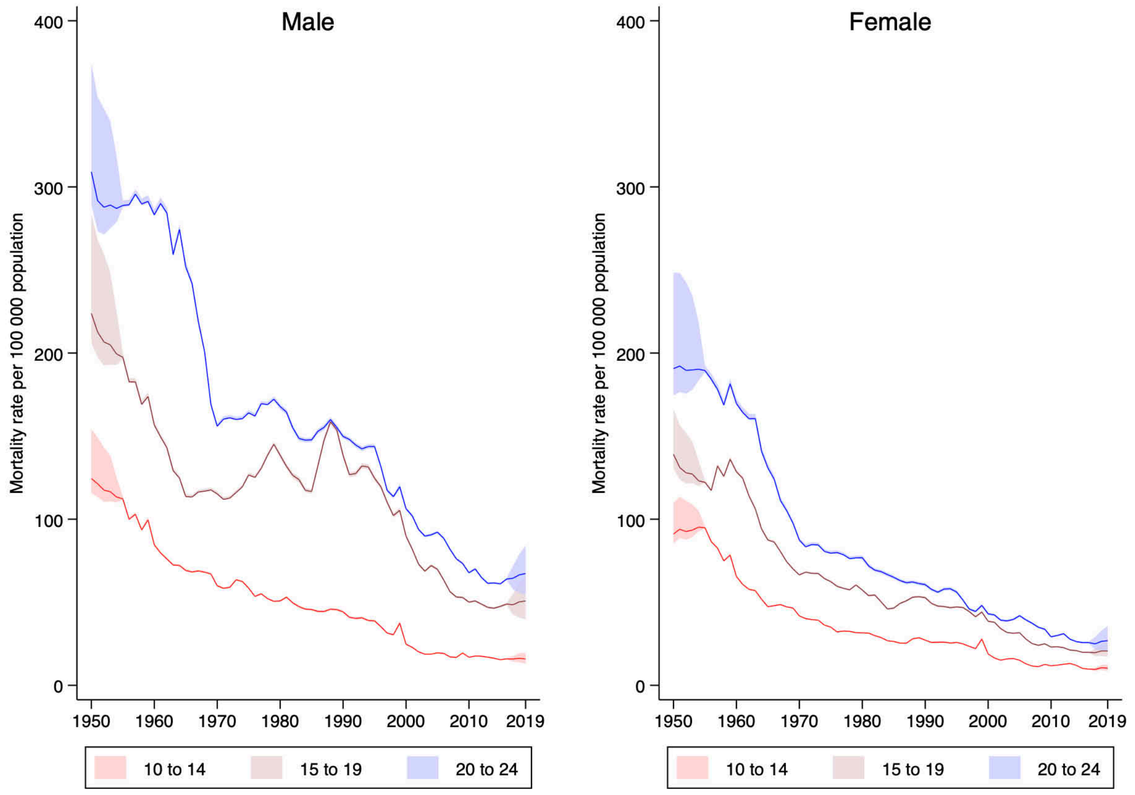

Figure S6b Number of deaths from all causes in 10-24 1950 – 2019: Taiwan (Province of China)

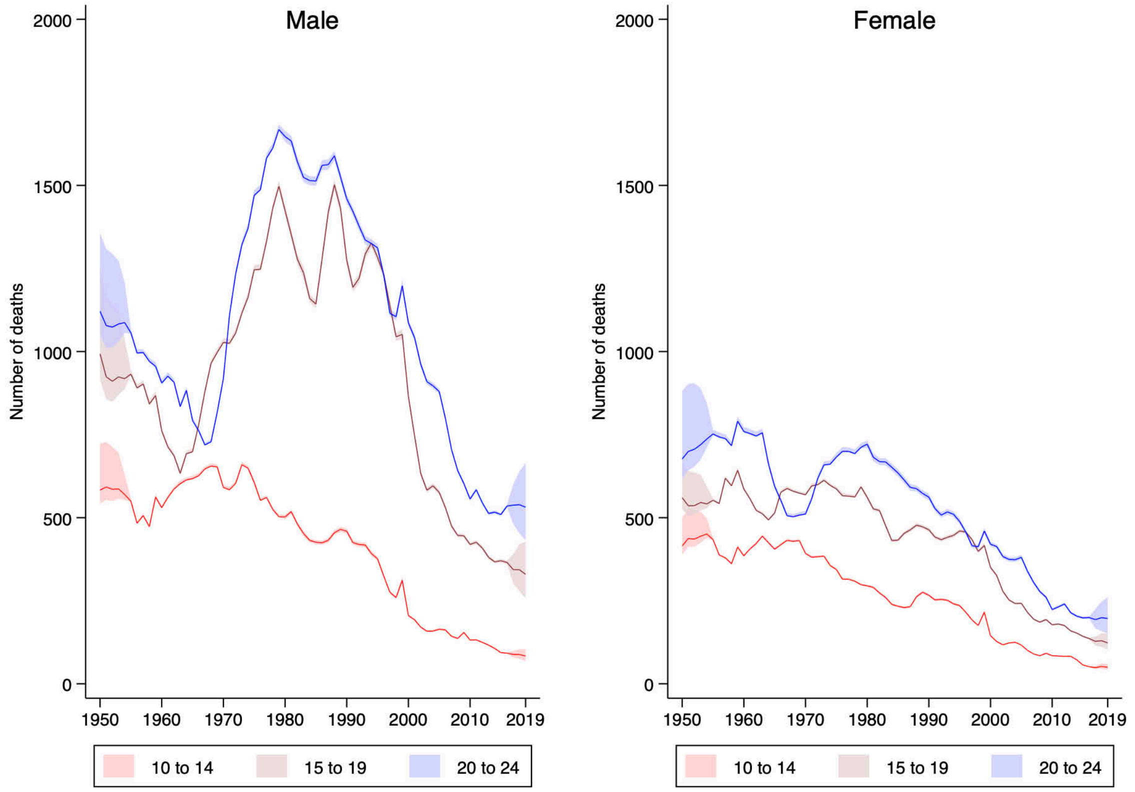

Figure S6c Mortality rate per 100,000 in 10-24 1980 – 2019 by cause: Taiwan (Province of China)

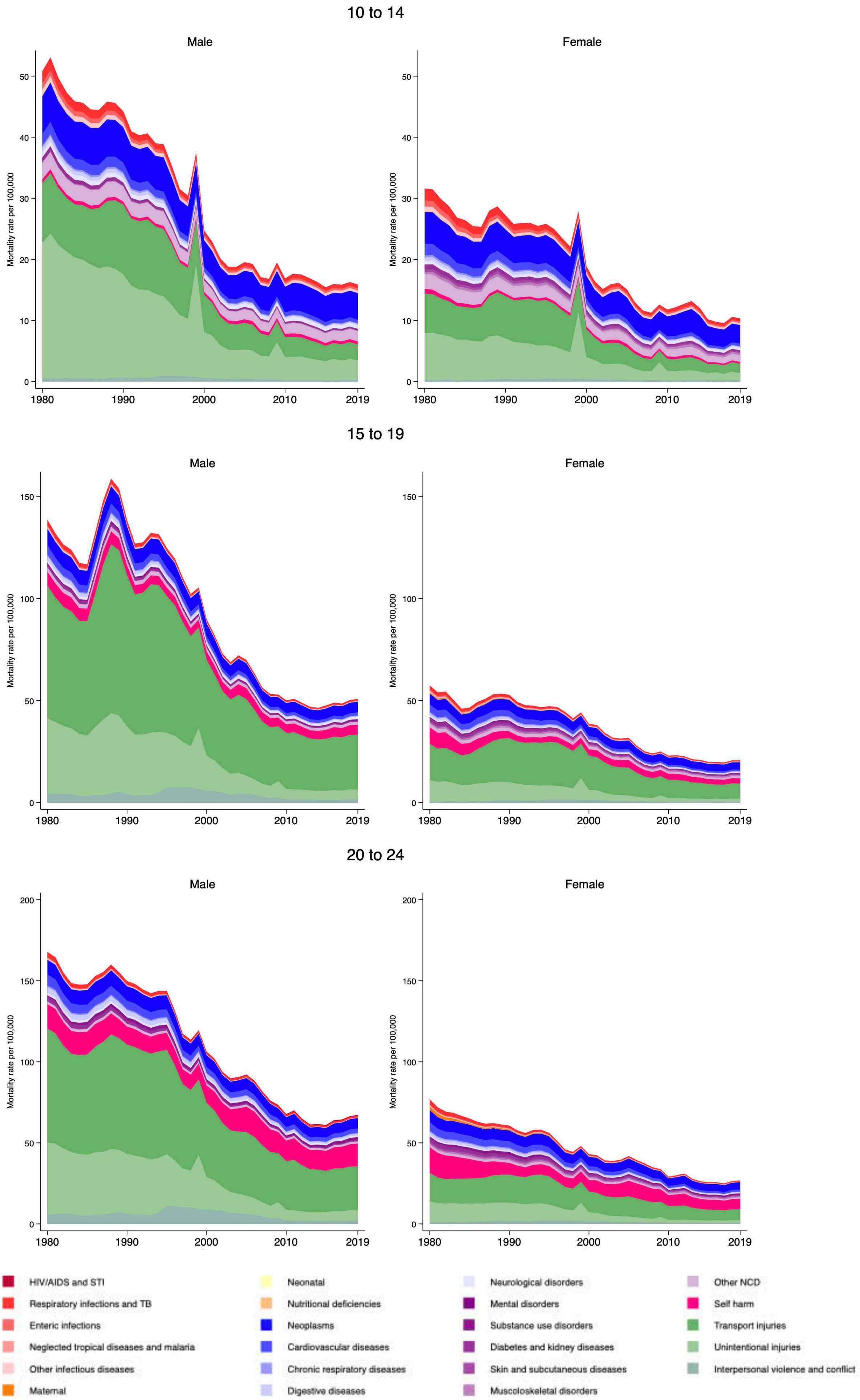

Figure S6d Number of deaths in 10-24 1980 – 2019 by cause: Taiwan (Province of China)

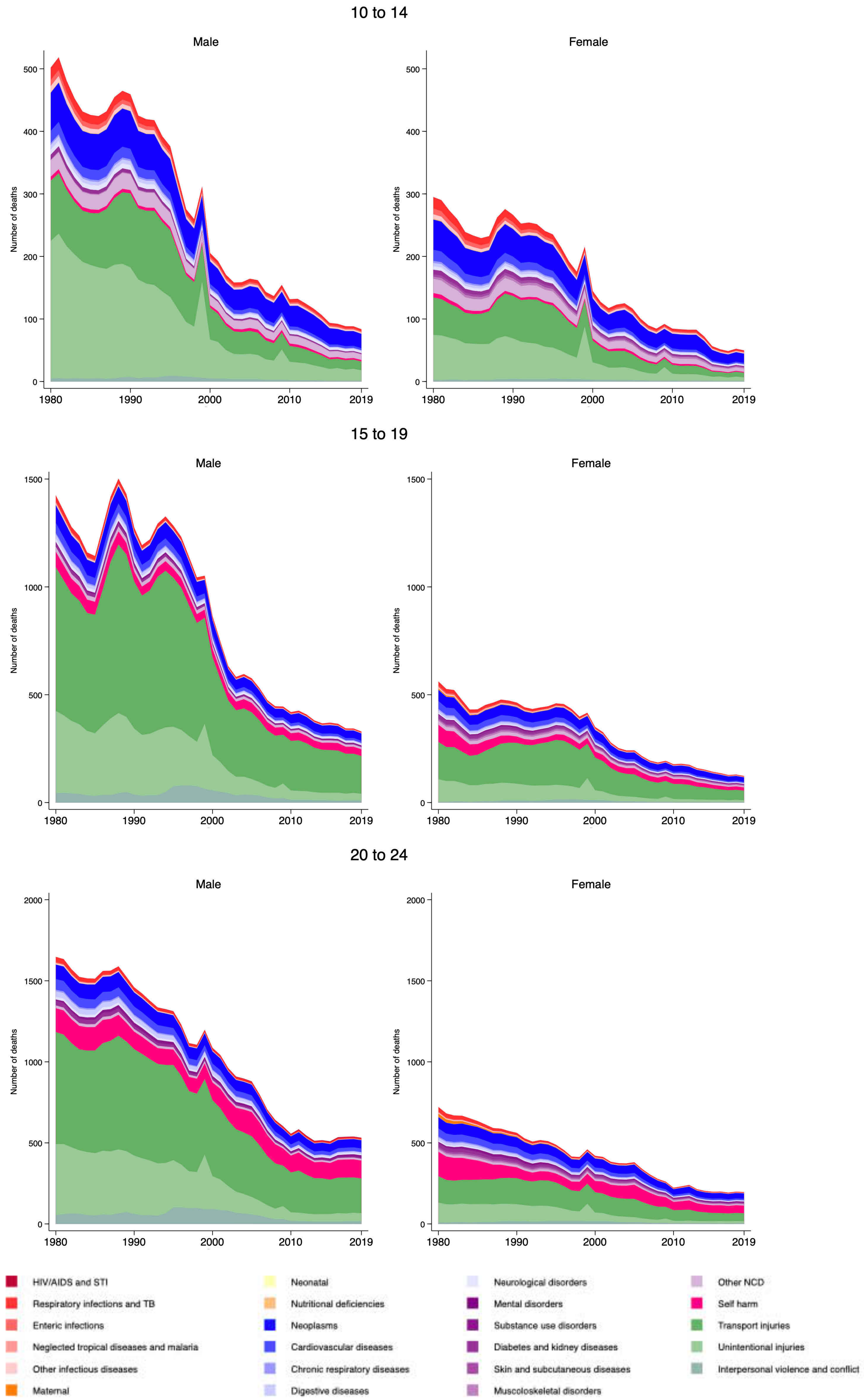

Figure S7: Southeast Asia

Figure S7a All-cause mortality rate per 100,000 in 10-24 1950 – 2019: Southeast Asia

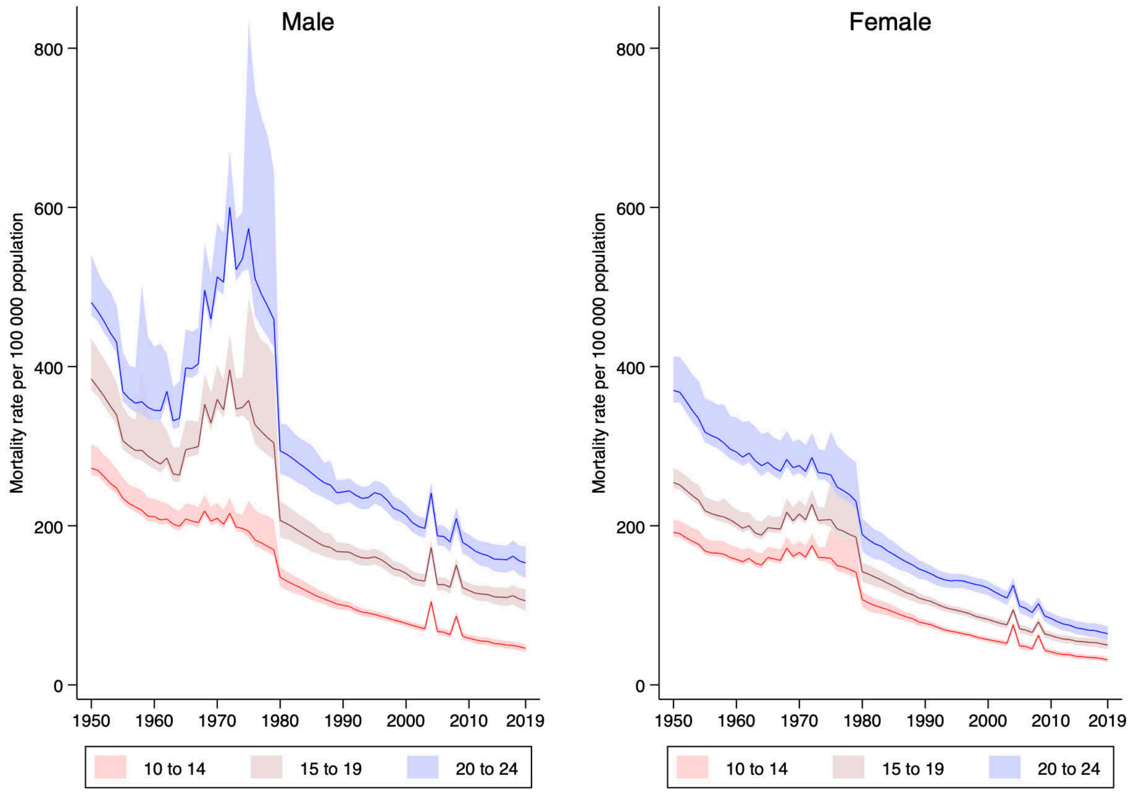

Figure S7b Number of deaths from all causes in 10-24 1950 – 2019: Southeast Asia

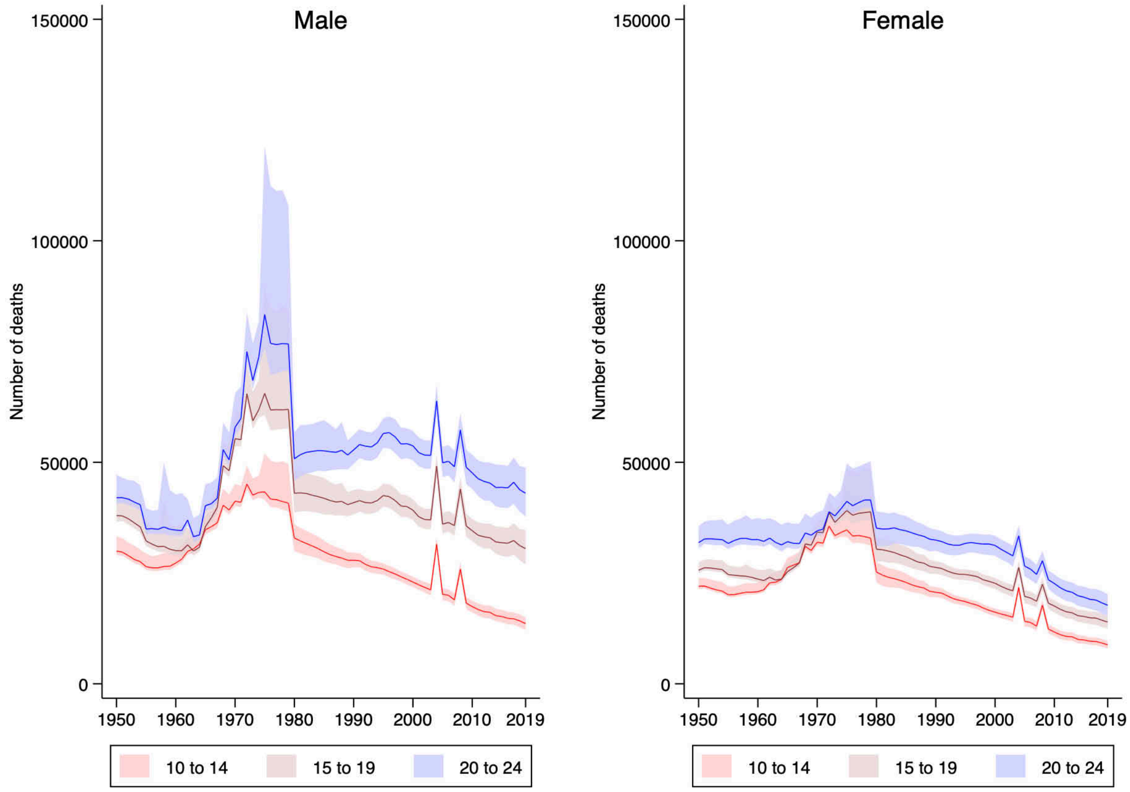

Figure S7c Mortality rate per 100,000 in 10-24 1980 – 2019 by cause: Southeast Asia

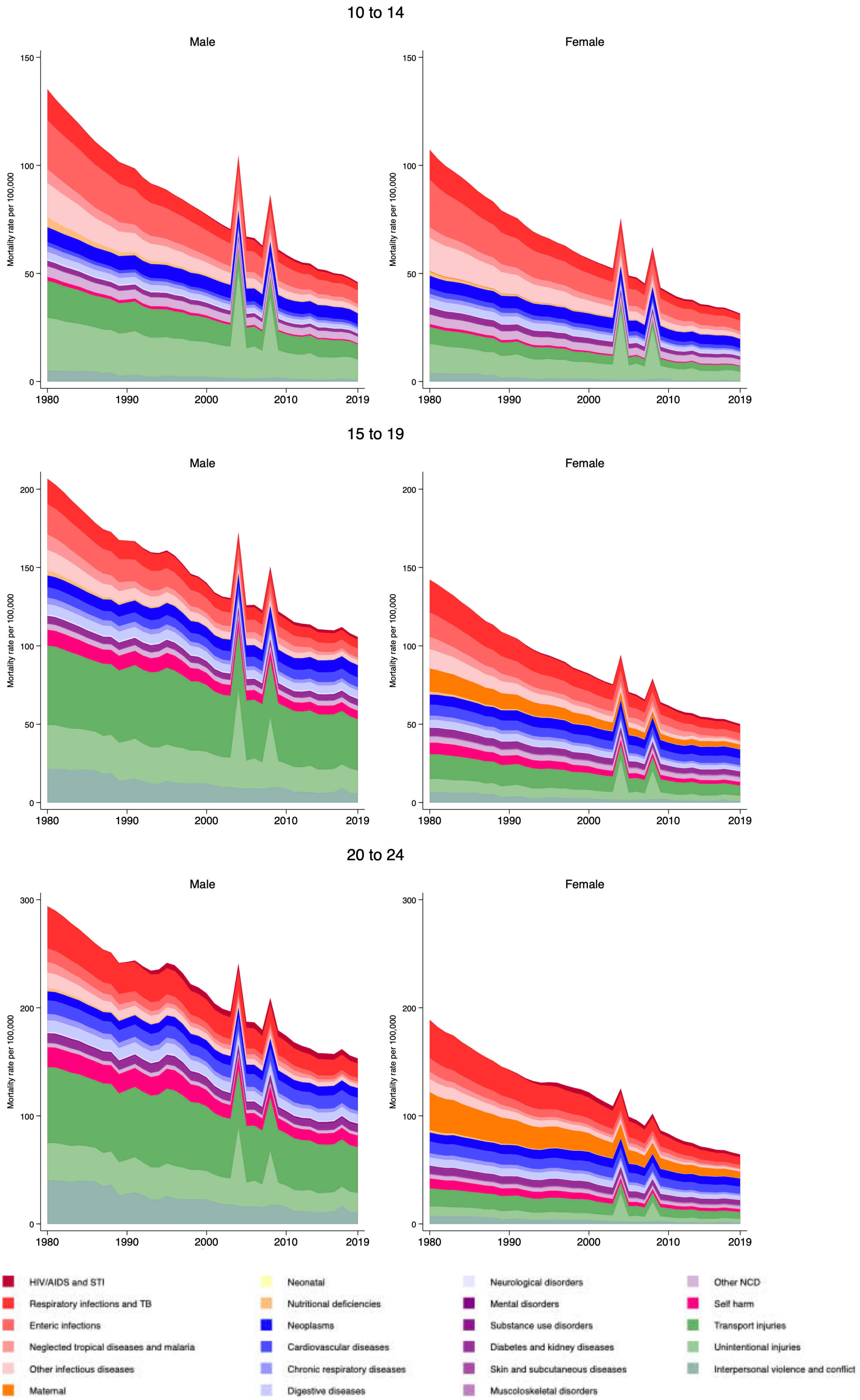

Figure S7d Number of deaths in 10-24 1980 – 2019 by cause: Southeast Asia

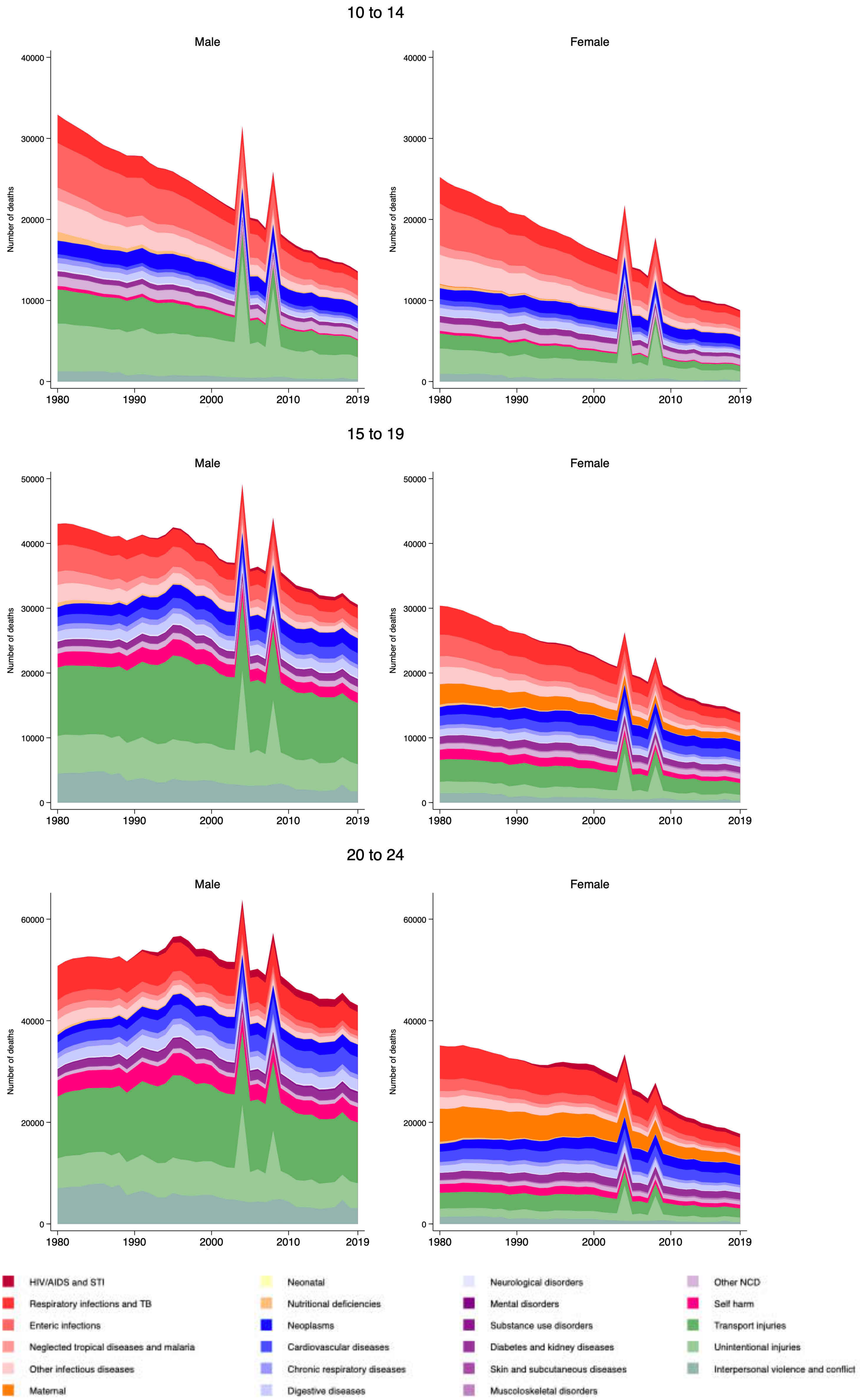

Figure S8: Cambodia

Figure S8a All-cause mortality rate per 100,000 in 10-24 1950 – 2019: Cambodia

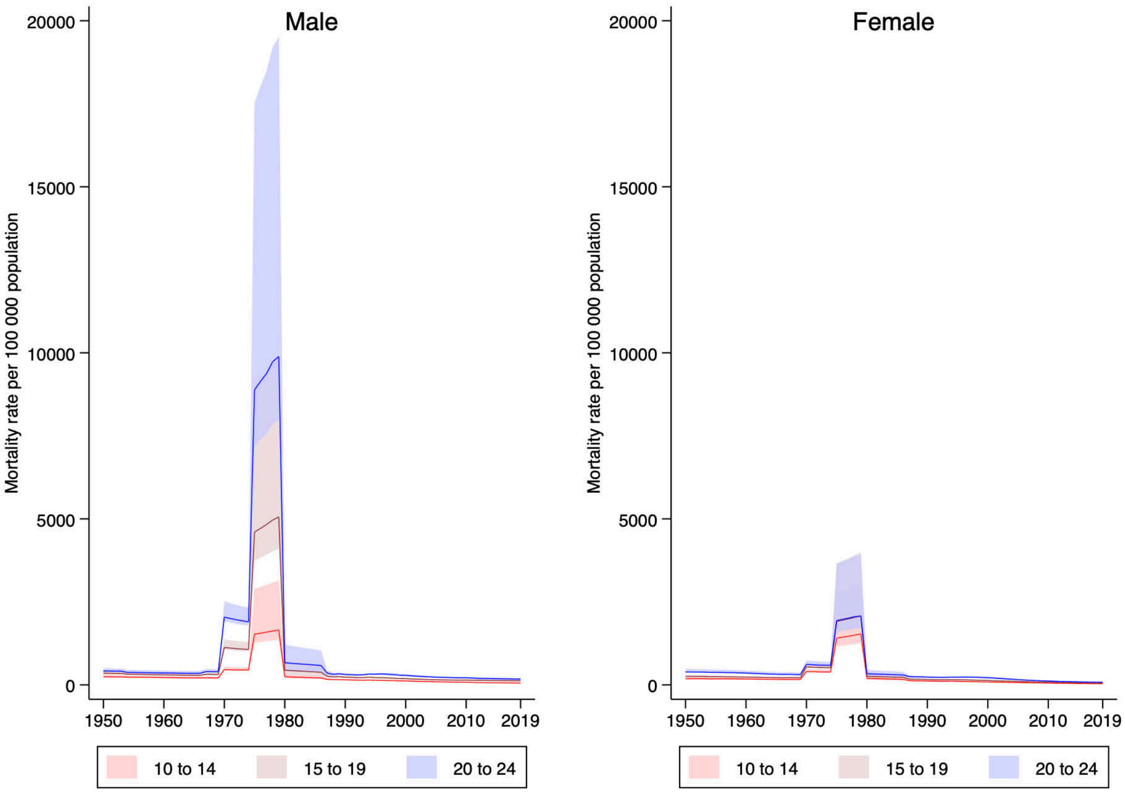

Figure S8b Number of deaths from all causes in 10-24 1950 – 2019: Cambodia

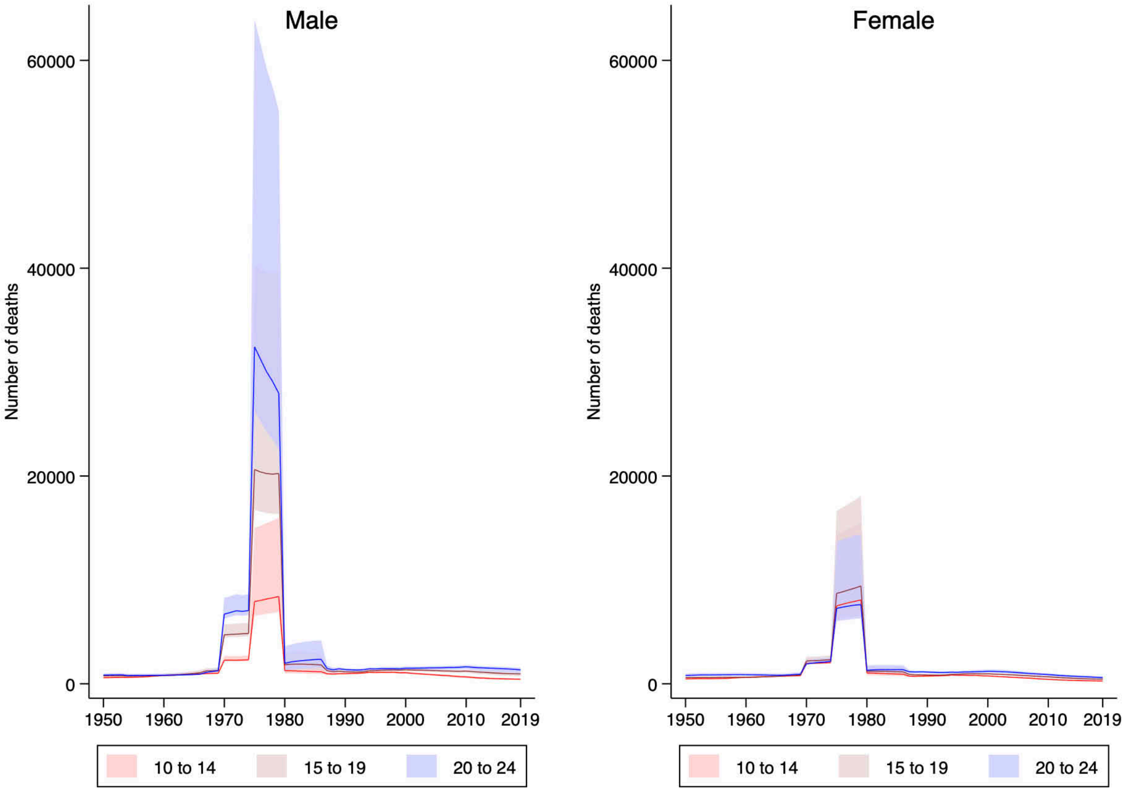

Figure S8c Mortality rate per 100,000 in 10-24 1980 – 2019 by cause: Cambodia

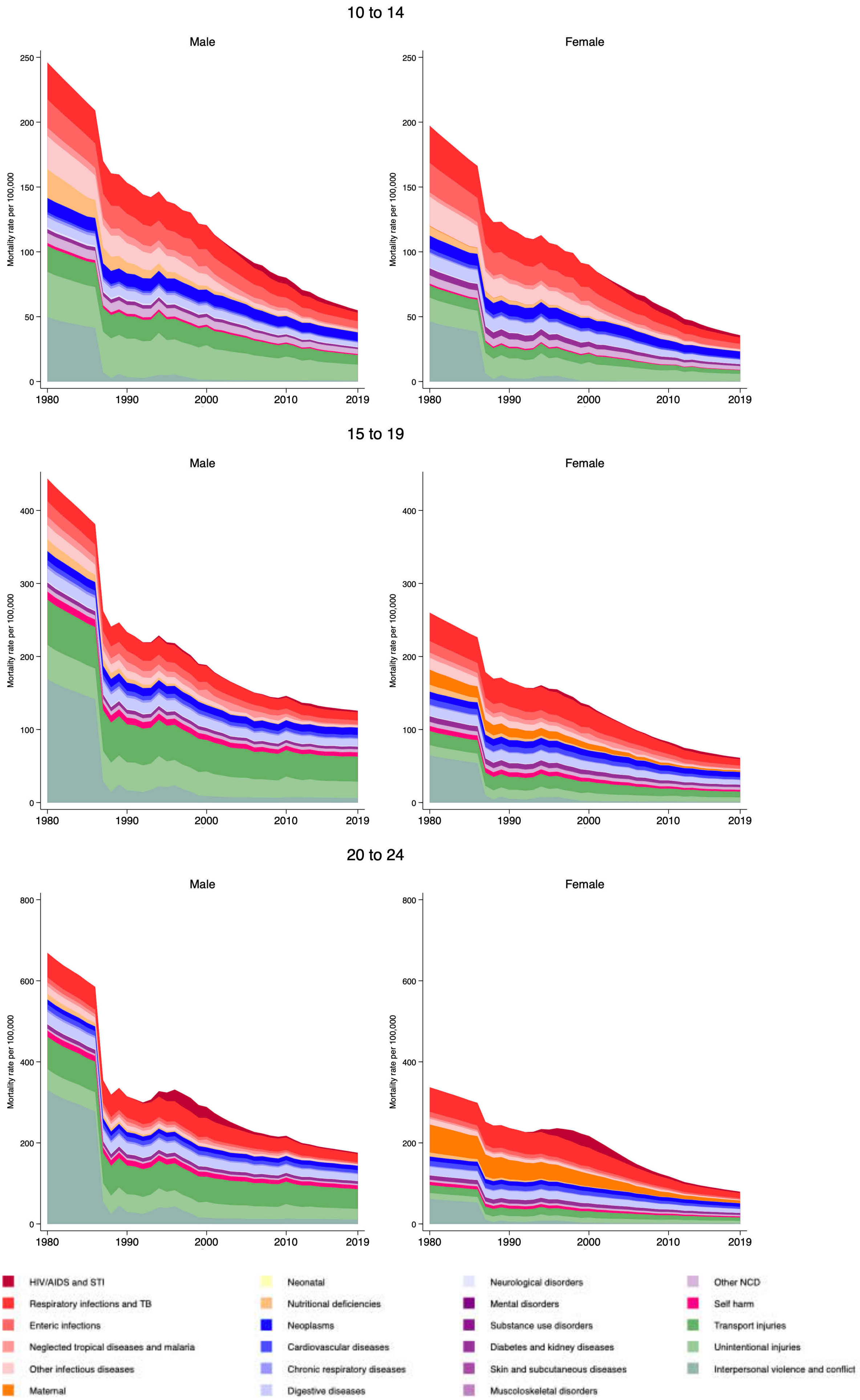

Figure S8d Number of deaths in 10-24 1980 – 2019 by cause: Cambodia

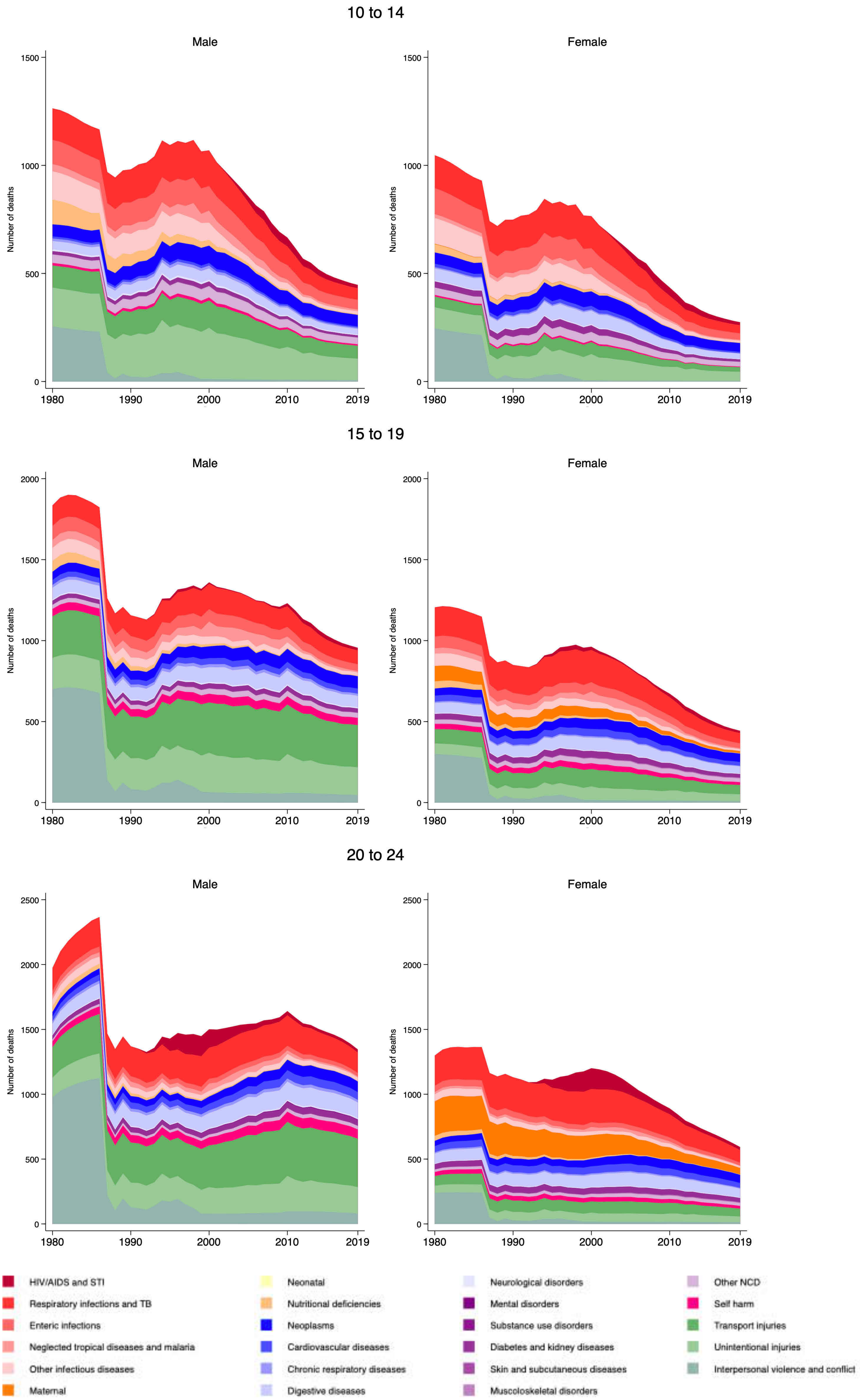

Figure S9: Indonesia

Figure S9a All-cause mortality rate per 100,000 in 10-24 1950 – 2019: Indonesia

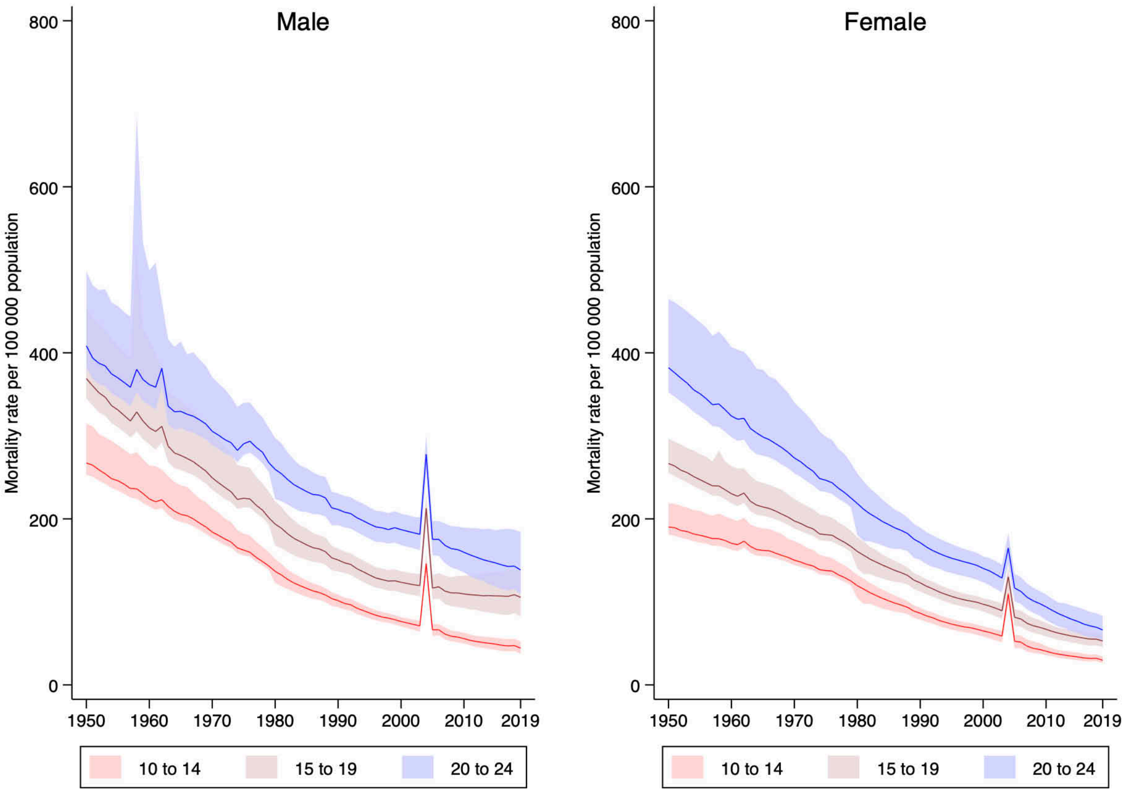

Figure S9b Number of deaths from all causes in 10-24 1950 – 2019: Indonesia

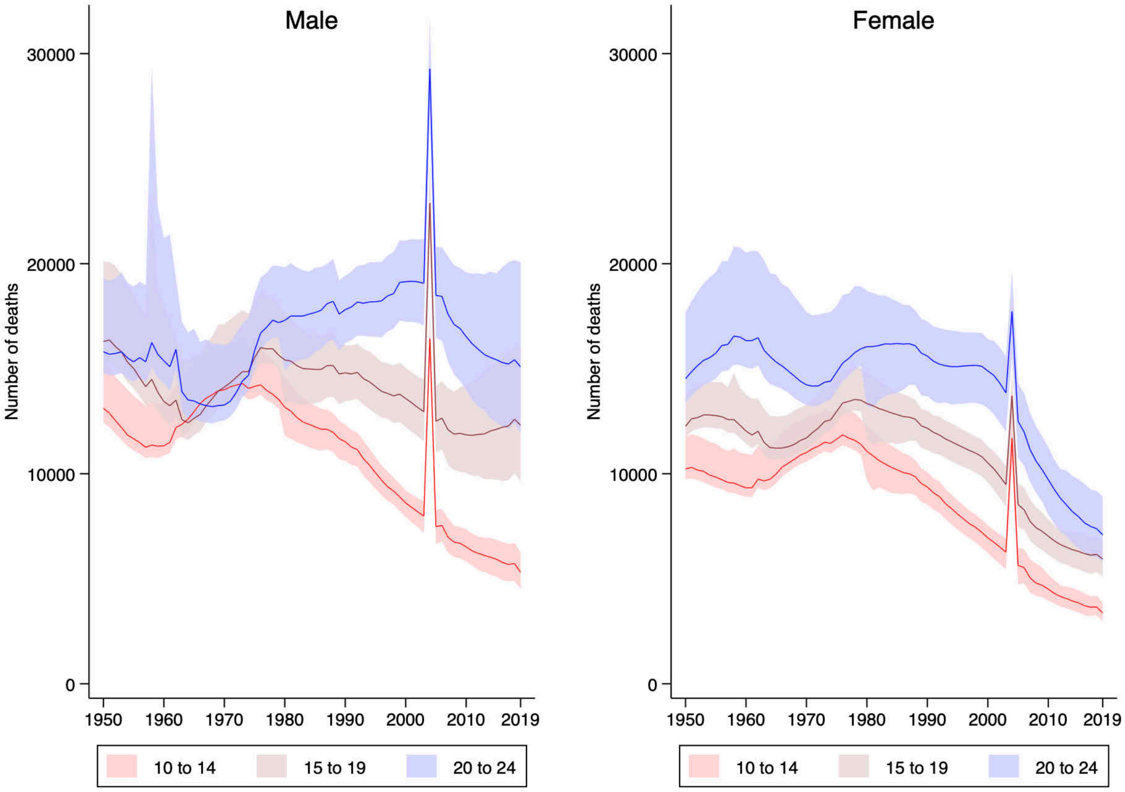

Figure S9c Mortality rate per 100,000 in 10-24 1980 – 2019 by cause: Indonesia

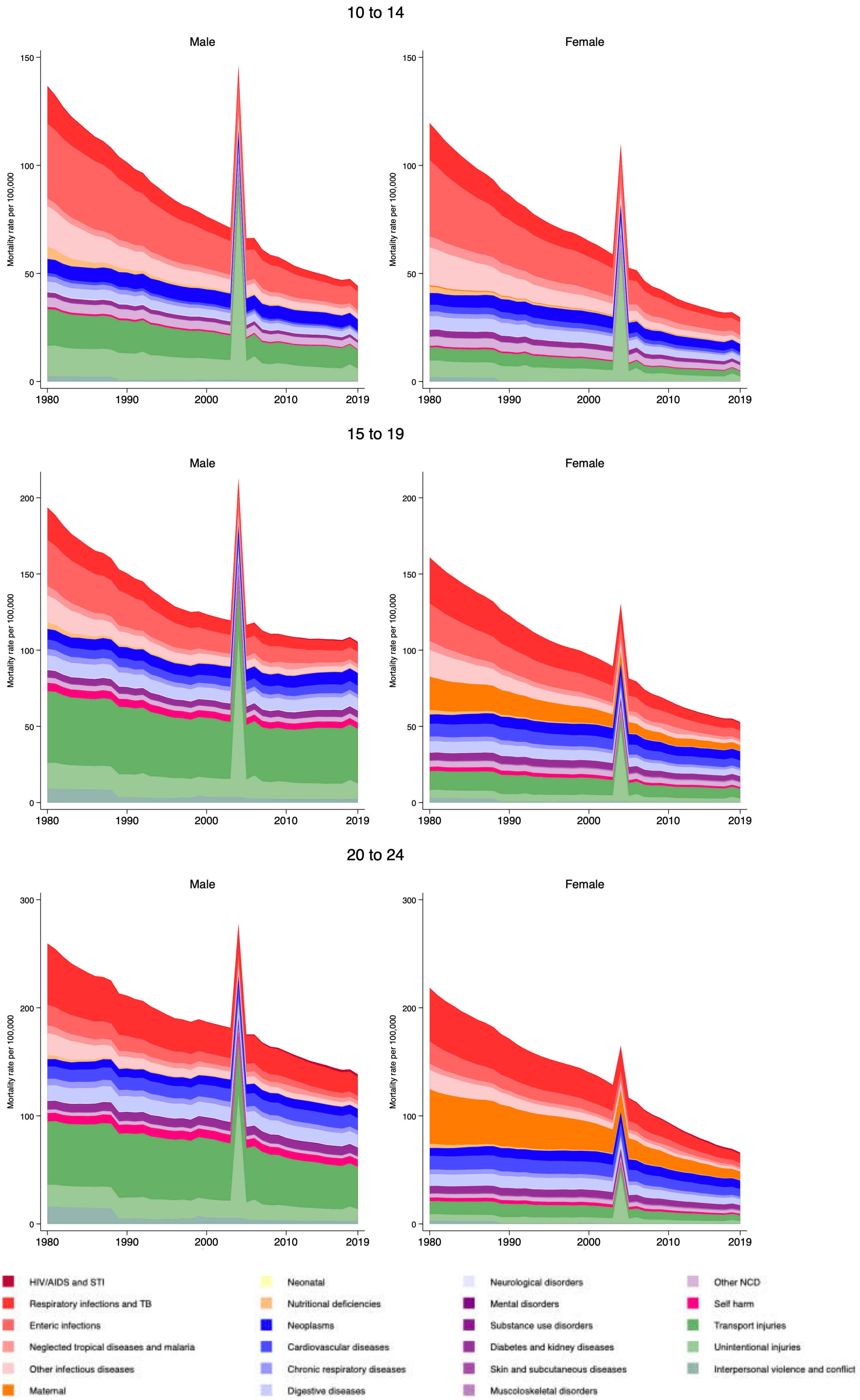

Figure S9d Number of deaths in 10-24 1980 – 2019 by cause: Indonesia

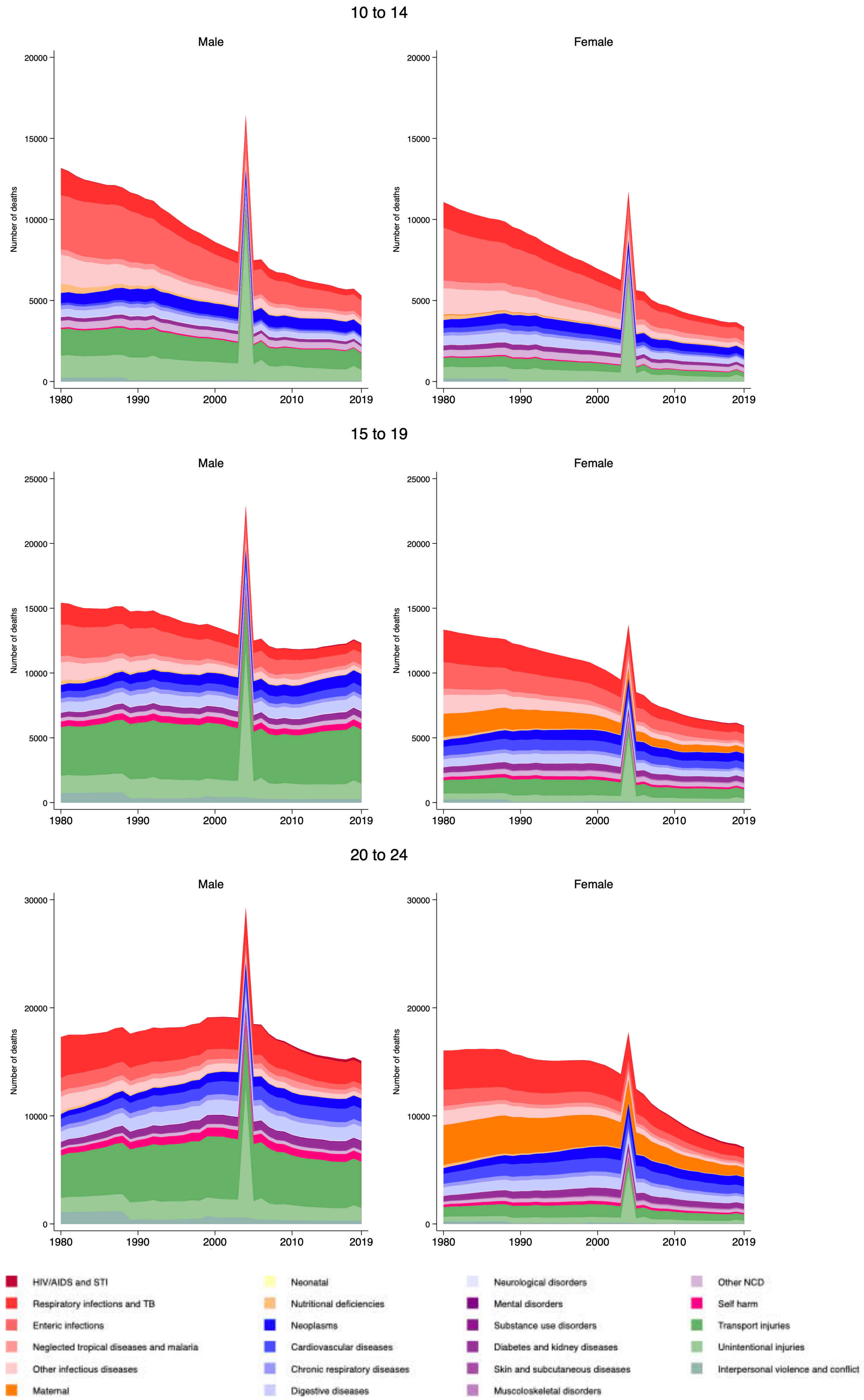

Figure S10: Lao People's Democratic Republic

Figure S10a All-cause mortality rate per 100,000 in 10-24 1950 – 2019: Lao People's Democratic Republic

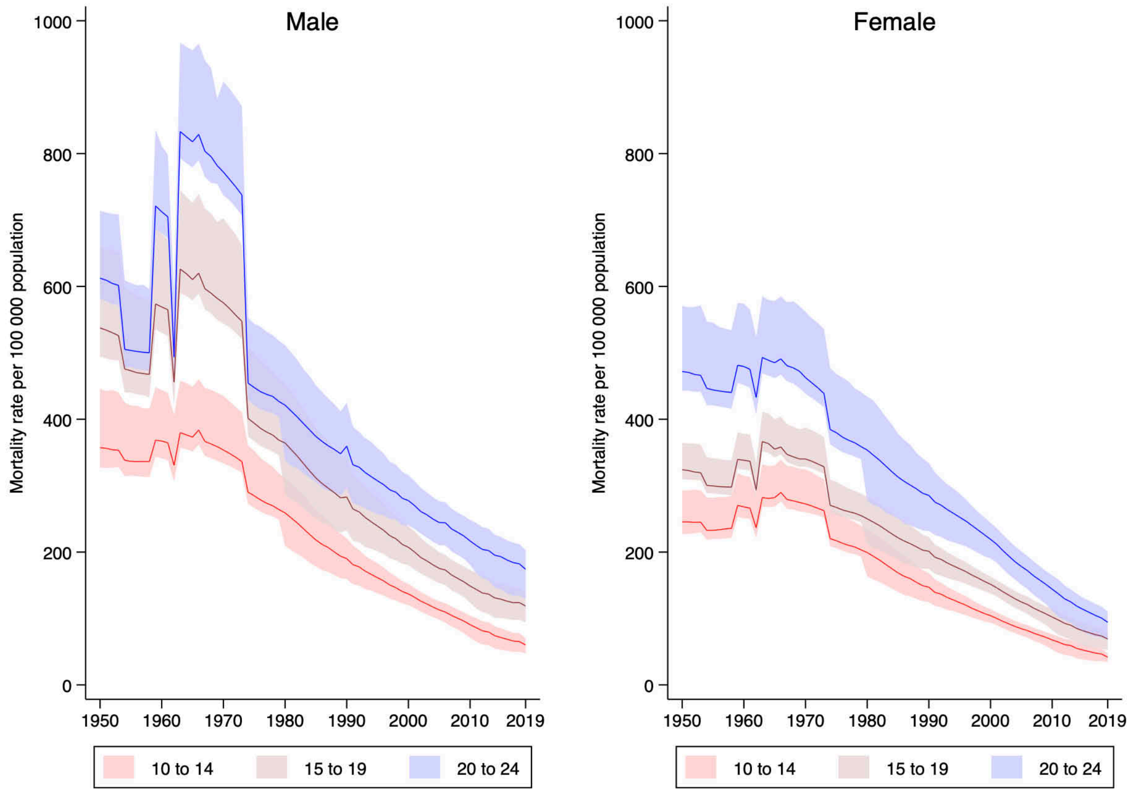

Figure S10b Number of deaths from all causes in 10-24 1950 – 2019: Lao People's Democratic Republic

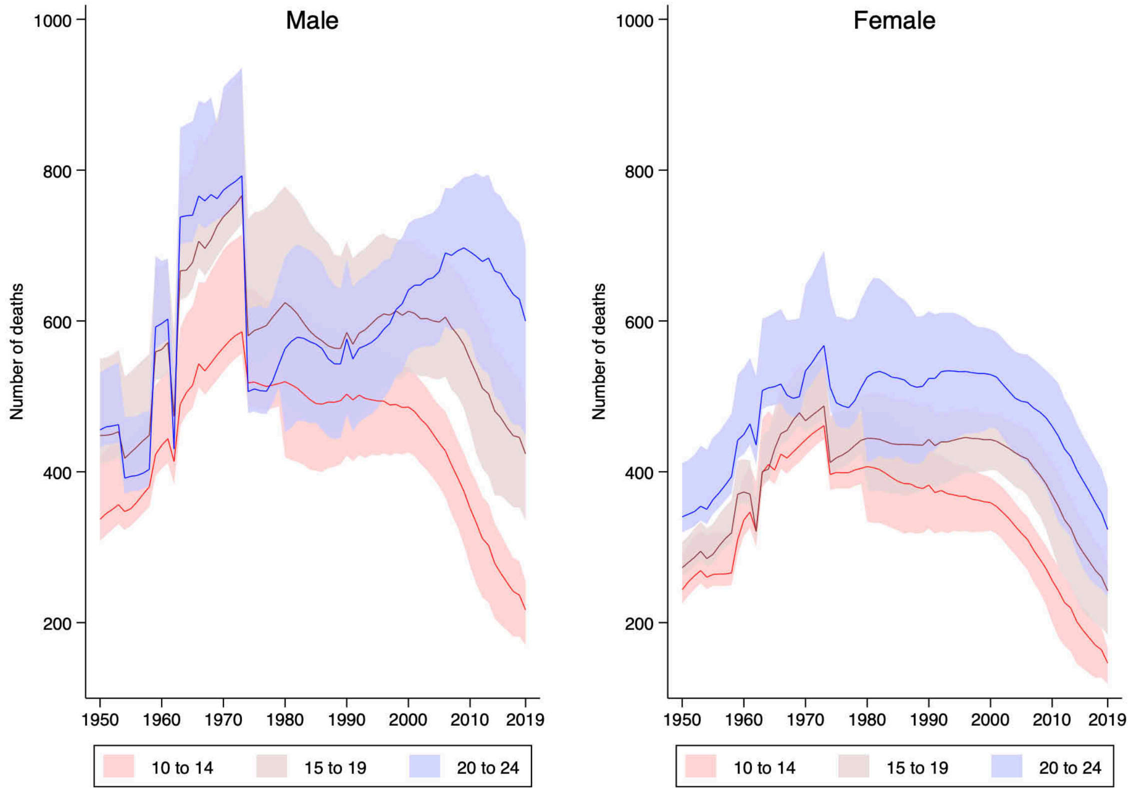

Figure S10c Mortality rate per 100,000 in 10-24 1980 – 2019 by cause: Lao People's Democratic Republic

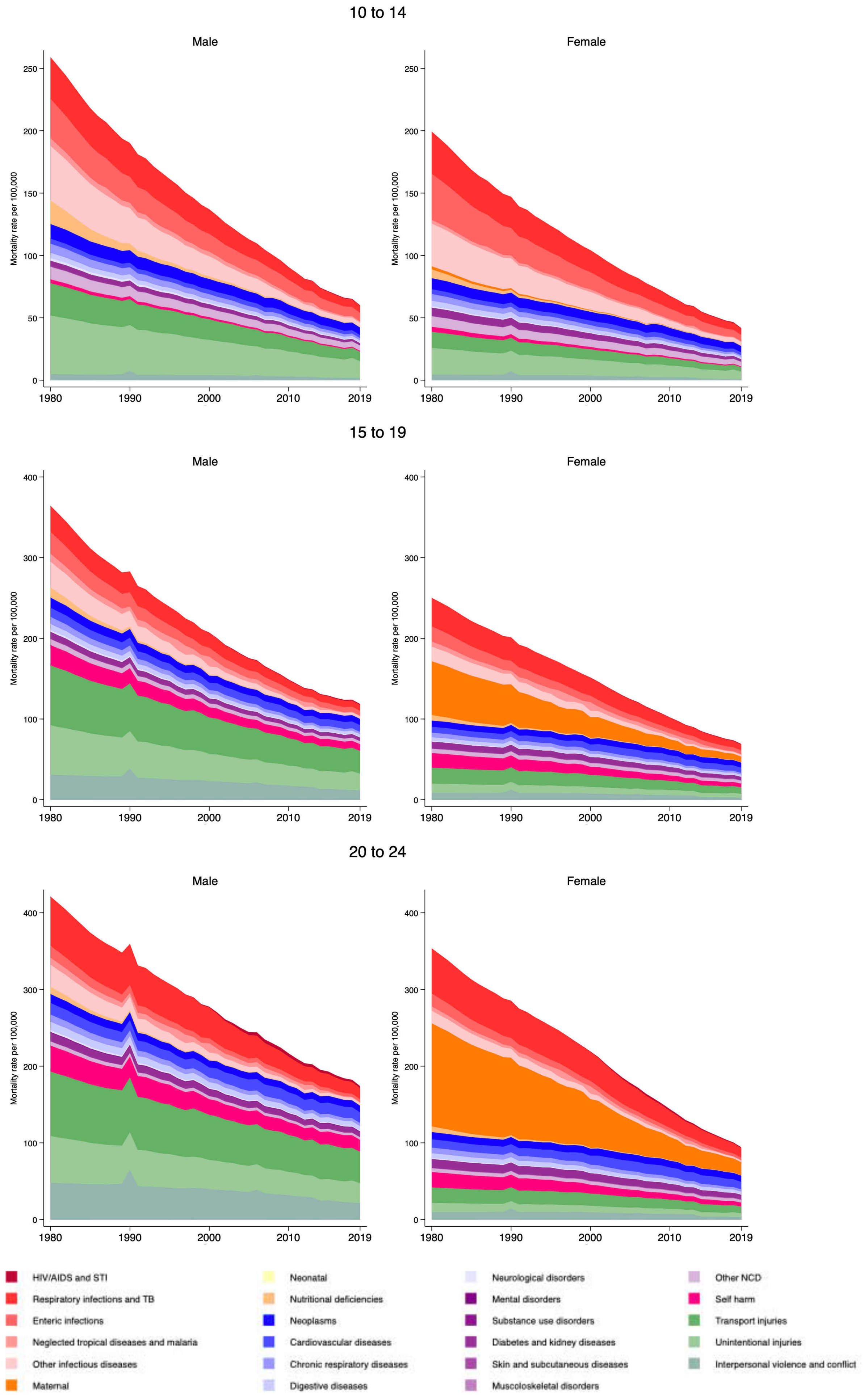

Figure S10d Number of deaths in 10-24 1980 – 2019 by cause: Lao People's Democratic Republic

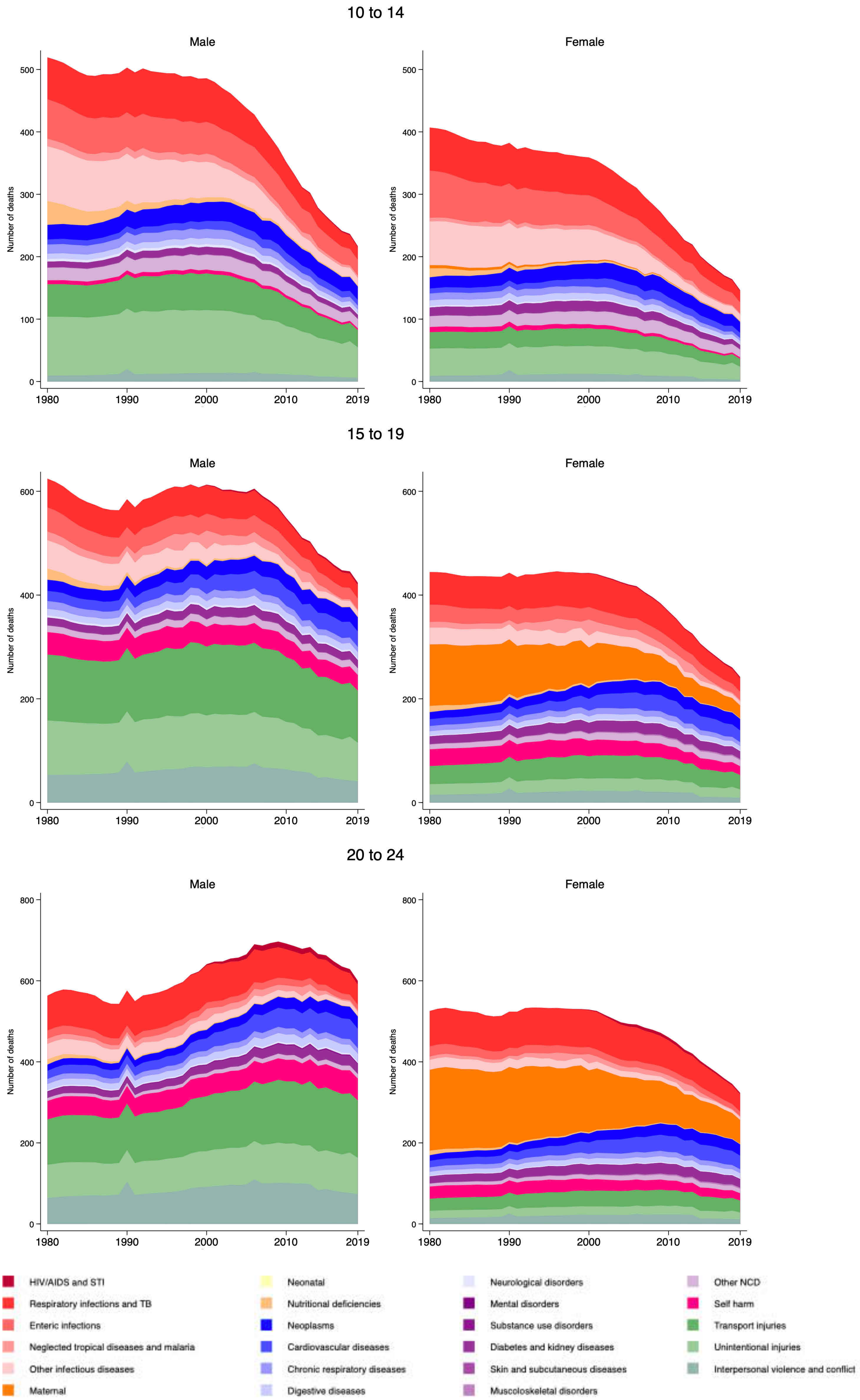

Figure S11: Malaysia

Figure S11a All-cause mortality rate per 100,000 in 10-24 1950 – 2019: Malaysia

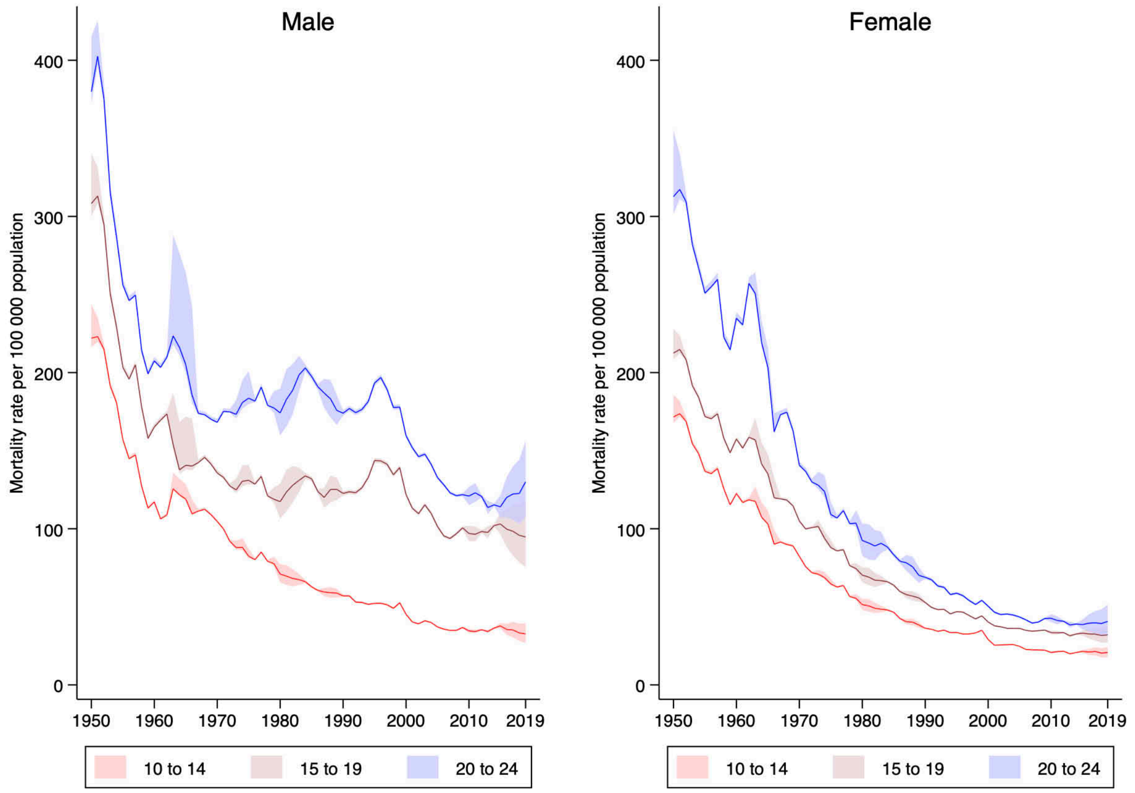

Figure S11b Number of deaths from all causes in 10-24 1950 – 2019: Malaysia

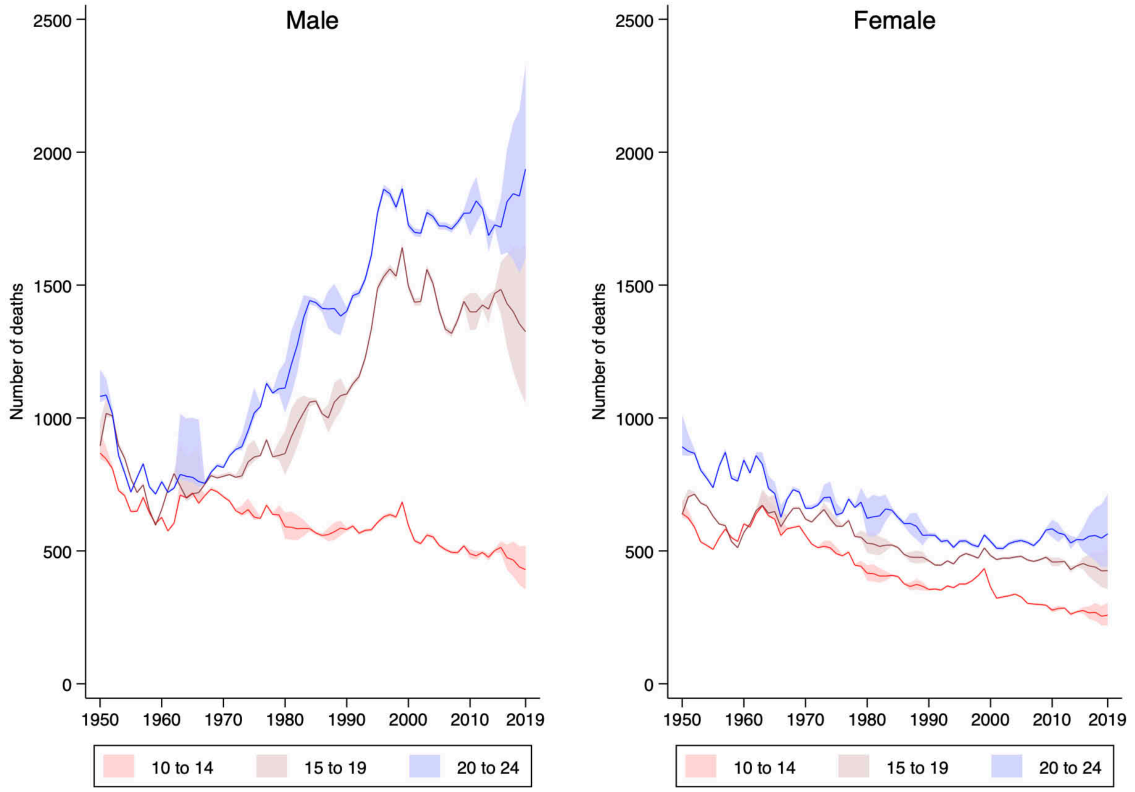

Figure S11c Mortality rate per 100,000 in 10-24 1980 – 2019 by cause: Malaysia

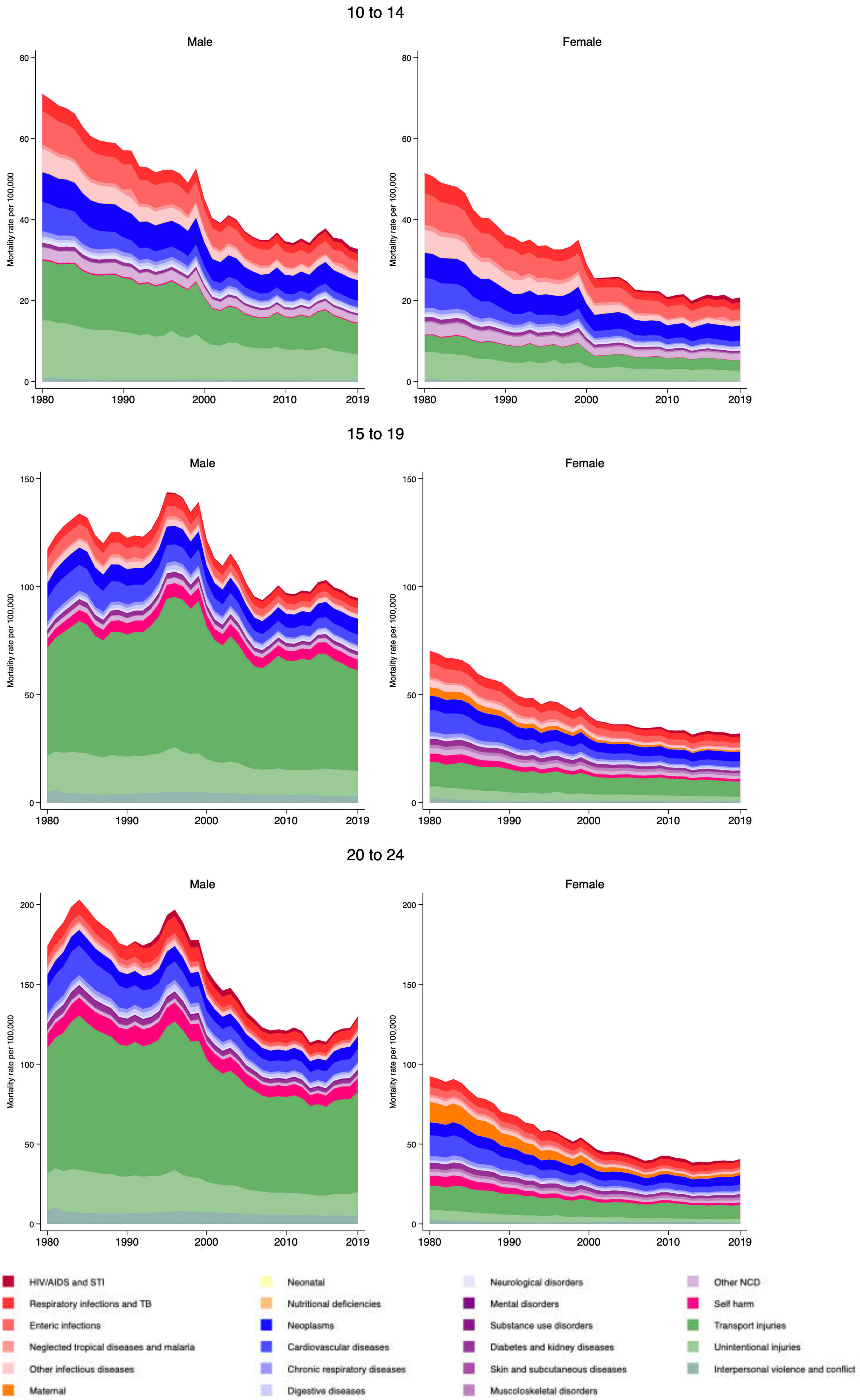

Figure S11d Number of deaths in 10-24 1980 – 2019 by cause: Malaysia

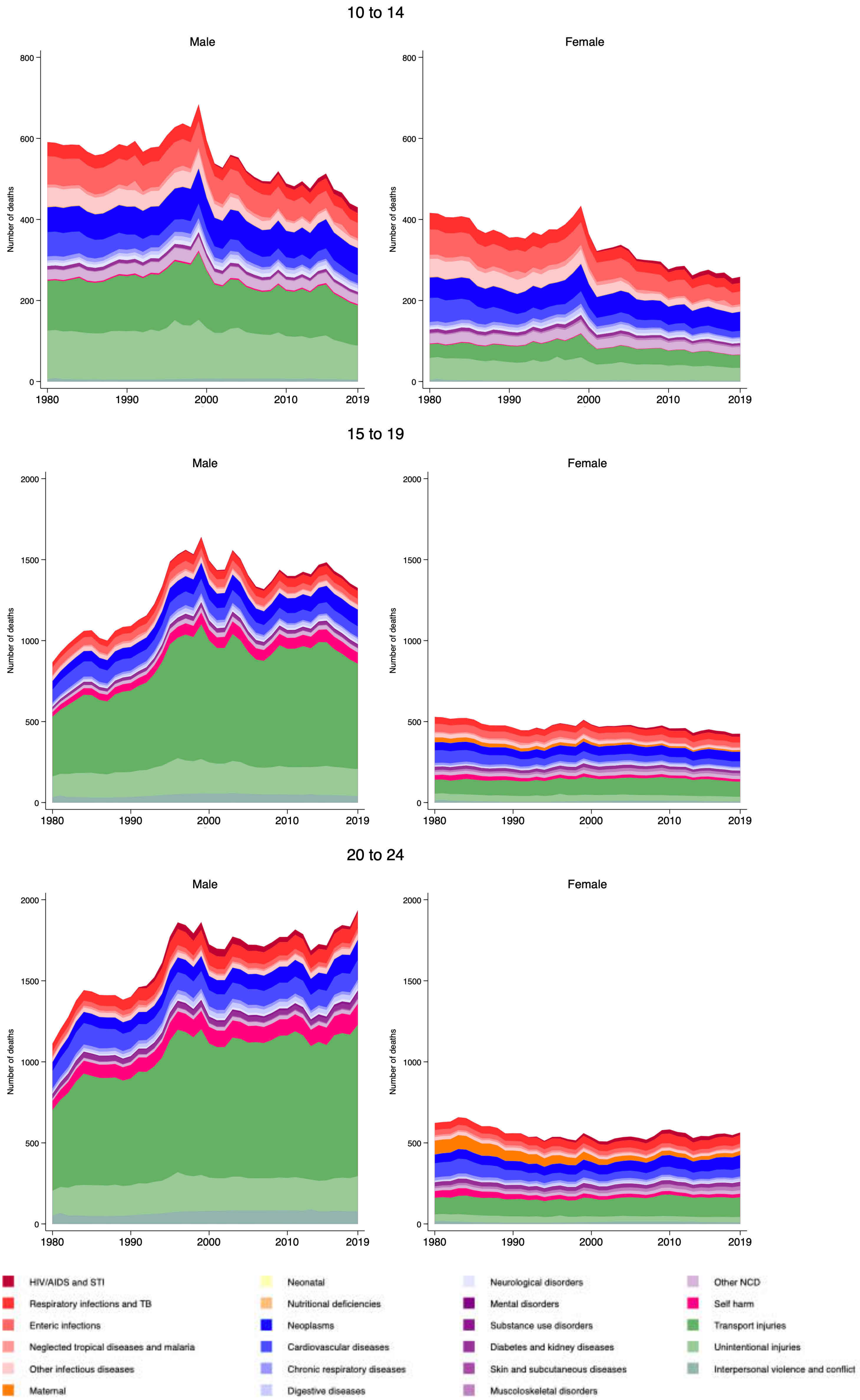

Figure S12: Maldives

Figure S12a All-cause mortality rate per 100,000 in 10-24 1950 – 2019: Maldives

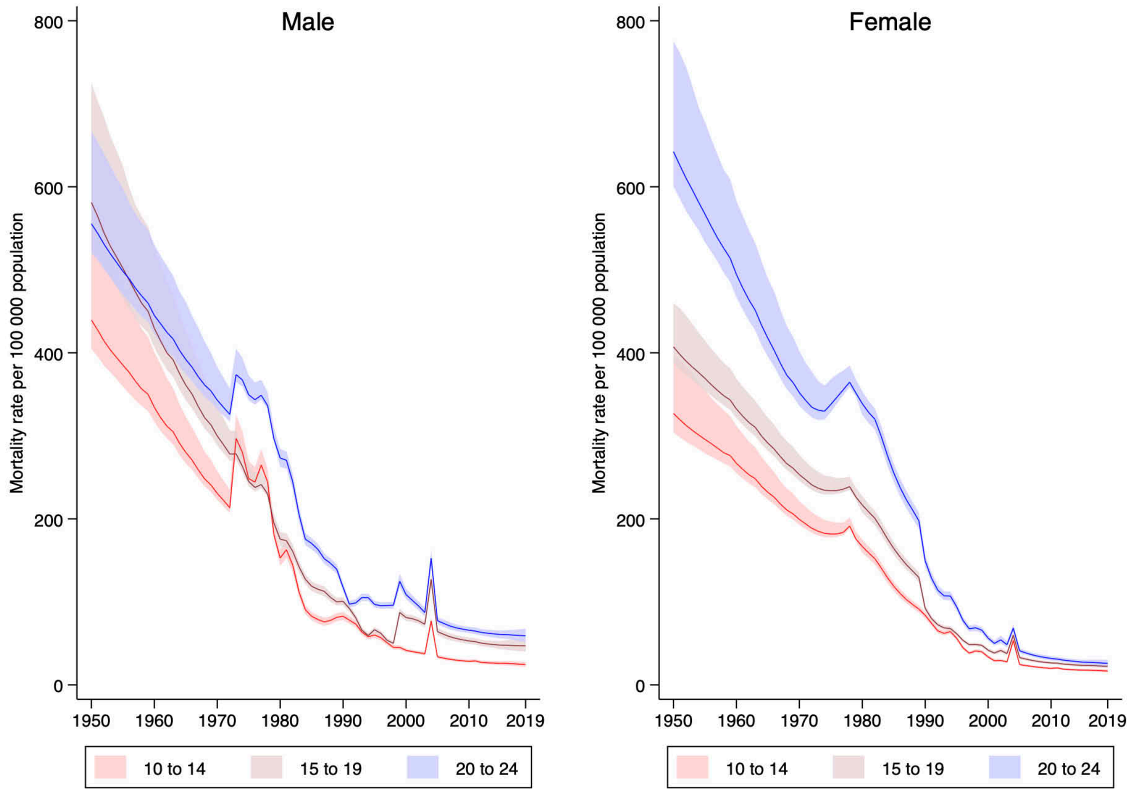

Figure S12b Number of deaths from all causes in 10-24 1950 – 2019: Maldives

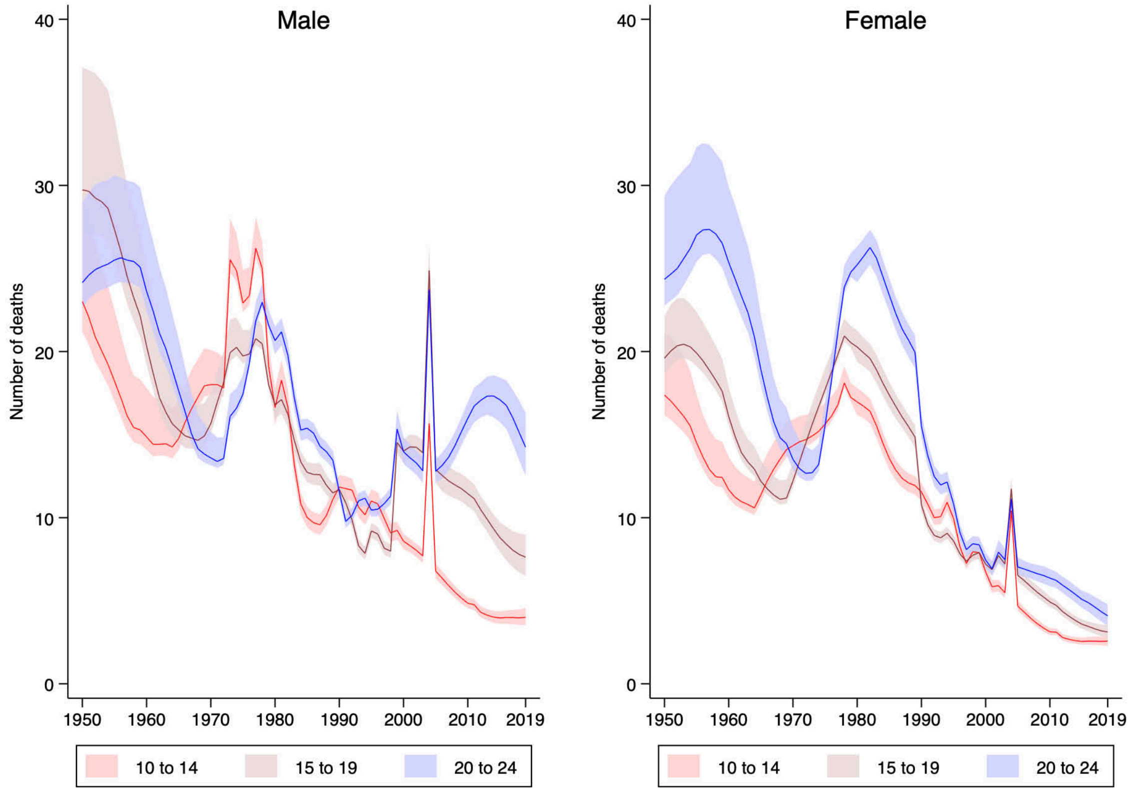

Figure S12c Mortality rate per 100,000 in 10-24 1980 – 2019 by cause: Maldives

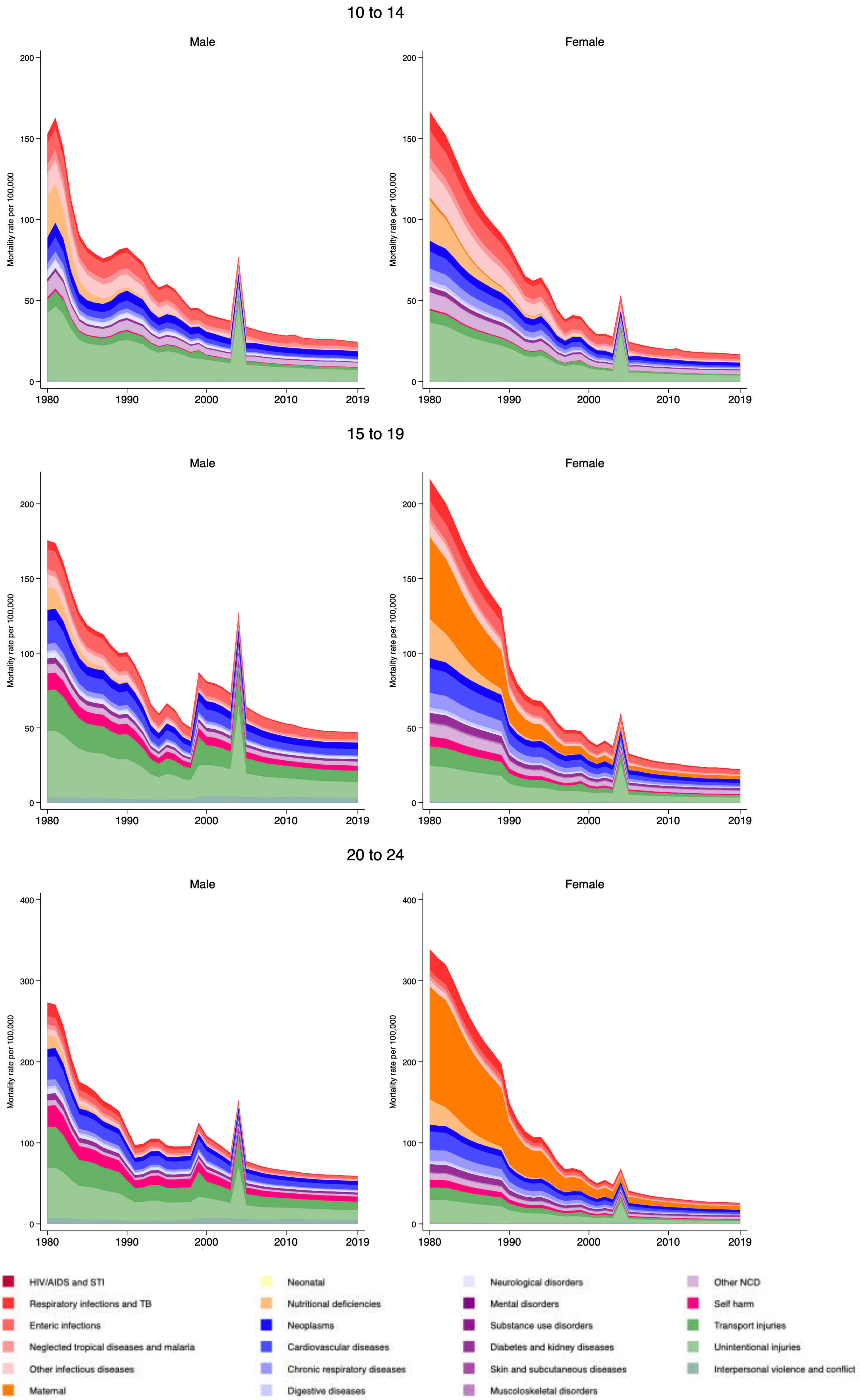

Figure S12d Number of deaths in 10-24 1980 – 2019 by cause: Maldives

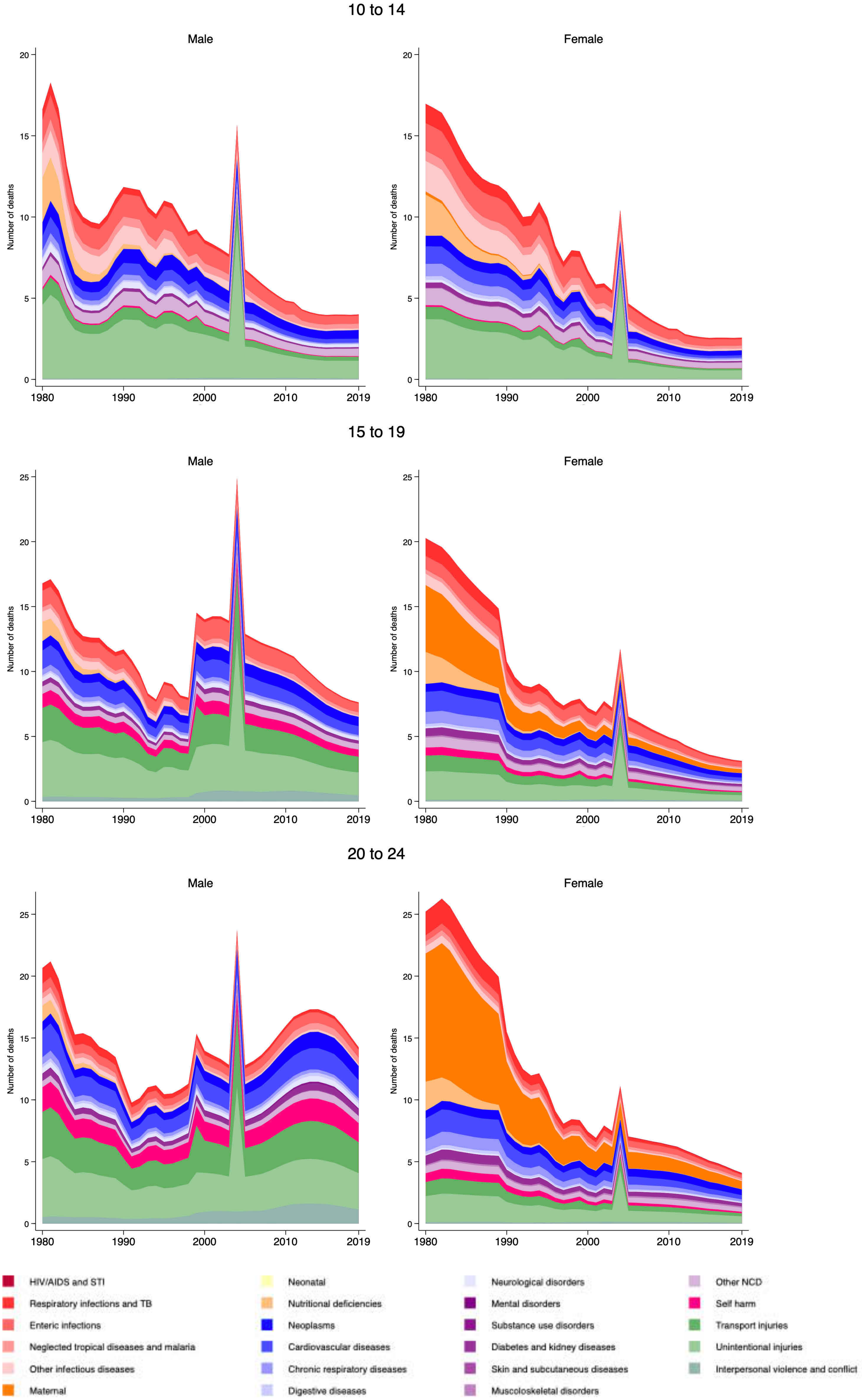

Figure S13: Myanmar

Figure S13a All-cause mortality rate per 100,000 in 10-24 1950 – 2019: Myanmar

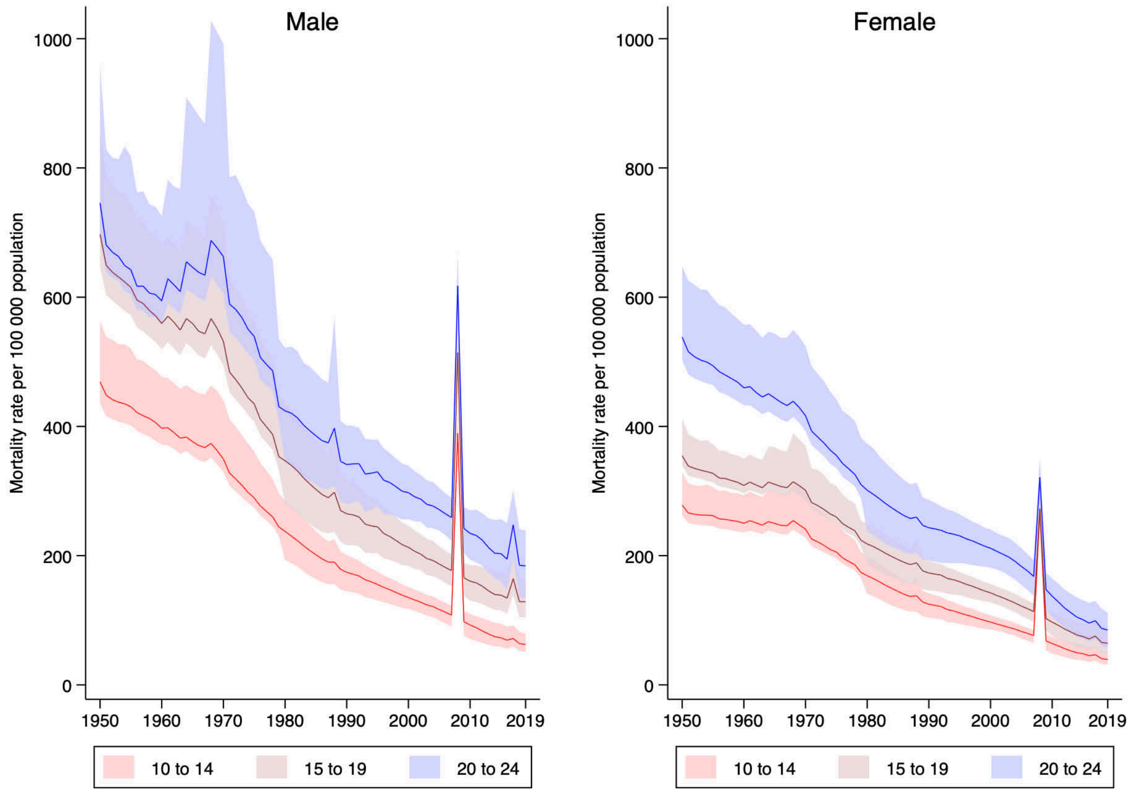

Figure S13b Number of deaths from all causes in 10-24 1950 – 2019: Myanmar

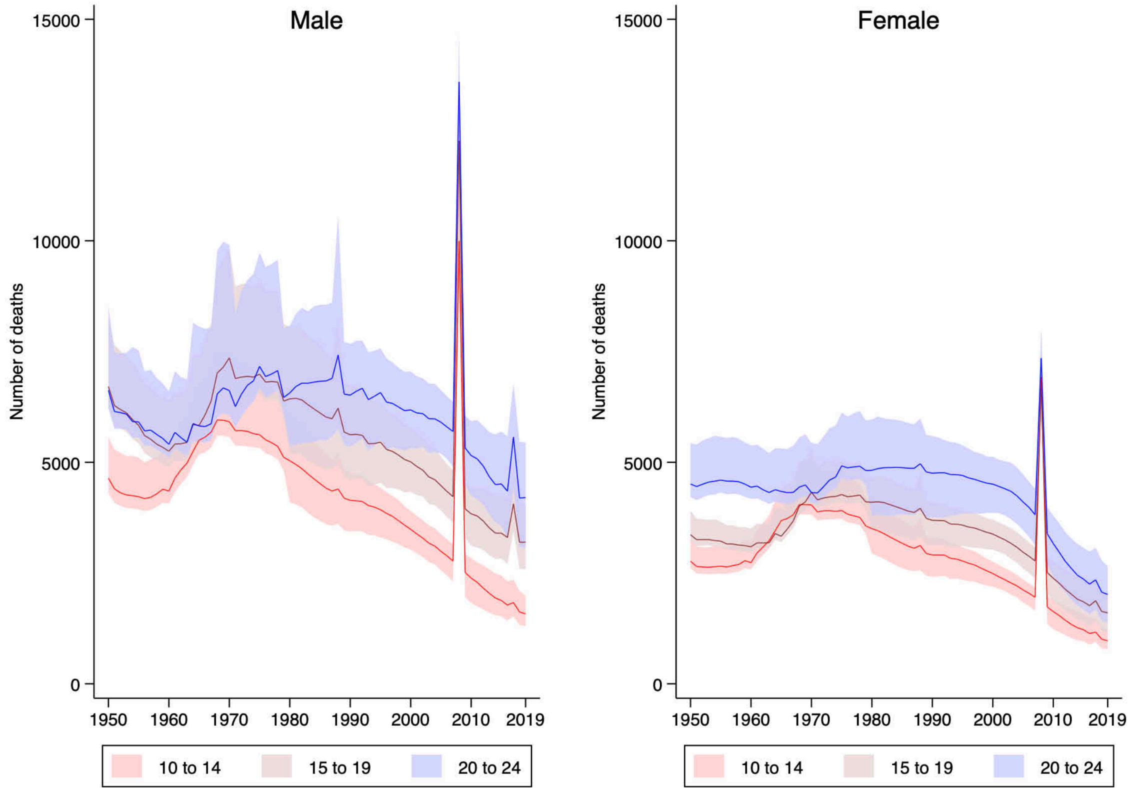

Figure S13c Mortality rate per 100,000 in 10-24 1980 – 2019 by cause: Myanmar

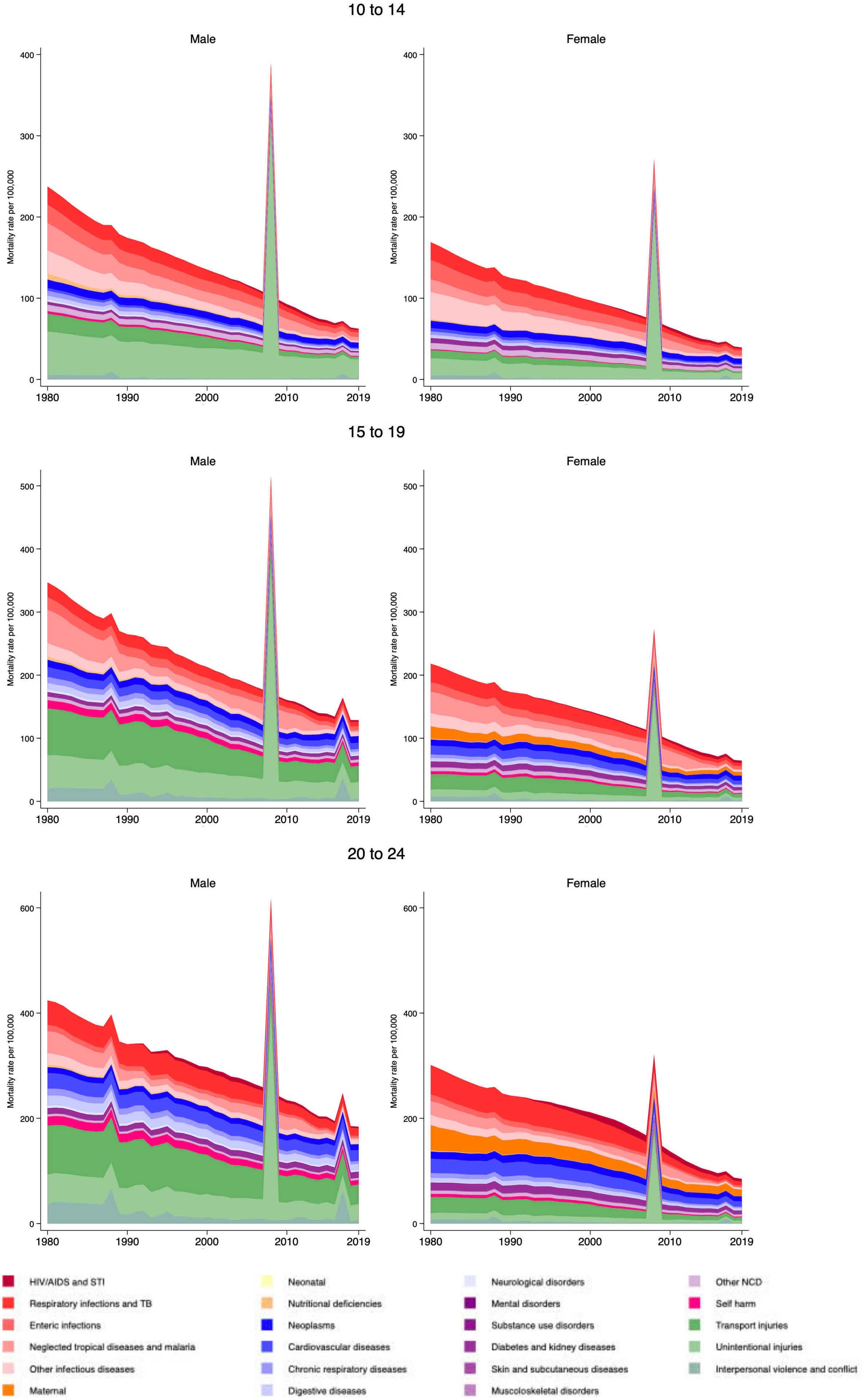

Figure S13d Number of deaths in 10-24 1980 – 2019 by cause: Myanmar

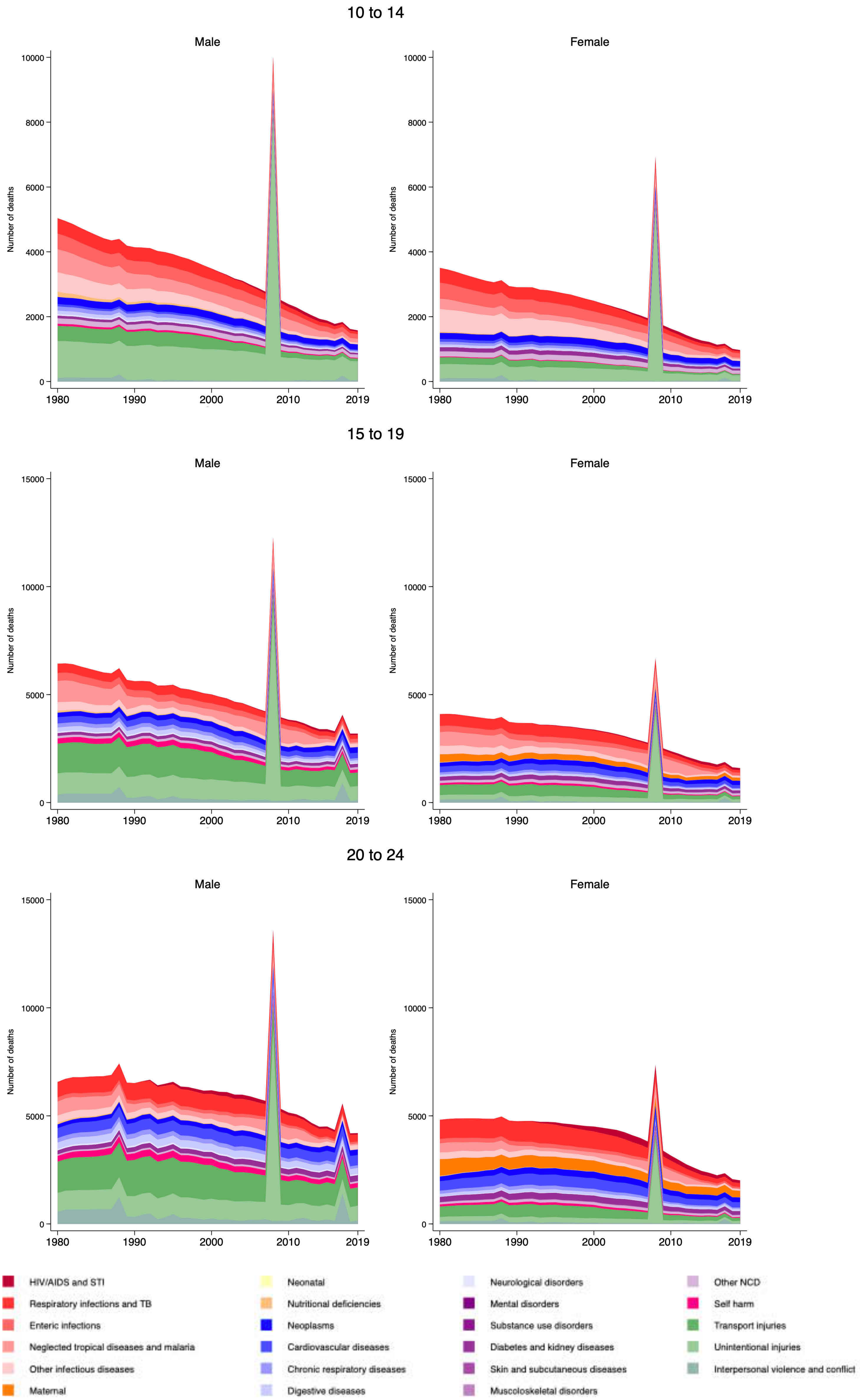

Figure S14: Philippines

Figure S14a All-cause mortality rate per 100,000 in 10-24 1950 – 2019: Philippines

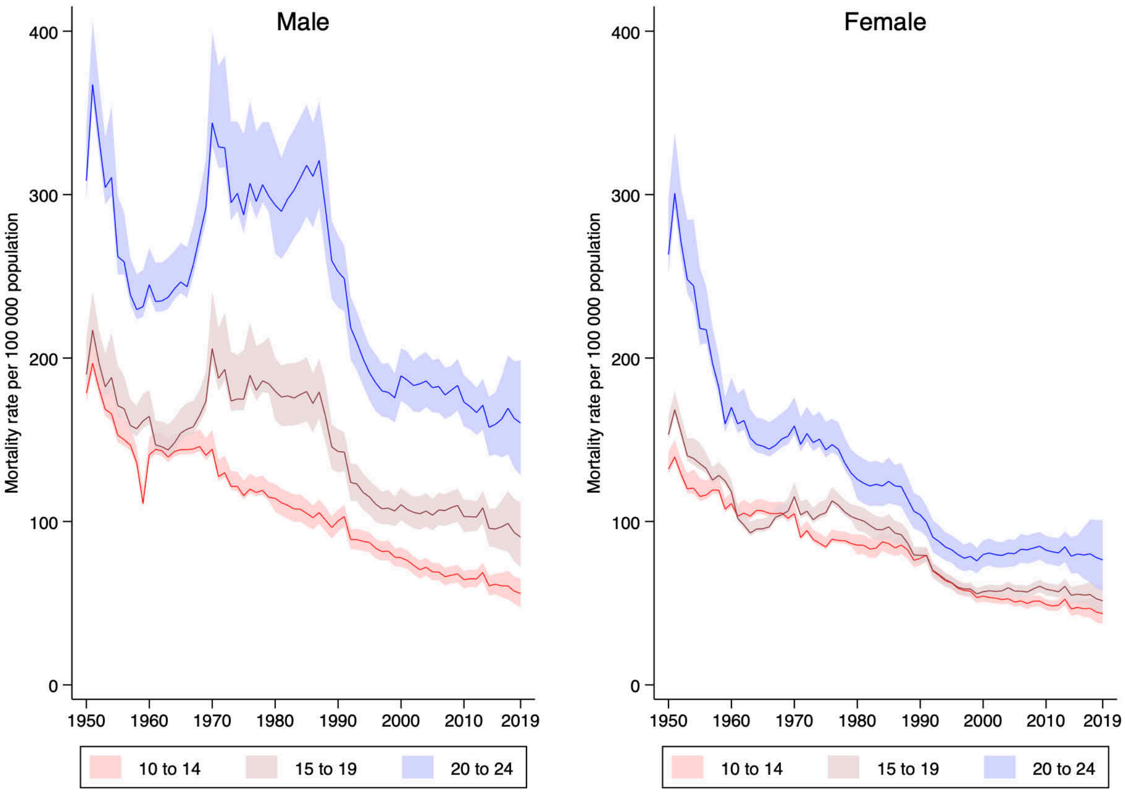

Figure S14b Number of deaths from all causes in 10-24 1950 – 2019: Philippines

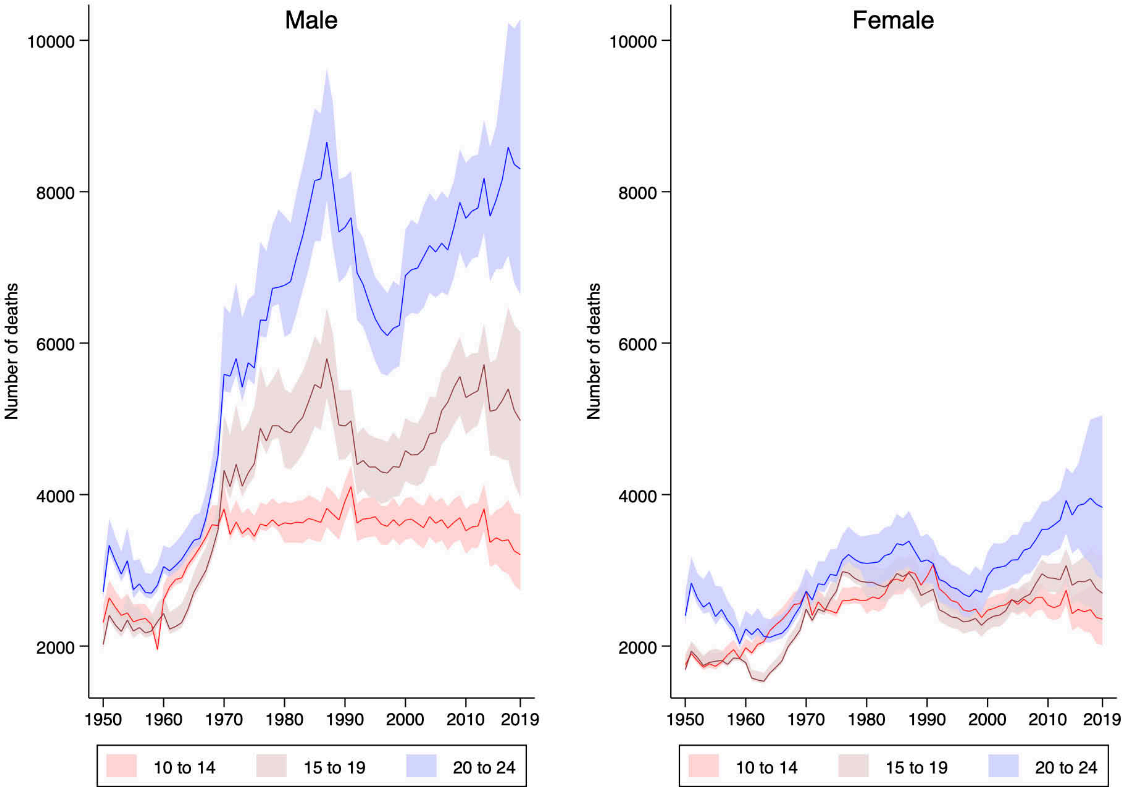

Figure S14c Mortality rate per 100,000 in 10-24 1980 – 2019 by cause: Philippines

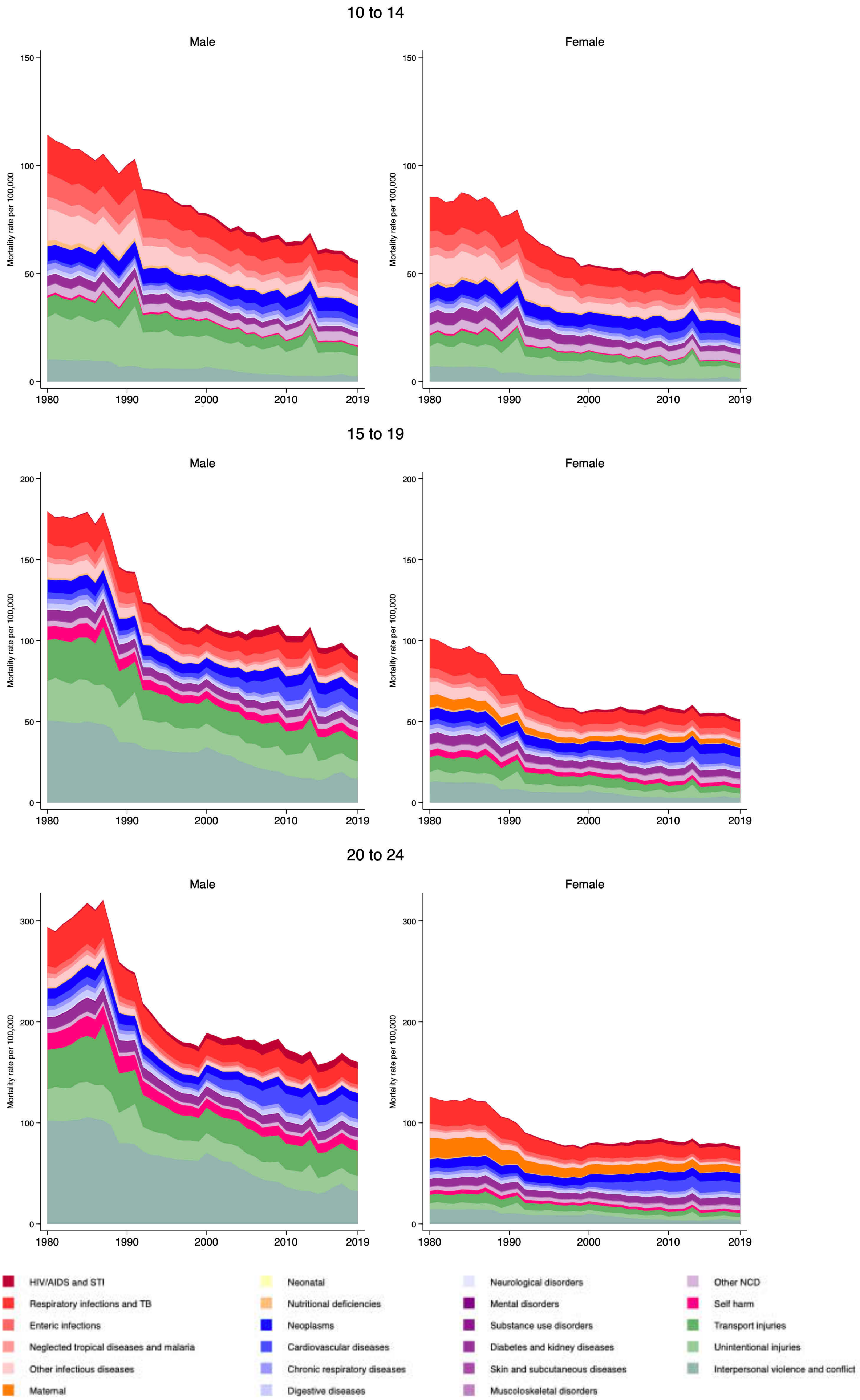

Figure S14d Number of deaths in 10-24 1980 – 2019 by cause: Philippines

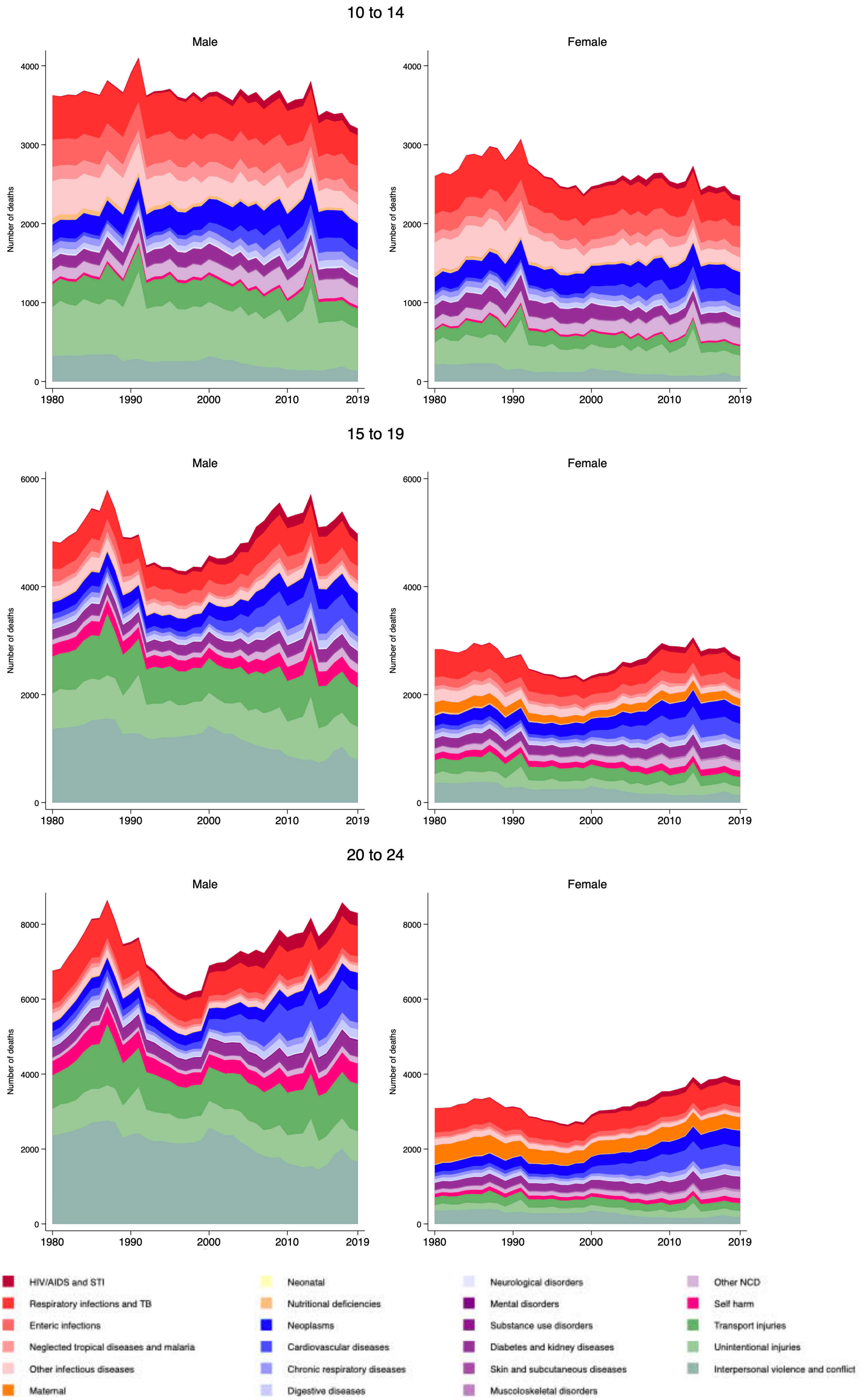

Figure S15: Sri Lanka

Figure S15a All-cause mortality rate per 100,000 in 10-24 1950 – 2019: Sri Lanka

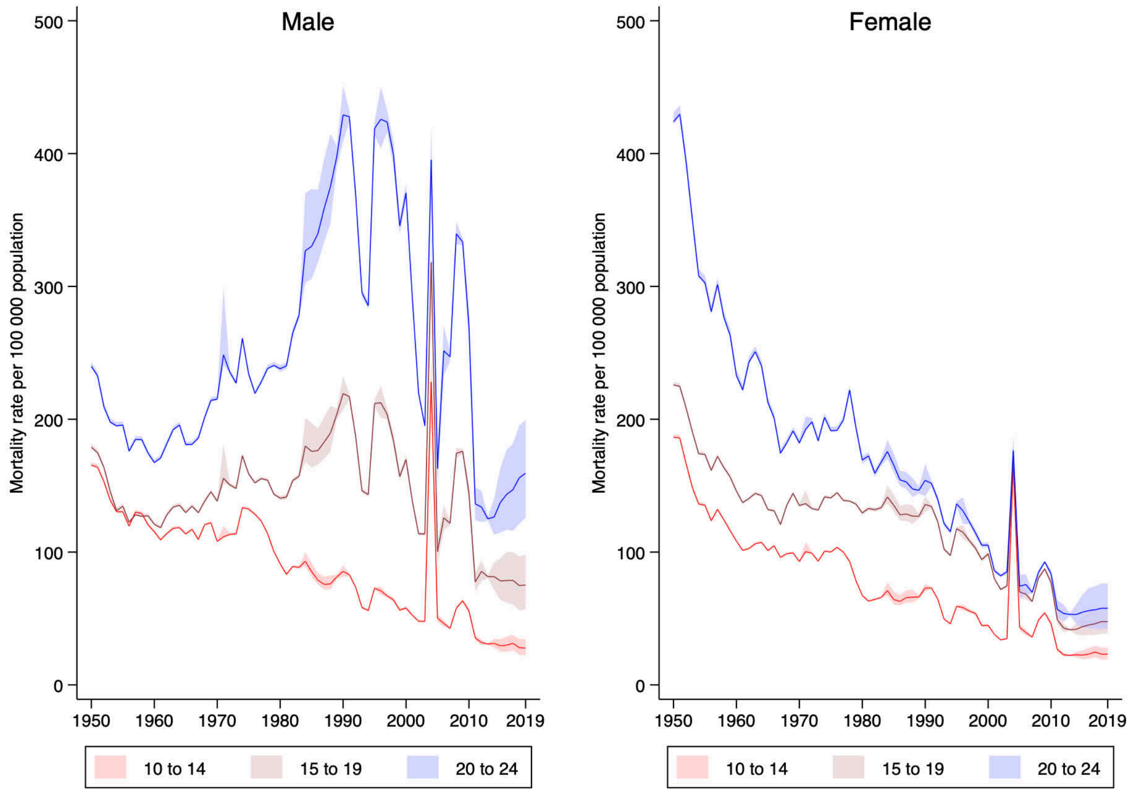

Figure S15b Number of deaths from all causes in 10-24 1950 – 2019: Sri Lanka

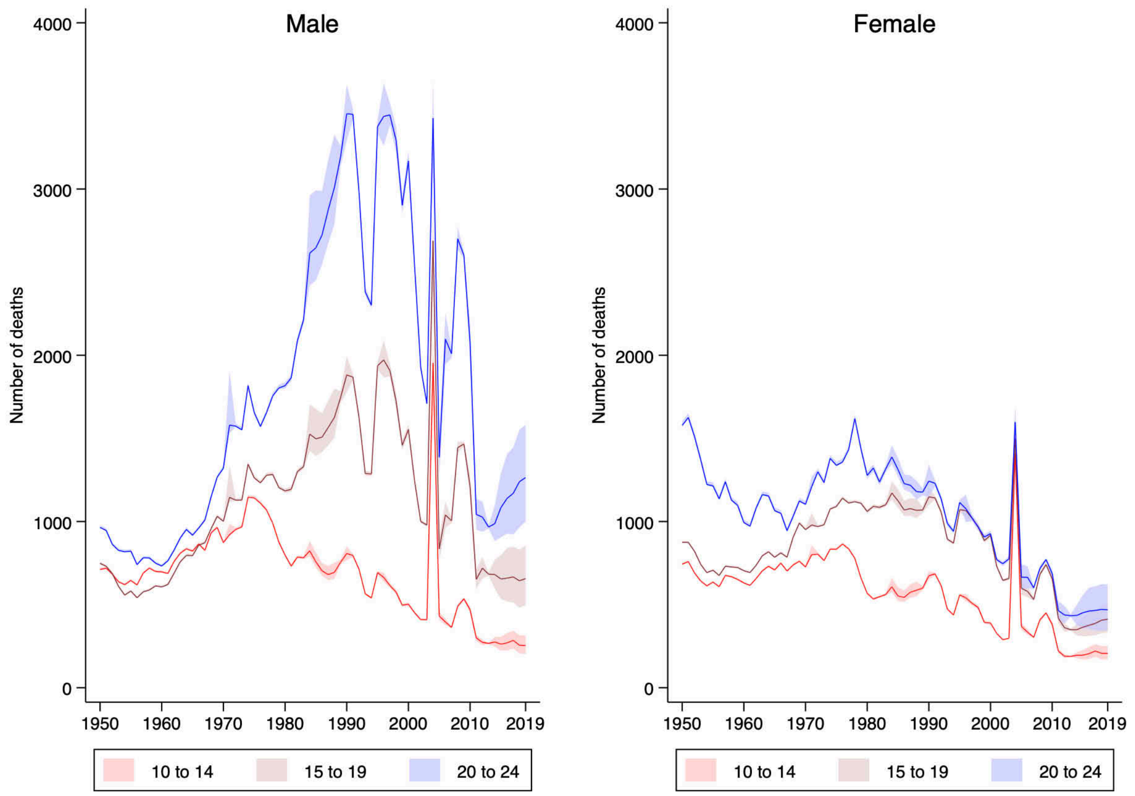

Figure S15c Mortality rate per 100,000 in 10-24 1980 – 2019 by cause: Sri Lanka

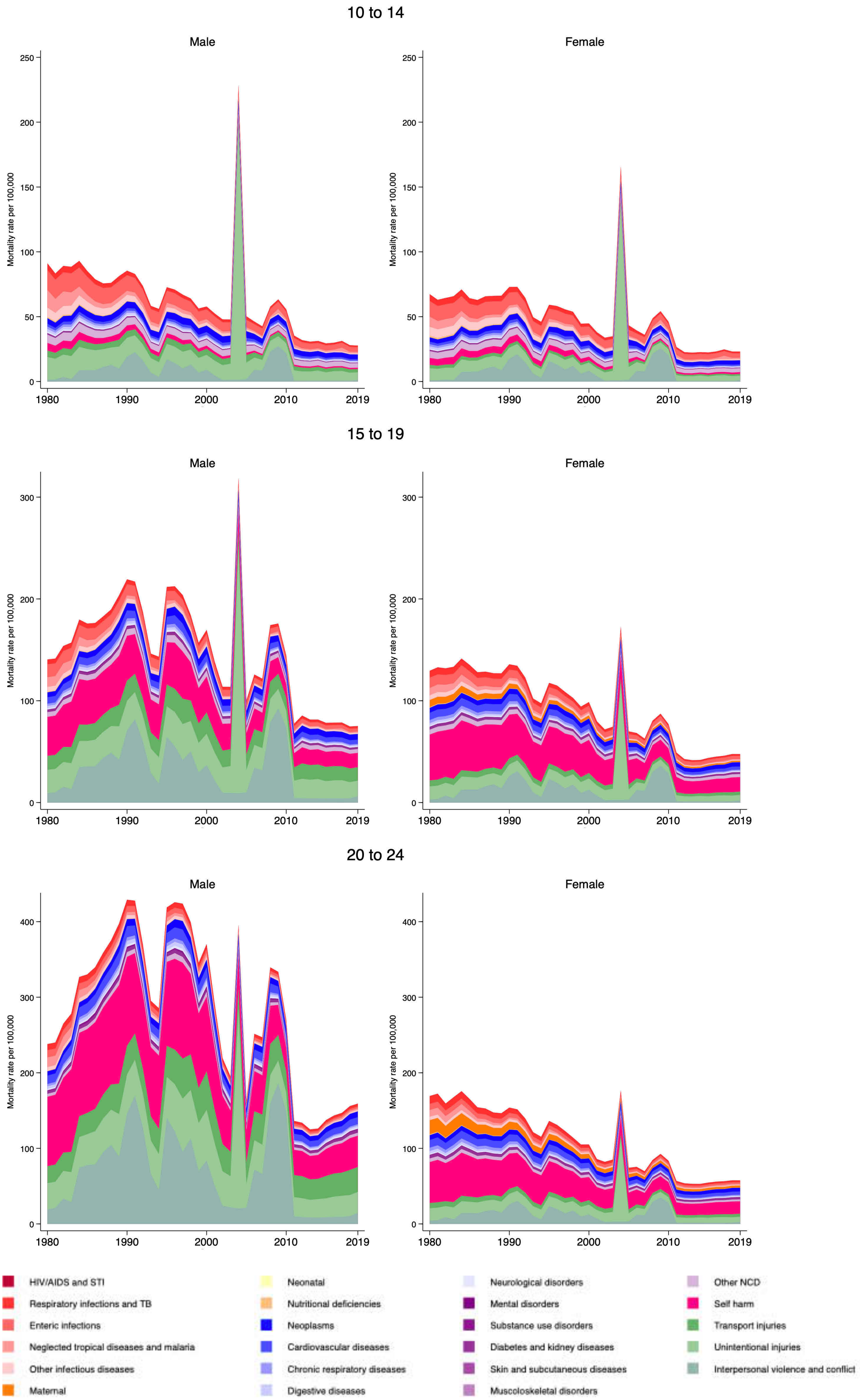

Figure S15d Number of deaths in 10-24 1980 – 2019 by cause: Sri Lanka

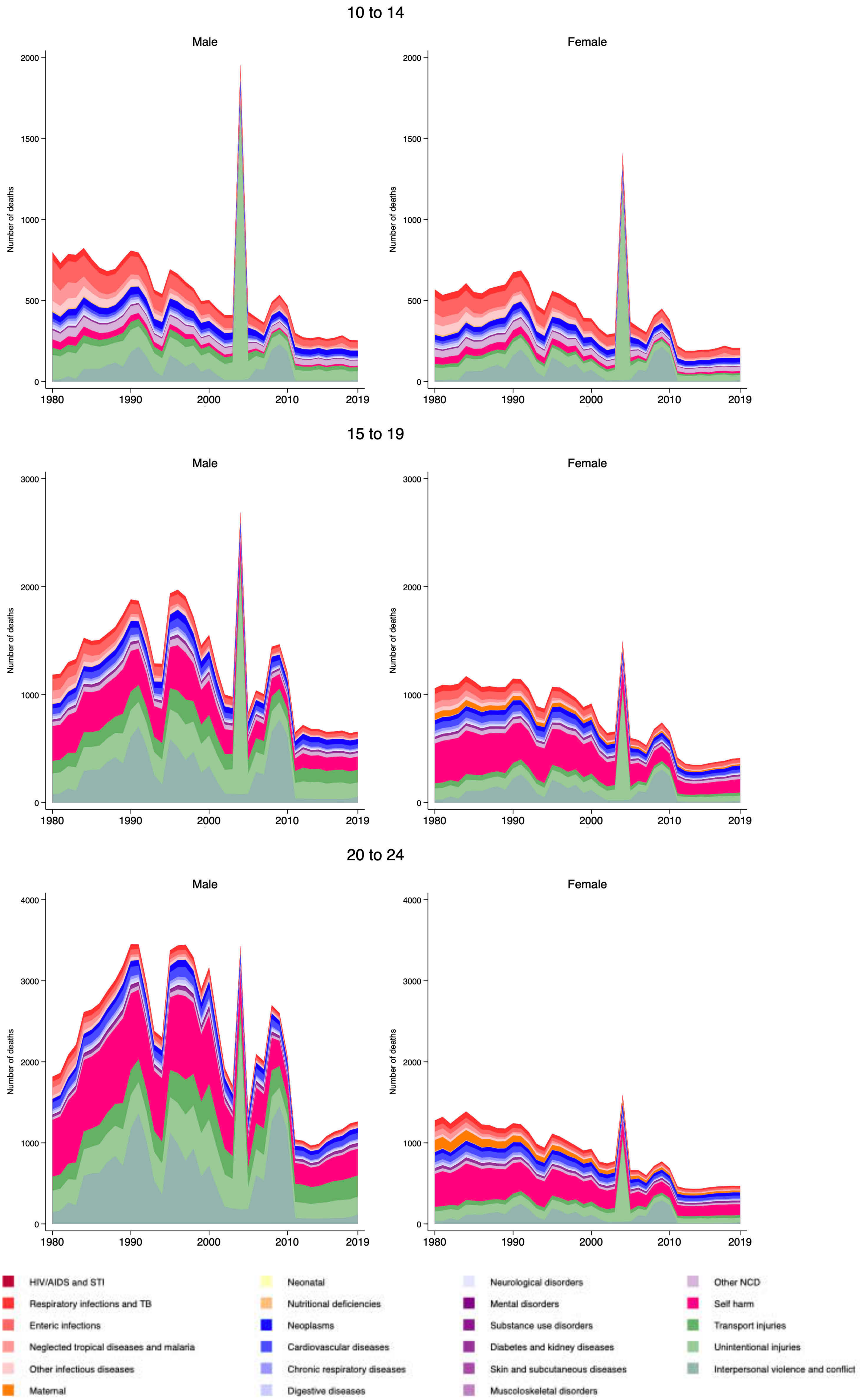

Figure S16: Thailand

Figure S16a All-cause mortality rate per 100,000 in 10-24 1950 – 2019: Thailand

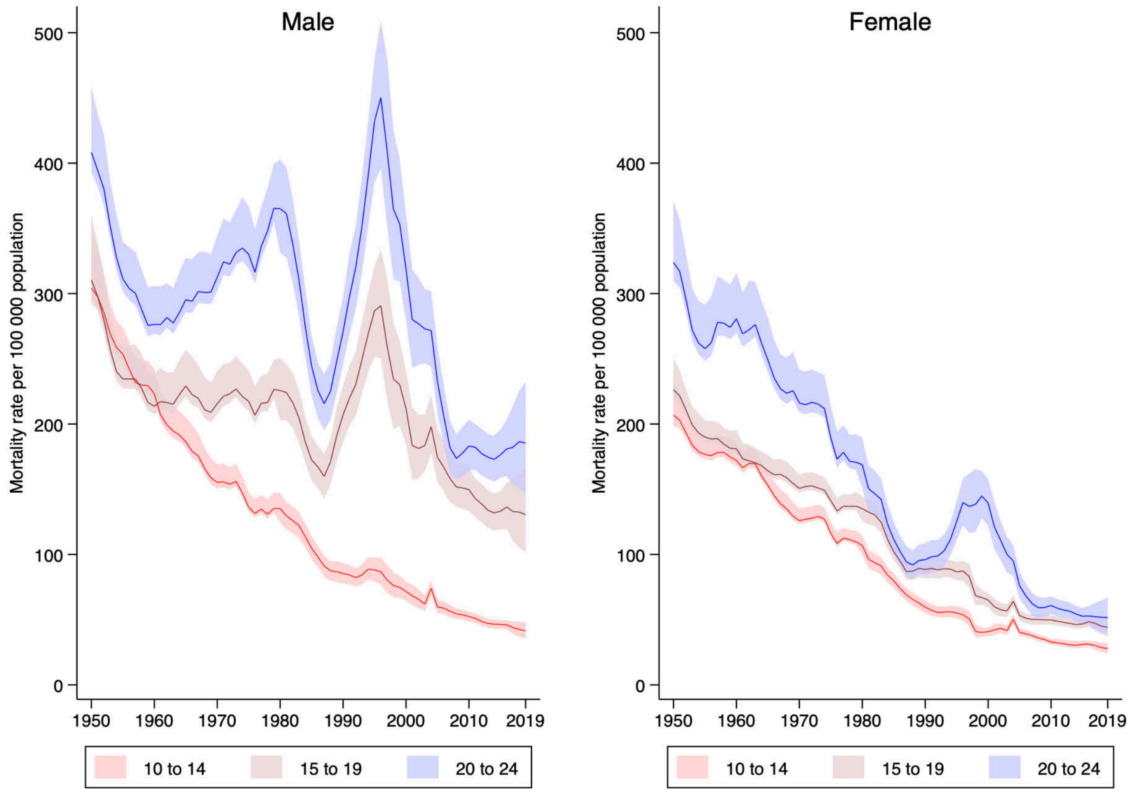

Figure S16b Number of deaths from all causes in 10-24 1950 – 2019: Thailand

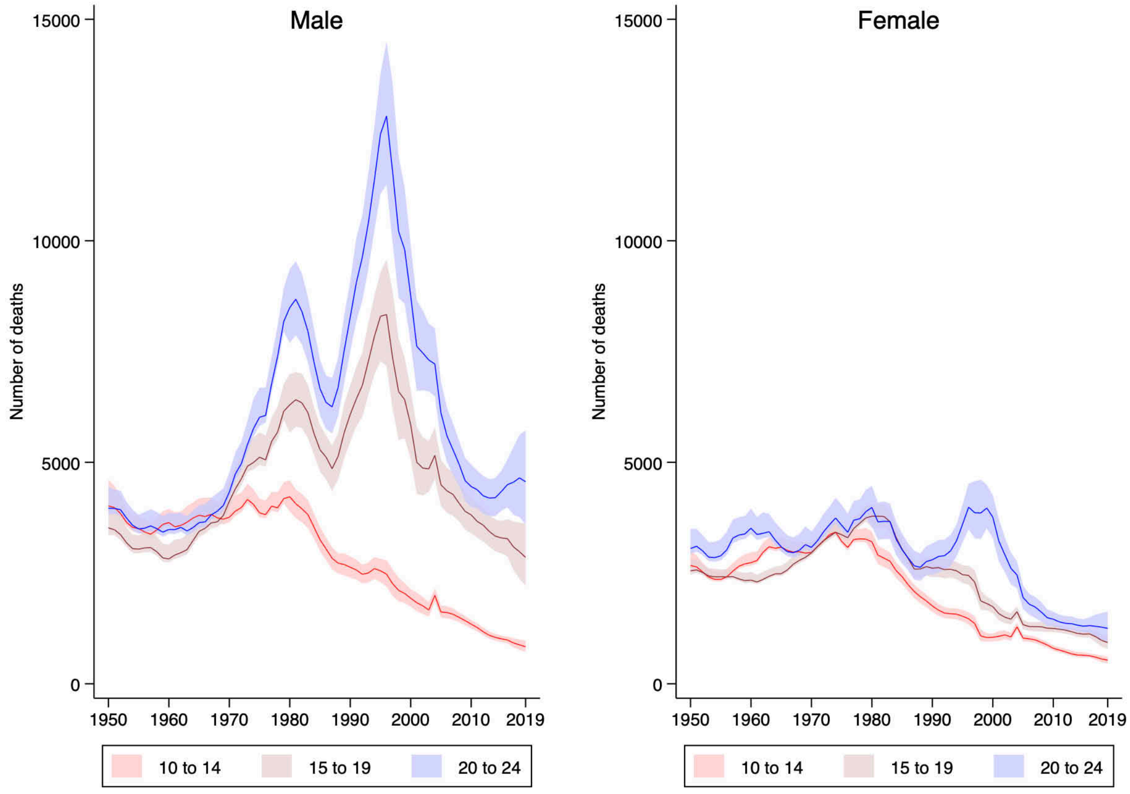

Figure S16c Mortality rate per 100,000 in 10-24 1980 – 2019 by cause: Thailand

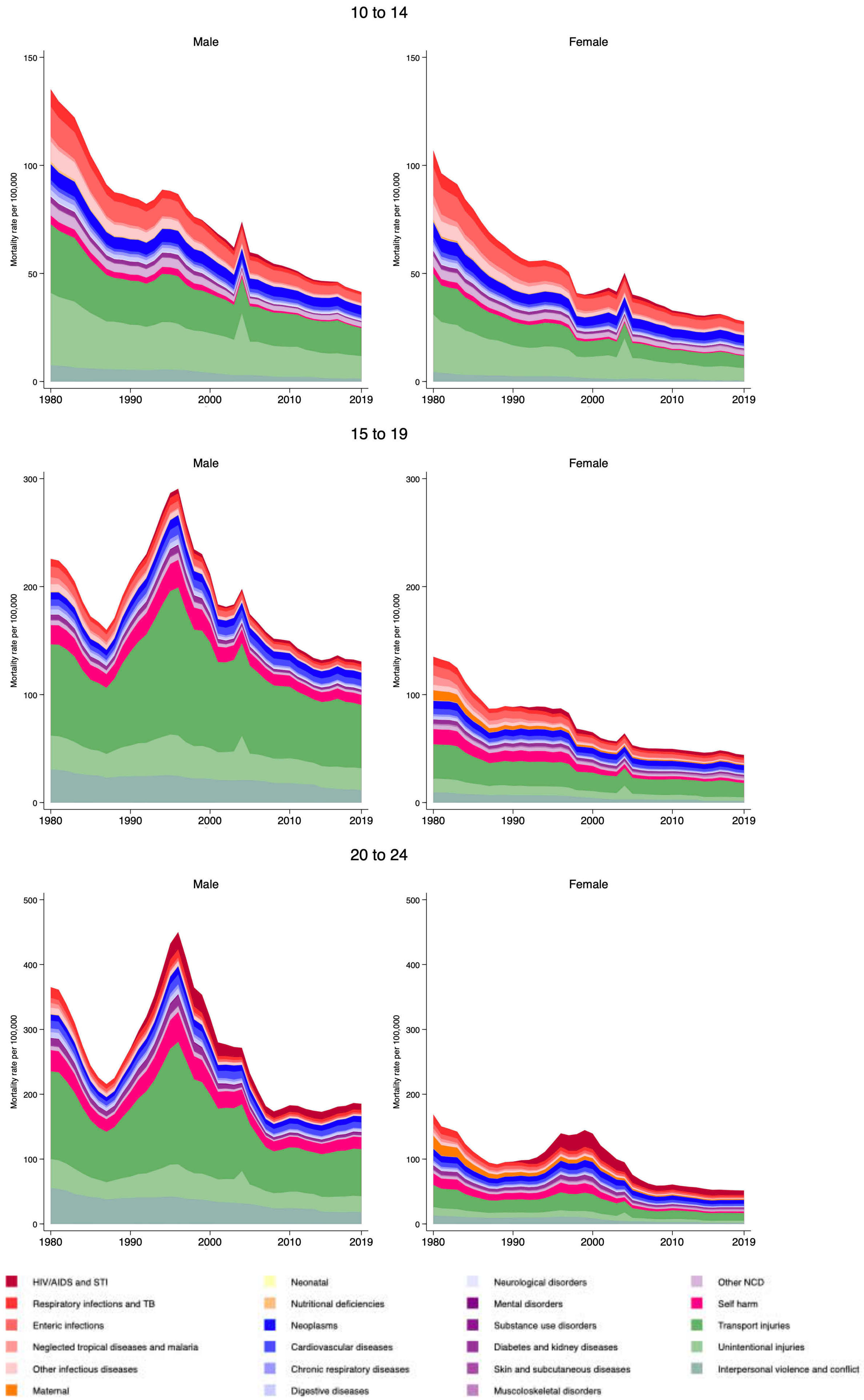

Figure S16d Number of deaths in 10-24 1980 – 2019 by cause: Thailand

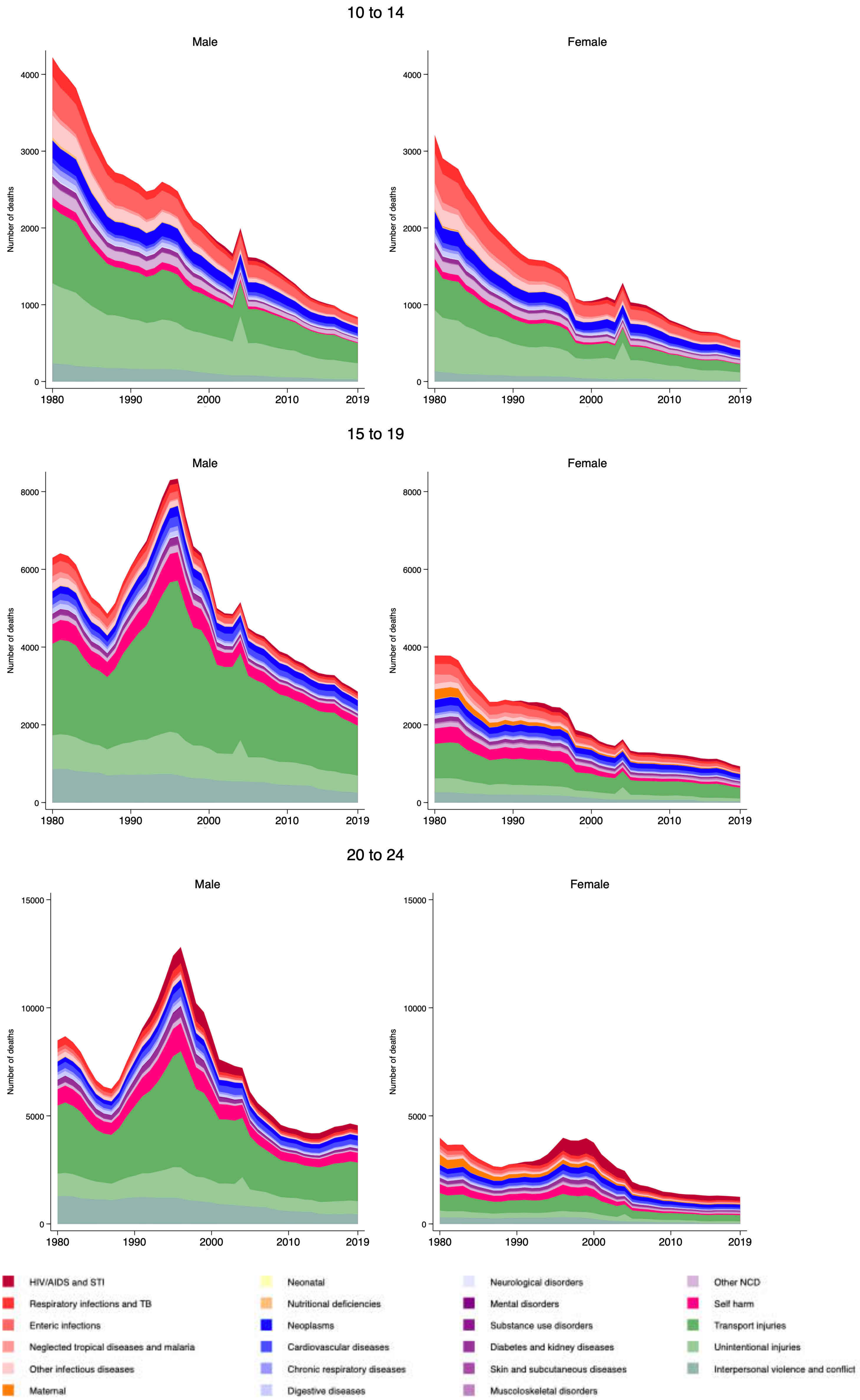

Figure S17: Timor-Leste

Figure S17a All-cause mortality rate per 100,000 in 10-24 1950 – 2019: Timor-Leste

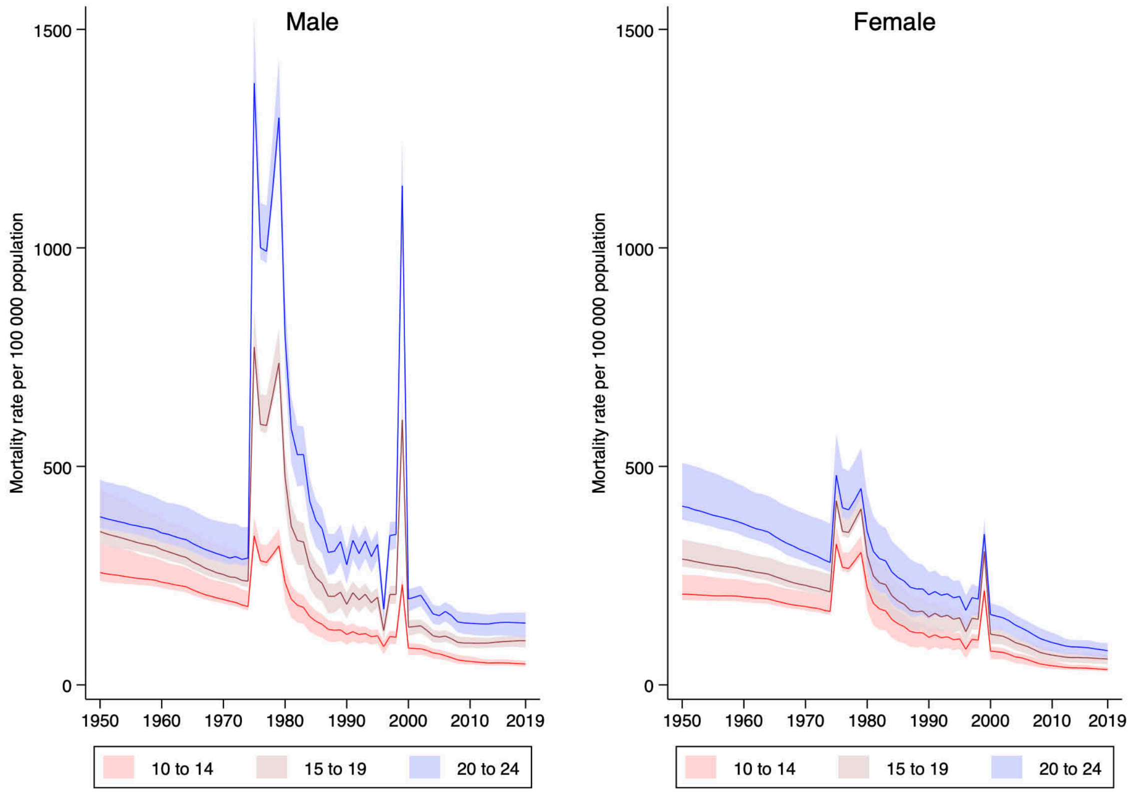

Figure S17b Number of deaths from all causes in 10-24 1950 – 2019: Timor-Leste

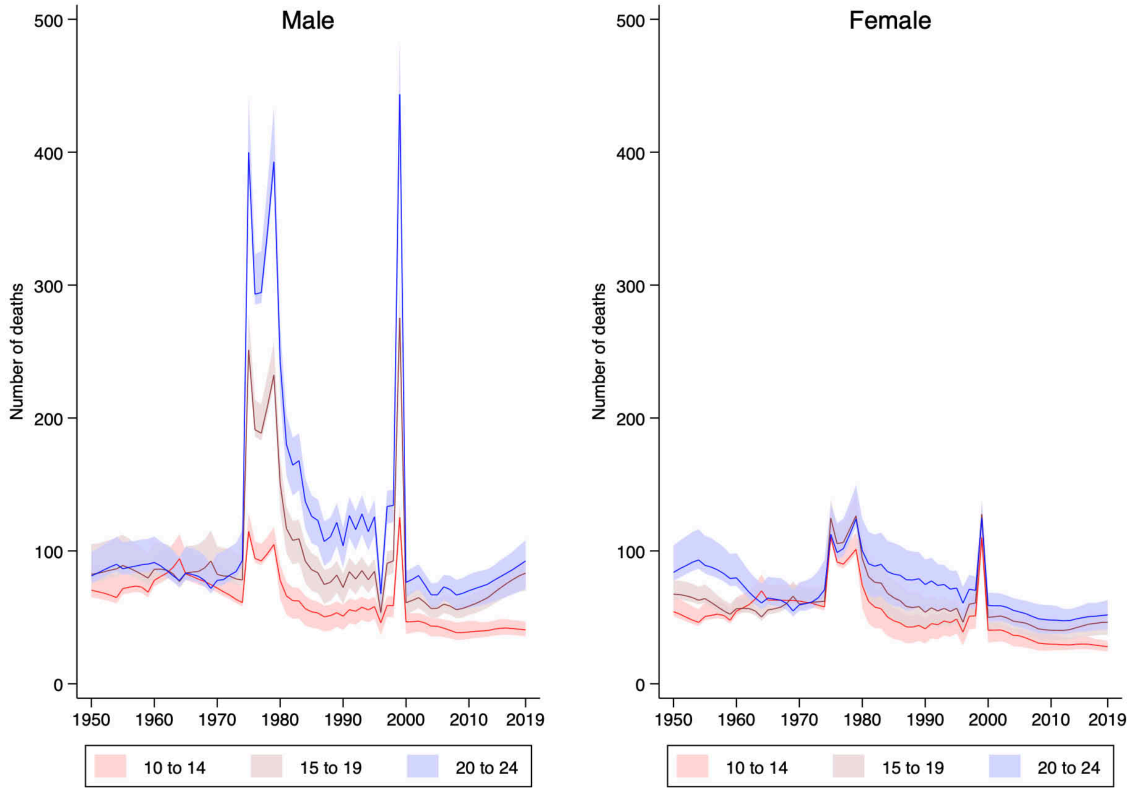

Figure S17c Mortality rate per 100,000 in 10-24 1980 – 2019 by cause: Timor-Leste

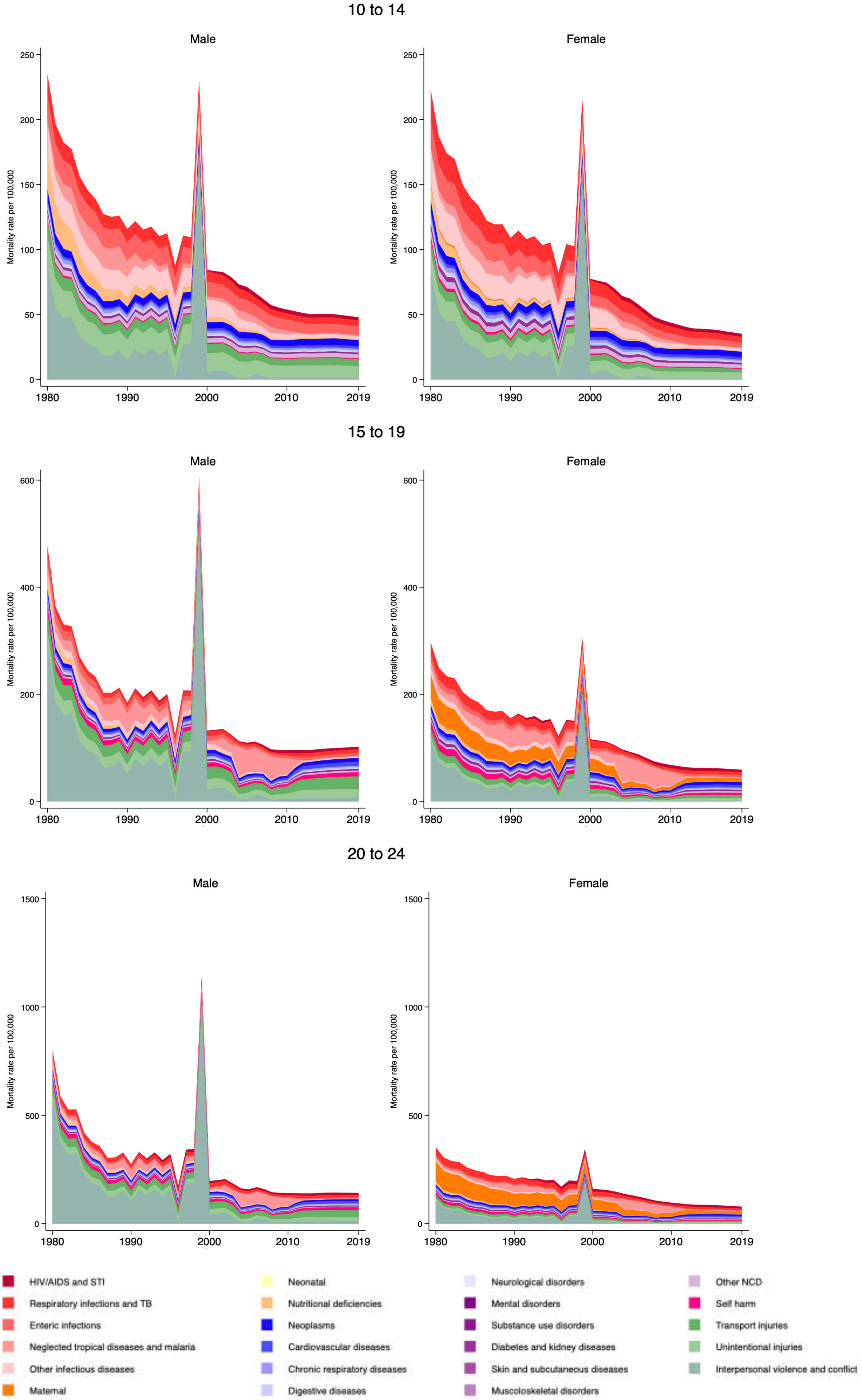

Figure S17d Number of deaths in 10-24 1980 – 2019 by cause: Timor-Leste

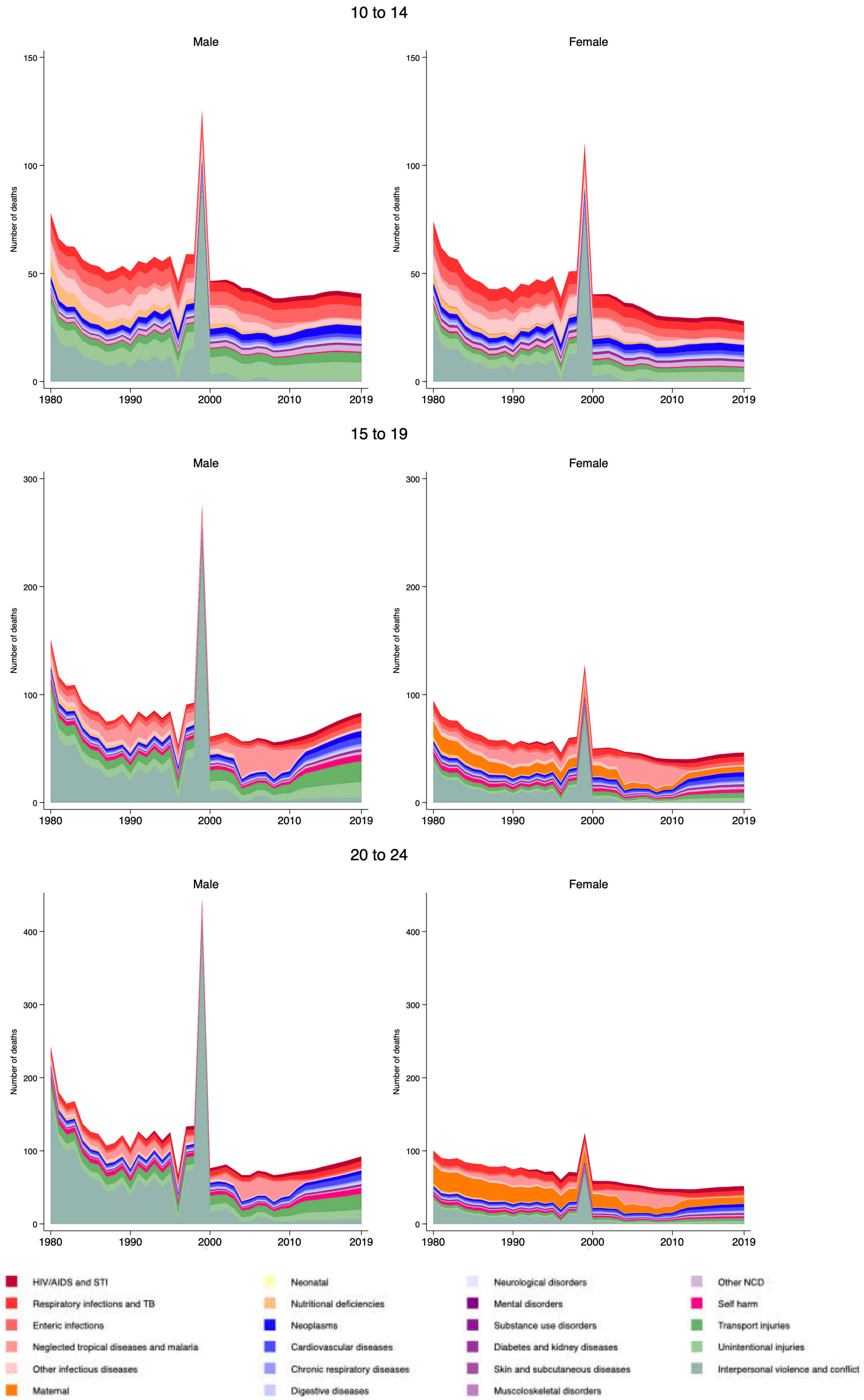

Figure S18: Viet Nam

Figure S18a All-cause mortality rate per 100,000 in 10-24 1950 – 2019: Viet Nam

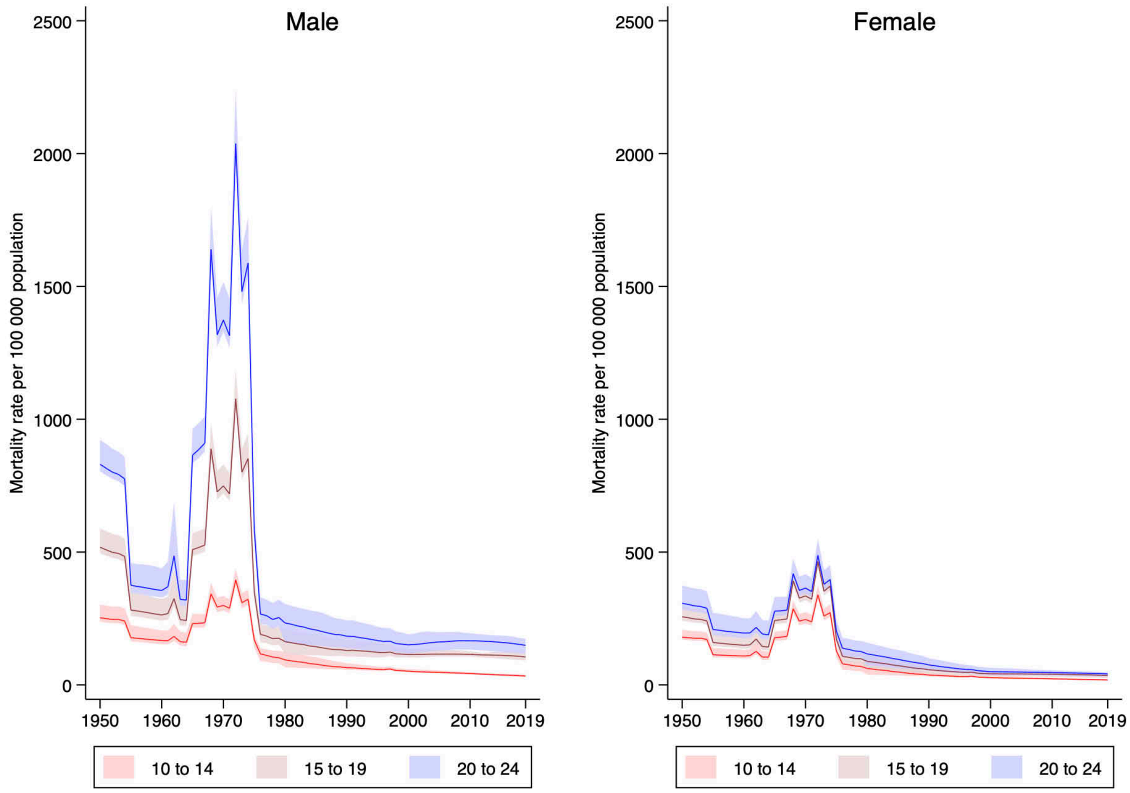

Figure S18b Number of deaths from all causes in 10-24 1950 – 2019: Viet Nam

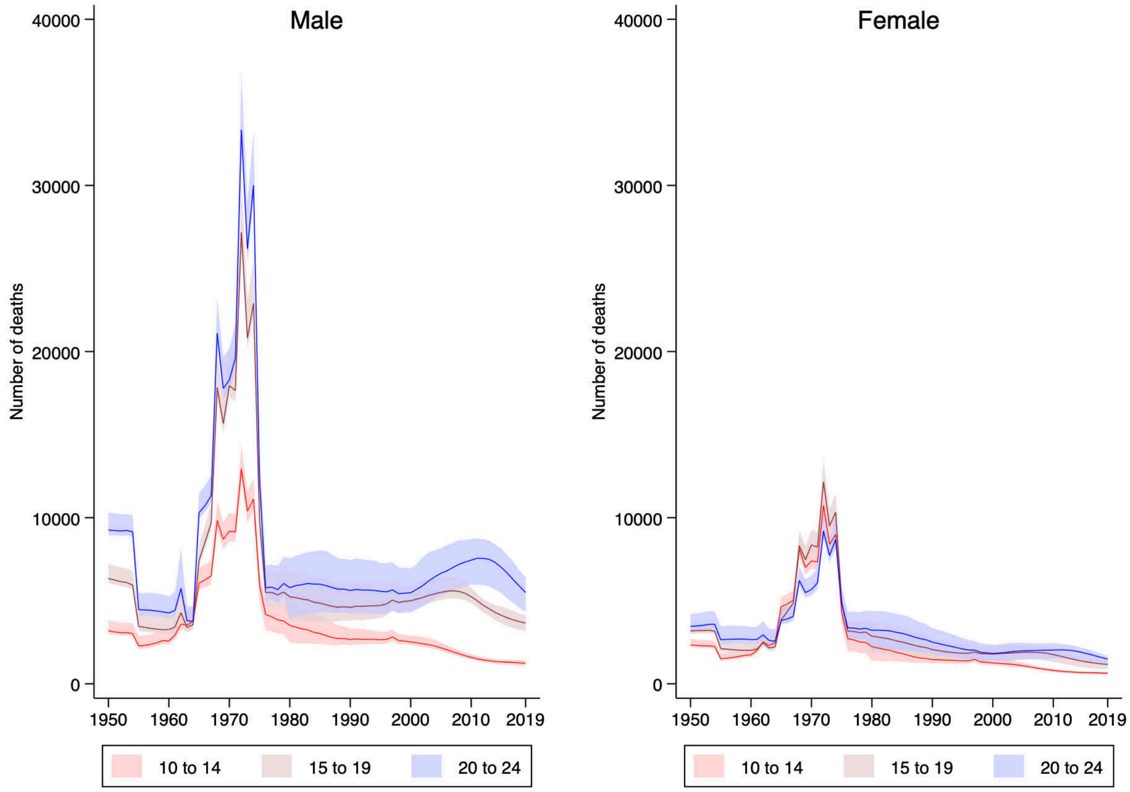

Figure S18c Mortality rate per 100,000 in 10-24 1980 – 2019 by cause: Viet Nam

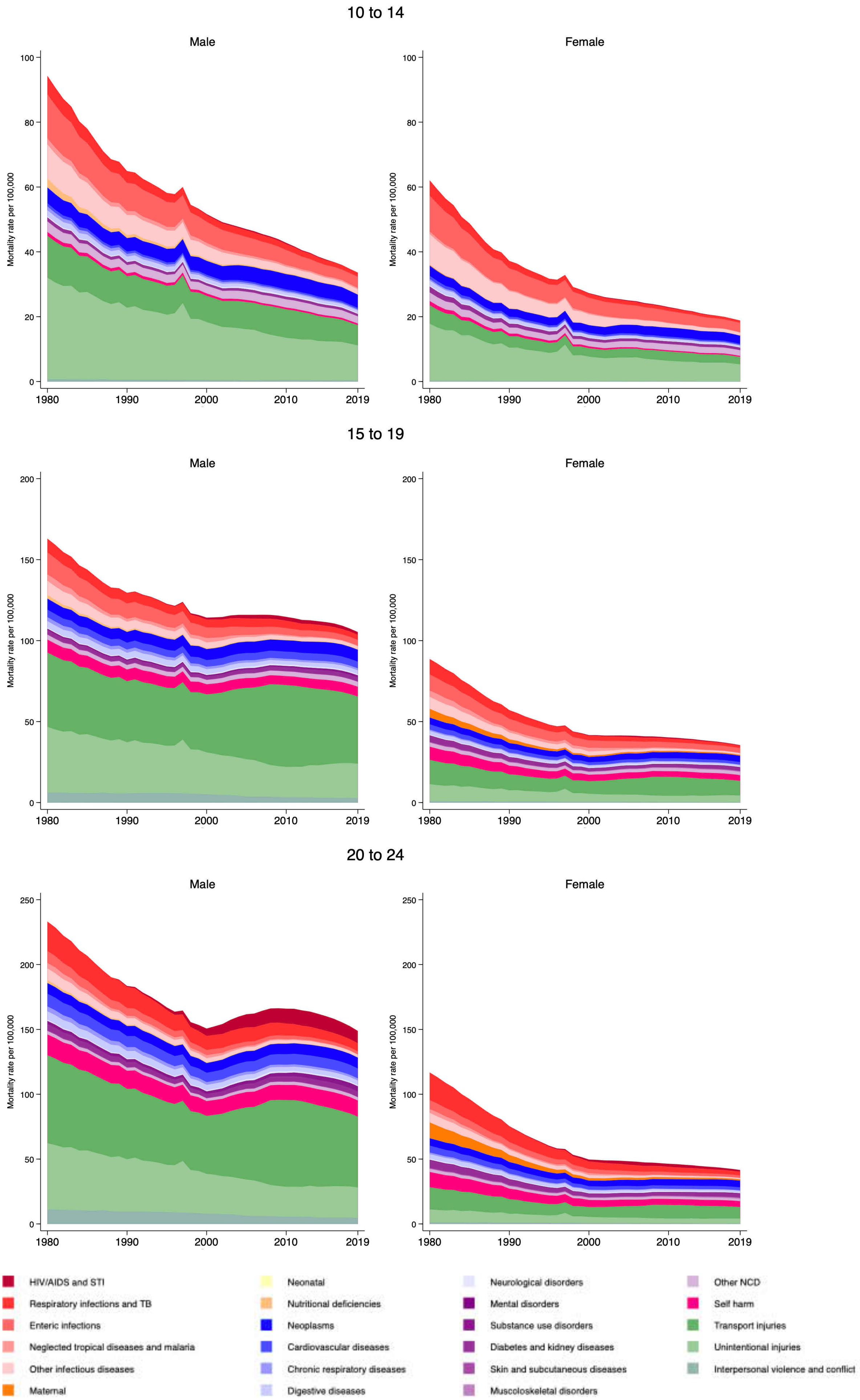

Figure S18d Number of deaths in 10-24 1980 – 2019 by cause: Viet Nam

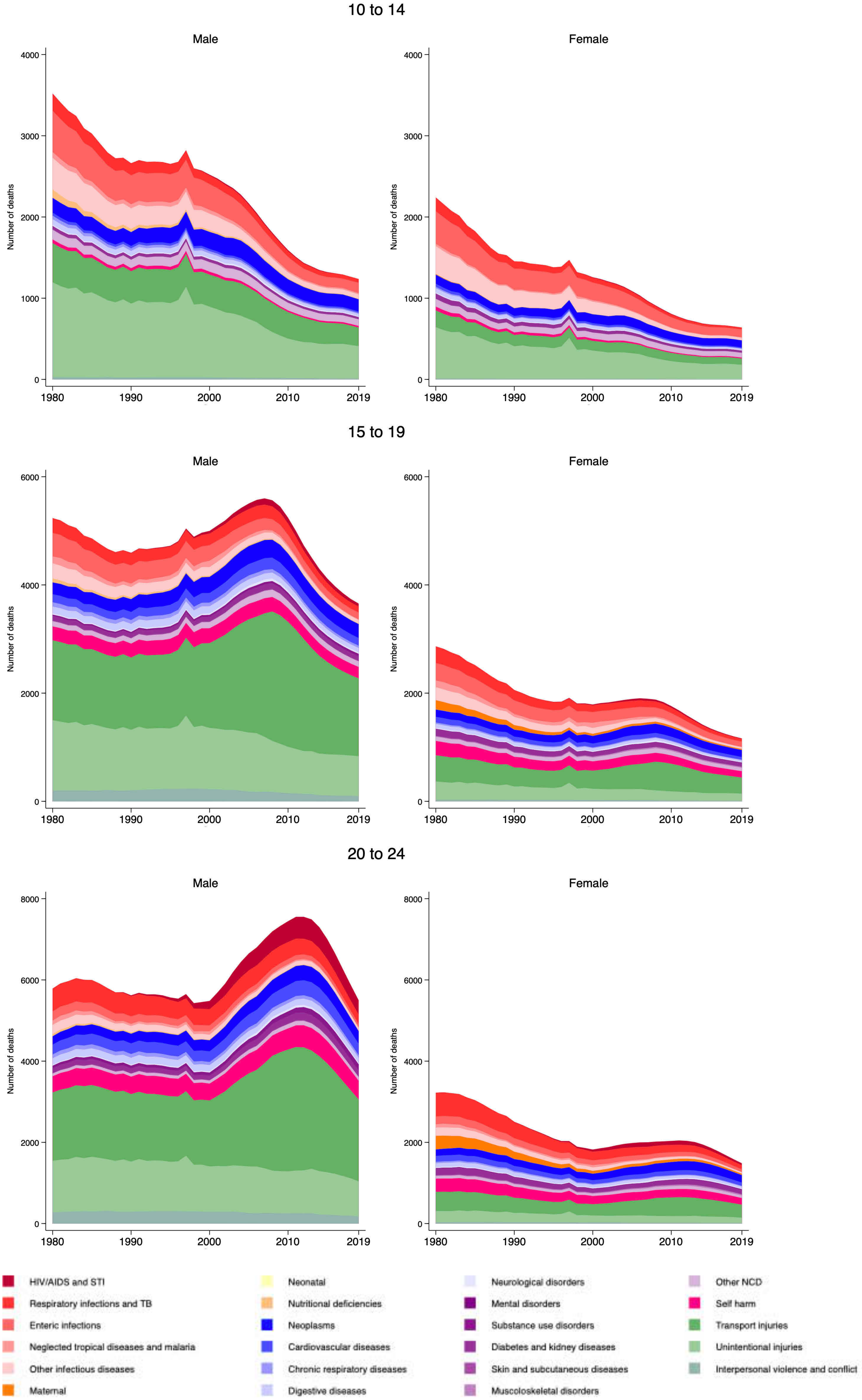

Figure S19: Oceania

Figure S19a All-cause mortality rate per 100,000 in 10-24 1950 – 2019: Oceania

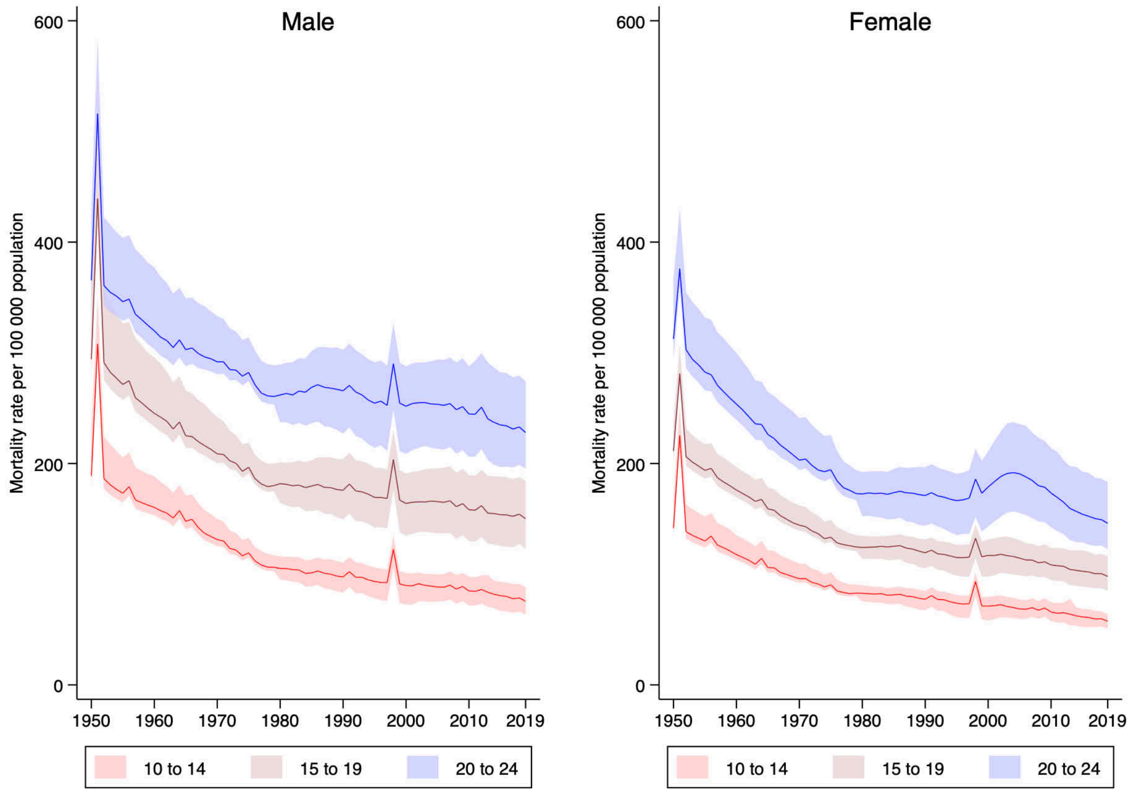

Figure S19b Number of deaths from all causes in 10-24 1950 – 2019: Oceania

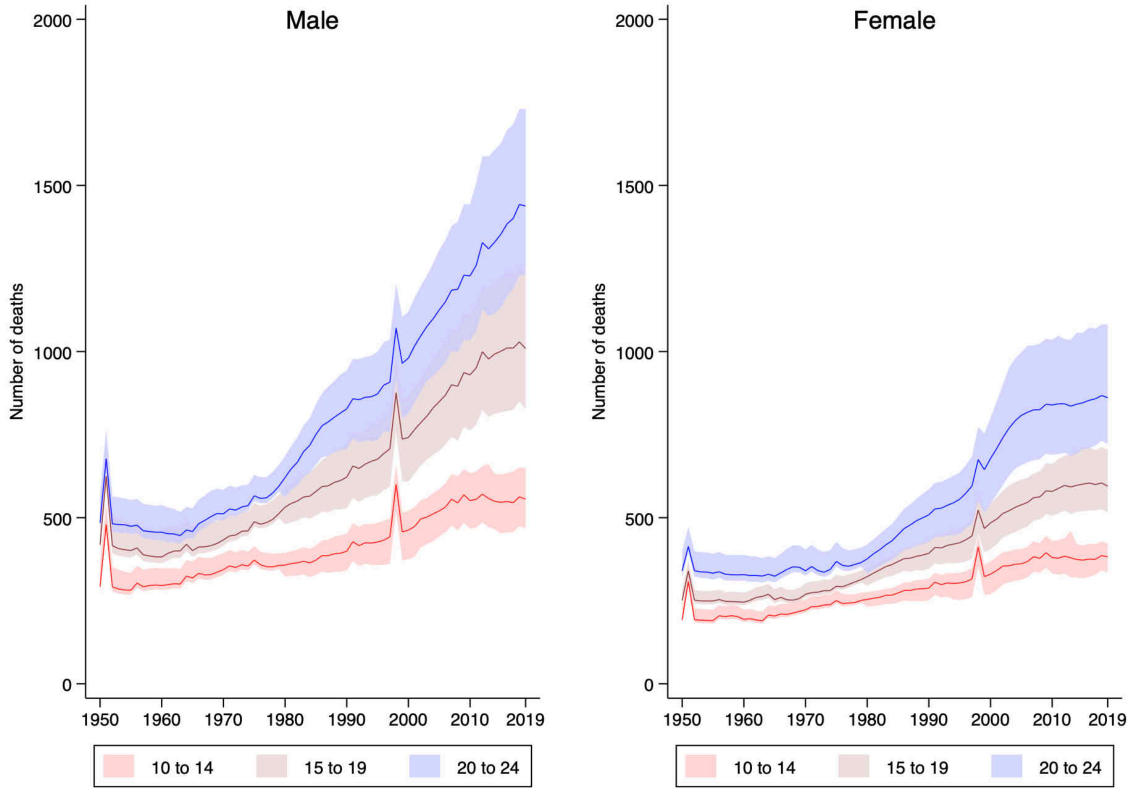

Figure S19c Mortality rate per 100,000 in 10-24 1980 – 2019 by cause: Oceania

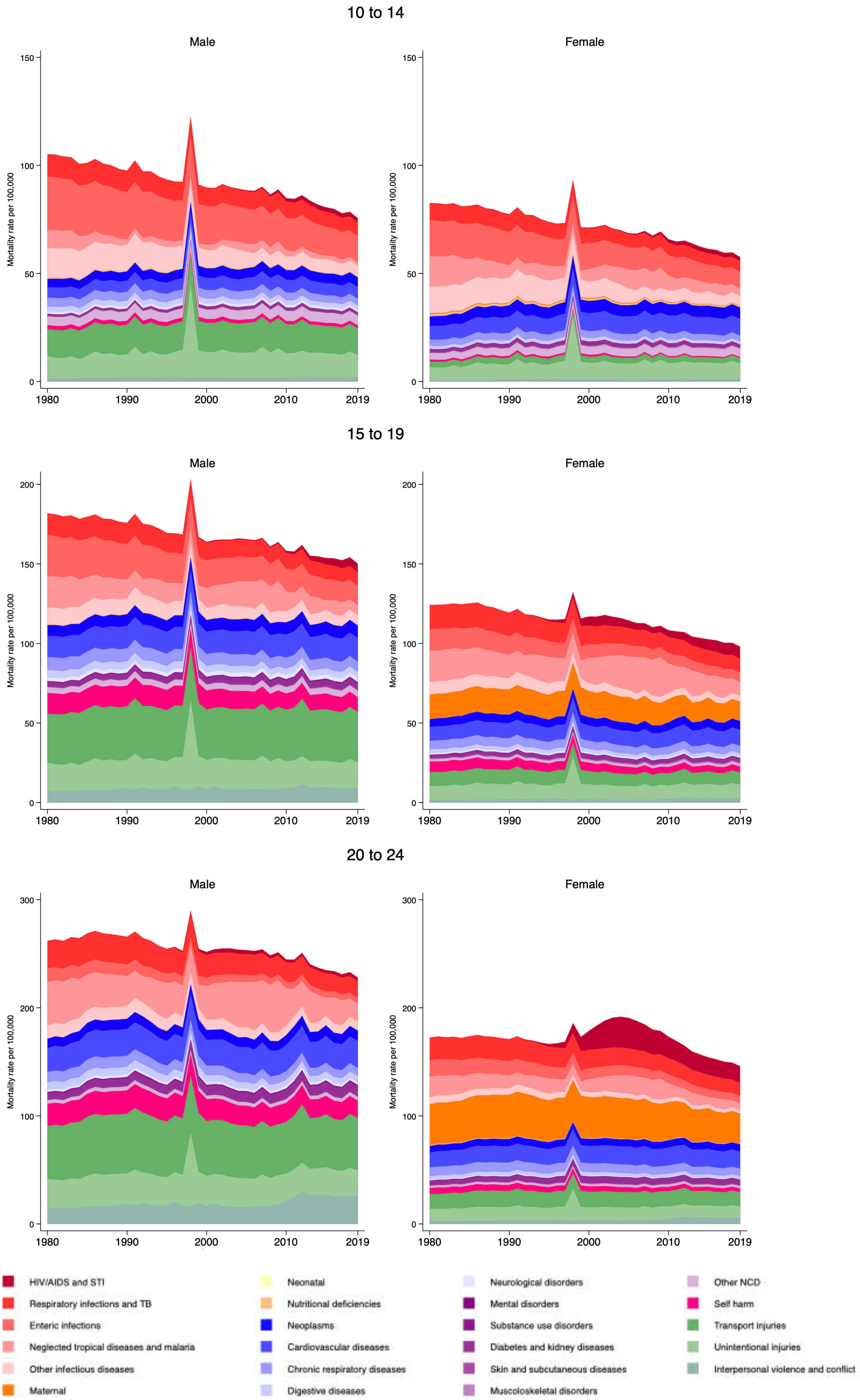

Figure S19d Number of deaths in 10-24 1980 – 2019 by cause: Oceania

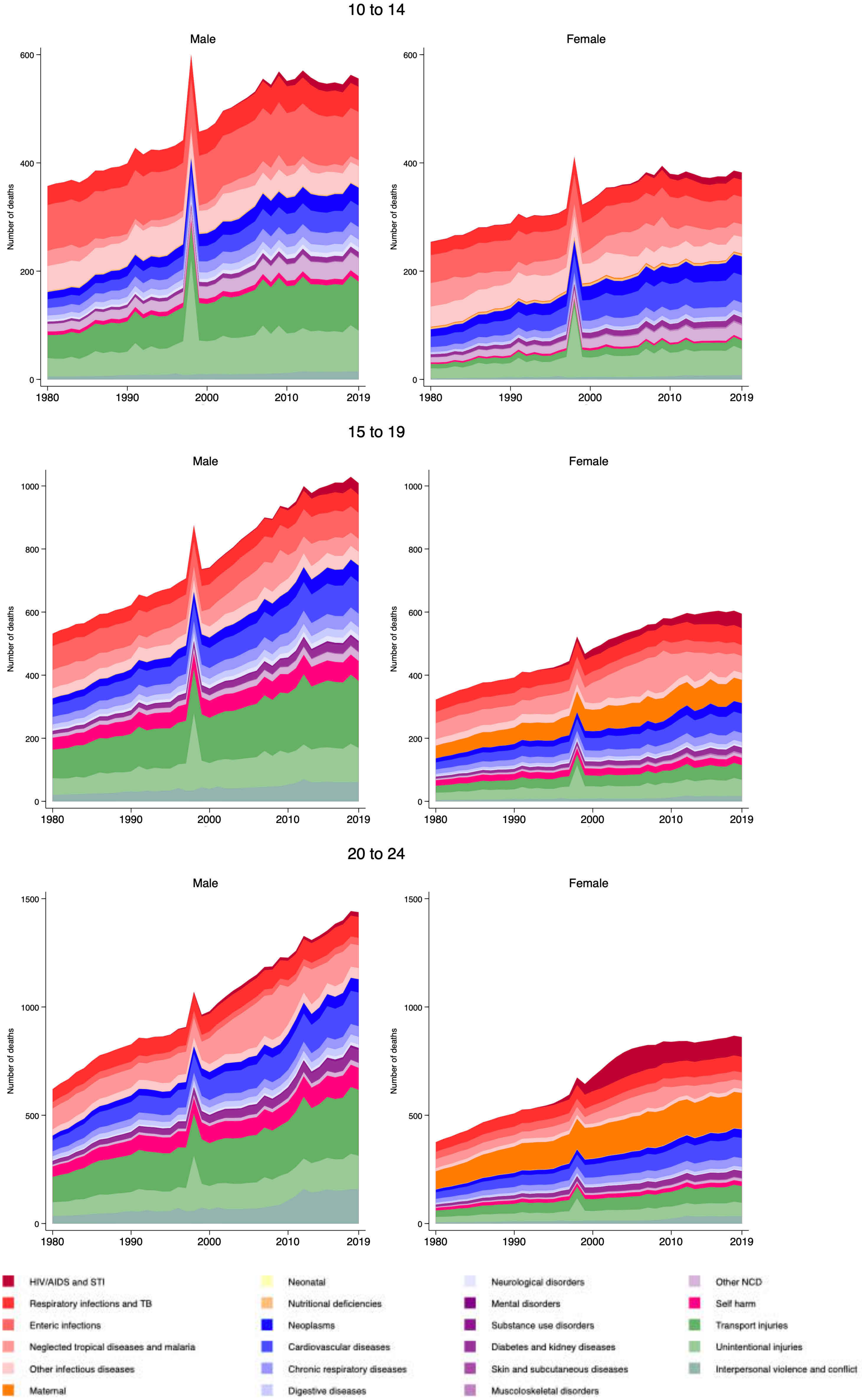

Figure S20: Fiji

Figure S20a All-cause mortality rate per 100,000 in 10-24 1950 – 2019: Fiji

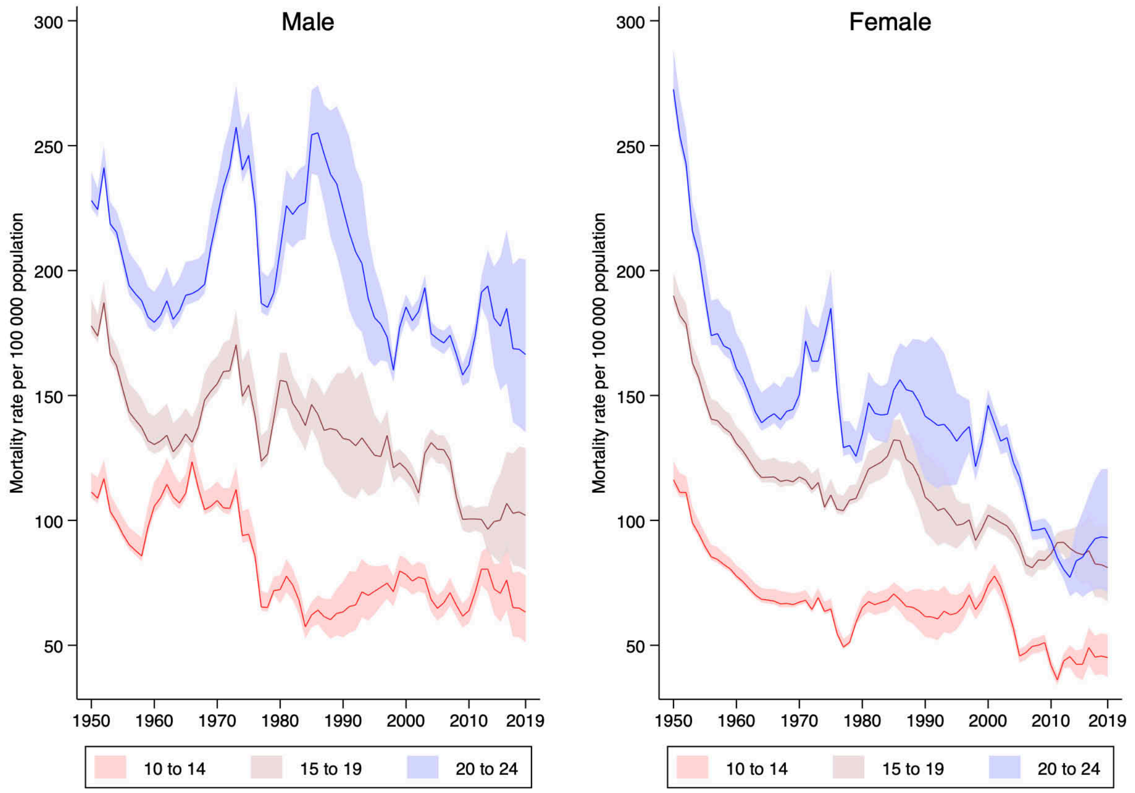

Figure S20b Number of deaths from all causes in 10-24 1950 – 2019: Fiji

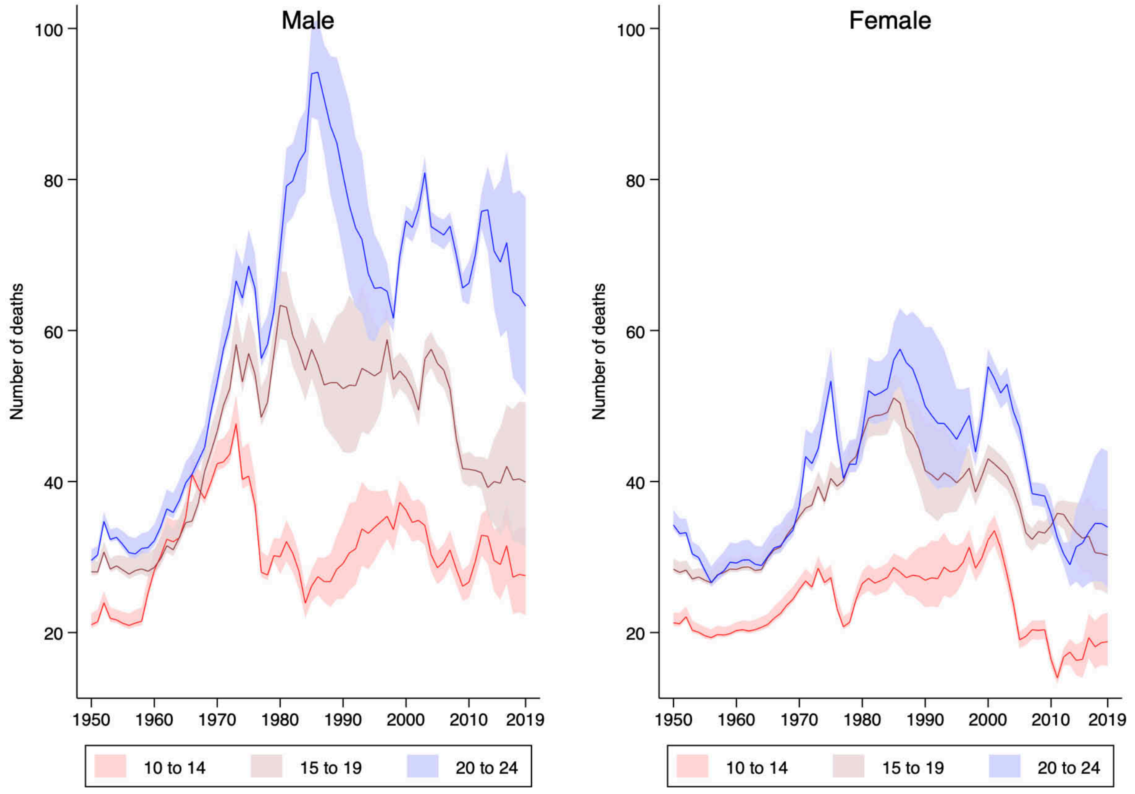

Figure S20c Mortality rate per 100,000 in 10-24 1980 – 2019 by cause: Fiji

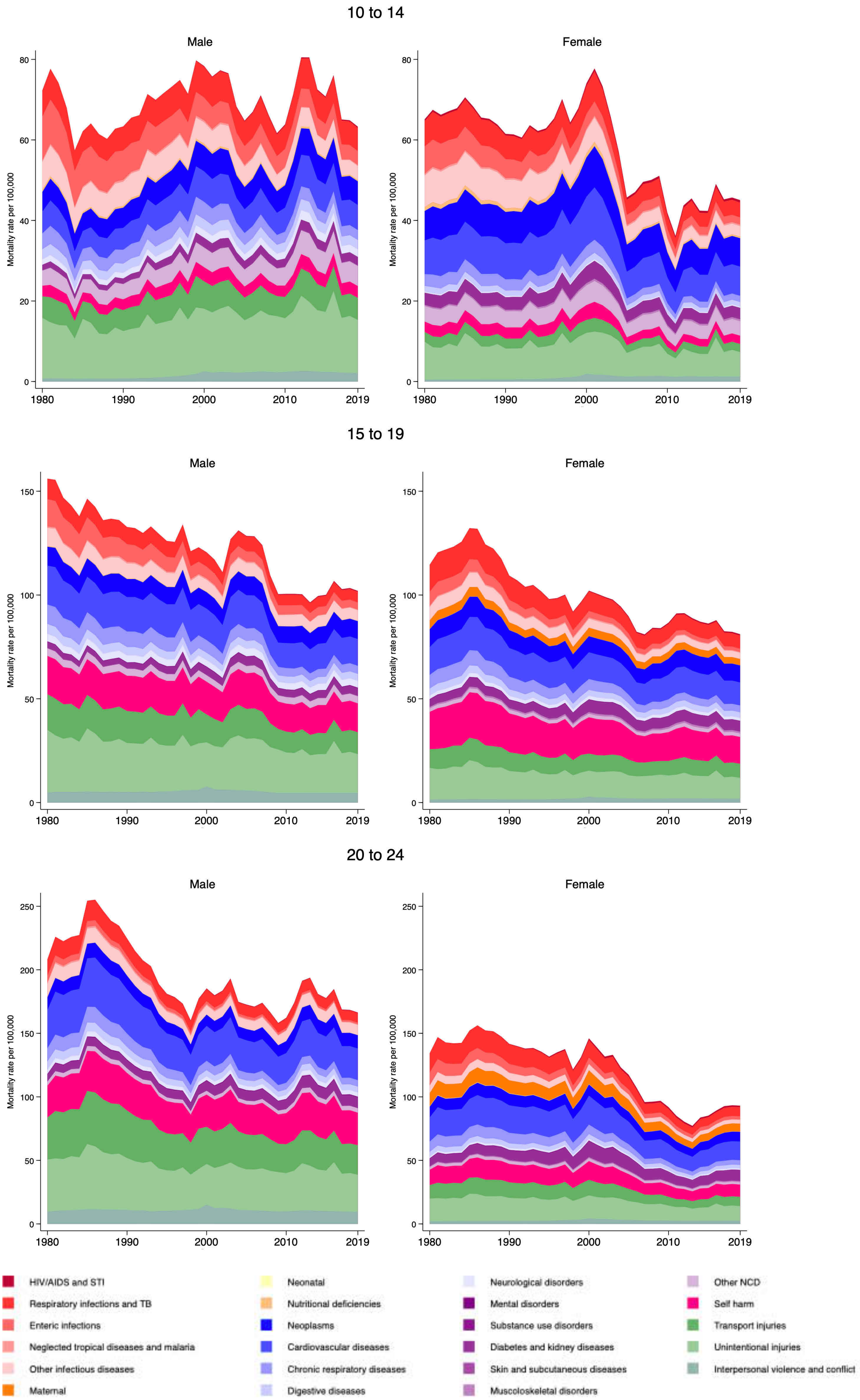

Figure S20d Number of deaths in 10-24 1980 – 2019 by cause: Fiji

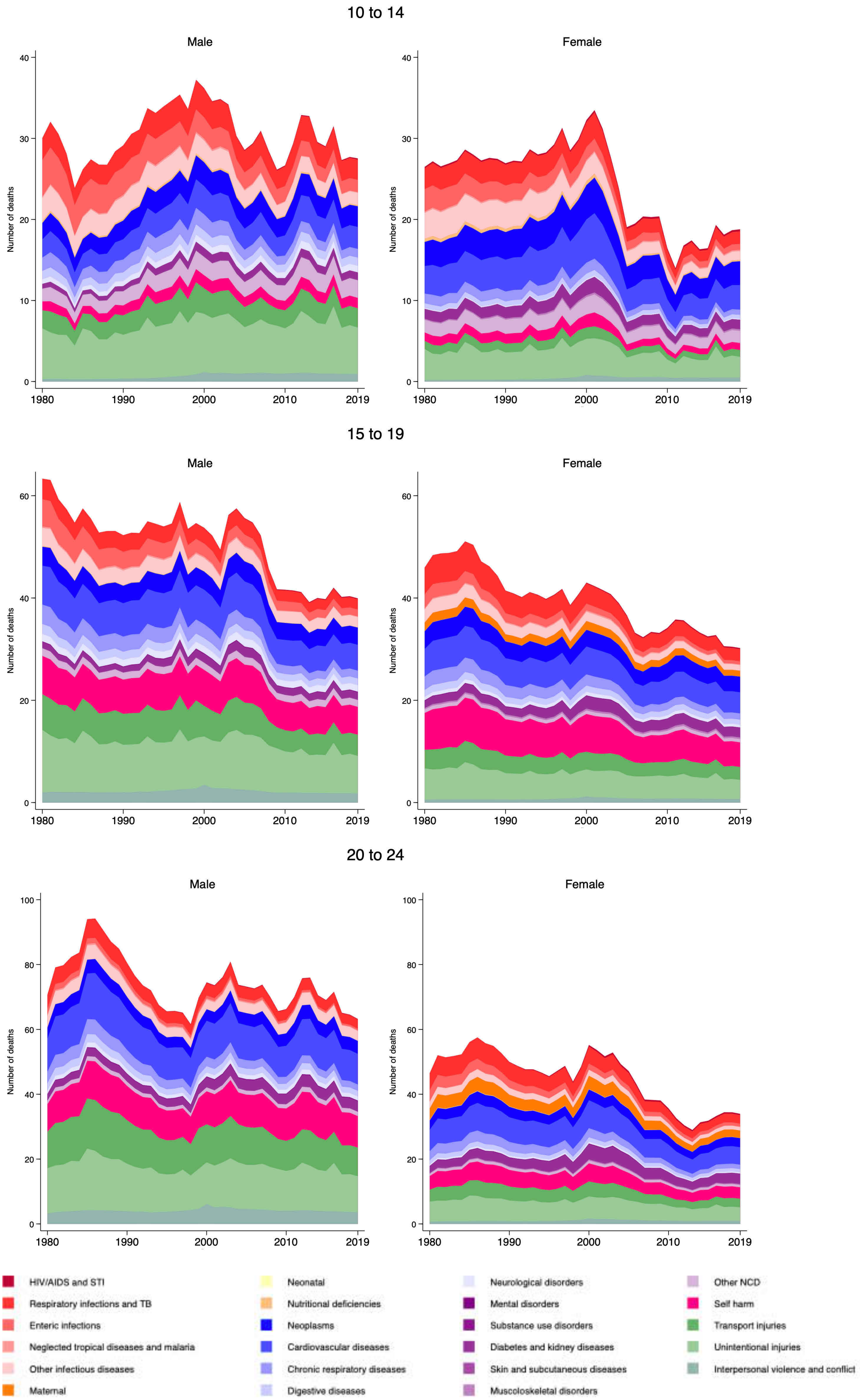

Figure S21: Kiribati

Figure S21a All-cause mortality rate per 100,000 in 10-24 1950 – 2019: Kiribati

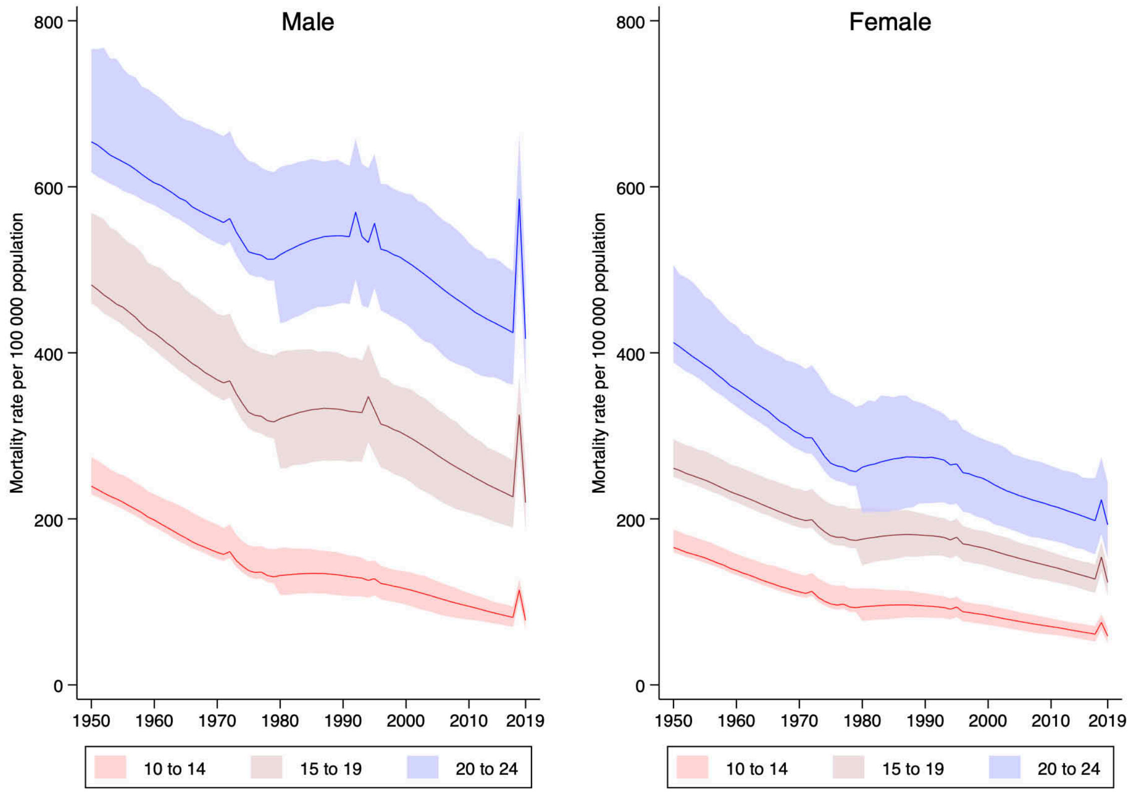

Figure S21b Number of deaths from all causes in 10-24 1950 – 2019: Kiribati

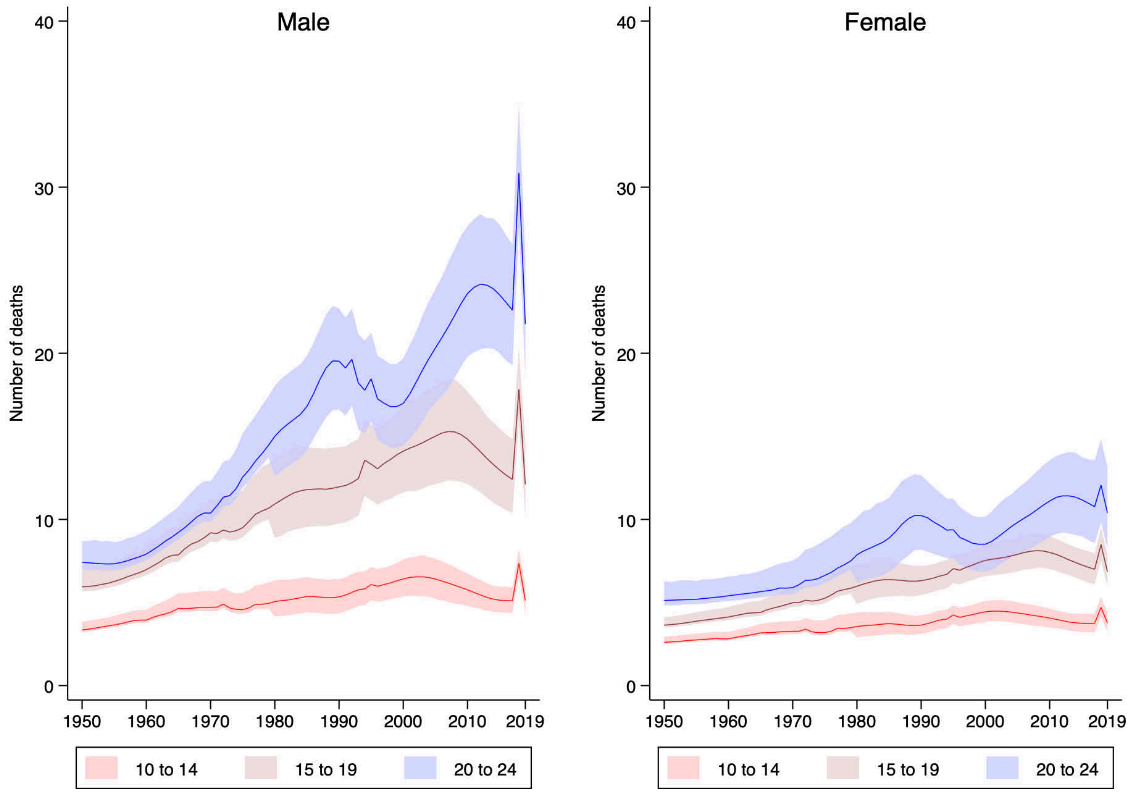

Figure S21c Mortality rate per 100,000 in 10-24 1980 – 2019 by cause: Kiribati

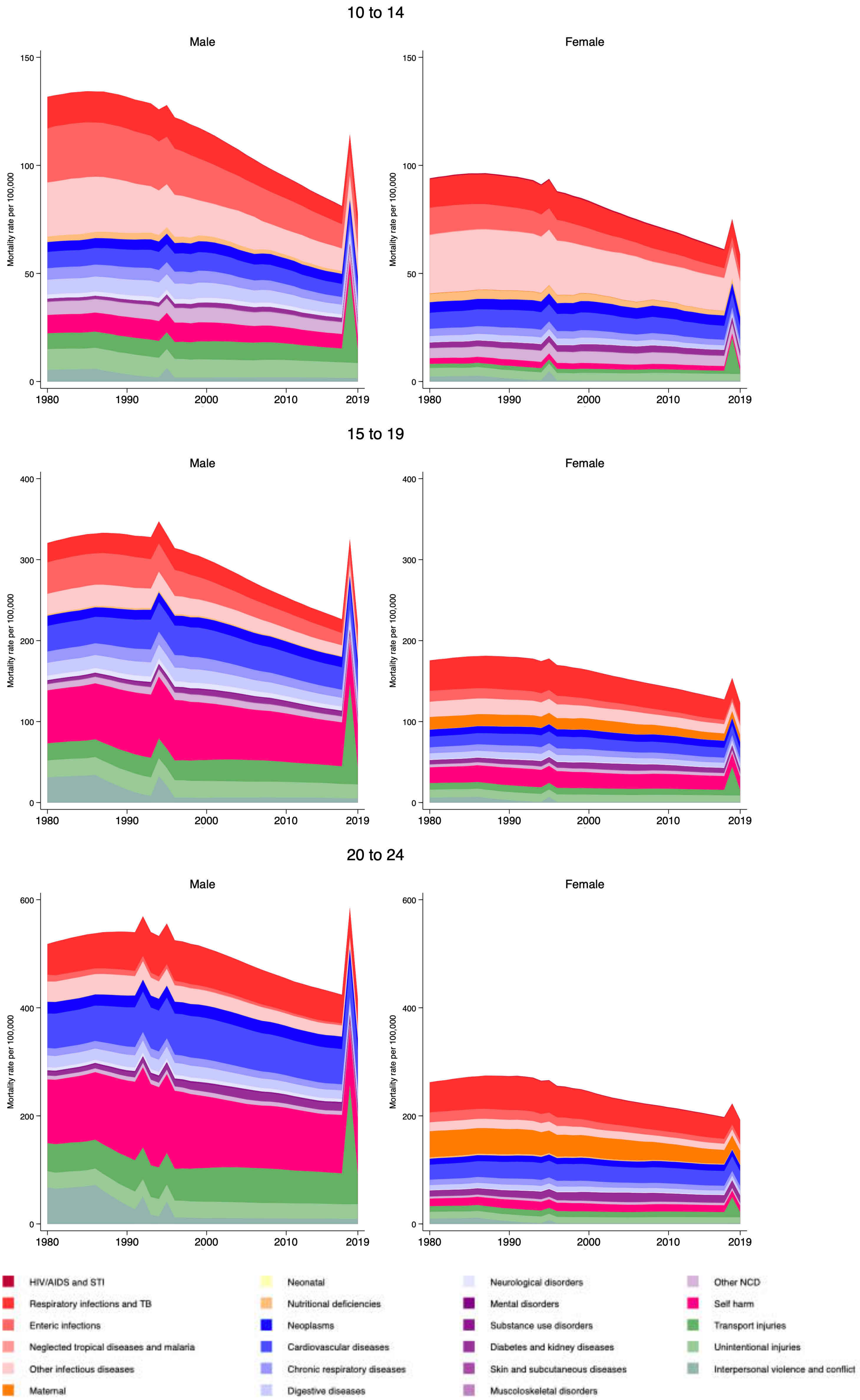

Figure S21d Number of deaths in 10-24 1980 – 2019 by cause: Kiribati

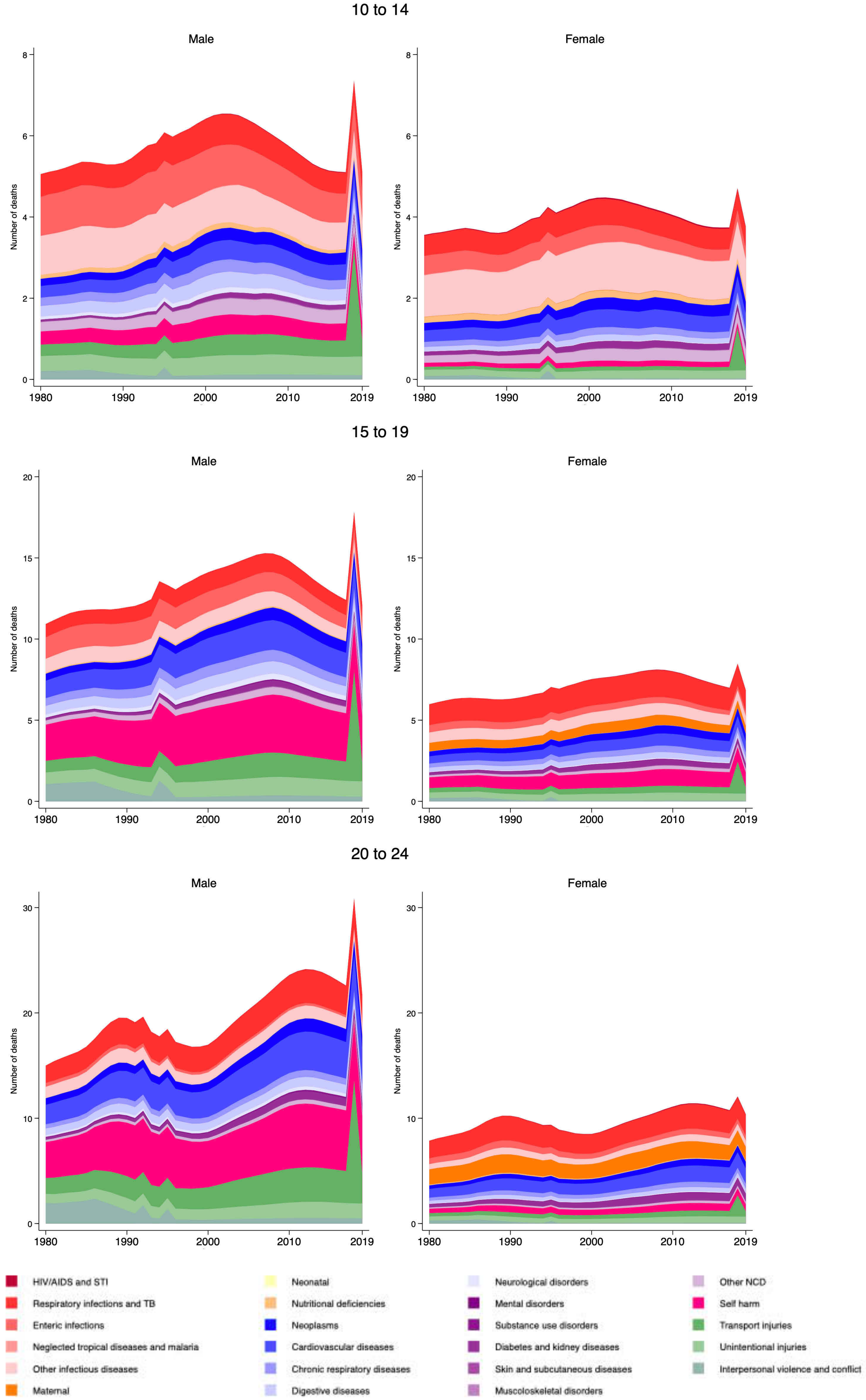

Figure S22: Marshall Islands

Figure S22a All-cause mortality rate per 100,000 in 10-24 1950 – 2019: Marshall Islands

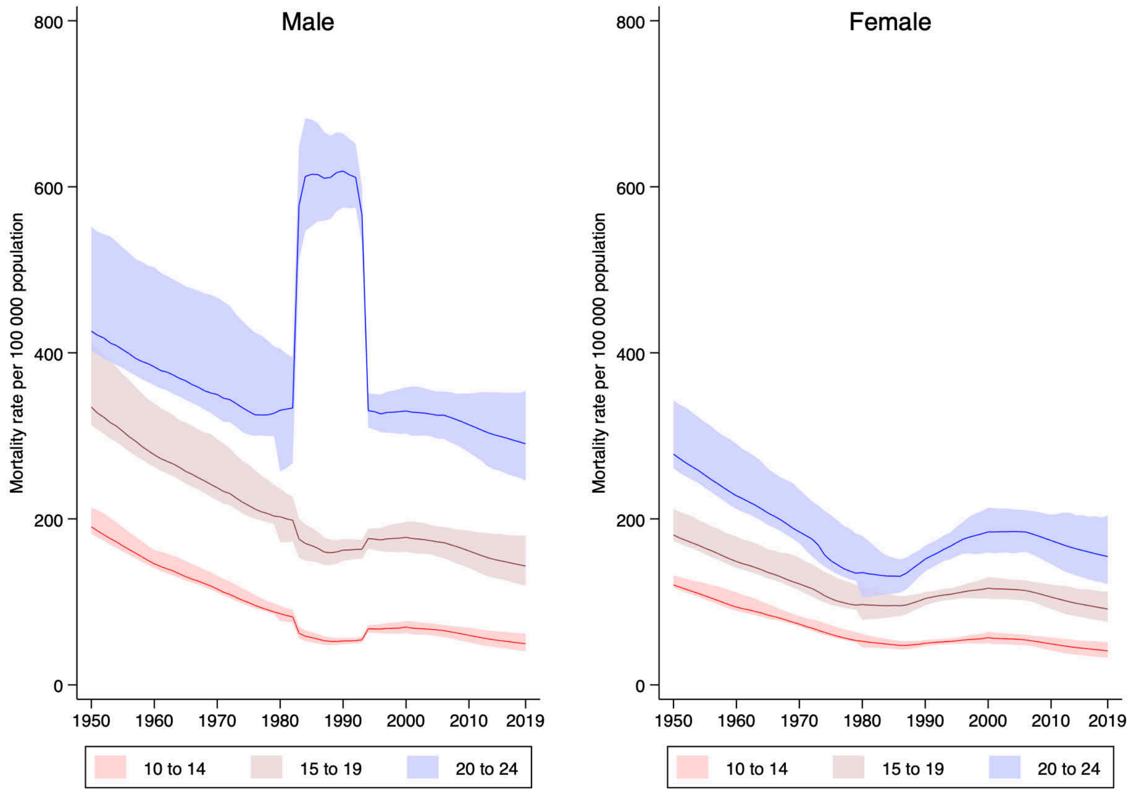

Figure S22b Number of deaths from all causes in 10-24 1950 – 2019: Marshall Islands

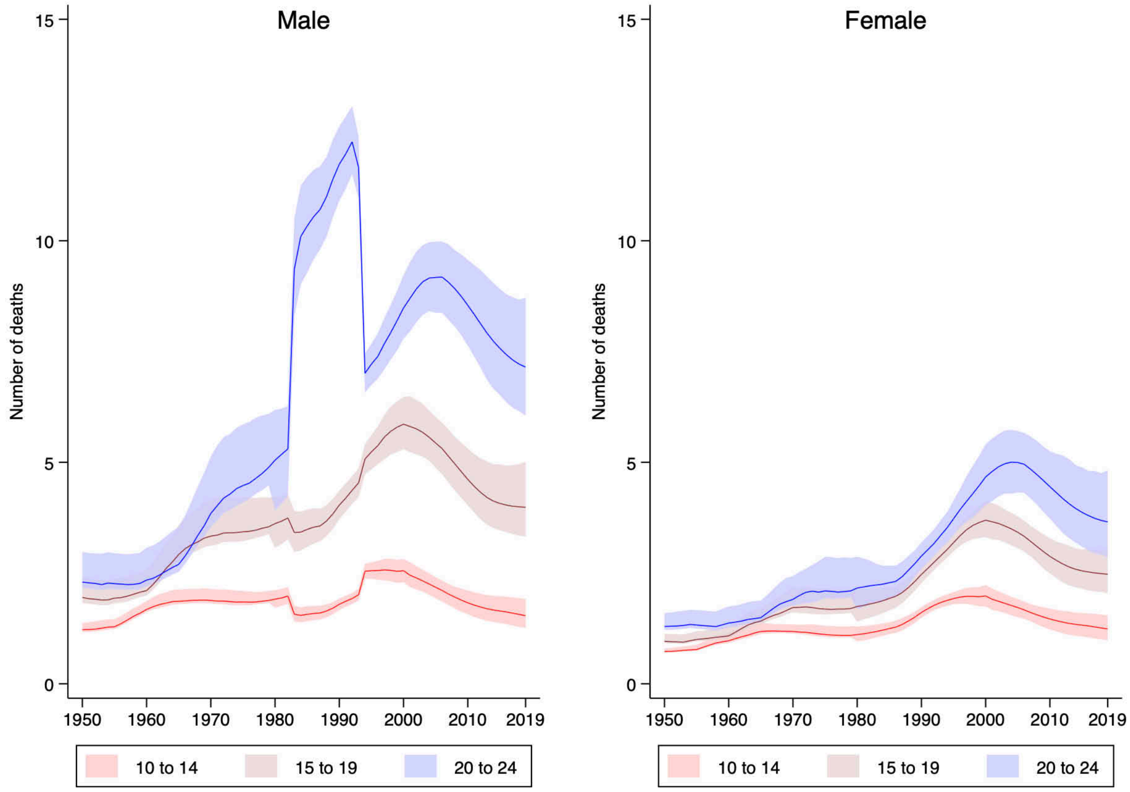

Figure S22c Mortality rate per 100,000 in 10-24 1980 – 2019 by cause: Marshall Islands

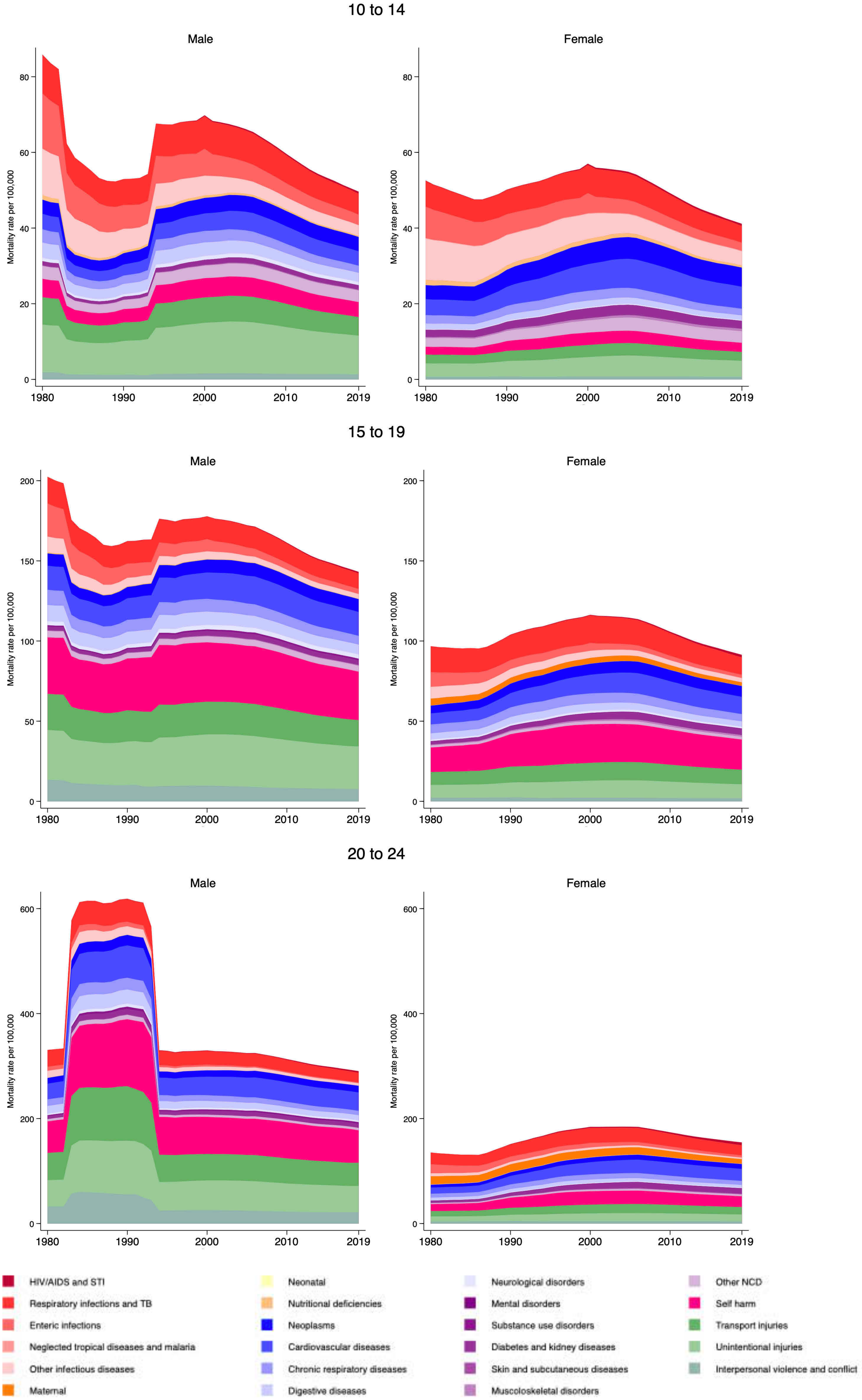

Figure S22d Number of deaths in 10-24 1980 – 2019 by cause: Marshall Islands

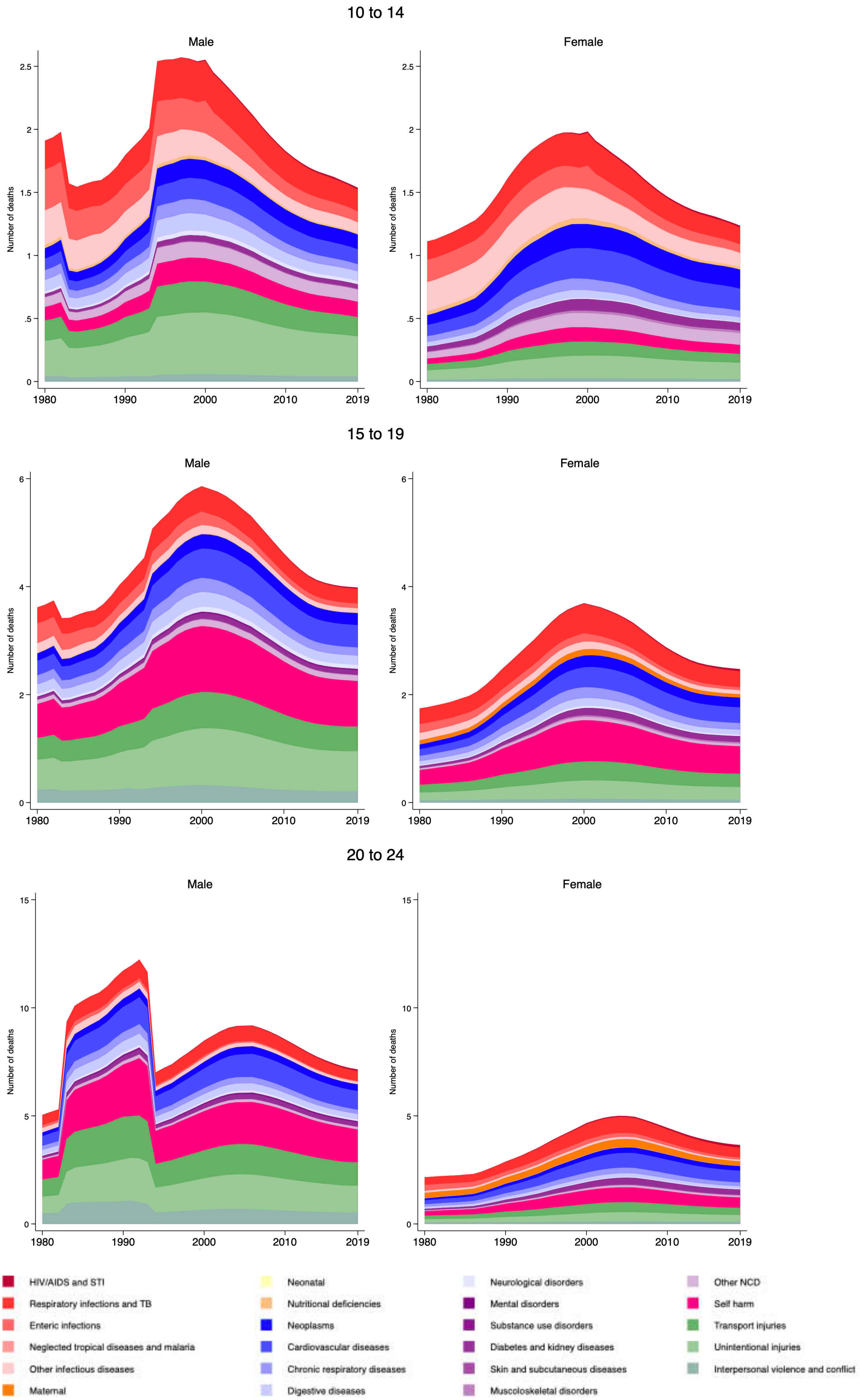

Figure S23: Micronesia (Federated States of)

Figure S23a All-cause mortality rate per 100,000 in 10-24 1950 – 2019: Micronesia (Federated States of)

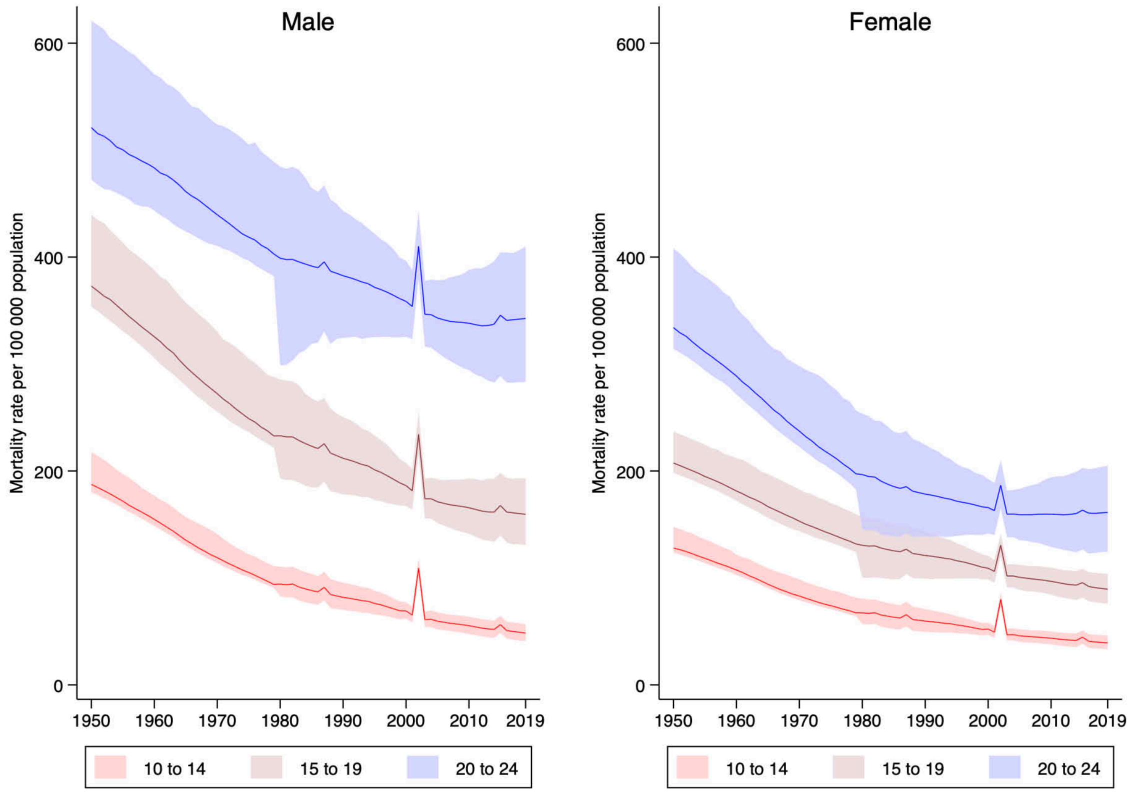

Figure S23b Number of deaths from all causes in 10-24 1950 – 2019: Micronesia (Federated States of)

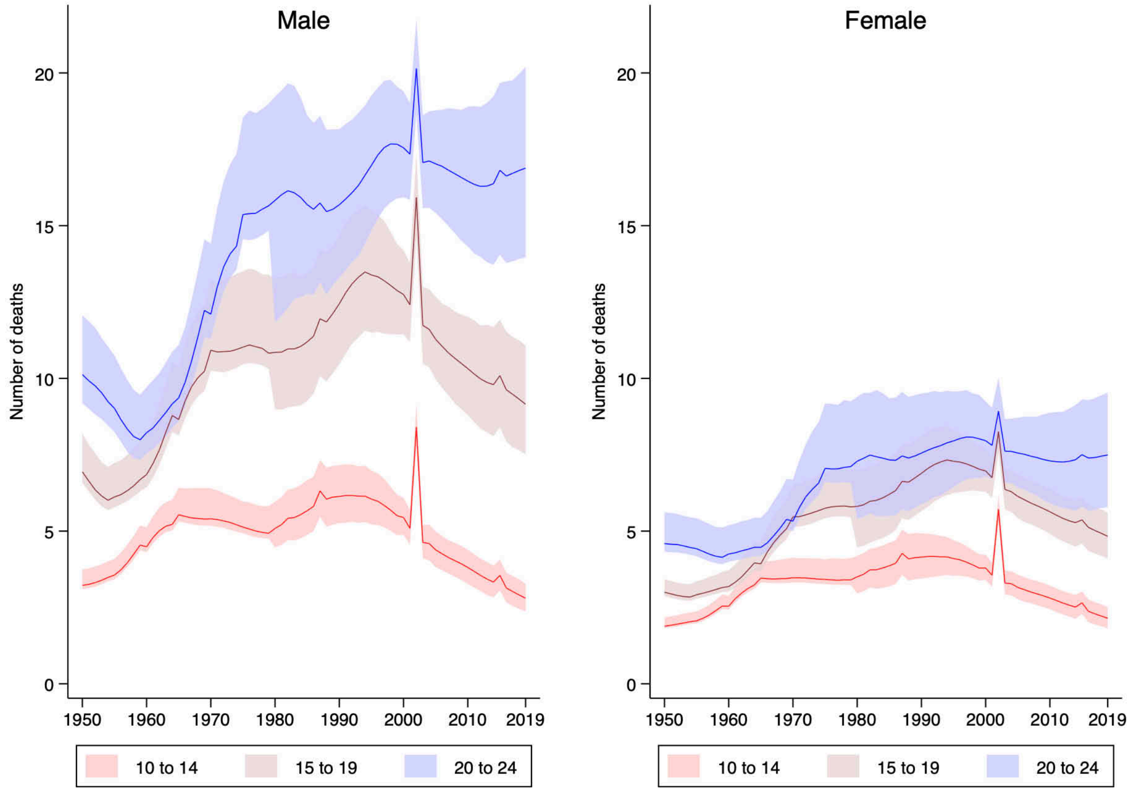

Figure S23c Mortality rate per 100,000 in 10-24 1980 – 2019 by cause: Micronesia (Federated States of)

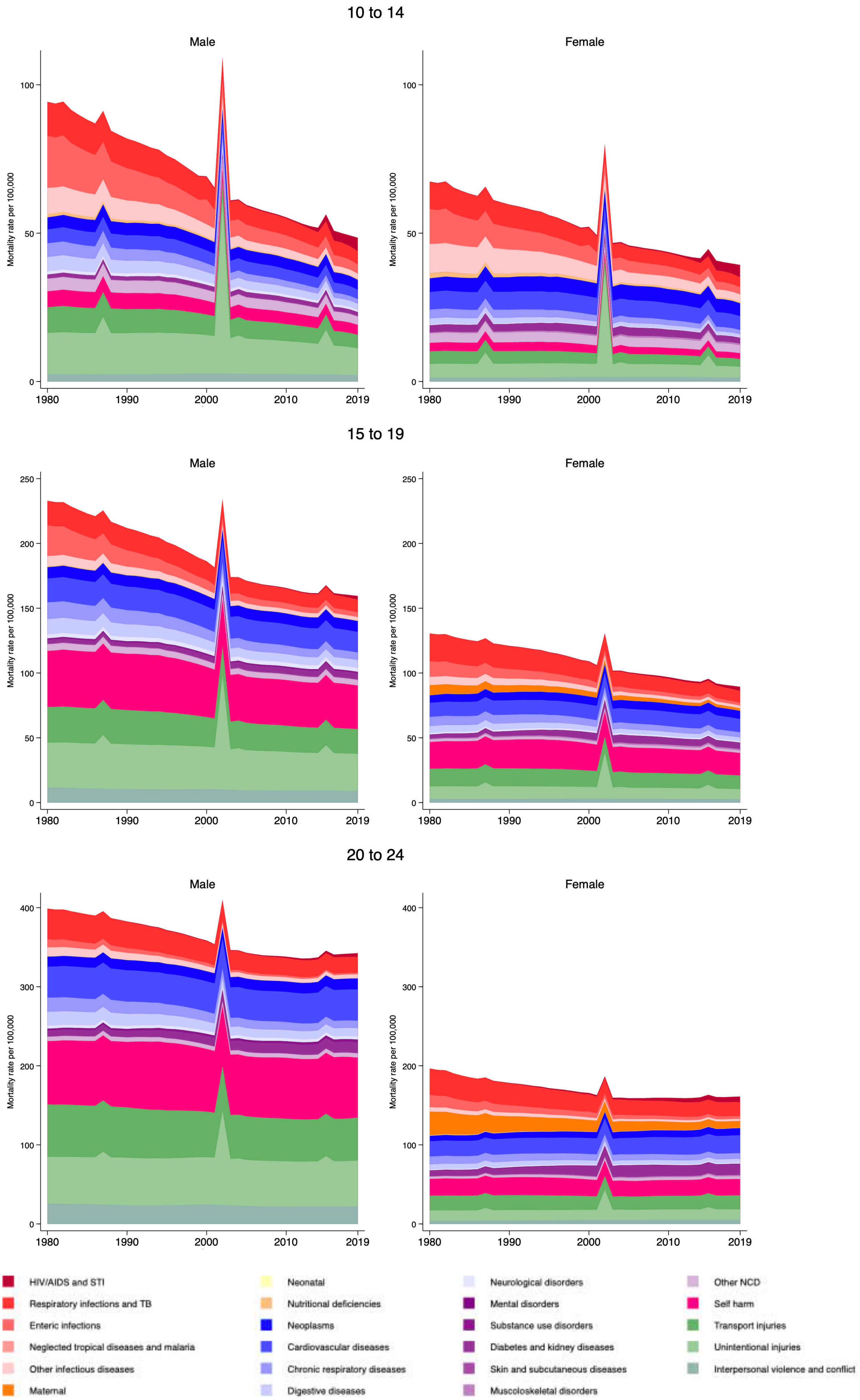

Figure S23d Number of deaths in 10-24 1980 – 2019 by cause: Micronesia (Federated States of)

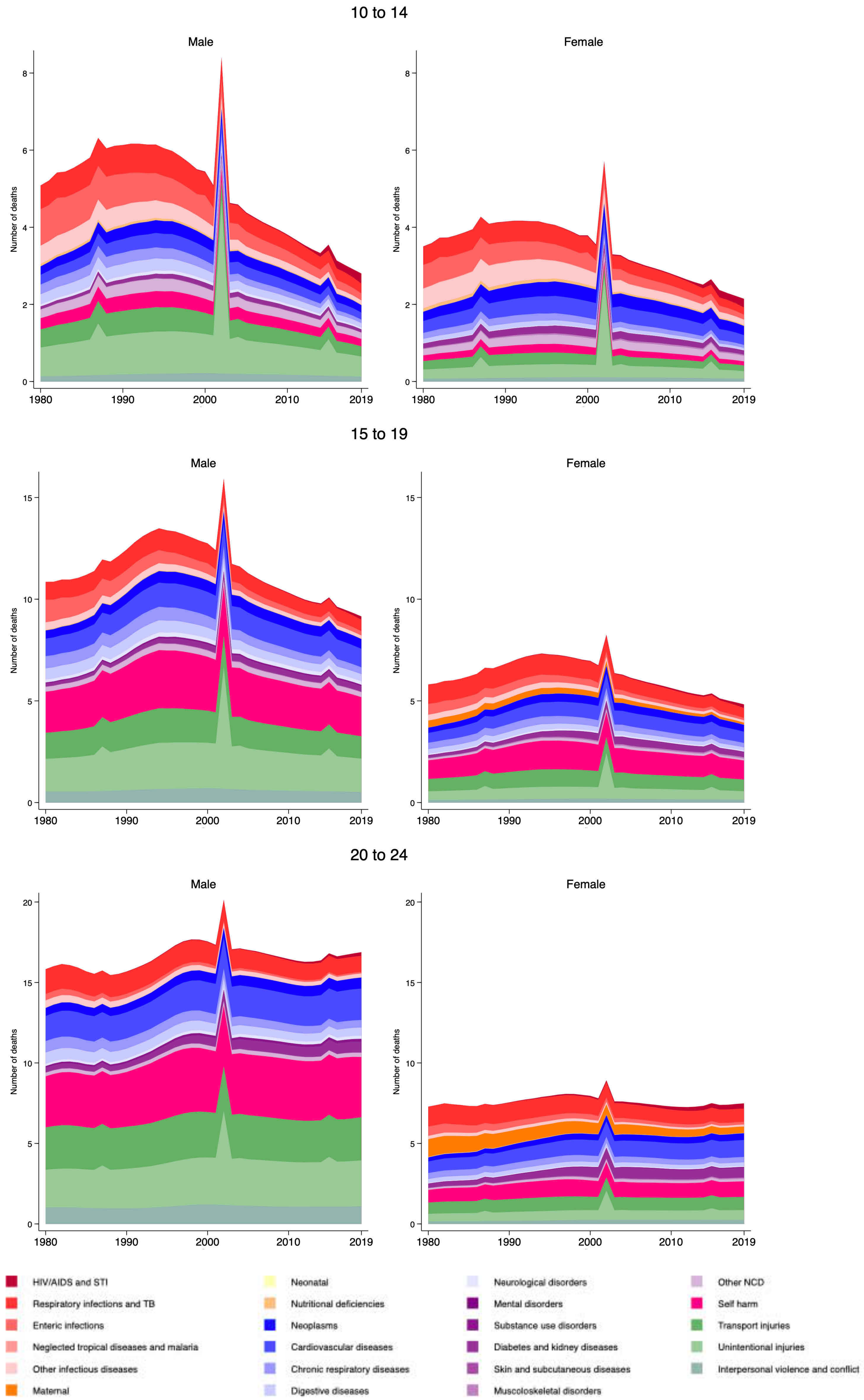

Figure S24: Papua New Guinea

Figure S24a All-cause mortality rate per 100,000 in 10-24 1950 – 2019: Papua New Guinea

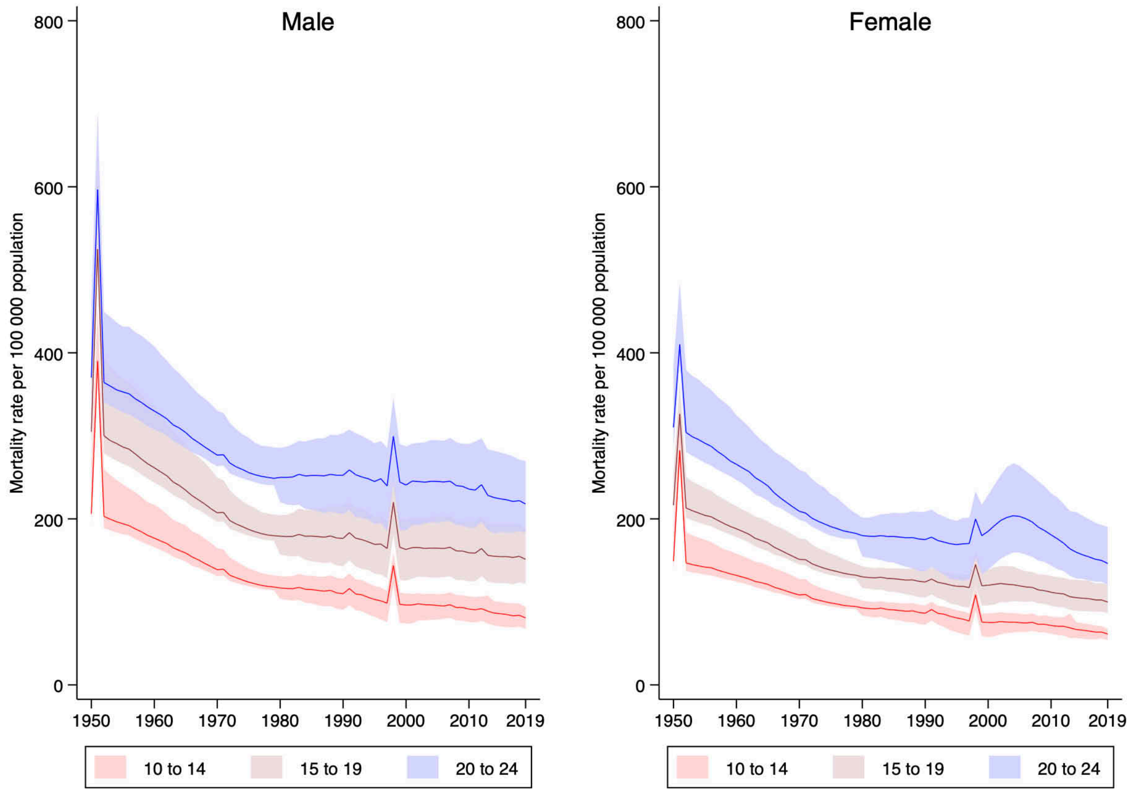

Figure S24b Number of deaths from all causes in 10-24 1950 – 2019: Papua New Guinea

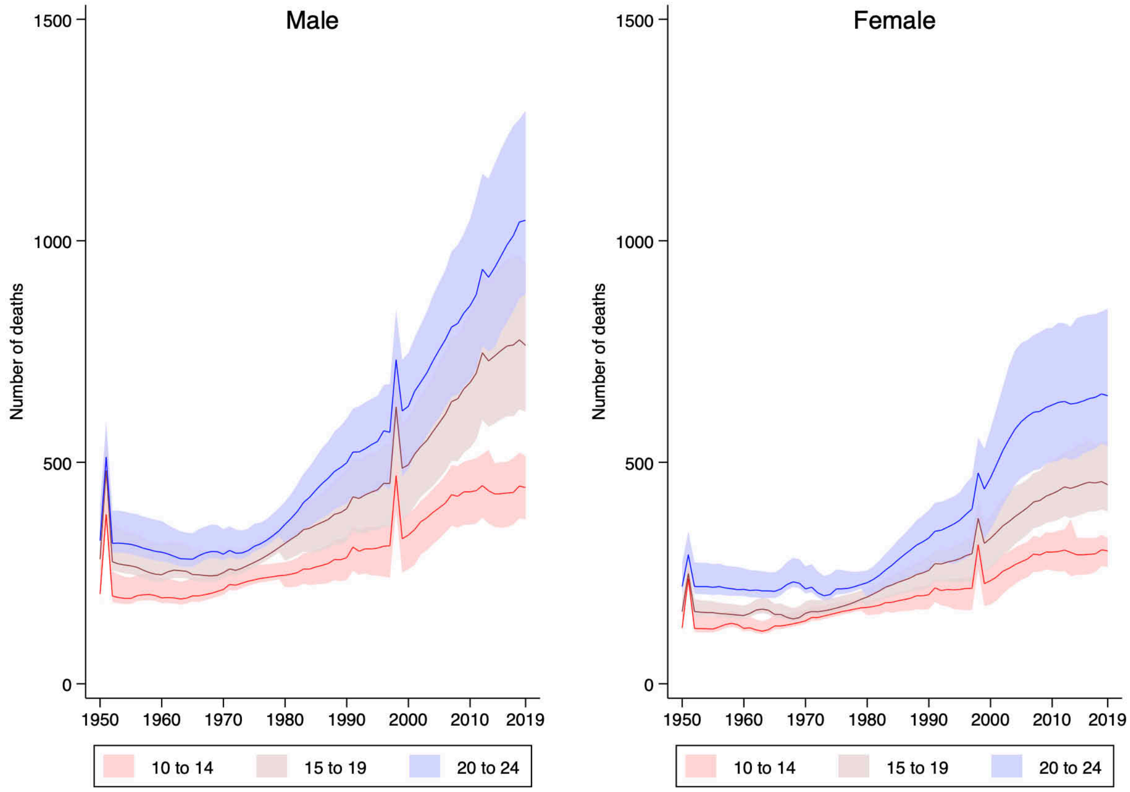

Figure S24c Mortality rate per 100,000 in 10-24 1980 – 2019 by cause: Papua New Guinea

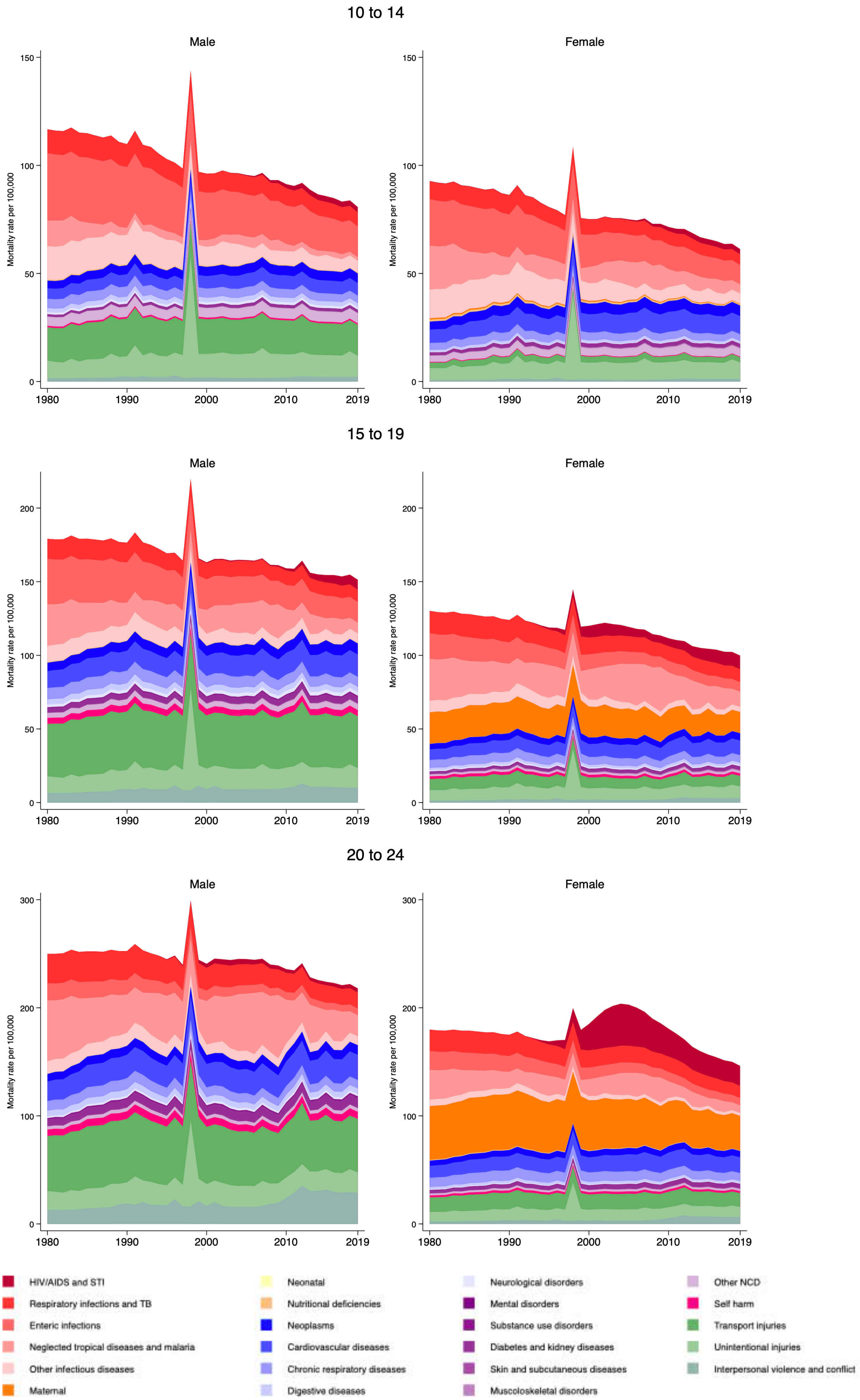

Figure S24d Number of deaths in 10-24 1980 – 2019 by cause: Papua New Guinea

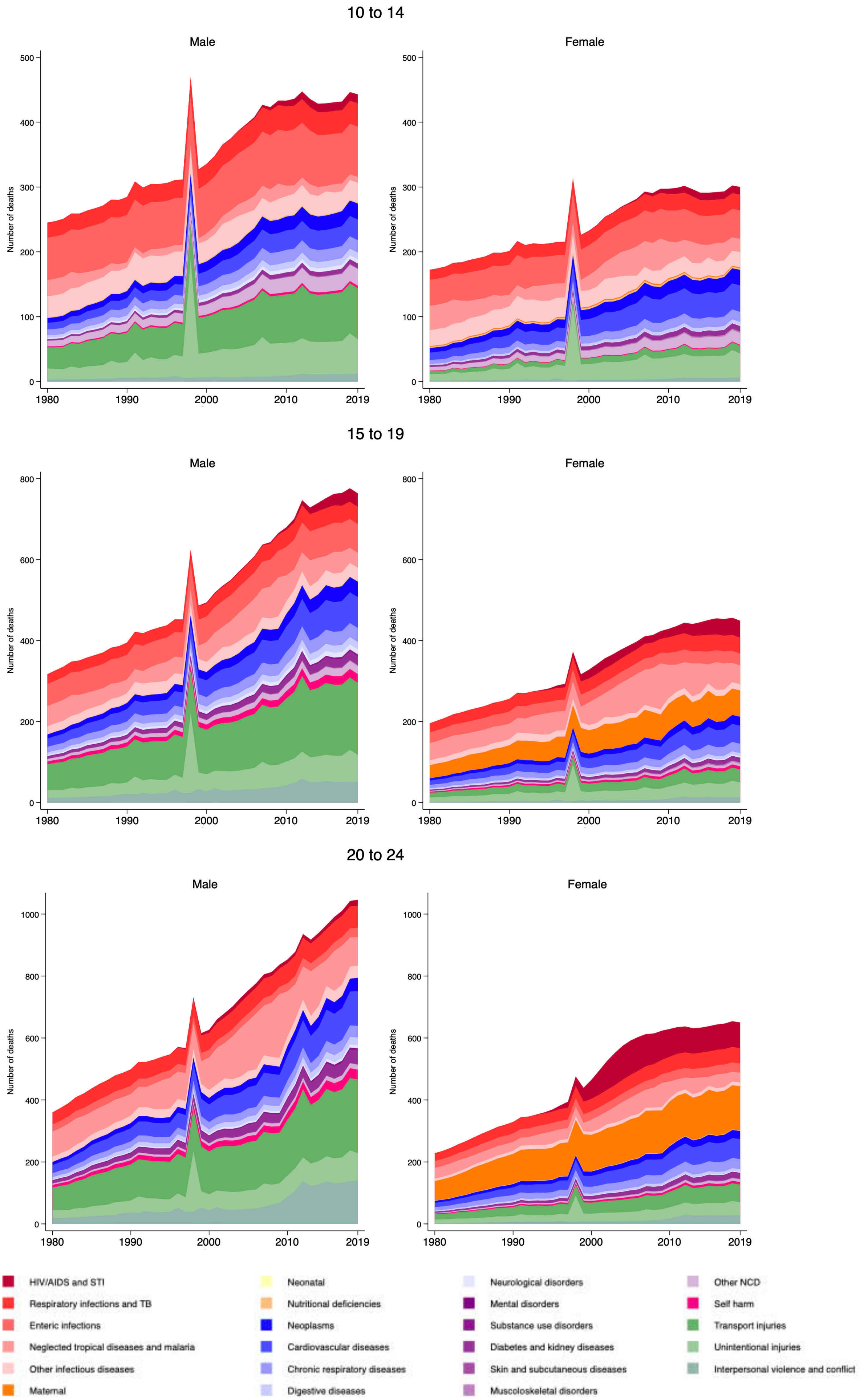

Figure S25: Samoa

Figure S25a All-cause mortality rate per 100,000 in 10-24 1950 – 2019: Samoa

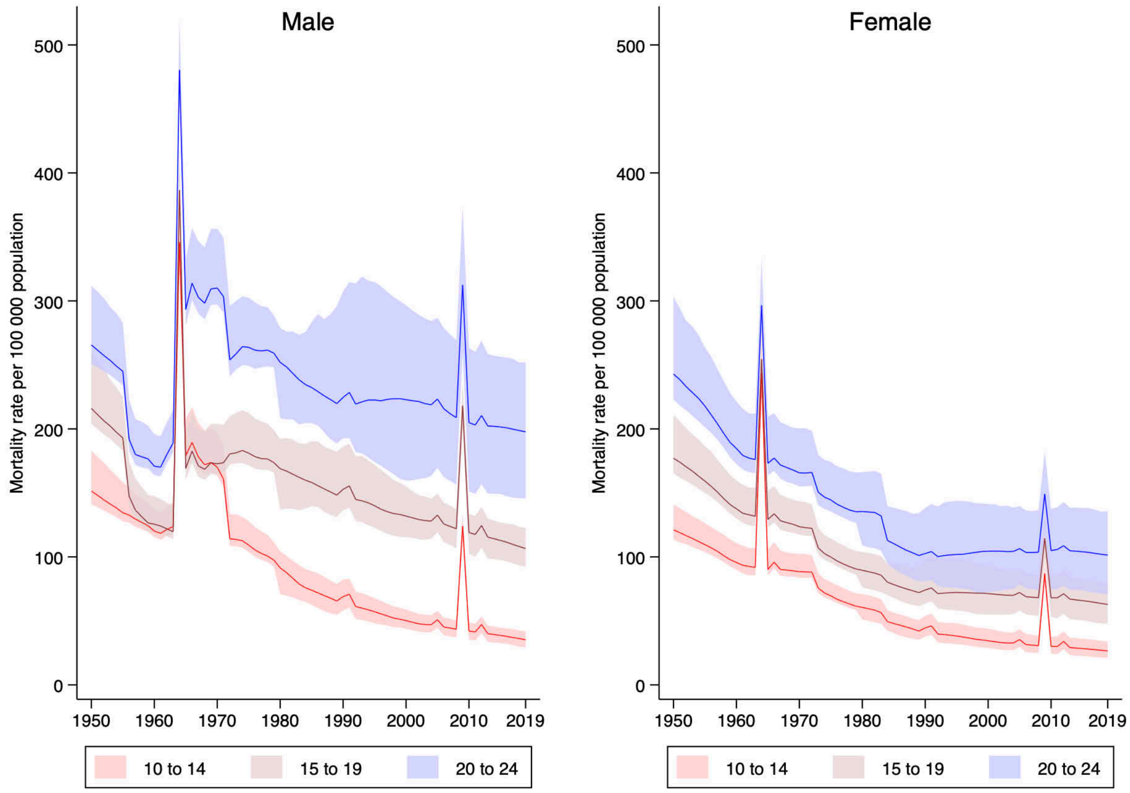

Figure S25b Number of deaths from all causes in 10-24 1950 – 2019: Samoa

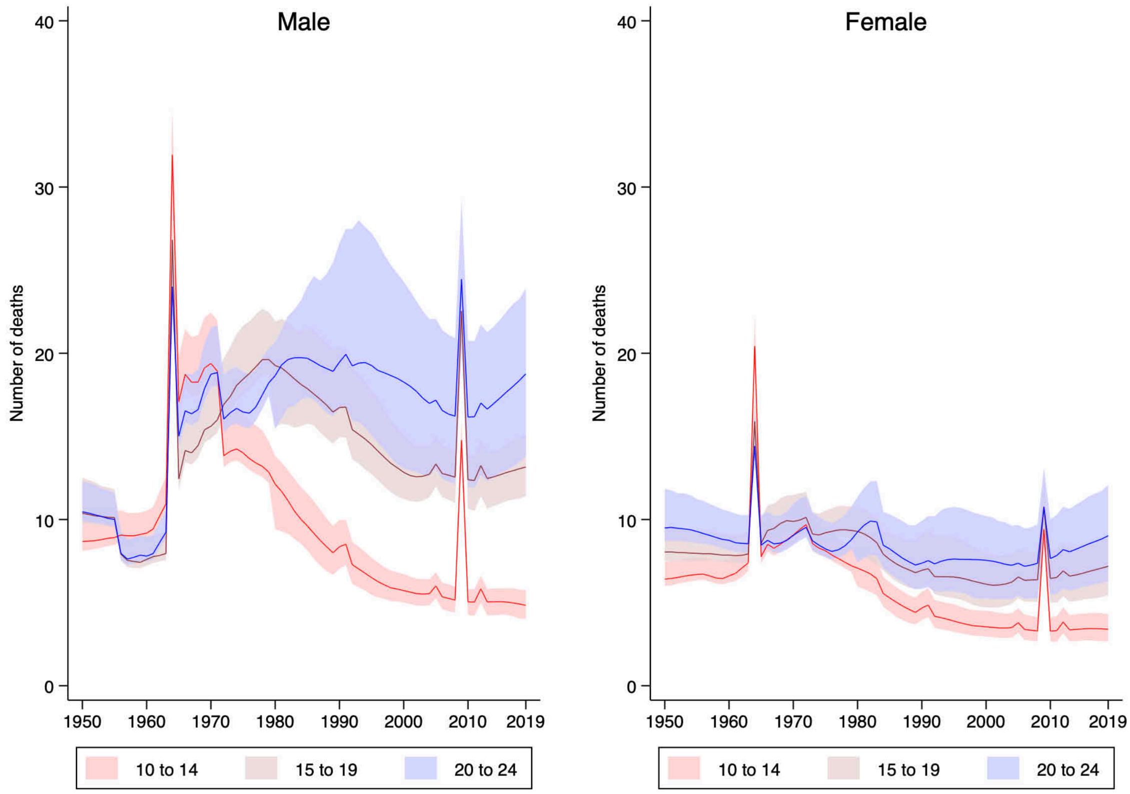

Figure S25c Mortality rate per 100,000 in 10-24 1980 – 2019 by cause: Samoa

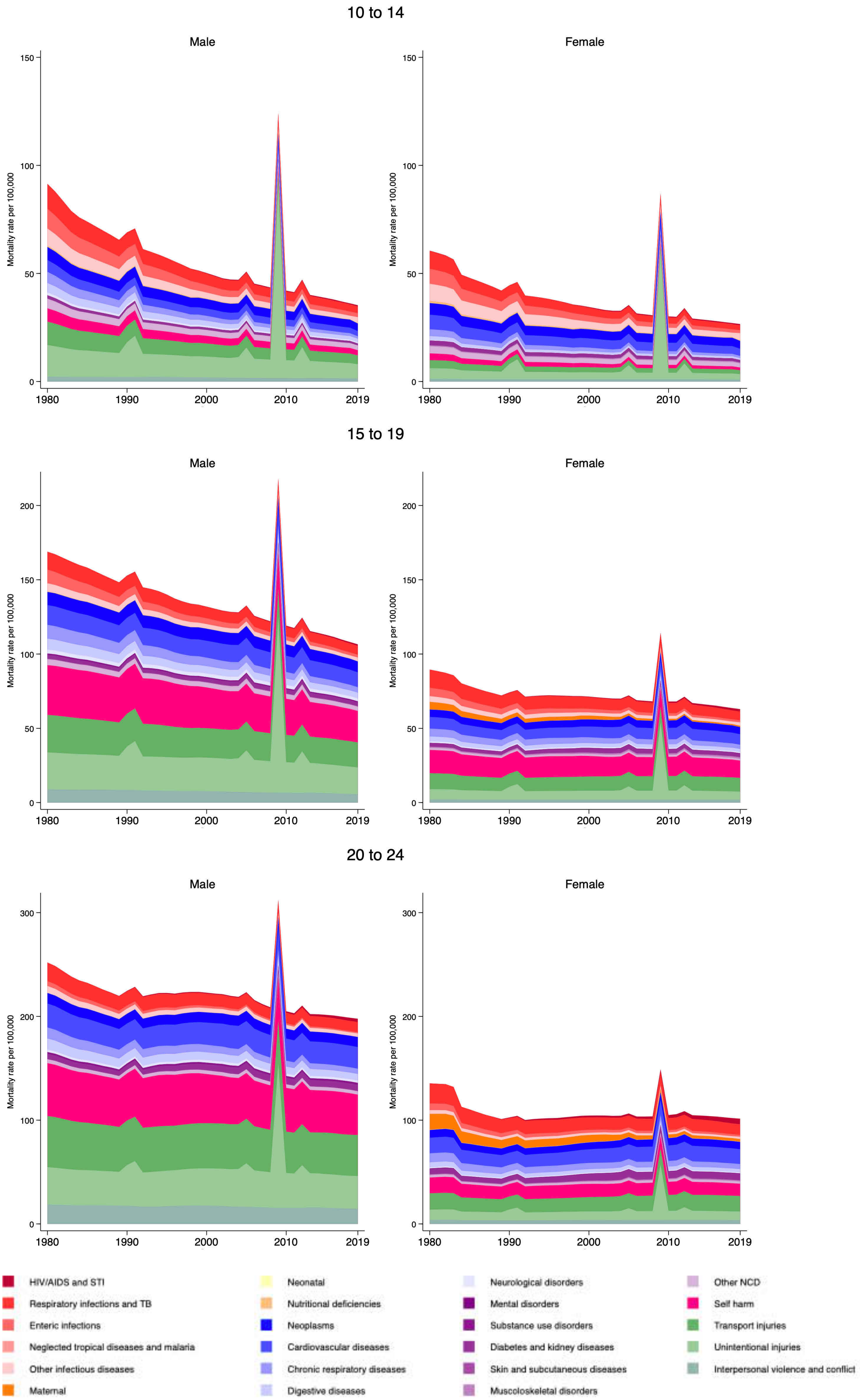

Figure S25d Number of deaths in 10-24 1980 – 2019 by cause: Samoa

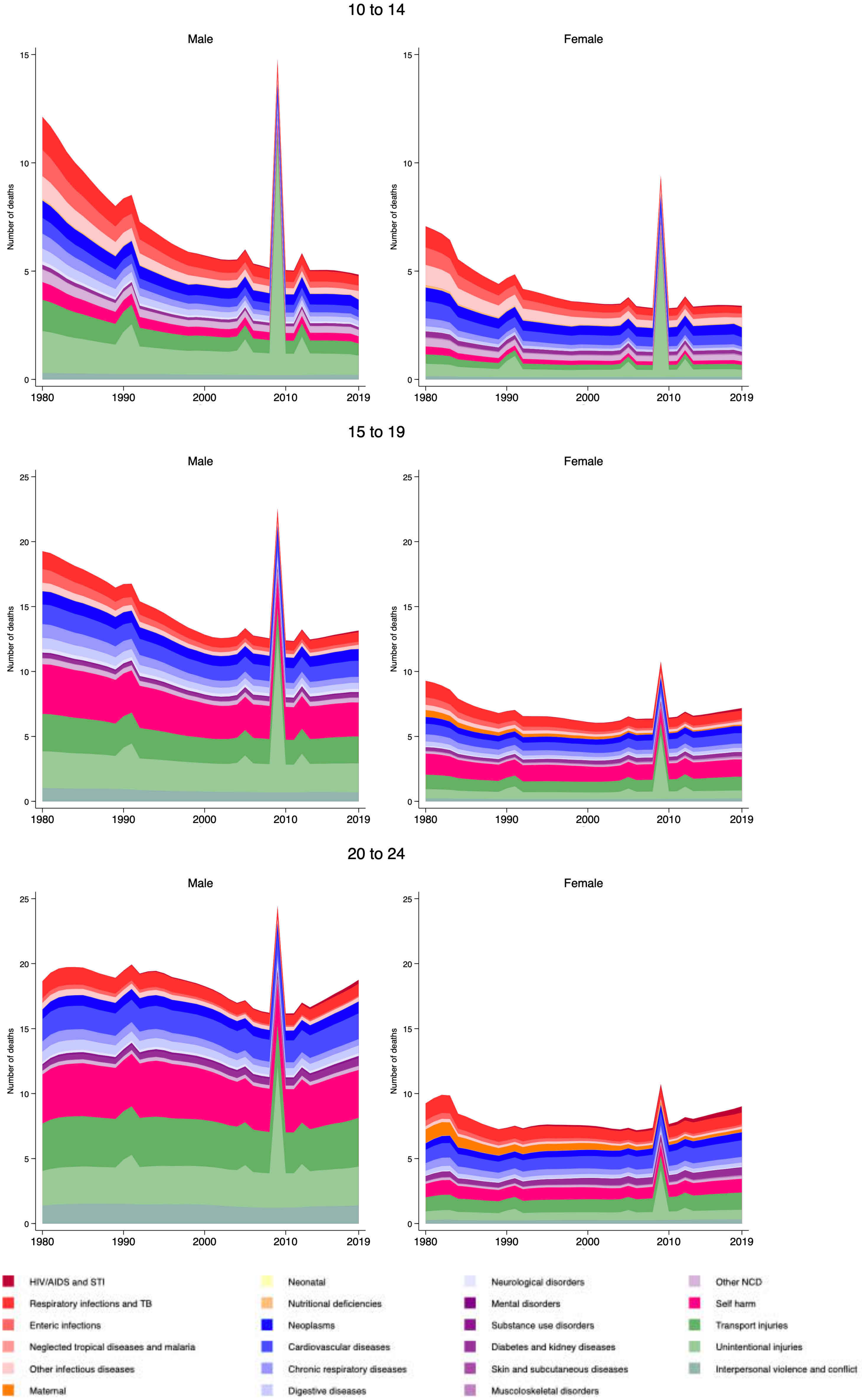

Figure S26: Solomon Islands

Figure S26a All-cause mortality rate per 100,000 in 10-24 1950 – 2019: Solomon Islands

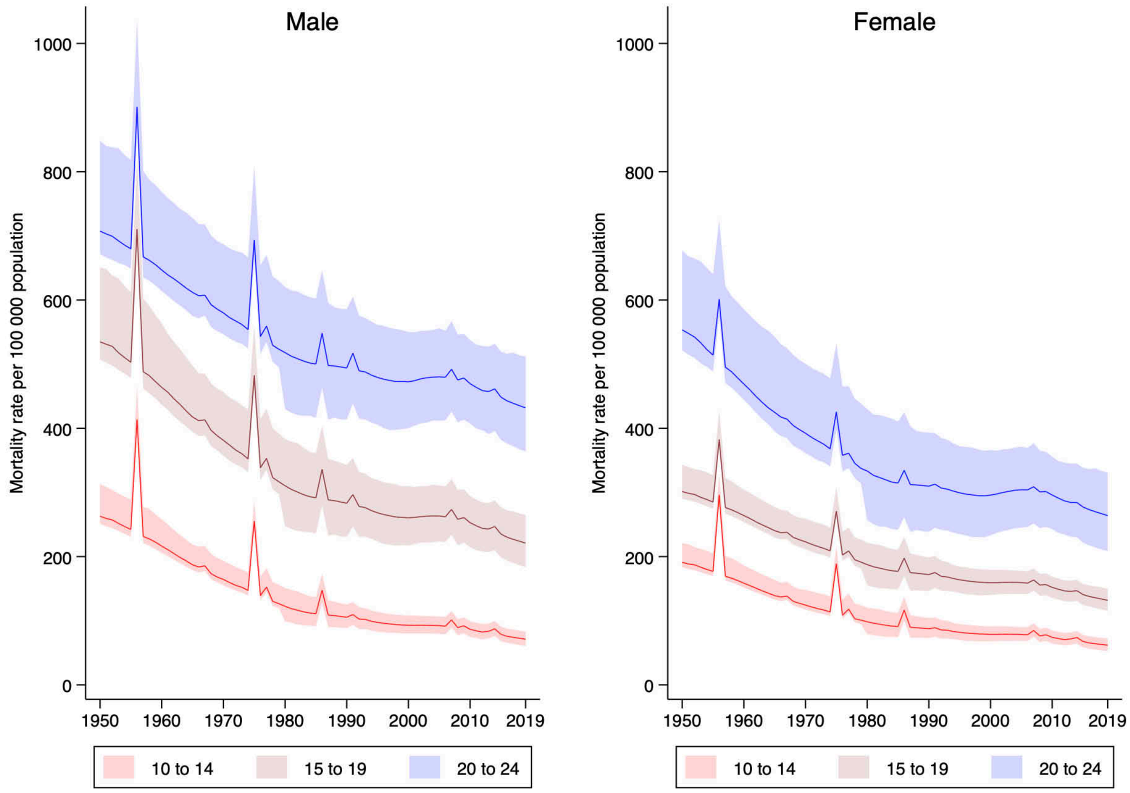

Figure S26b Number of deaths from all causes in 10-24 1950 – 2019: Solomon Islands

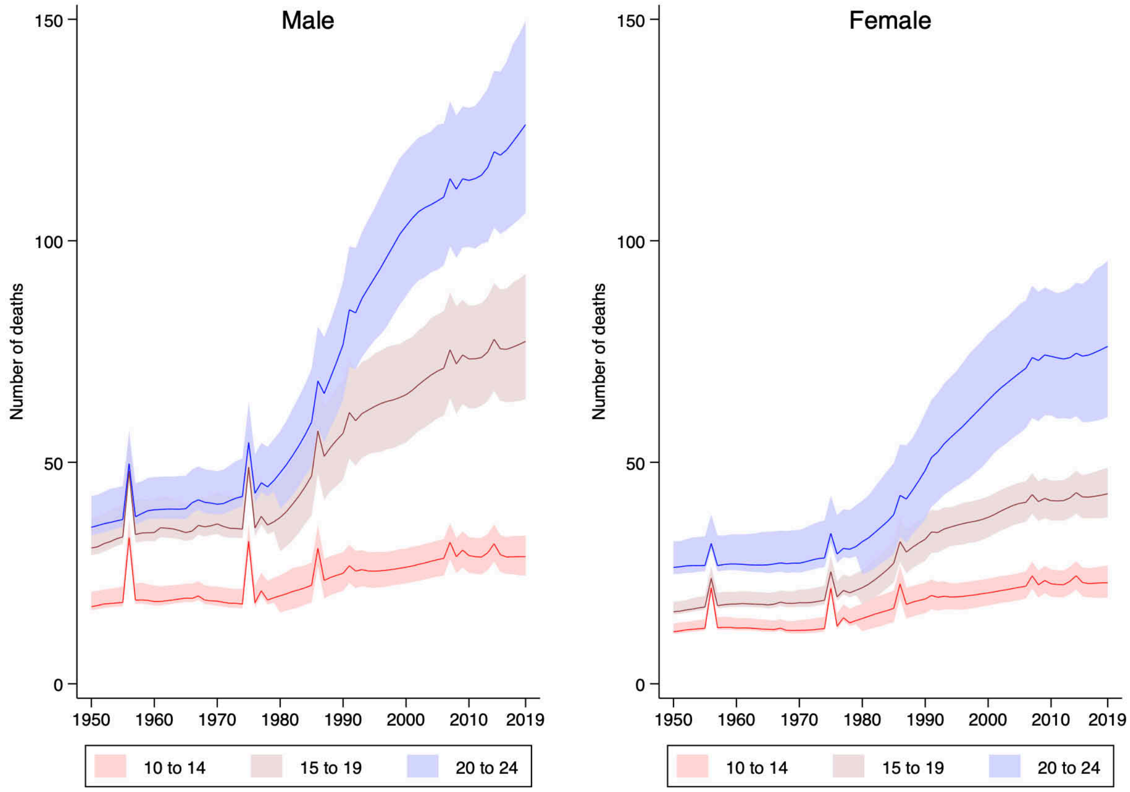

Figure S26c Mortality rate per 100,000 in 10-24 1980 – 2019 by cause: Solomon Islands

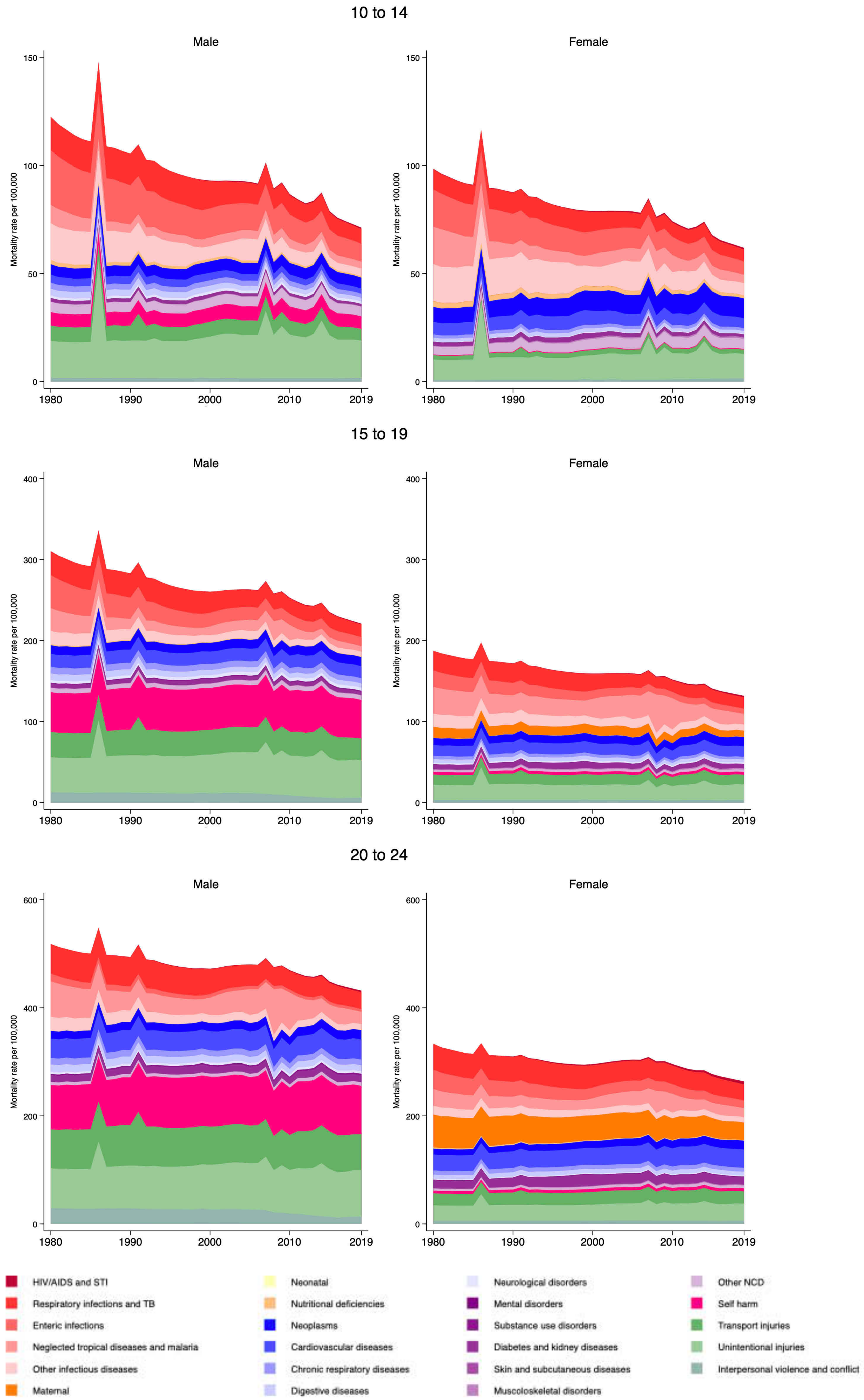

Figure S26d Number of deaths in 10-24 1980 – 2019 by cause: Solomon Islands

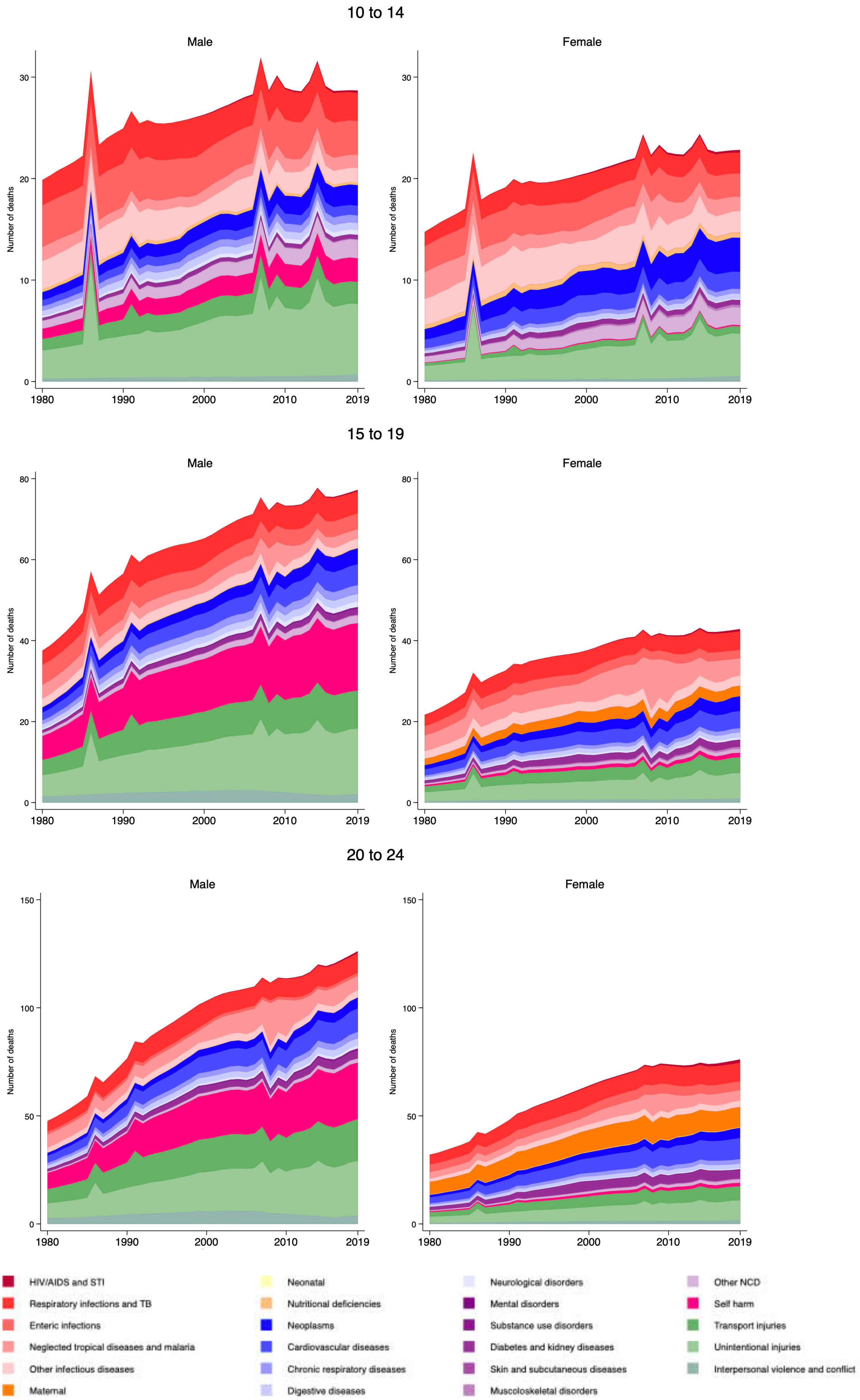

Figure S27: Tonga

Figure S27a All-cause mortality rate per 100,000 in 10-24 1950 – 2019: Tonga

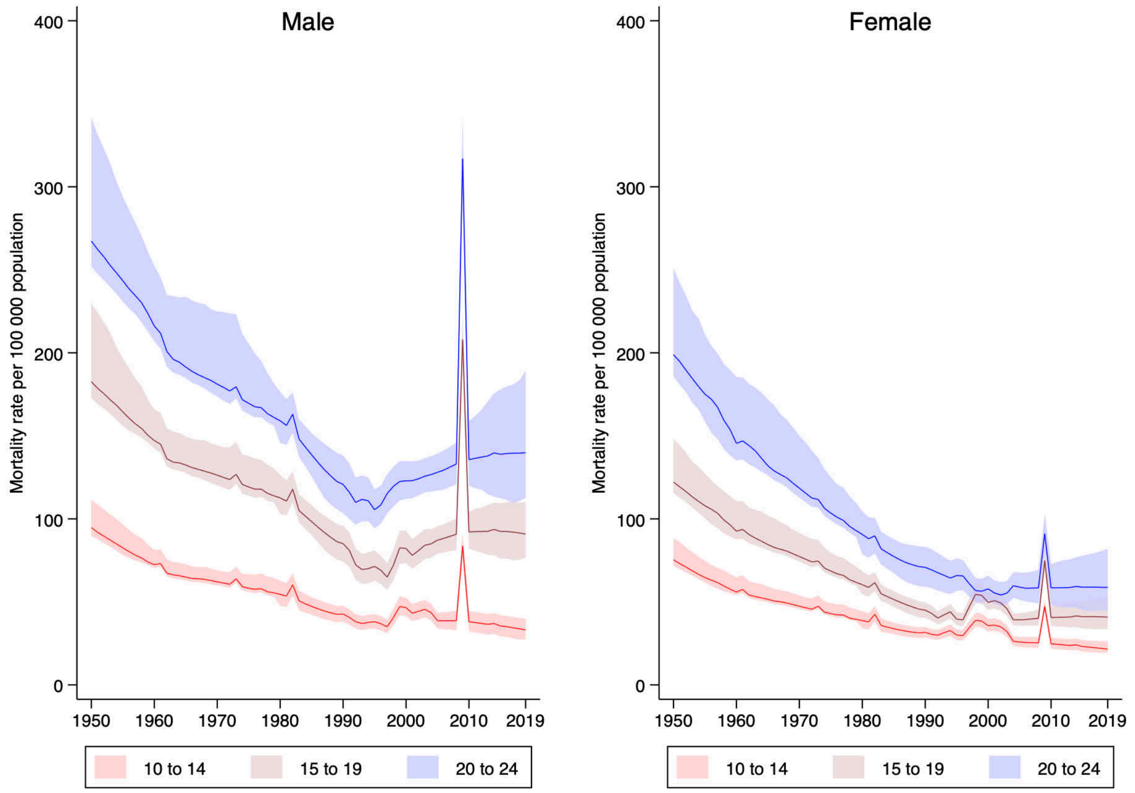

Figure S27b Number of deaths from all causes in 10-24 1950 – 2019: Tonga

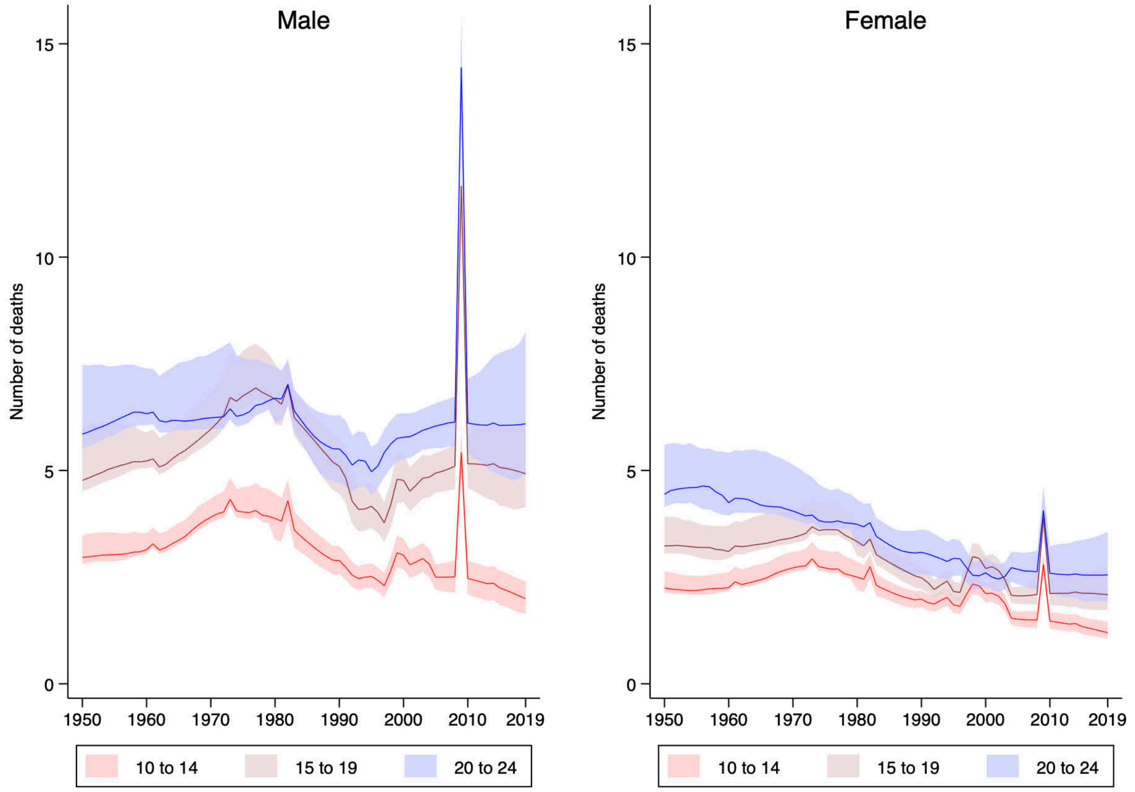

Figure S27c Mortality rate per 100,000 in 10-24 1980 – 2019 by cause: Tonga

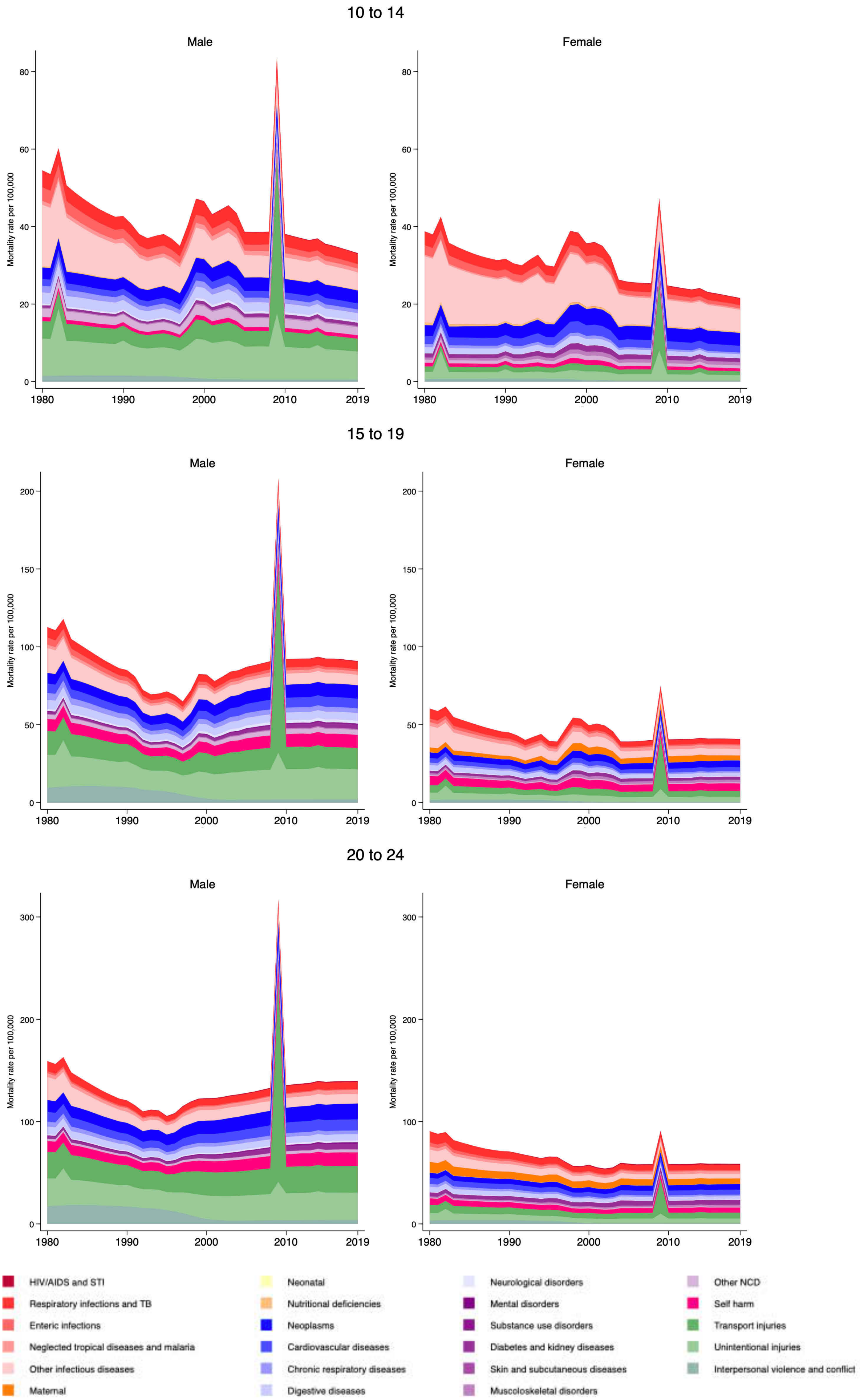

Figure S27d Number of deaths in 10-24 1980 – 2019 by cause: Tonga

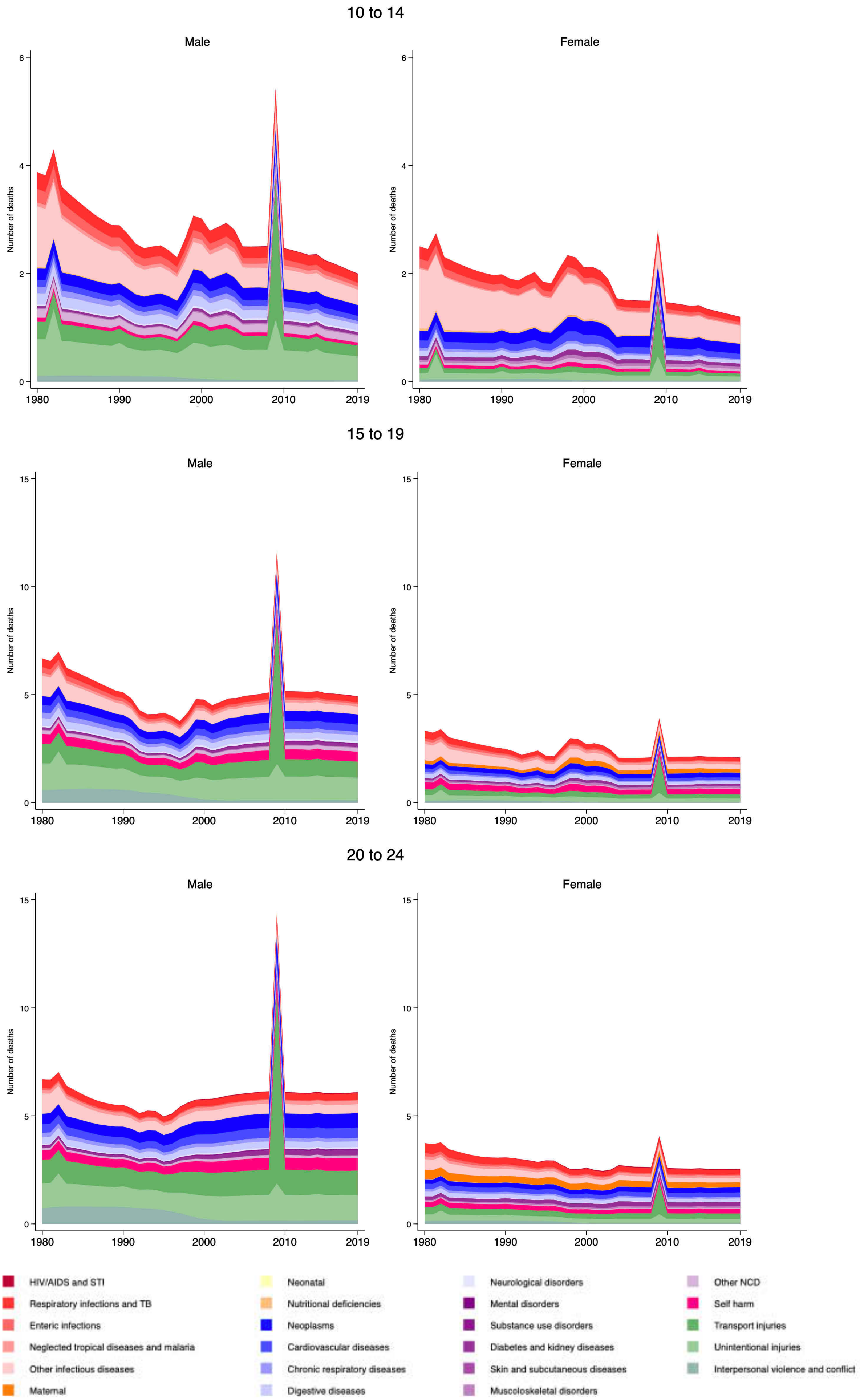

Figure S28: Vanuatu

Figure S28a All-cause mortality rate per 100,000 in 10-24 1950 – 2019: Vanuatu

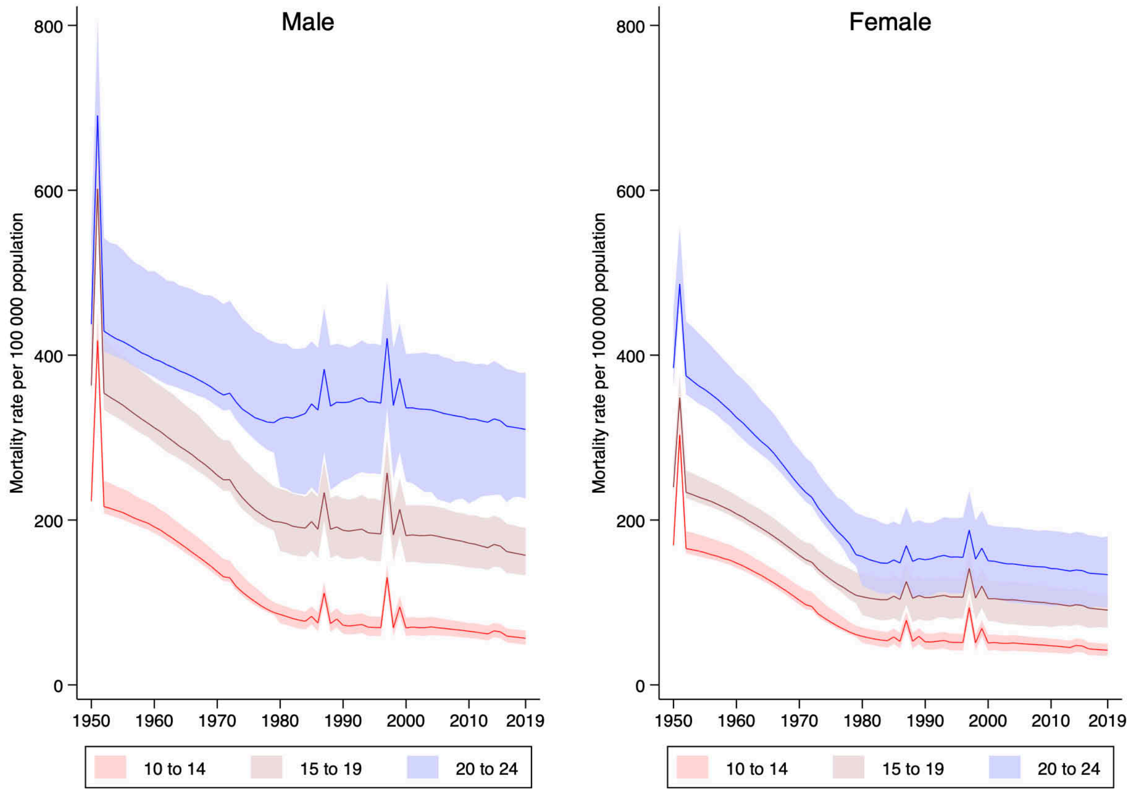

Figure S28b Number of deaths from all causes in 10-24 1950 – 2019: Vanuatu

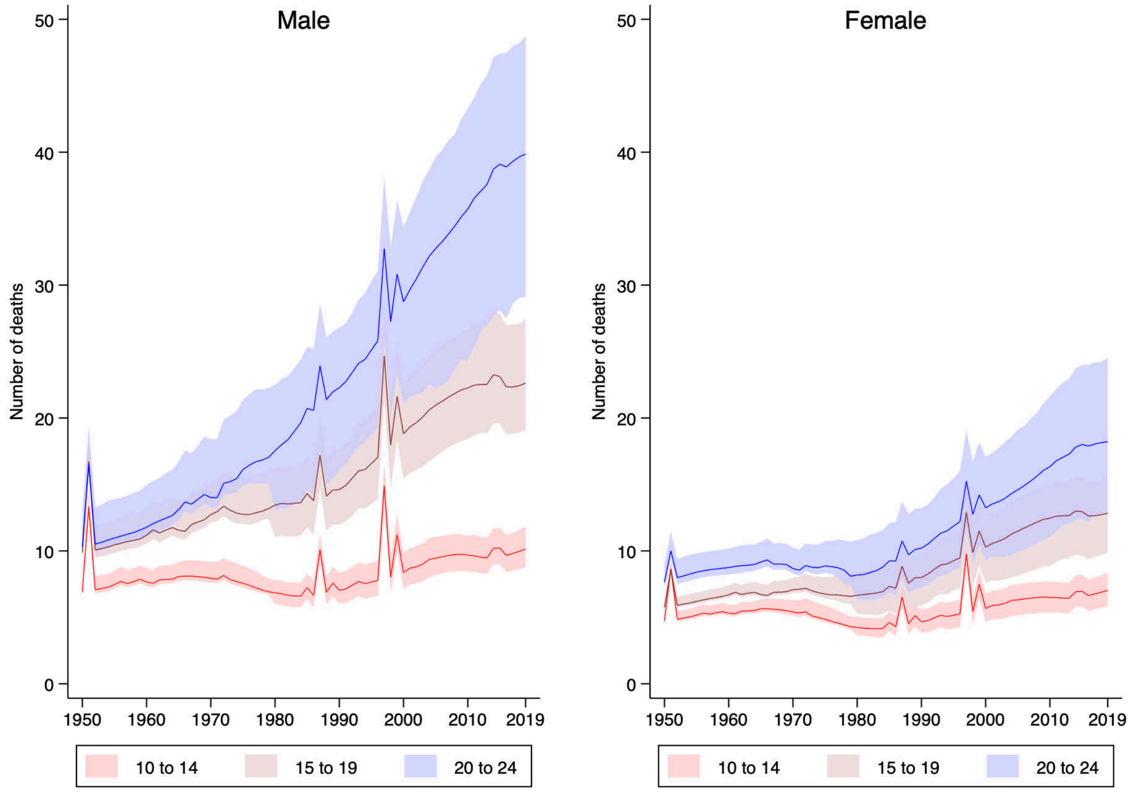

Figure S28c Mortality rate per 100,000 in 10-24 1980 – 2019 by cause: Vanuatu

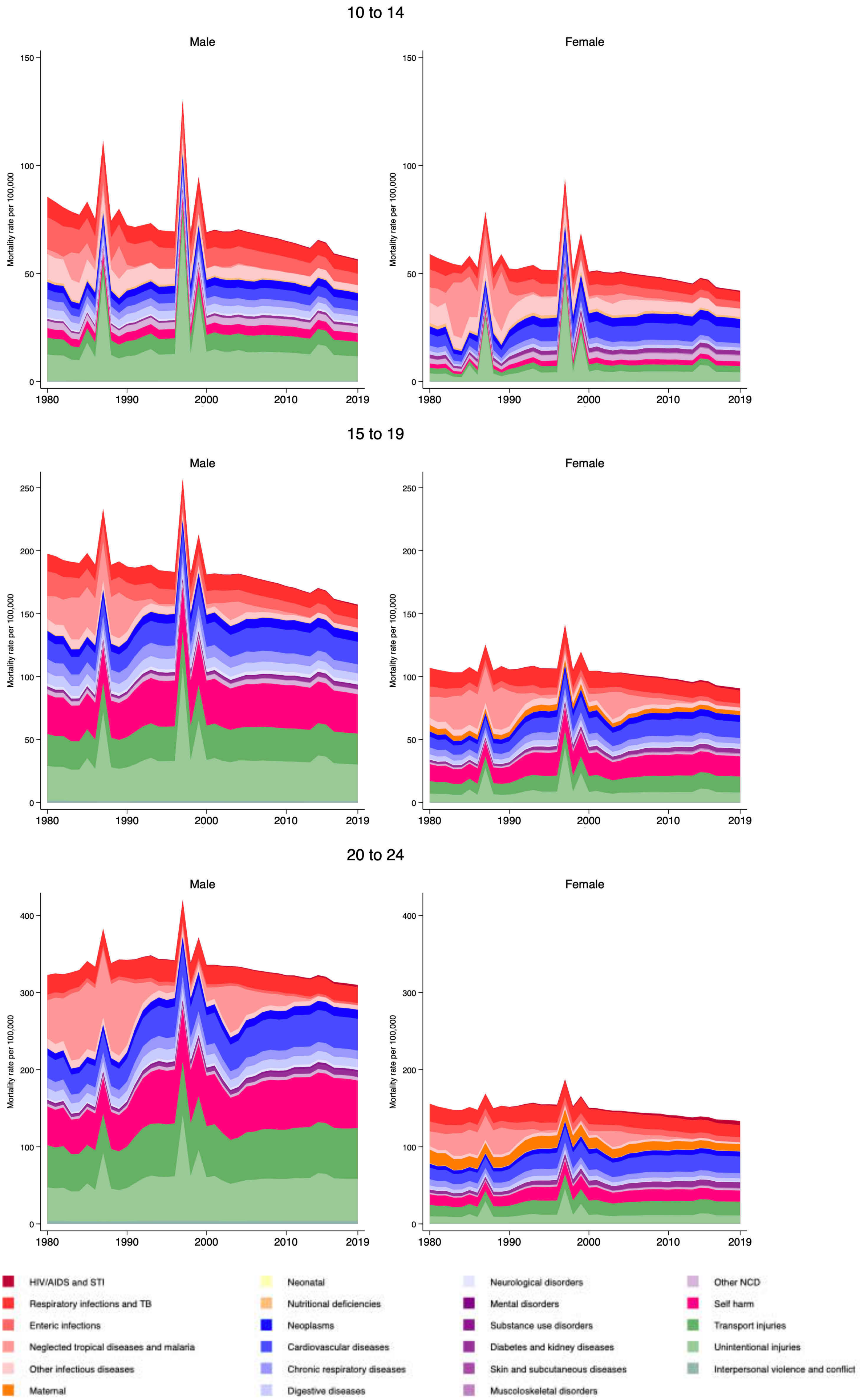

Figure S28d Number of deaths in 10-24 1980 – 2019 by cause: Vanuatu

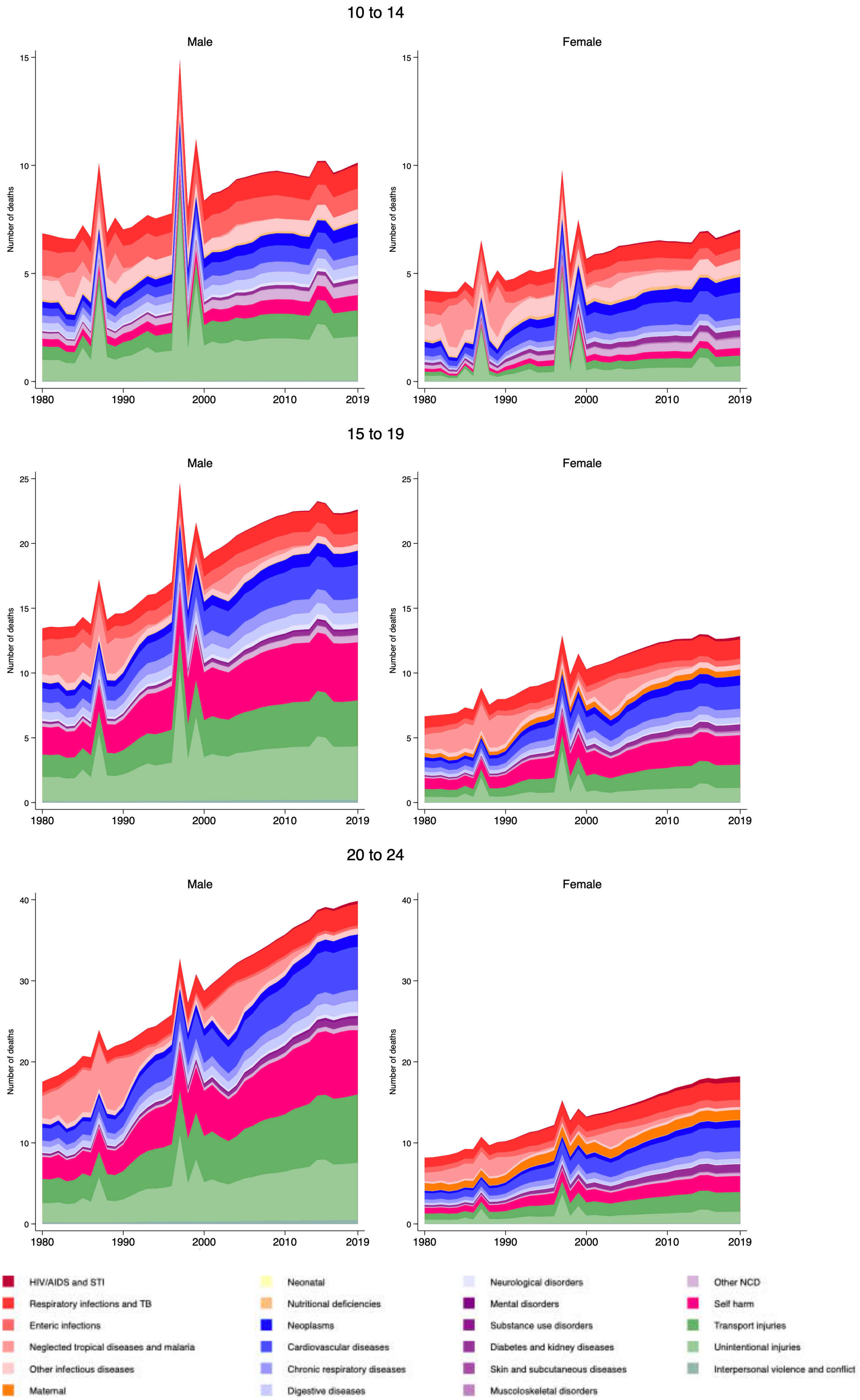

Figure S29: Central Europe, Eastern Europe, and Central Asia

Figure S29a All-cause mortality rate per 100,000 in 10-24 1950 – 2019: Central Europe, Eastern Europe, and Central Asia

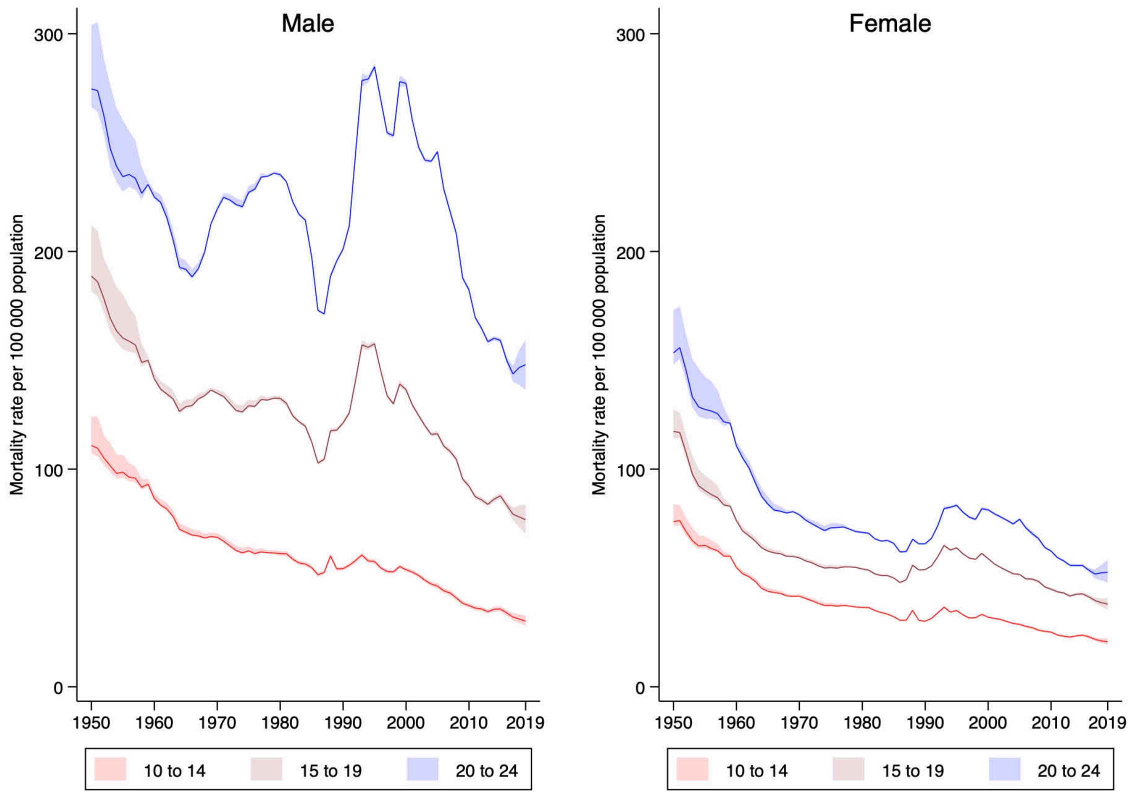

Figure S29b Number of deaths from all causes in 10-24 1950 – 2019: Central Europe, Eastern Europe, and Central

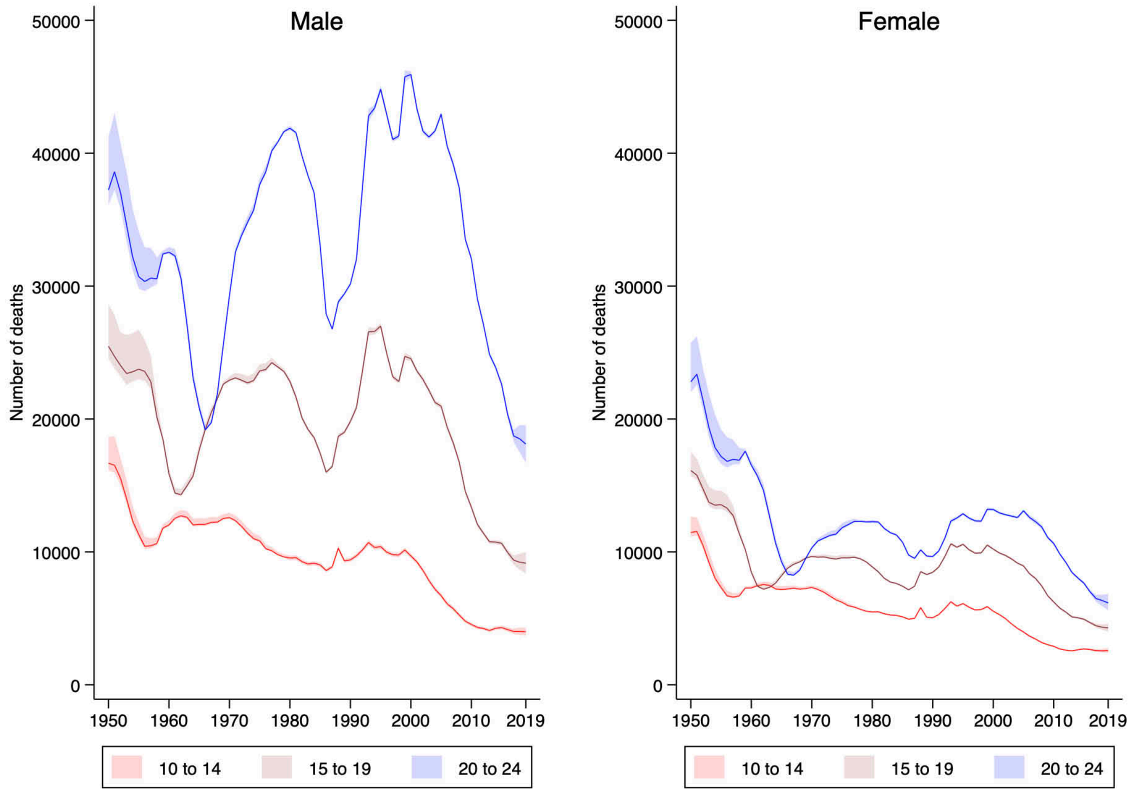

Asia

Figure S29c Mortality rate per 100,000 in 10-24 1980 – 2019 by cause: Central Europe, Eastern Europe, and Central Asia

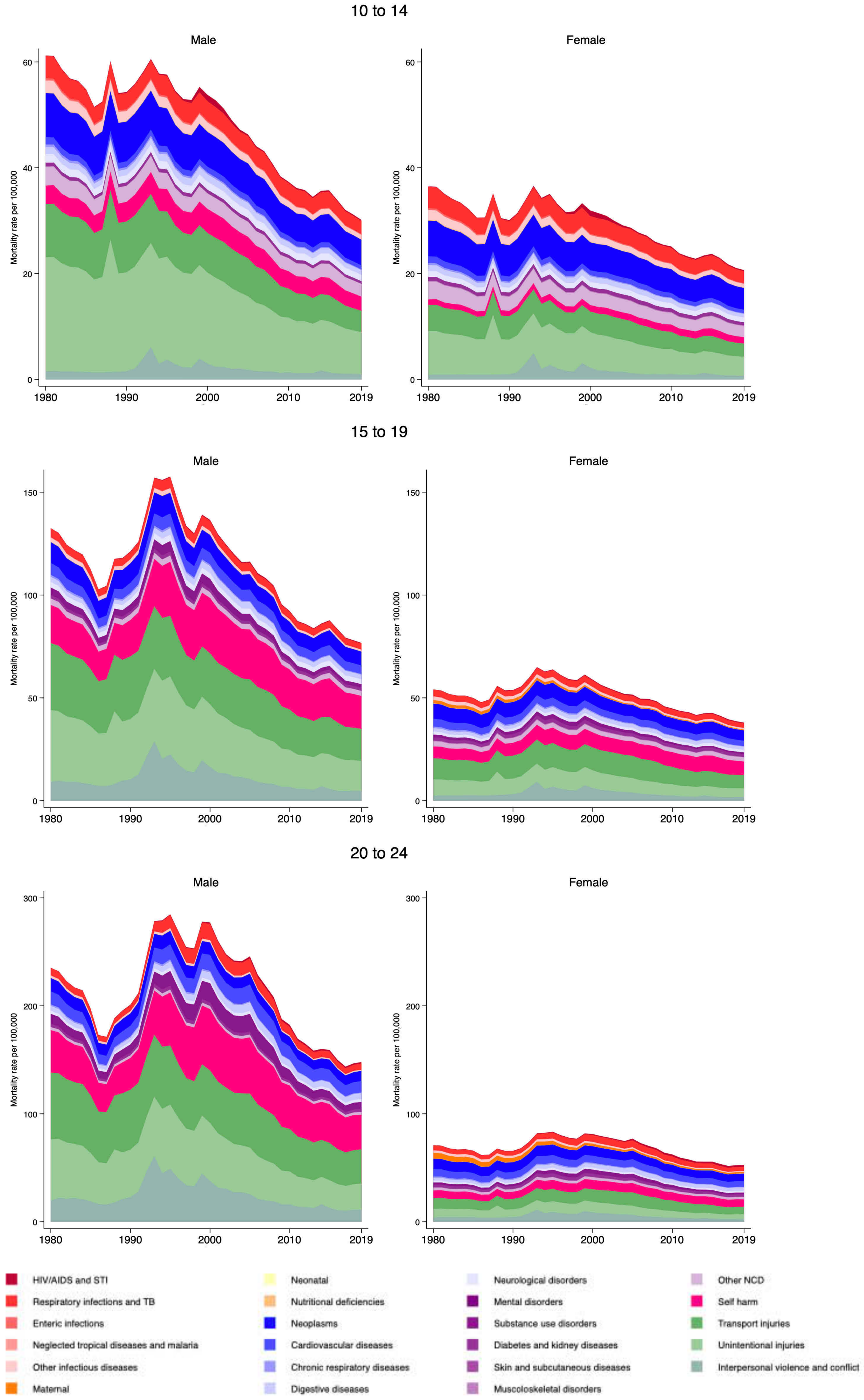

Figure S29d Number of deaths in 10-24 1980 – 2019 by cause: Central Europe, Eastern Europe, and Central Asia

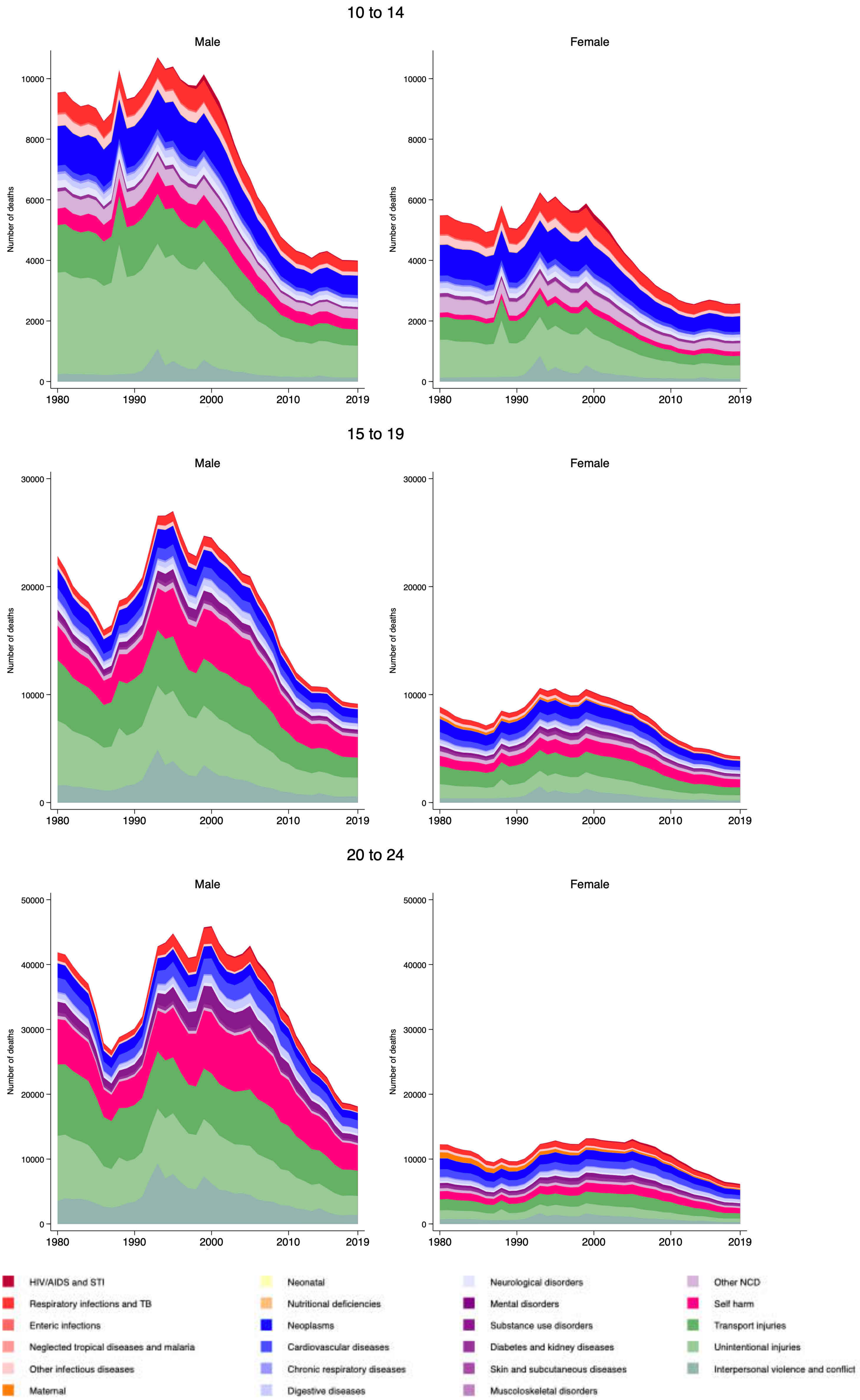

Figure S30: Central Asia

Figure S30a All-cause mortality rate per 100,000 in 10-24 1950 – 2019: Central Asia

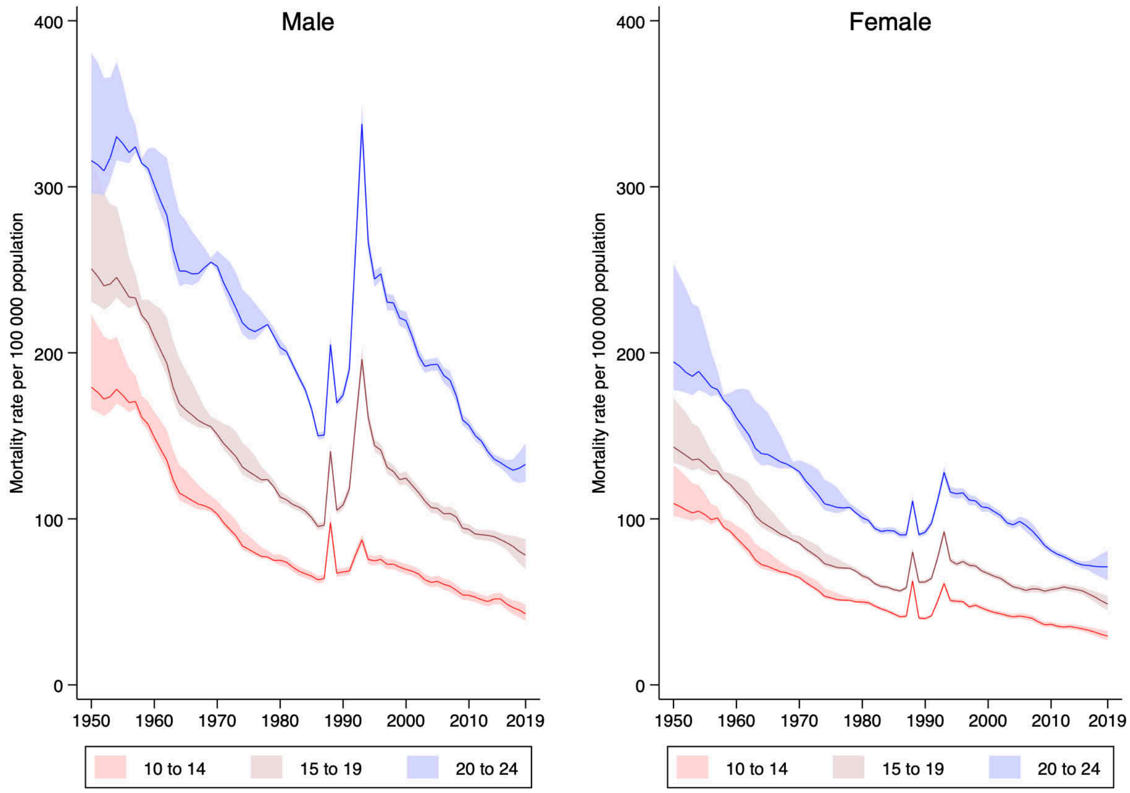

Figure S30b Number of deaths from all causes in 10-24 1950 – 2019: Central Asia

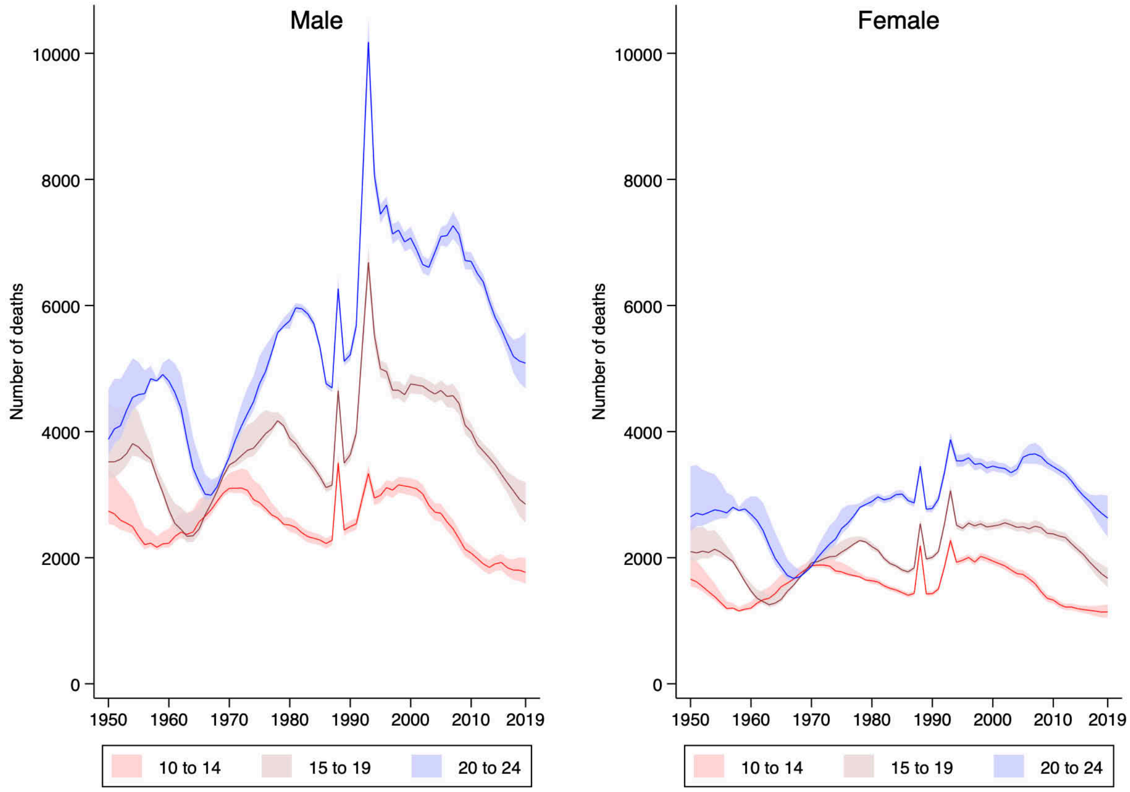

Figure S30c Mortality rate per 100,000 in 10-24 1980 – 2019 by cause: Central Asia

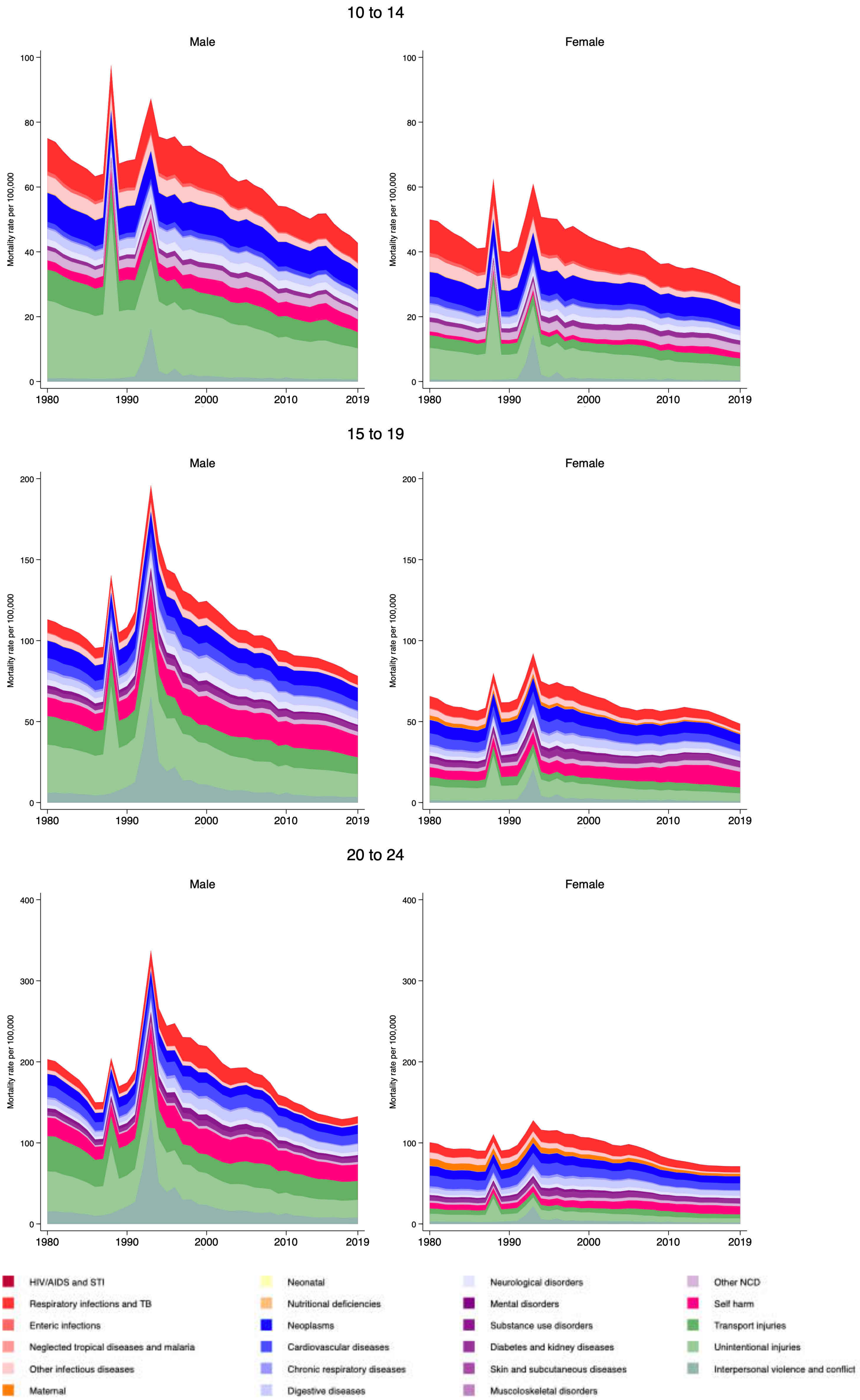

Figure S30d Number of deaths in 10-24 1980 – 2019 by cause: Central Asia

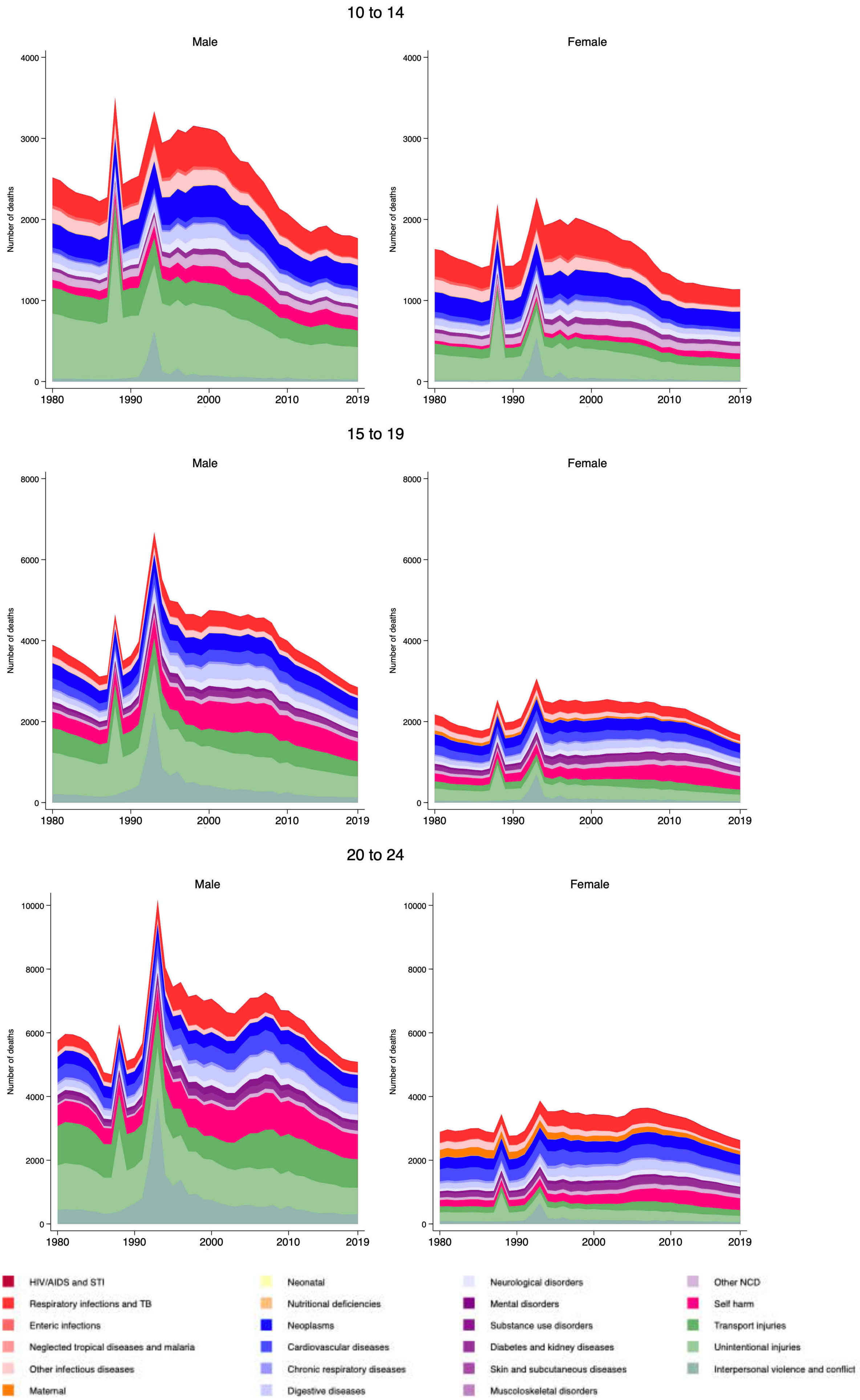

Figure S31: Armenia

Figure S31a All-cause mortality rate per 100,000 in 10-24 1950 – 2019: Armenia

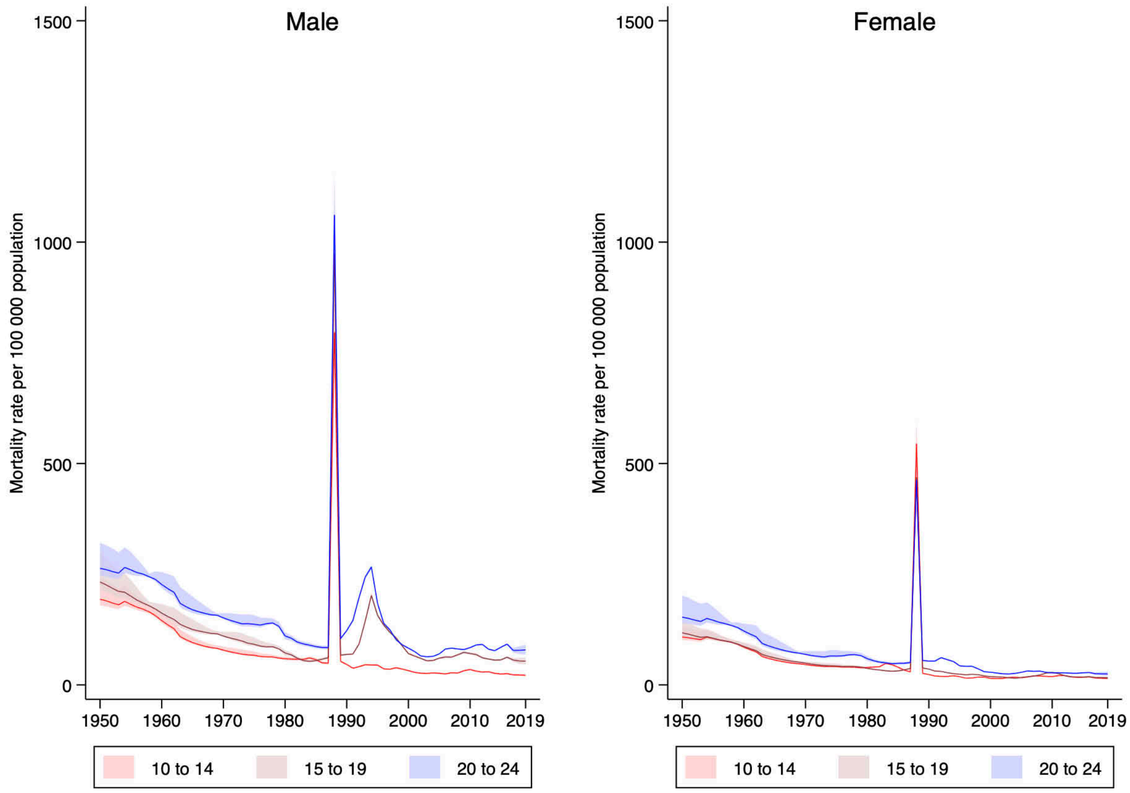

Figure S31b Number of deaths from all causes in 10-24 1950 – 2019: Armenia

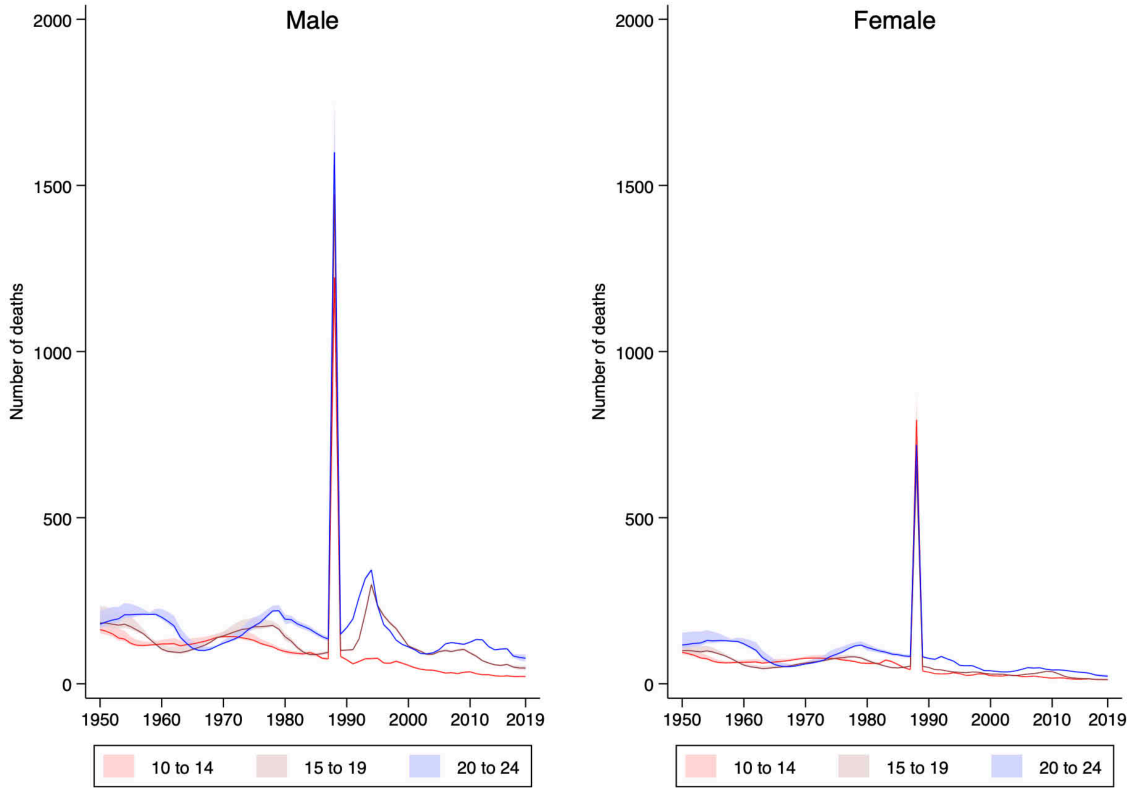

Figure S31c Mortality rate per 100,000 in 10-24 1980 – 2019 by cause: Armenia

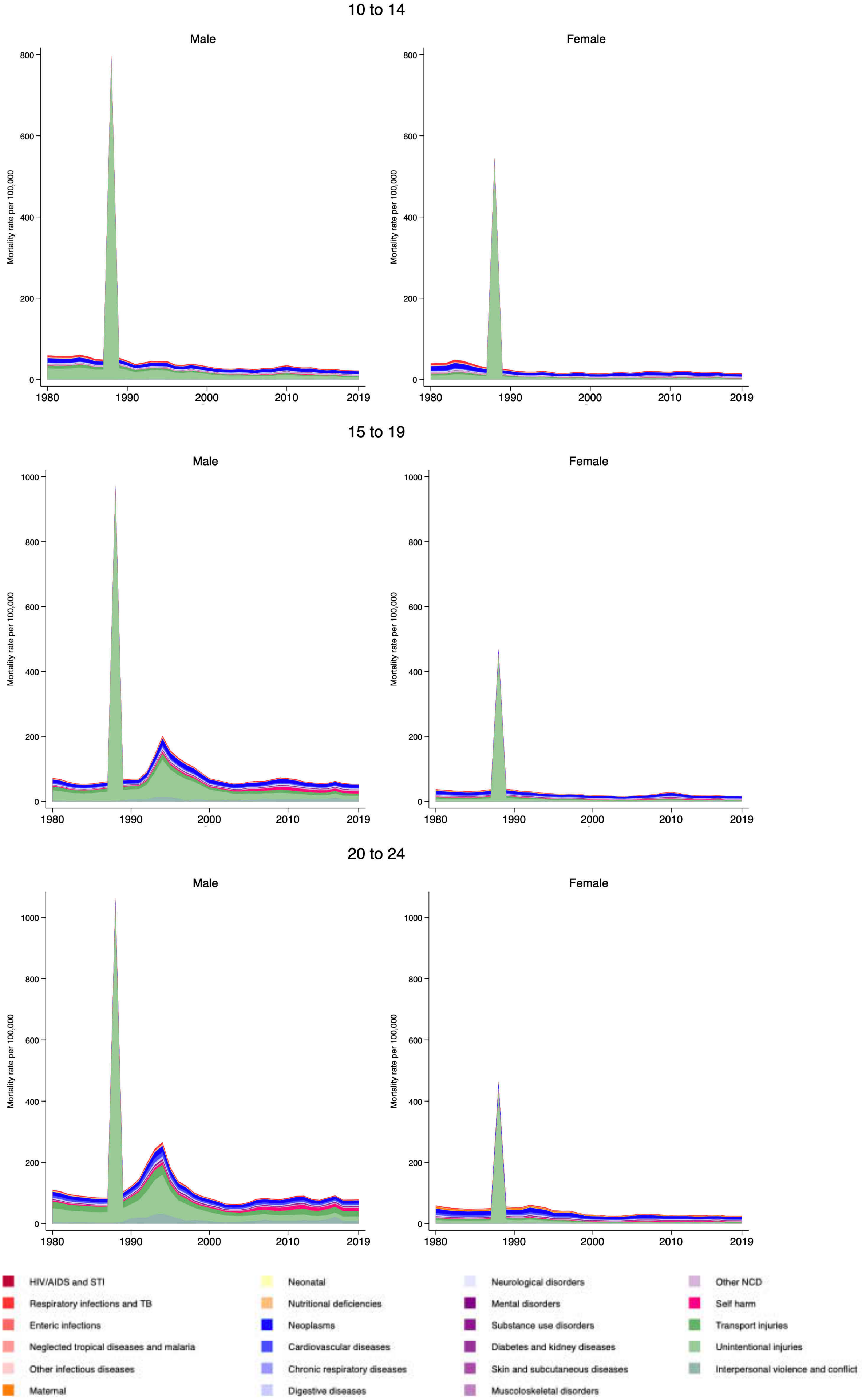

Figure S31d Number of deaths in 10-24 1980 – 2019 by cause: Armenia

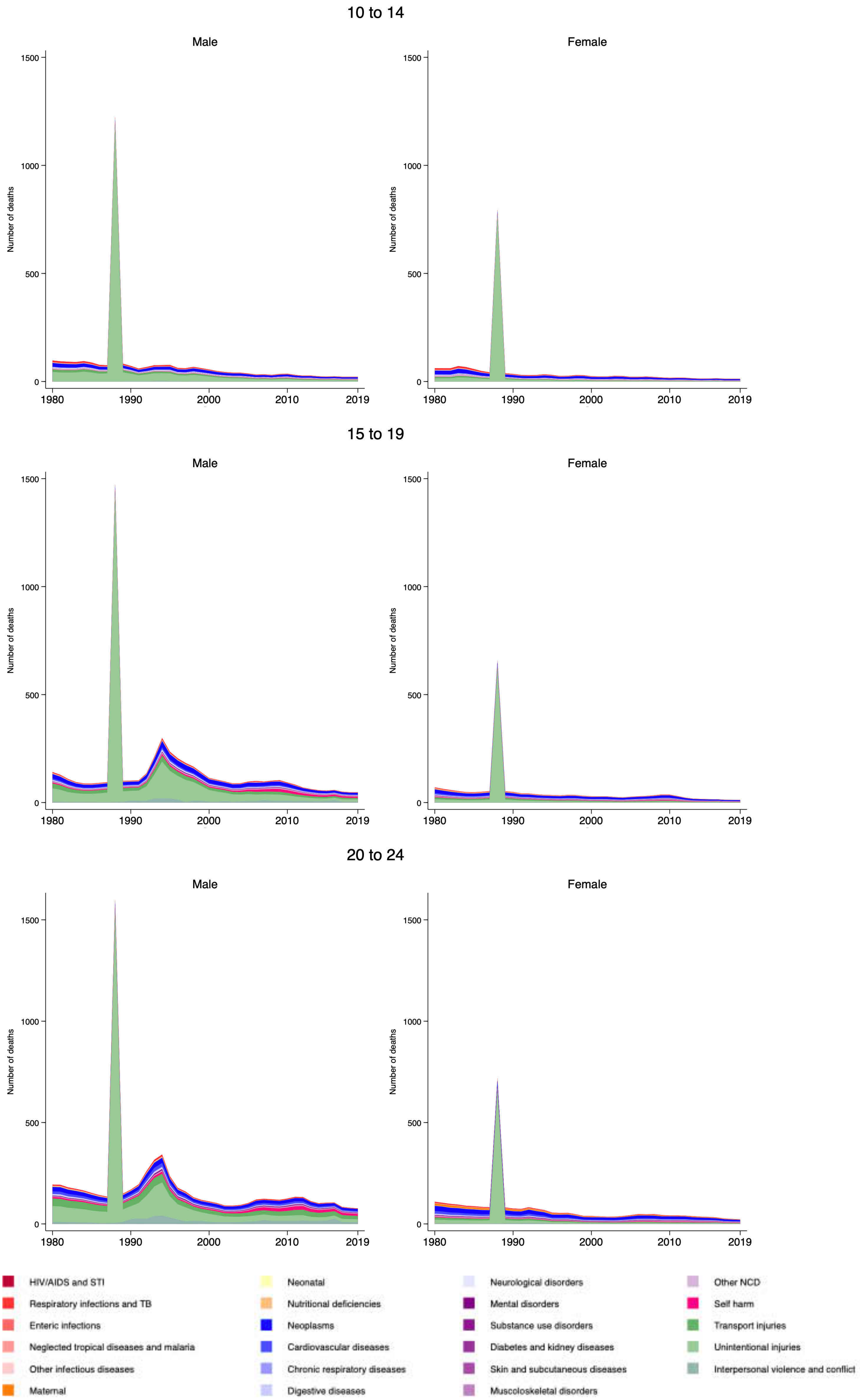

Figure S32: Azerbaijan

Figure S32a All-cause mortality rate per 100,000 in 10-24 1950 – 2019: Azerbaijan

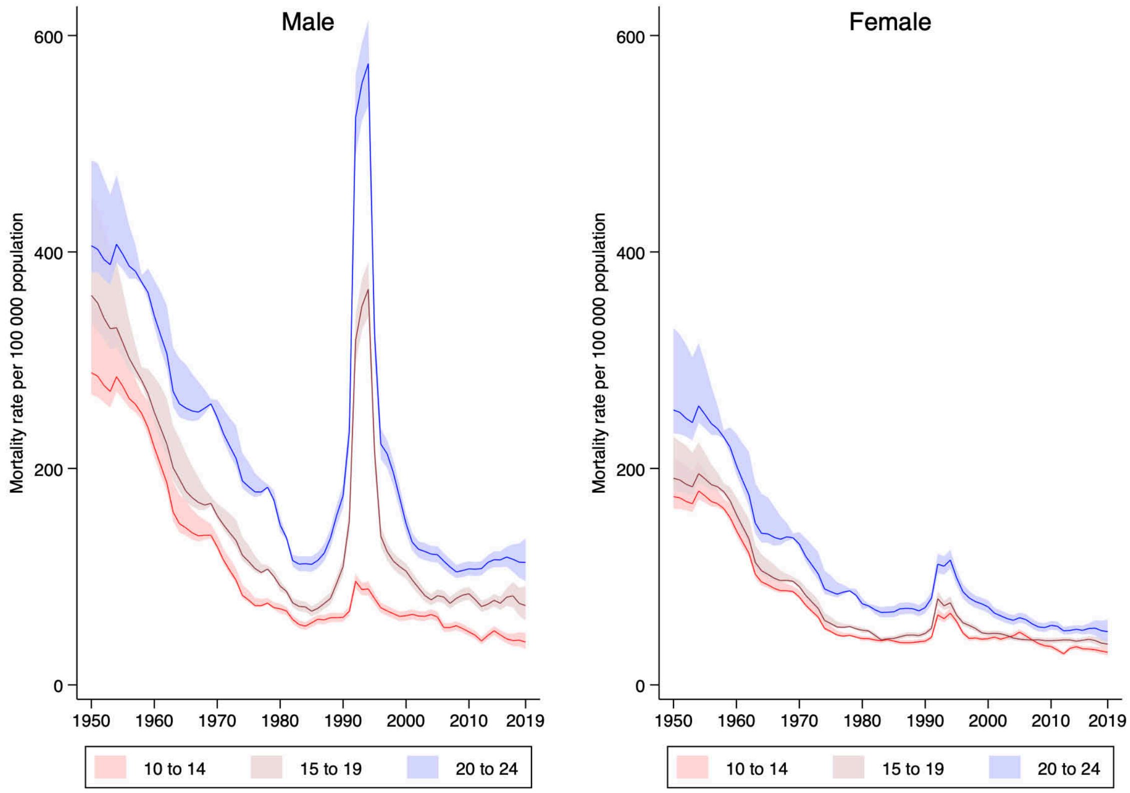

Figure S32b Number of deaths from all causes in 10-24 1950 – 2019: Azerbaijan

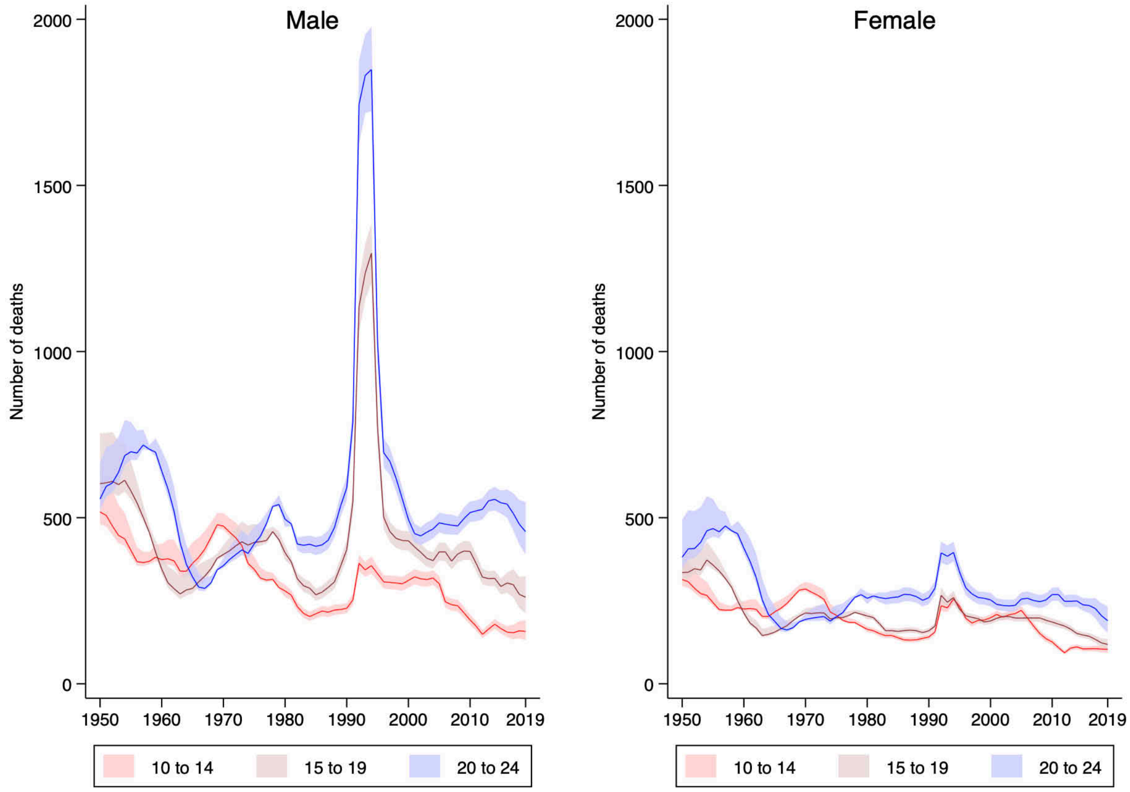

Figure S32c Mortality rate per 100,000 in 10-24 1980 – 2019 by cause: Azerbaijan

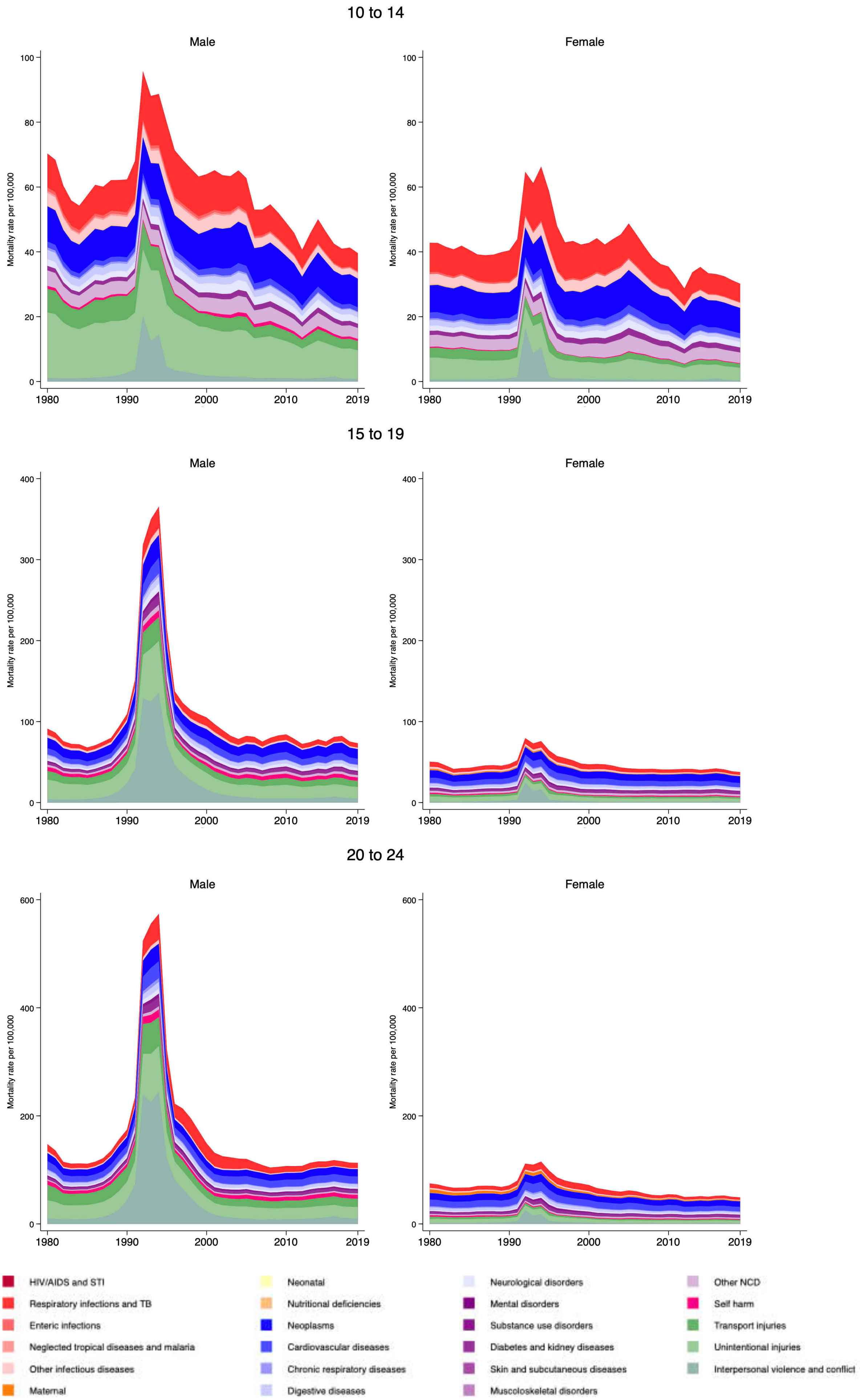

Figure S32d Number of deaths in 10-24 1980 – 2019 by cause: Azerbaijan

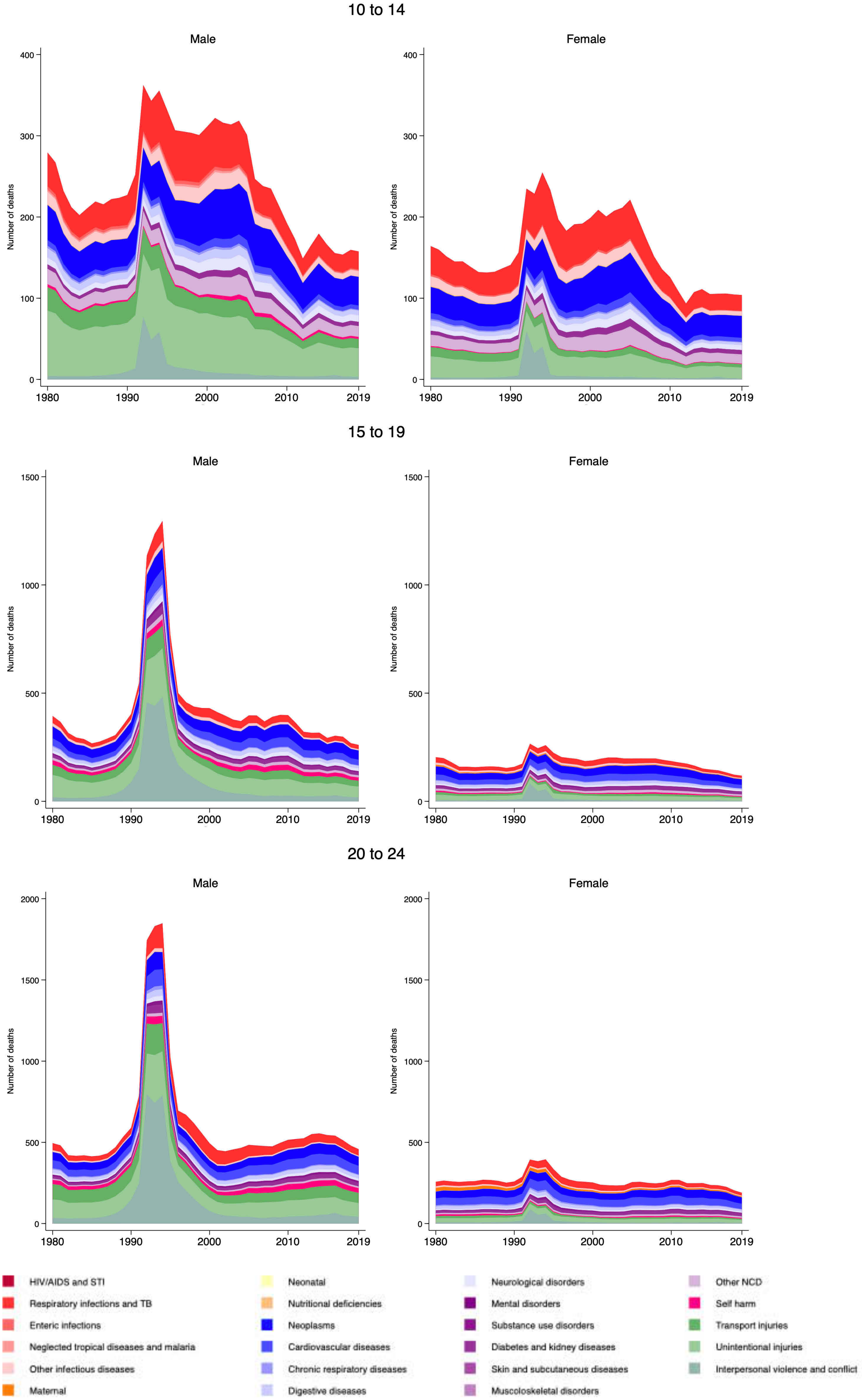

Figure S33: Georgia

Figure S33a All-cause mortality rate per 100,000 in 10-24 1950 – 2019: Georgia

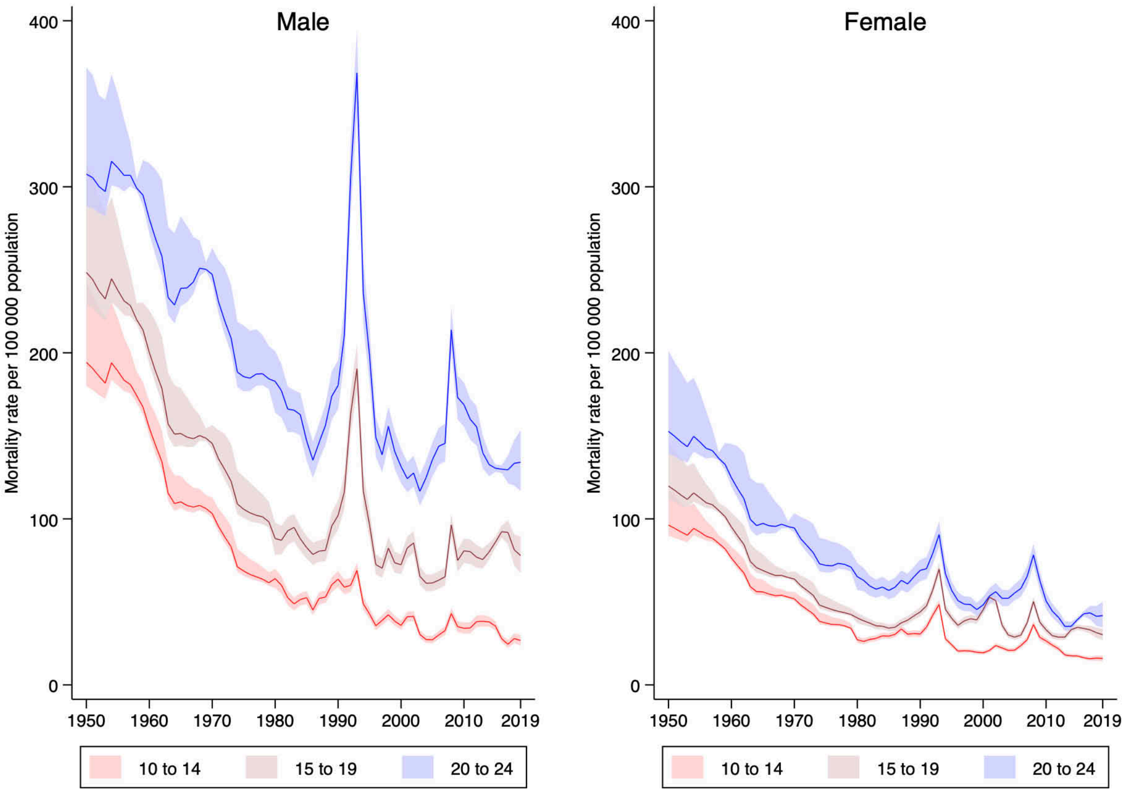

Figure S33b Number of deaths from all causes in 10-24 1950 – 2019: Georgia

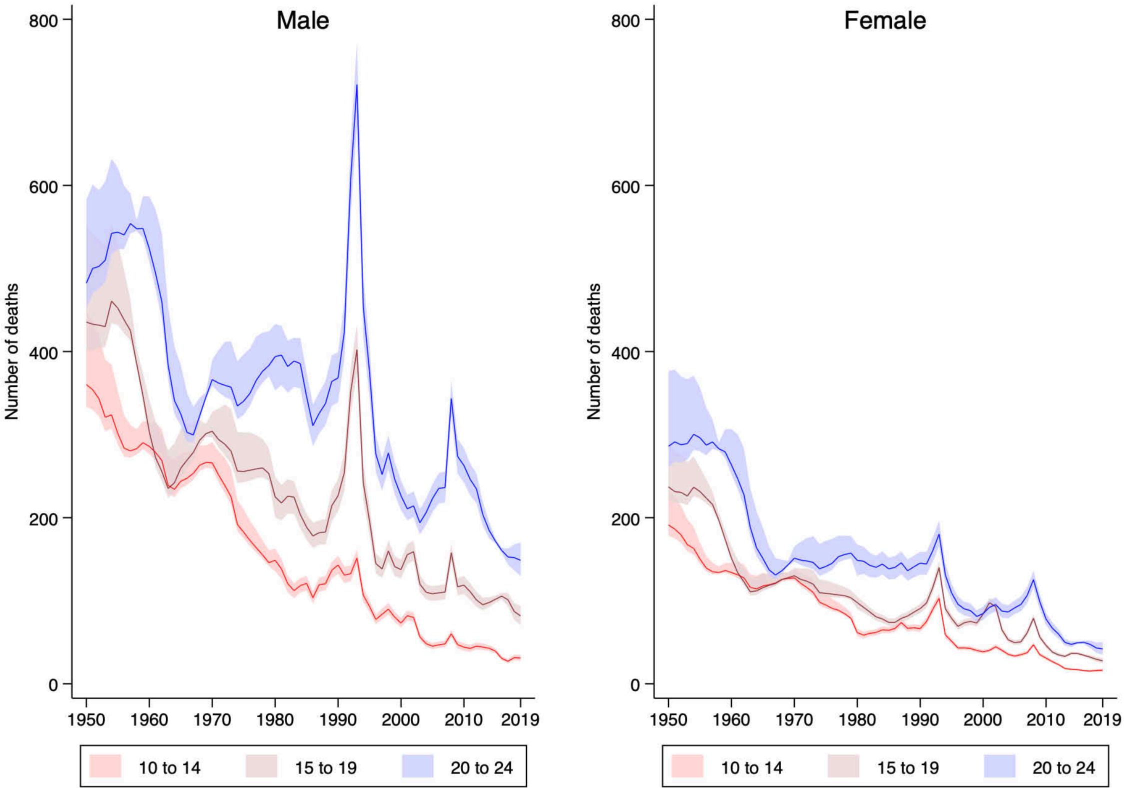

Figure S33c Mortality rate per 100,000 in 10-24 1980 – 2019 by cause: Georgia

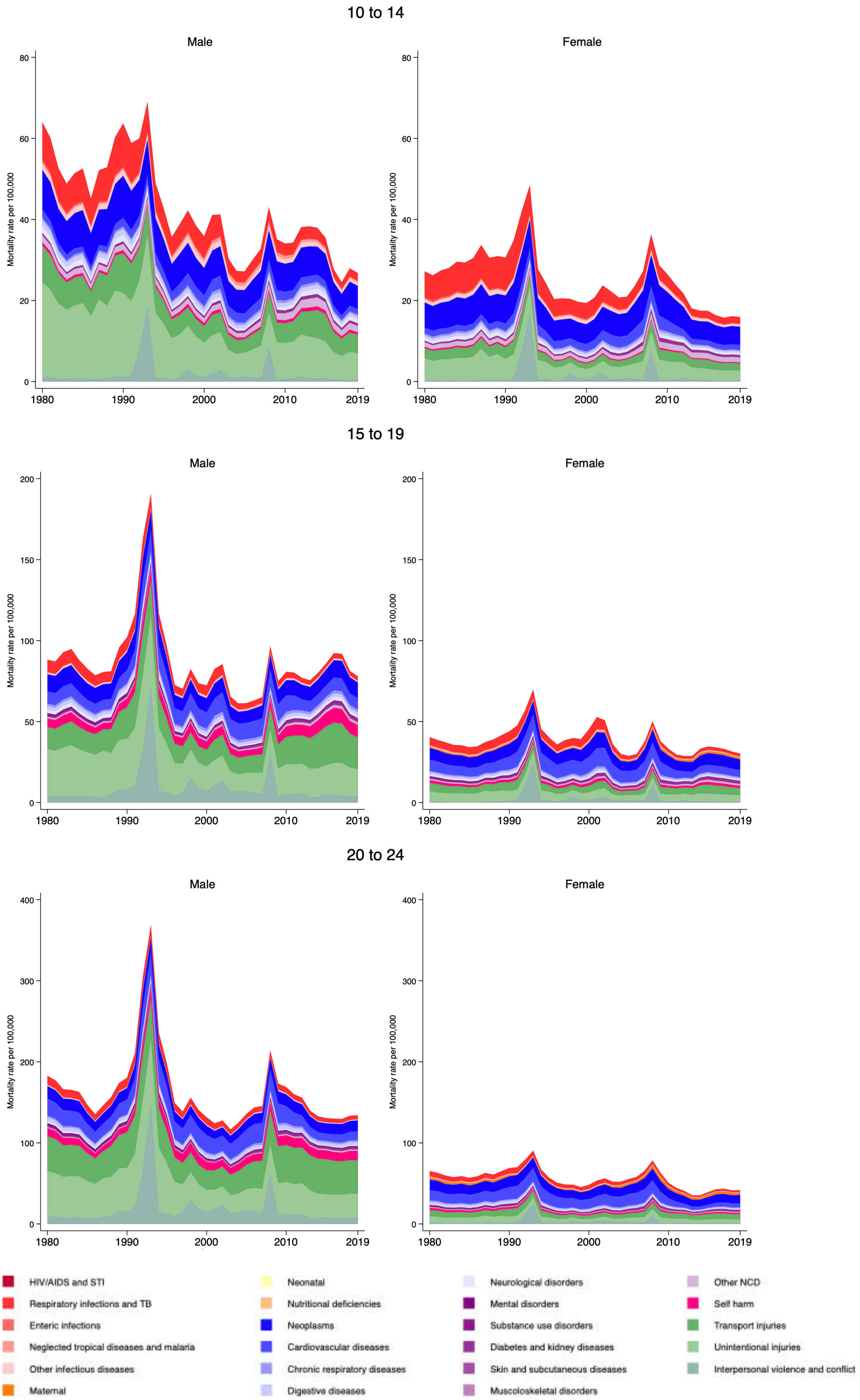

Figure S33d Number of deaths in 10-24 1980 – 2019 by cause: Georgia

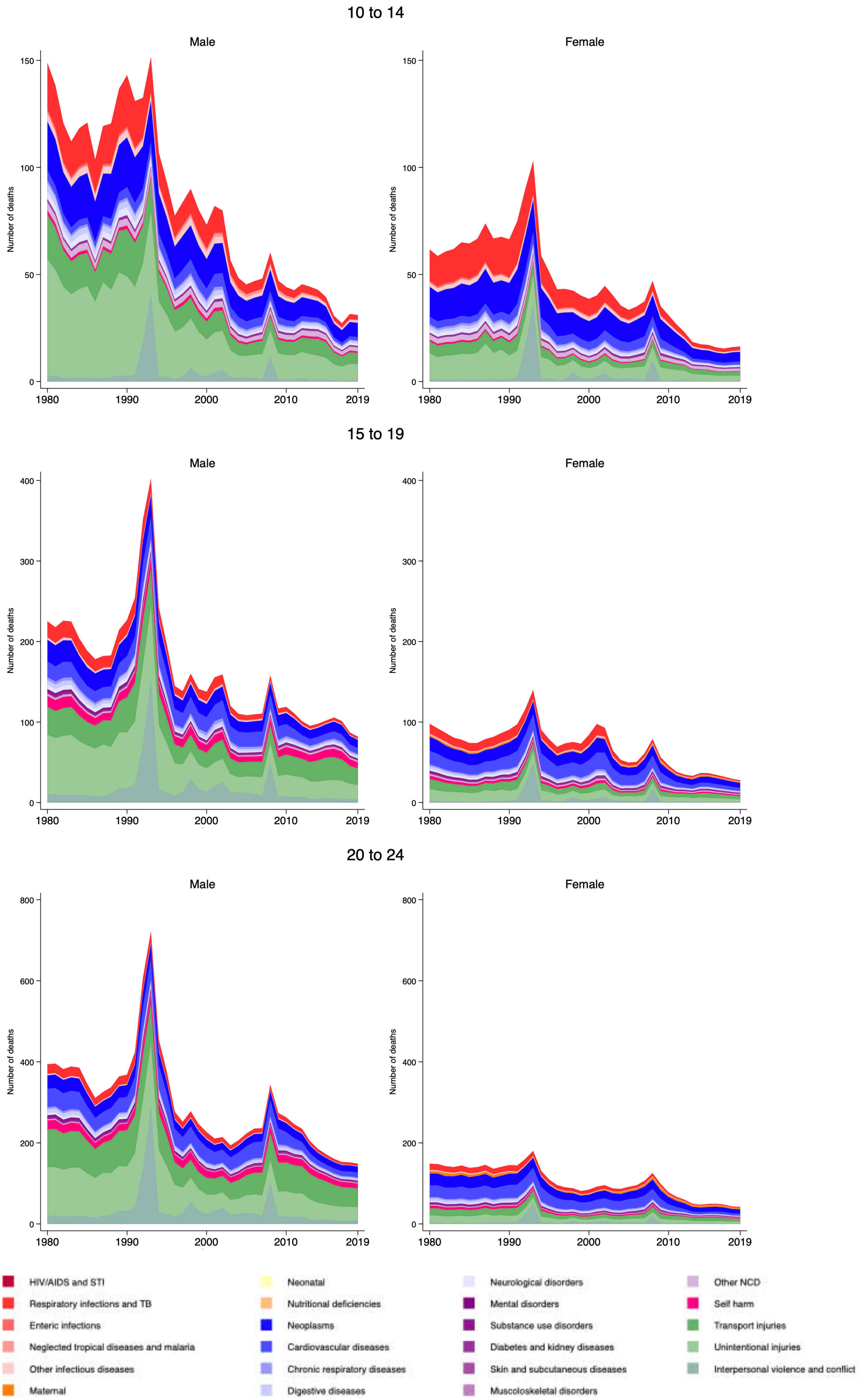

Figure S34: Kazakhstan

Figure S34a All-cause mortality rate per 100,000 in 10-24 1950 – 2019: Kazakhstan

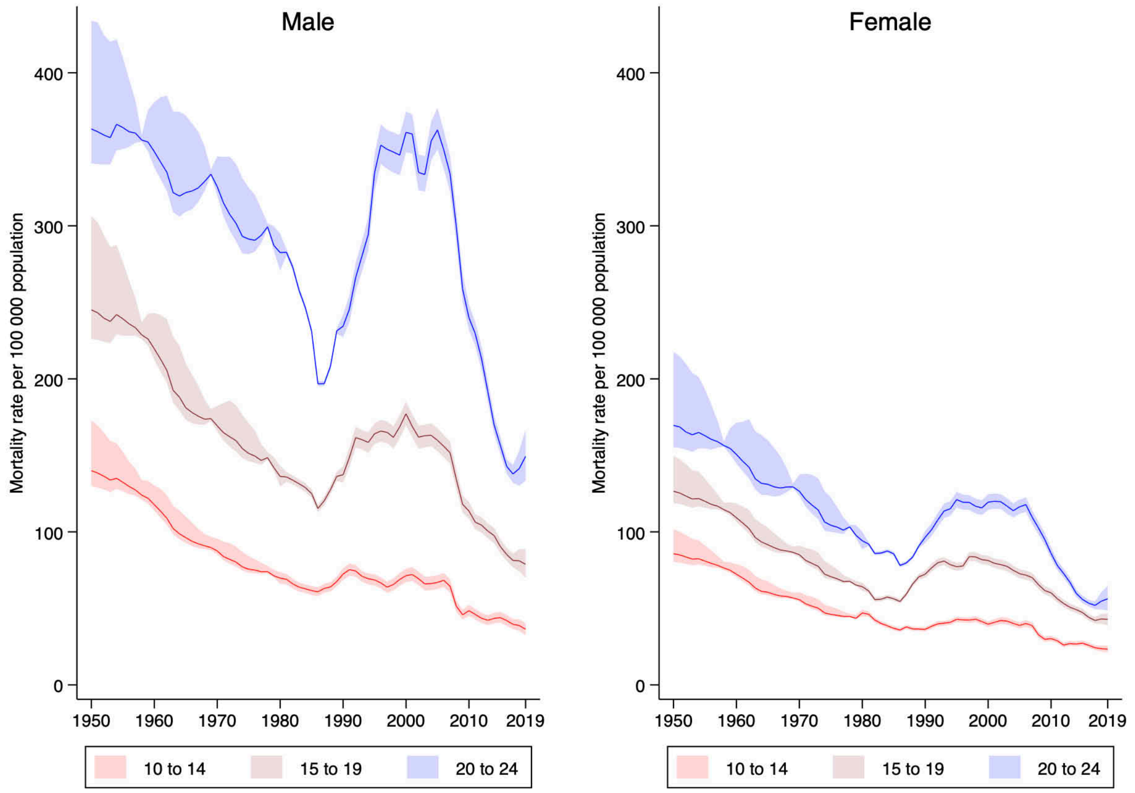

Figure S34b Number of deaths from all causes in 10-24 1950 – 2019: Kazakhstan

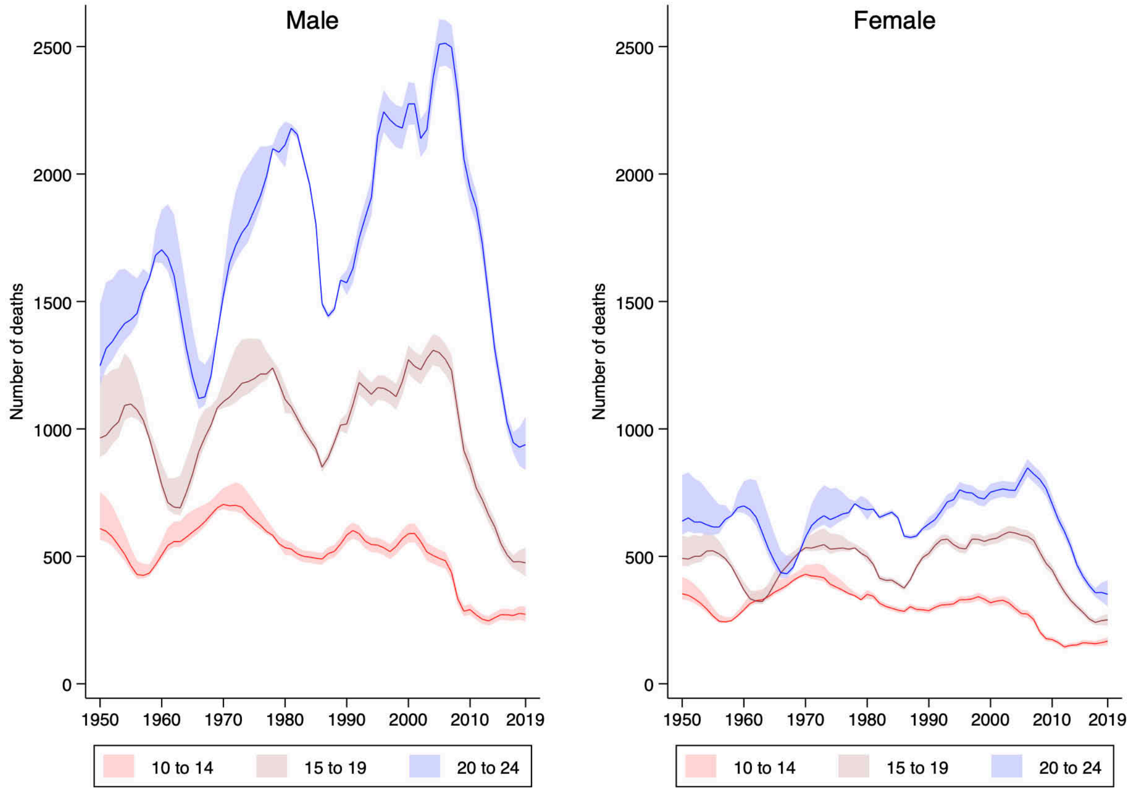

Figure S34c Mortality rate per 100,000 in 10-24 1980 – 2019 by cause: Kazakhstan

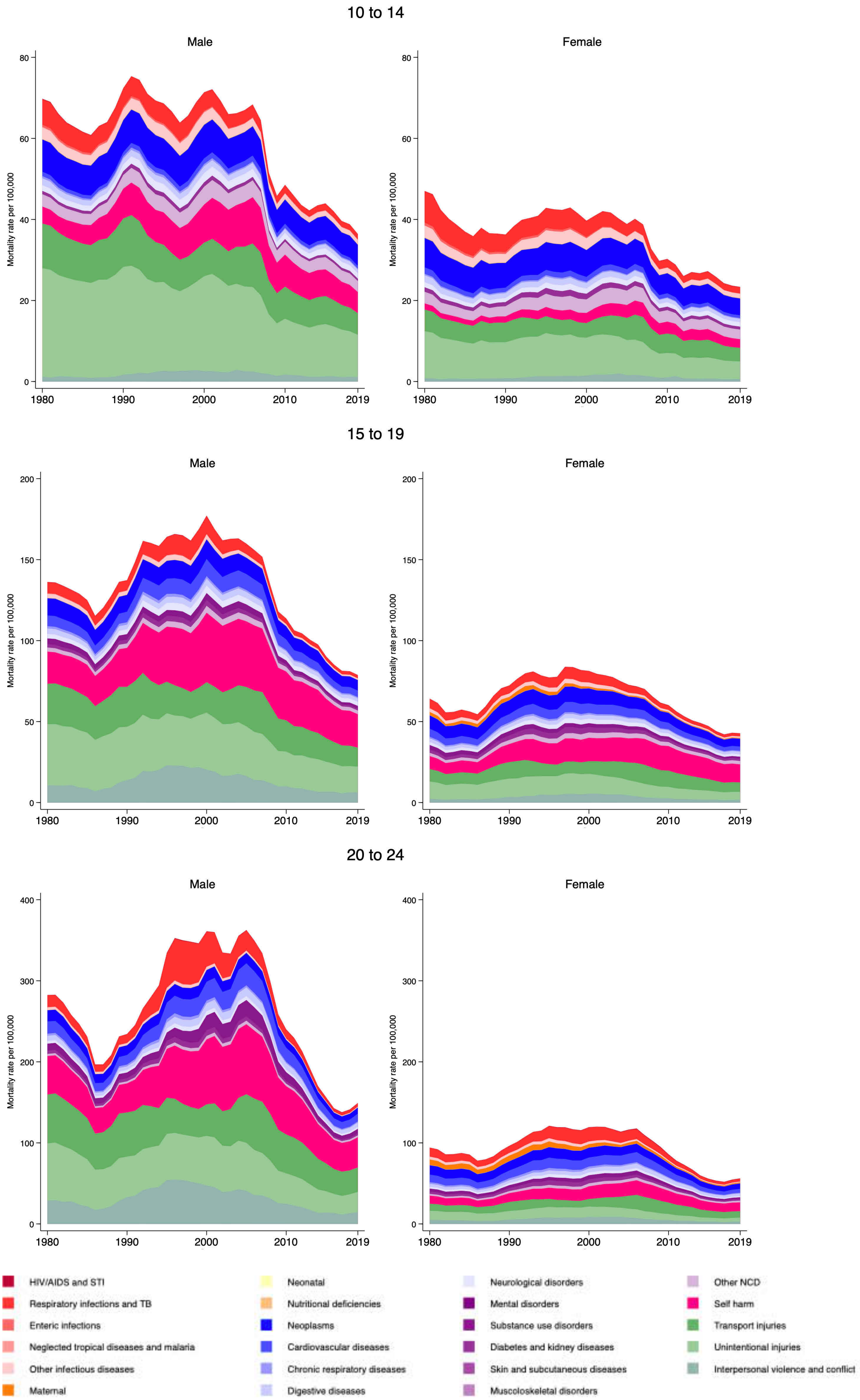

Figure S34d Number of deaths in 10-24 1980 – 2019 by cause: Kazakhstan

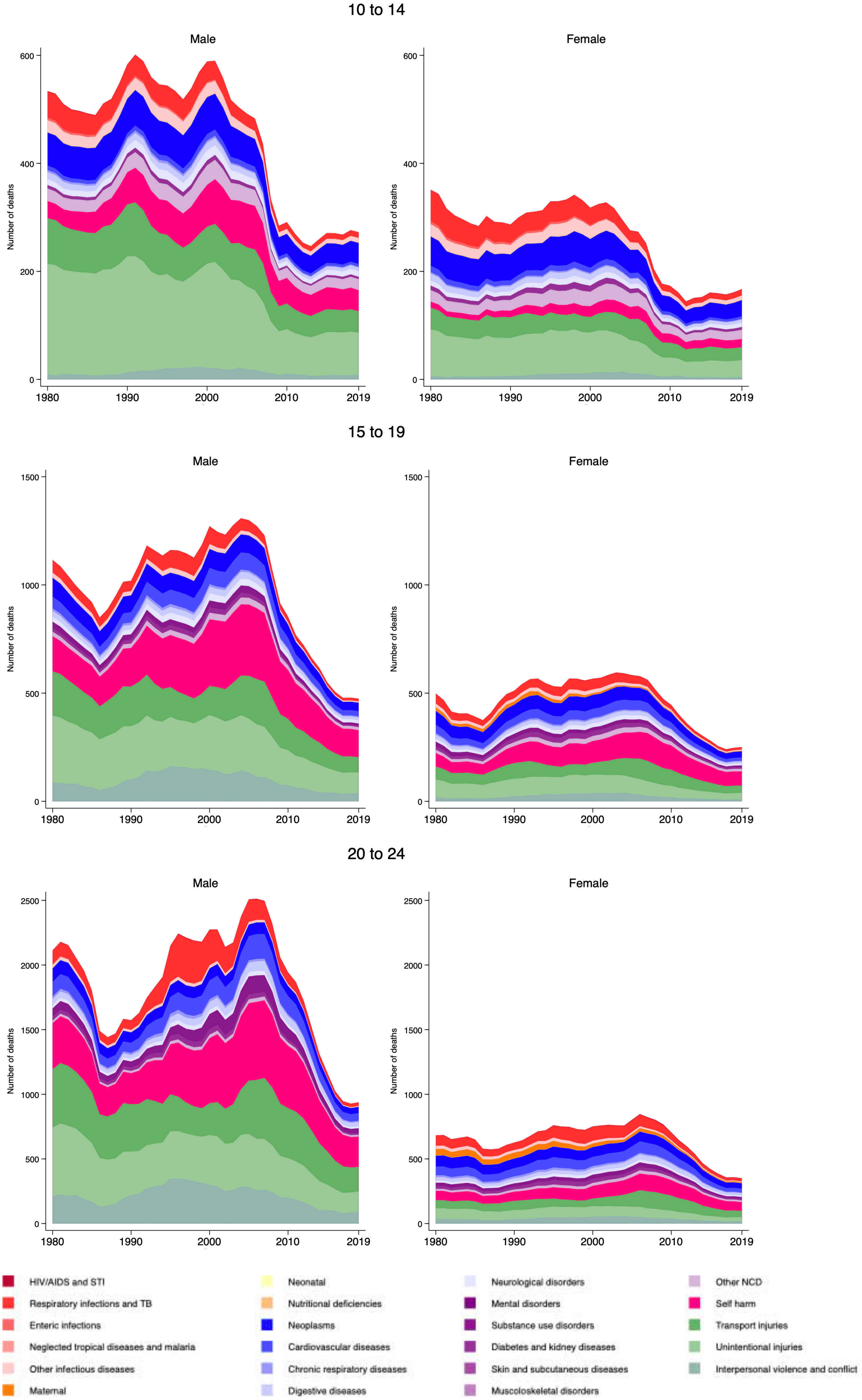

Figure S35: Kyrgyzstan

Figure S35a All-cause mortality rate per 100,000 in 10-24 1950 – 2019: Kyrgyzstan

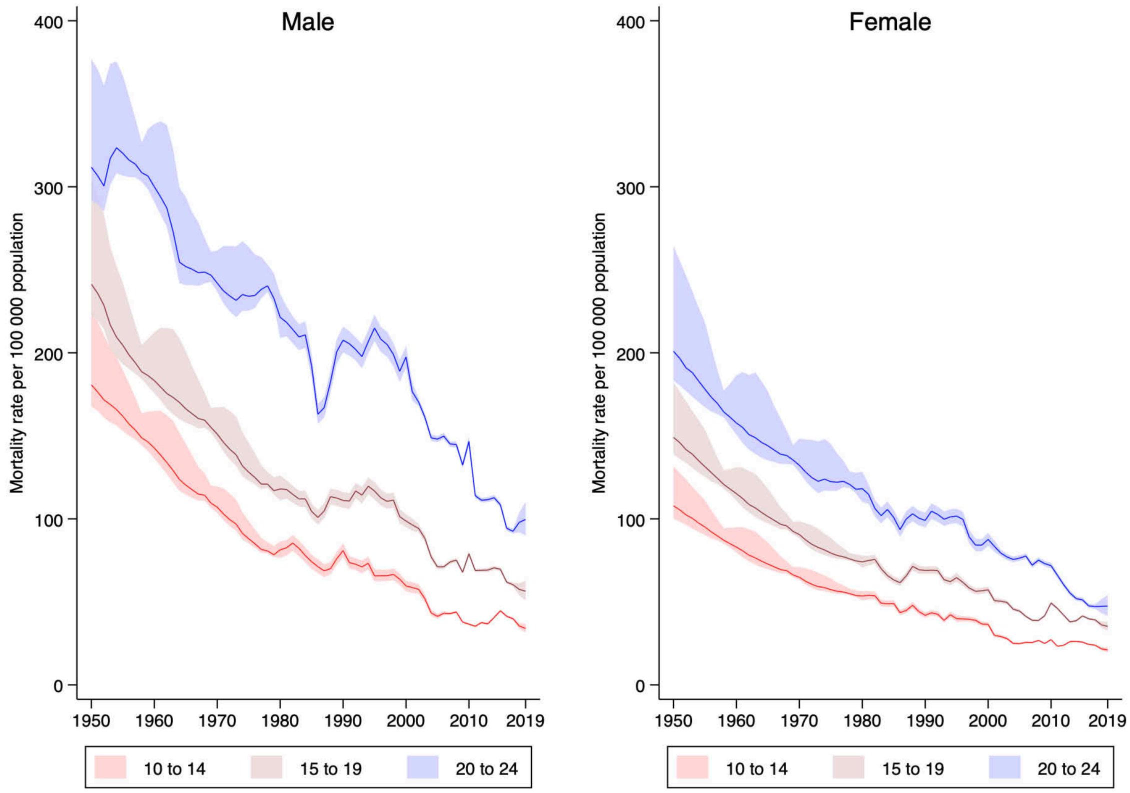

Figure S35b Number of deaths from all causes in 10-24 1950 – 2019: Kyrgyzstan

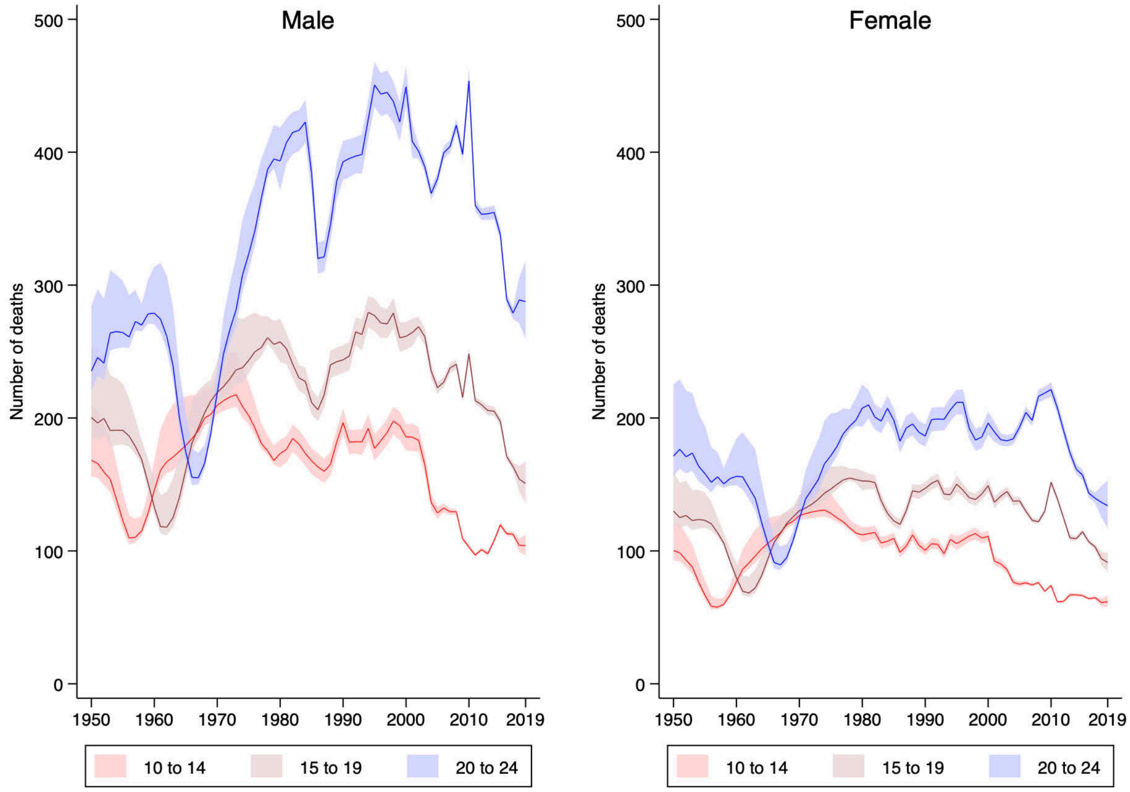

Figure S35c Mortality rate per 100,000 in 10-24 1980 – 2019 by cause: Kyrgyzstan

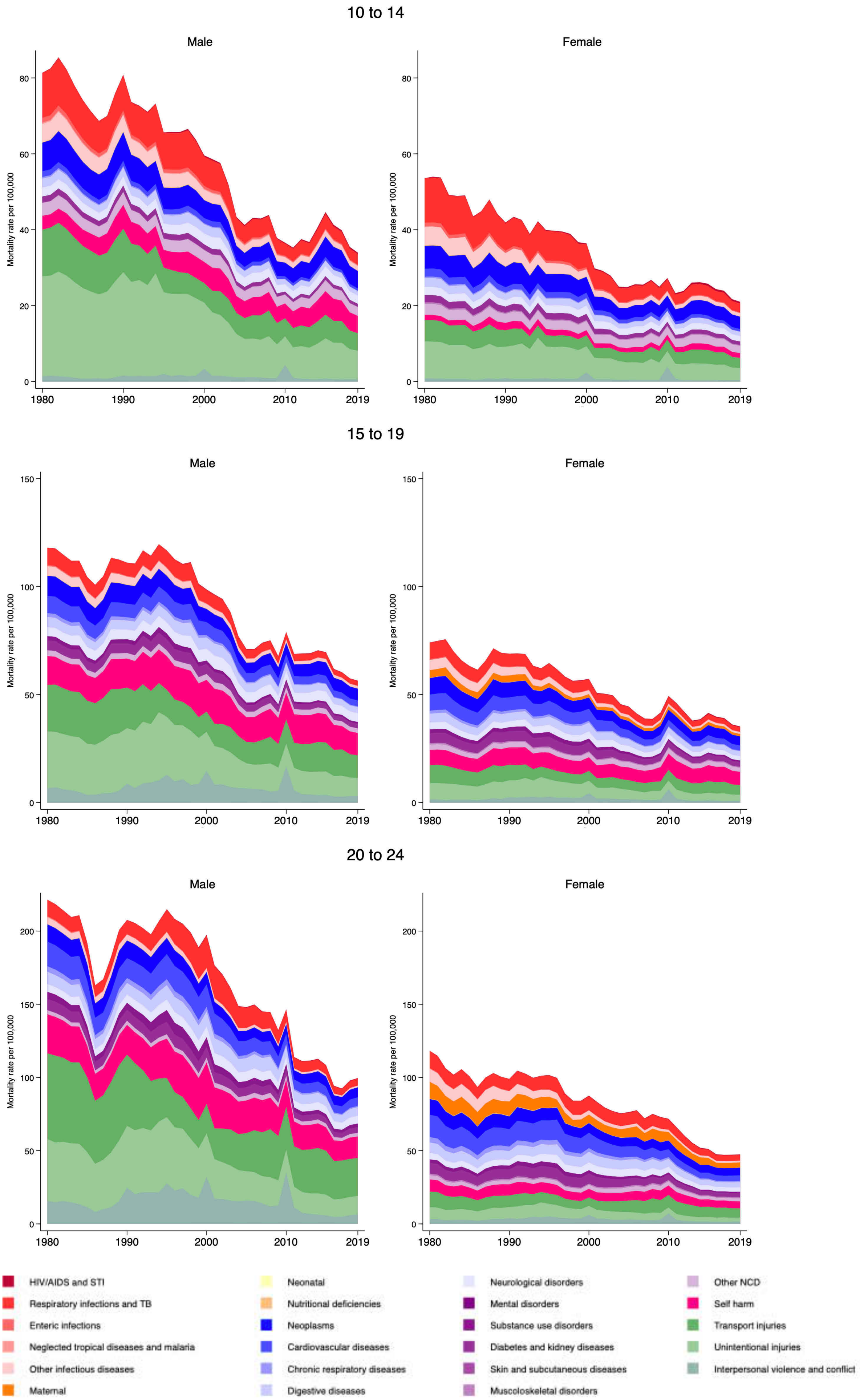

Figure S35d Number of deaths in 10-24 1980 – 2019 by cause: Kyrgyzstan

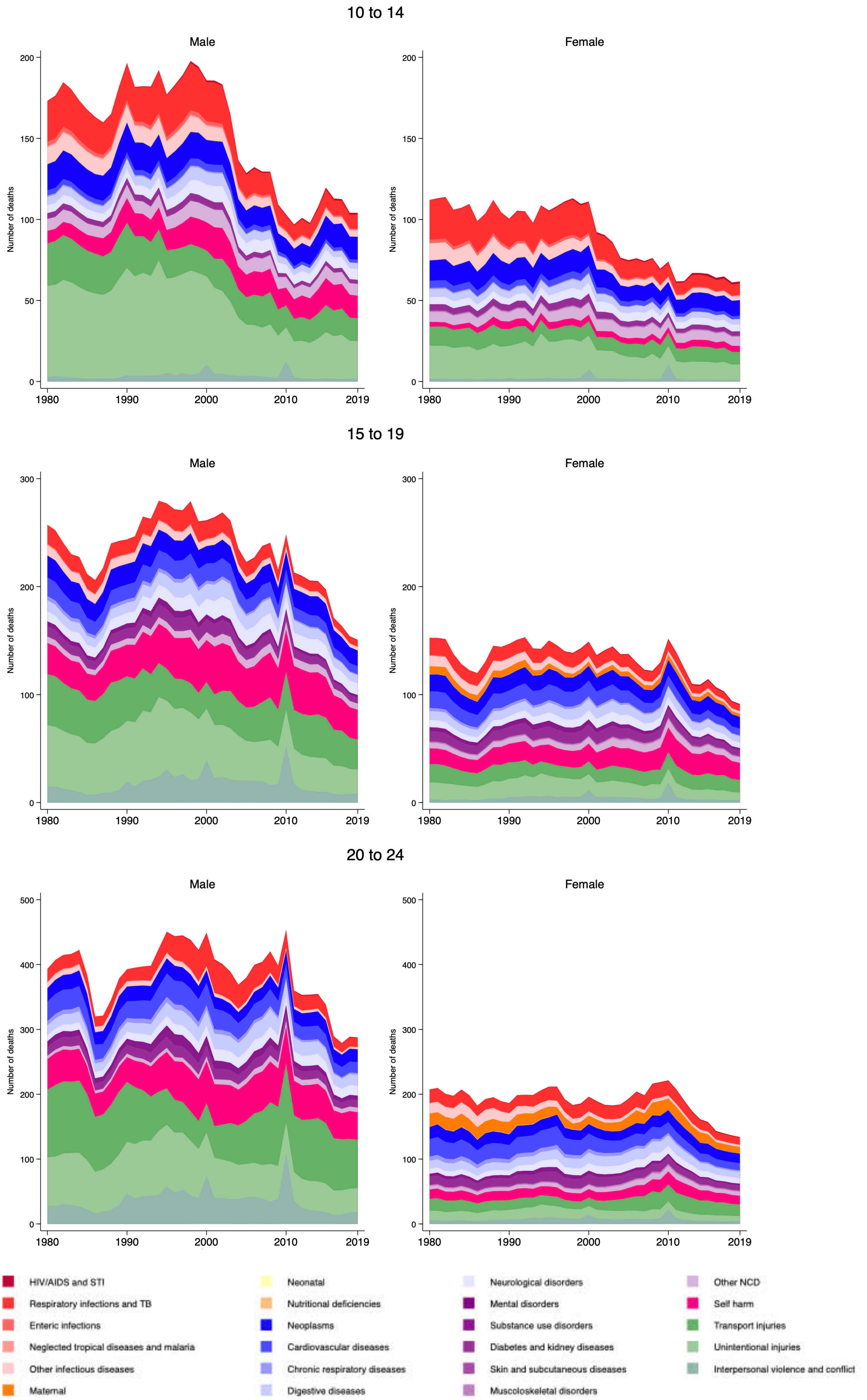

Figure S36: Mongolia

Figure S36a All-cause mortality rate per 100,000 in 10-24 1950 – 2019: Mongolia

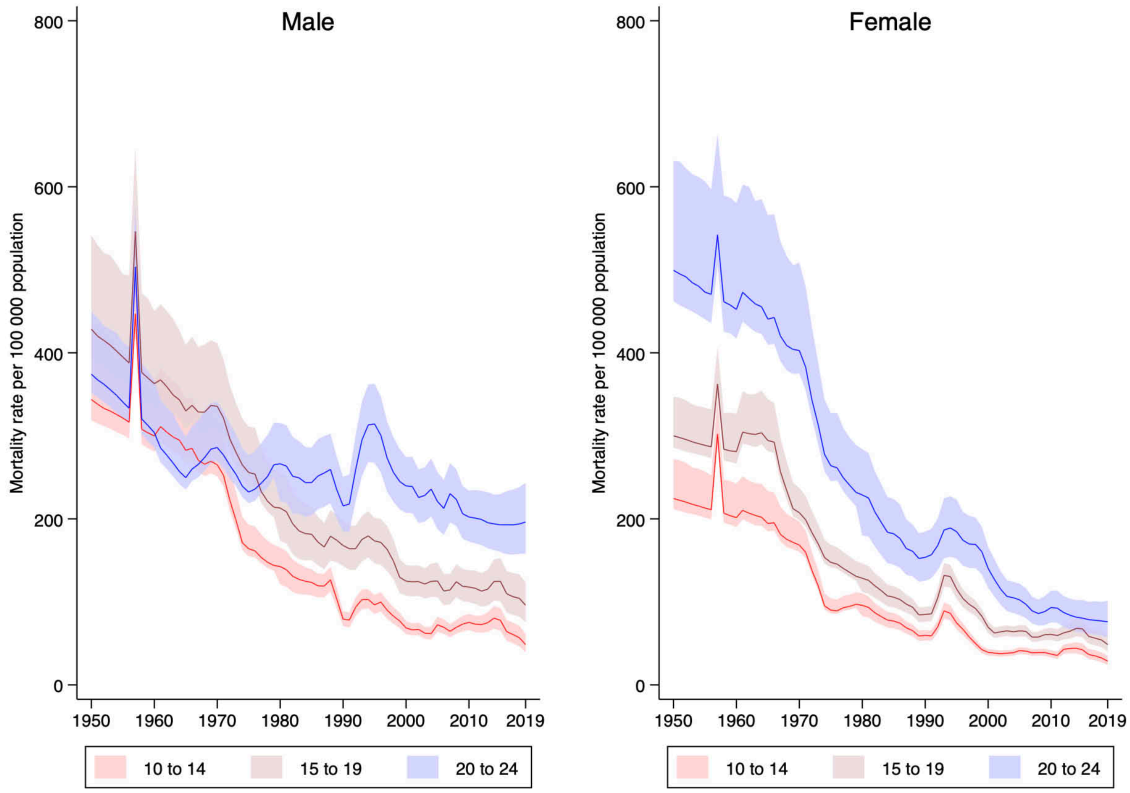

Figure S36b Number of deaths from all causes in 10-24 1950 – 2019: Mongolia

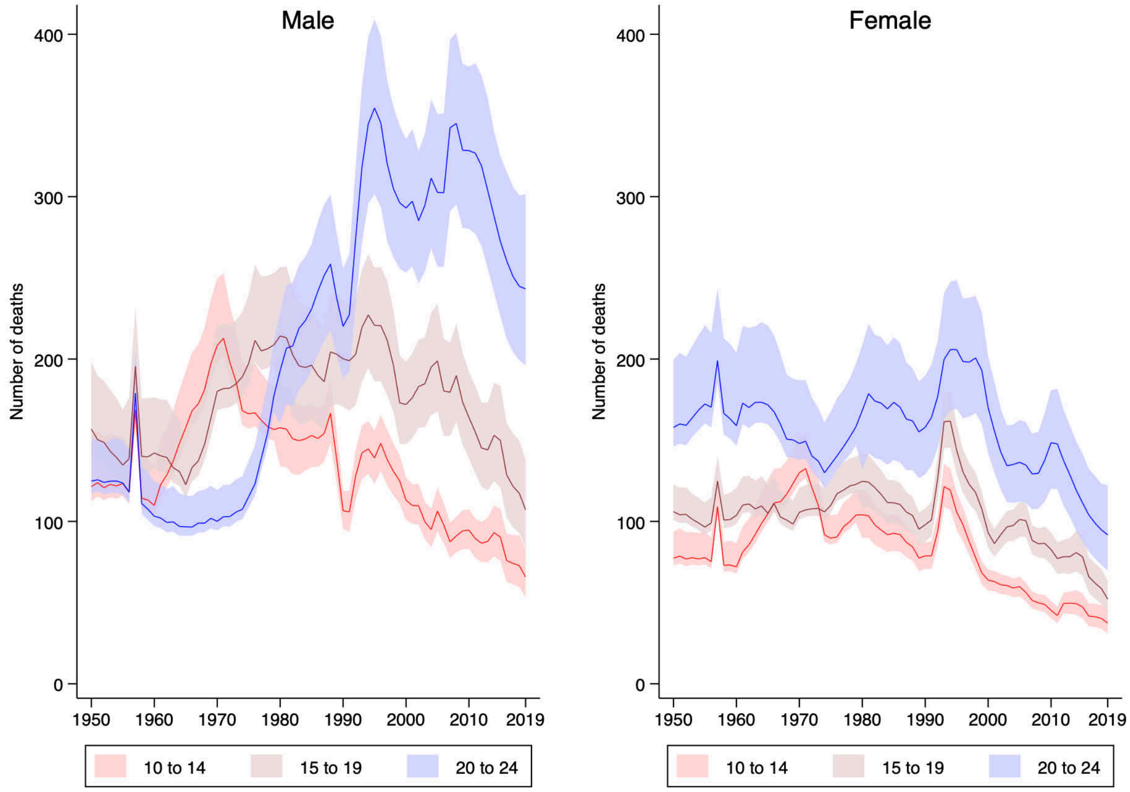

Figure S36c Mortality rate per 100,000 in 10-24 1980 – 2019 by cause: Mongolia

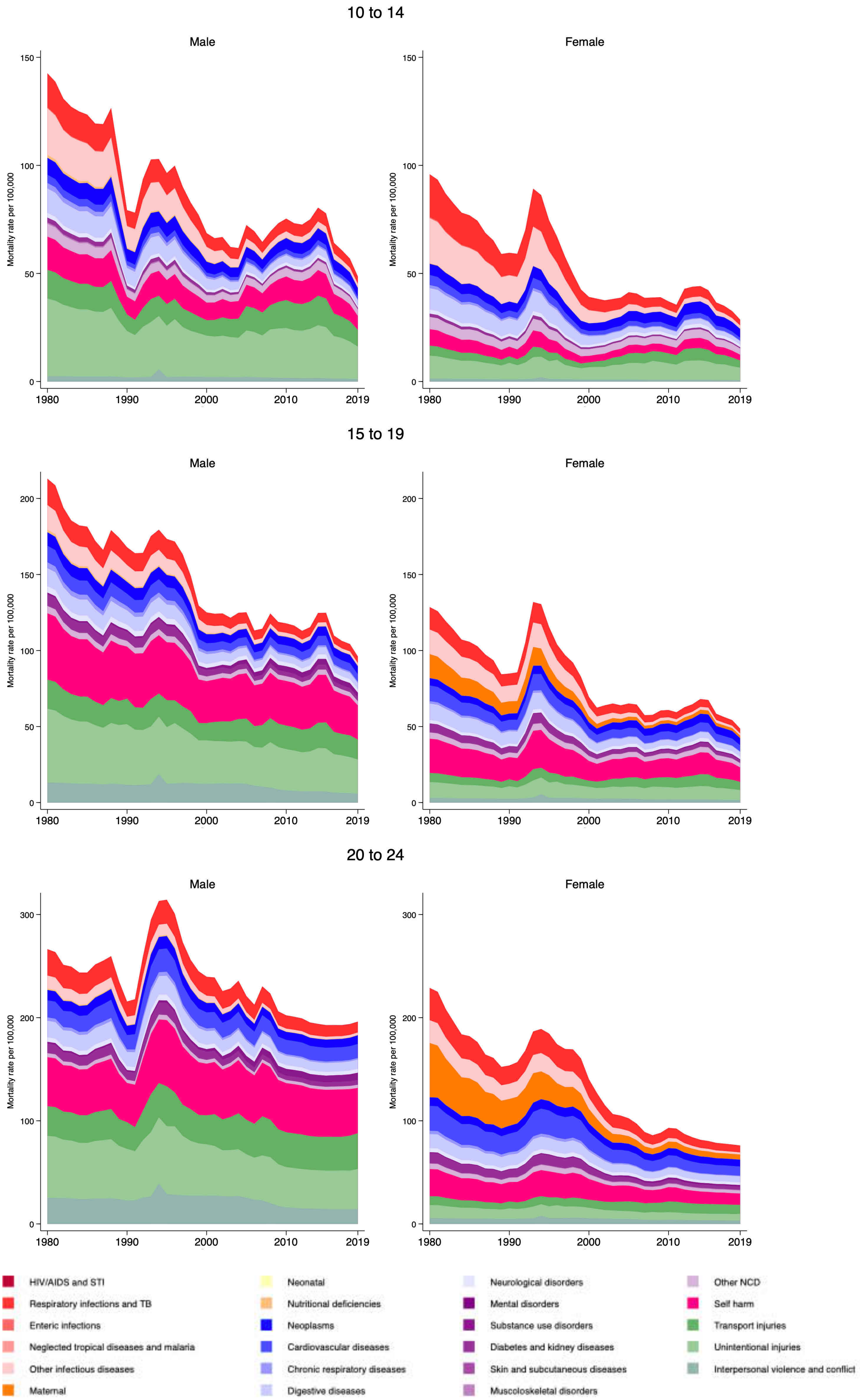

Figure S36d Number of deaths in 10-24 1980 – 2019 by cause: Mongolia

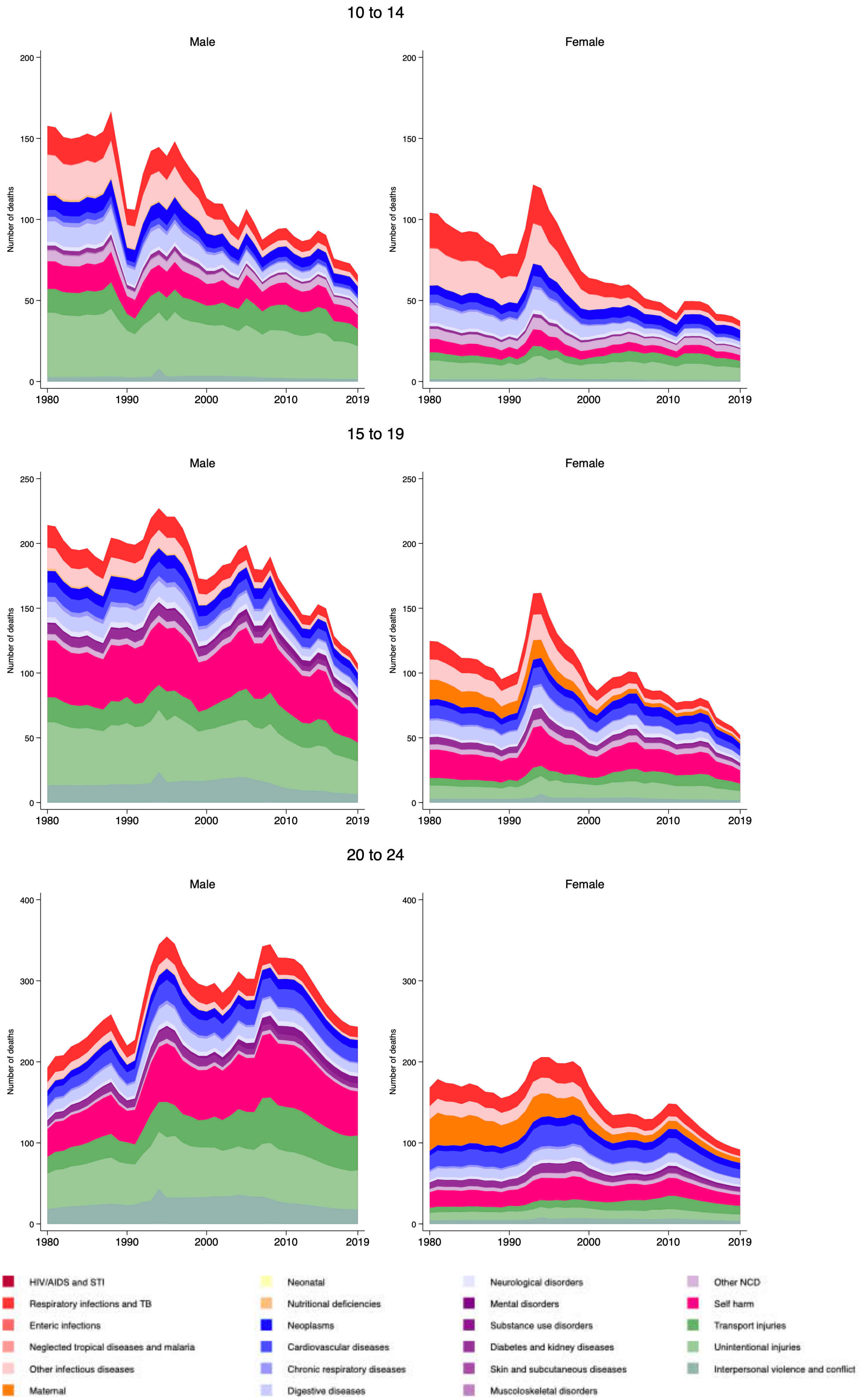

Figure S37: Tajikistan

Figure S37a All-cause mortality rate per 100,000 in 10-24 1950 – 2019: Tajikistan

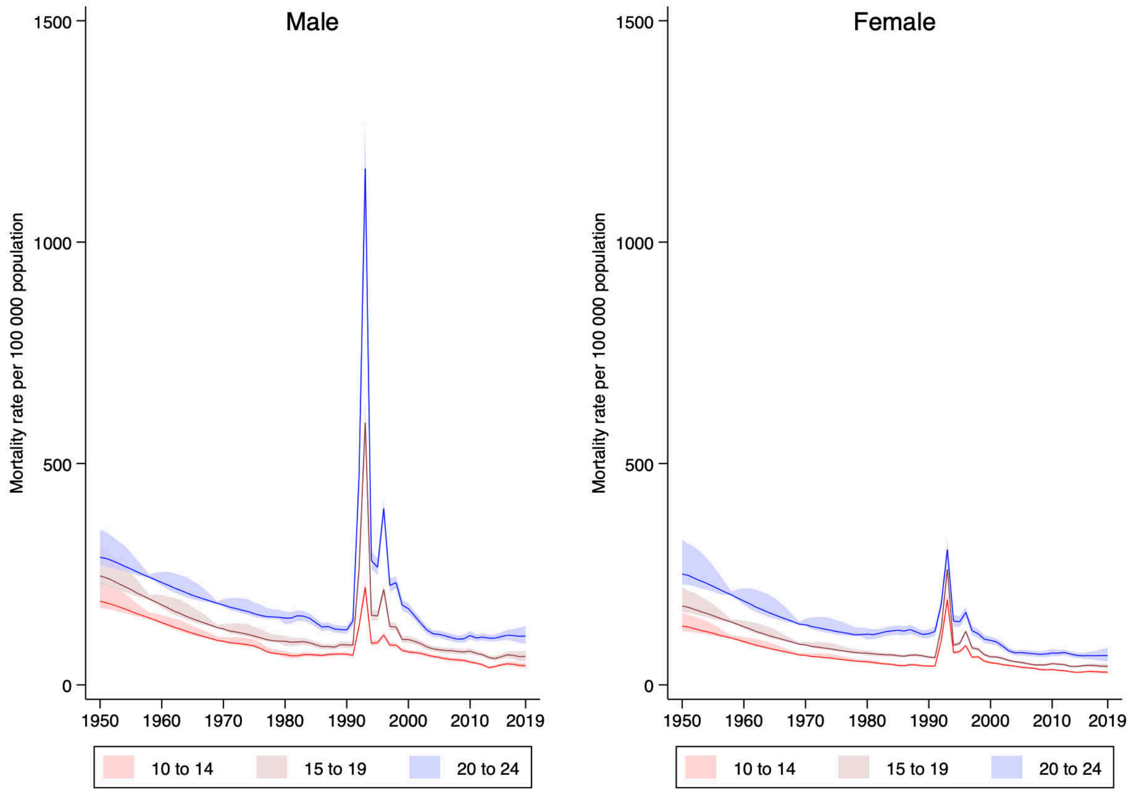

Figure S37b Number of deaths from all causes in 10-24 1950 – 2019: Tajikistan

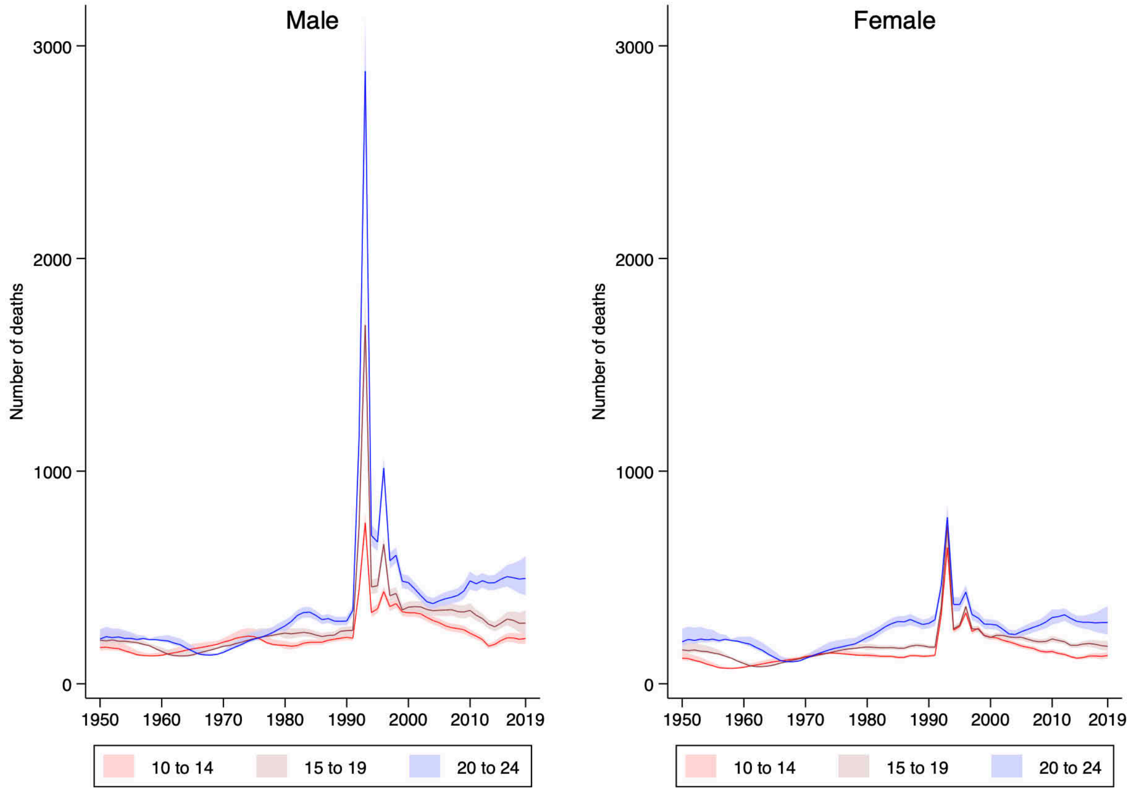

Figure S37c Mortality rate per 100,000 in 10-24 1980 – 2019 by cause: Tajikistan

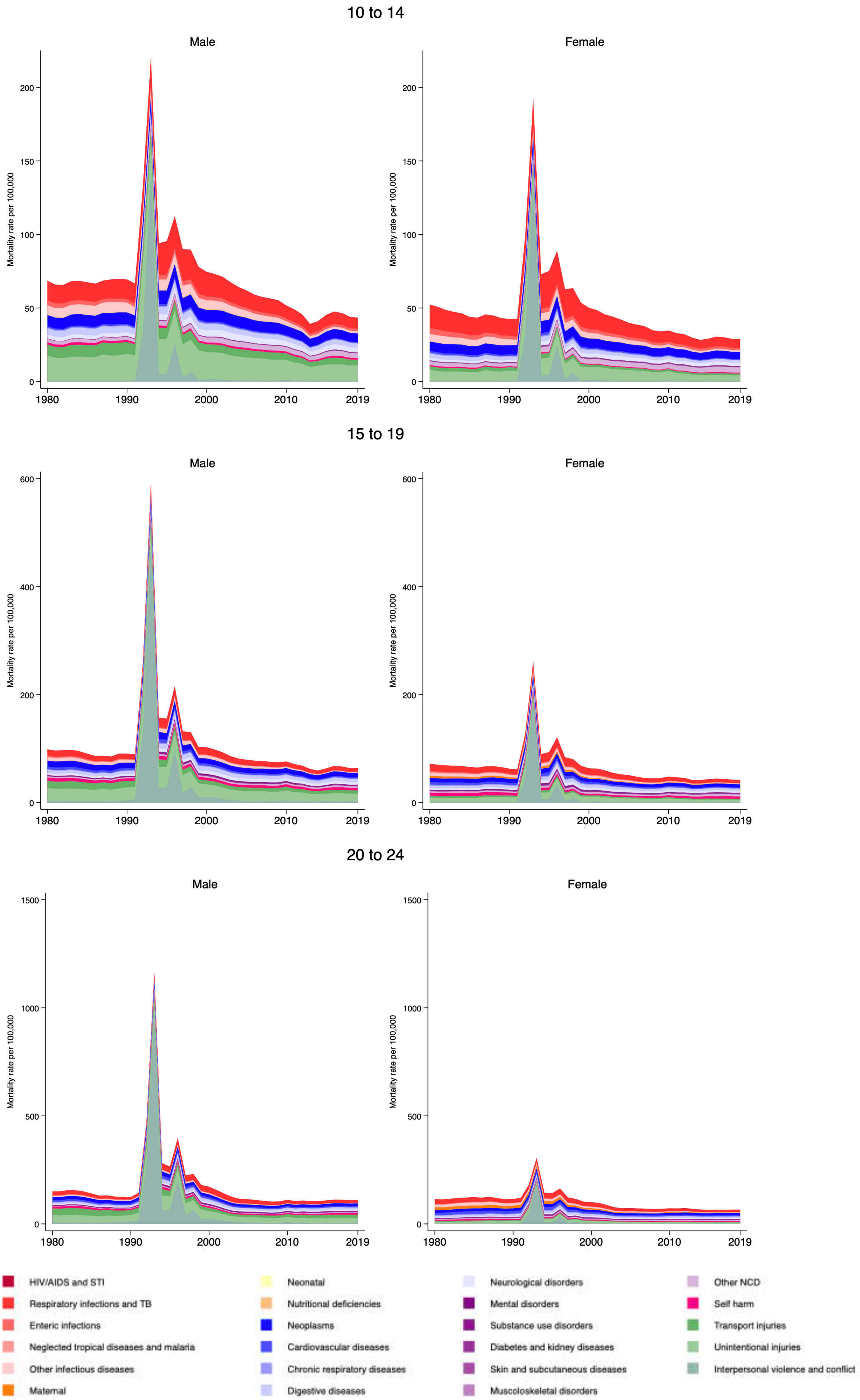

Figure S37d Number of deaths in 10-24 1980 – 2019 by cause: Tajikistan

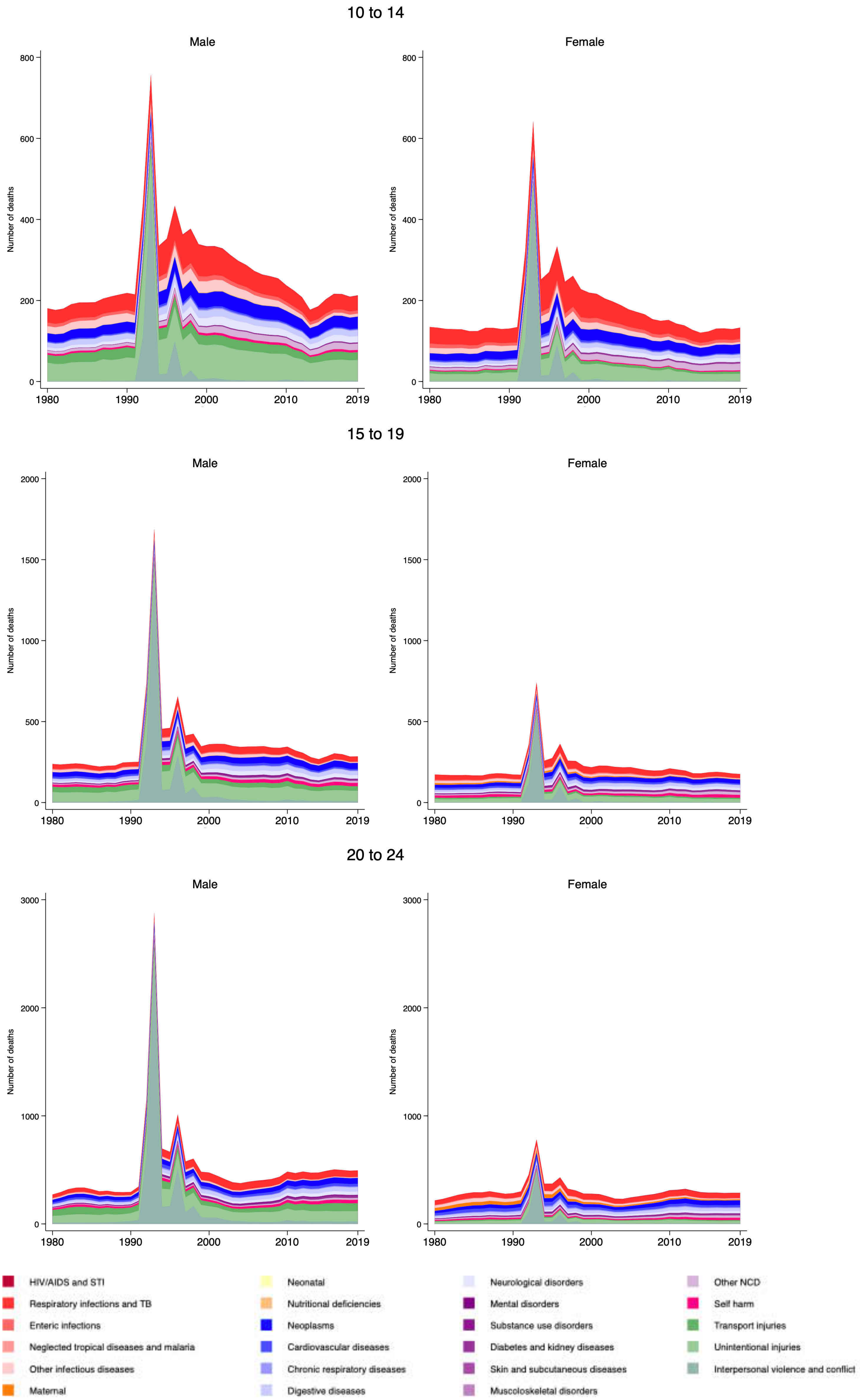

Figure S38: Turkmenistan

Figure S38a All-cause mortality rate per 100,000 in 10-24 1950 – 2019: Turkmenistan

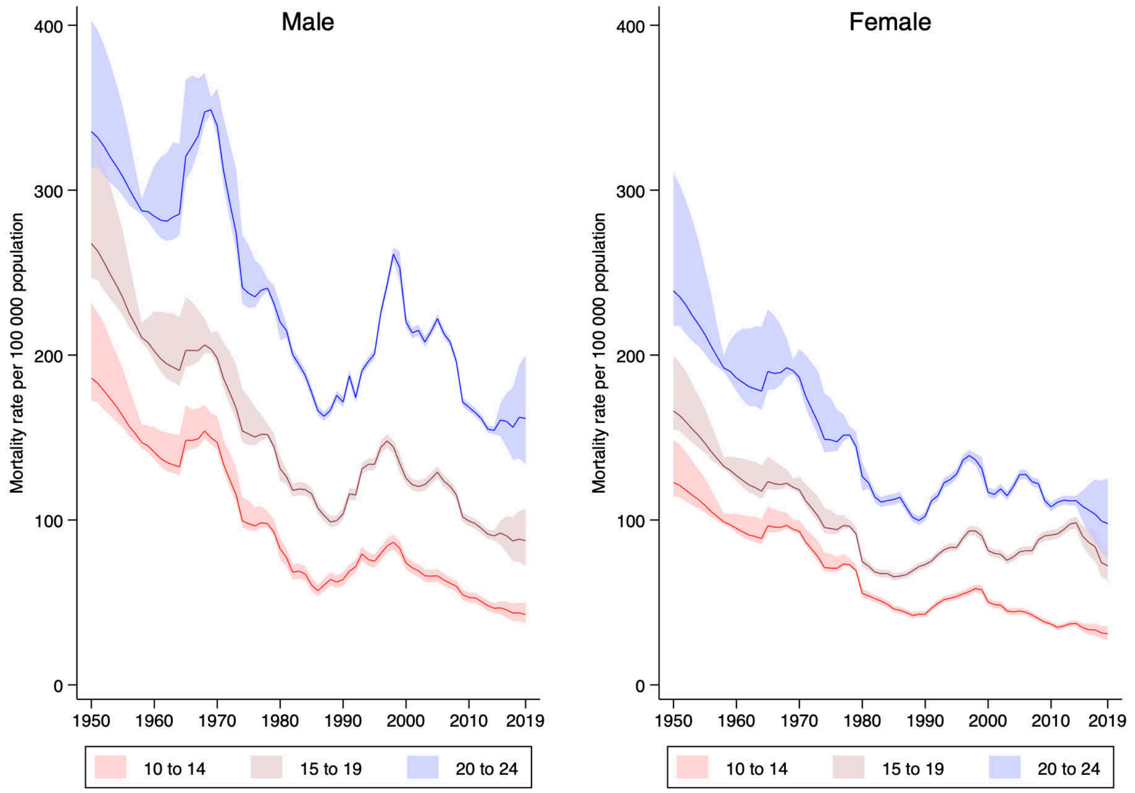

Figure S38b Number of deaths from all causes in 10-24 1950 – 2019: Turkmenistan

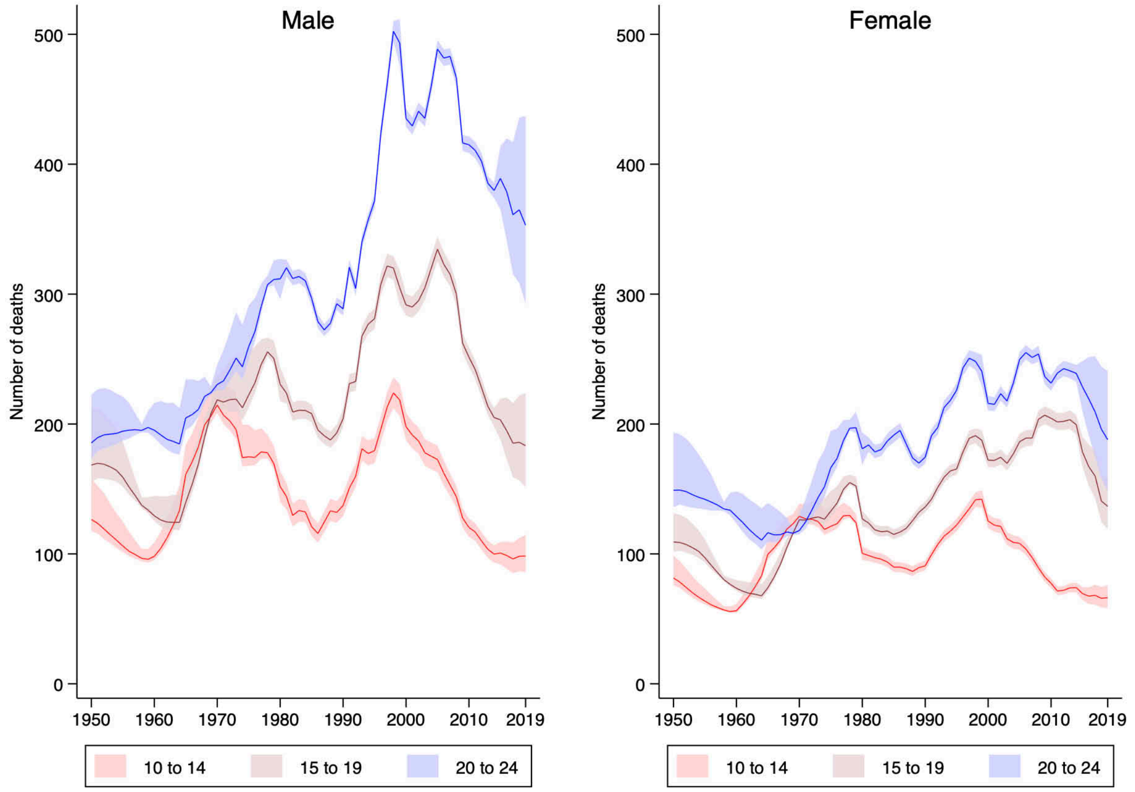

Figure S38c Mortality rate per 100,000 in 10-24 1980 – 2019 by cause: Turkmenistan

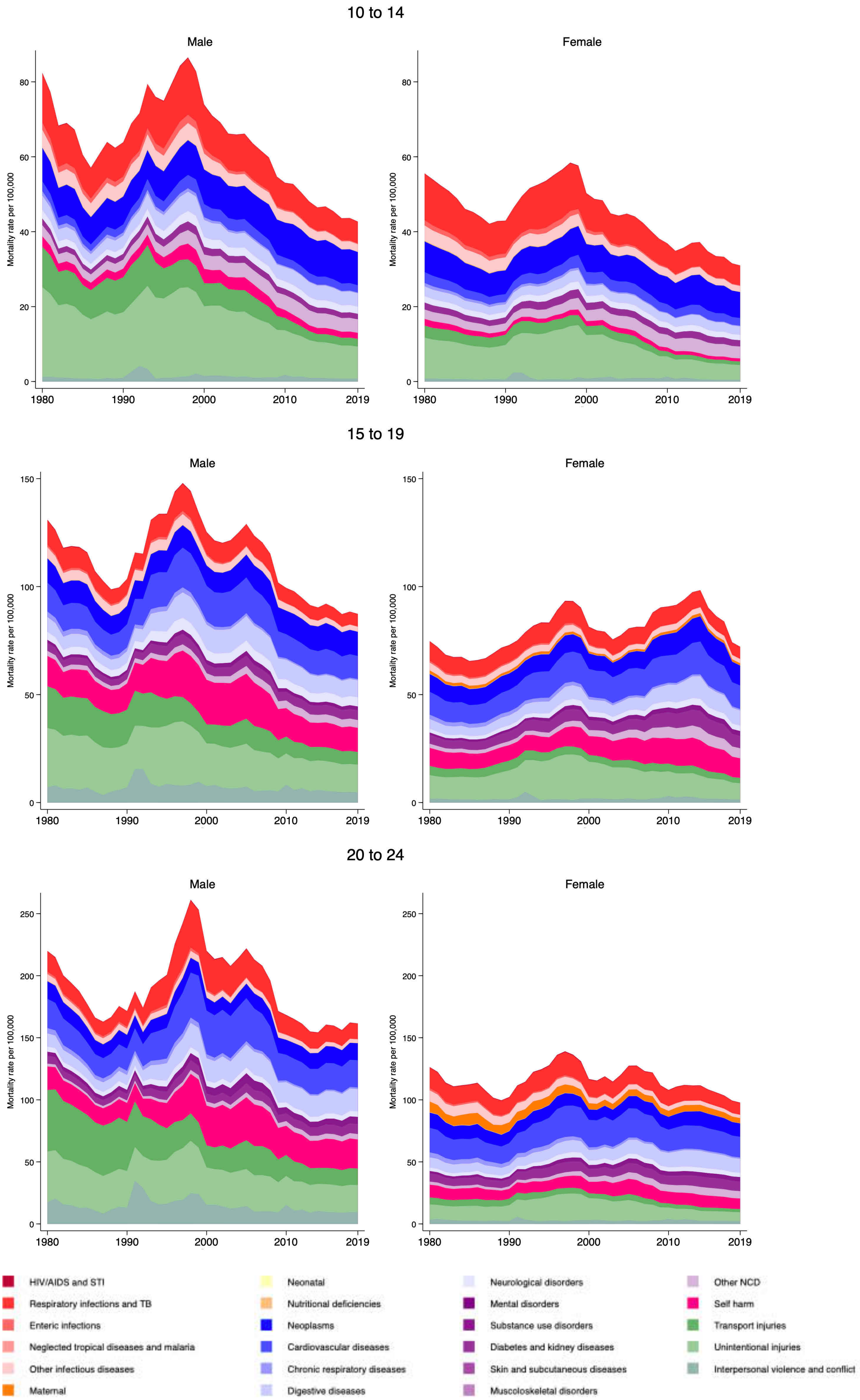

Figure S38d Number of deaths in 10-24 1980 – 2019 by cause: Turkmenistan

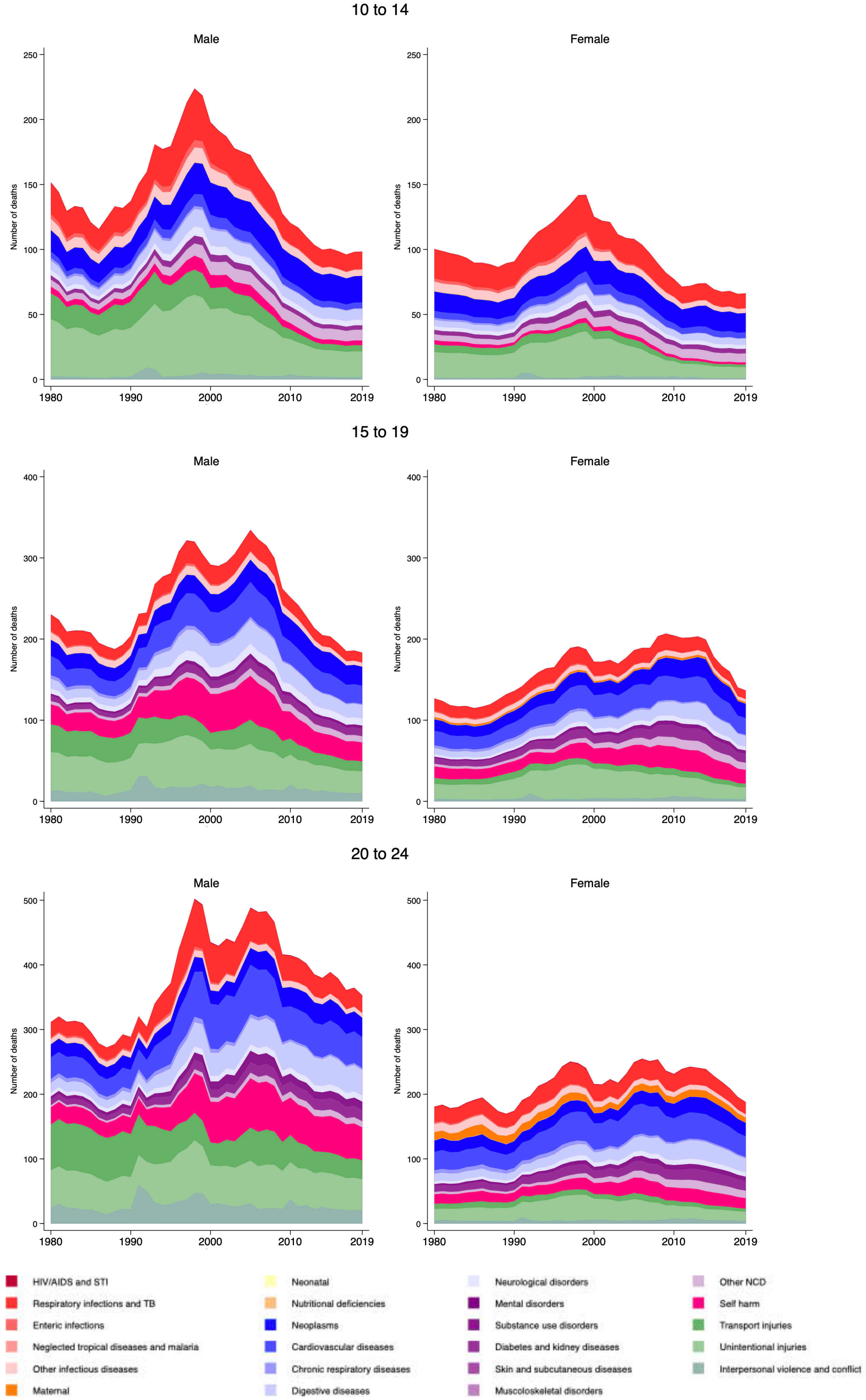

Figure S39: Uzbekistan

Figure S39a All-cause mortality rate per 100,000 in 10-24 1950 – 2019: Uzbekistan

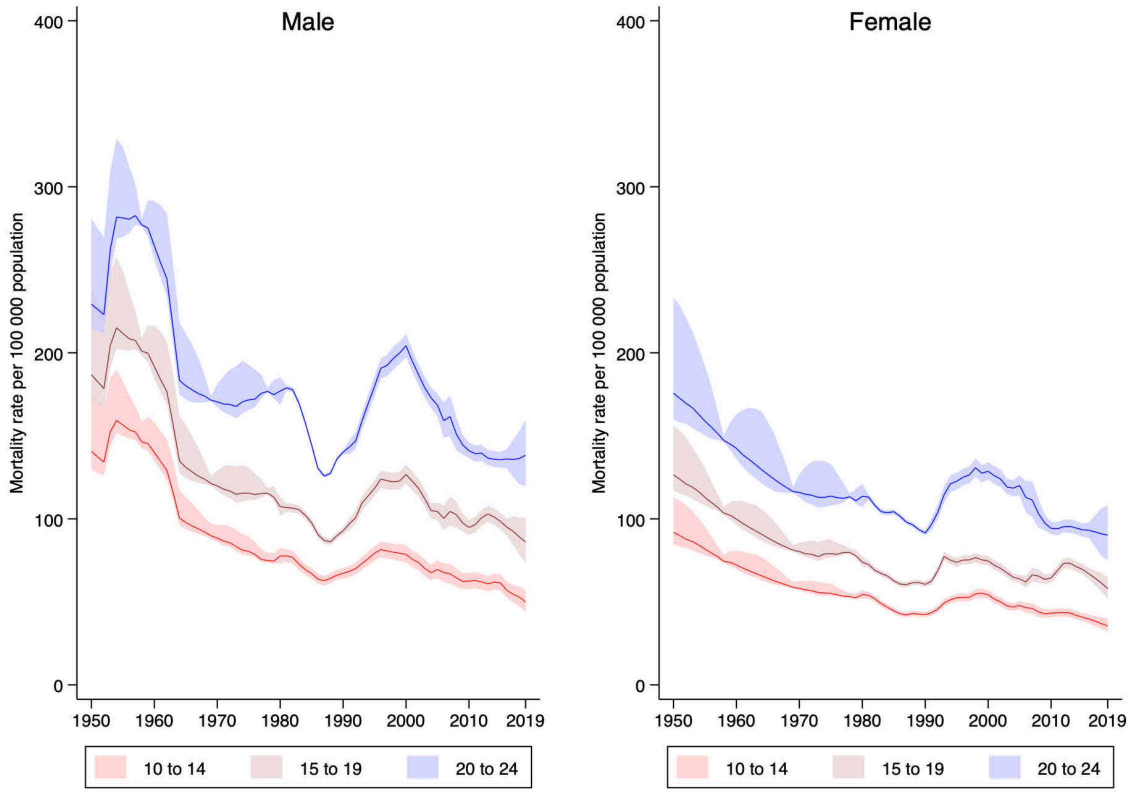

Figure S39b Number of deaths from all causes in 10-24 1950 – 2019: Uzbekistan

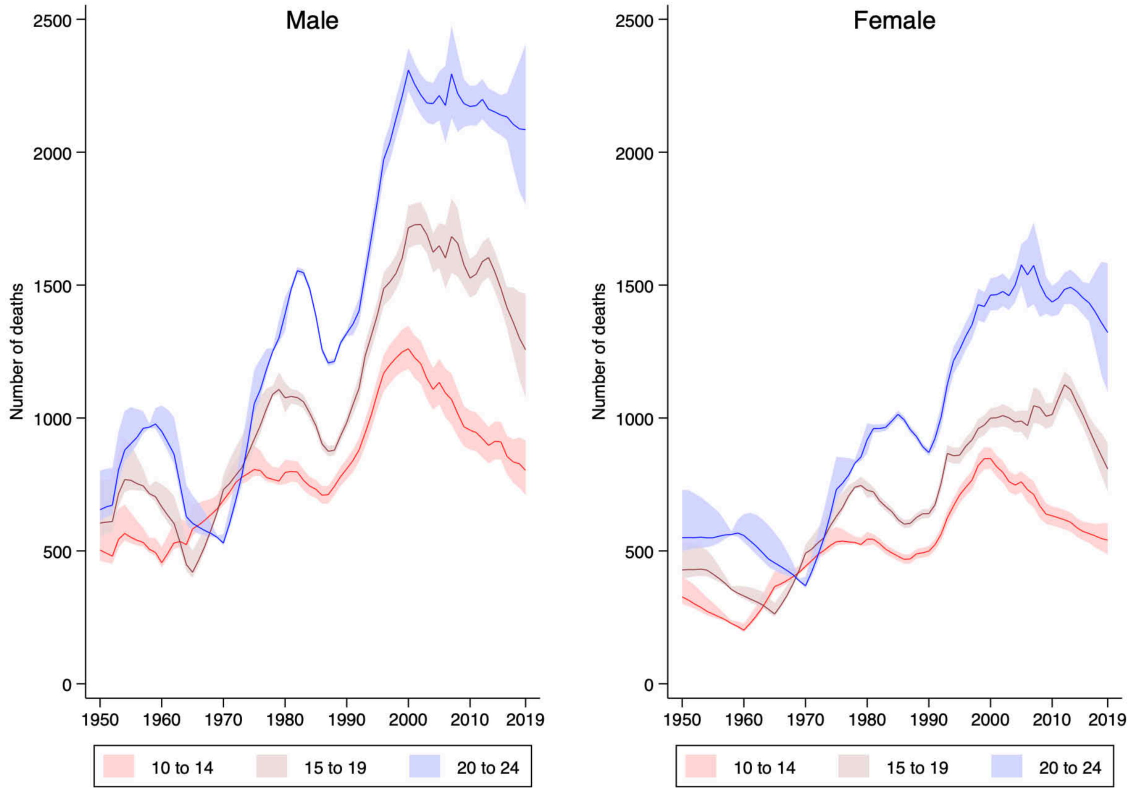

Figure S39c Mortality rate per 100,000 in 10-24 1980 – 2019 by cause: Uzbekistan

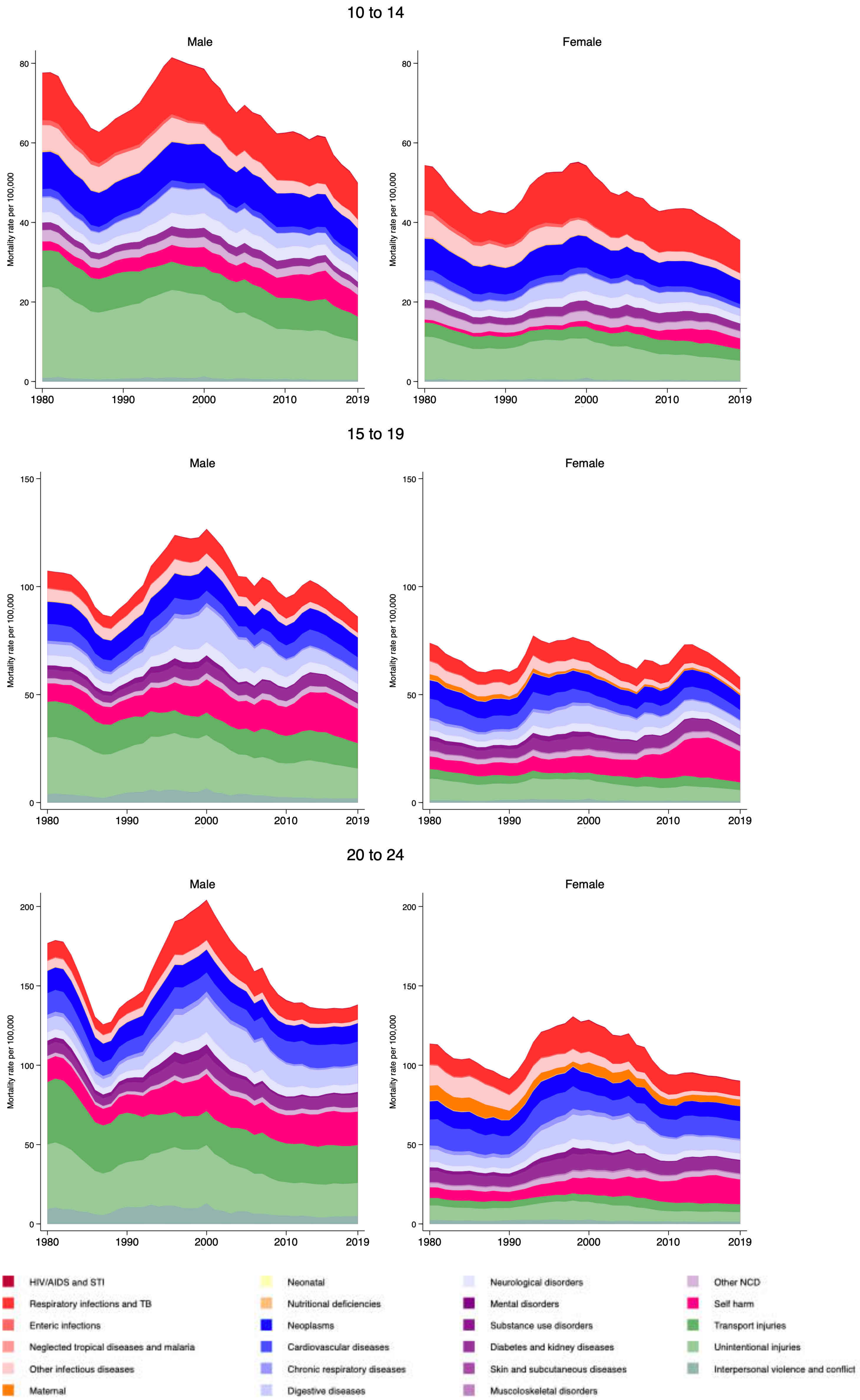

Figure S39d Number of deaths in 10-24 1980 – 2019 by cause: Uzbekistan

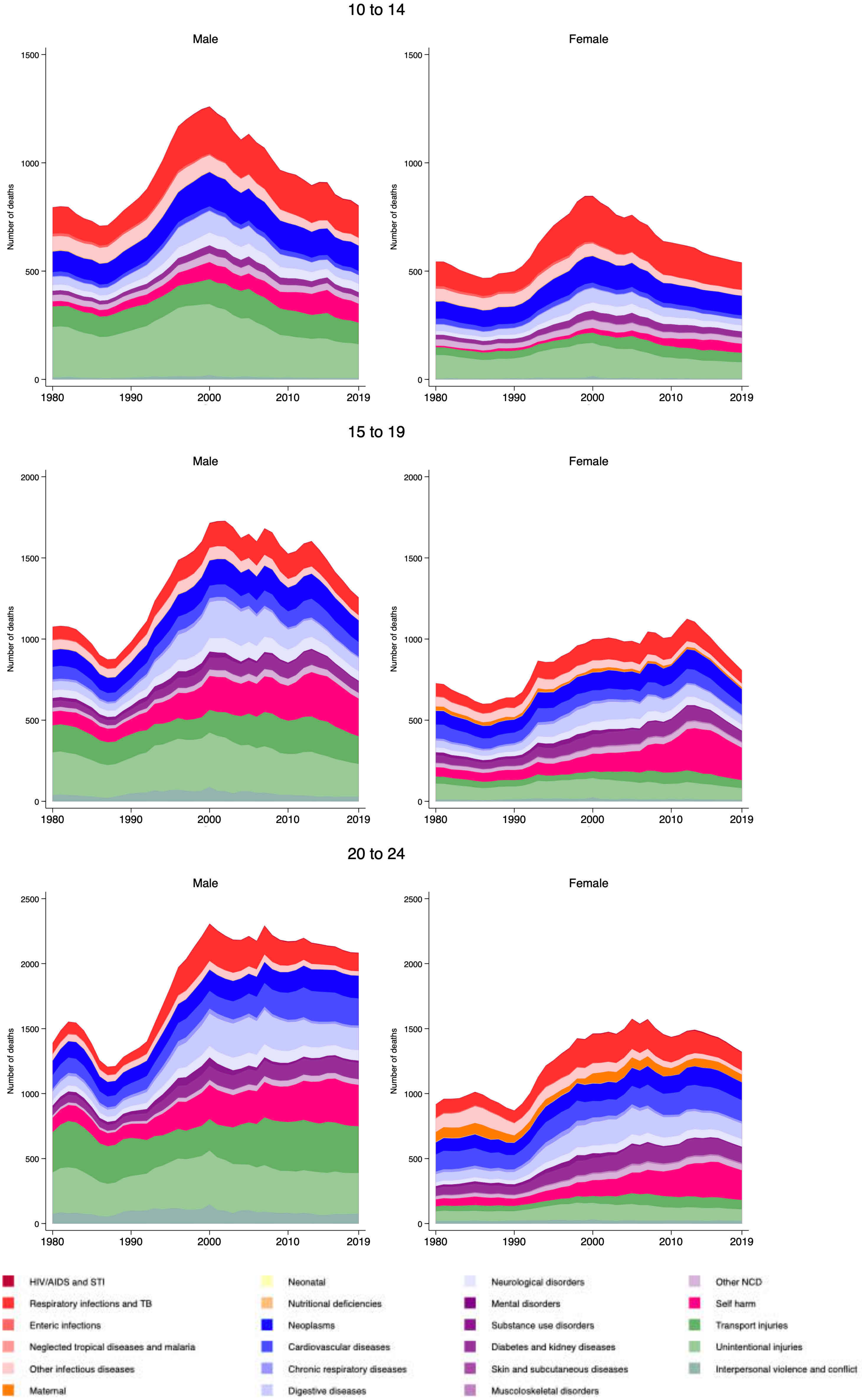

Figure S40: Central Europe

Figure S40a All-cause mortality rate per 100,000 in 10-24 1950 – 2019: Central Europe

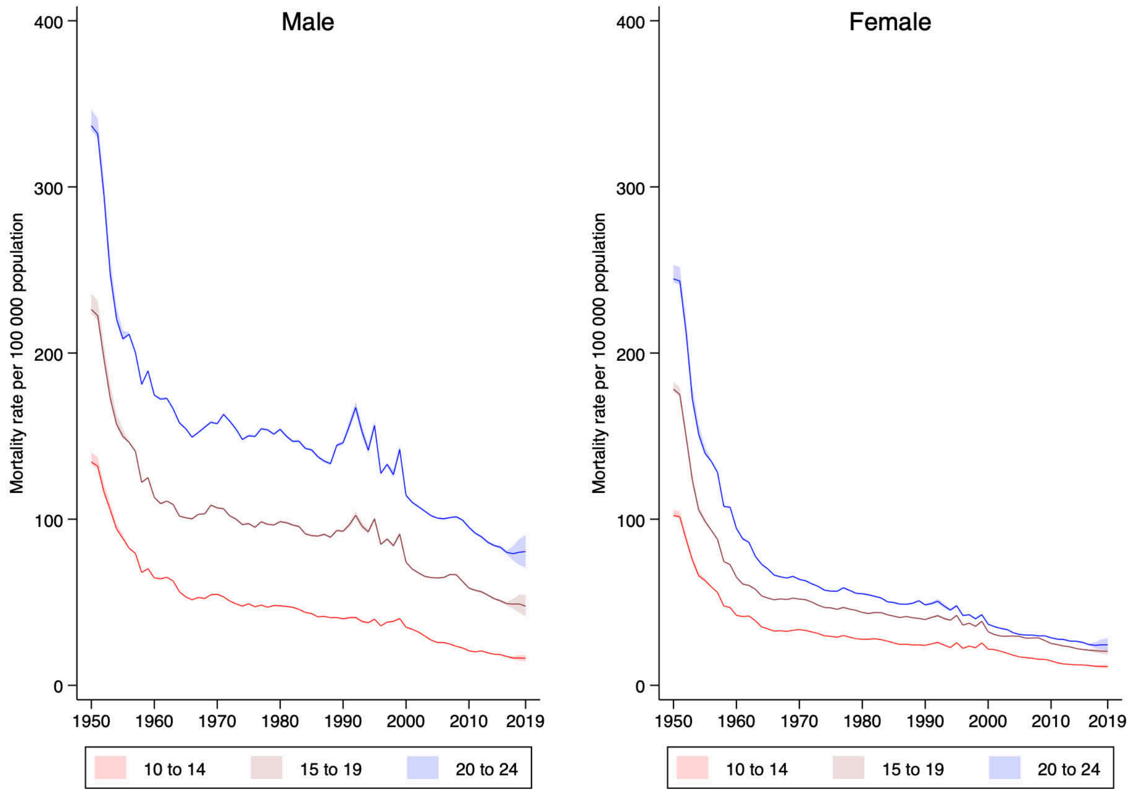

Figure S40b Number of deaths from all causes in 10-24 1950 – 2019: Central Europe

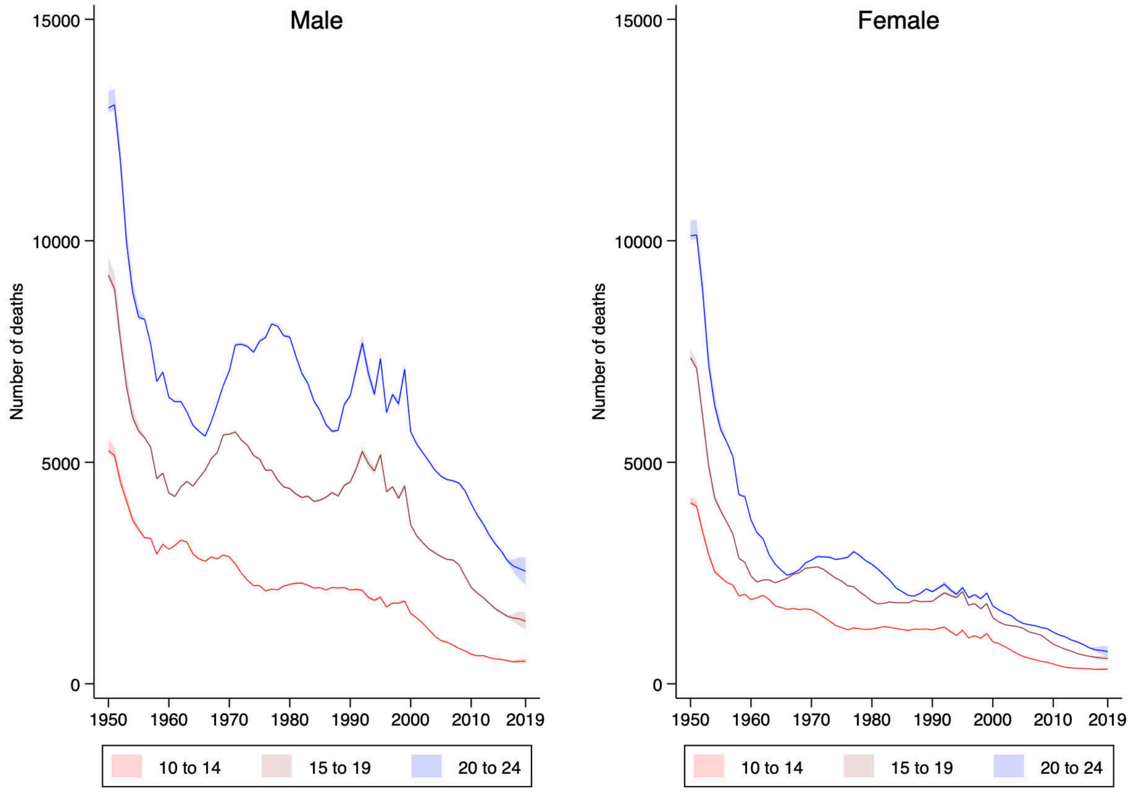

Figure S40c Mortality rate per 100,000 in 10-24 1980 – 2019 by cause: Central Europe

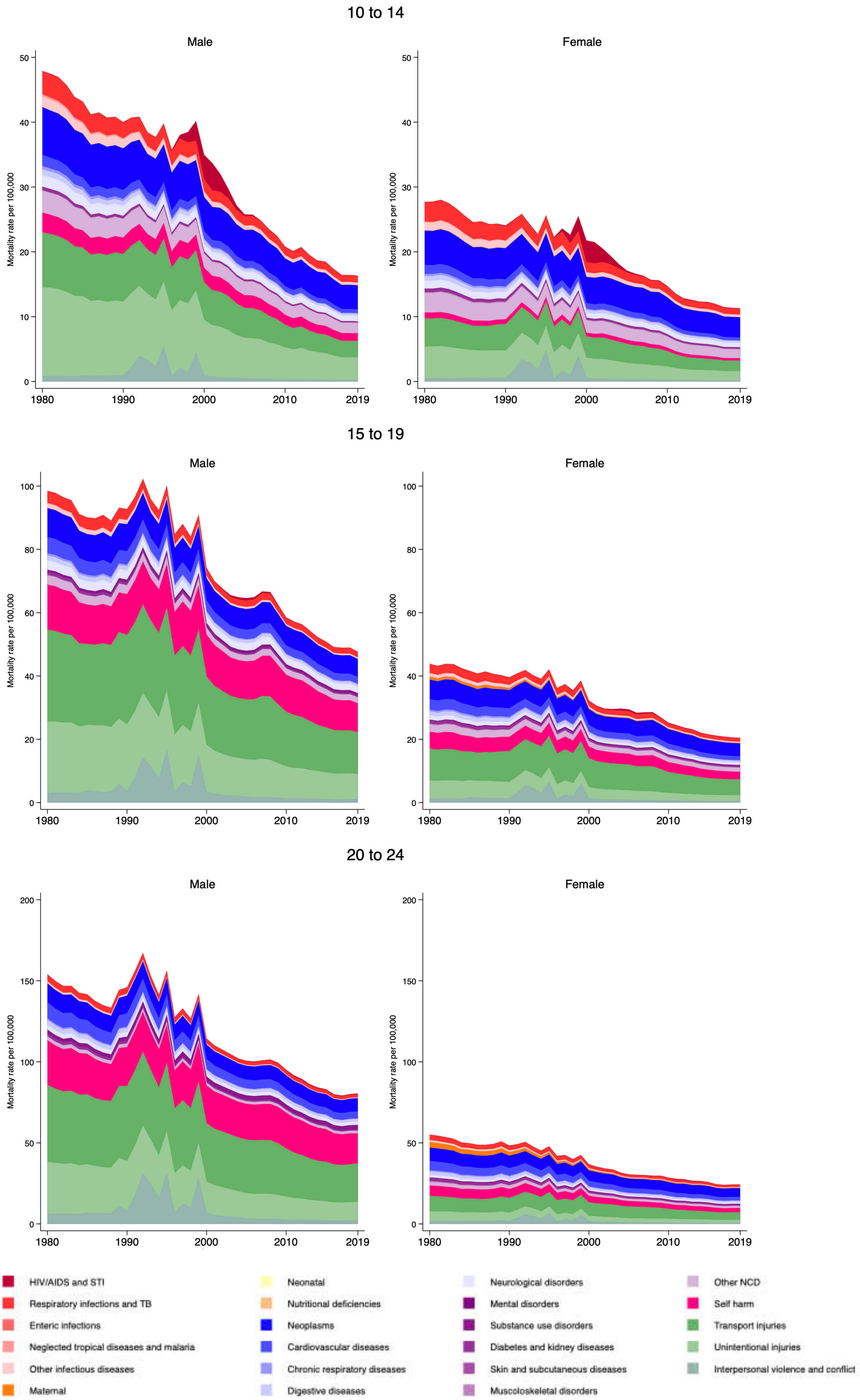

Figure S40d Number of deaths in 10-24 1980 – 2019 by cause: Central Europe

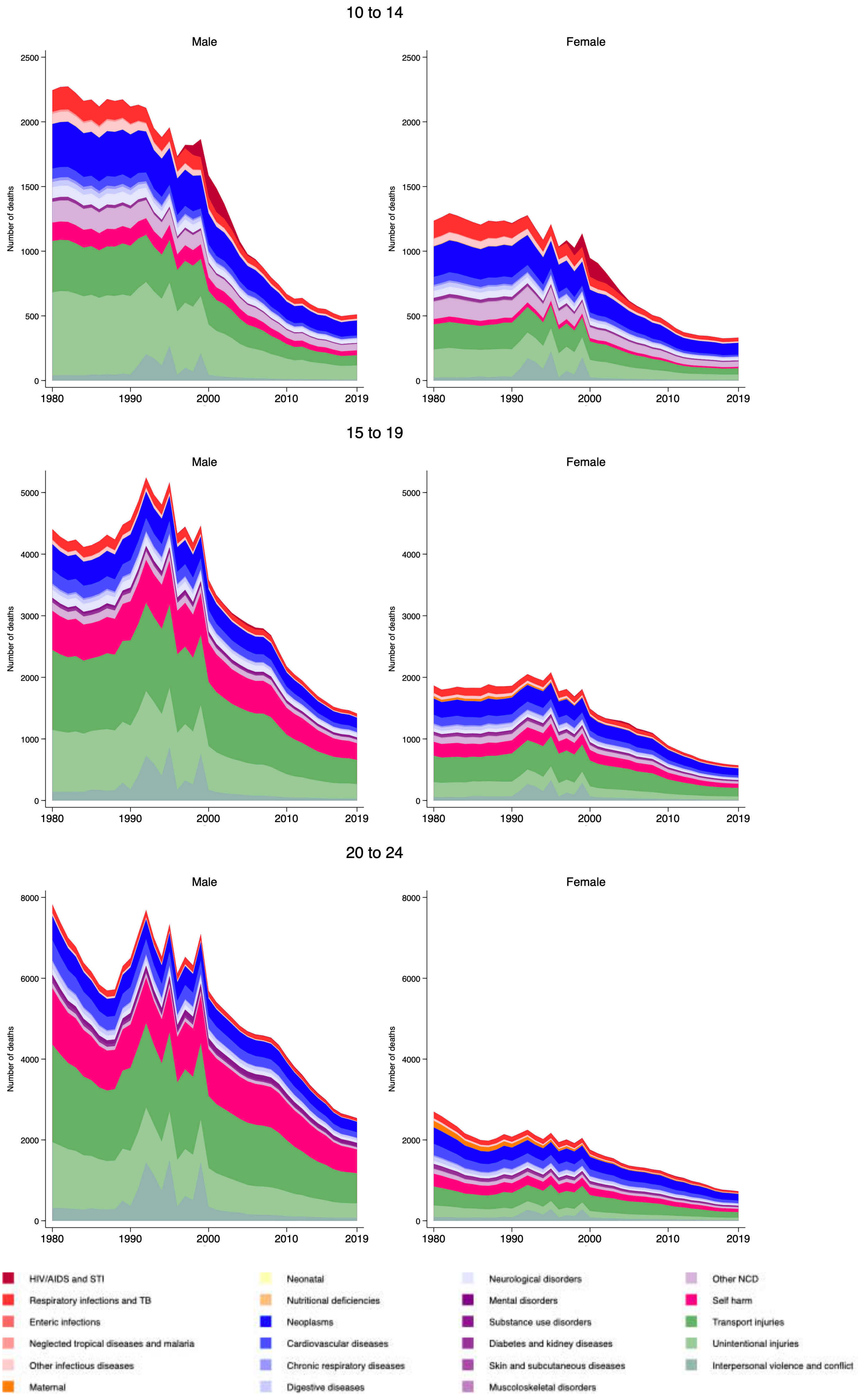

Figure S41: Albania

Figure S41a All-cause mortality rate per 100,000 in 10-24 1950 – 2019: Albania

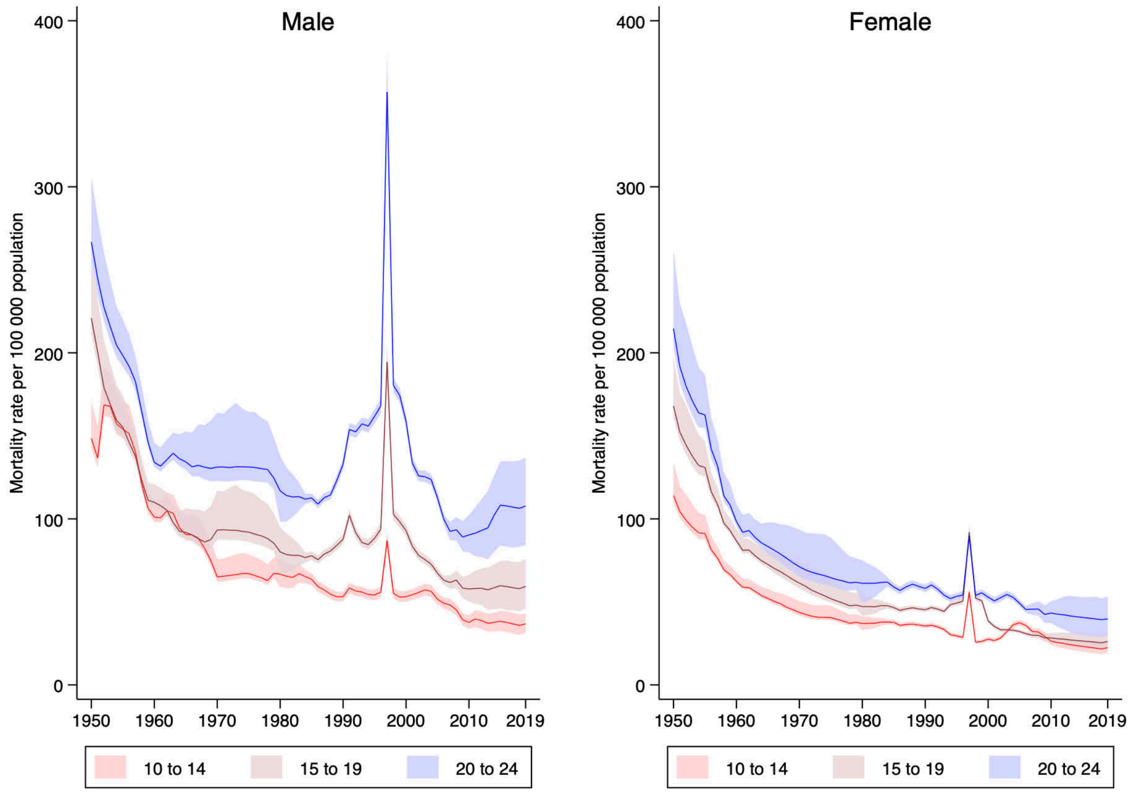

Figure S41b Number of deaths from all causes in 10-24 1950 – 2019: Albania

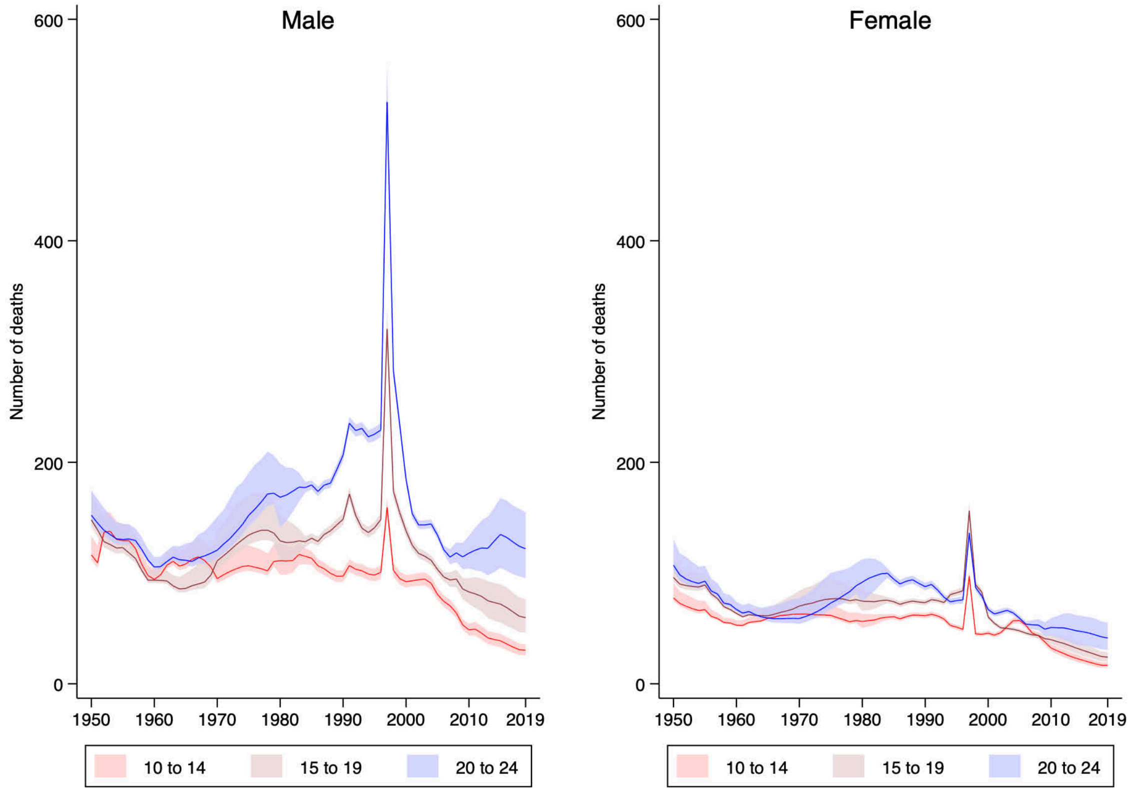

Figure S41c Mortality rate per 100,000 in 10-24 1980 – 2019 by cause: Albania

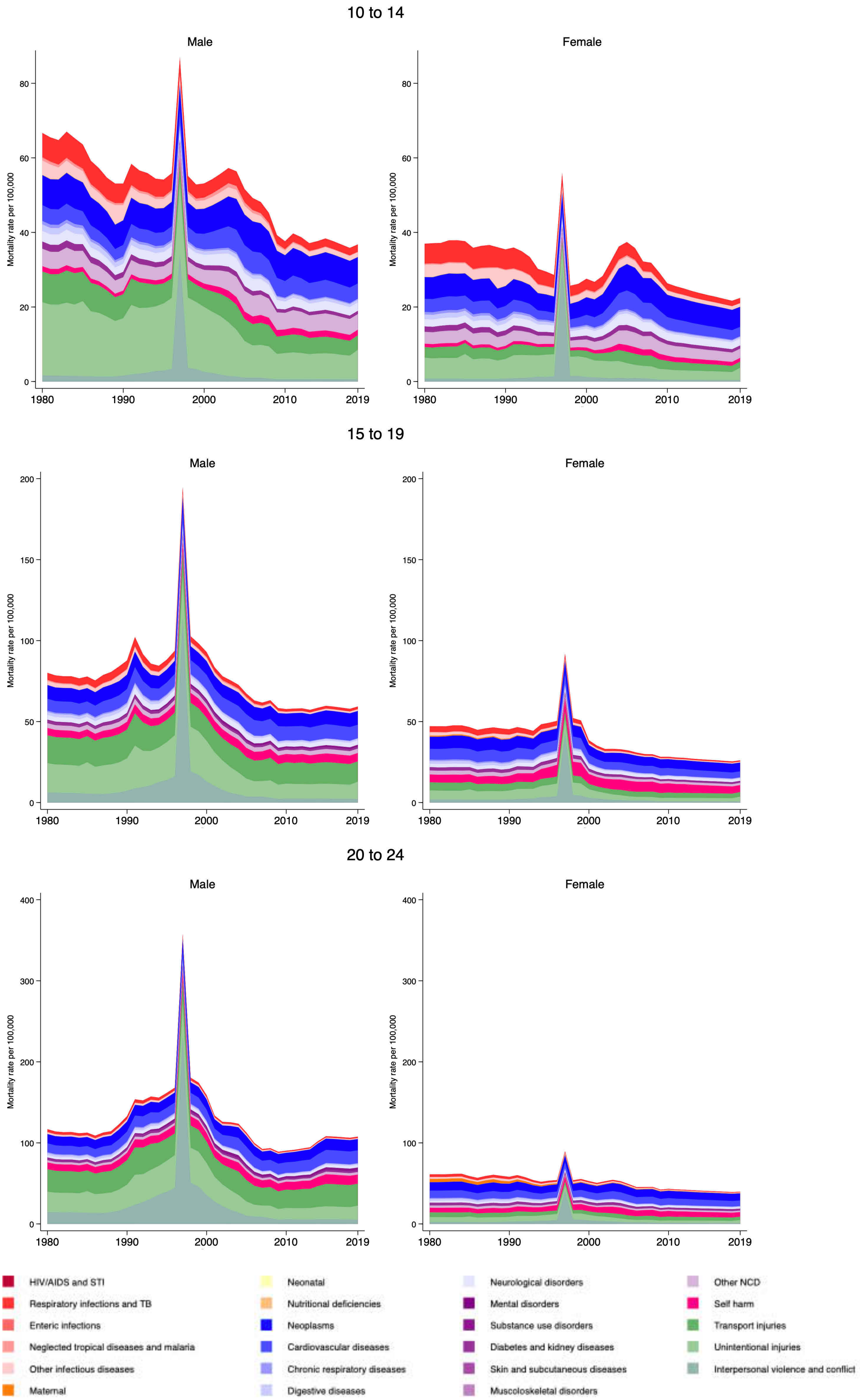

Figure S41d Number of deaths in 10-24 1980 – 2019 by cause: Albania

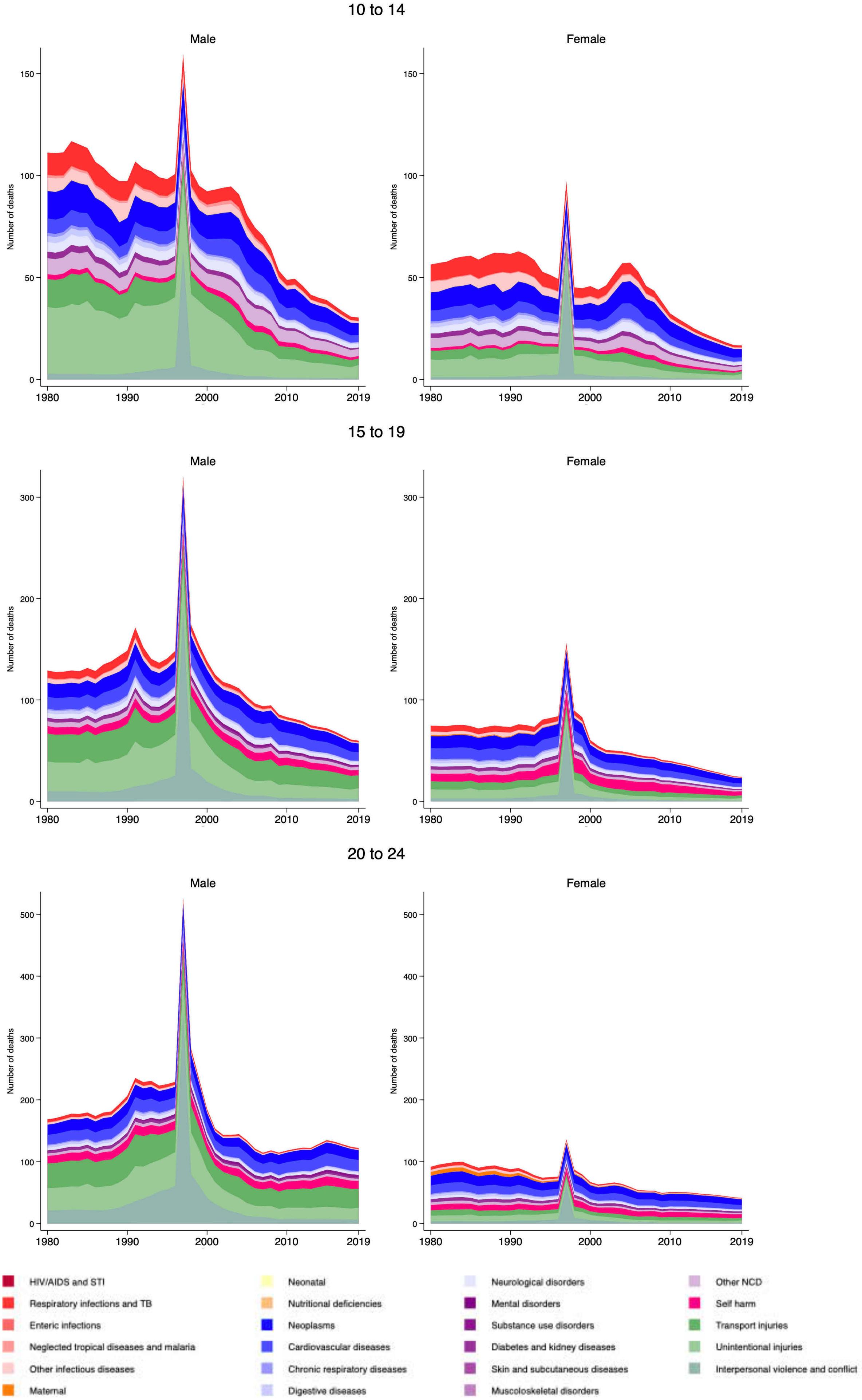

Figure S42: Bosnia and Herzegovina

Figure S42a All-cause mortality rate per 100,000 in 10-24 1950 – 2019: Bosnia and Herzegovina

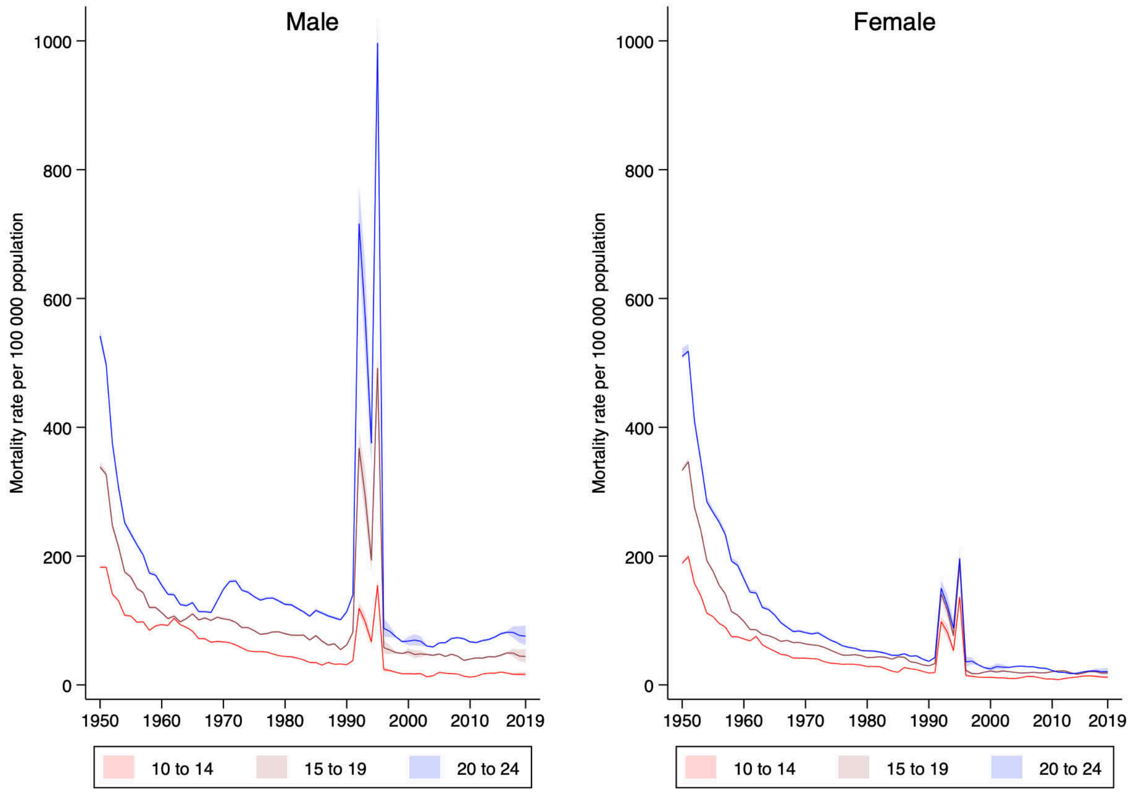

Figure S42b Number of deaths from all causes in 10-24 1950 – 2019: Bosnia and Herzegovina

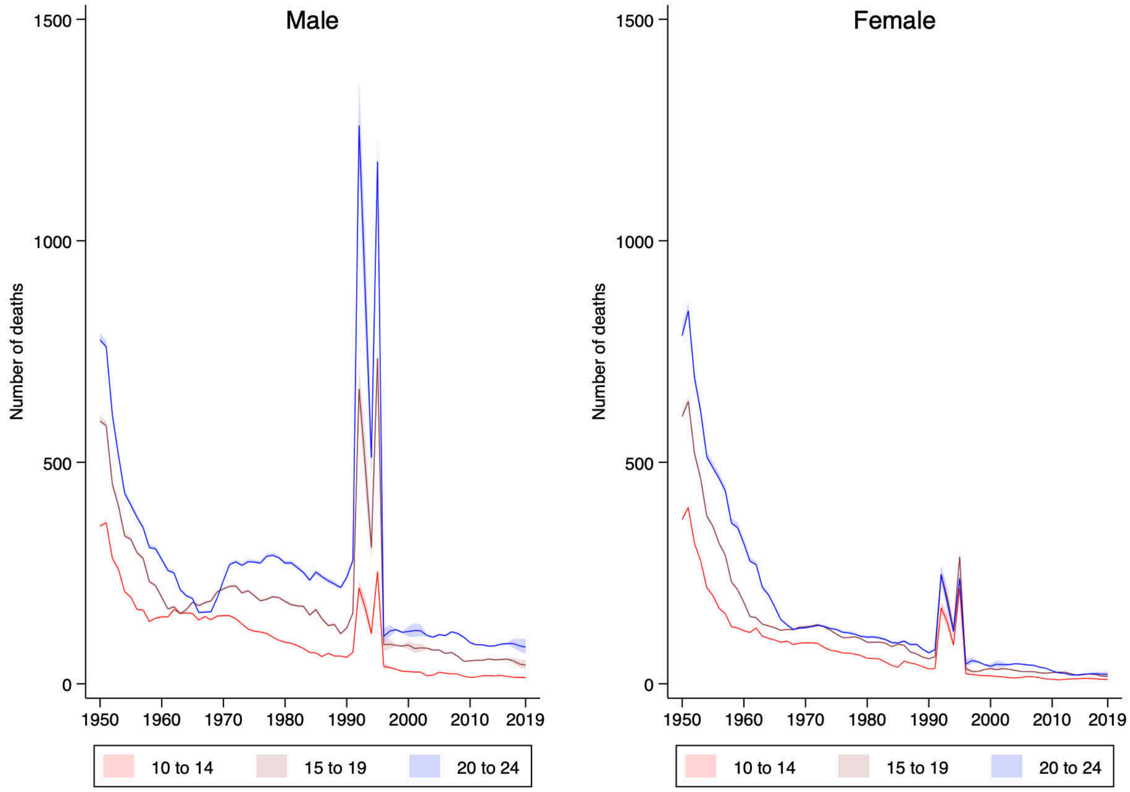

Figure S42c Mortality rate per 100,000 in 10-24 1980 – 2019 by cause: Bosnia and Herzegovina

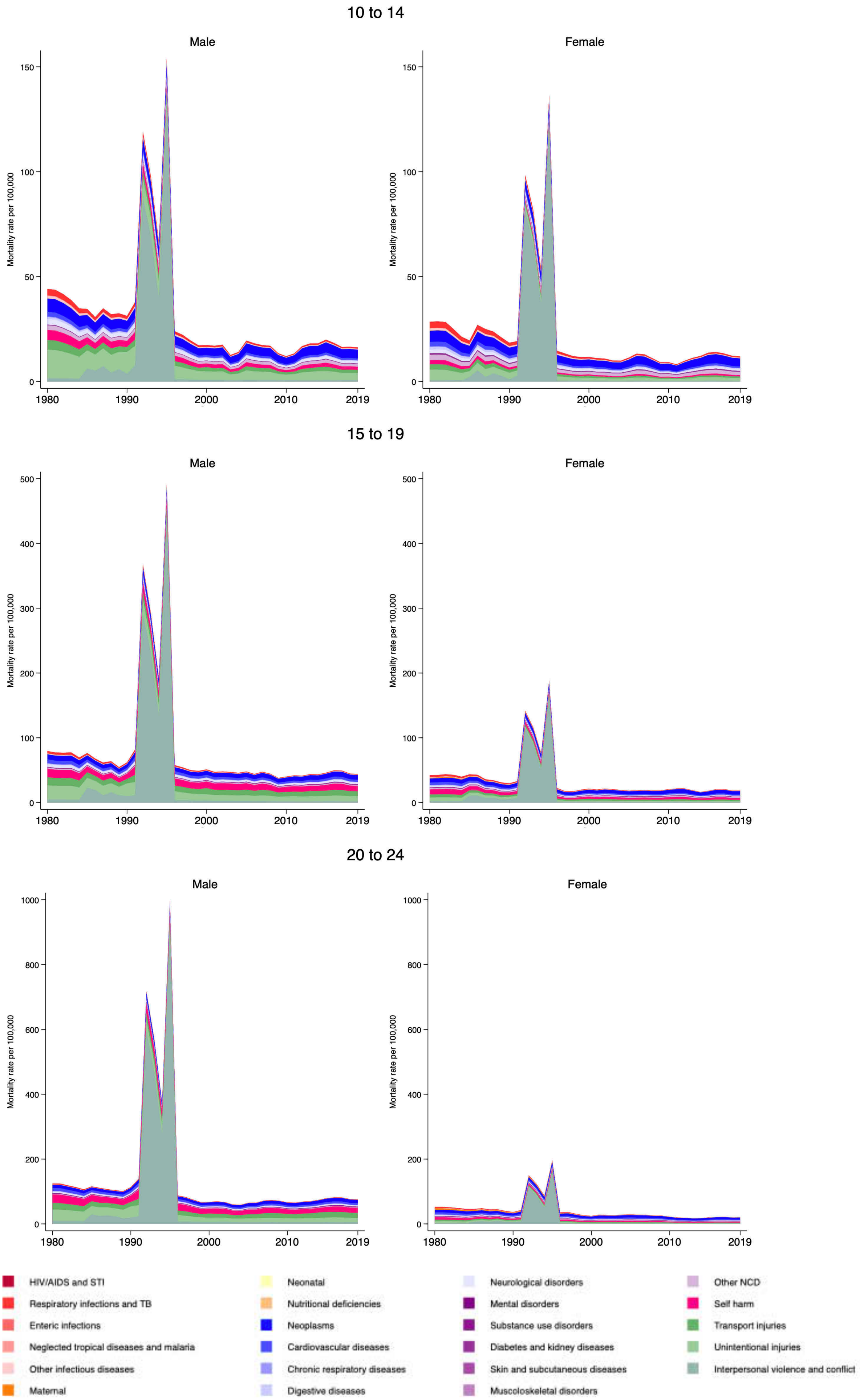

Figure S42d Number of deaths in 10-24 1980 – 2019 by cause: Bosnia and Herzegovina

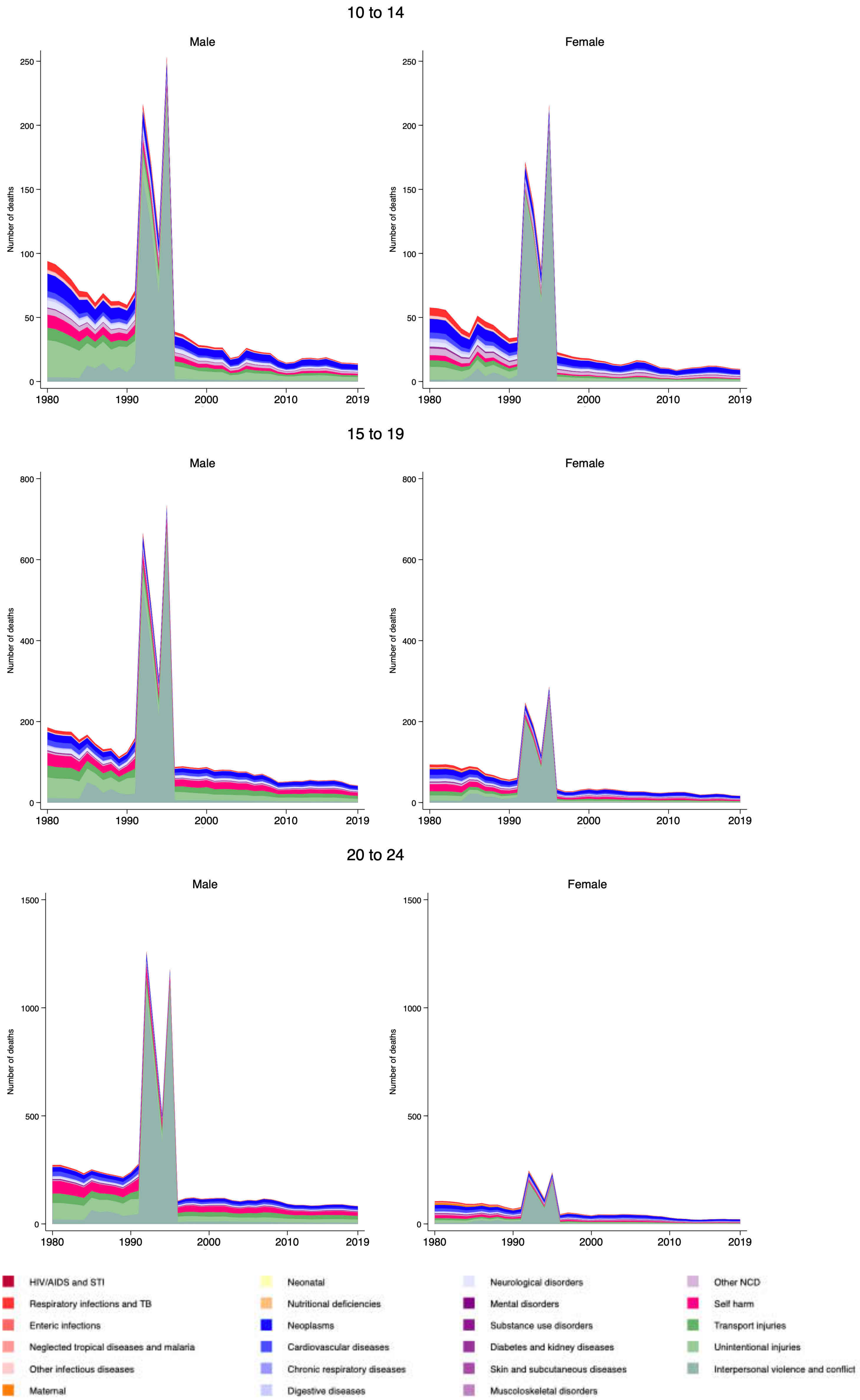

Figure S43: Bulgaria

Figure S43a All-cause mortality rate per 100,000 in 10-24 1950 – 2019: Bulgaria

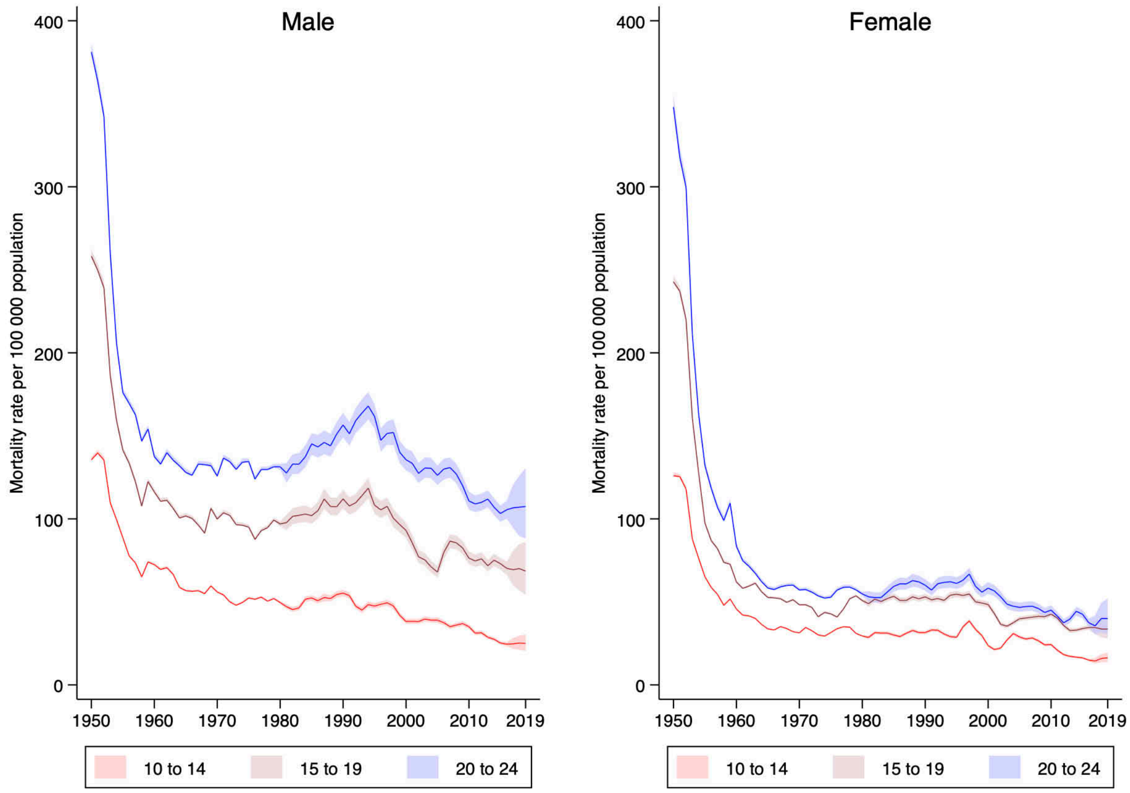

Figure S43b Number of deaths from all causes in 10-24 1950 – 2019: Bulgaria

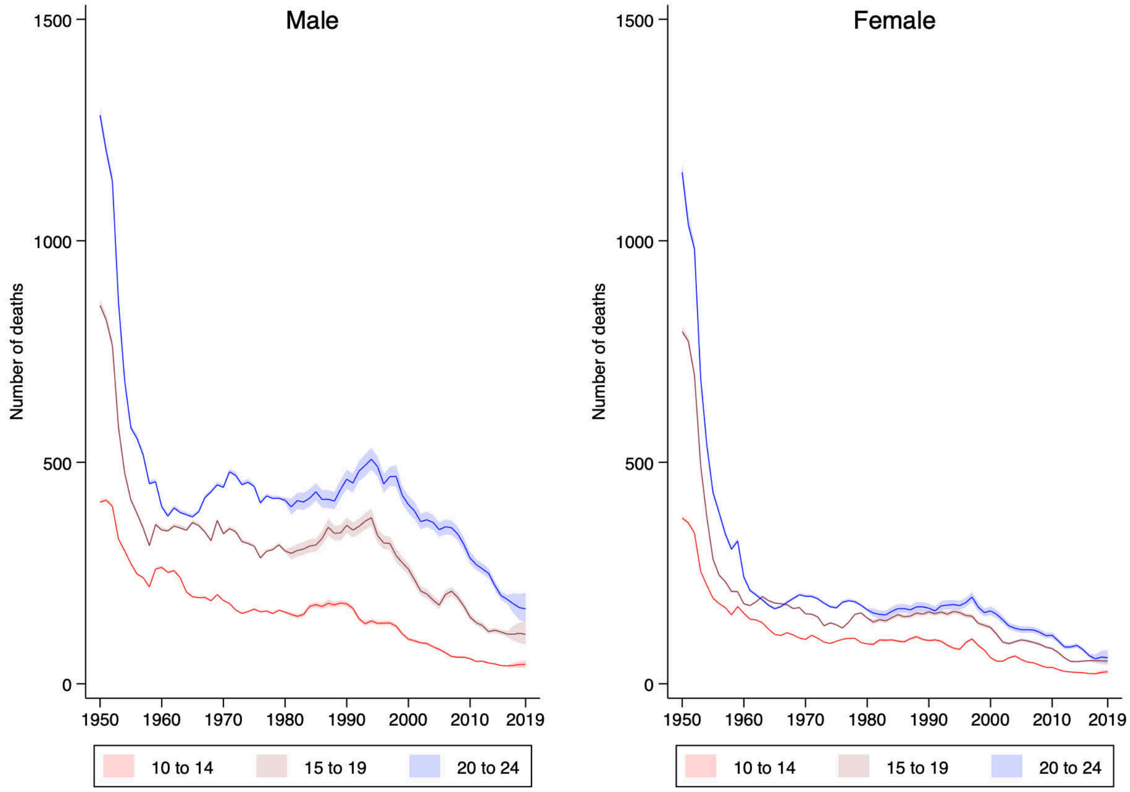

Figure S43c Mortality rate per 100,000 in 10-24 1980 – 2019 by cause: Bulgaria

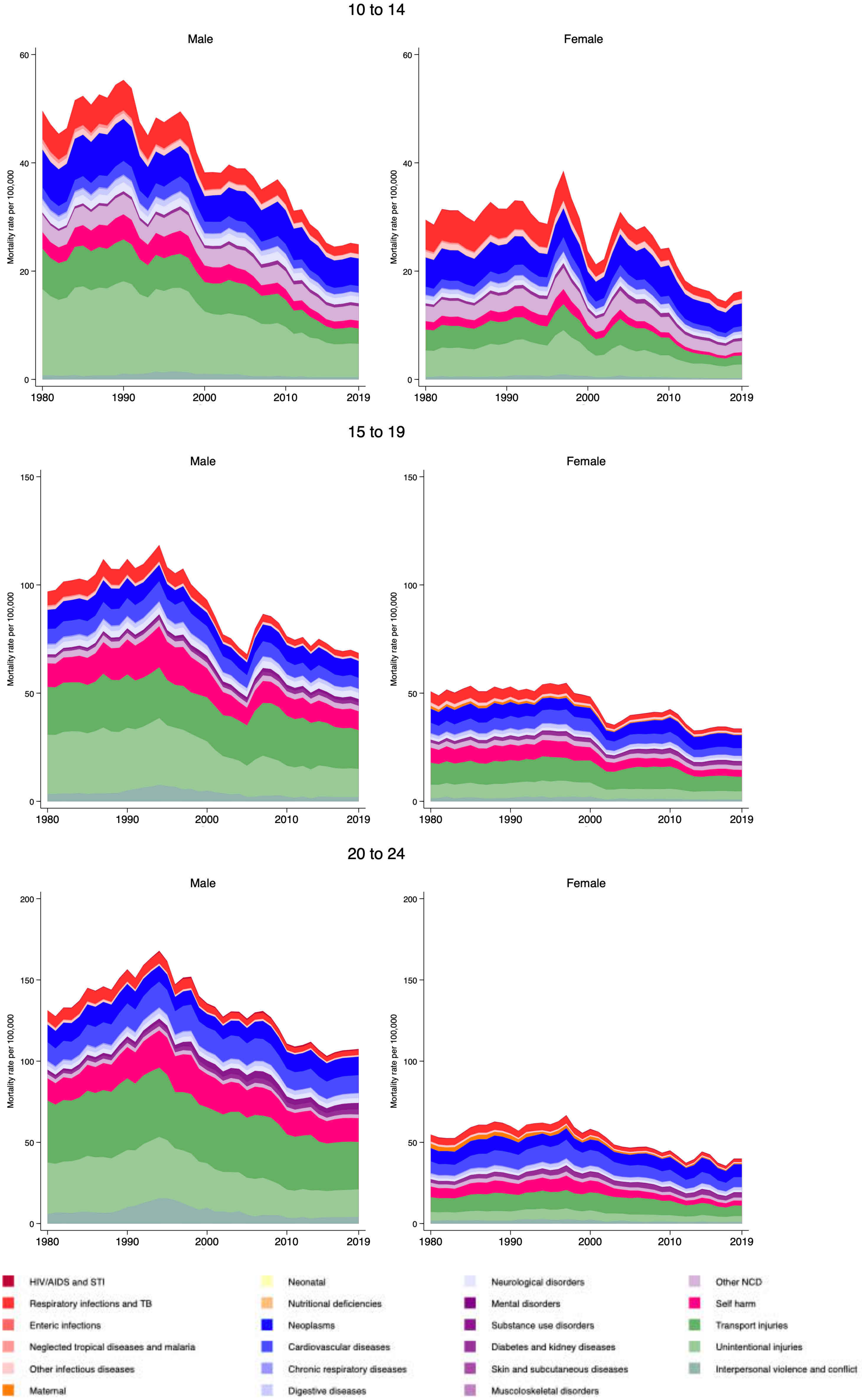

Figure S43d Number of deaths in 10-24 1980 – 2019 by cause: Bulgaria

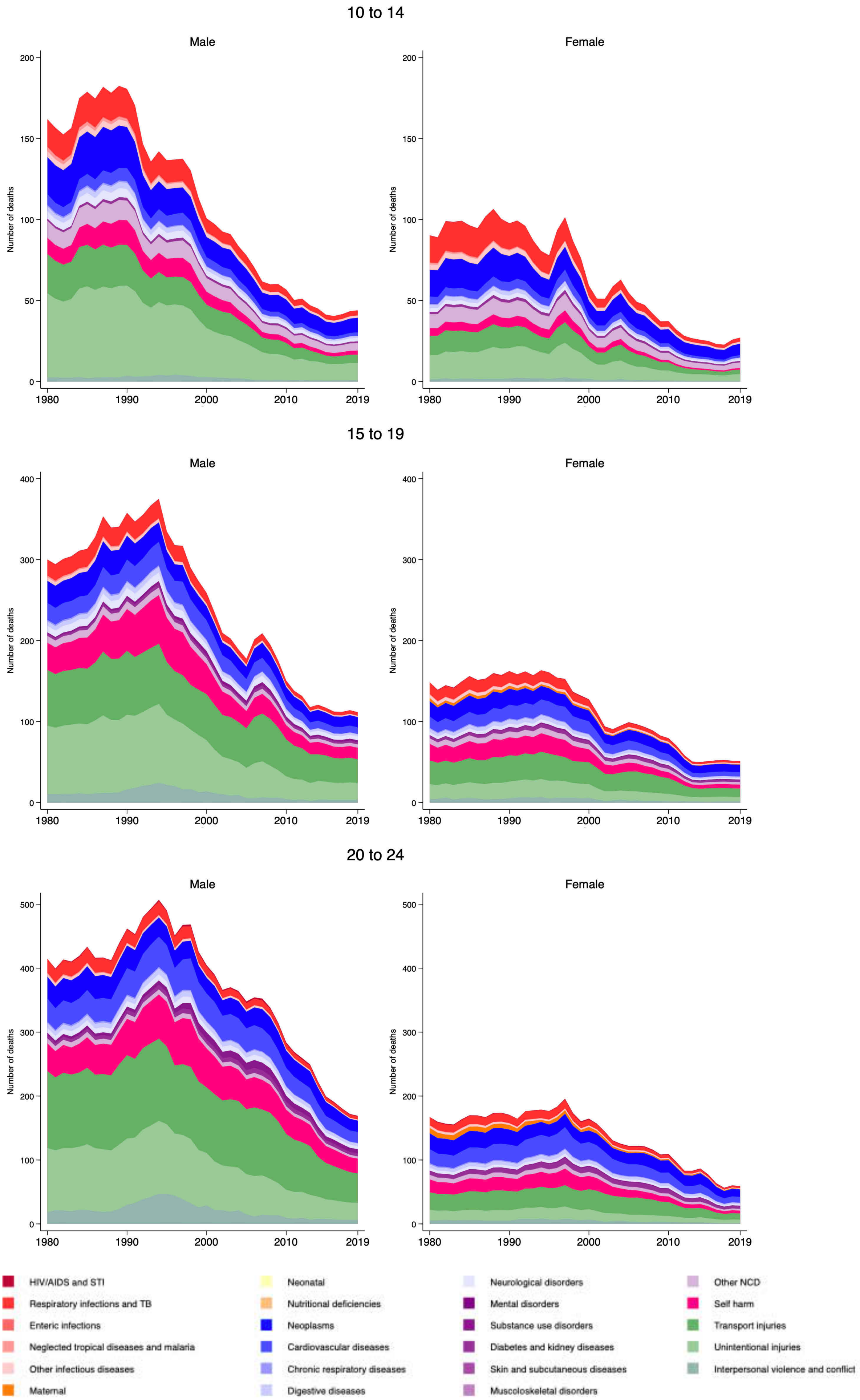

Figure S44: Croatia

Figure S44a All-cause mortality rate per 100,000 in 10-24 1950 – 2019: Croatia

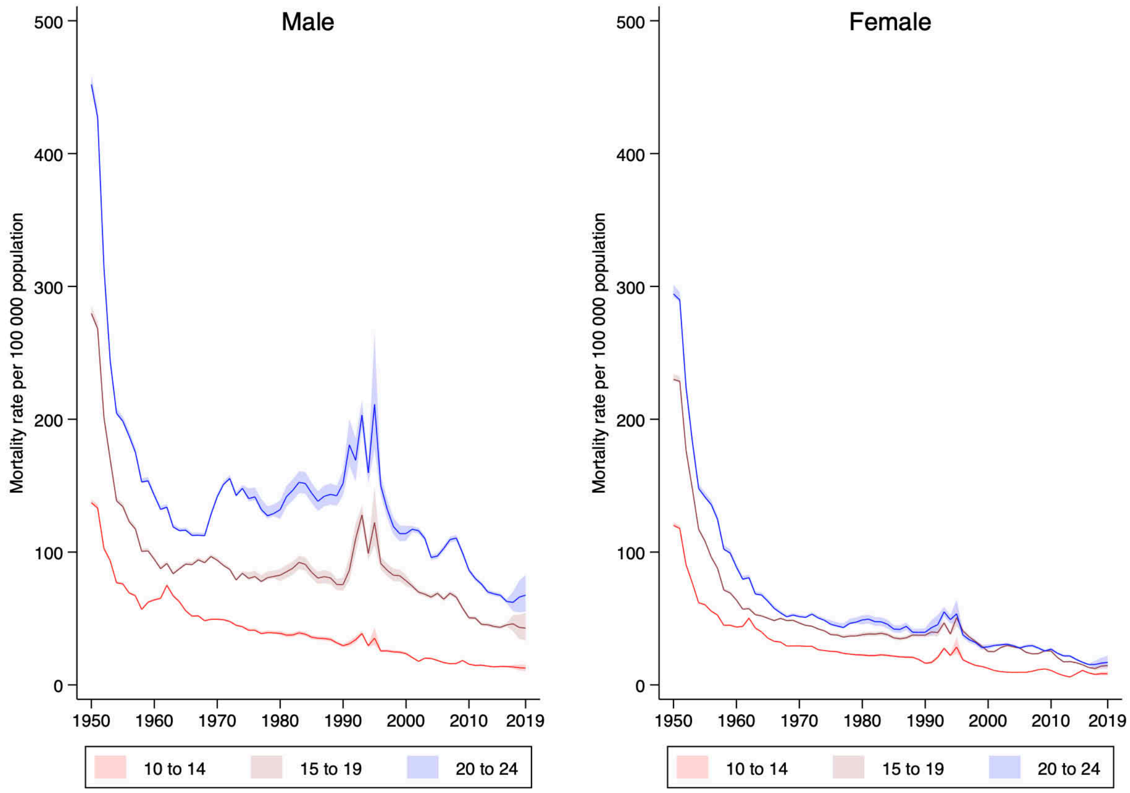

Figure S44b Number of deaths from all causes in 10-24 1950 – 2019: Croatia

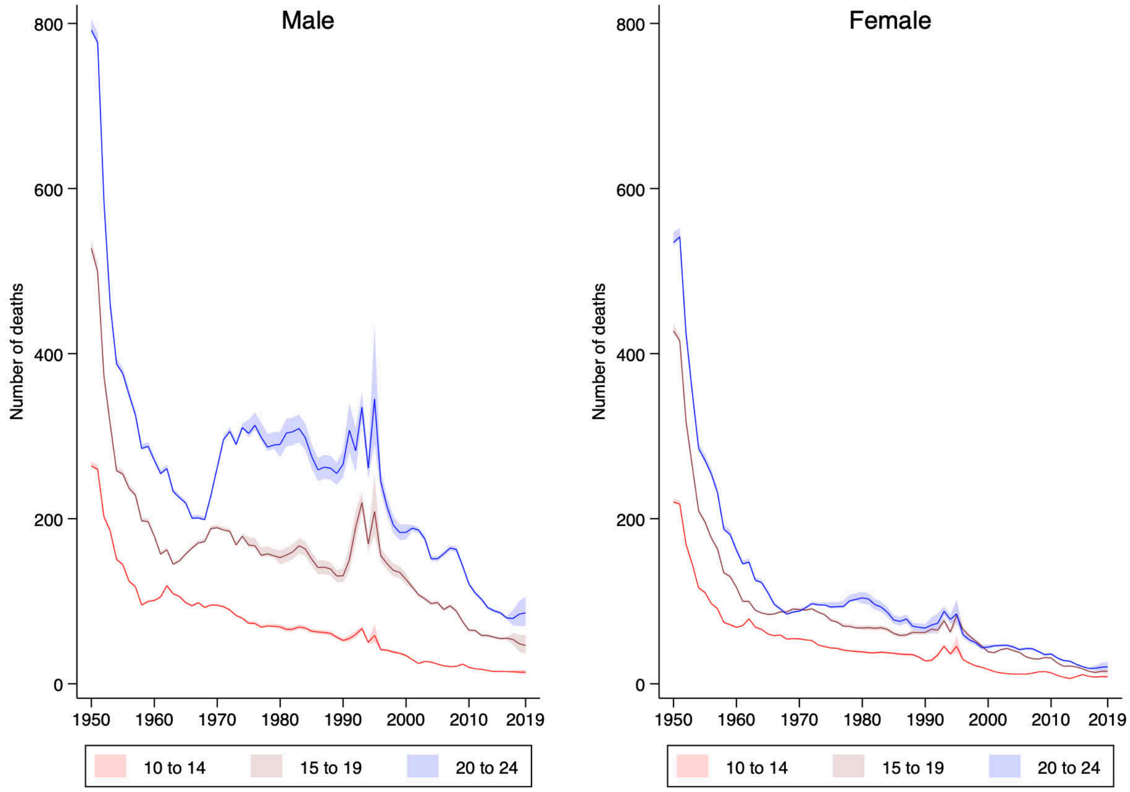

Figure S44c Mortality rate per 100,000 in 10-24 1980 – 2019 by cause: Croatia

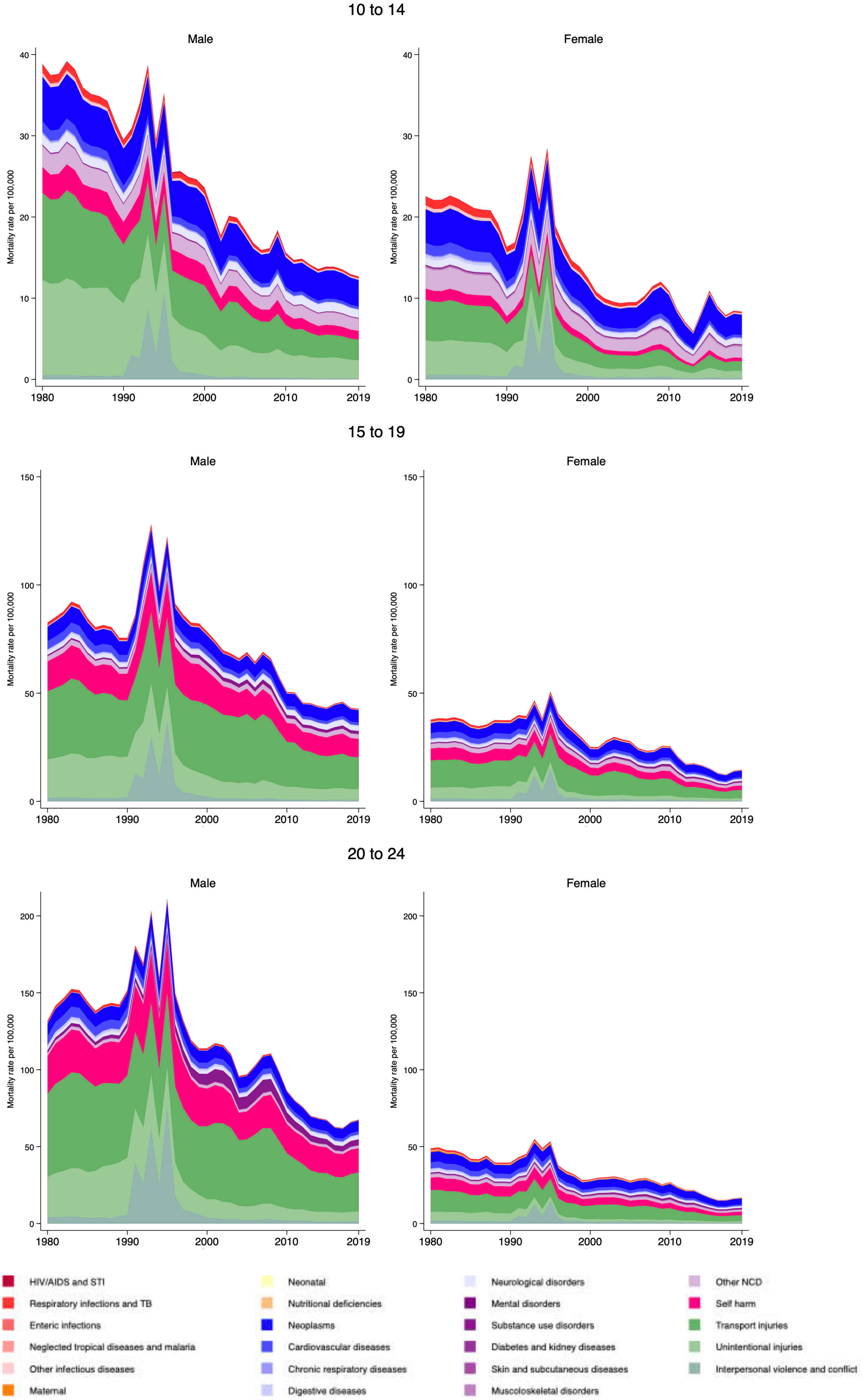

Figure S44d Number of deaths in 10-24 1980 – 2019 by cause: Croatia

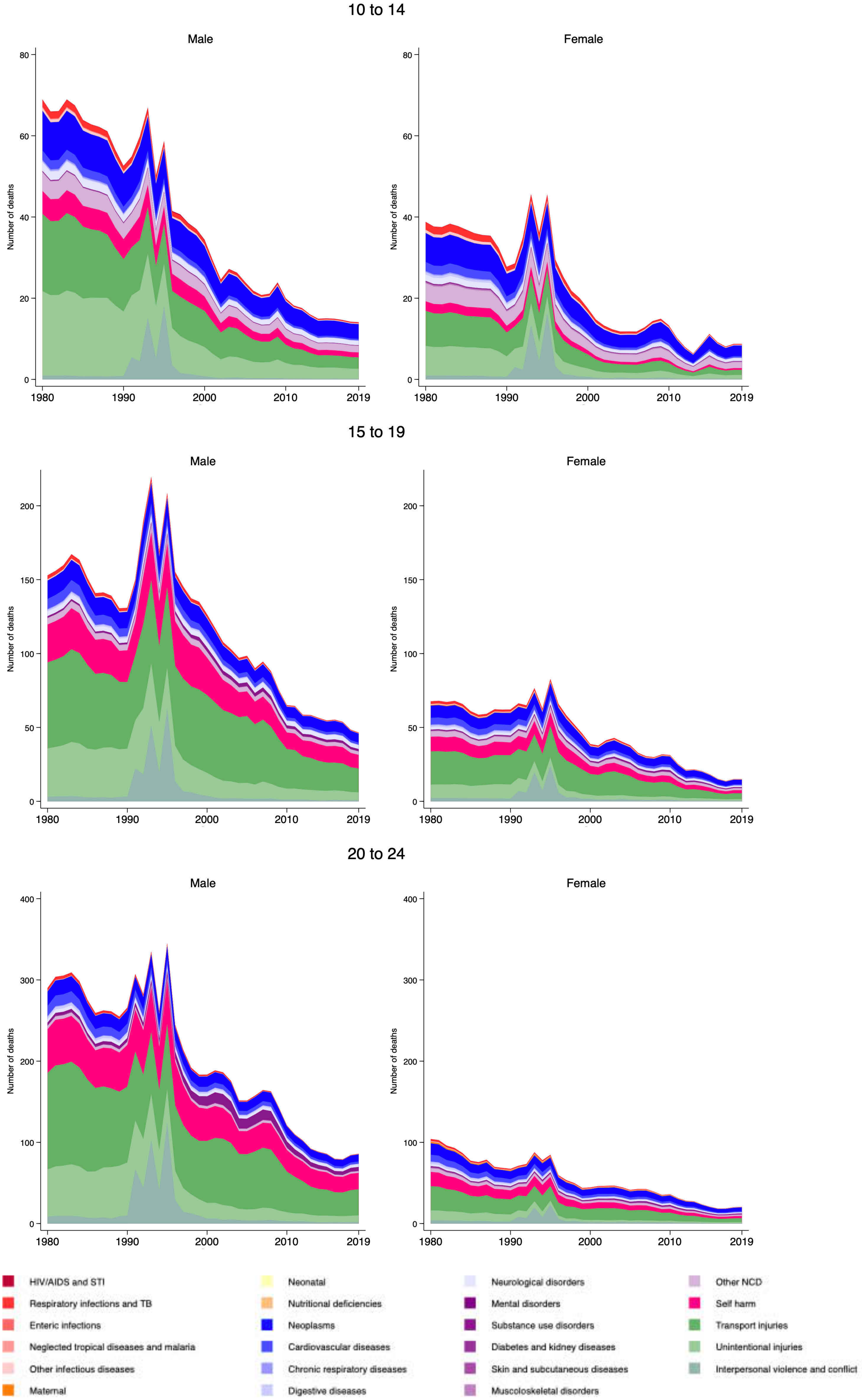

Figure S45: Czechia

Figure S45a All-cause mortality rate per 100,000 in 10-24 1950 – 2019: Czechia

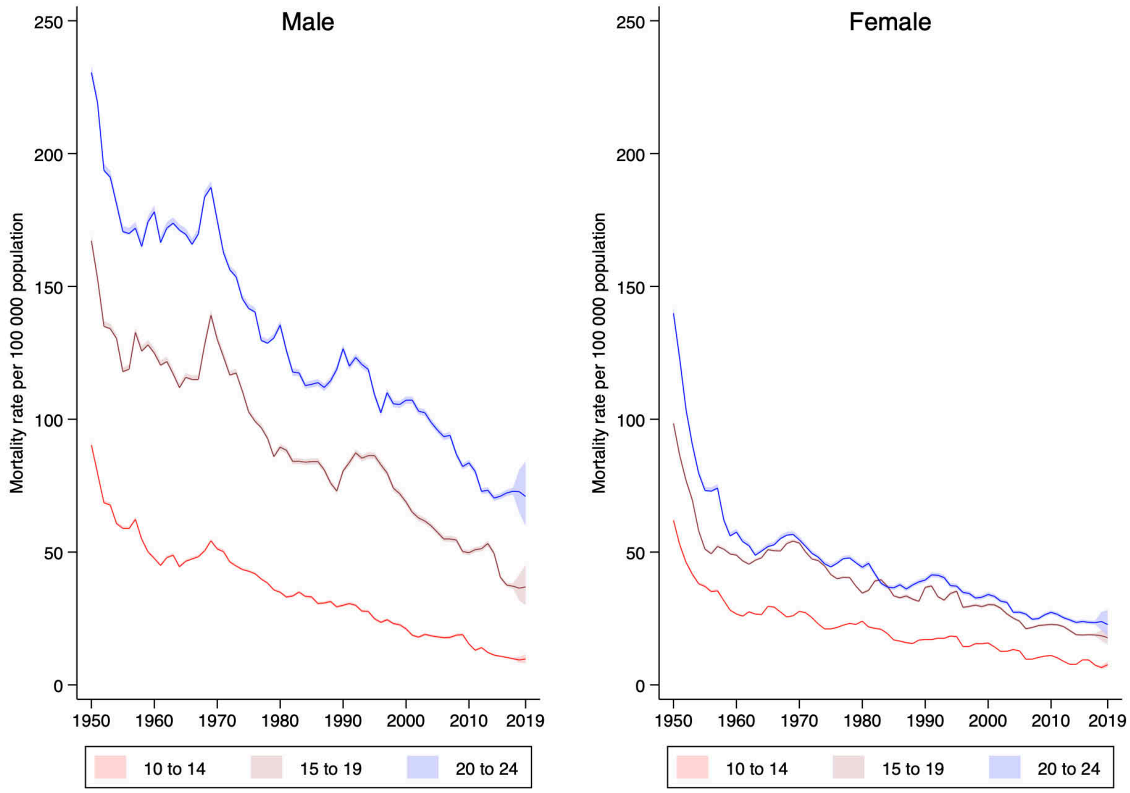

Figure S45b Number of deaths from all causes in 10-24 1950 – 2019: Czechia

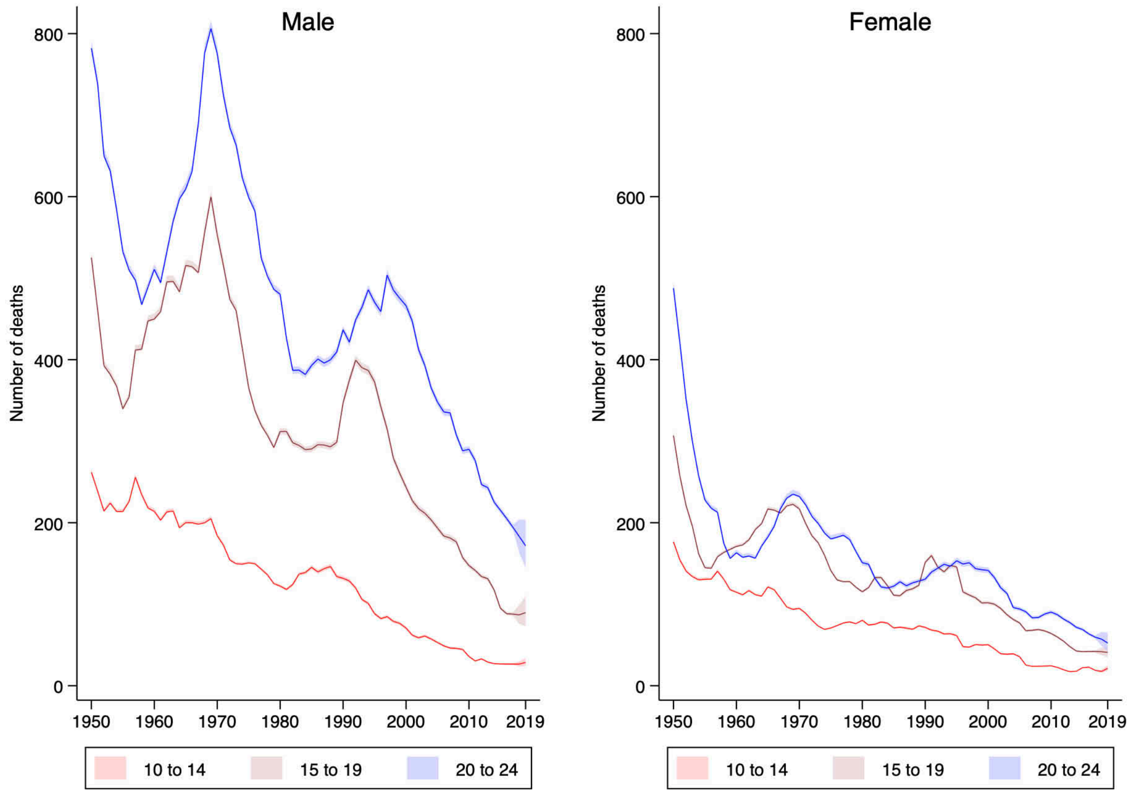

Figure S45c Mortality rate per 100,000 in 10-24 1980 – 2019 by cause: Czechia

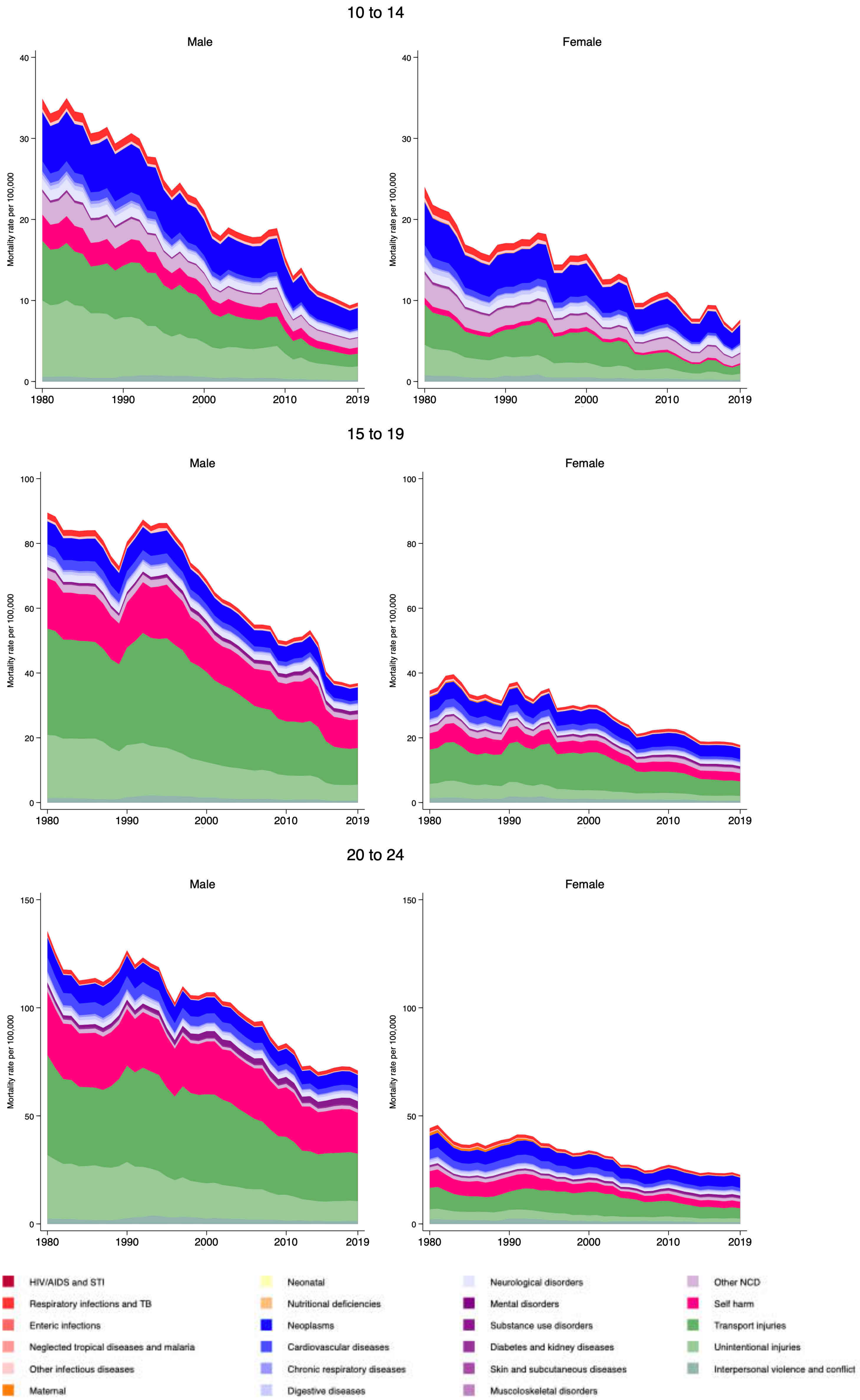

Figure S45d Number of deaths in 10-24 1980 – 2019 by cause: Czechia

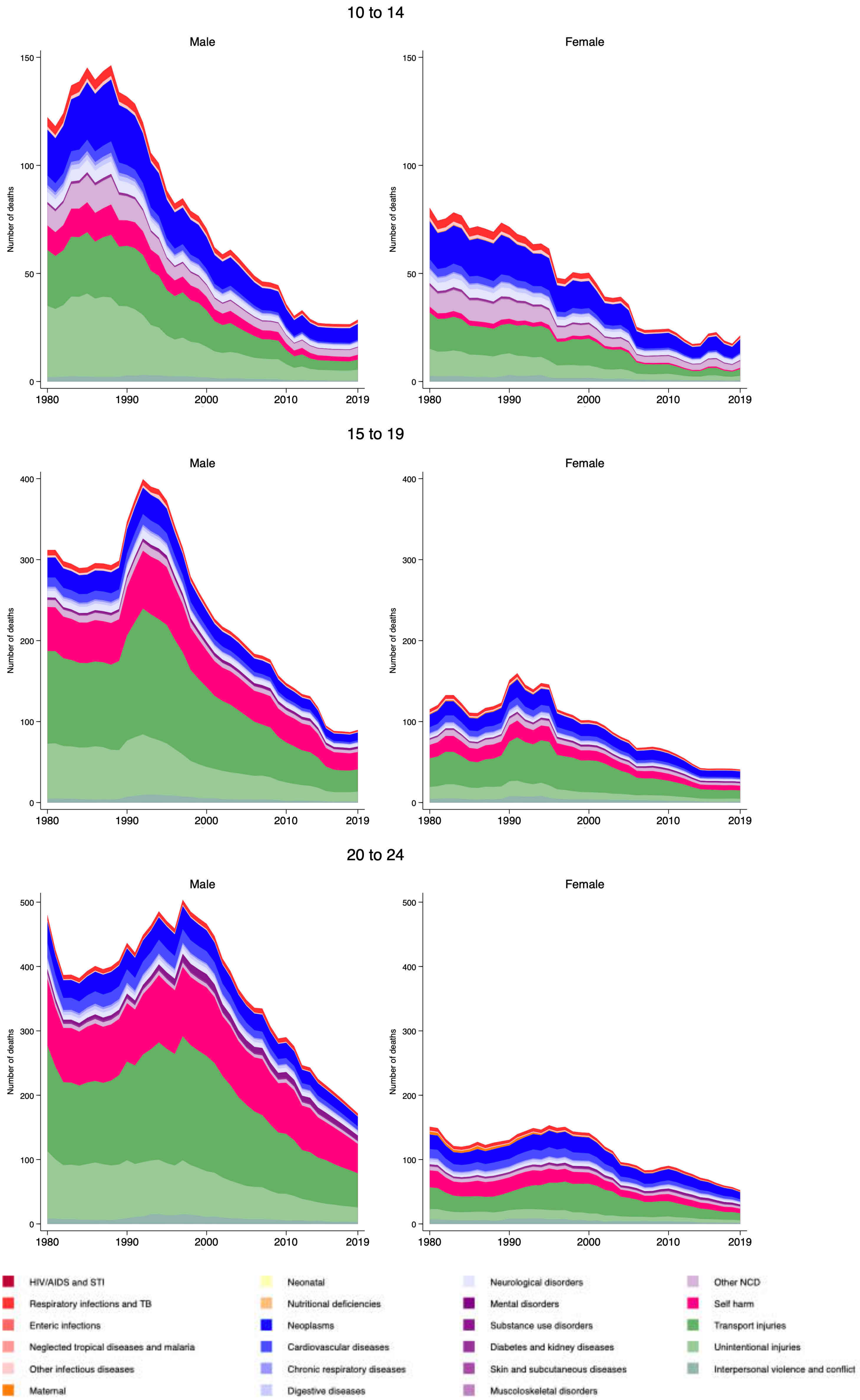

Figure S46: Hungary

Figure S46a All-cause mortality rate per 100,000 in 10-24 1950 – 2019: Hungary

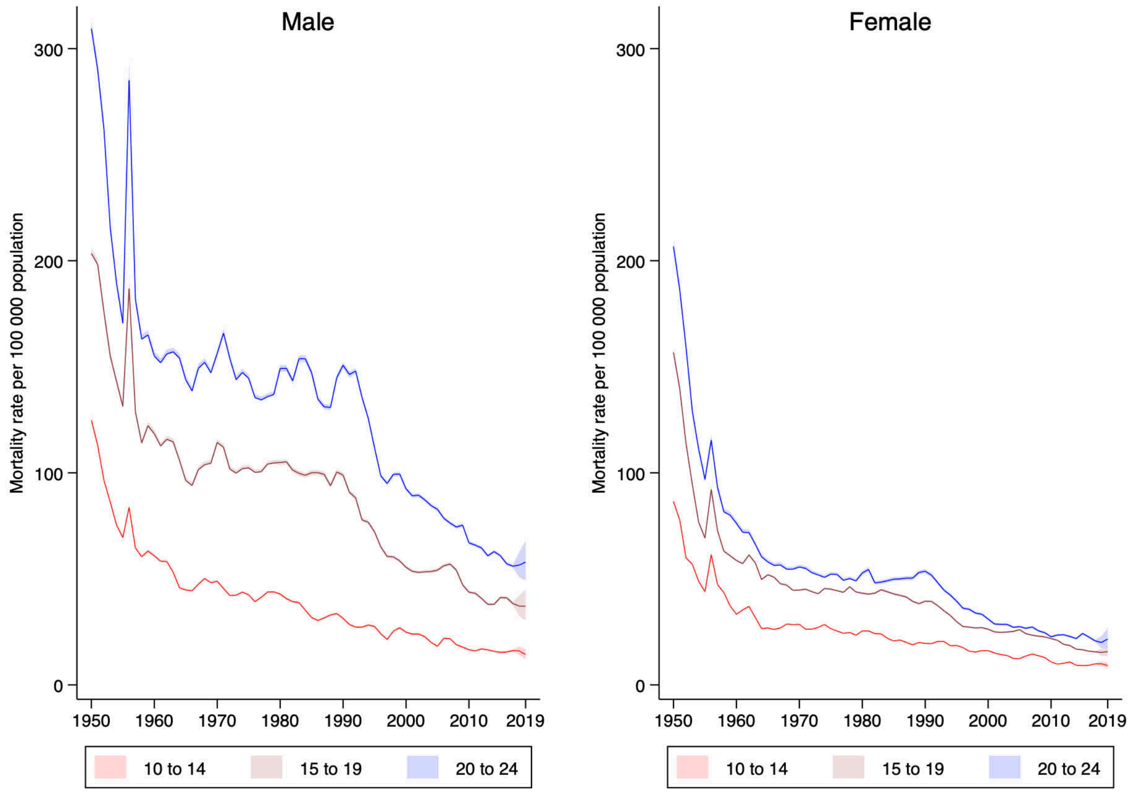

Figure S46b Number of deaths from all causes in 10-24 1950 – 2019: Hungary

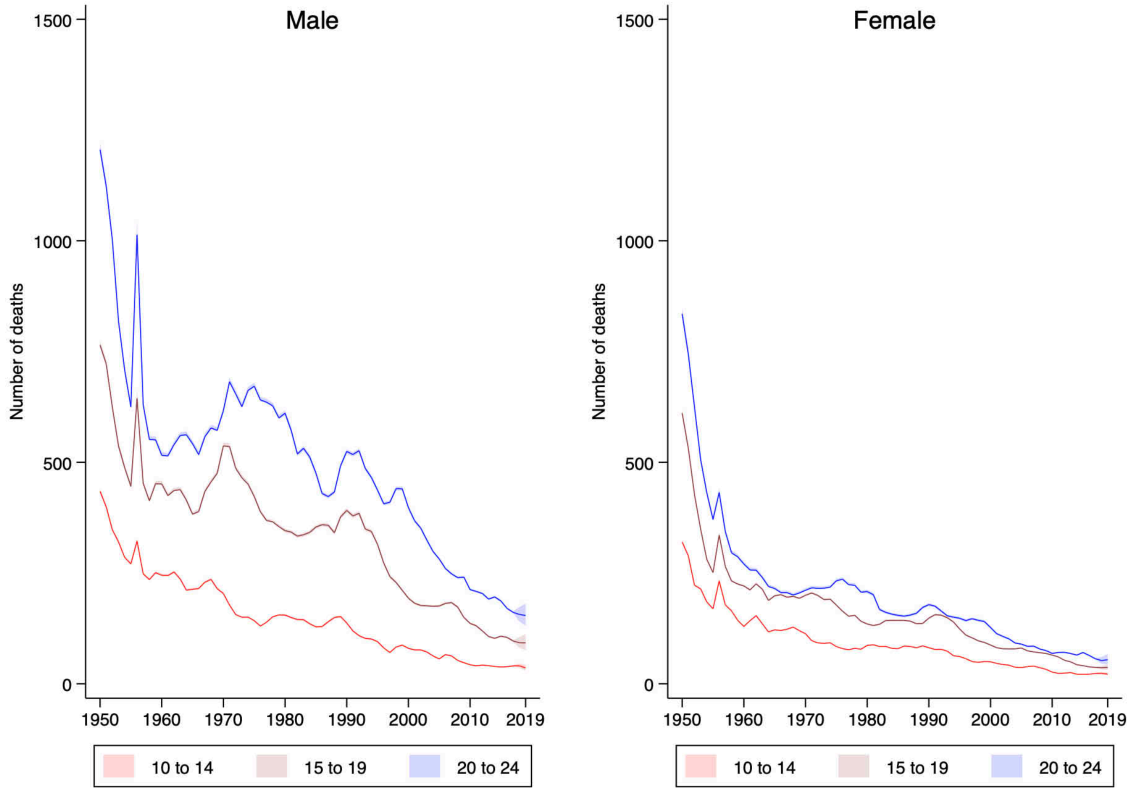

Figure S46c Mortality rate per 100,000 in 10-24 1980 – 2019 by cause: Hungary

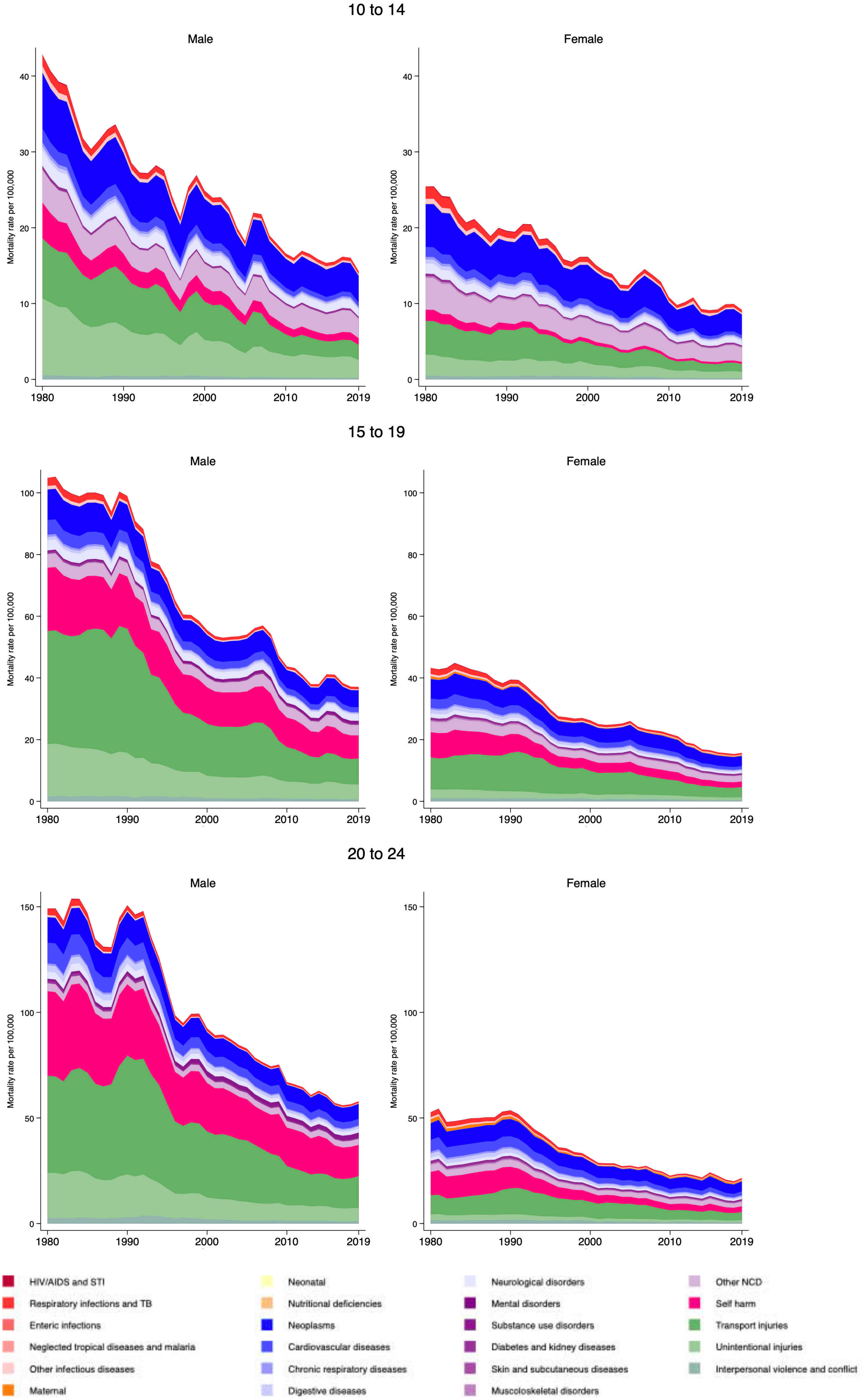

Figure S46d Number of deaths in 10-24 1980 – 2019 by cause: Hungary

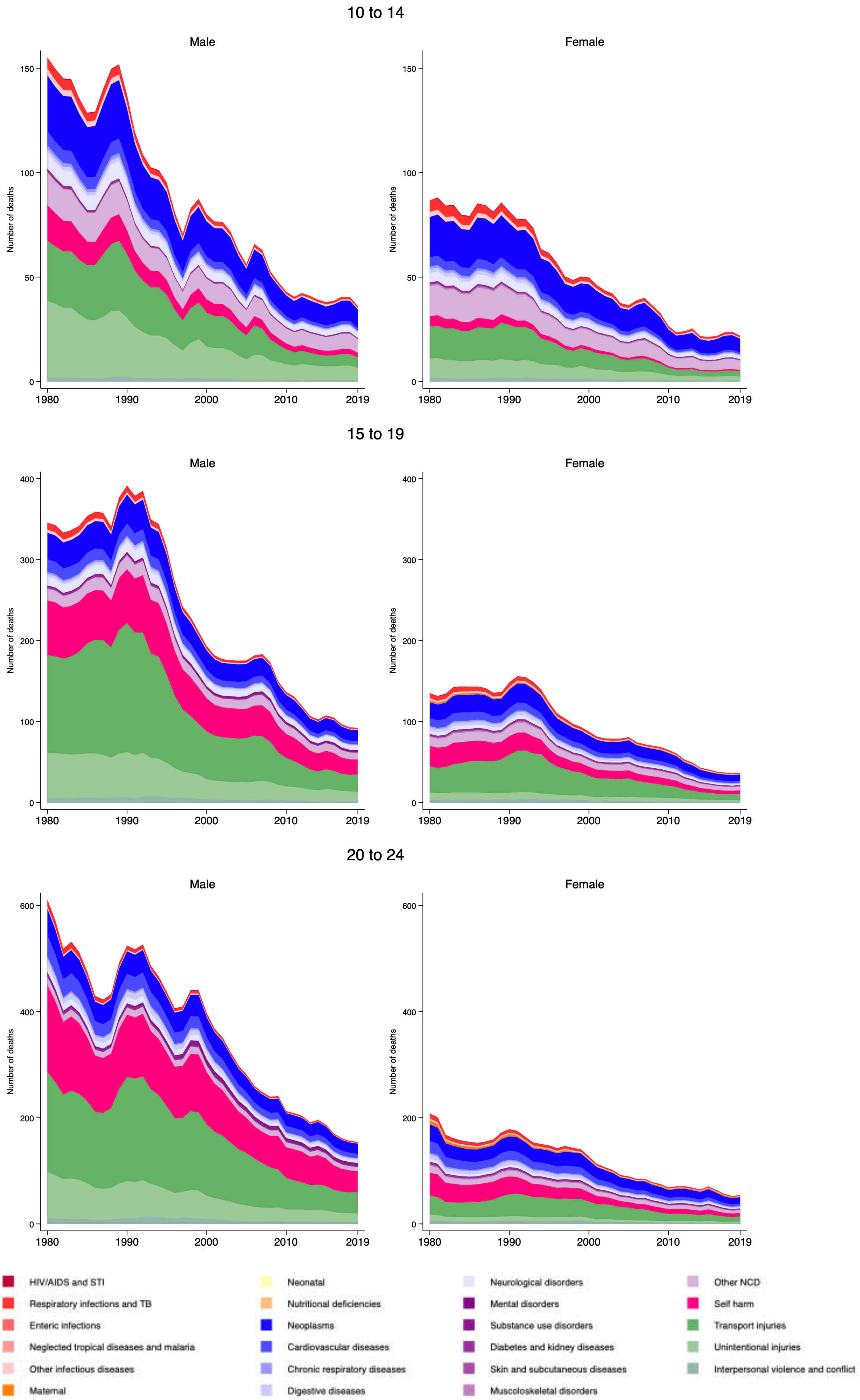

Figure S47: North Macedonia

Figure S47a All-cause mortality rate per 100,000 in 10-24 1950 – 2019: North Macedonia

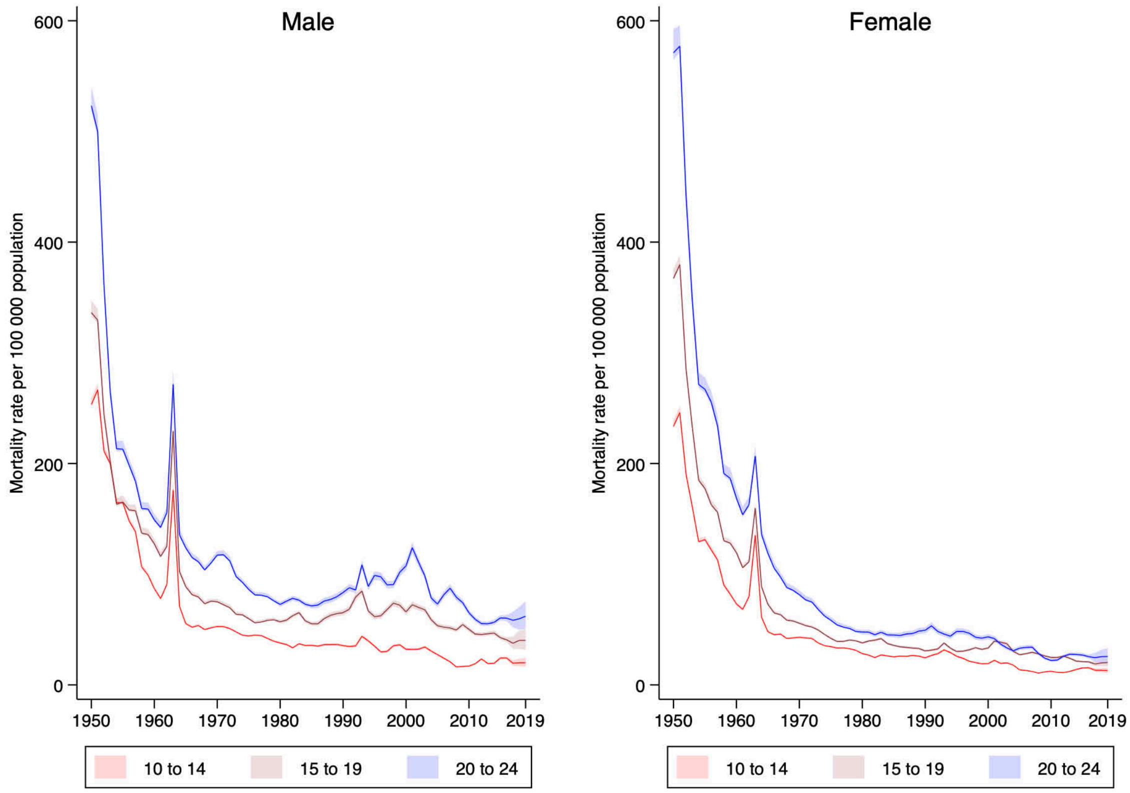

Figure S47b Number of deaths from all causes in 10-24 1950 – 2019: North Macedonia

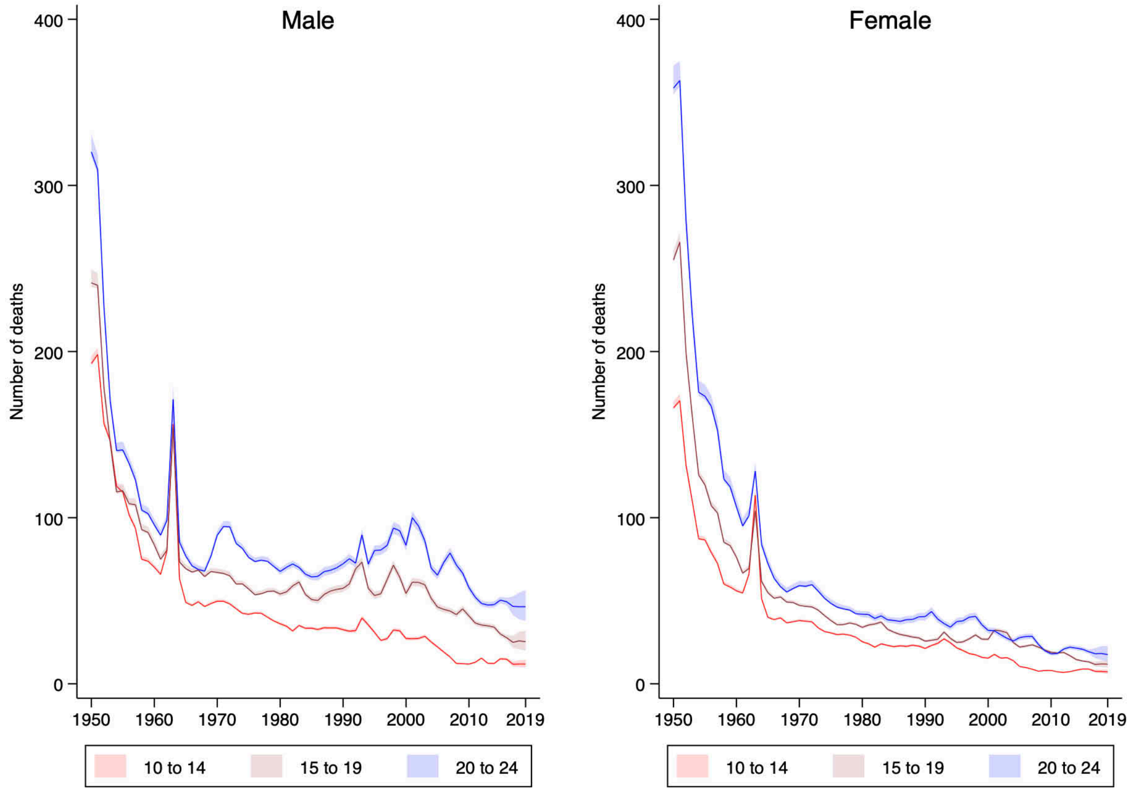

Figure S47c Mortality rate per 100,000 in 10-24 1980 – 2019 by cause: North Macedonia

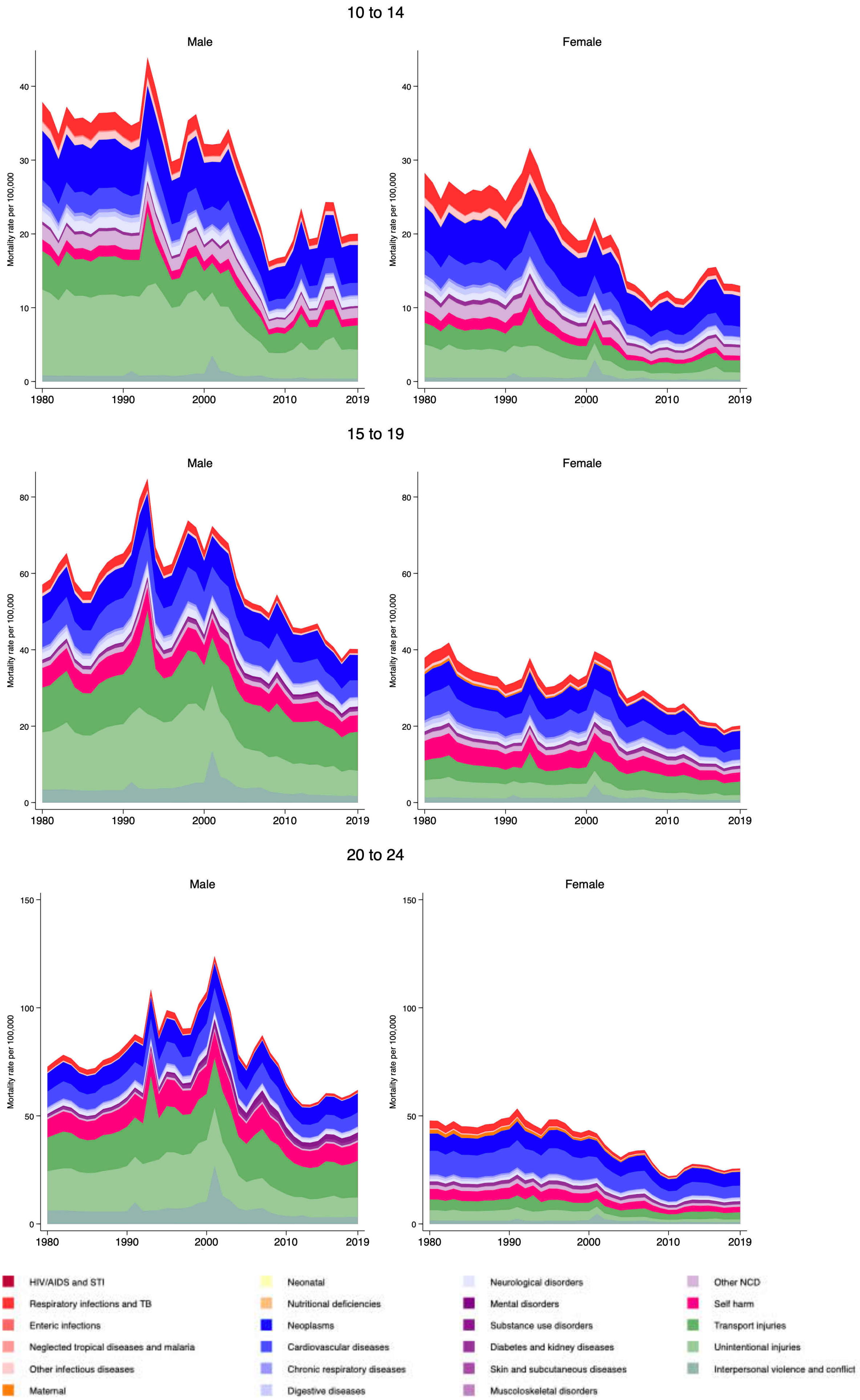

Figure S47d Number of deaths in 10-24 1980 – 2019 by cause: North Macedonia

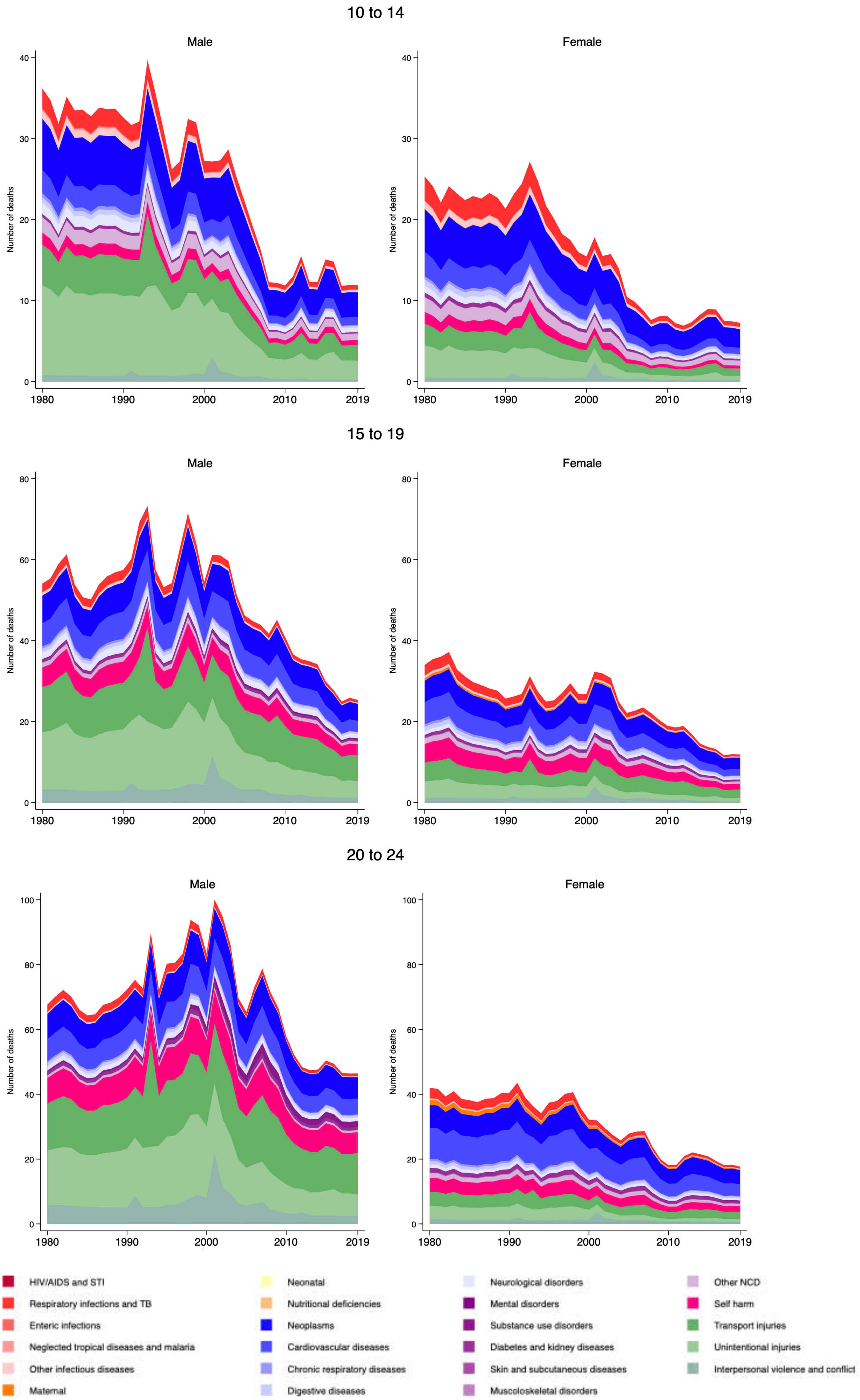

Figure S48: Montenegro

Figure S48a All-cause mortality rate per 100,000 in 10-24 1950 – 2019: Montenegro

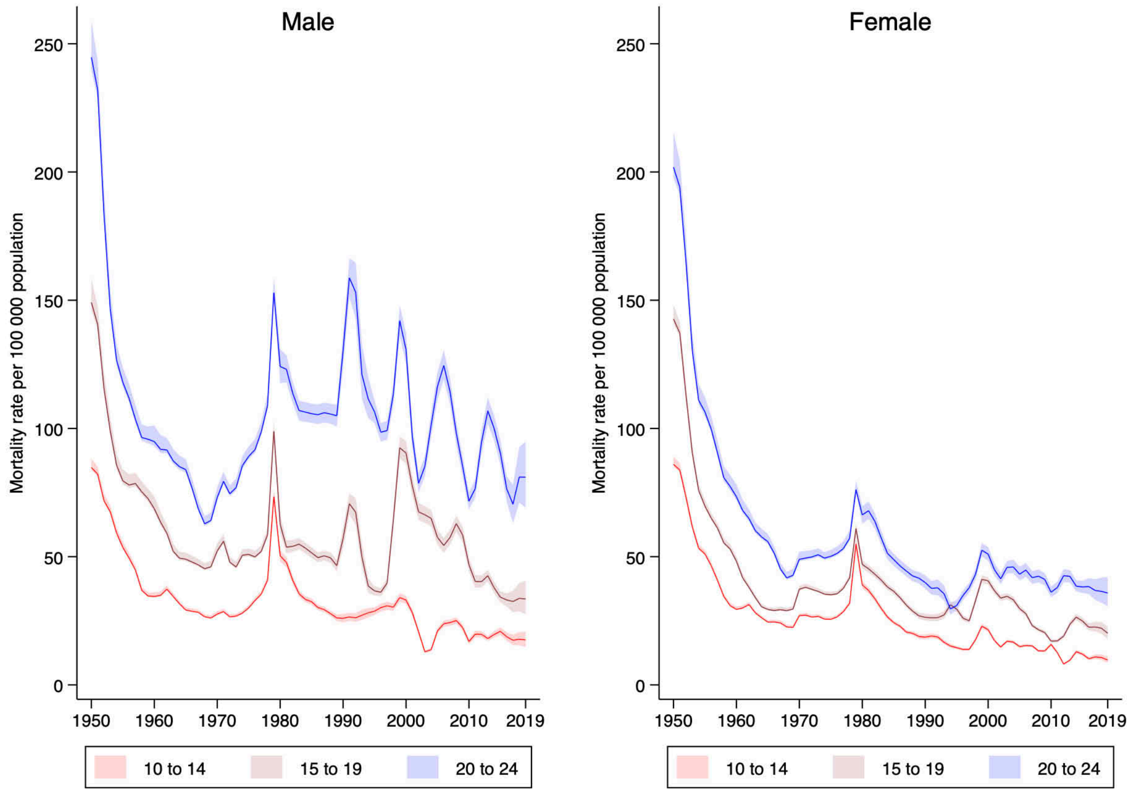

Figure S48b Number of deaths from all causes in 10-24 1950 – 2019: Montenegro

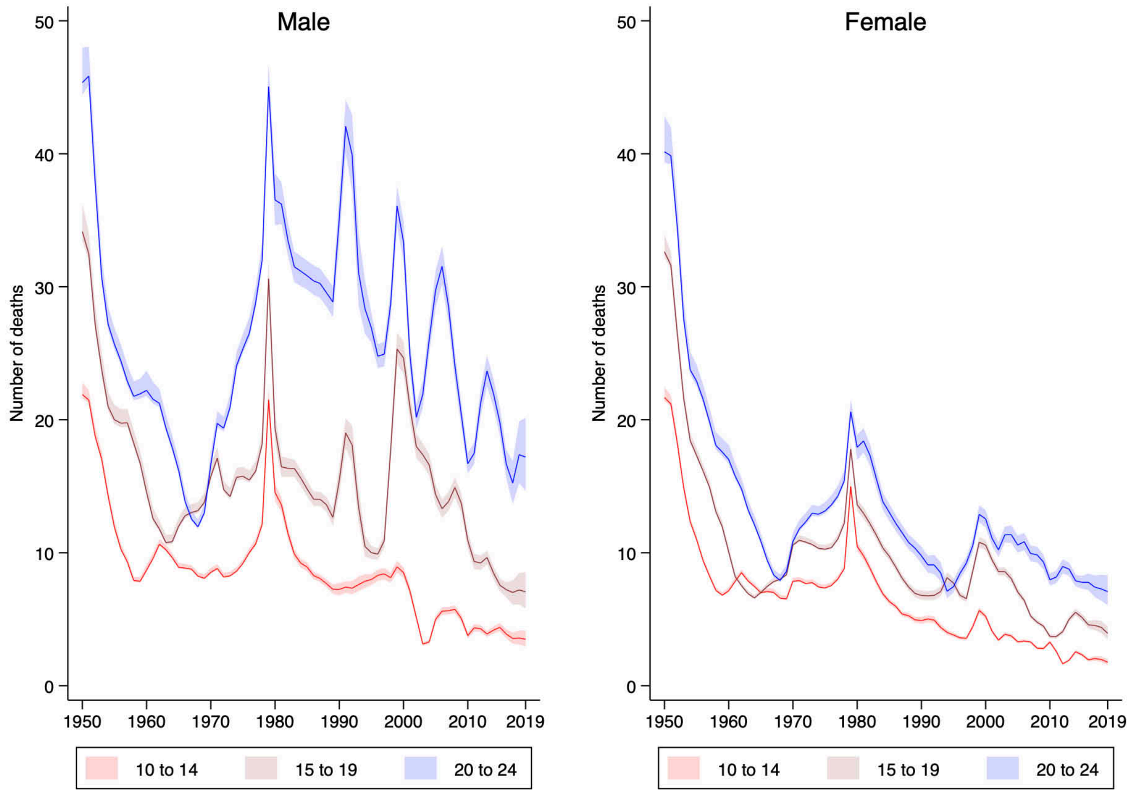

Figure S48c Mortality rate per 100,000 in 10-24 1980 – 2019 by cause: Montenegro

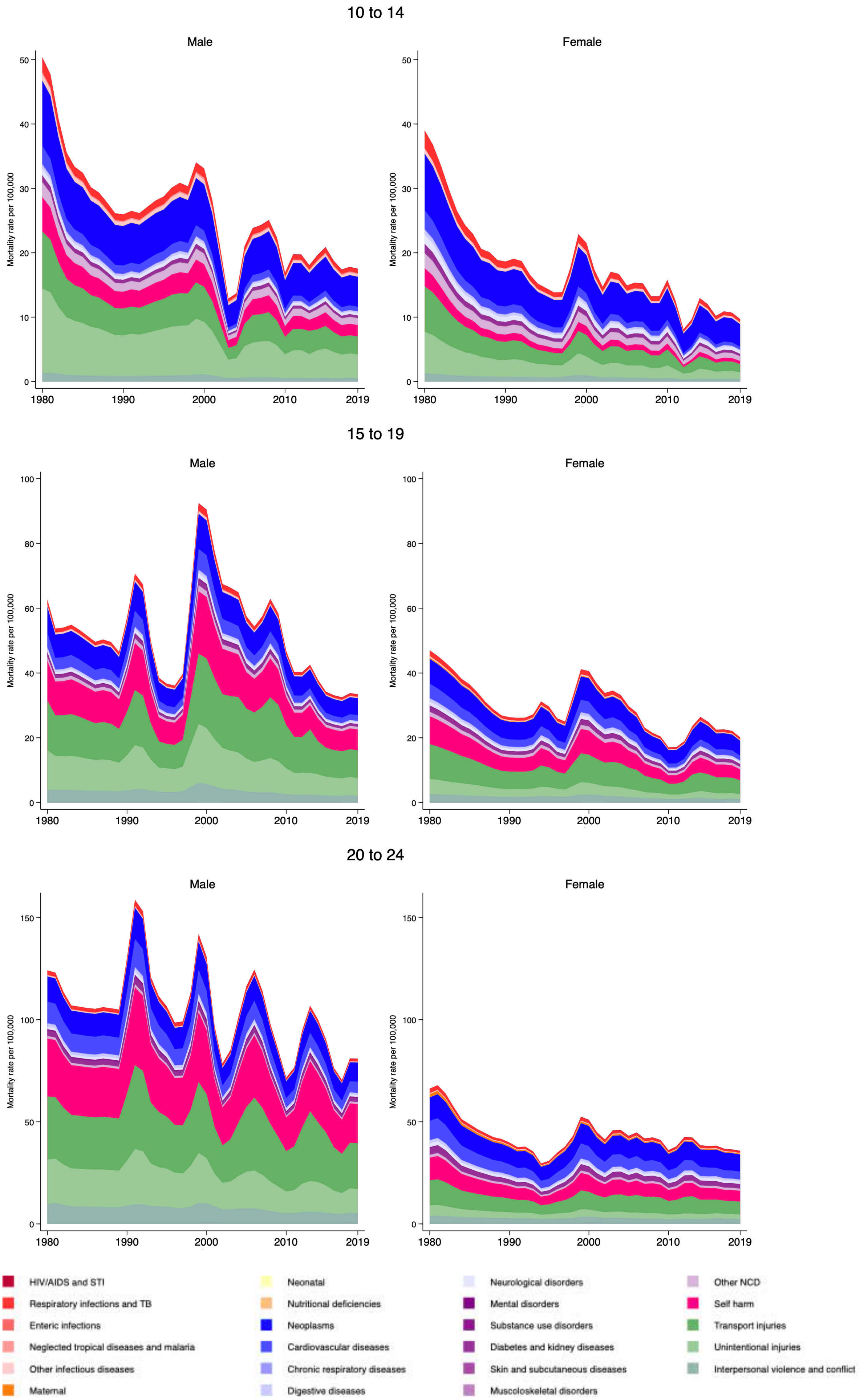

Figure S48d Number of deaths in 10-24 1980 – 2019 by cause: Montenegro

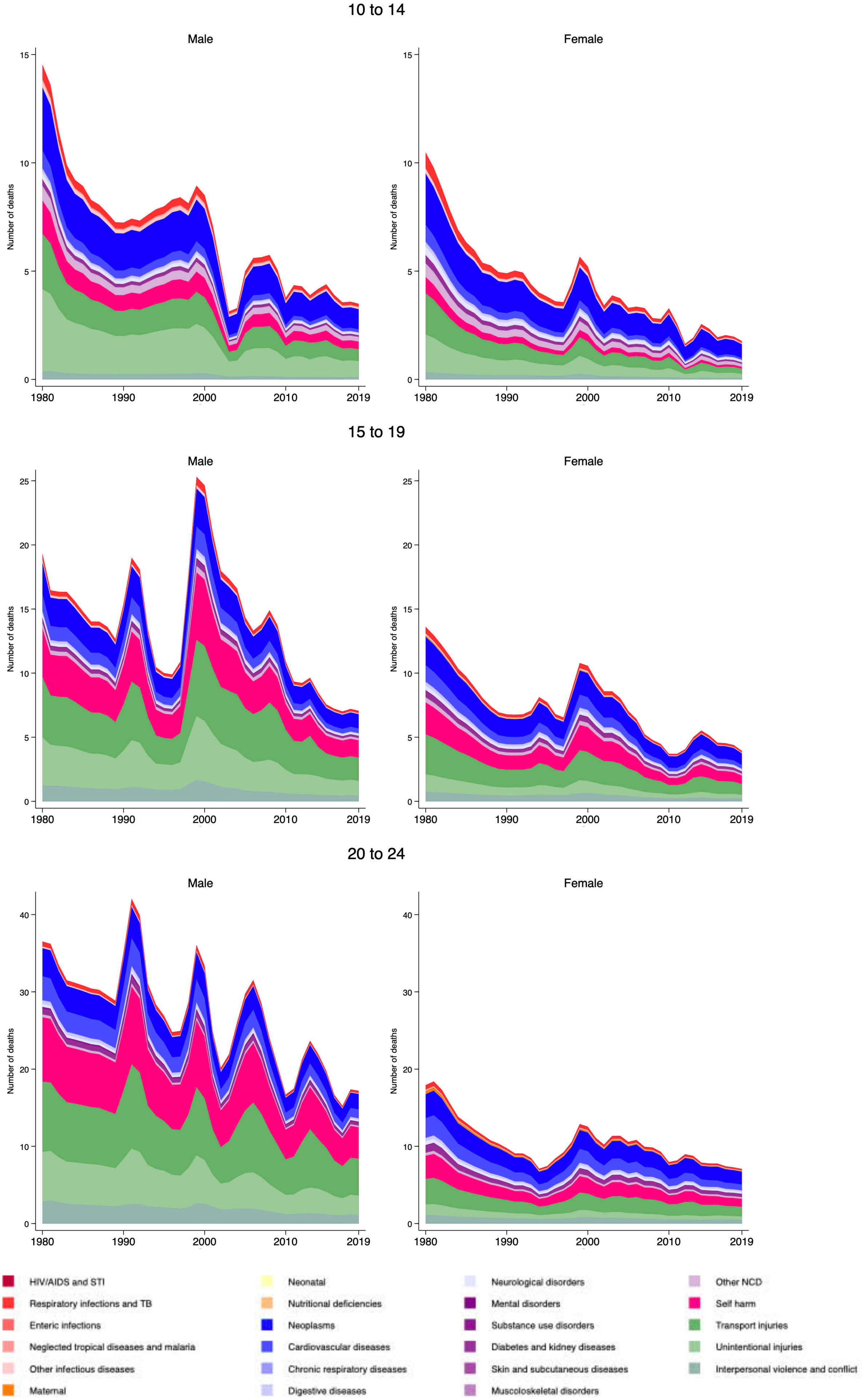

Figure S49: Poland

Figure S49a All-cause mortality rate per 100,000 in 10-24 1950 – 2019: Poland

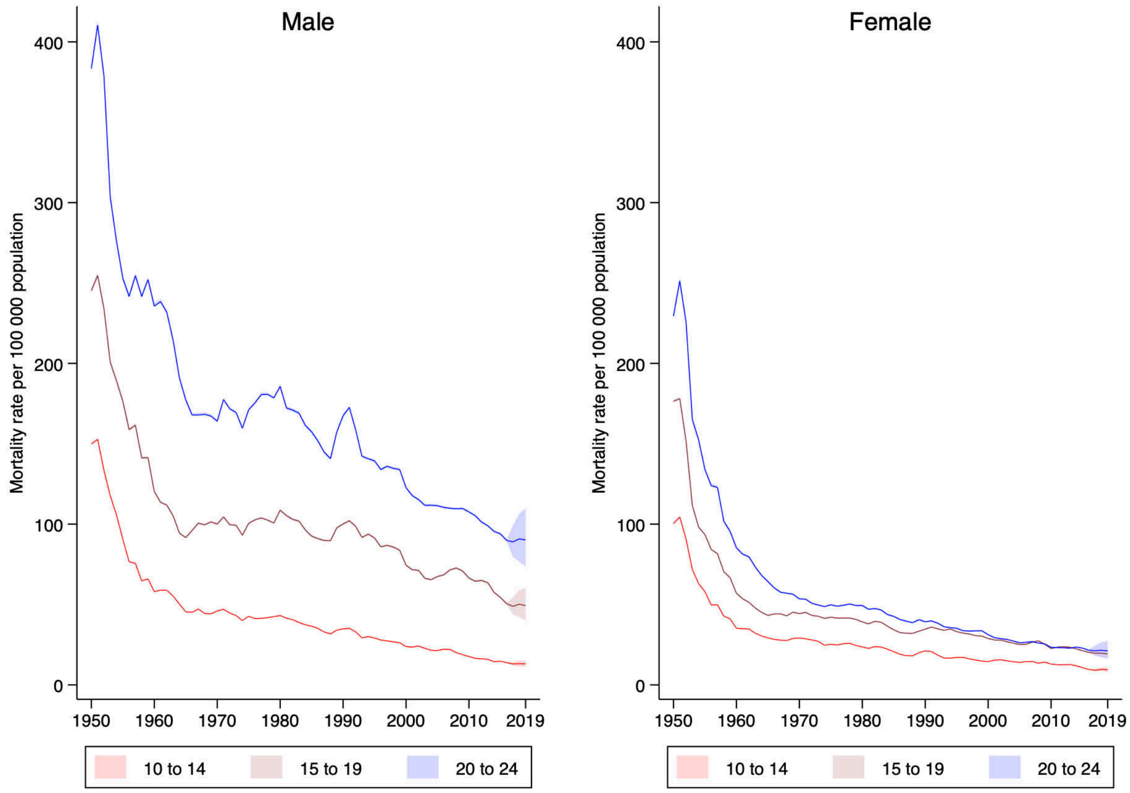

Figure S49b Number of deaths from all causes in 10-24 1950 – 2019: Poland

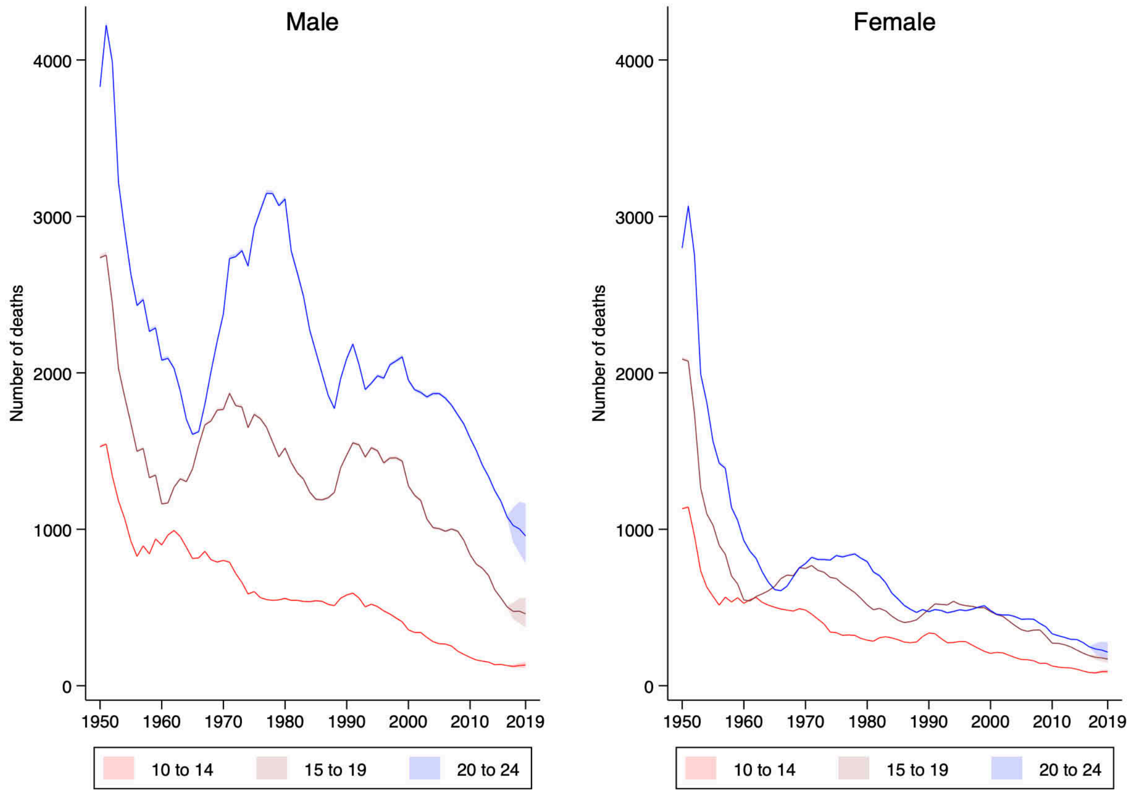

Figure S49c Mortality rate per 100,000 in 10-24 1980 – 2019 by cause: Poland

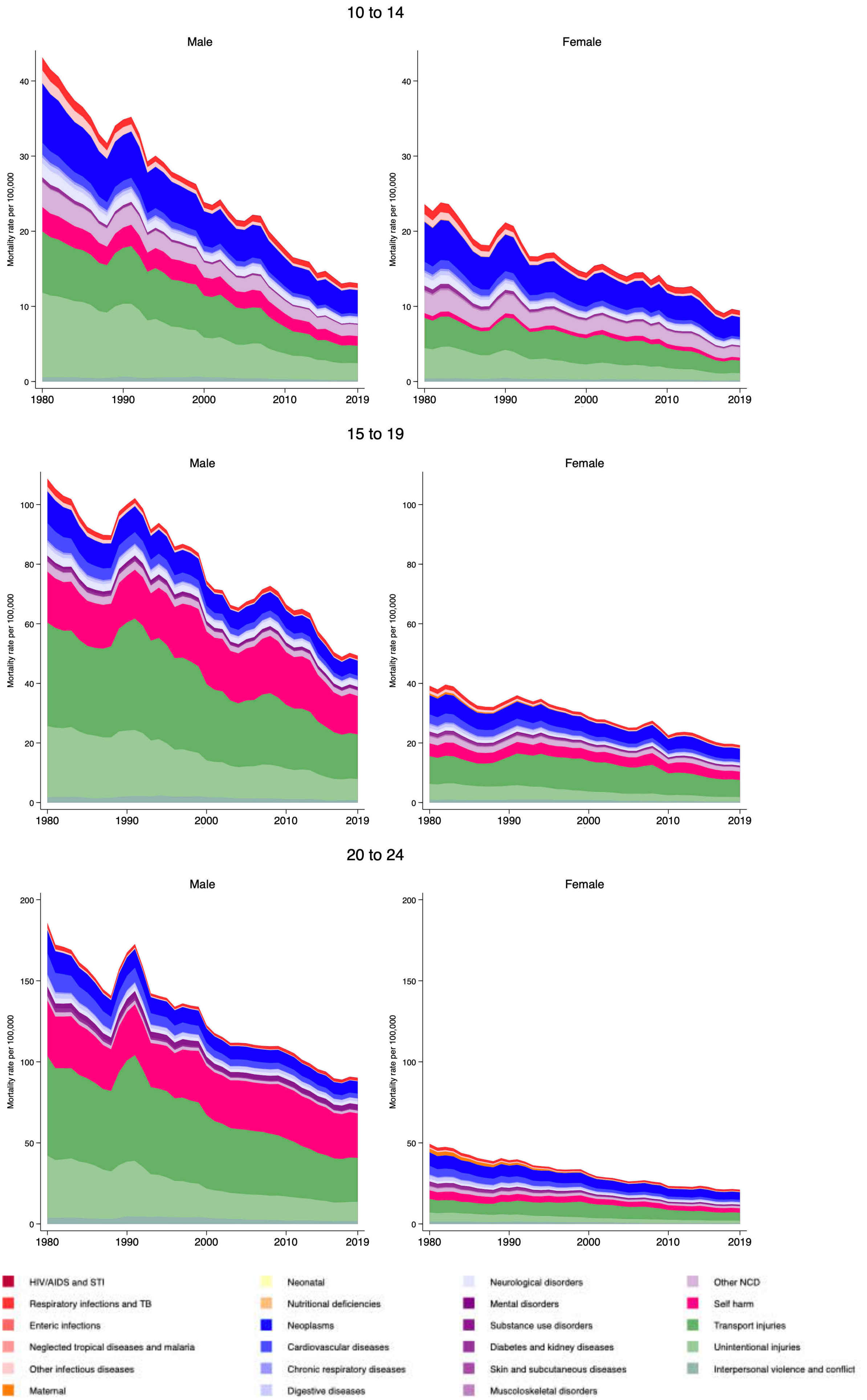

Figure S49d Number of deaths in 10-24 1980 – 2019 by cause: Poland

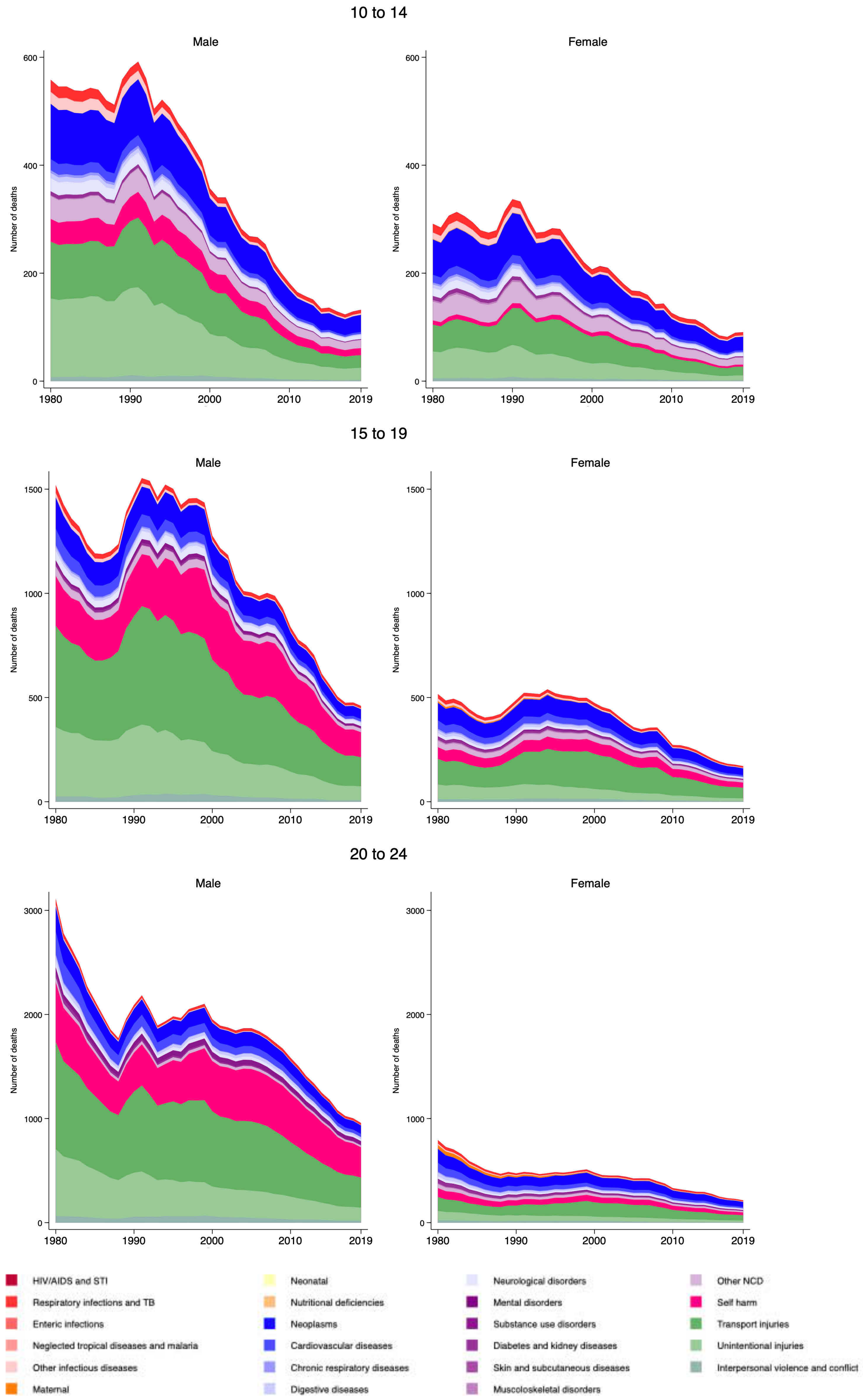

Figure S50: Romania

Figure S50a All-cause mortality rate per 100,000 in 10-24 1950 – 2019: Romania

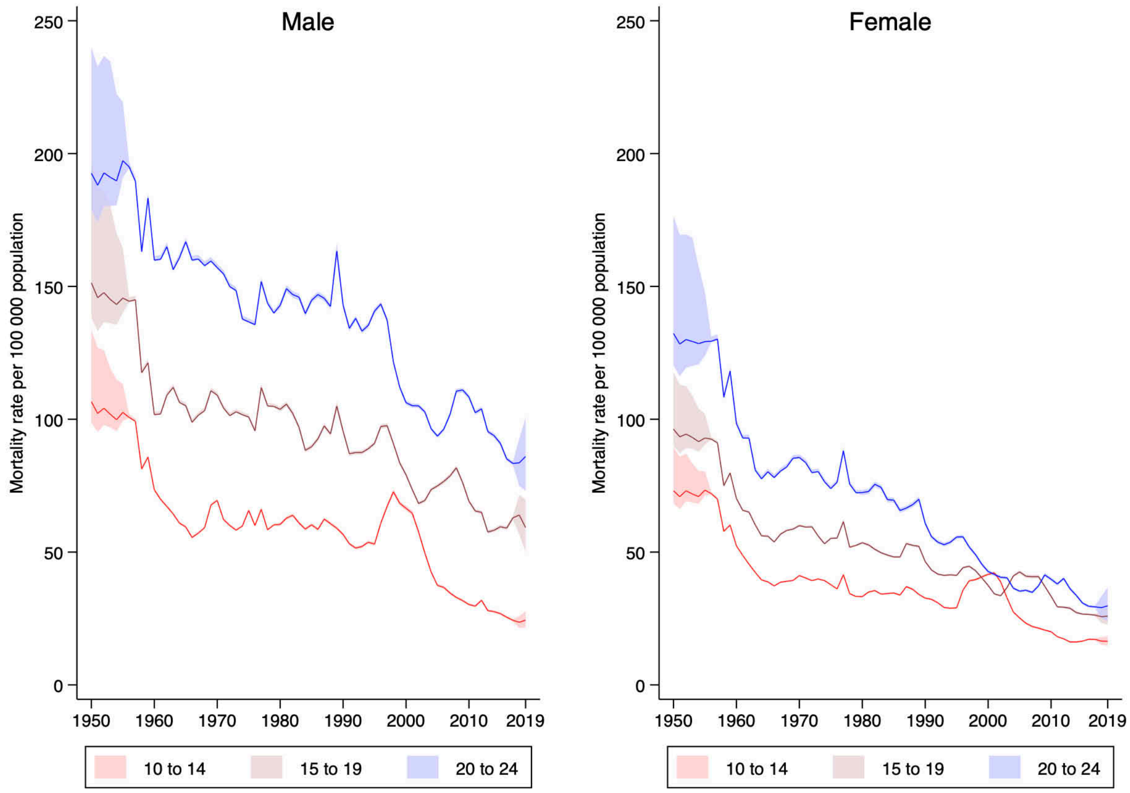

Figure S50b Number of deaths from all causes in 10-24 1950 – 2019: Romania

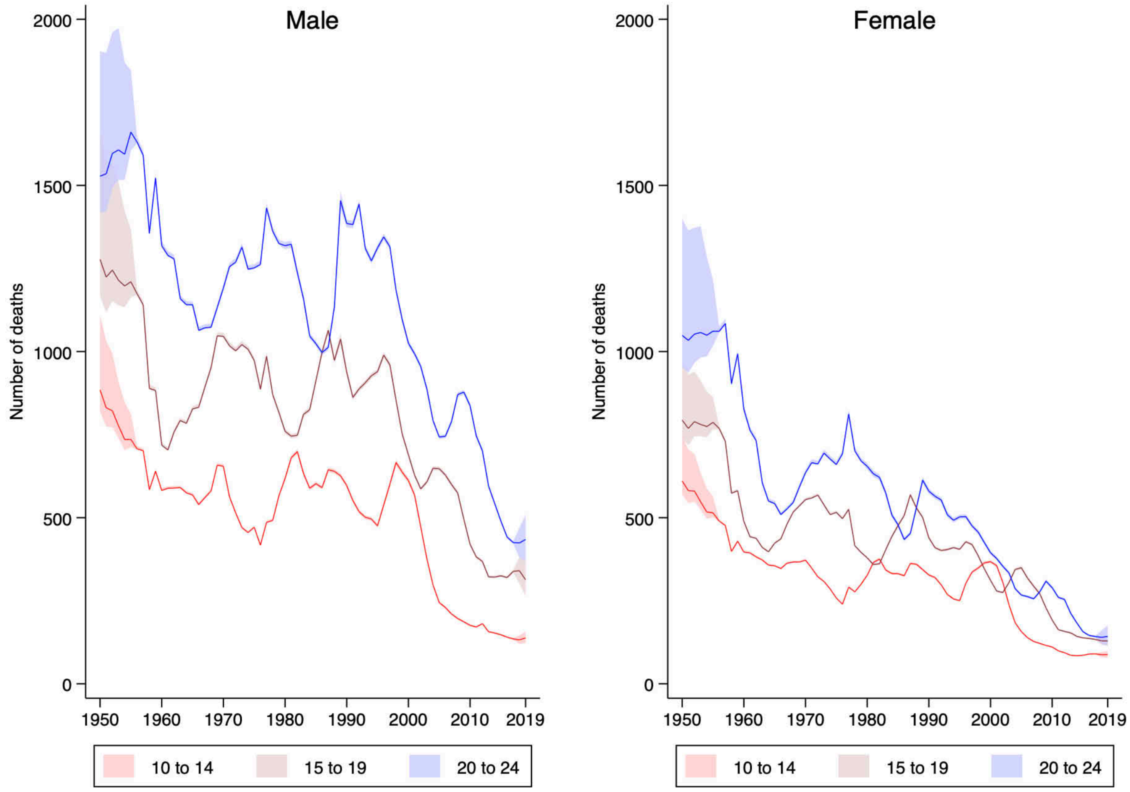

Figure S50c Mortality rate per 100,000 in 10-24 1980 – 2019 by cause: Romania

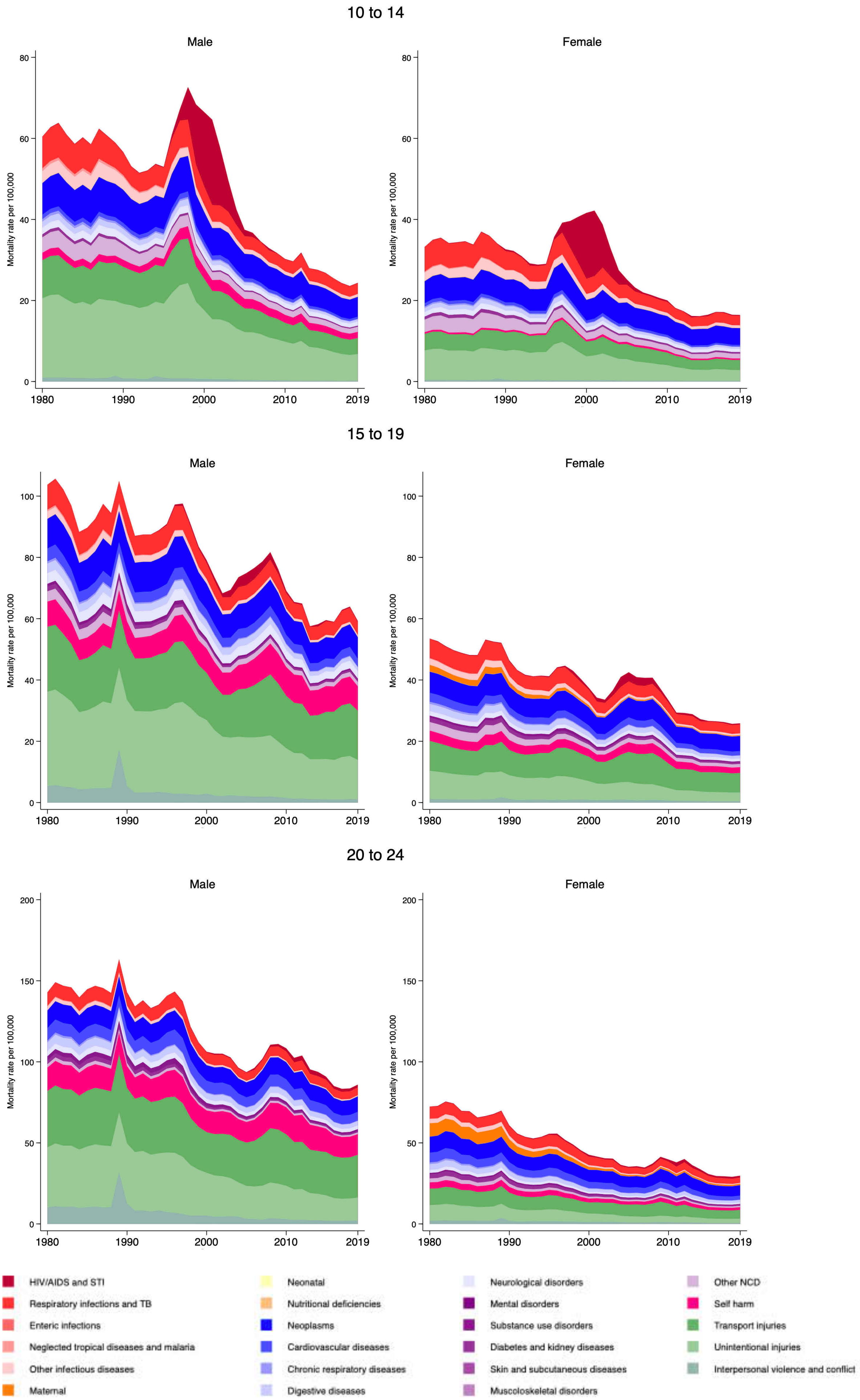

Figure S50d Number of deaths in 10-24 1980 – 2019 by cause: Romania

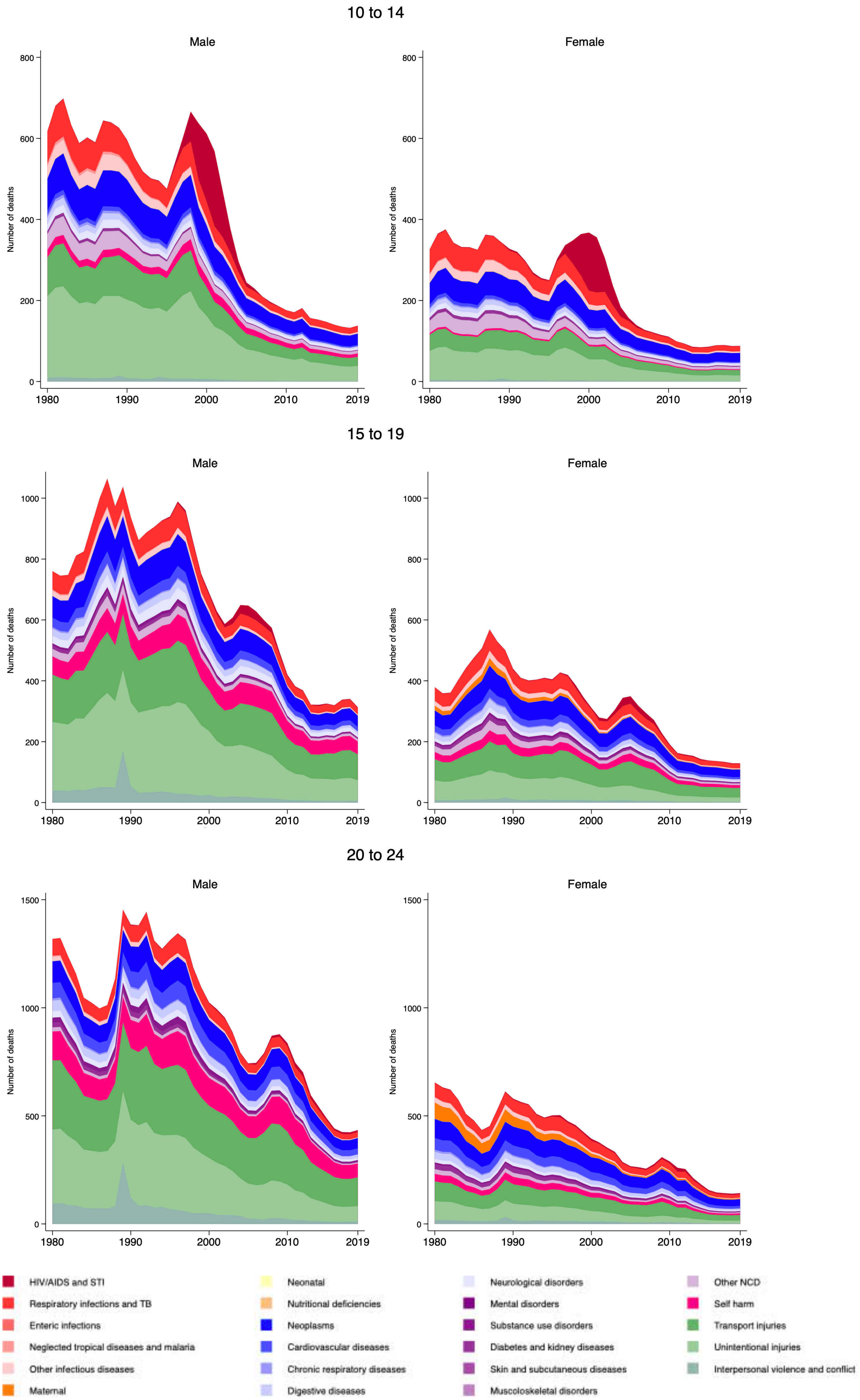

Figure S51: Serbia

Figure S51a All-cause mortality rate per 100,000 in 10-24 1950 – 2019: Serbia

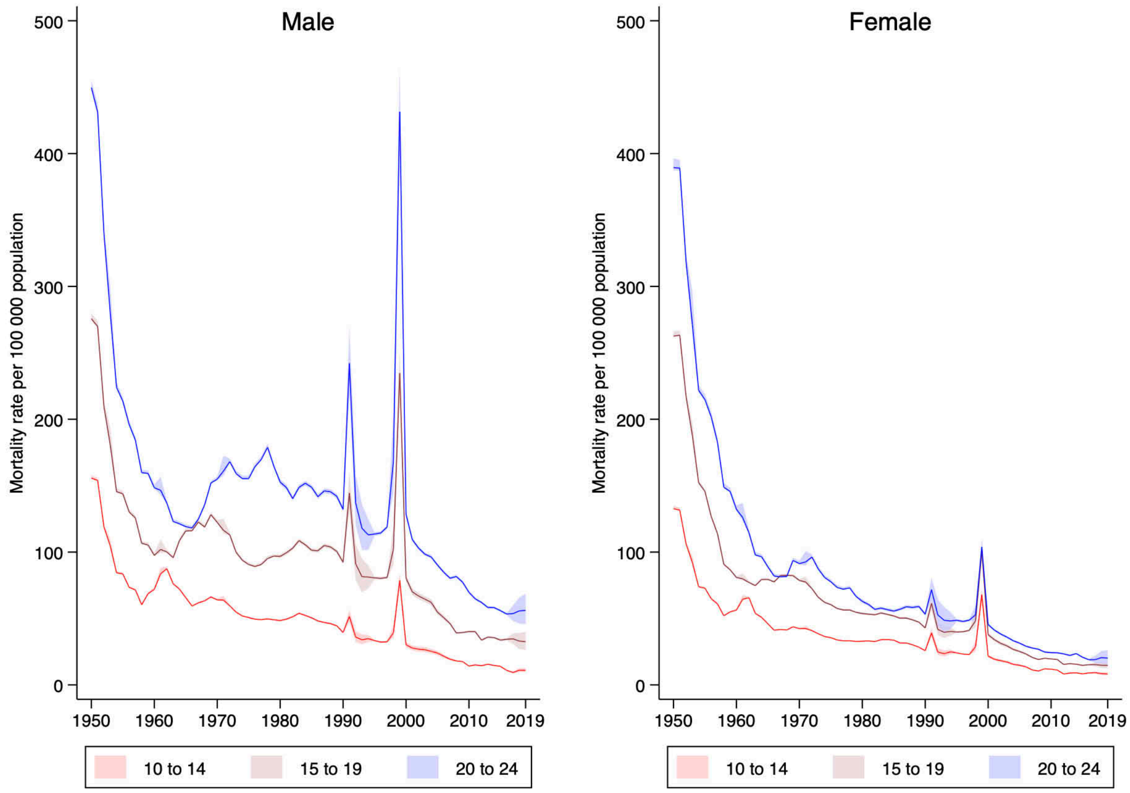

Figure S51b Number of deaths from all causes in 10-24 1950 – 2019: Serbia

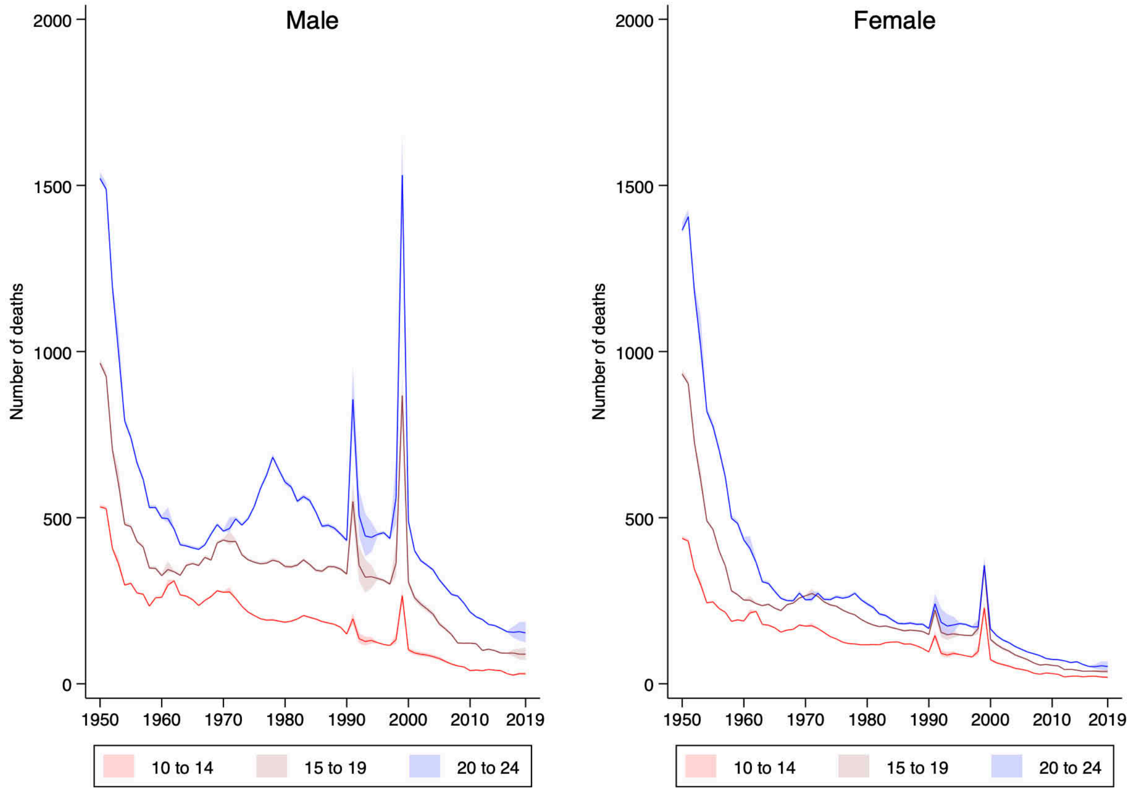

Figure S51c Mortality rate per 100,000 in 10-24 1980 – 2019 by cause: Serbia

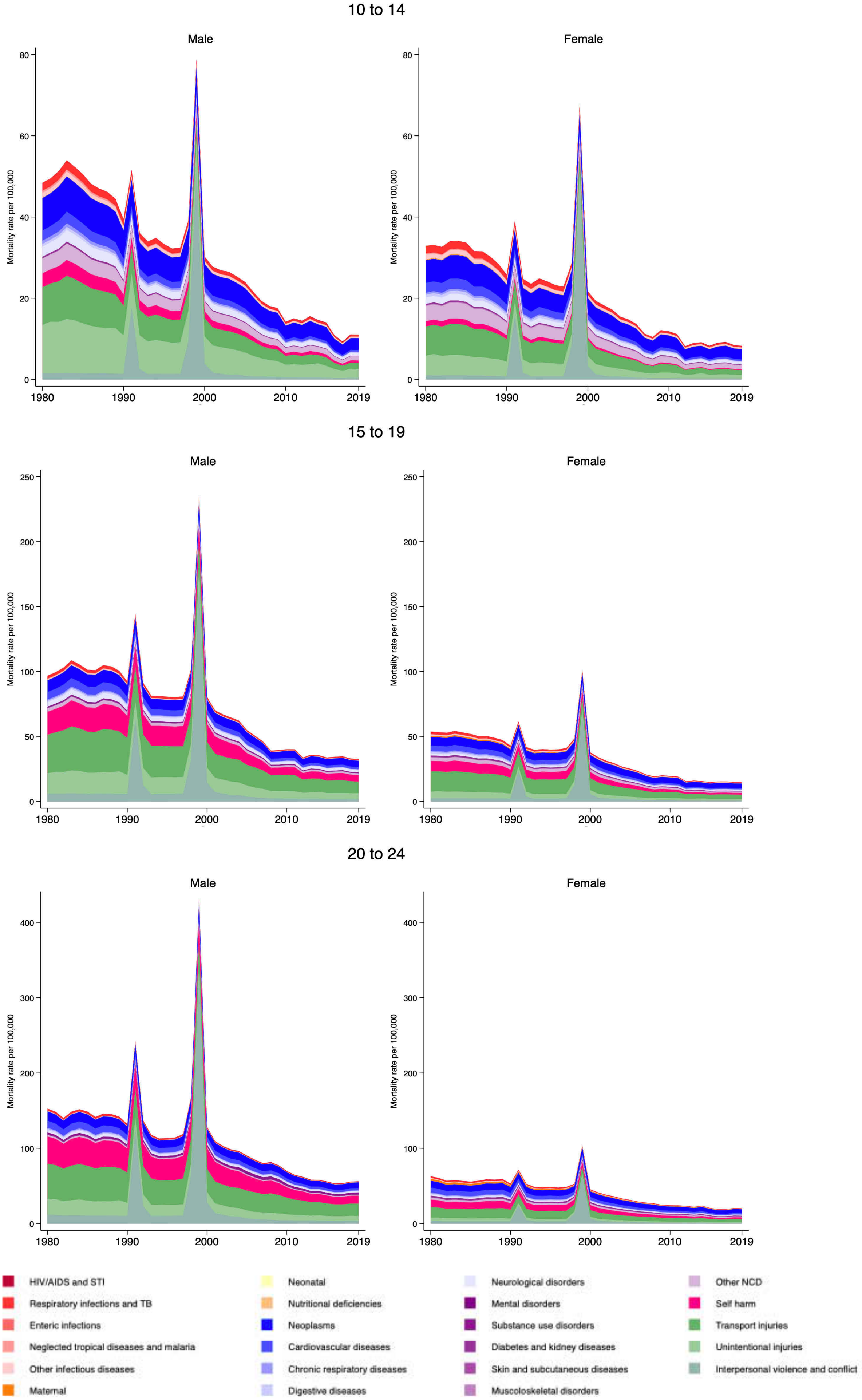

Figure S51d Number of deaths in 10-24 1980 – 2019 by cause: Serbia

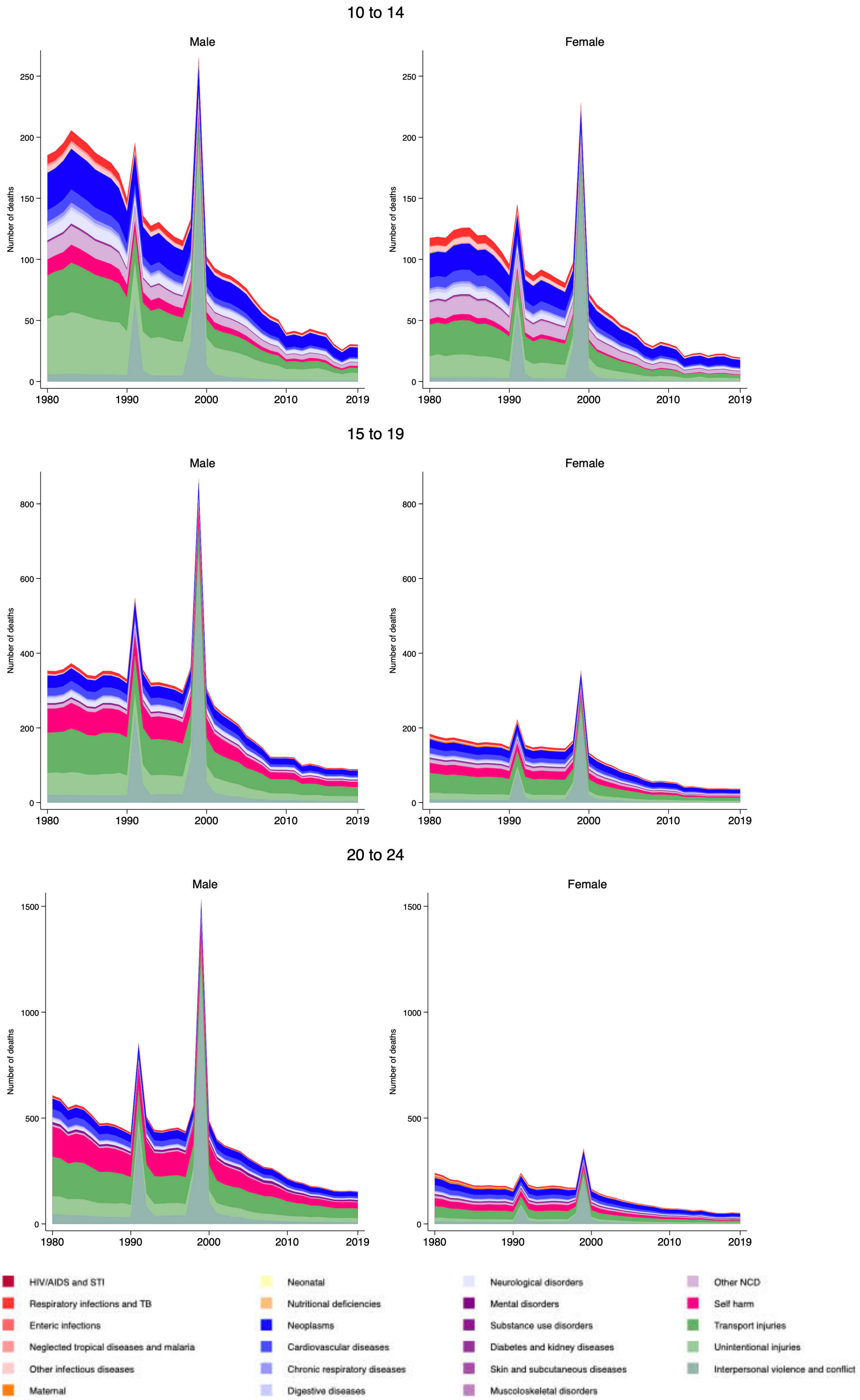

Figure S52: Slovakia

Figure S52a All-cause mortality rate per 100,000 in 10-24 1950 – 2019: Slovakia

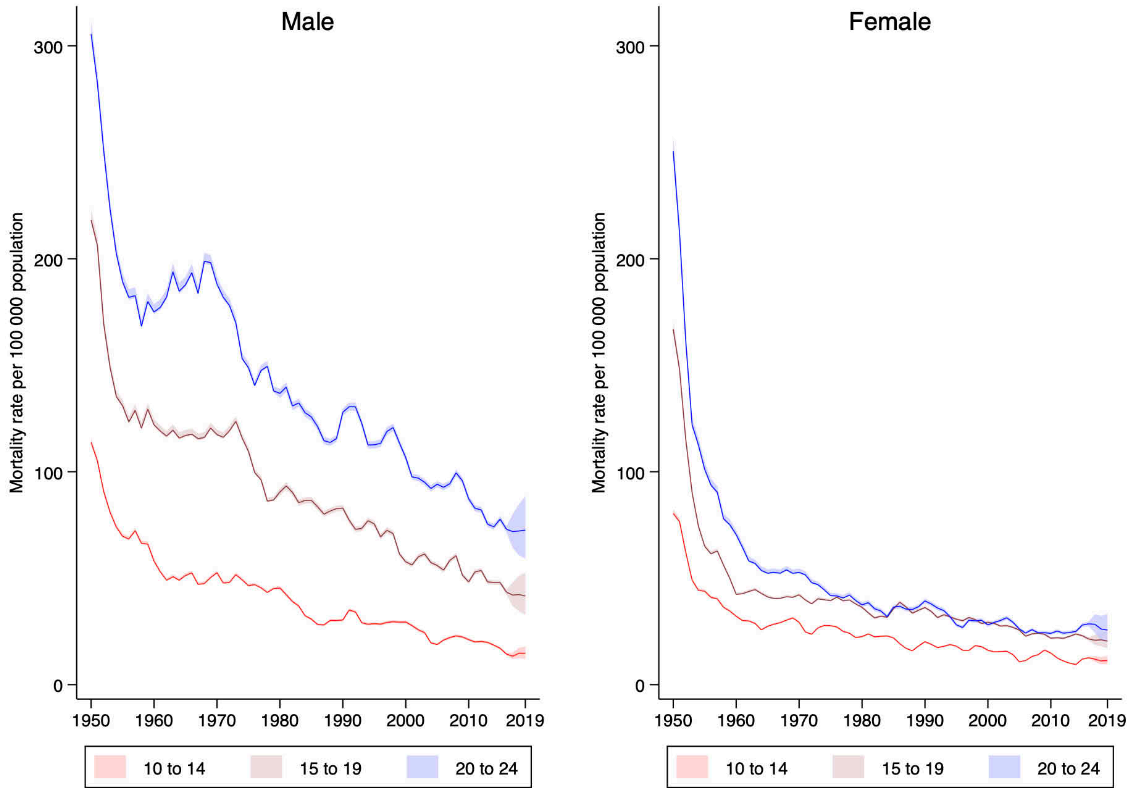

Figure S52b Number of deaths from all causes in 10-24 1950 – 2019: Slovakia

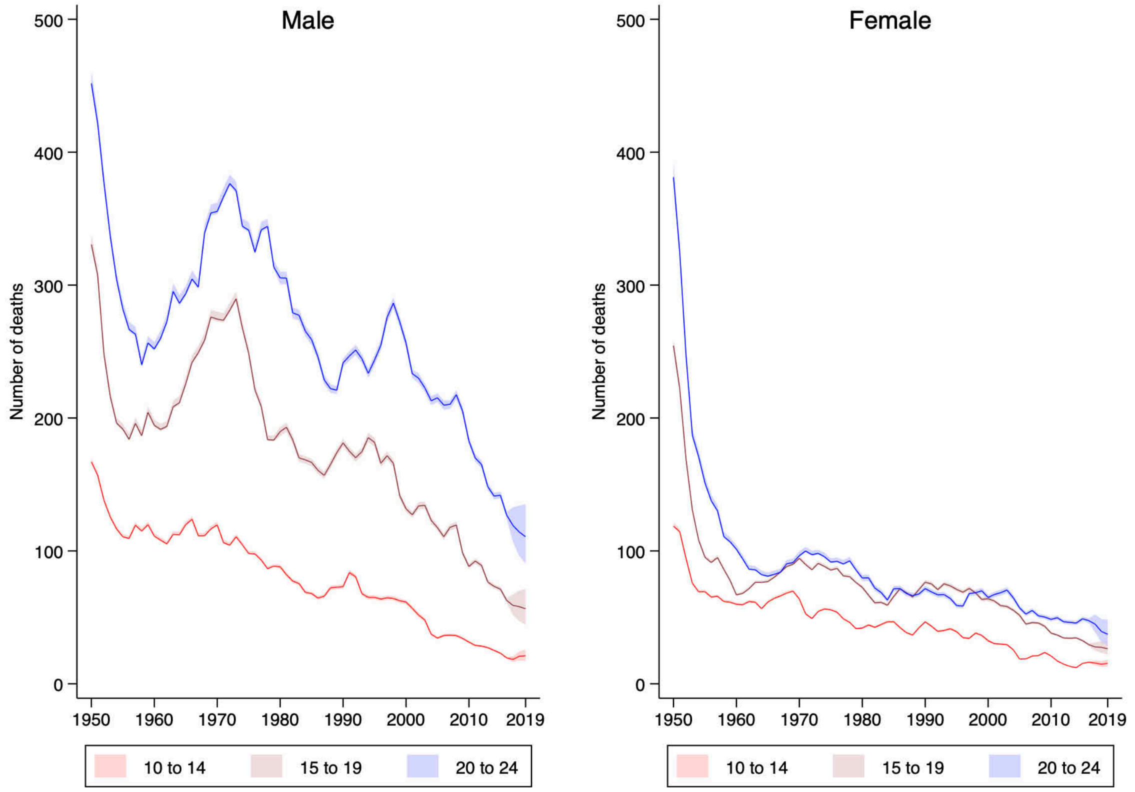

Figure S52c Mortality rate per 100,000 in 10-24 1980 – 2019 by cause: Slovakia

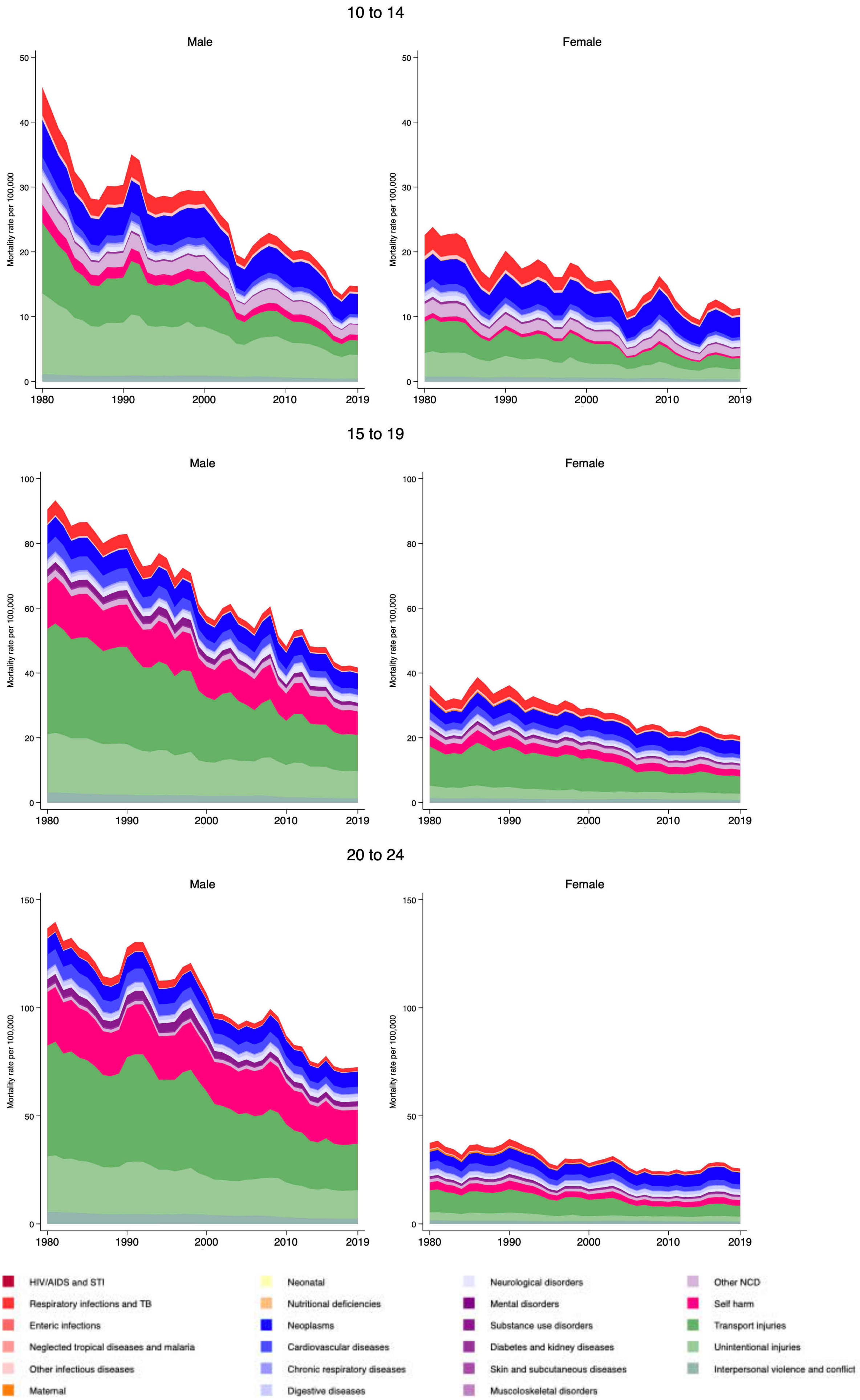

Figure S52d Number of deaths in 10-24 1980 – 2019 by cause: Slovakia

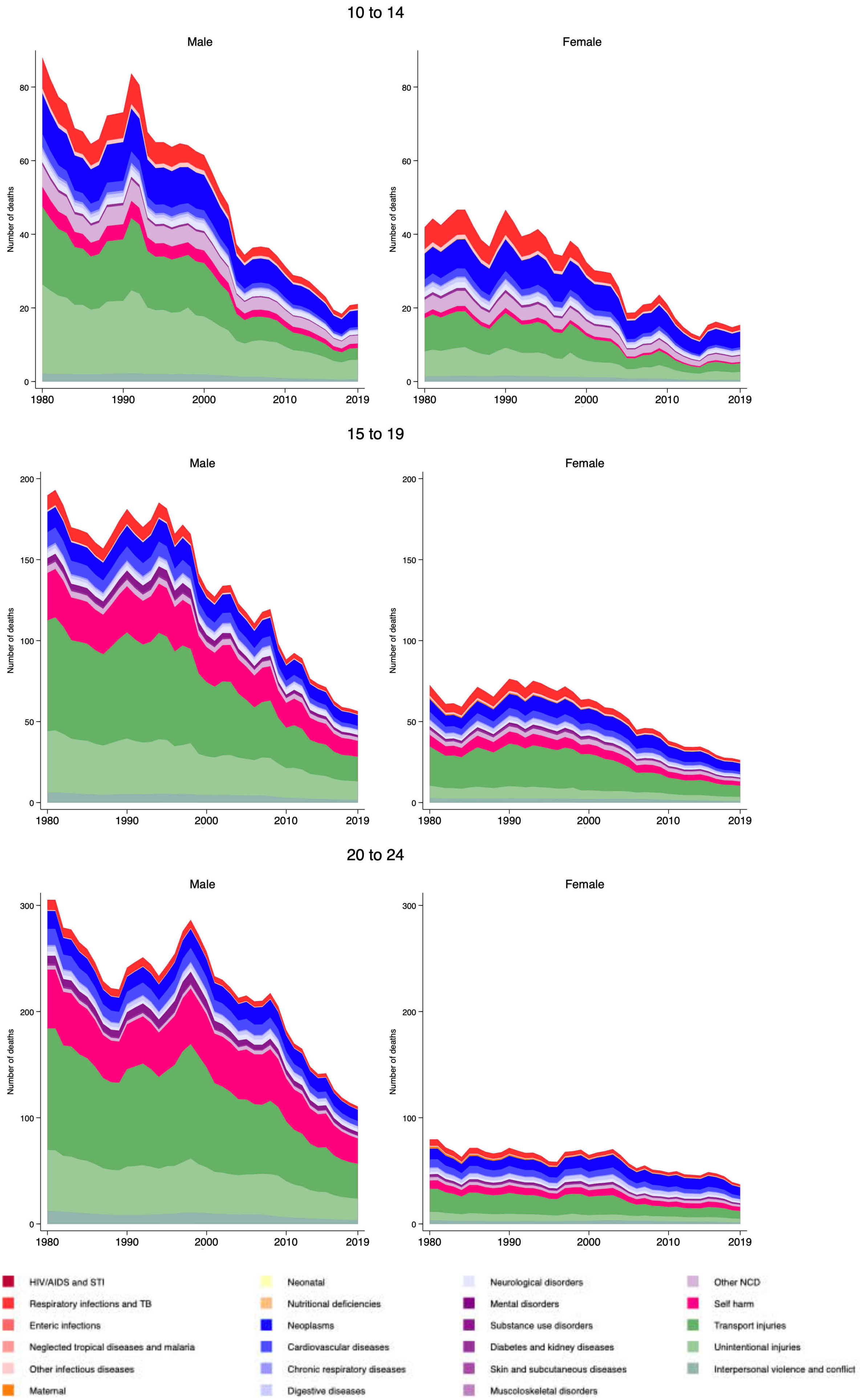

Figure S53: Slovenia

Figure S53a All-cause mortality rate per 100,000 in 10-24 1950 – 2019: Slovenia

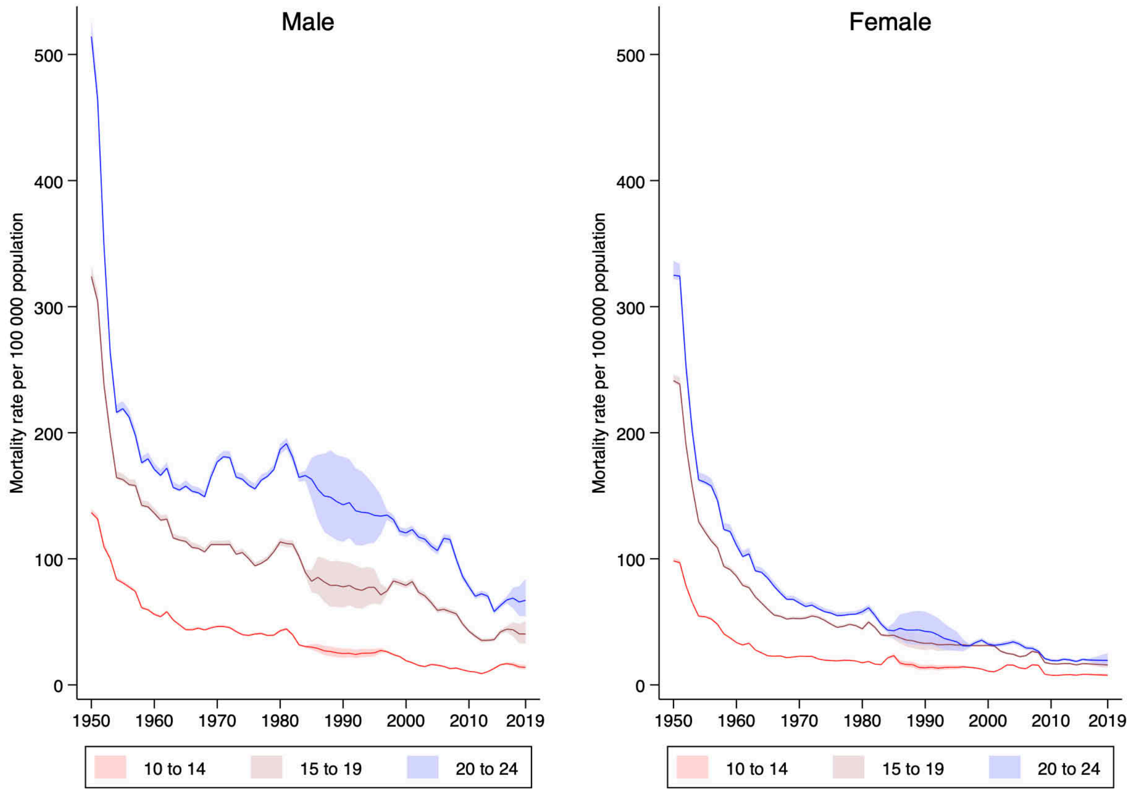

Figure S53b Number of deaths from all causes in 10-24 1950 – 2019: Slovenia

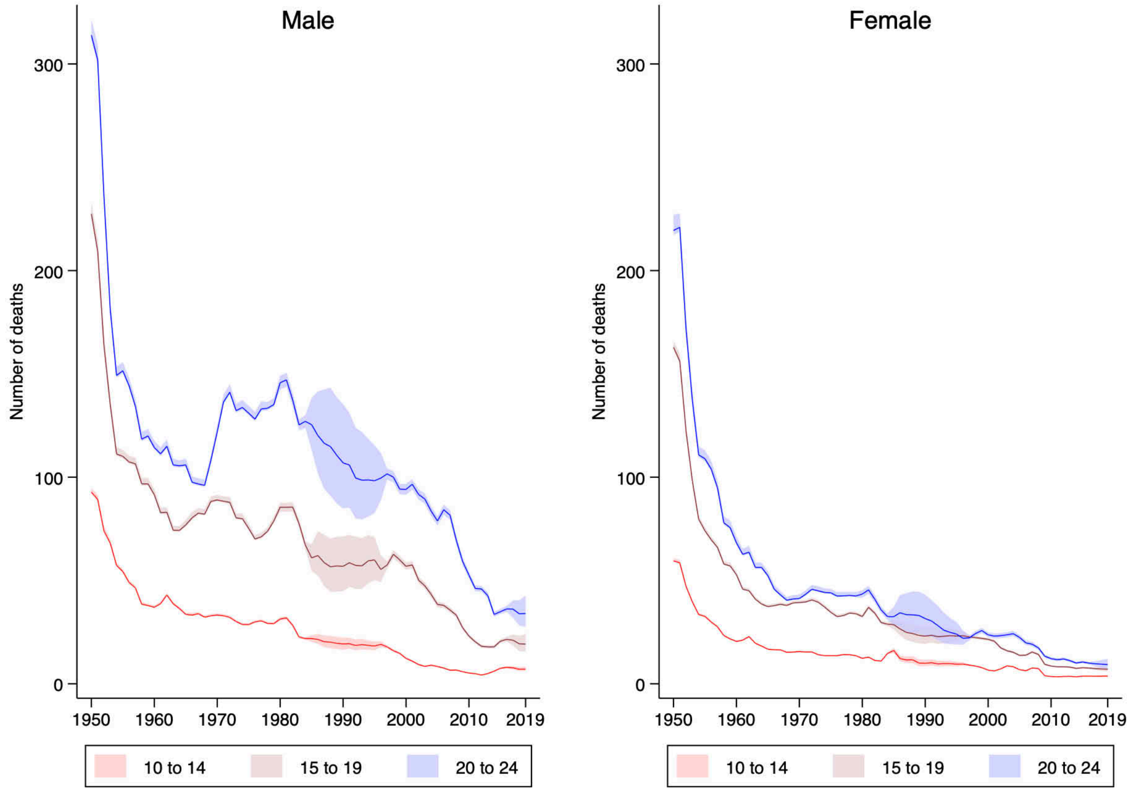

Figure S53c Mortality rate per 100,000 in 10-24 1980 – 2019 by cause: Slovenia

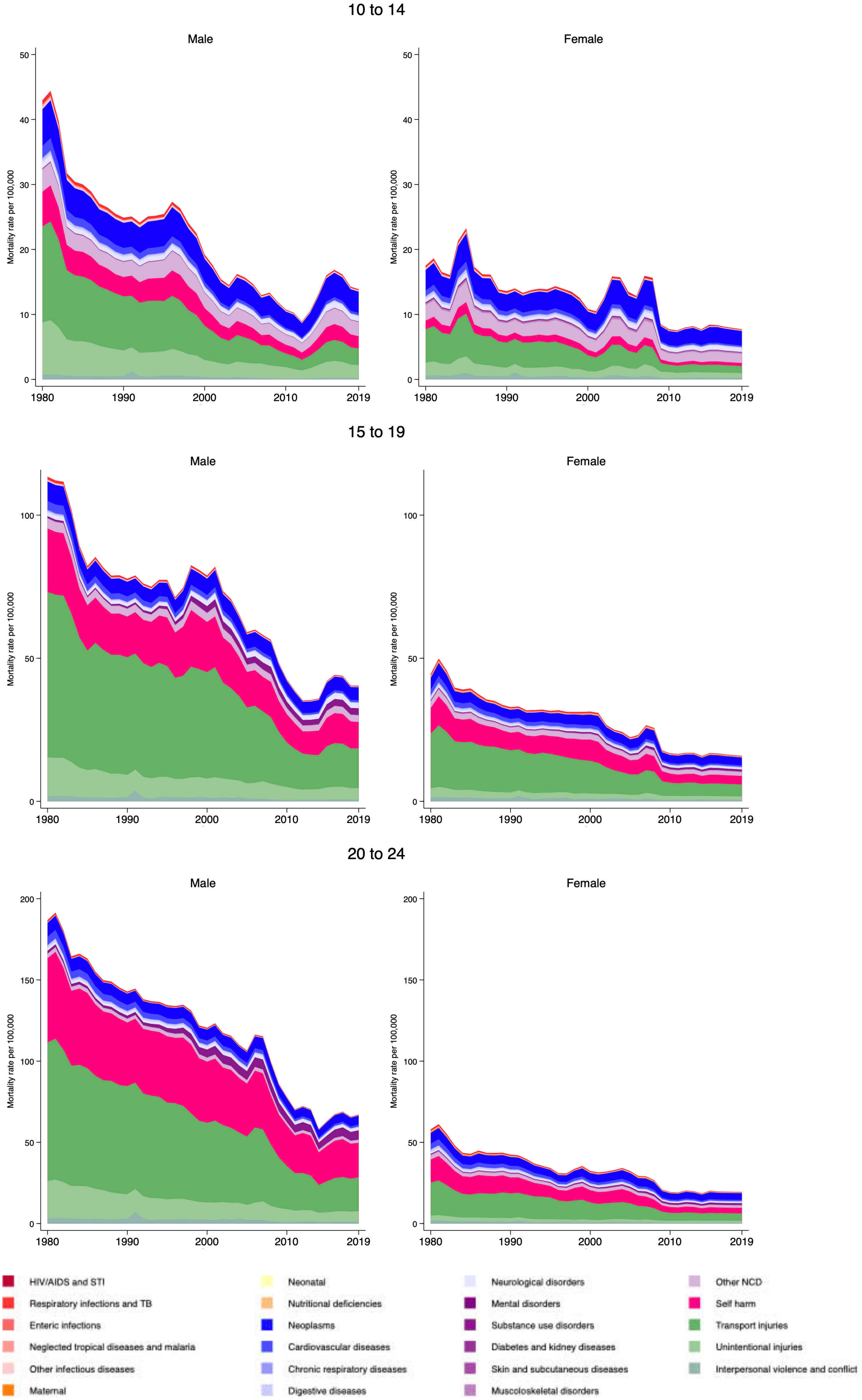

Figure S53d Number of deaths in 10-24 1980 – 2019 by cause: Slovenia

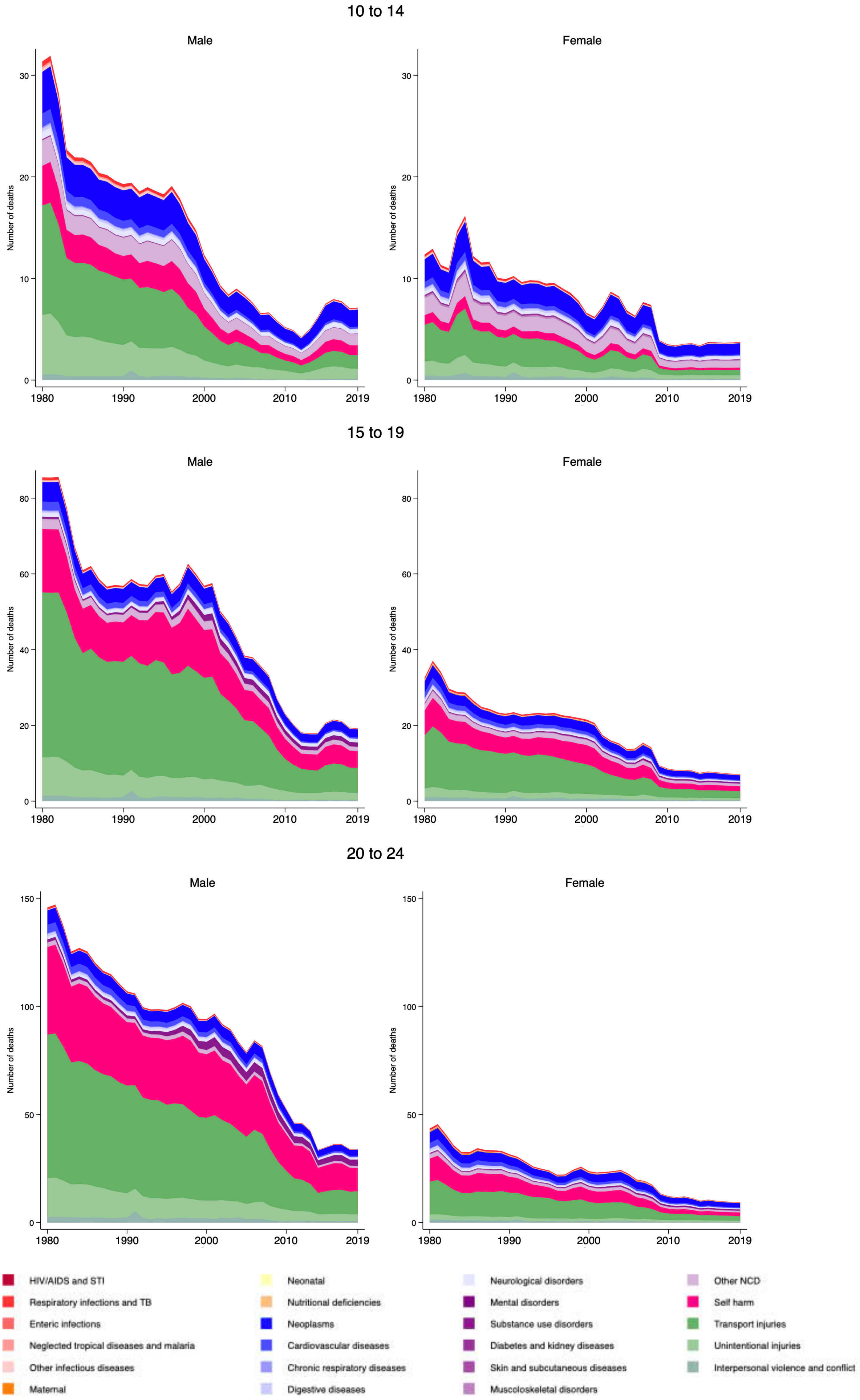

Figure S54: Eastern Europe

Figure S54a All-cause mortality rate per 100,000 in 10-24 1950 – 2019: Eastern Europe

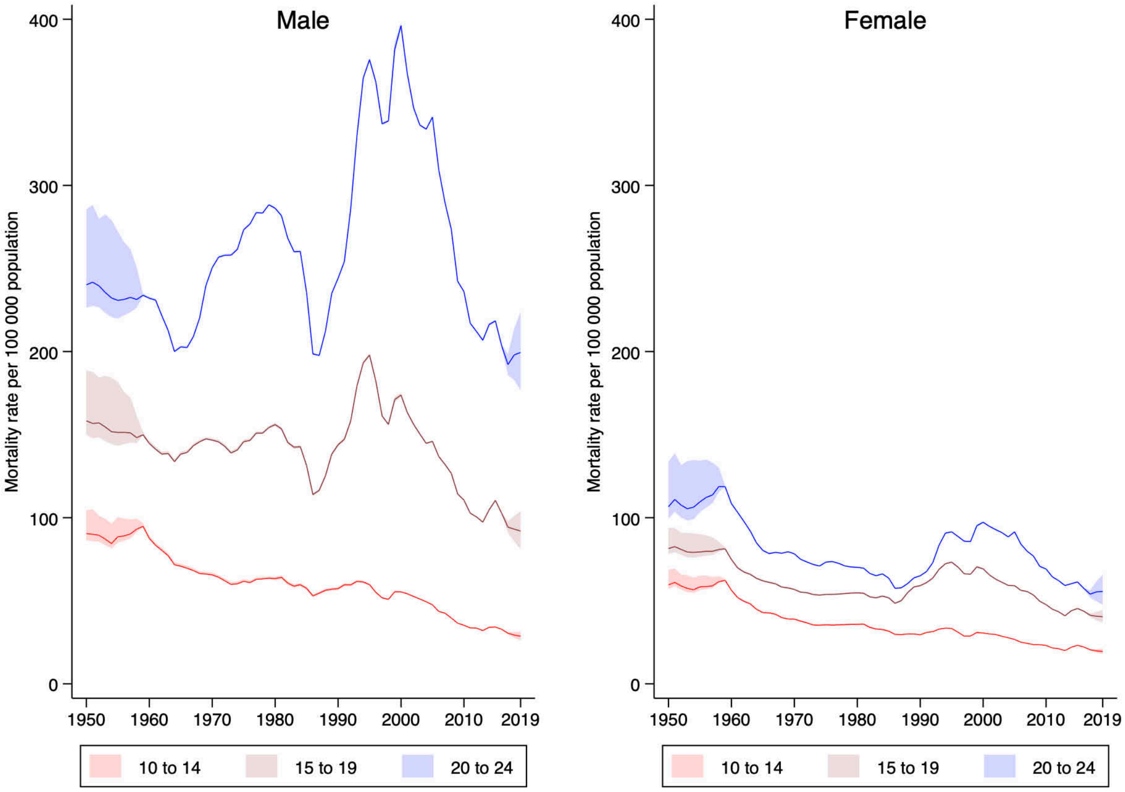

Figure S54b Number of deaths from all causes in 10-24 1950 – 2019: Eastern Europe

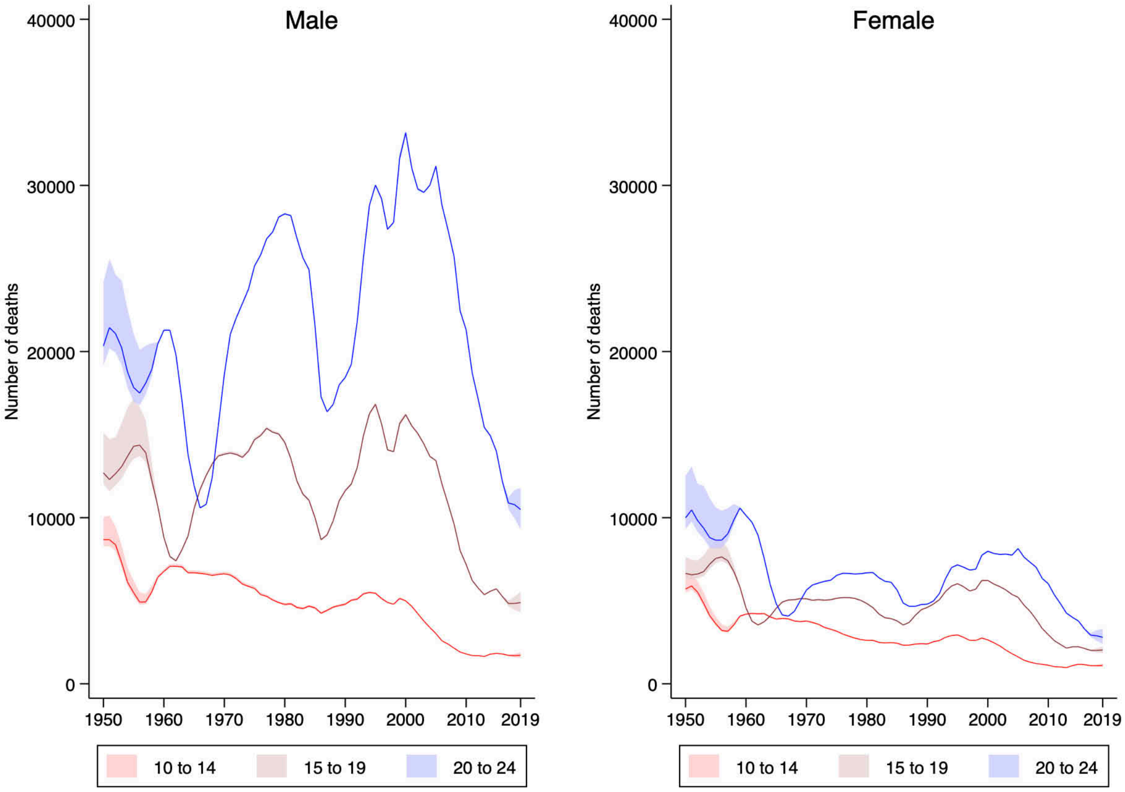

Figure S54c Mortality rate per 100,000 in 10-24 1980 – 2019 by cause: Eastern Europe

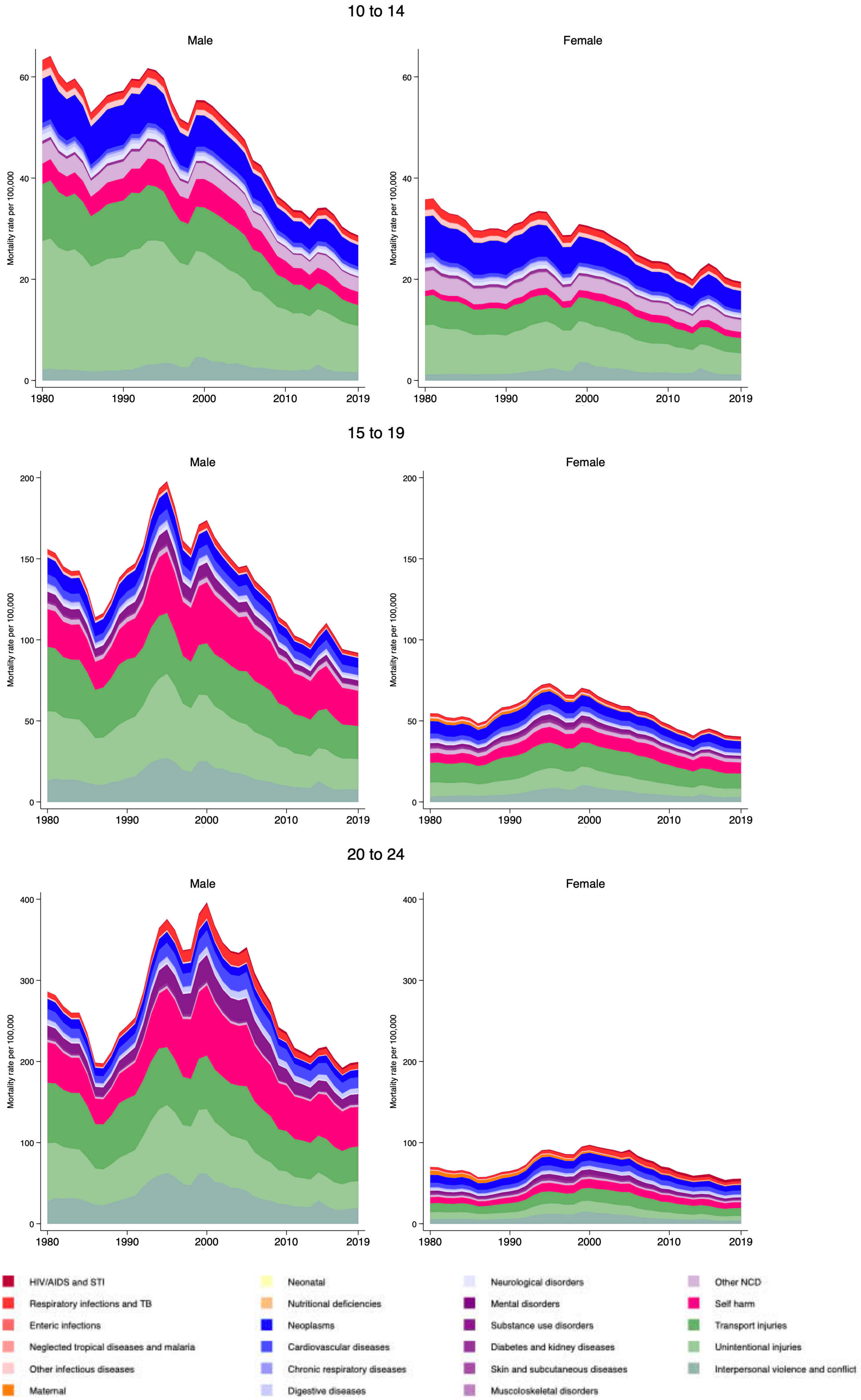

Figure S54d Number of deaths in 10-24 1980 – 2019 by cause: Eastern Europe

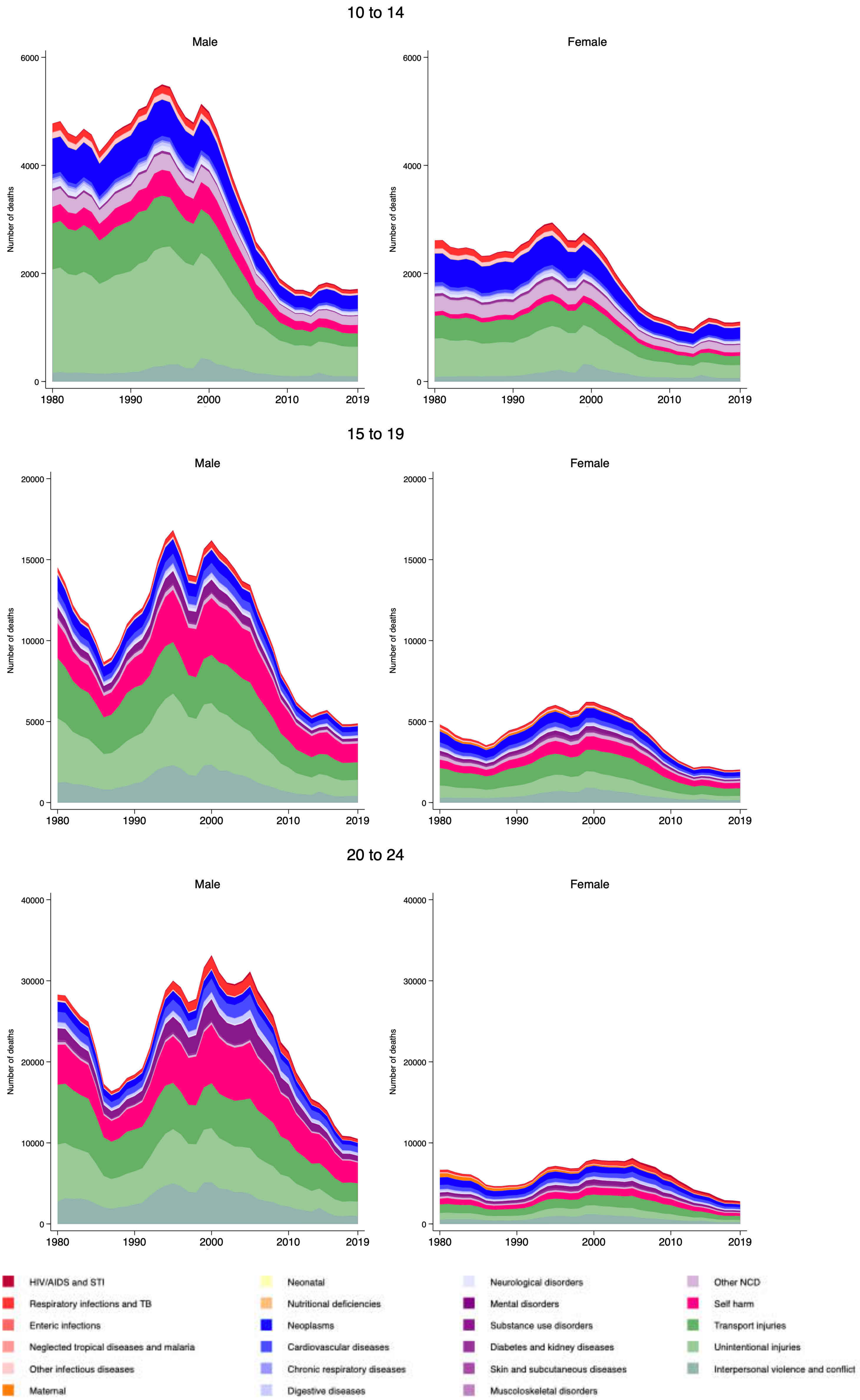

Figure S55: Belarus

Figure S55a All-cause mortality rate per 100,000 in 10-24 1950 – 2019: Belarus

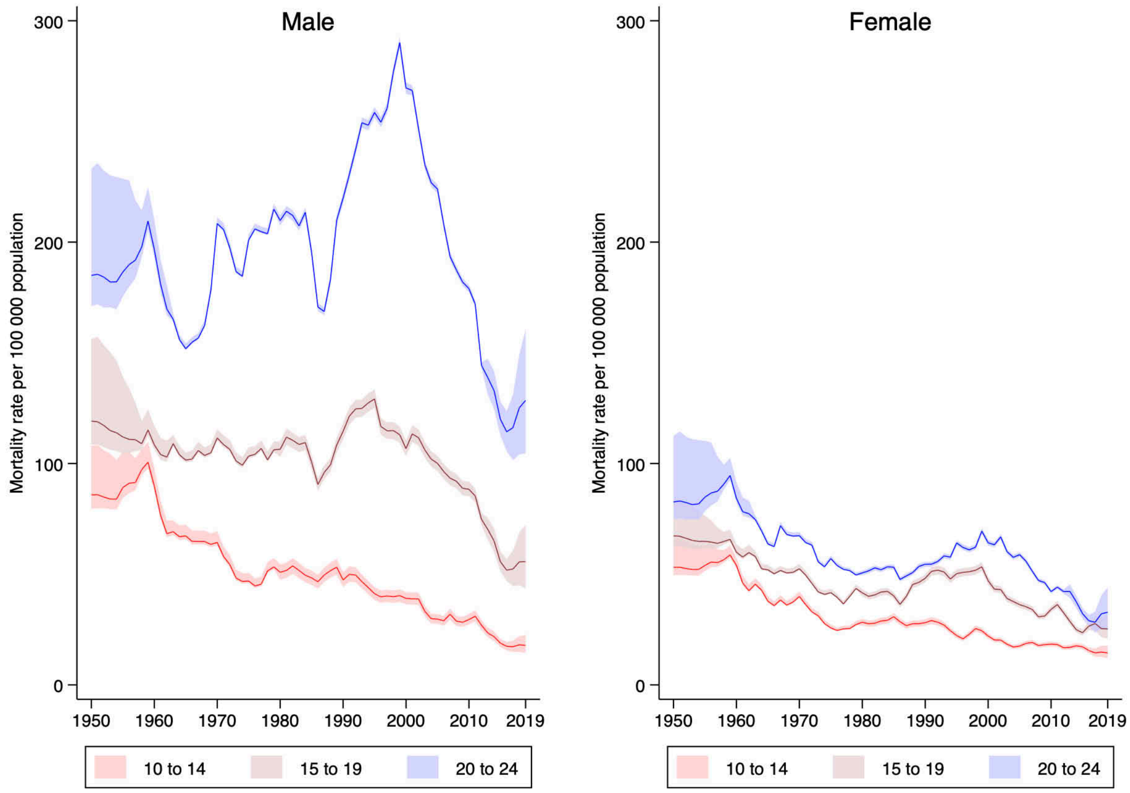

Figure S55b Number of deaths from all causes in 10-24 1950 – 2019: Belarus

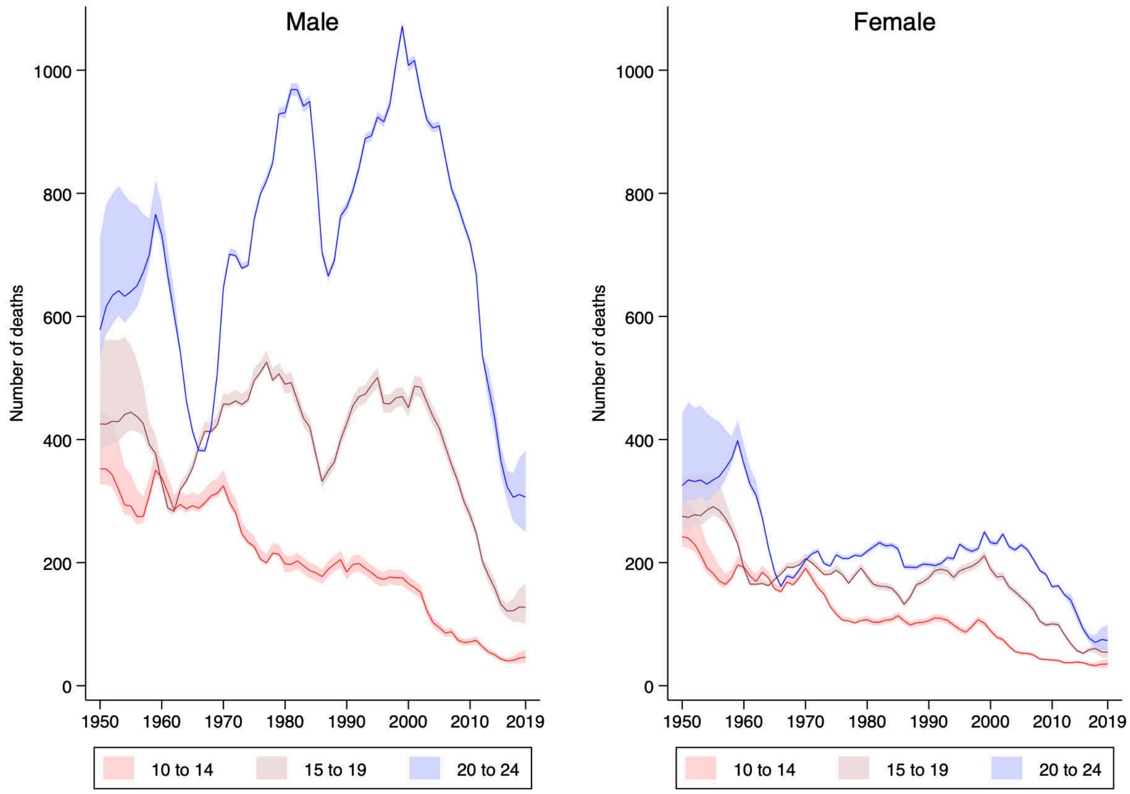

Figure S55c Mortality rate per 100,000 in 10-24 1980 – 2019 by cause: Belarus

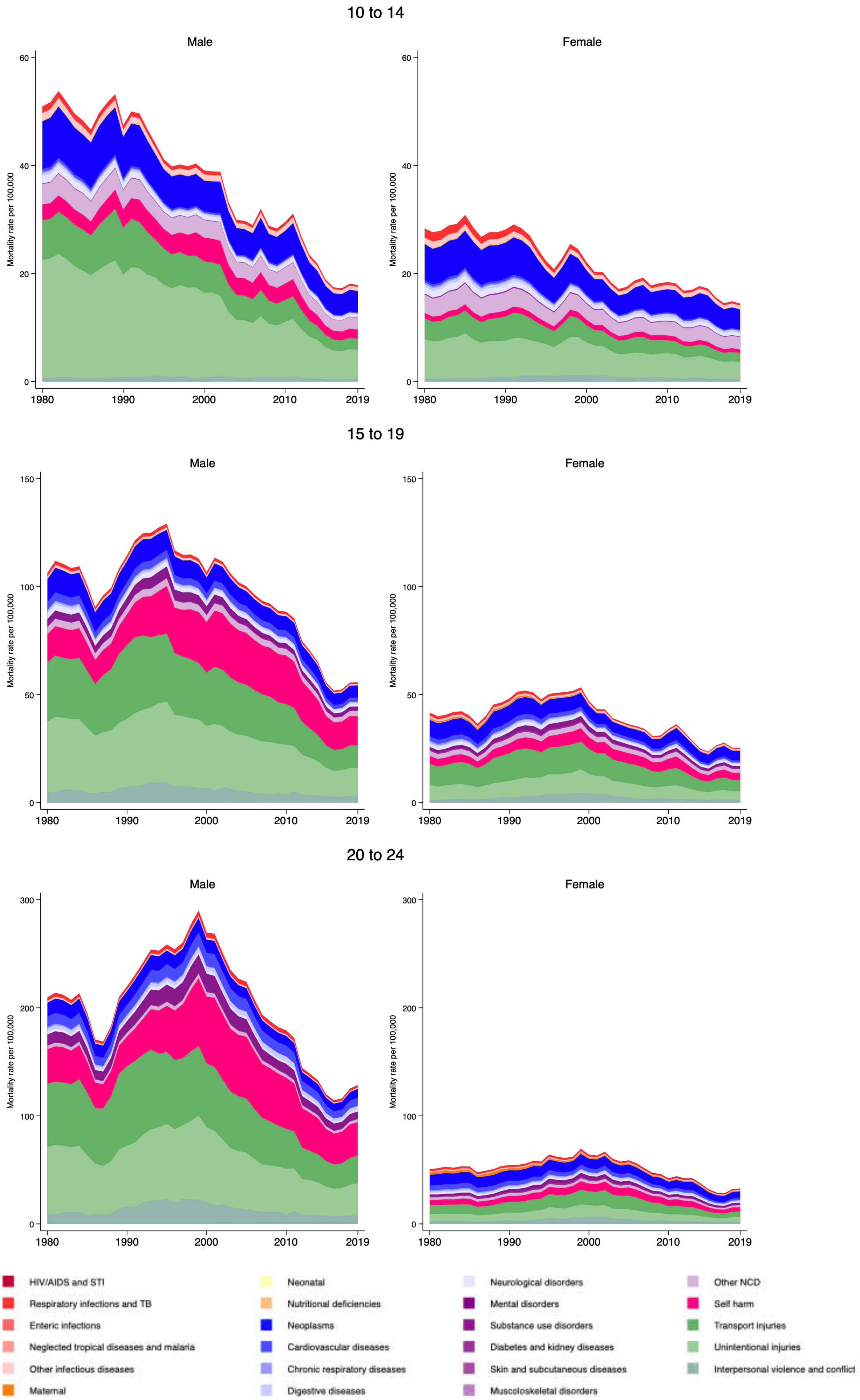

Figure S55d Number of deaths in 10-24 1980 – 2019 by cause: Belarus

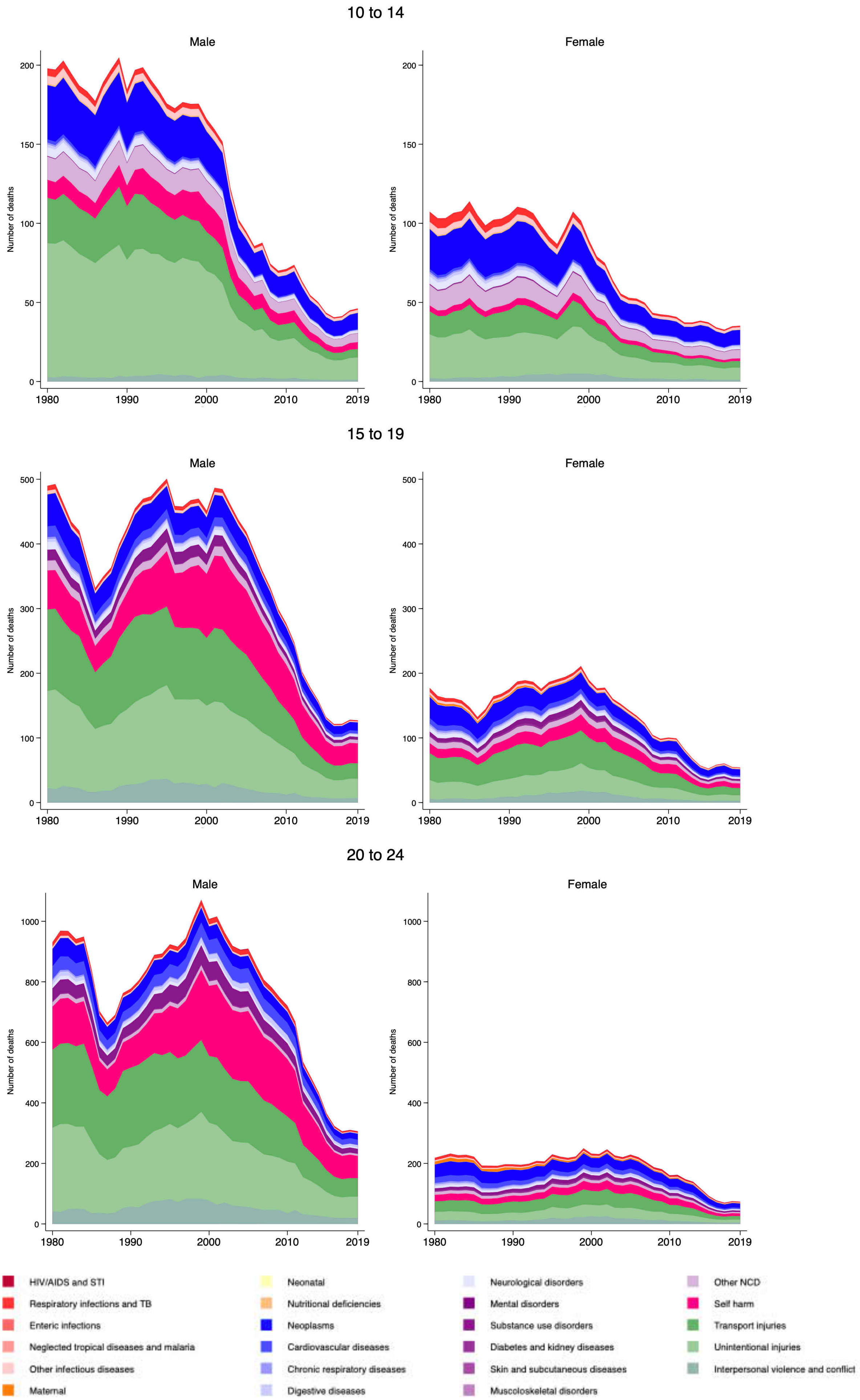

Figure S56: Estonia

Figure S56a All-cause mortality rate per 100,000 in 10-24 1950 – 2019: Estonia

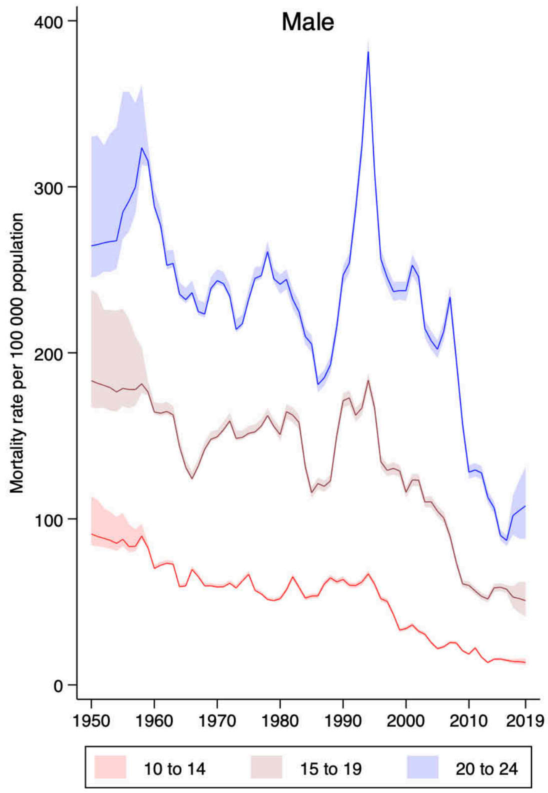

Figure S56b Number of deaths from all causes in 10-24 1950 – 2019: Estonia

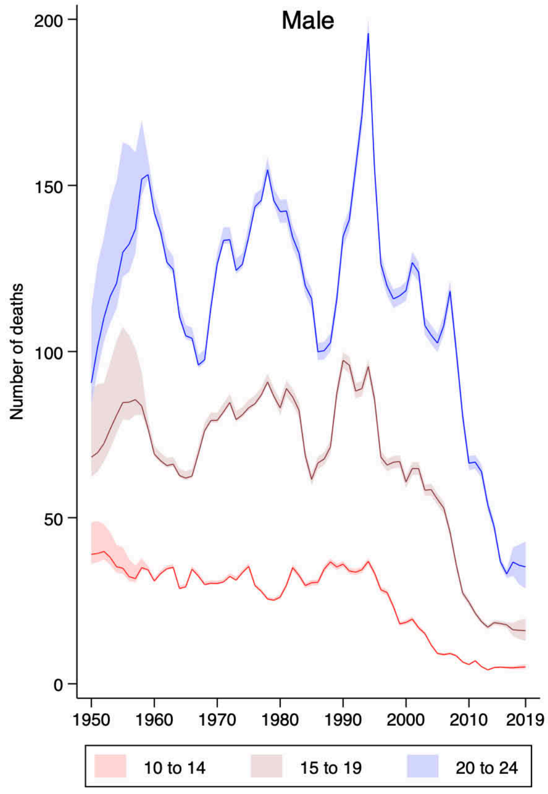

Figure S56c Mortality rate per 100,000 in 10-24 1980 – 2019 by cause: Estonia

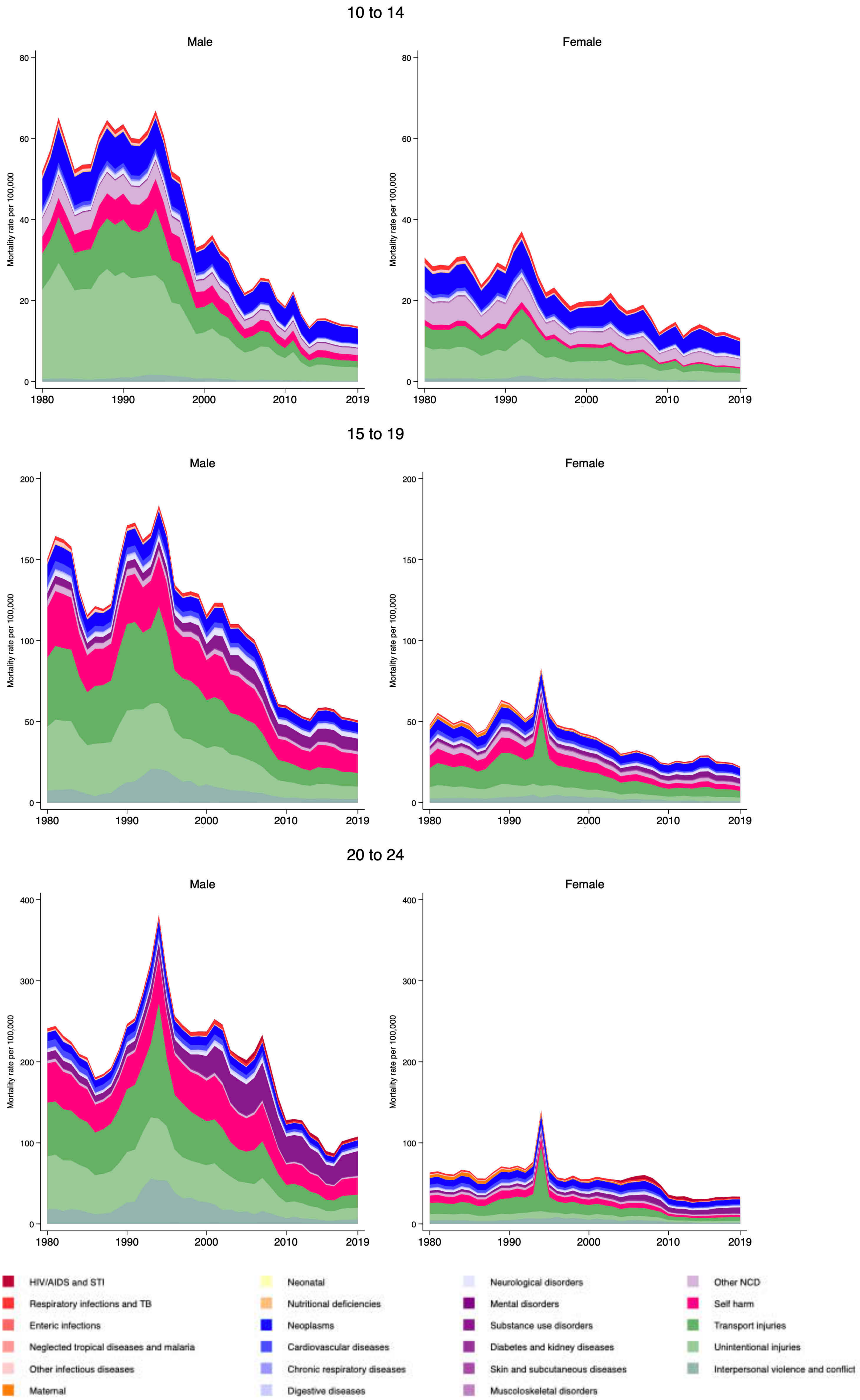

Figure S56d Number of deaths in 10-24 1980 – 2019 by cause: Estonia

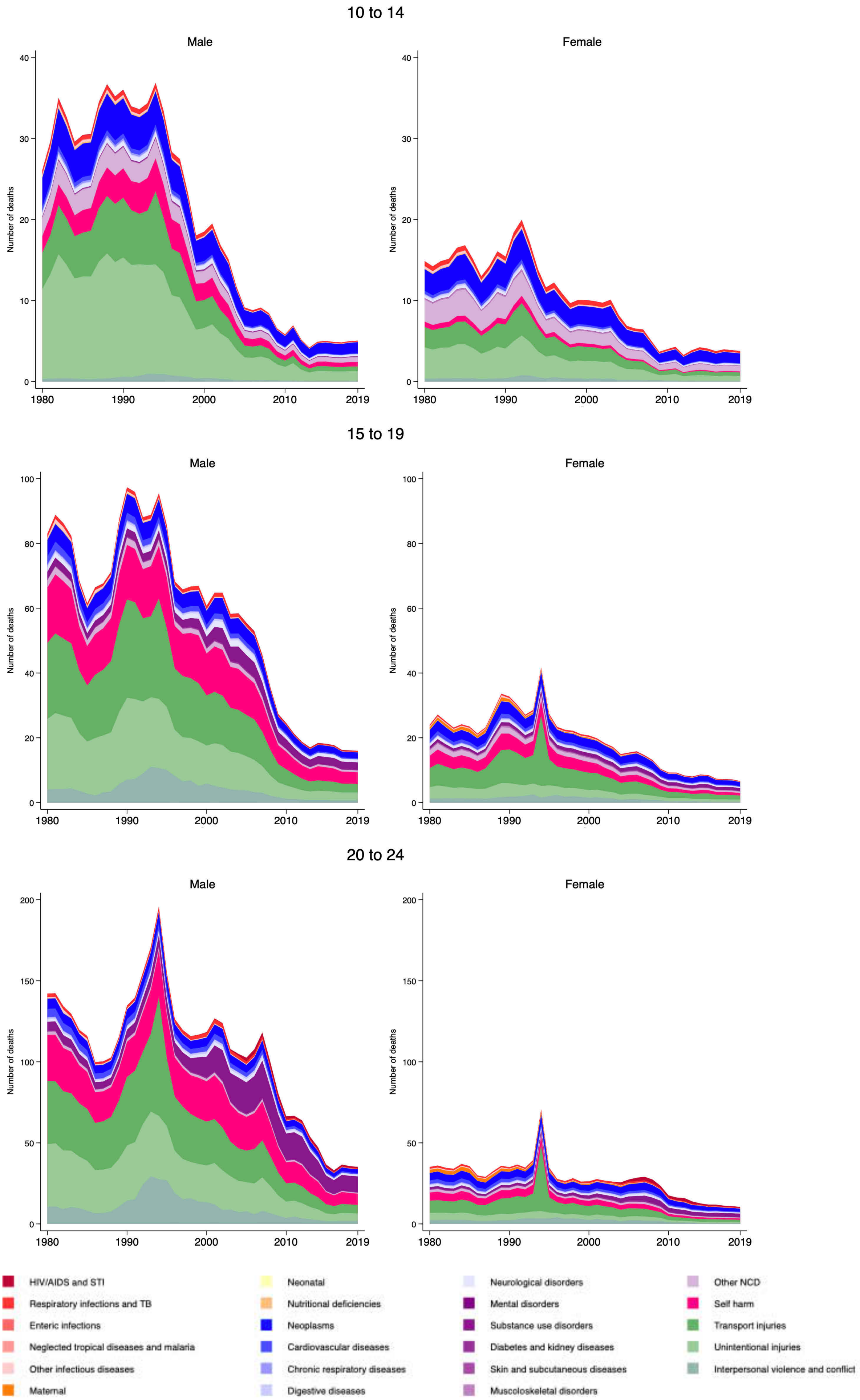

Figure S57: Latvia

Figure S57a All-cause mortality rate per 100,000 in 10-24 1950 – 2019: Latvia

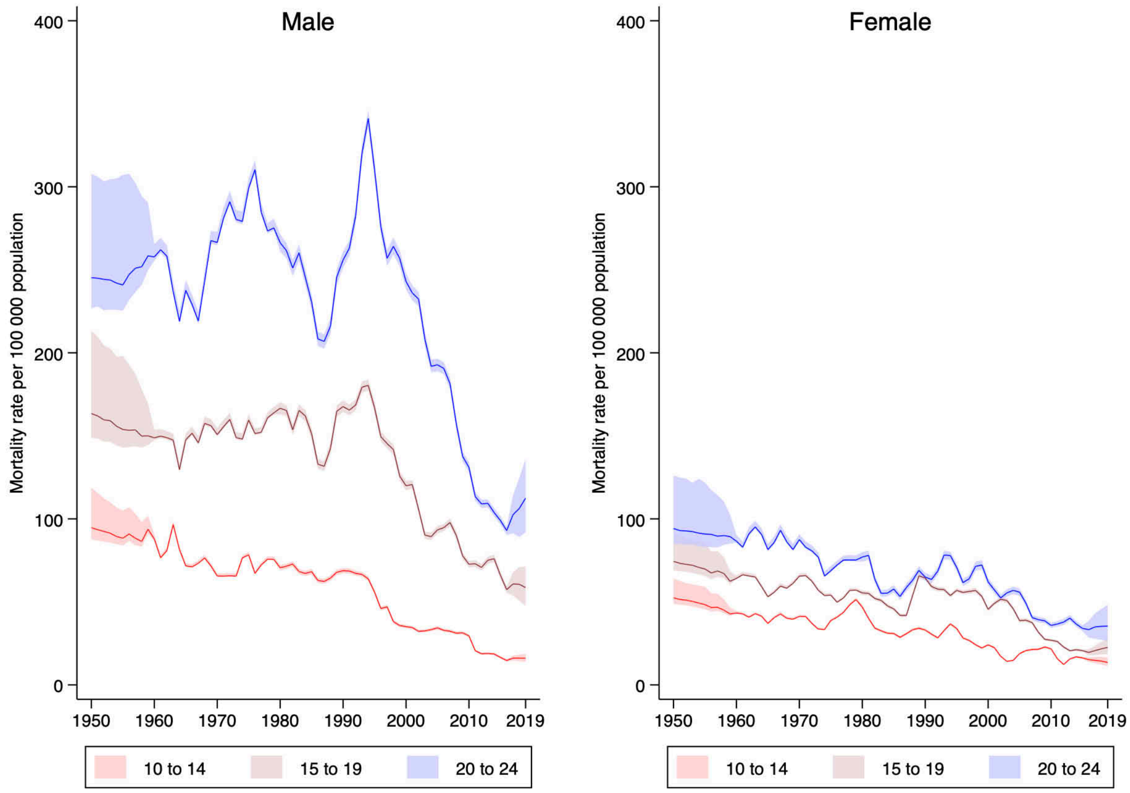

Figure S57b Number of deaths from all causes in 10-24 1950 – 2019: Latvia

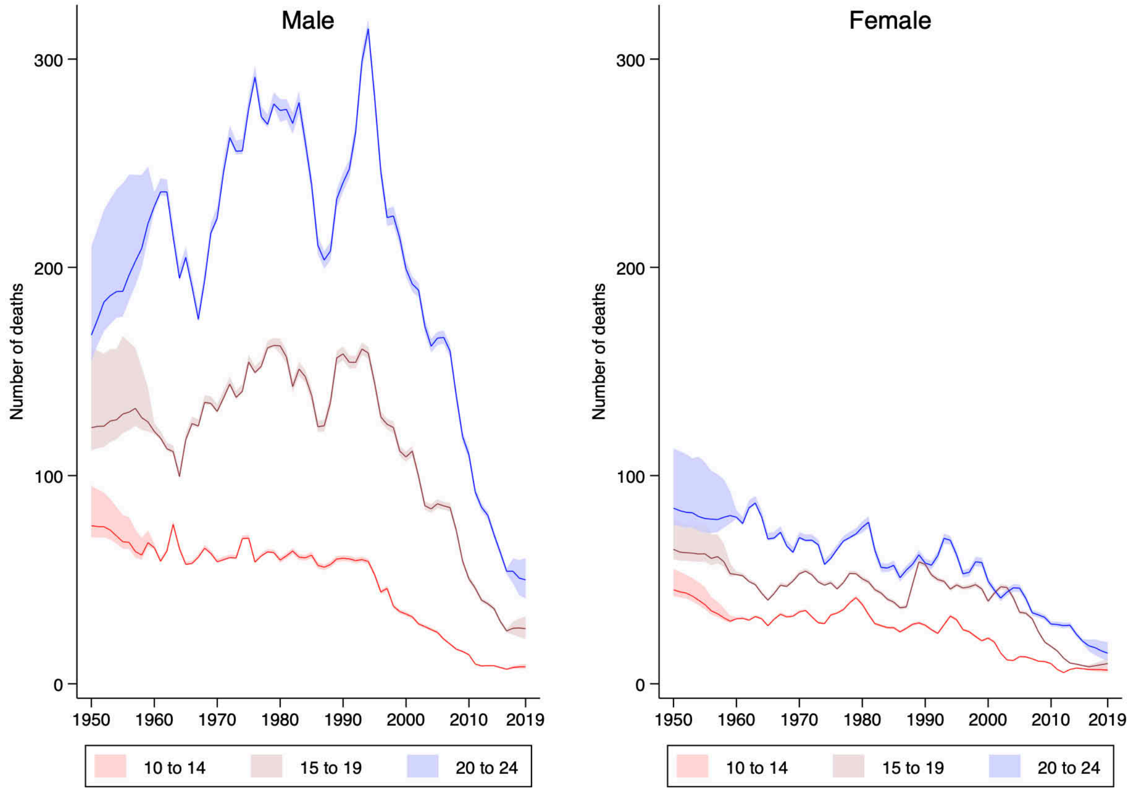

Figure S57c Mortality rate per 100,000 in 10-24 1980 – 2019 by cause: Latvia

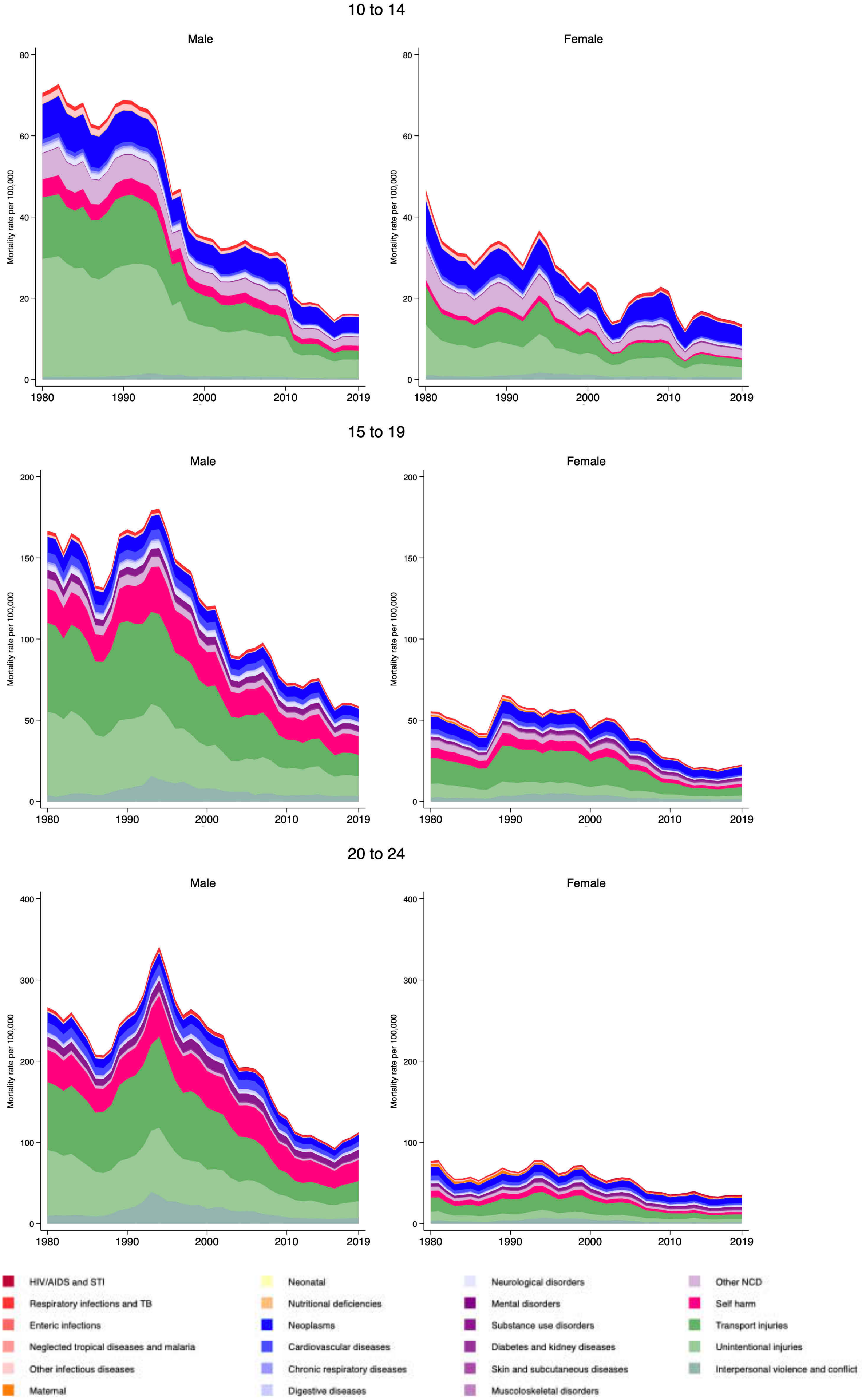

Figure S57d Number of deaths in 10-24 1980 – 2019 by cause: Latvia

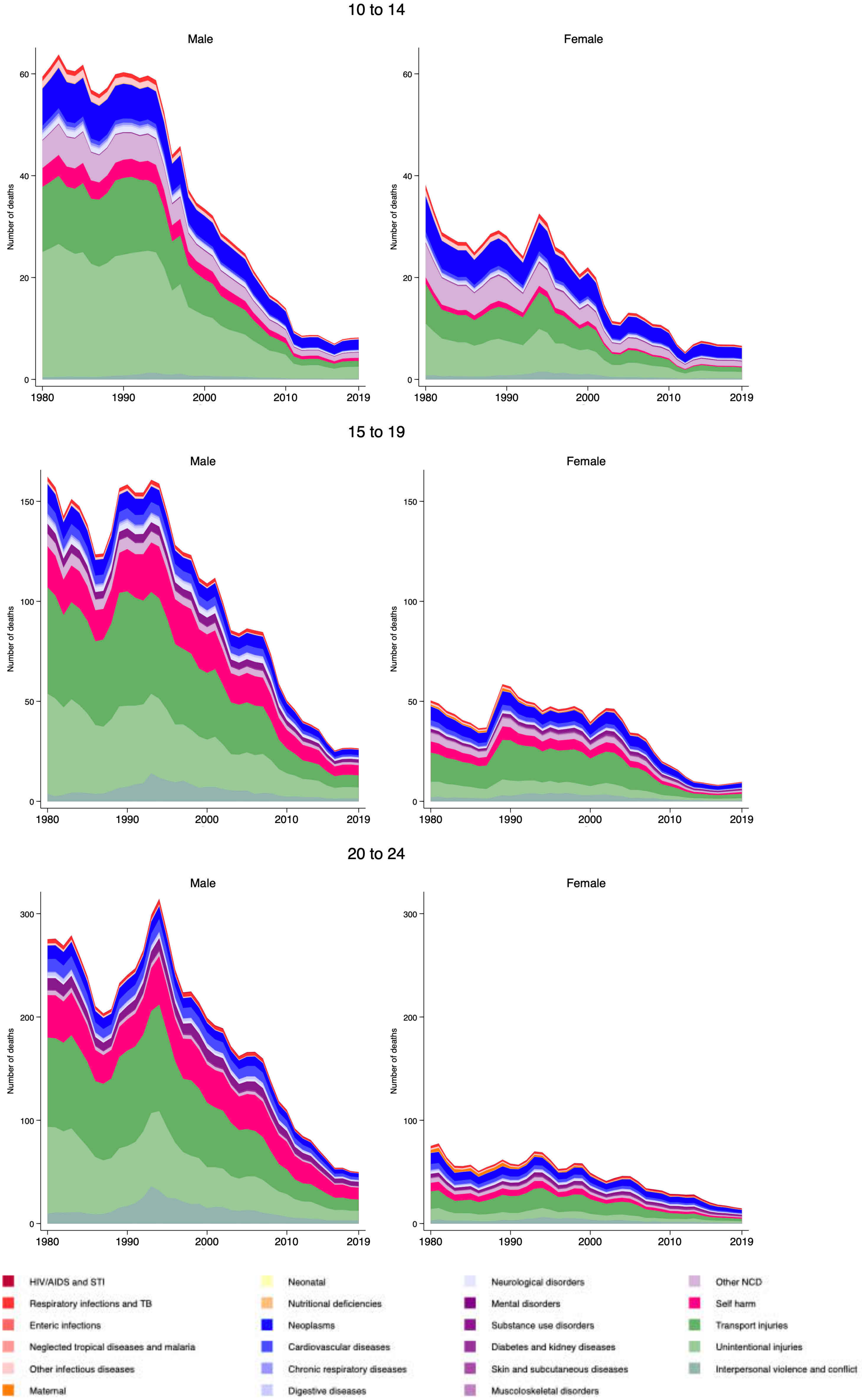

Figure S58: Lithuania

Figure S58a All-cause mortality rate per 100,000 in 10-24 1950 – 2019: Lithuania

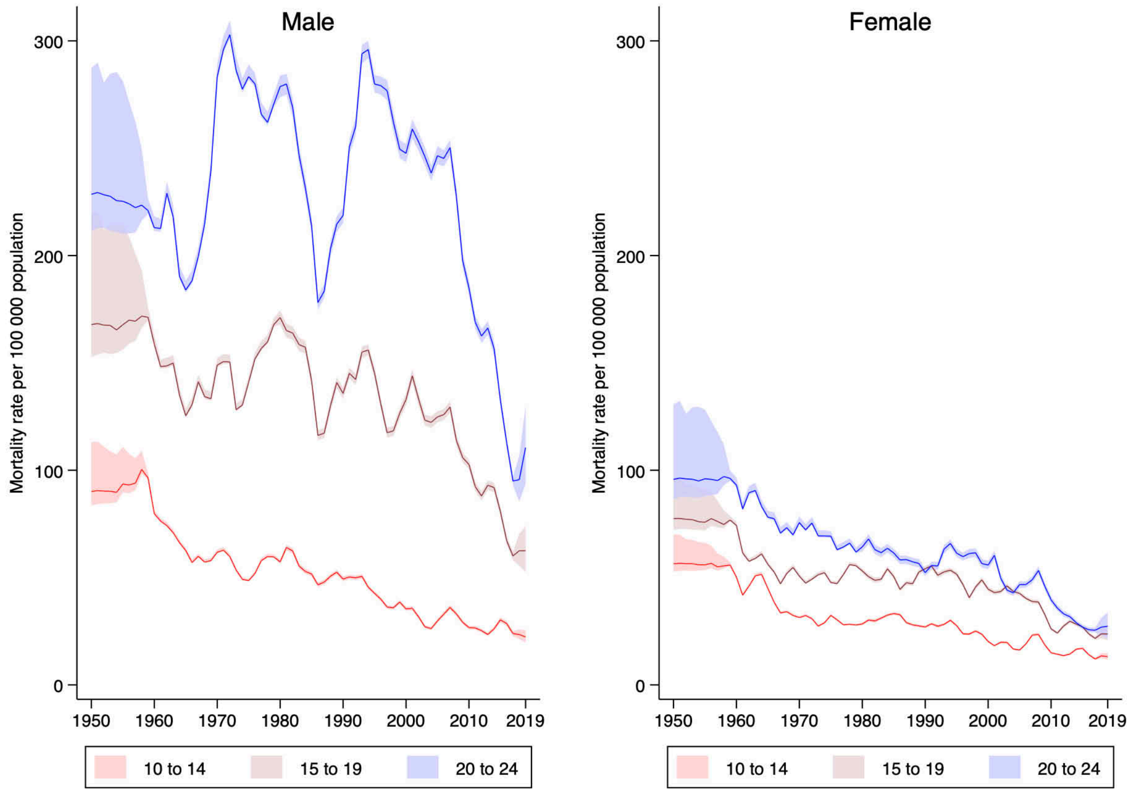

Figure S58b Number of deaths from all causes in 10-24 1950 – 2019: Lithuania

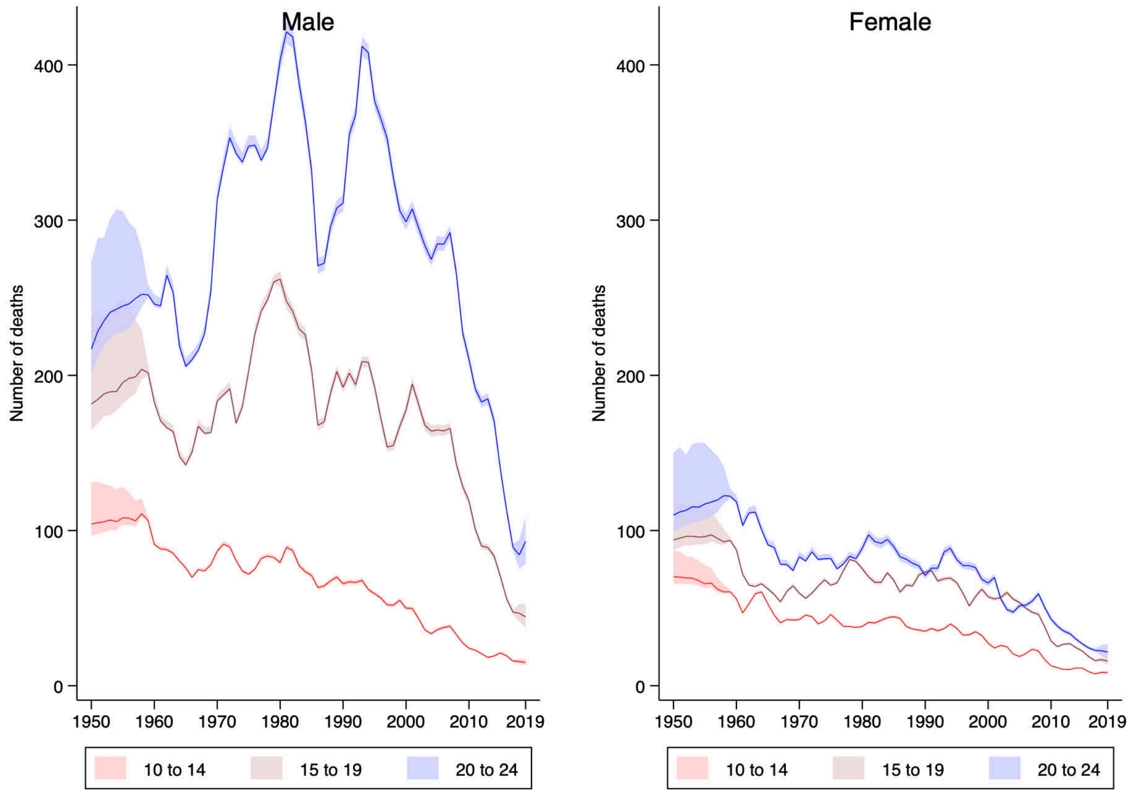

Figure S58c Mortality rate per 100,000 in 10-24 1980 – 2019 by cause: Lithuania

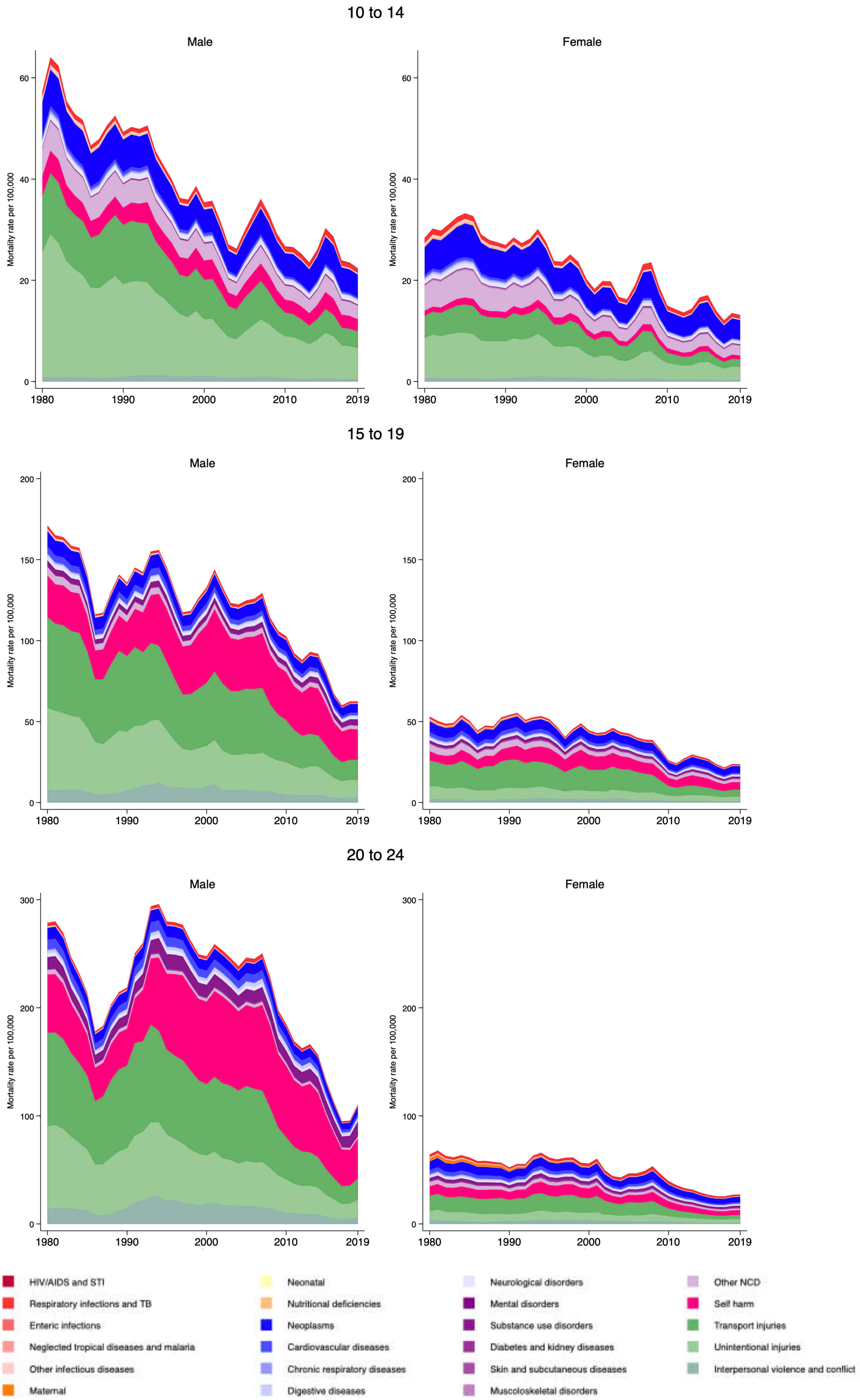

Figure S58d Number of deaths in 10-24 1980 – 2019 by cause: Lithuania

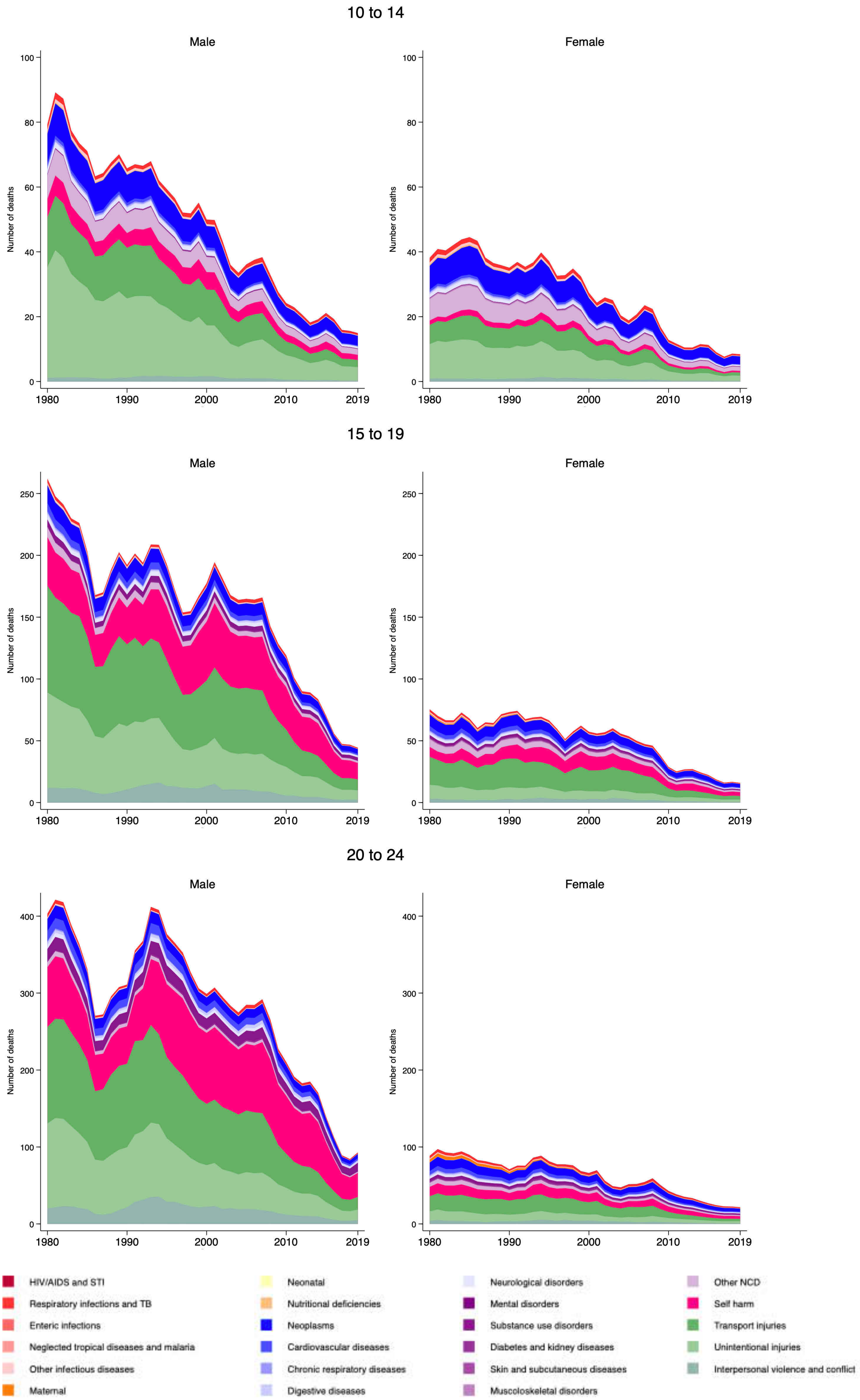

Figure S59: Republic of Moldova

Figure S59a All-cause mortality rate per 100,000 in 10-24 1950 – 2019: Republic of Moldova

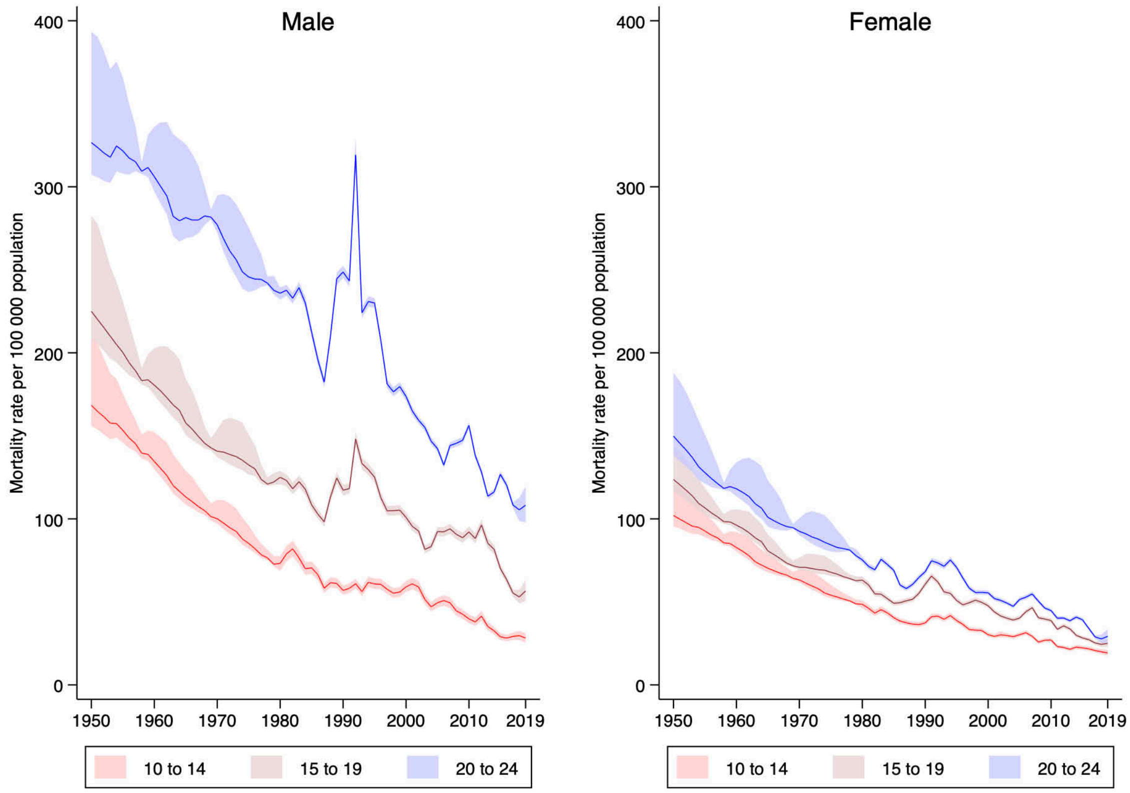

Figure S59b Number of deaths from all causes in 10-24 1950 – 2019: Republic of Moldova

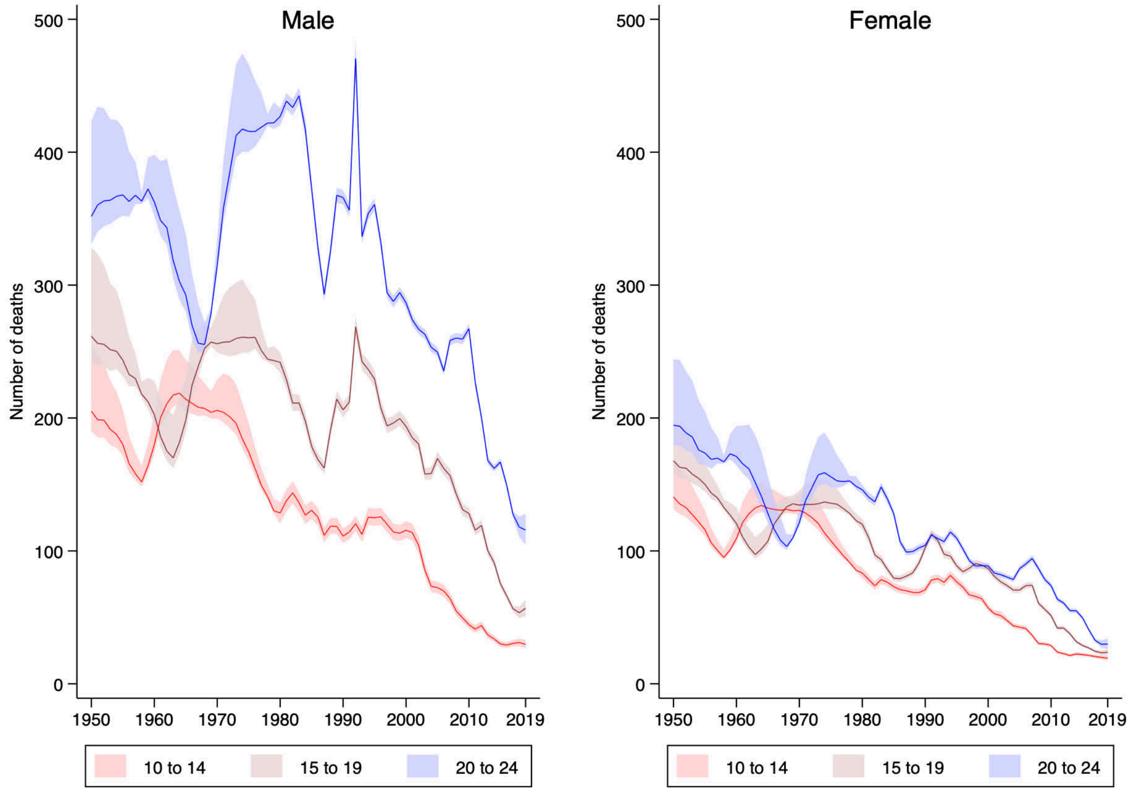

Figure S59c Mortality rate per 100,000 in 10-24 1980 – 2019 by cause: Republic of Moldova

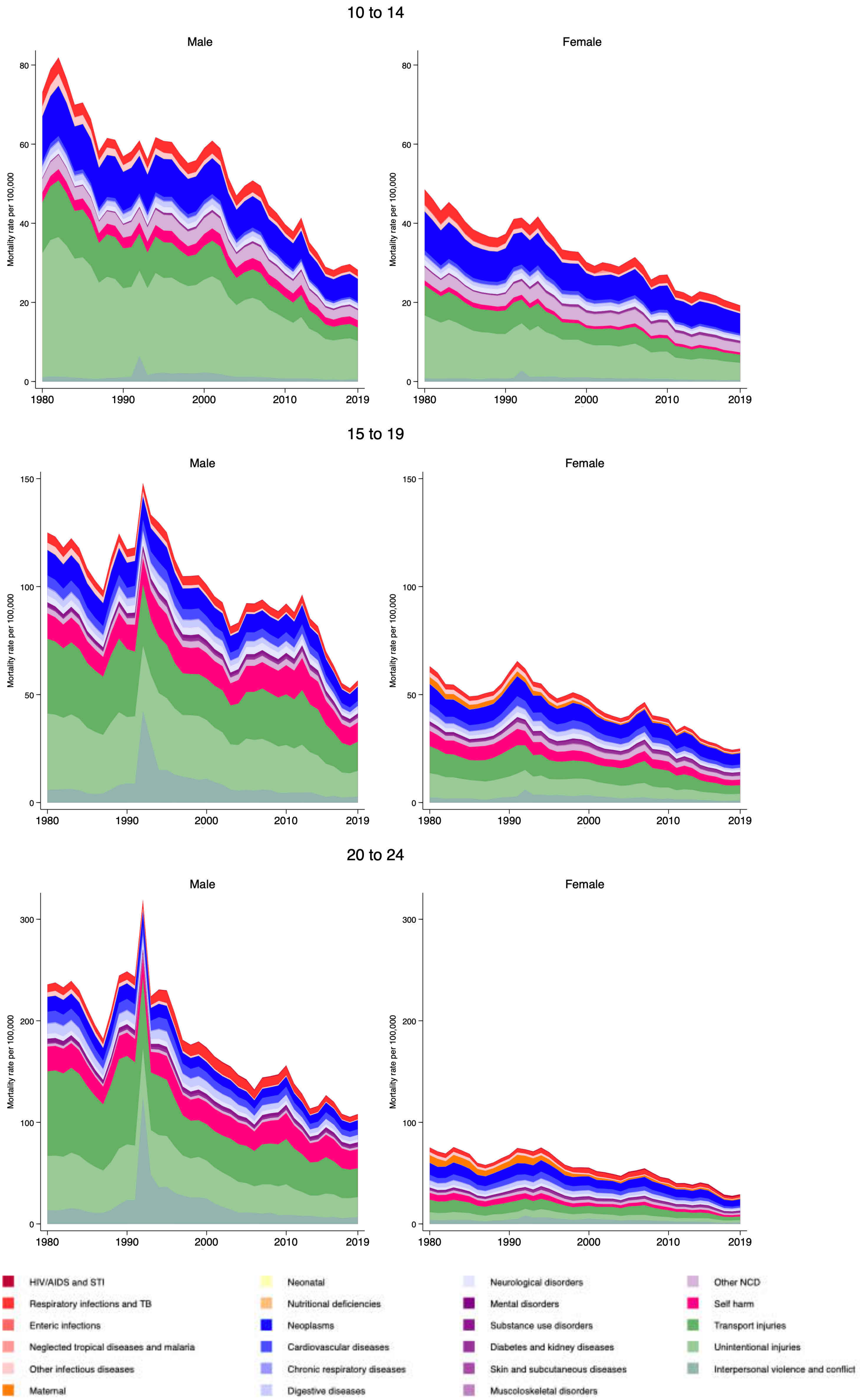

Figure S59d Number of deaths in 10-24 1980 – 2019 by cause: Republic of Moldova

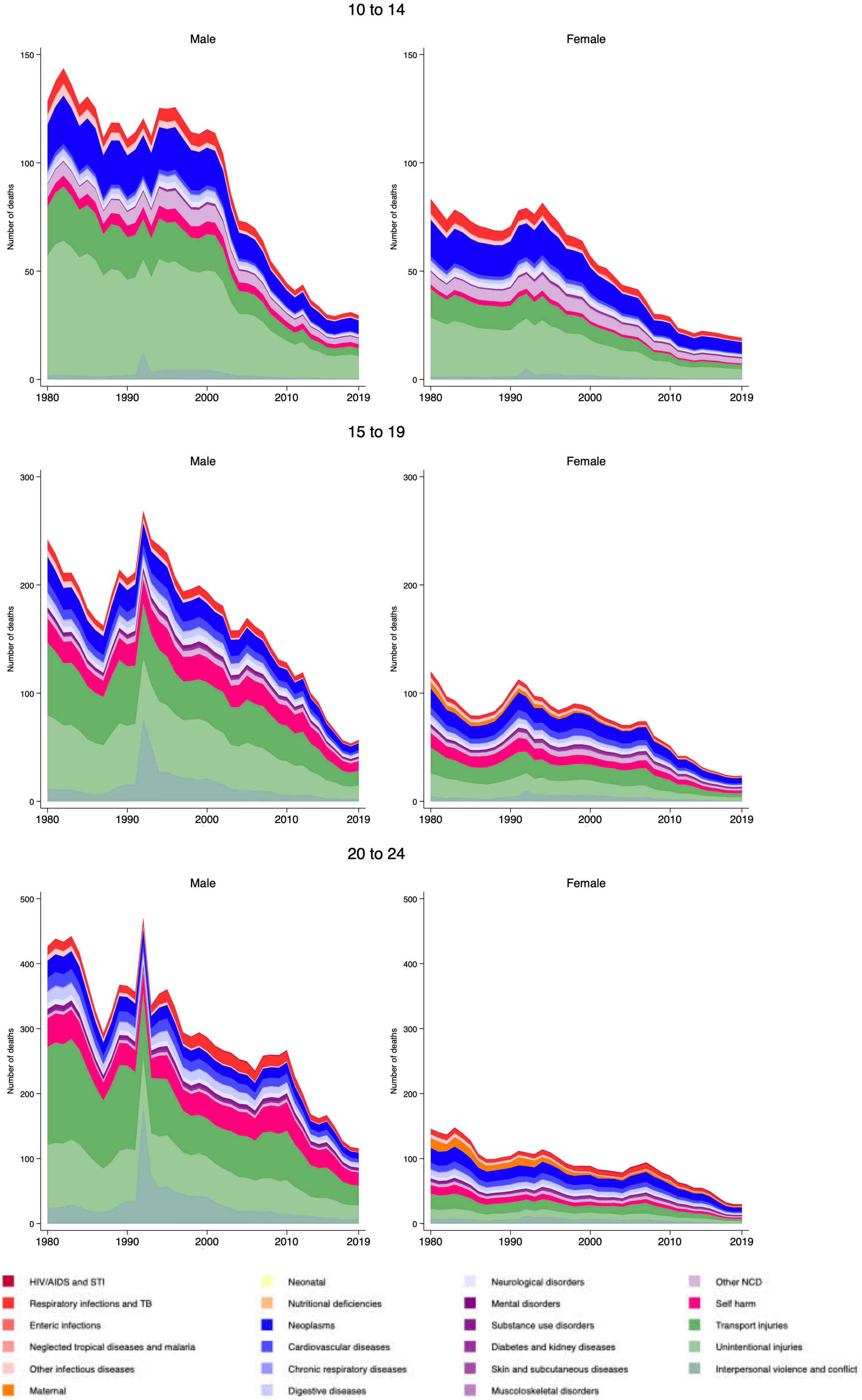

Figure S60: Russian Federation

Figure S60a All-cause mortality rate per 100,000 in 10-24 1950 – 2019: Russian Federation

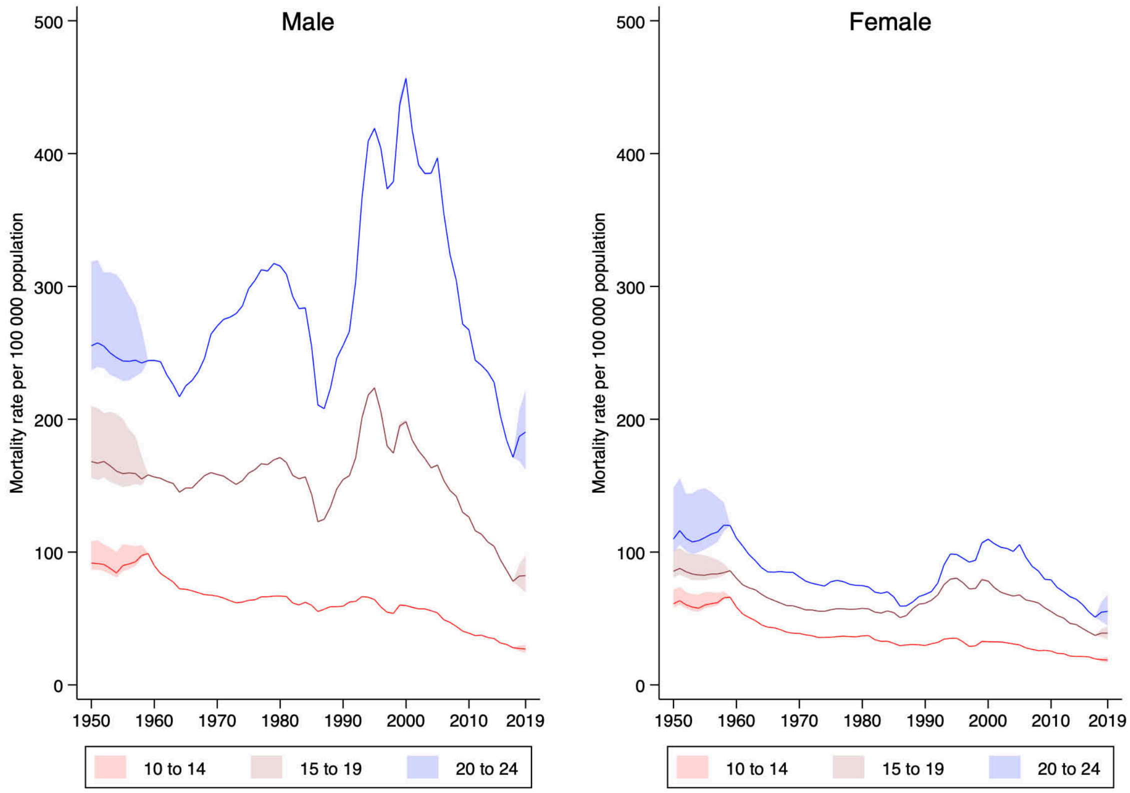

Figure S60b Number of deaths from all causes in 10-24 1950 – 2019: Russian Federation

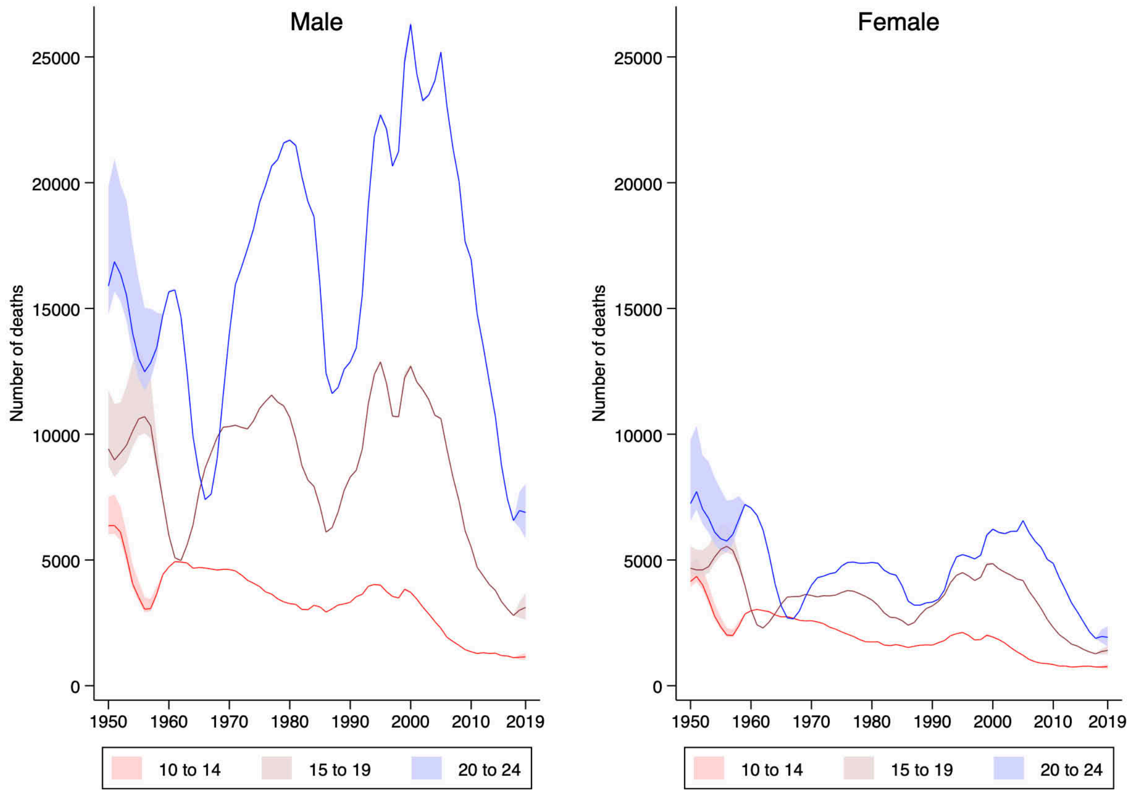

Figure S60c Mortality rate per 100,000 in 10-24 1980 – 2019 by cause: Russian Federation

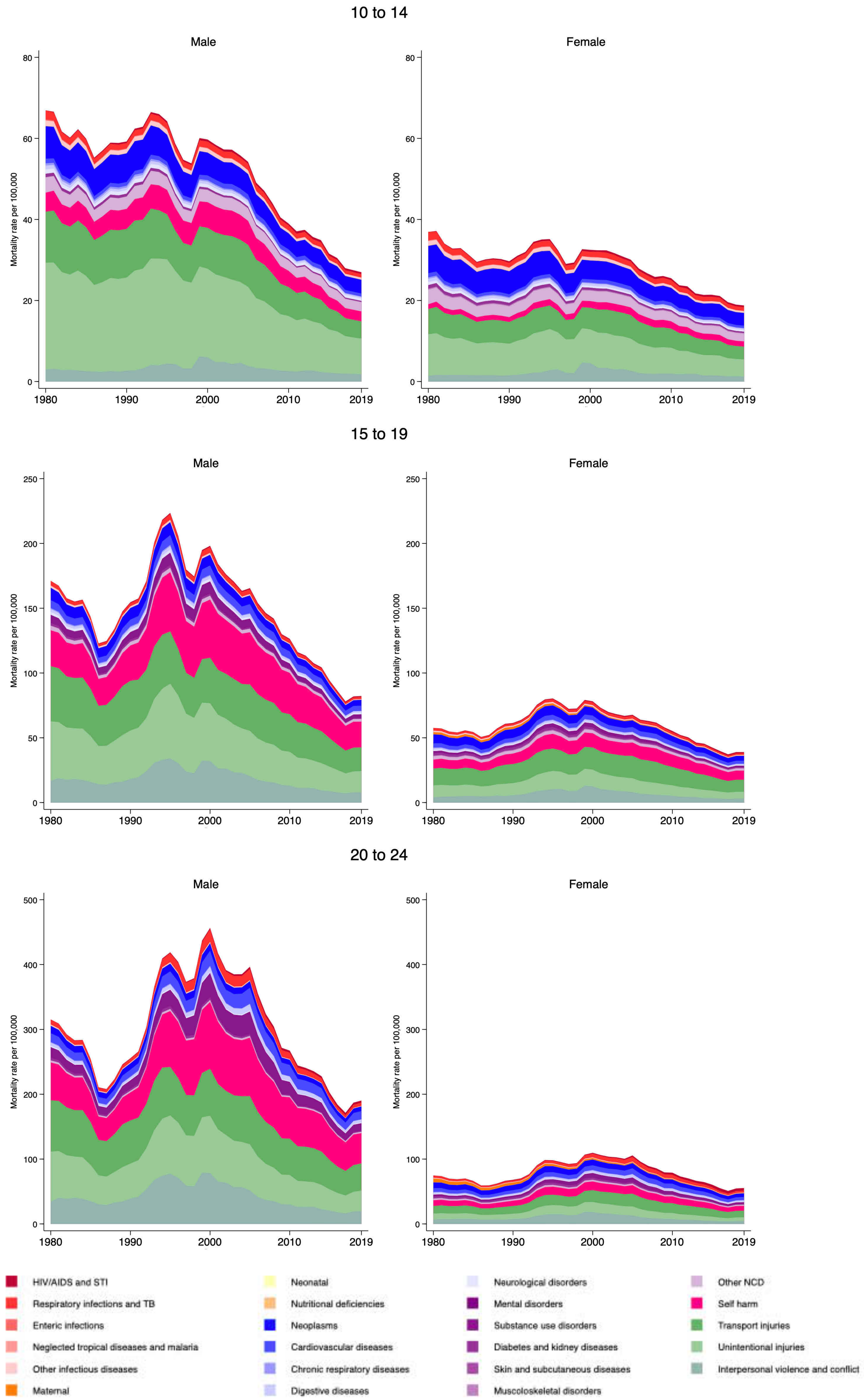

Figure S60d Number of deaths in 10-24 1980 – 2019 by cause: Russian Federation

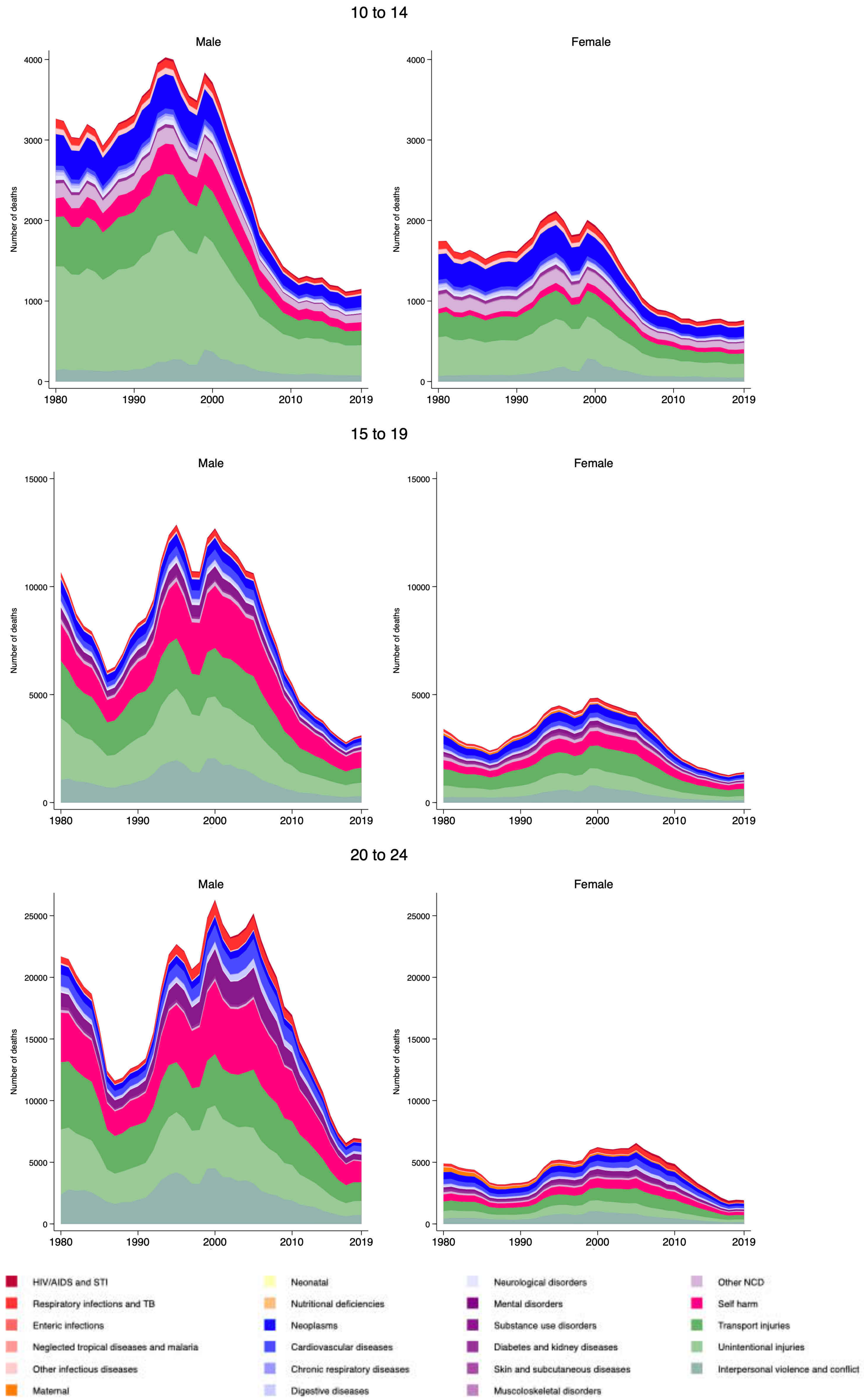

Figure S61: Ukraine

Figure S61a All-cause mortality rate per 100,000 in 10-24 1950 – 2019: Ukraine

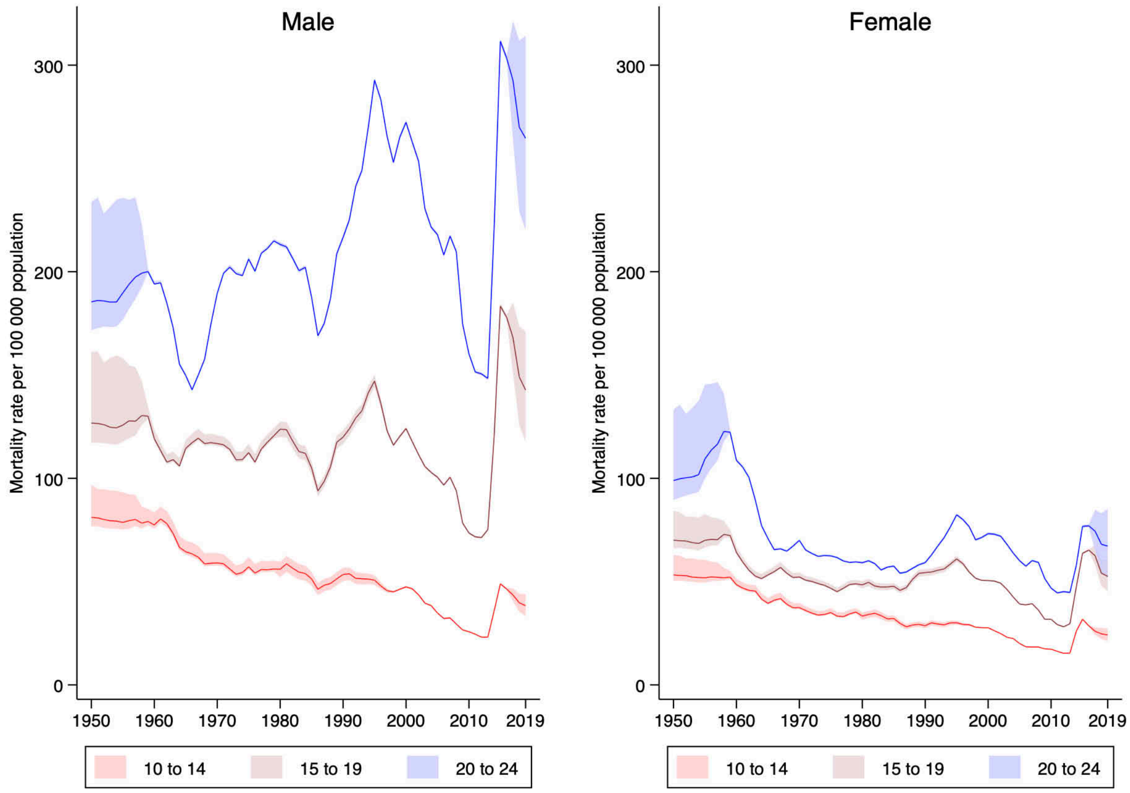

Figure S61b Number of deaths from all causes in 10-24 1950 – 2019: Ukraine

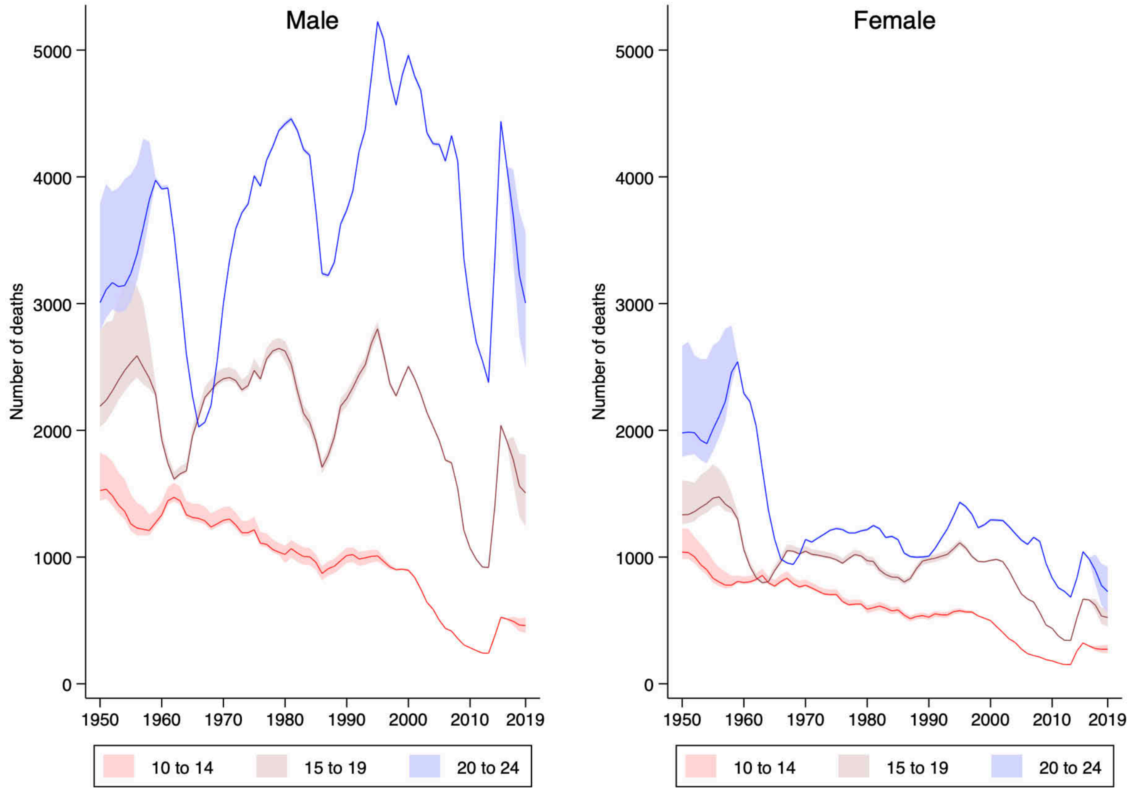

Figure S61c Mortality rate per 100,000 in 10-24 1980 – 2019 by cause: Ukraine

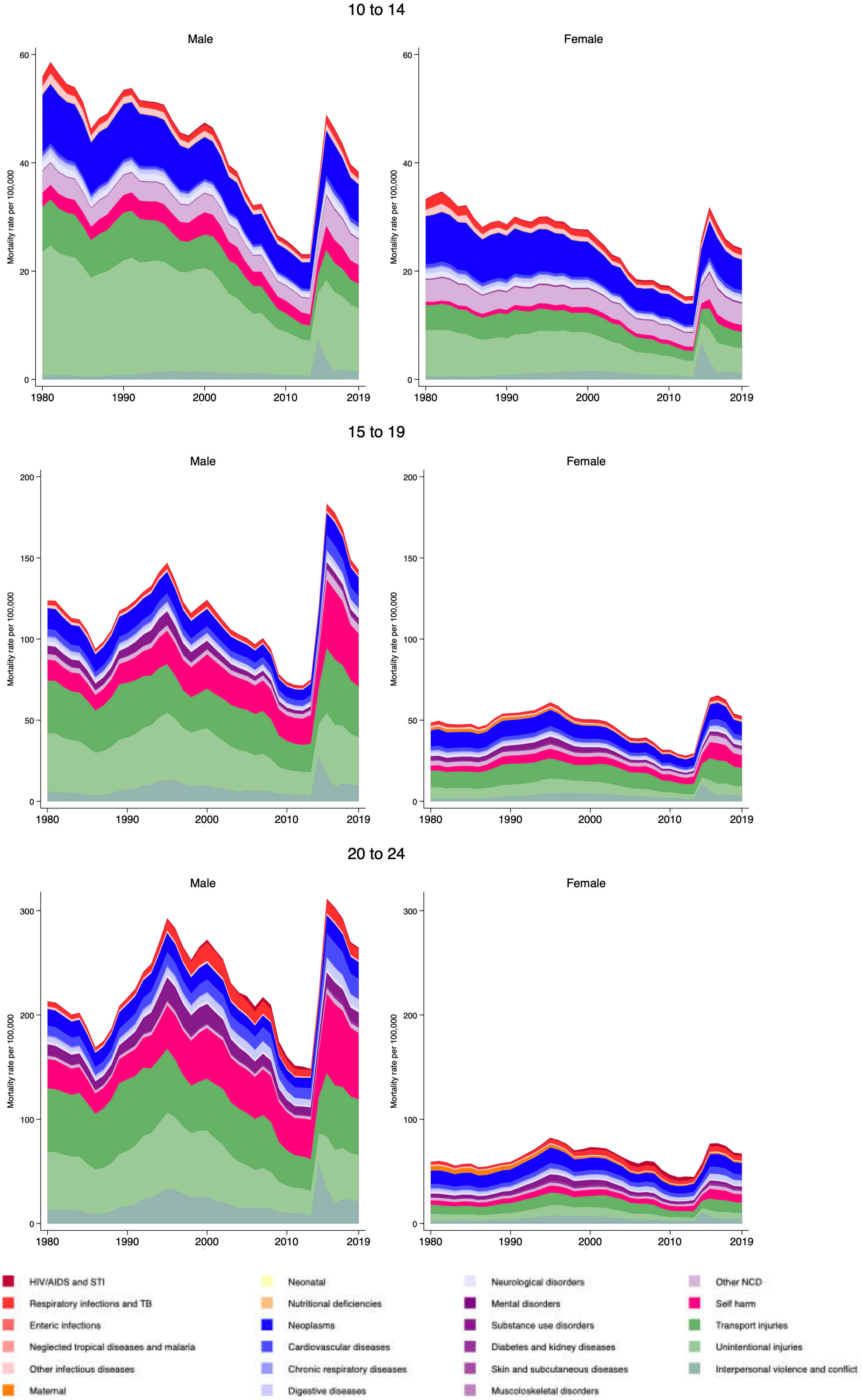

Figure S61d Number of deaths in 10-24 1980 – 2019 by cause: Ukraine

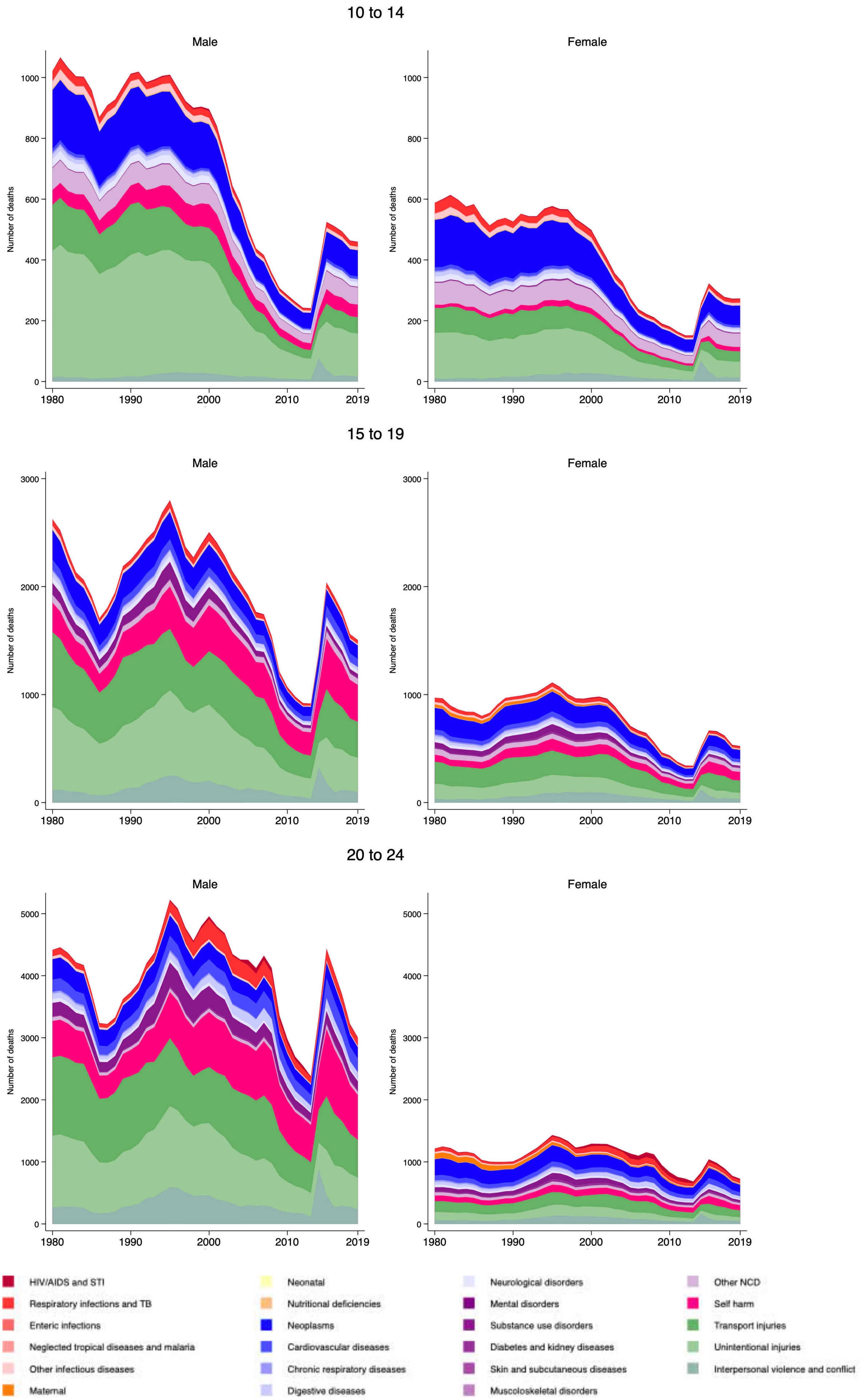

Figure S62: High-income

Figure S62a All-cause mortality rate per 100,000 in 10-24 1950 – 2019: High-income

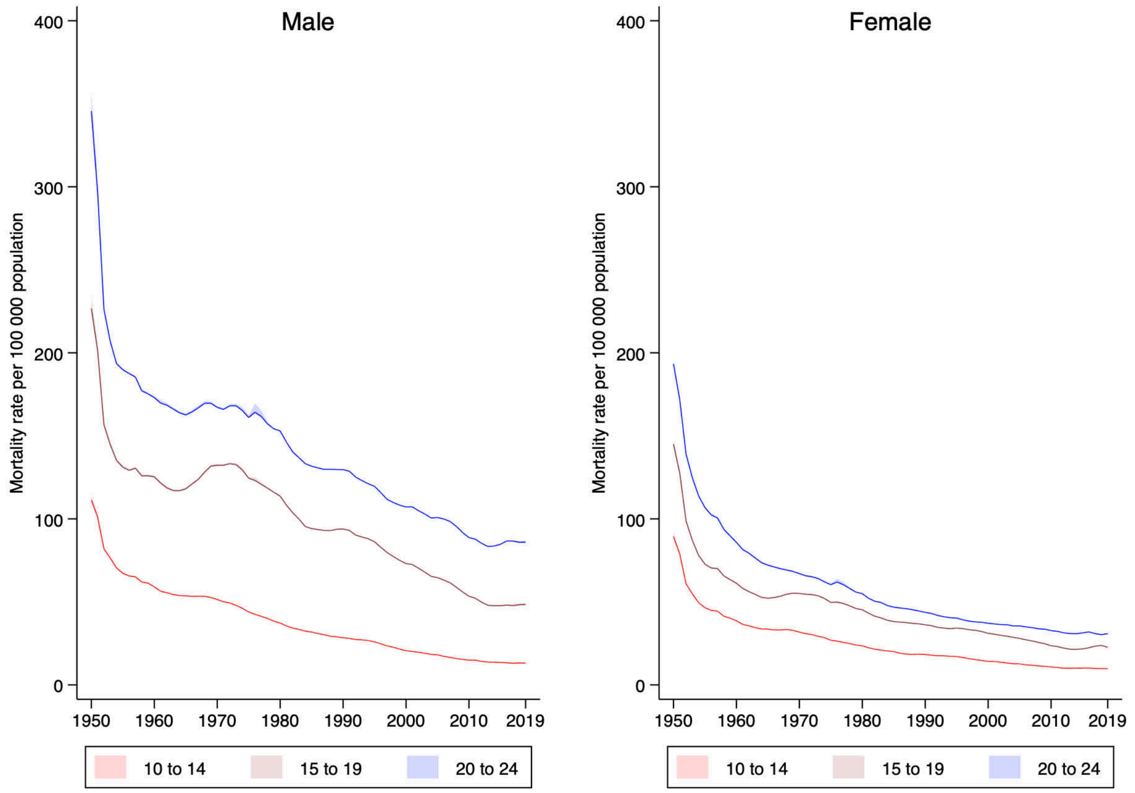

Figure S62b Number of deaths from all causes in 10-24 1950 – 2019: High-income

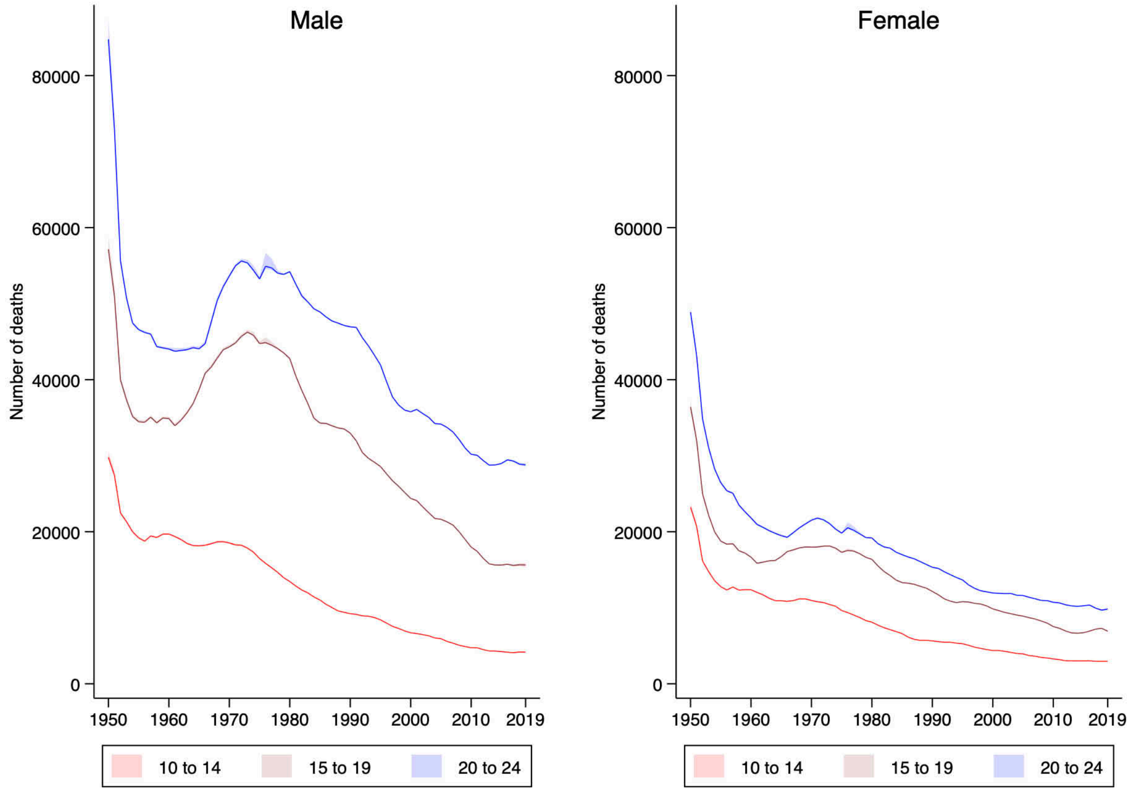

Figure S62c Mortality rate per 100,000 in 10-24 1980 – 2019 by cause: High-income

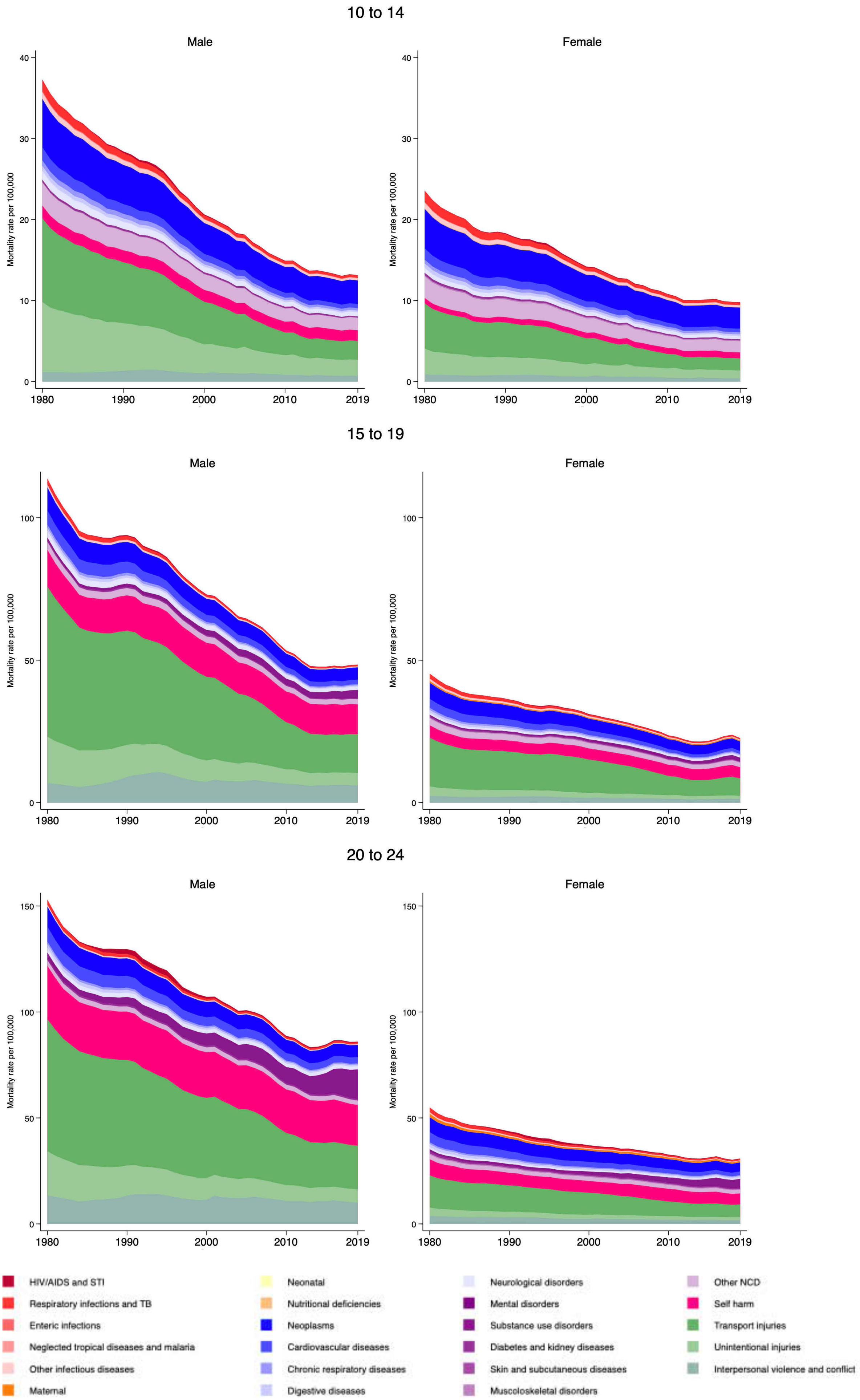

Figure S62d Number of deaths in 10-24 1980 – 2019 by cause: High-income

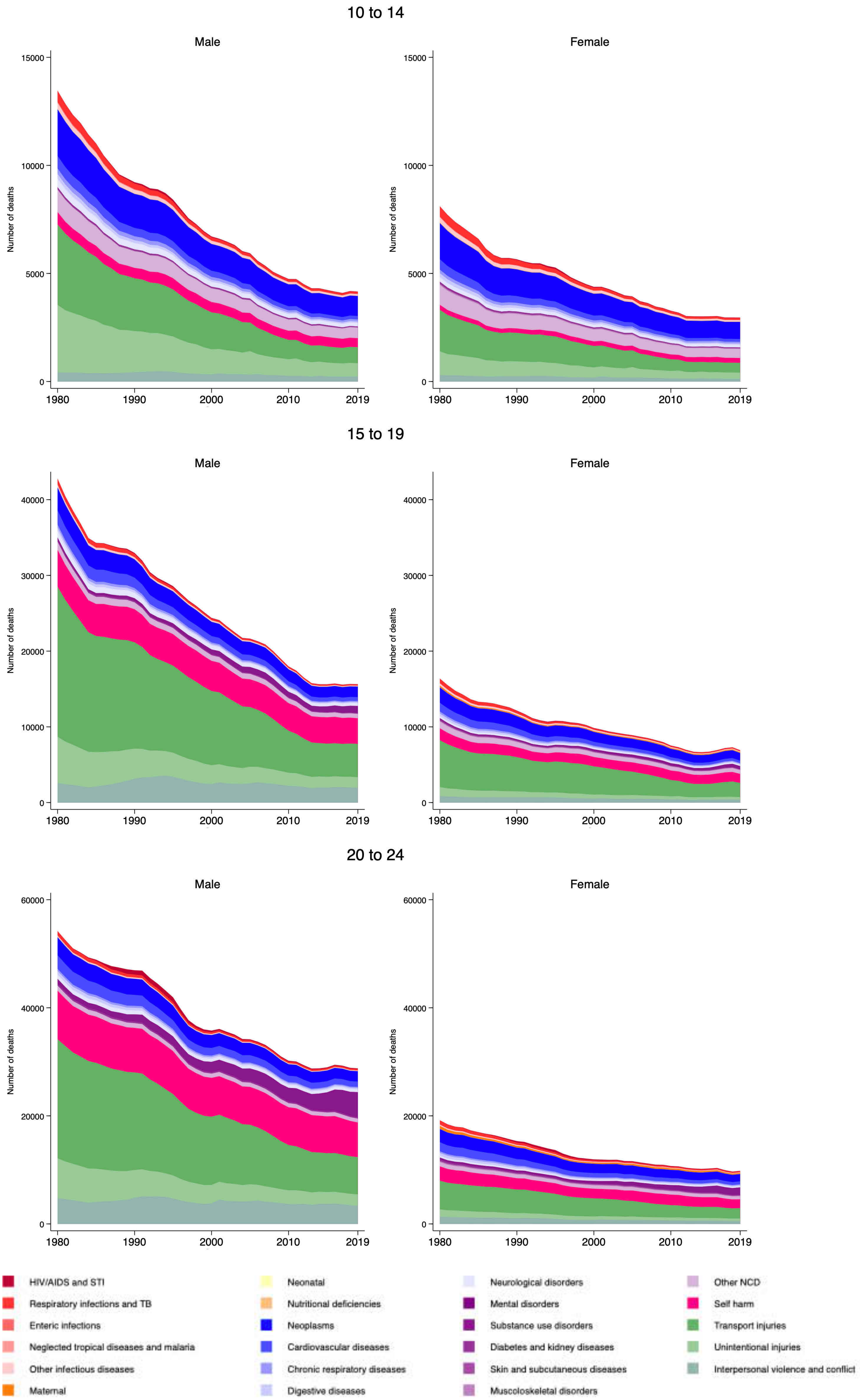

Figure S63: High-income Asia Pacific

Figure S63a All-cause mortality rate per 100,000 in 10-24 1950 – 2019: High-income Asia Pacific

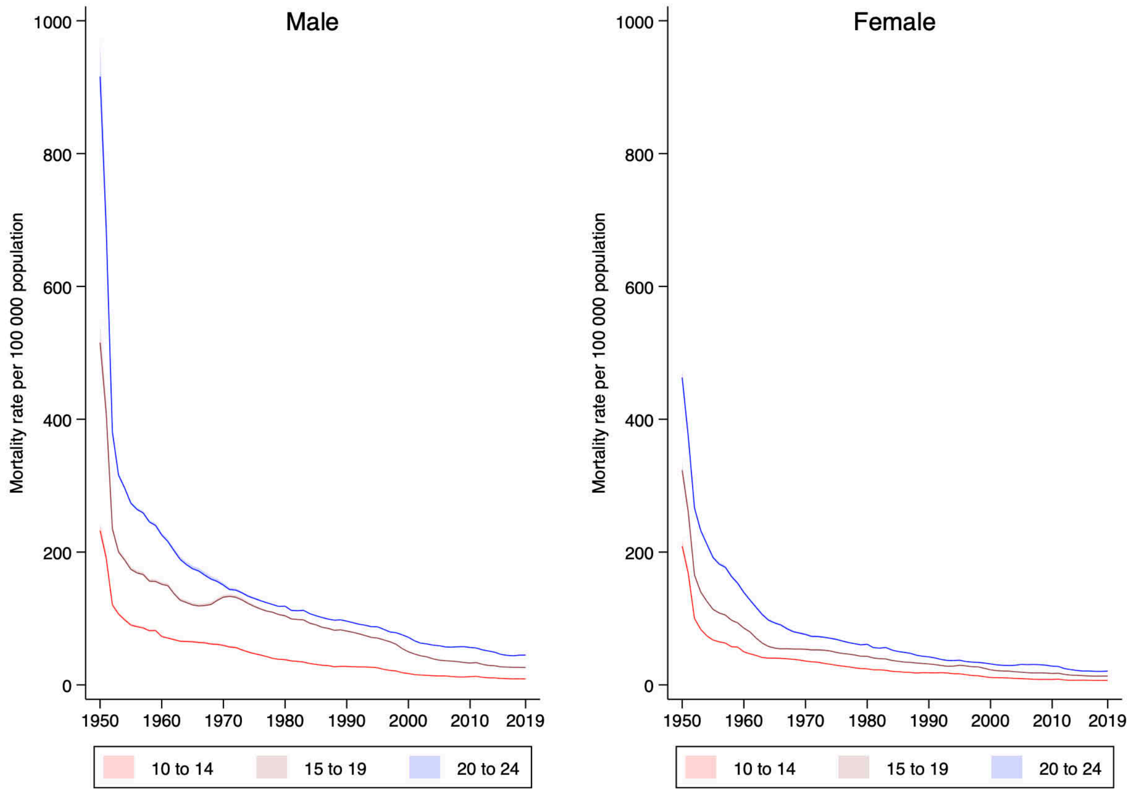

Figure S63b Number of deaths from all causes in 10-24 1950 – 2019: High-income Asia Pacific

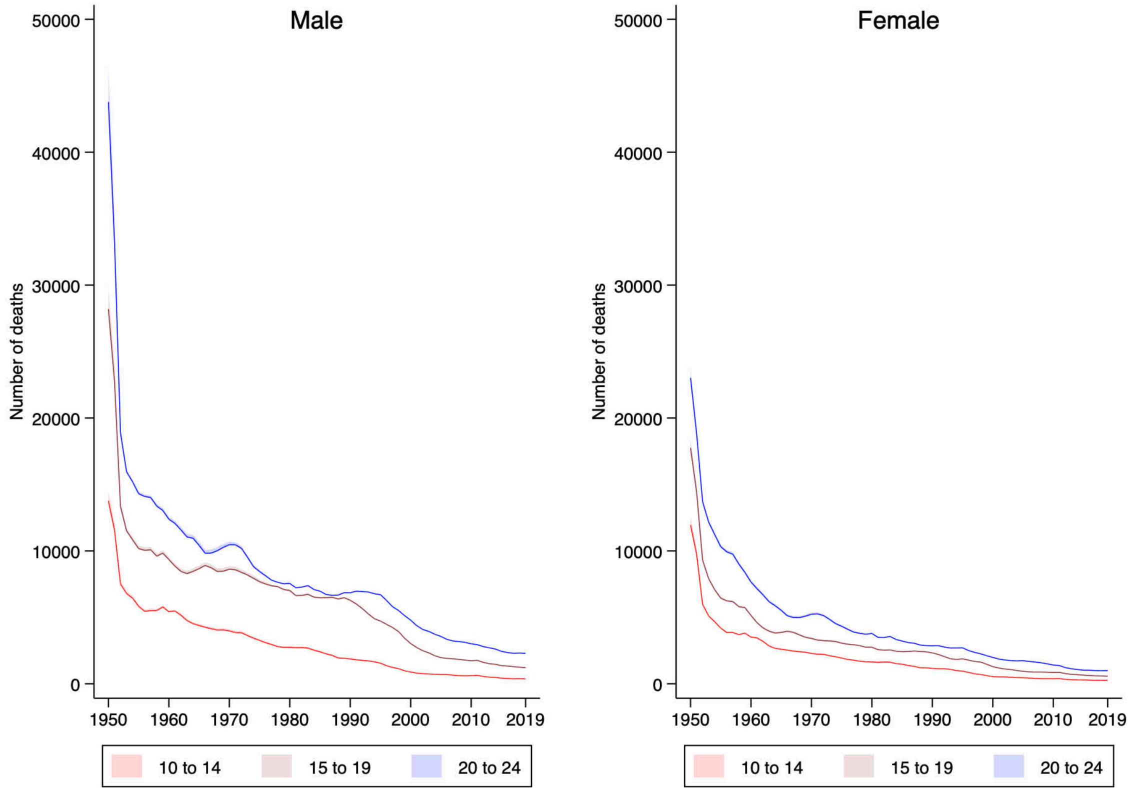

Figure S63c Mortality rate per 100,000 in 10-24 1980 – 2019 by cause: High-income Asia Pacific

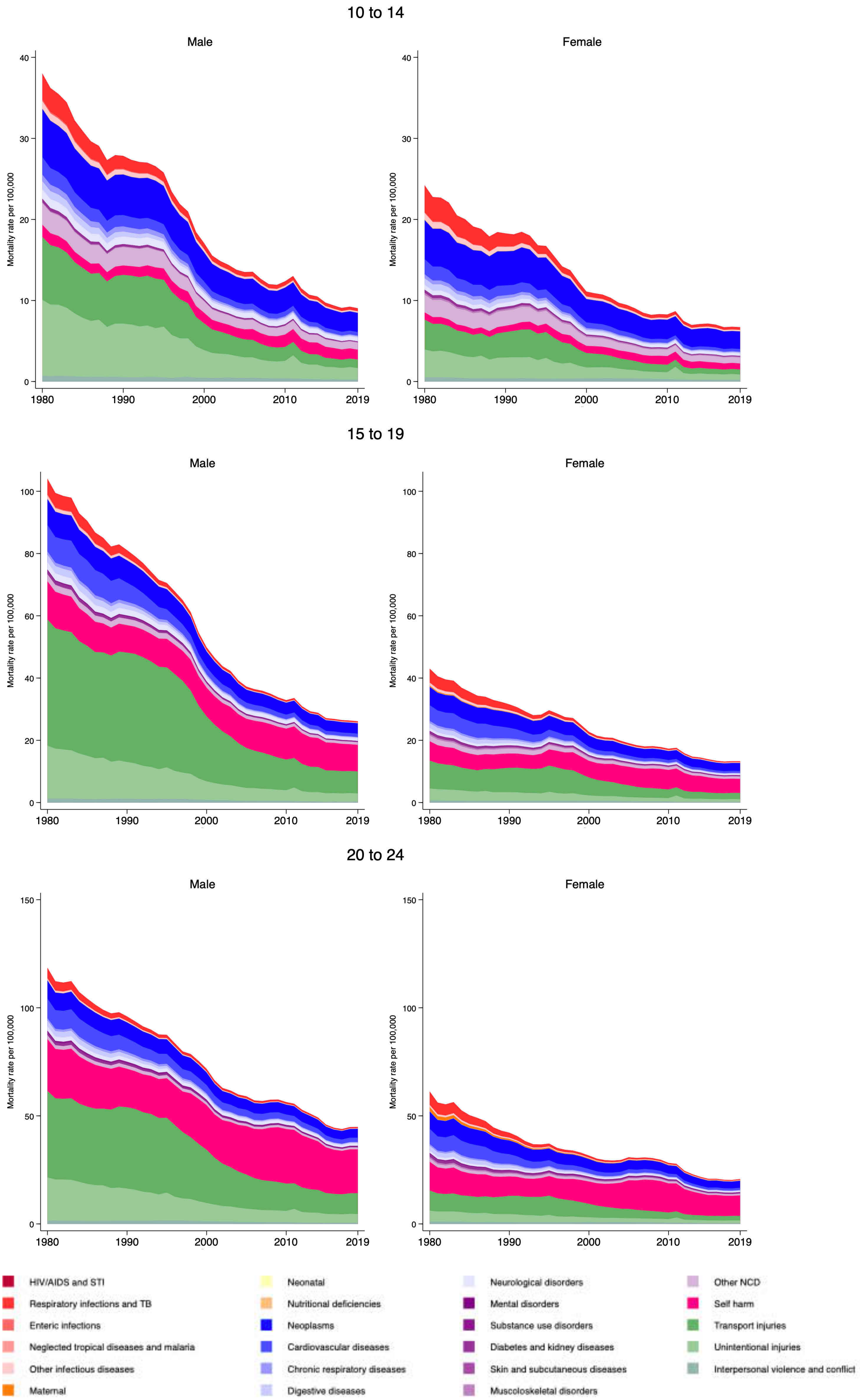

Figure S63d Number of deaths in 10-24 1980 – 2019 by cause: High-income Asia Pacific

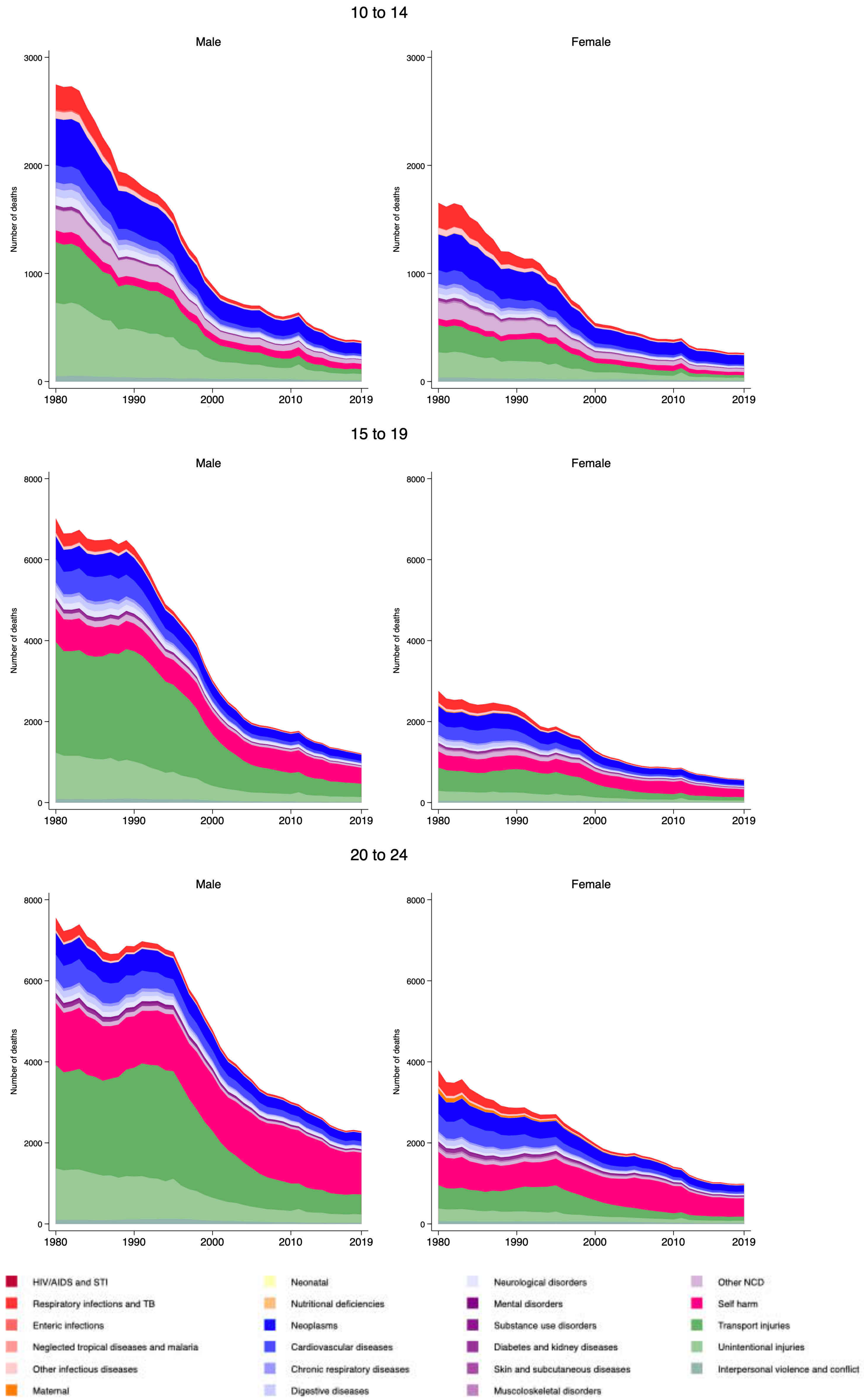

Figure S64: Brunei Darussalam

Figure S64a All-cause mortality rate per 100,000 in 10-24 1950 – 2019: Brunei Darussalam

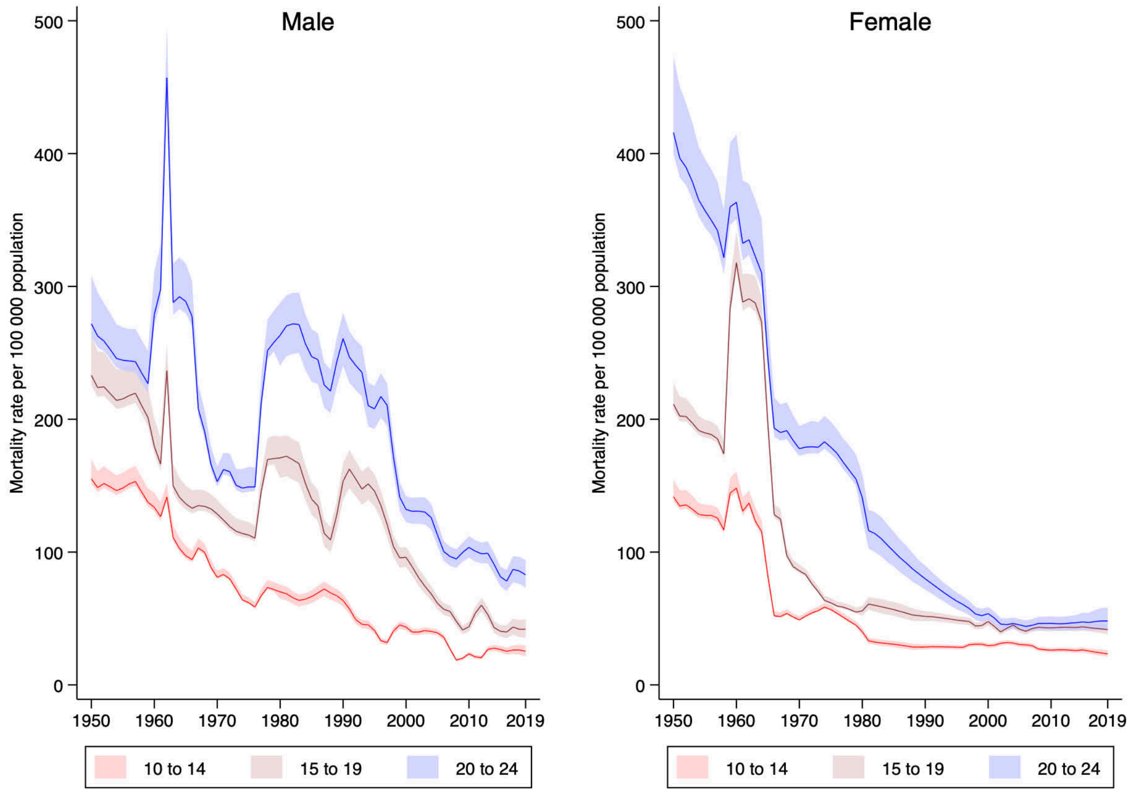

Figure S64b Number of deaths from all causes in 10-24 1950 – 2019: Brunei Darussalam

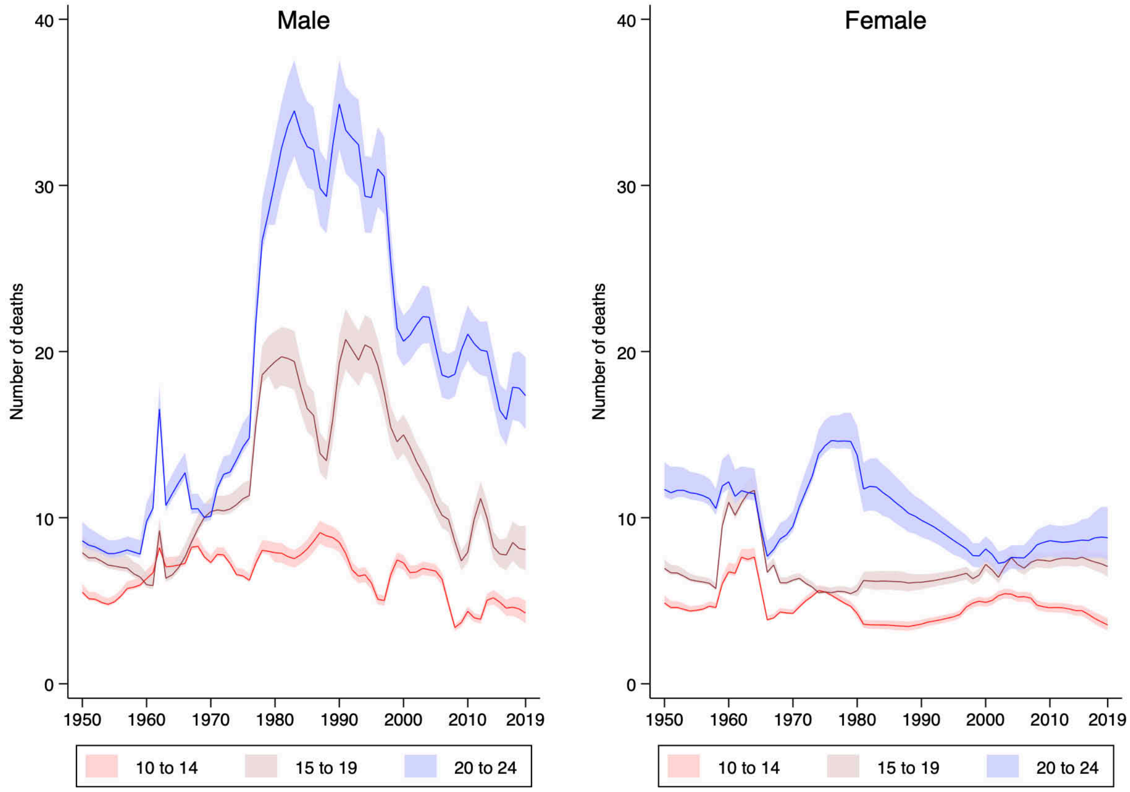

Figure S64c Mortality rate per 100,000 in 10-24 1980 – 2019 by cause: Brunei Darussalam

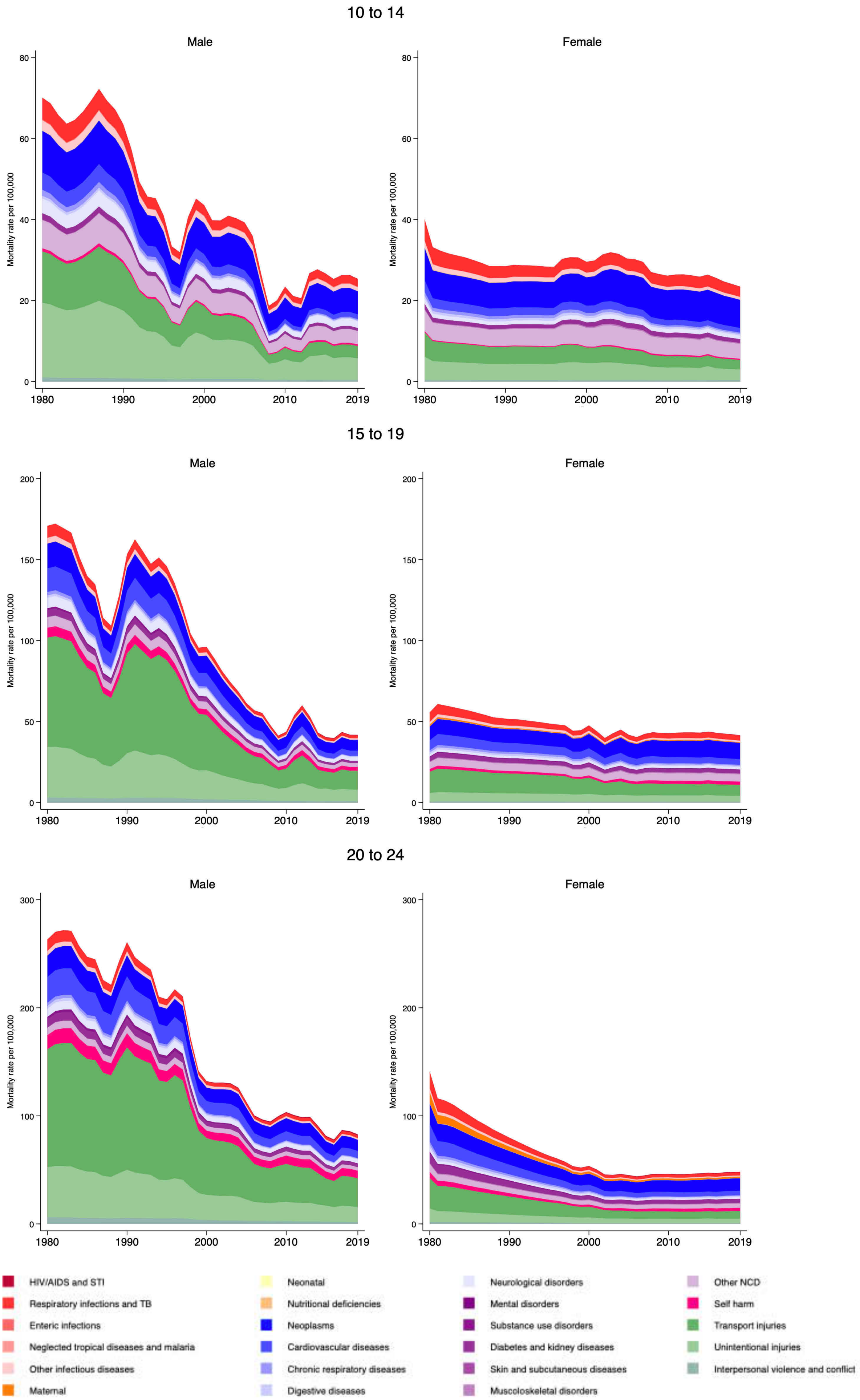

Figure S64d Number of deaths in 10-24 1980 – 2019 by cause: Brunei Darussalam

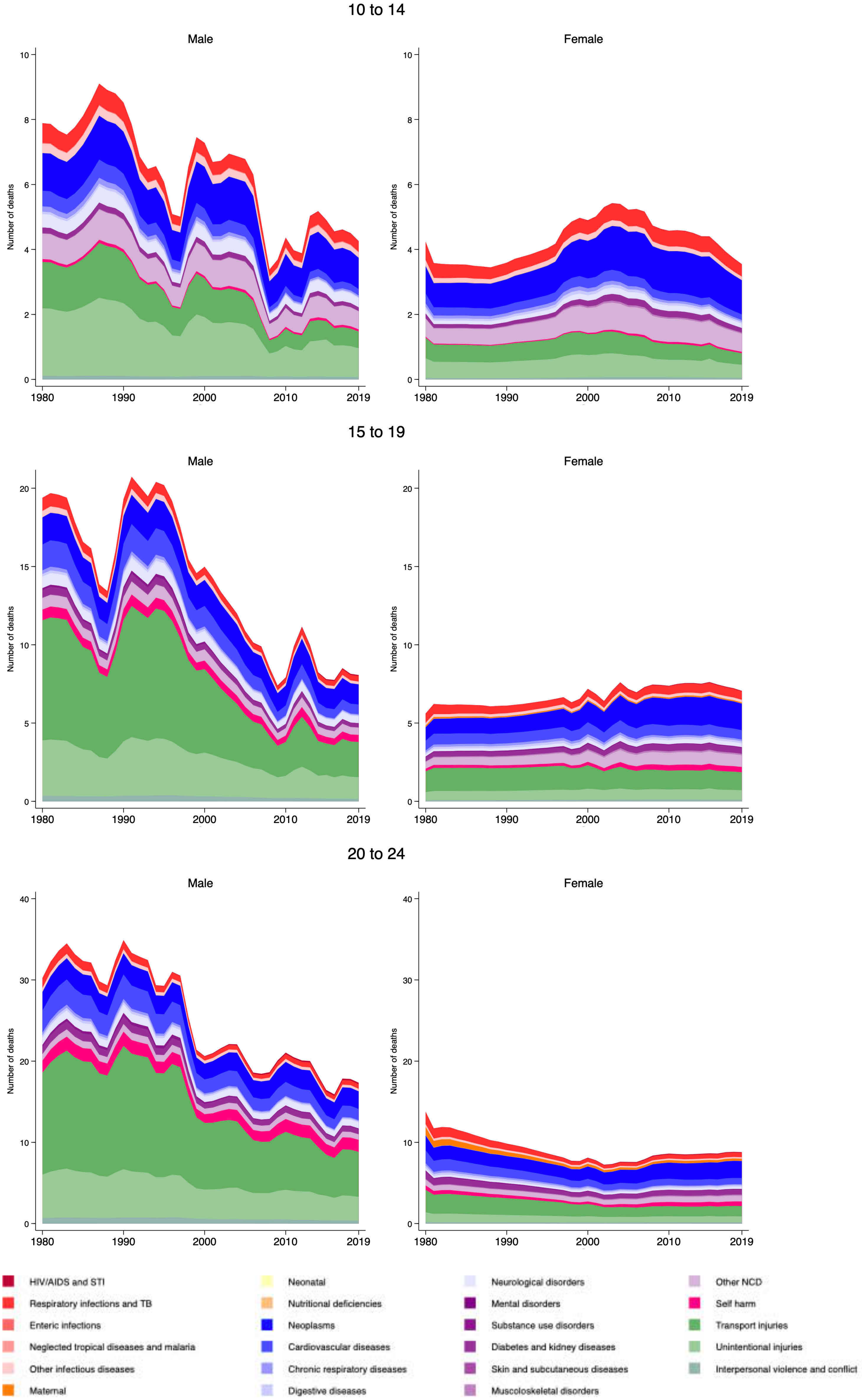

Figure S65: Japan

Figure S65a All-cause mortality rate per 100,000 in 10-24 1950 – 2019: Japan

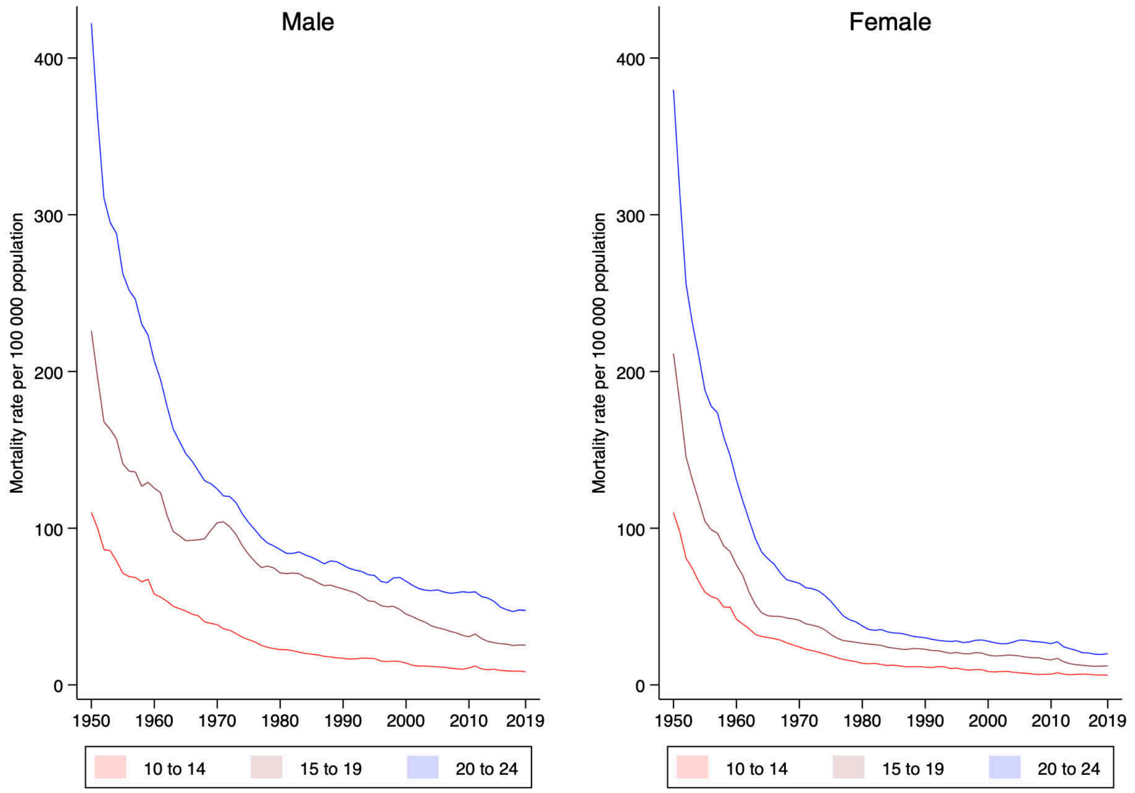

Figure S65b Number of deaths from all causes in 10-24 1950 – 2019: Japan

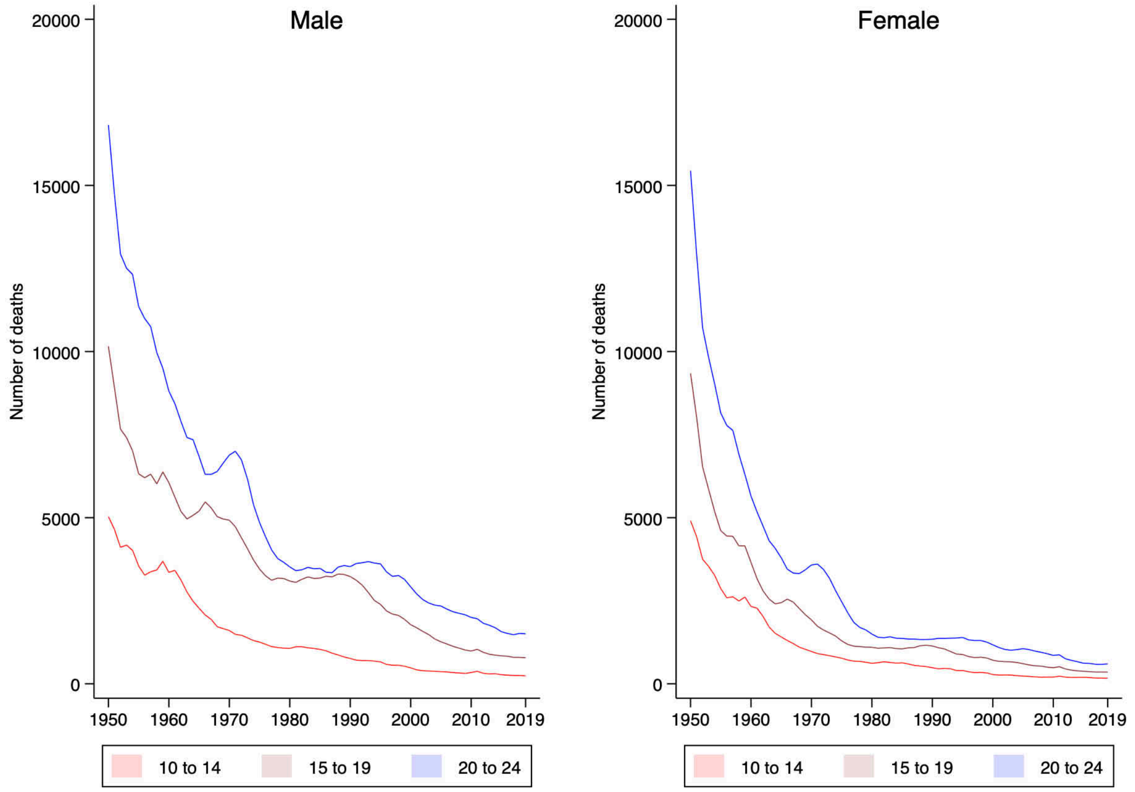

Figure S65c Mortality rate per 100,000 in 10-24 1980 – 2019 by cause: Japan

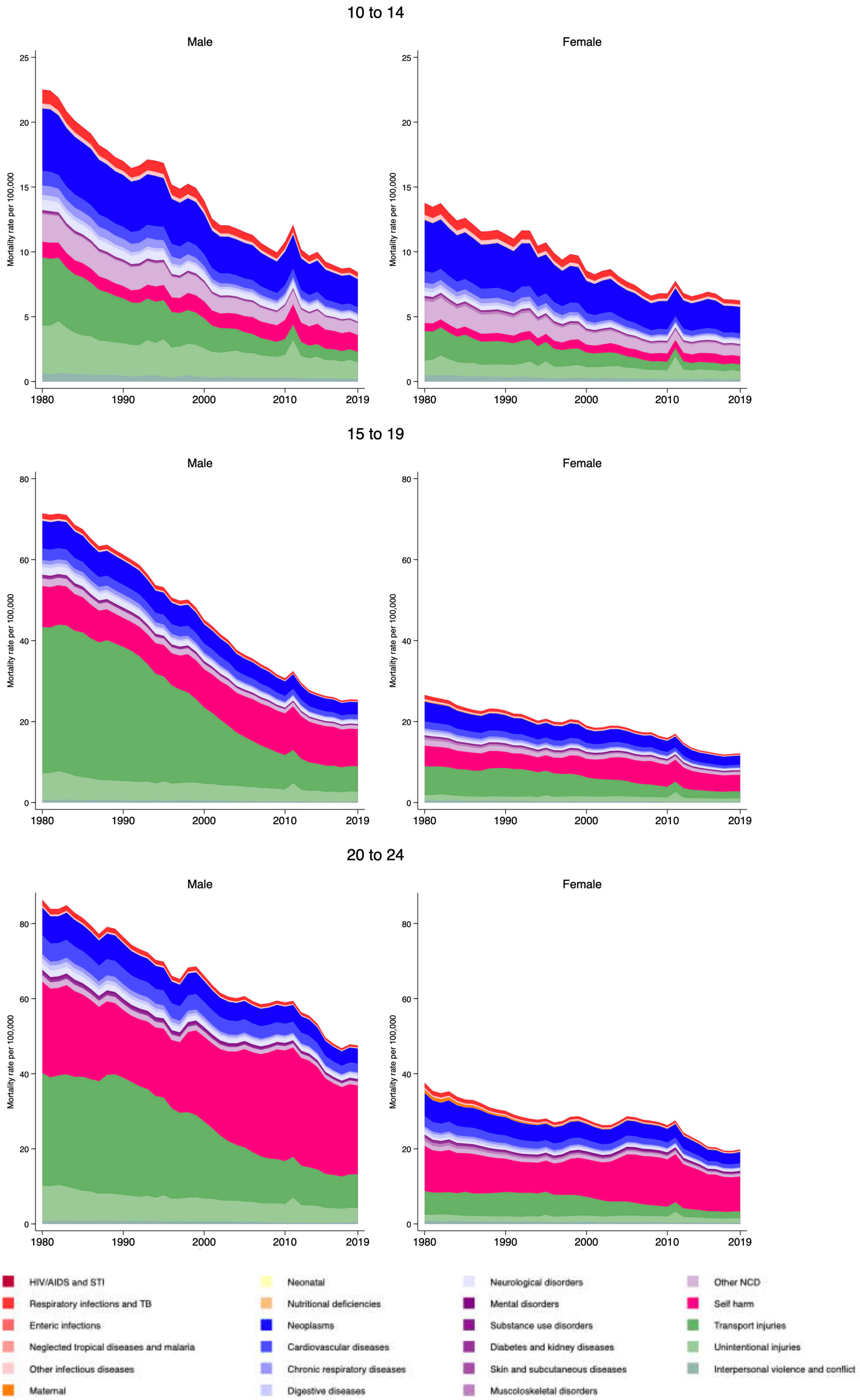

Figure S65d Number of deaths in 10-24 1980 – 2019 by cause: Japan

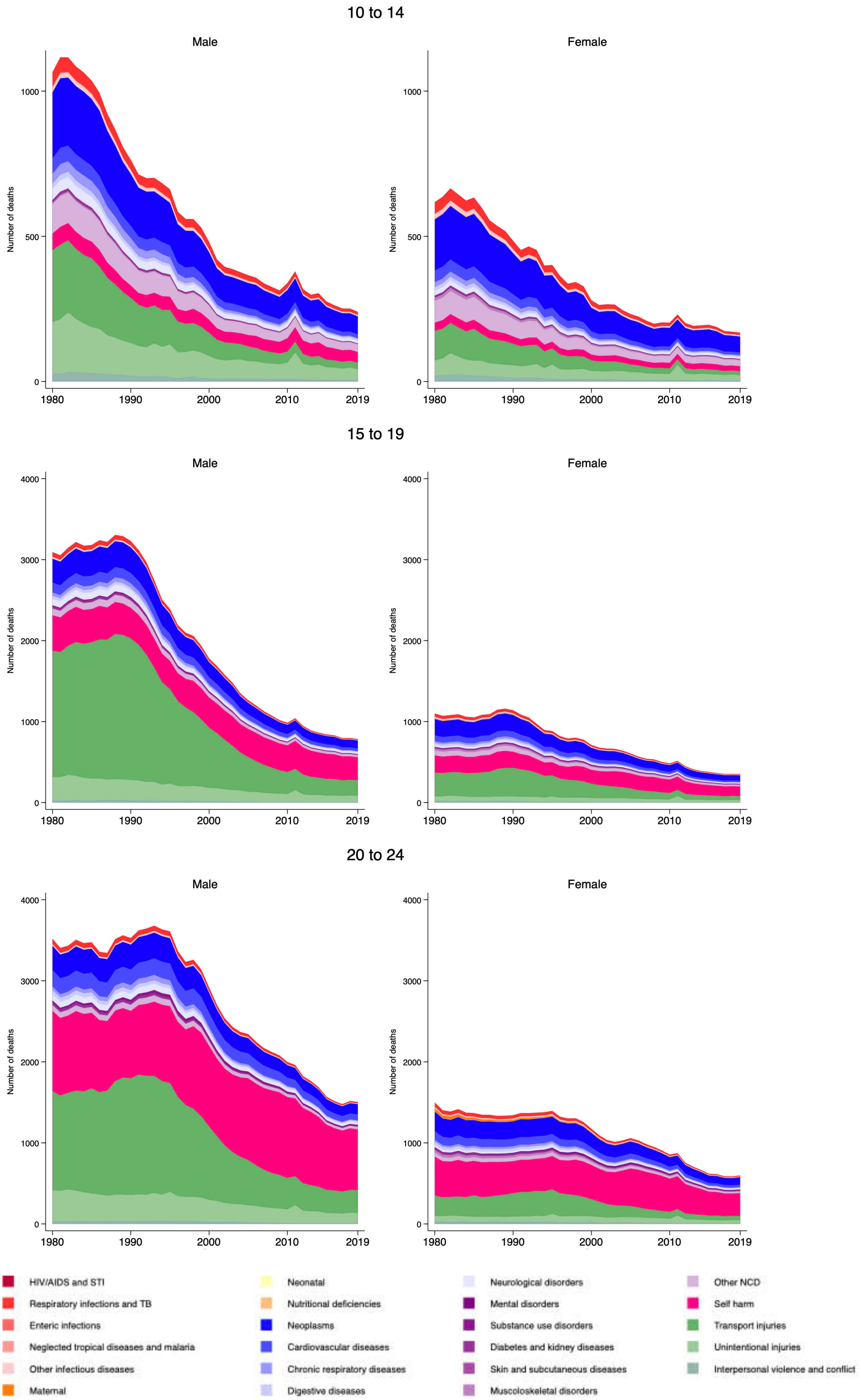

Figure S66: Republic of Korea

Figure S66a All-cause mortality rate per 100,000 in 10-24 1950 – 2019: Republic of Korea

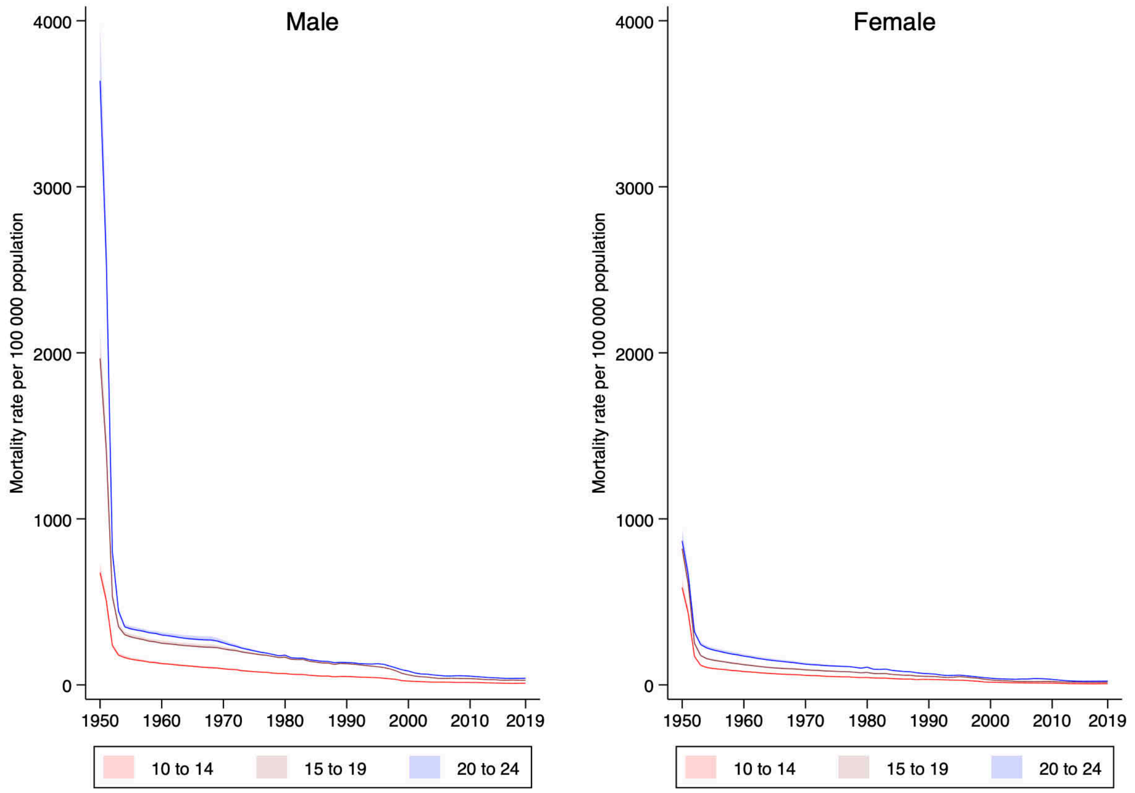

Figure S66b Number of deaths from all causes in 10-24 1950 – 2019: Republic of Korea

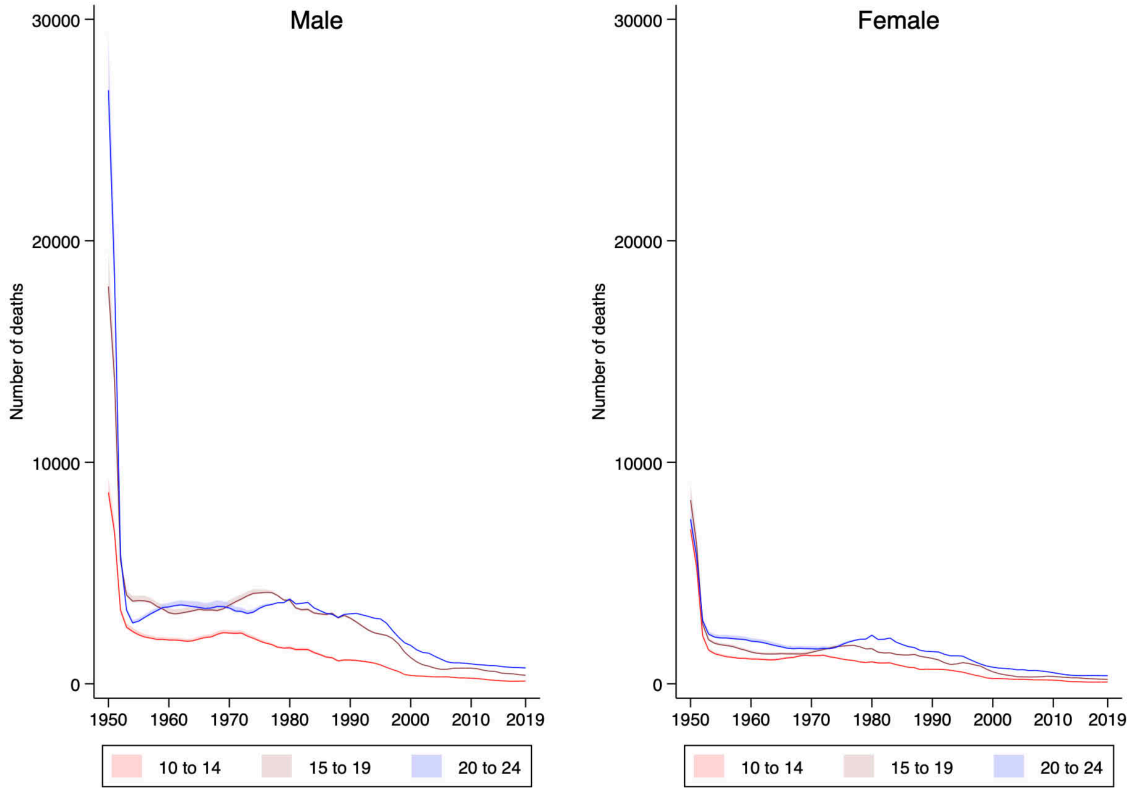

Figure S66c Mortality rate per 100,000 in 10-24 1980 – 2019 by cause: Republic of Korea

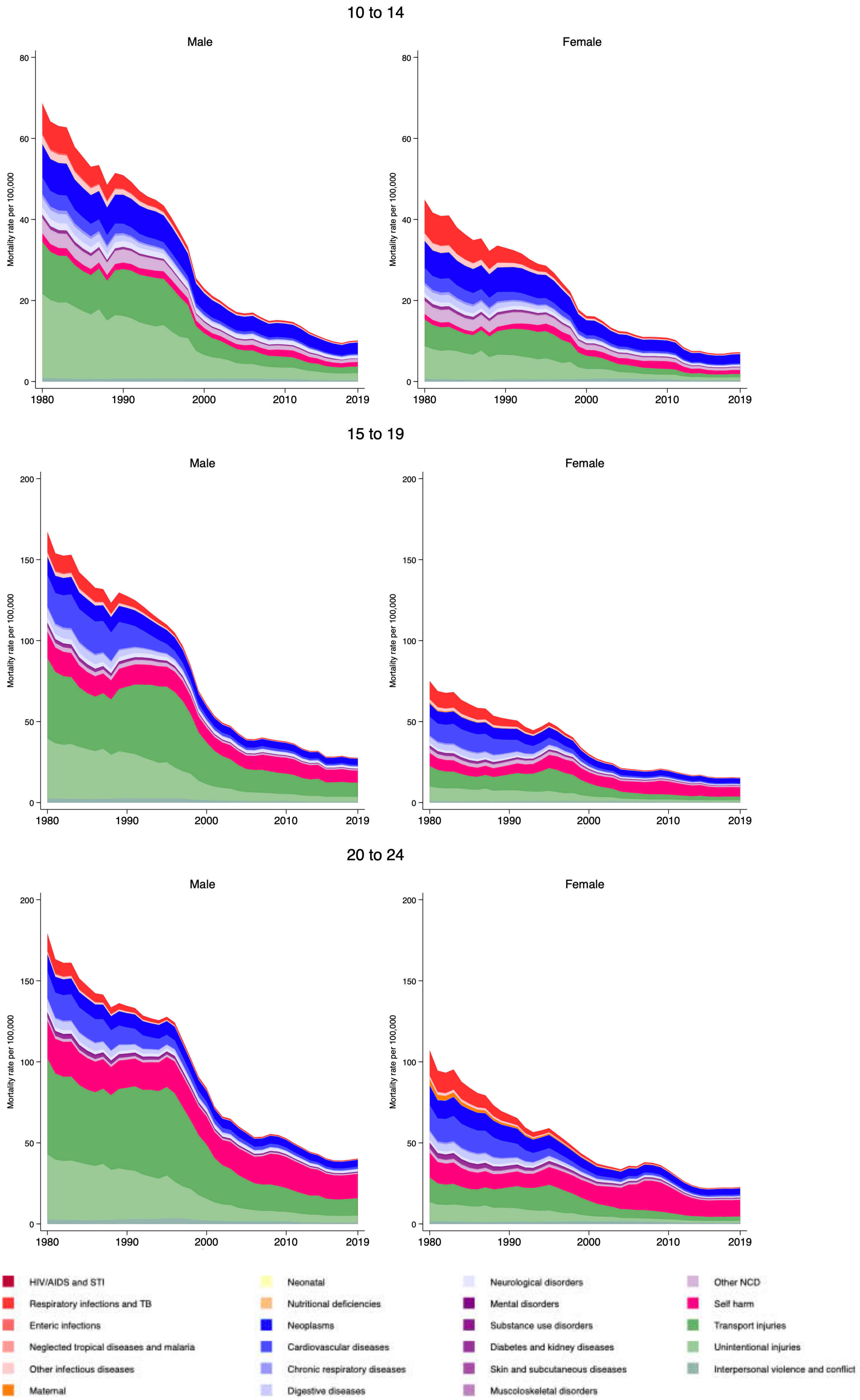

Figure S66d Number of deaths in 10-24 1980 – 2019 by cause: Republic of Korea

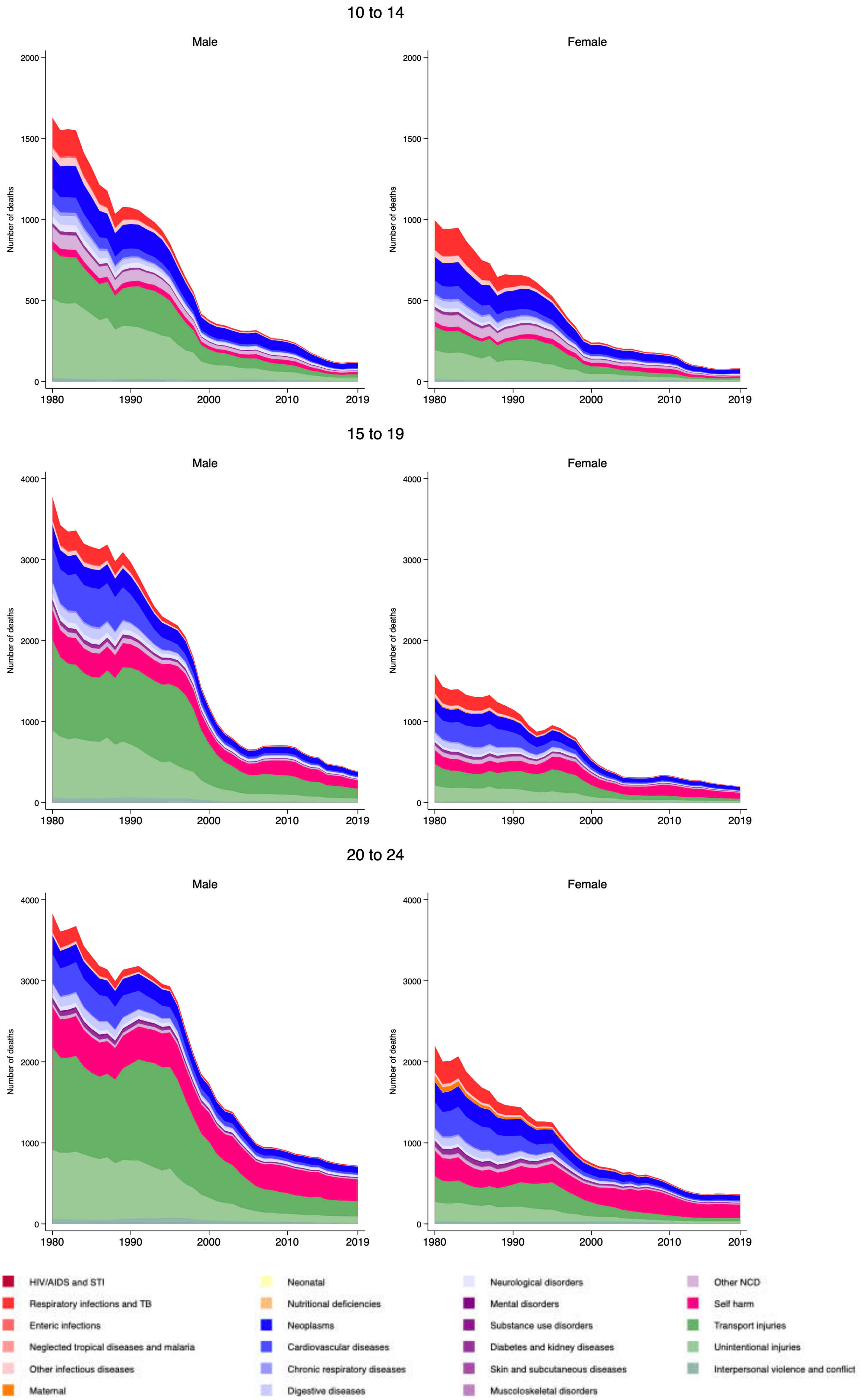

Figure S67: Singapore

Figure S67a All-cause mortality rate per 100,000 in 10-24 1950 – 2019: Singapore

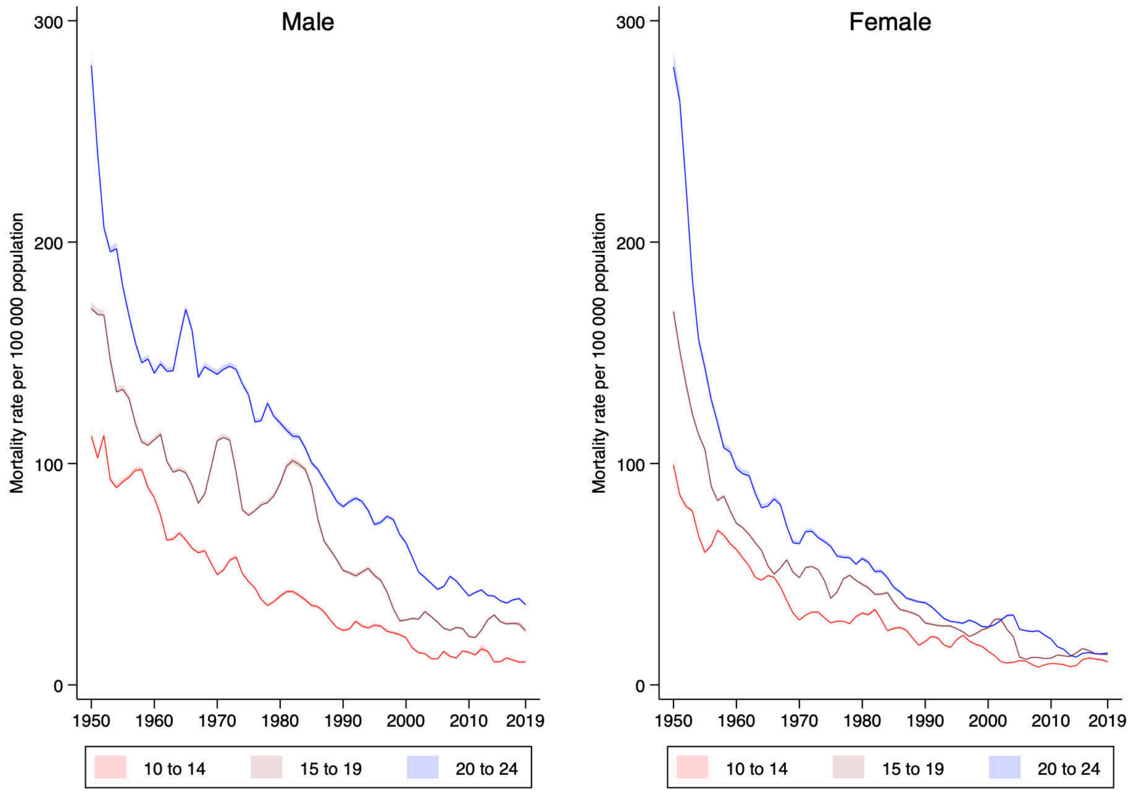

Figure S67b Number of deaths from all causes in 10-24 1950 – 2019: Singapore

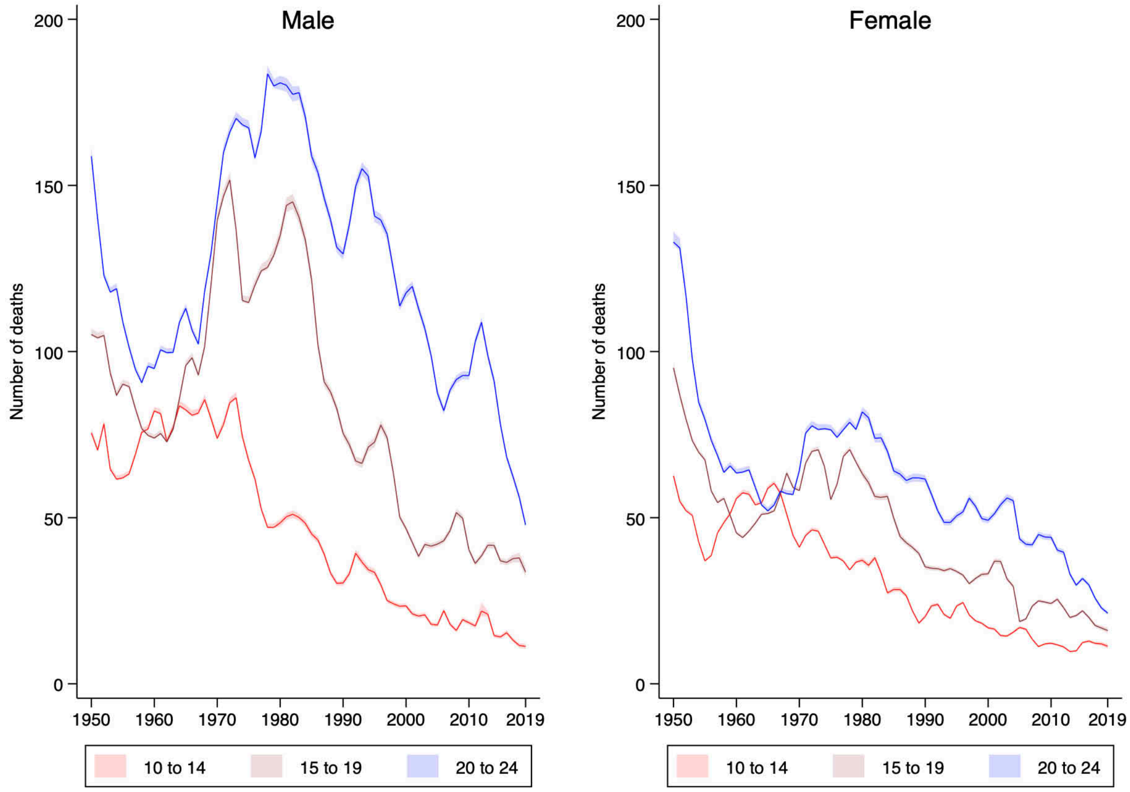

Figure S67c Mortality rate per 100,000 in 10-24 1980 – 2019 by cause: Singapore

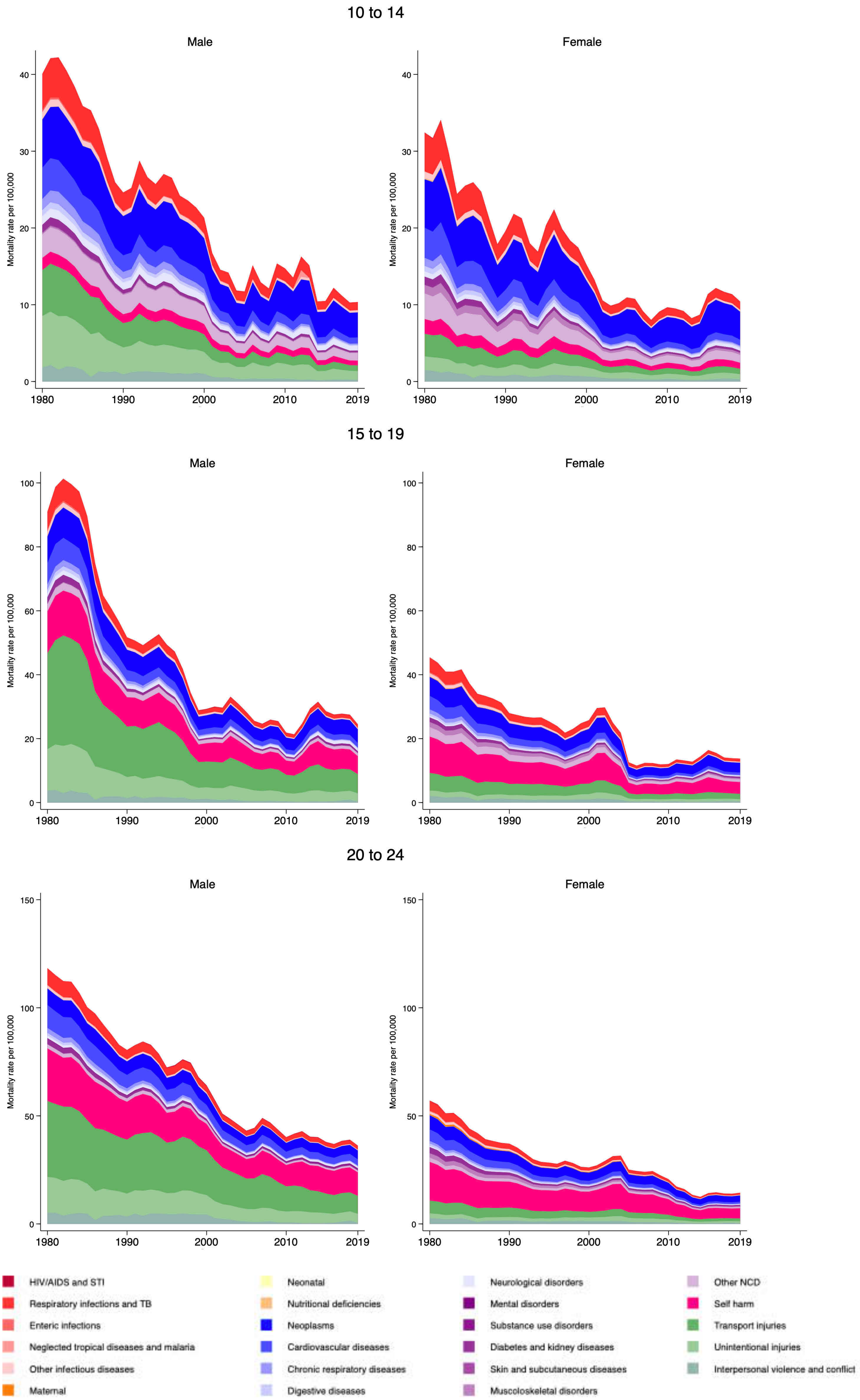

Figure S67d Number of deaths in 10-24 1980 – 2019 by cause: Singapore

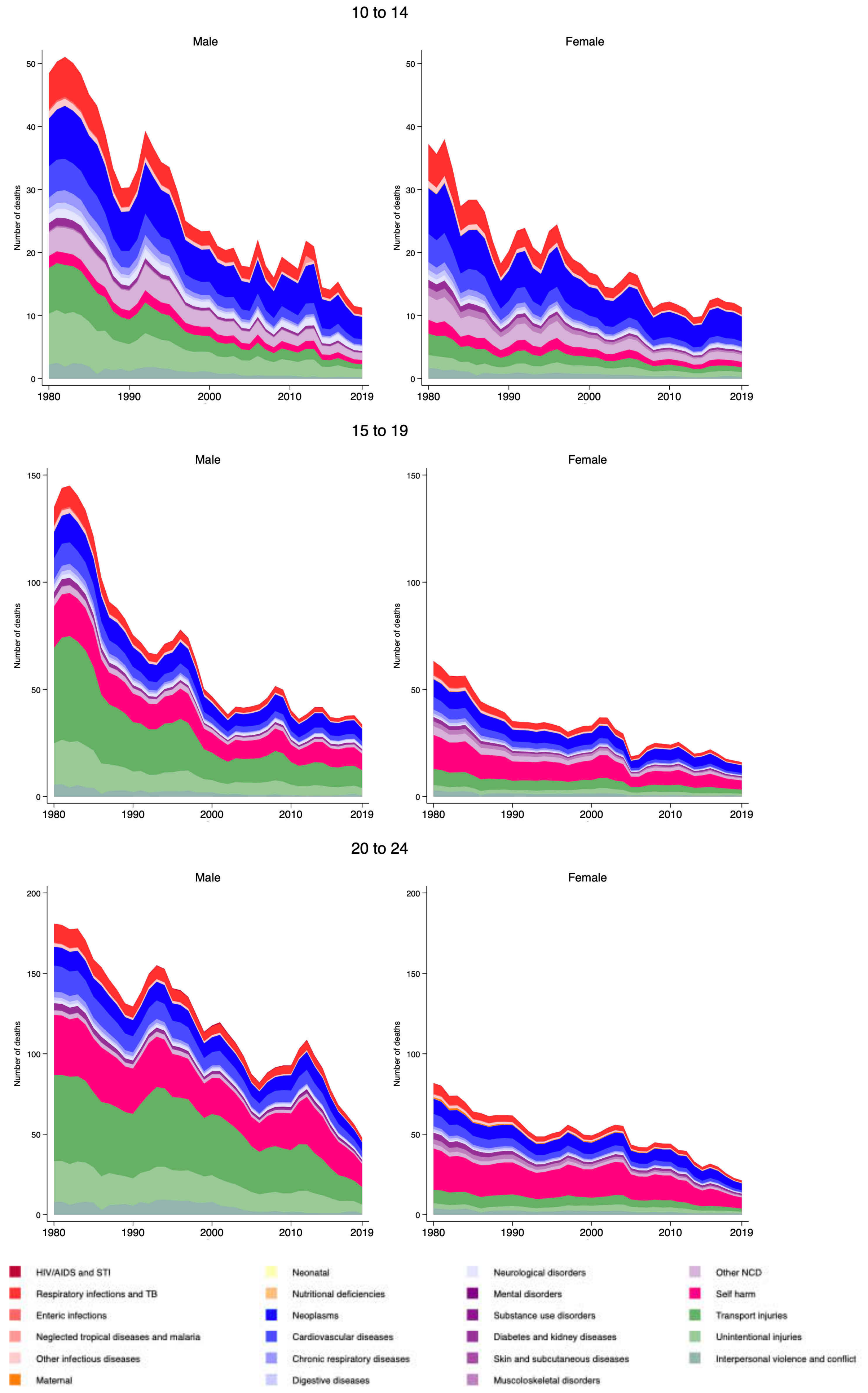

Figure S68: Australasia

Figure S68a All-cause mortality rate per 100,000 in 10-24 1950 – 2019: Australasia

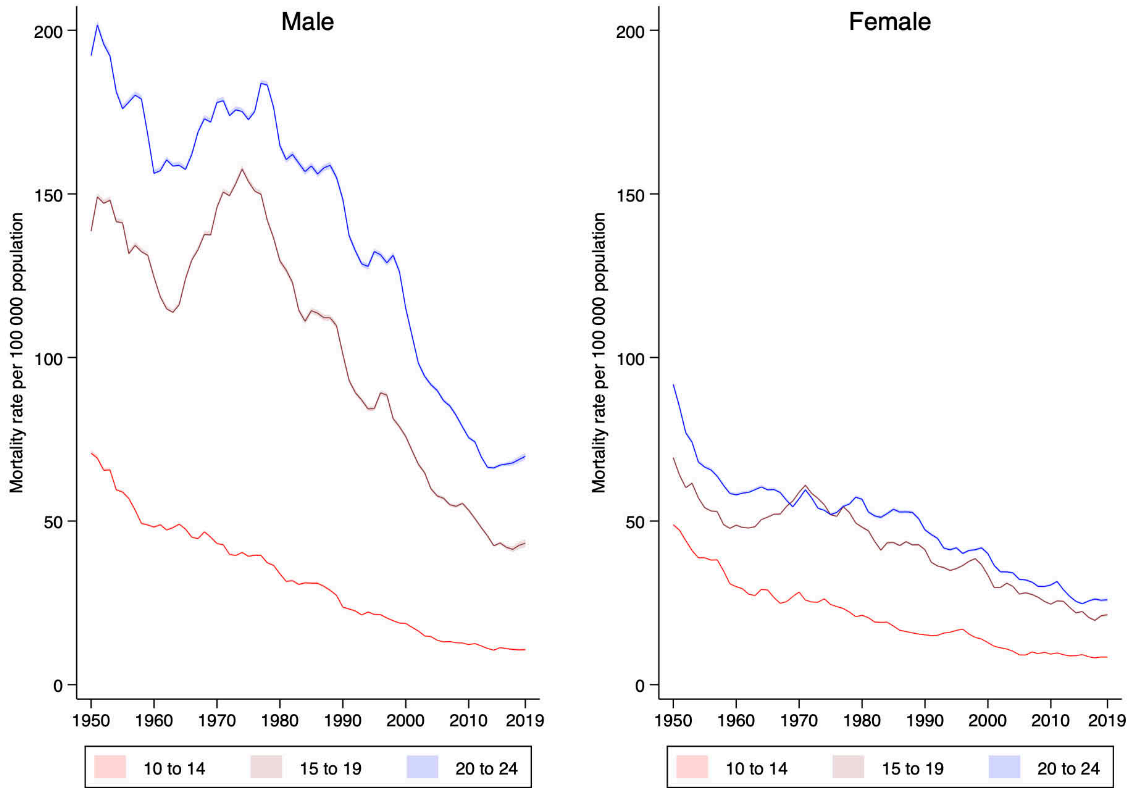

Figure S68b Number of deaths from all causes in 10-24 1950 – 2019: Australasia

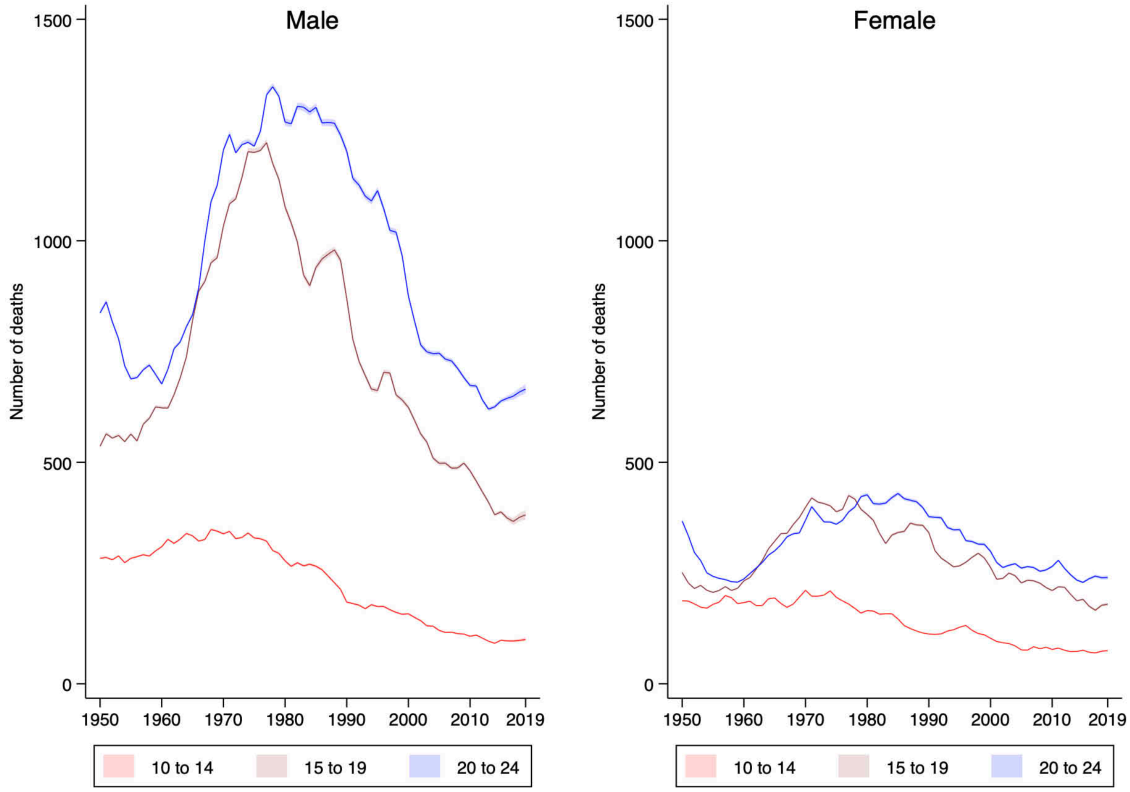

Figure S68c Mortality rate per 100,000 in 10-24 1980 – 2019 by cause: Australasia

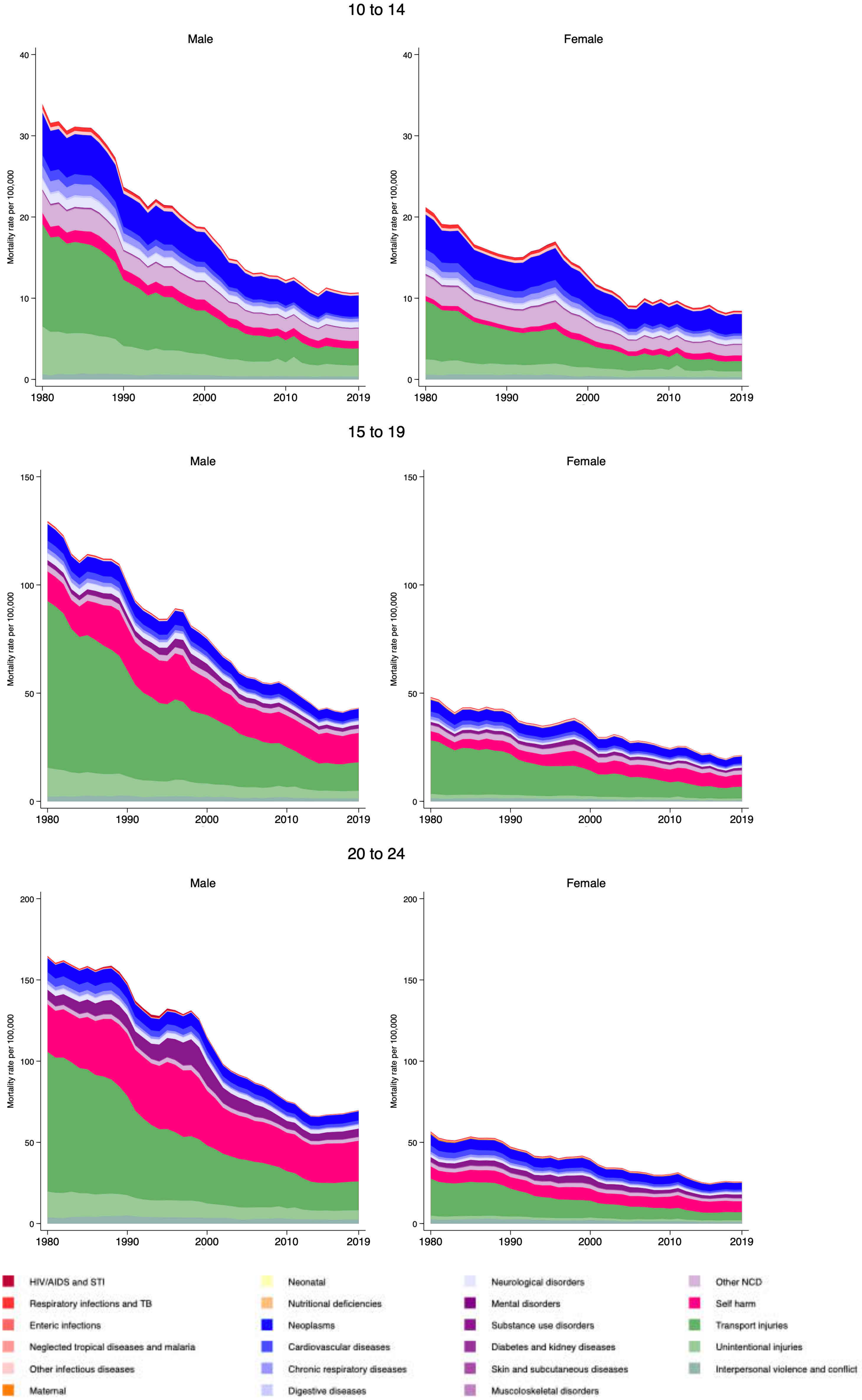

Figure S68d Number of deaths in 10-24 1980 – 2019 by cause: Australasia

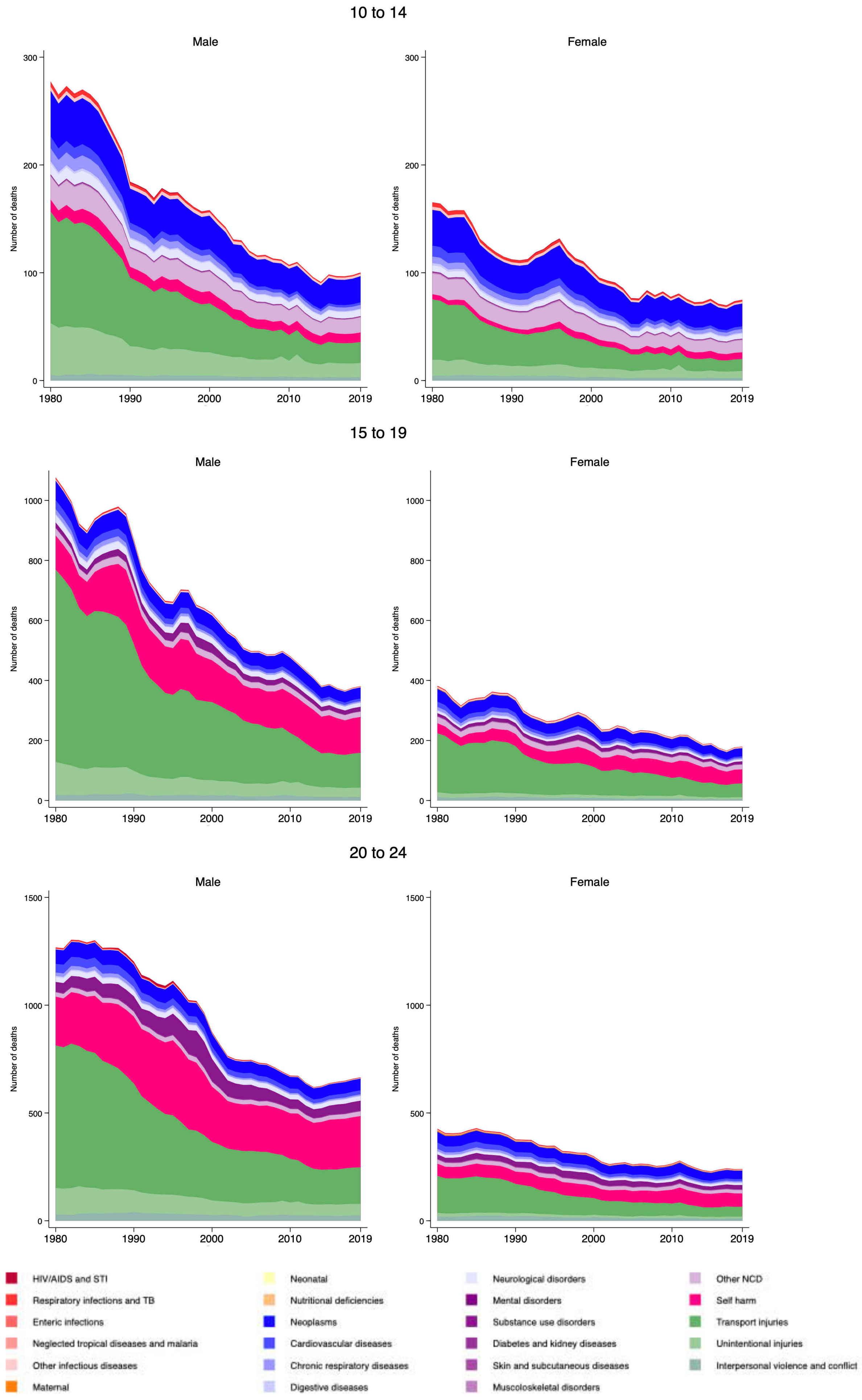

Figure S69: Australia

Figure S69a All-cause mortality rate per 100,000 in 10-24 1950 – 2019: Australia

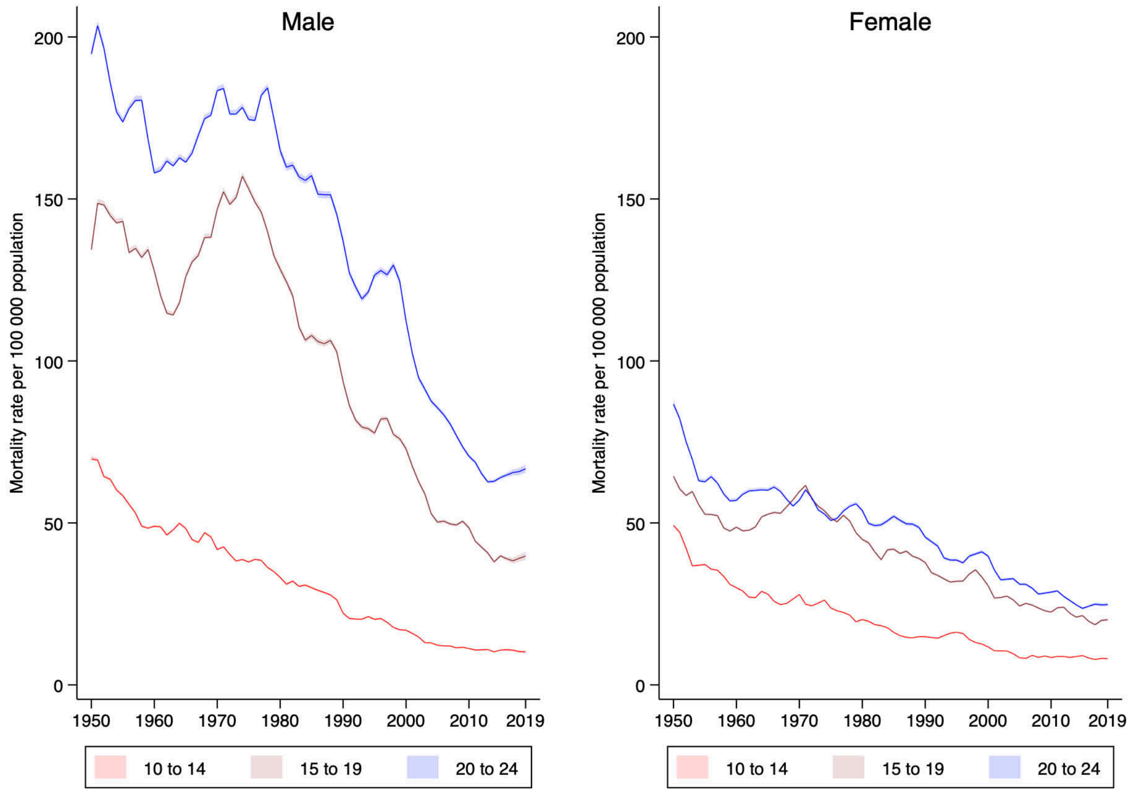

Figure S69b Number of deaths from all causes in 10-24 1950 – 2019: Australia

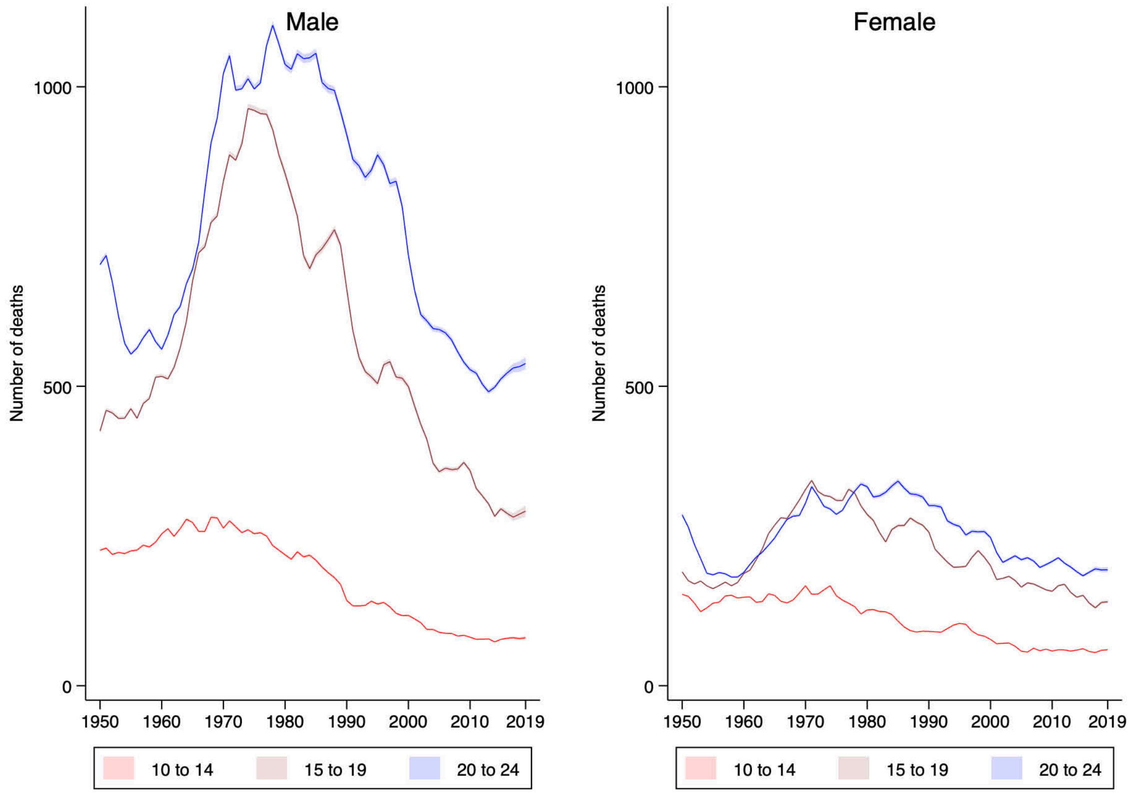

Figure S69c Mortality rate per 100,000 in 10-24 1980 – 2019 by cause: Australia

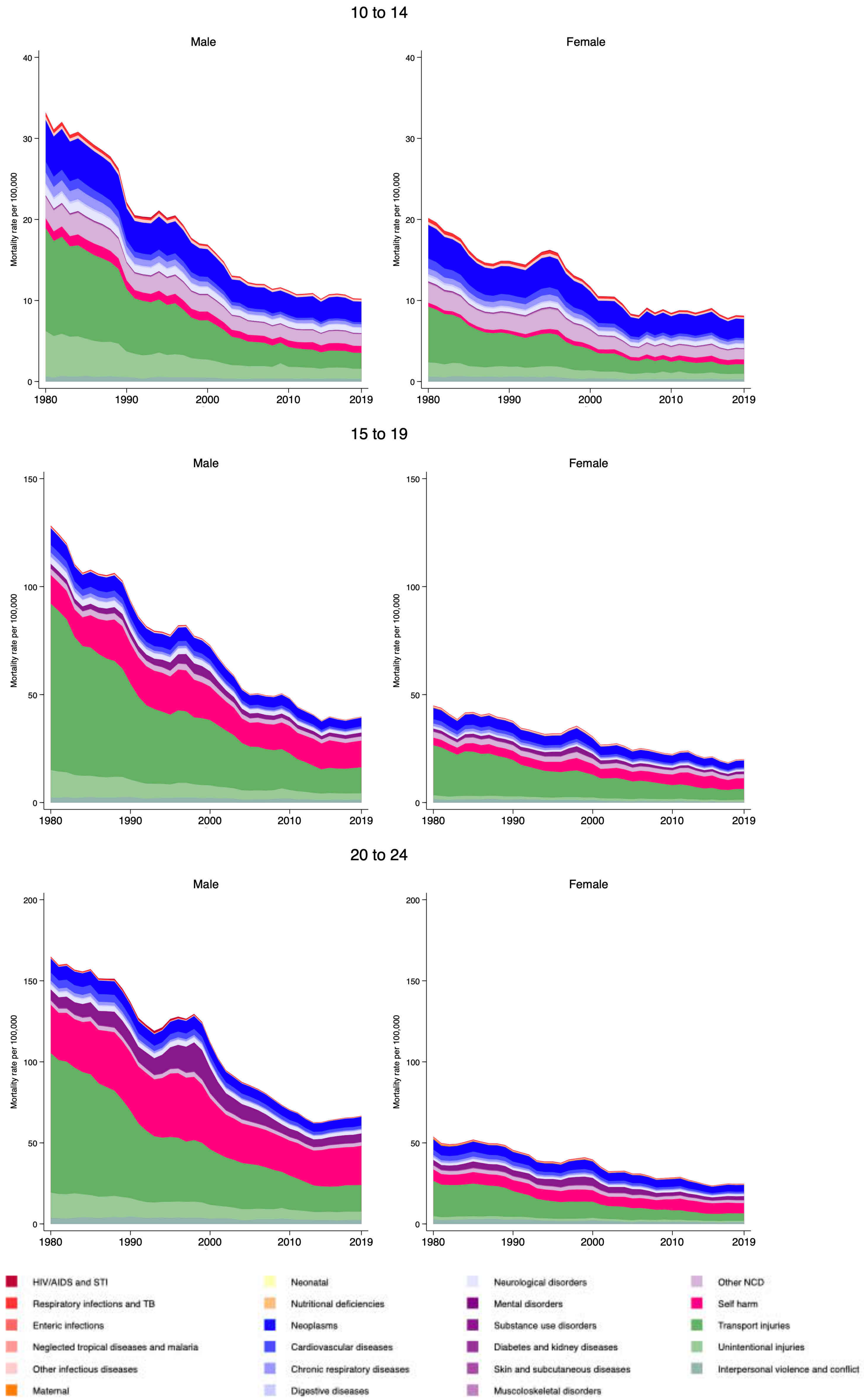

Figure S69d Number of deaths in 10-24 1980 – 2019 by cause: Australia

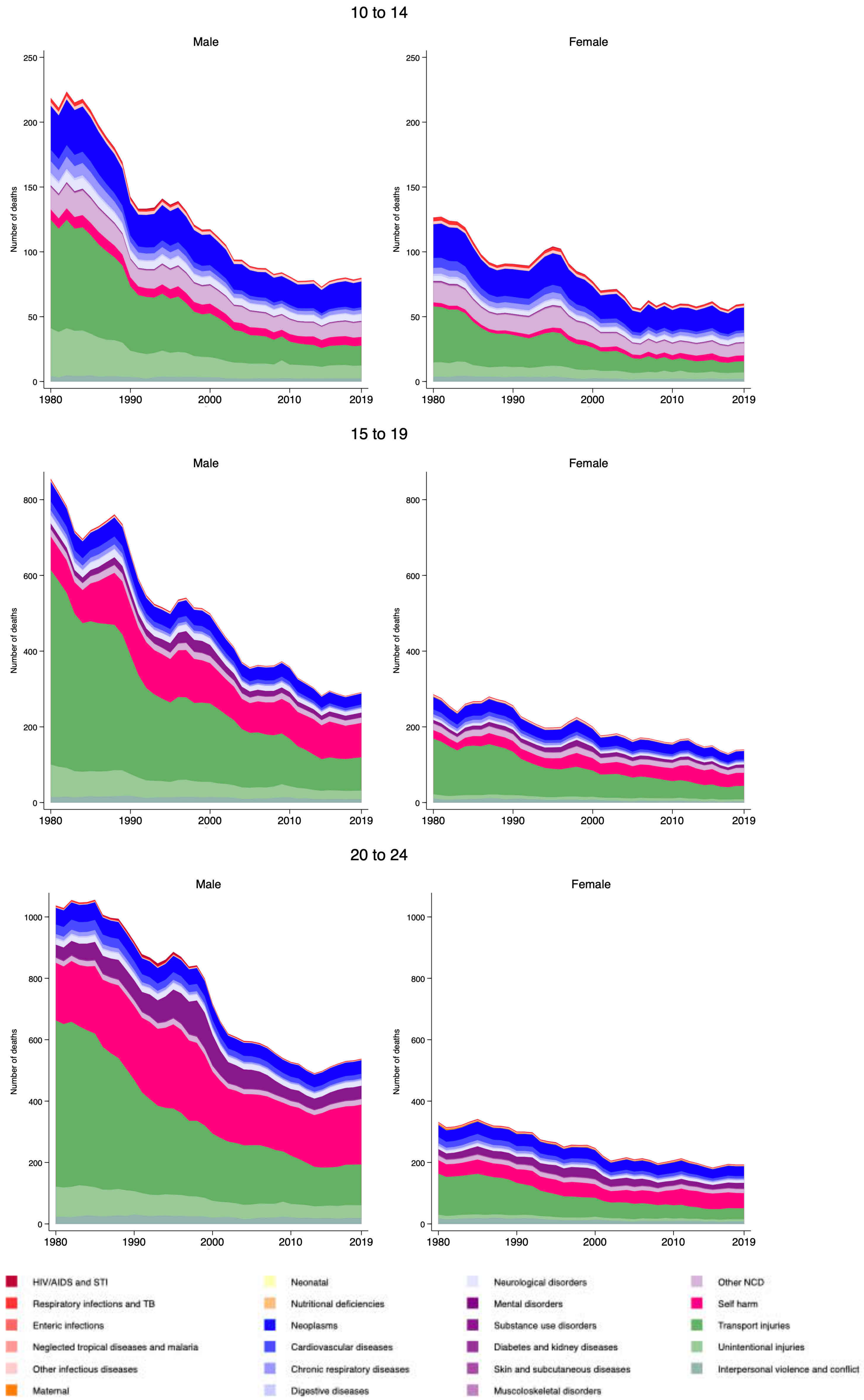

Figure S70: New Zealand

Figure S70a All-cause mortality rate per 100,000 in 10-24 1950 – 2019: New Zealand

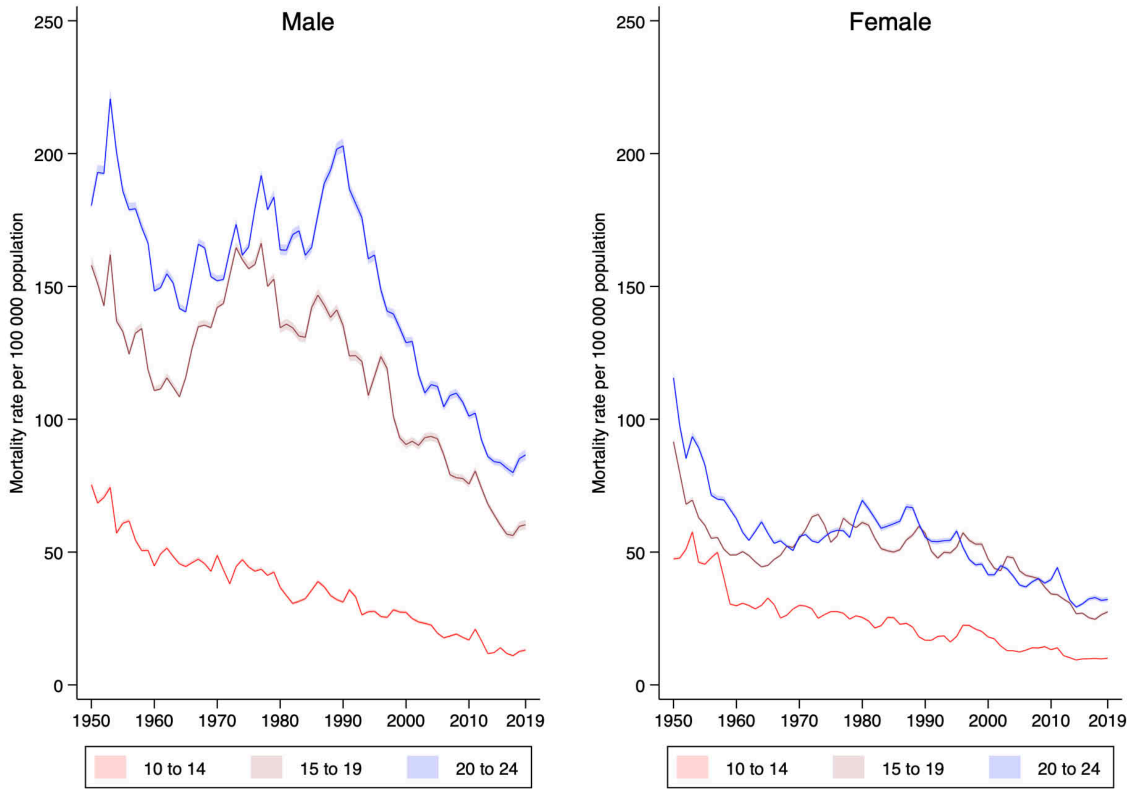

Figure S70b Number of deaths from all causes in 10-24 1950 – 2019: New Zealand

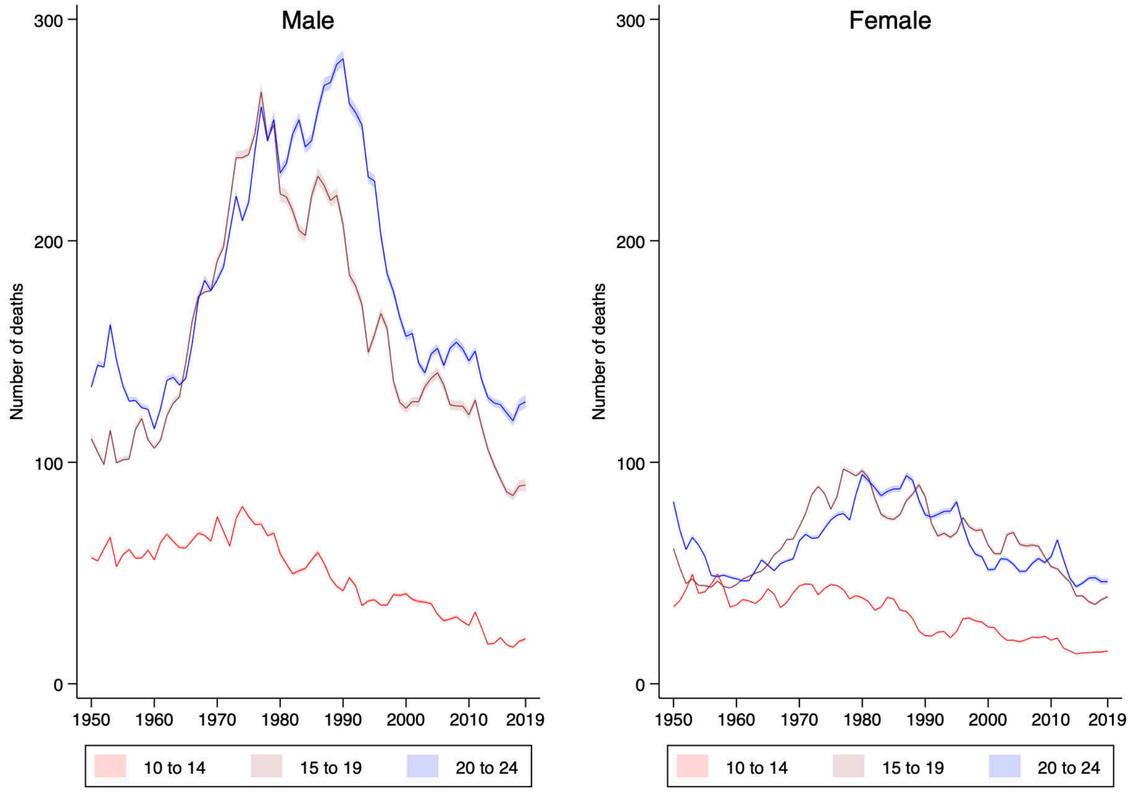

Figure S70c Mortality rate per 100,000 in 10-24 1980 – 2019 by cause: New Zealand

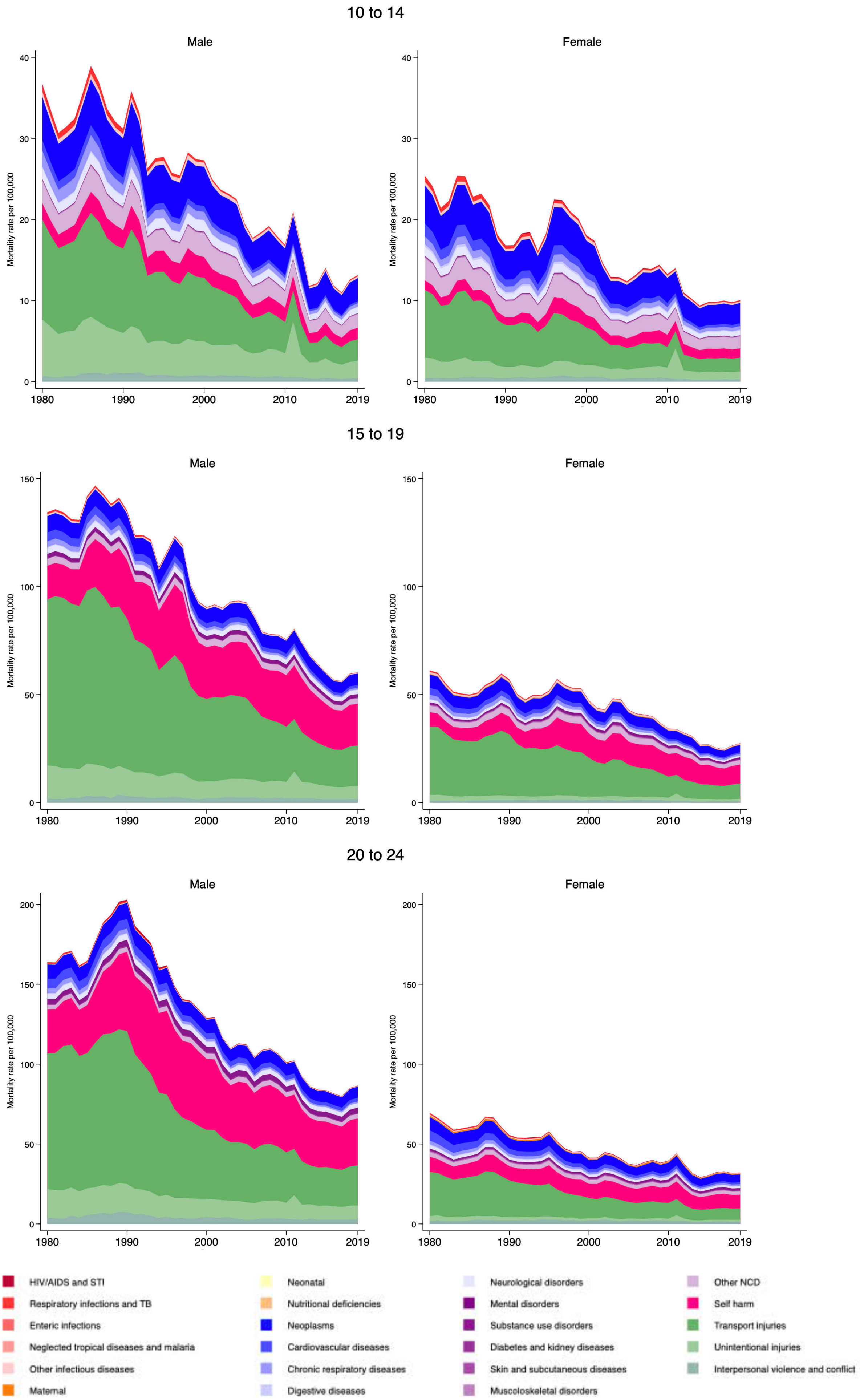

Figure S70d Number of deaths in 10-24 1980 – 2019 by cause: New Zealand

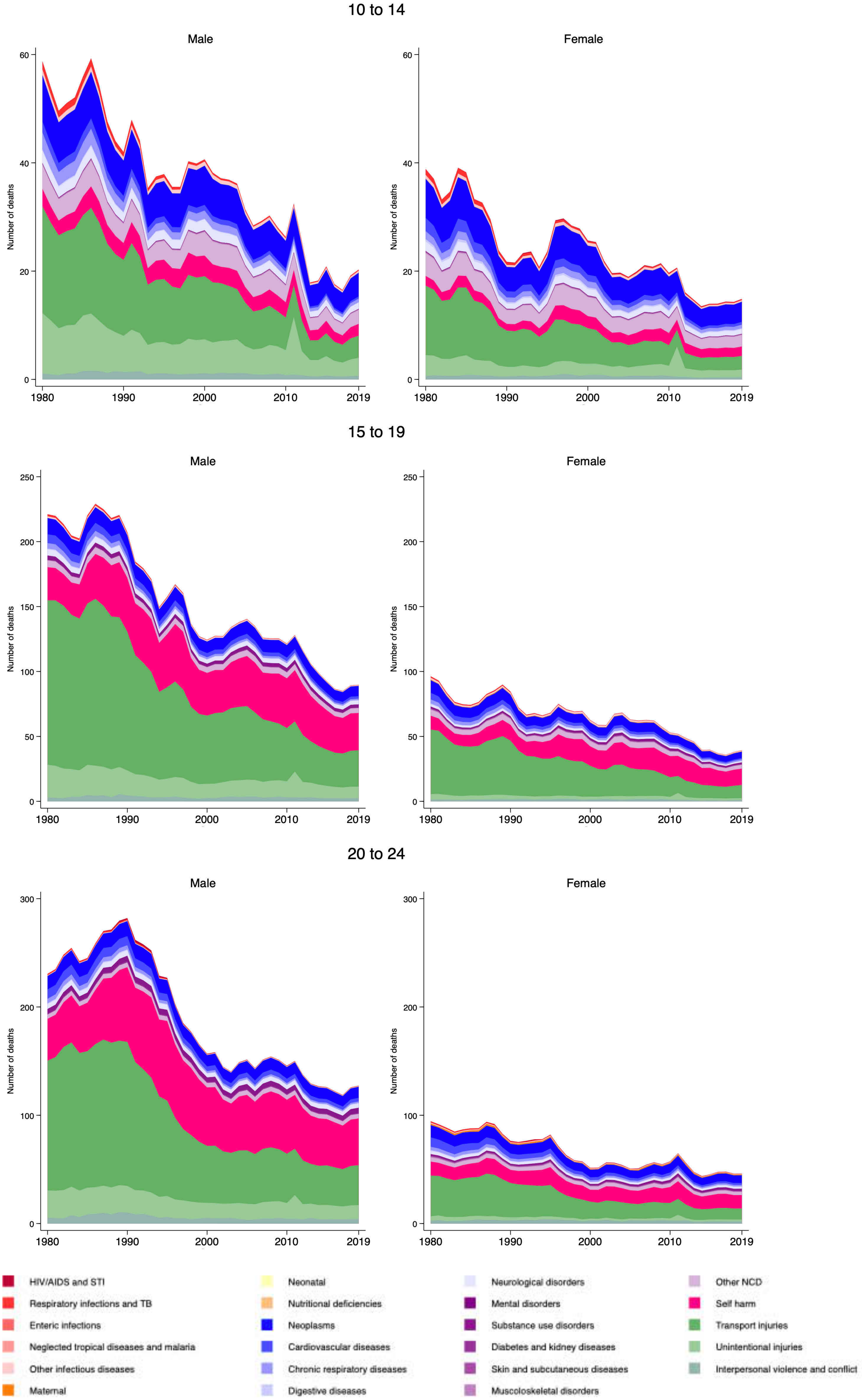

Figure S71: Western Europe

Figure S71a All-cause mortality rate per 100,000 in 10-24 1950 – 2019: Western Europe

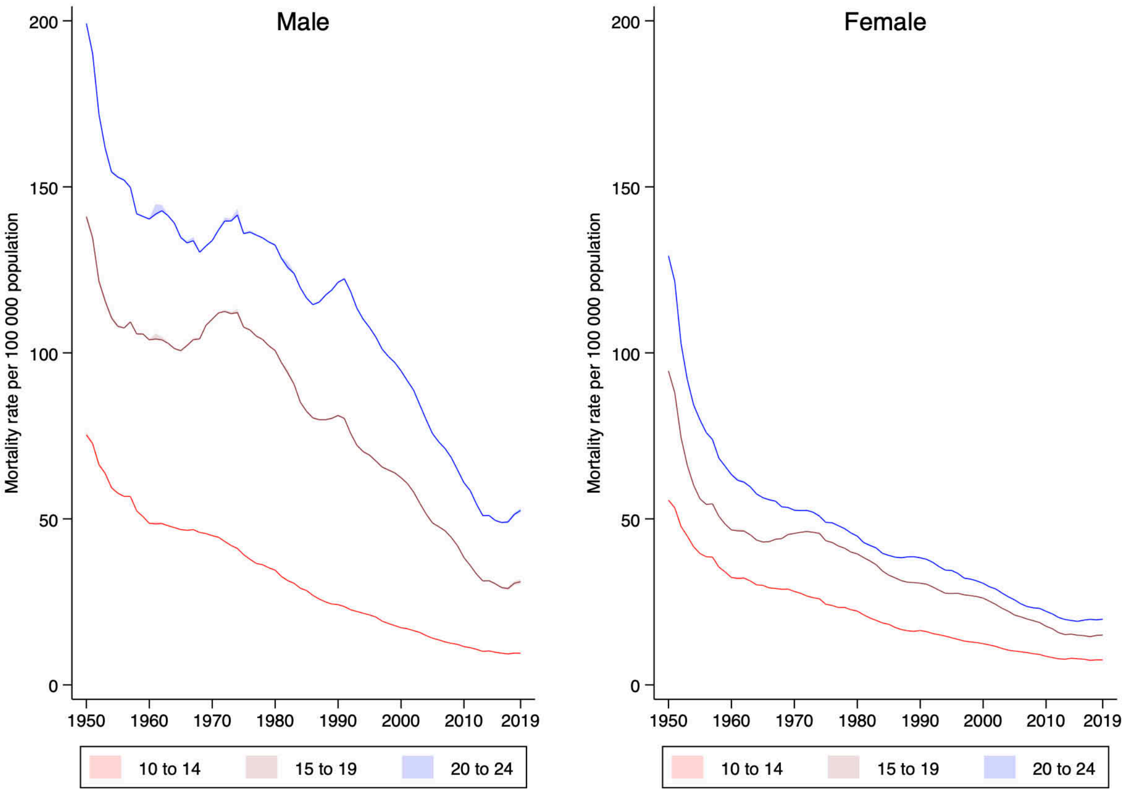

Figure S71b Number of deaths from all causes in 10-24 1950 – 2019: Western Europe

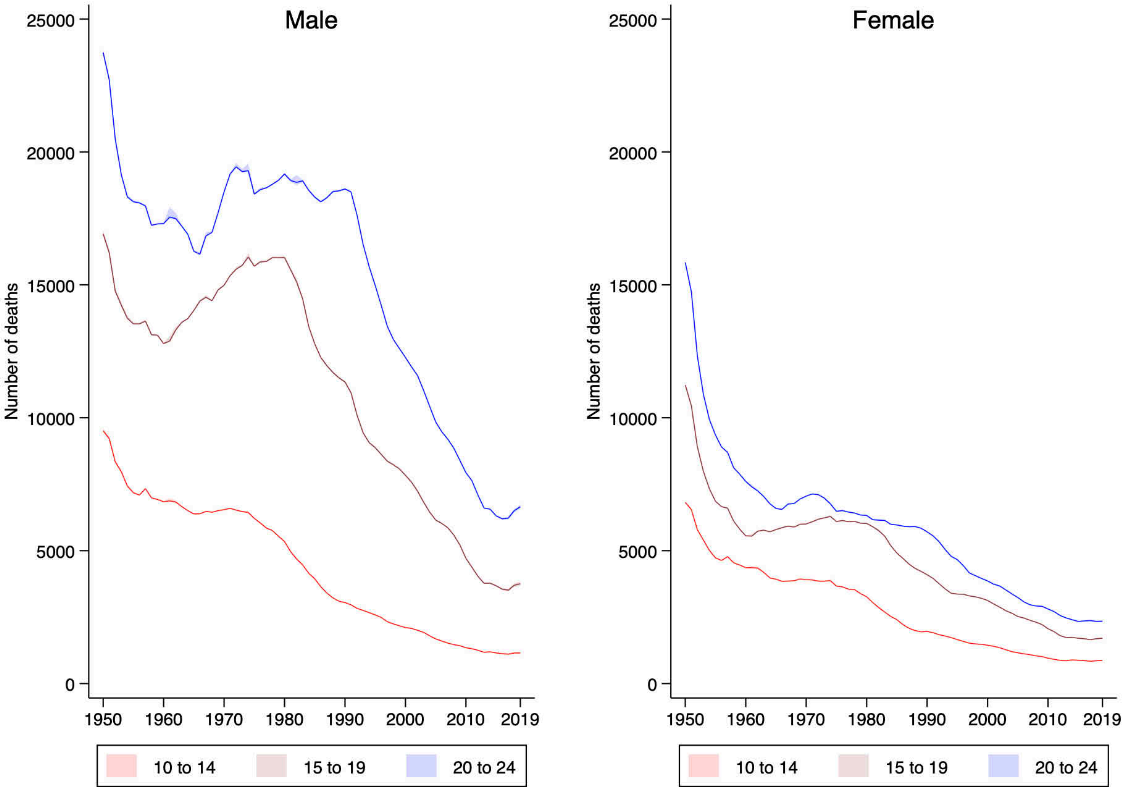

Figure S71c Mortality rate per 100,000 in 10-24 1980 – 2019 by cause: Western Europe

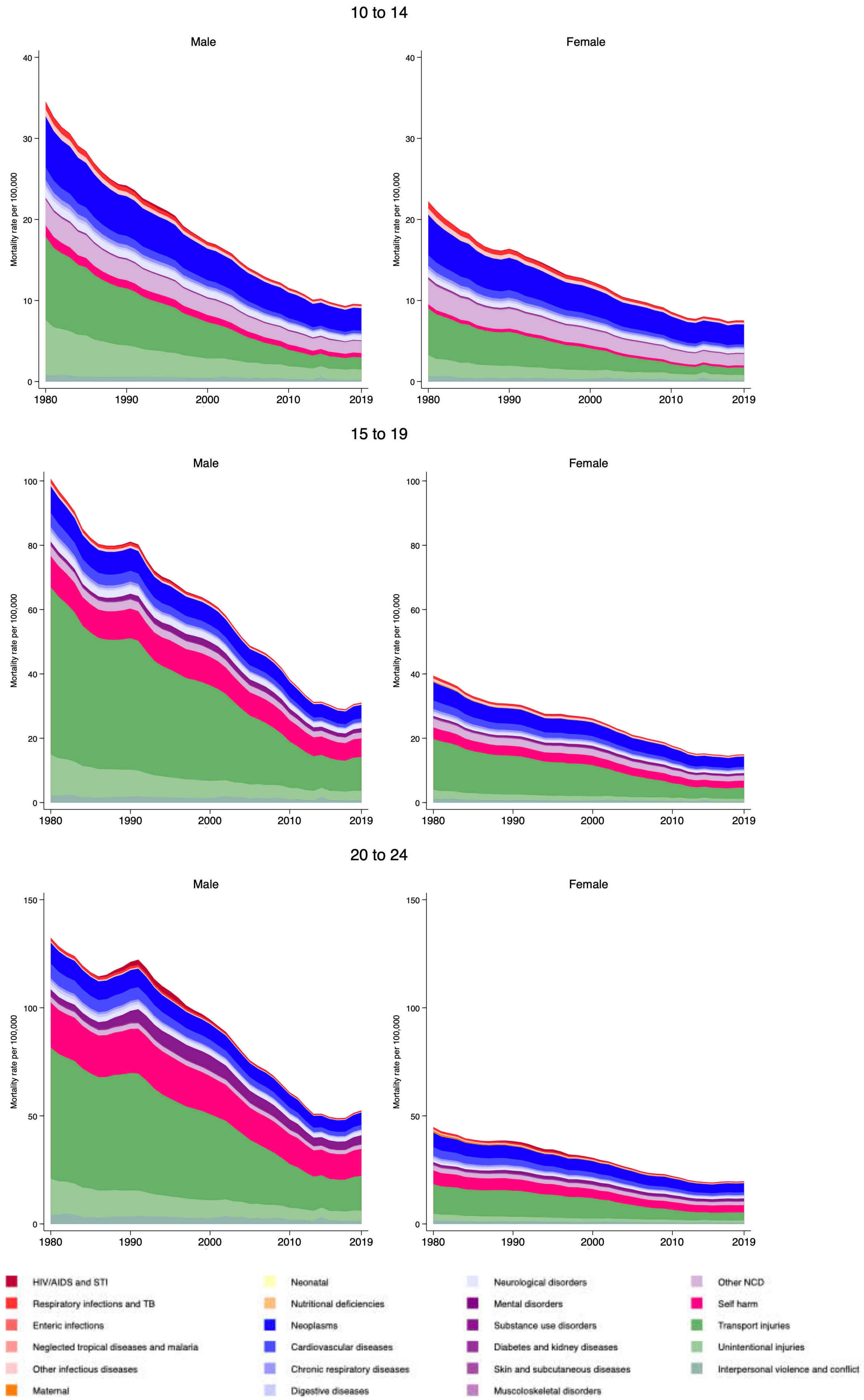

Figure S71d Number of deaths in 10-24 1980 – 2019 by cause: Western Europe

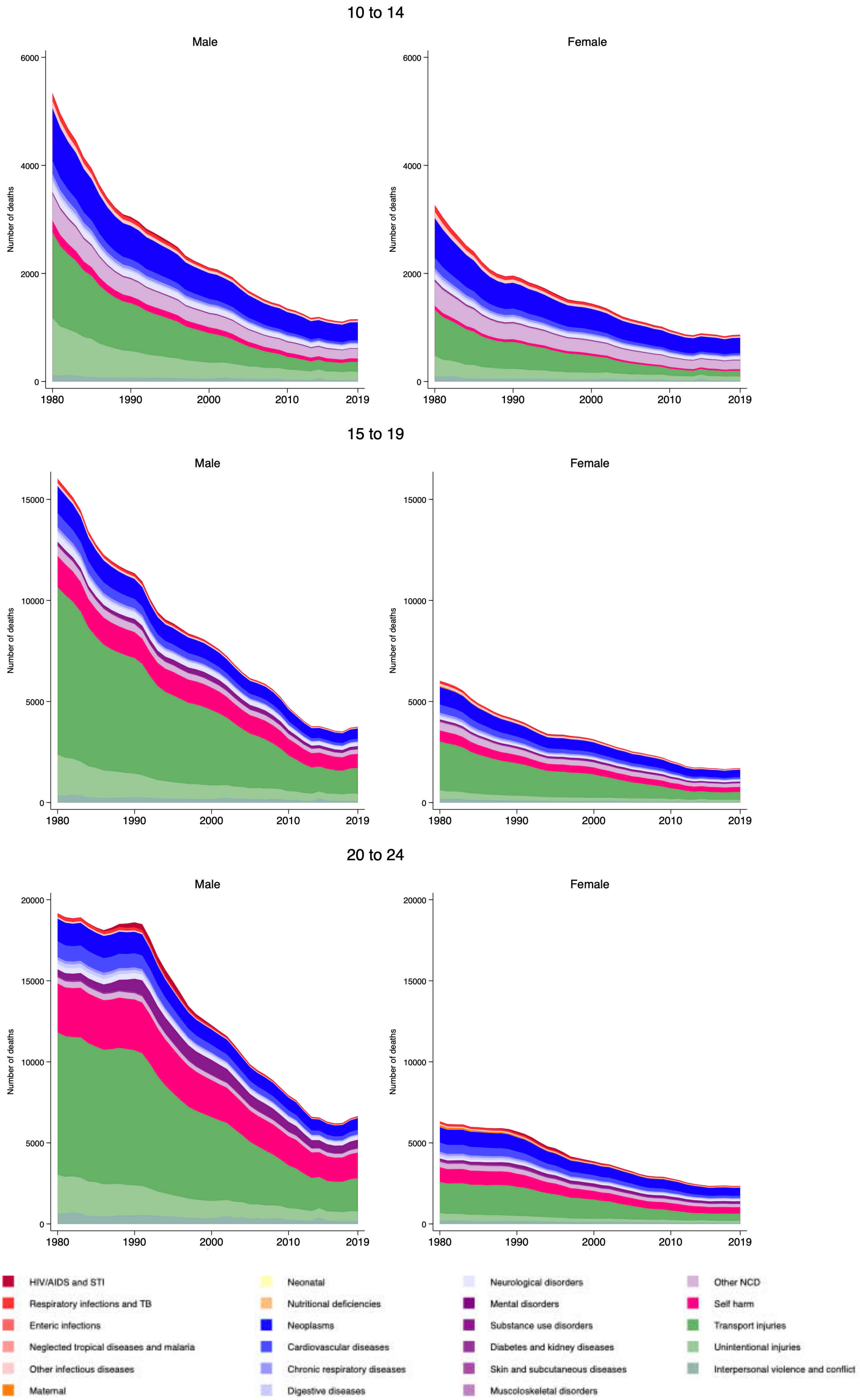

Figure S72: Andorra

Figure S72a All-cause mortality rate per 100,000 in 10-24 1950 – 2019: Andorra

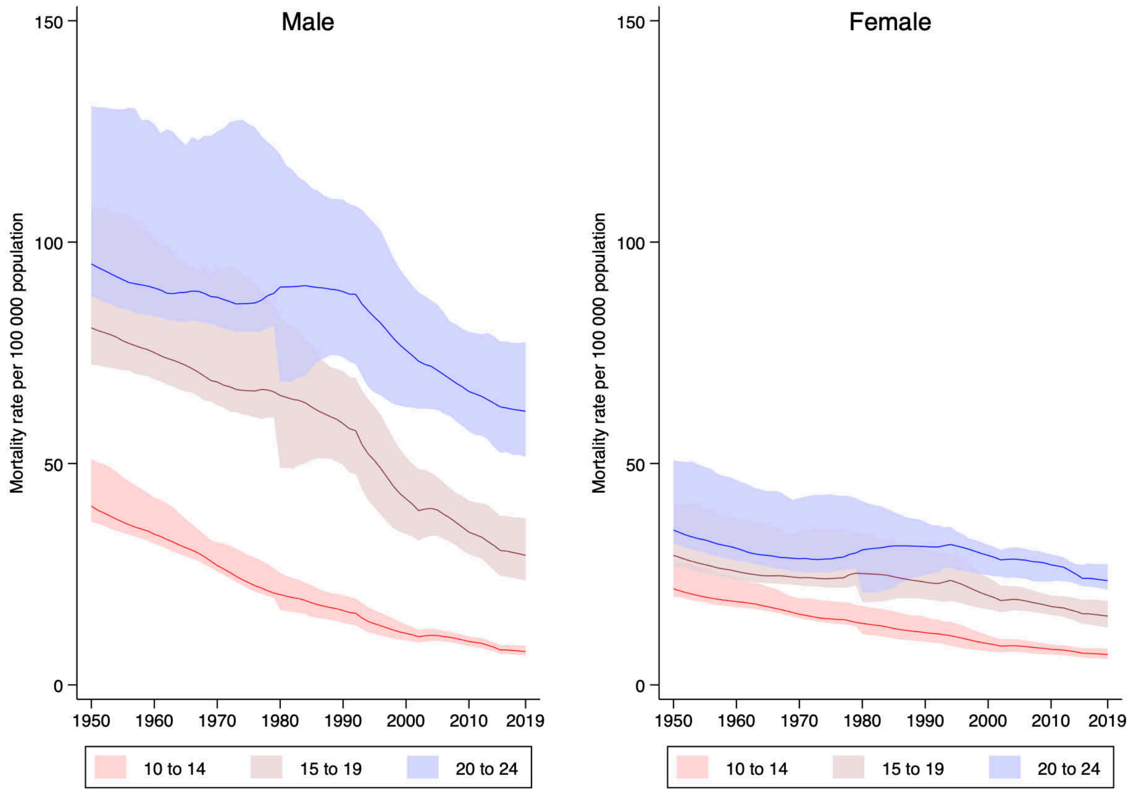

Figure S72b Number of deaths from all causes in 10-24 1950 – 2019: Andorra

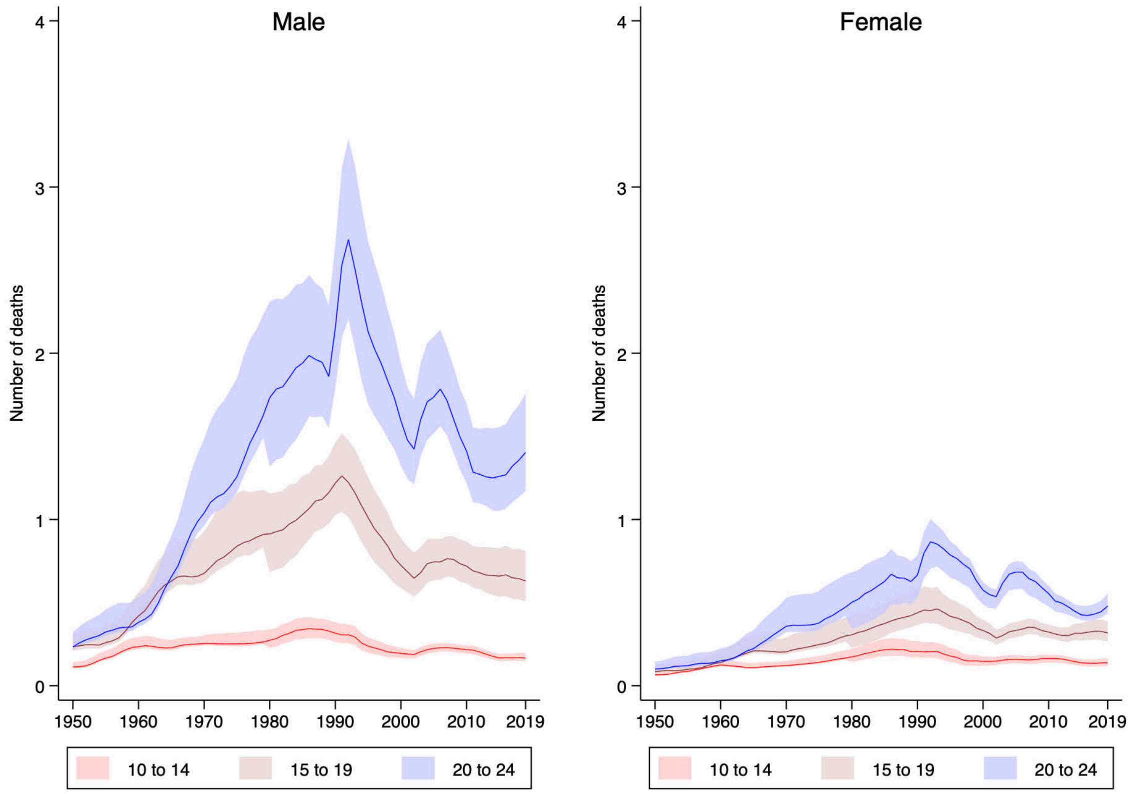

Figure S72c Mortality rate per 100,000 in 10-24 1980 – 2019 by cause: Andorra

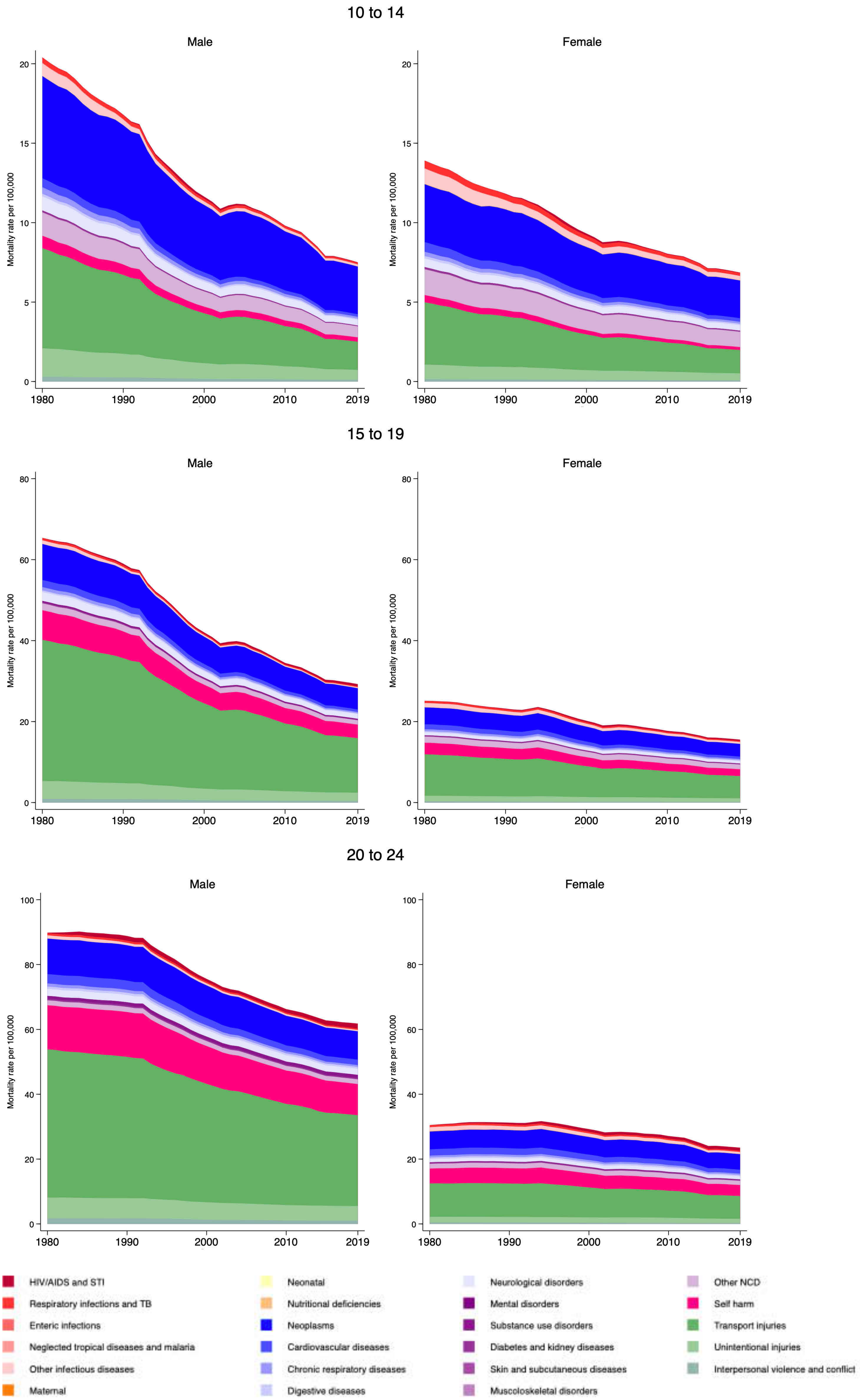

Figure S72d Number of deaths in 10-24 1980 – 2019 by cause: Andorra

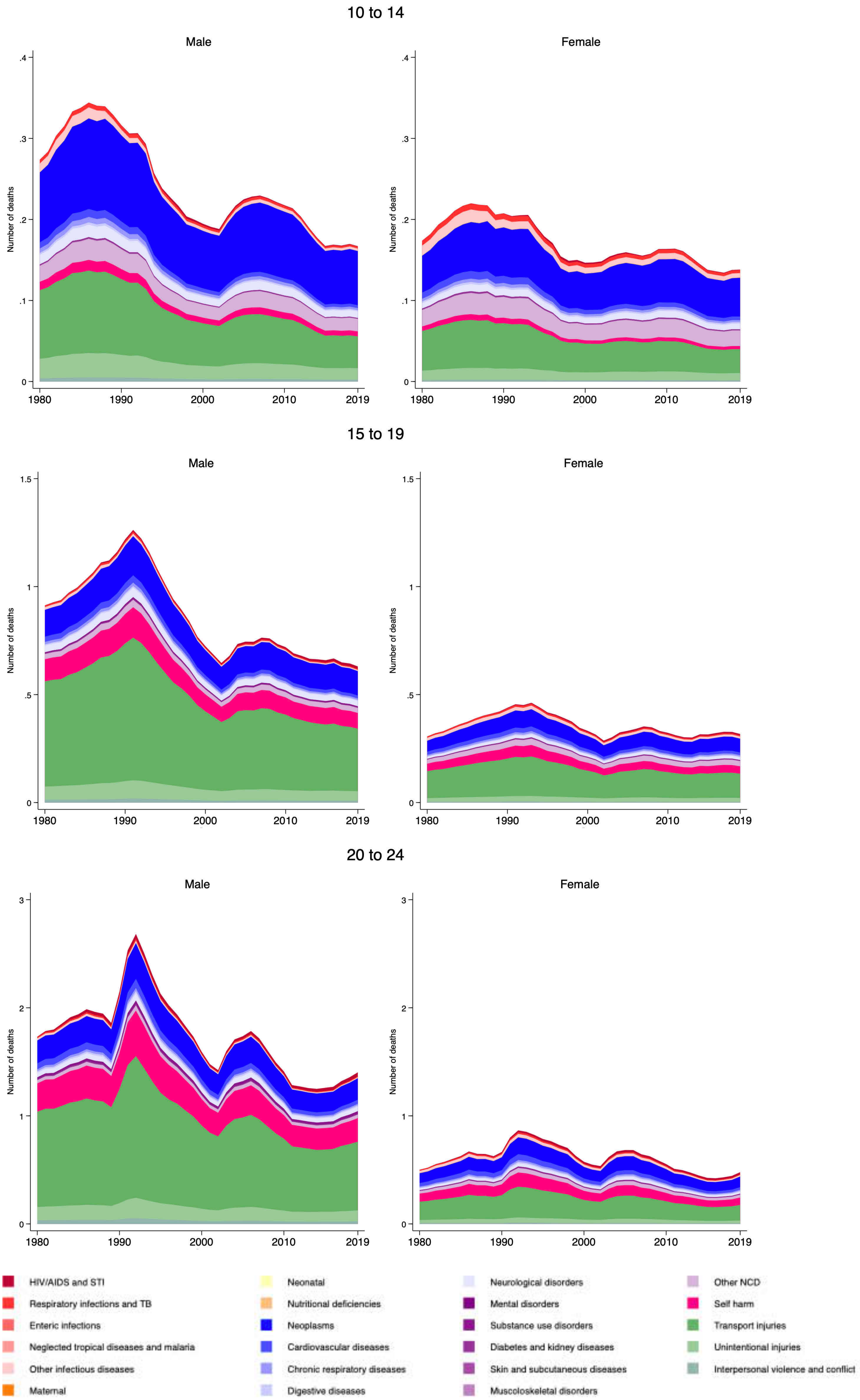

Figure S73: Austria

Figure S73a All-cause mortality rate per 100,000 in 10-24 1950 – 2019: Austria

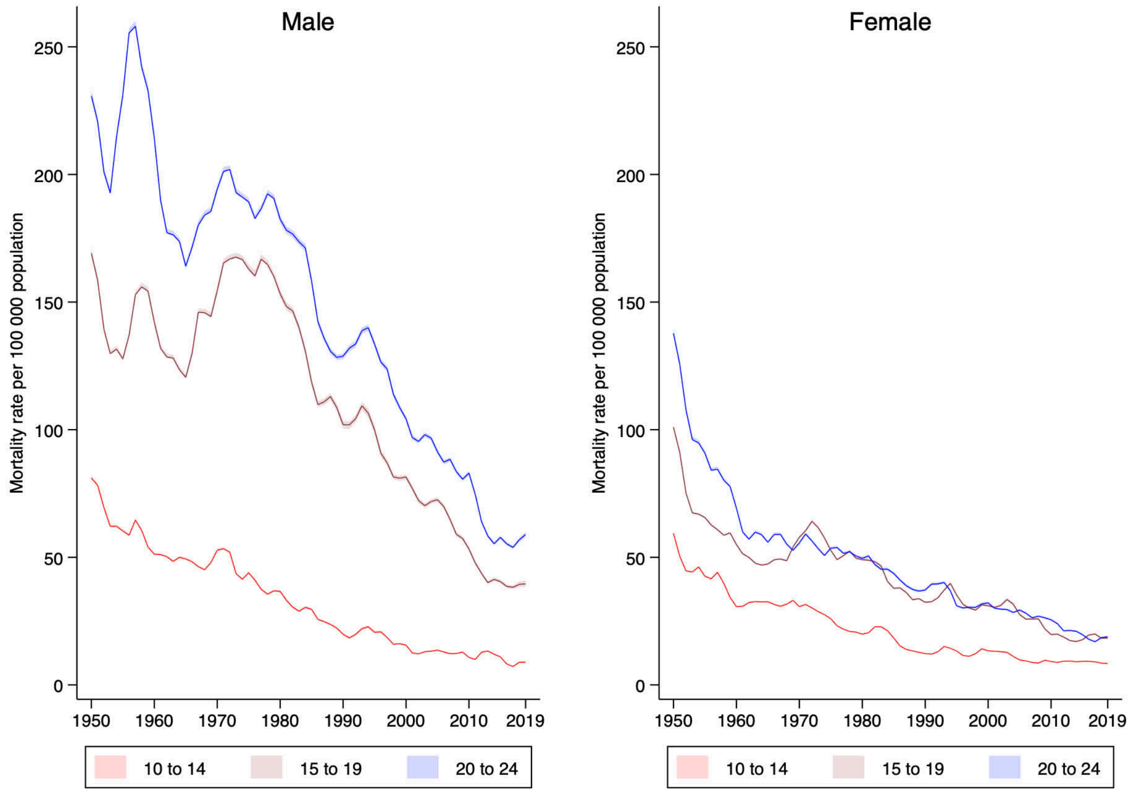

Figure S73b Number of deaths from all causes in 10-24 1950 – 2019: Austria

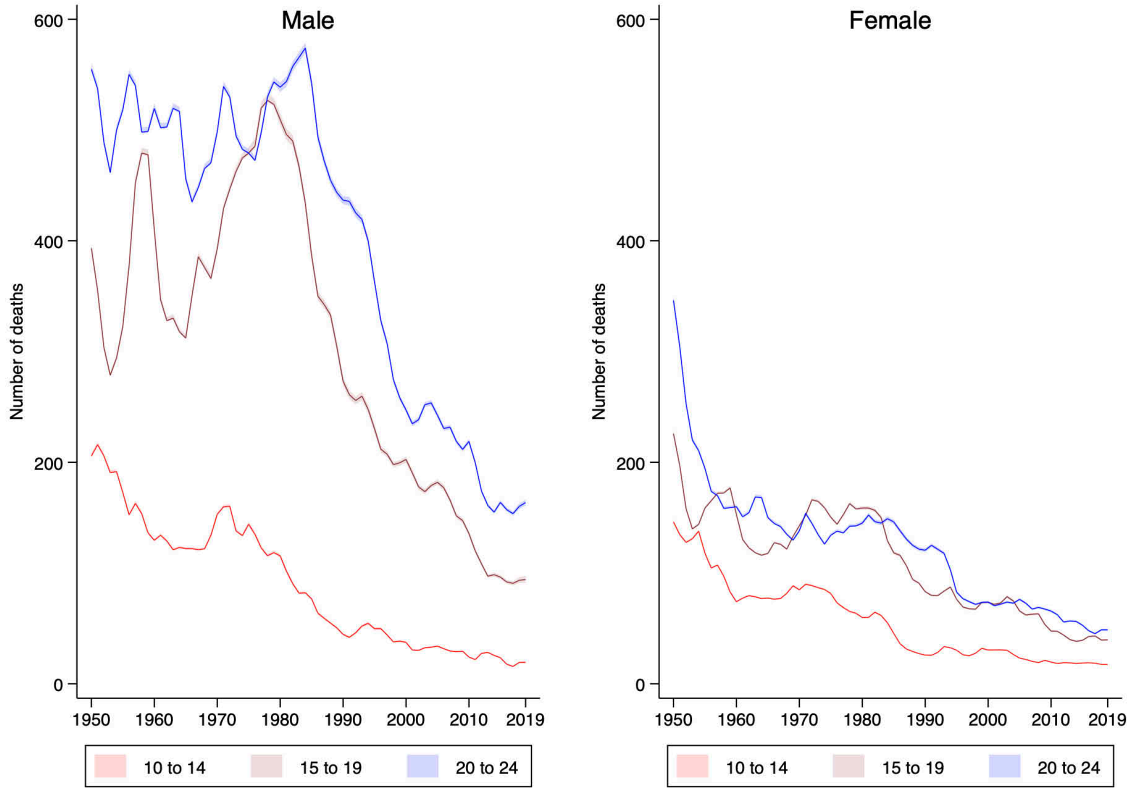

Figure S73c Mortality rate per 100,000 in 10-24 1980 – 2019 by cause: Austria

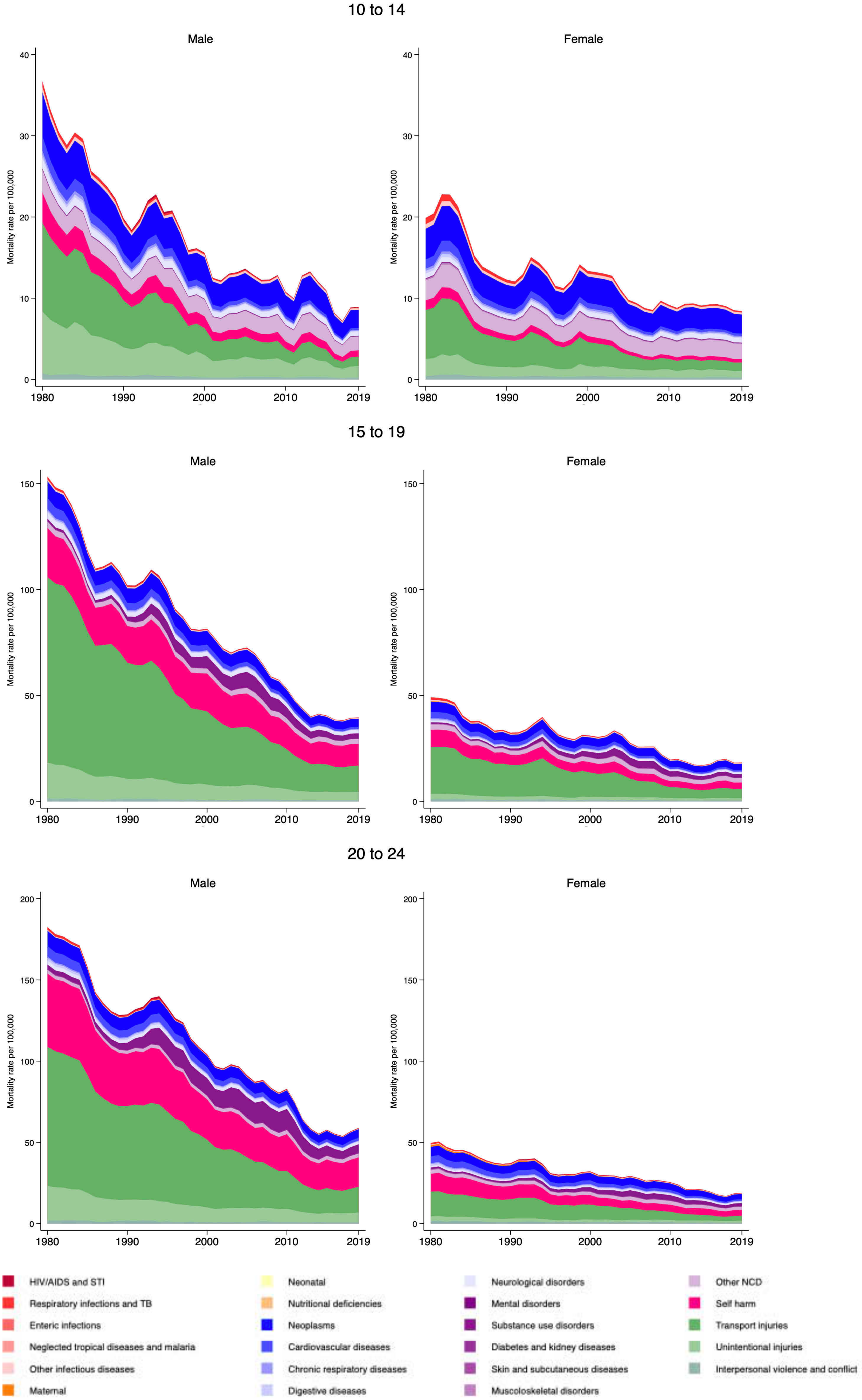

Figure S73d Number of deaths in 10-24 1980 – 2019 by cause: Austria

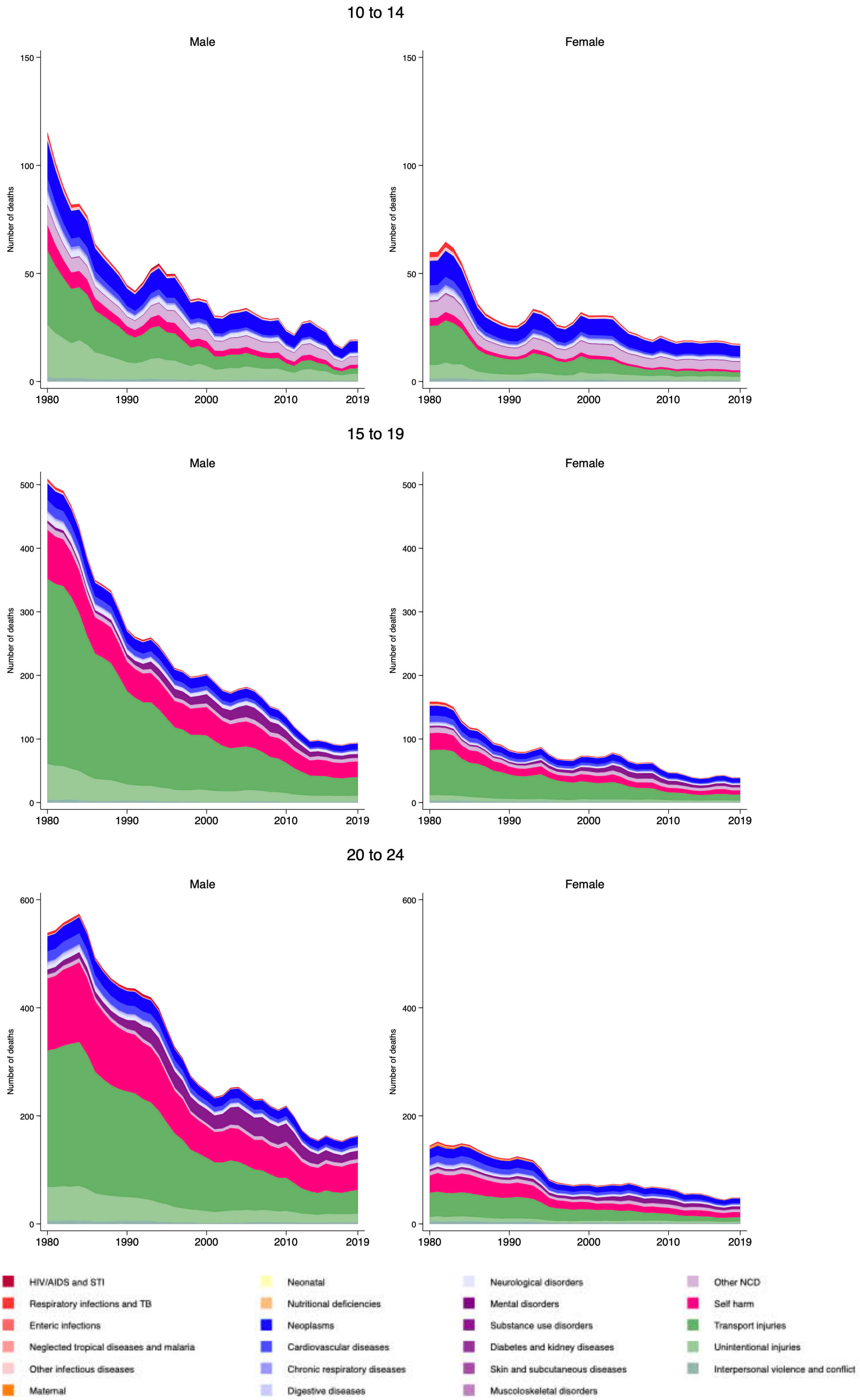

Figure S74: Belgium

Figure S74a All-cause mortality rate per 100,000 in 10-24 1950 – 2019: Belgium

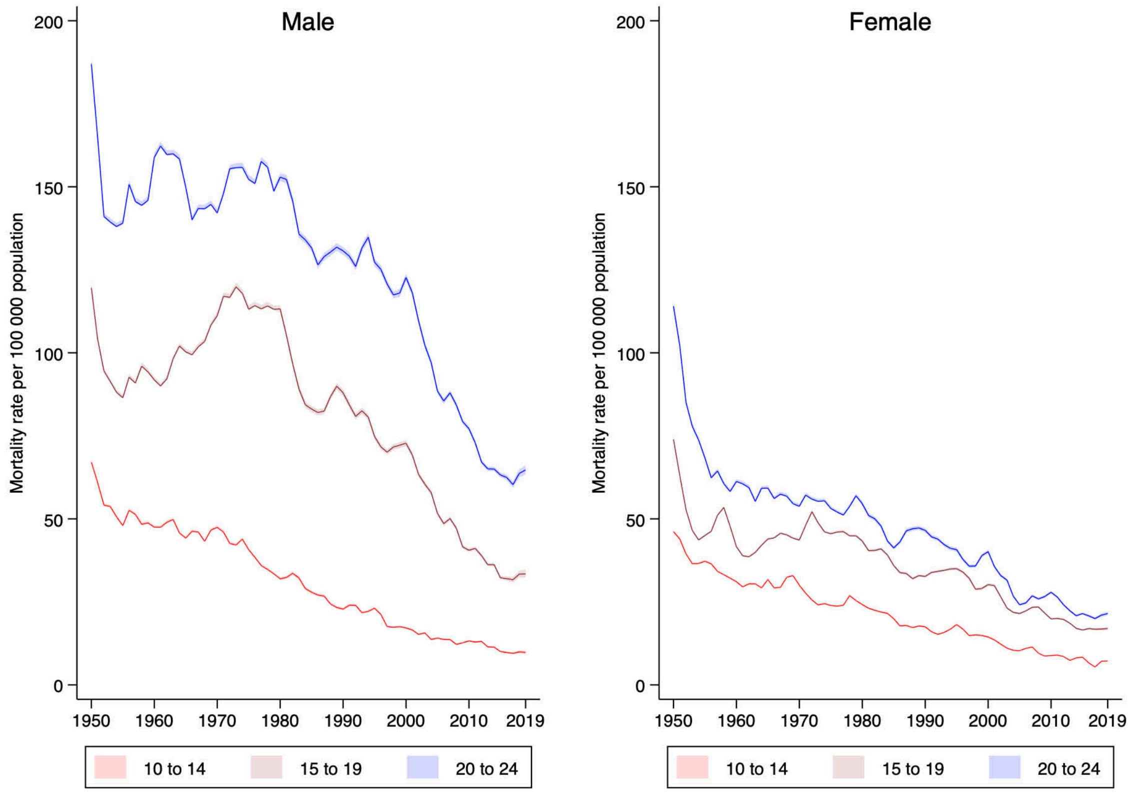

Figure S74b Number of deaths from all causes in 10-24 1950 – 2019: Belgium

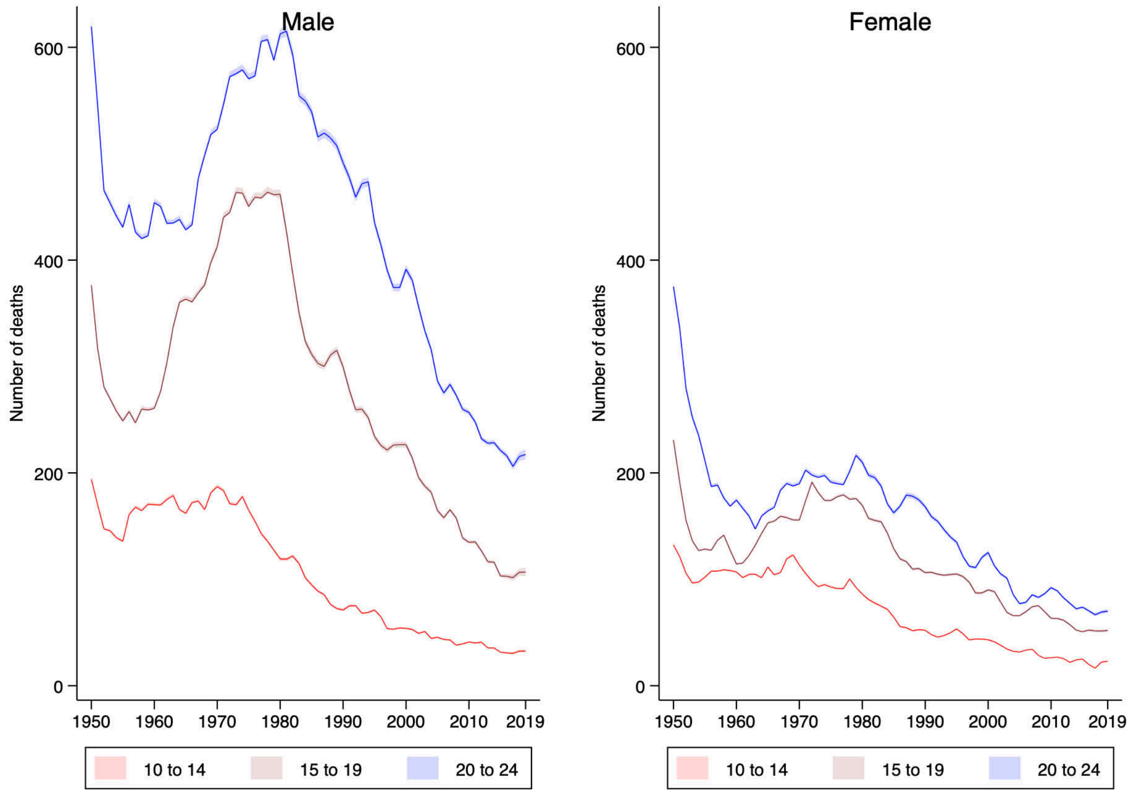

Figure S74c Mortality rate per 100,000 in 10-24 1980 – 2019 by cause: Belgium

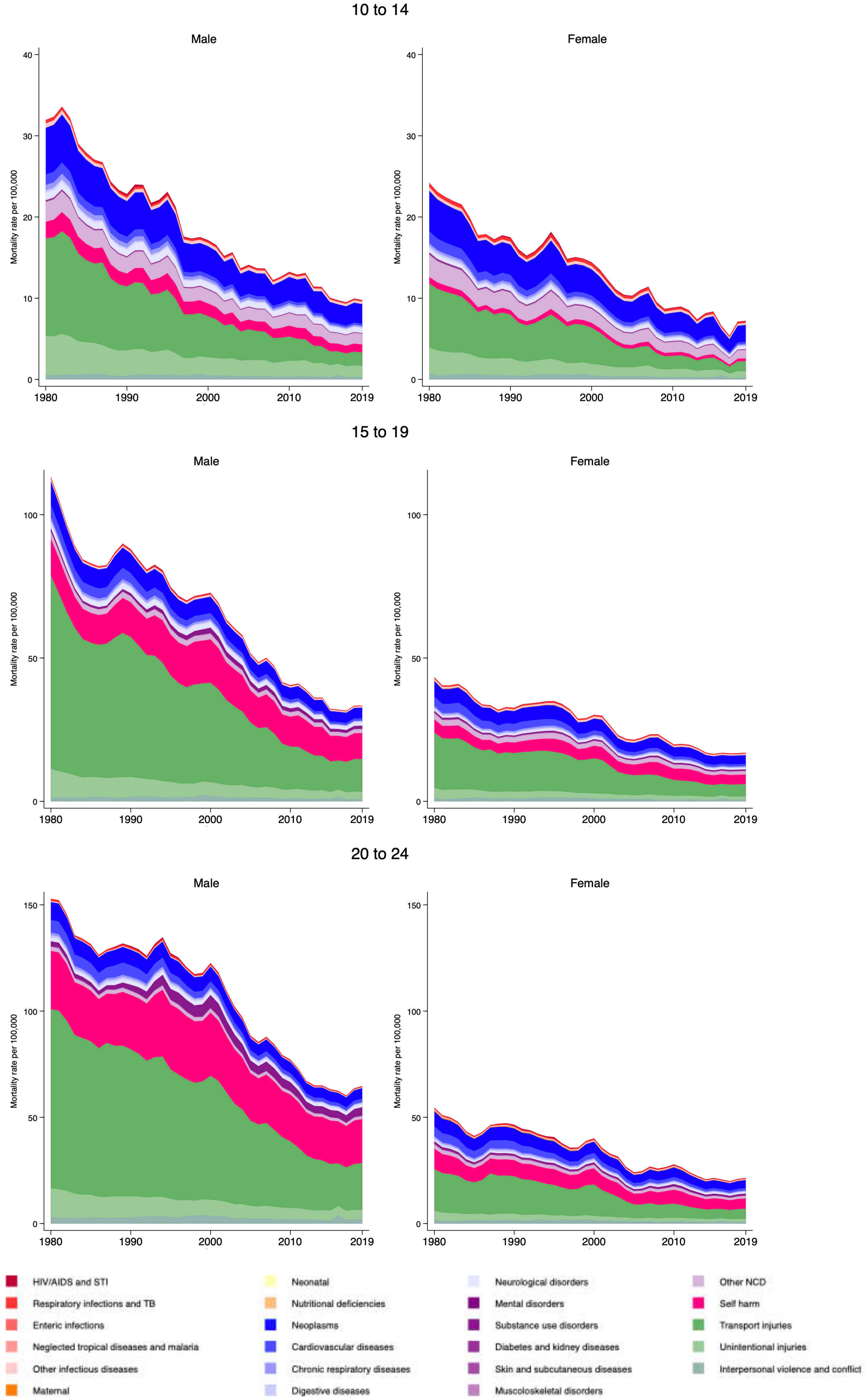

Figure S74d Number of deaths in 10-24 1980 – 2019 by cause: Belgium

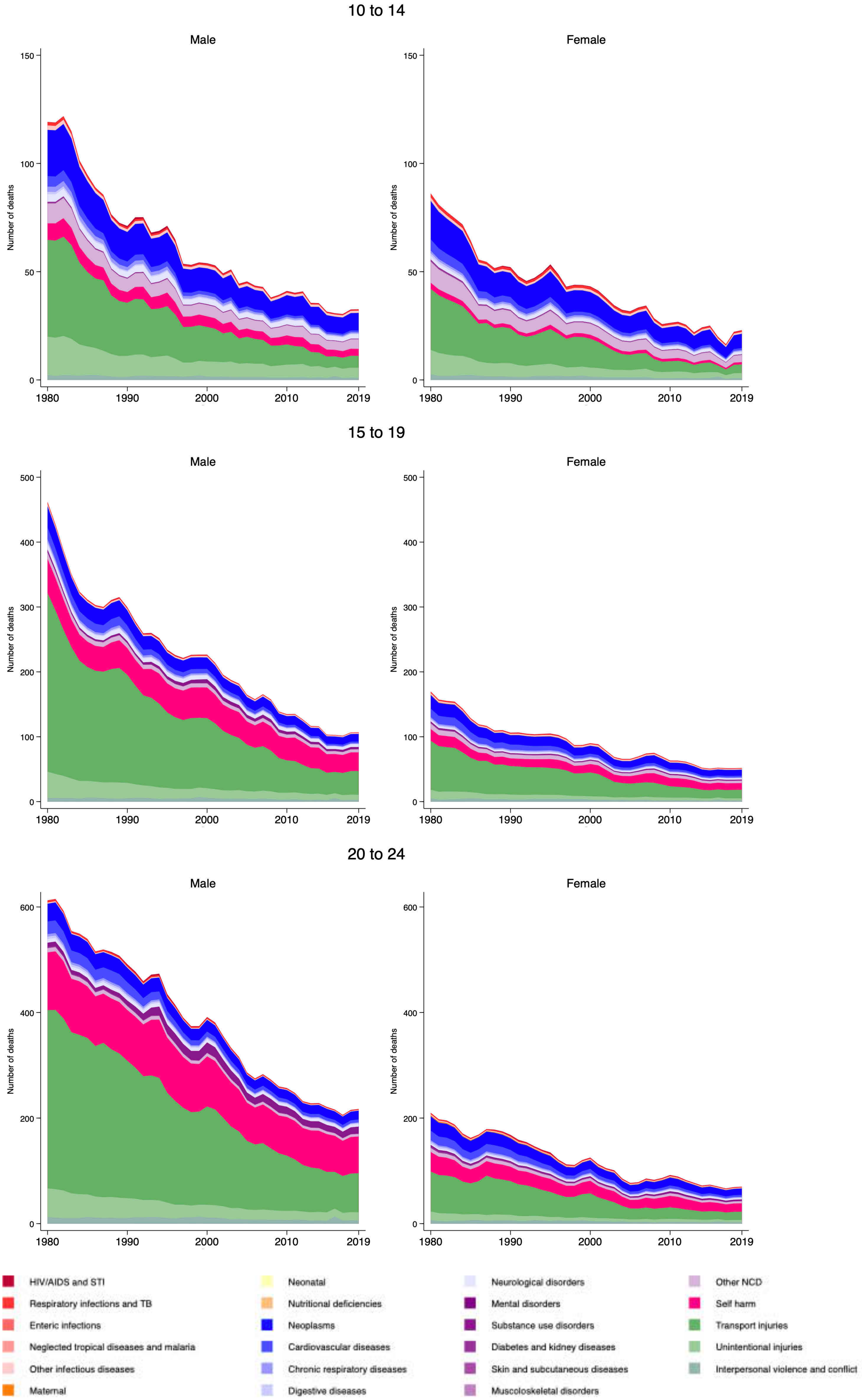

Figure S75: Cyprus

Figure S75a All-cause mortality rate per 100,000 in 10-24 1950 – 2019: Cyprus

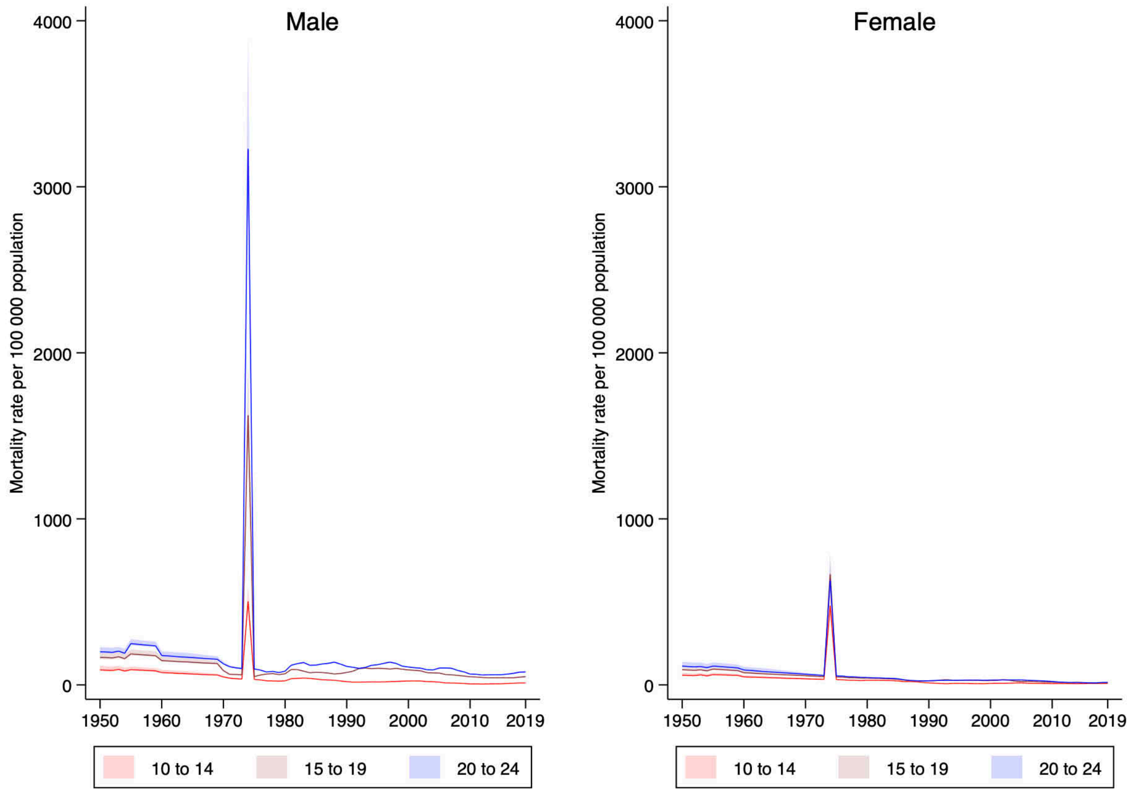

Figure S75b Number of deaths from all causes in 10-24 1950 – 2019: Cyprus

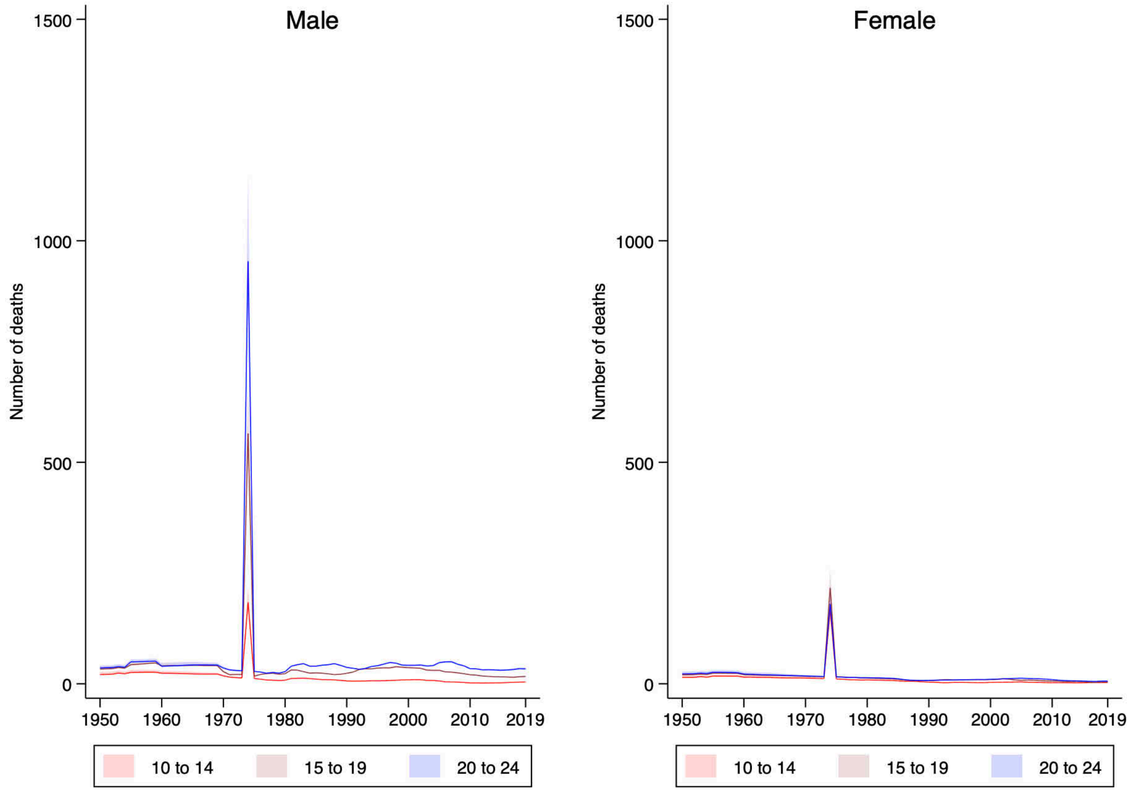

Figure S75c Mortality rate per 100,000 in 10-24 1980 – 2019 by cause: Cyprus

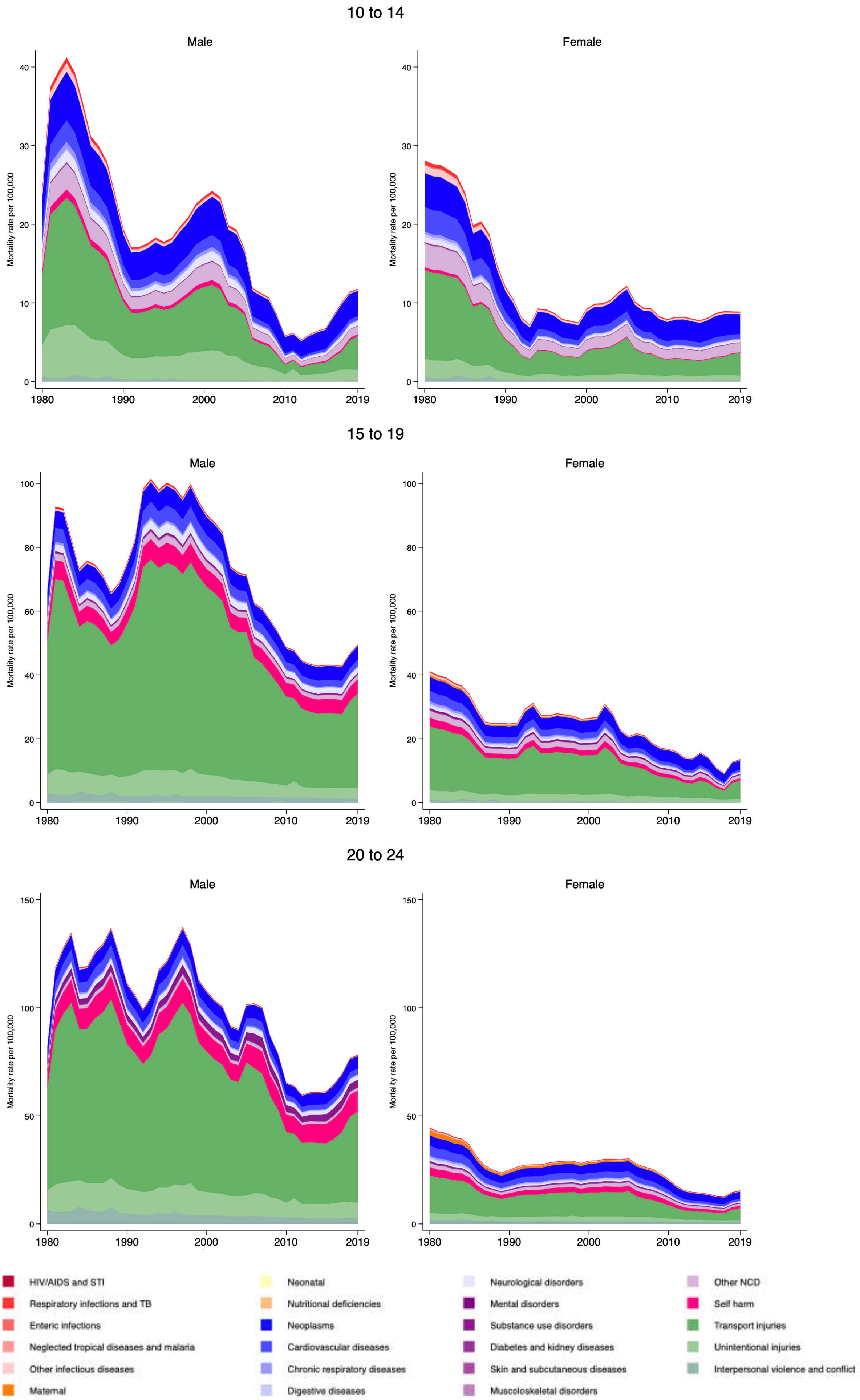

Figure S75d Number of deaths in 10-24 1980 – 2019 by cause: Cyprus

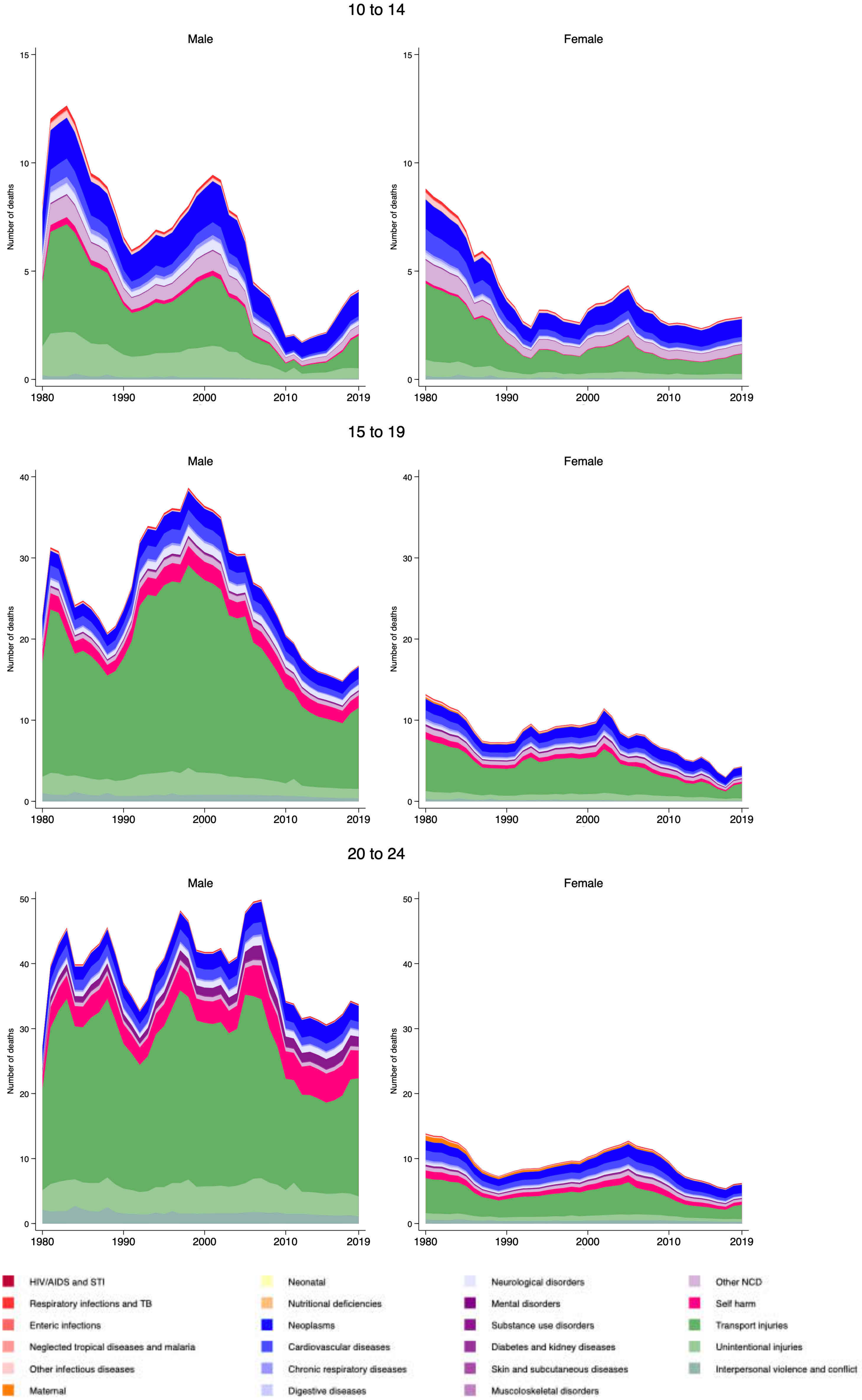

Figure S76: Denmark

Figure S76a All-cause mortality rate per 100,000 in 10-24 1950 – 2019: Denmark

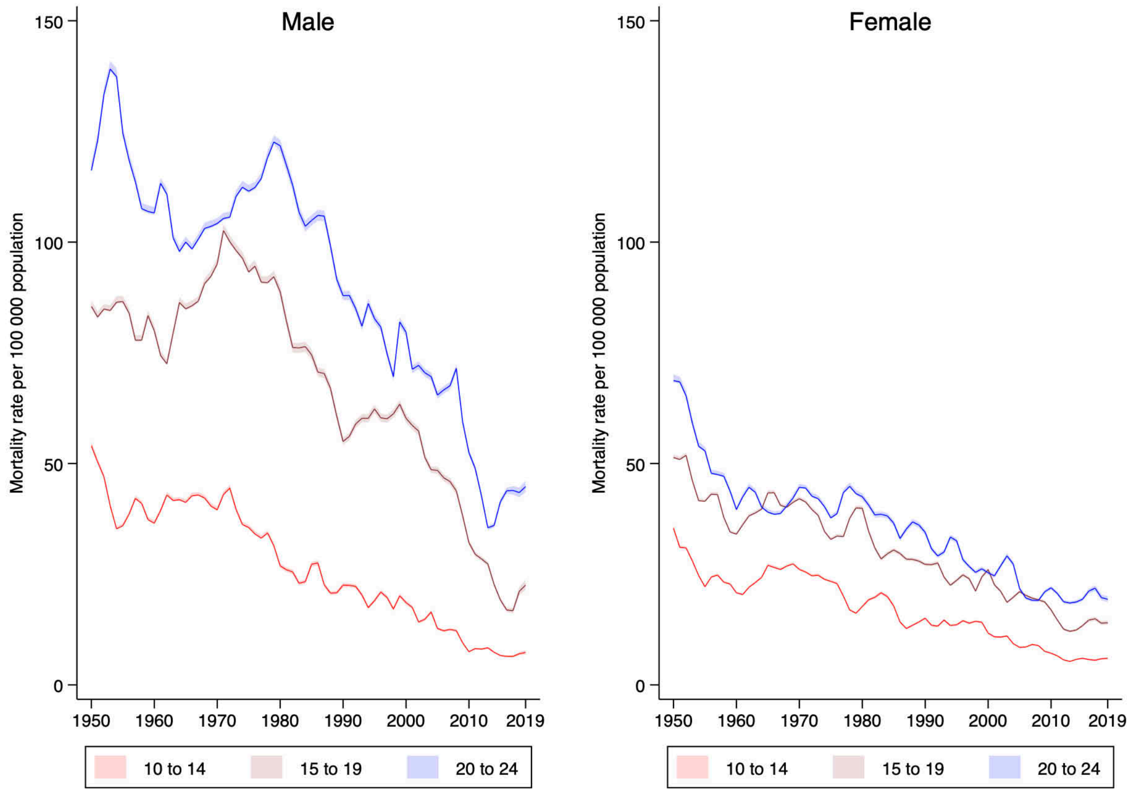

Figure S76b Number of deaths from all causes in 10-24 1950 – 2019: Denmark

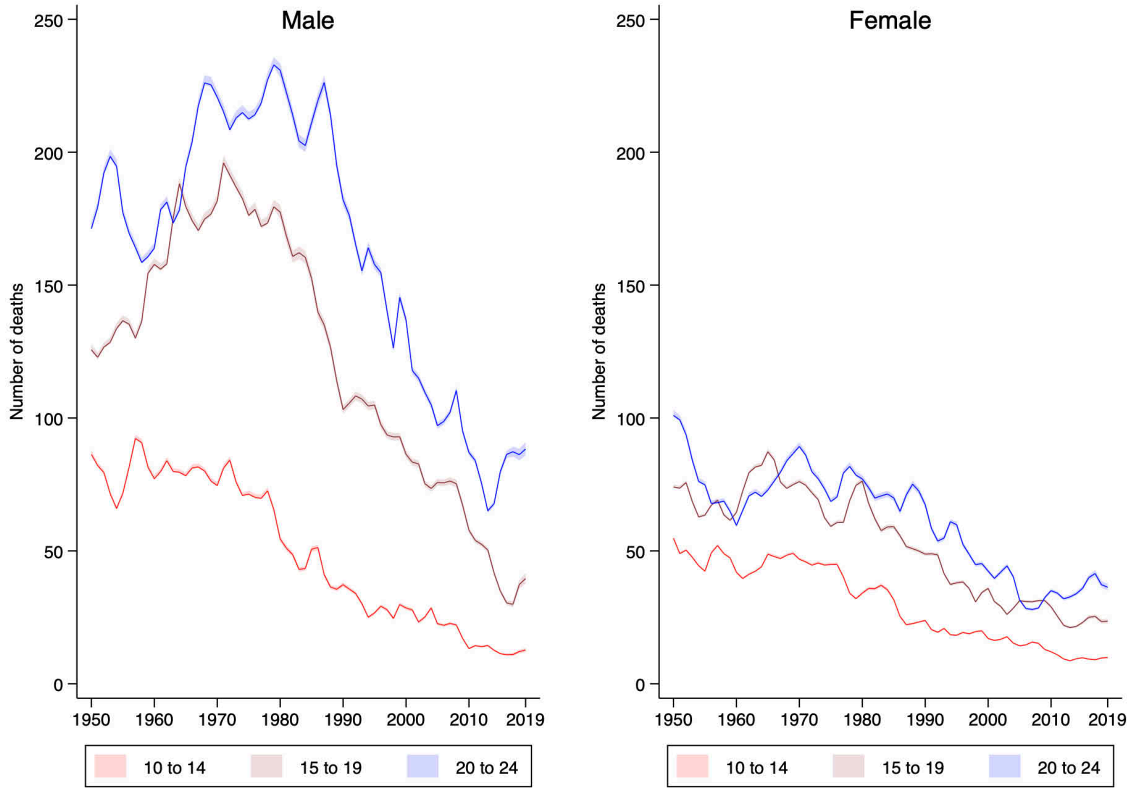

Figure S76c Mortality rate per 100,000 in 10-24 1980 – 2019 by cause: Denmark

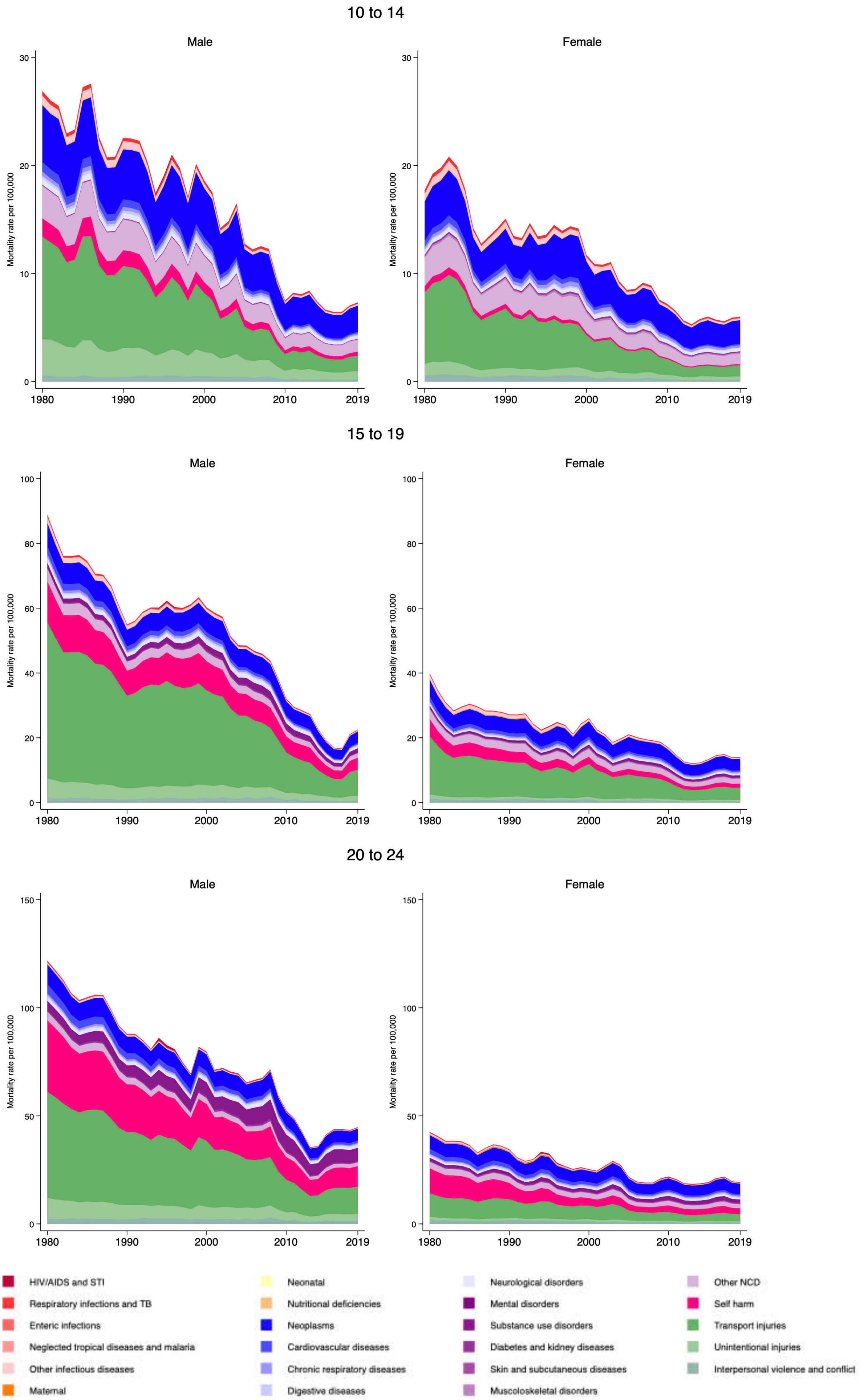

Figure S76d Number of deaths in 10-24 1980 – 2019 by cause: Denmark

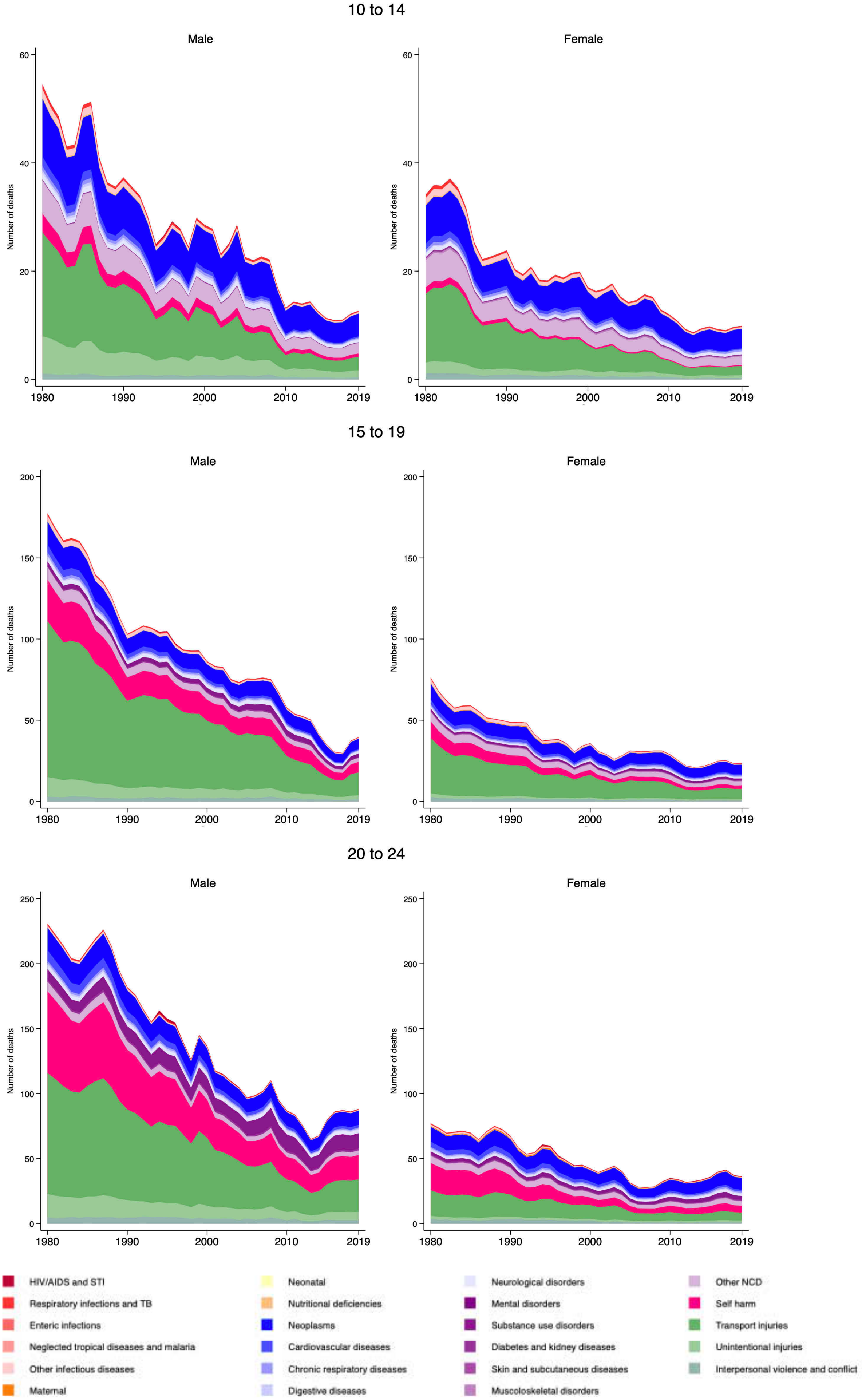

Figure S77: Finland

Figure S77a All-cause mortality rate per 100,000 in 10-24 1950 – 2019: Finland

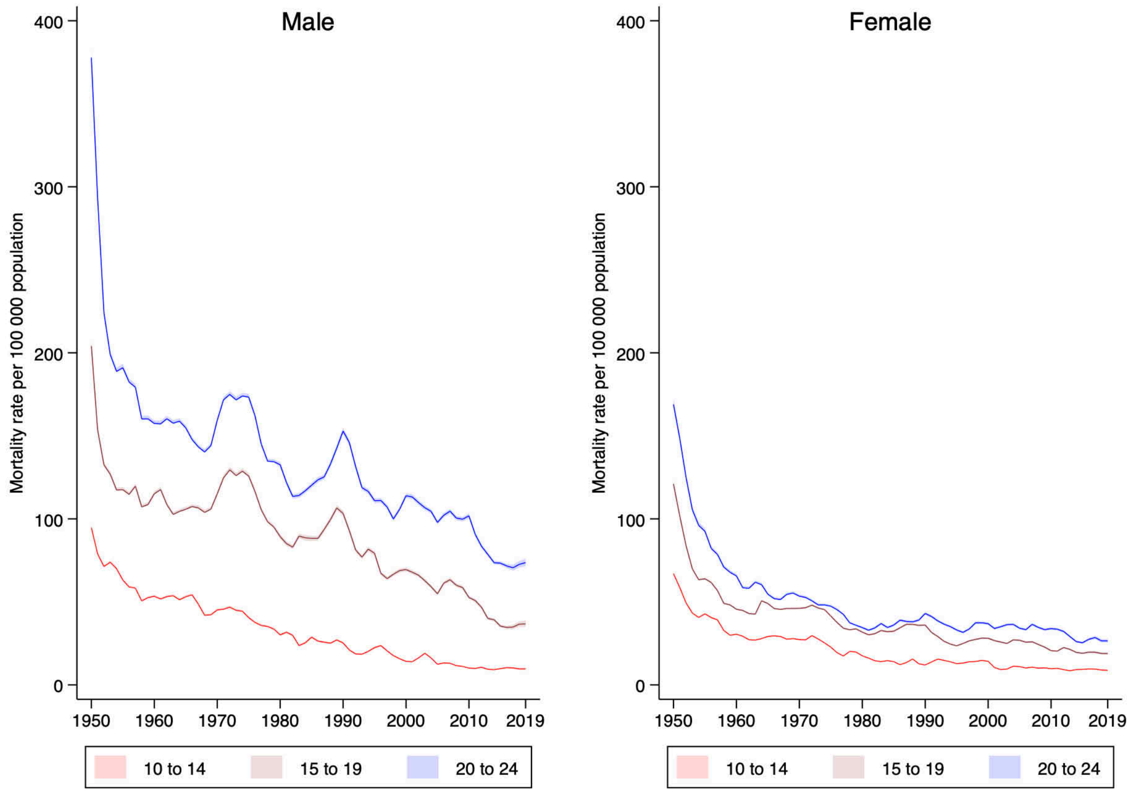

Figure S77b Number of deaths from all causes in 10-24 1950 – 2019: Finland

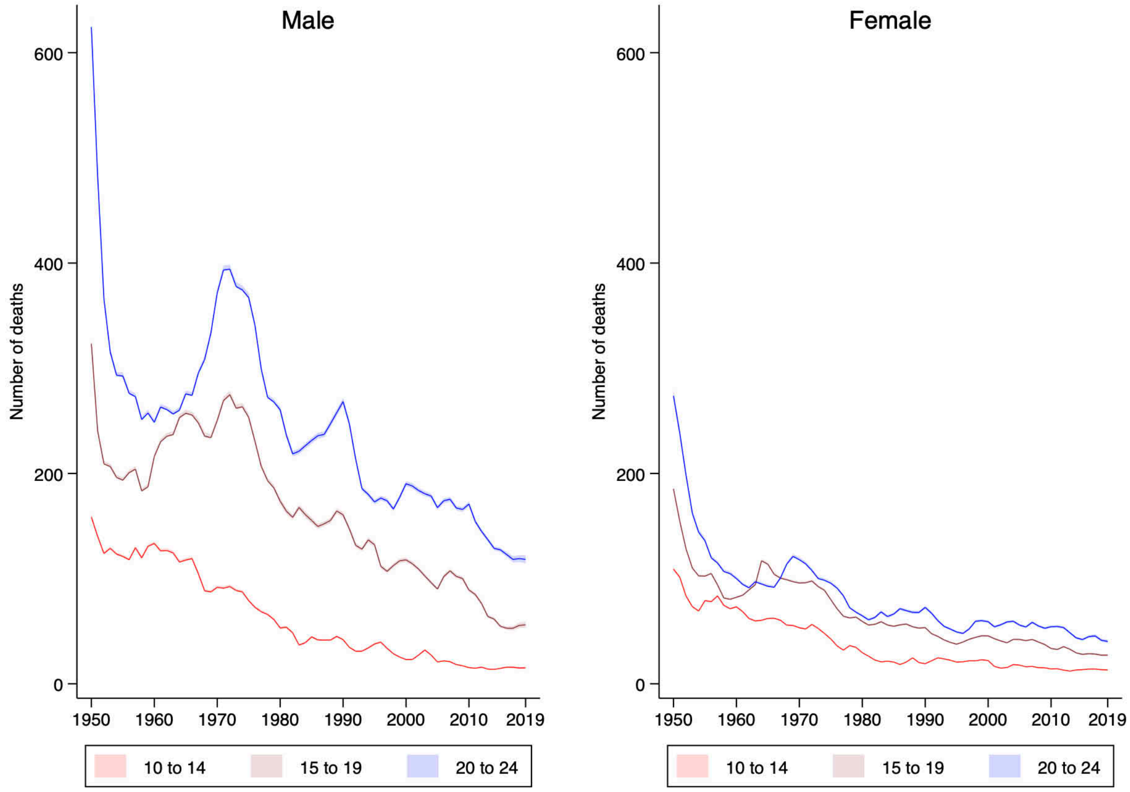

Figure S77c Mortality rate per 100,000 in 10-24 1980 – 2019 by cause: Finland

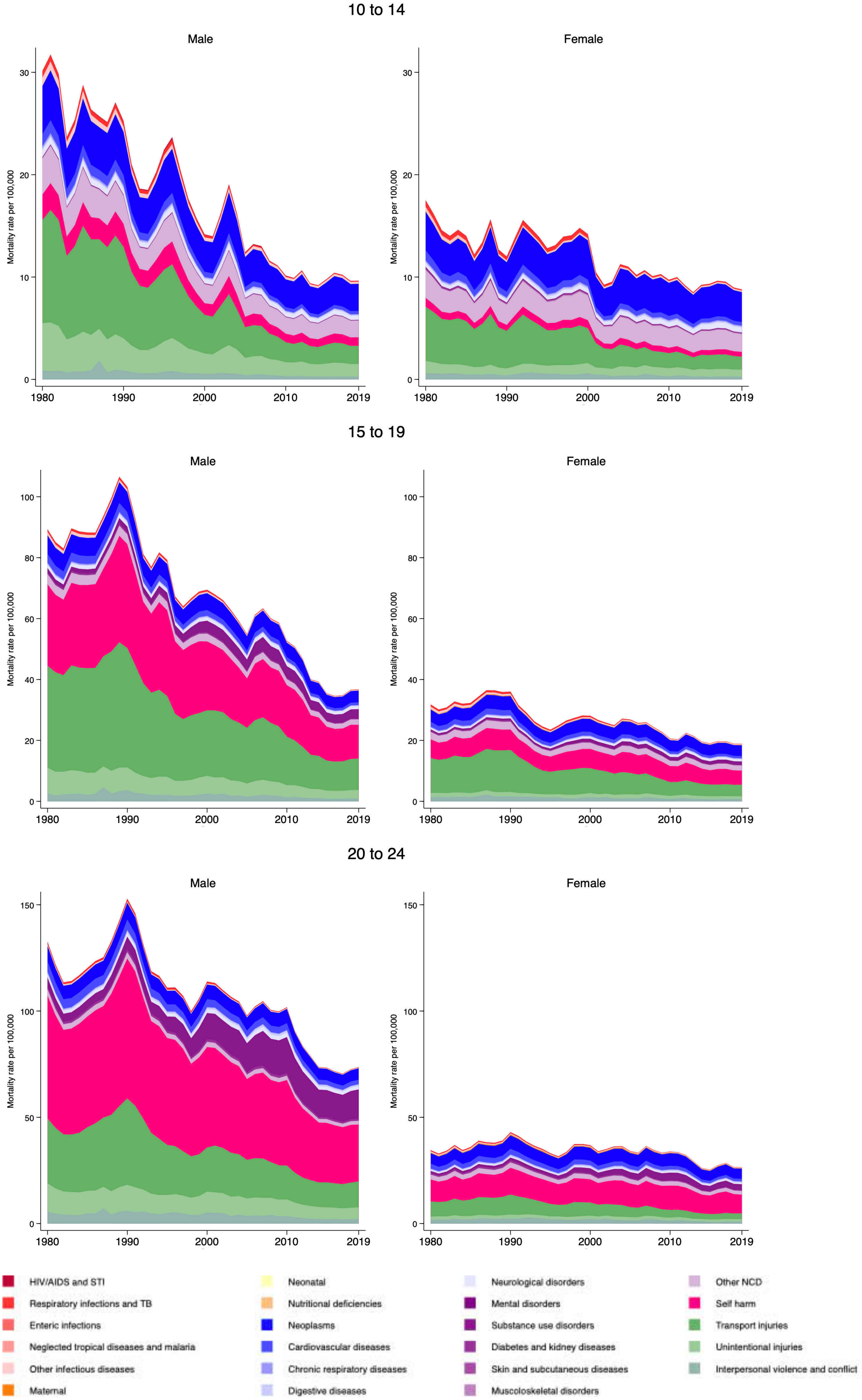

Figure S77d Number of deaths in 10-24 1980 – 2019 by cause: Finland

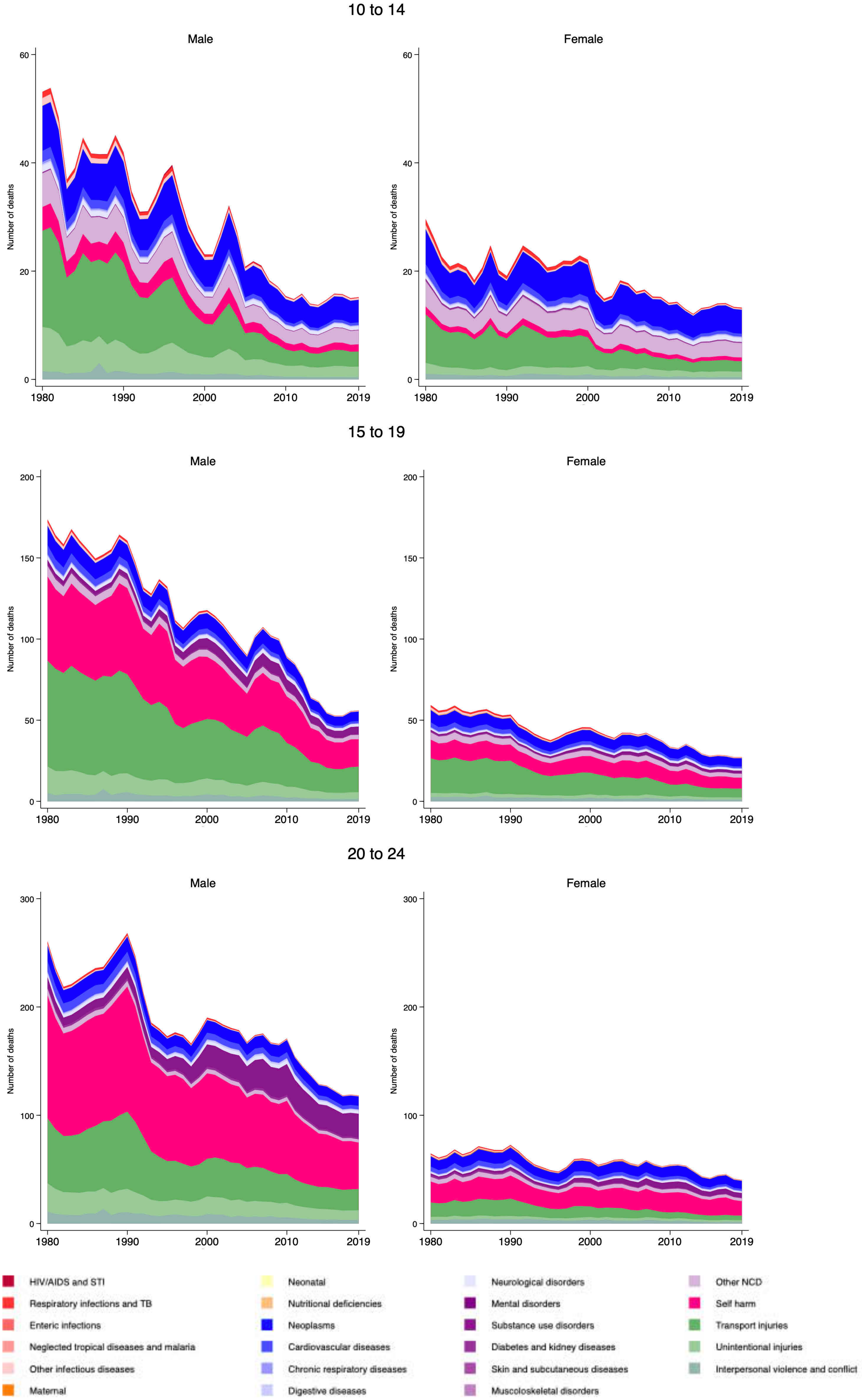

Figure S78: France

Figure S78a All-cause mortality rate per 100,000 in 10-24 1950 – 2019: France

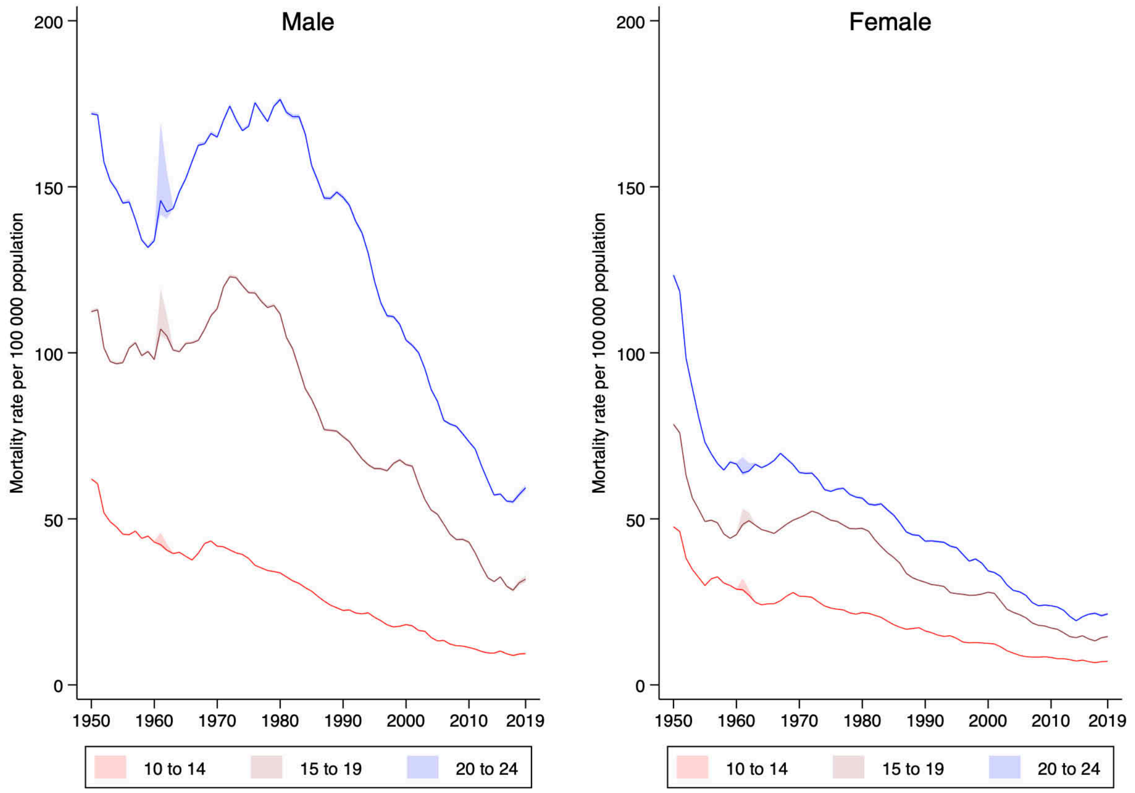

Figure S78b Number of deaths from all causes in 10-24 1950 – 2019: France

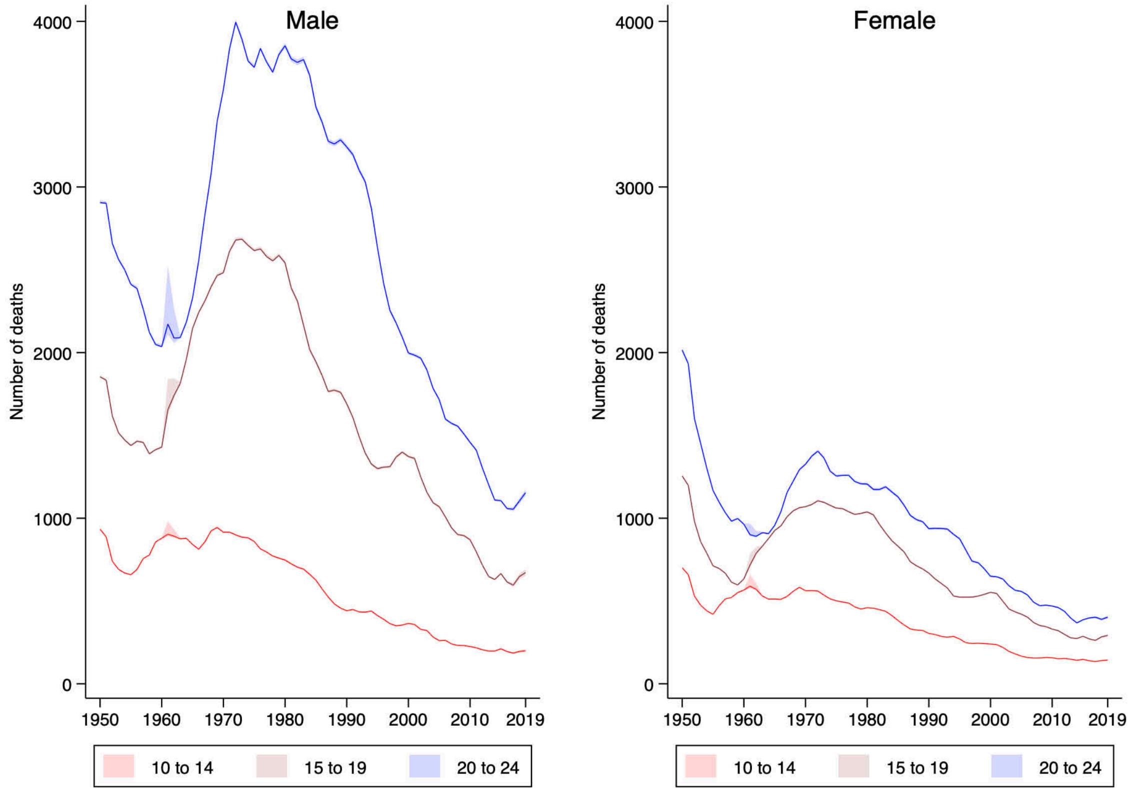

Figure S78c Mortality rate per 100,000 in 10-24 1980 – 2019 by cause: France

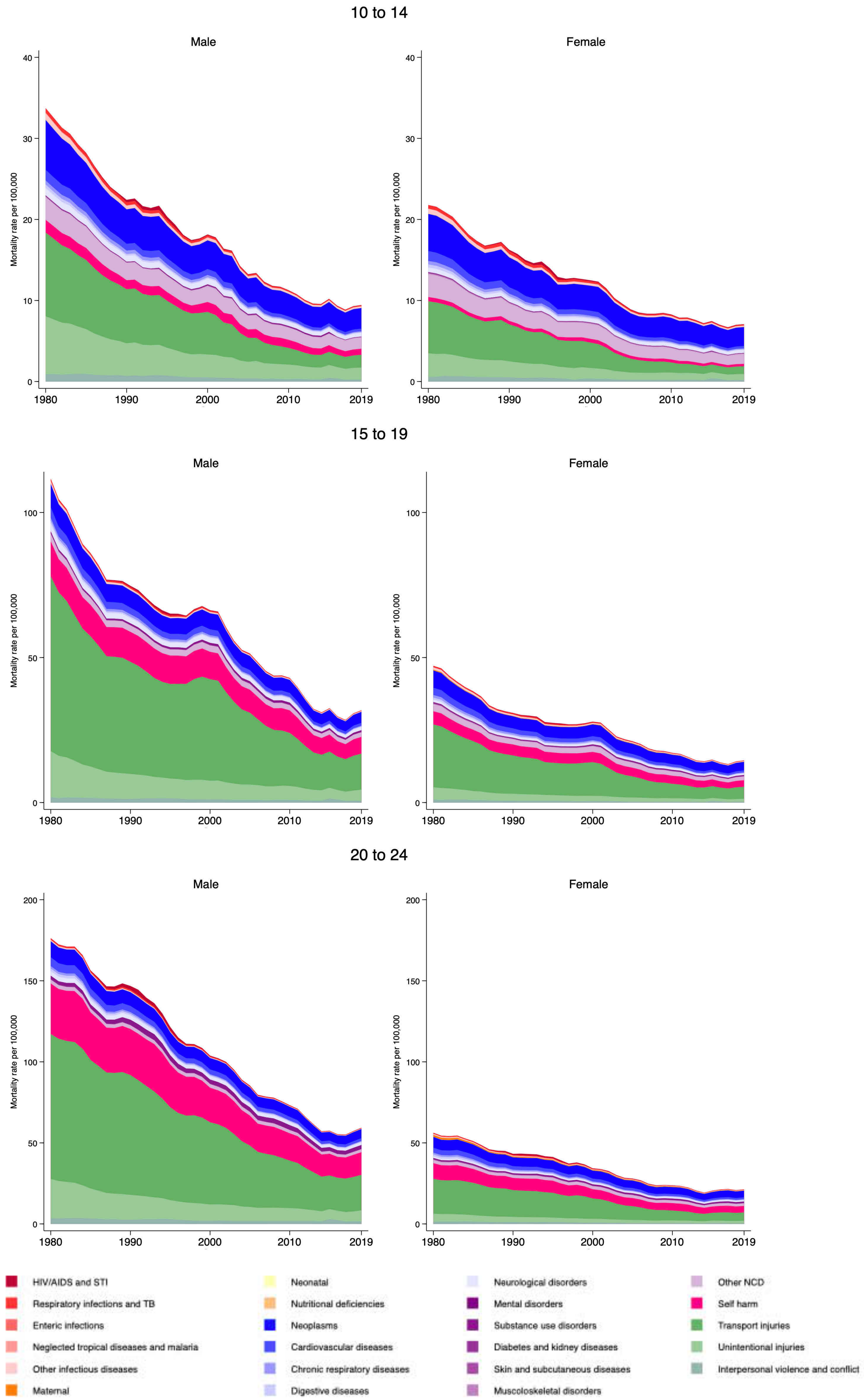

Figure S78d Number of deaths in 10-24 1980 – 2019 by cause: France

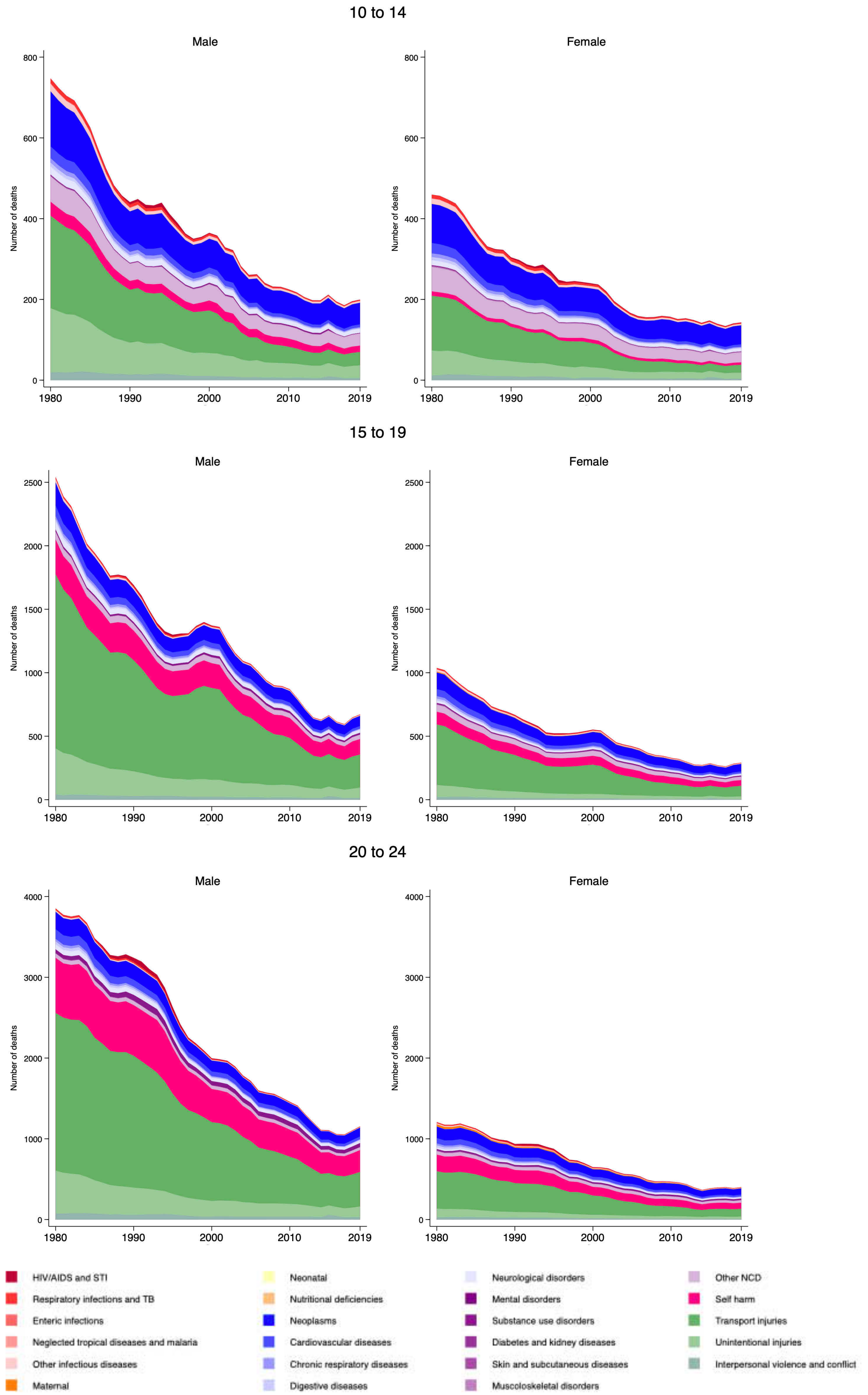

Figure S79: Germany

Figure S79a All-cause mortality rate per 100,000 in 10-24 1950 – 2019: Germany

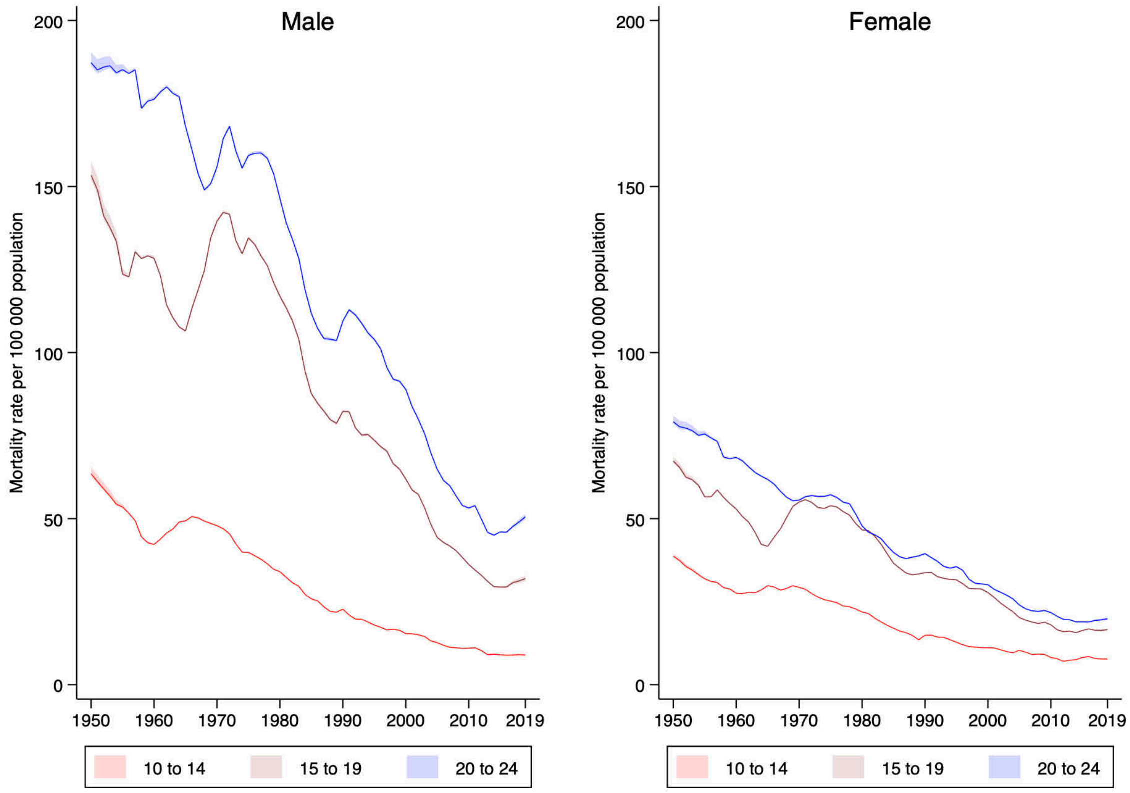

Figure S79b Number of deaths from all causes in 10-24 1950 – 2019: Germany

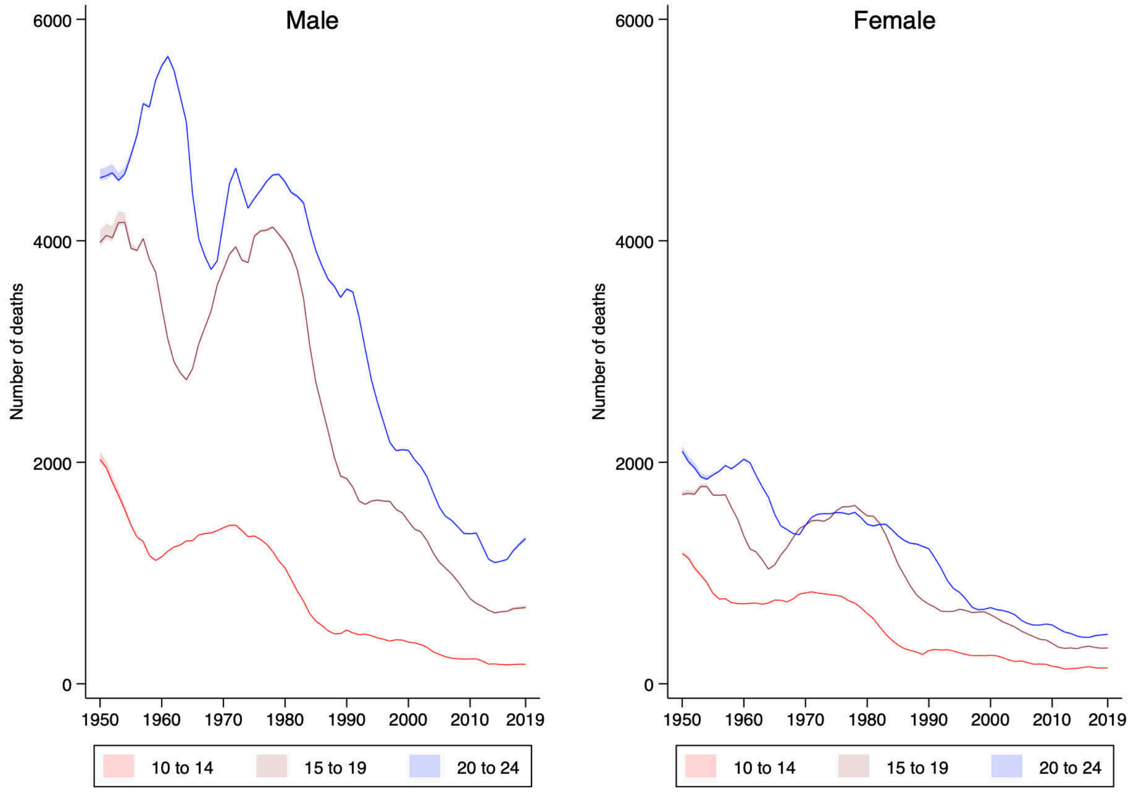

Figure S79c Mortality rate per 100,000 in 10-24 1980 – 2019 by cause: Germany

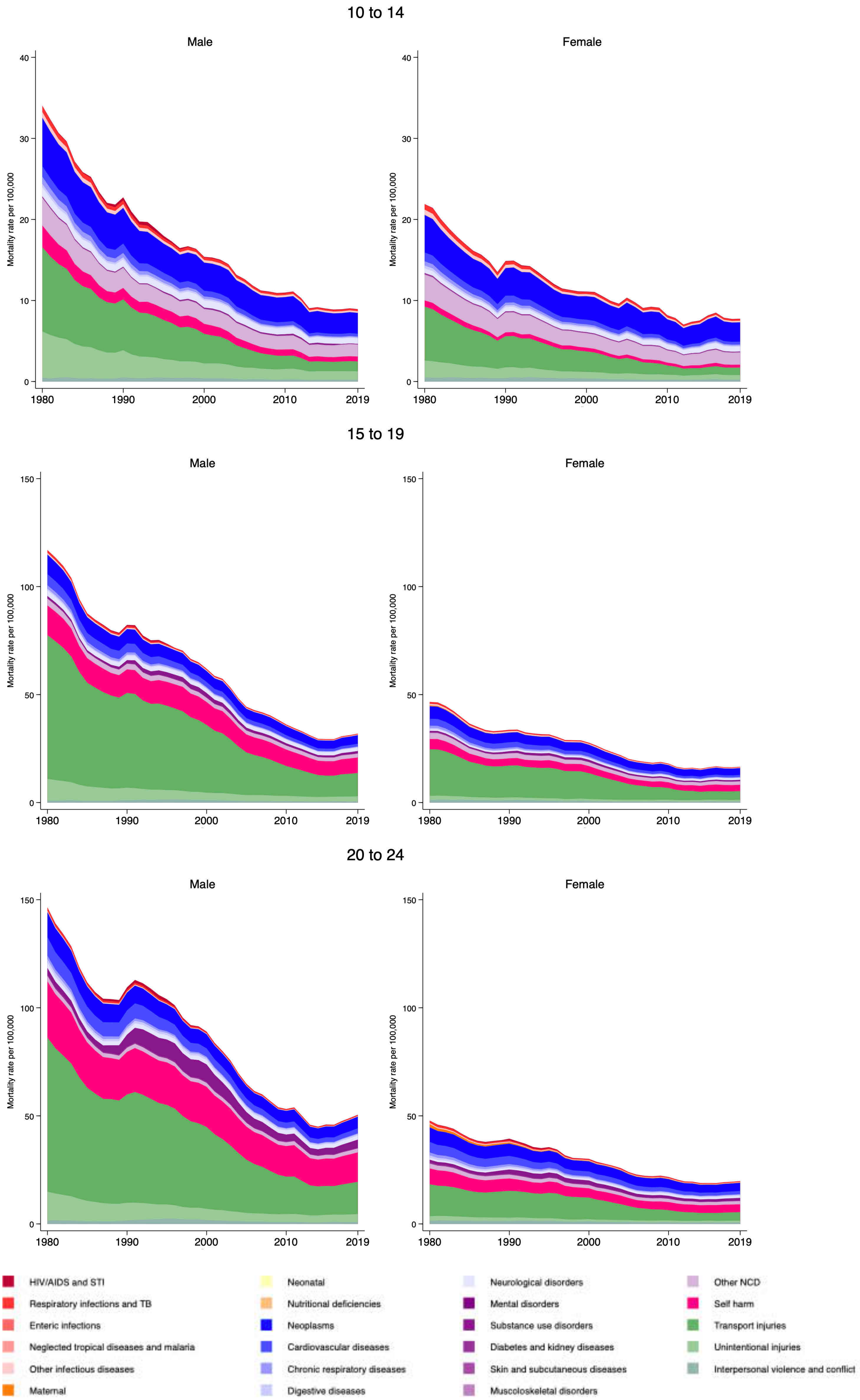

Figure S79d Number of deaths in 10-24 1980 – 2019 by cause: Germany

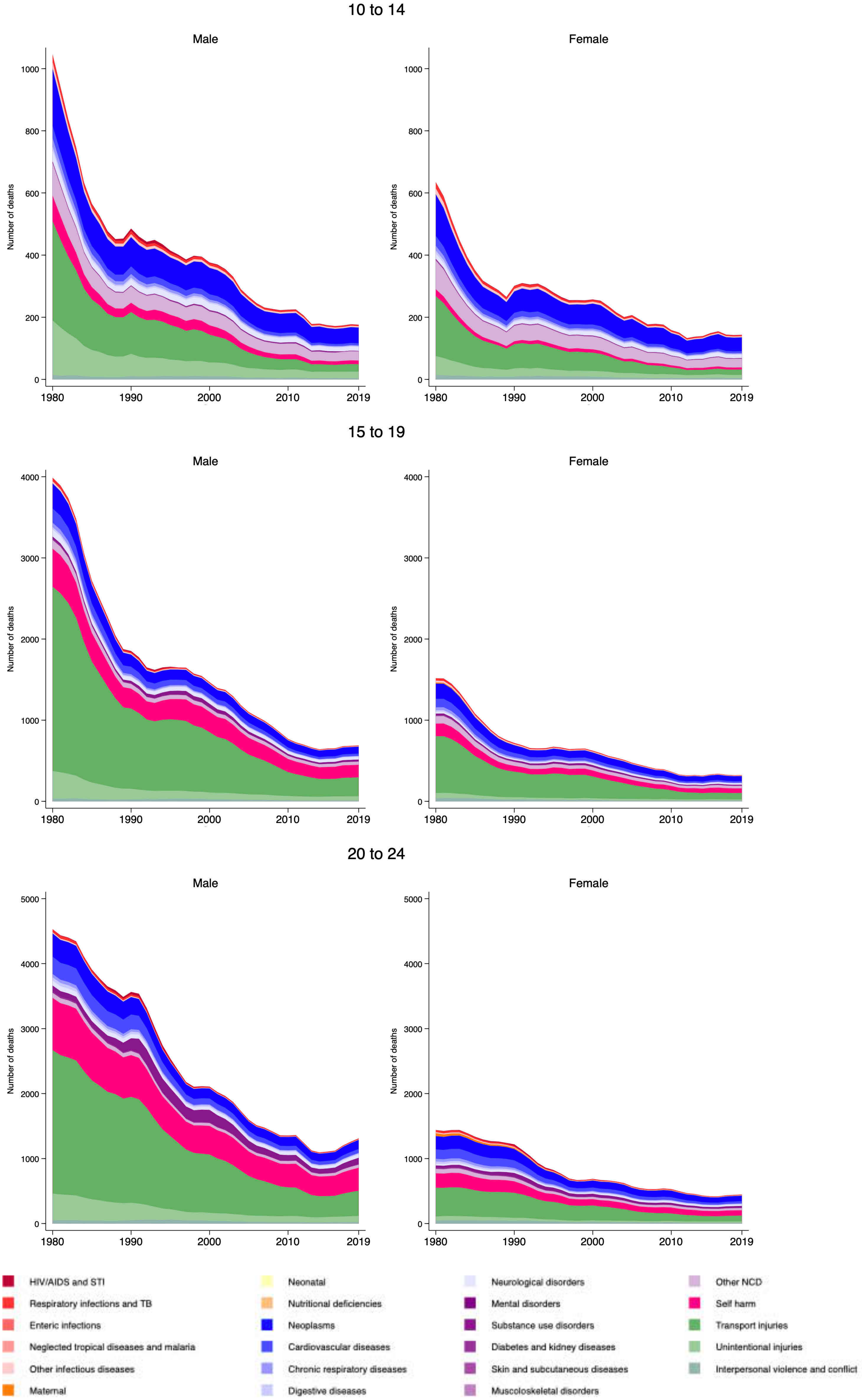

Figure S80: Greece

Figure S80a All-cause mortality rate per 100,000 in 10-24 1950 – 2019: Greece

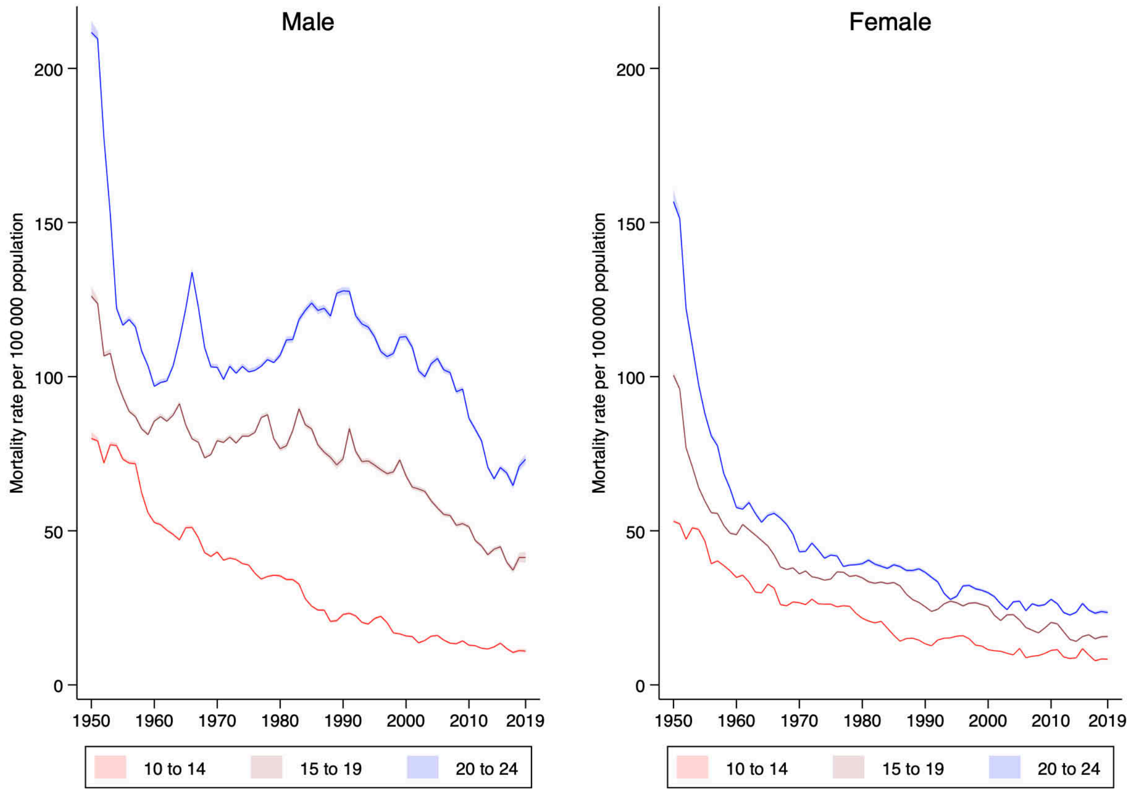

Figure S80b Number of deaths from all causes in 10-24 1950 – 2019: Greece

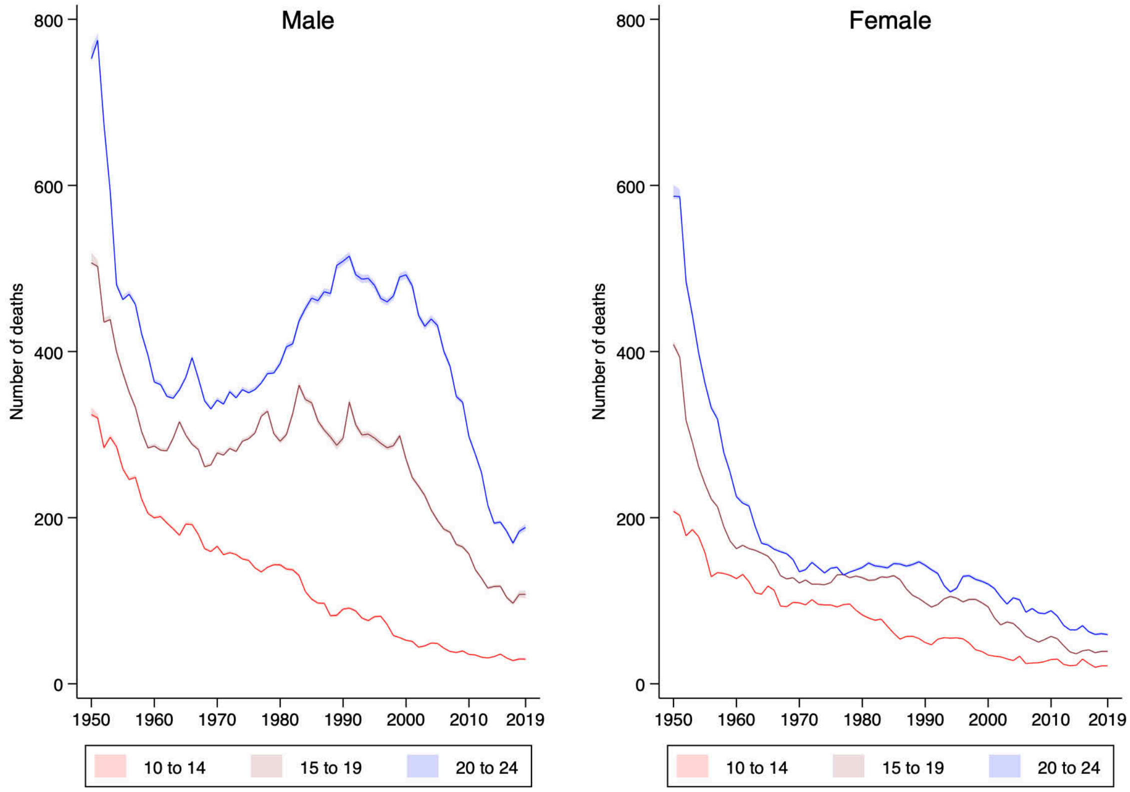

Figure S80c Mortality rate per 100,000 in 10-24 1980 – 2019 by cause: Greece

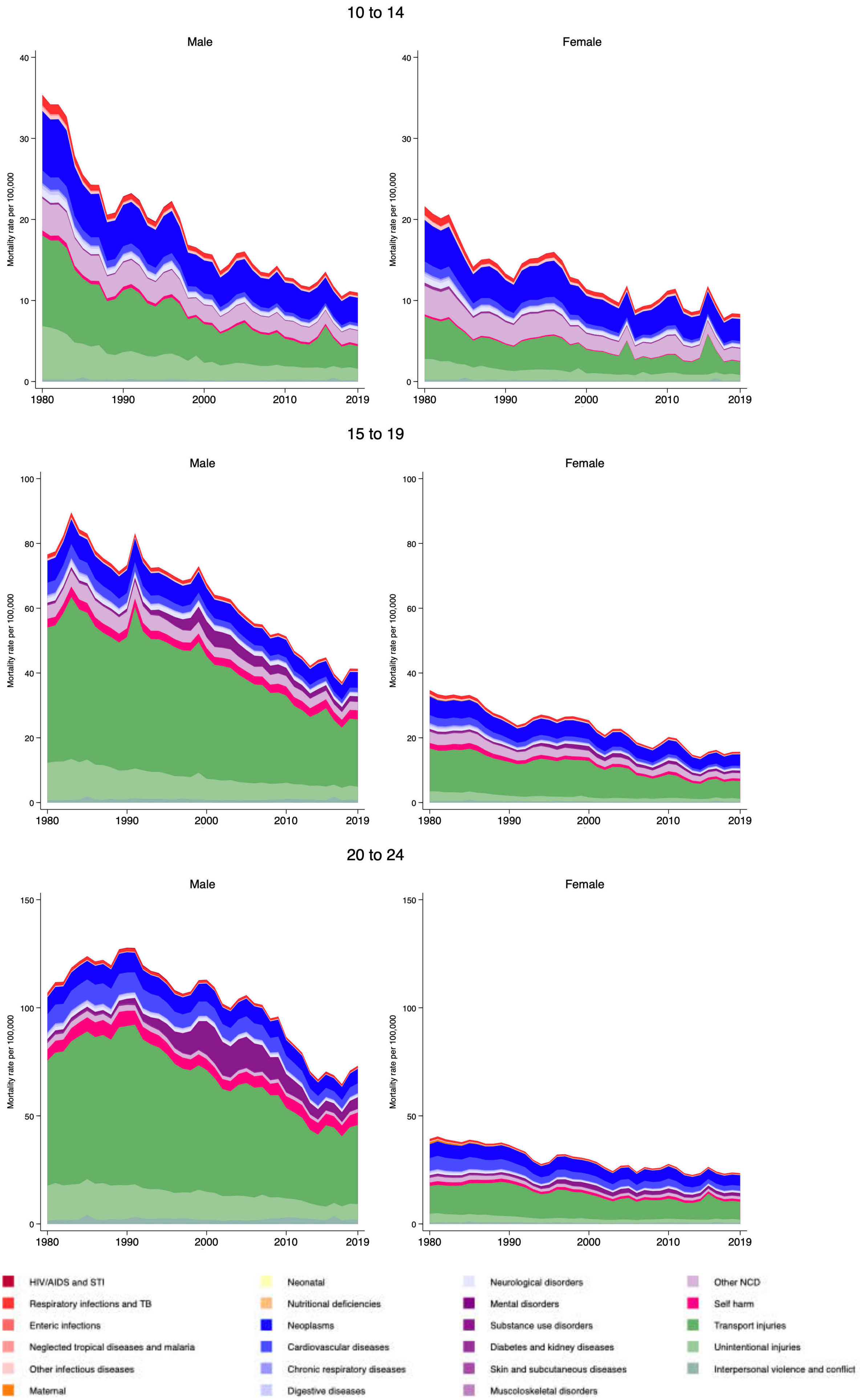

Figure S80d Number of deaths in 10-24 1980 – 2019 by cause: Greece

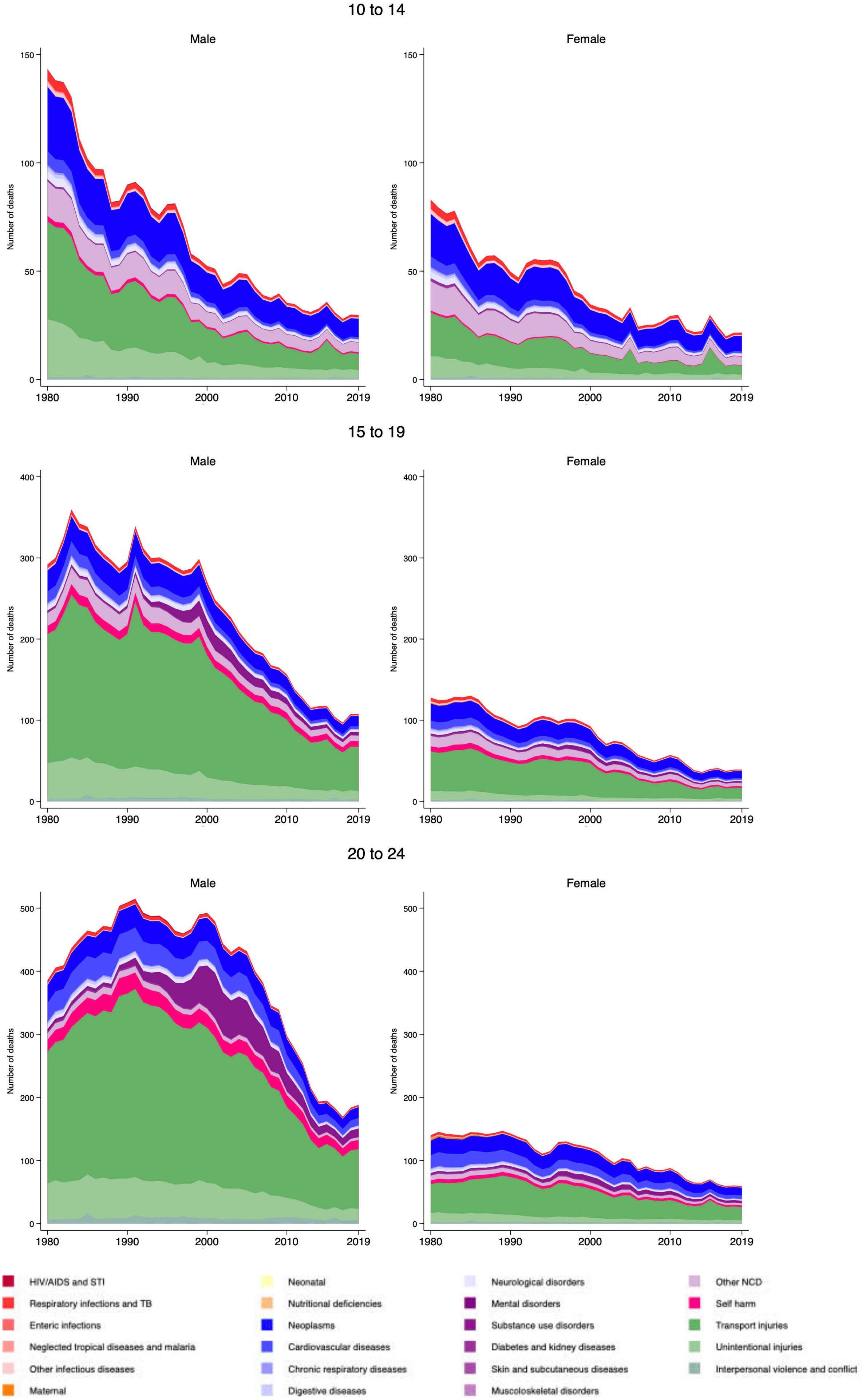

Figure S81: Iceland

Figure S81a All-cause mortality rate per 100,000 in 10-24 1950 – 2019: Iceland

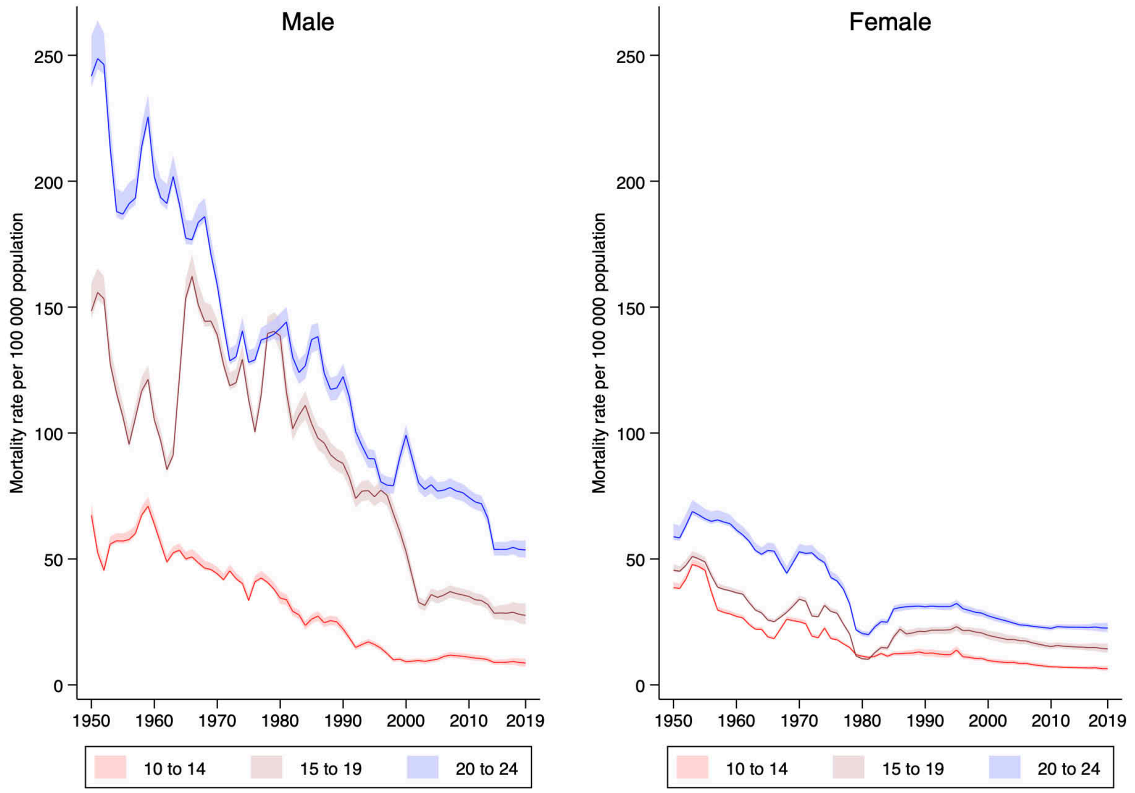

Figure S81b Number of deaths from all causes in 10-24 1950 – 2019: Iceland

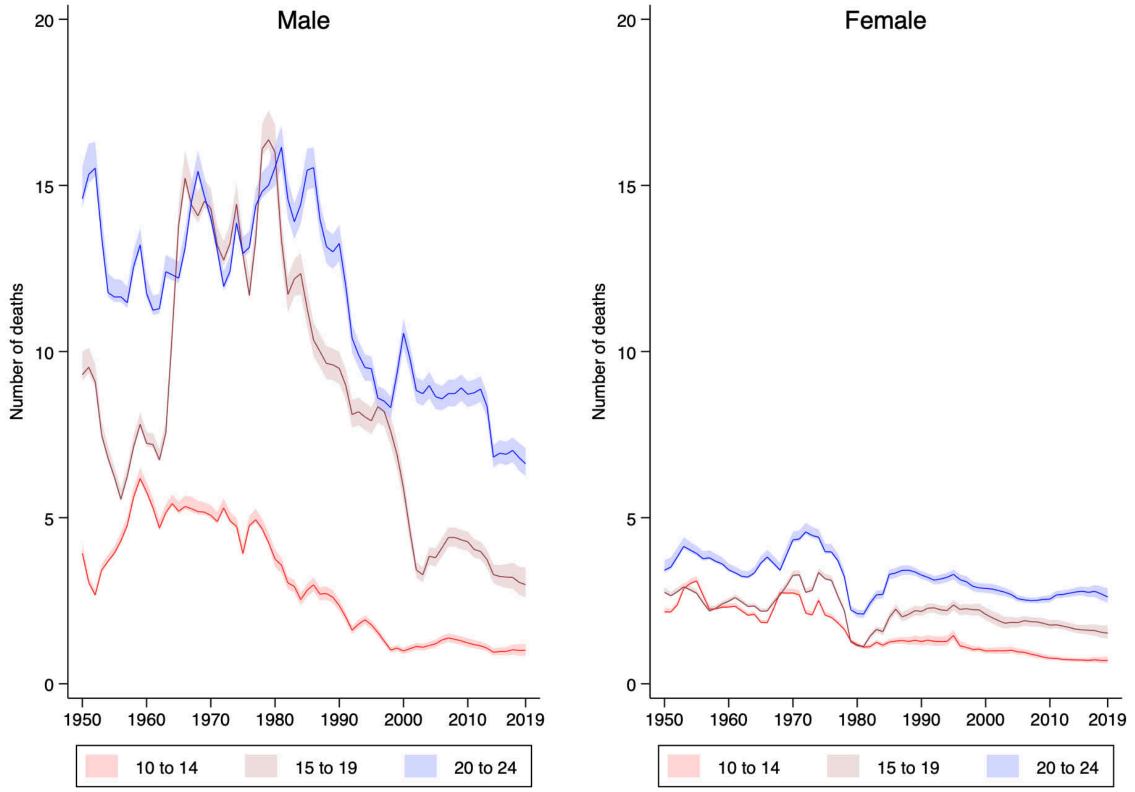

Figure S81c Mortality rate per 100,000 in 10-24 1980 – 2019 by cause: Iceland

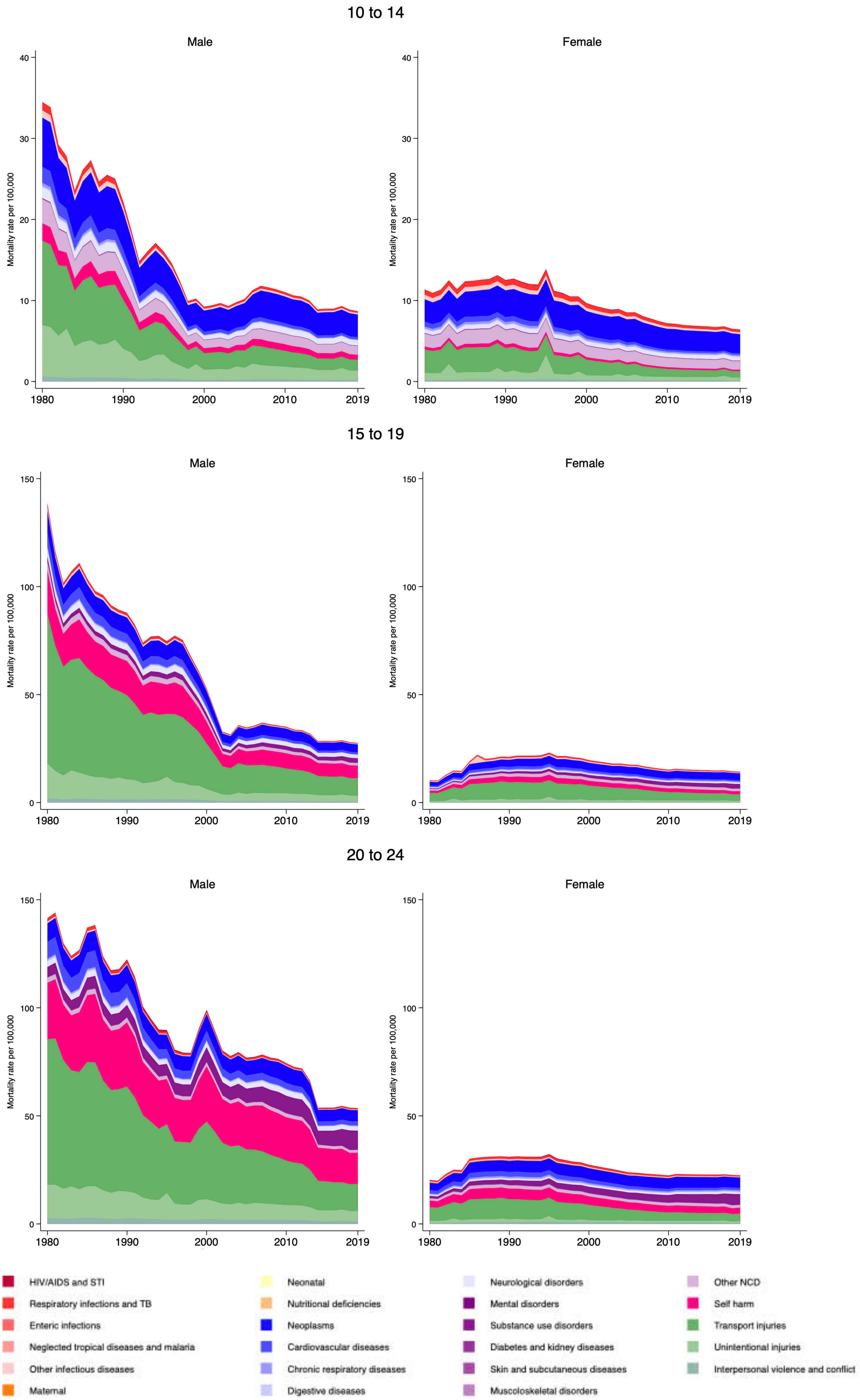

Figure S81d Number of deaths in 10-24 1980 – 2019 by cause: Iceland

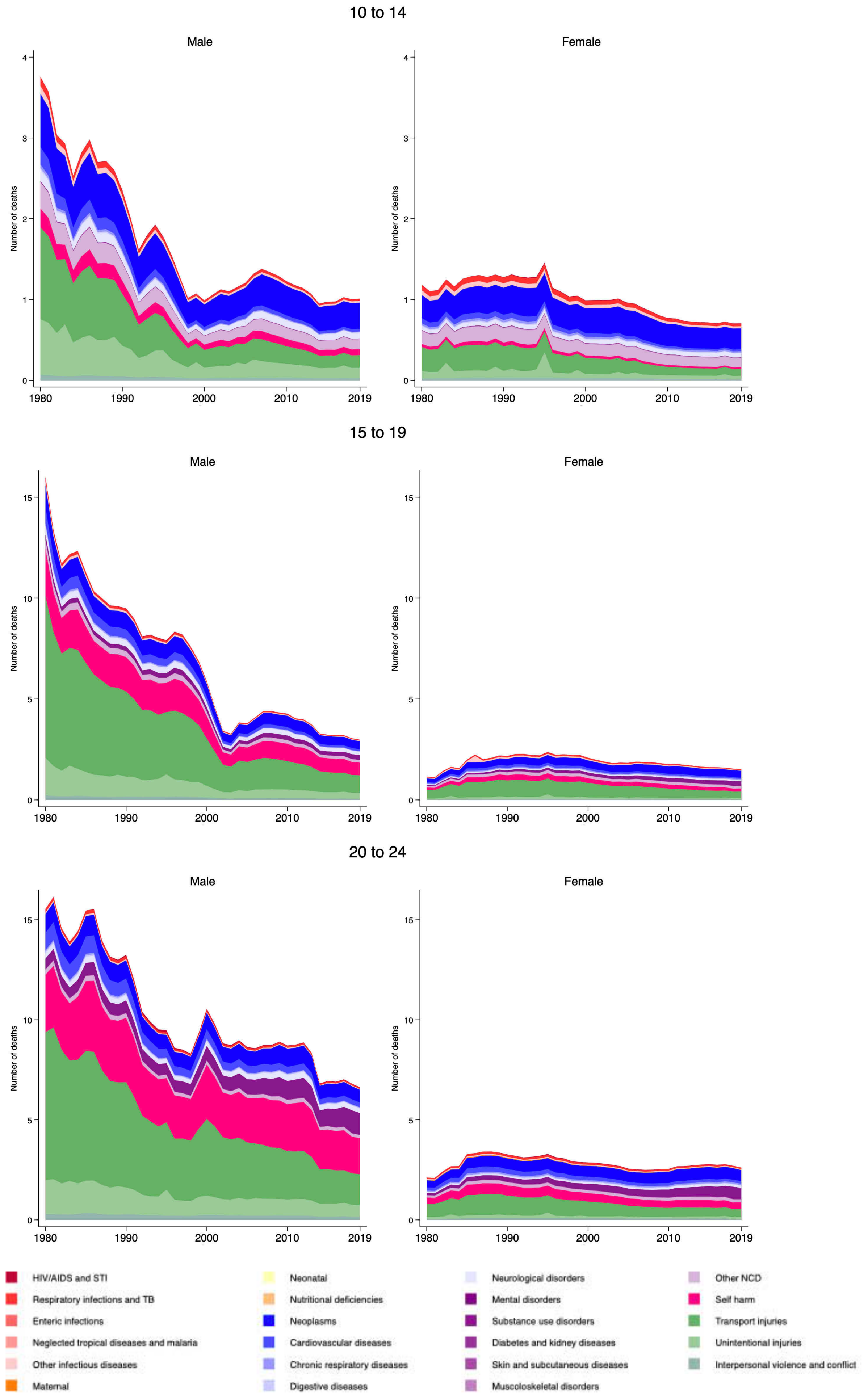

Figure S82: Ireland

Figure S82a All-cause mortality rate per 100,000 in 10-24 1950 – 2019: Ireland

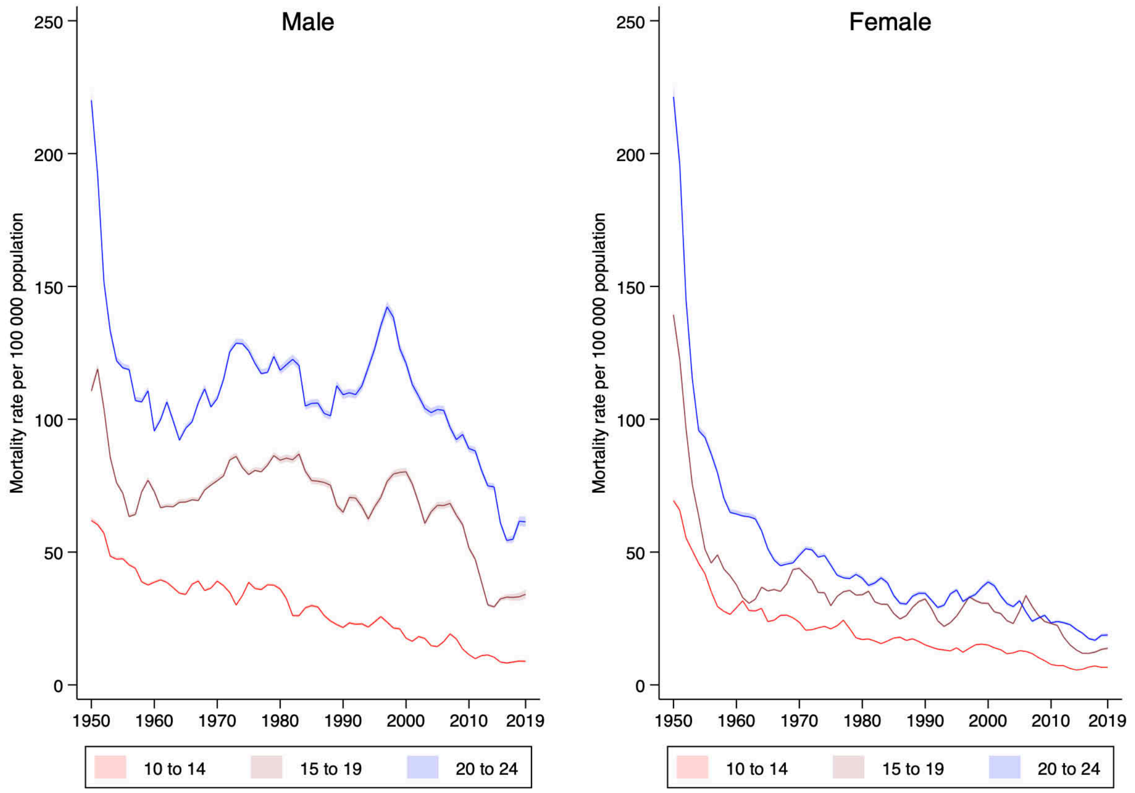

Figure S82b Number of deaths from all causes in 10-24 1950 – 2019: Ireland

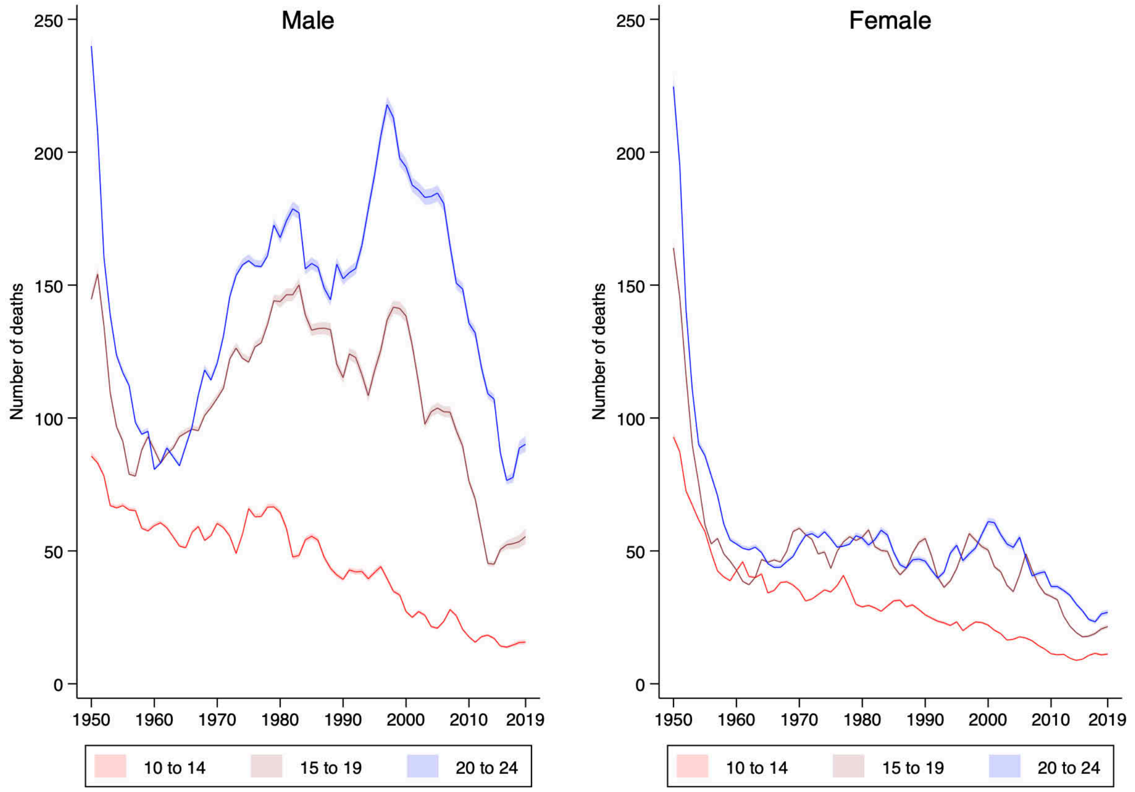

Figure S82c Mortality rate per 100,000 in 10-24 1980 – 2019 by cause: Ireland

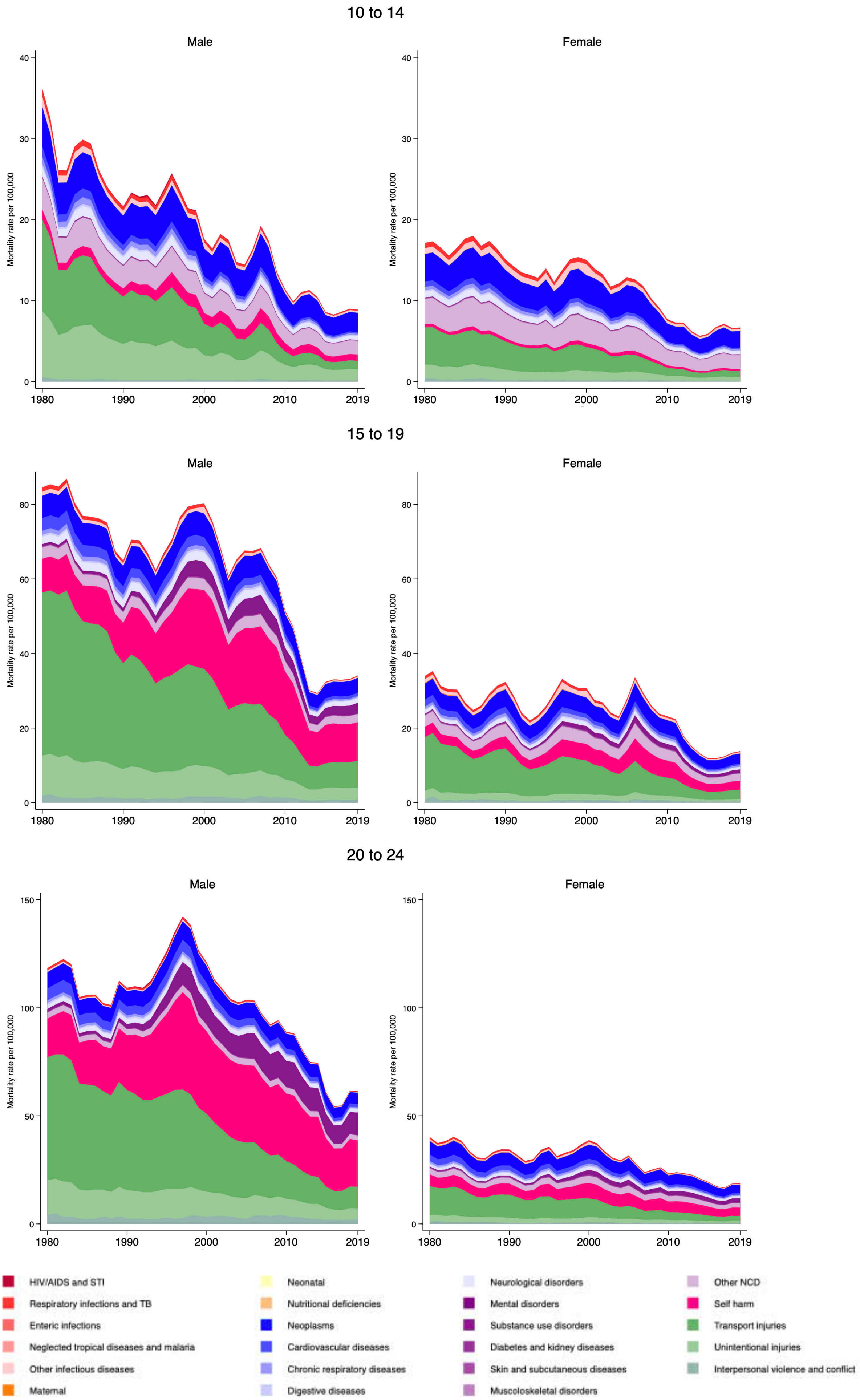

Figure S82d Number of deaths in 10-24 1980 – 2019 by cause: Ireland

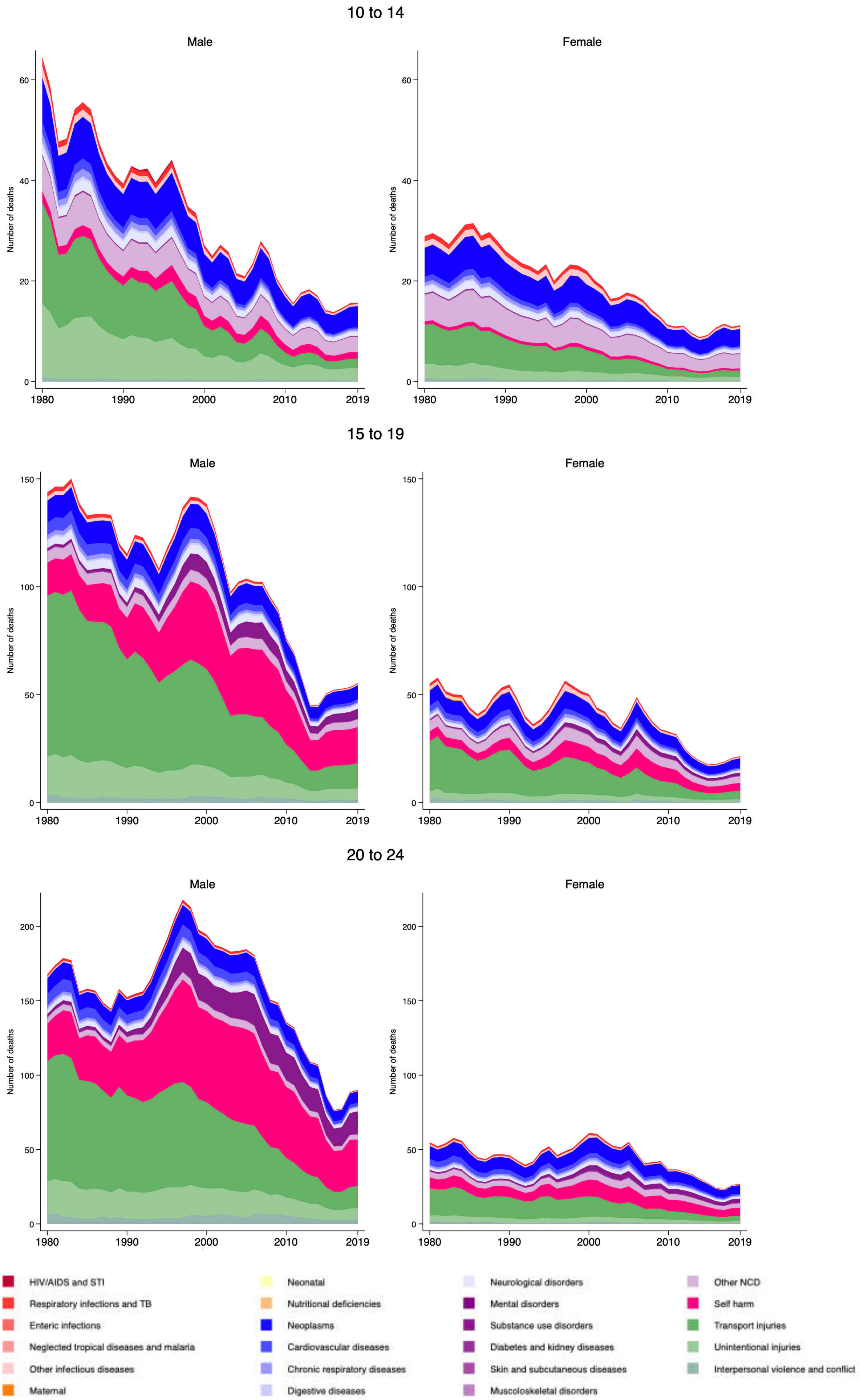

Figure S83: Israel

Figure S83a All-cause mortality rate per 100,000 in 10-24 1950 – 2019: Israel

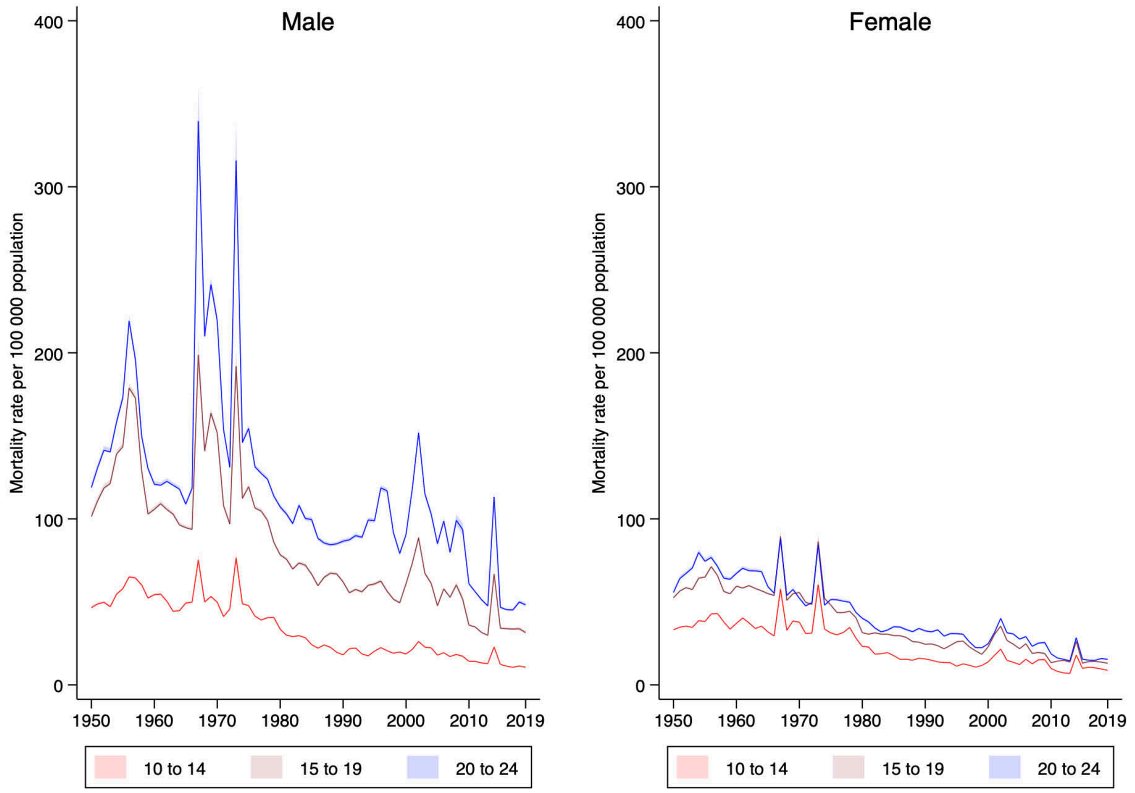

Figure S83b Number of deaths from all causes in 10-24 1950 – 2019: Israel

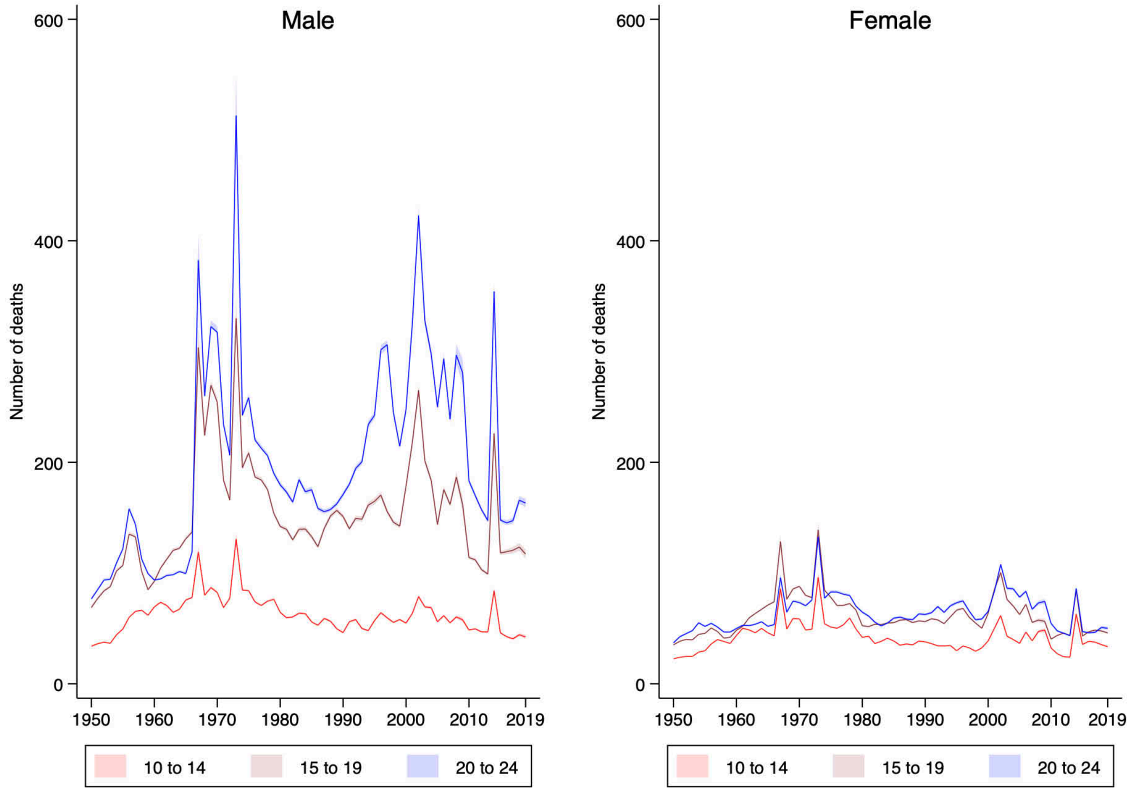

Figure S83c Mortality rate per 100,000 in 10-24 1980 – 2019 by cause: Israel

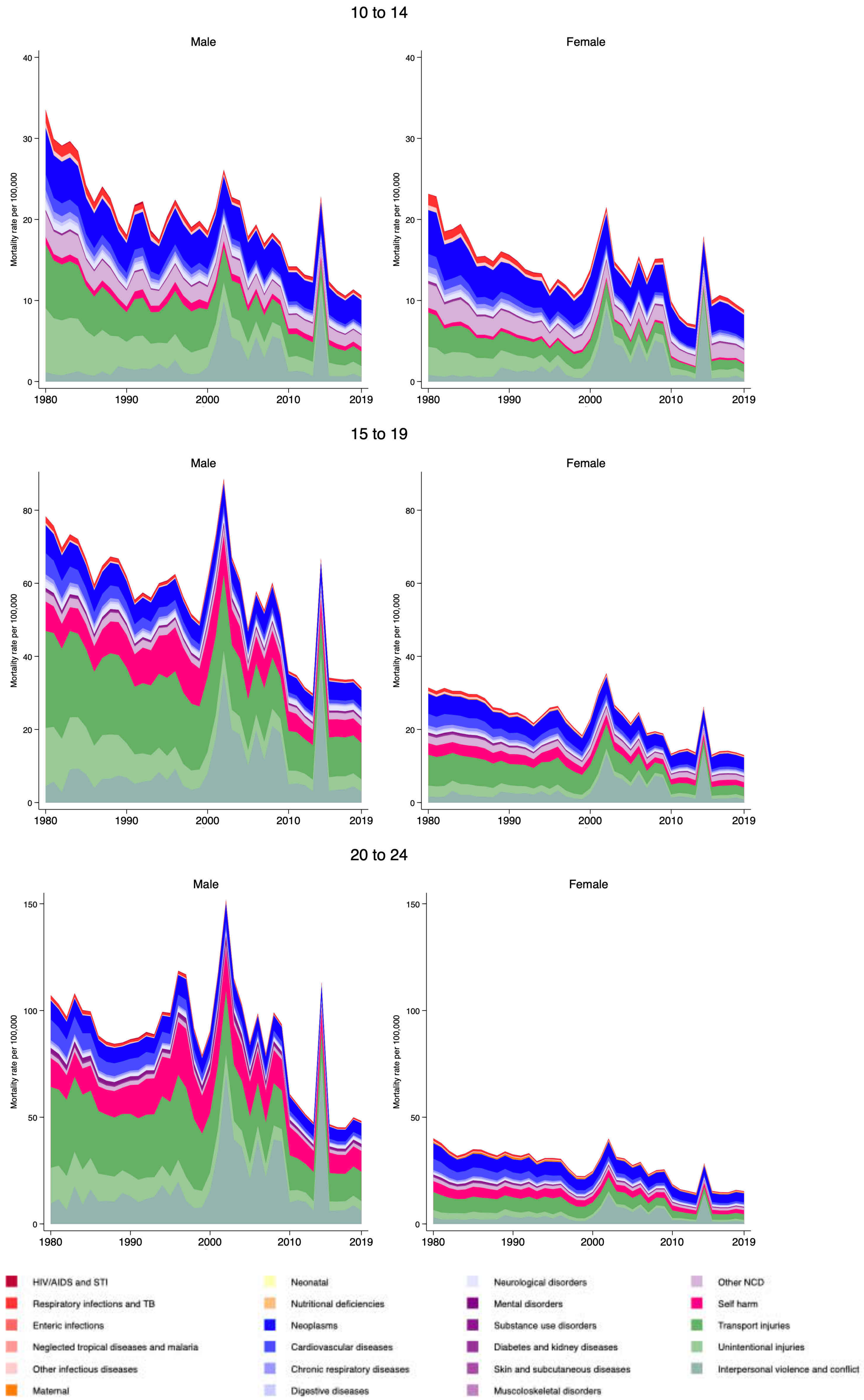

Figure S83d Number of deaths in 10-24 1980 – 2019 by cause: Israel

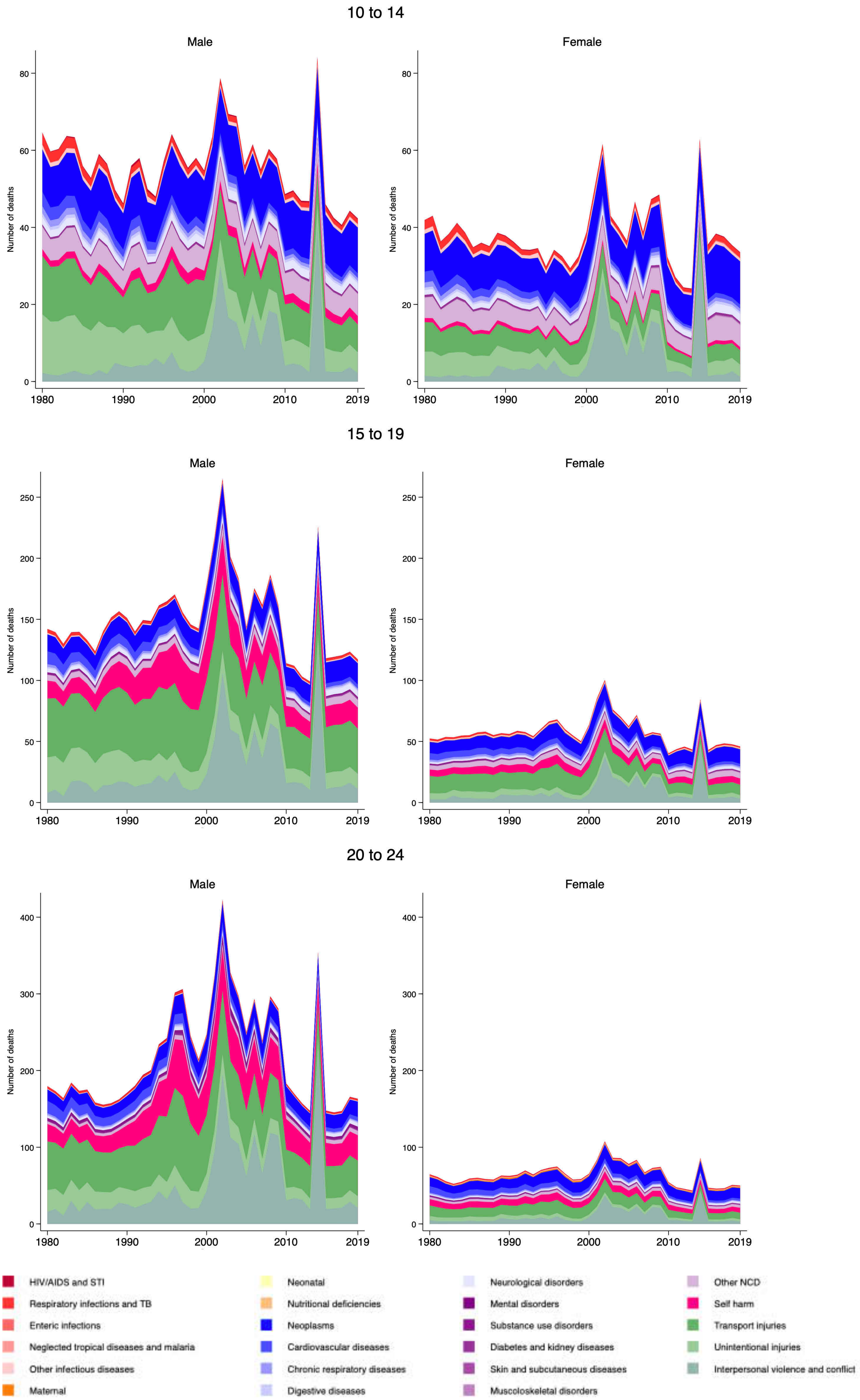

Figure S84: Italy

Figure S84a All-cause mortality rate per 100,000 in 10-24 1950 – 2019: Italy

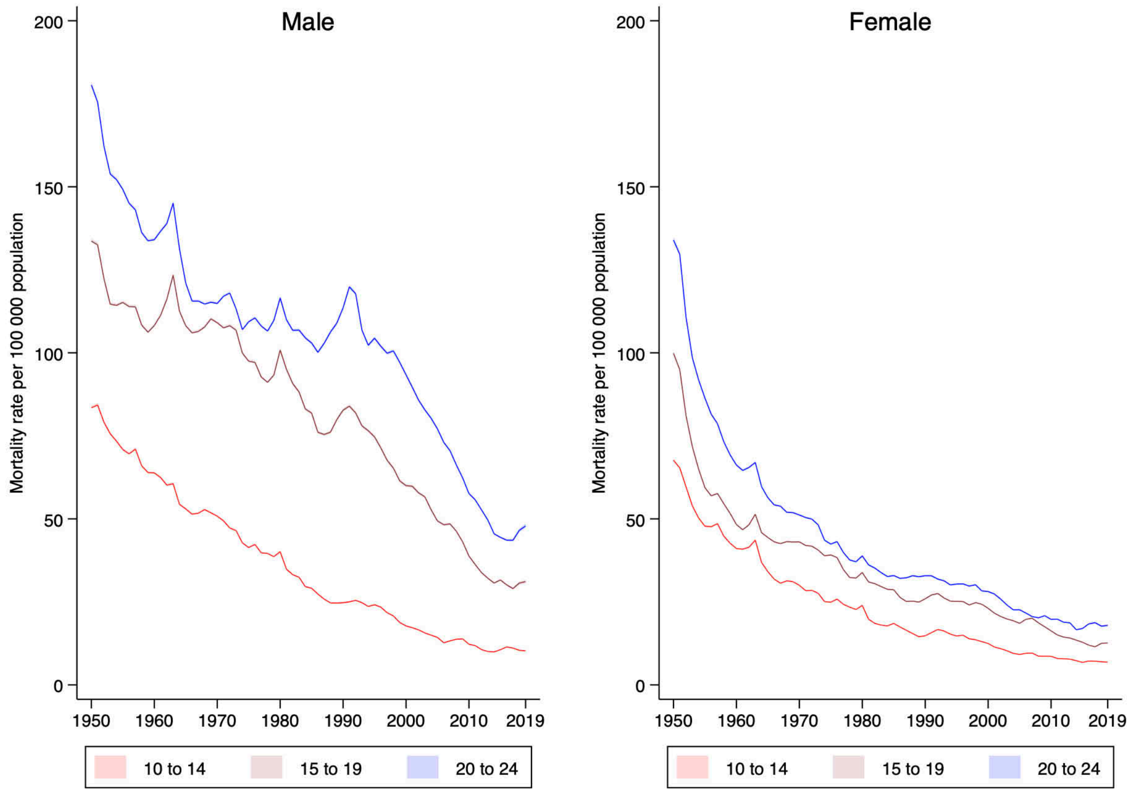

Figure S84b Number of deaths from all causes in 10-24 1950 – 2019: Italy

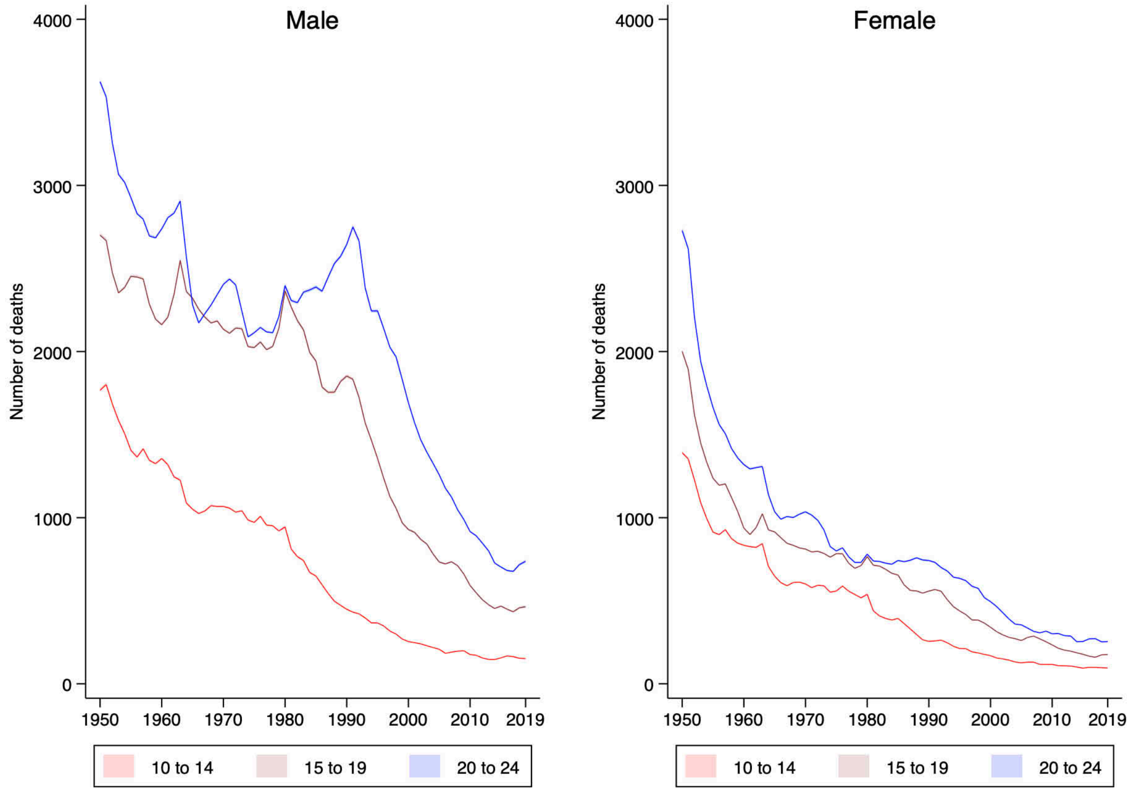

Figure S84c Mortality rate per 100,000 in 10-24 1980 – 2019 by cause: Italy

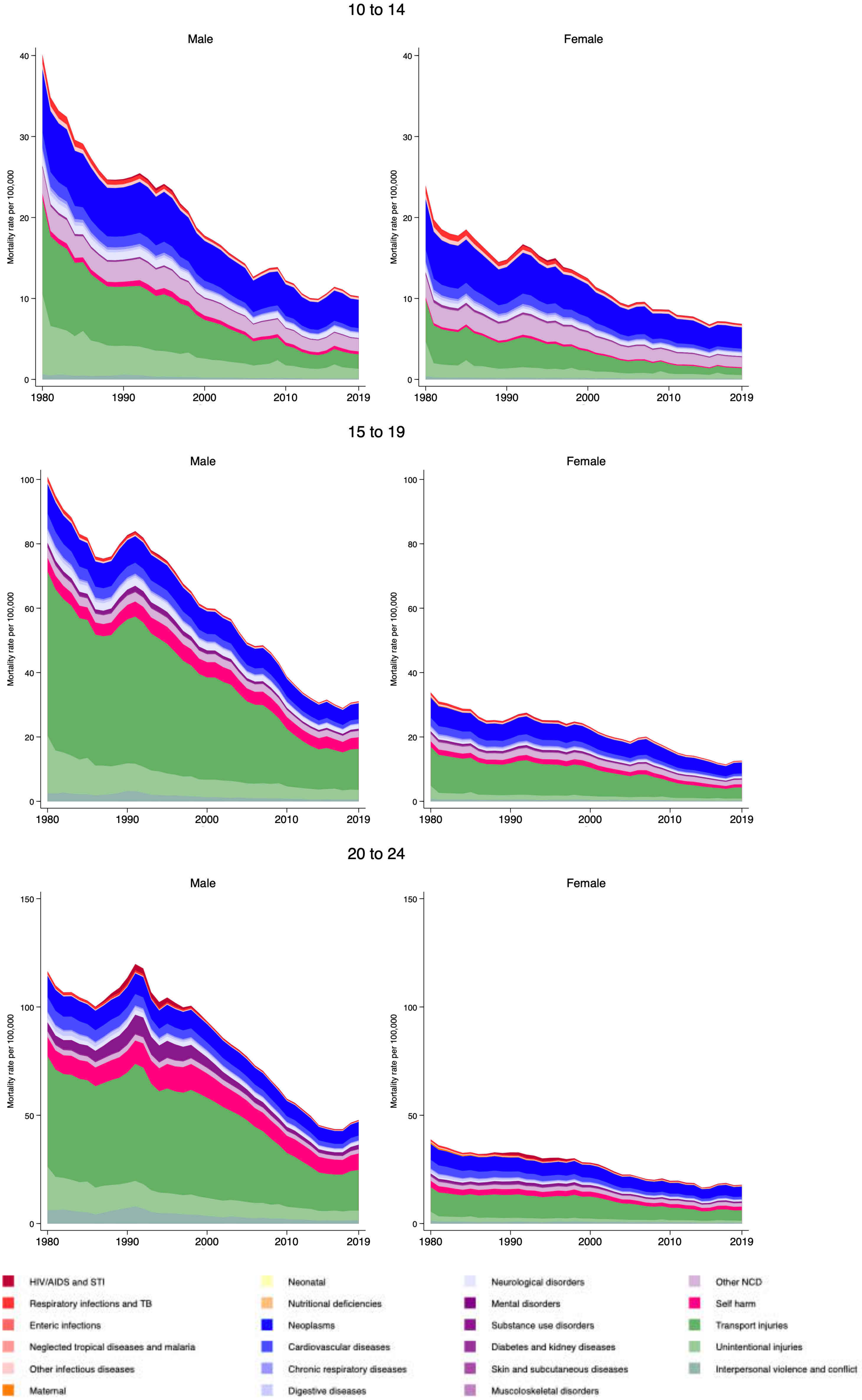

Figure S84d Number of deaths in 10-24 1980 – 2019 by cause: Italy

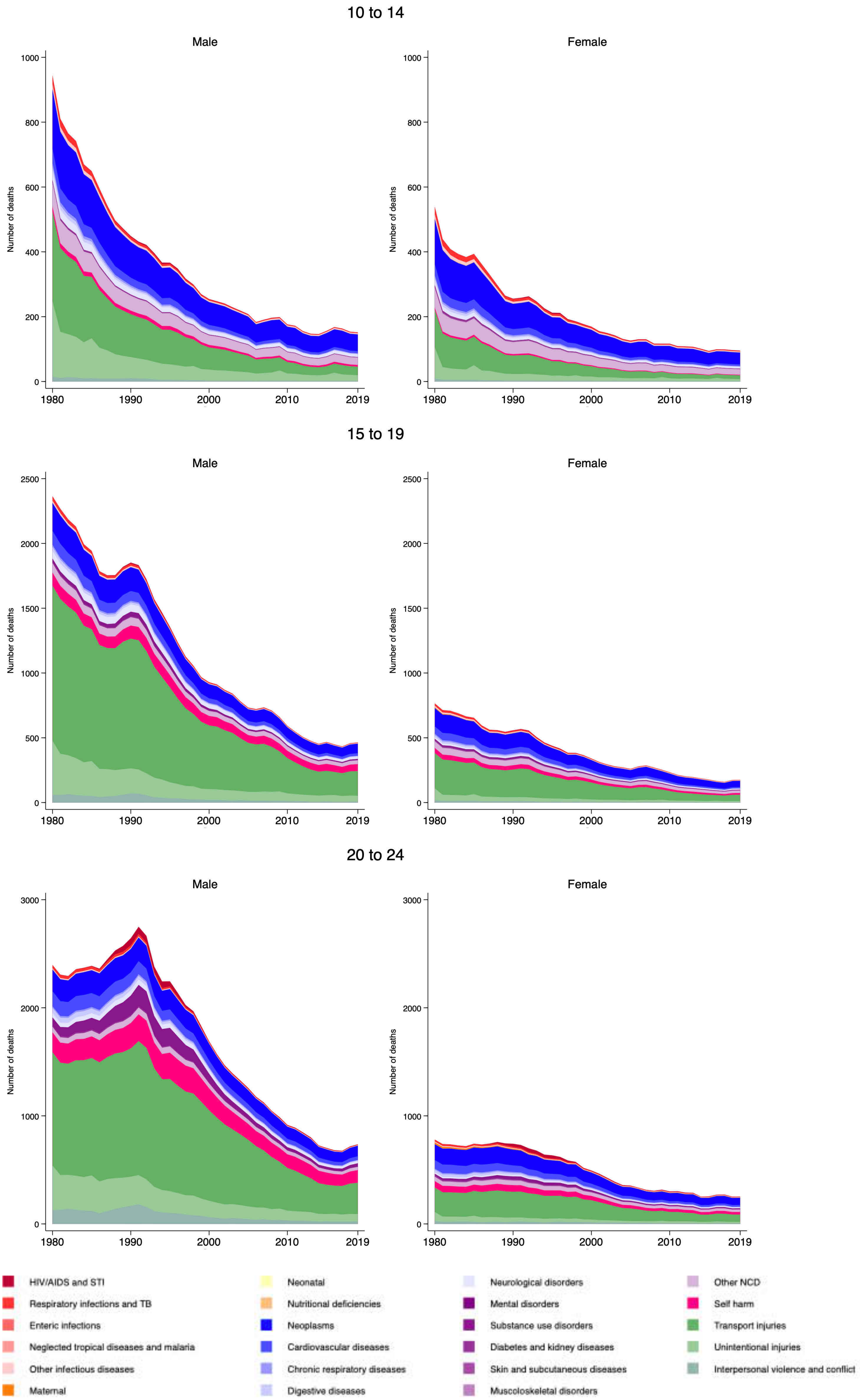

Figure S85: Luxembourg

Figure S85a All-cause mortality rate per 100,000 in 10-24 1950 – 2019: Luxembourg

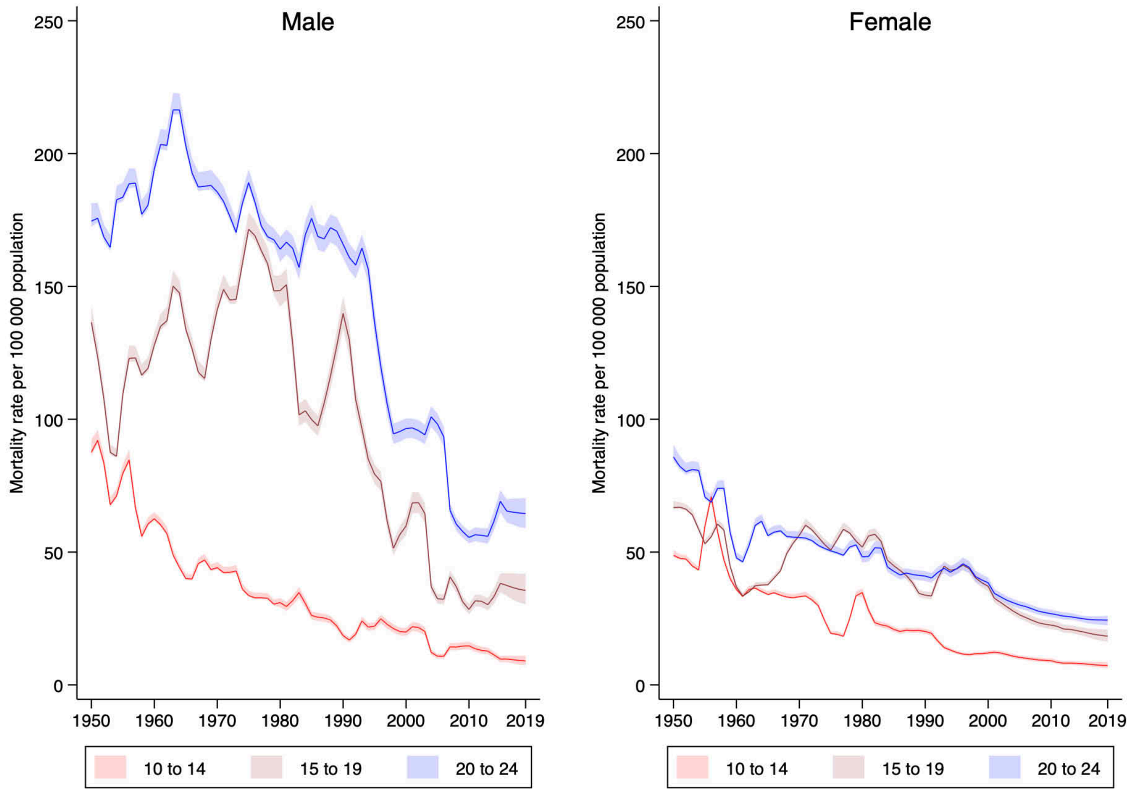

Figure S85b Number of deaths from all causes in 10-24 1950 – 2019: Luxembourg

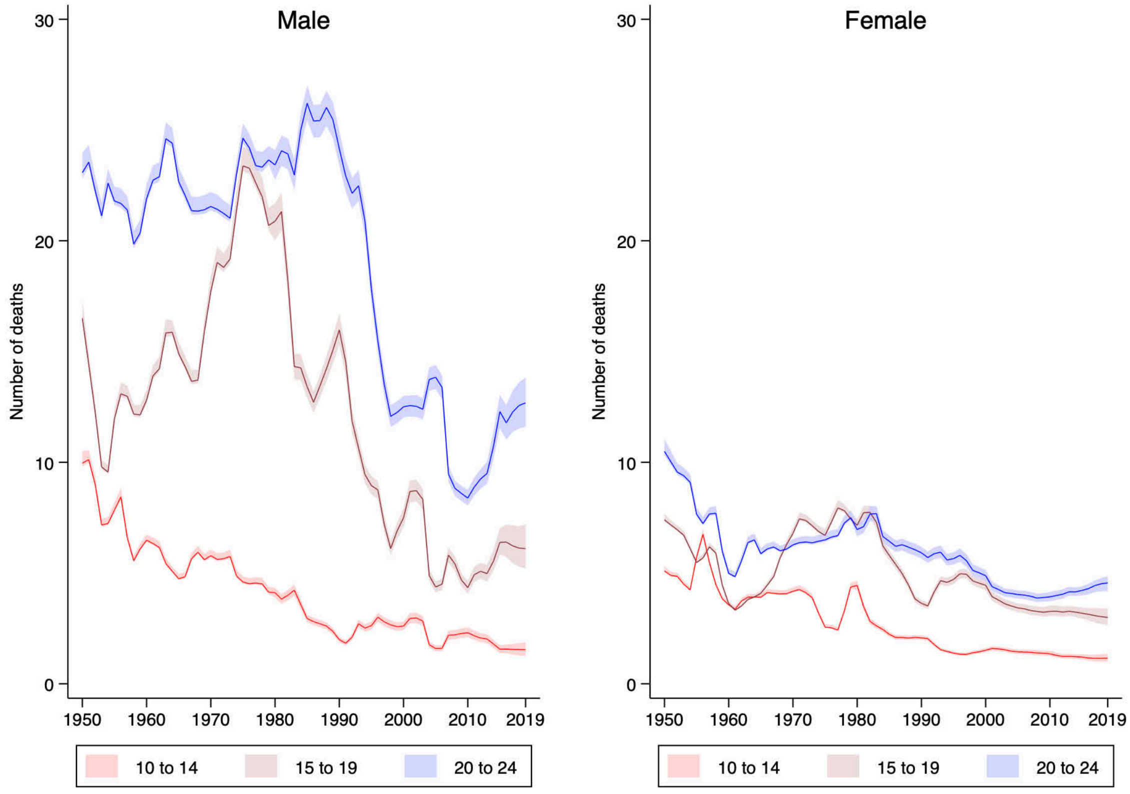

Figure S85c Mortality rate per 100,000 in 10-24 1980 – 2019 by cause: Luxembourg

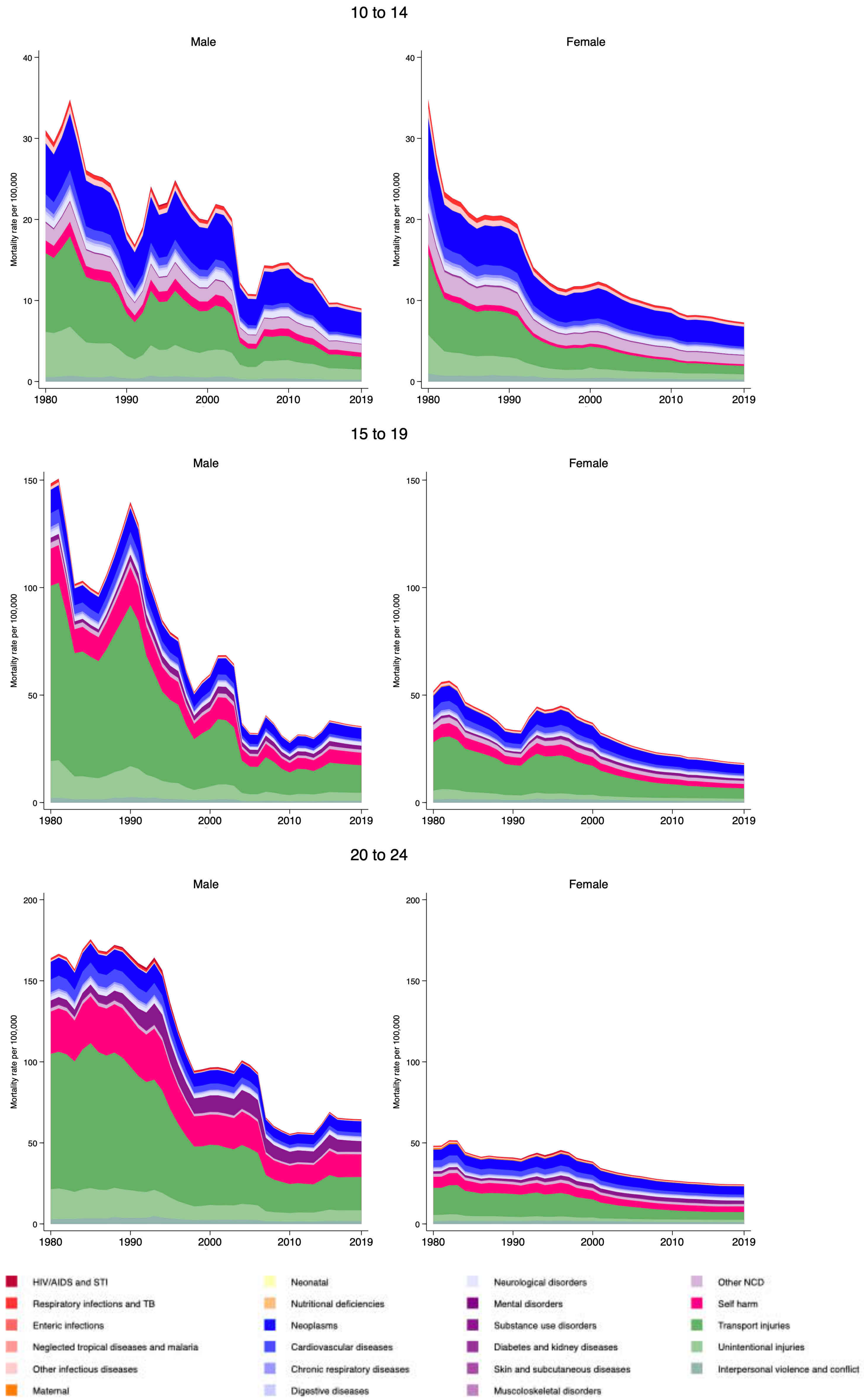

Figure S85d Number of deaths in 10-24 1980 – 2019 by cause: Luxembourg

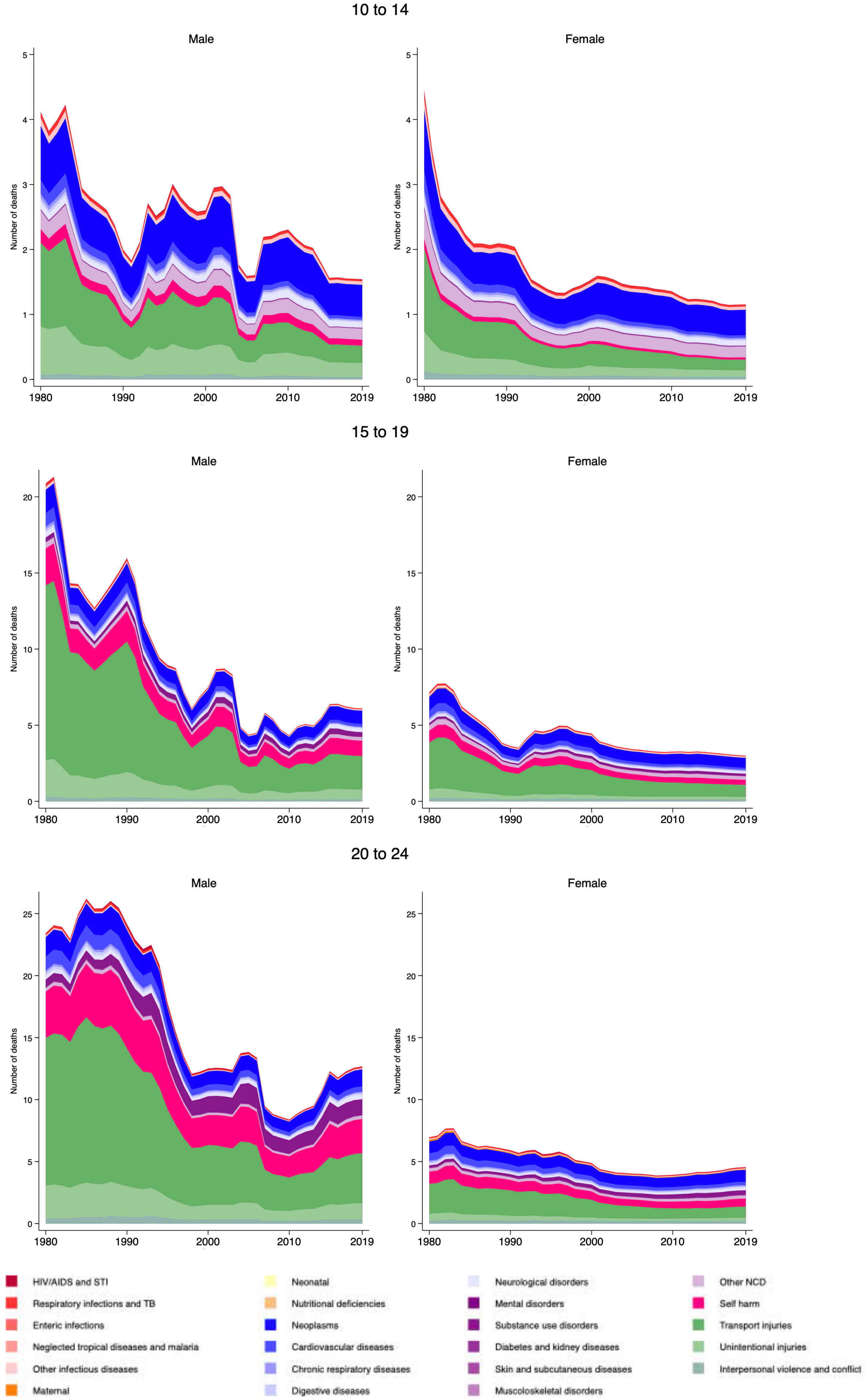

Figure S86: Malta

Figure S86a All-cause mortality rate per 100,000 in 10-24 1950 – 2019: Malta

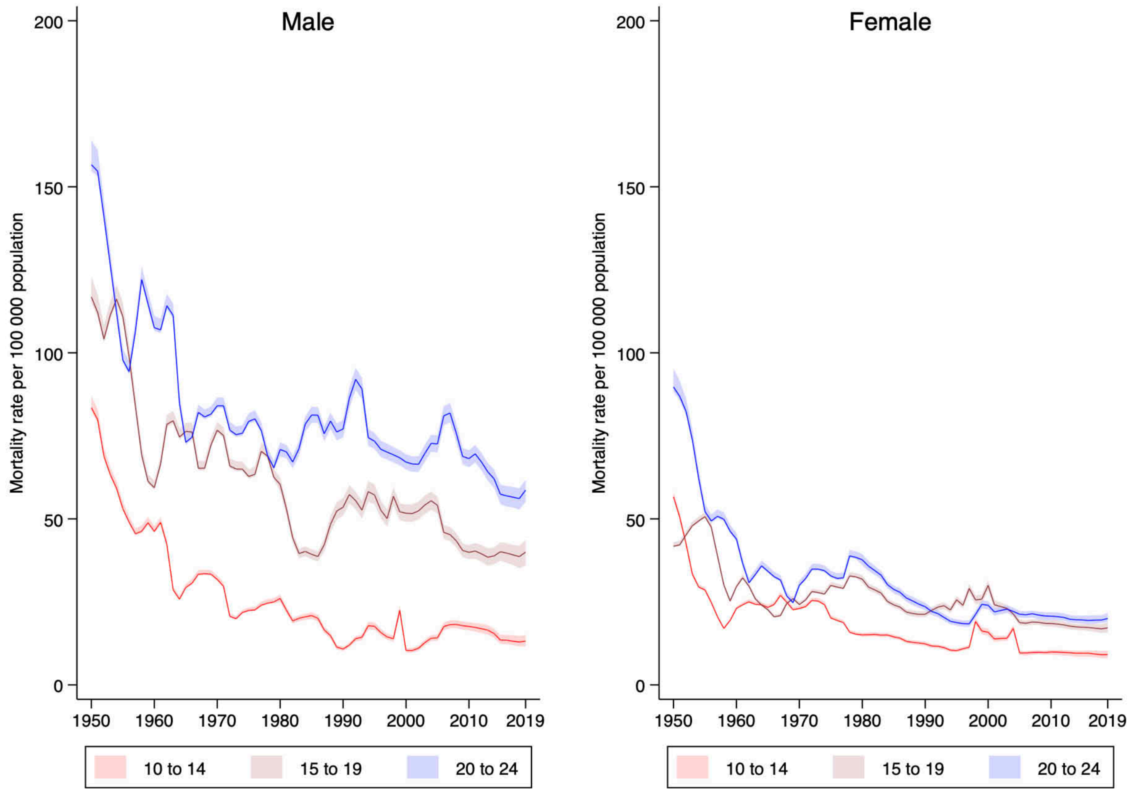

Figure S86b Number of deaths from all causes in 10-24 1950 – 2019: Malta

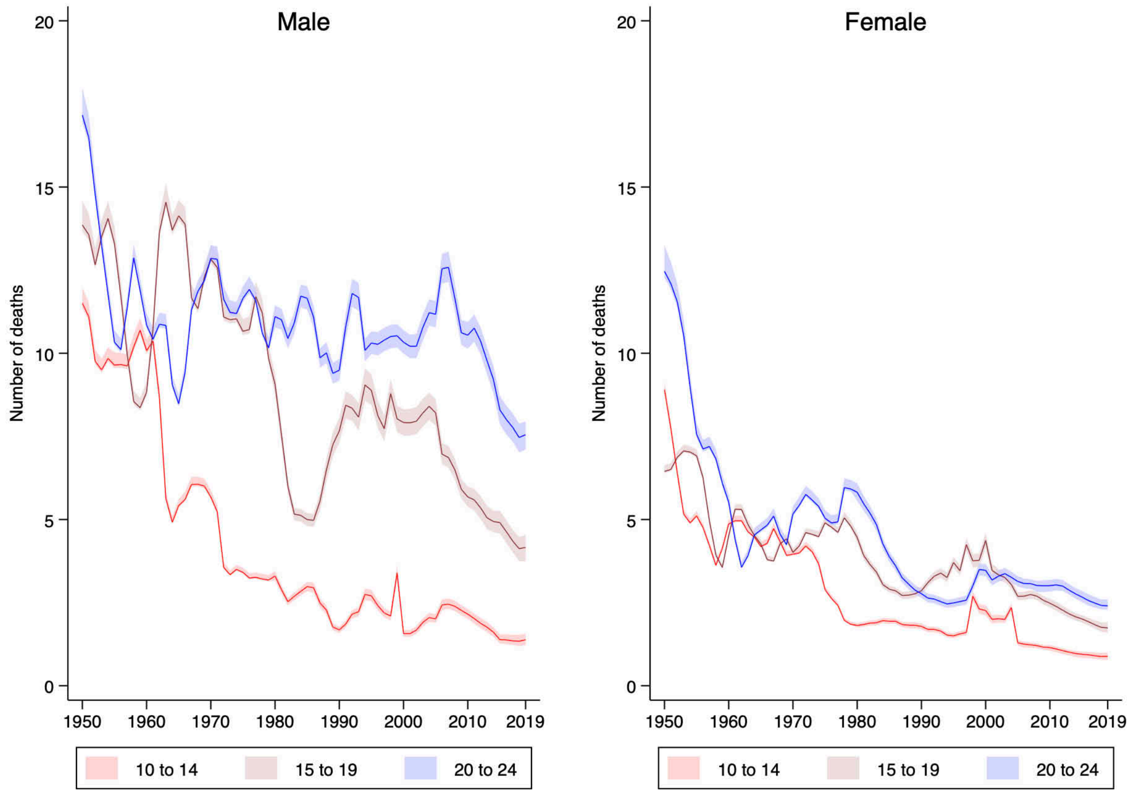

Figure S86c Mortality rate per 100,000 in 10-24 1980 – 2019 by cause: Malta

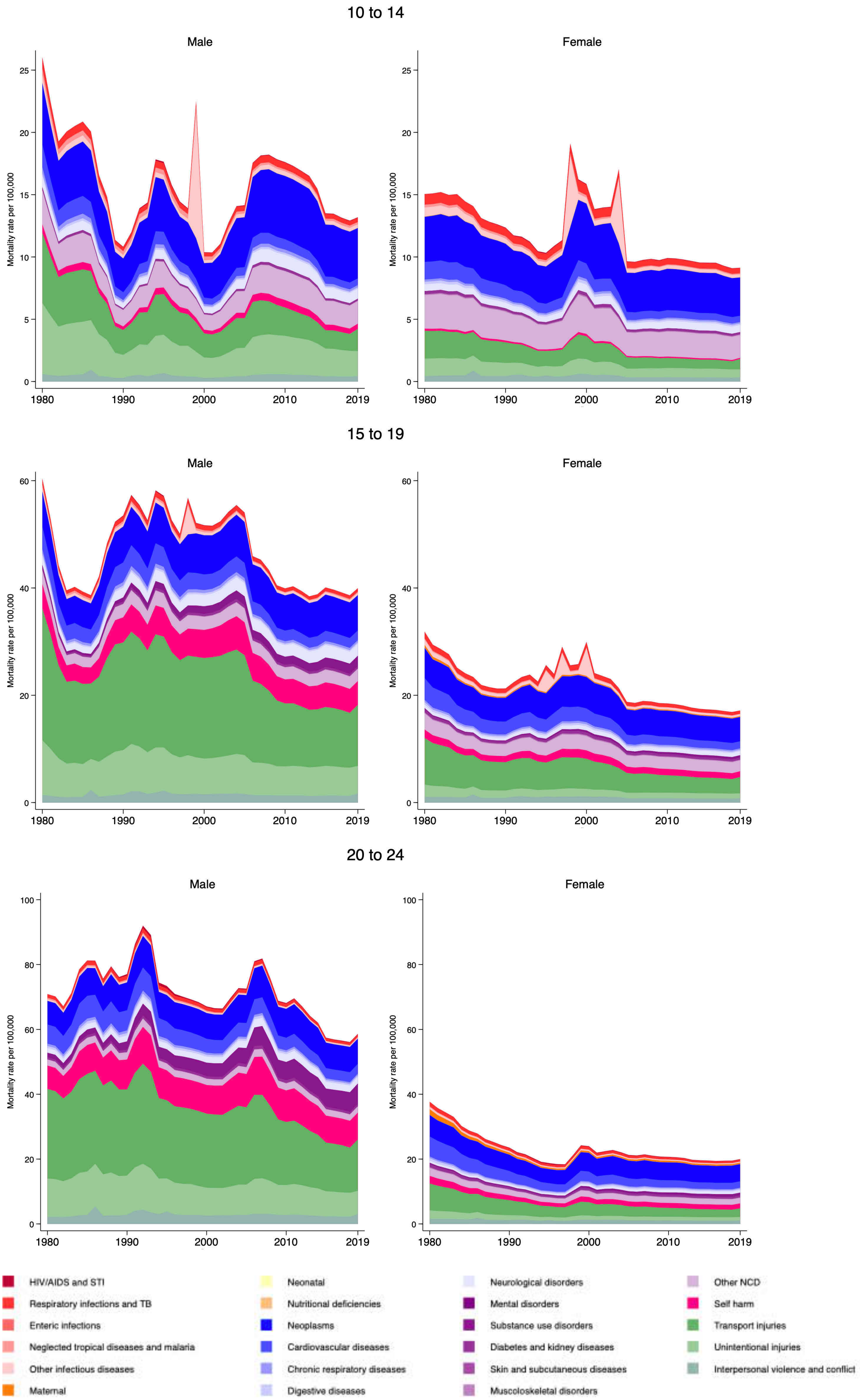

Figure S86d Number of deaths in 10-24 1980 – 2019 by cause: Malta

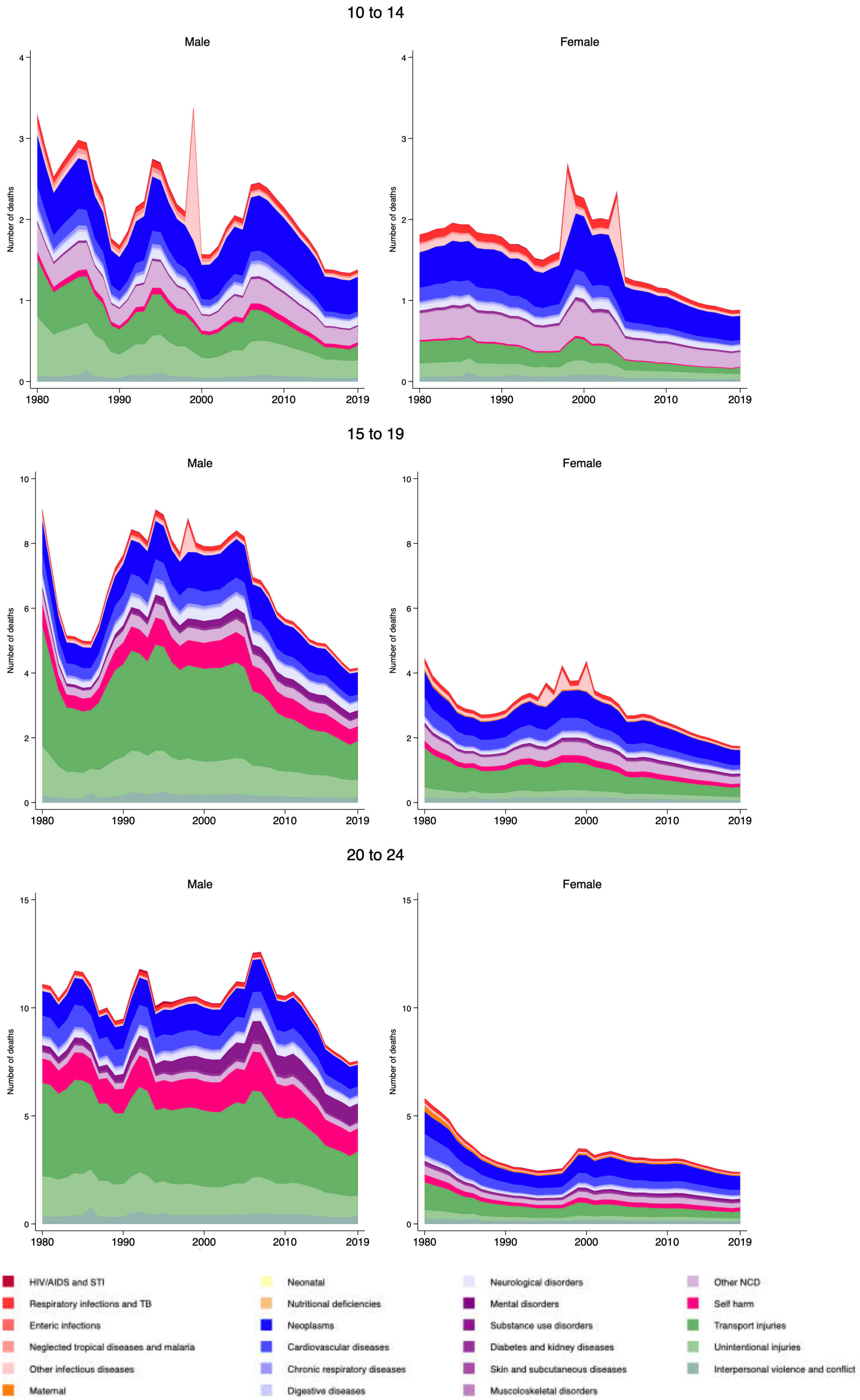

Figure S87: Netherlands

Figure S87a All-cause mortality rate per 100,000 in 10-24 1950 – 2019: Netherlands

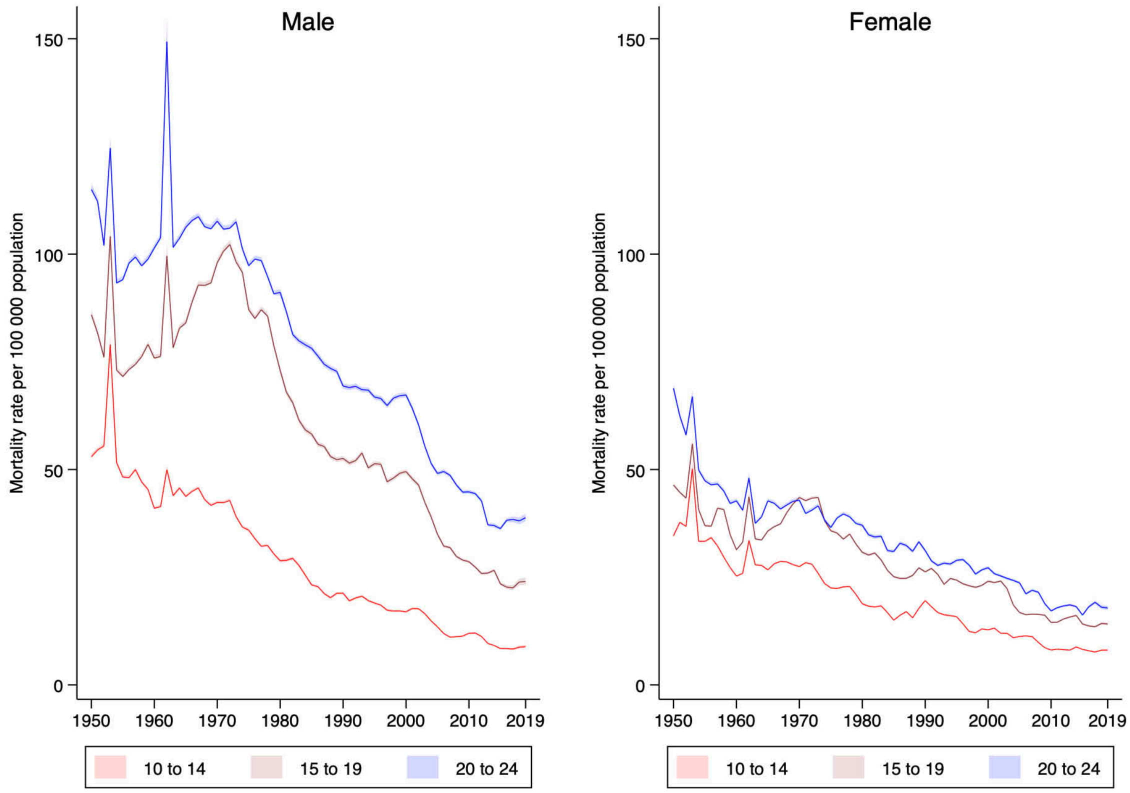

Figure S87b Number of deaths from all causes in 10-24 1950 – 2019: Netherlands

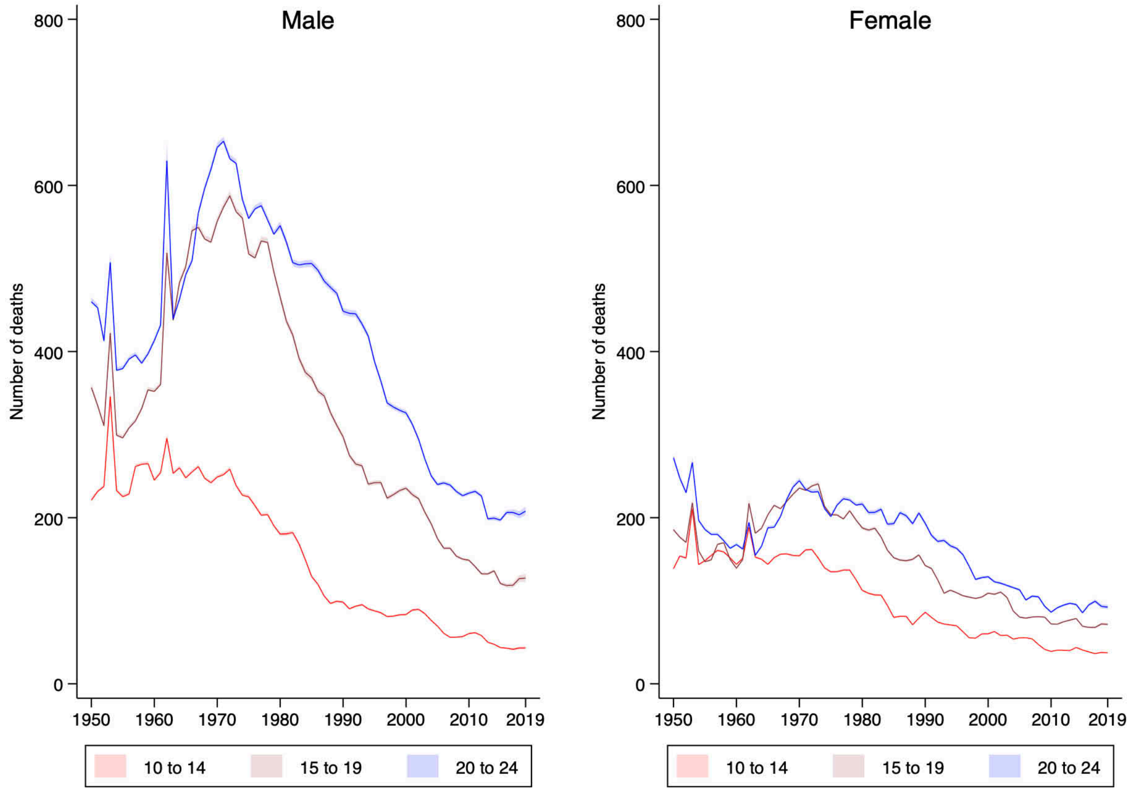

Figure S87c Mortality rate per 100,000 in 10-24 1980 – 2019 by cause: Netherlands

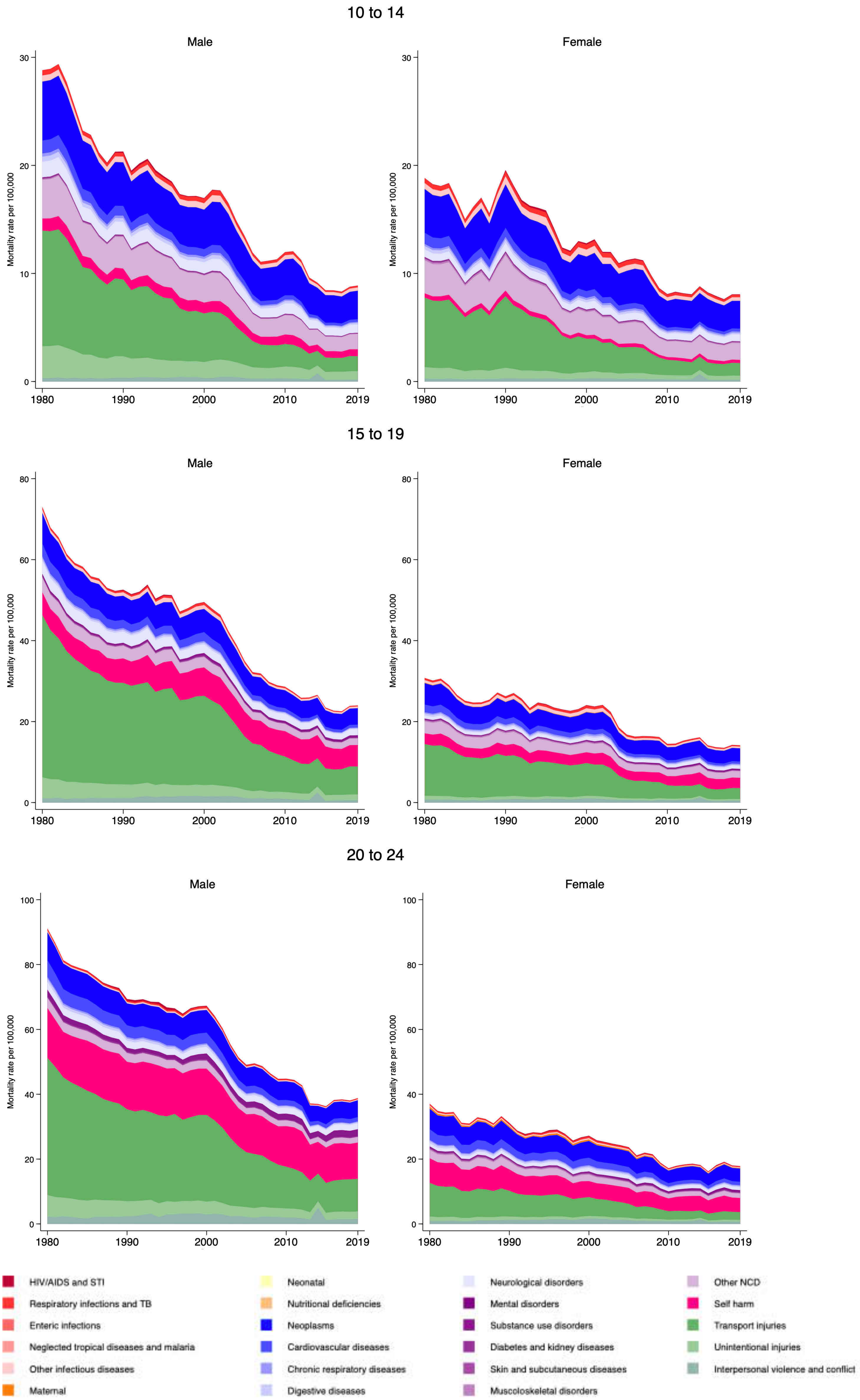

Figure S87d Number of deaths in 10-24 1980 – 2019 by cause: Netherlands

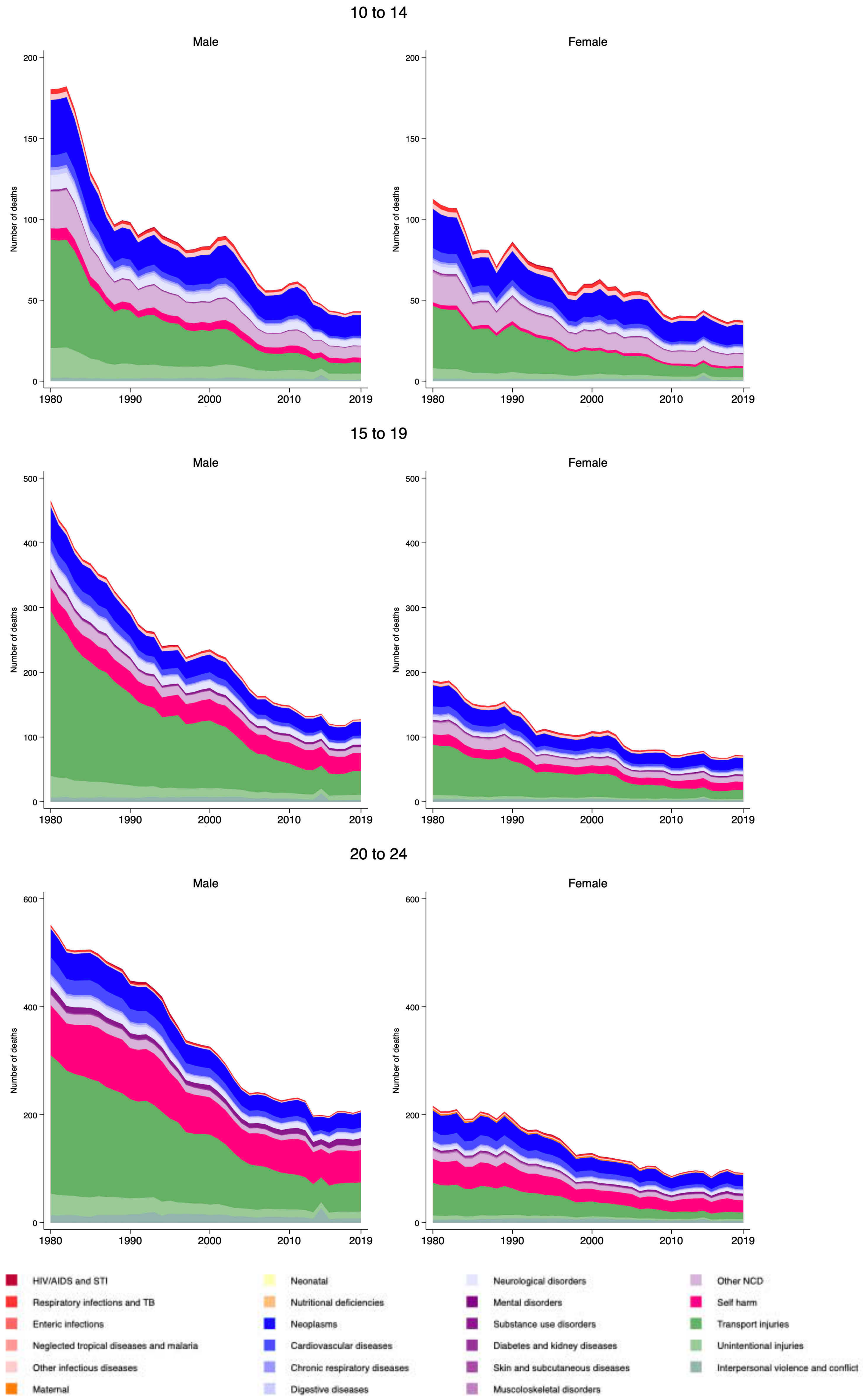

Figure S88: Norway

Figure S88a All-cause mortality rate per 100,000 in 10-24 1950 – 2019: Norway

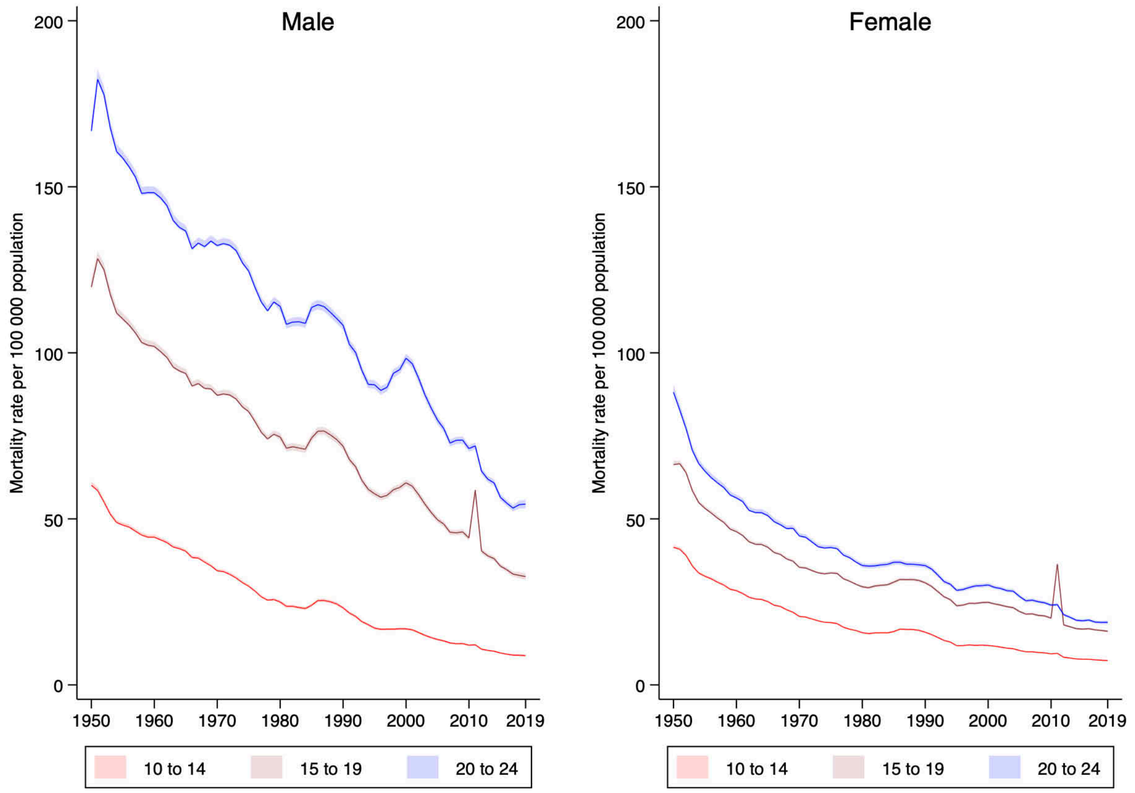

Figure S88b Number of deaths from all causes in 10-24 1950 – 2019: Norway

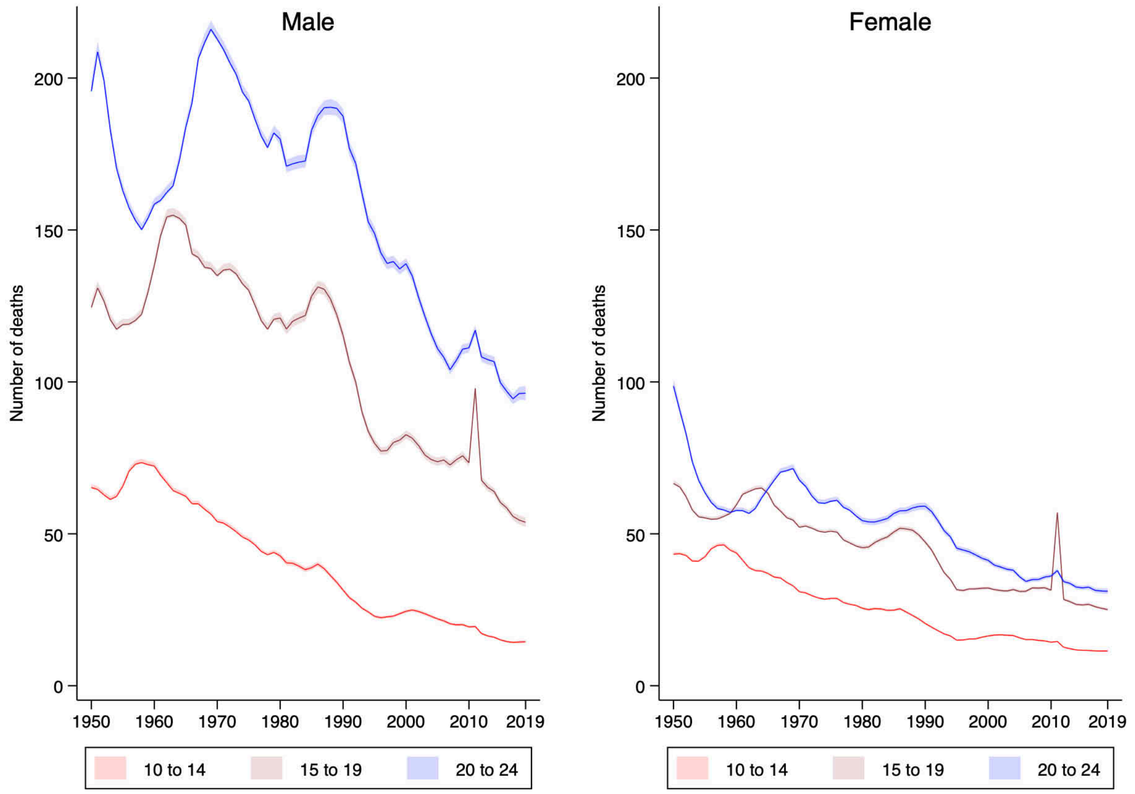

Figure S88c Mortality rate per 100,000 in 10-24 1980 – 2019 by cause: Norway

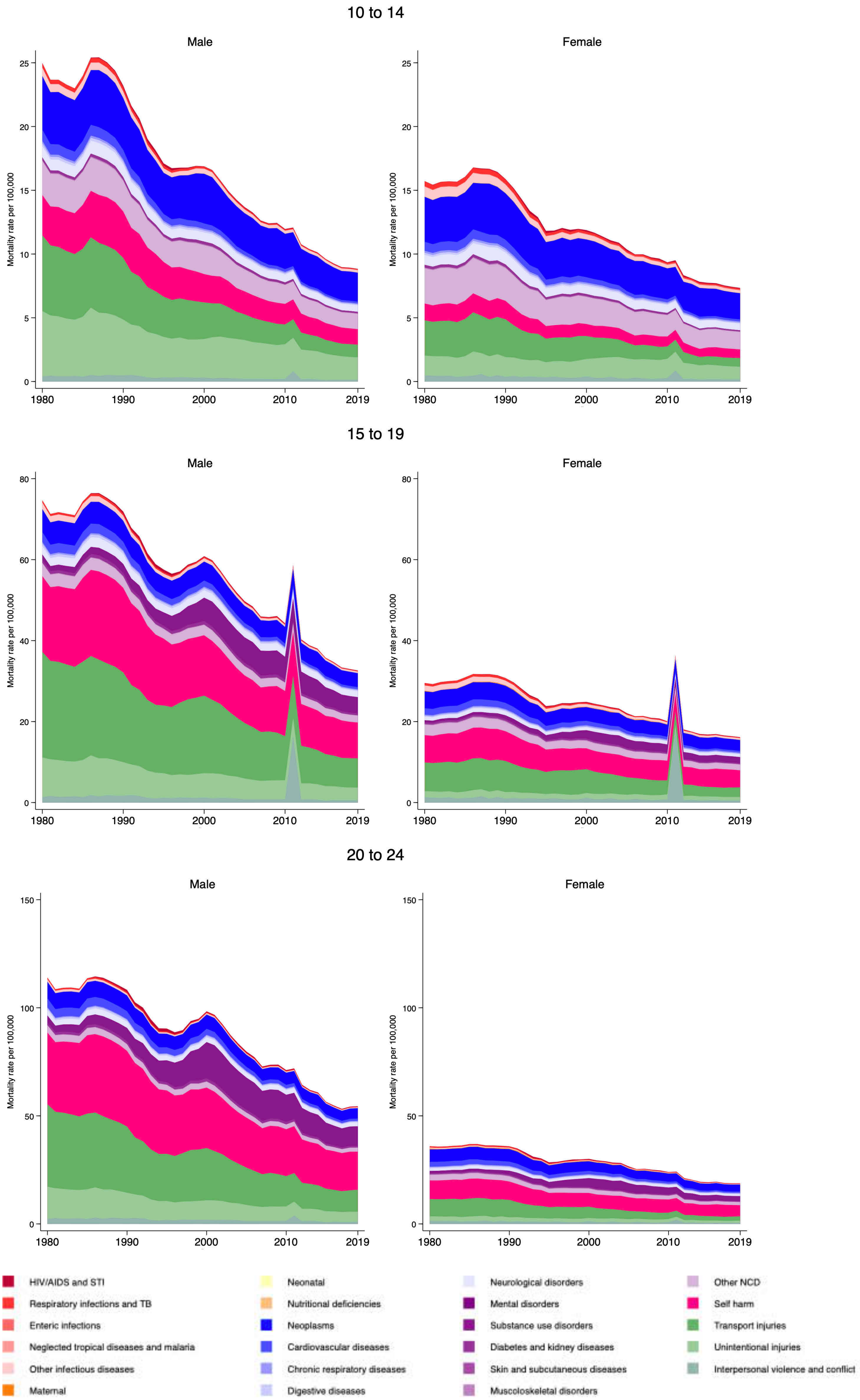

Figure S88d Number of deaths in 10-24 1980 – 2019 by cause: Norway

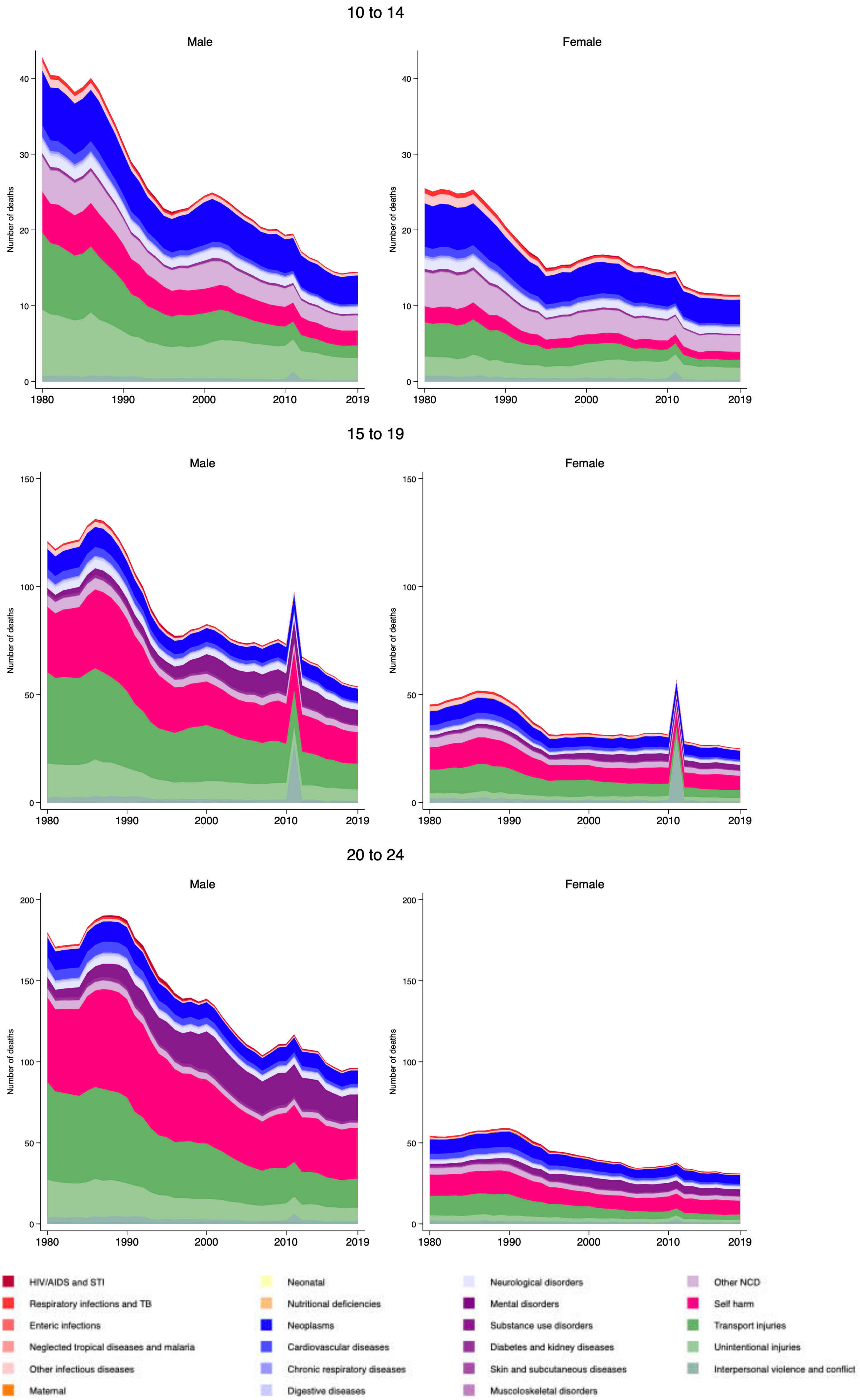

Figure S89: Portugal

Figure S89a All-cause mortality rate per 100,000 in 10-24 1950 – 2019: Portugal

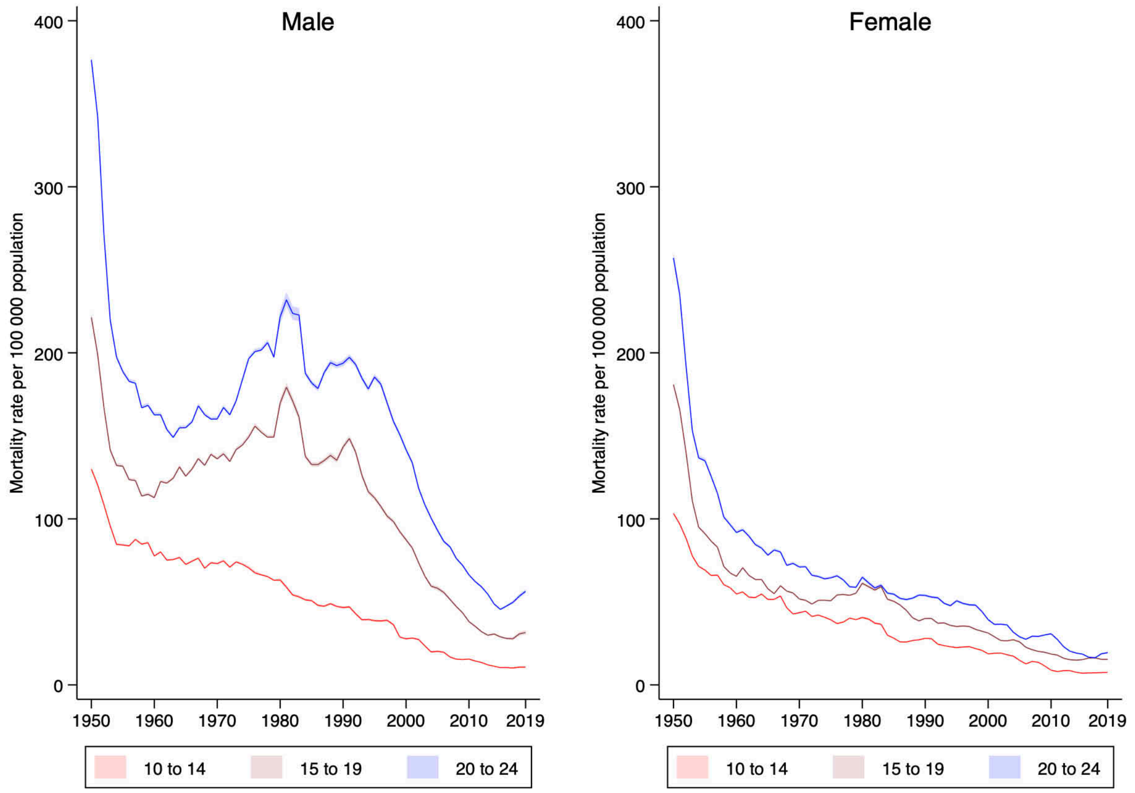

Figure S89b Number of deaths from all causes in 10-24 1950 – 2019: Portugal

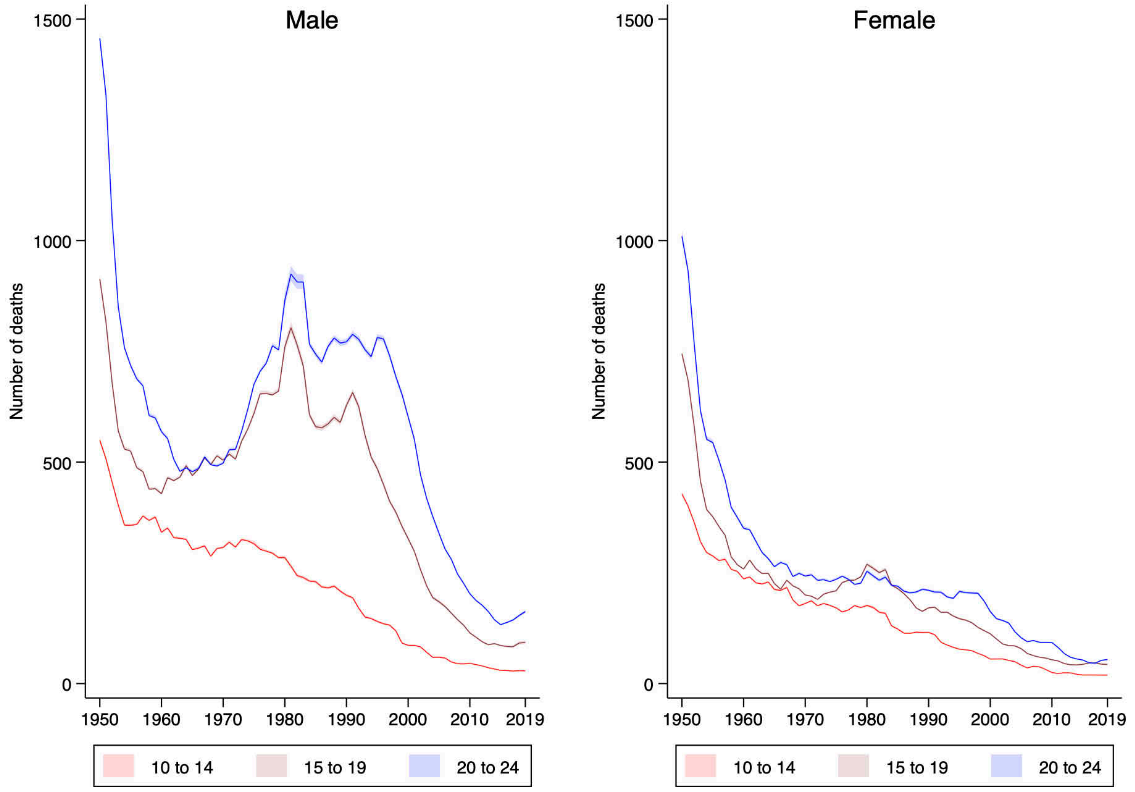

Figure S89c Mortality rate per 100,000 in 10-24 1980 – 2019 by cause: Portugal

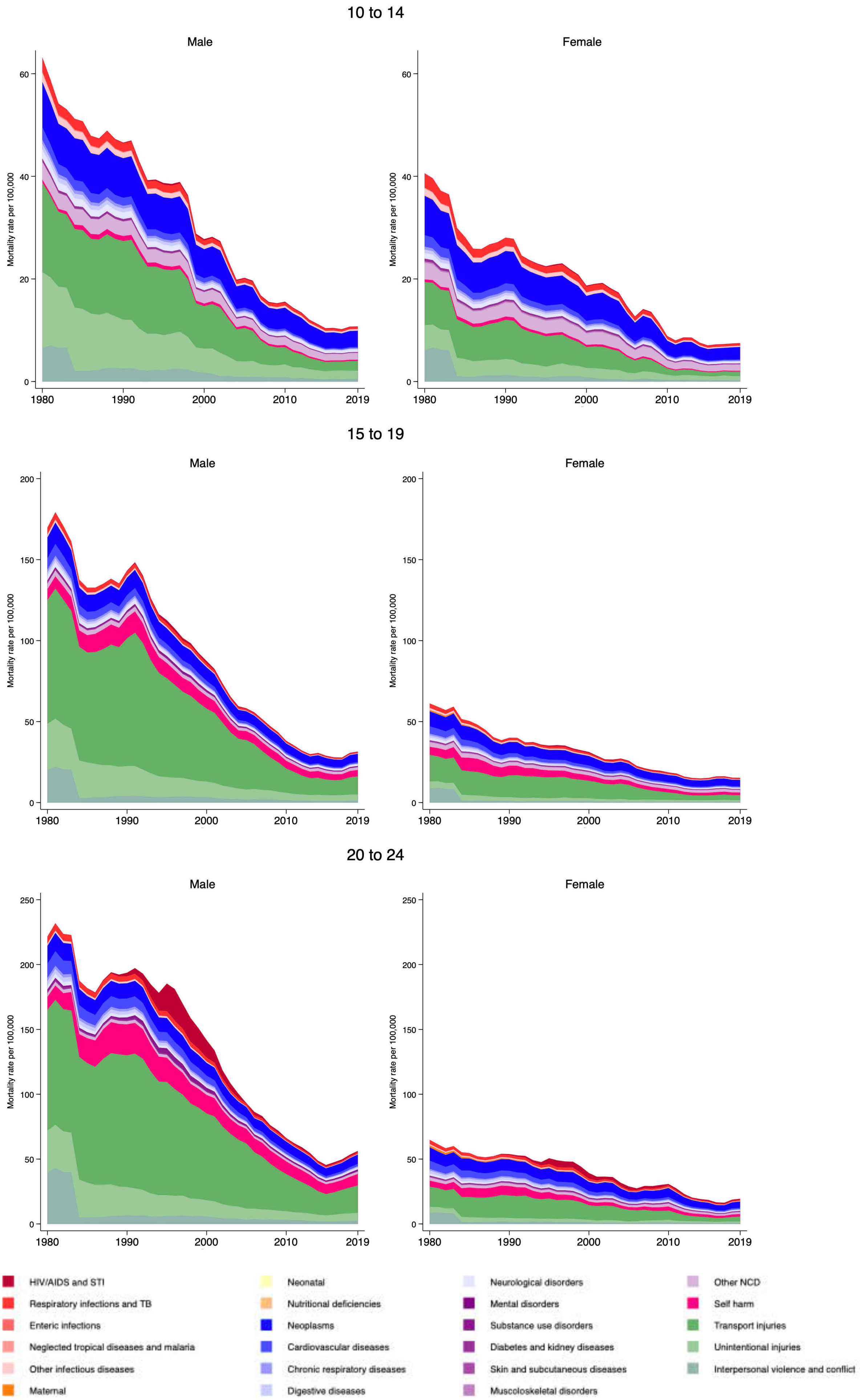

Figure S89d Number of deaths in 10-24 1980 – 2019 by cause: Portugal

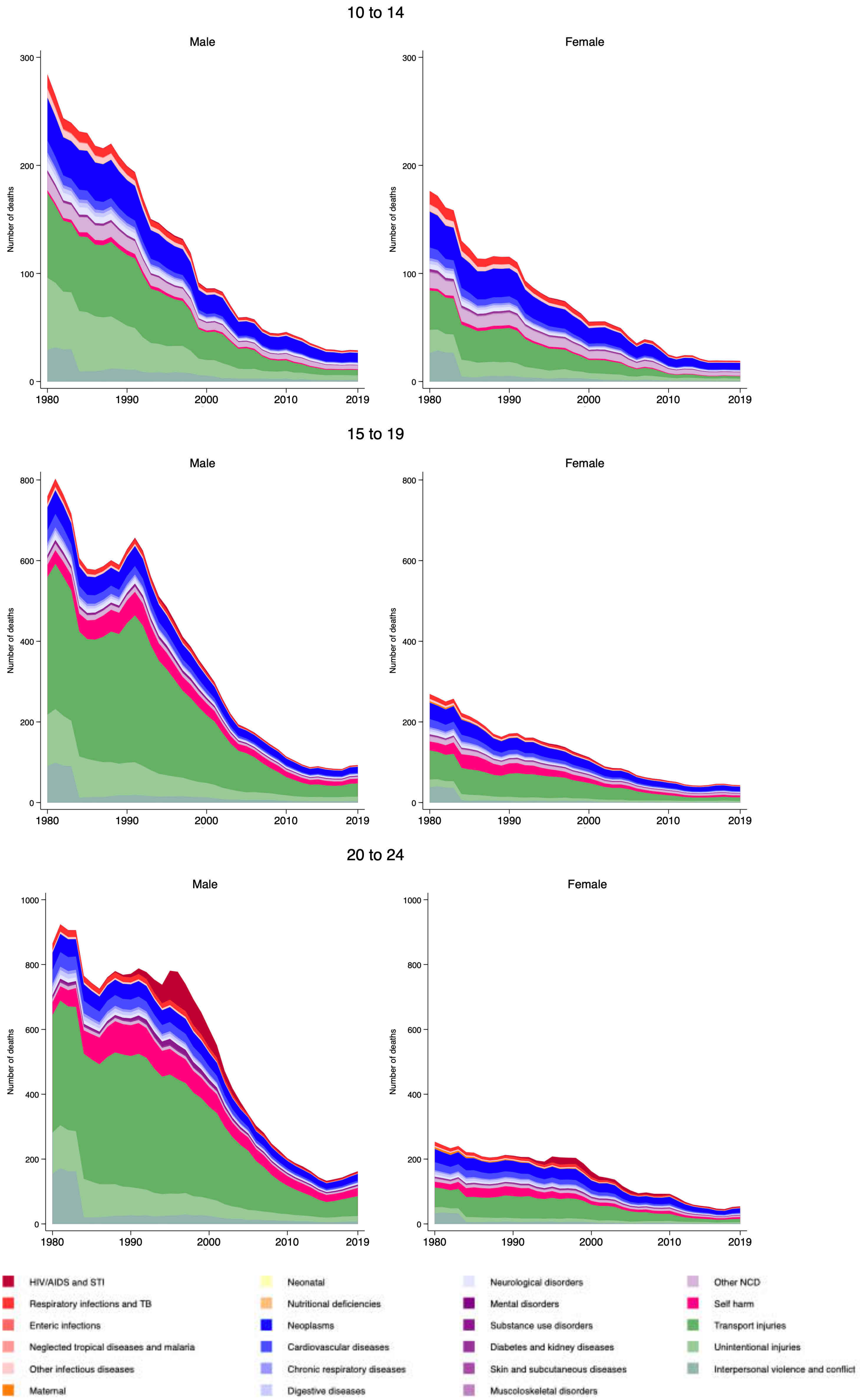

Figure S90: Spain

Figure S90a All-cause mortality rate per 100,000 in 10-24 1950 – 2019: Spain

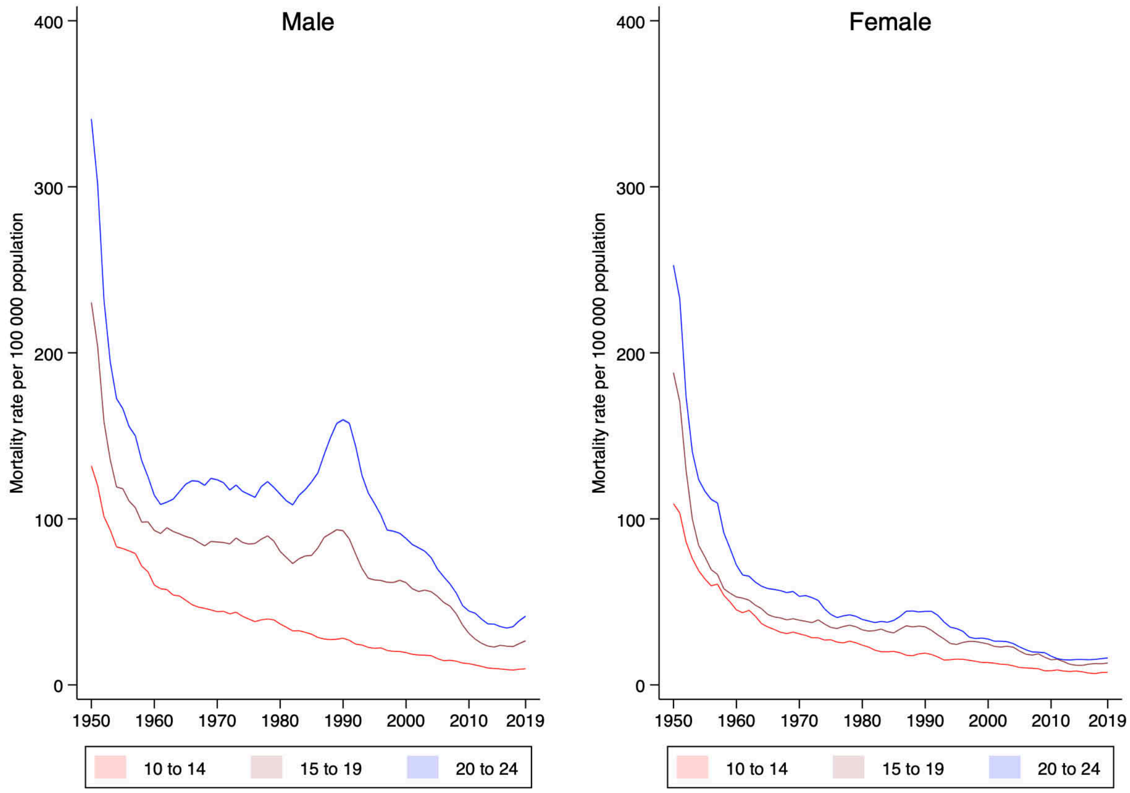

Figure S90b Number of deaths from all causes in 10-24 1950 – 2019: Spain

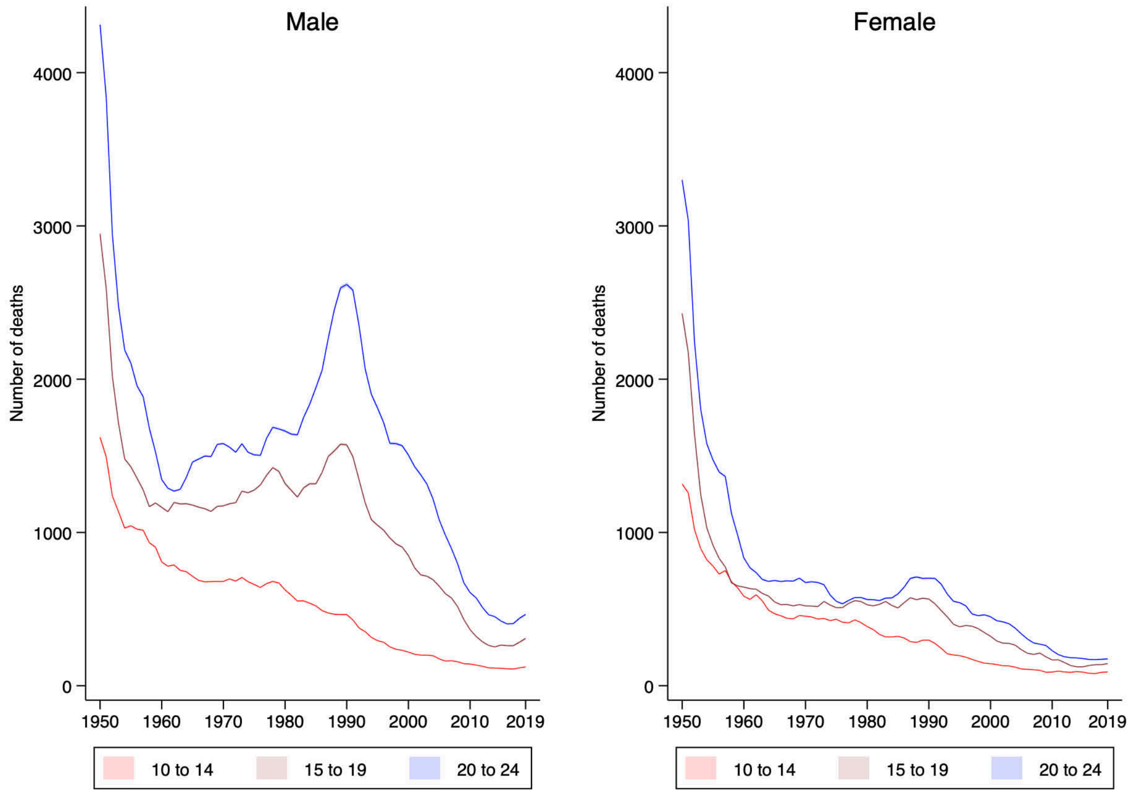

Figure S90c Mortality rate per 100,000 in 10-24 1980 – 2019 by cause: Spain

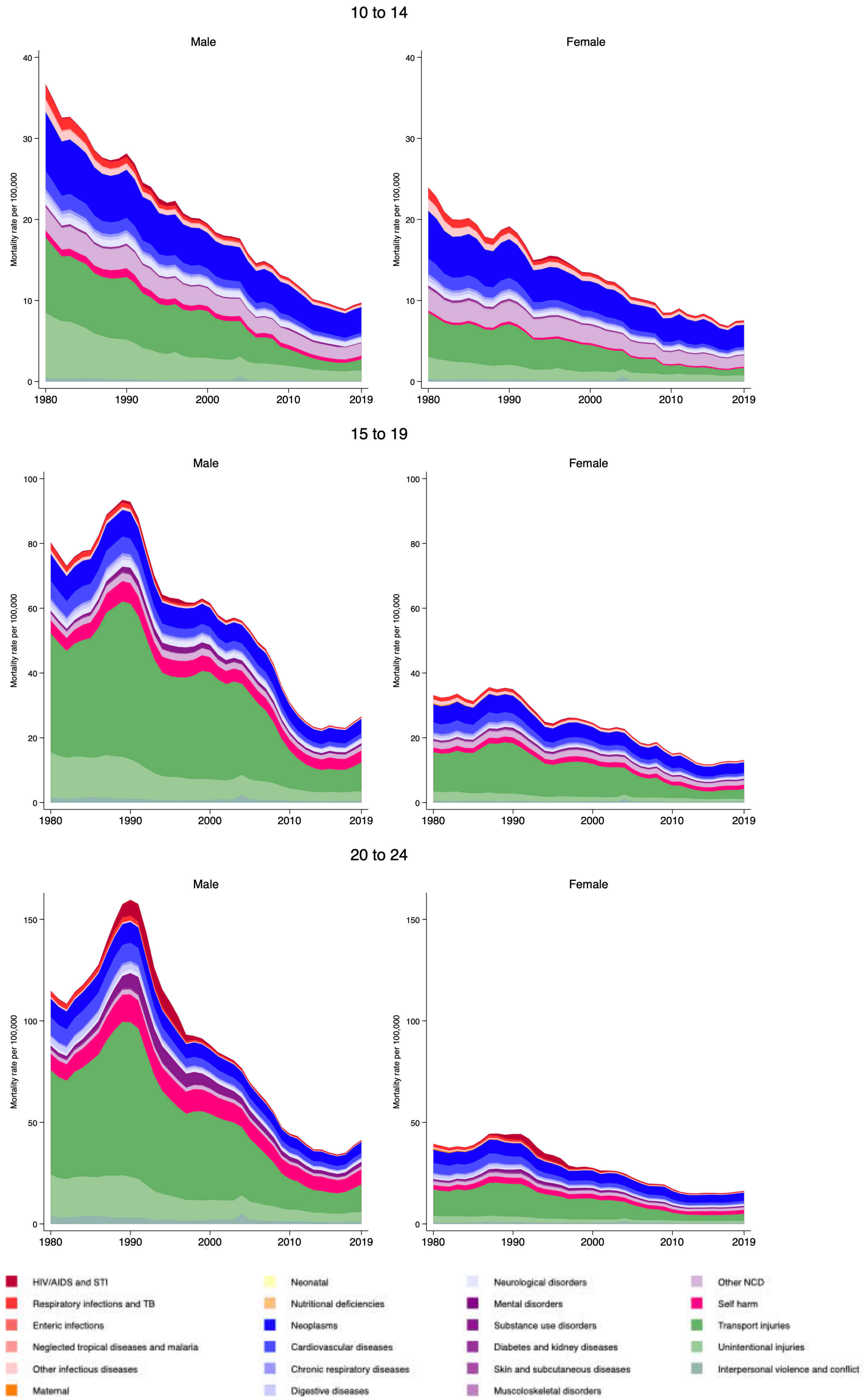

Figure S90d Number of deaths in 10-24 1980 – 2019 by cause: Spain

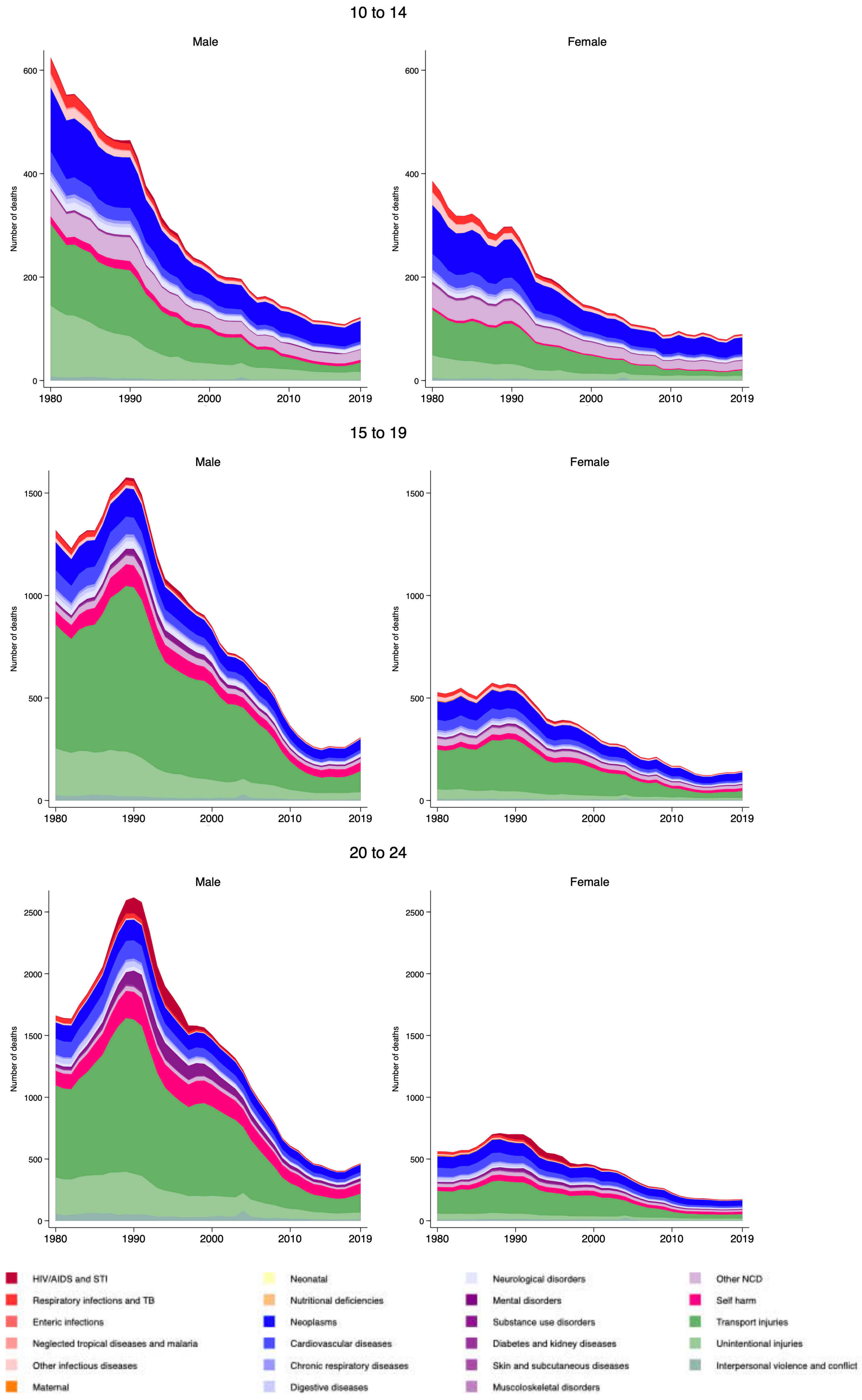

Figure S91: Sweden

Figure S91a All-cause mortality rate per 100,000 in 10-24 1950 – 2019: Sweden

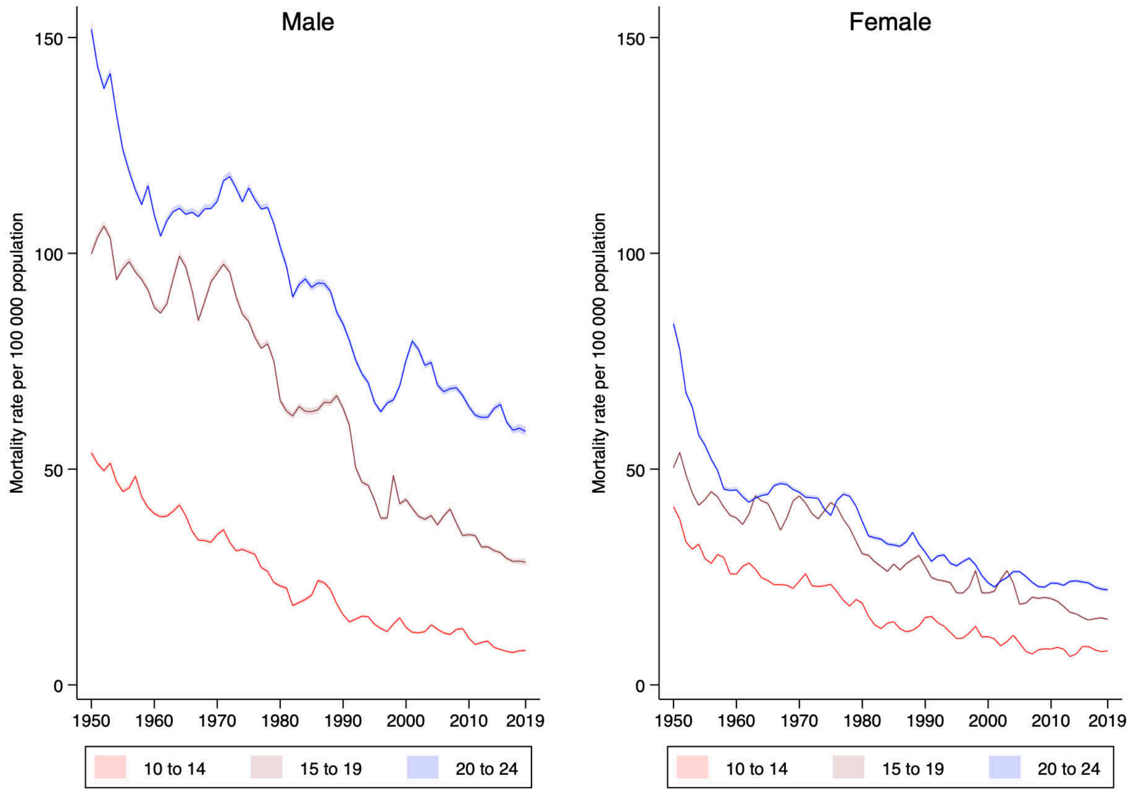

Figure S91b Number of deaths from all causes in 10-24 1950 – 2019: Sweden

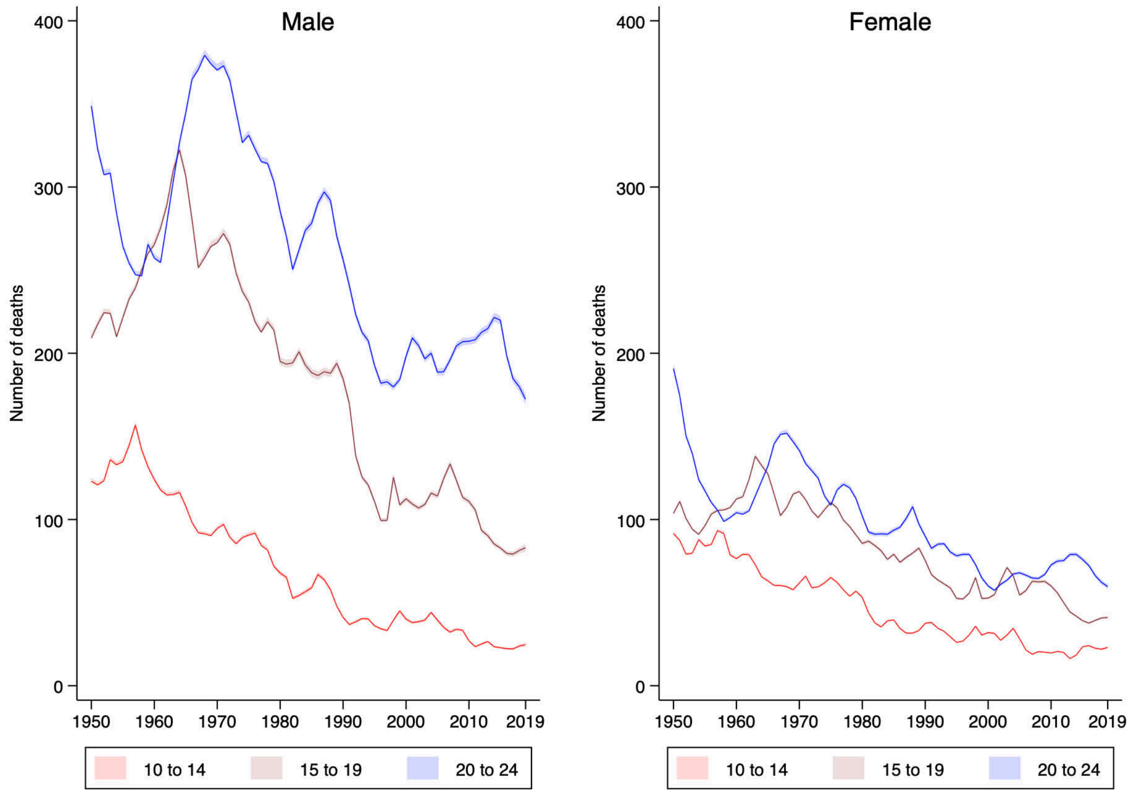

Figure S91c Mortality rate per 100,000 in 10-24 1980 – 2019 by cause: Sweden

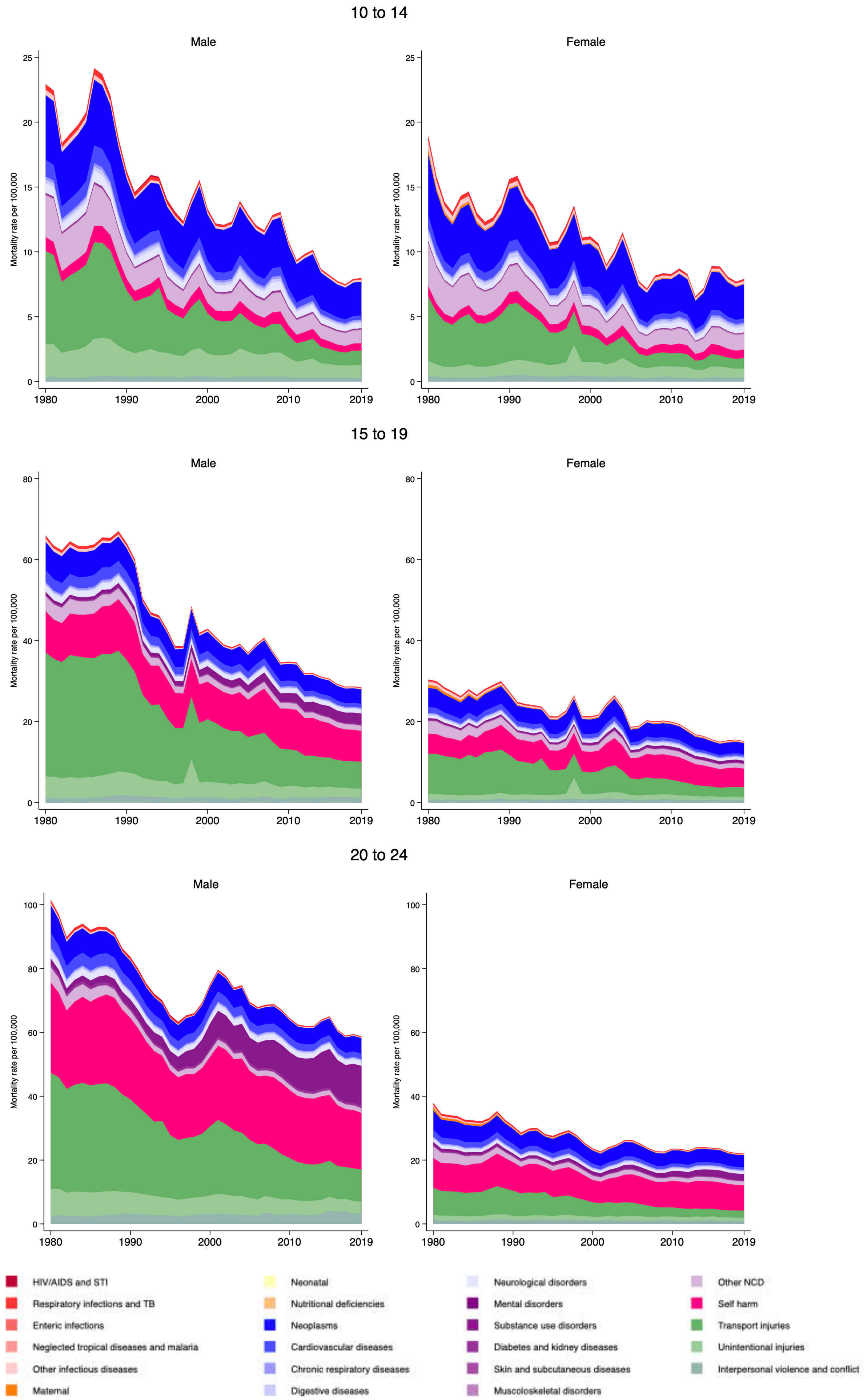

Figure S91d Number of deaths in 10-24 1980 – 2019 by cause: Sweden

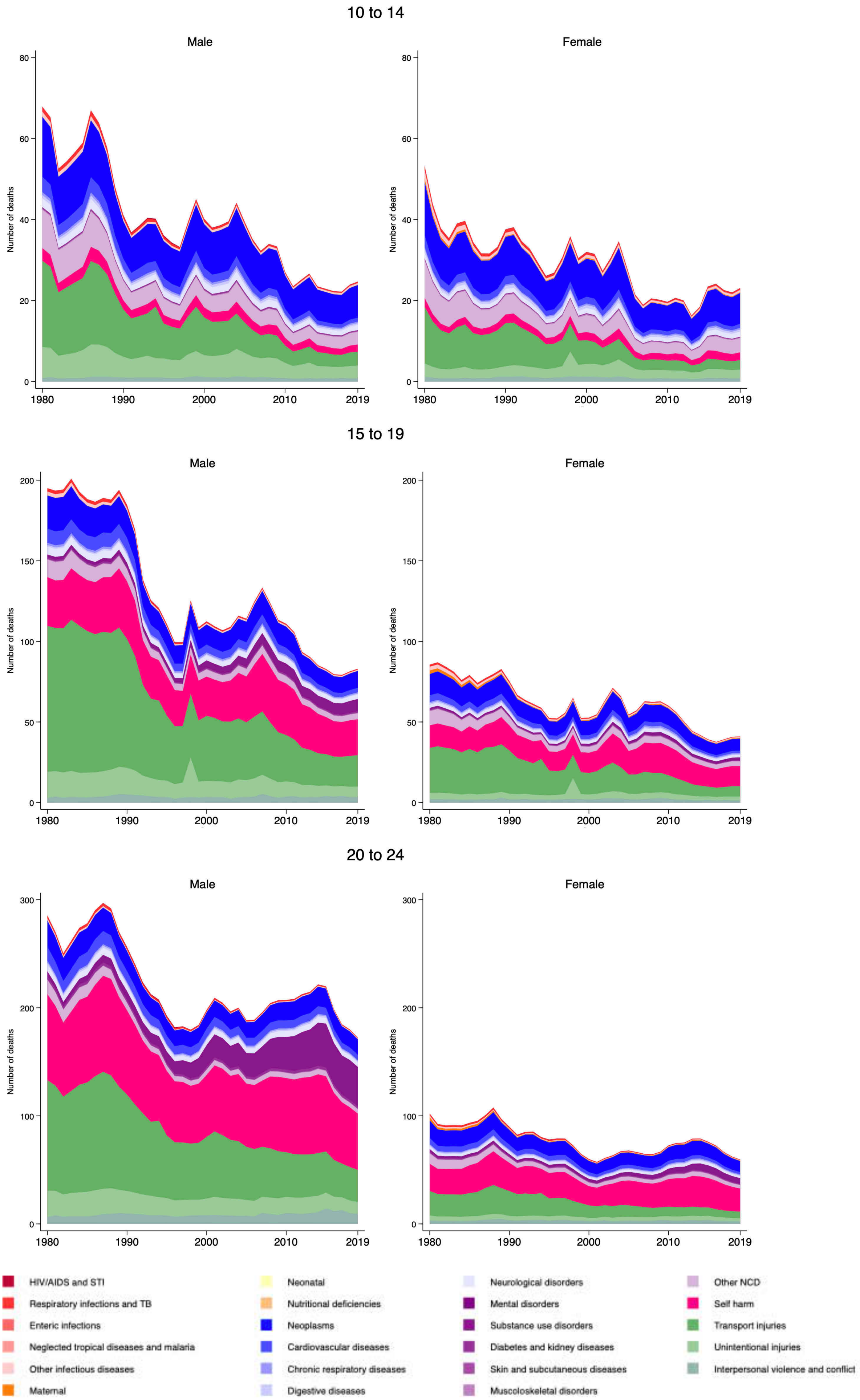

Figure S92: Switzerland

Figure S92a All-cause mortality rate per 100,000 in 10-24 1950 – 2019: Switzerland

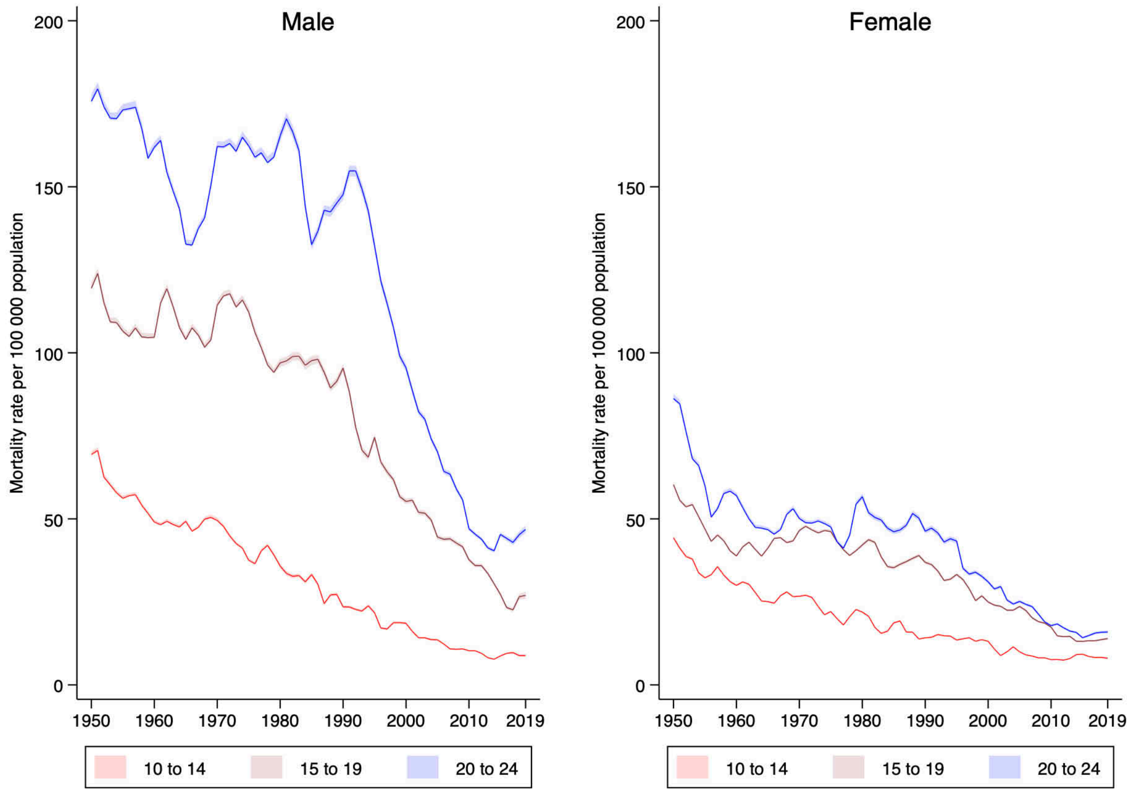

Figure S92b Number of deaths from all causes in 10-24 1950 – 2019: Switzerland

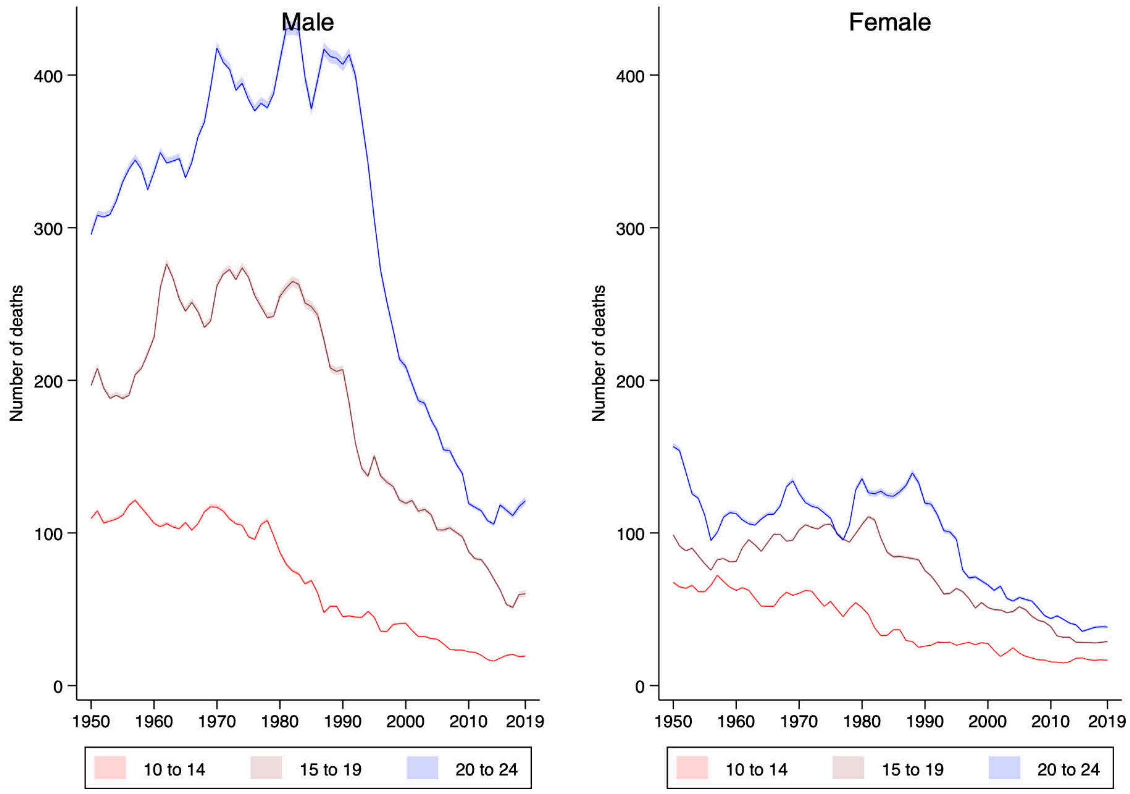

Figure S92c Mortality rate per 100,000 in 10-24 1980 – 2019 by cause: Switzerland

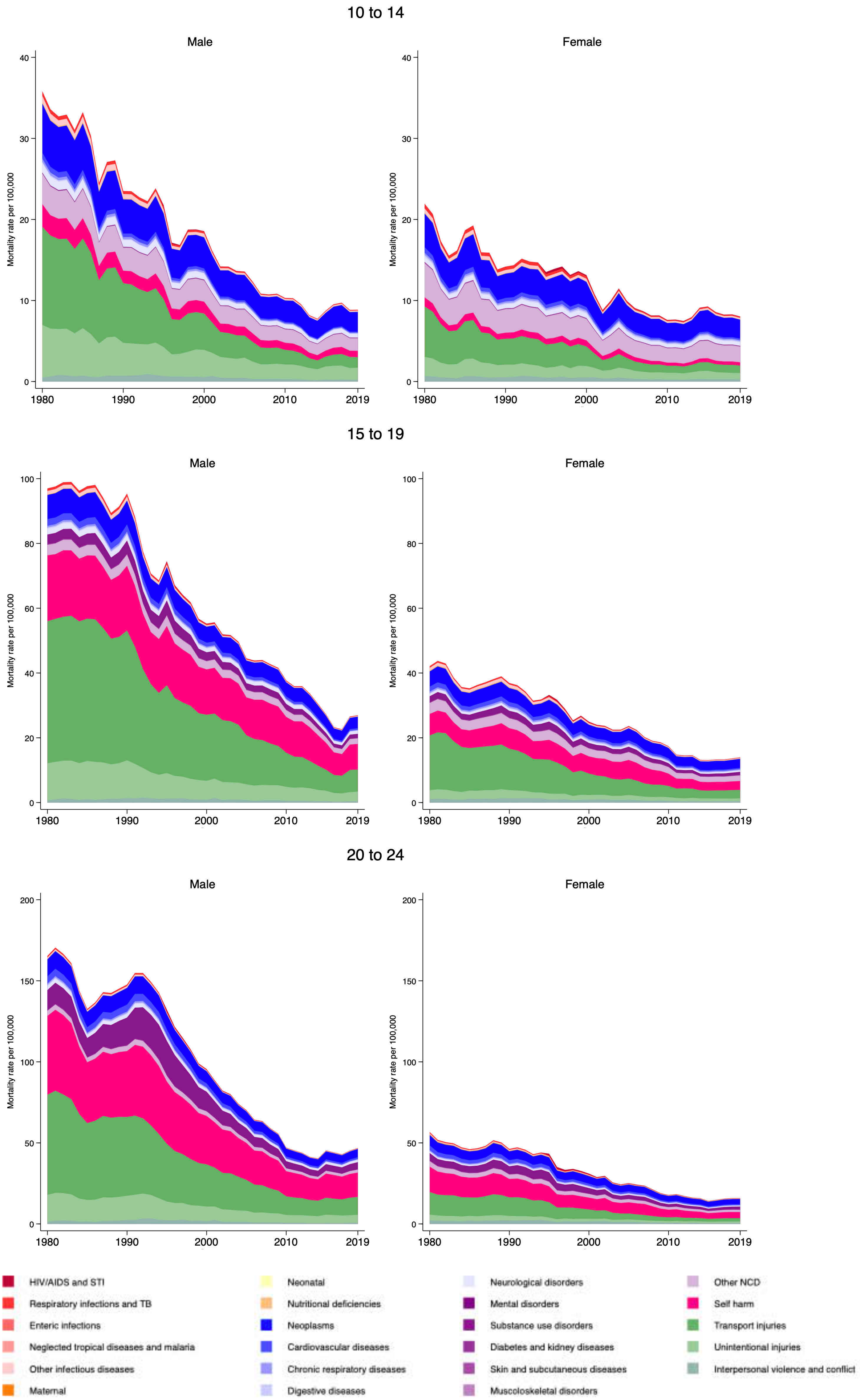

Figure S92d Number of deaths in 10-24 1980 – 2019 by cause: Switzerland

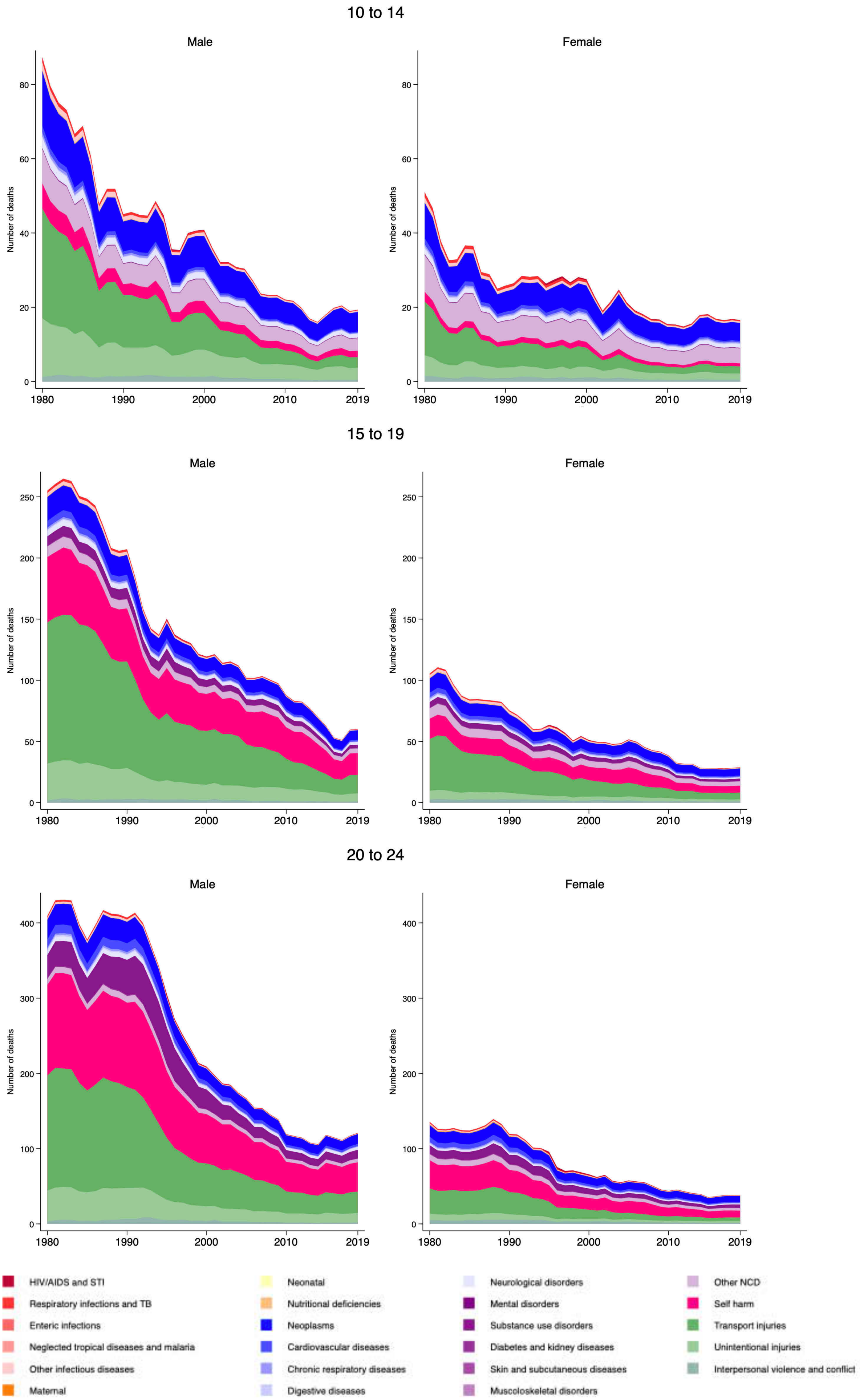

Figure S93: United Kingdom

Figure S93a All-cause mortality rate per 100,000 in 10-24 1950 – 2019: United Kingdom

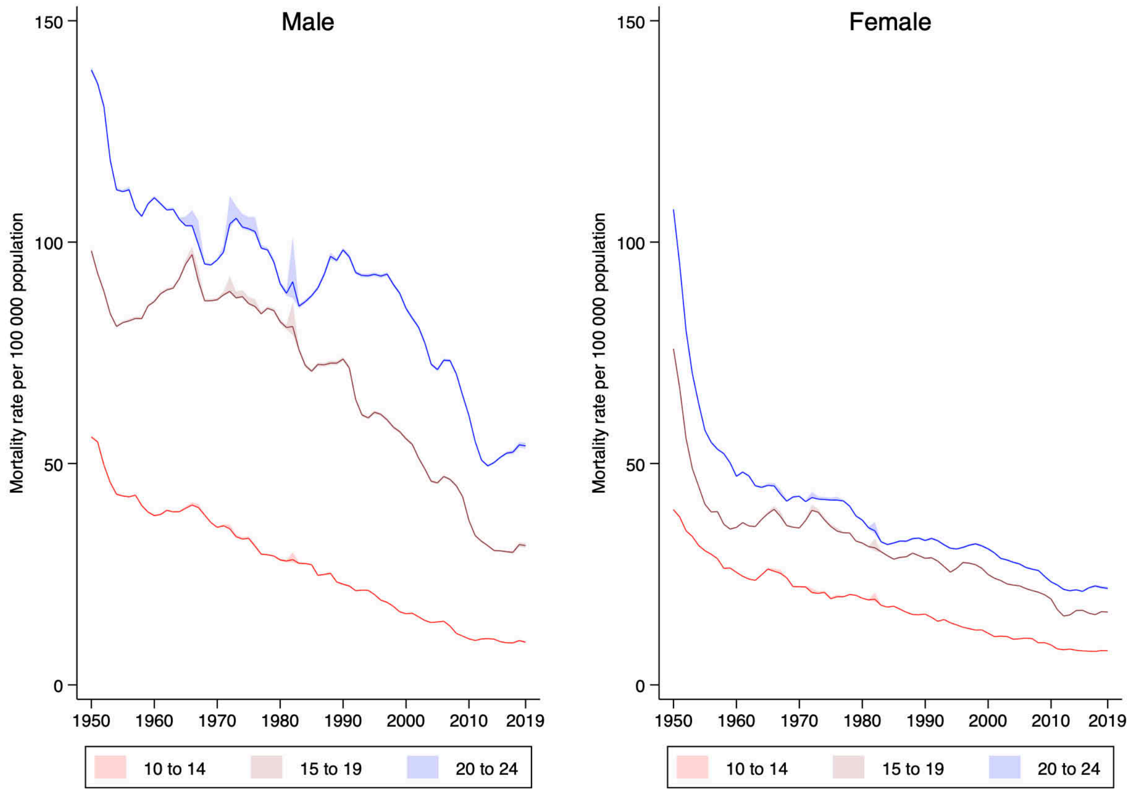

Figure S93b Number of deaths from all causes in 10-24 1950 – 2019: United Kingdom

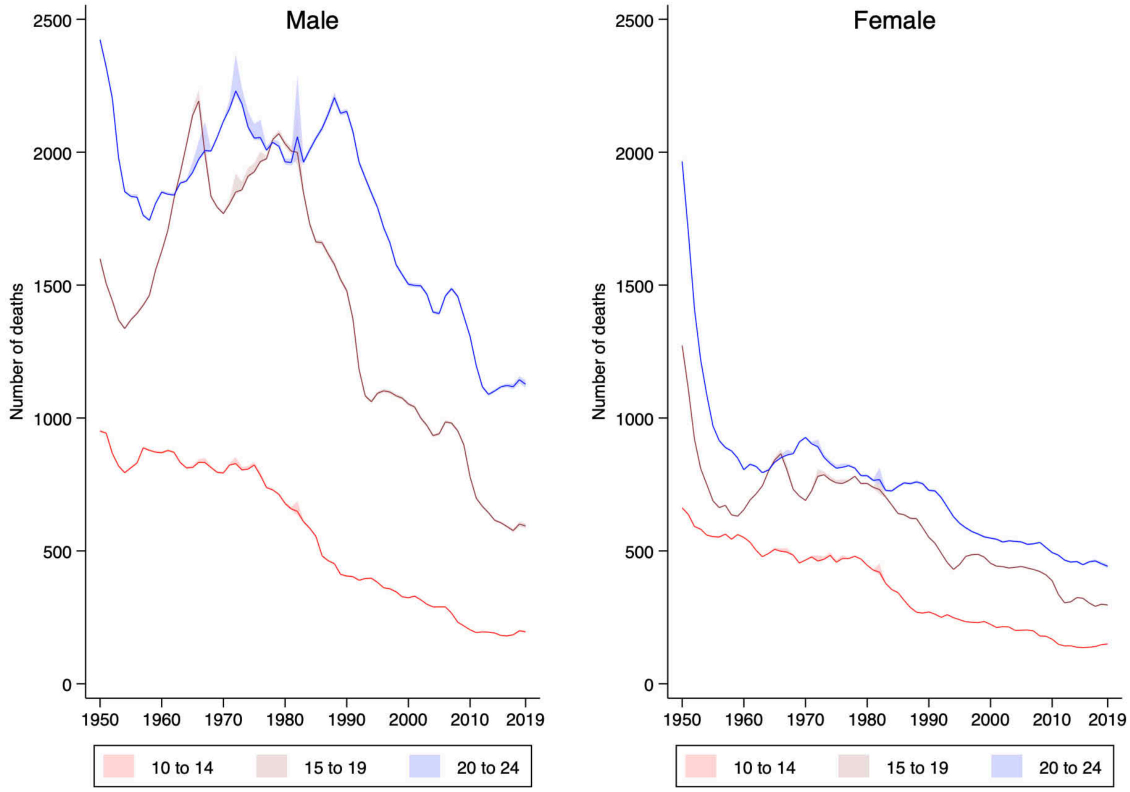

Figure S93c Mortality rate per 100,000 in 10-24 1980 – 2019 by cause: United Kingdom

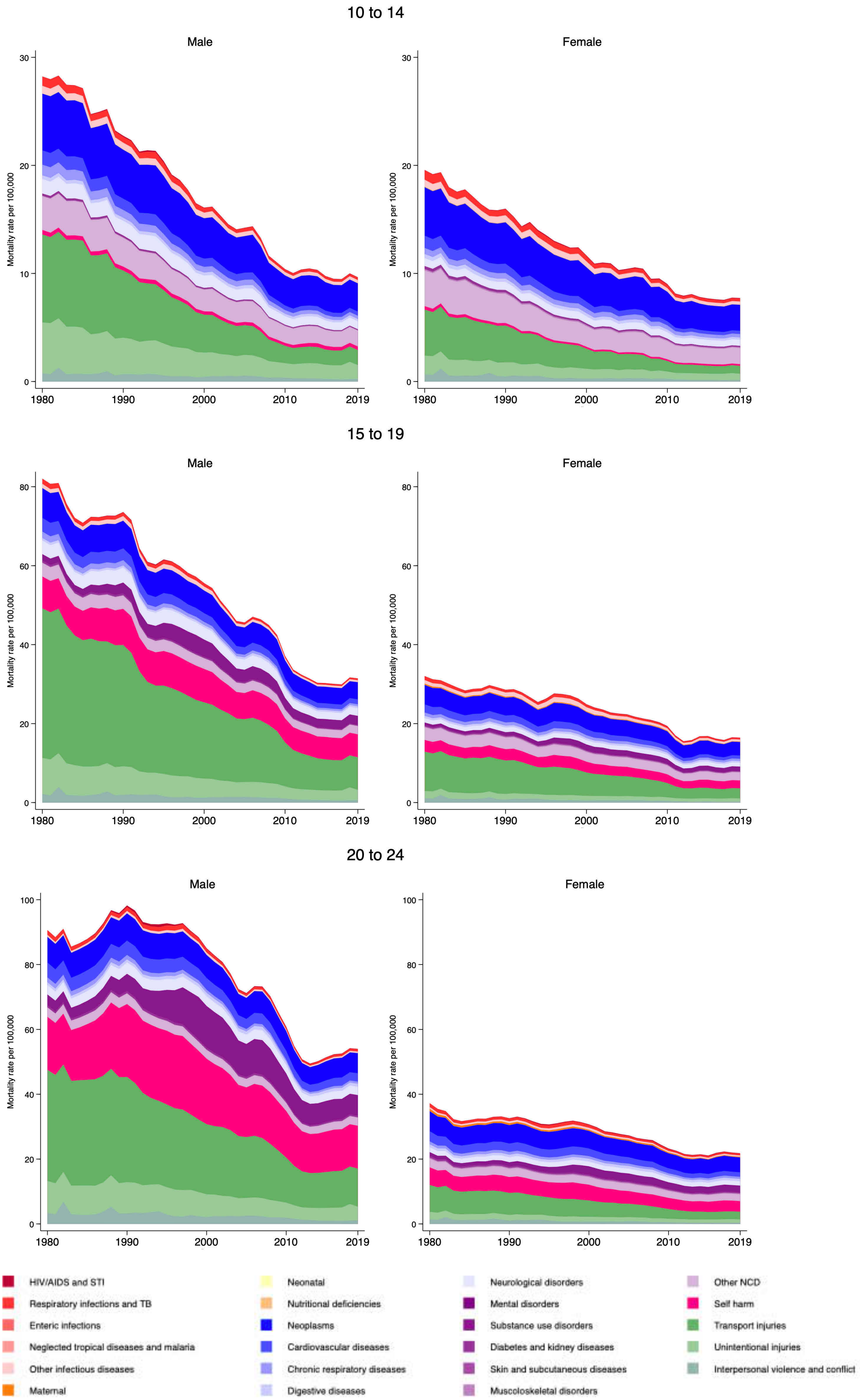

Figure S93d Number of deaths in 10-24 1980 – 2019 by cause: United Kingdom

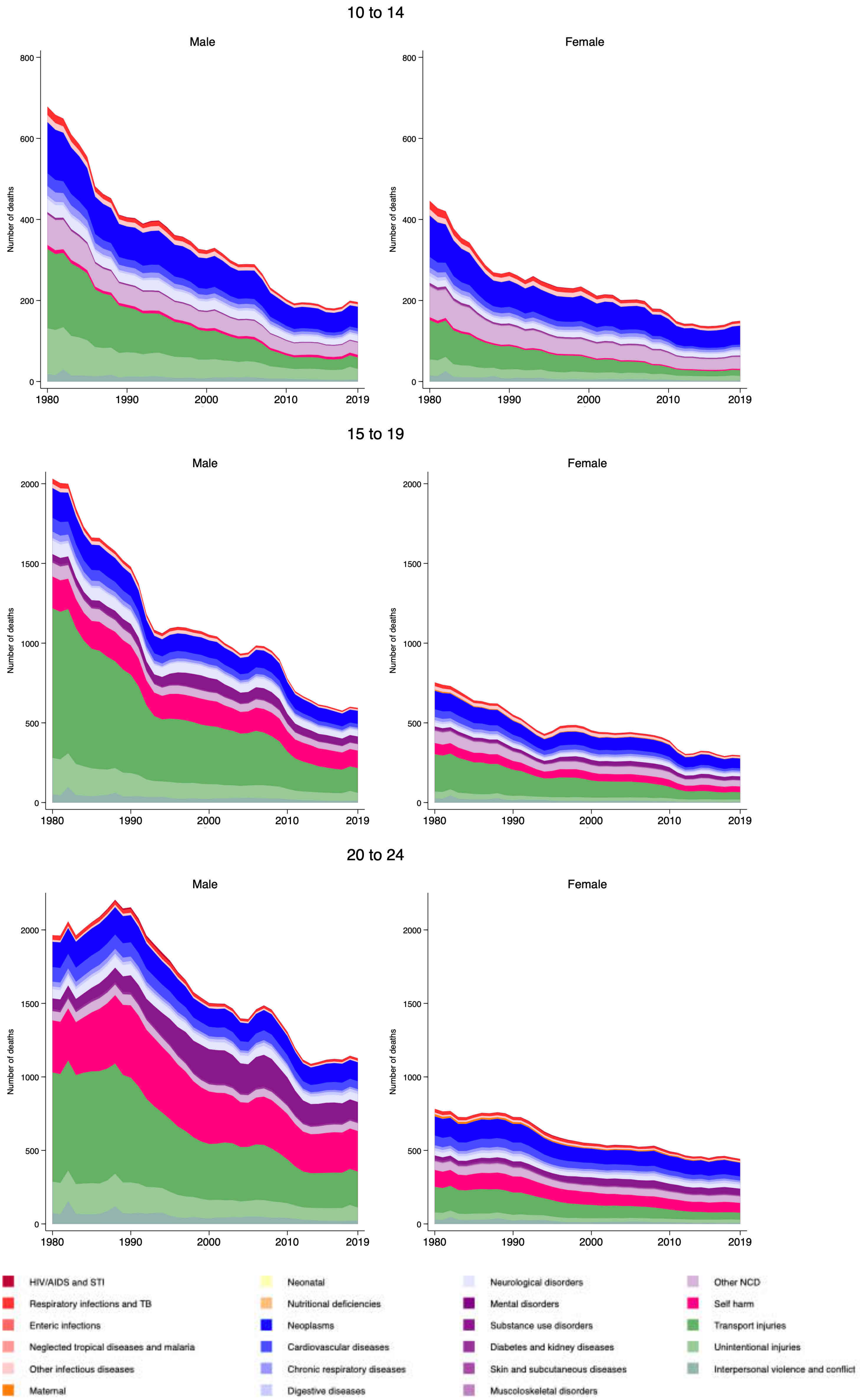

Figure S94: Southern Latin America

Figure S94a All-cause mortality rate per 100,000 in 10-24 1950 – 2019: Southern Latin America

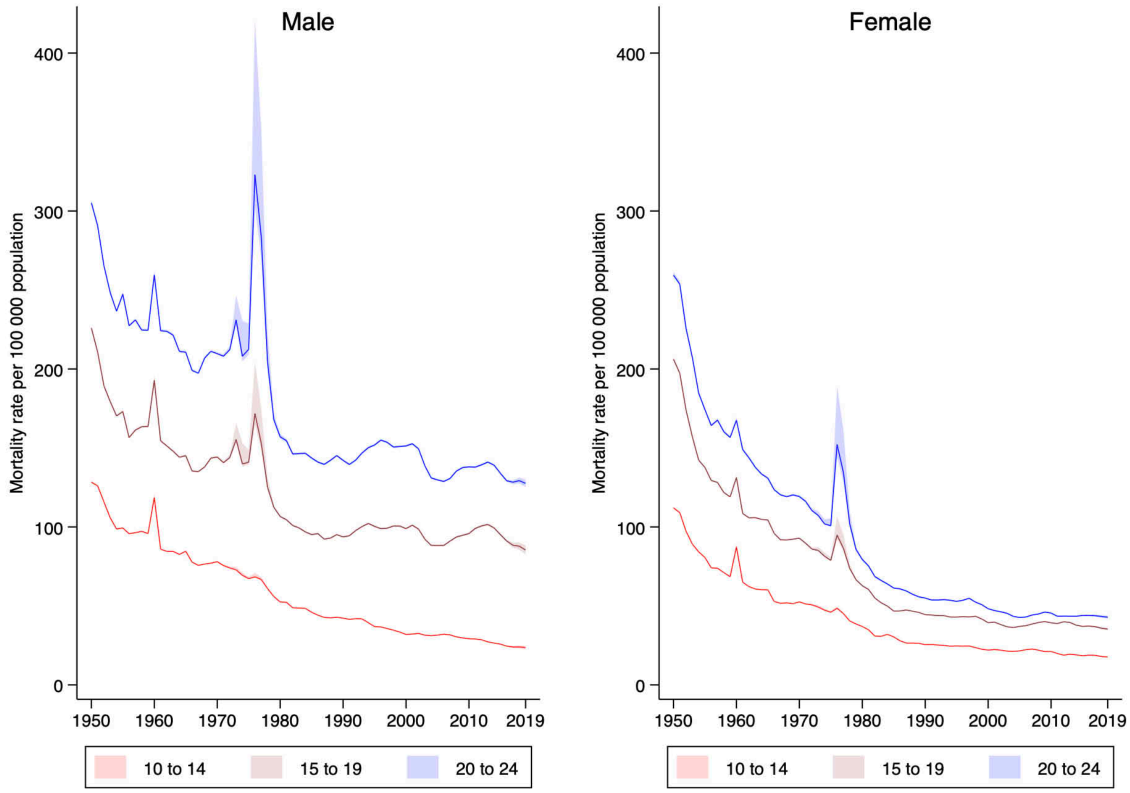

Figure S94b Number of deaths from all causes in 10-24 1950 – 2019: Southern Latin America

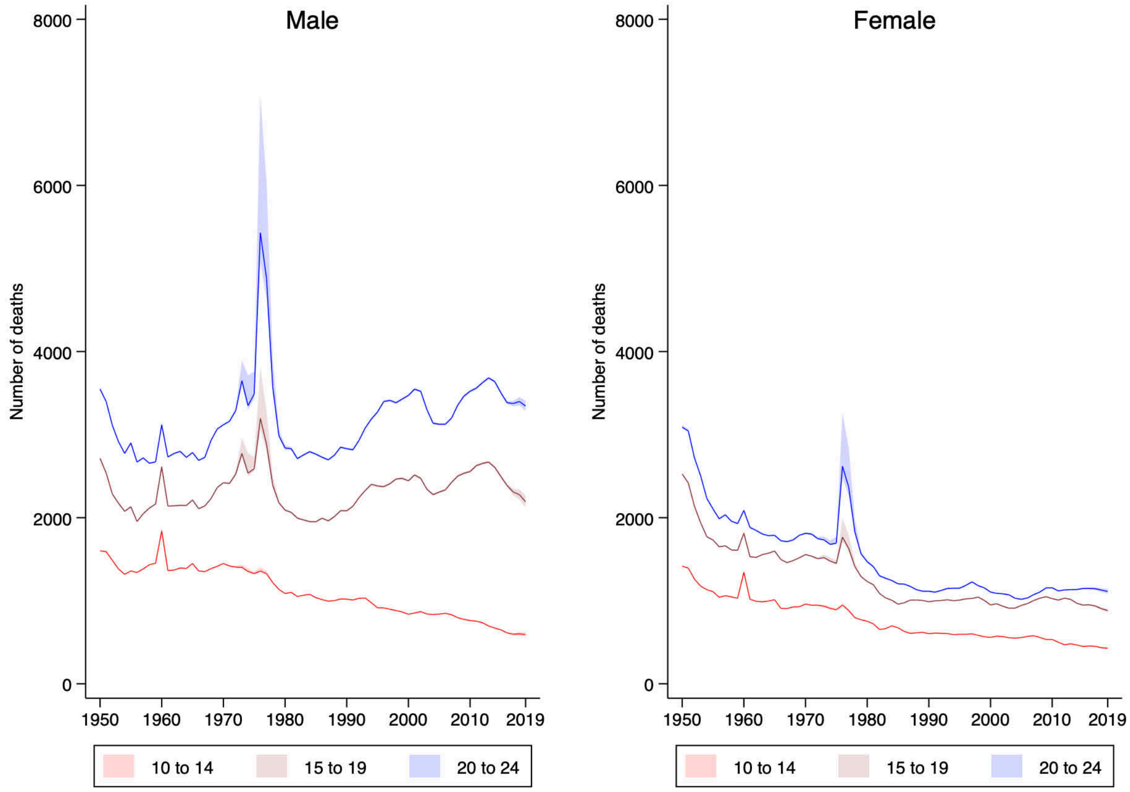

Figure S94c Mortality rate per 100,000 in 10-24 1980 – 2019 by cause: Southern Latin America

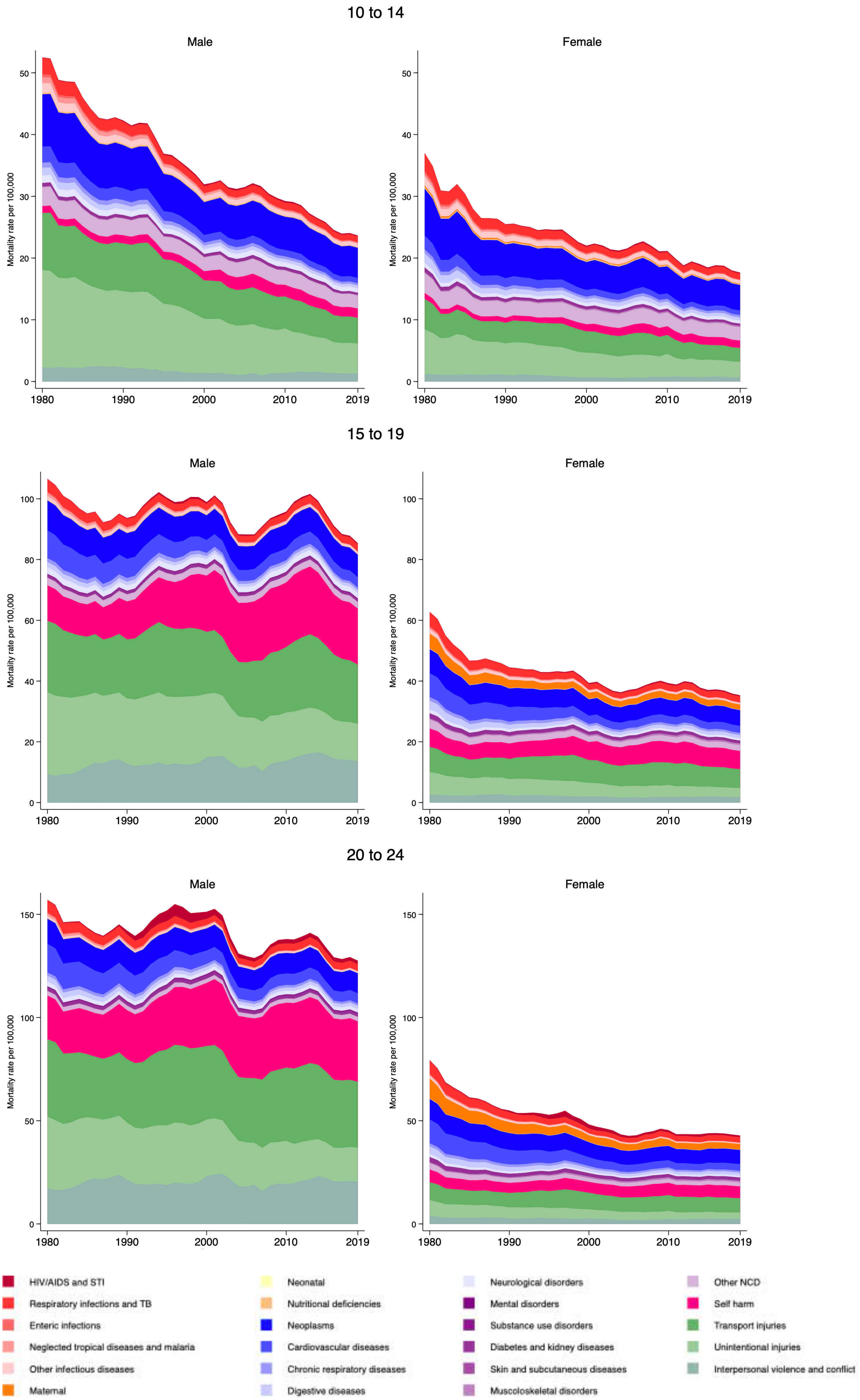

Figure S94d Number of deaths in 10-24 1980 – 2019 by cause: Southern Latin America

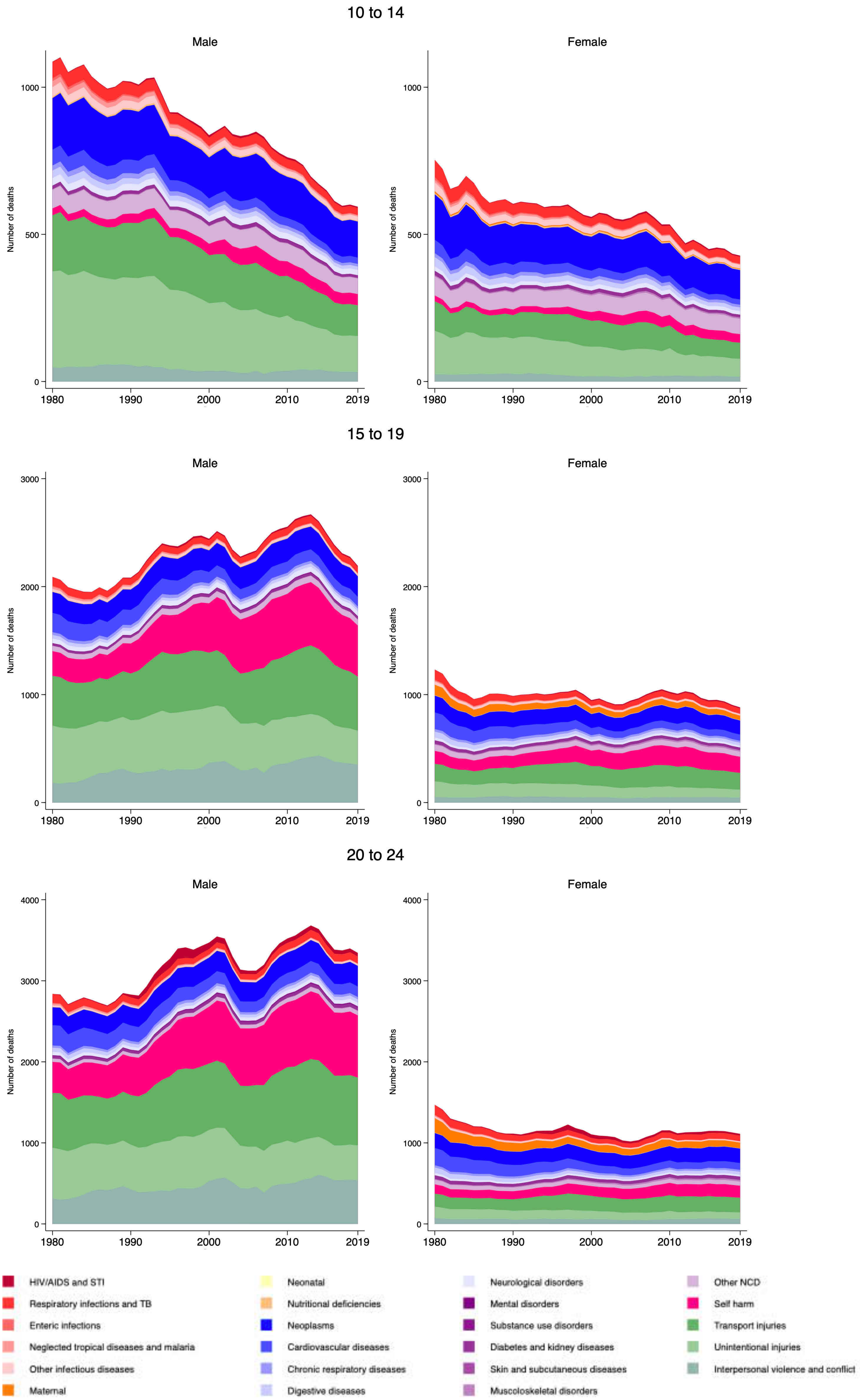

Figure S95: Argentina

Figure S95a All-cause mortality rate per 100,000 in 10-24 1950 – 2019: Argentina

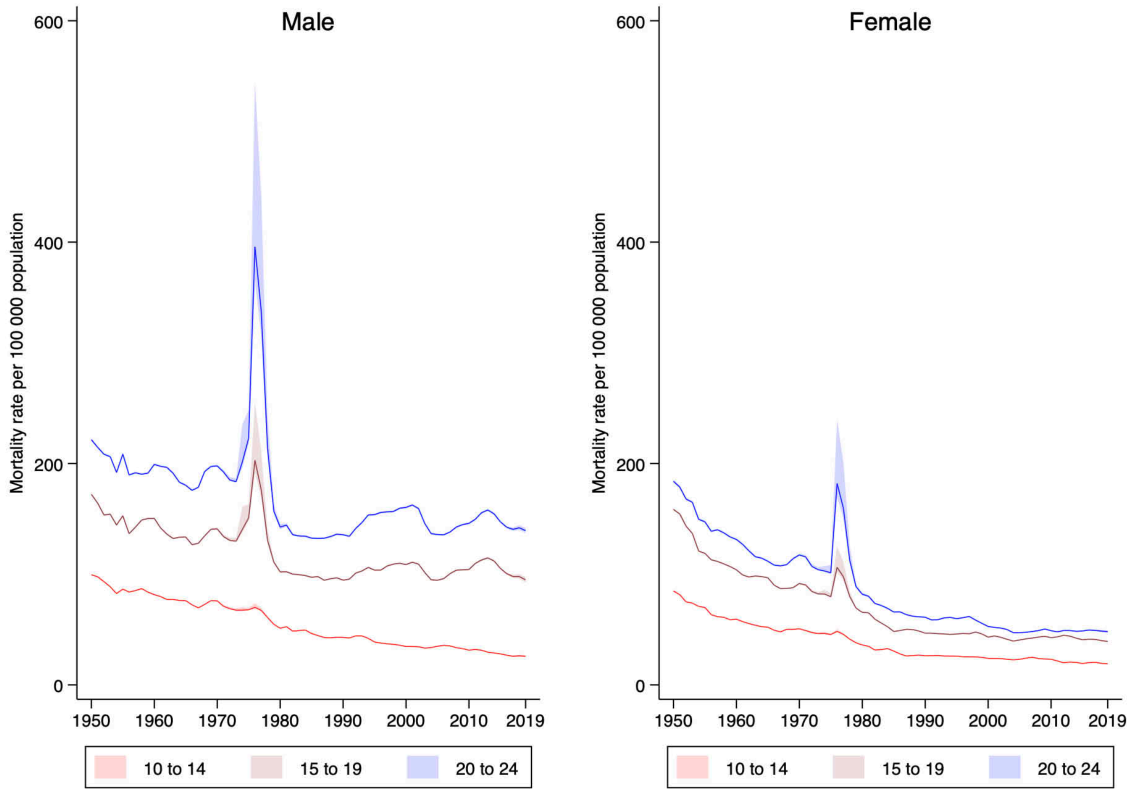

Figure S95b Number of deaths from all causes in 10-24 1950 – 2019: Argentina

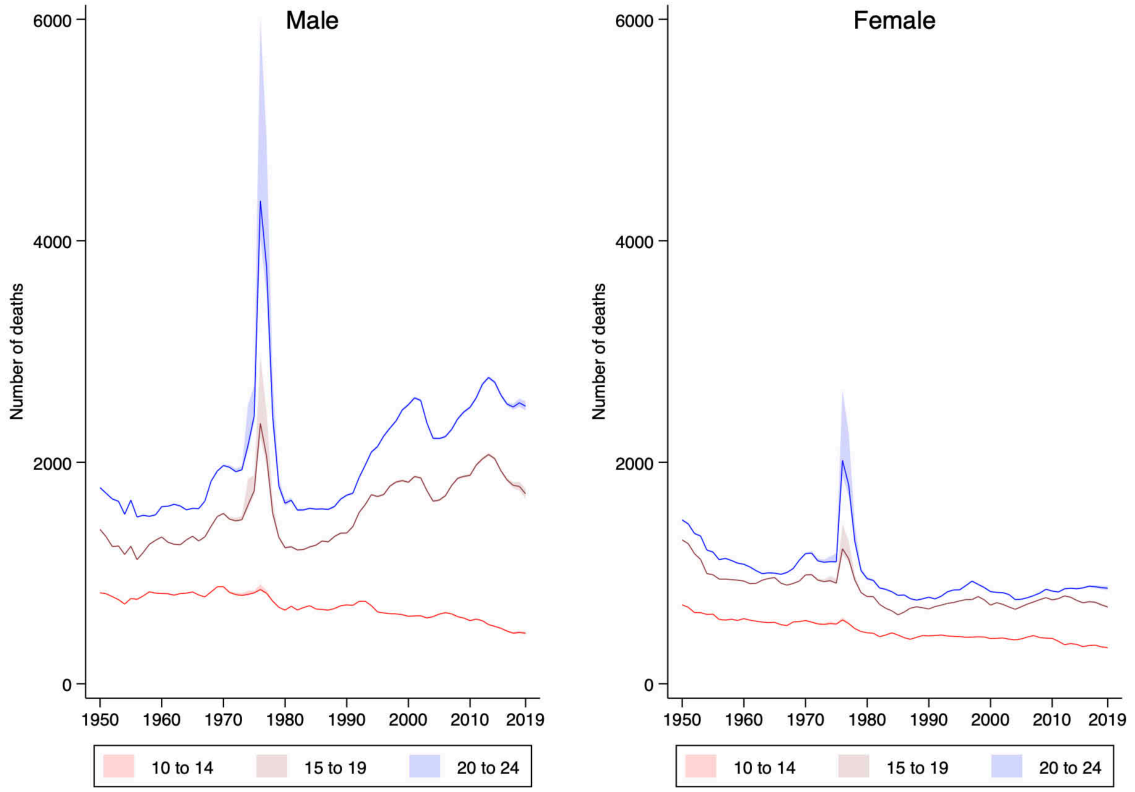

Figure S95c Mortality rate per 100,000 in 10-24 1980 – 2019 by cause: Argentina

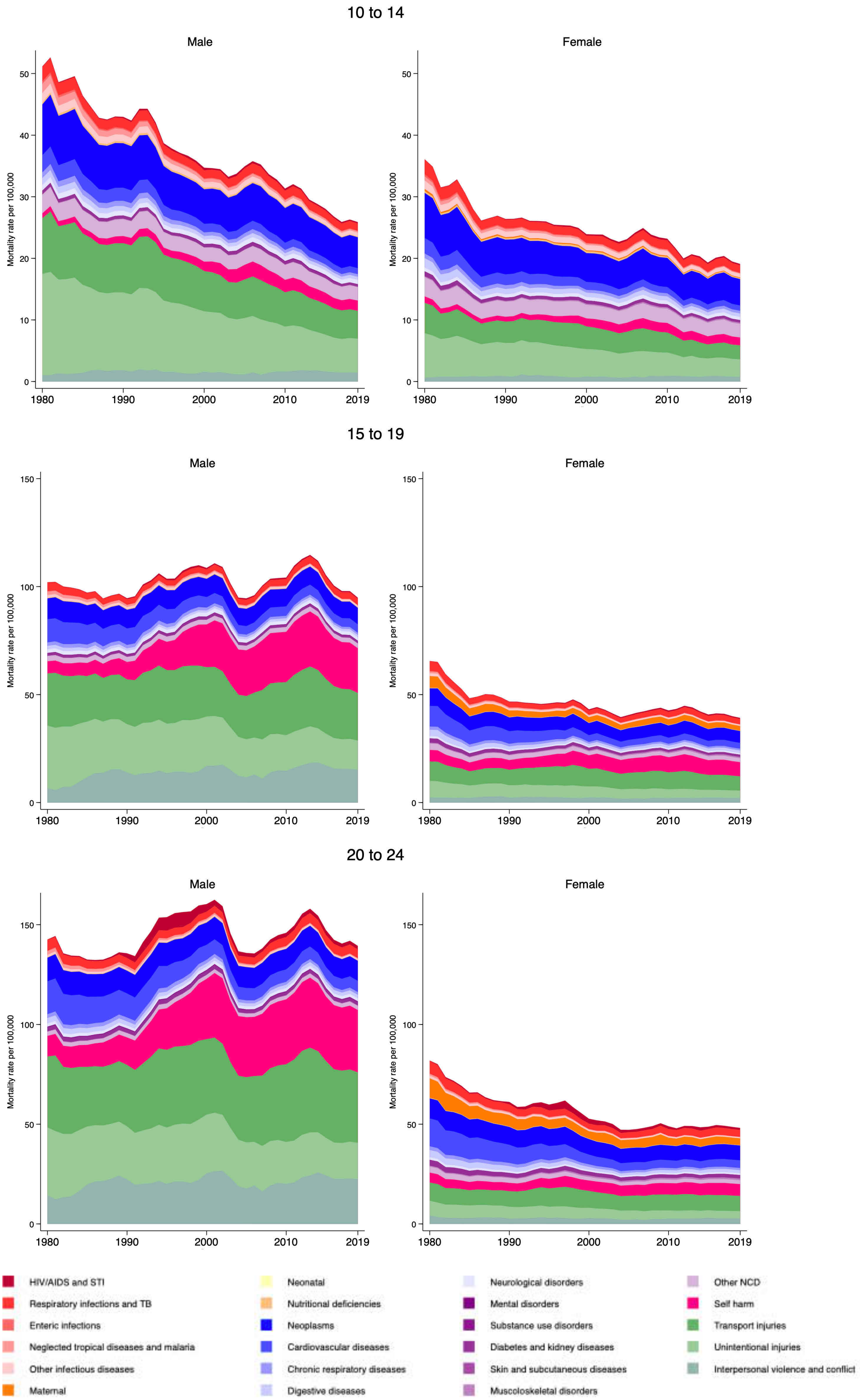

Figure S95d Number of deaths in 10-24 1980 – 2019 by cause: Argentina

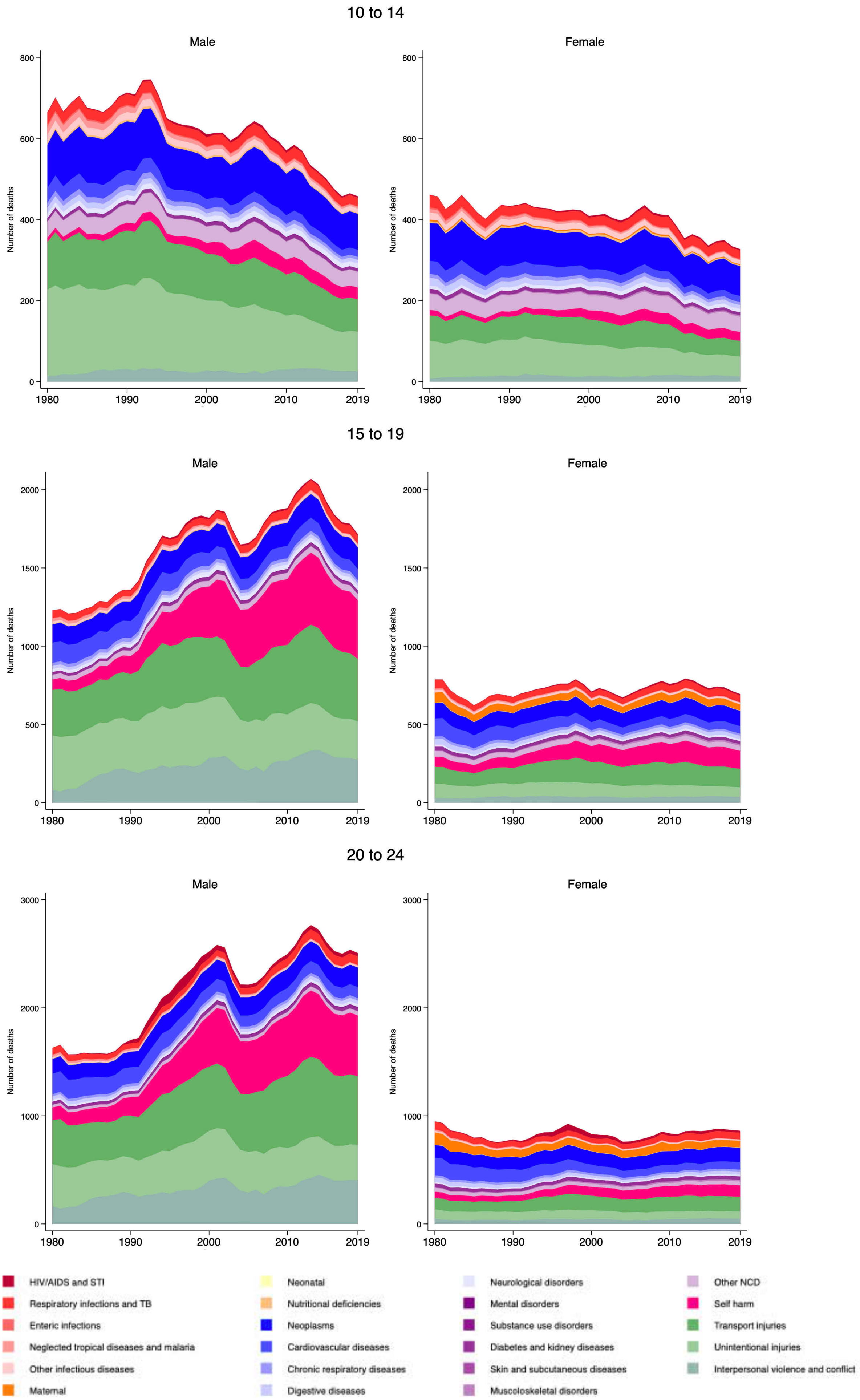

Figure S96: Chile

Figure S96a All-cause mortality rate per 100,000 in 10-24 1950 – 2019: Chile

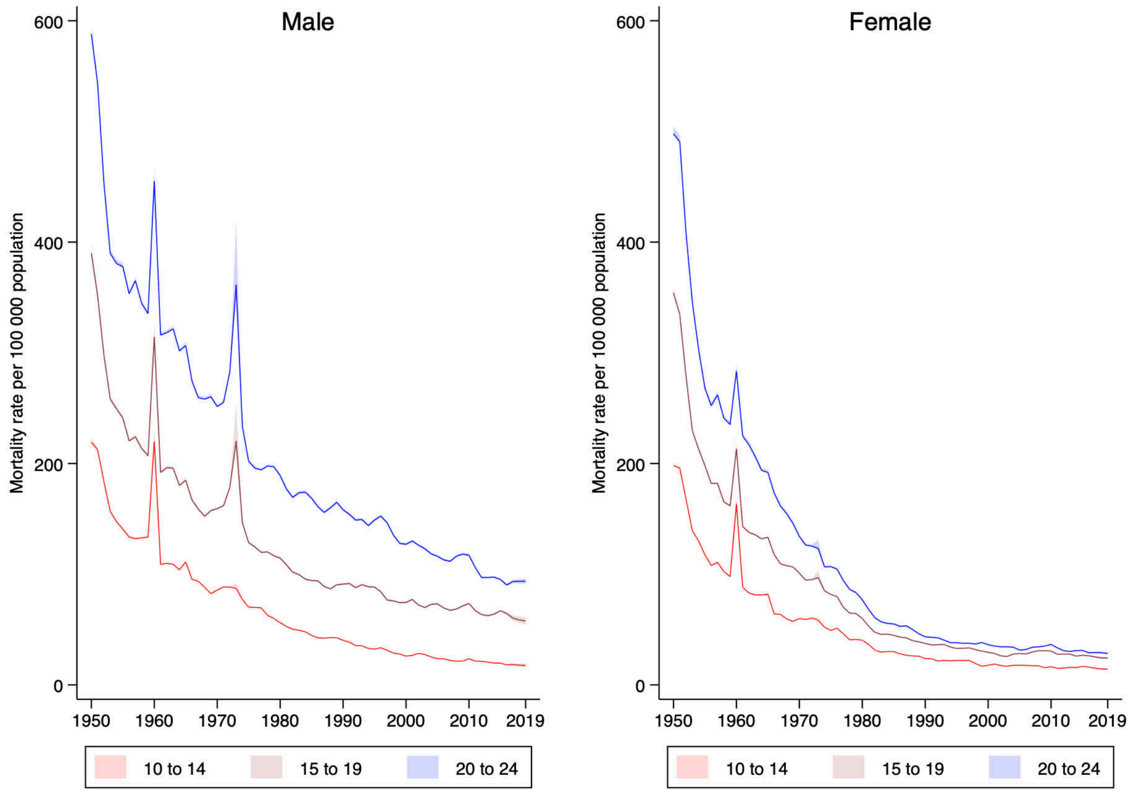

Figure S96b Number of deaths from all causes in 10-24 1950 – 2019: Chile

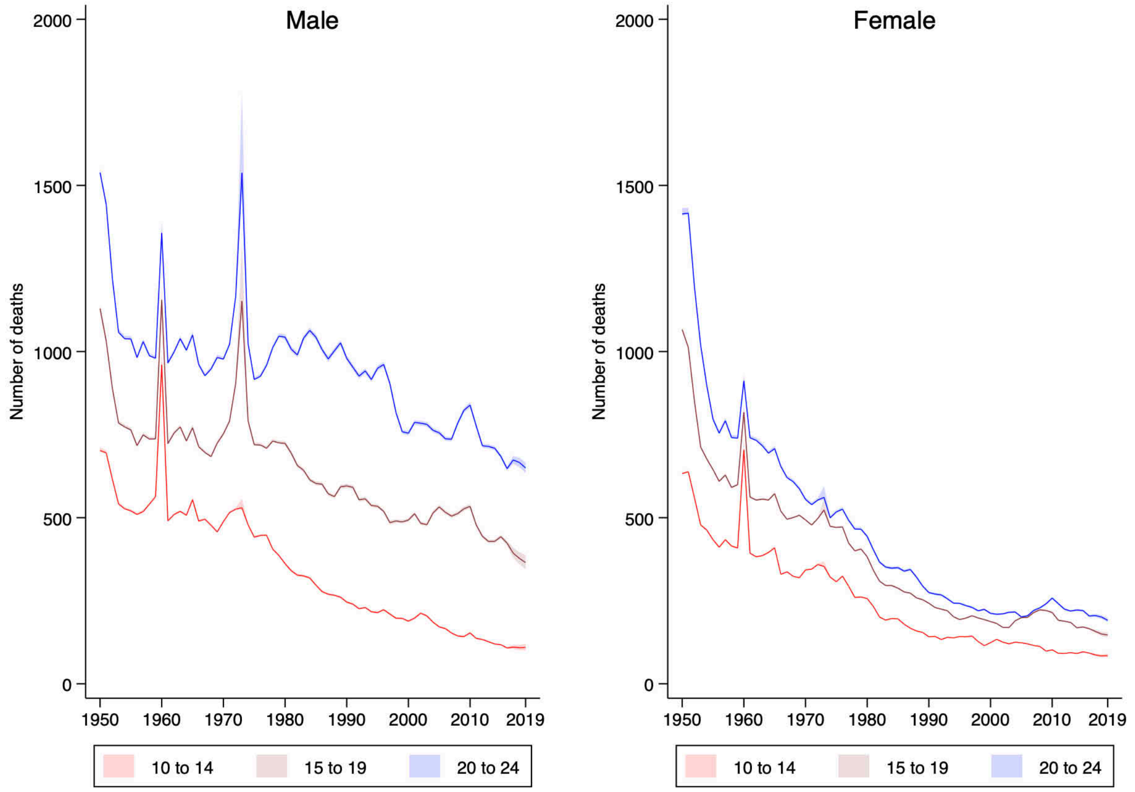

Figure S96c Mortality rate per 100,000 in 10-24 1980 – 2019 by cause: Chile

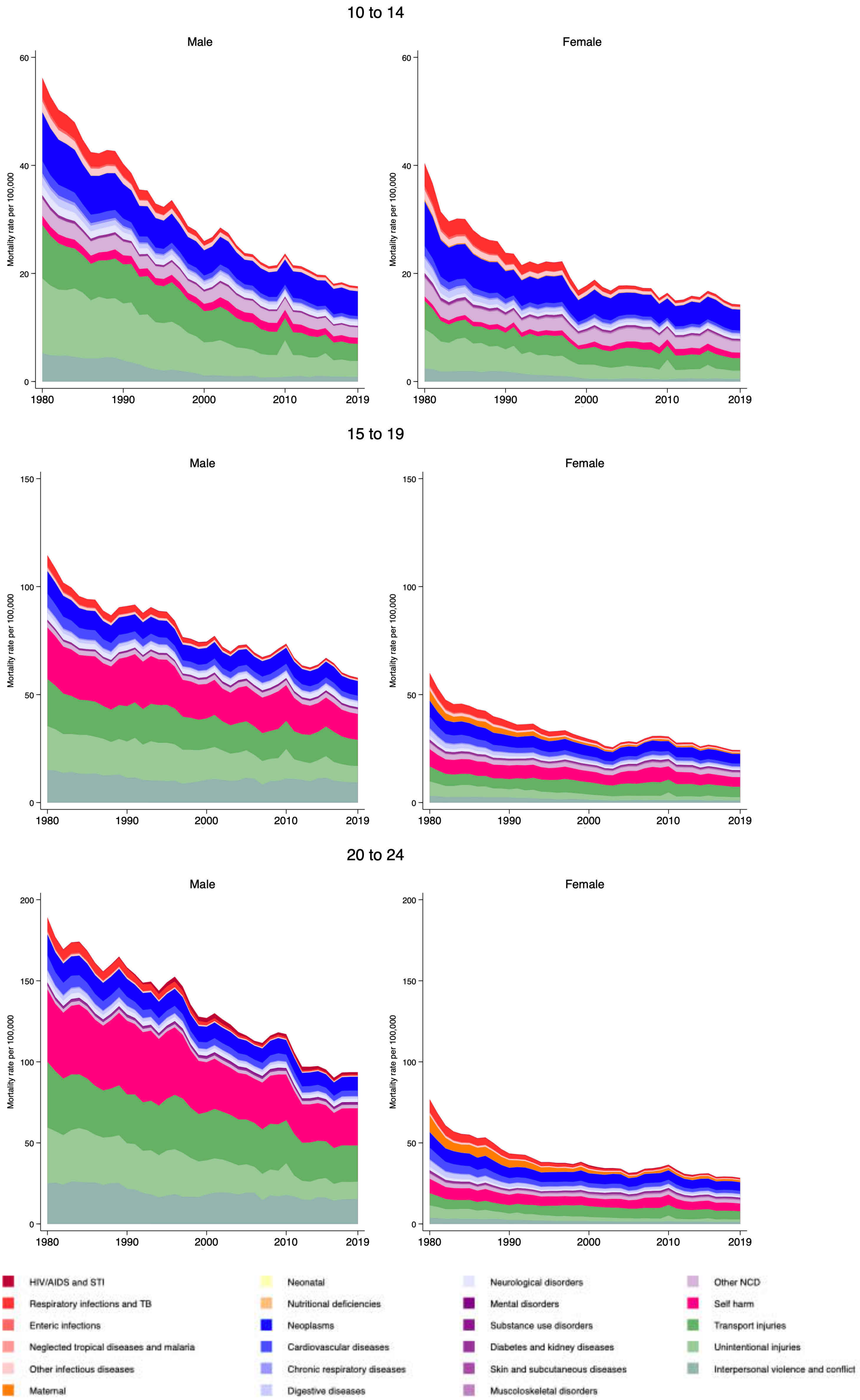

Figure S96d Number of deaths in 10-24 1980 – 2019 by cause: Chile

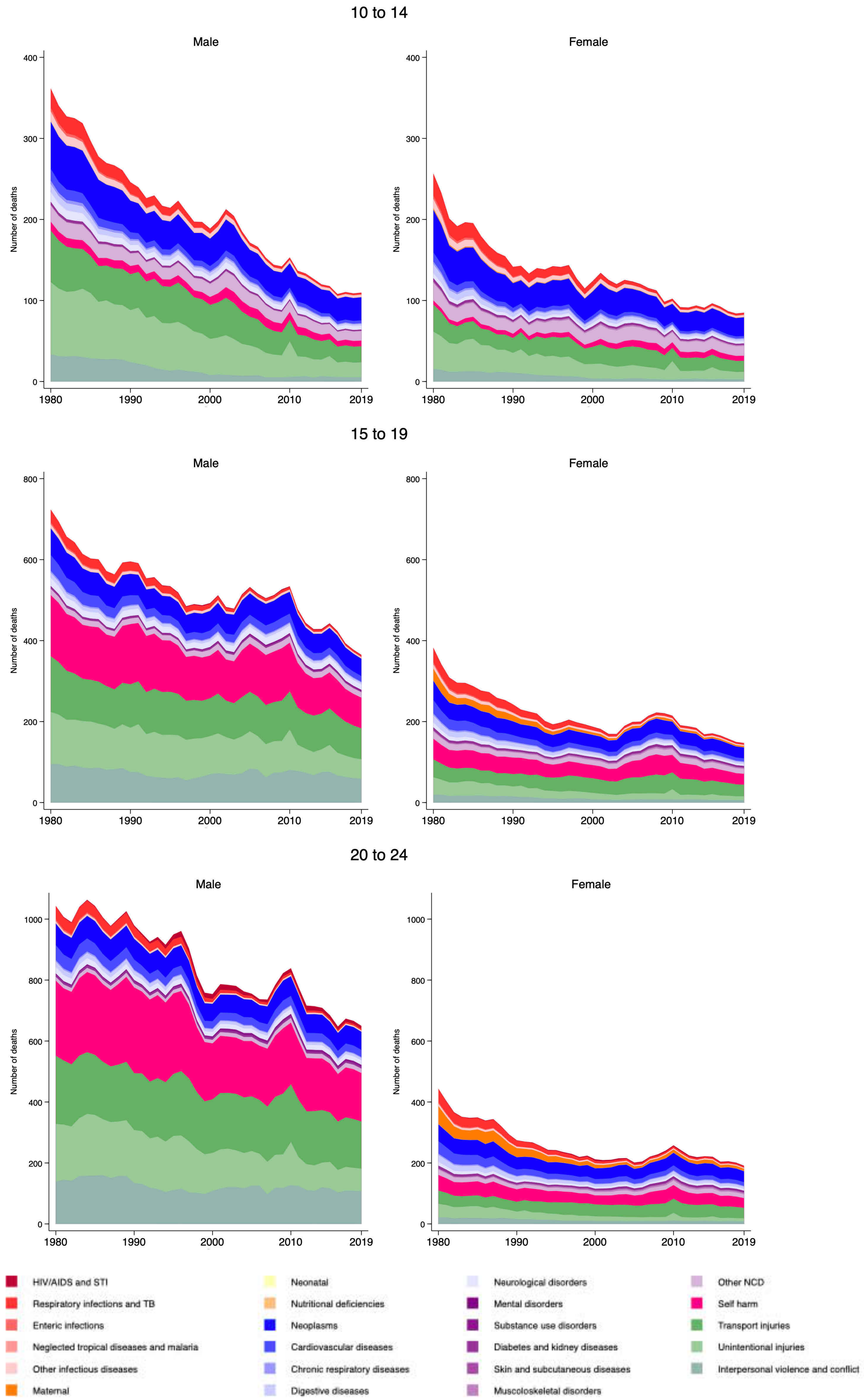

Figure S97: Uruguay

Figure S97a All-cause mortality rate per 100,000 in 10-24 1950 – 2019: Uruguay

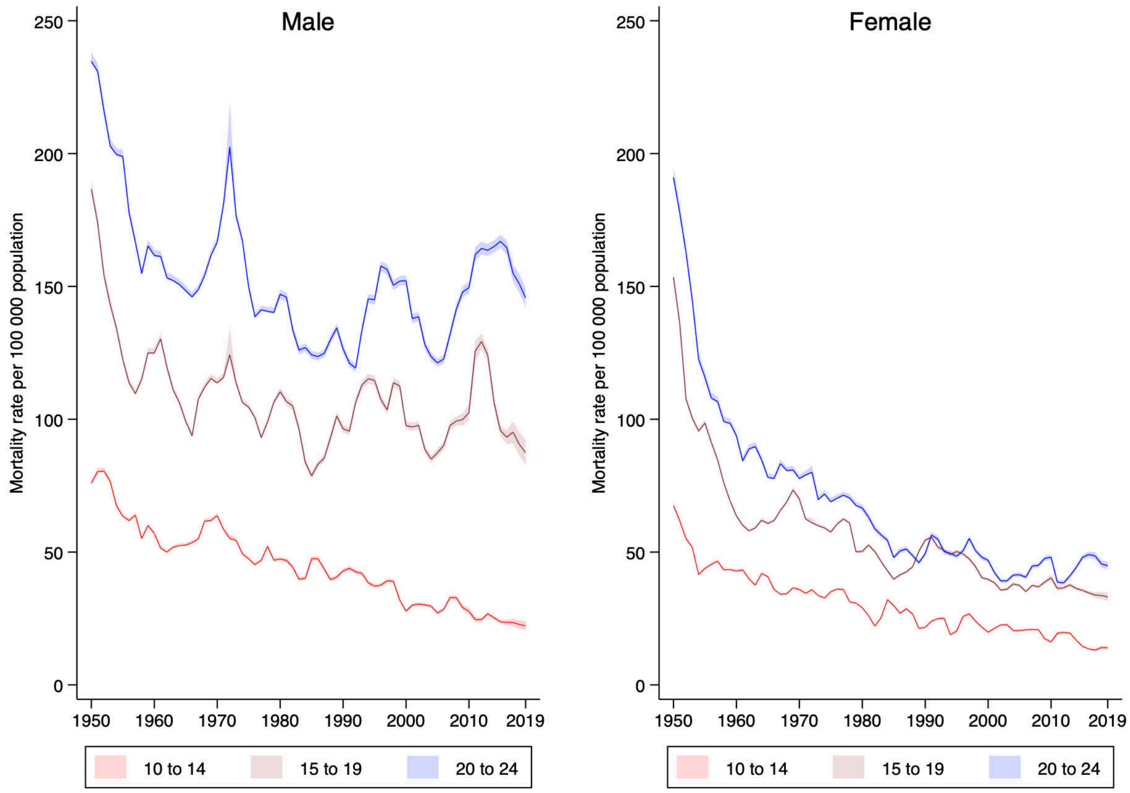

Figure S97b Number of deaths from all causes in 10-24 1950 – 2019: Uruguay

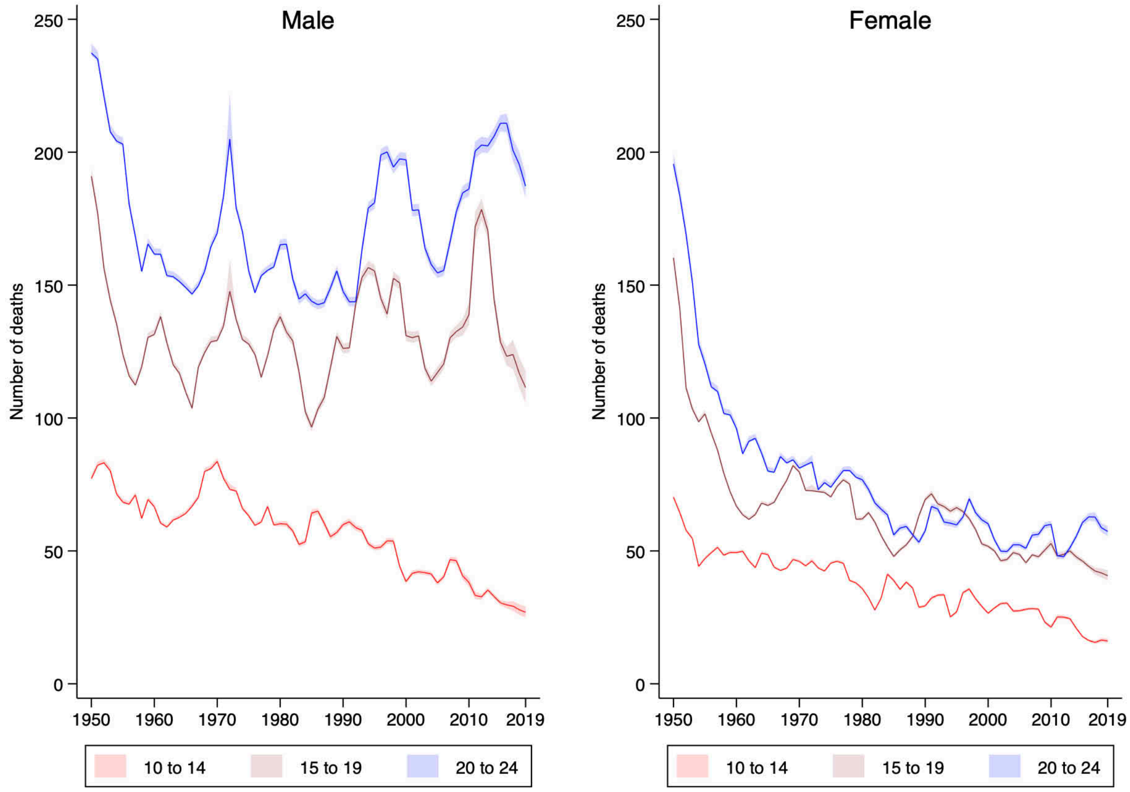

Figure S97c Mortality rate per 100,000 in 10-24 1980 – 2019 by cause: Uruguay

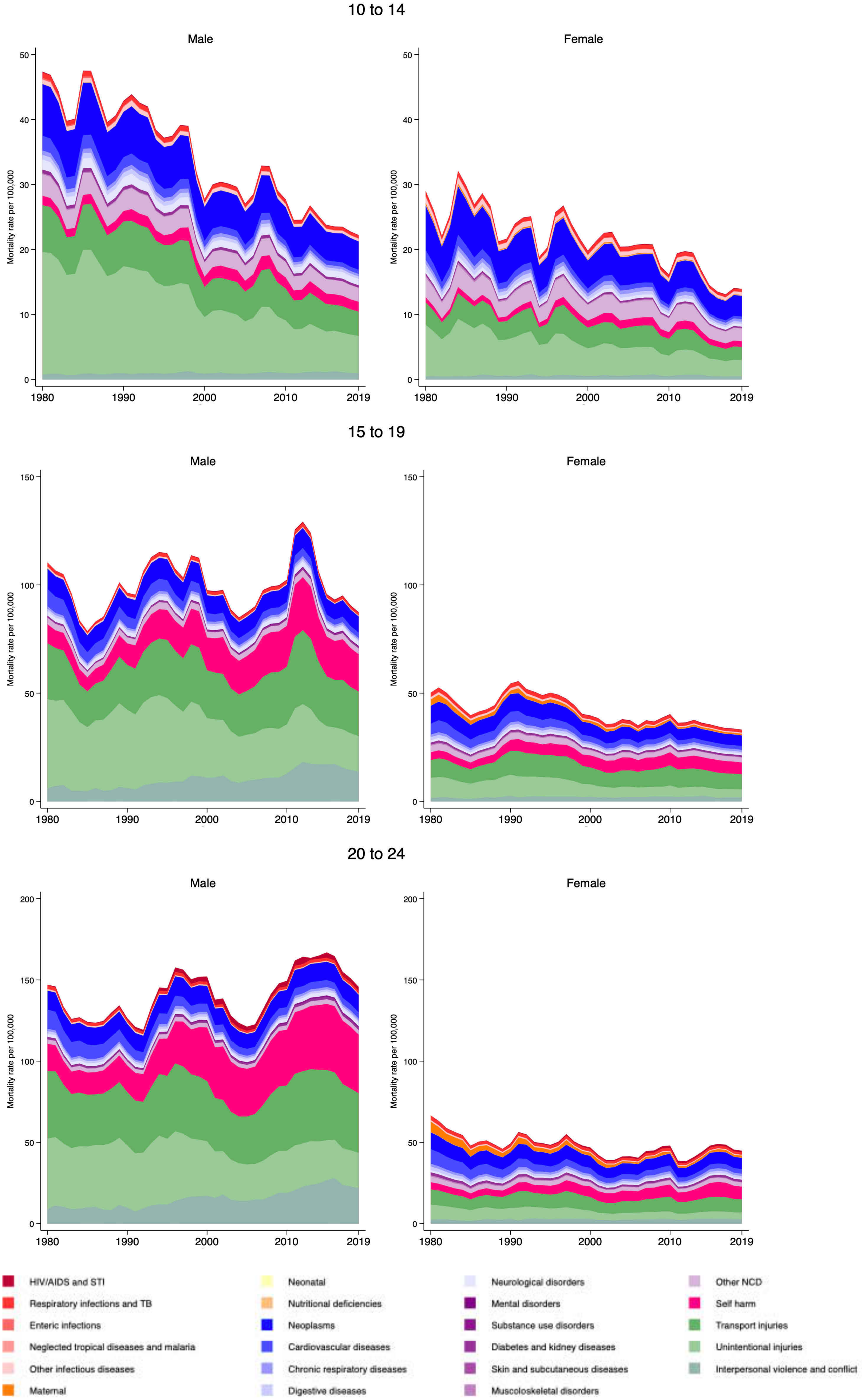

Figure S97d Number of deaths in 10-24 1980 – 2019 by cause: Uruguay

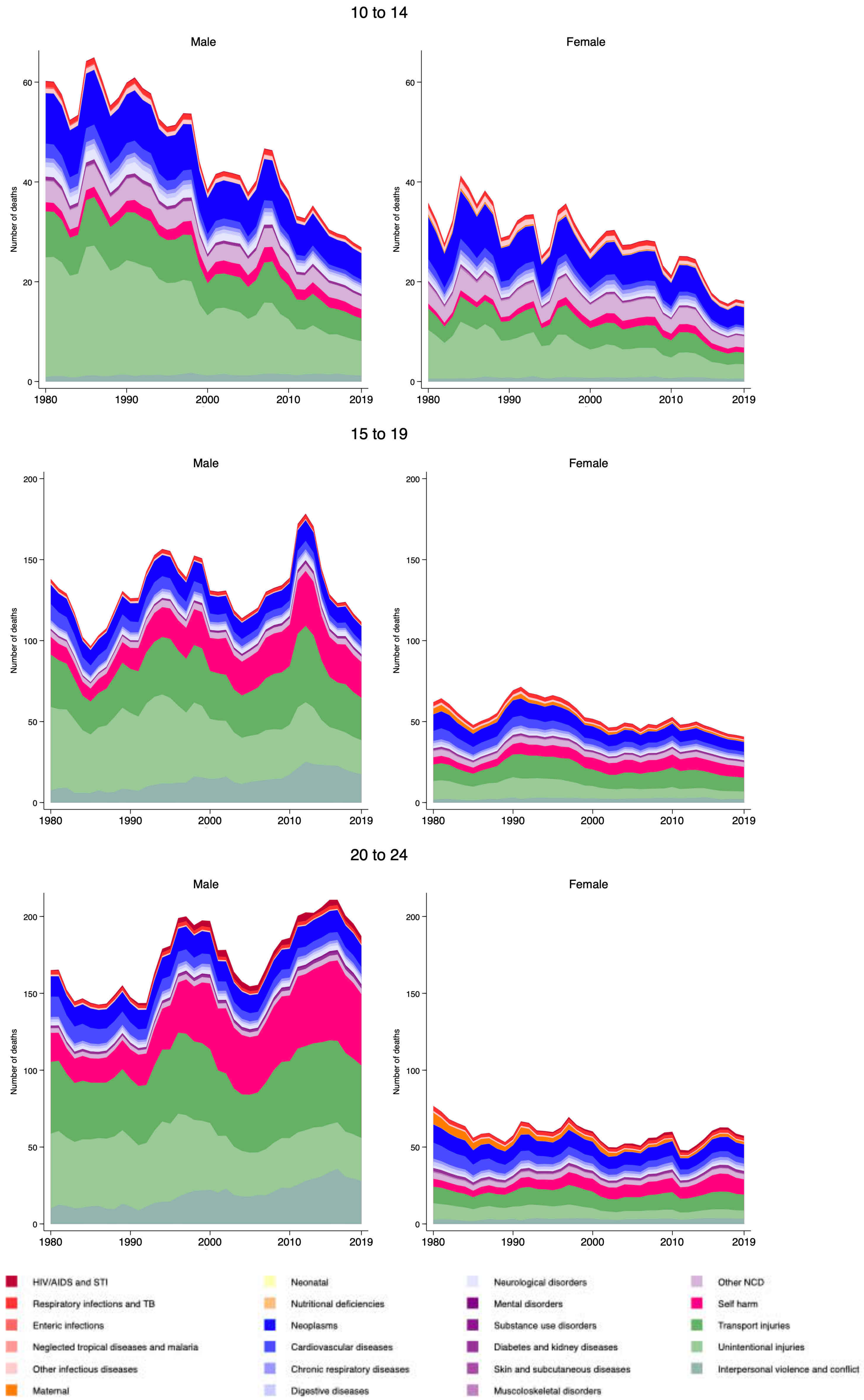

Figure S98: High-income North America

Figure S98a All-cause mortality rate per 100,000 in 10-24 1950 – 2019: High-income North America

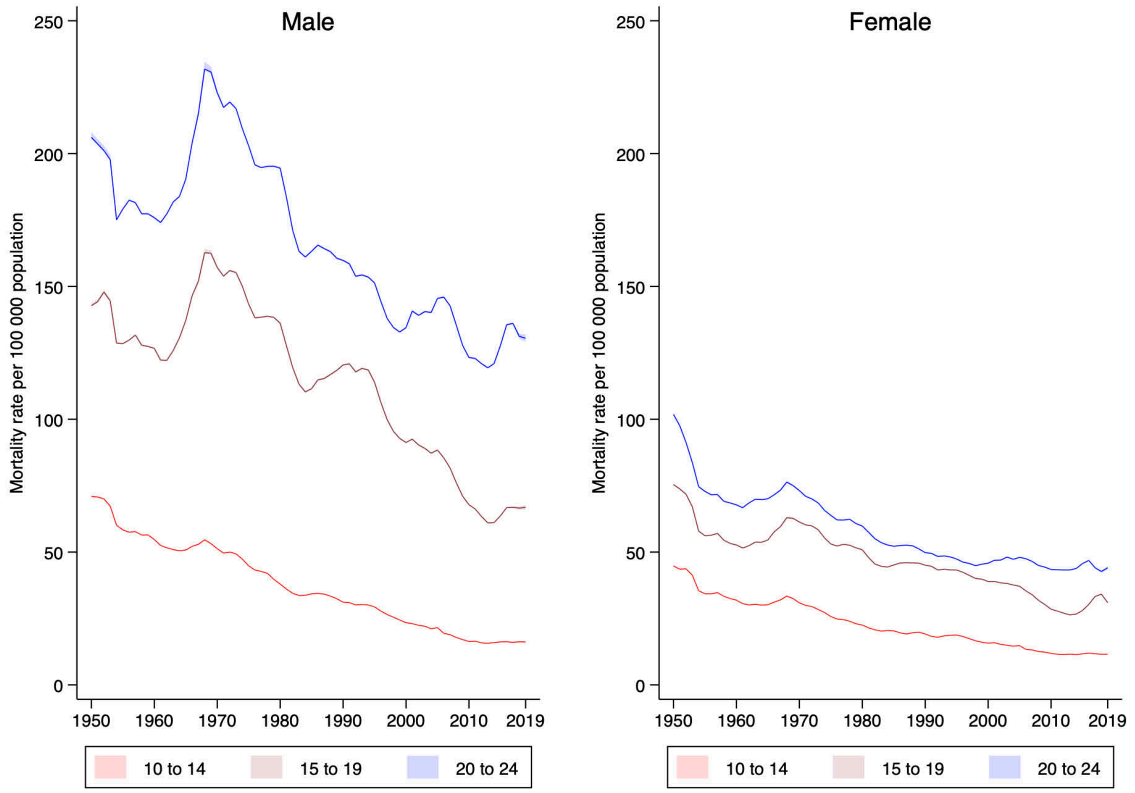

Figure S98b Number of deaths from all causes in 10-24 1950 – 2019: High-income North America

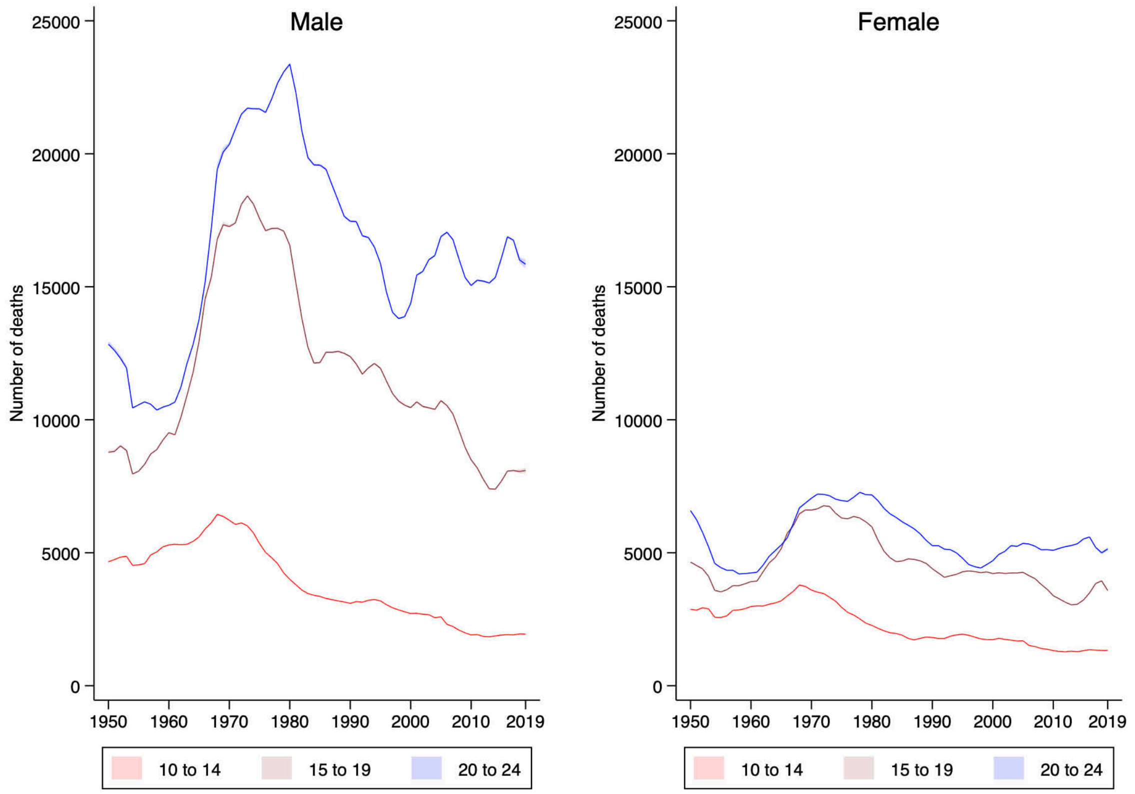

Figure S98c Mortality rate per 100,000 in 10-24 1980 – 2019 by cause: High-income North America

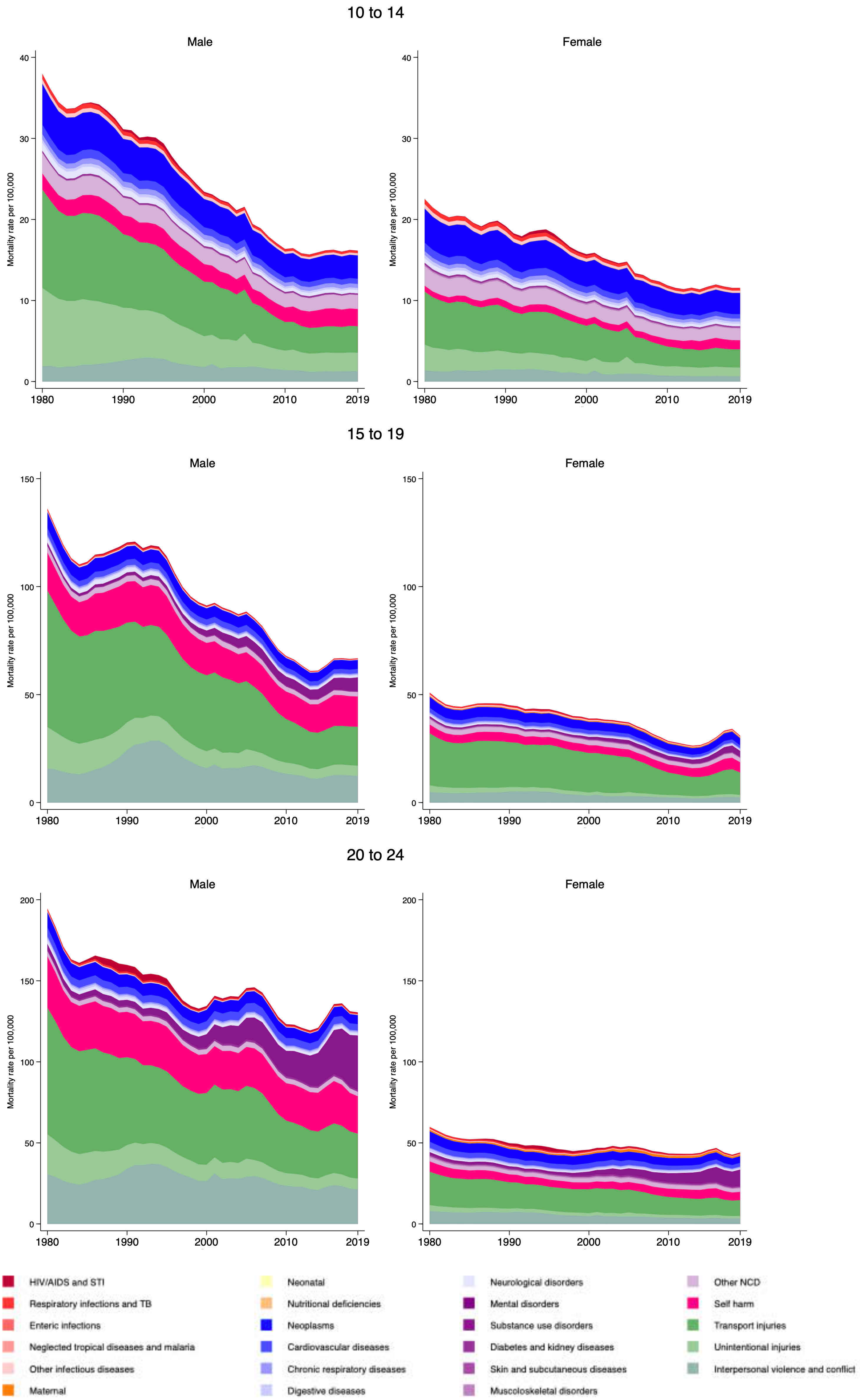

Figure S98d Number of deaths in 10-24 1980 – 2019 by cause: High-income North America

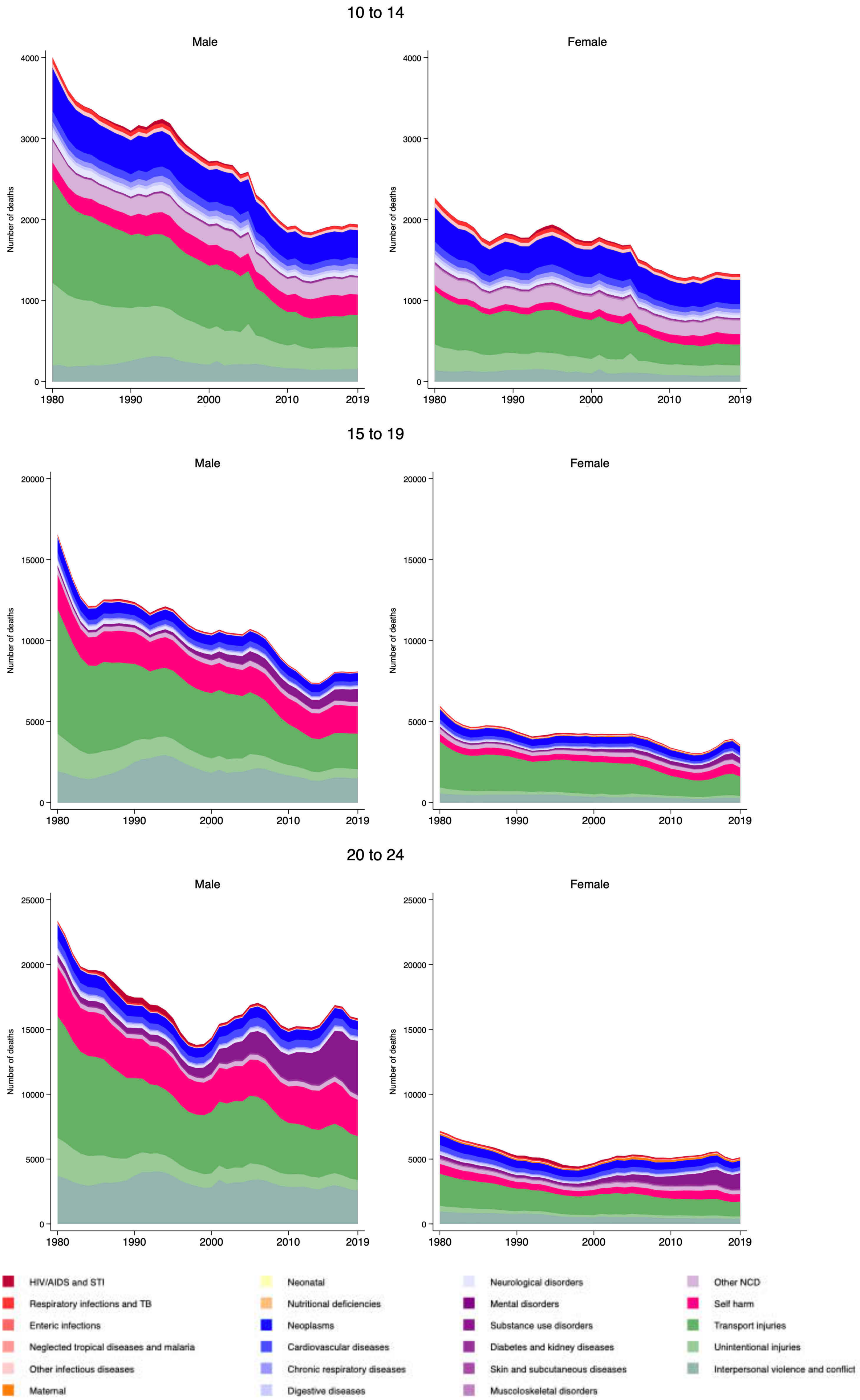

Figure S99: Canada

Figure S99a All-cause mortality rate per 100,000 in 10-24 1950 – 2019: Canada

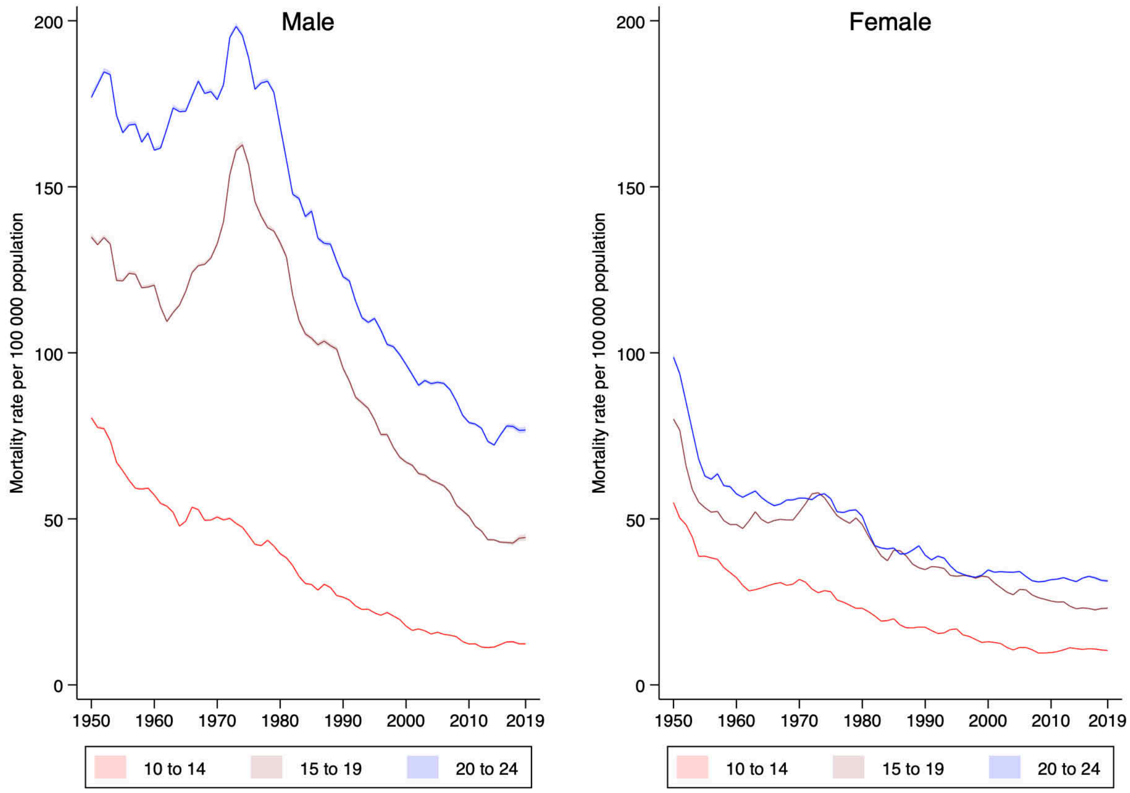

Figure S99b Number of deaths from all causes in 10-24 1950 – 2019: Canada

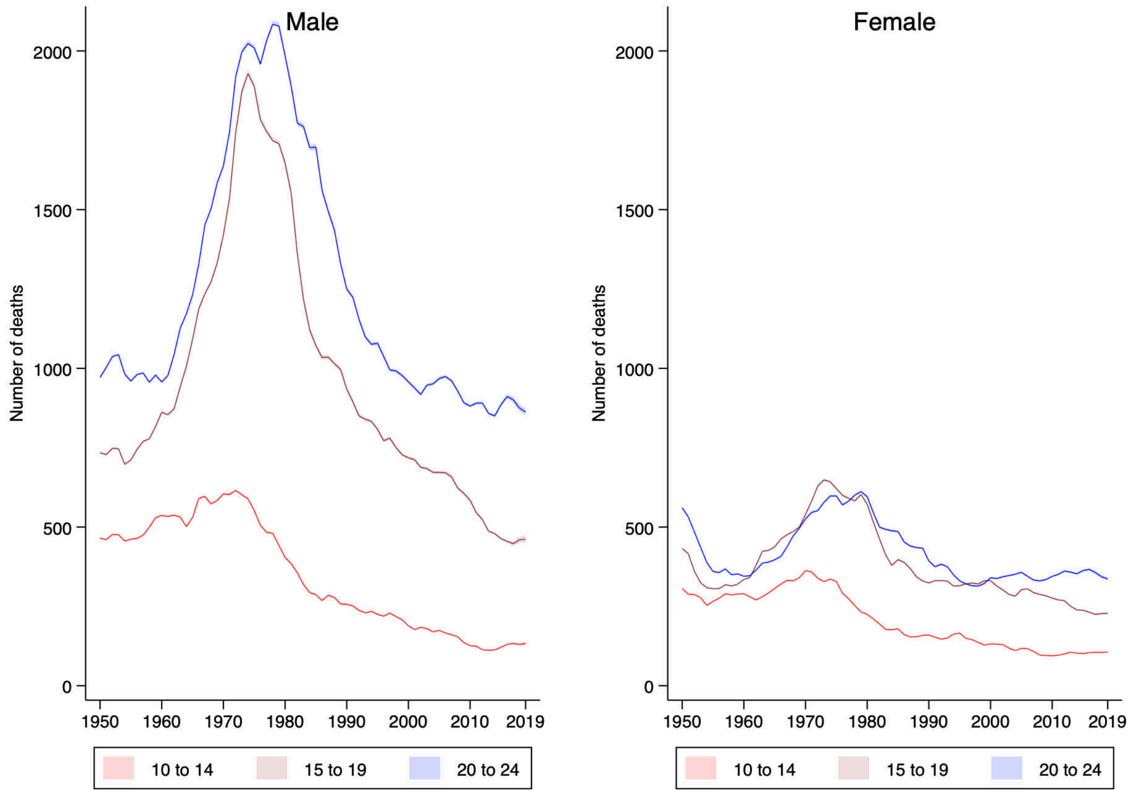

Figure S99c Mortality rate per 100,000 in 10-24 1980 – 2019 by cause: Canada

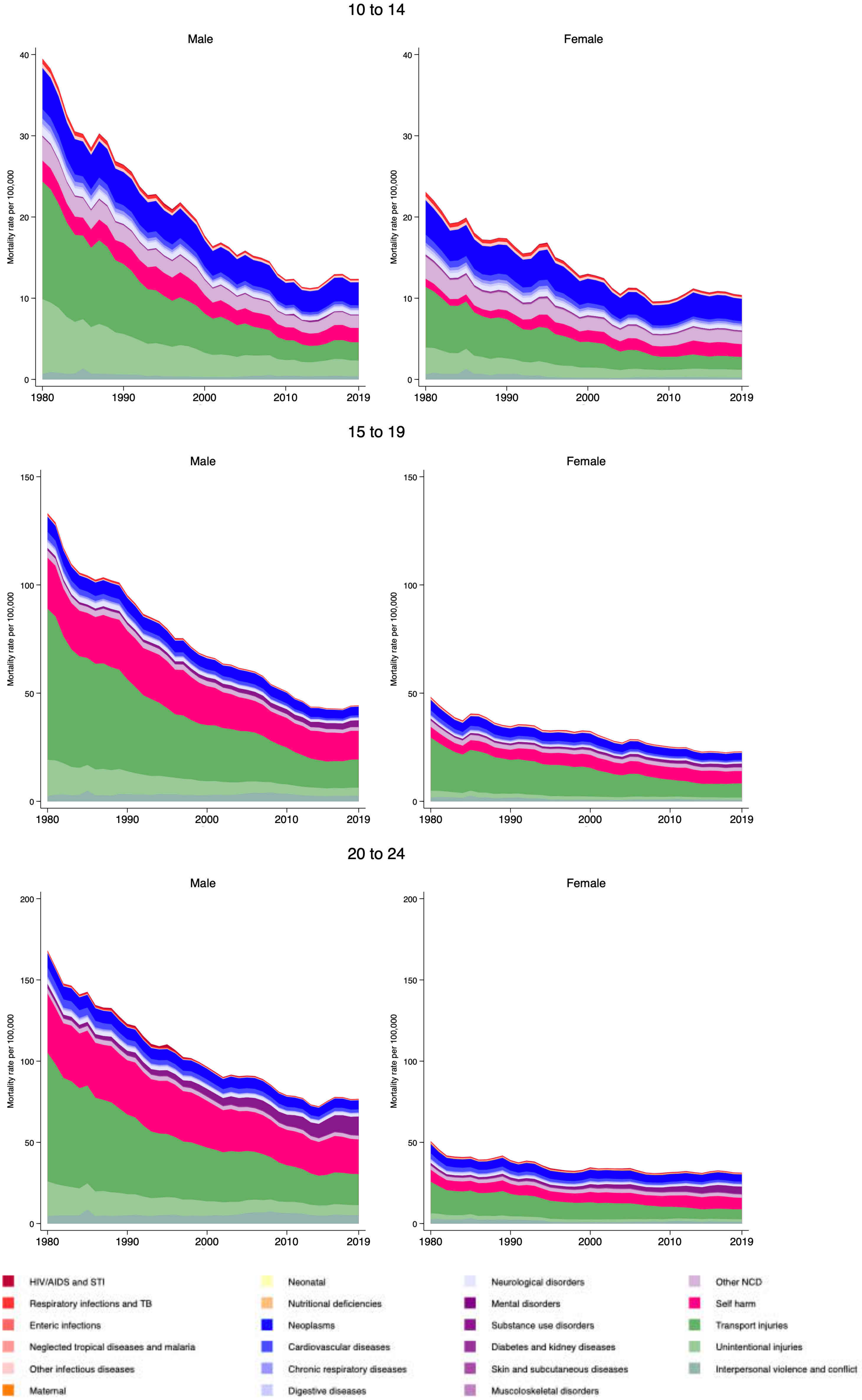

Figure S99d Number of deaths in 10-24 1980 – 2019 by cause: Canada

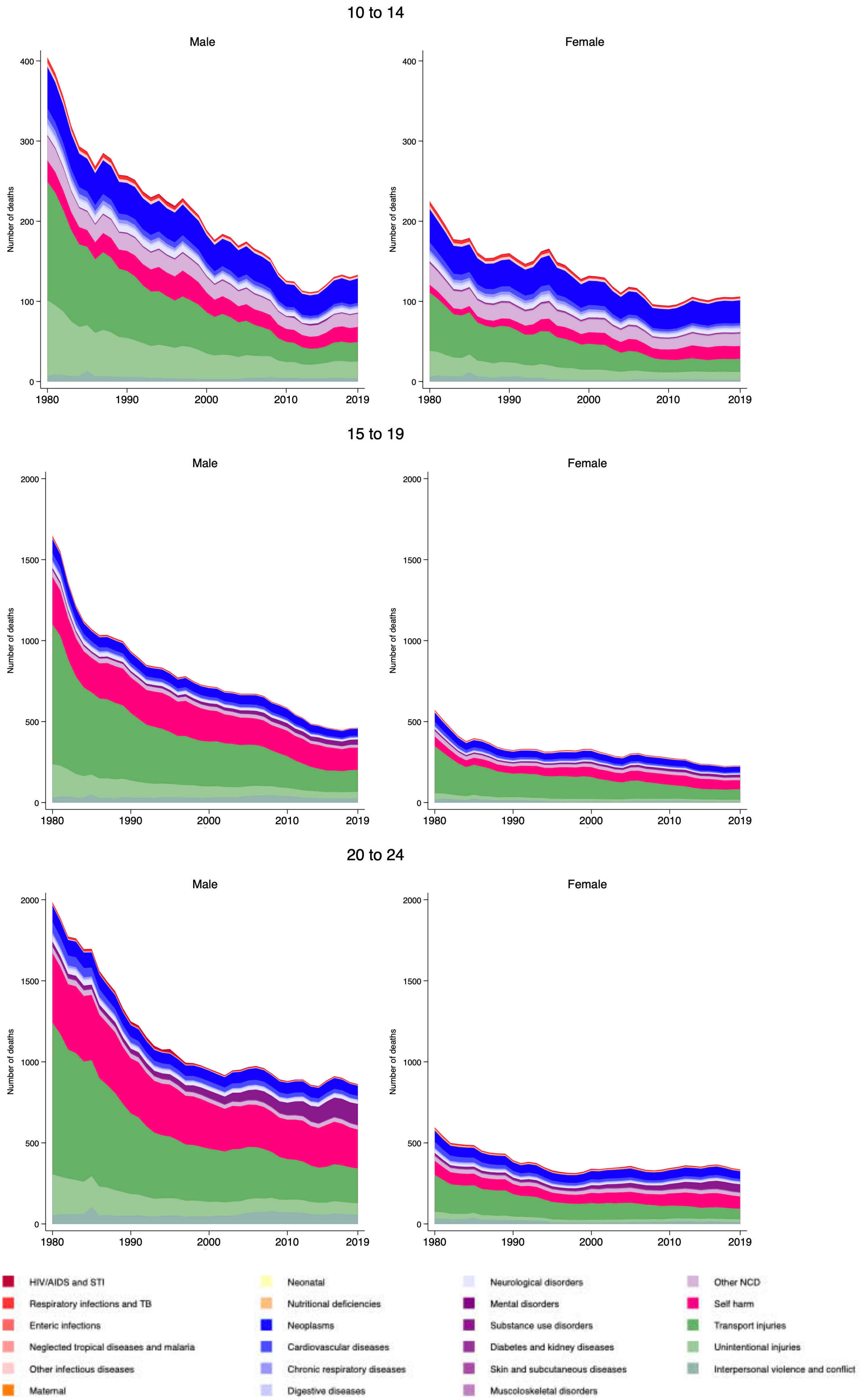

Figure S100: United States of America

Figure S100a All-cause mortality rate per 100,000 in 10-24 1950 – 2019: United States of America

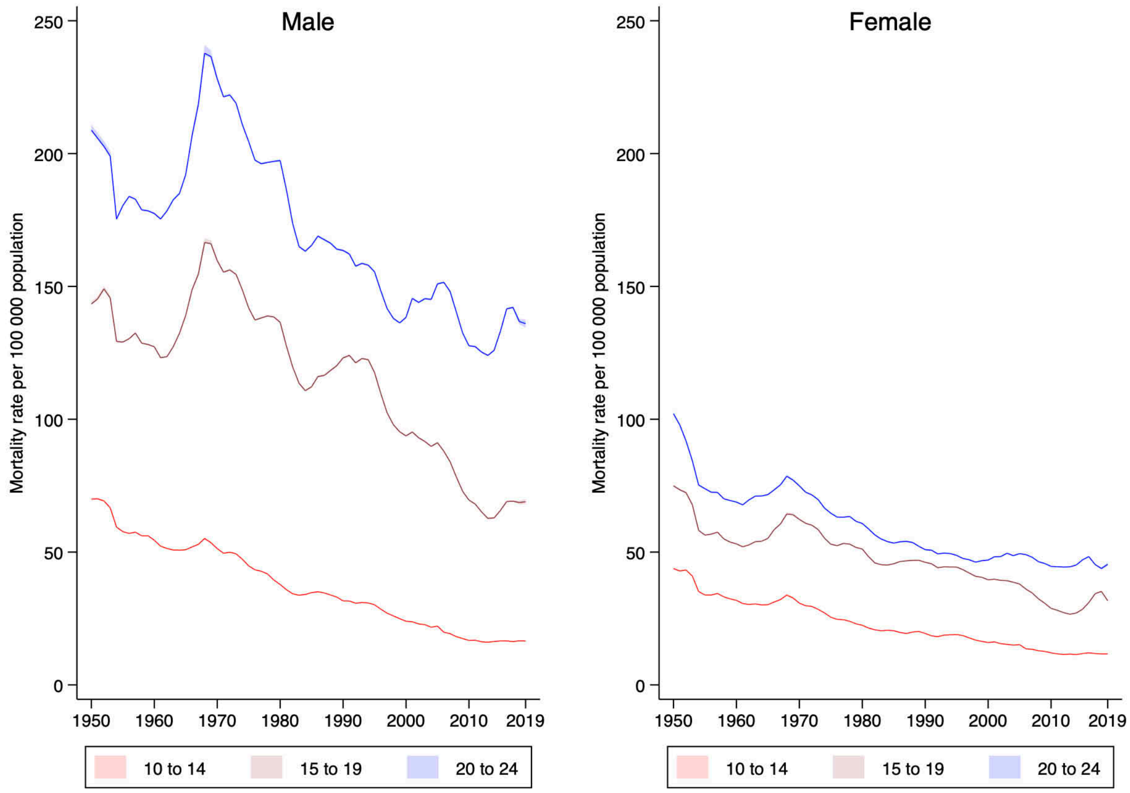

Figure S100b Number of deaths from all causes in 10-24 1950 – 2019: United States of America

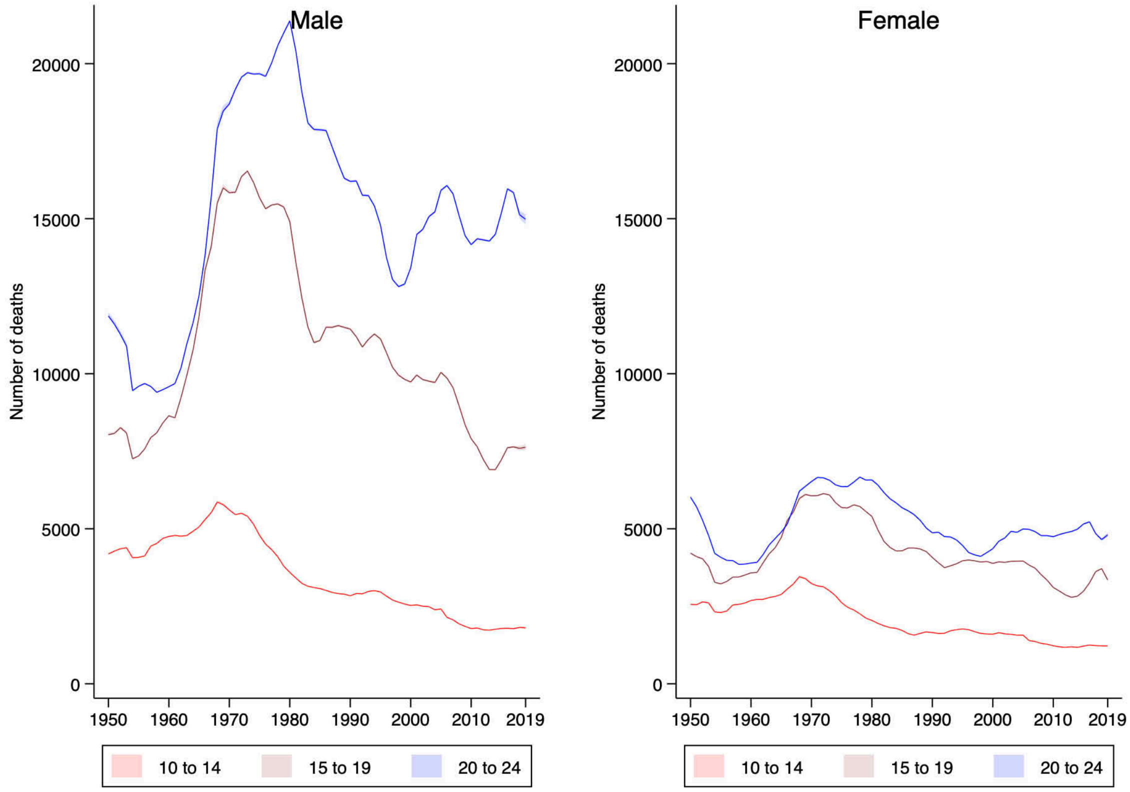

Figure S100c Mortality rate per 100,000 in 10-24 1980 – 2019 by cause: United States of America

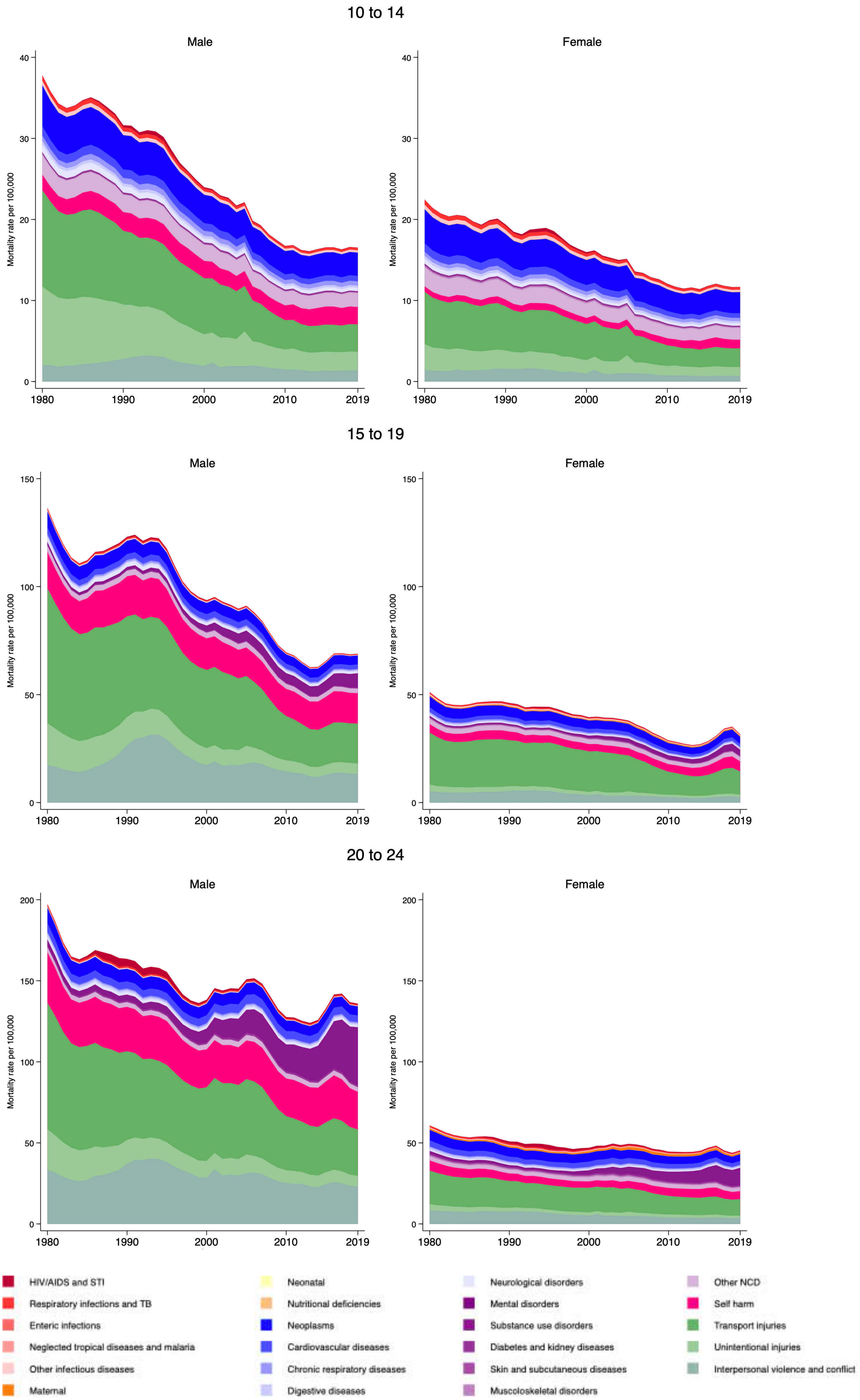

Figure S100d Number of deaths in 10-24 1980 – 2019 by cause: United States of America

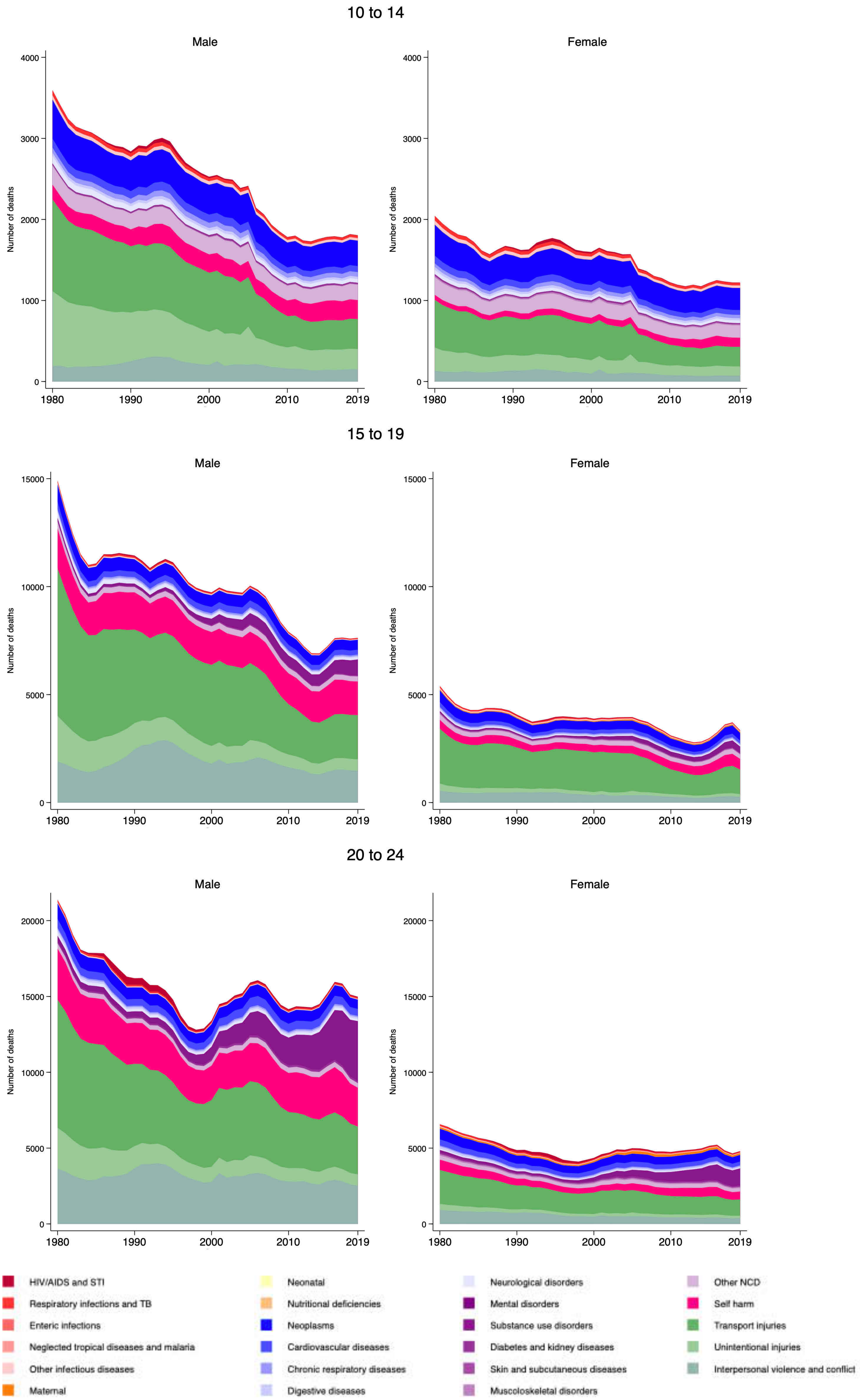

Figure S101: Latin America and Caribbean

Figure S101a All-cause mortality rate per 100,000 in 10-24 1950 – 2019: Latin America and Caribbean

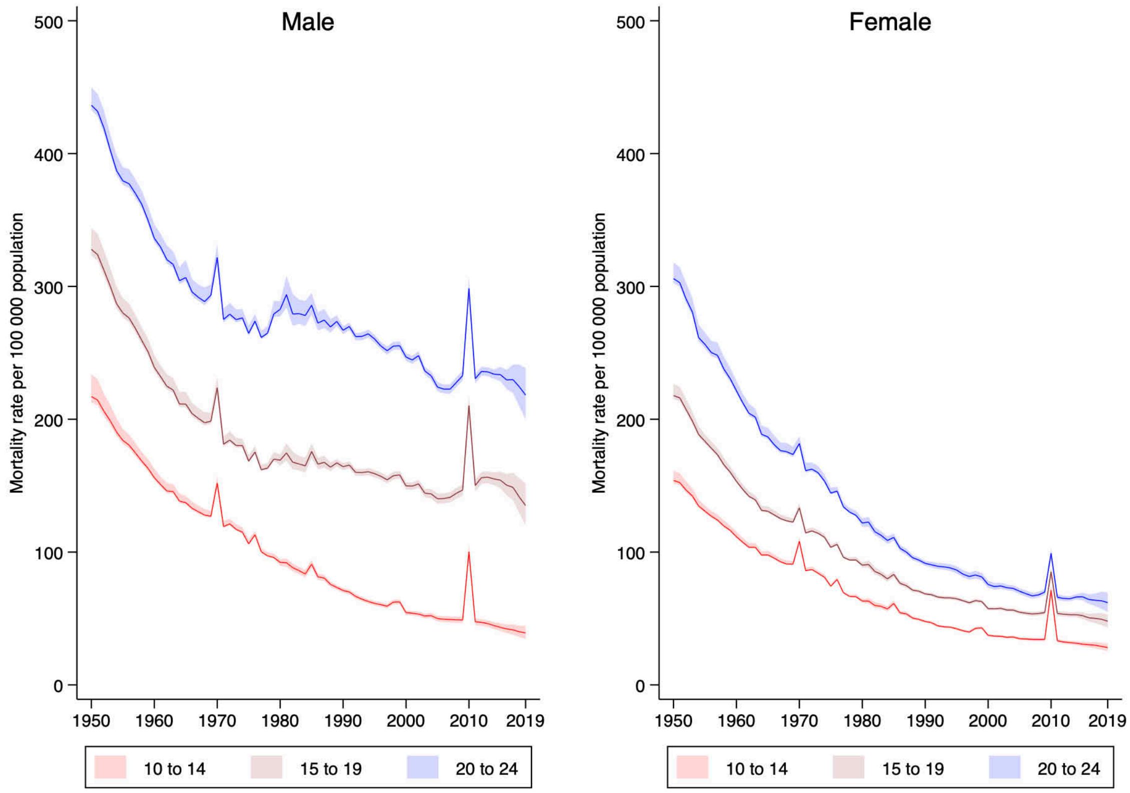

Figure S101b Number of deaths from all causes in 10-24 1950 – 2019: Latin America and Caribbean

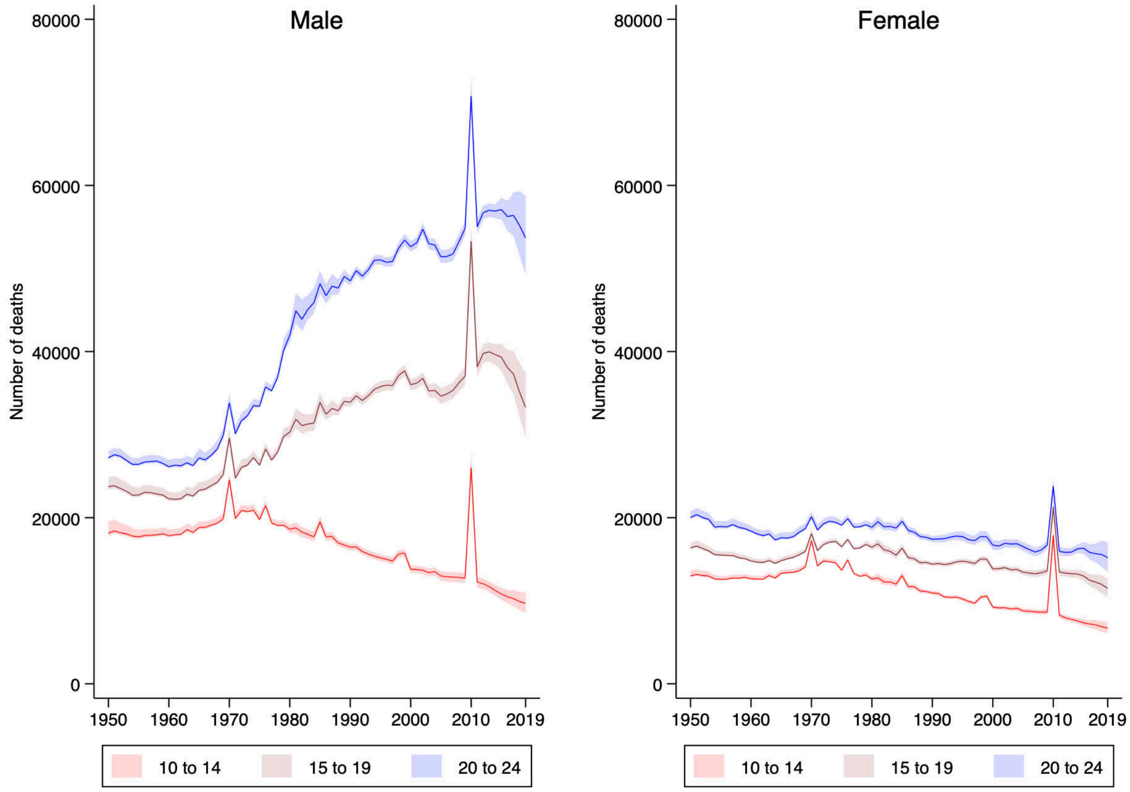

Figure S101c Mortality rate per 100,000 in 10-24 1980 – 2019 by cause: Latin America and Caribbean

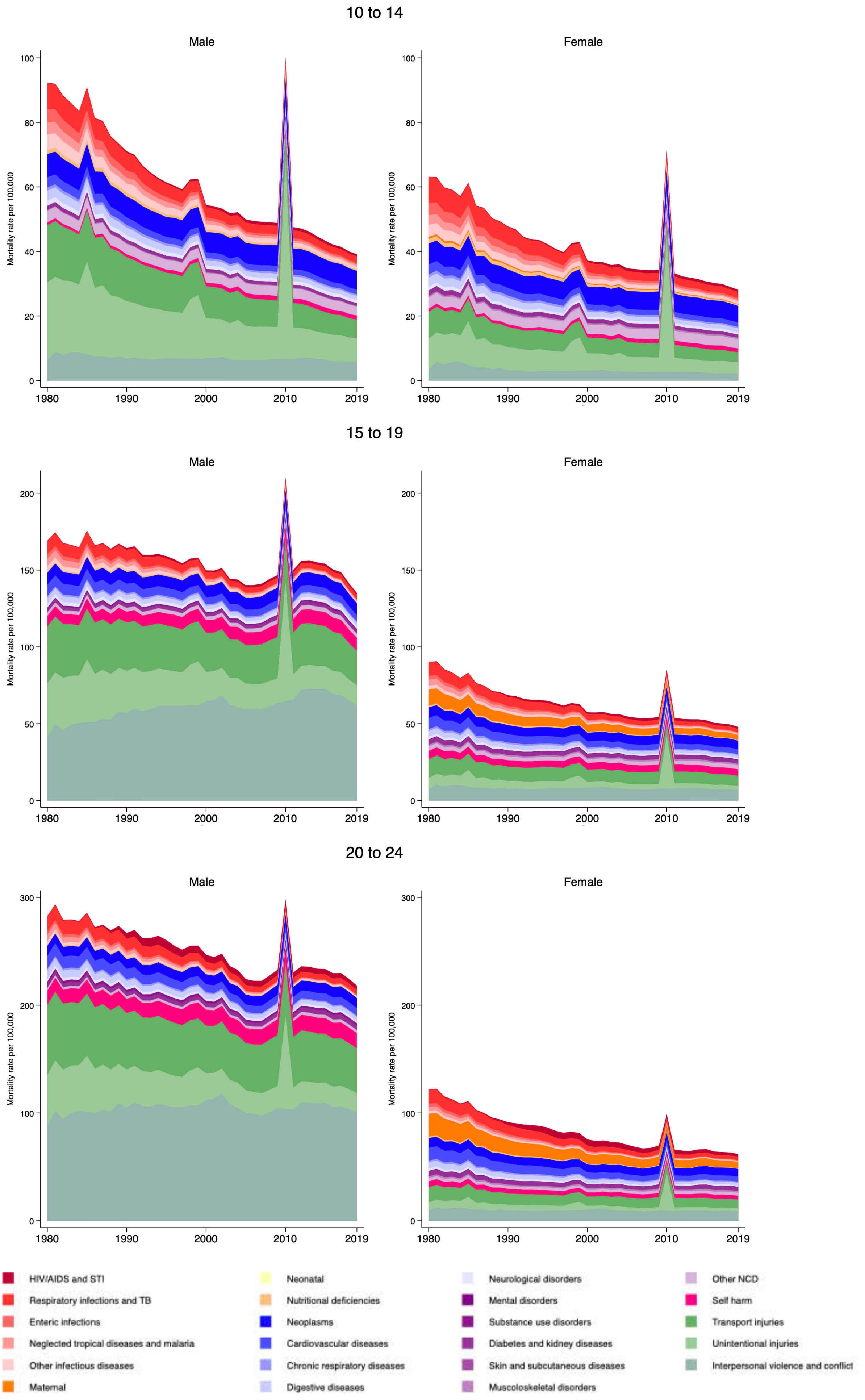

Figure S101d Number of deaths in 10-24 1980 – 2019 by cause: Latin America and Caribbean

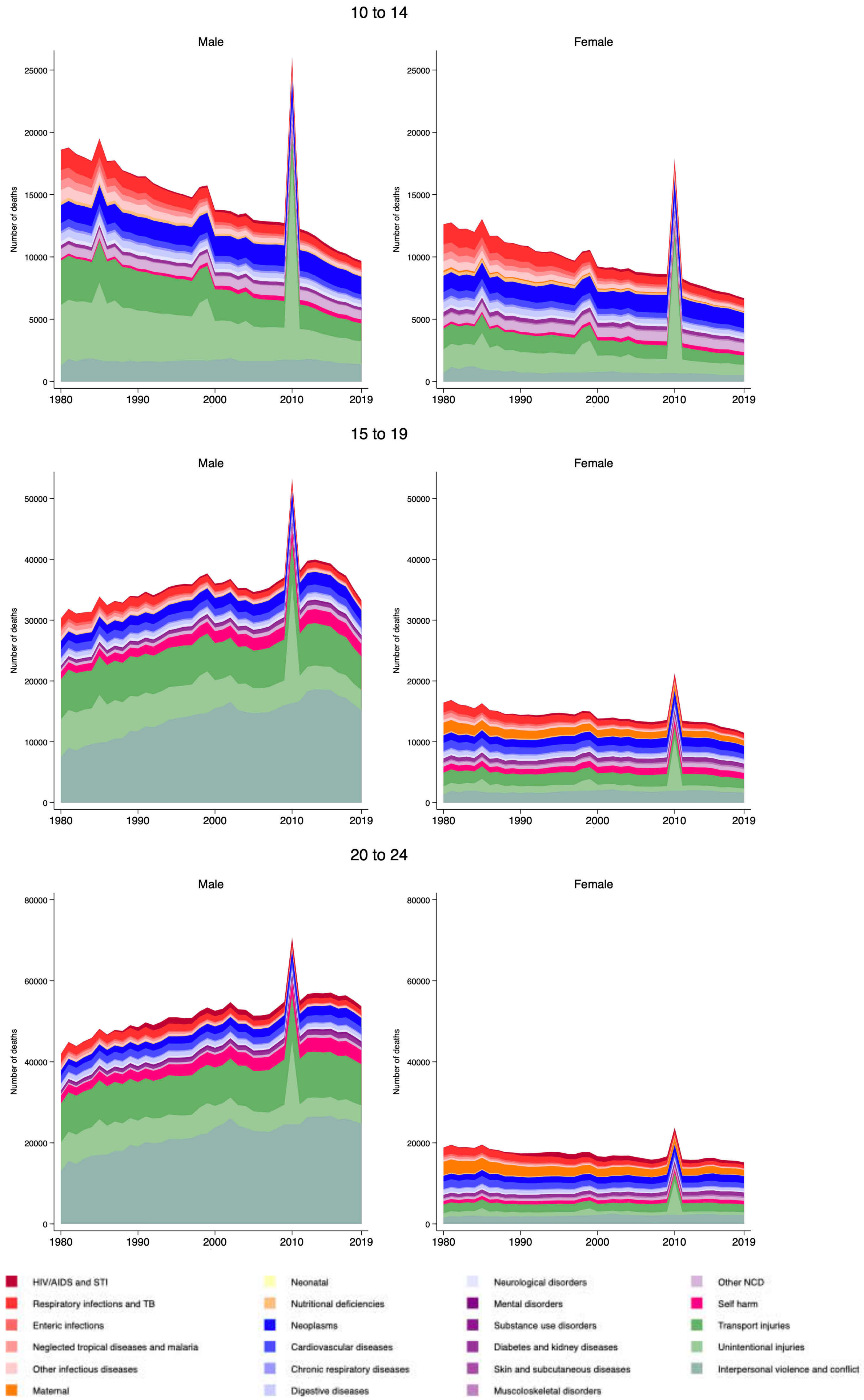

Figure S102: Caribbean

Figure S102a All-cause mortality rate per 100,000 in 10-24 1950 – 2019: Caribbean

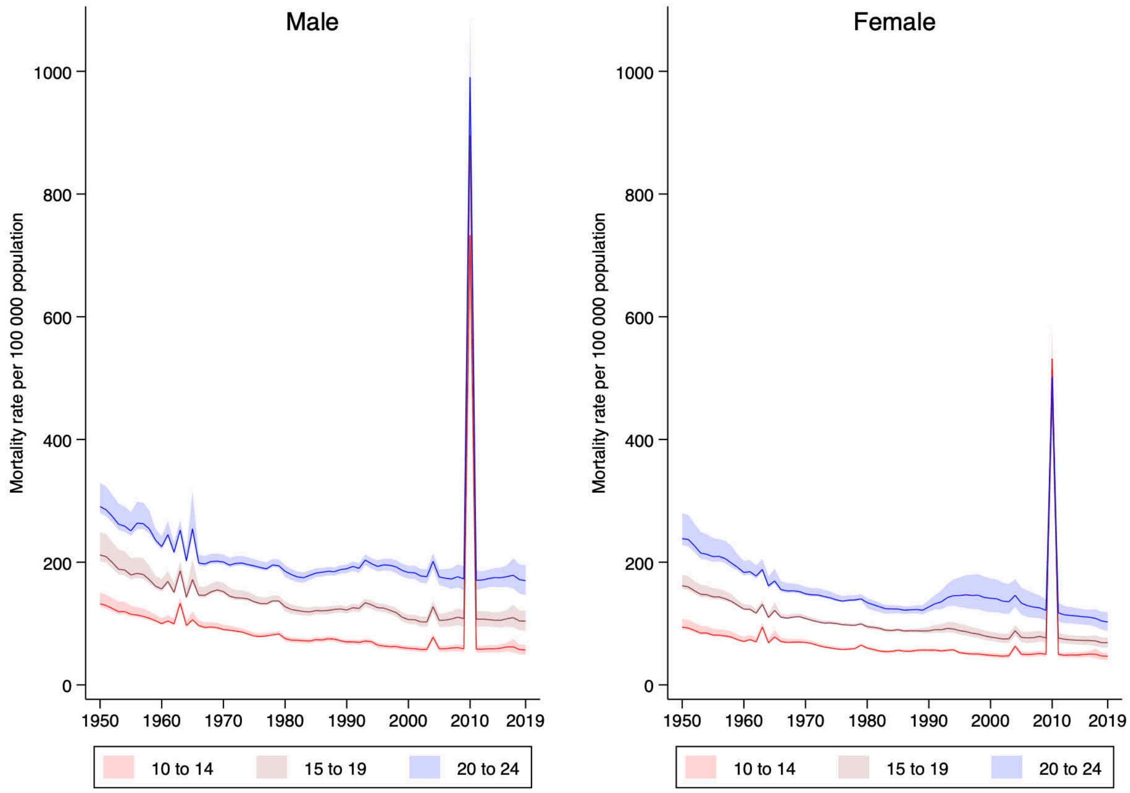

Figure S102b Number of deaths from all causes in 10-24 1950 – 2019: Caribbean

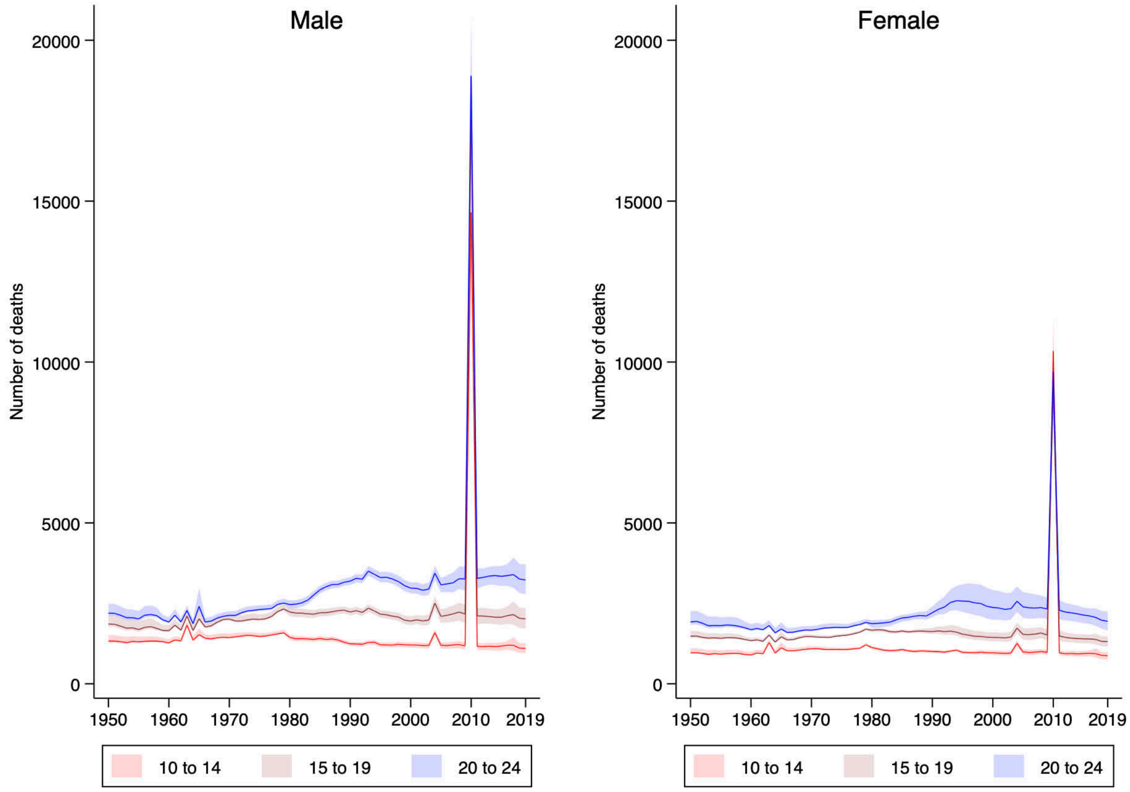

Figure S102c Mortality rate per 100,000 in 10-24 1980 – 2019 by cause: Caribbean

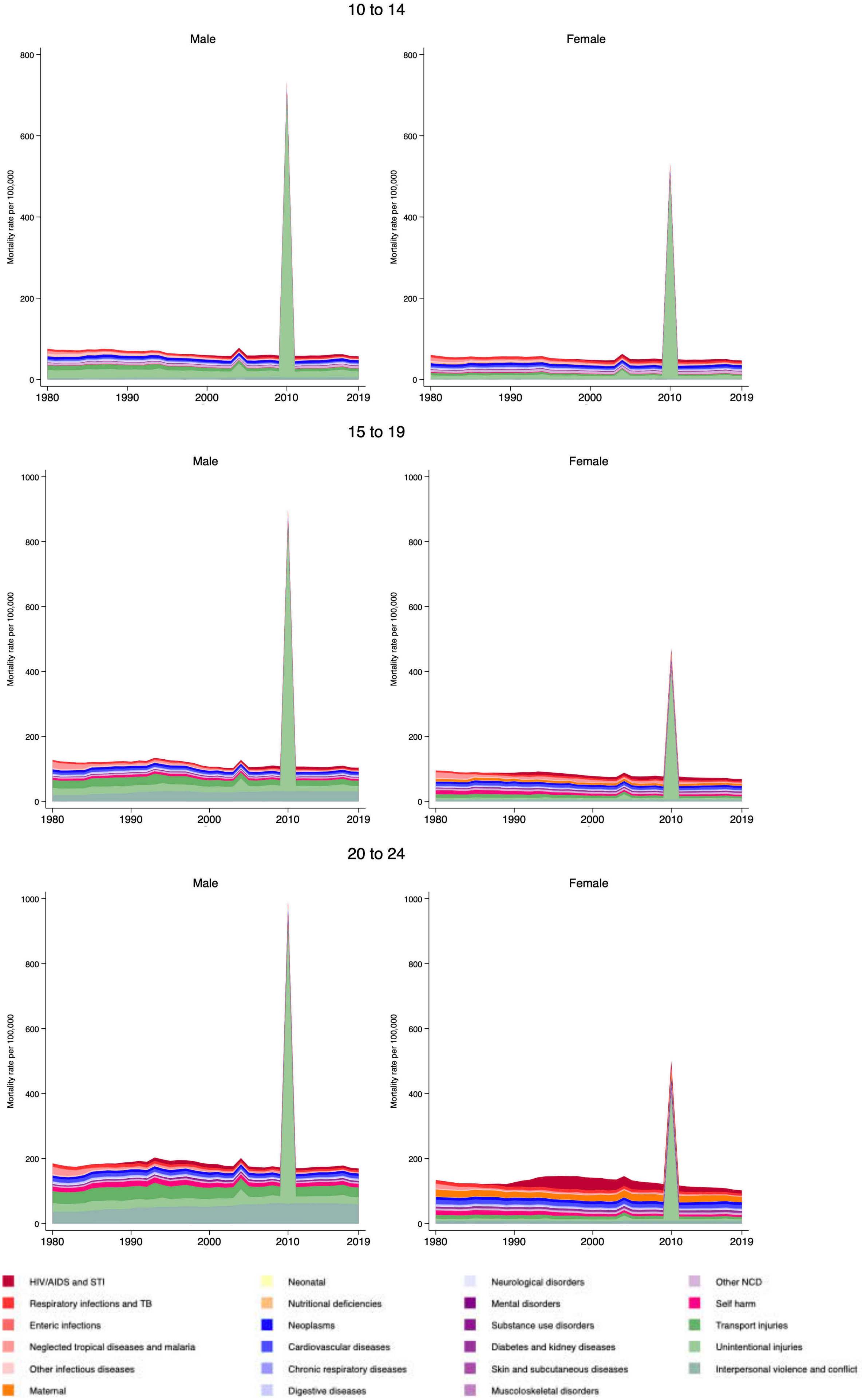

Figure S102d Number of deaths in 10-24 1980 – 2019 by cause: Caribbean

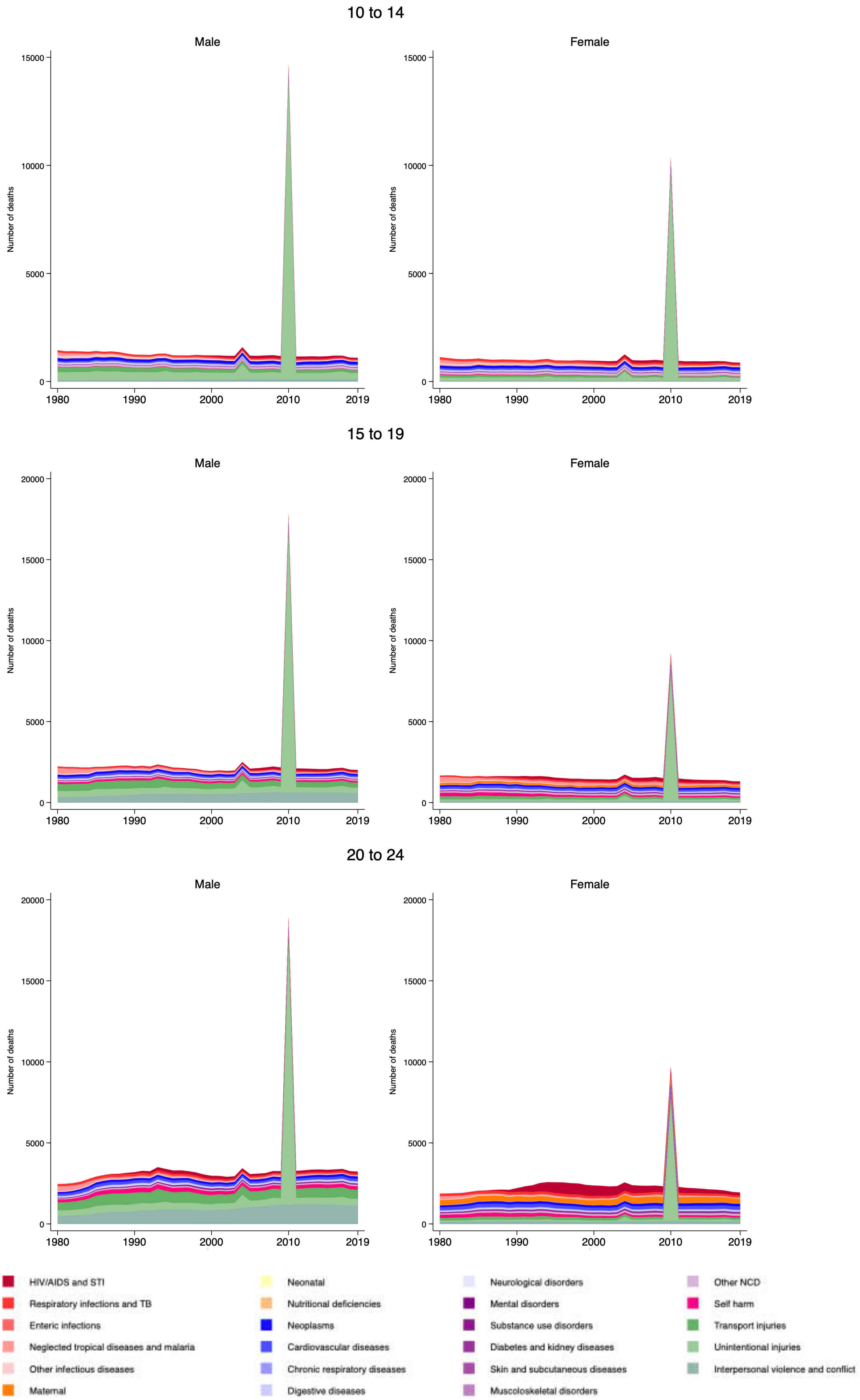

Figure S103: Antigua and Barbuda

Figure S103a All-cause mortality rate per 100,000 in 10-24 1950 – 2019: Antigua and Barbuda

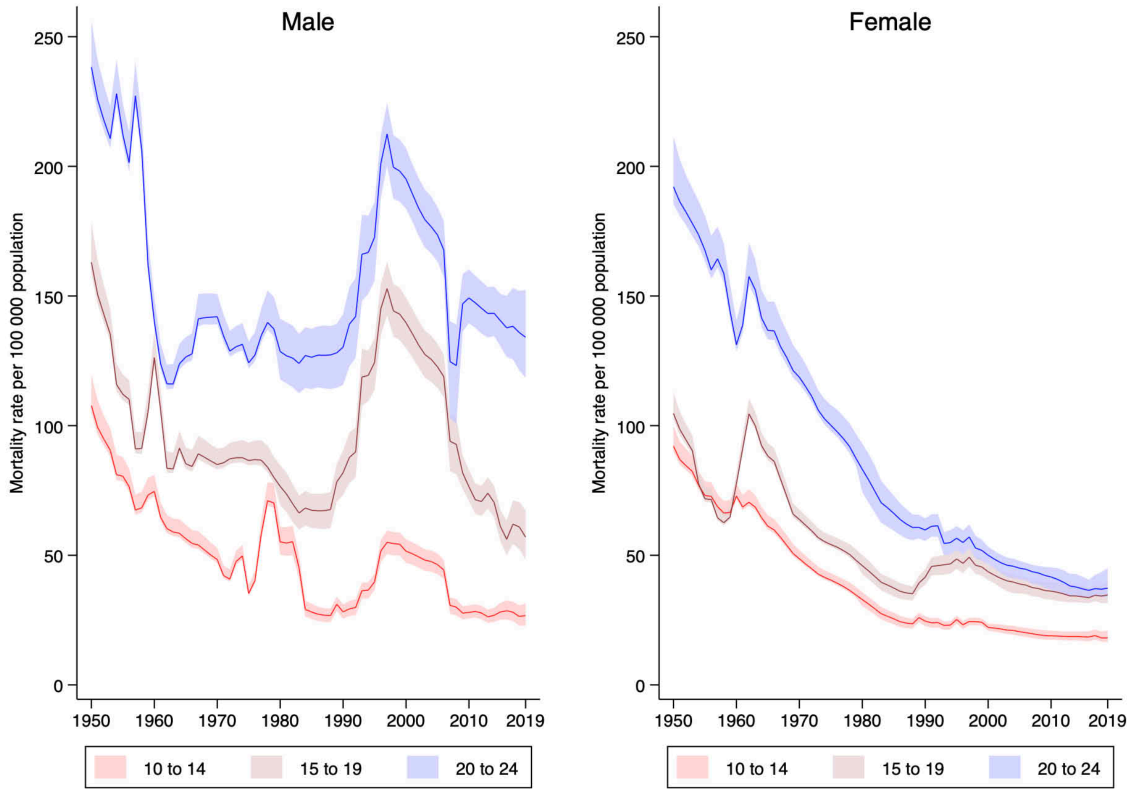

Figure S103b Number of deaths from all causes in 10-24 1950 – 2019: Antigua and Barbuda

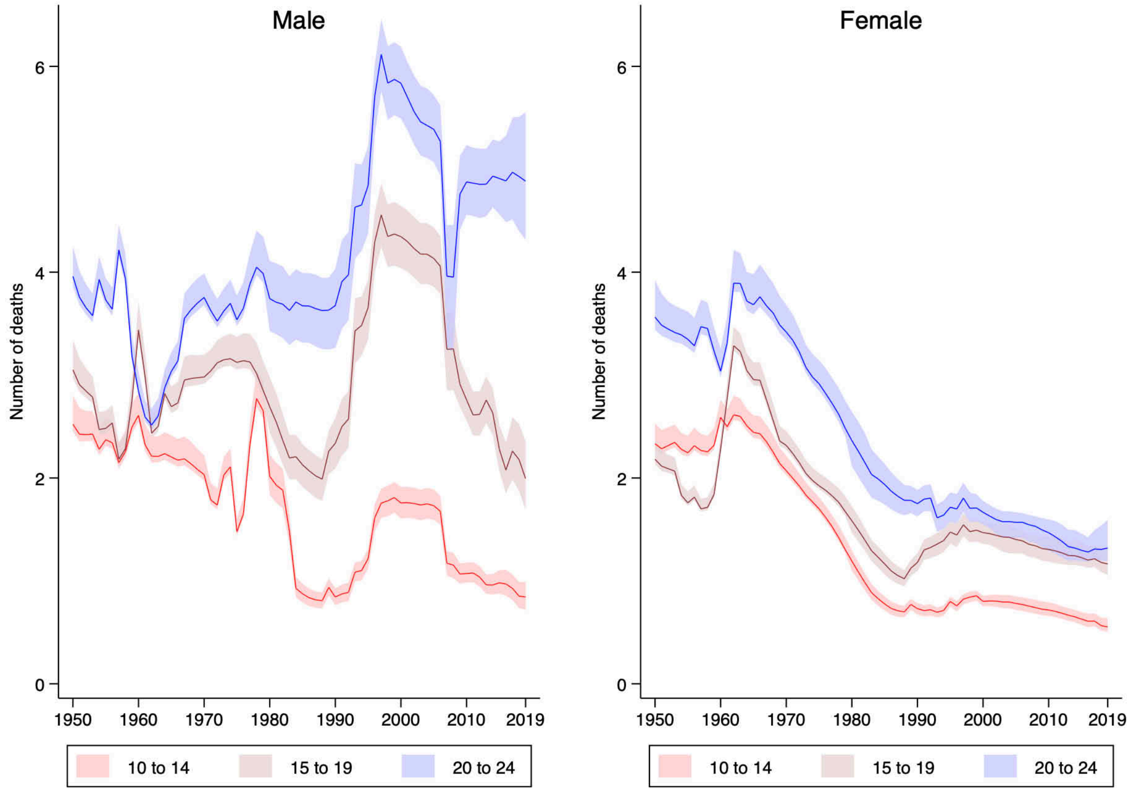

Figure S103c Mortality rate per 100,000 in 10-24 1980 – 2019 by cause: Antigua and Barbuda

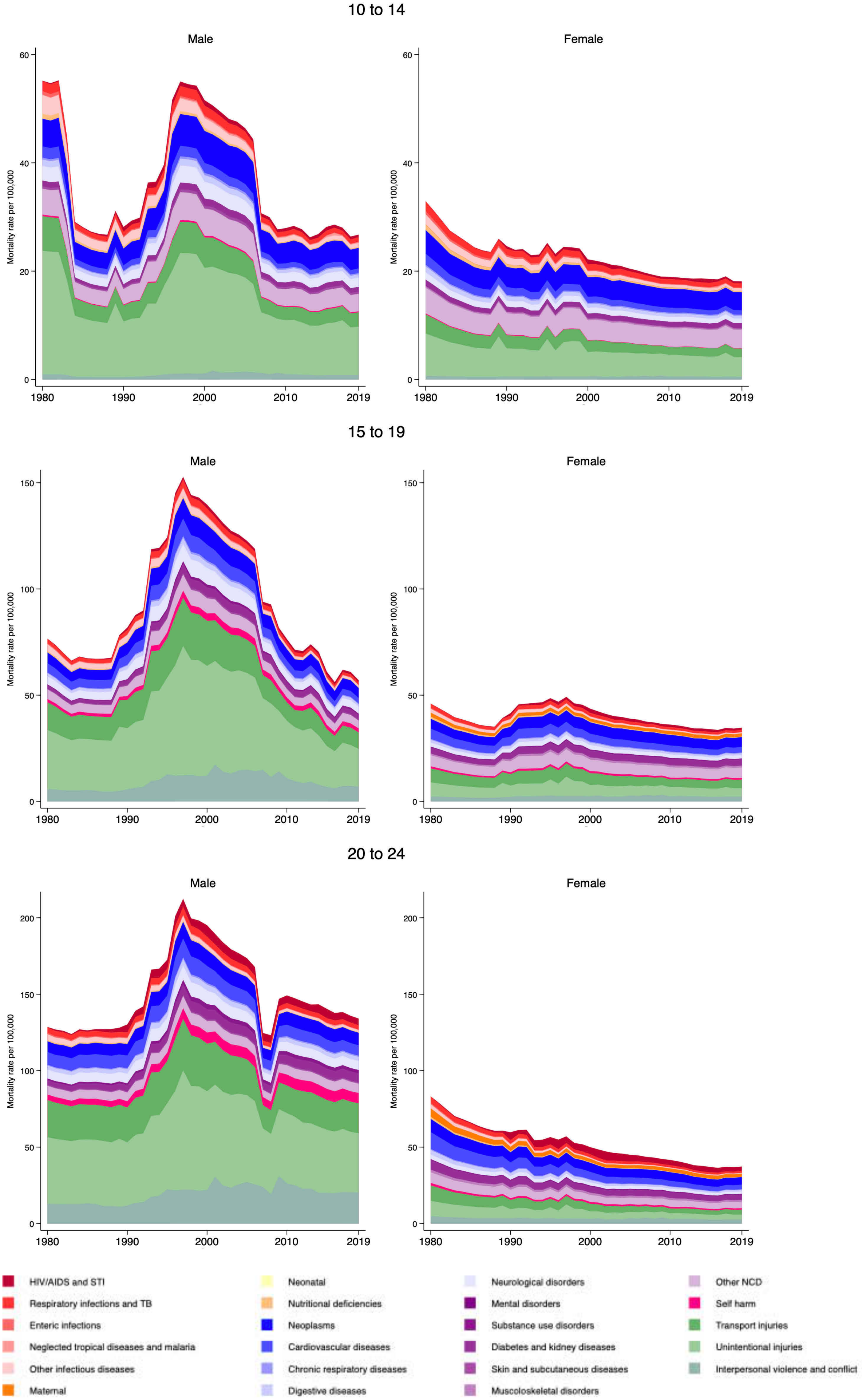

Figure S103d Number of deaths in 10-24 1980 – 2019 by cause: Antigua and Barbuda

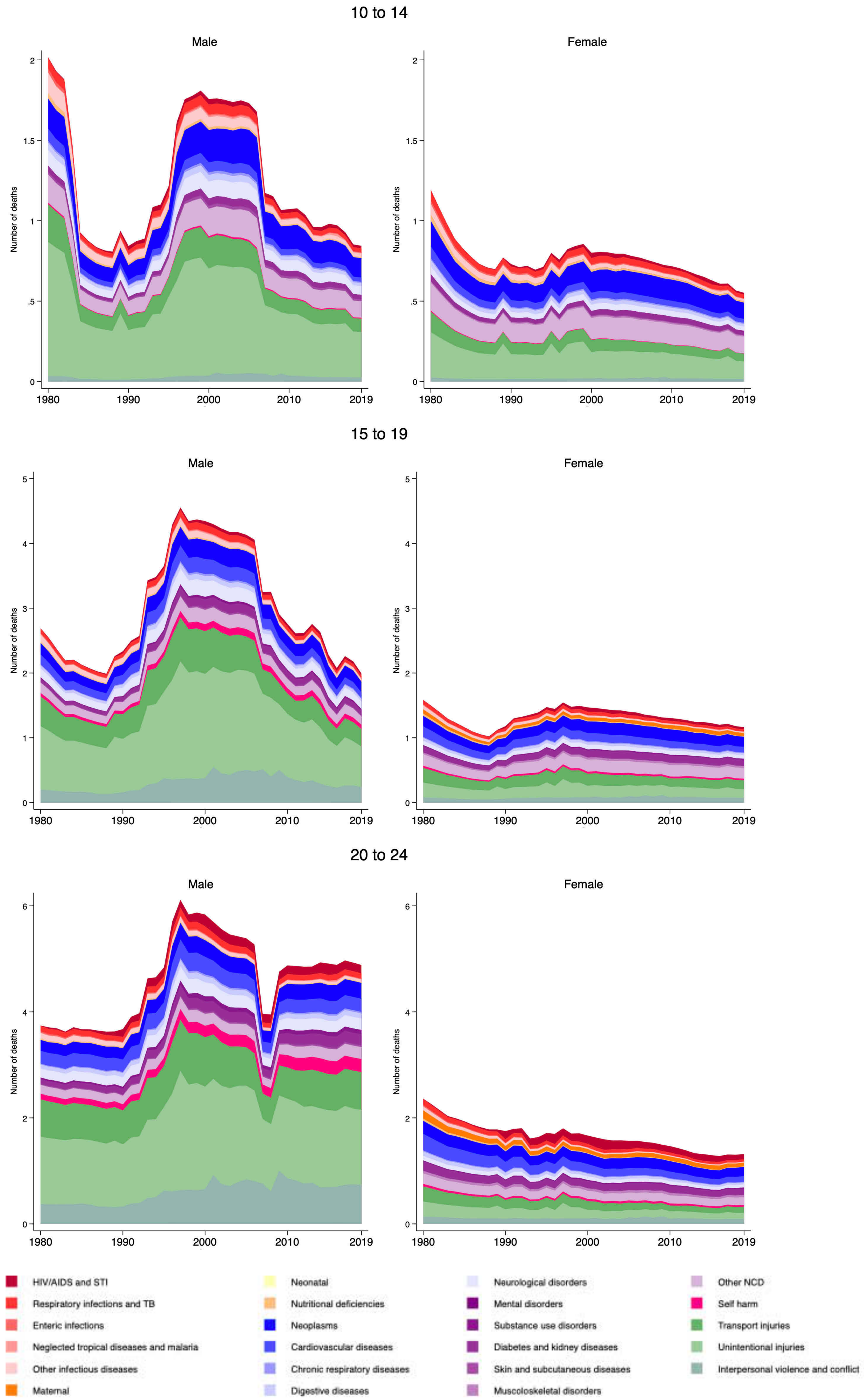

Figure S104: Bahamas

Figure S104a All-cause mortality rate per 100,000 in 10-24 1950 – 2019: Bahamas

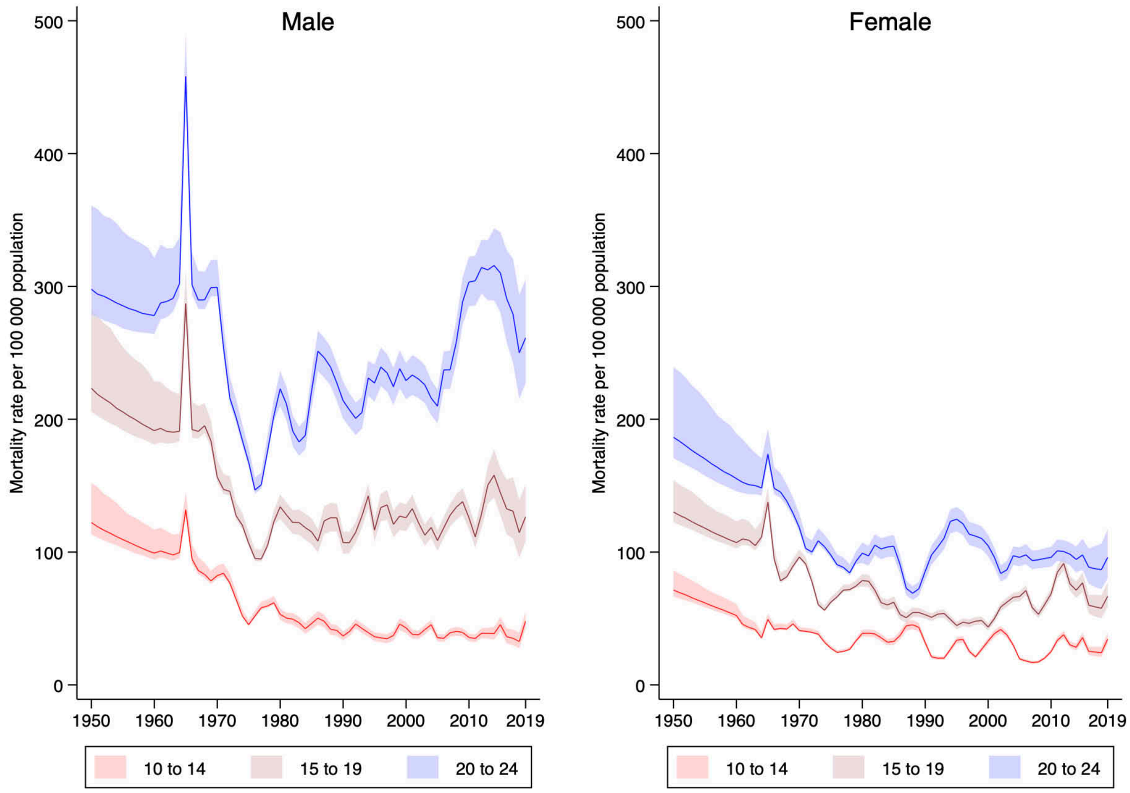

Figure S104b Number of deaths from all causes in 10-24 1950 – 2019: Bahamas

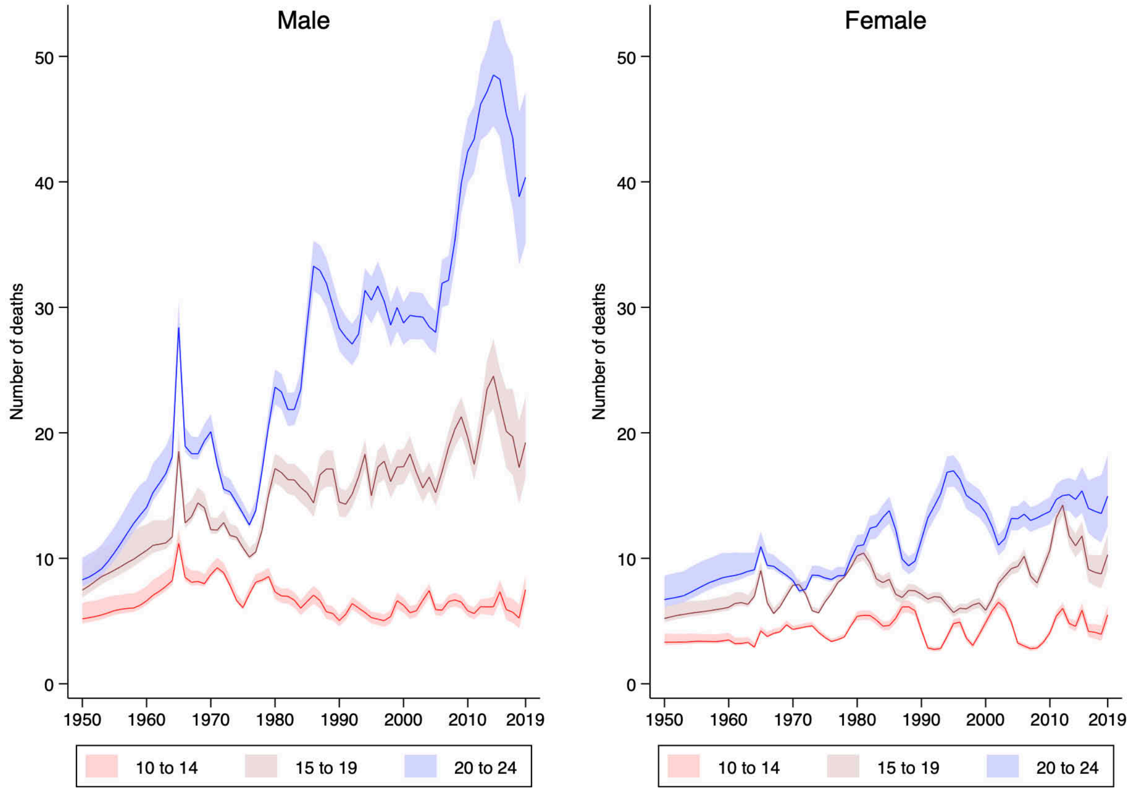

Figure S104c Mortality rate per 100,000 in 10-24 1980 – 2019 by cause: Bahamas

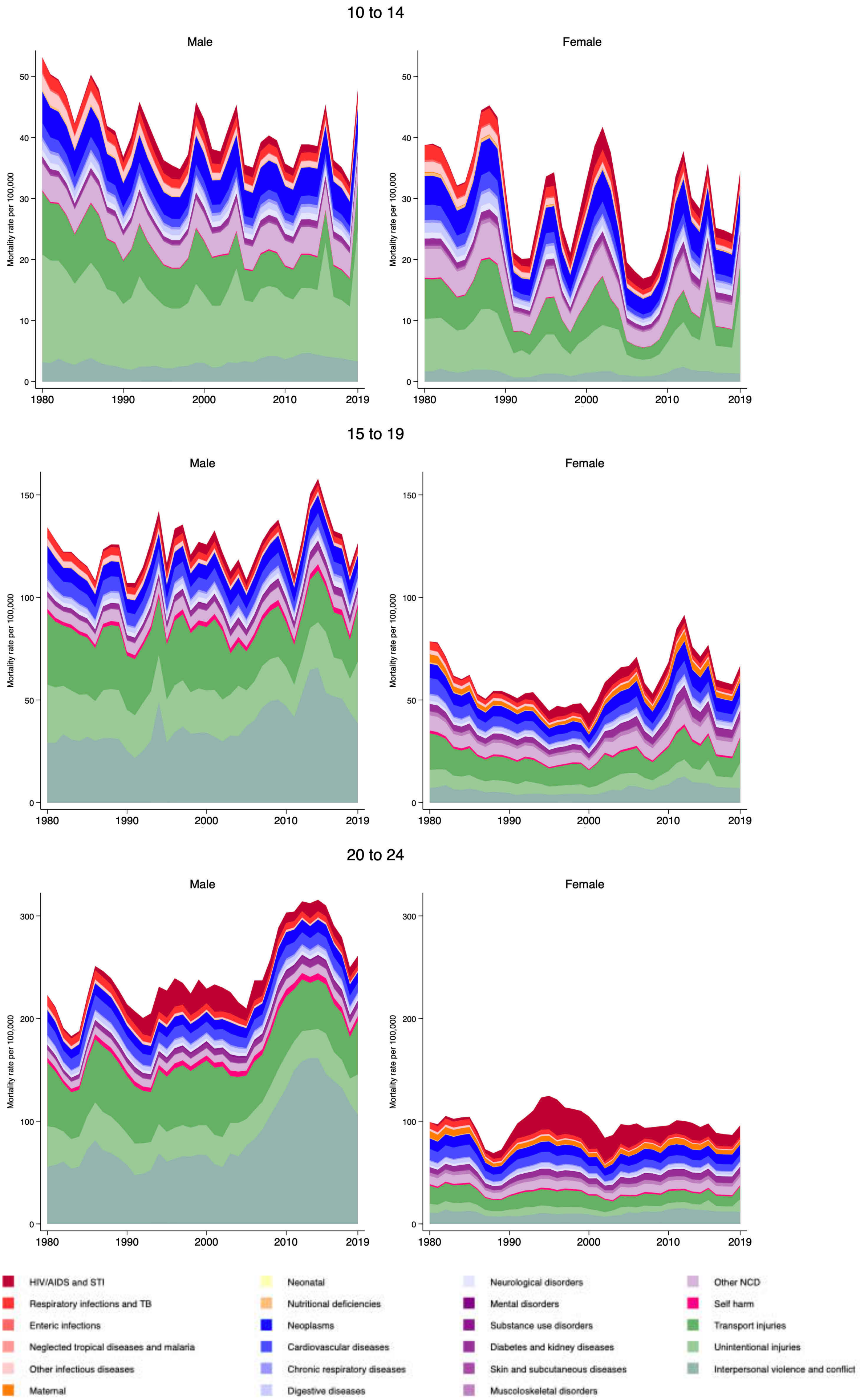

Figure S104d Number of deaths in 10-24 1980 – 2019 by cause: Bahamas

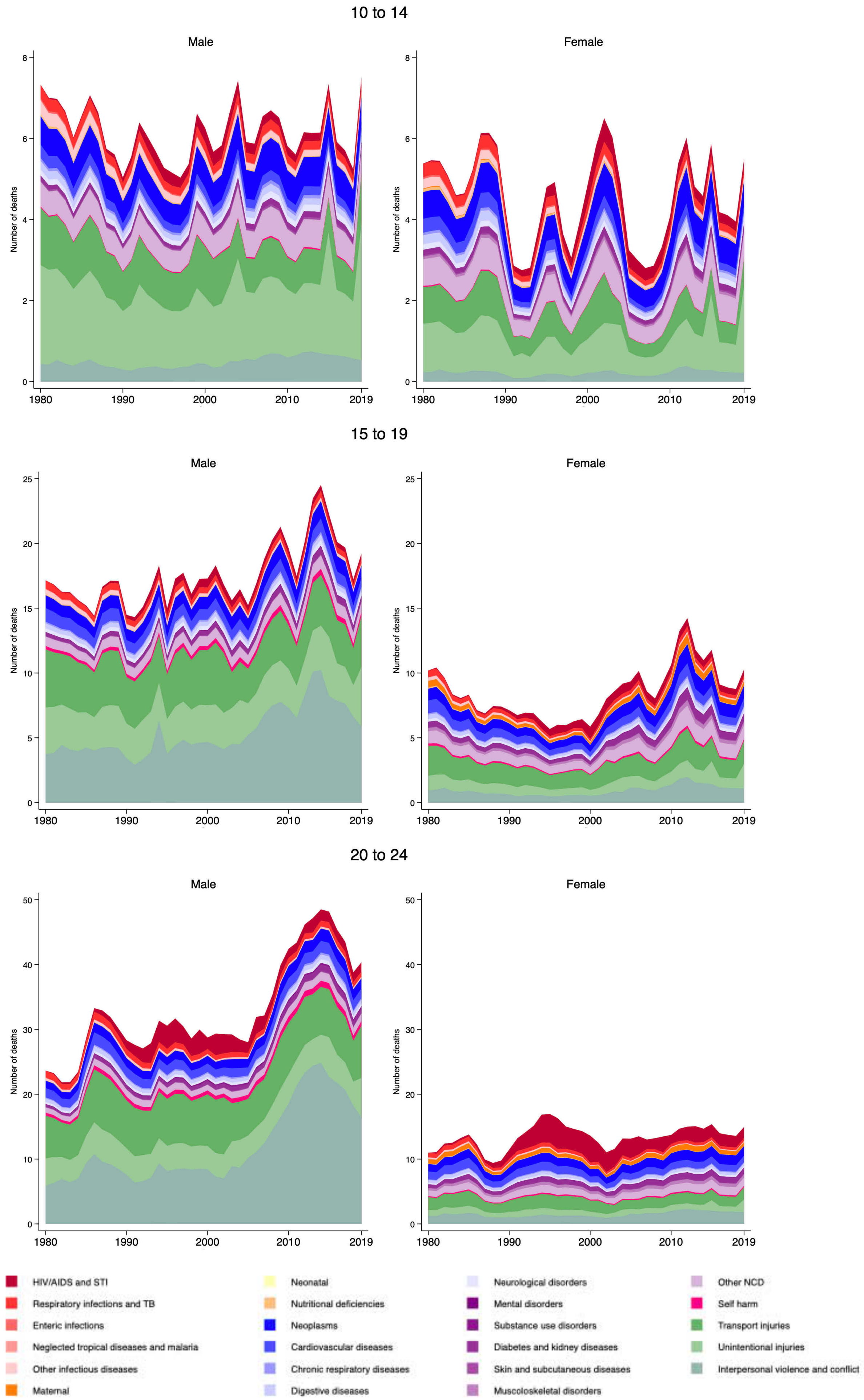

Figure S105: Barbados

Figure S105a All-cause mortality rate per 100,000 in 10-24 1950 – 2019: Barbados

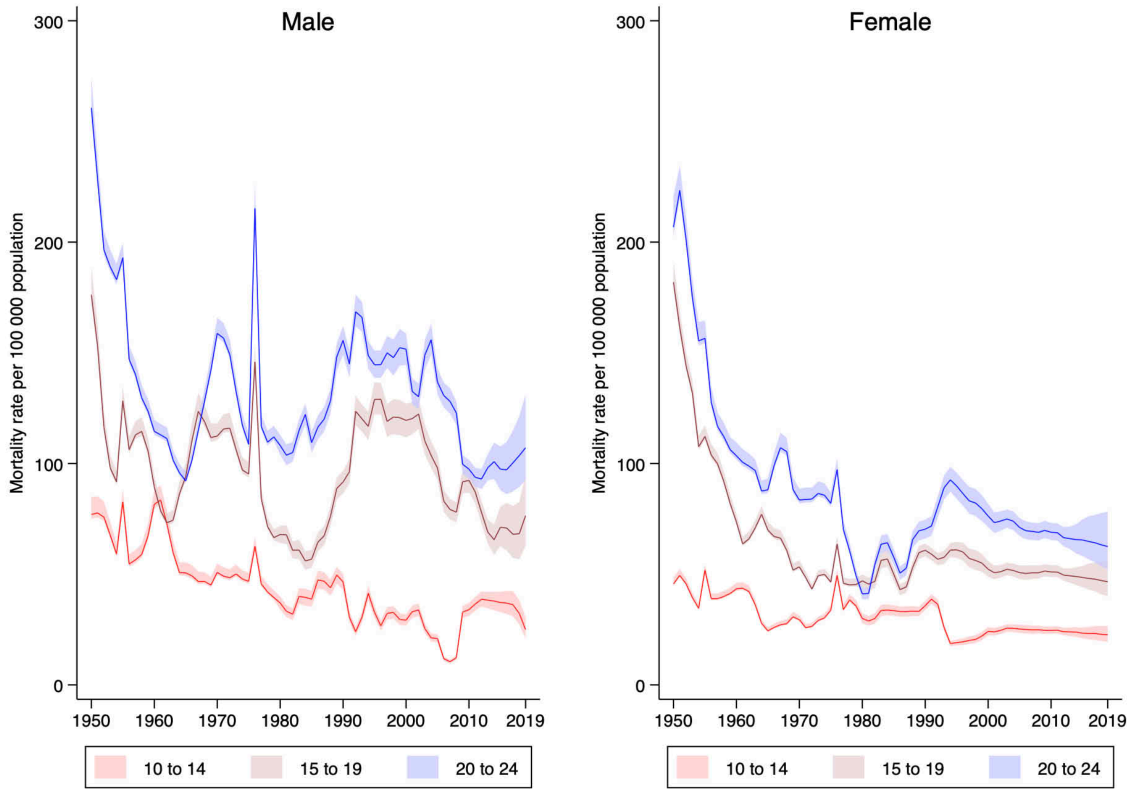

Figure S105b Number of deaths from all causes in 10-24 1950 – 2019: Barbados

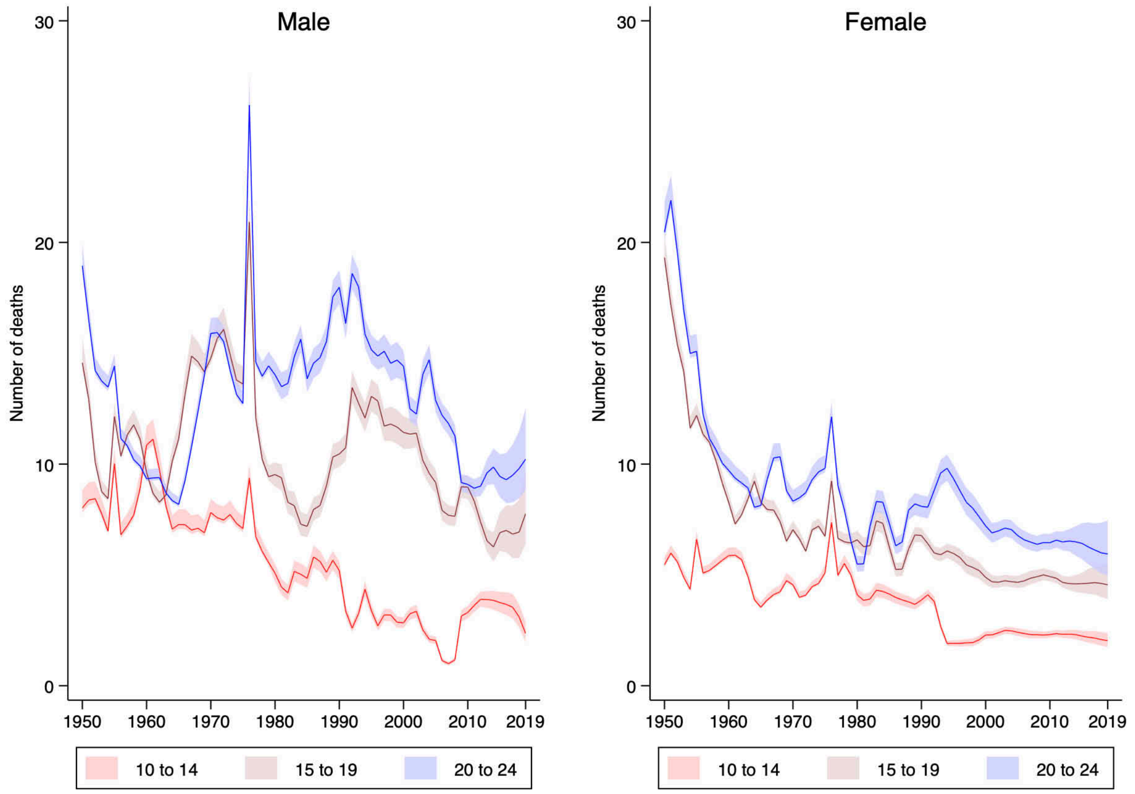

Figure S105c Mortality rate per 100,000 in 10-24 1980 – 2019 by cause: Barbados

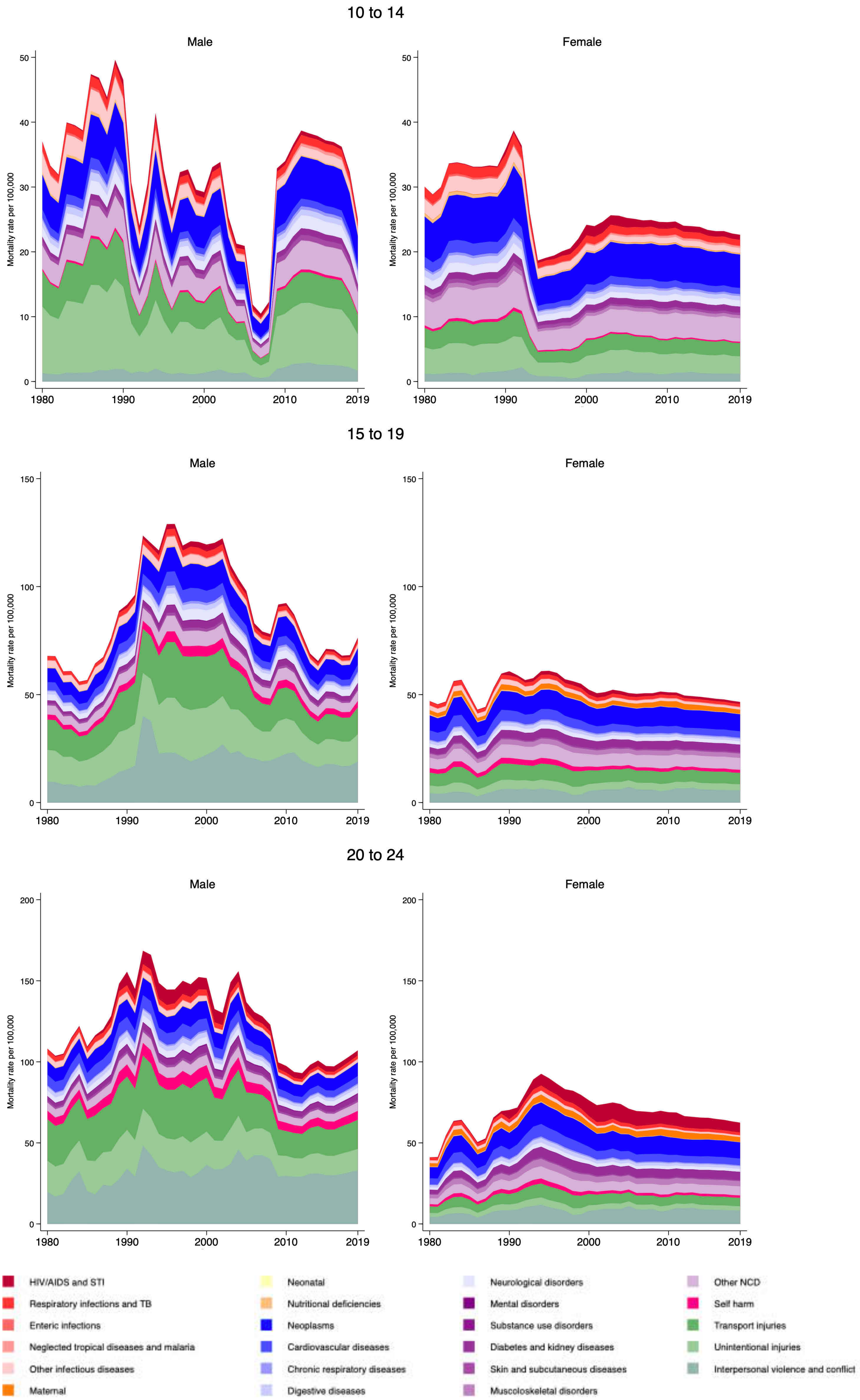

Figure S105d Number of deaths in 10-24 1980 – 2019 by cause: Barbados

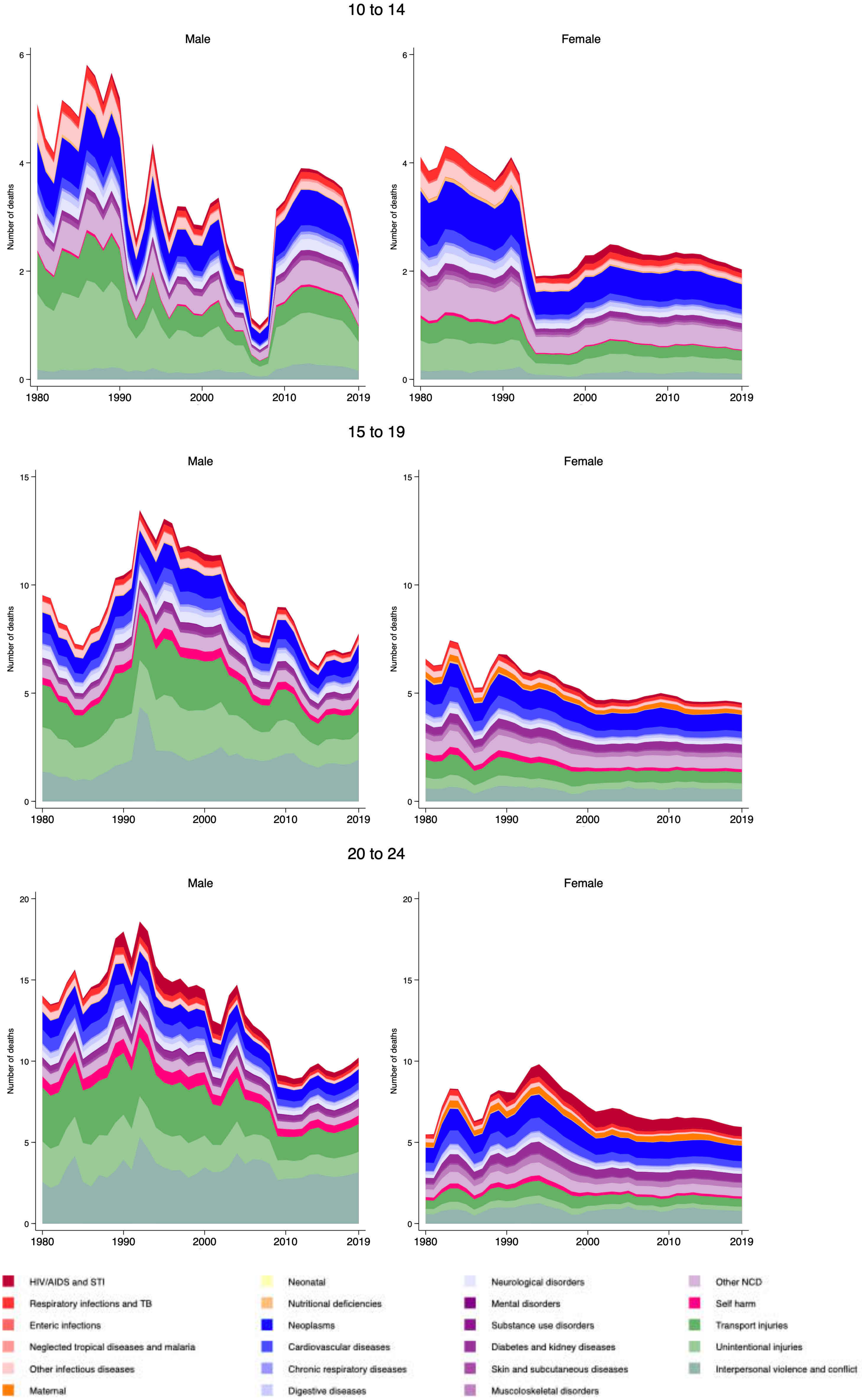

Figure S106: Belize

Figure S106a All-cause mortality rate per 100,000 in 10-24 1950 – 2019: Belize

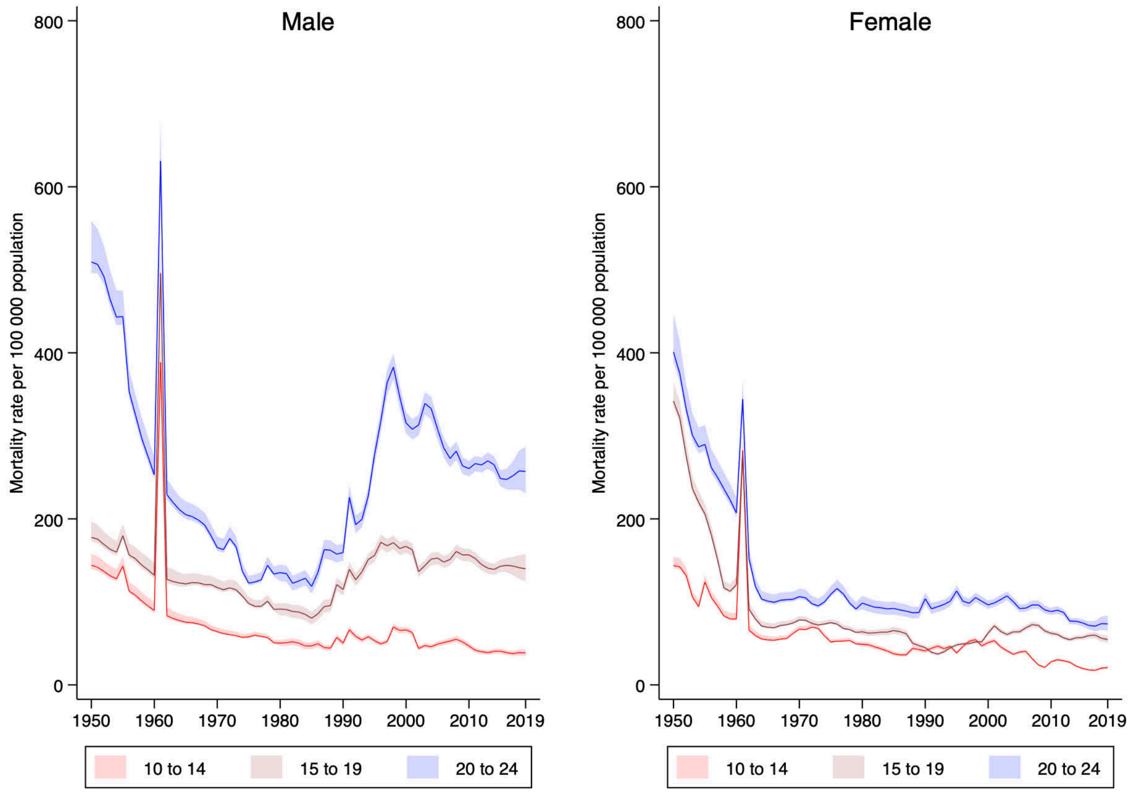

Figure S106b Number of deaths from all causes in 10-24 1950 – 2019: Belize

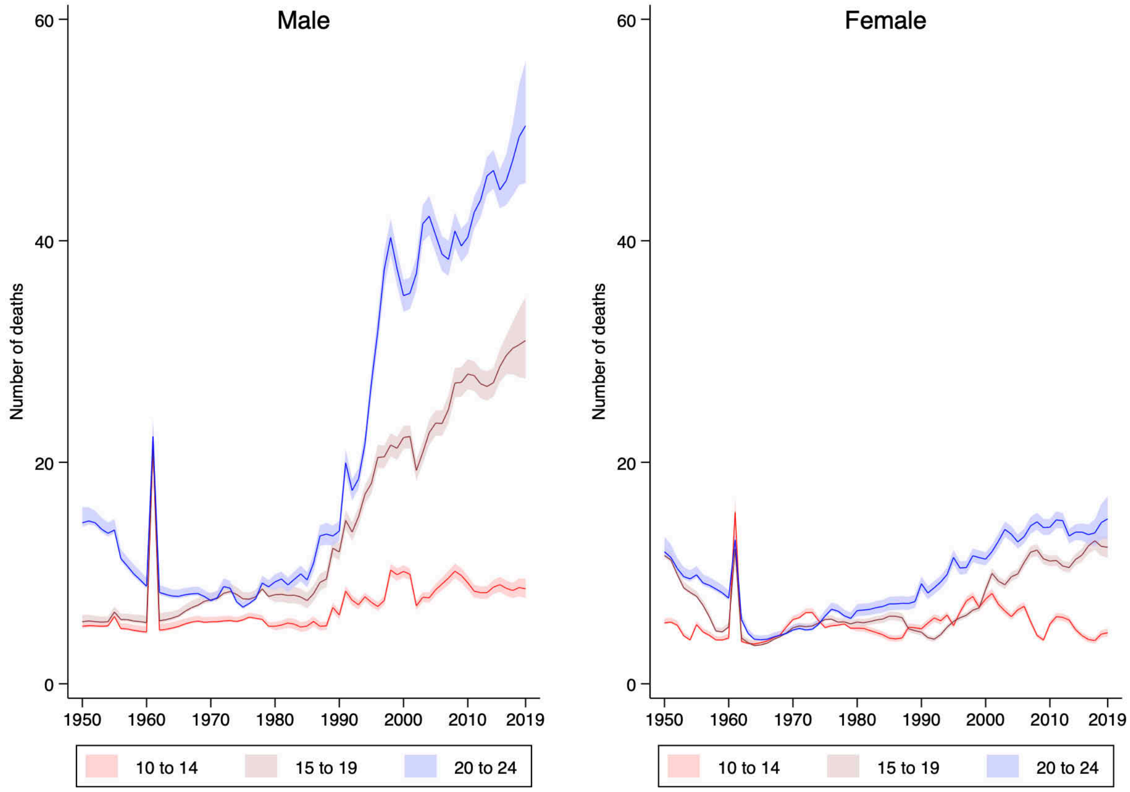

Figure S106c Mortality rate per 100,000 in 10-24 1980 – 2019 by cause: Belize

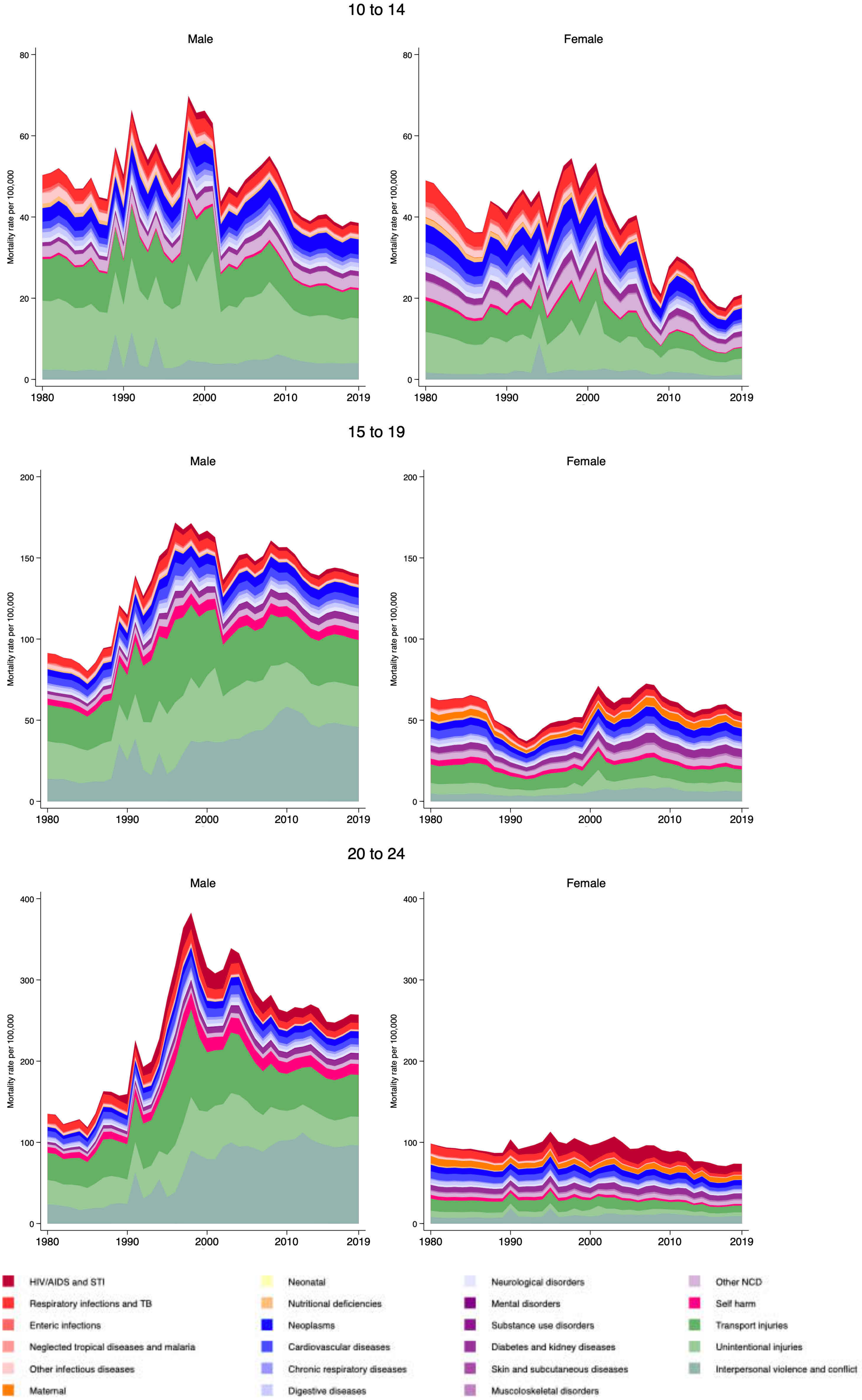

Figure S106d Number of deaths in 10-24 1980 – 2019 by cause: Belize

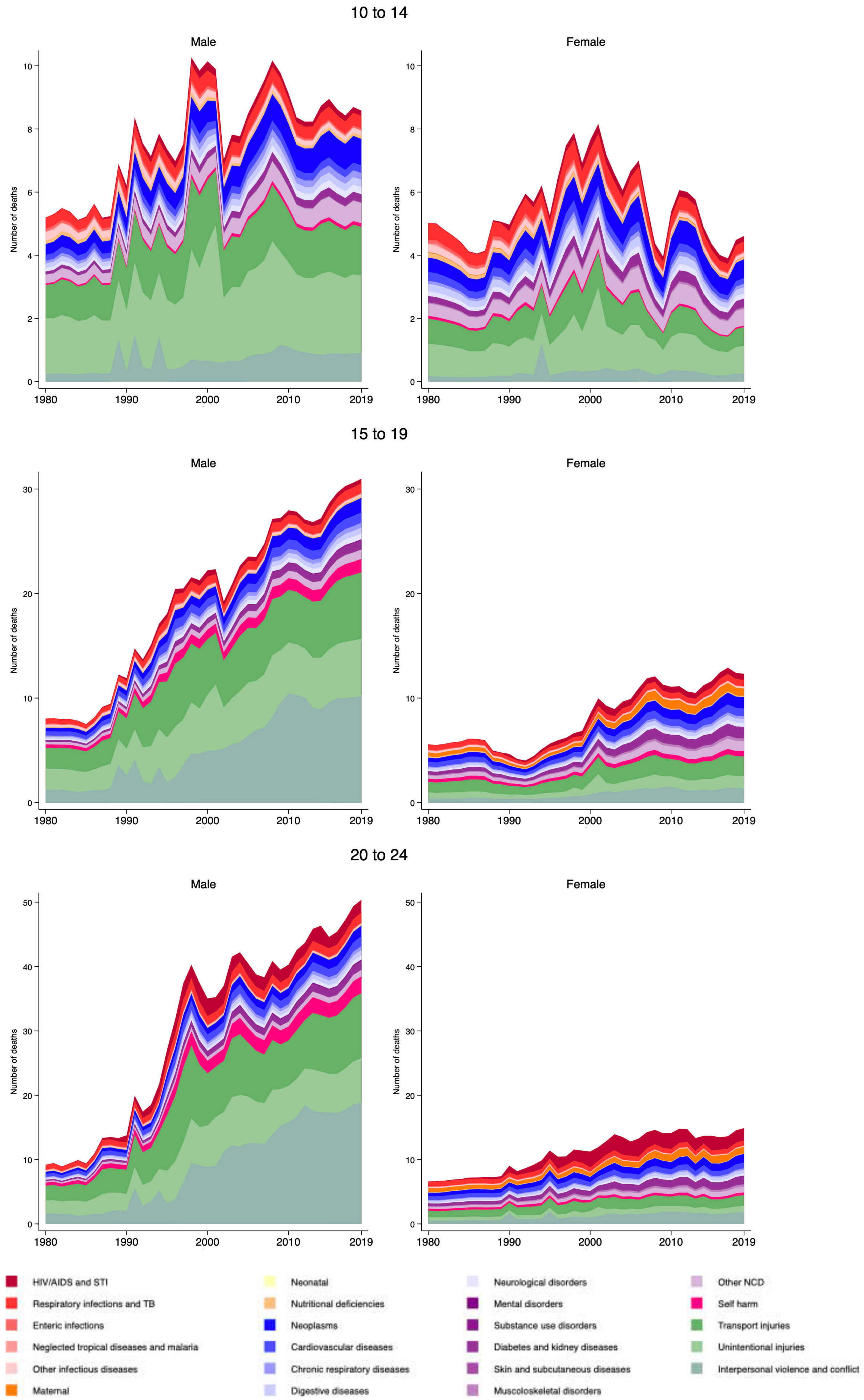

Figure S107: Cuba

Figure S107a All-cause mortality rate per 100,000 in 10-24 1950 – 2019: Cuba

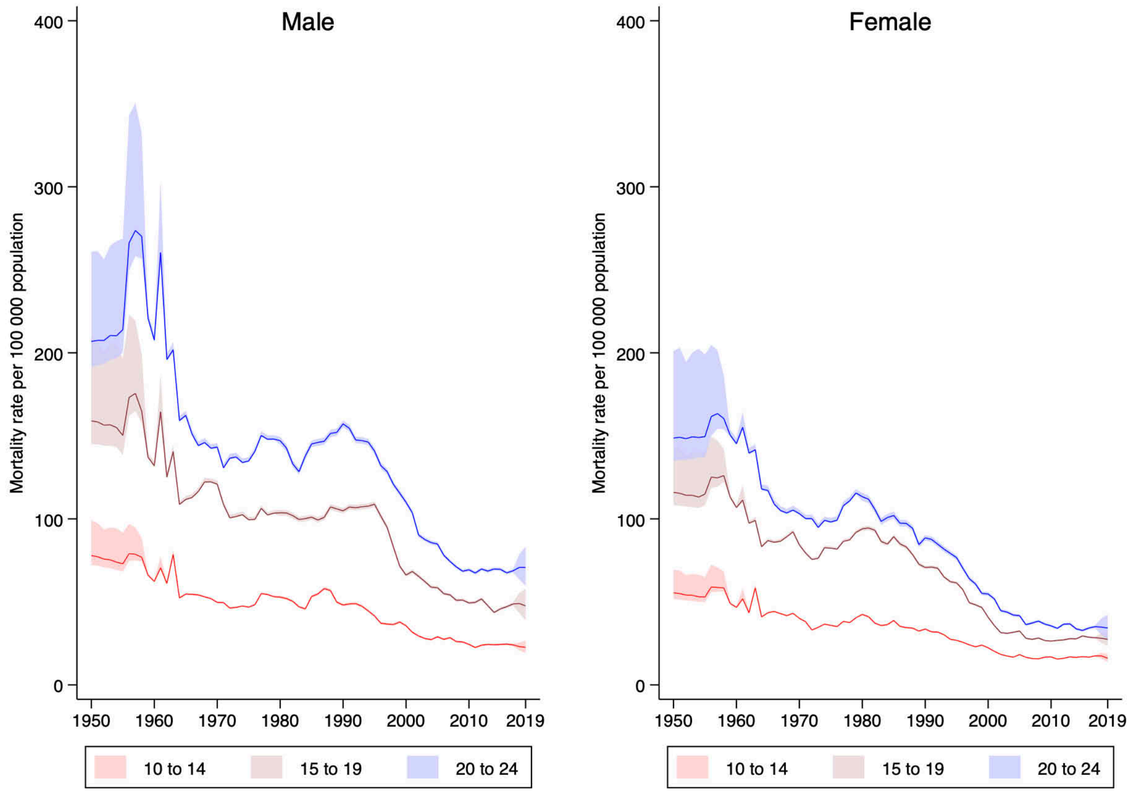

Figure S107b Number of deaths from all causes in 10-24 1950 – 2019: Cuba

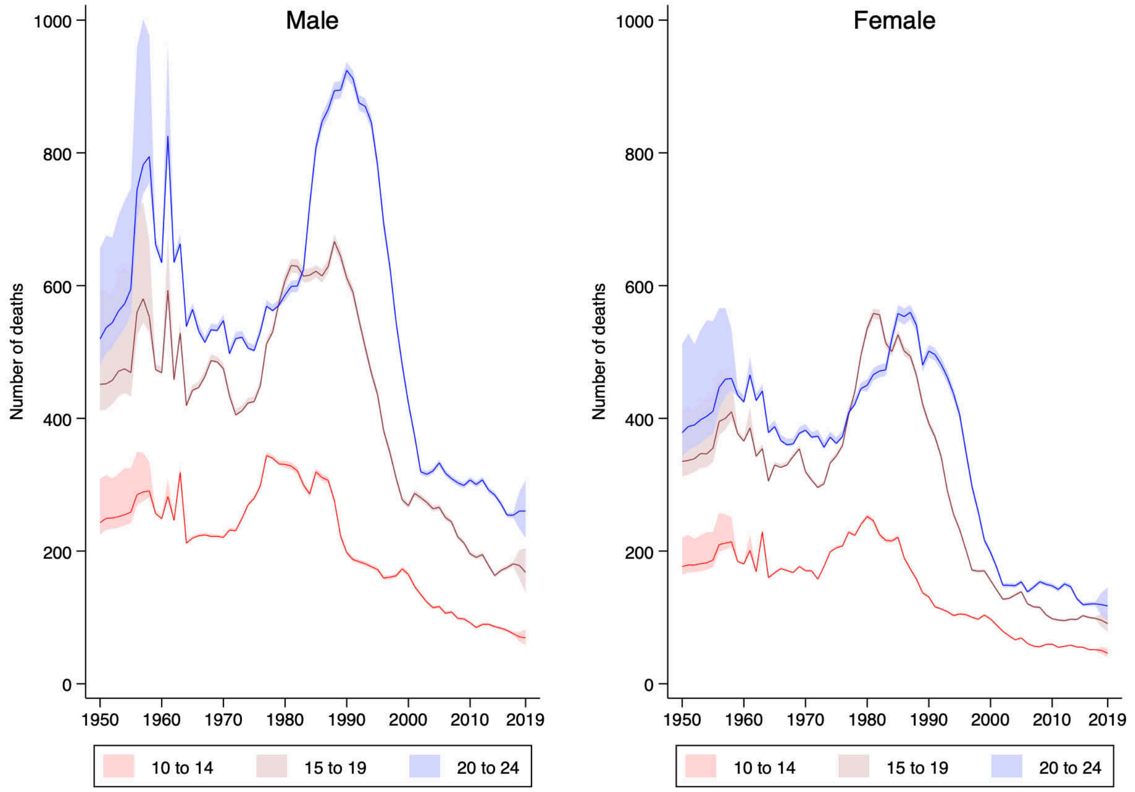

Figure S107c Mortality rate per 100,000 in 10-24 1980 – 2019 by cause: Cuba

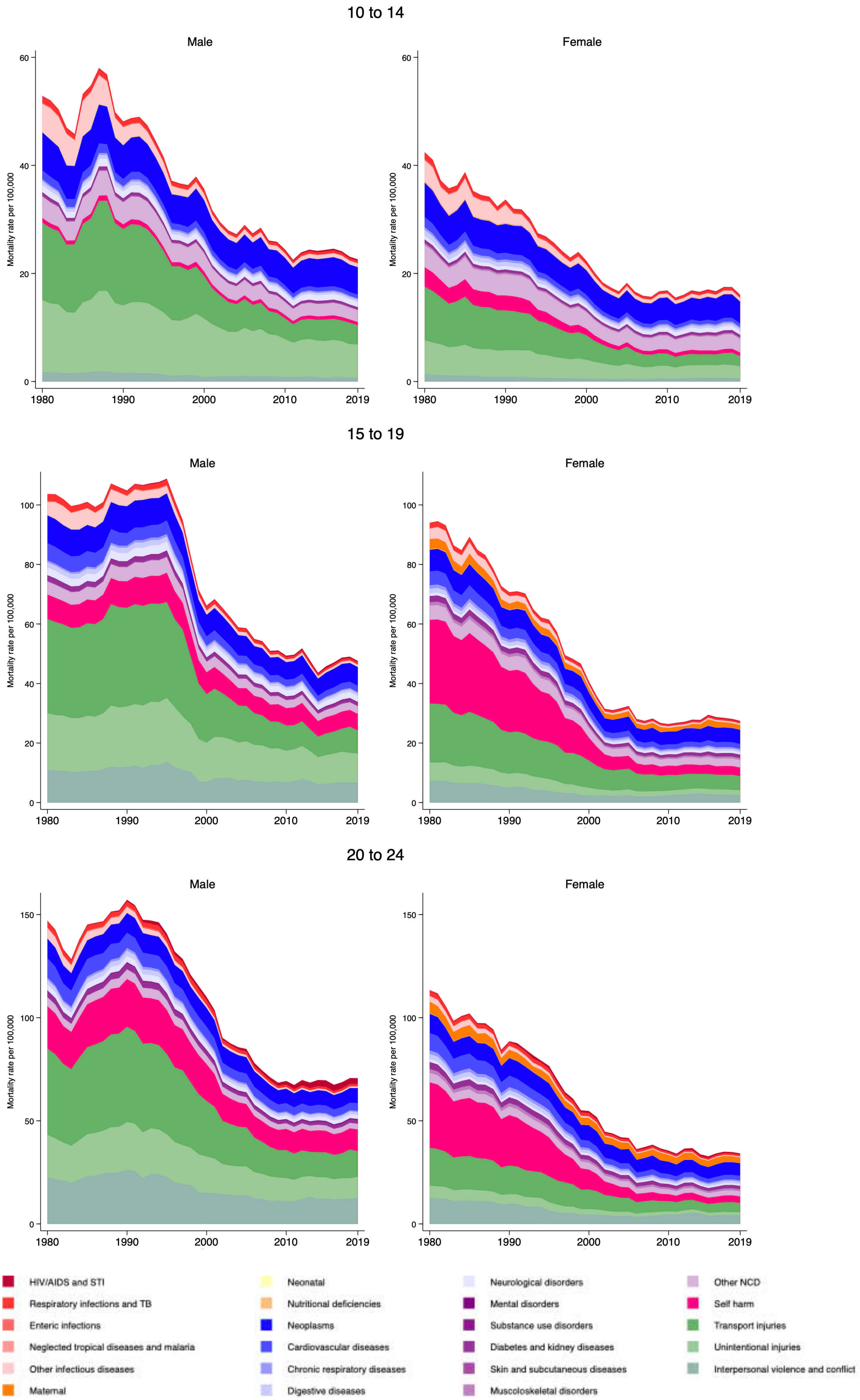

Figure S107d Number of deaths in 10-24 1980 – 2019 by cause: Cuba

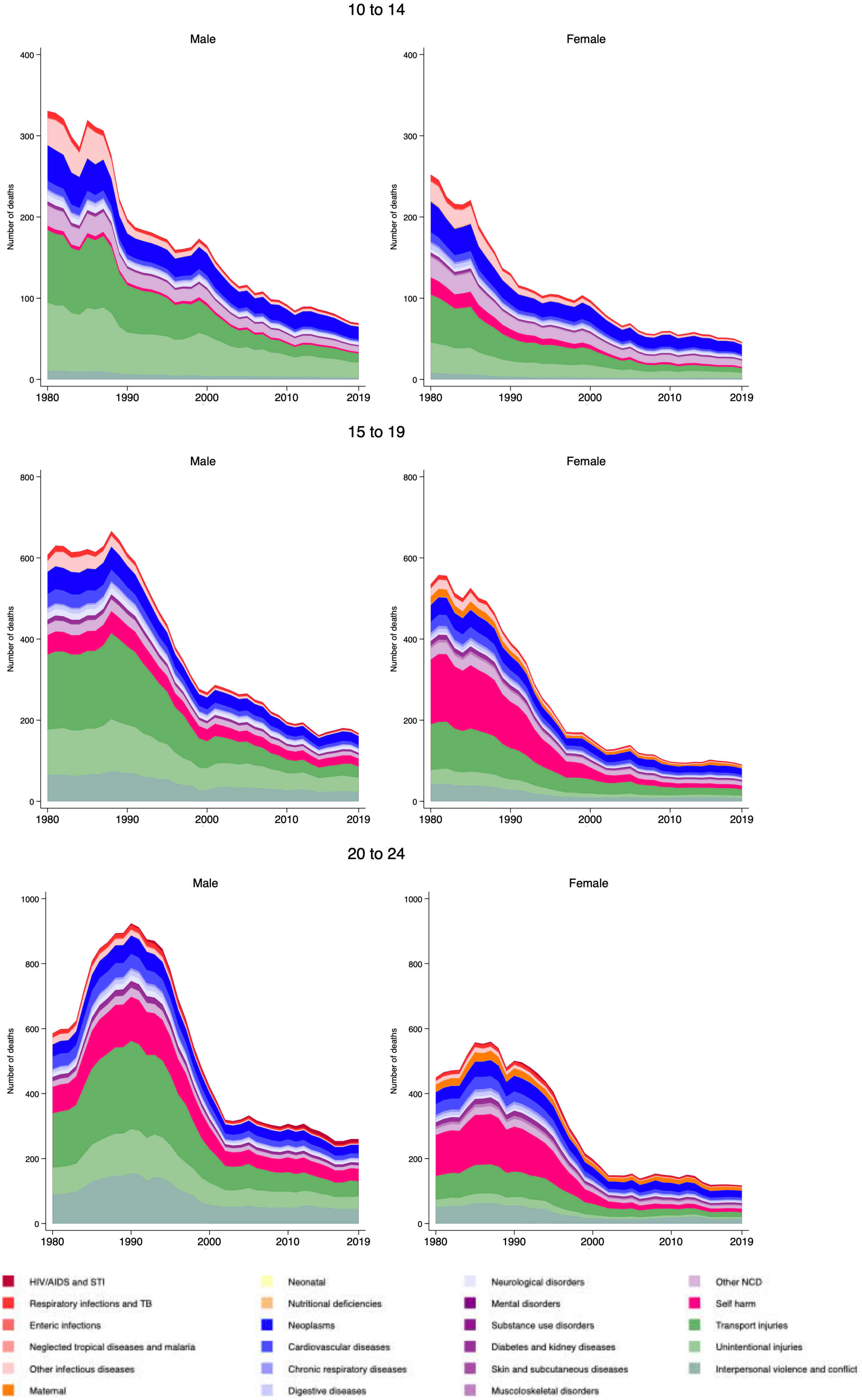

Figure S108: Dominica

Figure S108a All-cause mortality rate per 100,000 in 10-24 1950 – 2019: Dominica

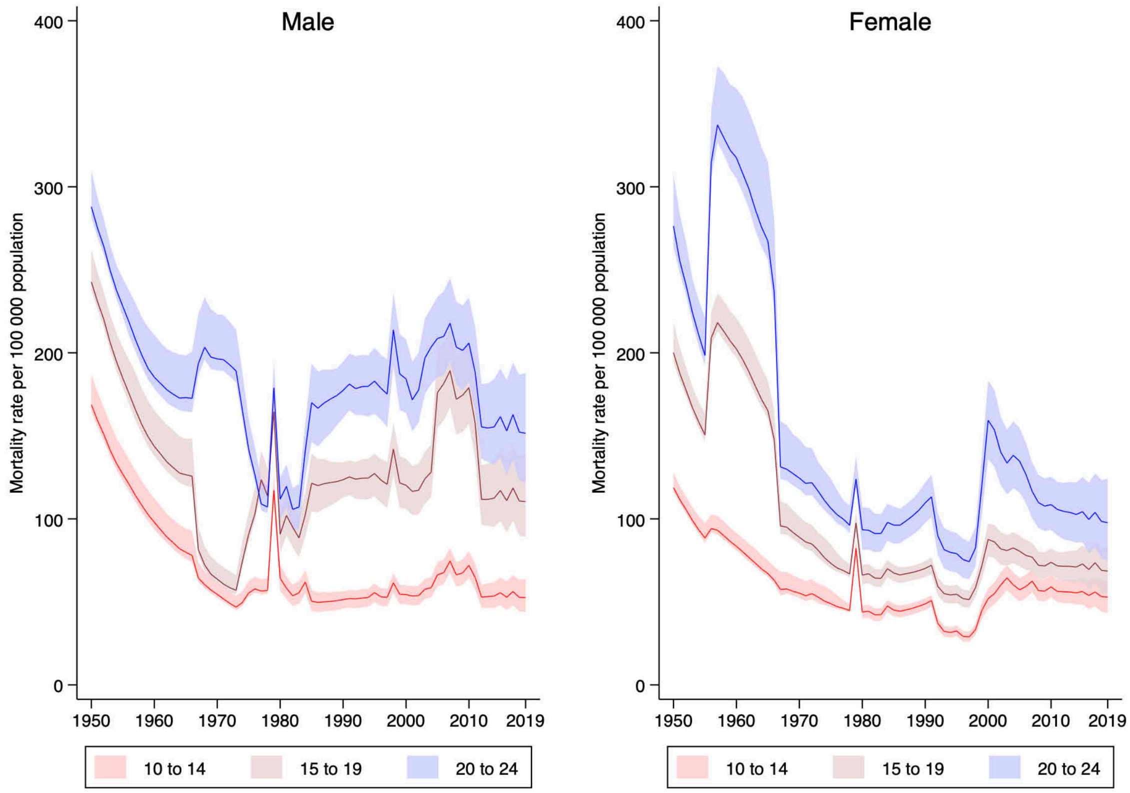

Figure S108b Number of deaths from all causes in 10-24 1950 – 2019: Dominica

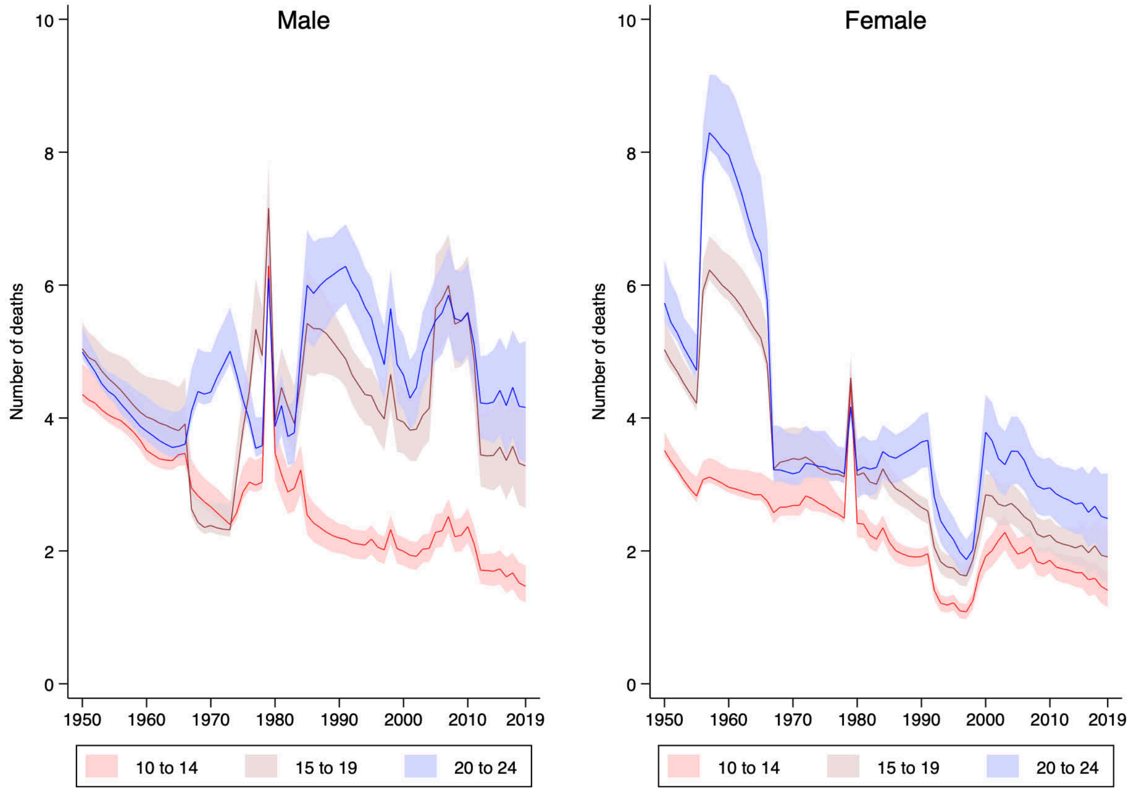

Figure S108c Mortality rate per 100,000 in 10-24 1980 – 2019 by cause: Dominica

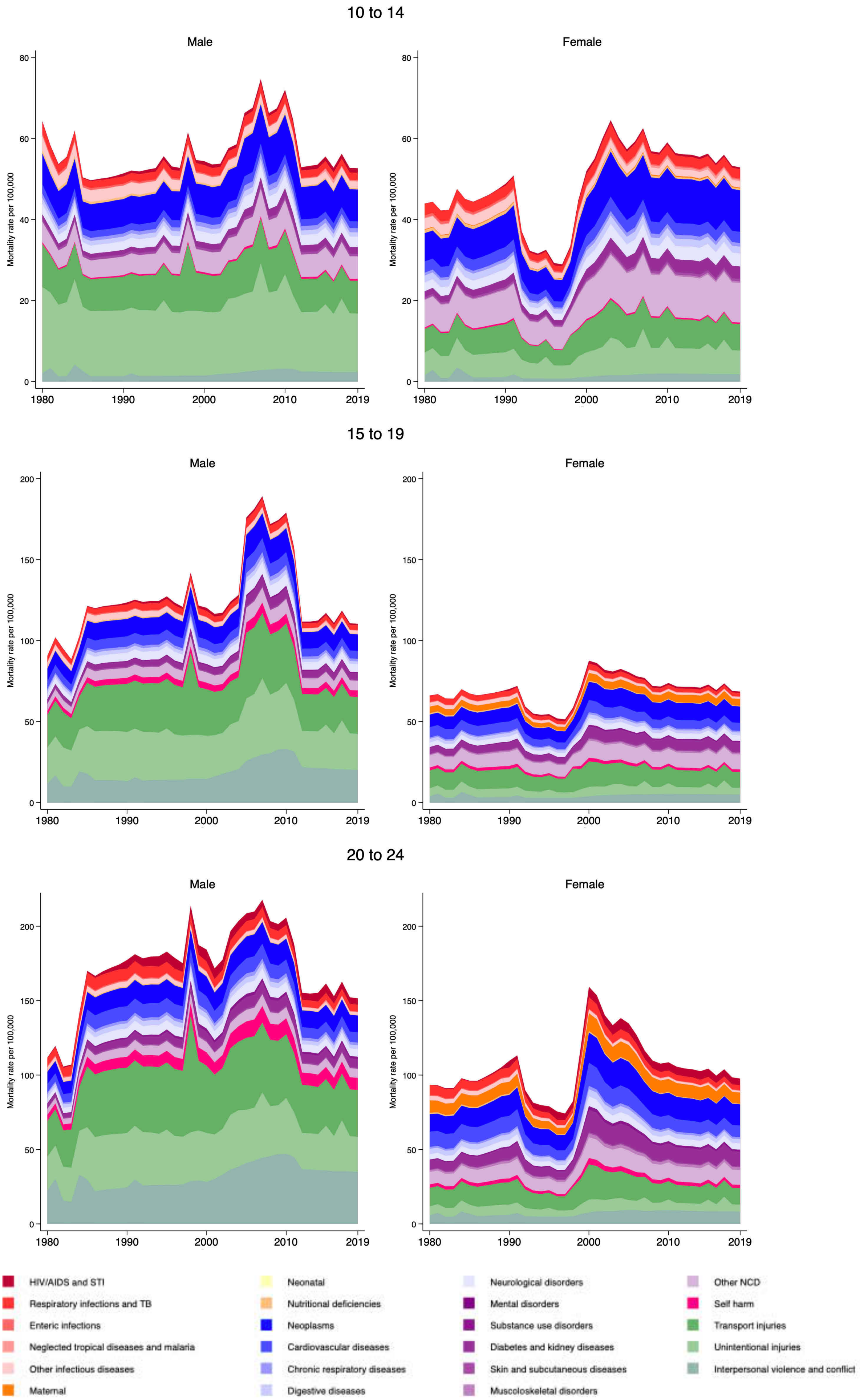

Figure S108d Number of deaths in 10-24 1980 – 2019 by cause: Dominica

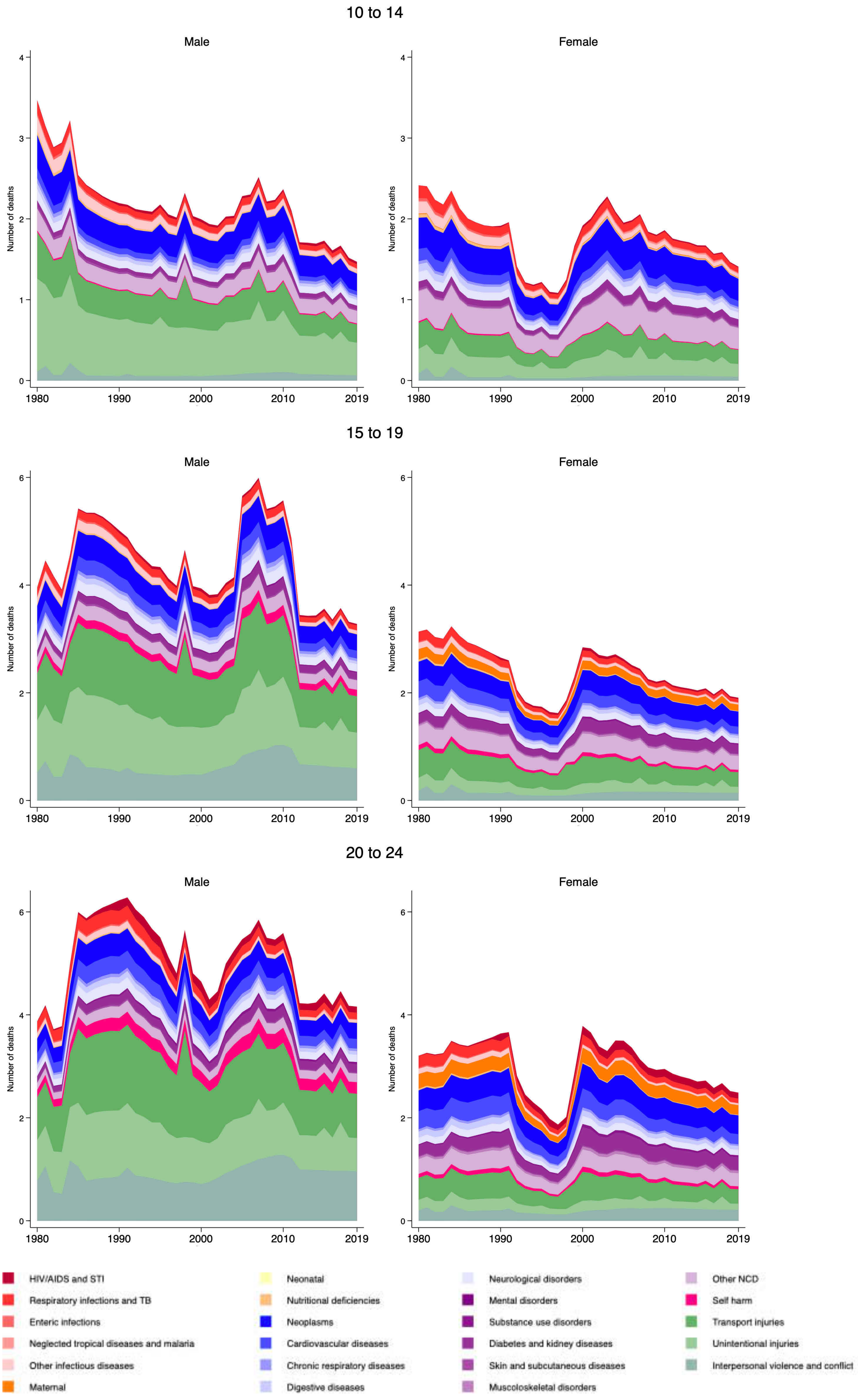

Figure S109: Dominican Republic

Figure S109a All-cause mortality rate per 100,000 in 10-24 1950 – 2019: Dominican Republic

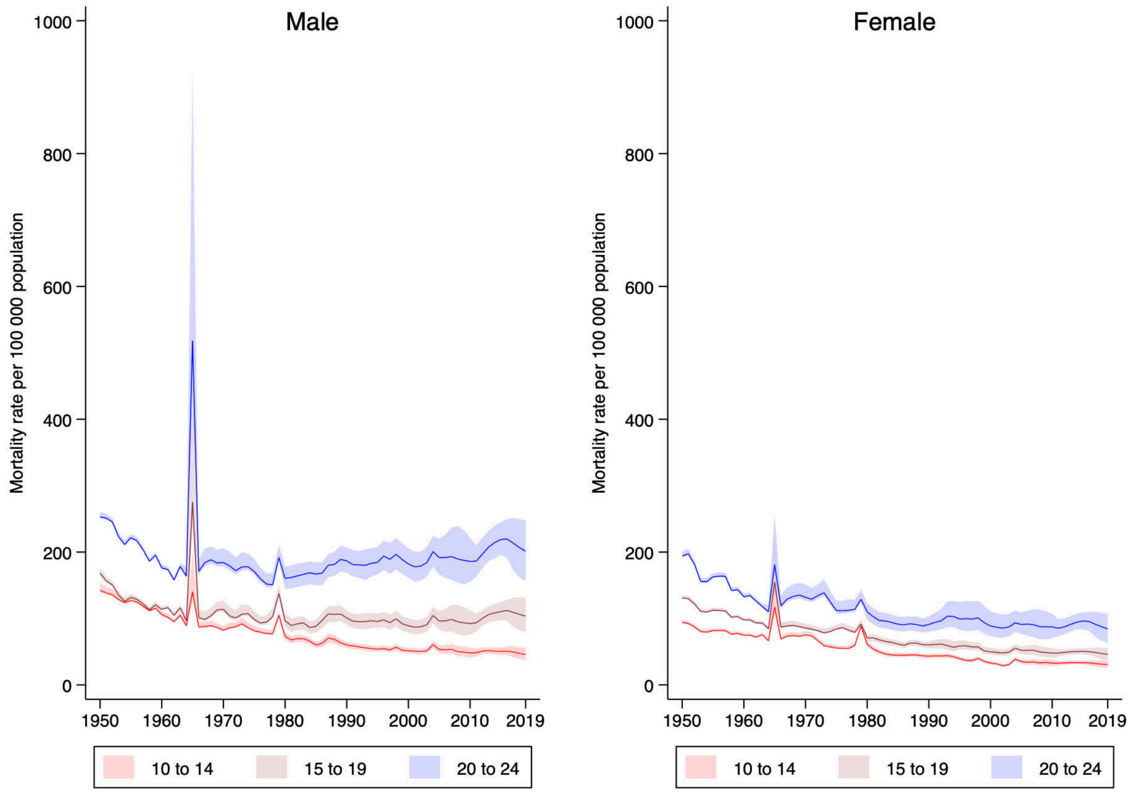

Figure S109b Number of deaths from all causes in 10-24 1950 – 2019: Dominican Republic

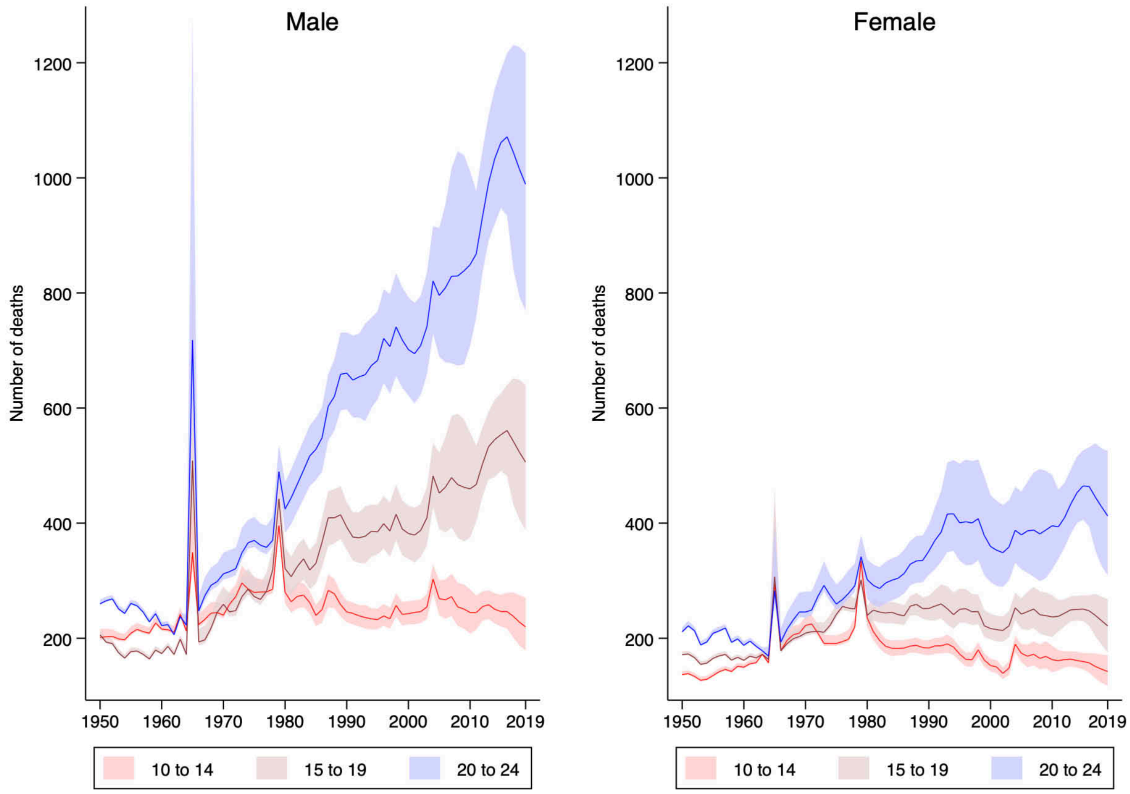

Figure S109c Mortality rate per 100,000 in 10-24 1980 – 2019 by cause: Dominican Republic

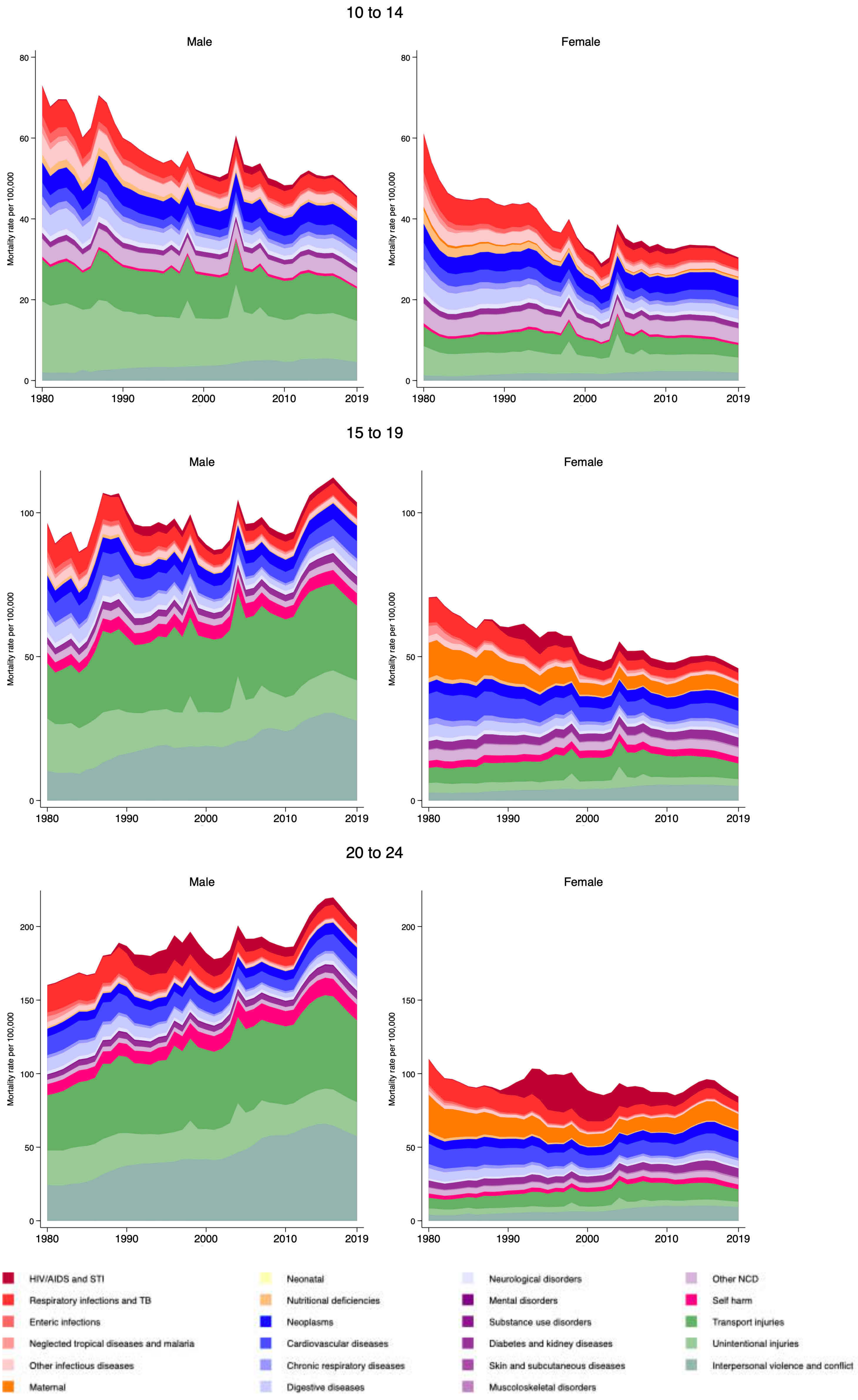

Figure S109d Number of deaths in 10-24 1980 – 2019 by cause: Dominican Republic

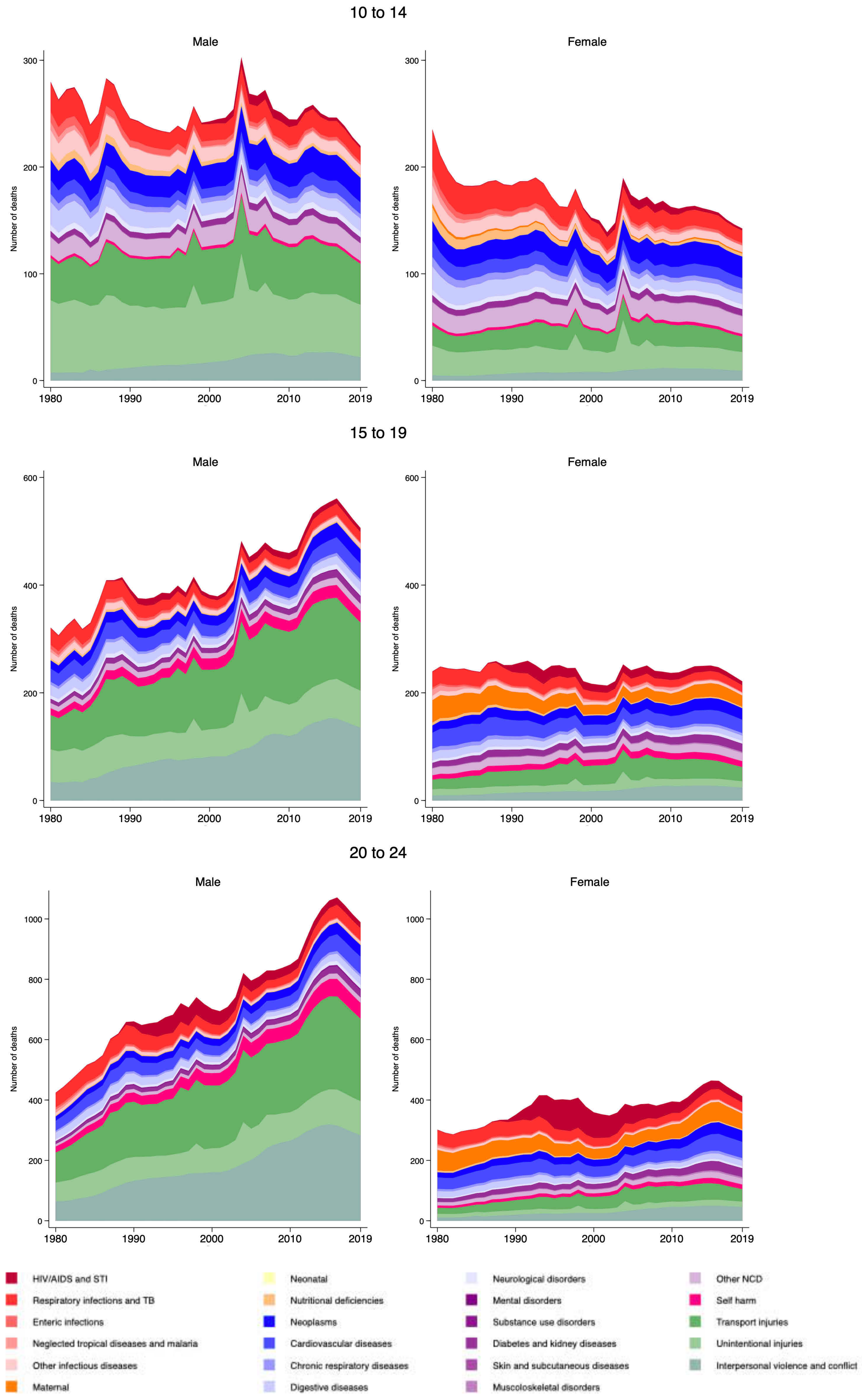

Figure S110: Grenada

Figure S110a All-cause mortality rate per 100,000 in 10-24 1950 – 2019: Grenada

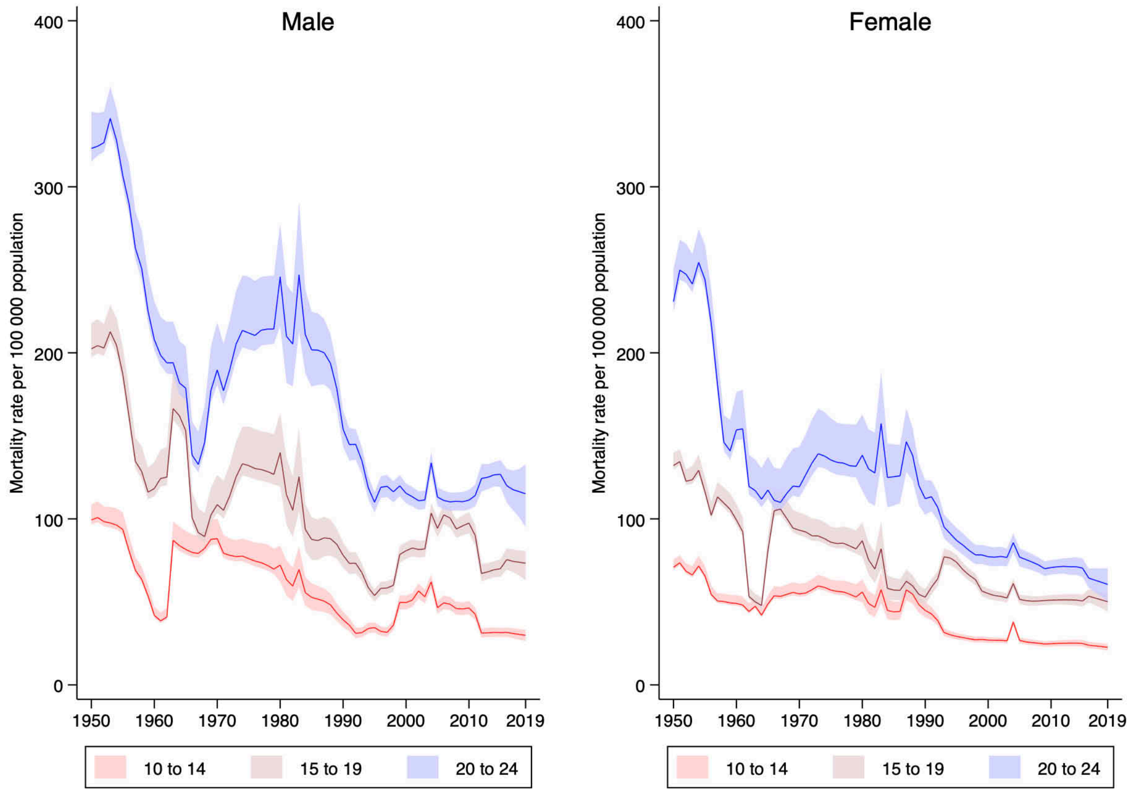

Figure S110b Number of deaths from all causes in 10-24 1950 – 2019: Grenada

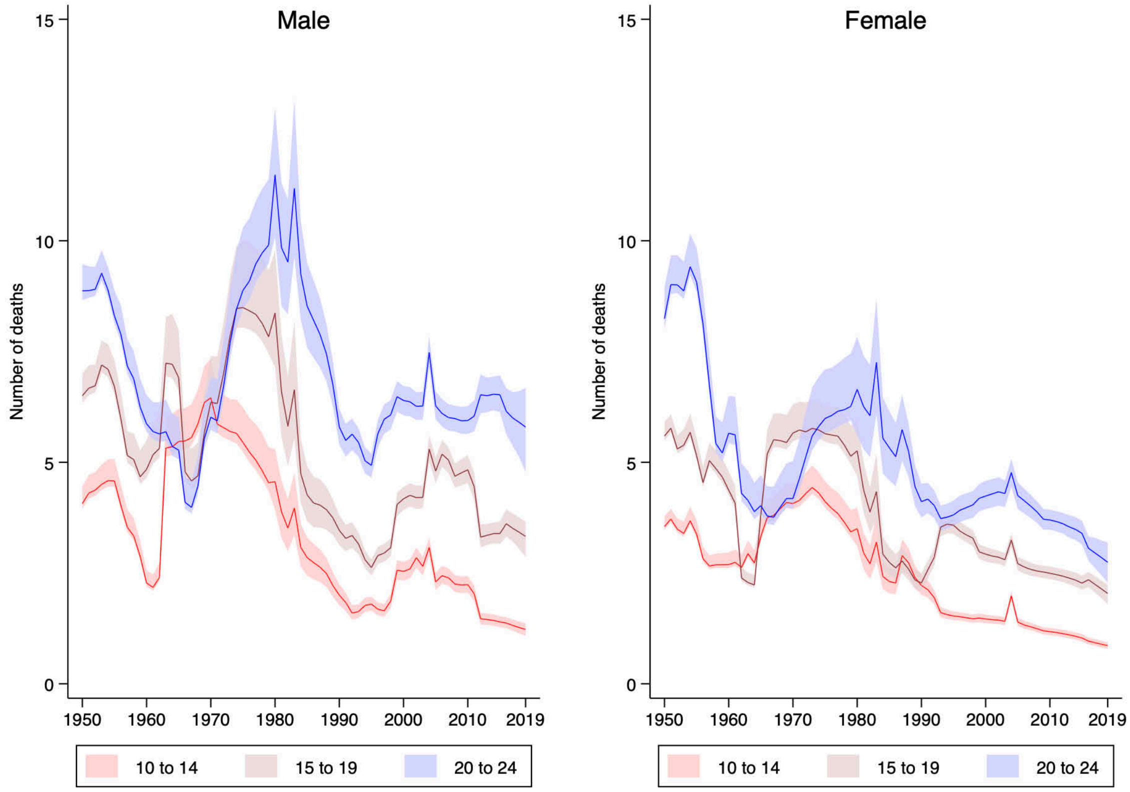

Figure S110c Mortality rate per 100,000 in 10-24 1980 – 2019 by cause: Grenada

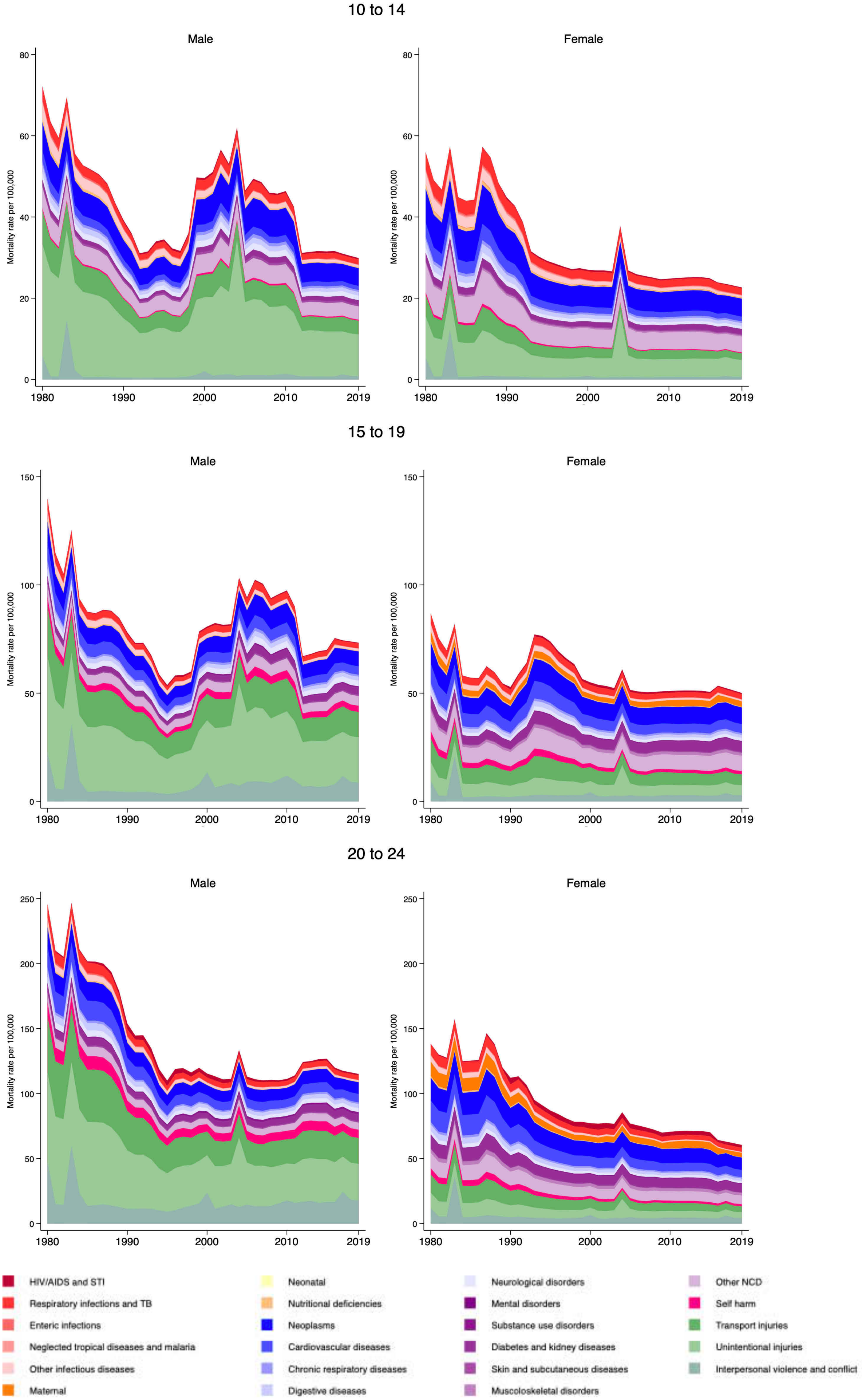

Figure S110d Number of deaths in 10-24 1980 – 2019 by cause: Grenada

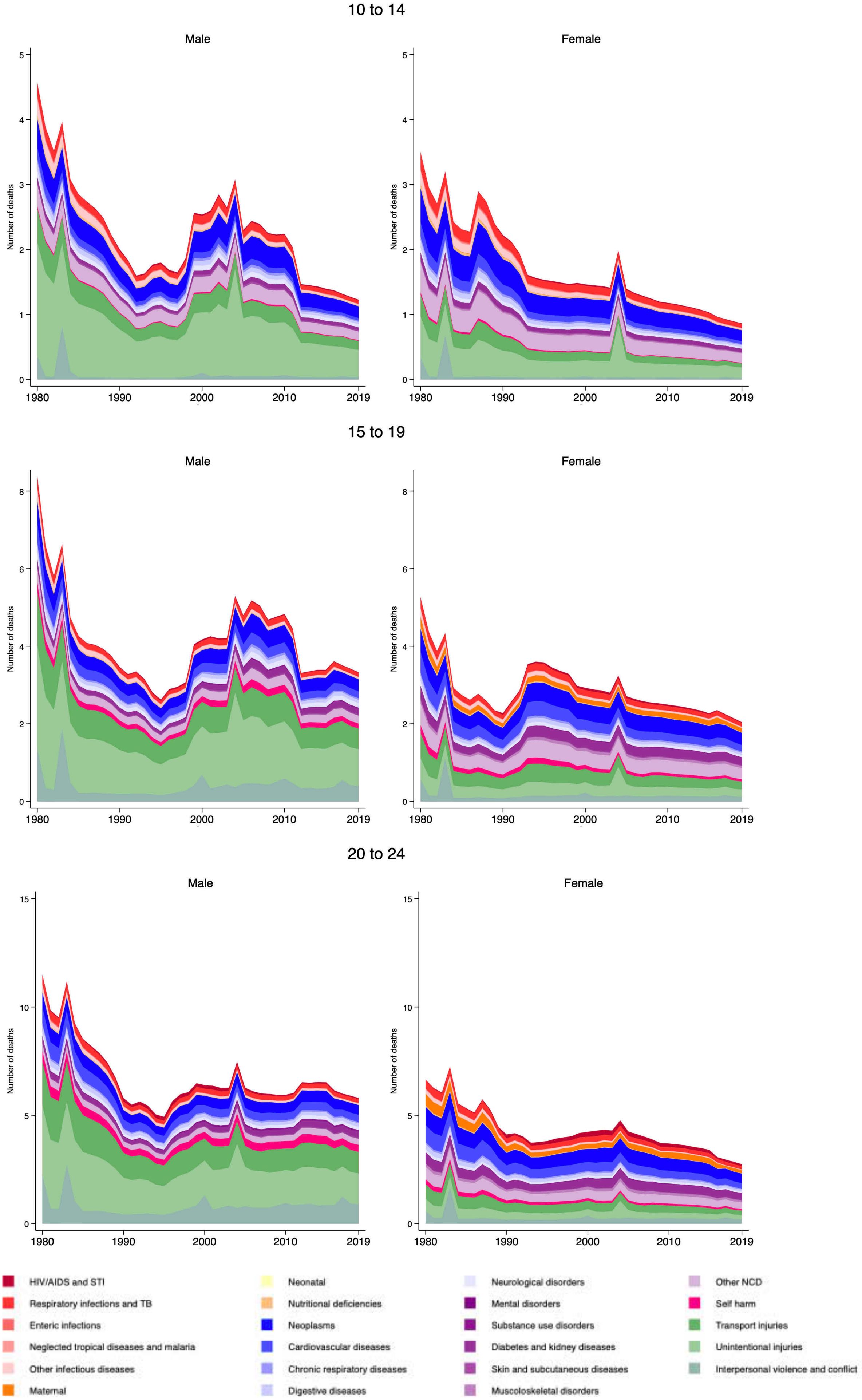

Figure S111: Guyana

Figure S111a All-cause mortality rate per 100,000 in 10-24 1950 – 2019: Guyana

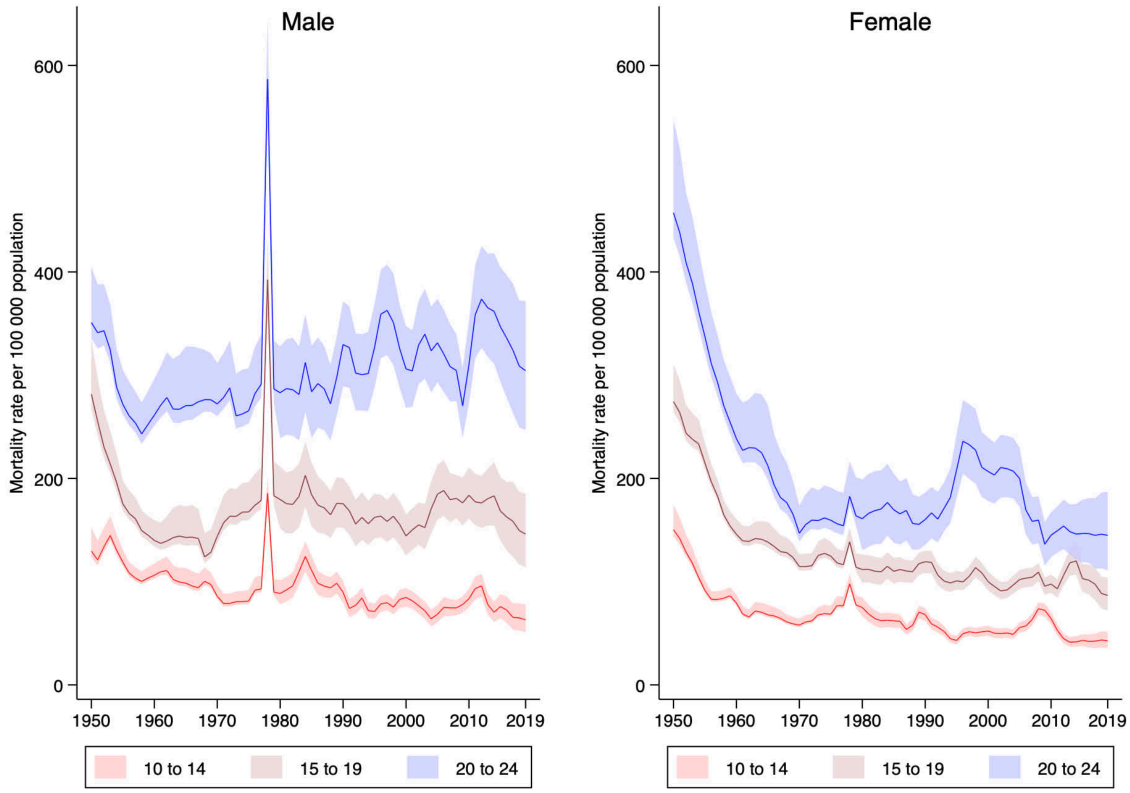

Figure S111b Number of deaths from all causes in 10-24 1950 – 2019: Guyana

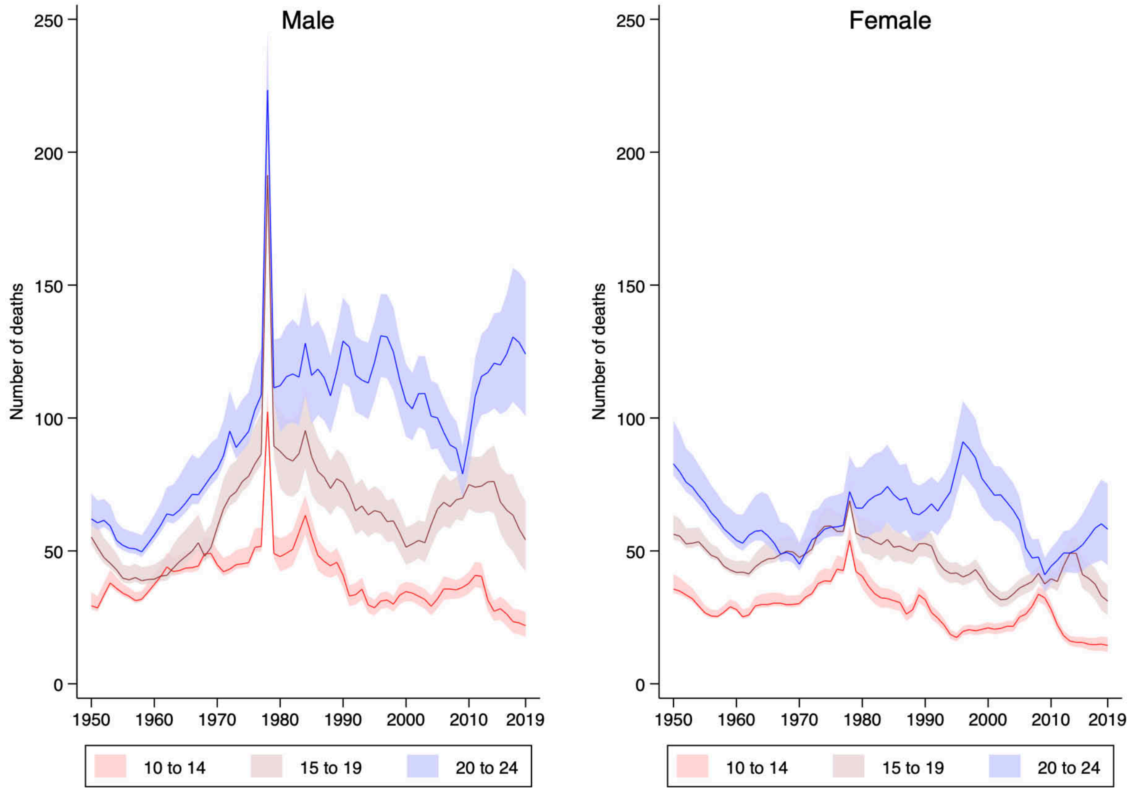

Figure S111c Mortality rate per 100,000 in 10-24 1980 – 2019 by cause: Guyana

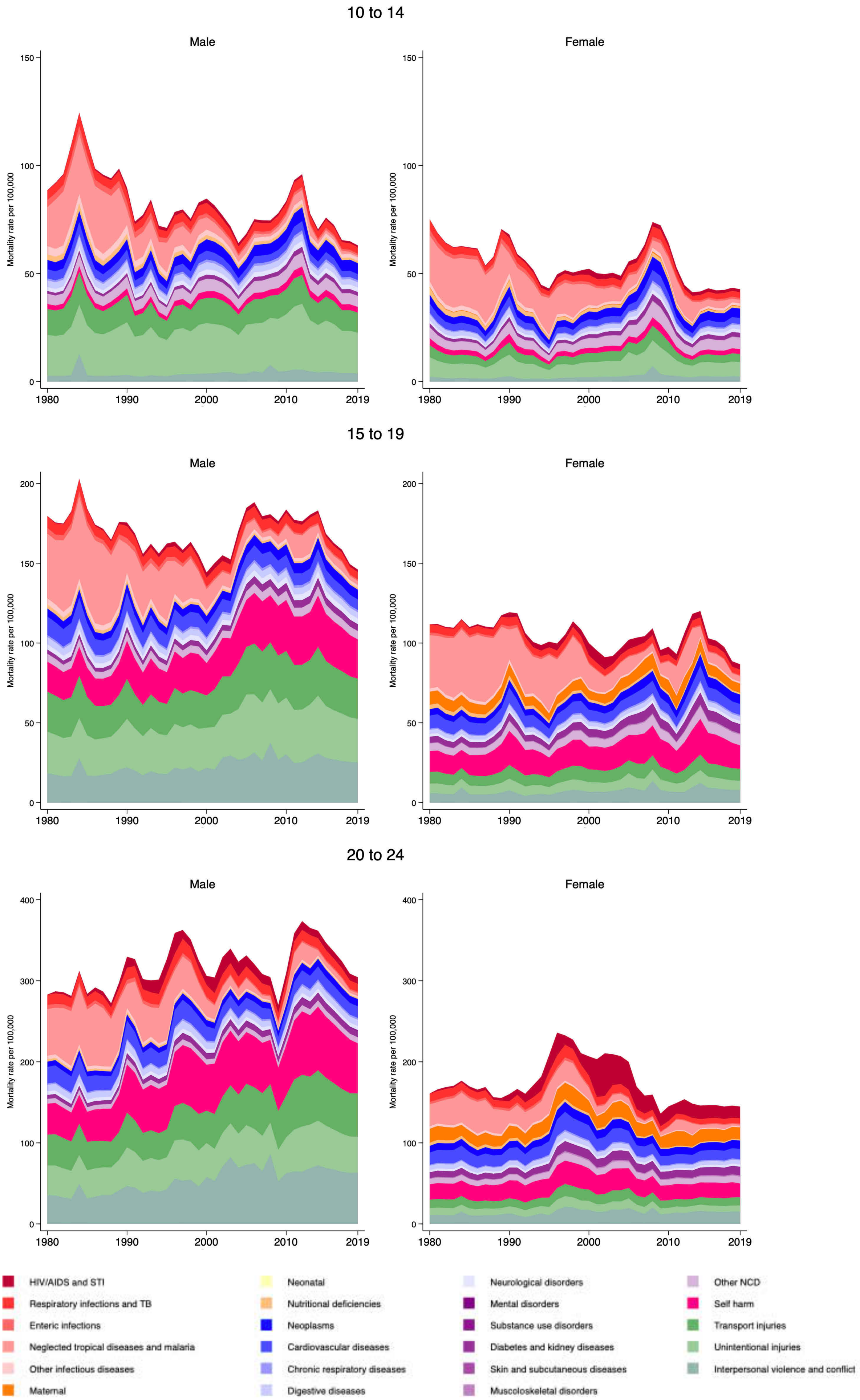

Figure S111d Number of deaths in 10-24 1980 – 2019 by cause: Guyana

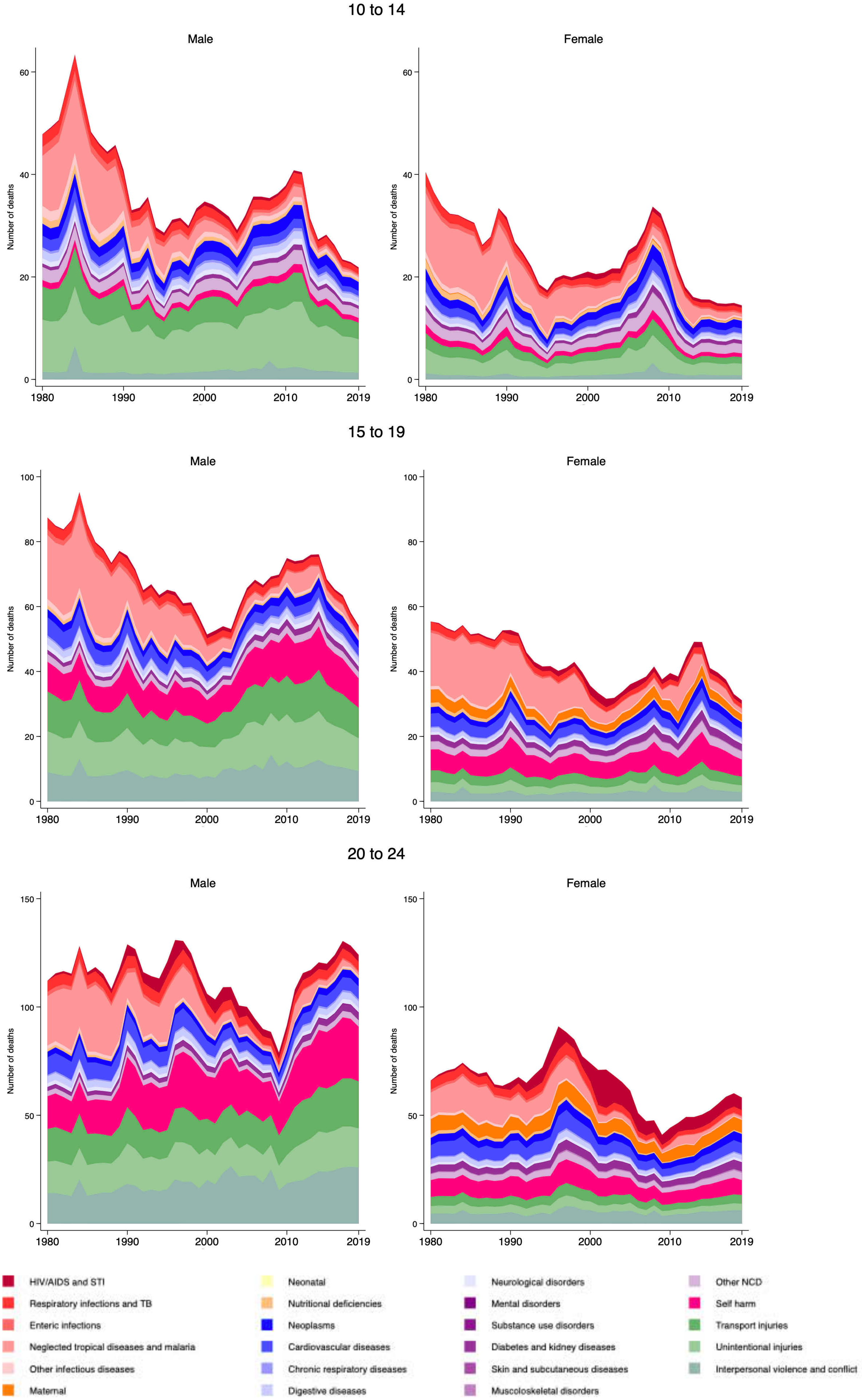

Figure S112: Haiti

Figure S112a All-cause mortality rate per 100,000 in 10-24 1950 – 2019: Haiti

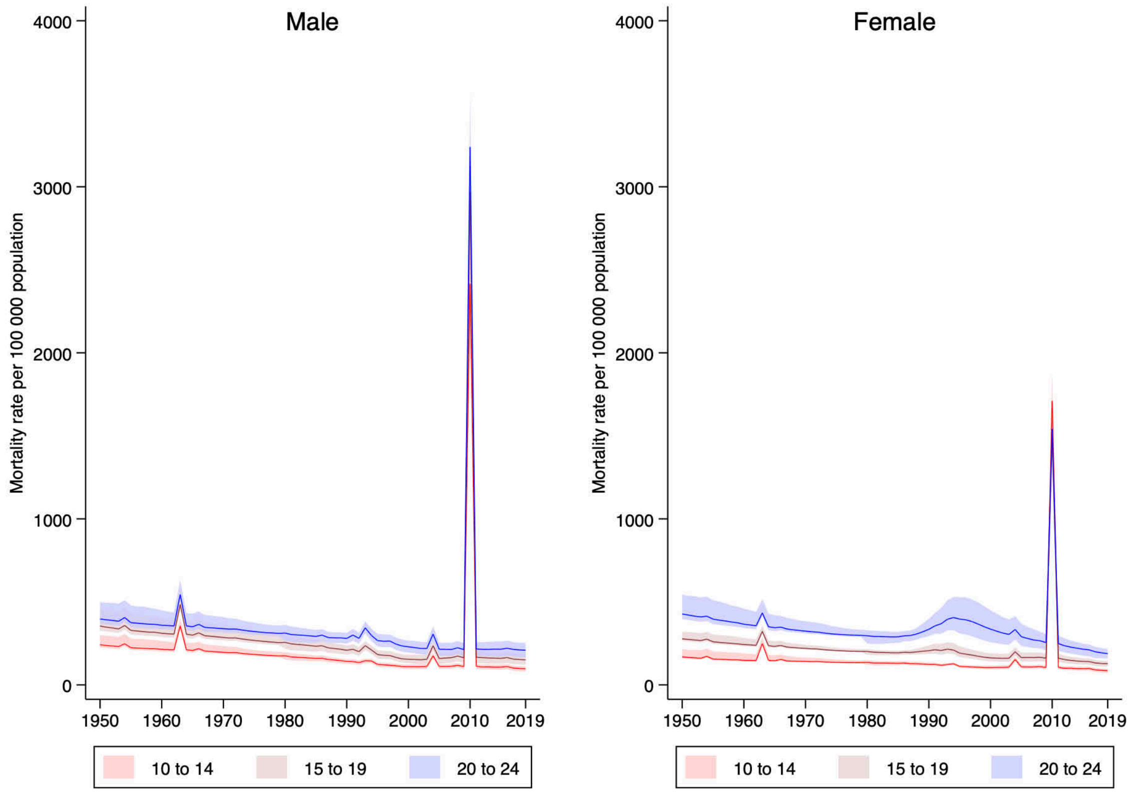

Figure S112b Number of deaths from all causes in 10-24 1950 – 2019: Haiti

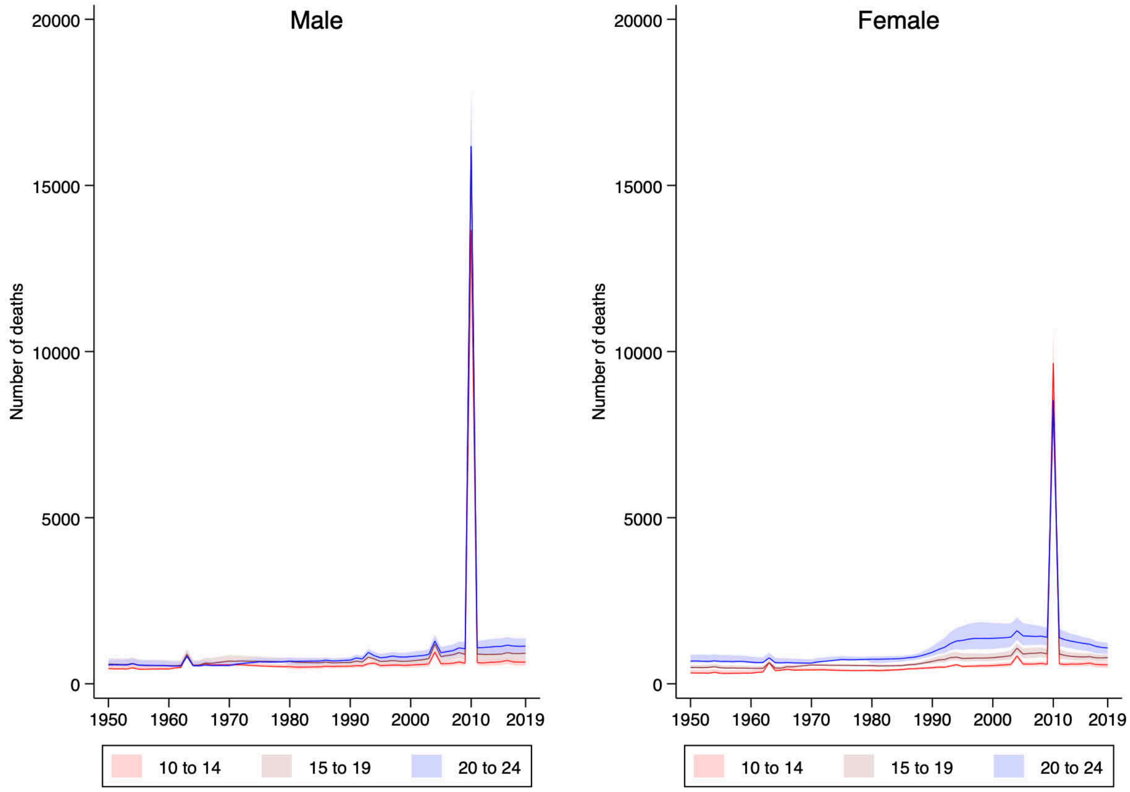

Figure S112c Mortality rate per 100,000 in 10-24 1980 – 2019 by cause: Haiti

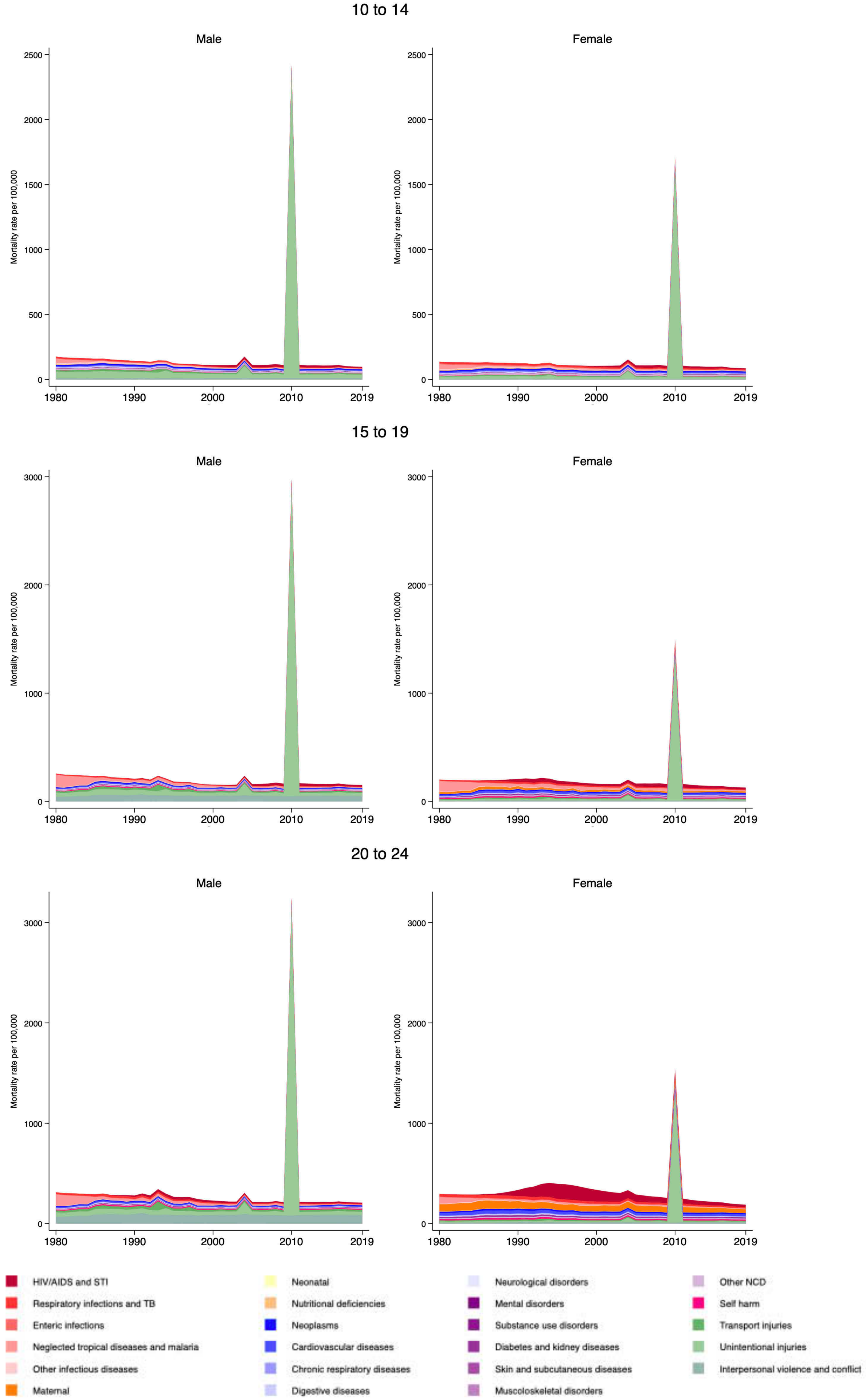

Figure S112d Number of deaths in 10-24 1980 – 2019 by cause: Haiti

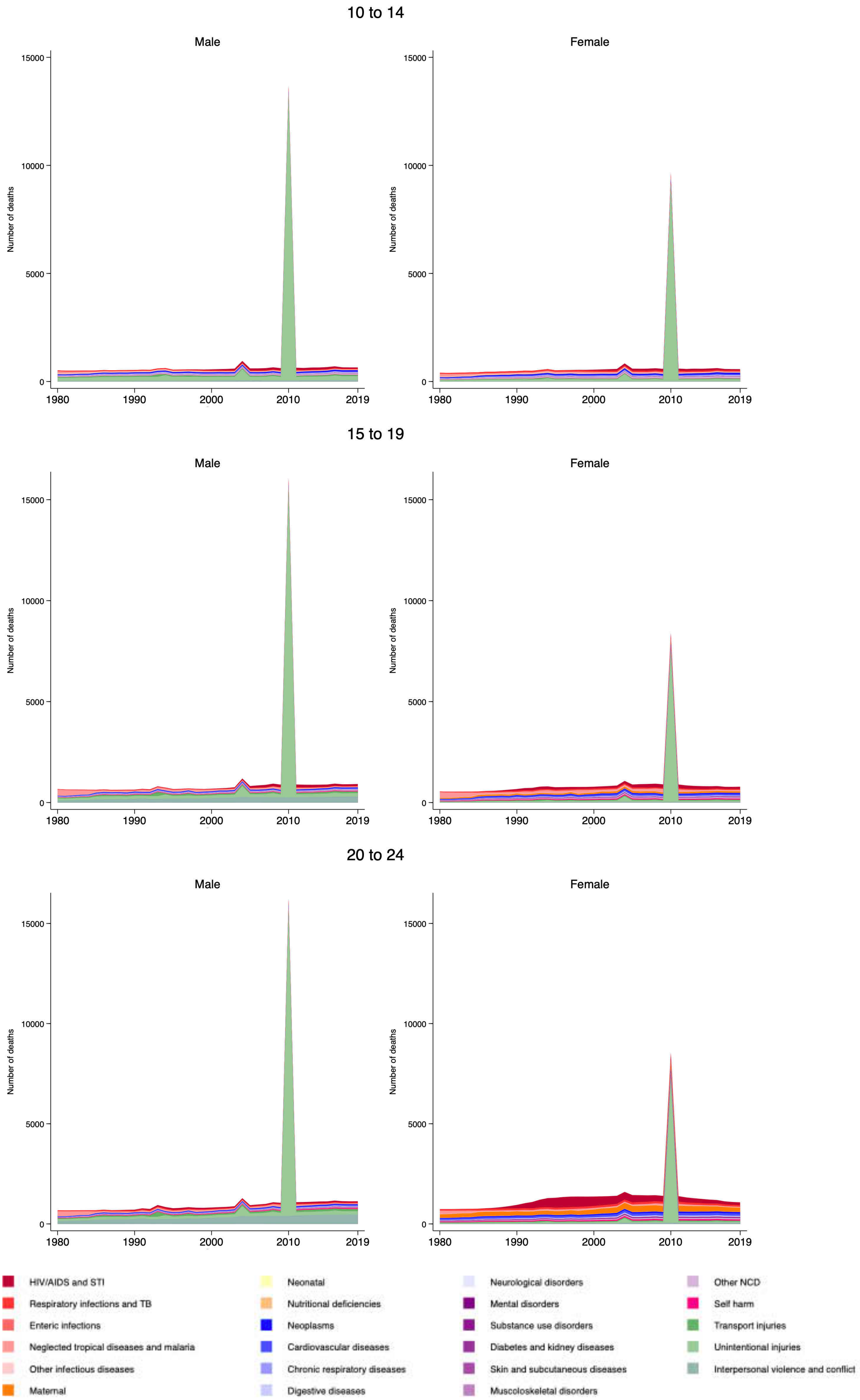

Figure S113: Jamaica

Figure S113a All-cause mortality rate per 100,000 in 10-24 1950 – 2019: Jamaica

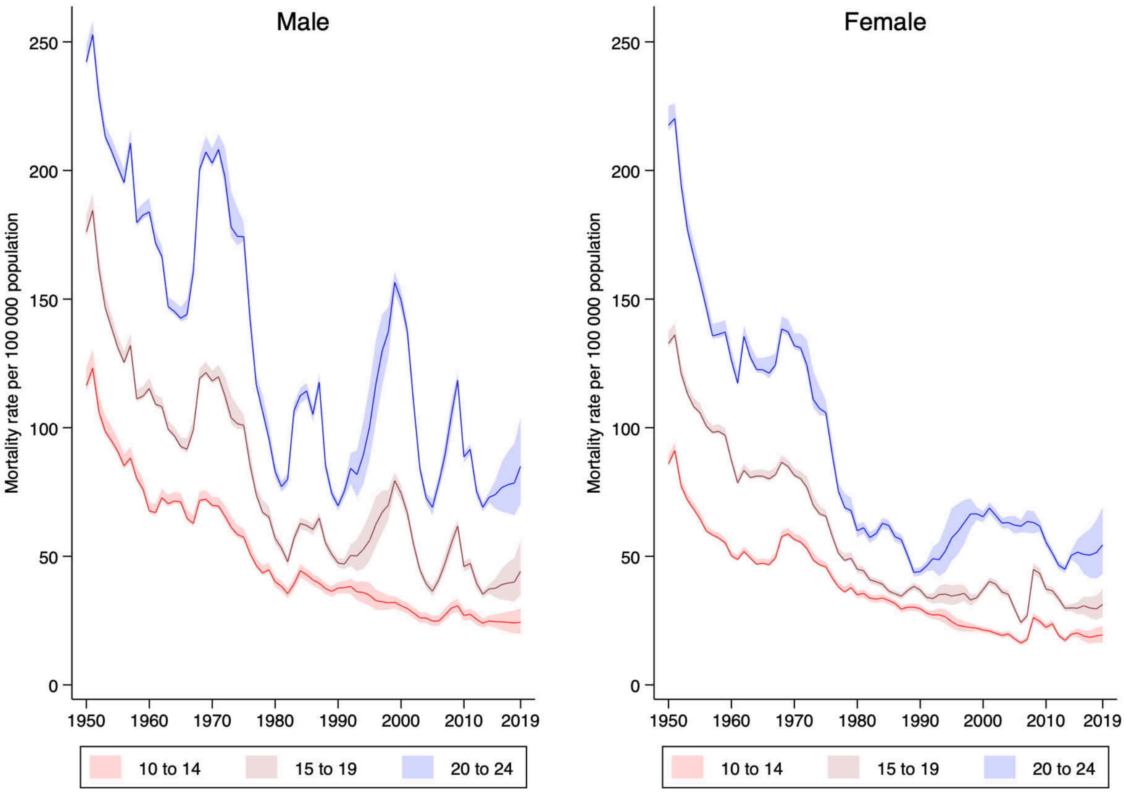

Figure S113b Number of deaths from all causes in 10-24 1950 – 2019: Jamaica

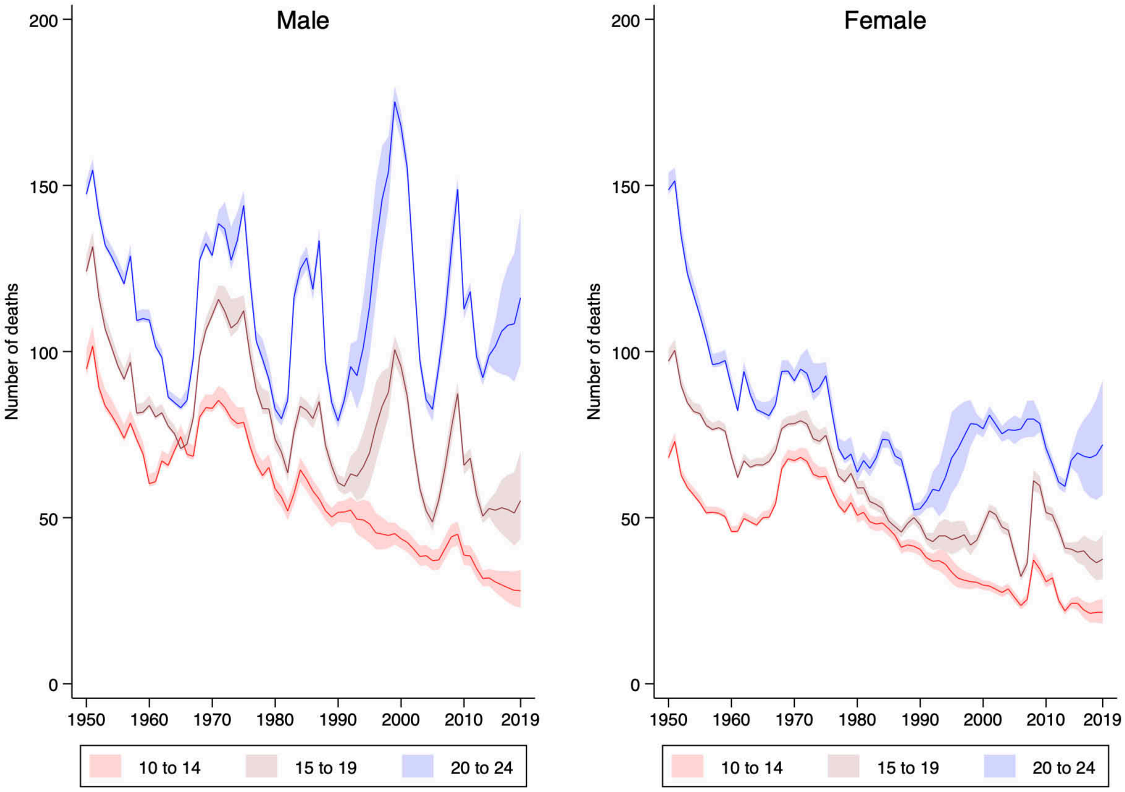

Figure S113c Mortality rate per 100,000 in 10-24 1980 – 2019 by cause: Jamaica

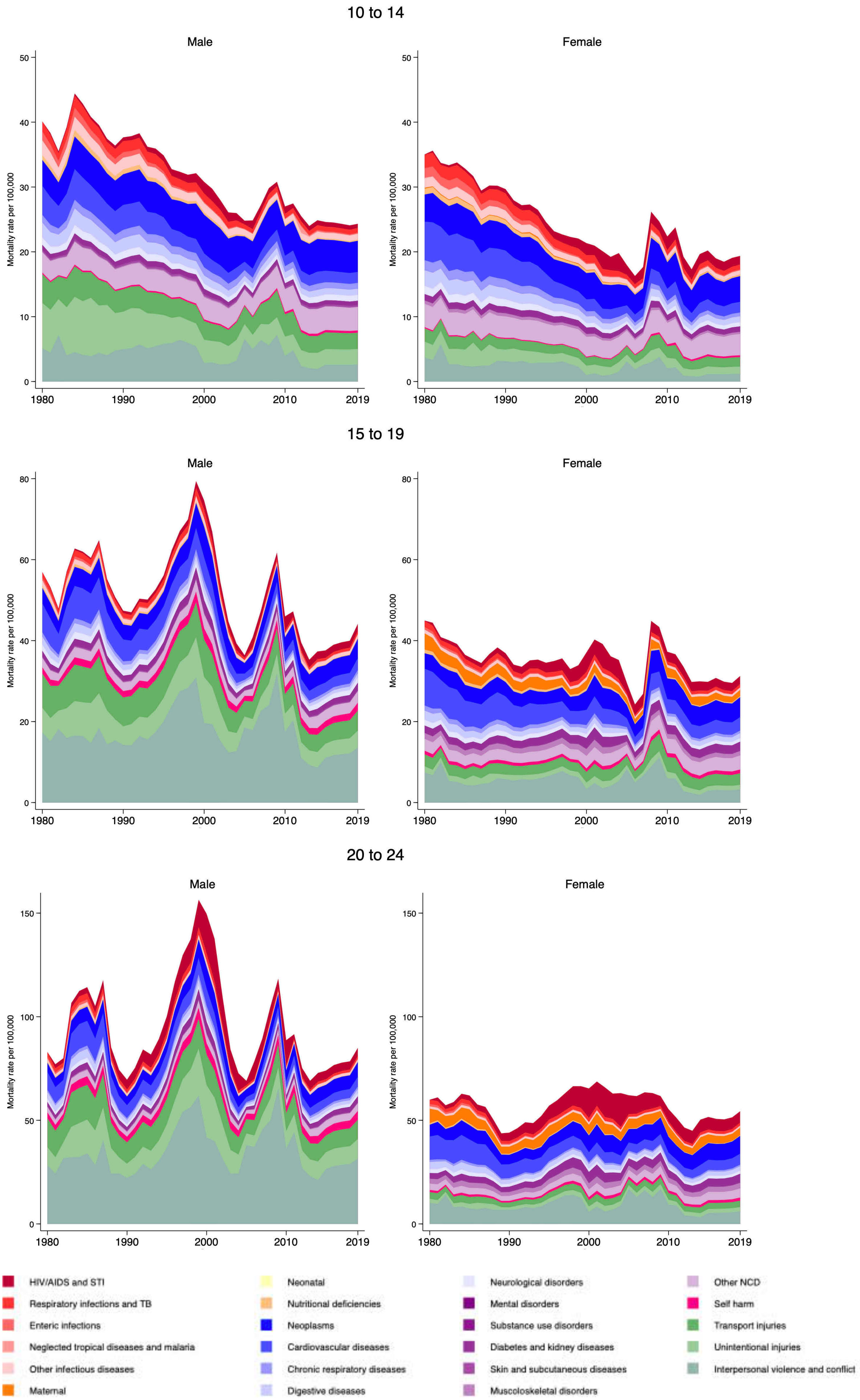

Figure S113d Number of deaths in 10-24 1980 – 2019 by cause: Jamaica

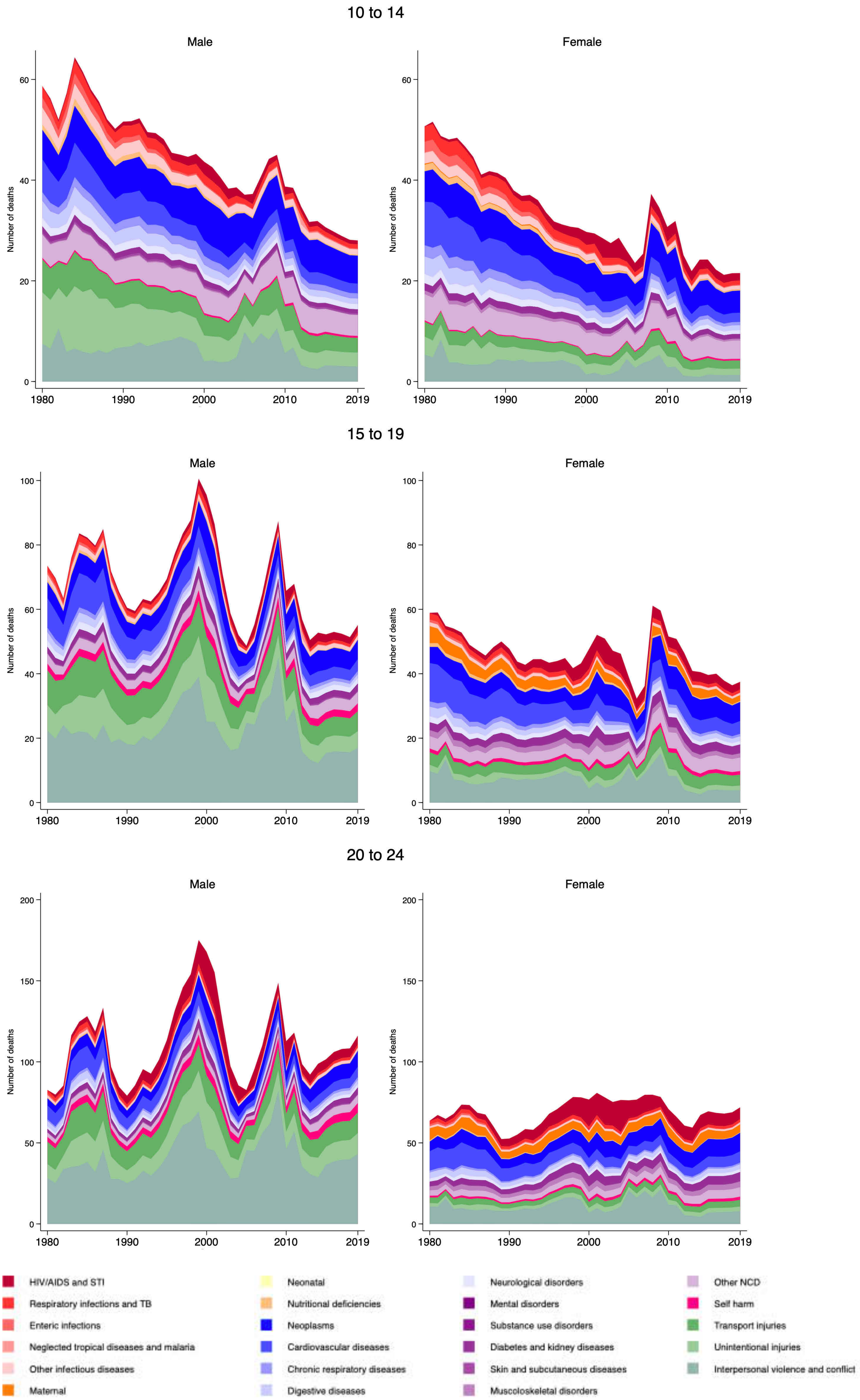

Figure S114: Saint Lucia

Figure S114a All-cause mortality rate per 100,000 in 10-24 1950 – 2019: Saint Lucia

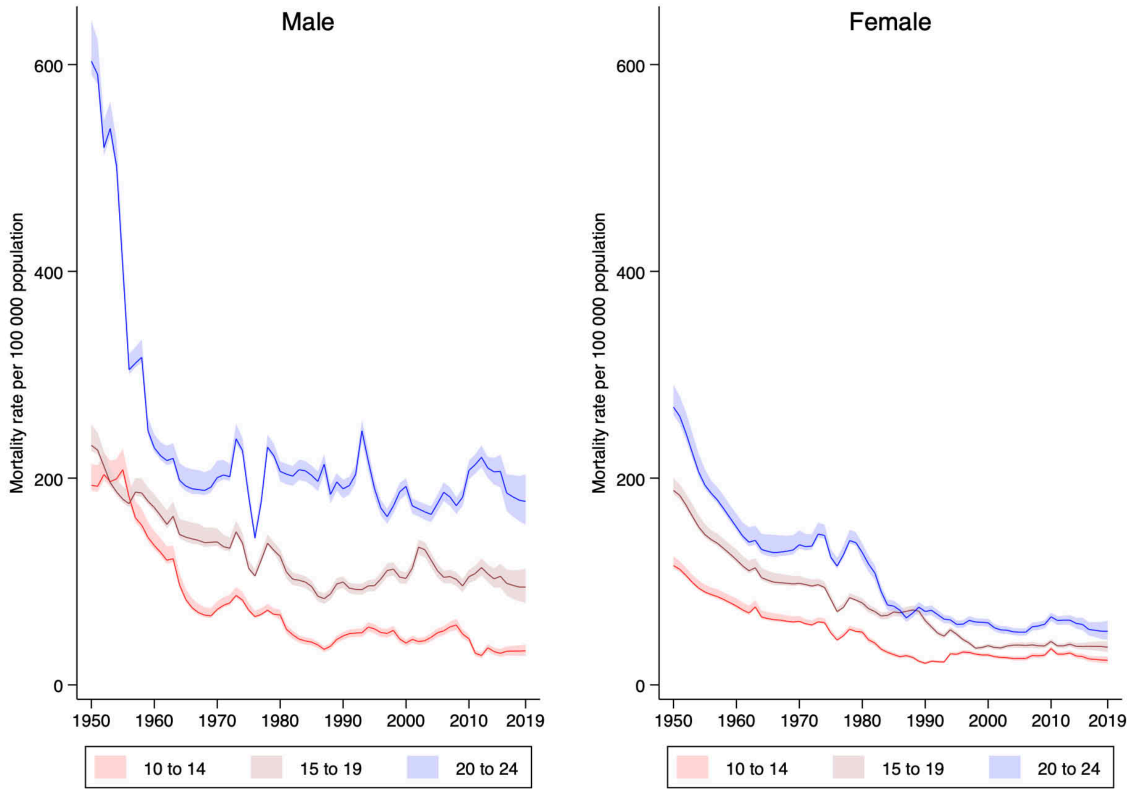

Figure S114b Number of deaths from all causes in 10-24 1950 – 2019: Saint Lucia

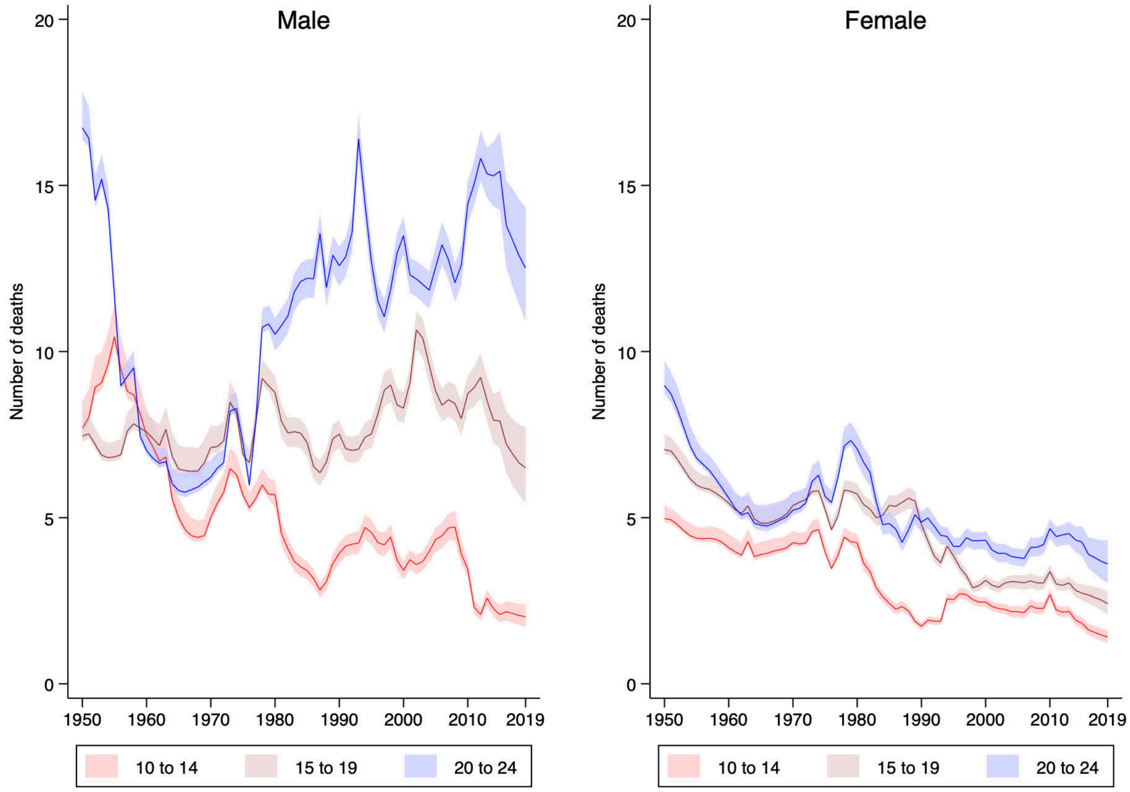

Figure S114c Mortality rate per 100,000 in 10-24 1980 – 2019 by cause: Saint Lucia

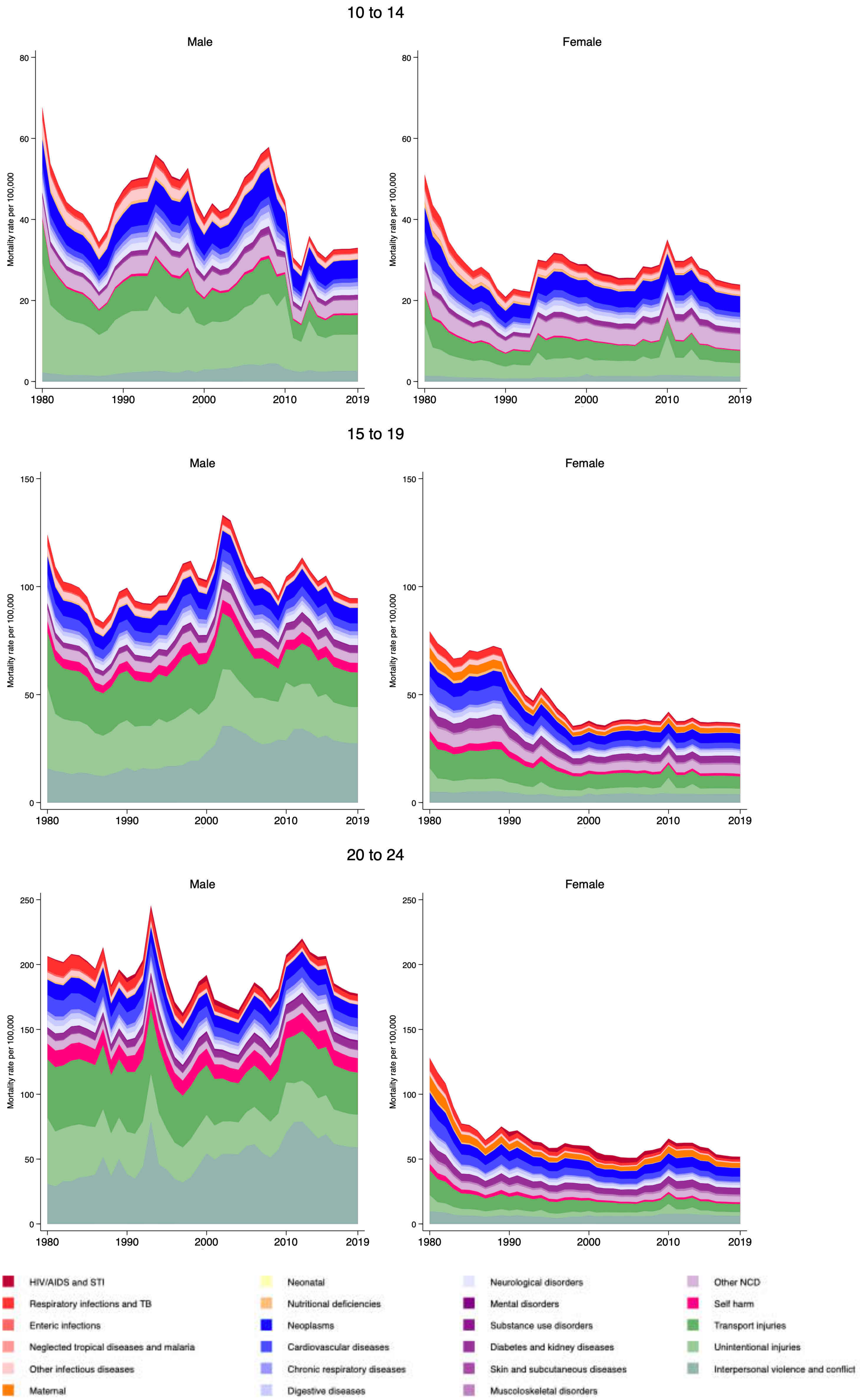

Figure S114d Number of deaths in 10-24 1980 – 2019 by cause: Saint Lucia

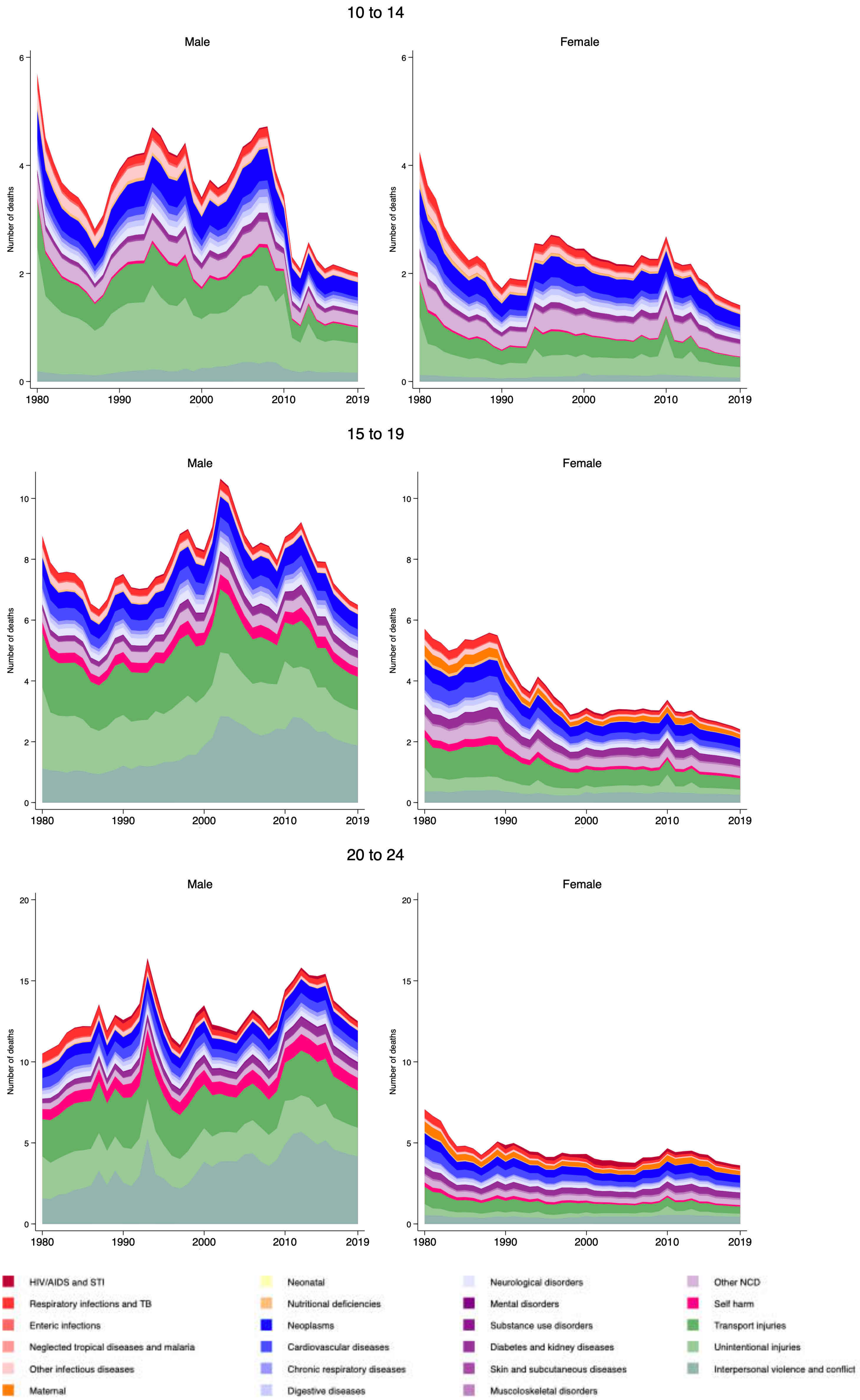

Figure S115: Saint Vincent and the Grenadines

Figure S115a All-cause mortality rate per 100,000 in 10-24 1950 – 2019: Saint Vincent and the Grenadines

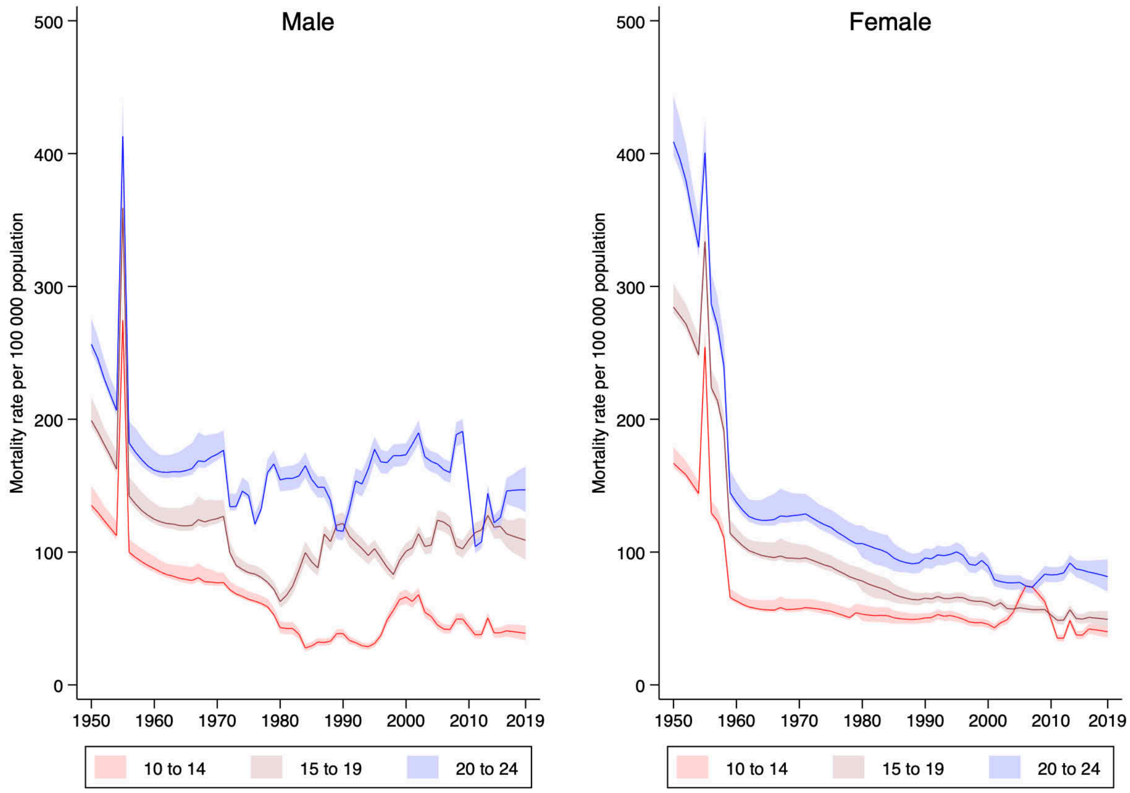

Figure S115b Number of deaths from all causes in 10-24 1950 – 2019: Saint Vincent and the Grenadines

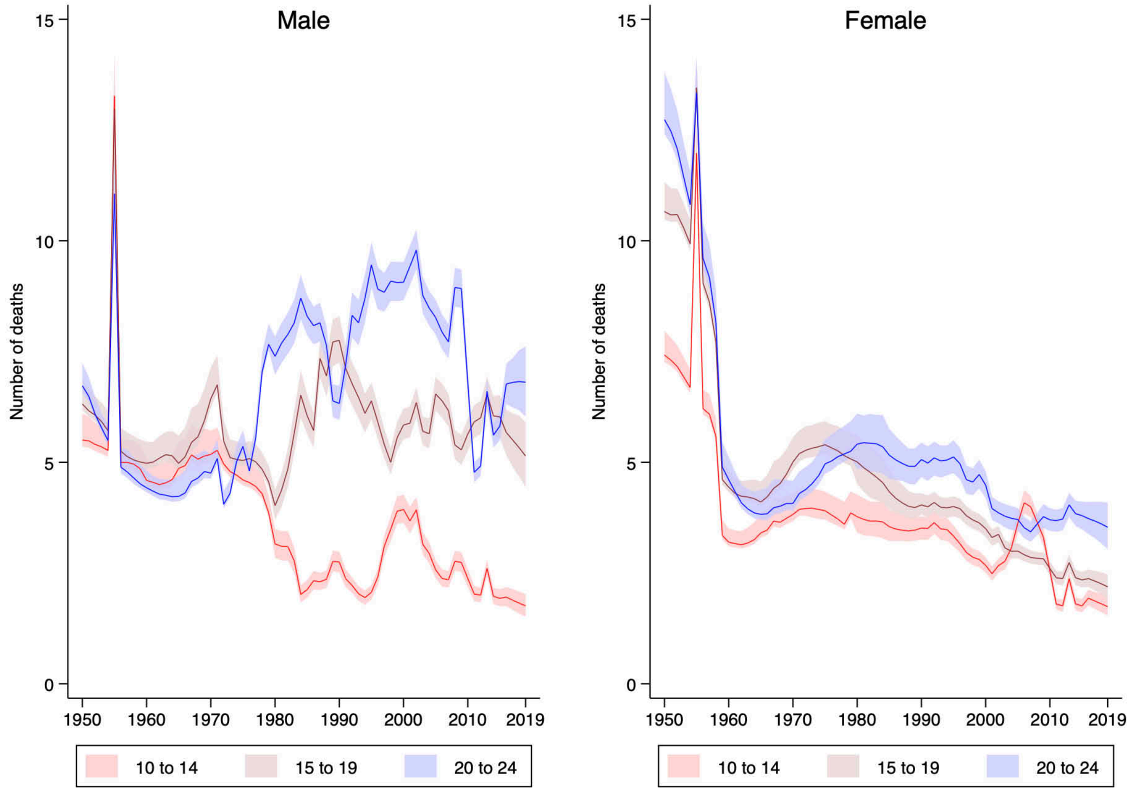

Figure S115c Mortality rate per 100,000 in 10-24 1980 – 2019 by cause: Saint Vincent and the Grenadines

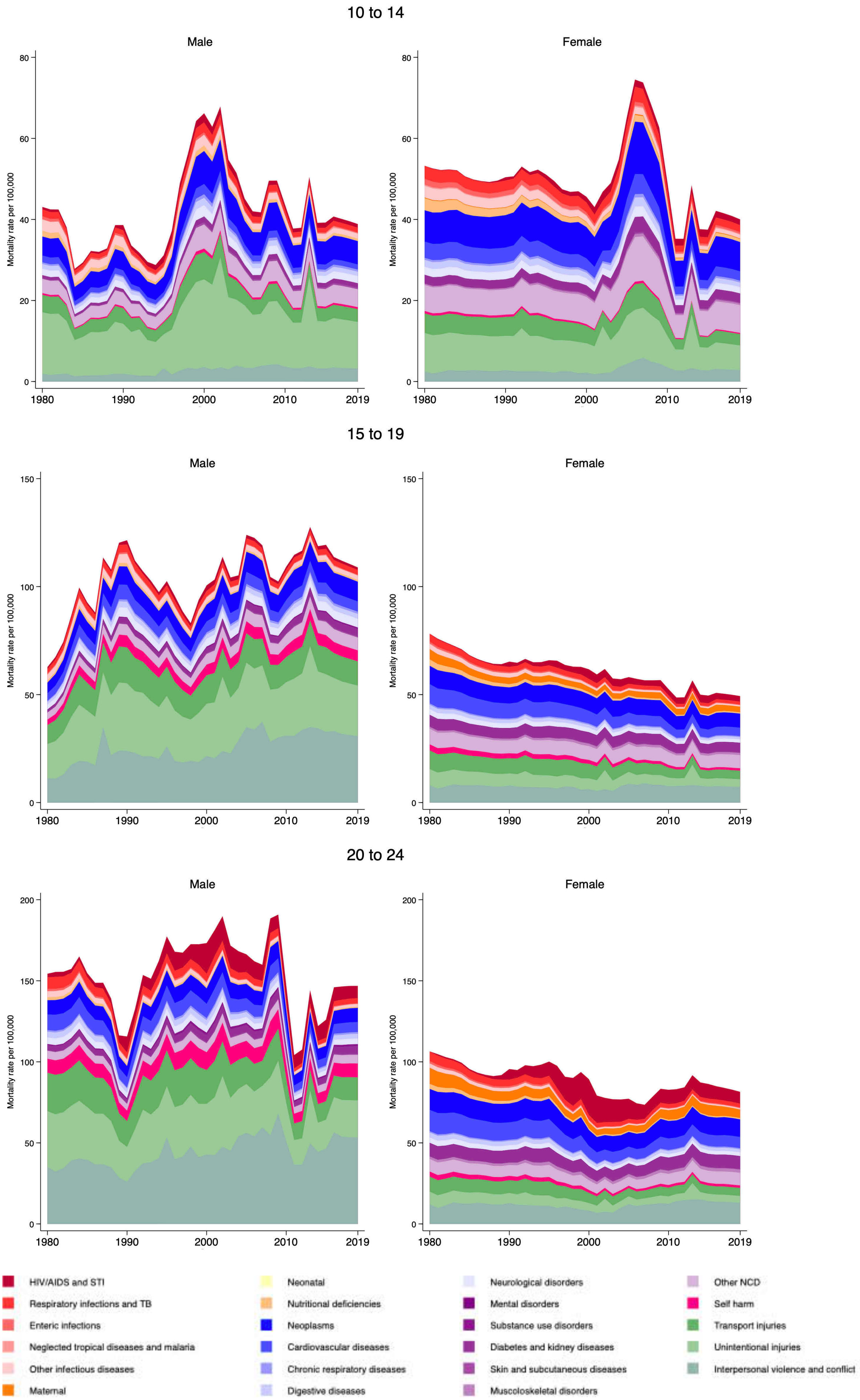

Figure S115d Number of deaths in 10-24 1980 – 2019 by cause: Saint Vincent and the Grenadines

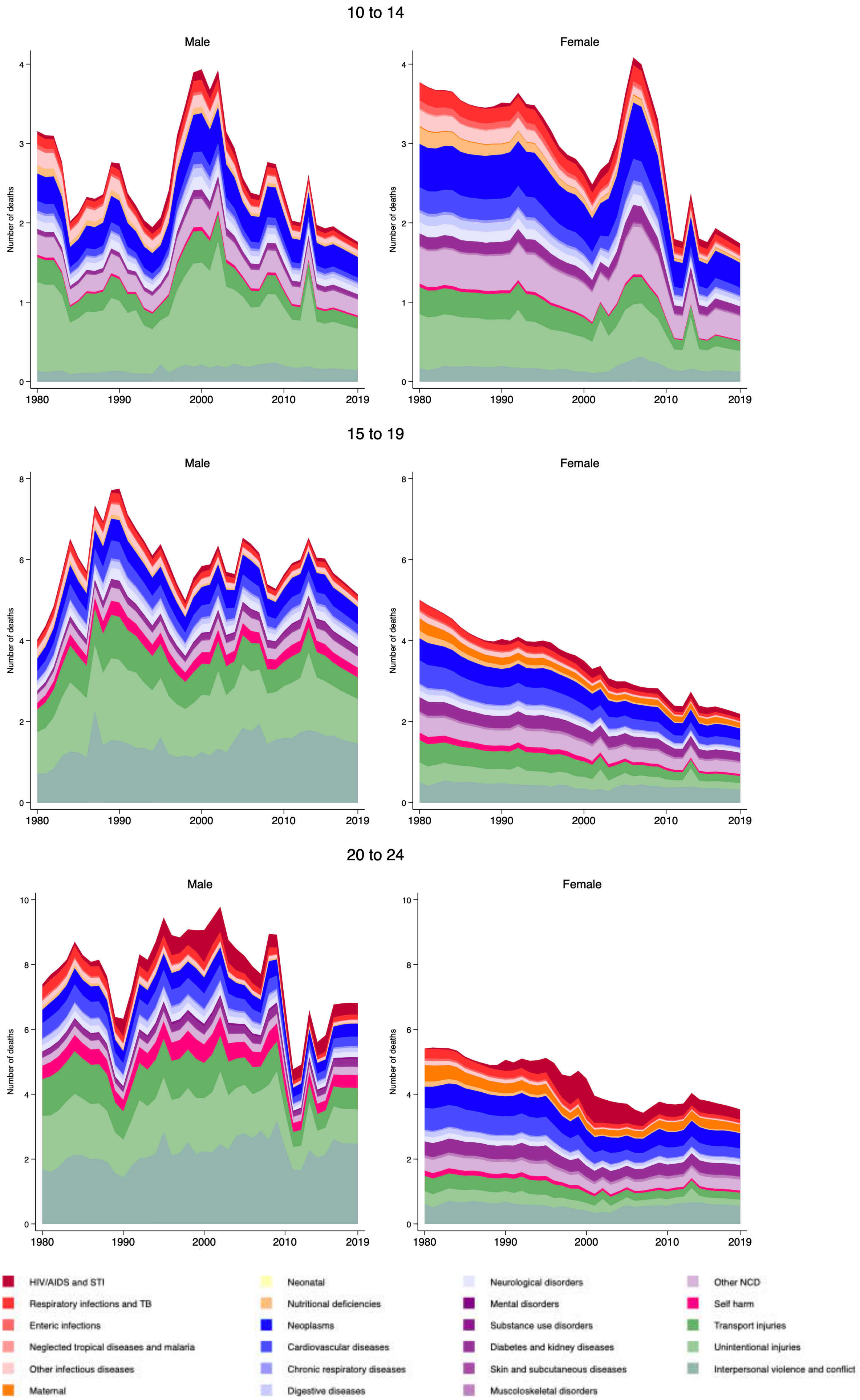

Figure S116: Suriname

Figure S116a All-cause mortality rate per 100,000 in 10-24 1950 – 2019: Suriname

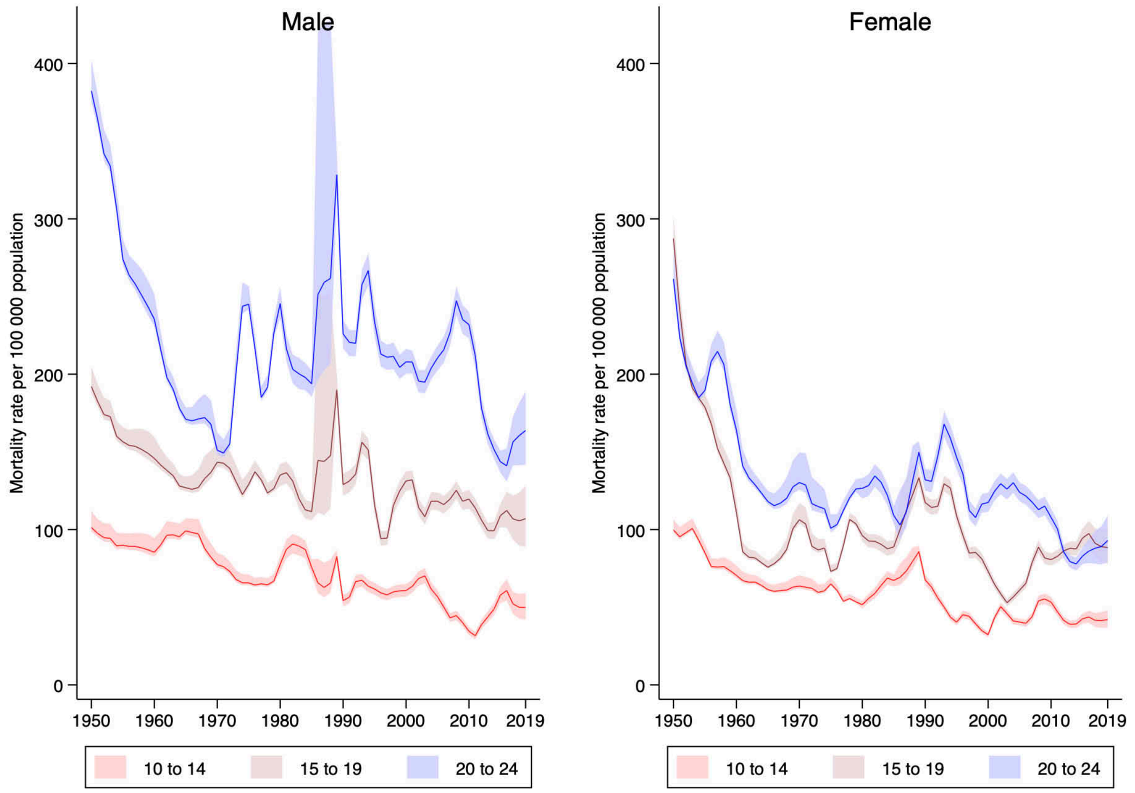

Figure S116b Number of deaths from all causes in 10-24 1950 – 2019: Suriname

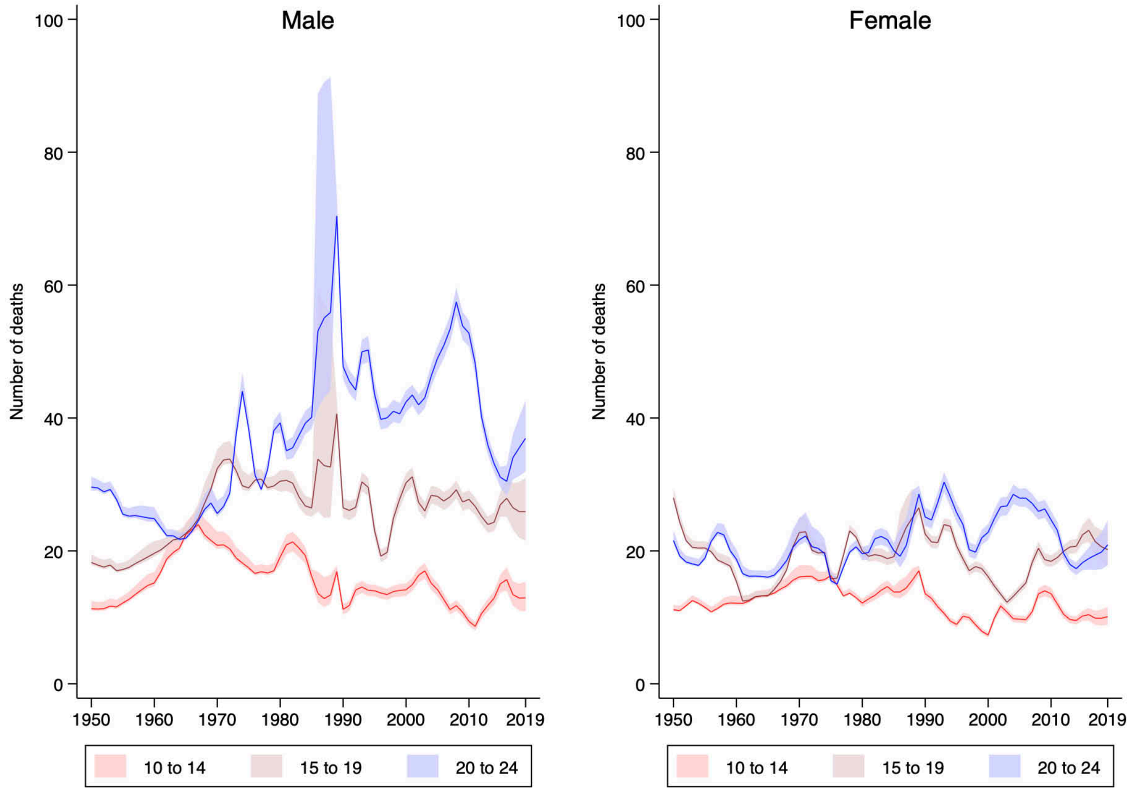

Figure S116c Mortality rate per 100,000 in 10-24 1980 – 2019 by cause: Suriname

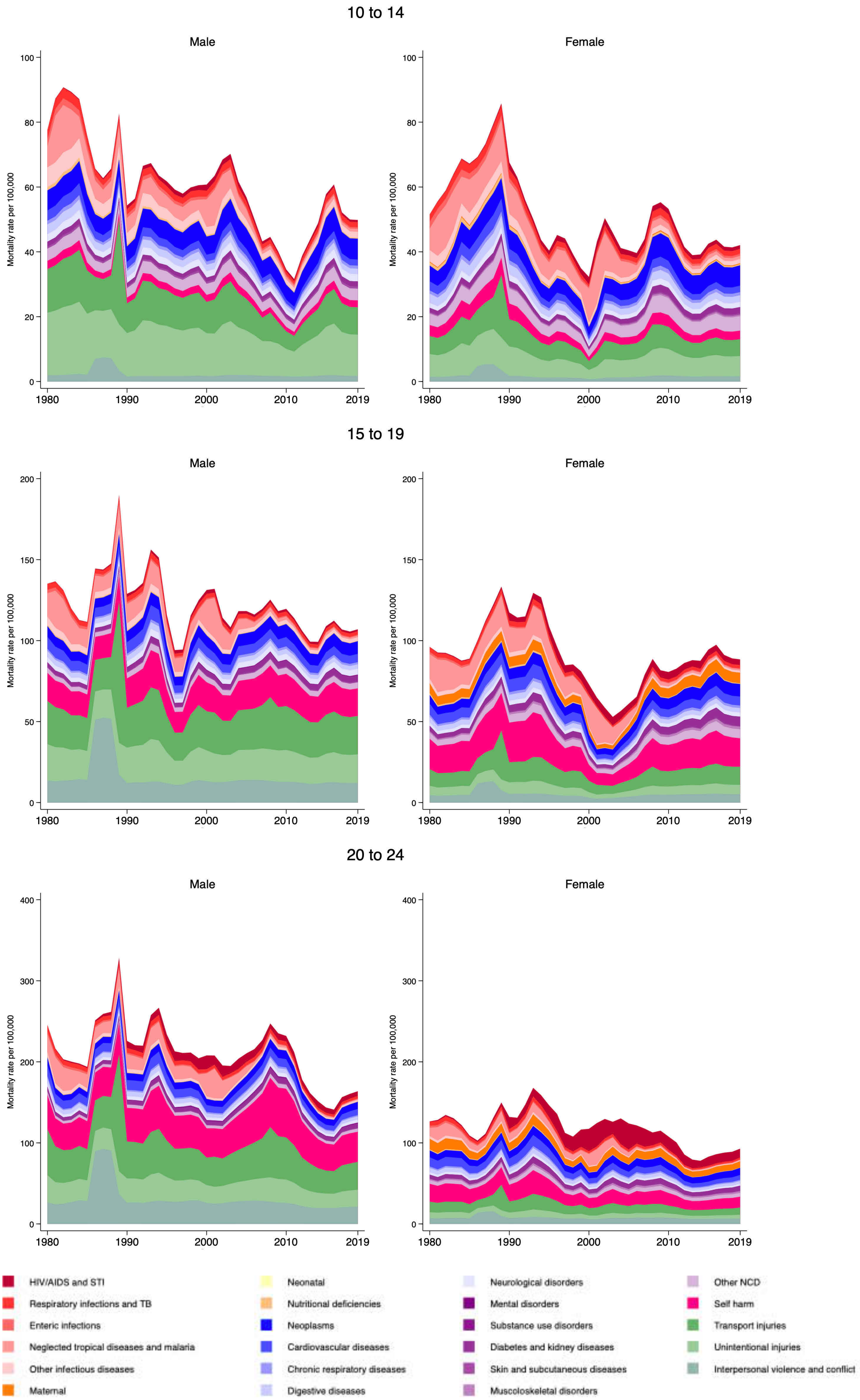

Figure S116d Number of deaths in 10-24 1980 – 2019 by cause: Suriname

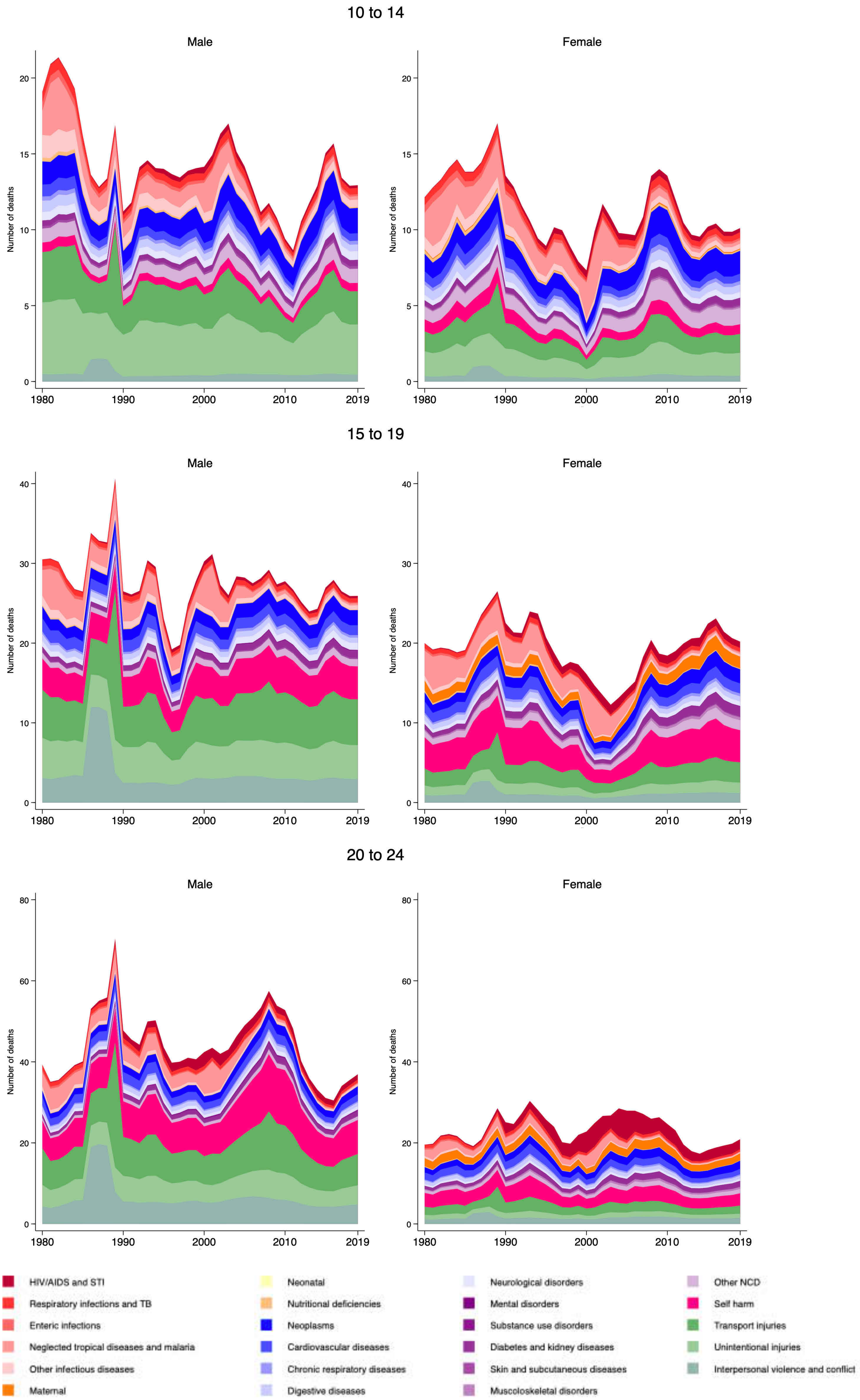

Figure S117: Trinidad and Tobago

Figure S117a All-cause mortality rate per 100,000 in 10-24 1950 – 2019: Trinidad and Tobago

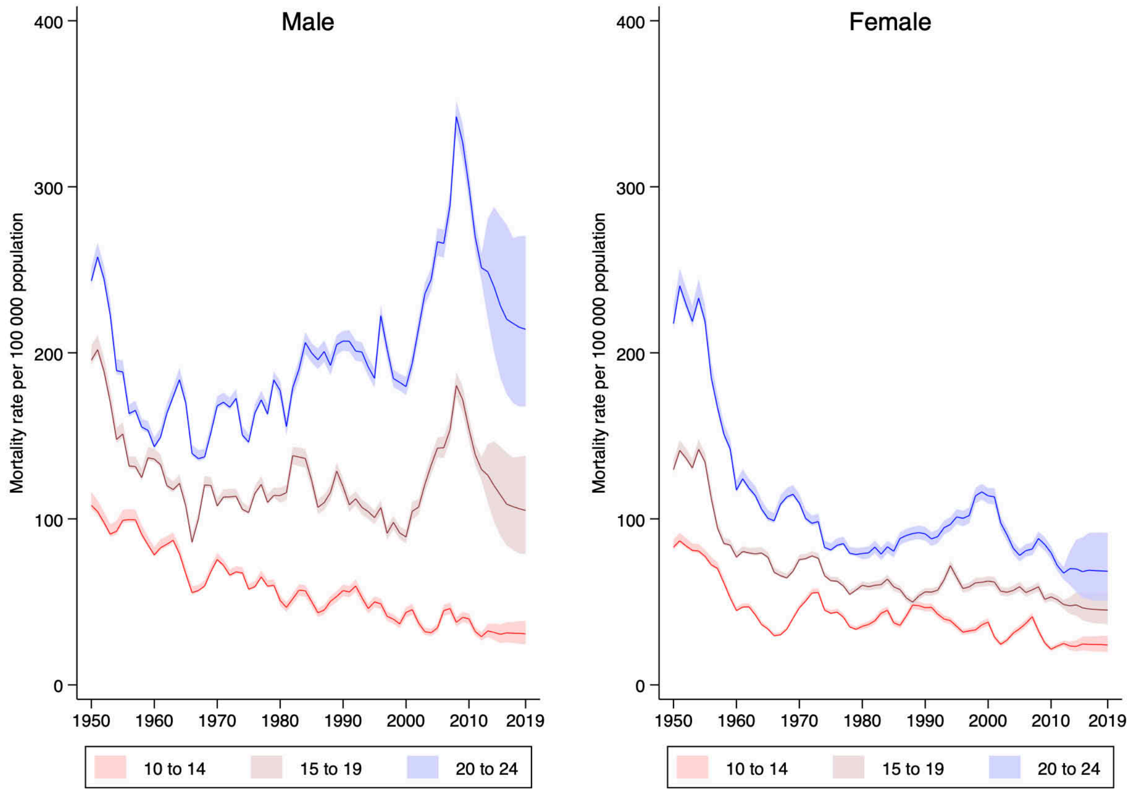

Figure S117b Number of deaths from all causes in 10-24 1950 – 2019: Trinidad and Tobago

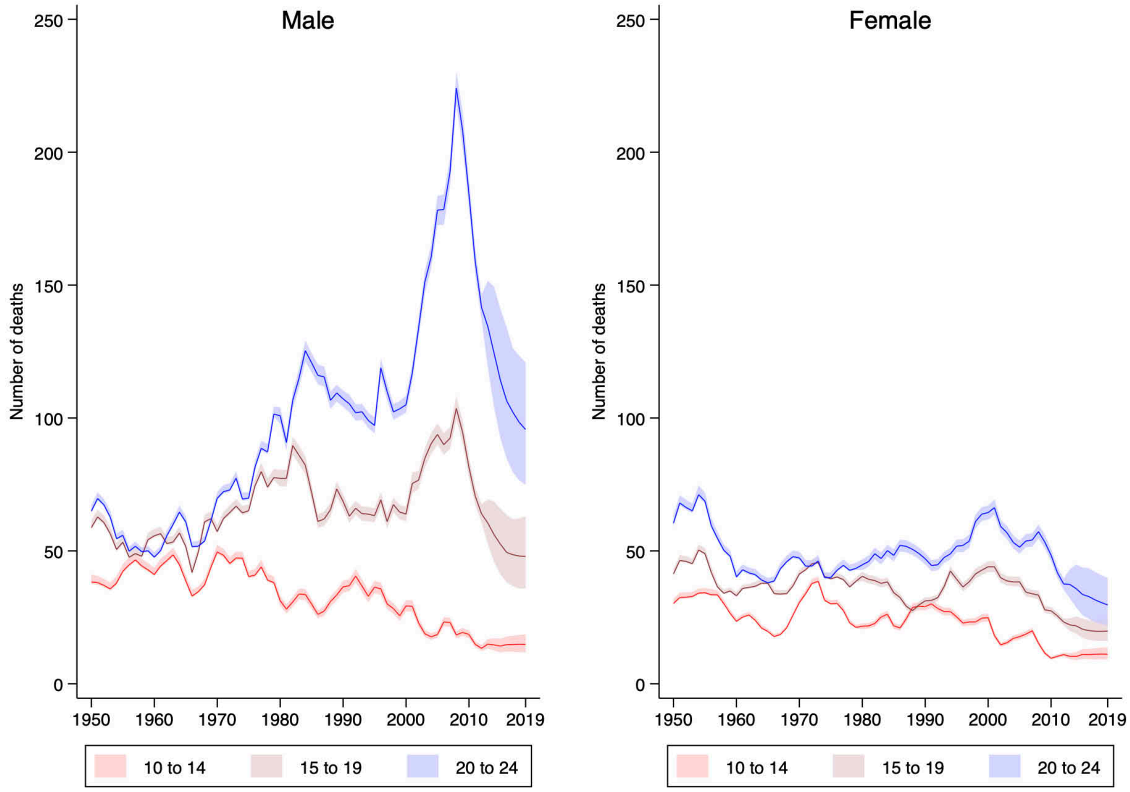

Figure S117c Mortality rate per 100,000 in 10-24 1980 – 2019 by cause: Trinidad and Tobago

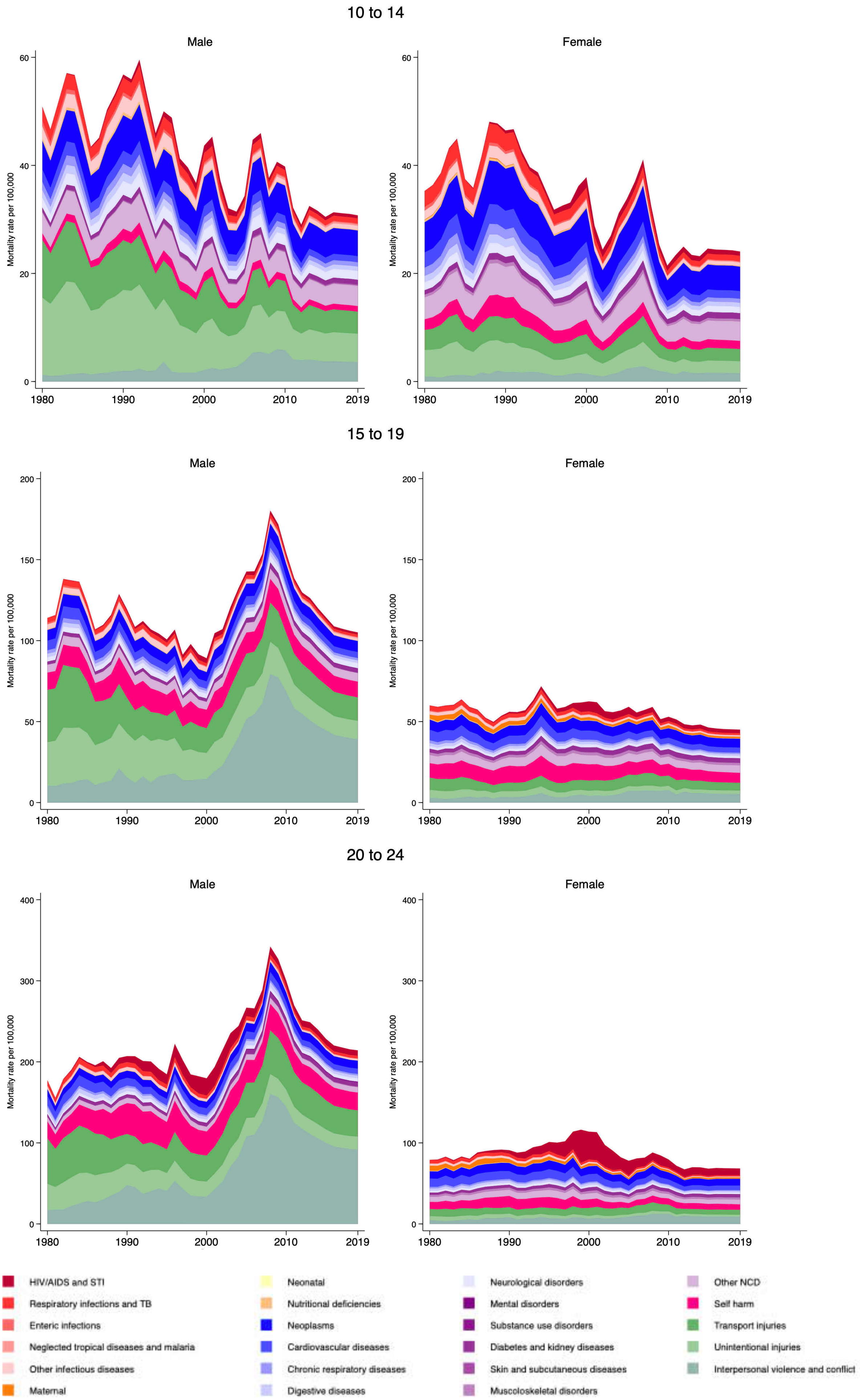

Figure S117d Number of deaths in 10-24 1980 – 2019 by cause: Trinidad and Tobago

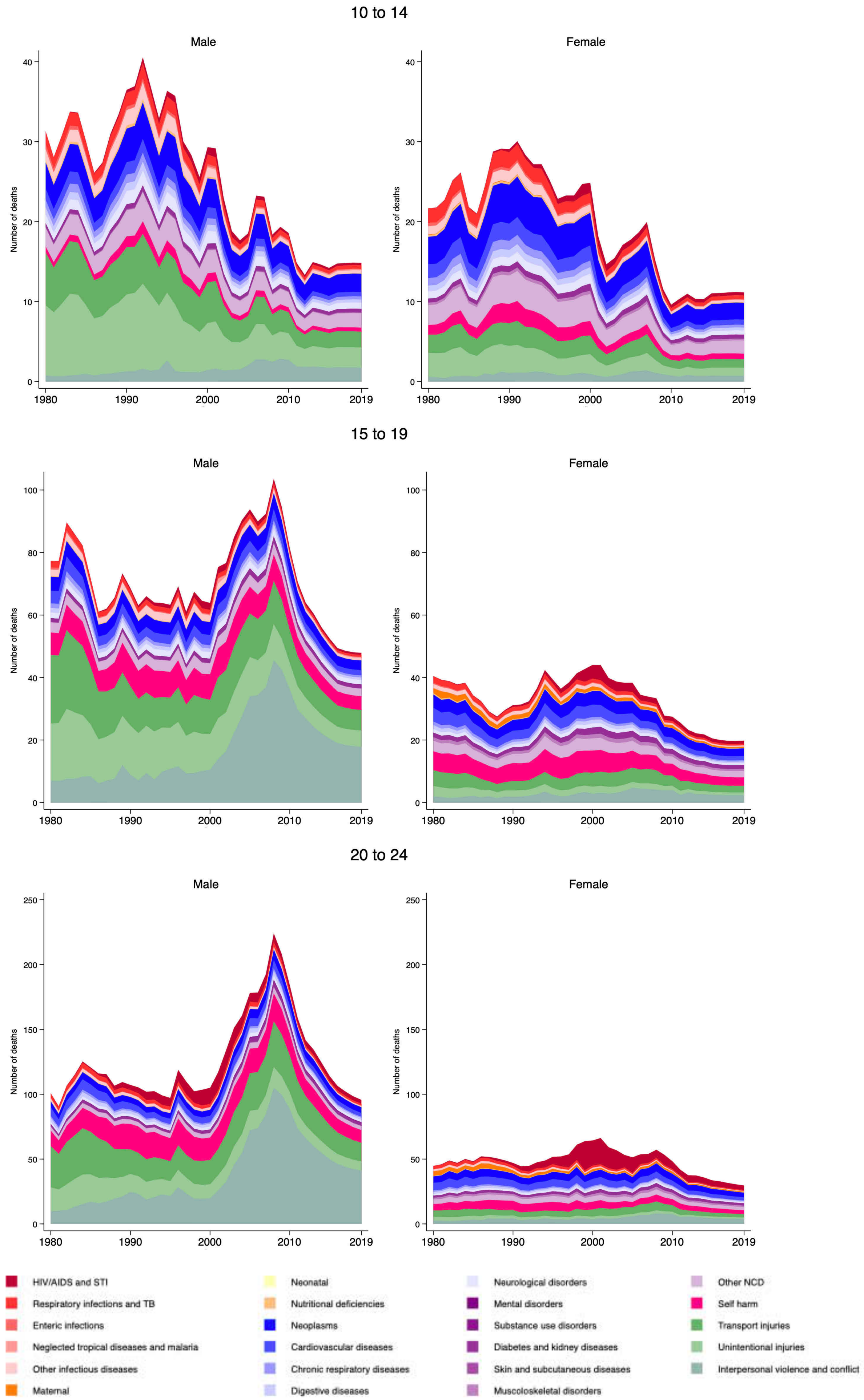

Figure S118: Andean Latin America

Figure S118a All-cause mortality rate per 100,000 in 10-24 1950 – 2019: Andean Latin America

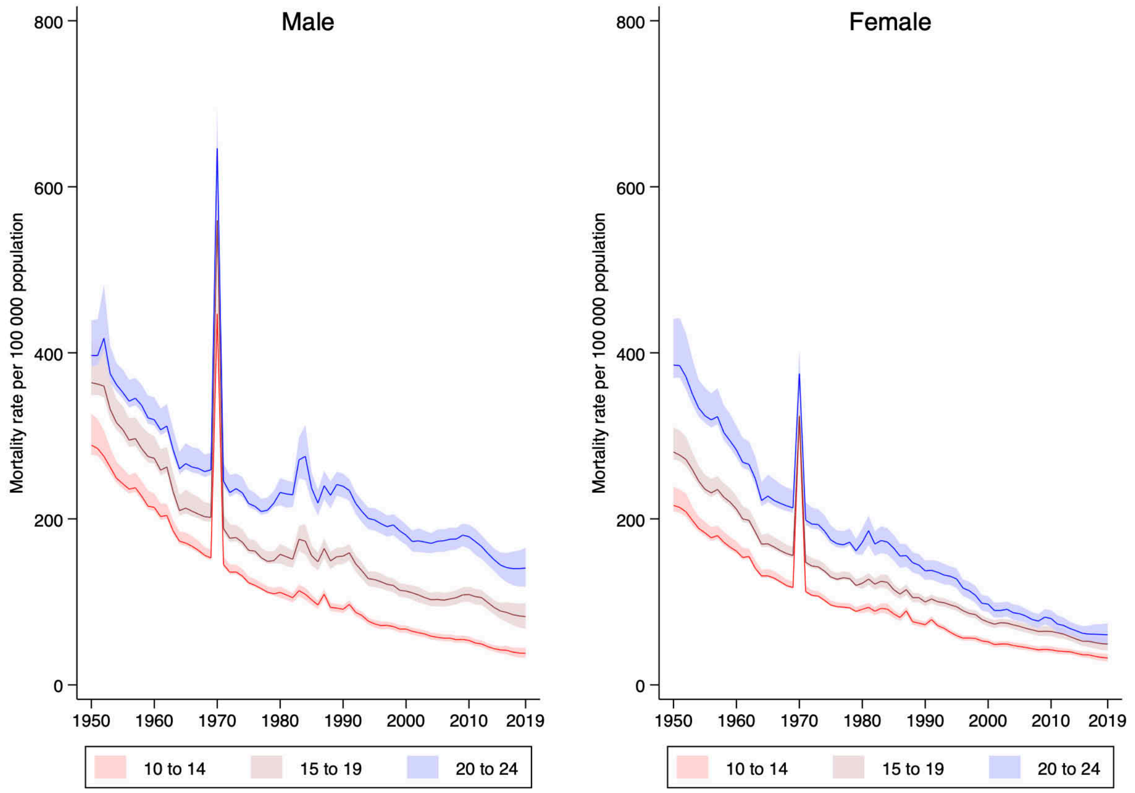

Figure S118b Number of deaths from all causes in 10-24 1950 – 2019: Andean Latin America

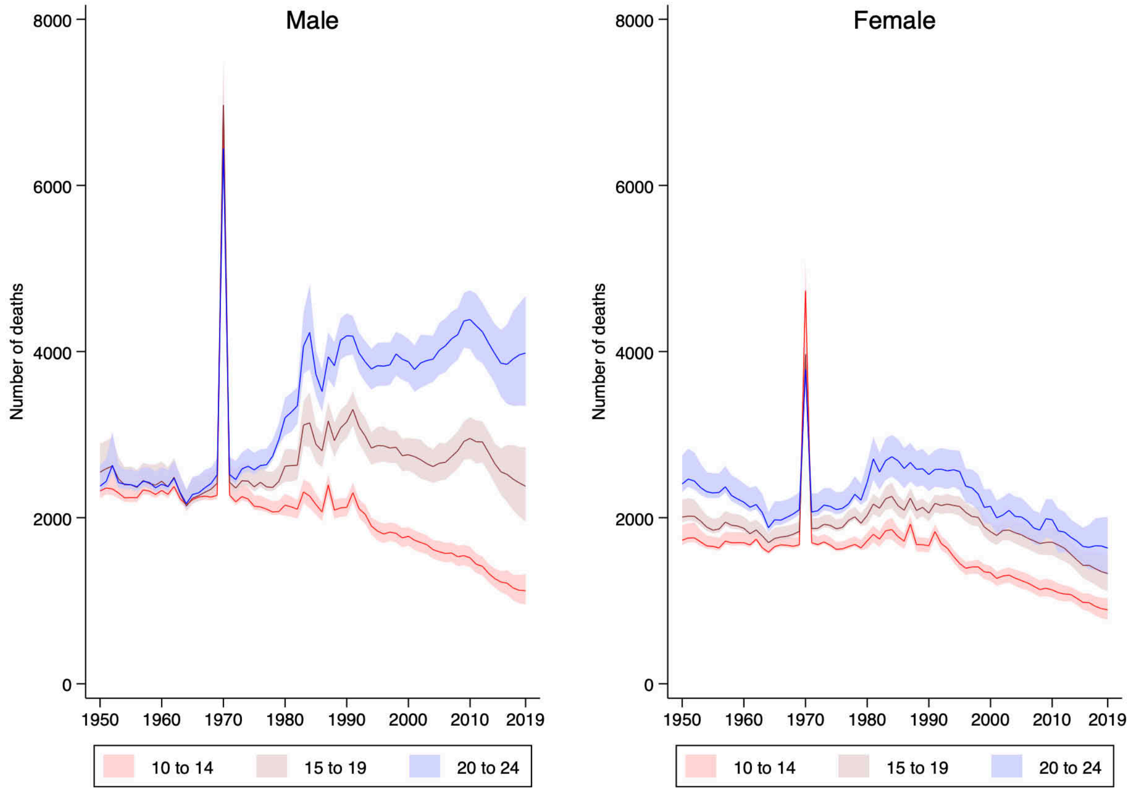

Figure S118c Mortality rate per 100,000 in 10-24 1980 – 2019 by cause: Andean Latin America

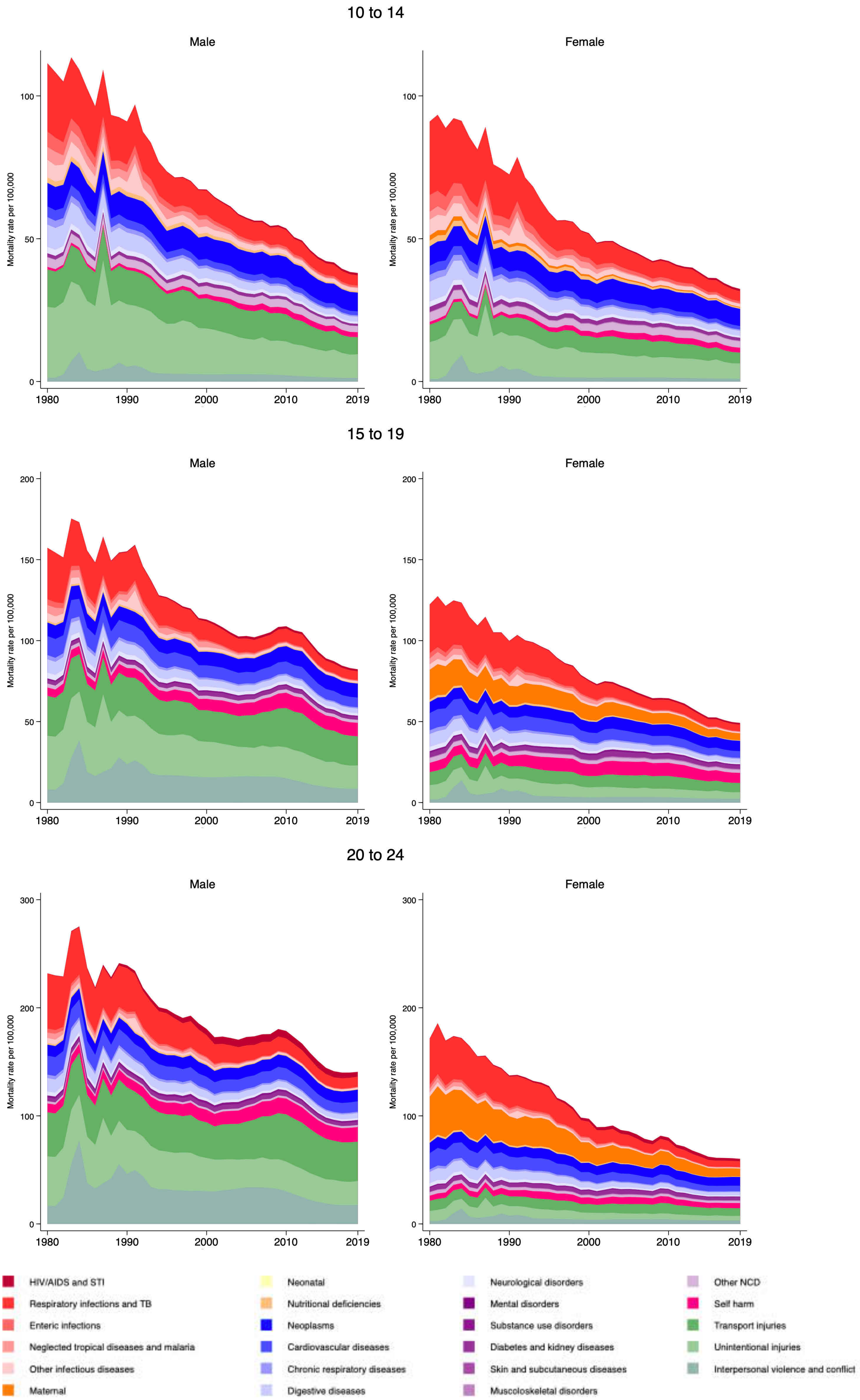

Figure S118d Number of deaths in 10-24 1980 – 2019 by cause: Andean Latin America

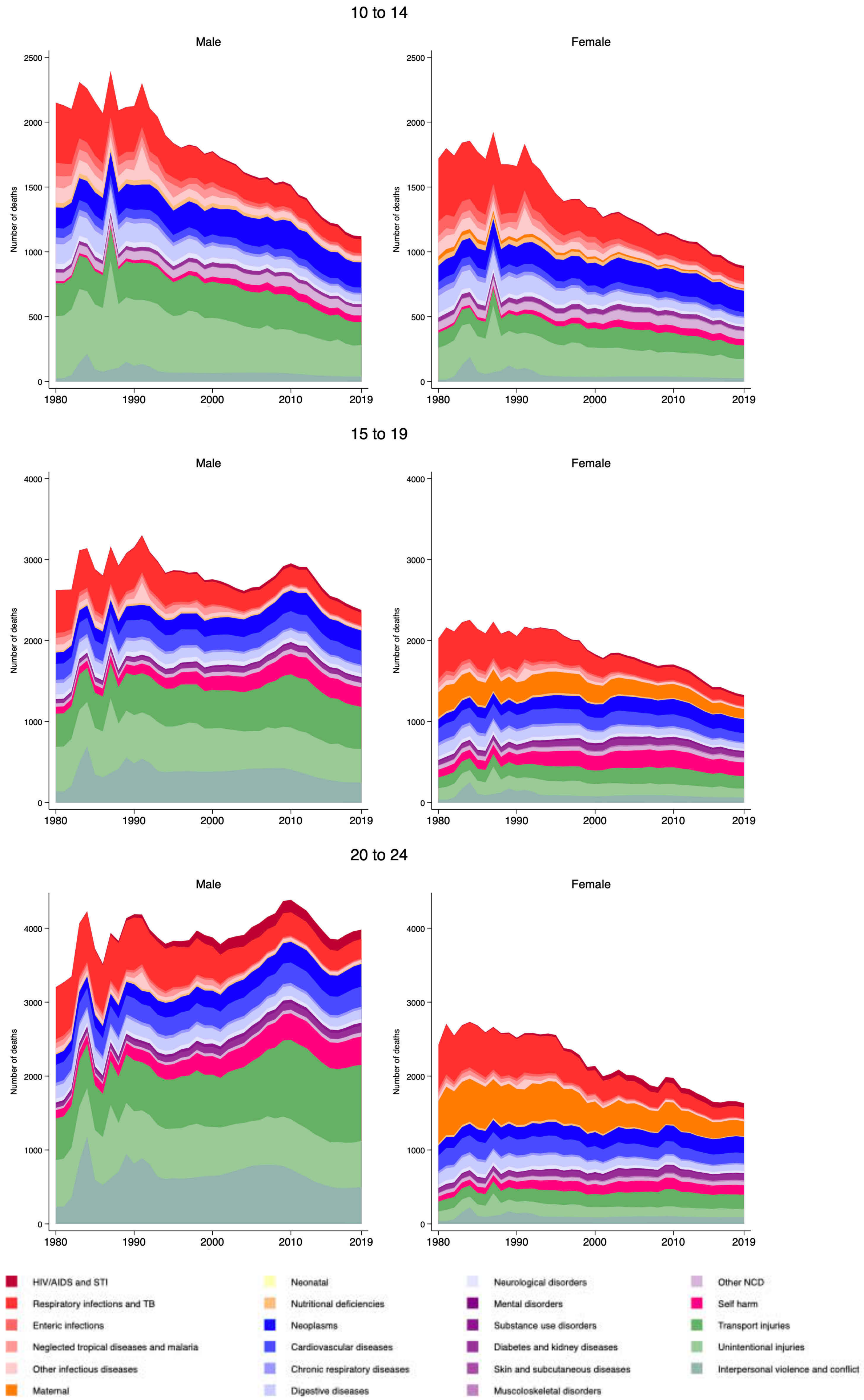

Figure S119: Bolivia (Plurinational State of)

Figure S119a All-cause mortality rate per 100,000 in 10-24 1950 – 2019: Bolivia (Plurinational State of)

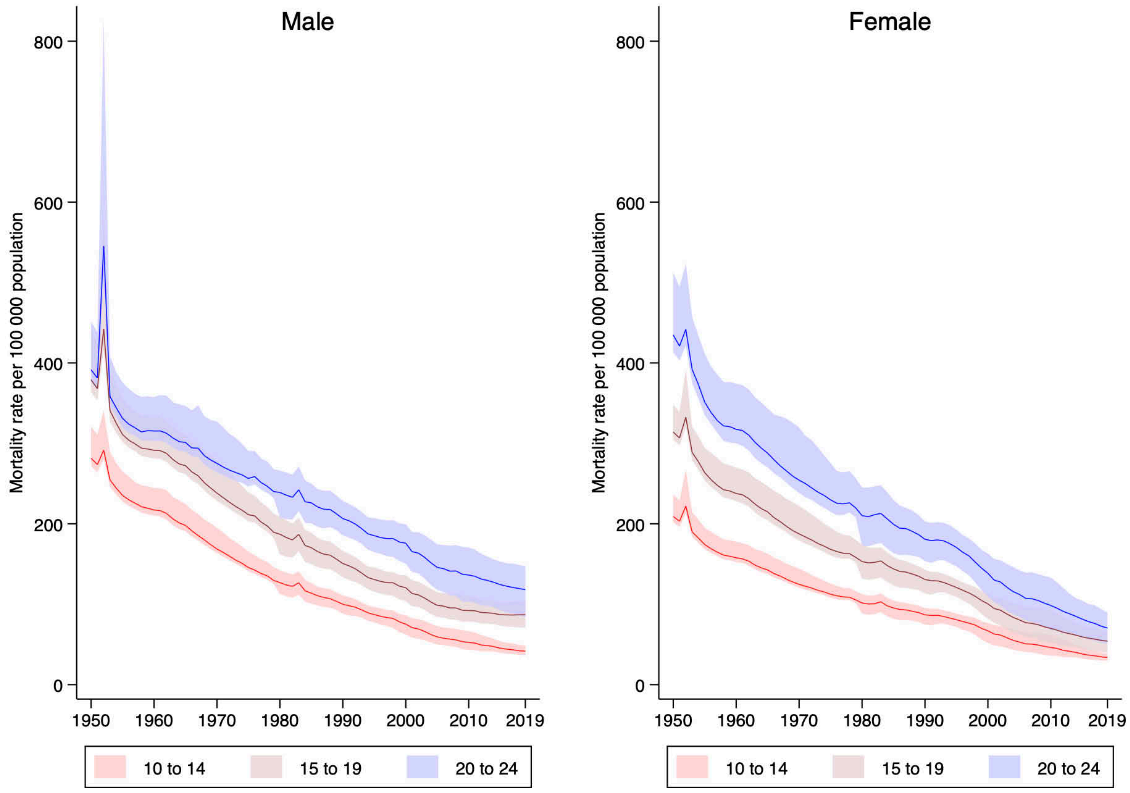

Figure S119b Number of deaths from all causes in 10-24 1950 – 2019: Bolivia (Plurinational State of)

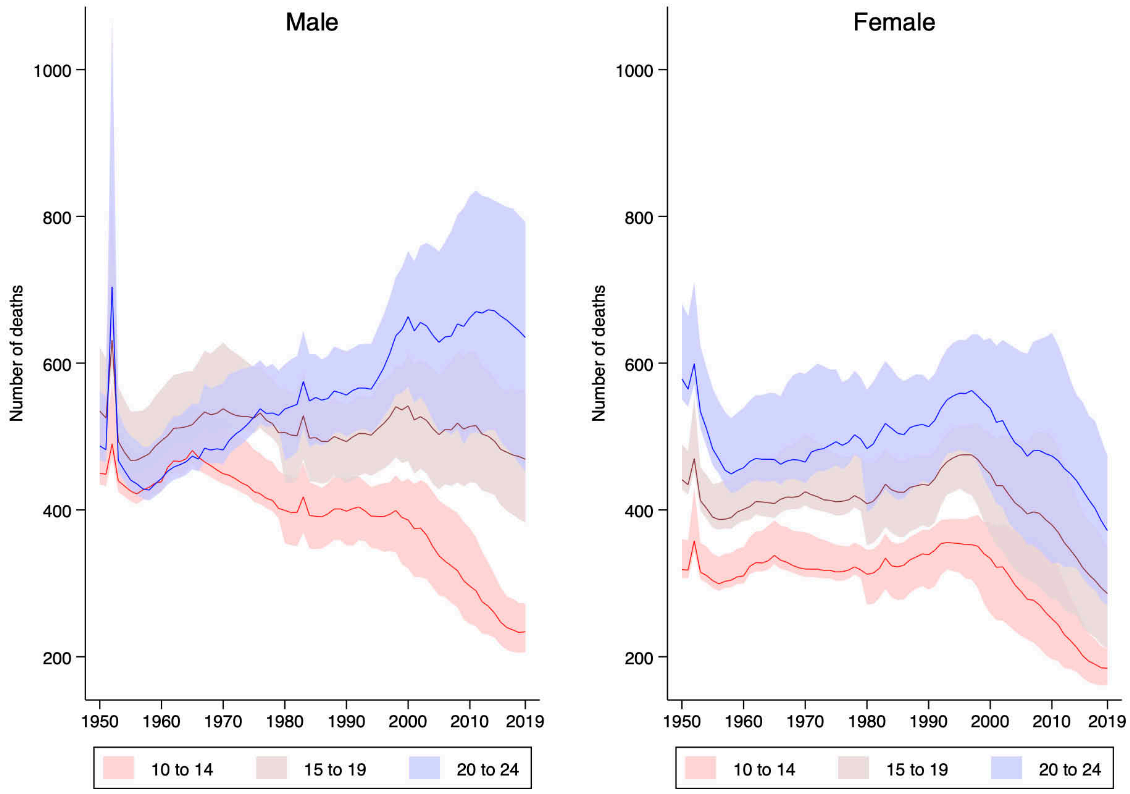

Figure S119c Mortality rate per 100,000 in 10-24 1980 – 2019 by cause: Bolivia (Plurinational State of)

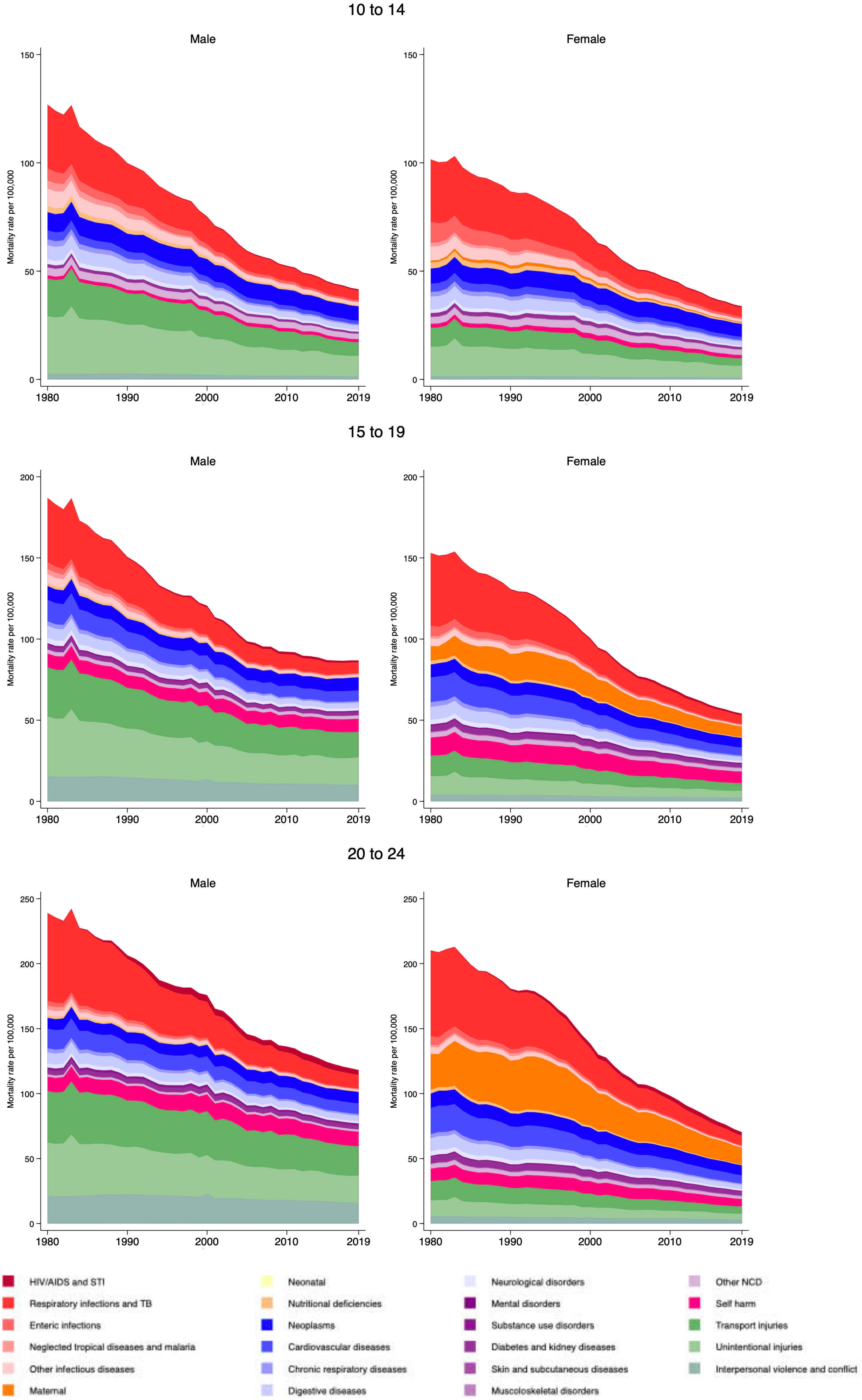

Figure S119d Number of deaths in 10-24 1980 – 2019 by cause: Bolivia (Plurinational State of)

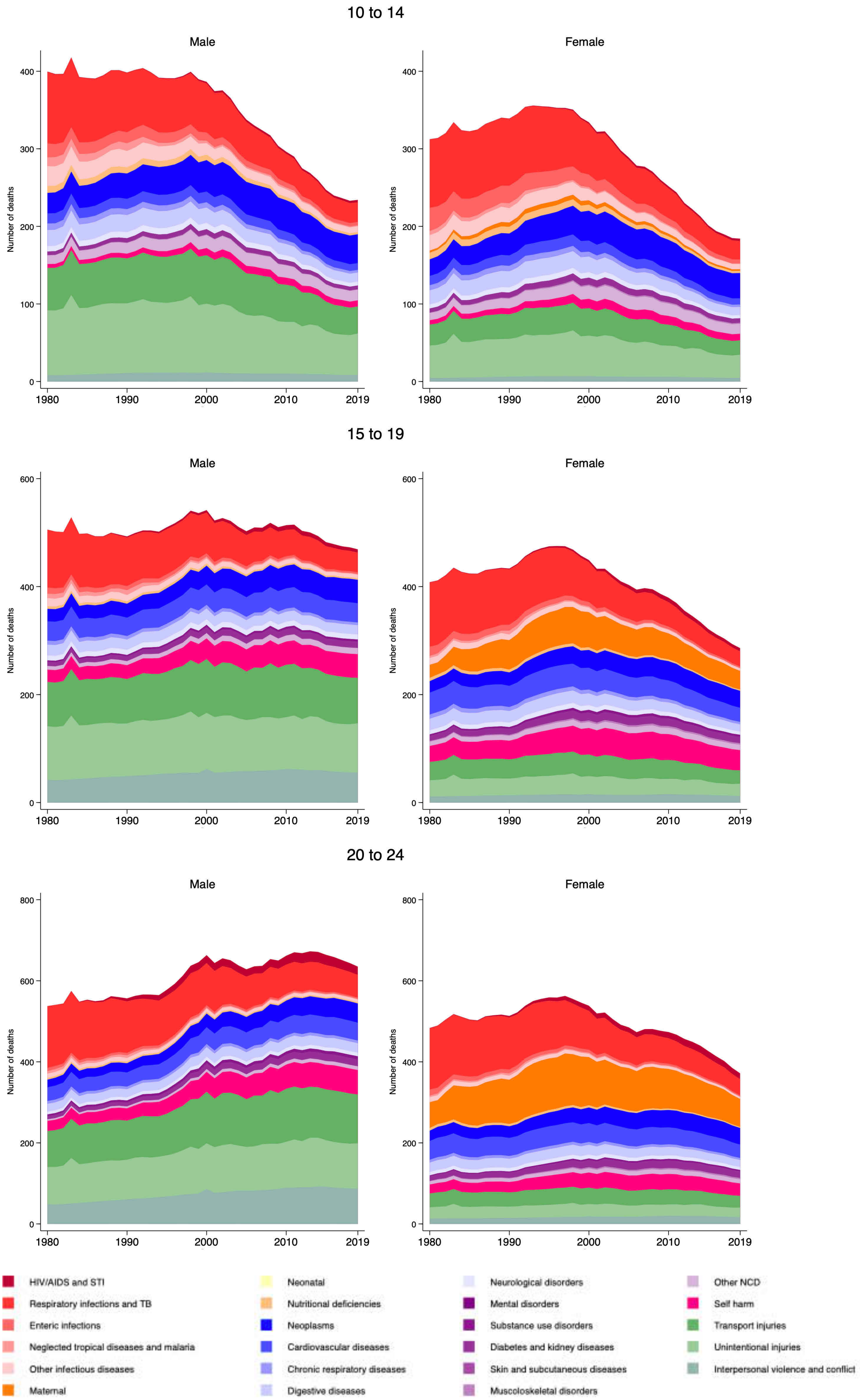

Figure S120: Ecuador

Figure S120a All-cause mortality rate per 100,000 in 10-24 1950 – 2019: Ecuador

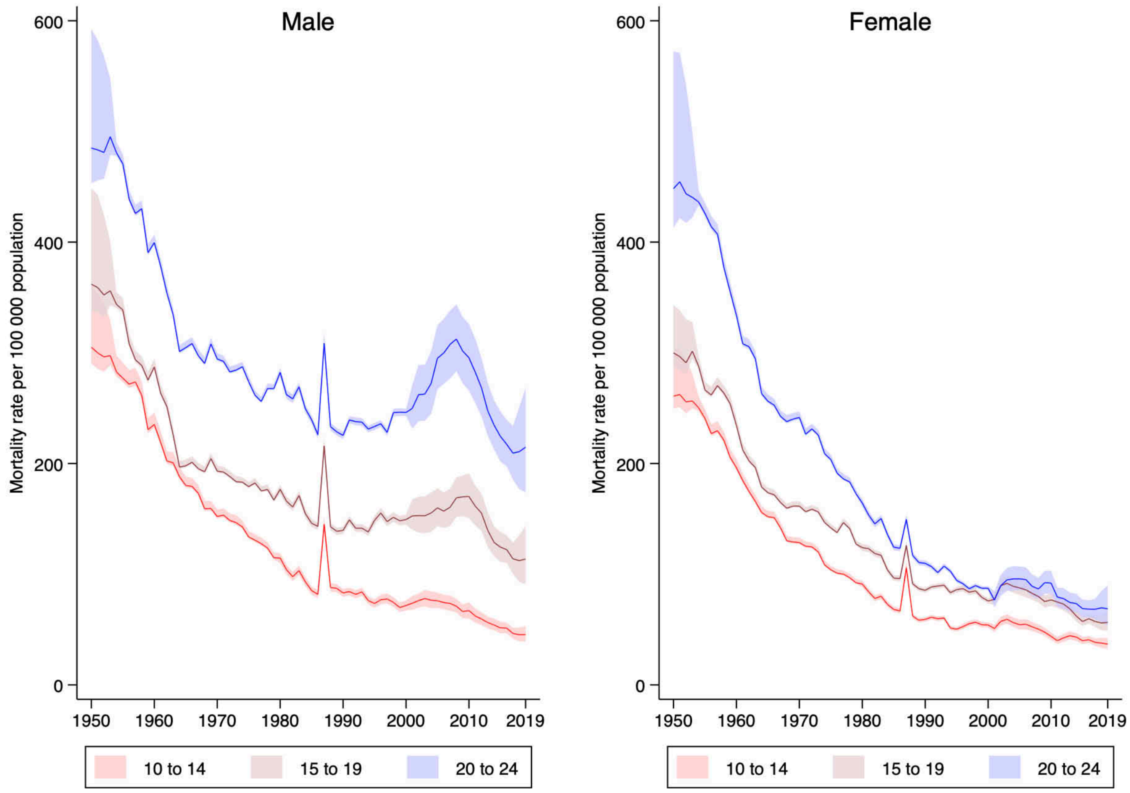

Figure S120b Number of deaths from all causes in 10-24 1950 – 2019: Ecuador

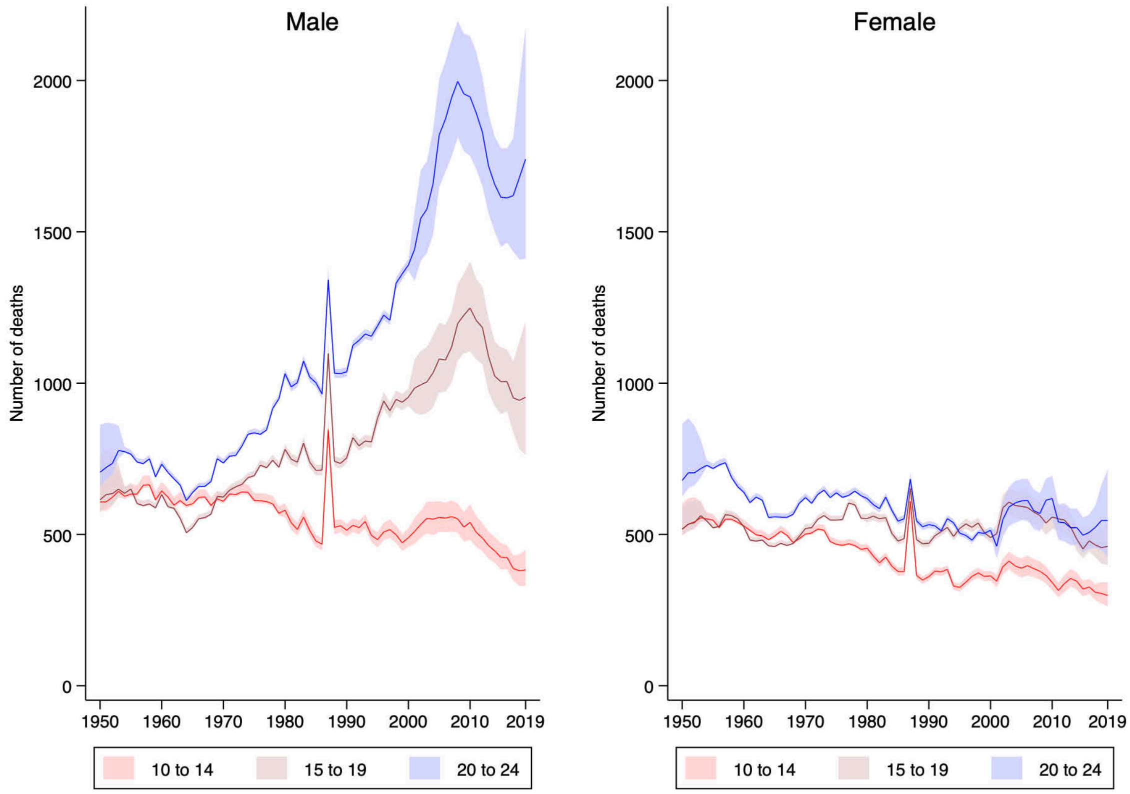

Figure S120c Mortality rate per 100,000 in 10-24 1980 – 2019 by cause: Ecuador

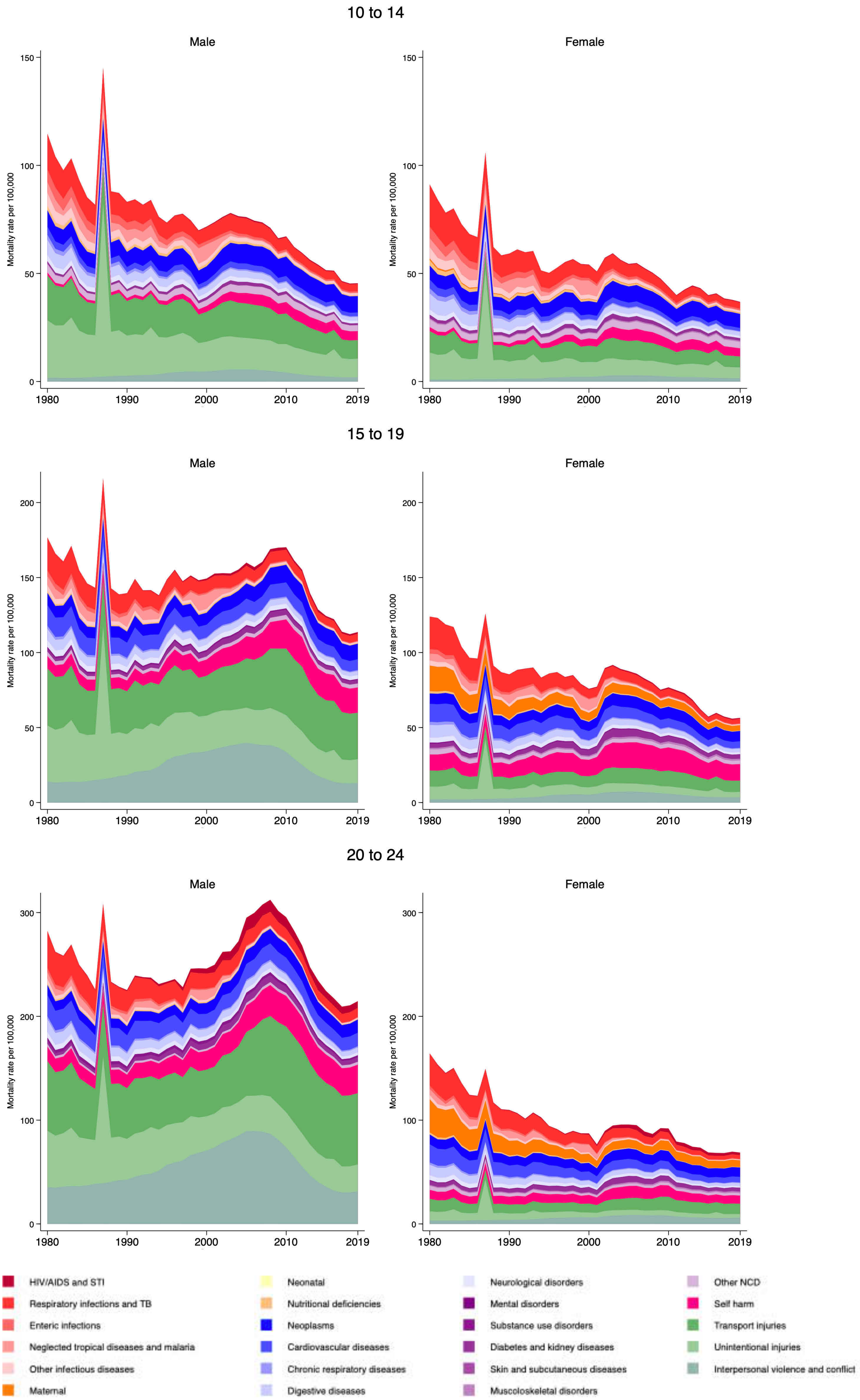

Figure S120d Number of deaths in 10-24 1980 – 2019 by cause: Ecuador

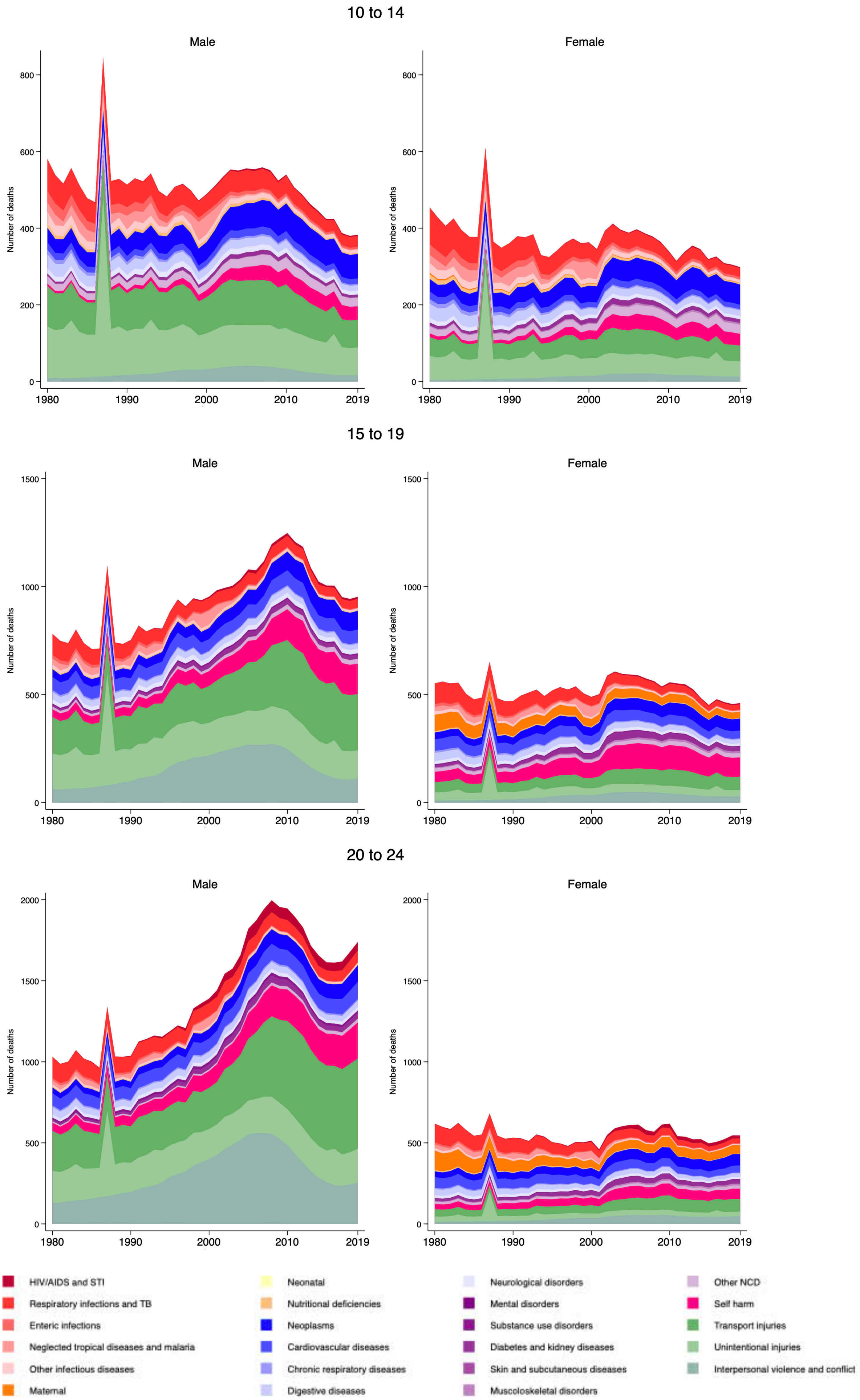

Figure S121: Peru

Figure S121a All-cause mortality rate per 100,000 in 10-24 1950 – 2019: Peru

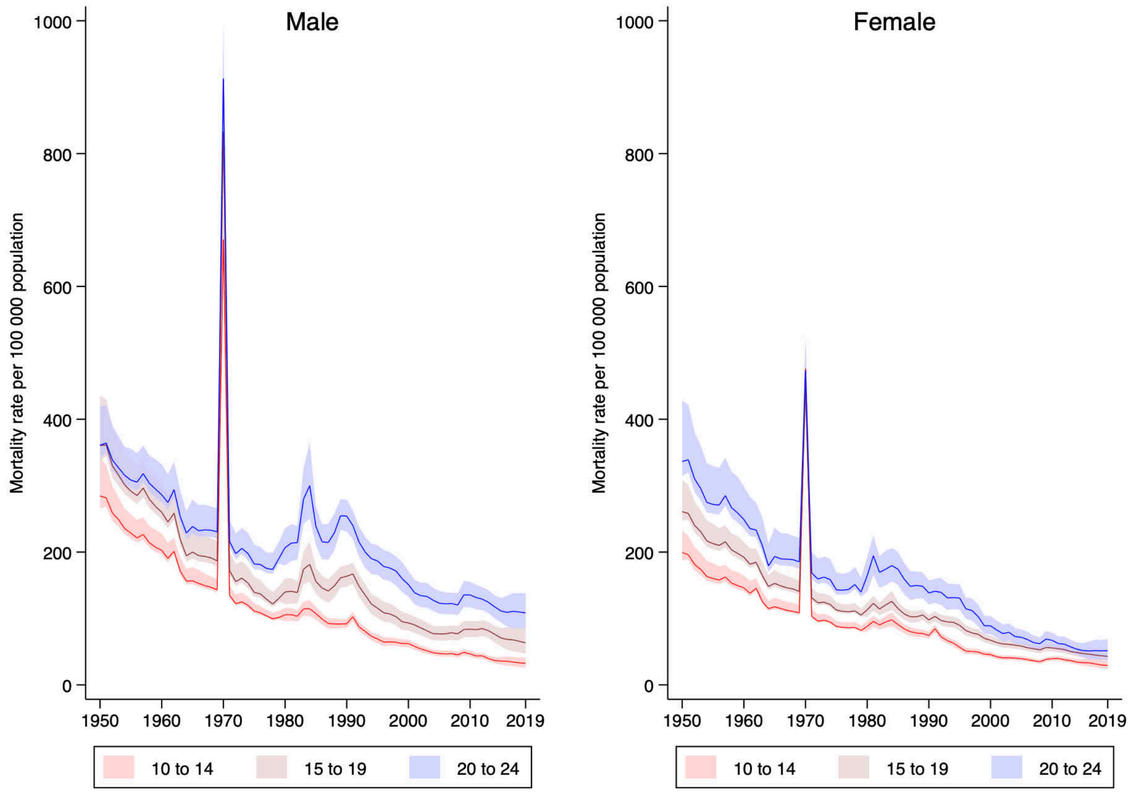

Figure S121b Number of deaths from all causes in 10-24 1950 – 2019: Peru

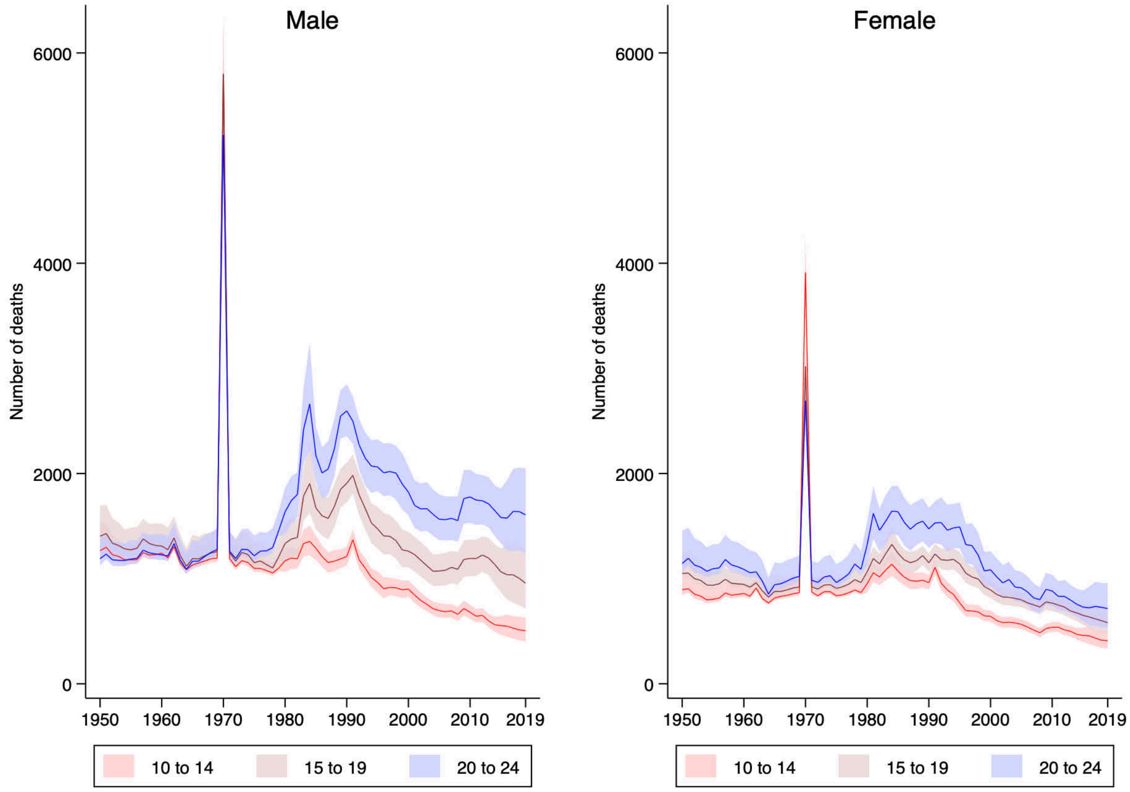

Figure S121c Mortality rate per 100,000 in 10-24 1980 – 2019 by cause: Peru

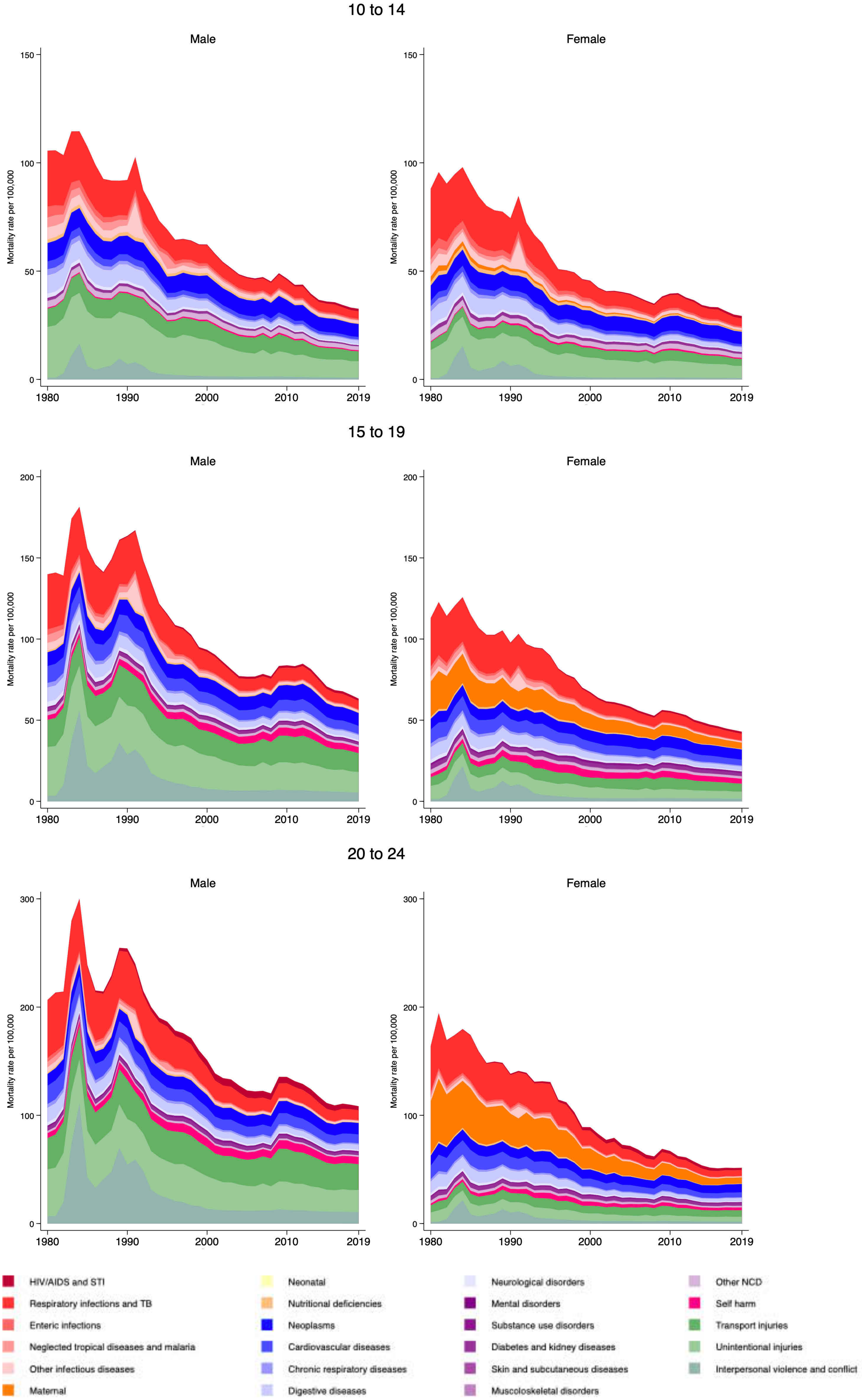

Figure S121d Number of deaths in 10-24 1980 – 2019 by cause: Peru

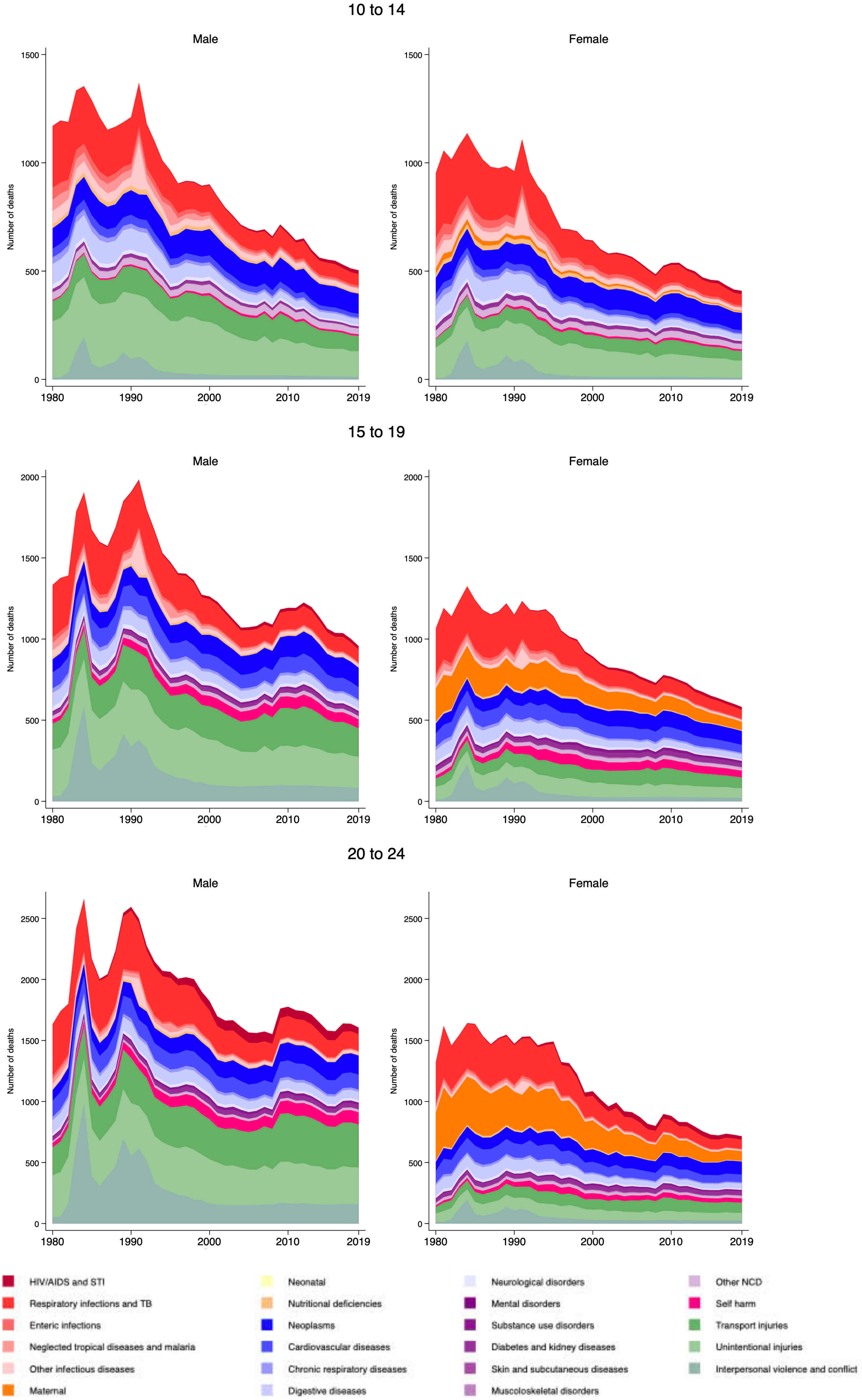

Figure S122: Central Latin America

Figure S122a All-cause mortality rate per 100,000 in 10-24 1950 – 2019: Central Latin America

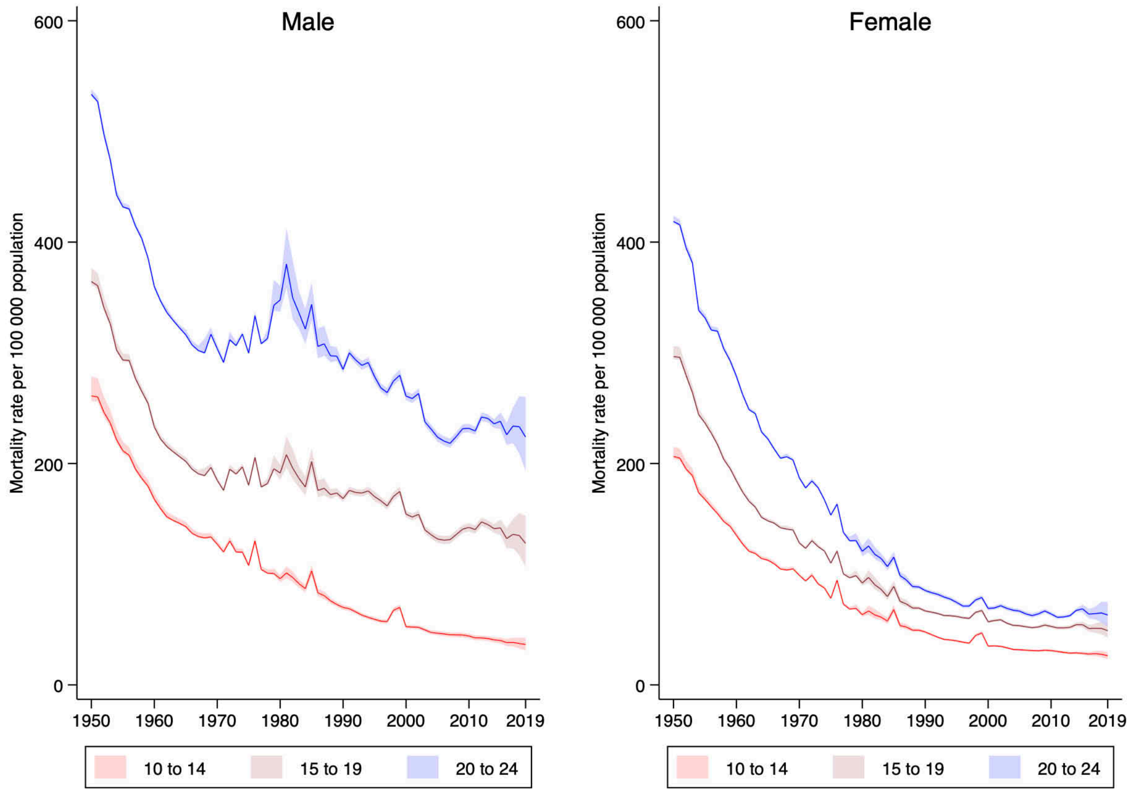

Figure S122b Number of deaths from all causes in 10-24 1950 – 2019: Central Latin America

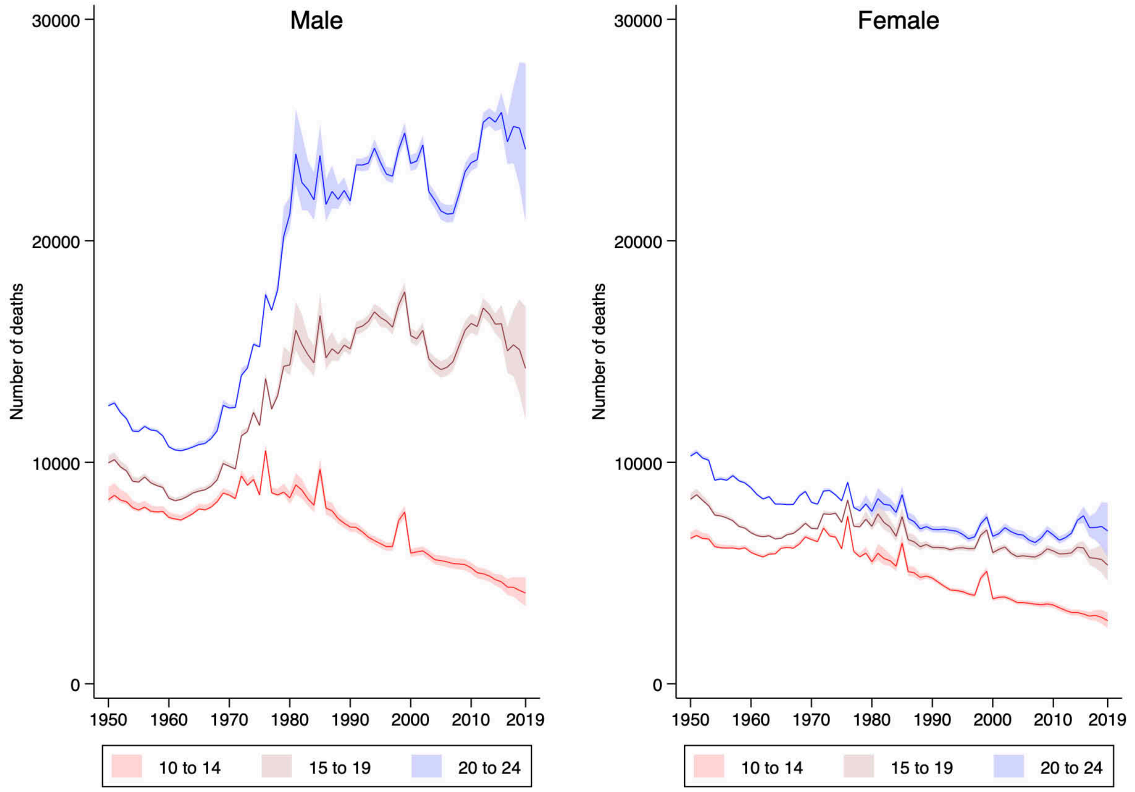

Figure S122c Mortality rate per 100,000 in 10-24 1980 – 2019 by cause: Central Latin America

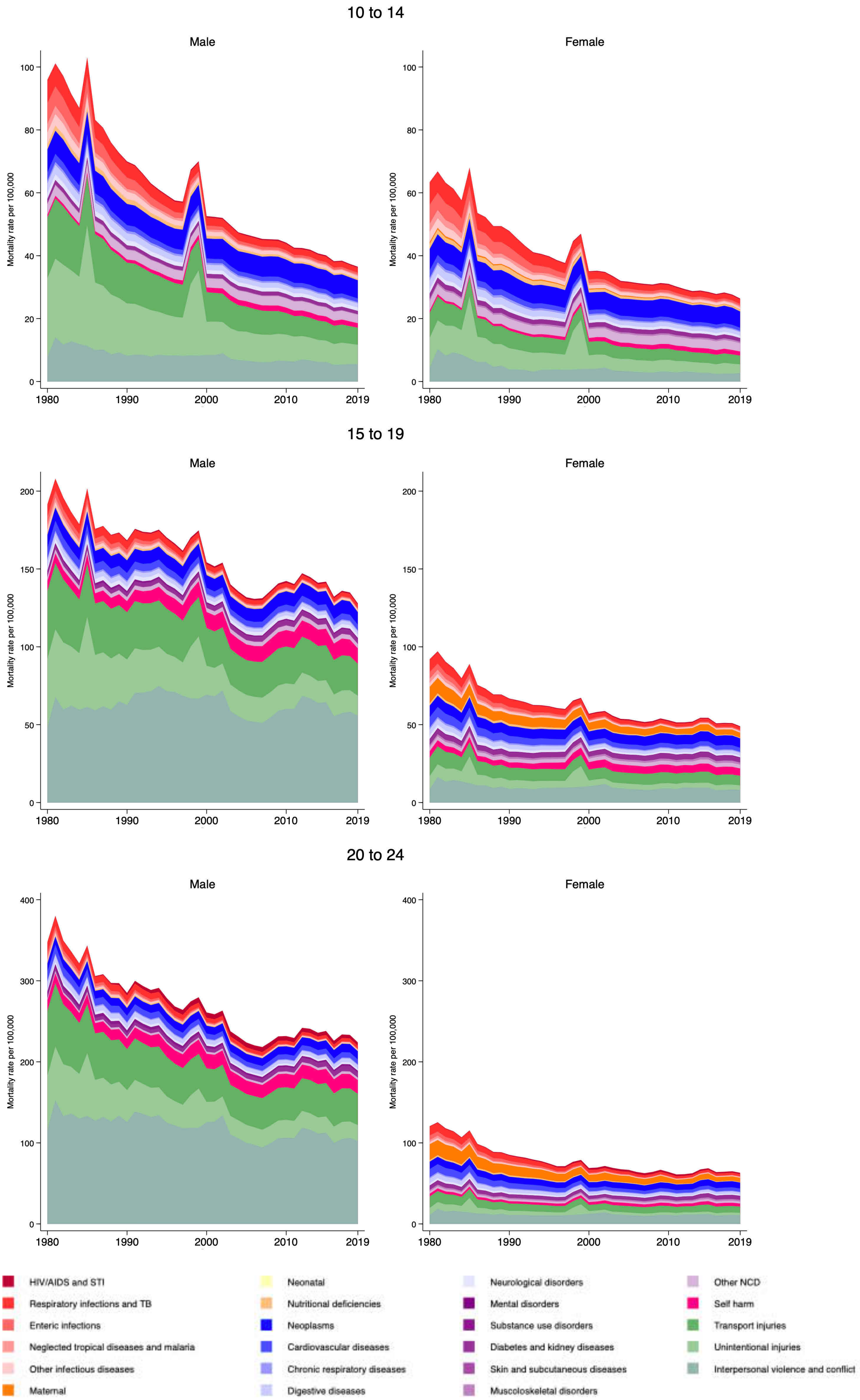

Figure S122d Number of deaths in 10-24 1980 – 2019 by cause: Central Latin America

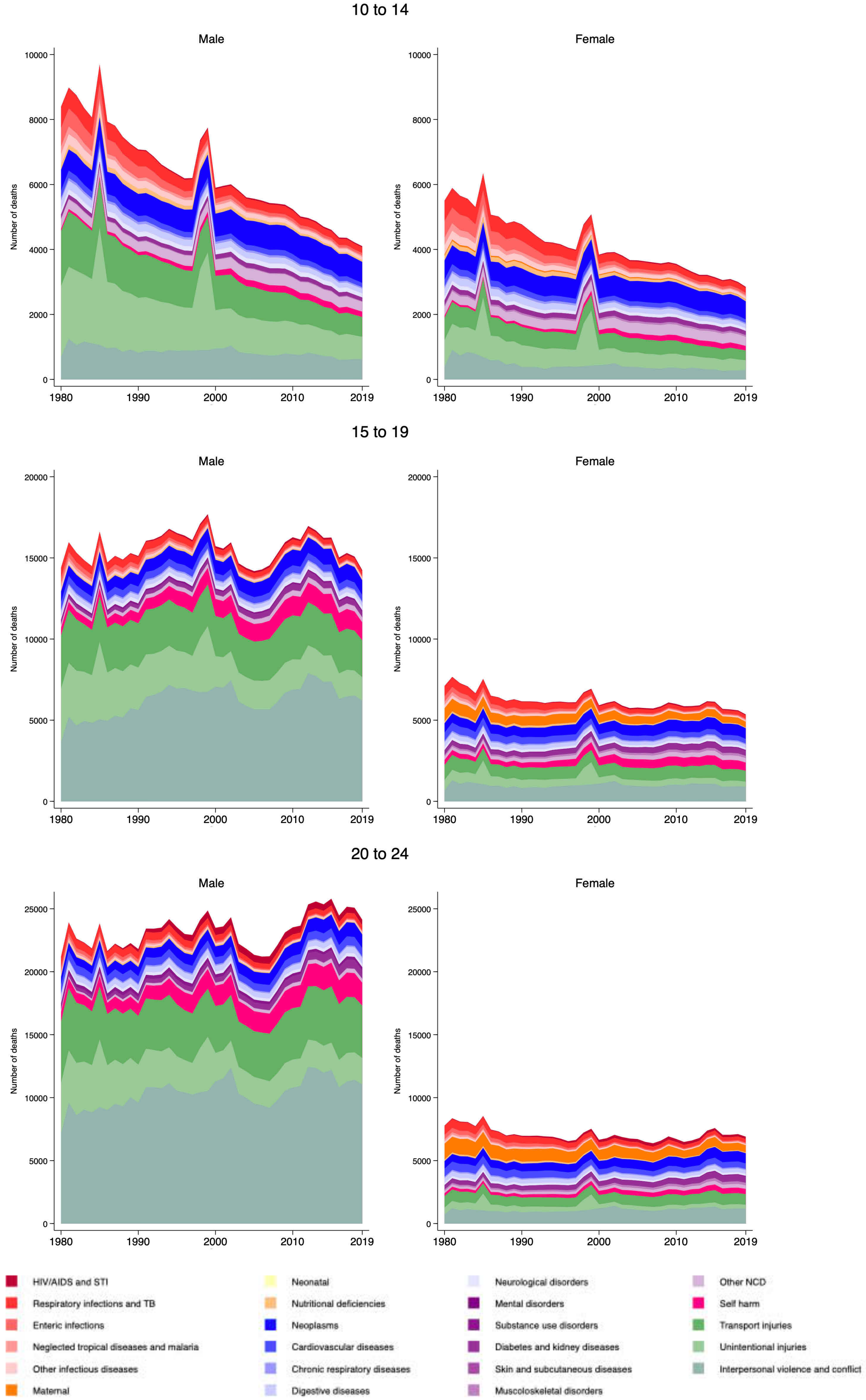

Figure S123: Colombia

Figure S123a All-cause mortality rate per 100,000 in 10-24 1950 – 2019: Colombia

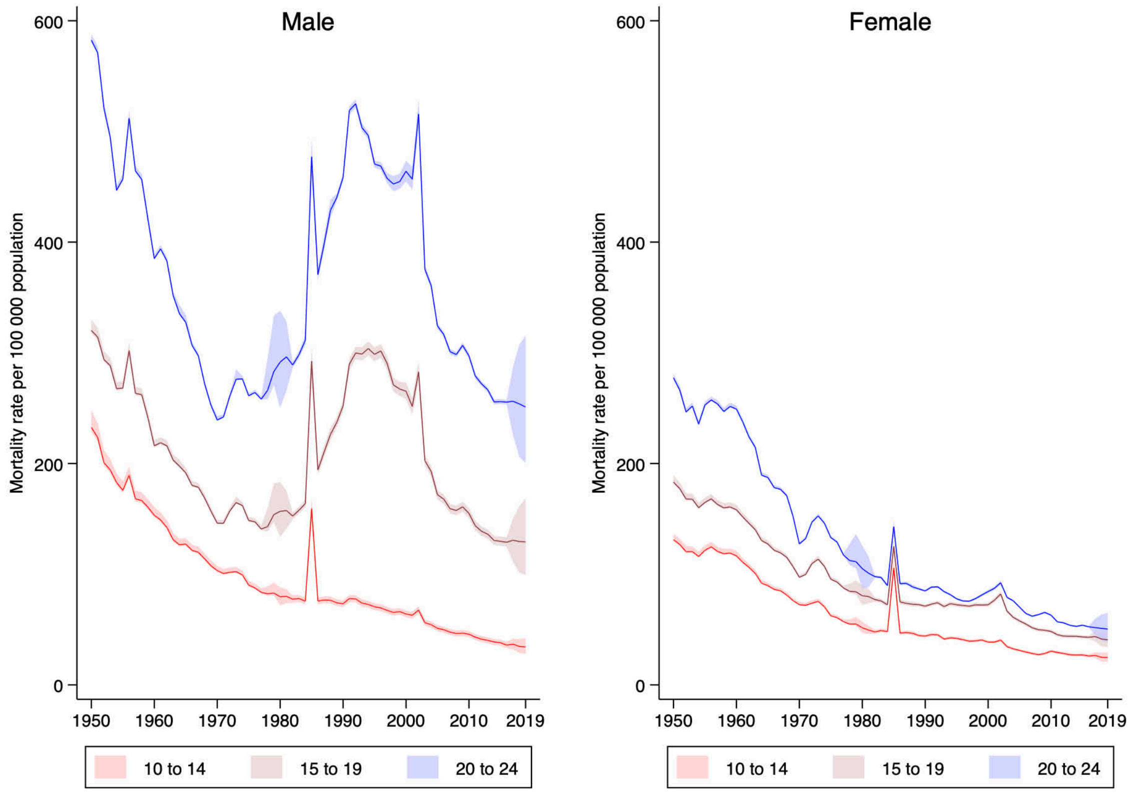

Figure S123b Number of deaths from all causes in 10-24 1950 – 2019: Colombia

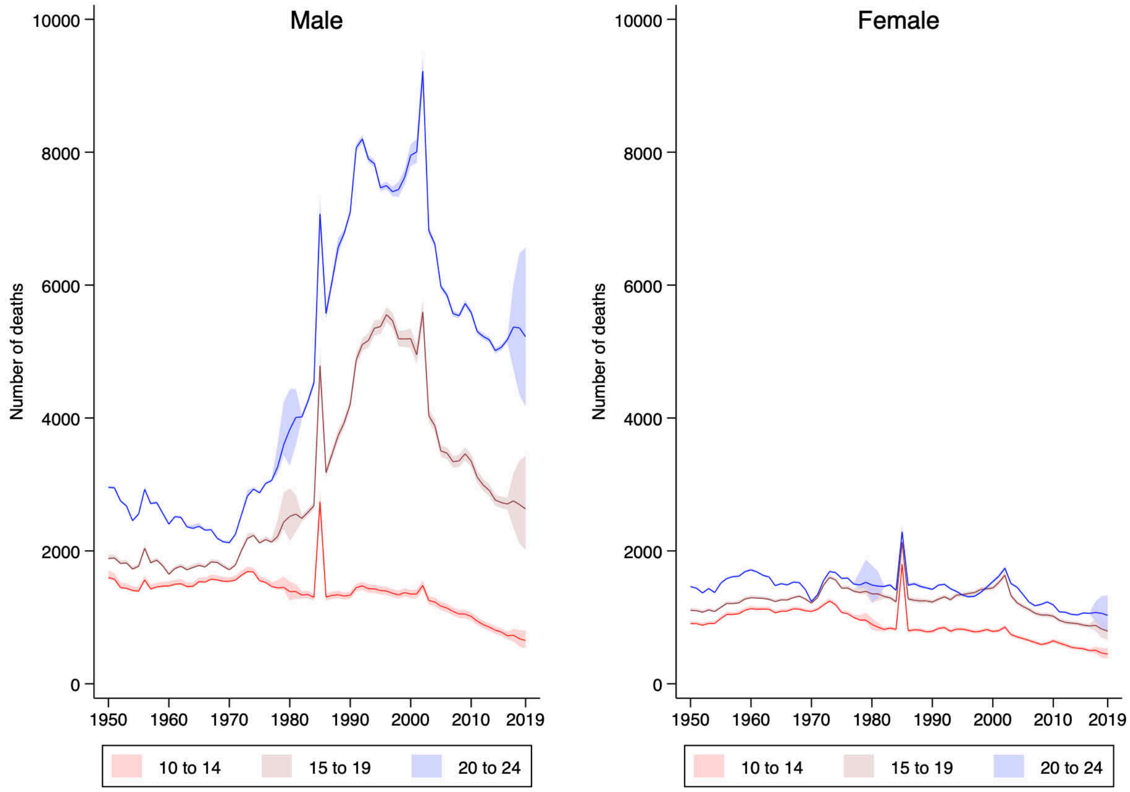

Figure S123c Mortality rate per 100,000 in 10-24 1980 – 2019 by cause: Colombia

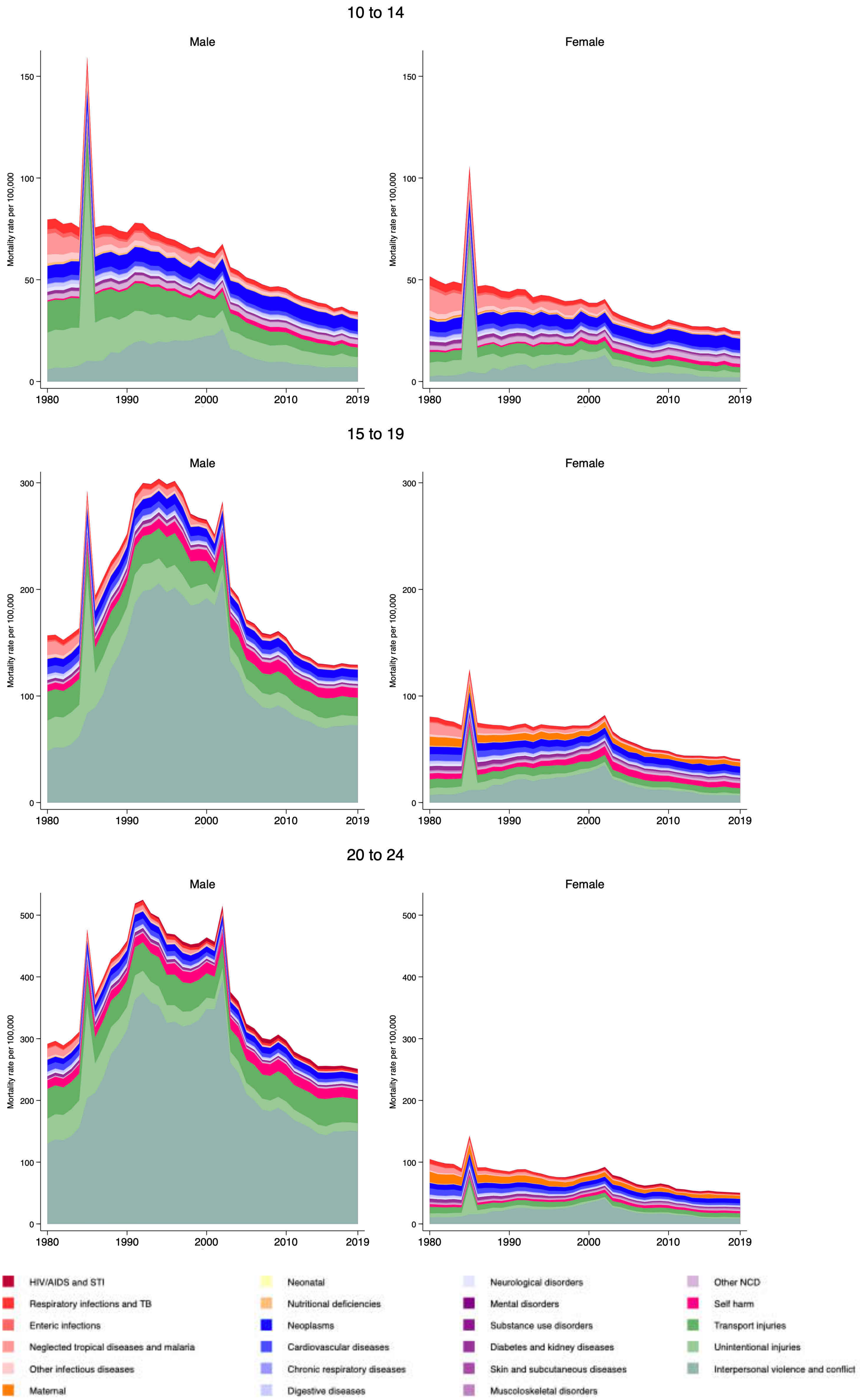

Figure S123d Number of deaths in 10-24 1980 – 2019 by cause: Colombia

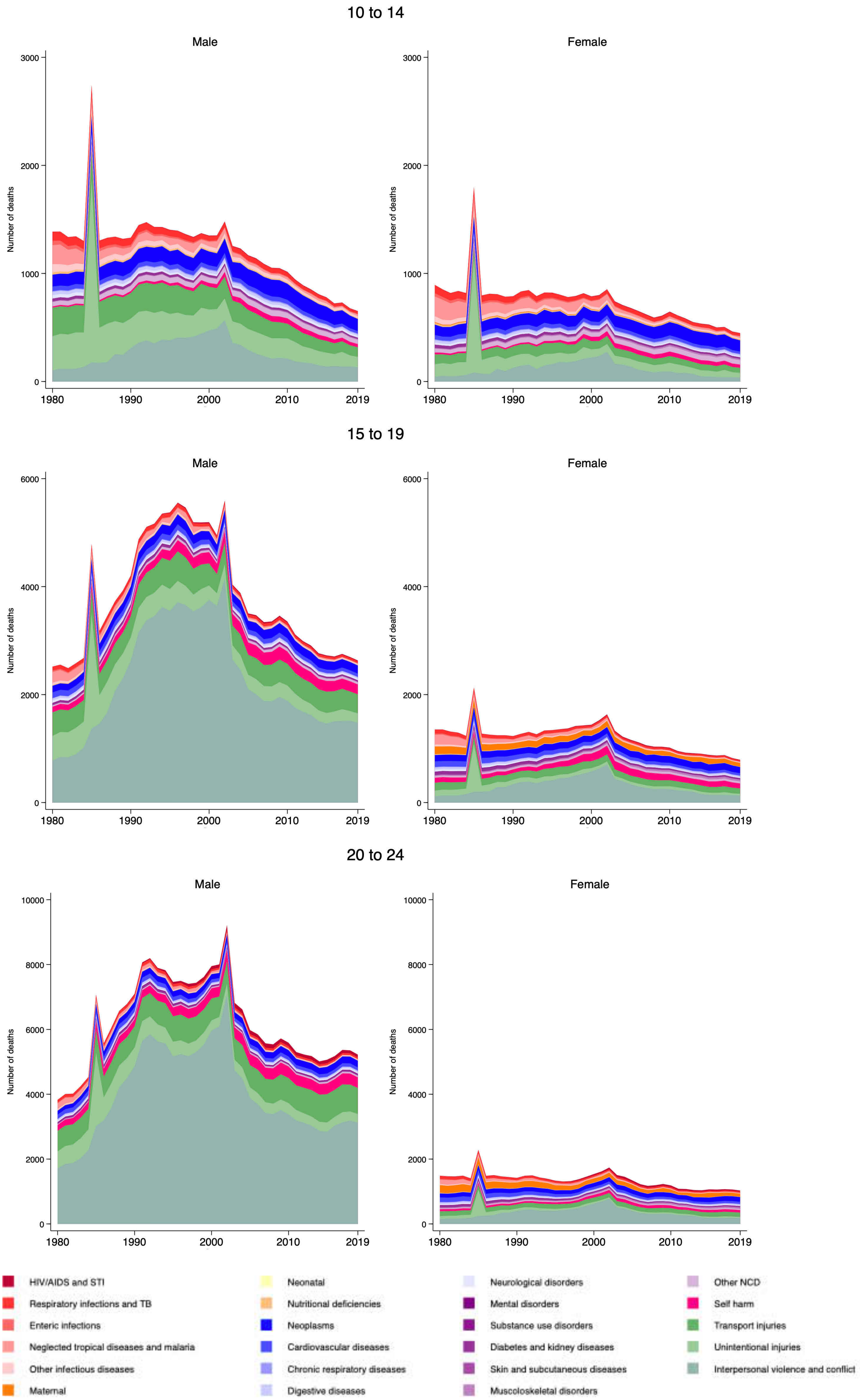

Figure S124: Costa Rica

Figure S124a All-cause mortality rate per 100,000 in 10-24 1950 – 2019: Costa Rica

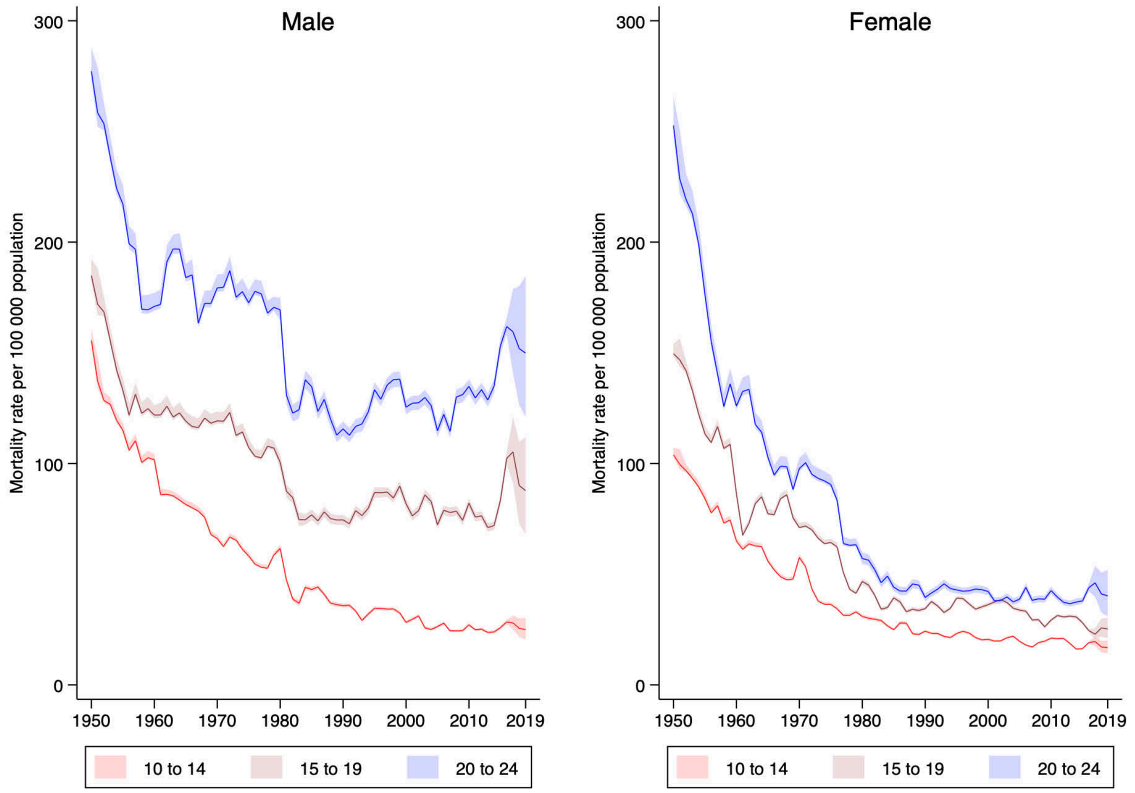

Figure S124b Number of deaths from all causes in 10-24 1950 – 2019: Costa Rica

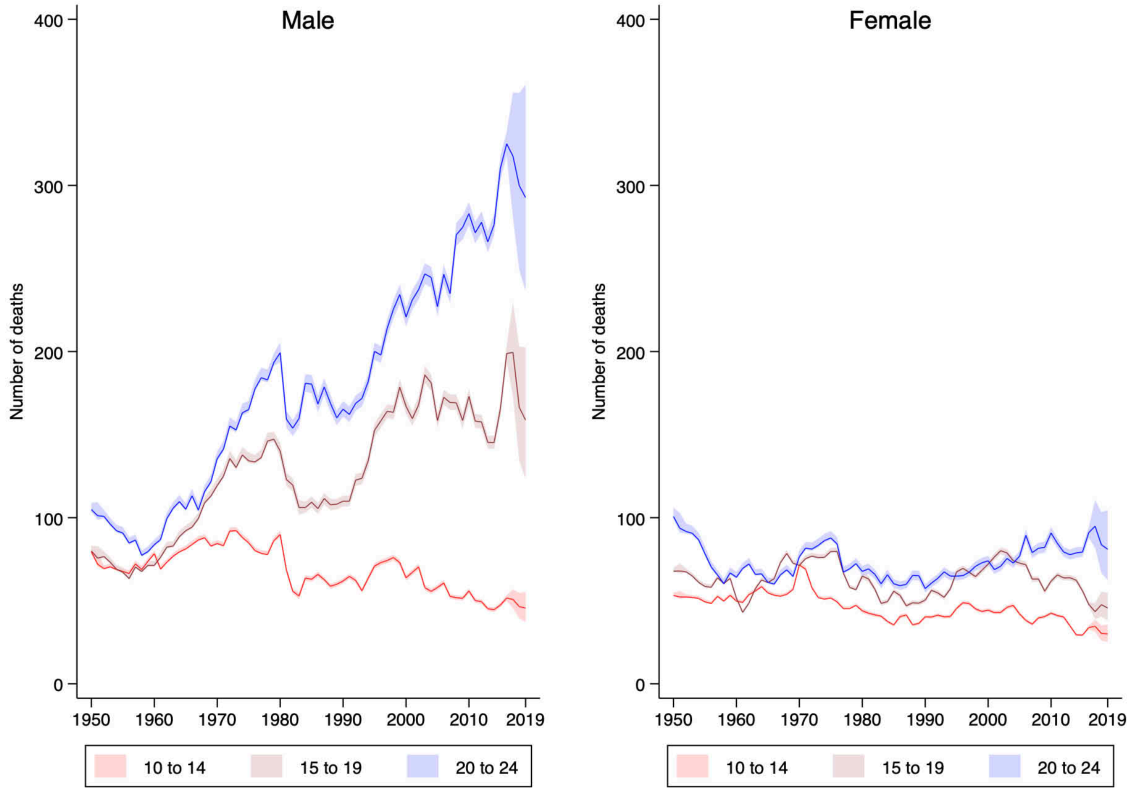

Figure S124c Mortality rate per 100,000 in 10-24 1980 – 2019 by cause: Costa Rica

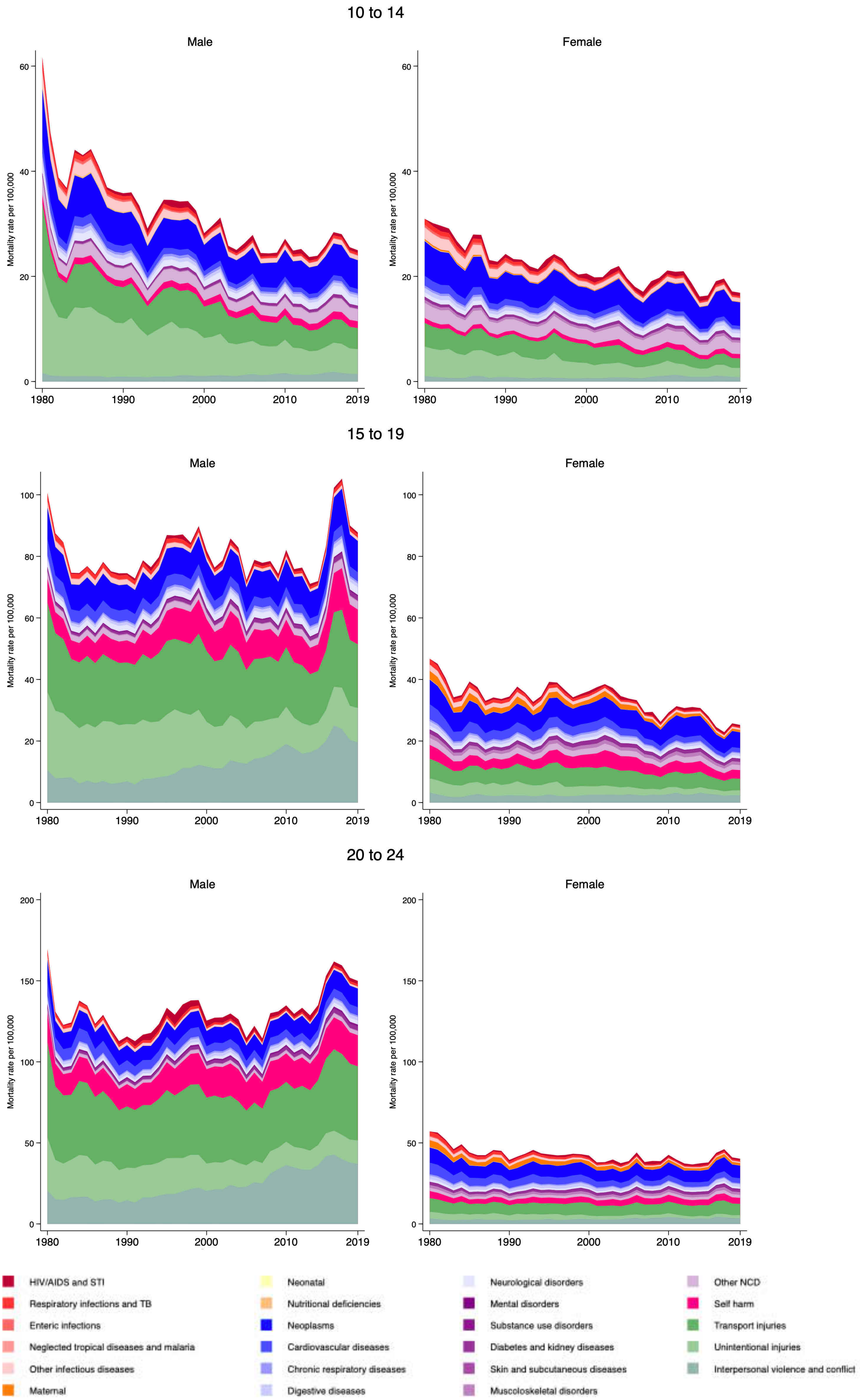

Figure S124d Number of deaths in 10-24 1980 – 2019 by cause: Costa Rica

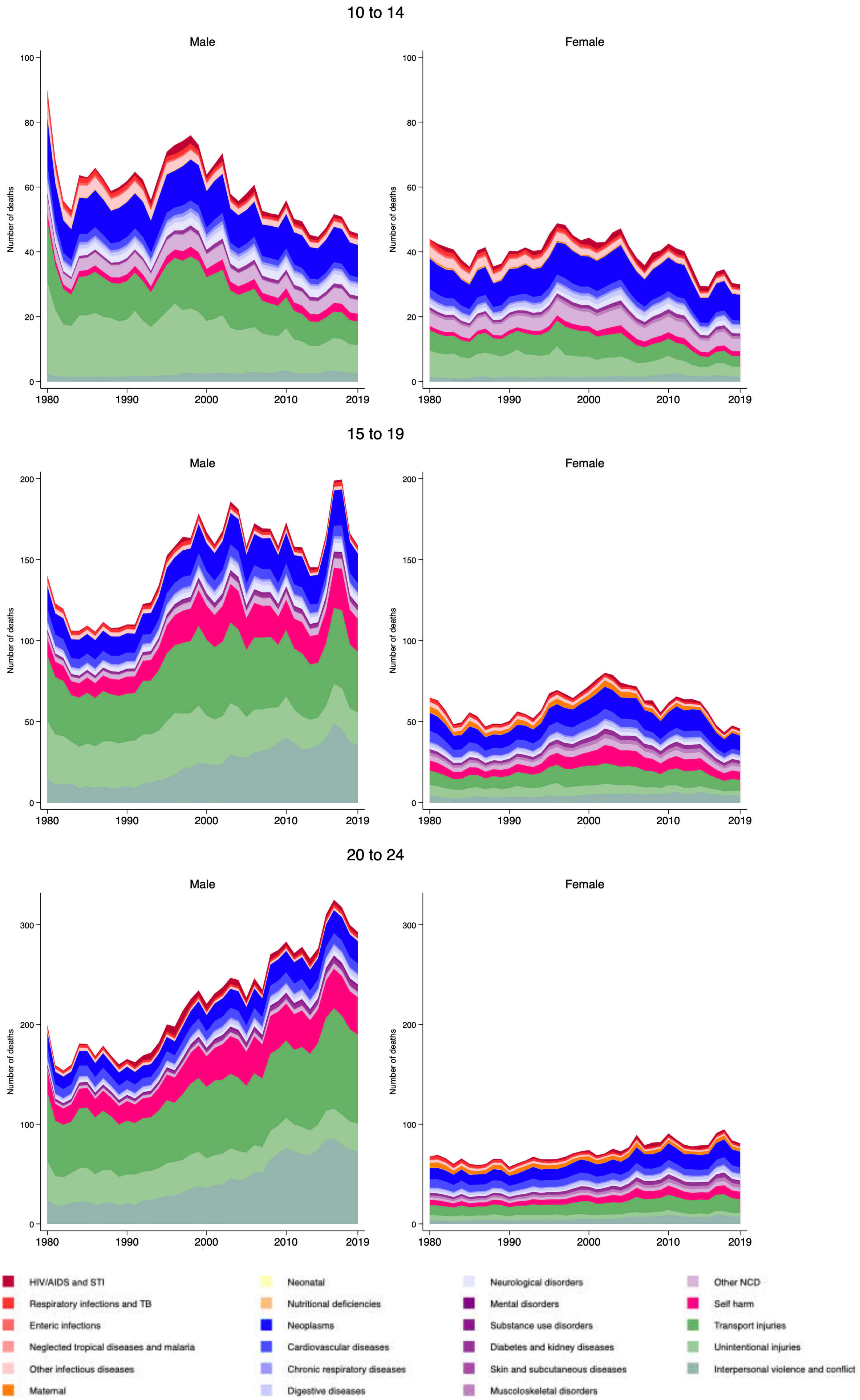

Figure S125: El Salvador

Figure S125a All-cause mortality rate per 100,000 in 10-24 1950 – 2019: El Salvador

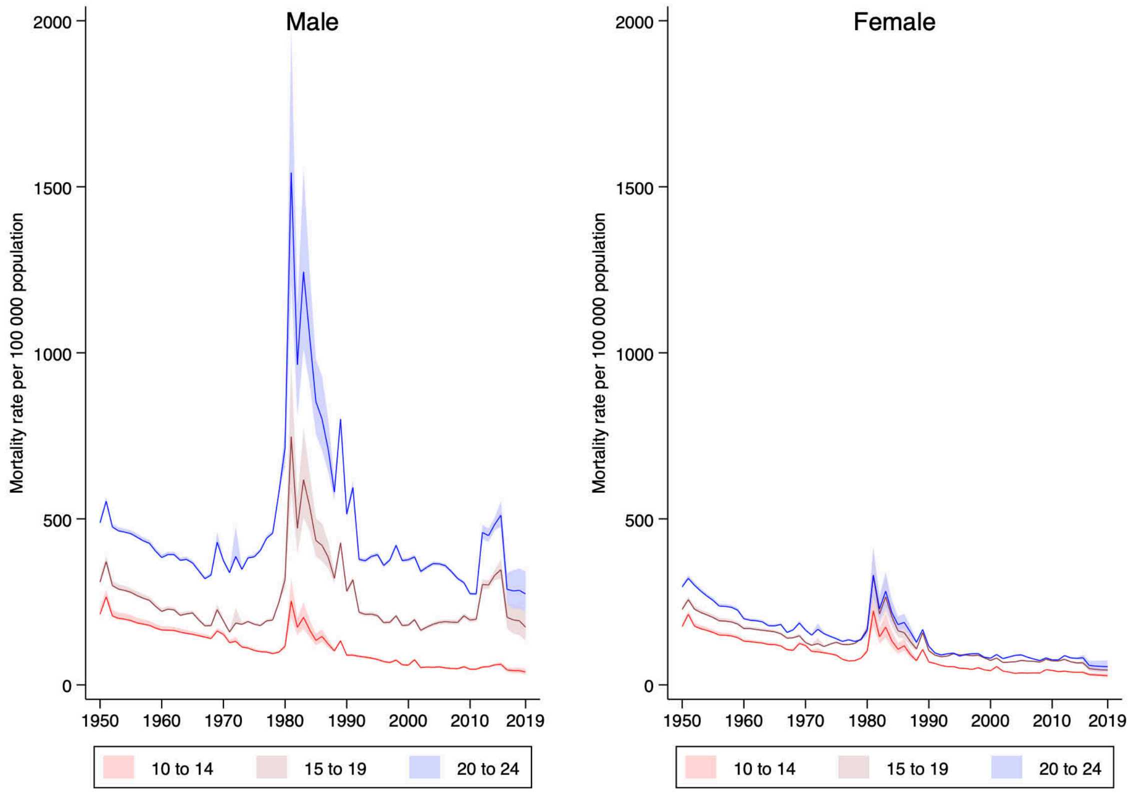

Figure S125b Number of deaths from all causes in 10-24 1950 – 2019: El Salvador

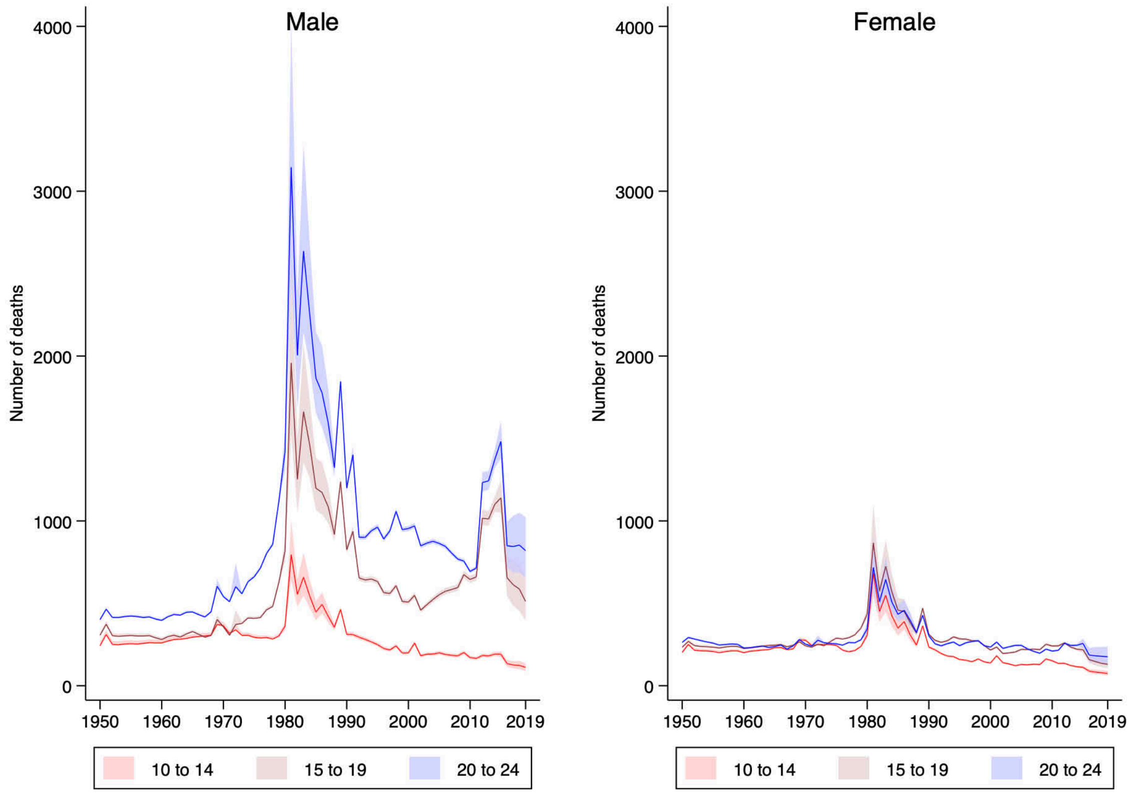

Figure S125c Mortality rate per 100,000 in 10-24 1980 – 2019 by cause: El Salvador

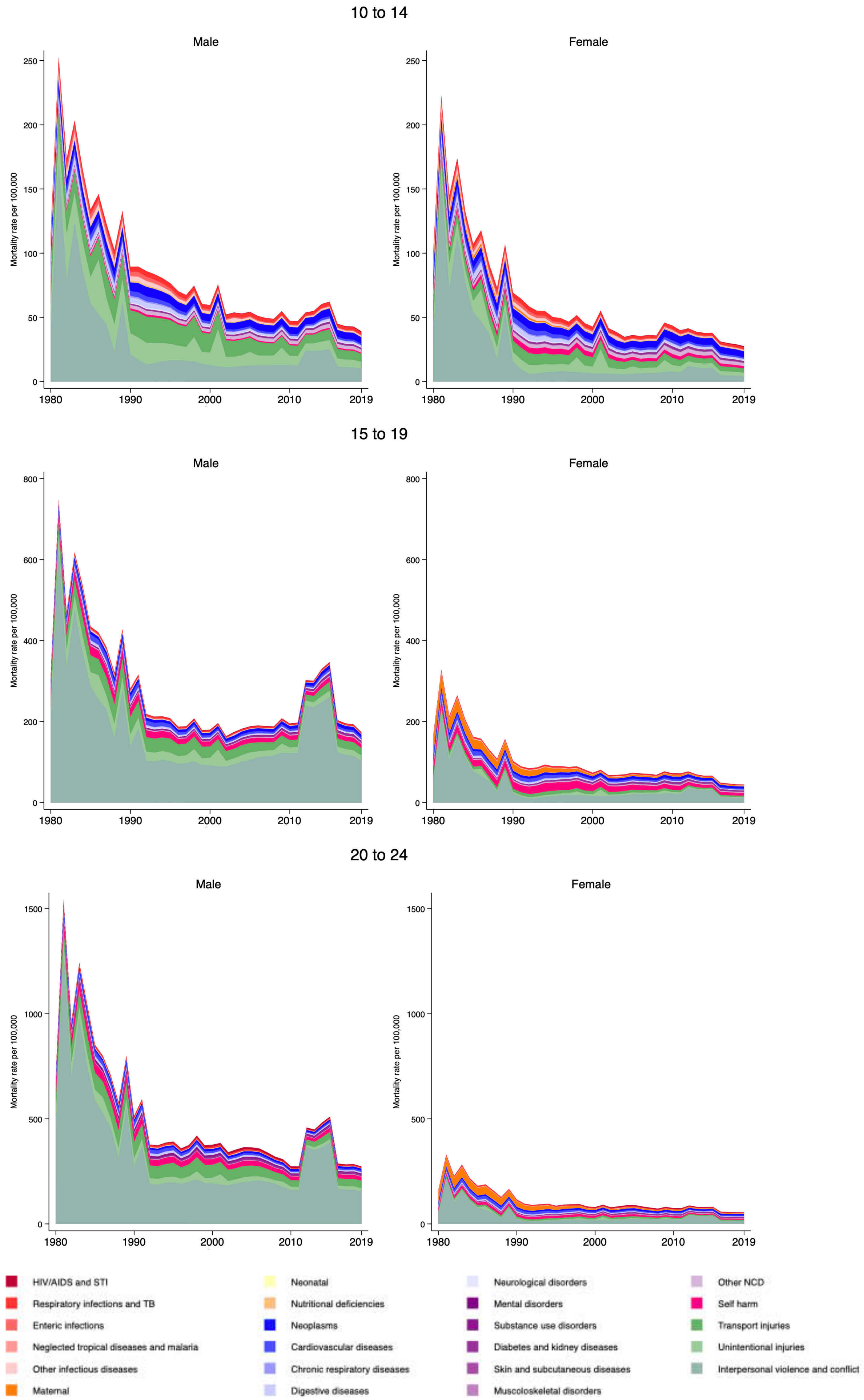

Figure S125d Number of deaths in 10-24 1980 – 2019 by cause: El Salvador

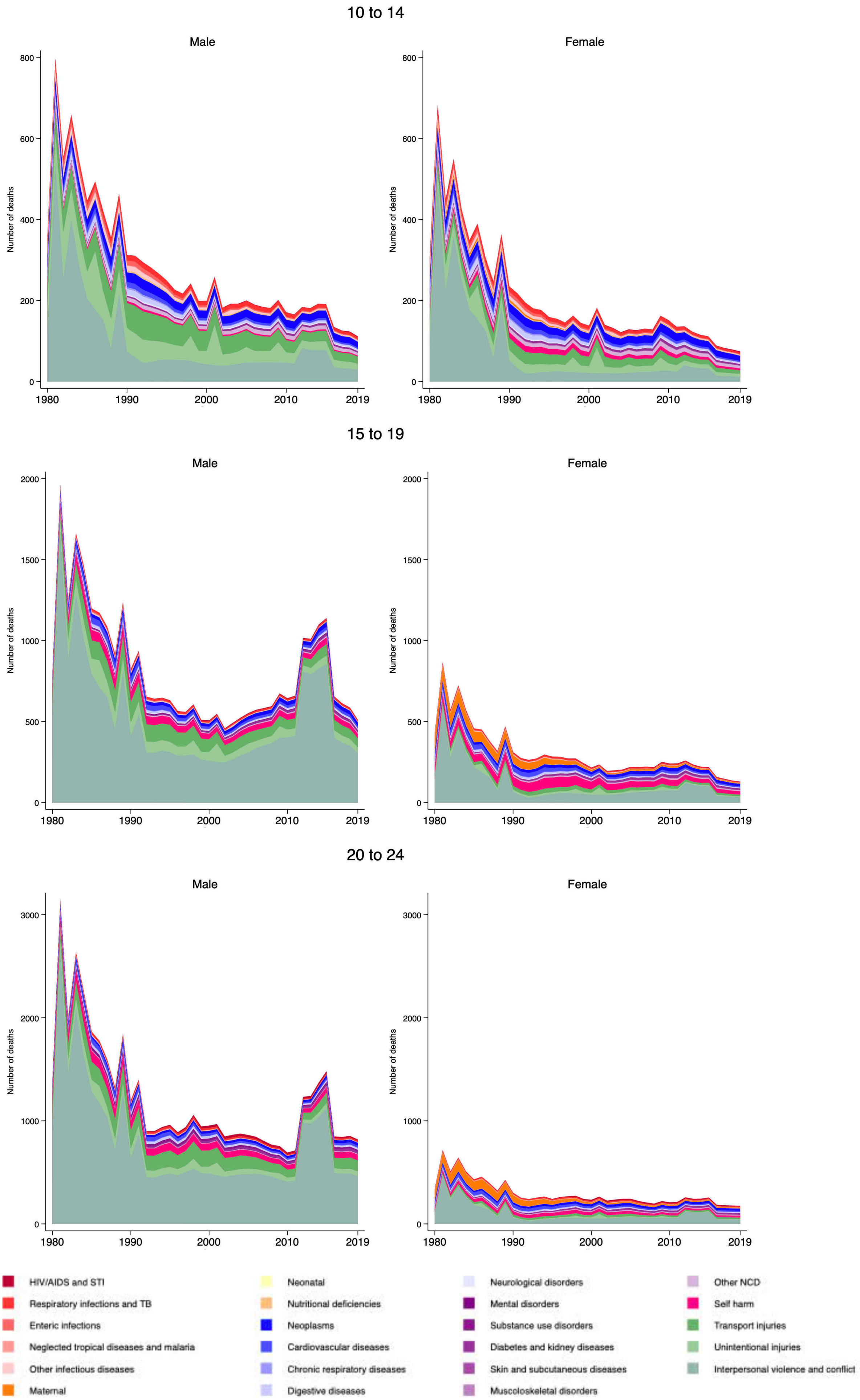

Figure S126: Guatemala

Figure S126a All-cause mortality rate per 100,000 in 10-24 1950 – 2019: Guatemala

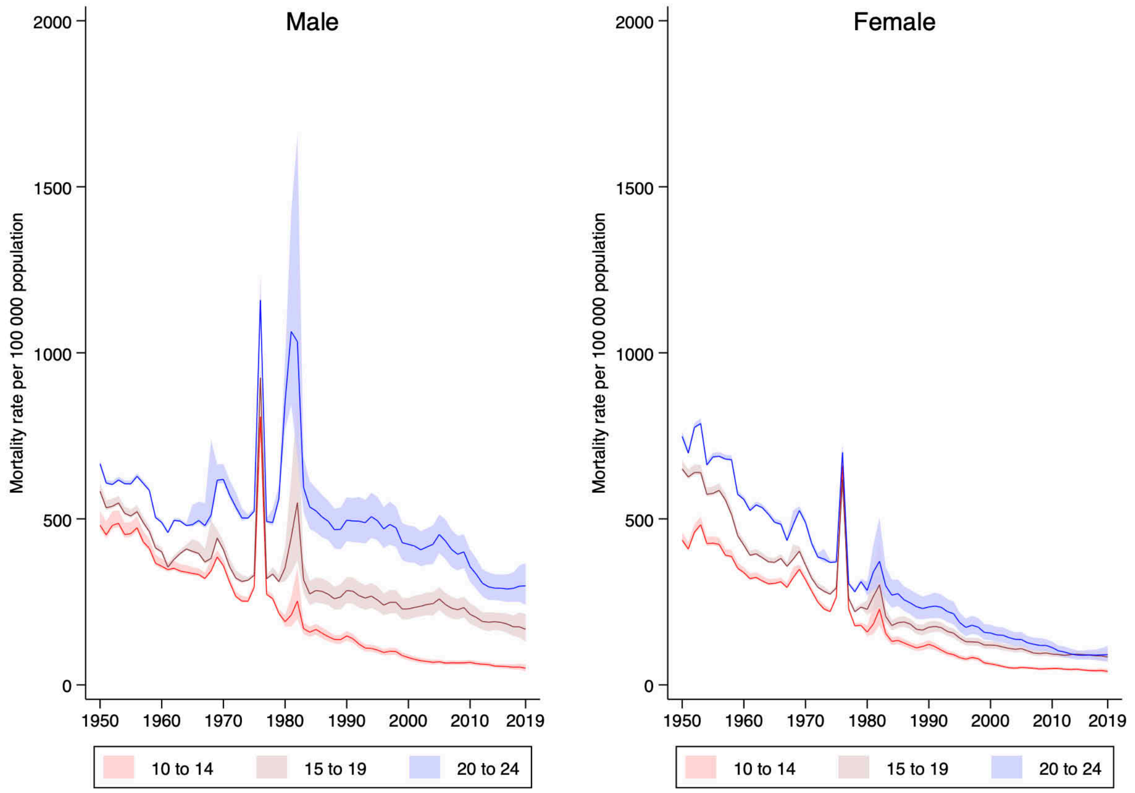

Figure S126b Number of deaths from all causes in 10-24 1950 – 2019: Guatemala

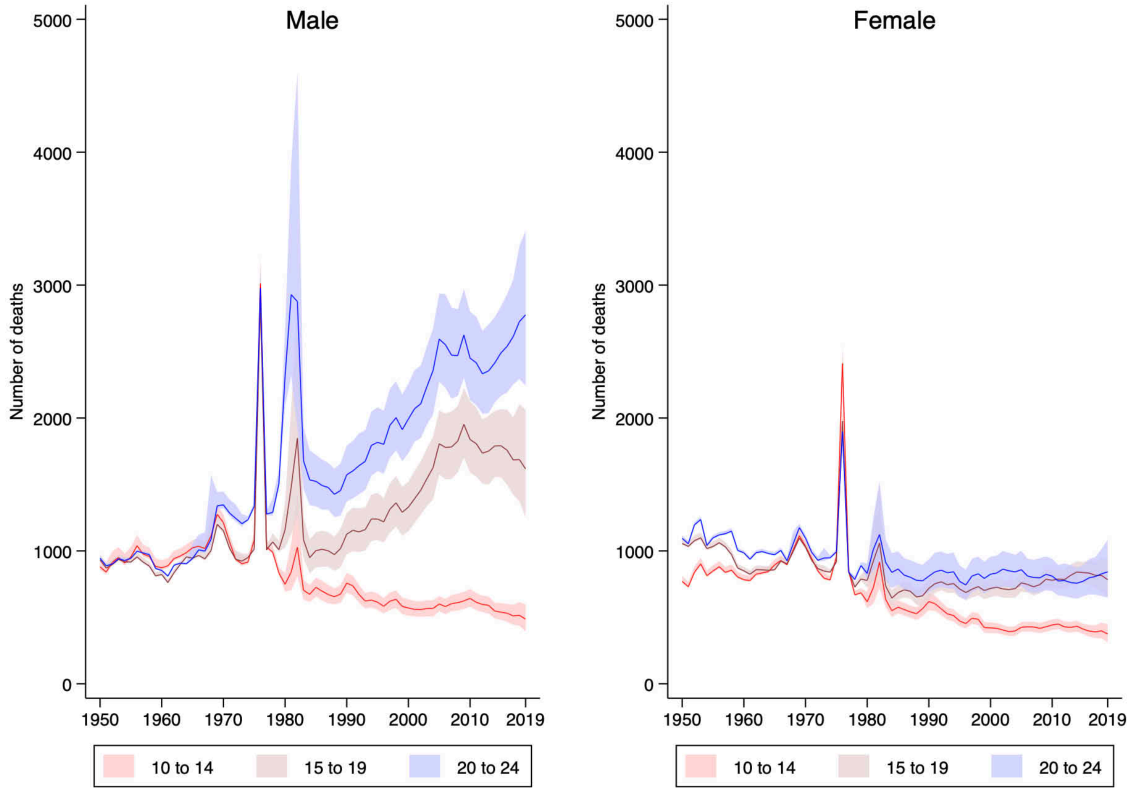

Figure S126c Mortality rate per 100,000 in 10-24 1980 – 2019 by cause: Guatemala

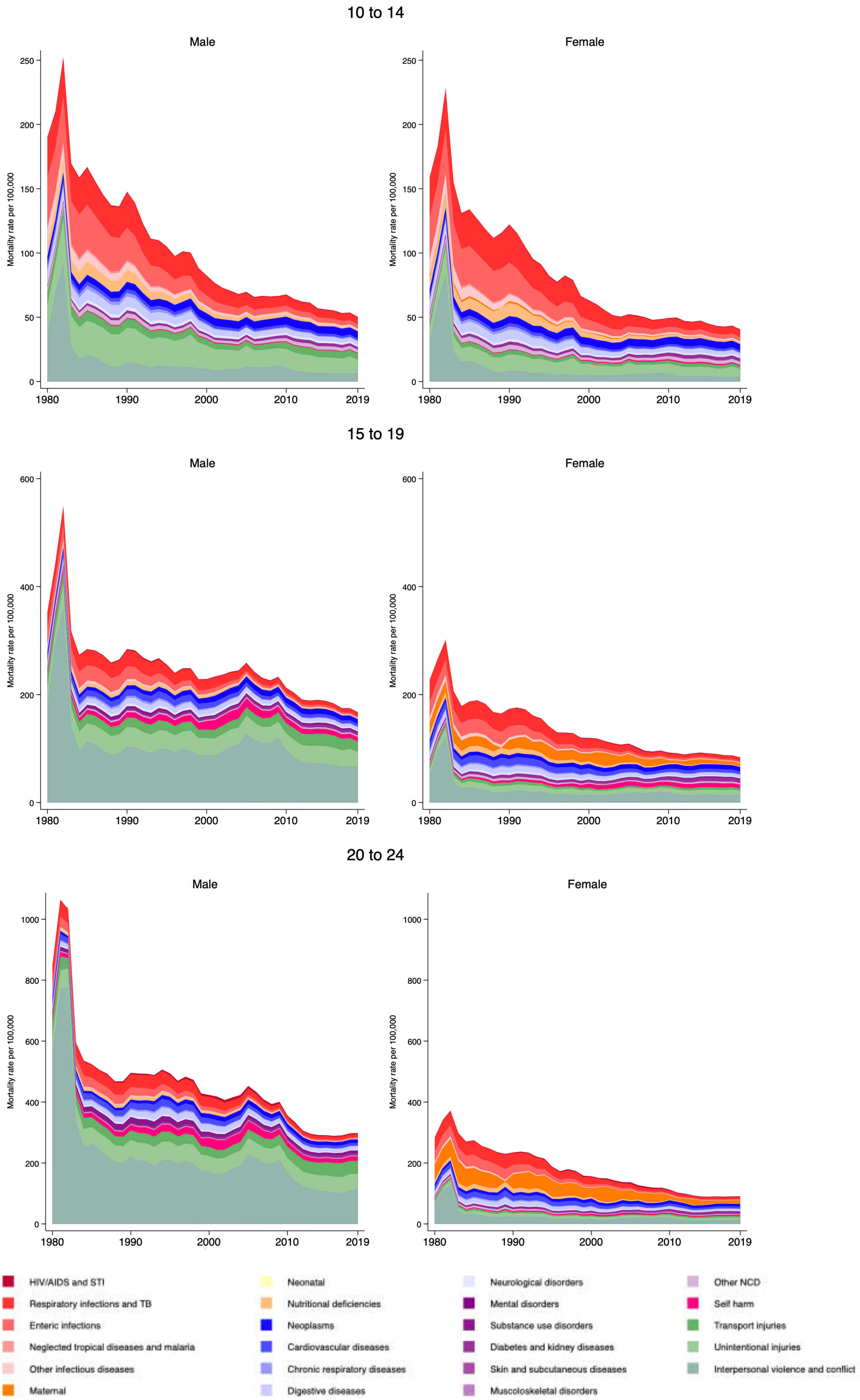

Figure S126d Number of deaths in 10-24 1980 – 2019 by cause: Guatemala

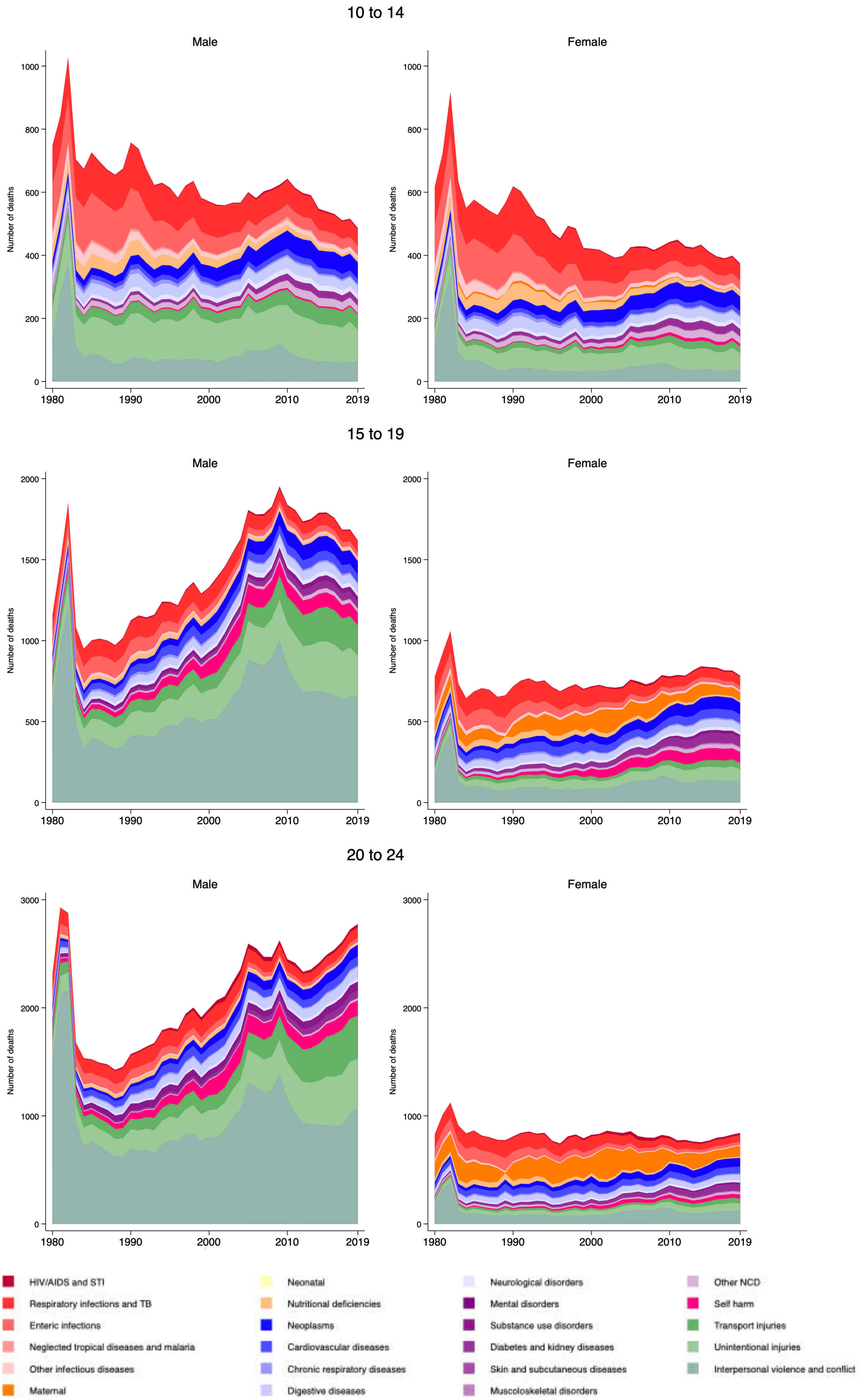

Figure S127: Honduras

Figure S127a All-cause mortality rate per 100,000 in 10-24 1950 – 2019: Honduras

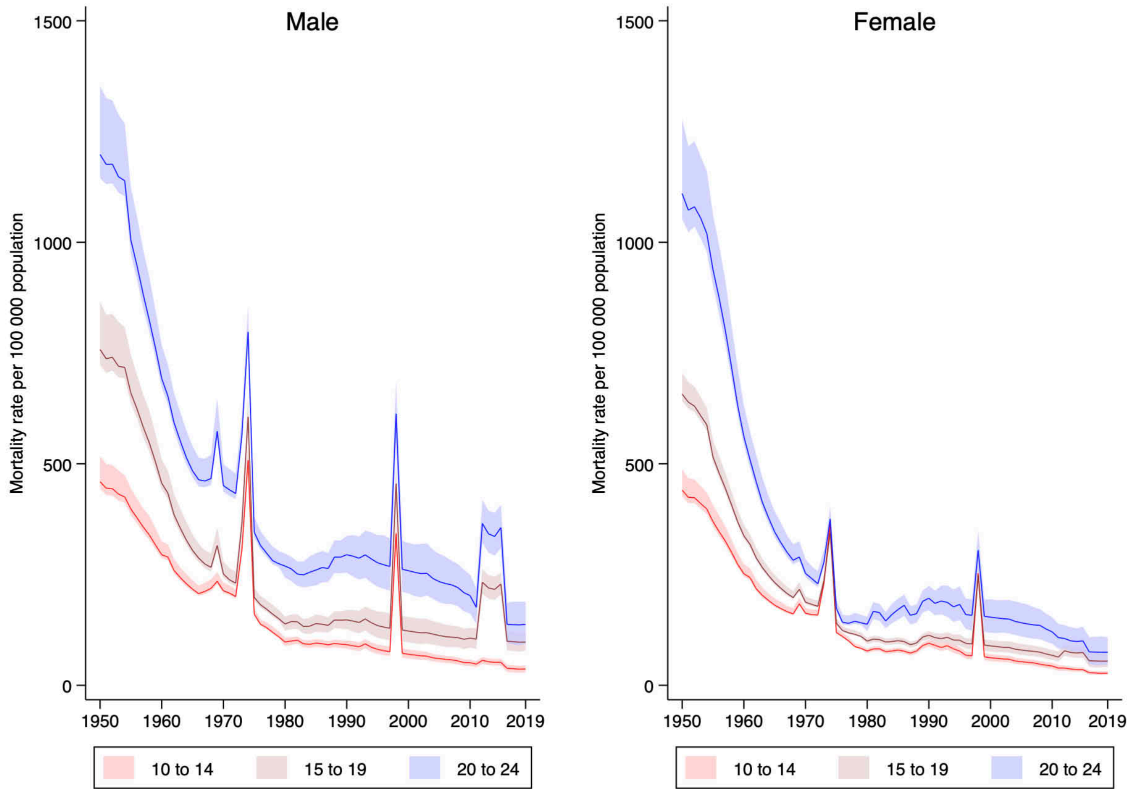

Figure S127b Number of deaths from all causes in 10-24 1950 – 2019: Honduras

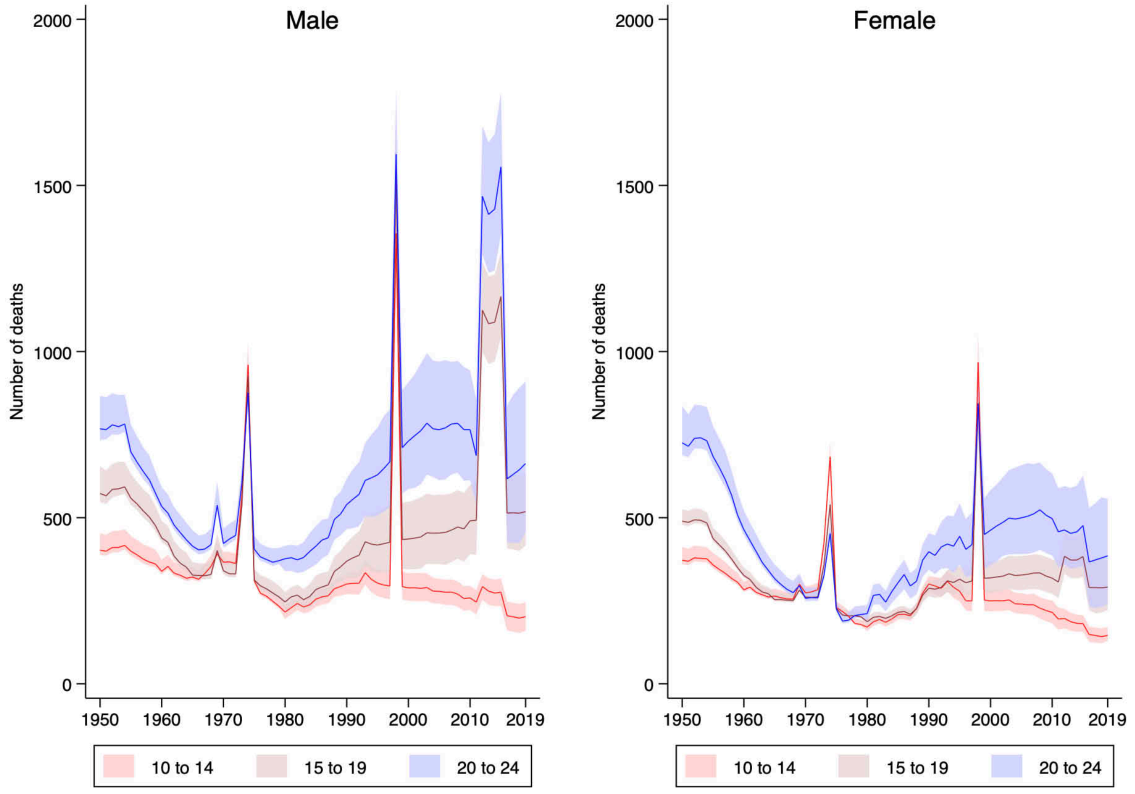

Figure S127c Mortality rate per 100,000 in 10-24 1980 – 2019 by cause: Honduras

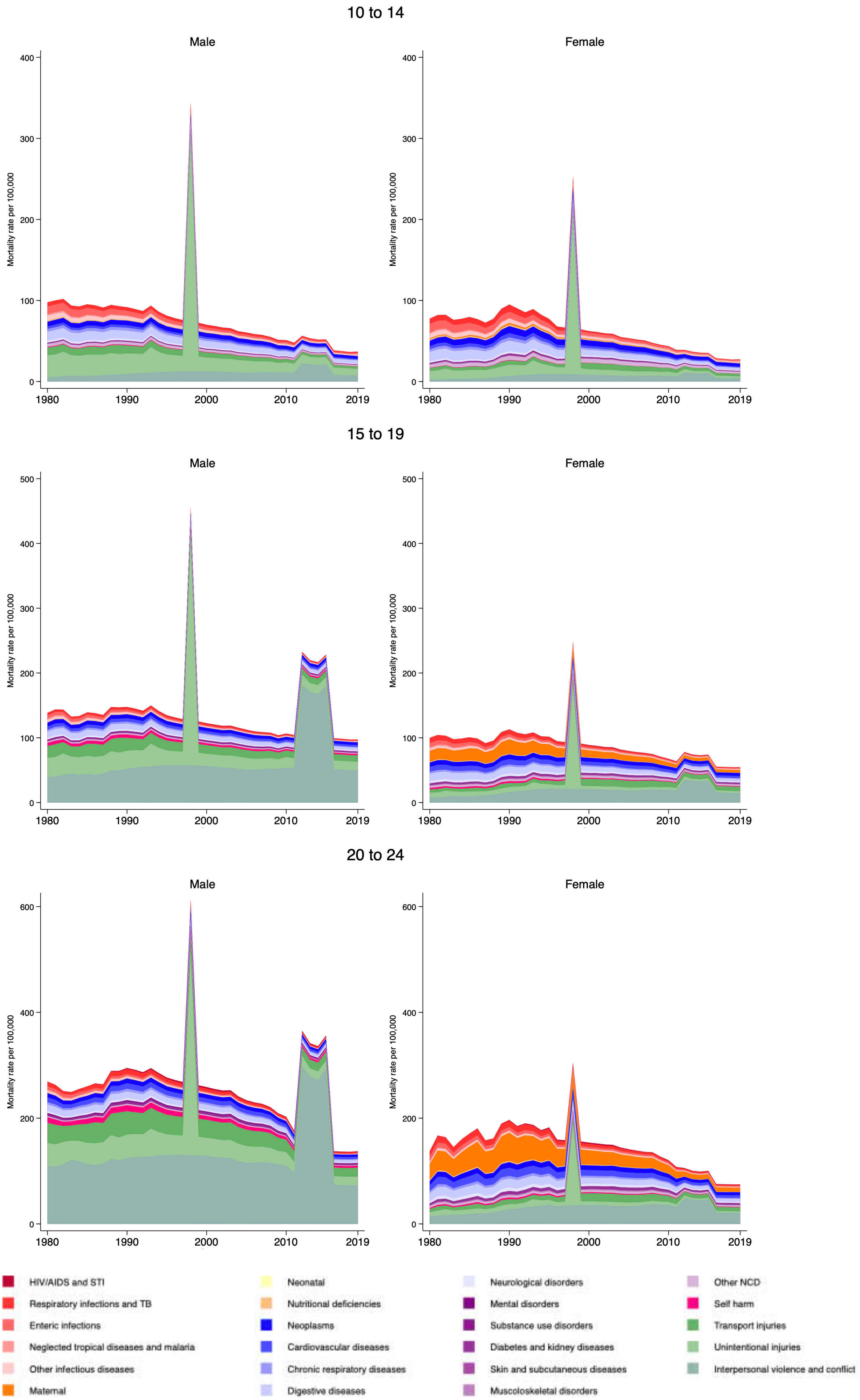

Figure S127d Number of deaths in 10-24 1980 – 2019 by cause: Honduras

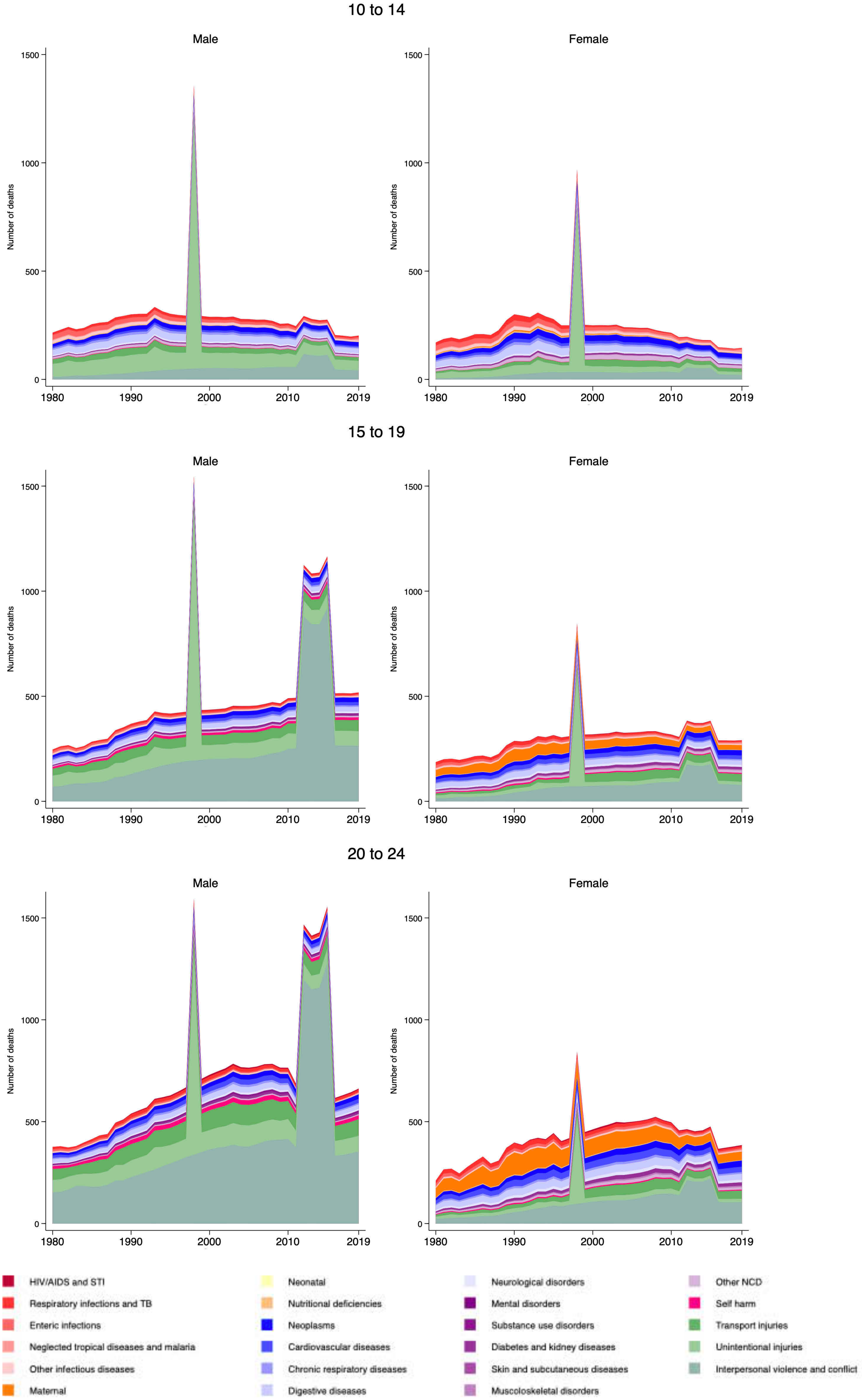

Figure S128: Mexico

Figure S128a All-cause mortality rate per 100,000 in 10-24 1950 – 2019: Mexico

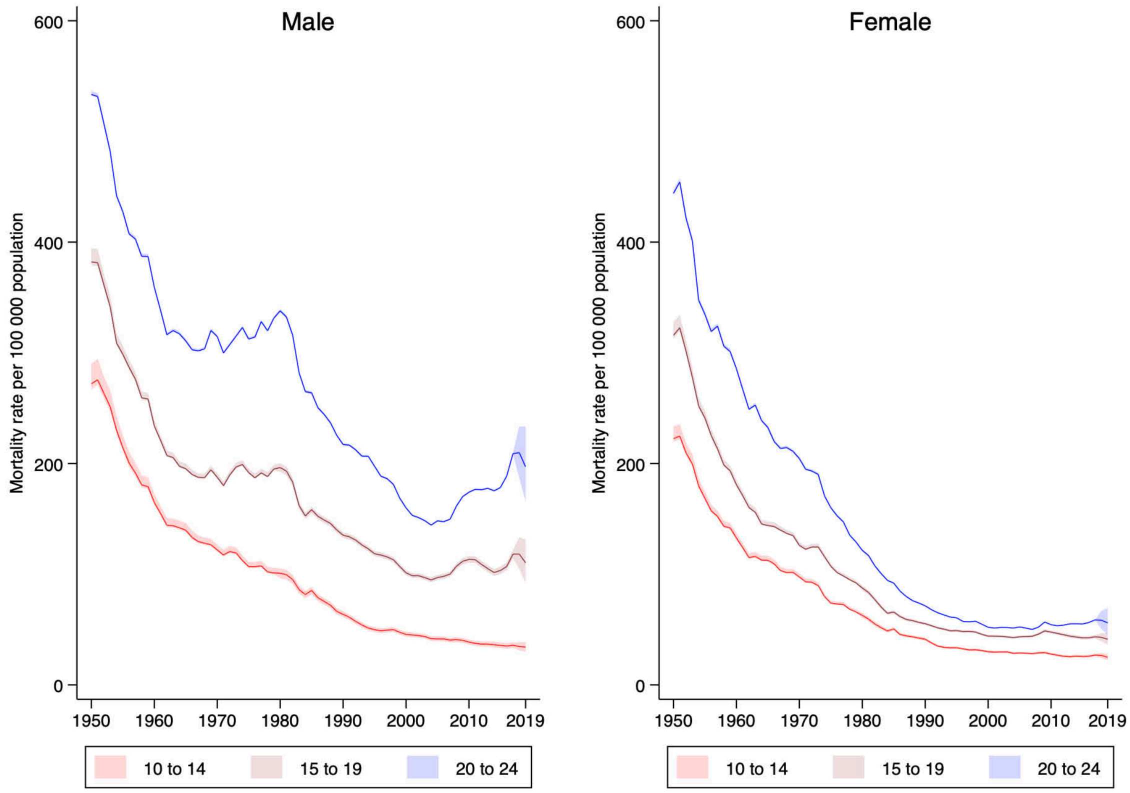

Figure S128b Number of deaths from all causes in 10-24 1950 – 2019: Mexico

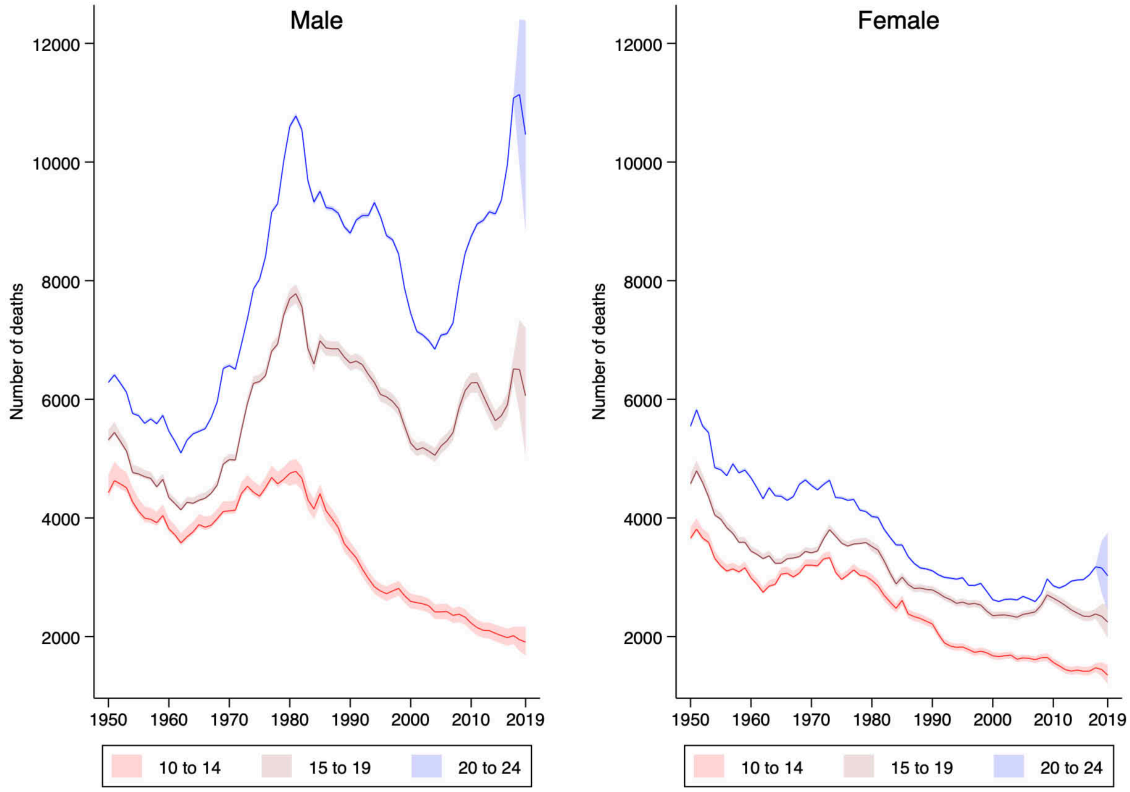

Figure S128c Mortality rate per 100,000 in 10-24 1980 – 2019 by cause: Mexico

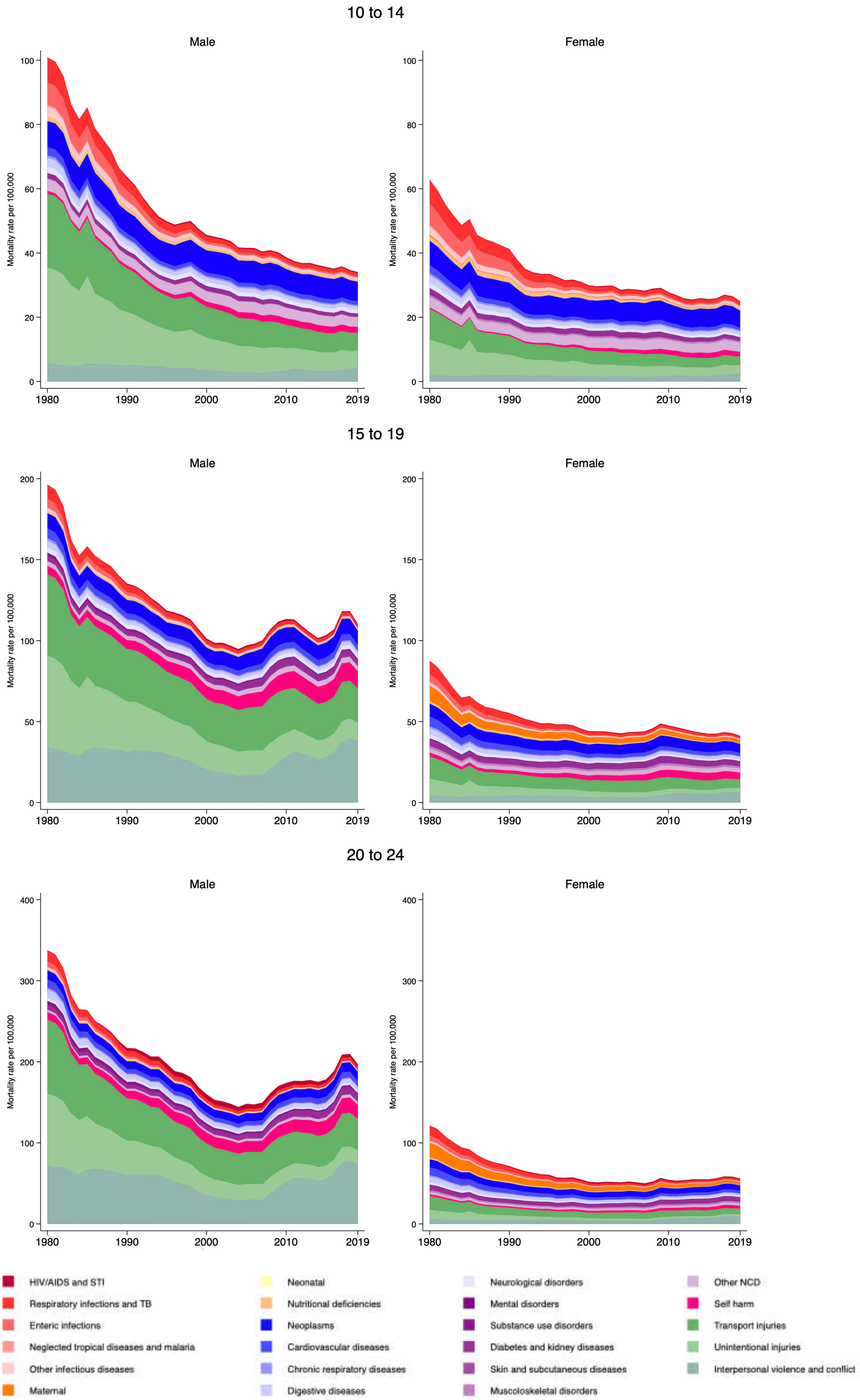

Figure S128d Number of deaths in 10-24 1980 – 2019 by cause: Mexico

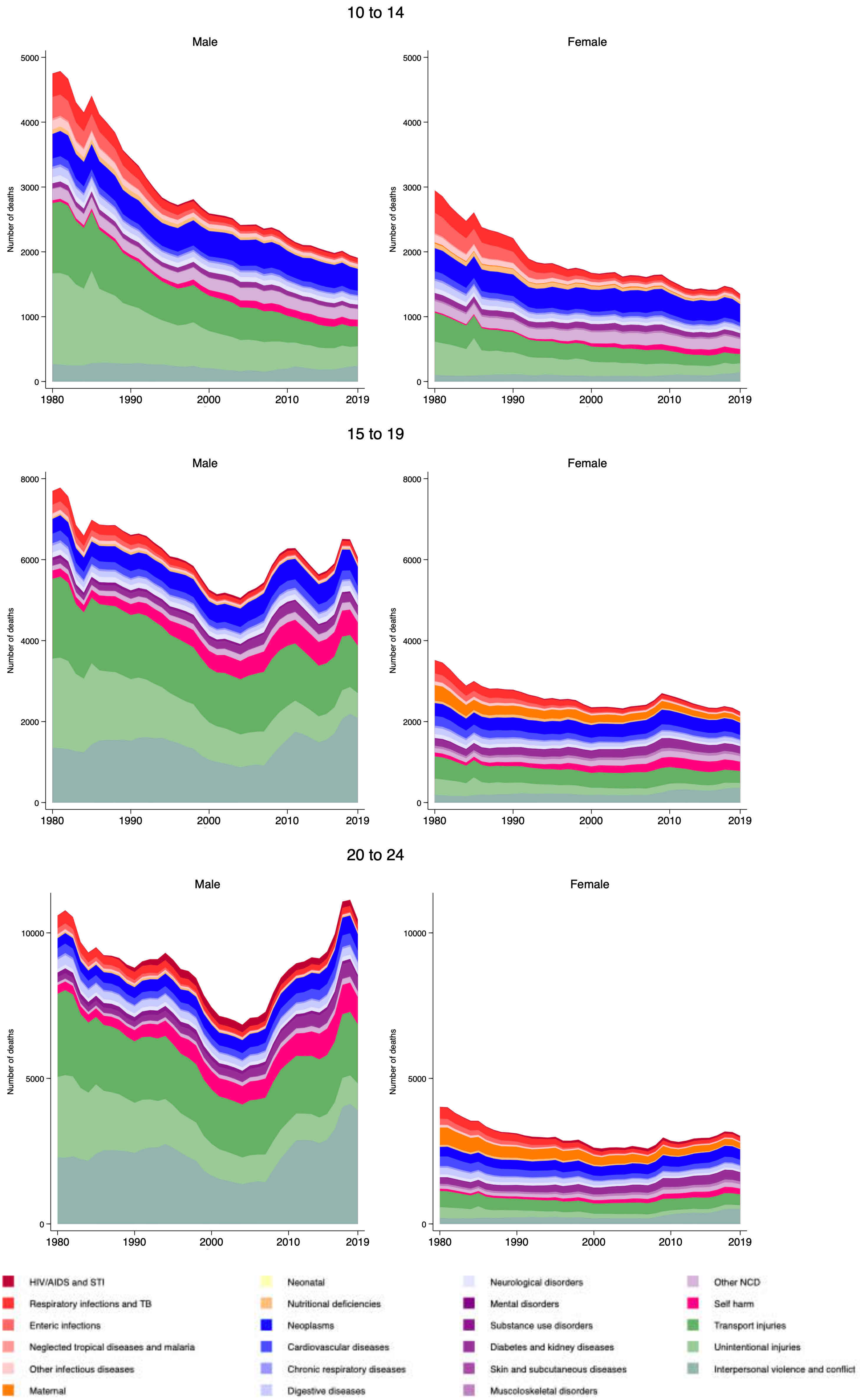

Figure S129: Nicaragua

Figure S129a All-cause mortality rate per 100,000 in 10-24 1950 – 2019: Nicaragua

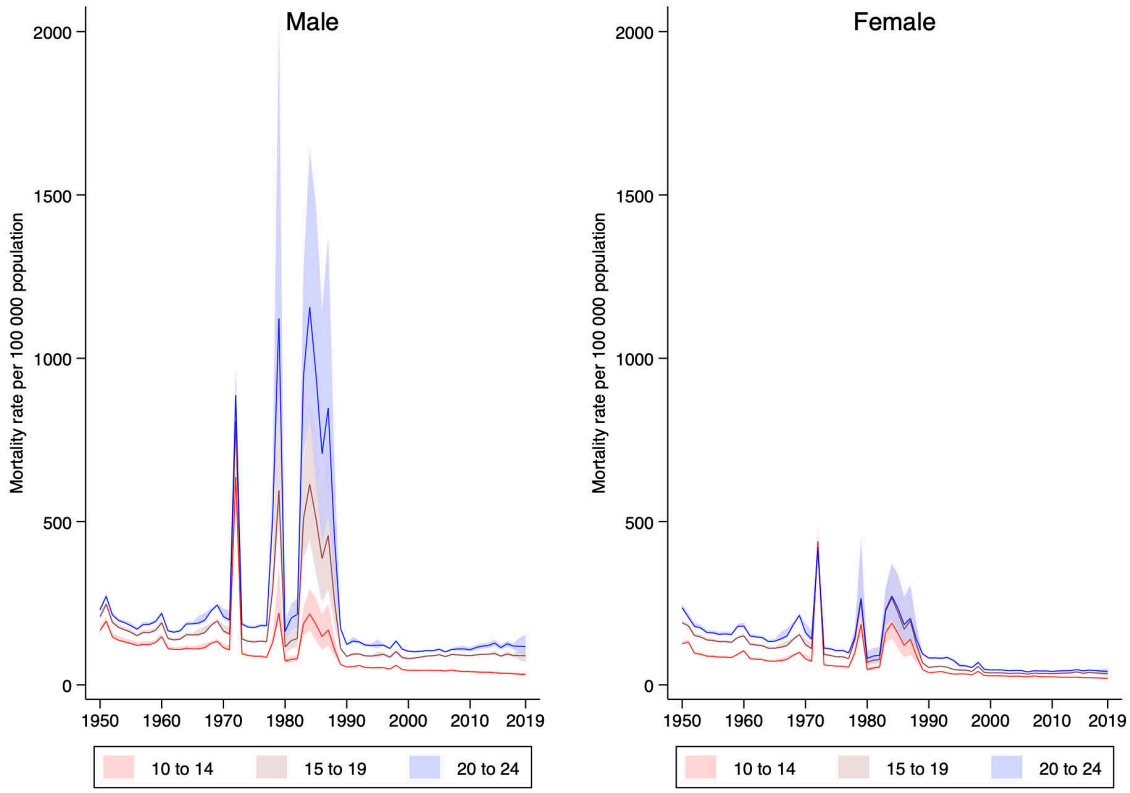

Figure S129b Number of deaths from all causes in 10-24 1950 – 2019: Nicaragua

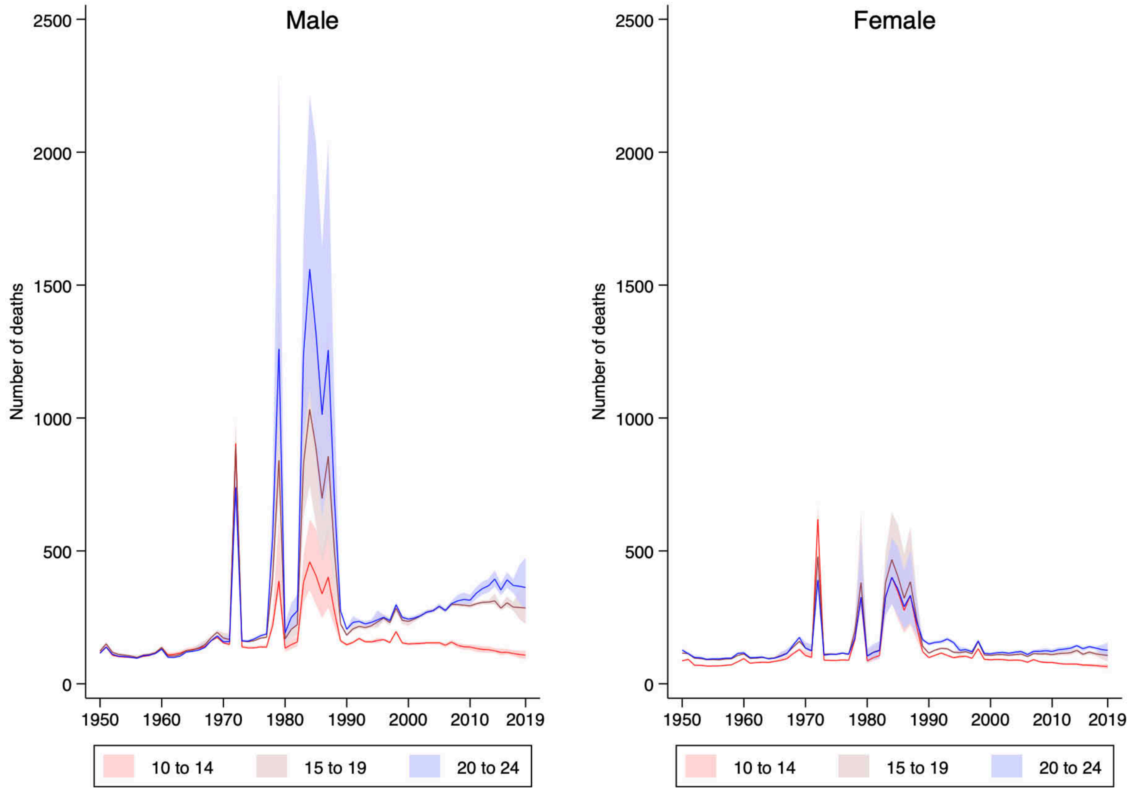

Figure S129c Mortality rate per 100,000 in 10-24 1980 – 2019 by cause: Nicaragua

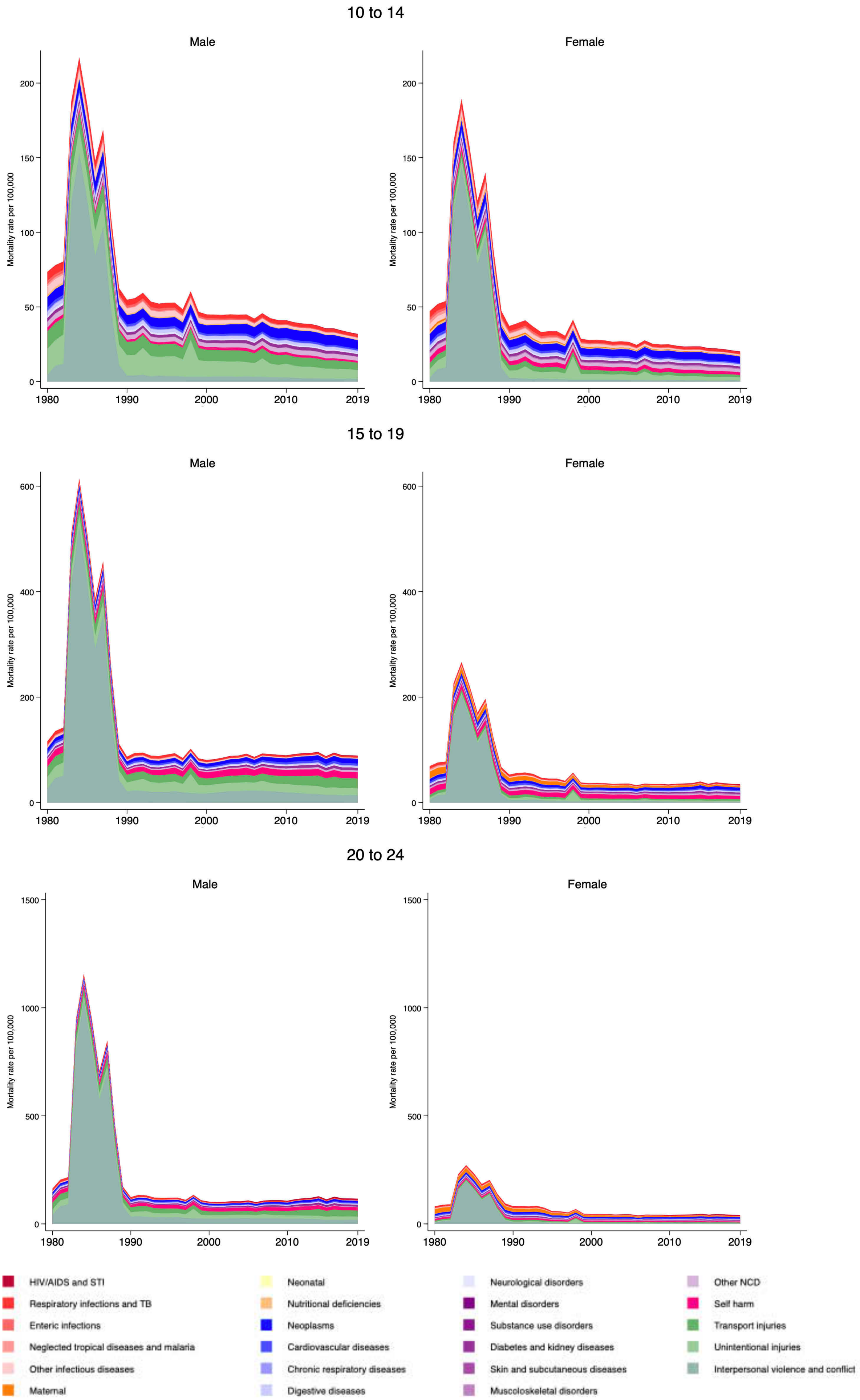

Figure S129d Number of deaths in 10-24 1980 – 2019 by cause: Nicaragua

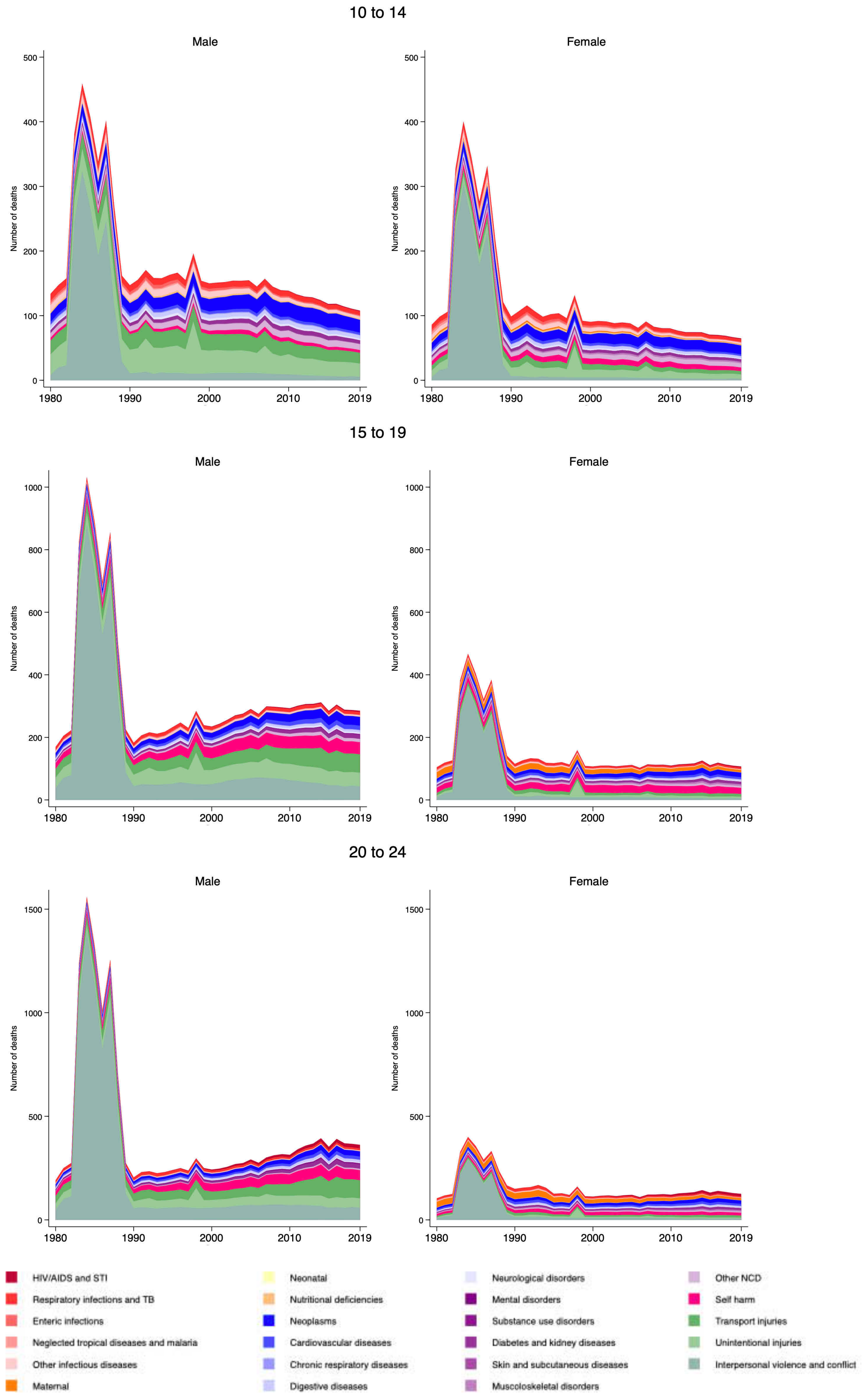

Figure S130: Panama

Figure S130a All-cause mortality rate per 100,000 in 10-24 1950 – 2019: Panama

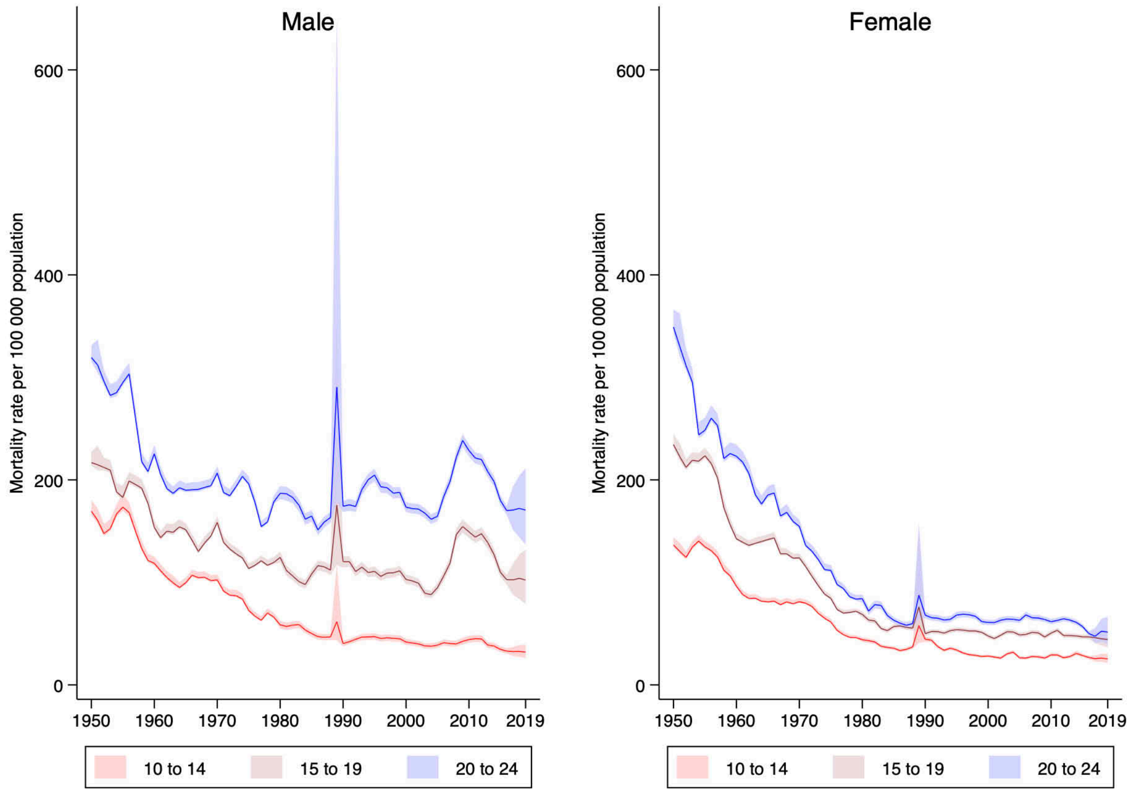

Figure S130b Number of deaths from all causes in 10-24 1950 – 2019: Panama

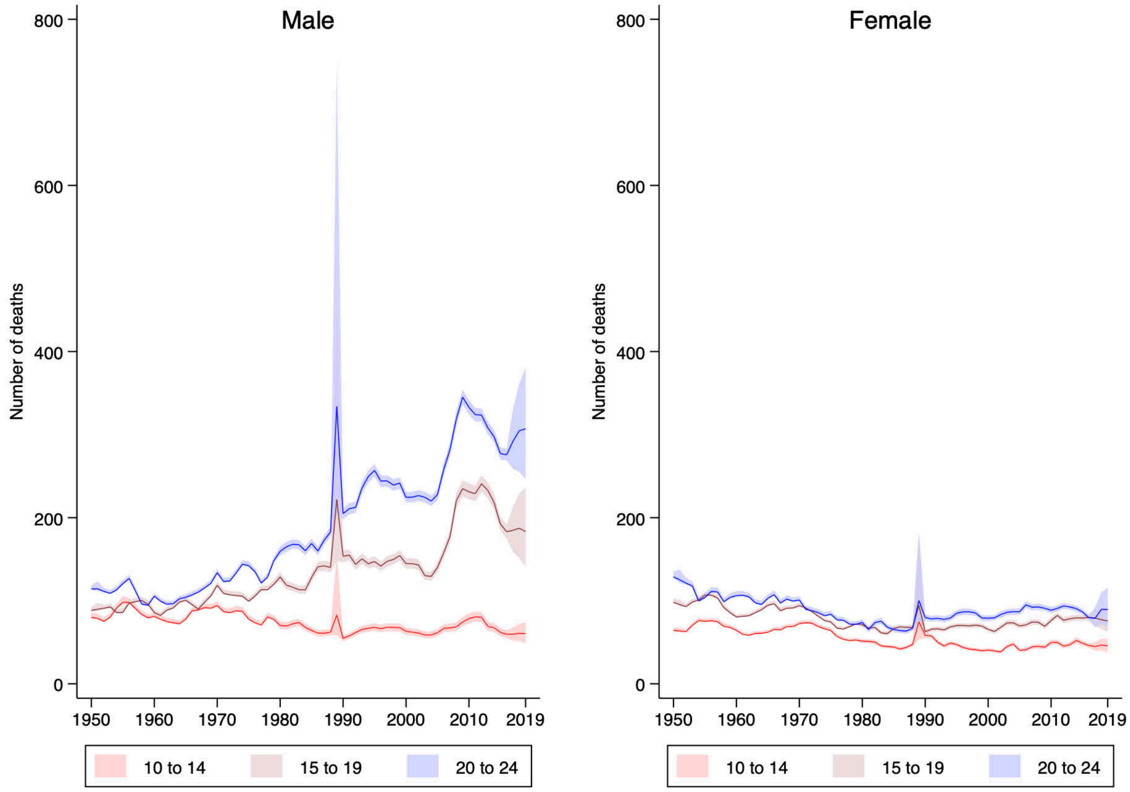

Figure S130c Mortality rate per 100,000 in 10-24 1980 – 2019 by cause: Panama

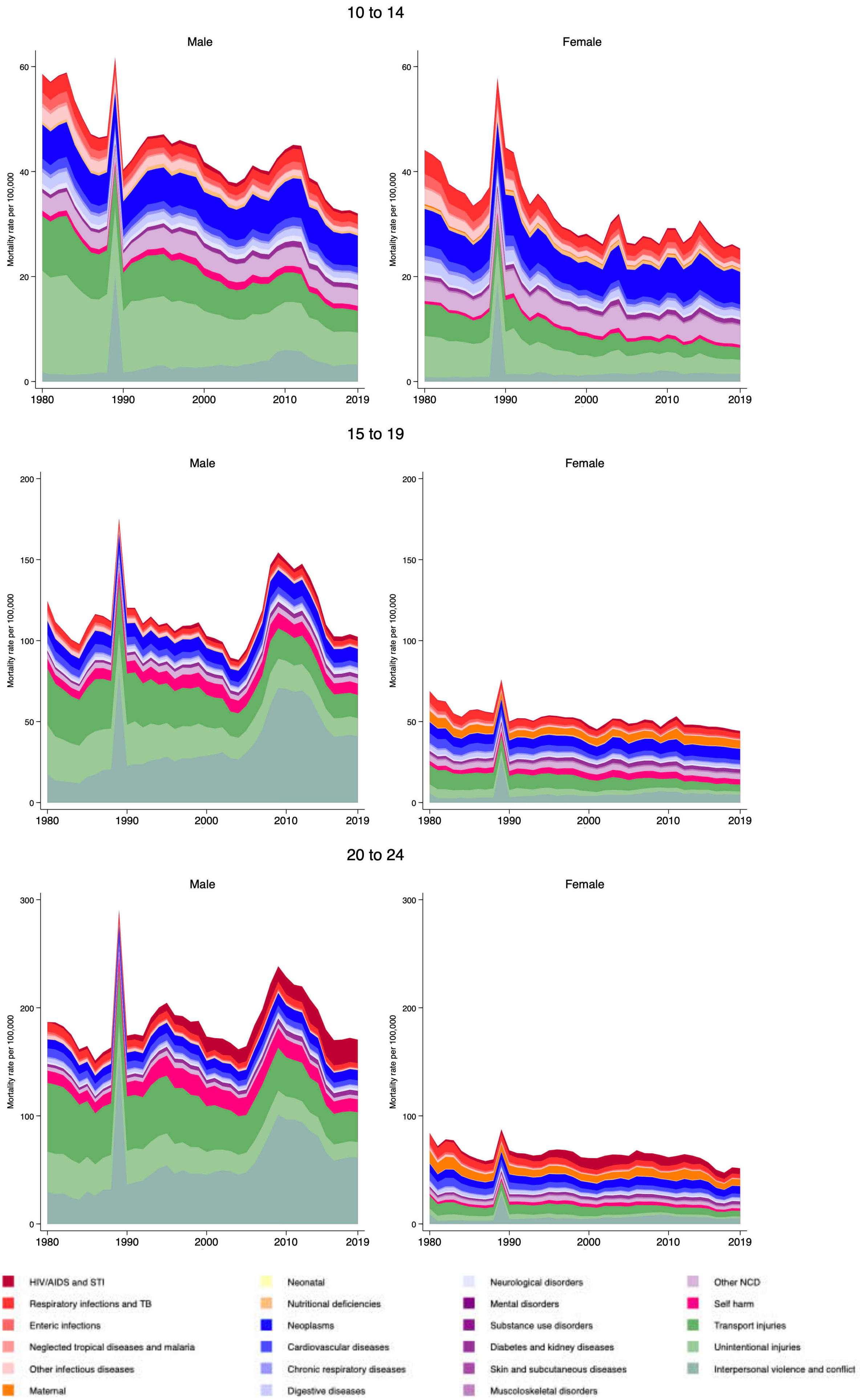

Figure S130d Number of deaths in 10-24 1980 – 2019 by cause: Panama

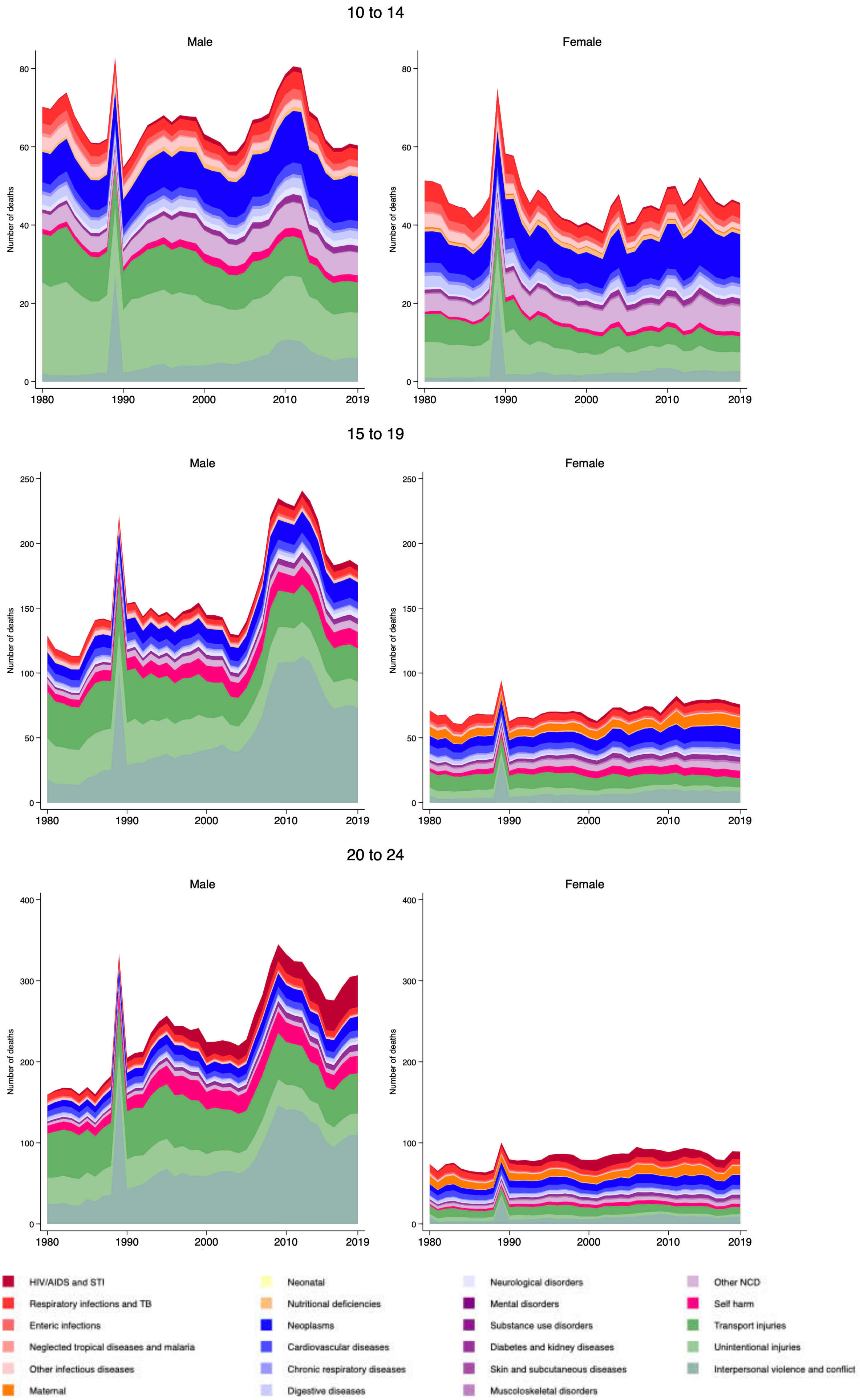

Figure S131: Venezuela (Bolivarian Republic of)

Figure S131a All-cause mortality rate per 100,000 in 10-24 1950 – 2019: Venezuela (Bolivarian Republic of)

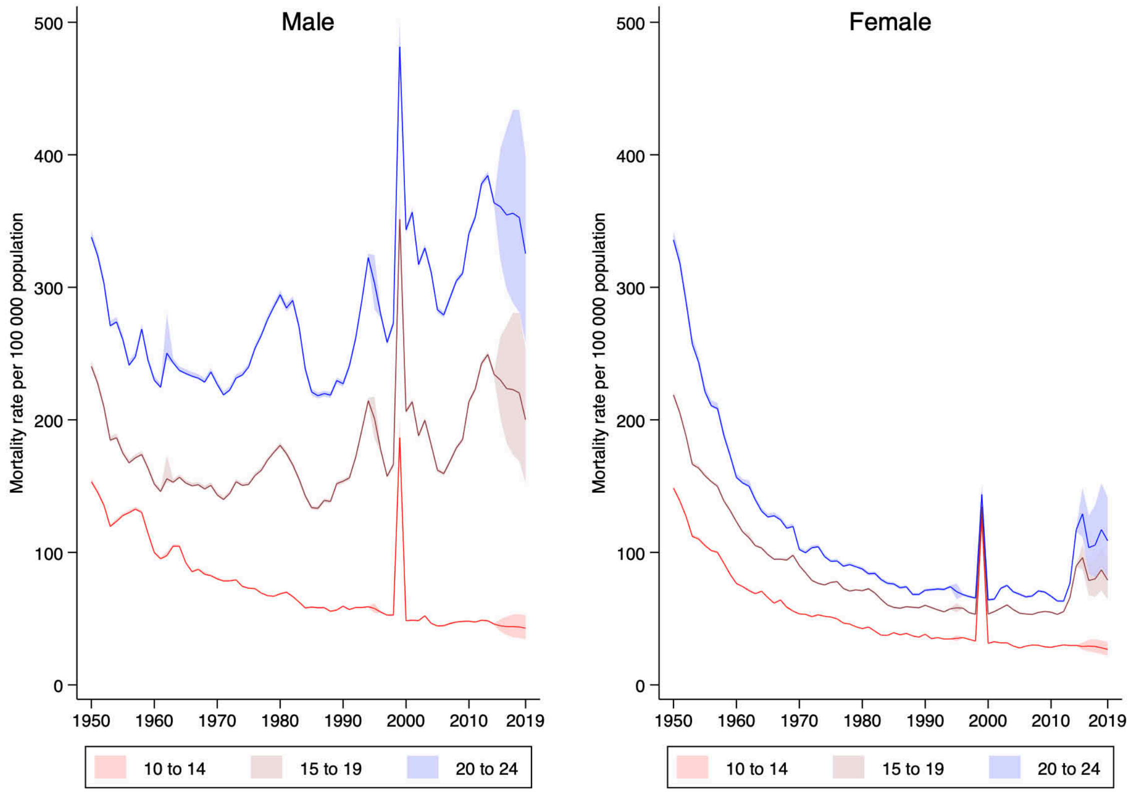

Figure S131b Number of deaths from all causes in 10-24 1950 – 2019: Venezuela (Bolivarian Republic of)

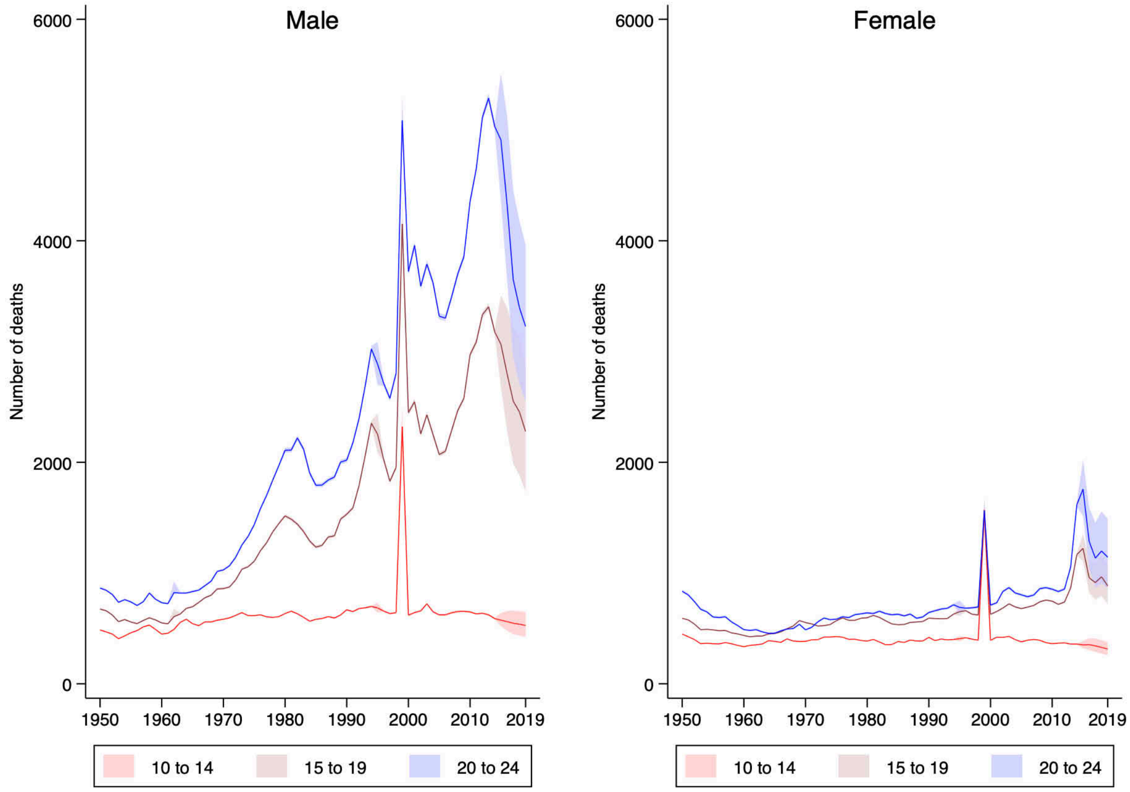

Figure S131c Mortality rate per 100,000 in 10-24 1980 – 2019 by cause: Venezuela (Bolivarian Republic of)

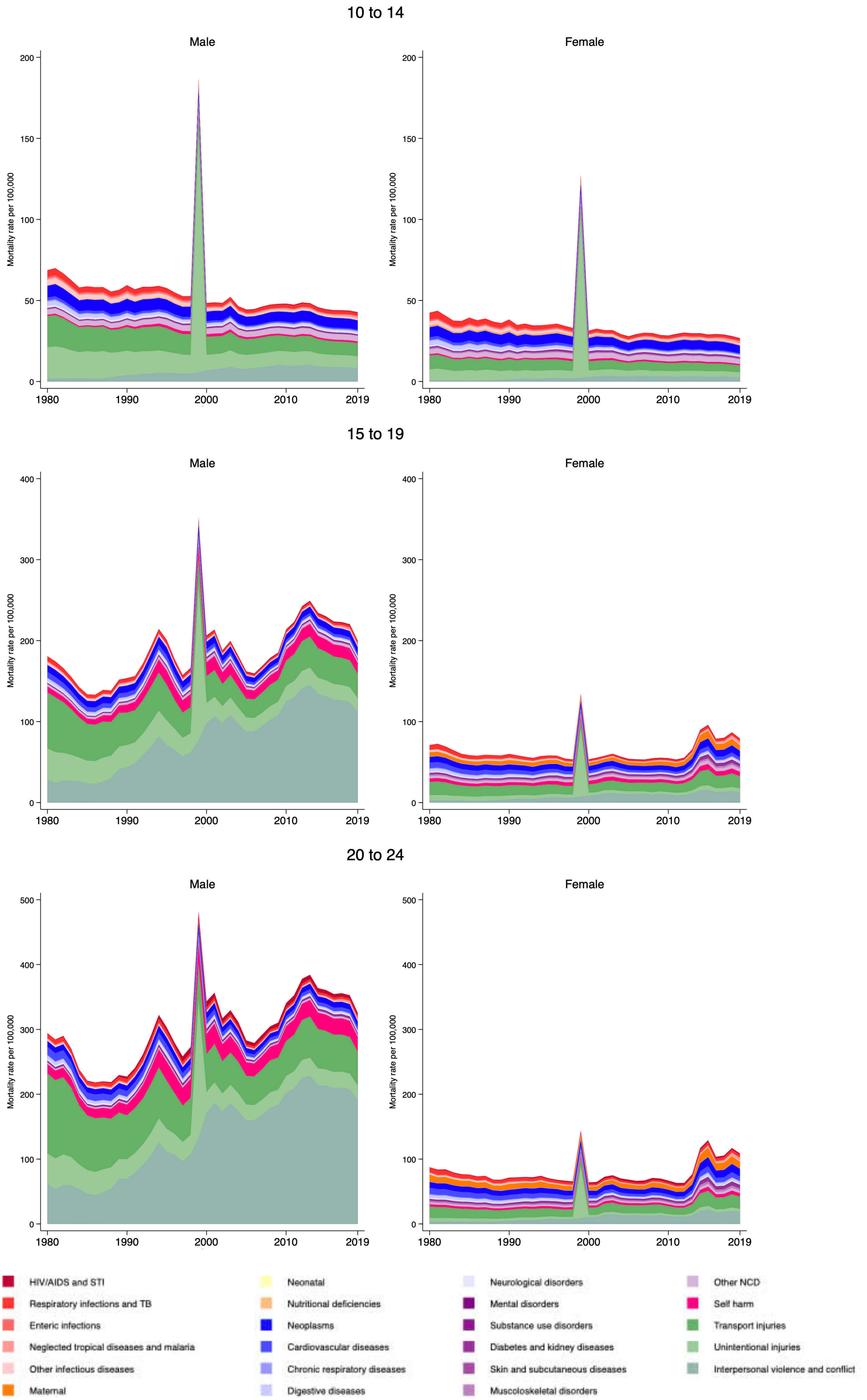

Figure S131d Number of deaths in 10-24 1980 – 2019 by cause: Venezuela (Bolivarian Republic of)

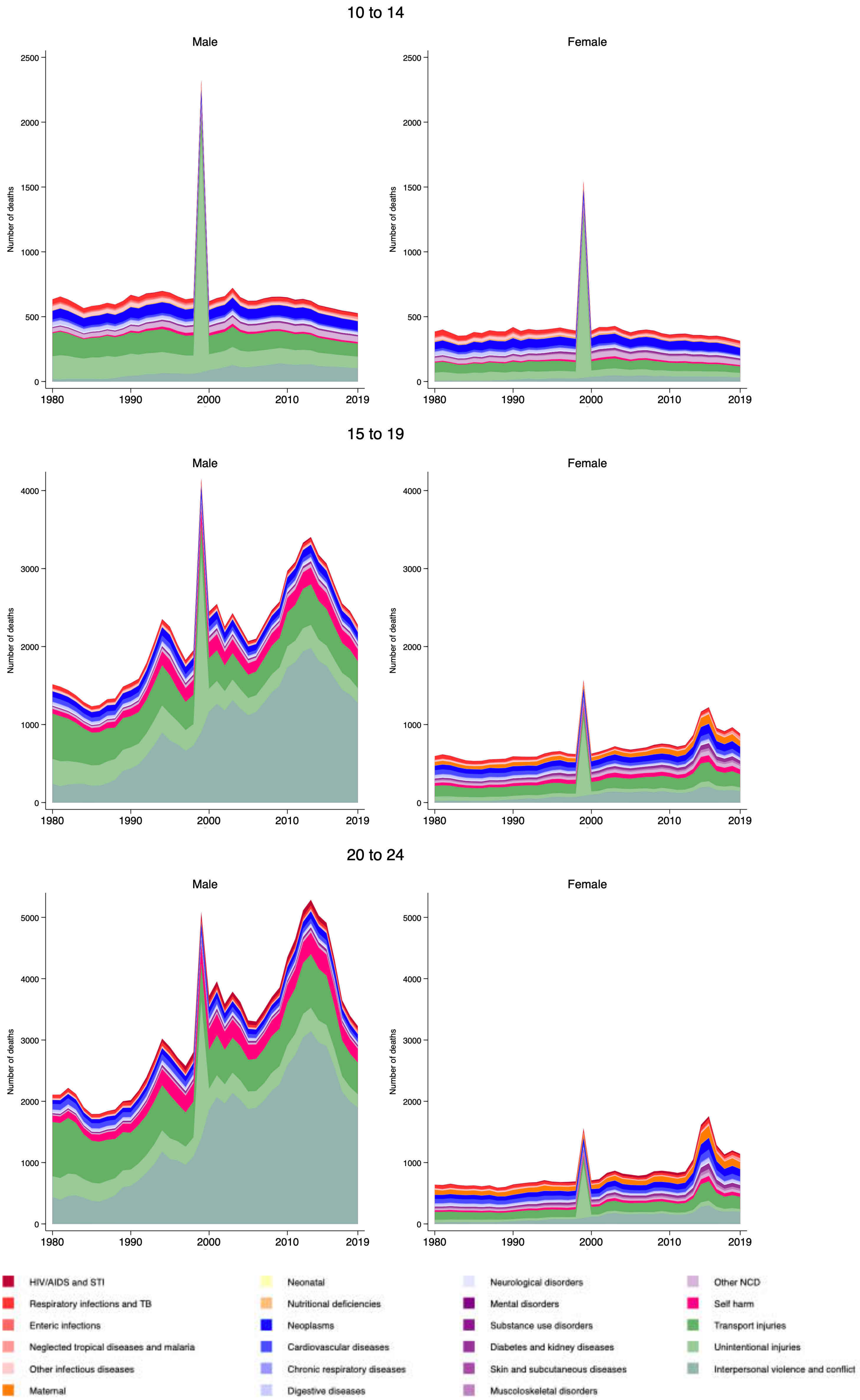

Figure S132: Tropical Latin America

Figure S132a All-cause mortality rate per 100,000 in 10-24 1950 – 2019: Tropical Latin America

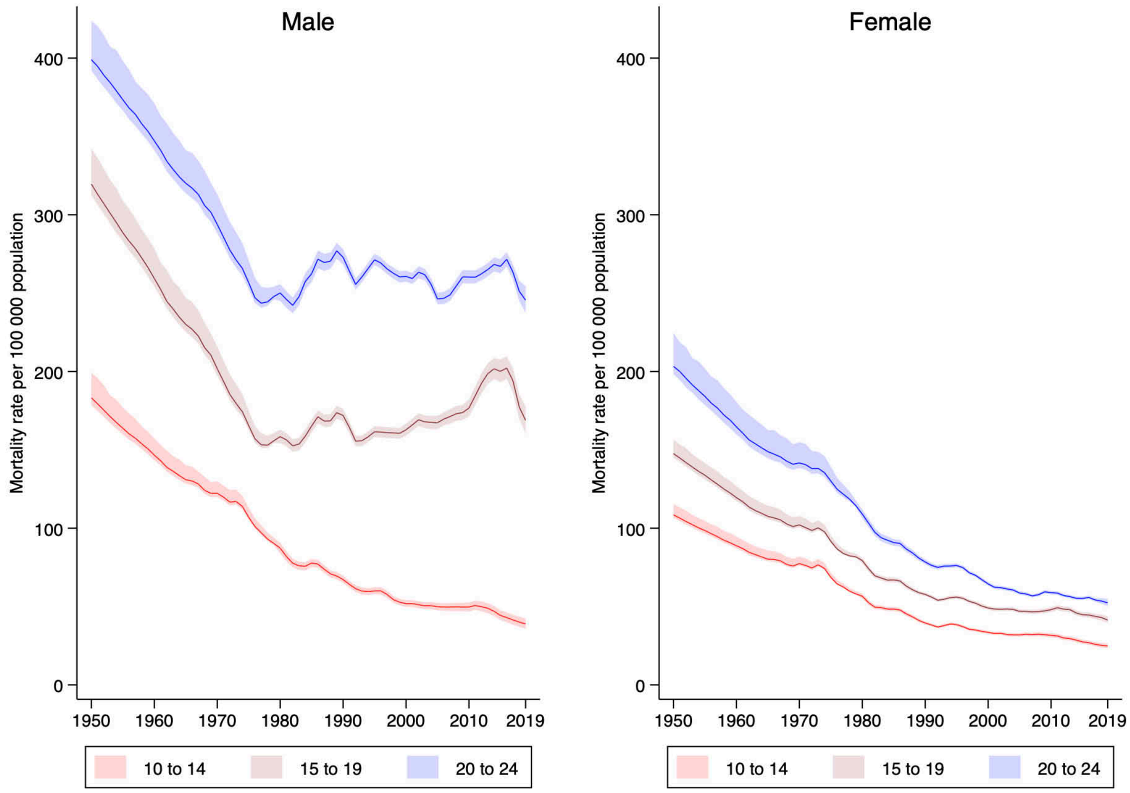

Figure S132b Number of deaths from all causes in 10-24 1950 – 2019: Tropical Latin America

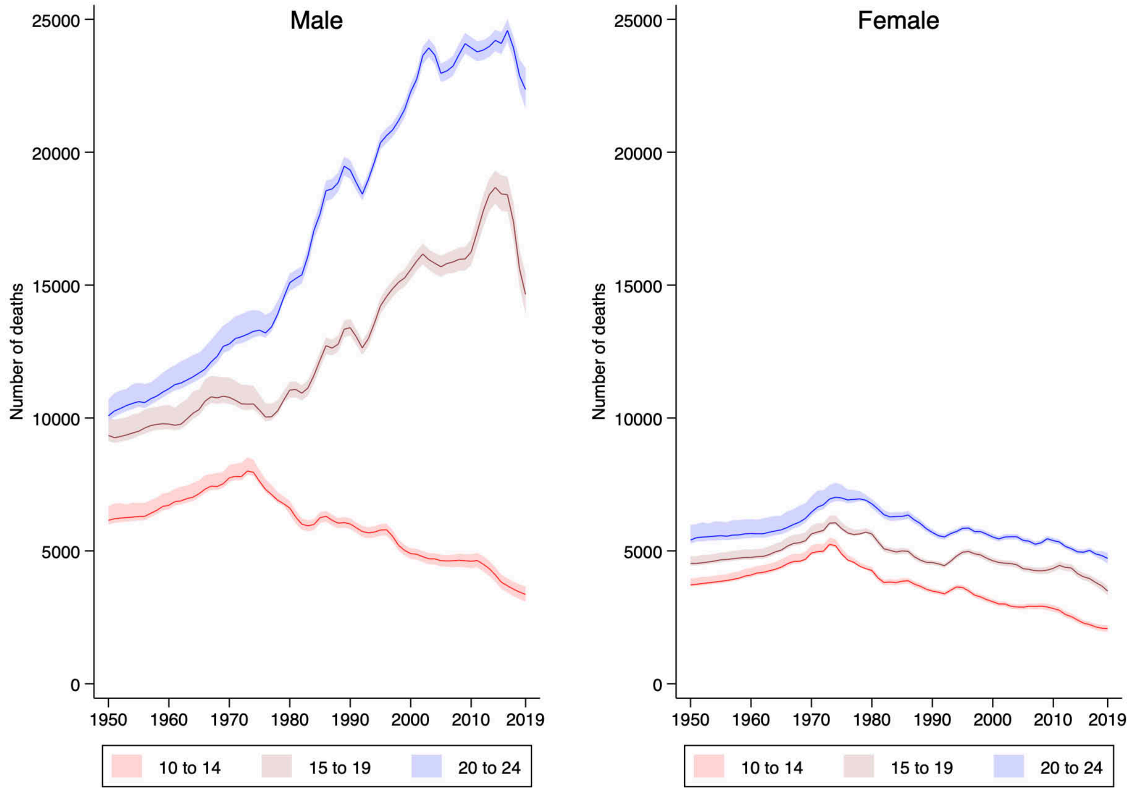

Figure S132c Mortality rate per 100,000 in 10-24 1980 – 2019 by cause: Tropical Latin America

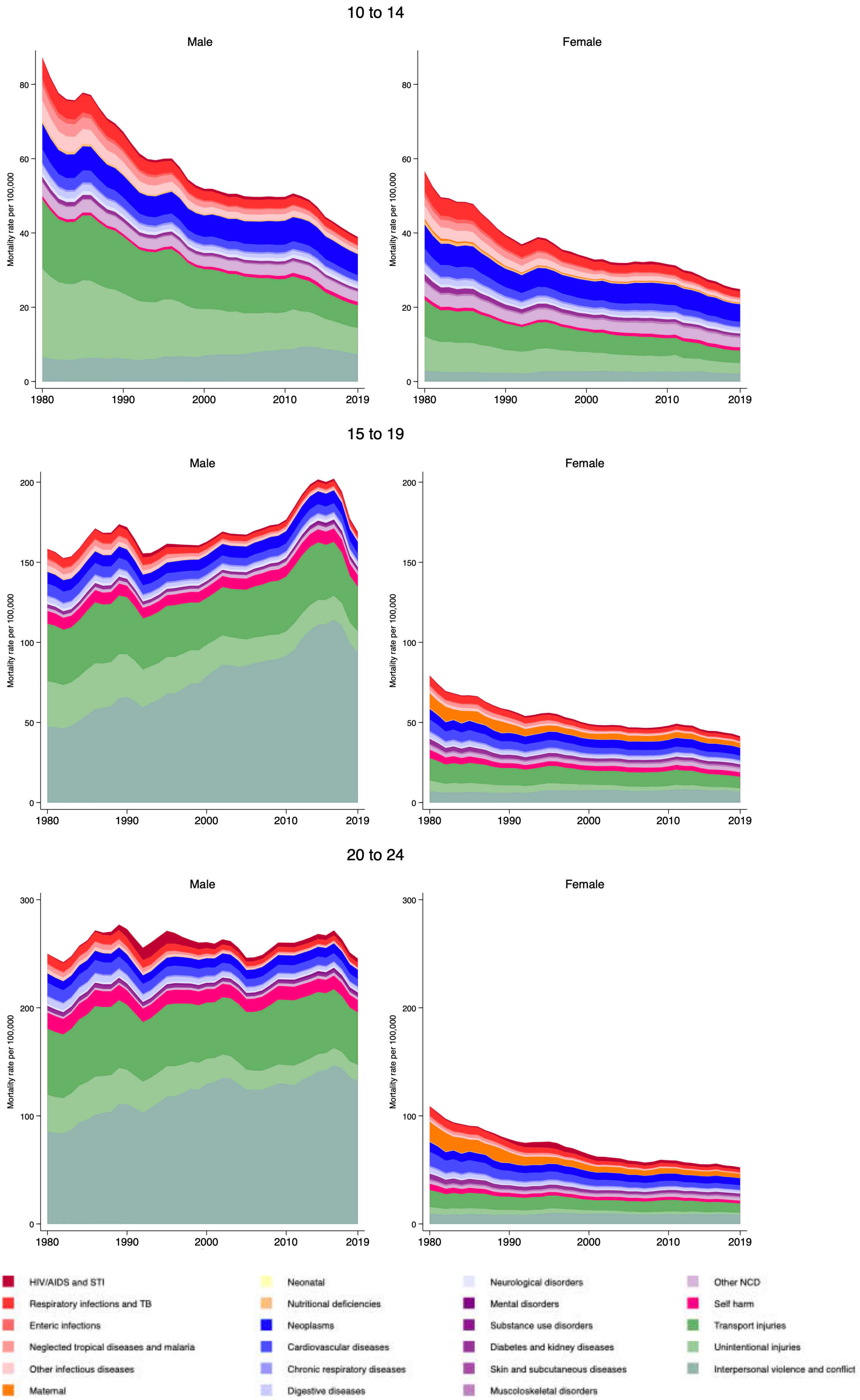

Figure S132d Number of deaths in 10-24 1980 – 2019 by cause: Tropical Latin America

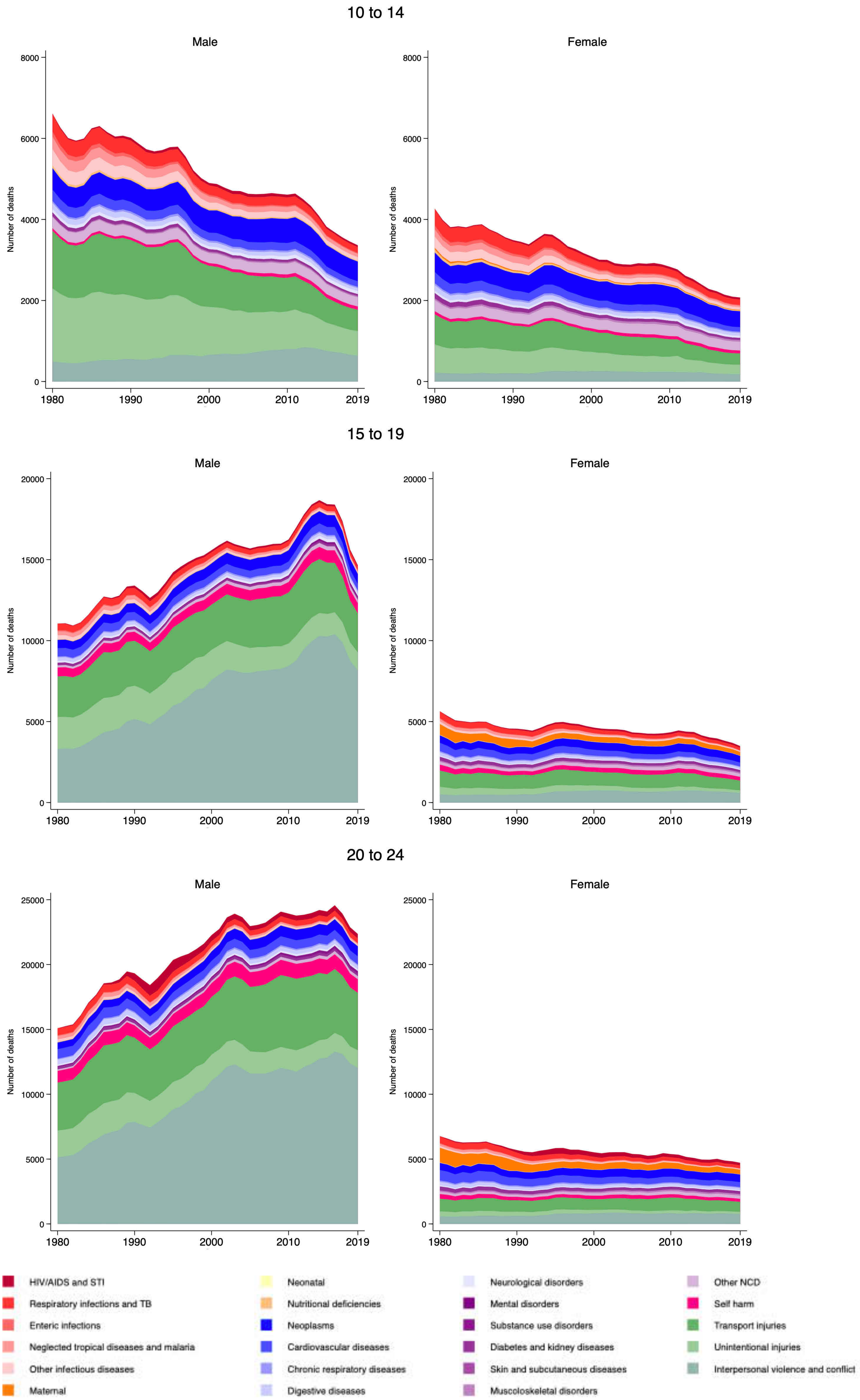

Figure S133: Brazil

Figure S133a All-cause mortality rate per 100,000 in 10-24 1950 – 2019: Brazil

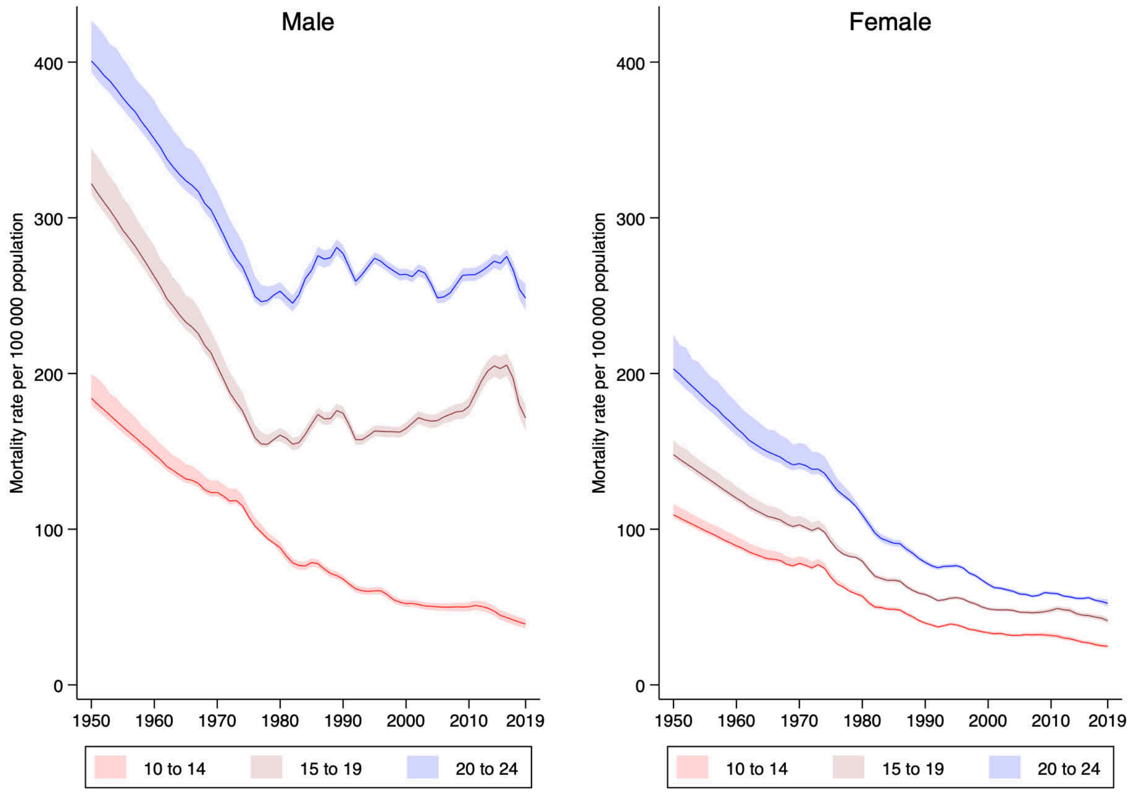

Figure S133b Number of deaths from all causes in 10-24 1950 – 2019: Brazil

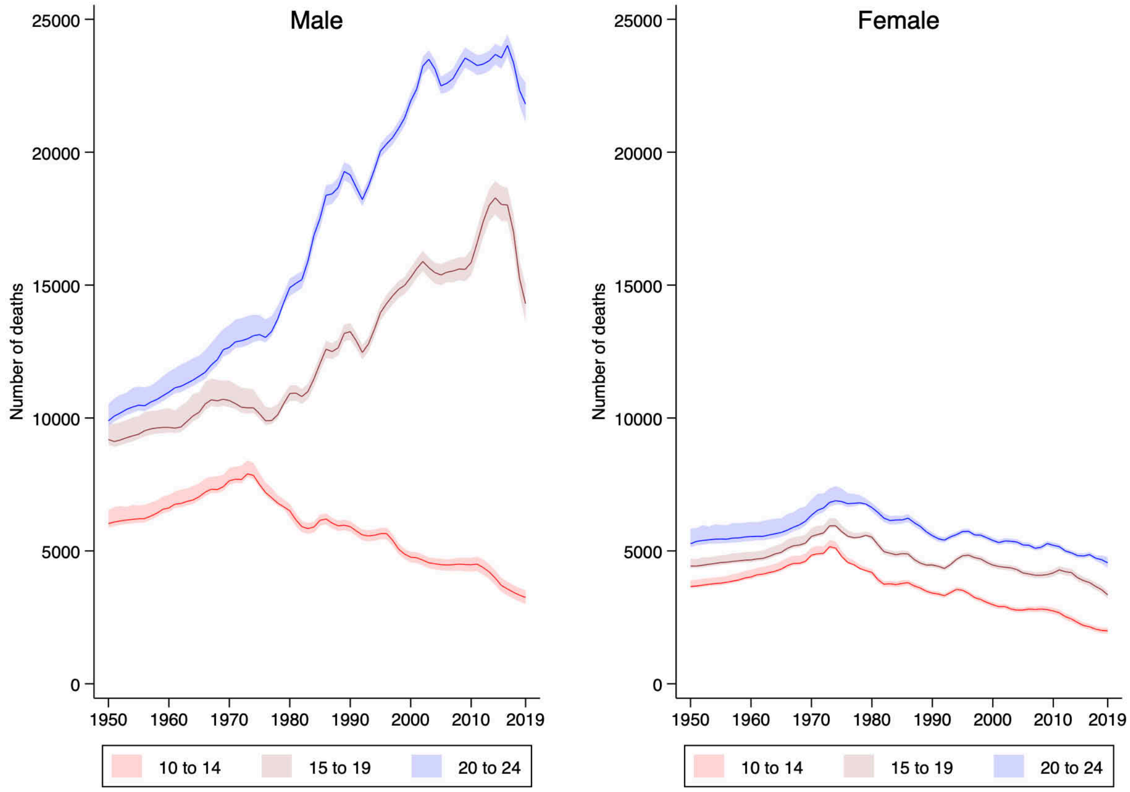

Figure S133c Mortality rate per 100,000 in 10-24 1980 – 2019 by cause: Brazil

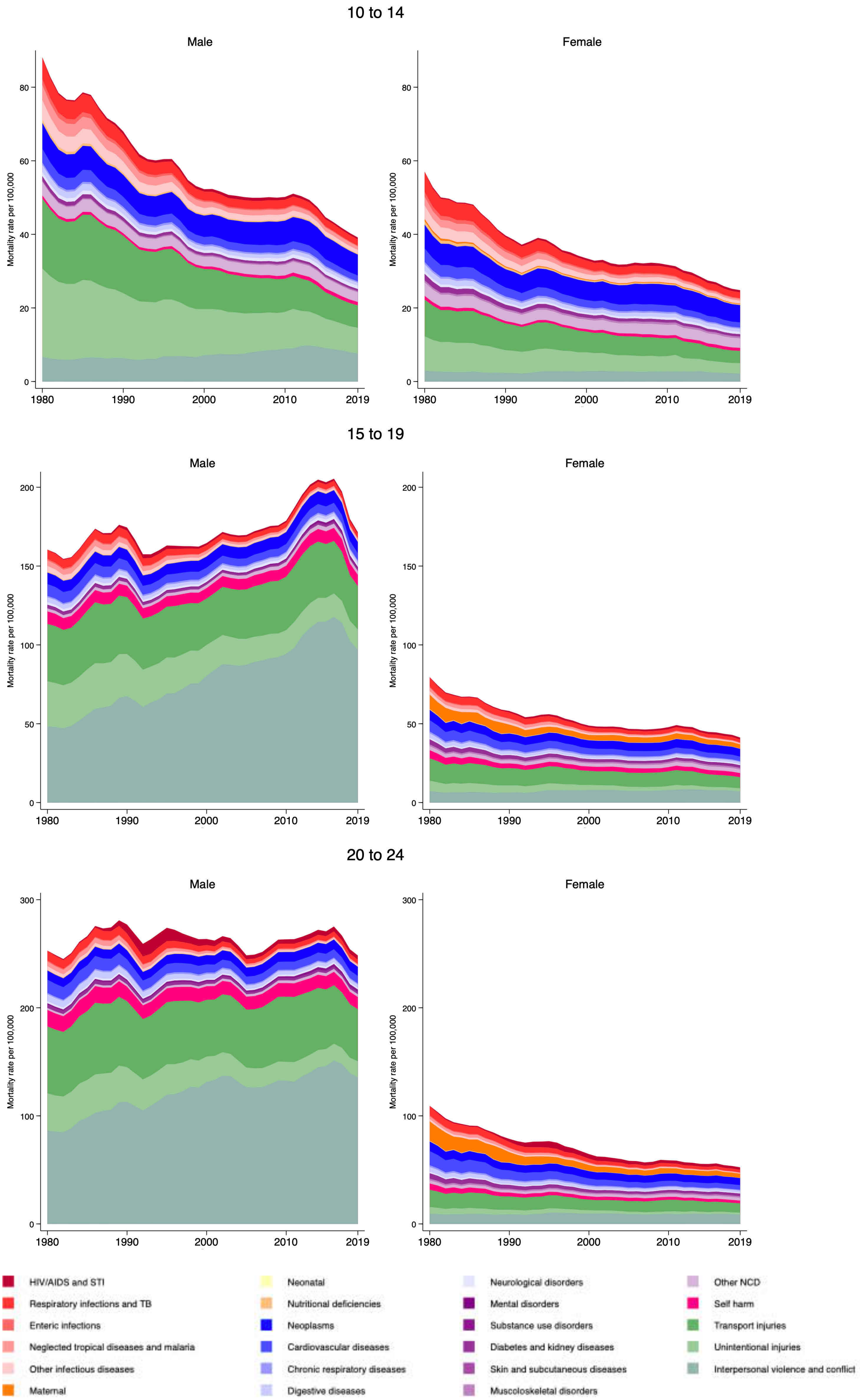

Figure S133d Number of deaths in 10-24 1980 – 2019 by cause: Brazil

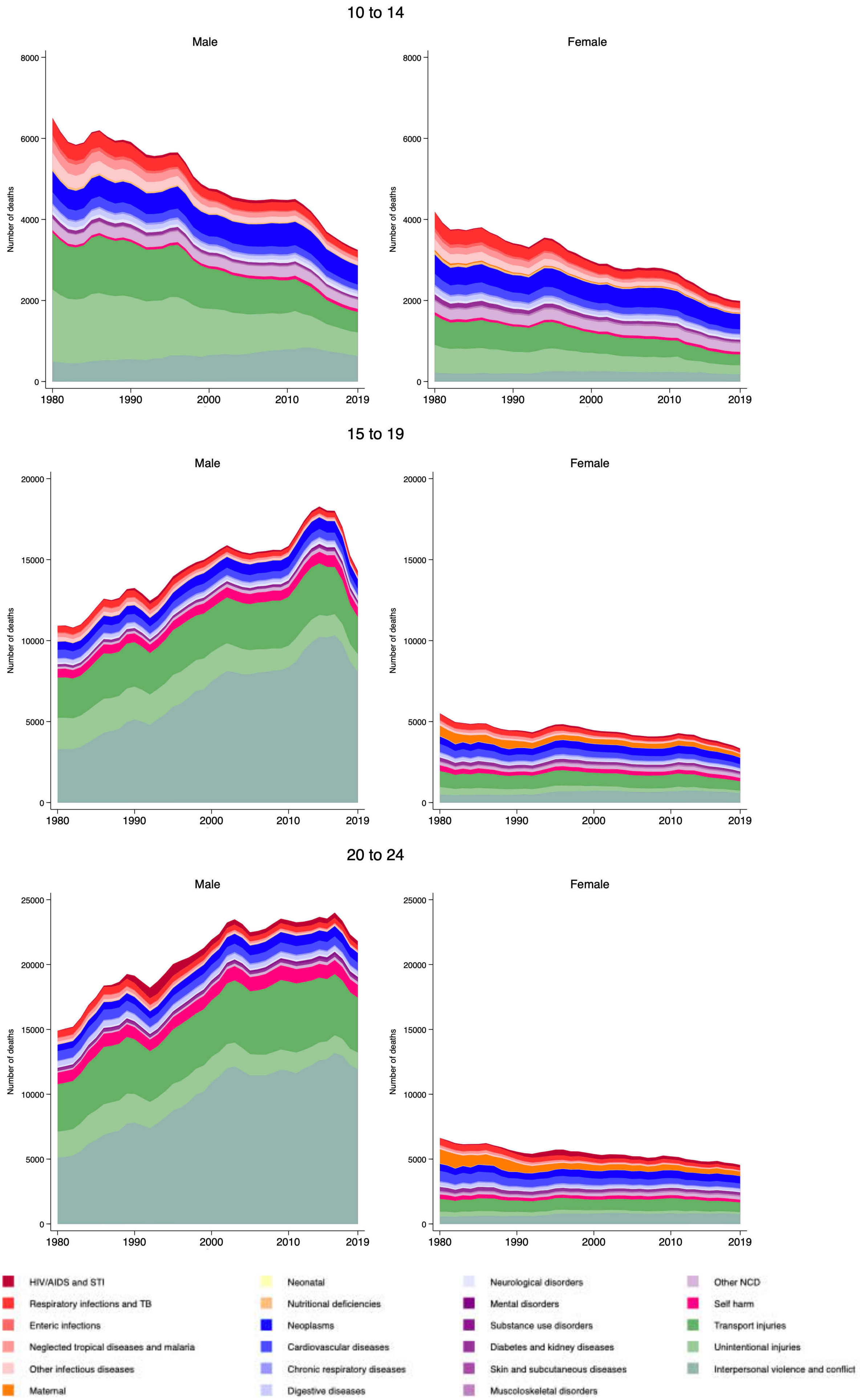

Figure S134: Paraguay

Figure S134a All-cause mortality rate per 100,000 in 10-24 1950 – 2019: Paraguay

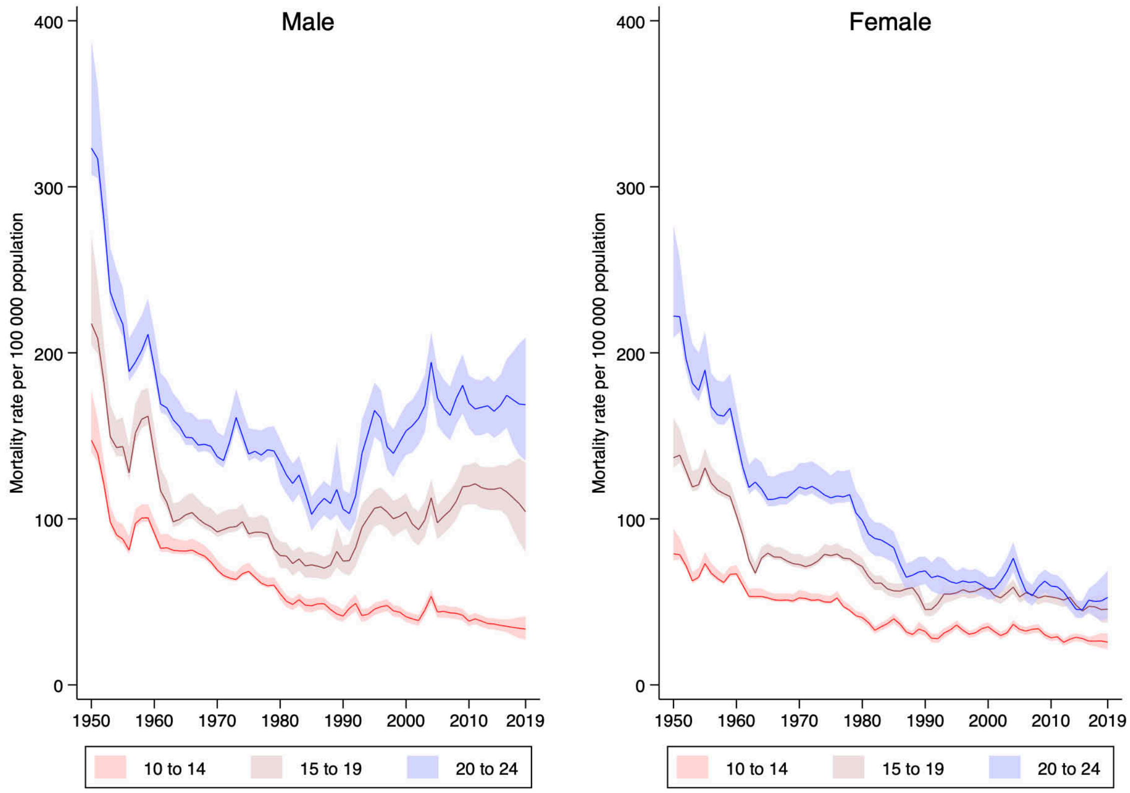

Figure S134b Number of deaths from all causes in 10-24 1950 – 2019: Paraguay

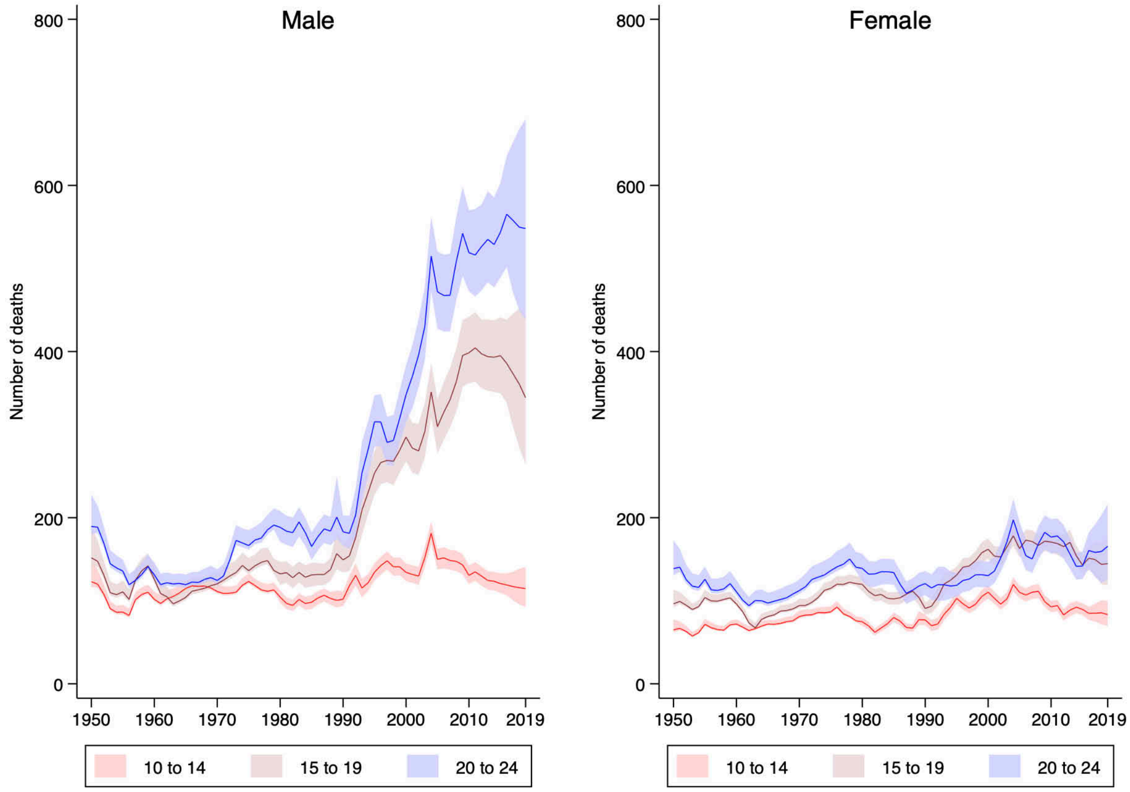

Figure S134c Mortality rate per 100,000 in 10-24 1980 – 2019 by cause: Paraguay

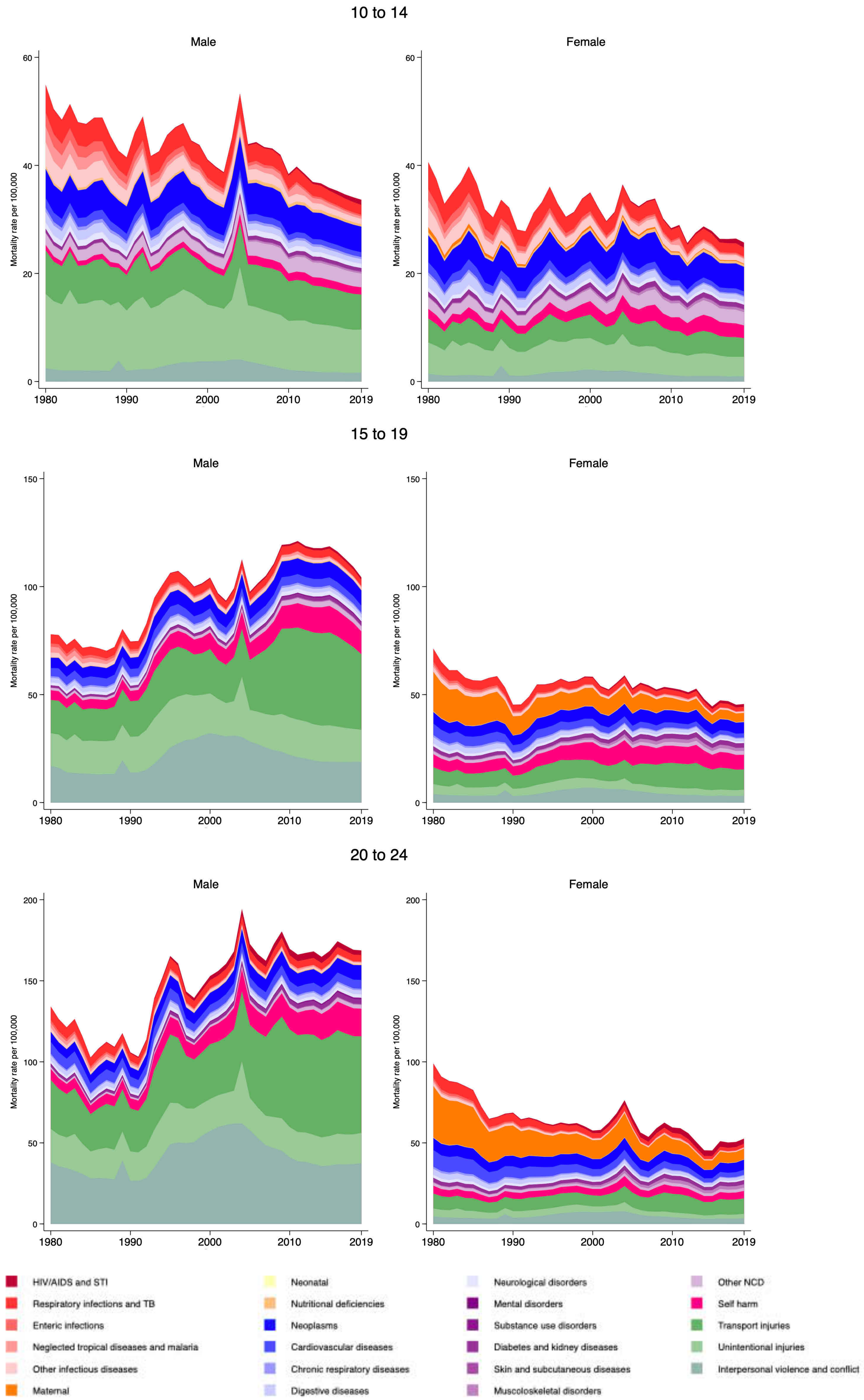

Figure S134d Number of deaths in 10-24 1980 – 2019 by cause: Paraguay

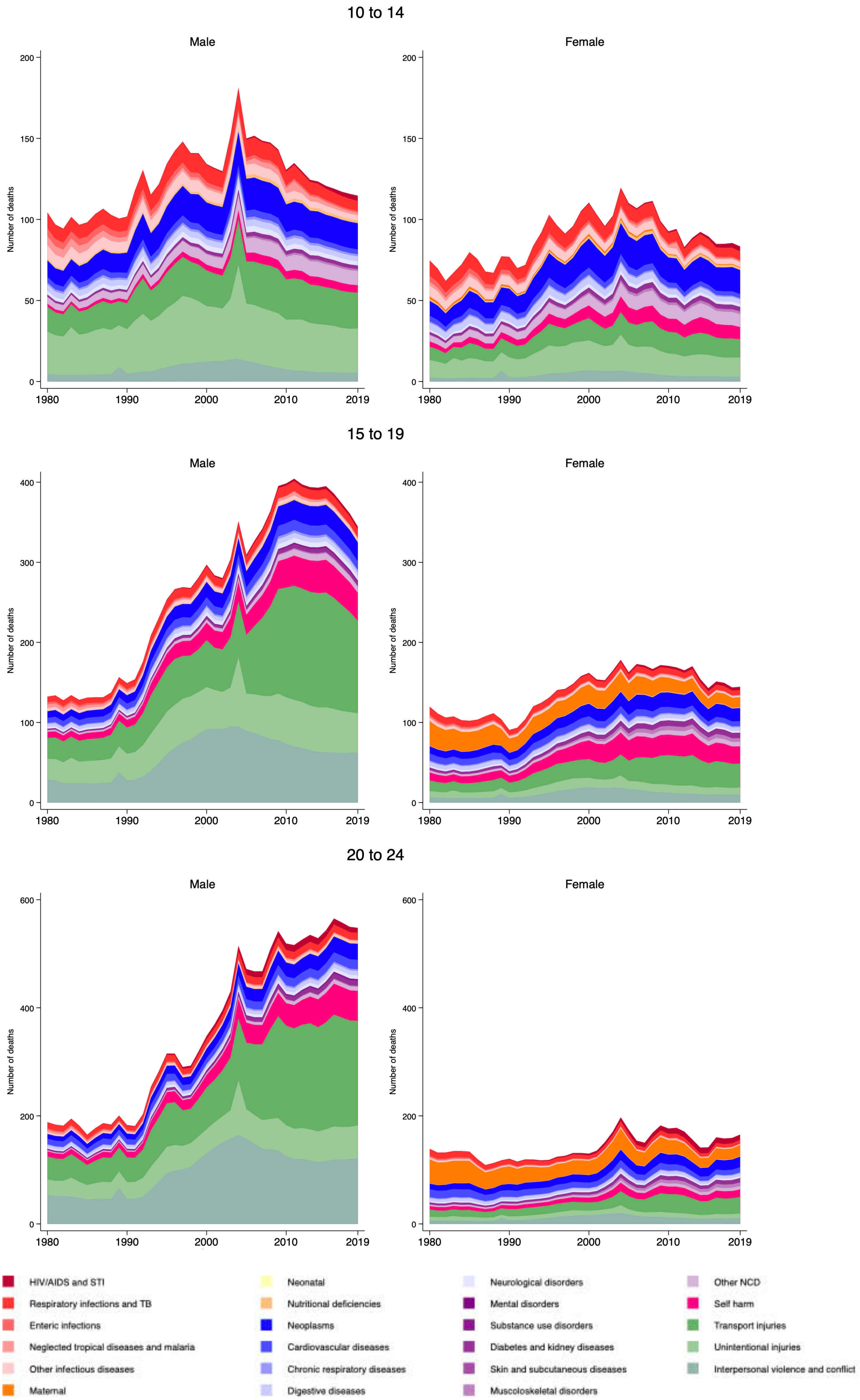

Figure S135: North Africa and Middle East

Figure S135a All-cause mortality rate per 100,000 in 10-24 1950 – 2019: North Africa and Middle East

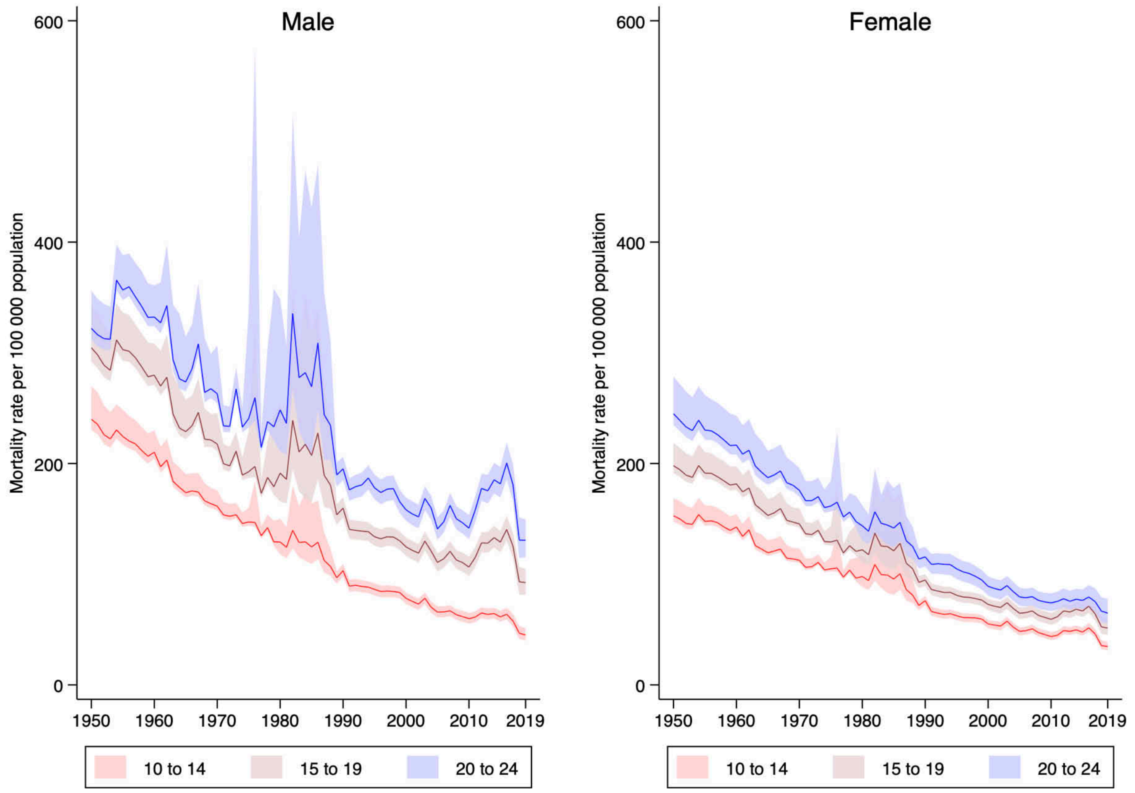

Figure S135b Number of deaths from all causes in 10-24 1950 – 2019: North Africa and Middle East

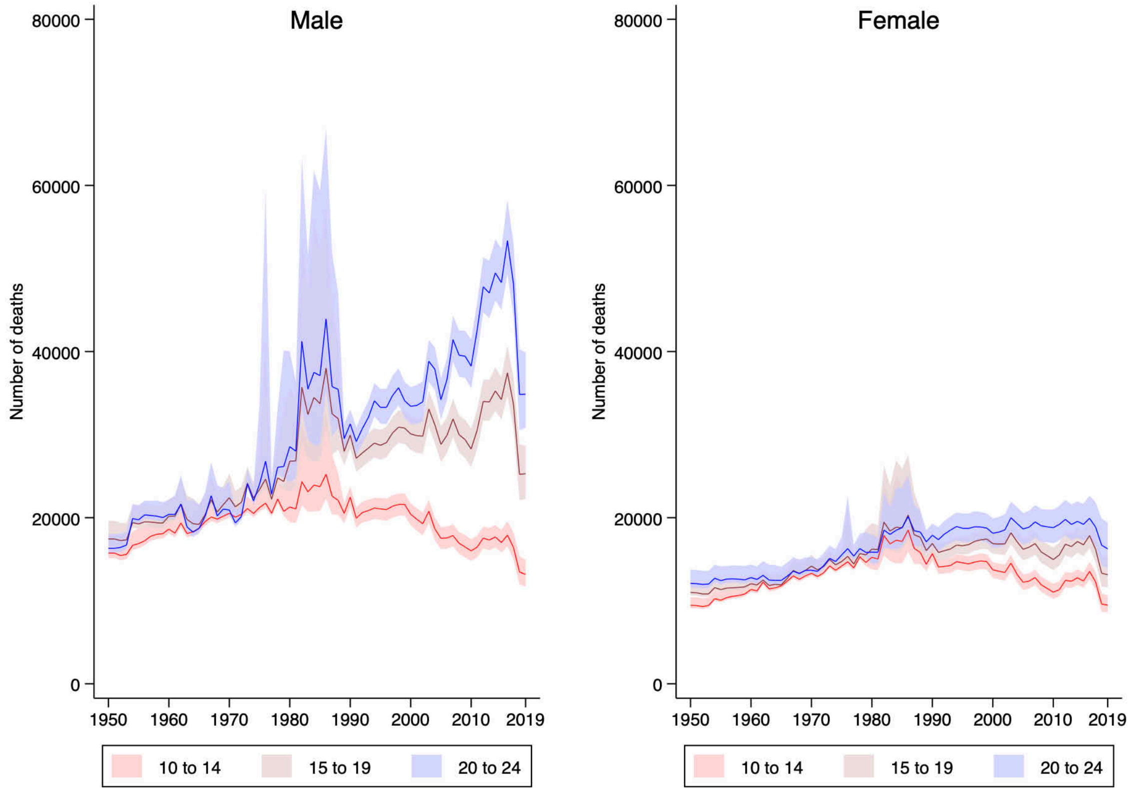

Figure S135c Mortality rate per 100,000 in 10-24 1980 – 2019 by cause: North Africa and Middle East

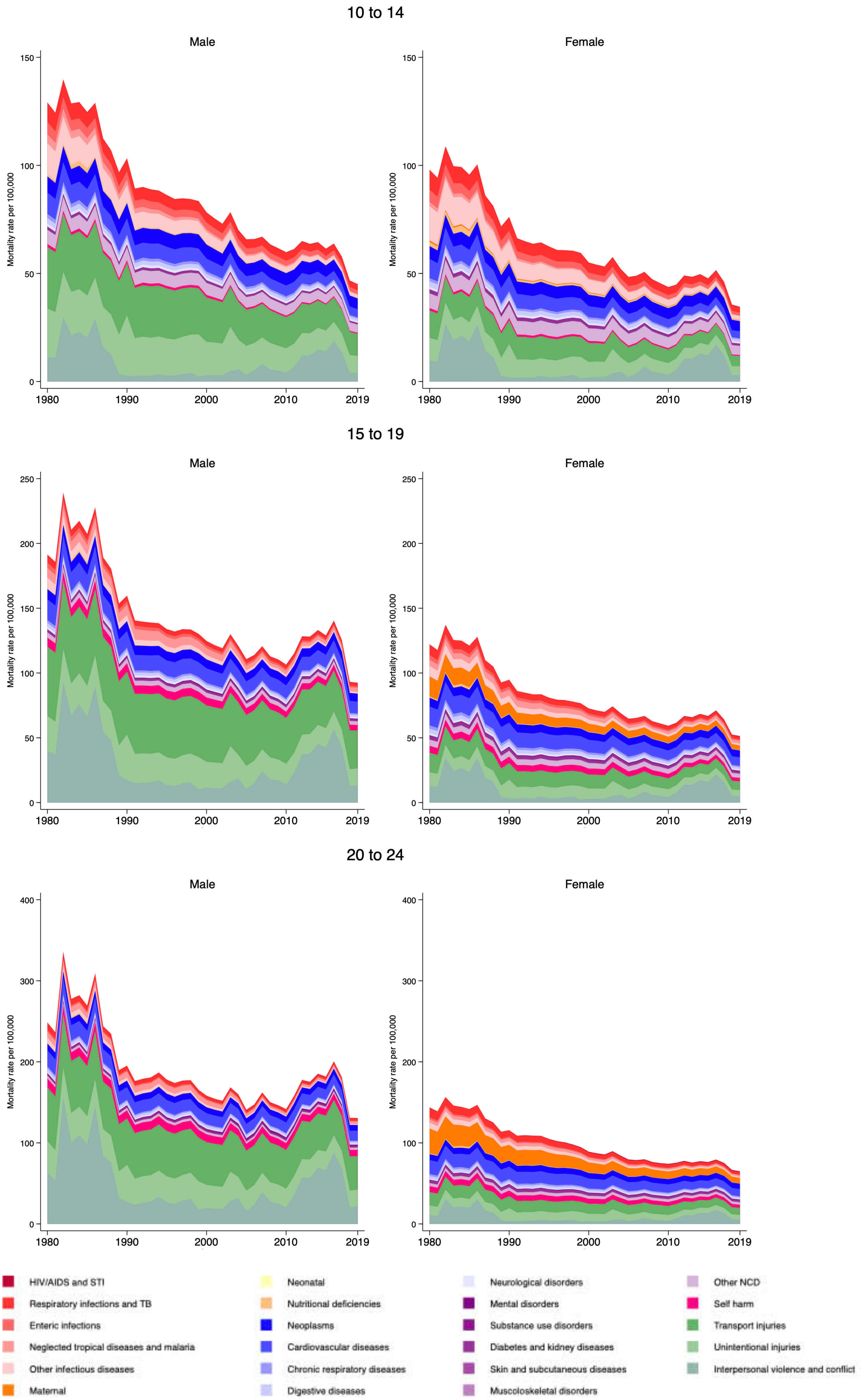

Figure S135d Number of deaths in 10-24 1980 – 2019 by cause: North Africa and Middle East

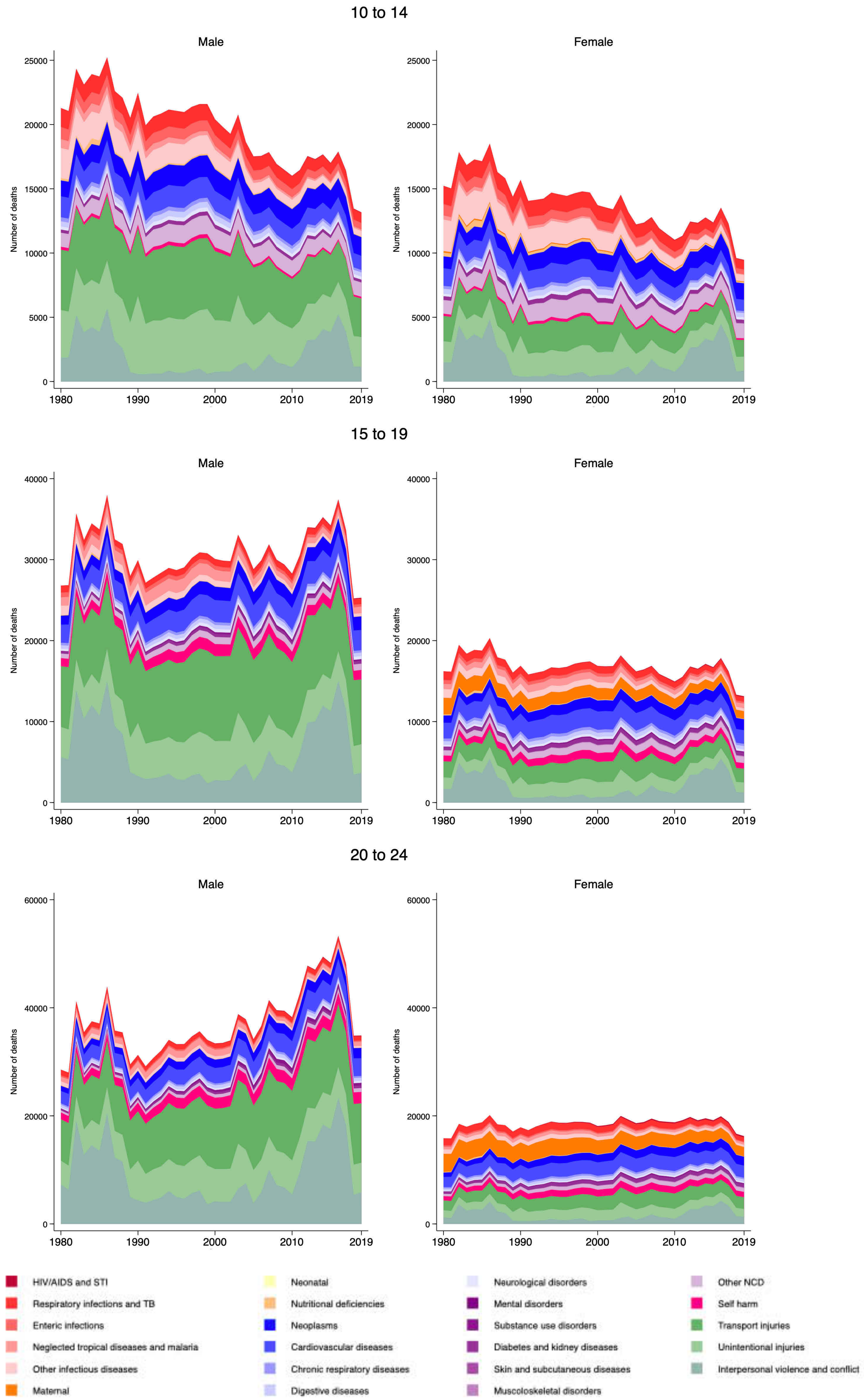

Figure S136: Algeria

Figure S137a All-cause mortality rate per 100,000 in 10-24 1950 – 2019: Algeria

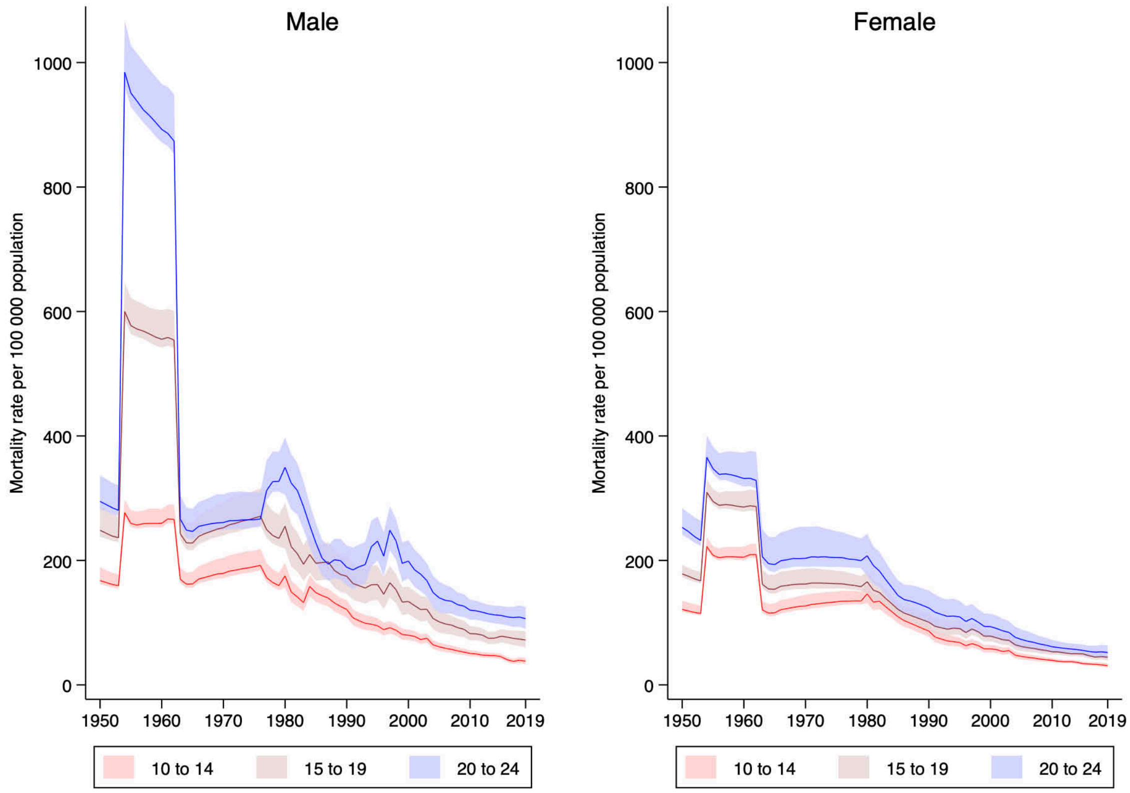

Figure S137b Number of deaths from all causes in 10-24 1950 – 2019: Algeria

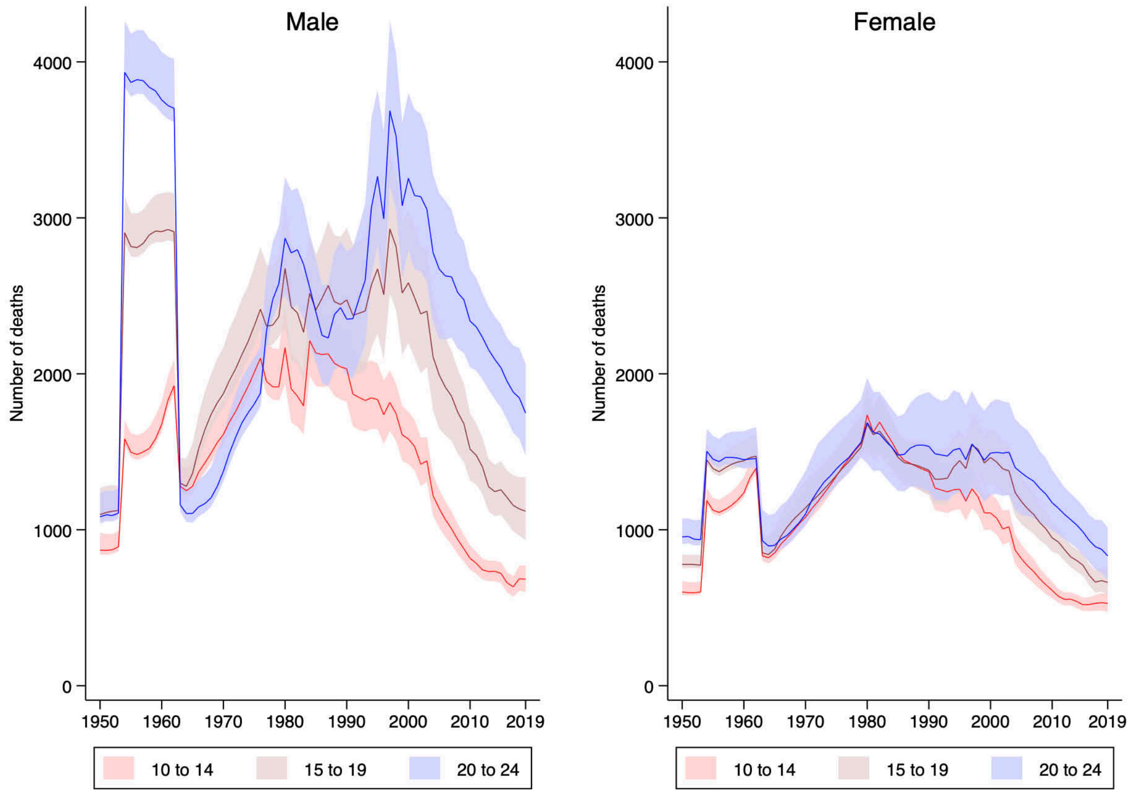

Figure S137c Mortality rate per 100,000 in 10-24 1980 – 2019 by cause: Algeria

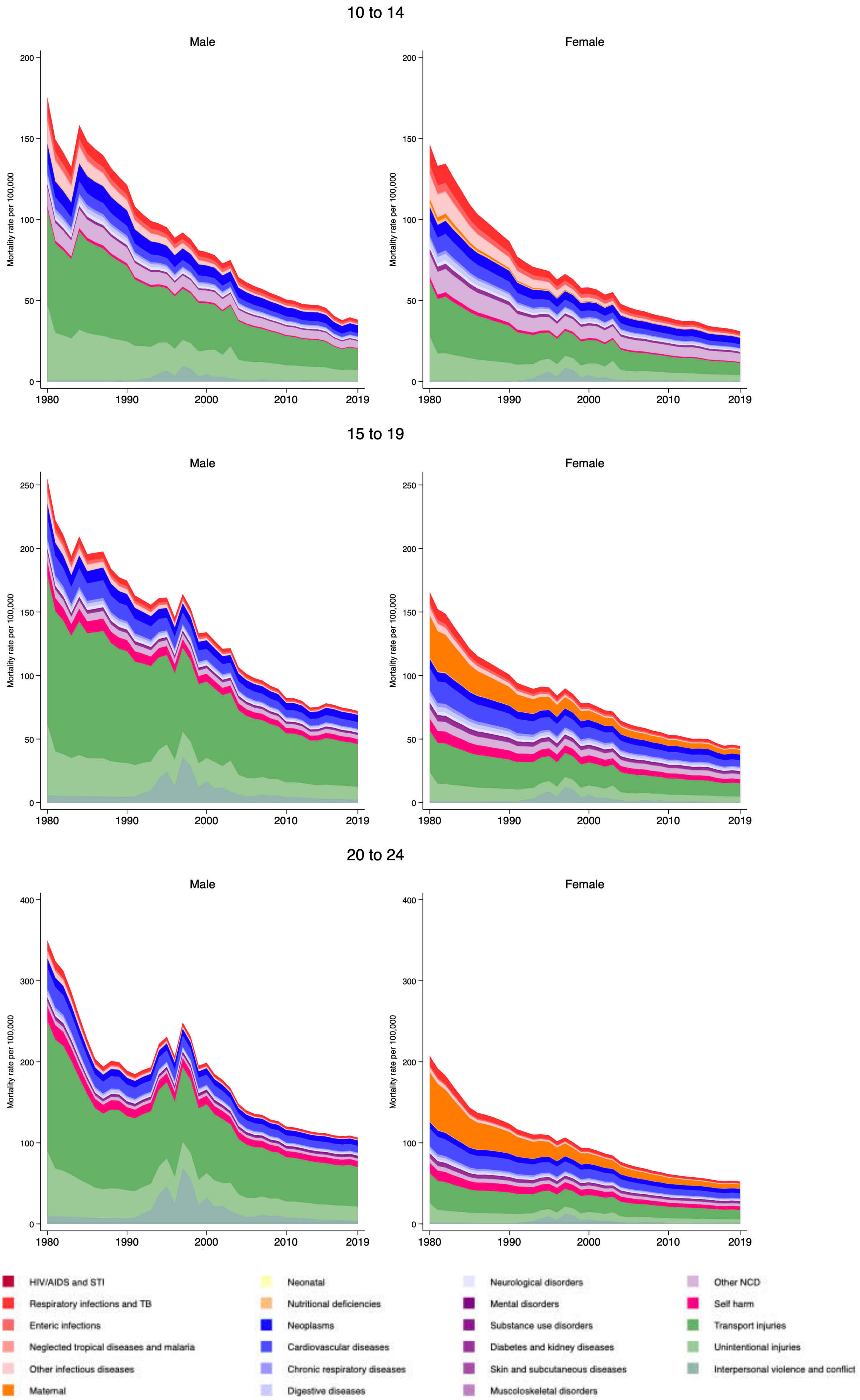

Figure S137d Number of deaths in 10-24 1980 – 2019 by cause: Algeria

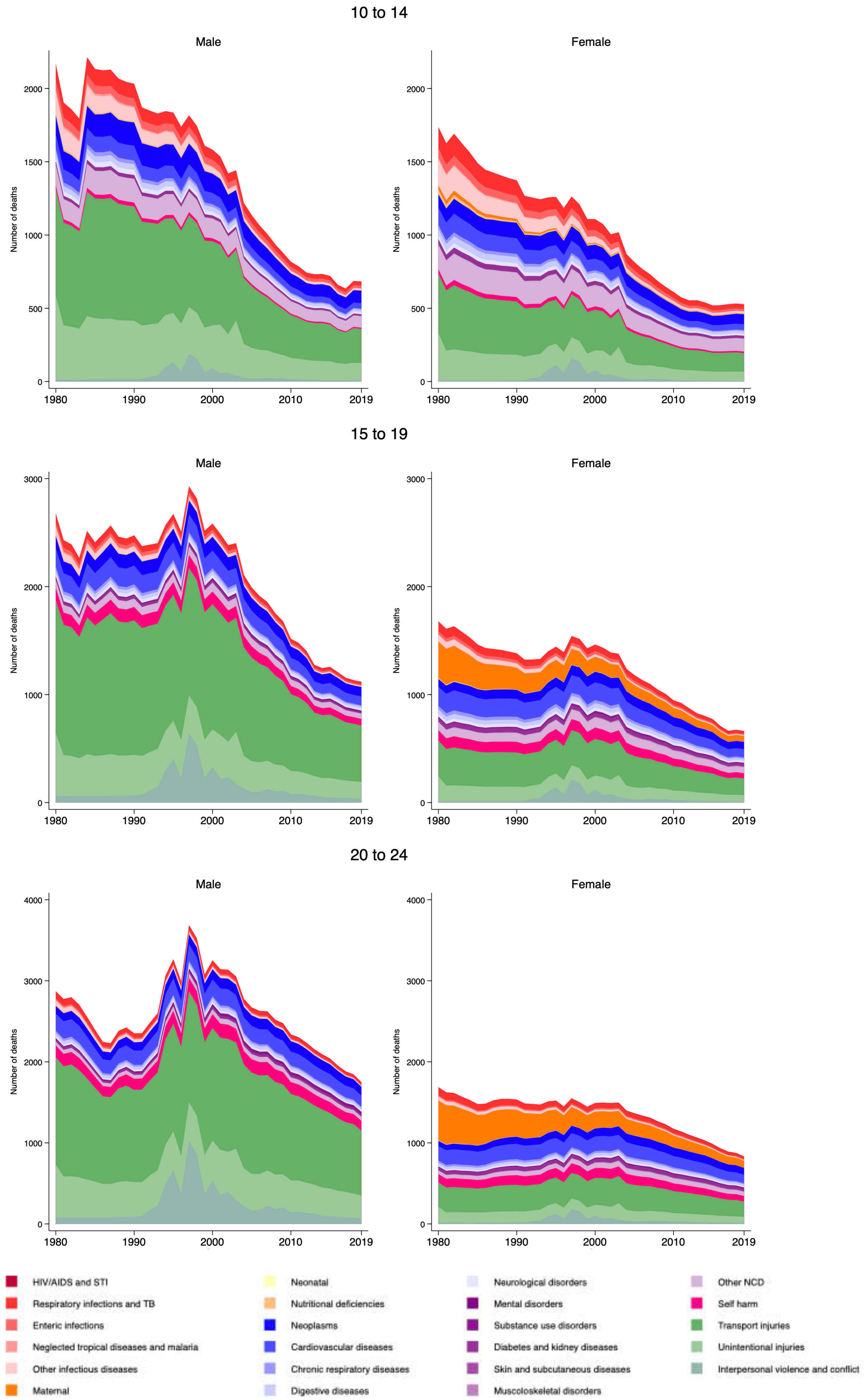

Figure S137: Bahrain

Figure S138a All-cause mortality rate per 100,000 in 10-24 1950 – 2019: Bahrain

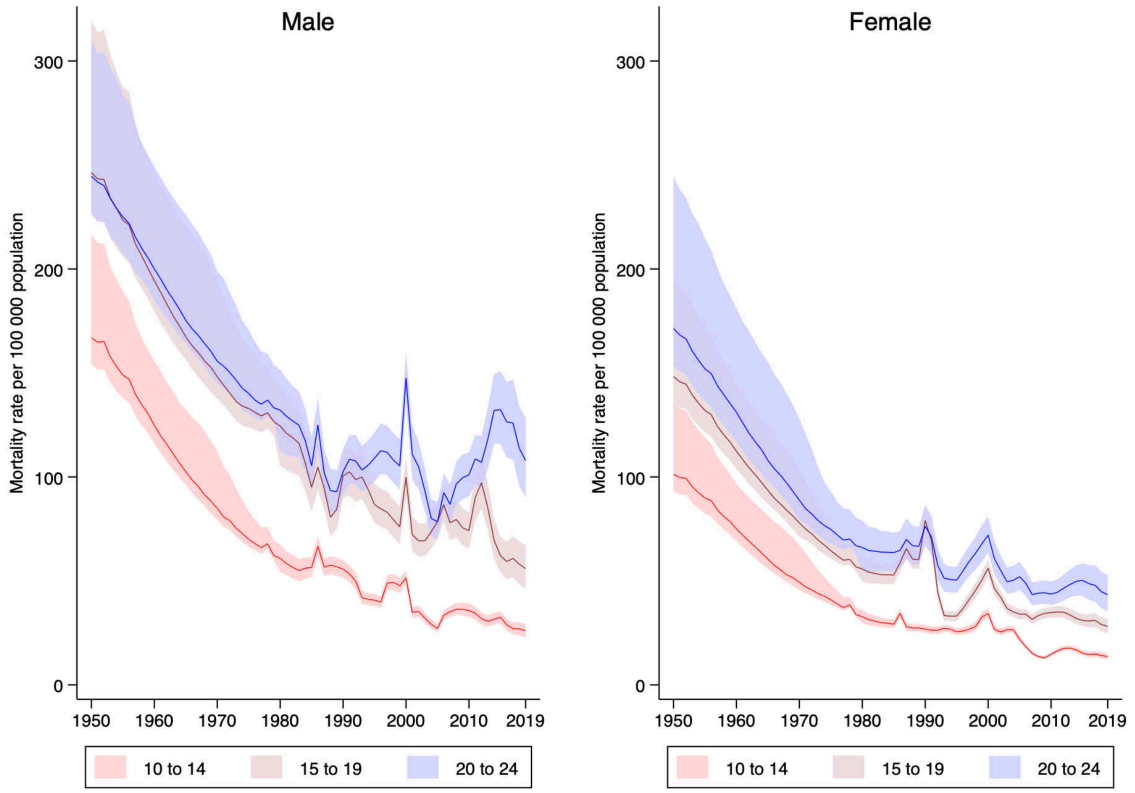

Figure S138b Number of deaths from all causes in 10-24 1950 – 2019: Bahrain

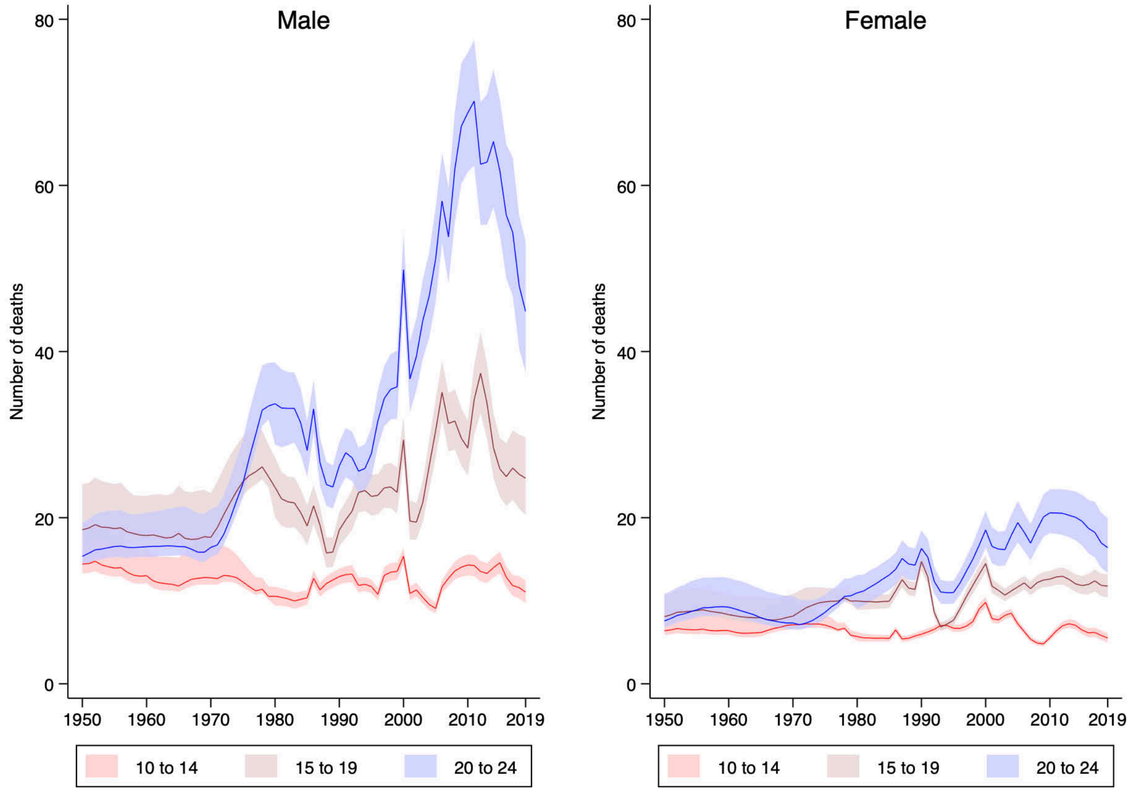

Figure S138c Mortality rate per 100,000 in 10-24 1980 – 2019 by cause: Bahrain

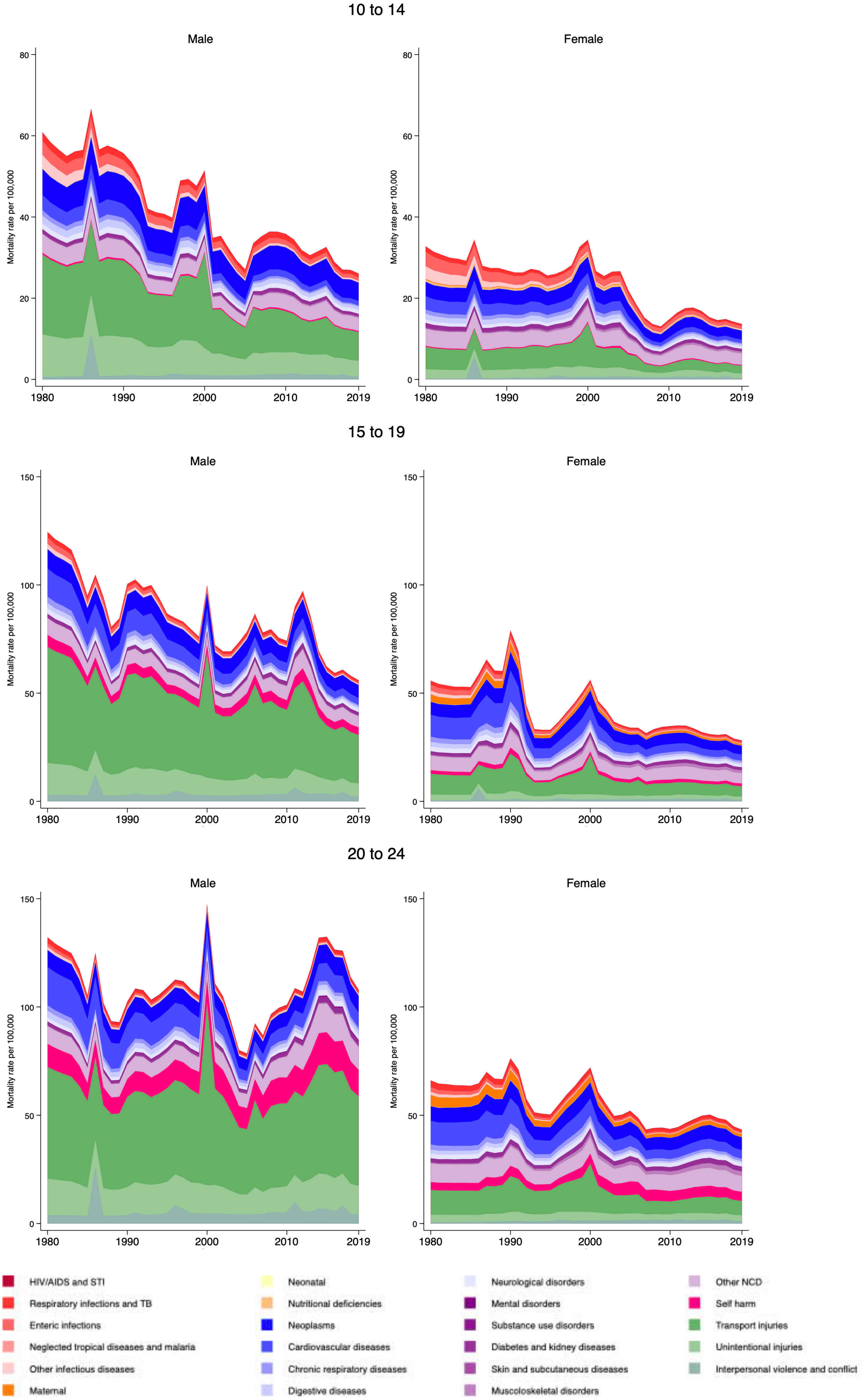

Figure S138d Number of deaths in 10-24 1980 – 2019 by cause: Bahrain

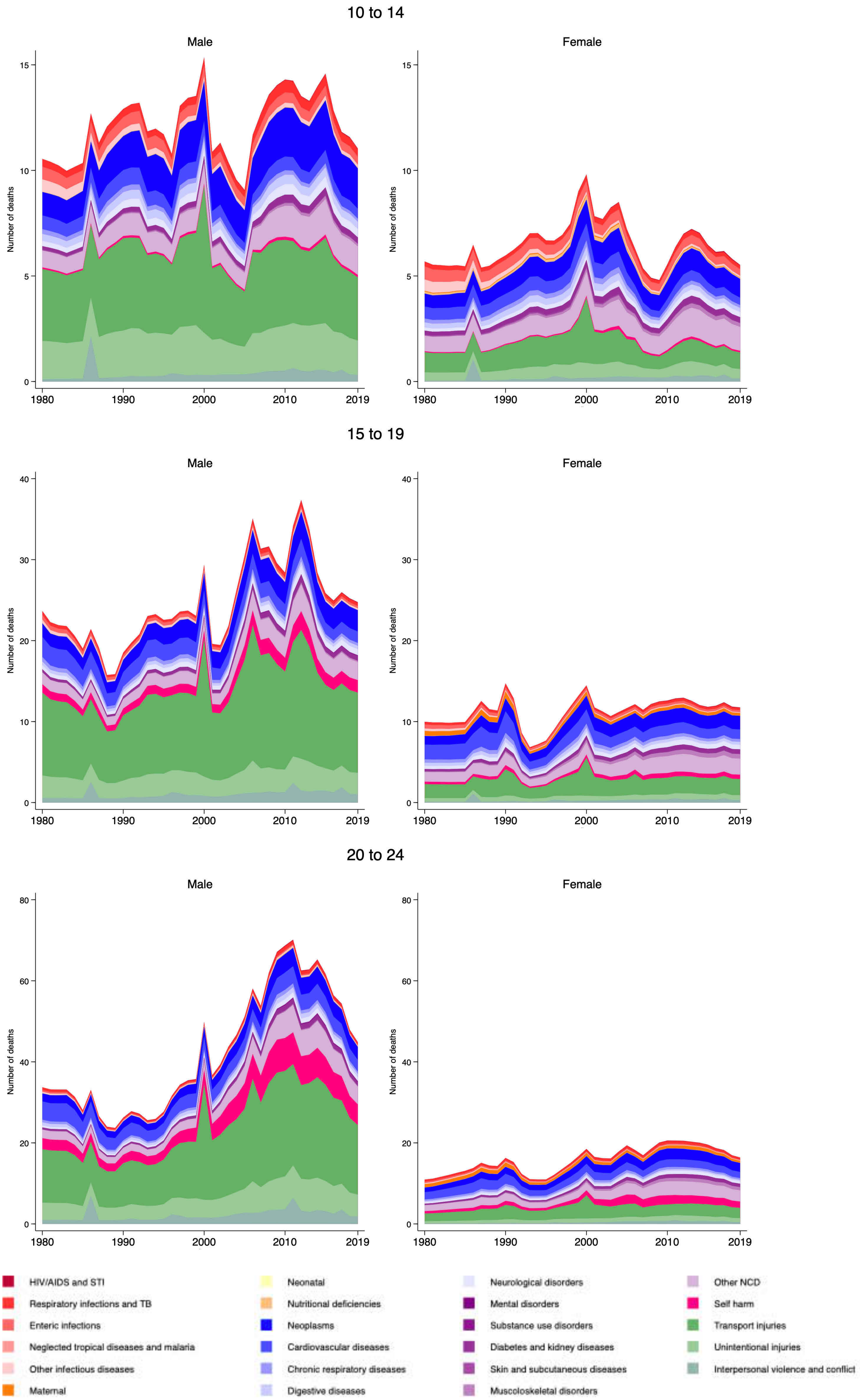

Figure S138: Egypt

Figure S139a All-cause mortality rate per 100,000 in 10-24 1950 – 2019: Egypt

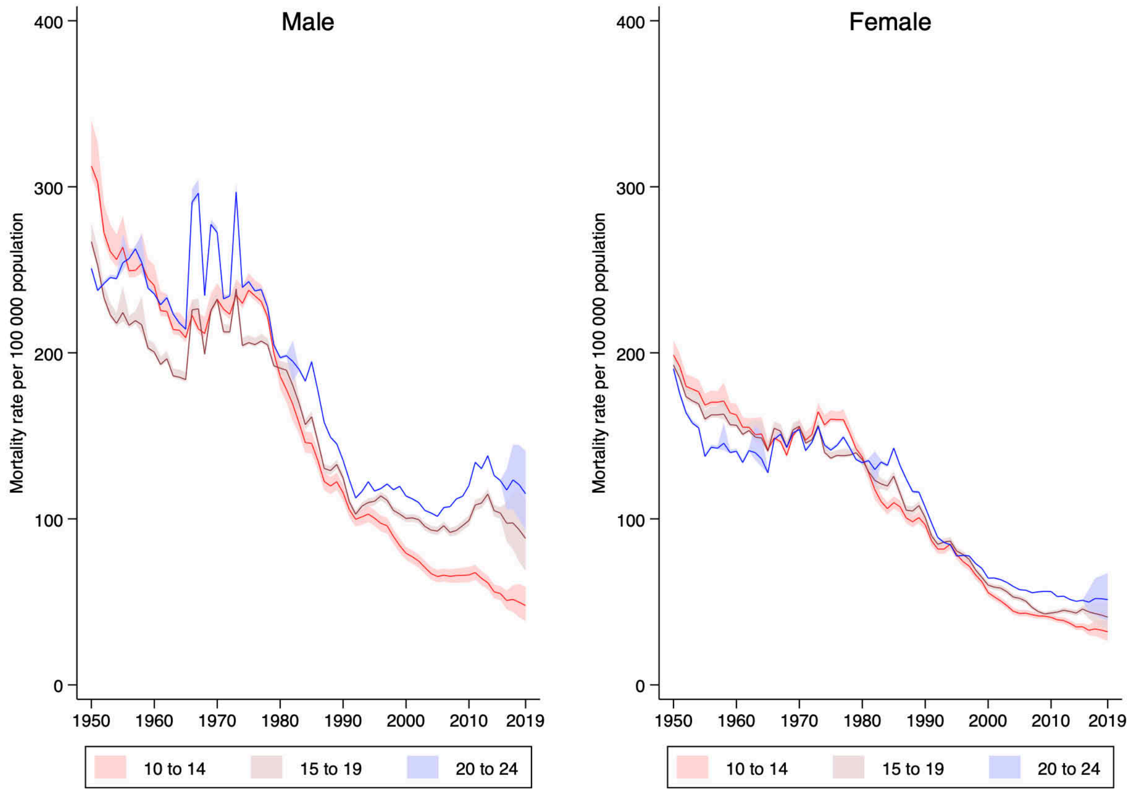

Figure S139b Number of deaths from all causes in 10-24 1950 – 2019: Egypt

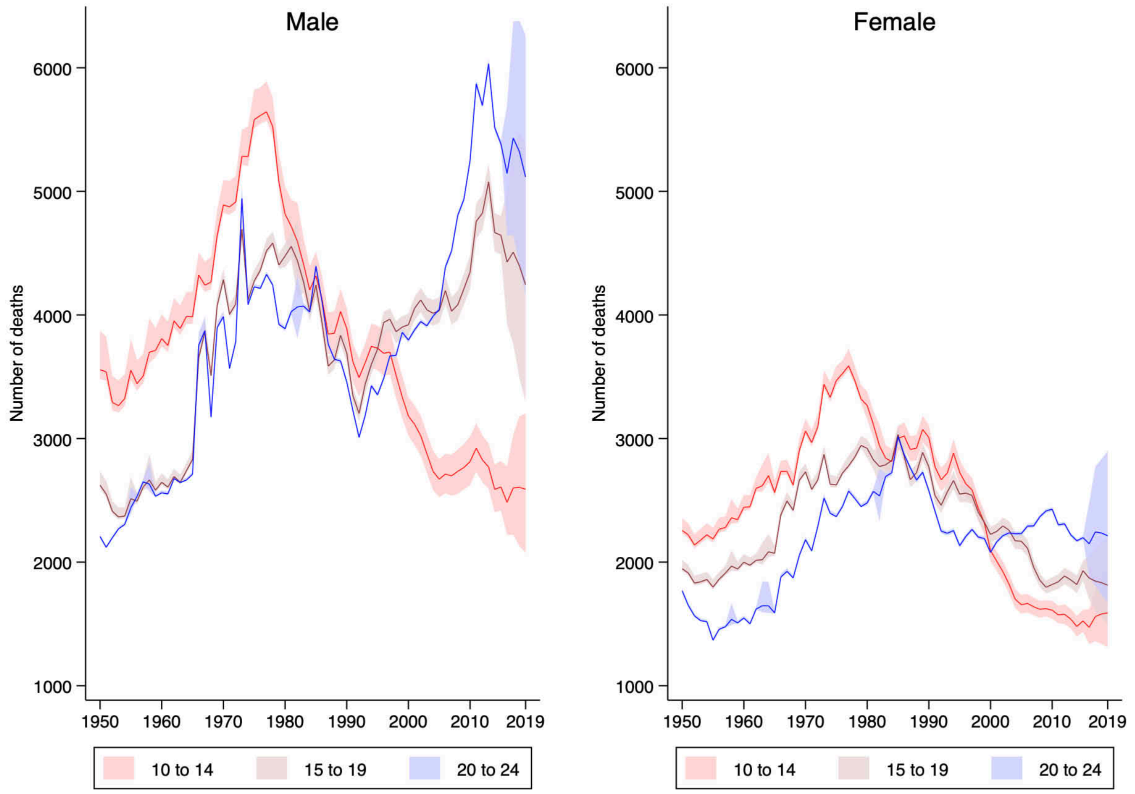

Figure S139c Mortality rate per 100,000 in 10-24 1980 – 2019 by cause: Egypt

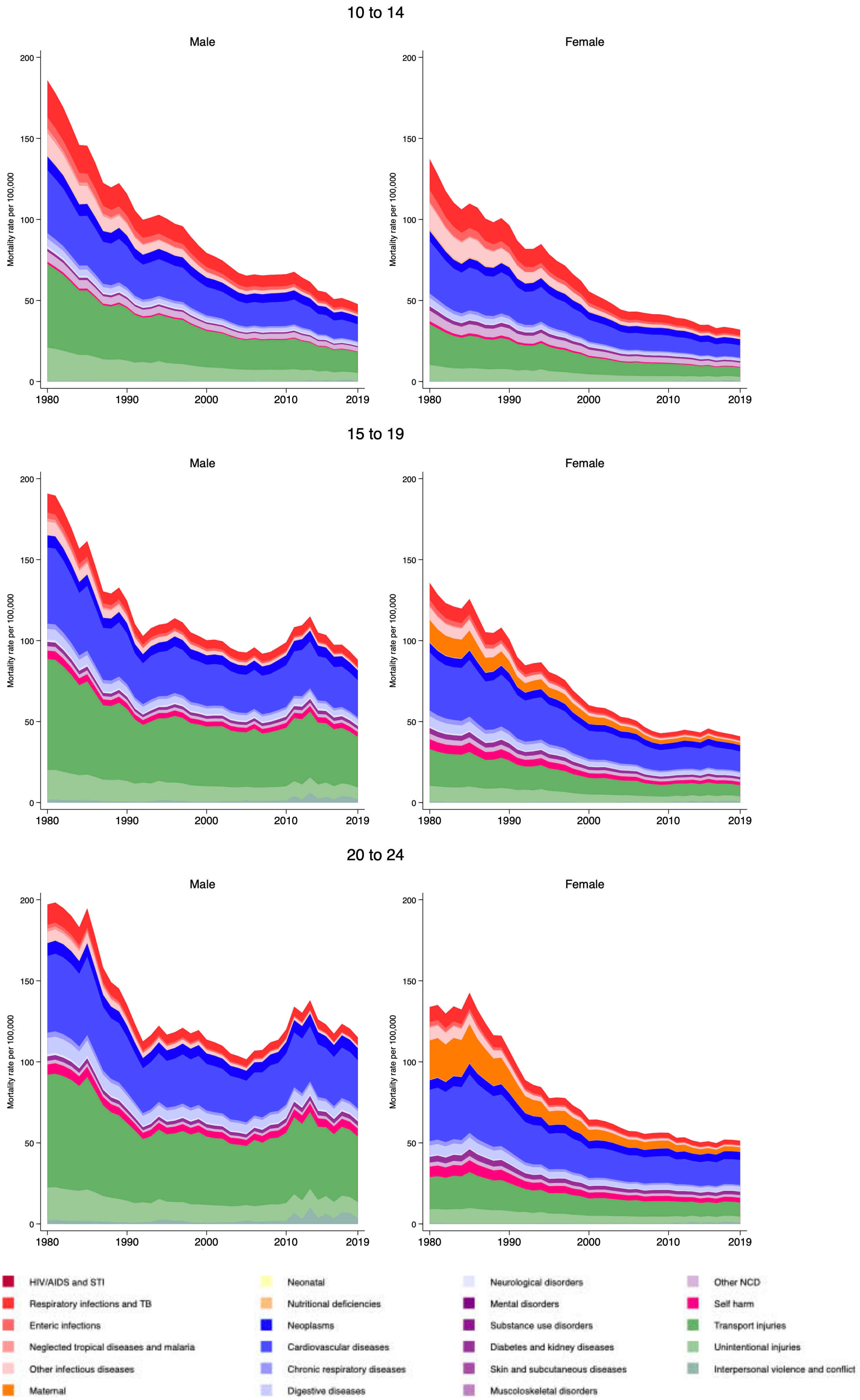

Figure S139d Number of deaths in 10-24 1980 – 2019 by cause: Egypt

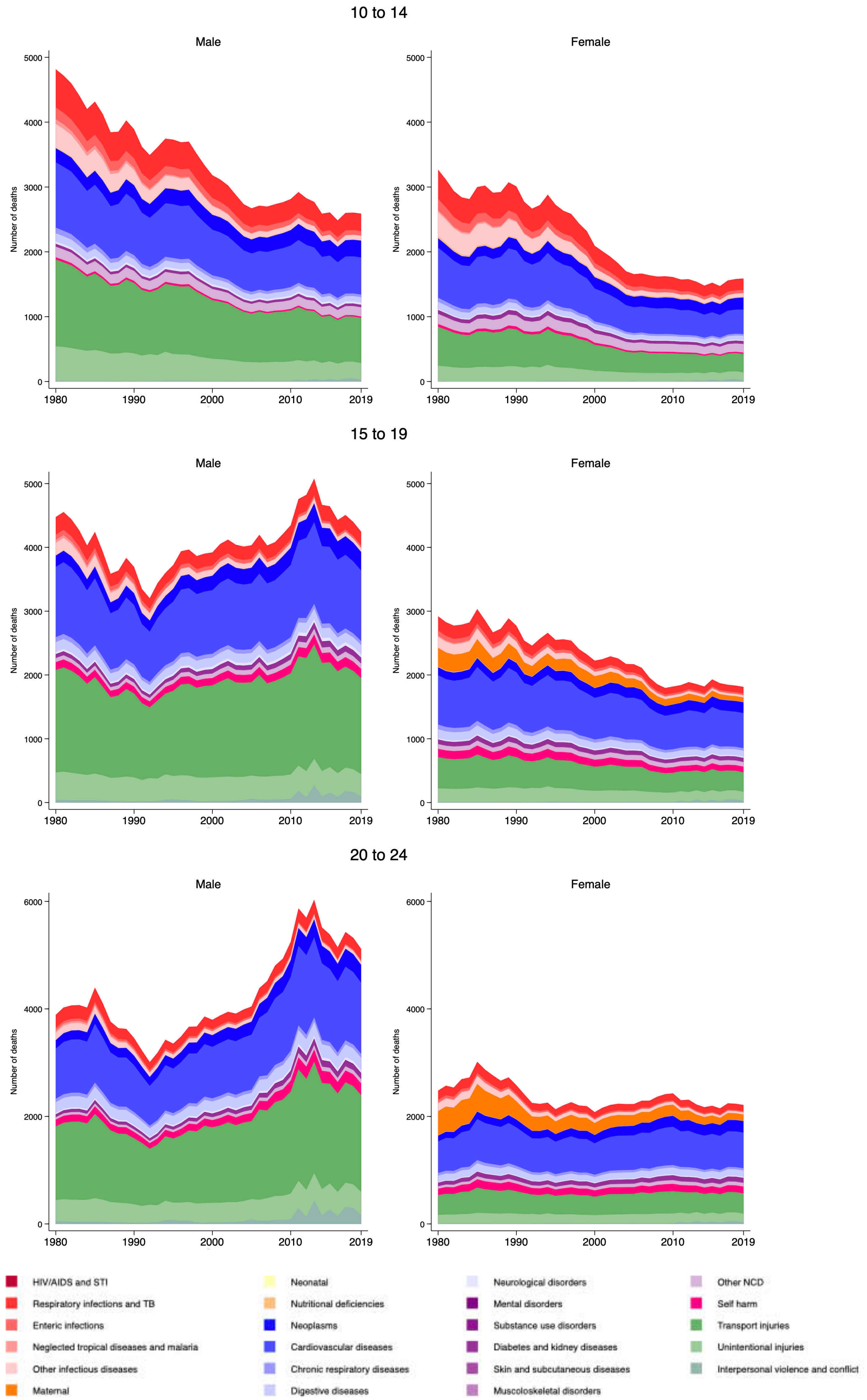

Figure S139: Iran (Islamic Republic of)

Figure S140a All-cause mortality rate per 100,000 in 10-24 1950 – 2019: Iran (Islamic Republic of)

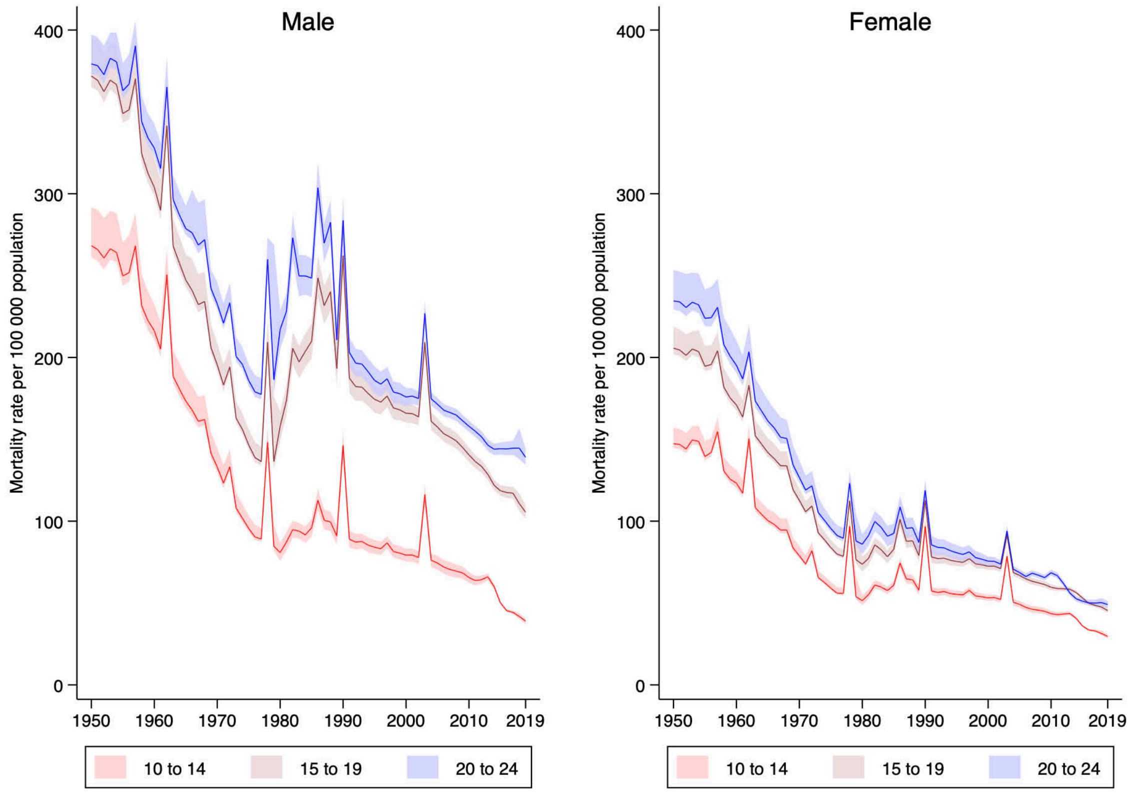

Figure S140b Number of deaths from all causes in 10-24 1950 – 2019: Iran (Islamic Republic of)

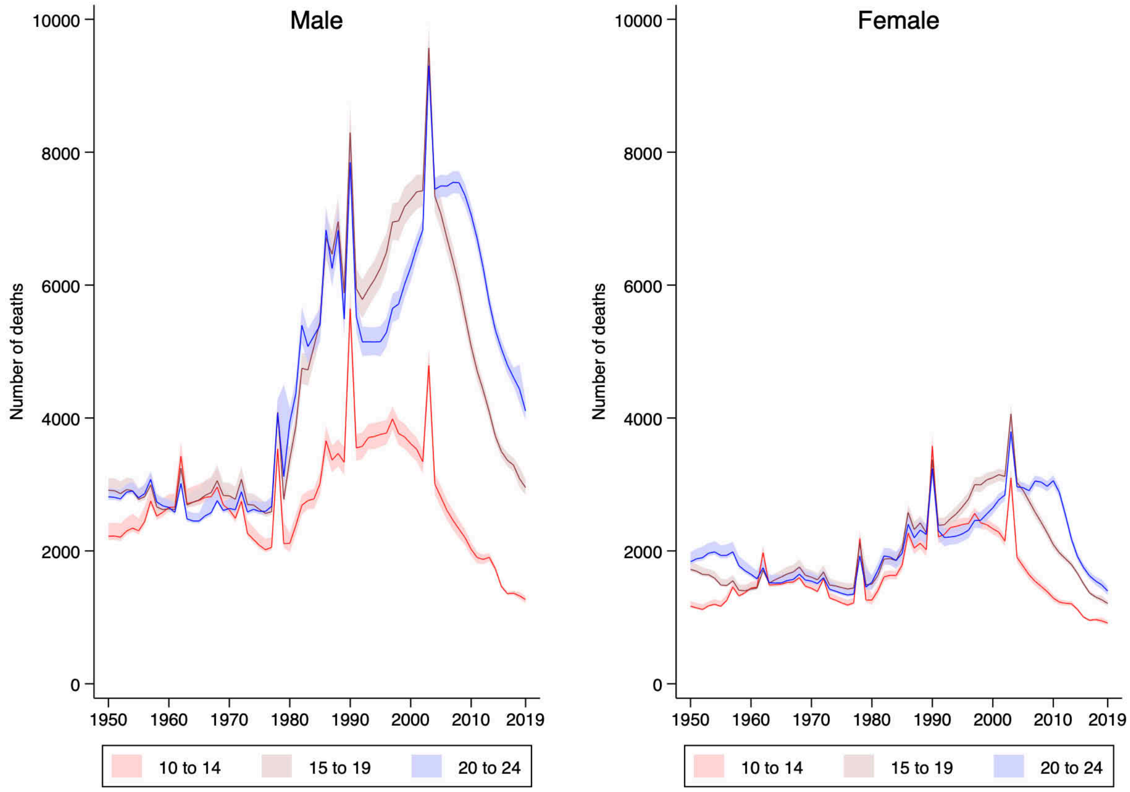

Figure S140c Mortality rate per 100,000 in 10-24 1980 – 2019 by cause: Iran (Islamic Republic of)

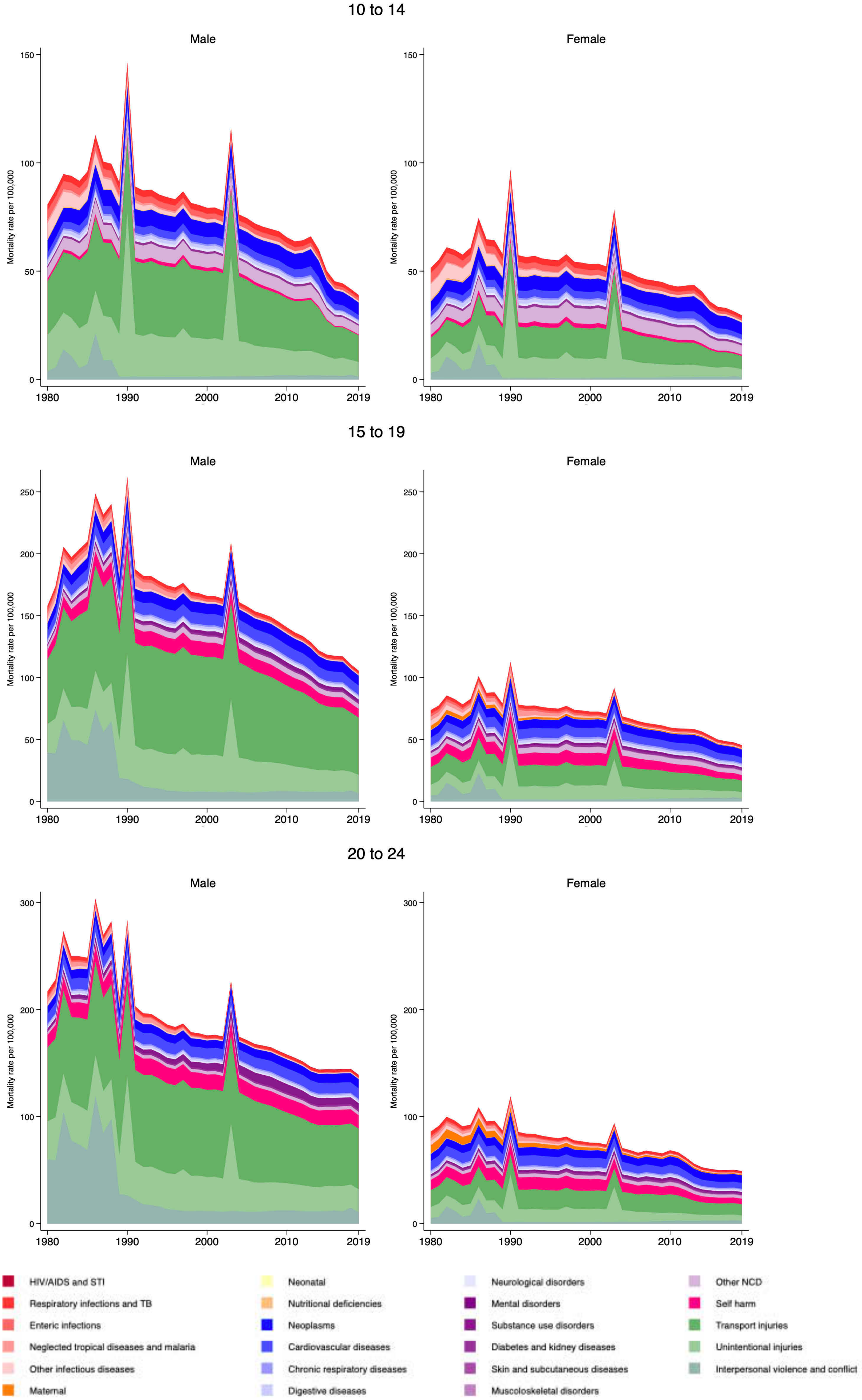

Figure S140d Number of deaths in 10-24 1980 – 2019 by cause: Iran (Islamic Republic of)

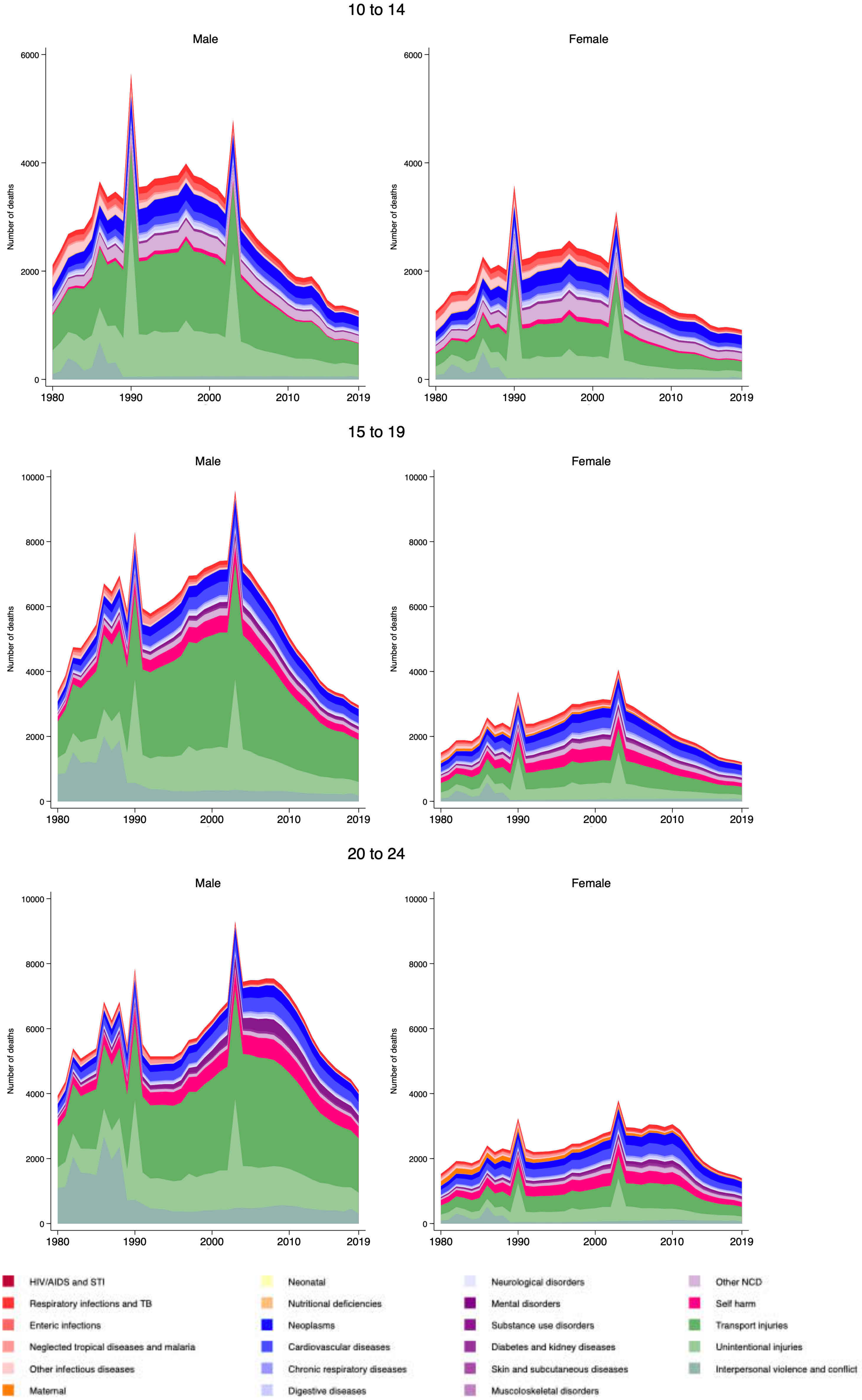

Figure S140: Iraq

Figure S141a All-cause mortality rate per 100,000 in 10-24 1950 – 2019: Iraq

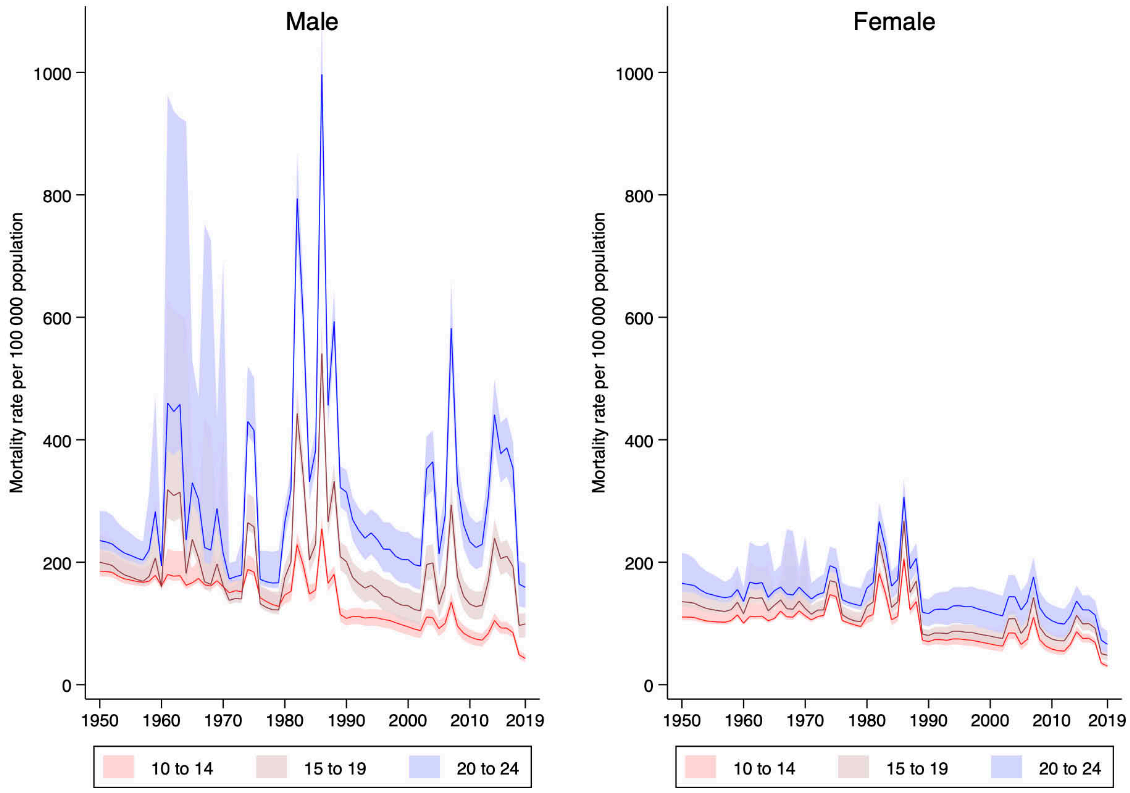

Figure S141b Number of deaths from all causes in 10-24 1950 – 2019: Iraq

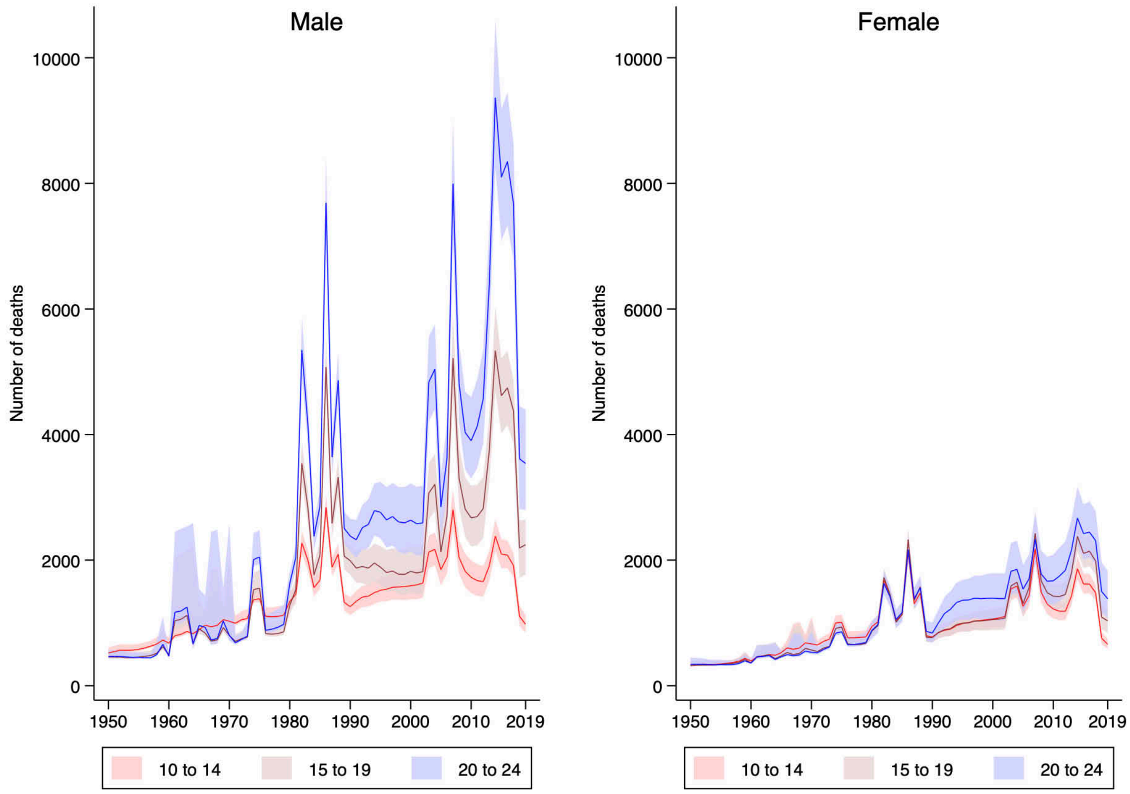

Figure S141c Mortality rate per 100,000 in 10-24 1980 – 2019 by cause: Iraq

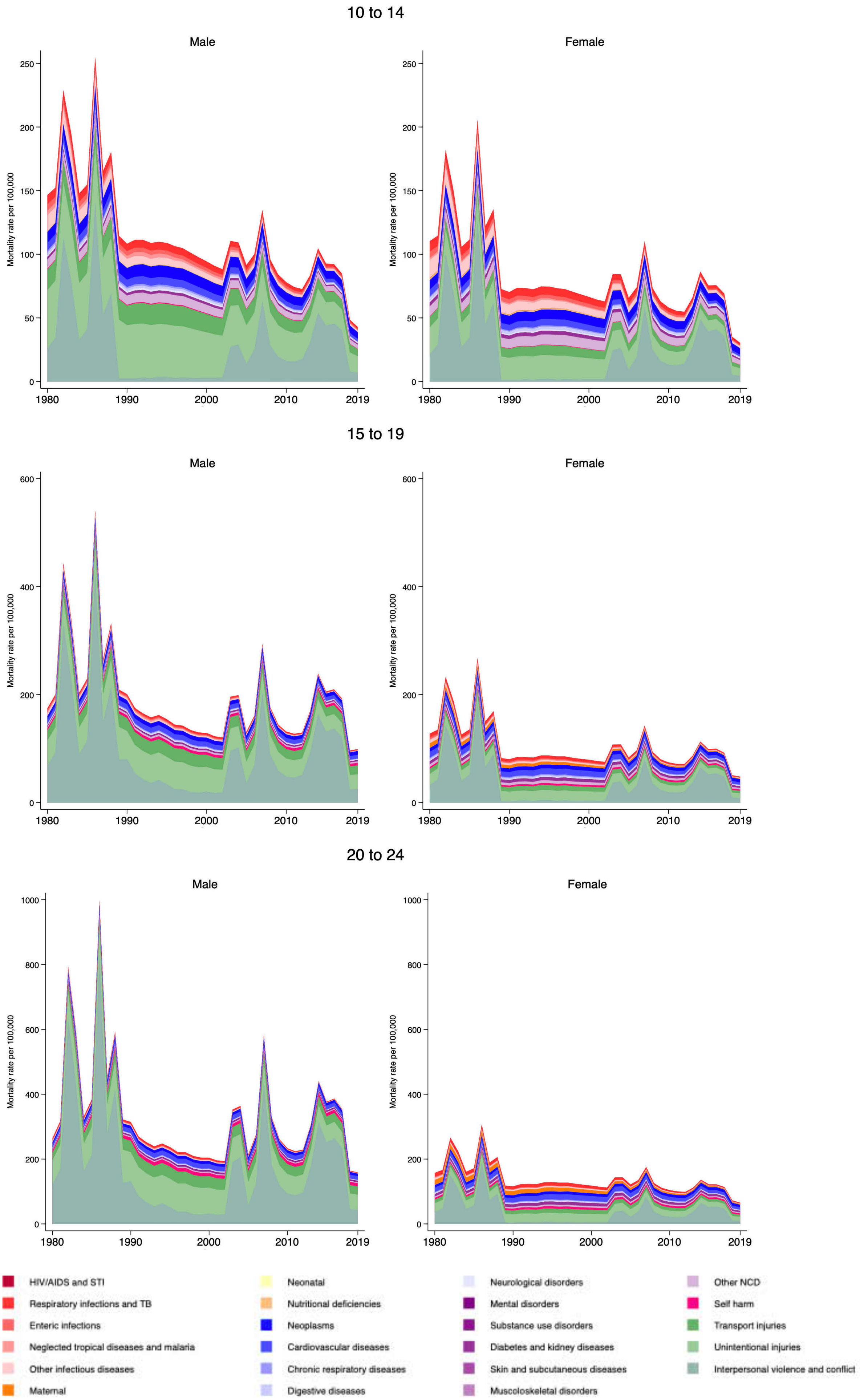

Figure S141d Number of deaths in 10-24 1980 – 2019 by cause: Iraq

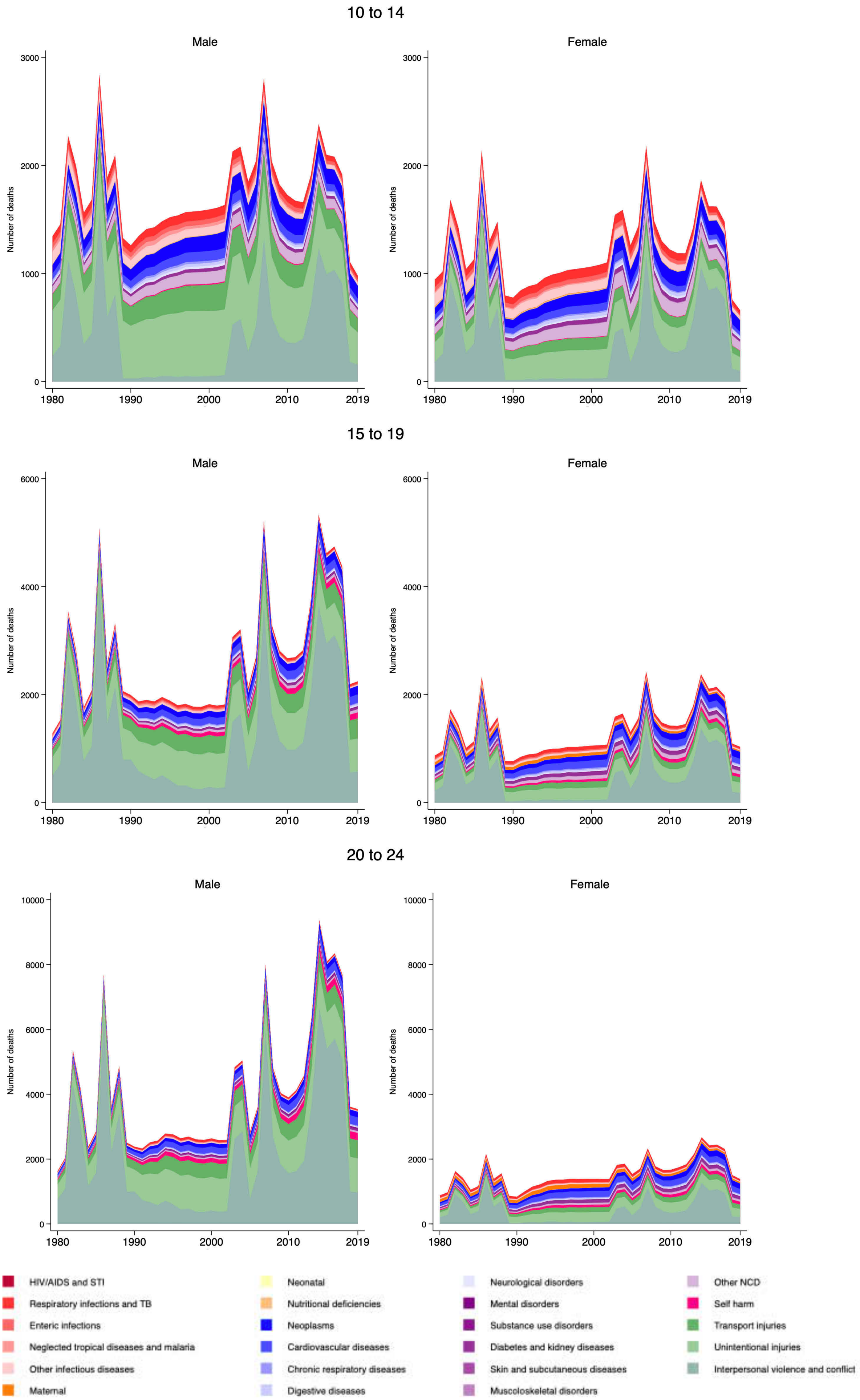

Figure S141: Jordan

Figure S142a All-cause mortality rate per 100,000 in 10-24 1950 – 2019: Jordan

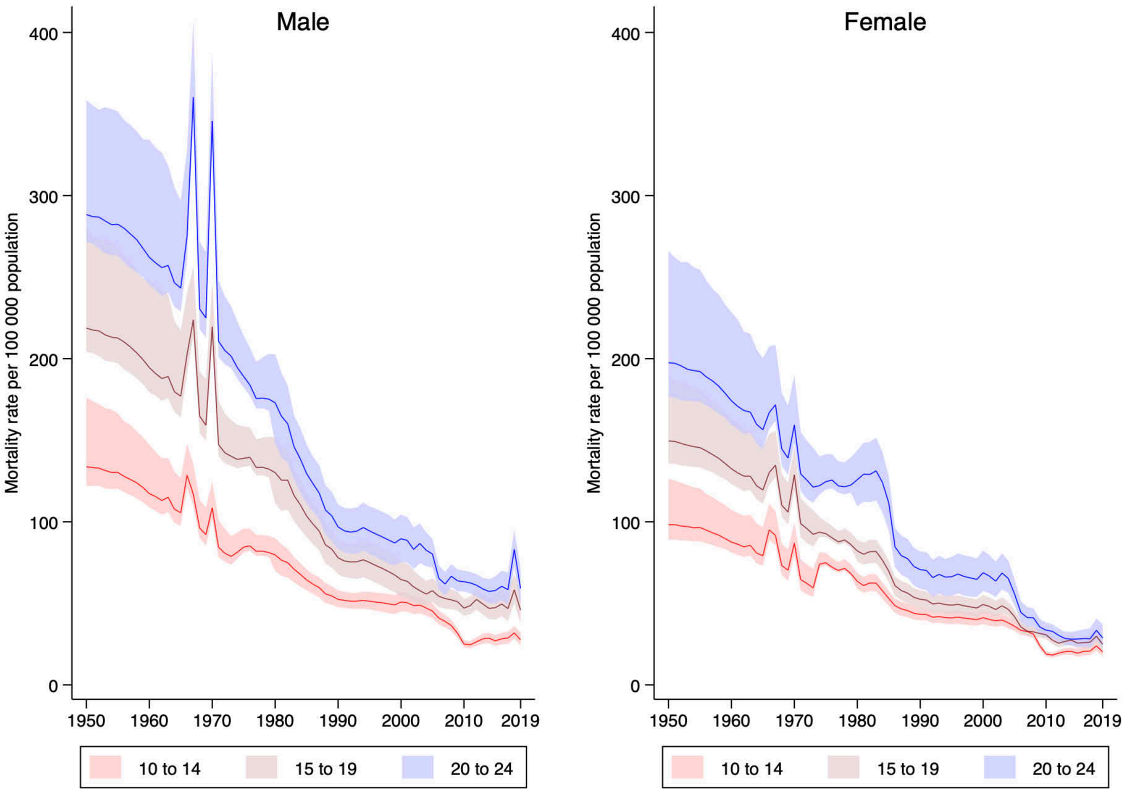

Figure S142b Number of deaths from all causes in 10-24 1950 – 2019: Jordan

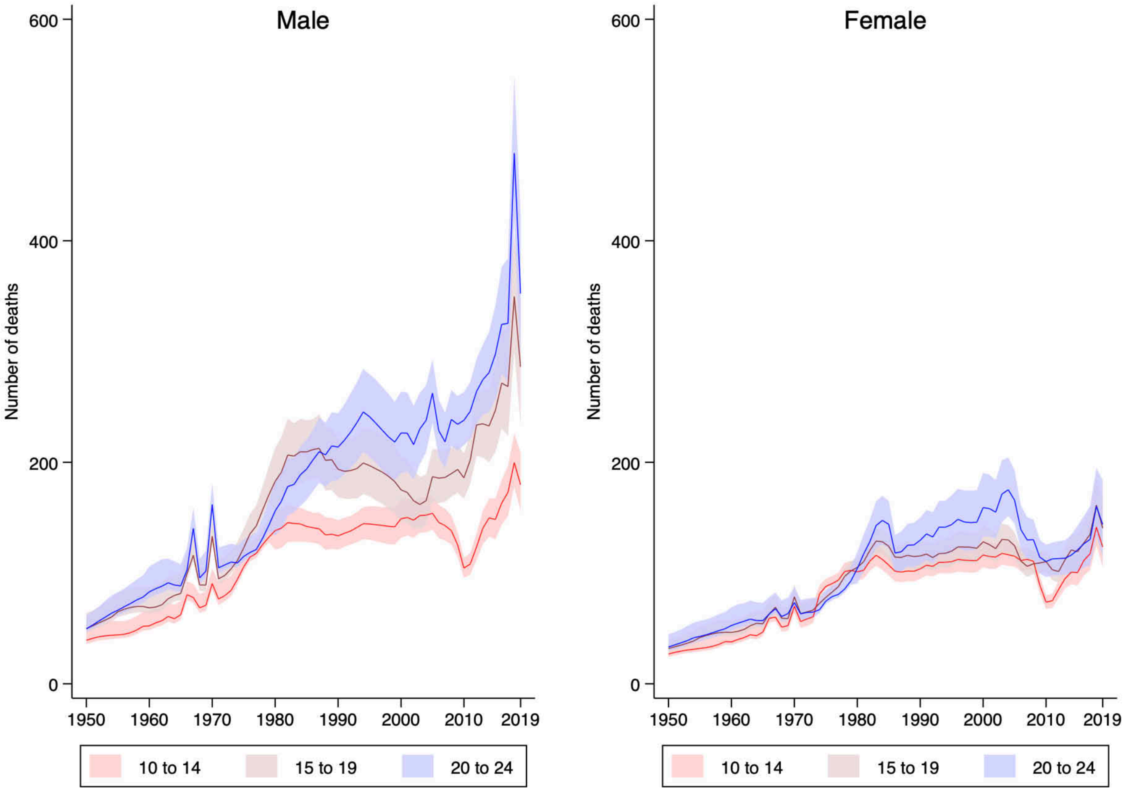

Figure S142c Mortality rate per 100,000 in 10-24 1980 – 2019 by cause: Jordan

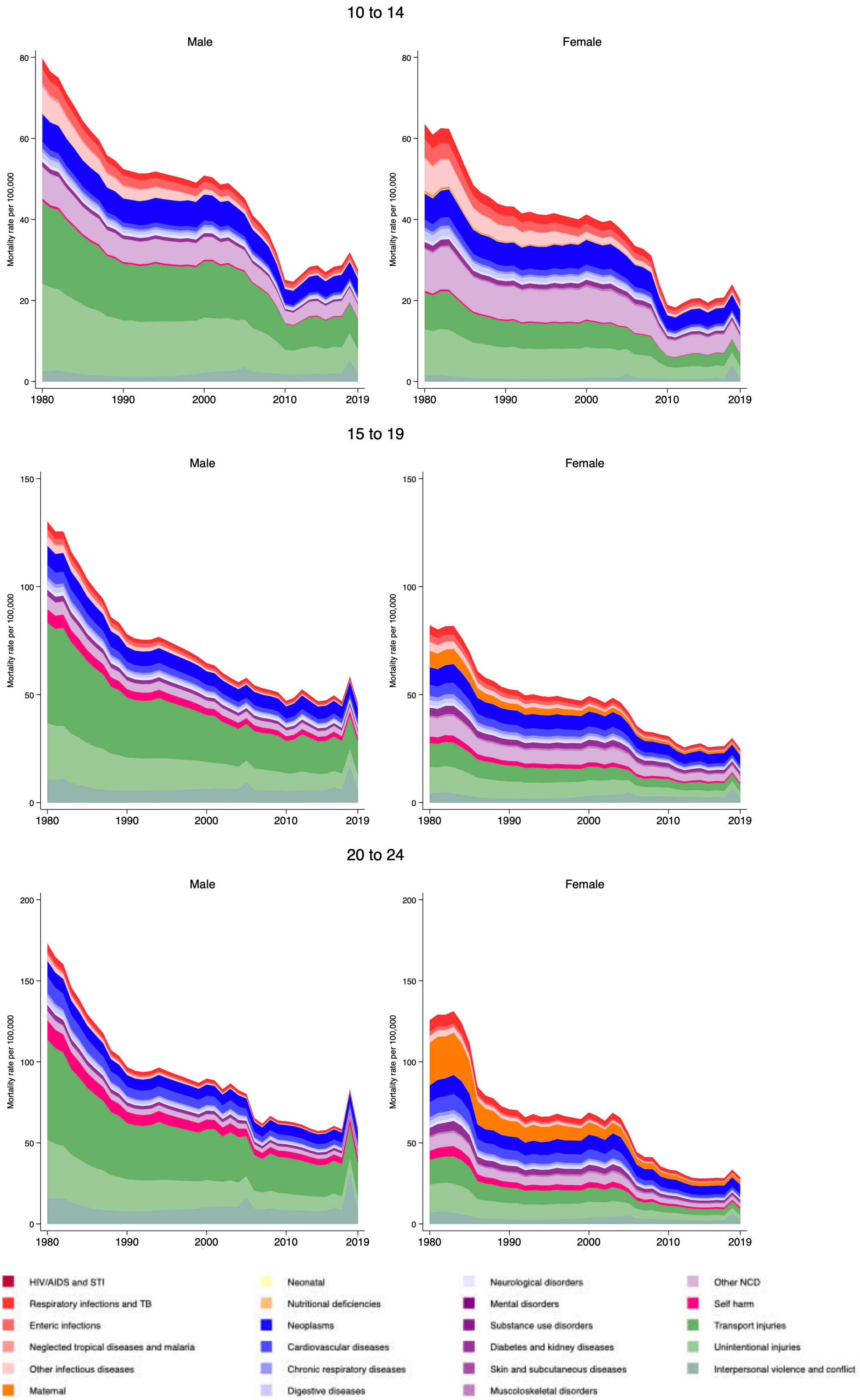

Figure S142d Number of deaths in 10-24 1980 – 2019 by cause: Jordan

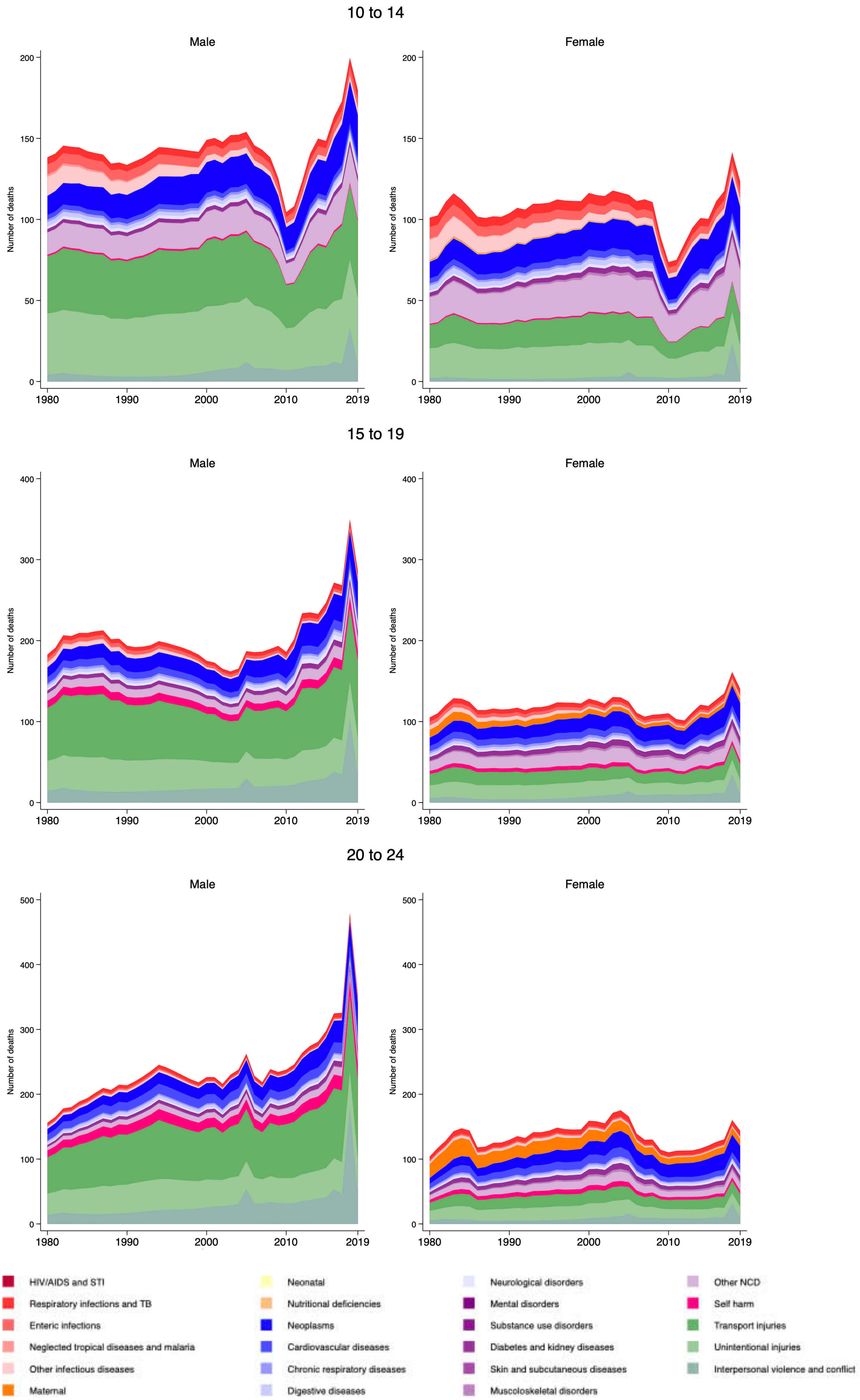

Figure S142: Kuwait

Figure S143a All-cause mortality rate per 100,000 in 10-24 1950 – 2019: Kuwait

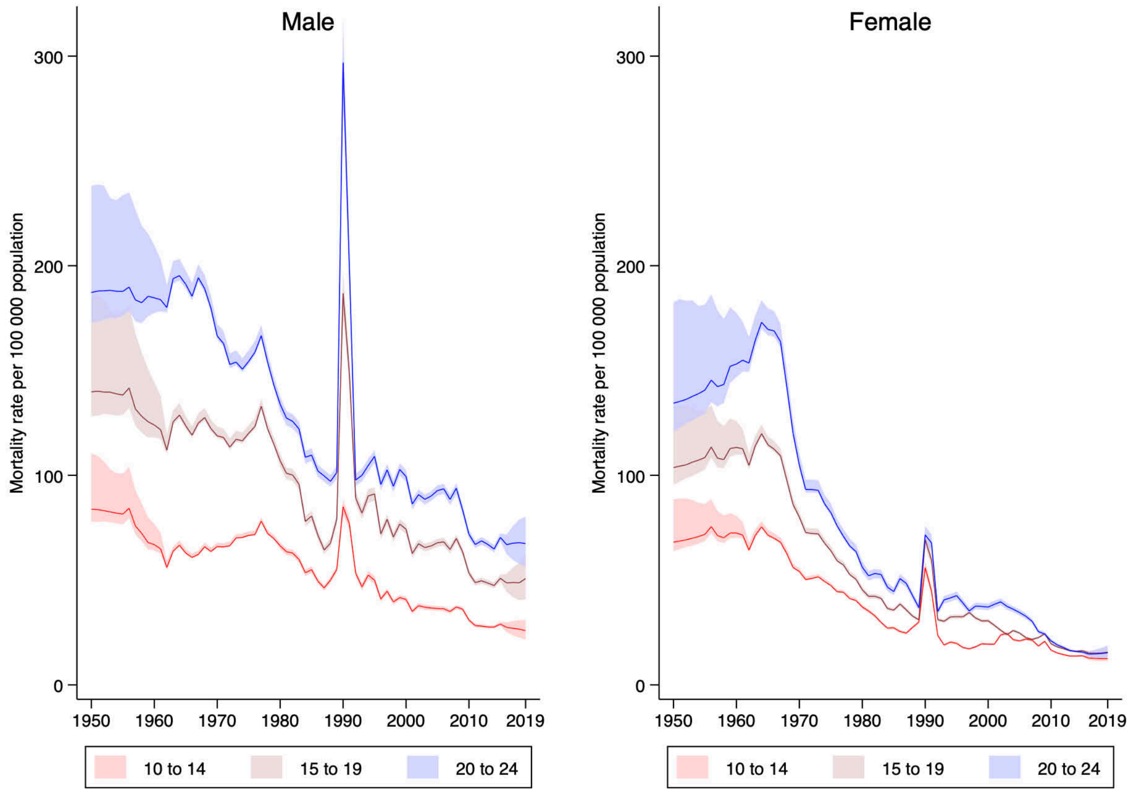

Figure S143b Number of deaths from all causes in 10-24 1950 – 2019: Kuwait

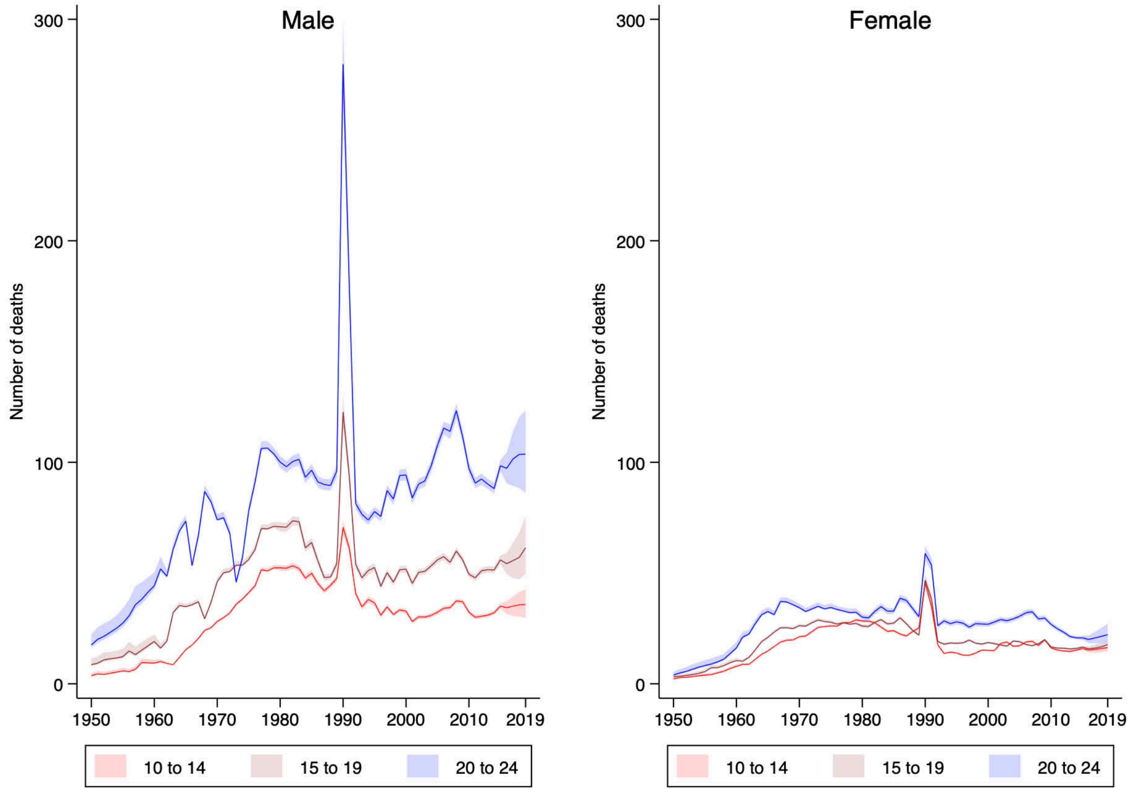

Figure S143c Mortality rate per 100,000 in 10-24 1980 – 2019 by cause: Kuwait

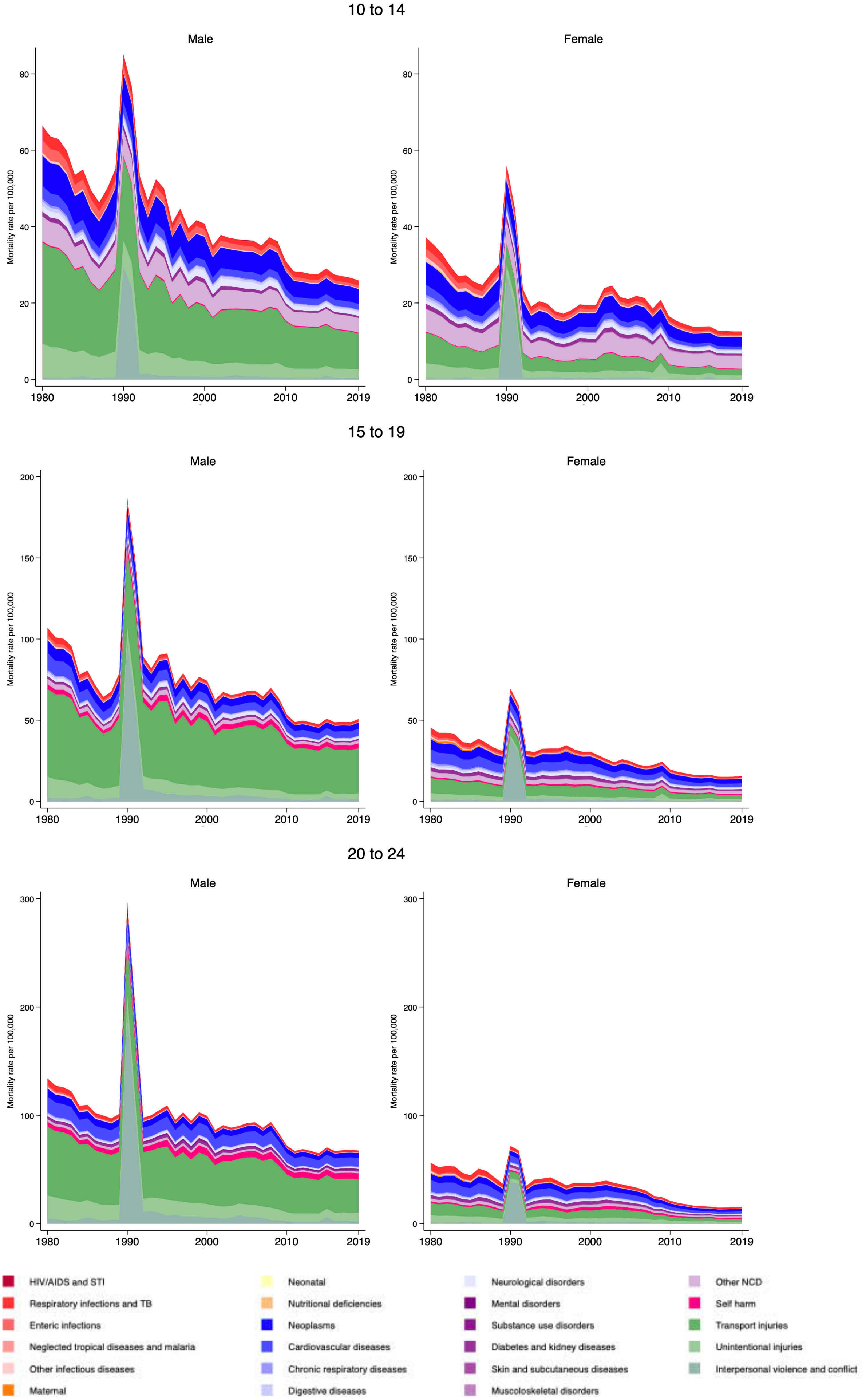

Figure S143d Number of deaths in 10-24 1980 – 2019 by cause: Kuwait

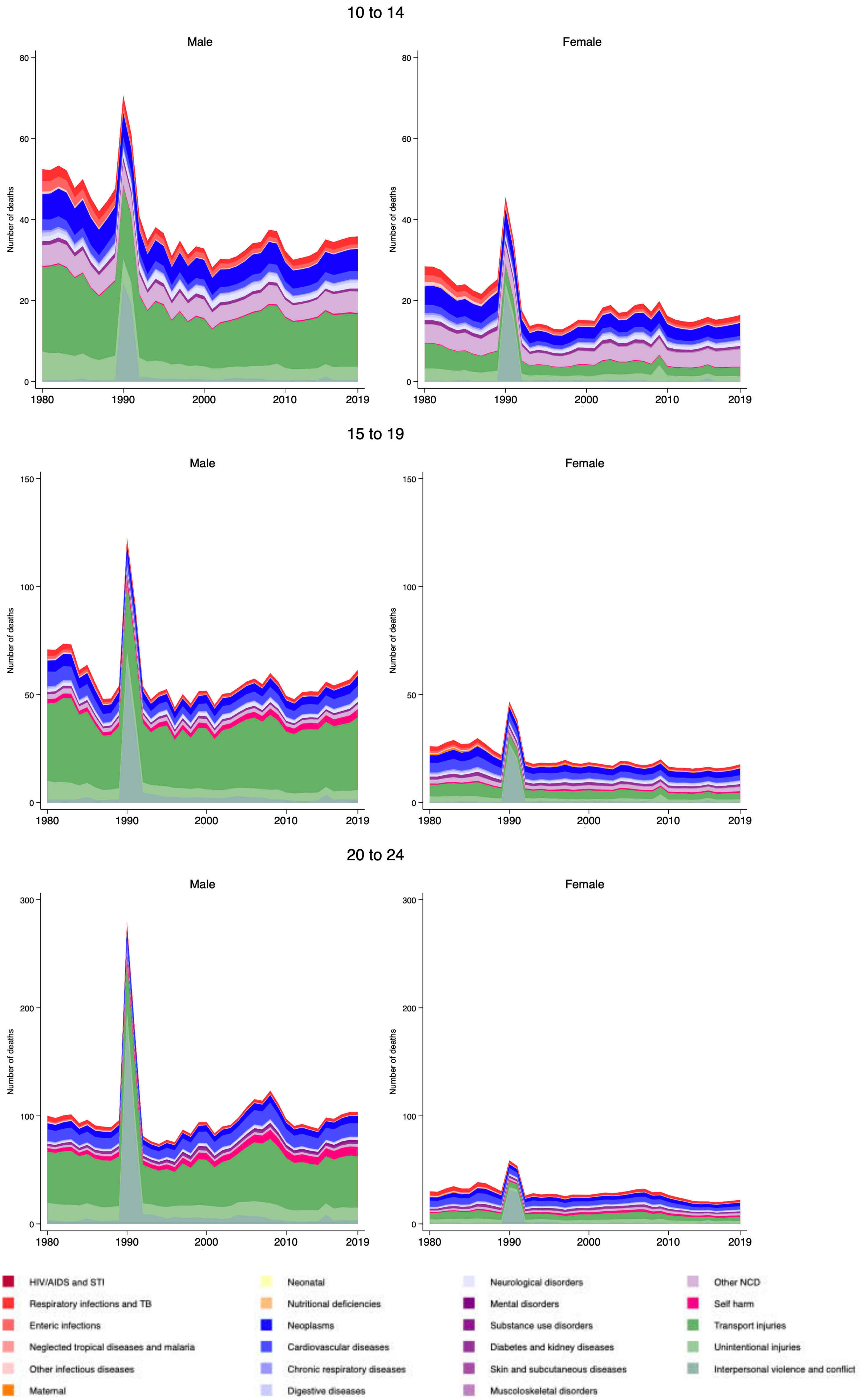

Figure S143: Lebanon

Figure S144a All-cause mortality rate per 100,000 in 10-24 1950 – 2019: Lebanon

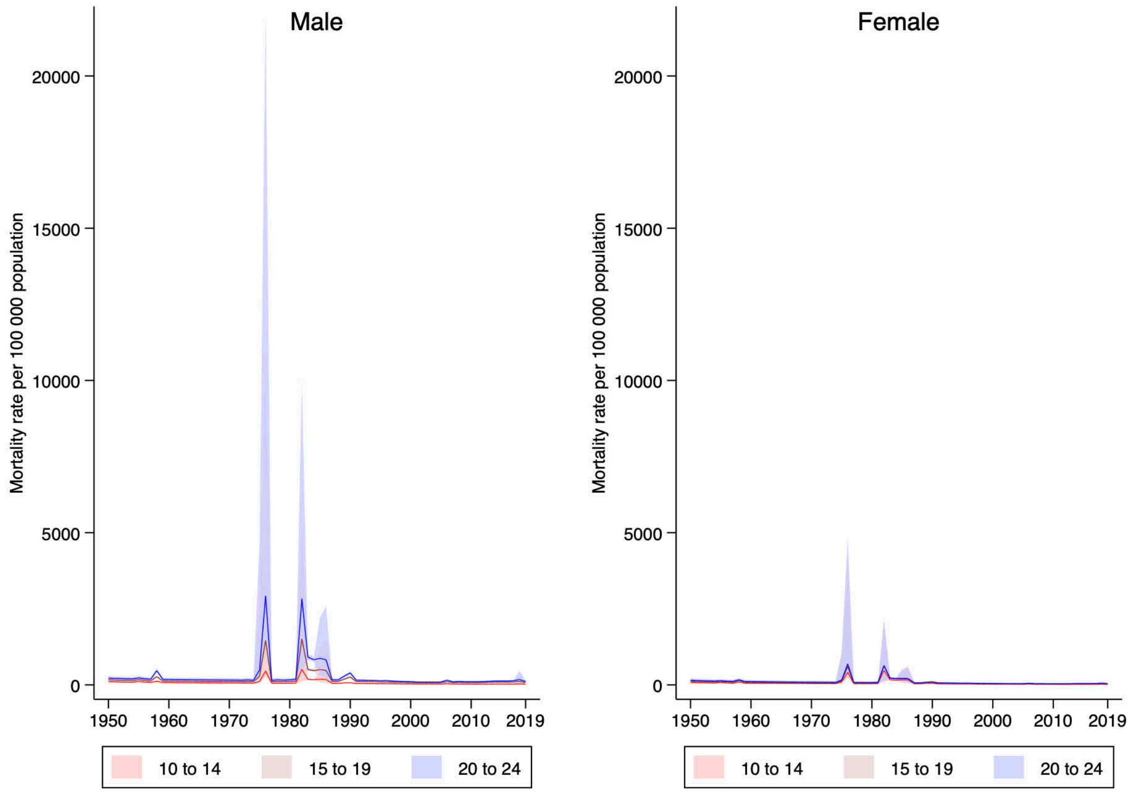

Figure S144b Number of deaths from all causes in 10-24 1950 – 2019: Lebanon

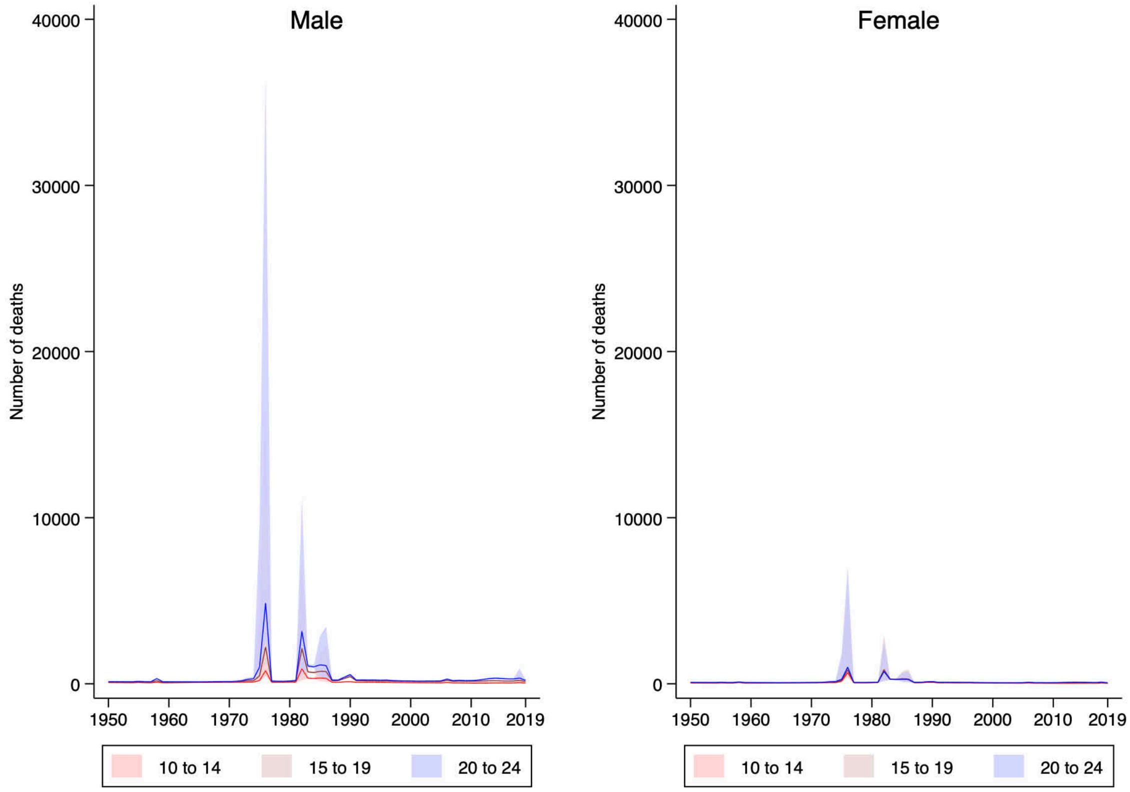

Figure S144c Mortality rate per 100,000 in 10-24 1980 – 2019 by cause: Lebanon

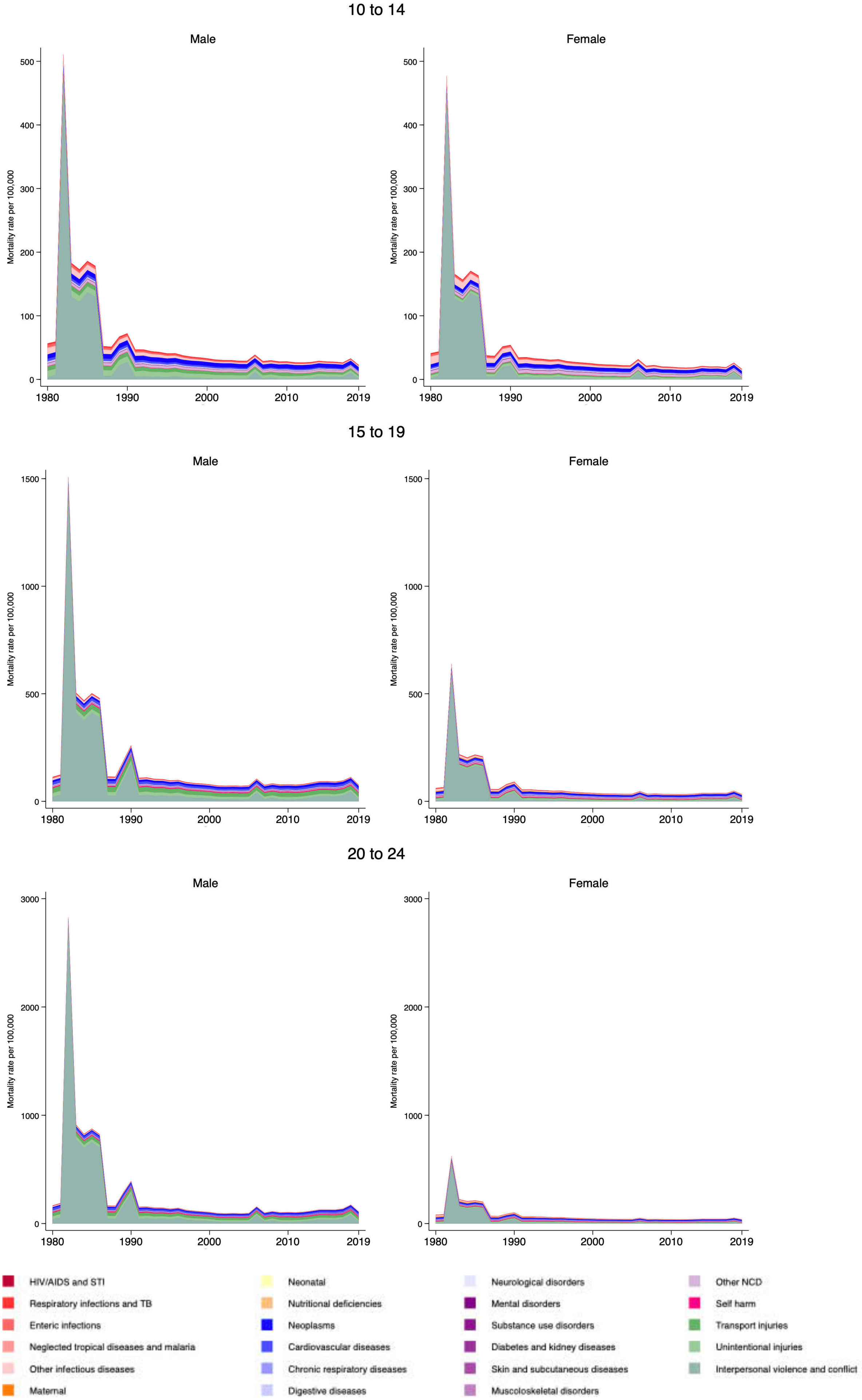

Figure S144d Number of deaths in 10-24 1980 – 2019 by cause: Lebanon

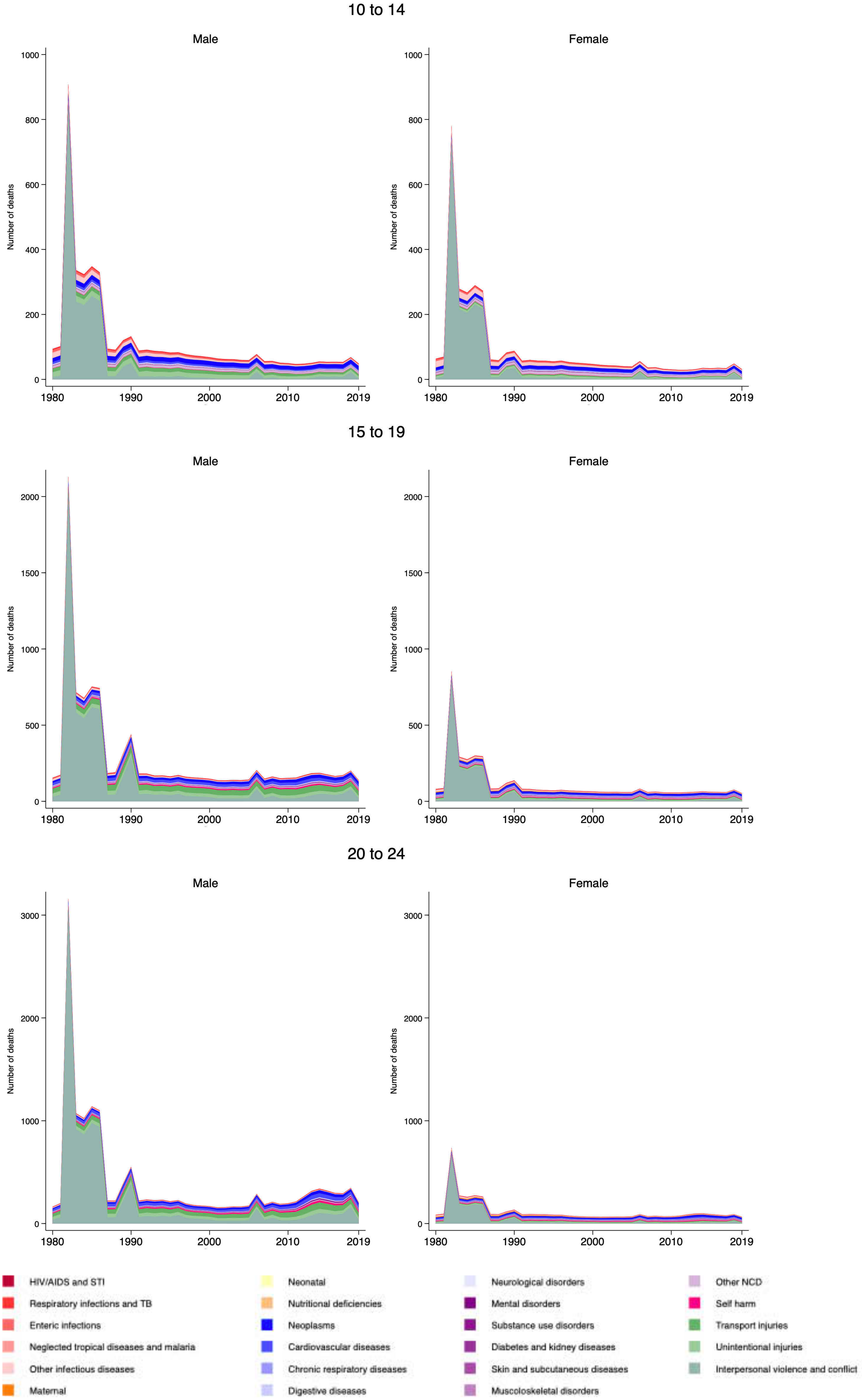

Figure S144: Libya

Figure S145a All-cause mortality rate per 100,000 in 10-24 1950 – 2019: Libya

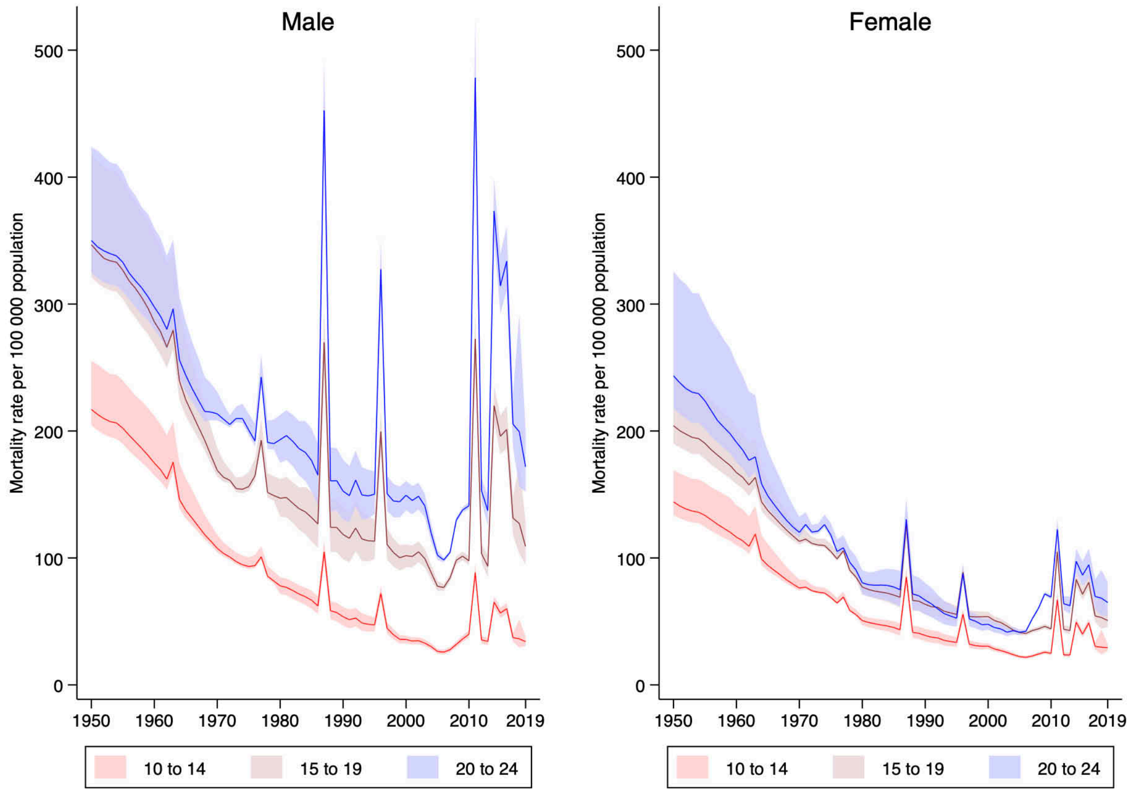

Figure S145b Number of deaths from all causes in 10-24 1950 – 2019: Libya

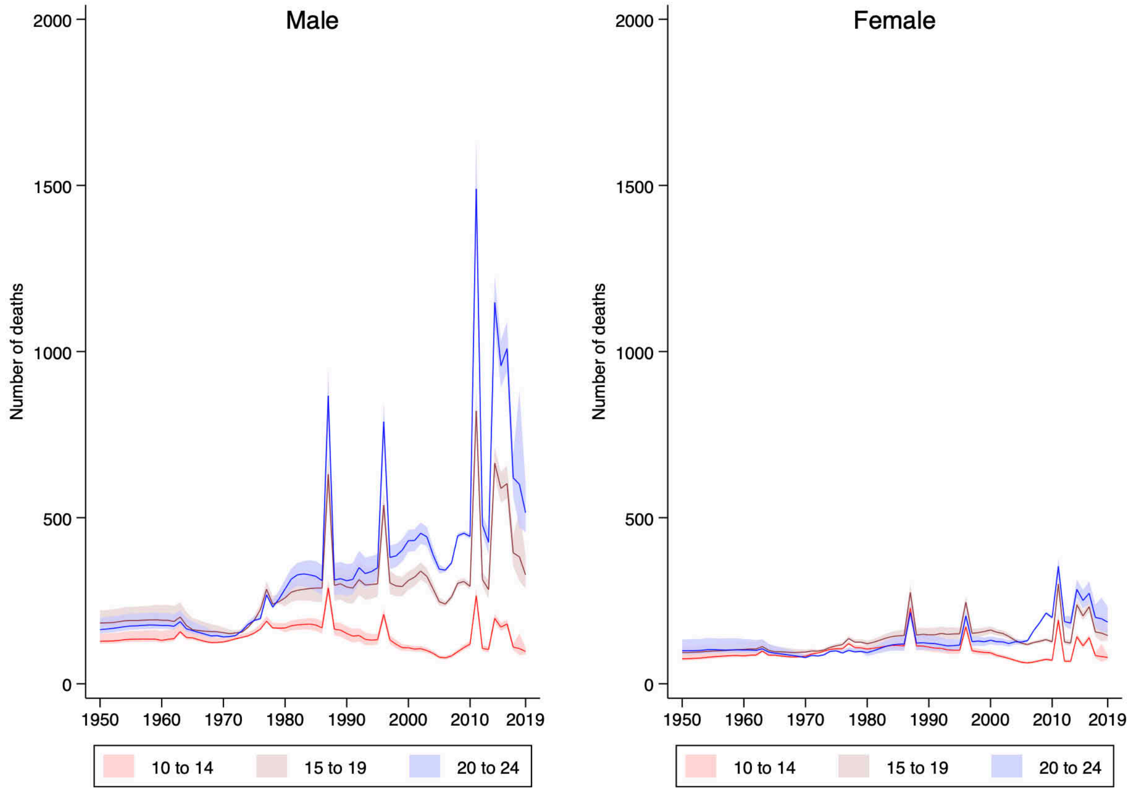

Figure S145c Mortality rate per 100,000 in 10-24 1980 – 2019 by cause: Libya

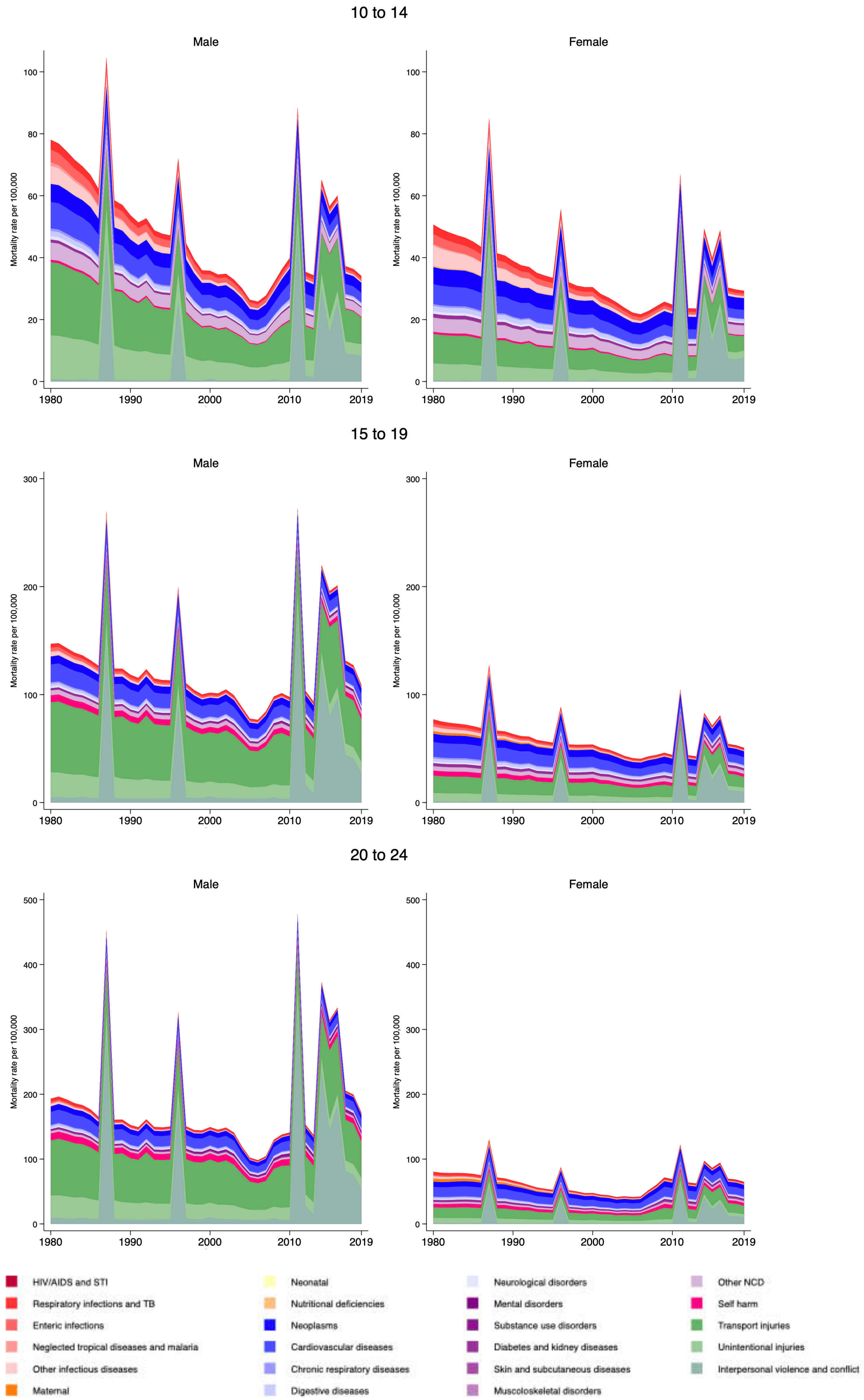

Figure S145d Number of deaths in 10-24 1980 – 2019 by cause: Libya

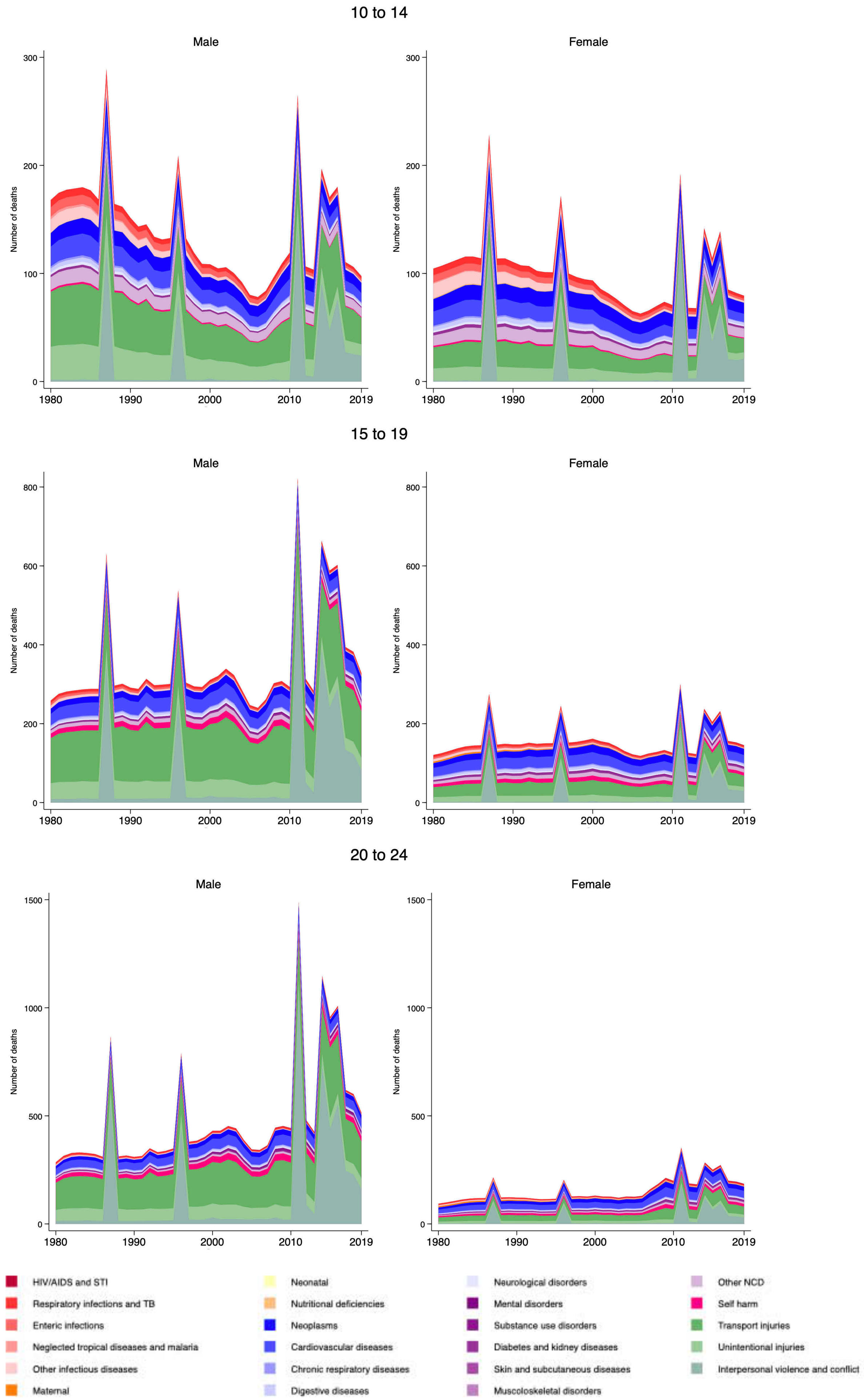

Figure S145: Morocco

Figure S146a All-cause mortality rate per 100,000 in 10-24 1950 – 2019: Morocco

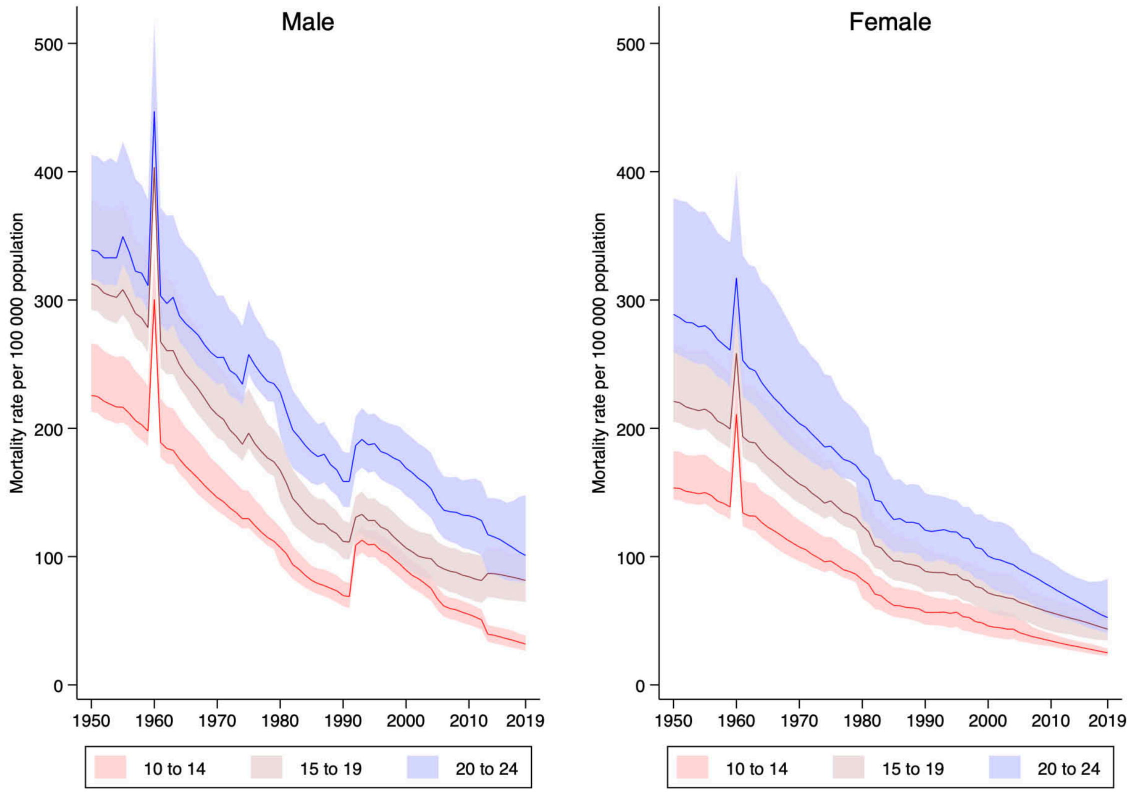

Figure S146b Number of deaths from all causes in 10-24 1950 – 2019: Morocco

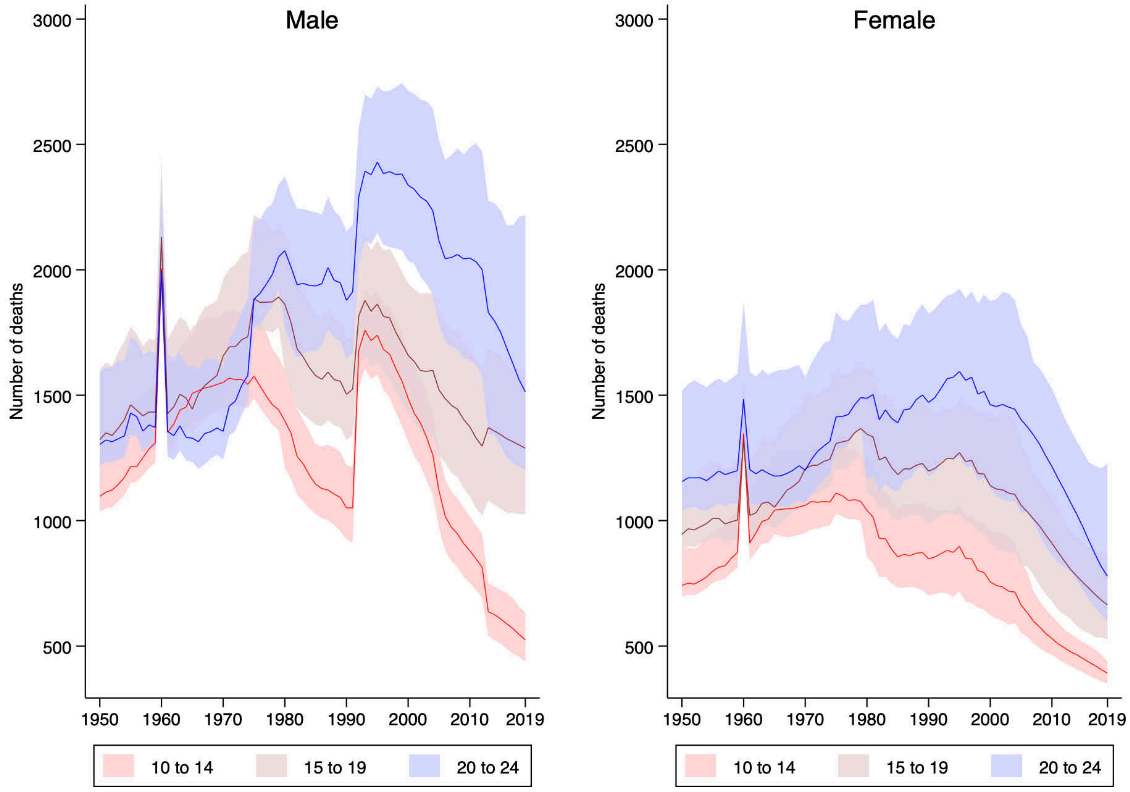

Figure S146c Mortality rate per 100,000 in 10-24 1980 – 2019 by cause: Morocco

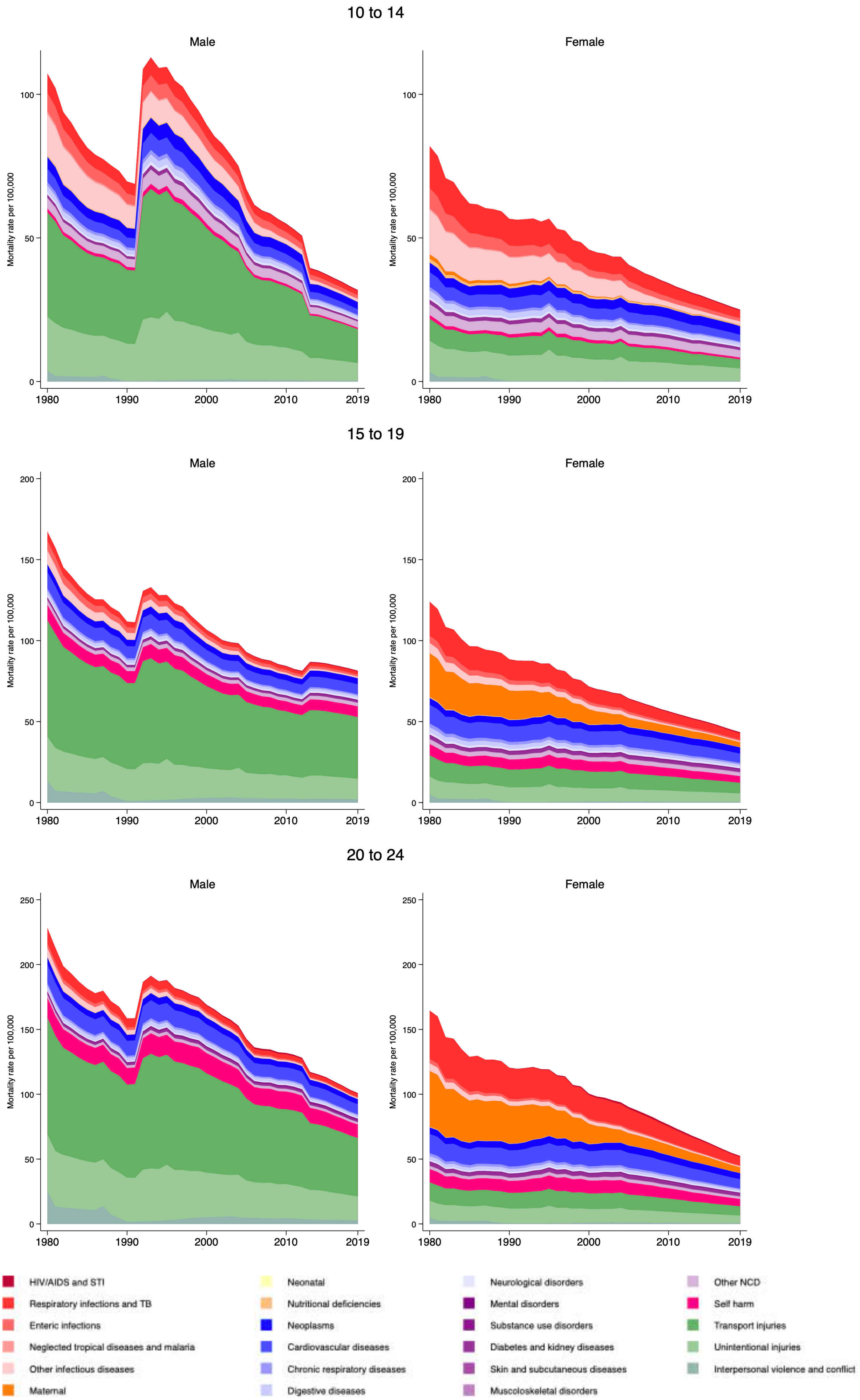

Figure S146d Number of deaths in 10-24 1980 – 2019 by cause: Morocco

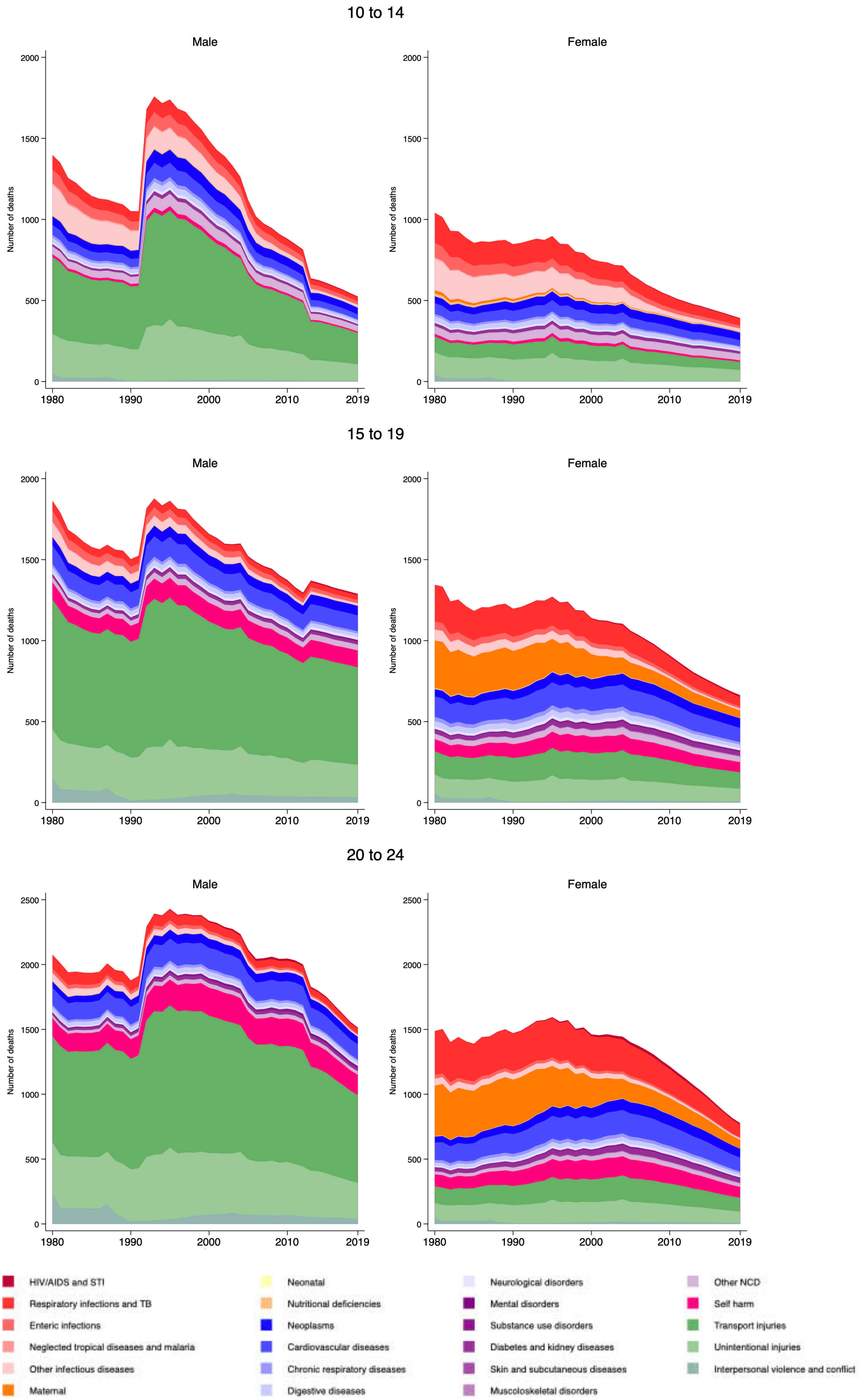

Figure S146: Palestine

Figure S147a All-cause mortality rate per 100,000 in 10-24 1950 – 2019: Palestine

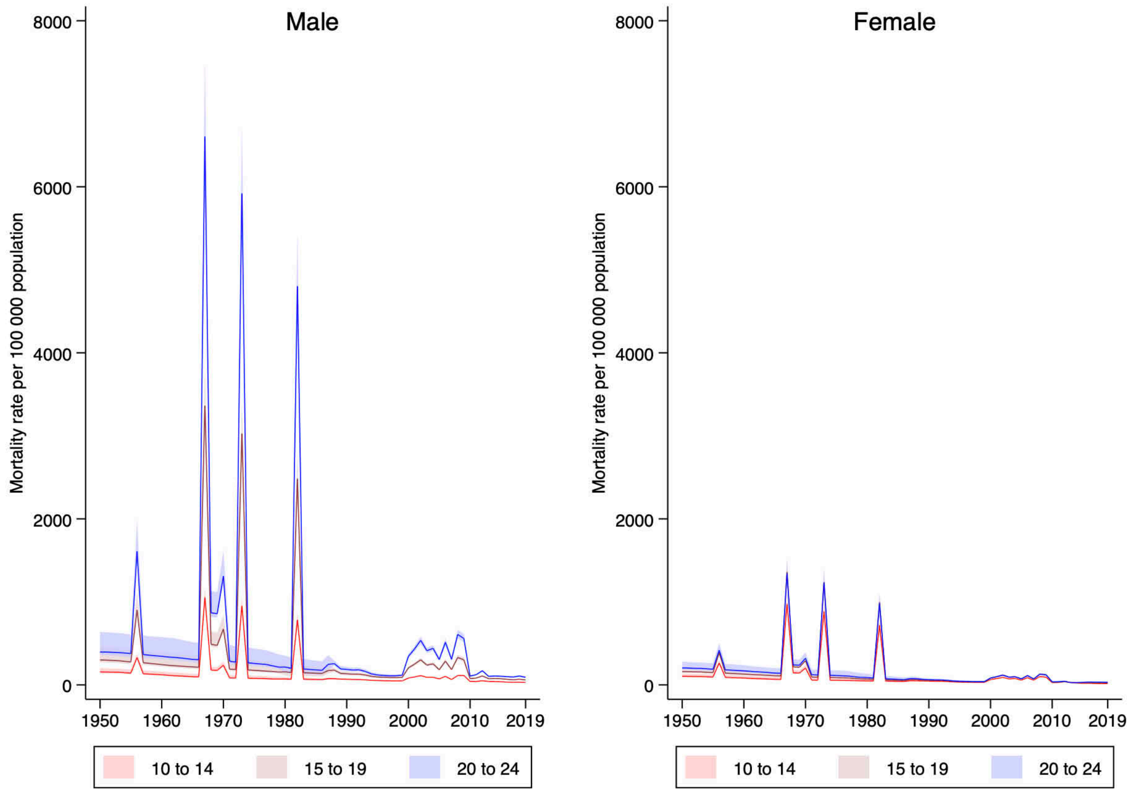

Figure S147b Number of deaths from all causes in 10-24 1950 – 2019: Palestine

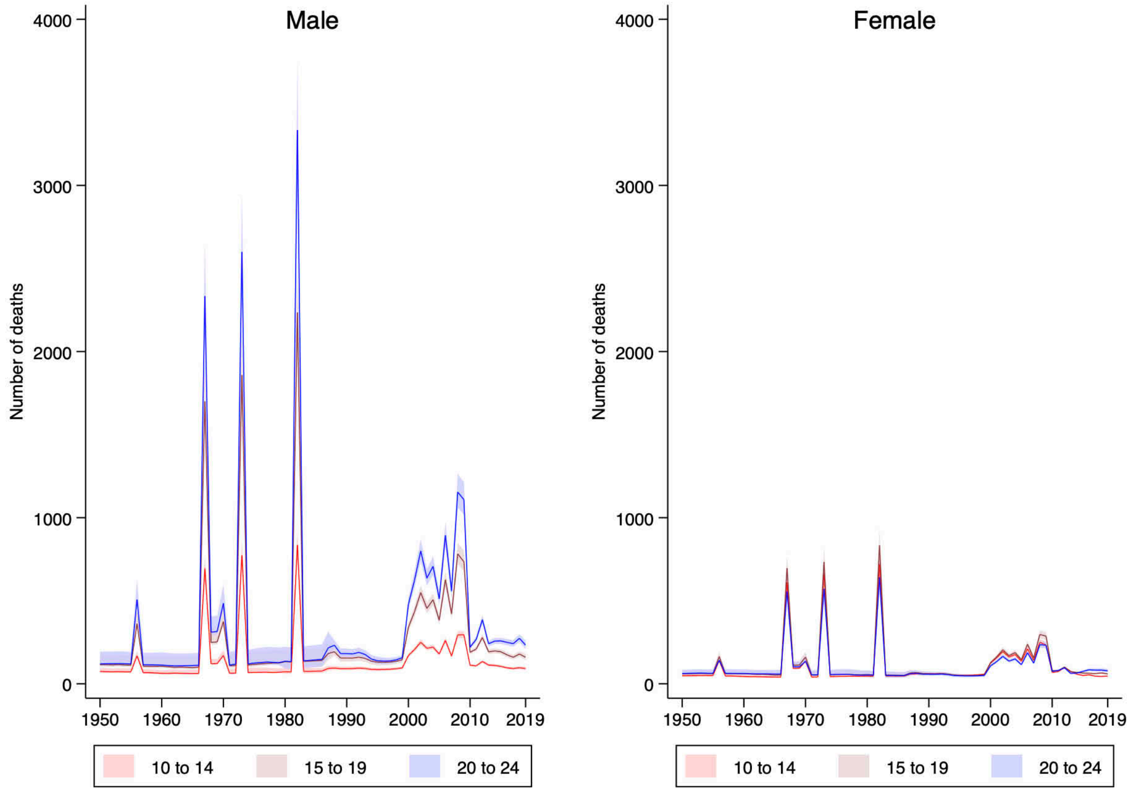

Figure S147c Mortality rate per 100,000 in 10-24 1980 – 2019 by cause: Palestine

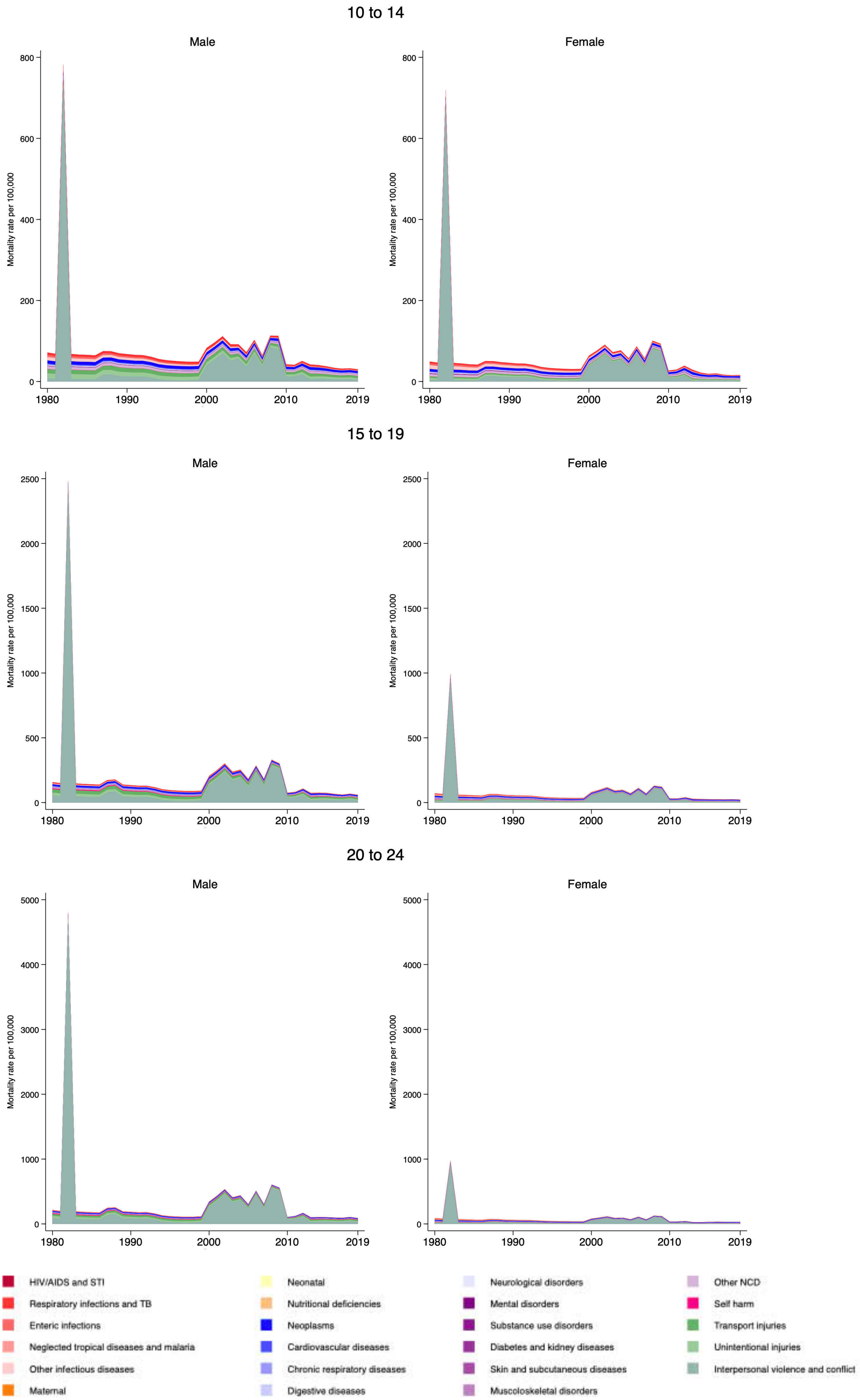

Figure S147d Number of deaths in 10-24 1980 – 2019 by cause: Palestine

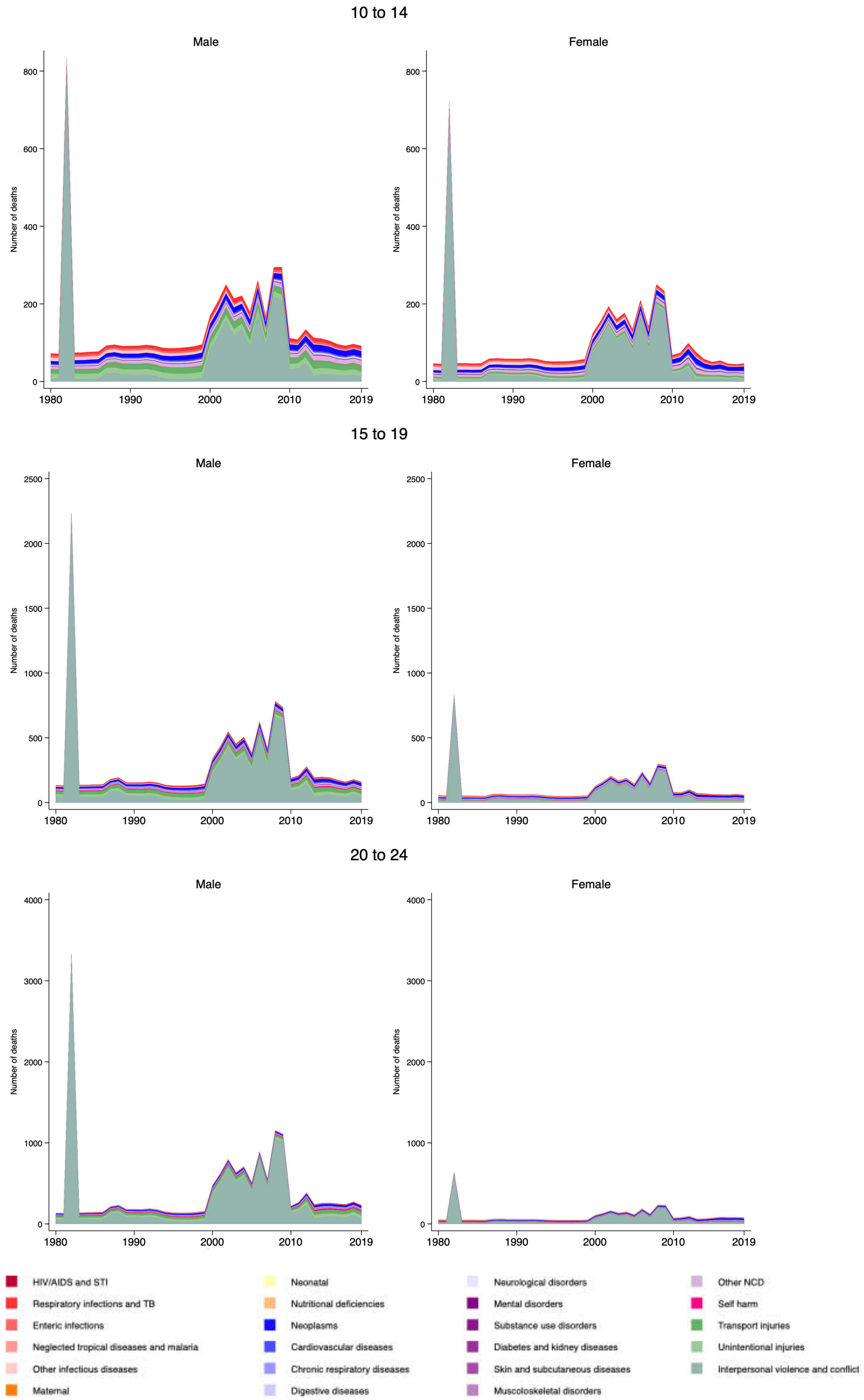

Figure S147: Oman

Figure S148a All-cause mortality rate per 100,000 in 10-24 1950 – 2019: Oman

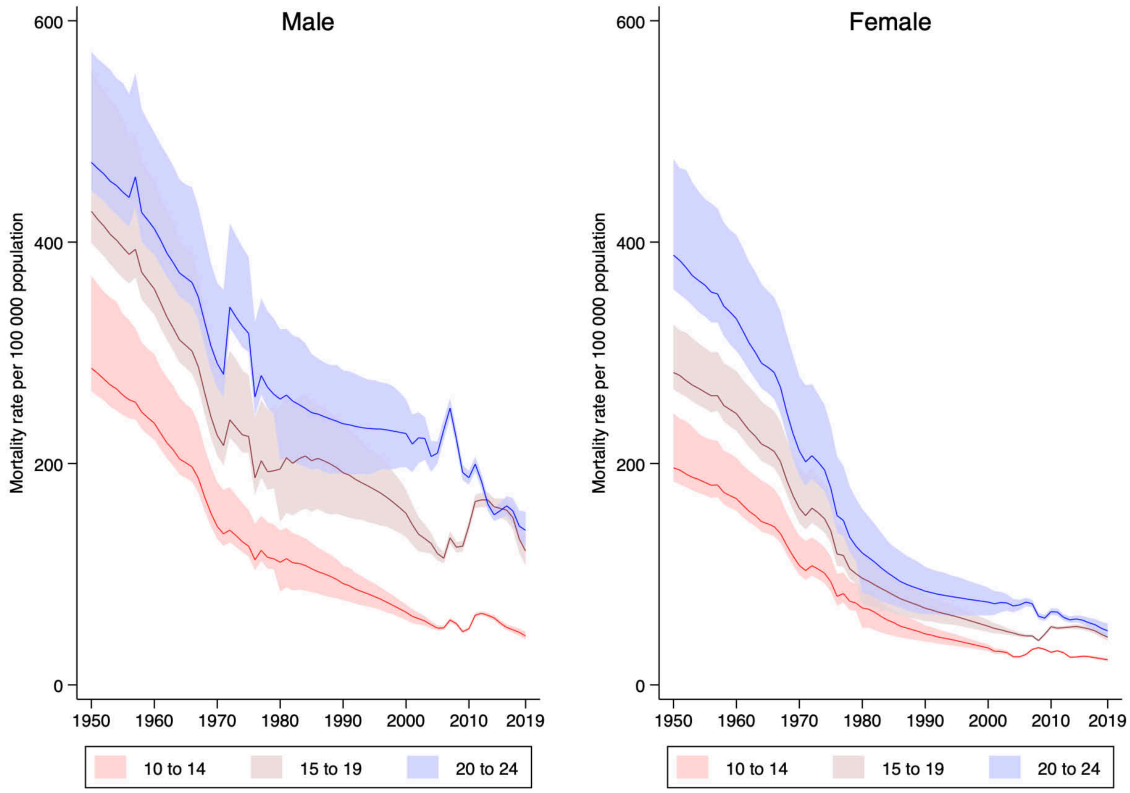

Figure S148b Number of deaths from all causes in 10-24 1950 – 2019: Oman

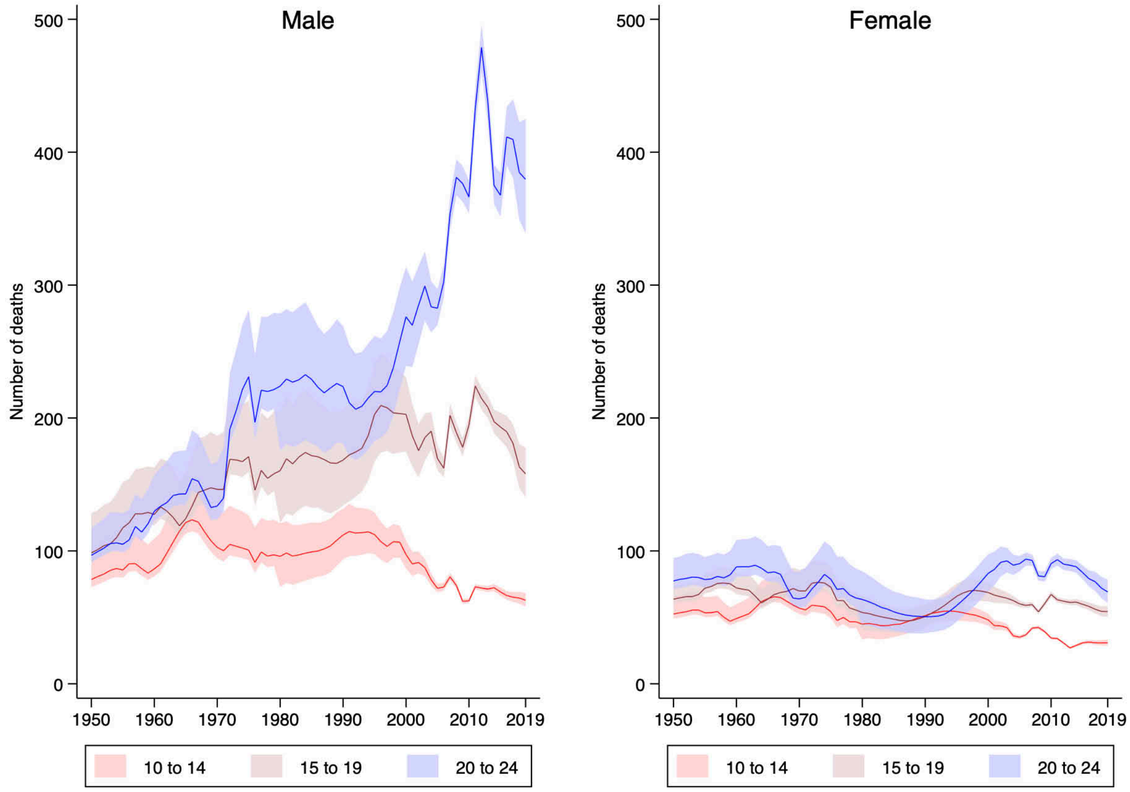

Figure S148c Mortality rate per 100,000 in 10-24 1980 – 2019 by cause: Oman

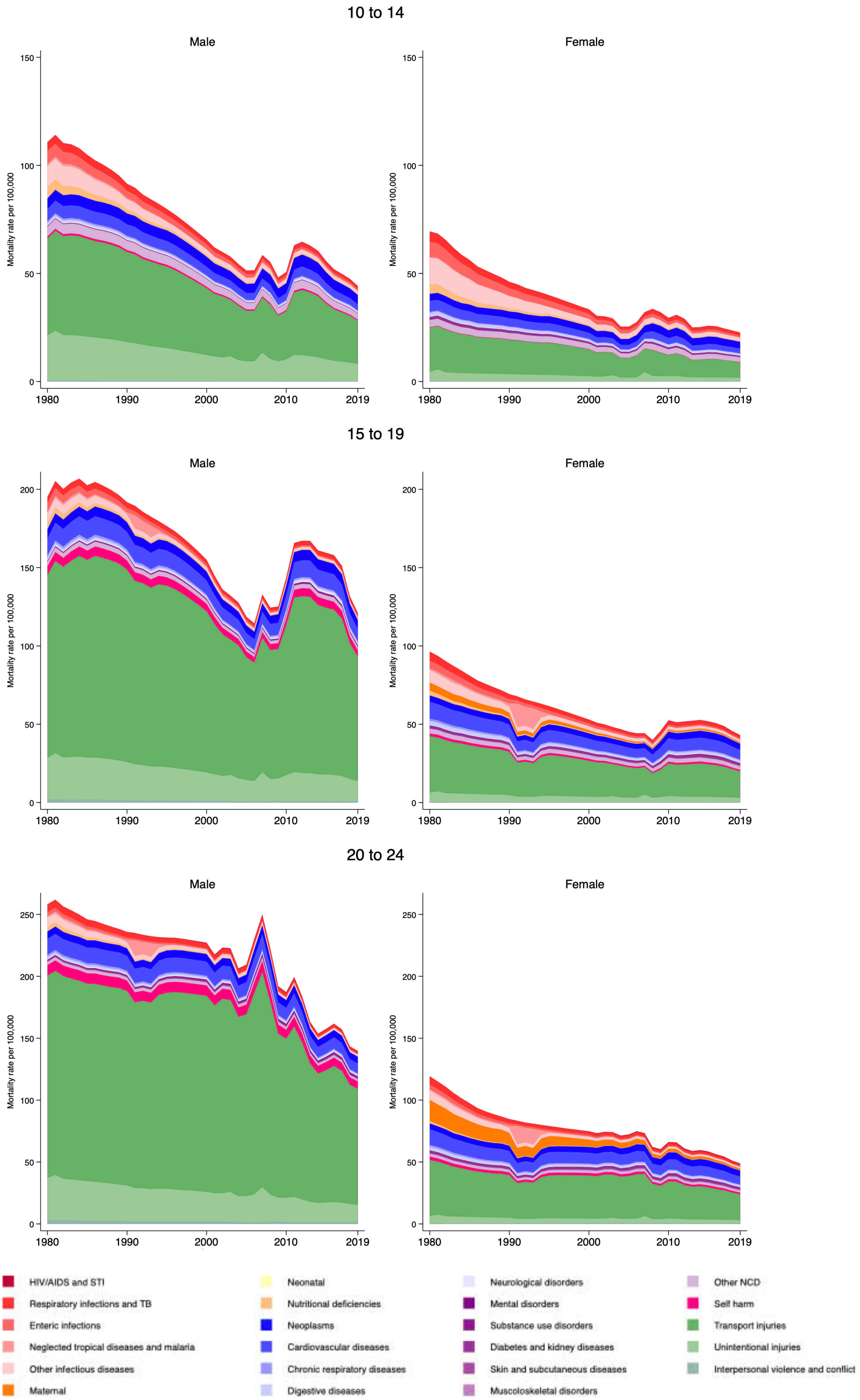

Figure S148d Number of deaths in 10-24 1980 – 2019 by cause: Oman

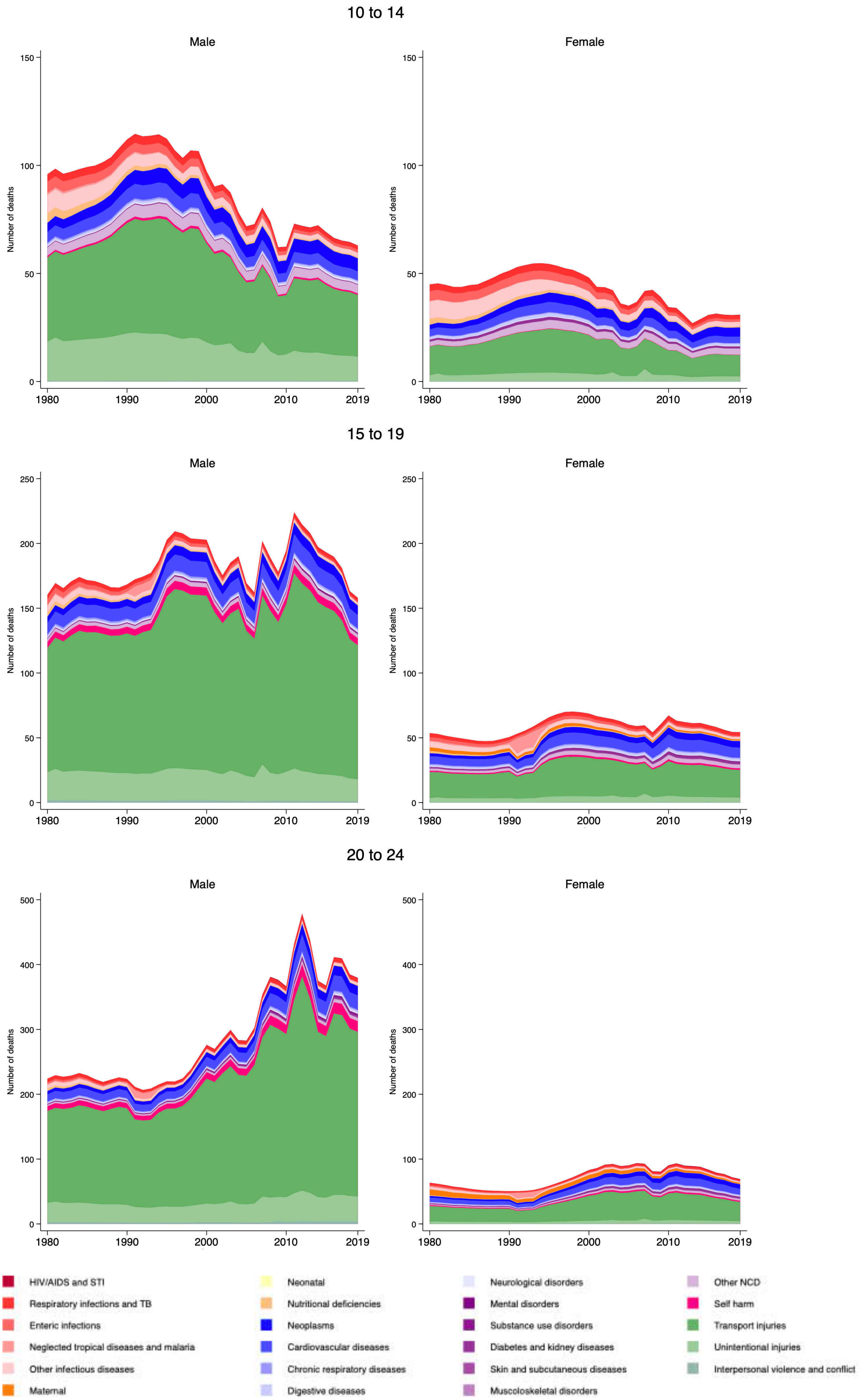

Figure S148: Qatar

Figure S149a All-cause mortality rate per 100,000 in 10-24 1950 – 2019: Qatar

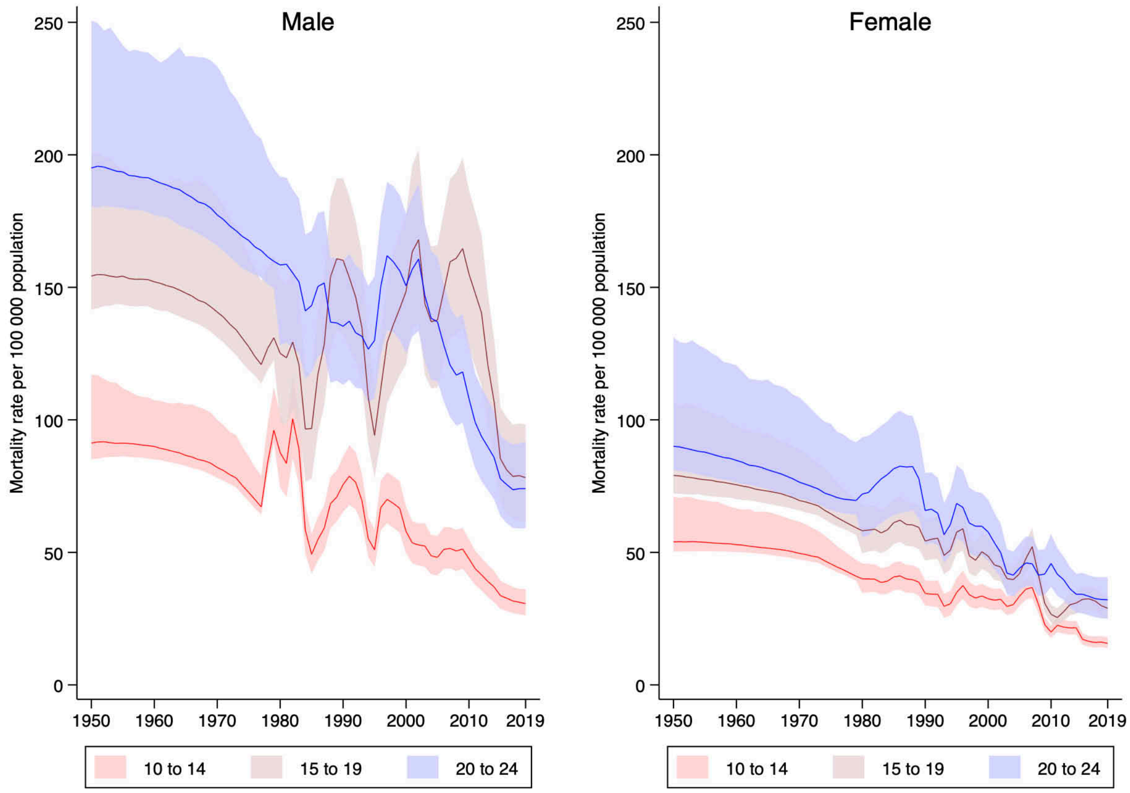

Figure S149b Number of deaths from all causes in 10-24 1950 – 2019: Qatar

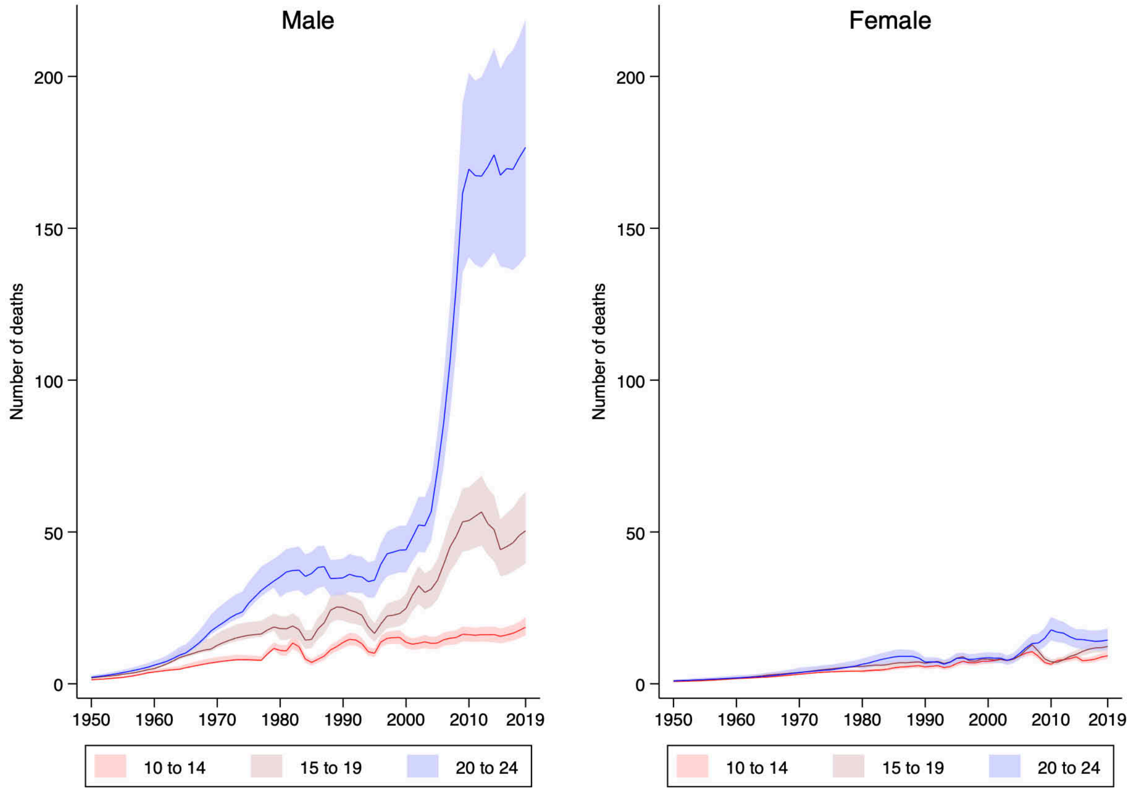

Figure S149c Mortality rate per 100,000 in 10-24 1980 – 2019 by cause: Qatar

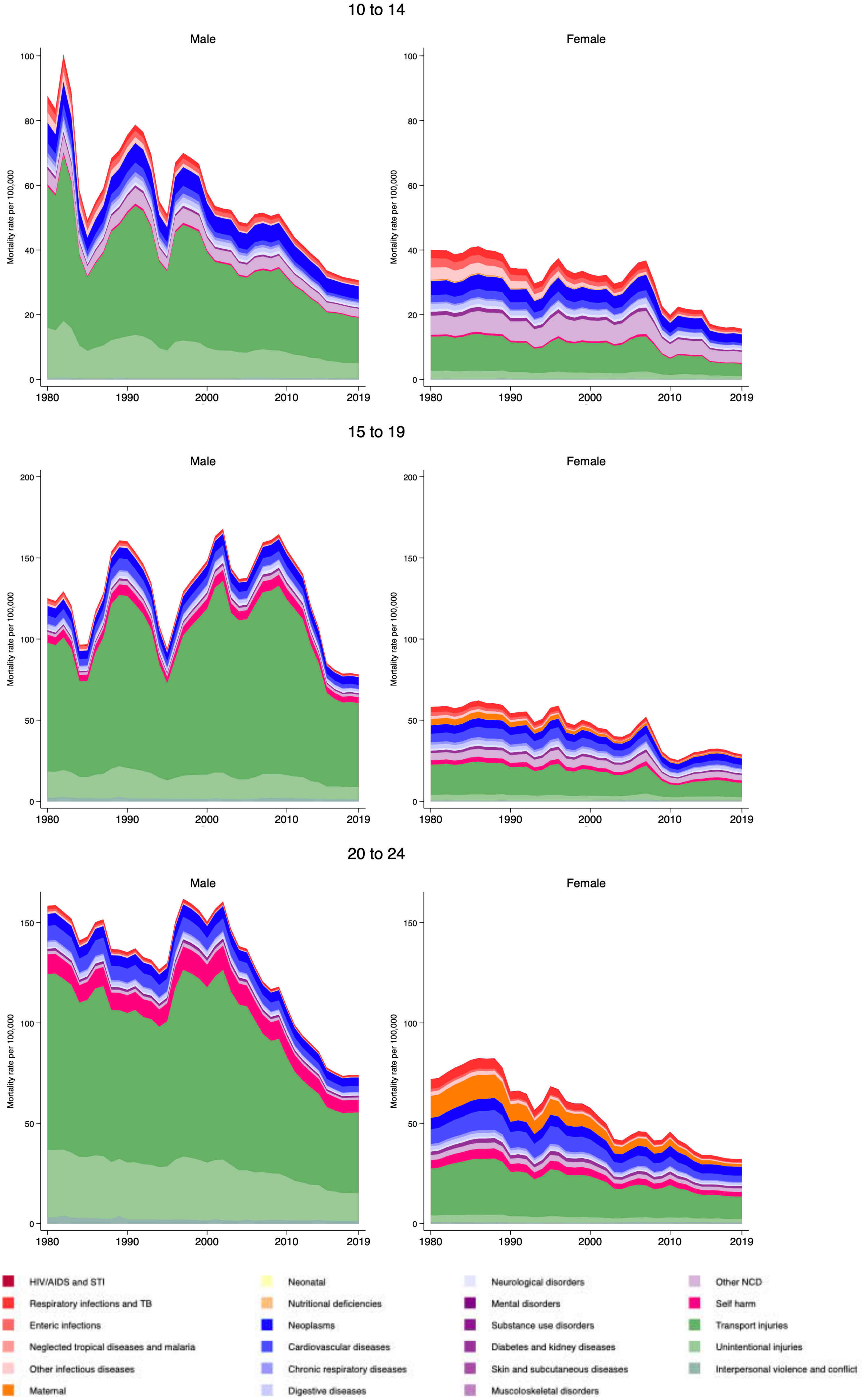

Figure S149d Number of deaths in 10-24 1980 – 2019 by cause: Qatar

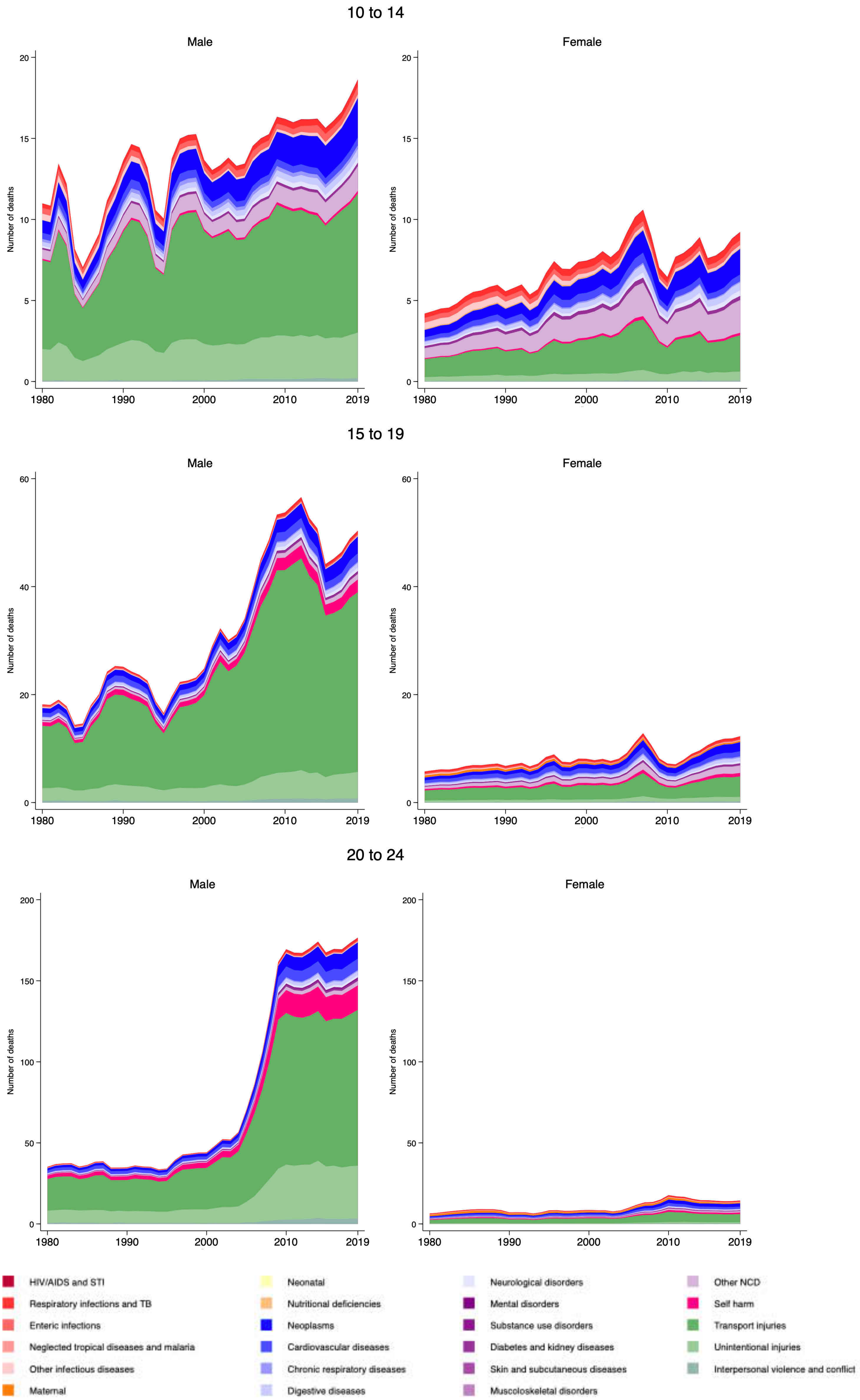

Figure S149: Saudi Arabia

Figure S150a All-cause mortality rate per 100,000 in 10-24 1950 – 2019: Saudi Arabia

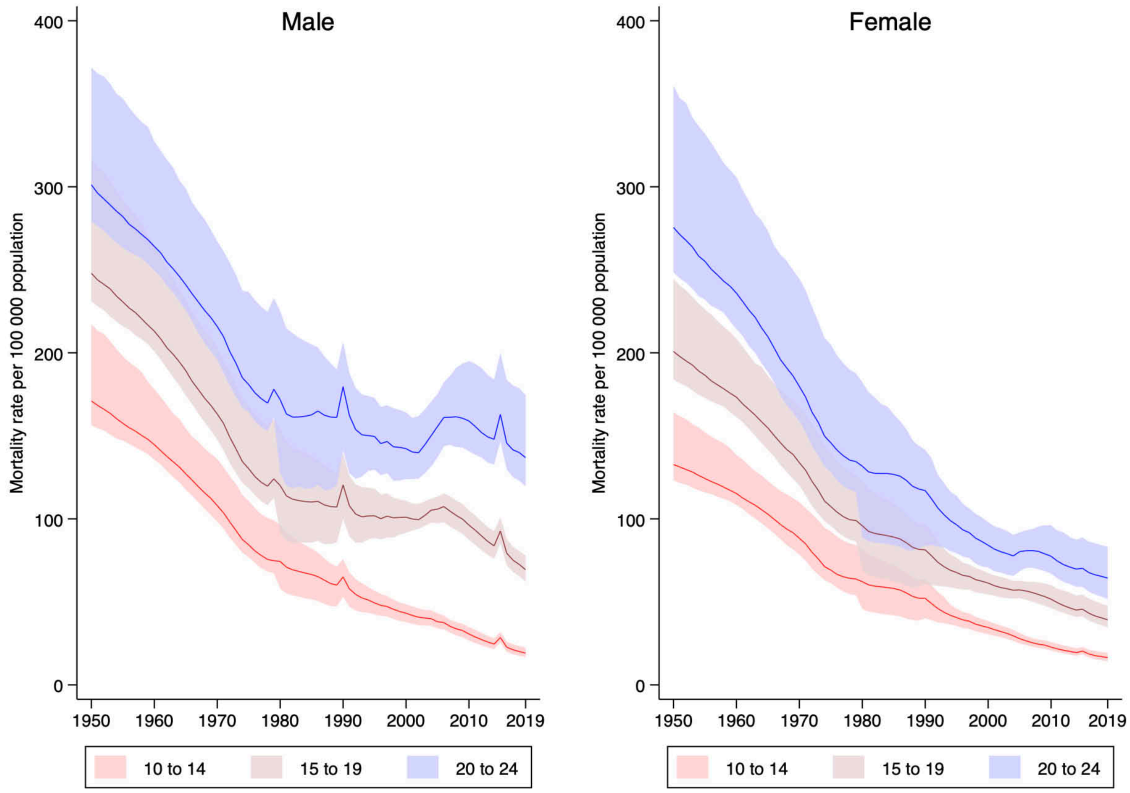

Figure S150b Number of deaths from all causes in 10-24 1950 – 2019: Saudi Arabia

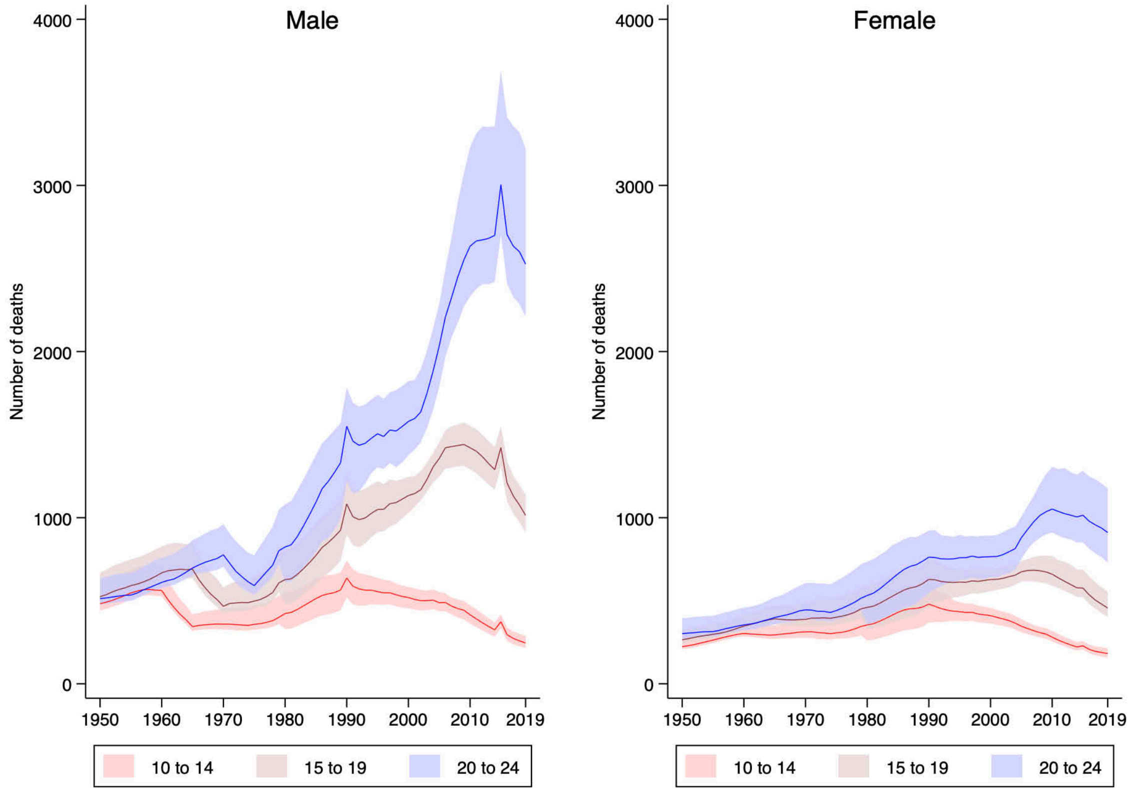

Figure S150c Mortality rate per 100,000 in 10-24 1980 – 2019 by cause: Saudi Arabia

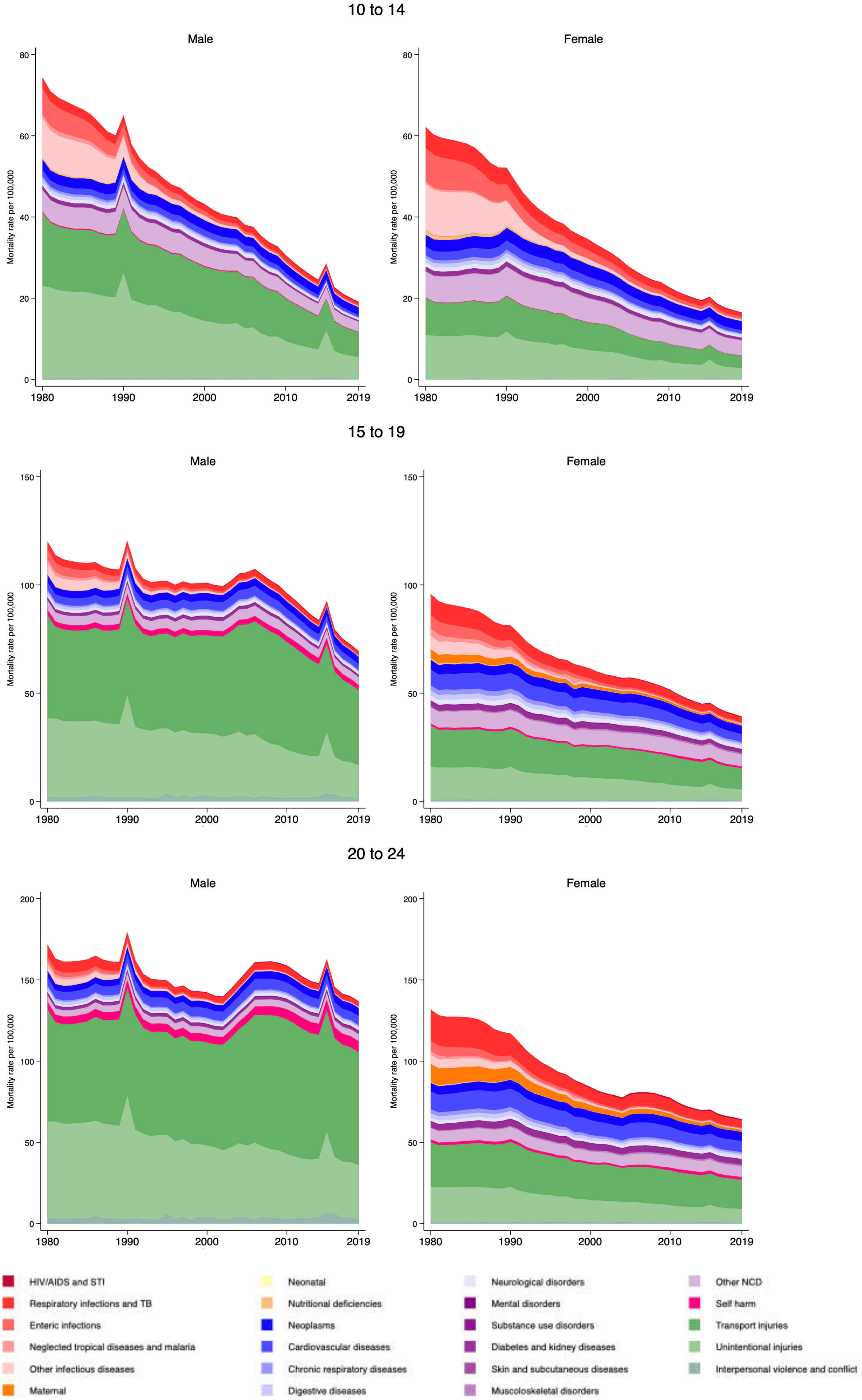

Figure S150d Number of deaths in 10-24 1980 – 2019 by cause: Saudi Arabia

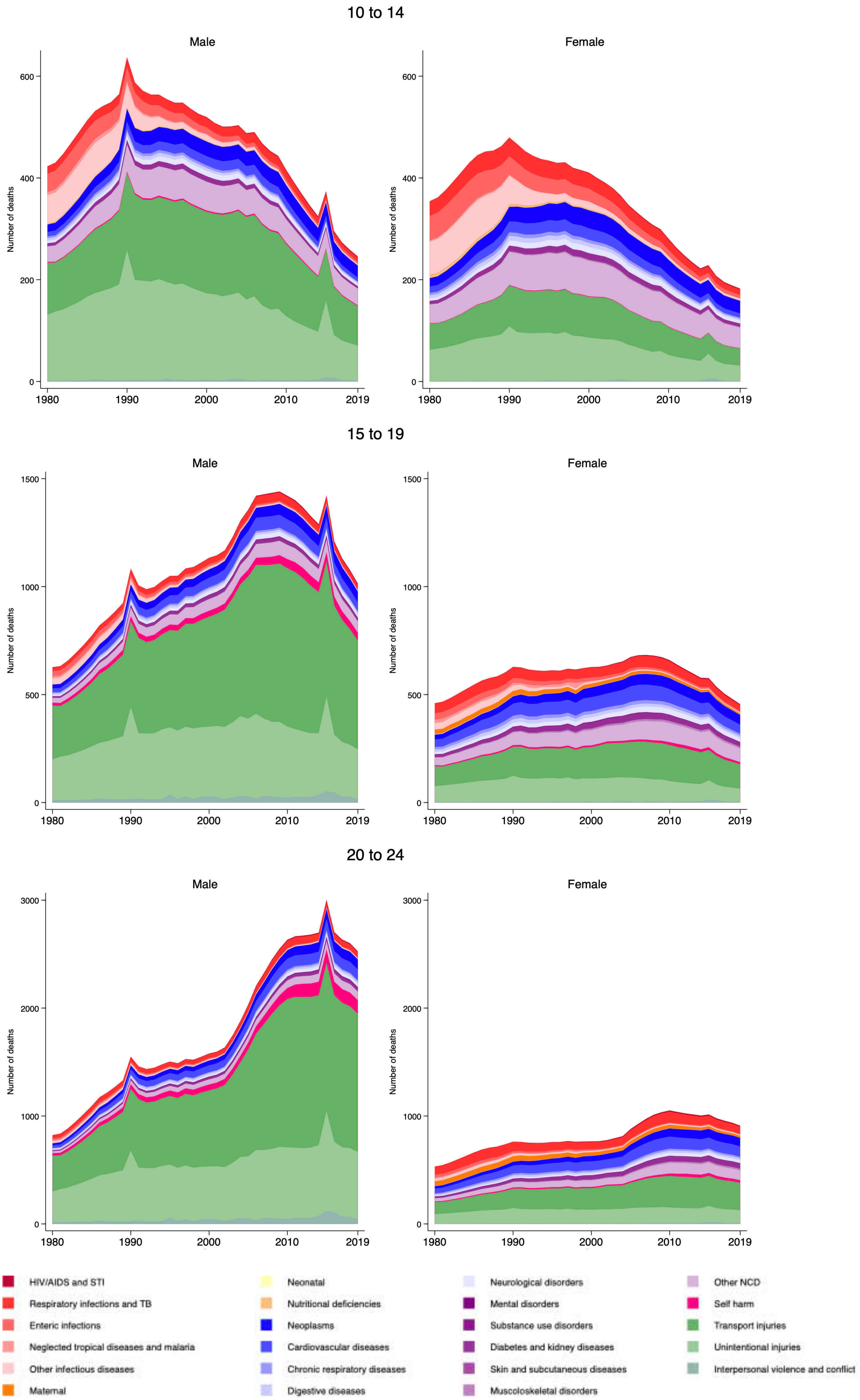

Figure S150: Syrian Arab Republic

Figure S151a All-cause mortality rate per 100,000 in 10-24 1950 – 2019: Syrian Arab Republic

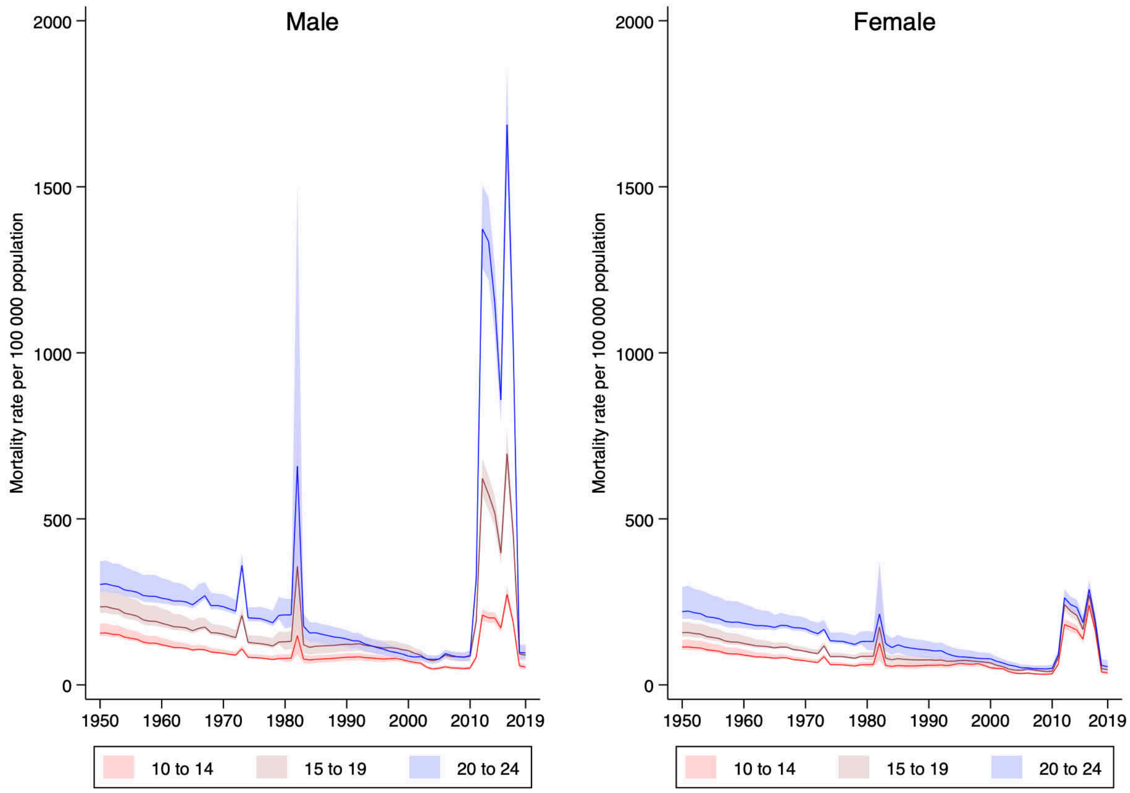

Figure S151b Number of deaths from all causes in 10-24 1950 – 2019: Syrian Arab Republic

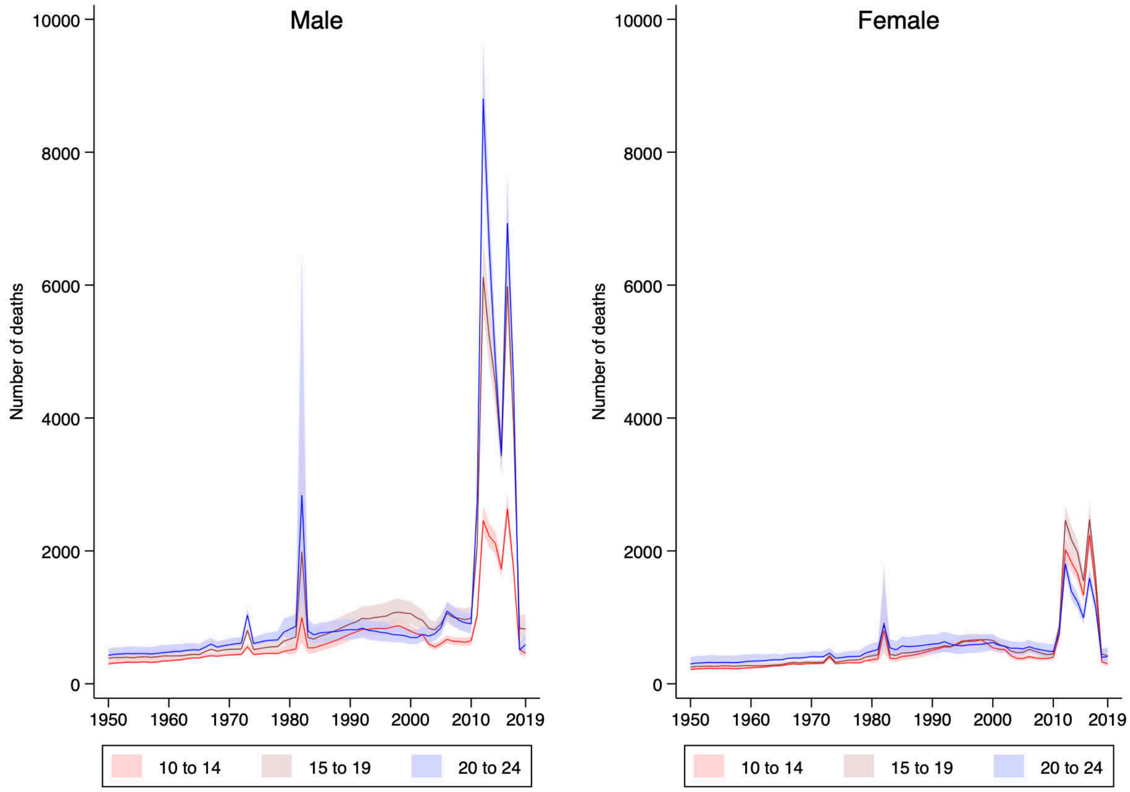

Figure S151c Mortality rate per 100,000 in 10-24 1980 – 2019 by cause: Syrian Arab Republic

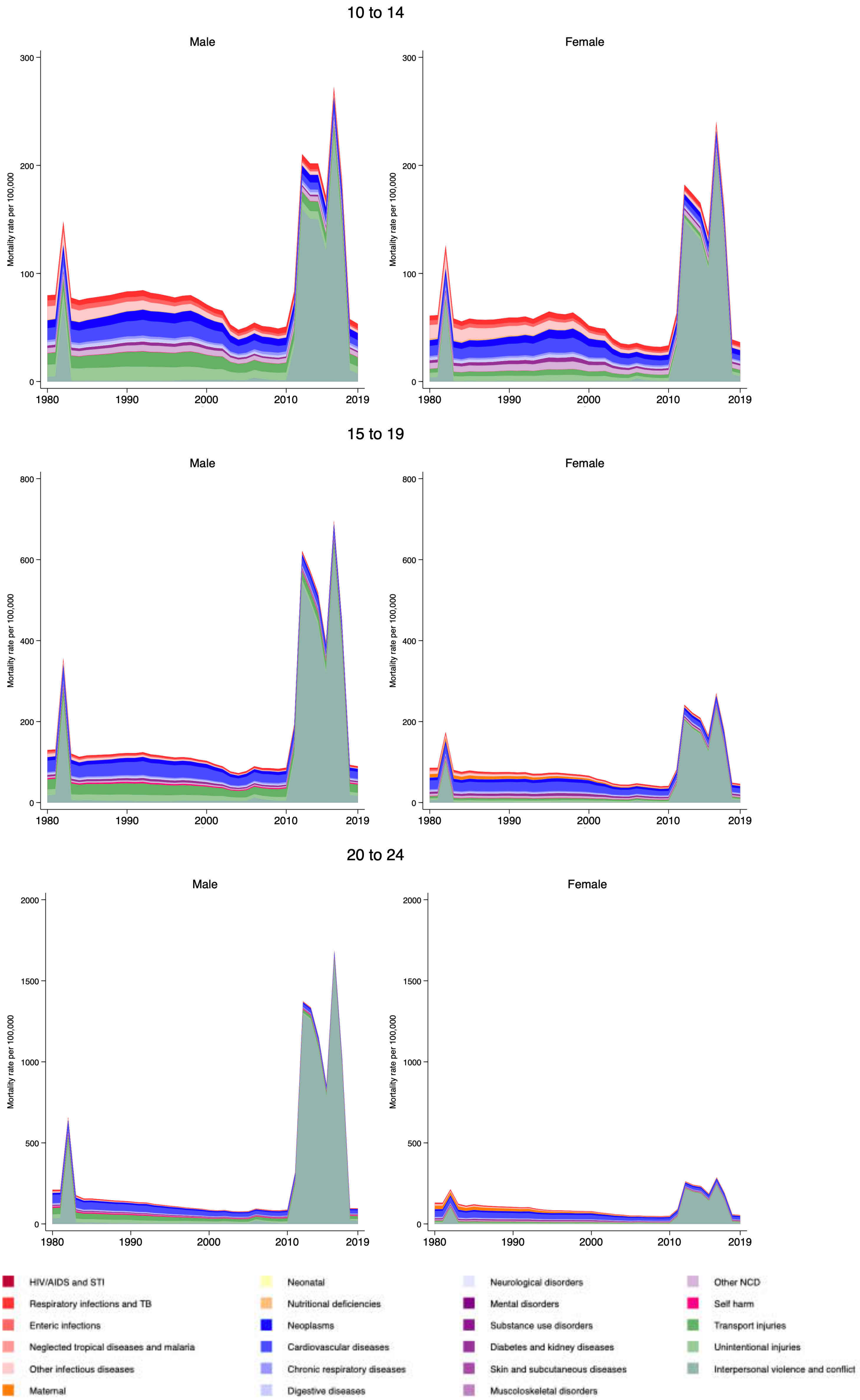

Figure S151d Number of deaths in 10-24 1980 – 2019 by cause: Syrian Arab Republic

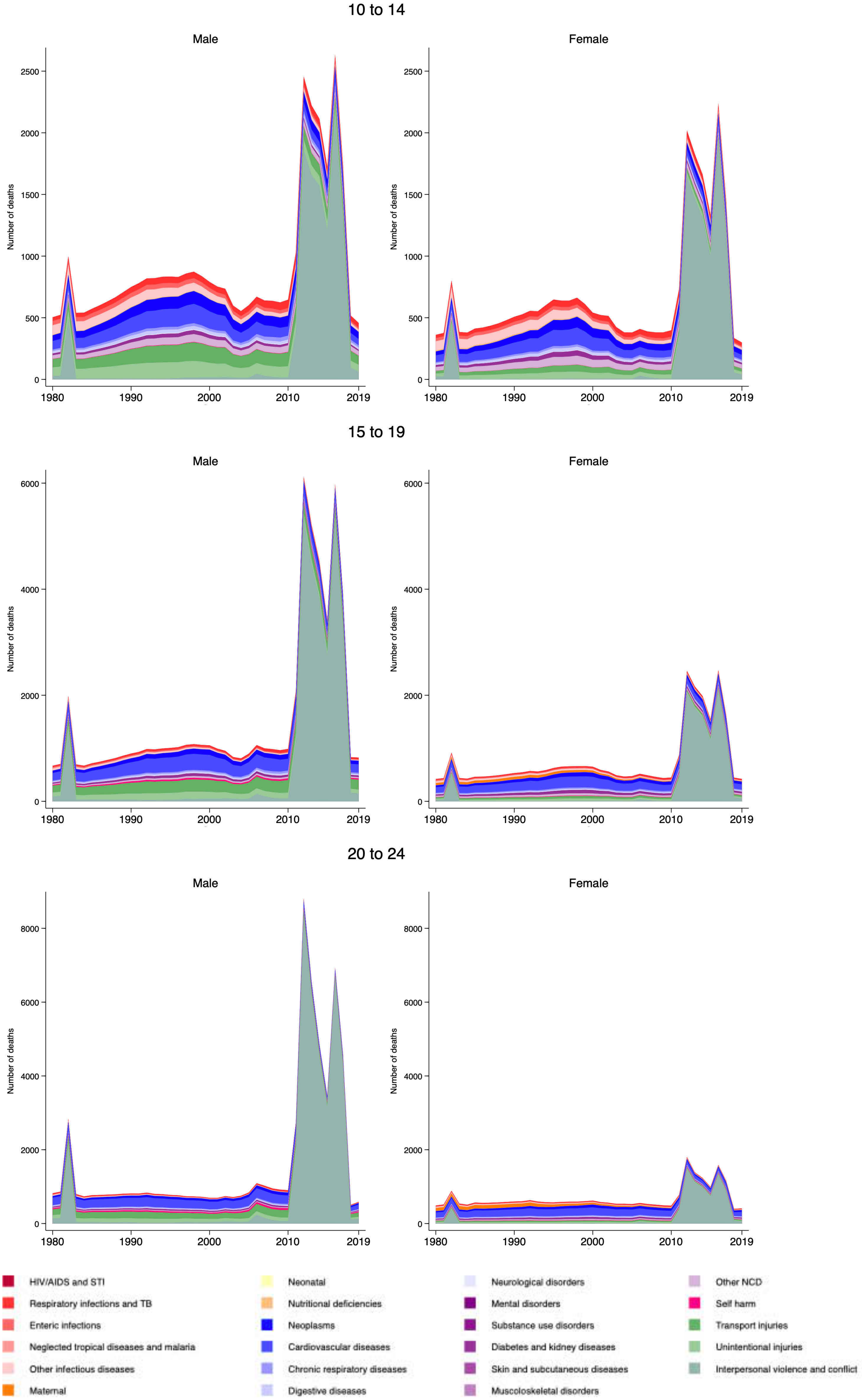

Figure S151: Tunisia

Figure S152a All-cause mortality rate per 100,000 in 10-24 1950 – 2019: Tunisia

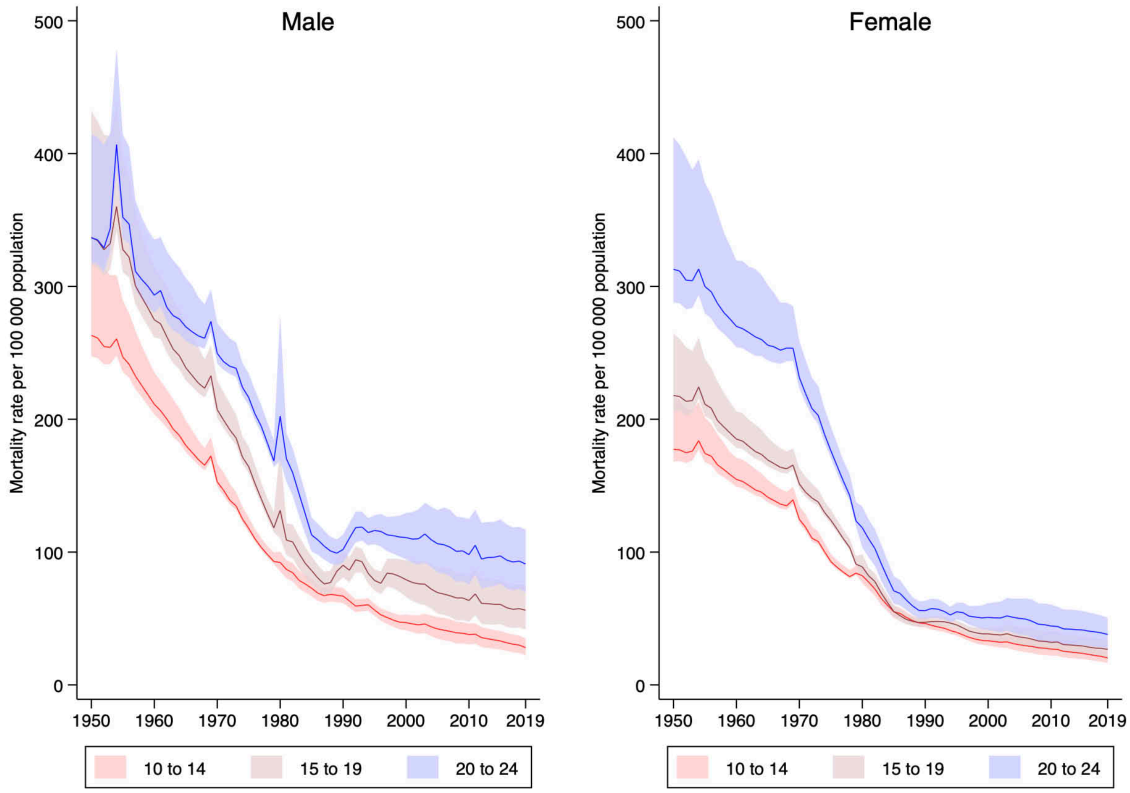

Figure S152b Number of deaths from all causes in 10-24 1950 – 2019: Tunisia

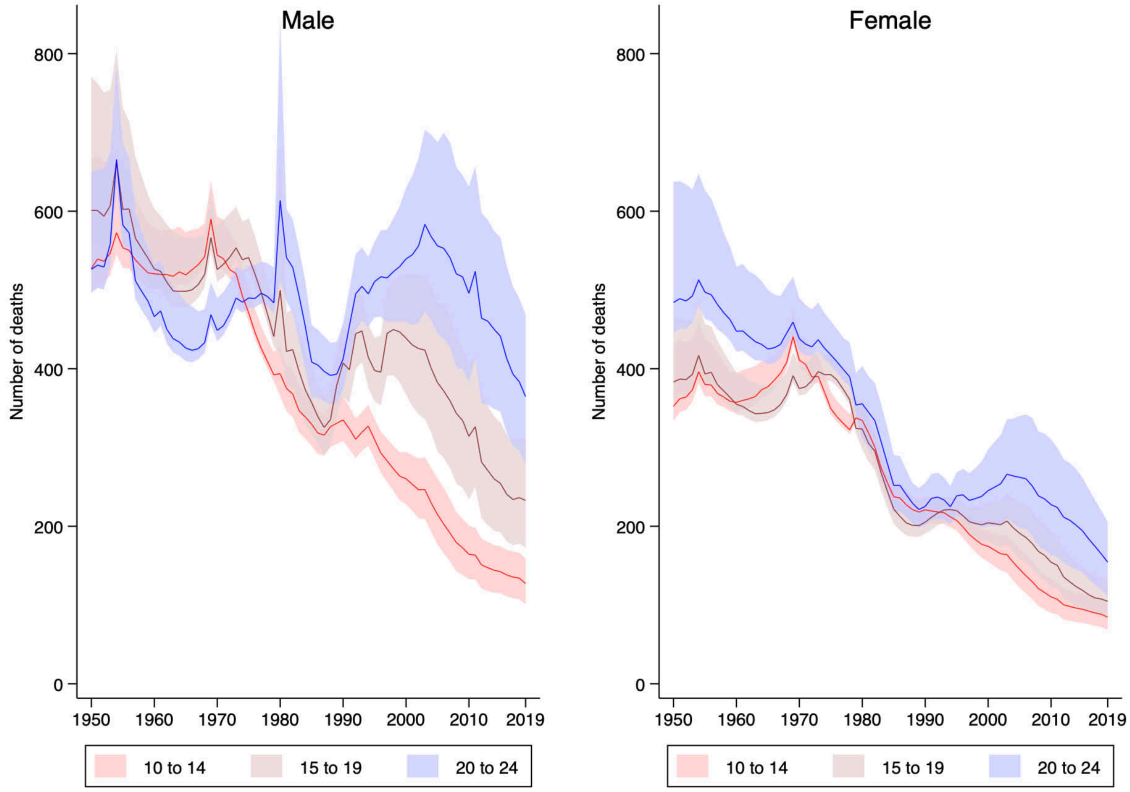

Figure S152c Mortality rate per 100,000 in 10-24 1980 – 2019 by cause: Tunisia

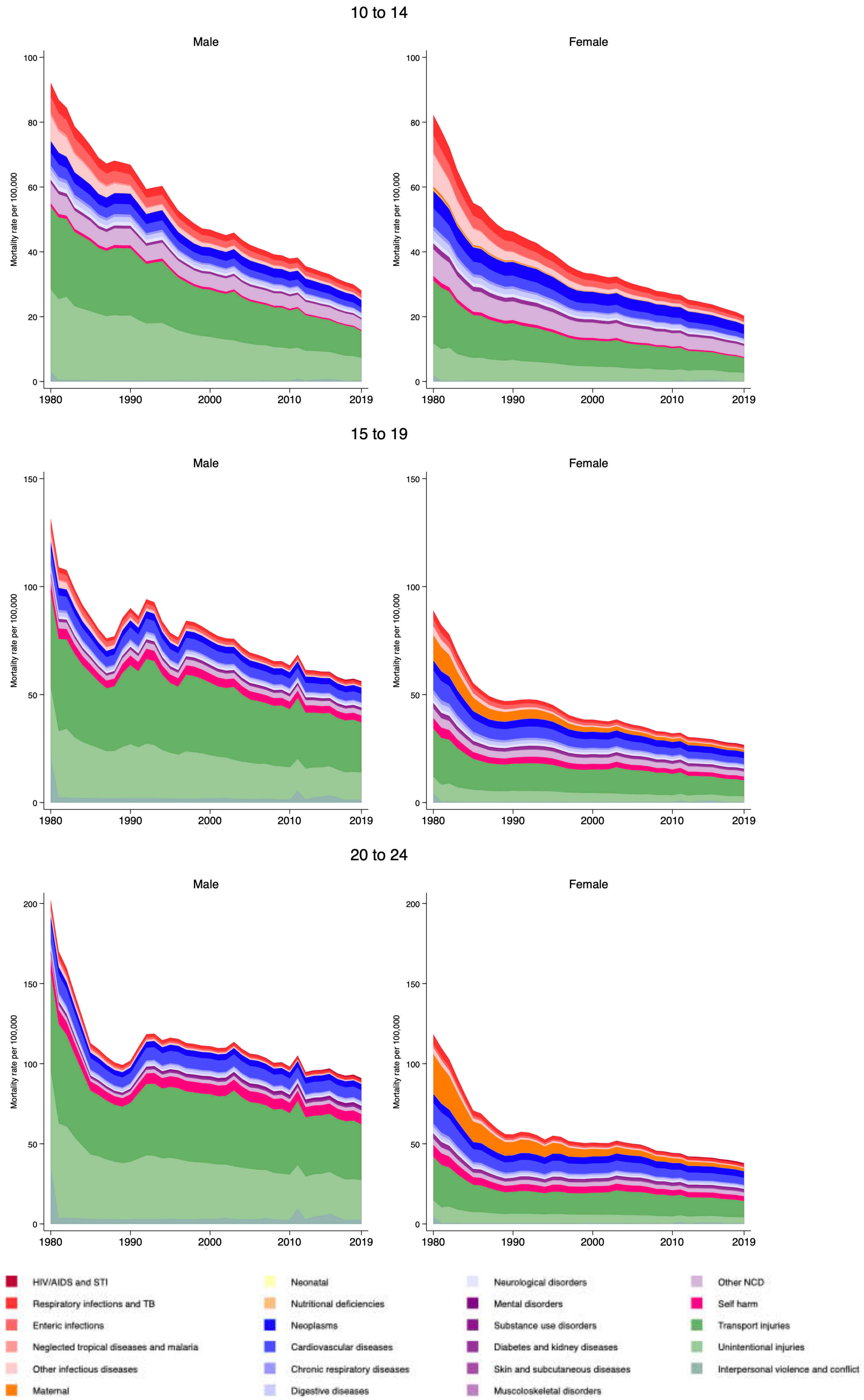

Figure S152d Number of deaths in 10-24 1980 – 2019 by cause: Tunisia

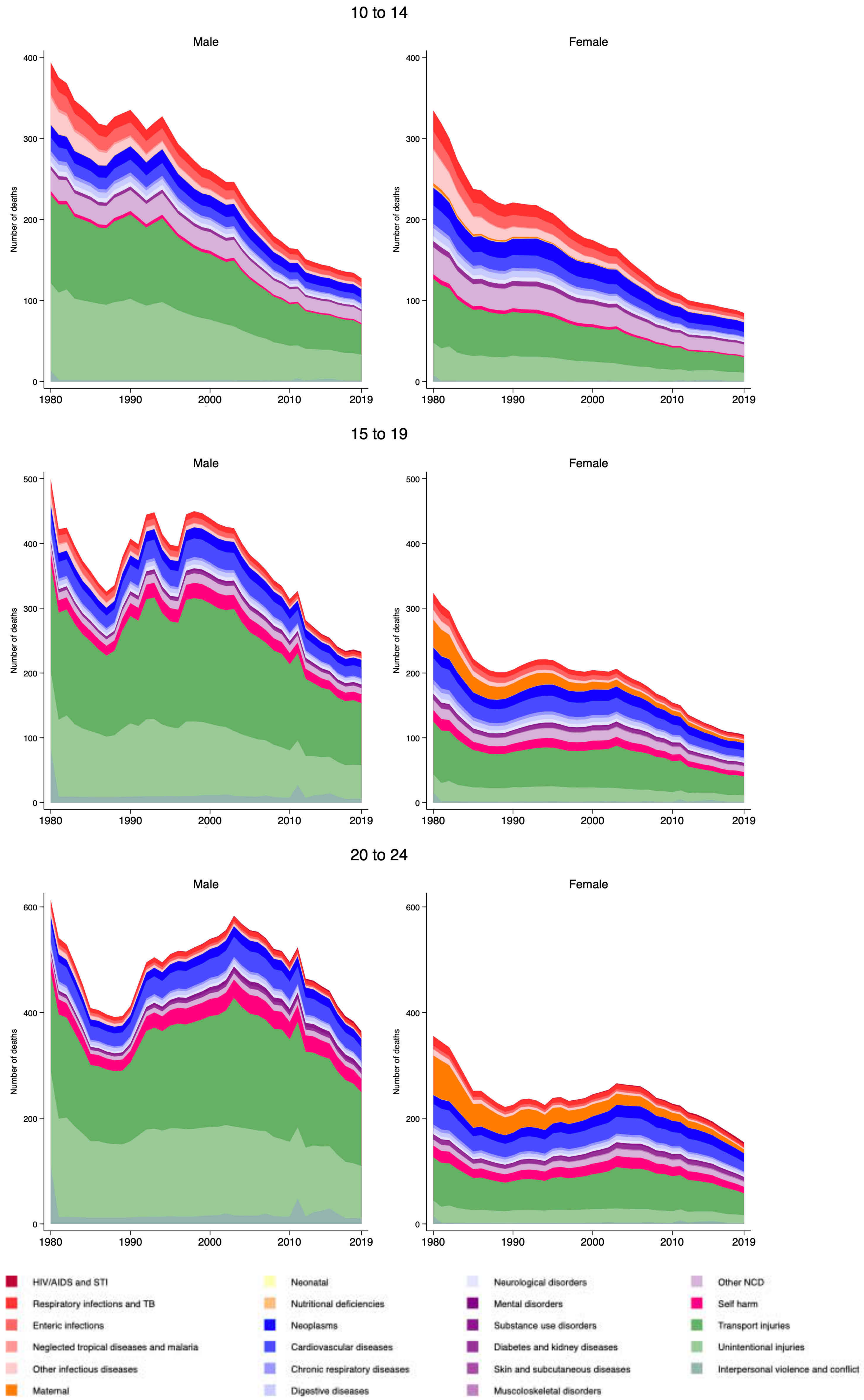

Figure S152: Turkey

Figure S153a All-cause mortality rate per 100,000 in 10-24 1950 – 2019: Turkey

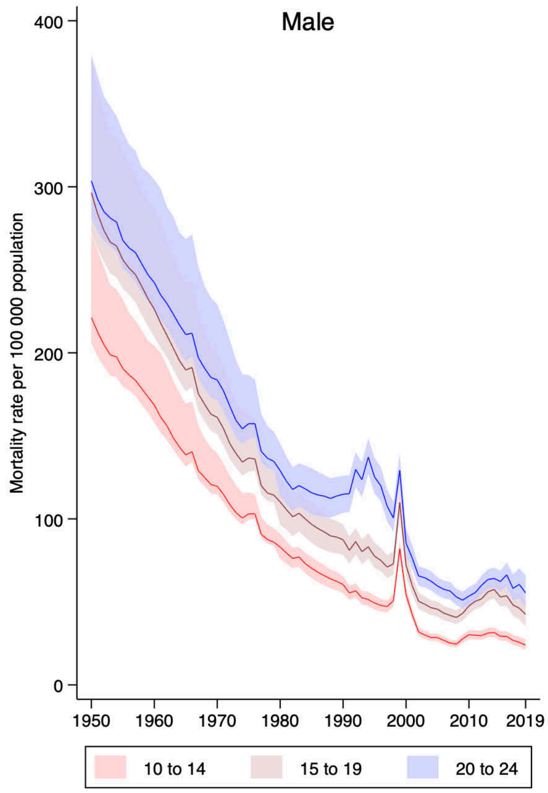

Figure S153b Number of deaths from all causes in 10-24 1950 – 2019: Turkey

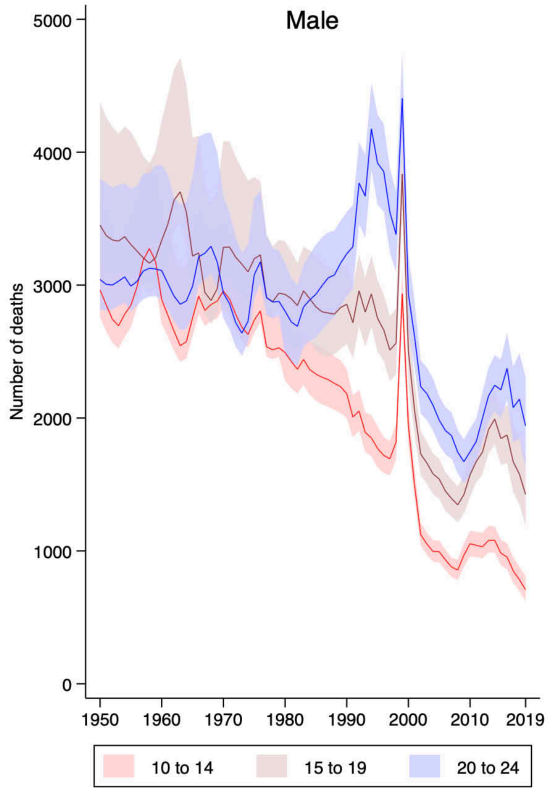

Figure S153c Mortality rate per 100,000 in 10-24 1980 – 2019 by cause: Turkey

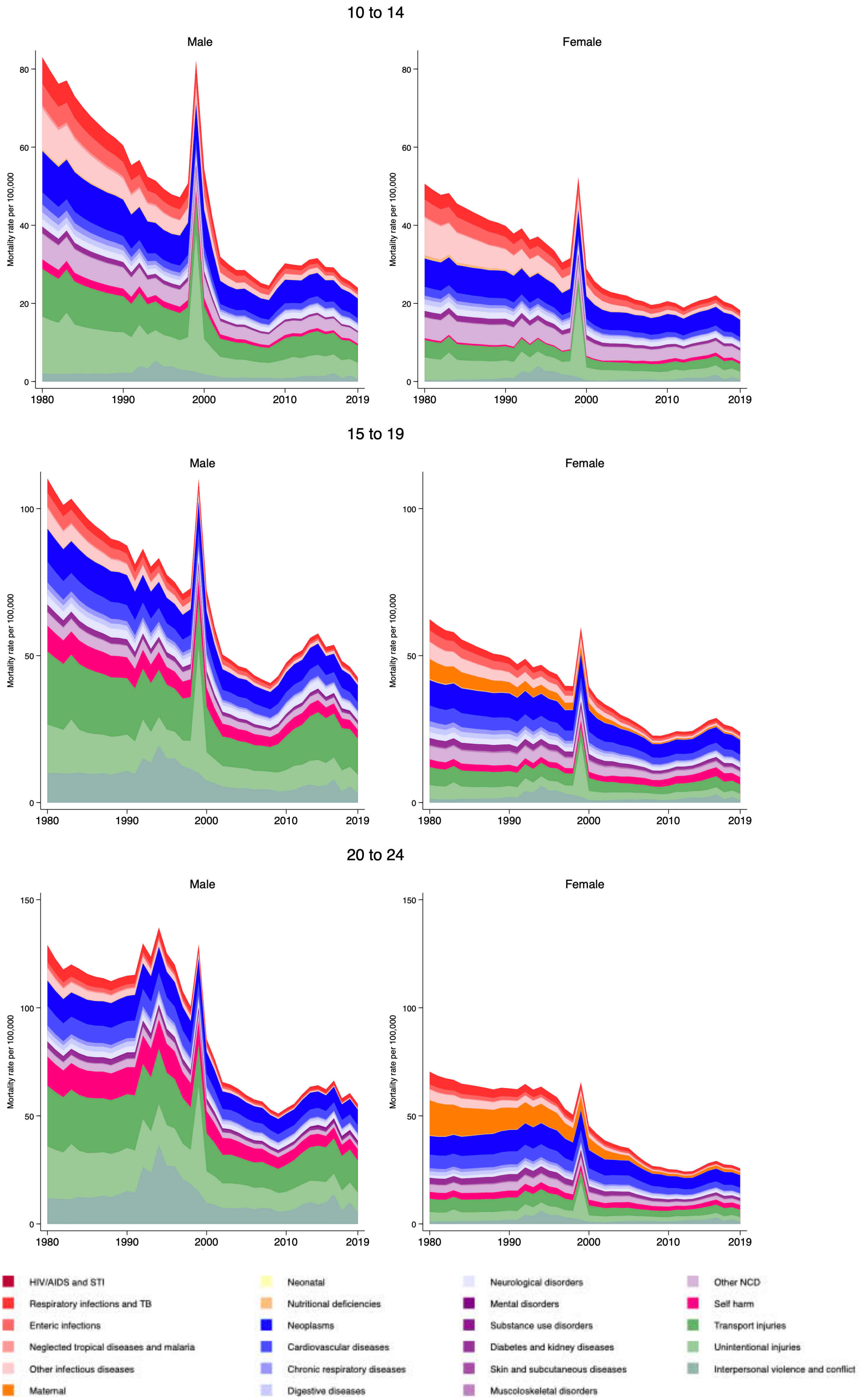

Figure S153d Number of deaths in 10-24 1980 – 2019 by cause: Turkey

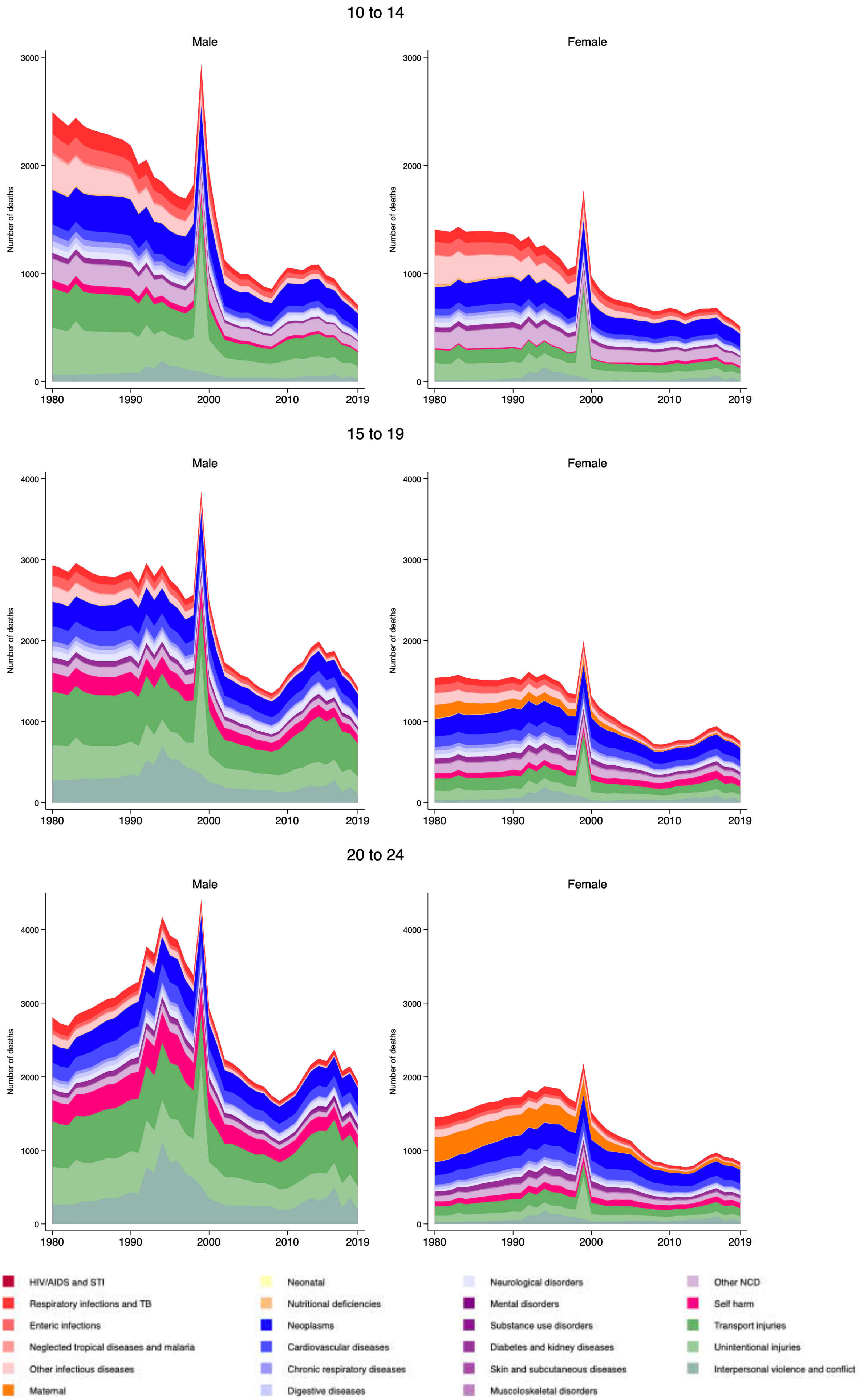

Figure S153: United Arab Emirates

Figure S154a All-cause mortality rate per 100,000 in 10-24 1950 – 2019: United Arab Emirates

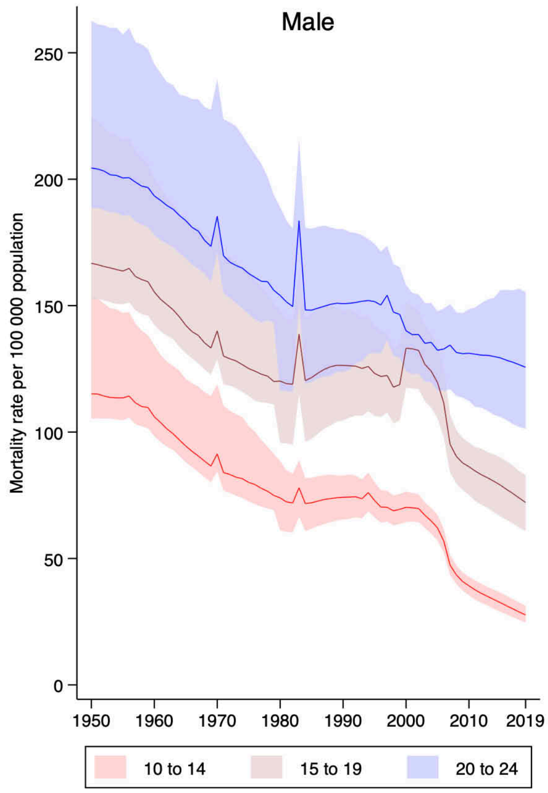

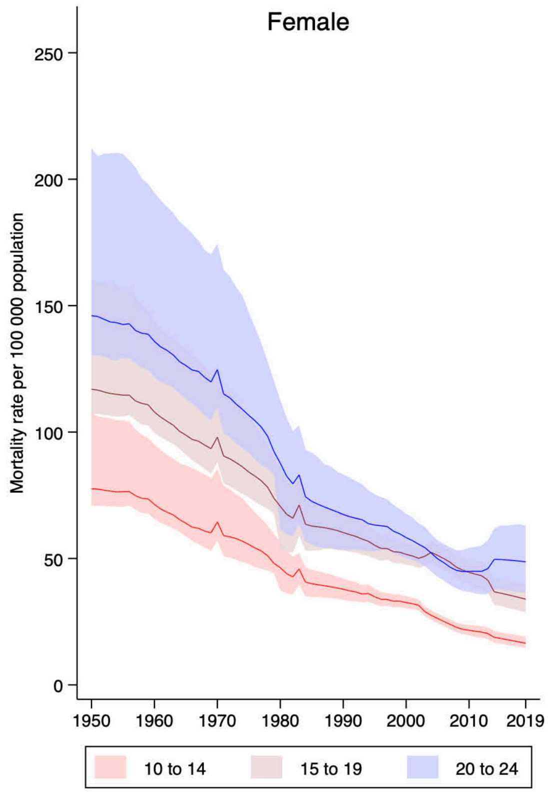

Figure S154b Number of deaths from all causes in 10-24 1950 – 2019: United Arab Emirates

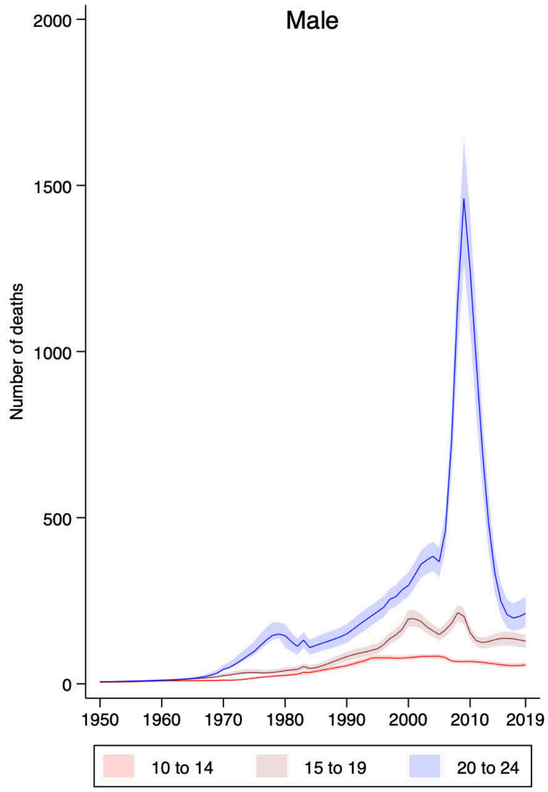

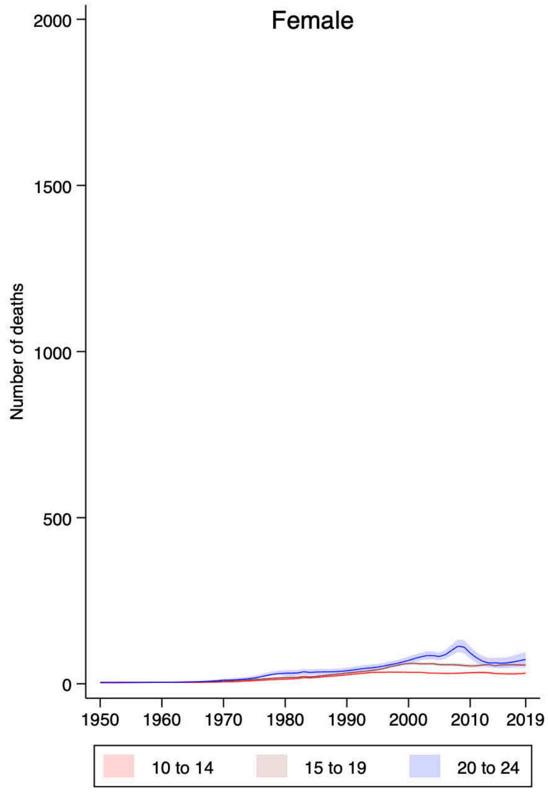

Figure S154c Mortality rate per 100,000 in 10-24 1980 – 2019 by cause: United Arab Emirates

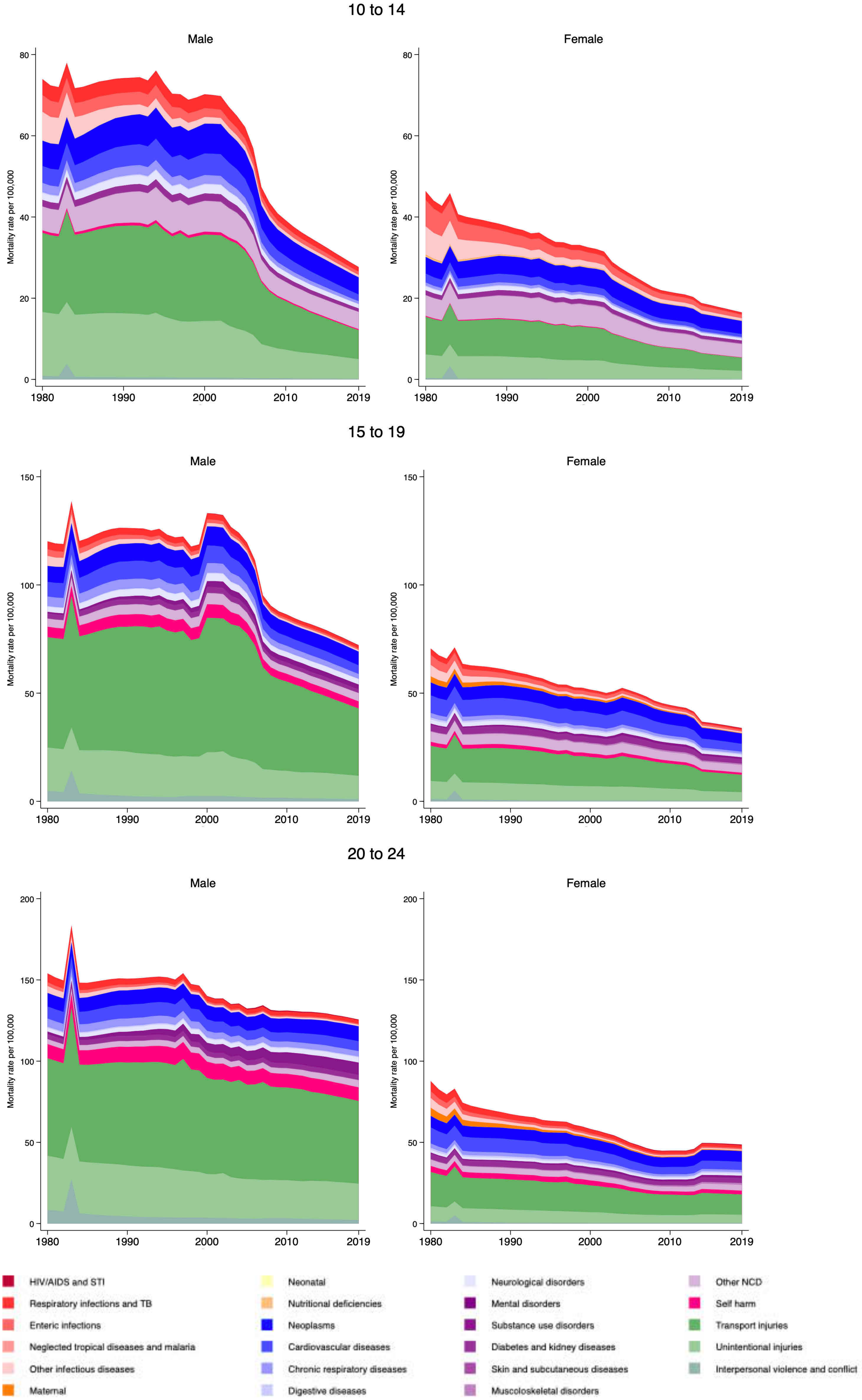

Figure S154d Number of deaths in 10-24 1980 – 2019 by cause: United Arab Emirates

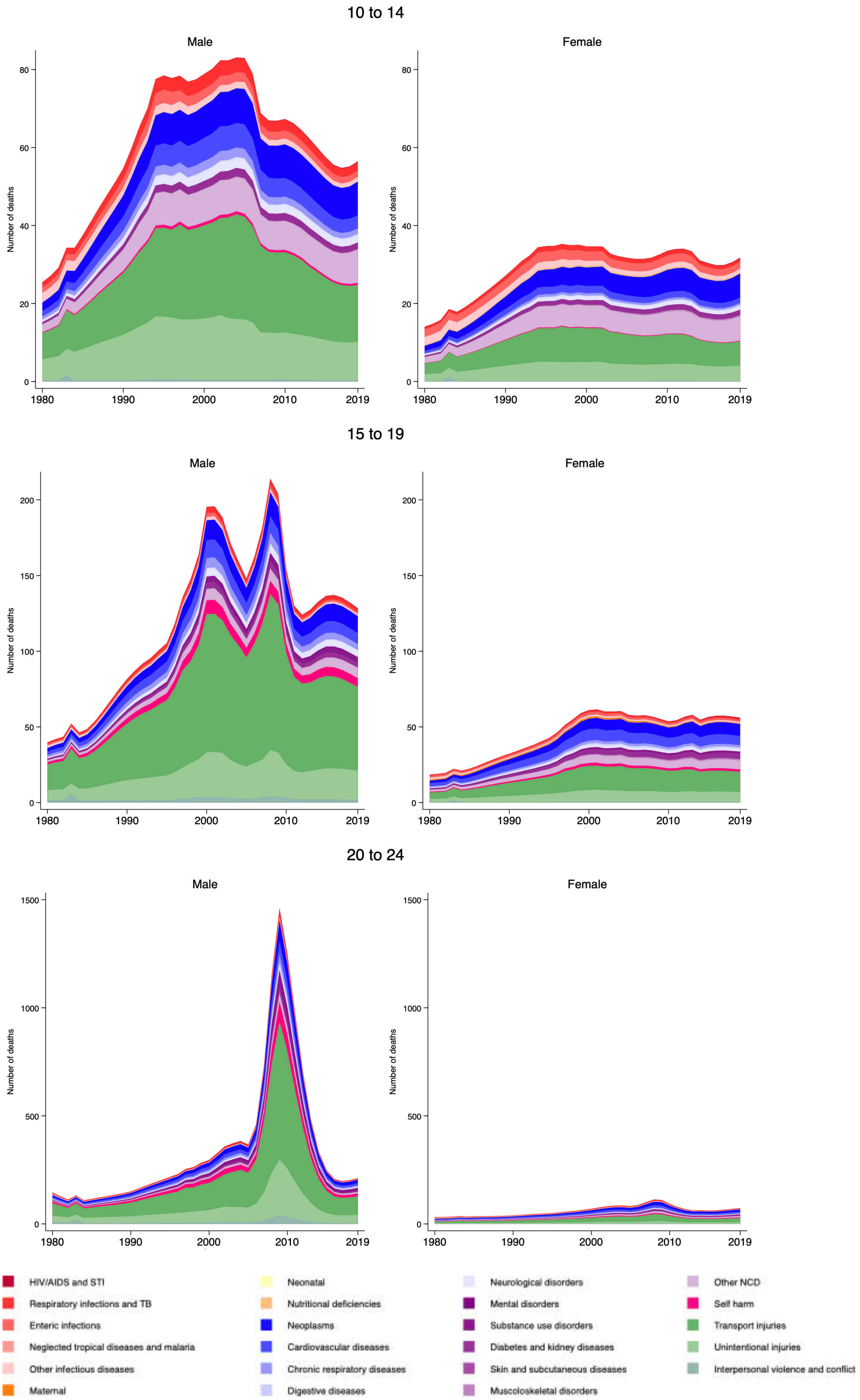

Figure S154: Yemen

Figure S155a All-cause mortality rate per 100,000 in 10-24 1950 – 2019: Yemen

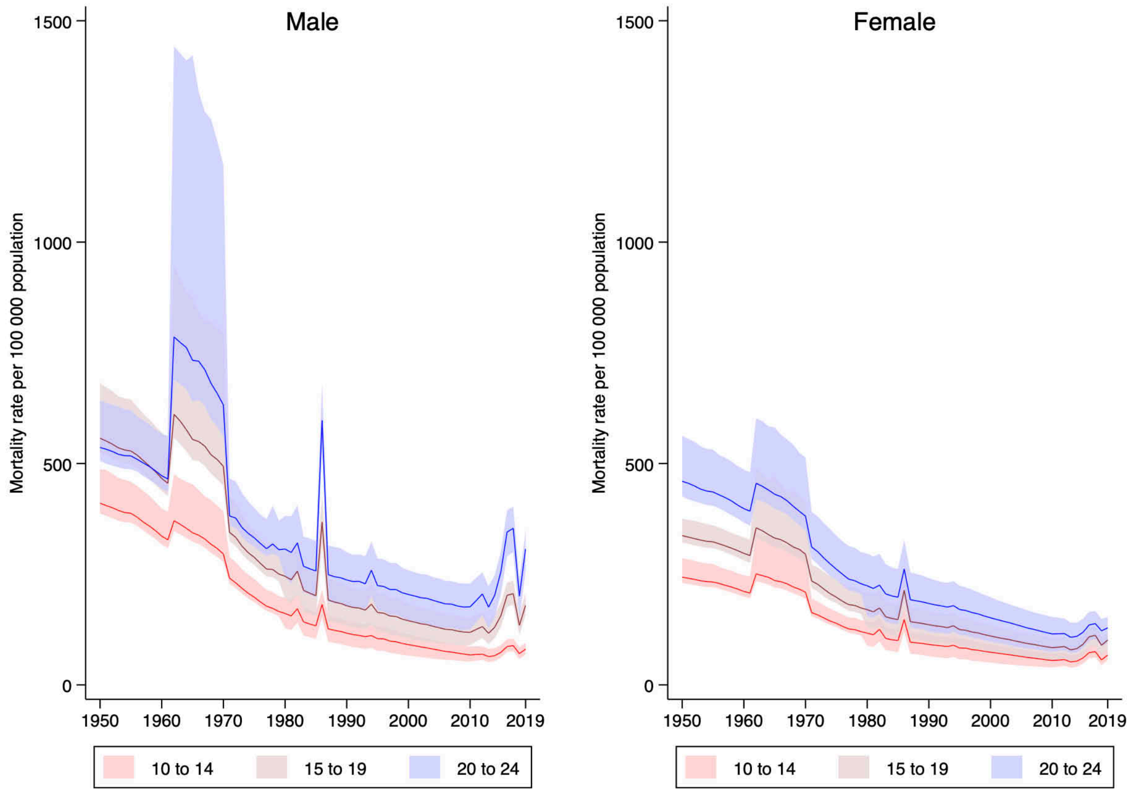

Figure S155b Number of deaths from all causes in 10-24 1950 – 2019: Yemen

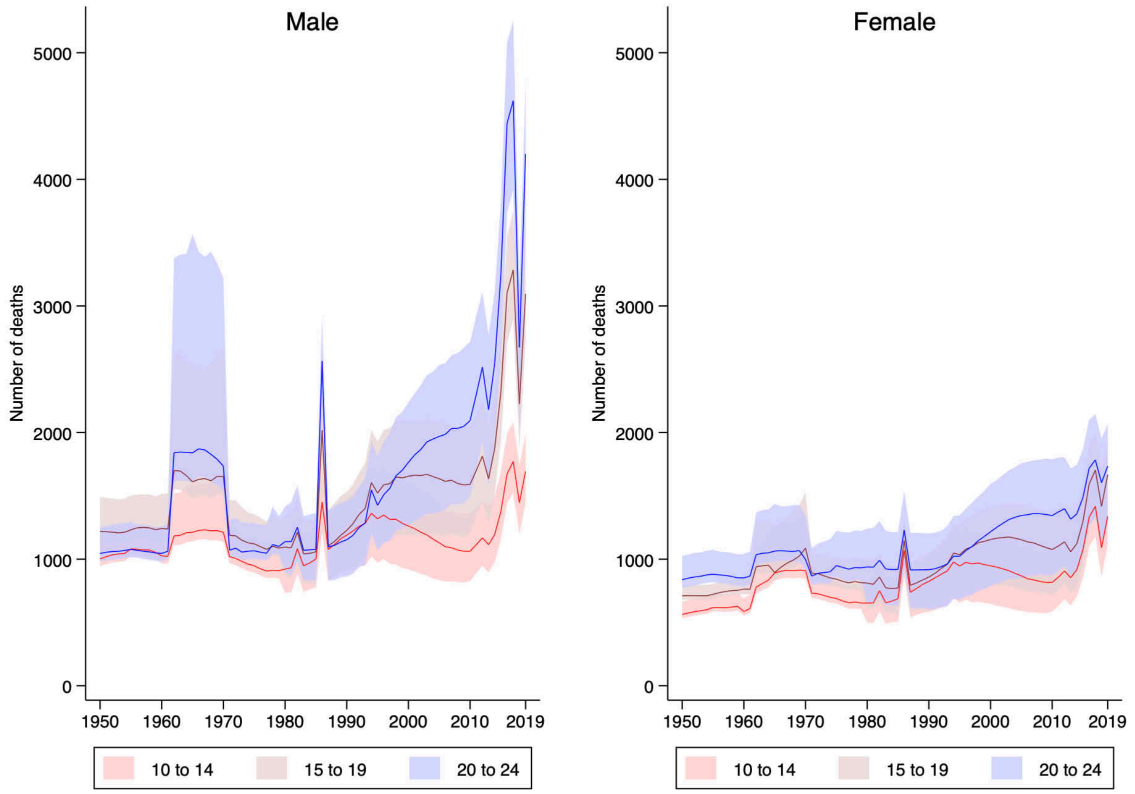

Figure S155c Mortality rate per 100,000 in 10-24 1980 – 2019 by cause: Yemen

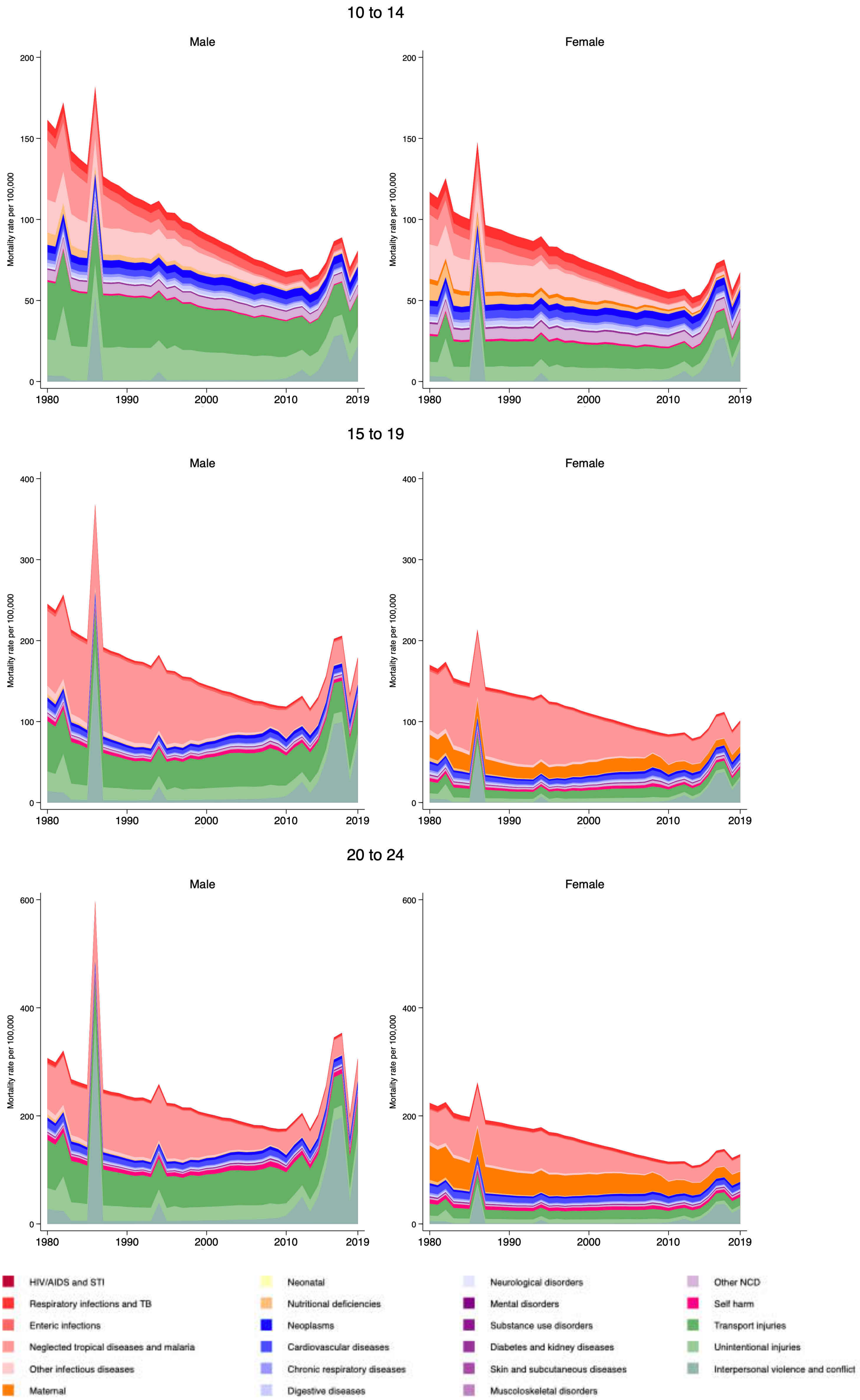

Figure S155d Number of deaths in 10-24 1980 – 2019 by cause: Yemen

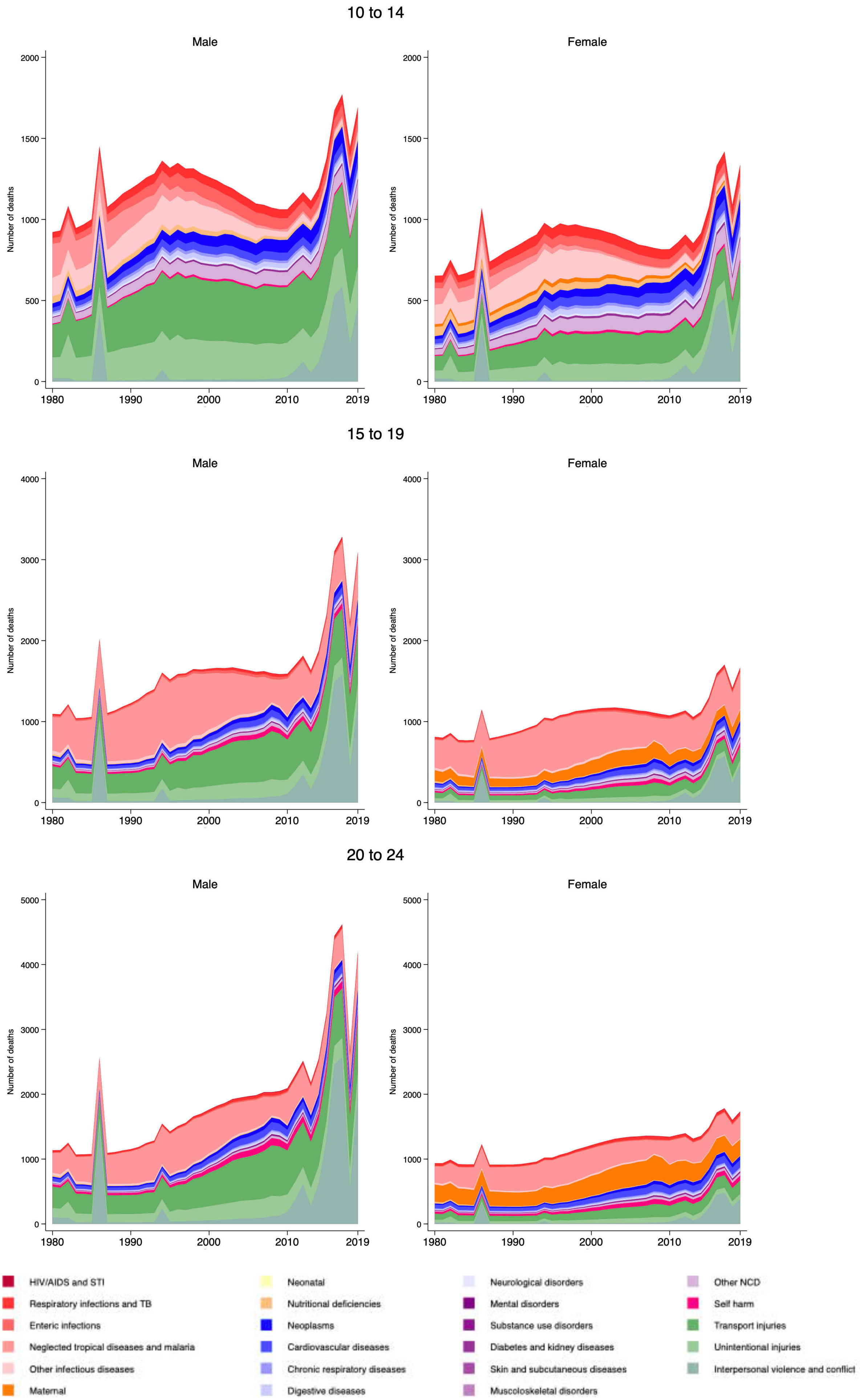

Figure S155: South Asia

Figure S156a All-cause mortality rate per 100,000 in 10-24 1950 – 2019: South Asia

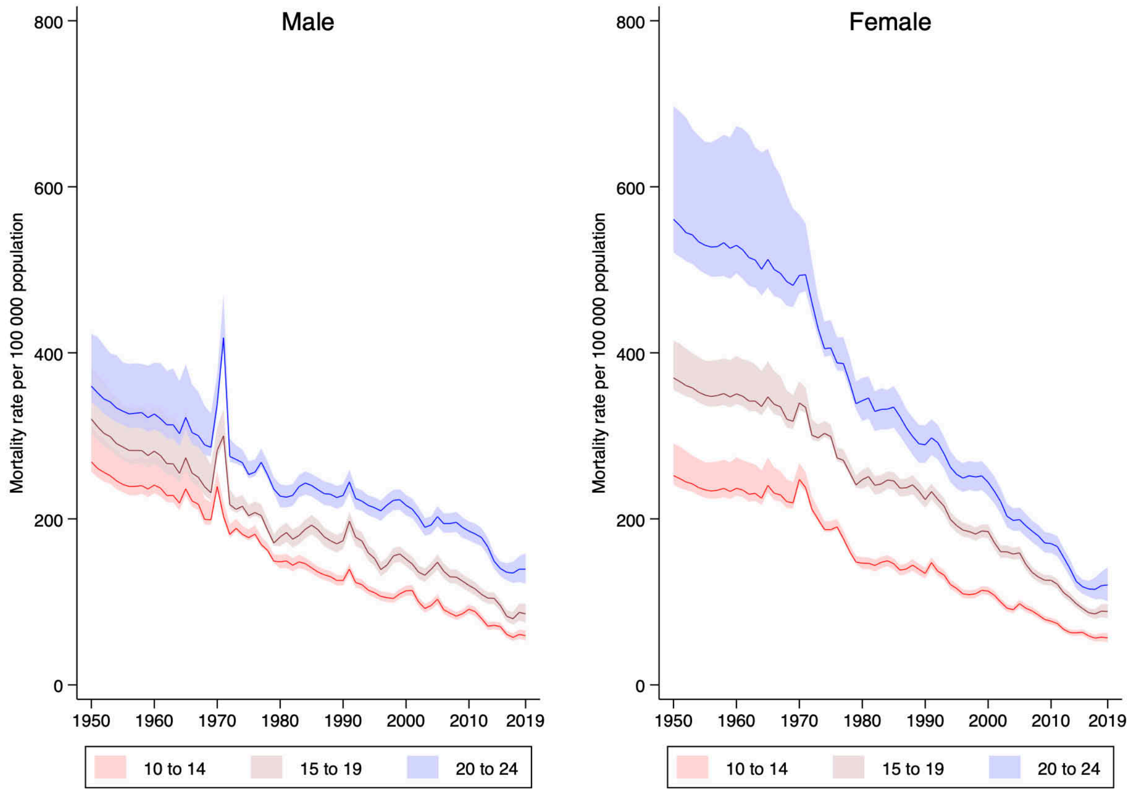

Figure S156b Number of deaths from all causes in 10-24 1950 – 2019: South Asia

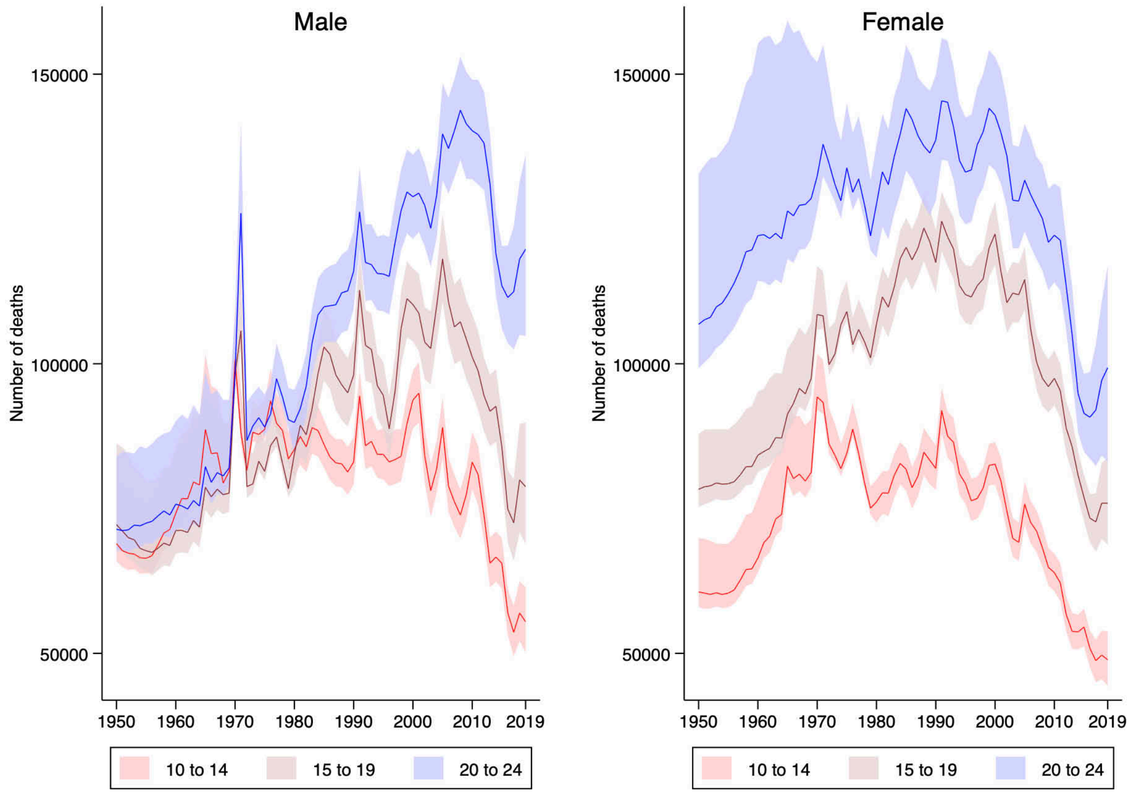

Figure S156c Mortality rate per 100,000 in 10-24 1980 – 2019 by cause: South Asia

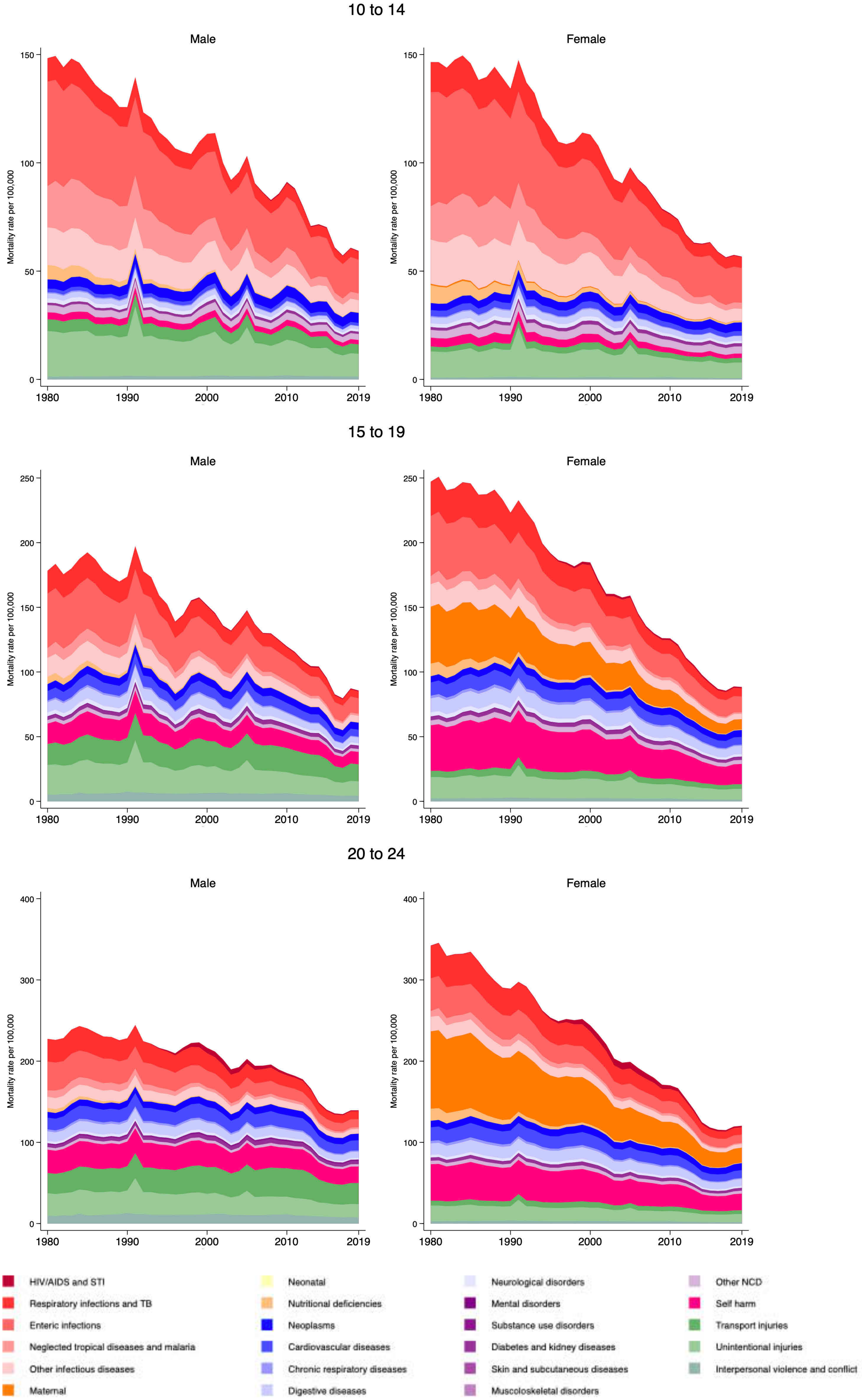

Figure S156d Number of deaths in 10-24 1980 – 2019 by cause: South Asia

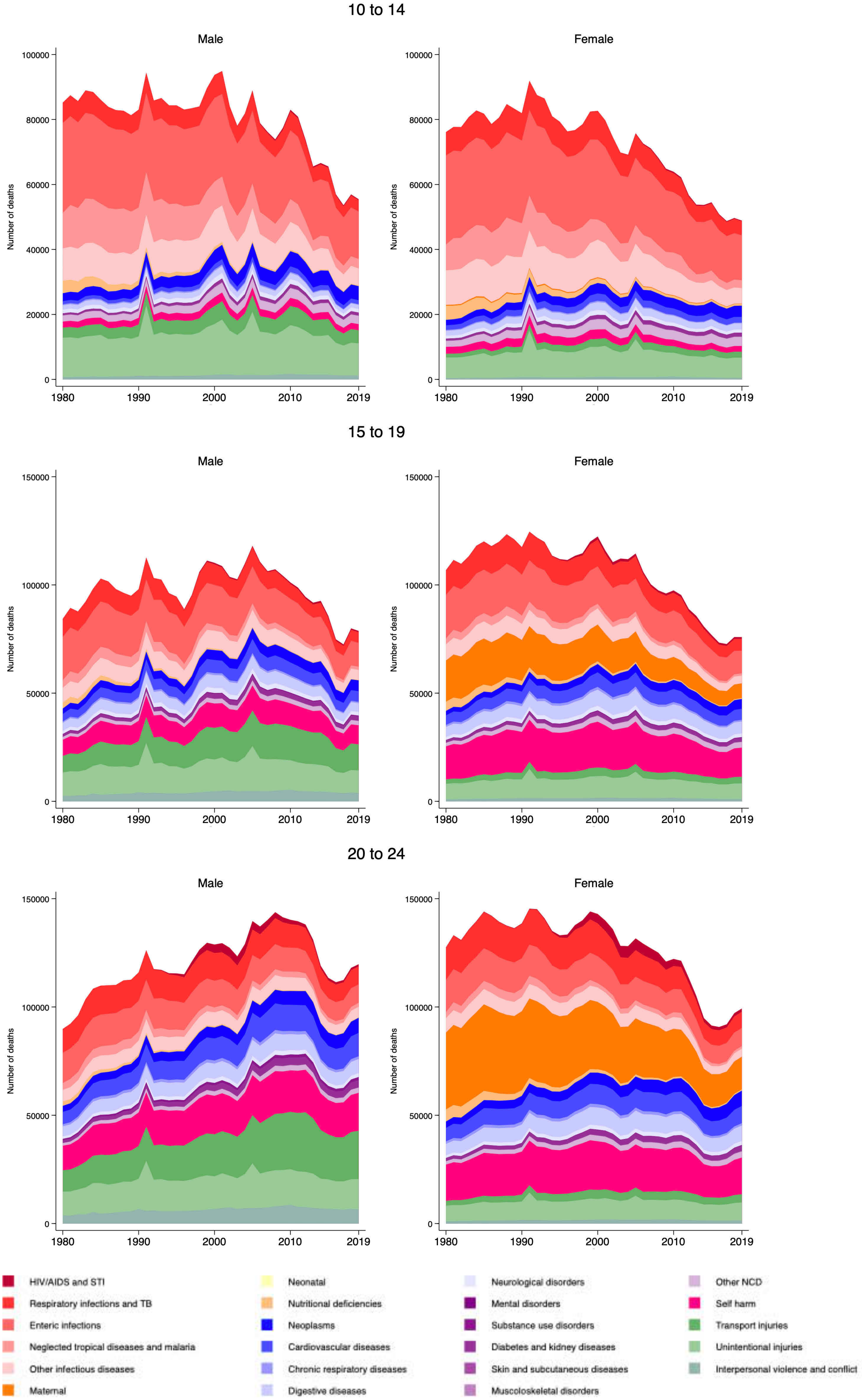

Figure S156: Afghanistan

Figure S158a All-cause mortality rate per 100,000 in 10-24 1950 – 2019: Afghanistan

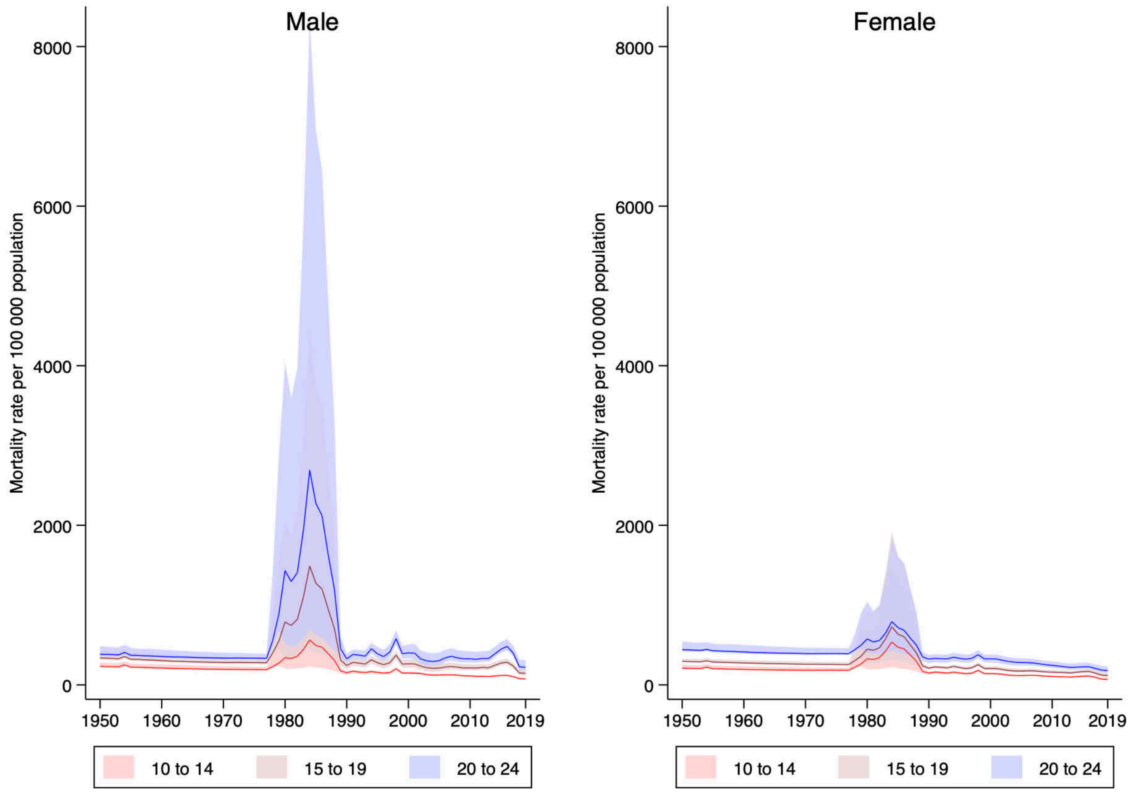

Figure S158b Number of deaths from all causes in 10-24 1950 – 2019: Afghanistan

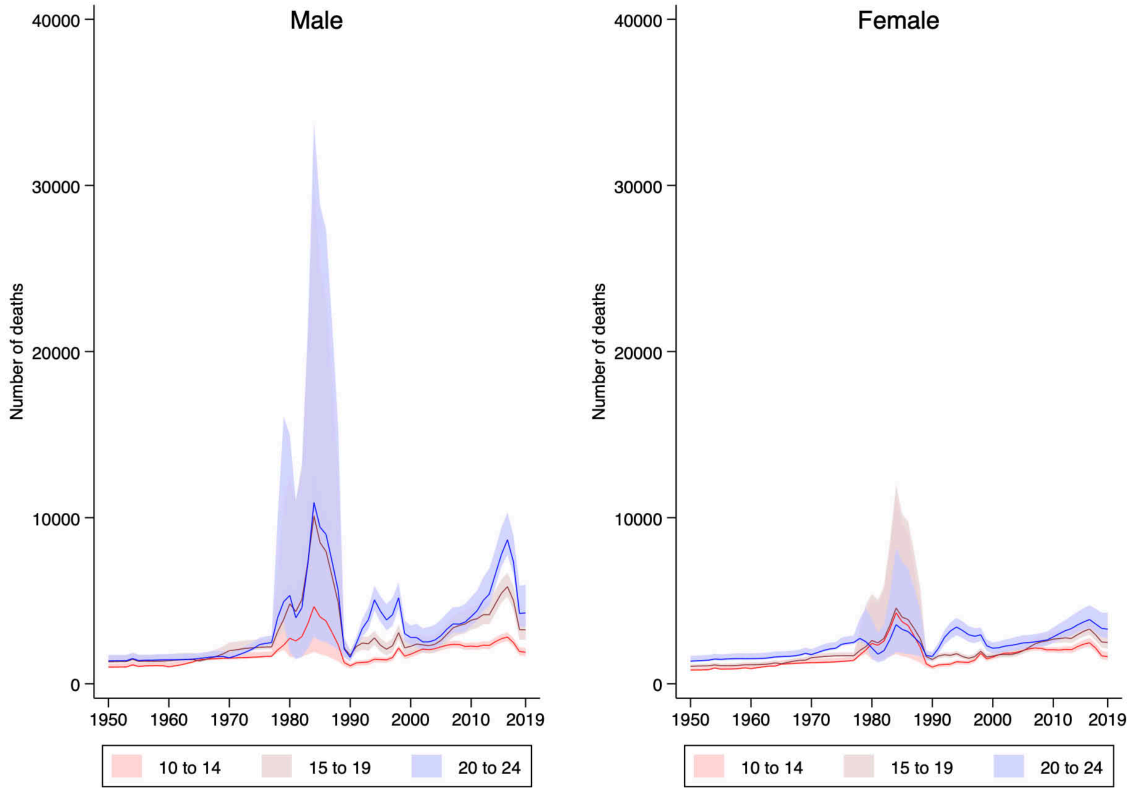

Figure S158c Mortality rate per 100,000 in 10-24 1980 – 2019 by cause: Afghanistan

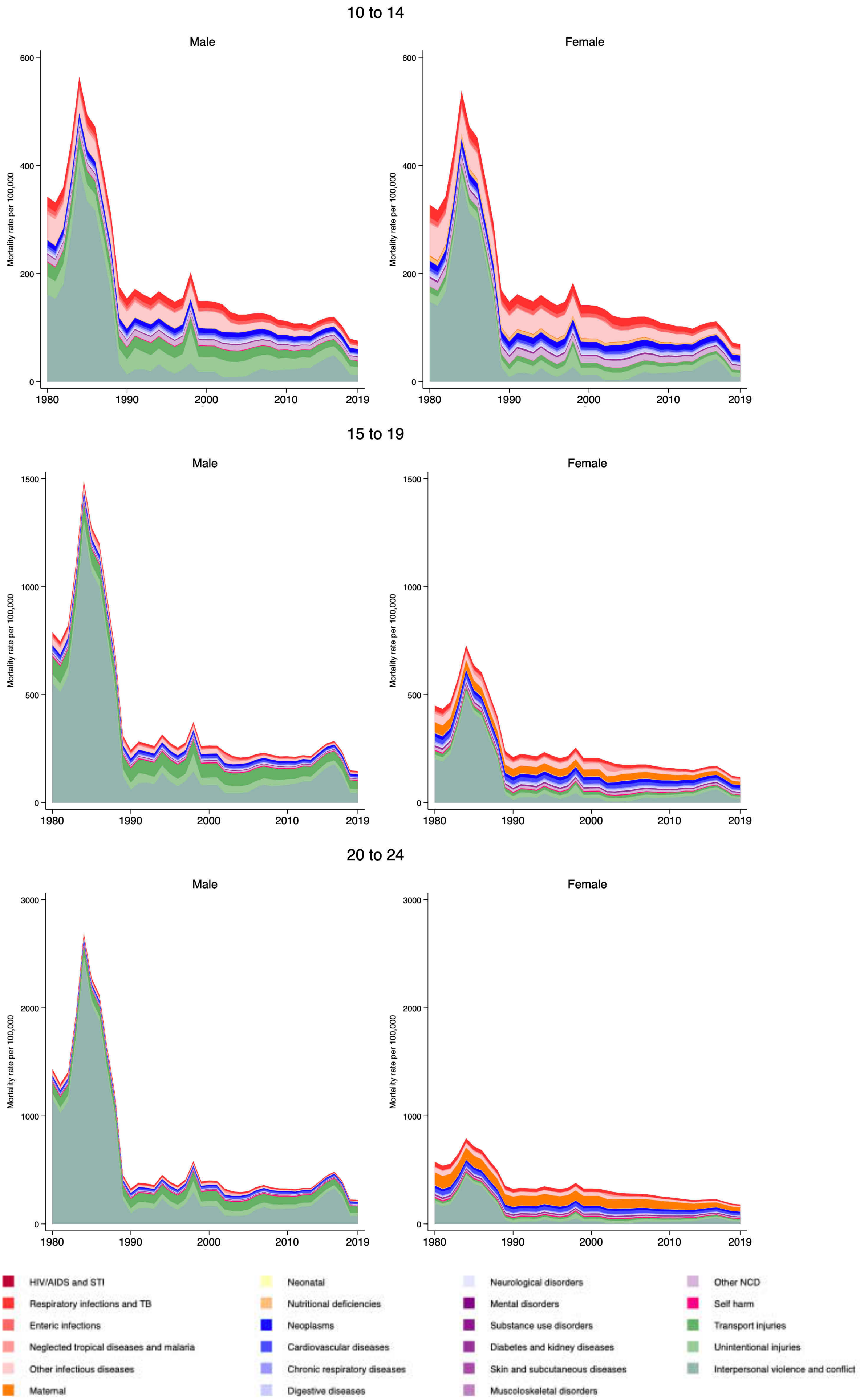

Figure S158d Number of deaths in 10-24 1980 – 2019 by cause: Afghanistan

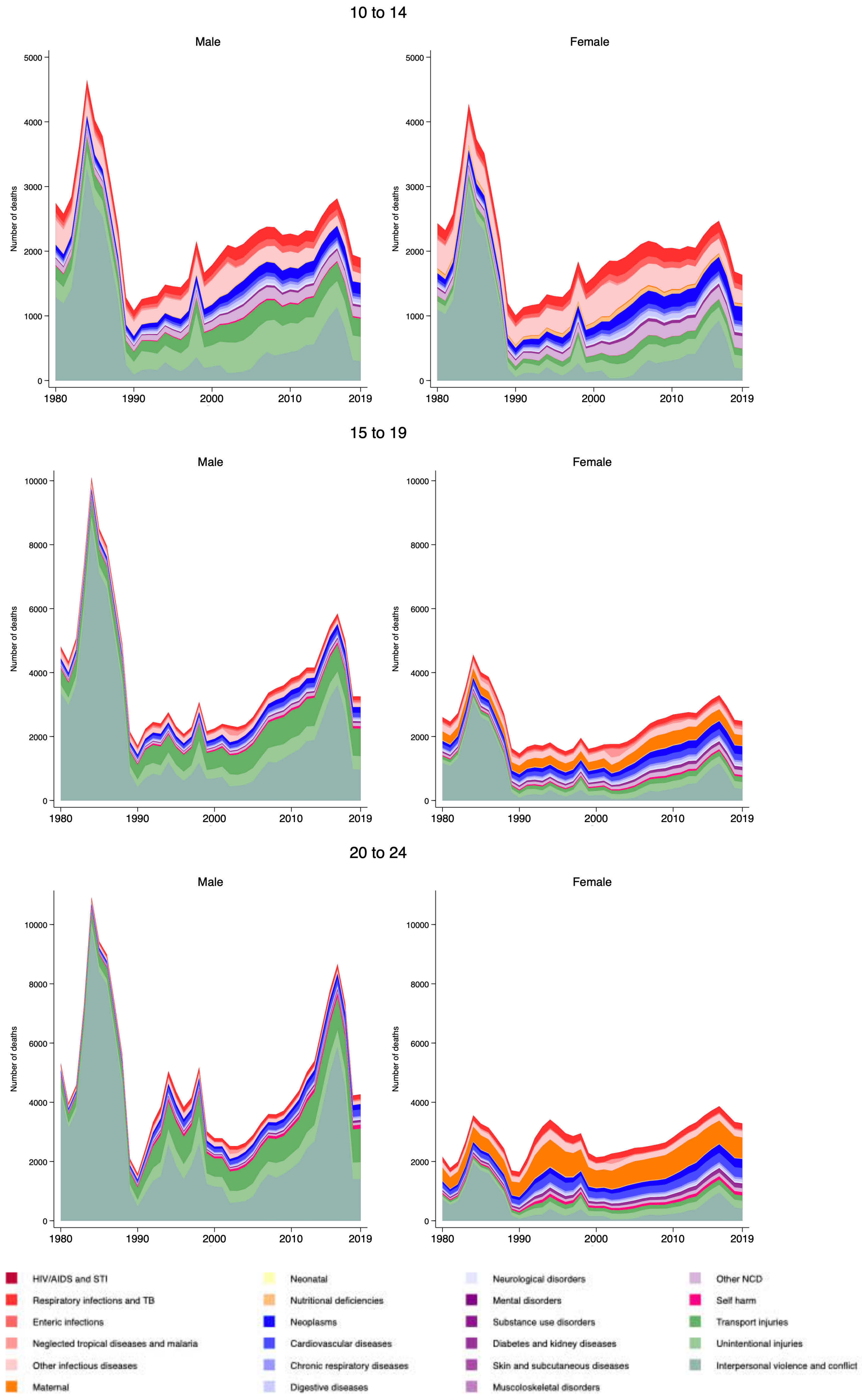

Figure S157: Bangladesh

Figure S159a All-cause mortality rate per 100,000 in 10-24 1950 – 2019: Bangladesh

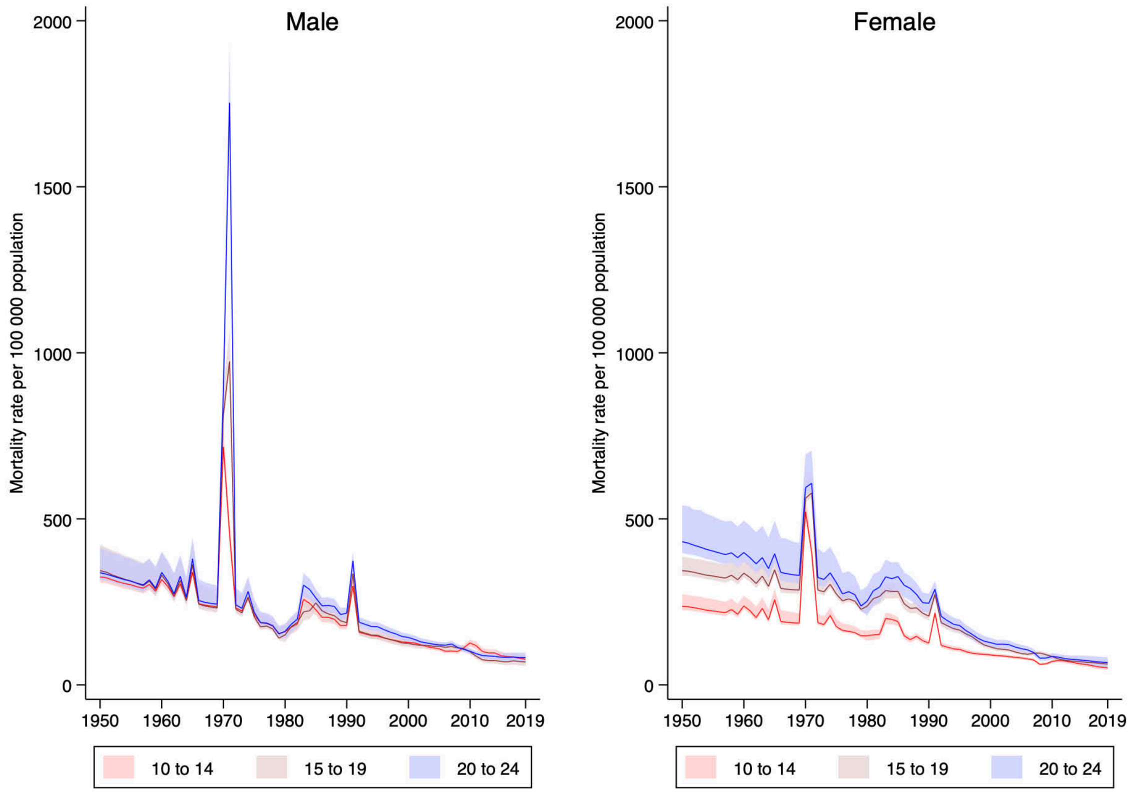

Figure S159b Number of deaths from all causes in 10-24 1950 – 2019: Bangladesh

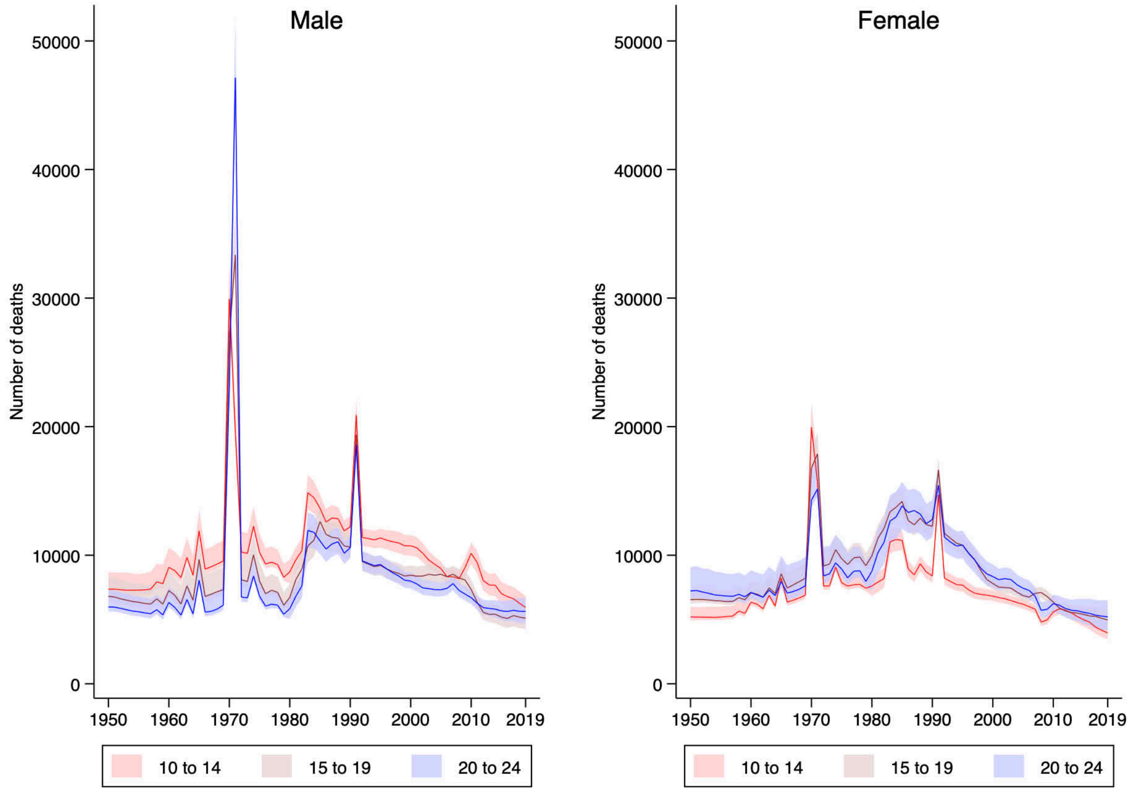

Figure S159c Mortality rate per 100,000 in 10-24 1980 – 2019 by cause: Bangladesh

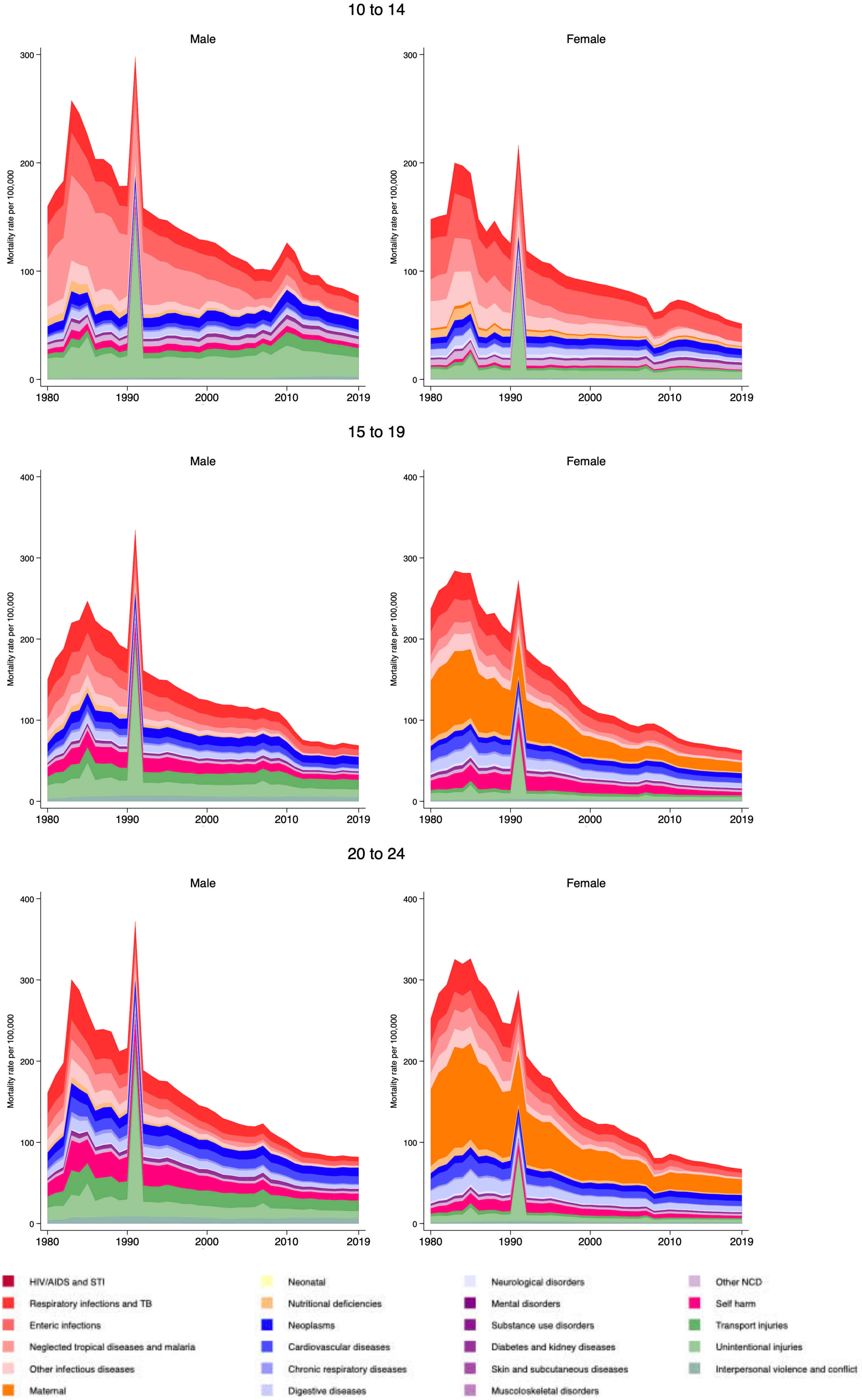

Figure S159d Number of deaths in 10-24 1980 – 2019 by cause: Bangladesh

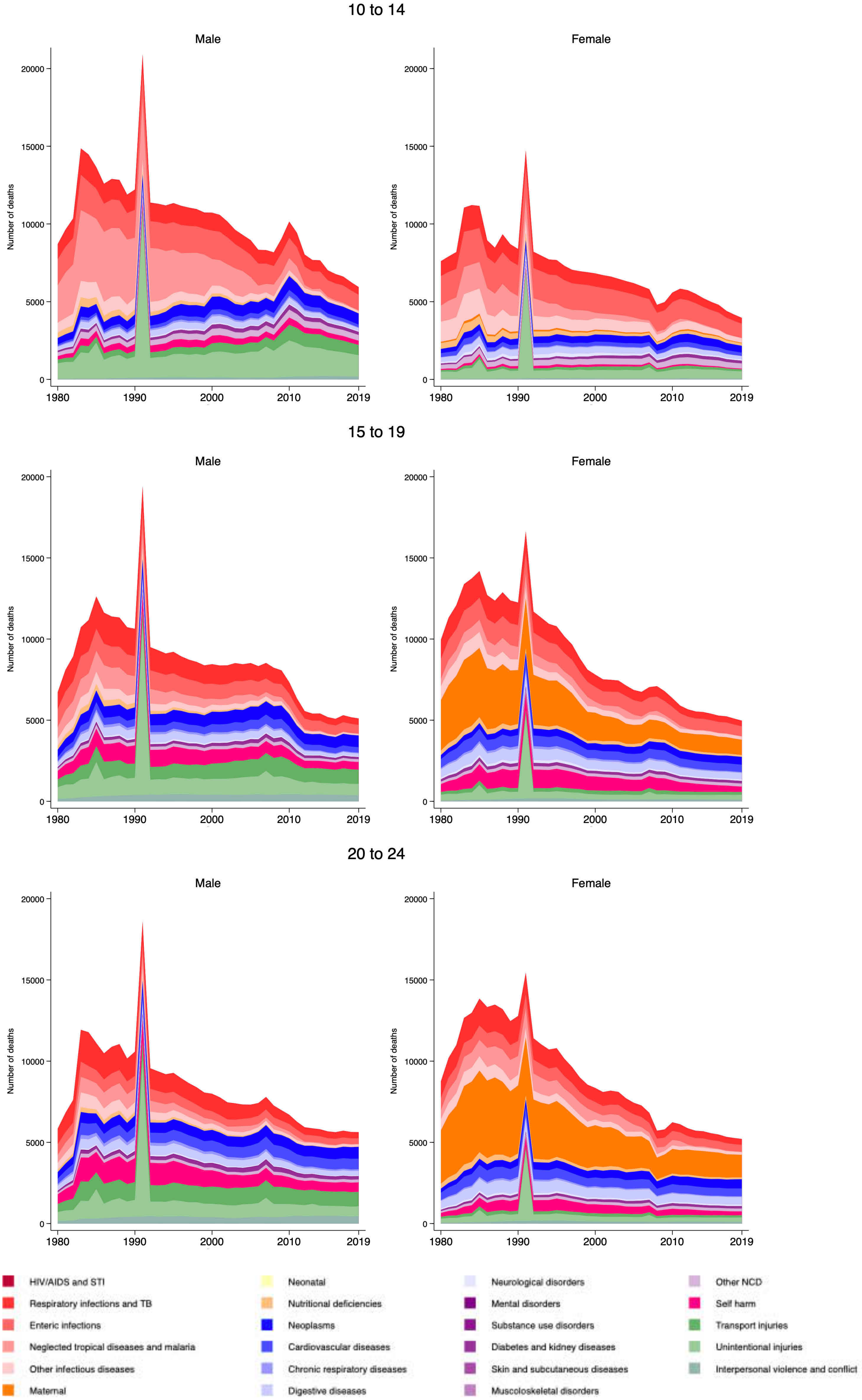

Figure S158: Bhutan

Figure S160a All-cause mortality rate per 100,000 in 10-24 1950 – 2019: Bhutan

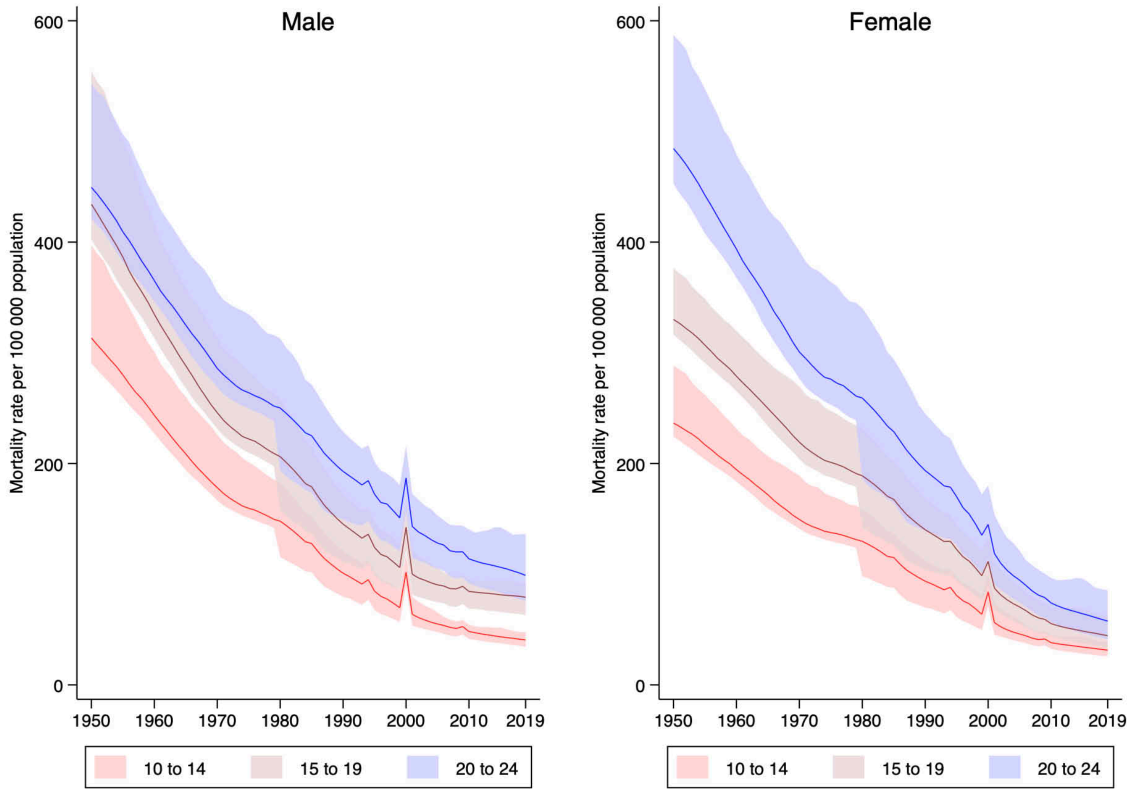

Figure S160b Number of deaths from all causes in 10-24 1950 – 2019: Bhutan

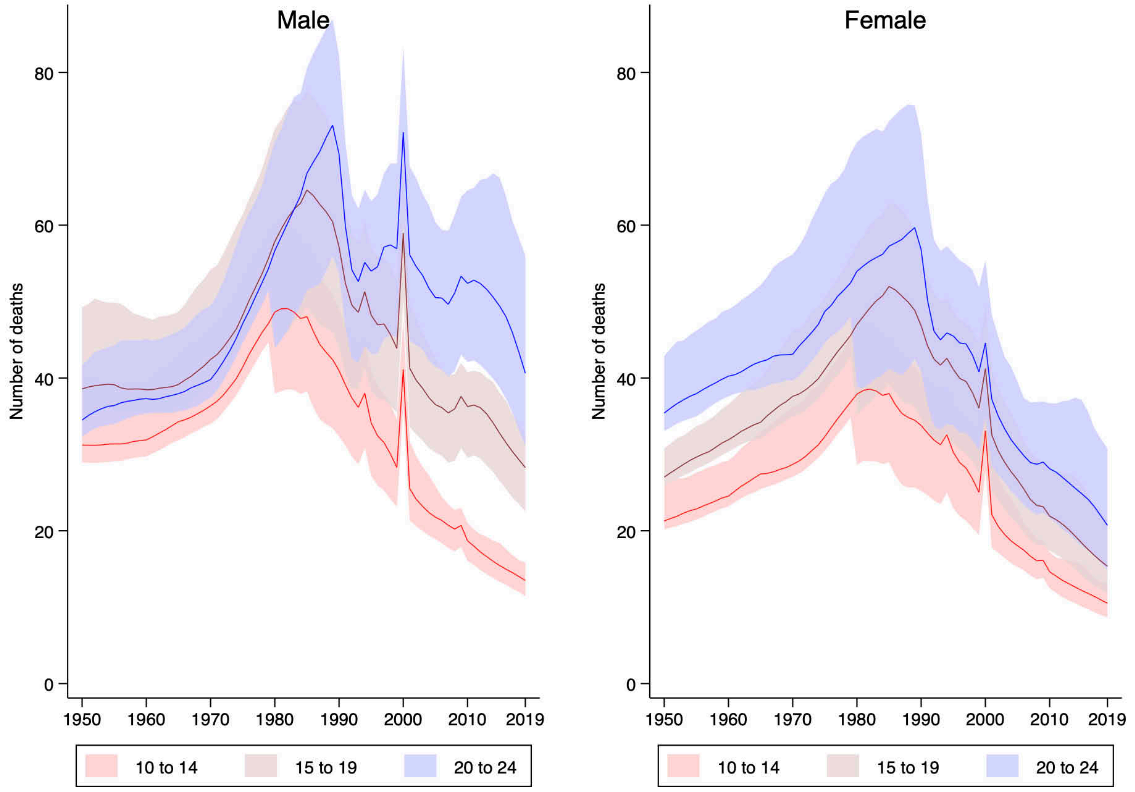

Figure S160c Mortality rate per 100,000 in 10-24 1980 – 2019 by cause: Bhutan

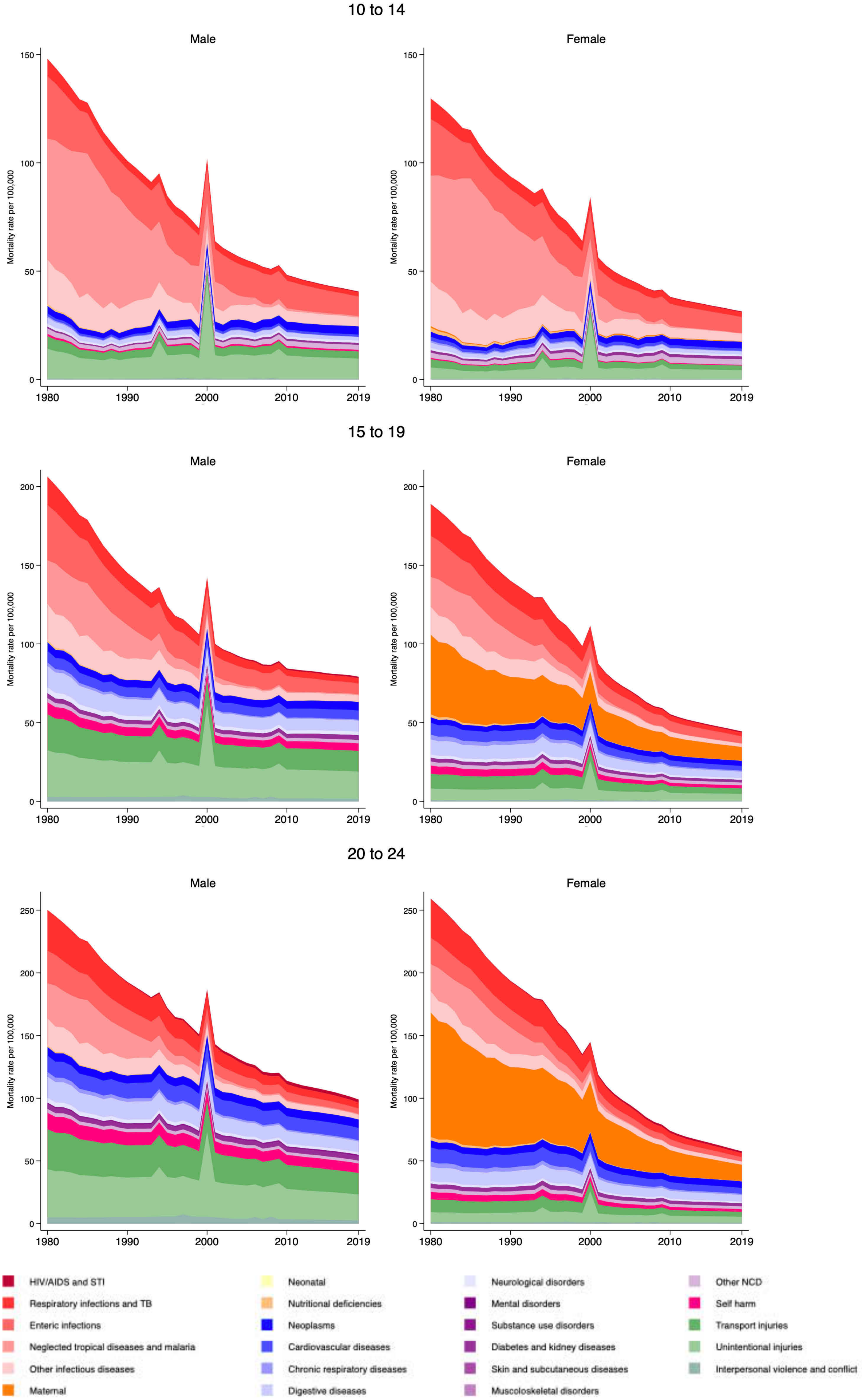

Figure S160d Number of deaths in 10-24 1980 – 2019 by cause: Bhutan

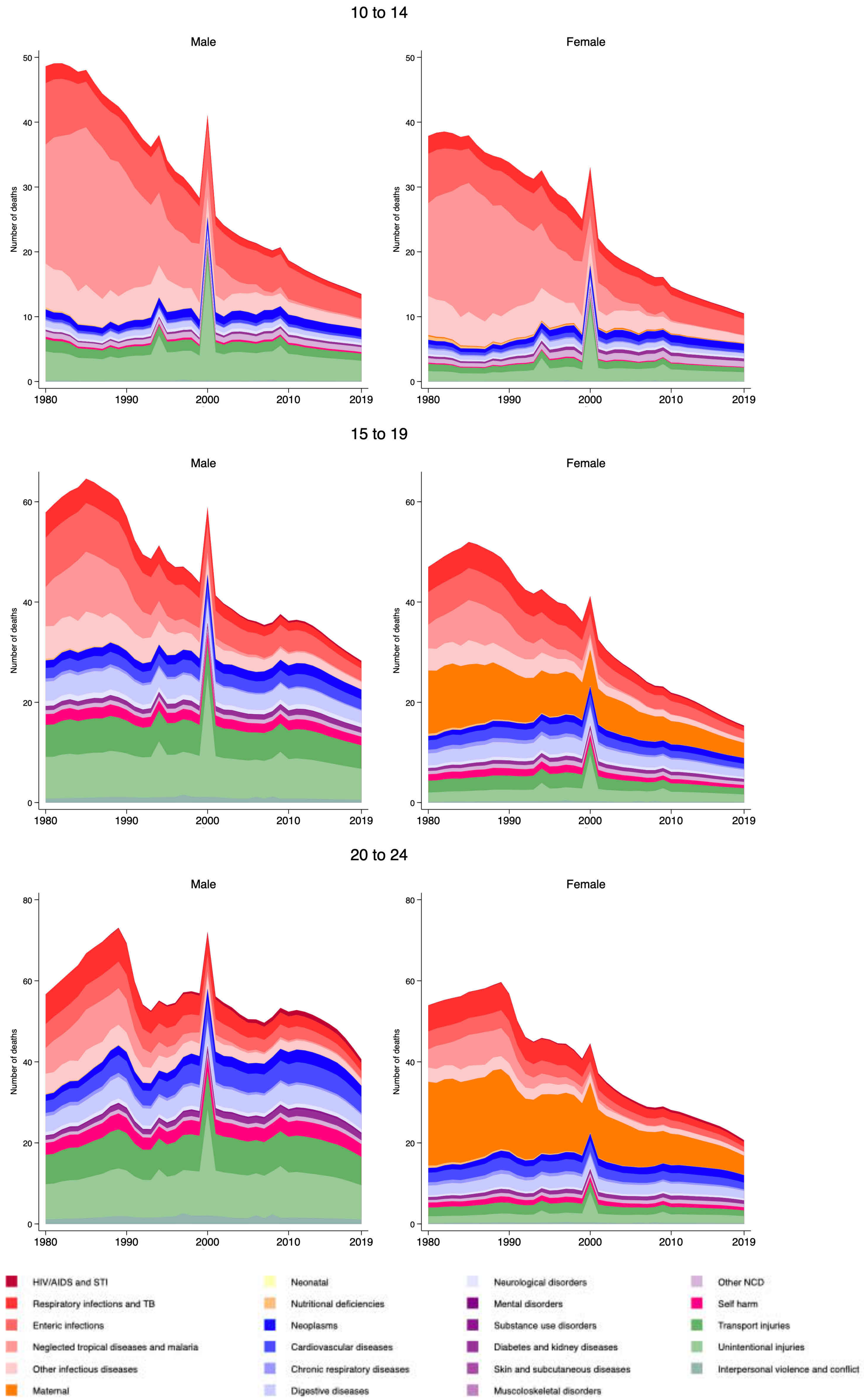

Figure S159: India

Figure S161a All-cause mortality rate per 100,000 in 10-24 1950 – 2019: India

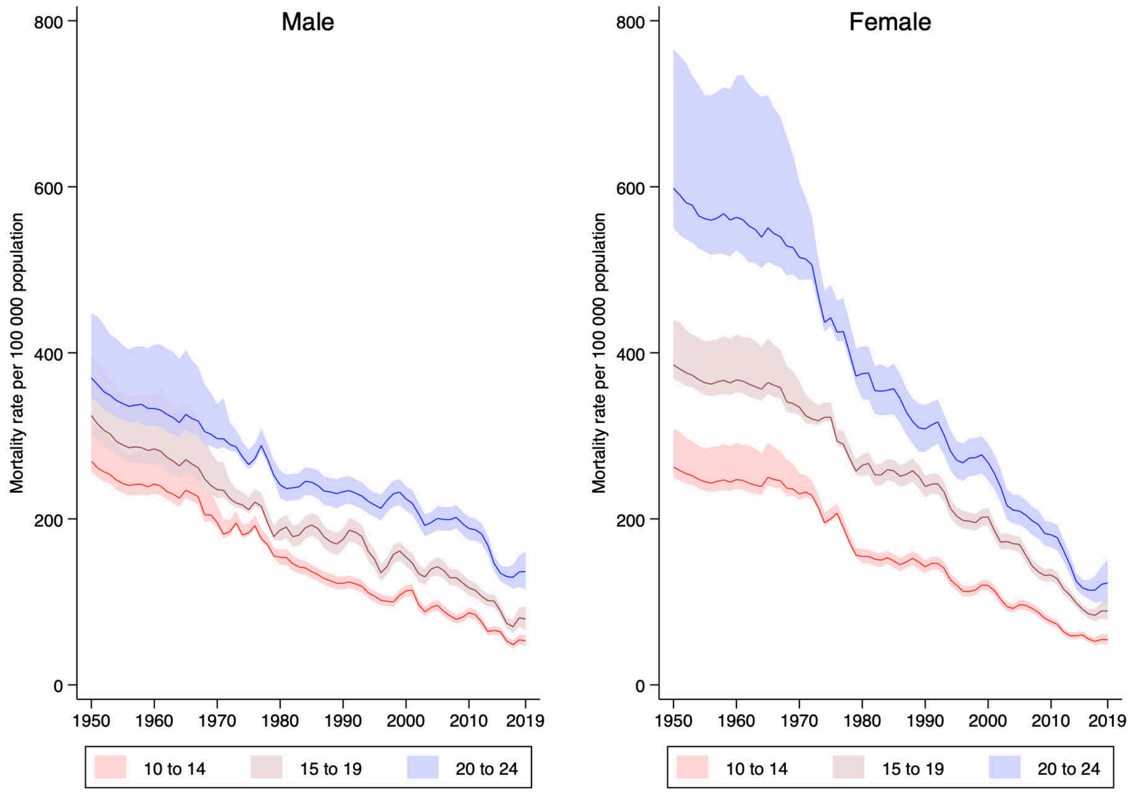

Figure S161b Number of deaths from all causes in 10-24 1950 – 2019: India

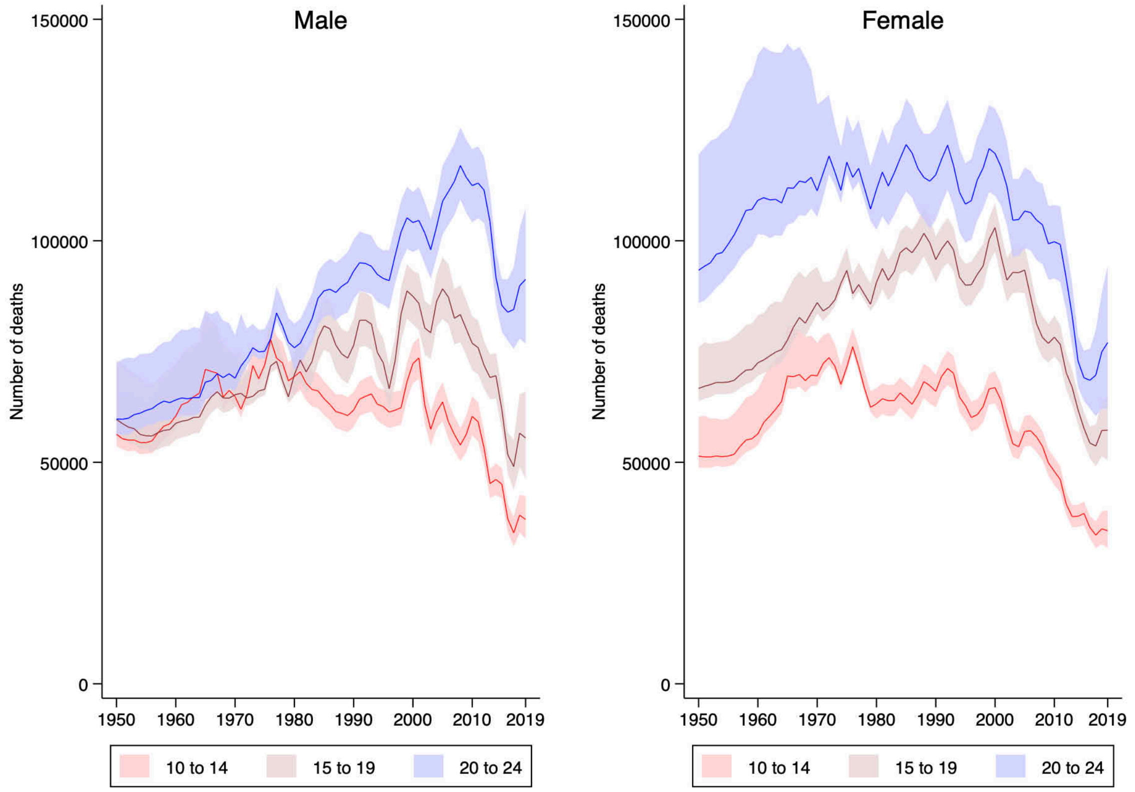

Figure S161c Mortality rate per 100,000 in 10-24 1980 – 2019 by cause: India

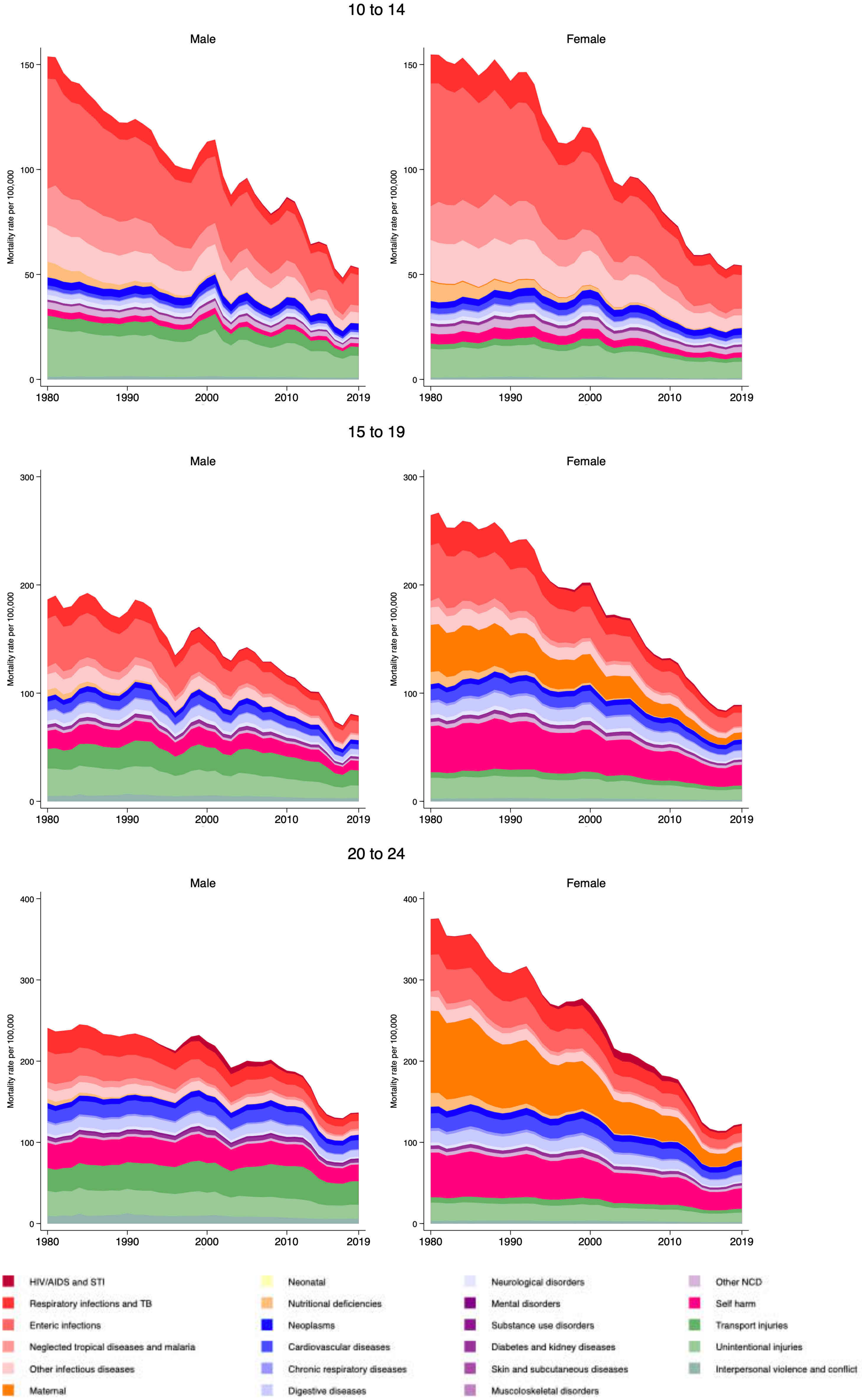

Figure S161d Number of deaths in 10-24 1980 – 2019 by cause: India

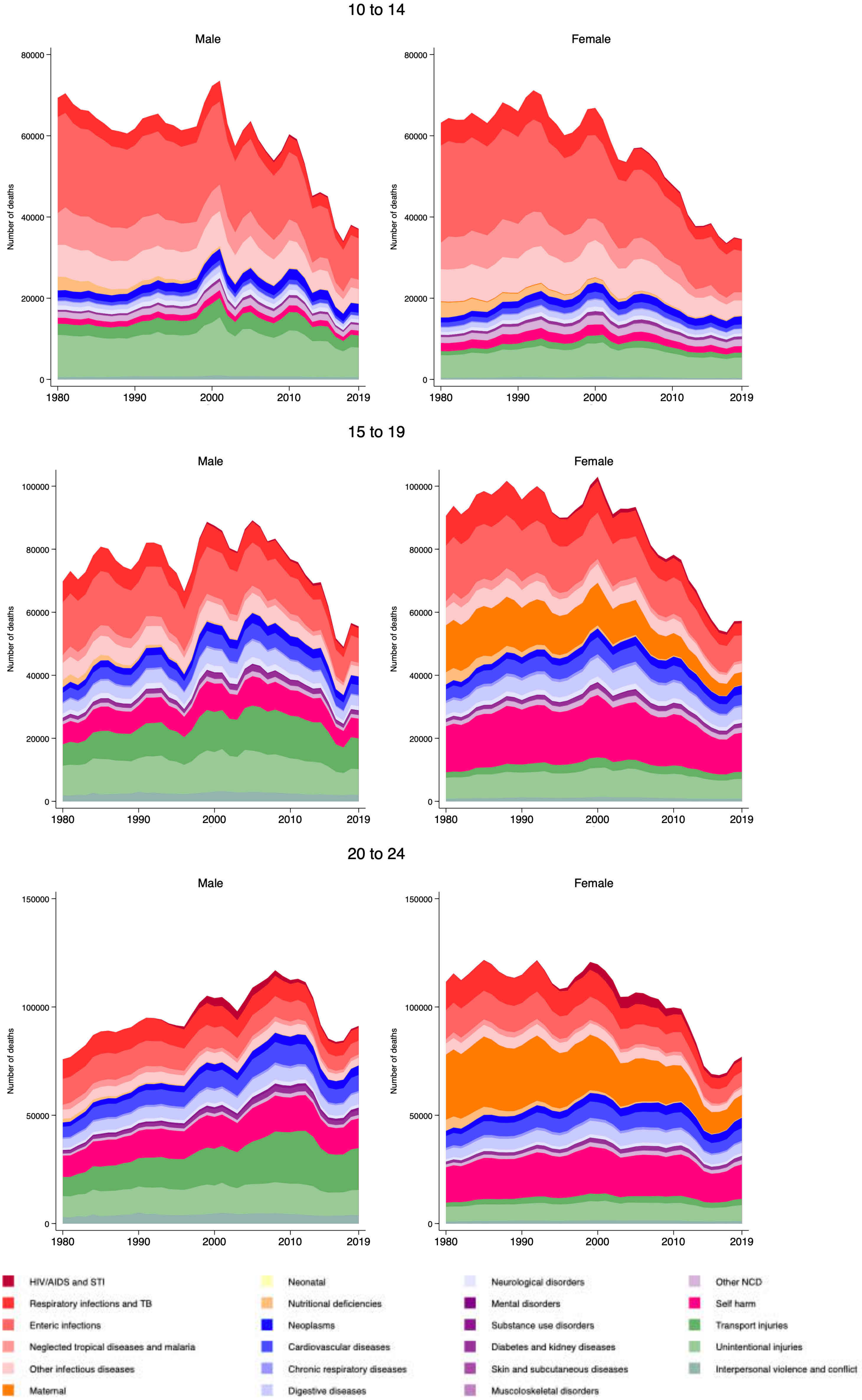

Figure S160: Nepal

Figure S162a All-cause mortality rate per 100,000 in 10-24 1950 – 2019: Nepal

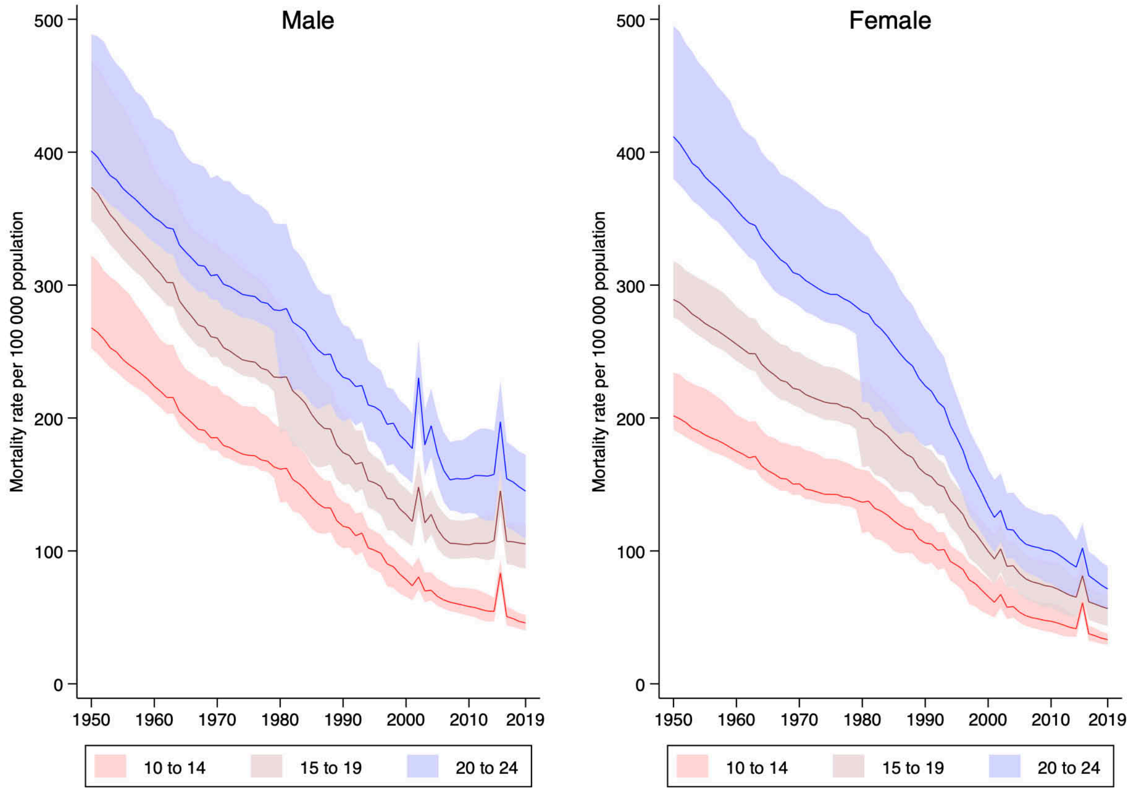

Figure S162b Number of deaths from all causes in 10-24 1950 – 2019: Nepal

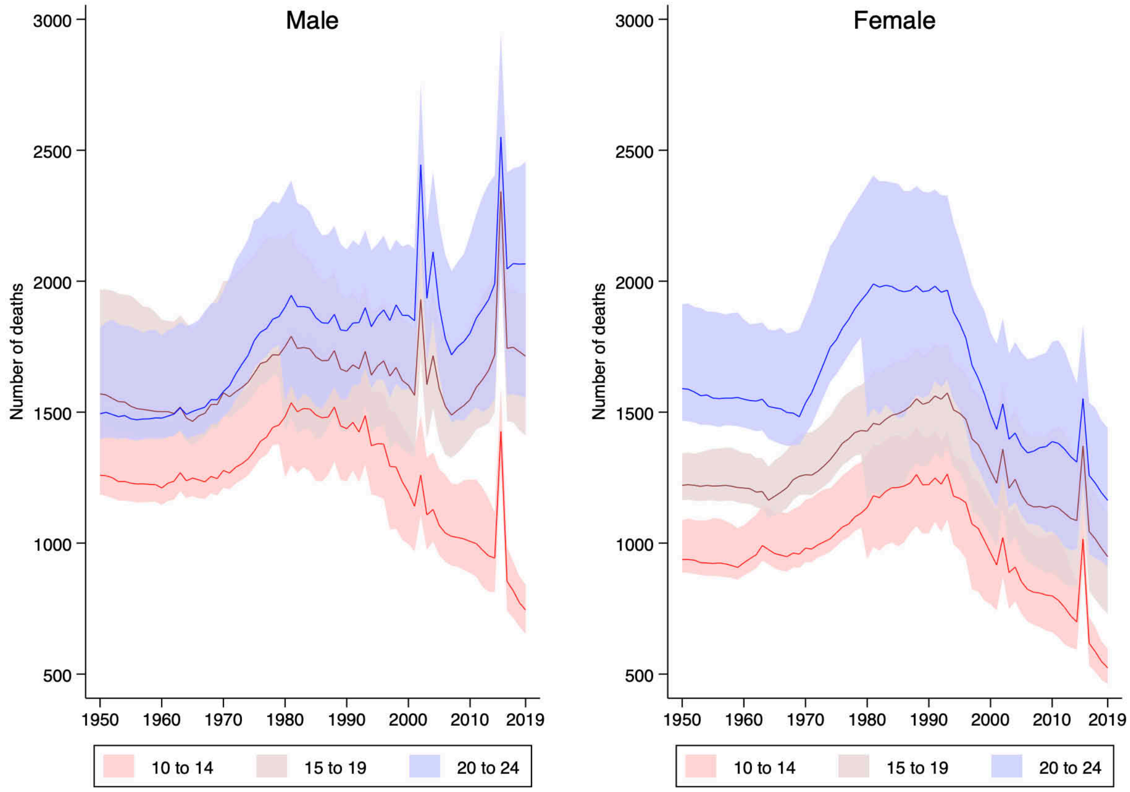

Figure S162c Mortality rate per 100,000 in 10-24 1980 – 2019 by cause: Nepal

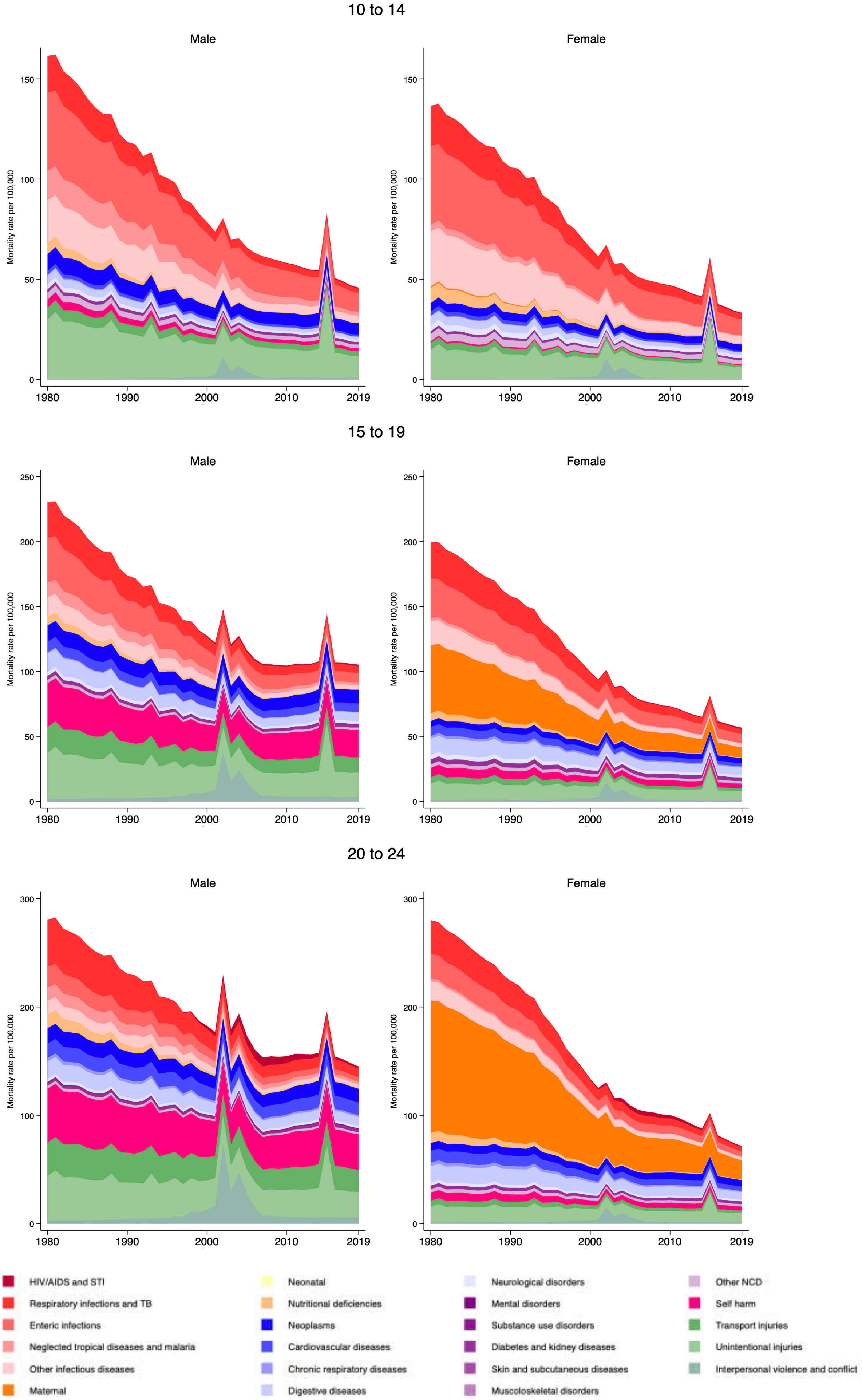

Figure S162d Number of deaths in 10-24 1980 – 2019 by cause: Nepal

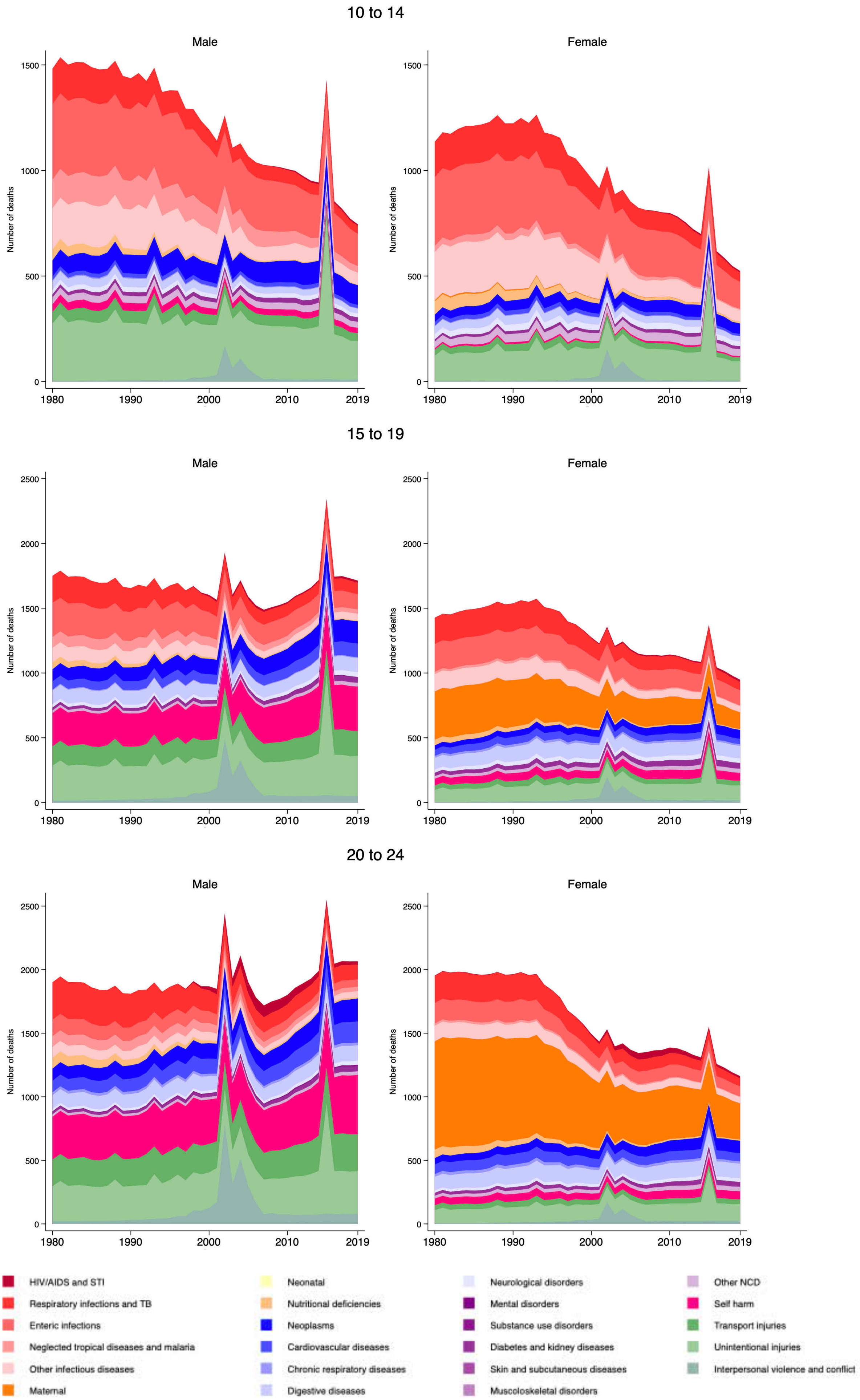

Figure S161: Pakistan

Figure S163a All-cause mortality rate per 100,000 in 10-24 1950 – 2019: Pakistan

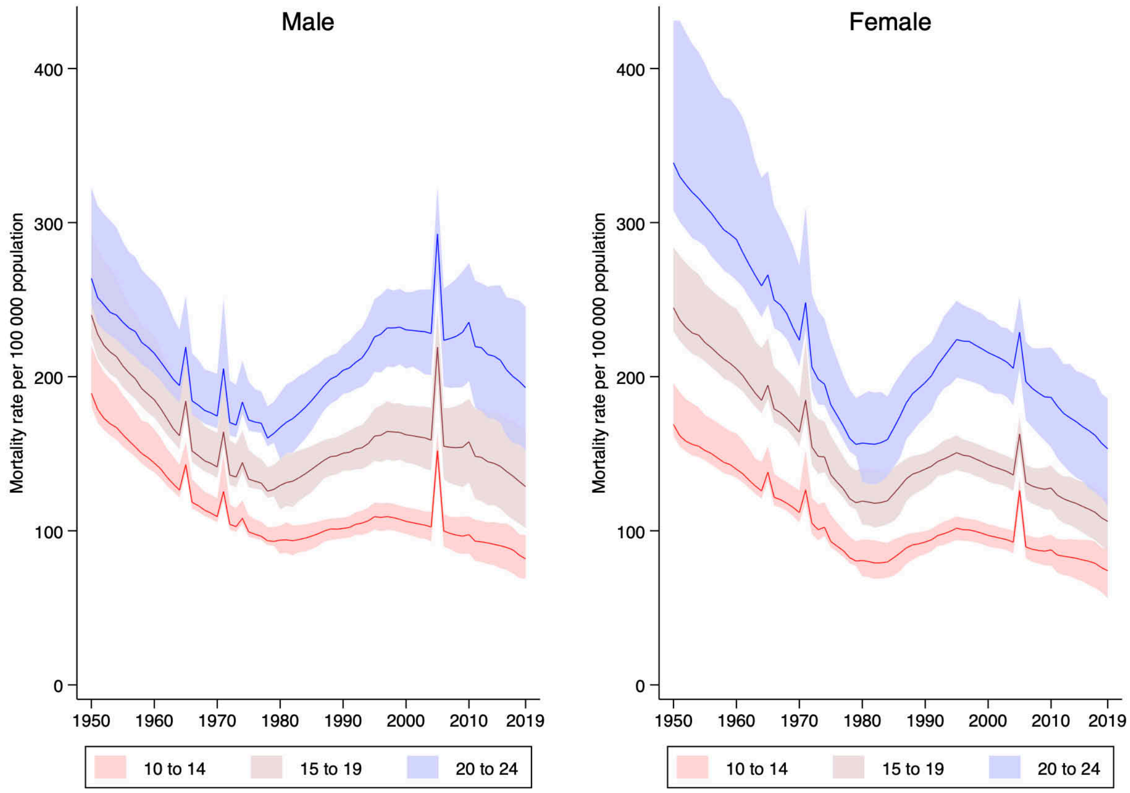

Figure S163b Number of deaths from all causes in 10-24 1950 – 2019: Pakistan

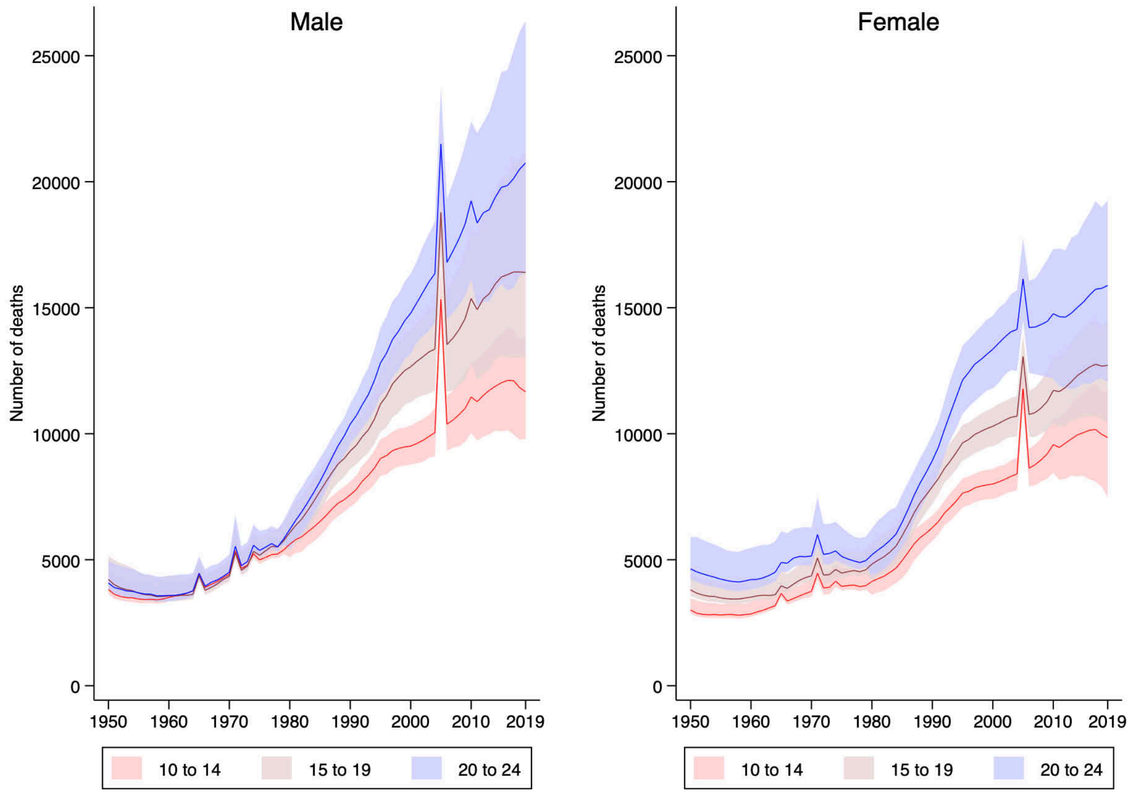

Figure S163c Mortality rate per 100,000 in 10-24 1980 – 2019 by cause: Pakistan

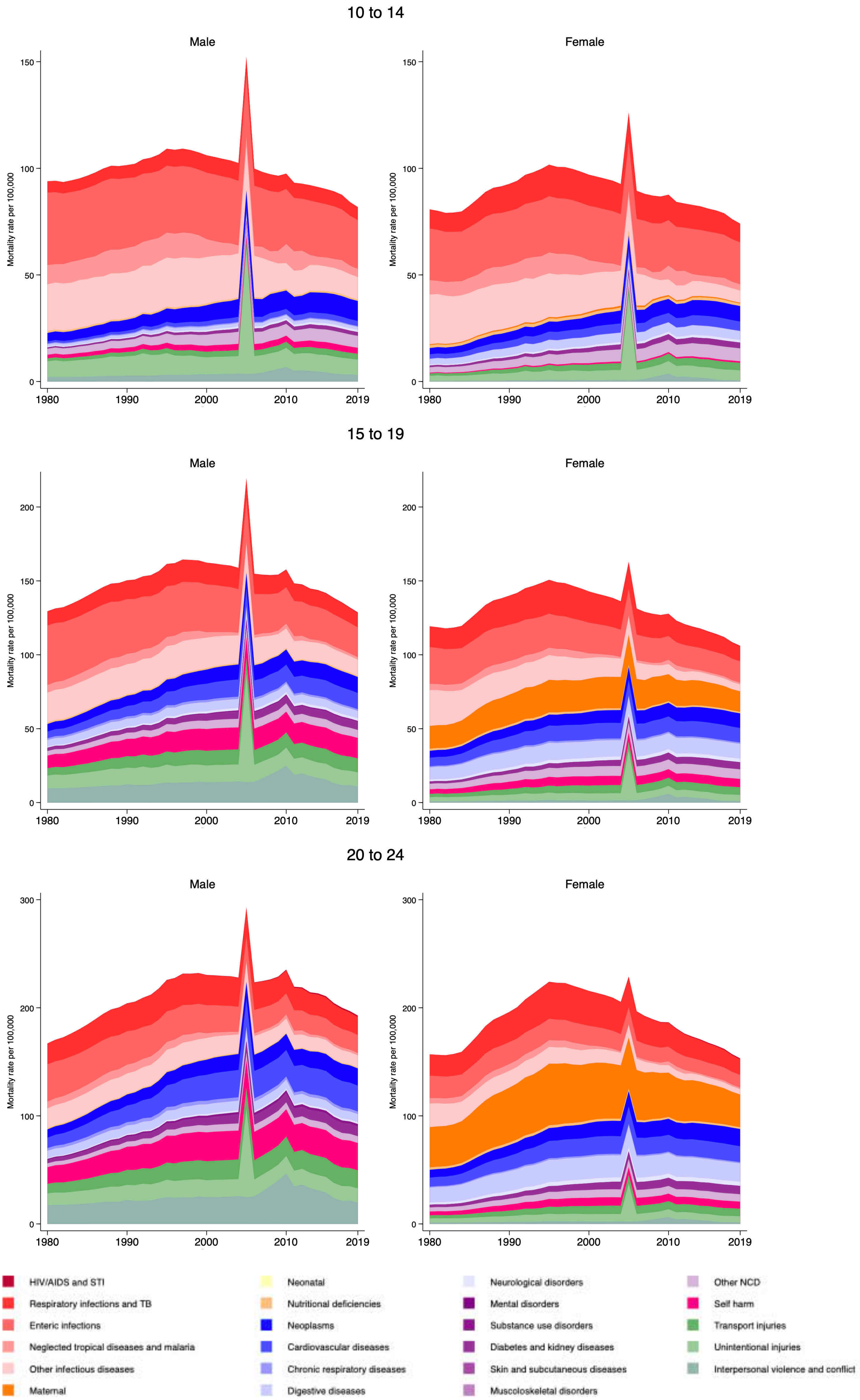

Figure S163d Number of deaths in 10-24 1980 – 2019 by cause: Pakistan

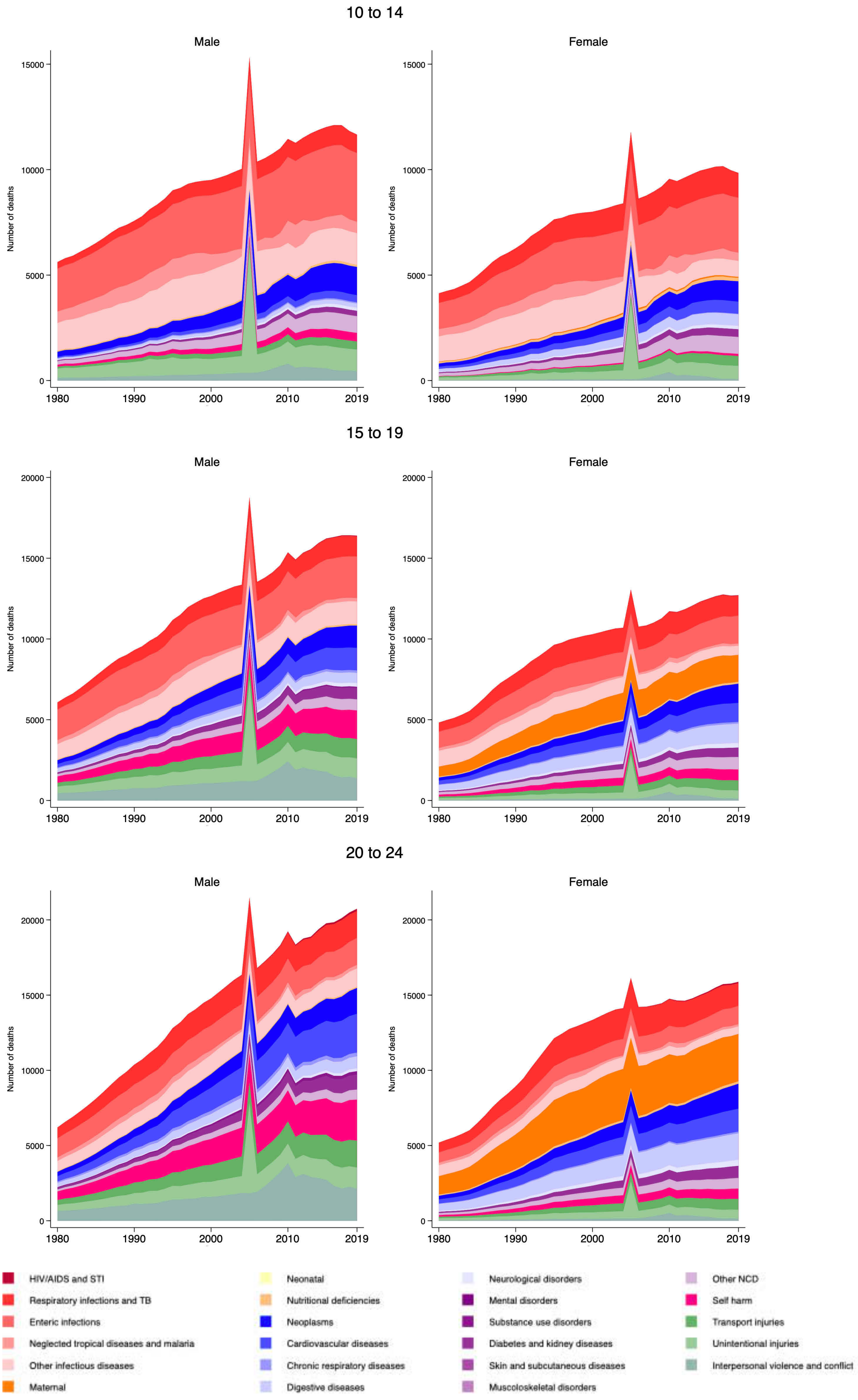

Figure S162: Sub-Saharan Africa

Figure S164a All-cause mortality rate per 100,000 in 10-24 1950 – 2019: Sub-Saharan Africa

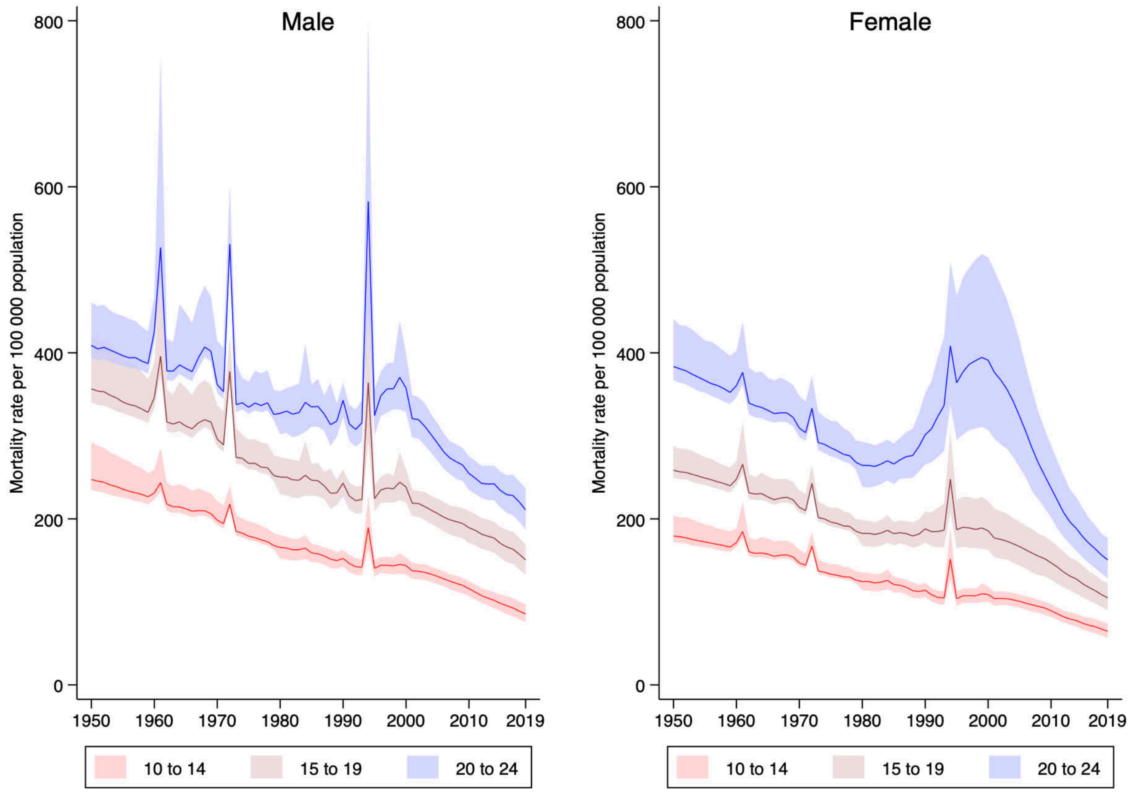

Figure S164b Number of deaths from all causes in 10-24 1950 – 2019: Sub-Saharan Africa

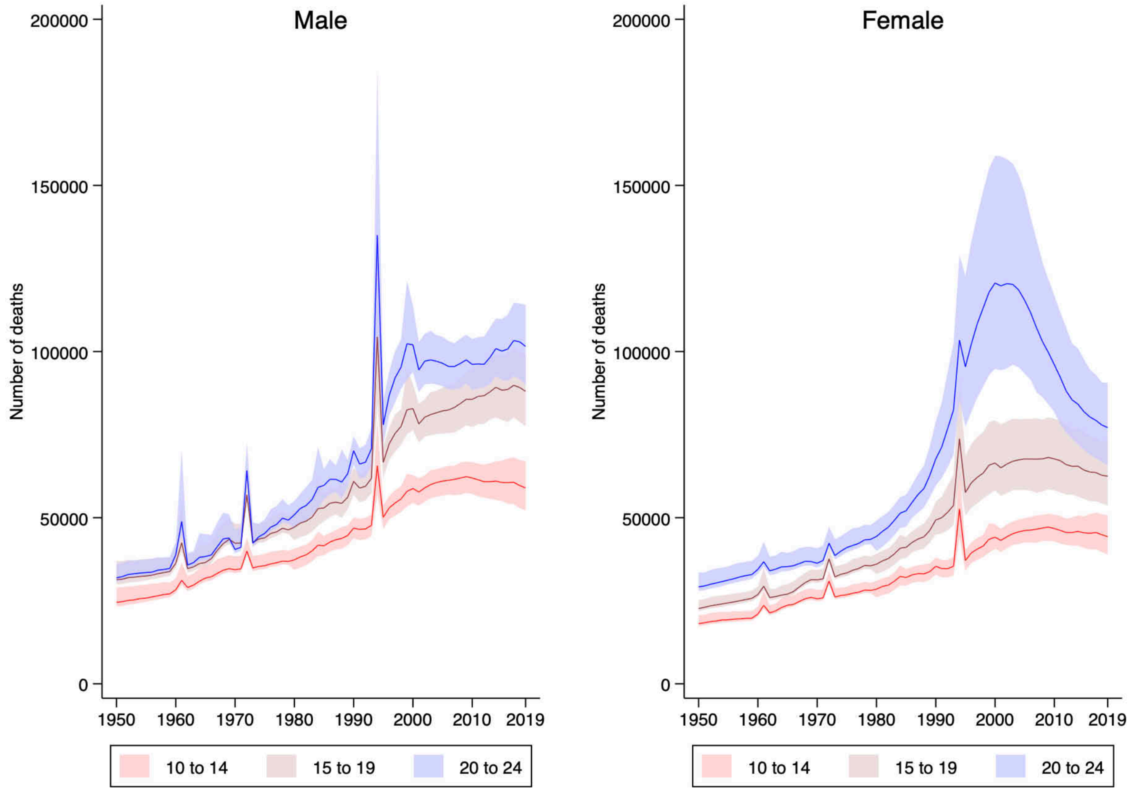

Figure S164c Mortality rate per 100,000 in 10-24 1980 – 2019 by cause: Sub-Saharan Africa

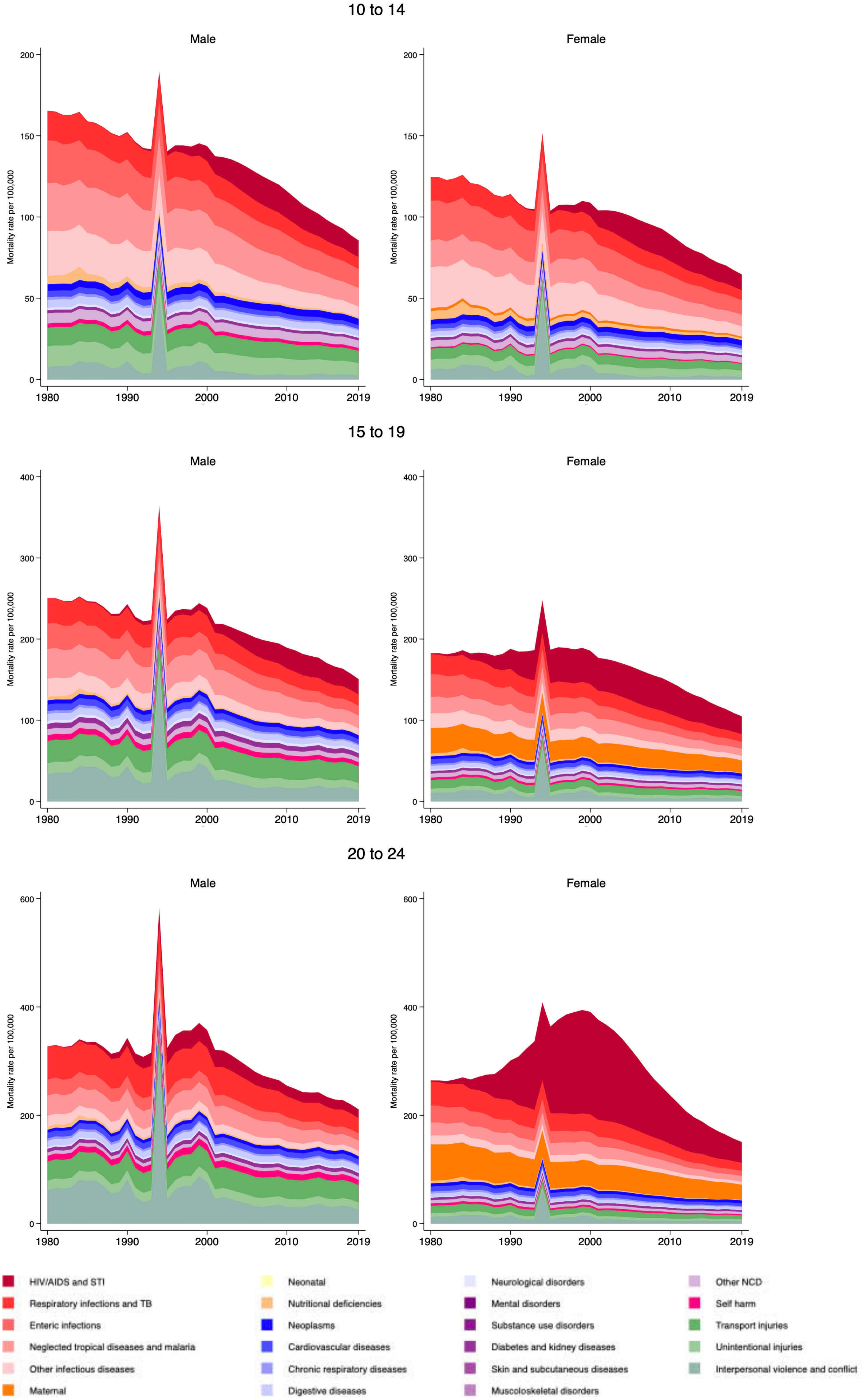

Figure S164d Number of deaths in 10-24 1980 – 2019 by cause: Sub-Saharan Africa

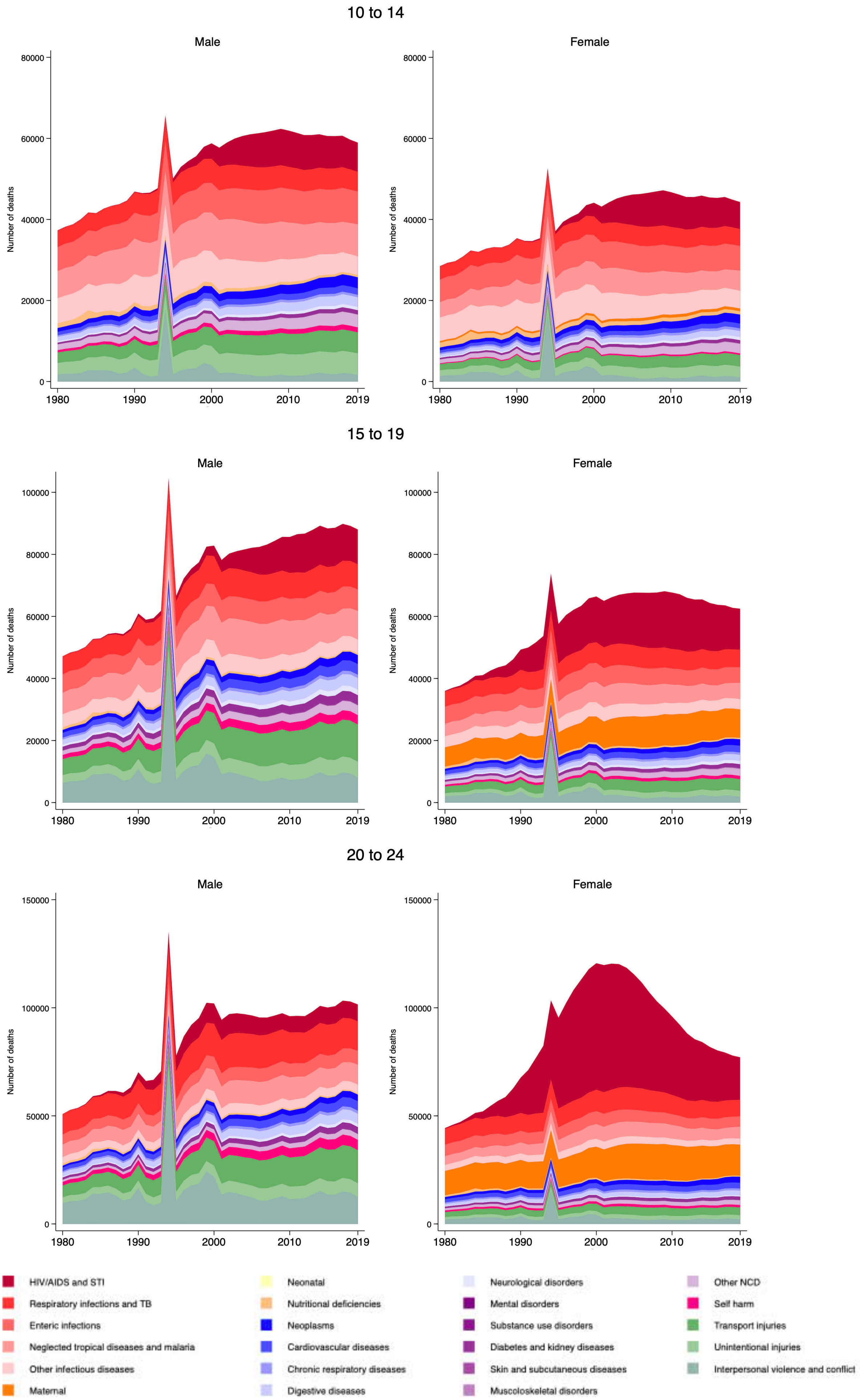

Figure S163: Central Sub-Saharan Africa

Figure S165a All-cause mortality rate per 100,000 in 10-24 1950 – 2019: Central Sub-Saharan Africa

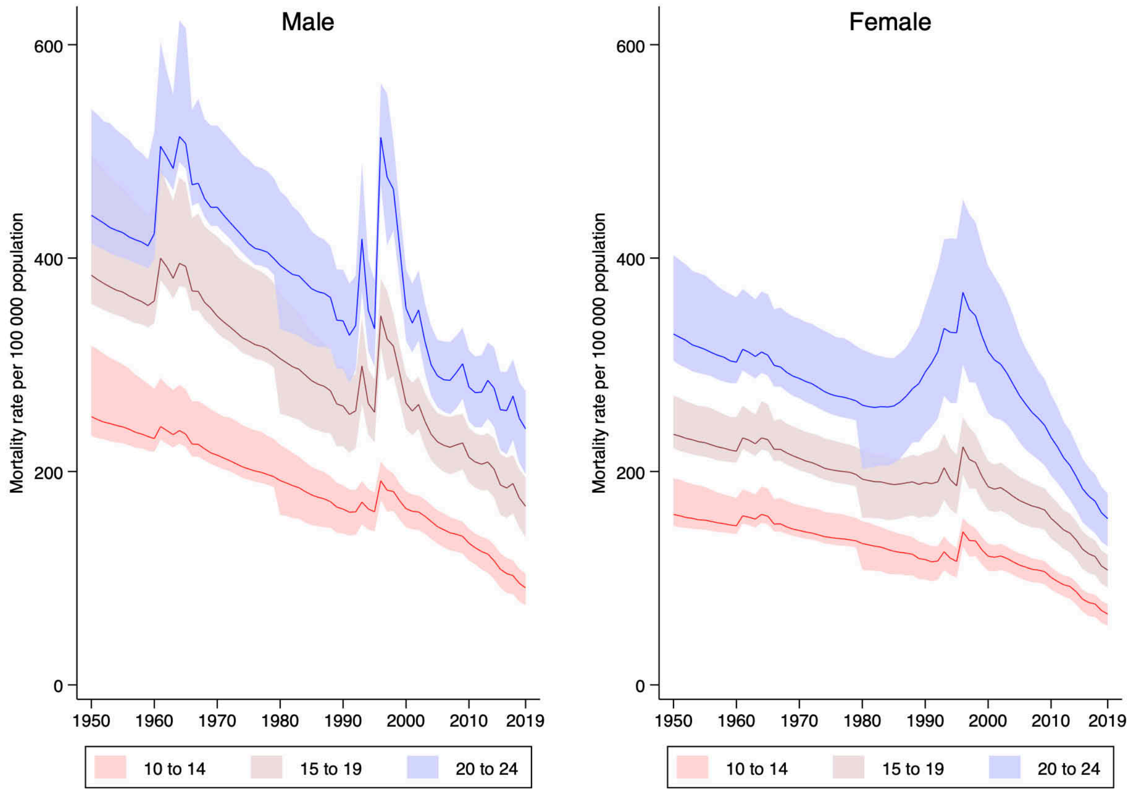

Figure S165b Number of deaths from all causes in 10-24 1950 – 2019: Central Sub-Saharan Africa

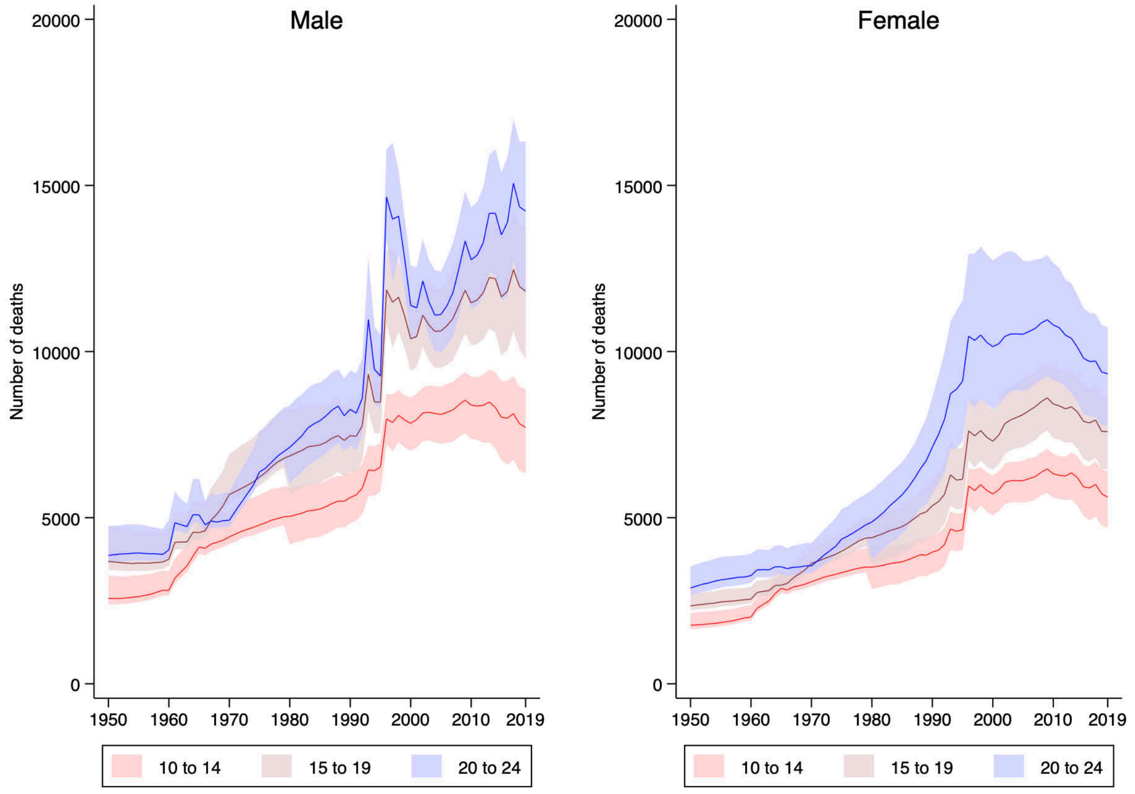

Figure S165c Mortality rate per 100,000 in 10-24 1980 – 2019 by cause: Central Sub-Saharan Africa

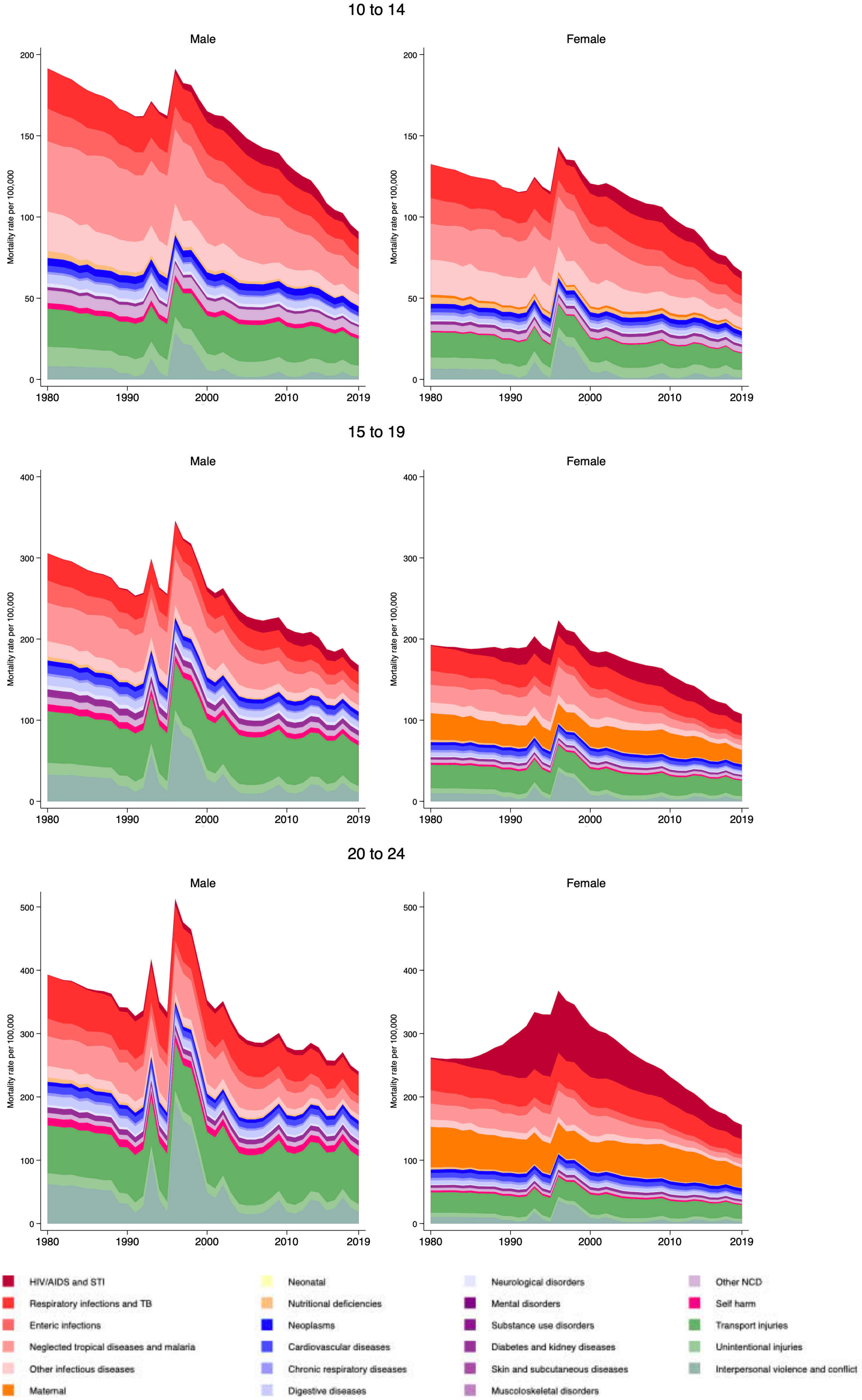

Figure S165d Number of deaths in 10-24 1980 – 2019 by cause: Central Sub-Saharan Africa

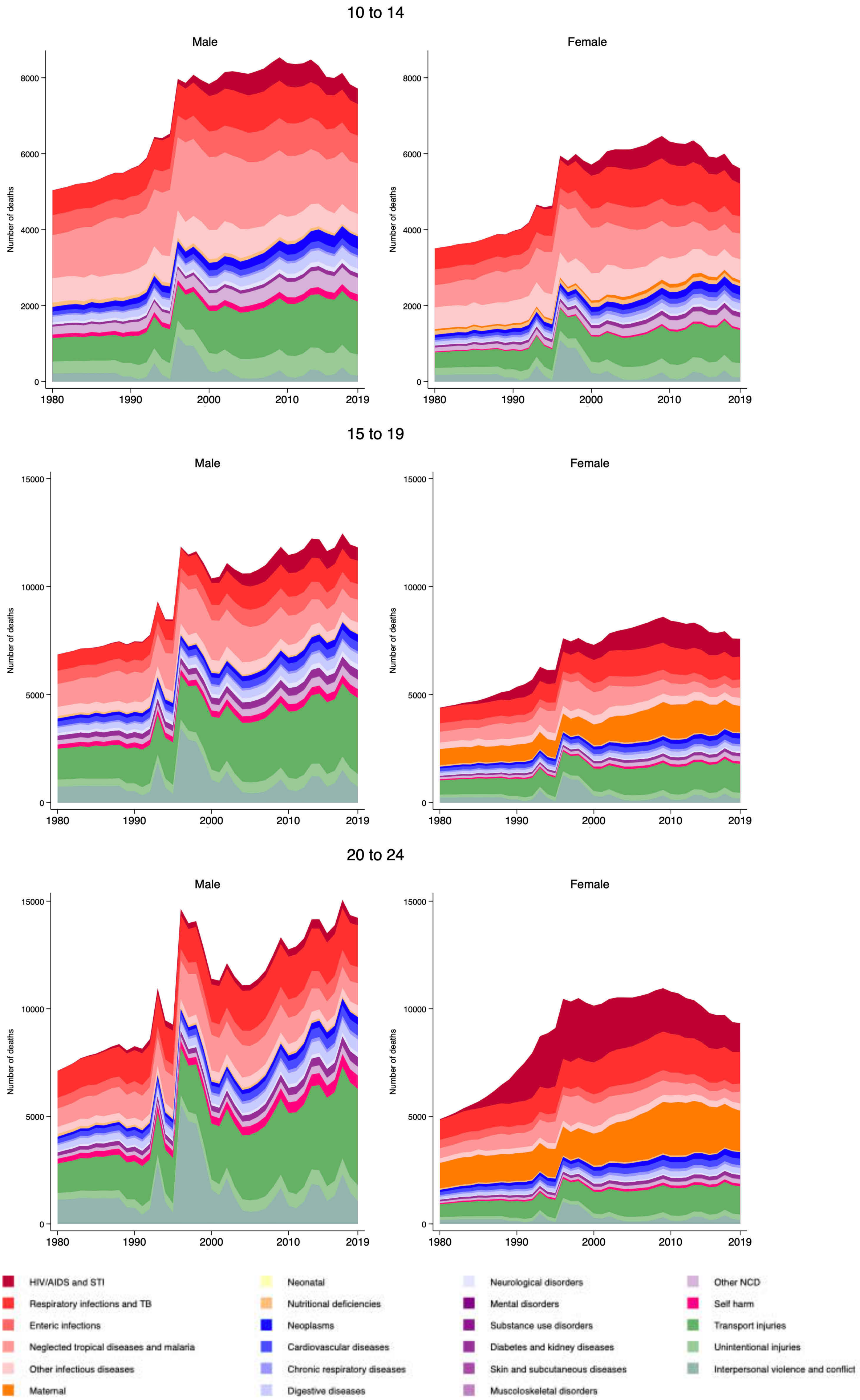

Figure S164: Angola

Figure S166a All-cause mortality rate per 100,000 in 10-24 1950 – 2019: Angola

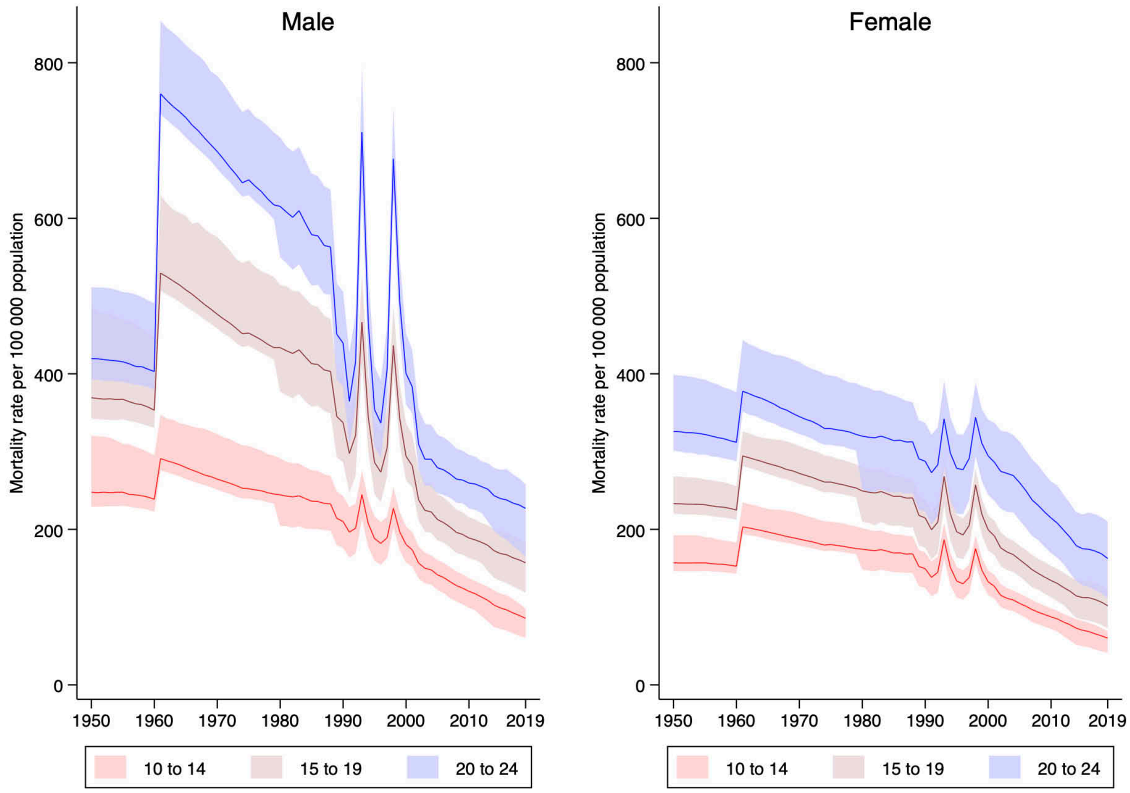

Figure S166b Number of deaths from all causes in 10-24 1950 – 2019: Angola

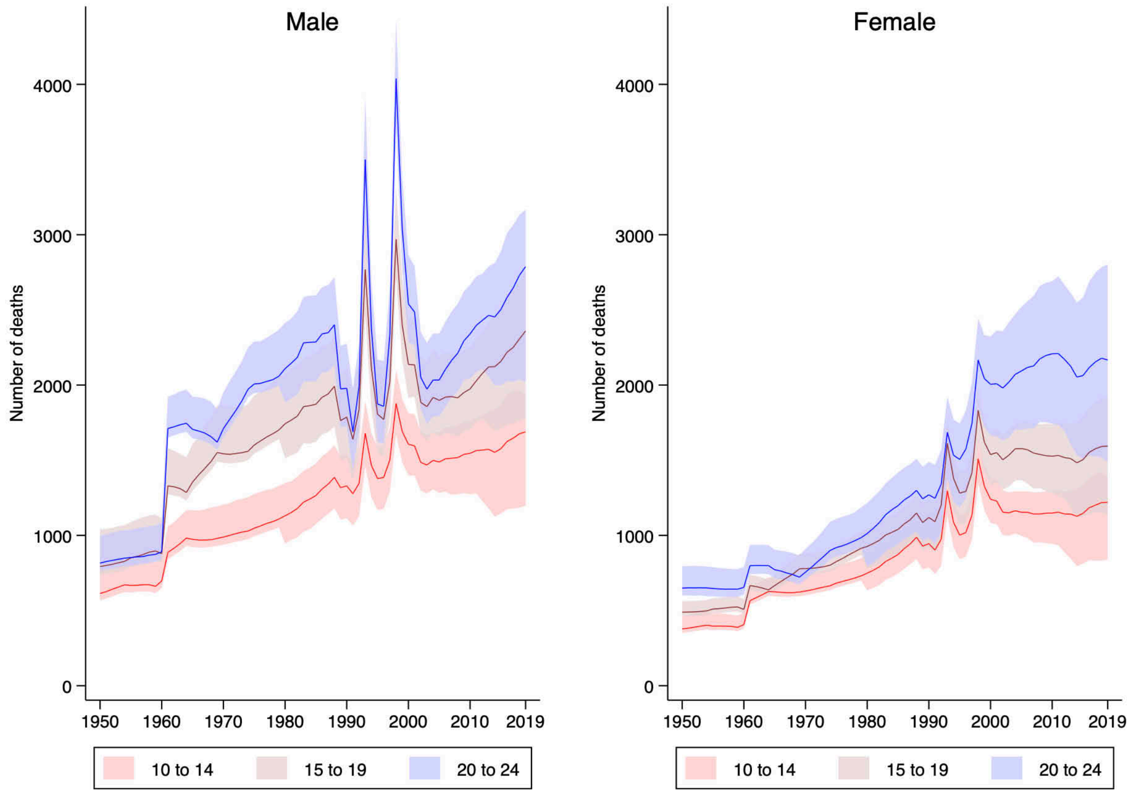

Figure S166c Mortality rate per 100,000 in 10-24 1980 – 2019 by cause: Angola

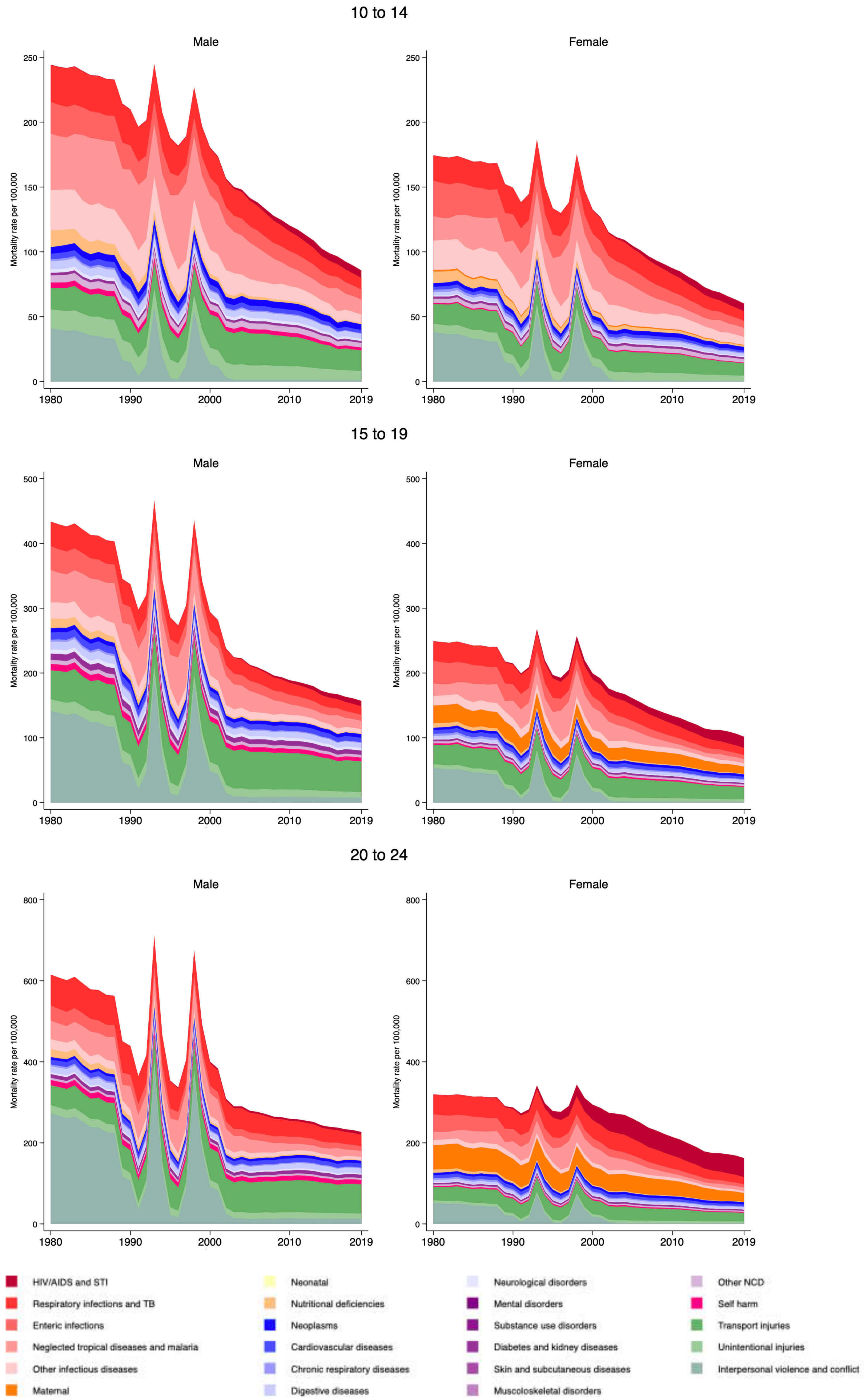

Figure S166d Number of deaths in 10-24 1980 – 2019 by cause: Angola

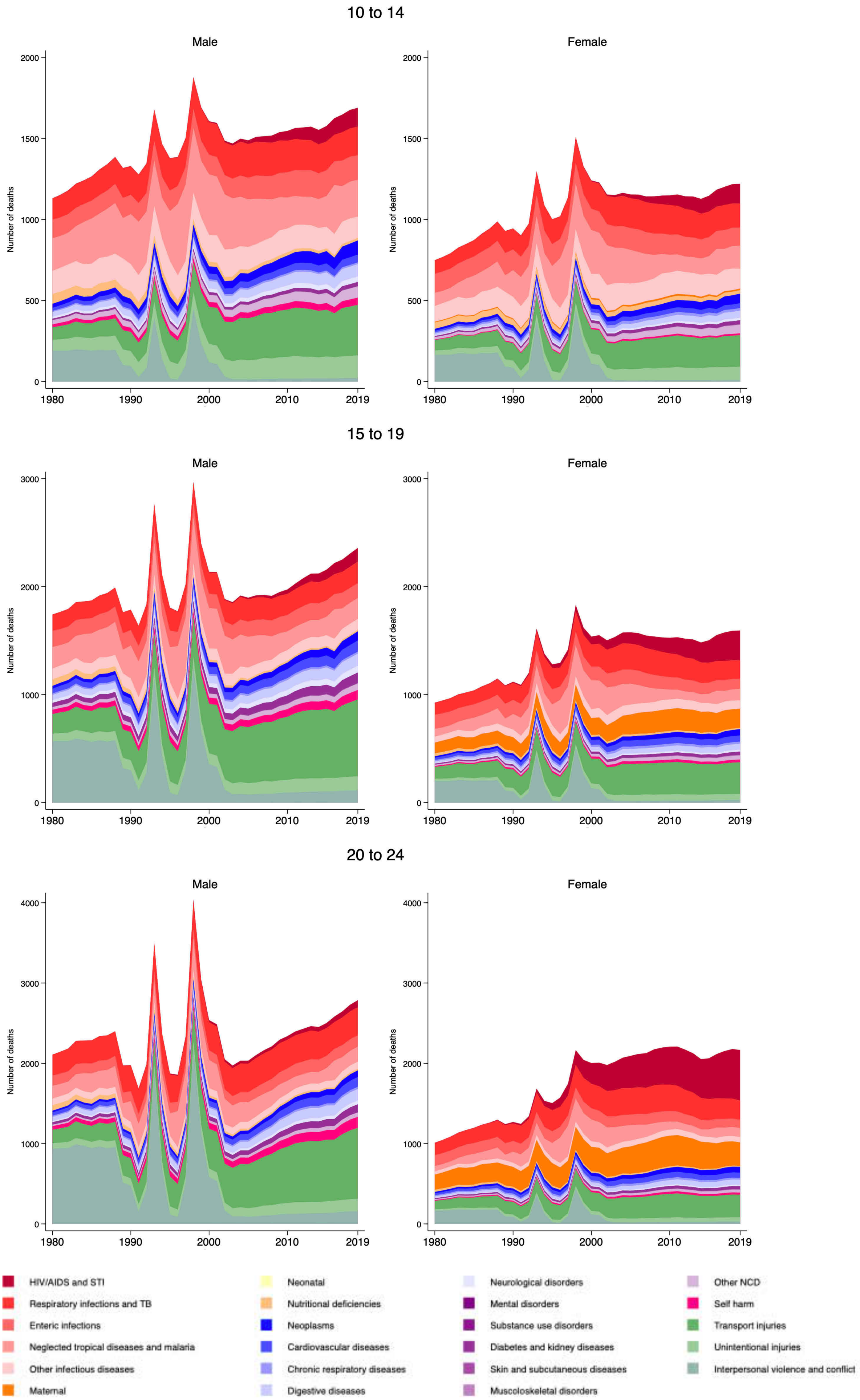

Figure S165: Central African Republic

Figure S167a All-cause mortality rate per 100,000 in 10-24 1950 – 2019: Central African Republic

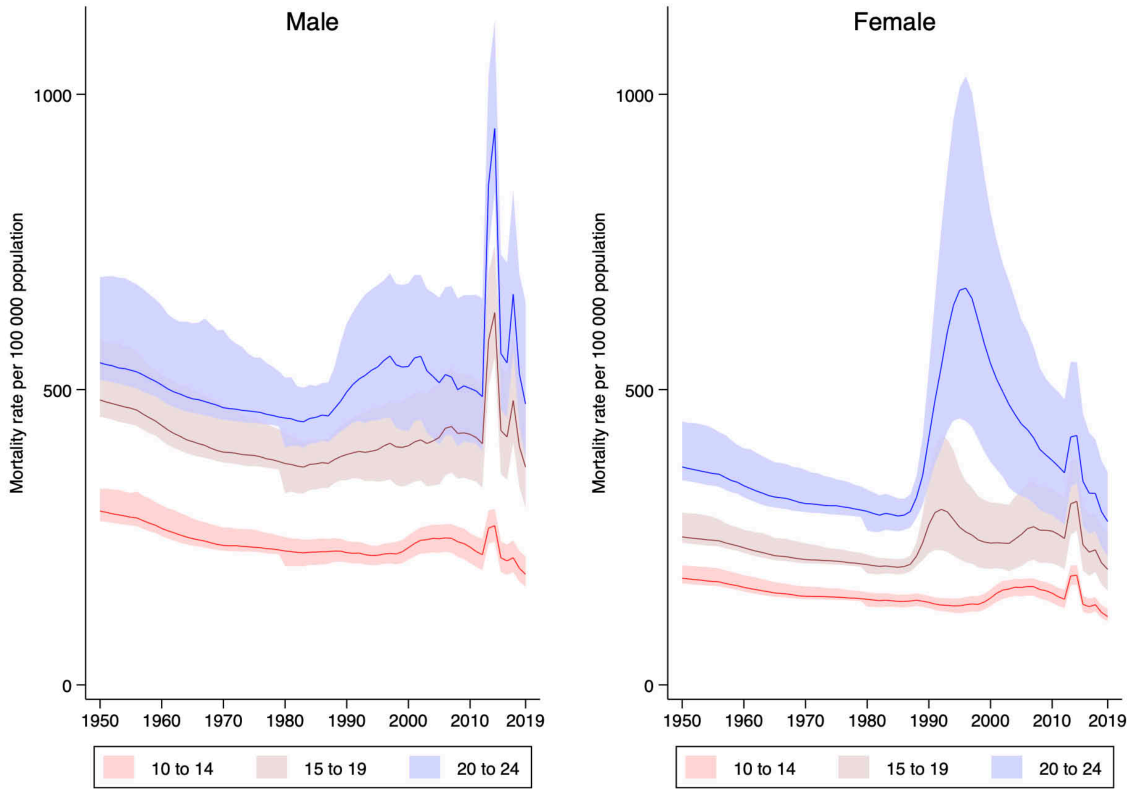

Figure S167b Number of deaths from all causes in 10-24 1950 – 2019: Central African Republic

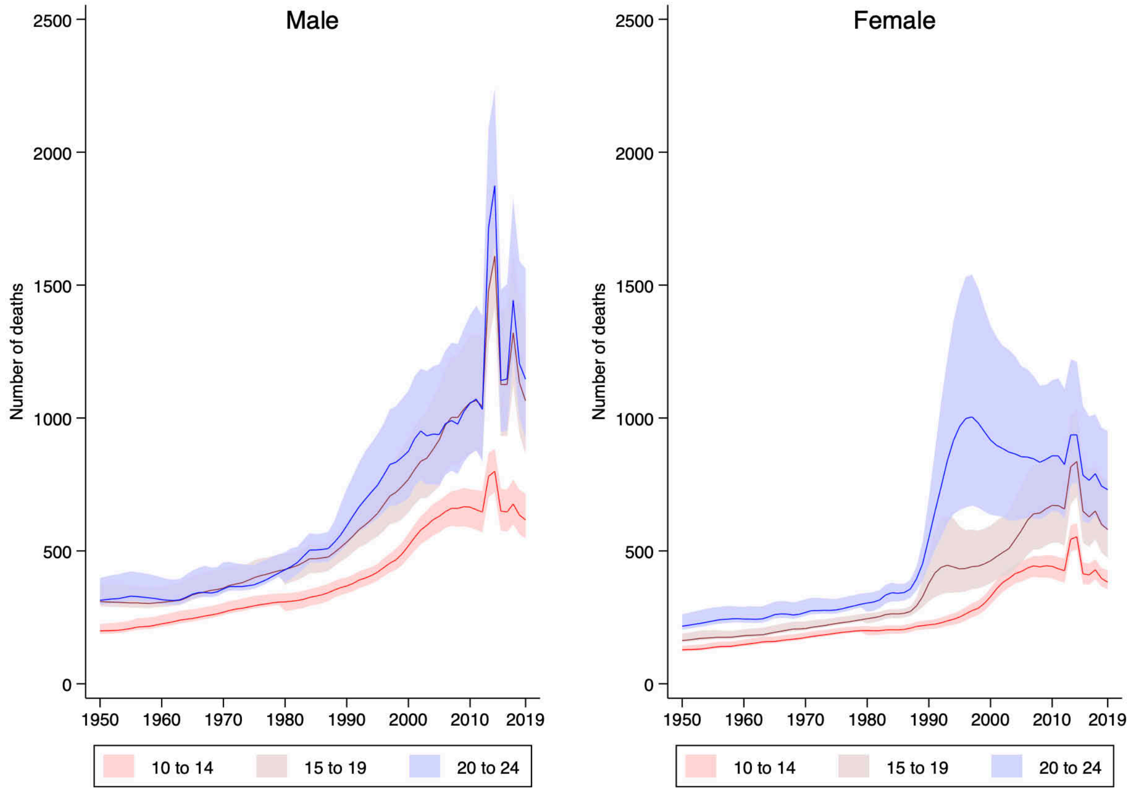

Figure S167c Mortality rate per 100,000 in 10-24 1980 – 2019 by cause: Central African Republic

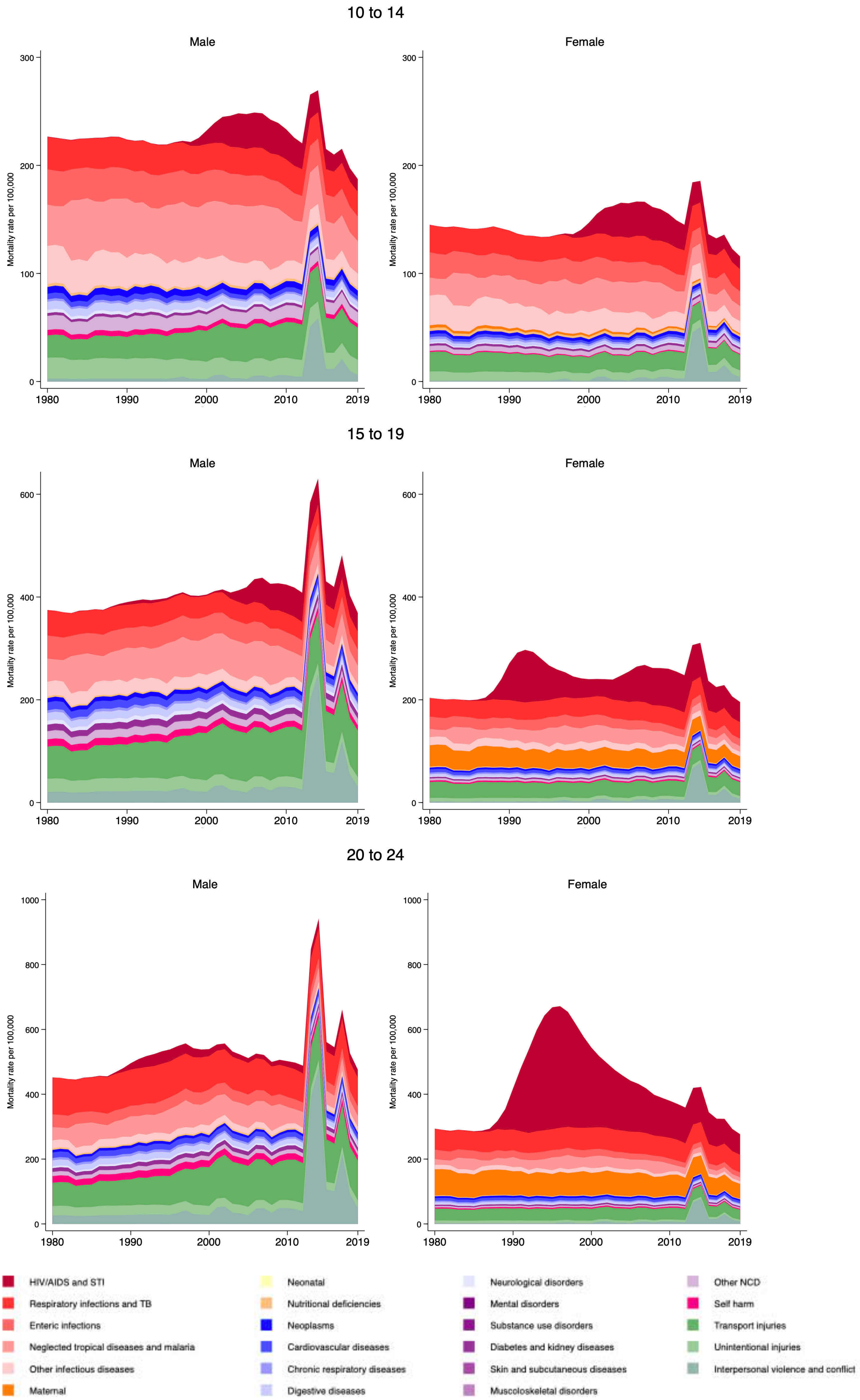

Figure S167d Number of deaths in 10-24 1980 – 2019 by cause: Central African Republic

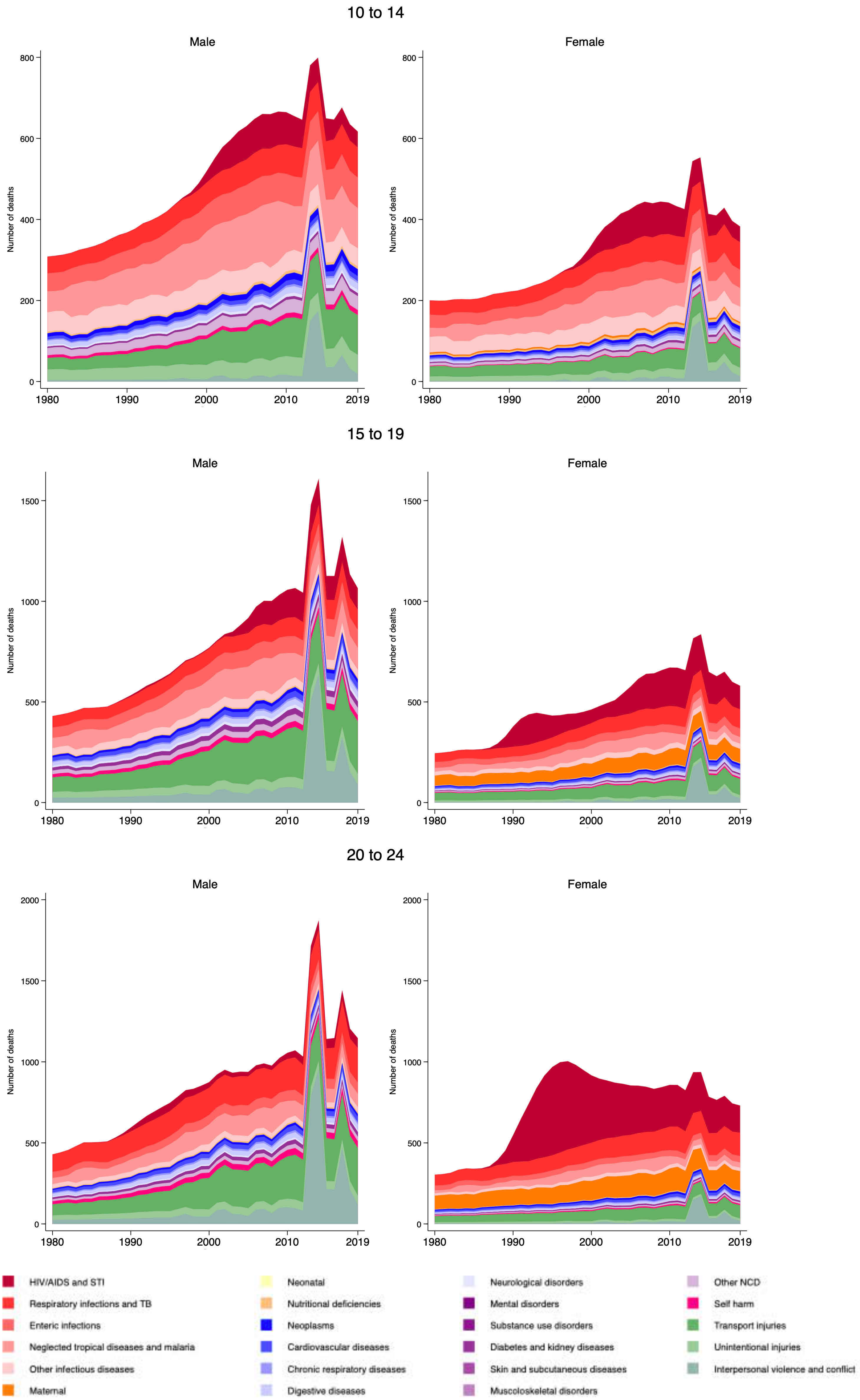

Figure S166: Congo

Figure S168a All-cause mortality rate per 100,000 in 10-24 1950 – 2019: Congo

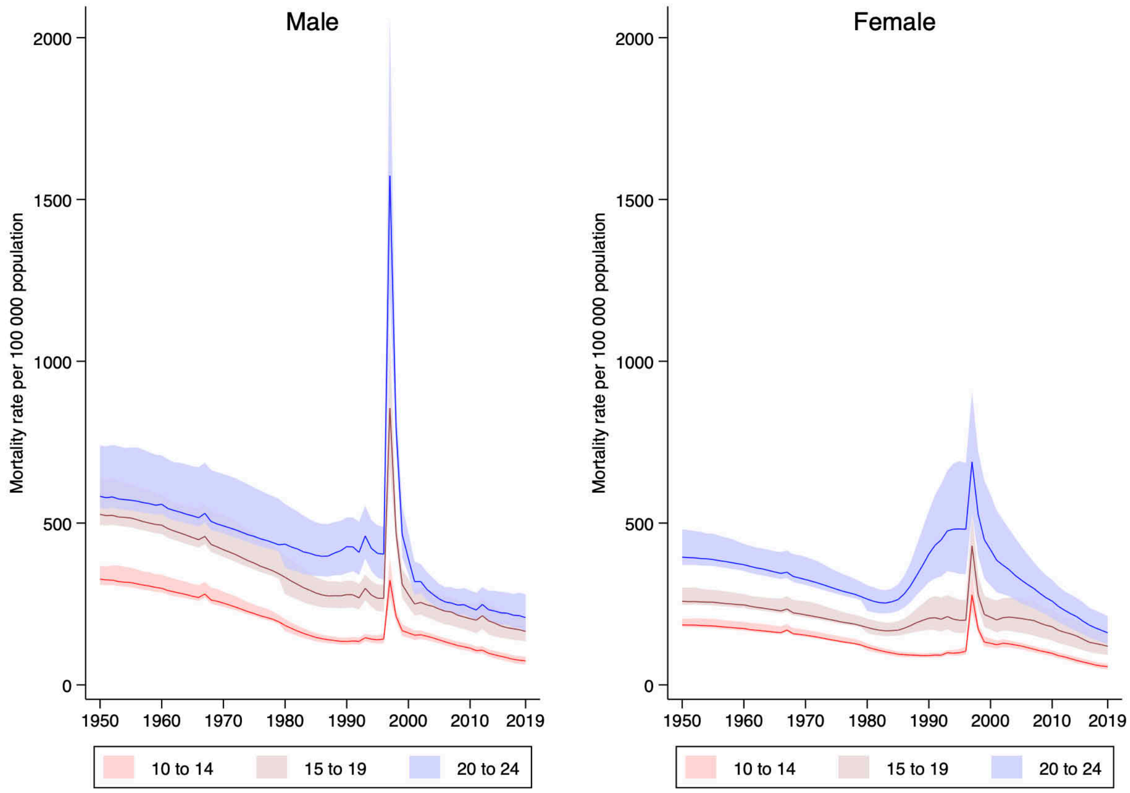

Figure S168b Number of deaths from all causes in 10-24 1950 – 2019: Congo

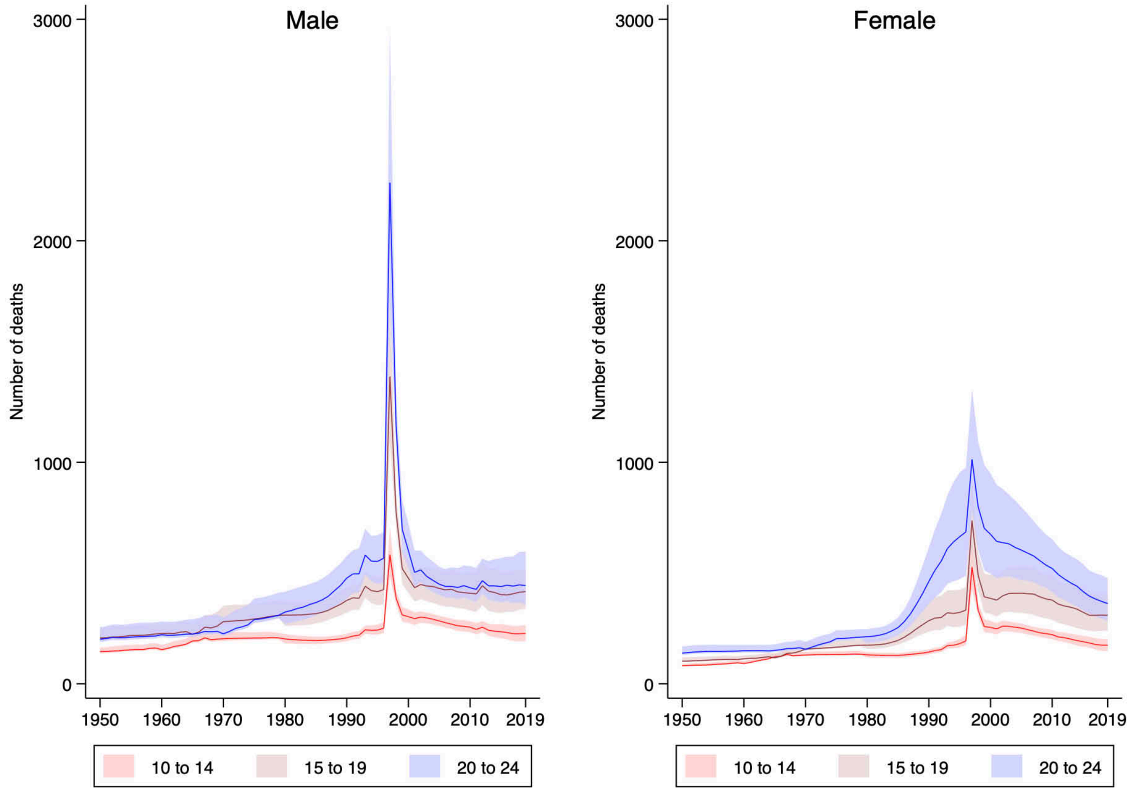

Figure S168c Mortality rate per 100,000 in 10-24 1980 – 2019 by cause: Congo

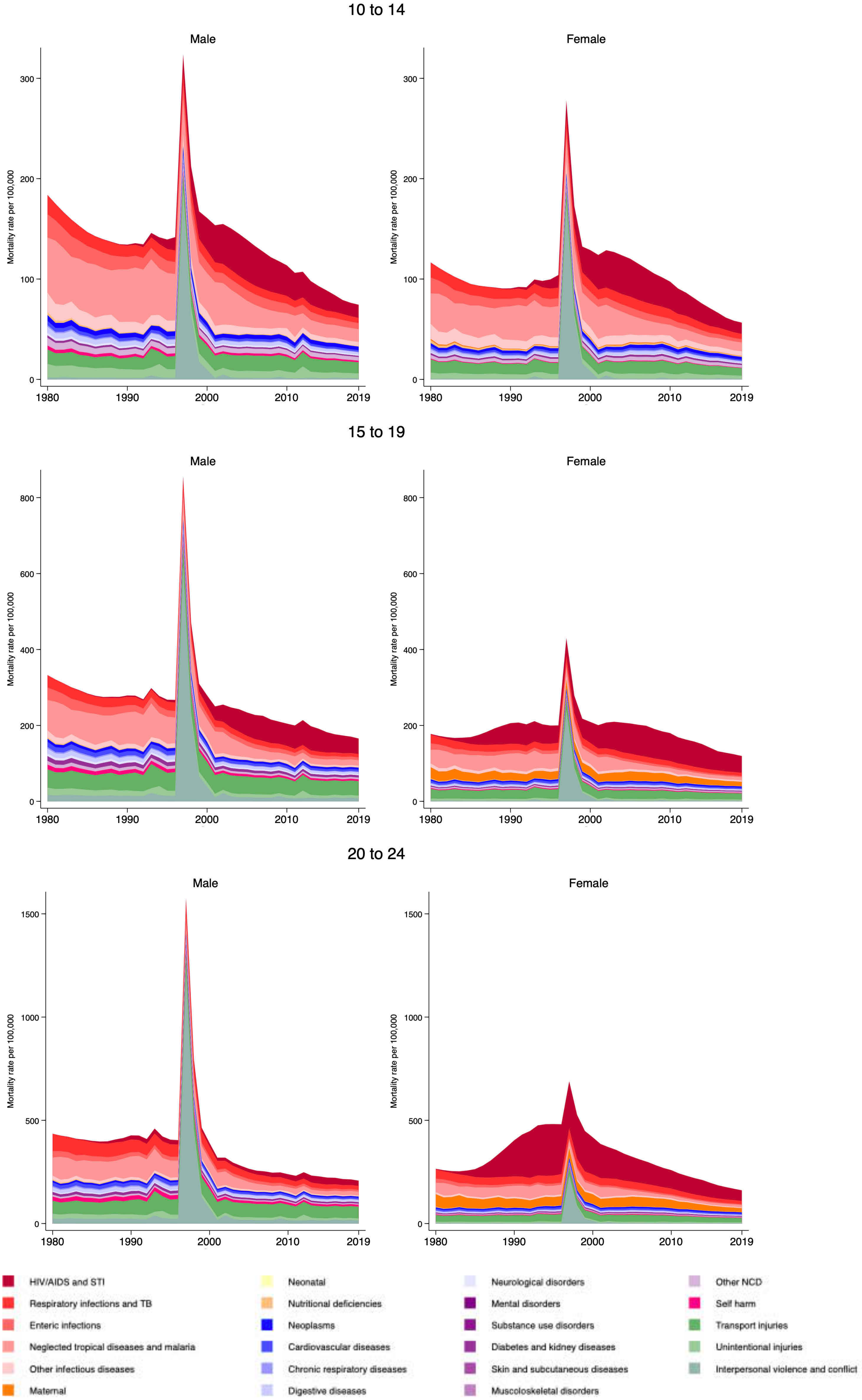

Figure S168d Number of deaths in 10-24 1980 – 2019 by cause: Congo

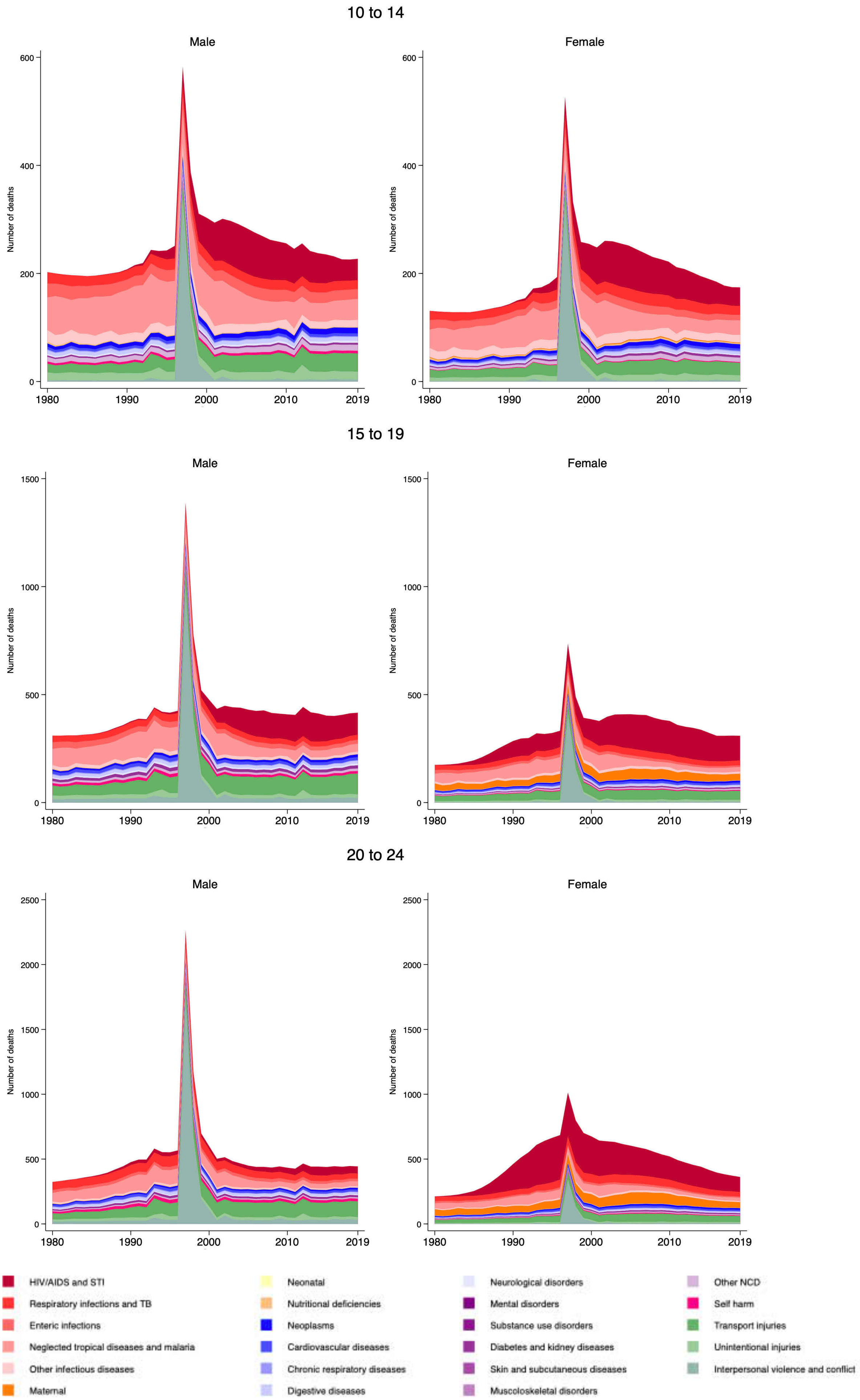

Figure S167: Democratic Republic of the Congo

Figure S169a All-cause mortality rate per 100,000 in 10-24 1950 – 2019: Democratic Republic of the Congo

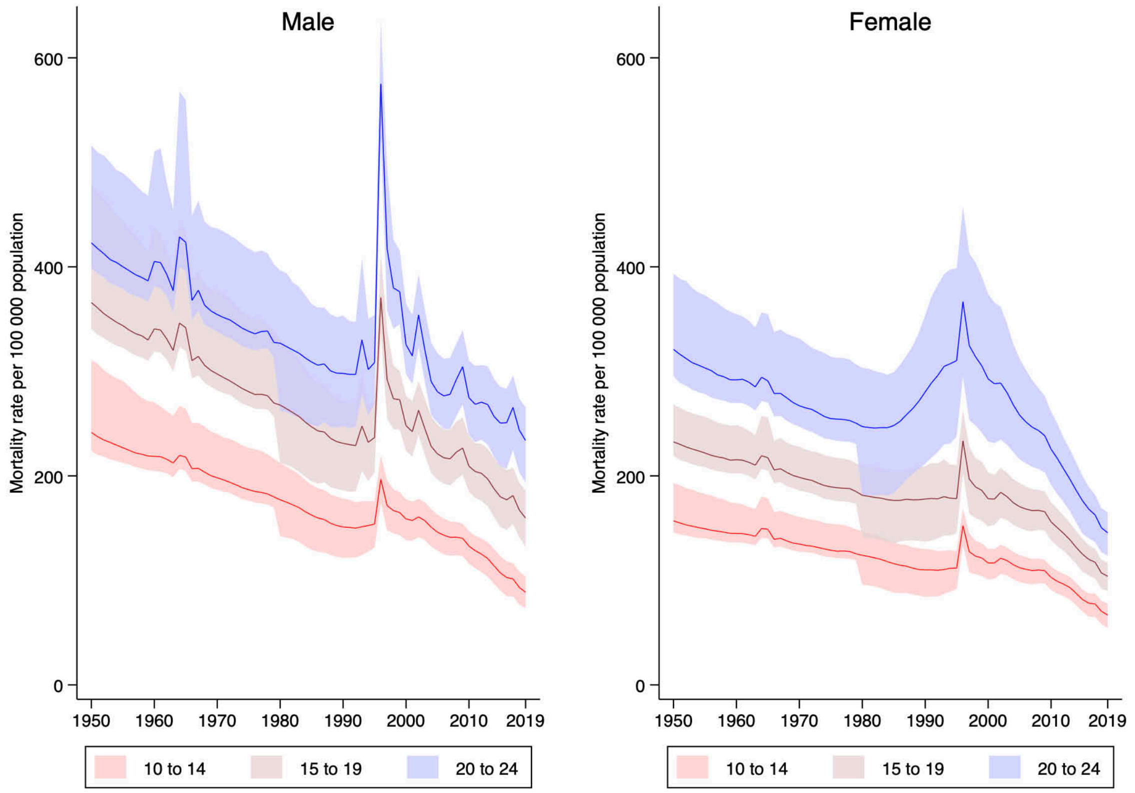

Figure S169b Number of deaths from all causes in 10-24 1950 – 2019: Democratic Republic of the Congo

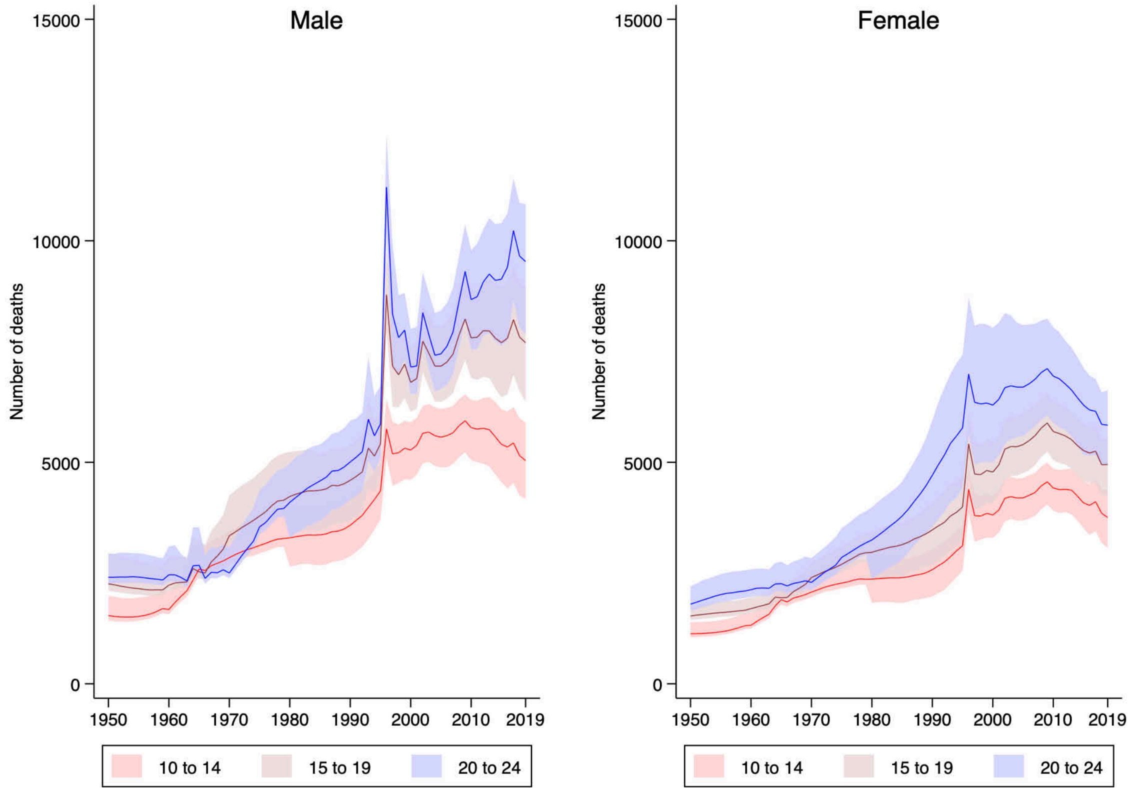

Figure S169c Mortality rate per 100,000 in 10-24 1980 – 2019 by cause: Democratic Republic of the Congo

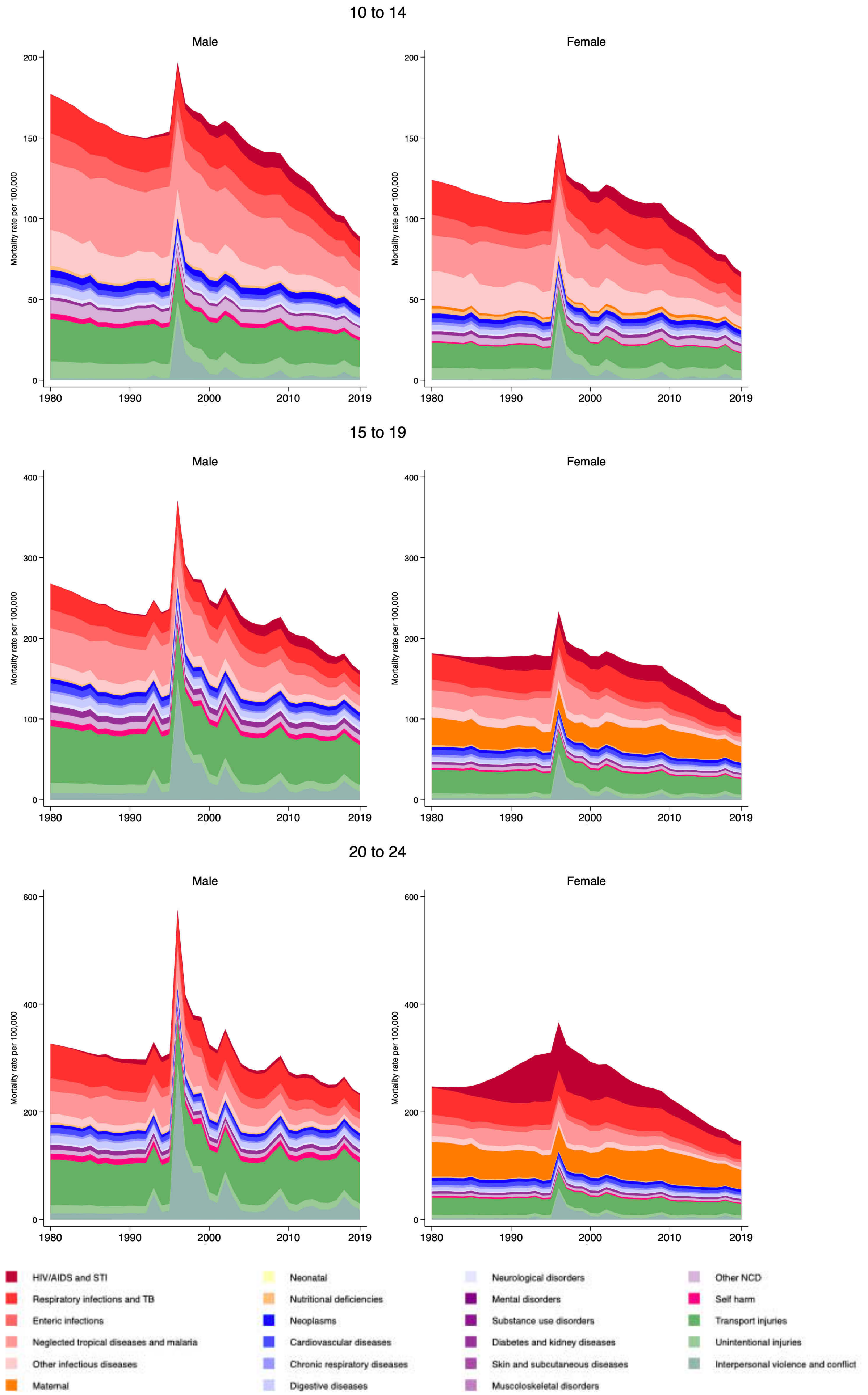

Figure S169d Number of deaths in 10-24 1980 – 2019 by cause: Democratic Republic of the Congo

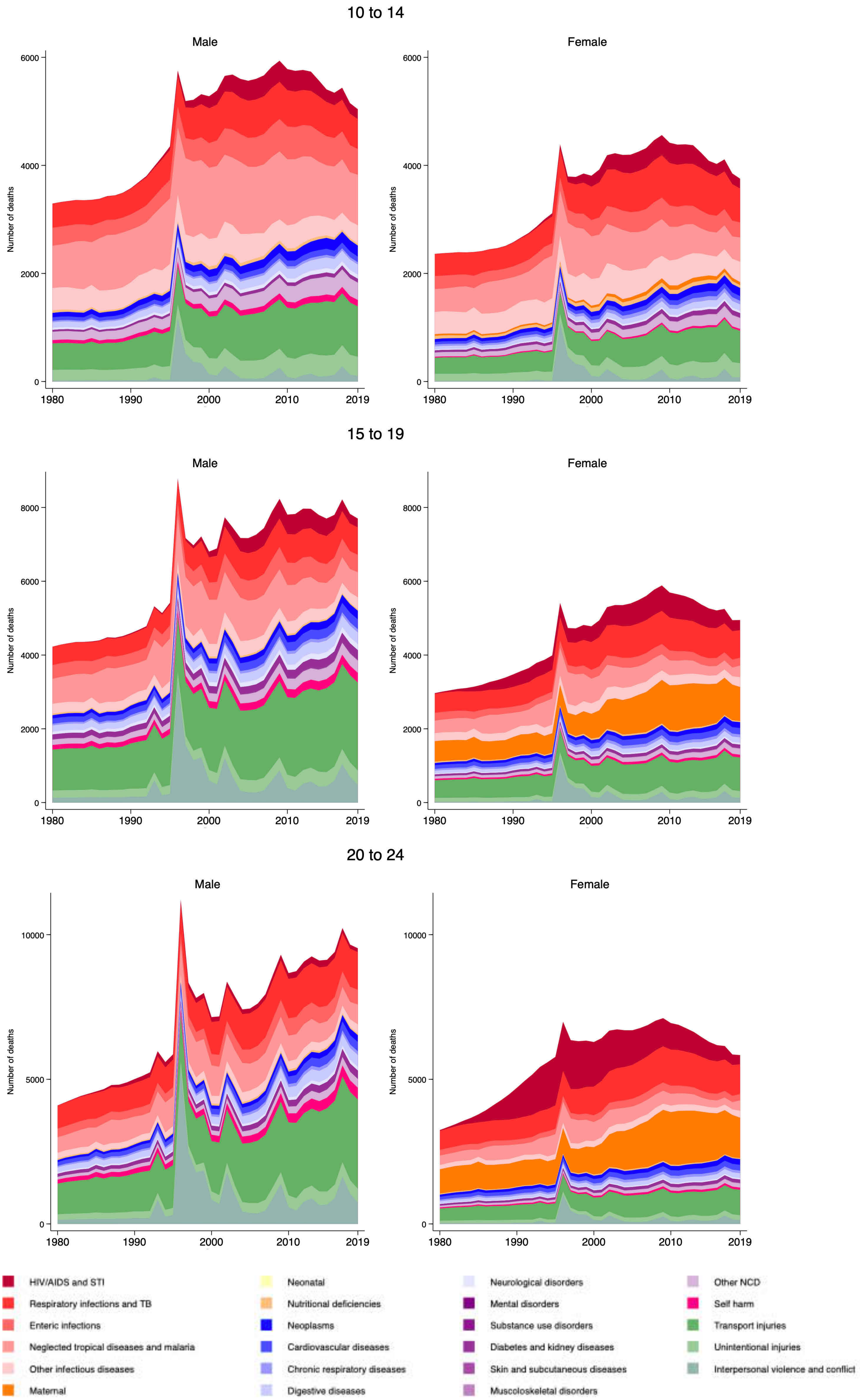

Figure S168: Equatorial Guinea

Figure S170a All-cause mortality rate per 100,000 in 10-24 1950 – 2019: Equatorial Guinea

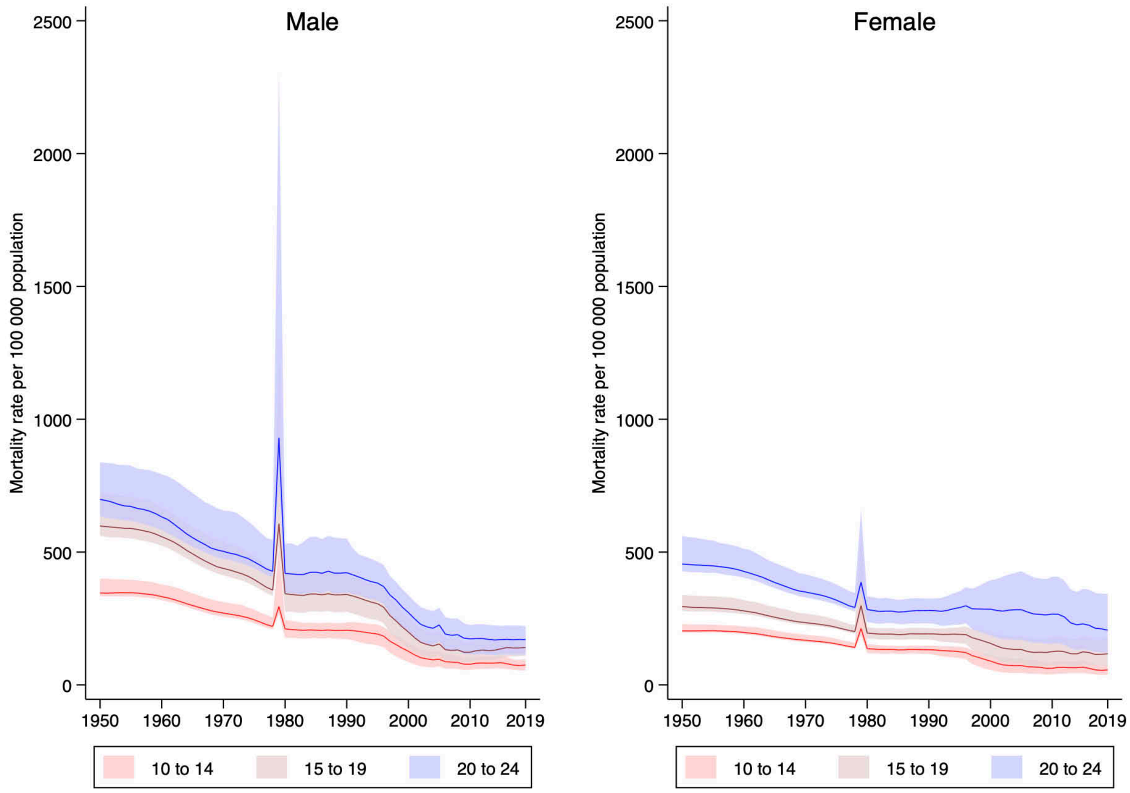

Figure S170b Number of deaths from all causes in 10-24 1950 – 2019: Equatorial Guinea

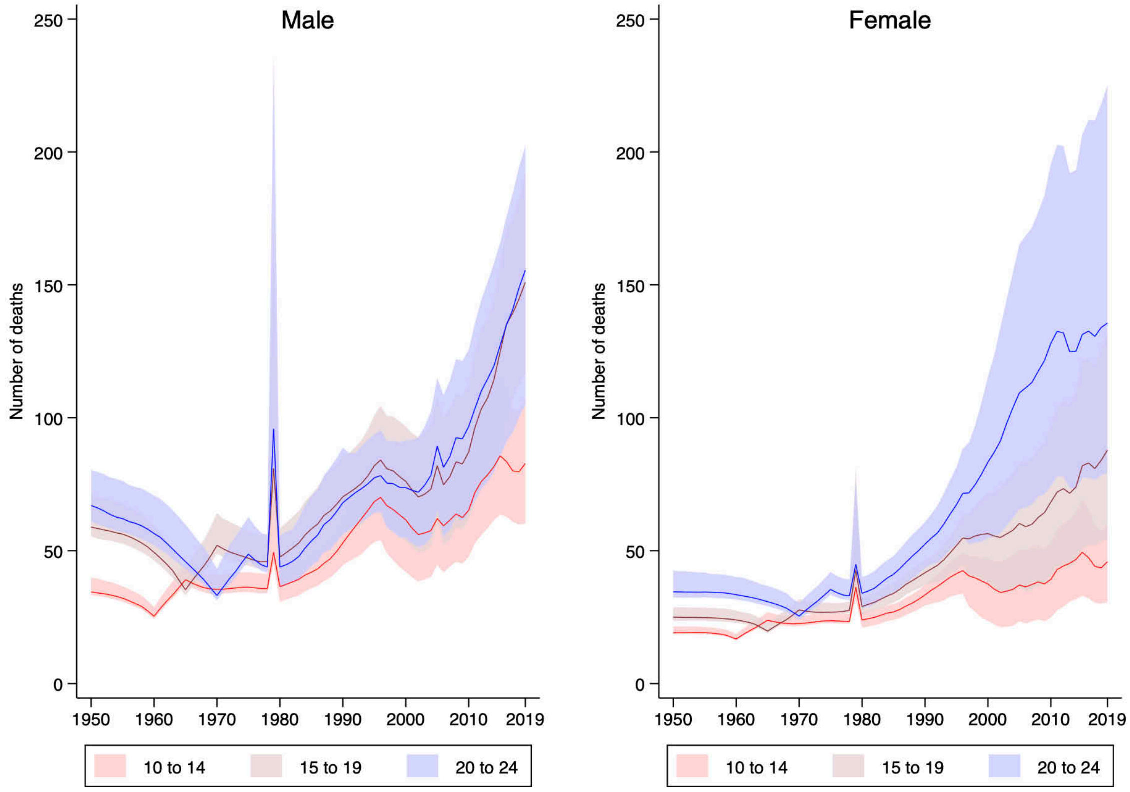

Figure S170c Mortality rate per 100,000 in 10-24 1980 – 2019 by cause: Equatorial Guinea

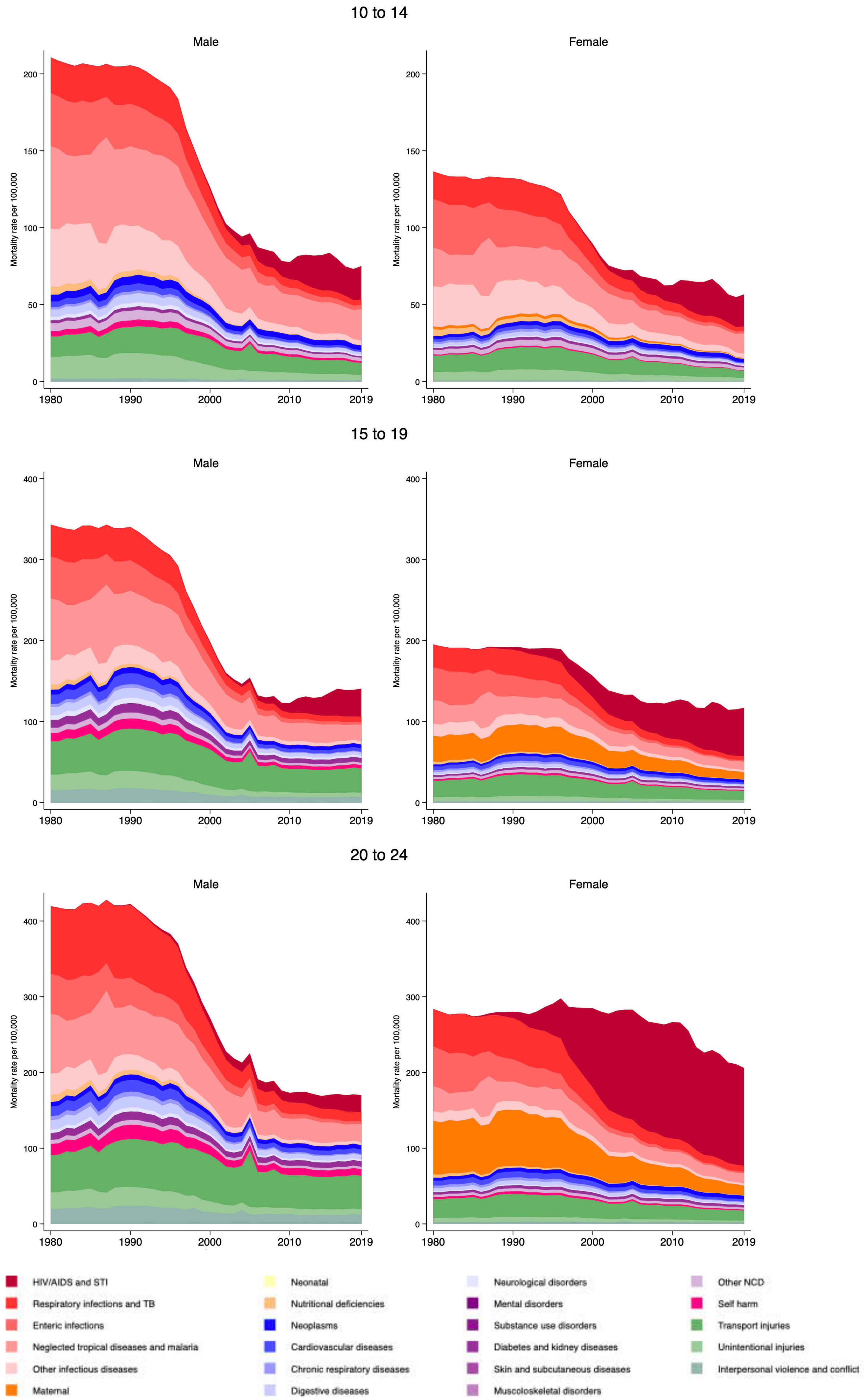

Figure S170d Number of deaths in 10-24 1980 – 2019 by cause: Equatorial Guinea

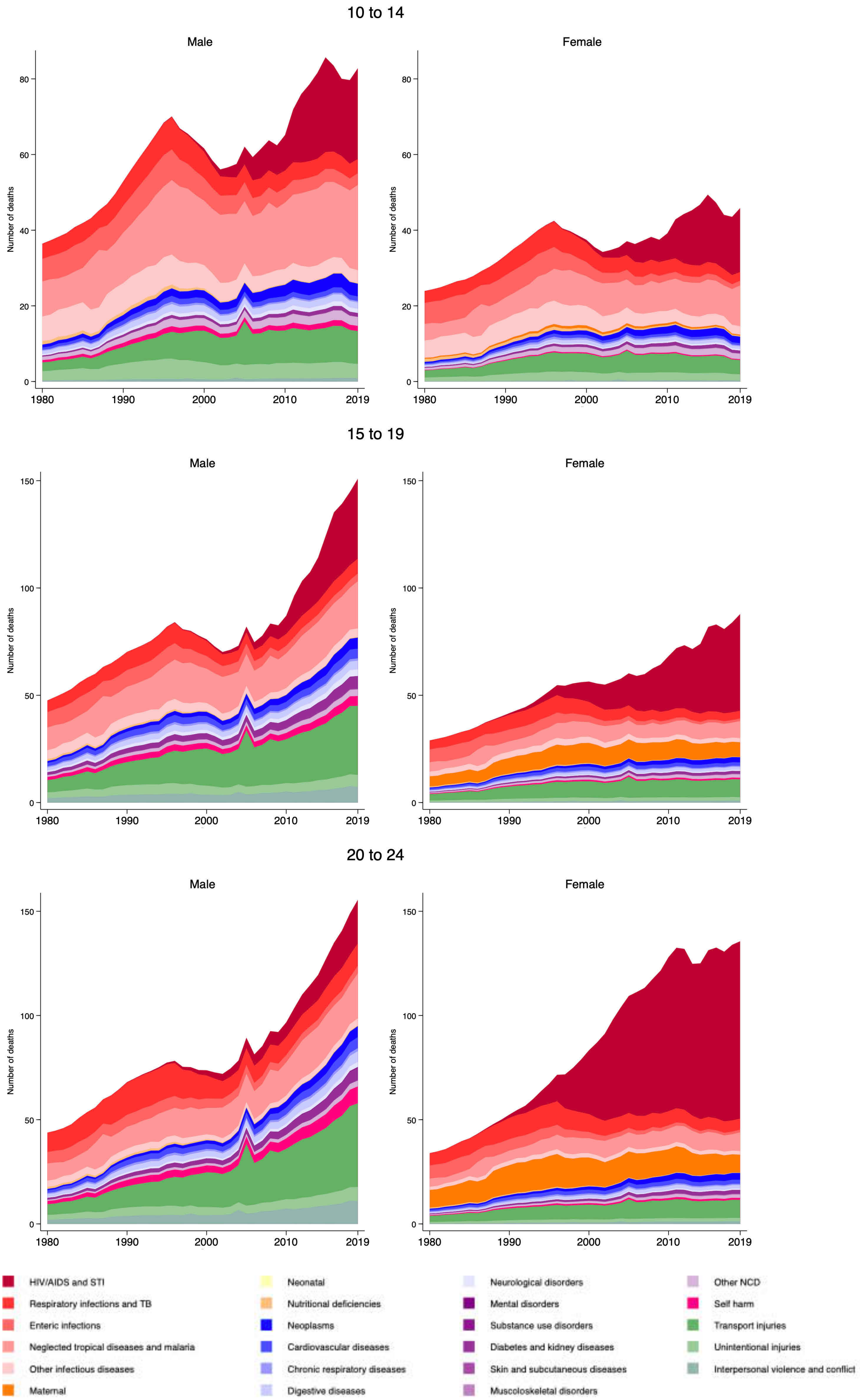

Figure S169: Gabon

Figure S171a All-cause mortality rate per 100,000 in 10-24 1950 – 2019: Gabon

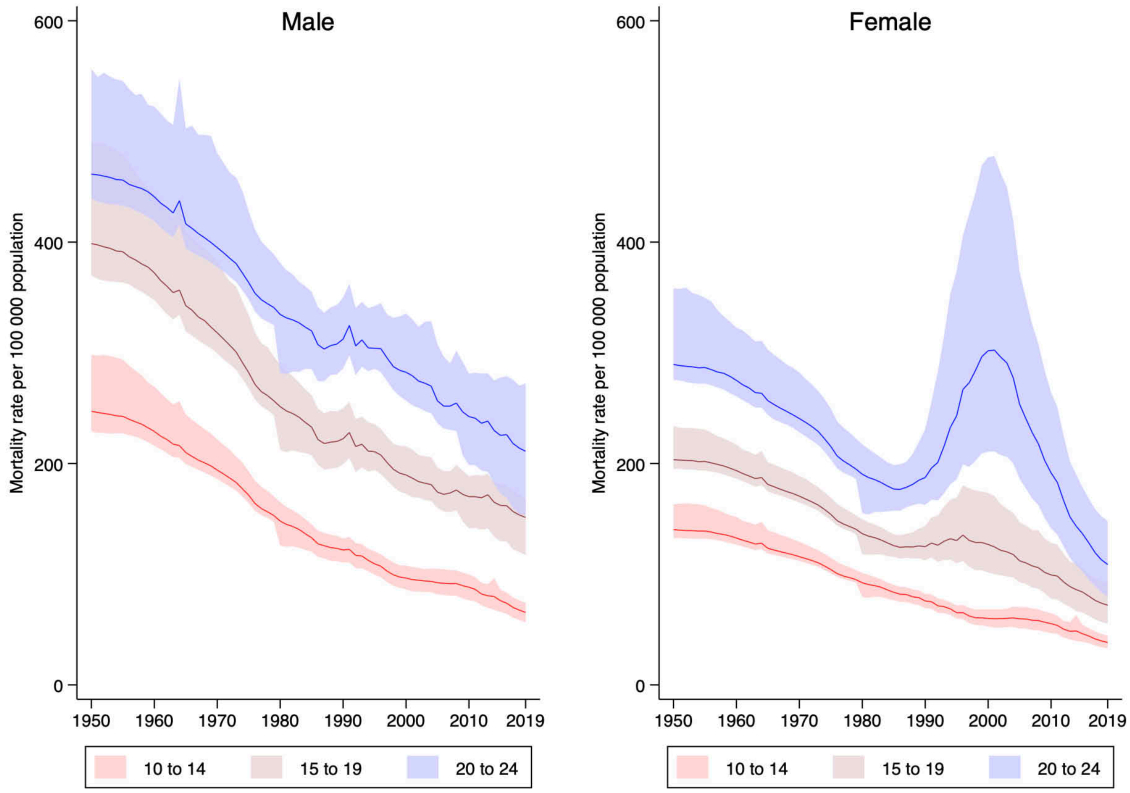

Figure S171b Number of deaths from all causes in 10-24 1950 – 2019: Gabon

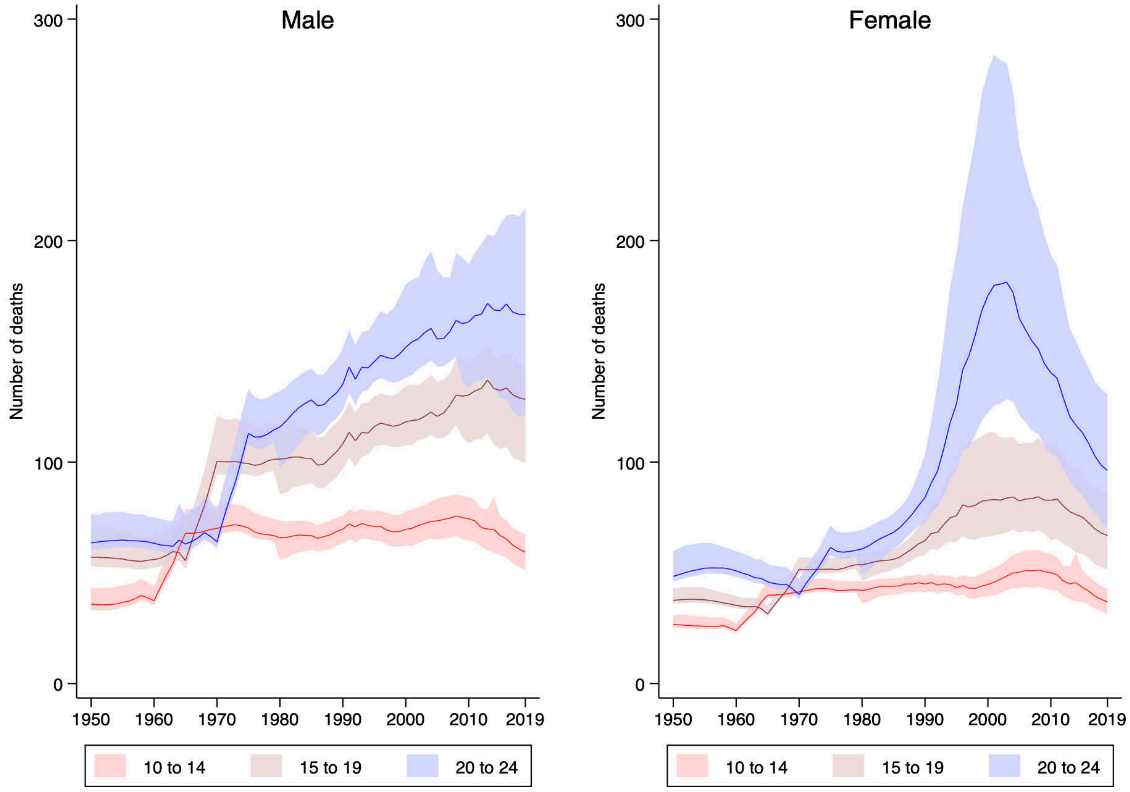

Figure S171c Mortality rate per 100,000 in 10-24 1980 – 2019 by cause: Gabon

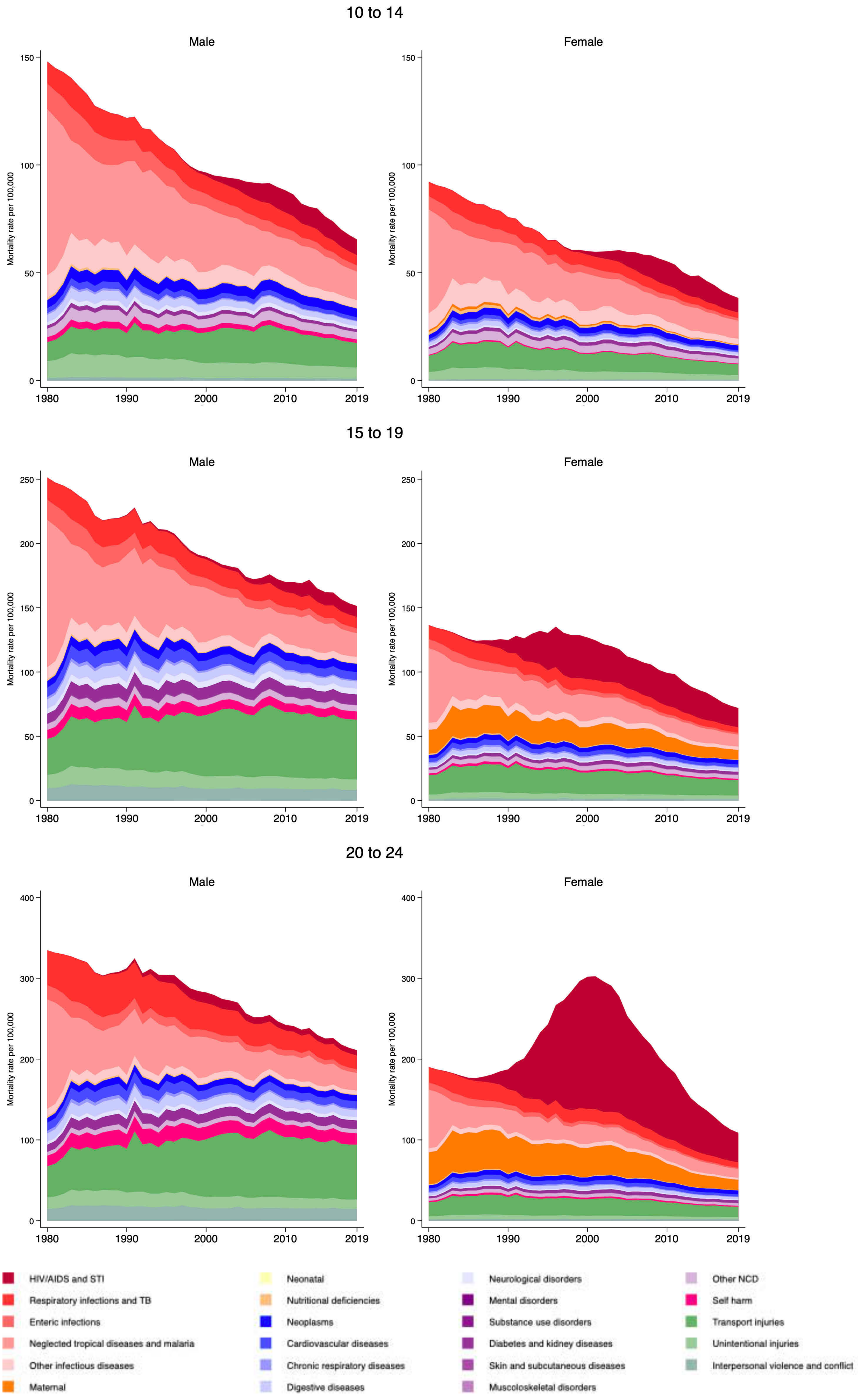

Figure S171d Number of deaths in 10-24 1980 – 2019 by cause: Gabon

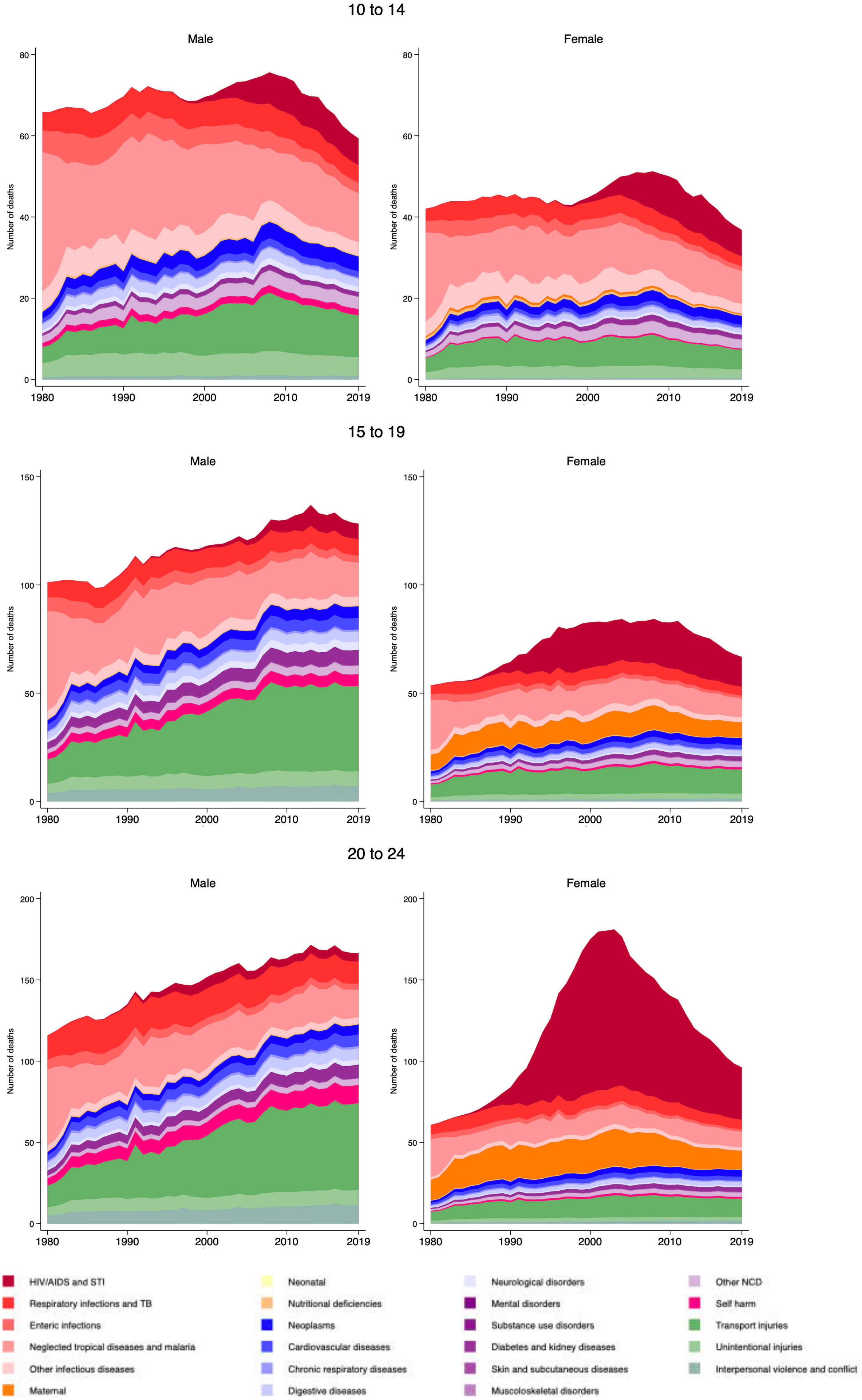

Figure S170: Eastern Sub-Saharan Africa

Figure S172a All-cause mortality rate per 100,000 in 10-24 1950 – 2019: Eastern Sub-Saharan Africa

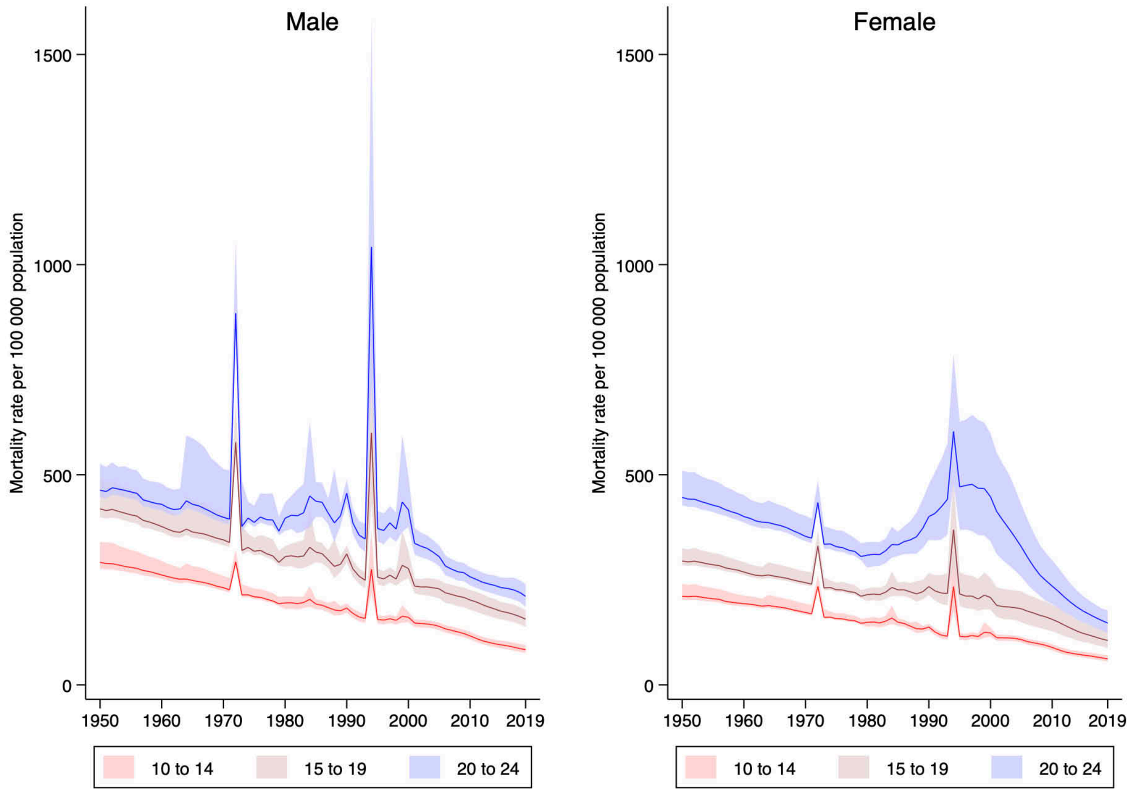

Figure S172b Number of deaths from all causes in 10-24 1950 – 2019: Eastern Sub-Saharan Africa

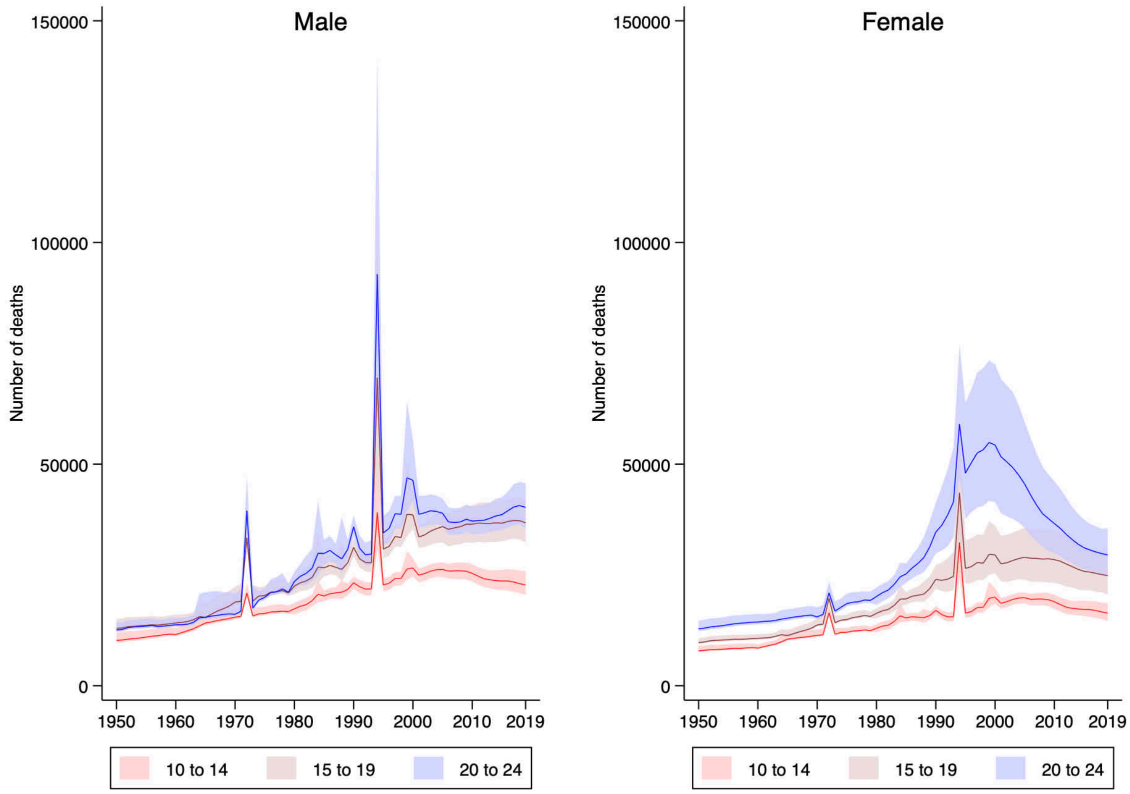

Figure S172c Mortality rate per 100,000 in 10-24 1980 – 2019 by cause: Eastern Sub-Saharan Africa

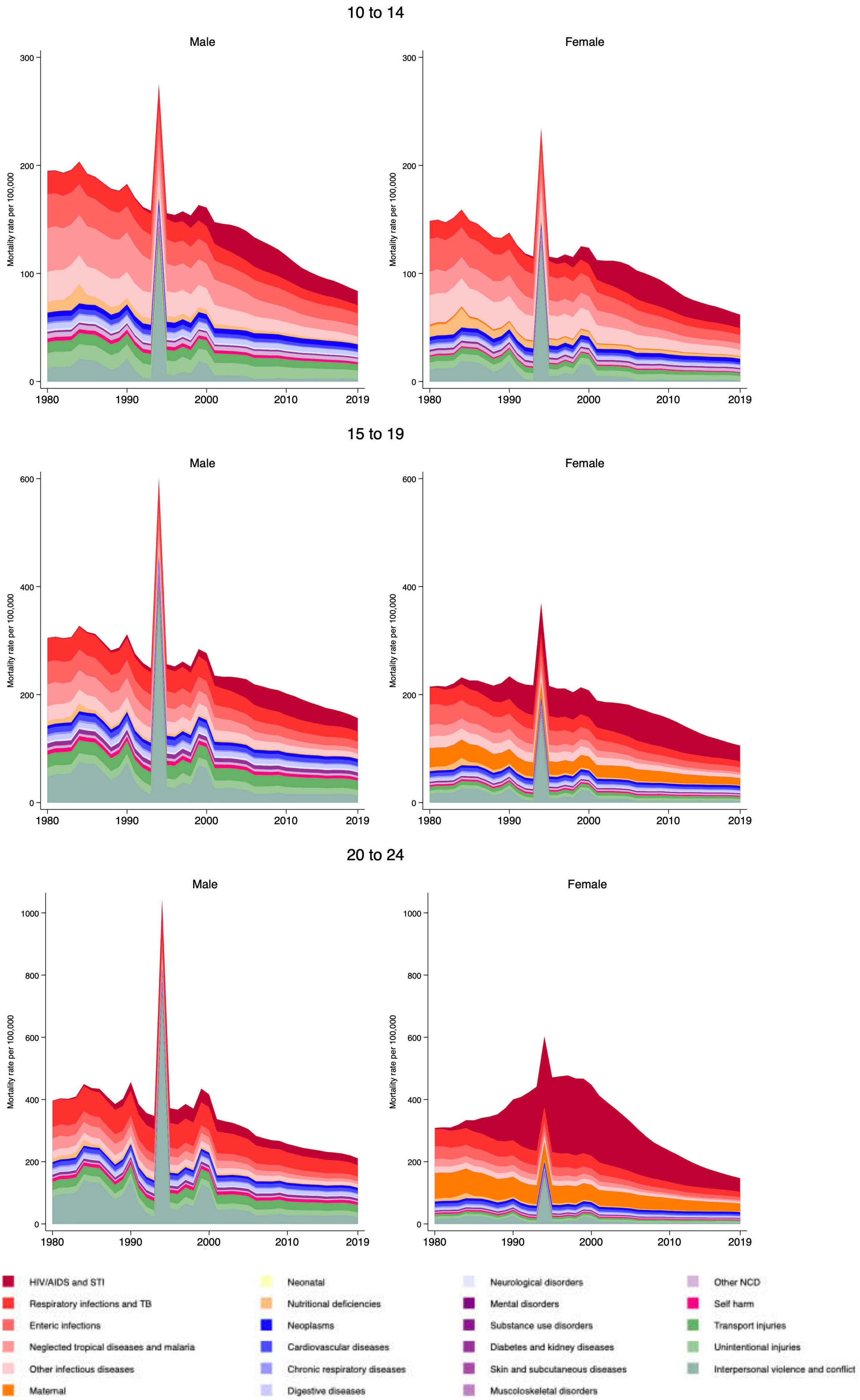

Figure S172d Number of deaths in 10-24 1980 – 2019 by cause: Eastern Sub-Saharan Africa

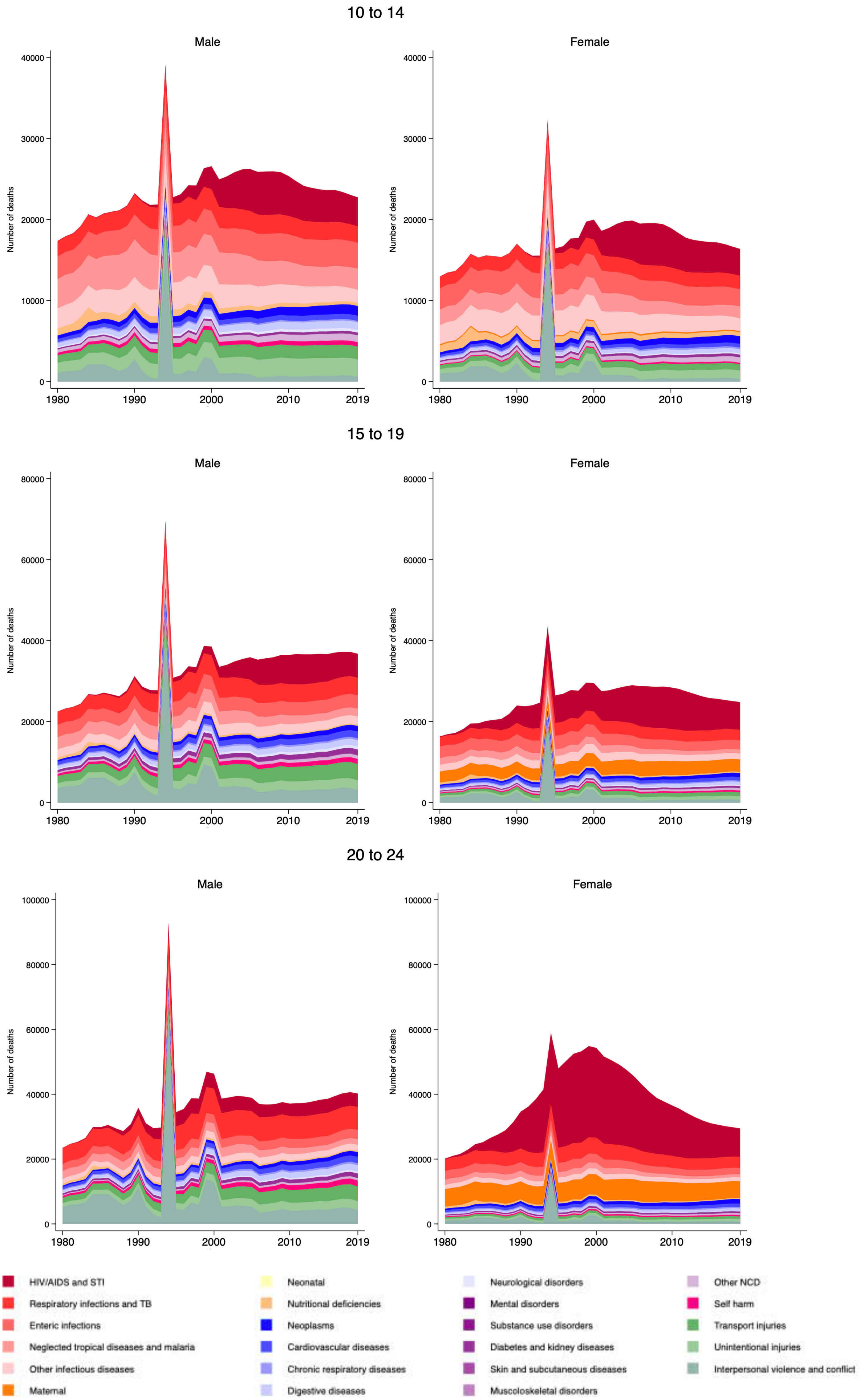

Figure S171: Burundi

Figure S173a All-cause mortality rate per 100,000 in 10-24 1950 – 2019: Burundi

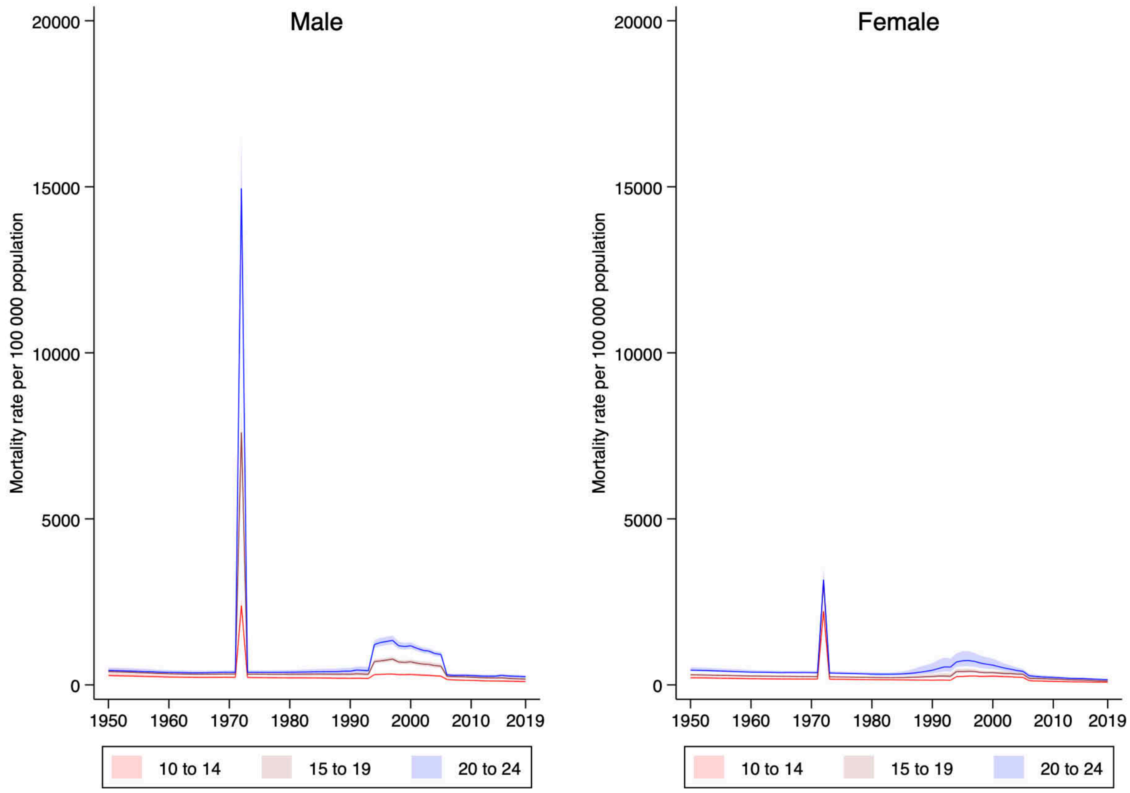

Figure S173b Number of deaths from all causes in 10-24 1950 – 2019: Burundi

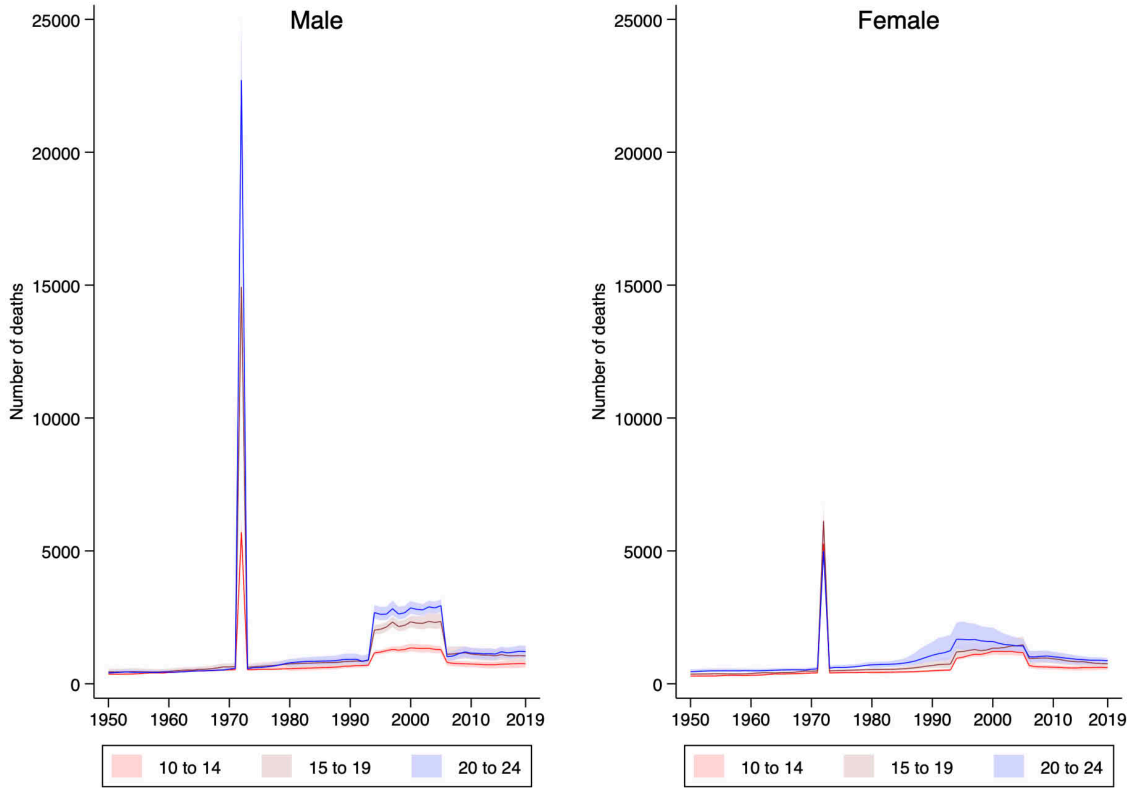

Figure S173c Mortality rate per 100,000 in 10-24 1980 – 2019 by cause: Burundi

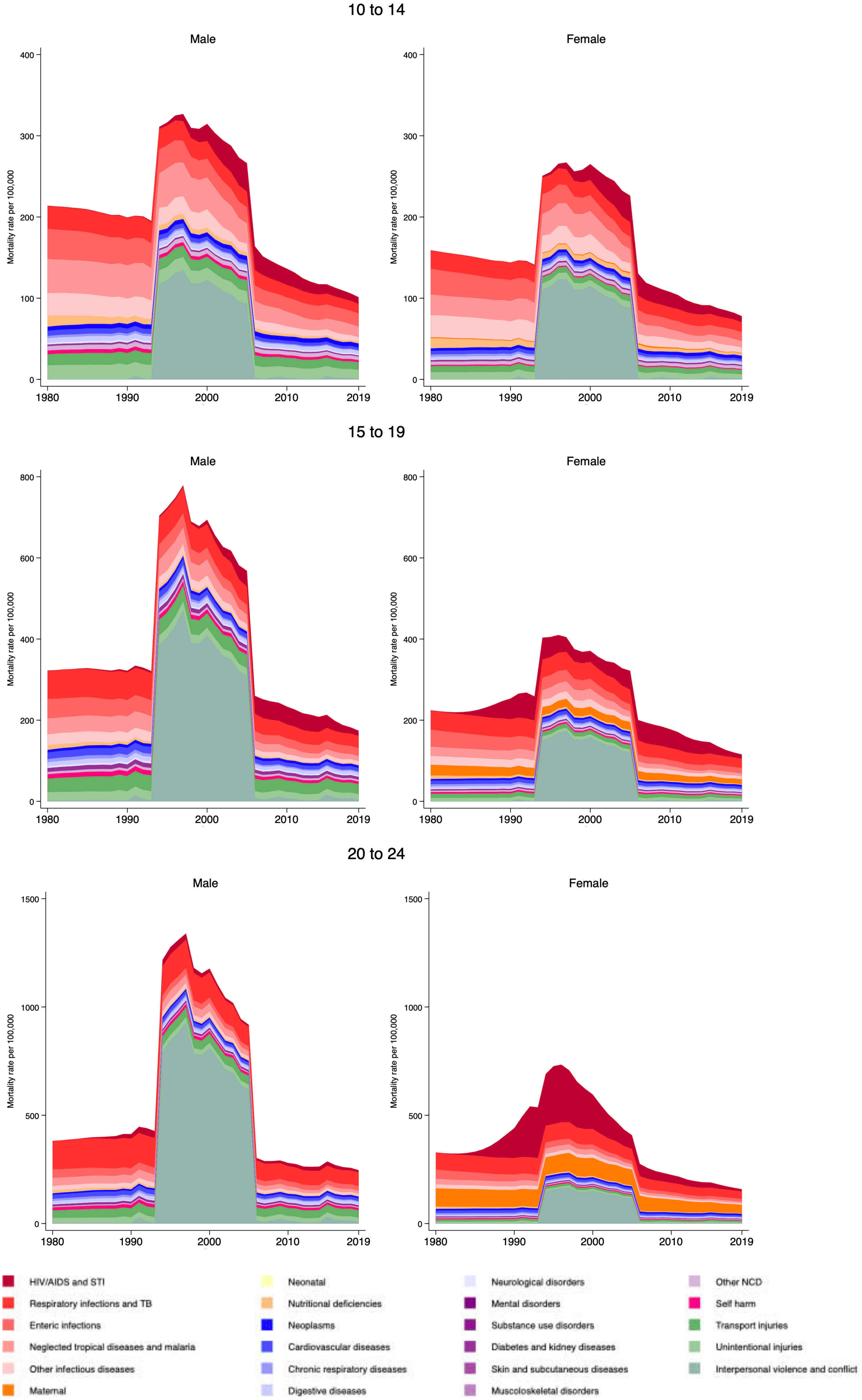

Figure S173d Number of deaths in 10-24 1980 – 2019 by cause: Burundi

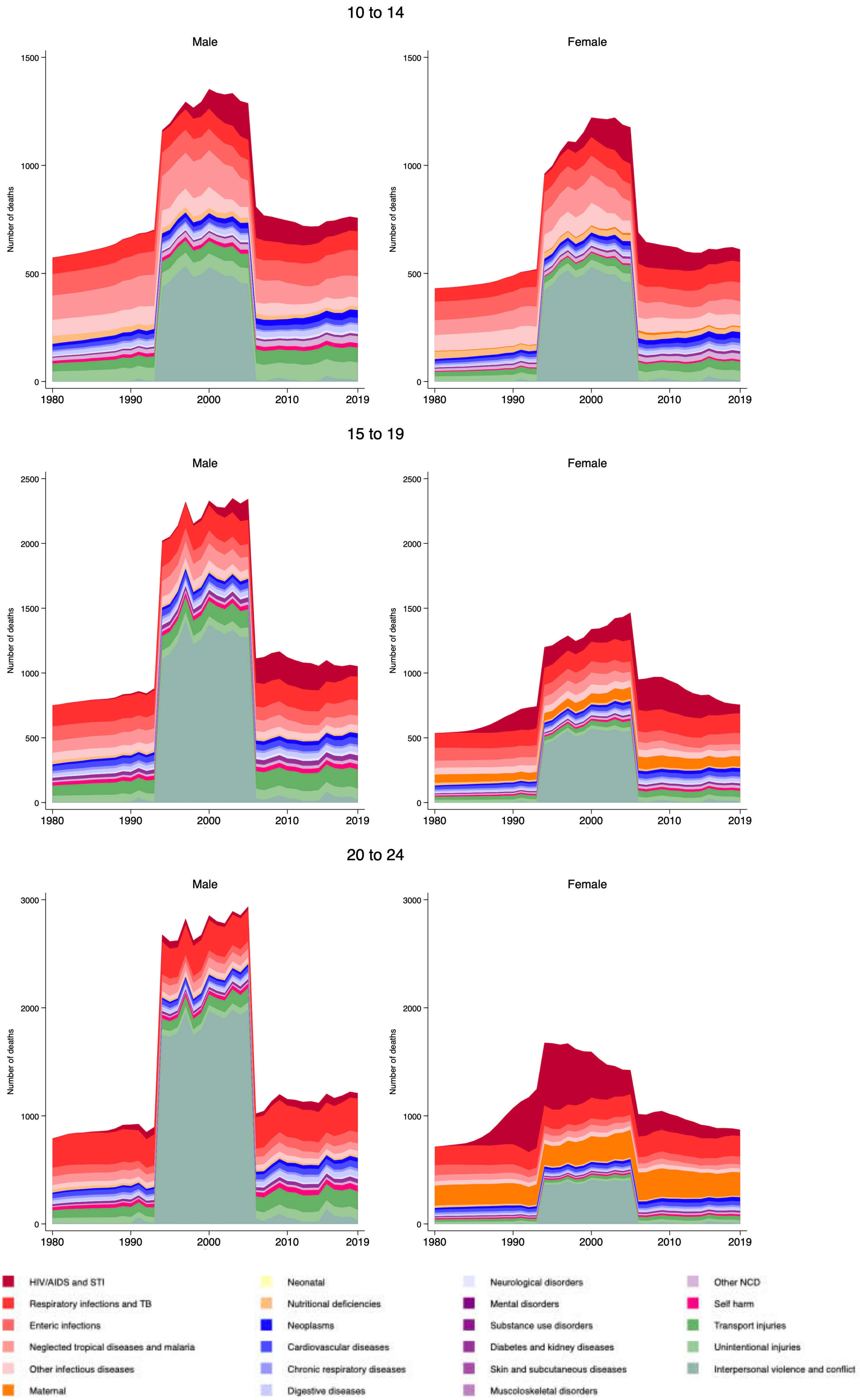

Figure S172: Comoros

Figure S174a All-cause mortality rate per 100,000 in 10-24 1950 – 2019: Comoros

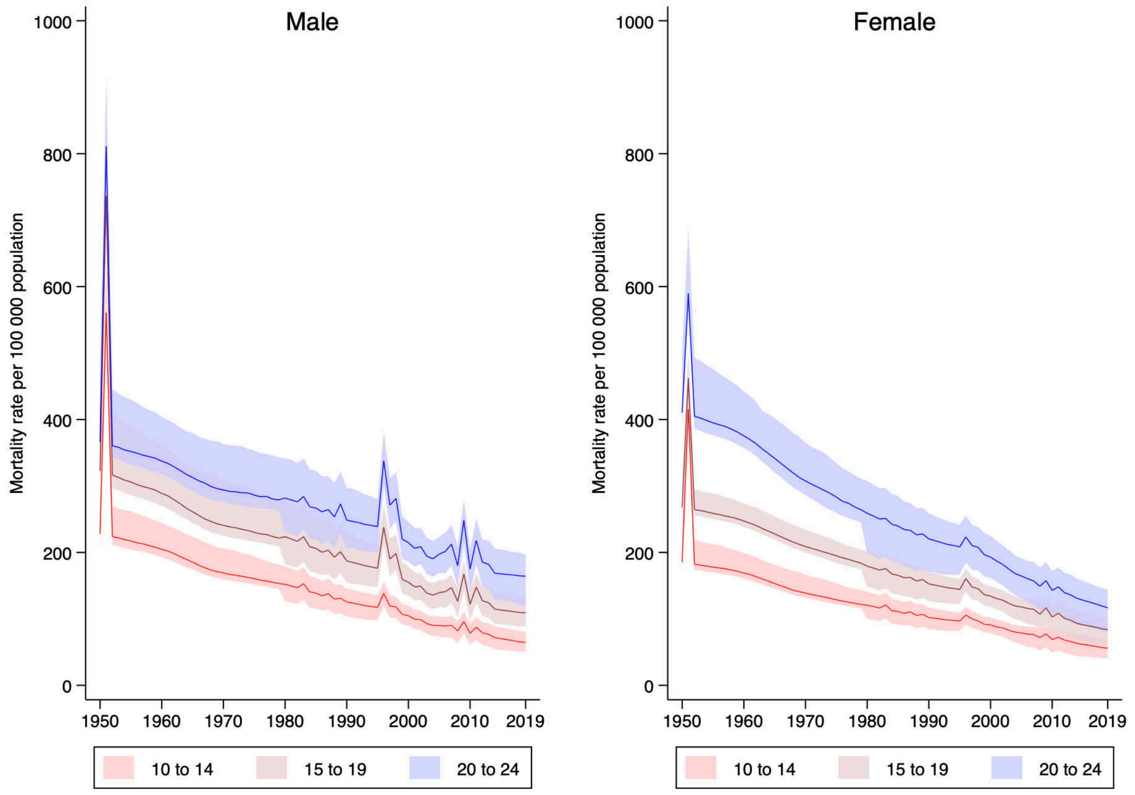

Figure S174b Number of deaths from all causes in 10-24 1950 – 2019: Comoros

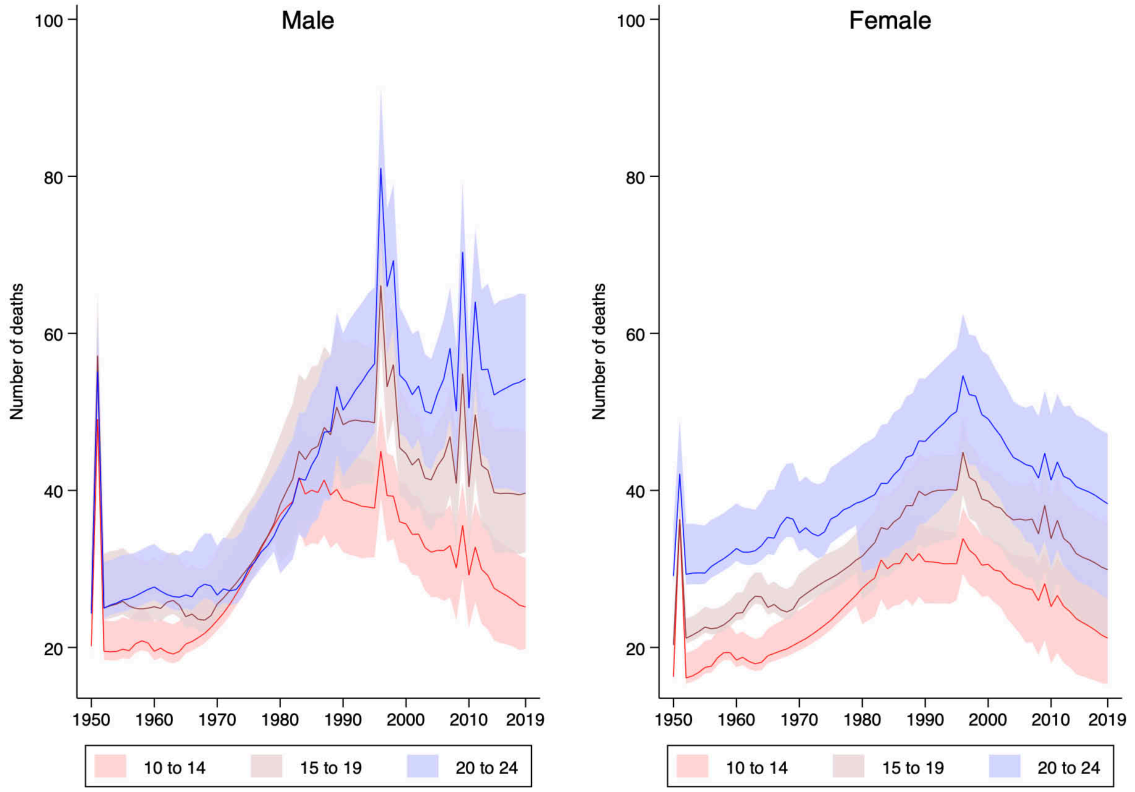

Figure S174c Mortality rate per 100,000 in 10-24 1980 – 2019 by cause: Comoros

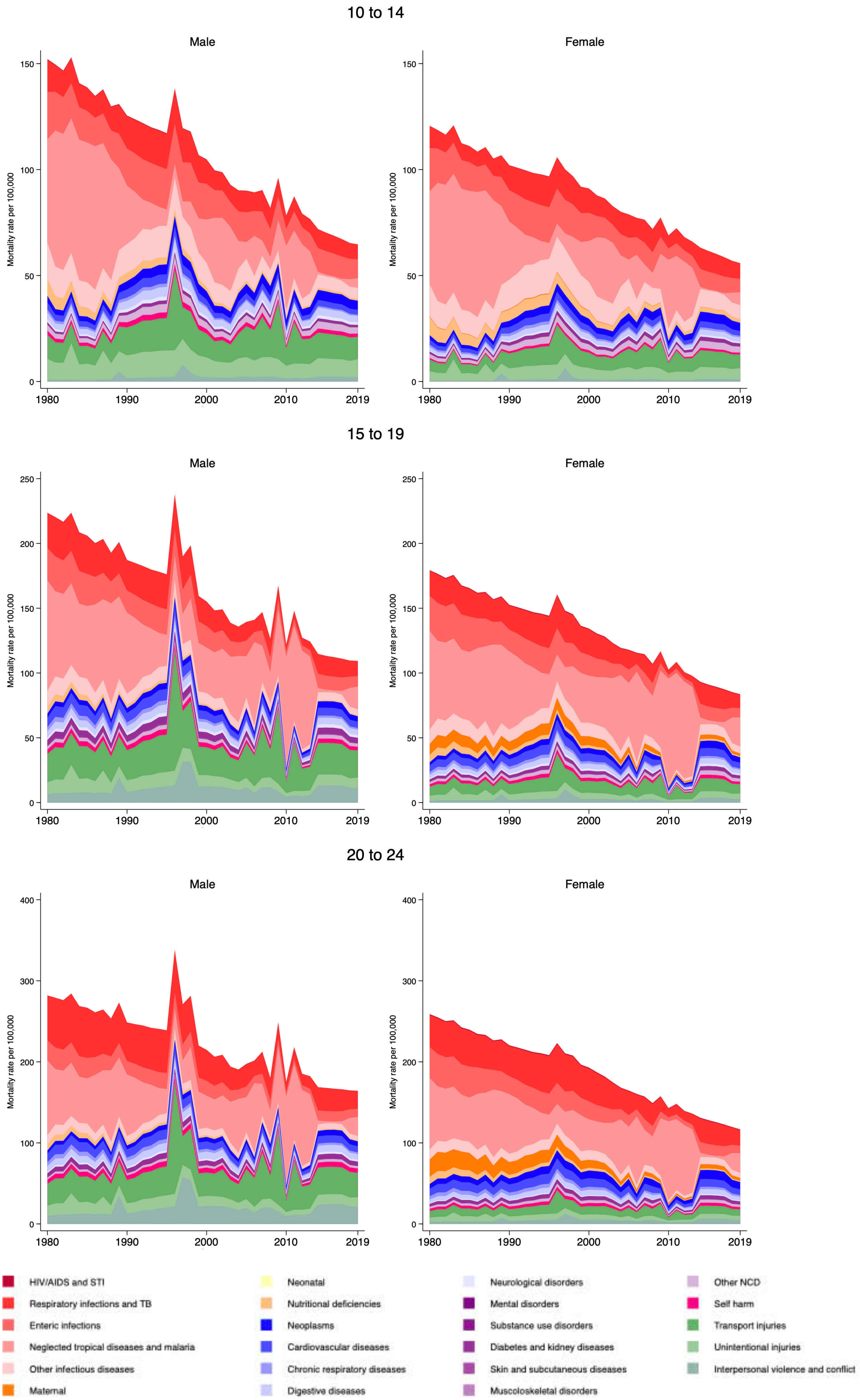

Figure S174d Number of deaths in 10-24 1980 – 2019 by cause: Comoros

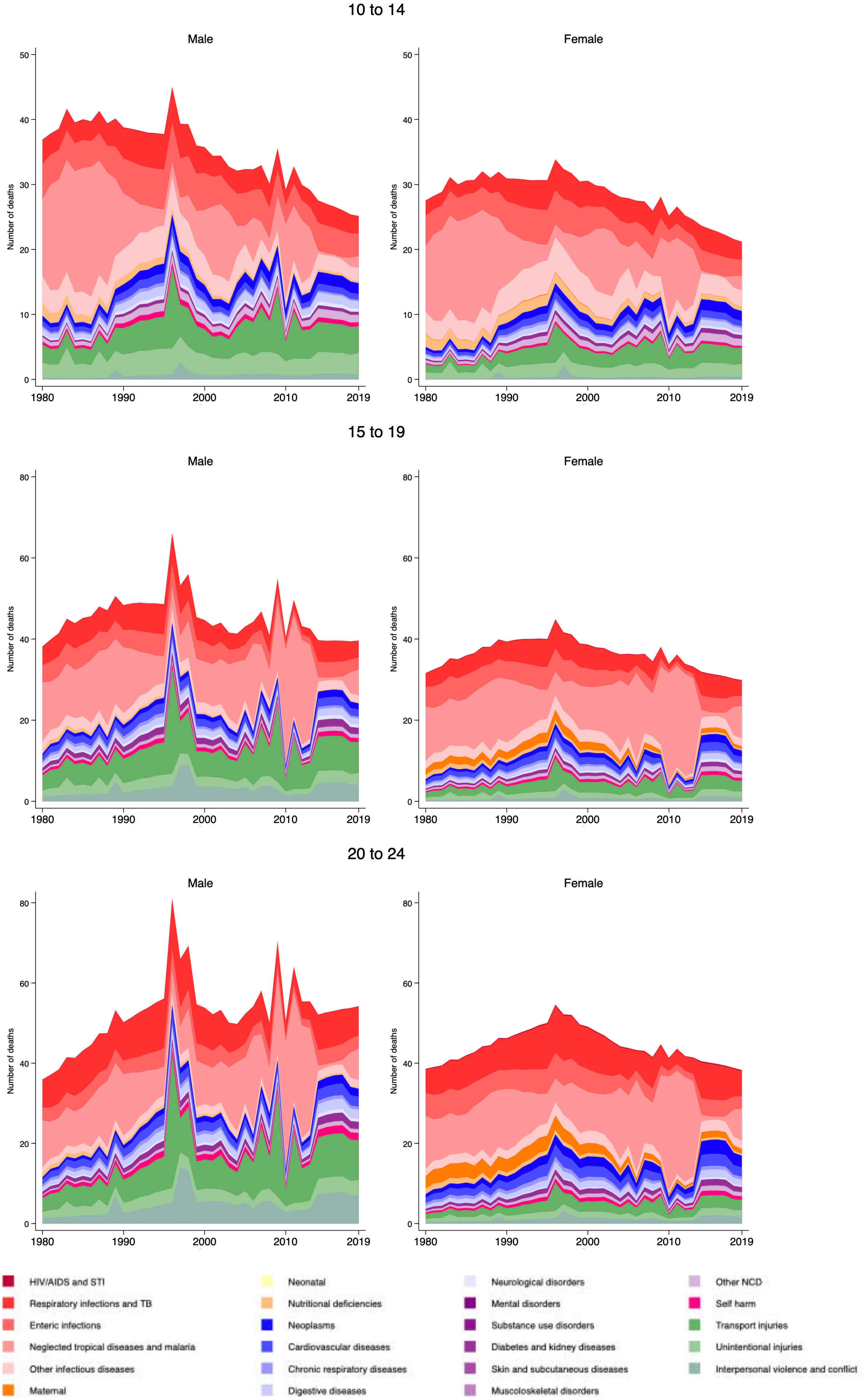

Figure S173: Djibouti

Figure S175a All-cause mortality rate per 100,000 in 10-24 1950 – 2019: Djibouti

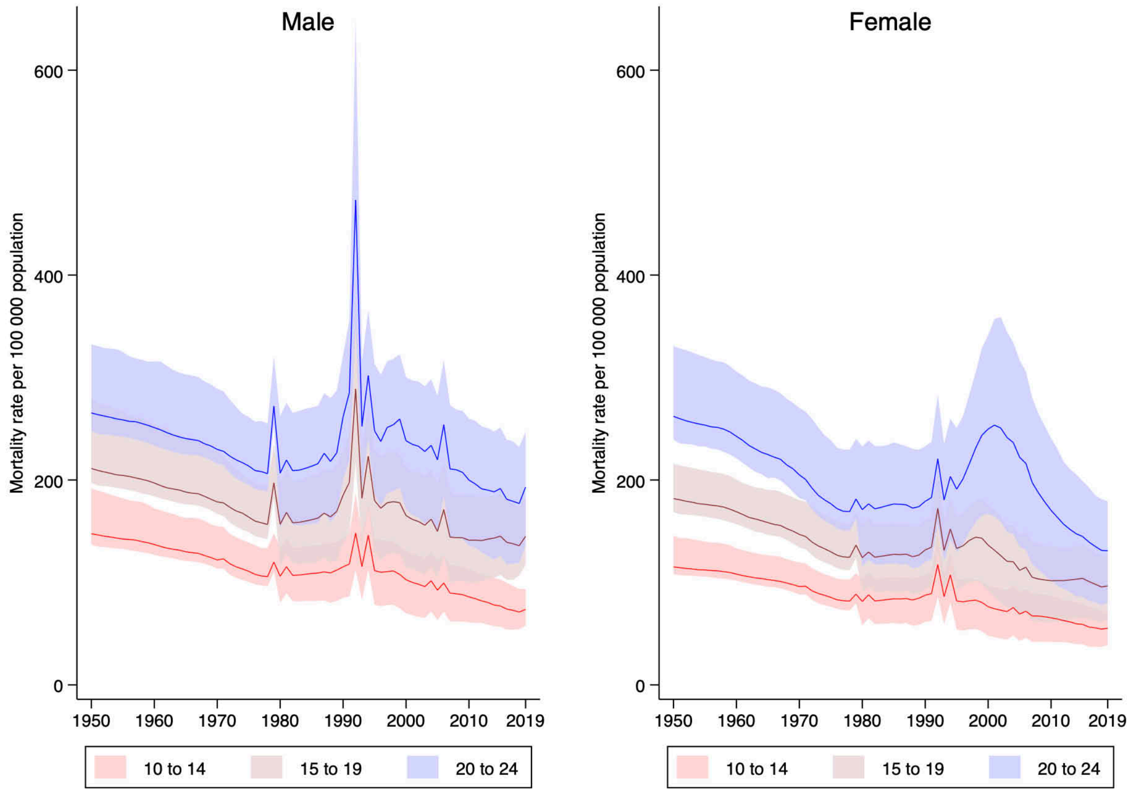

Figure S175b Number of deaths from all causes in 10-24 1950 – 2019: Djibouti

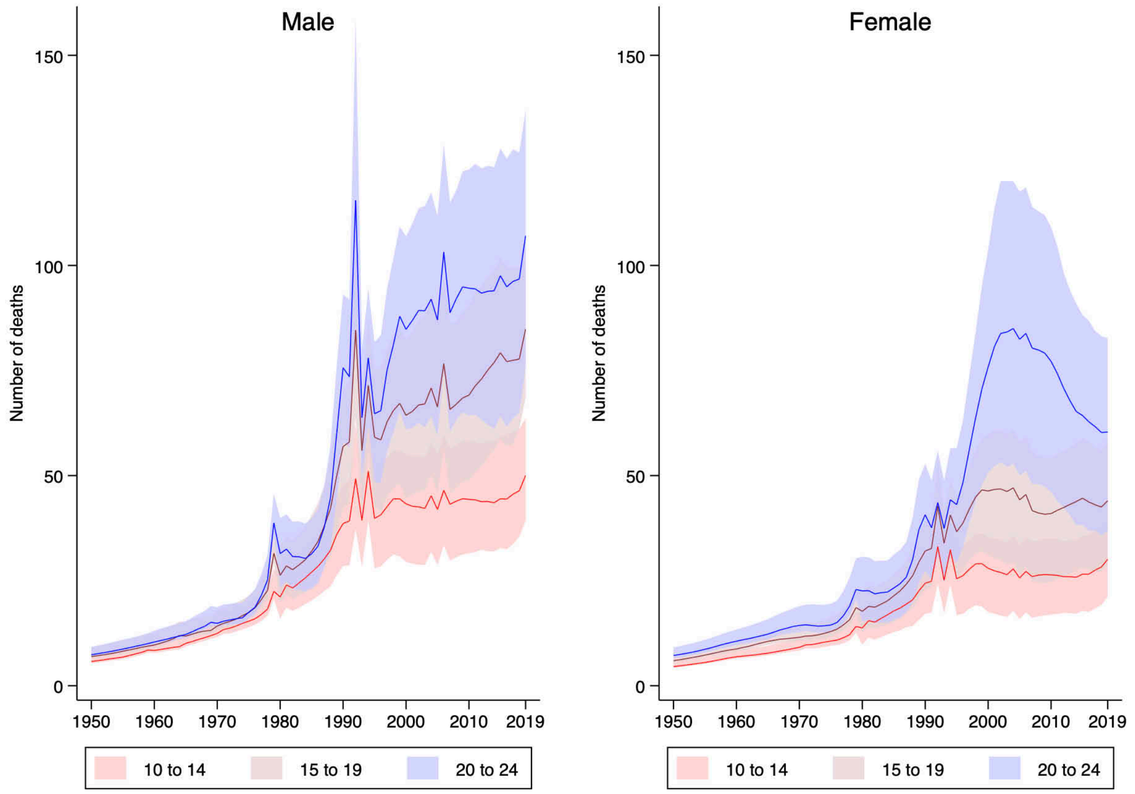

Figure S175c Mortality rate per 100,000 in 10-24 1980 – 2019 by cause: Djibouti

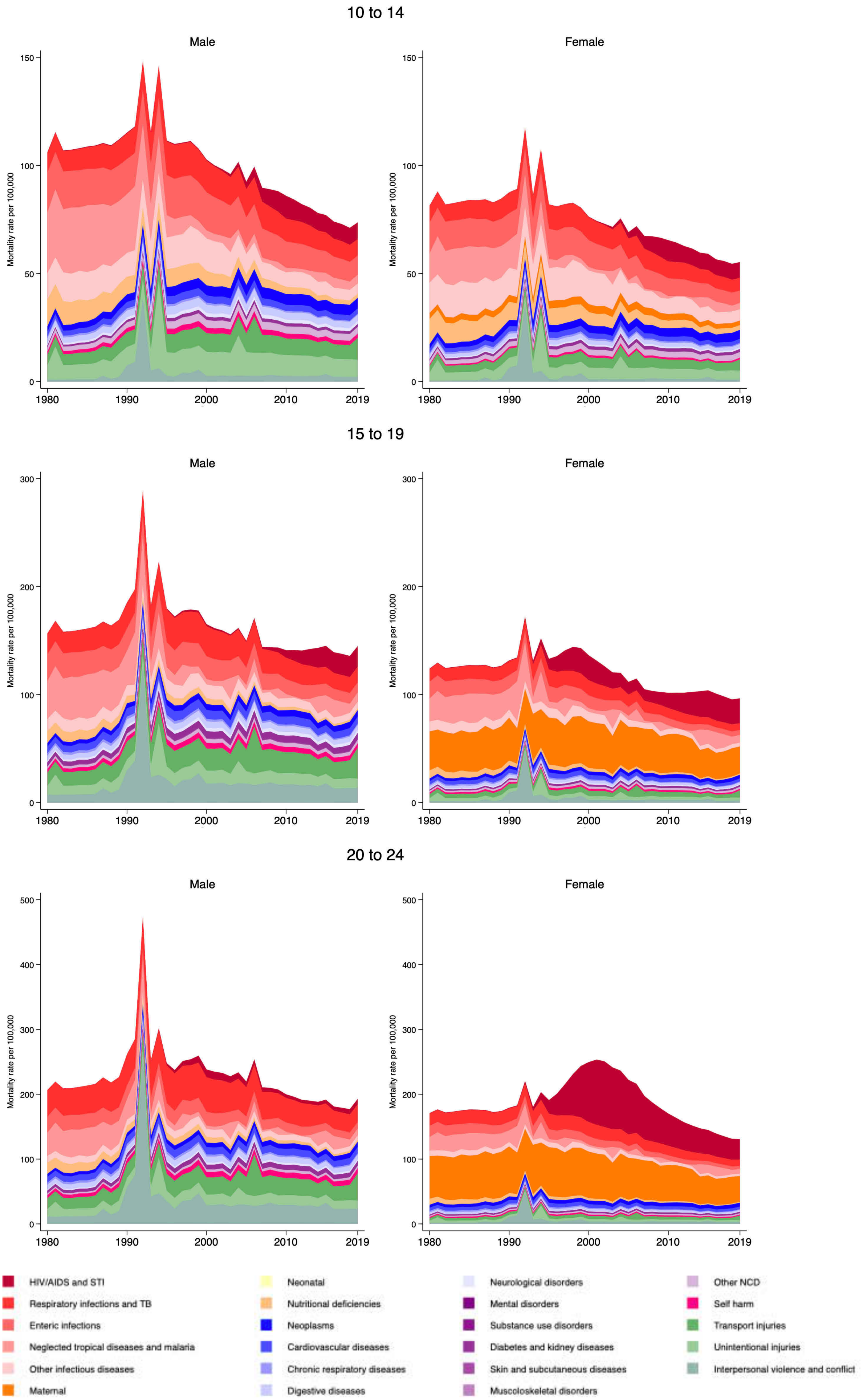

Figure S175d Number of deaths in 10-24 1980 – 2019 by cause: Djibouti

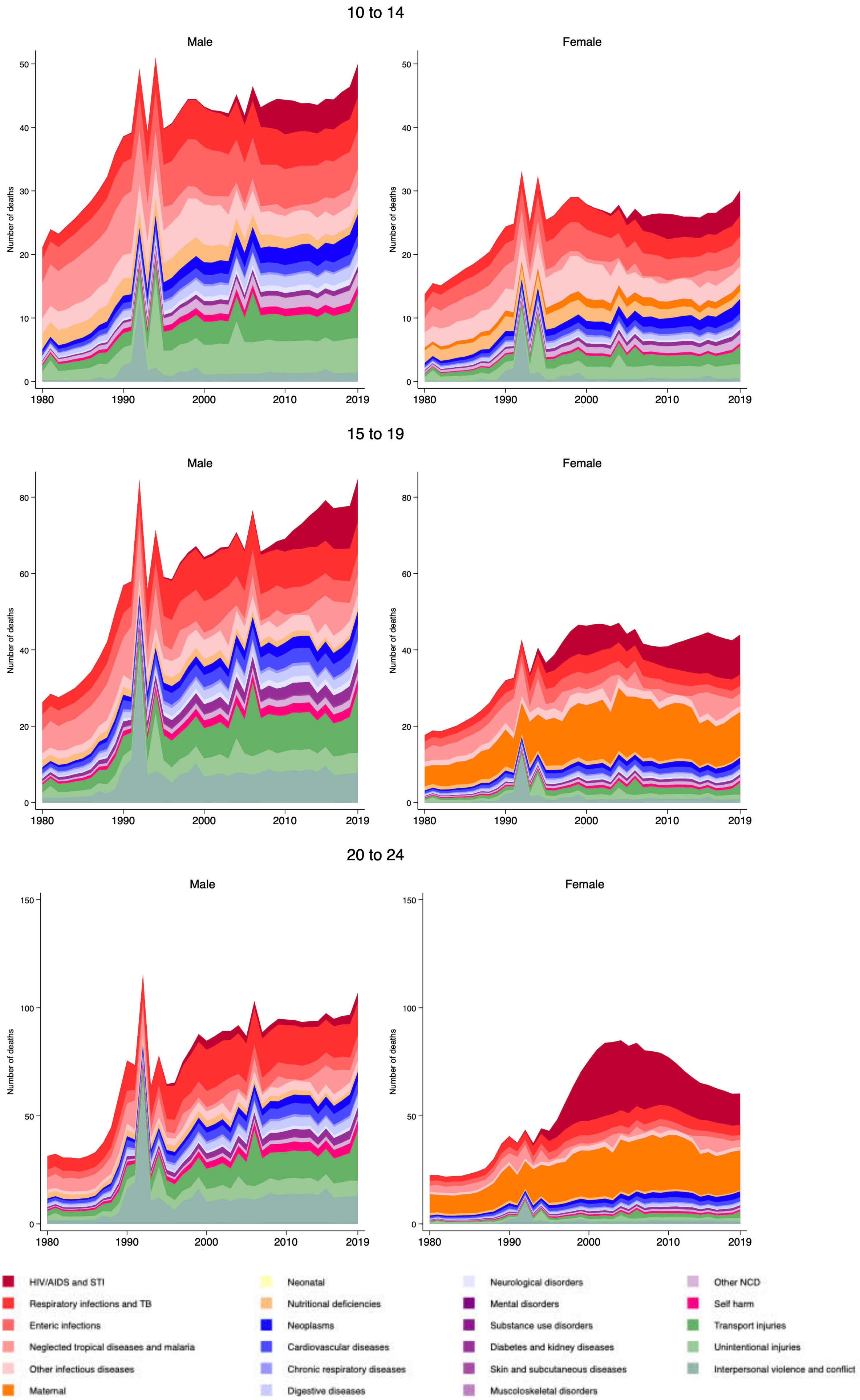

Figure S174: Eritrea

Figure S176a All-cause mortality rate per 100,000 in 10-24 1950 – 2019: Eritrea

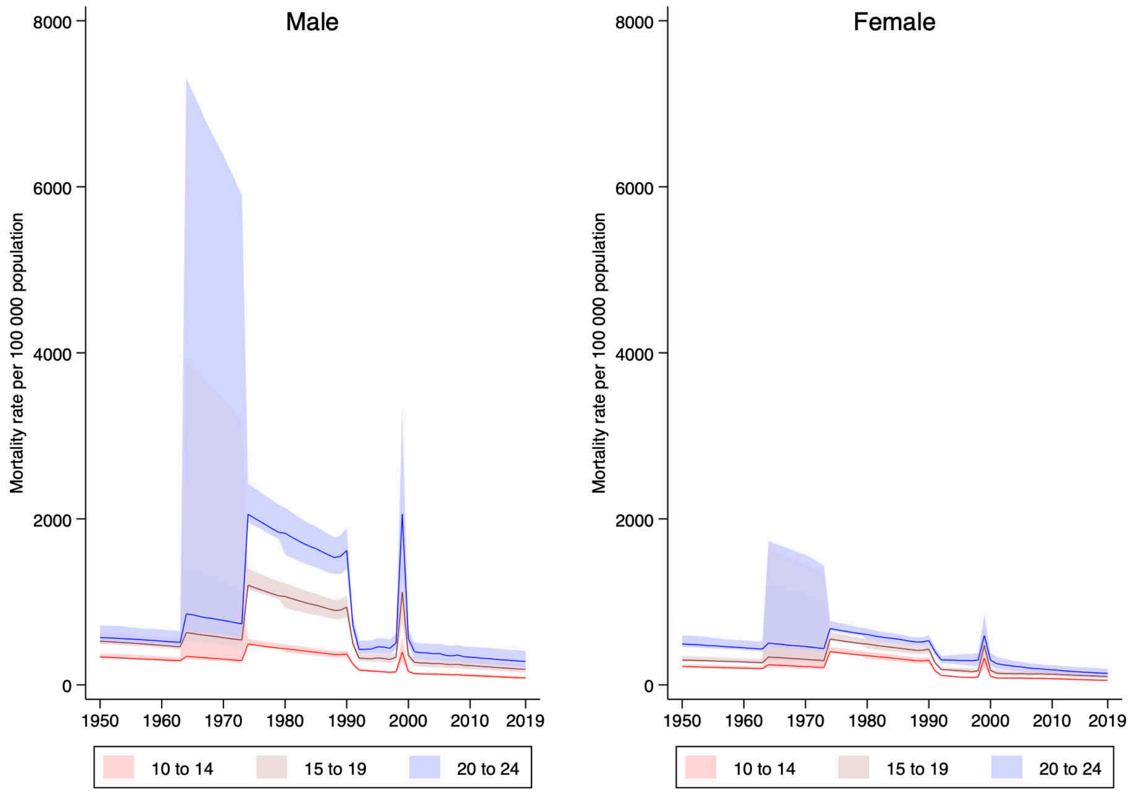

Figure S176b Number of deaths from all causes in 10-24 1950 – 2019: Eritrea

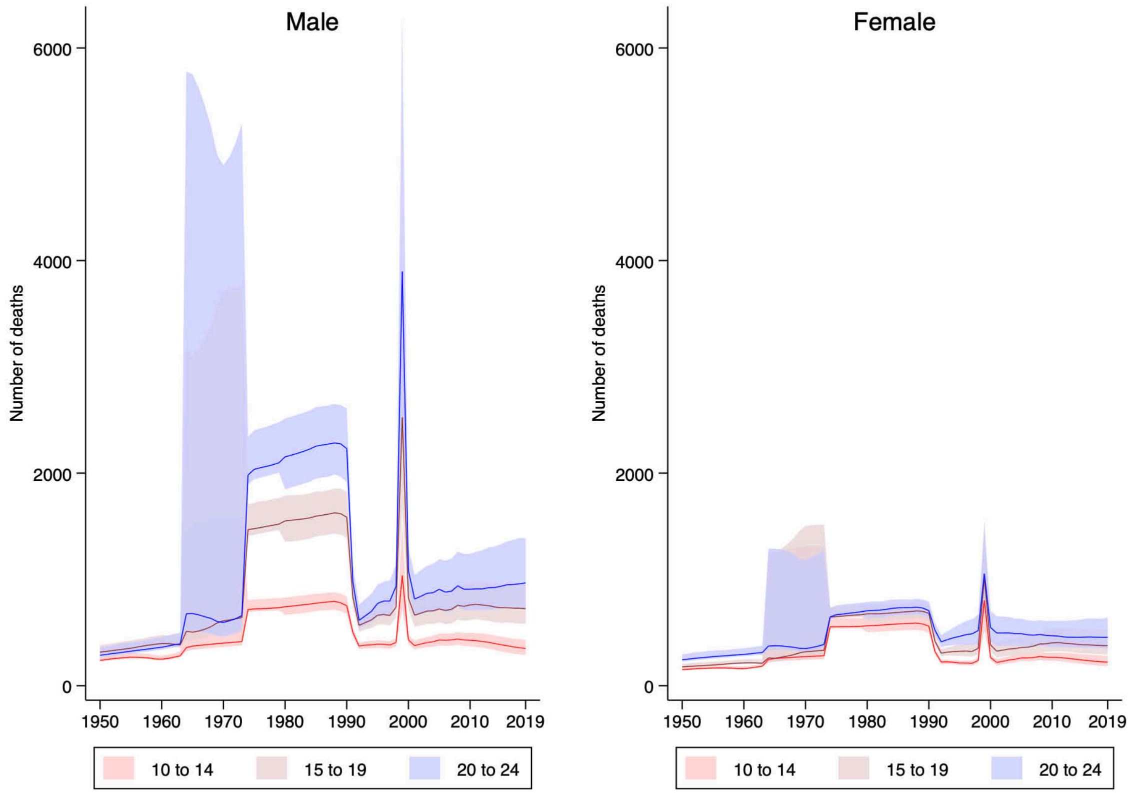

Figure S176c Mortality rate per 100,000 in 10-24 1980 – 2019 by cause: Eritrea

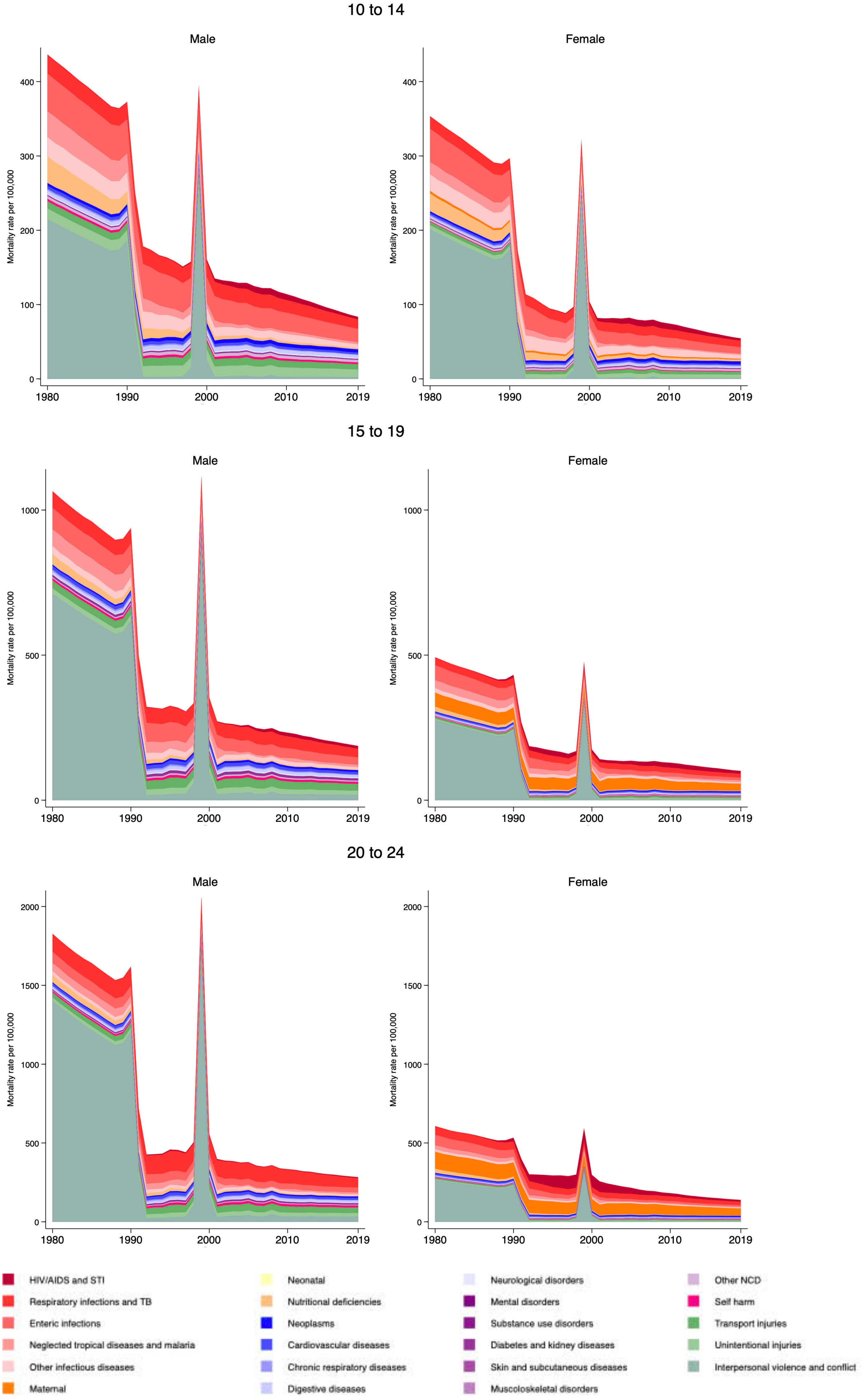

Figure S176d Number of deaths in 10-24 1980 – 2019 by cause: Eritrea

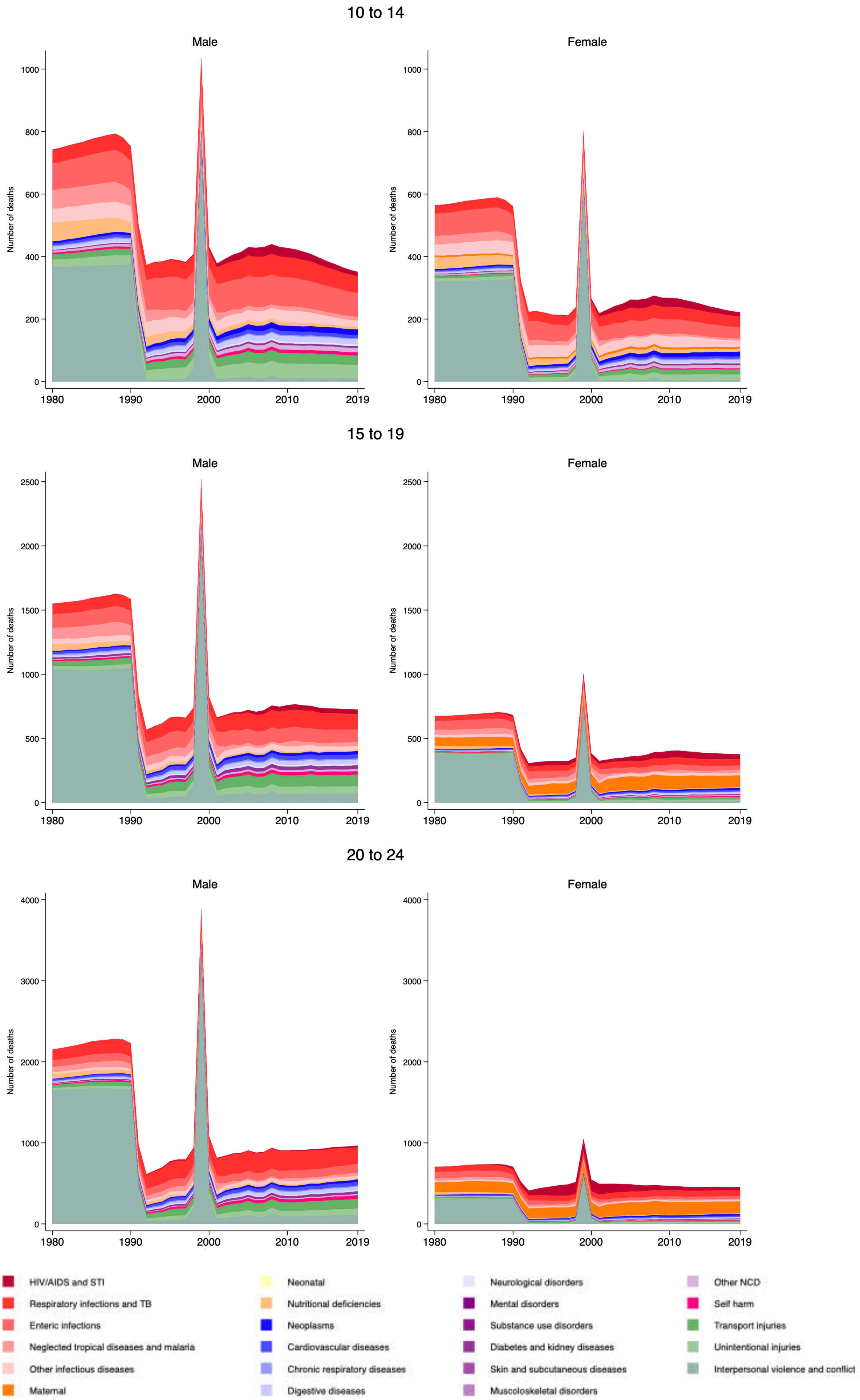

Figure S175: Ethiopia

Figure S177a All-cause mortality rate per 100,000 in 10-24 1950 – 2019: Ethiopia

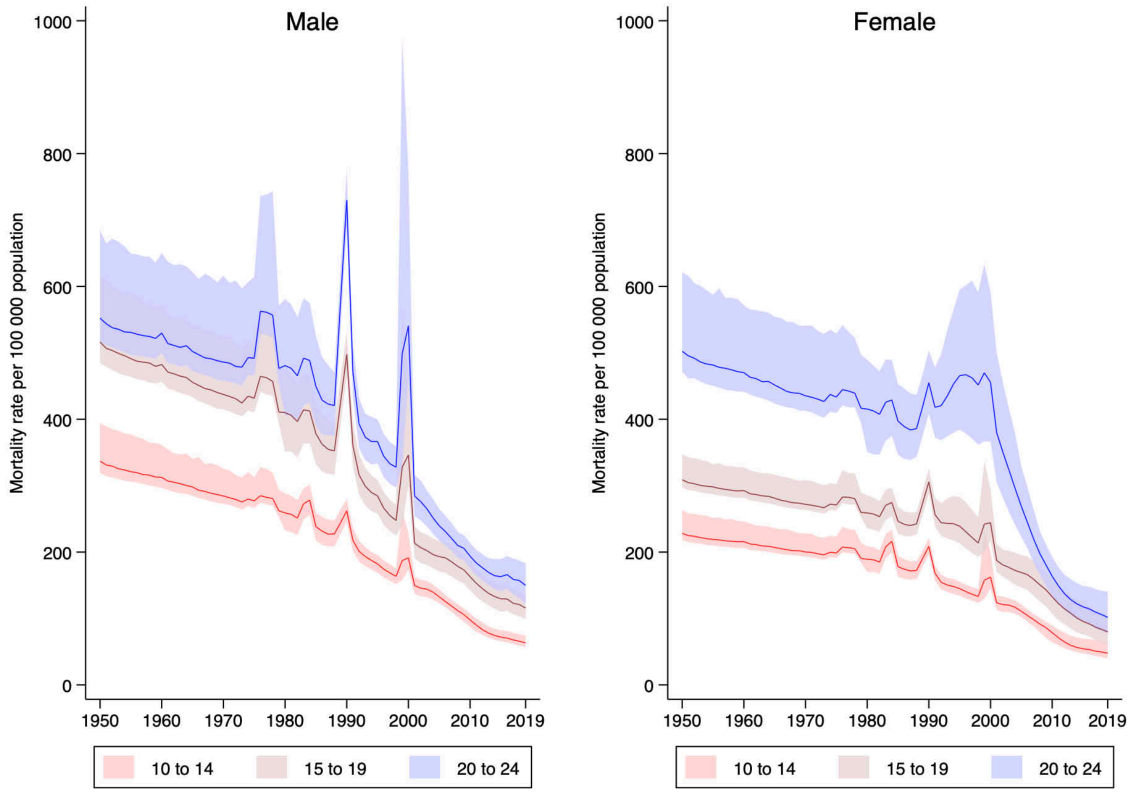

Figure S177b Number of deaths from all causes in 10-24 1950 – 2019: Ethiopia

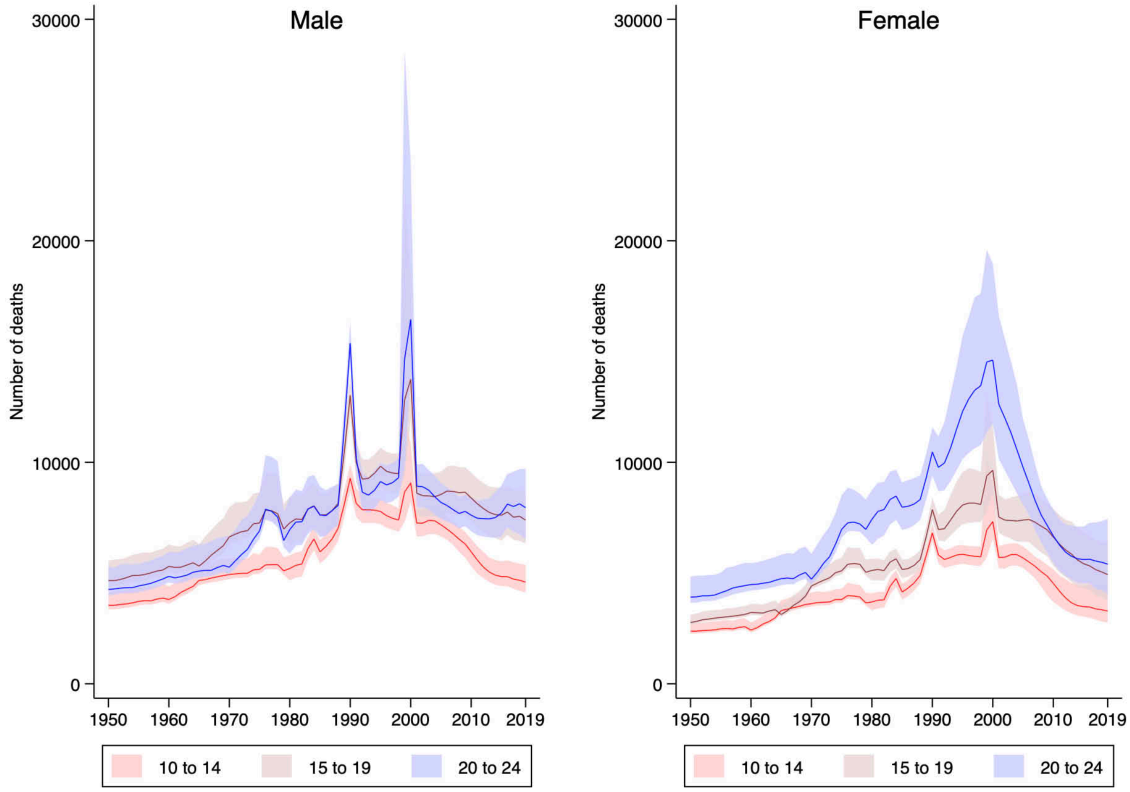

Figure S177c Mortality rate per 100,000 in 10-24 1980 – 2019 by cause: Ethiopia

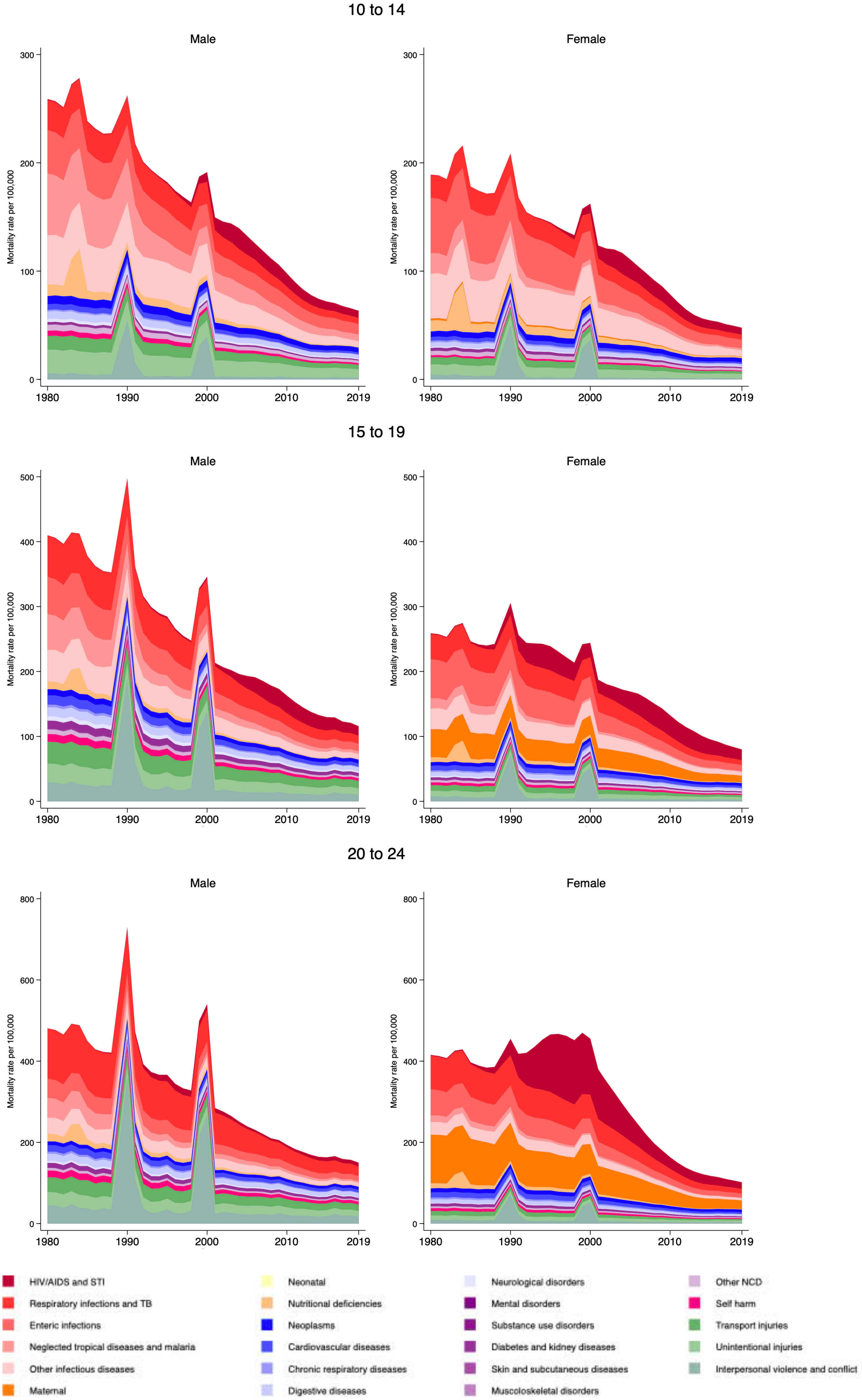

Figure S177d Number of deaths in 10-24 1980 – 2019 by cause: Ethiopia

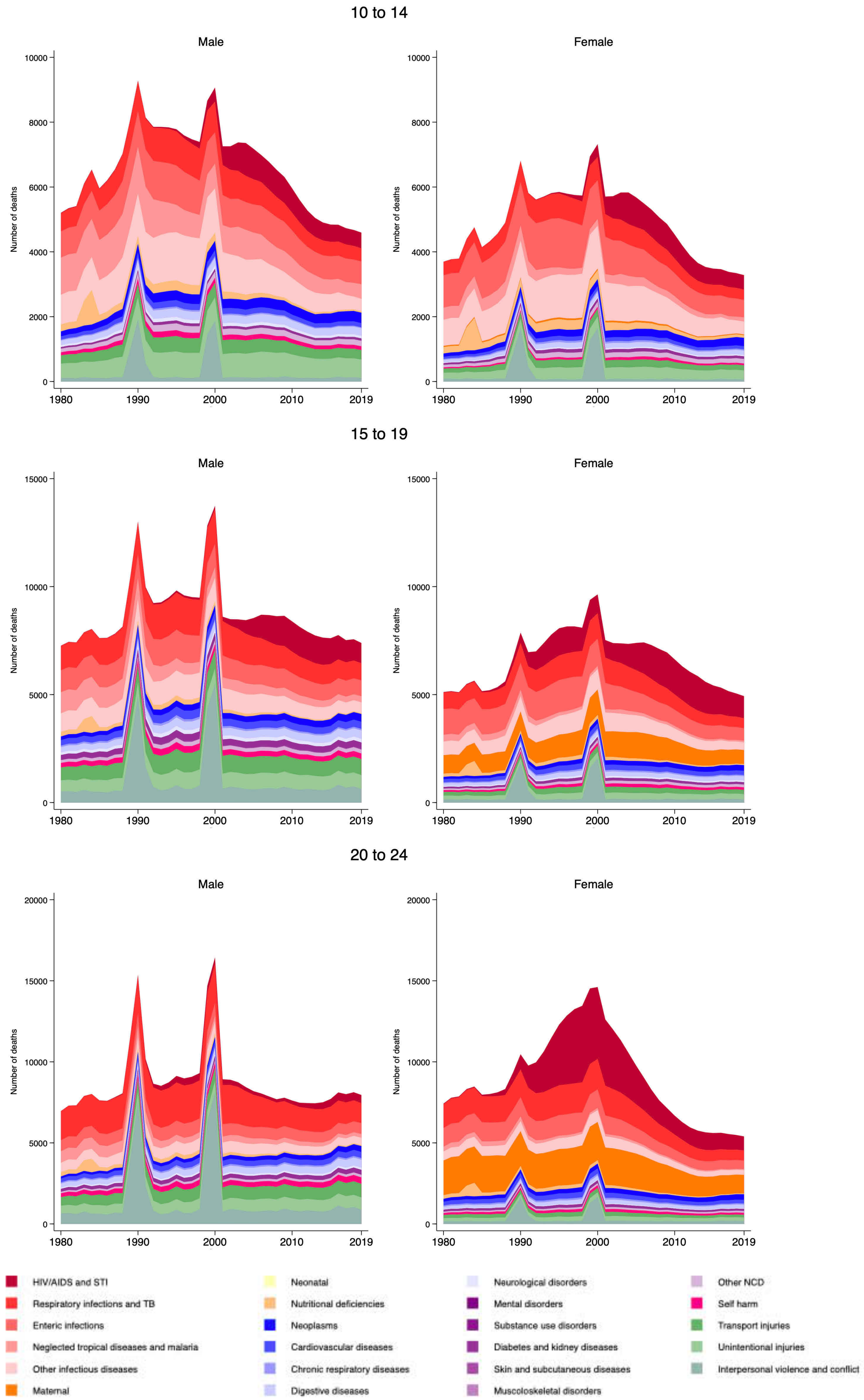

Figure S176: Kenya

Figure S178a All-cause mortality rate per 100,000 in 10-24 1950 – 2019: Kenya

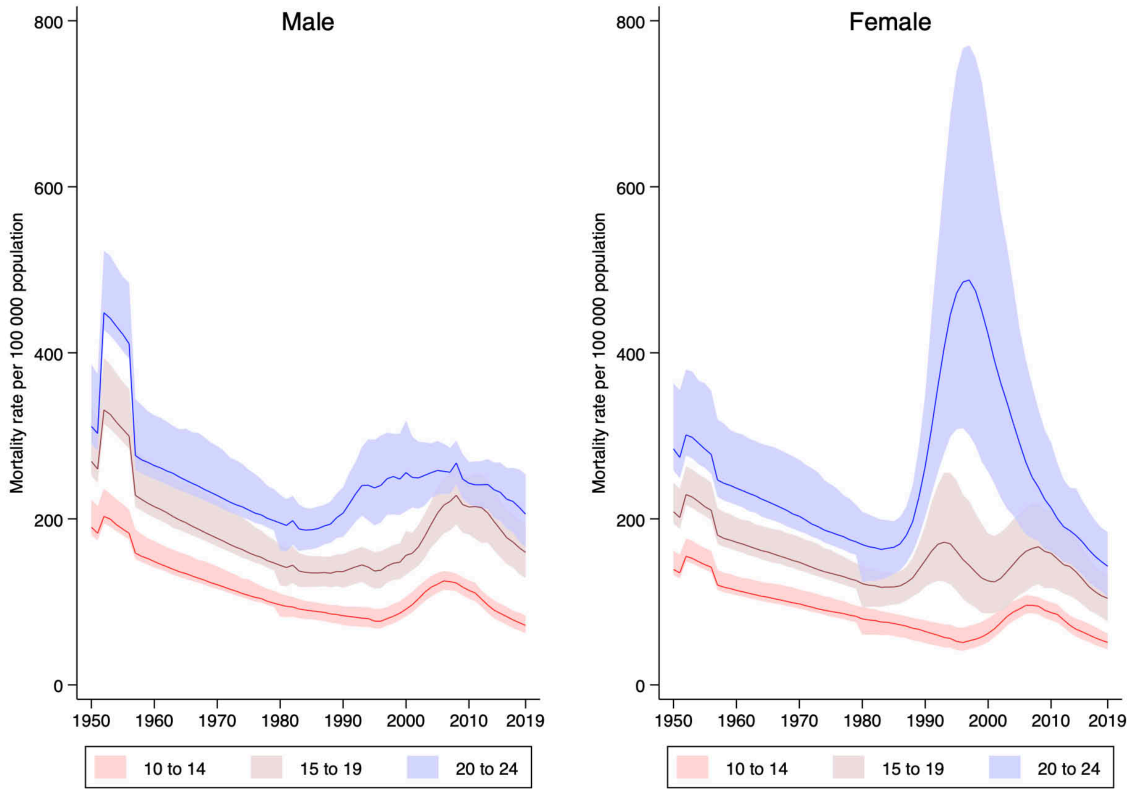

Figure S178b Number of deaths from all causes in 10-24 1950 – 2019: Kenya

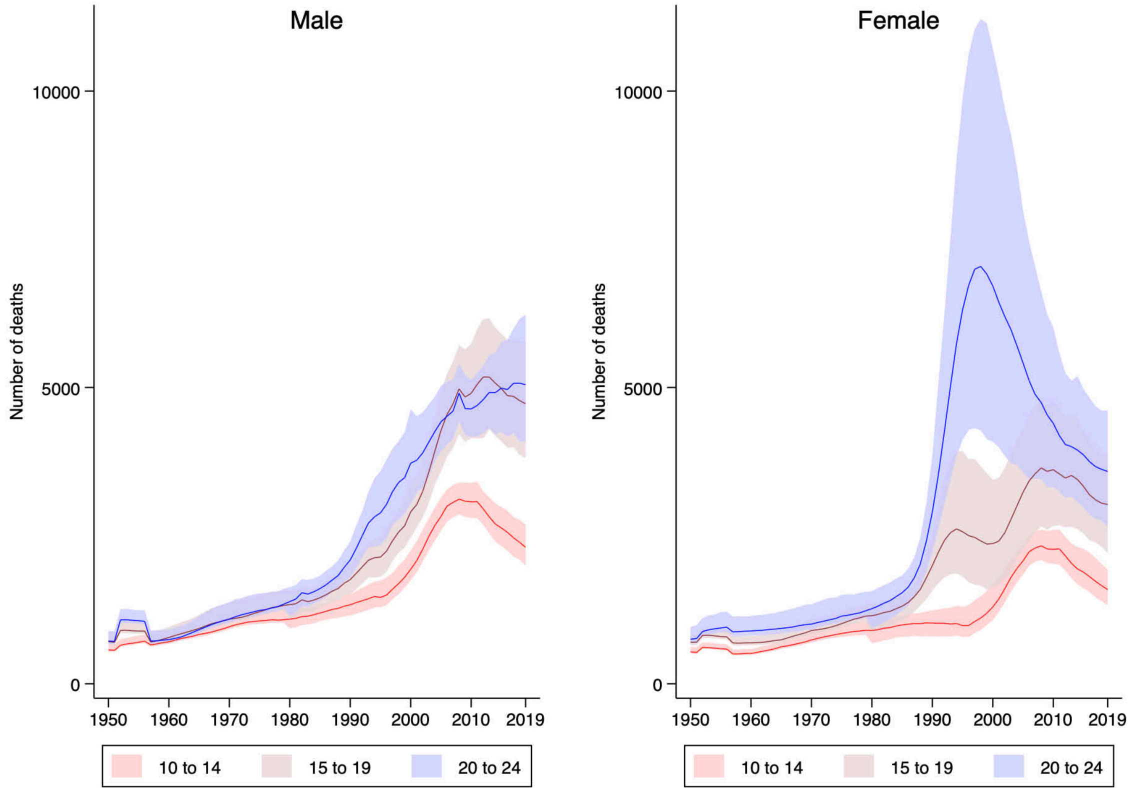

Figure S178c Mortality rate per 100,000 in 10-24 1980 – 2019 by cause: Kenya

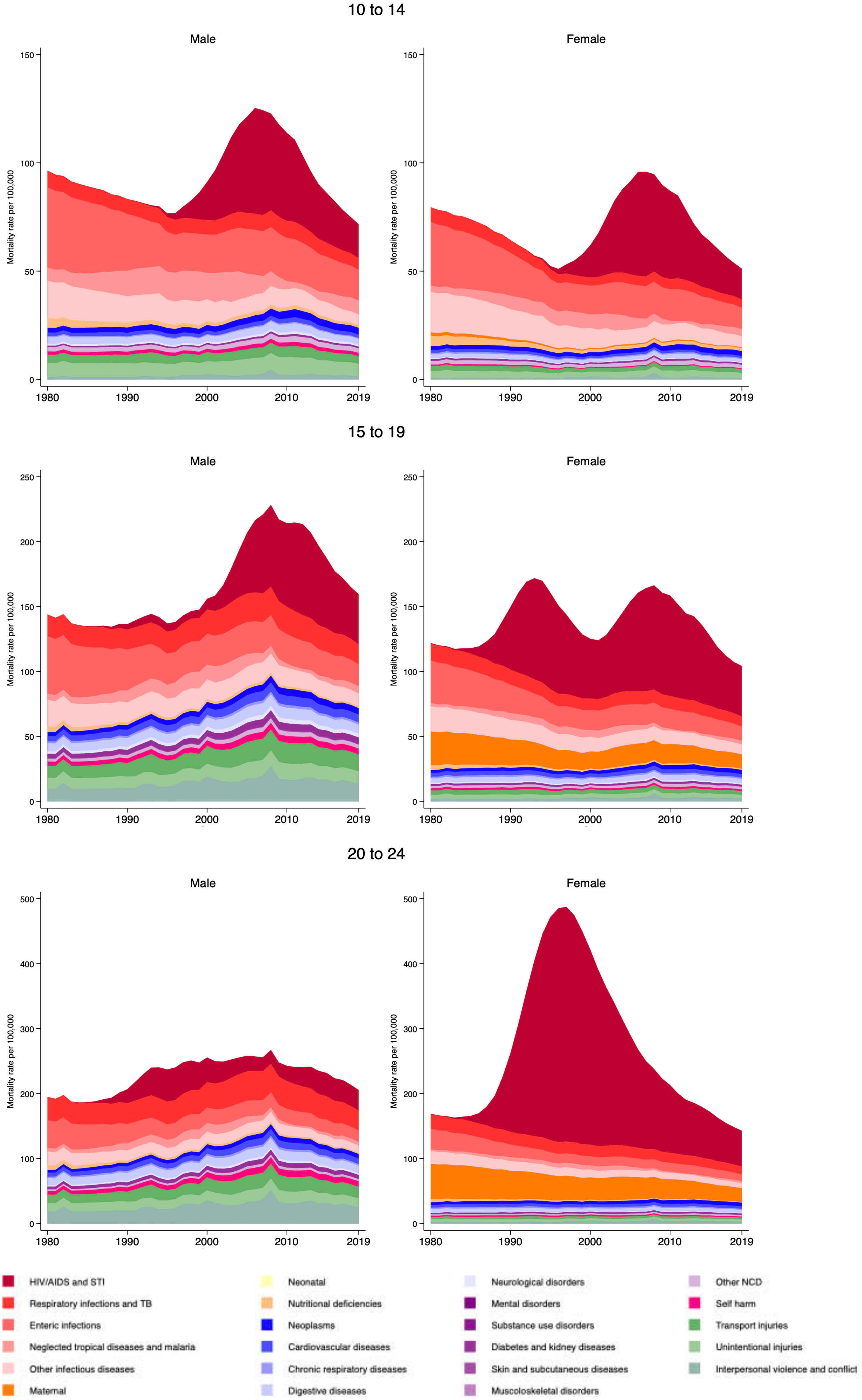

Figure S178d Number of deaths in 10-24 1980 – 2019 by cause: Kenya

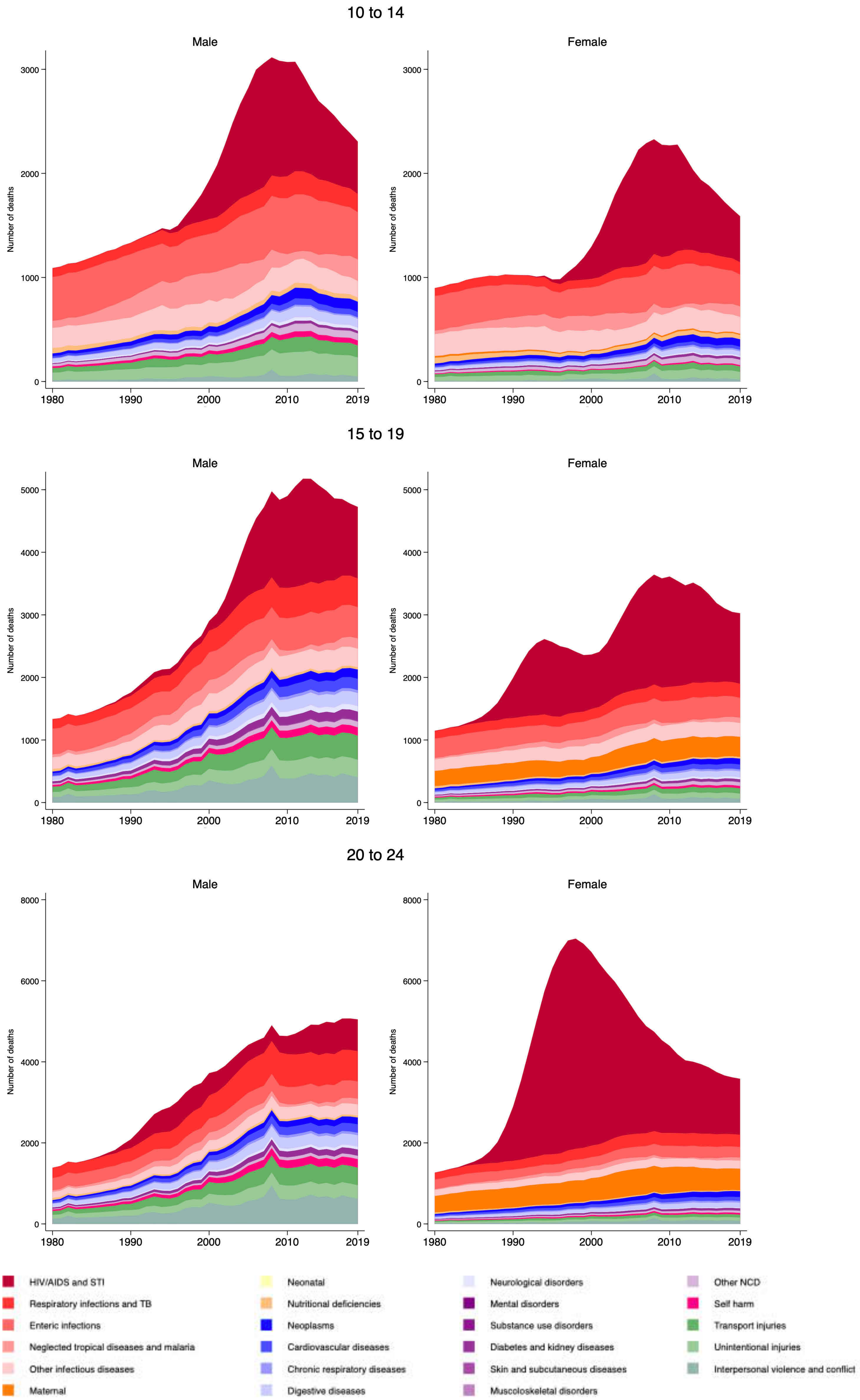

Figure S177: Madagascar

Figure S179a All-cause mortality rate per 100,000 in 10-24 1950 – 2019: Madagascar

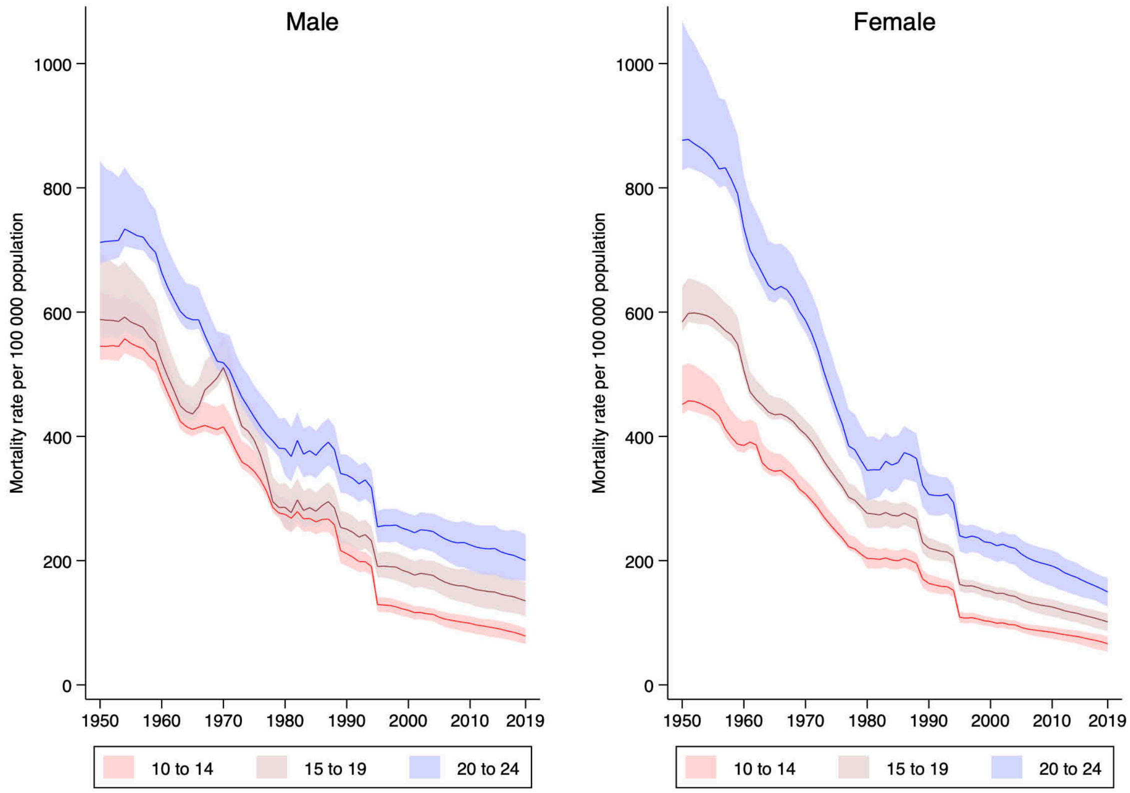

Figure S179b Number of deaths from all causes in 10-24 1950 – 2019: Madagascar

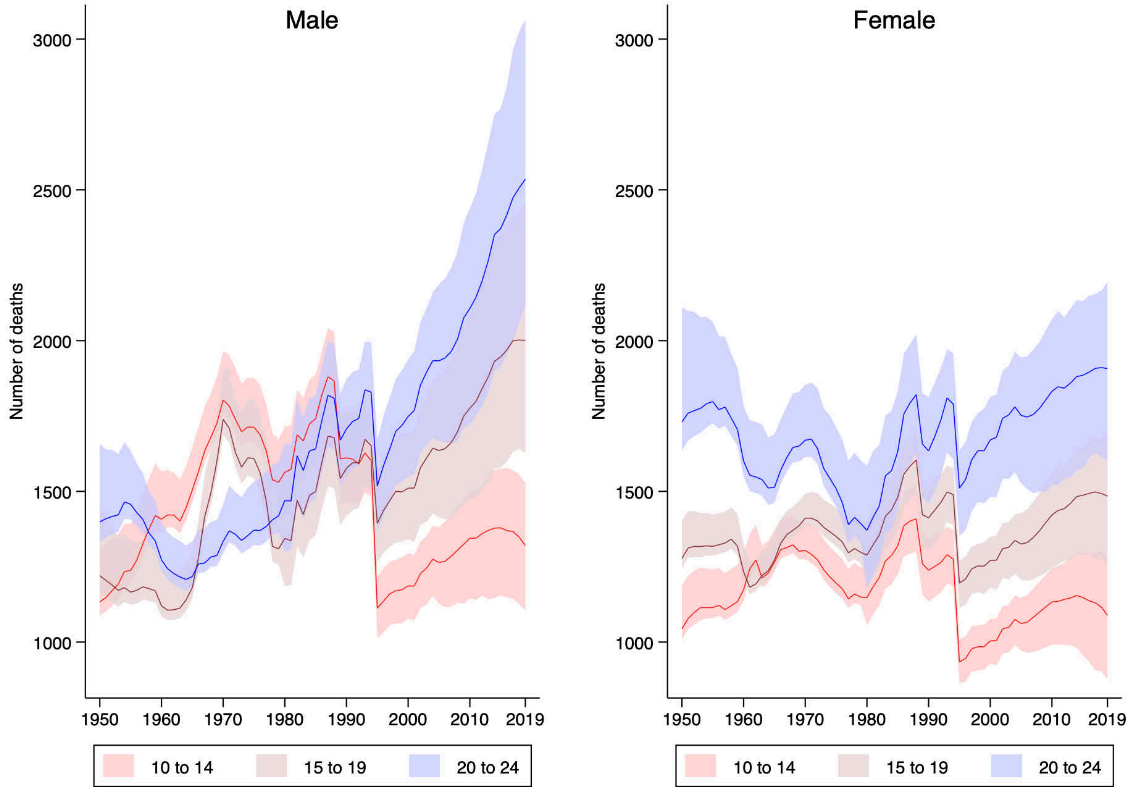

Figure S179c Mortality rate per 100,000 in 10-24 1980 – 2019 by cause: Madagascar

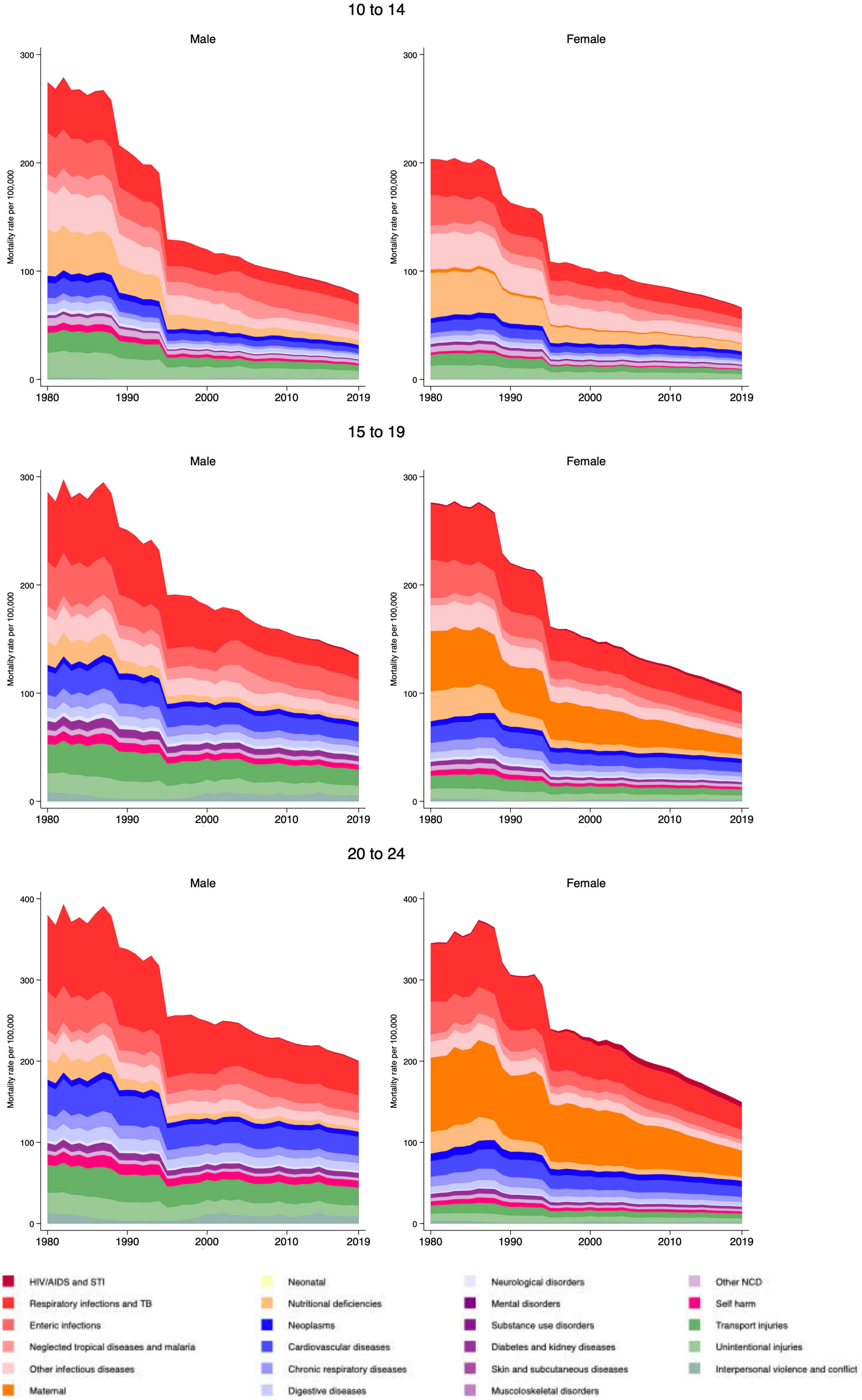

Figure S179d Number of deaths in 10-24 1980 – 2019 by cause: Madagascar

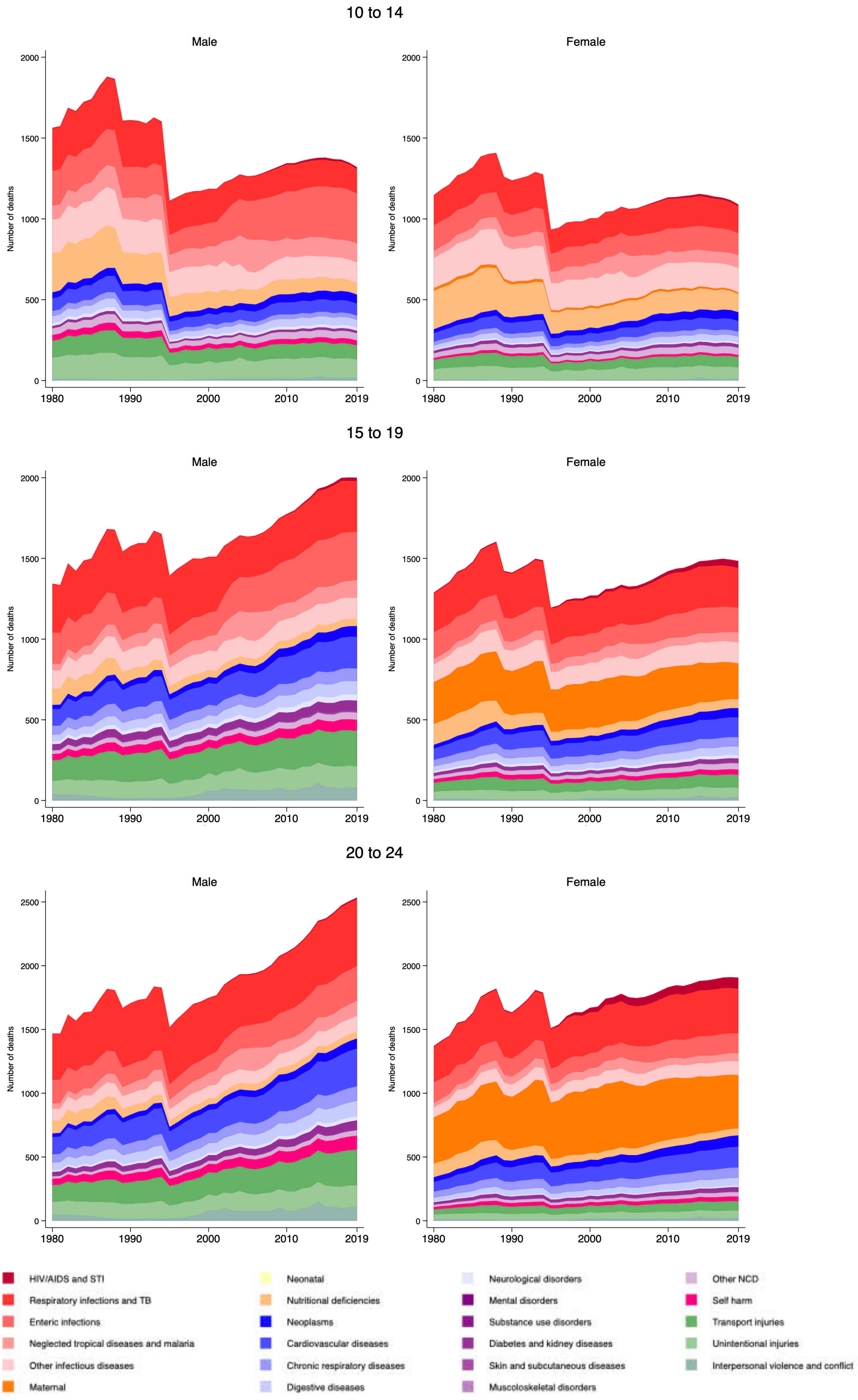

Figure S178: Malawi

Figure S180a All-cause mortality rate per 100,000 in 10-24 1950 – 2019: Malawi

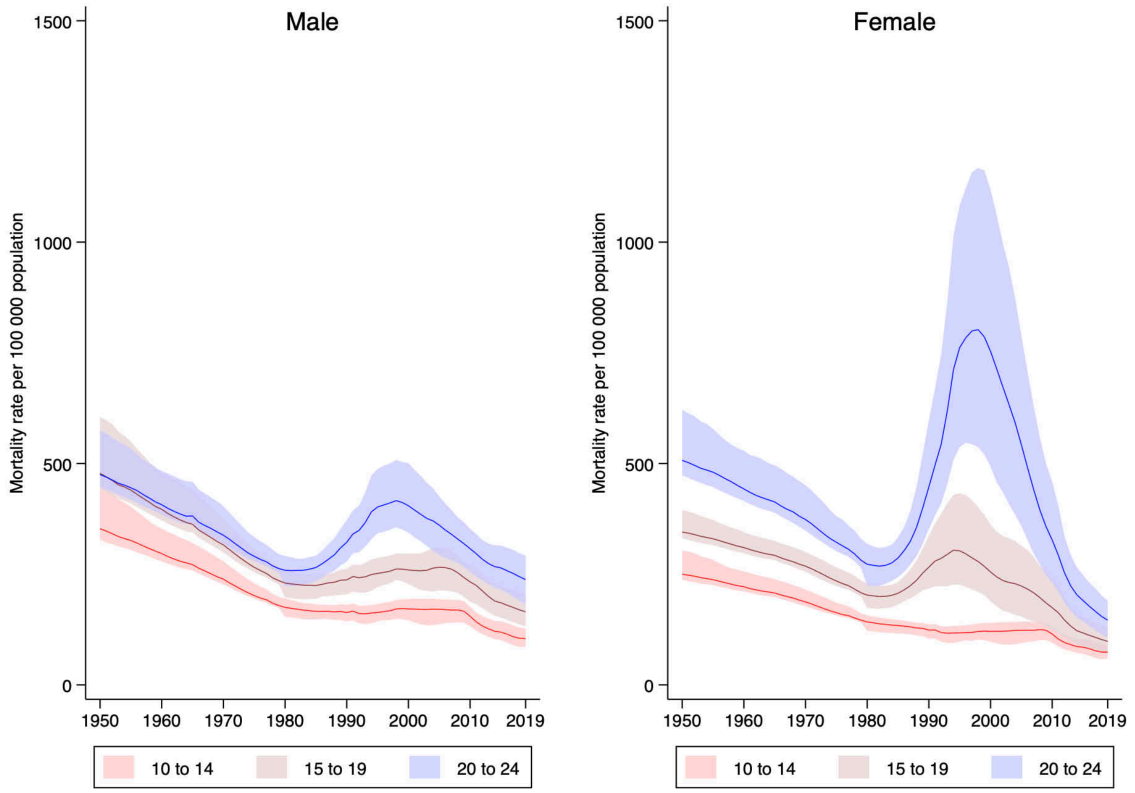

Figure S180b Number of deaths from all causes in 10-24 1950 – 2019: Malawi

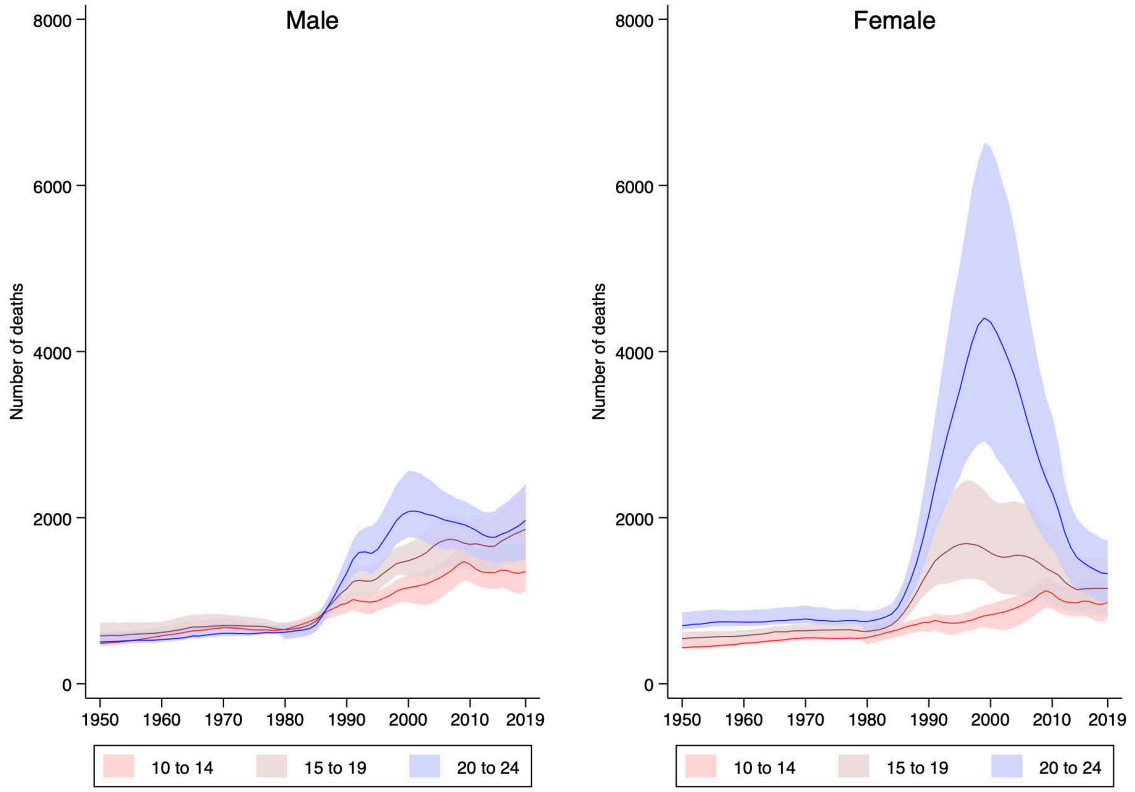

Figure S180c Mortality rate per 100,000 in 10-24 1980 – 2019 by cause: Malawi

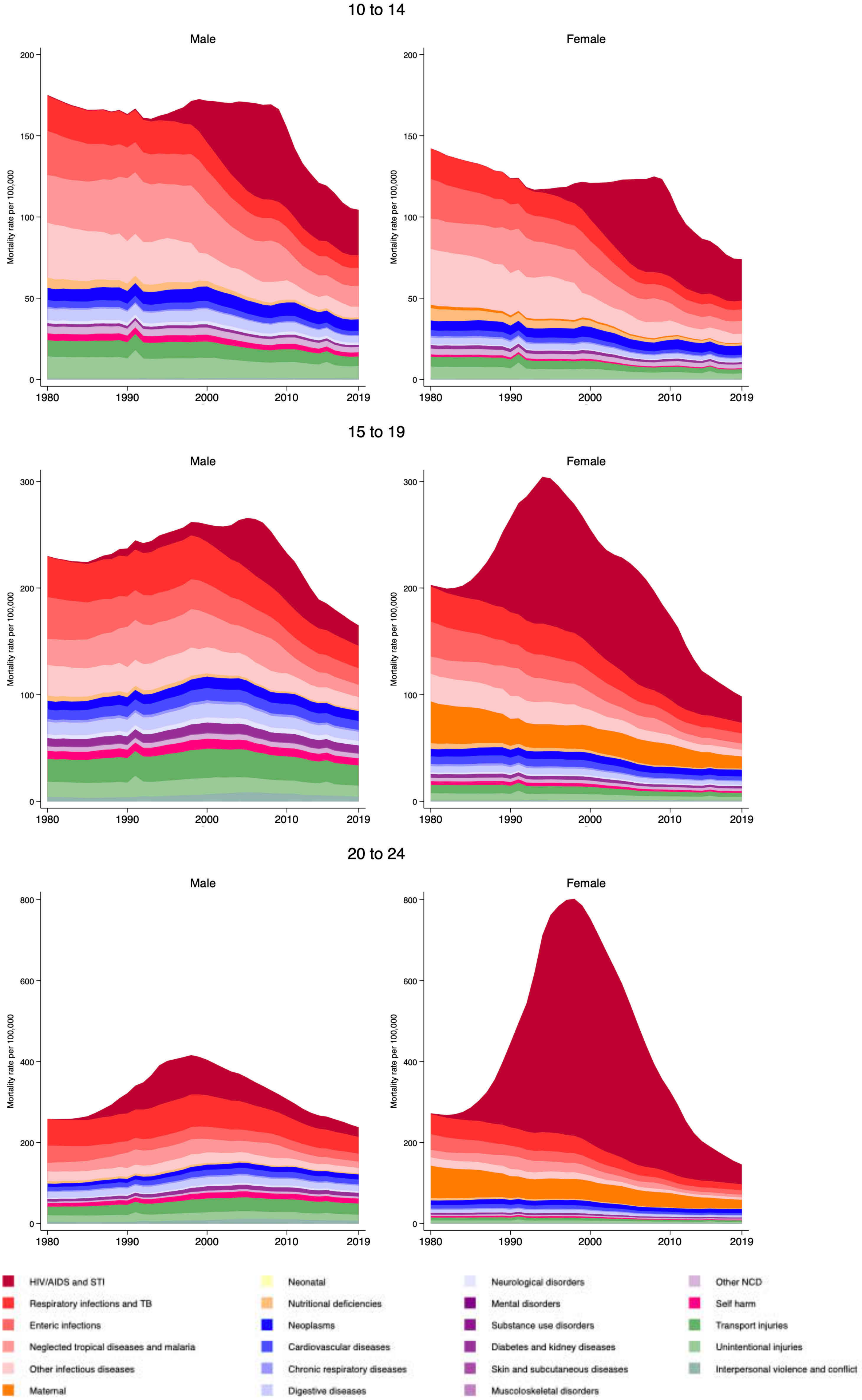

Figure S180d Number of deaths in 10-24 1980 – 2019 by cause: Malawi

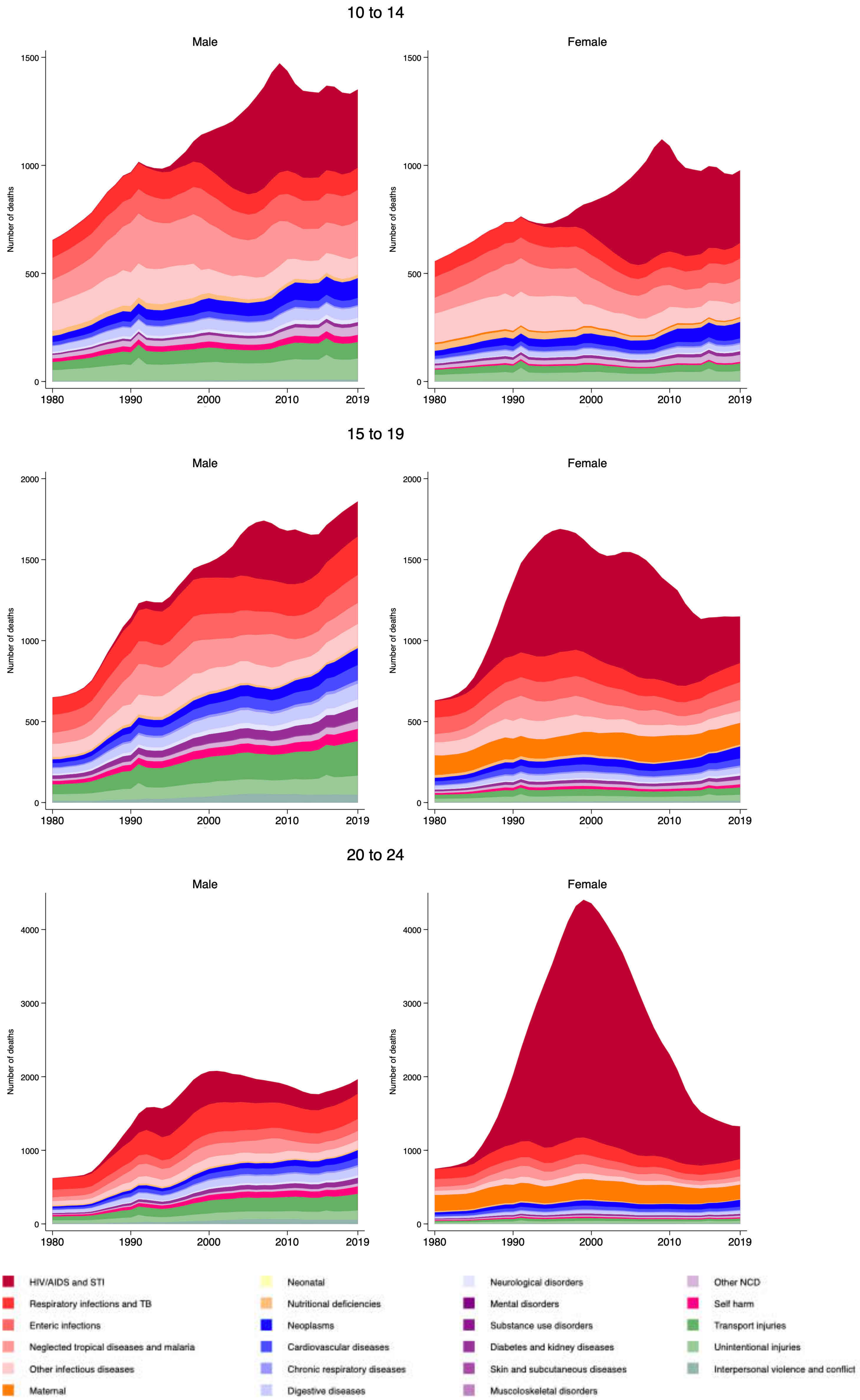

Figure S179: Mauritius

Figure S181a All-cause mortality rate per 100,000 in 10-24 1950 – 2019: Mauritius

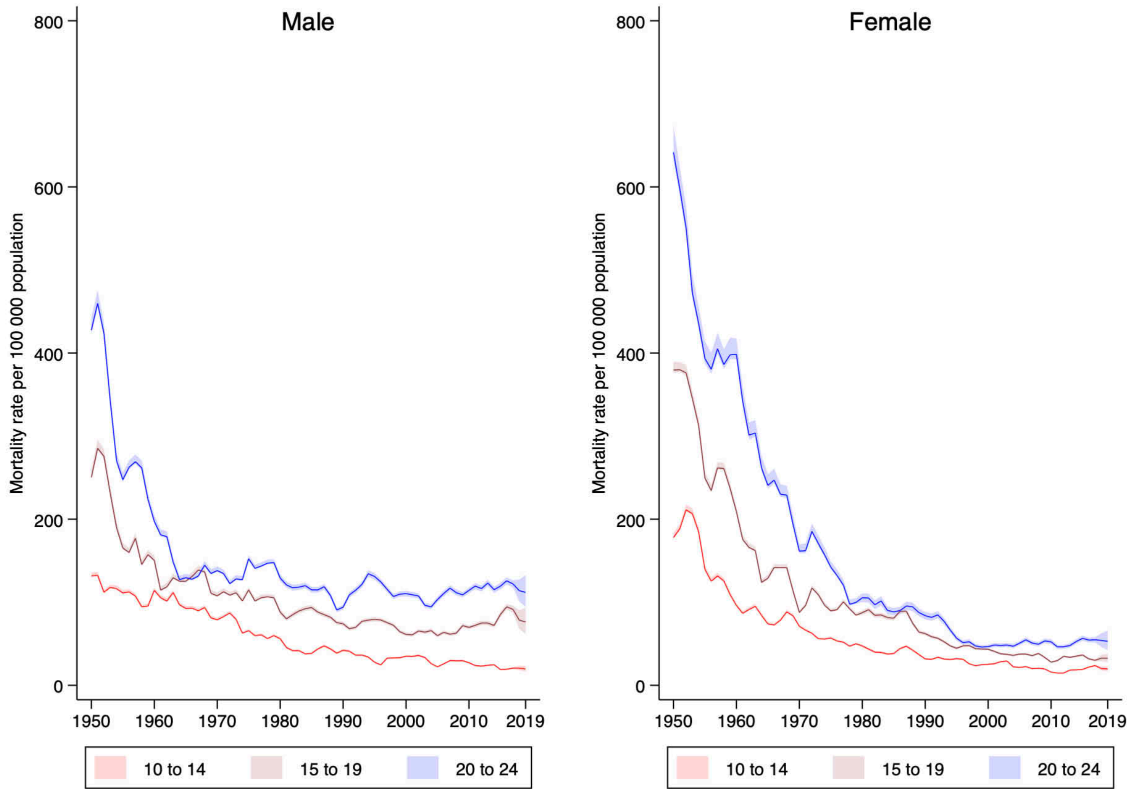

Figure S181b Number of deaths from all causes in 10-24 1950 – 2019: Mauritius

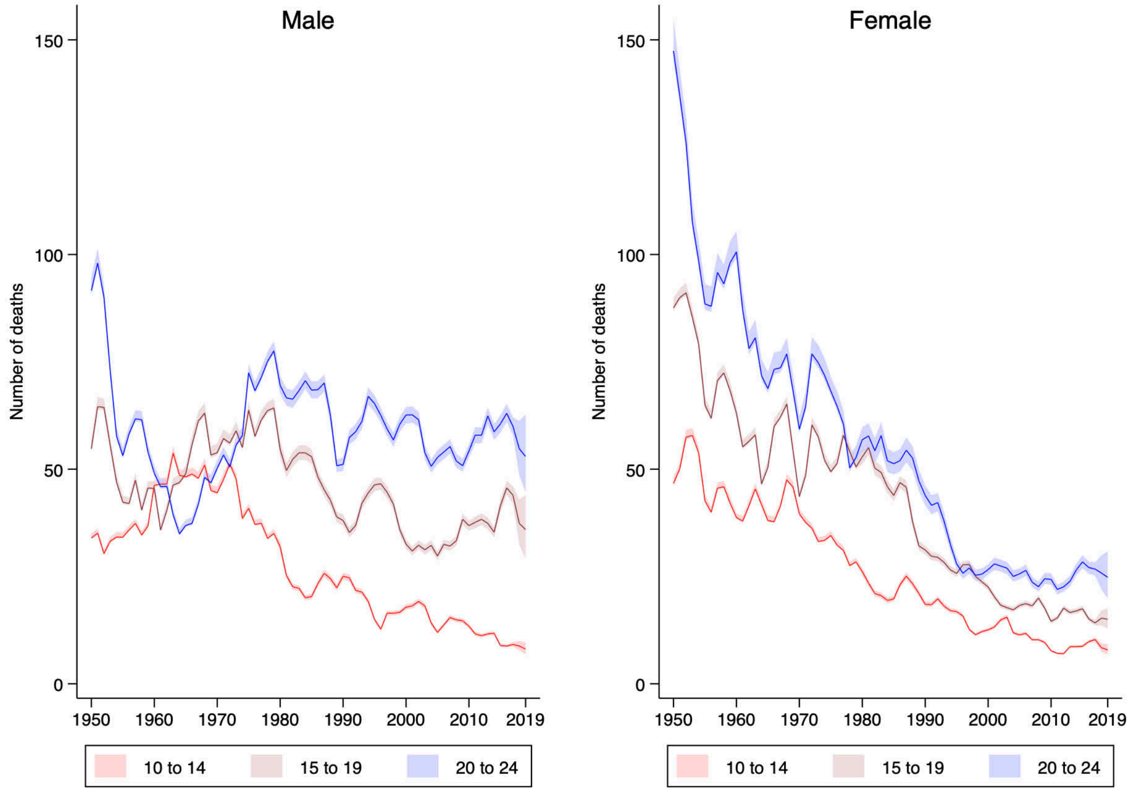

Figure S181c Mortality rate per 100,000 in 10-24 1980 – 2019 by cause: Mauritius

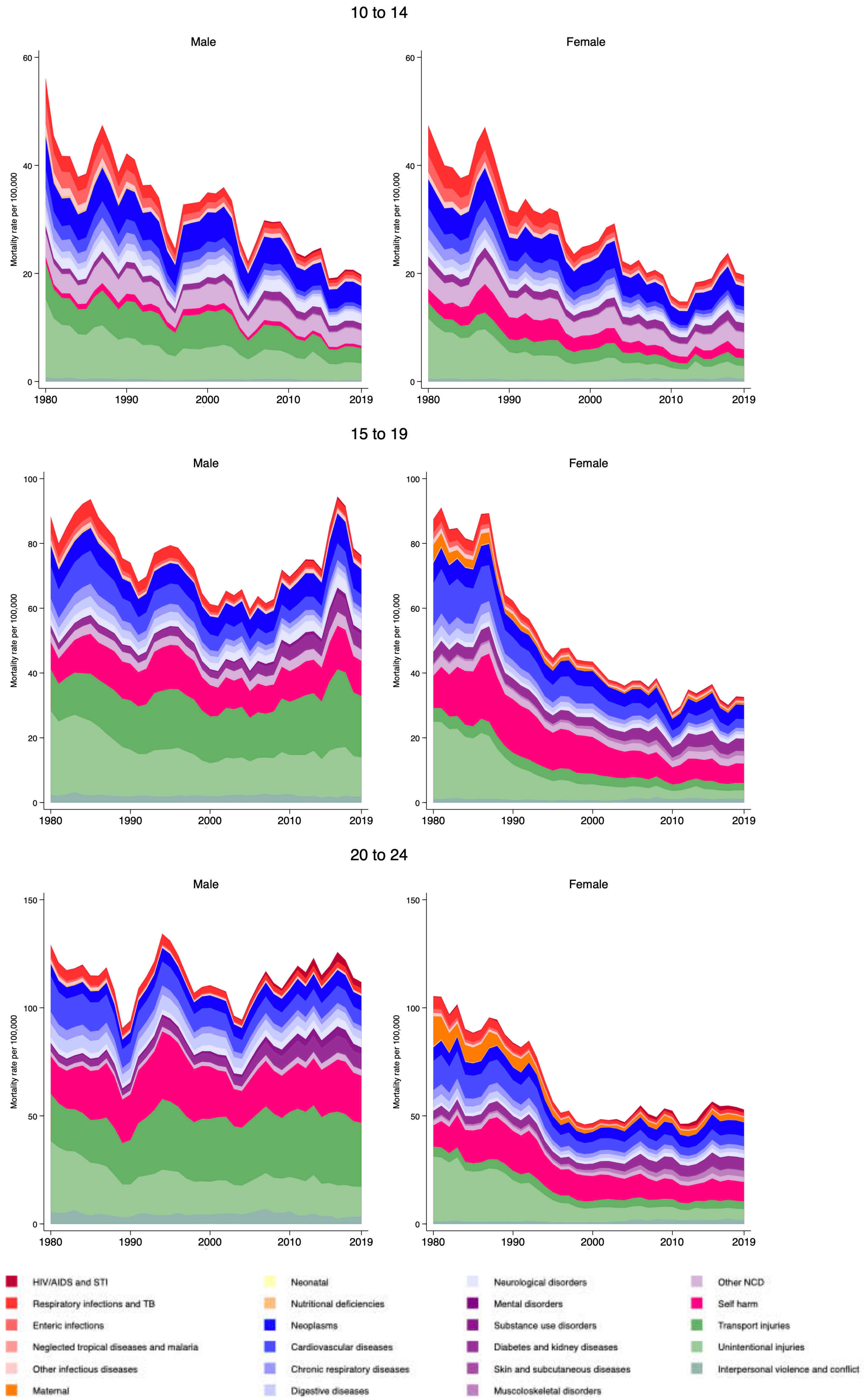

Figure S181d Number of deaths in 10-24 1980 – 2019 by cause: Mauritius

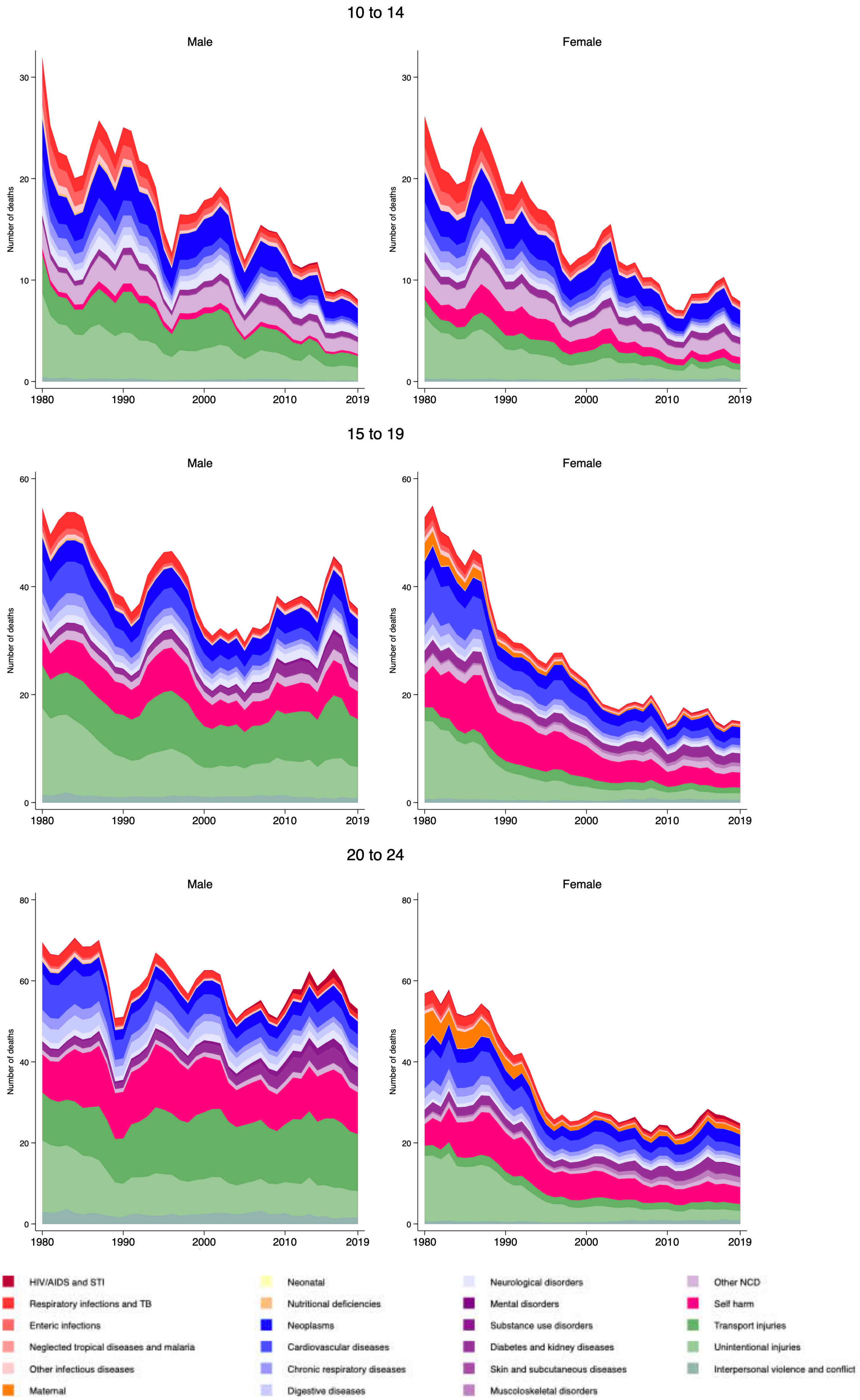

Figure S180: Mozambique

Figure S182a All-cause mortality rate per 100,000 in 10-24 1950 – 2019: Mozambique

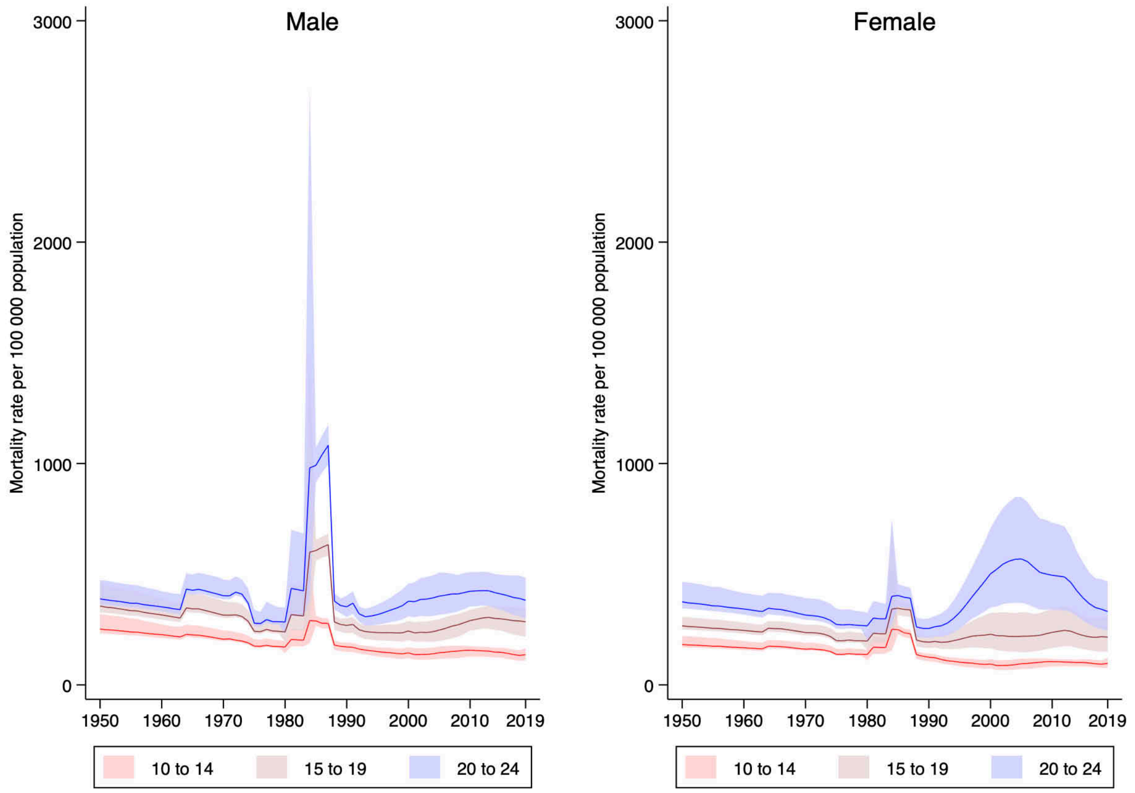

Figure S182b Number of deaths from all causes in 10-24 1950 – 2019: Mozambique

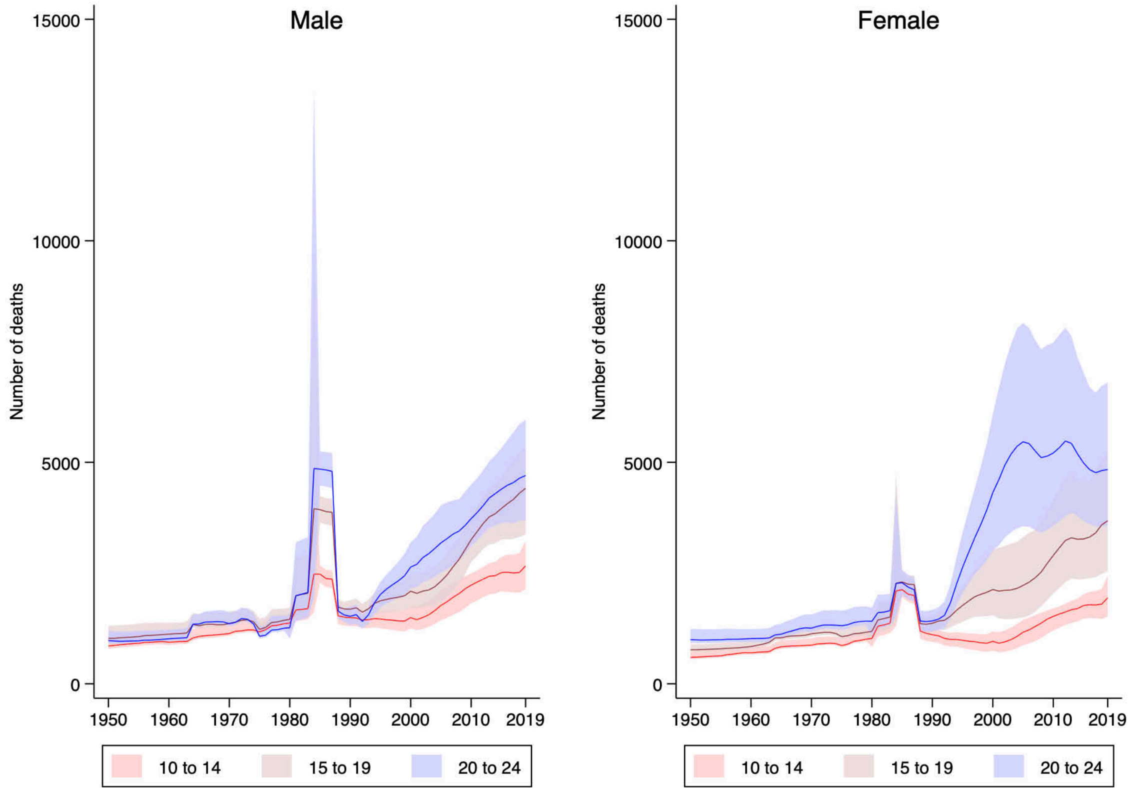

Figure S182c Mortality rate per 100,000 in 10-24 1980 – 2019 by cause: Mozambique

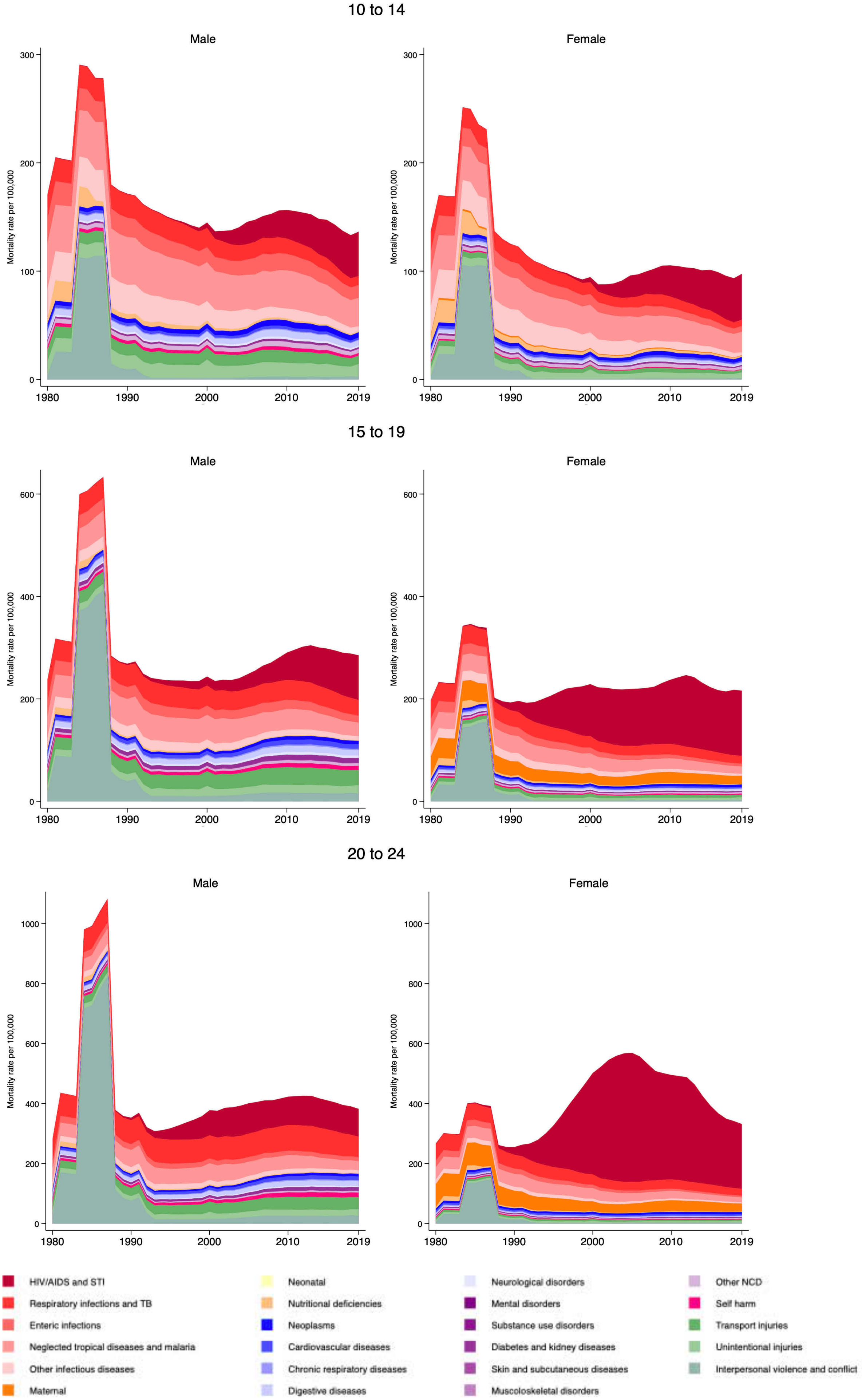

Figure S182d Number of deaths in 10-24 1980 – 2019 by cause: Mozambique

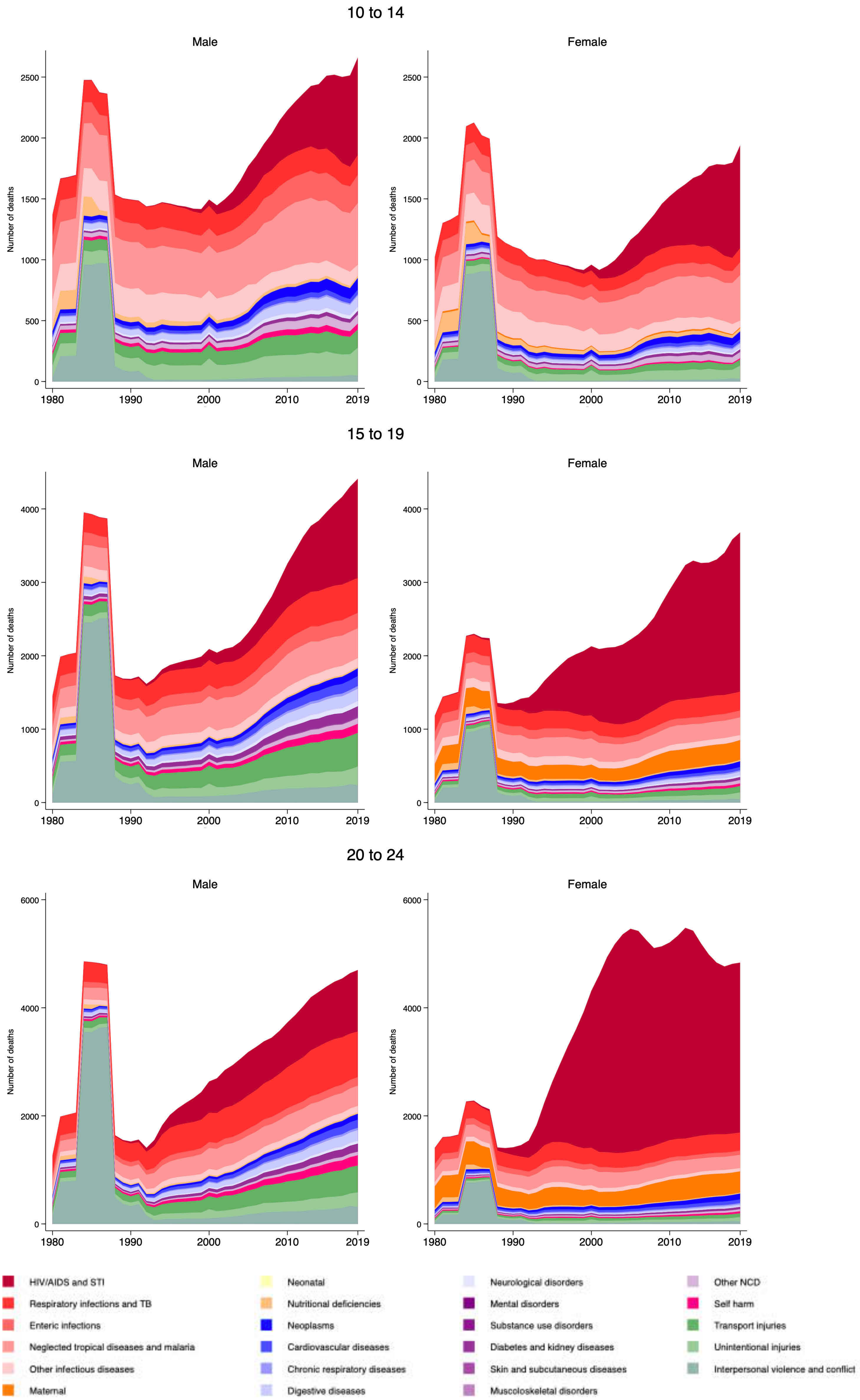

Figure S181: Rwanda

Figure S183a All-cause mortality rate per 100,000 in 10-24 1950 – 2019: Rwanda

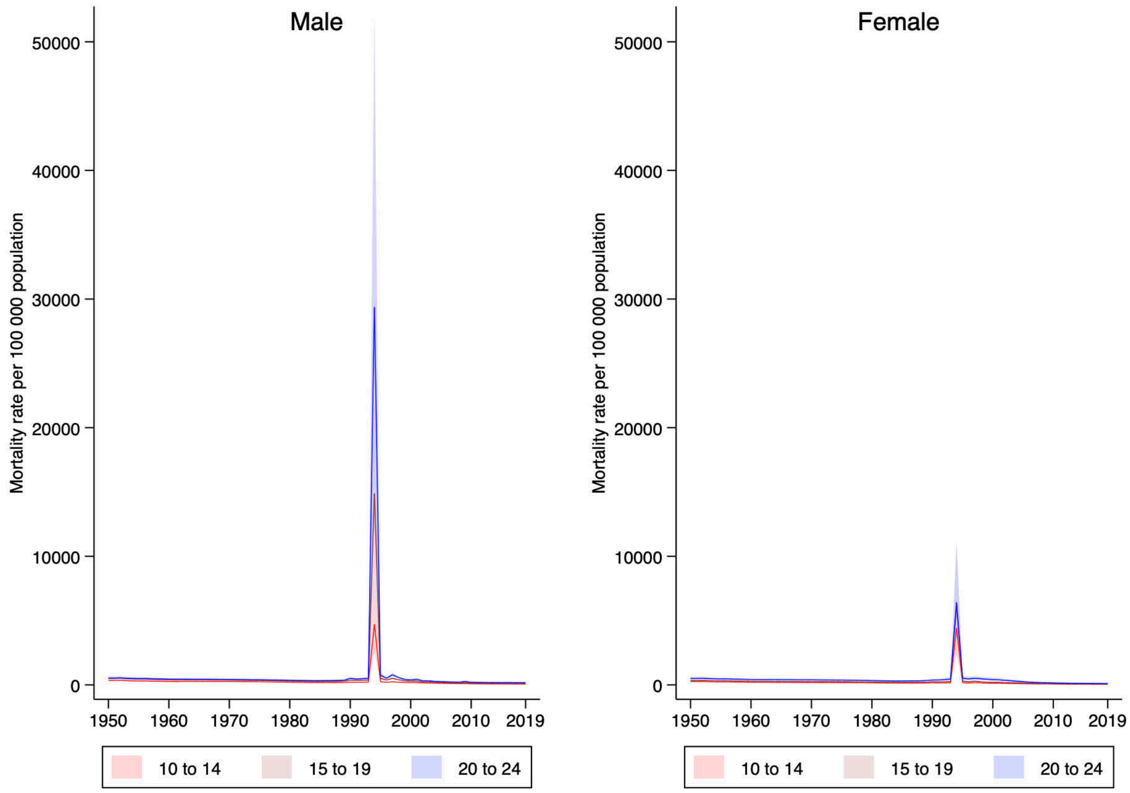

Figure S183b Number of deaths from all causes in 10-24 1950 – 2019: Rwanda

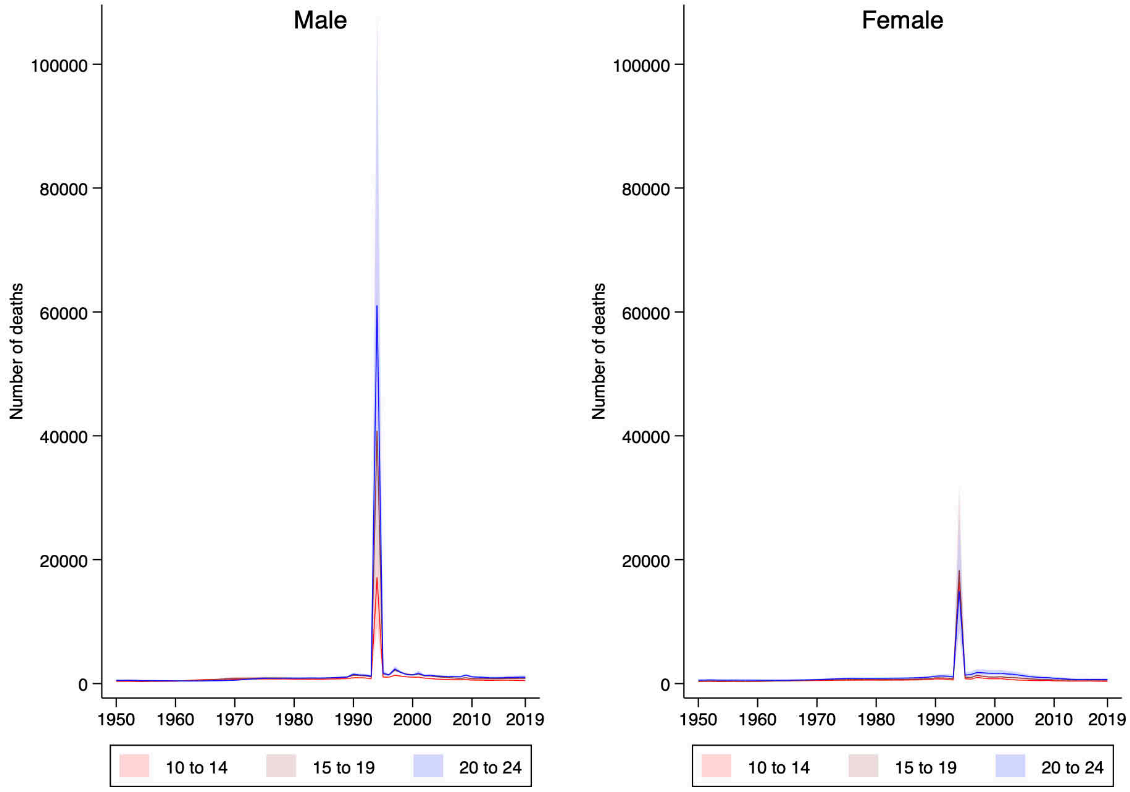

Figure S183c Mortality rate per 100,000 in 10-24 1980 – 2019 by cause: Rwanda

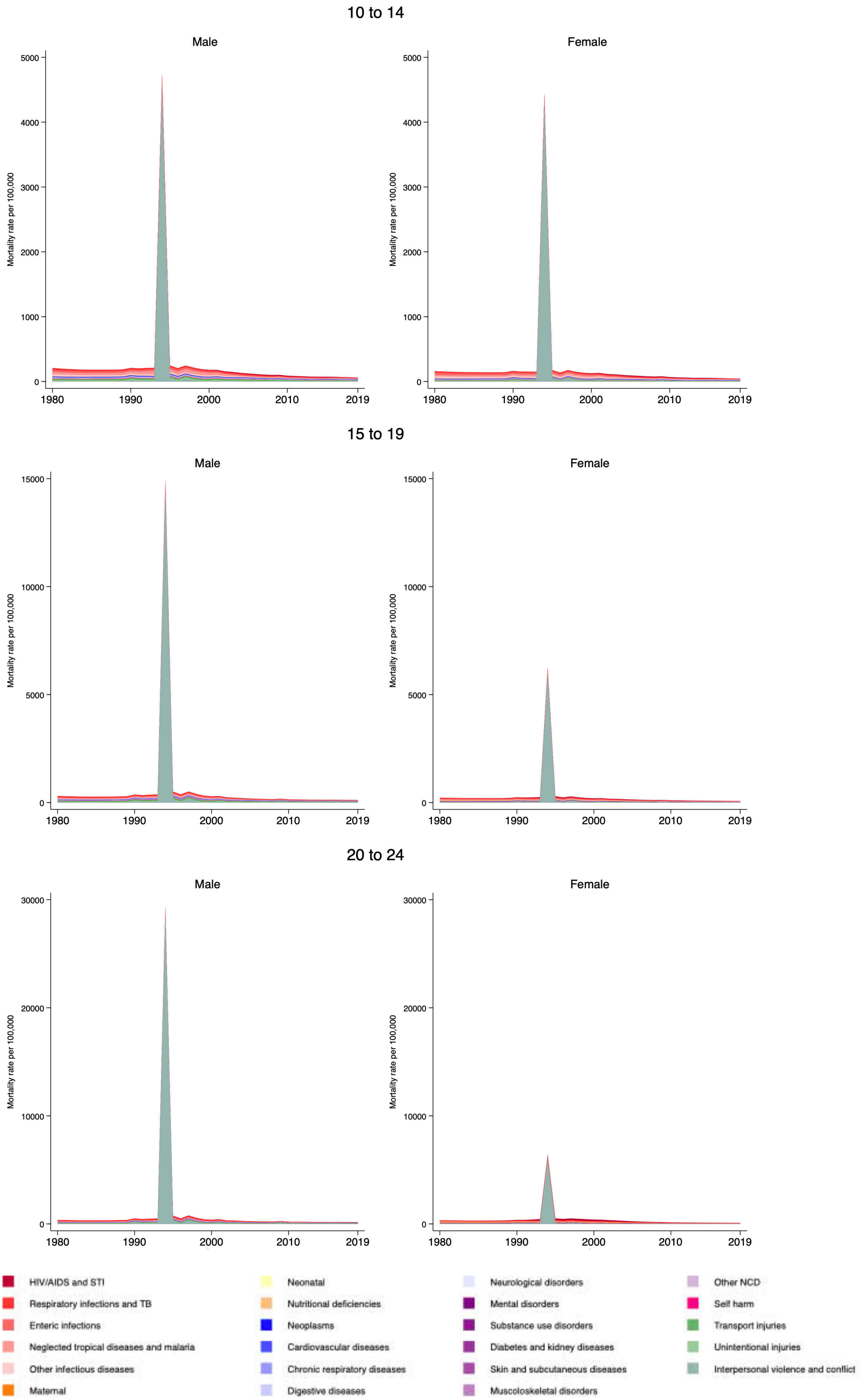

Figure S183d Number of deaths in 10-24 1980 – 2019 by cause: Rwanda

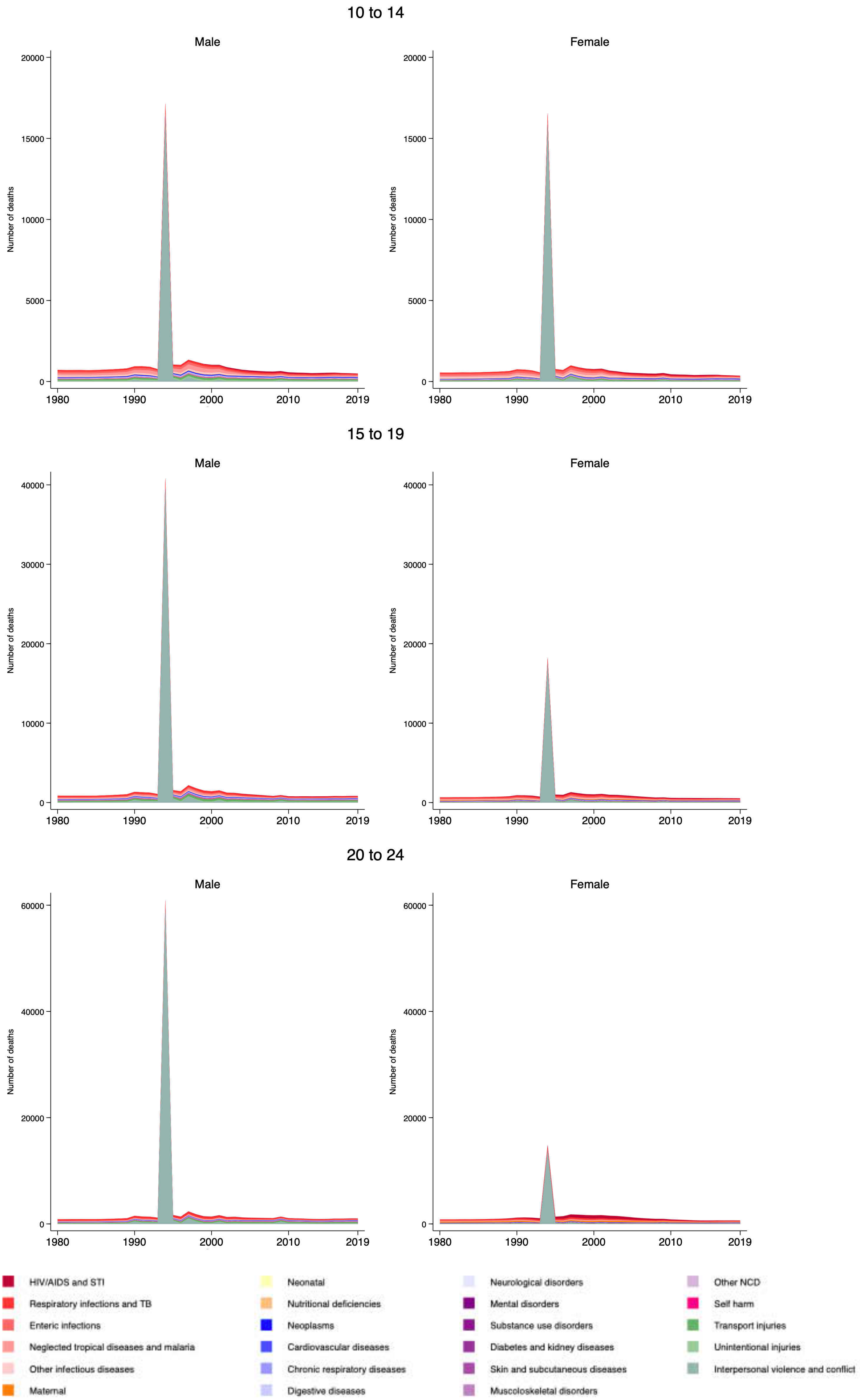

Figure S182: Seychelles

Figure S184a All-cause mortality rate per 100,000 in 10-24 1950 – 2019: Seychelles

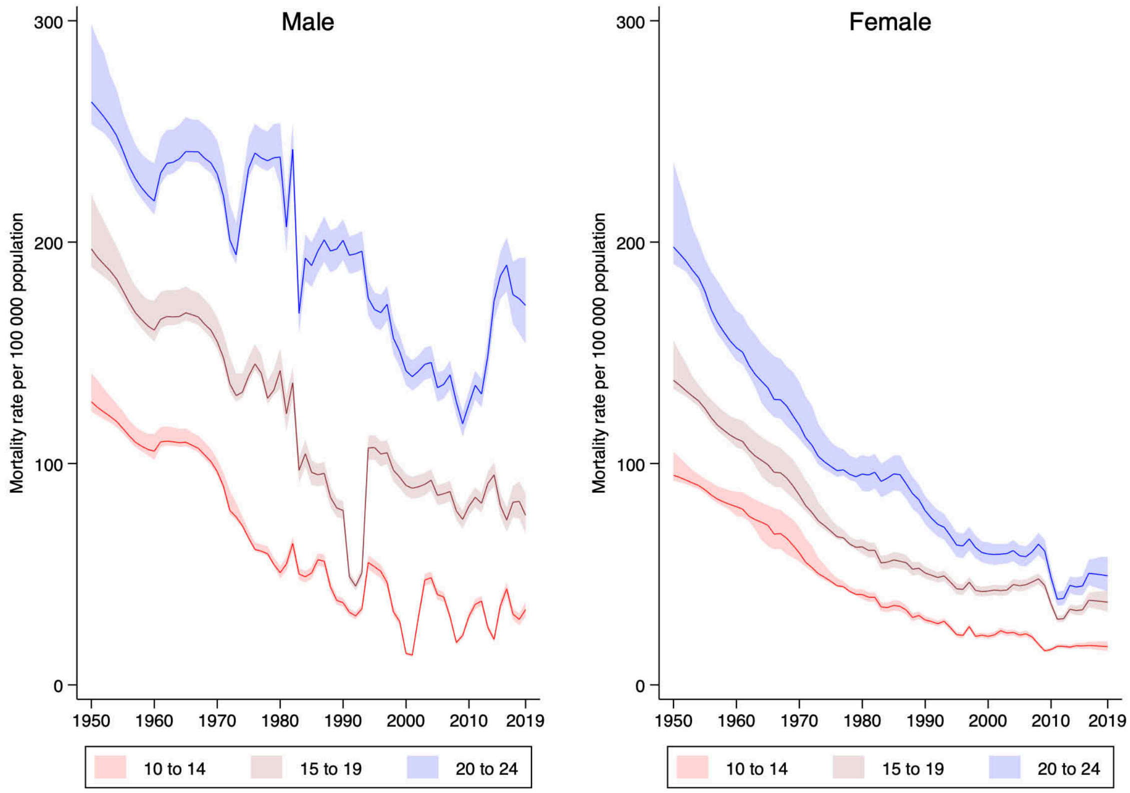

Figure S184b Number of deaths from all causes in 10-24 1950 – 2019: Seychelles

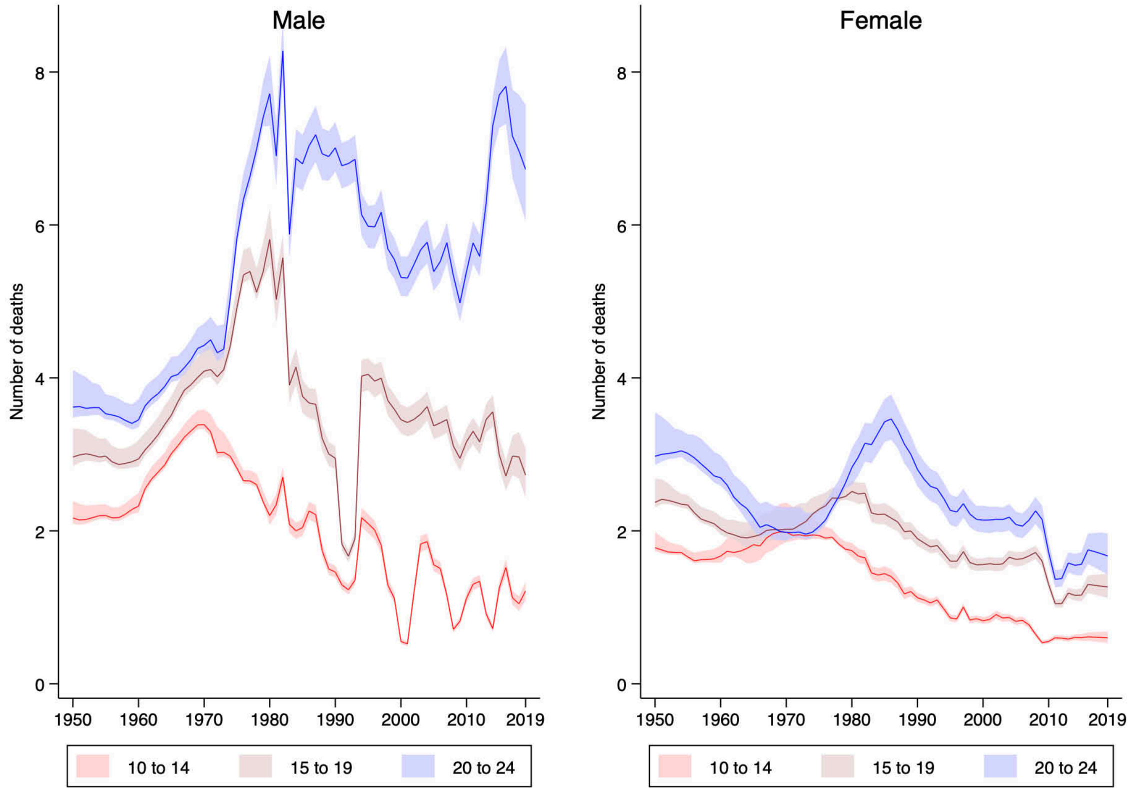

Figure S184c Mortality rate per 100,000 in 10-24 1980 – 2019 by cause: Seychelles

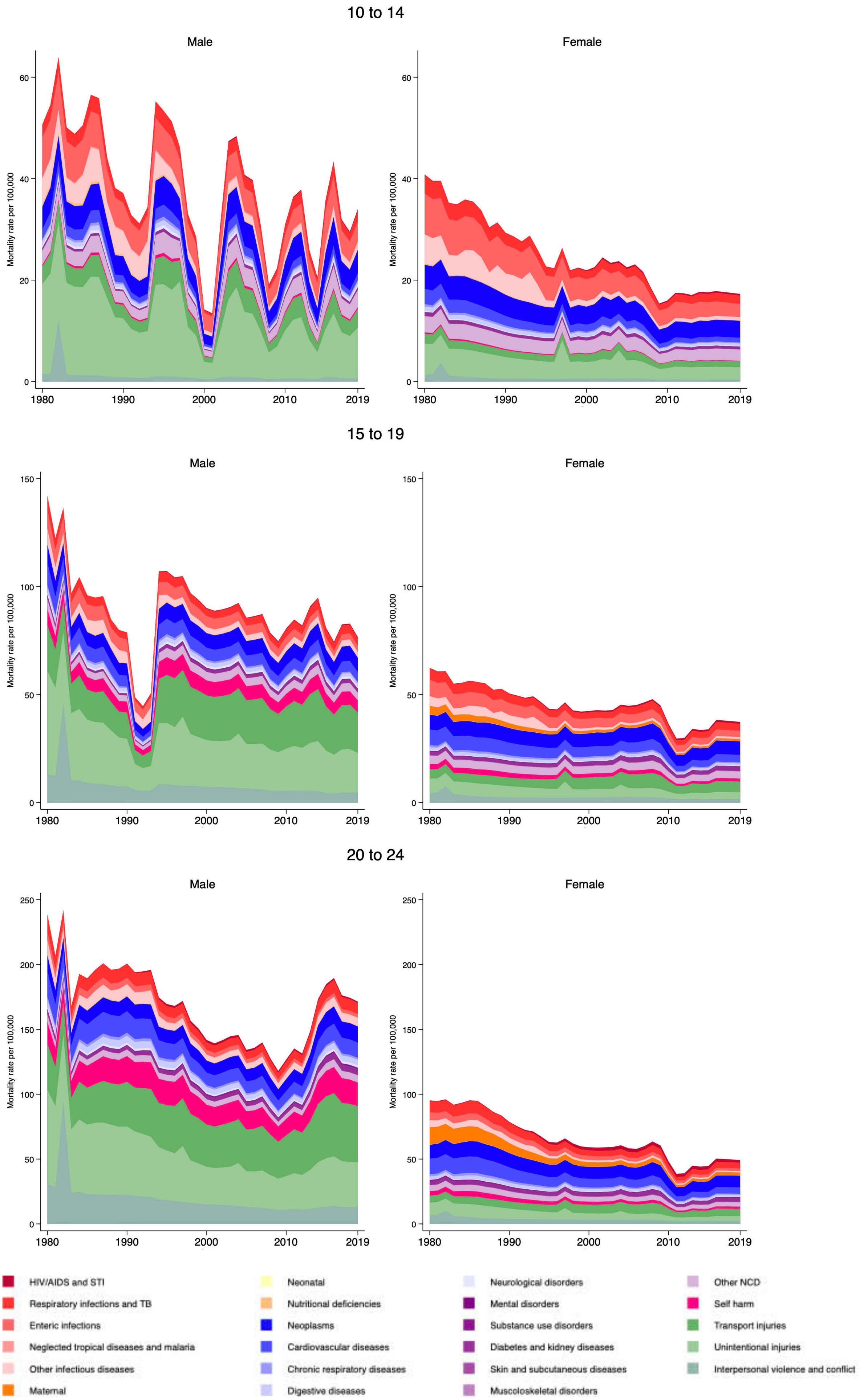

Figure S184d Number of deaths in 10-24 1980 – 2019 by cause: Seychelles

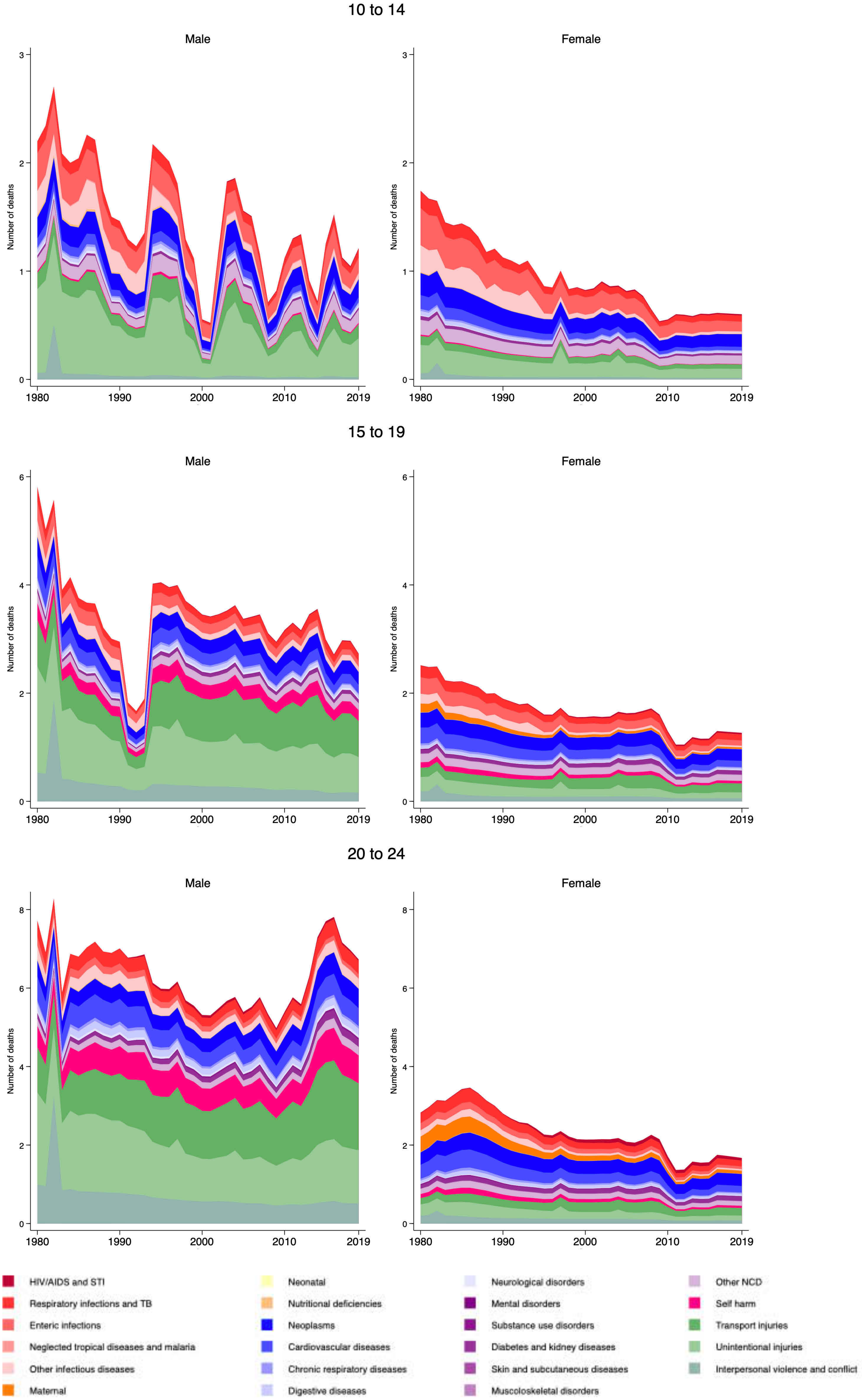

Figure S183: Somalia

Figure S185a All-cause mortality rate per 100,000 in 10-24 1950 – 2019: Somalia

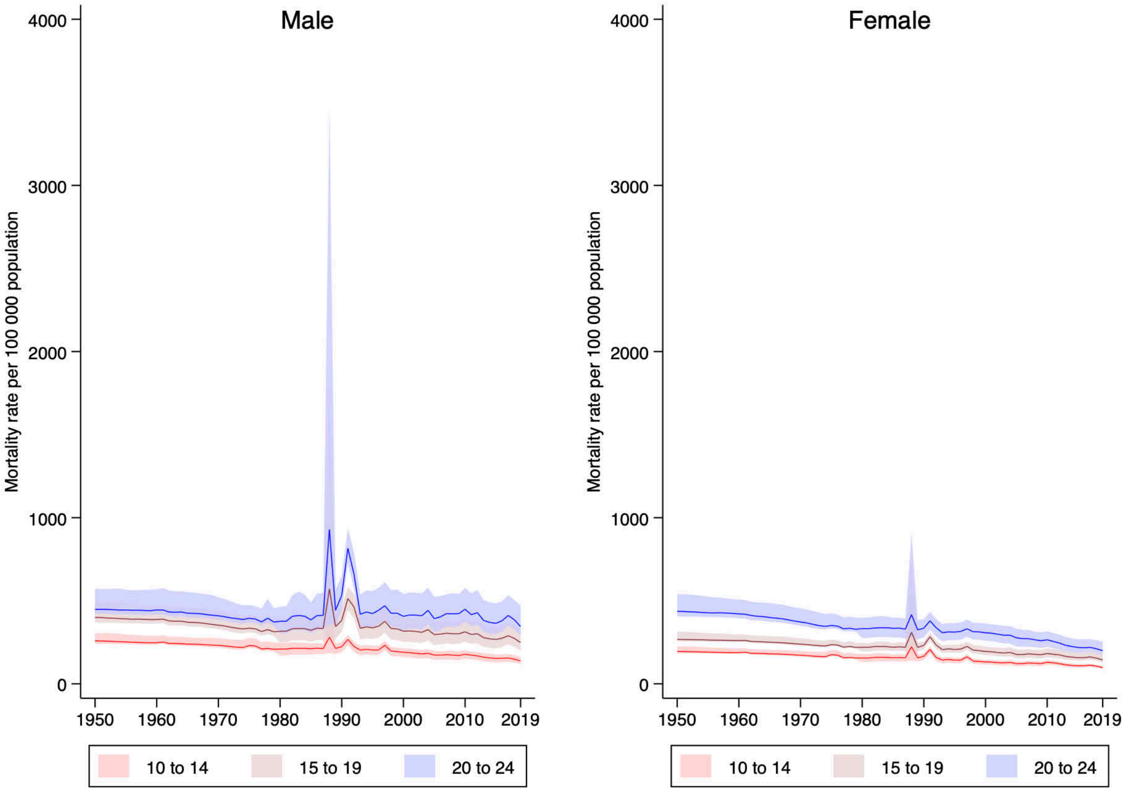

Figure S185b Number of deaths from all causes in 10-24 1950 – 2019: Somalia

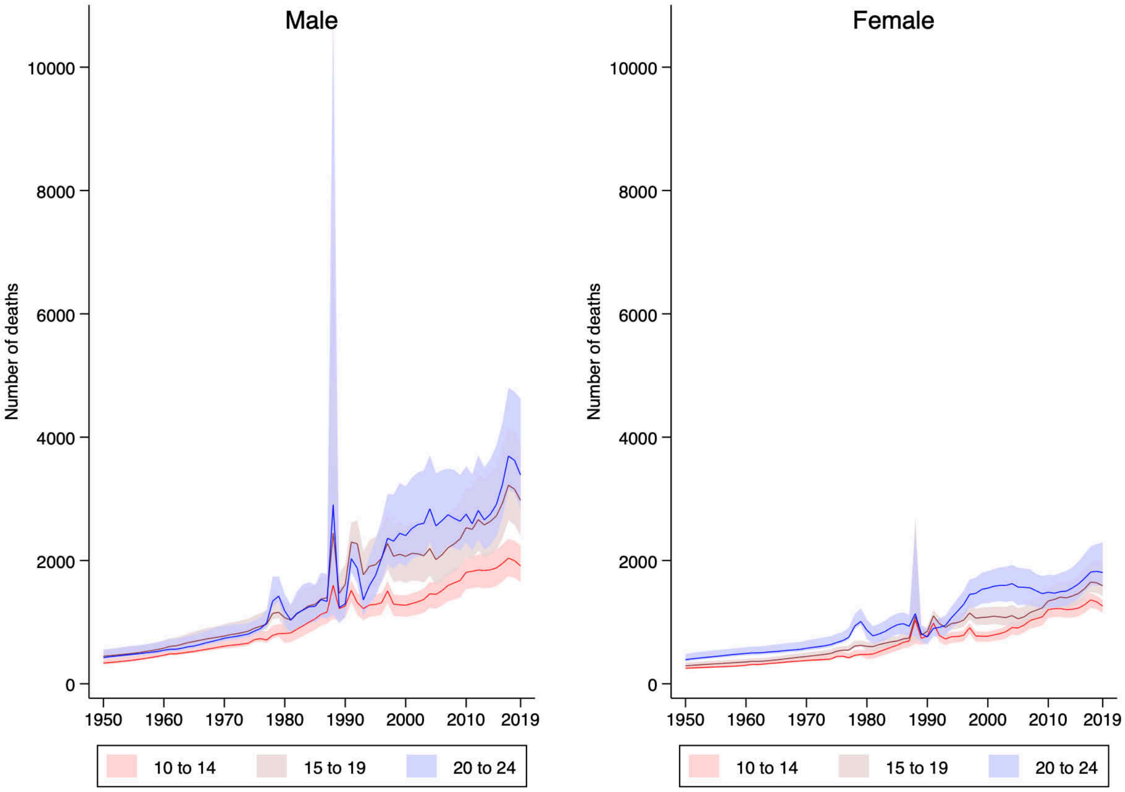

Figure S185c Mortality rate per 100,000 in 10-24 1980 – 2019 by cause: Somalia

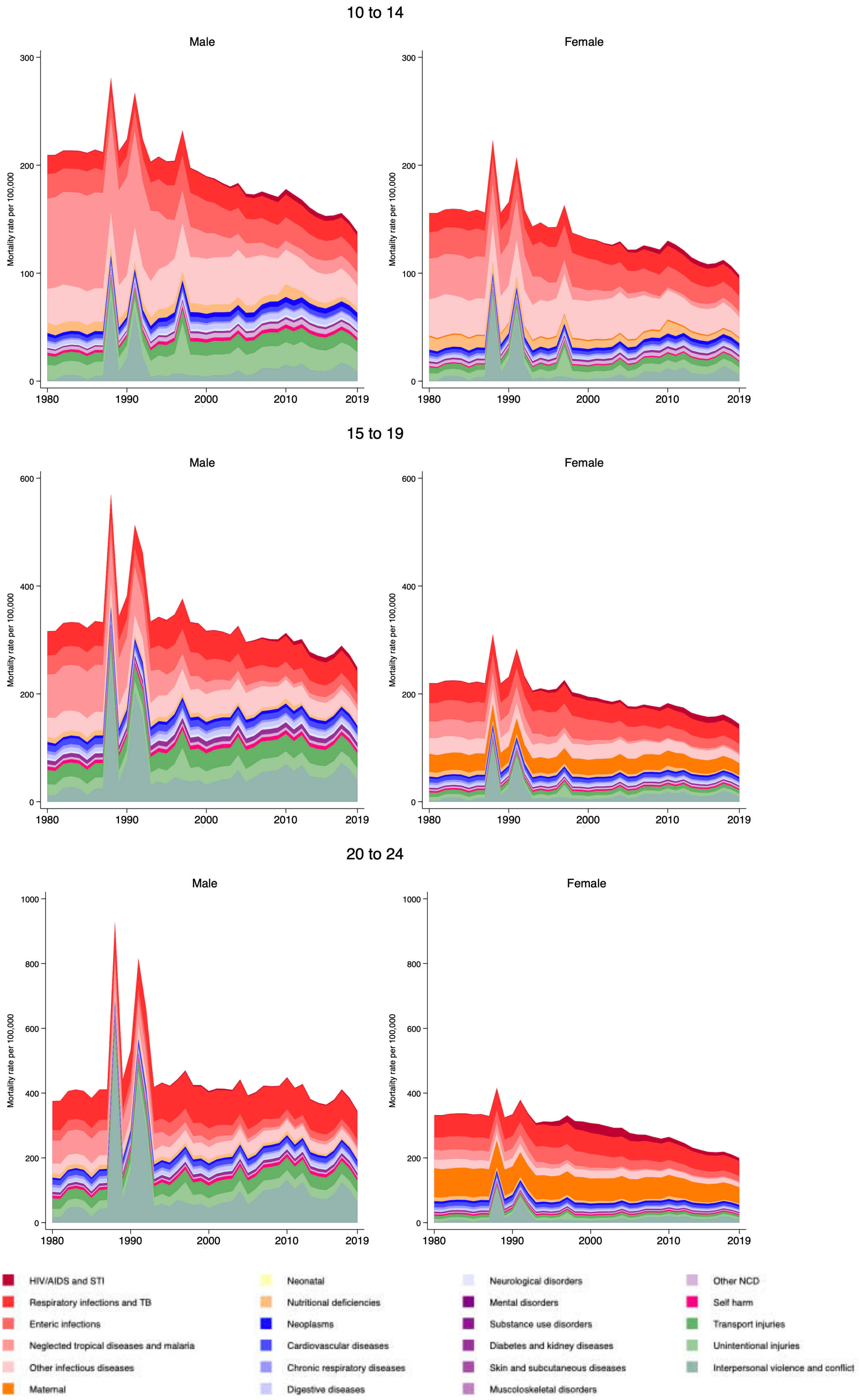

Figure S185d Number of deaths in 10-24 1980 – 2019 by cause: Somalia

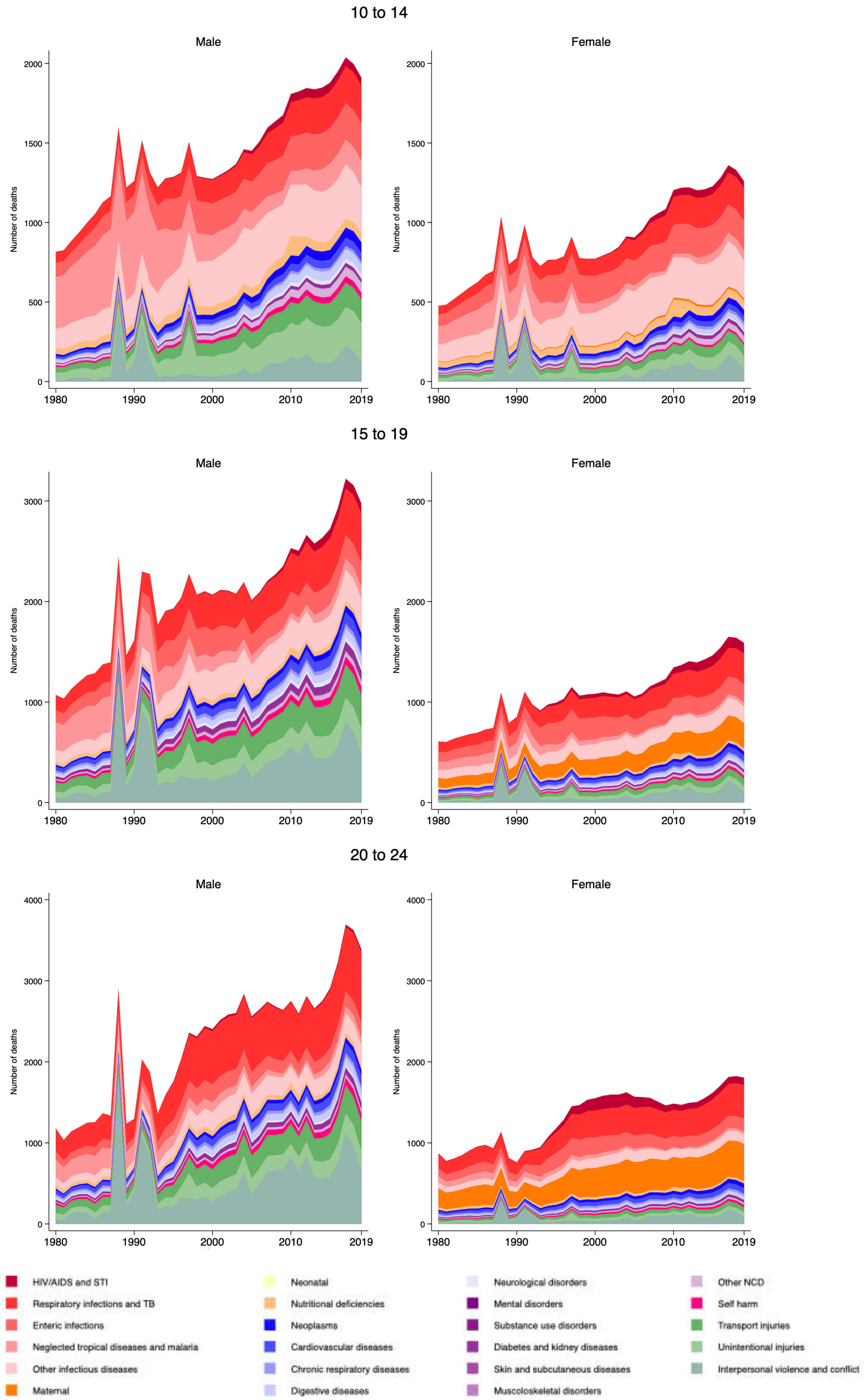

Figure S184: United Republic of Tanzania

Figure S186a All-cause mortality rate per 100,000 in 10-24 1950 – 2019: United Republic of Tanzania

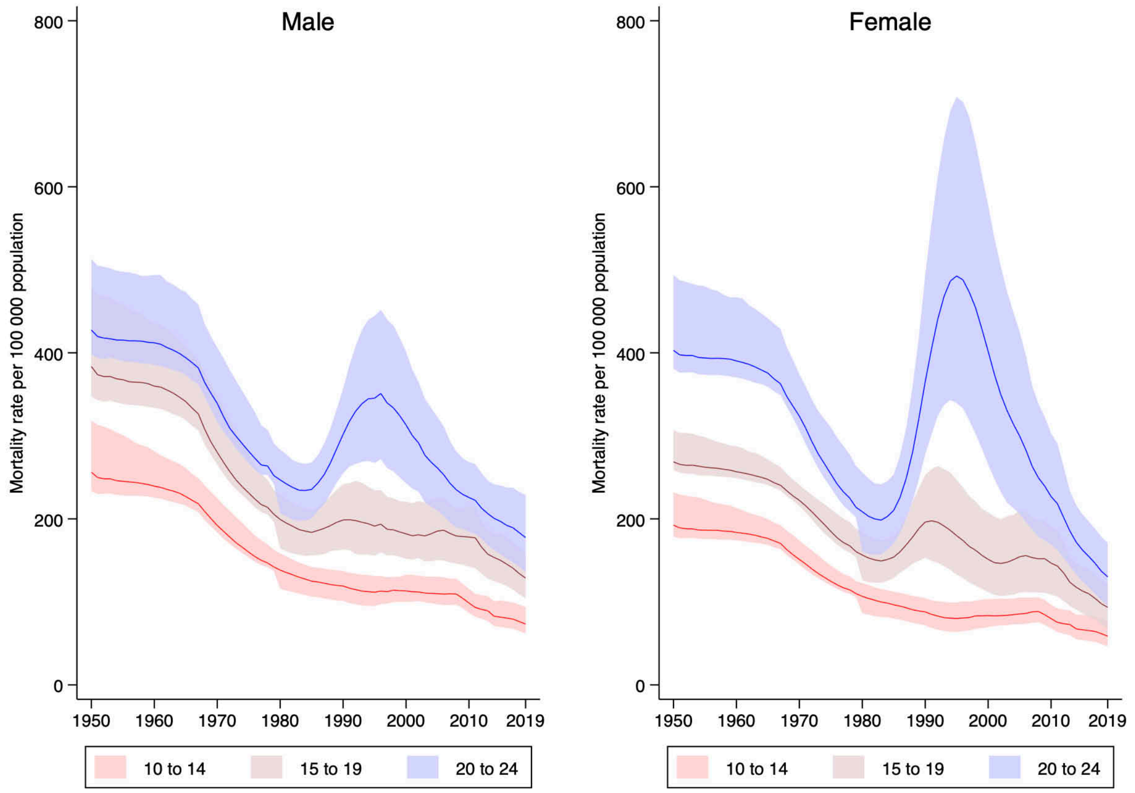

Figure S186b Number of deaths from all causes in 10-24 1950 – 2019: United Republic of Tanzania

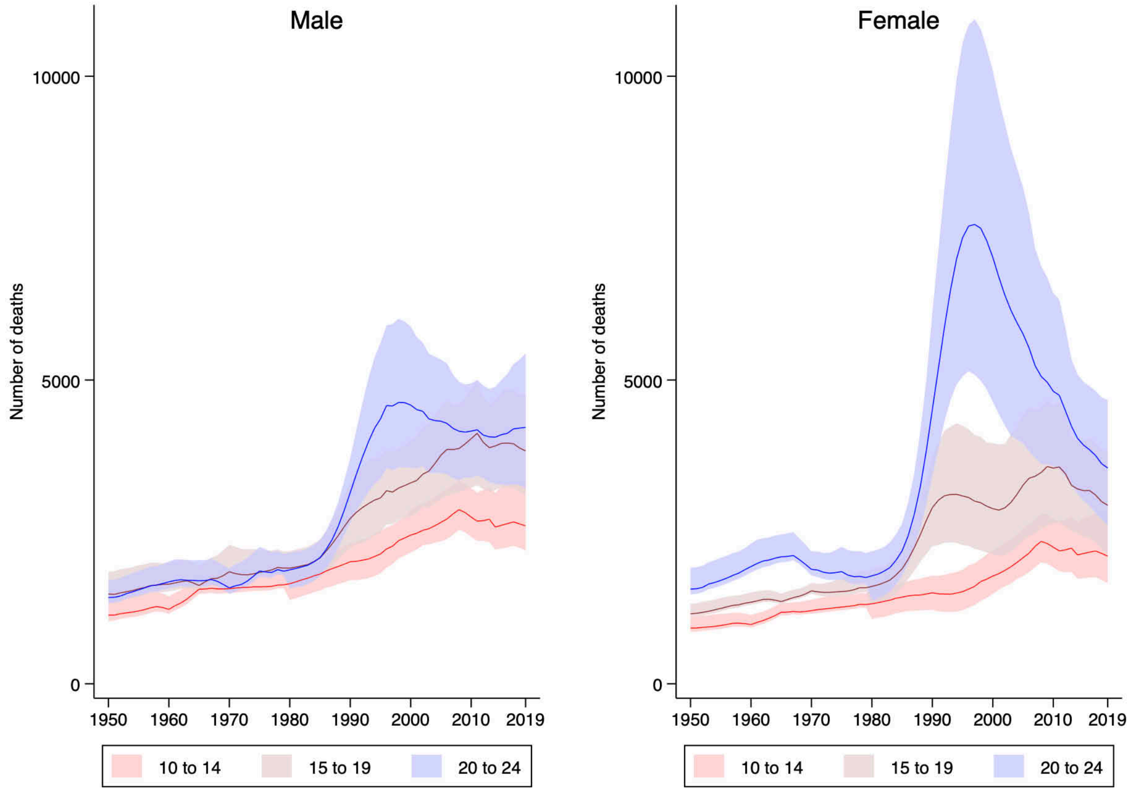

Figure S186c Mortality rate per 100,000 in 10-24 1980 – 2019 by cause: United Republic of Tanzania

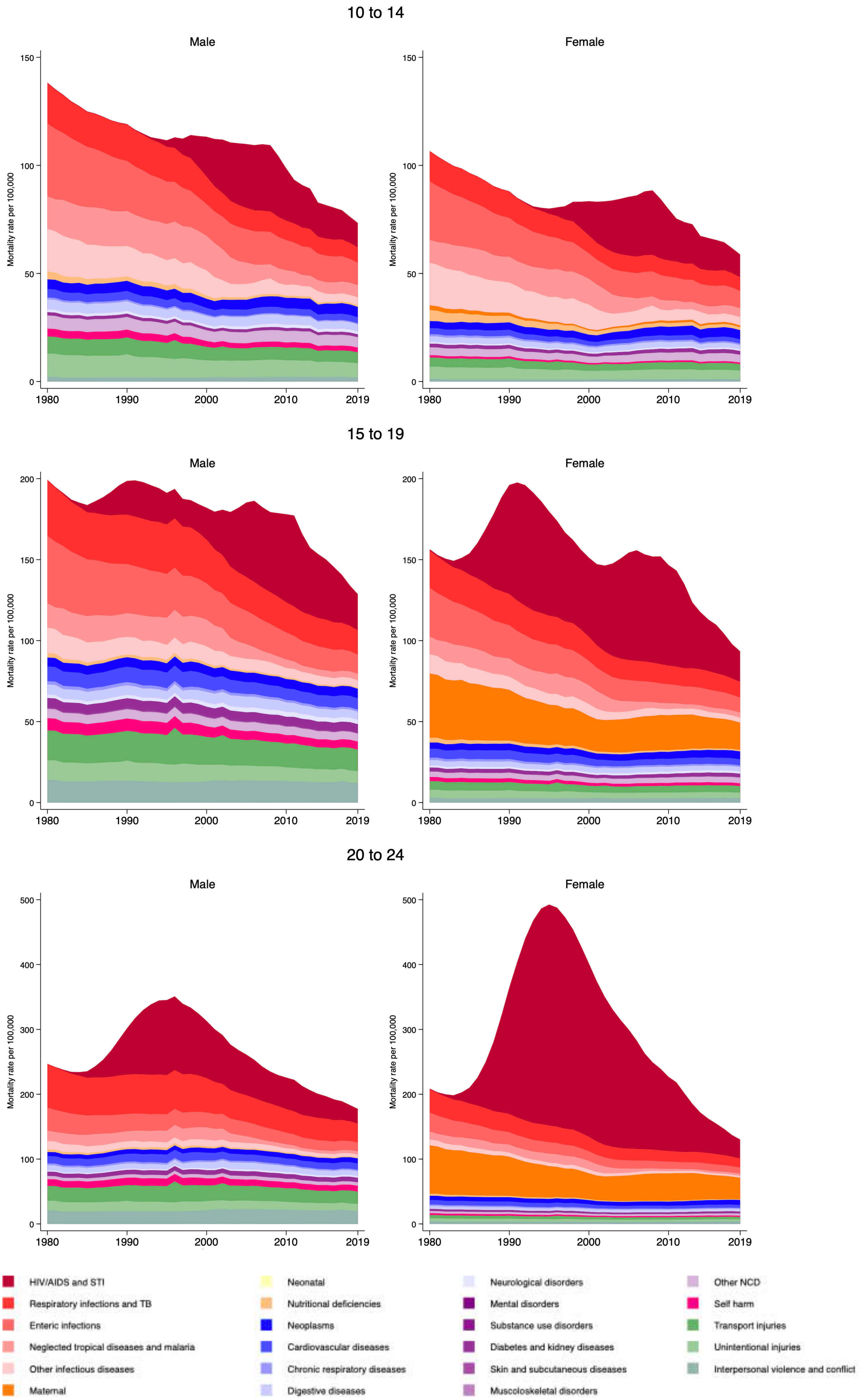

Figure S186d Number of deaths in 10-24 1980 – 2019 by cause: United Republic of Tanzania

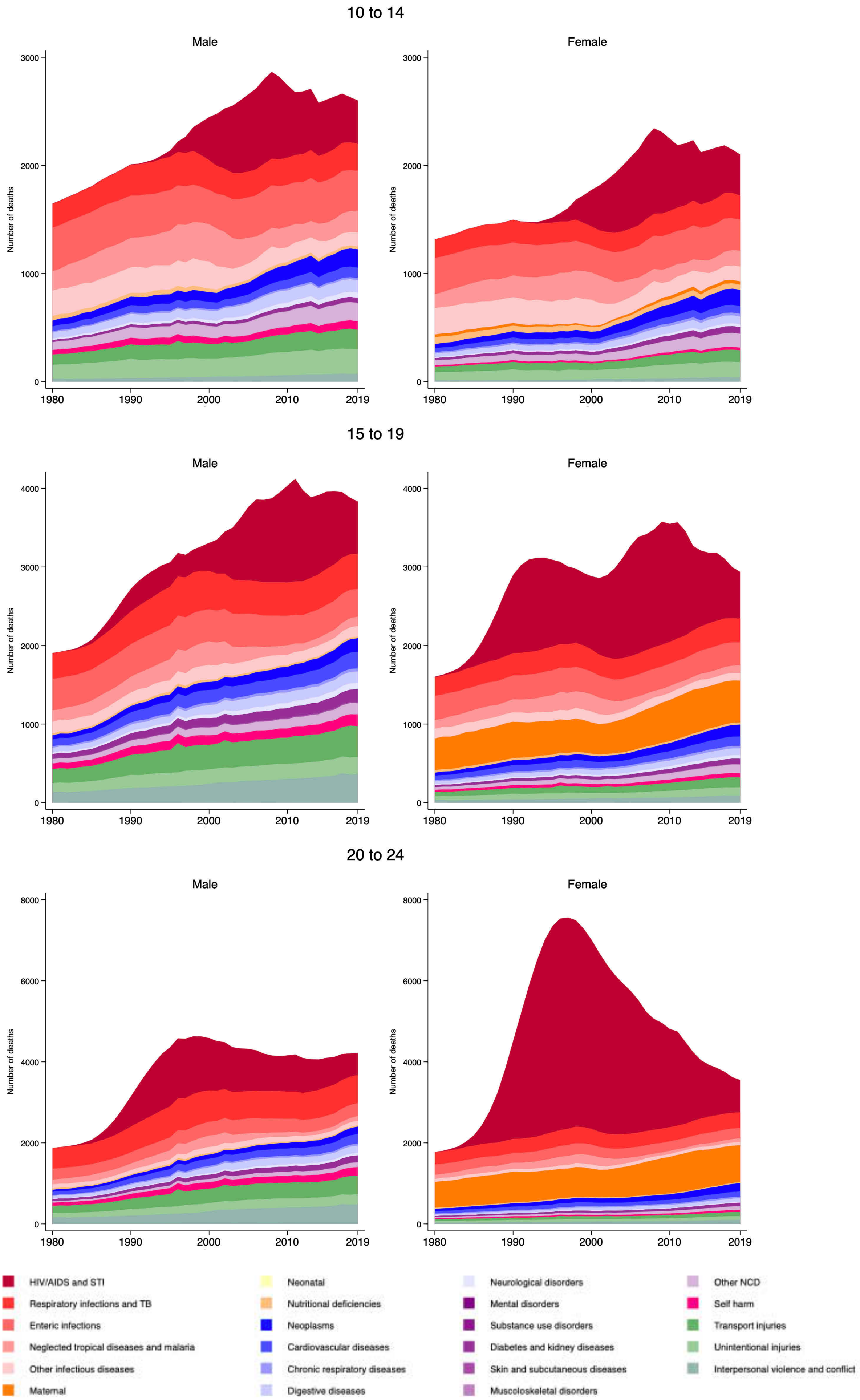

Figure S185: Uganda

Figure S187a All-cause mortality rate per 100,000 in 10-24 1950 – 2019: Uganda

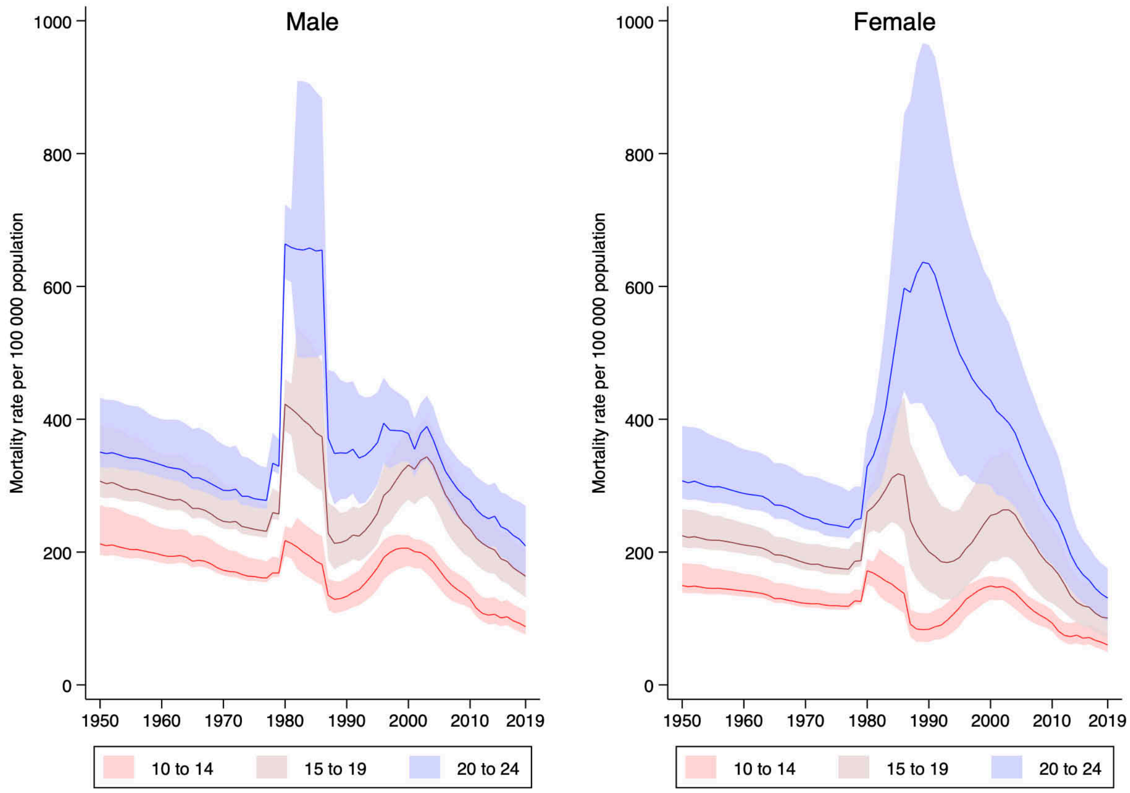

Figure S187b Number of deaths from all causes in 10-24 1950 – 2019: Uganda

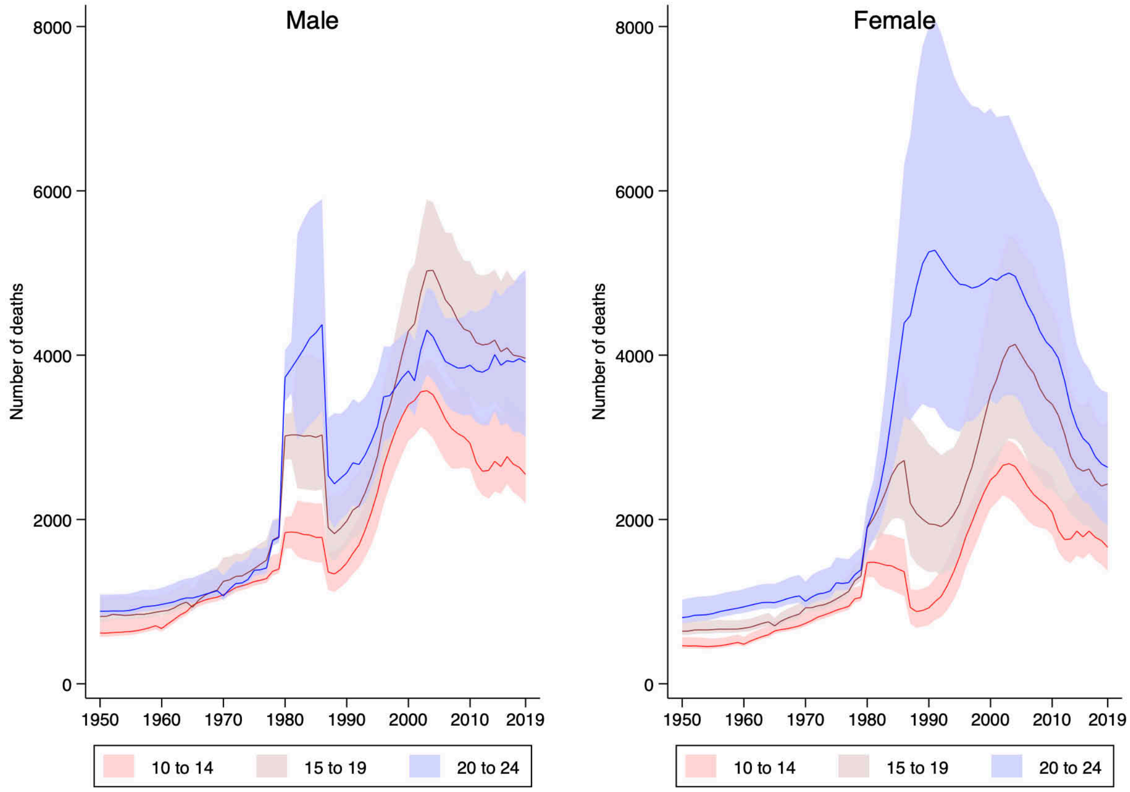

Figure S187c Mortality rate per 100,000 in 10-24 1980 – 2019 by cause: Uganda

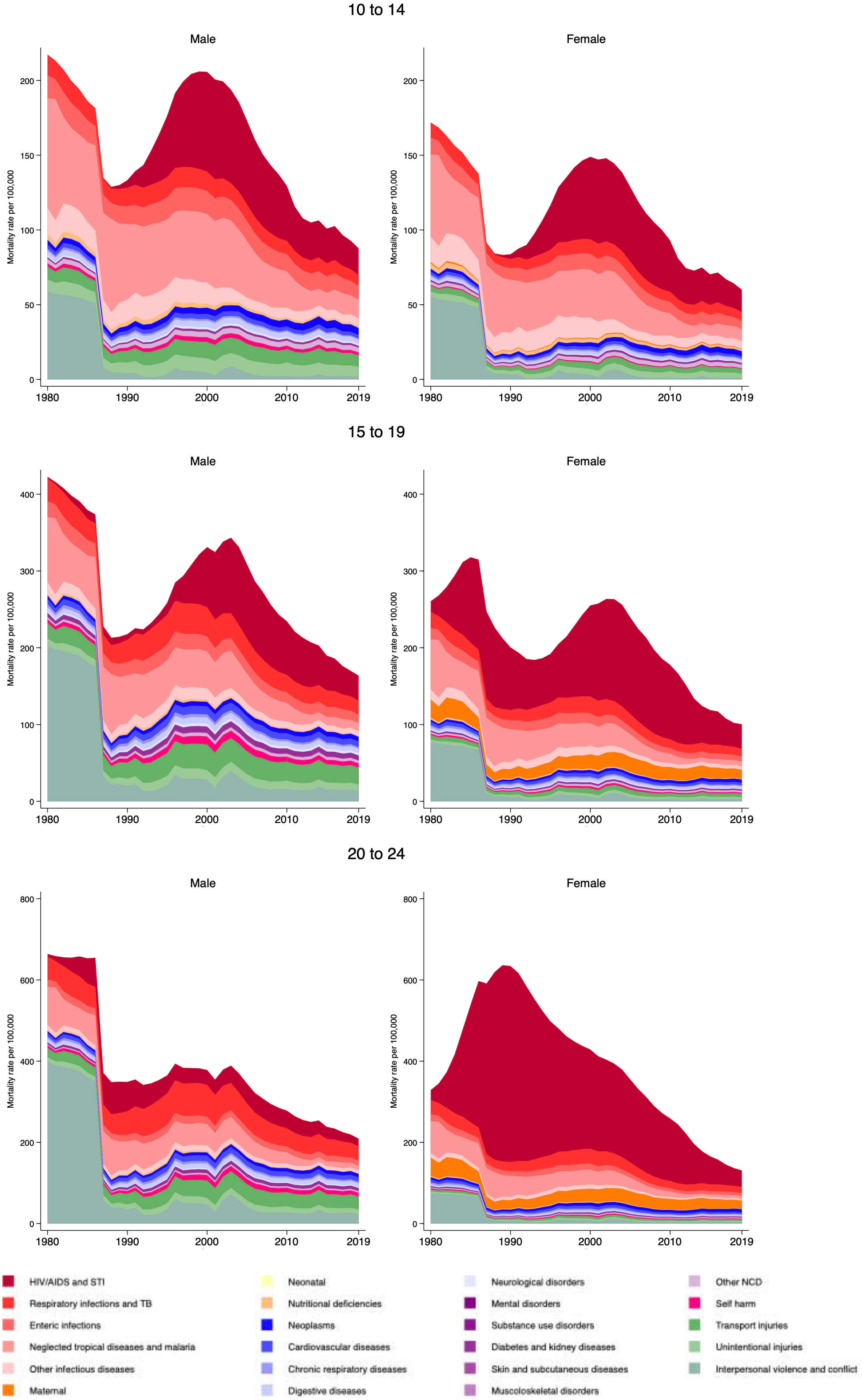

Figure S187d Number of deaths in 10-24 1980 – 2019 by cause: Uganda

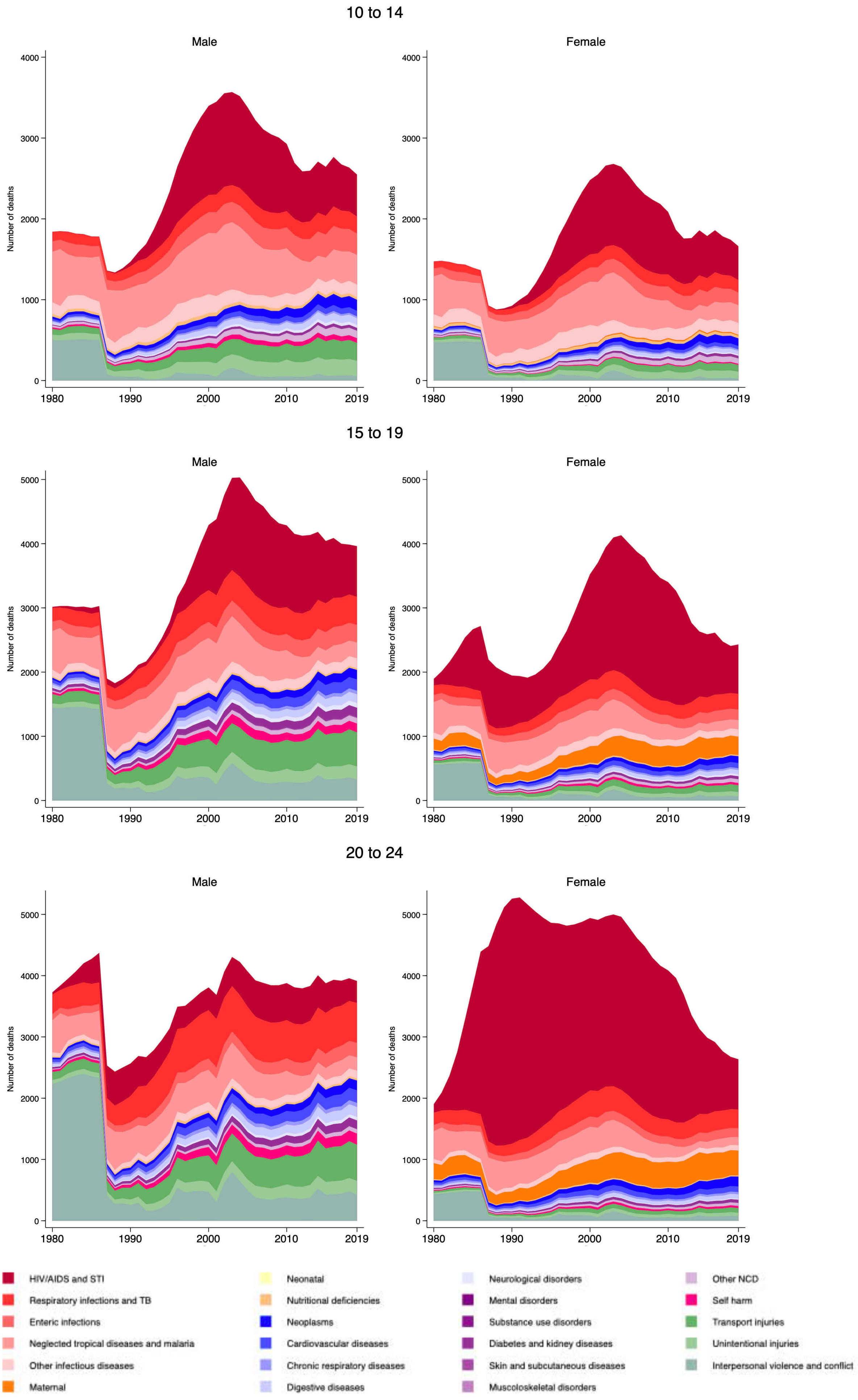

Figure S186: Zambia

Figure S188a All-cause mortality rate per 100,000 in 10-24 1950 – 2019: Zambia

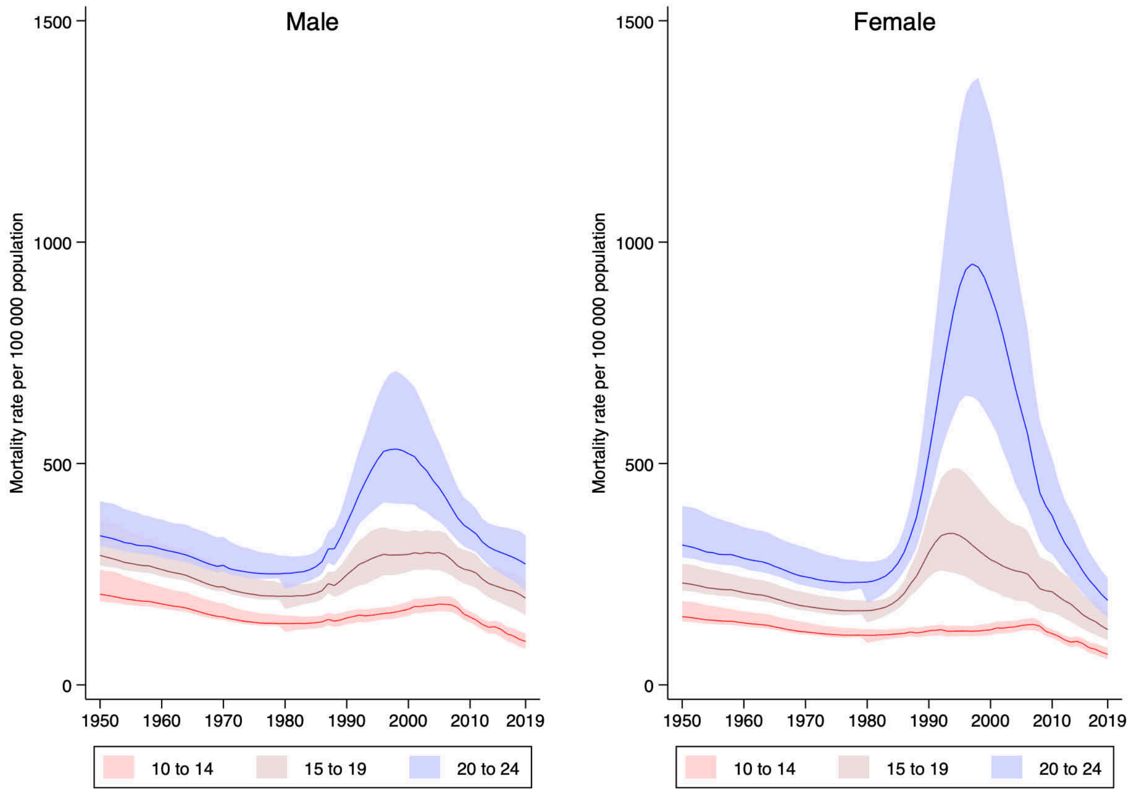

Figure S188b Number of deaths from all causes in 10-24 1950 – 2019: Zambia

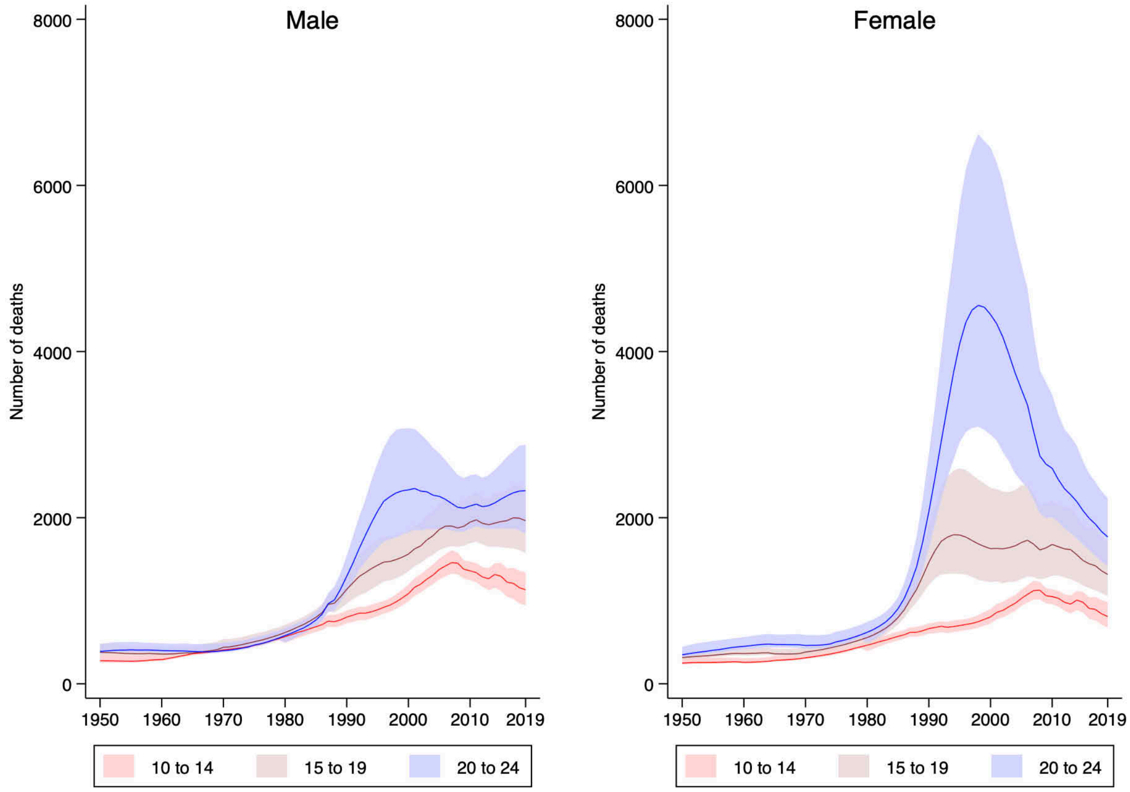

Figure S188c Mortality rate per 100,000 in 10-24 1980 – 2019 by cause: Zambia

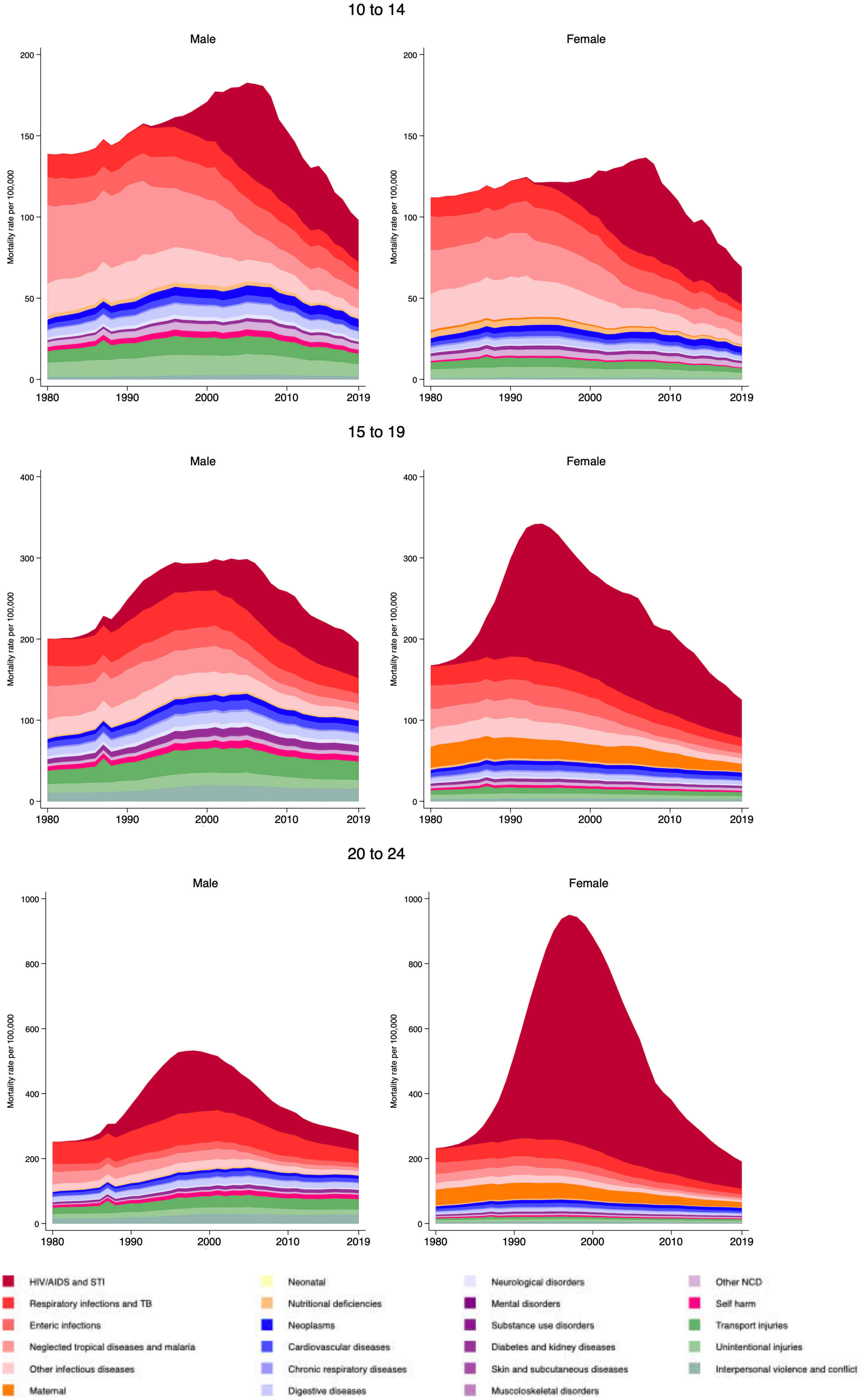

Figure S188d Number of deaths in 10-24 1980 – 2019 by cause: Zambia

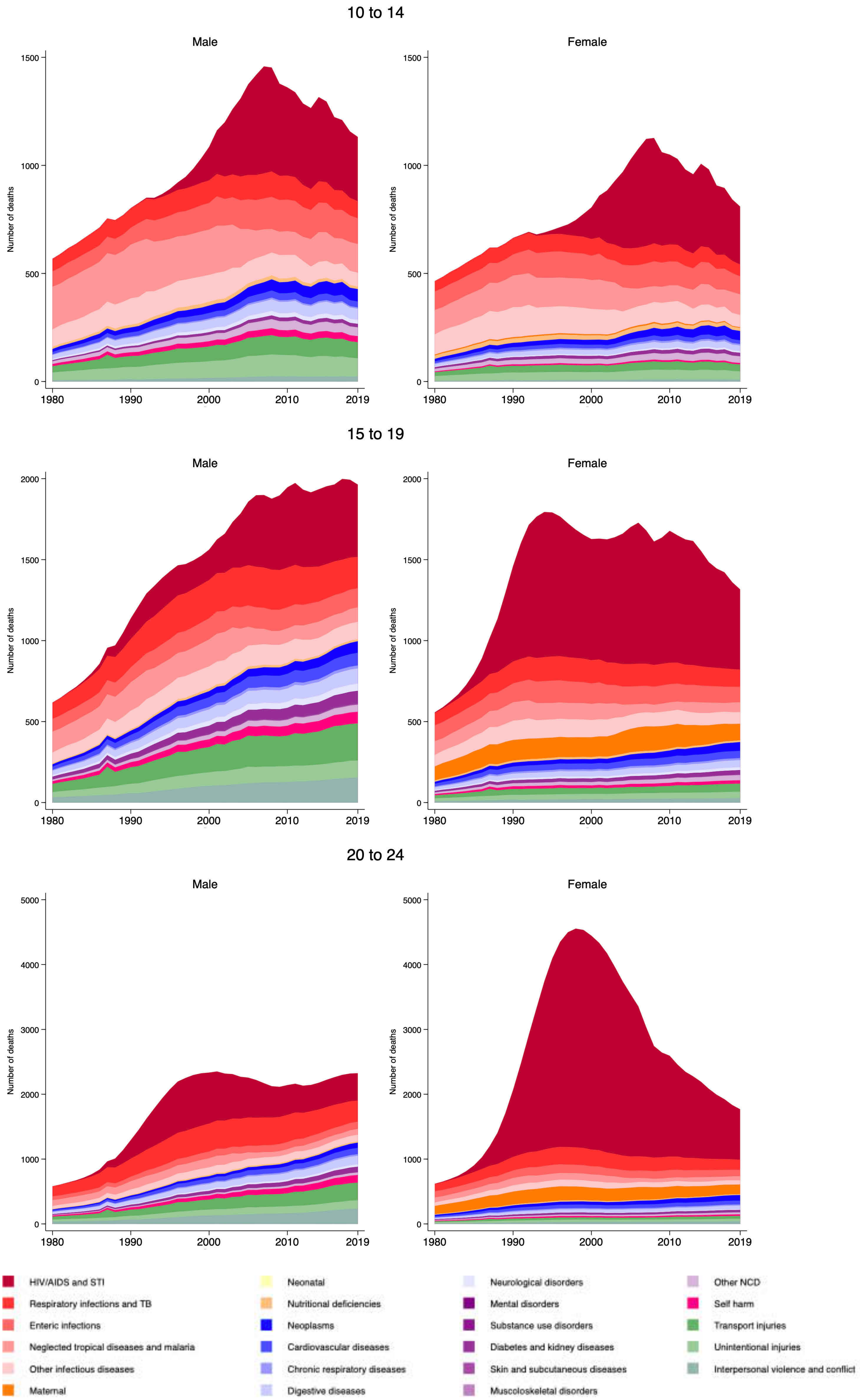

Figure S187: Southern Sub-Saharan Africa

Figure S189a All-cause mortality rate per 100,000 in 10-24 1950 – 2019: Southern Sub-Saharan Africa

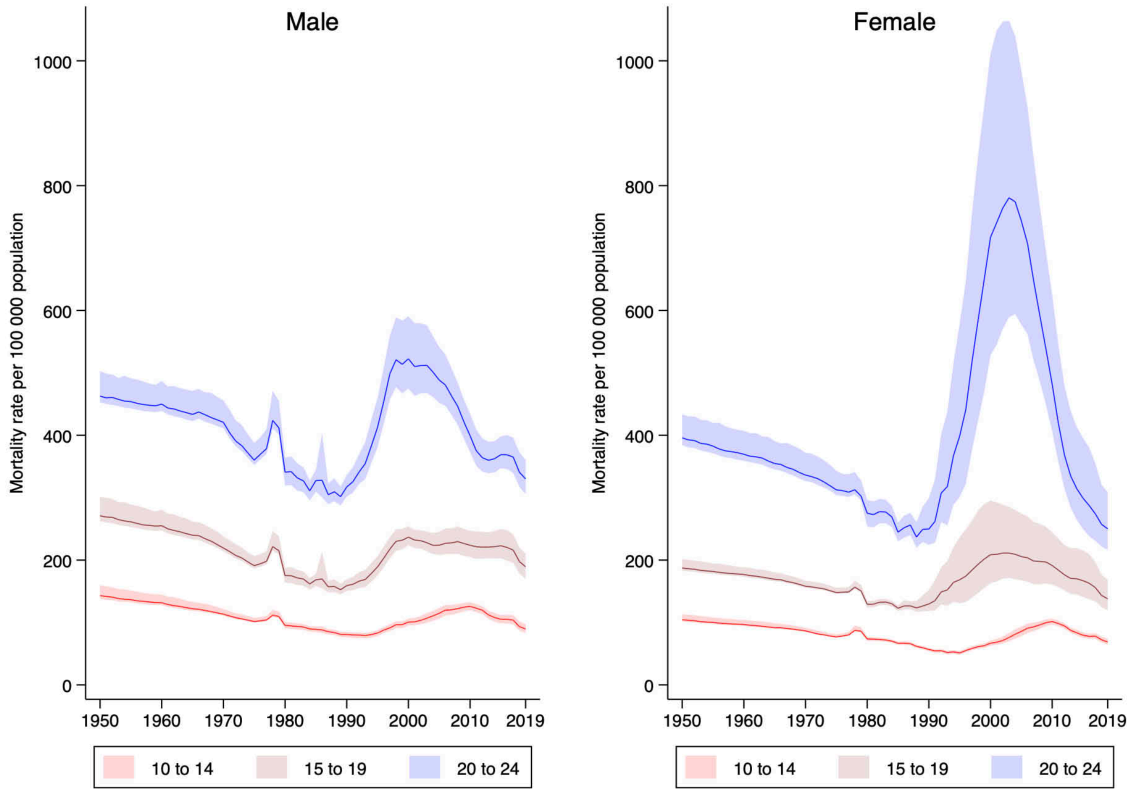

Figure S189b Number of deaths from all causes in 10-24 1950 – 2019: Southern Sub-Saharan Africa

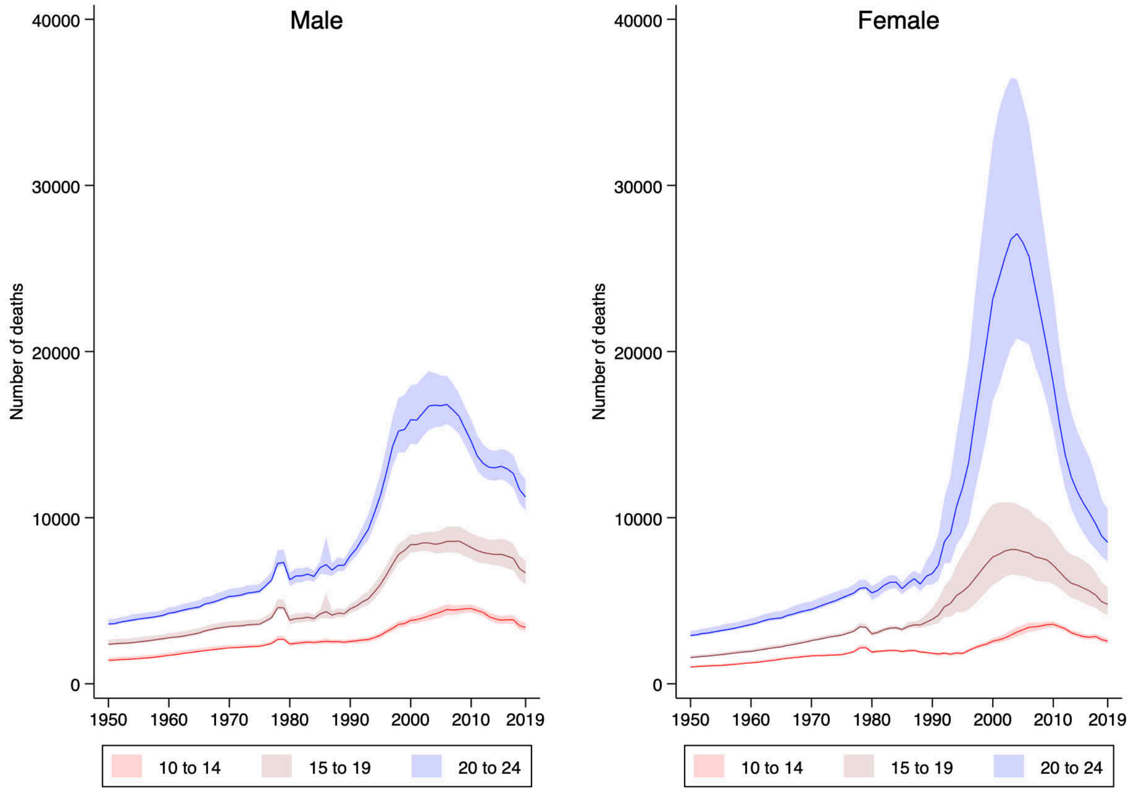

Figure S189c Mortality rate per 100,000 in 10-24 1980 – 2019 by cause: Southern Sub-Saharan Africa

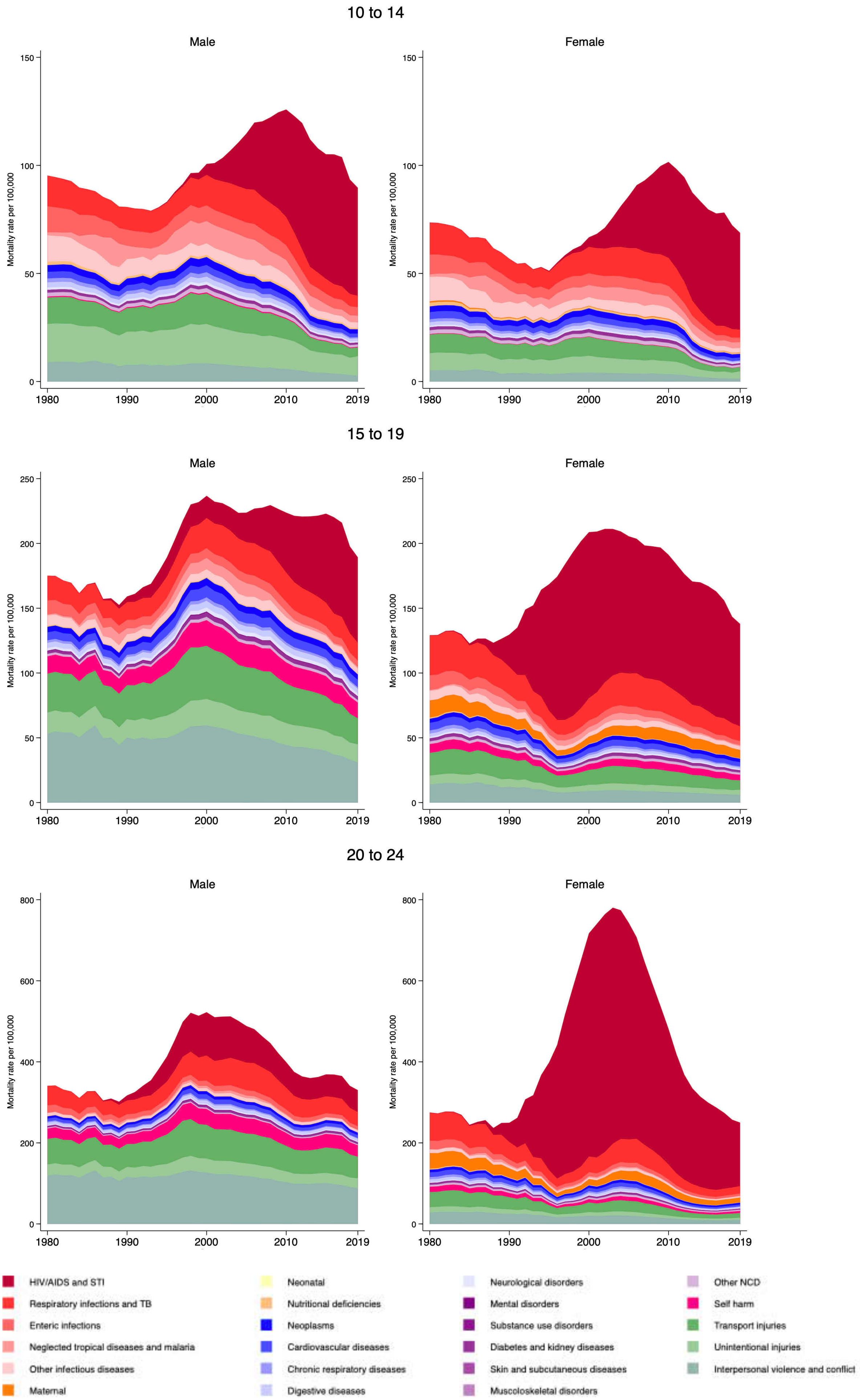

Figure S189d Number of deaths in 10-24 1980 – 2019 by cause: Southern Sub-Saharan Africa

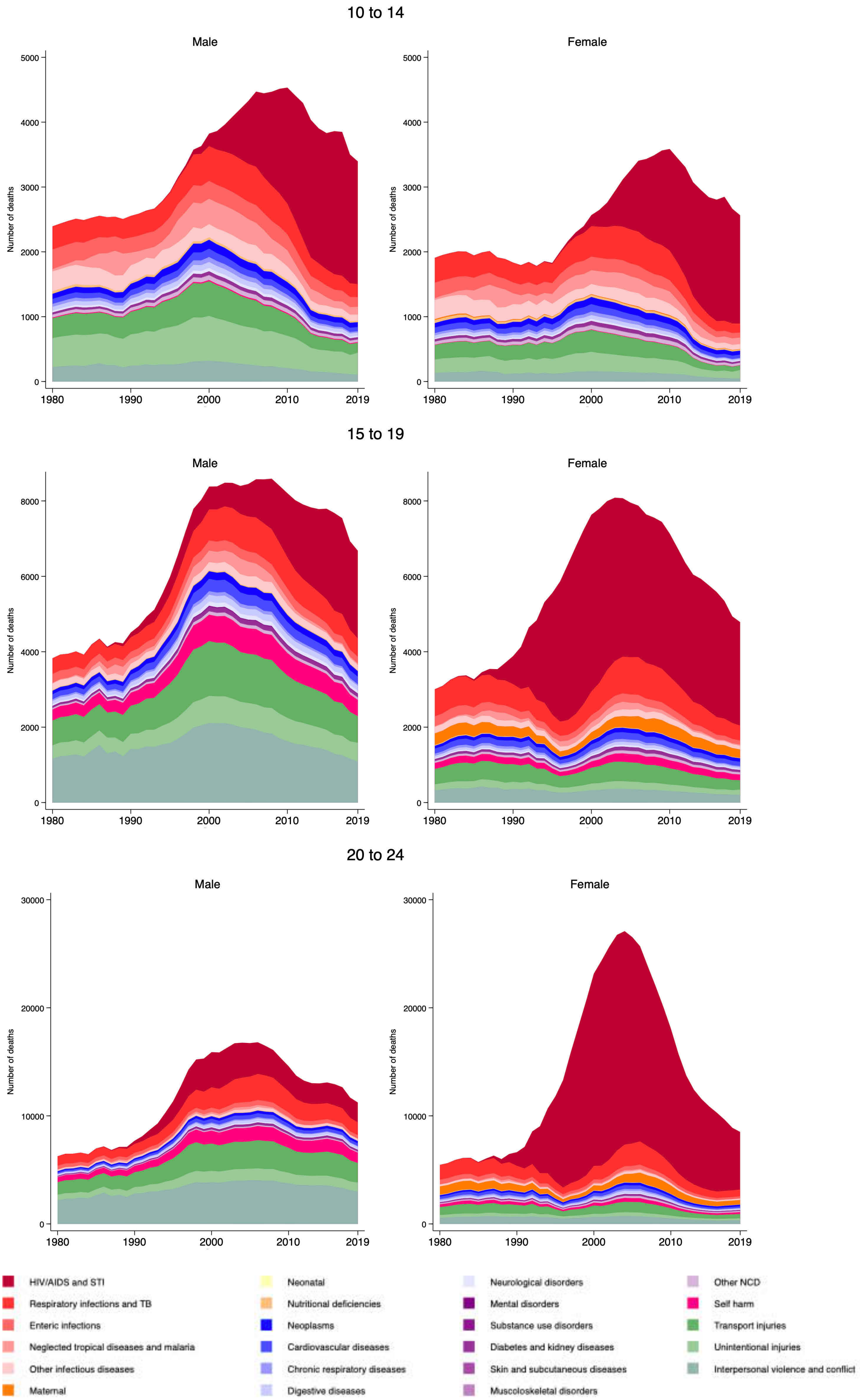

Figure S188: Botswana

Figure S190a All-cause mortality rate per 100,000 in 10-24 1950 – 2019: Botswana

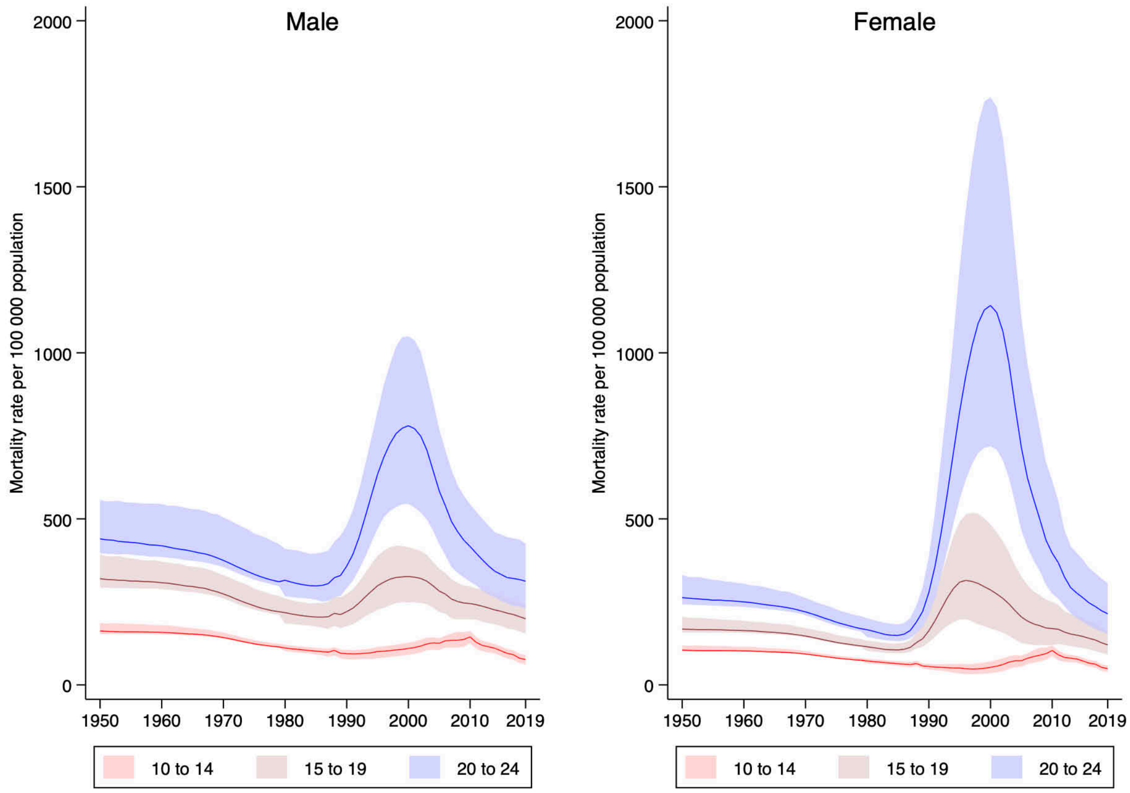

Figure S190b Number of deaths from all causes in 10-24 1950 – 2019: Botswana

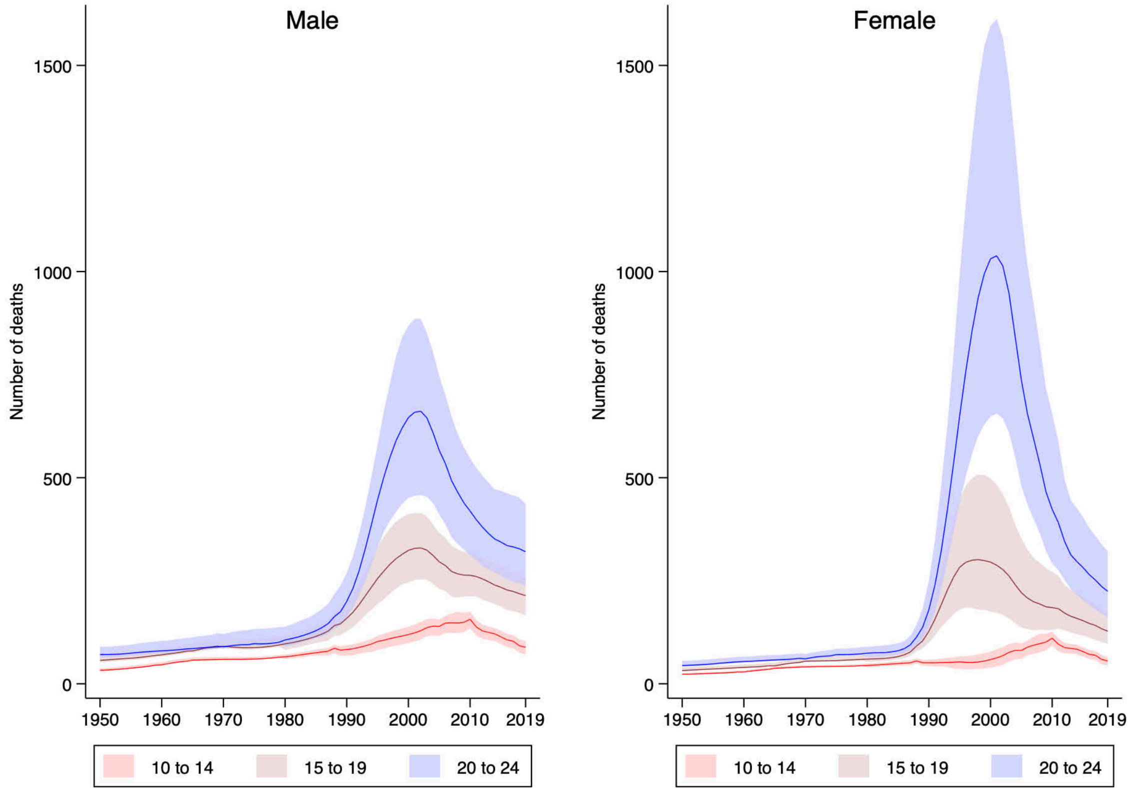

Figure S190c Mortality rate per 100,000 in 10-24 1980 – 2019 by cause: Botswana

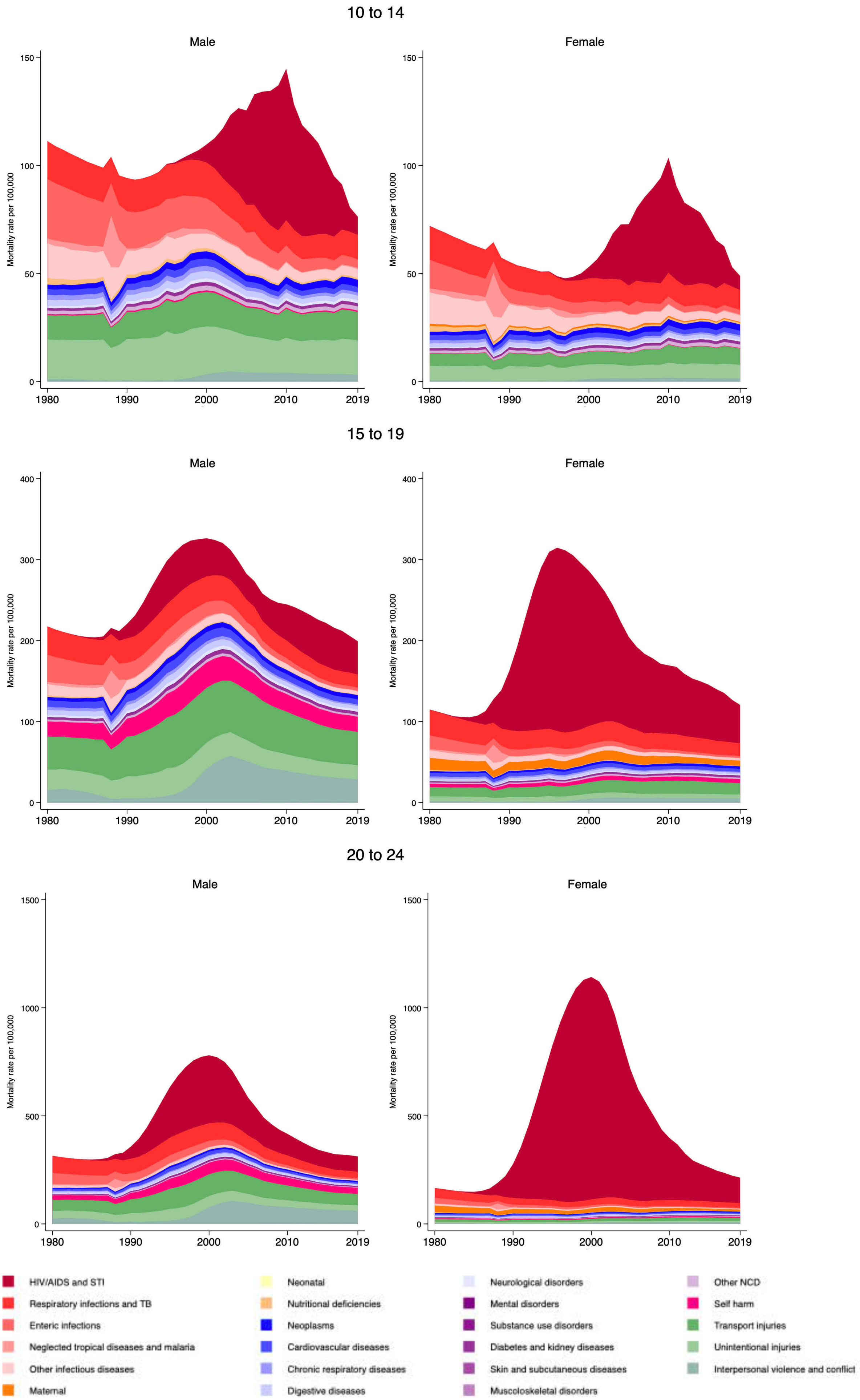

Figure S190d Number of deaths in 10-24 1980 – 2019 by cause: Botswana

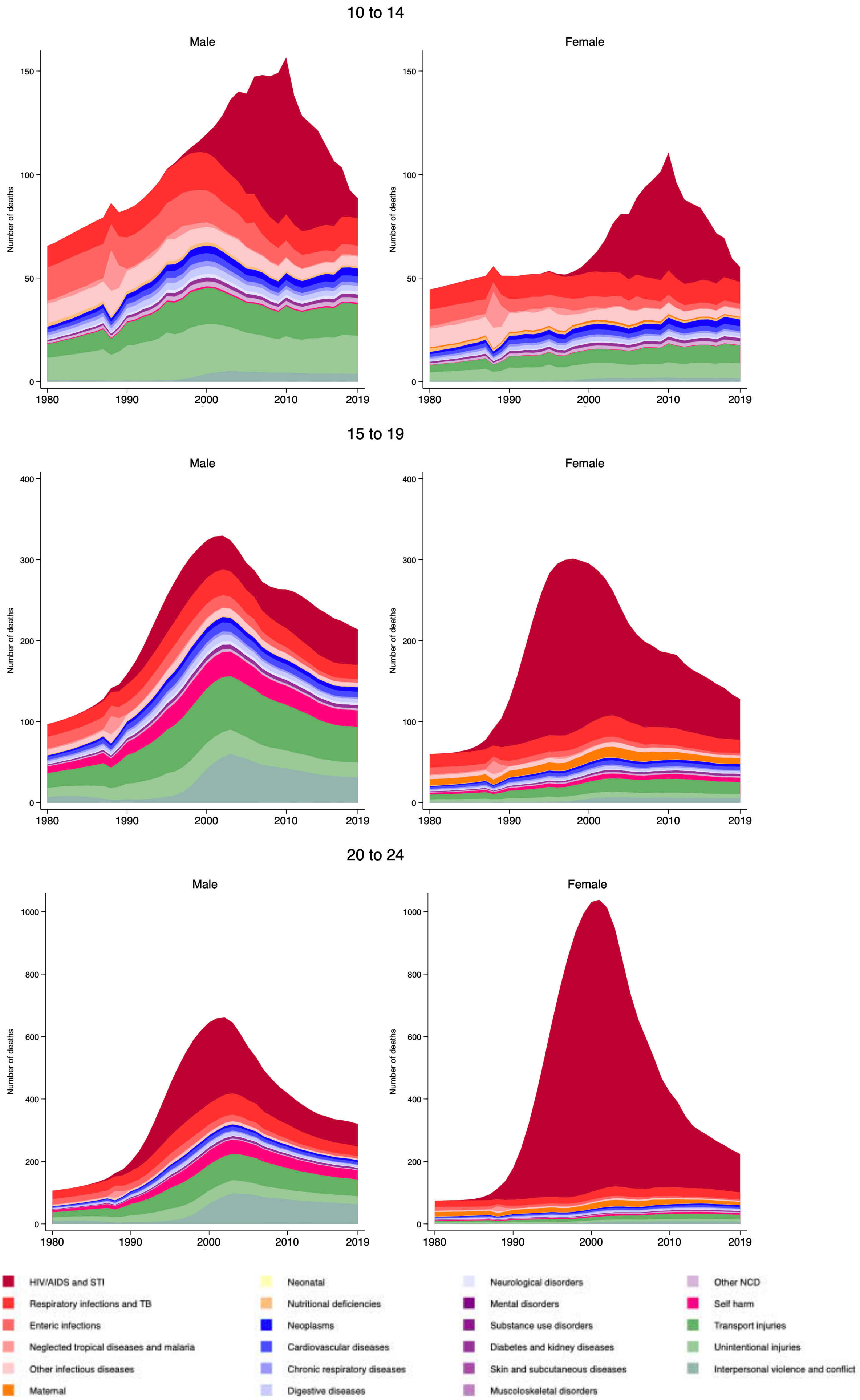

Figure S189: Lesotho

Figure S191a All-cause mortality rate per 100,000 in 10-24 1950 – 2019: Lesotho

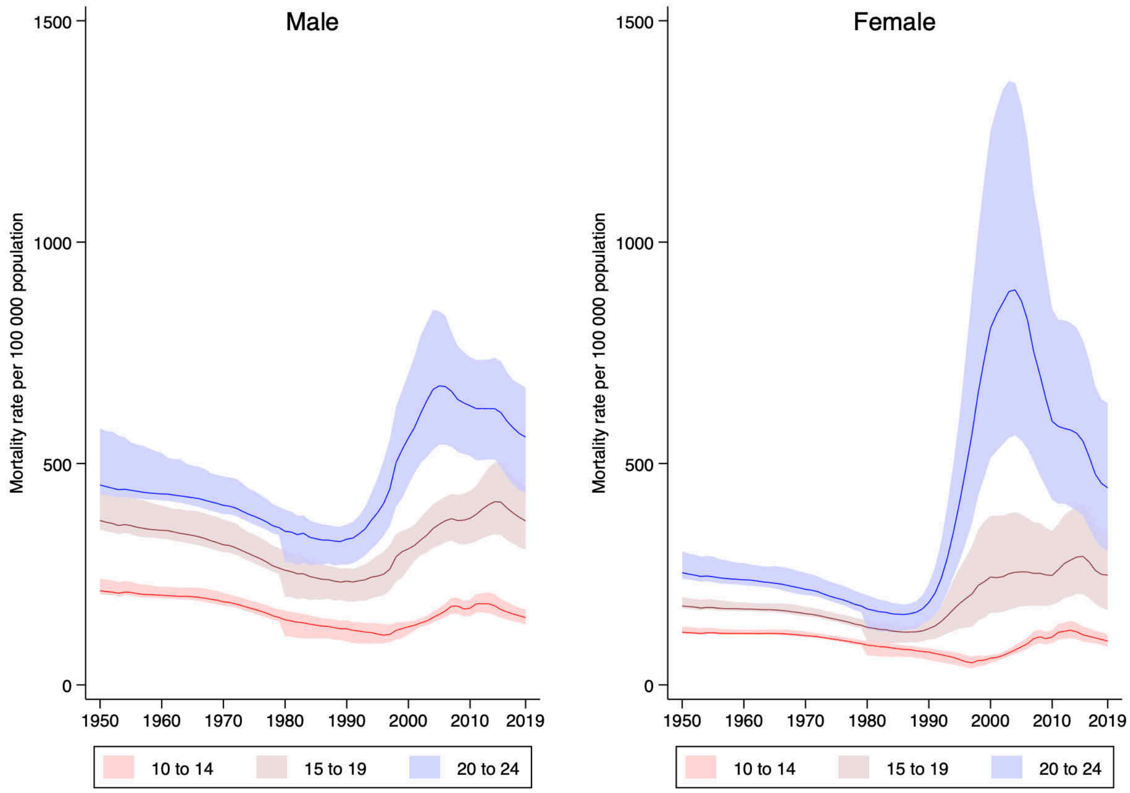

Figure S191b Number of deaths from all causes in 10-24 1950 – 2019: Lesotho

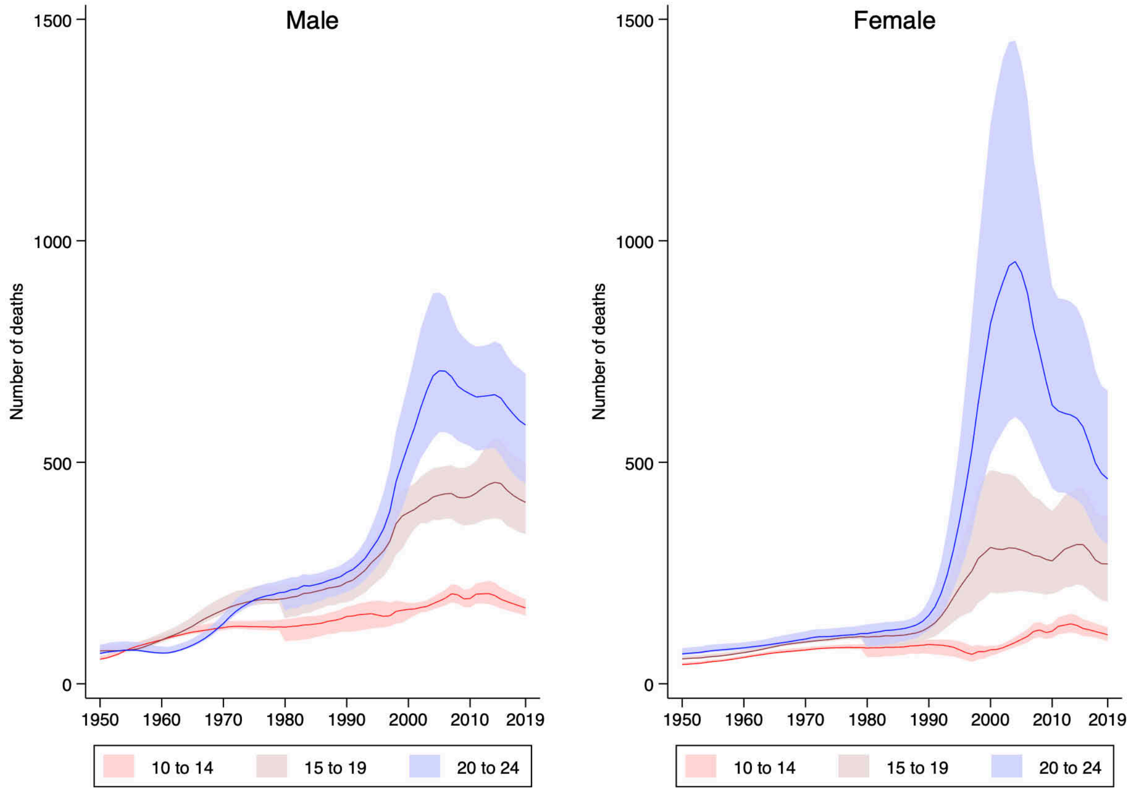

Figure S191c Mortality rate per 100,000 in 10-24 1980 – 2019 by cause: Lesotho

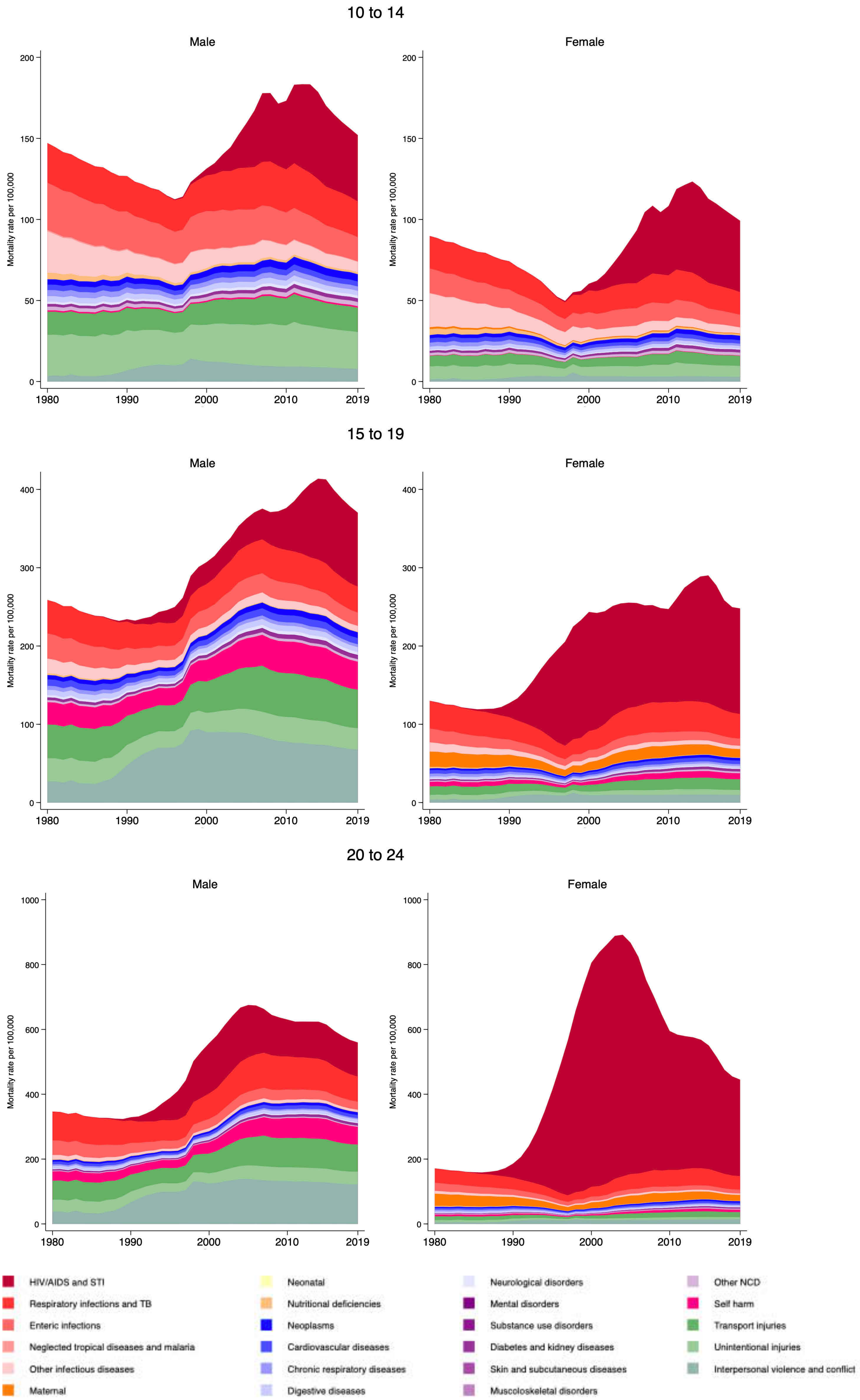

Figure S191d Number of deaths in 10-24 1980 – 2019 by cause: Lesotho

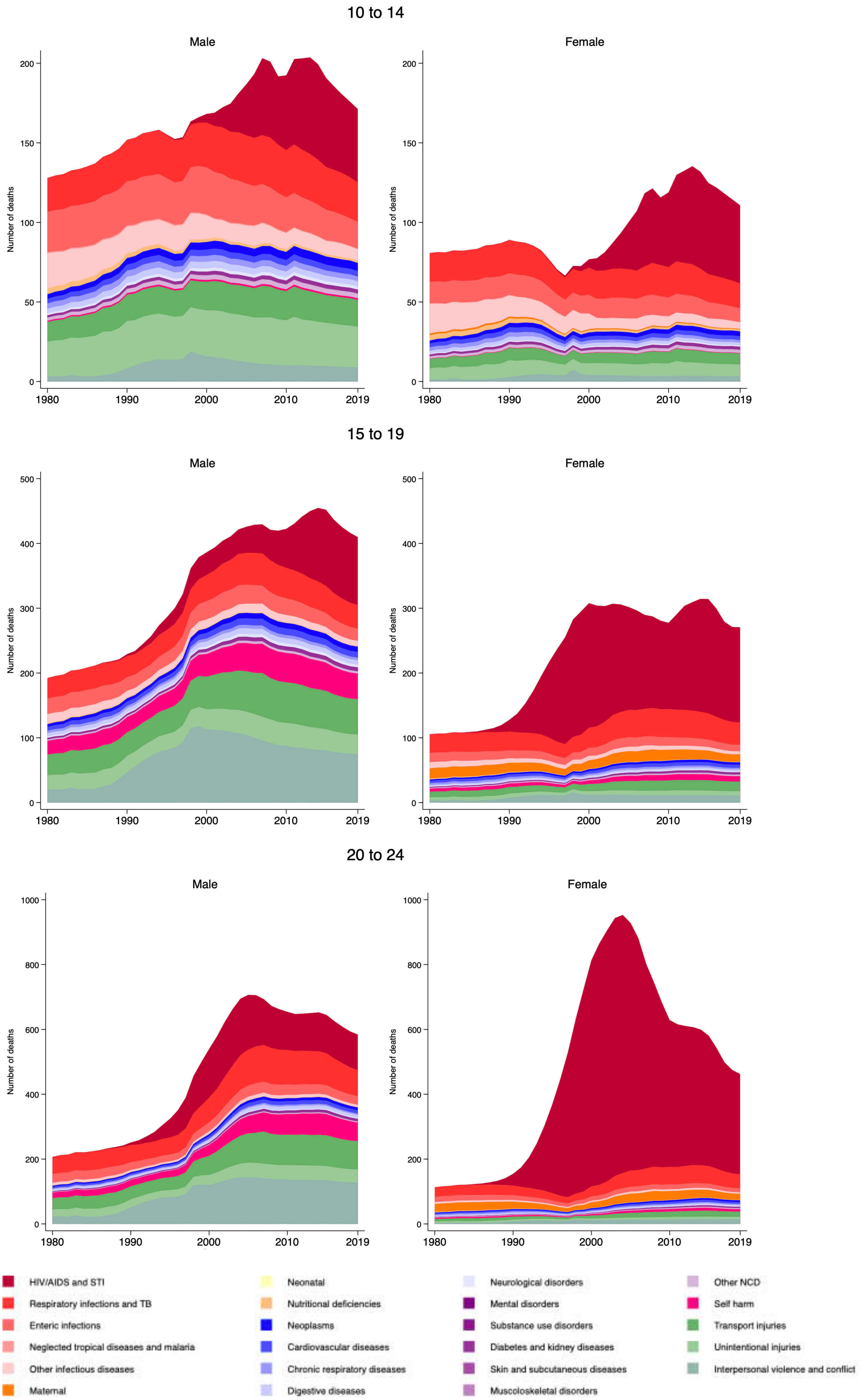

Figure S190: Namibia

Figure S192a All-cause mortality rate per 100,000 in 10-24 1950 – 2019: Namibia

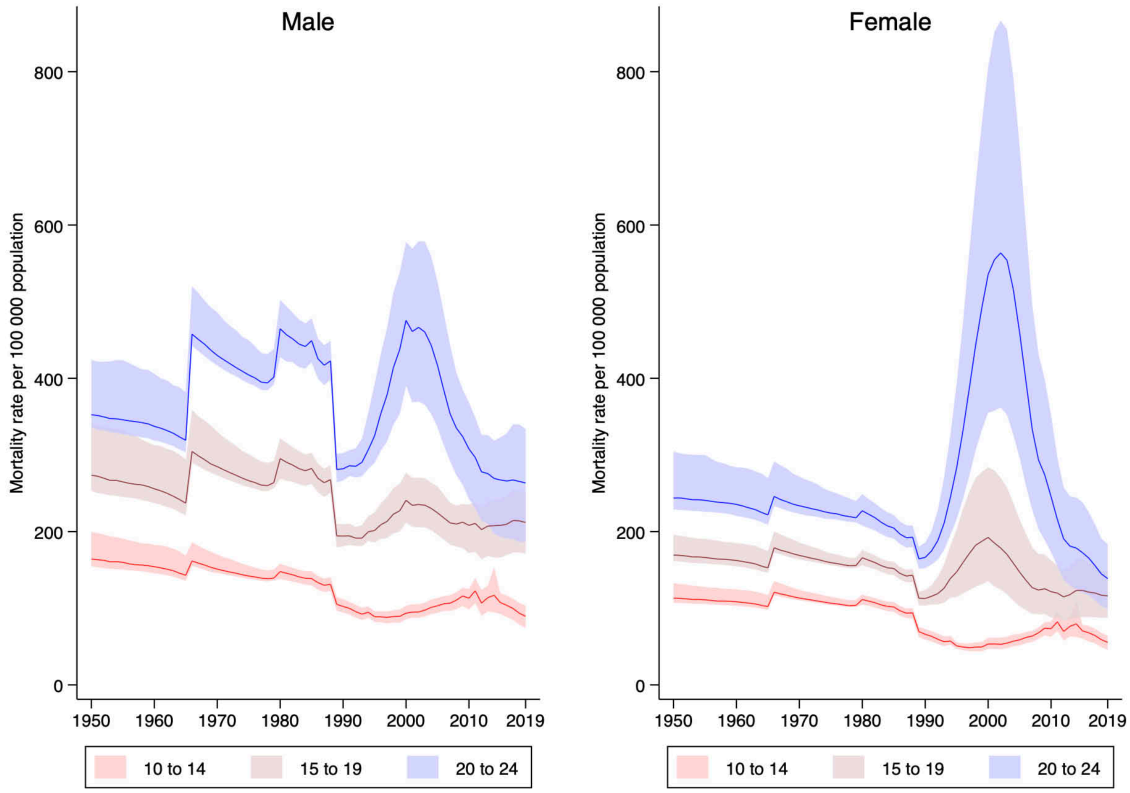

Figure S192b Number of deaths from all causes in 10-24 1950 – 2019: Namibia

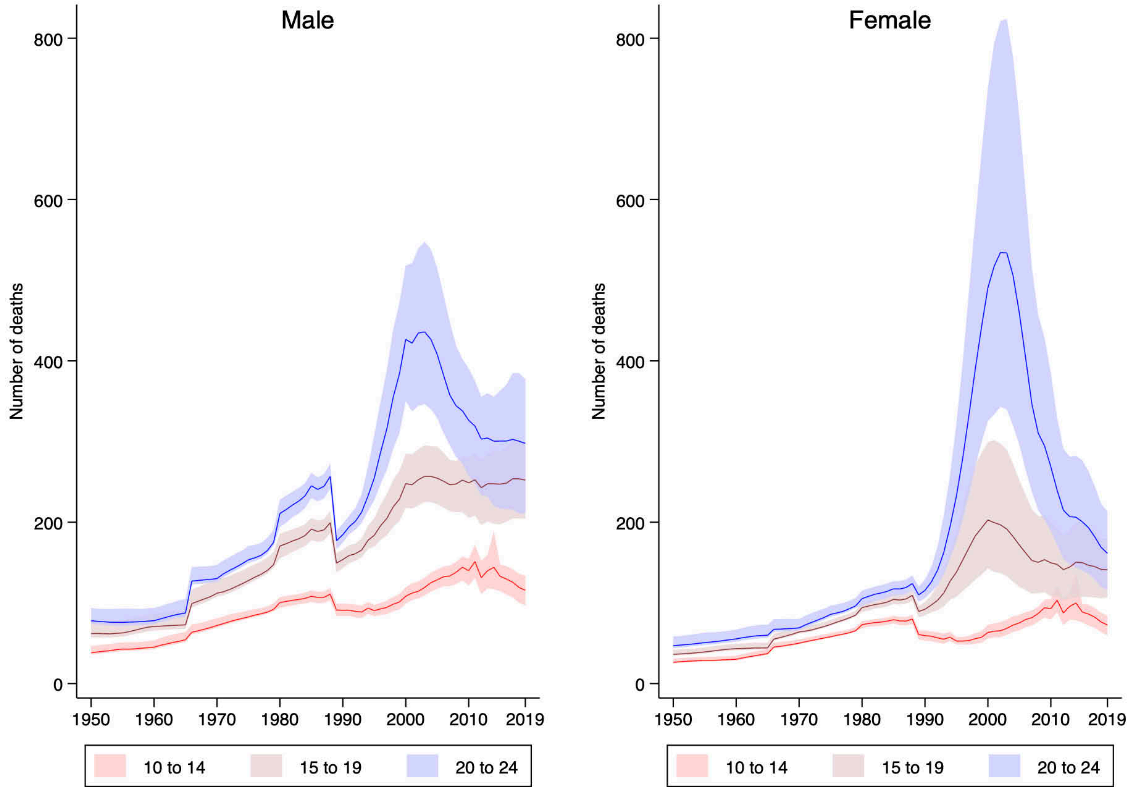

Figure S192c Mortality rate per 100,000 in 10-24 1980 – 2019 by cause: Namibia

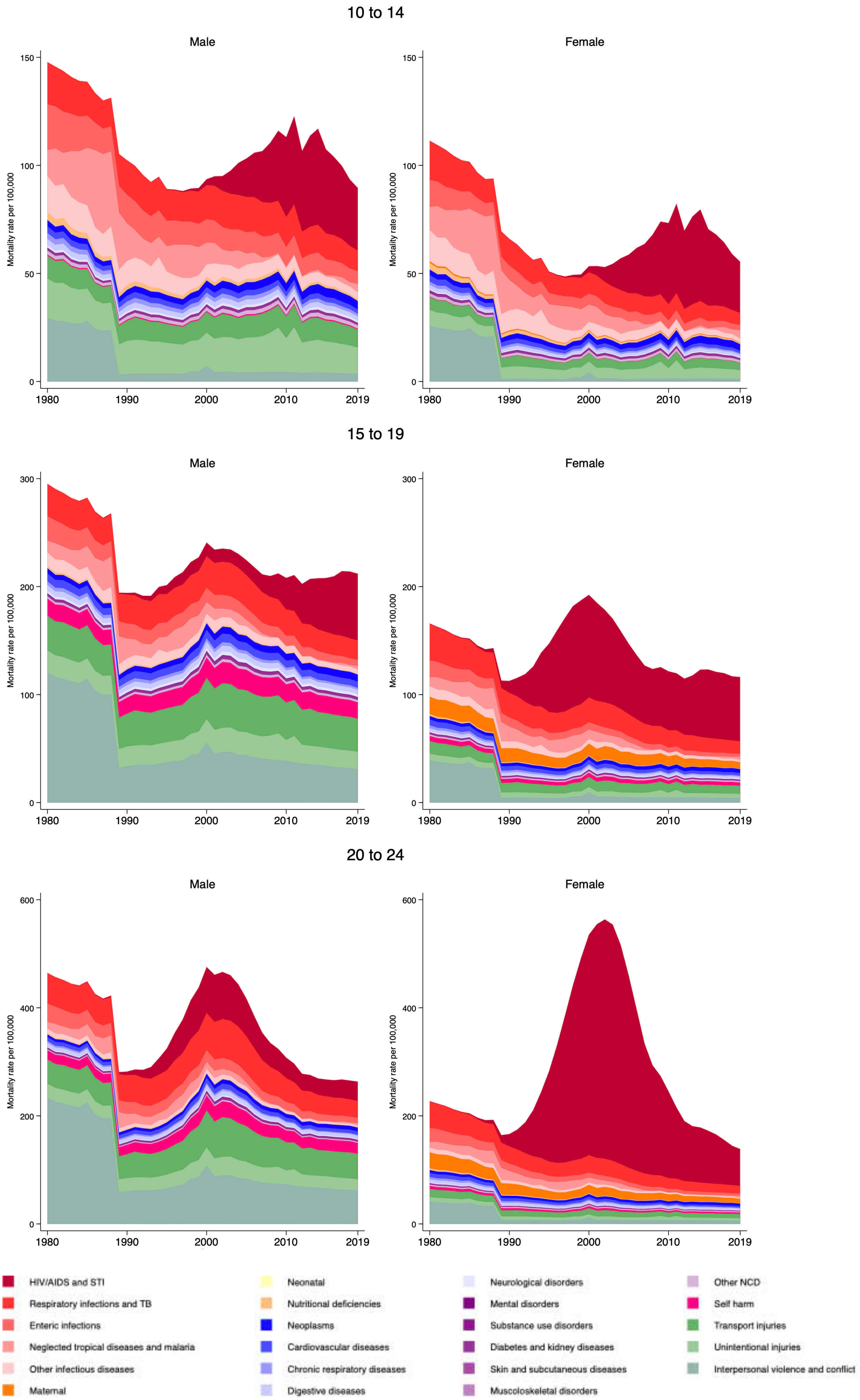

Figure S192d Number of deaths in 10-24 1980 – 2019 by cause: Namibia

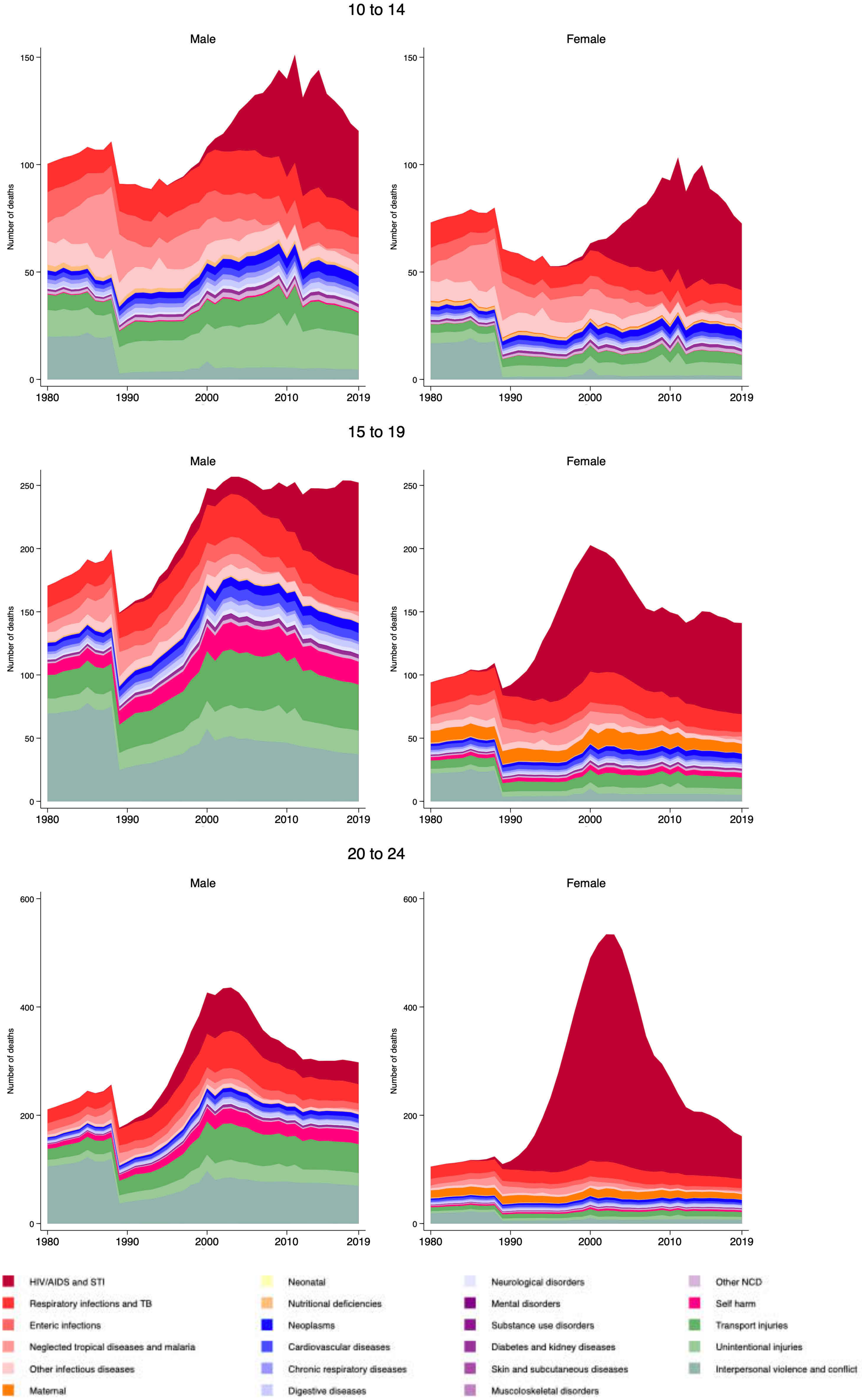

Figure S191: South Africa

Figure S193a All-cause mortality rate per 100,000 in 10-24 1950 – 2019: South Africa

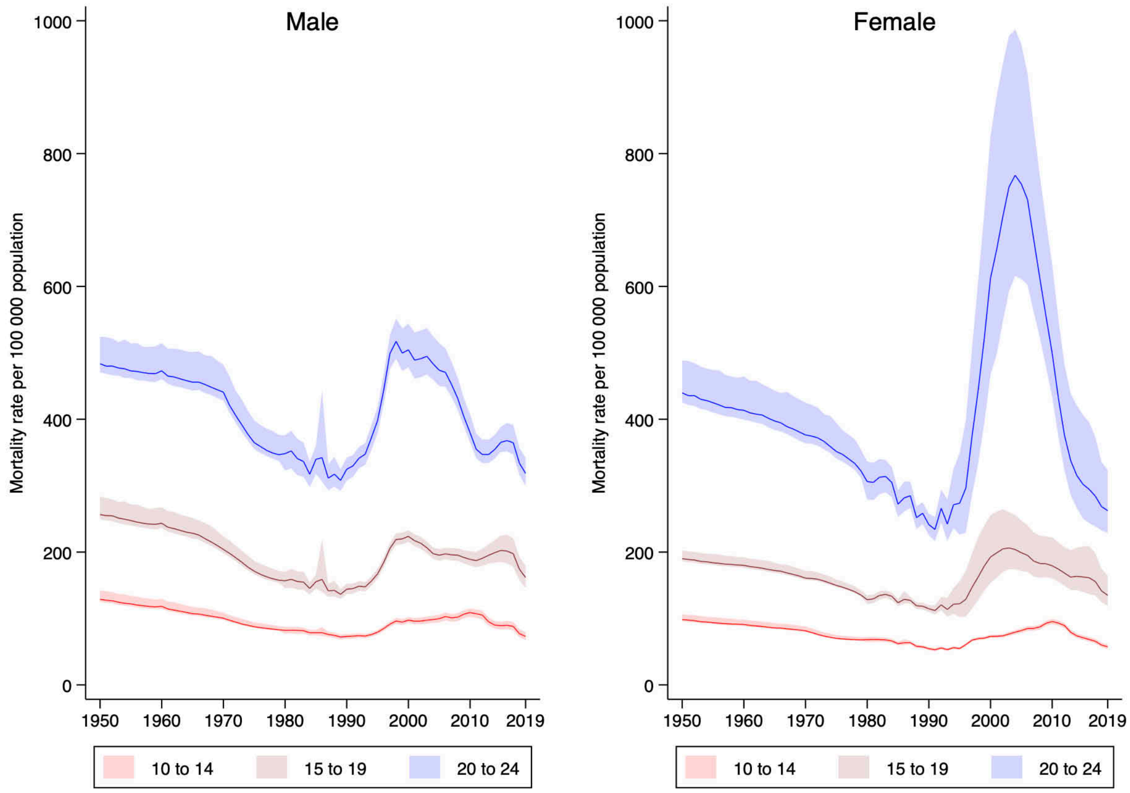

Figure S193b Number of deaths from all causes in 10-24 1950 – 2019: South Africa

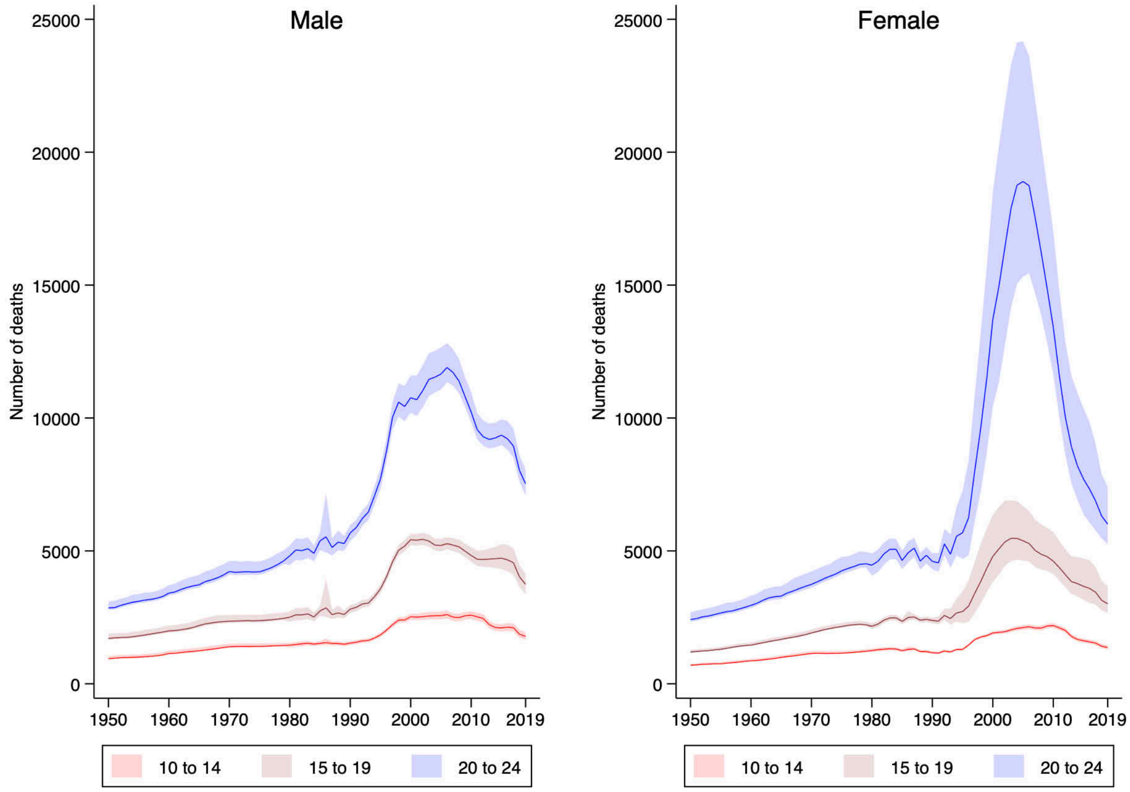

Figure S193c Mortality rate per 100,000 in 10-24 1980 – 2019 by cause: South Africa

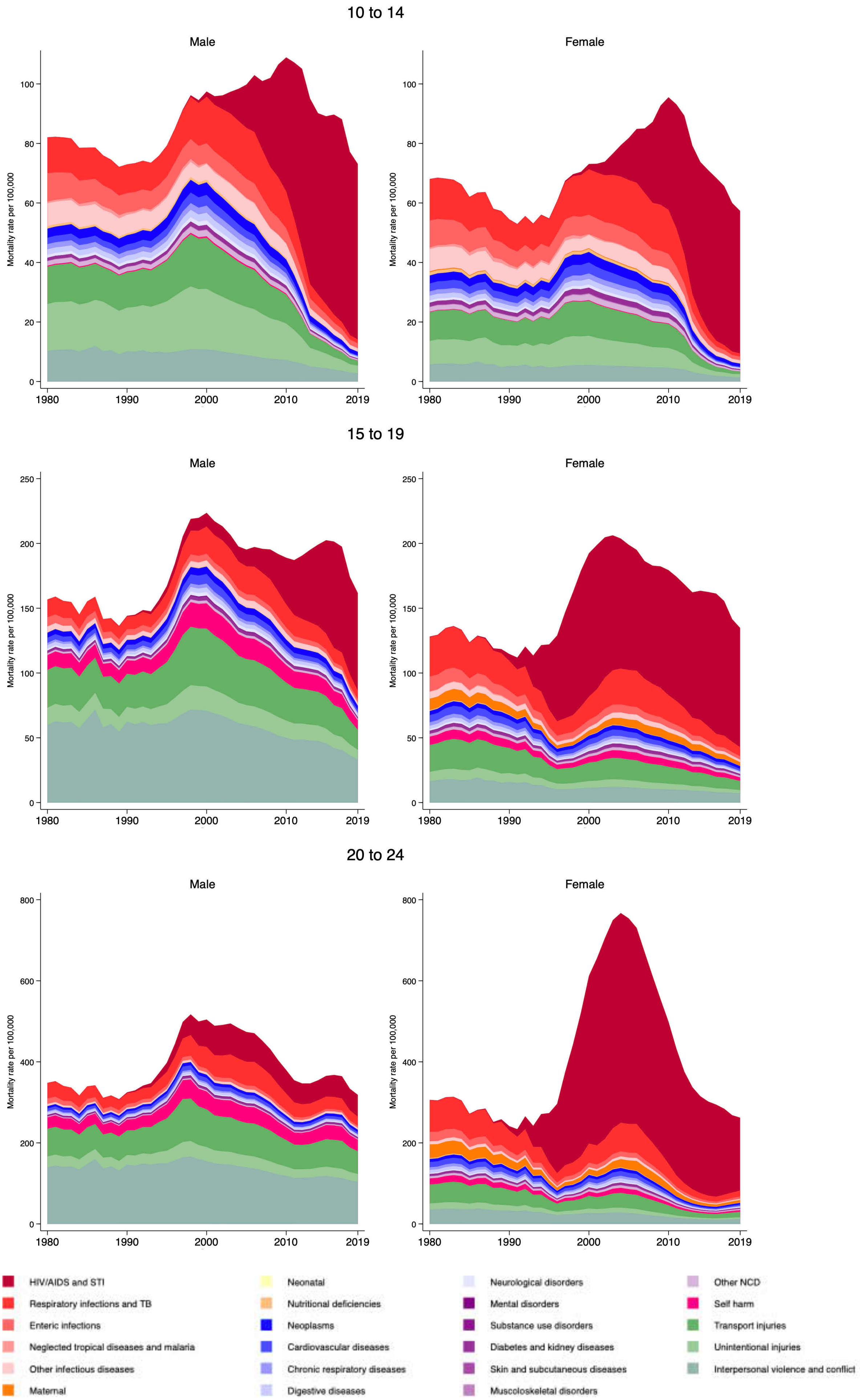

Figure S193d Number of deaths in 10-24 1980 – 2019 by cause: South Africa

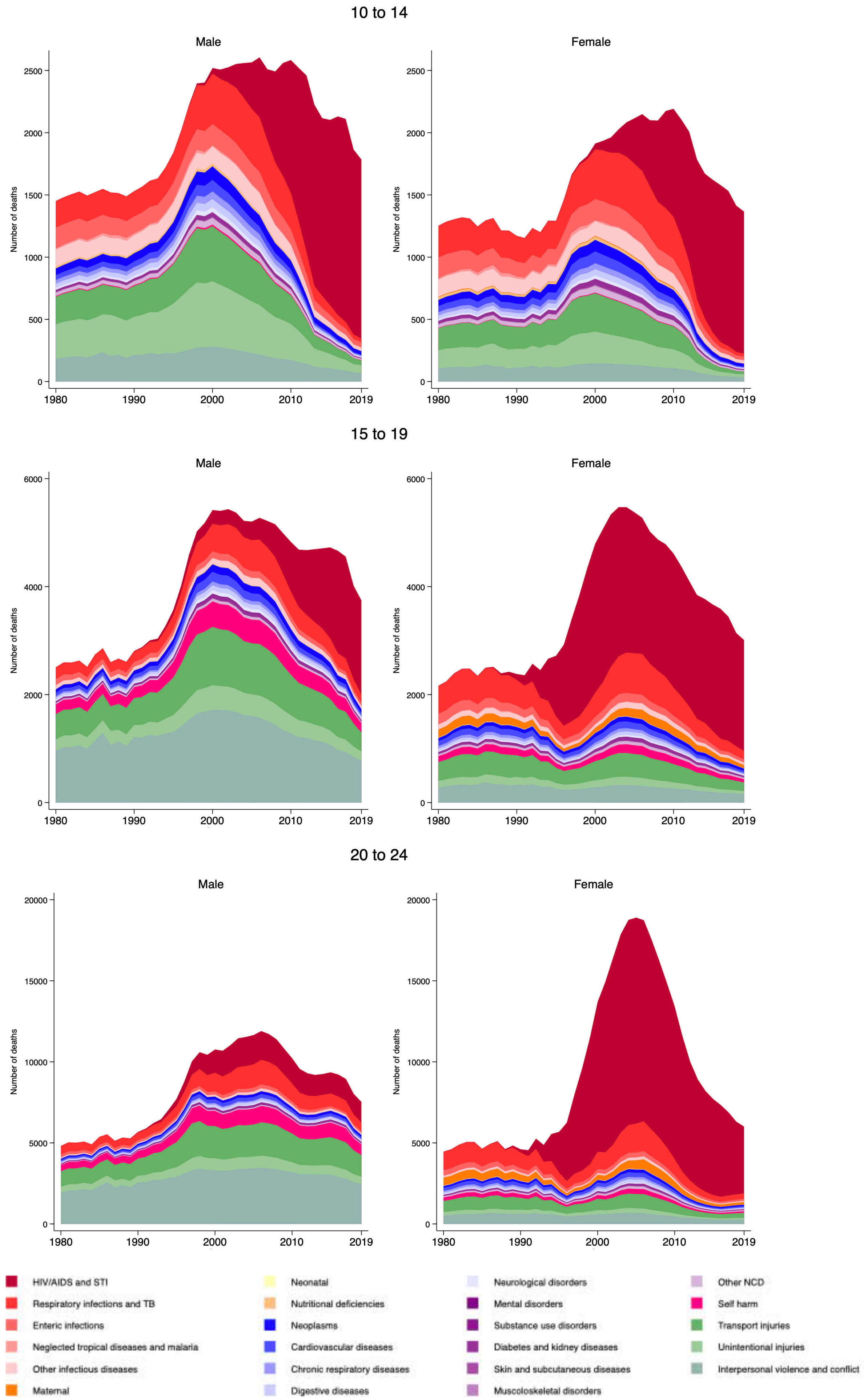

Figure S192: Eswatini

Figure S194a All-cause mortality rate per 100,000 in 10-24 1950 – 2019: Eswatini

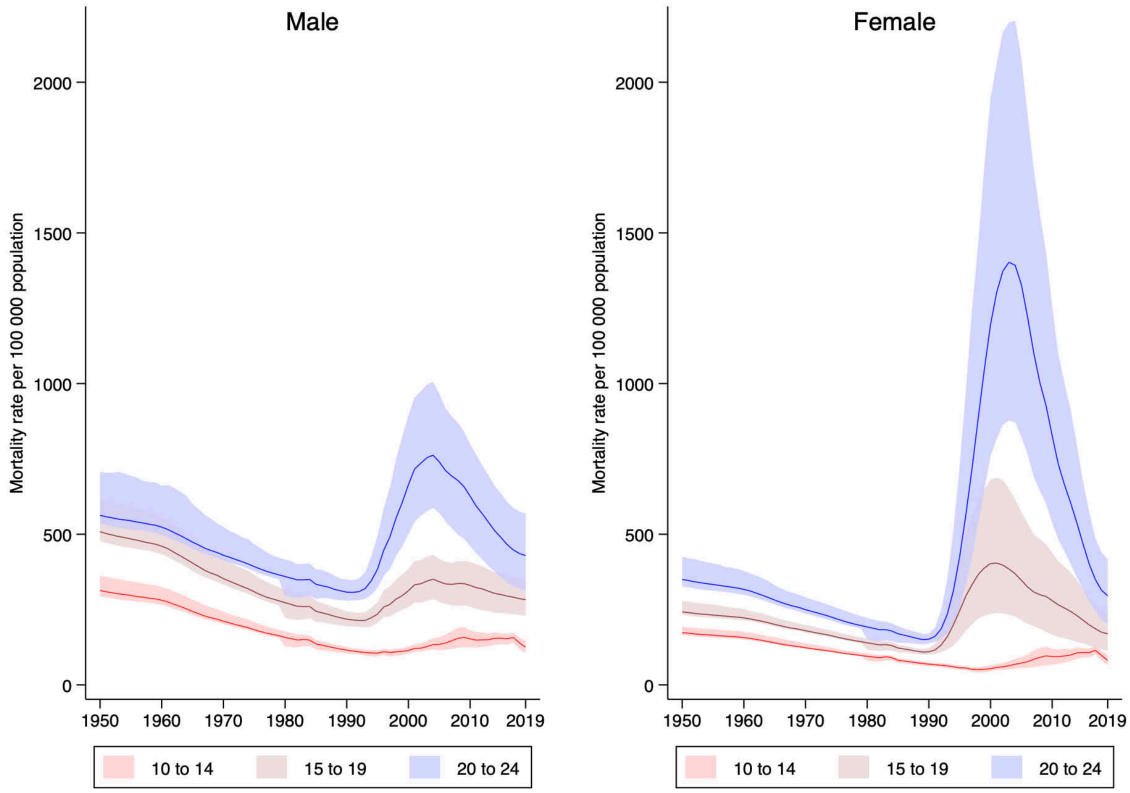

Figure S194b Number of deaths from all causes in 10-24 1950 – 2019: Eswatini

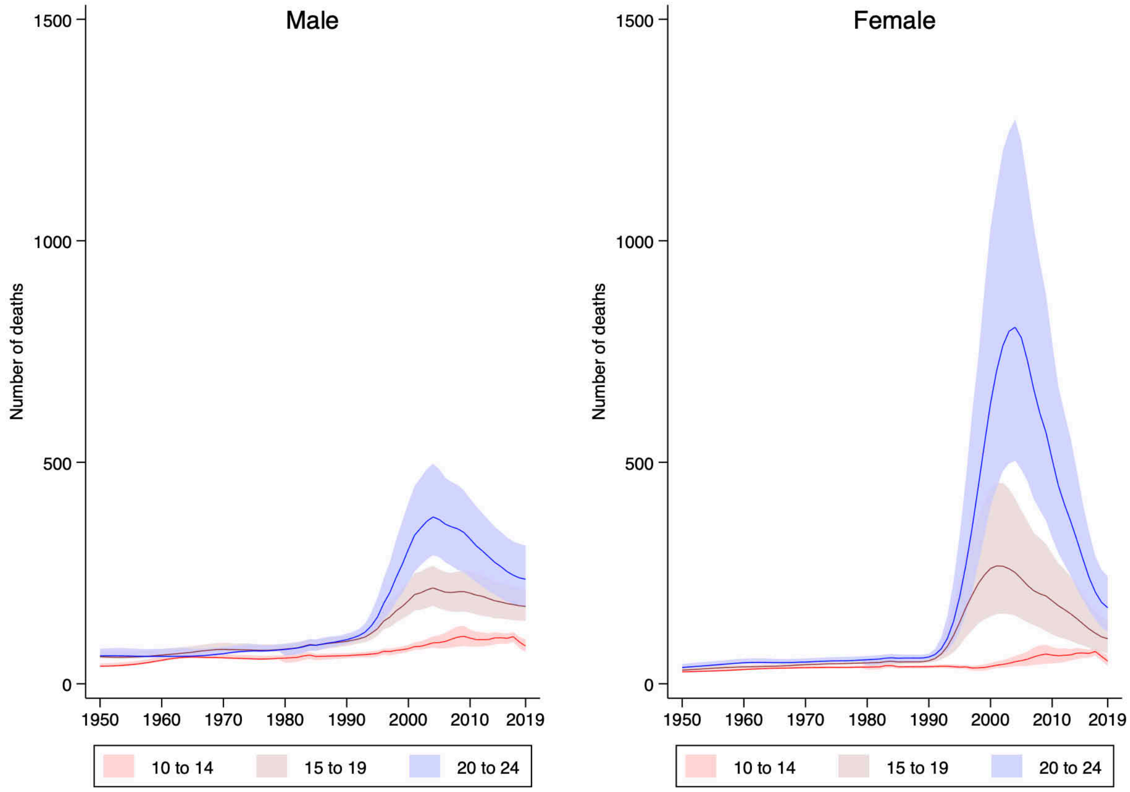

Figure S194c Mortality rate per 100,000 in 10-24 1980 – 2019 by cause: Eswatini

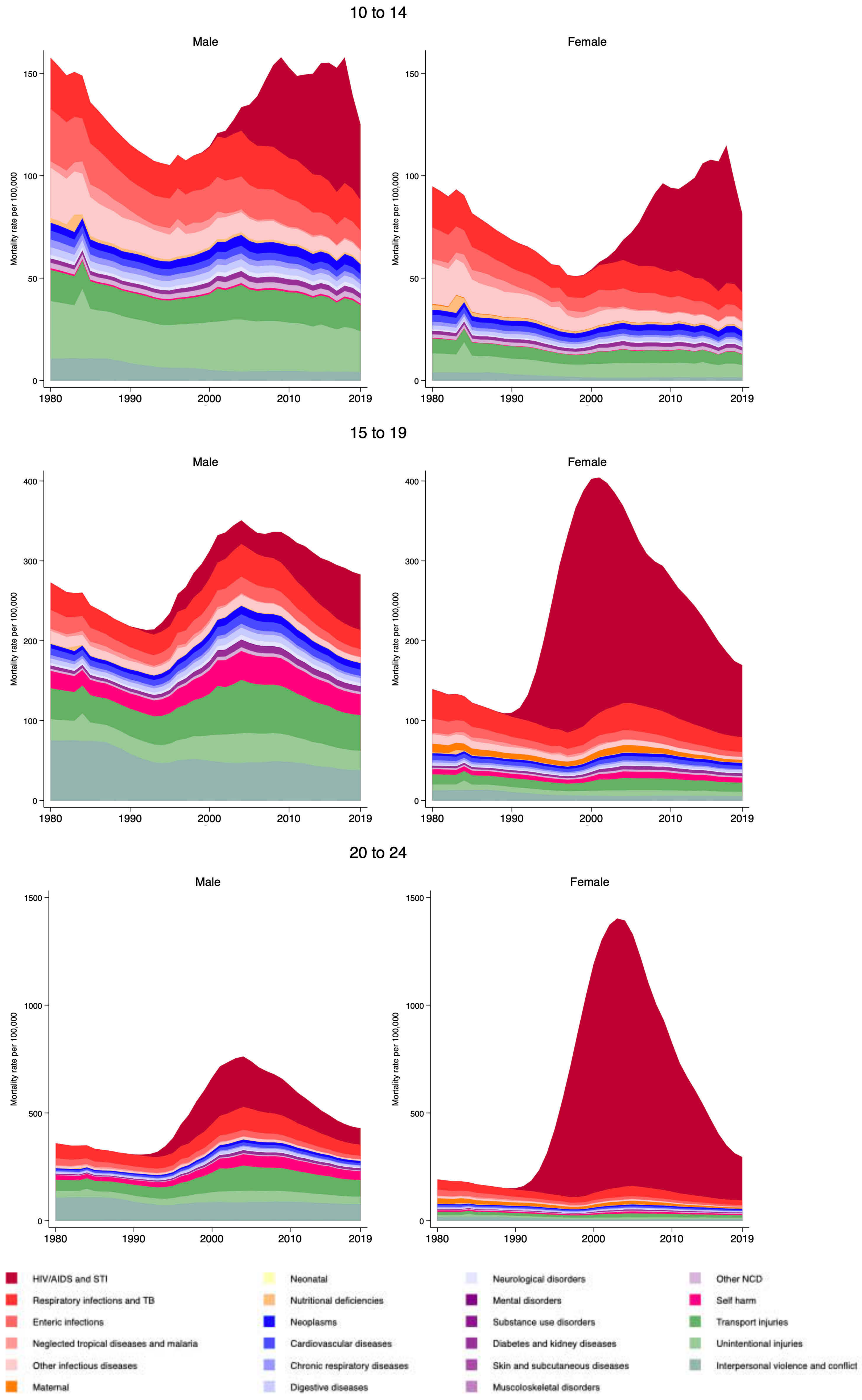

Figure S194d Number of deaths in 10-24 1980 – 2019 by cause: Eswatini

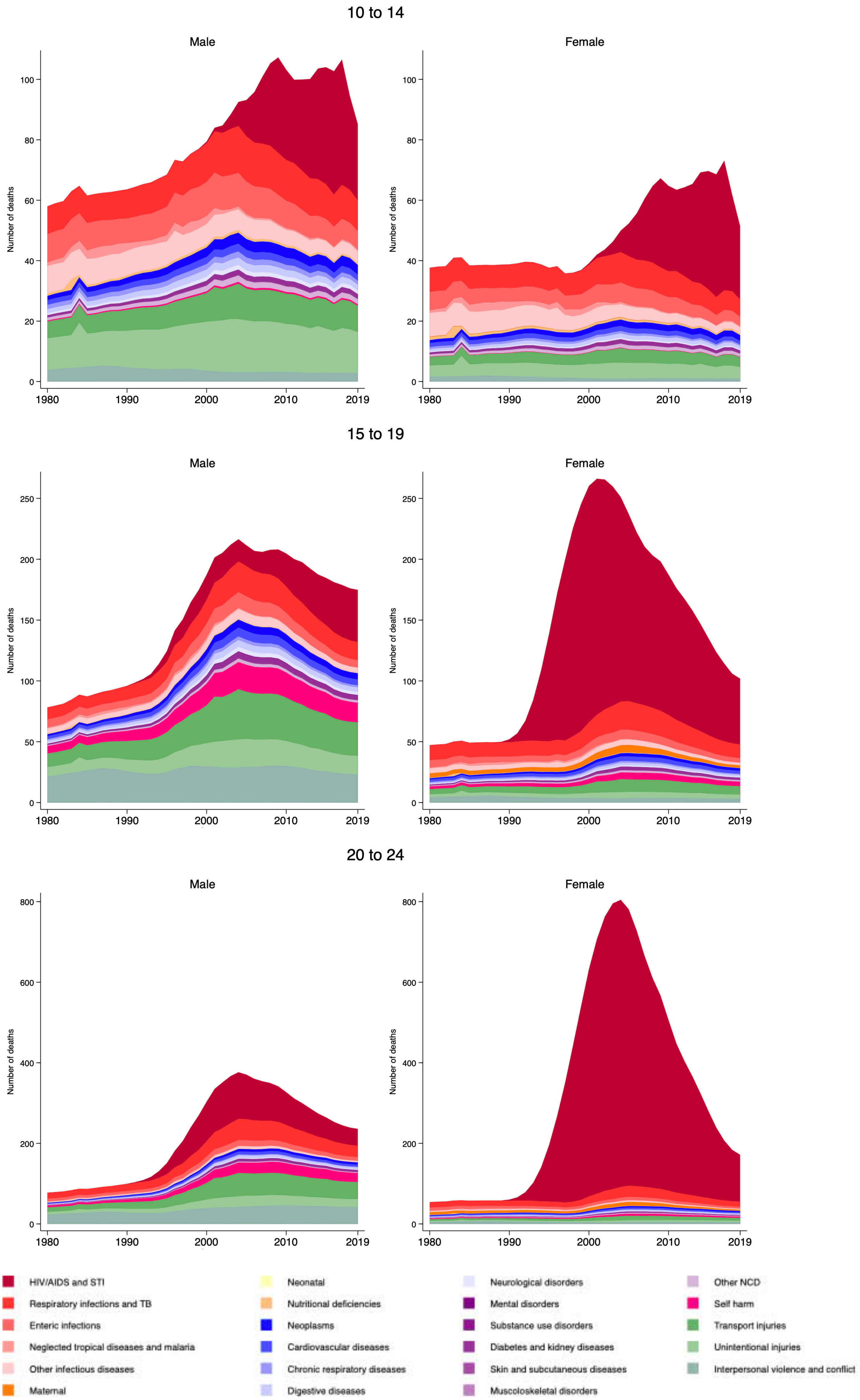

Figure S193: Zimbabwe

Figure S195a All-cause mortality rate per 100,000 in 10-24 1950 – 2019: Zimbabwe

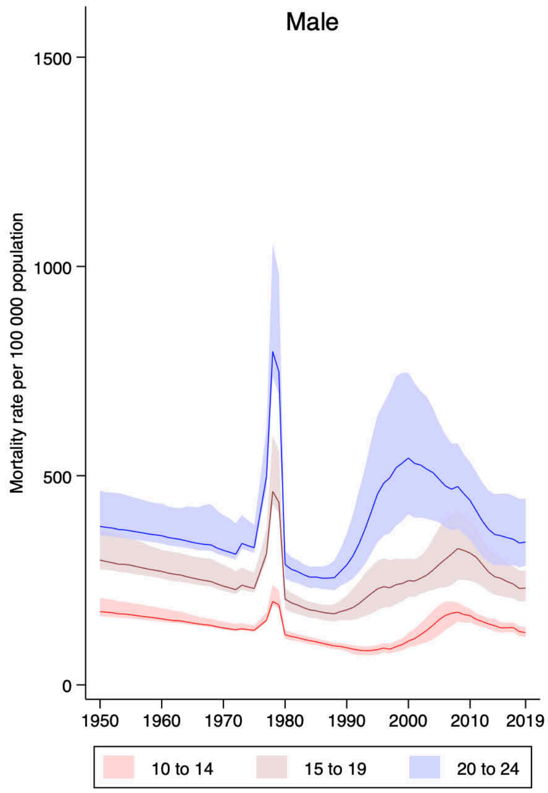

Figure S195b Number of deaths from all causes in 10-24 1950 – 2019: Zimbabwe

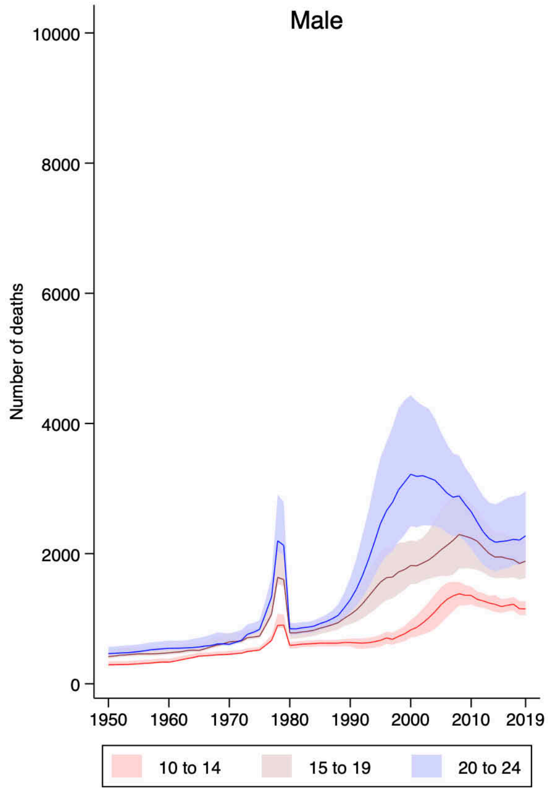

Figure S195c Mortality rate per 100,000 in 10-24 1980 – 2019 by cause: Zimbabwe

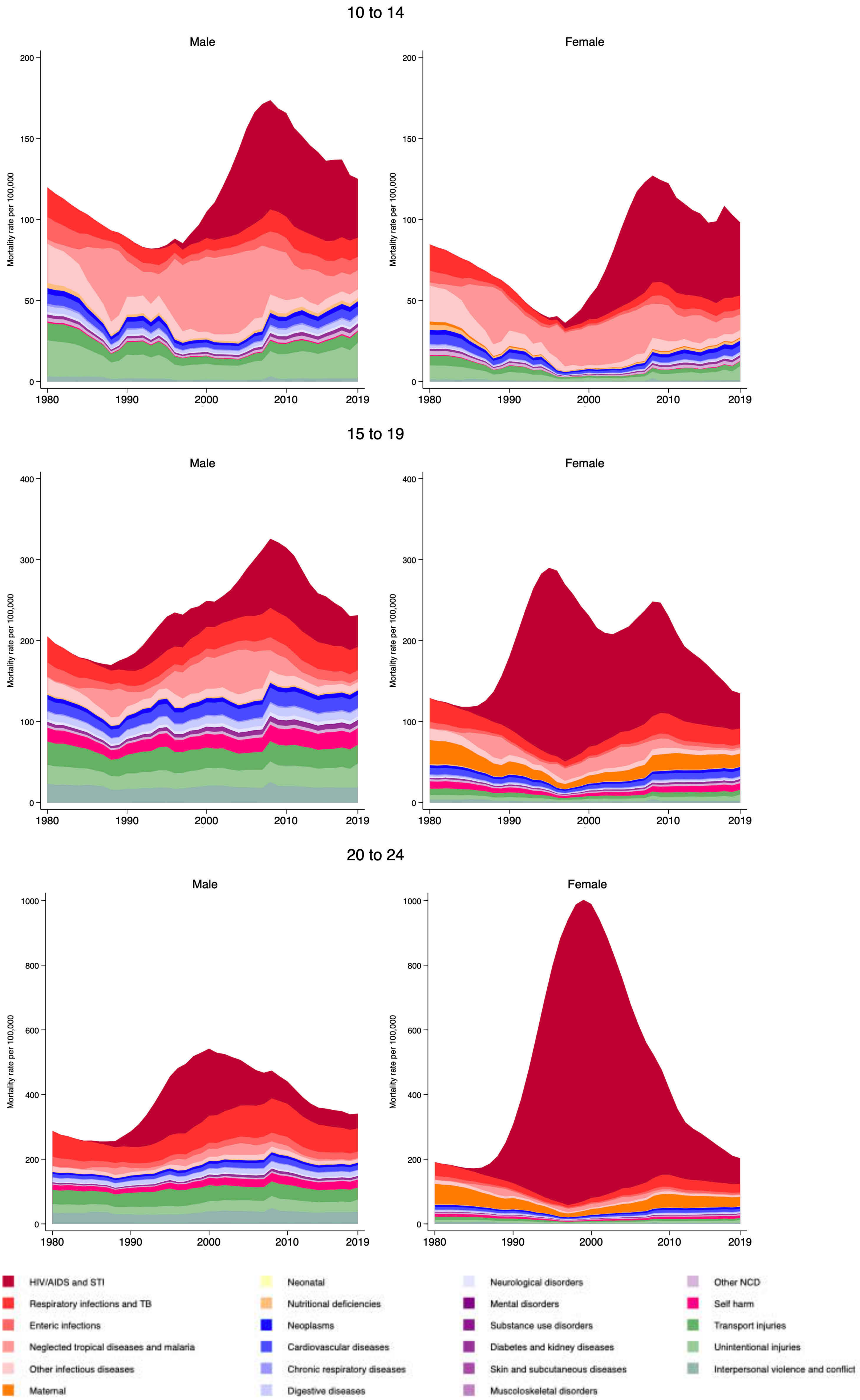

Figure S195d Number of deaths in 10-24 1980 – 2019 by cause: Zimbabwe

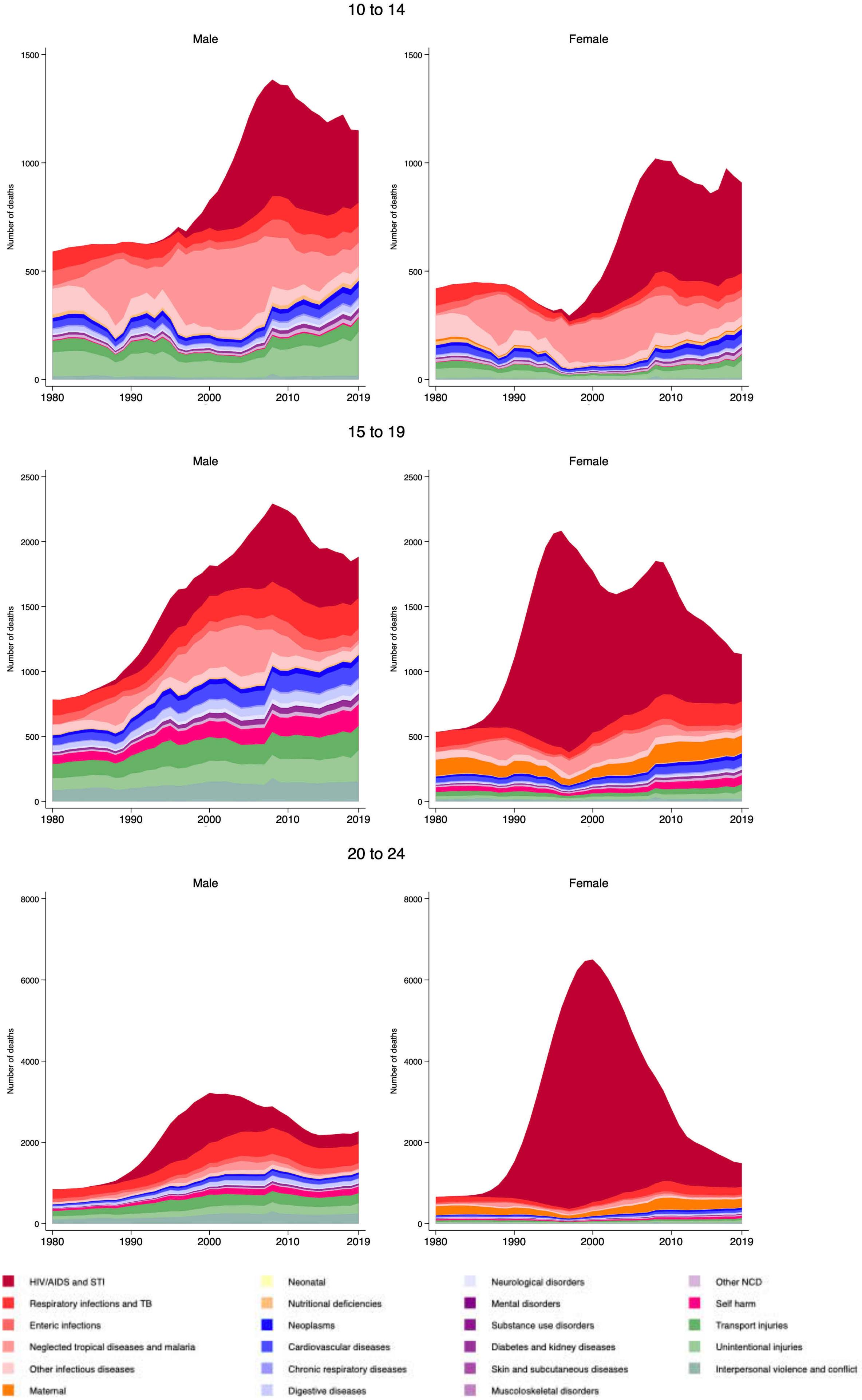

Figure S194: Western Sub-Saharan Africa

Figure S196a All-cause mortality rate per 100,000 in 10-24 1950 – 2019: Western Sub-Saharan Africa

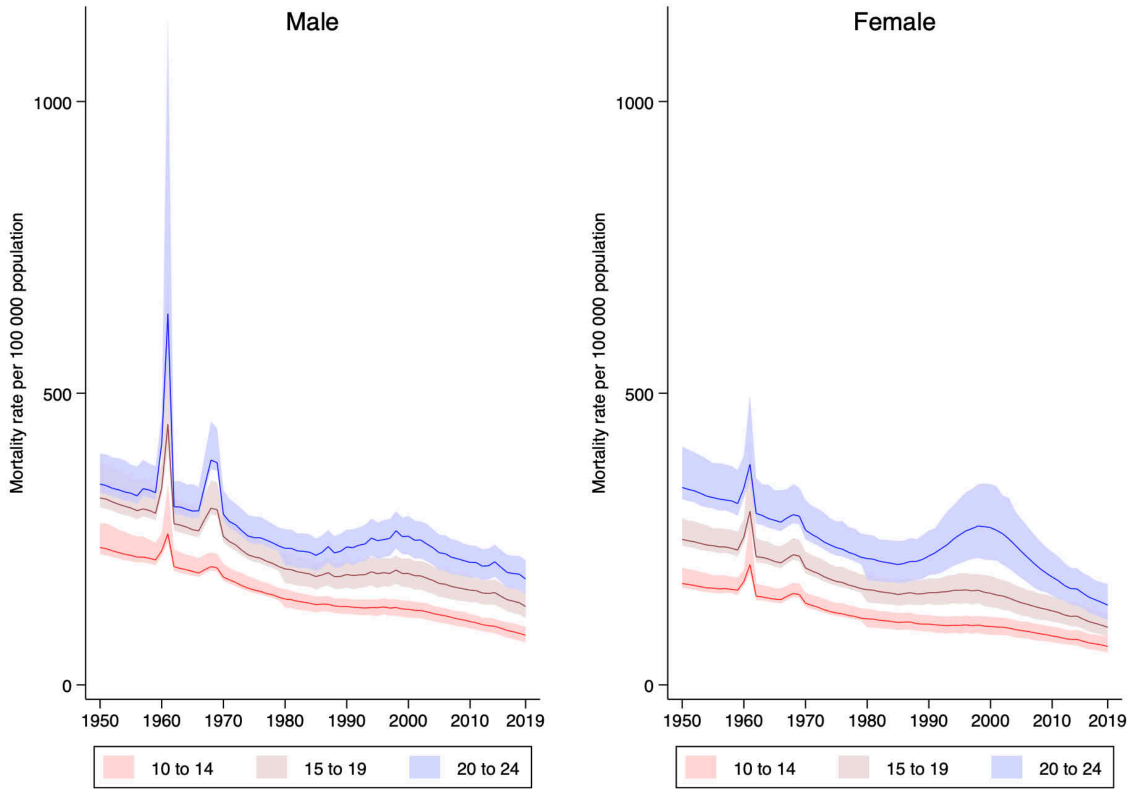

Figure S196b Number of deaths from all causes in 10-24 1950 – 2019: Western Sub-Saharan Africa

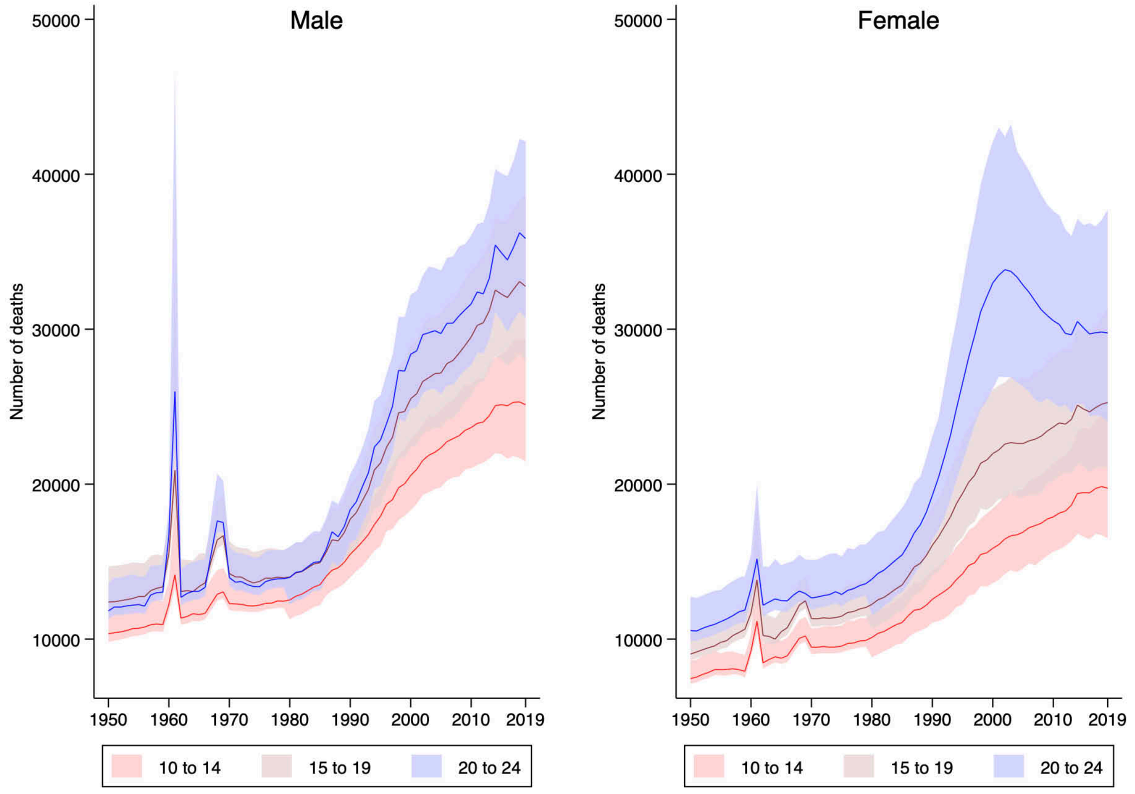

Figure S196c Mortality rate per 100,000 in 10-24 1980 – 2019 by cause: Western Sub-Saharan Africa

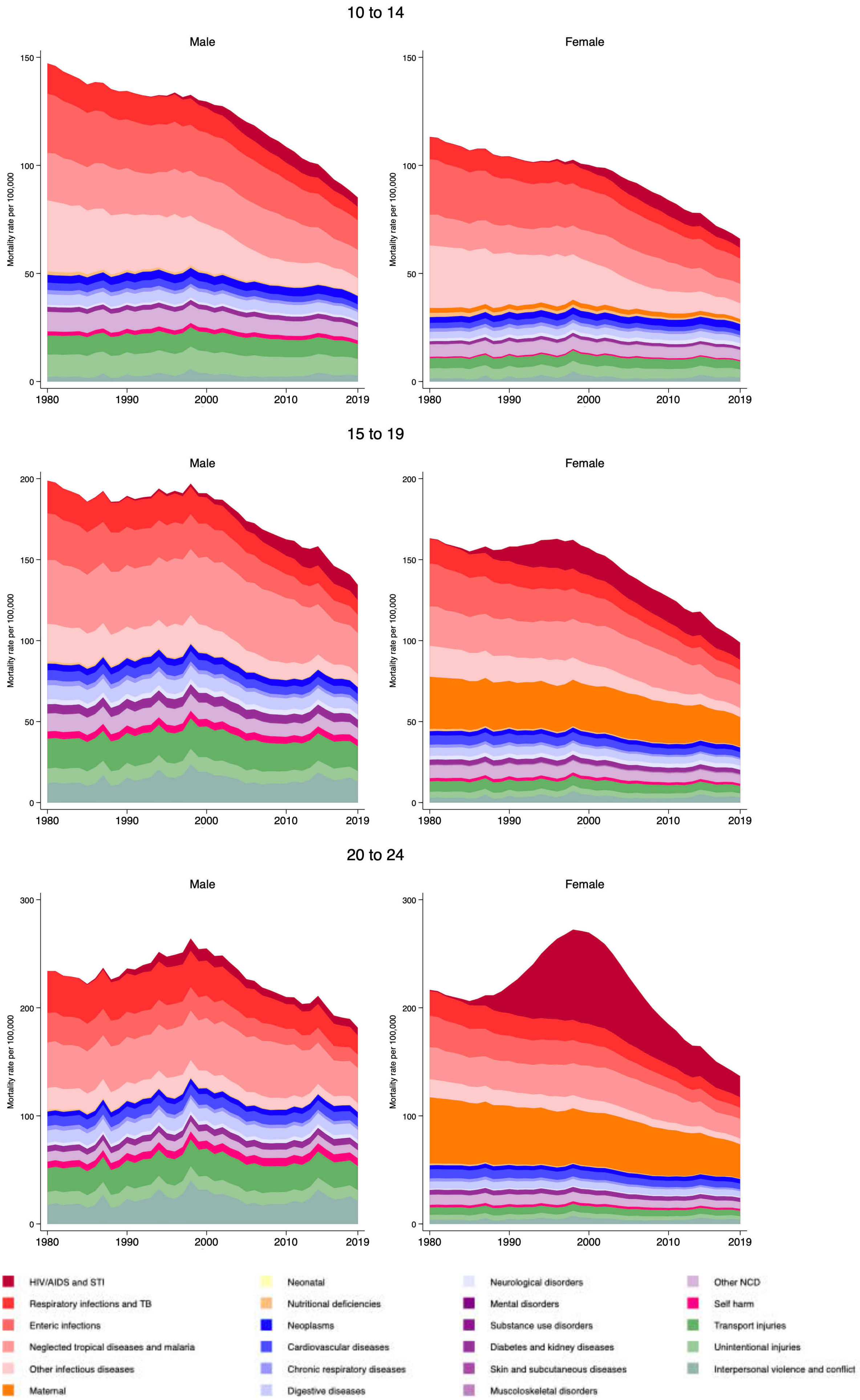

Figure S196d Number of deaths in 10-24 1980 – 2019 by cause: Western Sub-Saharan Africa

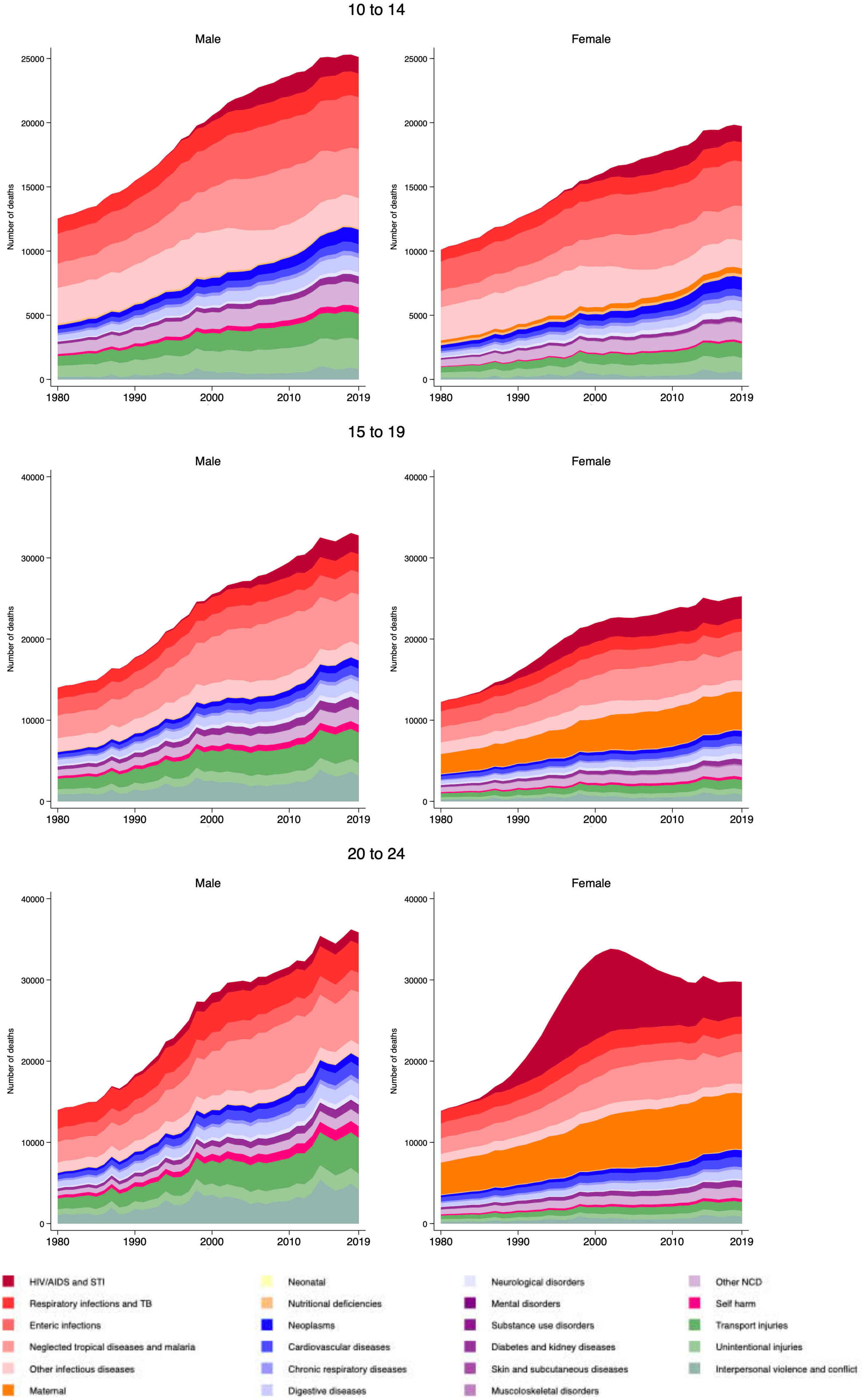

Figure S195: Benin

Figure S197a All-cause mortality rate per 100,000 in 10-24 1950 – 2019: Benin

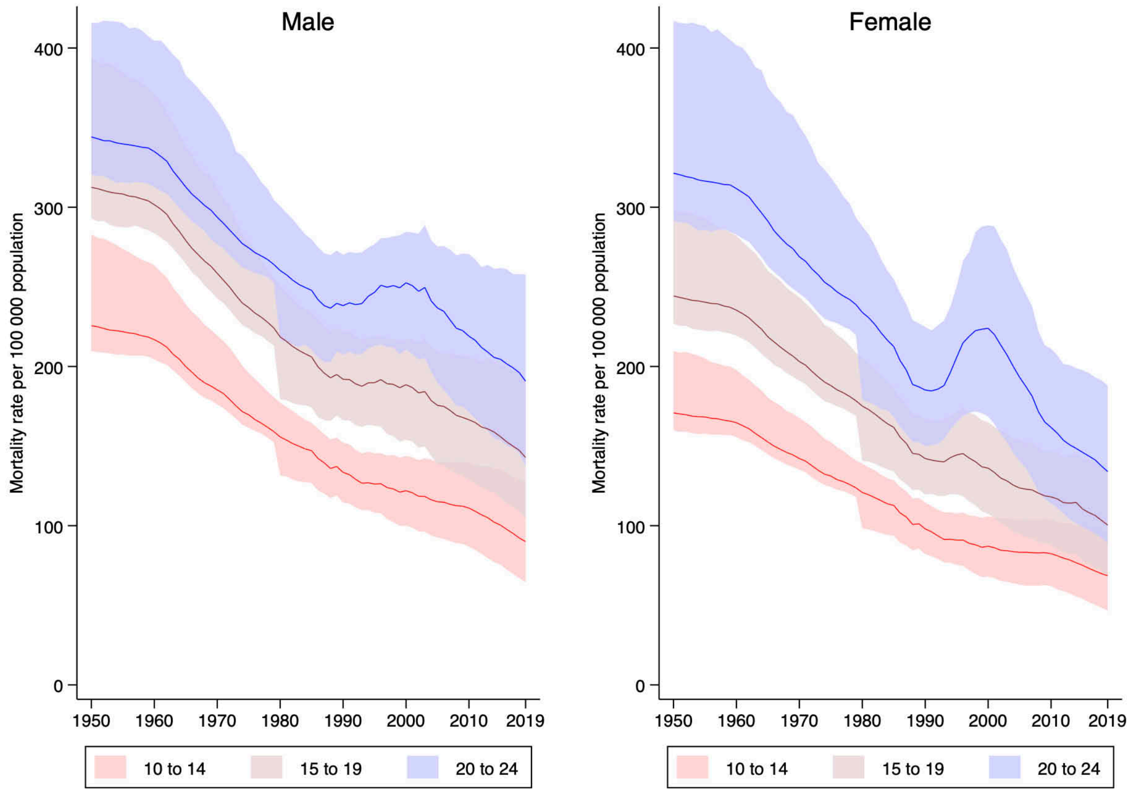

Figure S197b Number of deaths from all causes in 10-24 1950 – 2019: Benin

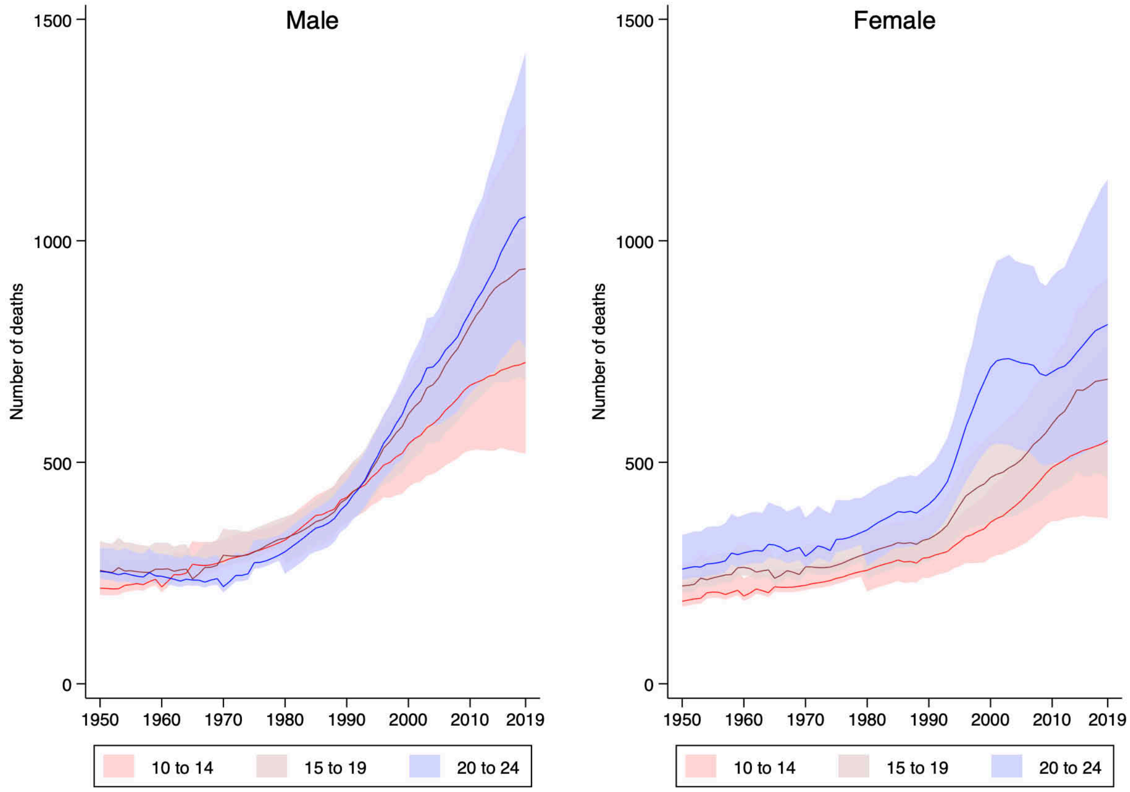

Figure S197c Mortality rate per 100,000 in 10-24 1980 – 2019 by cause: Benin

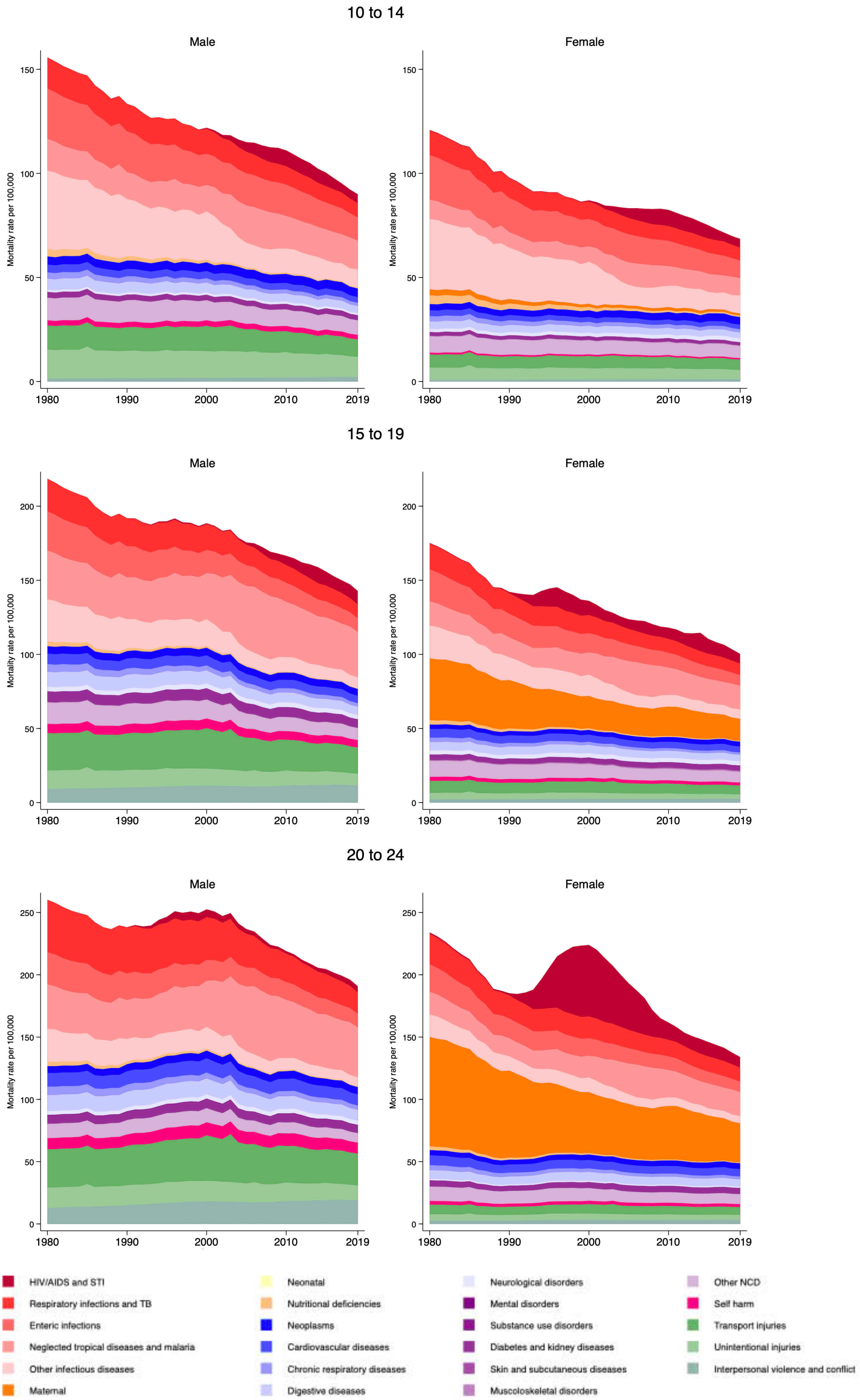

Figure S197d Number of deaths in 10-24 1980 – 2019 by cause: Benin

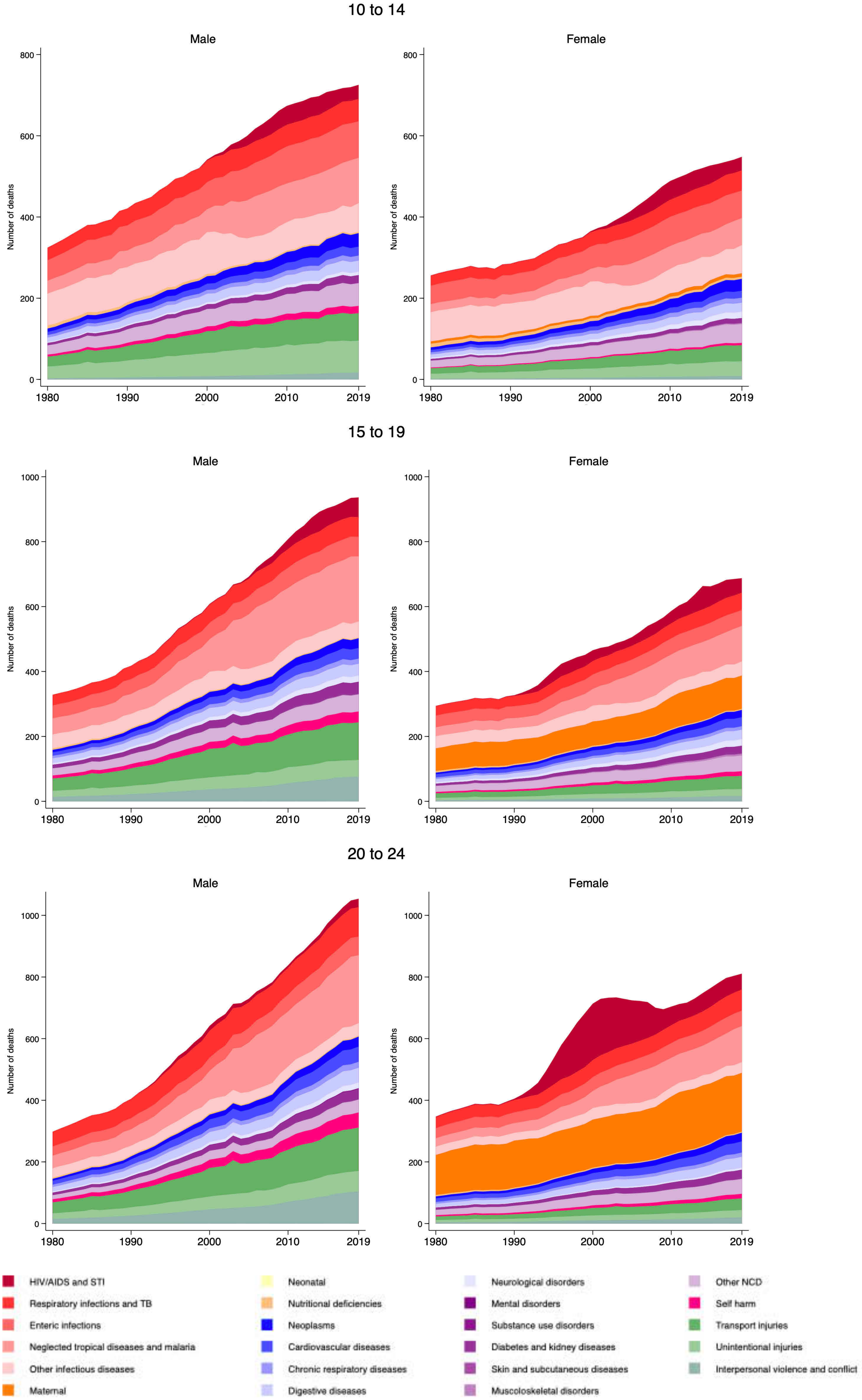

Figure S196: Burkina Faso

Figure S198a All-cause mortality rate per 100,000 in 10-24 1950 – 2019: Burkina Faso

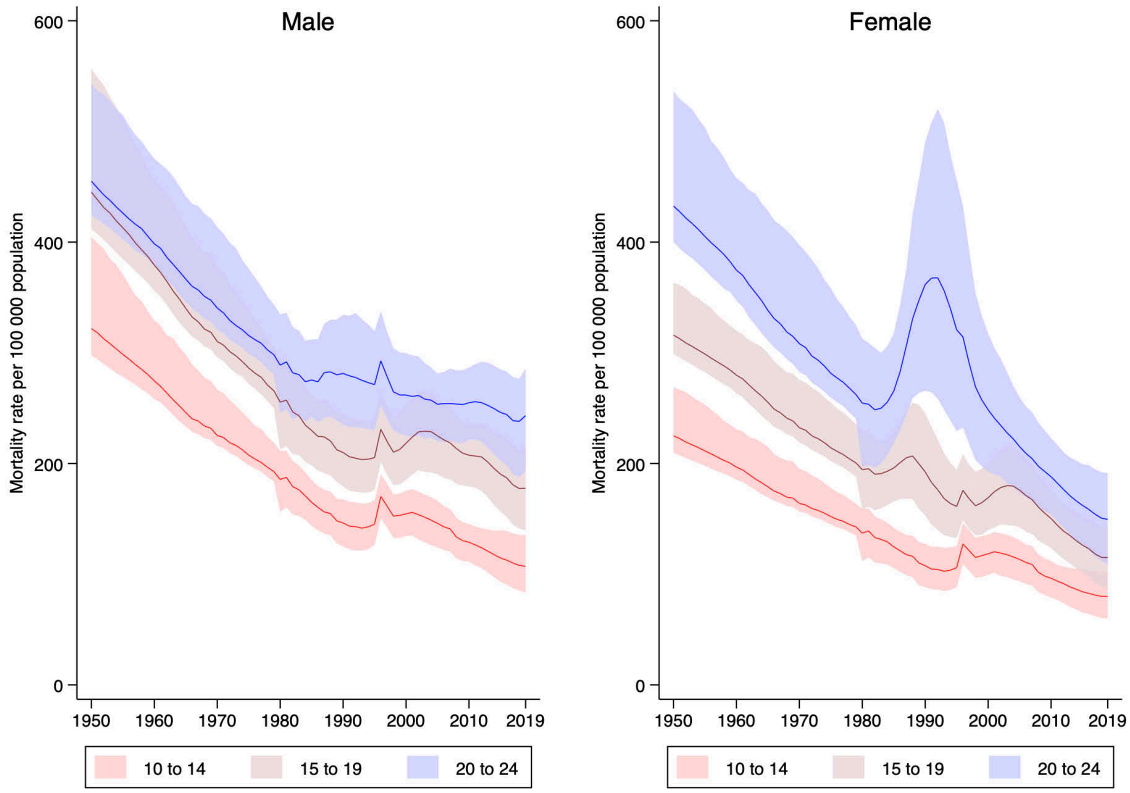

Figure S198b Number of deaths from all causes in 10-24 1950 – 2019: Burkina Faso

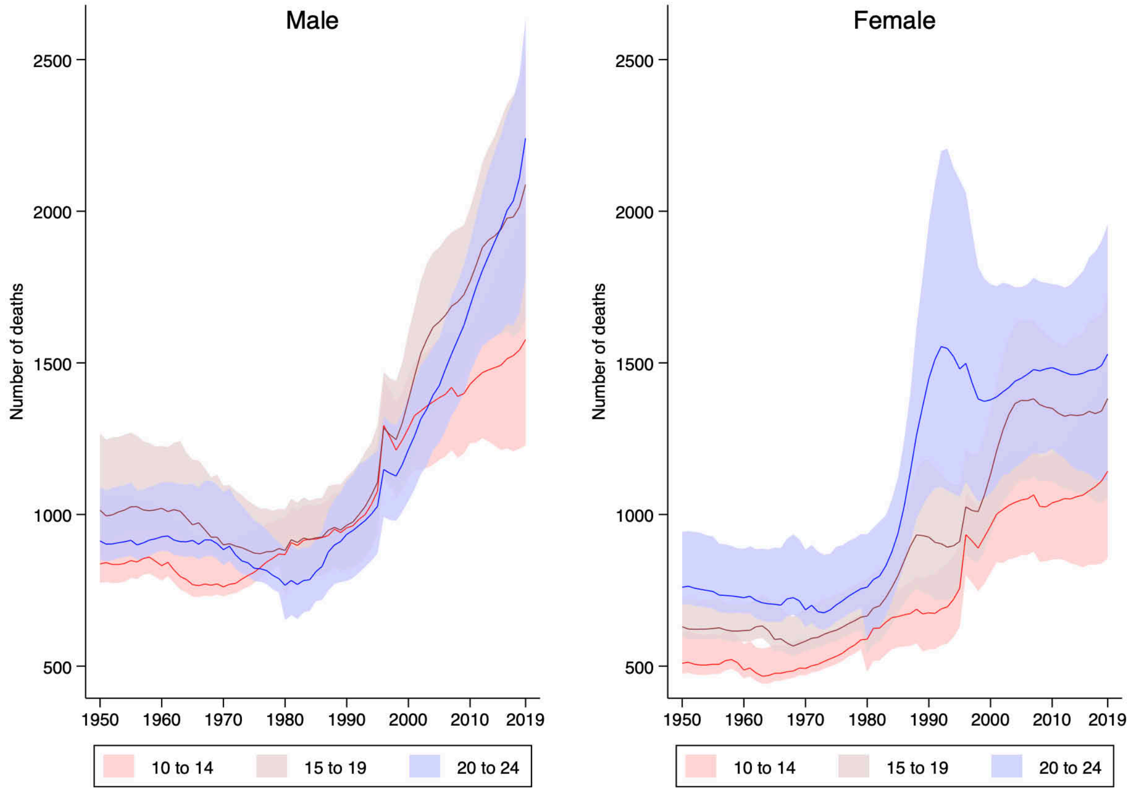

Figure S198c Mortality rate per 100,000 in 10-24 1980 – 2019 by cause: Burkina Faso

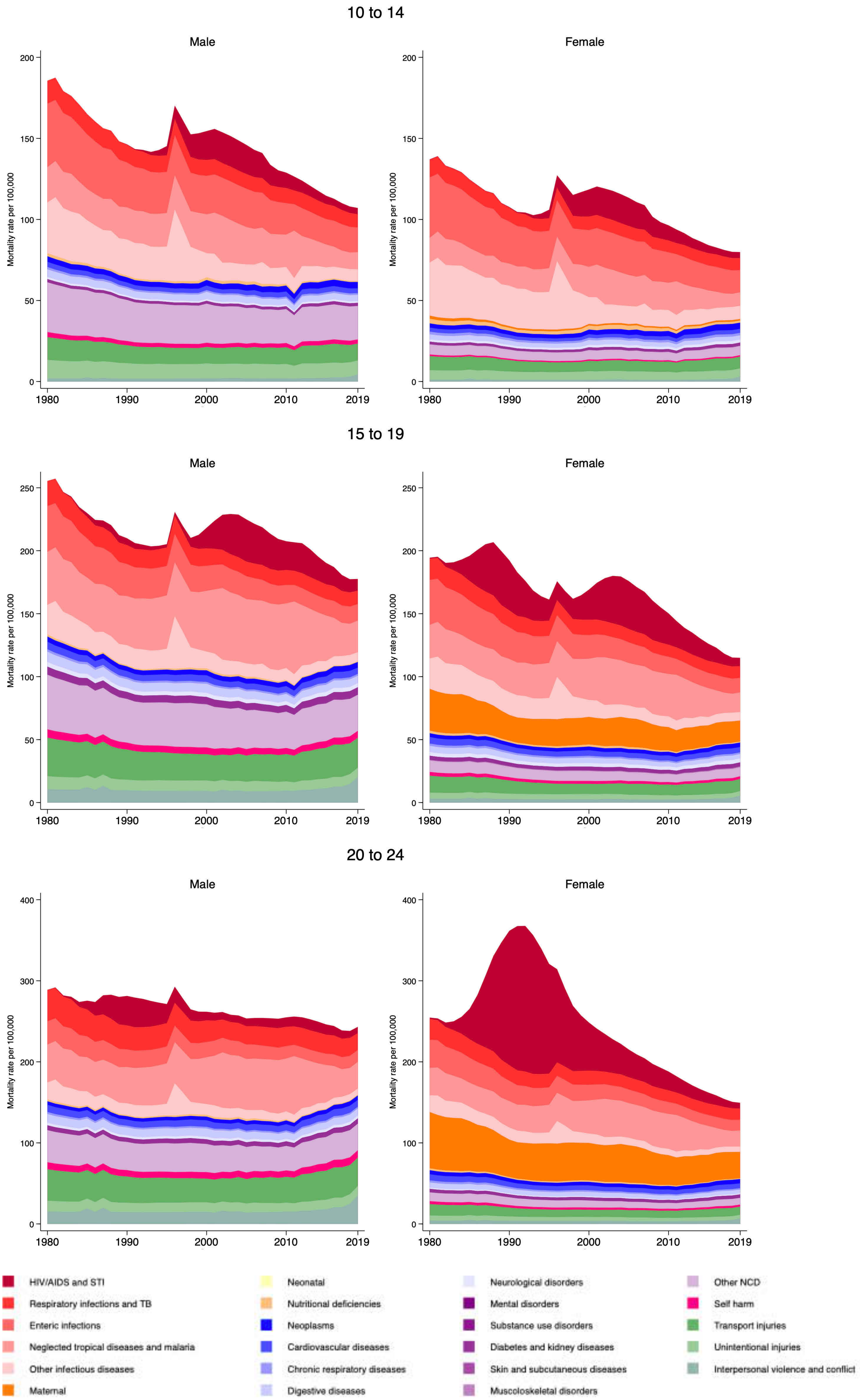

Figure S198d Number of deaths in 10-24 1980 – 2019 by cause: Burkina Faso

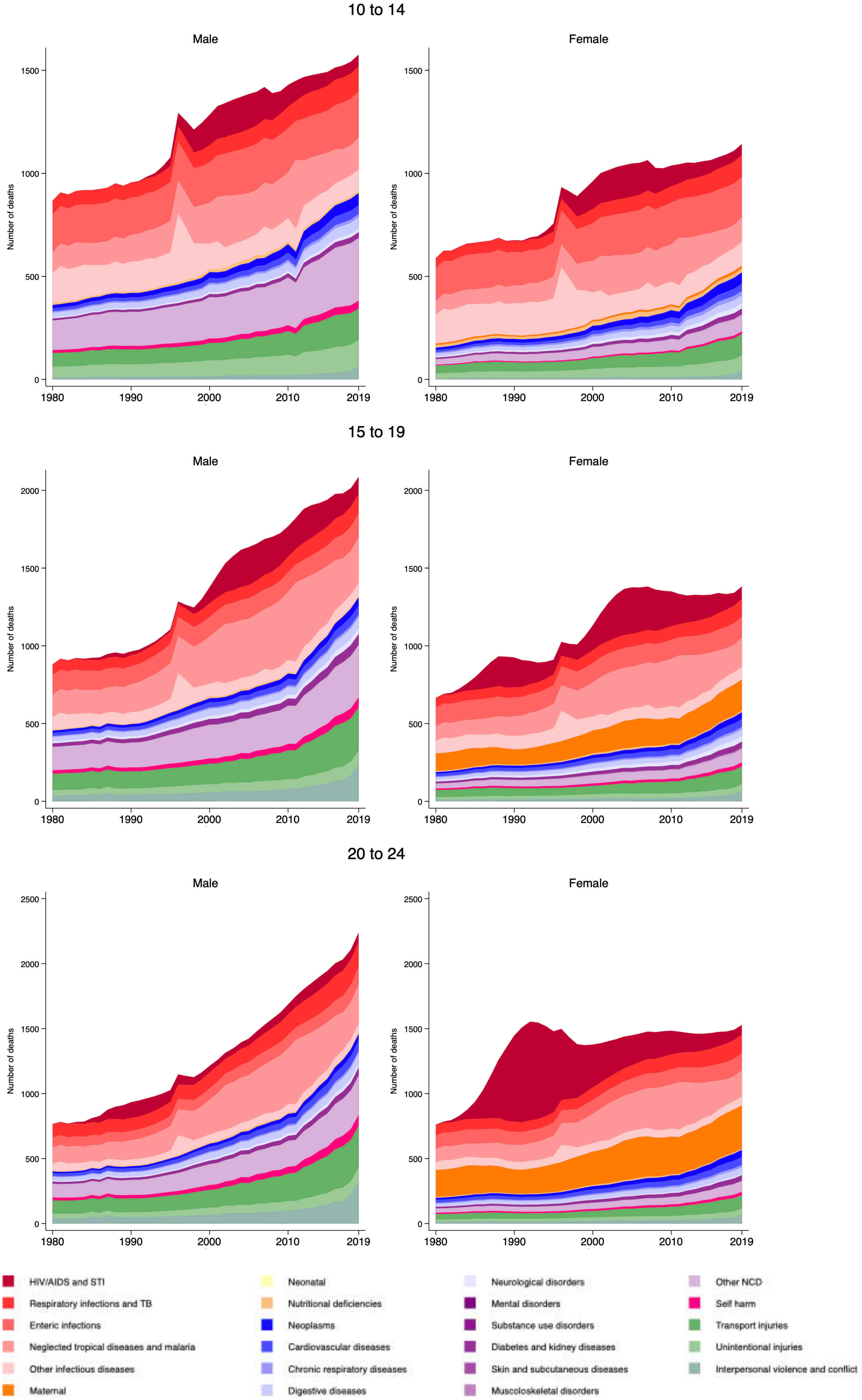

Figure S197: Cameroon

Figure S199a All-cause mortality rate per 100,000 in 10-24 1950 – 2019: Cameroon

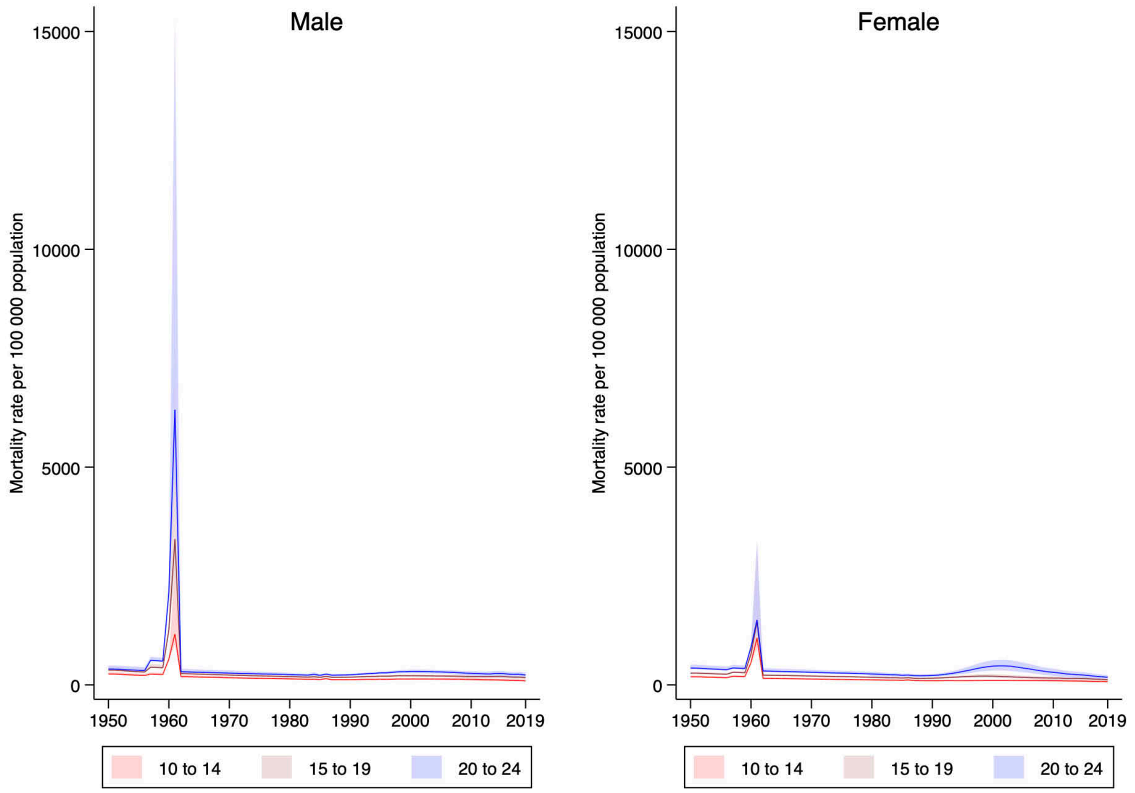

Figure S199b Number of deaths from all causes in 10-24 1950 – 2019: Cameroon

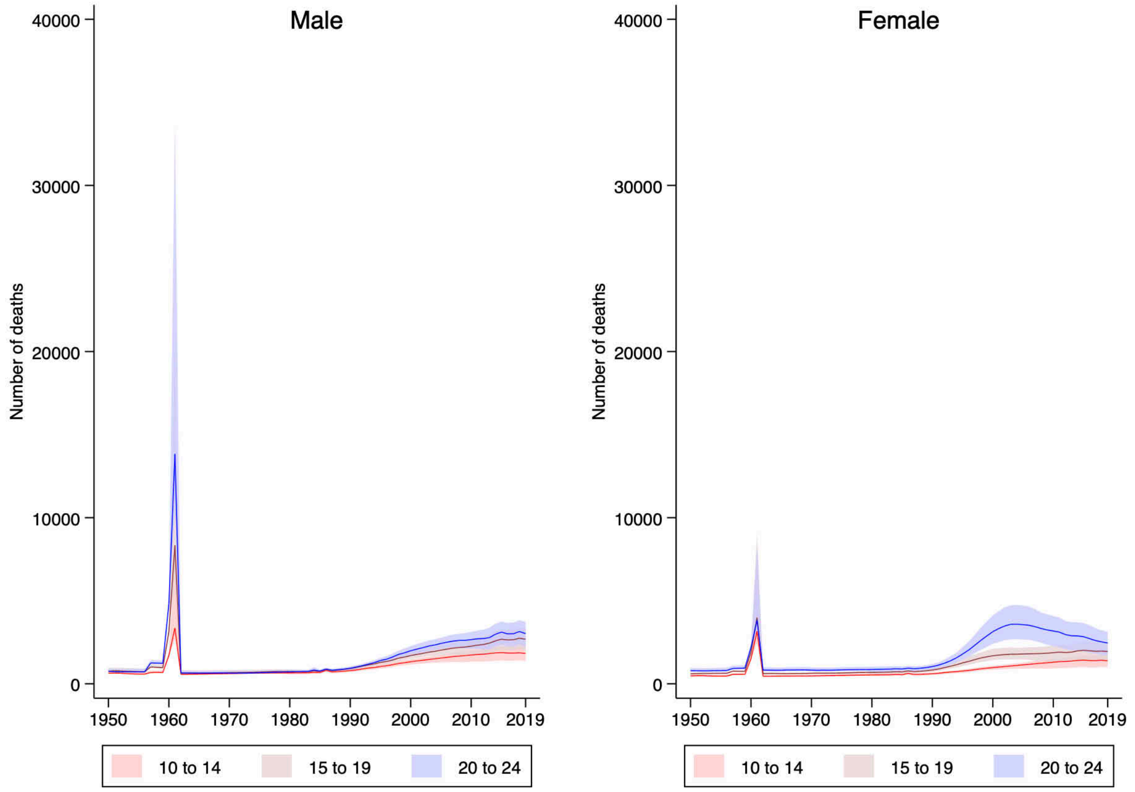

Figure S199c Mortality rate per 100,000 in 10-24 1980 – 2019 by cause: Cameroon

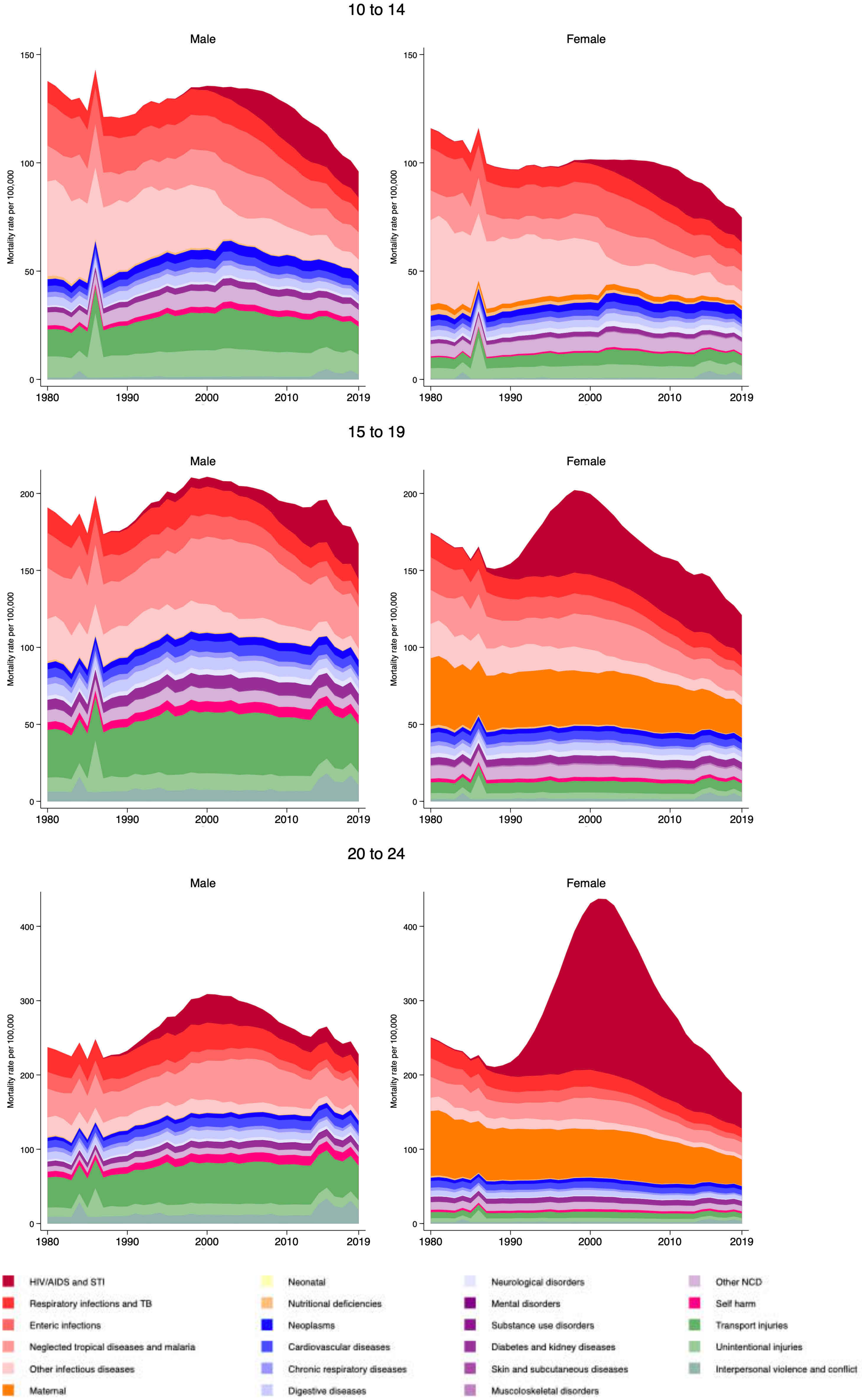

Figure S199d Number of deaths in 10-24 1980 – 2019 by cause: Cameroon

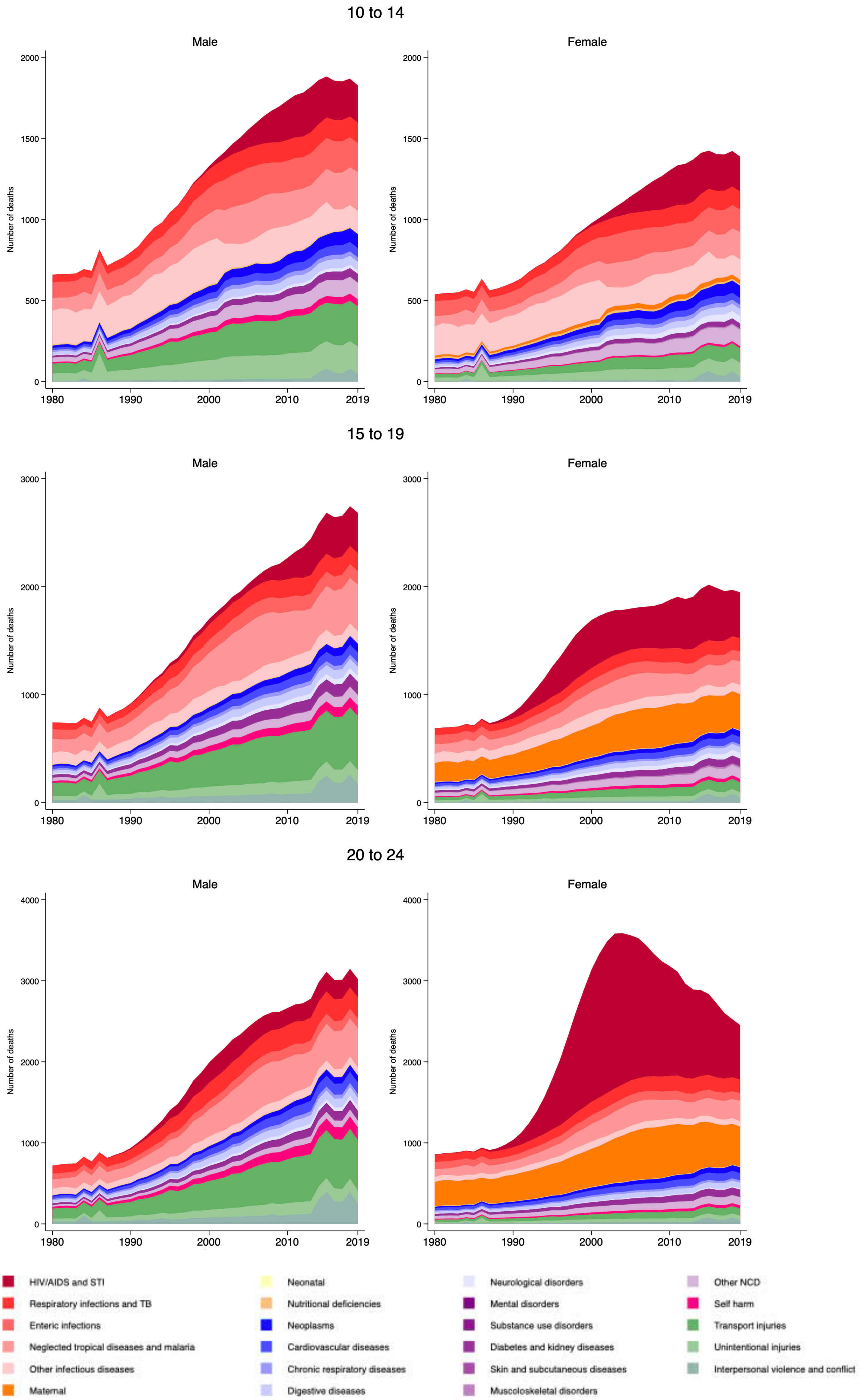

Figure S198: Cabo Verde

Figure S200a All-cause mortality rate per 100,000 in 10-24 1950 – 2019: Cabo Verde

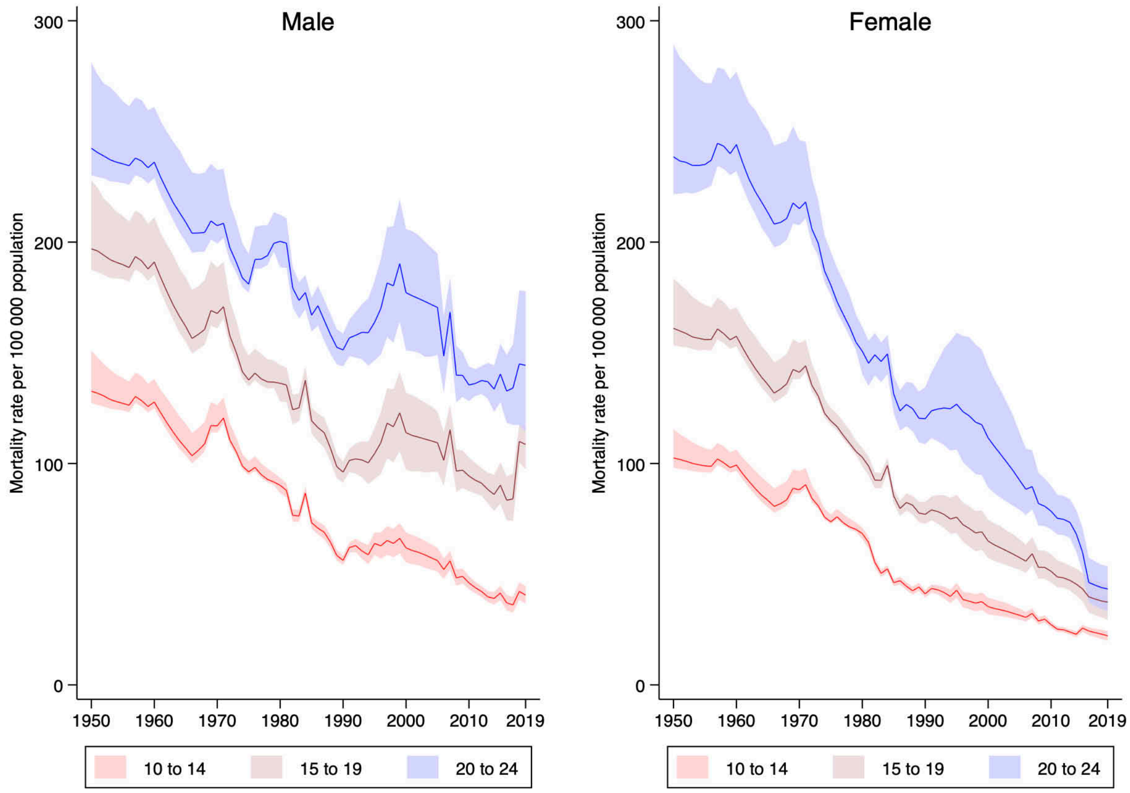

Figure S200b Number of deaths from all causes in 10-24 1950 – 2019: Cabo Verde

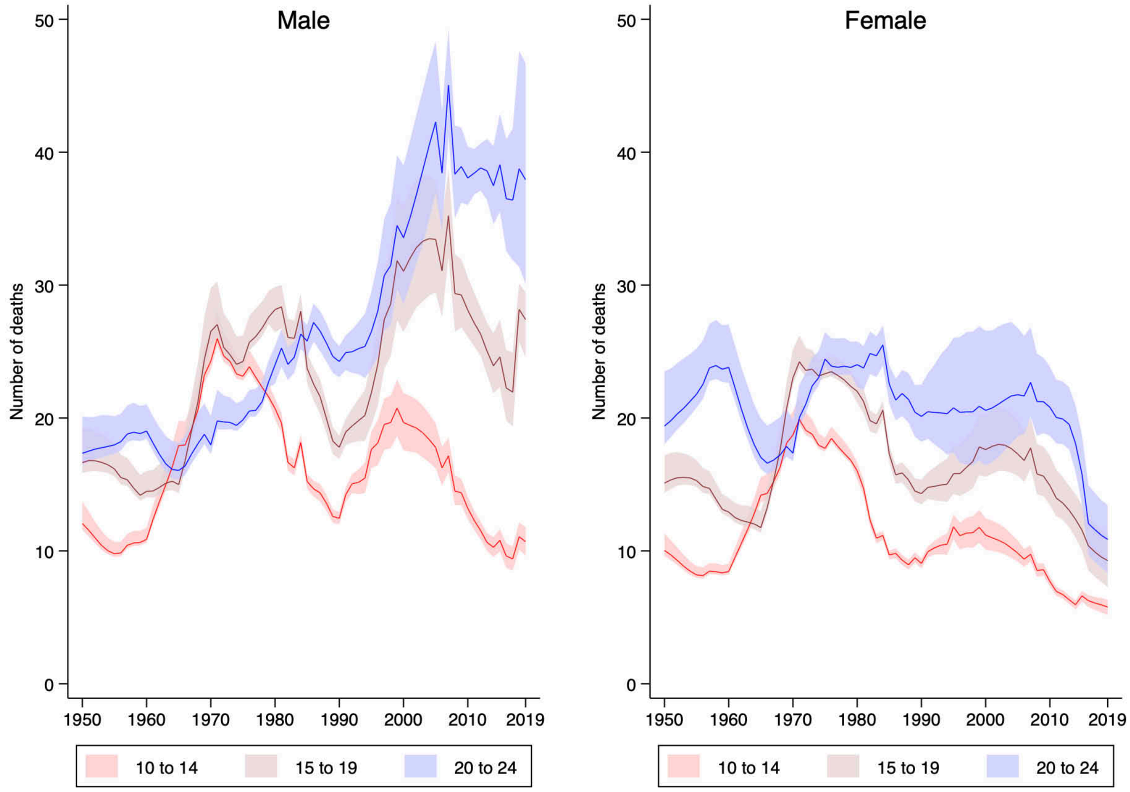

Figure S200c Mortality rate per 100,000 in 10-24 1980 – 2019 by cause: Cabo Verde

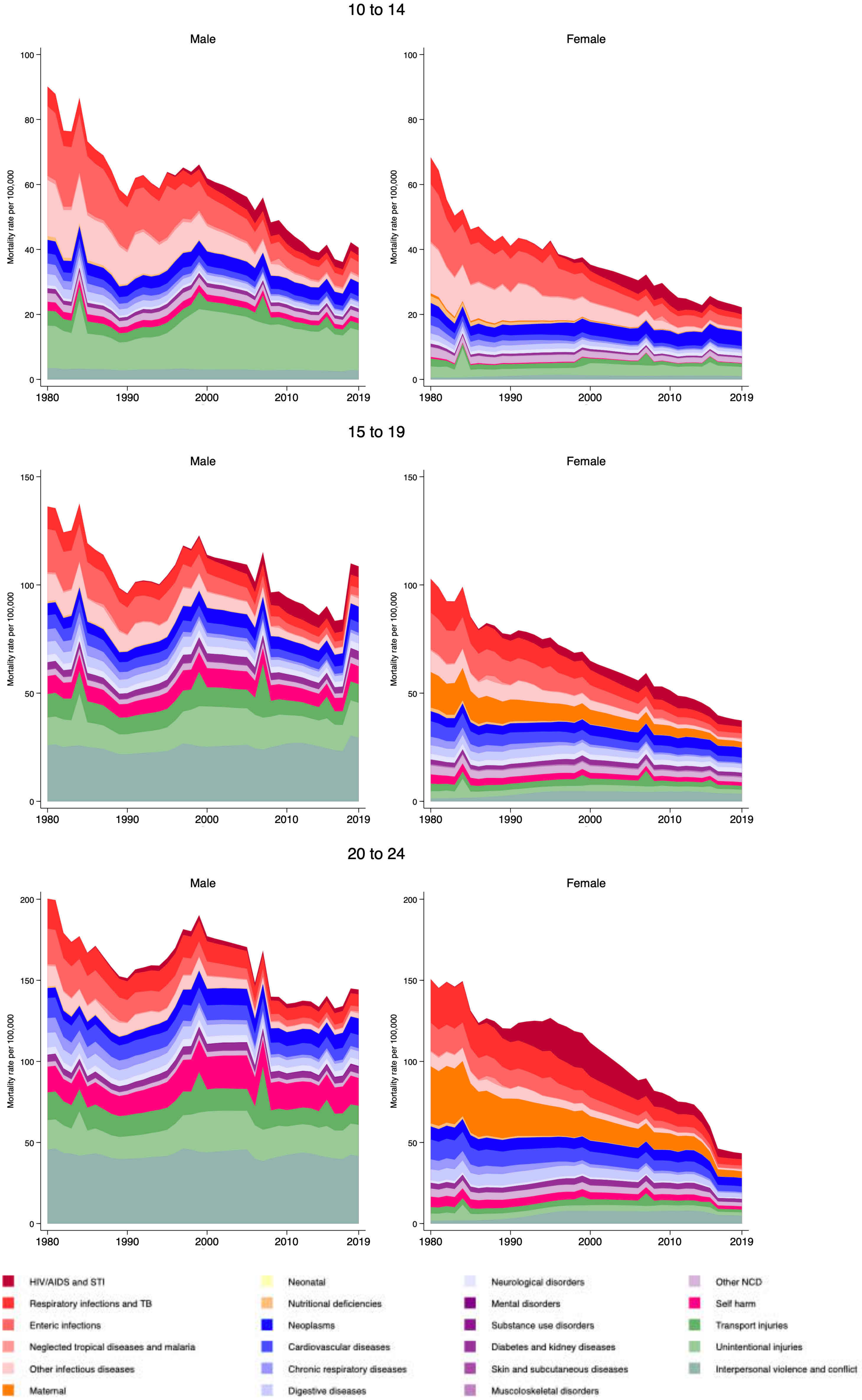

Figure S200d Number of deaths in 10-24 1980 – 2019 by cause: Cabo Verde

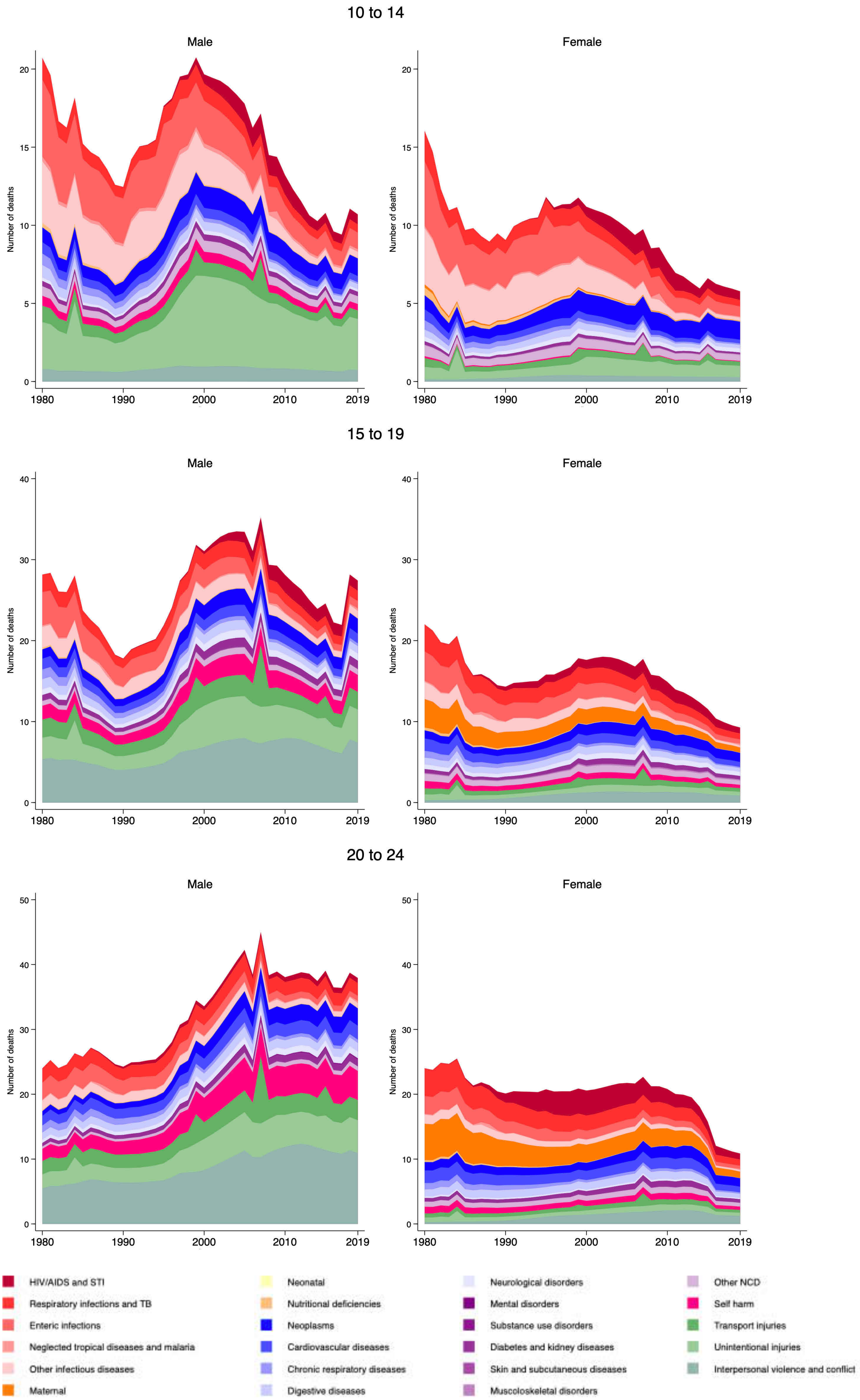

Figure S199: Chad

Figure S201a All-cause mortality rate per 100,000 in 10-24 1950 – 2019: Chad

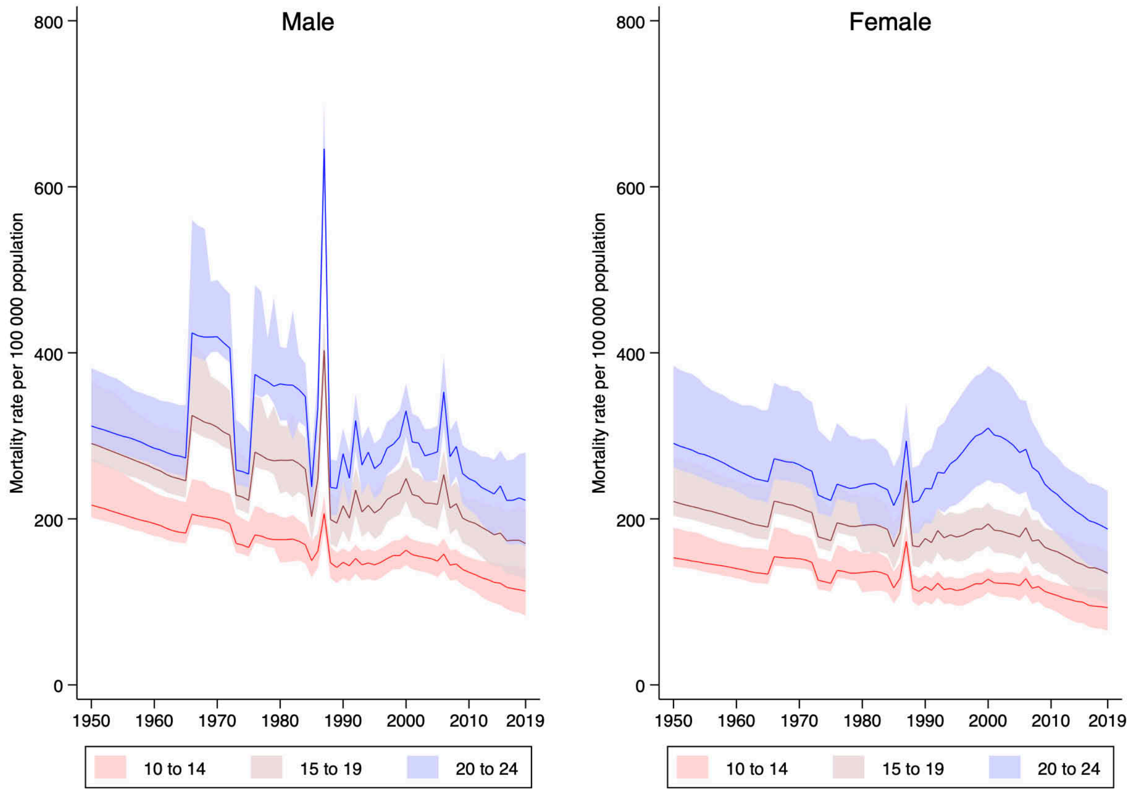

Figure S201b Number of deaths from all causes in 10-24 1950 – 2019: Chad

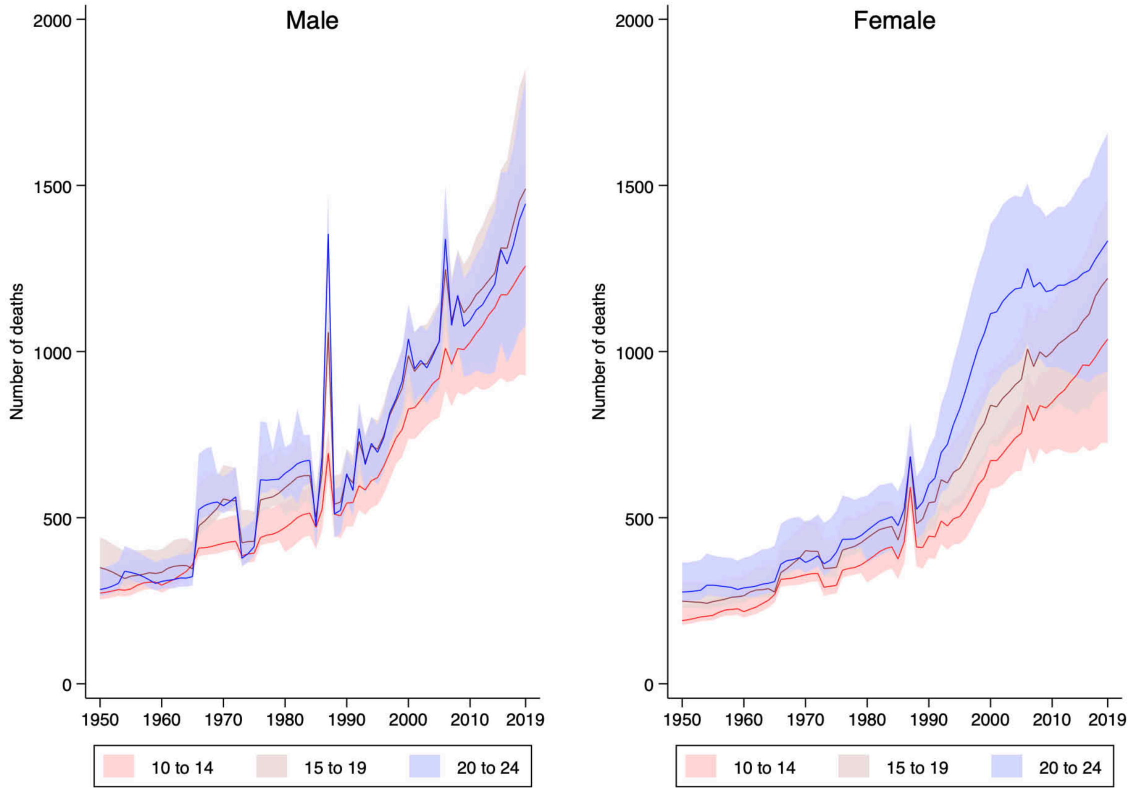

Figure S201c Mortality rate per 100,000 in 10-24 1980 – 2019 by cause: Chad

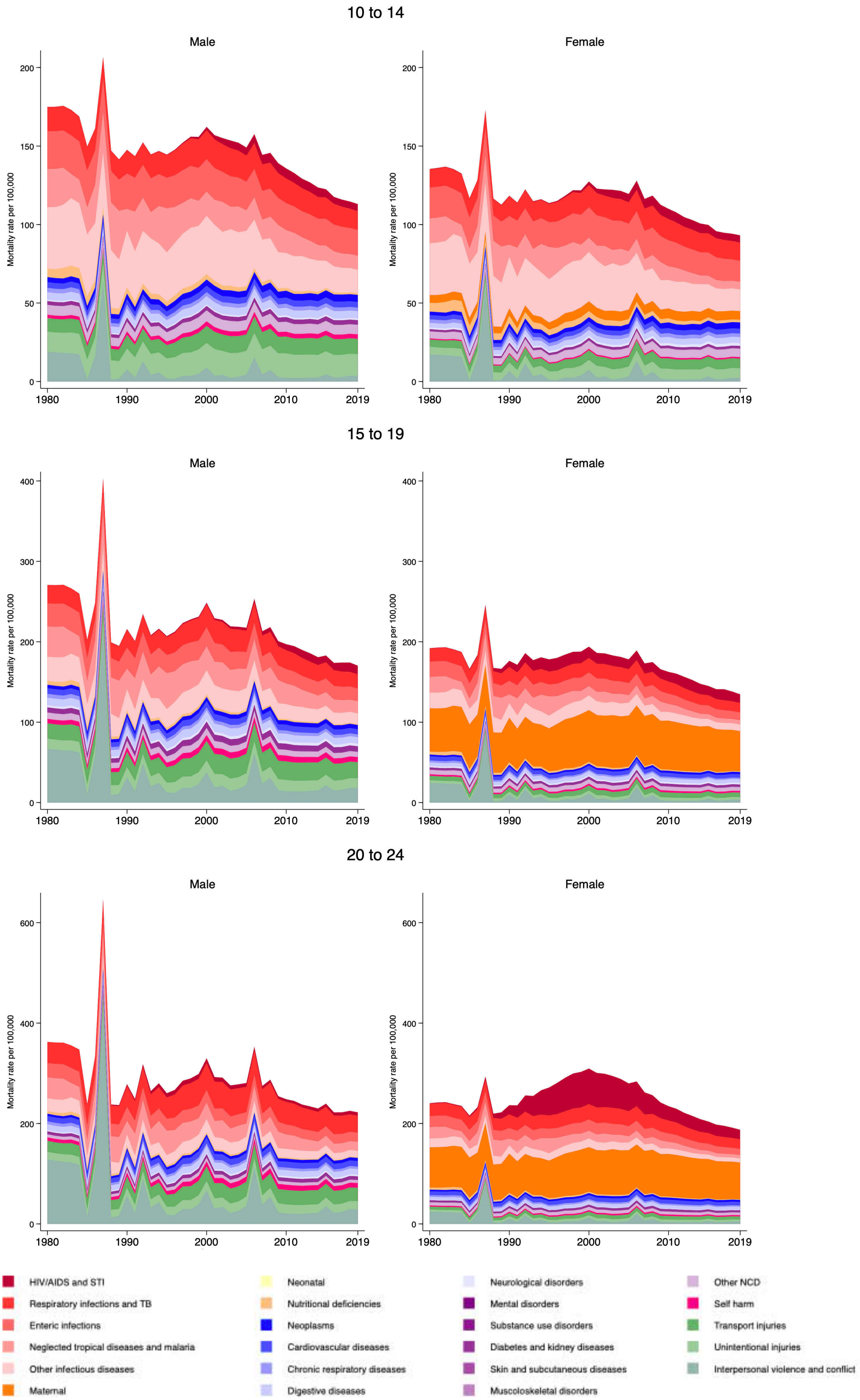

Figure S201d Number of deaths in 10-24 1980 – 2019 by cause: Chad

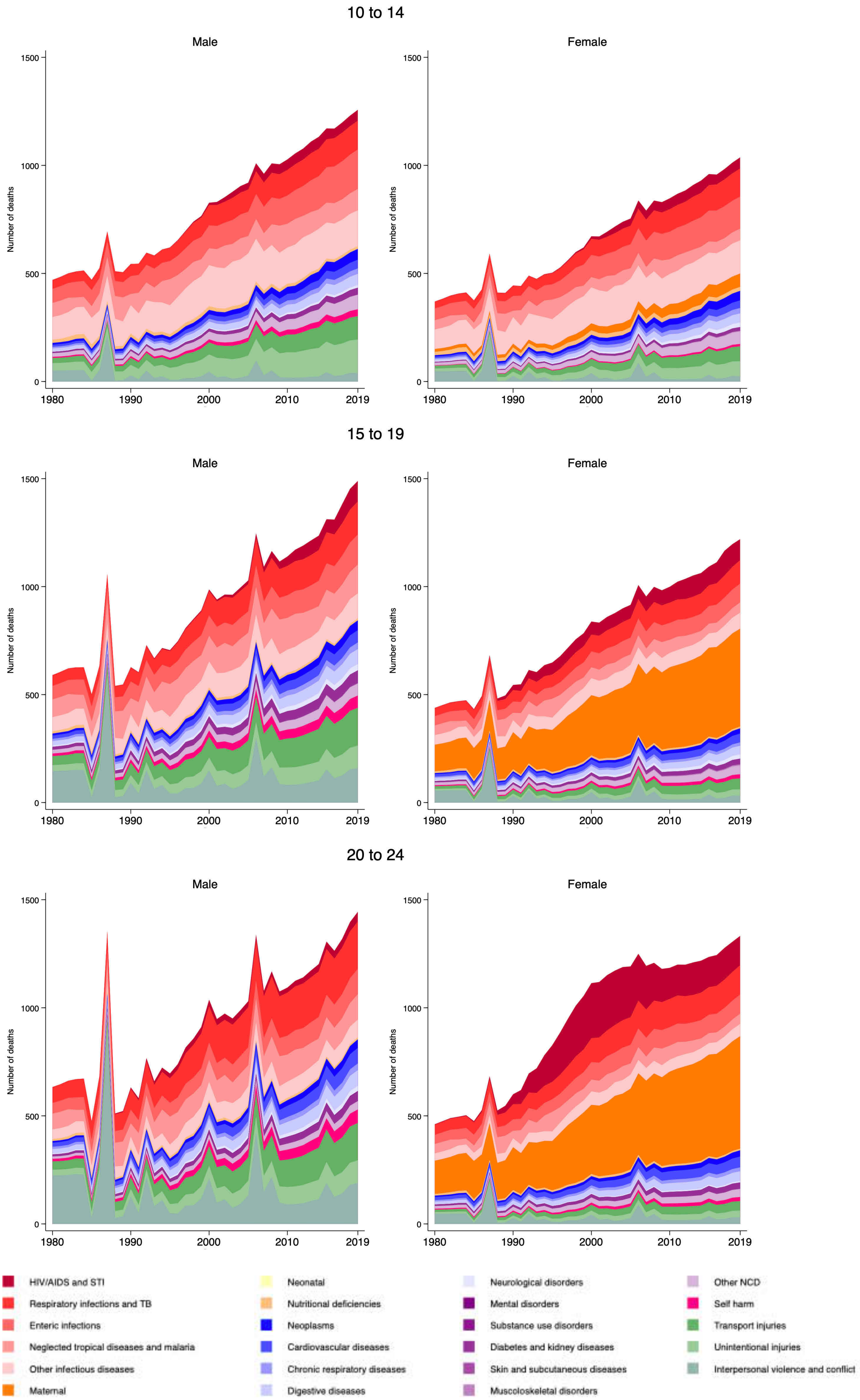

Figure S200: Côte d'Ivoire

Figure S202a All-cause mortality rate per 100,000 in 10-24 1950 – 2019: Côte d'Ivoire

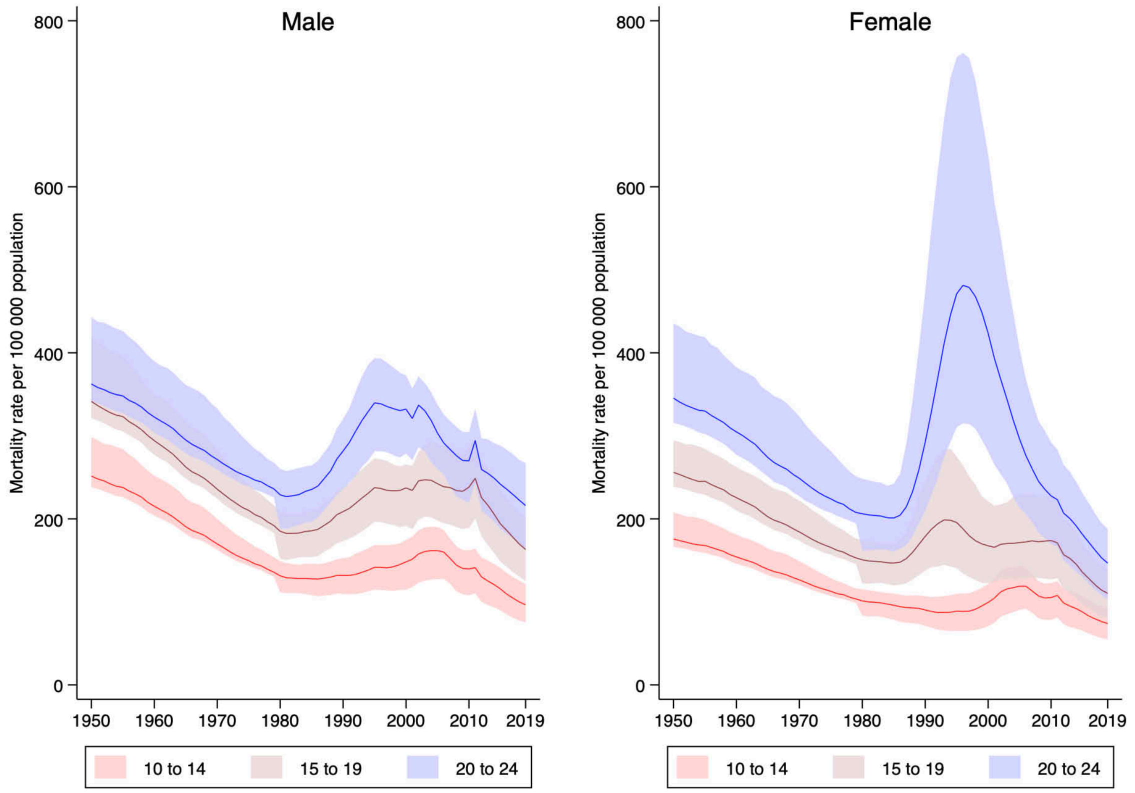

Figure S202b Number of deaths from all causes in 10-24 1950 – 2019: Côte d'Ivoire

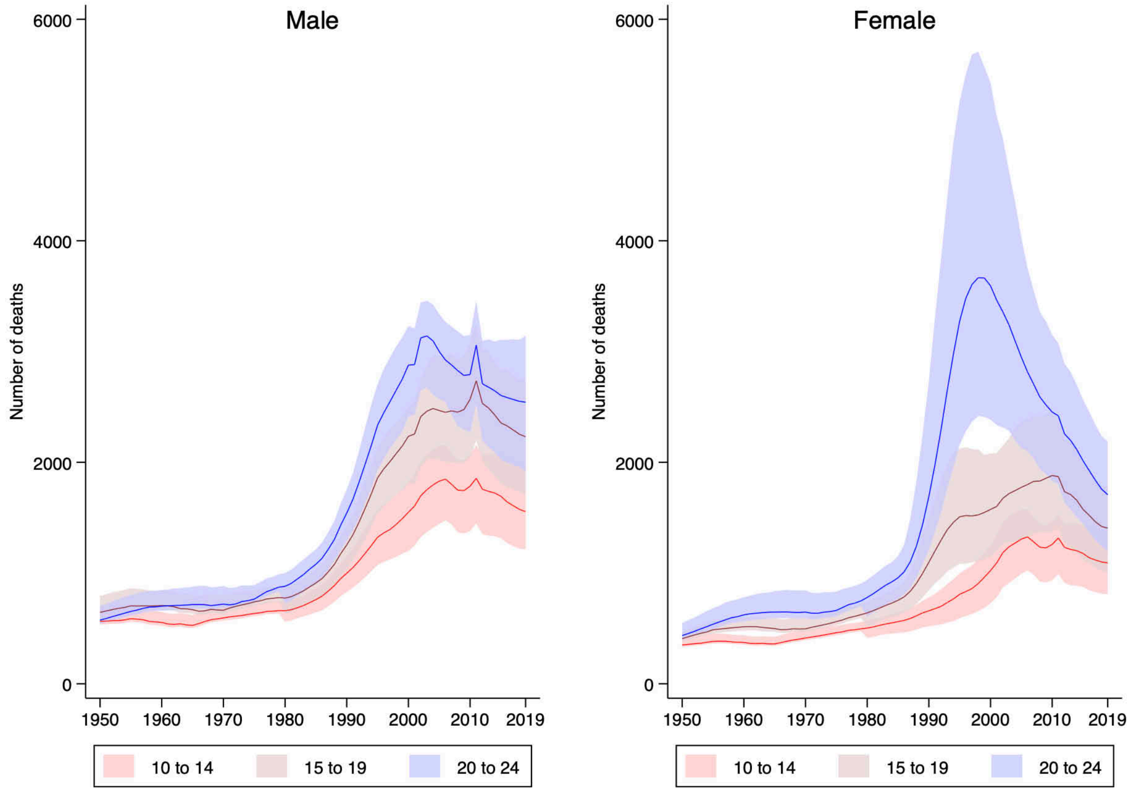

Figure S202c Mortality rate per 100,000 in 10-24 1980 – 2019 by cause: Côte d'Ivoire

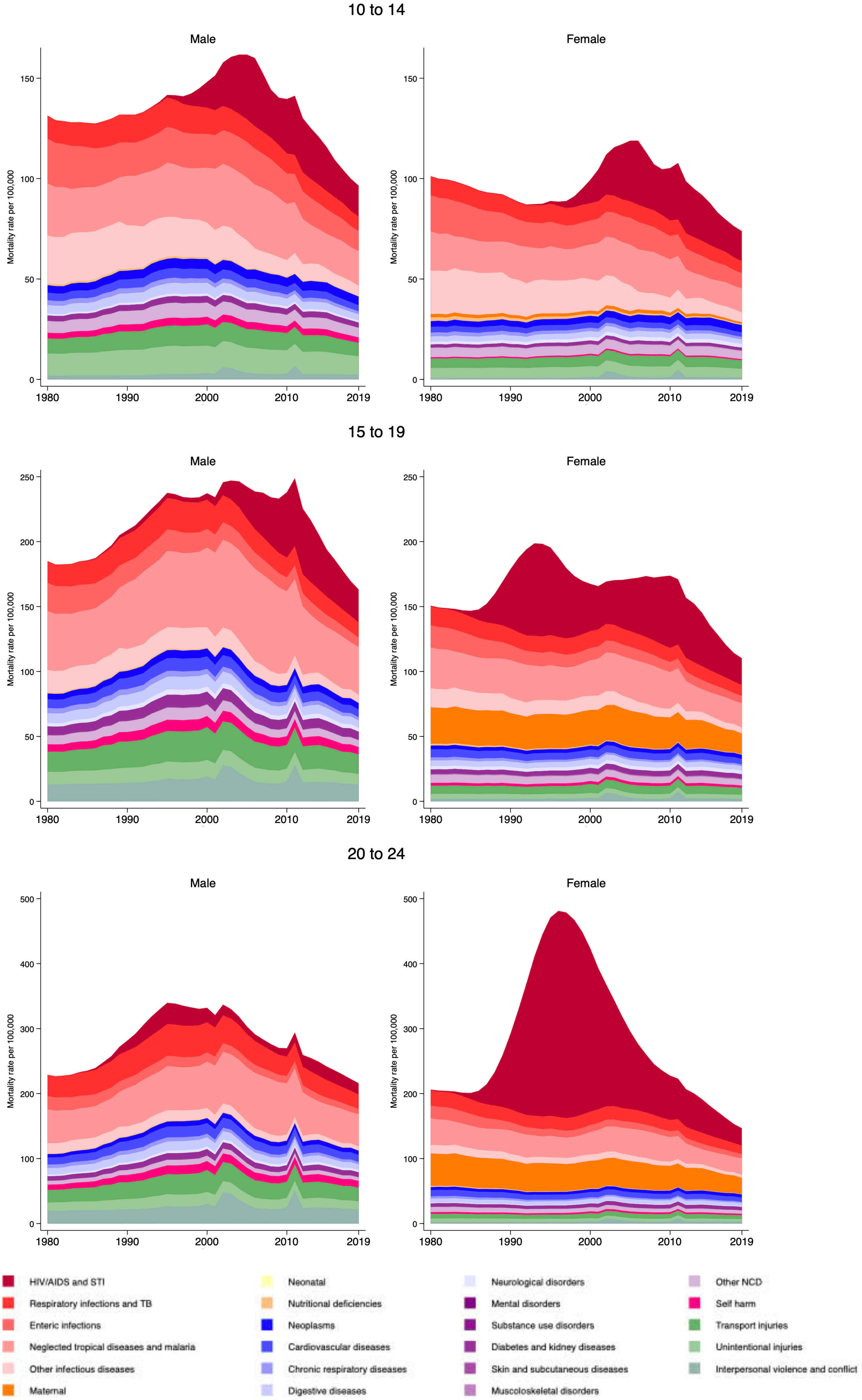

Figure S202d Number of deaths in 10-24 1980 – 2019 by cause: Côte d'Ivoire

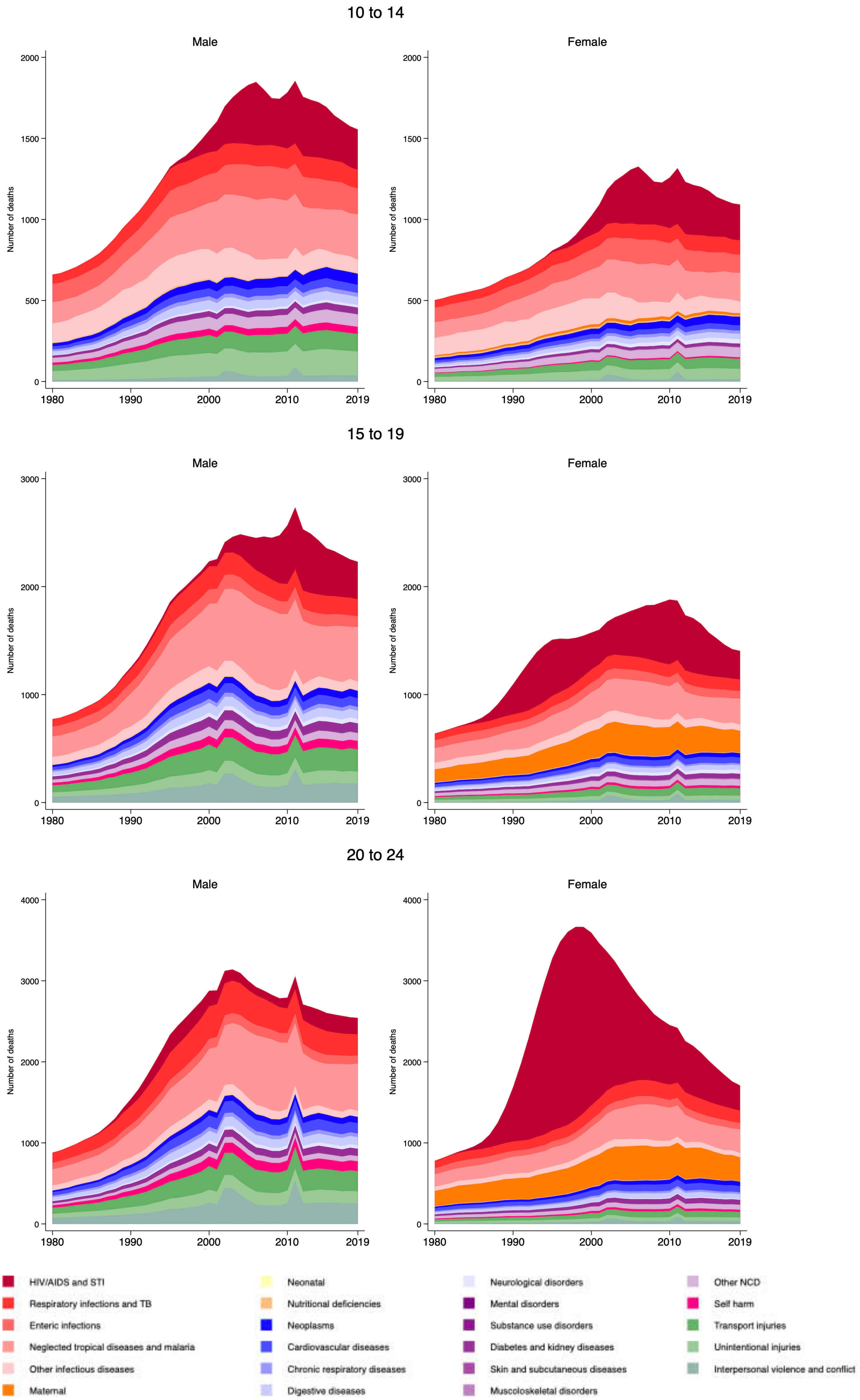

Figure S201: Gambia

Figure S203a All-cause mortality rate per 100,000 in 10-24 1950 – 2019: Gambia

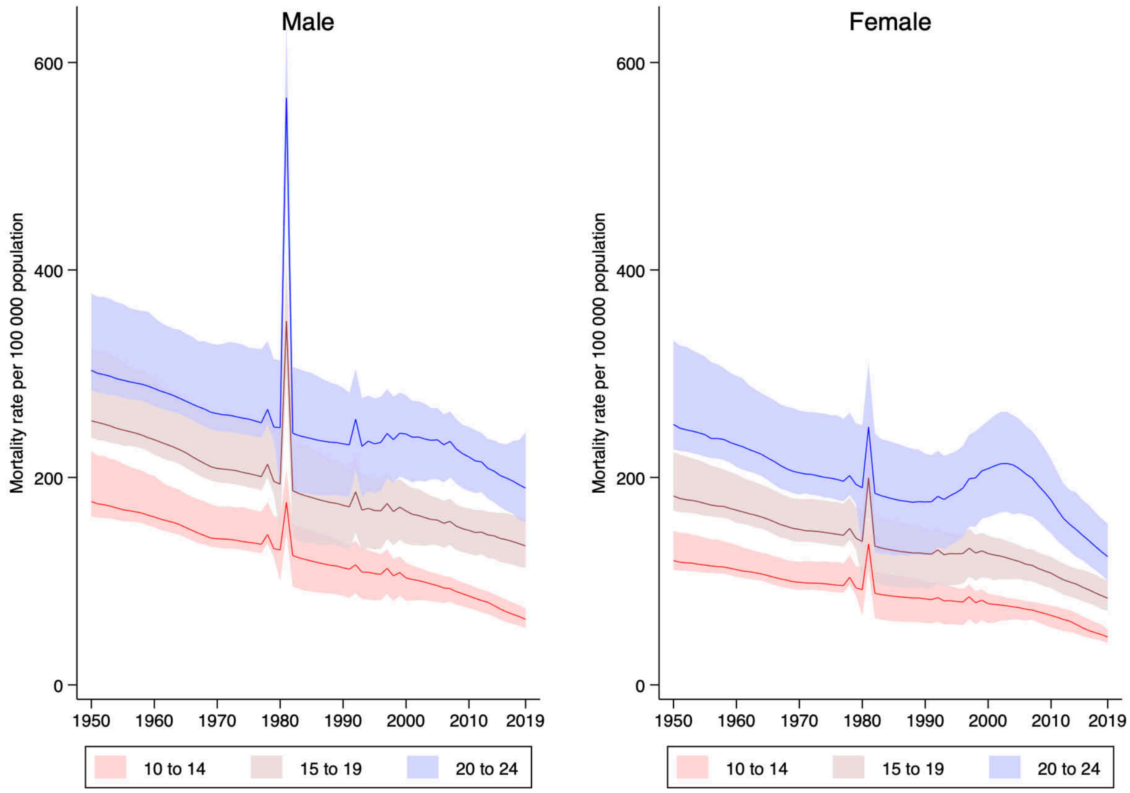

Figure S203b Number of deaths from all causes in 10-24 1950 – 2019: Gambia

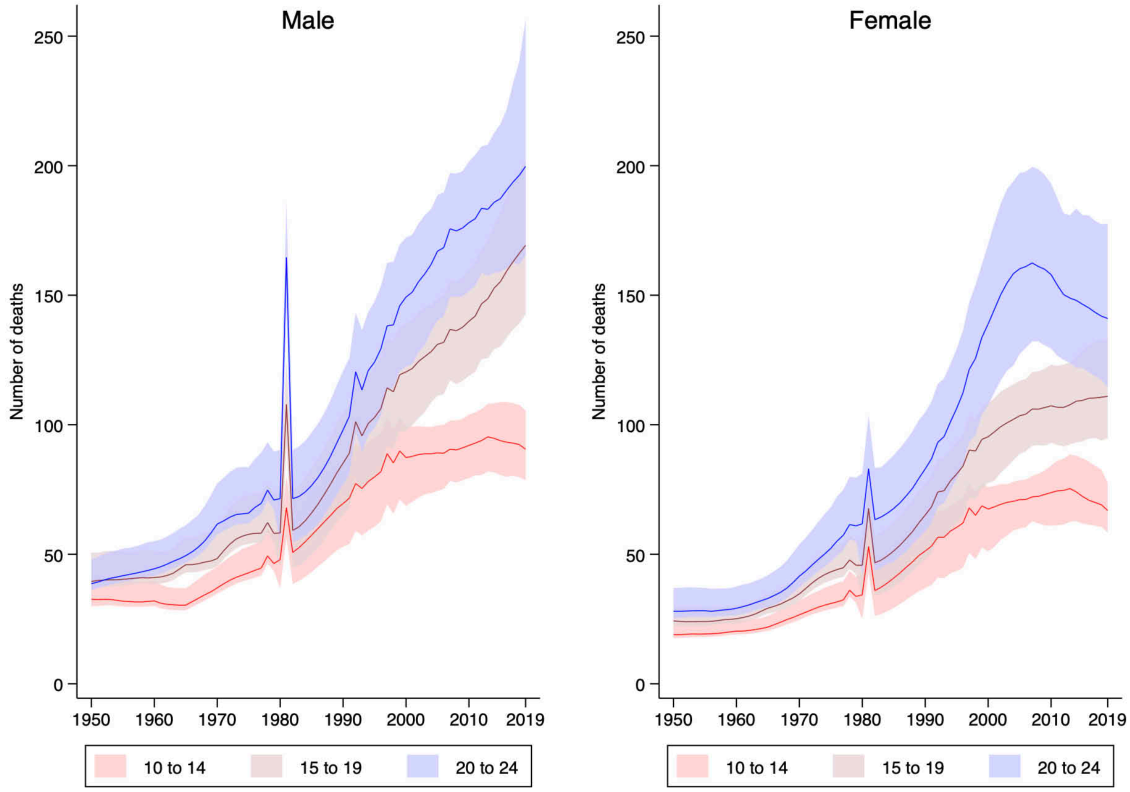

Figure S203c Mortality rate per 100,000 in 10-24 1980 – 2019 by cause: Gambia

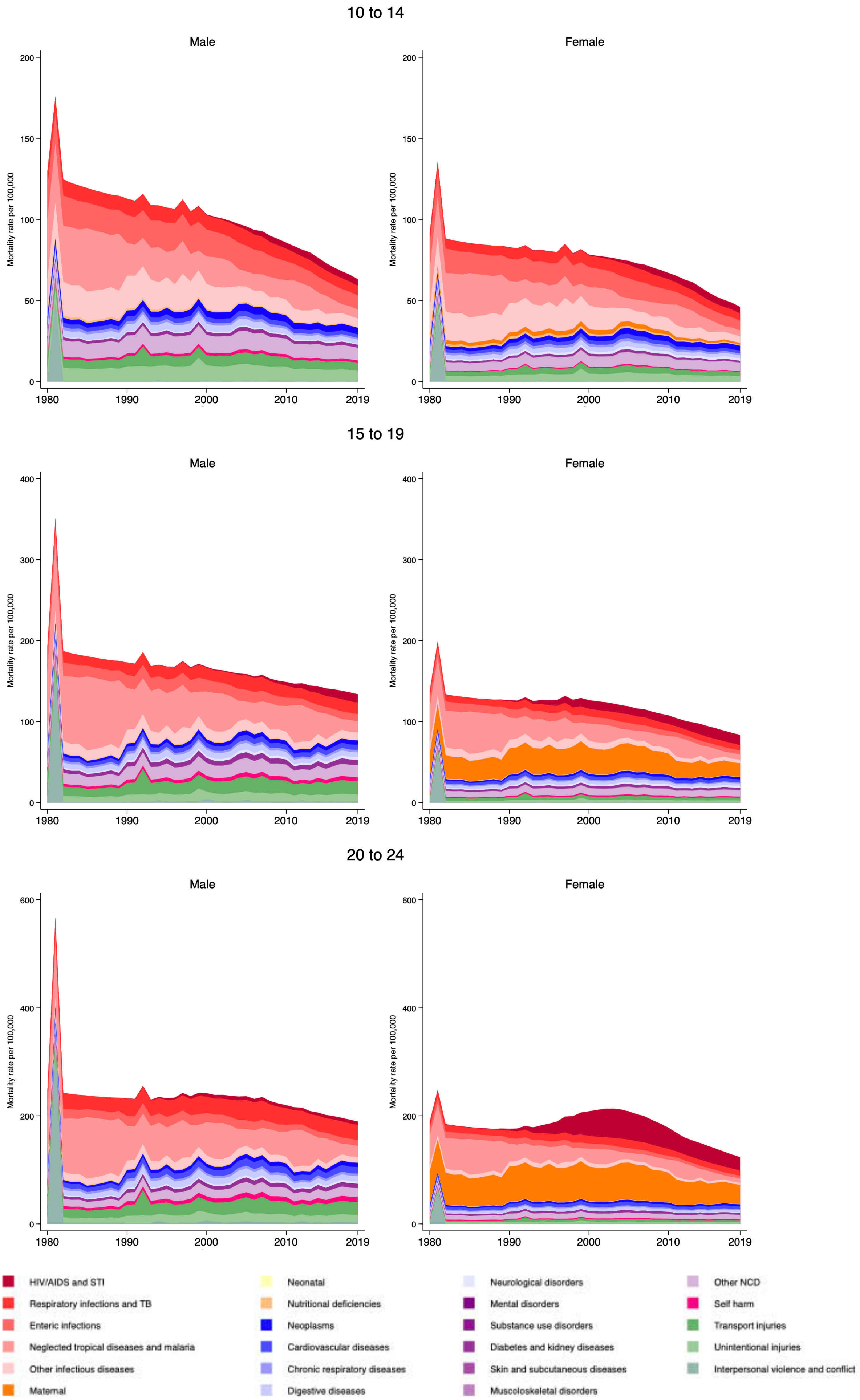

Figure S203d Number of deaths in 10-24 1980 – 2019 by cause: Gambia

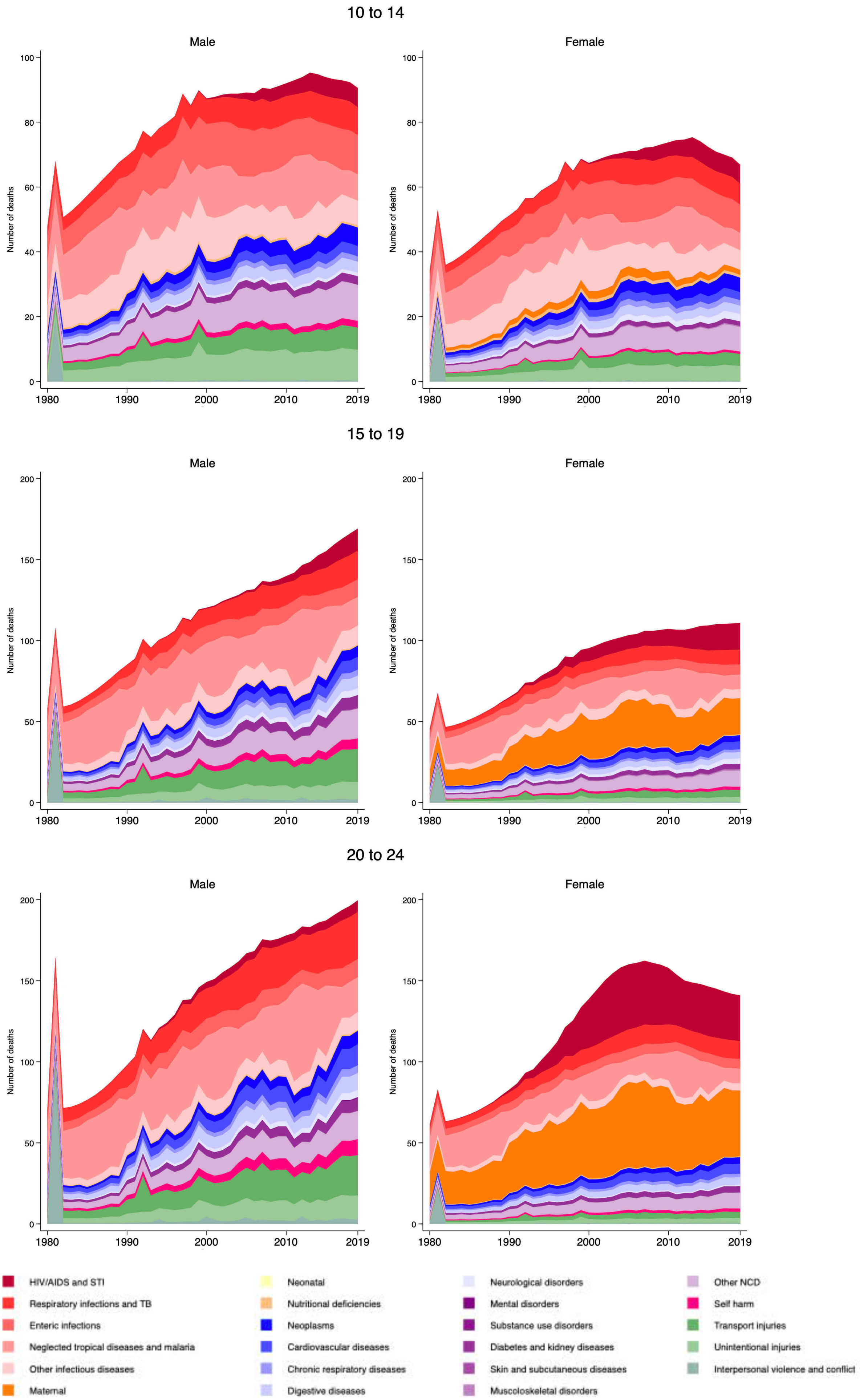

Figure S202: Ghana

Figure S204a All-cause mortality rate per 100,000 in 10-24 1950 – 2019: Ghana

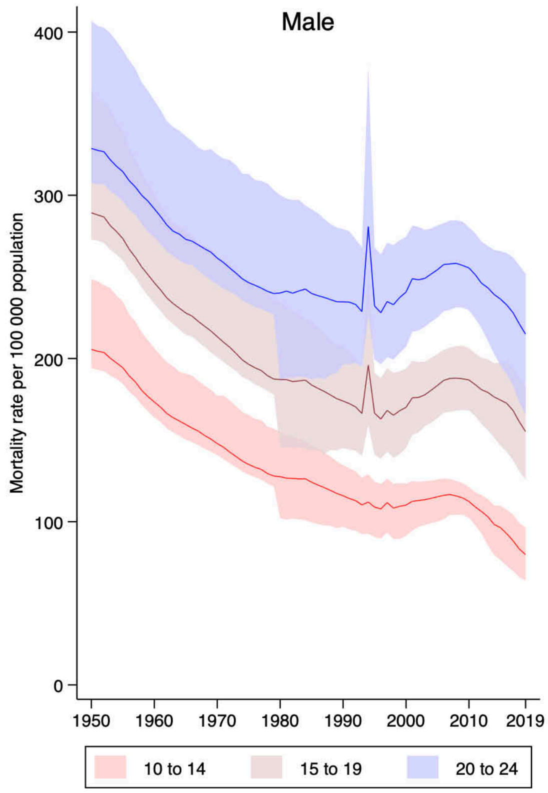

Figure S204b Number of deaths from all causes in 10-24 1950 – 2019: Ghana

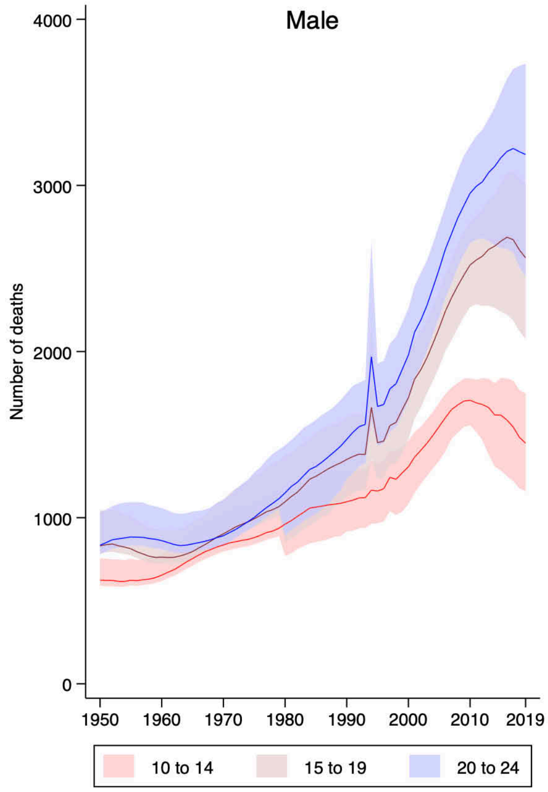

Figure S204c Mortality rate per 100,000 in 10-24 1980 – 2019 by cause: Ghana

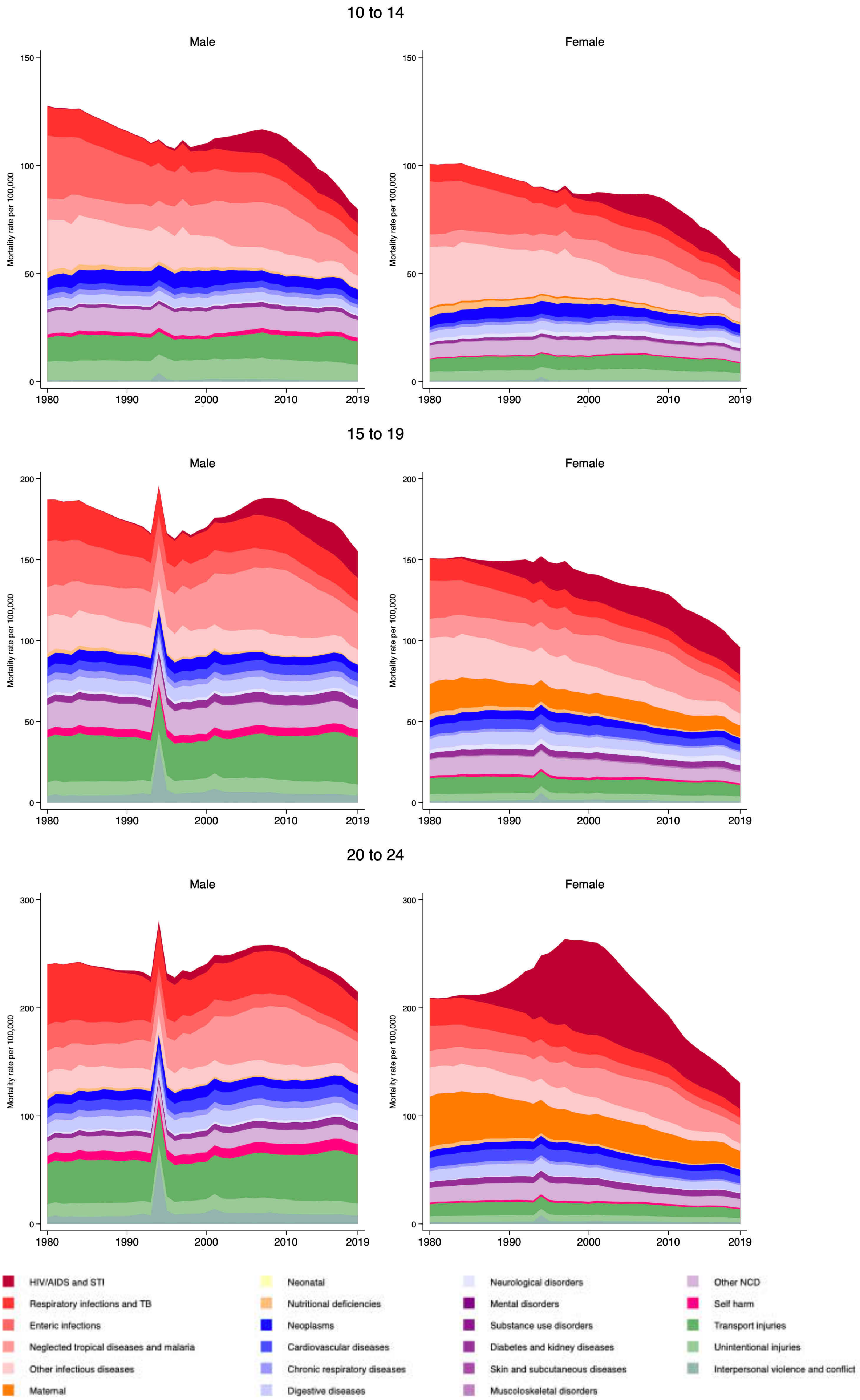

Figure S204d Number of deaths in 10-24 1980 – 2019 by cause: Ghana

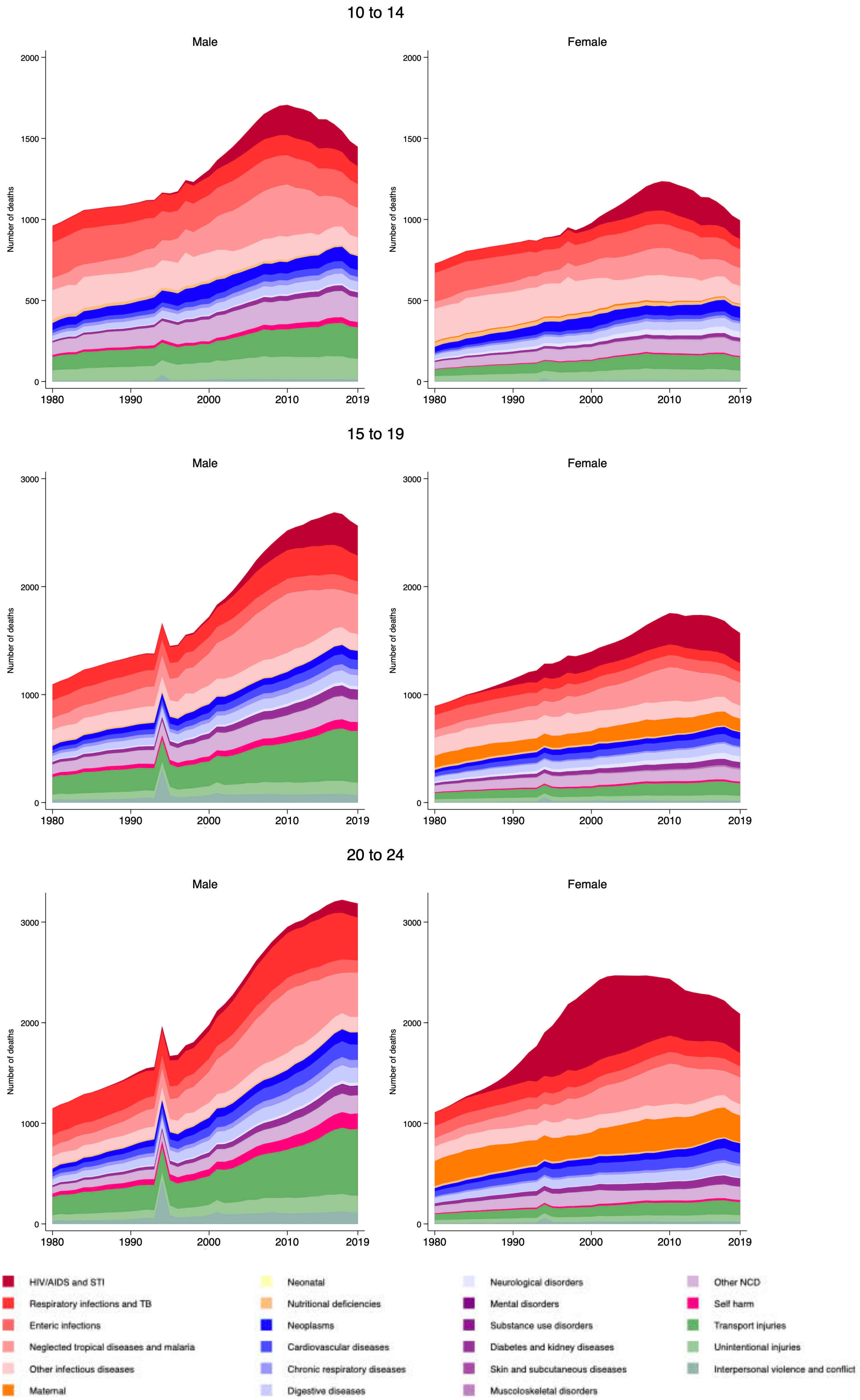

Figure S203: Guinea

Figure S205a All-cause mortality rate per 100,000 in 10-24 1950 – 2019: Guinea

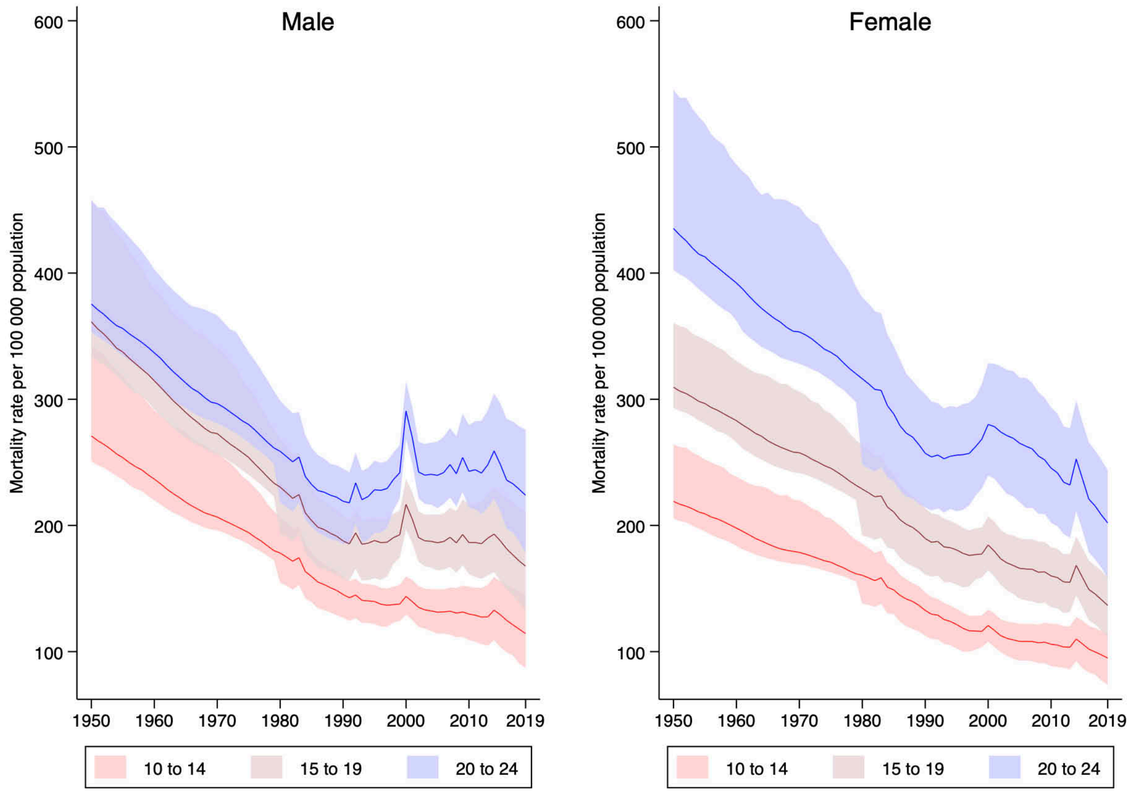

Figure S205b Number of deaths from all causes in 10-24 1950 – 2019: Guinea

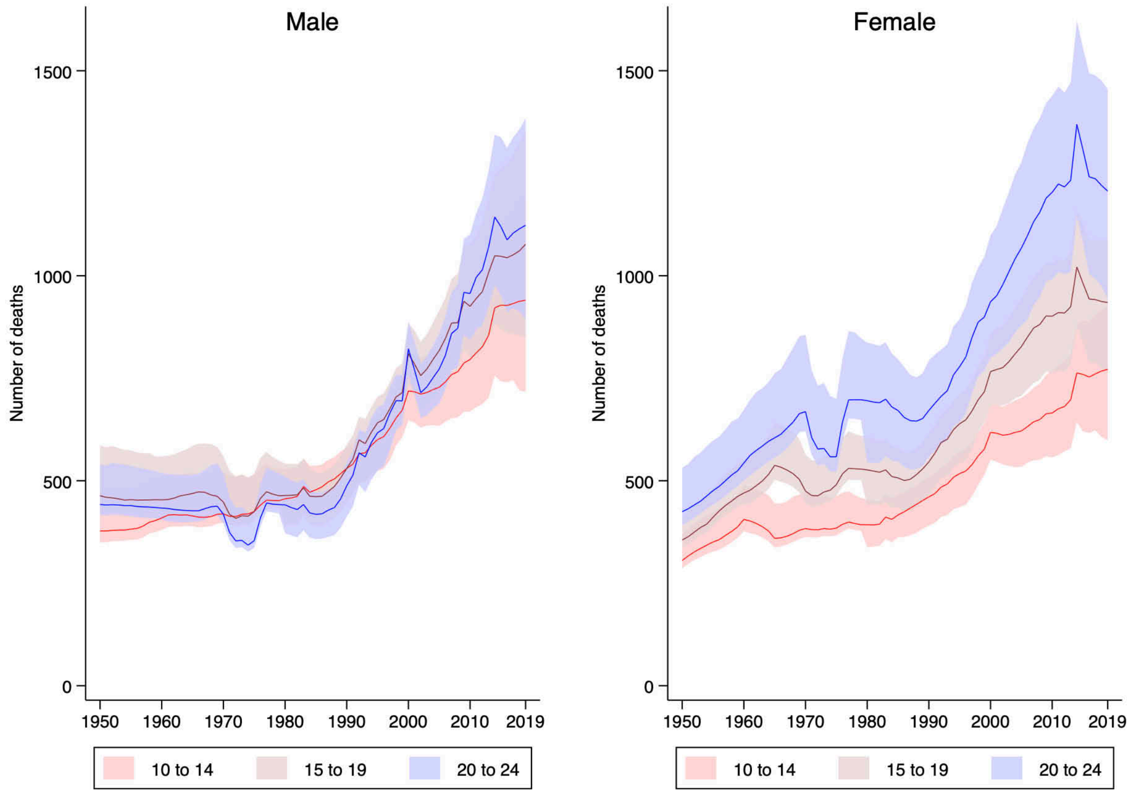

Figure S205c Mortality rate per 100,000 in 10-24 1980 – 2019 by cause: Guinea

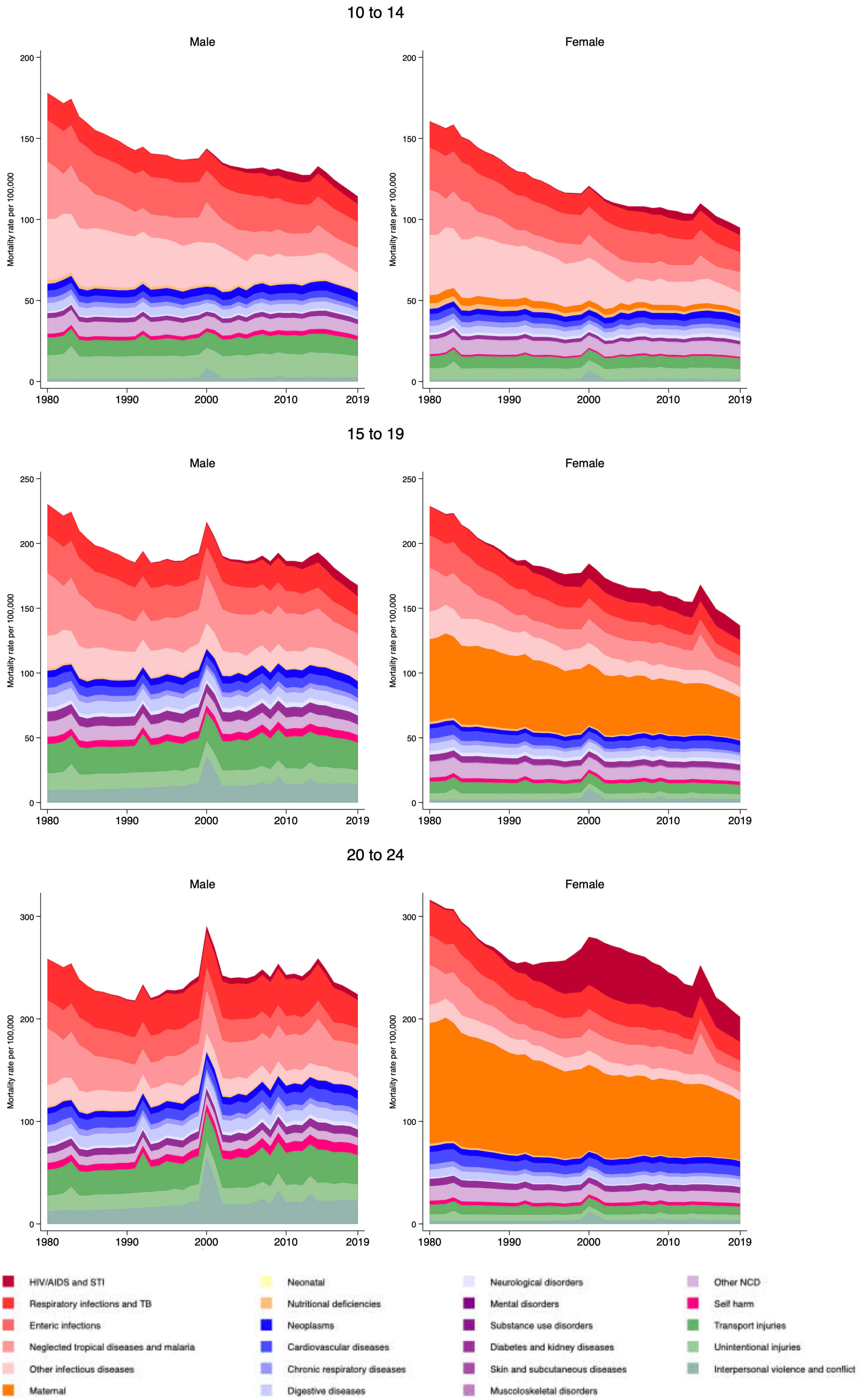

Figure S205d Number of deaths in 10-24 1980 – 2019 by cause: Guinea

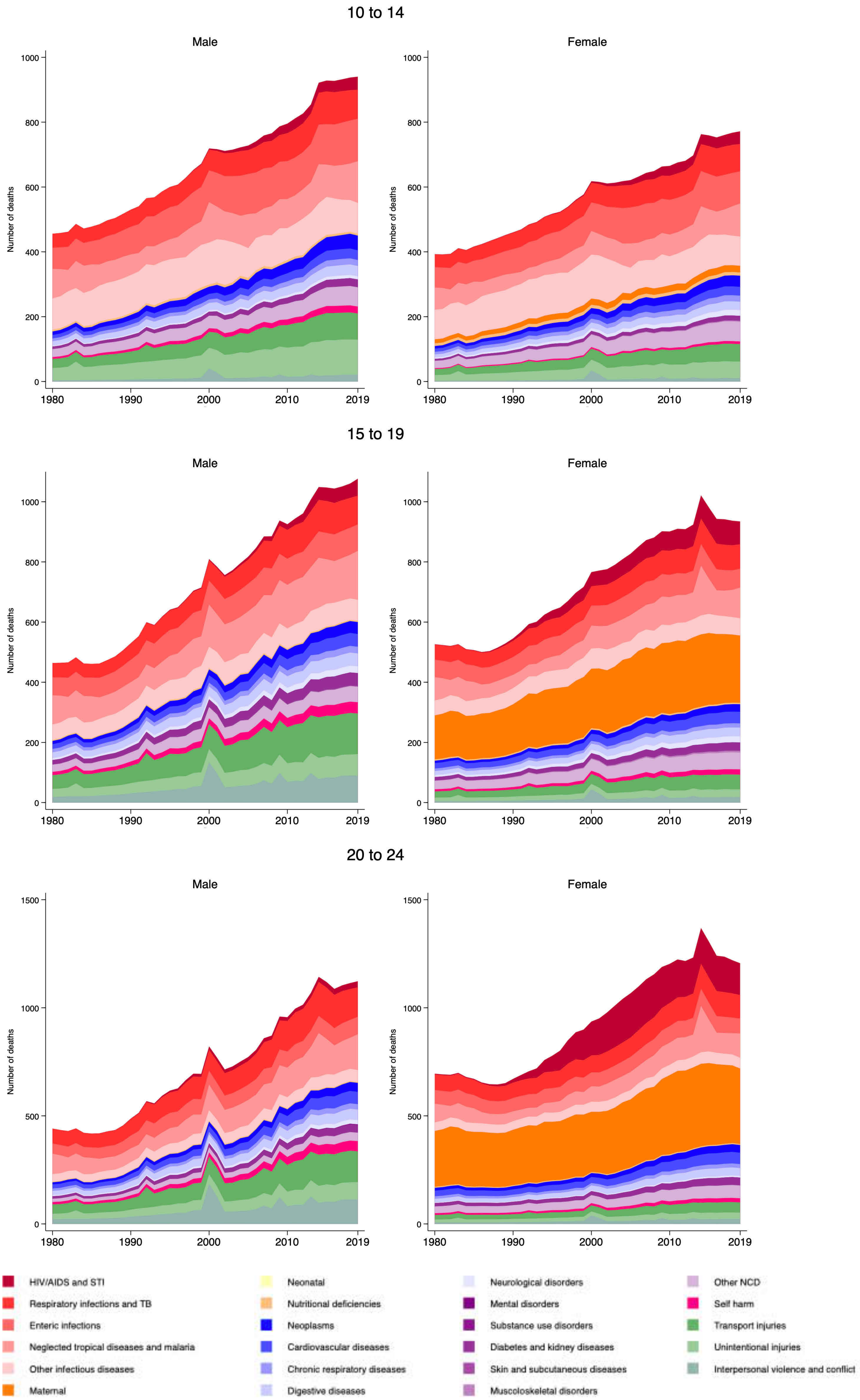

Figure S204: Guinea-Bissau

Figure S206a All-cause mortality rate per 100,000 in 10-24 1950 – 2019: Guinea-Bissau

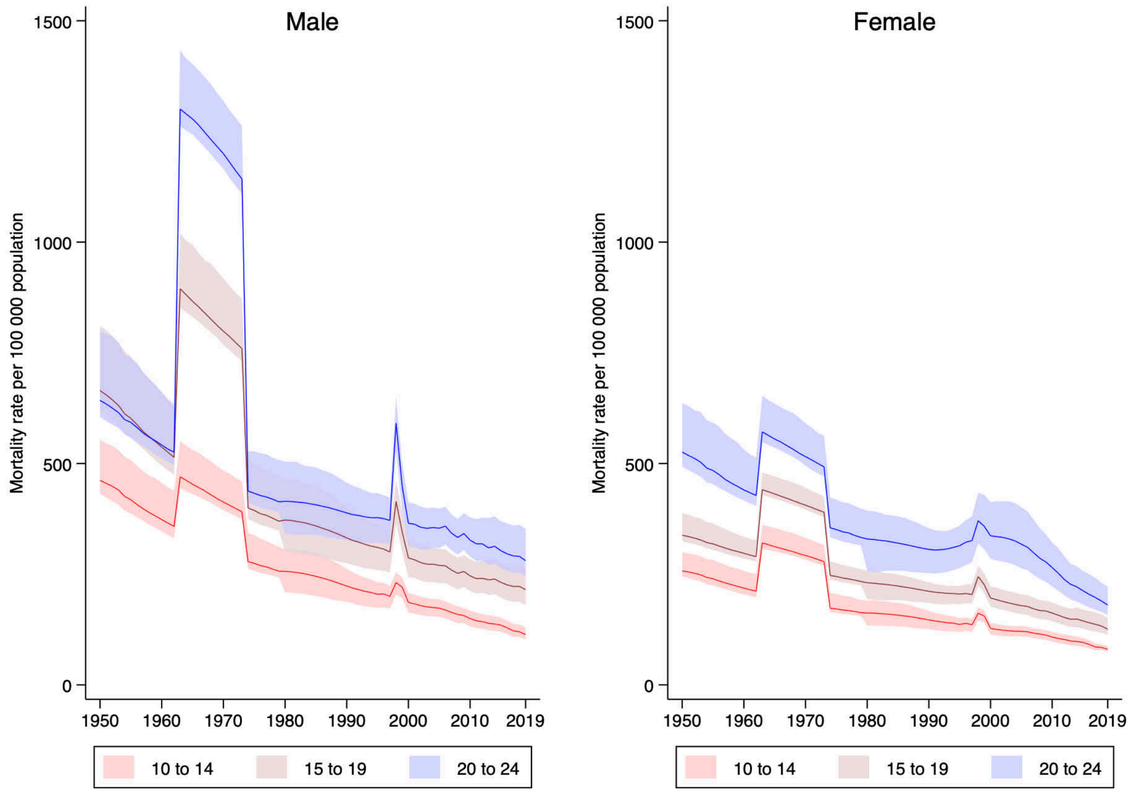

Figure S206b Number of deaths from all causes in 10-24 1950 – 2019: Guinea-Bissau

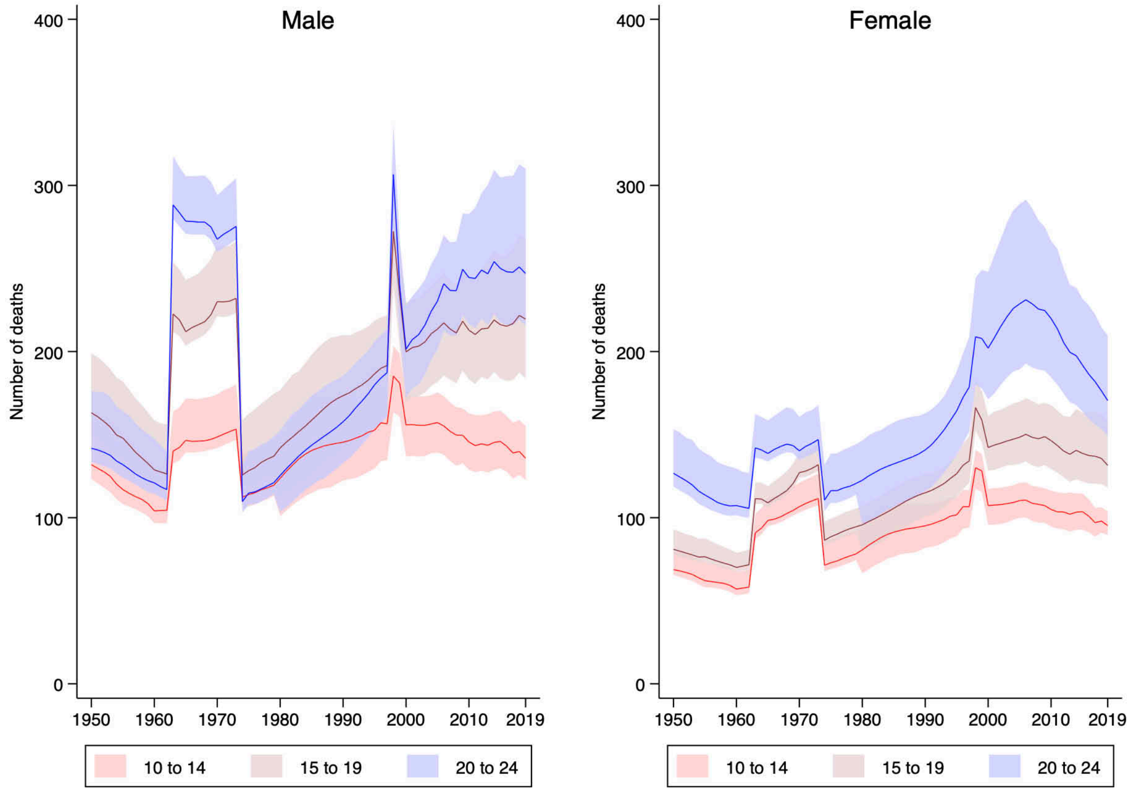

Figure S206c Mortality rate per 100,000 in 10-24 1980 – 2019 by cause: Guinea-Bissau

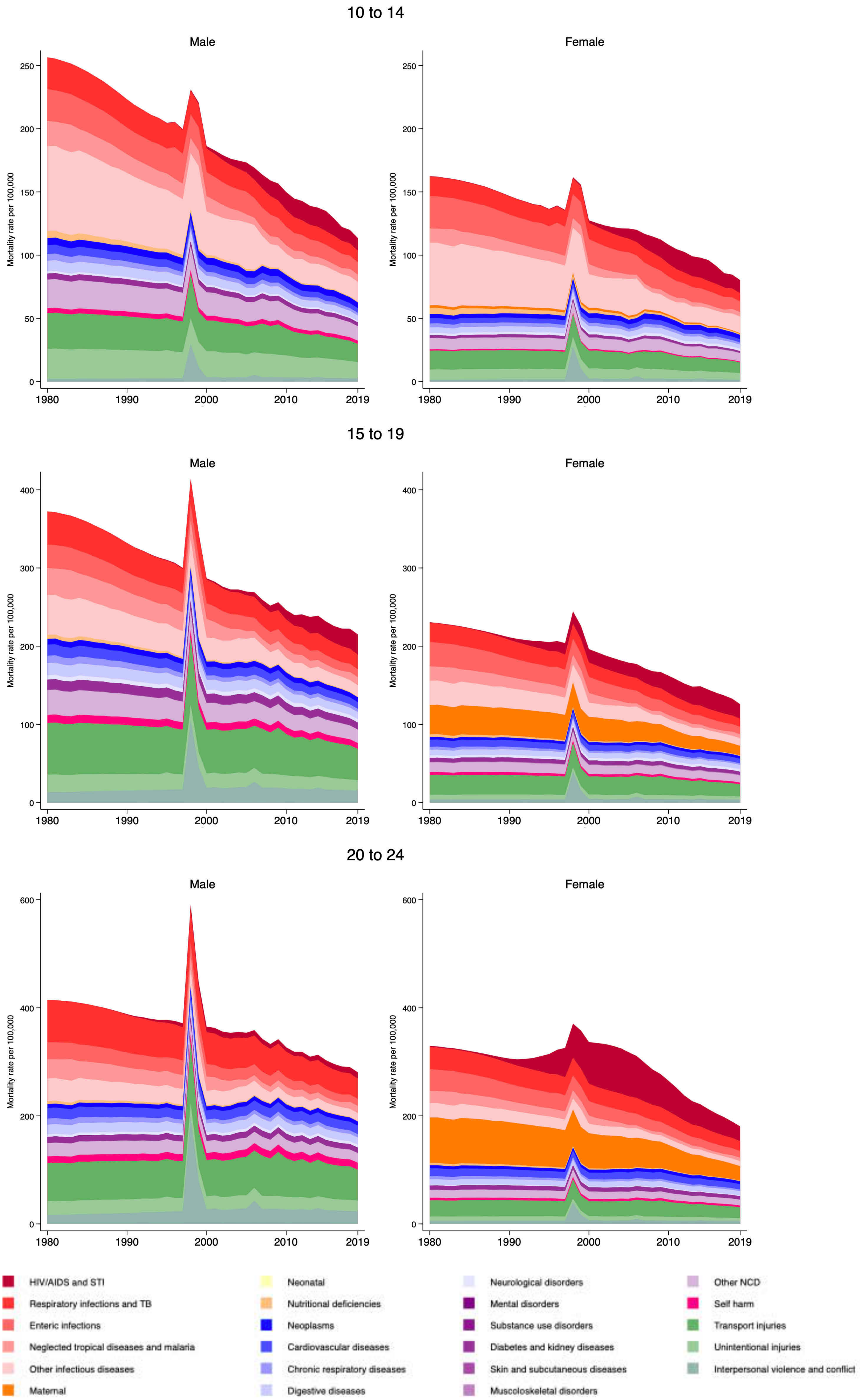

Figure S206d Number of deaths in 10-24 1980 – 2019 by cause: Guinea-Bissau

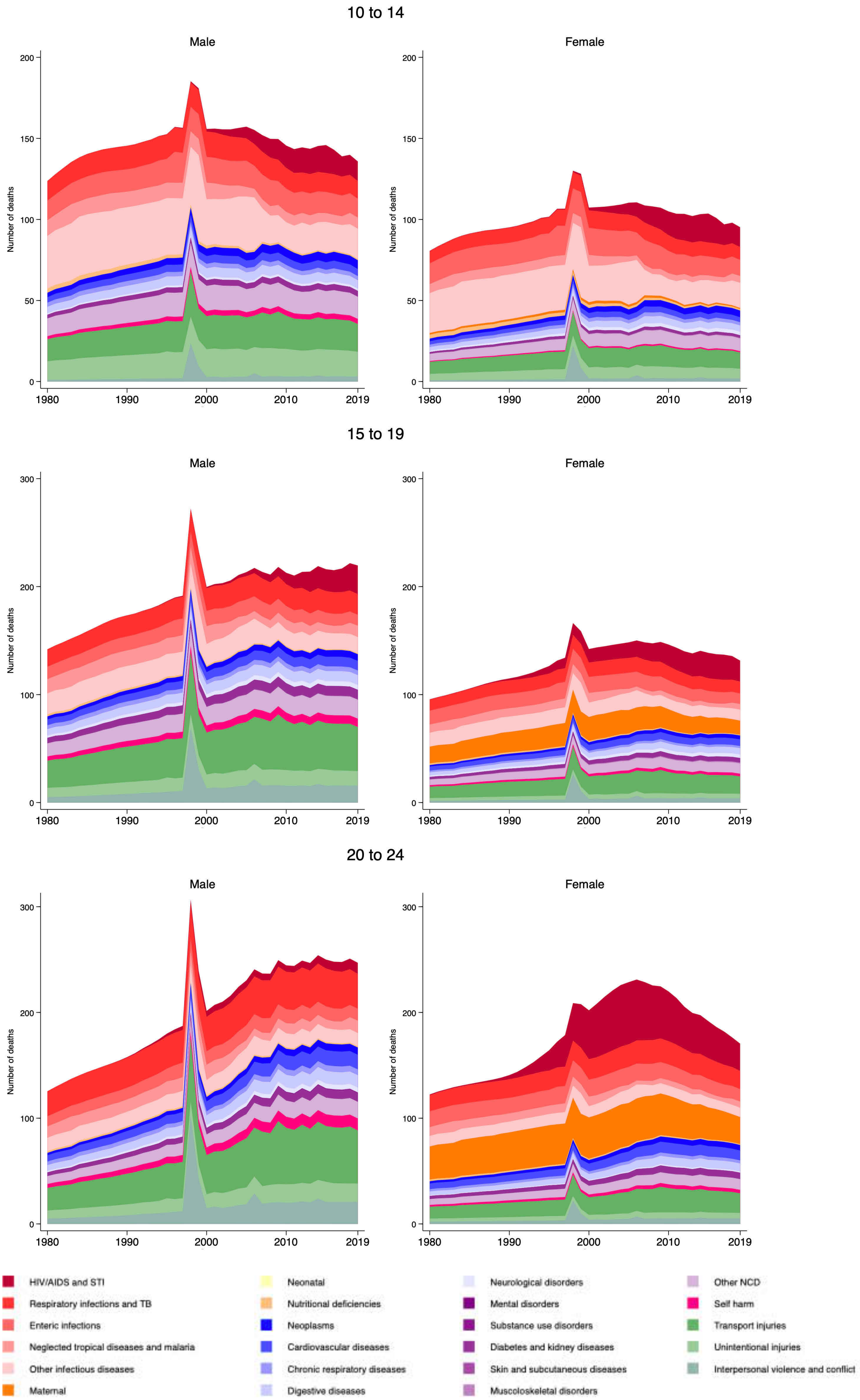

Figure S205: Liberia

Figure S207a All-cause mortality rate per 100,000 in 10-24 1950 – 2019: Liberia

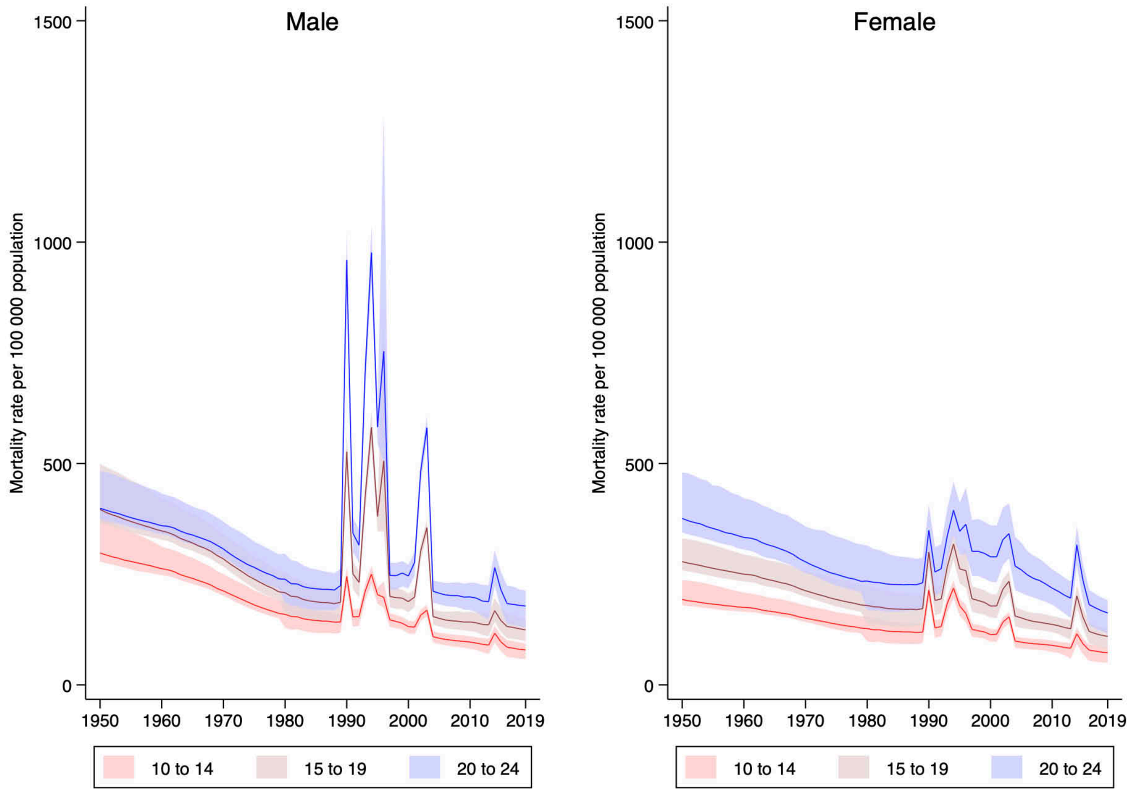

Figure S207b Number of deaths from all causes in 10-24 1950 – 2019: Liberia

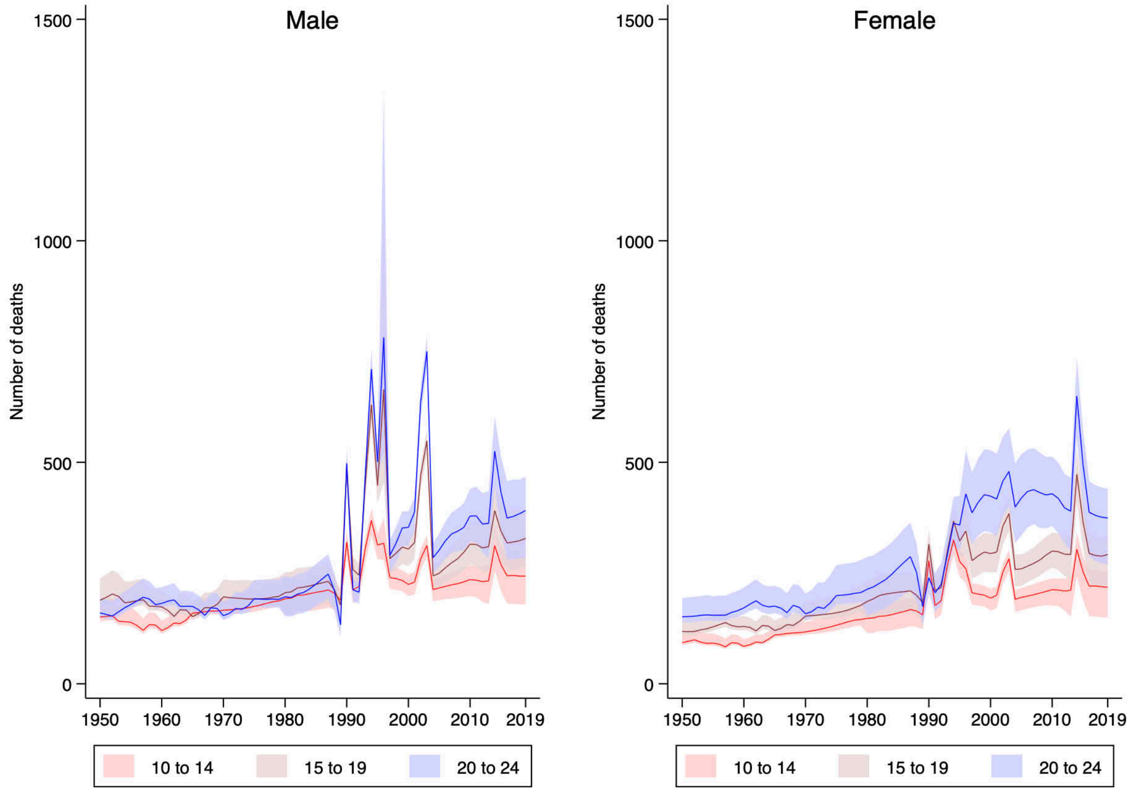

Figure S207c Mortality rate per 100,000 in 10-24 1980 – 2019 by cause: Liberia

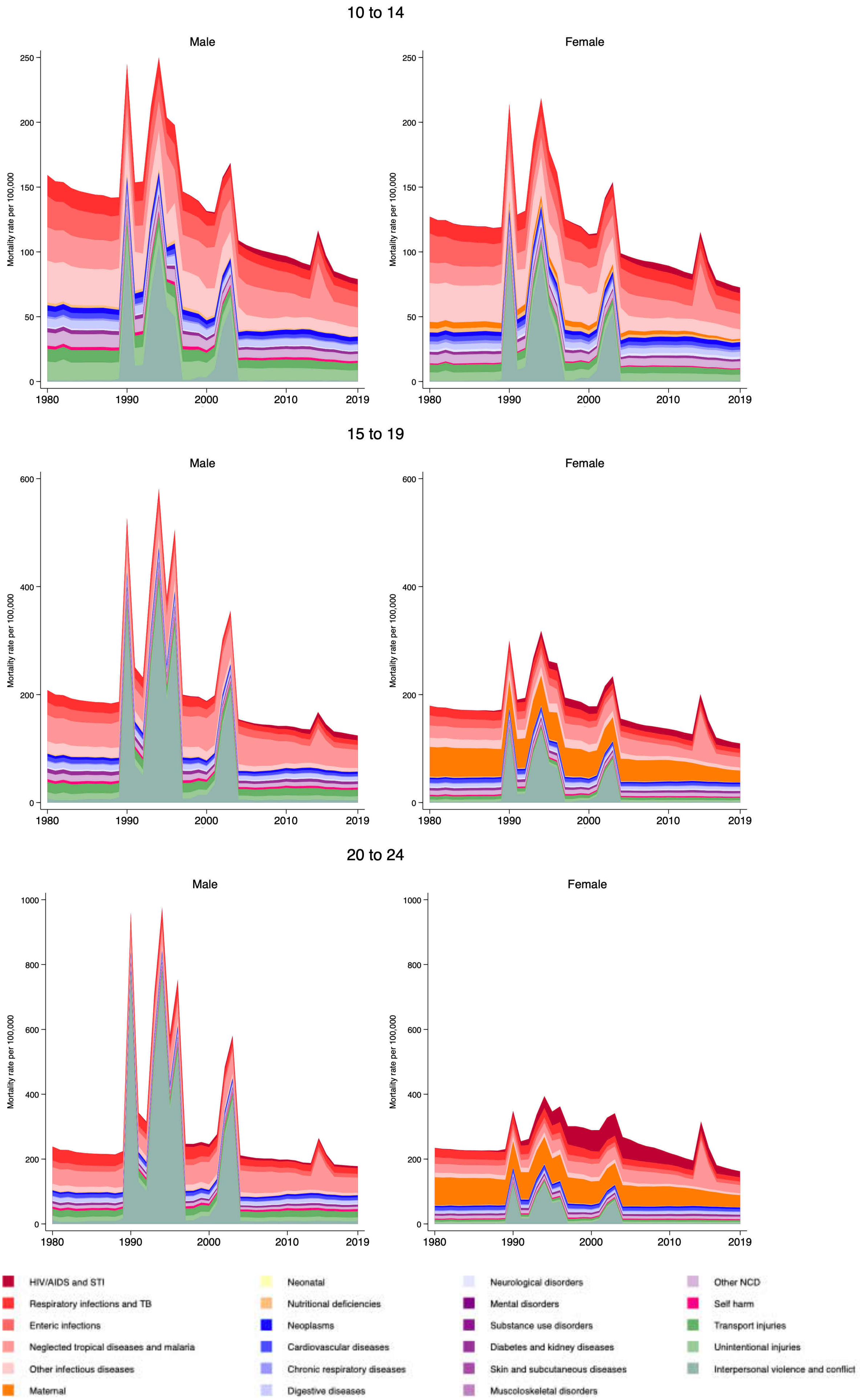

Figure S207d Number of deaths in 10-24 1980 – 2019 by cause: Liberia

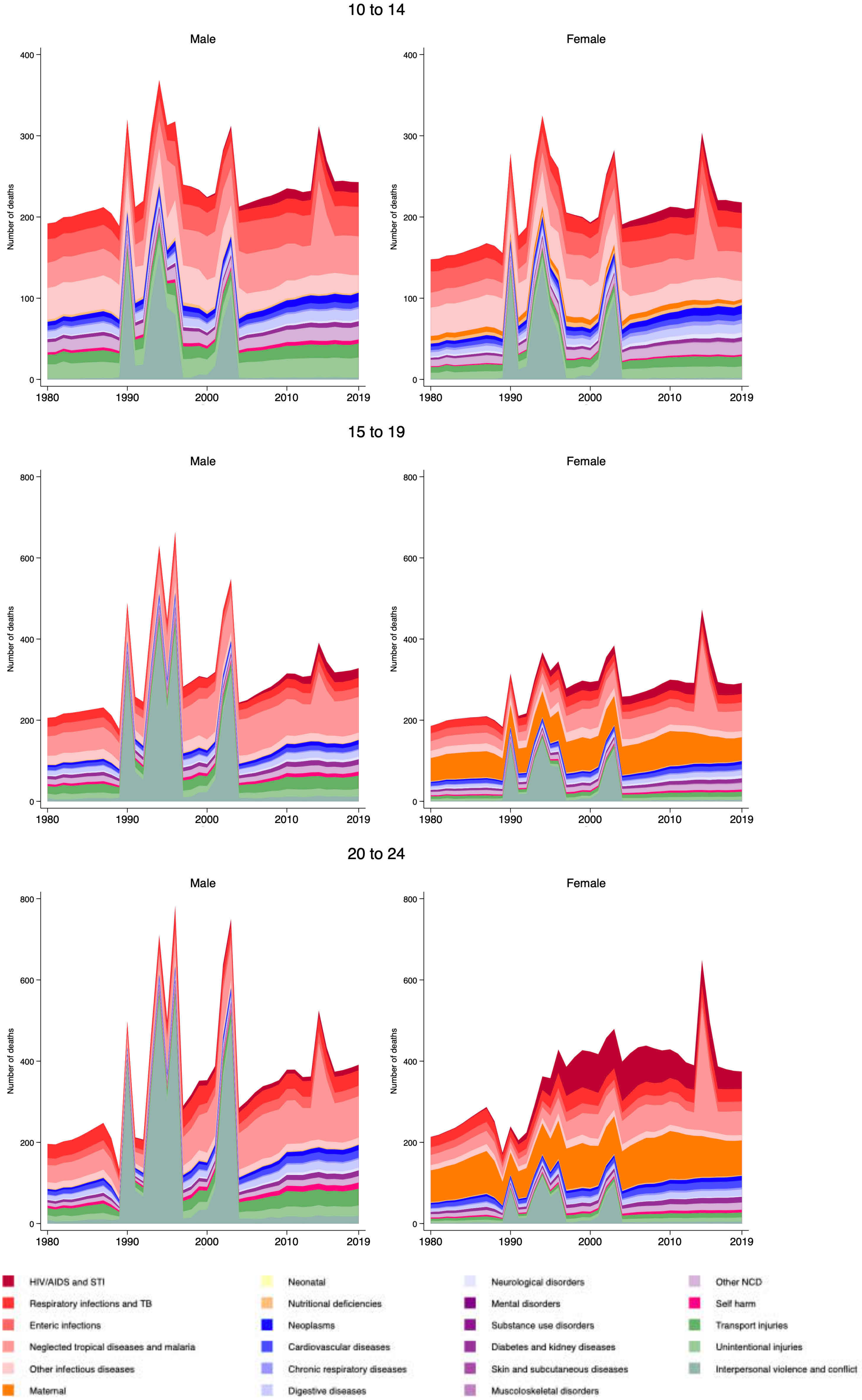

Figure S206: Mali

Figure S208a All-cause mortality rate per 100,000 in 10-24 1950 – 2019: Mali

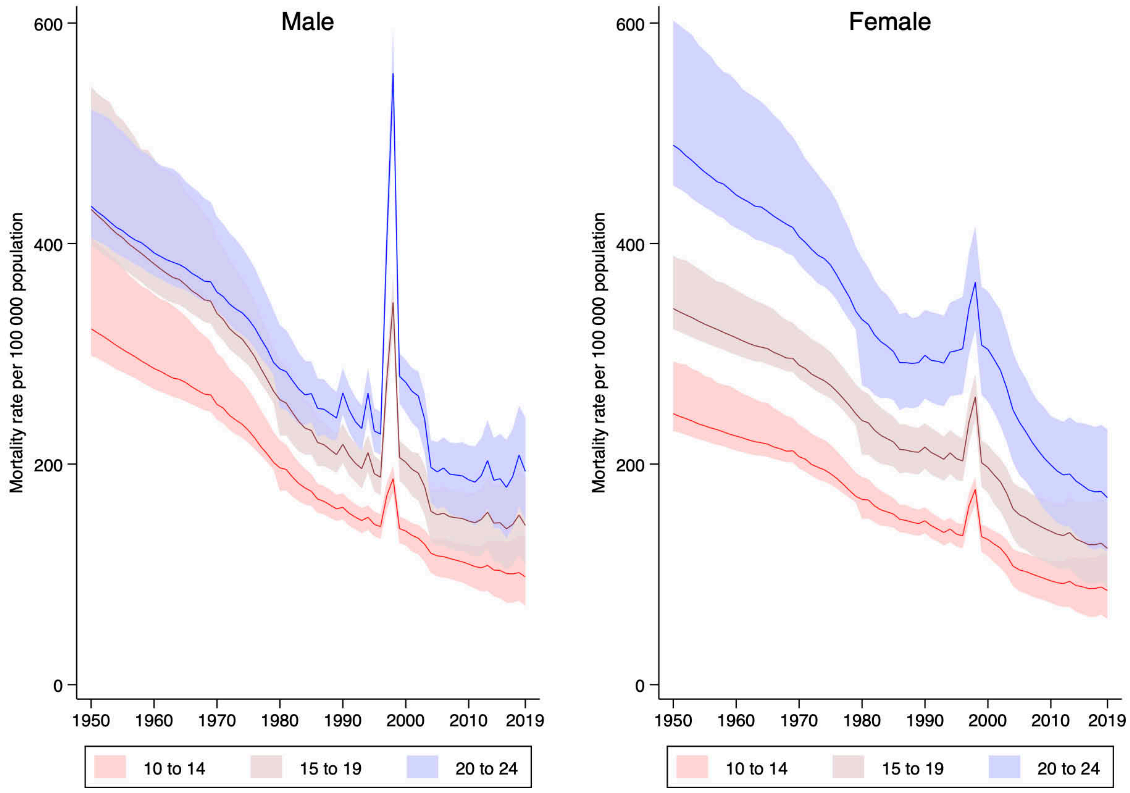

Figure S208b Number of deaths from all causes in 10-24 1950 – 2019: Mali

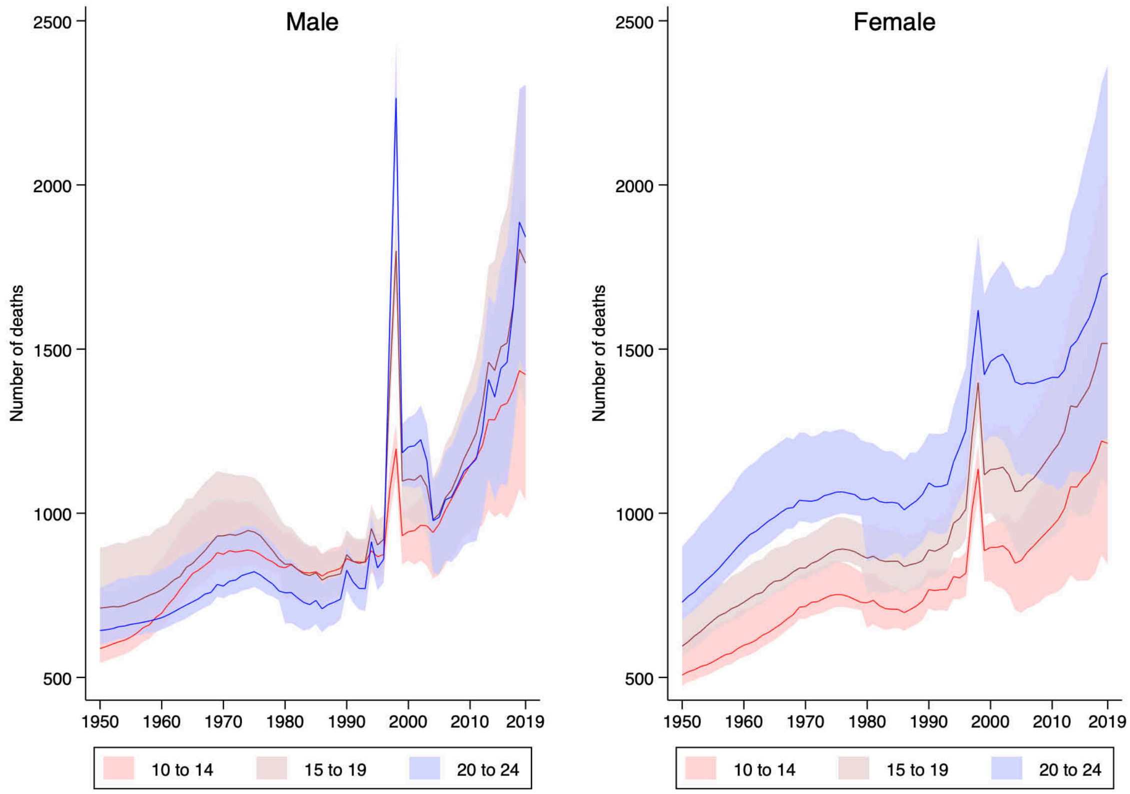

Figure S208c Mortality rate per 100,000 in 10-24 1980 – 2019 by cause: Mali

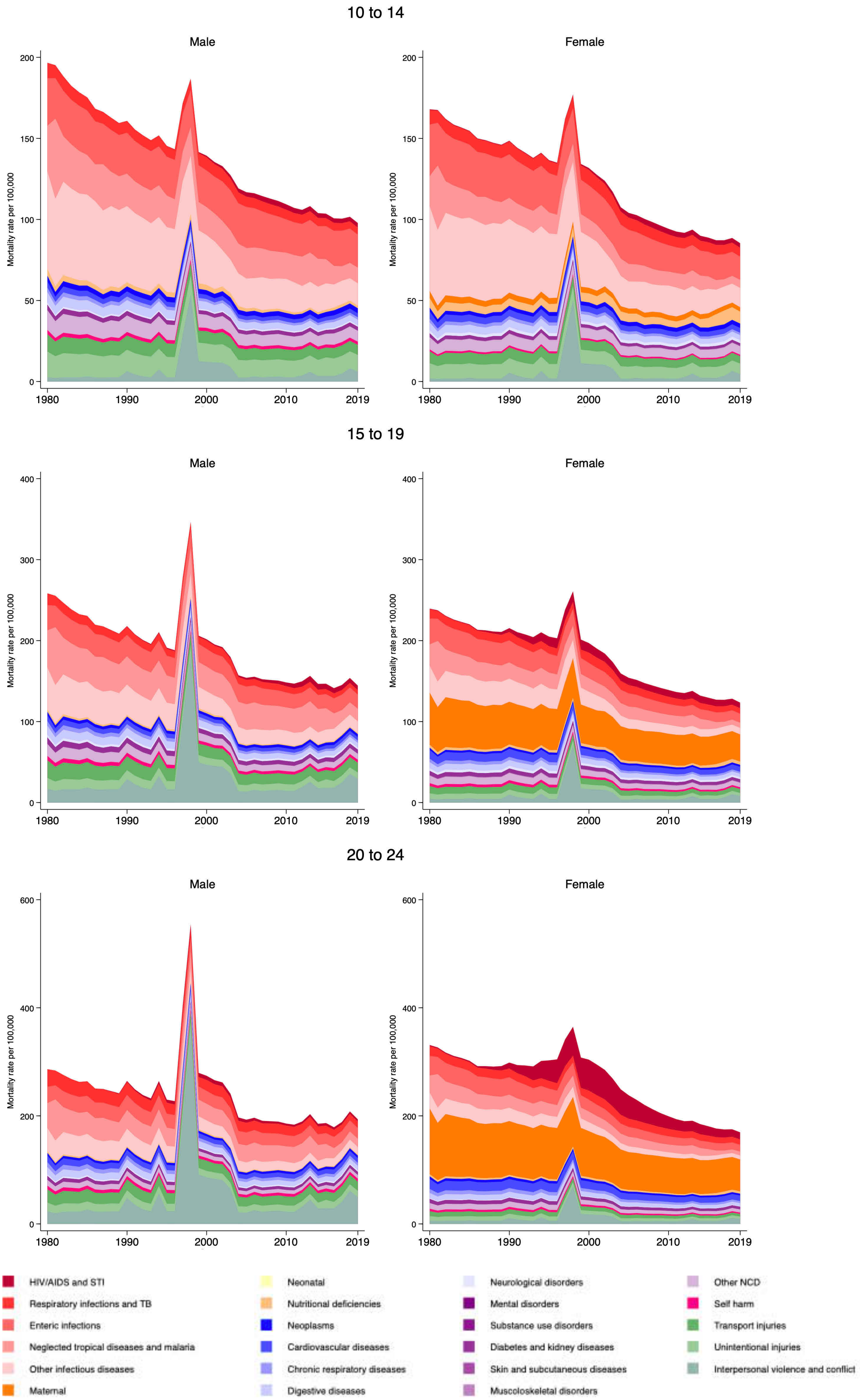

Figure S208d Number of deaths in 10-24 1980 – 2019 by cause: Mali

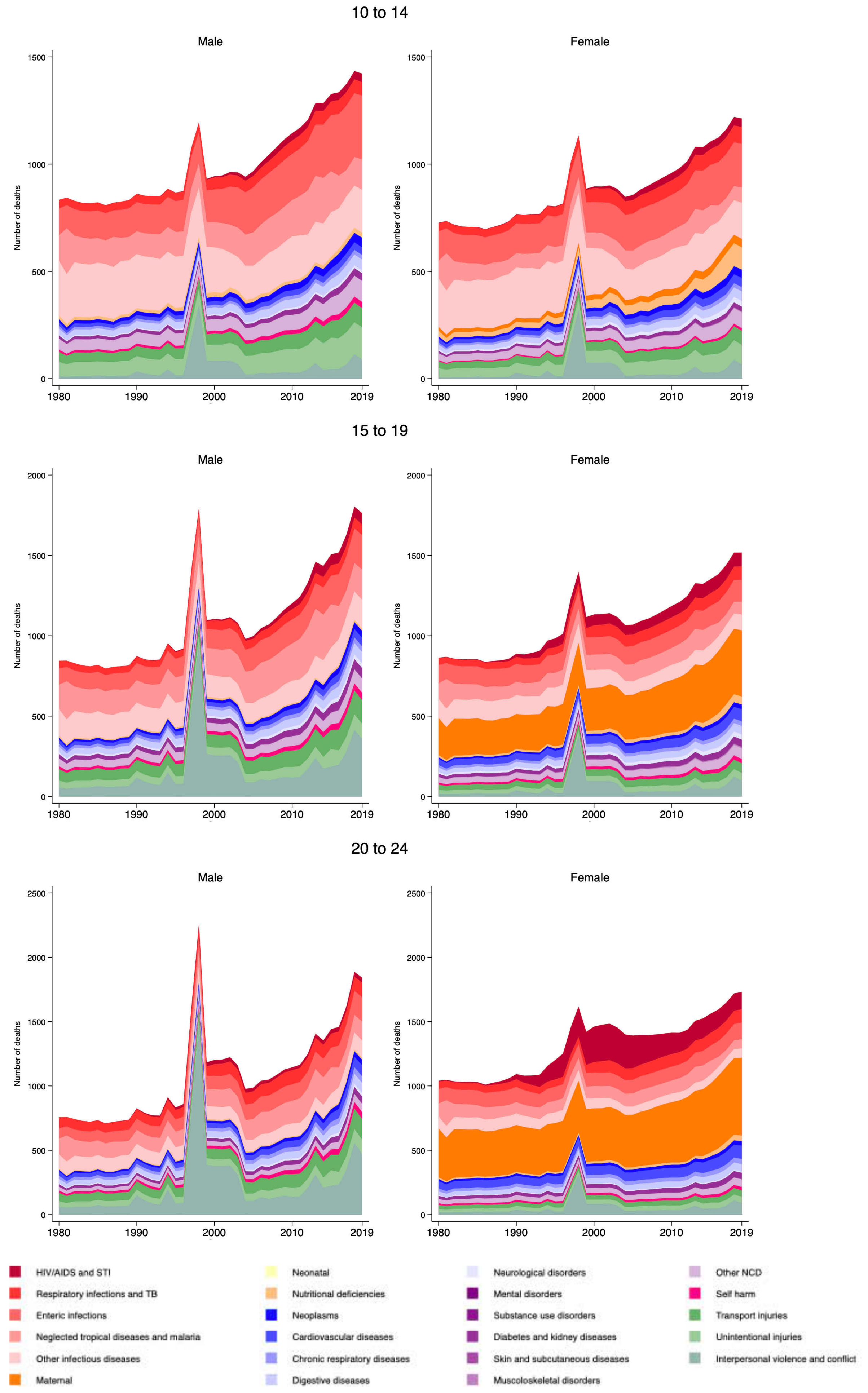

Figure S207: Mauritania

Figure S209a All-cause mortality rate per 100,000 in 10-24 1950 – 2019: Mauritania

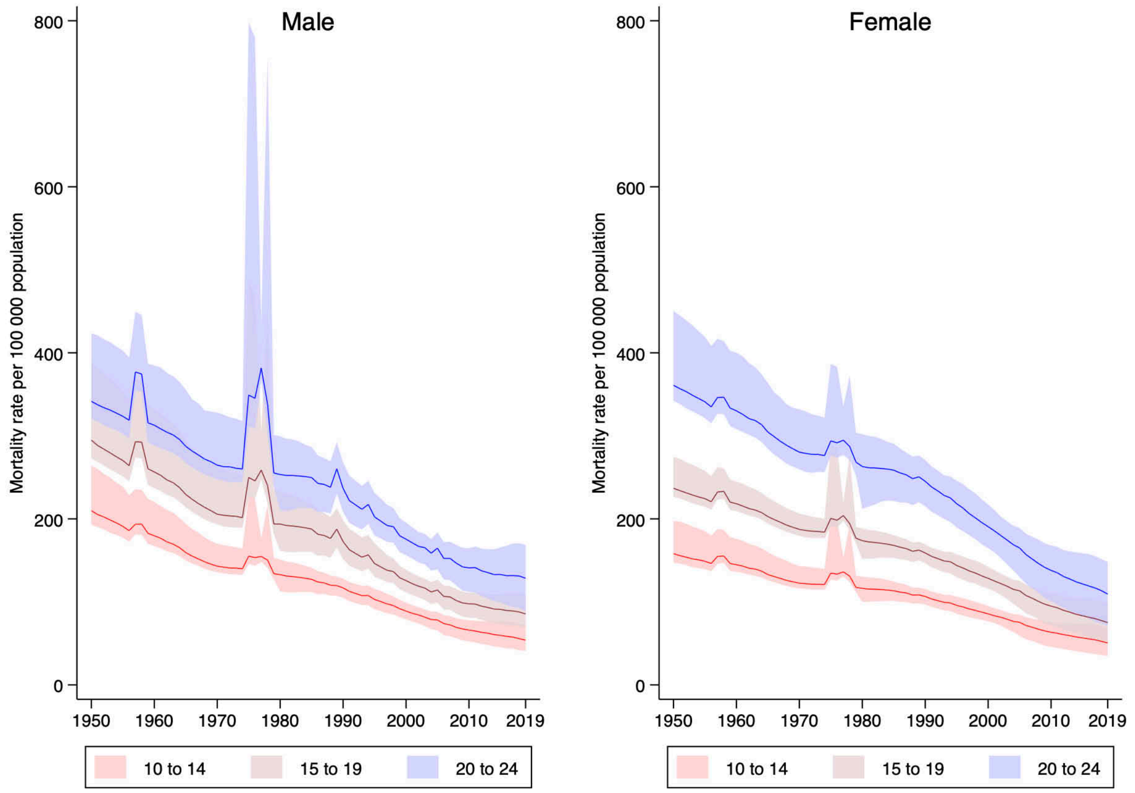

Figure S209b Number of deaths from all causes in 10-24 1950 – 2019: Mauritania

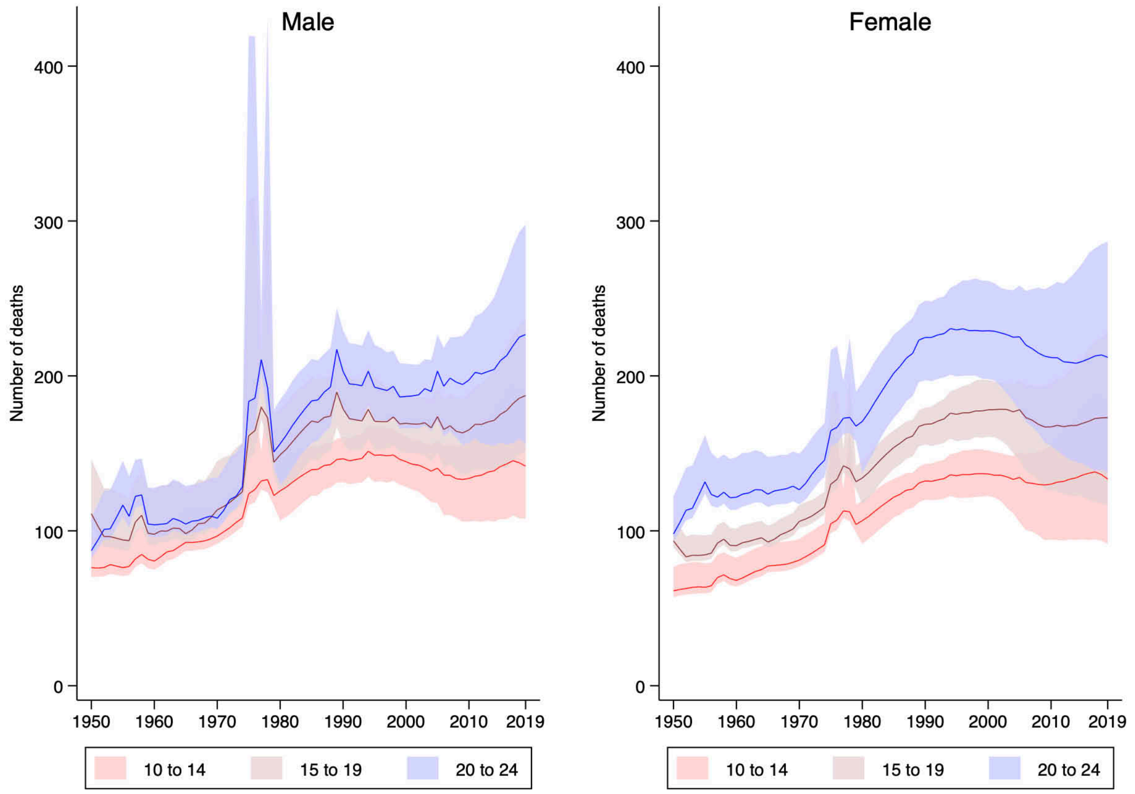

Figure S209c Mortality rate per 100,000 in 10-24 1980 – 2019 by cause: Mauritania

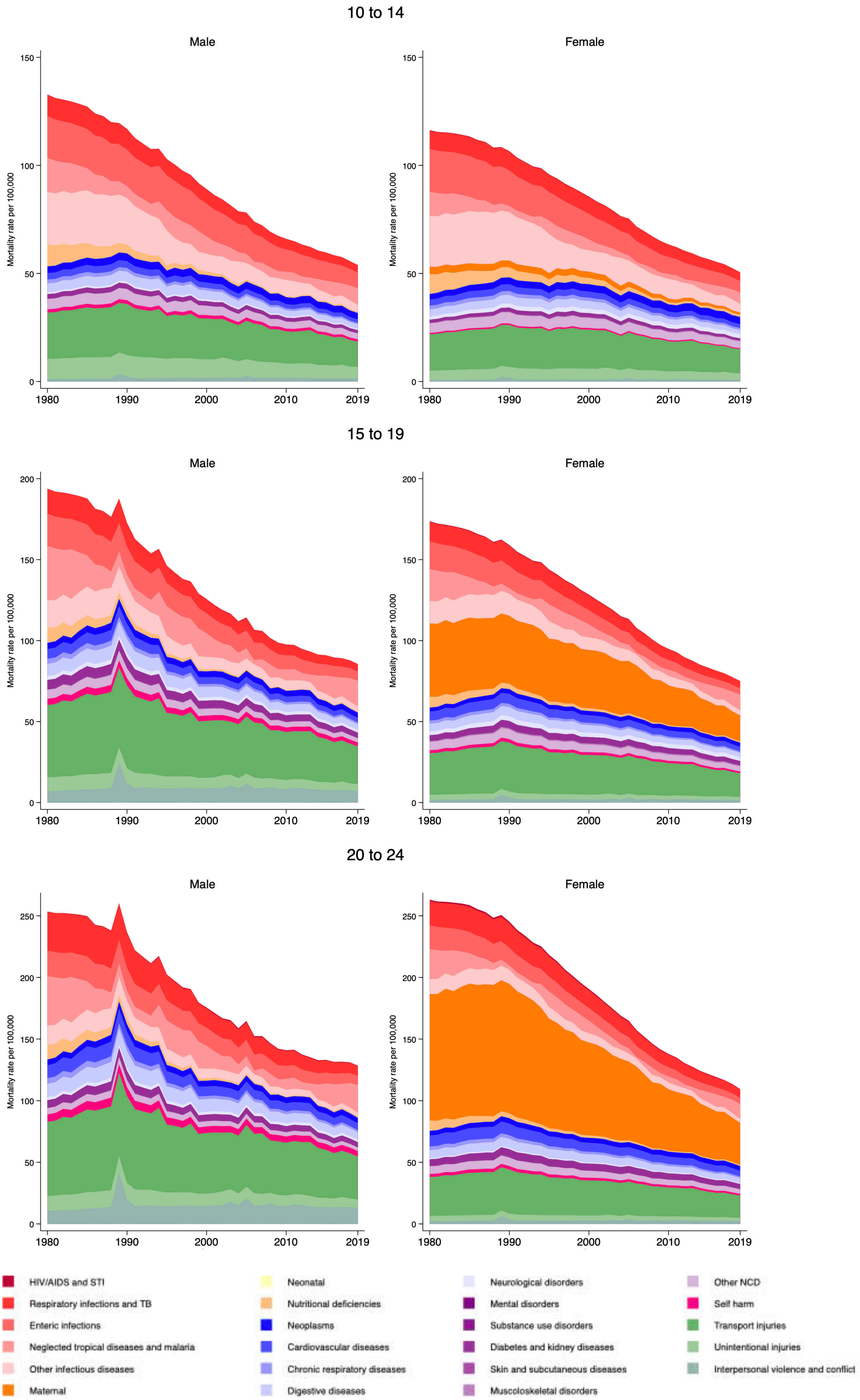

Figure S209d Number of deaths in 10-24 1980 – 2019 by cause: Mauritania

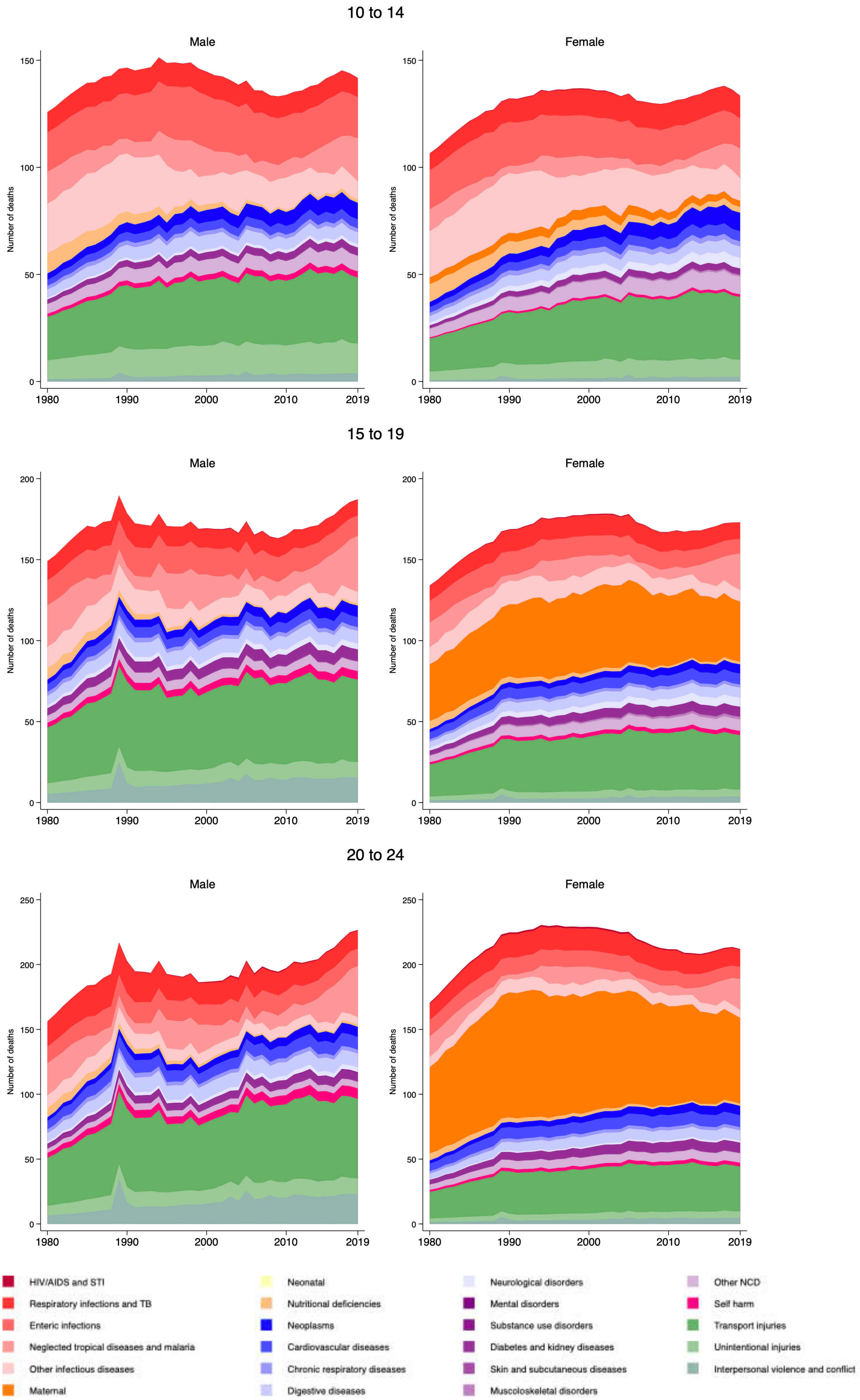

Figure S208: Niger

Figure S210a All-cause mortality rate per 100,000 in 10-24 1950 – 2019: Niger

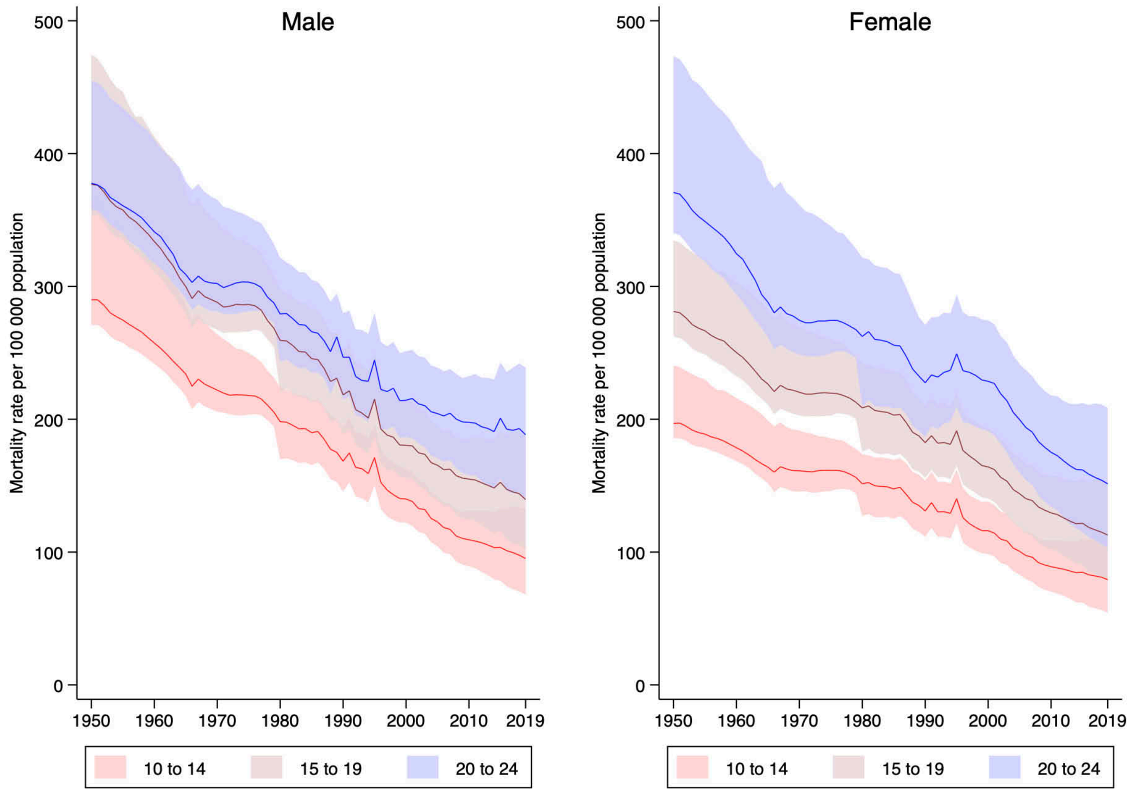

Figure S210b Number of deaths from all causes in 10-24 1950 – 2019: Niger

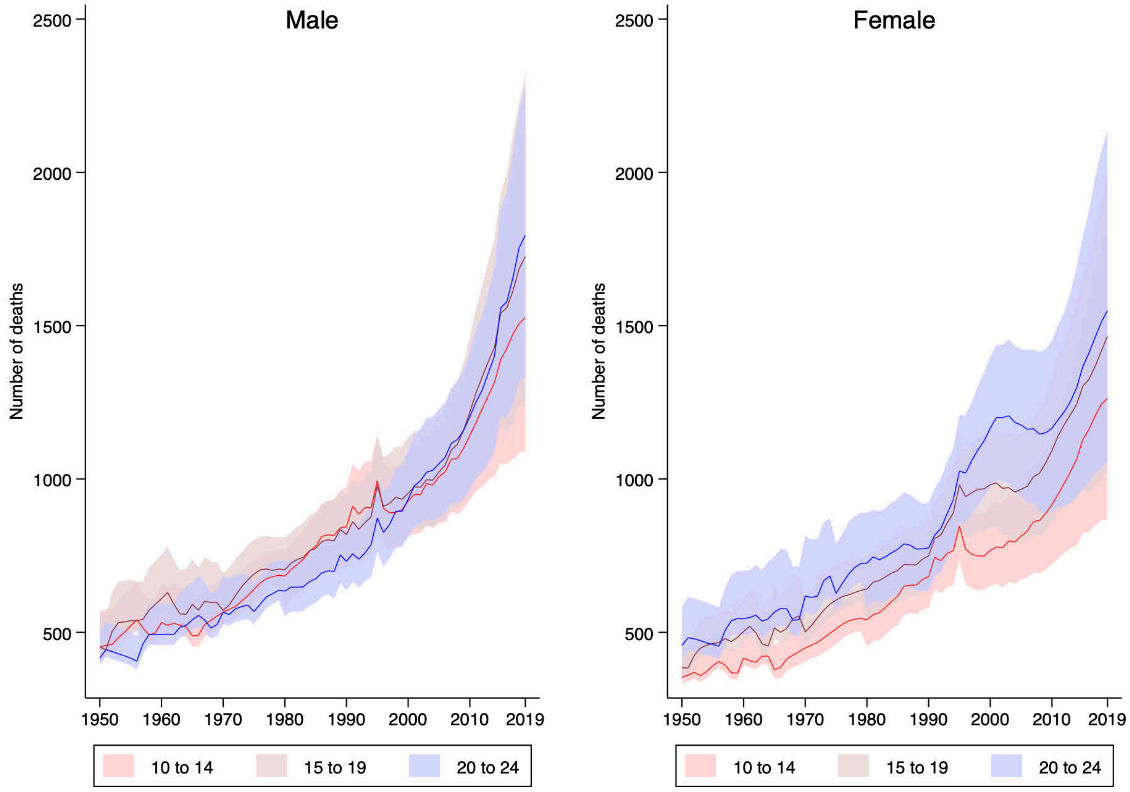

Figure S210c Mortality rate per 100,000 in 10-24 1980 – 2019 by cause: Niger

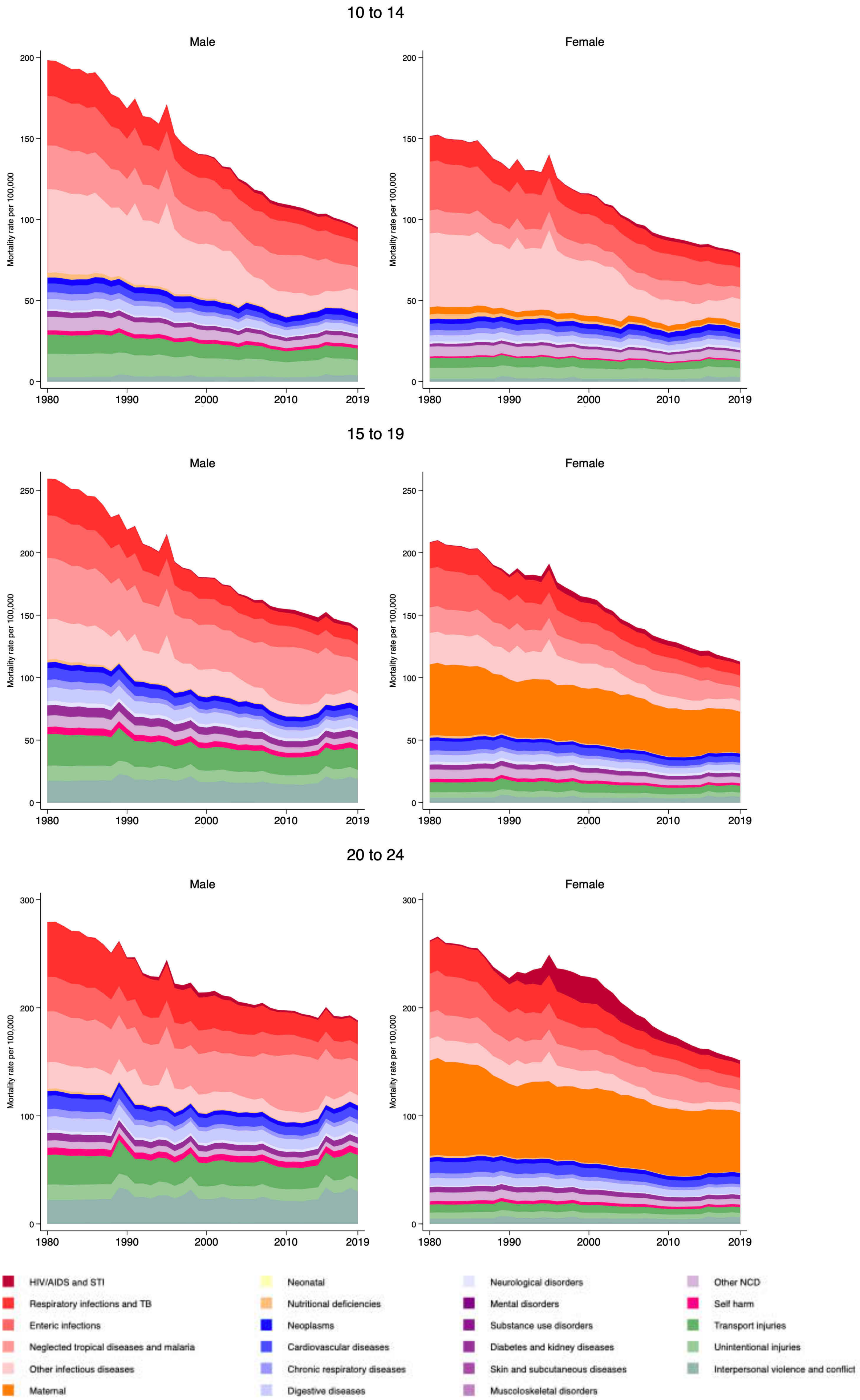

Figure S210d Number of deaths in 10-24 1980 – 2019 by cause: Niger

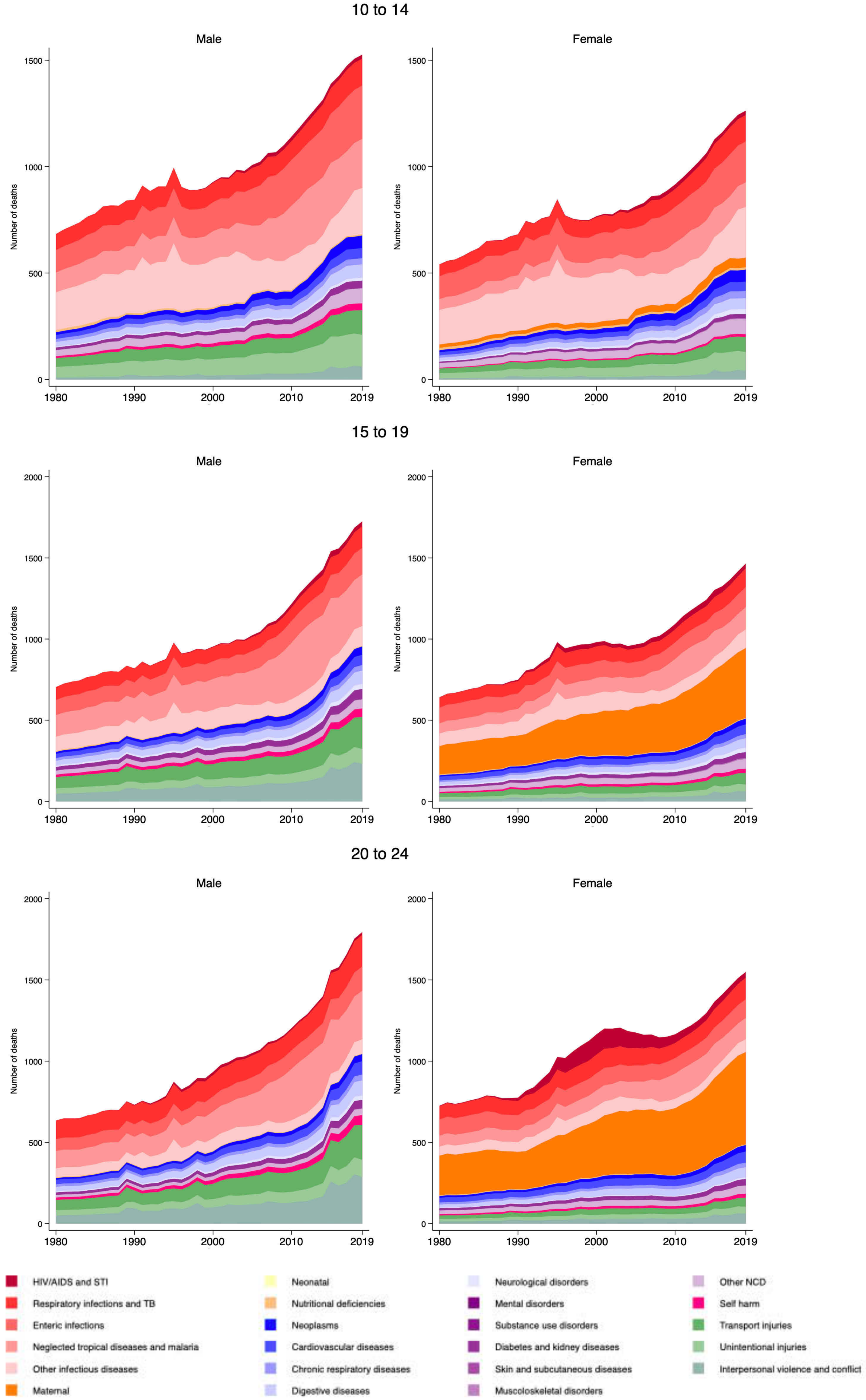

Figure S209: Nigeria

Figure S211a All-cause mortality rate per 100,000 in 10-24 1950 – 2019: Nigeria

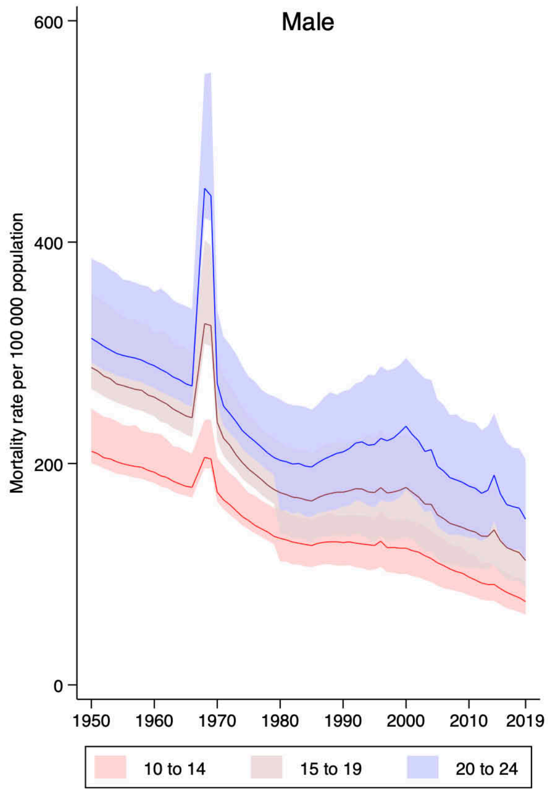

Figure S211b Number of deaths from all causes in 10-24 1950 – 2019: Nigeria

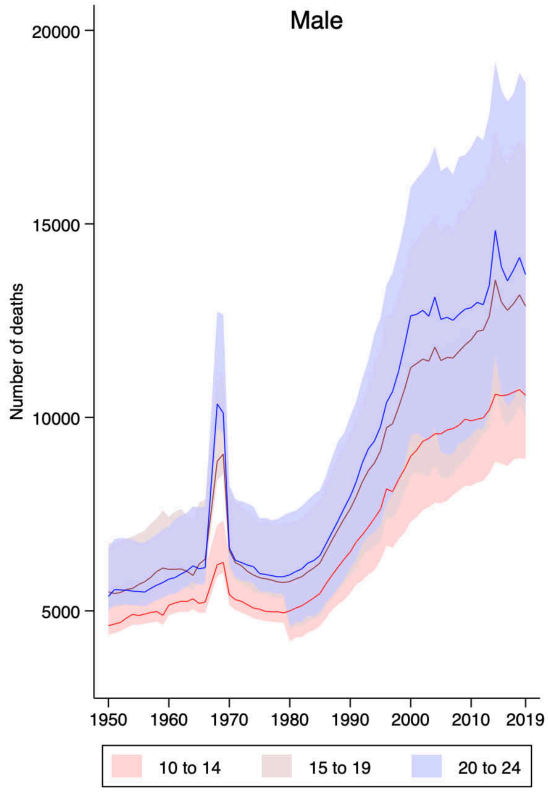

Figure S211c Mortality rate per 100,000 in 10-24 1980 – 2019 by cause: Nigeria

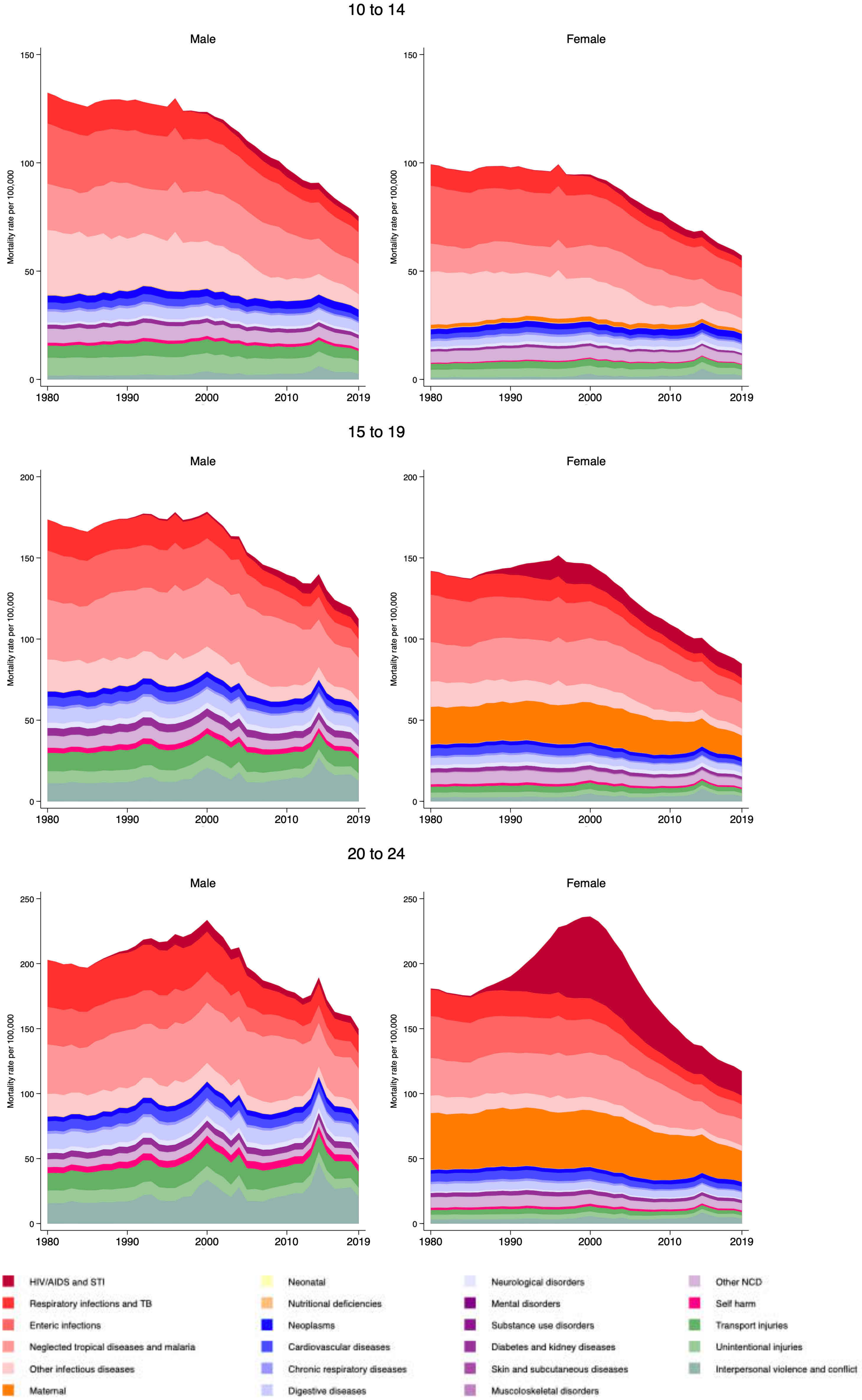

Figure S211d Number of deaths in 10-24 1980 – 2019 by cause: Nigeria

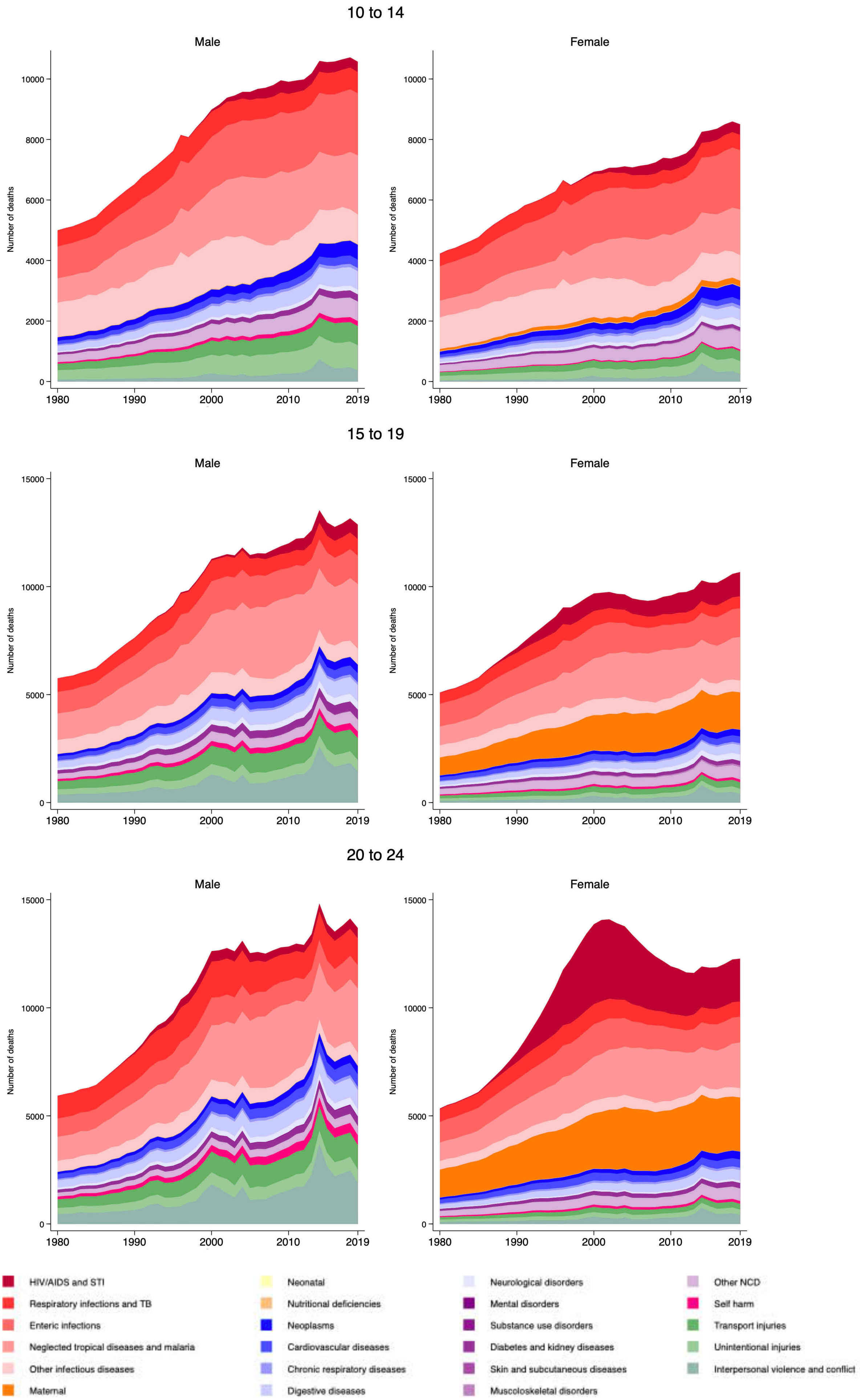

Figure S210: Sao Tome and Principe

Figure S212a All-cause mortality rate per 100,000 in 10-24 1950 – 2019: Sao Tome and Principe

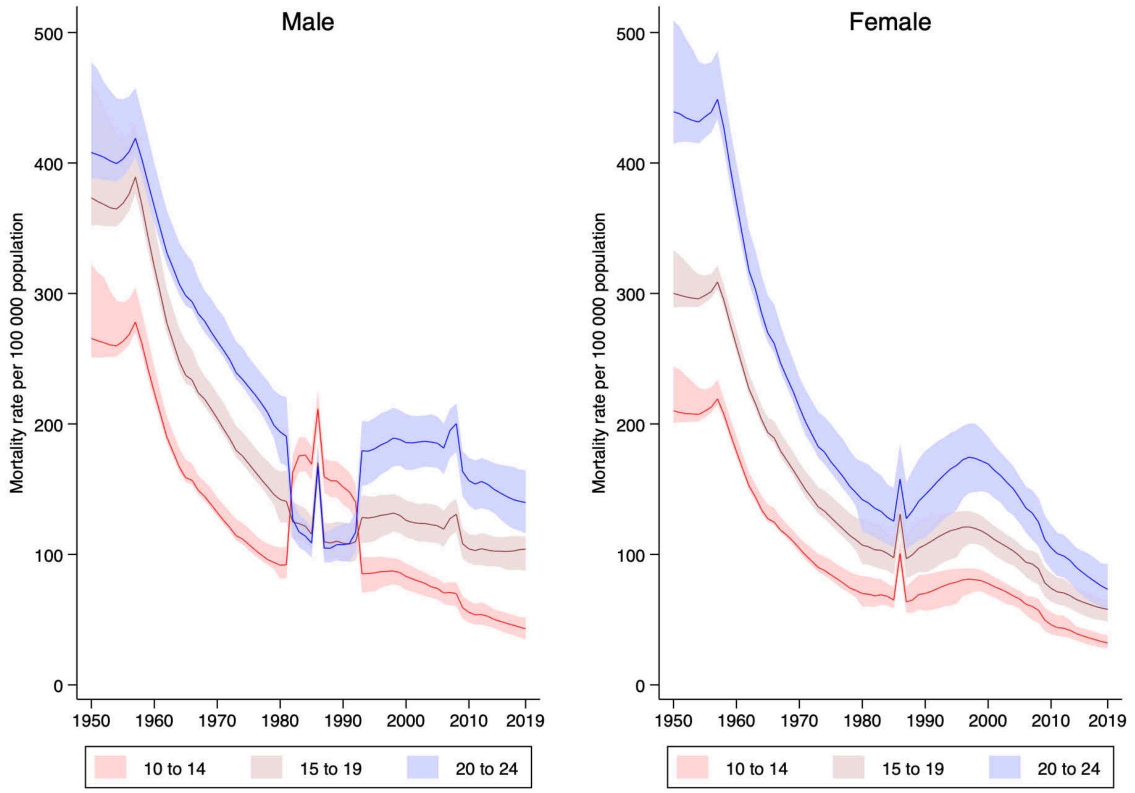

Figure S212b Number of deaths from all causes in 10-24 1950 – 2019: Sao Tome and Principe

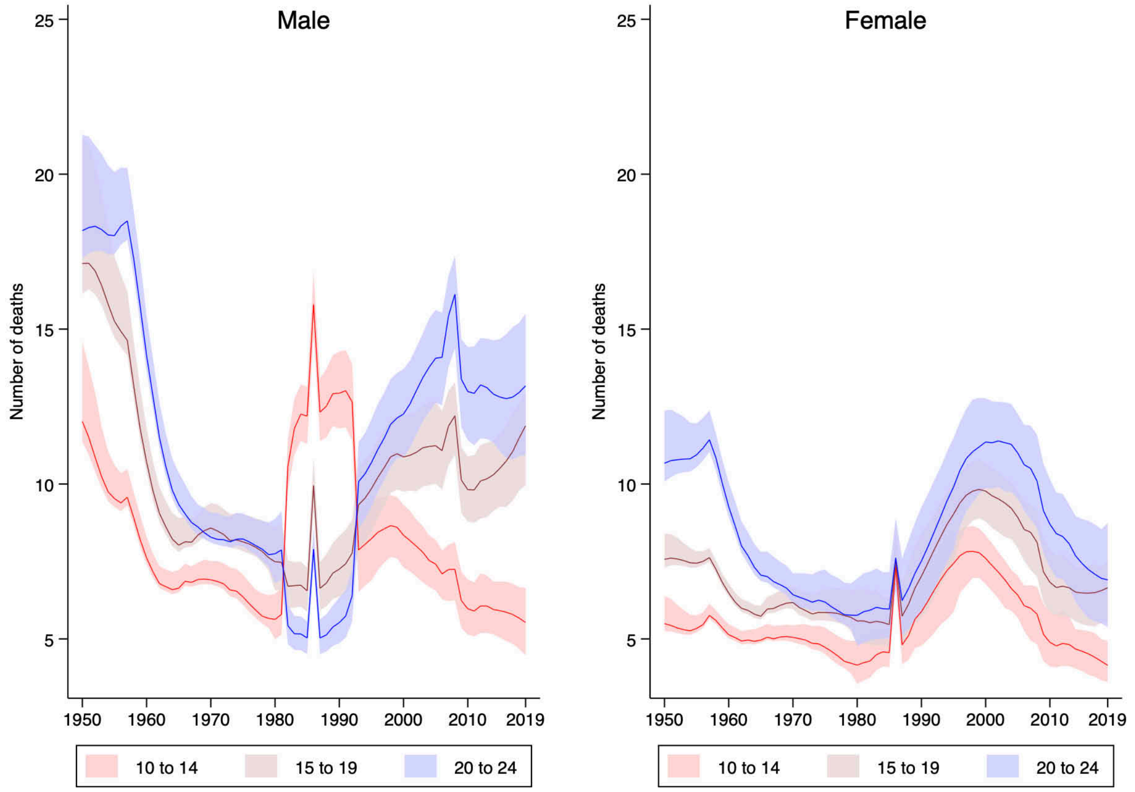

Figure S212c Mortality rate per 100,000 in 10-24 1980 – 2019 by cause: Sao Tome and Principe

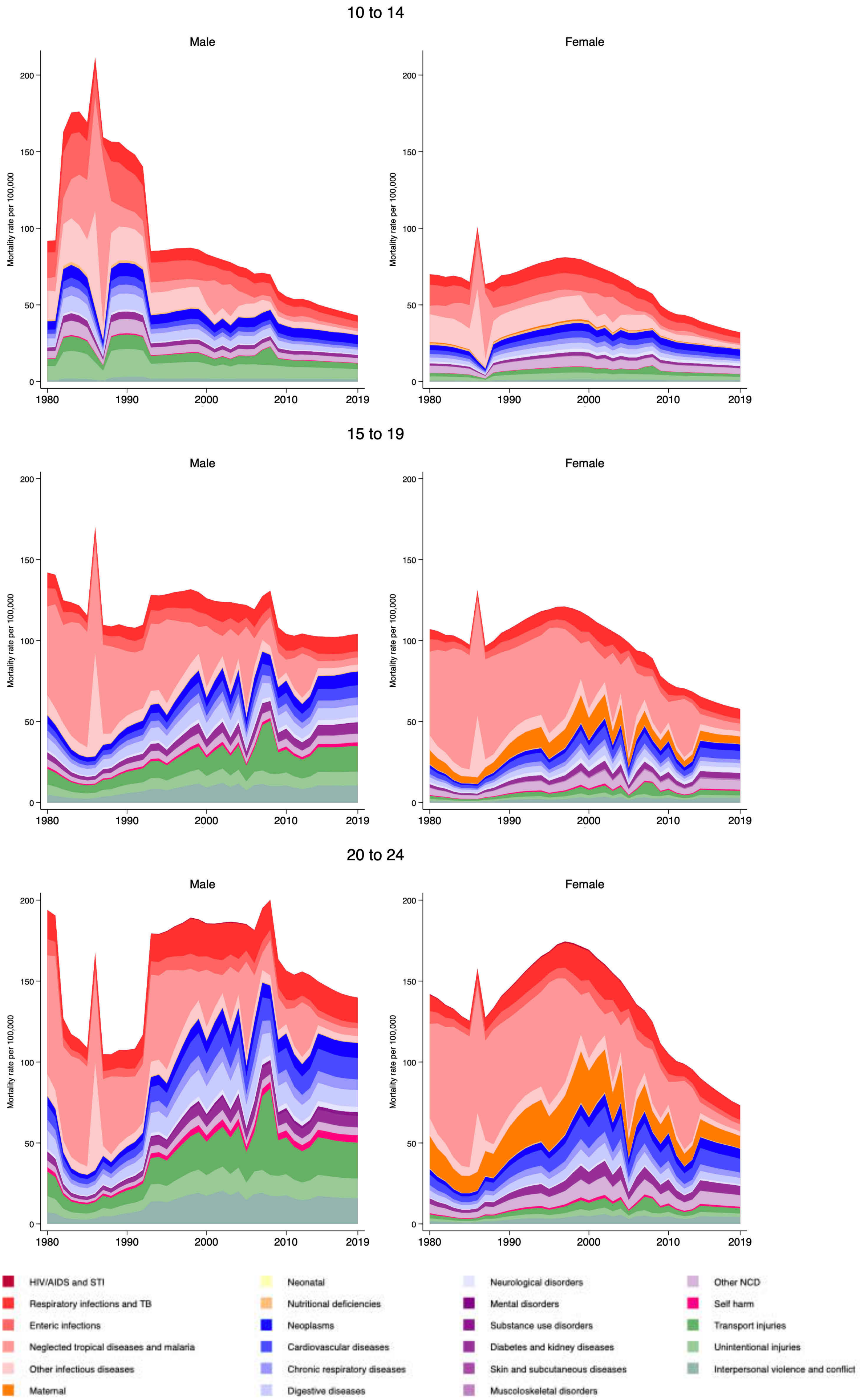

Figure S212d Number of deaths in 10-24 1980 – 2019 by cause: Sao Tome and Principe

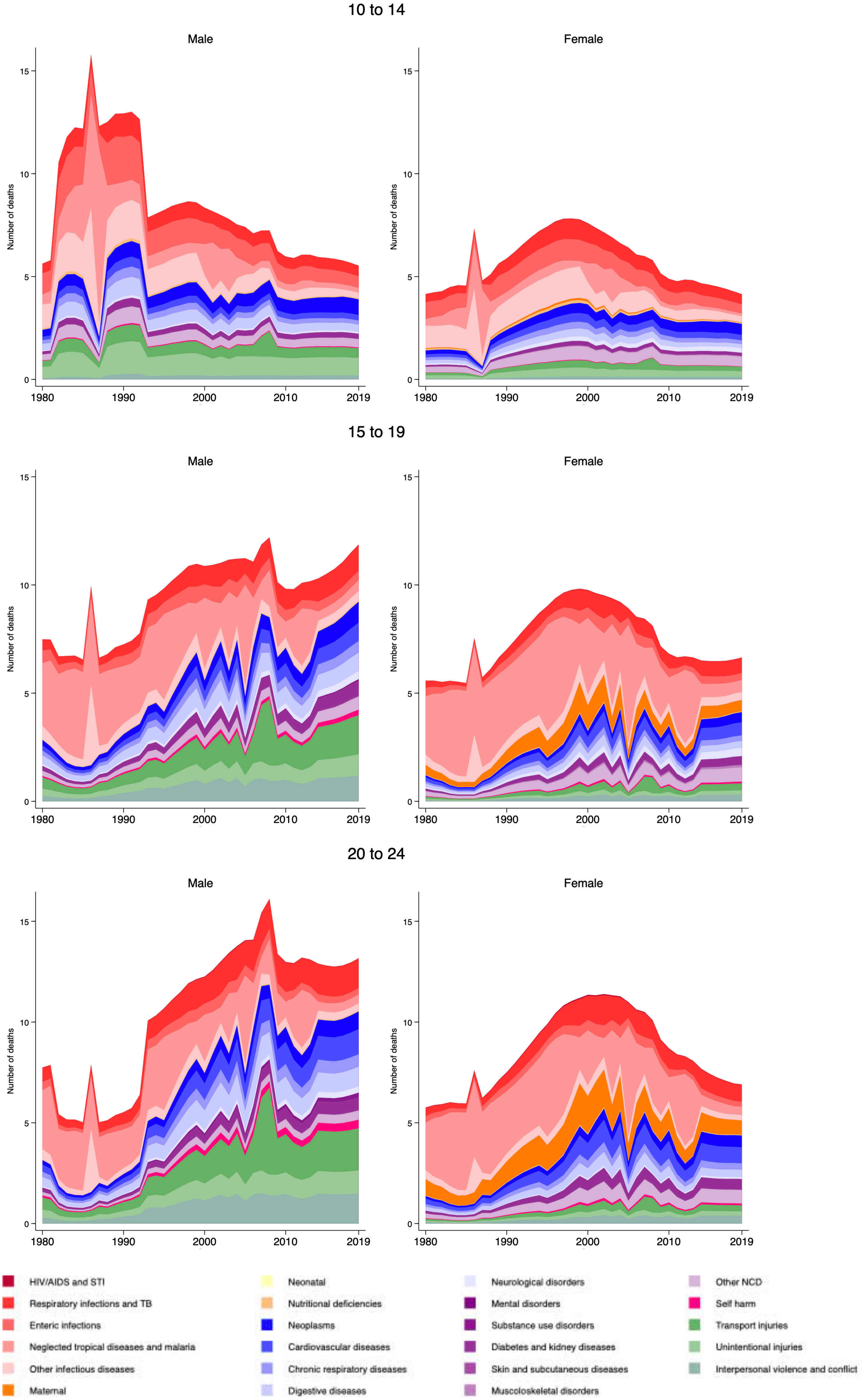

Figure S211: Senegal

Figure S213a All-cause mortality rate per 100,000 in 10-24 1950 – 2019: Senegal

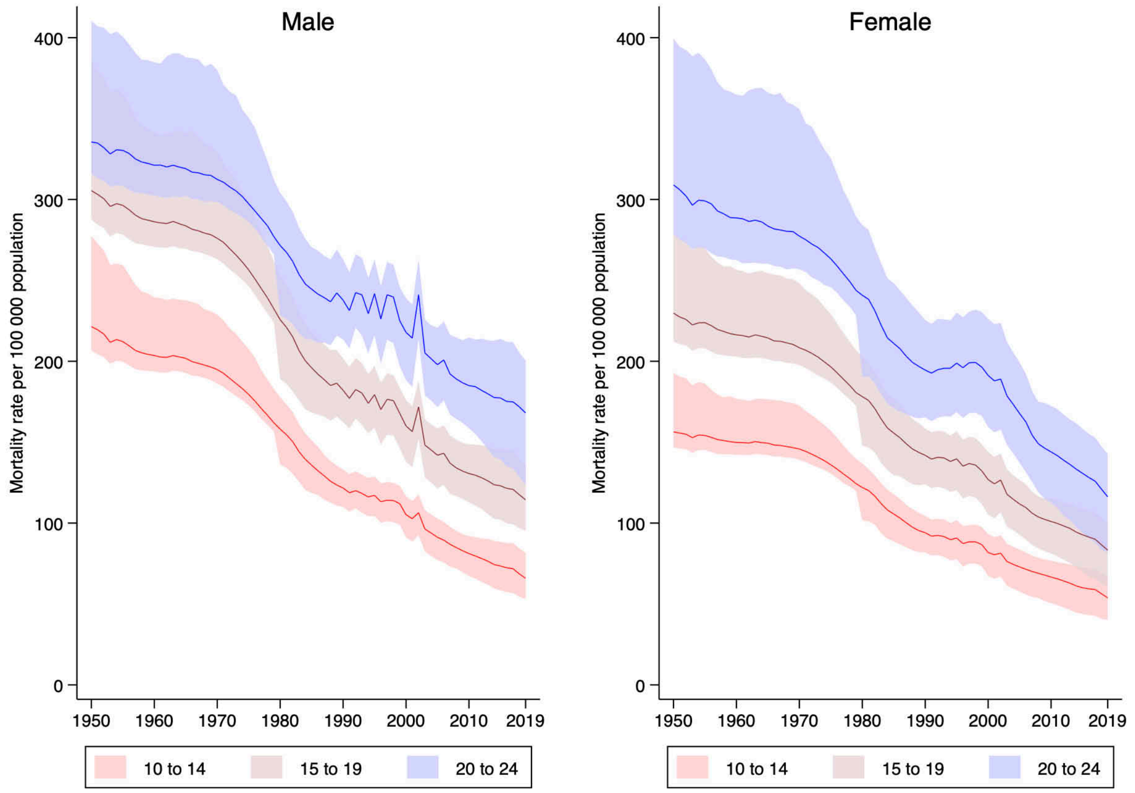

Figure S213b Number of deaths from all causes in 10-24 1950 – 2019: Senegal

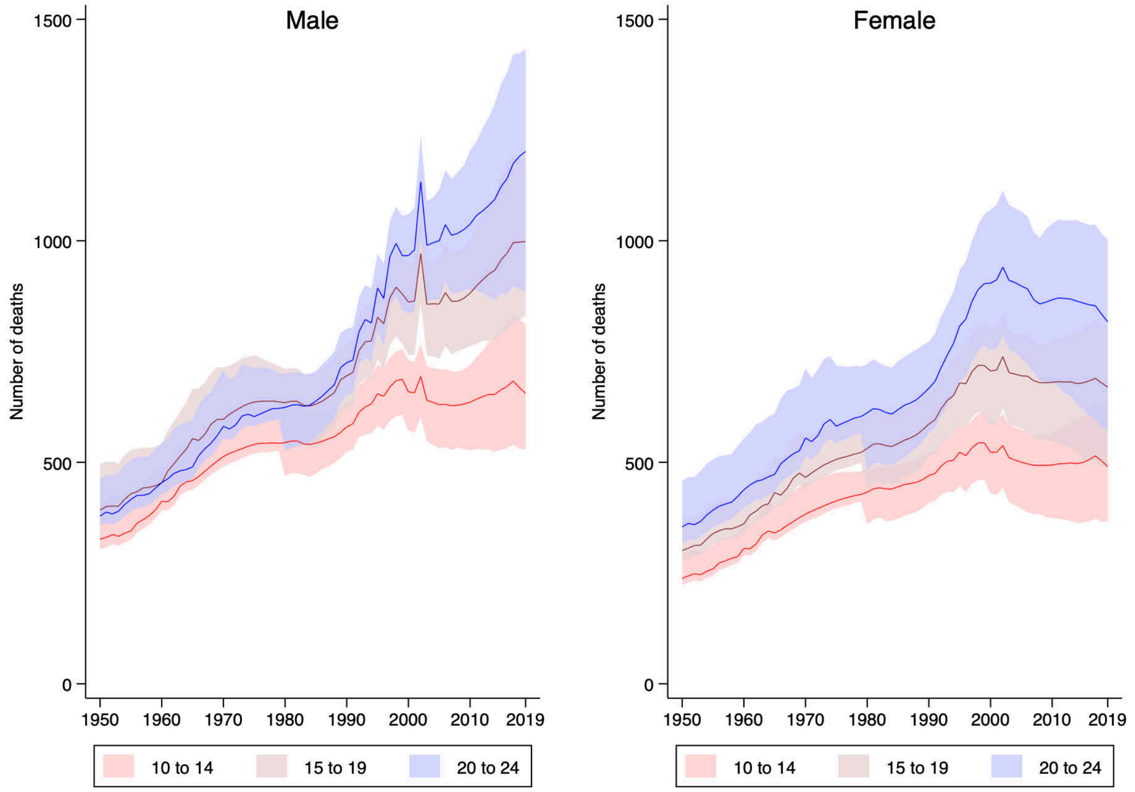

Figure S213c Mortality rate per 100,000 in 10-24 1980 – 2019 by cause: Senegal

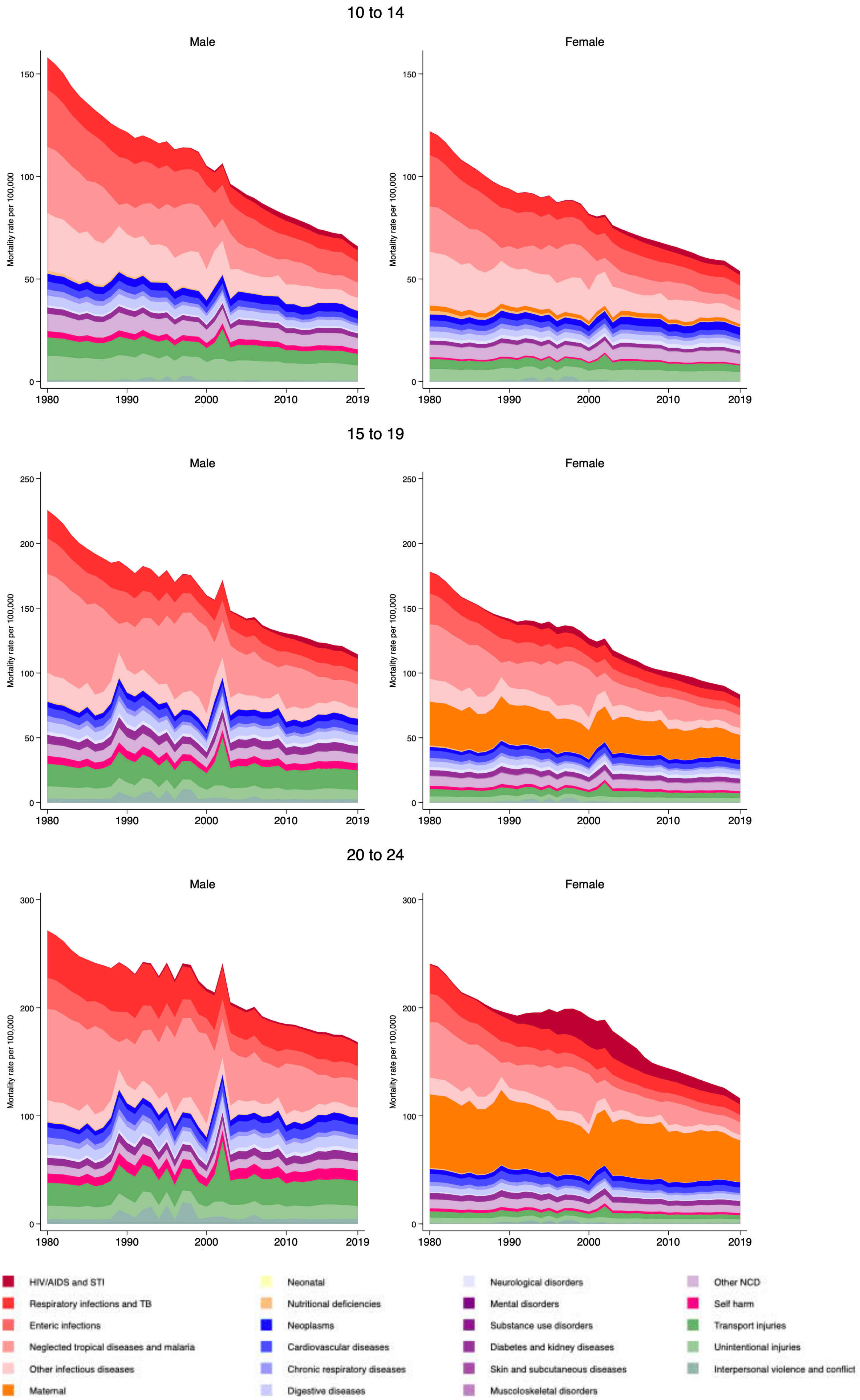

Figure S213d Number of deaths in 10-24 1980 – 2019 by cause: Senegal

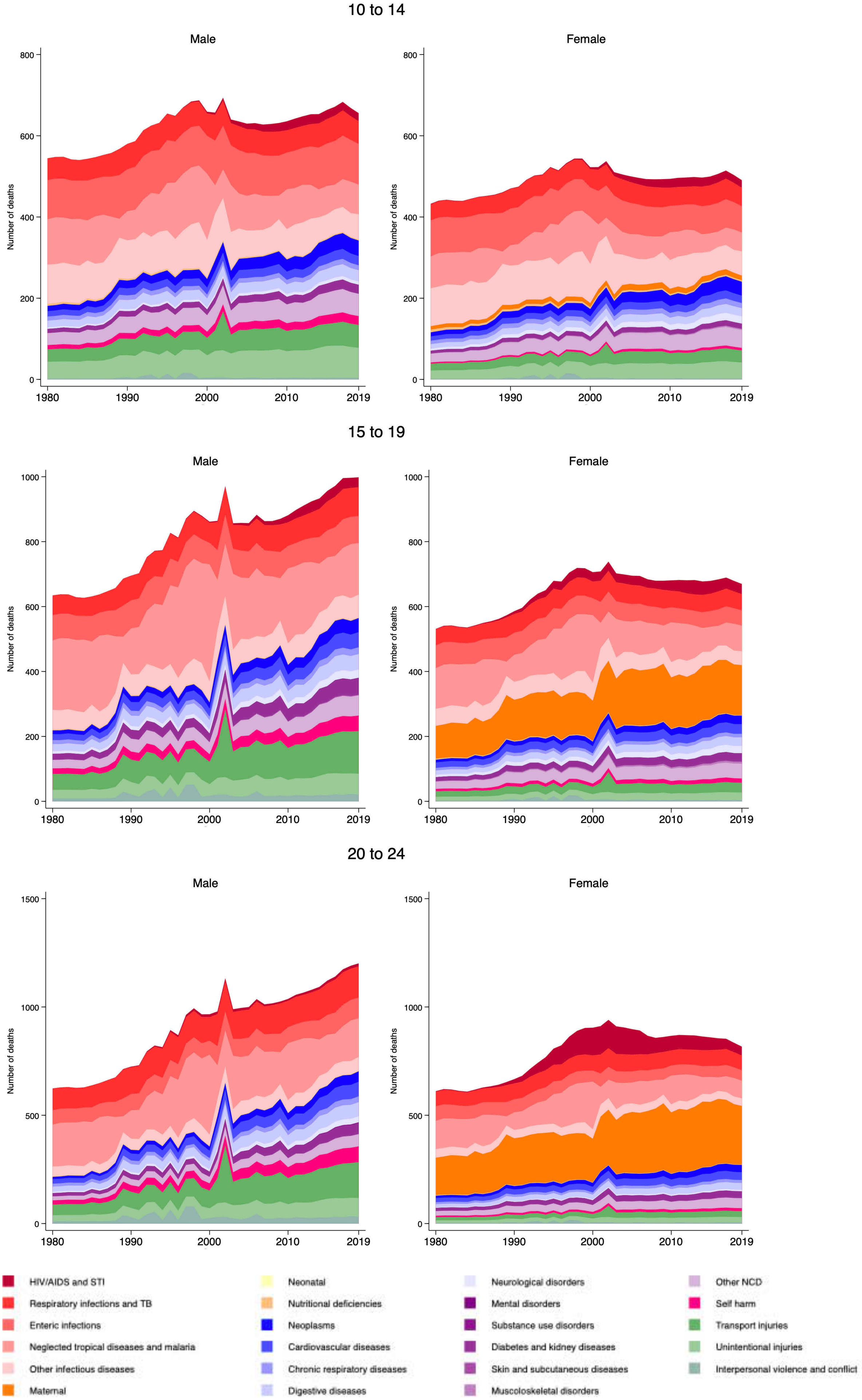

Figure S212: Sierra Leone

Figure S214a All-cause mortality rate per 100,000 in 10-24 1950 – 2019: Sierra Leone

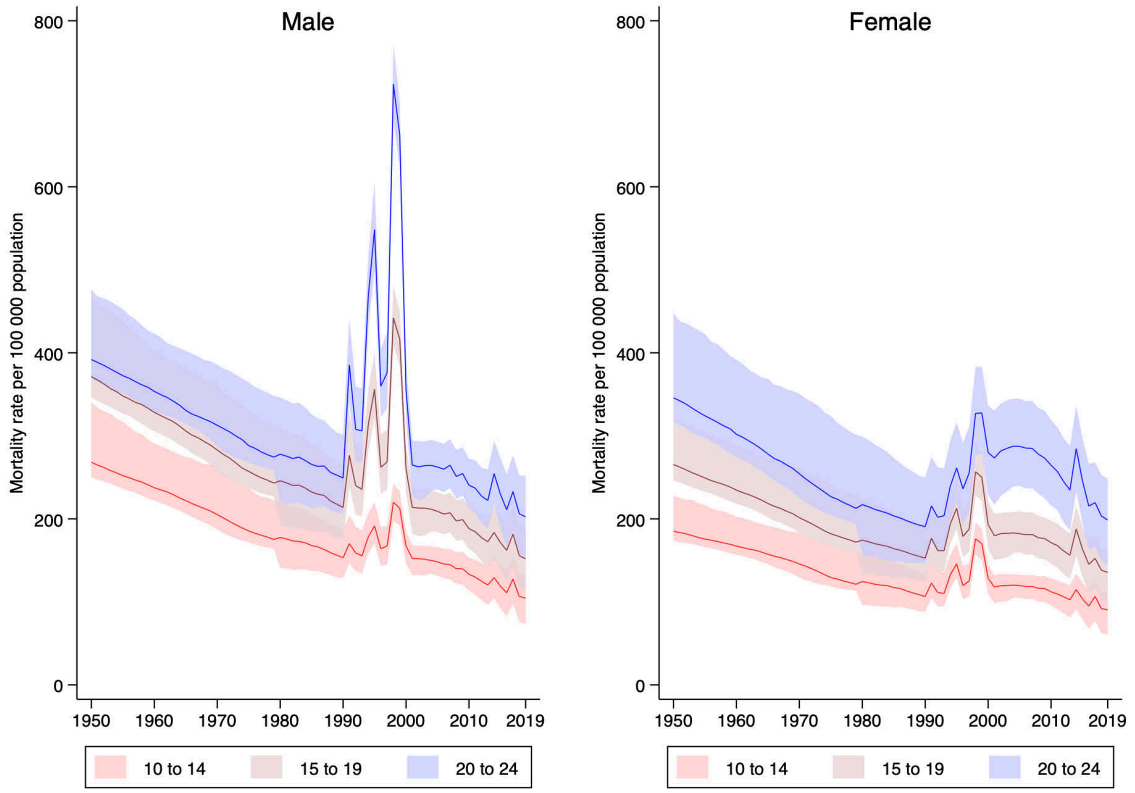

Figure S214b Number of deaths from all causes in 10-24 1950 – 2019: Sierra Leone

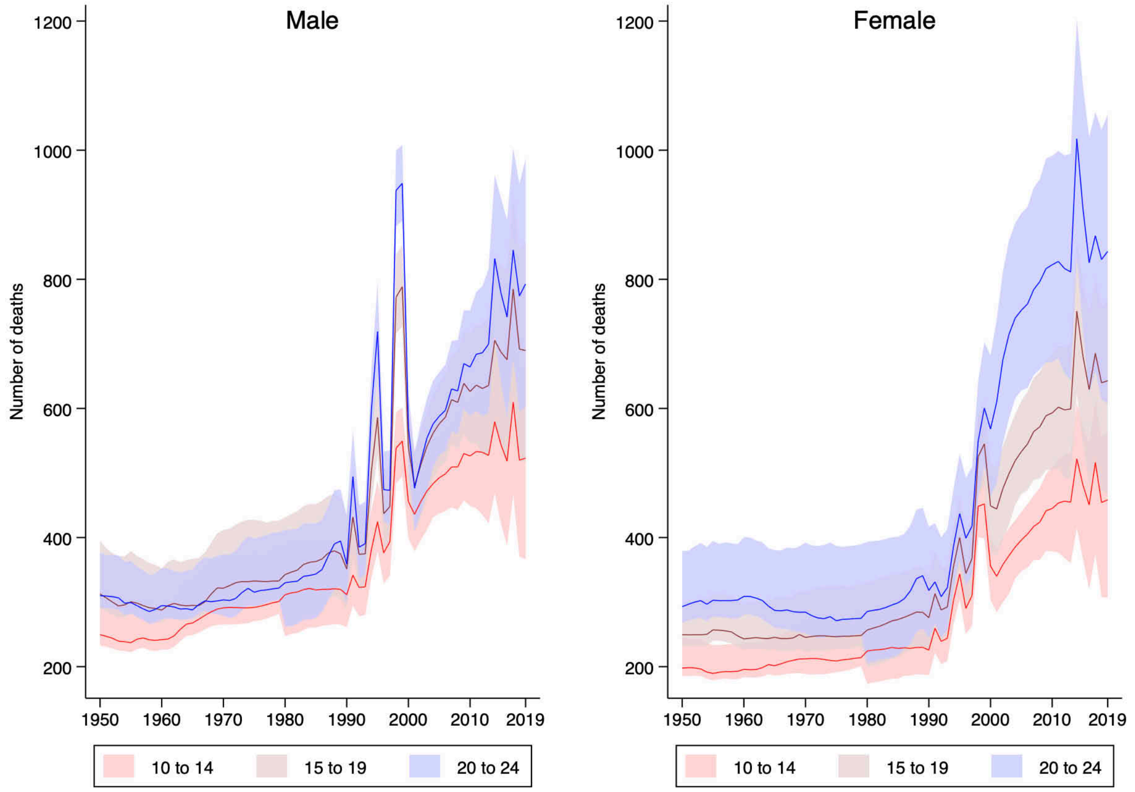

Figure S214c Mortality rate per 100,000 in 10-24 1980 – 2019 by cause: Sierra Leone

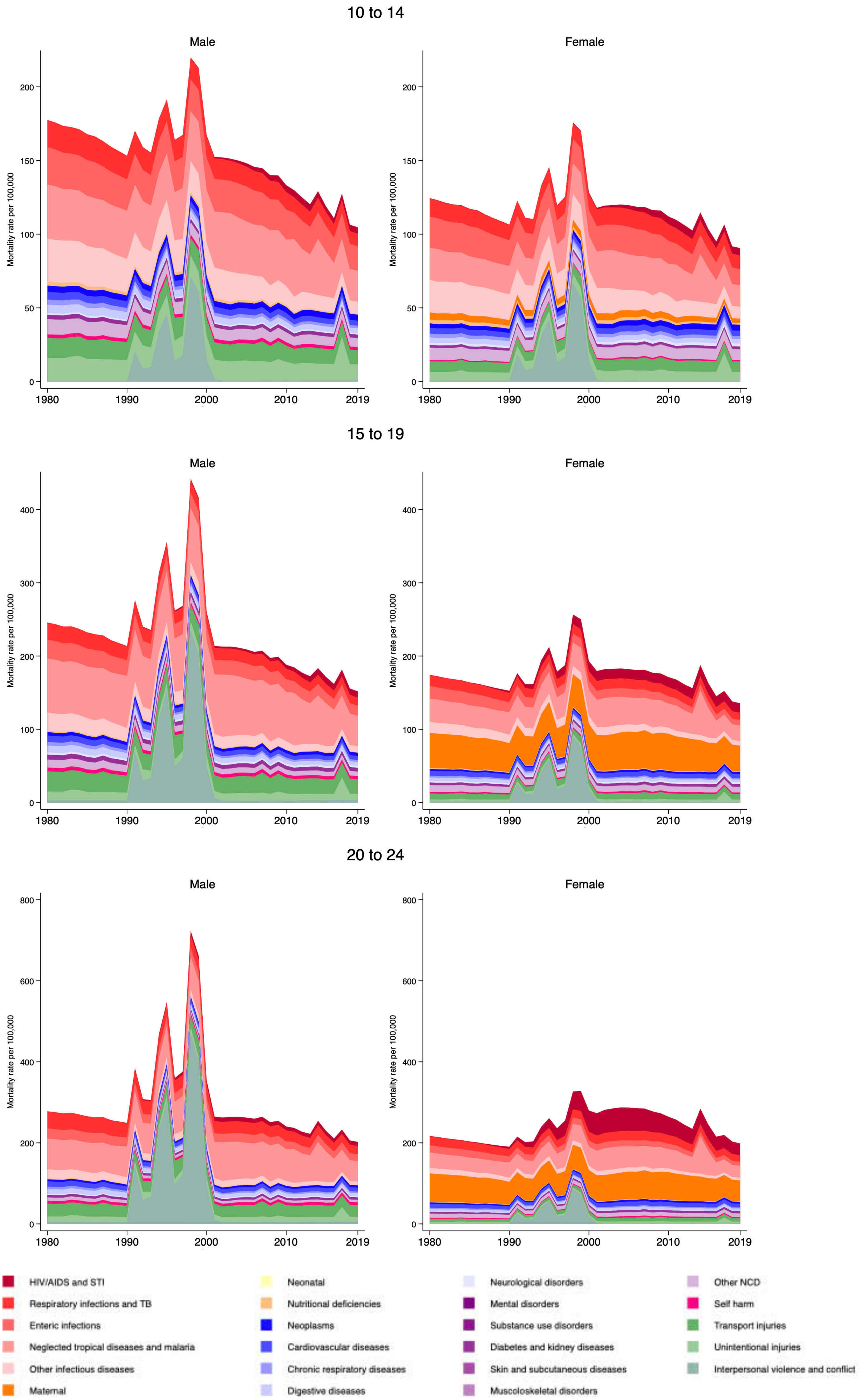

Figure S214d Number of deaths in 10-24 1980 – 2019 by cause: Sierra Leone

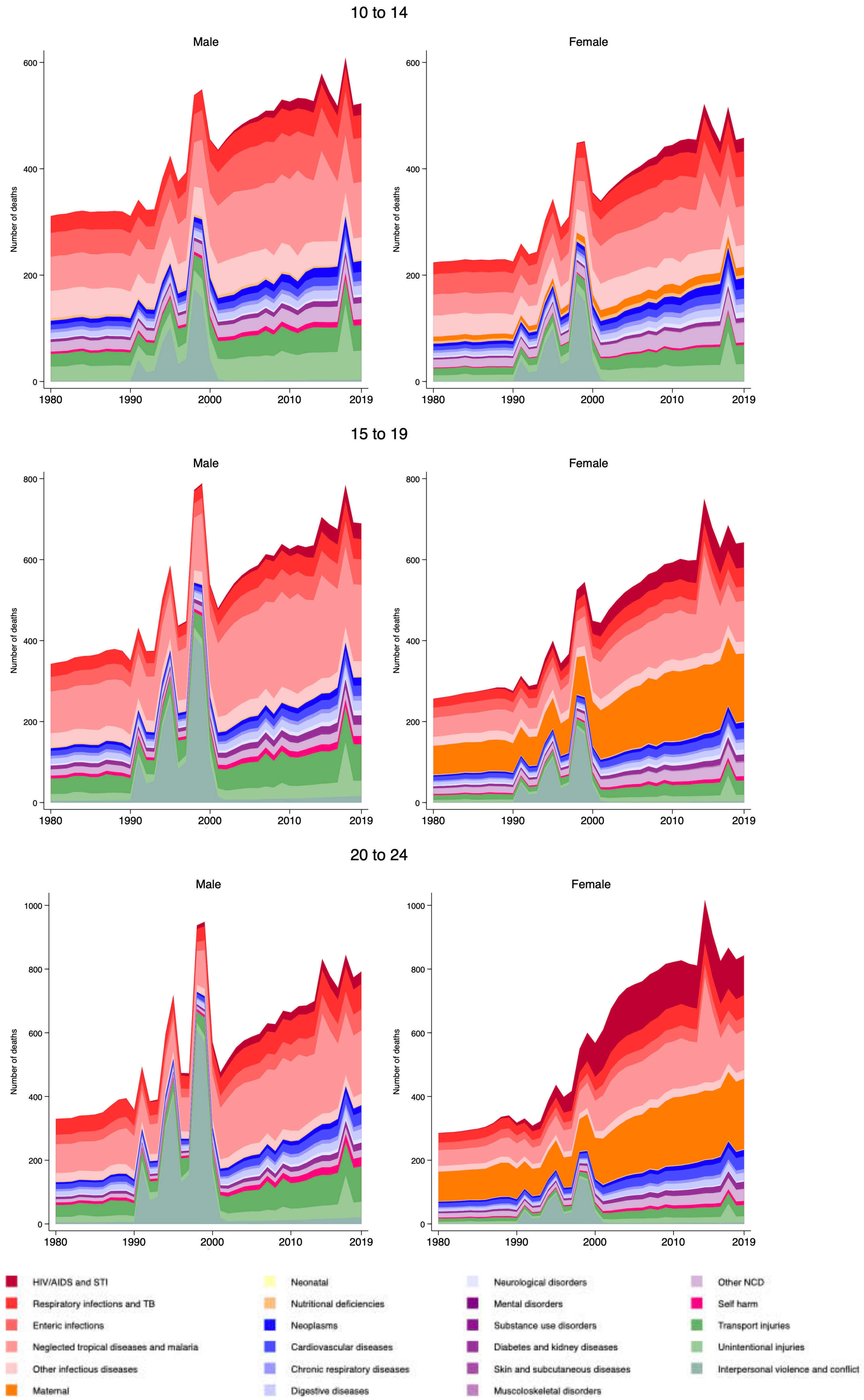

Figure S213: Togo

Figure S215a All-cause mortality rate per 100,000 in 10-24 1950 – 2019: Togo

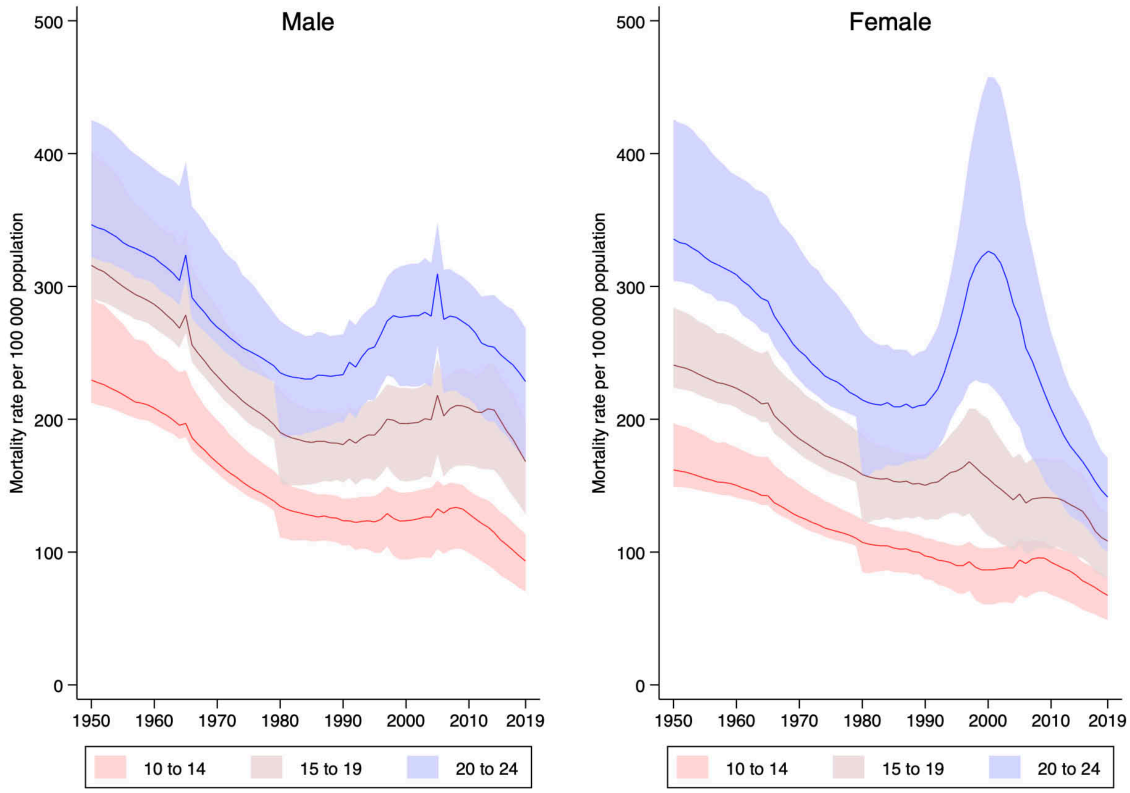

Figure S215b Number of deaths from all causes in 10-24 1950 – 2019: Togo

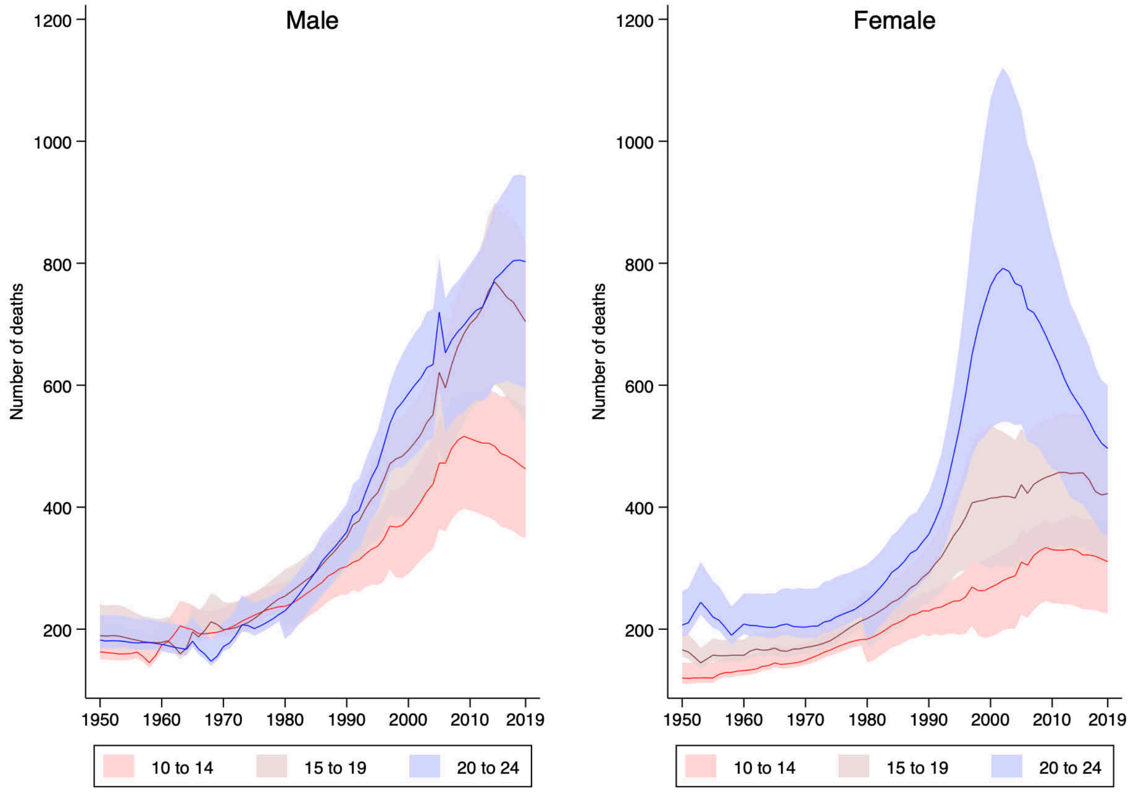

Figure S215c Mortality rate per 100,000 in 10-24 1980 – 2019 by cause: Togo

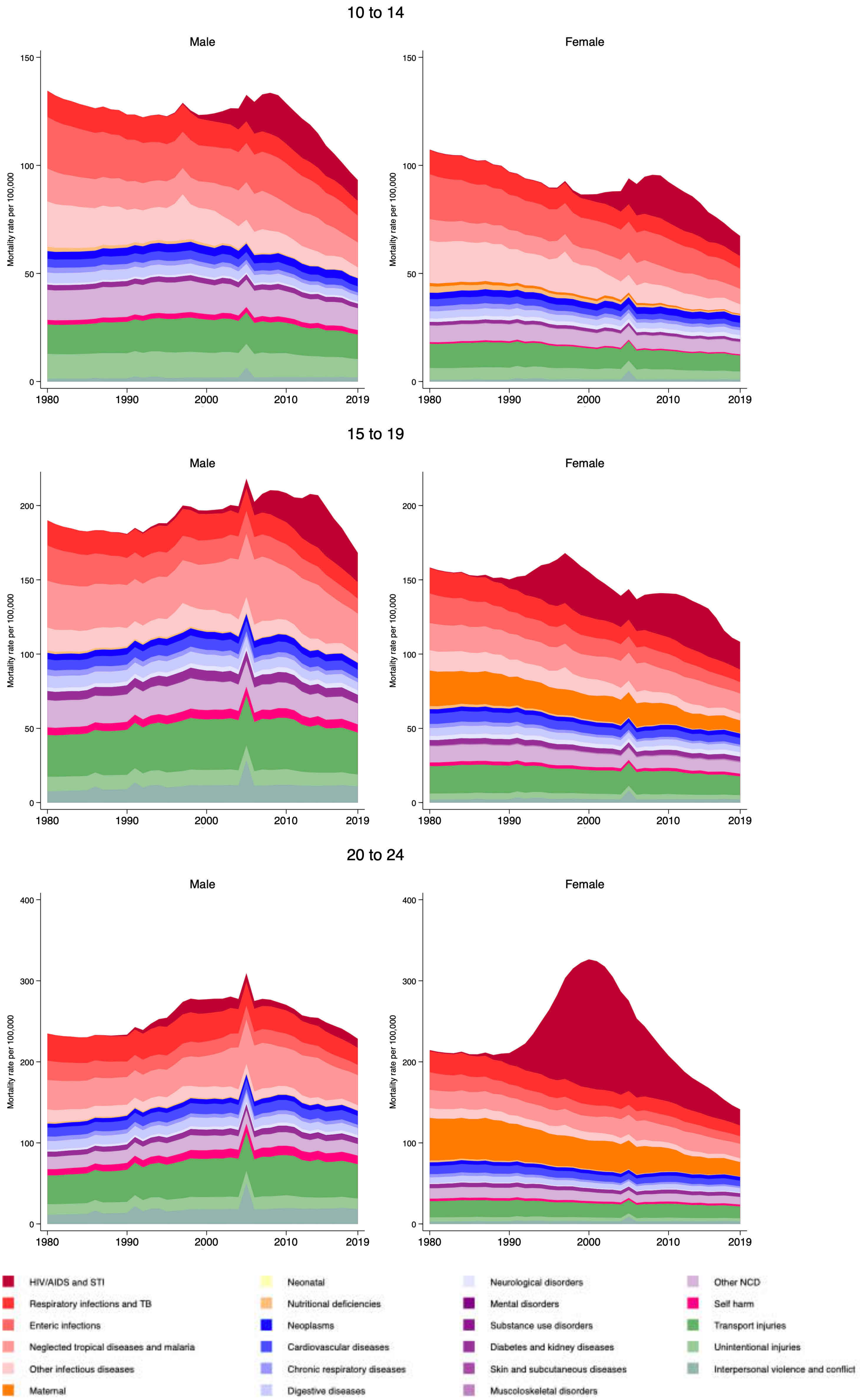

Figure S215d Number of deaths in 10-24 1980 – 2019 by cause: Togo

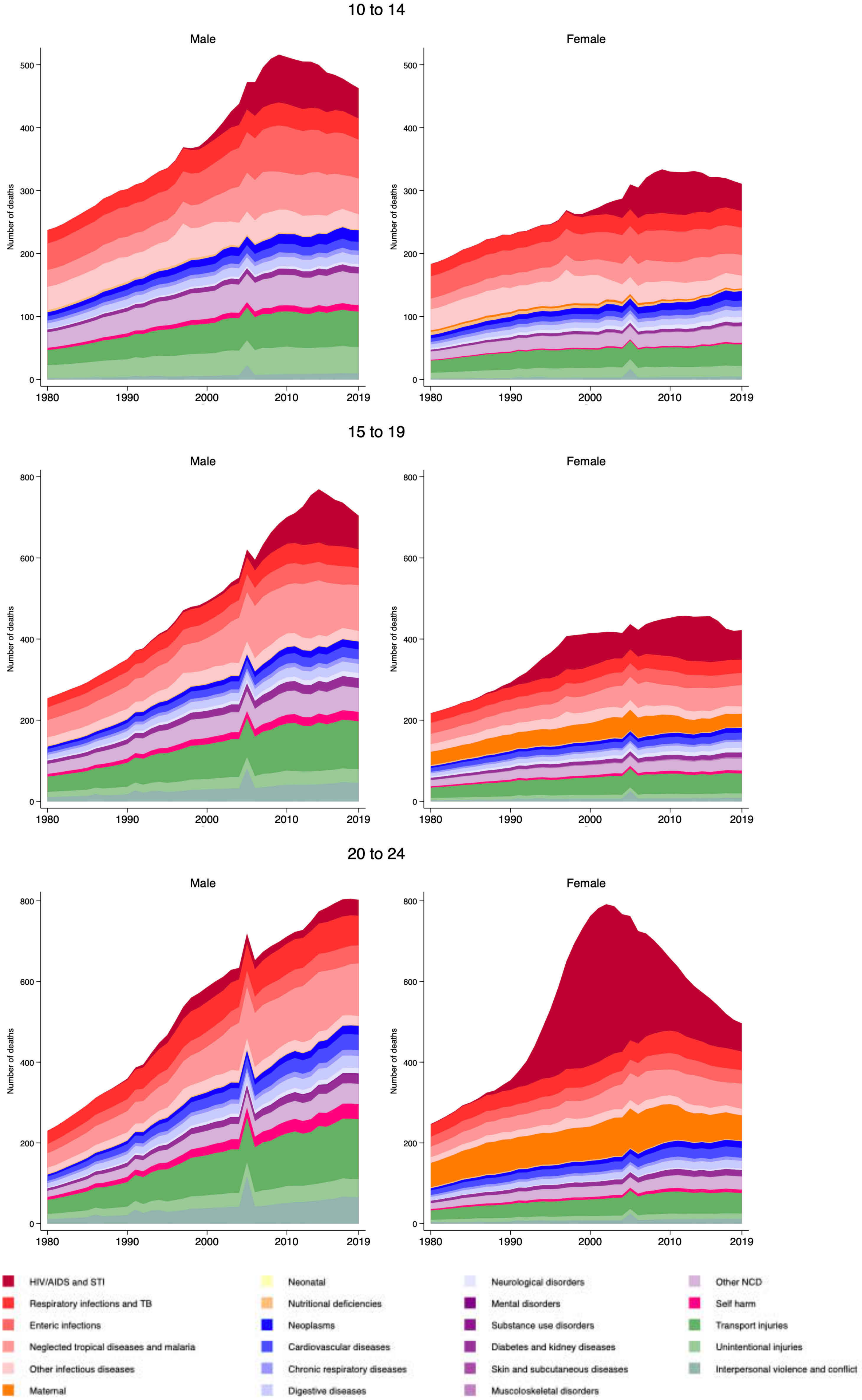

Figure S214: American Samoa

Figure S216a All-cause mortality rate per 100,000 in 10-24 1950 – 2019: American Samoa

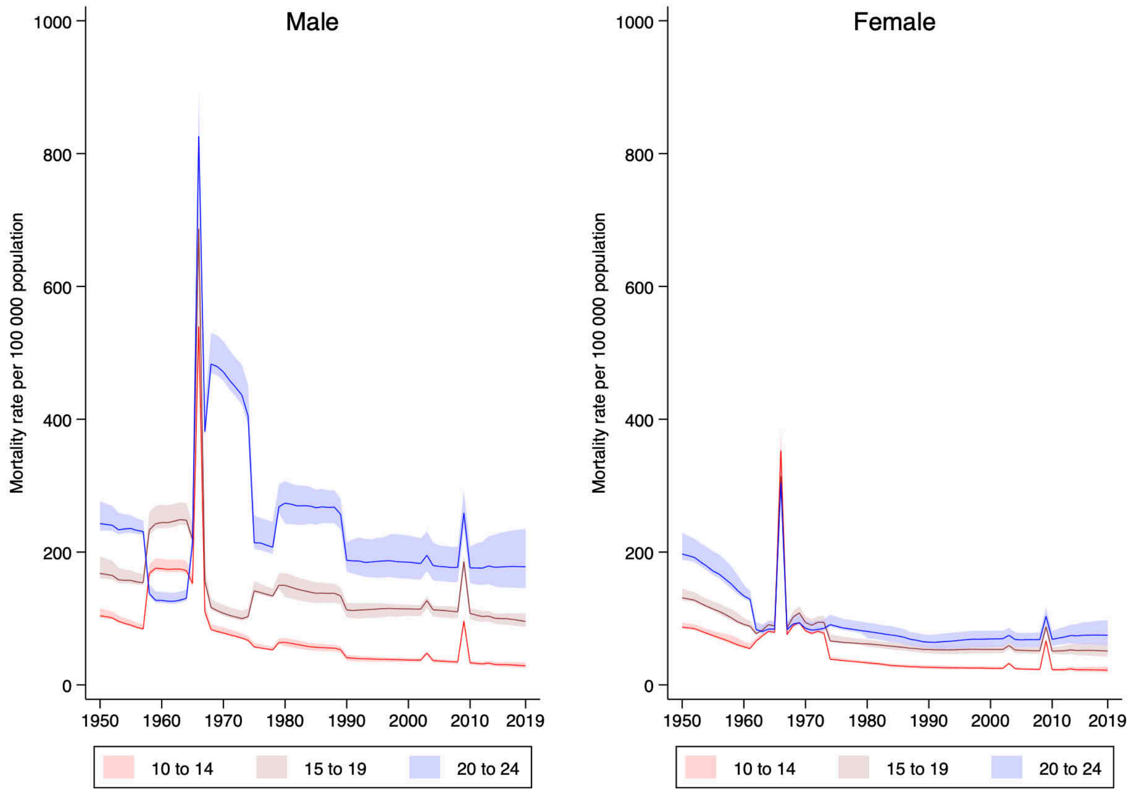

Figure S216b Number of deaths from all causes in 10-24 1950 – 2019: American Samoa

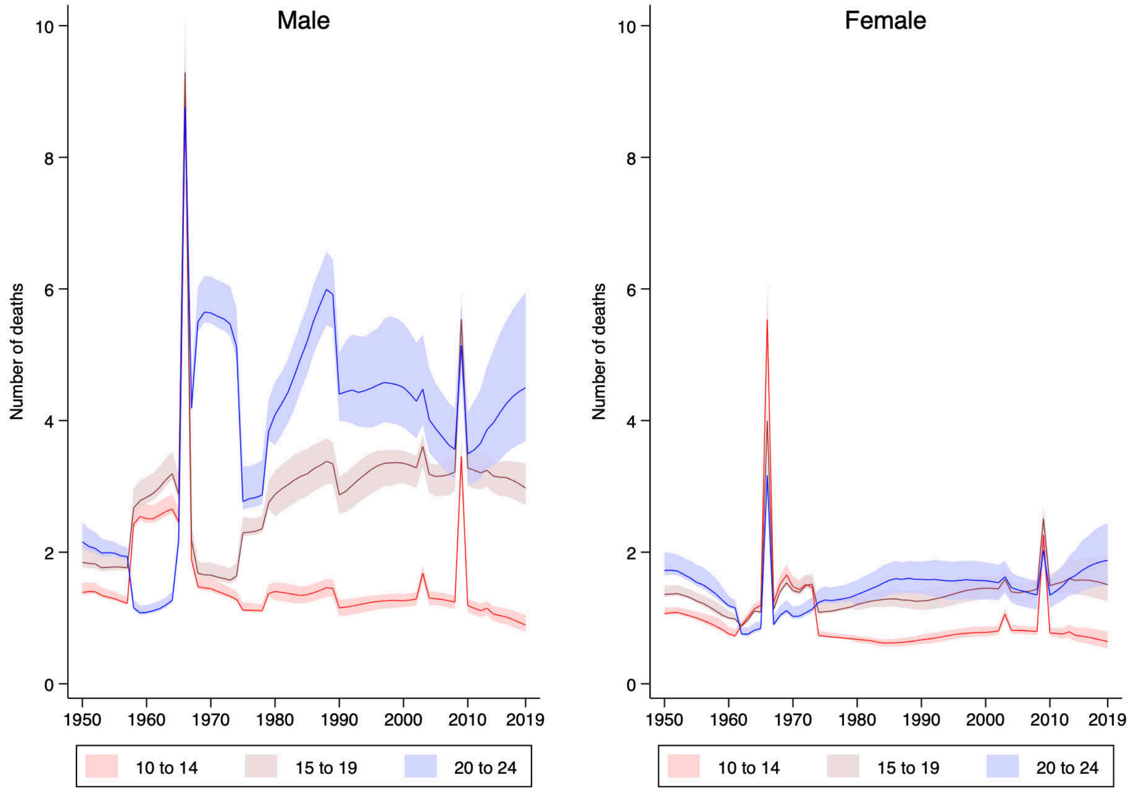

Figure S216c Mortality rate per 100,000 in 10-24 1980 – 2019 by cause: American Samoa

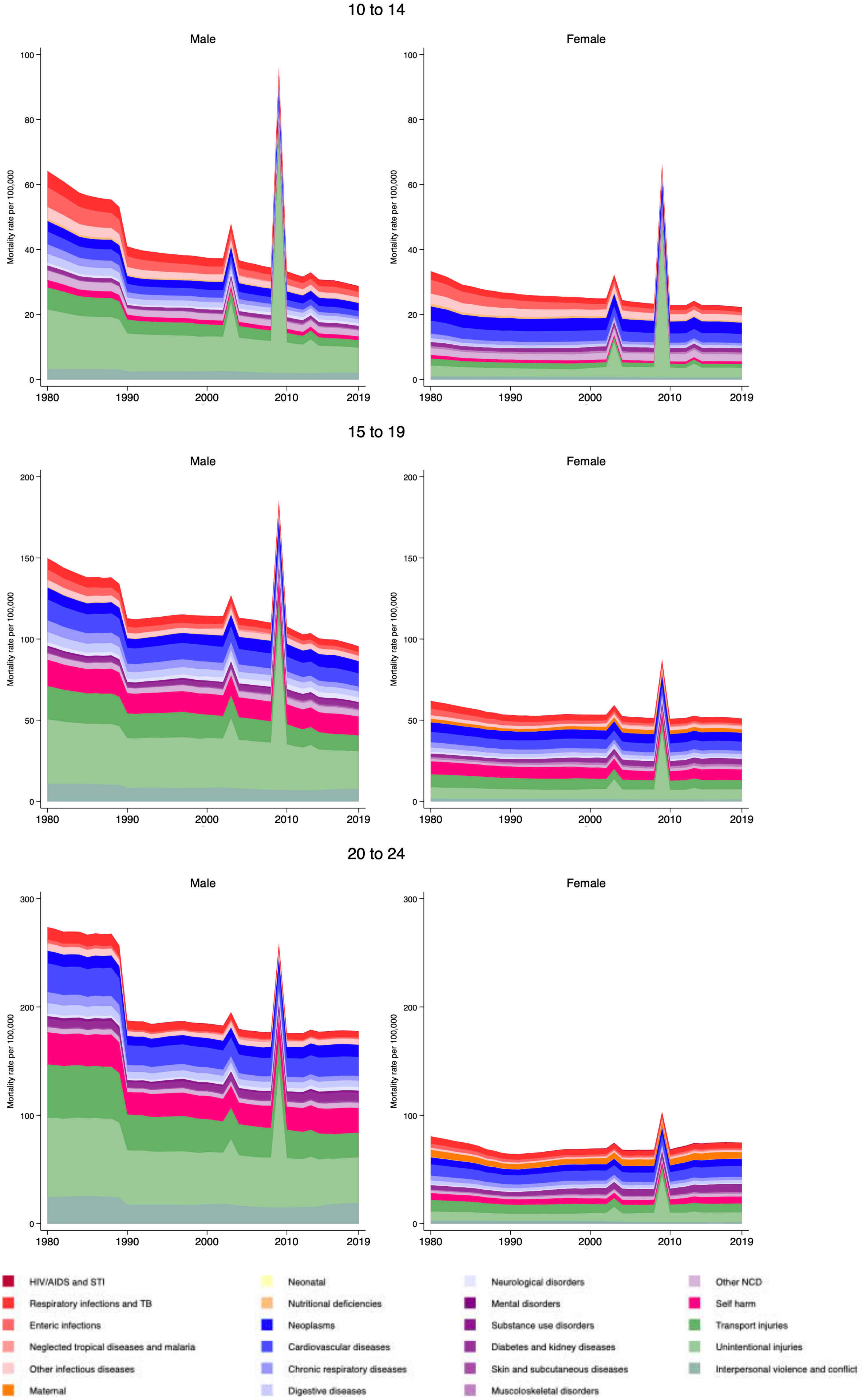

Figure S216d Number of deaths in 10-24 1980 – 2019 by cause: American Samoa

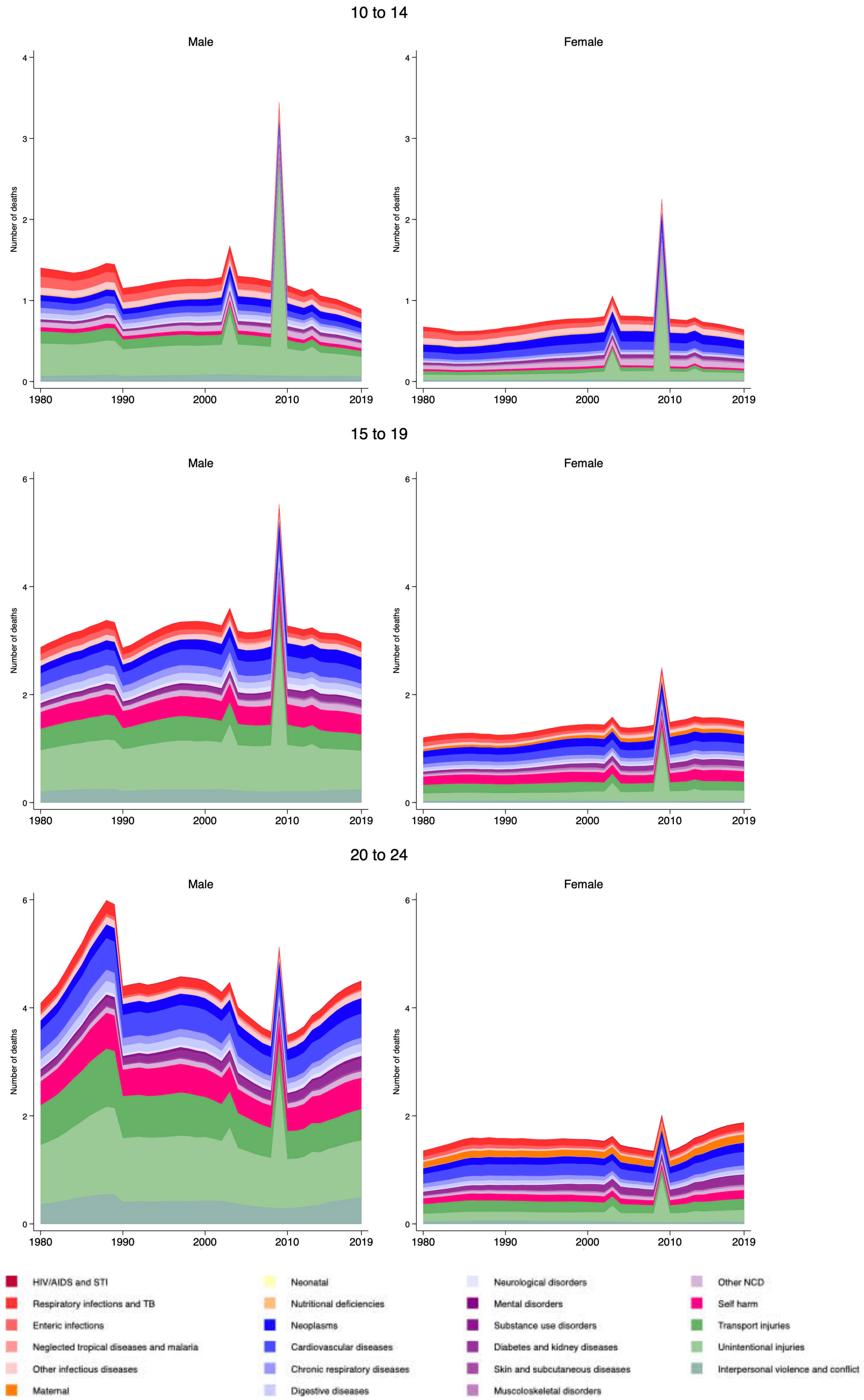

Figure S215: Bermuda

Figure S217a All-cause mortality rate per 100,000 in 10-24 1950 – 2019: Bermuda

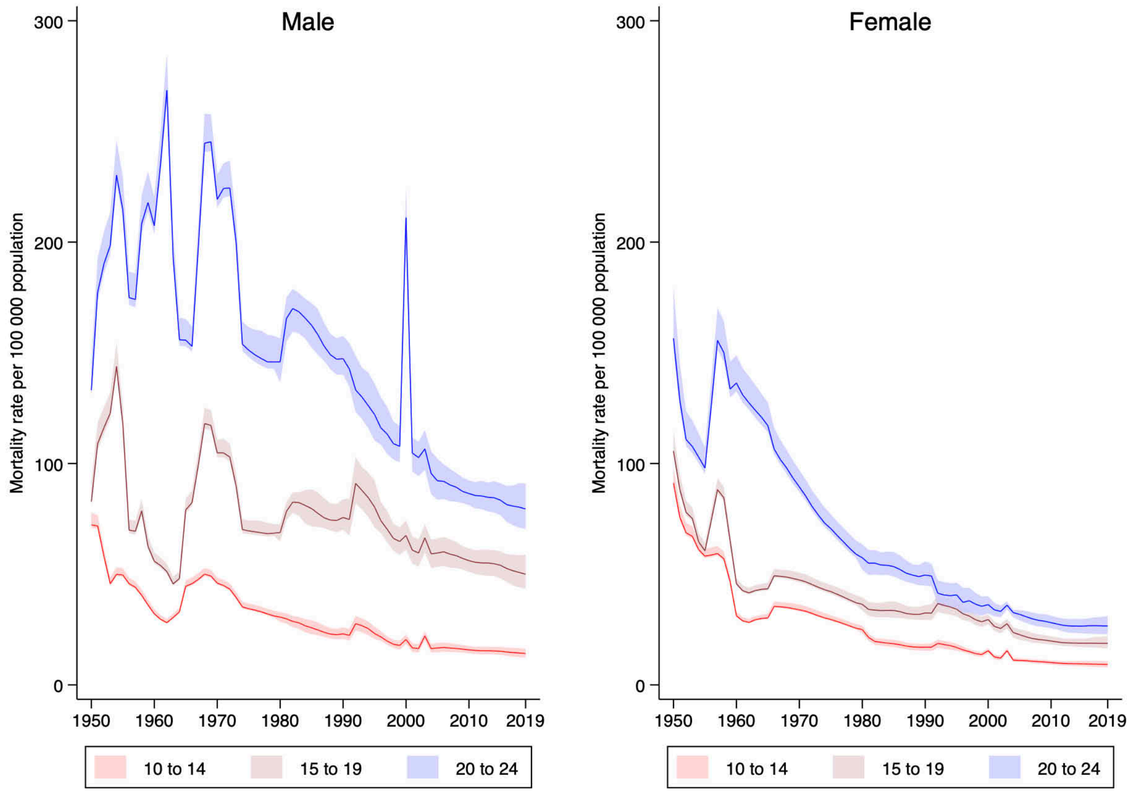

Figure S217b Number of deaths from all causes in 10-24 1950 – 2019: Bermuda

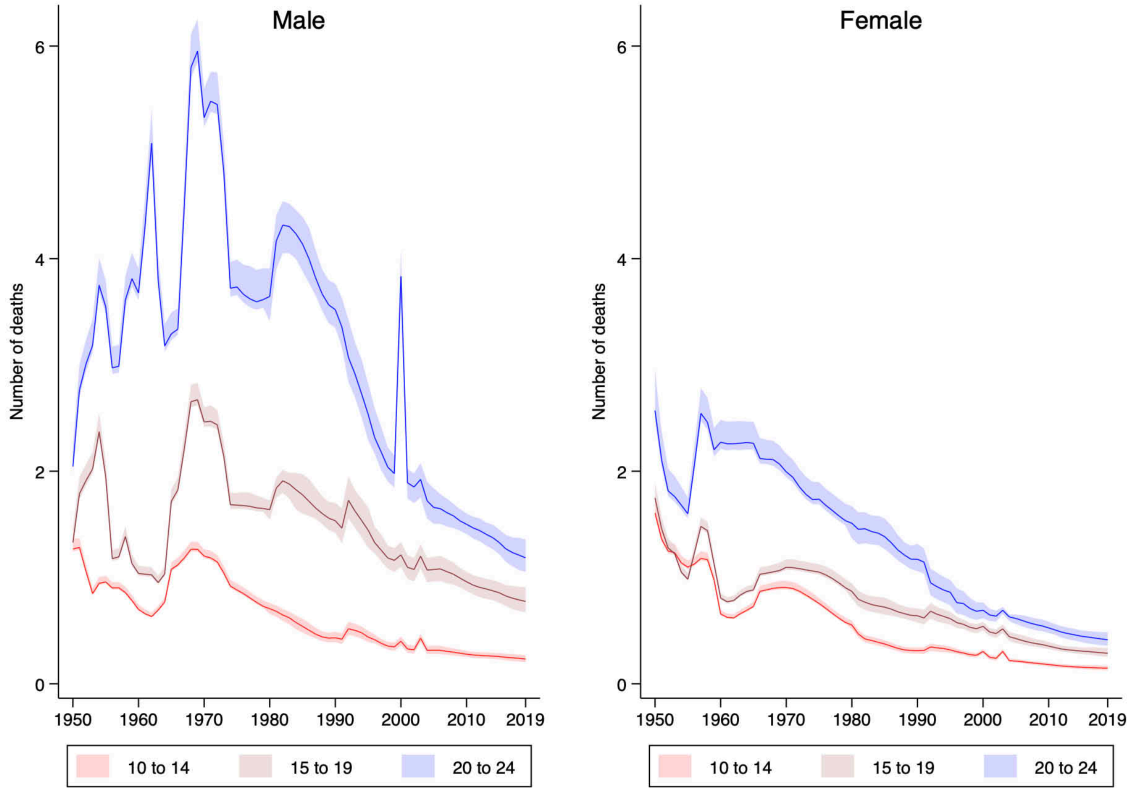

Figure S217c Mortality rate per 100,000 in 10-24 1980 – 2019 by cause: Bermuda

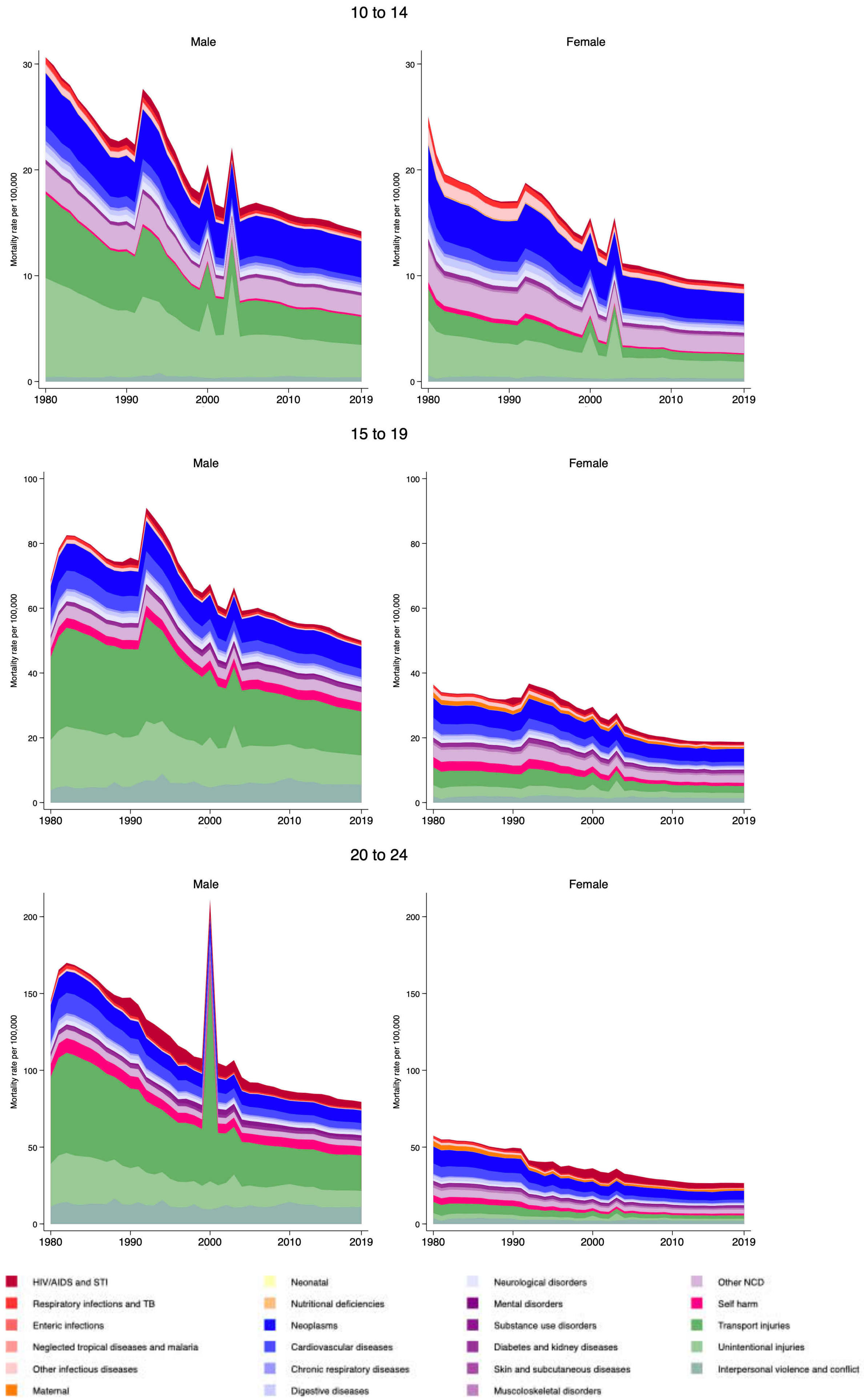

Figure S217d Number of deaths in 10-24 1980 – 2019 by cause: Bermuda

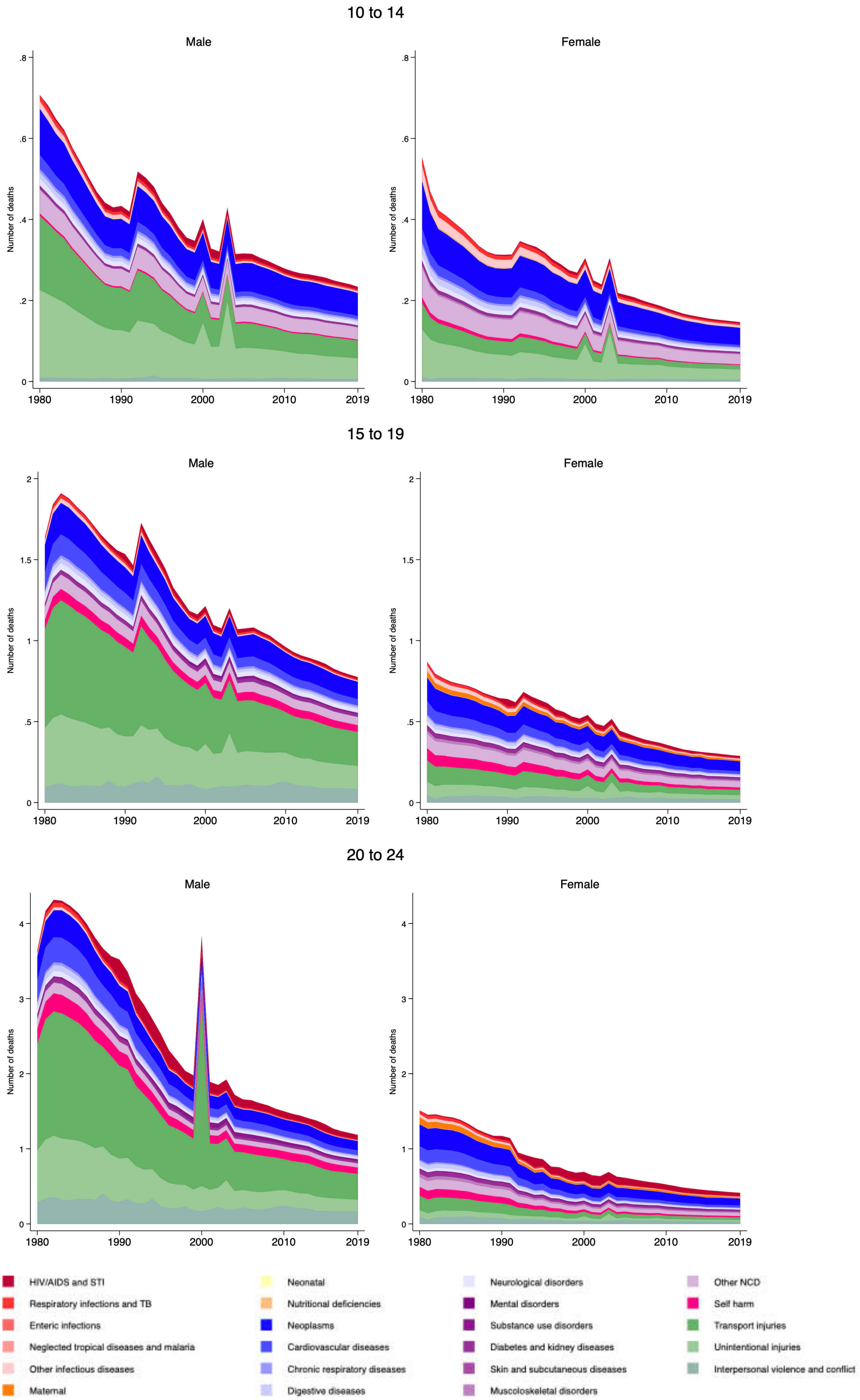

Figure S216: Cook Islands

Figure S218a All-cause mortality rate per 100,000 in 10-24 1950 – 2019: Cook Islands

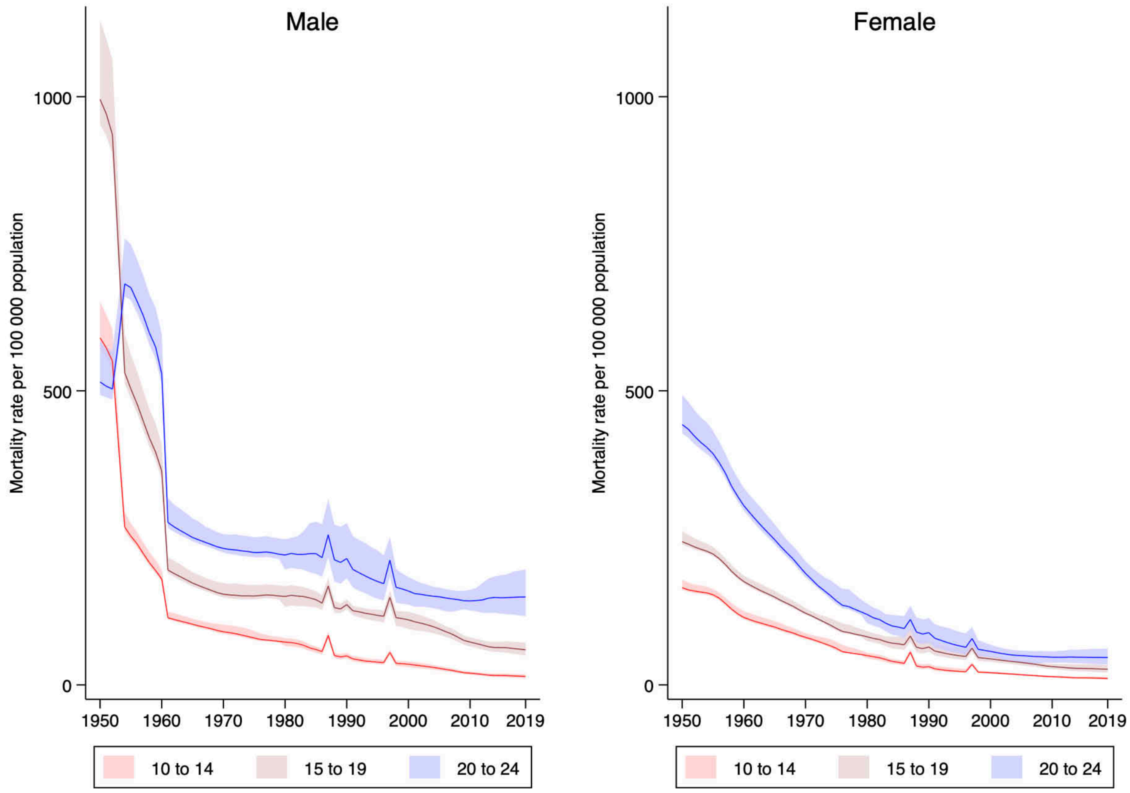

Figure S218b Number of deaths from all causes in 10-24 1950 – 2019: Cook Islands

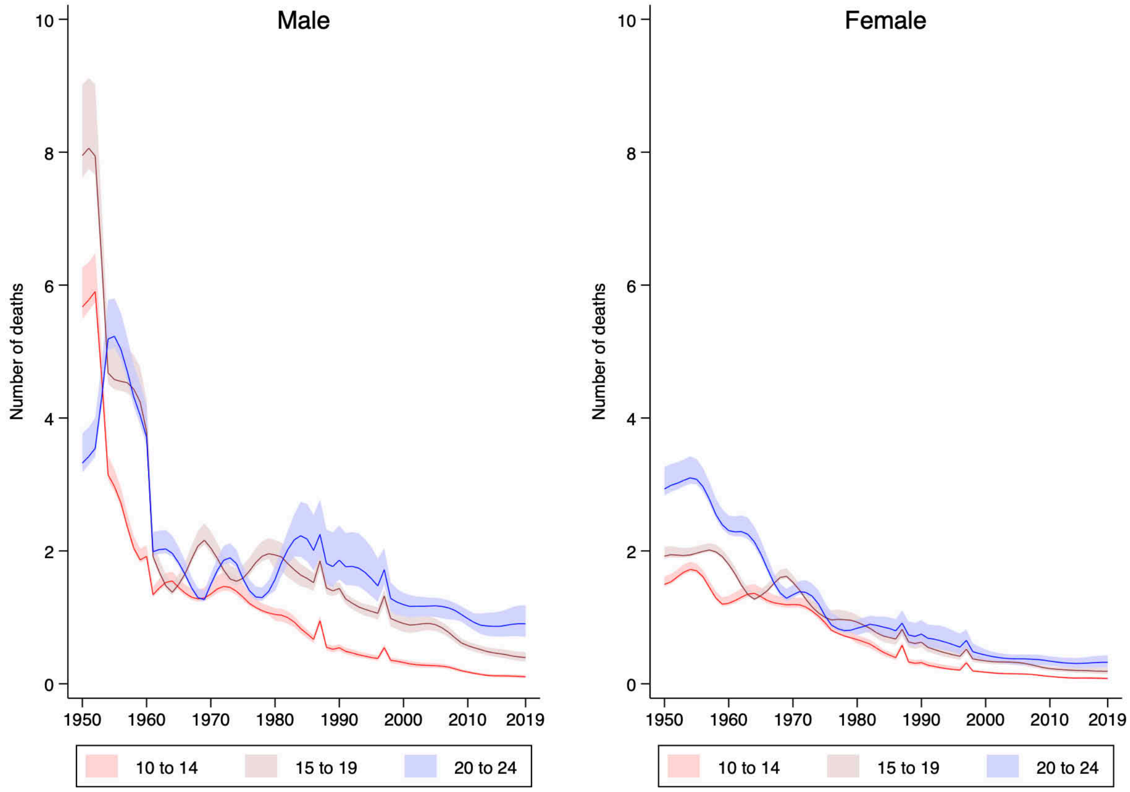

Figure S218c Mortality rate per 100,000 in 10-24 1980 – 2019 by cause: Cook Islands

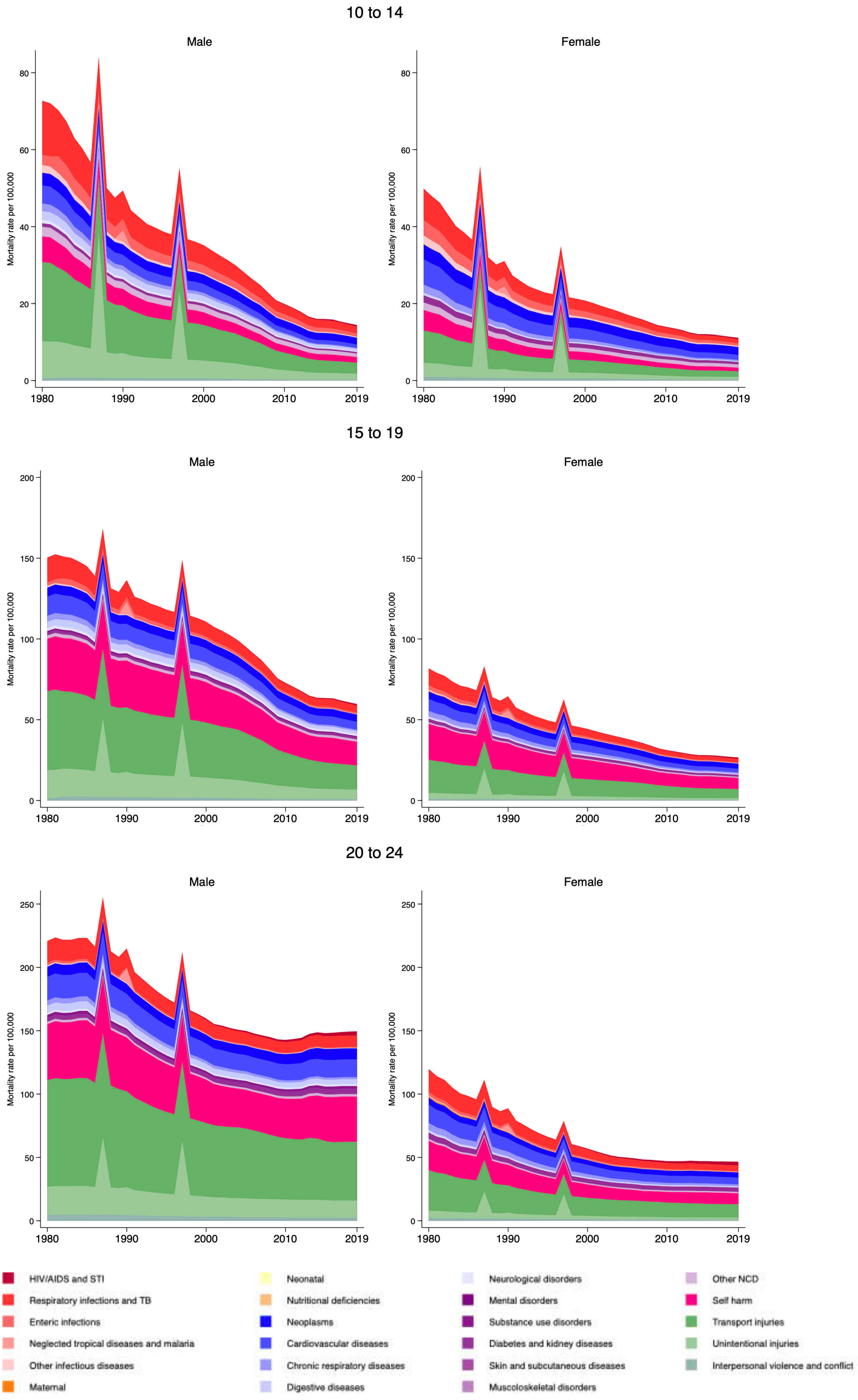

Figure S218d Number of deaths in 10-24 1980 – 2019 by cause: Cook Islands

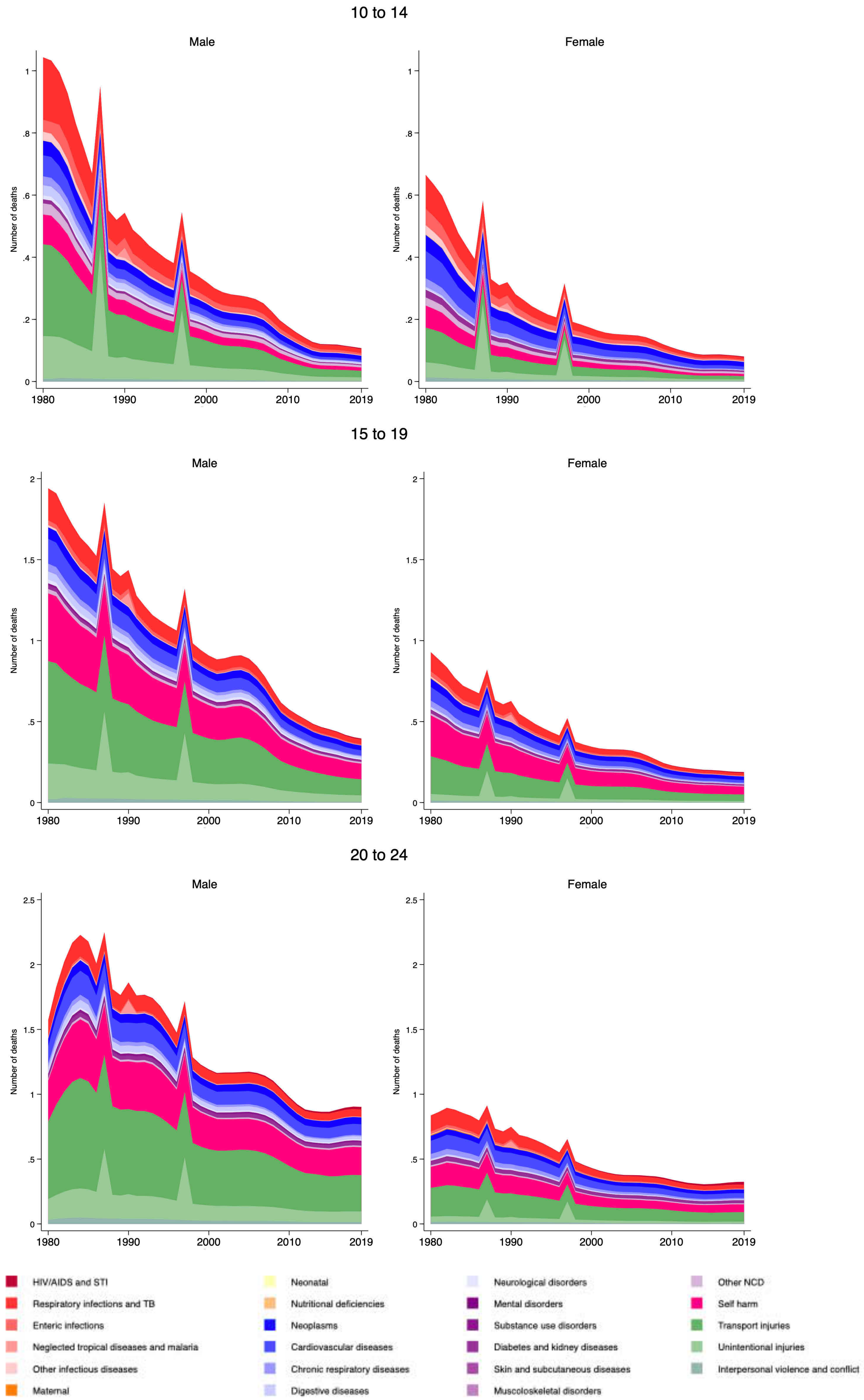

Figure S217: Greenland

Figure S219a All-cause mortality rate per 100,000 in 10-24 1950 – 2019: Greenland

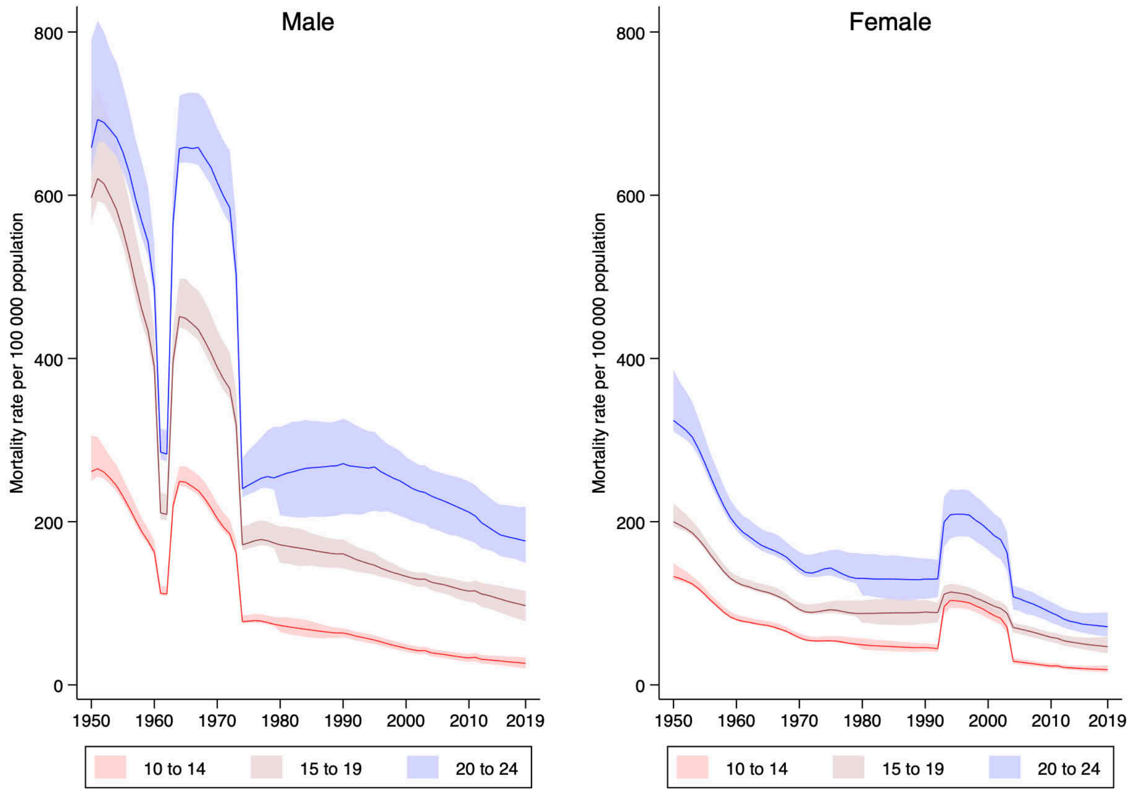

Figure S219b Number of deaths from all causes in 10-24 1950 – 2019: Greenland

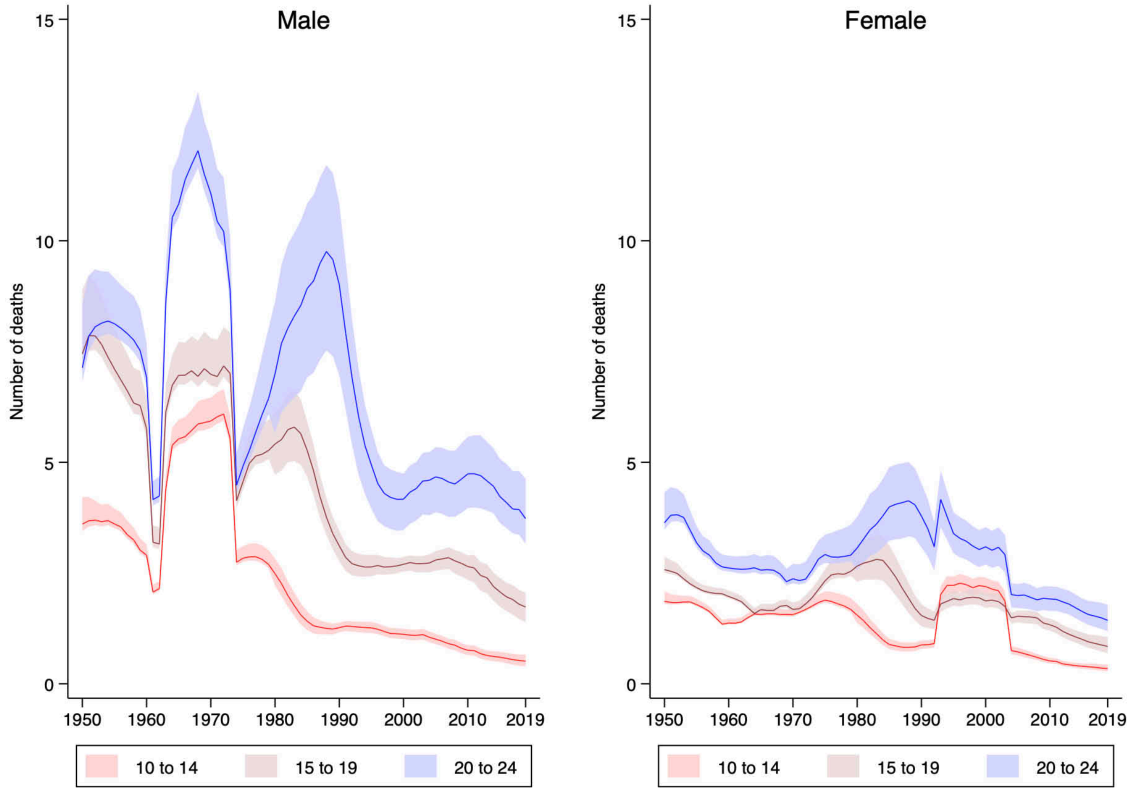

Figure S219c Mortality rate per 100,000 in 10-24 1980 – 2019 by cause: Greenland

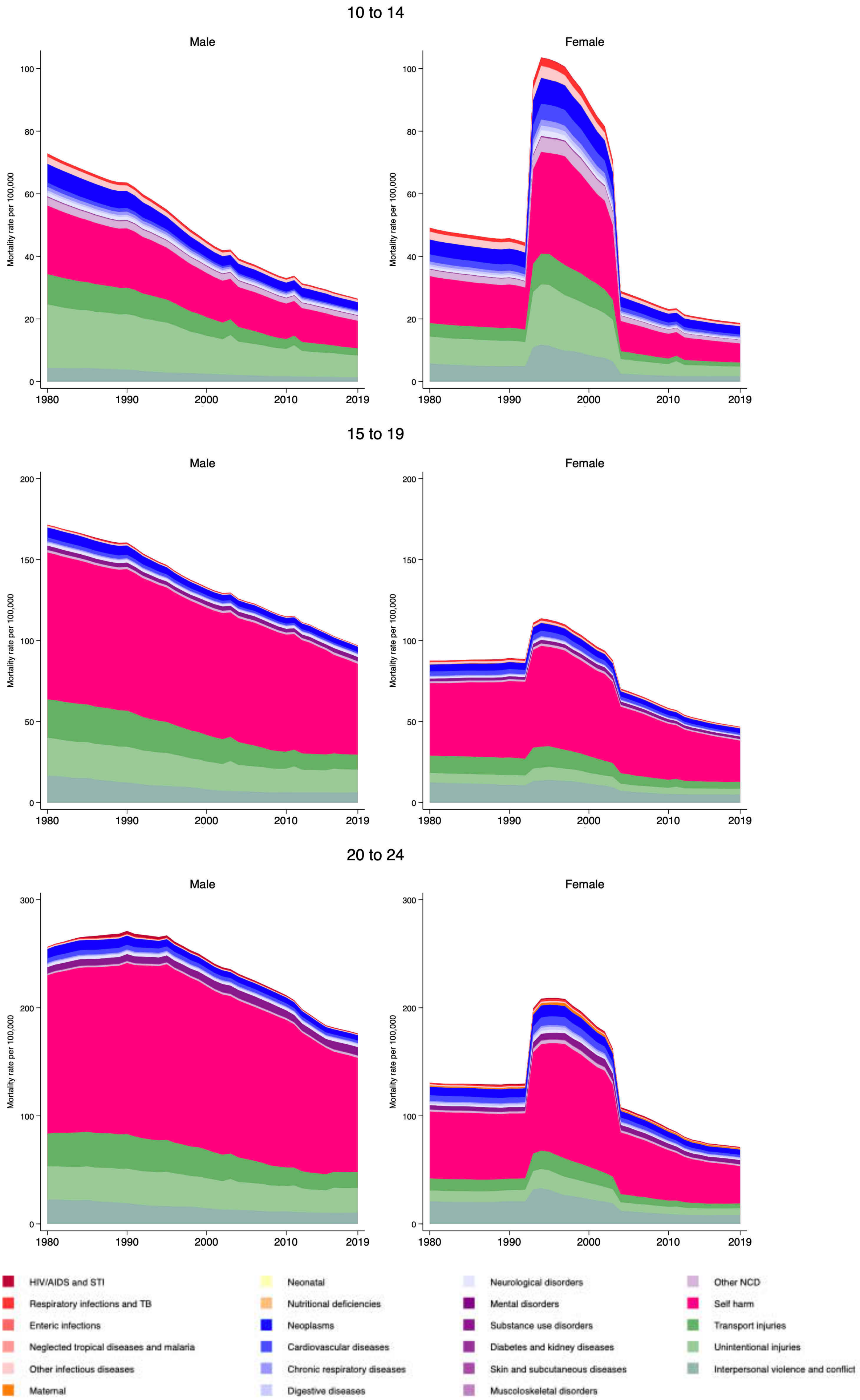

Figure S219d Number of deaths in 10-24 1980 – 2019 by cause: Greenland

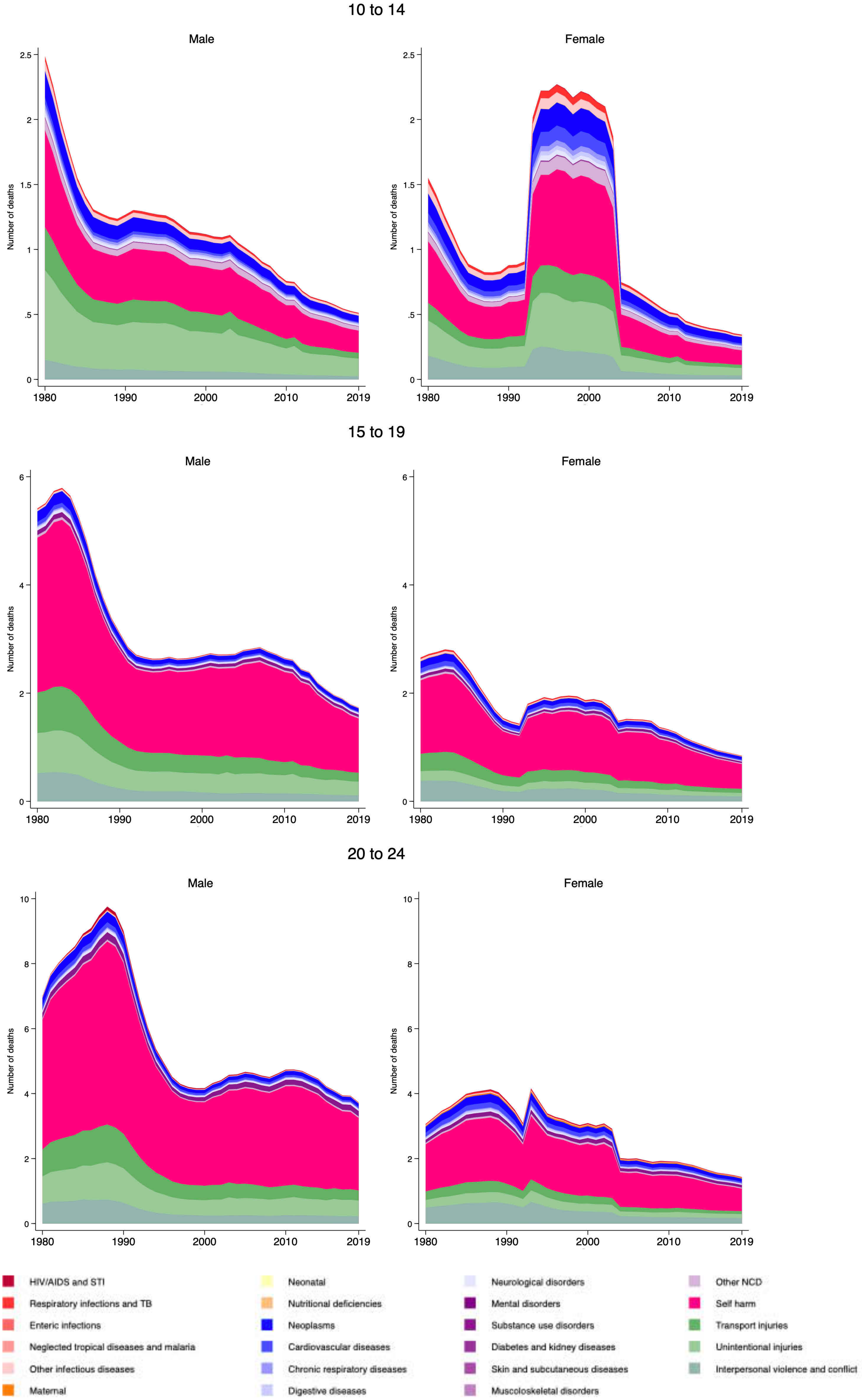

Figure S218: Guam

Figure S220a All-cause mortality rate per 100,000 in 10-24 1950 – 2019: Guam

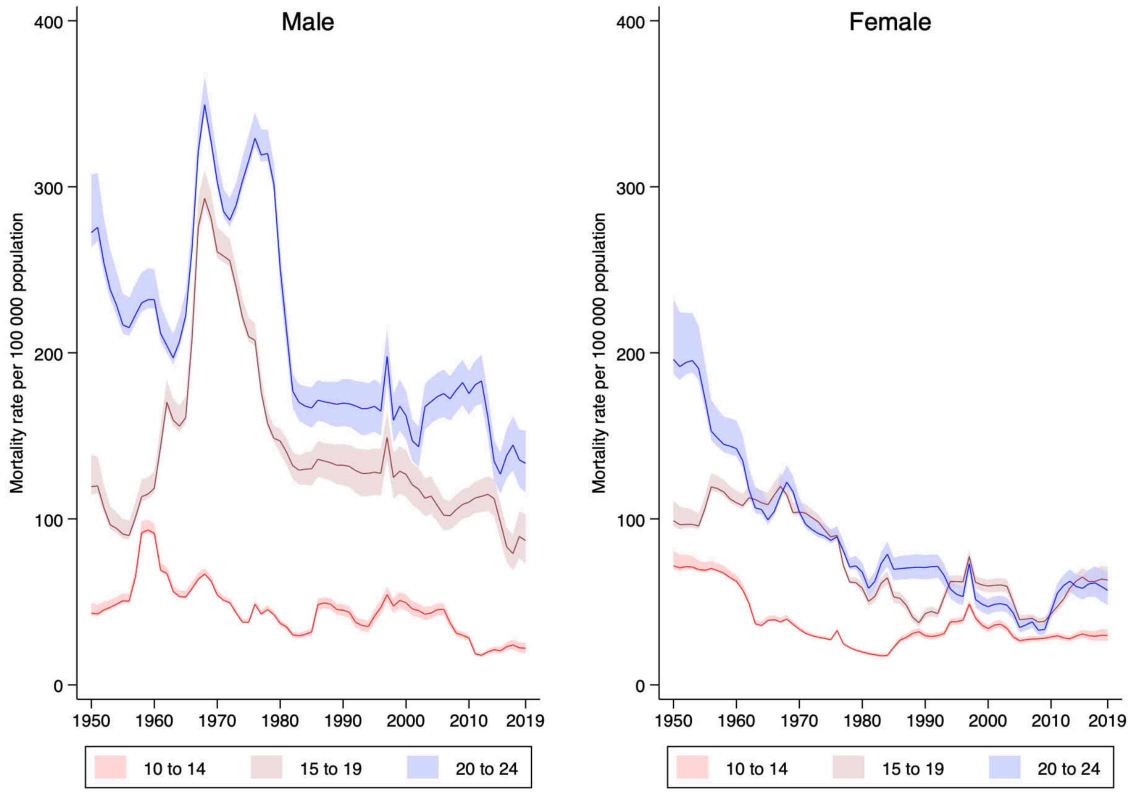

Figure S220b Number of deaths from all causes in 10-24 1950 – 2019: Guam

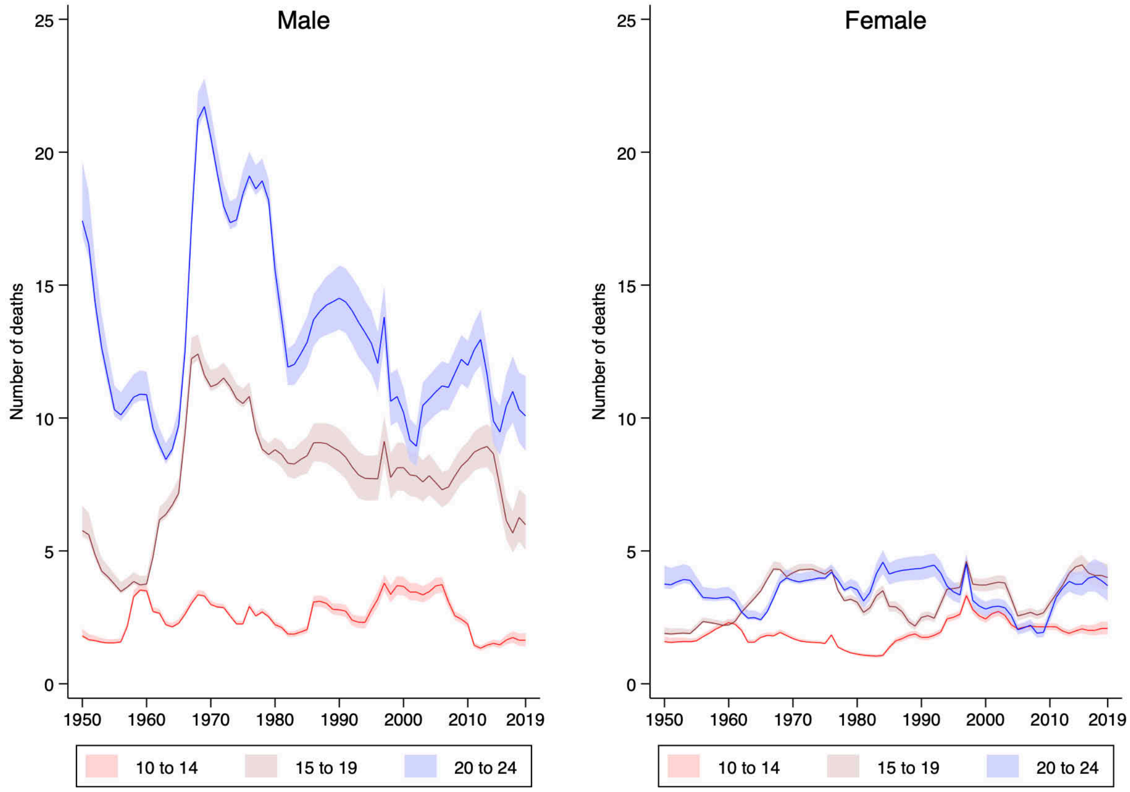

Figure S220c Mortality rate per 100,000 in 10-24 1980 – 2019 by cause: Guam

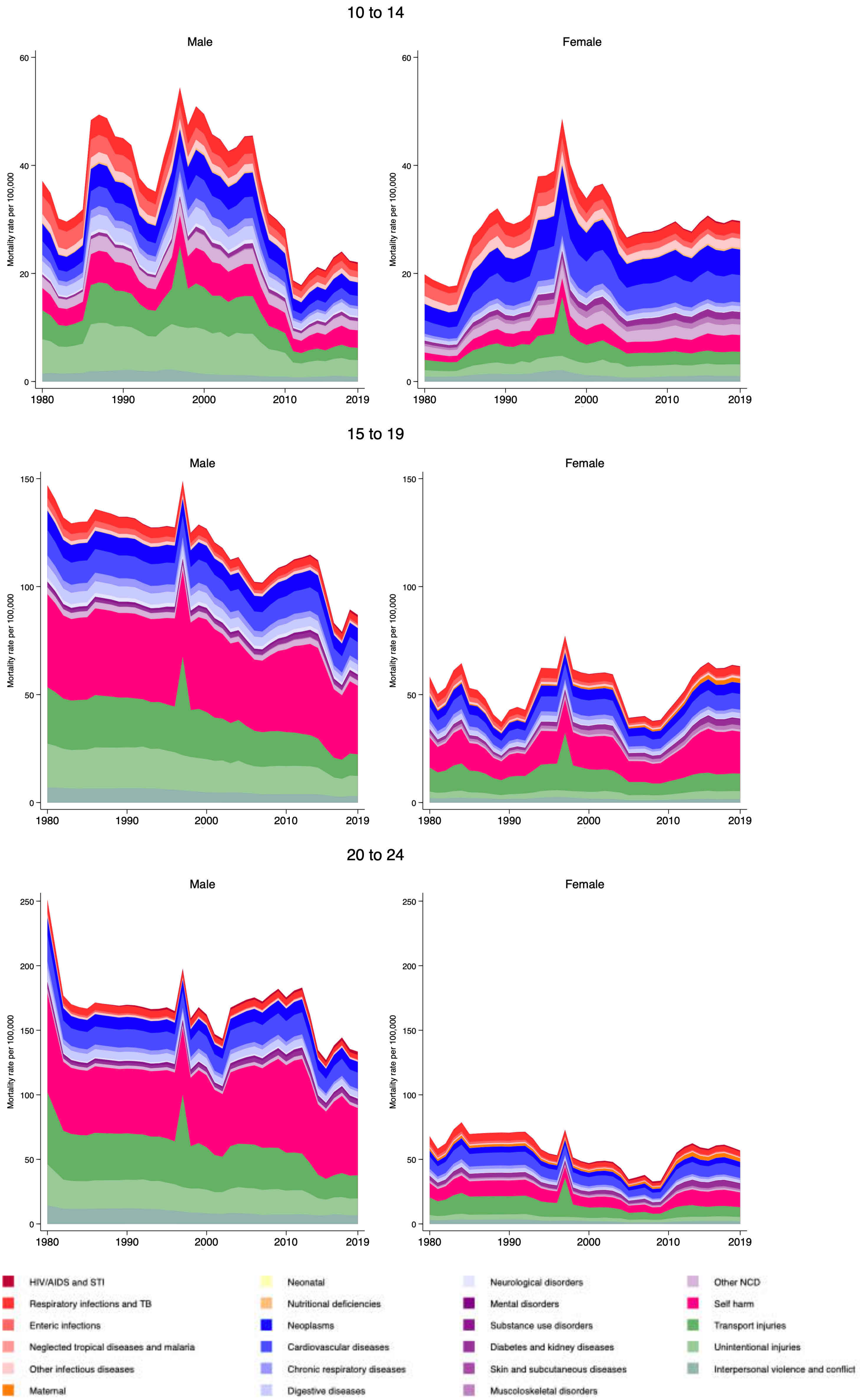

Figure S220d Number of deaths in 10-24 1980 – 2019 by cause: Guam

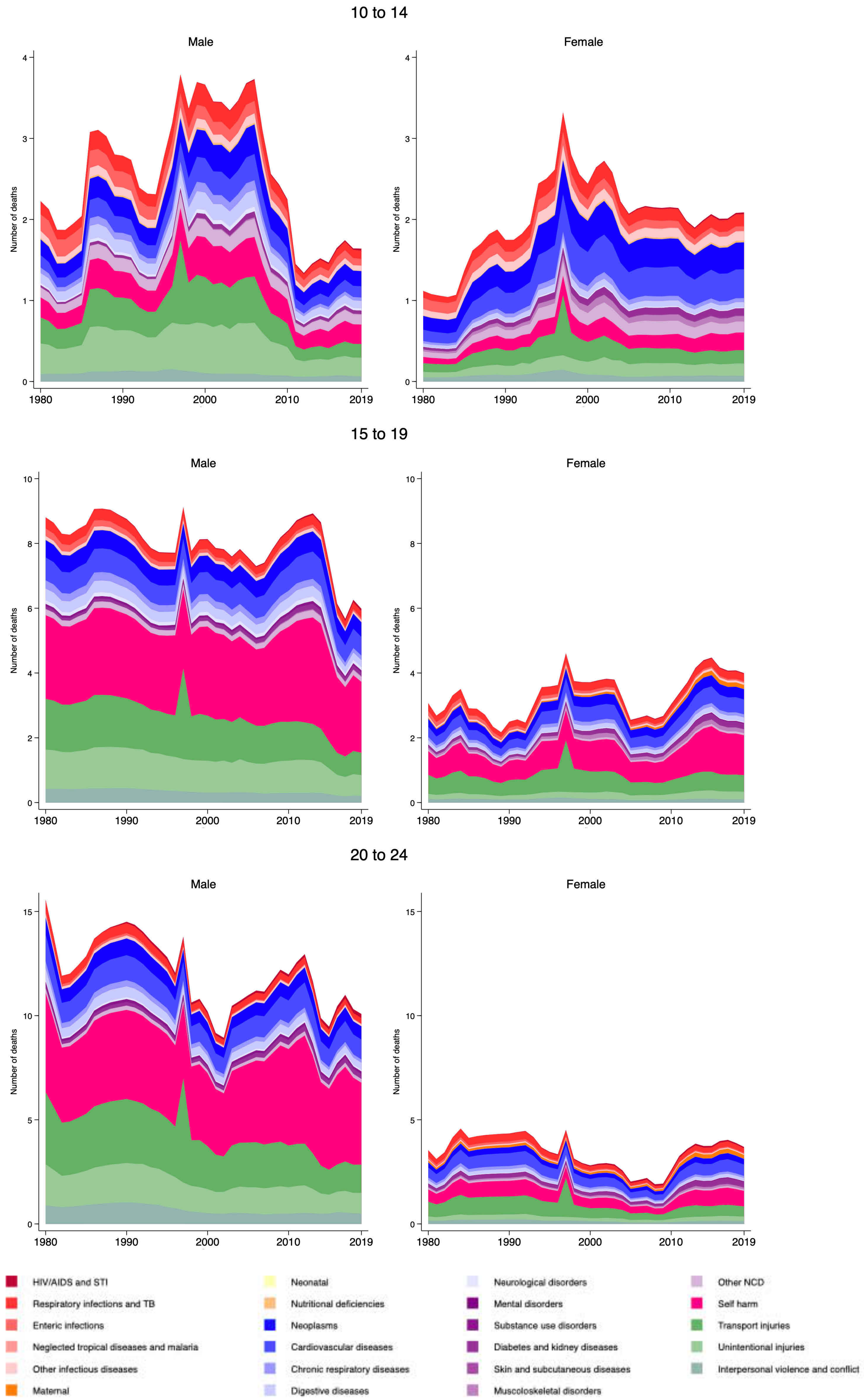

Figure S219: Monaco

Figure S221a All-cause mortality rate per 100,000 in 10-24 1950 – 2019: Monaco

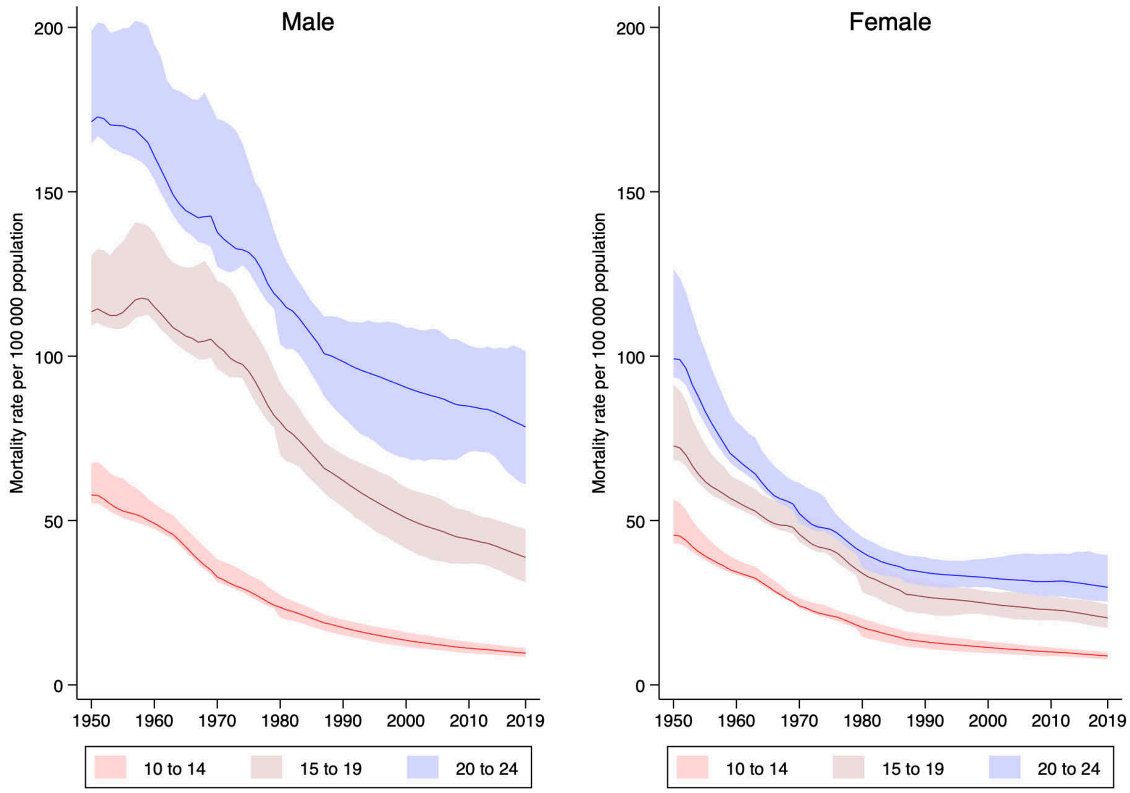

Figure S221b Number of deaths from all causes in 10-24 1950 – 2019: Monaco

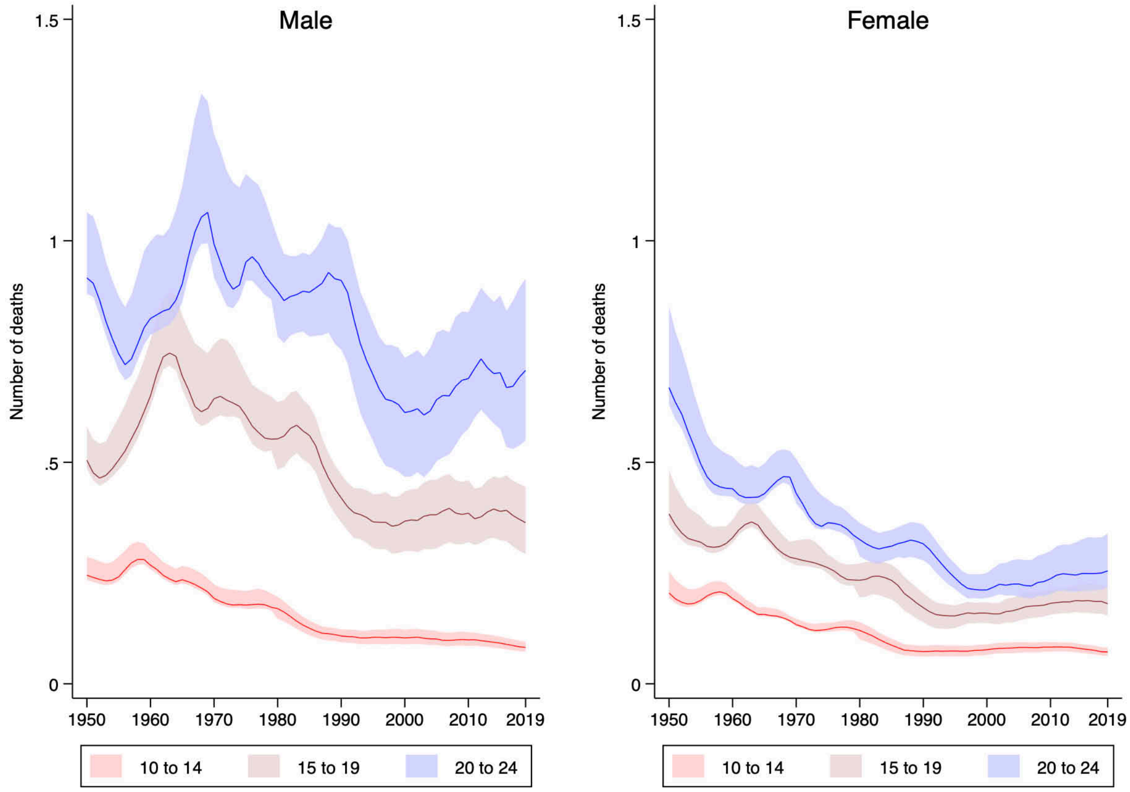

Figure S221c Mortality rate per 100,000 in 10-24 1980 – 2019 by cause: Monaco

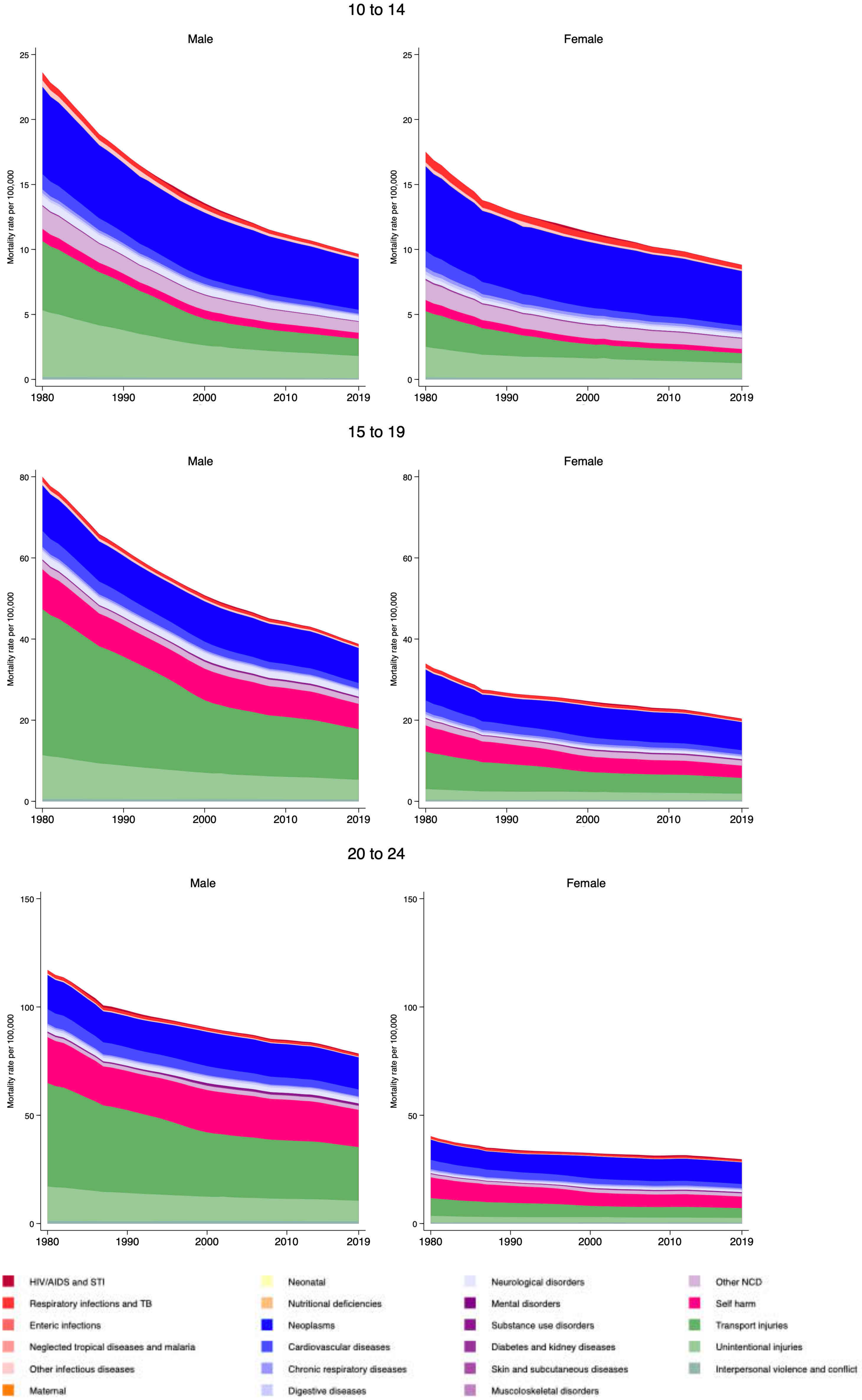

Figure S221d Number of deaths in 10-24 1980 – 2019 by cause: Monaco

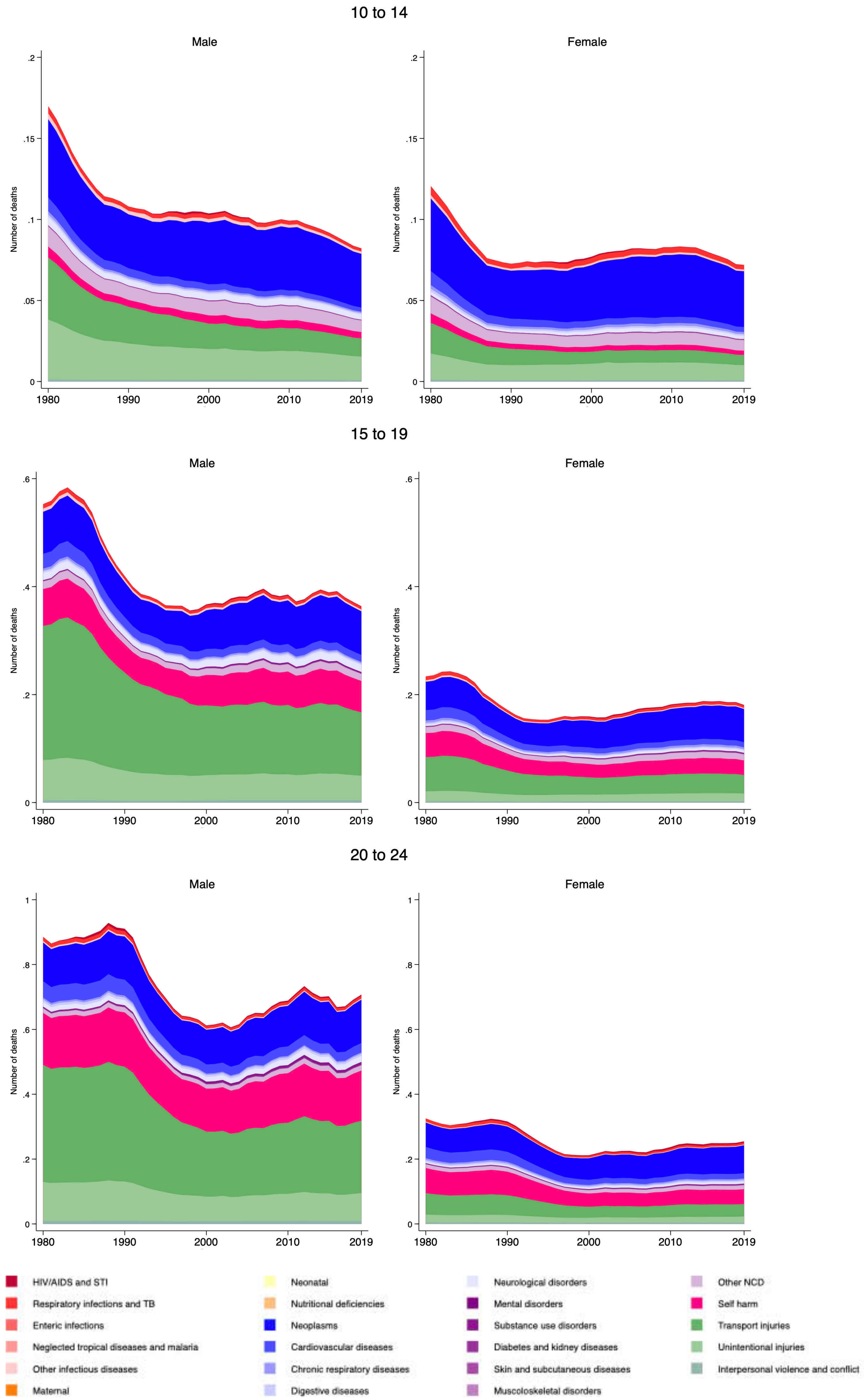

Figure S220: Nauru

Figure S222a All-cause mortality rate per 100,000 in 10-24 1950 – 2019: Nauru

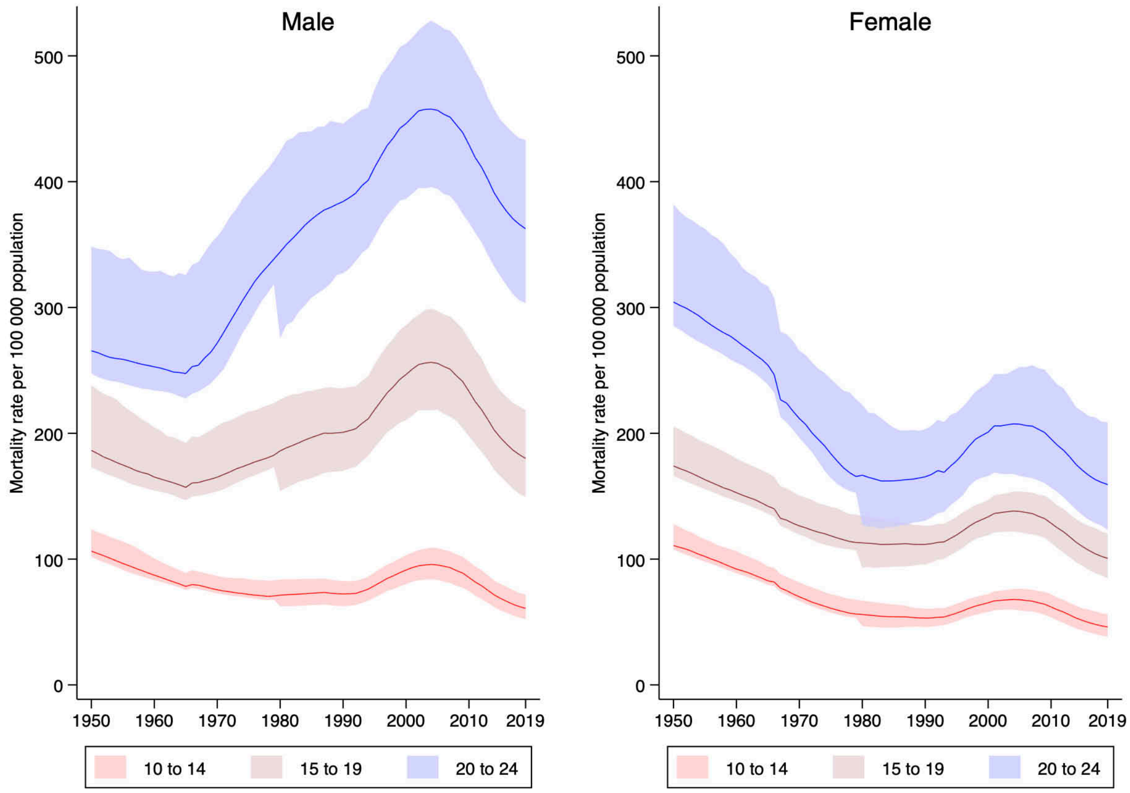

Figure S222b Number of deaths from all causes in 10-24 1950 – 2019: Nauru

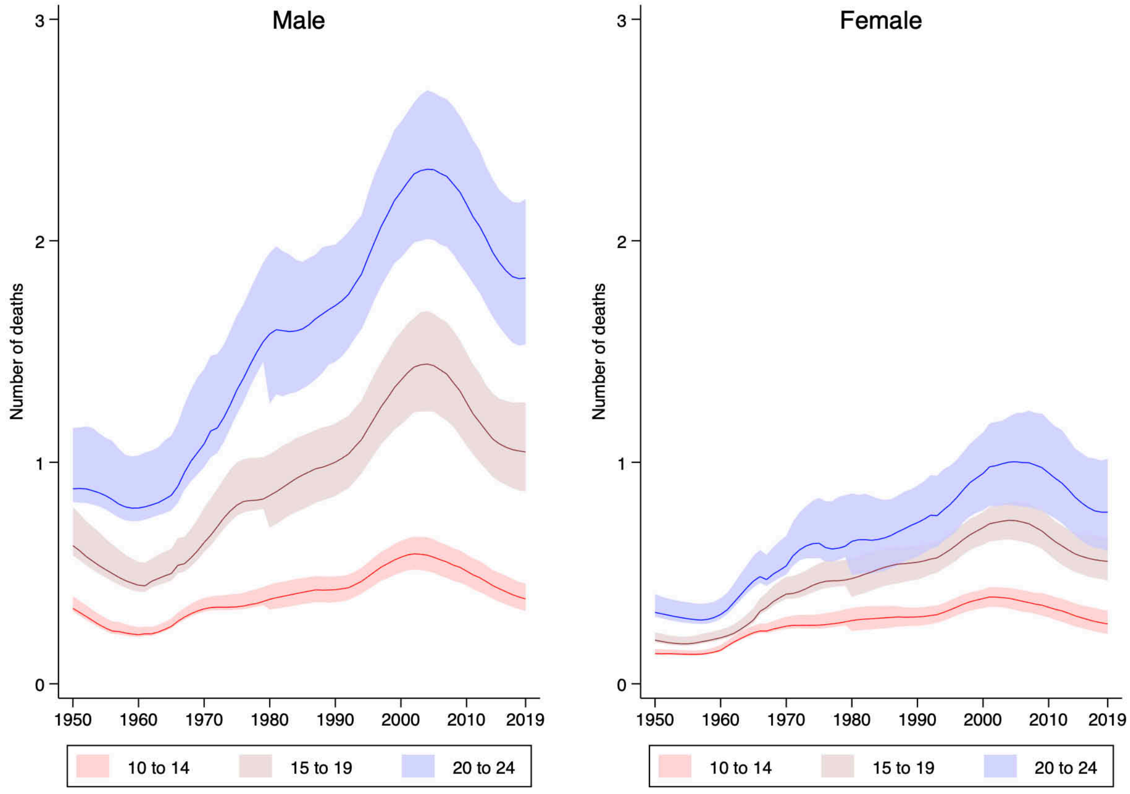

Figure S222c Mortality rate per 100,000 in 10-24 1980 – 2019 by cause: Nauru

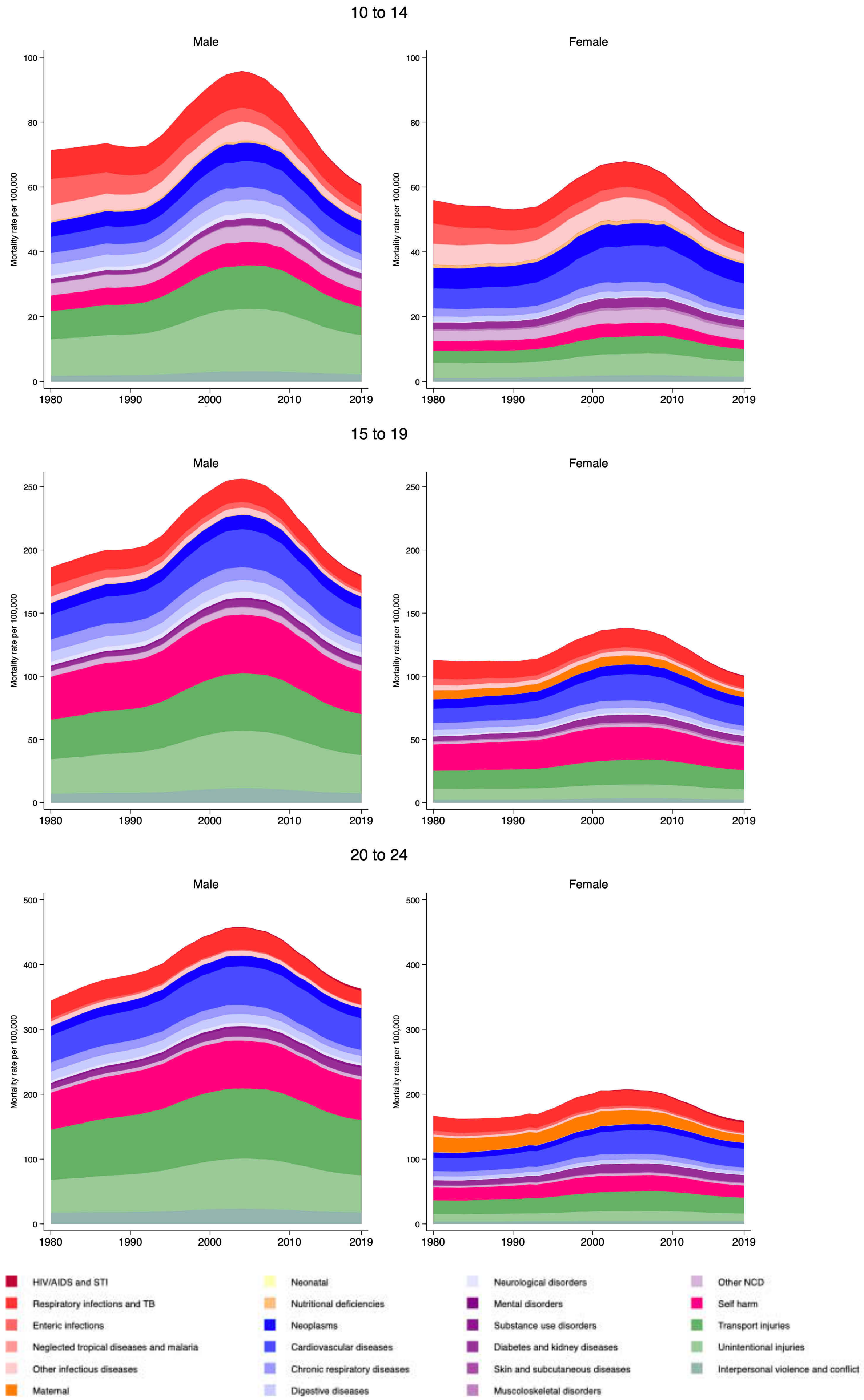

Figure S222d Number of deaths in 10-24 1980 – 2019 by cause: Nauru

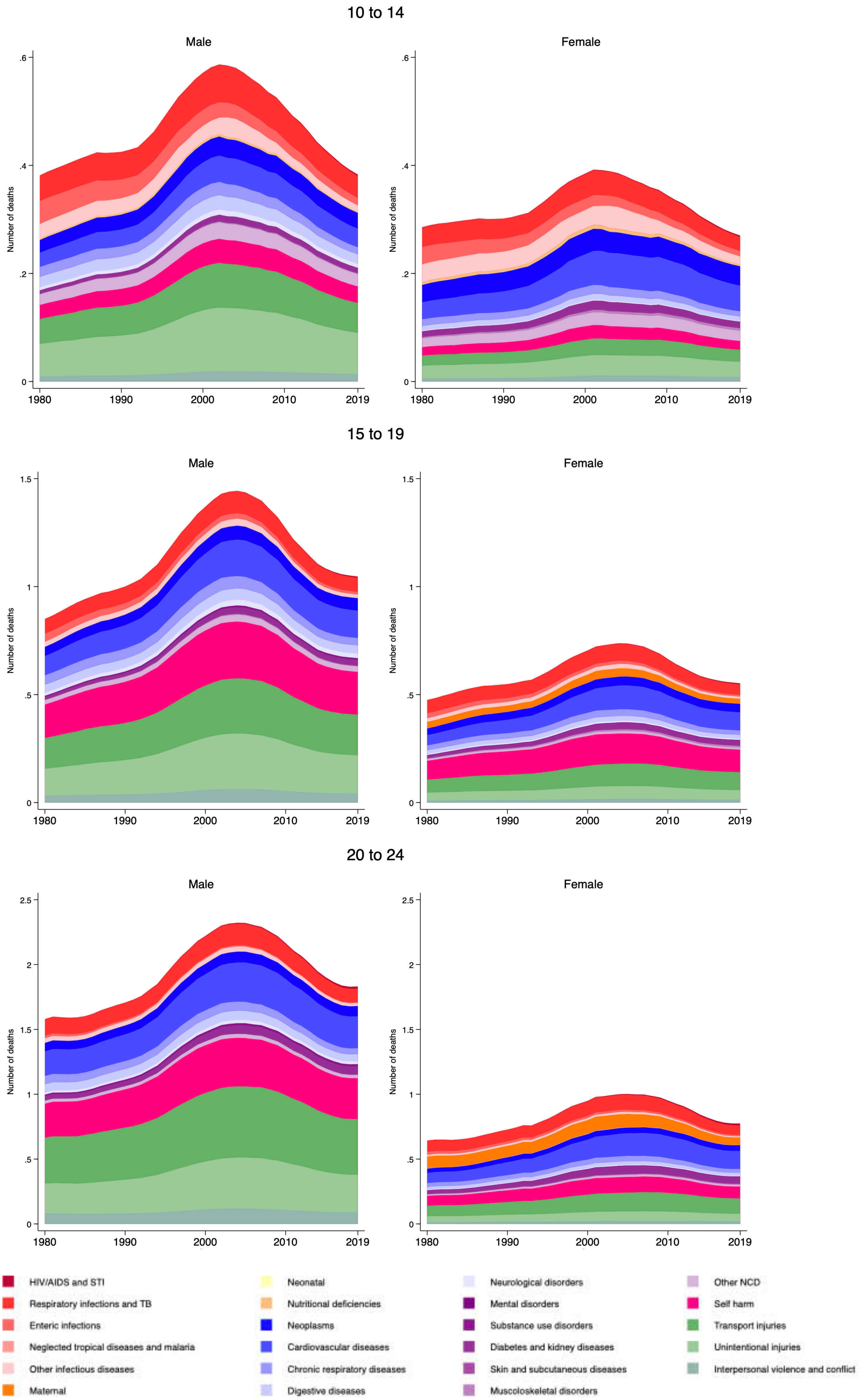

Figure S221: Niue

Figure S223a All-cause mortality rate per 100,000 in 10-24 1950 – 2019: Niue

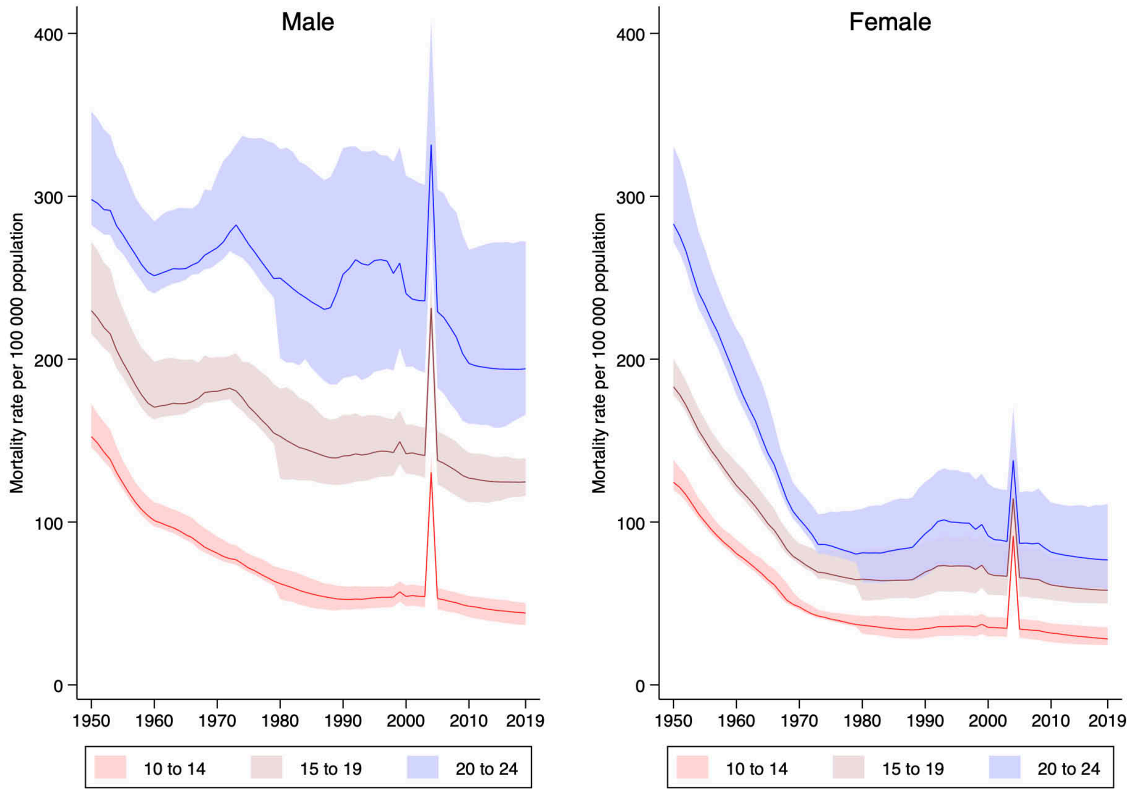

Figure S223b Number of deaths from all causes in 10-24 1950 – 2019: Niue

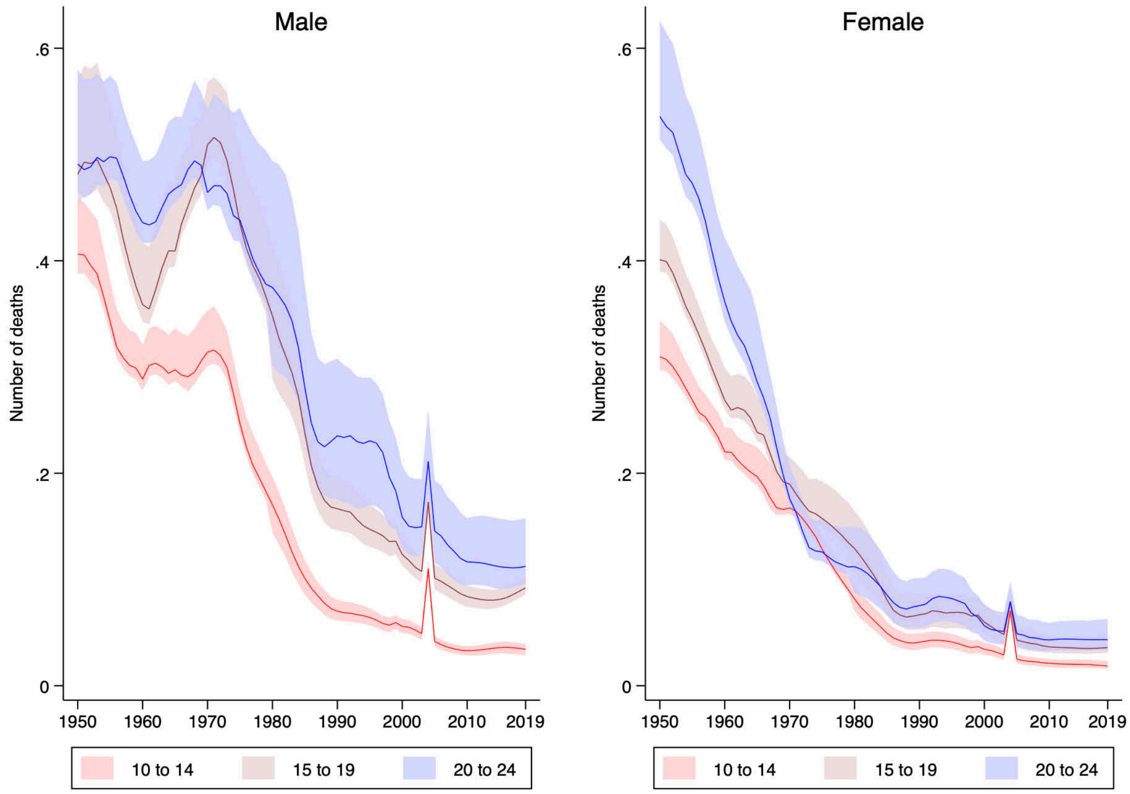

Figure S223c Mortality rate per 100,000 in 10-24 1980 – 2019 by cause: Niue

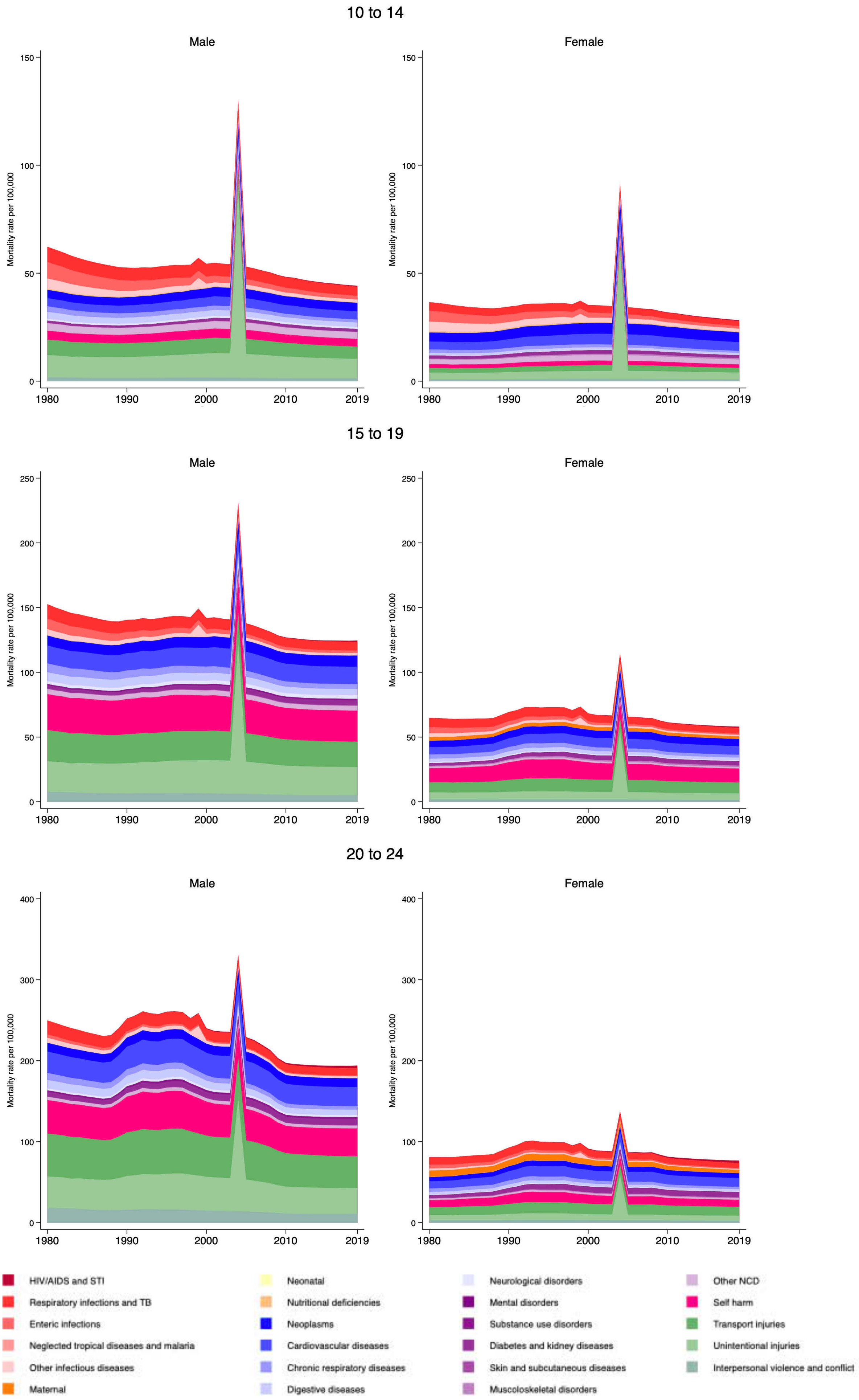

Figure S223d Number of deaths in 10-24 1980 – 2019 by cause: Niue

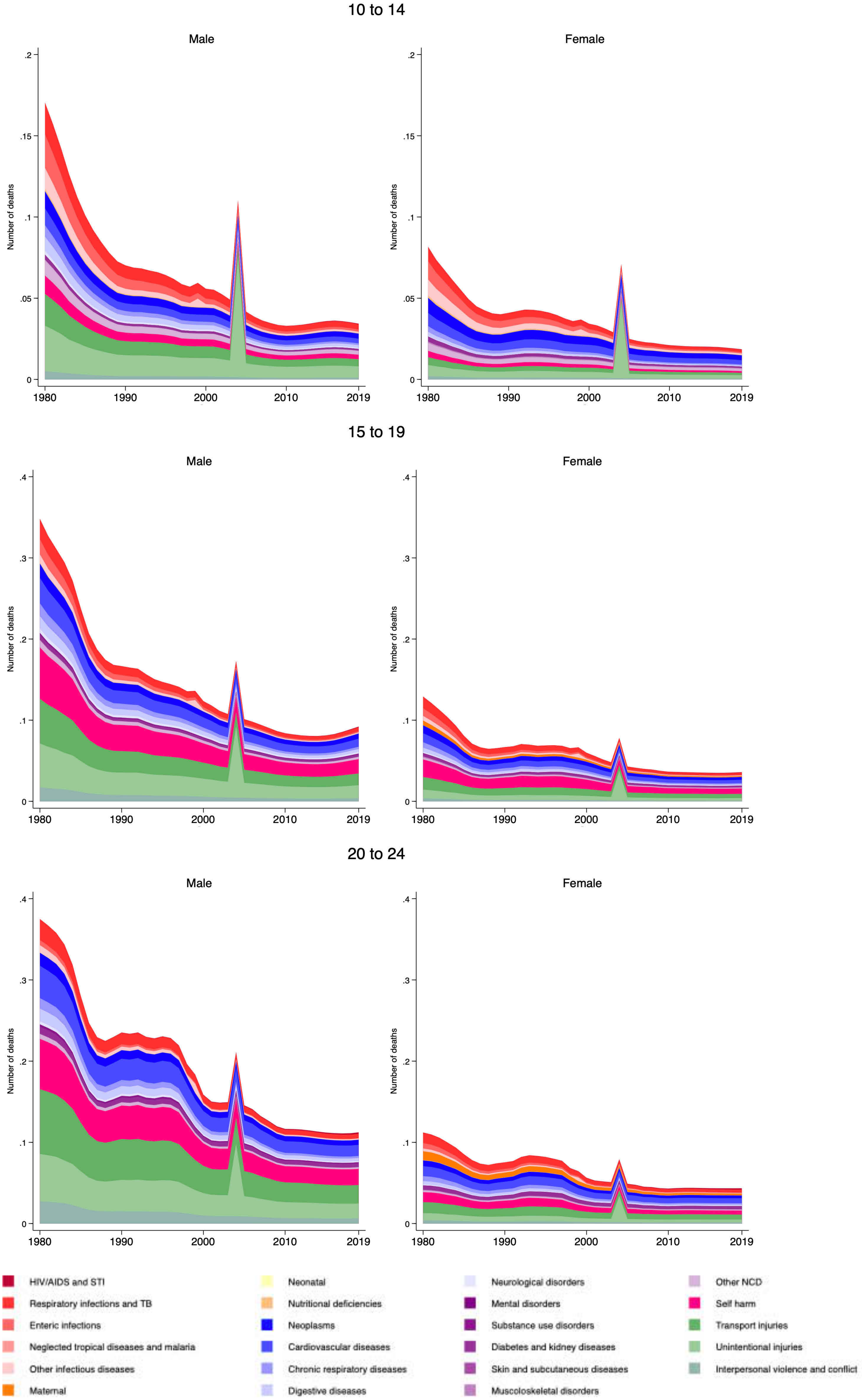

Figure S222: Northern Mariana Islands

Figure S224a All-cause mortality rate per 100,000 in 10-24 1950 – 2019: Northern Mariana Islands

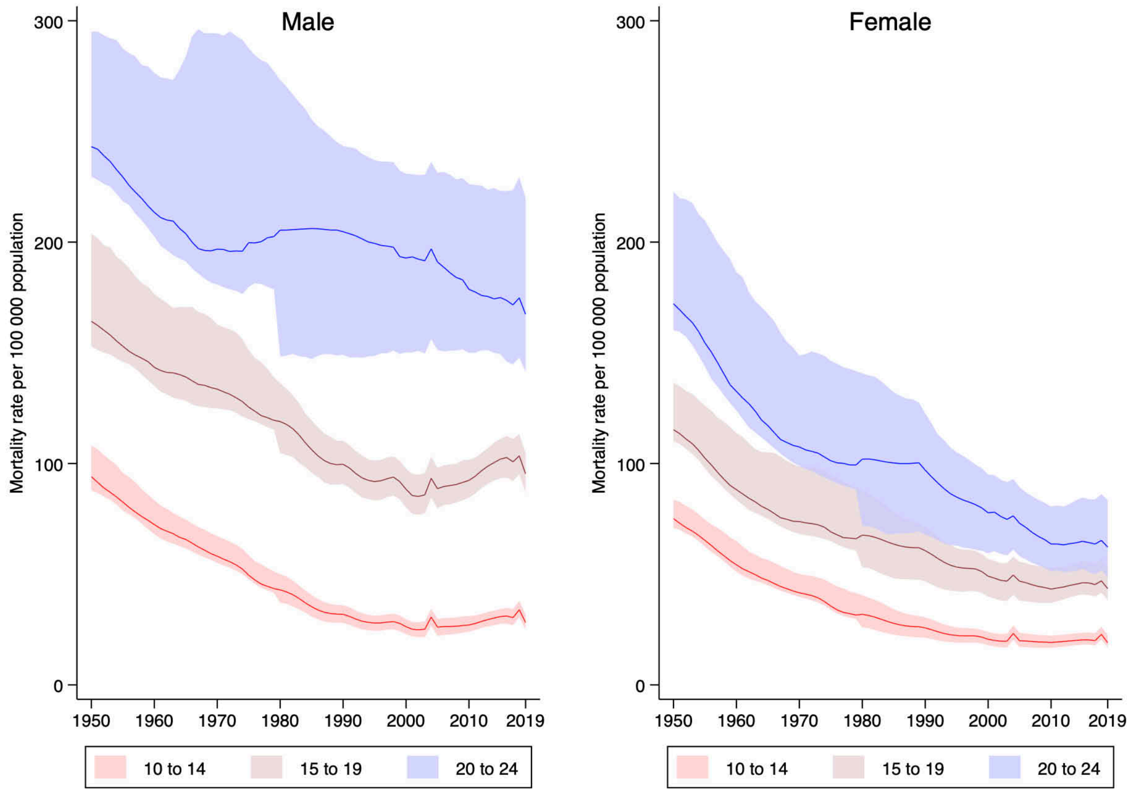

Figure S224b Number of deaths from all causes in 10-24 1950 – 2019: Northern Mariana Islands

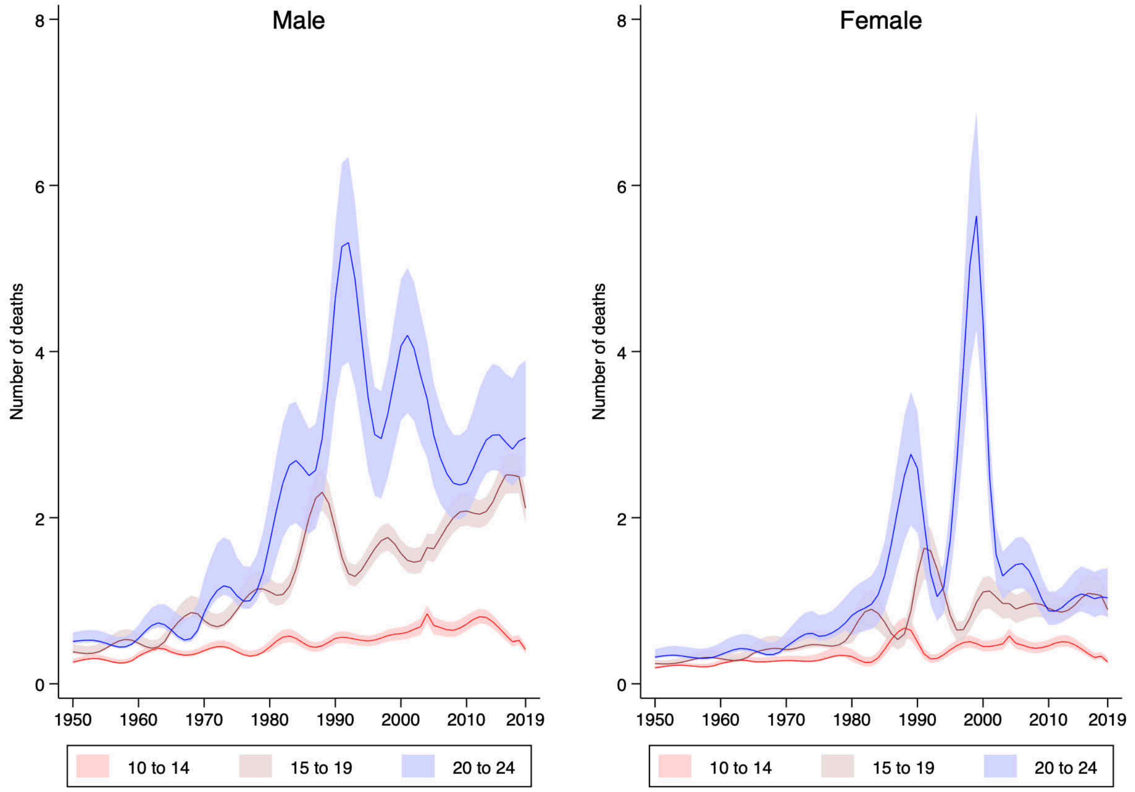

Figure S224c Mortality rate per 100,000 in 10-24 1980 – 2019 by cause: Northern Mariana Islands

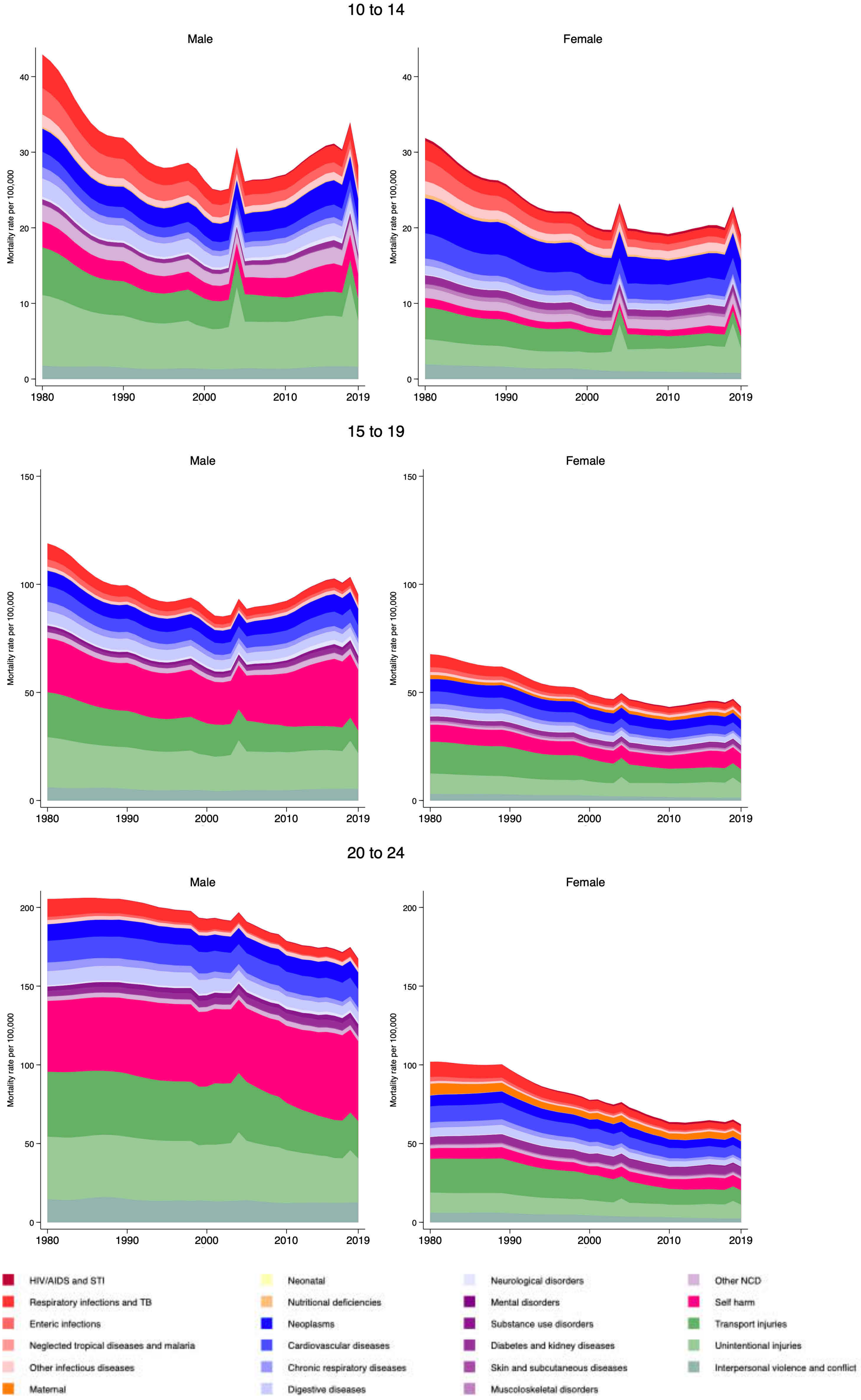

Figure S224d Number of deaths in 10-24 1980 – 2019 by cause: Northern Mariana Islands

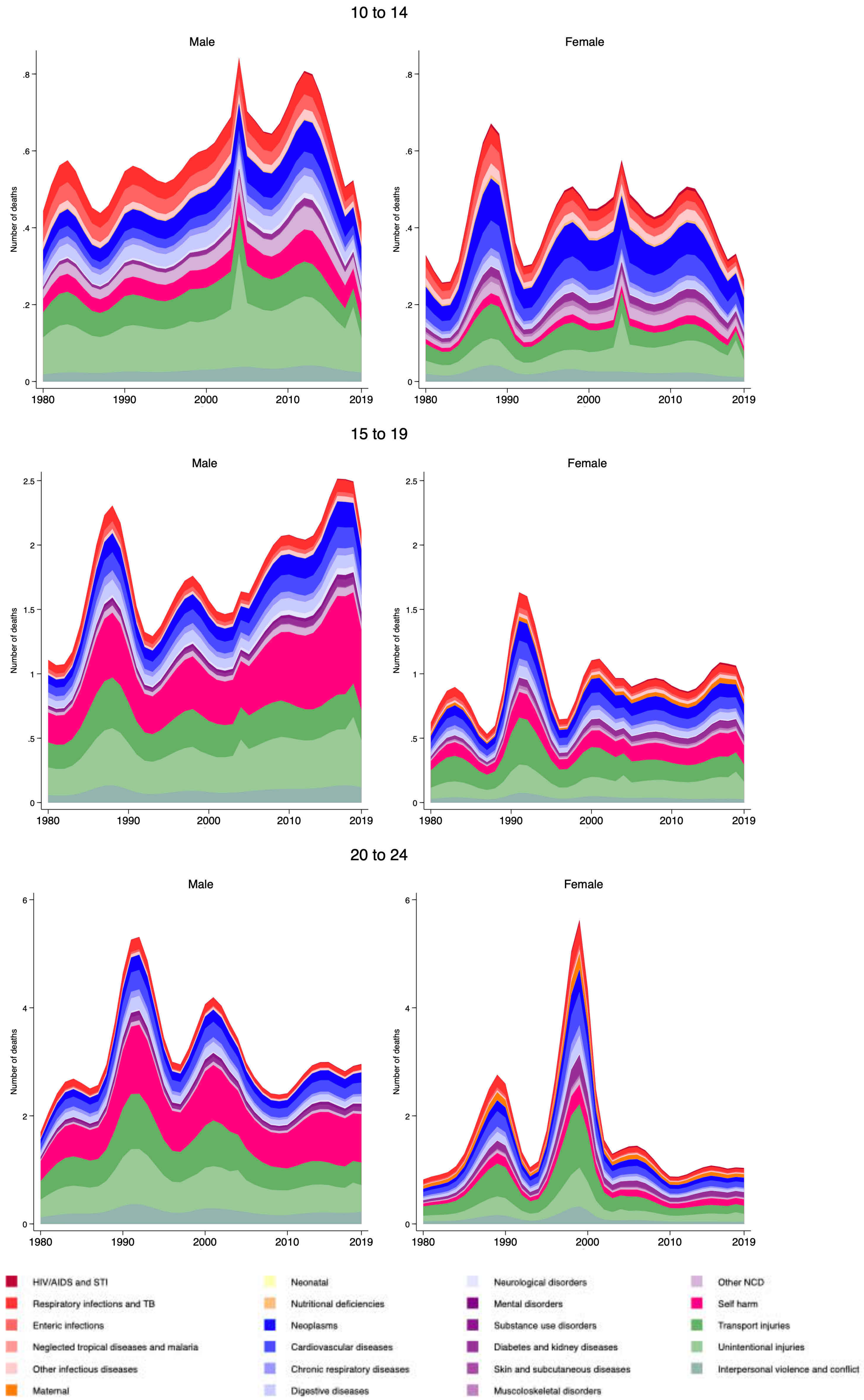

Figure S223: Palau

Figure S225a All-cause mortality rate per 100,000 in 10-24 1950 – 2019: Palau

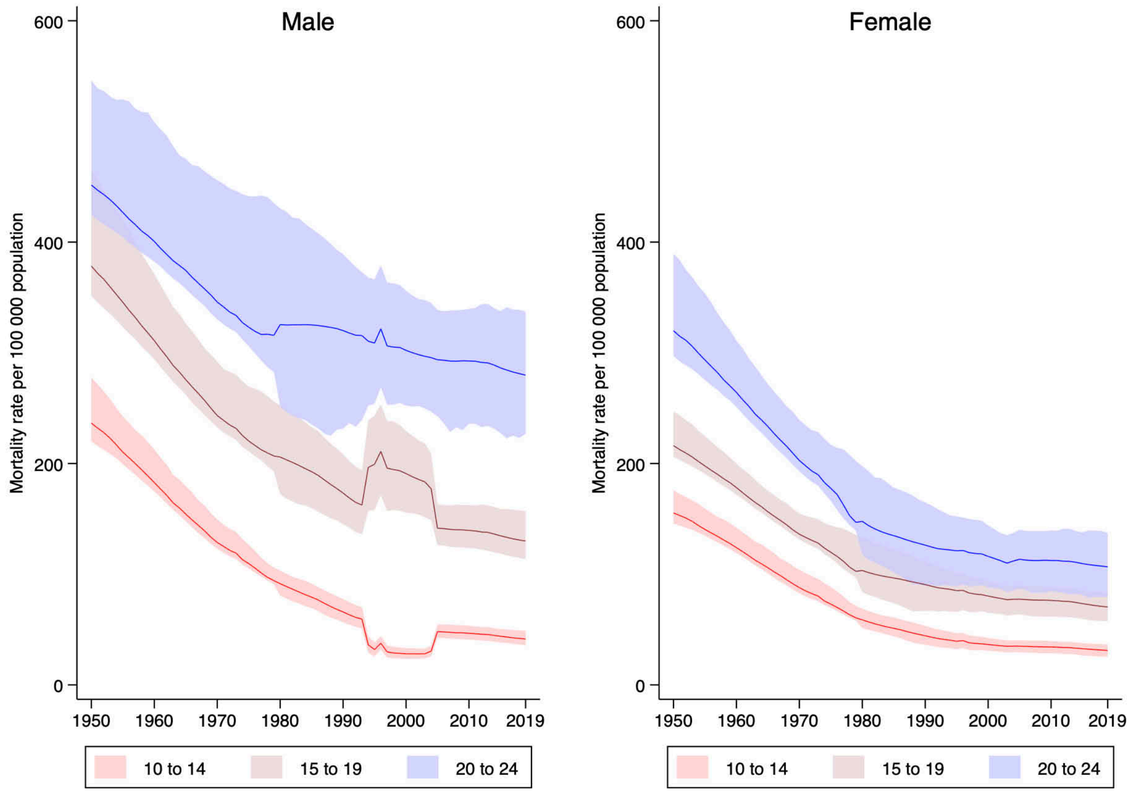

Figure S225b Number of deaths from all causes in 10-24 1950 – 2019: Palau

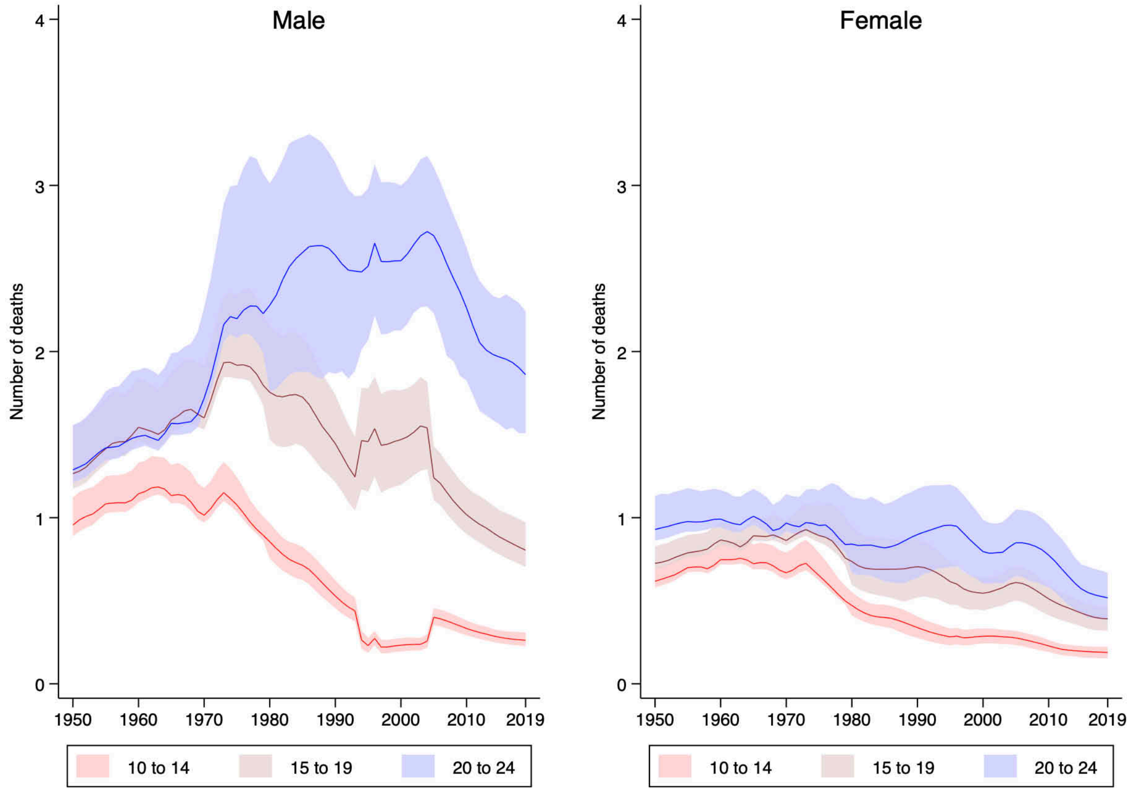

Figure S225c Mortality rate per 100,000 in 10-24 1980 – 2019 by cause: Palau

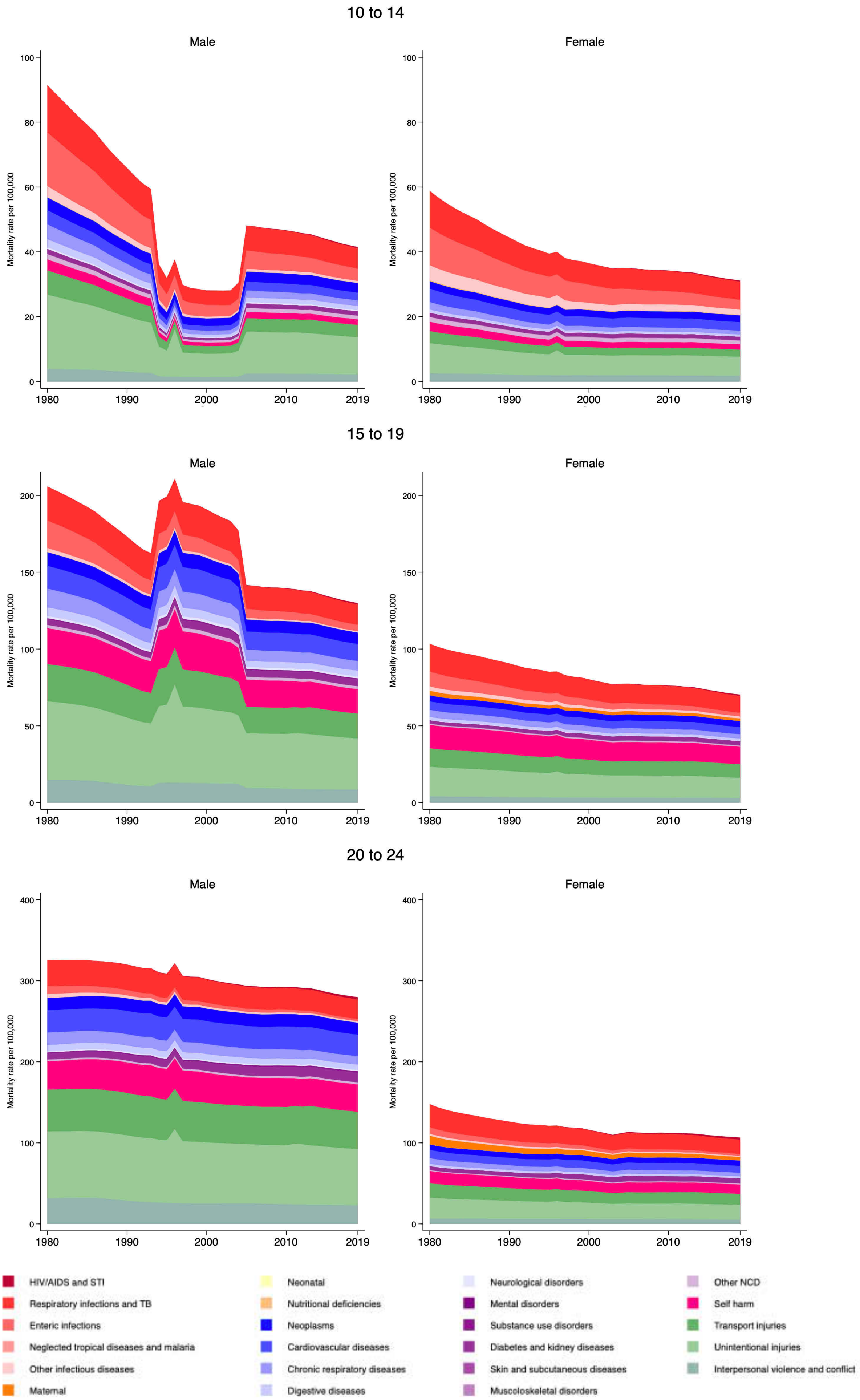

Figure S225d Number of deaths in 10-24 1980 – 2019 by cause: Palau

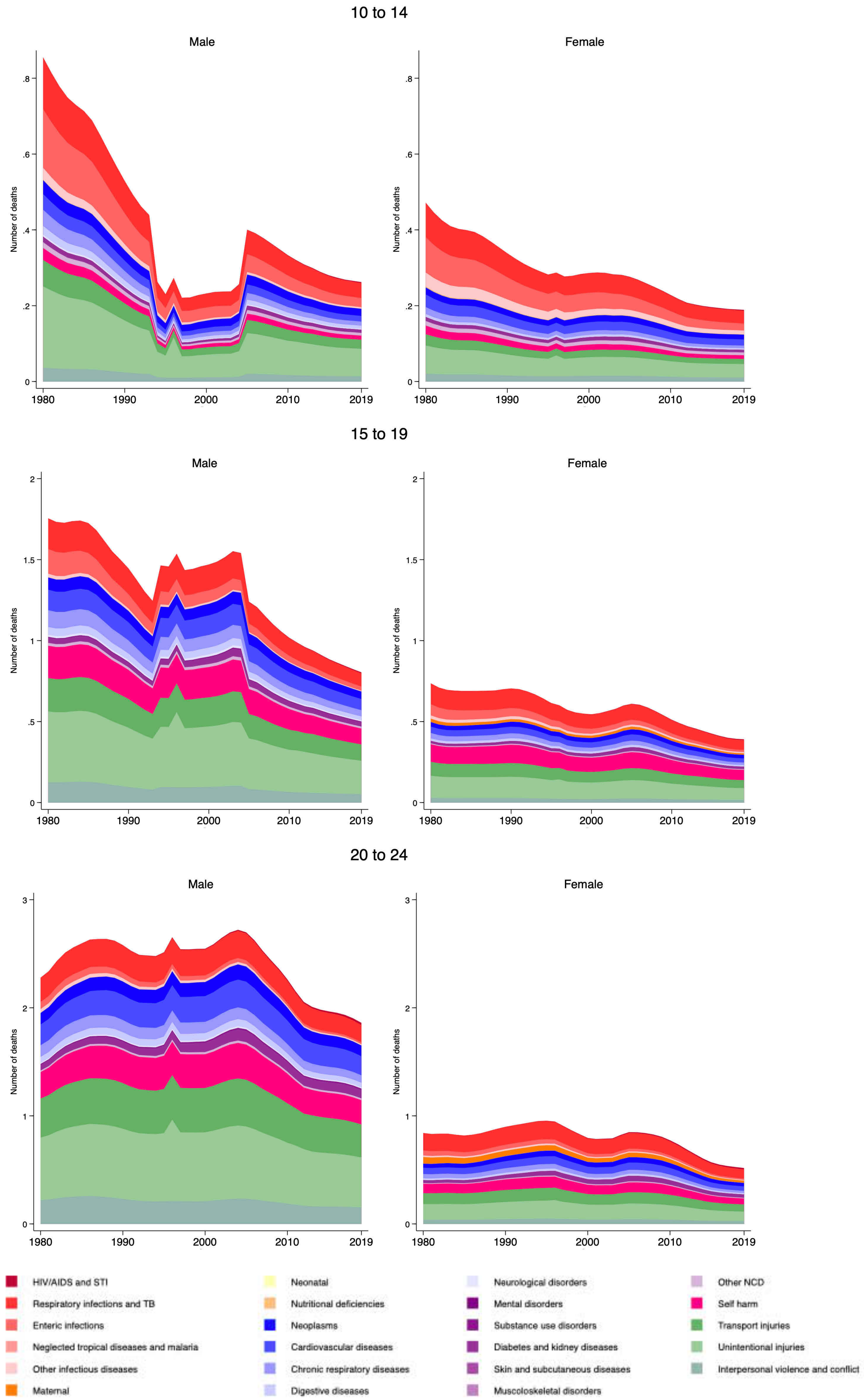

Figure S224: Puerto Rico

Figure S226a All-cause mortality rate per 100,000 in 10-24 1950 – 2019: Puerto Rico

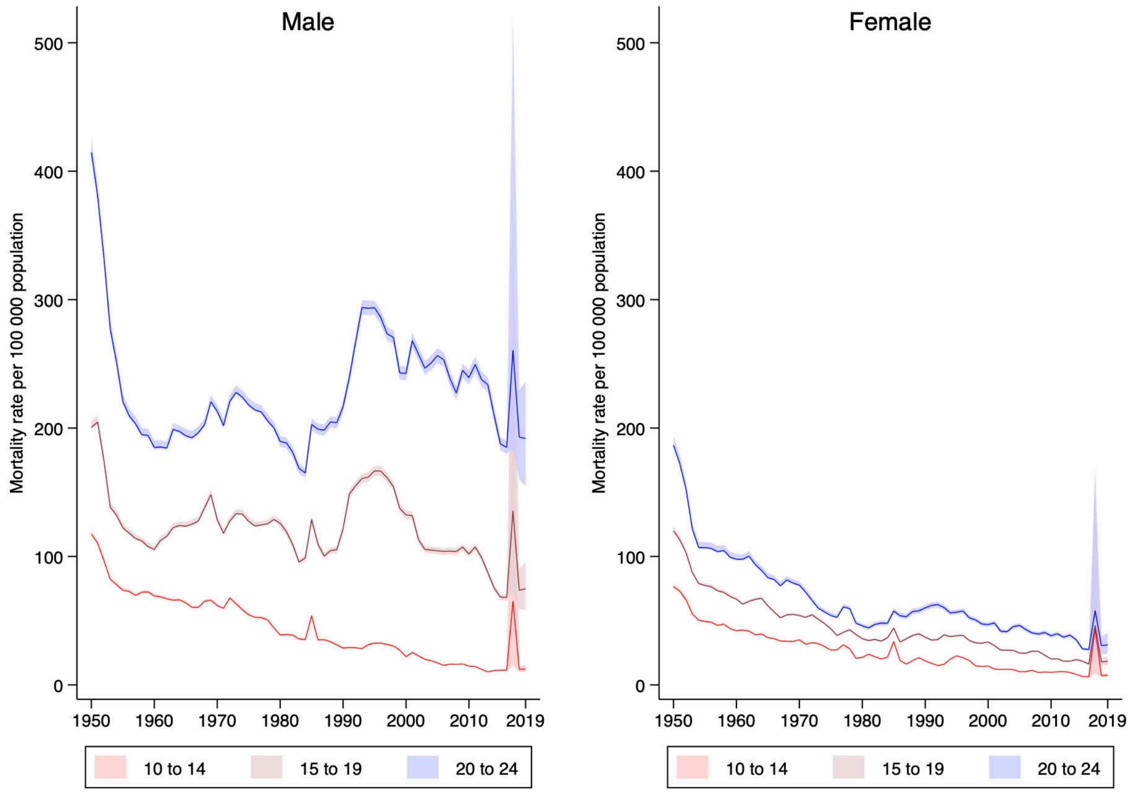

Figure S226b Number of deaths from all causes in 10-24 1950 – 2019: Puerto Rico

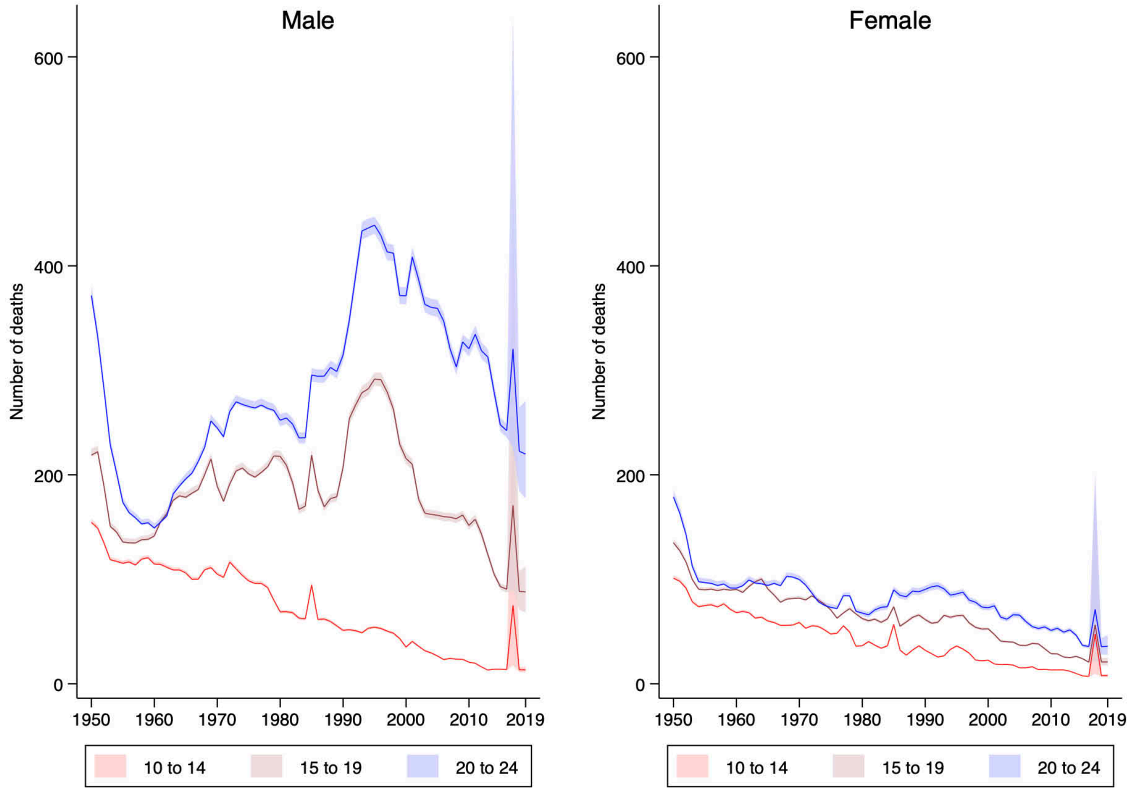

Figure S226c Mortality rate per 100,000 in 10-24 1980 – 2019 by cause: Puerto Rico

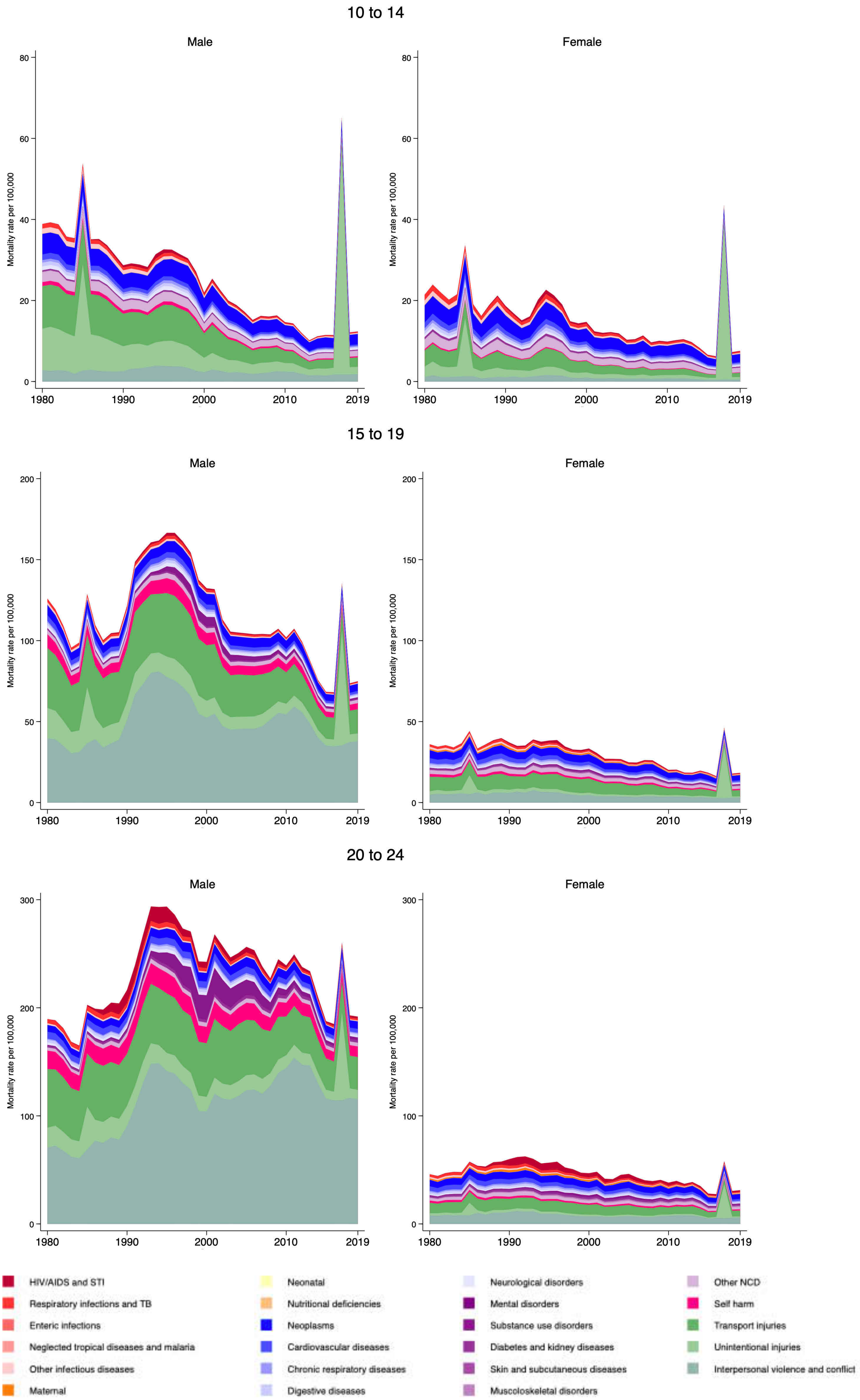

Figure S226d Number of deaths in 10-24 1980 – 2019 by cause: Puerto Rico

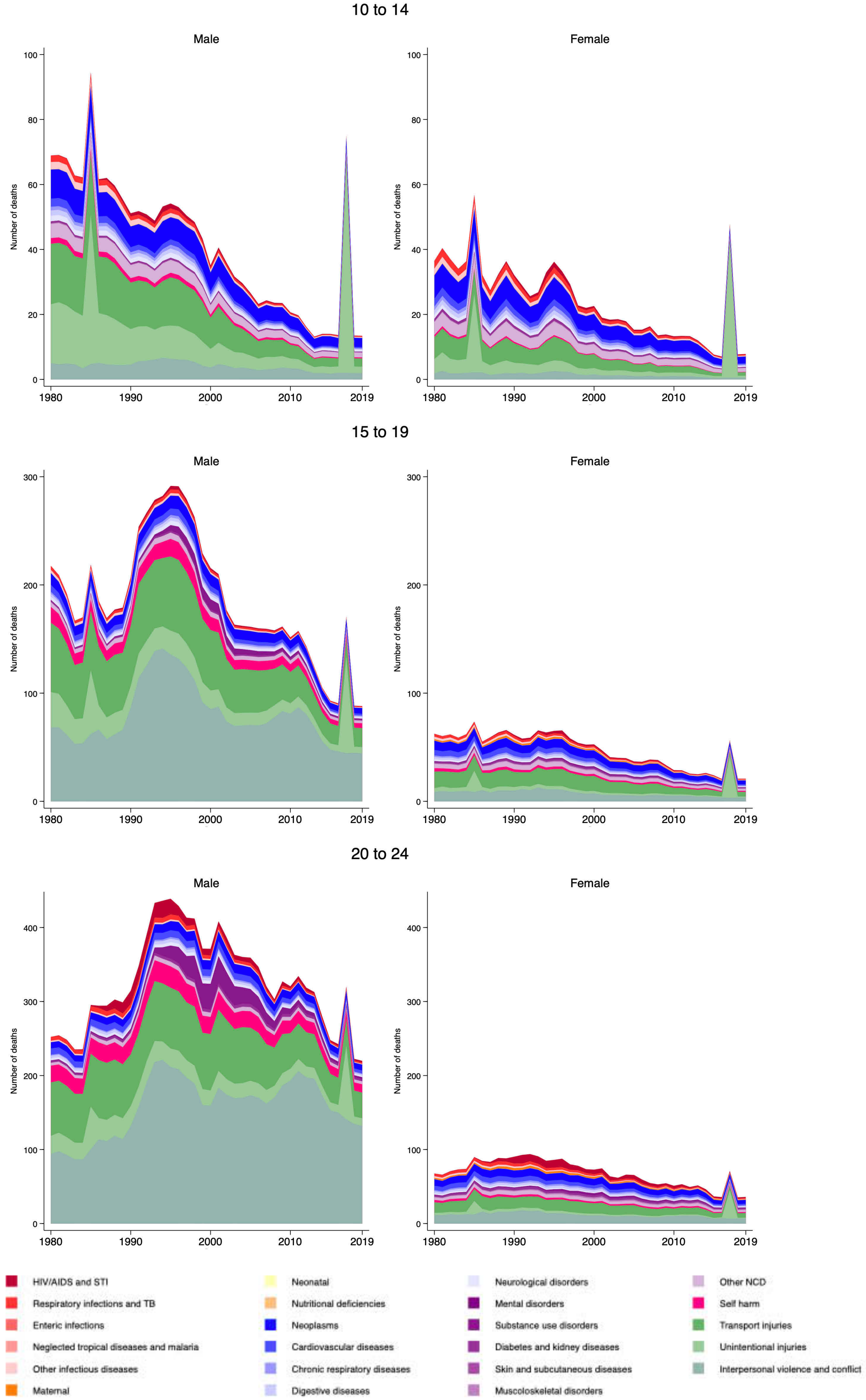

Figure S225: Saint Kitts and Nevis

Figure S227a All-cause mortality rate per 100,000 in 10-24 1950 – 2019: Saint Kitts and Nevis

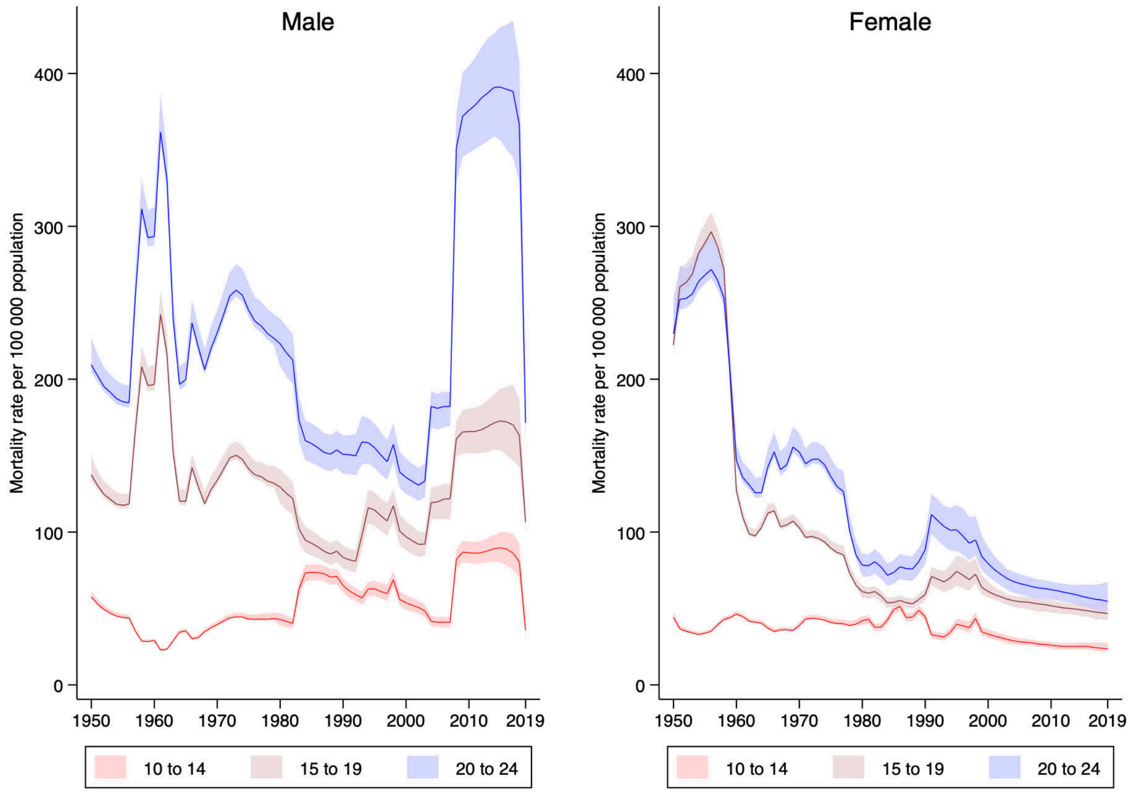

Figure S227b Number of deaths from all causes in 10-24 1950 – 2019: Saint Kitts and Nevis

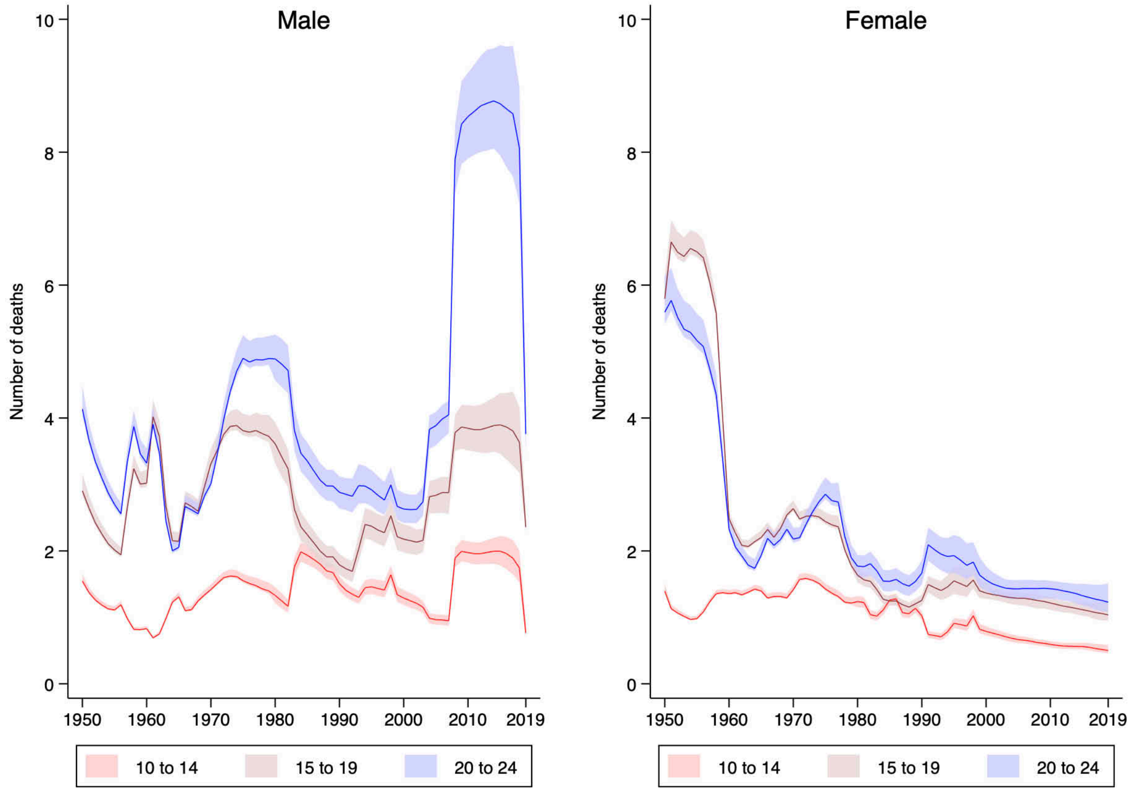

Figure S227c Mortality rate per 100,000 in 10-24 1980 – 2019 by cause: Saint Kitts and Nevis

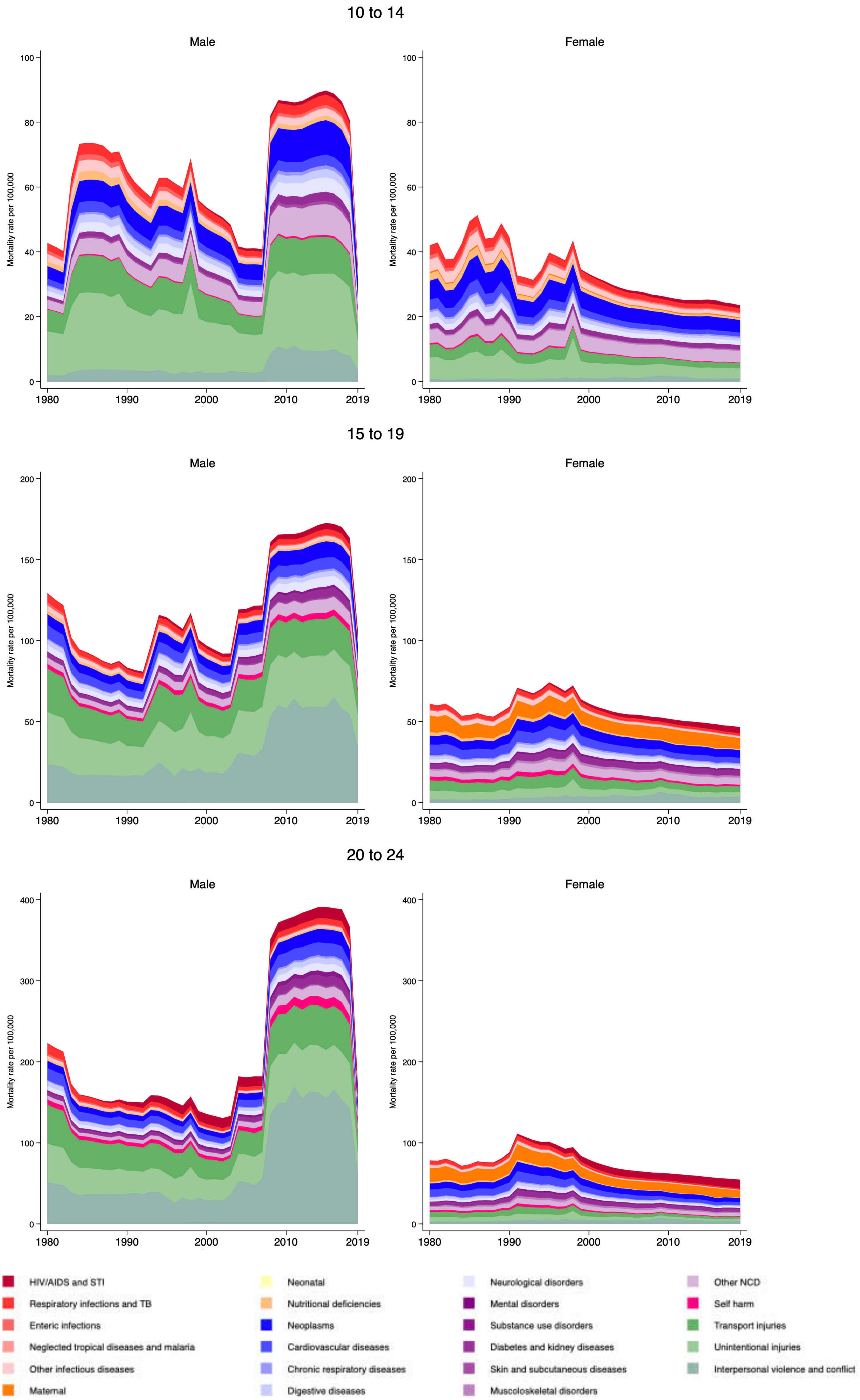

Figure S227d Number of deaths in 10-24 1980 – 2019 by cause: Saint Kitts and Nevis

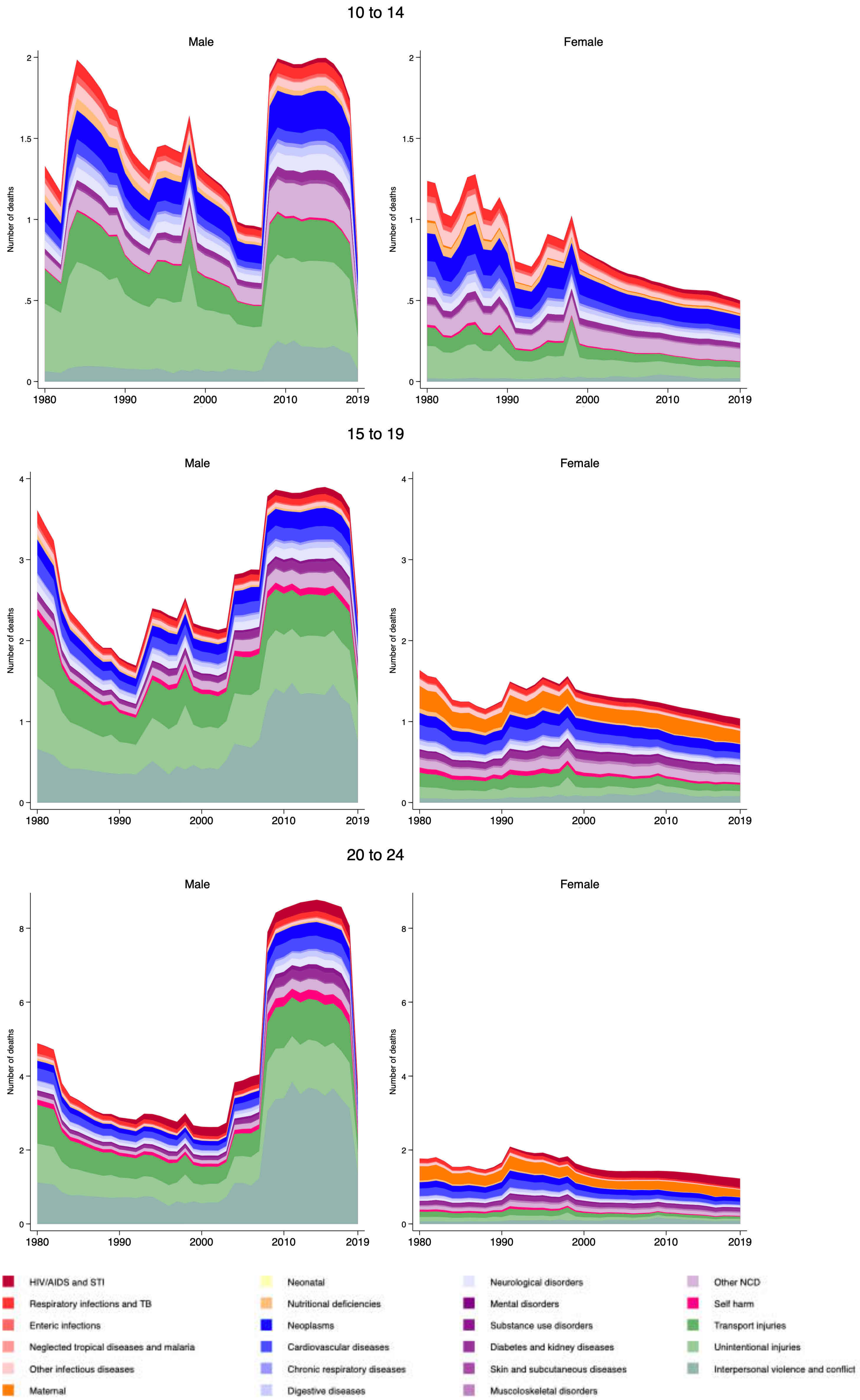

Figure S226: San Marino

Figure S228a All-cause mortality rate per 100,000 in 10-24 1950 – 2019: San Marino

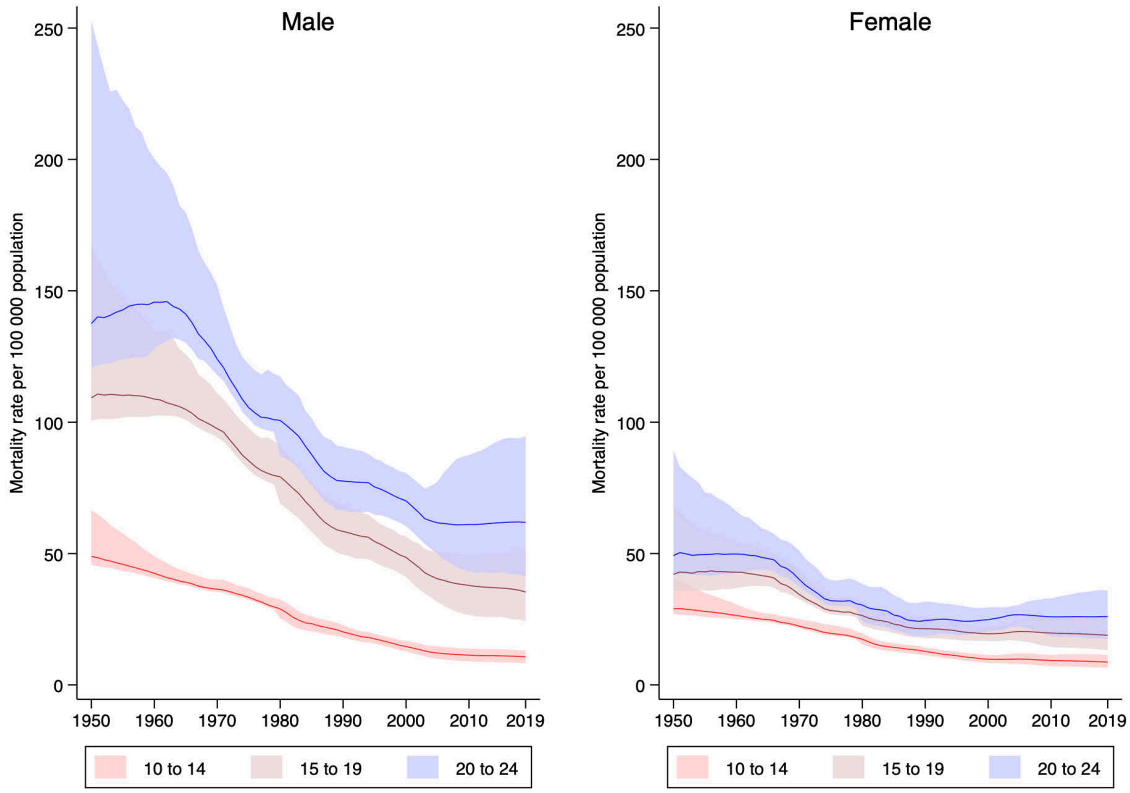

Figure S228b Number of deaths from all causes in 10-24 1950 – 2019: San Marino

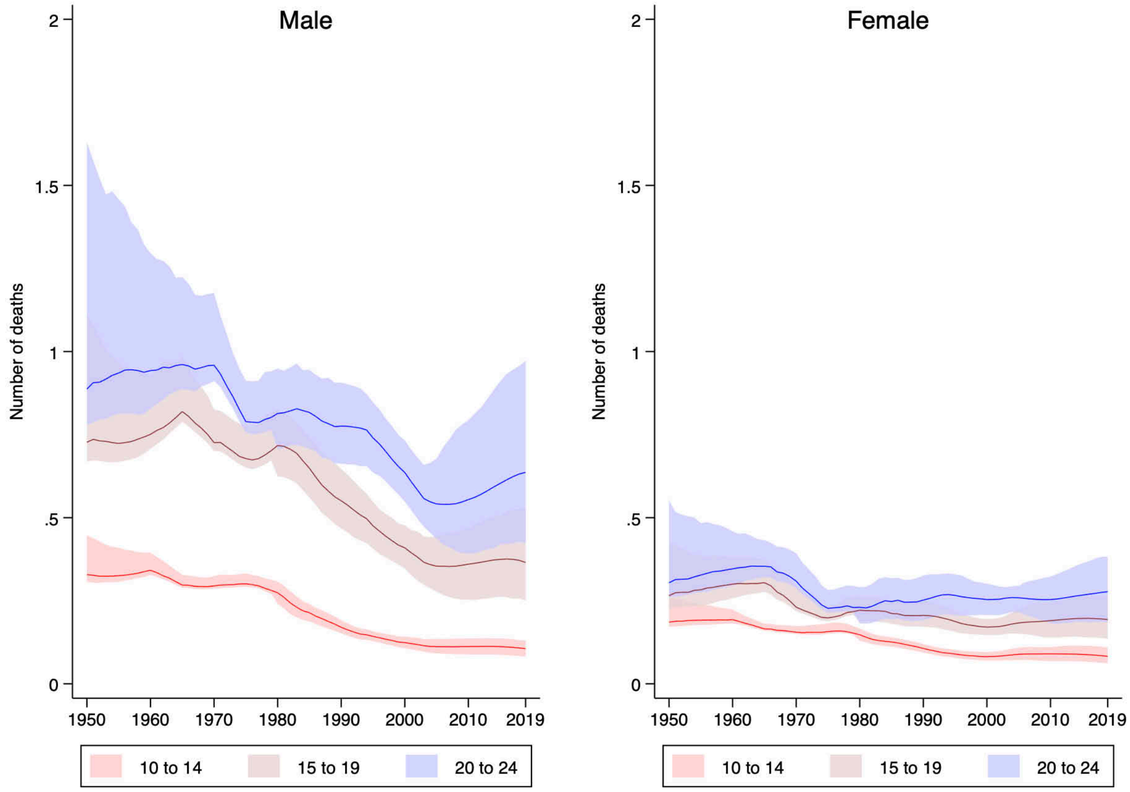

Figure S228c Mortality rate per 100,000 in 10-24 1980 – 2019 by cause: San Marino

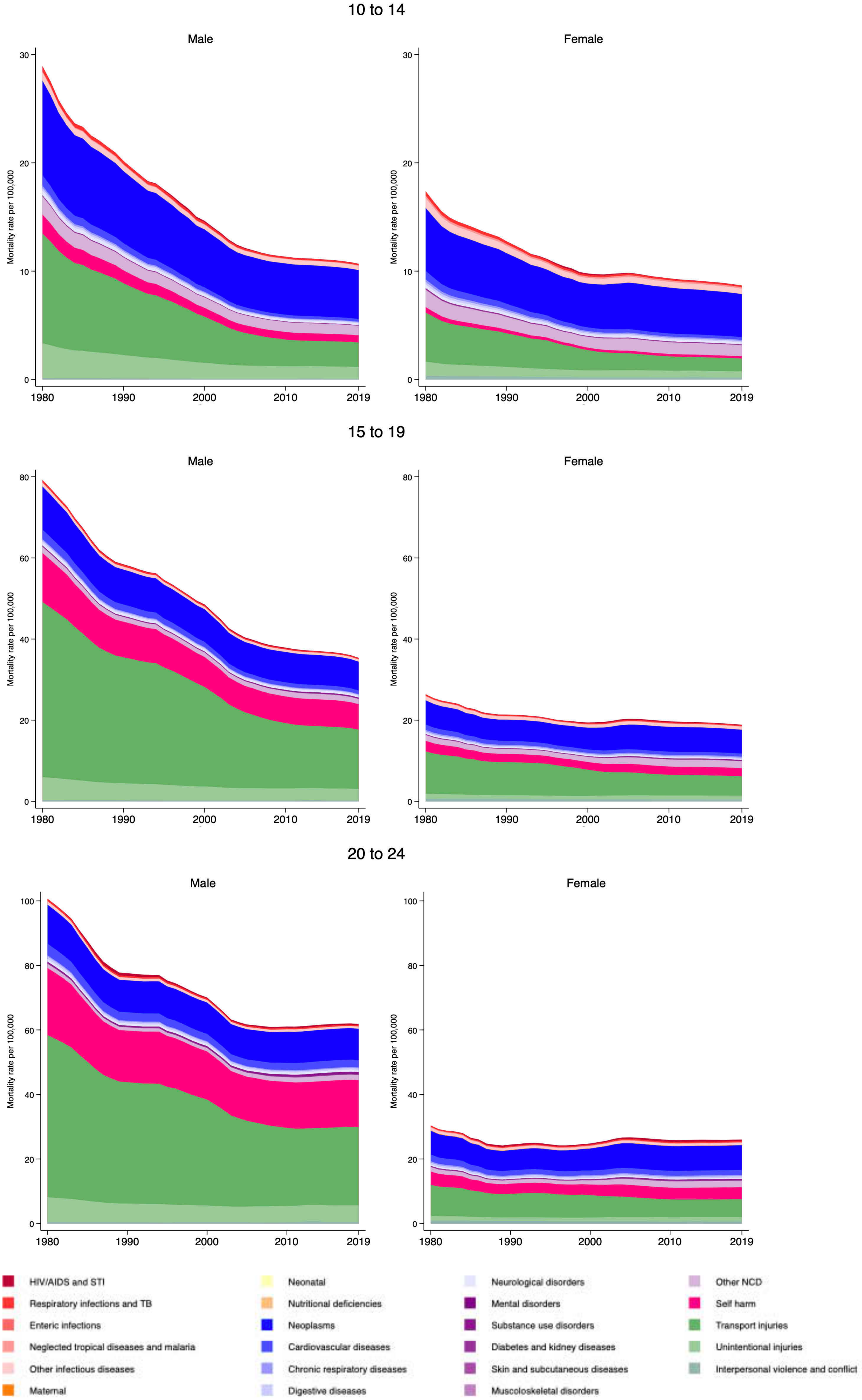

Figure S228d Number of deaths in 10-24 1980 – 2019 by cause: San Marino

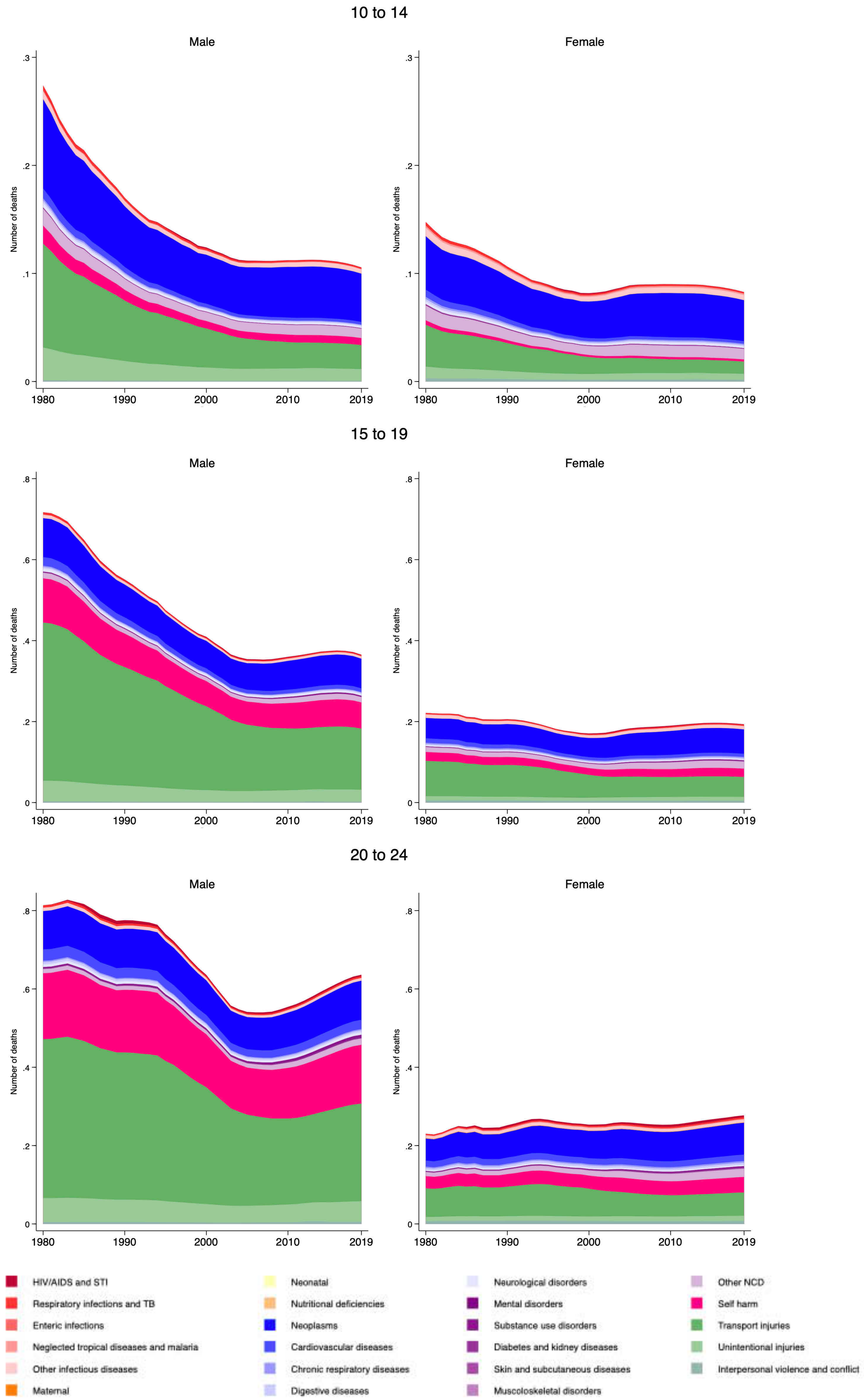

Figure S227: Tokelau

Figure S229a All-cause mortality rate per 100,000 in 10-24 1950 – 2019: Tokelau

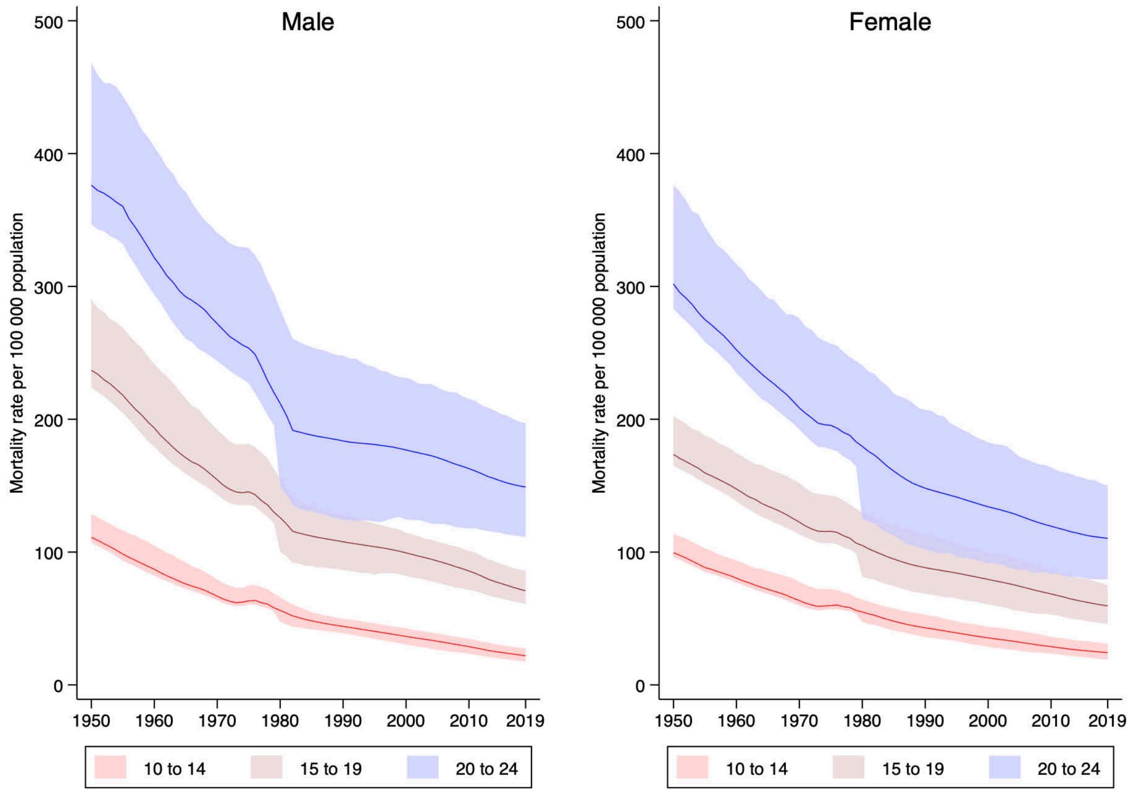

Figure S229b Number of deaths from all causes in 10-24 1950 – 2019: Tokelau

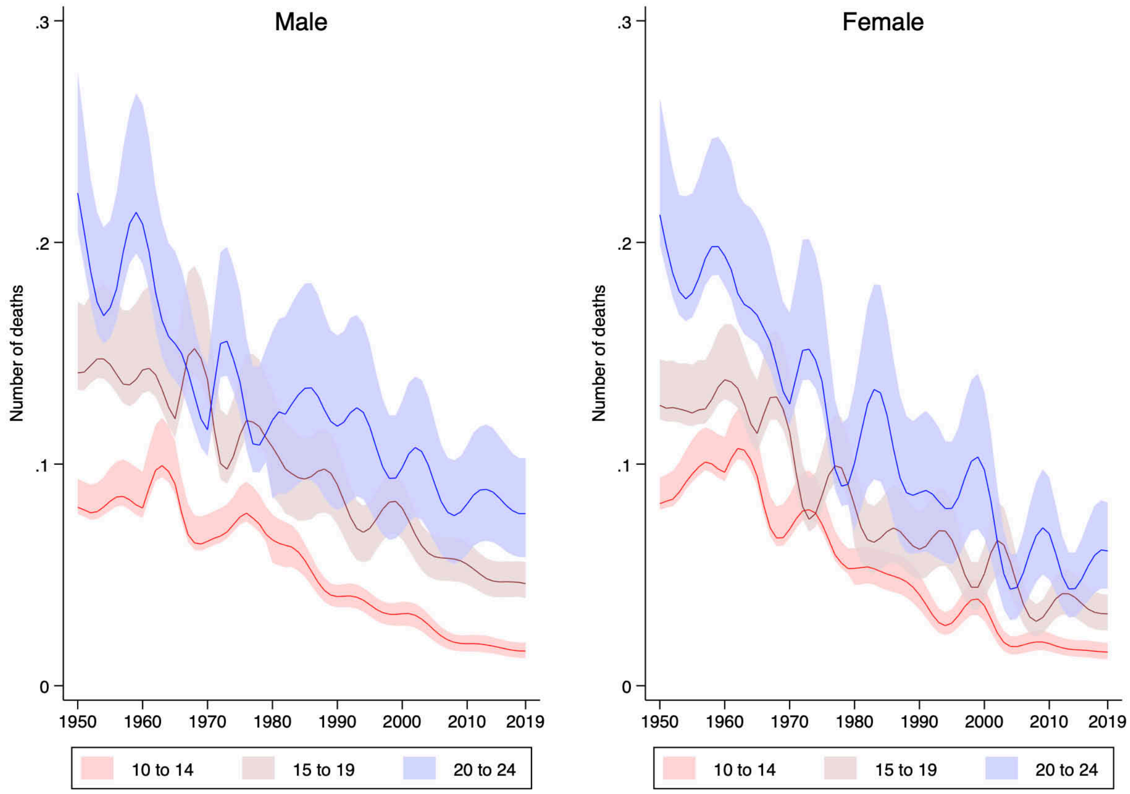

Figure S229c Mortality rate per 100,000 in 10-24 1980 – 2019 by cause: Tokelau

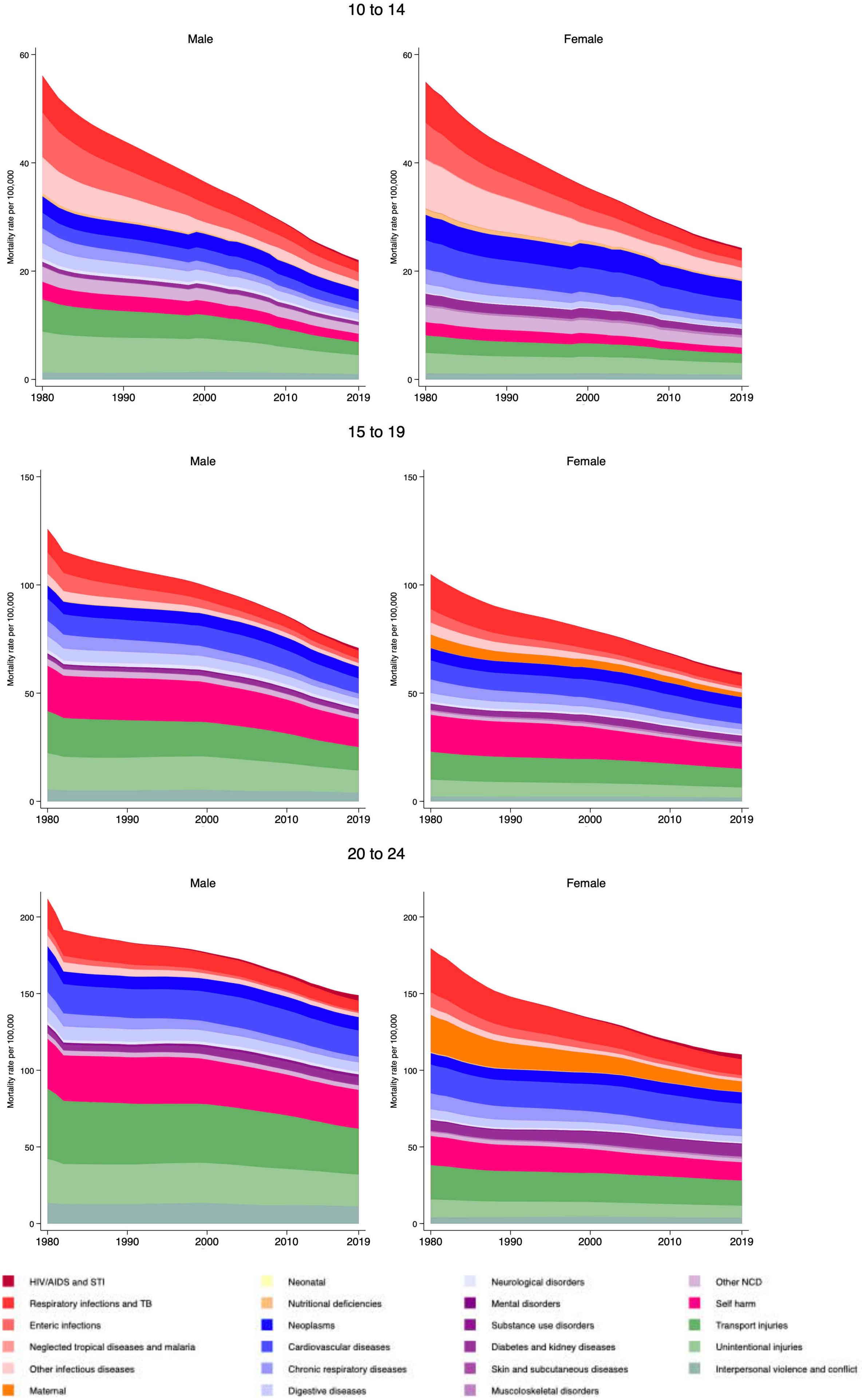

Figure S229d Number of deaths in 10-24 1980 – 2019 by cause: Tokelau

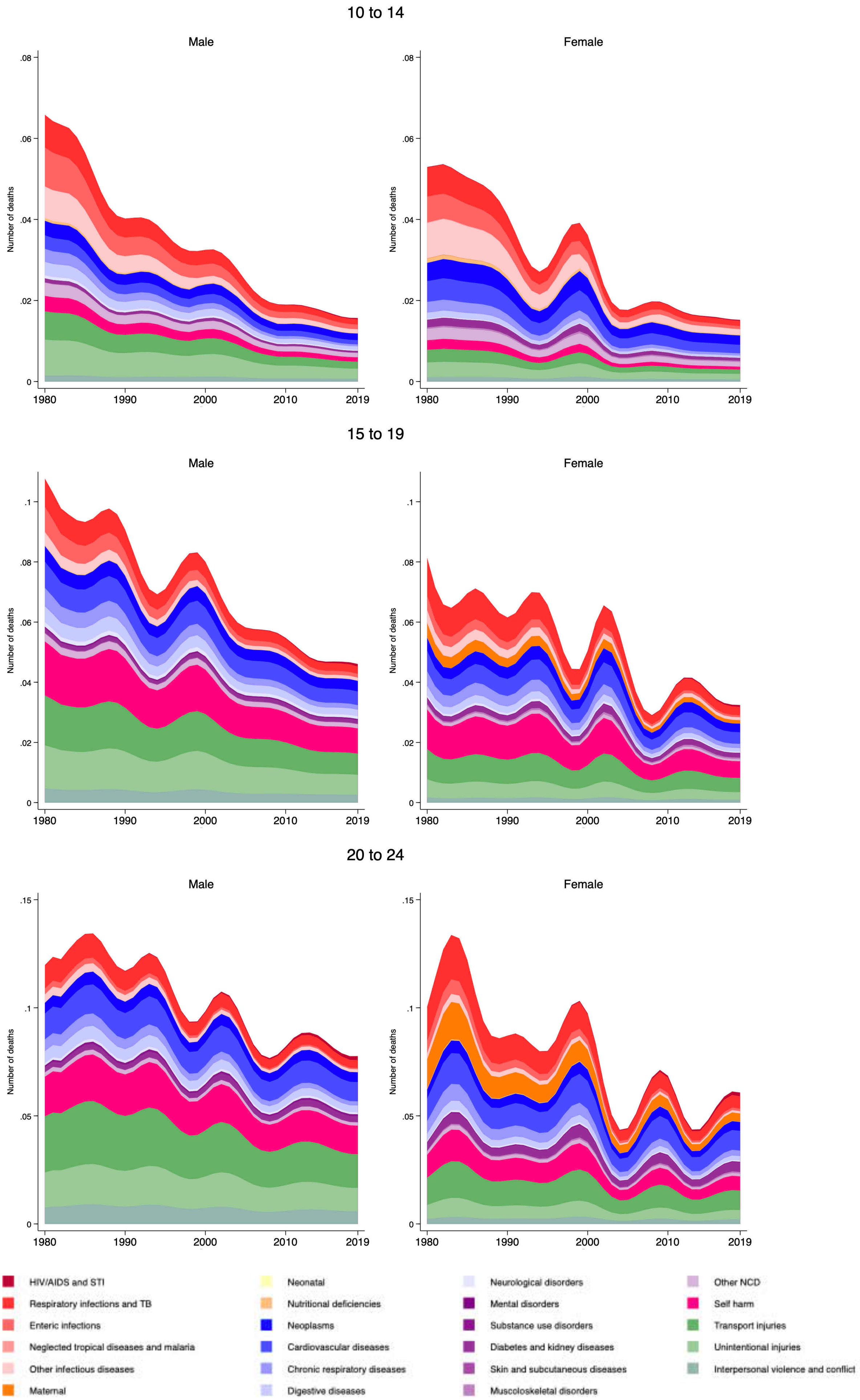

Figure S228: Tuvalu

Figure S230a All-cause mortality rate per 100,000 in 10-24 1950 – 2019: Tuvalu

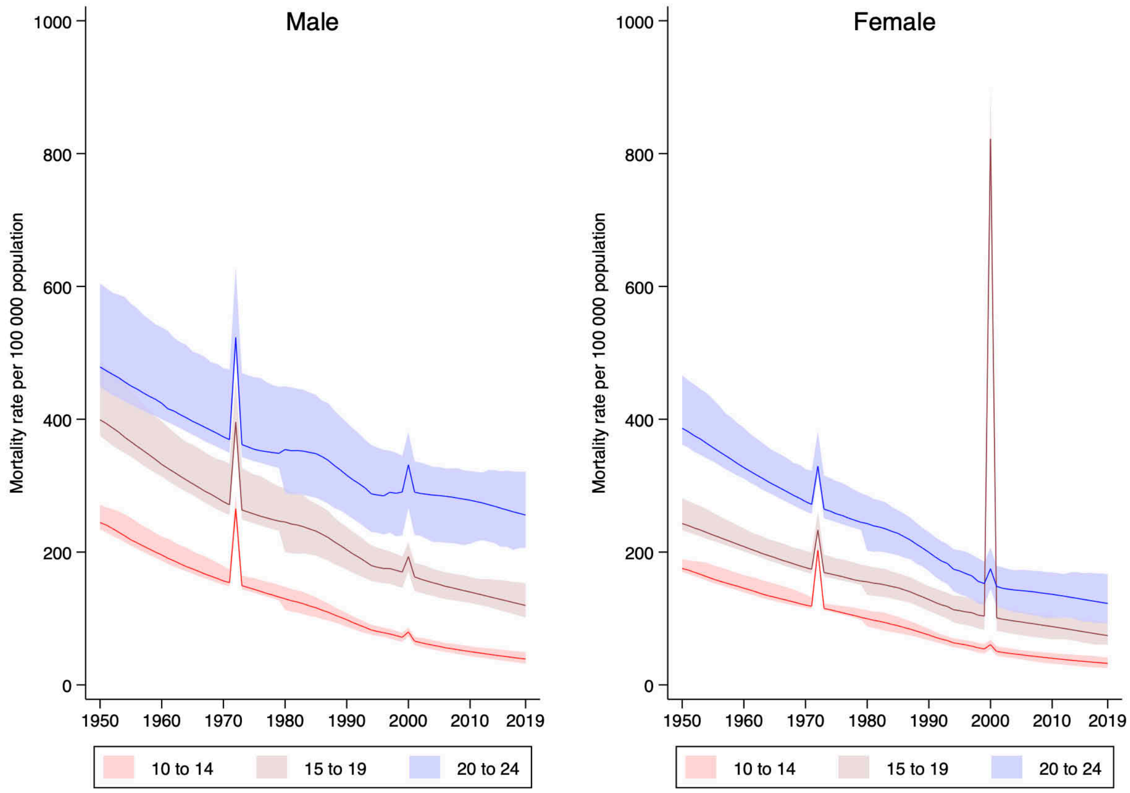

Figure S230b Number of deaths from all causes in 10-24 1950 – 2019: Tuvalu

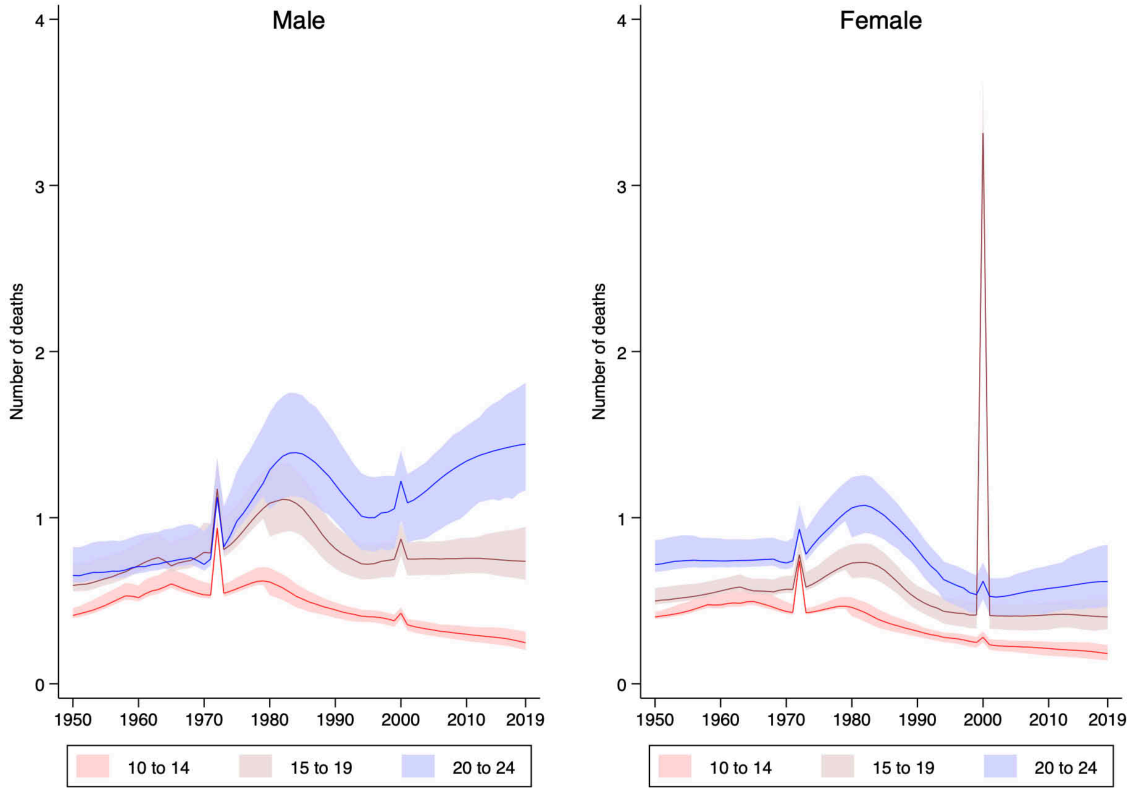

Figure S230c Mortality rate per 100,000 in 10-24 1980 – 2019 by cause: Tuvalu

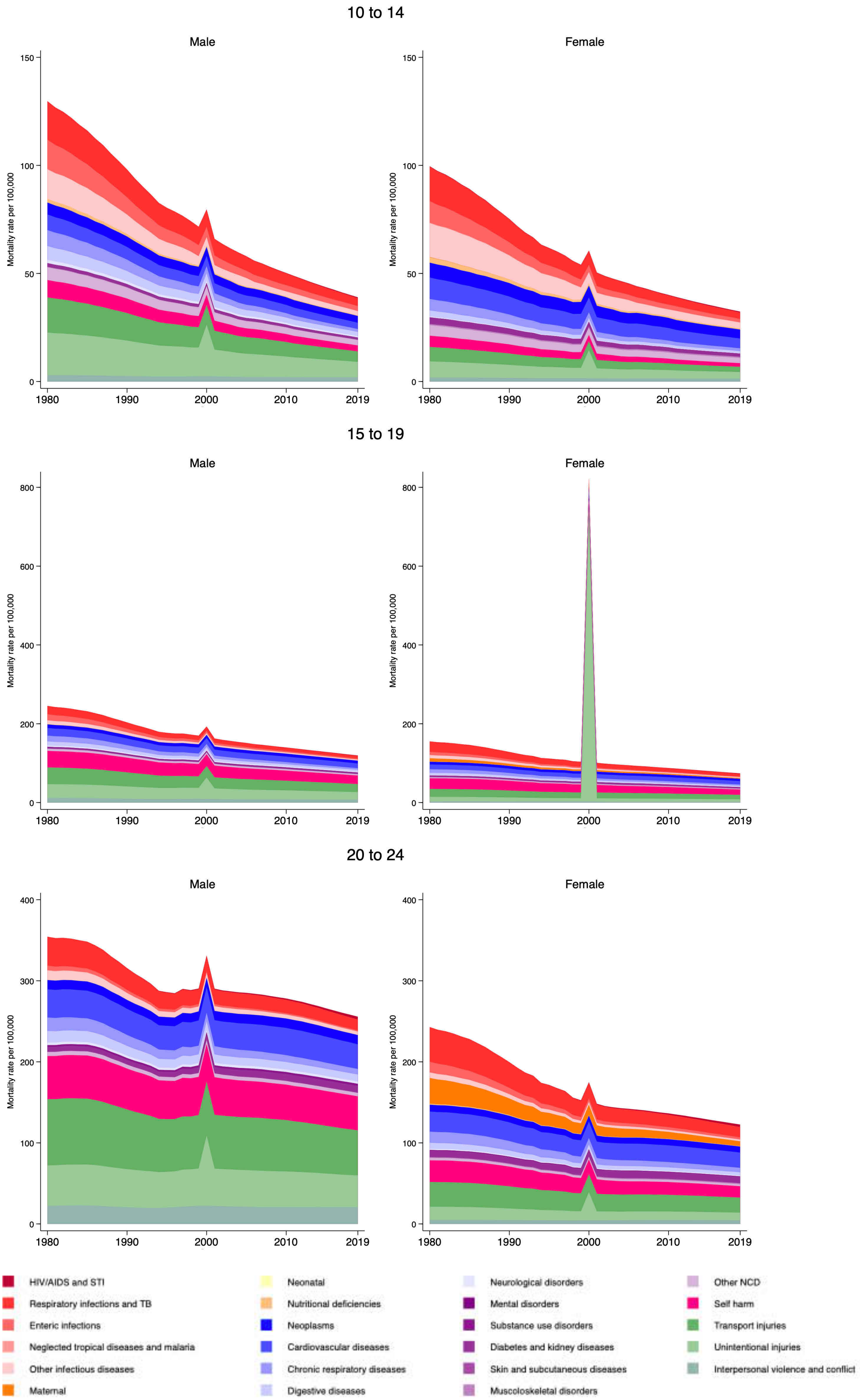

Figure S230d Number of deaths in 10-24 1980 – 2019 by cause: Tuvalu

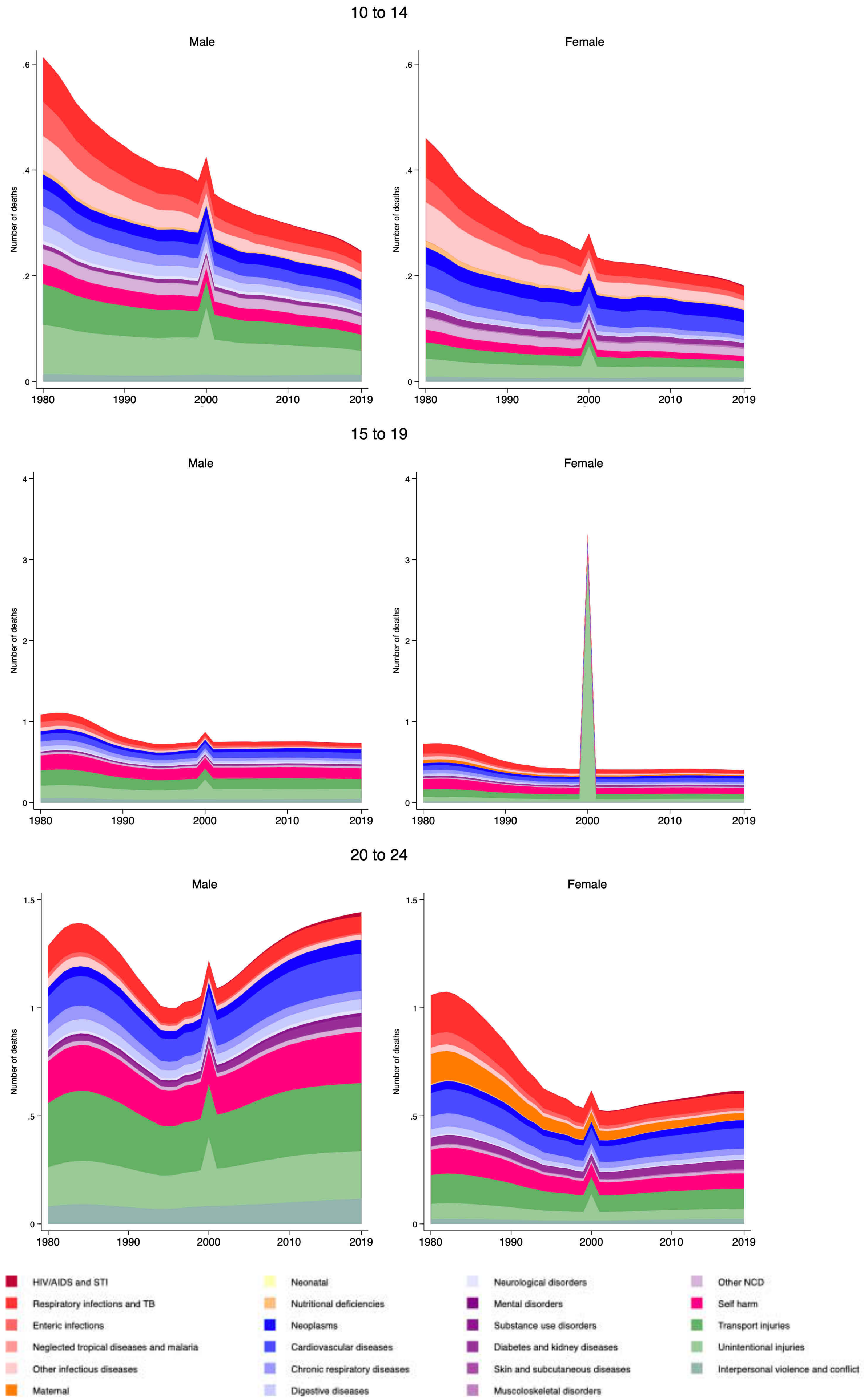

Figure S229: United States Virgin Islands

Figure S231a All-cause mortality rate per 100,000 in 10-24 1950 – 2019: United States Virgin Islands

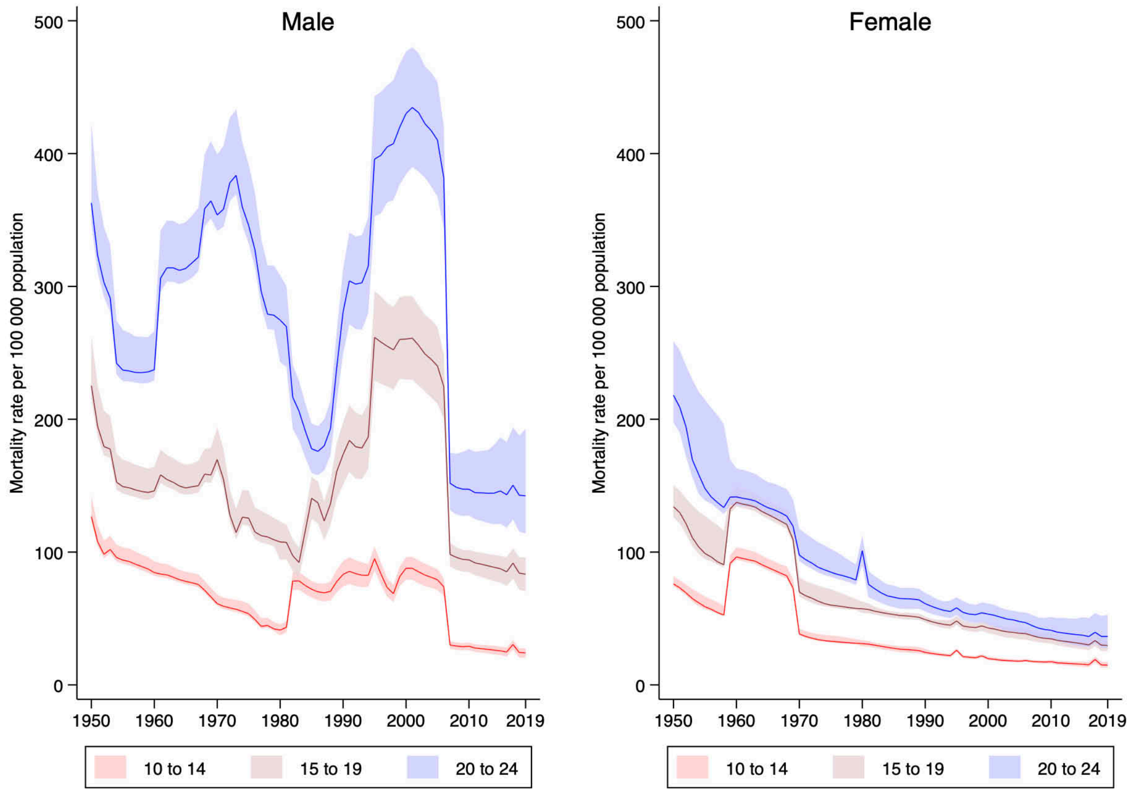

Figure S231b Number of deaths from all causes in 10-24 1950 – 2019: United States Virgin Islands

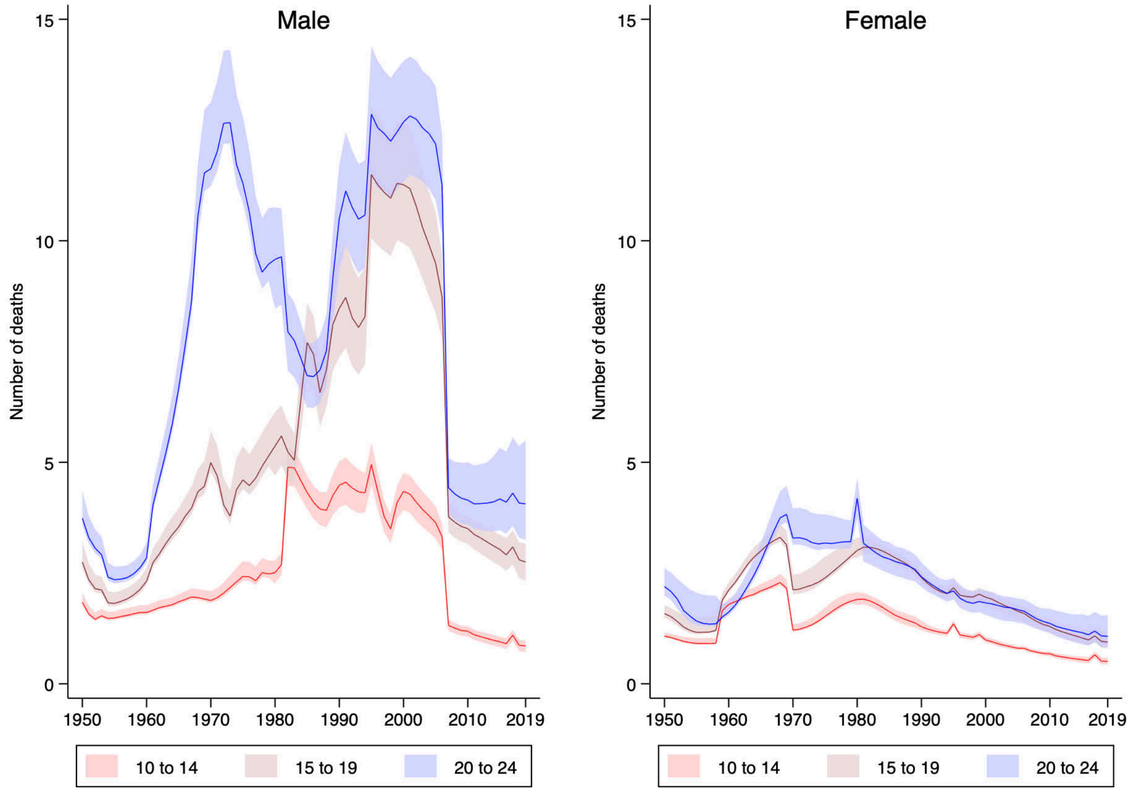

Figure S231c Mortality rate per 100,000 in 10-24 1980 – 2019 by cause: United States Virgin Islands

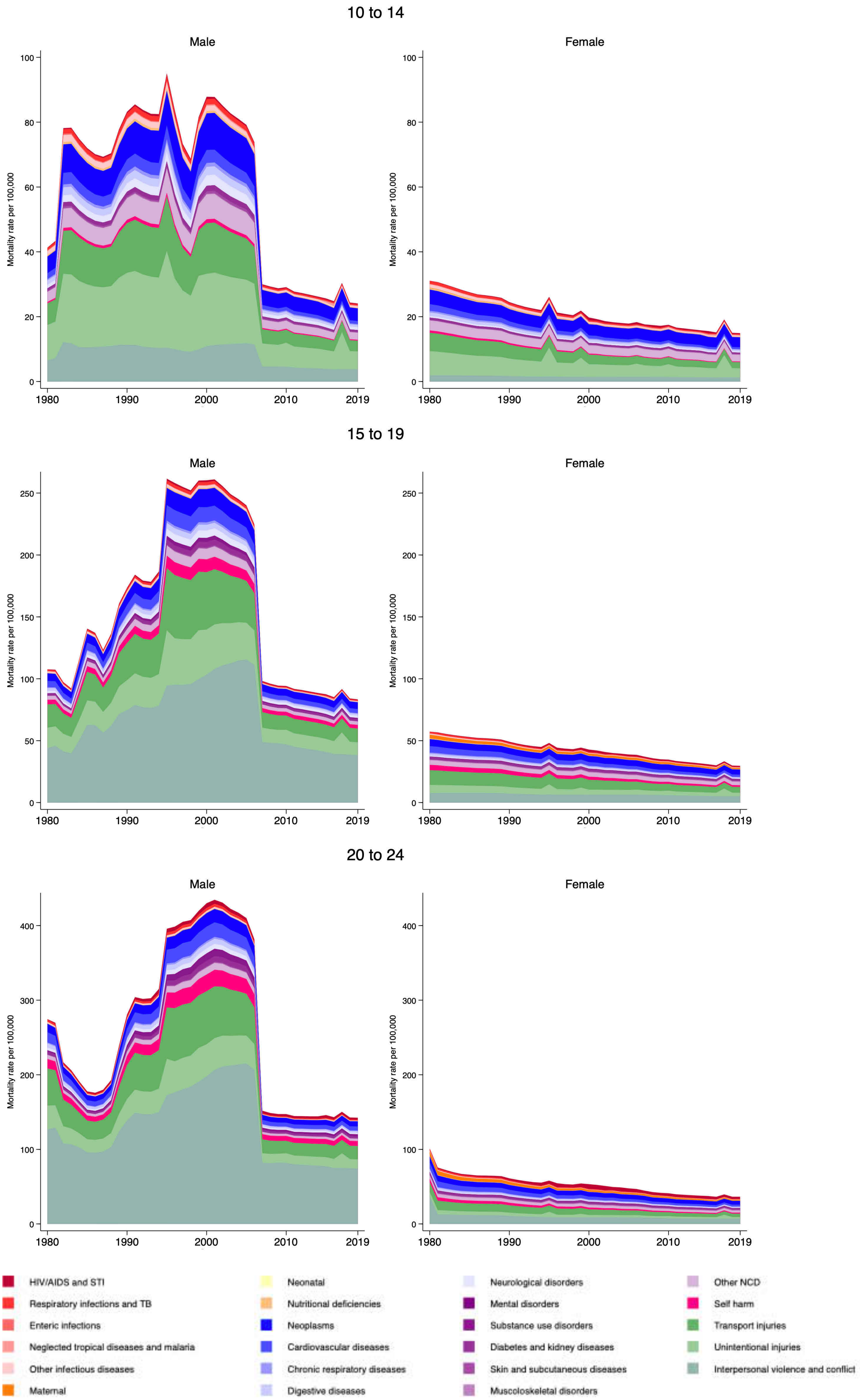

Figure S231d Number of deaths in 10-24 1980 – 2019 by cause: United States Virgin Islands

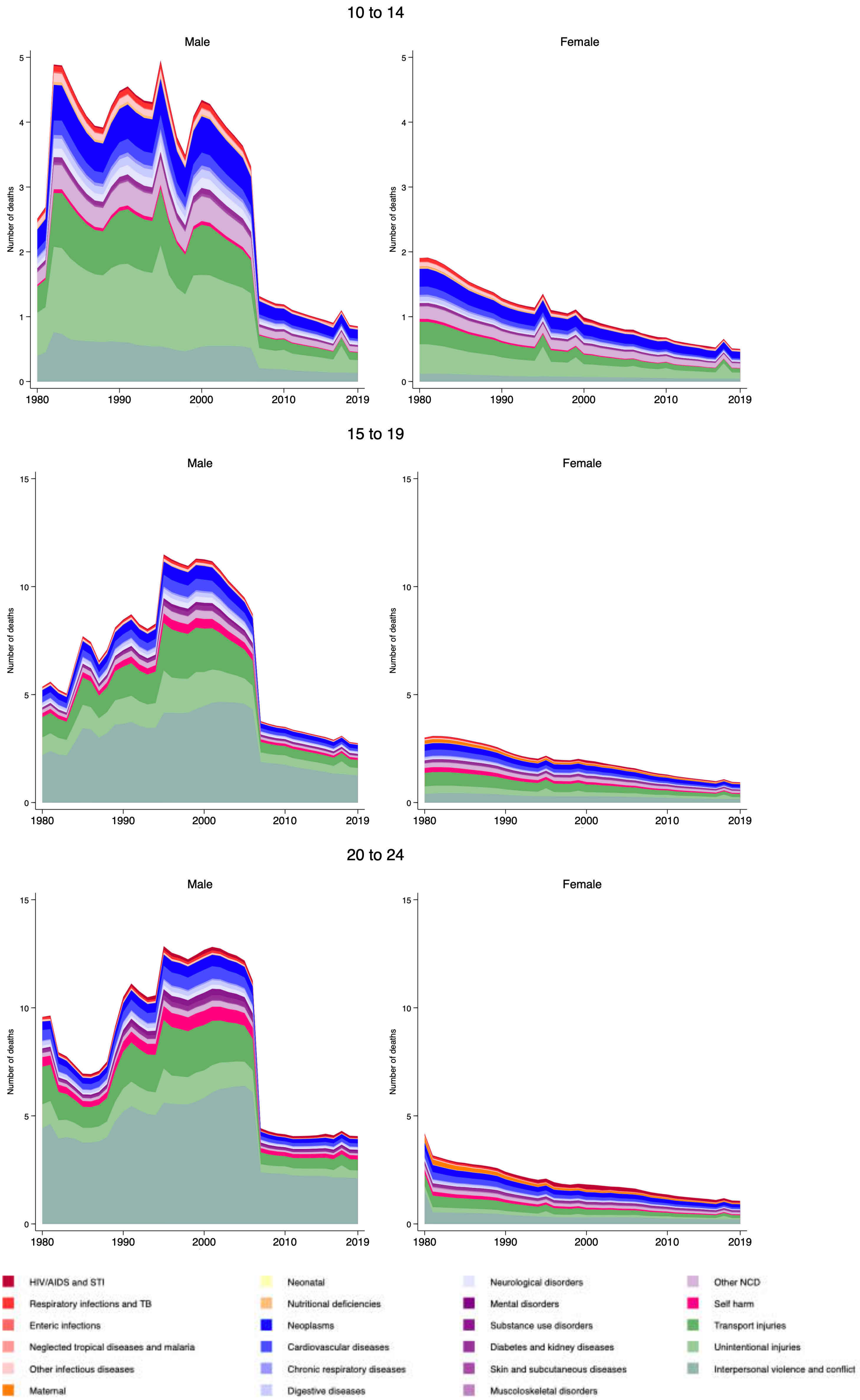

Figure S230: South Sudan

Figure S232a All-cause mortality rate per 100,000 in 10-24 1950 – 2019: South Sudan

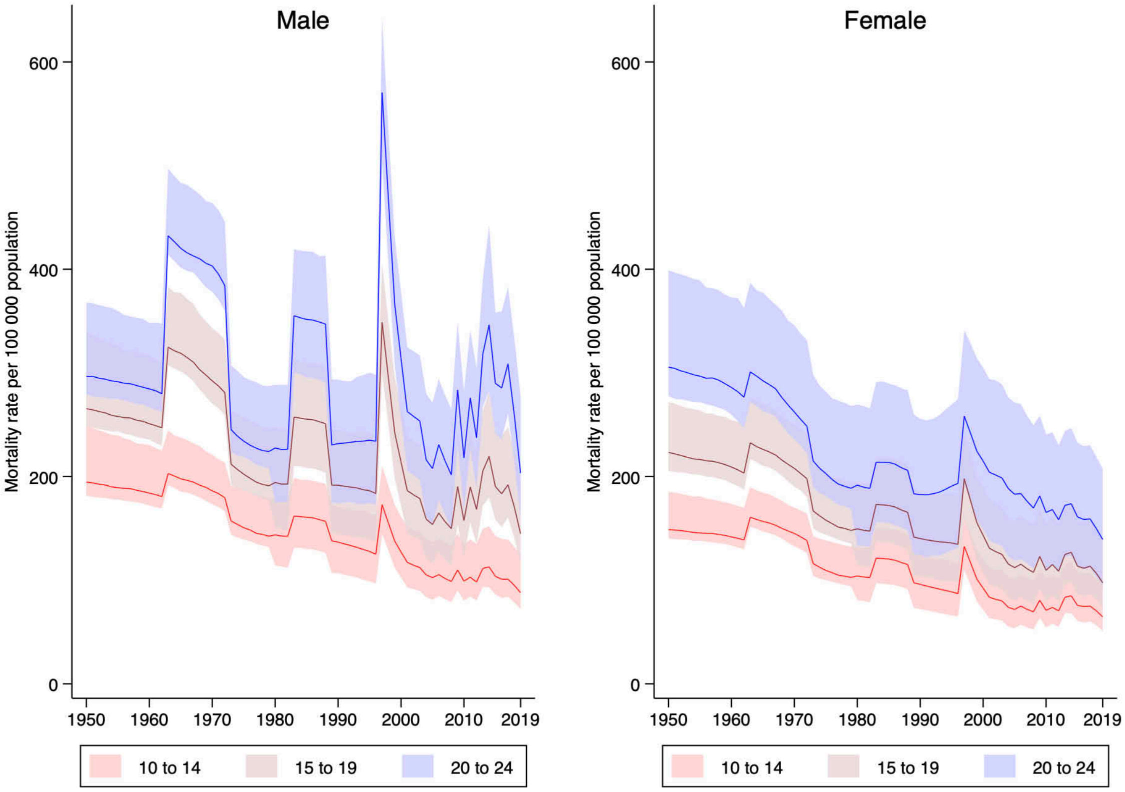

Figure S232b Number of deaths from all causes in 10-24 1950 – 2019: South Sudan

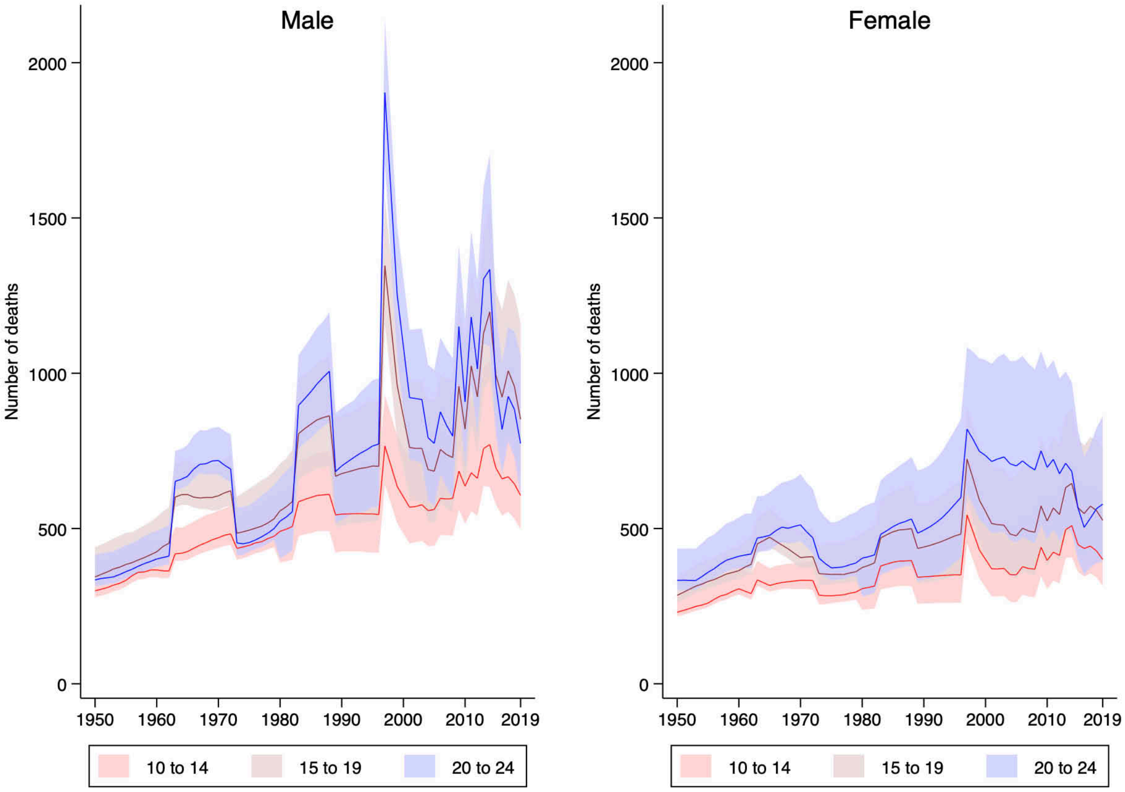

Figure S232c Mortality rate per 100,000 in 10-24 1980 – 2019 by cause: South Sudan

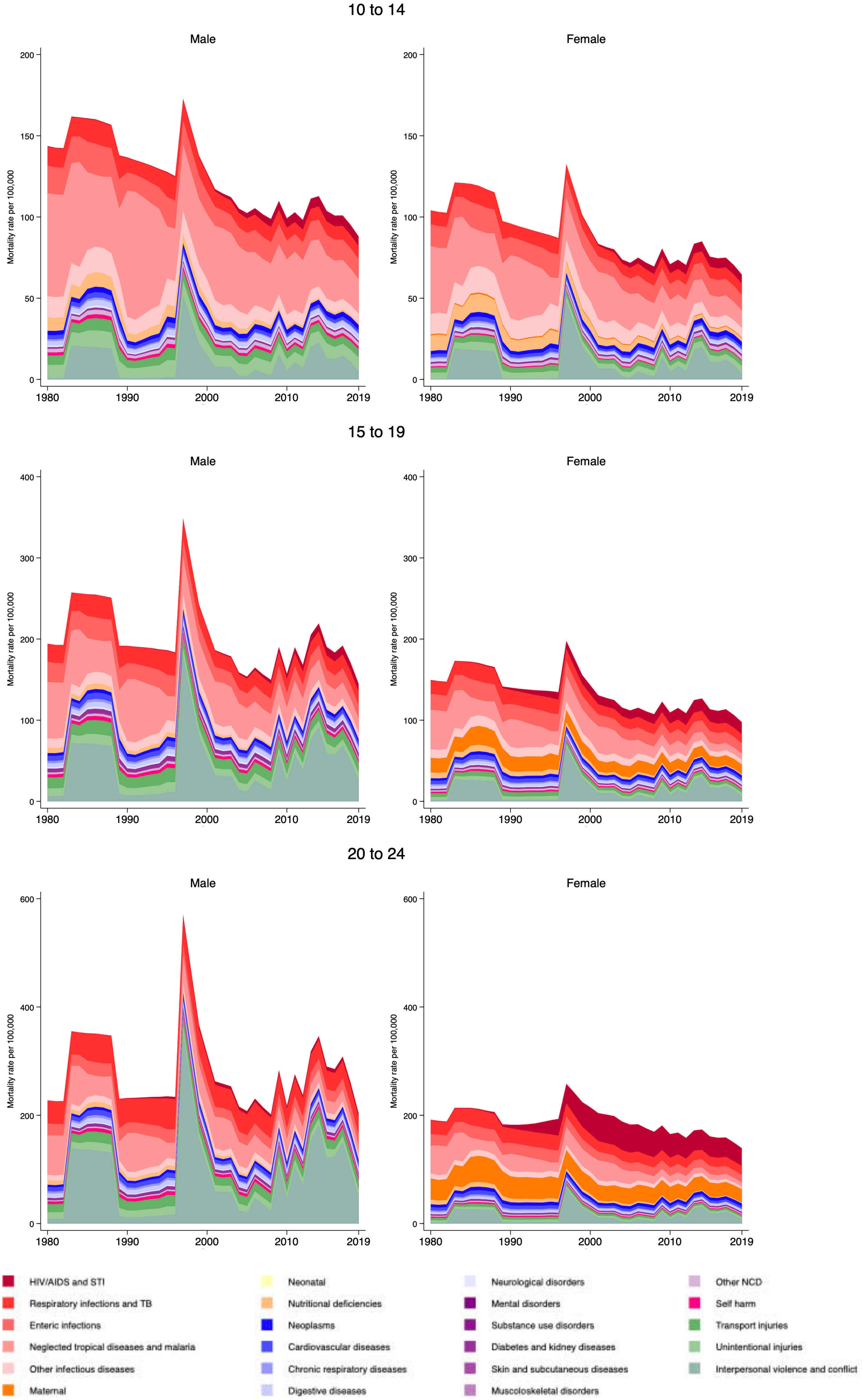

Figure S232d Number of deaths in 10-24 1980 – 2019 by cause: South Sudan

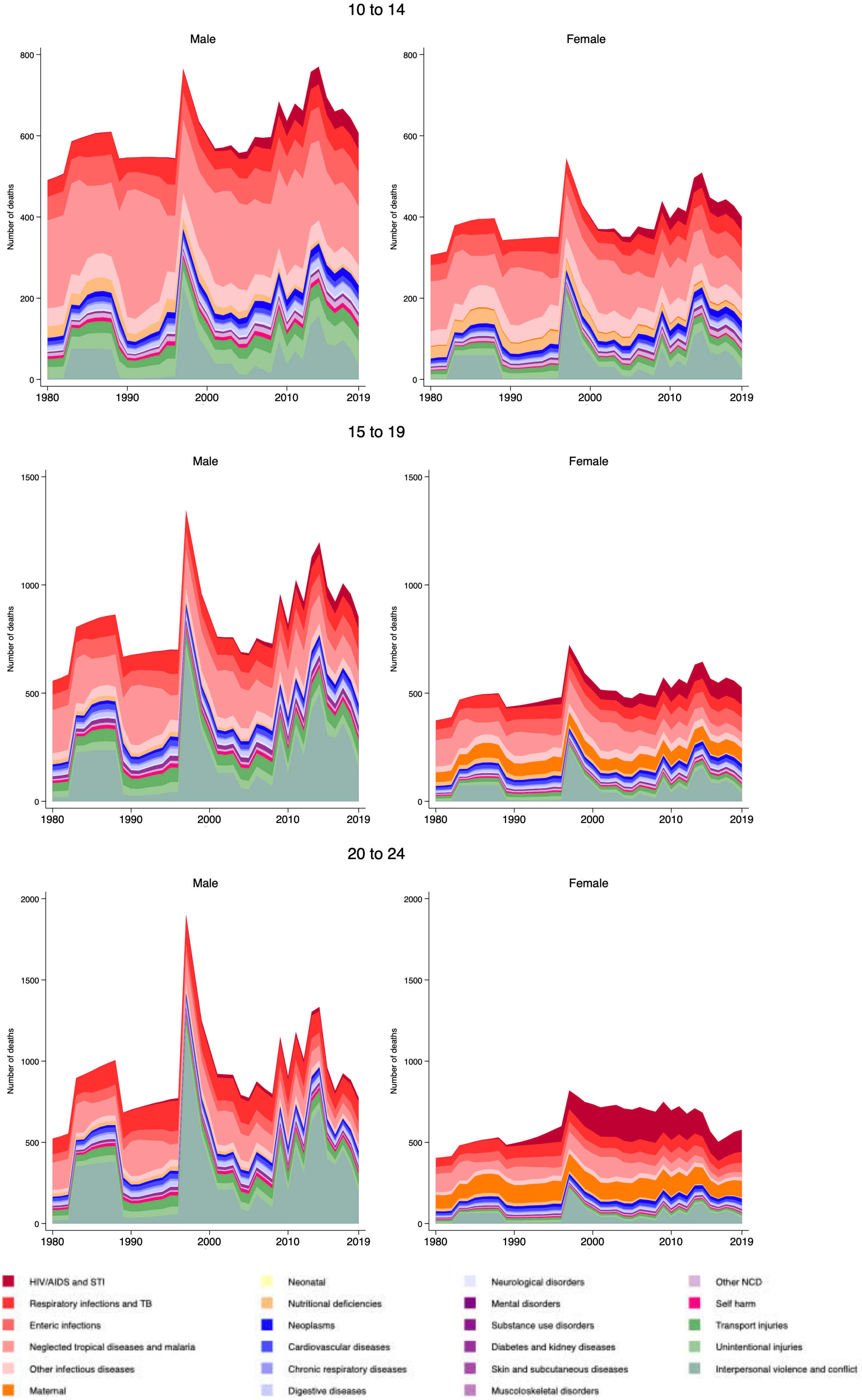

Figure S231: Sudan

Figure S233a All-cause mortality rate per 100,000 in 10-24 1950 – 2019: Sudan

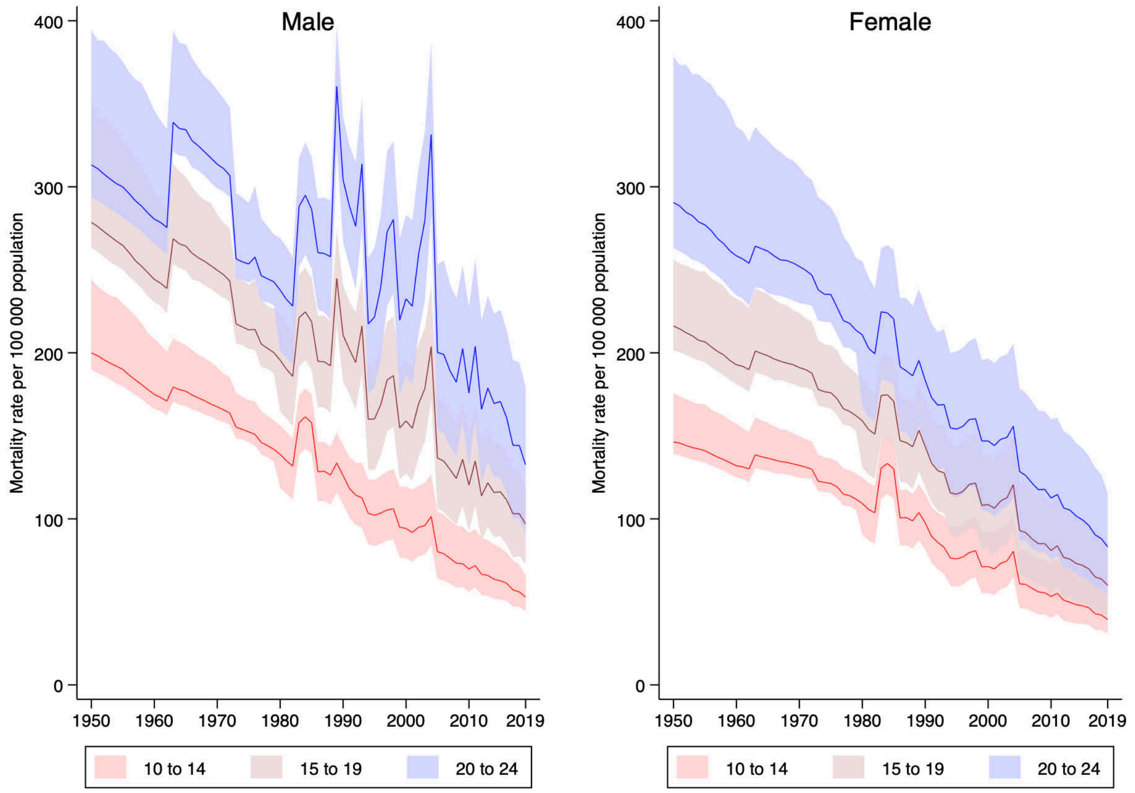

Figure S233b Number of deaths from all causes in 10-24 1950 – 2019: Sudan

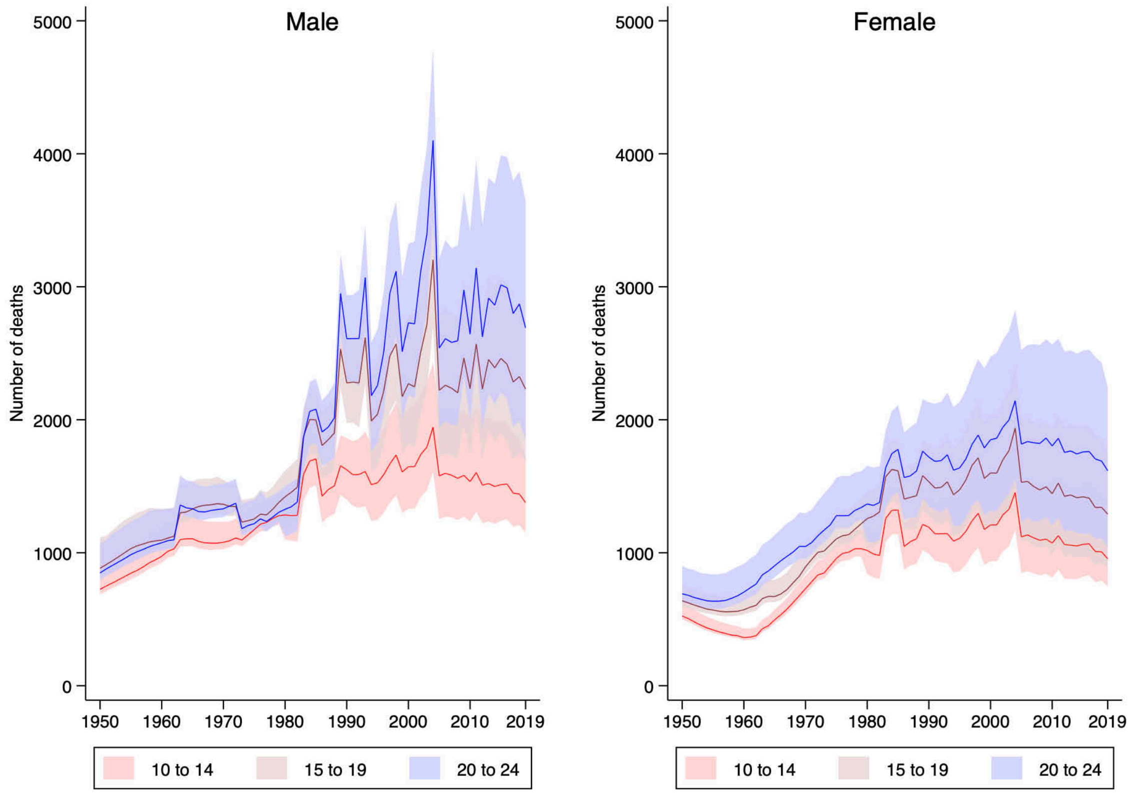

Figure S233c Mortality rate per 100,000 in 10-24 1980 – 2019 by cause: Sudan

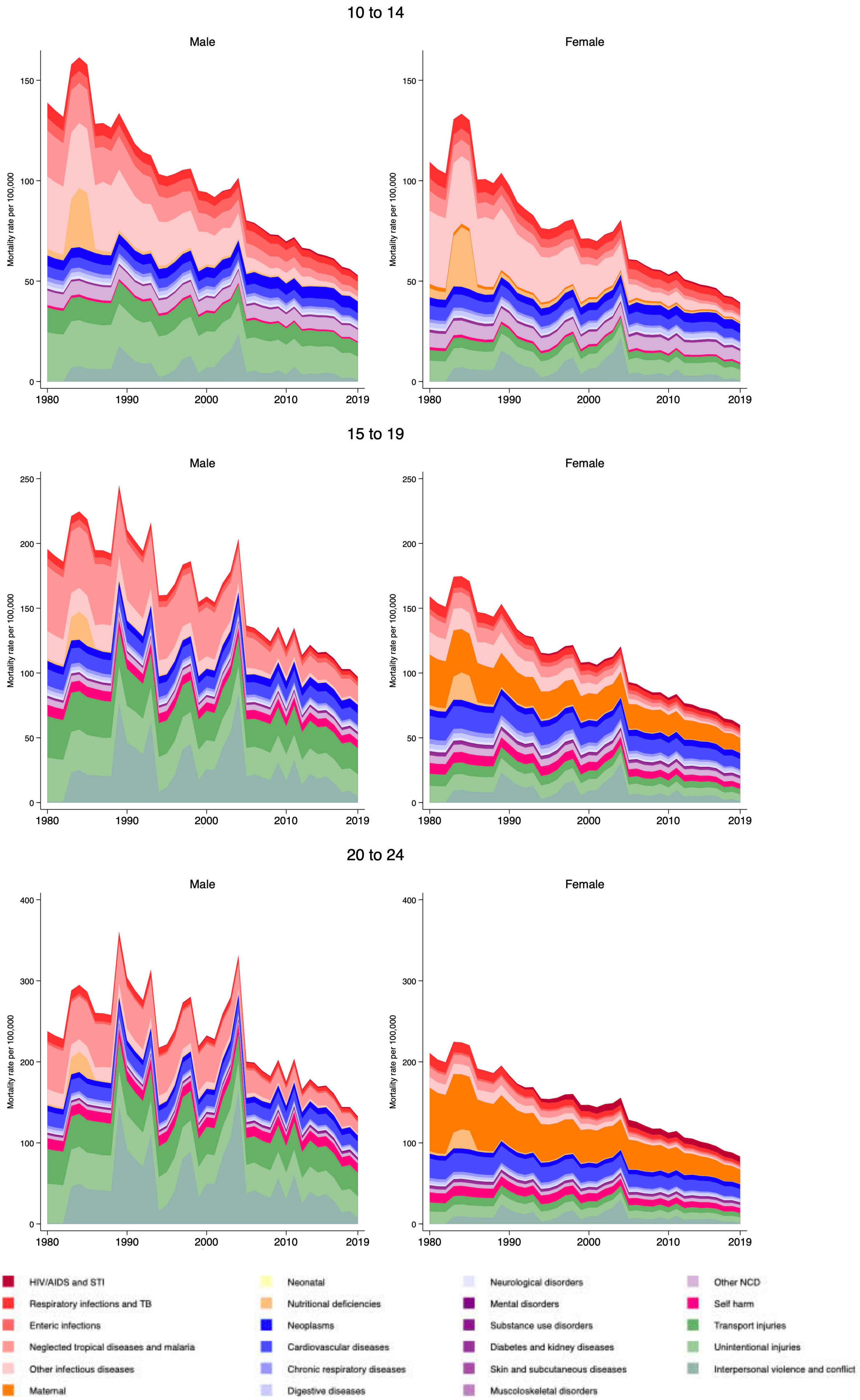

Figure S233d Number of deaths in 10-24 1980 – 2019 by cause: Sudan

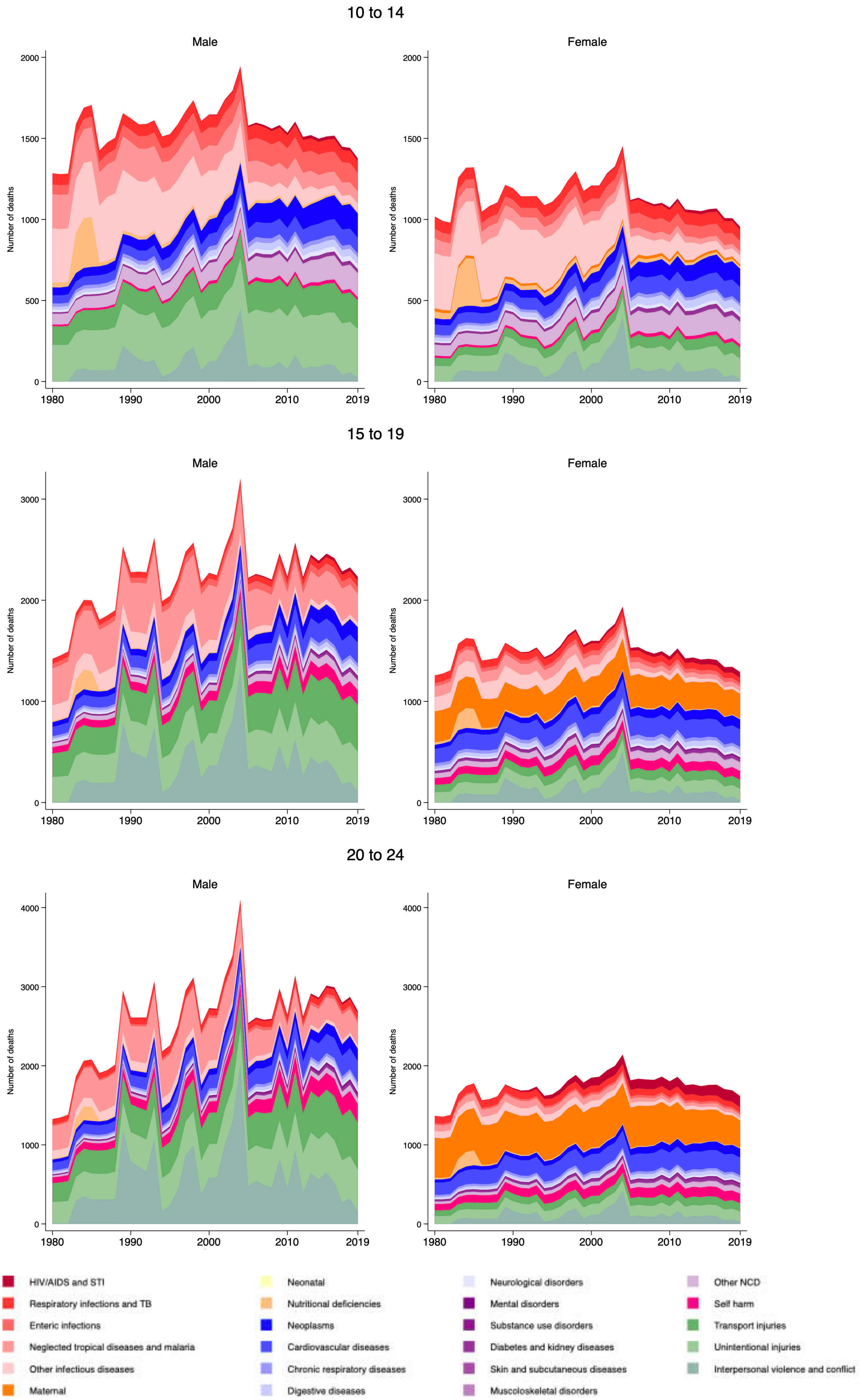

Table S1: All-cause mortality rate per 100,000 population and number of deaths in 10-24 by sex 1950 - 2019: Global

Location	Sex	Age	Year	Mortality per 100,000	Mortality per 100,000 uncertainty interval	Number	Number Uncertainty Interval
Global	Male	10 to 14	1950	202.32	[196.97 - 220.33]	272,090	[264,904 - 296,312]
			1985	100.42	[95.93 - 105.63]	273,112	[260,881 - 287,277]
			2019	51.33	[47.28 - 55.66]	170,079	[156,649 - 184,434]
		15 to 19	1950	304.35	[296.91 - 329.87]	367,938	[358,955 - 398,792]
			1985	156.09	[147.51 - 167.42]	405,068	[382,806 - 434,487]
			2019	95.14	[88.09 - 102.43]	302,328	[279,932 - 325,494]
		20 to 24	1950	392.55	[385.58 - 417.83]	433,760	[426,063 - 461,700]
			1985	209.99	[199.47 - 225.62]	467,059	[443,656 - 501,810]
			2019	143.67	[134.30 - 152.80]	437,271	[408,762 - 465,081]
	Female	10 to 14	1950	161.57	[157.70 - 172.98]	208,021	[203,049 - 222,722]
			1985	81.80	[77.65 - 86.93]	212,001	[201,242 - 225,294]
			2019	41.56	[38.29 - 45.13]	129,193	[119,022 - 140,301]
		15 to 19	1950	230.86	[225.94 - 245.20]	273,094	[267,273 - 290,053]
			1985	118.73	[113.15 - 124.90]	297,495	[283,519 - 312,944]
			2019	65.28	[60.15 - 71.38]	196,977	[181,494 - 215,401]
		20 to 24	1950	315.67	[305.92 - 344.37]	349,068	[338,285 - 380,804]
			1985	157.68	[149.02 - 167.04]	348,620	[329,481 - 369,326]
			2019	86.26	[77.70 - 95.07]	255,141	[229,825 - 281,183]

Table S2: All-cause mortality rate per 100,000 population and number of deaths in 10-24 by sex 1950 - 2019: Southeast Asia, East Asia, and Oceania

Location	Sex	Age	Year	Mortality per 100,000	Mortality per 100,000 uncertainty interval	Number	Number Uncertainty Interval
Southeast Asia, East Asia, and Oceania	Male	10 to 14	1950	232.00	[223.42 - 258.63]	97,918	[94,295 - 109,156]
			1985	88.16	[80.84 - 96.78]	82,187	[75,362 - 90,218]
			2019	35.21	[32.25 - 38.38]	24,644	[22,572 - 26,861]
		15 to 19	1950	368.85	[355.22 - 421.10]	139,611	[134,450 - 159,386]
			1985	133.75	[117.85 - 151.97]	129,882	[114,438 - 147,577]
			2019	73.26	[65.87 - 81.00]	52,172	[46,911 - 57,685]
		20 to 24	1950	491.91	[474.37 - 555.08]	164,598	[158,729 - 185,734]
			1985	175.13	[155.98 - 196.06]	130,245	[116,000 - 145,806]
			2019	109.74	[98.62 - 121.27]	80,455	[72,303 - 88,906]
	Female	10 to 14	1950	184.67	[179.53 - 203.81]	71,731	[69,737 - 79,166]
			1985	63.50	[56.75 - 71.73]	56,292	[50,312 - 63,587]
			2019	22.97	[21.17 - 24.74]	14,297	[13,177 - 15,402]
		15 to 19	1950	252.80	[244.21 - 279.10]	92,028	[88,903 - 101,604]
			1985	85.30	[75.38 - 96.03]	80,460	[71,101 - 90,575]
			2019	35.02	[32.05 - 37.92]	22,732	[20,807 - 24,618]
		20 to 24	1950	334.13	[317.16 - 391.48]	107,849	[102,371 - 126,360]
			1985	115.13	[97.87 - 134.96]	86,381	[73,430 - 101,254]
			2019	45.32	[39.99 - 50.94]	31,266	[27,586 - 35,144]

Table S3: All-cause mortality rate per 100,000 population and number of deaths in 10-24 by sex 1950 - 2019: East Asia

Location	Sex	Age	Year	Mortality per 100,000	Mortality per 100,000 uncertainty interval	Number	Number Uncertainty Interval
East Asia	Male	10 to 14	1950	217.55	[207.32 - 250.73]	67,540	[64,365 - 77,841]
			1985	77.72	[68.08 - 88.31]	51,999	[45,548 - 59,086]
			2019	26.44	[23.20 - 29.84]	10,497	[9,211 - 11,844]
		15 to 19	1950	364.44	[345.74 - 431.80]	101,424	[96,220 - 120,169]
			1985	118.20	[98.25 - 140.27]	87,422	[72,671 - 103,746]
			2019	49.53	[41.03 - 58.77]	20,638	[17,094 - 24,484]
		20 to 24	1950	497.63	[473.40 - 579.08]	122,341	[116,385 - 142,364]
			1985	141.55	[117.49 - 167.52]	76,909	[63,837 - 91,022]
			2019	80.70	[67.09 - 95.88]	35,993	[29,919 - 42,761]
	Female	10 to 14	1950	181.69	[175.04 - 206.23]	49,446	[47,638 - 56,127]
			1985	52.32	[43.66 - 63.74]	33,132	[27,650 - 40,361]
			2019	15.18	[13.41 - 16.99]	5,103	[4,510 - 5,713]
		15 to 19	1950	252.79	[241.25 - 286.28]	66,227	[63,204 - 75,000]
			1985	72.45	[59.64 - 87.06]	51,408	[42,315 - 61,771]
			2019	22.45	[19.51 - 25.48]	8,198	[7,123 - 9,304]
		20 to 24	1950	321.36	[297.85 - 394.92]	75,696	[70,157 - 93,023]
			1985	95.71	[72.84 - 123.87]	51,398	[39,115 - 66,524]
			2019	31.03	[24.78 - 37.89]	12,668	[10,118 - 15,472]

Table S4: All-cause mortality rate per 100,000 population and number of deaths in 10-24 by sex 1950 - 2019: China

Location	Sex	Age	Year	Mortality per 100,000	Mortality per 100,000 uncertainty interval	Number	Number Uncertainty Interval
China	Male	10 to 14	1950	184.32	[173.90 - 217.74]	55,043	[51,930 - 65,021]
			1985	78.12	[68.24 - 89.20]	50,839	[44,413 - 58,048]
			2019	26.40	[23.05 - 29.87]	10,117	[8,835 - 11,448]
		15 to 19	1950	280.09	[260.60 - 348.69]	75,309	[70,067 - 93,752]
			1985	117.93	[97.50 - 140.95]	85,018	[70,288 - 101,614]
			2019	48.11	[39.39 - 57.78]	19,289	[15,795 - 23,166]
		20 to 24	1950	350.41	[326.48 - 432.12]	83,474	[77,773 - 102,938]
			1985	140.48	[115.62 - 167.09]	73,752	[60,701 - 87,720]
			2019	78.92	[64.96 - 94.67]	33,750	[27,781 - 40,488]
	Female	10 to 14	1950	144.21	[137.41 - 168.25]	37,489	[35,721 - 43,737]
			1985	52.58	[43.63 - 64.25]	32,297	[26,797 - 39,465]
			2019	15.10	[13.29 - 16.97]	4,881	[4,298 - 5,488]
		15 to 19	1950	208.94	[196.86 - 241.93]	52,613	[49,572 - 60,920]
			1985	72.57	[59.25 - 87.47]	50,039	[40,855 - 60,314]
			2019	21.88	[18.92 - 25.03]	7,669	[6,628 - 8,769]
		20 to 24	1950	282.96	[258.48 - 358.69]	64,231	[58,673 - 81,421]
			1985	95.95	[72.14 - 125.12]	49,758	[37,408 - 64,883]
			2019	30.39	[23.94 - 37.78]	11,884	[9,361 - 14,773]

Table S5: All-cause mortality rate per 100,000 population and number of deaths in 10-24 by sex 1950 - 2019: Democratic People's Republic of Korea

Location	Sex	Age	Year	Mortality per 100,000	Mortality per 100,000 uncertainty interval	Number	Number Uncertainty Interval
Democratic People's Republic of Korea	Male	10 to 14	1950	1666.97	[1621.70 - 1832.08]	11,927	[11,603 - 13,108]
			1985	81.86	[62.49 - 113.28]	734	[560 - 1,015]
			2019	34.98	[30.20 - 40.46]	297	[256 - 343]
		15 to 19	1950	5049.28	[4915.48 - 5545.52]	25,214	[24,546 - 27,692]
			1985	141.12	[104.93 - 188.02]	1,261	[937 - 1,680]
			2019	110.98	[99.66 - 123.37]	1,019	[915 - 1,132]
		20 to 24	1950	9397.28	[9126.06 - 10336.19]	37,616	[36,531 - 41,375]
			1985	202.76	[145.55 - 274.06]	1,644	[1,180 - 2,222]
			2019	164.32	[131.32 - 216.79]	1,712	[1,368 - 2,258]
	Female	10 to 14	1950	1520.17	[1479.27 - 1667.02]	11,609	[11,297 - 12,731]
			1985	59.07	[41.91 - 84.70]	601	[427 - 862]
			2019	21.27	[19.10 - 24.54]	172	[154 - 198]
		15 to 19	1950	2144.11	[2086.71 - 2346.57]	13,167	[12,814 - 14,410]
			1985	87.34	[58.87 - 121.03]	937	[631 - 1,298]
			2019	45.81	[39.02 - 56.47]	406	[346 - 500]
		20 to 24	1950	2170.96	[2112.15 - 2385.68]	10,863	[10,569 - 11,938]
			1985	115.37	[76.64 - 166.72]	1,006	[668 - 1,453]
			2019	58.94	[45.02 - 83.62]	587	[449 - 833]

Table S6: All-cause mortality rate per 100,000 population and number of deaths in 10-24 by sex 1950 - 2019: Taiwan (Province of China)

Location	Sex	Age	Year	Mortality per 100,000	Mortality per 100,000 uncertainty interval	Number	Number Uncertainty Interval
Taiwan (Province of China)	Male	10 to 14	1950	124.46	[115.73 - 154.37]	583	[542 - 723]
			1985	45.64	[45.01 - 46.28]	426	[420 - 432]
			2019	15.88	[12.98 - 19.74]	84	[68 - 104]
		15 to 19	1950	223.85	[206.17 - 283.59]	993	[915 - 1,258]
			1985	116.64	[115.32 - 117.85]	1,143	[1,130 - 1,155]
			2019	50.75	[39.66 - 65.90]	329	[257 - 428]
		20 to 24	1950	309.06	[289.50 - 374.27]	1,121	[1,050 - 1,358]
			1985	147.77	[146.37 - 149.13]	1,513	[1,499 - 1,527]
			2019	67.31	[54.66 - 84.07]	532	[432 - 664]
	Female	10 to 14	1950	91.06	[85.33 - 109.80]	415	[389 - 500]
			1985	26.41	[26.05 - 26.79]	233	[230 - 237]
			2019	10.35	[8.67 - 12.54]	50	[42 - 60]
		15 to 19	1950	139.03	[130.50 - 166.16]	561	[526 - 670]
			1985	46.48	[45.93 - 47.07]	432	[427 - 438]
			2019	20.73	[17.22 - 25.40]	123	[102 - 151]
		20 to 24	1950	190.63	[174.46 - 248.35]	676	[619 - 881]
			1985	65.02	[63.99 - 66.09]	634	[624 - 645]
			2019	26.87	[20.59 - 35.60]	197	[151 - 261]

Table S7: All-cause mortality rate per 100,000 population and number of deaths in 10-24 by sex 1950 - 2019: Southeast Asia

Location	Sex	Age	Year	Mortality per 100,000	Mortality per 100,000 uncertainty interval	Number	Number Uncertainty Interval
Southeast Asia	Male	10 to 14	1950	272.26	[263.67 - 302.10]	29,964	[29,018 - 33,248]
			1985	114.93	[106.52 - 124.66]	29,817	[27,637 - 32,343]
			2019	45.98	[41.29 - 50.71]	13,591	[12,205 - 14,990]
		15 to 19	1950	384.34	[370.15 - 434.63]	37,965	[36,563 - 42,933]
			1985	183.51	[167.50 - 203.71]	41,881	[38,227 - 46,491]
			2019	105.68	[93.19 - 120.16]	30,525	[26,917 - 34,706]
		20 to 24	1950	480.51	[464.75 - 539.92]	42,015	[40,637 - 47,210]
			1985	266.18	[242.73 - 301.14]	52,585	[47,953 - 59,493]
			2019	153.20	[134.39 - 173.96]	43,024	[37,743 - 48,855]
	Female	10 to 14	1950	191.75	[186.19 - 208.02]	22,037	[21,398 - 23,906]
			1985	91.57	[84.44 - 99.59]	22,887	[21,105 - 24,892]
			2019	31.51	[28.48 - 34.62]	8,812	[7,966 - 9,684]
		15 to 19	1950	254.28	[247.27 - 272.54]	25,649	[24,941 - 27,491]
			1985	124.31	[115.07 - 134.59]	28,684	[26,552 - 31,058]
			2019	50.17	[44.89 - 55.25]	13,940	[12,472 - 15,352]
		20 to 24	1950	369.95	[354.42 - 412.99]	31,866	[30,529 - 35,573]
			1985	163.95	[148.29 - 179.82]	34,534	[31,236 - 37,879]
			2019	64.34	[55.61 - 73.40]	17,737	[15,331 - 20,235]

Table S8: All-cause mortality rate per 100,000 population and number of deaths in 10-24 by sex 1950 - 2019: Cambodia

Location	Sex	Age	Year	Mortality per 100,000	Mortality per 100,000 uncertainty interval	Number	Number Uncertainty Interval
Cambodia	Male	10 to 14	1950	244.15	[224.20 - 305.64]	590	[542 - 738]
			1985	215.03	[176.48 - 290.66]	1,180	[969 - 1,595]
			2019	54.95	[47.94 - 62.67]	447	[390 - 510]
		15 to 19	1950	353.88	[322.69 - 447.15]	779	[710 - 984]
			1985	391.49	[281.81 - 630.50]	1,854	[1,334 - 2,986]
			2019	125.84	[98.34 - 137.73]	956	[747 - 1,046]
		20 to 24	1950	420.43	[398.20 - 500.84]	833	[789 - 993]
			1985	598.73	[393.56 - 1061.46]	2,339	[1,538 - 4,147]
			2019	175.76	[134.50 - 199.81]	1,346	[1,030 - 1,530]
	Female	10 to 14	1950	185.53	[173.54 - 227.67]	475	[444 - 583]
			1985	170.95	[137.30 - 240.58]	945	[759 - 1,330]
			2019	35.73	[31.47 - 40.96]	274	[242 - 315]
		15 to 19	1950	257.74	[247.56 - 297.09]	598	[574 - 689]
			1985	231.47	[187.49 - 324.31]	1,166	[945 - 1,634]
			2019	61.36	[50.40 - 69.73]	444	[364 - 504]
		20 to 24	1950	389.69	[369.27 - 480.33]	799	[757 - 985]
			1985	304.96	[248.58 - 399.44]	1,364	[1,112 - 1,787]
			2019	79.62	[63.19 - 95.93]	593	[471 - 715]

Table S9: All-cause mortality rate per 100,000 population and number of deaths in 10-24 by sex 1950 - 2019: Indonesia

Location	Sex	Age	Year	Mortality per 100,000	Mortality per 100,000 uncertainty interval	Number	Number Uncertainty Interval
Indonesia	Male	10 to 14	1950	267.42	[253.28 - 314.97]	13,121	[12,427 - 15,454]
			1985	116.27	[106.18 - 125.70]	12,236	[11,174 - 13,228]
			2019	44.24	[37.54 - 52.14]	5,312	[4,507 - 6,260]
		15 to 19	1950	368.97	[344.66 - 455.09]	16,297	[15,223 - 20,101]
			1985	168.76	[150.73 - 188.70]	14,965	[13,366 - 16,733]
			2019	105.50	[82.53 - 137.22]	12,312	[9,632 - 16,013]
		20 to 24	1950	408.46	[382.13 - 498.24]	15,806	[14,787 - 19,280]
			1985	232.85	[208.23 - 260.88]	17,675	[15,806 - 19,802]
			2019	138.49	[109.47 - 184.14]	15,093	[11,930 - 20,068]
	Female	10 to 14	1950	190.26	[181.10 - 218.71]	10,230	[9,738 - 11,760]
			1985	101.32	[90.10 - 111.05]	10,186	[9,058 - 11,164]
			2019	29.73	[26.27 - 34.04]	3,376	[2,983 - 3,866]
		15 to 19	1950	266.67	[255.36 - 296.80]	12,260	[11,740 - 13,645]
			1985	141.15	[125.72 - 152.98]	12,816	[11,415 - 13,890]
			2019	52.89	[45.43 - 60.29]	5,939	[5,101 - 6,770]
		20 to 24	1950	382.12	[352.54 - 464.94]	14,527	[13,403 - 17,676]
			1985	193.07	[165.02 - 216.92]	16,203	[13,849 - 18,205]
			2019	66.13	[54.06 - 83.09]	7,096	[5,800 - 8,916]

Table S10: All-cause mortality rate per 100,000 population and number of deaths in 10-24 by sex 1950 - 2019: Lao People's Democratic Republic

Location	Sex	Age	Year	Mortality per 100,000	Mortality per 100,000 uncertainty interval	Number	Number Uncertainty Interval
Lao People's Democratic Republic	Male	10 to 14	1950	357.21	[326.96 - 445.79]	337	[308 - 421]
			1985	218.20	[178.49 - 263.87]	491	[401 - 593]
			2019	60.06	[47.17 - 70.55]	217	[170 - 255]
		15 to 19	1950	537.45	[494.26 - 659.24]	448	[412 - 550]
			1985	311.60	[250.37 - 387.17]	579	[465 - 720]
			2019	118.81	[94.01 - 139.48]	424	[335 - 497]
		20 to 24	1950	612.32	[581.52 - 713.58]	456	[433 - 531]
			1985	374.10	[305.67 - 450.87]	569	[465 - 686]
			2019	174.08	[130.21 - 202.90]	600	[449 - 699]
	Female	10 to 14	1950	245.40	[226.82 - 292.62]	244	[225 - 290]
			1985	168.73	[140.40 - 200.91]	387	[322 - 461]
			2019	41.81	[33.96 - 47.61]	146	[119 - 167]
		15 to 19	1950	323.91	[309.90 - 364.53]	273	[261 - 307]
			1985	221.51	[188.44 - 252.02]	436	[371 - 496]
			2019	69.15	[52.46 - 78.68]	242	[184 - 276]
		20 to 24	1950	471.91	[443.18 - 570.79]	340	[320 - 412]
			1985	313.08	[251.14 - 378.02]	524	[421 - 633]
			2019	94.29	[68.28 - 110.18]	323	[234 - 378]

Table S11: All-cause mortality rate per 100,000 population and number of deaths in 10-24 by sex 1950 - 2019: Malaysia

Location	Sex	Age	Year	Mortality per 100,000	Mortality per 100,000 uncertainty interval	Number	Number Uncertainty Interval
Malaysia	Male	10 to 14	1950	222.04	[216.27 - 243.75]	868	[845 - 953]
			1985	62.84	[62.09 - 63.51]	567	[561 - 573]
			2019	32.66	[26.95 - 39.44]	430	[355 - 519]
		15 to 19	1950	308.21	[300.13 - 340.17]	895	[872 - 988]
			1985	131.95	[130.53 - 133.42]	1,064	[1,052 - 1,076]
			2019	94.74	[75.48 - 117.88]	1,325	[1,056 - 1,649]
		20 to 24	1950	379.90	[371.81 - 415.20]	1,081	[1,058 - 1,182]
			1985	197.51	[195.58 - 199.53]	1,434	[1,420 - 1,449]
			2019	130.02	[107.60 - 156.28]	1,936	[1,602 - 2,327]
	Female	10 to 14	1950	171.57	[167.83 - 185.78]	641	[627 - 694]
			1985	46.52	[46.00 - 47.05]	404	[399 - 408]
			2019	20.76	[17.61 - 24.47]	258	[219 - 305]
		15 to 19	1950	212.51	[208.30 - 227.88]	637	[625 - 683]
			1985	63.85	[63.19 - 64.52]	512	[507 - 518]
			2019	32.03	[26.86 - 38.03]	425	[357 - 505]
		20 to 24	1950	312.72	[301.52 - 354.95]	892	[860 - 1,012]
			1985	83.34	[82.07 - 84.65]	626	[617 - 636]
			2019	40.62	[31.84 - 51.45]	564	[442 - 714]

Table S12: All-cause mortality rate per 100,000 population and number of deaths in 10-24 by sex 1950 - 2019: Maldives

Location	Sex	Age	Year	Mortality per 100,000	Mortality per 100,000 uncertainty interval	Number	Number Uncertainty Interval
Maldives	Male	10 to 14	1950	439.53	[404.85 - 554.03]	23	[21 - 29]
			1985	82.69	[77.76 - 87.88]	10	[9 - 11]
			2019	24.44	[21.60 - 27.86]	4	[4 - 5]
		15 to 19	1950	581.20	[532.47 - 725.46]	30	[27 - 37]
			1985	118.87	[113.21 - 124.61]	13	[12 - 13]
			2019	47.09	[40.10 - 55.39]	8	[6 - 9]
		20 to 24	1950	555.40	[520.25 - 666.60]	24	[23 - 29]
			1985	170.47	[163.31 - 176.84]	15	[15 - 16]
			2019	59.24	[52.06 - 67.83]	14	[13 - 16]
	Female	10 to 14	1950	327.00	[303.96 - 396.18]	17	[16 - 21]
			1985	118.90	[113.04 - 124.26]	14	[13 - 14]
			2019	16.76	[14.89 - 18.86]	3	[2 - 3]
		15 to 19	1950	407.22	[387.61 - 459.68]	20	[19 - 22]
			1985	164.41	[157.01 - 171.87]	17	[17 - 18]
			2019	22.33	[19.88 - 25.30]	3	[3 - 4]
		20 to 24	1950	642.34	[600.45 - 775.02]	24	[23 - 29]
			1985	255.47	[244.63 - 266.47]	23	[22 - 24]
			2019	25.98	[22.16 - 30.37]	4	[3 - 5]

Table S13: All-cause mortality rate per 100,000 population and number of deaths in 10-24 by sex 1950 - 2019: Myanmar

Location	Sex	Age	Year	Mortality per 100,000	Mortality per 100,000 uncertainty interval	Number	Number Uncertainty Interval
Myanmar	Male	10 to 14	1950	469.10	[435.85 - 562.86]	4,642	[4,313 - 5,570]
			1985	201.35	[163.84 - 238.09]	4,519	[3,677 - 5,344]
			2019	62.47	[51.43 - 78.73]	1,579	[1,300 - 1,990]
		15 to 19	1950	697.28	[646.45 - 855.20]	6,710	[6,221 - 8,230]
			1985	302.61	[242.39 - 380.22]	6,113	[4,896 - 7,681]
			2019	128.82	[104.15 - 163.35]	3,196	[2,584 - 4,053]
		20 to 24	1950	745.68	[700.52 - 959.53]	6,625	[6,224 - 8,525]
			1985	385.46	[310.54 - 483.09]	6,829	[5,501 - 8,558]
			2019	184.13	[134.71 - 238.68]	4,203	[3,075 - 5,448]
	Female	10 to 14	1950	278.40	[262.58 - 328.89]	2,767	[2,610 - 3,269]
			1985	144.70	[119.08 - 168.18]	3,173	[2,612 - 3,688]
			2019	39.30	[31.47 - 48.53]	971	[778 - 1,199]
		15 to 19	1950	354.66	[339.06 - 411.88]	3,365	[3,217 - 3,908]
			1985	194.00	[162.05 - 220.24]	3,948	[3,298 - 4,482]
			2019	64.52	[46.57 - 82.30]	1,601	[1,155 - 2,042]
		20 to 24	1950	538.32	[502.42 - 647.84]	4,511	[4,210 - 5,429]
			1985	267.71	[209.74 - 325.36]	4,881	[3,824 - 5,931]
			2019	84.93	[58.30 - 111.59]	2,017	[1,385 - 2,651]

Table S14: All-cause mortality rate per 100,000 population and number of deaths in 10-24 by sex 1950 - 2019: Philippines

Location	Sex	Age	Year	Mortality per 100,000	Mortality per 100,000 uncertainty interval	Number	Number Uncertainty Interval
Philippines	Male	10 to 14	1950	178.43	[172.65 - 195.97]	2,310	[2,235 - 2,537]
			1985	105.03	[97.21 - 113.40]	3,661	[3,388 - 3,953]
			2019	55.93	[47.58 - 65.19]	3,207	[2,728 - 3,737]
		15 to 19	1950	189.93	[182.90 - 211.79]	2,020	[1,945 - 2,253]
			1985	179.51	[161.33 - 200.58]	5,454	[4,901 - 6,094]
			2019	90.48	[71.99 - 111.76]	4,977	[3,960 - 6,148]
		20 to 24	1950	308.46	[296.84 - 341.82]	2,715	[2,613 - 3,009]
			1985	317.92	[286.67 - 355.18]	8,146	[7,345 - 9,101]
			2019	160.25	[128.25 - 198.50]	8,300	[6,643 - 10,281]
	Female	10 to 14	1950	132.11	[129.08 - 142.66]	1,750	[1,710 - 1,890]
			1985	86.48	[80.26 - 93.10]	2,885	[2,677 - 3,106]
			2019	43.64	[37.23 - 50.60]	2,354	[2,009 - 2,730]
		15 to 19	1950	153.20	[149.79 - 164.73]	1,687	[1,649 - 1,814]
			1985	96.73	[89.45 - 104.52]	2,959	[2,736 - 3,197]
			2019	51.45	[43.57 - 60.83]	2,697	[2,284 - 3,188]
		20 to 24	1950	263.31	[251.69 - 297.68]	2,400	[2,294 - 2,713]
			1985	124.56	[111.11 - 139.11]	3,352	[2,990 - 3,743]
			2019	76.51	[57.45 - 100.78]	3,831	[2,876 - 5,046]

Table S15: All-cause mortality rate per 100,000 population and number of deaths in 10-24 by sex 1950 - 2019: Sri Lanka

Location	Sex	Age	Year	Mortality per 100,000	Mortality per 100,000 uncertainty interval	Number	Number Uncertainty Interval
Sri Lanka	Male	10 to 14	1950	165.48	[164.93 - 167.60]	712	[709 - 721]
			1985	85.31	[81.41 - 91.73]	757	[722 - 814]
			2019	27.73	[22.24 - 34.44]	254	[204 - 315]
		15 to 19	1950	178.89	[178.15 - 181.48]	748	[745 - 759]
			1985	175.83	[163.67 - 197.00]	1,498	[1,394 - 1,678]
			2019	75.19	[57.01 - 98.01]	657	[498 - 856]
		20 to 24	1950	239.88	[238.94 - 243.03]	964	[961 - 977]
			1985	330.19	[305.70 - 373.28]	2,647	[2,450 - 2,992]
			2019	159.32	[125.94 - 199.28]	1,264	[999 - 1,581]
	Female	10 to 14	1950	186.38	[185.74 - 188.47]	743	[741 - 752]
			1985	64.30	[60.71 - 70.35]	552	[521 - 604]
			2019	23.12	[19.02 - 27.95]	207	[170 - 250]
		15 to 19	1950	225.85	[225.14 - 228.12]	874	[872 - 883]
			1985	135.31	[130.29 - 143.49]	1,125	[1,083 - 1,193]
			2019	47.67	[38.57 - 58.45]	413	[334 - 506]
		20 to 24	1950	424.00	[421.92 - 430.83]	1,578	[1,570 - 1,603]
			1985	165.47	[159.91 - 174.29]	1,313	[1,269 - 1,383]
			2019	57.68	[42.77 - 76.43]	469	[347 - 621]

Table S16: All-cause mortality rate per 100,000 population and number of deaths in 10-24 by sex 1950 - 2019: Thailand

Location	Sex	Age	Year	Mortality per 100,000	Mortality per 100,000 uncertainty interval	Number	Number Uncertainty Interval
Thailand	Male	10 to 14	1950	304.24	[291.87 - 349.04]	4,018	[3,855 - 4,610]
			1985	104.72	[96.34 - 113.79]	3,253	[2,993 - 3,535]
			2019	41.45	[35.56 - 48.17]	838	[719 - 974]
		15 to 19	1950	310.45	[296.37 - 359.80]	3,519	[3,359 - 4,078]
			1985	172.46	[156.35 - 190.55]	5,284	[4,790 - 5,838]
			2019	130.61	[101.55 - 165.69]	2,854	[2,219 - 3,621]
		20 to 24	1950	408.24	[393.16 - 457.97]	3,963	[3,816 - 4,446]
			1985	244.22	[221.96 - 267.79]	6,661	[6,054 - 7,304]
			2019	185.44	[146.30 - 232.46]	4,562	[3,599 - 5,719]
	Female	10 to 14	1950	206.78	[198.74 - 233.02]	2,671	[2,567 - 3,010]
			1985	79.88	[74.58 - 85.87]	2,418	[2,257 - 2,599]
			2019	27.88	[24.15 - 32.07]	535	[463 - 615]
		15 to 19	1950	226.35	[218.28 - 250.32]	2,552	[2,461 - 2,822]
			1985	102.12	[95.73 - 109.38]	3,036	[2,846 - 3,252]
			2019	44.18	[37.31 - 51.89]	932	[787 - 1,095]
		20 to 24	1950	323.90	[309.66 - 370.57]	3,056	[2,921 - 3,496]
			1985	111.63	[100.16 - 125.03]	3,026	[2,715 - 3,389]
			2019	51.58	[39.04 - 66.97]	1,252	[948 - 1,625]

Table S17: All-cause mortality rate per 100,000 population and number of deaths in 10-24 by sex 1950 - 2019: Timor-Leste

Location	Sex	Age	Year	Mortality per 100,000	Mortality per 100,000 uncertainty interval	Number	Number Uncertainty Interval
Timor-Leste	Male	10 to 14	1950	257.21	[237.86 - 325.07]	70	[65 - 89]
			1985	145.77	[113.39 - 169.55]	54	[42 - 63]
			2019	47.91	[42.01 - 55.36]	41	[36 - 47]
		15 to 19	1950	350.77	[324.05 - 447.59]	82	[76 - 105]
			1985	245.23	[199.36 - 280.94]	86	[70 - 98]
			2019	101.38	[85.44 - 113.68]	83	[70 - 93]
		20 to 24	1950	384.77	[359.36 - 470.07]	81	[76 - 99]
			1985	376.48	[315.80 - 423.21]	126	[106 - 142]
			2019	141.38	[109.45 - 165.50]	92	[72 - 108]
	Female	10 to 14	1950	207.67	[194.38 - 252.41]	54	[51 - 66]
			1985	139.93	[99.52 - 168.91]	47	[34 - 57]
			2019	35.14	[30.70 - 40.60]	28	[24 - 32]
		15 to 19	1950	287.70	[270.95 - 332.56]	67	[64 - 78]
			1985	192.05	[142.64 - 231.11]	65	[48 - 78]
			2019	59.19	[46.82 - 68.10]	46	[37 - 53]
		20 to 24	1950	408.84	[377.93 - 507.93]	84	[77 - 104]
			1985	245.73	[174.06 - 315.61]	83	[58 - 106]
			2019	78.31	[61.64 - 95.24]	52	[41 - 63]

Table S18: All-cause mortality rate per 100,000 population and number of deaths in 10-24 by sex 1950 - 2019: Viet Nam

Location	Sex	Age	Year	Mortality per 100,000	Mortality per 100,000 uncertainty interval	Number	Number Uncertainty Interval
Viet Nam	Male	10 to 14	1950	253.03	[238.07 - 302.33]	3,192	[3,003 - 3,813]
			1985	78.00	[61.82 - 104.29]	3,026	[2,398 - 4,046]
			2019	33.60	[29.22 - 38.35]	1,237	[1,075 - 1,411]
		15 to 19	1950	518.28	[495.89 - 588.65]	6,341	[6,067 - 7,202]
			1985	143.86	[111.63 - 188.81]	4,861	[3,772 - 6,379]
			2019	105.29	[92.06 - 119.09]	3,656	[3,196 - 4,135]
		20 to 24	1950	830.42	[803.63 - 922.10]	9,255	[8,957 - 10,277]
			1985	206.76	[148.55 - 276.64]	5,998	[4,310 - 8,026]
			2019	148.83	[117.50 - 173.05]	5,497	[4,340 - 6,392]
	Female	10 to 14	1950	179.73	[170.08 - 207.72]	2,339	[2,214 - 2,703]
			1985	48.78	[35.50 - 68.38]	1,825	[1,328 - 2,558]
			2019	18.82	[16.01 - 21.17]	639	[544 - 719]
		15 to 19	1950	256.36	[241.79 - 295.01]	3,183	[3,002 - 3,663]
			1985	72.79	[49.50 - 97.32]	2,519	[1,713 - 3,368]
			2019	35.48	[26.50 - 41.50]	1,164	[869 - 1,361]
		20 to 24	1950	307.54	[284.46 - 373.88]	3,456	[3,196 - 4,201]
			1985	96.28	[63.38 - 131.26]	3,037	[1,999 - 4,141]
			2019	41.62	[31.94 - 46.94]	1,486	[1,140 - 1,675]

Table S19: All-cause mortality rate per 100,000 population and number of deaths in 10-24 by sex 1950 - 2019: Oceania

Location	Sex	Age	Year	Mortality per 100,000	Mortality per 100,000 uncertainty interval	Number	Number Uncertainty Interval
Oceania	Male	10 to 14	1950	188.83	[178.55 - 230.20]	292	[276 - 356]
			1985	101.19	[89.05 - 113.22]	372	[327 - 416]
			2019	75.60	[63.67 - 88.30]	556	[468 - 649]
		15 to 19	1950	294.27	[278.27 - 364.13]	418	[396 - 518]
			1985	179.66	[157.24 - 202.86]	579	[507 - 654]
			2019	150.17	[122.96 - 184.33]	1,009	[826 - 1,238]
		20 to 24	1950	365.51	[346.93 - 430.27]	485	[460 - 570]
			1985	268.85	[236.37 - 301.60]	750	[660 - 842]
			2019	228.00	[195.42 - 274.29]	1,438	[1,232 - 1,730]
	Female	10 to 14	1950	141.73	[134.39 - 166.57]	192	[182 - 226]
			1985	81.32	[72.90 - 88.08]	273	[245 - 296]
			2019	57.63	[50.62 - 63.78]	382	[336 - 423]
		15 to 19	1950	211.26	[202.52 - 237.11]	251	[241 - 282]
			1985	125.26	[113.06 - 135.22]	368	[332 - 397]
			2019	98.07	[84.96 - 116.31]	595	[515 - 706]
		20 to 24	1950	312.49	[294.49 - 366.60]	340	[320 - 399]
			1985	173.68	[149.79 - 195.14]	449	[387 - 504]
			2019	146.02	[122.41 - 183.36]	861	[722 - 1,082]

Table S20: All-cause mortality rate per 100,000 population and number of deaths in 10-24 by sex 1950 - 2019: Fiji

Location	Sex	Age	Year	Mortality per 100,000	Mortality per 100,000 uncertainty interval	Number	Number Uncertainty Interval
Fiji	Male	10 to 14	1950	111.39	[108.80 - 119.16]	21	[21 - 23]
			1985	62.16	[57.17 - 67.28]	26	[24 - 28]
			2019	63.29	[51.21 - 77.90]	28	[22 - 34]
		15 to 19	1950	177.85	[174.92 - 188.81]	28	[28 - 30]
			1985	146.39	[136.63 - 157.32]	58	[54 - 62]
			2019	101.99	[80.12 - 128.90]	40	[31 - 50]
		20 to 24	1950	228.07	[225.04 - 239.40]	30	[29 - 31]
			1985	254.36	[238.78 - 272.21]	94	[88 - 101]
			2019	166.40	[135.22 - 204.39]	63	[51 - 78]
	Female	10 to 14	1950	116.27	[113.96 - 123.70]	21	[21 - 23]
			1985	70.58	[66.07 - 75.14]	29	[27 - 30]
			2019	45.07	[37.42 - 54.31]	19	[16 - 23]
		15 to 19	1950	189.90	[187.00 - 199.19]	28	[28 - 30]
			1985	132.23	[124.94 - 140.13]	51	[48 - 54]
			2019	81.14	[67.38 - 97.44]	30	[25 - 36]
		20 to 24	1950	272.57	[267.96 - 288.57]	34	[34 - 36]
			1985	152.22	[140.57 - 165.64]	56	[52 - 61]
			2019	93.11	[71.21 - 120.65]	34	[26 - 44]

Table S21: All-cause mortality rate per 100,000 population and number of deaths in 10-24 by sex 1950 - 2019: Kiribati

Location	Sex	Age	Year	Mortality per 100,000	Mortality per 100,000 uncertainty interval	Number	Number Uncertainty Interval
Kiribati	Male	10 to 14	1950	239.36	[229.67 - 274.25]	3	[3 - 4]
			1985	134.34	[110.82 - 163.60]	5	[4 - 7]
			2019	77.82	[66.41 - 90.87]	5	[4 - 6]
		15 to 19	1950	481.83	[459.67 - 568.50]	6	[6 - 7]
			1985	331.49	[268.30 - 404.23]	12	[10 - 14]
			2019	219.56	[184.59 - 263.60]	12	[10 - 15]
		20 to 24	1950	654.19	[617.47 - 765.97]	7	[7 - 9]
			1985	535.92	[452.34 - 633.38]	17	[14 - 20]
			2019	416.85	[356.68 - 492.31]	22	[19 - 26]
	Female	10 to 14	1950	165.53	[159.68 - 187.09]	3	[3 - 3]
			1985	96.33	[79.81 - 114.39]	4	[3 - 4]
			2019	58.87	[50.23 - 68.49]	4	[3 - 4]
		15 to 19	1950	261.00	[250.67 - 296.07]	4	[3 - 4]
			1985	180.55	[151.75 - 211.15]	6	[5 - 7]
			2019	123.47	[107.20 - 141.21]	7	[6 - 8]
		20 to 24	1950	412.33	[388.40 - 505.55]	5	[5 - 6]
			1985	272.28	[210.43 - 346.71]	9	[7 - 11]
			2019	192.87	[152.15 - 243.60]	10	[8 - 13]

Table S22: All-cause mortality rate per 100,000 population and number of deaths in 10-24 by sex 1950 - 2019: Marshall Islands

Location	Sex	Age	Year	Mortality per 100,000	Mortality per 100,000 uncertainty interval	Number	Number Uncertainty Interval
Marshall Islands	Male	10 to 14	1950	190.48	[181.95 - 213.18]	1	[1 - 1]
			1985	56.96	[51.16 - 63.71]	2	[1 - 2]
			2019	49.64	[40.73 - 61.87]	2	[1 - 2]
		15 to 19	1950	334.48	[313.05 - 412.54]	2	[2 - 2]
			1985	167.83	[148.83 - 189.27]	3	[3 - 4]
			2019	143.16	[119.46 - 180.05]	4	[3 - 5]
		20 to 24	1950	426.01	[402.19 - 551.80]	2	[2 - 3]
			1985	615.09	[552.53 - 681.13]	10	[9 - 11]
			2019	290.47	[245.95 - 354.17]	7	[6 - 9]
	Female	10 to 14	1950	120.46	[117.34 - 131.94]	1	[1 - 1]
			1985	48.39	[43.54 - 53.95]	1	[1 - 1]
			2019	41.14	[32.62 - 51.43]	1	[1 - 2]
		15 to 19	1950	180.64	[173.53 - 212.31]	1	[1 - 1]
			1985	95.62	[82.19 - 108.28]	2	[2 - 2]
			2019	91.43	[75.48 - 112.64]	2	[2 - 3]
		20 to 24	1950	277.93	[260.95 - 342.59]	1	[1 - 2]
			1985	131.10	[109.75 - 153.10]	2	[2 - 3]
			2019	154.68	[121.19 - 203.92]	4	[3 - 5]

Table S23: All-cause mortality rate per 100,000 population and number of deaths in 10-24 by sex 1950 - 2019: Micronesia (Federated States of)

Location	Sex	Age	Year	Mortality per 100,000	Mortality per 100,000 uncertainty interval	Number	Number Uncertainty Interval
Micronesia (Federated States of)	Male	10 to 14	1950	187.48	[180.09 - 217.57]	3	[3 - 4]
			1985	88.24	[75.86 - 103.86]	6	[5 - 7]
			2019	48.48	[41.13 - 56.50]	3	[2 - 3]
		15 to 19	1950	372.84	[353.48 - 439.51]	7	[7 - 8]
			1985	223.14	[184.64 - 267.75]	11	[9 - 13]
			2019	159.51	[130.76 - 193.13]	9	[8 - 11]
		20 to 24	1950	521.03	[472.52 - 620.99]	10	[9 - 12]
			1985	391.60	[318.86 - 464.60]	16	[13 - 19]
			2019	342.55	[283.21 - 409.74]	17	[14 - 20]
	Female	10 to 14	1950	127.94	[123.88 - 147.77]	2	[2 - 2]
			1985	63.31	[52.31 - 75.68]	4	[3 - 5]
			2019	39.32	[33.34 - 46.07]	2	[2 - 3]
		15 to 19	1950	207.49	[198.26 - 236.72]	3	[3 - 3]
			1985	125.40	[98.70 - 149.74]	6	[5 - 7]
			2019	89.42	[75.85 - 103.67]	5	[4 - 6]
		20 to 24	1950	334.04	[314.15 - 408.02]	5	[4 - 6]
			1985	185.47	[139.18 - 236.58]	7	[6 - 9]
			2019	161.22	[124.45 - 204.97]	7	[6 - 10]

Table S24: All-cause mortality rate per 100,000 population and number of deaths in 10-24 by sex 1950 - 2019: Papua New Guinea

Location	Sex	Age	Year	Mortality per 100,000	Mortality per 100,000 uncertainty interval	Number	Number Uncertainty Interval
Papua New Guinea	Male	10 to 14	1950	206.09	[190.81 - 265.90]	203	[188 - 262]
			1985	114.90	[97.93 - 131.50]	264	[225 - 302]
			2019	80.71	[67.60 - 93.66]	443	[371 - 514]
		15 to 19	1950	304.83	[282.34 - 403.38]	281	[261 - 372]
			1985	179.22	[148.86 - 211.01]	358	[298 - 422]
			2019	151.28	[121.77 - 188.13]	764	[615 - 950]
		20 to 24	1950	370.05	[345.52 - 461.20]	323	[302 - 403]
			1985	252.29	[210.92 - 295.34]	437	[366 - 512]
			2019	217.96	[183.32 - 269.50]	1,046	[880 - 1,294]
	Female	10 to 14	1950	148.84	[138.11 - 185.90]	126	[117 - 157]
			1985	90.41	[78.64 - 99.40]	187	[163 - 206]
			2019	61.29	[53.86 - 67.37]	300	[264 - 330]
		15 to 19	1950	216.57	[203.87 - 252.93]	163	[153 - 190]
			1985	127.97	[112.15 - 140.53]	229	[201 - 252]
			2019	99.87	[86.50 - 121.36]	449	[389 - 546]
		20 to 24	1950	310.29	[285.22 - 386.76]	220	[202 - 274]
			1985	178.82	[147.38 - 207.30]	280	[231 - 324]
			2019	146.30	[120.74 - 190.42]	650	[537 - 846]

Table S25: All-cause mortality rate per 100,000 population and number of deaths in 10-24 by sex 1950 - 2019: Samoa

Location	Sex	Age	Year	Mortality per 100,000	Mortality per 100,000 uncertainty interval	Number	Number Uncertainty Interval
Samoa	Male	10 to 14	1950	151.44	[141.34 - 182.97]	9	[8 - 10]
			1985	74.00	[62.65 - 88.91]	10	[8 - 12]
			2019	35.31	[29.32 - 41.93]	5	[4 - 6]
		15 to 19	1950	216.05	[203.83 - 260.51]	10	[10 - 13]
			1985	158.18	[139.51 - 190.44]	18	[16 - 22]
			2019	106.55	[92.38 - 122.61]	13	[11 - 15]
		20 to 24	1950	265.63	[250.90 - 311.69]	10	[10 - 12]
			1985	232.39	[204.48 - 283.37]	20	[17 - 24]
			2019	197.78	[145.59 - 252.01]	19	[14 - 24]
	Female	10 to 14	1950	121.13	[113.44 - 140.92]	6	[6 - 7]
			1985	47.99	[40.50 - 56.47]	5	[4 - 6]
			2019	26.62	[20.88 - 33.82]	3	[3 - 4]
		15 to 19	1950	177.09	[165.49 - 210.92]	8	[8 - 10]
			1985	78.41	[67.92 - 94.59]	8	[7 - 9]
			2019	62.92	[47.68 - 79.09]	7	[5 - 9]
		20 to 24	1950	242.87	[222.98 - 302.92]	9	[9 - 12]
			1985	110.39	[90.78 - 138.31]	8	[7 - 10]
			2019	101.48	[71.00 - 135.72]	9	[6 - 12]

Table S26: All-cause mortality rate per 100,000 population and number of deaths in 10-24 by sex 1950 - 2019: Solomon Islands

Location	Sex	Age	Year	Mortality per 100,000	Mortality per 100,000 uncertainty interval	Number	Number Uncertainty Interval
Solomon Islands	Male	10 to 14	1950	262.95	[250.66 - 313.27]	17	[17 - 21]
			1985	111.10	[91.60 - 136.92]	22	[18 - 27]
			2019	71.22	[60.61 - 83.04]	29	[24 - 33]
		15 to 19	1950	534.77	[507.14 - 650.77]	31	[29 - 37]
			1985	291.80	[236.41 - 361.37]	47	[38 - 58]
			2019	221.01	[183.72 - 264.80]	77	[64 - 93]
		20 to 24	1950	707.39	[671.59 - 848.24]	35	[34 - 42]
			1985	500.38	[416.38 - 602.32]	59	[49 - 71]
			2019	431.87	[363.64 - 511.98]	126	[106 - 150]
	Female	10 to 14	1950	191.09	[183.31 - 221.08]	12	[11 - 14]
			1985	91.07	[74.42 - 112.48]	17	[14 - 21]
			2019	61.89	[52.77 - 72.26]	23	[19 - 27]
		15 to 19	1950	301.47	[290.12 - 343.10]	16	[16 - 19]
			1985	177.00	[148.91 - 210.92]	27	[23 - 32]
			2019	131.90	[115.60 - 150.07]	43	[38 - 49]
		20 to 24	1950	553.43	[521.78 - 677.39]	26	[25 - 32]
			1985	314.69	[242.12 - 410.46]	38	[29 - 50]
			2019	263.92	[208.68 - 330.84]	76	[60 - 95]

Table S27: All-cause mortality rate per 100,000 population and number of deaths in 10-24 by sex 1950 - 2019: Tonga

Location	Sex	Age	Year	Mortality per 100,000	Mortality per 100,000 uncertainty interval	Number	Number Uncertainty Interval
Tonga	Male	10 to 14	1950	94.75	[89.69 - 111.30]	3	[3 - 3]
			1985	47.29	[42.26 - 52.80]	3	[3 - 4]
			2019	33.15	[27.24 - 39.75]	2	[2 - 2]
		15 to 19	1950	182.62	[172.91 - 229.58]	5	[5 - 6]
			1985	98.36	[90.37 - 108.21]	6	[5 - 6]
			2019	90.86	[76.53 - 109.95]	5	[4 - 6]
		20 to 24	1950	267.27	[252.09 - 341.61]	6	[6 - 7]
			1985	138.59	[125.60 - 151.63]	6	[5 - 7]
			2019	139.83	[112.54 - 189.10]	6	[5 - 8]
	Female	10 to 14	1950	75.35	[71.73 - 88.29]	2	[2 - 3]
			1985	33.86	[30.54 - 37.50]	2	[2 - 2]
			2019	21.56	[19.12 - 26.28]	1	[1 - 1]
		15 to 19	1950	122.20	[115.48 - 148.07]	3	[3 - 4]
			1985	51.48	[46.97 - 56.51]	3	[3 - 3]
			2019	40.84	[33.65 - 52.99]	2	[2 - 3]
		20 to 24	1950	198.88	[185.71 - 250.86]	4	[4 - 6]
			1985	77.39	[67.31 - 87.78]	3	[3 - 4]
			2019	58.73	[44.60 - 81.92]	3	[2 - 4]

Table S28: All-cause mortality rate per 100,000 population and number of deaths in 10-24 by sex 1950 - 2019: Vanuatu

Location	Sex	Age	Year	Mortality per 100,000	Mortality per 100,000 uncertainty interval	Number	Number Uncertainty Interval
Vanuatu	Male	10 to 14	1950	222.92	[213.11 - 254.35]	7	[7 - 8]
			1985	83.23	[73.43 - 95.30]	7	[6 - 8]
			2019	56.55	[48.96 - 65.93]	10	[9 - 12]
		15 to 19	1950	363.20	[342.28 - 429.73]	10	[9 - 12]
			1985	198.17	[162.97 - 235.32]	14	[12 - 17]
			2019	157.23	[132.98 - 190.86]	23	[19 - 27]
		20 to 24	1950	437.56	[410.32 - 550.01]	10	[10 - 13]
			1985	340.89	[239.66 - 416.77]	21	[15 - 25]
			2019	309.92	[226.21 - 378.89]	40	[29 - 49]
	Female	10 to 14	1950	169.42	[163.26 - 190.52]	5	[5 - 5]
			1985	58.17	[49.64 - 68.27]	5	[4 - 5]
			2019	42.06	[34.95 - 50.00]	7	[6 - 8]
		15 to 19	1950	239.84	[231.91 - 267.43]	6	[6 - 6]
			1985	107.63	[81.00 - 132.65]	7	[6 - 9]
			2019	90.71	[69.64 - 108.30]	13	[10 - 15]
		20 to 24	1950	384.45	[361.16 - 455.61]	8	[7 - 9]
			1985	151.58	[112.56 - 198.13]	9	[7 - 12]
			2019	133.73	[94.19 - 180.10]	18	[13 - 25]

Table S29: All-cause mortality rate per 100,000 population and number of deaths in 10-24 by sex 1950 - 2019: Central Europe, Eastern Europe, and Central Asia

Location	Sex	Age	Year	Mortality per 100,000	Mortality per 100,000 uncertainty interval	Number	Number Uncertainty Interval
Central Europe, Eastern Europe, and Central Asia	Male	10 to 14	1950	110.84	[107.24 - 124.05]	16,667	[16,125 - 18,653]
			1985	54.82	[53.90 - 55.70]	9,025	[8,874 - 9,171]
			2019	30.15	[27.97 - 32.65]	3,993	[3,705 - 4,324]
		15 to 19	1950	188.63	[181.71 - 211.97]	25,477	[24,542 - 28,629]
			1985	112.62	[111.72 - 113.52]	17,428	[17,290 - 17,567]
			2019	76.75	[70.27 - 83.69]	9,150	[8,377 - 9,977]
		20 to 24	1950	274.65	[266.29 - 303.92]	37,228	[36,095 - 41,195]
			1985	197.65	[197.11 - 198.22]	33,049	[32,958 - 33,145]
			2019	147.96	[136.40 - 159.45]	18,120	[16,704 - 19,526]
	Female	10 to 14	1950	75.92	[73.64 - 83.86]	11,462	[11,118 - 12,661]
			1985	32.20	[31.83 - 32.57]	5,117	[5,058 - 5,176]
			2019	20.66	[19.31 - 22.11]	2,580	[2,412 - 2,762]
		15 to 19	1950	117.28	[114.23 - 127.34]	16,124	[15,704 - 17,507]
			1985	49.99	[49.60 - 50.37]	7,422	[7,365 - 7,479]
			2019	37.99	[35.48 - 40.76]	4,287	[4,004 - 4,600]
		20 to 24	1950	153.38	[148.01 - 173.13]	22,803	[22,005 - 25,739]
			1985	65.89	[65.45 - 66.35]	10,732	[10,659 - 10,805]
			2019	52.55	[47.82 - 58.32]	6,165	[5,611 - 6,842]

Table S30: All-cause mortality rate per 100,000 population and number of deaths in 10-24 by sex 1950 - 2019: Central Asia

Location	Sex	Age	Year	Mortality per 100,000	Mortality per 100,000 uncertainty interval	Number	Number Uncertainty Interval
Central Asia	Male	10 to 14	1950	179.30	[166.01 - 222.92]	2,741	[2,538 - 3,407]
			1985	65.53	[63.10 - 67.94]	2,281	[2,197 - 2,365]
			2019	42.76	[38.42 - 48.22]	1,768	[1,588 - 1,994]
		15 to 19	1950	250.69	[230.87 - 315.63]	3,520	[3,242 - 4,432]
			1985	100.80	[98.42 - 102.96]	3,282	[3,204 - 3,352]
			2019	78.18	[70.05 - 87.76]	2,848	[2,552 - 3,197]
		20 to 24	1950	315.71	[295.94 - 380.36]	3,878	[3,635 - 4,672]
			1985	166.01	[163.91 - 168.11]	5,333	[5,265 - 5,401]
			2019	132.82	[122.31 - 145.51]	5,086	[4,684 - 5,572]
	Female	10 to 14	1950	109.26	[101.72 - 132.30]	1,660	[1,546 - 2,010]
			1985	42.77	[41.60 - 43.90]	1,446	[1,407 - 1,484]
			2019	29.44	[26.97 - 32.53]	1,140	[1,044 - 1,259]
		15 to 19	1950	143.26	[133.79 - 173.13]	2,097	[1,958 - 2,534]
			1985	57.41	[56.24 - 58.56]	1,806	[1,769 - 1,842]
			2019	48.70	[44.63 - 53.47]	1,675	[1,535 - 1,839]
		20 to 24	1950	194.55	[177.60 - 253.57]	2,648	[2,418 - 3,452]
			1985	92.81	[91.01 - 94.80]	3,009	[2,950 - 3,073]
			2019	71.14	[62.93 - 80.80]	2,630	[2,327 - 2,987]

Table S31: All-cause mortality rate per 100,000 population and number of deaths in 10-24 by sex 1950 - 2019: Armenia

Location	Sex	Age	Year	Mortality per 100,000	Mortality per 100,000 uncertainty interval	Number	Number Uncertainty Interval
Armenia	Male	10 to 14	1950	193.43	[179.77 - 242.28]	163	[152 - 204]
			1985	57.14	[53.98 - 60.33]	88	[83 - 93]
			2019	21.88	[19.34 - 24.60]	22	[20 - 25]
		15 to 19	1950	232.47	[214.32 - 296.75]	183	[169 - 234]
			1985	54.86	[51.92 - 58.07]	88	[83 - 93]
			2019	53.73	[46.18 - 61.92]	48	[41 - 55]
		20 to 24	1950	263.30	[246.36 - 321.02]	180	[168 - 219]
			1985	86.58	[82.46 - 90.93]	153	[145 - 160]
			2019	79.10	[68.48 - 90.96]	77	[67 - 89]
	Female	10 to 14	1950	108.28	[101.30 - 131.92]	95	[89 - 116]
			1985	40.01	[38.35 - 41.86]	58	[56 - 61]
			2019	14.54	[13.06 - 16.10]	13	[12 - 14]
		15 to 19	1950	117.63	[109.52 - 143.68]	100	[93 - 122]
			1985	31.60	[30.40 - 32.89]	48	[46 - 50]
			2019	16.28	[14.43 - 18.26]	13	[11 - 14]
		20 to 24	1950	153.14	[139.16 - 201.54]	117	[106 - 154]
			1985	48.71	[46.02 - 51.60]	88	[83 - 93]
			2019	24.86	[20.46 - 29.94]	23	[19 - 27]

Table S32: All-cause mortality rate per 100,000 population and number of deaths in 10-24 by sex 1950 - 2019: Azerbaijan

Location	Sex	Age	Year	Mortality per 100,000	Mortality per 100,000 uncertainty interval	Number	Number Uncertainty Interval
Azerbaijan	Male	10 to 14	1950	288.37	[268.06 - 357.42]	517	[481 - 641]
			1985	57.42	[53.61 - 61.40]	211	[197 - 226]
			2019	39.60	[33.07 - 48.11]	158	[132 - 191]
		15 to 19	1950	359.86	[333.07 - 450.17]	602	[558 - 754]
			1985	68.12	[63.58 - 72.82]	268	[250 - 286]
			2019	73.31	[59.75 - 91.13]	261	[212 - 324]
		20 to 24	1950	405.74	[381.35 - 484.59]	556	[523 - 664]
			1985	111.40	[104.30 - 118.70]	414	[388 - 441]
			2019	113.12	[95.96 - 135.20]	458	[388 - 547]
	Female	10 to 14	1950	173.95	[162.94 - 209.92]	314	[294 - 379]
			1985	40.58	[38.43 - 42.79]	139	[131 - 146]
			2019	30.17	[26.93 - 34.04]	104	[93 - 117]
		15 to 19	1950	190.89	[179.19 - 229.45]	335	[314 - 402]
			1985	42.93	[40.86 - 45.13]	158	[150 - 166]
			2019	37.69	[33.24 - 42.80]	119	[105 - 135]
		20 to 24	1950	253.92	[232.28 - 329.79]	381	[348 - 495]
			1985	67.51	[62.55 - 72.82]	261	[242 - 282]
			2019	49.20	[39.80 - 60.27]	190	[154 - 233]

Table S33: All-cause mortality rate per 100,000 population and number of deaths in 10-24 by sex 1950 - 2019: Georgia

Location	Sex	Age	Year	Mortality per 100,000	Mortality per 100,000 uncertainty interval	Number	Number Uncertainty Interval
Georgia	Male	10 to 14	1950	194.27	[179.86 - 242.32]	360	[334 - 449]
			1985	52.56	[48.83 - 56.44]	121	[112 - 130]
			2019	26.77	[23.67 - 30.08]	31	[27 - 35]
		15 to 19	1950	248.48	[228.94 - 314.67]	436	[401 - 552]
			1985	82.74	[76.04 - 89.63]	189	[173 - 204]
			2019	77.93	[67.36 - 89.33]	82	[71 - 94]
		20 to 24	1950	307.61	[288.02 - 371.75]	482	[452 - 583]
			1985	147.07	[135.16 - 159.37]	346	[318 - 375]
			2019	134.10	[116.83 - 153.42]	149	[130 - 170]
	Female	10 to 14	1950	96.27	[89.85 - 117.14]	191	[178 - 233]
			1985	29.37	[27.68 - 31.20]	64	[61 - 68]
			2019	15.92	[14.24 - 17.71]	16	[15 - 18]
		15 to 19	1950	119.76	[111.88 - 145.87]	237	[222 - 289]
			1985	34.19	[32.22 - 36.34]	74	[70 - 79]
			2019	30.23	[26.88 - 33.83]	28	[25 - 31]
		20 to 24	1950	152.68	[139.15 - 200.94]	286	[261 - 376]
			1985	56.91	[51.59 - 62.48]	138	[125 - 152]
			2019	41.75	[34.61 - 50.16]	42	[35 - 50]

Table S34: All-cause mortality rate per 100,000 population and number of deaths in 10-24 by sex 1950 - 2019: Kazakhstan

Location	Sex	Age	Year	Mortality per 100,000	Mortality per 100,000 uncertainty interval	Number	Number Uncertainty Interval
Kazakhstan	Male	10 to 14	1950	139.98	[129.91 - 172.91]	609	[565 - 752]
			1985	61.64	[58.74 - 64.59]	493	[470 - 516]
			2019	36.39	[32.56 - 40.45]	273	[244 - 303]
		15 to 19	1950	245.10	[226.02 - 306.23]	965	[889 - 1,205]
			1985	125.03	[122.33 - 127.76]	921	[901 - 941]
			2019	78.77	[69.71 - 88.69]	475	[420 - 535]
		20 to 24	1950	363.34	[340.88 - 434.02]	1,248	[1,171 - 1,491]
			1985	231.07	[229.37 - 232.87]	1,804	[1,791 - 1,818]
			2019	149.31	[133.45 - 166.73]	938	[839 - 1,048]
	Female	10 to 14	1950	85.74	[80.31 - 101.61]	353	[331 - 419]
			1985	36.99	[35.70 - 38.37]	289	[279 - 300]
			2019	23.35	[21.15 - 25.61]	167	[152 - 184]
		15 to 19	1950	126.56	[118.81 - 149.64]	492	[462 - 582]
			1985	56.28	[54.86 - 57.78]	392	[382 - 402]
			2019	42.81	[38.84 - 46.77]	251	[228 - 274]
		20 to 24	1950	169.59	[155.34 - 217.73]	638	[585 - 820]
			1985	85.65	[84.35 - 86.93]	651	[641 - 661]
			2019	56.24	[48.65 - 64.86]	351	[304 - 405]

Table S35: All-cause mortality rate per 100,000 population and number of deaths in 10-24 by sex 1950 - 2019: Kyrgyzstan

Location	Sex	Age	Year	Mortality per 100,000	Mortality per 100,000 uncertainty interval	Number	Number Uncertainty Interval
Kyrgyzstan	Male	10 to 14	1950	180.75	[167.55 - 225.48]	168	[156 - 210]
			1985	74.21	[69.86 - 78.45]	168	[158 - 177]
			2019	33.97	[31.53 - 36.66]	104	[97 - 112]
		15 to 19	1950	241.29	[222.53 - 304.51]	200	[185 - 253]
			1985	104.51	[100.18 - 109.17]	212	[203 - 221]
			2019	56.47	[50.83 - 62.68]	151	[136 - 167]
		20 to 24	1950	311.78	[291.79 - 376.91]	235	[220 - 285]
			1985	192.12	[185.27 - 199.21]	384	[370 - 398]
			2019	99.65	[89.85 - 110.24]	288	[259 - 318]
	Female	10 to 14	1950	107.88	[100.17 - 131.68]	100	[93 - 122]
			1985	49.05	[47.23 - 51.00]	110	[105 - 114]
			2019	21.02	[19.55 - 22.64]	62	[57 - 67]
		15 to 19	1950	149.10	[138.83 - 182.27]	130	[121 - 159]
			1985	63.27	[61.19 - 65.43]	123	[119 - 127]
			2019	35.25	[32.54 - 38.10]	91	[84 - 99]
		20 to 24	1950	200.93	[183.09 - 264.29]	171	[156 - 225]
			1985	100.83	[96.41 - 105.27]	199	[190 - 207]
			2019	47.51	[41.44 - 54.27]	134	[117 - 153]

Table S36: All-cause mortality rate per 100,000 population and number of deaths in 10-24 by sex 1950 - 2019: Mongolia

Location	Sex	Age	Year	Mortality per 100,000	Mortality per 100,000 uncertainty interval	Number	Number Uncertainty Interval
Mongolia	Male	10 to 14	1950	343.74	[318.59 - 432.96]	121	[113 - 153]
			1985	123.49	[107.78 - 139.09]	153	[133 - 172]
			2019	48.61	[39.36 - 61.23]	66	[53 - 83]
		15 to 19	1950	428.49	[396.72 - 541.45]	157	[145 - 198]
			1985	181.46	[150.39 - 213.32]	196	[163 - 231]
			2019	96.06	[75.03 - 123.80]	107	[84 - 138]
		20 to 24	1950	374.32	[351.80 - 449.51]	125	[117 - 150]
			1985	243.75	[204.42 - 285.85]	231	[194 - 271]
			2019	196.18	[158.17 - 243.24]	243	[196 - 302]
	Female	10 to 14	1950	224.52	[211.52 - 272.04]	77	[73 - 94]
			1985	76.92	[67.97 - 86.55]	93	[82 - 104]
			2019	28.65	[23.76 - 35.02]	37	[31 - 46]
		15 to 19	1950	300.04	[285.61 - 347.08]	106	[101 - 123]
			1985	105.73	[93.43 - 118.87]	111	[99 - 125]
			2019	48.64	[40.09 - 59.41]	52	[43 - 64]
		20 to 24	1950	499.44	[462.29 - 631.25]	158	[146 - 200]
			1985	182.06	[144.68 - 224.02]	173	[138 - 213]
			2019	76.11	[57.64 - 101.48]	92	[69 - 122]

Table S37: All-cause mortality rate per 100,000 population and number of deaths in 10-24 by sex 1950 - 2019: Tajikistan

Location	Sex	Age	Year	Mortality per 100,000	Mortality per 100,000 uncertainty interval	Number	Number Uncertainty Interval
Tajikistan	Male	10 to 14	1950	189.00	[173.53 - 234.60]	171	[157 - 212]
			1985	67.49	[63.19 - 72.06]	195	[183 - 208]
			2019	43.42	[38.32 - 50.12]	213	[188 - 246]
		15 to 19	1950	246.21	[225.40 - 309.87]	205	[187 - 257]
			1985	90.53	[84.04 - 97.27]	229	[213 - 246]
			2019	64.23	[53.66 - 77.84]	285	[238 - 346]
		20 to 24	1950	288.20	[270.27 - 350.56]	211	[198 - 256]
			1985	140.45	[130.71 - 150.19]	322	[299 - 344]
			2019	110.37	[92.55 - 133.44]	496	[416 - 599]
	Female	10 to 14	1950	132.71	[121.30 - 162.23]	121	[110 - 147]
			1985	44.00	[41.47 - 46.49]	124	[117 - 131]
			2019	28.95	[25.68 - 32.88]	133	[118 - 151]
		15 to 19	1950	178.07	[164.15 - 219.44]	160	[147 - 197]
			1985	65.91	[62.30 - 69.56]	167	[158 - 177]
			2019	41.94	[36.85 - 48.26]	176	[155 - 203]
		20 to 24	1950	250.37	[226.98 - 327.93]	198	[179 - 259]
			1985	123.37	[112.71 - 134.38]	293	[268 - 319]
			2019	66.42	[53.26 - 84.24]	288	[231 - 365]

Table S38: All-cause mortality rate per 100,000 population and number of deaths in 10-24 by sex 1950 - 2019: Turkmenistan

Location	Sex	Age	Year	Mortality per 100,000	Mortality per 100,000 uncertainty interval	Number	Number Uncertainty Interval
Turkmenistan	Male	10 to 14	1950	186.00	[172.80 - 231.66]	126	[118 - 158]
			1985	60.55	[56.93 - 64.68]	121	[114 - 129]
			2019	42.70	[37.37 - 49.75]	98	[86 - 115]
		15 to 19	1950	267.63	[246.91 - 336.07]	168	[155 - 211]
			1985	115.88	[112.30 - 119.66]	208	[202 - 215]
			2019	87.39	[72.14 - 106.69]	183	[151 - 224]
		20 to 24	1950	335.54	[313.91 - 402.74]	185	[174 - 223]
			1985	177.29	[173.89 - 180.46]	297	[291 - 302]
			2019	161.57	[133.92 - 199.96]	353	[293 - 437]
	Female	10 to 14	1950	122.77	[114.40 - 148.57]	82	[76 - 99]
			1985	46.08	[44.16 - 48.19]	90	[86 - 94]
			2019	31.01	[27.23 - 35.56]	66	[58 - 76]
		15 to 19	1950	166.08	[154.79 - 199.84]	109	[102 - 131]
			1985	65.56	[63.47 - 67.81]	115	[111 - 119]
			2019	72.18	[62.86 - 83.46]	137	[119 - 158]
		20 to 24	1950	238.96	[217.91 - 310.18]	149	[136 - 193]
			1985	112.47	[109.12 - 115.83]	191	[186 - 197]
			2019	97.82	[77.25 - 125.38]	188	[148 - 241]

Table S39: All-cause mortality rate per 100,000 population and number of deaths in 10-24 by sex 1950 - 2019: Uzbekistan

Location	Sex	Age	Year	Mortality per 100,000	Mortality per 100,000 uncertainty interval	Number	Number Uncertainty Interval
Uzbekistan	Male	10 to 14	1950	140.70	[129.53 - 175.65]	503	[463 - 628]
			1985	67.06	[64.05 - 70.33]	732	[699 - 768]
			2019	49.99	[44.19 - 56.94]	803	[710 - 915]
		15 to 19	1950	186.84	[170.92 - 237.39]	605	[553 - 768]
			1985	97.69	[95.39 - 100.00]	971	[948 - 994]
			2019	86.14	[73.80 - 100.61]	1,257	[1,076 - 1,468]
		20 to 24	1950	229.32	[214.65 - 280.86]	655	[613 - 802]
			1985	144.46	[143.20 - 145.76]	1,384	[1,372 - 1,396]
			2019	138.25	[119.58 - 159.64]	2,085	[1,803 - 2,407]
	Female	10 to 14	1950	91.87	[84.51 - 112.94]	327	[301 - 402]
			1985	44.72	[43.15 - 46.34]	480	[463 - 497]
			2019	35.56	[32.00 - 39.82]	540	[486 - 605]
		15 to 19	1950	126.58	[116.90 - 156.23]	429	[396 - 529]
			1985	62.72	[61.15 - 64.34]	618	[603 - 634]
			2019	58.03	[51.92 - 64.94]	808	[723 - 905]
		20 to 24	1950	175.70	[159.87 - 233.08]	550	[500 - 729]
			1985	104.37	[102.94 - 105.83]	1,014	[1,000 - 1,028]
			2019	90.23	[74.90 - 108.02]	1,322	[1,097 - 1,582]

Table S40: All-cause mortality rate per 100,000 population and number of deaths in 10-24 by sex 1950 - 2019: Central Europe

Location	Sex	Age	Year	Mortality per 100,000	Mortality per 100,000 uncertainty interval	Number	Number Uncertainty Interval
Central Europe	Male	10 to 14	1950	134.42	[132.69 - 140.31]	5,262	[5,195 - 5,493]
			1985	43.19	[42.84 - 43.53]	2,173	[2,155 - 2,190]
			2019	16.34	[14.51 - 18.27]	511	[454 - 572]
		15 to 19	1950	226.21	[223.49 - 235.82]	9,225	[9,114 - 9,617]
			1985	90.11	[89.48 - 90.72]	4,147	[4,118 - 4,175]
			2019	47.68	[41.44 - 54.27]	1,413	[1,228 - 1,608]
		20 to 24	1950	336.79	[333.94 - 346.50]	13,000	[12,890 - 13,375]
			1985	141.72	[140.89 - 142.48]	6,163	[6,127 - 6,197]
			2019	80.49	[70.73 - 90.36]	2,540	[2,232 - 2,851]
	Female	10 to 14	1950	102.21	[101.15 - 105.67]	4,084	[4,042 - 4,223]
			1985	25.54	[25.41 - 25.70]	1,225	[1,219 - 1,233]
			2019	11.30	[10.24 - 12.49]	332	[301 - 367]
		15 to 19	1950	178.25	[176.85 - 182.56]	7,361	[7,303 - 7,538]
			1985	41.66	[41.42 - 41.91]	1,830	[1,820 - 1,841]
			2019	20.46	[18.37 - 22.74]	572	[514 - 636]
		20 to 24	1950	244.63	[242.28 - 253.02]	10,115	[10,018 - 10,462]
			1985	49.83	[49.44 - 50.27]	2,081	[2,065 - 2,099]
			2019	24.40	[20.88 - 28.46]	731	[625 - 852]

Table S41: All-cause mortality rate per 100,000 population and number of deaths in 10-24 by sex 1950 - 2019: Albania

Location	Sex	Age	Year	Mortality per 100,000	Mortality per 100,000 uncertainty interval	Number	Number Uncertainty Interval
Albania	Male	10 to 14	1950	148.47	[142.64 - 170.39]	116	[112 - 134]
			1985	63.59	[60.12 - 67.19]	113	[107 - 120]
			2019	36.79	[31.29 - 43.00]	30	[26 - 35]
		15 to 19	1950	220.87	[211.24 - 257.75]	148	[142 - 173]
			1985	77.83	[75.21 - 80.38]	132	[127 - 136]
			2019	59.33	[45.93 - 75.82]	60	[46 - 76]
		20 to 24	1950	266.78	[256.64 - 306.01]	152	[146 - 175]
			1985	112.65	[110.09 - 115.23]	180	[175 - 184]
			2019	107.71	[84.18 - 136.88]	122	[95 - 155]
	Female	10 to 14	1950	114.10	[109.90 - 133.86]	78	[75 - 91]
			1985	37.65	[36.17 - 39.26]	61	[58 - 63]
			2019	22.42	[19.44 - 25.77]	17	[14 - 19]
		15 to 19	1950	168.01	[161.27 - 196.95]	96	[92 - 113]
			1985	46.61	[45.03 - 48.39]	74	[72 - 77]
			2019	26.10	[21.92 - 30.85]	24	[20 - 28]
		20 to 24	1950	214.57	[203.45 - 261.68]	107	[102 - 131]
			1985	58.96	[56.70 - 61.21]	95	[91 - 98]
			2019	39.78	[29.60 - 53.03]	41	[31 - 55]

Table S42: All-cause mortality rate per 100,000 population and number of deaths in 10-24 by sex 1950 - 2019: Bosnia and Herzegovina

Location	Sex	Age	Year	Mortality per 100,000	Mortality per 100,000 uncertainty interval	Number	Number Uncertainty Interval
Bosnia and Herzegovina	Male	10 to 14	1950	182.46	[181.62 - 185.35]	356	[354 - 361]
			1985	34.62	[33.89 - 35.40]	70	[68 - 71]
			2019	16.28	[13.33 - 19.82]	14	[11 - 17]
		15 to 19	1950	338.18	[336.04 - 345.16]	593	[589 - 605]
			1985	76.54	[74.49 - 78.83]	168	[163 - 173]
			2019	44.01	[34.54 - 55.69]	42	[33 - 53]
		20 to 24	1950	541.84	[538.66 - 552.06]	776	[772 - 791]
			1985	115.80	[113.19 - 118.58]	252	[247 - 258]
			2019	75.77	[61.77 - 92.44]	83	[68 - 101]
	Female	10 to 14	1950	188.70	[187.80 - 191.82]	371	[369 - 377]
			1985	19.59	[19.18 - 20.04]	38	[37 - 38]
			2019	11.98	[10.05 - 14.27]	10	[8 - 12]
		15 to 19	1950	332.88	[331.41 - 337.83]	603	[601 - 612]
			1985	43.60	[42.52 - 44.79]	90	[87 - 92]
			2019	18.56	[15.37 - 22.38]	17	[14 - 20]
		20 to 24	1950	510.06	[506.49 - 522.92]	786	[780 - 806]
			1985	45.93	[44.54 - 47.44]	92	[89 - 95]
			2019	20.79	[15.93 - 26.96]	22	[17 - 28]

Table S43: All-cause mortality rate per 100,000 population and number of deaths in 10-24 by sex 1950 - 2019: Bulgaria

Location	Sex	Age	Year	Mortality per 100,000	Mortality per 100,000 uncertainty interval	Number	Number Uncertainty Interval
Bulgaria	Male	10 to 14	1950	135.57	[135.05 - 137.30]	410	[408 - 415]
			1985	52.37	[50.72 - 54.02]	179	[173 - 184]
			2019	24.95	[20.41 - 30.34]	44	[36 - 53]
		15 to 19	1950	258.25	[257.16 - 262.11]	854	[851 - 867]
			1985	101.95	[97.12 - 106.49]	313	[299 - 327]
			2019	68.57	[54.20 - 86.01]	112	[88 - 140]
		20 to 24	1950	381.18	[379.56 - 386.27]	1,283	[1,278 - 1,301]
			1985	145.15	[138.53 - 151.52]	434	[414 - 453]
			2019	107.47	[88.11 - 130.57]	169	[139 - 205]
	Female	10 to 14	1950	125.98	[125.31 - 128.00]	374	[372 - 380]
			1985	30.04	[29.13 - 30.96]	96	[93 - 99]
			2019	16.34	[13.69 - 19.48]	27	[23 - 32]
		15 to 19	1950	242.77	[241.61 - 246.43]	795	[791 - 807]
			1985	53.42	[51.67 - 55.22]	156	[151 - 161]
			2019	33.65	[27.97 - 40.39]	52	[43 - 62]
		20 to 24	1950	348.00	[346.11 - 354.93]	1,155	[1,148 - 1,178]
			1985	59.32	[56.12 - 62.78]	170	[161 - 180]
			2019	40.03	[30.65 - 51.85]	59	[45 - 77]

Table S44: All-cause mortality rate per 100,000 population and number of deaths in 10-24 by sex 1950 - 2019: Croatia

Location	Sex	Age	Year	Mortality per 100,000	Mortality per 100,000 uncertainty interval	Number	Number Uncertainty Interval
Croatia	Male	10 to 14	1950	137.10	[136.34 - 139.91]	264	[262 - 269]
			1985	35.93	[34.56 - 37.37]	64	[61 - 66]
			2019	12.66	[10.36 - 15.45]	14	[12 - 17]
		15 to 19	1950	279.66	[277.95 - 285.32]	528	[524 - 538]
			1985	84.78	[80.29 - 89.43]	151	[143 - 159]
			2019	42.77	[33.53 - 54.21]	47	[37 - 59]
		20 to 24	1950	452.07	[449.79 - 459.53]	792	[788 - 805]
			1985	144.50	[136.96 - 152.24]	276	[261 - 290]
			2019	67.58	[55.14 - 82.69]	86	[70 - 105]
	Female	10 to 14	1950	120.05	[119.44 - 122.47]	220	[219 - 225]
			1985	21.66	[20.90 - 22.44]	37	[36 - 38]
			2019	8.35	[6.99 - 9.98]	9	[7 - 10]
		15 to 19	1950	229.92	[228.80 - 234.10]	427	[425 - 435]
			1985	35.47	[34.09 - 36.96]	61	[59 - 64]
			2019	14.60	[12.08 - 17.70]	15	[12 - 18]
		20 to 24	1950	294.30	[292.21 - 301.18]	535	[531 - 547]
			1985	42.04	[39.39 - 45.03]	77	[73 - 83]
			2019	16.91	[12.95 - 21.90]	21	[16 - 27]

Table S45: All-cause mortality rate per 100,000 population and number of deaths in 10-24 by sex 1950 - 2019: Czechia

Location	Sex	Age	Year	Mortality per 100,000	Mortality per 100,000 uncertainty interval	Number	Number Uncertainty Interval
Czechia	Male	10 to 14	1950	90.31	[89.97 - 91.50]	262	[261 - 265]
			1985	33.10	[32.49 - 33.68]	145	[143 - 148]
			2019	9.74	[8.23 - 11.54]	29	[24 - 34]
		15 to 19	1950	167.18	[166.57 - 169.53]	525	[523 - 533]
			1985	84.05	[82.90 - 85.27]	290	[286 - 295]
			2019	36.87	[30.02 - 45.13]	90	[73 - 110]
		20 to 24	1950	230.49	[229.69 - 233.45]	782	[779 - 792]
			1985	113.15	[111.86 - 114.55]	393	[389 - 398]
			2019	70.95	[59.78 - 84.13]	172	[145 - 204]
	Female	10 to 14	1950	61.94	[61.65 - 62.91]	176	[176 - 179]
			1985	16.92	[16.67 - 17.16]	71	[70 - 72]
			2019	7.63	[6.56 - 8.88]	21	[18 - 25]
		15 to 19	1950	98.41	[97.97 - 99.81]	307	[305 - 311]
			1985	33.53	[33.05 - 34.02]	111	[109 - 113]
			2019	17.79	[15.17 - 20.87]	41	[35 - 48]
		20 to 24	1950	139.89	[139.12 - 142.26]	488	[485 - 496]
			1985	36.56	[35.74 - 37.37]	122	[119 - 125]
			2019	22.74	[18.26 - 28.29]	52	[42 - 65]

Table S46: All-cause mortality rate per 100,000 population and number of deaths in 10-24 by sex 1950 - 2019: Hungary

Location	Sex	Age	Year	Mortality per 100,000	Mortality per 100,000 uncertainty interval	Number	Number Uncertainty Interval
Hungary	Male	10 to 14	1950	124.83	[124.43 - 126.33]	435	[433 - 440]
			1985	31.77	[31.29 - 32.25]	129	[127 - 131]
			2019	14.26	[12.03 - 16.90]	36	[30 - 43]
		15 to 19	1950	203.41	[202.66 - 206.09]	765	[762 - 775]
			1985	100.05	[98.87 - 101.26]	354	[350 - 358]
			2019	37.08	[30.49 - 44.92]	92	[76 - 112]
		20 to 24	1950	309.43	[308.36 - 313.15]	1,206	[1,202 - 1,220]
			1985	147.18	[145.65 - 148.71]	476	[471 - 481]
			2019	57.89	[49.22 - 67.93]	154	[131 - 181]
	Female	10 to 14	1950	86.47	[86.21 - 87.48]	320	[319 - 324]
			1985	20.69	[20.41 - 20.98]	79	[78 - 80]
			2019	9.18	[7.91 - 10.64]	22	[19 - 25]
		15 to 19	1950	156.81	[156.33 - 158.42]	611	[609 - 618]
			1985	42.87	[42.35 - 43.45]	143	[141 - 145]
			2019	15.71	[13.46 - 18.32]	37	[32 - 43]
		20 to 24	1950	206.69	[205.66 - 209.85]	835	[831 - 848]
			1985	49.84	[48.92 - 50.80]	155	[152 - 158]
			2019	21.59	[17.33 - 26.79]	54	[44 - 67]

Table S47: All-cause mortality rate per 100,000 population and number of deaths in 10-24 by sex 1950 - 2019: North Macedonia

Location	Sex	Age	Year	Mortality per 100,000	Mortality per 100,000 uncertainty interval	Number	Number Uncertainty Interval
North Macedonia	Male	10 to 14	1950	253.29	[251.52 - 259.17]	193	[191 - 197]
			1985	35.76	[34.90 - 36.68]	34	[33 - 34]
			2019	20.03	[16.41 - 24.37]	12	[10 - 14]
		15 to 19	1950	336.33	[332.89 - 347.54]	241	[239 - 249]
			1985	55.23	[53.53 - 56.86]	51	[49 - 52]
			2019	40.18	[31.64 - 50.62]	25	[20 - 32]
		20 to 24	1950	523.15	[518.39 - 540.21]	320	[317 - 331]
			1985	71.36	[69.22 - 73.47]	64	[62 - 66]
			2019	62.04	[50.79 - 75.34]	46	[38 - 56]
	Female	10 to 14	1950	233.57	[232.23 - 238.87]	166	[165 - 170]
			1985	25.36	[24.68 - 26.01]	22	[22 - 23]
			2019	12.97	[10.89 - 15.43]	7	[6 - 9]
		15 to 19	1950	367.30	[365.14 - 375.20]	255	[254 - 261]
			1985	35.74	[34.72 - 36.69]	31	[30 - 32]
			2019	20.15	[16.71 - 24.21]	12	[10 - 14]
		20 to 24	1950	571.06	[565.05 - 592.54]	359	[355 - 372]
			1985	45.05	[42.98 - 47.12]	38	[36 - 40]
			2019	25.66	[19.68 - 33.22]	18	[14 - 23]

Table S48: All-cause mortality rate per 100,000 population and number of deaths in 10-24 by sex 1950 - 2019: Montenegro

Location	Sex	Age	Year	Mortality per 100,000	Mortality per 100,000 uncertainty interval	Number	Number Uncertainty Interval
Montenegro	Male	10 to 14	1950	84.78	[83.68 - 88.35]	22	[22 - 23]
			1985	32.43	[31.29 - 33.70]	9	[9 - 9]
			2019	17.53	[14.77 - 20.82]	3	[3 - 4]
		15 to 19	1950	149.16	[146.13 - 158.42]	34	[33 - 36]
			1985	51.51	[49.54 - 53.61]	15	[14 - 15]
			2019	33.49	[27.53 - 40.54]	7	[6 - 9]
		20 to 24	1950	244.77	[239.76 - 258.98]	45	[44 - 48]
			1985	105.76	[102.19 - 109.61]	31	[30 - 32]
			2019	80.99	[69.09 - 94.90]	17	[15 - 20]
	Female	10 to 14	1950	86.03	[85.07 - 89.12]	22	[21 - 22]
			1985	24.31	[23.56 - 25.04]	6	[6 - 7]
			2019	9.71	[8.60 - 11.02]	2	[2 - 2]
		15 to 19	1950	142.70	[140.94 - 148.22]	33	[32 - 34]
			1985	36.52	[35.37 - 37.64]	10	[9 - 10]
			2019	20.14	[17.79 - 22.75]	4	[3 - 4]
		20 to 24	1950	201.89	[197.73 - 215.41]	40	[39 - 43]
			1985	48.87	[46.45 - 51.18]	13	[12 - 14]
			2019	35.83	[30.64 - 42.03]	7	[6 - 8]

Table S49: All-cause mortality rate per 100,000 population and number of deaths in 10-24 by sex 1950 - 2019: Poland

Location	Sex	Age	Year	Mortality per 100,000	Mortality per 100,000 uncertainty interval	Number	Number Uncertainty Interval
Poland	Male	10 to 14	1950	149.80	[149.48 - 150.94]	1,528	[1,525 - 1,540]
			1985	36.53	[36.25 - 36.81]	543	[539 - 547]
			2019	13.08	[11.23 - 15.10]	132	[114 - 153]
		15 to 19	1950	245.22	[244.66 - 247.02]	2,736	[2,730 - 2,757]
			1985	92.55	[91.90 - 93.19]	1,192	[1,183 - 1,200]
			2019	49.32	[40.04 - 60.30]	460	[373 - 562]
		20 to 24	1950	383.36	[382.63 - 385.92]	3,828	[3,821 - 3,854]
			1985	157.42	[156.43 - 158.37]	2,133	[2,120 - 2,146]
			2019	90.21	[73.79 - 109.84]	958	[783 - 1,166]
	Female	10 to 14	1950	100.38	[100.13 - 101.18]	1,131	[1,129 - 1,140]
			1985	20.69	[20.53 - 20.88]	295	[292 - 297]
			2019	9.45	[8.28 - 10.81]	91	[80 - 104]
		15 to 19	1950	176.34	[175.98 - 177.64]	2,088	[2,083 - 2,103]
			1985	34.27	[34.02 - 34.56]	419	[416 - 422]
			2019	19.28	[16.59 - 22.45]	171	[147 - 199]
		20 to 24	1950	229.45	[228.79 - 231.69]	2,797	[2,789 - 2,825]
			1985	42.60	[42.12 - 43.14]	555	[548 - 562]
			2019	21.16	[16.23 - 27.40]	215	[165 - 279]

Table S50: All-cause mortality rate per 100,000 population and number of deaths in 10-24 by sex 1950 - 2019: Romania

Location	Sex	Age	Year	Mortality per 100,000	Mortality per 100,000 uncertainty interval	Number	Number Uncertainty Interval
Romania	Male	10 to 14	1950	106.60	[98.74 - 133.66]	884	[819 - 1,109]
			1985	60.28	[59.72 - 60.93]	602	[597 - 609]
			2019	24.38	[21.59 - 27.73]	138	[122 - 157]
		15 to 19	1950	151.37	[138.14 - 196.90]	1,277	[1,166 - 1,662]
			1985	89.68	[88.93 - 90.48]	912	[904 - 920]
			2019	59.24	[50.02 - 69.83]	313	[264 - 369]
		20 to 24	1950	192.60	[178.81 - 239.97]	1,528	[1,418 - 1,904]
			1985	144.80	[143.77 - 145.90]	1,024	[1,016 - 1,031]
			2019	85.98	[73.08 - 100.59]	434	[369 - 508]
	Female	10 to 14	1950	73.11	[68.32 - 89.52]	610	[570 - 747]
			1985	34.63	[34.27 - 34.97]	332	[328 - 335]
			2019	16.43	[14.68 - 18.47]	88	[79 - 99]
		15 to 19	1950	96.27	[89.89 - 117.66]	794	[741 - 970]
			1985	48.16	[47.70 - 48.60]	476	[472 - 481]
			2019	25.87	[22.74 - 29.47]	129	[113 - 147]
		20 to 24	1950	132.29	[120.15 - 176.55]	1,049	[953 - 1,400]
			1985	69.55	[68.60 - 70.50]	479	[472 - 485]
			2019	29.80	[24.01 - 36.71]	143	[115 - 176]

Table S51: All-cause mortality rate per 100,000 population and number of deaths in 10-24 by sex 1950 - 2019: Serbia

Location	Sex	Age	Year	Mortality per 100,000	Mortality per 100,000 uncertainty interval	Number	Number Uncertainty Interval
Serbia	Male	10 to 14	1950	155.69	[155.14 - 157.69]	532	[531 - 539]
			1985	50.50	[49.87 - 51.21]	195	[192 - 198]
			2019	11.03	[9.46 - 12.74]	30	[26 - 35]
		15 to 19	1950	275.70	[274.60 - 279.60]	965	[961 - 979]
			1985	101.39	[100.06 - 102.75]	342	[337 - 346]
			2019	32.57	[26.26 - 39.86]	89	[72 - 109]
		20 to 24	1950	449.69	[447.91 - 455.47]	1,521	[1,515 - 1,540]
			1985	148.78	[147.05 - 150.54]	517	[511 - 523]
			2019	56.13	[45.71 - 68.51]	153	[125 - 187]
	Female	10 to 14	1950	132.68	[132.13 - 134.69]	438	[436 - 444]
			1985	33.71	[33.25 - 34.21]	126	[124 - 128]
			2019	8.26	[7.22 - 9.43]	20	[17 - 22]
		15 to 19	1950	262.65	[261.65 - 266.35]	932	[929 - 946]
			1985	51.93	[51.23 - 52.69]	165	[162 - 167]
			2019	14.72	[12.57 - 17.07]	37	[32 - 43]
		20 to 24	1950	389.44	[387.35 - 396.36]	1,365	[1,358 - 1,389]
			1985	55.60	[54.41 - 56.82]	182	[178 - 186]
			2019	20.22	[15.48 - 26.22]	52	[40 - 68]

Table S52: All-cause mortality rate per 100,000 population and number of deaths in 10-24 by sex 1950 - 2019: Slovakia

Location	Sex	Age	Year	Mortality per 100,000	Mortality per 100,000 uncertainty interval	Number	Number Uncertainty Interval
Slovakia	Male	10 to 14	1950	113.70	[113.11 - 115.61]	167	[166 - 170]
			1985	30.76	[30.21 - 31.30]	68	[67 - 69]
			2019	14.69	[12.03 - 17.92]	21	[17 - 26]
		15 to 19	1950	218.19	[216.80 - 223.06]	331	[329 - 338]
			1985	86.59	[85.09 - 88.01]	167	[164 - 169]
			2019	41.67	[32.71 - 52.71]	56	[44 - 71]
		20 to 24	1950	305.48	[303.60 - 311.88]	452	[449 - 461]
			1985	125.71	[123.76 - 127.64]	259	[255 - 263]
			2019	72.56	[59.23 - 88.53]	111	[90 - 135]
	Female	10 to 14	1950	80.40	[79.94 - 82.26]	119	[118 - 121]
			1985	21.99	[21.60 - 22.41]	47	[46 - 48]
			2019	11.34	[9.52 - 13.50]	15	[13 - 18]
		15 to 19	1950	166.91	[165.99 - 170.52]	254	[253 - 260]
			1985	35.61	[34.97 - 36.28]	66	[65 - 67]
			2019	20.51	[17.01 - 24.67]	26	[22 - 32]
		20 to 24	1950	250.54	[248.40 - 257.64]	381	[378 - 392]
			1985	36.42	[35.42 - 37.46]	72	[70 - 74]
			2019	25.65	[19.73 - 33.10]	37	[29 - 48]

Table S53: All-cause mortality rate per 100,000 population and number of deaths in 10-24 by sex 1950 - 2019: Slovenia

Location	Sex	Age	Year	Mortality per 100,000	Mortality per 100,000 uncertainty interval	Number	Number Uncertainty Interval
Slovenia	Male	10 to 14	1950	136.61	[135.60 - 139.64]	93	[92 - 95]
			1985	29.99	[27.83 - 32.20]	22	[20 - 24]
			2019	13.88	[12.11 - 16.07]	7	[6 - 8]
		15 to 19	1950	323.86	[321.32 - 332.36]	228	[226 - 233]
			1985	82.20	[73.45 - 91.12]	61	[54 - 68]
			2019	40.42	[32.54 - 50.69]	19	[15 - 24]
		20 to 24	1950	514.21	[510.36 - 526.77]	314	[312 - 322]
			1985	163.11	[147.13 - 179.98]	125	[113 - 138]
			2019	67.13	[54.37 - 84.15]	34	[28 - 43]
	Female	10 to 14	1950	98.33	[97.67 - 100.46]	60	[59 - 61]
			1985	23.26	[21.56 - 24.99]	16	[15 - 17]
			2019	7.73	[6.80 - 8.84]	4	[3 - 4]
		15 to 19	1950	241.30	[239.77 - 246.25]	163	[162 - 166]
			1985	39.44	[36.19 - 42.82]	29	[26 - 31]
			2019	15.89	[13.65 - 18.59]	7	[6 - 8]
		20 to 24	1950	324.92	[321.72 - 336.15]	219	[217 - 227]
			1985	42.93	[37.17 - 49.25]	32	[28 - 37]
			2019	19.48	[15.34 - 25.24]	9	[7 - 12]

Table S54: All-cause mortality rate per 100,000 population and number of deaths in 10-24 by sex 1950 - 2019: Eastern Europe

Location	Sex	Age	Year	Mortality per 100,000	Mortality per 100,000 uncertainty interval	Number	Number Uncertainty Interval
Eastern Europe	Male	10 to 14	1950	90.45	[86.30 - 104.59]	8,678	[8,279 - 10,034]
			1985	57.49	[56.56 - 58.44]	4,571	[4,497 - 4,647]
			2019	28.65	[26.17 - 31.44]	1,714	[1,565 - 1,881]
		15 to 19	1950	158.28	[149.91 - 188.53]	12,701	[12,029 - 15,128]
			1985	131.26	[130.33 - 132.19]	10,000	[9,929 - 10,070]
			2019	91.99	[81.06 - 103.90]	4,889	[4,308 - 5,522]
		20 to 24	1950	240.16	[226.32 - 285.61]	20,333	[19,161 - 24,181]
			1985	235.31	[234.83 - 235.79]	21,553	[21,509 - 21,597]
			2019	199.46	[176.56 - 224.07]	10,494	[9,290 - 11,789]
	Female	10 to 14	1950	59.59	[57.13 - 68.67]	5,710	[5,474 - 6,580]
			1985	31.71	[31.28 - 32.15]	2,445	[2,412 - 2,480]
			2019	19.51	[17.97 - 21.27]	1,108	[1,021 - 1,208]
		15 to 19	1950	81.44	[78.14 - 93.81]	6,642	[6,373 - 7,651]
			1985	51.79	[51.34 - 52.27]	3,786	[3,753 - 3,821]
			2019	40.40	[36.85 - 44.42]	2,040	[1,861 - 2,243]
		20 to 24	1950	106.61	[99.43 - 133.58]	9,990	[9,318 - 12,517]
			1985	63.62	[63.34 - 63.89]	5,642	[5,617 - 5,666]
			2019	55.64	[47.58 - 65.72]	2,804	[2,398 - 3,312]

Table S55: All-cause mortality rate per 100,000 population and number of deaths in 10-24 by sex 1950 - 2019: Belarus

Location	Sex	Age	Year	Mortality per 100,000	Mortality per 100,000 uncertainty interval	Number	Number Uncertainty Interval
Belarus	Male	10 to 14	1950	85.84	[79.61 - 107.97]	352	[327 - 443]
			1985	48.33	[45.16 - 51.65]	183	[171 - 196]
			2019	17.85	[14.40 - 22.52]	46	[37 - 58]
		15 to 19	1950	119.18	[108.46 - 156.26]	425	[387 - 558]
			1985	100.61	[97.33 - 103.88]	375	[363 - 387]
			2019	55.72	[43.56 - 72.34]	127	[99 - 165]
		20 to 24	1950	184.99	[171.15 - 233.32]	578	[535 - 729]
			1985	195.32	[193.29 - 197.20]	840	[832 - 848]
			2019	128.49	[104.63 - 160.29]	306	[249 - 382]
	Female	10 to 14	1950	53.11	[49.56 - 65.69]	242	[226 - 300]
			1985	30.84	[29.32 - 32.47]	114	[108 - 120]
			2019	14.44	[11.95 - 17.76]	35	[29 - 43]
		15 to 19	1950	67.34	[62.52 - 83.85]	275	[256 - 343]
			1985	40.25	[38.80 - 41.81]	148	[142 - 153]
			2019	25.32	[20.84 - 31.38]	54	[45 - 67]
		20 to 24	1950	82.65	[74.86 - 112.42]	325	[294 - 442]
			1985	53.19	[52.12 - 54.37]	222	[218 - 227]
			2019	32.80	[24.93 - 44.01]	74	[56 - 99]

Table S56: All-cause mortality rate per 100,000 population and number of deaths in 10-24 by sex 1950 - 2019: Estonia

Location	Sex	Age	Year	Mortality per 100,000	Mortality per 100,000 uncertainty interval	Number	Number Uncertainty Interval
Estonia	Male	10 to 14	1950	90.90	[84.16 - 113.51]	39	[36 - 49]
			1985	53.52	[52.00 - 55.01]	30	[30 - 31]
			2019	13.57	[11.72 - 15.83]	5	[4 - 6]
		15 to 19	1950	183.20	[167.06 - 237.99]	68	[62 - 89]
			1985	115.80	[112.34 - 119.19]	62	[60 - 63]
			2019	50.75	[41.08 - 62.19]	16	[13 - 20]
		20 to 24	1950	264.46	[245.24 - 330.15]	91	[84 - 113]
			1985	205.31	[200.15 - 210.52]	116	[113 - 119]
			2019	107.84	[87.84 - 131.16]	35	[29 - 43]
	Female	10 to 14	1950	53.65	[49.97 - 65.51]	22	[21 - 27]
			1985	31.01	[30.03 - 31.97]	17	[16 - 17]
			2019	10.72	[9.35 - 12.34]	4	[3 - 4]
		15 to 19	1950	80.78	[75.04 - 99.29]	33	[31 - 41]
			1985	48.75	[47.13 - 50.26]	23	[23 - 24]
			2019	22.23	[19.05 - 26.04]	7	[6 - 8]
		20 to 24	1950	98.23	[88.78 - 131.70]	47	[42 - 63]
			1985	65.25	[62.06 - 68.47]	36	[34 - 37]
			2019	33.76	[25.99 - 43.51]	11	[8 - 14]

Table S57: All-cause mortality rate per 100,000 population and number of deaths in 10-24 by sex 1950 - 2019: Latvia

Location	Sex	Age	Year	Mortality per 100,000	Mortality per 100,000 uncertainty interval	Number	Number Uncertainty Interval
Latvia	Male	10 to 14	1950	94.68	[87.69 - 118.60]	76	[70 - 95]
			1985	68.18	[66.52 - 69.86]	62	[60 - 63]
			2019	16.07	[13.90 - 18.70]	8	[7 - 10]
		15 to 19	1950	163.39	[148.77 - 213.35]	123	[112 - 161]
			1985	151.23	[147.87 - 154.53]	139	[135 - 142]
			2019	58.62	[47.73 - 71.30]	26	[22 - 32]
		20 to 24	1950	245.24	[226.82 - 307.76]	167	[155 - 210]
			1985	230.59	[226.14 - 234.95]	240	[235 - 244]
			2019	112.44	[91.99 - 136.03]	50	[41 - 60]
	Female	10 to 14	1950	52.45	[48.77 - 64.11]	45	[42 - 55]
			1985	30.94	[30.19 - 31.70]	27	[26 - 28]
			2019	13.54	[11.26 - 16.55]	7	[5 - 8]
		15 to 19	1950	74.42	[68.94 - 91.44]	65	[60 - 79]
			1985	45.77	[44.66 - 46.87]	39	[38 - 40]
			2019	22.59	[18.56 - 27.91]	10	[8 - 12]
		20 to 24	1950	94.17	[85.10 - 126.11]	84	[76 - 113]
			1985	57.72	[55.66 - 60.11]	57	[55 - 59]
			2019	35.41	[26.41 - 48.30]	15	[11 - 20]

Table S58: All-cause mortality rate per 100,000 population and number of deaths in 10-24 by sex 1950 - 2019: Lithuania

Location	Sex	Age	Year	Mortality per 100,000	Mortality per 100,000 uncertainty interval	Number	Number Uncertainty Interval
Lithuania	Male	10 to 14	1950	90.04	[83.66 - 113.08]	104	[97 - 131]
			1985	51.66	[50.54 - 52.75]	71	[70 - 73]
			2019	22.35	[19.77 - 25.42]	15	[13 - 17]
		15 to 19	1950	167.87	[152.70 - 219.37]	182	[165 - 237]
			1985	141.57	[138.90 - 144.24]	203	[200 - 207]
			2019	62.49	[52.58 - 73.88]	44	[37 - 52]
		20 to 24	1950	228.54	[211.47 - 287.33]	217	[201 - 273]
			1985	213.79	[210.18 - 217.51]	332	[326 - 337]
			2019	110.59	[93.58 - 129.82]	93	[79 - 109]
	Female	10 to 14	1950	56.40	[52.80 - 70.02]	70	[66 - 87]
			1985	33.24	[32.57 - 33.96]	45	[44 - 46]
			2019	13.15	[11.73 - 14.80]	8	[8 - 9]
		15 to 19	1950	77.49	[72.12 - 96.35]	94	[87 - 117]
			1985	50.50	[49.51 - 51.55]	68	[67 - 69]
			2019	23.72	[20.82 - 27.05]	16	[14 - 18]
		20 to 24	1950	95.75	[86.65 - 130.68]	110	[100 - 150]
			1985	61.47	[59.56 - 63.38]	90	[87 - 93]
			2019	27.33	[21.86 - 33.86]	22	[17 - 27]

Table S59: All-cause mortality rate per 100,000 population and number of deaths in 10-24 by sex 1950 - 2019: Republic of Moldova

Location	Sex	Age	Year	Mortality per 100,000	Mortality per 100,000 uncertainty interval	Number	Number Uncertainty Interval
Republic of Moldova	Male	10 to 14	1950	168.44	[155.95 - 209.33]	205	[190 - 255]
			1985	70.52	[66.91 - 74.34]	131	[124 - 138]
			2019	28.25	[25.44 - 31.24]	30	[27 - 33]
		15 to 19	1950	225.04	[208.29 - 282.43]	262	[242 - 328]
			1985	108.47	[105.44 - 111.77]	179	[174 - 184]
			2019	56.58	[50.64 - 63.03]	57	[51 - 63]
		20 to 24	1950	326.62	[307.21 - 393.44]	352	[331 - 424]
			1985	212.42	[209.38 - 215.50]	373	[367 - 378]
			2019	108.23	[97.85 - 119.55]	116	[105 - 128]
	Female	10 to 14	1950	102.06	[95.34 - 121.91]	141	[131 - 168]
			1985	40.51	[38.86 - 42.12]	73	[70 - 76]
			2019	19.26	[17.51 - 21.04]	19	[18 - 21]
		15 to 19	1950	123.73	[116.48 - 143.94]	168	[158 - 195]
			1985	49.18	[47.66 - 50.64]	79	[77 - 82]
			2019	25.00	[22.73 - 27.25]	24	[22 - 26]
		20 to 24	1950	149.82	[138.10 - 188.11]	195	[179 - 244]
			1985	69.05	[67.61 - 70.46]	128	[125 - 131]
			2019	29.23	[25.43 - 33.53]	30	[26 - 34]

Table S60: All-cause mortality rate per 100,000 population and number of deaths in 10-24 by sex 1950 - 2019: Russian Federation

Location	Sex	Age	Year	Mortality per 100,000	Mortality per 100,000 uncertainty interval	Number	Number Uncertainty Interval
Russian Federation	Male	10 to 14	1950	91.70	[86.74 - 108.21]	6,364	[6,020 - 7,510]
			1985	59.92	[59.66 - 60.20]	3,135	[3,121 - 3,149]
			2019	26.98	[23.96 - 30.40]	1,150	[1,021 - 1,296]
		15 to 19	1950	168.14	[155.88 - 209.96]	9,419	[8,732 - 11,762]
			1985	143.44	[143.00 - 143.89]	7,130	[7,108 - 7,152]
			2019	82.27	[69.50 - 97.49]	3,112	[2,629 - 3,688]
		20 to 24	1950	255.28	[236.96 - 318.80]	15,895	[14,755 - 19,850]
			1985	254.80	[254.17 - 255.46]	15,918	[15,879 - 15,959]
			2019	190.35	[161.86 - 221.85]	6,890	[5,859 - 8,030]
	Female	10 to 14	1950	61.09	[58.01 - 71.79]	4,145	[3,935 - 4,871]
			1985	31.27	[31.13 - 31.40]	1,586	[1,579 - 1,593]
			2019	18.77	[16.78 - 21.05]	762	[681 - 854]
		15 to 19	1950	85.57	[81.00 - 101.66]	4,671	[4,421 - 5,549]
			1985	54.44	[54.21 - 54.64]	2,588	[2,578 - 2,598]
			2019	39.02	[34.43 - 44.10]	1,406	[1,241 - 1,589]
		20 to 24	1950	109.85	[99.51 - 148.23]	7,244	[6,562 - 9,775]
			1985	66.32	[65.97 - 66.68]	3,971	[3,950 - 3,992]
			2019	55.38	[44.72 - 67.83]	1,926	[1,555 - 2,359]

Table S61: All-cause mortality rate per 100,000 population and number of deaths in 10-24 by sex 1950 - 2019: Ukraine

Location	Sex	Age	Year	Mortality per 100,000	Mortality per 100,000 uncertainty interval	Number	Number Uncertainty Interval
Ukraine	Male	10 to 14	1950	81.04	[76.81 - 96.94]	1,525	[1,445 - 1,824]
			1985	51.27	[48.08 - 54.62]	959	[899 - 1,022]
			2019	38.35	[33.41 - 43.79]	460	[401 - 525]
		15 to 19	1950	126.74	[117.18 - 161.29]	2,191	[2,025 - 2,788]
			1985	105.00	[101.92 - 107.94]	1,913	[1,857 - 1,966]
			2019	142.75	[117.64 - 171.07]	1,506	[1,241 - 1,805]
		20 to 24	1950	185.44	[171.70 - 233.80]	3,008	[2,785 - 3,792]
			1985	187.56	[186.70 - 188.44]	3,734	[3,717 - 3,752]
			2019	264.62	[220.11 - 314.18]	3,004	[2,499 - 3,567]
	Female	10 to 14	1950	53.25	[50.43 - 62.93]	1,040	[985 - 1,229]
			1985	32.17	[30.75 - 33.68]	583	[558 - 611]
			2019	24.17	[21.20 - 27.24]	273	[240 - 308]
		15 to 19	1950	70.07	[66.09 - 84.29]	1,334	[1,258 - 1,604]
			1985	47.78	[46.31 - 49.35]	840	[814 - 868]
			2019	52.55	[45.36 - 60.35]	524	[452 - 602]
		20 to 24	1950	98.93	[89.49 - 133.29]	1,980	[1,791 - 2,667]
			1985	57.53	[57.01 - 57.99]	1,138	[1,128 - 1,147]
			2019	67.17	[52.14 - 85.29]	728	[565 - 924]

Table S62: All-cause mortality rate per 100,000 population and number of deaths in 10-24 by sex 1950 - 2019: High-income

Location	Sex	Age	Year	Mortality per 100,000	Mortality per 100,000 uncertainty interval	Number	Number Uncertainty Interval
High-income	Male	10 to 14	1950	111.34	[110.51 - 114.13]	29,803	[29,581 - 30,548]
			1985	31.85	[31.67 - 32.04]	11,018	[10,955 - 11,084]
			2019	13.12	[12.81 - 13.44]	4,164	[4,064 - 4,266]
		15 to 19	1950	226.72	[224.85 - 233.30]	57,153	[56,682 - 58,812]
			1985	94.13	[93.90 - 94.38]	34,297	[34,213 - 34,388]
			2019	48.44	[47.68 - 49.23]	15,636	[15,389 - 15,889]
		20 to 24	1950	345.61	[342.42 - 356.36]	84,769	[83,985 - 87,405]
			1985	131.72	[131.53 - 131.91]	48,909	[48,840 - 48,980]
			2019	86.02	[85.34 - 86.71]	28,800	[28,573 - 29,031]
	Female	10 to 14	1950	89.25	[88.56 - 91.56]	23,237	[23,055 - 23,836]
			1985	20.06	[19.97 - 20.17]	6,605	[6,572 - 6,640]
			2019	9.80	[9.69 - 9.91]	2,962	[2,930 - 2,998]
		15 to 19	1950	144.91	[144.06 - 147.77]	36,407	[36,193 - 37,125]
			1985	38.20	[38.09 - 38.31]	13,315	[13,277 - 13,354]
			2019	22.64	[22.44 - 22.87]	6,921	[6,859 - 6,989]
		20 to 24	1950	193.29	[192.62 - 195.72]	48,888	[48,719 - 49,502]
			1985	46.83	[46.73 - 46.94]	16,987	[16,950 - 17,026]
			2019	30.89	[30.59 - 31.21]	9,833	[9,738 - 9,934]

Table S63: All-cause mortality rate per 100,000 population and number of deaths in 10-24 by sex 1950 - 2019: High-income Asia Pacific

Location	Sex	Age	Year	Mortality per 100,000	Mortality per 100,000 uncertainty interval	Number	Number Uncertainty Interval
High-income Asia Pacific	Male	10 to 14	1950	232.38	[228.81 - 244.12]	13,761	[13,550 - 14,457]
			1985	30.90	[30.19 - 31.66]	2,410	[2,354 - 2,469]
			2019	9.04	[8.85 - 9.24]	377	[369 - 385]
		15 to 19	1950	515.31	[507.04 - 544.70]	28,207	[27,754 - 29,815]
			1985	90.51	[89.67 - 91.34]	6,476	[6,416 - 6,536]
			2019	26.13	[25.70 - 26.59]	1,212	[1,192 - 1,233]
		20 to 24	1950	915.97	[900.39 - 968.78]	43,783	[43,039 - 46,308]
			1985	104.26	[103.87 - 104.68]	6,971	[6,945 - 6,999]
			2019	44.89	[44.22 - 45.56]	2,289	[2,255 - 2,323]
	Female	10 to 14	1950	208.85	[205.76 - 219.09]	11,945	[11,768 - 12,530]
			1985	20.03	[19.65 - 20.43]	1,476	[1,449 - 1,506]
			2019	6.71	[6.63 - 6.80]	264	[261 - 268]
		15 to 19	1950	323.27	[319.42 - 335.57]	17,752	[17,540 - 18,428]
			1985	35.62	[35.20 - 36.09]	2,415	[2,386 - 2,446]
			2019	13.30	[13.13 - 13.48]	574	[567 - 582]
		20 to 24	1950	462.73	[459.18 - 473.98]	23,017	[22,840 - 23,577]
			1985	50.32	[50.04 - 50.61]	3,219	[3,202 - 3,238]
			2019	20.77	[20.35 - 21.23]	993	[973 - 1,014]

Table S64: All-cause mortality rate per 100,000 population and number of deaths in 10-24 by sex 1950 - 2019: Brunei Darussalam

Location	Sex	Age	Year	Mortality per 100,000	Mortality per 100,000 uncertainty interval	Number	Number Uncertainty Interval
Brunei Darussalam	Male	10 to 14	1950	155.17	[151.41 - 169.54]	6	[5 - 6]
			1985	66.54	[61.67 - 71.18]	8	[8 - 9]
			2019	25.33	[21.59 - 29.72]	4	[4 - 5]
		15 to 19	1950	233.08	[225.38 - 263.92]	8	[8 - 9]
			1985	139.89	[128.09 - 152.13]	17	[15 - 18]
			2019	41.86	[35.44 - 49.36]	8	[7 - 10]
		20 to 24	1950	271.88	[262.17 - 308.19]	9	[8 - 10]
			1985	247.14	[228.24 - 267.82]	32	[30 - 35]
			2019	82.89	[73.19 - 93.86]	17	[15 - 20]
	Female	10 to 14	1950	141.70	[138.15 - 154.38]	5	[5 - 5]
			1985	30.64	[28.40 - 33.04]	4	[3 - 4]
			2019	23.43	[21.16 - 26.11]	4	[3 - 4]
		15 to 19	1950	211.34	[206.99 - 227.27]	7	[7 - 7]
			1985	56.70	[52.11 - 62.36]	6	[6 - 7]
			2019	41.62	[38.01 - 45.82]	7	[6 - 8]
		20 to 24	1950	415.81	[399.72 - 473.20]	12	[11 - 13]
			1985	100.55	[89.15 - 115.18]	11	[10 - 13]
			2019	48.19	[40.80 - 58.31]	9	[7 - 11]

Table S65: All-cause mortality rate per 100,000 population and number of deaths in 10-24 by sex 1950 - 2019: Japan

Location	Sex	Age	Year	Mortality per 100,000	Mortality per 100,000 uncertainty interval	Number	Number Uncertainty Interval
Japan	Male	10 to 14	1950	110.05	[109.82 - 110.91]	5,033	[5,023 - 5,072]
			1985	19.62	[19.47 - 19.76]	1,036	[1,028 - 1,044]
			2019	8.44	[8.26 - 8.62]	240	[235 - 245]
		15 to 19	1950	225.86	[225.56 - 227.03]	10,155	[10,142 - 10,208]
			1985	67.50	[67.16 - 67.83]	3,181	[3,165 - 3,196]
			2019	25.43	[25.00 - 25.86]	784	[771 - 798]
		20 to 24	1950	422.22	[421.79 - 423.82]	16,819	[16,802 - 16,883]
			1985	81.49	[81.19 - 81.79]	3,475	[3,463 - 3,488]
			2019	47.56	[46.89 - 48.26]	1,505	[1,484 - 1,528]
	Female	10 to 14	1950	109.97	[109.77 - 110.68]	4,908	[4,899 - 4,939]
			1985	12.63	[12.55 - 12.71]	634	[630 - 638]
			2019	6.26	[6.19 - 6.32]	169	[167 - 170]
		15 to 19	1950	211.42	[211.13 - 212.42]	9,346	[9,333 - 9,390]
			1985	23.46	[23.34 - 23.59]	1,052	[1,047 - 1,058]
			2019	12.15	[12.02 - 12.29]	353	[349 - 357]
		20 to 24	1950	379.71	[379.17 - 381.32]	15,443	[15,421 - 15,508]
			1985	33.14	[32.94 - 33.36]	1,368	[1,360 - 1,377]
			2019	19.86	[19.51 - 20.25]	598	[587 - 610]

Table S66: All-cause mortality rate per 100,000 population and number of deaths in 10-24 by sex 1950 - 2019: Republic of Korea

Location	Sex	Age	Year	Mortality per 100,000	Mortality per 100,000 uncertainty interval	Number	Number Uncertainty Interval
Republic of Korea	Male	10 to 14	1950	676.59	[660.57 - 729.46]	8,644	[8,440 - 9,320]
			1985	55.48	[53.19 - 57.96]	1,320	[1,266 - 1,380]
			2019	10.13	[9.74 - 10.53]	121	[117 - 126]
		15 to 19	1950	1966.47	[1916.73 - 2137.92]	17,939	[17,486 - 19,503]
			1985	137.54	[134.95 - 140.09]	3,157	[3,098 - 3,216]
			2019	27.65	[26.64 - 28.69]	386	[372 - 400]
		20 to 24	1950	3638.15	[3539.10 - 3973.23]	26,798	[26,068 - 29,266]
			1985	146.87	[145.86 - 147.94]	3,304	[3,282 - 3,329]
			2019	40.33	[38.95 - 41.79]	718	[694 - 744]
	Female	10 to 14	1950	585.45	[571.00 - 635.17]	6,969	[6,797 - 7,561]
			1985	36.34	[35.14 - 37.69]	811	[784 - 841]
			2019	7.23	[6.99 - 7.47]	81	[78 - 84]
		15 to 19	1950	820.57	[800.65 - 889.02]	8,298	[8,097 - 8,991]
			1985	60.78	[59.48 - 62.18]	1,307	[1,279 - 1,337]
			2019	15.49	[15.01 - 15.97]	198	[192 - 204]
		20 to 24	1950	867.07	[848.26 - 932.70]	7,429	[7,268 - 7,991]
			1985	84.03	[83.30 - 84.79]	1,776	[1,761 - 1,792]
			2019	22.75	[21.73 - 23.84]	364	[348 - 382]

Table S67: All-cause mortality rate per 100,000 population and number of deaths in 10-24 by sex 1950 - 2019: Singapore

Location	Sex	Age	Year	Mortality per 100,000	Mortality per 100,000 uncertainty interval	Number	Number Uncertainty Interval
Singapore	Male	10 to 14	1950	112.32	[111.79 - 114.26]	76	[75 - 77]
			1985	35.89	[35.04 - 36.71]	45	[44 - 46]
			2019	10.36	[9.66 - 11.14]	11	[11 - 12]
		15 to 19	1950	170.01	[169.26 - 172.98]	105	[105 - 107]
			1985	89.62	[88.24 - 90.98]	122	[120 - 123]
			2019	24.49	[23.36 - 25.74]	34	[32 - 35]
		20 to 24	1950	279.80	[278.61 - 284.13]	159	[158 - 161]
			1985	100.30	[99.13 - 101.41]	159	[157 - 161]
			2019	36.24	[35.48 - 37.05]	48	[47 - 49]
	Female	10 to 14	1950	99.37	[98.92 - 101.03]	63	[62 - 64]
			1985	25.51	[24.93 - 26.13]	28	[28 - 29]
			2019	10.43	[9.86 - 11.07]	11	[11 - 12]
		15 to 19	1950	168.64	[168.00 - 171.23]	95	[95 - 97]
			1985	37.29	[36.57 - 38.07]	50	[49 - 51]
			2019	13.76	[13.14 - 14.46]	16	[15 - 17]
		20 to 24	1950	279.13	[277.31 - 285.72]	133	[132 - 136]
			1985	44.07	[43.34 - 44.89]	64	[63 - 65]
			2019	14.44	[14.05 - 14.85]	21	[21 - 22]

Table S68: All-cause mortality rate per 100,000 population and number of deaths in 10-24 by sex 1950 - 2019: Australasia

Location	Sex	Age	Year	Mortality per 100,000	Mortality per 100,000 uncertainty interval	Number	Number Uncertainty Interval
Australasia	Male	10 to 14	1950	70.76	[70.51 - 71.67]	283	[282 - 287]
			1985	31.03	[30.68 - 31.36]	266	[263 - 268]
			2019	10.70	[10.29 - 11.14]	100	[96 - 104]
		15 to 19	1950	138.64	[138.23 - 139.96]	536	[534 - 541]
			1985	114.31	[113.39 - 115.16]	940	[932 - 947]
			2019	43.23	[42.03 - 44.50]	381	[371 - 392]
		20 to 24	1950	192.28	[191.81 - 193.88]	837	[835 - 844]
			1985	158.57	[157.53 - 159.61]	1,301	[1,293 - 1,310]
			2019	69.78	[68.72 - 70.85]	665	[655 - 676]
	Female	10 to 14	1950	48.86	[48.70 - 49.36]	187	[187 - 189]
			1985	17.91	[17.73 - 18.09]	146	[145 - 148]
			2019	8.44	[8.26 - 8.64]	75	[73 - 77]
		15 to 19	1950	69.38	[69.18 - 69.98]	251	[250 - 253]
			1985	43.45	[43.05 - 43.81]	342	[339 - 345]
			2019	21.37	[20.94 - 21.82]	180	[176 - 183]
		20 to 24	1950	91.76	[91.46 - 92.87]	367	[366 - 372]
			1985	53.63	[53.08 - 54.13]	430	[425 - 434]
			2019	25.93	[25.44 - 26.45]	240	[235 - 245]

Table S69: All-cause mortality rate per 100,000 population and number of deaths in 10-24 by sex 1950 - 2019: Australia

Location	Sex	Age	Year	Mortality per 100,000	Mortality per 100,000 uncertainty interval	Number	Number Uncertainty Interval
Australia	Male	10 to 14	1950	69.69	[69.46 - 70.54]	226	[225 - 229]
			1985	30.00	[29.63 - 30.33]	210	[207 - 212]
			2019	10.22	[9.84 - 10.63]	80	[77 - 83]
		15 to 19	1950	134.33	[133.93 - 135.74]	425	[424 - 430]
			1985	107.86	[106.92 - 108.80]	719	[713 - 726]
			2019	39.77	[38.53 - 41.09]	291	[282 - 301]
		20 to 24	1950	194.75	[194.20 - 196.55]	703	[701 - 710]
			1985	157.21	[156.04 - 158.38]	1,056	[1,048 - 1,064]
			2019	66.72	[65.51 - 68.02]	538	[528 - 548]
	Female	10 to 14	1950	49.22	[49.06 - 49.70]	153	[152 - 154]
			1985	16.23	[16.05 - 16.41]	108	[107 - 109]
			2019	8.12	[7.95 - 8.30]	60	[59 - 61]
		15 to 19	1950	64.38	[64.19 - 64.95]	190	[190 - 192]
			1985	41.92	[41.49 - 42.34]	267	[265 - 270]
			2019	20.11	[19.70 - 20.54]	140	[137 - 143]
		20 to 24	1950	86.66	[86.28 - 87.81]	285	[284 - 289]
			1985	52.07	[51.45 - 52.65]	342	[338 - 346]
			2019	24.79	[24.24 - 25.38]	194	[189 - 198]

Table S70: All-cause mortality rate per 100,000 population and number of deaths in 10-24 by sex 1950 - 2019: New Zealand

Location	Sex	Age	Year	Mortality per 100,000	Mortality per 100,000 uncertainty interval	Number	Number Uncertainty Interval
New Zealand	Male	10 to 14	1950	75.30	[74.88 - 76.89]	57	[57 - 58]
			1985	35.62	[34.92 - 36.33]	56	[55 - 57]
			2019	13.13	[12.54 - 13.73]	20	[19 - 21]
		15 to 19	1950	157.97	[157.20 - 161.34]	111	[110 - 113]
			1985	142.05	[139.93 - 144.16]	220	[217 - 224]
			2019	60.30	[58.46 - 62.20]	90	[87 - 93]
		20 to 24	1950	180.36	[179.47 - 183.60]	134	[133 - 136]
			1985	164.71	[162.65 - 166.69]	245	[242 - 248]
			2019	86.59	[84.58 - 88.68]	127	[124 - 130]
	Female	10 to 14	1950	47.38	[47.14 - 48.17]	35	[35 - 35]
			1985	25.34	[24.93 - 25.77]	38	[38 - 39]
			2019	10.05	[9.78 - 10.32]	15	[14 - 15]
		15 to 19	1950	91.49	[91.08 - 92.91]	61	[61 - 62]
			1985	50.00	[49.31 - 50.73]	74	[73 - 75]
			2019	27.50	[26.86 - 28.16]	39	[38 - 40]
		20 to 24	1950	115.58	[114.81 - 118.35]	82	[82 - 84]
			1985	60.66	[59.66 - 61.76]	88	[86 - 90]
			2019	32.14	[31.24 - 33.10]	46	[45 - 48]

Table S71: All-cause mortality rate per 100,000 population and number of deaths in 10-24 by sex 1950 - 2019: Western Europe

Location	Sex	Age	Year	Mortality per 100,000	Mortality per 100,000 uncertainty interval	Number	Number Uncertainty Interval
Western Europe	Male	10 to 14	1950	75.29	[75.05 - 75.98]	9,507	[9,476 - 9,594]
			1985	28.41	[28.28 - 28.55]	3,946	[3,928 - 3,964]
			2019	9.52	[9.25 - 9.81]	1,154	[1,121 - 1,189]
		15 to 19	1950	140.99	[140.61 - 142.03]	16,917	[16,871 - 17,042]
			1985	82.37	[82.15 - 82.61]	12,779	[12,744 - 12,817]
			2019	31.10	[30.45 - 31.77]	3,757	[3,679 - 3,838]
		20 to 24	1950	199.19	[198.95 - 200.03]	23,741	[23,713 - 23,841]
			1985	116.48	[116.26 - 116.71]	18,300	[18,266 - 18,337]
			2019	52.55	[52.00 - 53.11]	6,653	[6,583 - 6,725]
	Female	10 to 14	1950	55.62	[55.52 - 55.97]	6,816	[6,803 - 6,858]
			1985	18.26	[18.19 - 18.33]	2,406	[2,397 - 2,415]
			2019	7.53	[7.42 - 7.65]	866	[853 - 880]
		15 to 19	1950	94.55	[94.42 - 95.02]	11,235	[11,219 - 11,291]
			1985	33.06	[32.97 - 33.16]	4,911	[4,897 - 4,926]
			2019	15.02	[14.82 - 15.24]	1,705	[1,682 - 1,729]
		20 to 24	1950	129.20	[129.02 - 129.74]	15,842	[15,820 - 15,909]
			1985	39.01	[38.90 - 39.13]	5,967	[5,949 - 5,985]
			2019	19.77	[19.51 - 20.02]	2,347	[2,316 - 2,377]

Table S72: All-cause mortality rate per 100,000 population and number of deaths in 10-24 by sex 1950 - 2019: Andorra

Location	Sex	Age	Year	Mortality per 100,000	Mortality per 100,000 uncertainty interval	Number	Number Uncertainty Interval
Andorra	Male	10 to 14	1950	40.40	[36.85 - 50.97]	0	[0 - 0]
			1985	18.54	[15.37 - 22.51]	0	[0 - 0]
			2019	7.50	[6.65 - 8.87]	0	[0 - 0]
		15 to 19	1950	80.64	[72.29 - 107.14]	0	[0 - 0]
			1985	62.77	[50.80 - 76.92]	1	[1 - 1]
			2019	29.26	[23.56 - 37.57]	1	[1 - 1]
		20 to 24	1950	95.07	[87.59 - 130.76]	0	[0 - 0]
			1985	89.92	[71.64 - 112.18]	2	[2 - 2]
			2019	61.81	[51.55 - 77.44]	1	[1 - 2]
	Female	10 to 14	1950	21.70	[19.90 - 27.71]	0	[0 - 0]
			1985	12.74	[10.48 - 16.62]	0	[0 - 0]
			2019	6.86	[5.86 - 8.18]	0	[0 - 0]
		15 to 19	1950	29.26	[26.67 - 40.79]	0	[0 - 0]
			1985	24.43	[19.35 - 32.25]	0	[0 - 0]
			2019	15.55	[13.01 - 18.93]	0	[0 - 0]
		20 to 24	1950	34.95	[32.03 - 50.74]	0	[0 - 0]
			1985	31.38	[22.00 - 39.00]	1	[0 - 1]
			2019	23.53	[21.48 - 27.20]	0	[0 - 1]

Table S73: All-cause mortality rate per 100,000 population and number of deaths in 10-24 by sex 1950 - 2019: Austria

Location	Sex	Age	Year	Mortality per 100,000	Mortality per 100,000 uncertainty interval	Number	Number Uncertainty Interval
Austria	Male	10 to 14	1950	81.00	[80.73 - 81.97]	206	[205 - 208]
			1985	29.60	[29.20 - 30.01]	77	[76 - 78]
			2019	8.90	[8.54 - 9.30]	19	[19 - 20]
		15 to 19	1950	169.10	[168.53 - 170.85]	393	[392 - 397]
			1985	118.57	[117.34 - 119.86]	387	[383 - 391]
			2019	39.60	[38.36 - 40.93]	94	[91 - 97]
		20 to 24	1950	230.75	[230.08 - 232.95]	555	[553 - 560]
			1985	158.15	[156.84 - 159.52]	542	[538 - 547]
			2019	58.91	[57.97 - 59.93]	164	[161 - 166]
	Female	10 to 14	1950	59.36	[59.18 - 60.00]	146	[146 - 148]
			1985	18.39	[18.16 - 18.63]	46	[45 - 46]
			2019	8.41	[8.21 - 8.63]	17	[17 - 18]
		15 to 19	1950	101.00	[100.73 - 101.97]	226	[225 - 228]
			1985	37.88	[37.47 - 38.31]	118	[117 - 120]
			2019	18.35	[17.94 - 18.79]	40	[39 - 41]
		20 to 24	1950	137.75	[137.21 - 139.45]	346	[345 - 351]
			1985	43.68	[43.11 - 44.29]	146	[144 - 148]
			2019	18.87	[18.48 - 19.30]	49	[48 - 50]

Table S74: All-cause mortality rate per 100,000 population and number of deaths in 10-24 by sex 1950 - 2019: Belgium

Location	Sex	Age	Year	Mortality per 100,000	Mortality per 100,000 uncertainty interval	Number	Number Uncertainty Interval
Belgium	Male	10 to 14	1950	67.07	[66.83 - 67.86]	194	[193 - 196]
			1985	27.91	[27.49 - 28.33]	95	[93 - 96]
			2019	9.77	[9.36 - 10.21]	33	[31 - 34]
		15 to 19	1950	119.61	[119.22 - 120.77]	377	[375 - 380]
			1985	83.06	[82.14 - 83.98]	312	[308 - 315]
			2019	33.44	[32.29 - 34.67]	107	[103 - 111]
		20 to 24	1950	187.02	[186.45 - 188.65]	620	[618 - 625]
			1985	131.59	[130.50 - 132.68]	539	[535 - 544]
			2019	64.73	[63.43 - 66.13]	217	[213 - 222]
	Female	10 to 14	1950	46.15	[46.00 - 46.62]	132	[132 - 134]
			1985	19.92	[19.69 - 20.18]	65	[64 - 65]
			2019	7.22	[7.02 - 7.44]	23	[22 - 24]
		15 to 19	1950	73.94	[73.71 - 74.61]	231	[230 - 233]
			1985	35.95	[35.56 - 36.34]	129	[127 - 130]
			2019	16.96	[16.53 - 17.43]	52	[51 - 53]
		20 to 24	1950	114.03	[113.56 - 115.45]	375	[373 - 380]
			1985	41.26	[40.71 - 41.79]	163	[160 - 165]
			2019	21.49	[20.96 - 22.07]	70	[68 - 72]

Table S75: All-cause mortality rate per 100,000 population and number of deaths in 10-24 by sex 1950 - 2019: Cyprus

Location	Sex	Age	Year	Mortality per 100,000	Mortality per 100,000 uncertainty interval	Number	Number Uncertainty Interval
Cyprus	Male	10 to 14	1950	90.75	[83.91 - 115.37]	21	[19 - 27]
			1985	35.49	[34.25 - 36.69]	11	[10 - 11]
			2019	11.80	[10.45 - 13.34]	4	[4 - 5]
		15 to 19	1950	166.60	[156.18 - 199.36]	33	[31 - 40]
			1985	75.86	[73.54 - 78.29]	25	[24 - 26]
			2019	49.50	[43.85 - 56.12]	17	[15 - 19]
		20 to 24	1950	199.77	[191.46 - 228.22]	36	[34 - 41]
			1985	119.31	[116.15 - 122.70]	40	[39 - 41]
			2019	78.34	[72.74 - 85.04]	34	[31 - 37]
	Female	10 to 14	1950	58.58	[54.33 - 72.48]	15	[14 - 18]
			1985	24.03	[23.32 - 24.76]	7	[7 - 7]
			2019	8.89	[7.95 - 9.93]	3	[3 - 3]
		15 to 19	1950	91.82	[86.26 - 111.31]	20	[19 - 25]
			1985	34.02	[33.10 - 35.04]	10	[10 - 11]
			2019	13.63	[12.36 - 15.03]	4	[4 - 5]
		20 to 24	1950	113.58	[106.32 - 139.31]	22	[21 - 27]
			1985	36.76	[35.35 - 38.22]	11	[11 - 12]
			2019	15.52	[14.23 - 16.91]	6	[6 - 7]

Table S76: All-cause mortality rate per 100,000 population and number of deaths in 10-24 by sex 1950 - 2019: Denmark

Location	Sex	Age	Year	Mortality per 100,000	Mortality per 100,000 uncertainty interval	Number	Number Uncertainty Interval
Denmark	Male	10 to 14	1950	54.00	[53.74 - 54.89]	86	[86 - 88]
			1985	27.25	[26.67 - 27.82]	51	[50 - 52]
			2019	7.32	[6.90 - 7.78]	13	[12 - 13]
		15 to 19	1950	85.48	[85.06 - 86.89]	126	[125 - 128]
			1985	74.54	[73.43 - 75.68]	152	[150 - 155]
			2019	22.55	[21.50 - 23.72]	40	[38 - 42]
		20 to 24	1950	116.20	[115.64 - 117.93]	171	[170 - 174]
			1985	104.91	[103.75 - 106.14]	211	[209 - 214]
			2019	44.79	[43.58 - 46.11]	88	[86 - 91]
	Female	10 to 14	1950	35.39	[35.24 - 35.93]	55	[55 - 56]
			1985	17.82	[17.50 - 18.10]	32	[31 - 32]
			2019	6.01	[5.79 - 6.25]	10	[10 - 10]
		15 to 19	1950	51.35	[51.14 - 52.05]	74	[74 - 75]
			1985	30.49	[30.03 - 30.90]	59	[58 - 60]
			2019	14.05	[13.57 - 14.60]	24	[23 - 24]
		20 to 24	1950	68.71	[68.31 - 70.10]	101	[100 - 103]
			1985	36.52	[35.91 - 37.11]	70	[69 - 71]
			2019	19.34	[18.71 - 20.03]	36	[35 - 38]

Table S77: All-cause mortality rate per 100,000 population and number of deaths in 10-24 by sex 1950 - 2019: Finland

Location	Sex	Age	Year	Mortality per 100,000	Mortality per 100,000 uncertainty interval	Number	Number Uncertainty Interval
Finland	Male	10 to 14	1950	94.70	[94.30 - 96.02]	158	[158 - 161]
			1985	28.77	[28.05 - 29.48]	45	[44 - 46]
			2019	9.65	[9.05 - 10.31]	15	[14 - 16]
		15 to 19	1950	204.13	[203.20 - 206.68]	323	[322 - 327]
			1985	88.28	[86.57 - 89.82]	155	[152 - 158]
			2019	36.79	[34.91 - 38.83]	56	[53 - 59]
		20 to 24	1950	377.79	[376.17 - 382.26]	624	[622 - 632]
			1985	120.27	[118.76 - 121.64]	231	[228 - 234]
			2019	73.64	[71.40 - 76.04]	118	[115 - 122]
	Female	10 to 14	1950	67.00	[66.69 - 67.96]	109	[109 - 111]
			1985	14.03	[13.76 - 14.28]	21	[20 - 21]
			2019	8.79	[8.42 - 9.19]	13	[13 - 14]
		15 to 19	1950	121.01	[120.51 - 122.61]	185	[185 - 188]
			1985	32.34	[31.82 - 32.84]	55	[54 - 56]
			2019	18.89	[18.15 - 19.69]	27	[26 - 28]
		20 to 24	1950	168.90	[167.91 - 172.31]	274	[272 - 279]
			1985	36.04	[35.30 - 36.75]	66	[65 - 68]
			2019	26.48	[25.46 - 27.59]	40	[39 - 42]

Table S78: All-cause mortality rate per 100,000 population and number of deaths in 10-24 by sex 1950 - 2019: France

Location	Sex	Age	Year	Mortality per 100,000	Mortality per 100,000 uncertainty interval	Number	Number Uncertainty Interval
France	Male	10 to 14	1950	61.98	[61.84 - 62.40]	934	[932 - 940]
			1985	28.22	[28.00 - 28.44]	626	[621 - 631]
			2019	9.44	[9.09 - 9.82]	200	[192 - 208]
		15 to 19	1950	112.40	[112.19 - 113.03]	1,853	[1,850 - 1,864]
			1985	86.01	[85.51 - 86.49]	1,945	[1,934 - 1,956]
			2019	31.83	[30.91 - 32.83]	672	[653 - 693]
		20 to 24	1950	171.98	[171.67 - 172.79]	2,906	[2,901 - 2,920]
			1985	156.41	[155.73 - 157.11]	3,483	[3,468 - 3,498]
			2019	59.34	[58.42 - 60.32]	1,154	[1,136 - 1,173]
	Female	10 to 14	1950	47.62	[47.52 - 47.96]	701	[699 - 706]
			1985	18.13	[18.00 - 18.26]	382	[380 - 385]
			2019	7.08	[6.93 - 7.24]	143	[140 - 146]
		15 to 19	1950	78.47	[78.32 - 78.92]	1,255	[1,253 - 1,262]
			1985	38.37	[38.14 - 38.62]	831	[826 - 836]
			2019	14.56	[14.28 - 14.87]	292	[287 - 299]
		20 to 24	1950	123.38	[123.11 - 124.18]	2,015	[2,011 - 2,028]
			1985	51.12	[50.75 - 51.52]	1,129	[1,121 - 1,138]
			2019	21.37	[20.96 - 21.83]	402	[395 - 411]

Table S79: All-cause mortality rate per 100,000 population and number of deaths in 10-24 by sex 1950 - 2019: Germany

Location	Sex	Age	Year	Mortality per 100,000	Mortality per 100,000 uncertainty interval	Number	Number Uncertainty Interval
Germany	Male	10 to 14	1950	63.49	[62.74 - 65.61]	2,026	[2,002 - 2,093]
			1985	25.84	[25.63 - 26.02]	565	[561 - 569]
			2019	8.93	[8.65 - 9.25]	176	[171 - 182]
		15 to 19	1950	153.40	[151.95 - 157.54]	3,986	[3,948 - 4,094]
			1985	87.66	[87.21 - 88.09]	2,720	[2,707 - 2,734]
			2019	31.94	[31.11 - 32.90]	689	[671 - 710]
		20 to 24	1950	187.30	[186.28 - 190.40]	4,569	[4,545 - 4,645]
			1985	111.76	[111.34 - 112.17]	3,910	[3,895 - 3,924]
			2019	50.53	[49.76 - 51.43]	1,311	[1,291 - 1,334]
	Female	10 to 14	1950	38.69	[38.45 - 39.38]	1,177	[1,169 - 1,198]
			1985	16.98	[16.88 - 17.10]	351	[349 - 354]
			2019	7.75	[7.63 - 7.89]	144	[141 - 146]
		15 to 19	1950	67.34	[66.93 - 68.54]	1,710	[1,699 - 1,740]
			1985	36.57	[36.37 - 36.76]	1,080	[1,075 - 1,086]
			2019	16.58	[16.33 - 16.89]	323	[318 - 329]
		20 to 24	1950	79.16	[78.60 - 80.92]	2,100	[2,085 - 2,147]
			1985	39.82	[39.58 - 40.07]	1,337	[1,329 - 1,345]
			2019	19.84	[19.47 - 20.25]	447	[439 - 456]

Table S80: All-cause mortality rate per 100,000 population and number of deaths in 10-24 by sex 1950 - 2019: Greece

Location	Sex	Age	Year	Mortality per 100,000	Mortality per 100,000 uncertainty interval	Number	Number Uncertainty Interval
Greece	Male	10 to 14	1950	79.96	[79.33 - 82.10]	324	[322 - 333]
			1985	25.56	[25.19 - 25.93]	102	[101 - 103]
			2019	10.97	[10.36 - 11.61]	30	[28 - 31]
		15 to 19	1950	126.09	[125.34 - 129.25]	507	[504 - 519]
			1985	83.03	[82.10 - 83.97]	338	[335 - 342]
			2019	41.33	[39.60 - 43.17]	108	[103 - 113]
		20 to 24	1950	211.66	[210.65 - 215.45]	753	[749 - 766]
			1985	123.89	[122.64 - 125.10]	464	[460 - 469]
			2019	73.19	[71.67 - 74.87]	188	[184 - 193]
	Female	10 to 14	1950	53.04	[52.76 - 53.90]	208	[206 - 211]
			1985	16.14	[15.93 - 16.36]	61	[60 - 62]
			2019	8.35	[8.04 - 8.67]	22	[21 - 22]
		15 to 19	1950	100.42	[100.03 - 101.69]	408	[407 - 414]
			1985	33.23	[32.84 - 33.61]	130	[129 - 132]
			2019	15.69	[15.17 - 16.22]	39	[38 - 41]
		20 to 24	1950	156.79	[155.82 - 160.47]	587	[583 - 601]
			1985	38.96	[38.40 - 39.56]	145	[143 - 147]
			2019	23.49	[22.87 - 24.16]	59	[58 - 61]

Table S81: All-cause mortality rate per 100,000 population and number of deaths in 10-24 by sex 1950 - 2019: Iceland

Location	Sex	Age	Year	Mortality per 100,000	Mortality per 100,000 uncertainty interval	Number	Number Uncertainty Interval
Iceland	Male	10 to 14	1950	67.40	[66.02 - 72.55]	4	[4 - 4]
			1985	26.13	[24.28 - 28.02]	3	[3 - 3]
			2019	8.69	[7.17 - 10.36]	1	[1 - 1]
		15 to 19	1950	148.45	[145.54 - 159.60]	9	[9 - 10]
			1985	103.70	[98.64 - 108.95]	11	[11 - 12]
			2019	27.61	[23.99 - 32.34]	3	[3 - 3]
		20 to 24	1950	241.70	[237.41 - 257.84]	15	[14 - 16]
			1985	137.10	[131.86 - 142.88]	15	[15 - 16]
			2019	53.59	[50.57 - 57.36]	7	[6 - 7]
	Female	10 to 14	1950	38.62	[38.01 - 40.86]	2	[2 - 2]
			1985	12.37	[11.59 - 13.26]	1	[1 - 1]
			2019	6.42	[5.59 - 7.52]	1	[1 - 1]
		15 to 19	1950	45.51	[44.87 - 47.92]	3	[3 - 3]
			1985	19.12	[17.82 - 20.43]	2	[2 - 2]
			2019	14.30	[12.69 - 16.41]	2	[1 - 2]
		20 to 24	1950	58.82	[57.48 - 64.03]	3	[3 - 4]
			1985	30.15	[28.78 - 31.49]	3	[3 - 3]
			2019	22.55	[21.04 - 24.77]	3	[2 - 3]

Table S82: All-cause mortality rate per 100,000 population and number of deaths in 10-24 by sex 1950 - 2019: Ireland

Location	Sex	Age	Year	Mortality per 100,000	Mortality per 100,000 uncertainty interval	Number	Number Uncertainty Interval
Ireland	Male	10 to 14	1950	61.87	[61.56 - 63.00]	86	[85 - 87]
			1985	29.85	[29.23 - 30.47]	56	[54 - 57]
			2019	8.88	[8.36 - 9.44]	16	[15 - 17]
		15 to 19	1950	110.59	[109.97 - 112.59]	145	[144 - 147]
			1985	76.92	[75.64 - 78.19]	133	[131 - 135]
			2019	34.10	[32.38 - 35.97]	55	[53 - 58]
		20 to 24	1950	220.09	[218.97 - 223.54]	240	[239 - 244]
			1985	105.97	[104.48 - 107.50]	158	[156 - 160]
			2019	61.37	[59.46 - 63.40]	90	[87 - 93]
	Female	10 to 14	1950	69.34	[69.02 - 70.40]	93	[92 - 94]
			1985	17.64	[17.33 - 17.99]	31	[31 - 32]
			2019	6.65	[6.38 - 6.94]	11	[11 - 12]
		15 to 19	1950	139.26	[138.66 - 141.13]	164	[163 - 166]
			1985	26.87	[26.41 - 27.34]	44	[43 - 45]
			2019	13.78	[13.26 - 14.34]	22	[21 - 22]
		20 to 24	1950	221.39	[219.97 - 225.65]	225	[223 - 229]
			1985	33.66	[32.89 - 34.46]	49	[48 - 51]
			2019	18.78	[18.06 - 19.55]	27	[26 - 28]

Table S83: All-cause mortality rate per 100,000 population and number of deaths in 10-24 by sex 1950 - 2019: Israel

Location	Sex	Age	Year	Mortality per 100,000	Mortality per 100,000 uncertainty interval	Number	Number Uncertainty Interval
Israel	Male	10 to 14	1950	46.52	[46.19 - 47.66]	34	[34 - 35]
			1985	24.23	[23.89 - 24.56]	56	[55 - 57]
			2019	10.63	[10.24 - 11.03]	42	[41 - 44]
		15 to 19	1950	101.39	[100.70 - 104.06]	69	[68 - 71]
			1985	66.69	[65.68 - 67.65]	133	[131 - 135]
			2019	31.57	[30.50 - 32.70]	117	[113 - 121]
		20 to 24	1950	118.84	[118.14 - 121.49]	77	[76 - 78]
			1985	99.60	[98.17 - 101.15]	175	[173 - 178]
			2019	48.25	[47.16 - 49.42]	163	[160 - 167]
	Female	10 to 14	1950	33.16	[33.03 - 33.57]	23	[23 - 23]
			1985	17.63	[17.39 - 17.89]	39	[38 - 39]
			2019	8.86	[8.68 - 9.05]	34	[33 - 34]
		15 to 19	1950	52.45	[52.26 - 53.12]	35	[35 - 36]
			1985	29.70	[29.32 - 30.12]	55	[54 - 56]
			2019	13.01	[12.72 - 13.30]	46	[45 - 47]
		20 to 24	1950	55.66	[55.21 - 57.28]	37	[37 - 38]
			1985	35.02	[34.42 - 35.72]	59	[58 - 60]
			2019	15.39	[14.93 - 15.87]	50	[49 - 52]

Table S84: All-cause mortality rate per 100,000 population and number of deaths in 10-24 by sex 1950 - 2019: Italy

Location	Sex	Age	Year	Mortality per 100,000	Mortality per 100,000 uncertainty interval	Number	Number Uncertainty Interval
Italy	Male	10 to 14	1950	83.44	[83.23 - 84.07]	1,766	[1,762 - 1,780]
			1985	29.11	[28.87 - 29.35]	649	[644 - 654]
			2019	10.27	[10.04 - 10.50]	152	[148 - 155]
		15 to 19	1950	133.64	[133.41 - 134.44]	2,701	[2,696 - 2,717]
			1985	81.87	[81.41 - 82.33]	1,943	[1,933 - 1,954]
			2019	31.11	[30.60 - 31.61]	464	[457 - 472]
		20 to 24	1950	180.66	[180.41 - 181.47]	3,624	[3,619 - 3,640]
			1985	102.95	[102.47 - 103.44]	2,388	[2,377 - 2,400]
			2019	47.86	[47.25 - 48.49]	737	[728 - 747]
	Female	10 to 14	1950	67.63	[67.48 - 68.09]	1,391	[1,388 - 1,401]
			1985	18.52	[18.38 - 18.68]	394	[391 - 397]
			2019	6.86	[6.79 - 6.92]	96	[95 - 96]
		15 to 19	1950	99.86	[99.68 - 100.43]	2,001	[1,997 - 2,012]
			1985	28.66	[28.47 - 28.84]	655	[650 - 659]
			2019	12.64	[12.51 - 12.76]	176	[174 - 178]
		20 to 24	1950	133.93	[133.67 - 134.84]	2,729	[2,723 - 2,747]
			1985	32.94	[32.70 - 33.15]	743	[737 - 747]
			2019	17.92	[17.63 - 18.21]	254	[250 - 258]

Table S85: All-cause mortality rate per 100,000 population and number of deaths in 10-24 by sex 1950 - 2019: Luxembourg

Location	Sex	Age	Year	Mortality per 100,000	Mortality per 100,000 uncertainty interval	Number	Number Uncertainty Interval
Luxembourg	Male	10 to 14	1950	87.57	[86.21 - 92.37]	10	[10 - 11]
			1985	26.08	[24.63 - 27.58]	3	[3 - 3]
			2019	9.02	[7.34 - 10.94]	2	[1 - 2]
		15 to 19	1950	136.51	[134.60 - 142.98]	17	[16 - 17]
			1985	99.88	[96.10 - 103.85]	13	[13 - 14]
			2019	35.53	[30.25 - 41.93]	6	[5 - 7]
		20 to 24	1950	174.55	[172.45 - 181.27]	23	[23 - 24]
			1985	175.63	[170.47 - 180.99]	26	[25 - 27]
			2019	64.53	[59.06 - 70.38]	13	[12 - 14]
	Female	10 to 14	1950	48.77	[48.23 - 50.77]	5	[5 - 5]
			1985	20.89	[19.92 - 21.89]	2	[2 - 2]
			2019	7.29	[6.26 - 8.64]	1	[1 - 1]
		15 to 19	1950	66.79	[66.06 - 69.24]	7	[7 - 8]
			1985	44.88	[43.14 - 46.63]	6	[6 - 6]
			2019	18.34	[16.21 - 20.87]	3	[3 - 3]
		20 to 24	1950	85.77	[84.31 - 90.43]	10	[10 - 11]
			1985	42.79	[40.98 - 44.55]	6	[6 - 7]
			2019	24.38	[22.41 - 26.05]	5	[4 - 5]

Table S86: All-cause mortality rate per 100,000 population and number of deaths in 10-24 by sex 1950 - 2019: Malta

Location	Sex	Age	Year	Mortality per 100,000	Mortality per 100,000 uncertainty interval	Number	Number Uncertainty Interval
Malta	Male	10 to 14	1950	83.49	[82.42 - 86.96]	12	[11 - 12]
			1985	20.87	[19.90 - 21.87]	3	[3 - 3]
			2019	13.19	[11.64 - 14.96]	1	[1 - 2]
		15 to 19	1950	116.82	[115.14 - 122.95]	14	[14 - 15]
			1985	39.33	[37.79 - 40.87]	5	[5 - 5]
			2019	39.97	[35.96 - 43.63]	4	[4 - 5]
		20 to 24	1950	156.64	[154.68 - 163.98]	17	[17 - 18]
			1985	81.27	[78.89 - 83.81]	12	[11 - 12]
			2019	58.65	[55.17 - 61.77]	8	[7 - 8]
	Female	10 to 14	1950	56.65	[56.19 - 58.17]	9	[9 - 9]
			1985	14.47	[13.87 - 15.09]	2	[2 - 2]
			2019	9.13	[8.00 - 10.32]	1	[1 - 1]
		15 to 19	1950	41.77	[41.39 - 42.98]	6	[6 - 7]
			1985	24.12	[23.30 - 24.96]	3	[3 - 3]
			2019	17.17	[15.72 - 18.88]	2	[2 - 2]
		20 to 24	1950	89.74	[88.27 - 95.31]	12	[12 - 13]
			1985	28.76	[27.57 - 29.91]	4	[4 - 4]
			2019	20.00	[19.01 - 21.65]	2	[2 - 3]

Table S87: All-cause mortality rate per 100,000 population and number of deaths in 10-24 by sex 1950 - 2019: Netherlands

Location	Sex	Age	Year	Mortality per 100,000	Mortality per 100,000 uncertainty interval	Number	Number Uncertainty Interval
Netherlands	Male	10 to 14	1950	52.95	[52.76 - 53.63]	221	[220 - 224]
			1985	23.22	[22.92 - 23.51]	129	[127 - 131]
			2019	8.89	[8.53 - 9.27]	43	[41 - 45]
		15 to 19	1950	85.94	[85.62 - 87.06]	357	[355 - 361]
			1985	58.24	[57.62 - 58.82]	368	[364 - 372]
			2019	24.00	[23.15 - 24.92]	127	[123 - 132]
		20 to 24	1950	115.05	[114.63 - 116.31]	460	[458 - 465]
			1985	78.13	[77.50 - 78.82]	506	[502 - 510]
			2019	38.83	[37.96 - 39.76]	208	[203 - 213]
	Female	10 to 14	1950	34.60	[34.48 - 35.00]	139	[138 - 140]
			1985	15.03	[14.87 - 15.21]	80	[79 - 81]
			2019	8.08	[7.88 - 8.28]	37	[36 - 38]
		15 to 19	1950	46.40	[46.25 - 46.89]	185	[185 - 187]
			1985	25.13	[24.90 - 25.38]	152	[150 - 153]
			2019	14.16	[13.82 - 14.51]	72	[70 - 73]
		20 to 24	1950	68.86	[68.53 - 69.89]	272	[271 - 276]
			1985	30.98	[30.58 - 31.38]	193	[190 - 195]
			2019	17.85	[17.36 - 18.39]	92	[90 - 95]

Table S88: All-cause mortality rate per 100,000 population and number of deaths in 10-24 by sex 1950 - 2019: Norway

Location	Sex	Age	Year	Mortality per 100,000	Mortality per 100,000 uncertainty interval	Number	Number Uncertainty Interval
Norway	Male	10 to 14	1950	60.13	[59.79 - 61.17]	65	[65 - 66]
			1985	23.99	[23.53 - 24.49]	39	[38 - 40]
			2019	8.84	[8.50 - 9.18]	14	[14 - 15]
		15 to 19	1950	119.79	[119.19 - 122.04]	125	[124 - 127]
			1985	74.38	[73.25 - 75.54]	128	[126 - 130]
			2019	32.62	[31.73 - 33.59]	54	[52 - 55]
		20 to 24	1950	166.80	[165.97 - 169.82]	196	[195 - 199]
			1985	113.66	[112.28 - 115.06]	183	[181 - 185]
			2019	54.44	[53.14 - 55.79]	96	[94 - 99]
	Female	10 to 14	1950	41.42	[41.21 - 42.16]	43	[43 - 44]
			1985	16.07	[15.77 - 16.35]	25	[24 - 25]
			2019	7.35	[7.19 - 7.51]	11	[11 - 12]
		15 to 19	1950	66.37	[66.08 - 67.46]	67	[66 - 68]
			1985	30.94	[30.45 - 31.42]	51	[50 - 51]
			2019	16.16	[15.83 - 16.51]	25	[25 - 26]
		20 to 24	1950	88.14	[87.55 - 90.28]	99	[98 - 101]
			1985	36.94	[36.23 - 37.63]	57	[56 - 58]
			2019	18.83	[18.28 - 19.42]	31	[30 - 32]

Table S89: All-cause mortality rate per 100,000 population and number of deaths in 10-24 by sex 1950 - 2019: Portugal

Location	Sex	Age	Year	Mortality per 100,000	Mortality per 100,000 uncertainty interval	Number	Number Uncertainty Interval
Portugal	Male	10 to 14	1950	129.93	[129.47 - 131.34]	549	[547 - 555]
			1985	50.74	[50.09 - 51.43]	230	[227 - 233]
			2019	10.75	[10.19 - 11.34]	29	[27 - 30]
		15 to 19	1950	221.36	[220.69 - 223.59]	913	[910 - 922]
			1985	132.71	[131.36 - 134.13]	580	[574 - 586]
			2019	31.54	[30.28 - 32.85]	93	[89 - 97]
		20 to 24	1950	376.39	[375.42 - 379.84]	1,456	[1,453 - 1,470]
			1985	181.89	[180.30 - 183.48]	744	[737 - 750]
			2019	56.36	[55.14 - 57.64]	163	[159 - 166]
	Female	10 to 14	1950	103.25	[102.90 - 104.34]	428	[426 - 432]
			1985	28.16	[27.83 - 28.48]	123	[122 - 125]
			2019	7.50	[7.23 - 7.78]	19	[19 - 20]
		15 to 19	1950	180.81	[180.30 - 182.47]	745	[742 - 751]
			1985	50.37	[49.86 - 50.89]	214	[212 - 216]
			2019	15.32	[14.84 - 15.84]	43	[42 - 45]
		20 to 24	1950	257.11	[256.08 - 260.54]	1,010	[1,006 - 1,023]
			1985	54.69	[53.95 - 55.44]	220	[217 - 223]
			2019	19.43	[18.88 - 20.02]	54	[53 - 56]

Table S90: All-cause mortality rate per 100,000 population and number of deaths in 10-24 by sex 1950 - 2019: Spain

Location	Sex	Age	Year	Mortality per 100,000	Mortality per 100,000 uncertainty interval	Number	Number Uncertainty Interval
Spain	Male	10 to 14	1950	131.89	[131.58 - 133.05]	1,621	[1,617 - 1,635]
			1985	30.52	[30.24 - 30.80]	521	[516 - 526]
			2019	9.74	[9.44 - 10.05]	123	[119 - 127]
		15 to 19	1950	230.34	[229.87 - 231.97]	2,948	[2,942 - 2,969]
			1985	78.01	[77.51 - 78.54]	1,318	[1,309 - 1,327]
			2019	26.59	[25.89 - 27.32]	309	[300 - 317]
		20 to 24	1950	340.91	[340.29 - 342.92]	4,313	[4,305 - 4,338]
			1985	122.30	[121.60 - 122.96]	1,945	[1,934 - 1,956]
			2019	41.38	[40.70 - 42.09]	465	[457 - 473]
	Female	10 to 14	1950	109.11	[108.84 - 109.96]	1,316	[1,313 - 1,326]
			1985	20.18	[20.02 - 20.33]	323	[320 - 325]
			2019	7.54	[7.42 - 7.66]	90	[88 - 91]
		15 to 19	1950	188.01	[187.65 - 189.27]	2,429	[2,424 - 2,445]
			1985	31.33	[31.11 - 31.55]	508	[504 - 511]
			2019	13.16	[12.94 - 13.40]	145	[142 - 147]
		20 to 24	1950	252.74	[252.05 - 254.75]	3,299	[3,290 - 3,325]
			1985	38.84	[38.48 - 39.19]	600	[594 - 605]
			2019	16.26	[15.91 - 16.63]	175	[171 - 179]

Table S91: All-cause mortality rate per 100,000 population and number of deaths in 10-24 by sex 1950 - 2019: Sweden

Location	Sex	Age	Year	Mortality per 100,000	Mortality per 100,000 uncertainty interval	Number	Number Uncertainty Interval
Sweden	Male	10 to 14	1950	53.76	[53.51 - 54.61]	123	[122 - 125]
			1985	20.78	[20.30 - 21.26]	59	[58 - 60]
			2019	7.97	[7.62 - 8.34]	25	[24 - 26]
		15 to 19	1950	99.84	[99.46 - 101.23]	209	[208 - 212]
			1985	63.32	[62.47 - 64.16]	188	[186 - 191]
			2019	28.44	[27.63 - 29.24]	83	[81 - 85]
		20 to 24	1950	151.94	[151.40 - 153.89]	349	[347 - 353]
			1985	92.13	[91.26 - 93.00]	278	[276 - 281]
			2019	58.75	[57.70 - 59.86]	172	[169 - 176]
	Female	10 to 14	1950	41.28	[41.12 - 41.86]	92	[91 - 93]
			1985	14.65	[14.44 - 14.86]	40	[39 - 40]
			2019	7.91	[7.73 - 8.10]	23	[23 - 24]
		15 to 19	1950	50.32	[50.16 - 50.88]	104	[103 - 105]
			1985	27.99	[27.66 - 28.33]	79	[78 - 80]
			2019	15.19	[14.91 - 15.49]	41	[40 - 42]
		20 to 24	1950	83.64	[83.30 - 84.87]	191	[190 - 194]
			1985	32.43	[31.97 - 32.91]	94	[92 - 95]
			2019	22.04	[21.55 - 22.57]	60	[58 - 61]

Table S92: All-cause mortality rate per 100,000 population and number of deaths in 10-24 by sex 1950 - 2019: Switzerland

Location	Sex	Age	Year	Mortality per 100,000	Mortality per 100,000 uncertainty interval	Number	Number Uncertainty Interval
Switzerland	Male	10 to 14	1950	69.39	[69.10 - 70.34]	109	[109 - 111]
			1985	33.23	[32.69 - 33.79]	69	[68 - 70]
			2019	8.86	[8.48 - 9.28]	19	[19 - 20]
		15 to 19	1950	119.45	[118.99 - 121.09]	197	[196 - 199]
			1985	97.66	[96.29 - 99.00]	248	[245 - 252]
			2019	26.96	[25.98 - 28.03]	60	[58 - 63]
		20 to 24	1950	175.74	[175.06 - 177.90]	296	[294 - 299]
			1985	132.58	[131.11 - 134.03]	378	[374 - 382]
			2019	46.82	[45.80 - 47.93]	121	[118 - 124]
	Female	10 to 14	1950	44.28	[44.10 - 44.91]	68	[67 - 69]
			1985	18.68	[18.38 - 18.98]	37	[36 - 37]
			2019	8.03	[7.82 - 8.25]	17	[16 - 17]
		15 to 19	1950	60.29	[60.05 - 61.05]	99	[98 - 100]
			1985	35.27	[34.77 - 35.77]	84	[83 - 86]
			2019	13.96	[13.61 - 14.33]	29	[28 - 30]
		20 to 24	1950	86.24	[85.73 - 87.64]	157	[156 - 159]
			1985	46.12	[45.36 - 46.94]	124	[122 - 126]
			2019	15.94	[15.49 - 16.42]	38	[37 - 40]

Table S93: All-cause mortality rate per 100,000 population and number of deaths in 10-24 by sex 1950 - 2019: United Kingdom

Location	Sex	Age	Year	Mortality per 100,000	Mortality per 100,000 uncertainty interval	Number	Number Uncertainty Interval
United Kingdom	Male	10 to 14	1950	55.98	[55.87 - 56.34]	950	[948 - 956]
			1985	27.15	[26.96 - 27.36]	555	[551 - 559]
			2019	9.65	[9.44 - 9.86]	196	[192 - 200]
		15 to 19	1950	98.03	[97.87 - 98.56]	1,599	[1,596 - 1,607]
			1985	70.85	[70.47 - 71.23]	1,662	[1,653 - 1,671]
			2019	31.47	[30.96 - 31.98]	594	[584 - 604]
		20 to 24	1950	138.86	[138.65 - 139.51]	2,423	[2,419 - 2,434]
			1985	87.90	[87.48 - 88.30]	2,052	[2,042 - 2,062]
			2019	54.00	[53.34 - 54.71]	1,127	[1,113 - 1,142]
	Female	10 to 14	1950	39.57	[39.50 - 39.83]	662	[661 - 666]
			1985	17.78	[17.67 - 17.90]	343	[341 - 345]
			2019	7.73	[7.65 - 7.81]	150	[148 - 151]
		15 to 19	1950	75.91	[75.79 - 76.32]	1,273	[1,271 - 1,280]
			1985	28.40	[28.25 - 28.56]	641	[637 - 644]
			2019	16.45	[16.28 - 16.63]	296	[293 - 300]
		20 to 24	1950	107.38	[107.17 - 108.09]	1,965	[1,961 - 1,978]
			1985	32.06	[31.86 - 32.26]	743	[738 - 747]
			2019	21.81	[21.47 - 22.17]	442	[435 - 450]

Table S94: All-cause mortality rate per 100,000 population and number of deaths in 10-24 by sex 1950 - 2019: Southern Latin America

Location	Sex	Age	Year	Mortality per 100,000	Mortality per 100,000 uncertainty interval	Number	Number Uncertainty Interval
Southern Latin America	Male	10 to 14	1950	128.28	[128.01 - 129.21]	1,600	[1,597 - 1,612]
			1985	45.99	[45.74 - 46.25]	1,037	[1,031 - 1,043]
			2019	23.66	[22.67 - 24.71]	594	[569 - 620]
		15 to 19	1950	225.88	[225.41 - 227.36]	2,714	[2,708 - 2,732]
			1985	95.19	[94.70 - 95.72]	1,951	[1,941 - 1,962]
			2019	85.40	[82.65 - 88.28]	2,192	[2,122 - 2,266]
		20 to 24	1950	305.20	[304.69 - 306.68]	3,547	[3,541 - 3,565]
			1985	143.65	[142.98 - 144.36]	2,764	[2,752 - 2,778]
			2019	127.59	[125.29 - 130.03]	3,344	[3,284 - 3,408]
	Female	10 to 14	1950	112.09	[111.95 - 112.57]	1,416	[1,414 - 1,422]
			1985	30.40	[30.23 - 30.58]	675	[671 - 679]
			2019	17.66	[17.22 - 18.13]	428	[417 - 439]
		15 to 19	1950	206.22	[205.98 - 207.08]	2,526	[2,523 - 2,537]
			1985	46.72	[46.48 - 46.98]	958	[953 - 964]
			2019	35.34	[34.55 - 36.17]	882	[862 - 903]
		20 to 24	1950	259.43	[258.71 - 261.52]	3,090	[3,081 - 3,114]
			1985	61.31	[60.89 - 61.71]	1,204	[1,196 - 1,212]
			2019	42.89	[41.91 - 43.93]	1,112	[1,086 - 1,139]

Table S95: All-cause mortality rate per 100,000 population and number of deaths in 10-24 by sex 1950 - 2019: Argentina

Location	Sex	Age	Year	Mortality per 100,000	Mortality per 100,000 uncertainty interval	Number	Number Uncertainty Interval
Argentina	Male	10 to 14	1950	99.48	[99.27 - 100.18]	821	[820 - 827]
			1985	46.41	[46.11 - 46.73]	676	[671 - 680]
			2019	25.89	[25.11 - 26.68]	457	[444 - 471]
		15 to 19	1950	172.12	[171.77 - 173.26]	1,394	[1,391 - 1,403]
			1985	97.25	[96.63 - 97.96]	1,251	[1,243 - 1,261]
			2019	94.89	[92.45 - 97.39]	1,716	[1,672 - 1,761]
		20 to 24	1950	221.50	[221.13 - 222.83]	1,771	[1,768 - 1,782]
			1985	132.55	[131.83 - 133.38]	1,577	[1,569 - 1,587]
			2019	139.40	[137.16 - 141.78]	2,507	[2,467 - 2,549]
	Female	10 to 14	1950	84.89	[84.72 - 85.49]	713	[711 - 718]
			1985	30.60	[30.39 - 30.81]	440	[437 - 443]
			2019	19.10	[18.81 - 19.41]	327	[322 - 332]
		15 to 19	1950	158.54	[158.25 - 159.54]	1,299	[1,297 - 1,307]
			1985	48.33	[48.01 - 48.64]	623	[619 - 627]
			2019	39.25	[38.64 - 39.88]	694	[683 - 705]
		20 to 24	1950	183.87	[183.42 - 185.50]	1,479	[1,475 - 1,492]
			1985	65.86	[65.33 - 66.42]	799	[792 - 805]
			2019	48.17	[47.17 - 49.23]	863	[845 - 882]

Table S96: All-cause mortality rate per 100,000 population and number of deaths in 10-24 by sex 1950 - 2019: Chile

Location	Sex	Age	Year	Mortality per 100,000	Mortality per 100,000 uncertainty interval	Number	Number Uncertainty Interval
Chile	Male	10 to 14	1950	219.20	[218.25 - 222.10]	702	[699 - 711]
			1985	44.77	[44.25 - 45.32]	297	[294 - 301]
			2019	17.62	[16.15 - 19.25]	110	[101 - 120]
		15 to 19	1950	390.11	[388.47 - 394.86]	1,129	[1,125 - 1,143]
			1985	94.23	[93.31 - 95.16]	603	[597 - 609]
			2019	57.80	[54.53 - 61.32]	365	[344 - 387]
		20 to 24	1950	588.11	[586.46 - 593.35]	1,538	[1,534 - 1,552]
			1985	168.63	[167.30 - 170.04]	1,043	[1,035 - 1,052]
			2019	93.64	[91.39 - 96.01]	650	[634 - 666]
	Female	10 to 14	1950	198.10	[197.85 - 198.86]	633	[632 - 635]
			1985	30.08	[29.75 - 30.41]	195	[193 - 198]
			2019	14.26	[13.43 - 15.18]	85	[80 - 90]
		15 to 19	1950	354.33	[353.95 - 355.58]	1,067	[1,065 - 1,070]
			1985	44.80	[44.36 - 45.24]	288	[285 - 290]
			2019	24.35	[23.17 - 25.63]	147	[140 - 155]
		20 to 24	1950	497.99	[496.04 - 504.18]	1,414	[1,409 - 1,432]
			1985	55.06	[54.41 - 55.78]	350	[345 - 354]
			2019	28.43	[27.58 - 29.32]	191	[185 - 197]

Table S97: All-cause mortality rate per 100,000 population and number of deaths in 10-24 by sex 1950 - 2019: Uruguay

Location	Sex	Age	Year	Mortality per 100,000	Mortality per 100,000 uncertainty interval	Number	Number Uncertainty Interval
Uruguay	Male	10 to 14	1950	75.94	[75.51 - 77.26]	77	[77 - 79]
			1985	47.53	[46.56 - 48.56]	64	[63 - 66]
			2019	22.22	[20.55 - 24.05]	27	[25 - 29]
		15 to 19	1950	186.65	[185.73 - 189.69]	191	[190 - 194]
			1985	78.67	[77.36 - 80.05]	97	[95 - 98]
			2019	87.43	[82.81 - 92.36]	112	[106 - 118]
		20 to 24	1950	234.74	[233.73 - 238.16]	237	[236 - 241]
			1985	124.23	[122.69 - 125.68]	144	[142 - 146]
			2019	145.72	[142.26 - 149.34]	187	[183 - 192]
	Female	10 to 14	1950	67.49	[67.13 - 68.56]	70	[70 - 71]
			1985	29.86	[29.41 - 30.33]	39	[38 - 40]
			2019	13.95	[13.22 - 14.75]	16	[15 - 17]
		15 to 19	1950	153.44	[152.73 - 155.65]	160	[159 - 163]
			1985	39.80	[39.27 - 40.37]	48	[47 - 49]
			2019	33.13	[31.66 - 34.73]	41	[39 - 43]
		20 to 24	1950	190.97	[189.74 - 194.81]	196	[194 - 199]
			1985	47.97	[47.07 - 48.85]	56	[55 - 57]
			2019	44.87	[43.49 - 46.34]	57	[56 - 59]

Table S98: All-cause mortality rate per 100,000 population and number of deaths in 10-24 by sex 1950 - 2019: High-income North America

Location	Sex	Age	Year	Mortality per 100,000	Mortality per 100,000 uncertainty interval	Number	Number Uncertainty Interval
High-income North America	Male	10 to 14	1950	70.88	[70.77 - 71.27]	4,657	[4,650 - 4,683]
			1985	34.30	[34.17 - 34.43]	3,360	[3,347 - 3,373]
			2019	16.15	[15.88 - 16.44]	1,939	[1,907 - 1,973]
		15 to 19	1950	142.75	[142.47 - 143.78]	8,777	[8,759 - 8,840]
			1985	111.53	[111.21 - 111.84]	12,151	[12,117 - 12,185]
			2019	66.84	[65.97 - 67.70]	8,094	[7,989 - 8,198]
		20 to 24	1950	206.12	[205.59 - 208.06]	12,843	[12,810 - 12,964]
			1985	163.24	[162.87 - 163.63]	19,573	[19,528 - 19,619]
			2019	130.49	[129.17 - 131.88]	15,849	[15,689 - 16,018]
	Female	10 to 14	1950	44.76	[44.68 - 45.05]	2,871	[2,866 - 2,890]
			1985	20.37	[20.30 - 20.44]	1,902	[1,896 - 1,909]
			2019	11.56	[11.48 - 11.65]	1,328	[1,318 - 1,338]
		15 to 19	1950	75.40	[75.28 - 75.81]	4,648	[4,640 - 4,672]
			1985	45.14	[45.01 - 45.27]	4,689	[4,675 - 4,702]
			2019	30.97	[30.72 - 31.21]	3,580	[3,551 - 3,608]
		20 to 24	1950	101.81	[101.64 - 102.36]	6,582	[6,572 - 6,618]
			1985	52.19	[52.00 - 52.36]	6,167	[6,145 - 6,188]
			2019	44.09	[43.54 - 44.66]	5,142	[5,077 - 5,208]

Table S99: All-cause mortality rate per 100,000 population and number of deaths in 10-24 by sex 1950 - 2019: Canada

Location	Sex	Age	Year	Mortality per 100,000	Mortality per 100,000 uncertainty interval	Number	Number Uncertainty Interval
Canada	Male	10 to 14	1950	80.41	[80.21 - 81.12]	465	[463 - 469]
			1985	30.23	[29.95 - 30.51]	287	[284 - 290]
			2019	12.38	[11.99 - 12.79]	133	[129 - 138]
		15 to 19	1950	134.81	[134.48 - 135.96]	733	[732 - 740]
			1985	104.37	[103.70 - 105.11]	1,071	[1,064 - 1,079]
			2019	44.38	[43.37 - 45.46]	462	[451 - 473]
		20 to 24	1950	176.90	[176.54 - 178.32]	971	[969 - 979]
			1985	142.69	[141.84 - 143.58]	1,697	[1,687 - 1,708]
			2019	76.78	[75.90 - 77.74]	863	[853 - 873]
	Female	10 to 14	1950	54.90	[54.77 - 55.33]	307	[306 - 309]
			1985	19.92	[19.74 - 20.12]	179	[178 - 181]
			2019	10.39	[10.24 - 10.54]	106	[105 - 108]
		15 to 19	1950	80.02	[79.85 - 80.60]	432	[431 - 436]
			1985	40.56	[40.24 - 40.90]	397	[394 - 401]
			2019	23.10	[22.79 - 23.42]	228	[225 - 231]
		20 to 24	1950	98.67	[98.38 - 99.75]	561	[559 - 567]
			1985	41.22	[40.89 - 41.59]	486	[482 - 490]
			2019	31.31	[30.87 - 31.80]	336	[332 - 342]

Table S100: All-cause mortality rate per 100,000 population and number of deaths in 10-24 by sex 1950 - 2019: United States of America

Location	Sex	Age	Year	Mortality per 100,000	Mortality per 100,000 uncertainty interval	Number	Number Uncertainty Interval
United States of America	Male	10 to 14	1950	69.92	[69.80 - 70.34]	4,189	[4,182 - 4,214]
			1985	34.73	[34.59 - 34.86]	3,072	[3,060 - 3,084]
			2019	16.52	[16.26 - 16.80]	1,806	[1,777 - 1,835]
		15 to 19	1950	143.41	[143.13 - 144.53]	8,035	[8,019 - 8,098]
			1985	112.25	[111.93 - 112.58]	11,074	[11,042 - 11,106]
			2019	68.94	[68.06 - 69.87]	7,630	[7,532 - 7,732]
		20 to 24	1950	208.83	[208.29 - 210.93]	11,862	[11,832 - 11,982]
			1985	165.48	[165.09 - 165.87]	17,866	[17,825 - 17,909]
			2019	135.96	[134.49 - 137.50]	14,982	[14,821 - 15,152]
	Female	10 to 14	1950	43.77	[43.68 - 44.10]	2,563	[2,557 - 2,582]
			1985	20.41	[20.33 - 20.49]	1,722	[1,716 - 1,729]
			2019	11.67	[11.59 - 11.76]	1,222	[1,213 - 1,231]
		15 to 19	1950	74.93	[74.80 - 75.39]	4,213	[4,205 - 4,238]
			1985	45.60	[45.46 - 45.75]	4,289	[4,275 - 4,302]
			2019	31.70	[31.44 - 31.96]	3,351	[3,323 - 3,378]
		20 to 24	1950	102.07	[101.90 - 102.65]	6,018	[6,007 - 6,052]
			1985	53.39	[53.19 - 53.58]	5,677	[5,656 - 5,698]
			2019	45.38	[44.78 - 46.03]	4,804	[4,740 - 4,872]

Table S101: All-cause mortality rate per 100,000 population and number of deaths in 10-24 by sex 1950 - 2019: Latin America and Caribbean

Location	Sex	Age	Year	Mortality per 100,000	Mortality per 100,000 uncertainty interval	Number	Number Uncertainty Interval
Latin America and Caribbean	Male	10 to 14	1950	217.12	[212.45 - 233.69]	18,128	[17,738 - 19,511]
			1985	90.96	[88.06 - 94.14]	19,511	[18,890 - 20,193]
			2019	39.12	[34.51 - 44.41]	9,684	[8,543 - 10,995]
		15 to 19	1950	327.95	[322.93 - 343.78]	23,743	[23,380 - 24,889]
			1985	175.74	[170.67 - 181.82]	33,888	[32,911 - 35,061]
			2019	135.09	[120.26 - 151.62]	33,289	[29,635 - 37,364]
		20 to 24	1950	436.40	[432.86 - 449.88]	27,213	[26,992 - 28,053]
			1985	285.87	[278.74 - 294.99]	48,168	[46,967 - 49,705]
			2019	218.21	[199.89 - 238.55]	53,703	[49,195 - 58,710]
	Female	10 to 14	1950	153.96	[151.70 - 161.32]	12,994	[12,802 - 13,614]
			1985	61.34	[59.47 - 63.51]	13,047	[12,649 - 13,509]
			2019	28.15	[25.44 - 31.05]	6,690	[6,045 - 7,379]
		15 to 19	1950	217.80	[215.11 - 226.50]	16,357	[16,155 - 17,011]
			1985	83.19	[81.09 - 85.70]	16,337	[15,923 - 16,828]
			2019	47.96	[43.24 - 52.88]	11,489	[10,359 - 12,668]
		20 to 24	1950	305.90	[302.53 - 317.99]	20,025	[19,805 - 20,817]
			1985	111.07	[108.59 - 113.90]	19,573	[19,136 - 20,072]
			2019	61.93	[54.90 - 69.54]	15,197	[13,471 - 17,063]

Table S102: All-cause mortality rate per 100,000 population and number of deaths in 10-24 by sex 1950 - 2019: Caribbean

Location	Sex	Age	Year	Mortality per 100,000	Mortality per 100,000 uncertainty interval	Number	Number Uncertainty Interval
Caribbean	Male	10 to 14	1950	132.29	[127.20 - 150.77]	1,327	[1,276 - 1,512]
			1985	73.85	[70.00 - 77.65]	1,409	[1,336 - 1,482]
			2019	56.82	[48.93 - 65.14]	1,098	[946 - 1,259]
		15 to 19	1950	211.76	[201.23 - 249.00]	1,858	[1,766 - 2,185]
			1985	121.12	[115.05 - 127.87]	2,209	[2,098 - 2,332]
			2019	103.88	[88.27 - 121.08]	2,018	[1,715 - 2,352]
		20 to 24	1950	290.65	[279.80 - 328.78]	2,197	[2,115 - 2,485]
			1985	182.26	[175.44 - 189.86]	2,921	[2,812 - 3,043]
			2019	169.86	[146.50 - 195.31]	3,229	[2,785 - 3,712]
	Female	10 to 14	1950	94.18	[90.79 - 107.71]	964	[929 - 1,103]
			1985	56.53	[54.07 - 58.87]	1,065	[1,019 - 1,109]
			2019	46.59	[39.90 - 52.40]	875	[749 - 984]
		15 to 19	1950	161.56	[156.42 - 179.39]	1,480	[1,433 - 1,643]
			1985	90.02	[87.26 - 92.51]	1,642	[1,591 - 1,687]
			2019	68.86	[60.20 - 77.64]	1,312	[1,147 - 1,479]
		20 to 24	1950	238.36	[227.43 - 279.54]	1,923	[1,834 - 2,255]
			1985	123.68	[116.72 - 129.66]	2,047	[1,932 - 2,147]
			2019	102.48	[87.29 - 118.45]	1,943	[1,655 - 2,245]

Table S103: All-cause mortality rate per 100,000 population and number of deaths in 10-24 by sex 1950 - 2019: Antigua and Barbuda

Location	Sex	Age	Year	Mortality per 100,000	Mortality per 100,000 uncertainty interval	Number	Number Uncertainty Interval
Antigua and Barbuda	Male	10 to 14	1950	107.77	[104.87 - 119.61]	3	[2 - 3]
			1985	28.12	[25.39 - 31.26]	1	[1 - 1]
			2019	26.74	[22.80 - 31.44]	1	[1 - 1]
		15 to 19	1950	163.04	[158.57 - 178.53]	3	[3 - 3]
			1985	67.36	[60.52 - 73.72]	2	[2 - 2]
			2019	57.03	[48.31 - 67.39]	2	[2 - 2]
		20 to 24	1950	238.32	[232.55 - 256.16]	4	[4 - 4]
			1985	126.36	[114.05 - 137.34]	4	[3 - 4]
			2019	134.08	[118.41 - 152.38]	5	[4 - 6]
	Female	10 to 14	1950	92.11	[90.09 - 99.89]	2	[2 - 3]
			1985	25.48	[23.35 - 27.88]	1	[1 - 1]
			2019	18.11	[16.55 - 20.94]	1	[1 - 1]
		15 to 19	1950	104.71	[102.40 - 112.71]	2	[2 - 2]
			1985	37.39	[34.13 - 40.62]	1	[1 - 1]
			2019	34.71	[31.52 - 39.51]	1	[1 - 1]
		20 to 24	1950	192.11	[185.39 - 211.42]	4	[3 - 4]
			1985	66.18	[59.50 - 72.97]	2	[2 - 2]
			2019	37.31	[33.80 - 45.05]	1	[1 - 2]

Table S104: All-cause mortality rate per 100,000 population and number of deaths in 10-24 by sex 1950 - 2019: Bahamas

Location	Sex	Age	Year	Mortality per 100,000	Mortality per 100,000 uncertainty interval	Number	Number Uncertainty Interval
Bahamas	Male	10 to 14	1950	122.20	[113.19 - 152.20]	5	[5 - 6]
			1985	46.27	[41.85 - 51.23]	7	[6 - 7]
			2019	47.93	[42.54 - 55.13]	8	[7 - 9]
		15 to 19	1950	223.35	[205.55 - 282.74]	7	[7 - 9]
			1985	115.18	[105.80 - 125.40]	15	[14 - 17]
			2019	126.49	[108.05 - 150.68]	19	[16 - 23]
		20 to 24	1950	297.83	[278.61 - 360.87]	8	[8 - 10]
			1985	221.46	[208.72 - 235.37]	29	[27 - 30]
			2019	261.20	[227.21 - 305.26]	40	[35 - 47]
	Female	10 to 14	1950	71.43	[66.57 - 86.23]	3	[3 - 4]
			1985	32.68	[30.06 - 35.46]	5	[4 - 5]
			2019	34.47	[30.95 - 39.16]	5	[5 - 6]
		15 to 19	1950	130.19	[122.40 - 154.08]	5	[5 - 6]
			1985	62.27	[57.97 - 66.71]	8	[8 - 9]
			2019	66.75	[58.85 - 77.22]	10	[9 - 12]
		20 to 24	1950	186.40	[170.71 - 239.12]	7	[6 - 9]
			1985	104.38	[96.45 - 112.82]	14	[13 - 15]
			2019	96.00	[80.40 - 117.00]	15	[13 - 18]

Table S105: All-cause mortality rate per 100,000 population and number of deaths in 10-24 by sex 1950 - 2019: Barbados

Location	Sex	Age	Year	Mortality per 100,000	Mortality per 100,000 uncertainty interval	Number	Number Uncertainty Interval
Barbados	Male	10 to 14	1950	77.01	[74.91 - 84.78]	8	[8 - 9]
			1985	38.74	[35.34 - 42.48]	5	[4 - 5]
			2019	25.03	[20.90 - 30.49]	2	[2 - 3]
		15 to 19	1950	176.19	[172.62 - 189.24]	15	[14 - 16]
			1985	56.79	[52.90 - 61.03]	7	[7 - 8]
			2019	76.43	[62.90 - 94.92]	8	[6 - 10]
		20 to 24	1950	260.68	[256.33 - 274.34]	19	[19 - 20]
			1985	109.47	[104.81 - 114.46]	14	[13 - 14]
			2019	107.12	[92.49 - 130.95]	10	[9 - 12]
	Female	10 to 14	1950	45.63	[44.78 - 48.50]	5	[5 - 6]
			1985	33.56	[31.22 - 35.91]	4	[4 - 4]
			2019	22.64	[19.48 - 26.57]	2	[2 - 2]
		15 to 19	1950	181.95	[179.13 - 191.28]	19	[19 - 20]
			1985	50.04	[47.05 - 53.00]	6	[6 - 7]
			2019	46.63	[40.13 - 55.85]	5	[4 - 5]
		20 to 24	1950	206.80	[202.75 - 220.65]	20	[20 - 22]
			1985	57.70	[54.20 - 61.31]	7	[7 - 8]
			2019	62.51	[52.29 - 78.30]	6	[5 - 7]

Table S106: All-cause mortality rate per 100,000 population and number of deaths in 10-24 by sex 1950 - 2019: Belize

Location	Sex	Age	Year	Mortality per 100,000	Mortality per 100,000 uncertainty interval	Number	Number Uncertainty Interval
Belize	Male	10 to 14	1950	144.21	[139.63 - 157.87]	5	[5 - 6]
			1985	47.02	[43.02 - 51.06]	5	[5 - 6]
			2019	38.52	[34.72 - 42.58]	9	[8 - 9]
		15 to 19	1950	177.71	[172.72 - 196.90]	6	[5 - 6]
			1985	80.30	[72.76 - 87.94]	8	[7 - 8]
			2019	139.91	[124.45 - 157.46]	31	[28 - 35]
		20 to 24	1950	509.41	[496.36 - 559.07]	15	[14 - 16]
			1985	118.66	[110.06 - 127.62]	9	[9 - 10]
			2019	257.14	[230.72 - 286.79]	50	[45 - 56]
	Female	10 to 14	1950	143.73	[140.31 - 153.93]	6	[5 - 6]
			1985	37.45	[35.05 - 40.11]	4	[4 - 4]
			2019	20.89	[19.26 - 22.64]	5	[4 - 5]
		15 to 19	1950	341.86	[335.56 - 363.83]	12	[11 - 12]
			1985	65.49	[61.56 - 69.93]	6	[6 - 7]
			2019	54.69	[50.47 - 58.98]	12	[11 - 13]
		20 to 24	1950	401.06	[389.03 - 446.77]	12	[12 - 13]
			1985	92.16	[84.74 - 100.44]	7	[7 - 8]
			2019	73.58	[64.83 - 83.65]	15	[13 - 17]

Table S107: All-cause mortality rate per 100,000 population and number of deaths in 10-24 by sex 1950 - 2019: Cuba

Location	Sex	Age	Year	Mortality per 100,000	Mortality per 100,000 uncertainty interval	Number	Number Uncertainty Interval
Cuba	Male	10 to 14	1950	77.88	[72.11 - 99.08]	243	[225 - 309]
			1985	53.23	[52.38 - 54.06]	319	[314 - 324]
			2019	22.62	[19.11 - 26.65]	69	[59 - 82]
		15 to 19	1950	159.02	[144.93 - 209.61]	451	[411 - 595]
			1985	101.02	[99.50 - 102.46]	622	[612 - 631]
			2019	47.59	[38.77 - 57.90]	168	[137 - 204]
		20 to 24	1950	206.85	[191.40 - 261.05]	520	[481 - 656]
			1985	145.13	[143.25 - 147.14]	808	[797 - 819]
			2019	70.71	[59.61 - 83.42]	260	[220 - 307]
	Female	10 to 14	1950	55.48	[51.74 - 69.21]	177	[165 - 220]
			1985	38.78	[38.22 - 39.36]	221	[218 - 224]
			2019	16.05	[13.87 - 18.51]	46	[40 - 53]
		15 to 19	1950	115.98	[108.15 - 143.02]	335	[312 - 413]
			1985	89.31	[88.09 - 90.61]	526	[519 - 534]
			2019	27.45	[23.53 - 31.91]	91	[78 - 105]
		20 to 24	1950	148.68	[134.86 - 201.05]	379	[343 - 512]
			1985	102.04	[100.01 - 104.35]	558	[547 - 571]
			2019	34.22	[27.52 - 42.20]	117	[94 - 145]

Table S108: All-cause mortality rate per 100,000 population and number of deaths in 10-24 by sex 1950 - 2019: Dominica

Location	Sex	Age	Year	Mortality per 100,000	Mortality per 100,000 uncertainty interval	Number	Number Uncertainty Interval
Dominica	Male	10 to 14	1950	168.77	[164.53 - 186.70]	4	[4 - 5]
			1985	50.38	[44.56 - 57.09]	3	[2 - 3]
			2019	52.63	[43.93 - 63.71]	1	[1 - 2]
		15 to 19	1950	242.73	[237.64 - 261.97]	5	[5 - 5]
			1985	121.45	[104.47 - 139.18]	5	[5 - 6]
			2019	110.42	[89.13 - 138.57]	3	[3 - 4]
		20 to 24	1950	287.95	[281.45 - 309.57]	5	[5 - 5]
			1985	170.01	[146.37 - 193.50]	6	[5 - 7]
			2019	151.56	[121.47 - 187.79]	4	[3 - 5]
	Female	10 to 14	1950	118.79	[116.10 - 128.02]	4	[3 - 4]
			1985	45.07	[41.34 - 48.80]	2	[2 - 2]
			2019	52.84	[43.24 - 63.05]	1	[1 - 2]
		15 to 19	1950	200.11	[194.34 - 218.43]	5	[5 - 5]
			1985	67.41	[62.38 - 72.26]	3	[3 - 3]
			2019	68.57	[54.30 - 82.97]	2	[2 - 2]
		20 to 24	1950	276.33	[263.53 - 307.39]	6	[5 - 6]
			1985	96.24	[85.09 - 107.39]	3	[3 - 4]
			2019	97.69	[74.83 - 124.22]	2	[2 - 3]

Table S109: All-cause mortality rate per 100,000 population and number of deaths in 10-24 by sex 1950 - 2019: Dominican Republic

Location	Sex	Age	Year	Mortality per 100,000	Mortality per 100,000 uncertainty interval	Number	Number Uncertainty Interval
Dominican Republic	Male	10 to 14	1950	142.32	[139.32 - 152.60]	201	[197 - 216]
			1985	60.17	[55.49 - 64.77]	240	[221 - 258]
			2019	45.69	[37.09 - 56.20]	220	[178 - 270]
		15 to 19	1950	168.52	[166.32 - 175.64]	206	[203 - 215]
			1985	88.20	[78.78 - 97.67]	330	[295 - 366]
			2019	103.62	[79.37 - 131.12]	506	[388 - 640]
		20 to 24	1950	252.93	[251.06 - 260.07]	260	[258 - 267]
			1985	167.02	[150.15 - 184.31]	528	[475 - 583]
			2019	201.13	[156.46 - 247.45]	989	[769 - 1,217]
	Female	10 to 14	1950	94.08	[92.99 - 98.16]	137	[135 - 143]
			1985	44.80	[41.82 - 48.36]	182	[170 - 197]
			2019	30.46	[25.12 - 36.45]	142	[117 - 170]
		15 to 19	1950	131.01	[129.78 - 135.34]	172	[170 - 177]
			1985	61.56	[57.27 - 66.52]	241	[224 - 261]
			2019	45.93	[36.27 - 55.62]	221	[175 - 268]
		20 to 24	1950	193.92	[191.70 - 200.75]	211	[209 - 219]
			1985	91.40	[81.22 - 103.05]	304	[270 - 343]
			2019	84.39	[63.25 - 107.53]	413	[309 - 526]

Table S110: All-cause mortality rate per 100,000 population and number of deaths in 10-24 by sex 1950 - 2019: Grenada

Location	Sex	Age	Year	Mortality per 100,000	Mortality per 100,000 uncertainty interval	Number	Number Uncertainty Interval
Grenada	Male	10 to 14	1950	99.32	[96.50 - 108.62]	4	[4 - 4]
			1985	52.73	[45.17 - 61.11]	3	[2 - 3]
			2019	29.88	[26.45 - 33.21]	1	[1 - 1]
		15 to 19	1950	202.44	[197.40 - 218.01]	7	[6 - 7]
			1985	87.62	[75.31 - 100.89]	4	[4 - 5]
			2019	73.30	[63.05 - 80.63]	3	[3 - 4]
		20 to 24	1950	323.11	[315.30 - 345.22]	9	[9 - 9]
			1985	201.78	[179.61 - 224.75]	9	[8 - 9]
			2019	115.13	[94.98 - 132.76]	6	[5 - 7]
	Female	10 to 14	1950	70.86	[69.33 - 75.62]	4	[3 - 4]
			1985	43.98	[38.88 - 50.15]	2	[2 - 3]
			2019	22.66	[20.72 - 24.66]	1	[1 - 1]
		15 to 19	1950	132.11	[129.72 - 139.77]	6	[5 - 6]
			1985	57.13	[50.99 - 64.36]	3	[2 - 3]
			2019	50.08	[44.08 - 54.94]	2	[2 - 2]
		20 to 24	1950	230.85	[224.24 - 250.62]	8	[8 - 9]
			1985	125.39	[108.03 - 144.87]	5	[5 - 6]
			2019	60.54	[50.39 - 70.40]	3	[2 - 3]

Table S111: All-cause mortality rate per 100,000 population and number of deaths in 10-24 by sex 1950 - 2019: Guyana

Location	Sex	Age	Year	Mortality per 100,000	Mortality per 100,000 uncertainty interval	Number	Number Uncertainty Interval
Guyana	Male	10 to 14	1950	129.60	[123.50 - 152.18]	29	[28 - 34]
			1985	110.90	[97.64 - 124.81]	55	[49 - 62]
			2019	62.98	[50.81 - 77.80]	22	[18 - 27]
		15 to 19	1950	281.65	[267.11 - 335.24]	55	[52 - 66]
			1985	184.19	[153.48 - 215.18]	85	[71 - 100]
			2019	146.05	[113.87 - 185.28]	54	[42 - 69]
		20 to 24	1950	350.95	[335.93 - 405.08]	62	[59 - 72]
			1985	283.93	[239.06 - 328.59]	116	[98 - 134]
			2019	304.35	[246.88 - 371.37]	124	[101 - 151]
	Female	10 to 14	1950	150.43	[143.95 - 174.49]	36	[34 - 41]
			1985	62.07	[55.44 - 69.34]	31	[28 - 35]
			2019	42.71	[35.34 - 51.53]	14	[12 - 17]
		15 to 19	1950	274.44	[264.29 - 310.31]	56	[54 - 64]
			1985	110.04	[97.99 - 123.07]	51	[46 - 58]
			2019	86.69	[72.01 - 103.85]	31	[26 - 37]
		20 to 24	1950	457.37	[432.94 - 546.96]	83	[78 - 99]
			1985	169.67	[137.78 - 204.72]	71	[58 - 86]
			2019	144.91	[111.04 - 187.23]	58	[45 - 75]

Table S112: All-cause mortality rate per 100,000 population and number of deaths in 10-24 by sex 1950 - 2019: Haiti

Location	Sex	Age	Year	Mortality per 100,000	Mortality per 100,000 uncertainty interval	Number	Number Uncertainty Interval
Haiti	Male	10 to 14	1950	241.81	[225.45 - 299.21]	460	[429 - 569]
			1985	159.45	[141.67 - 177.18]	512	[455 - 569]
			2019	97.24	[81.29 - 110.73]	653	[546 - 744]
		15 to 19	1950	354.69	[326.13 - 456.34]	596	[548 - 767]
			1985	232.95	[199.30 - 270.15]	635	[543 - 736]
			2019	151.13	[120.61 - 185.84]	920	[735 - 1,132]
		20 to 24	1950	397.04	[369.85 - 498.38]	569	[530 - 714]
			1985	292.37	[253.80 - 330.96]	686	[596 - 777]
			2019	208.72	[163.37 - 252.23]	1,133	[887 - 1,369]
	Female	10 to 14	1950	169.25	[158.37 - 215.01]	329	[308 - 419]
			1985	129.90	[118.17 - 140.98]	430	[391 - 467]
			2019	85.99	[71.86 - 96.30]	572	[478 - 641]
		15 to 19	1950	277.61	[264.65 - 320.01]	488	[465 - 562]
			1985	193.20	[179.27 - 205.52]	552	[513 - 588]
			2019	127.73	[109.69 - 144.52]	787	[676 - 890]
		20 to 24	1950	427.27	[395.63 - 543.86]	686	[635 - 873]
			1985	289.71	[253.02 - 318.82]	763	[666 - 839]
			2019	188.46	[157.82 - 215.76]	1,078	[903 - 1,234]

Table S113: All-cause mortality rate per 100,000 population and number of deaths in 10-24 by sex 1950 - 2019: Jamaica

Location	Sex	Age	Year	Mortality per 100,000	Mortality per 100,000 uncertainty interval	Number	Number Uncertainty Interval
Jamaica	Male	10 to 14	1950	116.38	[113.95 - 123.56]	95	[93 - 101]
			1985	42.84	[40.41 - 45.19]	62	[58 - 65]
			2019	24.34	[19.89 - 29.74]	28	[23 - 34]
		15 to 19	1950	176.07	[173.99 - 182.79]	124	[123 - 129]
			1985	61.97	[59.78 - 64.14]	82	[79 - 85]
			2019	44.17	[34.89 - 55.99]	55	[44 - 70]
		20 to 24	1950	242.18	[240.19 - 248.62]	147	[146 - 151]
			1985	114.28	[111.21 - 117.39]	128	[125 - 132]
			2019	84.99	[70.38 - 103.75]	116	[96 - 142]
	Female	10 to 14	1950	85.99	[84.83 - 89.45]	68	[67 - 71]
			1985	32.84	[31.28 - 34.24]	47	[44 - 49]
			2019	19.38	[16.32 - 22.98]	22	[18 - 26]
		15 to 19	1950	132.77	[131.23 - 137.70]	97	[96 - 101]
			1985	36.50	[35.19 - 37.72]	49	[47 - 51]
			2019	31.27	[26.28 - 37.33]	38	[32 - 45]
		20 to 24	1950	217.59	[215.44 - 225.16]	149	[147 - 154]
			1985	62.01	[60.05 - 64.06]	73	[71 - 76]
			2019	54.40	[43.01 - 69.13]	72	[57 - 91]

Table S114: All-cause mortality rate per 100,000 population and number of deaths in 10-24 by sex 1950 - 2019: Saint Lucia

Location	Sex	Age	Year	Mortality per 100,000	Mortality per 100,000 uncertainty interval	Number	Number Uncertainty Interval
Saint Lucia	Male	10 to 14	1950	193.09	[187.79 - 213.88]	8	[7 - 9]
			1985	41.41	[38.10 - 44.82]	3	[3 - 4]
			2019	33.01	[27.99 - 39.07]	2	[2 - 2]
		15 to 19	1950	231.79	[226.45 - 252.07]	7	[7 - 8]
			1985	95.24	[89.63 - 100.89]	7	[7 - 8]
			2019	94.66	[79.31 - 112.47]	6	[5 - 8]
		20 to 24	1950	603.21	[590.65 - 642.42]	17	[16 - 18]
			1985	202.63	[193.03 - 212.05]	12	[12 - 13]
			2019	177.47	[154.75 - 203.46]	13	[11 - 14]
	Female	10 to 14	1950	115.41	[112.62 - 124.52]	5	[5 - 5]
			1985	29.31	[27.40 - 31.27]	2	[2 - 3]
			2019	23.91	[20.78 - 27.54]	1	[1 - 2]
		15 to 19	1950	188.11	[184.25 - 200.18]	7	[7 - 7]
			1985	70.57	[66.69 - 74.70]	5	[5 - 6]
			2019	36.47	[31.64 - 42.16]	2	[2 - 3]
		20 to 24	1950	268.66	[260.94 - 290.27]	9	[9 - 10]
			1985	76.24	[71.46 - 81.58]	5	[5 - 5]
			2019	51.90	[43.62 - 62.12]	4	[3 - 4]

Table S115: All-cause mortality rate per 100,000 population and number of deaths in 10-24 by sex 1950 - 2019: Saint Vincent and the Grenadines

Location	Sex	Age	Year	Mortality per 100,000	Mortality per 100,000 uncertainty interval	Number	Number Uncertainty Interval
Saint Vincent and the Grenadines	Male	10 to 14	1950	135.16	[131.50 - 149.91]	6	[5 - 6]
			1985	29.38	[26.84 - 32.31]	2	[2 - 2]
			2019	38.86	[33.52 - 44.66]	2	[2 - 2]
		15 to 19	1950	199.02	[194.60 - 216.89]	6	[6 - 7]
			1985	92.71	[86.31 - 100.03]	6	[6 - 7]
			2019	108.90	[94.01 - 124.97]	5	[4 - 6]
		20 to 24	1950	256.51	[250.58 - 276.08]	7	[7 - 7]
			1985	154.75	[146.78 - 163.42]	8	[8 - 9]
			2019	146.86	[130.12 - 164.29]	7	[6 - 8]
	Female	10 to 14	1950	166.86	[163.32 - 178.97]	7	[7 - 8]
			1985	50.63	[45.99 - 56.09]	4	[3 - 4]
			2019	40.02	[35.49 - 44.92]	2	[2 - 2]
		15 to 19	1950	284.41	[279.51 - 302.33]	11	[10 - 11]
			1985	68.28	[62.09 - 76.02]	4	[4 - 5]
			2019	49.32	[43.47 - 55.54]	2	[2 - 2]
		20 to 24	1950	408.89	[398.50 - 443.30]	13	[12 - 14]
			1985	95.55	[85.69 - 106.62]	5	[5 - 6]
			2019	81.50	[69.96 - 94.31]	4	[3 - 4]

Table S116: All-cause mortality rate per 100,000 population and number of deaths in 10-24 by sex 1950 - 2019: Suriname

Location	Sex	Age	Year	Mortality per 100,000	Mortality per 100,000 uncertainty interval	Number	Number Uncertainty Interval
Suriname	Male	10 to 14	1950	101.34	[98.83 - 111.92]	11	[11 - 12]
			1985	75.79	[71.08 - 80.79]	16	[15 - 17]
			2019	49.83	[41.98 - 59.05]	13	[11 - 15]
		15 to 19	1950	192.09	[188.25 - 204.86]	18	[18 - 19]
			1985	111.55	[105.95 - 117.71]	26	[25 - 28]
			2019	107.02	[88.89 - 128.19]	26	[22 - 31]
		20 to 24	1950	382.35	[375.20 - 402.46]	30	[29 - 31]
			1985	193.81	[185.43 - 202.48]	40	[38 - 42]
			2019	163.77	[141.71 - 188.98]	37	[32 - 43]
	Female	10 to 14	1950	99.76	[97.83 - 106.36]	11	[11 - 12]
			1985	67.24	[63.39 - 71.06]	14	[13 - 15]
			2019	42.09	[36.87 - 48.09]	10	[9 - 12]
		15 to 19	1950	287.35	[282.68 - 302.25]	28	[28 - 29]
			1985	88.84	[84.34 - 93.29]	19	[18 - 20]
			2019	88.41	[77.80 - 100.42]	20	[18 - 23]
		20 to 24	1950	261.36	[256.08 - 280.00]	22	[21 - 23]
			1985	109.13	[103.46 - 115.15]	20	[19 - 21]
			2019	92.91	[79.15 - 109.03]	21	[18 - 25]

Table S117: All-cause mortality rate per 100,000 population and number of deaths in 10-24 by sex 1950 - 2019: Trinidad and Tobago

Location	Sex	Age	Year	Mortality per 100,000	Mortality per 100,000 uncertainty interval	Number	Number Uncertainty Interval
Trinidad and Tobago	Male	10 to 14	1950	108.17	[105.86 - 116.16]	38	[38 - 41]
			1985	50.26	[47.21 - 53.26]	30	[28 - 32]
			2019	30.76	[24.44 - 38.54]	15	[12 - 19]
		15 to 19	1950	195.59	[192.83 - 204.74]	59	[58 - 62]
			1985	123.18	[118.27 - 128.02]	72	[69 - 75]
			2019	105.08	[78.68 - 138.10]	48	[36 - 63]
		20 to 24	1950	243.37	[240.90 - 251.75]	65	[64 - 67]
			1985	199.94	[194.12 - 205.89]	121	[117 - 124]
			2019	214.20	[167.70 - 270.49]	96	[75 - 121]
	Female	10 to 14	1950	82.95	[81.40 - 87.64]	30	[30 - 32]
			1985	37.46	[35.45 - 39.44]	22	[21 - 23]
			2019	24.06	[19.63 - 29.41]	11	[9 - 14]
		15 to 19	1950	129.73	[127.78 - 135.45]	41	[41 - 43]
			1985	59.10	[56.38 - 61.70]	34	[33 - 36]
			2019	45.06	[36.50 - 55.42]	20	[16 - 24]
		20 to 24	1950	217.68	[214.26 - 228.32]	60	[59 - 63]
			1985	80.49	[76.75 - 84.29]	48	[46 - 51]
			2019	68.46	[50.55 - 91.61]	30	[22 - 40]

Table S118: All-cause mortality rate per 100,000 population and number of deaths in 10-24 by sex 1950 - 2019: Andean Latin America

Location	Sex	Age	Year	Mortality per 100,000	Mortality per 100,000 uncertainty interval	Number	Number Uncertainty Interval
Andean Latin America	Male	10 to 14	1950	288.81	[277.54 - 326.49]	2,323	[2,232 - 2,626]
			1985	102.55	[96.71 - 108.78]	2,159	[2,036 - 2,290]
			2019	38.01	[32.39 - 44.82]	1,121	[956 - 1,323]
		15 to 19	1950	363.95	[348.82 - 413.10]	2,548	[2,442 - 2,892]
			1985	155.62	[144.08 - 167.88]	2,884	[2,670 - 3,111]
			2019	82.31	[67.46 - 98.61]	2,379	[1,950 - 2,850]
		20 to 24	1950	396.85	[384.05 - 439.16]	2,380	[2,303 - 2,634]
			1985	236.98	[220.66 - 255.72]	3,725	[3,469 - 4,020]
			2019	140.74	[118.23 - 165.09]	3,982	[3,345 - 4,671]
	Female	10 to 14	1950	216.26	[208.71 - 238.78]	1,730	[1,669 - 1,910]
			1985	85.58	[80.72 - 90.61]	1,773	[1,672 - 1,877]
			2019	32.41	[28.11 - 37.40]	892	[773 - 1,029]
		15 to 19	1950	280.49	[271.60 - 309.93]	2,007	[1,944 - 2,218]
			1985	114.69	[107.94 - 121.22]	2,144	[2,018 - 2,267]
			2019	49.20	[41.36 - 57.52]	1,328	[1,117 - 1,553]
		20 to 24	1950	385.26	[369.65 - 440.92]	2,406	[2,308 - 2,753]
			1985	164.74	[149.66 - 180.80]	2,685	[2,439 - 2,947]
			2019	60.27	[48.34 - 74.13]	1,634	[1,311 - 2,010]

Table S119: All-cause mortality rate per 100,000 population and number of deaths in 10-24 by sex 1950 - 2019: Bolivia (Plurinational State of)

Location	Sex	Age	Year	Mortality per 100,000	Mortality per 100,000 uncertainty interval	Number	Number Uncertainty Interval
Bolivia (Plurinational State of)	Male	10 to 14	1950	281.52	[271.75 - 321.18]	450	[435 - 514]
			1985	113.75	[100.79 - 126.22]	391	[347 - 434]
			2019	41.63	[36.71 - 48.47]	234	[207 - 273]
		15 to 19	1950	379.13	[363.68 - 440.56]	535	[513 - 621]
			1985	170.30	[149.68 - 188.97]	499	[438 - 553]
			2019	86.86	[70.86 - 104.44]	469	[383 - 564]
		20 to 24	1950	391.52	[375.85 - 451.57]	487	[468 - 562]
			1985	226.01	[200.41 - 250.02]	553	[491 - 612]
			2019	118.18	[84.21 - 147.50]	635	[452 - 792]
	Female	10 to 14	1950	209.09	[201.66 - 236.21]	319	[308 - 360]
			1985	95.37	[82.01 - 104.59]	323	[277 - 354]
			2019	33.79	[29.66 - 38.62]	184	[162 - 211]
		15 to 19	1950	314.07	[304.23 - 348.14]	441	[428 - 489]
			1985	143.96	[123.34 - 159.05]	424	[364 - 469]
			2019	54.08	[39.74 - 65.90]	286	[210 - 349]
		20 to 24	1950	434.77	[413.55 - 512.20]	579	[550 - 682]
			1985	199.40	[163.47 - 229.84]	504	[414 - 581]
			2019	70.37	[51.10 - 89.65]	372	[270 - 474]

Table S120: All-cause mortality rate per 100,000 population and number of deaths in 10-24 by sex 1950 - 2019: Ecuador

Location	Sex	Age	Year	Mortality per 100,000	Mortality per 100,000 uncertainty interval	Number	Number Uncertainty Interval
Ecuador	Male	10 to 14	1950	305.06	[290.30 - 360.47]	607	[578 - 717]
			1985	85.23	[81.58 - 88.86]	478	[458 - 499]
			2019	45.50	[39.24 - 53.23]	383	[330 - 448]
		15 to 19	1950	361.94	[337.03 - 448.77]	614	[572 - 762]
			1985	146.07	[142.72 - 149.72]	712	[696 - 730]
			2019	113.70	[90.94 - 143.42]	953	[762 - 1,202]
		20 to 24	1950	485.01	[453.18 - 592.60]	706	[659 - 862]
			1985	239.56	[236.01 - 243.22]	1,002	[987 - 1,017]
			2019	214.77	[174.07 - 268.41]	1,740	[1,410 - 2,175]
	Female	10 to 14	1950	260.87	[249.81 - 302.85]	518	[496 - 601]
			1985	68.23	[65.90 - 70.66]	378	[365 - 391]
			2019	36.86	[32.42 - 42.30]	298	[262 - 342]
		15 to 19	1950	299.95	[288.18 - 342.91]	517	[497 - 591]
			1985	96.54	[94.07 - 99.14]	478	[466 - 491]
			2019	56.47	[48.91 - 65.80]	461	[399 - 537]
		20 to 24	1950	448.31	[412.93 - 572.14]	678	[625 - 866]
			1985	124.41	[121.65 - 127.16]	544	[532 - 556]
			2019	68.79	[53.51 - 89.89]	547	[425 - 714]

Table S121: All-cause mortality rate per 100,000 population and number of deaths in 10-24 by sex 1950 - 2019: Peru

Location	Sex	Age	Year	Mortality per 100,000	Mortality per 100,000 uncertainty interval	Number	Number Uncertainty Interval
Peru	Male	10 to 14	1950	284.41	[265.94 - 342.05]	1,266	[1,184 - 1,523]
			1985	107.43	[98.29 - 117.62]	1,289	[1,180 - 1,412]
			2019	32.62	[26.01 - 40.68]	504	[402 - 629]
		15 to 19	1950	360.56	[335.47 - 435.79]	1,403	[1,306 - 1,696]
			1985	155.95	[136.97 - 176.13]	1,674	[1,470 - 1,890]
			2019	63.27	[47.09 - 83.94]	956	[712 - 1,269]
		20 to 24	1950	360.78	[341.27 - 419.22]	1,190	[1,126 - 1,383]
			1985	238.74	[210.47 - 269.00]	2,170	[1,913 - 2,446]
			2019	108.43	[84.33 - 138.29]	1,607	[1,250 - 2,049]
	Female	10 to 14	1950	199.40	[187.57 - 232.41]	895	[842 - 1,043]
			1985	90.91	[83.14 - 98.94]	1,072	[981 - 1,167]
			2019	29.30	[24.03 - 35.66]	409	[336 - 498]
		15 to 19	1950	260.82	[247.27 - 308.55]	1,050	[996 - 1,243]
			1985	115.02	[104.87 - 125.43]	1,242	[1,132 - 1,354]
			2019	42.92	[34.76 - 52.87]	582	[471 - 716]
		20 to 24	1950	336.44	[314.75 - 427.89]	1,144	[1,070 - 1,455]
			1985	174.17	[150.21 - 200.30]	1,637	[1,411 - 1,882]
			2019	51.56	[38.14 - 68.95]	716	[530 - 957]

Table S122: All-cause mortality rate per 100,000 population and number of deaths in 10-24 by sex 1950 - 2019: Central Latin America

Location	Sex	Age	Year	Mortality per 100,000	Mortality per 100,000 uncertainty interval	Number	Number Uncertainty Interval
Central Latin America	Male	10 to 14	1950	261.02	[256.18 - 278.47]	8,314	[8,160 - 8,870]
			1985	103.07	[98.51 - 108.28]	9,693	[9,264 - 10,183]
			2019	36.50	[31.12 - 42.88]	4,102	[3,497 - 4,818]
		15 to 19	1950	364.36	[361.27 - 376.53]	9,977	[9,893 - 10,310]
			1985	201.72	[192.78 - 213.59]	16,616	[15,879 - 17,593]
			2019	127.90	[106.94 - 152.80]	14,244	[11,910 - 17,018]
		20 to 24	1950	533.45	[531.84 - 538.04]	12,552	[12,514 - 12,660]
			1985	343.64	[329.14 - 363.60]	23,846	[22,841 - 25,232]
			2019	223.95	[193.09 - 259.95]	24,134	[20,808 - 28,013]
	Female	10 to 14	1950	206.39	[203.93 - 215.11]	6,566	[6,488 - 6,844]
			1985	68.02	[64.79 - 72.21]	6,349	[6,047 - 6,740]
			2019	26.40	[23.27 - 29.83]	2,850	[2,512 - 3,220]
		15 to 19	1950	296.58	[293.98 - 306.03]	8,335	[8,262 - 8,601]
			1985	89.05	[85.38 - 93.97]	7,549	[7,238 - 7,966]
			2019	49.04	[42.63 - 55.78]	5,356	[4,656 - 6,093]
		20 to 24	1950	418.64	[417.03 - 423.88]	10,280	[10,241 - 10,409]
			1985	115.43	[111.56 - 120.45]	8,542	[8,256 - 8,914]
			2019	63.16	[52.42 - 74.75]	6,902	[5,728 - 8,168]

Table S123: All-cause mortality rate per 100,000 population and number of deaths in 10-24 by sex 1950 - 2019: Colombia

Location	Sex	Age	Year	Mortality per 100,000	Mortality per 100,000 uncertainty interval	Number	Number Uncertainty Interval
Colombia	Male	10 to 14	1950	232.69	[228.51 - 248.43]	1,600	[1,572 - 1,709]
			1985	159.30	[150.56 - 169.06]	2,738	[2,587 - 2,905]
			2019	34.30	[28.11 - 42.19]	652	[534 - 802]
		15 to 19	1950	320.16	[317.49 - 330.20]	1,885	[1,869 - 1,944]
			1985	292.48	[280.61 - 305.92]	4,785	[4,591 - 5,005]
			2019	129.26	[99.12 - 168.39]	2,633	[2,019 - 3,430]
		20 to 24	1950	582.51	[580.61 - 587.95]	2,956	[2,947 - 2,984]
			1985	477.09	[463.40 - 493.10]	7,071	[6,868 - 7,308]
			2019	251.05	[200.50 - 315.69]	5,223	[4,171 - 6,568]
	Female	10 to 14	1950	131.24	[129.84 - 136.72]	907	[897 - 945]
			1985	105.72	[99.68 - 112.47]	1,798	[1,695 - 1,913]
			2019	24.76	[20.80 - 29.44]	449	[377 - 534]
		15 to 19	1950	183.30	[181.64 - 189.45]	1,107	[1,097 - 1,144]
			1985	124.94	[119.61 - 130.99]	2,129	[2,038 - 2,232]
			2019	40.68	[33.75 - 48.98]	793	[658 - 955]
		20 to 24	1950	277.44	[276.15 - 281.22]	1,462	[1,455 - 1,482]
			1985	142.99	[137.99 - 148.77]	2,286	[2,206 - 2,378]
			2019	50.47	[38.85 - 65.15]	1,031	[794 - 1,331]

Table S124: All-cause mortality rate per 100,000 population and number of deaths in 10-24 by sex 1950 - 2019: Costa Rica

Location	Sex	Age	Year	Mortality per 100,000	Mortality per 100,000 uncertainty interval	Number	Number Uncertainty Interval
Costa Rica	Male	10 to 14	1950	155.63	[154.26 - 160.69]	79	[79 - 82]
			1985	43.07	[42.03 - 44.16]	63	[61 - 65]
			2019	24.97	[20.49 - 30.31]	46	[37 - 55]
		15 to 19	1950	184.89	[182.47 - 192.34]	80	[79 - 83]
			1985	76.84	[74.51 - 79.25]	109	[106 - 113]
			2019	87.75	[68.15 - 111.97]	159	[123 - 203]
		20 to 24	1950	277.15	[273.57 - 287.76]	105	[104 - 109]
			1985	134.72	[130.63 - 138.82]	180	[175 - 186]
			2019	149.93	[121.05 - 184.71]	293	[236 - 361]
	Female	10 to 14	1950	103.94	[103.04 - 107.17]	53	[53 - 55]
			1985	24.92	[24.34 - 25.54]	35	[35 - 36]
			2019	16.89	[14.20 - 20.07]	30	[25 - 36]
		15 to 19	1950	149.62	[148.41 - 154.09]	68	[67 - 70]
			1985	39.34	[38.34 - 40.39]	56	[54 - 57]
			2019	25.25	[21.01 - 30.32]	46	[38 - 55]
		20 to 24	1950	252.69	[248.89 - 265.90]	101	[99 - 106]
			1985	44.12	[42.28 - 46.15]	60	[58 - 63]
			2019	40.23	[30.93 - 51.94]	81	[62 - 105]

Table S125: All-cause mortality rate per 100,000 population and number of deaths in 10-24 by sex 1950 - 2019: El Salvador

Location	Sex	Age	Year	Mortality per 100,000	Mortality per 100,000 uncertainty interval	Number	Number Uncertainty Interval
El Salvador	Male	10 to 14	1950	213.08	[208.05 - 233.06]	243	[237 - 266]
			1985	134.08	[119.12 - 154.08]	447	[397 - 514]
			2019	39.17	[31.55 - 48.29]	112	[90 - 138]
		15 to 19	1950	309.89	[305.67 - 325.46]	307	[303 - 322]
			1985	434.81	[386.06 - 500.81]	1,198	[1,064 - 1,380]
			2019	173.97	[134.22 - 221.42]	511	[395 - 651]
		20 to 24	1950	488.29	[485.43 - 498.18]	401	[399 - 409]
			1985	853.09	[755.49 - 981.53]	1,866	[1,653 - 2,147]
			2019	274.03	[219.56 - 341.70]	820	[657 - 1,022]
	Female	10 to 14	1950	175.62	[173.16 - 185.15]	202	[199 - 212]
			1985	107.20	[93.22 - 126.62]	349	[304 - 412]
			2019	27.58	[22.85 - 33.44]	74	[62 - 90]
		15 to 19	1950	227.15	[224.66 - 236.01]	233	[231 - 242]
			1985	162.82	[143.52 - 189.17]	458	[404 - 532]
			2019	44.78	[36.66 - 55.19]	130	[107 - 160]
		20 to 24	1950	295.01	[292.21 - 303.33]	262	[260 - 270]
			1985	181.57	[163.05 - 206.32]	433	[389 - 492]
			2019	55.17	[41.62 - 74.08]	176	[132 - 236]

Table S126: All-cause mortality rate per 100,000 population and number of deaths in 10-24 by sex 1950 - 2019: Guatemala

Location	Sex	Age	Year	Mortality per 100,000	Mortality per 100,000 uncertainty interval	Number	Number Uncertainty Interval
Guatemala	Male	10 to 14	1950	480.91	[469.53 - 523.67]	880	[859 - 958]
			1985	166.92	[150.96 - 183.58]	726	[657 - 799]
			2019	50.32	[40.79 - 61.79]	487	[395 - 598]
		15 to 19	1950	582.90	[576.04 - 609.03]	933	[922 - 975]
			1985	284.27	[248.20 - 323.31]	1,002	[875 - 1,140]
			2019	167.59	[129.83 - 213.89]	1,617	[1,253 - 2,064]
		20 to 24	1950	665.65	[662.39 - 677.05]	945	[940 - 961]
			1985	524.26	[459.39 - 592.99]	1,524	[1,335 - 1,724]
			2019	298.38	[241.10 - 366.47]	2,776	[2,243 - 3,410]
	Female	10 to 14	1950	436.33	[430.31 - 459.01]	771	[760 - 811]
			1985	134.03	[123.08 - 146.16]	577	[530 - 629]
			2019	40.59	[33.84 - 48.62]	375	[312 - 449]
		15 to 19	1950	650.45	[643.88 - 676.06]	1,056	[1,046 - 1,098]
			1985	187.42	[172.35 - 204.10]	686	[631 - 747]
			2019	83.90	[69.79 - 100.55]	784	[652 - 939]
		20 to 24	1950	748.08	[743.71 - 763.28]	1,095	[1,089 - 1,117]
			1985	274.73	[237.38 - 317.27]	864	[747 - 998]
			2019	90.84	[69.98 - 117.00]	843	[649 - 1,085]

Table S127: All-cause mortality rate per 100,000 population and number of deaths in 10-24 by sex 1950 - 2019: Honduras

Location	Sex	Age	Year	Mortality per 100,000	Mortality per 100,000 uncertainty interval	Number	Number Uncertainty Interval
Honduras	Male	10 to 14	1950	459.63	[443.22 - 517.78]	402	[388 - 453]
			1985	95.32	[86.83 - 104.63]	255	[232 - 280]
			2019	36.97	[29.16 - 44.87]	202	[160 - 246]
		15 to 19	1950	758.06	[723.62 - 866.06]	573	[547 - 654]
			1985	139.38	[120.70 - 160.15]	284	[246 - 326]
			2019	97.53	[78.49 - 119.19]	518	[417 - 633]
		20 to 24	1950	1198.08	[1144.09 - 1352.62]	767	[733 - 866]
			1985	260.11	[225.39 - 298.06]	414	[359 - 475]
			2019	137.49	[94.44 - 188.77]	663	[455 - 910]
	Female	10 to 14	1950	440.66	[426.10 - 487.74]	372	[360 - 412]
			1985	79.67	[73.97 - 85.52]	208	[194 - 224]
			2019	27.52	[24.52 - 32.38]	146	[130 - 171]
		15 to 19	1950	657.53	[641.74 - 705.23]	490	[478 - 525]
			1985	100.82	[93.20 - 108.58]	215	[199 - 232]
			2019	54.63	[41.68 - 69.79]	291	[222 - 371]
		20 to 24	1950	1110.00	[1052.65 - 1277.74]	725	[688 - 835]
			1985	170.92	[148.04 - 195.36]	304	[263 - 348]
			2019	74.80	[46.56 - 108.21]	385	[240 - 557]

Table S128: All-cause mortality rate per 100,000 population and number of deaths in 10-24 by sex 1950 - 2019: Mexico

Location	Sex	Age	Year	Mortality per 100,000	Mortality per 100,000 uncertainty interval	Number	Number Uncertainty Interval
Mexico	Male	10 to 14	1950	272.05	[266.91 - 290.15]	4,428	[4,344 - 4,723]
			1985	85.40	[81.87 - 88.53]	4,410	[4,228 - 4,572]
			2019	34.10	[30.00 - 38.74]	1,908	[1,679 - 2,168]
		15 to 19	1950	382.12	[378.66 - 394.37]	5,317	[5,268 - 5,487]
			1985	158.33	[155.29 - 161.16]	6,986	[6,852 - 7,110]
			2019	110.34	[91.67 - 131.23]	6,058	[5,033 - 7,205]
		20 to 24	1950	533.38	[532.38 - 536.61]	6,283	[6,271 - 6,321]
			1985	263.88	[262.46 - 265.25]	9,506	[9,455 - 9,556]
			2019	197.11	[165.94 - 233.30]	10,463	[8,808 - 12,384]
	Female	10 to 14	1950	222.43	[219.46 - 233.72]	3,660	[3,611 - 3,845]
			1985	50.57	[48.99 - 52.14]	2,610	[2,529 - 2,692]
			2019	24.99	[22.27 - 28.07]	1,351	[1,204 - 1,517]
		15 to 19	1950	315.79	[312.57 - 327.70]	4,575	[4,529 - 4,748]
			1985	65.82	[64.35 - 67.33]	3,001	[2,934 - 3,070]
			2019	41.29	[36.35 - 47.01]	2,246	[1,977 - 2,557]
		20 to 24	1950	444.05	[442.98 - 447.67]	5,546	[5,533 - 5,591]
			1985	91.98	[91.29 - 92.68]	3,544	[3,518 - 3,571]
			2019	56.13	[45.26 - 69.50]	3,027	[2,441 - 3,748]

Table S129: All-cause mortality rate per 100,000 population and number of deaths in 10-24 by sex 1950 - 2019: Nicaragua

Location	Sex	Age	Year	Mortality per 100,000	Mortality per 100,000 uncertainty interval	Number	Number Uncertainty Interval
Nicaragua	Male	10 to 14	1950	166.42	[162.82 - 179.82]	117	[114 - 126]
			1985	185.71	[135.72 - 265.03]	408	[298 - 582]
			2019	31.90	[27.24 - 36.87]	108	[92 - 125]
		15 to 19	1950	208.42	[205.45 - 219.02]	123	[122 - 130]
			1985	511.25	[343.36 - 774.77]	889	[597 - 1,348]
			2019	89.08	[70.30 - 113.90]	285	[225 - 365]
		20 to 24	1950	229.94	[227.61 - 238.65]	115	[114 - 119]
			1985	953.70	[617.32 - 1468.41]	1,323	[857 - 2,037]
			2019	117.03	[89.74 - 153.16]	362	[278 - 474]
	Female	10 to 14	1950	126.06	[123.86 - 132.00]	87	[85 - 91]
			1985	157.22	[110.04 - 231.68]	345	[242 - 509]
			2019	20.16	[17.49 - 22.85]	65	[56 - 74]
		15 to 19	1950	190.10	[187.55 - 197.27]	116	[114 - 120]
			1985	221.46	[155.11 - 327.16]	403	[282 - 595]
			2019	34.80	[27.17 - 43.43]	107	[84 - 134]
		20 to 24	1950	236.58	[232.47 - 247.93]	127	[124 - 133]
			1985	234.02	[166.93 - 337.81]	356	[254 - 514]
			2019	41.37	[31.99 - 50.77]	126	[97 - 155]

Table S130: All-cause mortality rate per 100,000 population and number of deaths in 10-24 by sex 1950 - 2019: Panama

Location	Sex	Age	Year	Mortality per 100,000	Mortality per 100,000 uncertainty interval	Number	Number Uncertainty Interval
Panama	Male	10 to 14	1950	169.38	[165.95 - 180.36]	80	[78 - 85]
			1985	50.10	[47.10 - 53.18]	64	[60 - 68]
			2019	32.01	[26.00 - 39.29]	60	[49 - 74]
		15 to 19	1950	216.92	[213.70 - 227.66]	88	[87 - 93]
			1985	108.18	[103.80 - 112.82]	128	[123 - 134]
			2019	102.33	[78.71 - 131.82]	183	[141 - 236]
		20 to 24	1950	319.35	[315.35 - 331.20]	114	[113 - 118]
			1985	164.69	[159.29 - 170.36]	169	[163 - 175]
			2019	170.56	[136.97 - 211.28]	307	[246 - 380]
	Female	10 to 14	1950	136.54	[134.39 - 143.82]	64	[63 - 68]
			1985	35.82	[34.21 - 37.57]	44	[42 - 46]
			2019	25.34	[21.14 - 30.36]	46	[38 - 55]
		15 to 19	1950	234.46	[231.37 - 244.53]	98	[97 - 102]
			1985	57.00	[54.81 - 59.33]	67	[64 - 69]
			2019	44.27	[36.63 - 53.44]	76	[63 - 91]
		20 to 24	1950	349.10	[343.46 - 366.17]	129	[127 - 135]
			1985	63.16	[60.17 - 66.24]	65	[62 - 69]
			2019	51.58	[39.61 - 66.72]	89	[69 - 116]

Table S131: All-cause mortality rate per 100,000 population and number of deaths in 10-24 by sex 1950 - 2019: Venezuela (Bolivarian Republic of)

Location	Sex	Age	Year	Mortality per 100,000	Mortality per 100,000 uncertainty interval	Number	Number Uncertainty Interval
Venezuela (Bolivarian Republic of)	Male	10 to 14	1950	153.27	[152.66 - 155.54]	486	[484 - 493]
			1985	58.72	[58.10 - 59.31]	582	[576 - 588]
			2019	42.80	[34.33 - 52.60]	527	[423 - 648]
		15 to 19	1950	240.37	[239.26 - 244.40]	674	[671 - 685]
			1985	133.78	[132.25 - 135.30]	1,234	[1,220 - 1,248]
			2019	200.04	[152.30 - 253.68]	2,279	[1,735 - 2,890]
		20 to 24	1950	337.93	[336.41 - 342.87]	864	[860 - 877]
			1985	221.05	[218.68 - 223.38]	1,792	[1,773 - 1,811]
			2019	325.49	[257.63 - 399.47]	3,227	[2,554 - 3,961]
	Female	10 to 14	1950	148.49	[147.89 - 150.47]	449	[447 - 455]
			1985	39.43	[38.97 - 39.87]	382	[377 - 386]
			2019	26.77	[22.05 - 32.15]	314	[259 - 378]
		15 to 19	1950	218.79	[218.00 - 221.33]	591	[589 - 598]
			1985	58.51	[57.88 - 59.13]	534	[528 - 539]
			2019	79.12	[64.34 - 95.21]	885	[720 - 1,065]
		20 to 24	1950	335.84	[333.82 - 341.89]	836	[831 - 851]
			1985	76.20	[74.97 - 77.48]	629	[618 - 639]
			2019	108.77	[81.84 - 141.71]	1,144	[860 - 1,490]

Table S132: All-cause mortality rate per 100,000 population and number of deaths in 10-24 by sex 1950 - 2019: Tropical Latin America

Location	Sex	Age	Year	Mortality per 100,000	Mortality per 100,000 uncertainty interval	Number	Number Uncertainty Interval
Tropical Latin America	Male	10 to 14	1950	183.21	[178.51 - 199.08]	6,150	[5,992 - 6,683]
			1985	77.80	[75.23 - 80.38]	6,249	[6,042 - 6,456]
			2019	38.93	[35.80 - 42.39]	3,363	[3,092 - 3,661]
		15 to 19	1950	319.61	[312.52 - 342.40]	9,346	[9,138 - 10,012]
			1985	165.26	[161.17 - 169.39]	12,179	[11,878 - 12,483]
			2019	168.90	[160.45 - 177.77]	14,648	[13,915 - 15,417]
		20 to 24	1950	398.98	[392.09 - 423.86]	10,083	[9,909 - 10,712]
			1985	262.42	[257.04 - 268.05]	17,675	[17,313 - 18,054]
			2019	245.58	[237.61 - 254.43]	22,359	[21,634 - 23,165]
	Female	10 to 14	1950	108.54	[106.56 - 115.28]	3,728	[3,660 - 3,959]
			1985	48.37	[47.04 - 49.77]	3,860	[3,754 - 3,972]
			2019	24.87	[23.44 - 26.44]	2,074	[1,954 - 2,204]
		15 to 19	1950	147.61	[144.92 - 156.60]	4,529	[4,447 - 4,805]
			1985	66.99	[65.51 - 68.57]	5,001	[4,891 - 5,119]
			2019	41.43	[39.50 - 43.57]	3,492	[3,329 - 3,672]
		20 to 24	1950	203.30	[198.19 - 224.76]	5,407	[5,271 - 5,978]
			1985	90.81	[88.82 - 93.06]	6,299	[6,161 - 6,456]
			2019	52.41	[50.19 - 54.84]	4,718	[4,519 - 4,937]

Table S133: All-cause mortality rate per 100,000 population and number of deaths in 10-24 by sex 1950 - 2019: Brazil

Location	Sex	Age	Year	Mortality per 100,000	Mortality per 100,000 uncertainty interval	Number	Number Uncertainty Interval
Brazil	Male	10 to 14	1950	184.13	[179.42 - 199.67]	6,028	[5,873 - 6,536]
			1985	78.60	[75.99 - 81.31]	6,151	[5,947 - 6,364]
			2019	39.15	[36.14 - 42.41]	3,248	[2,998 - 3,519]
		15 to 19	1950	321.95	[314.90 - 344.63]	9,190	[8,989 - 9,837]
			1985	167.61	[163.37 - 171.84]	12,048	[11,743 - 12,352]
			2019	171.46	[163.18 - 179.85]	14,303	[13,612 - 15,003]
		20 to 24	1950	400.75	[393.63 - 426.57]	9,893	[9,717 - 10,530]
			1985	266.33	[260.77 - 272.18]	17,510	[17,145 - 17,895]
			2019	248.42	[240.52 - 257.66]	21,811	[21,117 - 22,622]
	Female	10 to 14	1950	109.25	[107.27 - 115.98]	3,662	[3,596 - 3,888]
			1985	48.59	[47.23 - 50.04]	3,780	[3,674 - 3,893]
			2019	24.84	[23.53 - 26.26]	1,991	[1,886 - 2,105]
		15 to 19	1950	147.87	[145.20 - 157.11]	4,433	[4,353 - 4,710]
			1985	67.25	[65.71 - 68.87]	4,899	[4,787 - 5,017]
			2019	41.27	[39.53 - 43.15]	3,347	[3,207 - 3,500]
		20 to 24	1950	202.94	[197.52 - 224.83]	5,271	[5,130 - 5,839]
			1985	91.00	[89.01 - 93.27]	6,165	[6,030 - 6,319]
			2019	52.40	[50.10 - 54.89]	4,553	[4,353 - 4,769]

Table S134: All-cause mortality rate per 100,000 population and number of deaths in 10-24 by sex 1950 - 2019: Paraguay

Location	Sex	Age	Year	Mortality per 100,000	Mortality per 100,000 uncertainty interval	Number	Number Uncertainty Interval
Paraguay	Male	10 to 14	1950	147.42	[140.05 - 177.46]	123	[117 - 148]
			1985	47.68	[43.67 - 51.77]	98	[90 - 107]
			2019	33.65	[27.20 - 41.29]	115	[93 - 141]
		15 to 19	1950	217.58	[204.55 - 271.07]	152	[143 - 189]
			1985	72.29	[64.66 - 80.34]	131	[117 - 146]
			2019	104.26	[80.01 - 133.98]	345	[264 - 443]
		20 to 24	1950	323.28	[307.26 - 388.36]	189	[180 - 228]
			1985	102.69	[92.67 - 113.37]	165	[149 - 182]
			2019	168.72	[135.09 - 209.25]	548	[439 - 680]
	Female	10 to 14	1950	78.91	[75.04 - 93.84]	65	[62 - 77]
			1985	39.81	[37.05 - 42.79]	80	[74 - 86]
			2019	25.72	[21.41 - 30.83]	83	[69 - 100]
		15 to 19	1950	136.83	[130.56 - 160.59]	96	[92 - 113]
			1985	56.64	[52.72 - 61.02]	102	[95 - 110]
			2019	45.65	[37.55 - 55.26]	145	[119 - 175]
		20 to 24	1950	222.14	[209.03 - 276.50]	139	[131 - 173]
			1985	82.63	[73.58 - 92.37]	134	[119 - 150]
			2019	52.67	[39.96 - 68.90]	165	[126 - 216]

Table S135: All-cause mortality rate per 100,000 population and number of deaths in 10-24 by sex 1950 - 2019: North Africa and Middle East

Location	Sex	Age	Year	Mortality per 100,000	Mortality per 100,000 uncertainty interval	Number	Number Uncertainty Interval
North Africa and Middle East	Male	10 to 14	1950	240.01	[230.26 - 269.90]	15,743	[15,103 - 17,703]
			1985	124.67	[109.34 - 163.96]	23,737	[20,818 - 31,220]
			2019	45.05	[40.10 - 51.08]	13,167	[11,720 - 14,929]
		15 to 19	1950	304.54	[291.97 - 342.27]	17,445	[16,725 - 19,607]
			1985	207.29	[164.79 - 321.15]	33,735	[26,819 - 52,265]
			2019	92.48	[81.36 - 104.72]	25,297	[22,255 - 28,643]
		20 to 24	1950	322.03	[311.80 - 356.04]	16,302	[15,784 - 18,024]
			1985	269.44	[208.96 - 431.00]	37,102	[28,774 - 59,349]
			2019	130.65	[115.48 - 149.53]	34,871	[30,822 - 39,910]
	Female	10 to 14	1950	152.82	[147.51 - 168.61]	9,460	[9,132 - 10,438]
			1985	95.68	[81.14 - 133.85]	17,146	[14,541 - 23,986]
			2019	34.60	[31.34 - 38.88]	9,472	[8,580 - 10,642]
		15 to 19	1950	198.01	[191.41 - 218.51]	11,003	[10,636 - 12,142]
			1985	121.15	[103.25 - 167.09]	18,765	[15,991 - 25,880]
			2019	51.32	[45.23 - 59.37]	13,137	[11,580 - 15,199]
		20 to 24	1950	244.99	[234.66 - 278.40]	12,104	[11,594 - 13,755]
			1985	141.72	[124.66 - 177.89]	18,804	[16,540 - 23,603]
			2019	64.91	[55.49 - 77.65]	16,253	[13,892 - 19,441]

Table S136: All-cause mortality rate per 100,000 population and number of deaths in 10-24 by sex 1950 - 2019: Algeria

Location	Sex	Age	Year	Mortality per 100,000	Mortality per 100,000 uncertainty interval	Number	Number Uncertainty Interval
Algeria	Male	10 to 14	1950	167.57	[162.01 - 189.21]	869	[840 - 981]
			1985	148.22	[133.49 - 163.82]	2,133	[1,921 - 2,357]
			2019	38.15	[33.52 - 43.09]	683	[600 - 771]
		15 to 19	1950	248.45	[238.20 - 286.92]	1,098	[1,052 - 1,268]
			1985	195.59	[167.62 - 224.12]	2,410	[2,065 - 2,762]
			2019	72.11	[60.20 - 86.21]	1,119	[934 - 1,338]
		20 to 24	1950	294.83	[282.80 - 336.45]	1,084	[1,039 - 1,237]
			1985	228.14	[197.69 - 260.27]	2,396	[2,076 - 2,733]
			2019	106.48	[89.82 - 125.48]	1,749	[1,475 - 2,061]
	Female	10 to 14	1950	121.32	[117.31 - 135.36]	601	[581 - 671]
			1985	109.42	[99.22 - 119.67]	1,491	[1,352 - 1,630]
			2019	30.97	[27.74 - 34.69]	528	[473 - 591]
		15 to 19	1950	178.42	[172.82 - 192.66]	778	[754 - 840]
			1985	121.34	[109.61 - 133.02]	1,464	[1,323 - 1,605]
			2019	44.51	[39.30 - 50.56]	663	[585 - 753]
		20 to 24	1950	253.19	[240.81 - 284.09]	955	[908 - 1,071]
			1985	143.75	[121.51 - 167.84]	1,478	[1,250 - 1,726]
			2019	52.22	[42.52 - 63.61]	833	[678 - 1,015]

Table S137: All-cause mortality rate per 100,000 population and number of deaths in 10-24 by sex 1950 - 2019: Bahrain

Location	Sex	Age	Year	Mortality per 100,000	Mortality per 100,000 uncertainty interval	Number	Number Uncertainty Interval
Bahrain	Male	10 to 14	1950	166.98	[154.05 - 216.44]	14	[13 - 19]
			1985	56.51	[51.99 - 61.41]	10	[10 - 11]
			2019	26.08	[22.91 - 29.41]	11	[10 - 12]
		15 to 19	1950	246.32	[225.84 - 319.38]	19	[17 - 24]
			1985	95.03	[83.45 - 107.47]	19	[17 - 22]
			2019	55.97	[45.98 - 67.09]	25	[20 - 30]
		20 to 24	1950	244.61	[227.19 - 309.78]	15	[14 - 19]
			1985	105.39	[93.43 - 118.81]	28	[25 - 32]
			2019	107.92	[90.18 - 128.25]	45	[37 - 53]
	Female	10 to 14	1950	101.30	[93.27 - 135.57]	6	[6 - 9]
			1985	29.21	[27.18 - 31.34]	5	[5 - 6]
			2019	13.64	[12.25 - 15.08]	6	[5 - 6]
		15 to 19	1950	148.25	[135.66 - 194.67]	8	[7 - 11]
			1985	52.81	[48.47 - 57.19]	10	[9 - 11]
			2019	28.15	[24.89 - 31.61]	12	[10 - 13]
		20 to 24	1950	171.39	[153.73 - 244.33]	8	[7 - 11]
			1985	63.63	[54.90 - 73.15]	13	[11 - 15]
			2019	43.40	[35.40 - 52.80]	16	[13 - 20]

Table S138: All-cause mortality rate per 100,000 population and number of deaths in 10-24 by sex 1950 - 2019: Egypt

Location	Sex	Age	Year	Mortality per 100,000	Mortality per 100,000 uncertainty interval	Number	Number Uncertainty Interval
Egypt	Male	10 to 14	1950	312.58	[305.95 - 340.14]	3,557	[3,482 - 3,871]
			1985	145.48	[139.40 - 152.01]	4,317	[4,137 - 4,511]
			2019	47.75	[38.31 - 59.05]	2,590	[2,078 - 3,202]
		15 to 19	1950	266.99	[264.15 - 278.31]	2,622	[2,594 - 2,733]
			1985	161.45	[158.35 - 164.81]	4,243	[4,162 - 4,331]
			2019	88.18	[68.53 - 112.09]	4,246	[3,299 - 5,397]
		20 to 24	1950	250.85	[250.25 - 252.72]	2,206	[2,201 - 2,223]
			1985	194.67	[193.71 - 195.64]	4,395	[4,373 - 4,417]
			2019	115.12	[93.52 - 140.95]	5,117	[4,157 - 6,265]
	Female	10 to 14	1950	198.68	[196.42 - 207.53]	2,256	[2,231 - 2,357]
			1985	109.77	[106.13 - 113.54]	3,000	[2,901 - 3,103]
			2019	31.99	[26.45 - 38.54]	1,590	[1,315 - 1,915]
		15 to 19	1950	192.64	[190.79 - 199.64]	1,947	[1,928 - 2,018]
			1985	125.69	[122.65 - 128.83]	3,033	[2,960 - 3,109]
			2019	40.84	[33.58 - 49.40]	1,813	[1,491 - 2,194]
		20 to 24	1950	190.40	[189.76 - 192.56]	1,768	[1,762 - 1,788]
			1985	142.64	[141.50 - 143.69]	3,016	[2,992 - 3,038]
			2019	51.36	[38.86 - 67.30]	2,213	[1,675 - 2,901]

Table S139: All-cause mortality rate per 100,000 population and number of deaths in 10-24 by sex 1950 - 2019: Iran (Islamic Republic of)

Location	Sex	Age	Year	Mortality per 100,000	Mortality per 100,000 uncertainty interval	Number	Number Uncertainty Interval
Iran (Islamic Republic of)	Male	10 to 14	1950	268.24	[261.18 - 291.47]	2,221	[2,163 - 2,414]
			1985	96.00	[90.58 - 101.51]	3,012	[2,842 - 3,185]
			2019	38.97	[37.35 - 40.53]	1,267	[1,214 - 1,317]
		15 to 19	1950	371.89	[365.05 - 395.47]	2,913	[2,859 - 3,097]
			1985	209.90	[199.52 - 220.76]	5,452	[5,182 - 5,734]
			2019	105.54	[101.44 - 109.91]	2,953	[2,839 - 3,076]
		20 to 24	1950	379.28	[372.94 - 397.30]	2,815	[2,768 - 2,948]
			1985	248.46	[236.96 - 260.41]	5,394	[5,144 - 5,653]
			2019	139.02	[134.35 - 144.32]	4,106	[3,968 - 4,263]
	Female	10 to 14	1950	147.35	[144.70 - 157.12]	1,167	[1,146 - 1,245]
			1985	60.88	[58.24 - 63.62]	1,787	[1,709 - 1,867]
			2019	29.57	[28.49 - 30.65]	915	[881 - 948]
		15 to 19	1950	205.73	[201.79 - 218.75]	1,722	[1,689 - 1,831]
			1985	82.78	[79.09 - 86.84]	2,038	[1,947 - 2,138]
			2019	45.39	[43.91 - 46.89]	1,209	[1,170 - 1,249]
		20 to 24	1950	234.54	[229.20 - 253.31]	1,837	[1,795 - 1,984]
			1985	92.52	[86.62 - 98.56]	1,964	[1,839 - 2,093]
			2019	49.11	[46.98 - 51.36]	1,397	[1,336 - 1,461]

Table S140: All-cause mortality rate per 100,000 population and number of deaths in 10-24 by sex 1950 - 2019: Iraq

Location	Sex	Age	Year	Mortality per 100,000	Mortality per 100,000 uncertainty interval	Number	Number Uncertainty Interval
Iraq	Male	10 to 14	1950	185.58	[176.70 - 217.96]	522	[497 - 613]
			1985	154.81	[142.27 - 168.15]	1,682	[1,546 - 1,827]
			2019	42.76	[36.86 - 49.17]	979	[844 - 1,126]
		15 to 19	1950	199.90	[187.40 - 245.37]	460	[431 - 564]
			1985	229.88	[208.37 - 252.81]	2,078	[1,884 - 2,285]
			2019	99.13	[78.00 - 117.03]	2,246	[1,767 - 2,652]
		20 to 24	1950	235.34	[220.65 - 283.71]	467	[438 - 563]
			1985	383.33	[349.91 - 418.29]	2,859	[2,610 - 3,120]
			2019	158.86	[125.61 - 197.47]	3,540	[2,799 - 4,401]
	Female	10 to 14	1950	110.42	[104.76 - 132.29]	320	[304 - 384]
			1985	111.58	[103.31 - 120.32]	1,131	[1,047 - 1,220]
			2019	30.44	[27.00 - 34.16]	660	[586 - 741]
		15 to 19	1950	135.54	[127.51 - 164.62]	325	[306 - 395]
			1985	135.42	[125.17 - 146.43]	1,135	[1,049 - 1,227]
			2019	48.10	[39.26 - 56.97]	1,033	[843 - 1,224]
		20 to 24	1950	165.69	[151.83 - 215.73]	342	[314 - 446]
			1985	171.71	[149.89 - 196.31]	1,168	[1,020 - 1,335]
			2019	65.87	[51.22 - 86.73]	1,385	[1,077 - 1,824]

Table S141: All-cause mortality rate per 100,000 population and number of deaths in 10-24 by sex 1950 - 2019: Jordan

Location	Sex	Age	Year	Mortality per 100,000	Mortality per 100,000 uncertainty interval	Number	Number Uncertainty Interval
Jordan	Male	10 to 14	1950	133.73	[121.96 - 176.15]	39	[36 - 52]
			1985	64.32	[58.02 - 70.81]	142	[128 - 156]
			2019	27.69	[24.06 - 32.09]	180	[156 - 208]
		15 to 19	1950	218.76	[204.10 - 280.30]	50	[47 - 64]
			1985	103.53	[90.68 - 117.71]	209	[183 - 238]
			2019	45.90	[37.47 - 55.84]	286	[234 - 348]
		20 to 24	1950	288.37	[271.68 - 358.73]	50	[47 - 62]
			1985	129.56	[111.05 - 147.35]	194	[166 - 220]
			2019	59.12	[48.72 - 71.19]	352	[290 - 424]
	Female	10 to 14	1950	98.23	[89.02 - 126.37]	27	[24 - 35]
			1985	53.01	[48.39 - 57.57]	107	[98 - 116]
			2019	20.21	[17.22 - 24.03]	124	[105 - 147]
		15 to 19	1950	149.59	[135.70 - 189.44]	32	[29 - 40]
			1985	69.82	[63.09 - 76.01]	124	[112 - 135]
			2019	24.91	[21.09 - 29.85]	141	[119 - 168]
		20 to 24	1950	197.51	[176.59 - 266.12]	33	[30 - 45]
			1985	111.64	[94.73 - 127.98]	144	[122 - 165]
			2019	28.84	[23.00 - 36.85]	144	[115 - 184]

Table S142: All-cause mortality rate per 100,000 population and number of deaths in 10-24 by sex 1950 - 2019: Kuwait

Location	Sex	Age	Year	Mortality per 100,000	Mortality per 100,000 uncertainty interval	Number	Number Uncertainty Interval
Kuwait	Male	10 to 14	1950	83.82	[77.82 - 110.41]	4	[3 - 5]
			1985	54.97	[53.43 - 56.59]	50	[49 - 51]
			2019	25.90	[21.57 - 30.92]	36	[30 - 43]
		15 to 19	1950	139.81	[128.16 - 185.92]	9	[8 - 12]
			1985	80.55	[78.20 - 83.13]	64	[62 - 66]
			2019	50.71	[40.86 - 62.46]	61	[50 - 76]
		20 to 24	1950	187.22	[172.89 - 238.15]	18	[16 - 22]
			1985	109.65	[106.86 - 112.74]	96	[94 - 99]
			2019	67.45	[56.12 - 80.22]	104	[86 - 123]
	Female	10 to 14	1950	68.12	[63.73 - 88.55]	2	[2 - 3]
			1985	27.23	[26.52 - 27.97]	24	[23 - 25]
			2019	12.55	[11.28 - 14.17]	16	[15 - 19]
		15 to 19	1950	103.70	[95.54 - 132.66]	3	[3 - 4]
			1985	35.67	[34.78 - 36.64]	27	[27 - 28]
			2019	15.58	[13.76 - 17.80]	18	[16 - 20]
		20 to 24	1950	134.42	[121.15 - 182.53]	4	[4 - 5]
			1985	44.54	[42.86 - 46.40]	33	[32 - 34]
			2019	15.34	[12.75 - 18.73]	22	[18 - 27]

Table S143: All-cause mortality rate per 100,000 population and number of deaths in 10-24 by sex 1950 - 2019: Lebanon

Location	Sex	Age	Year	Mortality per 100,000	Mortality per 100,000 uncertainty interval	Number	Number Uncertainty Interval
Lebanon	Male	10 to 14	1950	106.94	[98.34 - 147.47]	74	[68 - 102]
			1985	186.32	[84.27 - 432.64]	348	[158 - 809]
			2019	22.75	[19.55 - 26.93]	49	[42 - 58]
		15 to 19	1950	168.30	[154.46 - 229.27]	108	[99 - 147]
			1985	502.53	[202.58 - 1232.28]	753	[304 - 1,848]
			2019	76.90	[62.62 - 90.70]	139	[113 - 164]
		20 to 24	1950	222.46	[205.95 - 288.64]	132	[122 - 171]
			1985	876.11	[328.13 - 2210.08]	1,140	[427 - 2,876]
			2019	111.05	[87.04 - 130.96]	208	[163 - 246]
	Female	10 to 14	1950	76.69	[70.17 - 110.23]	53	[48 - 76]
			1985	170.73	[69.08 - 414.18]	290	[117 - 704]
			2019	16.45	[14.29 - 18.92]	32	[28 - 37]
		15 to 19	1950	117.89	[107.90 - 165.99]	74	[67 - 104]
			1985	217.62	[94.84 - 517.12]	301	[131 - 715]
			2019	33.71	[26.64 - 40.00]	54	[43 - 64]
		20 to 24	1950	152.08	[135.13 - 221.21]	84	[74 - 122]
			1985	214.21	[103.56 - 485.04]	275	[133 - 623]
			2019	39.87	[33.34 - 47.99]	66	[55 - 80]

Table S144: All-cause mortality rate per 100,000 population and number of deaths in 10-24 by sex 1950 - 2019: Libya

Location	Sex	Age	Year	Mortality per 100,000	Mortality per 100,000 uncertainty interval	Number	Number Uncertainty Interval
Libya	Male	10 to 14	1950	216.99	[204.38 - 255.45]	128	[121 - 151]
			1985	66.69	[59.96 - 74.19]	177	[159 - 197]
			2019	34.08	[30.68 - 38.08]	98	[88 - 109]
		15 to 19	1950	346.78	[321.25 - 418.30]	183	[170 - 221]
			1985	131.80	[112.77 - 152.19]	288	[247 - 333]
			2019	109.07	[94.95 - 127.90]	328	[286 - 385]
		20 to 24	1950	350.12	[324.91 - 423.67]	163	[151 - 197]
			1985	176.64	[152.39 - 201.74]	323	[279 - 369]
			2019	171.81	[152.16 - 200.45]	515	[456 - 601]
	Female	10 to 14	1950	144.21	[133.60 - 169.25]	76	[70 - 89]
			1985	45.18	[40.55 - 50.11]	116	[104 - 128]
			2019	29.33	[26.80 - 32.20]	79	[73 - 87]
		15 to 19	1950	204.18	[189.99 - 242.82]	94	[88 - 112]
			1985	70.78	[63.20 - 78.89]	144	[129 - 161]
			2019	50.72	[45.60 - 57.16]	145	[131 - 164]
		20 to 24	1950	243.55	[218.09 - 325.81]	100	[89 - 133]
			1985	77.15	[64.28 - 91.07]	119	[99 - 141]
			2019	64.92	[53.95 - 81.00]	186	[154 - 232]

Table S145: All-cause mortality rate per 100,000 population and number of deaths in 10-24 by sex 1950 - 2019: Morocco

Location	Sex	Age	Year	Mortality per 100,000	Mortality per 100,000 uncertainty interval	Number	Number Uncertainty Interval
Morocco	Male	10 to 14	1950	225.60	[212.92 - 265.99]	1,097	[1,036 - 1,294]
			1985	81.47	[73.06 - 92.84]	1,145	[1,027 - 1,305]
			2019	31.87	[26.51 - 38.47]	525	[436 - 633]
		15 to 19	1950	312.60	[292.16 - 378.50]	1,323	[1,237 - 1,602]
			1985	129.00	[113.00 - 148.73]	1,578	[1,383 - 1,820]
			2019	81.46	[64.81 - 100.93]	1,289	[1,025 - 1,597]
		20 to 24	1950	338.88	[316.28 - 412.90]	1,304	[1,217 - 1,589]
			1985	181.49	[157.17 - 208.98]	1,936	[1,676 - 2,229]
			2019	100.94	[80.14 - 147.83]	1,514	[1,202 - 2,218]
	Female	10 to 14	1950	153.54	[144.29 - 182.13]	741	[697 - 879]
			1985	61.98	[53.15 - 76.09]	856	[734 - 1,050]
			2019	25.03	[22.41 - 28.13]	391	[350 - 440]
		15 to 19	1950	221.01	[205.10 - 264.49]	945	[877 - 1,131]
			1985	96.55	[81.52 - 116.79]	1,183	[999 - 1,431]
			2019	43.48	[34.54 - 56.61]	664	[527 - 864]
		20 to 24	1950	288.83	[259.41 - 379.41]	1,156	[1,038 - 1,518]
			1985	128.87	[103.42 - 159.86]	1,390	[1,116 - 1,724]
			2019	52.48	[40.34 - 82.83]	778	[598 - 1,228]

Table S146: All-cause mortality rate per 100,000 population and number of deaths in 10-24 by sex 1950 - 2019: Palestine

Location	Sex	Age	Year	Mortality per 100,000	Mortality per 100,000 uncertainty interval	Number	Number Uncertainty Interval
Palestine	Male	10 to 14	1950	156.77	[139.84 - 202.40]	74	[66 - 96]
			1985	65.81	[55.42 - 81.40]	77	[64 - 95]
			2019	30.27	[27.19 - 33.54]	92	[83 - 102]
		15 to 19	1950	301.07	[271.31 - 457.90]	116	[104 - 176]
			1985	141.33	[102.99 - 212.56]	139	[101 - 209]
			2019	60.21	[53.41 - 67.83]	160	[142 - 180]
		20 to 24	1950	396.32	[360.43 - 637.69]	120	[109 - 193]
			1985	180.47	[125.09 - 292.28]	144	[100 - 234]
			2019	91.28	[81.70 - 101.81]	233	[209 - 260]
	Female	10 to 14	1950	103.99	[91.29 - 132.29]	49	[43 - 62]
			1985	42.50	[35.85 - 53.70]	46	[39 - 59]
			2019	16.19	[14.71 - 17.69]	47	[43 - 51]
		15 to 19	1950	160.77	[143.76 - 209.15]	60	[54 - 78]
			1985	55.63	[43.68 - 73.82]	52	[41 - 68]
			2019	23.52	[21.36 - 25.73]	60	[54 - 65]
		20 to 24	1950	205.09	[181.89 - 279.21]	62	[55 - 85]
			1985	66.44	[49.35 - 96.01]	50	[37 - 72]
			2019	32.32	[27.89 - 37.36]	79	[68 - 91]

Table S147: All-cause mortality rate per 100,000 population and number of deaths in 10-24 by sex 1950 - 2019: Oman

Location	Sex	Age	Year	Mortality per 100,000	Mortality per 100,000 uncertainty interval	Number	Number Uncertainty Interval
Oman	Male	10 to 14	1950	286.09	[265.15 - 369.93]	78	[73 - 101]
			1985	104.91	[83.69 - 128.23]	99	[79 - 121]
			2019	44.20	[40.85 - 48.04]	63	[58 - 68]
		15 to 19	1950	427.81	[399.08 - 556.38]	99	[92 - 128]
			1985	202.56	[156.32 - 255.62]	172	[132 - 217]
			2019	120.99	[107.45 - 135.97]	158	[140 - 178]
		20 to 24	1950	472.05	[446.41 - 572.00]	97	[91 - 117]
			1985	245.97	[194.28 - 298.96]	229	[181 - 278]
			2019	139.69	[124.70 - 156.43]	380	[339 - 425]
	Female	10 to 14	1950	196.12	[183.84 - 245.23]	53	[49 - 66]
			1985	56.26	[44.35 - 66.97]	45	[35 - 53]
			2019	22.69	[21.07 - 24.41]	31	[29 - 33]
		15 to 19	1950	282.22	[266.94 - 325.43]	64	[60 - 73]
			1985	81.08	[64.54 - 96.30]	49	[39 - 58]
			2019	43.10	[39.81 - 46.44]	54	[50 - 59]
		20 to 24	1950	388.28	[357.39 - 474.91]	77	[71 - 95]
			1985	97.58	[71.86 - 124.89]	54	[40 - 69]
			2019	48.89	[43.04 - 55.43]	69	[61 - 78]

Table S148: All-cause mortality rate per 100,000 population and number of deaths in 10-24 by sex 1950 - 2019: Qatar

Location	Sex	Age	Year	Mortality per 100,000	Mortality per 100,000 uncertainty interval	Number	Number Uncertainty Interval
Qatar	Male	10 to 14	1950	91.27	[85.23 - 117.03]	1	[1 - 2]
			1985	49.24	[42.00 - 56.95]	7	[6 - 8]
			2019	30.65	[26.20 - 36.15]	19	[16 - 22]
		15 to 19	1950	154.29	[141.66 - 200.14]	2	[2 - 3]
			1985	96.71	[77.77 - 117.44]	15	[12 - 18]
			2019	78.21	[61.62 - 98.20]	50	[40 - 63]
		20 to 24	1950	195.04	[180.60 - 250.60]	2	[2 - 3]
			1985	143.14	[117.91 - 171.29]	36	[30 - 43]
			2019	74.06	[59.07 - 91.75]	177	[141 - 219]
	Female	10 to 14	1950	53.98	[50.32 - 71.04]	1	[1 - 1]
			1985	40.74	[35.73 - 46.15]	5	[5 - 6]
			2019	15.68	[13.79 - 17.96]	9	[8 - 11]
		15 to 19	1950	79.01	[72.12 - 106.65]	1	[1 - 1]
			1985	61.23	[53.09 - 70.07]	7	[6 - 8]
			2019	28.91	[24.86 - 33.61]	12	[11 - 14]
		20 to 24	1950	90.04	[81.01 - 131.18]	1	[1 - 1]
			1985	81.57	[64.08 - 102.15]	9	[7 - 11]
			2019	32.13	[24.98 - 40.68]	14	[11 - 18]

Table S149: All-cause mortality rate per 100,000 population and number of deaths in 10-24 by sex 1950 - 2019: Saudi Arabia

Location	Sex	Age	Year	Mortality per 100,000	Mortality per 100,000 uncertainty interval	Number	Number Uncertainty Interval
Saudi Arabia	Male	10 to 14	1950	170.99	[156.45 - 217.27]	482	[441 - 613]
			1985	66.48	[51.57 - 82.08]	513	[398 - 634]
			2019	19.14	[16.72 - 22.53]	246	[215 - 289]
		15 to 19	1950	247.90	[231.08 - 316.70]	525	[489 - 670]
			1985	110.18	[85.70 - 137.59]	773	[601 - 965]
			2019	69.38	[62.25 - 77.92]	1,015	[910 - 1,139]
		20 to 24	1950	301.17	[279.13 - 371.79]	513	[475 - 633]
			1985	162.64	[118.40 - 204.97]	1,093	[795 - 1,377]
			2019	136.87	[119.63 - 174.51]	2,527	[2,208 - 3,221]
	Female	10 to 14	1950	132.70	[123.25 - 164.29]	224	[208 - 277]
			1985	58.12	[41.87 - 72.54]	431	[310 - 537]
			2019	16.48	[14.16 - 19.33]	183	[157 - 214]
		15 to 19	1950	200.81	[183.81 - 244.49]	265	[243 - 323]
			1985	89.04	[62.01 - 109.47]	546	[380 - 671]
			2019	39.21	[34.59 - 47.76]	456	[402 - 556]
		20 to 24	1950	275.50	[248.42 - 360.99]	302	[273 - 396]
			1985	126.79	[84.08 - 162.99]	662	[439 - 851]
			2019	64.30	[51.58 - 83.14]	911	[731 - 1,178]

Table S150: All-cause mortality rate per 100,000 population and number of deaths in 10-24 by sex 1950 - 2019: Syrian Arab Republic

Location	Sex	Age	Year	Mortality per 100,000	Mortality per 100,000 uncertainty interval	Number	Number Uncertainty Interval
Syrian Arab Republic	Male	10 to 14	1950	156.01	[146.51 - 183.82]	300	[282 - 354]
			1985	76.94	[65.80 - 88.93]	573	[490 - 662]
			2019	53.41	[46.22 - 62.27]	458	[396 - 534]
		15 to 19	1950	235.14	[216.10 - 288.61]	386	[355 - 474]
			1985	116.66	[94.05 - 141.71]	718	[578 - 872]
			2019	89.85	[72.34 - 113.02]	826	[665 - 1,039]
		20 to 24	1950	302.72	[279.50 - 372.34]	429	[396 - 528]
			1985	156.90	[129.11 - 188.23]	767	[632 - 921]
			2019	96.35	[78.94 - 121.02]	590	[483 - 741]
	Female	10 to 14	1950	113.95	[105.34 - 135.75]	217	[201 - 258]
			1985	58.30	[50.80 - 67.25]	413	[360 - 476]
			2019	36.41	[31.69 - 42.05]	300	[261 - 346]
		15 to 19	1950	157.53	[145.40 - 188.93]	253	[234 - 304]
			1985	79.26	[68.84 - 91.69]	464	[403 - 536]
			2019	46.29	[39.25 - 55.10]	424	[359 - 504]
		20 to 24	1950	220.92	[198.93 - 295.00]	300	[270 - 400]
			1985	120.82	[97.02 - 150.94]	570	[458 - 712]
			2019	54.90	[42.11 - 73.52]	412	[316 - 551]

Table S151: All-cause mortality rate per 100,000 population and number of deaths in 10-24 by sex 1950 - 2019: Tunisia

Location	Sex	Age	Year	Mortality per 100,000	Mortality per 100,000 uncertainty interval	Number	Number Uncertainty Interval
Tunisia	Male	10 to 14	1950	263.13	[247.37 - 331.80]	527	[496 - 665]
			1985	72.79	[66.71 - 79.72]	330	[302 - 361]
			2019	28.09	[22.34 - 35.06]	127	[101 - 159]
		15 to 19	1950	336.93	[315.99 - 432.03]	601	[564 - 771]
			1985	86.07	[76.41 - 96.86]	357	[317 - 402]
			2019	56.22	[41.73 - 74.96]	233	[173 - 311]
		20 to 24	1950	336.61	[317.71 - 414.74]	526	[497 - 648]
			1985	112.86	[100.33 - 125.89]	408	[363 - 456]
			2019	91.04	[69.62 - 116.99]	365	[279 - 469]
	Female	10 to 14	1950	177.34	[168.09 - 216.56]	352	[334 - 430]
			1985	55.10	[50.66 - 59.60]	238	[218 - 257]
			2019	20.30	[16.48 - 24.81]	85	[69 - 103]
		15 to 19	1950	217.95	[205.82 - 264.33]	383	[362 - 465]
			1985	55.33	[50.55 - 60.34]	222	[203 - 242]
			2019	26.72	[20.99 - 33.81]	105	[82 - 132]
		20 to 24	1950	312.93	[287.82 - 411.96]	484	[445 - 637]
			1985	70.84	[61.17 - 81.75]	252	[218 - 291]
			2019	37.98	[27.42 - 50.52]	154	[112 - 205]

Table S152: All-cause mortality rate per 100,000 population and number of deaths in 10-24 by sex 1950 - 2019: Turkey

Location	Sex	Age	Year	Mortality per 100,000	Mortality per 100,000 uncertainty interval	Number	Number Uncertainty Interval
Turkey	Male	10 to 14	1950	221.22	[206.02 - 271.13]	2,963	[2,759 - 3,631]
			1985	70.27	[64.26 - 76.94]	2,330	[2,131 - 2,552]
			2019	24.00	[21.05 - 27.44]	708	[621 - 810]
		15 to 19	1950	296.48	[271.92 - 376.09]	3,451	[3,165 - 4,378]
			1985	96.70	[85.58 - 109.83]	2,836	[2,510 - 3,221]
			2019	42.54	[35.37 - 50.91]	1,425	[1,185 - 1,705]
		20 to 24	1950	303.54	[281.00 - 379.24]	3,042	[2,816 - 3,800]
			1985	115.71	[102.90 - 130.45]	2,931	[2,607 - 3,305]
			2019	55.48	[46.43 - 66.01]	1,941	[1,624 - 2,310]
	Female	10 to 14	1950	112.75	[104.15 - 143.40]	1,367	[1,262 - 1,738]
			1985	44.47	[41.16 - 48.06]	1,390	[1,287 - 1,502]
			2019	18.27	[16.17 - 20.77]	510	[452 - 580]
		15 to 19	1950	153.31	[141.52 - 198.05]	1,640	[1,514 - 2,118]
			1985	54.26	[49.58 - 59.14]	1,529	[1,397 - 1,666]
			2019	23.95	[20.82 - 27.54]	760	[661 - 874]
		20 to 24	1950	176.29	[158.69 - 249.46]	1,659	[1,493 - 2,347]
			1985	64.32	[55.43 - 73.90]	1,583	[1,364 - 1,819]
			2019	25.72	[20.42 - 32.06]	839	[666 - 1,046]

Table S153: All-cause mortality rate per 100,000 population and number of deaths in 10-24 by sex 1950 - 2019: United Arab Emirates

Location	Sex	Age	Year	Mortality per 100,000	Mortality per 100,000 uncertainty interval	Number	Number Uncertainty Interval
United Arab Emirates	Male	10 to 14	1950	115.10	[105.32 - 153.88]	5	[4 - 6]
			1985	72.05	[61.85 - 81.90]	38	[32 - 43]
			2019	27.68	[24.71 - 31.31]	56	[50 - 64]
		15 to 19	1950	166.74	[152.50 - 224.37]	6	[5 - 8]
			1985	121.57	[97.26 - 146.70]	49	[39 - 59]
			2019	72.18	[60.80 - 83.06]	128	[108 - 148]
		20 to 24	1950	204.45	[188.61 - 262.68]	6	[6 - 8]
			1985	148.22	[119.84 - 180.39]	116	[94 - 141]
			2019	125.63	[101.32 - 155.39]	212	[171 - 262]
	Female	10 to 14	1950	77.57	[70.80 - 107.61]	3	[3 - 4]
			1985	40.04	[34.79 - 45.57]	19	[17 - 22]
			2019	16.51	[14.56 - 19.06]	32	[28 - 37]
		15 to 19	1950	116.93	[107.18 - 160.91]	4	[4 - 5]
			1985	62.85	[53.01 - 72.32]	22	[19 - 26]
			2019	33.90	[28.72 - 39.83]	56	[47 - 66]
		20 to 24	1950	146.04	[130.38 - 212.33]	4	[4 - 6]
			1985	72.60	[55.47 - 91.85]	35	[27 - 45]
			2019	48.68	[36.50 - 62.97]	73	[55 - 95]

Table S154: All-cause mortality rate per 100,000 population and number of deaths in 10-24 by sex 1950 - 2019: Yemen

Location	Sex	Age	Year	Mortality per 100,000	Mortality per 100,000 uncertainty interval	Number	Number Uncertainty Interval
Yemen	Male	10 to 14	1950	410.42	[387.28 - 487.42]	1,001	[945 - 1,189]
			1985	133.50	[103.69 - 164.13]	1,001	[778 - 1,231]
			2019	80.77	[68.75 - 94.91]	1,693	[1,441 - 1,990]
		15 to 19	1950	557.06	[525.62 - 681.60]	1,220	[1,151 - 1,492]
			1985	201.53	[154.06 - 253.01]	1,058	[809 - 1,328]
			2019	179.70	[157.43 - 207.68]	3,095	[2,711 - 3,577]
		20 to 24	1950	536.32	[505.40 - 642.33]	1,045	[985 - 1,251]
			1985	257.22	[197.43 - 325.09]	1,079	[828 - 1,364]
			2019	306.95	[256.69 - 348.49]	4,202	[3,514 - 4,770]
	Female	10 to 14	1950	243.30	[230.34 - 286.19]	563	[533 - 662]
			1985	100.09	[73.77 - 124.33]	689	[508 - 855]
			2019	67.42	[56.21 - 80.72]	1,338	[1,115 - 1,602]
		15 to 19	1950	337.10	[321.67 - 375.65]	711	[679 - 793]
			1985	147.54	[108.93 - 181.59]	772	[570 - 950]
			2019	101.35	[79.13 - 117.94]	1,667	[1,302 - 1,940]
		20 to 24	1950	459.87	[425.76 - 562.47]	838	[776 - 1,025]
			1985	198.07	[135.85 - 261.91]	919	[630 - 1,215]
			2019	128.90	[94.90 - 153.56]	1,735	[1,278 - 2,067]

Table S155: All-cause mortality rate per 100,000 population and number of deaths in 10-24 by sex 1950 - 2019: South Asia

Location	Sex	Age	Year	Mortality per 100,000	Mortality per 100,000 uncertainty interval	Number	Number Uncertainty Interval
South Asia	Male	10 to 14	1950	268.66	[256.77 - 311.87]	68,953	[65,902 - 80,042]
			1985	141.01	[134.41 - 147.95]	86,078	[82,048 - 90,312]
			2019	59.34	[53.64 - 65.70]	55,467	[50,132 - 61,412]
		15 to 19	1950	320.19	[302.89 - 382.08]	72,239	[68,336 - 86,201]
			1985	192.47	[181.63 - 204.44]	102,911	[97,116 - 109,313]
			2019	85.69	[74.89 - 97.78]	78,767	[68,836 - 89,881]
		20 to 24	1950	359.76	[340.76 - 422.91]	71,428	[67,656 - 83,966]
			1985	240.21	[226.96 - 254.00]	109,845	[103,783 - 116,150]
			2019	139.47	[122.03 - 158.41]	119,765	[104,790 - 136,024]
	Female	10 to 14	1950	252.16	[241.25 - 290.77]	60,616	[57,995 - 69,899]
			1985	145.91	[139.24 - 152.35]	81,803	[78,059 - 85,414]
			2019	56.72	[51.50 - 62.38]	48,902	[44,407 - 53,786]
		15 to 19	1950	369.77	[355.29 - 414.67]	78,330	[75,261 - 87,840]
			1985	245.82	[235.30 - 256.14]	120,118	[114,977 - 125,163]
			2019	88.44	[80.14 - 97.48]	75,919	[68,800 - 83,687]
		20 to 24	1950	560.82	[520.80 - 697.06]	106,814	[99,191 - 132,763]
			1985	334.63	[309.82 - 359.99]	144,071	[133,388 - 154,987]
			2019	120.42	[100.77 - 141.72]	99,315	[83,110 - 116,886]

Table S156: All-cause mortality rate per 100,000 population and number of deaths in 10-24 by sex 1950 - 2019: Afghanistan

Location	Sex	Age	Year	Mortality per 100,000	Mortality per 100,000 uncertainty interval	Number	Number Uncertainty Interval
Afghanistan	Male	10 to 14	1950	232.69	[220.35 - 277.97]	1,010	[956 - 1,207]
			1985	494.03	[218.33 - 1286.21]	4,031	[1,781 - 10,494]
			2019	75.53	[66.04 - 85.81]	1,899	[1,660 - 2,157]
		15 to 19	1950	338.85	[314.99 - 428.34]	1,332	[1,239 - 1,684]
			1985	1273.98	[407.95 - 3728.96]	8,504	[2,723 - 24,892]
			2019	145.64	[118.53 - 184.49]	3,258	[2,651 - 4,126]
		20 to 24	1950	384.79	[361.86 - 484.69]	1,383	[1,301 - 1,743]
			1985	2274.52	[623.91 - 6940.83]	9,433	[2,588 - 28,787]
			2019	221.79	[179.04 - 310.30]	4,269	[3,446 - 5,973]
	Female	10 to 14	1950	209.92	[199.63 - 250.00]	832	[791 - 991]
			1985	472.02	[209.86 - 1218.94]	3,733	[1,660 - 9,640]
			2019	68.75	[59.89 - 80.53]	1,634	[1,424 - 1,914]
		15 to 19	1950	295.53	[286.09 - 330.24]	1,053	[1,019 - 1,176]
			1985	635.55	[293.39 - 1614.00]	4,014	[1,853 - 10,194]
			2019	117.93	[100.16 - 149.68]	2,486	[2,112 - 3,155]
		20 to 24	1950	441.81	[419.70 - 542.01]	1,365	[1,297 - 1,675]
			1985	717.87	[405.69 - 1602.52]	3,279	[1,853 - 7,319]
			2019	179.13	[149.16 - 233.34]	3,290	[2,740 - 4,286]

Table S157: All-cause mortality rate per 100,000 population and number of deaths in 10-24 by sex 1950 - 2019: Bangladesh

Location	Sex	Age	Year	Mortality per 100,000	Mortality per 100,000 uncertainty interval	Number	Number Uncertainty Interval
Bangladesh	Male	10 to 14	1950	325.37	[308.16 - 384.82]	7,356	[6,967 - 8,699]
			1985	226.30	[209.76 - 243.51]	13,661	[12,662 - 14,700]
			2019	77.28	[67.02 - 88.69]	5,943	[5,154 - 6,819]
		15 to 19	1950	343.99	[320.87 - 421.83]	6,805	[6,348 - 8,345]
			1985	247.14	[223.33 - 272.11]	12,623	[11,407 - 13,898]
			2019	69.04	[57.36 - 82.85]	5,107	[4,243 - 6,128]
		20 to 24	1950	337.78	[317.30 - 411.78]	5,978	[5,615 - 7,288]
			1985	261.54	[237.78 - 287.03]	11,115	[10,105 - 12,198]
			2019	82.13	[68.66 - 97.69]	5,630	[4,707 - 6,697]
	Female	10 to 14	1950	236.77	[224.89 - 273.65]	5,201	[4,940 - 6,011]
			1985	190.50	[177.96 - 203.40]	11,161	[10,426 - 11,916]
			2019	51.49	[45.00 - 58.83]	3,962	[3,463 - 4,527]
		15 to 19	1950	343.89	[328.54 - 385.61]	6,548	[6,255 - 7,342]
			1985	281.47	[262.06 - 300.55]	14,189	[13,211 - 15,151]
			2019	62.79	[54.34 - 72.66]	4,974	[4,305 - 5,756]
		20 to 24	1950	431.39	[396.99 - 540.91]	7,226	[6,650 - 9,060]
			1985	326.39	[284.31 - 369.84]	13,849	[12,063 - 15,692]
			2019	67.17	[53.45 - 83.73]	5,210	[4,146 - 6,495]

Table S158: All-cause mortality rate per 100,000 population and number of deaths in 10-24 by sex 1950 - 2019: Bhutan

Location	Sex	Age	Year	Mortality per 100,000	Mortality per 100,000 uncertainty interval	Number	Number Uncertainty Interval
Bhutan	Male	10 to 14	1950	313.41	[290.54 - 397.09]	31	[29 - 40]
			1985	127.75	[101.73 - 152.27]	48	[38 - 57]
			2019	40.57	[34.33 - 47.65]	14	[11 - 16]
		15 to 19	1950	434.26	[402.31 - 554.39]	39	[36 - 49]
			1985	178.94	[136.73 - 215.30]	65	[49 - 78]
			2019	79.17	[63.01 - 91.78]	28	[23 - 33]
		20 to 24	1950	449.44	[421.44 - 542.89]	34	[32 - 42]
			1985	225.10	[173.54 - 271.41]	67	[51 - 81]
			2019	98.95	[75.80 - 136.42]	41	[31 - 56]
	Female	10 to 14	1950	236.58	[224.23 - 288.47]	21	[20 - 26]
			1985	115.11	[87.82 - 137.48]	38	[29 - 45]
			2019	31.41	[25.91 - 39.16]	11	[9 - 13]
		15 to 19	1950	330.16	[316.55 - 376.44]	27	[26 - 31]
			1985	167.63	[126.43 - 202.70]	52	[39 - 63]
			2019	44.44	[34.08 - 62.57]	15	[12 - 22]
		20 to 24	1950	484.55	[452.65 - 587.03]	35	[33 - 43]
			1985	228.85	[164.59 - 294.13]	57	[41 - 74]
			2019	57.65	[41.30 - 85.60]	21	[15 - 31]

Table S159: All-cause mortality rate per 100,000 population and number of deaths in 10-24 by sex 1950 - 2019: India

Location	Sex	Age	Year	Mortality per 100,000	Mortality per 100,000 uncertainty interval	Number	Number Uncertainty Interval
India	Male	10 to 14	1950	269.46	[256.37 - 316.67]	56,333	[53,597 - 66,202]
			1985	136.49	[128.59 - 144.86]	64,361	[60,638 - 68,309]
			2019	53.11	[46.98 - 60.61]	37,104	[32,825 - 42,343]
		15 to 19	1950	324.39	[302.79 - 396.35]	59,680	[55,706 - 72,918]
			1985	192.53	[179.70 - 206.90]	80,794	[75,410 - 86,822]
			2019	79.18	[66.04 - 94.10]	55,519	[46,306 - 65,977]
		20 to 24	1950	369.94	[346.09 - 447.50]	59,783	[55,928 - 72,318]
			1985	244.13	[228.10 - 261.03]	88,693	[82,868 - 94,834]
			2019	136.67	[114.95 - 160.71]	91,282	[76,779 - 107,341]
	Female	10 to 14	1950	262.33	[249.15 - 307.89]	51,393	[48,813 - 60,319]
			1985	149.26	[141.13 - 157.23]	64,424	[60,916 - 67,865]
			2019	54.31	[48.13 - 61.58]	34,559	[30,627 - 39,183]
		15 to 19	1950	385.51	[368.90 - 439.24]	66,674	[63,801 - 75,966]
			1985	257.87	[244.41 - 271.11]	98,429	[93,291 - 103,482]
			2019	89.15	[78.58 - 100.75]	57,271	[50,482 - 64,725]
		20 to 24	1950	598.10	[550.57 - 765.30]	93,352	[85,933 - 119,449]
			1985	356.74	[325.64 - 386.98]	121,708	[111,096 - 132,022]
			2019	122.88	[98.98 - 150.51]	77,034	[62,049 - 94,353]

Table S160: All-cause mortality rate per 100,000 population and number of deaths in 10-24 by sex 1950 - 2019: Nepal

Location	Sex	Age	Year	Mortality per 100,000	Mortality per 100,000 uncertainty interval	Number	Number Uncertainty Interval
Nepal	Male	10 to 14	1950	267.83	[252.29 - 322.40]	1,259	[1,186 - 1,515]
			1985	140.31	[116.56 - 164.26]	1,489	[1,237 - 1,744]
			2019	45.82	[40.19 - 51.73]	745	[653 - 841]
		15 to 19	1950	373.48	[348.40 - 468.24]	1,570	[1,464 - 1,968]
			1985	202.79	[165.61 - 241.81]	1,711	[1,397 - 2,040]
			2019	105.30	[86.70 - 120.11]	1,714	[1,411 - 1,955]
		20 to 24	1950	400.88	[374.68 - 488.68]	1,494	[1,396 - 1,821]
			1985	257.06	[210.36 - 305.59]	1,861	[1,523 - 2,212]
			2019	145.02	[109.14 - 172.32]	2,066	[1,555 - 2,455]
	Female	10 to 14	1950	201.70	[190.96 - 233.99]	937	[887 - 1,087]
			1985	122.73	[100.00 - 142.84]	1,212	[988 - 1,411]
			2019	33.31	[29.42 - 37.94]	524	[463 - 597]
		15 to 19	1950	289.17	[275.76 - 317.99]	1,221	[1,164 - 1,342]
			1985	181.41	[146.68 - 205.90]	1,494	[1,208 - 1,696]
			2019	56.71	[43.64 - 66.61]	948	[730 - 1,114]
		20 to 24	1950	411.68	[379.85 - 495.10]	1,590	[1,467 - 1,912]
			1985	254.69	[191.47 - 307.40]	1,966	[1,478 - 2,373]
			2019	71.41	[55.75 - 88.40]	1,163	[908 - 1,440]

Table S161: All-cause mortality rate per 100,000 population and number of deaths in 10-24 by sex 1950 - 2019: Pakistan

Location	Sex	Age	Year	Mortality per 100,000	Mortality per 100,000 uncertainty interval	Number	Number Uncertainty Interval
Pakistan	Male	10 to 14	1950	189.20	[180.53 - 220.18]	3,820	[3,645 - 4,446]
			1985	96.53	[88.09 - 105.06]	6,518	[5,949 - 7,094]
			2019	81.82	[68.73 - 97.13]	11,662	[9,796 - 13,843]
		15 to 19	1950	239.99	[224.77 - 293.75]	4,215	[3,947 - 5,159]
			1985	139.90	[125.09 - 155.06]	7,719	[6,902 - 8,556]
			2019	128.68	[101.82 - 166.22]	16,399	[12,977 - 21,183]
		20 to 24	1950	263.92	[247.46 - 322.84]	4,074	[3,820 - 4,984]
			1985	184.51	[165.12 - 205.68]	8,109	[7,257 - 9,039]
			2019	192.85	[152.39 - 245.28]	20,746	[16,394 - 26,387]
	Female	10 to 14	1950	169.00	[161.53 - 195.69]	3,004	[2,871 - 3,479]
			1985	82.51	[70.95 - 94.42]	4,969	[4,272 - 5,686]
			2019	74.08	[56.28 - 88.05]	9,846	[7,480 - 11,703]
		15 to 19	1950	244.74	[229.38 - 283.84]	3,804	[3,565 - 4,411]
			1985	124.05	[106.32 - 140.99]	5,954	[5,103 - 6,767]
			2019	106.12	[86.86 - 120.75]	12,711	[10,403 - 14,462]
		20 to 24	1950	338.81	[307.79 - 431.00]	4,640	[4,215 - 5,902]
			1985	166.55	[141.39 - 194.90]	6,491	[5,511 - 7,596]
			2019	153.25	[116.16 - 185.84]	15,888	[12,042 - 19,267]

Table S162: All-cause mortality rate per 100,000 population and number of deaths in 10-24 by sex 1950 - 2019: Sub-Saharan Africa

Location	Sex	Age	Year	Mortality per 100,000	Mortality per 100,000 uncertainty interval	Number	Number Uncertainty Interval
Sub-Saharan Africa	Male	10 to 14	1950	247.41	[234.99 - 292.72]	24,500	[23,270 - 28,986]
			1985	158.92	[147.94 - 170.01]	41,555	[38,684 - 44,455]
			2019	85.56	[75.80 - 97.21]	58,958	[52,234 - 66,984]
		15 to 19	1950	356.62	[340.11 - 419.46]	31,384	[29,931 - 36,914]
			1985	246.53	[227.96 - 266.54]	52,927	[48,940 - 57,224]
			2019	150.58	[132.53 - 169.32]	88,017	[77,466 - 98,969]
		20 to 24	1950	409.00	[393.85 - 460.44]	31,915	[30,733 - 35,929]
			1985	334.76	[311.09 - 361.91]	59,740	[55,515 - 64,586]
			2019	210.88	[187.01 - 236.87]	101,557	[90,061 - 114,073]
	Female	10 to 14	1950	179.33	[171.96 - 204.35]	18,128	[17,383 - 20,656]
			1985	120.87	[111.69 - 130.26]	31,990	[29,560 - 34,476]
			2019	64.65	[56.87 - 74.00]	44,290	[38,964 - 50,696]
		15 to 19	1950	258.59	[249.46 - 287.70]	22,670	[21,870 - 25,222]
			1985	182.24	[168.28 - 196.07]	41,078	[37,933 - 44,196]
			2019	104.86	[90.02 - 123.02]	62,491	[53,649 - 73,311]
		20 to 24	1950	383.43	[367.36 - 440.66]	29,174	[27,951 - 33,529]
			1985	266.09	[241.84 - 290.66]	52,072	[47,326 - 56,881]
			2019	150.67	[128.92 - 176.88]	77,112	[65,979 - 90,524]

Table S163: All-cause mortality rate per 100,000 population and number of deaths in 10-24 by sex 1950 - 2019: Central Sub-Saharan Africa

Location	Sex	Age	Year	Mortality per 100,000	Mortality per 100,000 uncertainty interval	Number	Number Uncertainty Interval
Central Sub-Saharan Africa	Male	10 to 14	1950	251.46	[233.40 - 317.84]	2,571	[2,386 - 3,249]
			1985	178.04	[149.82 - 204.33]	5,262	[4,428 - 6,039]
			2019	90.89	[74.68 - 104.39]	7,714	[6,338 - 8,860]
		15 to 19	1950	383.93	[357.18 - 497.00]	3,684	[3,427 - 4,769]
			1985	285.15	[240.09 - 330.92]	7,189	[6,053 - 8,343]
			2019	167.45	[138.35 - 194.44]	11,819	[9,765 - 13,724]
		20 to 24	1950	440.10	[414.15 - 539.55]	3,864	[3,636 - 4,737]
			1985	371.26	[319.07 - 425.06]	7,919	[6,805 - 9,066]
			2019	240.03	[197.45 - 275.38]	14,230	[11,706 - 16,326]
	Female	10 to 14	1950	159.81	[149.16 - 193.60]	1,763	[1,645 - 2,135]
			1985	125.26	[103.65 - 143.42]	3,673	[3,039 - 4,205]
			2019	66.32	[55.48 - 75.51]	5,614	[4,696 - 6,392]
		15 to 19	1950	234.84	[221.87 - 271.01]	2,347	[2,218 - 2,709]
			1985	187.80	[154.95 - 212.15]	4,730	[3,903 - 5,344]
			2019	107.61	[91.29 - 121.96]	7,589	[6,438 - 8,601]
		20 to 24	1950	328.86	[303.78 - 402.68]	2,885	[2,665 - 3,532]
			1985	261.37	[207.80 - 306.29]	5,689	[4,523 - 6,666]
			2019	155.80	[129.78 - 179.30]	9,331	[7,772 - 10,738]

Table S164: All-cause mortality rate per 100,000 population and number of deaths in 10-24 by sex 1950 - 2019: Angola

Location	Sex	Age	Year	Mortality per 100,000	Mortality per 100,000 uncertainty interval	Number	Number Uncertainty Interval
Angola	Male	10 to 14	1950	247.79	[229.23 - 320.51]	614	[568 - 794]
			1985	236.28	[200.56 - 276.02]	1,266	[1,074 - 1,479]
			2019	85.69	[60.75 - 98.30]	1,689	[1,197 - 1,938]
		15 to 19	1950	369.23	[342.23 - 484.57]	793	[735 - 1,041]
			1985	413.07	[358.06 - 488.24]	1,872	[1,623 - 2,213]
			2019	157.03	[118.52 - 183.87]	2,359	[1,781 - 2,763]
		20 to 24	1950	419.69	[392.87 - 511.35]	816	[764 - 994]
			1985	578.90	[513.30 - 657.33]	2,287	[2,027 - 2,596]
			2019	226.96	[164.84 - 257.86]	2,788	[2,025 - 3,167]
	Female	10 to 14	1950	157.02	[146.26 - 192.51]	378	[352 - 464]
			1985	169.62	[144.96 - 193.36]	873	[746 - 995]
			2019	60.17	[41.45 - 68.36]	1,220	[840 - 1,386]
		15 to 19	1950	233.18	[220.37 - 267.89]	489	[463 - 562]
			1985	242.42	[204.56 - 268.64]	1,040	[877 - 1,152]
			2019	101.81	[72.75 - 122.34]	1,594	[1,139 - 1,916]
		20 to 24	1950	325.86	[300.98 - 398.89]	649	[600 - 795]
			1985	314.18	[247.04 - 371.99]	1,198	[942 - 1,418]
			2019	162.54	[111.72 - 210.13]	2,167	[1,489 - 2,801]

Table S165: All-cause mortality rate per 100,000 population and number of deaths in 10-24 by sex 1950 - 2019: Central African Republic

Location	Sex	Age	Year	Mortality per 100,000	Mortality per 100,000 uncertainty interval	Number	Number Uncertainty Interval
Central African Republic	Male	10 to 14	1950	294.27	[277.25 - 331.84]	199	[188 - 225]
			1985	224.99	[203.02 - 247.00]	330	[297 - 362]
			2019	187.25	[165.74 - 217.44]	617	[546 - 716]
		15 to 19	1950	482.48	[454.10 - 584.88]	310	[291 - 375]
			1985	374.25	[330.78 - 414.26]	471	[416 - 521]
			2019	368.87	[298.87 - 477.51]	1,065	[863 - 1,378]
		20 to 24	1950	545.45	[516.40 - 690.35]	314	[298 - 398]
			1985	453.02	[409.90 - 506.47]	504	[456 - 563]
			2019	475.76	[384.71 - 648.22]	1,146	[927 - 1,561]
	Female	10 to 14	1950	180.70	[171.44 - 201.98]	128	[121 - 143]
			1985	141.36	[131.22 - 152.61]	203	[188 - 219]
			2019	115.73	[107.50 - 129.23]	383	[355 - 427]
		15 to 19	1950	250.62	[240.49 - 291.44]	163	[156 - 189]
			1985	199.19	[186.87 - 212.58]	263	[247 - 281]
			2019	195.65	[159.43 - 256.95]	580	[473 - 762]
		20 to 24	1950	369.11	[346.25 - 446.29]	217	[203 - 262]
			1985	285.93	[259.89 - 315.48]	341	[310 - 376]
			2019	276.70	[218.58 - 360.20]	731	[577 - 951]

Table S166: All-cause mortality rate per 100,000 population and number of deaths in 10-24 by sex 1950 - 2019: Congo

Location	Sex	Age	Year	Mortality per 100,000	Mortality per 100,000 uncertainty interval	Number	Number Uncertainty Interval
Congo	Male	10 to 14	1950	327.05	[309.82 - 367.54]	145	[138 - 163]
			1985	146.84	[134.34 - 160.08]	195	[179 - 213]
			2019	74.41	[63.30 - 86.68]	227	[193 - 265]
		15 to 19	1950	526.98	[494.14 - 640.89]	207	[194 - 252]
			1985	284.09	[244.22 - 333.36]	317	[272 - 372]
			2019	165.44	[134.60 - 203.71]	416	[339 - 513]
		20 to 24	1950	582.69	[546.61 - 739.13]	201	[189 - 256]
			1985	400.49	[336.70 - 502.35]	367	[309 - 460]
			2019	207.96	[167.39 - 279.90]	444	[357 - 597]
	Female	10 to 14	1950	185.34	[178.73 - 204.84]	82	[79 - 90]
			1985	95.13	[87.16 - 103.08]	128	[117 - 139]
			2019	56.59	[48.00 - 65.68]	174	[148 - 202]
		15 to 19	1950	258.27	[247.83 - 300.42]	102	[98 - 119]
			1985	169.19	[152.26 - 196.68]	196	[176 - 228]
			2019	119.44	[92.31 - 156.89]	309	[239 - 406]
		20 to 24	1950	394.85	[370.72 - 480.38]	139	[130 - 169]
			1985	263.60	[228.33 - 310.48]	254	[220 - 299]
			2019	161.14	[126.43 - 211.84]	362	[284 - 476]

Table S167: All-cause mortality rate per 100,000 population and number of deaths in 10-24 by sex 1950 - 2019: Democratic Republic of the Congo

Location	Sex	Age	Year	Mortality per 100,000	Mortality per 100,000 uncertainty interval	Number	Number Uncertainty Interval
Democratic Republic of the Congo	Male	10 to 14	1950	241.54	[223.66 - 310.87]	1,541	[1,427 - 1,983]
			1985	162.47	[129.28 - 191.41]	3,363	[2,676 - 3,962]
			2019	88.71	[73.40 - 103.68]	5,039	[4,170 - 5,890]
		15 to 19	1950	365.83	[340.64 - 479.33]	2,258	[2,102 - 2,958]
			1985	246.86	[194.82 - 293.90]	4,370	[3,448 - 5,202]
			2019	159.62	[131.99 - 185.89]	7,699	[6,366 - 8,966]
		20 to 24	1950	423.01	[398.56 - 516.29]	2,403	[2,264 - 2,933]
			1985	308.94	[248.89 - 364.38]	4,580	[3,690 - 5,402]
			2019	233.84	[193.27 - 265.69]	9,531	[7,877 - 10,829]
	Female	10 to 14	1950	156.95	[145.91 - 193.01]	1,128	[1,049 - 1,387]
			1985	116.06	[88.39 - 136.60]	2,398	[1,826 - 2,822]
			2019	66.79	[54.42 - 77.74]	3,754	[3,059 - 4,370]
		15 to 19	1950	232.56	[218.94 - 268.42]	1,531	[1,441 - 1,767]
			1985	176.54	[135.20 - 204.17]	3,142	[2,406 - 3,634]
			2019	103.94	[89.79 - 116.98]	4,950	[4,276 - 5,571]
		20 to 24	1950	321.08	[295.39 - 393.73]	1,797	[1,654 - 2,204]
			1985	248.14	[183.74 - 299.16]	3,787	[2,804 - 4,566]
			2019	145.52	[123.60 - 165.07]	5,839	[4,960 - 6,624]

Table S168: All-cause mortality rate per 100,000 population and number of deaths in 10-24 by sex 1950 - 2019: Equatorial Guinea

Location	Sex	Age	Year	Mortality per 100,000	Mortality per 100,000 uncertainty interval	Number	Number Uncertainty Interval
Equatorial Guinea	Male	10 to 14	1950	345.96	[334.01 - 398.59]	34	[33 - 40]
			1985	205.84	[176.30 - 232.86]	42	[36 - 47]
			2019	75.14	[54.72 - 97.39]	83	[60 - 107]
		15 to 19	1950	598.10	[561.55 - 722.81]	59	[55 - 71]
			1985	342.18	[276.74 - 418.92]	58	[47 - 71]
			2019	140.81	[108.84 - 178.80]	151	[117 - 192]
		20 to 24	1950	697.94	[636.22 - 837.43]	67	[61 - 80]
			1985	424.34	[349.73 - 558.35]	54	[44 - 71]
			2019	170.25	[115.04 - 221.53]	156	[105 - 202]
	Female	10 to 14	1950	203.43	[197.23 - 228.39]	19	[19 - 21]
			1985	131.58	[116.89 - 145.98]	27	[24 - 30]
			2019	56.72	[37.85 - 74.39]	46	[31 - 60]
		15 to 19	1950	294.96	[278.69 - 337.87]	25	[24 - 29]
			1985	189.43	[170.39 - 211.39]	34	[31 - 38]
			2019	117.09	[72.77 - 175.60]	88	[55 - 132]
		20 to 24	1950	455.11	[428.07 - 560.59]	34	[32 - 42]
			1985	274.00	[229.31 - 320.11]	41	[34 - 48]
			2019	206.08	[120.49 - 341.91]	136	[79 - 225]

Table S169: All-cause mortality rate per 100,000 population and number of deaths in 10-24 by sex 1950 - 2019: Gabon

Location	Sex	Age	Year	Mortality per 100,000	Mortality per 100,000 uncertainty interval	Number	Number Uncertainty Interval
Gabon	Male	10 to 14	1950	247.26	[229.01 - 298.32]	36	[33 - 43]
			1985	132.92	[120.32 - 145.09]	67	[60 - 73]
			2019	65.46	[56.59 - 74.19]	59	[51 - 67]
		15 to 19	1950	398.67	[369.89 - 490.48]	57	[53 - 70]
			1985	232.89	[207.45 - 261.79]	102	[90 - 114]
			2019	151.36	[117.26 - 168.26]	128	[99 - 143]
		20 to 24	1950	461.44	[439.62 - 556.49]	64	[61 - 77]
			1985	319.77	[285.29 - 354.98]	128	[114 - 142]
			2019	211.08	[153.34 - 272.36]	166	[121 - 215]
	Female	10 to 14	1950	140.32	[132.73 - 163.27]	27	[25 - 31]
			1985	83.61	[76.51 - 90.39]	44	[40 - 47]
			2019	38.30	[32.97 - 44.40]	37	[32 - 43]
		15 to 19	1950	203.48	[195.20 - 233.86]	38	[36 - 43]
			1985	125.79	[115.70 - 136.04]	56	[52 - 61]
			2019	71.97	[55.28 - 92.38]	67	[51 - 86]
		20 to 24	1950	289.56	[275.52 - 358.01]	48	[46 - 60]
			1985	176.94	[157.37 - 198.61]	68	[61 - 77]
			2019	108.80	[79.38 - 147.98]	96	[70 - 131]

Table S170: All-cause mortality rate per 100,000 population and number of deaths in 10-24 by sex 1950 - 2019: Eastern Sub-Saharan Africa

Location	Sex	Age	Year	Mortality per 100,000	Mortality per 100,000 uncertainty interval	Number	Number Uncertainty Interval
Eastern Sub-Saharan Africa	Male	10 to 14	1950	291.59	[276.37 - 340.37]	10,231	[9,697 - 11,942]
			1985	192.41	[180.64 - 204.36]	20,255	[19,016 - 21,512]
			2019	83.76	[75.41 - 95.27]	22,728	[20,462 - 25,851]
		15 to 19	1950	418.96	[398.81 - 487.28]	12,956	[12,333 - 15,069]
			1985	316.05	[291.79 - 341.64]	26,607	[24,565 - 28,761]
			2019	156.46	[137.77 - 177.77]	36,751	[32,361 - 41,756]
		20 to 24	1950	463.22	[447.22 - 525.69]	12,574	[12,140 - 14,270]
			1985	436.98	[399.41 - 482.71]	29,842	[27,277 - 32,965]
			2019	210.84	[185.76 - 239.47]	40,234	[35,447 - 45,697]
	Female	10 to 14	1950	210.71	[202.38 - 240.22]	7,893	[7,581 - 8,999]
			1985	149.11	[140.54 - 158.60]	15,300	[14,421 - 16,274]
			2019	61.91	[55.08 - 70.56]	16,371	[14,564 - 18,658]
		15 to 19	1950	294.71	[284.74 - 325.21]	9,725	[9,396 - 10,731]
			1985	226.21	[211.47 - 243.30]	19,582	[18,305 - 21,061]
			2019	105.71	[87.57 - 128.04]	24,846	[20,582 - 30,095]
		20 to 24	1950	446.15	[427.24 - 510.32]	12,841	[12,297 - 14,689]
			1985	333.19	[306.78 - 364.47]	25,216	[23,217 - 27,583]
			2019	147.27	[123.83 - 177.24]	29,503	[24,807 - 35,505]

Table S171: All-cause mortality rate per 100,000 population and number of deaths in 10-24 by sex 1950 - 2019: Burundi

Location	Sex	Age	Year	Mortality per 100,000	Mortality per 100,000 uncertainty interval	Number	Number Uncertainty Interval
Burundi	Male	10 to 14	1950	281.98	[261.12 - 356.21]	364	[337 - 459]
			1985	209.49	[181.16 - 234.74]	607	[525 - 680]
			2019	101.25	[81.87 - 116.33]	758	[613 - 870]
		15 to 19	1950	402.33	[371.86 - 513.47]	450	[416 - 575]
			1985	328.39	[271.07 - 385.88]	793	[655 - 932]
			2019	174.23	[140.20 - 216.15]	1,053	[847 - 1,306]
		20 to 24	1950	435.94	[408.38 - 520.47]	410	[384 - 489]
			1985	398.69	[332.65 - 480.15]	854	[713 - 1,029]
			2019	246.67	[200.12 - 289.05]	1,212	[983 - 1,420]
	Female	10 to 14	1950	213.96	[201.26 - 260.25]	293	[276 - 357]
			1985	151.67	[133.71 - 166.60]	447	[394 - 491]
			2019	77.97	[66.83 - 82.98]	612	[525 - 651]
		15 to 19	1950	303.00	[289.77 - 341.76]	366	[350 - 413]
			1985	222.17	[202.37 - 252.88]	563	[513 - 641]
			2019	115.56	[100.00 - 126.91]	756	[655 - 831]
		20 to 24	1950	445.06	[418.70 - 530.90]	455	[428 - 543]
			1985	332.30	[284.73 - 402.56]	770	[660 - 933]
			2019	158.96	[134.77 - 177.04]	873	[740 - 972]

Table S172: All-cause mortality rate per 100,000 population and number of deaths in 10-24 by sex 1950 - 2019: Comoros

Location	Sex	Age	Year	Mortality per 100,000	Mortality per 100,000 uncertainty interval	Number	Number Uncertainty Interval
Comoros	Male	10 to 14	1950	227.89	[214.61 - 274.72]	20	[19 - 24]
			1985	138.77	[115.44 - 161.64]	40	[33 - 47]
			2019	64.71	[50.95 - 80.72]	25	[20 - 31]
		15 to 19	1950	322.53	[301.20 - 411.91]	25	[23 - 32]
			1985	206.01	[169.25 - 254.06]	45	[37 - 56]
			2019	109.30	[88.58 - 131.07]	40	[32 - 48]
		20 to 24	1950	366.03	[345.70 - 454.09]	24	[23 - 30]
			1985	266.75	[220.31 - 323.04]	43	[36 - 52]
			2019	164.17	[119.63 - 196.76]	54	[40 - 65]
	Female	10 to 14	1950	185.19	[175.98 - 222.90]	16	[15 - 20]
			1985	111.11	[91.78 - 128.52]	31	[25 - 35]
			2019	55.87	[40.51 - 69.83]	21	[15 - 26]
		15 to 19	1950	267.55	[258.56 - 301.81]	20	[20 - 23]
			1985	165.52	[136.57 - 184.84]	36	[30 - 40]
			2019	83.65	[59.65 - 100.57]	30	[21 - 36]
		20 to 24	1950	410.33	[390.78 - 502.65]	29	[28 - 36]
			1985	239.61	[185.55 - 284.97]	42	[33 - 50]
			2019	116.66	[80.10 - 143.96]	38	[26 - 47]

Table S173: All-cause mortality rate per 100,000 population and number of deaths in 10-24 by sex 1950 - 2019: Djibouti

Location	Sex	Age	Year	Mortality per 100,000	Mortality per 100,000 uncertainty interval	Number	Number Uncertainty Interval
Djibouti	Male	10 to 14	1950	147.55	[136.87 - 191.80]	6	[5 - 8]
			1985	108.74	[81.73 - 136.54]	27	[20 - 34]
			2019	73.67	[57.88 - 93.66]	50	[39 - 64]
		15 to 19	1950	211.23	[196.93 - 278.54]	7	[6 - 9]
			1985	161.23	[118.61 - 204.20]	32	[24 - 41]
			2019	145.05	[117.24 - 183.14]	85	[69 - 107]
		20 to 24	1950	265.46	[247.94 - 332.28]	7	[7 - 9]
			1985	213.46	[157.37 - 270.75]	31	[23 - 40]
			2019	193.10	[136.69 - 247.04]	107	[76 - 137]
	Female	10 to 14	1950	115.21	[108.07 - 145.49]	5	[4 - 6]
			1985	84.03	[60.36 - 105.64]	18	[13 - 22]
			2019	55.30	[38.92 - 71.08]	30	[21 - 39]
		15 to 19	1950	181.84	[168.71 - 215.93]	6	[6 - 7]
			1985	127.41	[91.02 - 158.00]	21	[15 - 27]
			2019	96.59	[63.45 - 127.51]	44	[29 - 58]
		20 to 24	1950	262.08	[239.13 - 330.83]	7	[7 - 9]
			1985	176.59	[118.65 - 236.68]	23	[16 - 31]
			2019	130.91	[80.10 - 179.34]	60	[37 - 83]

Table S174: All-cause mortality rate per 100,000 population and number of deaths in 10-24 by sex 1950 - 2019: Eritrea

Location	Sex	Age	Year	Mortality per 100,000	Mortality per 100,000 uncertainty interval	Number	Number Uncertainty Interval
Eritrea	Male	10 to 14	1950	336.41	[319.36 - 377.64]	239	[227 - 268]
			1985	393.51	[355.43 - 438.22]	777	[701 - 865]
			2019	83.46	[68.66 - 102.00]	351	[289 - 429]
		15 to 19	1950	526.03	[491.04 - 636.01]	317	[296 - 383]
			1985	960.72	[845.74 - 1099.66]	1,596	[1,405 - 1,826]
			2019	187.02	[149.72 - 245.05]	725	[580 - 950]
		20 to 24	1950	572.04	[532.18 - 712.24]	287	[267 - 357]
			1985	1641.56	[1419.49 - 1908.66]	2,254	[1,949 - 2,620]
			2019	283.70	[212.45 - 407.56]	968	[725 - 1,390]
	Female	10 to 14	1950	221.14	[209.72 - 246.48]	152	[144 - 169]
			1985	315.22	[281.03 - 356.16]	581	[518 - 657]
			2019	54.58	[45.09 - 68.86]	222	[183 - 280]
		15 to 19	1950	299.46	[284.41 - 345.78]	178	[169 - 206]
			1985	444.55	[398.18 - 499.80]	690	[618 - 776]
			2019	101.07	[78.43 - 140.89]	377	[293 - 526]
		20 to 24	1950	493.00	[457.38 - 595.20]	245	[227 - 296]
			1985	551.46	[497.16 - 610.22]	733	[661 - 811]
			2019	139.25	[106.27 - 195.76]	456	[348 - 641]

Table S175: All-cause mortality rate per 100,000 population and number of deaths in 10-24 by sex 1950 - 2019: Ethiopia

Location	Sex	Age	Year	Mortality per 100,000	Mortality per 100,000 uncertainty interval	Number	Number Uncertainty Interval
Ethiopia	Male	10 to 14	1950	336.87	[319.02 - 394.17]	3,545	[3,358 - 4,149]
			1985	238.44	[217.46 - 259.25]	5,958	[5,434 - 6,478]
			2019	63.44	[56.99 - 74.17]	4,594	[4,127 - 5,371]
		15 to 19	1950	516.27	[484.34 - 616.10]	4,662	[4,373 - 5,563]
			1985	378.10	[332.51 - 427.56]	7,620	[6,701 - 8,617]
			2019	115.77	[99.10 - 135.69]	7,392	[6,328 - 8,664]
		20 to 24	1950	552.22	[516.96 - 685.14]	4,262	[3,990 - 5,288]
			1985	449.64	[390.48 - 525.40]	7,623	[6,620 - 8,907]
			2019	149.85	[122.64 - 182.84]	7,955	[6,511 - 9,706]
	Female	10 to 14	1950	228.19	[218.10 - 263.17]	2,375	[2,270 - 2,739]
			1985	178.11	[162.89 - 194.12]	4,144	[3,790 - 4,517]
			2019	47.74	[40.04 - 64.77]	3,283	[2,754 - 4,455]
		15 to 19	1950	308.89	[297.43 - 347.29]	2,771	[2,669 - 3,116]
			1985	246.42	[227.14 - 268.21]	5,160	[4,756 - 5,616]
			2019	79.93	[60.96 - 104.32]	4,930	[3,760 - 6,435]
		20 to 24	1950	502.39	[471.25 - 620.98]	3,912	[3,670 - 4,836]
			1985	397.12	[346.32 - 452.69]	7,974	[6,954 - 9,089]
			2019	101.75	[76.78 - 140.17]	5,401	[4,075 - 7,440]

Table S176: All-cause mortality rate per 100,000 population and number of deaths in 10-24 by sex 1950 - 2019: Kenya

Location	Sex	Age	Year	Mortality per 100,000	Mortality per 100,000 uncertainty interval	Number	Number Uncertainty Interval
Kenya	Male	10 to 14	1950	189.98	[180.16 - 223.37]	572	[543 - 673]
			1985	89.24	[79.11 - 100.28]	1,194	[1,059 - 1,342]
			2019	71.69	[61.85 - 83.45]	2,305	[1,989 - 2,683]
		15 to 19	1950	269.13	[252.19 - 334.56]	713	[668 - 886]
			1985	134.99	[117.96 - 155.04]	1,453	[1,270 - 1,669]
			2019	159.58	[128.61 - 195.30]	4,725	[3,808 - 5,783]
		20 to 24	1950	311.39	[290.66 - 386.21]	721	[673 - 895]
			1985	186.88	[163.35 - 212.39]	1,607	[1,405 - 1,827]
			2019	205.72	[166.05 - 253.60]	5,048	[4,074 - 6,222]
	Female	10 to 14	1950	139.07	[132.58 - 161.90]	540	[514 - 628]
			1985	74.03	[59.08 - 85.82]	993	[793 - 1,151]
			2019	51.16	[42.74 - 62.00]	1,589	[1,327 - 1,925]
		15 to 19	1950	208.66	[194.51 - 243.35]	703	[655 - 819]
			1985	117.99	[96.61 - 134.40]	1,311	[1,073 - 1,493]
			2019	104.28	[75.96 - 133.61]	3,026	[2,205 - 3,878]
		20 to 24	1950	284.26	[258.14 - 363.49]	752	[683 - 961]
			1985	165.72	[133.41 - 197.11]	1,537	[1,237 - 1,828]
			2019	142.89	[105.47 - 183.98]	3,583	[2,644 - 4,613]

Table S177: All-cause mortality rate per 100,000 population and number of deaths in 10-24 by sex 1950 - 2019: Madagascar

Location	Sex	Age	Year	Mortality per 100,000	Mortality per 100,000 uncertainty interval	Number	Number Uncertainty Interval
Madagascar	Male	10 to 14	1950	545.15	[523.06 - 632.43]	1,133	[1,087 - 1,315]
			1985	262.50	[242.63 - 284.49]	1,741	[1,609 - 1,887]
			2019	78.61	[65.73 - 90.98]	1,320	[1,104 - 1,528]
		15 to 19	1950	588.18	[559.24 - 695.99]	1,220	[1,160 - 1,444]
			1985	279.52	[252.61 - 309.52]	1,500	[1,355 - 1,661]
			2019	135.14	[109.84 - 165.69]	2,001	[1,626 - 2,453]
		20 to 24	1950	712.25	[677.73 - 843.71]	1,399	[1,331 - 1,657]
			1985	369.56	[332.68 - 408.73]	1,641	[1,477 - 1,815]
			2019	200.38	[167.42 - 242.36]	2,536	[2,119 - 3,067]
	Female	10 to 14	1950	451.32	[435.88 - 513.95]	1,044	[1,008 - 1,189]
			1985	199.79	[185.56 - 216.12]	1,320	[1,226 - 1,428]
			2019	66.27	[53.36 - 77.84]	1,089	[877 - 1,279]
		15 to 19	1950	584.15	[568.91 - 641.56]	1,277	[1,244 - 1,403]
			1985	271.89	[251.56 - 291.73]	1,481	[1,370 - 1,589]
			2019	101.41	[86.16 - 115.29]	1,485	[1,261 - 1,688]
		20 to 24	1950	876.52	[828.14 - 1068.96]	1,730	[1,635 - 2,110]
			1985	357.80	[315.28 - 401.58]	1,632	[1,438 - 1,832]
			2019	149.65	[125.73 - 172.24]	1,907	[1,602 - 2,195]

Table S178: All-cause mortality rate per 100,000 population and number of deaths in 10-24 by sex 1950 - 2019: Malawi

Location	Sex	Age	Year	Mortality per 100,000	Mortality per 100,000 uncertainty interval	Number	Number Uncertainty Interval
Malawi	Male	10 to 14	1950	352.78	[327.35 - 445.48]	487	[452 - 615]
			1985	166.00	[148.26 - 181.72]	783	[699 - 857]
			2019	104.45	[86.58 - 127.62]	1,352	[1,121 - 1,652]
		15 to 19	1950	477.85	[443.03 - 605.53]	577	[535 - 731]
			1985	224.39	[194.70 - 247.21]	755	[655 - 831]
			2019	165.02	[132.30 - 203.37]	1,861	[1,492 - 2,293]
		20 to 24	1950	475.25	[447.18 - 574.47]	504	[474 - 610]
			1985	265.16	[233.77 - 291.76]	711	[627 - 782]
			2019	238.01	[182.11 - 291.82]	1,967	[1,505 - 2,412]
	Female	10 to 14	1950	250.07	[237.39 - 303.25]	437	[415 - 530]
			1985	133.91	[116.15 - 146.64]	652	[565 - 714]
			2019	74.08	[59.55 - 91.05]	978	[786 - 1,202]
		15 to 19	1950	345.27	[331.59 - 395.08]	543	[521 - 621]
			1985	207.09	[181.08 - 232.35]	773	[676 - 867]
			2019	98.33	[70.95 - 128.32]	1,149	[829 - 1,500]
		20 to 24	1950	507.12	[473.22 - 621.70]	699	[653 - 858]
			1985	286.60	[243.28 - 333.47]	922	[782 - 1,072]
			2019	146.06	[107.07 - 190.29]	1,327	[973 - 1,728]

Table S179: All-cause mortality rate per 100,000 population and number of deaths in 10-24 by sex 1950 - 2019: Mauritius

Location	Sex	Age	Year	Mortality per 100,000	Mortality per 100,000 uncertainty interval	Number	Number Uncertainty Interval
Mauritius	Male	10 to 14	1950	131.61	[130.30 - 135.93]	34	[34 - 35]
			1985	38.48	[37.28 - 39.70]	20	[20 - 21]
			2019	19.77	[16.61 - 23.53]	8	[7 - 10]
		15 to 19	1950	250.34	[247.41 - 259.98]	55	[54 - 57]
			1985	93.68	[90.71 - 96.71]	53	[51 - 55]
			2019	76.29	[62.01 - 93.42]	36	[29 - 44]
		20 to 24	1950	427.73	[422.92 - 443.04]	92	[91 - 95]
			1985	114.95	[111.64 - 118.34]	68	[66 - 70]
			2019	111.88	[94.20 - 132.51]	53	[45 - 63]
	Female	10 to 14	1950	177.99	[176.09 - 183.64]	47	[46 - 48]
			1985	38.27	[37.08 - 39.49]	20	[19 - 20]
			2019	19.69	[16.88 - 22.97]	8	[7 - 9]
		15 to 19	1950	379.53	[376.23 - 389.87]	88	[87 - 90]
			1985	80.75	[78.27 - 83.32]	44	[43 - 45]
			2019	32.60	[27.80 - 38.19]	15	[13 - 18]
		20 to 24	1950	641.72	[631.74 - 673.78]	147	[145 - 155]
			1985	88.46	[84.09 - 93.08]	51	[49 - 54]
			2019	52.97	[42.39 - 65.93]	25	[20 - 31]

Table S180: All-cause mortality rate per 100,000 population and number of deaths in 10-24 by sex 1950 - 2019: Mozambique

Location	Sex	Age	Year	Mortality per 100,000	Mortality per 100,000 uncertainty interval	Number	Number Uncertainty Interval
Mozambique	Male	10 to 14	1950	252.33	[232.77 - 320.02]	856	[789 - 1,085]
			1985	289.12	[267.02 - 311.95]	2,477	[2,288 - 2,673]
			2019	136.48	[109.62 - 165.53]	2,662	[2,138 - 3,229]
		15 to 19	1950	356.58	[327.67 - 455.00]	1,027	[944 - 1,311]
			1985	606.93	[561.33 - 653.99]	3,930	[3,635 - 4,235]
			2019	285.21	[218.06 - 344.61]	4,412	[3,374 - 5,331]
		20 to 24	1950	388.53	[362.79 - 473.33]	976	[911 - 1,189]
			1985	991.81	[915.44 - 1073.12]	4,846	[4,473 - 5,243]
			2019	382.35	[299.48 - 484.58]	4,701	[3,682 - 5,958]
	Female	10 to 14	1950	182.41	[169.88 - 220.86]	598	[557 - 724]
			1985	249.84	[229.81 - 270.29]	2,127	[1,956 - 2,301]
			2019	97.58	[76.41 - 122.59]	1,940	[1,519 - 2,438]
		15 to 19	1950	266.88	[249.95 - 308.12]	769	[720 - 888]
			1985	346.32	[316.13 - 378.20]	2,299	[2,099 - 2,511]
			2019	216.21	[149.31 - 306.06]	3,682	[2,542 - 5,212]
		20 to 24	1950	375.28	[345.23 - 465.36]	1,001	[921 - 1,241]
			1985	403.81	[355.56 - 454.60]	2,283	[2,010 - 2,570]
			2019	331.78	[244.32 - 466.15]	4,837	[3,562 - 6,796]

Table S181: All-cause mortality rate per 100,000 population and number of deaths in 10-24 by sex 1950 - 2019: Rwanda

Location	Sex	Age	Year	Mortality per 100,000	Mortality per 100,000 uncertainty interval	Number	Number Uncertainty Interval
Rwanda	Male	10 to 14	1950	343.82	[315.34 - 419.20]	355	[326 - 433]
			1985	182.47	[161.67 - 200.17]	707	[627 - 776]
			2019	63.22	[53.07 - 80.93]	493	[414 - 632]
		15 to 19	1950	512.37	[467.63 - 631.91]	513	[468 - 632]
			1985	271.98	[232.55 - 312.43]	869	[743 - 999]
			2019	120.80	[101.13 - 149.15]	841	[704 - 1,038]
		20 to 24	1950	533.73	[500.90 - 643.59]	504	[473 - 608]
			1985	333.84	[287.79 - 378.75]	898	[774 - 1,019]
			2019	172.61	[126.54 - 219.50]	1,034	[758 - 1,314]
	Female	10 to 14	1950	260.42	[239.95 - 306.03]	339	[312 - 398]
			1985	143.60	[128.53 - 157.73]	567	[508 - 623]
			2019	45.74	[35.69 - 61.08]	361	[281 - 482]
		15 to 19	1950	341.96	[327.26 - 387.44]	415	[397 - 470]
			1985	207.28	[193.86 - 220.99]	690	[646 - 736]
			2019	76.35	[54.89 - 97.03]	543	[390 - 690]
		20 to 24	1950	502.01	[472.05 - 607.75]	558	[524 - 675]
			1985	312.65	[278.68 - 348.66]	889	[793 - 992]
			2019	106.10	[74.20 - 137.65]	657	[460 - 853]

Table S182: All-cause mortality rate per 100,000 population and number of deaths in 10-24 by sex 1950 - 2019: Seychelles

Location	Sex	Age	Year	Mortality per 100,000	Mortality per 100,000 uncertainty interval	Number	Number Uncertainty Interval
Seychelles	Male	10 to 14	1950	127.89	[123.38 - 140.81]	2	[2 - 2]
			1985	50.56	[47.90 - 53.27]	2	[2 - 2]
			2019	34.04	[30.76 - 37.47]	1	[1 - 1]
		15 to 19	1950	196.91	[188.89 - 221.75]	3	[3 - 3]
			1985	96.00	[90.44 - 101.52]	4	[4 - 4]
			2019	76.62	[68.37 - 86.49]	3	[2 - 3]
		20 to 24	1950	263.35	[253.27 - 298.53]	4	[3 - 4]
			1985	189.41	[179.63 - 199.97]	7	[6 - 7]
			2019	171.45	[154.16 - 192.95]	7	[6 - 8]
	Female	10 to 14	1950	94.69	[92.31 - 105.38]	2	[2 - 2]
			1985	35.90	[33.63 - 38.30]	1	[1 - 2]
			2019	17.29	[15.29 - 19.61]	1	[1 - 1]
		15 to 19	1950	137.58	[133.67 - 155.65]	2	[2 - 3]
			1985	56.45	[53.05 - 60.08]	2	[2 - 2]
			2019	37.36	[33.02 - 42.36]	1	[1 - 1]
		20 to 24	1950	197.80	[190.19 - 235.85]	3	[3 - 4]
			1985	95.31	[87.93 - 103.64]	3	[3 - 4]
			2019	49.28	[42.03 - 57.83]	2	[1 - 2]

Table S183: All-cause mortality rate per 100,000 population and number of deaths in 10-24 by sex 1950 - 2019: Somalia

Location	Sex	Age	Year	Mortality per 100,000	Mortality per 100,000 uncertainty interval	Number	Number Uncertainty Interval
Somalia	Male	10 to 14	1950	259.14	[241.36 - 304.32]	337	[313 - 395]
			1985	211.51	[176.23 - 242.51]	1,053	[877 - 1,208]
			2019	138.36	[119.54 - 162.43]	1,911	[1,651 - 2,244]
		15 to 19	1950	399.65	[370.40 - 498.76]	447	[415 - 558]
			1985	322.43	[254.01 - 397.47]	1,287	[1,014 - 1,587]
			2019	248.77	[200.54 - 327.29]	2,976	[2,399 - 3,915]
		20 to 24	1950	447.75	[423.82 - 572.67]	426	[403 - 545]
			1985	385.07	[306.39 - 491.10]	1,255	[999 - 1,601]
			2019	345.07	[289.04 - 471.18]	3,388	[2,838 - 4,626]
	Female	10 to 14	1950	195.05	[184.10 - 225.03]	253	[238 - 291]
			1985	156.95	[134.72 - 176.64]	625	[536 - 703]
			2019	98.21	[89.73 - 106.21]	1,260	[1,151 - 1,363]
		15 to 19	1950	267.41	[256.41 - 313.04]	293	[280 - 342]
			1985	220.14	[197.76 - 244.84]	695	[624 - 773]
			2019	144.13	[128.85 - 177.18]	1,590	[1,422 - 1,955]
		20 to 24	1950	435.94	[406.66 - 538.59]	392	[366 - 484]
			1985	334.12	[278.34 - 397.62]	957	[797 - 1,139]
			2019	199.82	[175.49 - 253.95]	1,806	[1,586 - 2,295]

Table S184: All-cause mortality rate per 100,000 population and number of deaths in 10-24 by sex 1950 - 2019: United Republic of Tanzania

Location	Sex	Age	Year	Mortality per 100,000	Mortality per 100,000 uncertainty interval	Number	Number Uncertainty Interval
United Republic of Tanzania	Male	10 to 14	1950	256.16	[233.11 - 318.14]	1,132	[1,030 - 1,405]
			1985	124.98	[106.25 - 141.95]	1,807	[1,536 - 2,052]
			2019	73.36	[61.86 - 93.82]	2,601	[2,193 - 3,326]
		15 to 19	1950	383.49	[347.27 - 479.16]	1,478	[1,338 - 1,846]
			1985	183.73	[155.93 - 210.85]	2,072	[1,759 - 2,378]
			2019	128.70	[104.29 - 159.74]	3,834	[3,107 - 4,758]
		20 to 24	1950	427.40	[398.07 - 512.88]	1,422	[1,324 - 1,706]
			1985	235.48	[200.76 - 267.89]	2,076	[1,770 - 2,362]
			2019	177.49	[135.62 - 228.73]	4,221	[3,225 - 5,439]
	Female	10 to 14	1950	192.44	[178.57 - 231.64]	917	[851 - 1,104]
			1985	96.53	[79.79 - 112.19]	1,426	[1,179 - 1,657]
			2019	58.84	[46.31 - 77.20]	2,101	[1,654 - 2,757]
		15 to 19	1950	268.65	[258.57 - 306.93]	1,153	[1,110 - 1,317]
			1985	153.93	[128.83 - 179.84]	1,894	[1,585 - 2,213]
			2019	93.43	[68.34 - 121.84]	2,938	[2,149 - 3,832]
		20 to 24	1950	402.90	[380.84 - 493.76]	1,559	[1,474 - 1,911]
			1985	210.26	[170.53 - 253.49]	2,189	[1,776 - 2,639]
			2019	130.22	[95.50 - 171.21]	3,552	[2,605 - 4,670]

Table S185: All-cause mortality rate per 100,000 population and number of deaths in 10-24 by sex 1950 - 2019: Uganda

Location	Sex	Age	Year	Mortality per 100,000	Mortality per 100,000 uncertainty interval	Number	Number Uncertainty Interval
Uganda	Male	10 to 14	1950	212.24	[196.27 - 270.88]	618	[572 - 789]
			1985	186.27	[154.82 - 229.08]	1,783	[1,482 - 2,193]
			2019	87.72	[75.63 - 111.18]	2,548	[2,197 - 3,230]
		15 to 19	1950	306.83	[282.72 - 393.13]	820	[756 - 1,051]
			1985	379.51	[297.27 - 501.50]	3,000	[2,350 - 3,965]
			2019	163.67	[131.52 - 204.78]	3,962	[3,184 - 4,958]
		20 to 24	1950	350.48	[328.13 - 432.27]	883	[827 - 1,089]
			1985	653.32	[492.69 - 893.57]	4,273	[3,222 - 5,844]
			2019	209.14	[160.36 - 269.23]	3,914	[3,001 - 5,038]
	Female	10 to 14	1950	149.67	[139.11 - 183.68]	464	[432 - 570]
			1985	144.16	[113.80 - 184.09]	1,396	[1,102 - 1,783]
			2019	60.03	[49.49 - 77.73]	1,662	[1,370 - 2,152]
		15 to 19	1950	224.59	[207.88 - 264.81]	640	[592 - 754]
			1985	317.95	[240.88 - 421.21]	2,668	[2,021 - 3,534]
			2019	100.51	[71.43 - 131.79]	2,432	[1,728 - 3,189]
		20 to 24	1950	307.20	[280.60 - 390.05]	806	[736 - 1,023]
			1985	538.98	[406.55 - 758.77]	3,840	[2,896 - 5,405]
			2019	130.85	[95.77 - 175.81]	2,635	[1,929 - 3,541]

Table S186: All-cause mortality rate per 100,000 population and number of deaths in 10-24 by sex 1950 - 2019: Zambia

Location	Sex	Age	Year	Mortality per 100,000	Mortality per 100,000 uncertainty interval	Number	Number Uncertainty Interval
Zambia	Male	10 to 14	1950	204.81	[189.00 - 260.79]	278	[257 - 354]
			1985	140.60	[127.40 - 154.99]	687	[623 - 758]
			2019	98.09	[81.84 - 116.46]	1,132	[944 - 1,344]
		15 to 19	1950	292.70	[269.60 - 372.83]	379	[350 - 483]
			1985	207.04	[187.57 - 232.00]	800	[725 - 897]
			2019	195.92	[157.01 - 233.66]	1,964	[1,574 - 2,342]
		20 to 24	1950	336.90	[313.82 - 414.96]	390	[363 - 481]
			1985	267.05	[241.65 - 300.75]	768	[695 - 865]
			2019	272.92	[212.16 - 337.69]	2,325	[1,807 - 2,877]
	Female	10 to 14	1950	154.20	[143.51 - 189.09]	250	[232 - 306]
			1985	115.40	[105.80 - 126.97]	571	[524 - 629]
			2019	69.13	[57.70 - 84.16]	810	[676 - 986]
		15 to 19	1950	230.28	[213.09 - 273.44]	316	[292 - 375]
			1985	192.76	[172.47 - 215.19]	798	[714 - 891]
			2019	125.17	[100.46 - 161.85]	1,317	[1,057 - 1,703]
		20 to 24	1950	316.08	[288.08 - 403.72]	350	[319 - 447]
			1985	275.02	[237.28 - 317.16]	904	[780 - 1,042]
			2019	191.11	[153.03 - 241.62]	1,769	[1,416 - 2,236]

Table S187: All-cause mortality rate per 100,000 population and number of deaths in 10-24 by sex 1950 - 2019: Southern Sub-Saharan Africa

Location	Sex	Age	Year	Mortality per 100,000	Mortality per 100,000 uncertainty interval	Number	Number Uncertainty Interval
Southern Sub-Saharan Africa	Male	10 to 14	1950	143.11	[137.73 - 159.84]	1,408	[1,355 - 1,572]
			1985	89.00	[84.82 - 93.50]	2,527	[2,409 - 2,655]
			2019	89.66	[82.97 - 97.32]	3,395	[3,142 - 3,685]
		15 to 19	1950	270.97	[262.15 - 301.15]	2,382	[2,305 - 2,648]
			1985	168.43	[158.49 - 178.00]	4,199	[3,951 - 4,437]
			2019	189.29	[170.24 - 210.40]	6,679	[6,007 - 7,424]
		20 to 24	1950	462.74	[452.57 - 502.76]	3,599	[3,520 - 3,911]
			1985	327.49	[309.59 - 344.78]	6,987	[6,605 - 7,356]
			2019	330.18	[305.69 - 360.92]	11,238	[10,405 - 12,284]
	Female	10 to 14	1950	104.42	[101.57 - 113.21]	1,013	[985 - 1,098]
			1985	66.66	[64.13 - 69.13]	1,946	[1,872 - 2,019]
			2019	68.83	[64.55 - 73.77]	2,565	[2,406 - 2,749]
		15 to 19	1950	187.39	[183.09 - 201.56]	1,580	[1,544 - 1,700]
			1985	122.84	[118.36 - 127.30]	3,272	[3,152 - 3,390]
			2019	137.93	[119.78 - 168.22]	4,787	[4,157 - 5,839]
		20 to 24	1950	395.88	[384.07 - 433.67]	2,914	[2,827 - 3,192]
			1985	244.82	[230.00 - 260.21]	5,730	[5,383 - 6,090]
			2019	250.13	[215.98 - 308.94]	8,519	[7,356 - 10,522]

Table S188: All-cause mortality rate per 100,000 population and number of deaths in 10-24 by sex 1950 - 2019: Botswana

Location	Sex	Age	Year	Mortality per 100,000	Mortality per 100,000 uncertainty interval	Number	Number Uncertainty Interval
Botswana	Male	10 to 14	1950	162.56	[153.15 - 185.96]	33	[31 - 38]
			1985	101.71	[91.79 - 111.65]	75	[68 - 83]
			2019	76.27	[61.16 - 89.30]	89	[71 - 104]
		15 to 19	1950	319.79	[292.39 - 391.89]	57	[52 - 70]
			1985	204.44	[175.54 - 246.39]	115	[99 - 139]
			2019	199.26	[154.52 - 241.39]	214	[166 - 259]
		20 to 24	1950	439.87	[396.08 - 557.49]	71	[64 - 90]
			1985	298.17	[256.40 - 396.02]	129	[111 - 171]
			2019	312.34	[230.48 - 425.77]	320	[236 - 437]
	Female	10 to 14	1950	104.90	[99.70 - 118.89]	23	[22 - 26]
			1985	63.79	[57.36 - 71.08]	49	[44 - 55]
			2019	48.85	[38.60 - 58.07]	55	[44 - 66]
		15 to 19	1950	168.06	[158.01 - 204.86]	32	[30 - 39]
			1985	105.34	[95.56 - 125.19]	66	[60 - 78]
			2019	120.54	[91.32 - 160.99]	128	[97 - 171]
		20 to 24	1950	262.81	[242.86 - 331.19]	44	[41 - 56]
			1985	148.96	[131.40 - 182.54]	80	[71 - 98]
			2019	214.26	[154.52 - 306.78]	225	[162 - 321]

Table S189: All-cause mortality rate per 100,000 population and number of deaths in 10-24 by sex 1950 - 2019: Lesotho

Location	Sex	Age	Year	Mortality per 100,000	Mortality per 100,000 uncertainty interval	Number	Number Uncertainty Interval
Lesotho	Male	10 to 14	1950	212.55	[205.13 - 240.01]	56	[54 - 63]
			1985	135.05	[105.36 - 153.50]	135	[105 - 154]
			2019	152.02	[136.28 - 169.78]	171	[153 - 191]
		15 to 19	1950	371.29	[350.44 - 434.17]	74	[70 - 87]
			1985	241.66	[193.22 - 276.66]	209	[167 - 239]
			2019	370.28	[305.42 - 448.95]	410	[338 - 497]
		20 to 24	1950	451.41	[431.78 - 578.95]	69	[66 - 88]
			1985	330.10	[272.20 - 366.46]	224	[185 - 249]
			2019	559.77	[434.53 - 670.69]	584	[454 - 700]
	Female	10 to 14	1950	118.88	[115.60 - 131.22]	43	[42 - 48]
			1985	81.78	[64.07 - 91.01]	83	[65 - 92]
			2019	99.21	[85.81 - 114.42]	111	[96 - 128]
		15 to 19	1950	178.45	[173.05 - 198.14]	56	[55 - 62]
			1985	120.33	[94.75 - 134.14]	109	[86 - 122]
			2019	248.02	[169.99 - 348.35]	271	[186 - 380]
		20 to 24	1950	253.37	[239.90 - 301.64]	68	[64 - 81]
			1985	159.55	[121.74 - 183.47]	123	[94 - 141]
			2019	445.22	[303.38 - 636.53]	463	[315 - 661]

Table S190: All-cause mortality rate per 100,000 population and number of deaths in 10-24 by sex 1950 - 2019: Namibia

Location	Sex	Age	Year	Mortality per 100,000	Mortality per 100,000 uncertainty interval	Number	Number Uncertainty Interval
Namibia	Male	10 to 14	1950	164.12	[154.77 - 199.68]	38	[36 - 47]
			1985	138.72	[129.79 - 148.29]	108	[101 - 116]
			2019	89.56	[74.64 - 103.59]	116	[96 - 134]
		15 to 19	1950	273.57	[252.90 - 341.47]	62	[57 - 77]
			1985	282.41	[262.13 - 302.91]	191	[178 - 205]
			2019	211.99	[171.67 - 251.20]	252	[204 - 299]
		20 to 24	1950	352.57	[336.11 - 423.76]	78	[74 - 94]
			1985	449.13	[420.78 - 478.40]	245	[230 - 261]
			2019	263.47	[186.82 - 333.87]	298	[211 - 377]
	Female	10 to 14	1950	113.28	[107.11 - 133.06]	26	[25 - 31]
			1985	101.69	[95.40 - 108.13]	79	[74 - 84]
			2019	55.45	[45.60 - 64.16]	73	[60 - 84]
		15 to 19	1950	169.43	[161.57 - 195.98]	36	[35 - 42]
			1985	151.94	[143.30 - 160.84]	104	[98 - 110]
			2019	116.34	[87.81 - 147.71]	141	[107 - 179]
		20 to 24	1950	243.89	[228.63 - 304.36]	47	[44 - 59]
			1985	204.74	[190.88 - 221.66]	117	[109 - 127]
			2019	138.69	[99.61 - 183.52]	161	[116 - 214]

Table S191: All-cause mortality rate per 100,000 population and number of deaths in 10-24 by sex 1950 - 2019: South Africa

Location	Sex	Age	Year	Mortality per 100,000	Mortality per 100,000 uncertainty interval	Number	Number Uncertainty Interval
South Africa	Male	10 to 14	1950	128.88	[125.10 - 142.41]	946	[919 - 1,046]
			1985	78.60	[74.60 - 82.79]	1,522	[1,445 - 1,603]
			2019	73.09	[67.56 - 79.43]	1,785	[1,649 - 1,939]
		15 to 19	1950	256.49	[249.30 - 283.28]	1,709	[1,661 - 1,887]
			1985	155.35	[145.14 - 166.11]	2,741	[2,561 - 2,931]
			2019	161.70	[145.44 - 178.98]	3,744	[3,368 - 4,144]
		20 to 24	1950	483.62	[470.40 - 524.14]	2,857	[2,779 - 3,097]
			1985	339.64	[317.72 - 360.39]	5,376	[5,030 - 5,705]
			2019	318.56	[299.66 - 341.88]	7,526	[7,080 - 8,077]
	Female	10 to 14	1950	98.40	[95.74 - 106.24]	698	[679 - 753]
			1985	62.06	[59.28 - 65.08]	1,247	[1,191 - 1,308]
			2019	57.34	[53.95 - 61.09]	1,366	[1,285 - 1,456]
		15 to 19	1950	190.18	[186.04 - 202.37]	1,196	[1,170 - 1,273]
			1985	123.70	[118.47 - 129.17]	2,349	[2,250 - 2,453]
			2019	134.82	[119.03 - 164.33]	3,011	[2,659 - 3,670]
		20 to 24	1950	439.64	[425.22 - 488.81]	2,422	[2,342 - 2,693]
			1985	272.09	[252.83 - 293.11]	4,643	[4,314 - 5,001]
			2019	262.38	[228.64 - 323.65]	6,008	[5,236 - 7,411]

Table S192: All-cause mortality rate per 100,000 population and number of deaths in 10-24 by sex 1950 - 2019: Eswatini

Location	Sex	Age	Year	Mortality per 100,000	Mortality per 100,000 uncertainty interval	Number	Number Uncertainty Interval
Eswatini	Male	10 to 14	1950	313.35	[294.23 - 361.74]	40	[37 - 46]
			1985	135.92	[117.52 - 149.37]	62	[53 - 68]
			2019	125.09	[104.55 - 146.94]	85	[71 - 100]
		15 to 19	1950	508.70	[477.11 - 617.21]	61	[57 - 74]
			1985	244.53	[209.05 - 279.40]	87	[75 - 100]
			2019	282.96	[230.32 - 345.76]	175	[142 - 214]
		20 to 24	1950	563.03	[534.85 - 706.36]	63	[60 - 79]
			1985	333.98	[285.53 - 384.31]	87	[74 - 100]
			2019	429.29	[314.06 - 567.30]	236	[173 - 312]
	Female	10 to 14	1950	173.62	[165.01 - 193.56]	27	[25 - 30]
			1985	81.74	[73.07 - 88.39]	38	[34 - 42]
			2019	81.40	[64.57 - 100.89]	51	[41 - 64]
		15 to 19	1950	242.33	[232.36 - 278.33]	31	[30 - 35]
			1985	122.55	[107.89 - 133.19]	49	[43 - 54]
			2019	169.50	[114.12 - 239.23]	102	[69 - 144]
		20 to 24	1950	349.89	[327.41 - 425.74]	37	[34 - 45]
			1985	169.73	[139.87 - 194.70]	58	[48 - 66]
			2019	295.80	[202.77 - 417.64]	172	[118 - 243]

Table S193: All-cause mortality rate per 100,000 population and number of deaths in 10-24 by sex 1950 - 2019: Zimbabwe

Location	Sex	Age	Year	Mortality per 100,000	Mortality per 100,000 uncertainty interval	Number	Number Uncertainty Interval
Zimbabwe	Male	10 to 14	1950	175.35	[163.92 - 207.66]	293	[274 - 347]
			1985	103.17	[95.31 - 111.52]	625	[577 - 676]
			2019	125.08	[114.42 - 137.82]	1,150	[1,052 - 1,267]
		15 to 19	1950	298.18	[276.11 - 369.51]	419	[388 - 519]
			1985	177.32	[159.27 - 195.52]	855	[768 - 943]
			2019	231.44	[200.00 - 273.12]	1,884	[1,628 - 2,224]
		20 to 24	1950	378.84	[357.32 - 464.46]	464	[437 - 568]
			1985	257.94	[232.57 - 282.79]	926	[835 - 1,015]
			2019	341.31	[284.24 - 444.70]	2,274	[1,893 - 2,962]
	Female	10 to 14	1950	119.72	[113.45 - 136.65]	196	[186 - 223]
			1985	74.08	[68.90 - 79.08]	450	[418 - 480]
			2019	98.24	[90.35 - 108.27]	909	[836 - 1,002]
		15 to 19	1950	177.23	[169.41 - 205.75]	230	[219 - 267]
			1985	118.29	[109.88 - 130.56]	594	[552 - 655]
			2019	134.94	[109.00 - 170.13]	1,134	[916 - 1,430]
		20 to 24	1950	261.14	[243.08 - 324.36]	292	[272 - 362]
			1985	172.06	[154.18 - 196.94]	708	[635 - 811]
			2019	203.39	[159.89 - 260.74]	1,490	[1,172 - 1,911]

Table S194: All-cause mortality rate per 100,000 population and number of deaths in 10-24 by sex 1950 - 2019: Western Sub-Saharan Africa

Location	Sex	Age	Year	Mortality per 100,000	Mortality per 100,000 uncertainty interval	Number	Number Uncertainty Interval
Western Sub-Saharan Africa	Male	10 to 14	1950	235.65	[223.62 - 276.70]	10,340	[9,812 - 12,141]
			1985	137.49	[124.09 - 151.74]	13,511	[12,195 - 14,912]
			2019	85.15	[72.79 - 99.52]	25,120	[21,474 - 29,360]
		15 to 19	1950	320.47	[304.25 - 379.70]	12,400	[11,772 - 14,692]
			1985	185.81	[163.04 - 209.34]	14,932	[13,103 - 16,823]
			2019	134.43	[114.39 - 158.31]	32,769	[27,883 - 38,591]
		20 to 24	1950	344.41	[329.26 - 396.43]	11,823	[11,303 - 13,609]
			1985	222.11	[194.43 - 250.97]	14,993	[13,124 - 16,941]
			2019	181.60	[155.66 - 213.38]	35,855	[30,732 - 42,129]
	Female	10 to 14	1950	173.75	[165.89 - 201.11]	7,453	[7,116 - 8,626]
			1985	106.92	[93.88 - 123.48]	11,071	[9,720 - 12,785]
			2019	66.07	[55.23 - 82.61]	19,740	[16,502 - 24,681]
		15 to 19	1950	249.46	[238.32 - 285.85]	9,040	[8,637 - 10,359]
			1985	155.06	[135.80 - 179.54]	13,494	[11,819 - 15,625]
			2019	98.84	[82.42 - 122.83]	25,269	[21,071 - 31,404]
		20 to 24	1950	338.43	[318.07 - 408.63]	10,550	[9,915 - 12,738]
			1985	206.26	[174.16 - 246.69]	15,438	[13,035 - 18,464]
			2019	136.82	[110.61 - 173.36]	29,760	[24,058 - 37,708]

Table S195: All-cause mortality rate per 100,000 population and number of deaths in 10-24 by sex 1950 - 2019: Benin

Location	Sex	Age	Year	Mortality per 100,000	Mortality per 100,000 uncertainty interval	Number	Number Uncertainty Interval
Benin	Male	10 to 14	1950	225.58	[209.62 - 282.89]	216	[201 - 271]
			1985	147.09	[126.80 - 164.55]	380	[328 - 425]
			2019	89.99	[64.49 - 127.80]	726	[520 - 1,030]
		15 to 19	1950	312.51	[292.75 - 393.64]	256	[240 - 323]
			1985	205.95	[174.20 - 234.04]	366	[310 - 416]
			2019	142.80	[104.42 - 192.70]	936	[685 - 1,264]
		20 to 24	1950	344.17	[320.54 - 415.82]	254	[236 - 307]
			1985	247.82	[210.45 - 279.76]	351	[298 - 397]
			2019	190.79	[137.37 - 257.81]	1,054	[759 - 1,424]
	Female	10 to 14	1950	170.84	[159.70 - 209.74]	186	[174 - 229]
			1985	112.78	[93.65 - 128.49]	280	[232 - 319]
			2019	68.55	[46.62 - 95.23]	548	[373 - 762]
		15 to 19	1950	244.32	[226.92 - 298.15]	221	[205 - 270]
			1985	161.80	[133.99 - 187.28]	319	[264 - 369]
			2019	100.38	[67.65 - 133.39]	688	[463 - 914]
		20 to 24	1950	321.40	[291.44 - 417.05]	259	[235 - 336]
			1985	212.55	[168.50 - 254.83]	389	[308 - 466]
			2019	134.02	[89.13 - 188.10]	811	[539 - 1,138]

Table S196: All-cause mortality rate per 100,000 population and number of deaths in 10-24 by sex 1950 - 2019: Burkina Faso

Location	Sex	Age	Year	Mortality per 100,000	Mortality per 100,000 uncertainty interval	Number	Number Uncertainty Interval
Burkina Faso	Male	10 to 14	1950	321.91	[297.69 - 404.80]	837	[774 - 1,053]
			1985	164.98	[143.57 - 184.99]	919	[800 - 1,030]
			2019	107.06	[83.40 - 135.45]	1,577	[1,228 - 1,995]
		15 to 19	1950	445.17	[411.73 - 556.71]	1,014	[938 - 1,268]
			1985	229.88	[198.52 - 262.02]	923	[797 - 1,052]
			2019	177.62	[139.65 - 215.31]	2,087	[1,641 - 2,530]
		20 to 24	1950	455.02	[424.61 - 542.83]	913	[852 - 1,089]
			1985	275.50	[242.26 - 312.02]	812	[714 - 919]
			2019	243.22	[193.03 - 285.83]	2,240	[1,778 - 2,633]
	Female	10 to 14	1950	225.02	[209.70 - 268.93]	510	[475 - 609]
			1985	124.75	[105.64 - 141.83]	664	[562 - 754]
			2019	79.87	[59.84 - 102.35]	1,143	[856 - 1,464]
		15 to 19	1950	316.11	[298.74 - 363.09]	630	[595 - 723]
			1985	196.00	[165.69 - 228.13]	798	[675 - 929]
			2019	114.99	[87.73 - 143.42]	1,382	[1,054 - 1,724]
		20 to 24	1950	432.65	[400.19 - 536.40]	760	[703 - 942]
			1985	265.32	[218.74 - 317.41]	938	[773 - 1,122]
			2019	149.50	[108.96 - 191.30]	1,529	[1,114 - 1,956]

Table S197: All-cause mortality rate per 100,000 population and number of deaths in 10-24 by sex 1950 - 2019: Cameroon

Location	Sex	Age	Year	Mortality per 100,000	Mortality per 100,000 uncertainty interval	Number	Number Uncertainty Interval
Cameroon	Male	10 to 14	1950	250.70	[236.65 - 299.37]	637	[602 - 761]
			1985	123.94	[109.74 - 137.35]	683	[604 - 757]
			2019	95.98	[72.49 - 118.91]	1,827	[1,380 - 2,263]
		15 to 19	1950	340.58	[318.69 - 424.92]	763	[714 - 952]
			1985	173.97	[153.48 - 192.88]	749	[661 - 831]
			2019	167.35	[126.71 - 208.63]	2,684	[2,032 - 3,347]
		20 to 24	1950	365.24	[343.74 - 446.68]	754	[709 - 922]
			1985	221.19	[193.70 - 245.99]	770	[674 - 856]
			2019	227.86	[168.00 - 281.90]	3,022	[2,228 - 3,739]
	Female	10 to 14	1950	185.18	[175.16 - 215.40]	477	[451 - 555]
			1985	104.44	[90.40 - 115.36]	553	[479 - 611]
			2019	74.82	[53.20 - 91.50]	1,388	[987 - 1,697]
		15 to 19	1950	270.21	[257.64 - 300.85]	603	[574 - 671]
			1985	157.30	[135.47 - 175.50]	715	[616 - 798]
			2019	121.01	[84.91 - 150.46]	1,948	[1,367 - 2,422]
		20 to 24	1950	388.04	[358.96 - 469.14]	790	[731 - 955]
			1985	221.75	[182.73 - 257.03]	893	[735 - 1,035]
			2019	176.20	[122.50 - 224.84]	2,454	[1,706 - 3,132]

Table S198: All-cause mortality rate per 100,000 population and number of deaths in 10-24 by sex 1950 - 2019: Cabo Verde

Location	Sex	Age	Year	Mortality per 100,000	Mortality per 100,000 uncertainty interval	Number	Number Uncertainty Interval
Cabo Verde	Male	10 to 14	1950	132.67	[127.30 - 150.98]	12	[12 - 14]
			1985	73.28	[70.56 - 76.00]	15	[15 - 16]
			2019	40.55	[36.63 - 44.62]	11	[10 - 12]
		15 to 19	1950	196.97	[187.46 - 227.91]	17	[16 - 19]
			1985	119.29	[113.57 - 125.23]	24	[23 - 25]
			2019	108.61	[97.27 - 116.73]	27	[25 - 29]
		20 to 24	1950	242.39	[230.19 - 281.05]	17	[16 - 20]
			1985	167.00	[159.52 - 174.96]	26	[25 - 27]
			2019	144.33	[114.48 - 177.75]	38	[30 - 47]
	Female	10 to 14	1950	102.50	[98.11 - 115.59]	10	[10 - 11]
			1985	46.17	[44.59 - 47.72]	10	[9 - 10]
			2019	22.18	[19.94 - 24.25]	6	[5 - 6]
		15 to 19	1950	161.06	[153.49 - 183.52]	15	[14 - 17]
			1985	85.33	[82.09 - 88.45]	17	[17 - 18]
			2019	37.36	[29.40 - 45.78]	9	[7 - 11]
		20 to 24	1950	238.54	[221.68 - 289.16]	19	[18 - 24]
			1985	131.30	[122.73 - 139.71]	23	[21 - 24]
			2019	43.35	[33.33 - 53.65]	11	[8 - 13]

Table S199: All-cause mortality rate per 100,000 population and number of deaths in 10-24 by sex 1950 - 2019: Chad

Location	Sex	Age	Year	Mortality per 100,000	Mortality per 100,000 uncertainty interval	Number	Number Uncertainty Interval
Chad	Male	10 to 14	1950	216.71	[201.79 - 272.49]	273	[254 - 343]
			1985	149.69	[129.03 - 172.45]	471	[406 - 543]
			2019	113.05	[83.45 - 140.77]	1,257	[928 - 1,566]
		15 to 19	1950	290.70	[269.55 - 365.96]	350	[324 - 440]
			1985	202.54	[171.08 - 238.82]	502	[424 - 591]
			2019	170.31	[125.97 - 211.56]	1,490	[1,102 - 1,851]
		20 to 24	1950	311.85	[290.30 - 381.56]	284	[264 - 347]
			1985	238.70	[203.70 - 277.04]	475	[405 - 551]
			2019	222.35	[165.88 - 279.60]	1,445	[1,078 - 1,816]
	Female	10 to 14	1950	153.12	[142.61 - 189.10]	190	[177 - 235]
			1985	116.84	[97.98 - 136.40]	376	[315 - 439]
			2019	93.14	[65.05 - 112.95]	1,037	[724 - 1,258]
		15 to 19	1950	220.98	[203.76 - 273.53]	249	[229 - 308]
			1985	166.38	[139.39 - 194.09]	433	[363 - 505]
			2019	134.76	[97.51 - 160.95]	1,220	[883 - 1,457]
		20 to 24	1950	290.94	[262.20 - 384.48]	276	[249 - 365]
			1985	216.07	[174.10 - 262.85]	476	[384 - 579]
			2019	187.68	[132.41 - 233.39]	1,333	[940 - 1,658]

Table S200: All-cause mortality rate per 100,000 population and number of deaths in 10-24 by sex 1950 - 2019: Côte d'Ivoire

Location	Sex	Age	Year	Mortality per 100,000	Mortality per 100,000 uncertainty interval	Number	Number Uncertainty Interval
Côte d'Ivoire	Male	10 to 14	1950	251.53	[238.03 - 298.91]	564	[534 - 670]
			1985	127.75	[108.23 - 145.72]	760	[644 - 867]
			2019	96.51	[75.38 - 121.42]	1,555	[1,215 - 1,957]
		15 to 19	1950	341.43	[321.23 - 419.74]	645	[607 - 793]
			1985	185.64	[154.01 - 210.83]	910	[755 - 1,034]
			2019	163.14	[124.98 - 202.02]	2,231	[1,709 - 2,762]
		20 to 24	1950	362.48	[341.46 - 442.93]	575	[542 - 703]
			1985	235.41	[196.37 - 264.46]	1,080	[901 - 1,213]
			2019	216.06	[162.96 - 267.14]	2,543	[1,918 - 3,144]
	Female	10 to 14	1950	175.77	[166.20 - 207.73]	351	[331 - 414]
			1985	95.96	[78.61 - 109.96]	561	[460 - 643]
			2019	73.89	[54.78 - 92.25]	1,092	[809 - 1,363]
		15 to 19	1950	256.01	[238.73 - 294.52]	408	[380 - 469]
			1985	146.76	[119.56 - 169.99]	754	[614 - 873]
			2019	110.27	[79.02 - 139.76]	1,405	[1,007 - 1,781]
		20 to 24	1950	345.50	[315.59 - 435.25]	436	[398 - 549]
			1985	201.07	[160.56 - 242.23]	957	[764 - 1,153]
			2019	146.72	[102.80 - 187.95]	1,708	[1,197 - 2,188]

Table S201: All-cause mortality rate per 100,000 population and number of deaths in 10-24 by sex 1950 - 2019: Gambia

Location	Sex	Age	Year	Mortality per 100,000	Mortality per 100,000 uncertainty interval	Number	Number Uncertainty Interval
Gambia	Male	10 to 14	1950	176.69	[162.43 - 225.10]	33	[30 - 42]
			1985	119.41	[89.83 - 149.26]	57	[43 - 72]
			2019	63.17	[54.86 - 73.56]	91	[79 - 105]
		15 to 19	1950	254.54	[238.42 - 325.61]	40	[37 - 51]
			1985	180.72	[136.85 - 228.38]	66	[50 - 83]
			2019	133.93	[112.90 - 159.76]	169	[143 - 202]
		20 to 24	1950	303.27	[283.75 - 377.32]	39	[36 - 48]
			1985	237.44	[183.95 - 300.82]	77	[59 - 97]
			2019	189.75	[157.07 - 243.69]	200	[165 - 257]
	Female	10 to 14	1950	119.85	[111.09 - 148.56]	19	[18 - 24]
			1985	85.50	[61.37 - 105.96]	41	[29 - 51]
			2019	46.10	[40.30 - 53.51]	67	[58 - 78]
		15 to 19	1950	182.21	[168.01 - 224.64]	24	[22 - 30]
			1985	129.69	[94.31 - 158.44]	51	[37 - 63]
			2019	83.61	[71.42 - 100.38]	111	[95 - 133]
		20 to 24	1950	251.13	[227.00 - 332.08]	28	[25 - 37]
			1985	179.17	[124.38 - 233.58]	68	[47 - 88]
			2019	123.66	[100.26 - 155.66]	141	[114 - 178]

Table S202: All-cause mortality rate per 100,000 population and number of deaths in 10-24 by sex 1950 - 2019: Ghana

Location	Sex	Age	Year	Mortality per 100,000	Mortality per 100,000 uncertainty interval	Number	Number Uncertainty Interval
Ghana	Male	10 to 14	1950	205.56	[194.11 - 248.45]	625	[590 - 755]
			1985	124.23	[99.59 - 146.93]	1,063	[853 - 1,258]
			2019	79.85	[63.96 - 96.34]	1,449	[1,160 - 1,748]
		15 to 19	1950	289.28	[272.76 - 364.55]	830	[783 - 1,046]
			1985	183.78	[143.48 - 222.33]	1,249	[975 - 1,511]
			2019	155.29	[125.63 - 182.54]	2,564	[2,075 - 3,014]
		20 to 24	1950	328.71	[307.58 - 406.95]	834	[781 - 1,033]
			1985	239.83	[187.20 - 291.40]	1,308	[1,021 - 1,589]
			2019	214.91	[165.57 - 251.86]	3,186	[2,454 - 3,733]
	Female	10 to 14	1950	149.30	[141.12 - 176.10]	492	[465 - 580]
			1985	99.68	[76.54 - 118.93]	813	[624 - 970]
			2019	56.82	[43.58 - 69.79]	995	[763 - 1,222]
		15 to 19	1950	222.98	[207.30 - 255.94]	699	[650 - 802]
			1985	150.77	[113.94 - 175.33]	1,018	[769 - 1,184]
			2019	96.02	[73.22 - 115.13]	1,571	[1,198 - 1,884]
		20 to 24	1950	304.36	[277.03 - 380.58]	789	[718 - 987]
			1985	211.71	[153.19 - 263.04]	1,299	[940 - 1,614]
			2019	130.77	[94.97 - 158.12]	2,088	[1,517 - 2,525]

Table S203: All-cause mortality rate per 100,000 population and number of deaths in 10-24 by sex 1950 - 2019: Guinea

Location	Sex	Age	Year	Mortality per 100,000	Mortality per 100,000 uncertainty interval	Number	Number Uncertainty Interval
Guinea	Male	10 to 14	1950	270.94	[250.80 - 342.12]	378	[350 - 477]
			1985	159.54	[139.20 - 178.95]	478	[417 - 536]
			2019	114.25	[87.05 - 144.33]	940	[717 - 1,188]
		15 to 19	1950	361.43	[334.42 - 457.64]	463	[429 - 587]
			1985	203.87	[172.09 - 230.53]	461	[389 - 521]
			2019	167.78	[132.49 - 211.21]	1,077	[850 - 1,356]
		20 to 24	1950	375.45	[353.40 - 457.92]	442	[416 - 539]
			1985	232.46	[199.05 - 261.58]	418	[358 - 471]
			2019	223.94	[178.11 - 275.91]	1,123	[893 - 1,384]
	Female	10 to 14	1950	219.06	[205.56 - 264.35]	305	[287 - 368]
			1985	148.93	[129.53 - 167.10]	417	[362 - 467]
			2019	94.91	[73.63 - 114.07]	772	[599 - 928]
		15 to 19	1950	309.56	[293.24 - 360.47]	355	[337 - 414]
			1985	210.69	[181.06 - 240.94]	507	[436 - 580]
			2019	136.67	[111.50 - 159.06]	935	[763 - 1,088]
		20 to 24	1950	435.39	[402.33 - 545.30]	425	[392 - 532]
			1985	288.44	[235.06 - 342.09]	671	[547 - 796]
			2019	202.09	[158.12 - 243.70]	1,206	[944 - 1,455]

Table S204: All-cause mortality rate per 100,000 population and number of deaths in 10-24 by sex 1950 - 2019: Guinea-Bissau

Location	Sex	Age	Year	Mortality per 100,000	Mortality per 100,000 uncertainty interval	Number	Number Uncertainty Interval
Guinea-Bissau	Male	10 to 14	1950	462.02	[431.62 - 554.21]	132	[123 - 158]
			1985	245.24	[200.56 - 297.85]	140	[115 - 171]
			2019	113.58	[102.60 - 129.86]	136	[123 - 155]
		15 to 19	1950	664.81	[621.34 - 810.81]	163	[152 - 199]
			1985	359.13	[291.01 - 448.24]	159	[129 - 198]
			2019	215.09	[180.05 - 262.56]	220	[184 - 268]
		20 to 24	1950	642.61	[604.84 - 798.50]	142	[133 - 176]
			1985	406.83	[337.96 - 491.41]	144	[119 - 173]
			2019	280.63	[245.35 - 352.31]	247	[216 - 310]
	Female	10 to 14	1950	257.33	[245.20 - 299.73]	69	[65 - 80]
			1985	157.54	[131.92 - 185.16]	91	[76 - 107]
			2019	80.31	[75.60 - 87.60]	95	[90 - 104]
		15 to 19	1950	338.06	[325.02 - 387.75]	81	[78 - 93]
			1985	223.29	[191.42 - 249.02]	106	[91 - 118]
			2019	125.90	[113.13 - 151.72]	132	[118 - 158]
		20 to 24	1950	525.96	[492.87 - 635.98]	127	[119 - 153]
			1985	320.23	[257.68 - 380.60]	132	[107 - 157]
			2019	180.56	[158.07 - 221.99]	171	[149 - 210]

Table S205: All-cause mortality rate per 100,000 population and number of deaths in 10-24 by sex 1950 - 2019: Liberia

Location	Sex	Age	Year	Mortality per 100,000	Mortality per 100,000 uncertainty interval	Number	Number Uncertainty Interval
Liberia	Male	10 to 14	1950	298.09	[278.15 - 376.57]	151	[141 - 191]
			1985	145.48	[117.46 - 168.08]	206	[167 - 239]
			2019	79.02	[58.32 - 93.17]	243	[179 - 286]
		15 to 19	1950	396.53	[366.34 - 499.14]	189	[175 - 238]
			1985	187.74	[146.55 - 222.33]	225	[175 - 266]
			2019	124.32	[98.92 - 149.45]	328	[261 - 395]
		20 to 24	1950	398.85	[373.97 - 482.69]	160	[150 - 194]
			1985	217.11	[168.99 - 260.82]	225	[175 - 270]
			2019	178.27	[129.69 - 213.22]	391	[285 - 468]
	Female	10 to 14	1950	192.78	[179.75 - 236.68]	93	[87 - 114]
			1985	120.30	[93.11 - 141.55]	160	[124 - 188]
			2019	72.60	[49.77 - 84.34]	218	[149 - 253]
		15 to 19	1950	278.39	[258.87 - 331.27]	118	[110 - 141]
			1985	171.31	[132.60 - 206.90]	207	[160 - 250]
			2019	109.69	[82.33 - 126.10]	292	[219 - 335]
		20 to 24	1950	375.96	[344.30 - 479.80]	151	[139 - 193]
			1985	226.87	[165.50 - 290.16]	261	[191 - 334]
			2019	162.55	[116.88 - 191.42]	374	[269 - 441]

Table S206: All-cause mortality rate per 100,000 population and number of deaths in 10-24 by sex 1950 - 2019: Mali

Location	Sex	Age	Year	Mortality per 100,000	Mortality per 100,000 uncertainty interval	Number	Number Uncertainty Interval
Mali	Male	10 to 14	1950	322.78	[298.36 - 405.73]	588	[544 - 739]
			1985	175.30	[162.11 - 190.98]	822	[760 - 895]
			2019	97.79	[71.40 - 134.49]	1,422	[1,038 - 1,956]
		15 to 19	1950	431.06	[398.28 - 542.45]	711	[657 - 895]
			1985	230.53	[208.54 - 253.98]	819	[741 - 902]
			2019	144.43	[108.38 - 188.88]	1,762	[1,322 - 2,305]
		20 to 24	1950	433.96	[405.14 - 521.38]	643	[600 - 773]
			1985	263.98	[238.30 - 292.98]	734	[663 - 815]
			2019	193.50	[148.63 - 242.12]	1,842	[1,415 - 2,304]
	Female	10 to 14	1950	245.72	[229.95 - 293.03]	507	[475 - 605]
			1985	154.65	[142.28 - 169.29]	707	[651 - 774]
			2019	85.37	[59.48 - 118.66]	1,213	[845 - 1,686]
		15 to 19	1950	341.07	[322.02 - 388.86]	595	[562 - 678]
			1985	220.14	[197.65 - 243.06]	854	[767 - 943]
			2019	123.48	[88.08 - 164.84]	1,517	[1,082 - 2,026]
		20 to 24	1950	489.22	[452.67 - 602.30]	729	[675 - 898]
			1985	301.97	[258.18 - 345.35]	1,030	[881 - 1,178]
			2019	169.58	[120.49 - 231.67]	1,731	[1,230 - 2,365]

Table S207: All-cause mortality rate per 100,000 population and number of deaths in 10-24 by sex 1950 - 2019: Mauritania

Location	Sex	Age	Year	Mortality per 100,000	Mortality per 100,000 uncertainty interval	Number	Number Uncertainty Interval
Mauritania	Male	10 to 14	1950	209.92	[192.94 - 264.04]	76	[70 - 96]
			1985	127.21	[111.82 - 141.21]	139	[123 - 155]
			2019	53.88	[41.00 - 71.71]	142	[108 - 189]
		15 to 19	1950	294.79	[272.49 - 387.75]	111	[103 - 146]
			1985	187.74	[160.33 - 218.98]	171	[146 - 199]
			2019	85.52	[69.19 - 108.37]	187	[152 - 237]
		20 to 24	1950	341.60	[320.67 - 423.44]	87	[82 - 108]
			1985	249.80	[212.93 - 286.53]	184	[157 - 211]
			2019	128.44	[88.40 - 168.58]	227	[156 - 298]
	Female	10 to 14	1950	158.12	[147.31 - 197.50]	61	[57 - 77]
			1985	113.42	[101.34 - 124.50]	122	[109 - 134]
			2019	50.56	[34.72 - 68.74]	133	[92 - 181]
		15 to 19	1950	236.85	[226.46 - 274.64]	93	[89 - 108]
			1985	168.10	[153.08 - 180.25]	154	[140 - 165]
			2019	75.05	[50.71 - 99.03]	173	[117 - 228]
		20 to 24	1950	360.97	[342.11 - 450.56]	98	[93 - 122]
			1985	258.70	[221.62 - 290.58]	202	[173 - 227]
			2019	109.44	[71.07 - 147.98]	212	[138 - 287]

Table S208: All-cause mortality rate per 100,000 population and number of deaths in 10-24 by sex 1950 - 2019: Niger

Location	Sex	Age	Year	Mortality per 100,000	Mortality per 100,000 uncertainty interval	Number	Number Uncertainty Interval
Niger	Male	10 to 14	1950	290.00	[270.78 - 365.93]	451	[421 - 570]
			1985	189.95	[165.55 - 214.47]	780	[680 - 881]
			2019	95.18	[68.07 - 133.05]	1,527	[1,092 - 2,135]
		15 to 19	1950	376.74	[353.49 - 474.67]	452	[424 - 569]
			1985	245.63	[212.71 - 282.96]	774	[670 - 891]
			2019	139.54	[101.56 - 187.46]	1,725	[1,256 - 2,318]
		20 to 24	1950	377.63	[357.88 - 454.65]	416	[394 - 501]
			1985	265.89	[235.04 - 304.97]	675	[596 - 774]
			2019	188.35	[140.12 - 238.92]	1,795	[1,335 - 2,277]
	Female	10 to 14	1950	196.91	[185.76 - 240.50]	353	[333 - 431]
			1985	147.52	[125.18 - 169.49]	618	[525 - 710]
			2019	79.29	[54.50 - 111.59]	1,263	[868 - 1,778]
		15 to 19	1950	281.14	[262.05 - 334.47]	385	[359 - 458]
			1985	203.11	[172.66 - 235.60]	702	[597 - 814]
			2019	112.83	[78.75 - 153.14]	1,466	[1,023 - 1,989]
		20 to 24	1950	370.72	[340.30 - 473.26]	457	[420 - 584]
			1985	255.78	[208.12 - 310.20]	771	[627 - 935]
			2019	151.36	[103.28 - 208.58]	1,550	[1,057 - 2,135]

Table S209: All-cause mortality rate per 100,000 population and number of deaths in 10-24 by sex 1950 - 2019: Nigeria

Location	Sex	Age	Year	Mortality per 100,000	Mortality per 100,000 uncertainty interval	Number	Number Uncertainty Interval
Nigeria	Male	10 to 14	1950	211.08	[200.05 - 249.71]	4,617	[4,376 - 5,462]
			1985	125.92	[106.48 - 149.32]	5,452	[4,610 - 6,465]
			2019	75.32	[63.56 - 92.81]	10,571	[8,920 - 13,026]
		15 to 19	1950	286.74	[267.28 - 353.45]	5,491	[5,118 - 6,768]
			1985	166.14	[129.09 - 209.81]	6,240	[4,849 - 7,881]
			2019	112.39	[87.05 - 148.45]	12,874	[9,972 - 17,005]
		20 to 24	1950	313.10	[291.33 - 385.34]	5,372	[4,999 - 6,611]
			1985	196.89	[152.13 - 248.58]	6,433	[4,970 - 8,122]
			2019	149.72	[113.12 - 203.83]	13,692	[10,346 - 18,640]
	Female	10 to 14	1950	160.84	[152.50 - 189.51]	3,285	[3,115 - 3,871]
			1985	95.94	[75.66 - 125.83]	4,770	[3,762 - 6,257]
			2019	57.17	[46.21 - 88.18]	8,505	[6,873 - 13,118]
		15 to 19	1950	231.33	[216.60 - 276.13]	3,874	[3,627 - 4,624]
			1985	137.25	[106.43 - 180.34]	5,790	[4,490 - 7,607]
			2019	84.72	[65.10 - 128.95]	10,678	[8,205 - 16,253]
		20 to 24	1950	305.04	[274.37 - 399.34]	4,370	[3,931 - 5,721]
			1985	175.26	[122.71 - 248.75]	6,103	[4,273 - 8,662]
			2019	117.24	[83.03 - 183.08]	12,277	[8,695 - 19,173]

Table S210: All-cause mortality rate per 100,000 population and number of deaths in 10-24 by sex 1950 - 2019: Sao Tome and Principe

Location	Sex	Age	Year	Mortality per 100,000	Mortality per 100,000 uncertainty interval	Number	Number Uncertainty Interval
Sao Tome and Principe	Male	10 to 14	1950	265.52	[251.06 - 322.28]	12	[11 - 15]
			1985	169.13	[156.76 - 182.18]	12	[11 - 13]
			2019	43.04	[34.79 - 51.66]	6	[4 - 7]
		15 to 19	1950	373.31	[352.10 - 461.06]	17	[16 - 21]
			1985	115.51	[103.53 - 128.70]	7	[6 - 7]
			2019	104.12	[87.36 - 113.46]	12	[10 - 13]
		20 to 24	1950	408.00	[387.96 - 477.43]	18	[17 - 21]
			1985	108.75	[97.56 - 120.45]	5	[5 - 6]
			2019	139.83	[116.11 - 164.54]	13	[11 - 15]
	Female	10 to 14	1950	210.03	[200.76 - 244.05]	5	[5 - 6]
			1985	65.00	[58.32 - 75.20]	5	[4 - 5]
			2019	32.11	[27.87 - 38.16]	4	[4 - 5]
		15 to 19	1950	300.09	[289.10 - 333.08]	8	[7 - 8]
			1985	97.47	[84.42 - 113.61]	5	[5 - 6]
			2019	57.87	[48.35 - 65.27]	7	[6 - 8]
		20 to 24	1950	439.27	[414.83 - 509.04]	11	[10 - 12]
			1985	125.48	[105.69 - 150.86]	6	[5 - 7]
			2019	73.30	[57.10 - 92.65]	7	[5 - 9]

Table S211: All-cause mortality rate per 100,000 population and number of deaths in 10-24 by sex 1950 - 2019: Senegal

Location	Sex	Age	Year	Mortality per 100,000	Mortality per 100,000 uncertainty interval	Number	Number Uncertainty Interval
Senegal	Male	10 to 14	1950	221.46	[206.61 - 277.52]	326	[304 - 409]
			1985	135.79	[118.61 - 151.16]	543	[475 - 605]
			2019	65.91	[53.09 - 81.64]	656	[528 - 812]
		15 to 19	1950	305.47	[287.63 - 386.24]	392	[369 - 496]
			1985	196.01	[168.29 - 217.84]	631	[542 - 702]
			2019	114.42	[95.16 - 135.93]	998	[830 - 1,186]
		20 to 24	1950	335.57	[316.16 - 410.48]	379	[357 - 463]
			1985	244.59	[213.57 - 270.55]	638	[557 - 706]
			2019	168.10	[123.83 - 200.39]	1,202	[885 - 1,432]
	Female	10 to 14	1950	156.39	[146.77 - 192.81]	238	[224 - 294]
			1985	105.63	[88.70 - 117.90]	445	[374 - 496]
			2019	53.79	[40.16 - 67.05]	491	[366 - 612]
		15 to 19	1950	229.80	[212.07 - 278.70]	301	[278 - 365]
			1985	155.68	[129.68 - 176.87]	542	[452 - 616]
			2019	83.35	[60.54 - 100.07]	670	[487 - 804]
		20 to 24	1950	309.04	[278.52 - 399.34]	354	[319 - 458]
			1985	211.11	[171.05 - 248.32]	619	[501 - 728]
			2019	116.28	[81.75 - 142.75]	817	[574 - 1,003]

Table S212: All-cause mortality rate per 100,000 population and number of deaths in 10-24 by sex 1950 - 2019: Sierra Leone

Location	Sex	Age	Year	Mortality per 100,000	Mortality per 100,000 uncertainty interval	Number	Number Uncertainty Interval
Sierra Leone	Male	10 to 14	1950	268.21	[250.44 - 340.20]	249	[233 - 316]
			1985	167.87	[137.20 - 206.04]	319	[261 - 391]
			2019	104.68	[73.37 - 133.21]	523	[367 - 666]
		15 to 19	1950	371.33	[345.97 - 469.44]	313	[291 - 395]
			1985	232.31	[183.92 - 288.99]	363	[287 - 451]
			2019	151.83	[113.55 - 188.80]	690	[516 - 858]
		20 to 24	1950	391.88	[368.66 - 476.15]	310	[292 - 376]
			1985	265.51	[212.68 - 326.05]	344	[275 - 422]
			2019	202.48	[153.79 - 251.91]	793	[602 - 986]
	Female	10 to 14	1950	185.01	[173.63 - 228.28]	198	[186 - 244]
			1985	117.29	[93.28 - 147.40]	228	[182 - 287]
			2019	90.56	[60.62 - 111.46]	458	[307 - 564]
		15 to 19	1950	265.37	[246.12 - 319.94]	250	[231 - 301]
			1985	164.08	[128.85 - 205.86]	273	[214 - 342]
			2019	135.66	[98.79 - 161.76]	643	[468 - 767]
		20 to 24	1950	345.75	[316.51 - 447.39]	293	[268 - 379]
			1985	203.53	[148.54 - 273.27]	299	[218 - 401]
			2019	198.63	[143.17 - 248.50]	843	[608 - 1,055]

Table S213: All-cause mortality rate per 100,000 population and number of deaths in 10-24 by sex 1950 - 2019: Togo

Location	Sex	Age	Year	Mortality per 100,000	Mortality per 100,000 uncertainty interval	Number	Number Uncertainty Interval
Togo	Male	10 to 14	1950	229.52	[212.35 - 291.61]	163	[151 - 207]
			1985	127.48	[107.91 - 145.00]	270	[228 - 307]
			2019	93.21	[70.27 - 113.55]	463	[349 - 564]
		15 to 19	1950	315.88	[290.62 - 401.22]	189	[174 - 241]
			1985	182.57	[150.93 - 206.50]	294	[243 - 332]
			2019	168.13	[128.29 - 200.08]	704	[537 - 838]
		20 to 24	1950	346.45	[321.86 - 425.21]	182	[169 - 223]
			1985	230.36	[190.43 - 263.10]	295	[244 - 337]
			2019	228.41	[169.06 - 268.41]	803	[594 - 943]
	Female	10 to 14	1950	161.81	[149.12 - 196.96]	120	[110 - 146]
			1985	103.10	[84.91 - 116.76]	210	[173 - 238]
			2019	67.37	[48.88 - 80.15]	311	[226 - 370]
		15 to 19	1950	240.78	[223.81 - 283.98]	166	[154 - 196]
			1985	153.15	[124.20 - 176.13]	249	[202 - 286]
			2019	108.26	[79.55 - 130.02]	422	[310 - 507]
		20 to 24	1950	335.65	[304.09 - 425.71]	207	[187 - 262]
			1985	209.41	[164.70 - 252.40]	300	[236 - 362]
			2019	141.40	[100.26 - 170.90]	496	[352 - 600]

Table S214: All-cause mortality rate per 100,000 population and number of deaths in 10-24 by sex 1950 - 2019: American Samoa

Location	Sex	Age	Year	Mortality per 100,000	Mortality per 100,000 uncertainty interval	Number	Number Uncertainty Interval
American Samoa	Male	10 to 14	1950	104.20	[101.00 - 115.40]	1	[1 - 2]
			1985	56.66	[51.52 - 62.22]	1	[1 - 1]
			2019	28.74	[25.50 - 33.73]	1	[1 - 1]
		15 to 19	1950	167.75	[160.39 - 193.69]	2	[2 - 2]
			1985	137.97	[123.28 - 154.29]	3	[3 - 4]
			2019	95.56	[87.34 - 107.38]	3	[3 - 3]
		20 to 24	1950	242.86	[231.99 - 276.35]	2	[2 - 2]
			1985	266.74	[241.12 - 293.98]	5	[5 - 6]
			2019	177.78	[145.61 - 235.00]	4	[4 - 6]
	Female	10 to 14	1950	86.82	[84.70 - 94.16]	1	[1 - 1]
			1985	28.69	[26.10 - 31.40]	1	[1 - 1]
			2019	22.26	[18.55 - 27.45]	1	[1 - 1]
		15 to 19	1950	131.15	[127.05 - 145.01]	1	[1 - 2]
			1985	57.31	[52.29 - 63.50]	1	[1 - 1]
			2019	51.03	[42.56 - 63.34]	2	[1 - 2]
		20 to 24	1950	197.13	[188.25 - 228.85]	2	[2 - 2]
			1985	73.66	[63.43 - 85.91]	2	[1 - 2]
			2019	74.83	[59.57 - 97.39]	2	[1 - 2]

Table S215: All-cause mortality rate per 100,000 population and number of deaths in 10-24 by sex 1950 - 2019: Bermuda

Location	Sex	Age	Year	Mortality per 100,000	Mortality per 100,000 uncertainty interval	Number	Number Uncertainty Interval
Bermuda	Male	10 to 14	1950	72.29	[70.90 - 77.86]	1	[1 - 1]
			1985	25.81	[22.98 - 29.18]	1	[0 - 1]
			2019	14.18	[12.34 - 16.27]	0	[0 - 0]
		15 to 19	1950	82.82	[80.74 - 91.26]	1	[1 - 1]
			1985	79.64	[71.62 - 88.36]	2	[2 - 2]
			2019	50.01	[43.45 - 58.80]	1	[1 - 1]
		20 to 24	1950	133.13	[129.79 - 146.05]	2	[2 - 2]
			1985	162.44	[152.33 - 172.46]	4	[4 - 4]
			2019	79.45	[70.47 - 90.97]	1	[1 - 1]
	Female	10 to 14	1950	91.18	[89.50 - 98.59]	2	[2 - 2]
			1985	18.60	[17.06 - 20.34]	0	[0 - 0]
			2019	9.21	[7.99 - 10.67]	0	[0 - 0]
		15 to 19	1950	105.62	[103.54 - 114.40]	2	[2 - 2]
			1985	33.63	[30.31 - 37.76]	1	[1 - 1]
			2019	18.74	[16.48 - 21.95]	0	[0 - 0]
		20 to 24	1950	156.55	[150.78 - 179.42]	3	[2 - 3]
			1985	53.45	[48.96 - 59.98]	1	[1 - 2]
			2019	26.62	[22.88 - 31.10]	0	[0 - 0]

Table S216: All-cause mortality rate per 100,000 population and number of deaths in 10-24 by sex 1950 - 2019: Cook Islands

Location	Sex	Age	Year	Mortality per 100,000	Mortality per 100,000 uncertainty interval	Number	Number Uncertainty Interval
Cook Islands	Male	10 to 14	1950	590.01	[570.61 - 651.80]	6	[5 - 6]
			1985	60.24	[55.04 - 65.30]	1	[1 - 1]
			2019	14.44	[11.36 - 17.85]	0	[0 - 0]
		15 to 19	1950	995.42	[952.83 - 1129.15]	8	[8 - 9]
			1985	144.97	[131.67 - 159.05]	2	[1 - 2]
			2019	59.75	[50.43 - 71.89]	0	[0 - 0]
		20 to 24	1950	515.08	[493.37 - 584.70]	3	[3 - 4]
			1985	223.26	[188.75 - 277.46]	2	[2 - 3]
			2019	149.57	[116.91 - 196.09]	1	[1 - 1]
	Female	10 to 14	1950	165.31	[161.49 - 178.66]	1	[1 - 2]
			1985	38.50	[34.70 - 42.70]	0	[0 - 0]
			2019	11.11	[8.86 - 13.46]	0	[0 - 0]
		15 to 19	1950	243.53	[238.48 - 261.50]	2	[2 - 2]
			1985	69.96	[61.09 - 82.45]	1	[1 - 1]
			2019	26.63	[20.86 - 33.91]	0	[0 - 0]
		20 to 24	1950	442.63	[427.56 - 492.79]	3	[3 - 3]
			1985	98.40	[83.49 - 118.19]	1	[1 - 1]
			2019	46.62	[34.73 - 61.87]	0	[0 - 0]

Table S217: All-cause mortality rate per 100,000 population and number of deaths in 10-24 by sex 1950 - 2019: Greenland

Location	Sex	Age	Year	Mortality per 100,000	Mortality per 100,000 uncertainty interval	Number	Number Uncertainty Interval
Greenland	Male	10 to 14	1950	261.52	[249.98 - 305.67]	4	[3 - 4]
			1985	67.20	[57.70 - 77.57]	1	[1 - 2]
			2019	26.47	[20.19 - 34.09]	1	[0 - 1]
		15 to 19	1950	596.96	[567.17 - 712.71]	7	[7 - 9]
			1985	165.15	[146.31 - 185.61]	5	[5 - 6]
			2019	97.06	[77.83 - 114.91]	2	[1 - 2]
		20 to 24	1950	658.15	[628.44 - 790.60]	7	[7 - 9]
			1985	266.03	[206.17 - 323.36]	9	[7 - 11]
			2019	176.24	[149.32 - 218.62]	4	[3 - 5]
	Female	10 to 14	1950	132.85	[128.51 - 149.17]	2	[2 - 2]
			1985	46.86	[41.19 - 53.65]	1	[1 - 1]
			2019	18.70	[15.30 - 23.69]	0	[0 - 0]
		15 to 19	1950	199.95	[194.03 - 222.22]	3	[3 - 3]
			1985	88.30	[73.53 - 105.51]	3	[2 - 3]
			2019	46.83	[38.59 - 58.67]	1	[1 - 1]
		20 to 24	1950	324.17	[310.35 - 385.86]	4	[3 - 4]
			1985	129.66	[104.84 - 158.82]	4	[3 - 5]
			2019	71.25	[58.93 - 88.75]	1	[1 - 2]

Table S218: All-cause mortality rate per 100,000 population and number of deaths in 10-24 by sex 1950 - 2019: Guam

Location	Sex	Age	Year	Mortality per 100,000	Mortality per 100,000 uncertainty interval	Number	Number Uncertainty Interval
Guam	Male	10 to 14	1950	43.24	[41.64 - 49.59]	2	[2 - 2]
			1985	31.87	[29.83 - 34.08]	2	[2 - 2]
			2019	22.08	[18.94 - 25.69]	2	[1 - 2]
		15 to 19	1950	119.41	[114.52 - 138.69]	6	[6 - 7]
			1985	130.14	[119.96 - 140.89]	9	[8 - 9]
			2019	86.88	[73.00 - 102.97]	6	[5 - 7]
		20 to 24	1950	272.46	[263.52 - 307.46]	17	[17 - 20]
			1985	166.77	[154.89 - 179.33]	13	[12 - 14]
			2019	133.46	[115.94 - 153.24]	10	[9 - 12]
	Female	10 to 14	1950	71.76	[69.48 - 80.88]	2	[2 - 2]
			1985	22.72	[21.25 - 24.15]	1	[1 - 1]
			2019	29.70	[26.34 - 33.40]	2	[2 - 2]
		15 to 19	1950	98.90	[95.79 - 111.15]	2	[2 - 2]
			1985	53.04	[49.56 - 56.48]	3	[3 - 3]
			2019	63.16	[55.80 - 71.00]	4	[4 - 4]
		20 to 24	1950	196.05	[187.13 - 232.53]	4	[4 - 4]
			1985	69.62	[62.90 - 76.69]	4	[4 - 5]
			2019	56.92	[47.92 - 67.14]	4	[3 - 4]

Table S219: All-cause mortality rate per 100,000 population and number of deaths in 10-24 by sex 1950 - 2019: Monaco

Location	Sex	Age	Year	Mortality per 100,000	Mortality per 100,000 uncertainty interval	Number	Number Uncertainty Interval
Monaco	Male	10 to 14	1950	57.76	[55.19 - 67.48]	0	[0 - 0]
			1985	20.29	[17.72 - 23.13]	0	[0 - 0]
			2019	9.65	[8.46 - 11.20]	0	[0 - 0]
		15 to 19	1950	113.51	[109.33 - 130.56]	1	[0 - 1]
			1985	70.21	[62.21 - 78.64]	1	[0 - 1]
			2019	38.78	[31.23 - 47.39]	0	[0 - 0]
		20 to 24	1950	171.31	[164.58 - 198.90]	1	[1 - 1]
			1985	106.46	[94.43 - 116.59]	1	[1 - 1]
			2019	78.49	[60.98 - 101.47]	1	[1 - 1]
	Female	10 to 14	1950	45.57	[43.05 - 56.37]	0	[0 - 0]
			1985	14.90	[12.42 - 17.37]	0	[0 - 0]
			2019	8.82	[7.73 - 10.01]	0	[0 - 0]
		15 to 19	1950	72.70	[68.42 - 91.46]	0	[0 - 0]
			1985	29.50	[24.06 - 33.95]	0	[0 - 0]
			2019	20.38	[17.35 - 24.52]	0	[0 - 0]
		20 to 24	1950	99.19	[93.52 - 126.37]	1	[1 - 1]
			1985	36.39	[32.81 - 40.70]	0	[0 - 0]
			2019	29.69	[25.32 - 39.46]	0	[0 - 0]

Table S220: All-cause mortality rate per 100,000 population and number of deaths in 10-24 by sex 1950 - 2019: Nauru

Location	Sex	Age	Year	Mortality per 100,000	Mortality per 100,000 uncertainty interval	Number	Number Uncertainty Interval
Nauru	Male	10 to 14	1950	106.30	[101.85 - 123.76]	0	[0 - 0]
			1985	72.86	[63.43 - 84.21]	0	[0 - 0]
			2019	60.79	[52.22 - 71.71]	0	[0 - 0]
		15 to 19	1950	186.42	[172.84 - 238.30]	1	[1 - 1]
			1985	196.70	[164.34 - 233.72]	1	[1 - 1]
			2019	180.08	[149.49 - 218.40]	1	[1 - 1]
		20 to 24	1950	265.55	[247.63 - 348.35]	1	[1 - 1]
			1985	370.13	[307.85 - 440.03]	2	[1 - 2]
			2019	362.60	[303.34 - 433.06]	2	[2 - 2]
	Female	10 to 14	1950	110.77	[107.78 - 127.79]	0	[0 - 0]
			1985	54.18	[45.54 - 63.96]	0	[0 - 0]
			2019	46.02	[38.28 - 56.23]	0	[0 - 0]
		15 to 19	1950	174.08	[166.25 - 205.16]	0	[0 - 0]
			1985	111.87	[94.01 - 130.33]	1	[0 - 1]
			2019	100.55	[84.68 - 120.29]	1	[0 - 1]
		20 to 24	1950	304.36	[285.29 - 382.06]	0	[0 - 0]
			1985	162.22	[125.54 - 204.48]	1	[1 - 1]
			2019	159.25	[123.02 - 208.62]	1	[1 - 1]

Table S221: All-cause mortality rate per 100,000 population and number of deaths in 10-24 by sex 1950 - 2019: Niue

Location	Sex	Age	Year	Mortality per 100,000	Mortality per 100,000 uncertainty interval	Number	Number Uncertainty Interval
Niue	Male	10 to 14	1950	152.49	[145.64 - 172.40]	0	[0 - 0]
			1985	55.86	[47.34 - 65.18]	0	[0 - 0]
			2019	44.19	[36.88 - 50.36]	0	[0 - 0]
		15 to 19	1950	229.80	[215.38 - 271.95]	0	[0 - 1]
			1985	143.34	[125.47 - 172.14]	0	[0 - 0]
			2019	124.60	[116.06 - 139.18]	0	[0 - 0]
		20 to 24	1950	298.03	[282.27 - 352.04]	0	[0 - 1]
			1985	235.10	[194.60 - 316.37]	0	[0 - 0]
			2019	194.11	[165.82 - 272.35]	0	[0 - 0]
	Female	10 to 14	1950	124.41	[119.51 - 138.10]	0	[0 - 0]
			1985	34.36	[28.80 - 41.66]	0	[0 - 0]
			2019	28.21	[24.43 - 35.50]	0	[0 - 0]
		15 to 19	1950	182.99	[177.82 - 200.10]	0	[0 - 0]
			1985	64.12	[53.28 - 84.47]	0	[0 - 0]
			2019	58.10	[49.93 - 77.57]	0	[0 - 0]
		20 to 24	1950	283.07	[271.68 - 330.67]	1	[1 - 1]
			1985	82.62	[64.00 - 113.53]	0	[0 - 0]
			2019	76.77	[58.44 - 111.02]	0	[0 - 0]

Table S222: All-cause mortality rate per 100,000 population and number of deaths in 10-24 by sex 1950 - 2019: Northern Mariana Islands

Location	Sex	Age	Year	Mortality per 100,000	Mortality per 100,000 uncertainty interval	Number	Number Uncertainty Interval
Northern Mariana Islands	Male	10 to 14	1950	94.04	[87.77 - 108.09]	0	[0 - 0]
			1985	35.34	[30.82 - 40.59]	0	[0 - 1]
			2019	28.20	[24.86 - 31.78]	0	[0 - 0]
		15 to 19	1950	164.23	[152.87 - 203.71]	0	[0 - 0]
			1985	106.27	[95.83 - 119.46]	2	[2 - 2]
			2019	95.39	[86.92 - 104.63]	2	[2 - 2]
		20 to 24	1950	243.12	[229.60 - 295.02]	1	[0 - 1]
			1985	206.14	[147.15 - 255.39]	3	[2 - 3]
			2019	167.42	[141.39 - 220.06]	3	[3 - 4]
	Female	10 to 14	1950	75.10	[70.80 - 83.63]	0	[0 - 0]
			1985	27.83	[22.66 - 34.19]	0	[0 - 1]
			2019	19.09	[16.91 - 22.57]	0	[0 - 0]
		15 to 19	1950	115.26	[110.03 - 136.45]	0	[0 - 0]
			1985	63.60	[48.12 - 78.21]	1	[1 - 1]
			2019	43.51	[38.30 - 53.35]	1	[1 - 1]
		20 to 24	1950	172.16	[160.22 - 222.82]	0	[0 - 0]
			1985	100.26	[68.20 - 132.17]	1	[1 - 2]
			2019	62.27	[48.52 - 83.46]	1	[1 - 1]

Table S223: All-cause mortality rate per 100,000 population and number of deaths in 10-24 by sex 1950 - 2019: Palau

Location	Sex	Age	Year	Mortality per 100,000	Mortality per 100,000 uncertainty interval	Number	Number Uncertainty Interval
Palau	Male	10 to 14	1950	236.50	[219.93 - 277.72]	1	[1 - 1]
			1985	79.23	[69.37 - 91.53]	1	[1 - 1]
			2019	41.49	[35.86 - 48.85]	0	[0 - 0]
		15 to 19	1950	378.36	[351.43 - 464.58]	1	[1 - 2]
			1985	192.63	[159.65 - 232.75]	2	[1 - 2]
			2019	129.93	[113.40 - 156.95]	1	[1 - 1]
		20 to 24	1950	451.56	[424.75 - 546.28]	1	[1 - 2]
			1985	325.22	[235.12 - 412.12]	3	[2 - 3]
			2019	279.80	[226.67 - 337.19]	2	[2 - 2]
	Female	10 to 14	1950	155.33	[145.90 - 175.87]	1	[1 - 1]
			1985	51.12	[43.82 - 61.03]	0	[0 - 0]
			2019	31.17	[25.51 - 36.67]	0	[0 - 0]
		15 to 19	1950	216.20	[206.20 - 246.70]	1	[1 - 1]
			1985	96.60	[73.74 - 122.52]	1	[1 - 1]
			2019	70.32	[57.68 - 83.06]	0	[0 - 0]
		20 to 24	1950	319.92	[297.02 - 389.44]	1	[1 - 1]
			1985	134.82	[101.59 - 178.10]	1	[1 - 1]
			2019	106.71	[79.85 - 137.43]	1	[0 - 1]

Table S224: All-cause mortality rate per 100,000 population and number of deaths in 10-24 by sex 1950 - 2019: Puerto Rico

Location	Sex	Age	Year	Mortality per 100,000	Mortality per 100,000 uncertainty interval	Number	Number Uncertainty Interval
Puerto Rico	Male	10 to 14	1950	117.37	[116.59 - 119.86]	154	[153 - 158]
			1985	53.80	[52.01 - 55.81]	95	[91 - 98]
			2019	12.36	[10.13 - 15.06]	13	[11 - 16]
		15 to 19	1950	200.62	[198.97 - 206.33]	219	[217 - 225]
			1985	128.79	[125.79 - 132.05]	219	[214 - 224]
			2019	74.94	[58.12 - 95.76]	88	[68 - 112]
		20 to 24	1950	414.78	[411.85 - 425.71]	372	[369 - 381]
			1985	202.78	[198.60 - 207.38]	295	[289 - 302]
			2019	191.87	[154.83 - 236.30]	220	[177 - 271]
	Female	10 to 14	1950	76.53	[75.92 - 78.73]	101	[100 - 104]
			1985	33.64	[32.45 - 34.93]	57	[55 - 59]
			2019	7.57	[6.35 - 9.03]	8	[7 - 9]
		15 to 19	1950	119.91	[119.08 - 123.05]	135	[134 - 139]
			1985	44.21	[43.03 - 45.49]	74	[72 - 76]
			2019	18.40	[15.27 - 22.14]	21	[17 - 25]
		20 to 24	1950	186.75	[185.00 - 193.29]	179	[177 - 185]
			1985	57.58	[55.49 - 59.64]	90	[87 - 93]
			2019	31.24	[23.99 - 40.36]	36	[28 - 47]

Table S225: All-cause mortality rate per 100,000 population and number of deaths in 10-24 by sex 1950 - 2019: Saint Kitts and Nevis

Location	Sex	Age	Year	Mortality per 100,000	Mortality per 100,000 uncertainty interval	Number	Number Uncertainty Interval
Saint Kitts and Nevis	Male	10 to 14	1950	57.59	[56.42 - 61.48]	2	[2 - 2]
			1985	73.67	[68.34 - 78.97]	2	[2 - 2]
			2019	35.68	[31.24 - 42.93]	1	[1 - 1]
		15 to 19	1950	137.41	[133.93 - 149.38]	3	[3 - 3]
			1985	92.72	[84.59 - 100.99]	2	[2 - 2]
			2019	106.46	[103.21 - 114.94]	2	[2 - 3]
		20 to 24	1950	209.29	[204.24 - 226.58]	4	[4 - 4]
			1985	157.86	[145.12 - 170.99]	3	[3 - 4]
			2019	171.40	[155.57 - 195.07]	4	[3 - 4]
	Female	10 to 14	1950	44.05	[43.36 - 47.37]	1	[1 - 2]
			1985	49.37	[46.42 - 52.34]	1	[1 - 1]
			2019	23.53	[21.58 - 27.36]	1	[0 - 1]
		15 to 19	1950	222.35	[218.22 - 234.41]	6	[6 - 6]
			1985	54.03	[50.94 - 57.34]	1	[1 - 1]
			2019	46.62	[42.60 - 55.83]	1	[1 - 1]
		20 to 24	1950	229.80	[222.46 - 252.05]	6	[5 - 6]
			1985	73.55	[66.10 - 81.54]	2	[1 - 2]
			2019	54.71	[48.30 - 67.37]	1	[1 - 2]

Table S226: All-cause mortality rate per 100,000 population and number of deaths in 10-24 by sex 1950 - 2019: San Marino

Location	Sex	Age	Year	Mortality per 100,000	Mortality per 100,000 uncertainty interval	Number	Number Uncertainty Interval
San Marino	Male	10 to 14	1950	48.89	[45.66 - 66.50]	0	[0 - 0]
			1985	23.32	[20.45 - 26.14]	0	[0 - 0]
			2019	10.68	[8.24 - 13.21]	0	[0 - 0]
		15 to 19	1950	109.29	[100.55 - 167.07]	1	[1 - 1]
			1985	67.36	[58.21 - 77.17]	1	[1 - 1]
			2019	35.37	[24.29 - 51.16]	0	[0 - 1]
		20 to 24	1950	137.55	[120.84 - 252.79]	1	[1 - 2]
			1985	87.68	[76.01 - 101.44]	1	[1 - 1]
			2019	61.86	[41.21 - 94.52]	1	[0 - 1]
	Female	10 to 14	1950	29.00	[26.91 - 40.53]	0	[0 - 0]
			1985	14.29	[12.69 - 15.87]	0	[0 - 0]
			2019	8.67	[6.50 - 11.46]	0	[0 - 0]
		15 to 19	1950	42.07	[35.87 - 67.28]	0	[0 - 0]
			1985	23.13	[19.71 - 27.24]	0	[0 - 0]
			2019	18.88	[13.23 - 25.80]	0	[0 - 0]
		20 to 24	1950	49.21	[42.18 - 89.32]	0	[0 - 1]
			1985	26.61	[20.84 - 34.16]	0	[0 - 0]
			2019	26.04	[17.48 - 35.89]	0	[0 - 0]

Table S227: All-cause mortality rate per 100,000 population and number of deaths in 10-24 by sex 1950 - 2019: Tokelau

Location	Sex	Age	Year	Mortality per 100,000	Mortality per 100,000 uncertainty interval	Number	Number Uncertainty Interval
Tokelau	Male	10 to 14	1950	111.09	[106.88 - 128.77]	0	[0 - 0]
			1985	48.20	[41.92 - 54.58]	0	[0 - 0]
			2019	22.04	[17.54 - 27.52]	0	[0 - 0]
		15 to 19	1950	236.79	[223.86 - 290.31]	0	[0 - 0]
			1985	112.08	[89.63 - 134.70]	0	[0 - 0]
			2019	70.88	[60.78 - 85.86]	0	[0 - 0]
		20 to 24	1950	376.21	[346.86 - 468.38]	0	[0 - 0]
			1985	188.07	[130.65 - 254.30]	0	[0 - 0]
			2019	148.98	[111.19 - 197.04]	0	[0 - 0]
	Female	10 to 14	1950	99.54	[96.08 - 113.74]	0	[0 - 0]
			1985	47.97	[40.97 - 57.57]	0	[0 - 0]
			2019	24.29	[18.92 - 30.98]	0	[0 - 0]
		15 to 19	1950	173.51	[164.85 - 202.02]	0	[0 - 0]
			1985	94.80	[74.38 - 119.99]	0	[0 - 0]
			2019	59.50	[45.79 - 75.13]	0	[0 - 0]
		20 to 24	1950	301.83	[283.12 - 376.53]	0	[0 - 0]
			1985	160.96	[112.59 - 219.13]	0	[0 - 0]
			2019	110.33	[79.57 - 150.03]	0	[0 - 0]

Table S228: All-cause mortality rate per 100,000 population and number of deaths in 10-24 by sex 1950 - 2019: Tuvalu

Location	Sex	Age	Year	Mortality per 100,000	Mortality per 100,000 uncertainty interval	Number	Number Uncertainty Interval
Tuvalu	Male	10 to 14	1950	244.49	[234.38 - 271.31]	0	[0 - 0]
			1985	116.16	[102.11 - 132.05]	1	[0 - 1]
			2019	38.99	[32.04 - 49.47]	0	[0 - 0]
		15 to 19	1950	399.11	[375.18 - 486.42]	1	[1 - 1]
			1985	231.70	[194.80 - 273.11]	1	[1 - 1]
			2019	119.63	[101.40 - 153.25]	1	[1 - 1]
		20 to 24	1950	478.68	[449.23 - 604.38]	1	[1 - 1]
			1985	348.24	[281.90 - 436.71]	1	[1 - 2]
			2019	255.71	[206.43 - 321.05]	1	[1 - 2]
	Female	10 to 14	1950	175.28	[170.00 - 188.97]	0	[0 - 0]
			1985	89.11	[77.84 - 102.74]	0	[0 - 0]
			2019	32.38	[25.15 - 41.64]	0	[0 - 0]
		15 to 19	1950	242.92	[232.62 - 281.09]	0	[0 - 1]
			1985	146.26	[127.72 - 168.24]	1	[1 - 1]
			2019	74.26	[60.49 - 94.12]	0	[0 - 1]
		20 to 24	1950	386.40	[361.83 - 466.27]	1	[1 - 1]
			1985	228.11	[195.42 - 263.16]	1	[1 - 1]
			2019	122.64	[92.83 - 166.52]	1	[0 - 1]

Table S229: All-cause mortality rate per 100,000 population and number of deaths in 10-24 by sex 1950 - 2019: United States Virgin Islands

Location	Sex	Age	Year	Mortality per 100,000	Mortality per 100,000 uncertainty interval	Number	Number Uncertainty Interval
United States Virgin Islands	Male	10 to 14	1950	126.85	[123.04 - 142.20]	2	[2 - 2]
			1985	72.01	[65.83 - 78.16]	4	[4 - 5]
			2019	24.07	[20.23 - 27.52]	1	[1 - 1]
		15 to 19	1950	225.33	[215.11 - 263.15]	3	[3 - 3]
			1985	140.53	[124.94 - 156.70]	8	[7 - 9]
			2019	83.39	[70.58 - 96.01]	3	[2 - 3]
		20 to 24	1950	362.91	[347.81 - 422.54]	4	[4 - 4]
			1985	177.73	[159.45 - 196.60]	7	[6 - 8]
			2019	142.45	[113.92 - 192.71]	4	[3 - 5]
	Female	10 to 14	1950	76.01	[74.36 - 81.90]	1	[1 - 1]
			1985	27.55	[25.41 - 29.63]	2	[2 - 2]
			2019	14.93	[12.83 - 17.55]	1	[0 - 1]
		15 to 19	1950	134.16	[126.12 - 150.27]	2	[1 - 2]
			1985	52.96	[50.37 - 55.22]	3	[3 - 3]
			2019	29.71	[25.28 - 36.86]	1	[1 - 1]
		20 to 24	1950	218.05	[197.50 - 258.88]	2	[2 - 3]
			1985	66.04	[58.45 - 74.79]	3	[2 - 3]
			2019	36.49	[27.92 - 52.81]	1	[1 - 2]

Table S230: All-cause mortality rate per 100,000 population and number of deaths in 10-24 by sex 1950 - 2019: South Sudan

Location	Sex	Age	Year	Mortality per 100,000	Mortality per 100,000 uncertainty interval	Number	Number Uncertainty Interval
South Sudan	Male	10 to 14	1950	194.65	[181.43 - 248.73]	300	[279 - 383]
			1985	160.75	[129.98 - 196.74]	600	[485 - 735]
			2019	88.00	[71.95 - 124.96]	607	[496 - 862]
		15 to 19	1950	265.48	[248.46 - 339.50]	344	[322 - 440]
			1985	255.31	[208.22 - 309.34]	836	[682 - 1,013]
			2019	144.86	[115.48 - 197.70]	852	[679 - 1,162]
		20 to 24	1950	296.55	[279.29 - 367.97]	334	[315 - 415]
			1985	351.60	[295.61 - 417.34]	941	[791 - 1,117]
			2019	203.32	[159.31 - 277.41]	774	[607 - 1,056]
	Female	10 to 14	1950	148.77	[140.21 - 185.20]	231	[217 - 287]
			1985	120.42	[95.96 - 149.72]	391	[312 - 487]
			2019	64.58	[50.95 - 95.31]	400	[316 - 591]
		15 to 19	1950	223.31	[205.24 - 271.65]	285	[262 - 347]
			1985	172.12	[136.37 - 218.01]	490	[388 - 620]
			2019	97.53	[72.65 - 140.17]	526	[392 - 756]
		20 to 24	1950	305.51	[277.75 - 399.10]	333	[303 - 435]
			1985	213.69	[155.27 - 289.64]	506	[368 - 686]
			2019	139.31	[95.19 - 207.46]	578	[395 - 861]

Table S231: All-cause mortality rate per 100,000 population and number of deaths in 10-24 by sex 1950 - 2019: Sudan

Location	Sex	Age	Year	Mortality per 100,000	Mortality per 100,000 uncertainty interval	Number	Number Uncertainty Interval
Sudan	Male	10 to 14	1950	199.99	[189.70 - 244.20]	725	[688 - 885]
			1985	157.88	[139.42 - 174.96]	1,706	[1,506 - 1,891]
			2019	52.95	[44.30 - 66.02]	1,377	[1,152 - 1,717]
		15 to 19	1950	278.57	[263.24 - 351.95]	883	[835 - 1,116]
			1985	218.68	[189.56 - 245.16]	1,999	[1,733 - 2,241]
			2019	97.04	[72.98 - 119.05]	2,231	[1,678 - 2,737]
		20 to 24	1950	313.22	[294.07 - 394.53]	848	[796 - 1,068]
			1985	286.50	[250.79 - 318.09]	2,080	[1,820 - 2,309]
			2019	132.63	[91.06 - 179.72]	2,691	[1,848 - 3,647]
	Female	10 to 14	1950	146.28	[138.95 - 175.46]	525	[498 - 629]
			1985	130.11	[112.15 - 146.35]	1,321	[1,139 - 1,486]
			2019	39.38	[30.85 - 52.94]	955	[748 - 1,283]
		15 to 19	1950	216.17	[201.56 - 256.36]	639	[596 - 758]
			1985	170.87	[144.67 - 195.37]	1,616	[1,368 - 1,848]
			2019	59.95	[41.01 - 81.26]	1,291	[883 - 1,750]
		20 to 24	1950	290.50	[263.04 - 378.36]	691	[626 - 900]
			1985	220.41	[180.54 - 261.73]	1,778	[1,456 - 2,111]
			2019	83.19	[53.95 - 115.48]	1,617	[1,049 - 2,245]
